# Genome-Wide Co-Expression Distributions as a Metric to Prioritize Genes of Functional Importance

**DOI:** 10.3390/genes11101231

**Published:** 2020-10-20

**Authors:** Pâmela A. Alexandre, Nicholas J. Hudson, Sigrid A. Lehnert, Marina R. S. Fortes, Marina Naval-Sánchez, Loan T. Nguyen, Laercio R. Porto-Neto, Antonio Reverter

**Affiliations:** 1 CSIRO Agriculture & Food, St Lucia, QLD 4067, Australia; Sigrid.lehnert@csiro.au (S.A.L.); laercio.portoneto@csiro.au (L.R.P.-N.); toni.reverter-gomez@csiro.au (A.R.); 2 School of Agriculture and Food Sciences, The University of Queensland, Brisbane, QLD 4072, Australia; n.hudson@uq.edu.au; 3 School of Chemistry and Molecular Biosciences, The University of Queensland, Brisbane, QLD 4072, Australia; m.fortes@uq.edu.au; 4 Institute for Molecular Bioscience, The University of Queensland, Brisbane, QLD 4072, Australia; m.navalsanchez@imb.uq.edu.au; 5 Queensland Alliance for Agriculture and Food Innovation, The University of Queensland, Brisbane, QLD 4072, Australia; t.nguyen3@uq.edu.au

**Keywords:** transcriptome analysis, correlated gene expression, gene regulation

## Abstract

Genome-wide gene expression analysis are routinely used to gain a systems-level understanding of complex processes, including network connectivity. Network connectivity tends to be built on a small subset of extremely high co-expression signals that are deemed significant, but this overlooks the vast majority of pairwise signals. Here, we developed a computational pipeline to assign to every gene its pair-wise genome-wide co-expression distribution to one of 8 template distributions shapes varying between unimodal, bimodal, skewed, or symmetrical, representing different proportions of positive and negative correlations. We then used a hypergeometric test to determine if specific genes (regulators versus non-regulators) and properties (differentially expressed or not) are associated with a particular distribution shape. We applied our methodology to five publicly available RNA sequencing (RNA-seq) datasets from four organisms in different physiological conditions and tissues. Our results suggest that genes can be assigned consistently to pre-defined distribution shapes, regarding the enrichment of differential expression and regulatory genes, in situations involving contrasting phenotypes, time-series, or physiological baseline data. There is indeed a striking additional biological signal present in the genome-wide distribution of co-expression values which would be overlooked by currently adopted approaches. Our method can be applied to extract further information from transcriptomic data and help uncover the molecular mechanisms involved in the regulation of complex biological process and phenotypes.

## 1. Introduction

Uncovering the genetic architecture behind complex phenotypes involves analyzing a large variety of genes that interact with each other to respond to environmental stimuli [1]. Therefore, gene co-expression studies are becoming increasingly popular in the quest of going beyond differential expression (DE) and recovering the functional information from relevant tissues [2]. A gene co-expression study requires the computation of the co-expression correlation coefficient between a given gene and all the other genes under scrutiny, potentially numbering in the thousands. However, defining which gene-level features are relevant is not a simple task and main strategies include focusing on highly connected genes, overlaying other information onto the network, such as patterns of DE and exploiting variation in expression [3]. There is merit in developing additional data-driven metrics to dissect co-expression networks and prioritize genes.

One system-wide topological feature of those co-expression networks is a structure comprising many nodes with few connections and few central nodes (hubs) with many connections, in a non-random fashion characterized by a scale-free power-law distribution [4]. The implication is, within the network, different genes present different “behaviors” (i.e., different abilities to influence other molecules in the network), represented by the strength and number of their correlation coefficients. With this in mind, we propose that individual genes possess genome-wide distributions of co-expression values that may be different from those observed when examining all pairs. Furthermore, these gene-specific distributions may respond to environmental condition and/or physiological state, producing different distributions in different biological circumstances. 

Hudson et al. [5] demonstrated these differences certainly exist at a whole system level by plotting frequency distributions of all pairwise correlation coefficients for six transcriptional landscapes of bovine skeletal muscle, considering different cattle breeds, nutrition, and physiological states, each one yielding a distinct distribution. Similarly, Remondini et al. [6] explored the dynamic properties of all pairwise gene correlation distributions, such as skewness, using expression data of rat fibroblasts expressing a conditional Myc-estrogen receptor oncoprotein. Their analysis has identified the cascade of c-myc-activated genes within the network. Therefore, current evidence supports the concept that correlation dynamics in co-expression networks have biological meaning. In the approach described here, we aimed to determine if genome-wide patterns of within-gene co-expression values are the source of untapped biological information. We anticipate this new metric will improve our ability to extract further biologically relevant insights from co-expression networks.

## 2. Material & Methods

### 2.1. Algorithm

**Figure 1:**
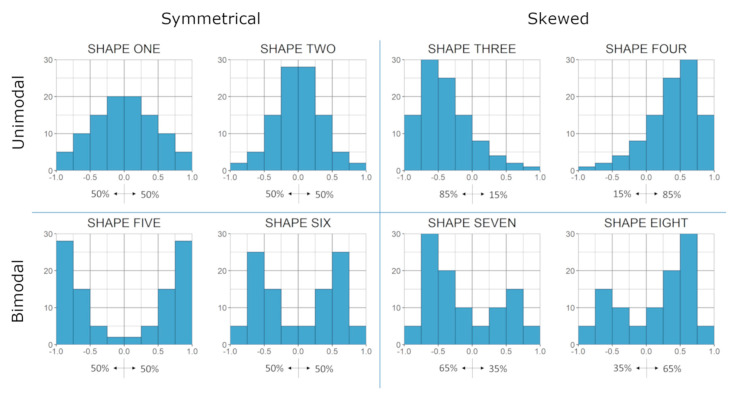
Distribution shapes template. Individual genes were assigned to one of these eight distributions shapes based on their correlation coefficients to all other expressed genes. Distributions were based on the proportion of correlations falling in each of the eight 0.25-bins of the −1 to +1 range.

We aimed to cluster genes based on them sharing a density distribution of genome-wide correlation coefficients. For that, eight shapes were used as templates, varying between unimodal, bimodal, skewed or symmetrical, representing different proportions of positive and negative correlations (Figure 1). Shapes were determinate considering specific proportions of each 0.25-bins as a nominal value, obtained in an ad-hoc basis, to add to 100 across the 8 bins while producing the desired distribution shape in terms of symmetry and uni- or bi-modality.

Starting with a normalized expression matrix, the Pearson correlation coefficient was computed for each possible gene pair across all the samples. For each gene, the number of correlations within the eight 0.25-bins of the distributions was recorded and summary statistics calculated (i.e., mean, Standard Deviation (SD), skewness, and kurtosis). Then, based on the proportion of correlations falling in each bin and the summary statistics, the Euclidian distance between each gene distribution to each of the eight template distribution shapes was computed. We did this by comparing the observed values for a given gene to the expected values for each template shape listed in Supplementary File 1: Table S1. In algebraic terms, the distance of the *i*-th gene to the *j*-th template shape ($D_{i,j}$) was computed as follows:

(1) $$D_{i,j}={\left[\sum_{k=1}^{8}{\left(OBP_{i,k}-EBP_{j,k}\right)}^{2}+{\left(OMN_{i}-EMN_{j}\right)}^{2}+\;{\left(OSD_{i}-ESD_{j}\right)}^{2}+\;{\left(OSK_{i}-ESK_{j}\right)}^{2}+\;{\left(OKU_{i}-EKU_{j}\right)}^{2}\;\right]}^{1/2},$$

where: subscripts *i*, *j*, and *k* indicate gene, template distribution shape, and 0.25-bin within a shape, respectively; $OBP_{i,k}$ is the observed bin proportion of the *i*-th gene in the *k*-th bin, which is the proportion of all the co-expression correlation coefficients from the *i*-th gene that fall within the *k*-th 0.25 bin; $EBP_{j,k}$ is the expected bin proportion of the *k*-th bin in the *j*-th template distribution shape; $OMN_{i}$ is the observed mean of all the co-expression coefficients from the *i*-th gene; $EMN_{j}$ is the expected mean of all the co-expression coefficients in the *j*-th template distribution shape; $OSD_{i}$ is the observed SD of all the co-expression coefficients from the *i*-th gene; $ESD_{j}$ is the expected SD of all the co-expression coefficients in the *j*-th template distribution shape; $OSK_{i}$ is the observed skewness of all the co-expression coefficients from the *i*-th gene; $ESK_{j}$ is the expected skewness of all the co-expression coefficients in *j*-th template distribution shape; $OKU_{i}$ is the observed kurtosis of all the co-expression coefficients from the *i*-th gene; $EKU_{j}$ is the expected kurtosis of all the co-expression correlation coefficients in *j*-th template distribution shape.

Distances were transformed into similarities ($S_{i,j}$) of a given gene to belong to each of the template distribution shape as follows:

(2) $$S_{i,j}=1.0-\;\frac{D_{i,j}-Min\left\{D_{i,j}\right\}}{Max\left\{D_{i,j}\right\}-Min\left\{D_{i,j}\right\}},$$

where $Min\left\{D_{i,j}\right\}$ and $Max\left\{D_{i,j}\right\}$ are the minimum and maximum $D_{i,j}$, respectively. Finally, similarities were transformed to probabilities ($P_{i,j}$) of a given gene to belong to each template distribution shape so that the sum of all probabilities for a given gene added to one:

(3) $$P_{i,j}=\frac{S_{i,j}}{\sum_{j=1}^{8}S_{i,j}}.$$

Finally, a gene was assigned to the *j*-th distribution template shape if its $P_{i,j}$ was the largest across all *j*’s.

### 2.2. Assessing Biological Relevance

To gain insight into biological drivers of gene co-expression distribution, genes were categorized according to their reported biological relevance (e.g., DE, transcription factors). A hypergeometric test was applied to identify enriched or depleted categories in each shape, using the function “phyper” in the R environment [7]. Therefore, we compared the within-shape proportion of genes in a given category to the proportion of overall genes in that category. To test the association between categories and types of distribution, a chi-square test of independence was applied. Results were considered significant if *p*-value ≤ 0.05.

To investigate the relationship between the number of connections per gene (degree) and distribution shapes, we used the same datasets as input to a Partial Correlation and Information Theory (PCIT) analysis [8]. The PCIT algorithm determines significant correlations (connections) between two genes after accounting for all the other genes under scrutiny. We then evaluate: (1) if there was a relationship between distribution shape and the average number of connections (significant correlations) per gene using a one-way ANOVA; (2) if the top and the bottom 5% genes based on degree would be enriched in specific shapes. 

Finally, we evaluated if the different distribution shapes would capture some general biological process. For that purpose, we used the online platform GOrilla (http://cbl-gorilla.cs.technion.ac.il/) to test a list of genes falling in each shape for each dataset against all genes considered for analysis in that dataset. GOrilla uses the hypergeometric test and false discovery rate (FDR) correction to determine significantly enriched gene ontology terms (Padj < 0.05). For this analysis, we focused on cell components.

### 2.3. Data Resources

We applied our methodology to five publicly available RNA sequencing (RNA-seq) datasets from four organisms in different physiological conditions, from different tissues. These five datasets focused on different biological questions and were chosen so that we could better explore the utility of the new metric. Supplementary File 1: Table S2 summarizes the characteristics of each dataset. 

The Cattle Feed Efficiency dataset corresponds to Reference [9,10]. In brief, the data represents 11,662 genes with average log_2_(FPKM; fragments per kilobase of gene per million mapped reads) > 1 across all five tissues (adrenal gland, hypothalamus, liver, skeletal muscle, and pituitary) from 18 Nellore bulls from extremes of feed efficiency. We classified genes as DE (382 genes) and regulators (REG, 1072 genes) according to Reference [9]. 

The Cattle Puberty dataset is comprised of five tissues (hypothalamus, pituitary, ovary, uterus, and liver) from 6 pre- and 6 post-pubertal Brahman heifers, corresponding to Reference [11,12,13,14]. A total of 16,978 genes that presented average FPKM ≥ 0.2 in at least 1 tissue were used for analysis. Genes were classified as DE (2335 genes) based on the four aforementioned works, and as REG (1584 genes) as described before.

The Duck Subcutaneous Preadipocyte Differentiation dataset is available in Additional File 3 of the source article [15]. Data represents preadipocytes cultured in differentiation medium and collected at −48 h, 0 h, 12 h, 24 h, 48 h, and 72 h. We kept for analysis only genes presenting FPKM > 0 in all samples (13,322 genes), which were then log2-transformed. Genes were classified as DE (3321 genes) based on the list provided by the authors (additional file 4 [15]) and as REG (675 genes) based on the Animal Transcription Factor Database 3.0 [16].

The Drosophila Embryogenesis dataset corresponds to [17] investigating a time-course experiment with 14 time points during *Drosophila melanogaster* embryogenesis. Data were averaged within each time point and log2 transformed prior to implementation. Genes were classified in the categories defined by Reference [17], based on pairwise comparison of genes up- or down-regulated (relative to the first time point, 0 h) in mRNA and protein data, respectively, namely: up/up (511 genes), down/up (1770 genes), down/down (1048 genes), and up/down (326 genes). Genes were also classified as regulators (791 genes) based on the list provided by [18] consisting of essential genes involved in replication and transcription, splicing, DNA repair, and cell division. 

The human dataset was downloaded from The Genotype-Tissue Expression Project V8 (https://www.gtexportal.org/) which contain data of non-diseased individuals [19]. We used liver RNA-seq data from 15 individuals, provided as TPM counts. We kept for analysis only genes presenting non-zero counts in all samples, which were then log2-transformed. Genes were classified as REG (1153 genes) and tissue enriched (TE, 231 genes) according to information provided by The Human Protein Atlas [20]. Moreover, genes were defined as DE (793 genes) if they were identified by [21] as having high probability (>0.95) of being DE in any experiment, based on a meta-analysis of over 600 DE studies.

## 3. Results and Discussion

### 3.1. Overall Co-Expression Distribution

Although our methodology aims to evaluate co-expression distributions at the individual gene-level, we did calculate all correlations across all genes for each of the five RNA-Seq datasets we evaluated: Cattle Feed Efficiency, Cattle Puberty, Drosophila Embryogenesis, Duck Preadipocyte, and Human. The overall frequency distributions in each dataset give us an overview of co-expression patterns (Supplementary File 1: Figure S1). The higher number of positive correlations, even though discrete in some datasets, was already expected and documented in previous research [5,22]. The number of positive correlations is especially elevated in the Cattle Feed Efficiency dataset. This can be due to the high inflammatory response found in the liver of those animals, which remains strong even when analyzing all five tissues together and results in a set of highly positively co-expressed genes [9]. 

On the other hand, among the 5 tissues analyzed in the Cattle Puberty Dataset, only ovary and uterus showed great differences between pre- and post-puberty and the effect of the coordinated mechanisms regulating those differences are not so strong in the overall frequency distribution. Both Drosophila Embryogenesis and Duck Preadipocyte datasets representing developmental processes through time-series data present similar shapes. They show strong bimodal positive and negative correlations because of the tightly coordinated processes the datasets represent. The Human dataset is the one with the frequency distribution closer to a bell shape, but still, the higher presence of positive correlations can be observed.

It is important to mention that, while the choice of datasets was somehow arbitrary, we selected datasets that we were familiar with, and, therefore, we were confident about data generation and bioinformatics analysis. Both cattle datasets and the drosophila dataset are associated with previous publications of the authors. The human dataset was chosen based on the credibility of the Genotype-Tissue Expression Project and the possibility of including a physiological baseline dataset, which is not associated to any particular disease or phenotype, something not often found in animal studies. The duck dataset was chosen for being particularly well-designed and unique, giving us the possibility to draw comparisons among time-series datasets. We used the expression values reported in the original studies because we aimed to develop a method that could be incorporated into any pipeline and still produce consistent results.

### 3.2. Co-Expression Distribution in Datasets With Contrasting Phenotypes

**Figure 2:**
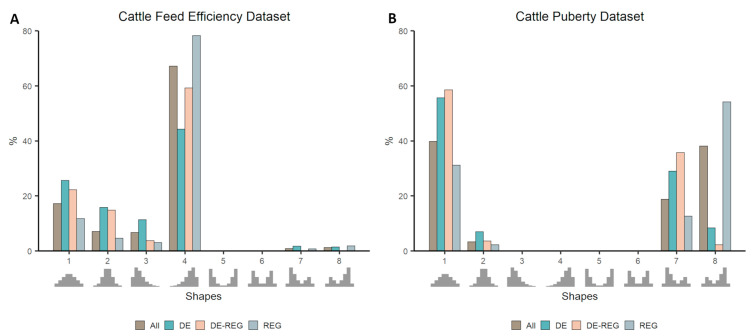
Proportion of genes falling in each template shape in datasets with contrasting phenotypes: cattle feed efficiency (**A**) and cattle puberty (**B**). The proportion of genes classified as differentially expressed (DE), regulator (REG), or both (DE-REG) are compared to the overall (All) proportion of genes within each shape.

The two cattle datasets represented contrasting phenotypes, i.e., high versus low feed efficiency, and pre- versus post-puberty. Considering co-expression distributions at gene-level, the proportion of genes falling in each distribution shape can be found in Figure 2. When comparing the proportion of each category of genes (DE, DE-REG, or REG) with that of all genes within individual distributions, for the Cattle Feed Efficiency dataset, we identified an over-representation of REG in negatively skewed distributions (i.e., with an overabundance of positive co-expression (Shapes 4 and 8) and an under-representation of those genes in either null distributions (Shapes 1 and 2) or in a positively skewed distribution (Shape 3, Figure 2A). In contrast, DE genes were under-represented in Shape 4 and over-represented in null distributions (Shapes 1 and 2) and in bimodal skewed distributions (Shapes 7 and 8). The exact number of genes falling in each distribution shape for both cattle datasets and the significance of enrichment analysis can be found in Supplementary File 1: Table S3.

Similarly, the results using the Cattle Puberty dataset showed an over-representation of REG in a bimodal negative skewed distribution (Shape 8) and an under-representation of those genes in null distributions (Shapes 1 and 2), as well as in a positively skewed distribution (Shape 7, Figure 2B). DE genes also behave similarly to the previous analysis, being over-represented in null distributions (Shapes 1 and 2) and in a bimodal positively skewed distribution (Shape 7). 

**Figure 3:**
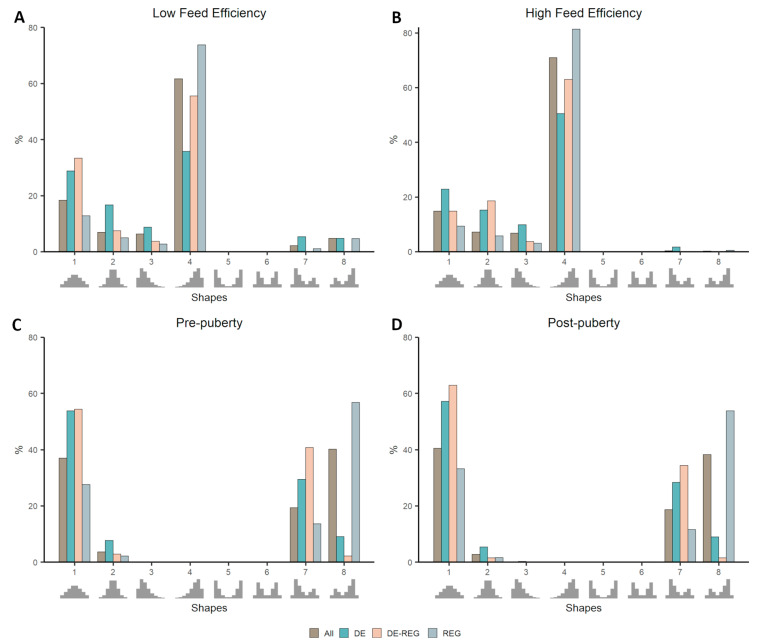
Proportion of genes falling in each template shape in contrasting phenotypic conditions within cattle datasets: low feed efficiency (**A**), high feed efficiency (**B**), pre-puberty (**C**), and post-puberty (**D**). The proportion of genes classified as DE, REG, or DE-REG are compared to the overall (All) proportion of genes within each shape.

Considering that several genes are expected to present different behavior according to the contrasting condition tested, we applied our pipeline again using the two cattle datasets split by phenotype. We then identified genes that were assigned to different shapes by comparing high to low feed efficiency and pre- to post-puberty. From the 11,662 genes tested using the Cattle Feed Efficiency dataset, 2032 genes were assigned to different shapes depending on the condition, among which 133 are DE and 158 are REG. Likewise, from the 16,978 genes tested using the Cattle Puberty dataset, 2740 were assigned to different shapes depending on the phenotype, among which 620 were DE and 216 were REG. The shift in the proportion of each class of gene depending on the phenotypic condition can be seen in Figure 3. 

The enrichment analysis for DE and REG among the genes changing behavior according to the phenotype were again concordant, with REG being under-represented and DE being over-represented (*p*-value < 0.01) in both datasets. Most REG genes, such as transcription factors and co-factors, are expected to be central genes in the network, presenting co-expression with many genes as a consequence of their regulatory role in highly coordinated biological processes. This can be one reason why they are not particularly enriched among the genes changing co-expression distribution between conditions. Nevertheless, the few REG that do change co-expression distribution between conditions are definitely worth further exploration as potential key regulators. Conversely, a gene identified as DE, being either central to a specific function or the final product of an altered pathway, are more prone to be condition-specific/enriched, not only regarding their expression level but also regarding their relationship with other genes. By identifying DE genes of central or peripheral function in the network based on their enrichment in null or non-null distributions, and exploring changes in their behavior, one can gain insight into the molecular dynamics behind the phenotype regulation. 

In general, genes changing behavior between conditions might be DE genes that fail the significance threshold in the DE analysis. They can also be genes that, although not DE between conditions, play different roles depending on the overall gene expression pattern, a feature already widely explored using differential connectivity measures [23]. The advantage of comparing distribution shapes is that it considers the direction (positive or negative) of the correlations and the proportion of the correlation falling in each bin of the distribution, corresponding to the correlation’s strength. One can explore all genes changing behavior or focus on specific distribution changes, for instance, from unimodal to bimodal or from symmetric to skewed. In our datasets, 38% of genes changing shapes in Feed Efficiency and 77% in Puberty represents a change from null to non-null distributions or vice-versa (which, in this case, also represents changes from symmetrical to skewed). Respectively, 37% and 76% of genes changing shapes are moving from unimodal to bimodal distributions, or vice-versa. Although percentages are similar, different sets of genes are selected depending on the criteria. 

The enrichment analysis for DE and REG in each shape show similar results between phenotypic conditions (Supplementary File 1: Tables S4 and S5). Nevertheless, it is possible to observe a shift in the proportion of genes falling in each shape, particularly from bimodal to unimodal shapes, implicating in a loss/gain of genes presenting both positive and negative correlations at the same time.

### 3.3. Co-Expression Distribution in Time-Series Datasets

**Figure 4:**
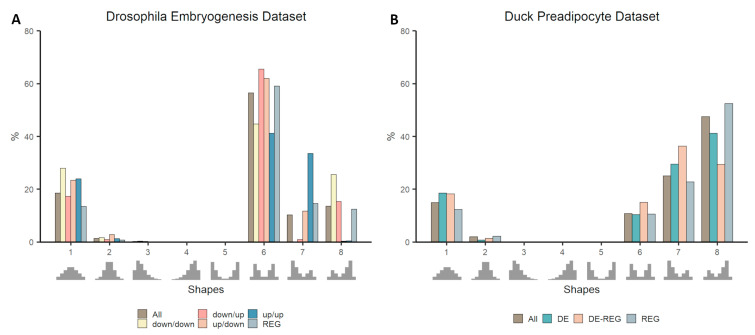
Proportion of genes falling in each template shape in time-series datasets: Drosophila embryogenesis (**A**) and Duck Preadipocyte (**B**). The proportion of genes classified as DE, REG, or DE-REG are compared to the overall (All) proportion of genes within each shape. In the Drosophila dataset, DE genes were clustered into four groups (down/down, down/up, up/down, and up/up); refer to methods for more information.

Both time-series datasets, Duck Fat differentiation and Drosophila Embryogenesis, follow the same pattern identified in the cattle datasets (Figure 4), with REG being under-represented in a null distribution (Shape 1) and over-represented in a bimodal skewed distribution (Shape 7 or 8; Supplementary File 1: Table S6). Although, in the Drosophila dataset, the DE genes were subdivided into different classes according to their concordance between mRNA and protein expression data, it is possible to observe in both datasets the enrichment of DE genes being split between null and non-null distributions. Quite remarkably, considering non-null distribution in the Drosophila dataset, genes consistently down-regulated (down-down) are enriched in the negatively-skewed bimodal distribution (Shape 8), while genes consistently up-regulated (up/up) are enriched in the opposite shape - the positively-skewed bimodal distribution (Shape 7, Figure 4A). Moreover, those two classes are under-represented in the symmetrical bimodal distribution (Shape 6) where up/down and down/up are both enriched. 

Another curious observation is that, while both cattle datasets representing contrasting phenotypes present no genes in Shape 6, both time-series datasets not only present gene falling in this shape but also there is an enrichment of DE genes. It is important to notice at this point the impact of species, tissues and even filtering criteria in the proportion of genes assigned to each shape in each dataset. Although some patterns can be identified, each dataset has its idiosyncrasies, and different aspects may be worth investigating. 

### 3.4. Co-Expression Distribution in a Physiological Baseline Dataset

**Figure 5:**
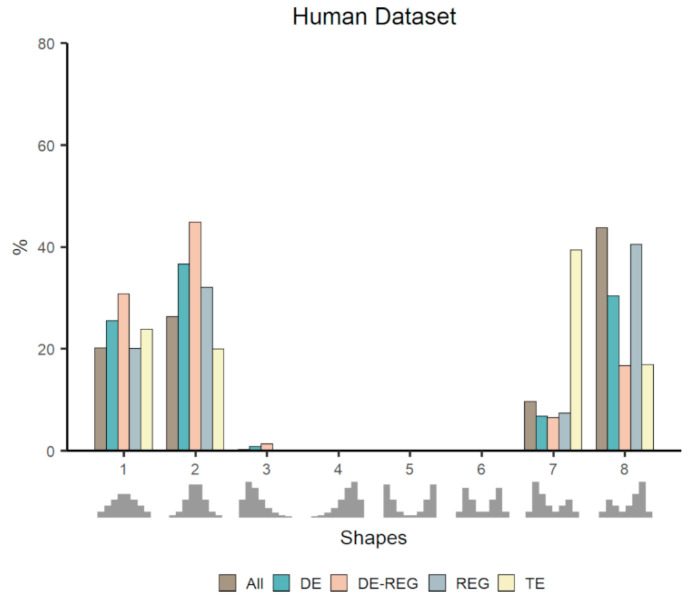
Proportion of genes falling in each template shape in the human dataset. The proportion of genes classified as DE, REG, or DE-REG are compared to the overall (All) proportion of genes within each shape.

In contrast to the other datasets, the Human dataset consists of several data points all representing a single “non-disease” baseline state and this fact is reflected in the results (Figure 5). The REG, found in other datasets to be over-represented in skewed distributions, are over-represented in a null distribution (Shape 2) and under-represented in skewed bimodal distributions (Shapes 7 and 8; Supplementary File 1: Table S7). In the previous datasets, DE genes already showed enrichment in null distributions, but they also showed enrichment in other non-null shapes. Without the effect of contrasting conditions, genes with a high probability of being DE were enriched in null distributions (Shapes 1 and 2) and in Shape 3 but with very few genes falling in this last shape (43 genes—0.2% of the total). Tissue enriched genes (TE) was the only category enriched in non-null distribution (Shape 7). That is probably because genes particular to tissue’s specific activities tend to be tightly correlated, as they need to be expressed in a coordinated pattern to keep tissues’ functions despite physiological conditions. That behavior can be clearly observed in studies that constructed co-expression networks using multiple tissue transcriptomic data, where tissue-specific genes push genes to cluster by tissue [9,24].

### 3.5. Relationship between Gene Categories and Distribution Shapes 

Considering the similarities observed between the first four datasets and the fact that without contrasting phenotypes genes with regulatory potential appear enriched in null distributions (i.e., Shapes 1 and 2), we evaluated the dependence between DE or REG genes and null versus non-null (Shapes 3 to 8), unimodal (Shapes 1 to 4) versus bimodal (Shapes 5 to 8), and symmetric (Shapes 1, 2, 5, and 6) versus skewed (Shapes 3, 4, 7, and 8) distributions. Again, we found a consistent pattern among most of the datasets, with DE genes being found more frequently than expected (*p*-value < 0.05) in null, unimodal, and symmetrical distributions, as well as REG being found more frequently than expected (*p*-value < 0.05) in non-null, bimodal, and skewed distributions (Supplementary File 1: Figure S2). In contrast, for the human dataset, both DE and REG were found more frequently than expected (*p*-value < 0.05) in null, unimodal, and symmetrical distributions.

### 3.6. Relationship between Gene Degree and Distribution Shapes 

Because genes that are expected to be highly connected to others (e.g., REG and TE) appeared enriched in non-null distributions, we explored the relationship between the number of significant correlations and the distributions shapes. For all datasets, there was a significant difference in the average number of connections per gene in each distribution shape (*p*-value < 2 × 10^−16^; Supplementary File 1: Table S8).

As anticipated, the bottom 5% genes regarding degree (least connected genes) were enriched in both null distributions (Shapes 1 and 2) for all datasets (Supplementary File 1: Table S9). The few enrichments out of those 2 shapes were due to the low number of genes assigned to that shape. Conversely, bottom genes were depleted in non-null distributions, particularly in bimodal shapes. The top 5% genes (corresponding to genes with the higher number of significant correlations) were enriched in different shapes depending on the dataset, but exclusively in non-null distribution. Except for the Human dataset, those enrichments were found in shapes that were also enriched for functionally important categories of genes (DE or REG). This overlap, particularly between transcription factors and highly connected genes have been reported before [5]. For all datasets, hub genes were depleted in null distributions. These results reinforce that genes related to tightly coordinated processes are more often found in non-null distribution.

### 3.7. Functional Enrichment within Distribution Shape

The functional enrichment of genes falling in each shape, although not consistently among the different datasets, demonstrate strong biological signals (Table 1), with some of the enrichments as significant as FDR of 1.51 × 10^−153^ (nucleoplasm). This result demonstrates genes grouped according to the distribution of their co-expression correlations can capture specific gene functions. Here, across all datasets, we only reported enrichment results at Cellular Component level. That is because it is not the focus of this work to discuss particular biological mechanisms behind each dataset, but rather a proof of principle that coherent biological signals are contained in the distribution data—something that is clearly illustrated by the Cellular Component enrichment statistics. 

Nevertheless, we also have found shape-specific enrichment for Biological Process and Molecular Function in all our test datasets. As an example, for the cattle feed efficiency dataset, we found significant enrichment for genes falling in shapes 1 to 4 (Supplementary Files 2 to 5), each shape capturing specific Biological Processes. The animals on the feed efficiency dataset have been already extensively characterized [9,10,25,26,27,28], with low feed efficiency being associated with inflammatory/immune response and altered lipid metabolism in liver. Interestingly, shape 4, the one presenting the higher number of genes and reflecting a higher number of positive correlations, is not enriched specifically for inflammatory/immune response, but for transcription-related terms, such as mRNA processing (FDR = 7.13 × 10^−19^) and mRNA splicing (FDR = 4.54 × 10^−15^). Acute inflammatory response (FDR = 2.0 × 10^−13^), regulation of humoral immune response (FDR = 1.35 × 10^−8^), and other related terms were identified in shape 3, together with terms, such as lipid catabolic process (FDR = 3.97 × 10^−18^), lipid transport (FDR = 6.88 × 10^−8^) and lipid homeostasis (FDR = 5.34 × 10^−7^). The fact that both biological processes (immune response and lipid metabolism) were enriched in the same distribution shape, particularly one with higher number of negative correlations, indicate a possible negative regulation supported by the literature in humans [29,30,31] and are worth further investigation. These high significance levels (up to FDR = 2.91 × 10^−30^, Supplementary File 4) were only possible because we are considering all expressed genes as opposed to a limited list of differentially expressed genes. This more holistic approach reflects the paradigm shift in biological research introduced by high throughput technologies, in which one understands that the whole is greater than the sum of its parts, and information processing and knowledge ordering strategies focus on assessing molecular phenotypes more comprehensively first and then determining which aspects would be important to focus on [32]. 

## 4. Conclusions

We started with the premise that, in a co-expression network, different genes present different co-expression distributions and can be grouped according to those. Our underlying null hypotheses is that a random gene, presenting no key role to the biological questions being examined, will present a null distribution with most of its co-expression correlations around zero and only a few extremes, likely false positives, on either boundary, ±1. On the other hand, a gene of relevance will reveal a distribution skewed towards the extremes reflecting the (higher than average number of) genes to which it significantly interacts. There is no benchmark dataset of this type of analysis, which makes it difficult to compare our proposed approach to existing gene clustering methods. Indeed, considering the five vastly different datasets we analyzed, genes were assigned consistently to pre-defined distribution shapes, regarding the enrichment of DE and regulatory genes, in situations involving contrasting phenotypes, time-series, or physiological baseline data.

Admittedly, there is some subjectivity in the creation of the proposed 8 template shapes. However, we believe 8 is the minimum number of shapes required to capture in a balanced way the symmetry versus skewness contrast in one dimension, as well as the uni- versus multi-modality contrast in another dimension. Similarly, the use of more or less bins within distributions (e.g., having 10 0.10-bins instead of 8 0.25-bins) is worthy of further research. Indeed, across the five datasets analyzed, no gene was allocated to Shape 5.

Similarly, caution should be taken when drawing general rules because the five datasets selected differ vastly not only in biological aspects, such as organism, tissue, and phenotype but also in numerical intricacies, including data filtering criteria, transformation, and normalization methods. Conversely, this diversity could be a test of robustness of the proposed approach. 

Despite these potential limitations, the results clearly highlight that the distribution shape of correlation coefficients can be used as a novel metric to prioritize genes of functional importance and to further explore topological characteristics of gene networks. By considering that highly connected genes will be assigned to particular distribution shapes according to the experimental design underlying the gene co-expression networks, regulatory genes can even be identified in datasets that do not represent physiological contrasts or time-series.

## Figures and Tables

**Table 1:** Hypergeometric gene set enrichments for each distribution within each data set (false discovery rate q-values) based on Cellular Component.

	Feed Efficiency	Puberty	Drosophila	Duck	Human
**Shape 1**	Ribosomal unit (7.84 × 10^−15^) Mitochondrial part (1.61 × 10^−14^)	Membrane part (9.17 × 10^−32^)	Cytoplasmic part (1.67 × 10^−8^) Mitochondrion (1.89 × 10^−4^)	Condensed chromatin outer kinetochore (3.42 × 10^−3^)	Plasma membrane part (1.95 × 10^−5^)
**Shape 2**	Mitochondrial part (3.82 × 10^−13^) ribosomal unit (1.09 × 10^−3^)	Condensed chromatin outer kinetochore (3.91 × 10^−2^)	No functional enrichment	No functional enrichment	Immunoglobulin complex (2.98 × 10^−21^)
**Shape 3**	Contractile fiber (7.55 × 10^−24^)	No functional enrichment	No functional enrichment	No functional enrichment	No functional enrichment
**Shape 4**	Nucleoplasm (2.31 × 10^−27^)	No functional enrichment			No functional enrichment
**Shape 5**	-	-	-	-	-
**Shape 6**	-	-	Nuclear part (7.64 × 10^−26^)	Proteasome complex (6.71 × 10^−3^)	-
**Shape 7**	No functional enrichment	-	Cytosolic part (9.75 × 10^−27^)	Ribosomal subunit (5.04 × 10^−16^)	Peroxisomal matrix (3.83 × 10^−8^)
**Shape 8**	Neuron projection (2.51 × 10^−2^)	Nucleoplasm (1.51 × 10^−153^)	Cytoplasm (1.45 × 10^−6^)	Golgi apparatus (2.4 × 10^−2^)	Intracellular organelle part (1.29 × 10^−108^)

## Data Availability

We implemented this method in a FORTRAN95 code. The code and example dataset are available in a Supplementary information (Supplementary File 6). All RNA-seq datasets used in this work were previously publicly available.

## Supplementary Materials

The following are available online at https://www.mdpi.com/2073-4425/11/10/1231/s1, Supplementary File 1 (SupplementaryFile1_TablesAndFigures.pdf) contain supplementary tables (Tables S1–S9) and figures (Figures S1 and S2). Supplementary Files 2 to 5 (SupplementaryFile2_FE_shape1.html, SupplementaryFile3_FE_shape2.html, SupplementaryFile4_FE_shape3.html, SupplementaryFile5_FE_shape4.html) contain the functional enrichment for genes in cattle feed efficiency dataset falling in shapes 1 to 4. Supplementary File 6 (SupplementaryFile6.zip) contain the FORTRAN95 code, README.txt and example data.

## References

[1] Swami M. (2009). Networking complex traits. Nat. Rev. Genet..

[2] Hudson N.J., Dalrymple B.P., Reverter A. (2012). Beyond differential expression: The quest for causal mutations and effector molecules. BMC Genom..

[3] Mar J.C., Matigian N.A., Mackay-Sim A., Mellick G.D., Sue C.M., Silburn P.A., McGrath J.J., Quackenbush J., Wells C.A. (2011). Variance of Gene Expression Identifies Altered Network Constraints in Neurological Disease. PLoS Genet..

[4] Barabási A.-L., Oltvai Z.N. (2004). Network biology: Understanding the cell’s functional organization. Nat. Rev. Genet..

[5] Hudson N.J., Reverter A., Wang Y.H., Greenwood P.L., Dalrymple B.P. (2009). Inferring the Transcriptional Landscape of Bovine Skeletal Muscle by Integrating Co-Expression Networks. PLoS ONE.

[6] Remondini D., O’Connell B., Intrator N., Sedivy J.M., Neretti N., Castellani G.C., Cooper L.N. (2005). Targeting c-Myc-activated genes with a correlation method: Detection of global changes in large gene expression network dynamics. Proc. Natl. Acad. Sci. USA.

[7] R Core Team (2020). R: A Language and Environment for Statistical Computing.

[8] Reverter A., Chan E.K.F. (2008). Combining partial correlation and an information theory approach to the reversed engineering of gene co-expression networks. Bioinformatics.

[9] Alexandre P.A., Naval-Sanchez M., Porto-Neto L.R., Ferraz J.B.S., Reverter A., Fukumasu H. (2019). Systems Biology Reveals *NR2F6* and *TGFB1* as Key Regulators of Feed Efficiency in Beef Cattle. Front. Genet..

[10] Alexandre P.A., Kogelman L.J., Santana M.H., Passarelli D., Pulz L.H., Fantinato-Neto P., Silva P.L., Leme P.R., Strefezzi R.F., Coutinho L.L. (2015). Liver transcriptomic networks reveal main biological processes associated with feed efficiency in beef cattle. BMC Genom..

[11] Nguyen L.T., Reverter A., Cánovas A., Venus B., Anderson S.T., Islas-Trejo A., Dias M.M., Crawford N.F., Lehnert S.A., Medrano J.F. (2018). *STAT6*, *PBX2*, and *PBRM1* Emerge as Predicted Regulators of 452 Differentially Expressed Genes Associated with Puberty in Brahman Heifers. Front. Genet..

[12] Fortes M.R.S., Zacchi L.F., Nguyen L.T., Raidan F., Weller M.M.D.C.A., Choo J.J.Y., Reverter A., Rego J.P.A., Boe-Hansen G.B., Porto-Neto L.R. (2018). Pre- and post-puberty expression of genes and proteins in the uterus of *Bos indicus* heifers: The luteal phase effect post-puberty. Anim. Genet..

[13] Nguyen L.T., Reverter A., Cánovas A., Venus B., Islas-Trejo A., Porto-Neto L.R., Lehnert S.A., Medrano J.F., Moore S.S., Fortes M.R.S. (2017). Global differential gene expression in the pituitary gland and the ovaries of pre- and postpubertal Brahman heifers. J. Anim. Sci..

[14] Fortes M.R.S., Nguyen L.T., Weller M.M.D.C.A., Cánovas A., Islas-Trejo A., Porto-Neto L.R., Reverter A., Lehnert S.A., Boe-Hansen G.B., Thomas M.G. (2016). Transcriptome analyses identify five transcription factors differentially expressed in the hypothalamus of post-versus prepubertal Brahman heifers. J. Anim. Sci..

[15] Wang Z., Yin Z.-T., Zhang F., Li X.-Q., Chen S.-R., Yang N., Porter T.E., Hou Z.-C. (2019). Dynamics of transcriptome changes during subcutaneous preadipocyte differentiation in ducks. BMC Genom..

[16] Hu H., Miao Y.-R., Jia L.-H., Yu Q.-Y., Zhang Q., Guo A.-Y. (2018). AnimalTFDB 3.0: A comprehensive resource for annotation and prediction of animal transcription factors. Nucleic Acids Res..

[17] Becker K., Bluhm A., Casas-Vila N., Dinges N., DeJung M., Sayols S., Kreutz C., Roignant J.-Y., Butter F., Legewie S. (2018). Quantifying post-transcriptional regulation in the development of *Drosophila melanogaster*. Nat. Commun..

[18] Rhee D.Y., Cho D.Y., Zhai B., Slattery M., Ma L., Mintseris J., Wong C.Y., White K.P., Celniker S.E., Przytycka T.M. (2014). Transcription Factor Networks in *Drosophila melanogaster*. Cell Rep..

[19] Lonsdale J., Thomas J., Salvatore M., Phillips R., Lo E., Shad S., Hasz R., Walters G., Garcia F., Young N. (2013). The Genotype-Tissue Expression (GTEx) project. Nat. Genet..

[20] Uhlen M., Fagerberg L., Hallstrom B.M., Lindskog C., Oksvold P., Mardinoglu A., Sivertsson A., Kampf C., Sjöstedt E., Asplund A. (2015). Tissue-based map of the human proteome. Science.

[21] Crow M., Lim N., Ballouz S., Pavlidis P., Gillis J. (2019). Predictability of human differential gene expression. Proc. Natl. Acad. Sci. USA.

[22] Lee H.K., Hsu A.K., Sajdak J., Qin J., Pavlidis P. (2004). Coexpression Analysis of Human Genes Across Many Microarray Data Sets. Genome Res..

[23] Reverter A., Hudson N.J., Nagaraj S.H., Pérez-Enciso M., Dalrymple B.P. (2010). Regulatory impact factors: Unraveling the transcriptional regulation of complex traits from expression data. Bioinformatics.

[24] Cánovas A., Reverter A., DeAtley K.L., Ashley R.L., Colgrave M.L., Fortes M.R.S., Islas-Trejo A., Lehnert S., Porto-Neto L., Rincón G. (2014). Multi-Tissue Omics Analyses Reveal Molecular Regulatory Networks for Puberty in Composite Beef Cattle. PLoS ONE.

[25] Mota L.F.M., Bonafé C.M., Alexandre P.A., Santana M.H., Novais F.J., Toriyama E., Pires A.V., da Luz Silva S., Leme P.R., Ferraz J.B.S. (2017). Circulating leptin and its muscle gene expression in Nellore cattle with divergent feed efficiency. J. Anim. Sci. Biotechnol..

[26] Fonseca L.D., Eler J.P., Pereira M.A., Rosa A.F., Alexandre P.A., Moncau C.T., Salvato F., Rosa-Fernandes L., Palmisano G., Ferraz J.B.S. (2019). Liver proteomics unravel the metabolic pathways related to Feed Efficiency in beef cattle. Sci. Rep..

[27] Novais F.J., Pires P.R.L., Alexandre P.A., Dromms R.A., Iglesias A.H., Ferraz J.B.S., Styczynski M.P.-W., Fukumasu H. (2019). Identification of a metabolomic signature associated with feed efficiency in beef cattle. BMC Genom..

[28] Alexandre P.A., Reverter A., Berezin R.B., Porto-Neto L.R., Ribeiro G., Santana M.H.A., Ferraz J.B.S., Fukumasu H. (2020). Exploring the Regulatory Potential of Long Non-Coding RNA in Feed Efficiency of Indicine Cattle. Genes.

[29] Wree A., Kahraman A., Gerken G., Canbay A. (2010). Obesity Affects the Liver—The Link between Adipocytes and Hepatocytes. Digestion.

[30] Fernández-Sánchez A., Madrigal-Santillán E., Bautista M., Esquivel-Soto J., Morales-González A., Esquivel-Chirino C., Durante-Montiel I., Sánchez-Rivera G., Valadez-Vega C., Morales-González J.A. (2011). Inflammation, Oxidative Stress, and Obesity. Int. J. Mol. Sci..

[31] Hotamisligil G.S. (2006). Inflammation and metabolic disorders. Nat. Cell Biol..

[32] Noble D. (2006). Systems biology and the heart. Biosystems.

