# Supplementary material for: Genome-Wide Co-Expression Distributions as a Metric to Prioritize Genes of Functional Importance
Source: Genes (Basel). 2020 Oct 20;11(10):1231. doi: 10.3390/genes11101231 (PMC7593939; doi:10.3390/genes11101231)
Supplement: Supplementary file 1 [file genes-11-01231-s001.zip › SupplementaryFile1_TablesAndFigures.pdf]

Genome-wide co-expression distributions as a metric to prioritize genes of functional importance

Additional File 1

Table S1. Expected bin proportions, mean, standard deviation (SD), skewness and kurtosis for the 8 shapes.

	Expected 0.25-Bin Proportions, %								Mean	SD	Skewness	Kurtosis
	Bin 1	Bin 2	Bin 3	Bin 4	Bin 5	Bin 6	Bin 7	Bin 8				
**Shape 1**	5	10	15	20	20	15	10	5	0	0.456	0	-0.660
**Shape 2**	2	5	15	28	28	15	5	2	0	0.354	0	0.065
**Shape 3**	15	30	25	15	9	3	2	1	-0.387	0.382	0.857	0.640
**Shape 4**	1	2	3	9	15	25	30	15	0.387	0.382	-0.857	0.640
**Shape 5**	28	15	5	2	2	5	15	28	0	0.752	0	-1.771
**Shape 6**	5	25	15	5	5	15	25	5	0	0.566	0	-1.535
**Shape 7**	5	30	20	10	5	10	15	5	-0.137	0.532	0.5	-1.111
**Shape 8**	5	15	10	5	10	20	30	5	0.137	0.532	-0.5	-1.111

Table S2. Datasets summary.

Dataset	Number of tissues	Number of libraries	Number of genes	Gene filtering criteria	Reference
**Cattle feed efficiency**	5	86	11,662	Average log2FPKM > 1 across all tissues	Alexandre et al. (2015)
**Cattle puberty**	5	60	16,978	Average FPKM ≥ 0.2 in at least 1 tissue	Fortes et al. (2016)<br>Nguyen et al. (2017)<br>Fortes et al. (2018)<br>Nguyen et al. (2018)
**Drosophila embryogenesis**	1 (14 time-points)	68 averaged by time point	7,771	More than 0 counts in at least 20 samples and more than 100 counts on average	Becker et al. (2018)
**Duck preadipocyte**	1 (6 time-points)	36 averaged by time point	13,322	FPKM > 0 in all samples	Wang et al. (2019)
**Human**	1	15	19,206	TPM > 0 in all samples	Lonsdale et al. (2013)

FPKM - fragments per kilobase of gene per million mapped reads
TPM – transcripts per million reads

Table S3. Number of genes assigned to each distribution shape in datasets with contrasting phenotypes. Numbers in parentheses represent the P-values for enrichment (above) and depletion (below). The categories include differentially expressed (DE) and regulator (REG) genes.

	Cattle Feed Efficiency Dataset					Cattle Puberty Dataset				
	**REG**	**DE**	**DE-REG**	**Others**	**Total**	**REG**	**DE**	**DE-REG**	**Others**	**Total**
**Shape 1**	(1)<br>122<br>(1.62E-07)	(2.93E-05)<br>91<br>(1)	(0.31)<br>6<br>(0.83)	(0.03)<br>1,784<br>(0.98)	2,003	(1)<br>449<br>(4.87E-13)	(8.70E-59)<br>1,223<br>(1)	(5.14E-06)<br>82<br>(1)	(1)<br>5,003<br>(4.19E-21)	6,757
**Shape 2**	(1)<br>48<br>(3.61E-04)	(9.20E-09)<br>56<br>(1)	(0.12)<br>4<br>(0.96)	(0.80)<br>717<br>(0.23)	825	(1)<br>31<br>(4.39E-03)	(2.11E-20)<br>153<br>(1)	(0.49)<br>5<br>(0.68)	(1)<br>372<br>(1.82E-10)	561
**Shape 3**	(1)<br>31<br>(2.10E-08)	(0.08)<br>40<br>(0.95)	(0.85)<br>1<br>(0.45)	(3.73E-03)<br>708<br>(1)	780	(1)<br>0<br>(0.84)	(1)<br>0<br>(0.76)	(1)<br>0<br>(0.98)	(0.60)<br>2<br>(1)	2
**Shape 4**	(5.94E-17)<br>818<br>(1)	(1)<br>157<br>(1.94E-19)	(0.86)<br>16<br>(0.25)	(0.98)<br>6,835<br>(0.02)	7,826	(0.16)<br>1<br>(0.99)	(1)<br>0<br>(0.76)	(1)<br>0<br>(0.98)	(0.95)<br>1<br>(0.40)	2
**Shape 5**	(1)<br>0<br>(1)	(1)<br>0<br>(1)	(1)<br>0<br>(1)	(1)<br>0<br>(1)	0	(1)<br>0<br>(1)	(1)<br>0<br>(1)	(1)<br>0<br>(1)	(1)<br>0<br>(1)	0
**Shape 6**	(1)<br>0<br>(1)	(1)<br>0<br>(1)	(1)<br>0<br>(1)	(1)<br>0<br>(1)	0	(1)<br>0<br>(1)	(1)<br>0<br>(1)	(1)<br>0<br>(1)	(1)<br>0<br>(1)	0
**Shape 7**	(0.74)<br>7<br>(0.40)	(7.63E-111)<br>6<br>(1)	(1)<br>0<br>(1)	(0.76)<br>80<br>(0.35)	93	(1)<br>182<br>(4.22E-11)	(3.11E-36)<br>636<br>(1)	(1.52E-06)<br>50<br>(1)	(1)<br>2,315<br>(7.78E-14)	3,183
**Shape 8**	(0.03)<br>19<br>(0.98)	(2.47E-08)<br>5<br>(1)	(1)<br>0<br>(1)	(0.98)<br>111<br>(0.04)	135	(5.86E-38)<br>781<br>(1)	(1)<br>183<br>(4.99E-251)	(1)<br>3<br>(5.12E-25)	(1.43E-75)<br>5,506<br>(1)	6,473
**Total**	1,045	355	27	10,235	11,662	346	236	9	1,952	16,978

Table S4. Number of genes assigned to each distribution shape in the cattle feed efficiency dataset split by phenotype. Numbers in parentheses represent the P-values for enrichment (above) and depletion (below). The categories include differentially expressed (DE) and regulator (REG) genes.

	**Low Feed Efficiency**					**High Feed Efficiency**				
	**REG**	**DE**	**DE-REG**	**Others**	**Total**	**REG**	**DE**	**DE-REG**	**Others**	**Total**
**Shape 1**	(1)<br>134<br>(3.49E-07)	(7.67E-07)<br>102<br>(1)	(0.05)<br>9<br>(0.98)	(0.12)<br>1,892<br>(0.89)	2,137	(1)<br>98<br>(2.97E-08)	(3.18E-05)<br>81<br>(1)	(0.58)<br>4<br>(0.63)	(0.01)<br>1,546<br>(0.99)	1,729
**Shape 2**	(1)<br>52<br>(0.01)	(1.39E-10)<br>59<br>(1)	(0.56)<br>2<br>(0.72)	(0.96)<br>687<br>(0.05)	800	(0.97)<br>60<br>(0.04)	(7.37E-08)<br>54<br>(1)	(0.04)<br>5<br>(0.99)	(0.97)<br>711<br>(0.03)	830
**Shape 3**	(1)<br>28<br>(2.14E-08)	(0.04)<br>31<br>(0.98)	(0.83)<br>1<br>(0.49)	(1.96E-04)<br>672<br>(1)	732	1<br>32<br>(3.66E-08)	0.01<br>35<br>(0.99)	0.85<br>1<br>(0.45)	5.89E-04<br>718<br>(1)	786
**Shape 4**	(3.75E-18)<br>771<br>(1)	(1)<br>127<br>(1.16E-23)	(0.80)<br>15<br>(0.32)	(0.97)<br>6,279<br>(0.03)	7,192	(3.66E-16)<br>850<br>(1)	(1)<br>179<br>(1.42E-16)	(0.87)<br>17<br>(0.24)	(0.99)<br>7,221<br>(0.02)	8,267
**Shape 5**	(1)<br>0<br>(1)	(1)<br>0<br>(1)	(1)<br>0<br>(1)	(1)<br>0<br>(1)	0	(1)<br>0<br>(1)	(1)<br>0<br>(1)	(1)<br>0<br>(1)	(1)<br>0<br>(1)	0
**Shape 6**	(1)<br>0<br>(1)	(1)<br>0<br>(1)	(1)<br>0<br>(1)	(1)<br>0<br>(1)	0	(1)<br>0<br>(1)	(1)<br>0<br>(1)	(1)<br>0<br>(1)	(1)<br>0<br>(1)	0
**Shape 7**	(1)<br>11<br>(4.71E-03)	(2.08E-04)<br>19<br>(1)	(1)<br>0<br>(0.56)	(0.52)<br>218<br>(0.56)	248	(1)<br>0<br>(0.04)	(4.21E-04)<br>6<br>(1)	(1)<br>0<br>(0.93)	(0.90)<br>27<br>(0.21)	33
**Shape 8**	(0.56)<br>49<br>(0.50)	(0.52)<br>17<br>(0.58)	(1)<br>0<br>(0.27)	(0.44)<br>487<br>(0.61)	553	(0.01)<br>5<br>(1)	(1)<br>0<br>(0.59)	(1)<br>0<br>(0.96)	(0.99)<br>12<br>(0.05)	17
**Total**	1,045	355	27	10,235	11,662	1,045	355	27	10,235	11,662

Table S5. Number of genes assigned to each distribution shape in the cattle puberty dataset split by phenotype. Numbers in parentheses represent the P-values for enrichment (above) and depletion (below). The categories include differentially expressed (DE) and regulator (REG) genes.

	**Pre-puberty**					**Post-puberty**				
	**REG**	**DE**	**DE-REG**	**Others**	**Total**	**REG**	**DE**	**DE-REG**	**Others**	**Total**
	(1)	(6.66E-66)	(2.05E-05)	(1)		(1)	(4.66E-64)	(6.50E-08)	(1)	
**Shape 1**	397	1,179	76	4,620	6,272	478	1,254	88	5,046	6,866
	(1.61E-15)	(1)	(1)	(1.61E-22)		(1.32E-09)	(1)	(1)	(6.50E-28)	
	(1)	(9.98E-23)	(0.75)	(1)		(1)	(4.12E-14)	(0.89)	(1)	
**Shape 2**	31	169	4	412	616	23	118	2	314	457
	(5.45E-04)	(1)	(0.42)	(1.86E-10)		(2.81E-03)	(1)	(0.27)	(3.77E-06)	
	(1)	(0.13)	(1)	(0.92)		(1)	(0.01)	(1)	(0.99)	
**Shape 3**	0	2	0	3	5	0	4	0	3	7
	(0.64)	(0.98)	(0.96)	(0.31)		(0.54)	(1)	(0.94)	(0.05)	
	(1)	(0.02)	(1)	(1)		(1)	(0.13)	(1)	(1)	
**Shape 4**	0	2	0	0	2	0	1	0	0	1
	(0.84)	(1)	(0.98)	(0.05)		(0.92)	(1)	(0.99)	(0.22)	
	(1)	(1)	(1)	(1)		(1)	(1)	(1)	(1)	
**Shape 5**	0	0	0	0	0	0	0	0	0	0
	(1)	(1)	(1)	(1)		(1)	(1)	(1)	(1)	
	(1)	(1)	(1)	(1)		(1)	(1)	(1)	(1)	
**Shape 6**	0	0	0	0	0	0	0	0	0	0
	(1)	(1)	(1)	(1)		(1)	(1)	(1)	(1)	
	(1)	(3.96E-35)	(3.58E-09)	(1)		(1)	(3.76E-33)	(7.22E-06)	(1)	
**Shape 7**	196	646	57	2,373	3,272	167	622	48	2,323	3,160
	(1.70E-09)	(1)	(1)	(2.75E-15)		(2.62E-14)	(1)	(1)	(2.57E-10)	
	(1.17E-40)	(1)	(1)	(7.56E-81)		(7.00E-36)	(1)	(1)	(2.93E-74)	
**Shape 8**	819	198	3	5,791	6,811	775	197	2	5,513	6,487
	(1)	(5.71E-265)	(6.54E-27)	(1)		(1)	(7.66E-240)	(1.47E-26)	(1)	
**Total**	1,443	2,196	140	13,199	16,978	1,443	2,196	140	13,199	16,978

Table S6. Number of genes assigned to each distribution shape in time-series datasets. Numbers in parentheses represent the P-values for enrichment (above) and depletion (below). The categories include differentially expressed (DE) and regulator (REG) genes – refer to methods for specific categories in Drosophila dataset.

	**Drosophila Embryogenesis Dataset**							**Duck Preadipocyte Dataset**				
	**Down/down**	**Down/up**	**Up/down**	**Up/up**	**REG**	**Others**	**Total**	**REG**	**DE**	**DE-REG**	**Others**	**Total**
	(4.29E-16)	(0.94)	(0.01)	(8.74E-04)	(1)	(1)		(0.97)	(1.17E-10)	(0.15)	(1)	
**Shape 1**	292	305	76	122	106	638	1,433	63	584	29	1,307	1,983
	(1)	(0.07)	(0.99)	(1)	(4.17E-05)	(8.45E-13)		(0.05)	(1)	(0.90)	(1.47E-08)	
	(0.16)	(0.96)	(0.02)	(0.63)	(0.98)	(0.69)		(0.43)	(1)	(0.83)	(6.66E-10)	
**Shape 2**	17	16	9	6	5	50	98	11	21	2	228	262
	(0.90)	(0.08)	(0.99)	(0.53)	(0.06)	(0.39)		(0.69)	(1.71E-11)	(0.39)	(1)	
	(0.14)	(0.41)	(1)	(1)	(1)	(0.87)		1	1	1	(0.26)	
**Shape 3**	3	3	0	0	0	4	10	0	0	0	4	4
	(0.96)	(0.83)	(0.65)	(0.51)	(0.34)	(0.31)		(0.85)	(0.34)	(0.95)	1	
	(1)	(1)	(1)	(1)	(1)	(1)		(1)	(1)	(1)	(1)	
**Shape 4**	0	0	0	0	0	0	0	0	0	0	0	0
	(1)	(1)	(1)	(1)	(1)	(1)		(1)	(1)	(1)	(1)	
	(1)	(1)	(1)	(1)	(1)	(1)		(1)	(1)	(1)	(1)	
**Shape 5**	0	0	0	0	0	0	0	0	0	0	0	0
	(1)	(1)	(1)	(1)	(1)	(1)		(1)	(1)	(1)	(1)	
	(1)	(1.38E-18)	(0.02)	(1)	(0.07)	(0.12)		(0.58)	(0.83)	(0.06)	(0.32)	
**Shape 6**	468	1,159	202	210	467	2,351	4,390	54	324	24	1,023	1,425
	(8.76E-17)	(1)	(0.98)	(3.46E-13)	(0.94)	(0.89)		(0.47)	(0.18)	(0.97)	(0.71)	
	(1)	(1)	(0.20)	(4.75E-51)	(2.62E-05)	(6.07E-29)		(0.90)	(5.42E-11)	(9.71E-04)	(1)	
**Shape 7**	1	16	38	171	115	564	790	117	930	58	2,227	3,332
	(3.27E-51)	(1.6E-69)	(0.84)	(1)	(1)	(1)		(0.12)	(1)	(1)	(1.06E-10)	
	(1.43E-29)	(0.01)	(1)	(1)	(0.85)	(1)		(0.01)	(1)	(1)	(1.05E-14)	
**Shape 8**	267	271	1	2	98	509	1,050	270	1,302	47	4,697	6,316
	(1)	(0.99)	(5.10E-20)	(1.48E-30)	(0.18)	(9.72E-04)		(0.99)	(5.3E-16)	(2.2E-06)	(1)	
**Total**	1,048	1,770	326	511	791	4,116	7,771	515	3,161	160	9,486	13,322

Table S7. Number of genes assigned to each distribution shape in the human dataset. Numbers in parentheses represent the P-values for enrichment (above) and depletion (below). The categories include differentially expressed (DE), regulator (REG) and tissue enriched (TE) genes.

	Human Dataset					
	**REG**	**DE**	**DE-REG**	**TE**	**Others**	**Total**
	(0.55)	(2.48E-04)	(0.02)	(0.09)	(1)	
**Shape 1**	215	182	24	55	3,411	3,881
	(0.48)	(1)	(0.99)	(0.93)	(1.53E-03)	
	(9.31E-06)	(3.28E-10)	(2.92E-04)	(0.99)	(1)	
**Shape 2**	344	262	35	46	4,382	5,065
	(1)	(1)	(1)	(0.01)	(6.34E-13)	
	(0.92)	(4.82E-03)	(0.16)	(1)	(0.96)	
**Shape 3**	1	6	1	0	35	43
	(0.30)	(1)	(0.99)	(0.60)	(0.09)	
	(0.37)	(1)	(1)	(1)	(0.79)	
**Shape 4**	1	0	0	0	7	8
	(0.93)	(0.74)	(0.97)	(0.91)	(0.60)	
	(1)	(1)	(1)	(1)	(1)	
**Shape 5**	0	0	0	0	0	0
	(1)	(1)	(1)	(1)	(1)	
	(1)	(1)	(1)	(1)	(1)	
**Shape 6**	0	0	0	0	0	0
	(1)	(1)	(1)	(1)	(1)	
	(1)	(1)	(0.88)	(7.21E-34)	(9.40E-01)	
**Shape 7**	79	48	5	91	1,640	1,860
	(4.00E-03)	(2.94E-03)	(0.22)	(1)	(0.07)	
	(0.99)	(1)	(1)	(1)	(3.58E-24)	
**Shape 8**	435	217	13	39	7,733	8,427
	(0.01)	(5.79E-14)	(3.59E-07)	(2.65E-18)	(1)	
**Total**	1,075	715	78	231	17,208	19,284

Table S8. Average number of connections per gene (Log10) falling in each shape according to the dataset and P-value for the one-way ANOVA.

**Dataset**	**Average number of connections per gene (Log10)**								**P-value**
	**Shape 1**	**Shape 2**	**Shape 3**	**Shape 4**	**Shape 5**	**Shape 6**	**Shape 7**	**Shape 8**	
**Cattle feed efficiency**	3.04	2.81	3.30	3.49	-	-	3.15	3.20	<2E-16
**Cattle puberty**	3.14	2.52	1.40	1.87	-	-	3.57	3.58	<2E-16
**Drosophila embryogenesis**	2.58	2.26	2.39	-	-	3.03	2.92	2.87	<2E-16
**Duck preadipocyte**	2.33	2.06	1.82	-	-	2.60	2.60	2.66	<2E-16
**Human**	2.60	2.34	2.46	2.40	-	-	3.17	3.44	<2E-16

Table S9. Number of genes assigned to each distribution shape in all datasets based on genes being on the top or bottom 5% when ranked by degree (number of significant correlations to other genes). Numbers in parentheses represent the P-values for enrichment (above) and depletion (below).

	Feed Efficiency		Puberty		Drosophila		Duck		Human	
	**Top**	**Bottom**	**Top**	**Bottom**	**Top**	**Bottom**	**Top**	**Bottom**	**Top**	**Bottom**
	(1)	(4.72E-24)	(1)	(1.56E-37)	(1)	(7.1E-126)	(1)	(9.3E-110)	(1)	(1)
**Shape 1**	0	198	0	518	0	280	0	335	0	61
	(8.44E-50)	(1)	(3.27E-194)	(1)	(3.74E-36)	(1)	(1.18E-48)	(1)	(9.50E-98)	(3.78E-35)
	(1)	(4.27E-252)	(1)	(8.64E-275)	(1)	(8.84E-44)	(1)	(2.3E-280)	(1)	(<1E-999)
**Shape 2**	0	335	0	314	0	53	0	231	0	894
	(8.41E-20)	(1)	(1.93E-13)	(1)	(6.31E-03)	(1)	(1.27E-06)	(1)	(1.90E-132)	(1)
	(1)	(1)	(1)	(2.50E-03)	(1)	(2.68E-06)	(1)	(6.19E-06)	(1)	(4.02E-05)
**Shape 3**	5	18	0	2	0	6	0	4	0	10
	(1.54E-12)	(6.76E-05)	(0.90)	(1)	(5.98E-01)	(1)	(0.81)	(1)	(0.11)	(1)
	(1.38E-94)	(1)	(1)	(2.50E-03)	(1)	(1)	(1)	(1)	(1)	(0.34)
**Shape 4**	578	31	0	2	0	0	0	0	0	1
	(1)	(6.97E-233)	(0.90)	(1)	(1)	(1)	(1)	(1)	(0.66)	(0.94)
	(1)	(1)	(1)	(1)	(1)	(1)	(1)	(1)	(1)	(1)
**Shape 5**	0	0	0	0	0	0	0	0	0	0
	(1)	(1)	(1)	(1)	(1)	(1)	(1)	(1)	(1)	(1)
	(1)	(1)	(1)	(1)	(1.31E-100)	(1)	(1)	(1)	(1)	(1)
**Shape 6**	0	0	0	0	389	27	0	1	0	0
	(1)	(1)	(1)	(1)	(1)	(3.3E-101)	(2.41E-34)	(2.06E-32)	(1)	(1)
	(1)	(0.99)	(1.54E-31)	(1)	(1)	(1)	(0.99)	(1)	(1)	(1)
**Shape 7**	0	1	299	5	0	13	142	45	1	0
	(8.32E-03)	(0.05)	(1)	(5.83E-70)	(2.48E-19)	(1.99E-07)	(0.01)	(2.35E-36)	(2.13E-42)	(1.94E-44)
	(1)	(1)	(1.77E-58)	(1)	(1)	(1)	(1.31E-64)	(1)	(<1E-999)	(1)
**Shape 8**	0	0	550	8	0	10	524	50	965	0
	(9.45E-04)	(9.45E-04)	(1)	(3.03E-166)	(6.20E-26)	(2.14E-14)	(1)	(8.6E-118)	(1)	(2.81E-250)
**Total**	583	583	849	849	389	389	666	666	966	966

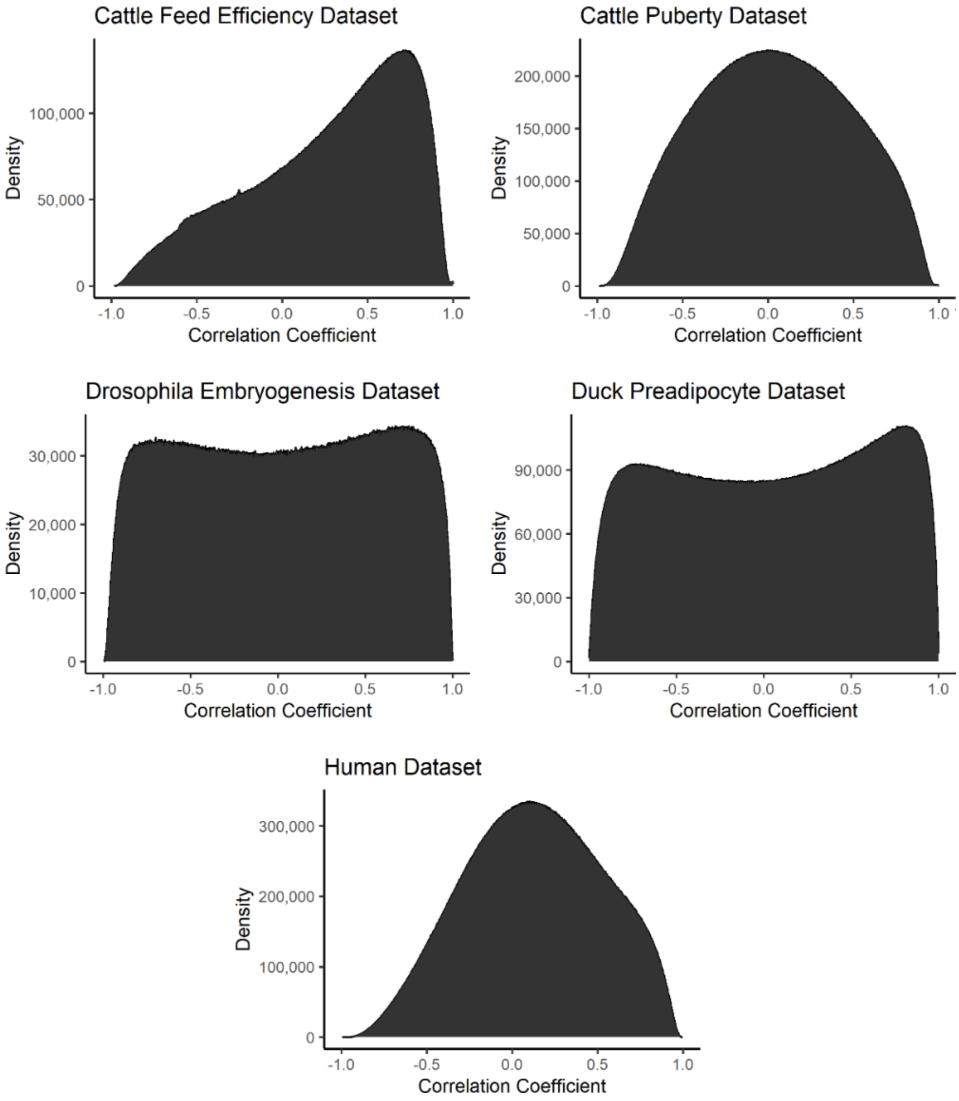

Figure S1. Frequency distributions of all co-expression correlation coefficients in each of the five RNA-Sequence datasets. Datasets include: 1) Cattle Feed Efficiency (11,662 genes and 67,995,291 correlations); 2) Cattle Puberty (16,978 genes and 144,117,753 correlations); 3) Drosophila Embryogenesis (7,771 genes and 30,190,335 correlations); 4) Duck Preadipocyte (13,322 genes and 88,731,181 correlations); and 5) Human (19,311 genes and 186,447,705 correlations).

Cattle Feed Efficiency Dataset		Null		Unimodal		Symmetric	
		Yes	No	Yes	No	Yes	No
DE	Yes	157	225	371	11	157	225
	No	2,671	8,609	11,063	217	2,671	8,609
REG	Yes	180	892	1,046	26	180	892
	No	2,648	7,942	10,388	202	2,648	7,942

Cattle Puberty Dataset		Null		Unimodal		Symmetric	
		Yes	No	Yes	No	Yes	No
DE	Yes	1,463	872	1,463	872	1,463	872
	No	5,855	8,788	5,859	8,784	5,855	8,788
REG	Yes	567	1,017	568	1,016	567	1,017
	No	6,751	8,643	6,754	8,640	6,751	8,643

Drosophyla Embryogenesis Dataset		Null		Unimodal		Symmetric	
		Yes	No	Yes	No	Yes	No
DE	Yes	842	2,805	842	2,805	2,881	766
	No	689	3,435	699	3,425	3,040	1,084
REG	Yes	111	686	117	680	578	219
	No	1,420	5,554	1,424	5,550	5,343	1,631

Duck Preadipocyte Dataset		Null		Unimodal		Symmetric	
		Yes	No	Yes	No	Yes	No
DE	Yes	636	2685	636	2685	984	2686
	No	1,609	8,392	1,613	8,388	2,337	7,315
REG	Yes	105	570	105	570	183	492
	No	2,140	10,507	2,144	10,503	3,487	9,160

Human Non-diseased Dataset		Null		Unimodal		Symmetric	
		Yes	No	Yes	No	Yes	No
DE	Yes	503	290	510	283	503	290
	No	8,443	10,048	8,487	10,004	8,443	10,048
REG	Yes	618	535	621	532	618	535
	No	8,328	9,803	8,376	9,755	8,328	9,803

Figure S2. Number of genes assigned to different distribution shapes. The tables show the number of differentially expressed genes (DE) and regulator (REG) genes assigned to null (Shapes 1 and 2), unimodal (Shapes 1 to 4) and symmetric (Shapes 1, 2, 5 and 6) distributions. Blue cells represent the type of distribution in which DE or REG were found in higher number than expected by chance where variables were found to be dependent (P<0.05).
