# Supplementary material for: Genome-Wide Co-Expression Distributions as a Metric to Prioritize Genes of Functional Importance
Source: Genes (Basel). 2020 Oct 20;11(10):1231. doi: 10.3390/genes11101231 (PMC7593939; doi:10.3390/genes11101231)
Supplement: Supplementary file 1 [file genes-11-01231-s001.zip › SupplementaryFile2_FE_shape1.html]

Results


*P-value color scale*

|  |  |  |  |  |
| --- | --- | --- | --- | --- |
| > 10-3 | 10-3 to 10-5 | 10-5 to 10-7 | 10-7 to 10-9 | < 10-9 |


|  |  |  |  |  |  |
| --- | --- | --- | --- | --- | --- |
| **GO term** | **Description** | **P-value** | **FDR q-value** | **Enrichment (N, B, n, b)** | **Genes** |
| GO:0006614 | SRP-dependent cotranslational protein targeting to membrane | 7.36E-19 | 1.02E-14 | 3.49 (10334,85,1776,51) | [+] Show genes  RPL24 - ribosomal protein l24  RPL23A - ribosomal protein l23a  RPL21 - ribosomal protein l21  RPL19 - ribosomal protein l19  RPL18A - ribosomal protein l18a  RPL18 - ribosomal protein l18  RPL17 - ribosomal protein l17  RPL13 - ribosomal protein l13  RPL12 - ribosomal protein l12  RPL10 - ribosomal protein l10  RPL7A - ribosomal protein l7a  RPL5 - ribosomal protein l5  RPL4 - ribosomal protein l4  RPL35 - ribosomal protein l35  RPS5 - ribosomal protein s5  RPS2 - ribosomal protein s2  RPLP2 - ribosomal protein, large, p2  RPL36A - ribosomal protein l36a  RPL37A - ribosomal protein l37a  RPL38 - ribosomal protein l38  RPL34 - ribosomal protein l34  RPL37 - ribosomal protein l37  RPL29 - ribosomal protein l29  RPL31 - ribosomal protein l31  RPL36 - ribosomal protein l36  RPL27 - ribosomal protein l27  RPL30 - ribosomal protein l30  RPL27A - ribosomal protein l27a  RPL28 - ribosomal protein l28  RPS24 - ribosomal protein s24  RPS21 - ribosomal protein s21  RPS27 - ribosomal protein s27  RPS26 - ribosomal protein s26  RPS25 - ribosomal protein s25  RPS17 - ribosomal protein s17  RPS16 - ribosomal protein s16  RPS15A - ribosomal protein s15a  RPS20 - ribosomal protein s20  RPS19 - ribosomal protein s19  RPS18 - ribosomal protein s18  RPS11 - ribosomal protein s11  RPS8 - ribosomal protein s8  RPS9 - ribosomal protein s9  RPS14 - ribosomal protein s14  RPS13 - ribosomal protein s13  RPS7 - ribosomal protein s7  RPSA - ribosomal protein sa  RPL10A - ribosomal protein l10a  RPL23 - ribosomal protein l23  RPS28 - ribosomal protein s28  RPS29 - ribosomal protein s29 |
| GO:0006613 | cotranslational protein targeting to membrane | 2.29E-17 | 1.59E-13 | 3.30 (10334,90,1776,51) | [+] Show genes  RPL24 - ribosomal protein l24  RPL23A - ribosomal protein l23a  RPL21 - ribosomal protein l21  RPL19 - ribosomal protein l19  RPL18A - ribosomal protein l18a  RPL18 - ribosomal protein l18  RPL17 - ribosomal protein l17  RPL13 - ribosomal protein l13  RPL12 - ribosomal protein l12  RPL10 - ribosomal protein l10  RPL7A - ribosomal protein l7a  RPL5 - ribosomal protein l5  RPL4 - ribosomal protein l4  RPL35 - ribosomal protein l35  RPS5 - ribosomal protein s5  RPS2 - ribosomal protein s2  RPLP2 - ribosomal protein, large, p2  RPL36A - ribosomal protein l36a  RPL37A - ribosomal protein l37a  RPL38 - ribosomal protein l38  RPL34 - ribosomal protein l34  RPL37 - ribosomal protein l37  RPL29 - ribosomal protein l29  RPL31 - ribosomal protein l31  RPL36 - ribosomal protein l36  RPL27 - ribosomal protein l27  RPL30 - ribosomal protein l30  RPL27A - ribosomal protein l27a  RPL28 - ribosomal protein l28  RPS24 - ribosomal protein s24  RPS21 - ribosomal protein s21  RPS27 - ribosomal protein s27  RPS26 - ribosomal protein s26  RPS25 - ribosomal protein s25  RPS17 - ribosomal protein s17  RPS16 - ribosomal protein s16  RPS15A - ribosomal protein s15a  RPS20 - ribosomal protein s20  RPS19 - ribosomal protein s19  RPS18 - ribosomal protein s18  RPS11 - ribosomal protein s11  RPS8 - ribosomal protein s8  RPS9 - ribosomal protein s9  RPS14 - ribosomal protein s14  RPS13 - ribosomal protein s13  RPS7 - ribosomal protein s7  RPSA - ribosomal protein sa  RPL10A - ribosomal protein l10a  RPL23 - ribosomal protein l23  RPS28 - ribosomal protein s28  RPS29 - ribosomal protein s29 |
| GO:0006412 | translation | 1.38E-16 | 6.4E-13 | 2.53 (10334,177,1776,77) | [+] Show genes  DRG2 - developmentally regulated gtp binding protein 2  RPL24 - ribosomal protein l24  RPL23A - ribosomal protein l23a  RPL21 - ribosomal protein l21  MRPL35 - mitochondrial ribosomal protein l35  RPL19 - ribosomal protein l19  RPL18A - ribosomal protein l18a  RPL18 - ribosomal protein l18  RPL17 - ribosomal protein l17  RPL13 - ribosomal protein l13  GFM2 - g elongation factor, mitochondrial 2  RPL12 - ribosomal protein l12  HBS1L - hbs1-like (s. cerevisiae)  RPL10 - ribosomal protein l10  RPL7A - ribosomal protein l7a  RPL5 - ribosomal protein l5  RPL4 - ribosomal protein l4  RPL35 - ribosomal protein l35  RPS5 - ribosomal protein s5  EIF4B - eukaryotic translation initiation factor 4b  EIF4EBP2 - eukaryotic translation initiation factor 4e binding protein 2  RPS2 - ribosomal protein s2  RPLP2 - ribosomal protein, large, p2  AGO2 - argonaute risc catalytic component 2  MRPS12 - mitochondrial ribosomal protein s12  RPL36A - ribosomal protein l36a  PET112 - pet112 homolog (yeast)  MRPS2 - mitochondrial ribosomal protein s2  RPL37A - ribosomal protein l37a  RPL38 - ribosomal protein l38  RPL34 - ribosomal protein l34  MRPL27 - mitochondrial ribosomal protein l27  RPL37 - ribosomal protein l37  RPL29 - ribosomal protein l29  EIF4G1 - eukaryotic translation initiation factor 4 gamma, 1  RPL31 - ribosomal protein l31  MRPS36 - mitochondrial ribosomal protein s36  RPL36 - ribosomal protein l36  RPL27 - ribosomal protein l27  RPL30 - ribosomal protein l30  GGT5 - gamma-glutamyltransferase 5  RPL27A - ribosomal protein l27a  RPL28 - ribosomal protein l28  RPS24 - ribosomal protein s24  RPS21 - ribosomal protein s21  MRPS18C - mitochondrial ribosomal protein s18c  RPS27 - ribosomal protein s27  RPS26 - ribosomal protein s26  RPS25 - ribosomal protein s25  MRPL52 - mitochondrial ribosomal protein l52  RPS17 - ribosomal protein s17  RPS16 - ribosomal protein s16  RPS15A - ribosomal protein s15a  RPS20 - ribosomal protein s20  RPS19 - ribosomal protein s19  RPS18 - ribosomal protein s18  RPS11 - ribosomal protein s11  RPS8 - ribosomal protein s8  RPS9 - ribosomal protein s9  RPS14 - ribosomal protein s14  RWDD1 - rwd domain containing 1  GUF1 - guf1 gtpase homolog (s. cerevisiae)  RPS13 - ribosomal protein s13  MRPS18B - mitochondrial ribosomal protein s18b  MRPS5 - mitochondrial ribosomal protein s5  MRPL41 - mitochondrial ribosomal protein l41  RPS7 - ribosomal protein s7  MRPL34 - mitochondrial ribosomal protein l34  RPSA - ribosomal protein sa  PABPC4 - poly(a) binding protein, cytoplasmic 4 (inducible form)  MRPL28 - mitochondrial ribosomal protein l28  MRPS17 - mitochondrial ribosomal protein s17  RPL10A - ribosomal protein l10a  RPL23 - ribosomal protein l23  IARS2 - isoleucyl-trna synthetase 2, mitochondrial  RPS28 - ribosomal protein s28  RPS29 - ribosomal protein s29 |
| GO:0045047 | protein targeting to ER | 1.51E-15 | 5.25E-12 | 3.03 (10334,100,1776,52) | [+] Show genes  RPL24 - ribosomal protein l24  RPL23A - ribosomal protein l23a  RPL21 - ribosomal protein l21  RPL19 - ribosomal protein l19  RPL18A - ribosomal protein l18a  RPL18 - ribosomal protein l18  RPL17 - ribosomal protein l17  RPL13 - ribosomal protein l13  RPL12 - ribosomal protein l12  RPL10 - ribosomal protein l10  RPL7A - ribosomal protein l7a  RPL5 - ribosomal protein l5  PMM2 - phosphomannomutase 2  RPL4 - ribosomal protein l4  RPL35 - ribosomal protein l35  RPS5 - ribosomal protein s5  RPS2 - ribosomal protein s2  RPLP2 - ribosomal protein, large, p2  RPL36A - ribosomal protein l36a  RPL37A - ribosomal protein l37a  RPL38 - ribosomal protein l38  RPL34 - ribosomal protein l34  RPL37 - ribosomal protein l37  RPL29 - ribosomal protein l29  RPL31 - ribosomal protein l31  RPL36 - ribosomal protein l36  RPL27 - ribosomal protein l27  RPL30 - ribosomal protein l30  RPL27A - ribosomal protein l27a  RPL28 - ribosomal protein l28  RPS24 - ribosomal protein s24  RPS21 - ribosomal protein s21  RPS27 - ribosomal protein s27  RPS26 - ribosomal protein s26  RPS25 - ribosomal protein s25  RPS17 - ribosomal protein s17  RPS16 - ribosomal protein s16  RPS15A - ribosomal protein s15a  RPS20 - ribosomal protein s20  RPS19 - ribosomal protein s19  RPS18 - ribosomal protein s18  RPS11 - ribosomal protein s11  RPS8 - ribosomal protein s8  RPS9 - ribosomal protein s9  RPS14 - ribosomal protein s14  RPS13 - ribosomal protein s13  RPS7 - ribosomal protein s7  RPSA - ribosomal protein sa  RPL10A - ribosomal protein l10a  RPL23 - ribosomal protein l23  RPS28 - ribosomal protein s28  RPS29 - ribosomal protein s29 |
| GO:0043043 | peptide biosynthetic process | 2.55E-15 | 7.09E-12 | 2.39 (10334,192,1776,79) | [+] Show genes  DRG2 - developmentally regulated gtp binding protein 2  RPL24 - ribosomal protein l24  RPL23A - ribosomal protein l23a  RPL21 - ribosomal protein l21  MRPL35 - mitochondrial ribosomal protein l35  RPL19 - ribosomal protein l19  RPL18A - ribosomal protein l18a  RPL18 - ribosomal protein l18  CNDP2 - cndp dipeptidase 2 (metallopeptidase m20 family)  RPL17 - ribosomal protein l17  RPL13 - ribosomal protein l13  GFM2 - g elongation factor, mitochondrial 2  RPL12 - ribosomal protein l12  HBS1L - hbs1-like (s. cerevisiae)  RPL10 - ribosomal protein l10  RPL7A - ribosomal protein l7a  RPL5 - ribosomal protein l5  RPL4 - ribosomal protein l4  RPL35 - ribosomal protein l35  RPS5 - ribosomal protein s5  EIF4B - eukaryotic translation initiation factor 4b  EIF4EBP2 - eukaryotic translation initiation factor 4e binding protein 2  RPS2 - ribosomal protein s2  RPLP2 - ribosomal protein, large, p2  AGO2 - argonaute risc catalytic component 2  MRPS12 - mitochondrial ribosomal protein s12  RPL36A - ribosomal protein l36a  PET112 - pet112 homolog (yeast)  MRPS2 - mitochondrial ribosomal protein s2  RPL37A - ribosomal protein l37a  RPL38 - ribosomal protein l38  RPL34 - ribosomal protein l34  MRPL27 - mitochondrial ribosomal protein l27  RPL37 - ribosomal protein l37  RPL29 - ribosomal protein l29  EIF4G1 - eukaryotic translation initiation factor 4 gamma, 1  RPL31 - ribosomal protein l31  MRPS36 - mitochondrial ribosomal protein s36  RPL36 - ribosomal protein l36  RPL27 - ribosomal protein l27  RPL30 - ribosomal protein l30  GGT5 - gamma-glutamyltransferase 5  RPL27A - ribosomal protein l27a  RPL28 - ribosomal protein l28  RPS24 - ribosomal protein s24  RPS21 - ribosomal protein s21  MRPS18C - mitochondrial ribosomal protein s18c  RPS27 - ribosomal protein s27  RPS26 - ribosomal protein s26  RPS25 - ribosomal protein s25  MRPL52 - mitochondrial ribosomal protein l52  RPS17 - ribosomal protein s17  RPS16 - ribosomal protein s16  RPS15A - ribosomal protein s15a  RPS20 - ribosomal protein s20  RPS19 - ribosomal protein s19  RPS18 - ribosomal protein s18  RPS11 - ribosomal protein s11  RPS8 - ribosomal protein s8  RPS9 - ribosomal protein s9  RPS14 - ribosomal protein s14  RWDD1 - rwd domain containing 1  RPS13 - ribosomal protein s13  GUF1 - guf1 gtpase homolog (s. cerevisiae)  MRPS18B - mitochondrial ribosomal protein s18b  MRPS5 - mitochondrial ribosomal protein s5  MRPL41 - mitochondrial ribosomal protein l41  RPS7 - ribosomal protein s7  MRPL34 - mitochondrial ribosomal protein l34  RPSA - ribosomal protein sa  PABPC4 - poly(a) binding protein, cytoplasmic 4 (inducible form)  MRPL28 - mitochondrial ribosomal protein l28  MRPS17 - mitochondrial ribosomal protein s17  RPL10A - ribosomal protein l10a  DMD - dystrophin  RPL23 - ribosomal protein l23  IARS2 - isoleucyl-trna synthetase 2, mitochondrial  RPS28 - ribosomal protein s28  RPS29 - ribosomal protein s29 |
| GO:0070972 | protein localization to endoplasmic reticulum | 3.22E-15 | 7.48E-12 | 2.86 (10334,114,1776,56) | [+] Show genes  RPL24 - ribosomal protein l24  RPL23A - ribosomal protein l23a  RPL21 - ribosomal protein l21  RPL19 - ribosomal protein l19  RPL18A - ribosomal protein l18a  RPL18 - ribosomal protein l18  RPL17 - ribosomal protein l17  RPL13 - ribosomal protein l13  RPL12 - ribosomal protein l12  RPL10 - ribosomal protein l10  RPL7A - ribosomal protein l7a  RPL5 - ribosomal protein l5  PMM2 - phosphomannomutase 2  RPL4 - ribosomal protein l4  RPL35 - ribosomal protein l35  RPS5 - ribosomal protein s5  RPS2 - ribosomal protein s2  RPLP2 - ribosomal protein, large, p2  RPL36A - ribosomal protein l36a  RPL37A - ribosomal protein l37a  RPL38 - ribosomal protein l38  RPL34 - ribosomal protein l34  RPL37 - ribosomal protein l37  RPL29 - ribosomal protein l29  UBAC2 - uba domain containing 2  RPL31 - ribosomal protein l31  RPL36 - ribosomal protein l36  RPL27 - ribosomal protein l27  RPL30 - ribosomal protein l30  RPL27A - ribosomal protein l27a  RPL28 - ribosomal protein l28  RPS24 - ribosomal protein s24  RPS21 - ribosomal protein s21  RPS27 - ribosomal protein s27  RPS26 - ribosomal protein s26  RPS25 - ribosomal protein s25  RPS17 - ribosomal protein s17  RPS16 - ribosomal protein s16  RPS15A - ribosomal protein s15a  VAPA - vamp (vesicle-associated membrane protein)-associated protein a, 33kda  RPS20 - ribosomal protein s20  RPS19 - ribosomal protein s19  RPS18 - ribosomal protein s18  RPS11 - ribosomal protein s11  RPS8 - ribosomal protein s8  RPS9 - ribosomal protein s9  RPS14 - ribosomal protein s14  RPS13 - ribosomal protein s13  RPS7 - ribosomal protein s7  RPSA - ribosomal protein sa  DDRGK1 - ddrgk domain containing 1  PPP1R15A - protein phosphatase 1, regulatory subunit 15a  RPL10A - ribosomal protein l10a  RPL23 - ribosomal protein l23  RPS28 - ribosomal protein s28  RPS29 - ribosomal protein s29 |
| GO:0072599 | establishment of protein localization to endoplasmic reticulum | 7.47E-15 | 1.48E-11 | 2.94 (10334,103,1776,52) | [+] Show genes  RPL24 - ribosomal protein l24  RPL23A - ribosomal protein l23a  RPL21 - ribosomal protein l21  RPL19 - ribosomal protein l19  RPL18A - ribosomal protein l18a  RPL18 - ribosomal protein l18  RPL17 - ribosomal protein l17  RPL13 - ribosomal protein l13  RPL12 - ribosomal protein l12  RPL10 - ribosomal protein l10  RPL7A - ribosomal protein l7a  RPL5 - ribosomal protein l5  PMM2 - phosphomannomutase 2  RPL4 - ribosomal protein l4  RPL35 - ribosomal protein l35  RPS5 - ribosomal protein s5  RPS2 - ribosomal protein s2  RPLP2 - ribosomal protein, large, p2  RPL36A - ribosomal protein l36a  RPL37A - ribosomal protein l37a  RPL38 - ribosomal protein l38  RPL34 - ribosomal protein l34  RPL37 - ribosomal protein l37  RPL29 - ribosomal protein l29  RPL31 - ribosomal protein l31  RPL36 - ribosomal protein l36  RPL27 - ribosomal protein l27  RPL30 - ribosomal protein l30  RPL27A - ribosomal protein l27a  RPL28 - ribosomal protein l28  RPS24 - ribosomal protein s24  RPS21 - ribosomal protein s21  RPS27 - ribosomal protein s27  RPS26 - ribosomal protein s26  RPS25 - ribosomal protein s25  RPS17 - ribosomal protein s17  RPS16 - ribosomal protein s16  RPS15A - ribosomal protein s15a  RPS20 - ribosomal protein s20  RPS19 - ribosomal protein s19  RPS18 - ribosomal protein s18  RPS11 - ribosomal protein s11  RPS8 - ribosomal protein s8  RPS9 - ribosomal protein s9  RPS14 - ribosomal protein s14  RPS13 - ribosomal protein s13  RPS7 - ribosomal protein s7  RPSA - ribosomal protein sa  RPL10A - ribosomal protein l10a  RPL23 - ribosomal protein l23  RPS28 - ribosomal protein s28  RPS29 - ribosomal protein s29 |
| GO:0006413 | translational initiation | 8.05E-15 | 1.4E-11 | 2.71 (10334,129,1776,60) | [+] Show genes  RPL24 - ribosomal protein l24  RPL23A - ribosomal protein l23a  PAIP1 - poly(a) binding protein interacting protein 1  RPL21 - ribosomal protein l21  RPL19 - ribosomal protein l19  RPL18A - ribosomal protein l18a  RPL18 - ribosomal protein l18  RPL17 - ribosomal protein l17  RPL13 - ribosomal protein l13  RPL12 - ribosomal protein l12  RPL10 - ribosomal protein l10  RPL7A - ribosomal protein l7a  RPL5 - ribosomal protein l5  RPL4 - ribosomal protein l4  EIF3J - eukaryotic translation initiation factor 3, subunit j  EIF3H - eukaryotic translation initiation factor 3, subunit h  EIF3I - eukaryotic translation initiation factor 3, subunit i  RPL35 - ribosomal protein l35  RPS5 - ribosomal protein s5  EIF4B - eukaryotic translation initiation factor 4b  RPS2 - ribosomal protein s2  RPLP2 - ribosomal protein, large, p2  AGO2 - argonaute risc catalytic component 2  RPL36A - ribosomal protein l36a  RPL37A - ribosomal protein l37a  RPL38 - ribosomal protein l38  RPL34 - ribosomal protein l34  RPL37 - ribosomal protein l37  RPL29 - ribosomal protein l29  EIF4G1 - eukaryotic translation initiation factor 4 gamma, 1  RPL31 - ribosomal protein l31  RPL36 - ribosomal protein l36  RPL27 - ribosomal protein l27  RPL30 - ribosomal protein l30  RPL27A - ribosomal protein l27a  EIF1 - eukaryotic translation initiation factor 1  RPL28 - ribosomal protein l28  RPS24 - ribosomal protein s24  RPS21 - ribosomal protein s21  RPS27 - ribosomal protein s27  RPS26 - ribosomal protein s26  RPS25 - ribosomal protein s25  RPS17 - ribosomal protein s17  RPS16 - ribosomal protein s16  RPS15A - ribosomal protein s15a  RPS20 - ribosomal protein s20  RPS19 - ribosomal protein s19  MCTS1 - malignant t cell amplified sequence 1  RPS18 - ribosomal protein s18  RPS11 - ribosomal protein s11  RPS8 - ribosomal protein s8  RPS9 - ribosomal protein s9  RPS14 - ribosomal protein s14  RPS13 - ribosomal protein s13  RPS7 - ribosomal protein s7  RPSA - ribosomal protein sa  RPL10A - ribosomal protein l10a  RPL23 - ribosomal protein l23  RPS28 - ribosomal protein s28  RPS29 - ribosomal protein s29 |
| GO:0019083 | viral transcription | 1.15E-14 | 1.77E-11 | 2.88 (10334,107,1776,53) | [+] Show genes  RPL24 - ribosomal protein l24  RPL23A - ribosomal protein l23a  RPL21 - ribosomal protein l21  RPL19 - ribosomal protein l19  RPL18A - ribosomal protein l18a  USF2 - upstream transcription factor 2, c-fos interacting  RPL18 - ribosomal protein l18  RPL17 - ribosomal protein l17  RPL13 - ribosomal protein l13  RPL12 - ribosomal protein l12  RPL10 - ribosomal protein l10  RPL7A - ribosomal protein l7a  RPL5 - ribosomal protein l5  RPL4 - ribosomal protein l4  RPL35 - ribosomal protein l35  RPS5 - ribosomal protein s5  RPS2 - ribosomal protein s2  RPLP2 - ribosomal protein, large, p2  RPL36A - ribosomal protein l36a  RPL37A - ribosomal protein l37a  RPL38 - ribosomal protein l38  RPL34 - ribosomal protein l34  RPL37 - ribosomal protein l37  RPL29 - ribosomal protein l29  RPL31 - ribosomal protein l31  RPL36 - ribosomal protein l36  RPL27 - ribosomal protein l27  RPL30 - ribosomal protein l30  RPL27A - ribosomal protein l27a  RPL28 - ribosomal protein l28  RPS24 - ribosomal protein s24  RPS21 - ribosomal protein s21  RPS27 - ribosomal protein s27  RPS26 - ribosomal protein s26  RPS25 - ribosomal protein s25  RPS17 - ribosomal protein s17  RPS16 - ribosomal protein s16  RPS15A - ribosomal protein s15a  RPS20 - ribosomal protein s20  RPS19 - ribosomal protein s19  RPS18 - ribosomal protein s18  RPS11 - ribosomal protein s11  RPS8 - ribosomal protein s8  RPS9 - ribosomal protein s9  RPS14 - ribosomal protein s14  RPS13 - ribosomal protein s13  NUP37 - nucleoporin 37kda  RPS7 - ribosomal protein s7  RPSA - ribosomal protein sa  RPL10A - ribosomal protein l10a  RPL23 - ribosomal protein l23  RPS28 - ribosomal protein s28  RPS29 - ribosomal protein s29 |
| GO:0000184 | nuclear-transcribed mRNA catabolic process, nonsense-mediated decay | 7.89E-14 | 1.1E-10 | 2.78 (10334,111,1776,53) | [+] Show genes  RPL24 - ribosomal protein l24  RPL23A - ribosomal protein l23a  RPL21 - ribosomal protein l21  RPL19 - ribosomal protein l19  RPL18A - ribosomal protein l18a  RPL18 - ribosomal protein l18  RPL17 - ribosomal protein l17  RPL13 - ribosomal protein l13  RPL12 - ribosomal protein l12  RPL10 - ribosomal protein l10  RPL7A - ribosomal protein l7a  RPL5 - ribosomal protein l5  RPL4 - ribosomal protein l4  RPL35 - ribosomal protein l35  RPS5 - ribosomal protein s5  RPS2 - ribosomal protein s2  RPLP2 - ribosomal protein, large, p2  RPL36A - ribosomal protein l36a  RPL37A - ribosomal protein l37a  RPL38 - ribosomal protein l38  RPL34 - ribosomal protein l34  RPL37 - ribosomal protein l37  RPL29 - ribosomal protein l29  EIF4G1 - eukaryotic translation initiation factor 4 gamma, 1  RPL31 - ribosomal protein l31  RPL36 - ribosomal protein l36  RPL27 - ribosomal protein l27  RPL30 - ribosomal protein l30  RPL27A - ribosomal protein l27a  RPL28 - ribosomal protein l28  RPS24 - ribosomal protein s24  RPS21 - ribosomal protein s21  RPS27 - ribosomal protein s27  RPS26 - ribosomal protein s26  RPS25 - ribosomal protein s25  RPS17 - ribosomal protein s17  RPS16 - ribosomal protein s16  RPS15A - ribosomal protein s15a  RPS20 - ribosomal protein s20  RPS19 - ribosomal protein s19  RPS18 - ribosomal protein s18  RPS11 - ribosomal protein s11  RPS8 - ribosomal protein s8  RPS9 - ribosomal protein s9  RPS14 - ribosomal protein s14  RPS13 - ribosomal protein s13  RPS7 - ribosomal protein s7  RPSA - ribosomal protein sa  SMG5 - smg5 nonsense mediated mrna decay factor  RPL10A - ribosomal protein l10a  RPL23 - ribosomal protein l23  RPS28 - ribosomal protein s28  RPS29 - ribosomal protein s29 |
| GO:0006091 | generation of precursor metabolites and energy | 1.52E-13 | 1.92E-10 | 2.02 (10334,294,1776,102) | [+] Show genes  COX6B1 - cytochrome c oxidase subunit vib polypeptide 1 (ubiquitous)  SDHD - succinate dehydrogenase complex, subunit d, integral membrane protein  SDHC - succinate dehydrogenase complex, subunit c, integral membrane protein, 15kda  COX4I1 - cytochrome c oxidase subunit iv isoform 1  NDUFA1 - nadh dehydrogenase (ubiquinone) 1 alpha subcomplex, 1, 7.5kda  SDHA - succinate dehydrogenase complex, subunit a, flavoprotein (fp)  NDUFA5 - nadh dehydrogenase (ubiquinone) 1 alpha subcomplex, 5  BPGM - 2,3-bisphosphoglycerate mutase  COX10 - cytochrome c oxidase assembly homolog 10 (yeast)  AOX1 - aldehyde oxidase 1  COX7C - cytochrome c oxidase subunit viic  OXA1L - oxidase (cytochrome c) assembly 1-like  OXCT1 - 3-oxoacid coa transferase 1  COX7A1 - cytochrome c oxidase subunit viia polypeptide 1 (muscle)  COX6C - cytochrome c oxidase subunit vic  CYCS - cytochrome c, somatic  ACAT1 - acetyl-coa acetyltransferase 1  NHLRC1 - nhl repeat containing 1  ACO2 - aconitase 2, mitochondrial  AKR1A1 - aldo-keto reductase family 1, member a1 (aldehyde reductase)  BID - bh3 interacting domain death agonist  OGDH - oxoglutarate (alpha-ketoglutarate) dehydrogenase (lipoamide)  GYS1 - glycogen synthase 1 (muscle)  TSTA3 - tissue specific transplantation antigen p35b  GYG1 - glycogenin 1  NR1D1 - nuclear receptor subfamily 1, group d, member 1  ATP5L - atp synthase, h+ transporting, mitochondrial fo complex, subunit g  ACSS1 - acyl-coa synthetase short-chain family member 1  TPI1 - triosephosphate isomerase 1  GNMT - glycine n-methyltransferase  MDH2 - malate dehydrogenase 2, nad (mitochondrial)  NDUFA12 - nadh dehydrogenase (ubiquinone) 1 alpha subcomplex, 12  GLDC - glycine dehydrogenase (decarboxylating)  FECH - ferrochelatase  FDX1 - ferredoxin 1  POMC - proopiomelanocortin  GYG2 - glycogenin 2  POR - p450 (cytochrome) oxidoreductase  TXNRD3 - thioredoxin reductase 3  COQ10A - coenzyme q10 homolog a (s. cerevisiae)  UQCRC2 - ubiquinol-cytochrome c reductase core protein ii  XDH - xanthine dehydrogenase  NQO2 - nad(p)h dehydrogenase, quinone 2  UQCRB - ubiquinol-cytochrome c reductase binding protein  PGK1 - phosphoglycerate kinase 1  CROT - carnitine o-octanoyltransferase  UQCRFS1 - ubiquinol-cytochrome c reductase, rieske iron-sulfur polypeptide 1  ATP5S - atp synthase, h+ transporting, mitochondrial fo complex, subunit s (factor b)  ADSL - adenylosuccinate lyase  AGL - amylo-alpha-1, 6-glucosidase, 4-alpha-glucanotransferase  PHKA1 - phosphorylase kinase, alpha 1 (muscle)  PHKB - phosphorylase kinase, beta  COX1 - cytochrome c oxidase subunit i  MTFR1L - mitochondrial fission regulator 1-like  CYTB - cytochrome b  PINK1 - pten induced putative kinase 1  AKT2 - v-akt murine thymoma viral oncogene homolog 2  ATP5J - atp synthase, h+ transporting, mitochondrial fo complex, subunit f6  PYGL - phosphorylase, glycogen, liver  PYGM - phosphorylase, glycogen, muscle  ATP5C1 - atp synthase, h+ transporting, mitochondrial f1 complex, gamma polypeptide 1  NCF1 - neutrophil cytosolic factor 1  ATP5F1 - atp synthase, h+ transporting, mitochondrial fo complex, subunit b1  ATP5G3 - atp synthase, h+ transporting, mitochondrial fo complex, subunit c3 (subunit 9)  NDUFA11 - nadh dehydrogenase (ubiquinone) 1 alpha subcomplex, 11, 14.7kda  CYC1 - cytochrome c-1  ATP5A1 - atp synthase, h+ transporting, mitochondrial f1 complex, alpha subunit 1, cardiac muscle  IDH3B - isocitrate dehydrogenase 3 (nad+) beta  CYBA - cytochrome b-245, alpha polypeptide  ATP5B - atp synthase, h+ transporting, mitochondrial f1 complex, beta polypeptide  NDUFB11 - nadh dehydrogenase (ubiquinone) 1 beta subcomplex, 11, 17.3kda  DLD - dihydrolipoamide dehydrogenase  GALK1 - galactokinase 1  TEFM - transcription elongation factor, mitochondrial  DLST - dihydrolipoamide s-succinyltransferase (e2 component of 2-oxo-glutarate complex)  ALDOA - aldolase a, fructose-bisphosphate  COX5A - cytochrome c oxidase subunit va  AVPR1A - arginine vasopressin receptor 1a  GLRX5 - glutaredoxin 5  ETFA - electron-transfer-flavoprotein, alpha polypeptide  GBAS - glioblastoma amplified sequence  BDH1 - 3-hydroxybutyrate dehydrogenase, type 1  NDUFC1 - nadh dehydrogenase (ubiquinone) 1, subcomplex unknown, 1, 6kda  NDUFB8 - nadh dehydrogenase (ubiquinone) 1 beta subcomplex, 8, 19kda  NDUFB9 - nadh dehydrogenase (ubiquinone) 1 beta subcomplex, 9, 22kda  NDUFB6 - nadh dehydrogenase (ubiquinone) 1 beta subcomplex, 6, 17kda  NDUFB4 - nadh dehydrogenase (ubiquinone) 1 beta subcomplex, 4, 15kda  NDUFB5 - nadh dehydrogenase (ubiquinone) 1 beta subcomplex, 5, 16kda  NDUFB3 - nadh dehydrogenase (ubiquinone) 1 beta subcomplex, 3, 12kda  UGP2 - udp-glucose pyrophosphorylase 2  NDUFAB1 - nadh dehydrogenase (ubiquinone) 1, alpha/beta subcomplex, 1, 8kda  NDUFB1 - nadh dehydrogenase (ubiquinone) 1 beta subcomplex, 1, 7kda  NDUFA9 - nadh dehydrogenase (ubiquinone) 1 alpha subcomplex, 9, 39kda  NDUFA10 - nadh dehydrogenase (ubiquinone) 1 alpha subcomplex, 10, 42kda  HMGCL - 3-hydroxymethyl-3-methylglutaryl-coa lyase  GAPDH - glyceraldehyde-3-phosphate dehydrogenase  NDUFV2 - nadh dehydrogenase (ubiquinone) flavoprotein 2, 24kda  NDUFS4 - nadh dehydrogenase (ubiquinone) fe-s protein 4, 18kda (nadh-coenzyme q reductase)  AIFM2 - apoptosis-inducing factor, mitochondrion-associated, 2  NDUFS3 - nadh dehydrogenase (ubiquinone) fe-s protein 3, 30kda (nadh-coenzyme q reductase)  NDUFS2 - nadh dehydrogenase (ubiquinone) fe-s protein 2, 49kda (nadh-coenzyme q reductase)  NDUFS1 - nadh dehydrogenase (ubiquinone) fe-s protein 1, 75kda (nadh-coenzyme q reductase) |
| GO:0043604 | amide biosynthetic process | 2.51E-13 | 2.91E-10 | 2.03 (10334,284,1776,99) | [+] Show genes  MRPL35 - mitochondrial ribosomal protein l35  GFM2 - g elongation factor, mitochondrial 2  FA2H - fatty acid 2-hydroxylase  GCH1 - gtp cyclohydrolase 1  RPL35 - ribosomal protein l35  ACAT1 - acetyl-coa acetyltransferase 1  EIF4B - eukaryotic translation initiation factor 4b  EIF4EBP2 - eukaryotic translation initiation factor 4e binding protein 2  MRPL27 - mitochondrial ribosomal protein l27  EIF4G1 - eukaryotic translation initiation factor 4 gamma, 1  MRPS36 - mitochondrial ribosomal protein s36  RPL36 - ribosomal protein l36  GGT5 - gamma-glutamyltransferase 5  MRPS18C - mitochondrial ribosomal protein s18c  ACSS1 - acyl-coa synthetase short-chain family member 1  PDHB - pyruvate dehydrogenase (lipoamide) beta  MRPL52 - mitochondrial ribosomal protein l52  PDHA1 - pyruvate dehydrogenase (lipoamide) alpha 1  TECR - trans-2,3-enoyl-coa reductase  RWDD1 - rwd domain containing 1  RPSA - ribosomal protein sa  ASL - argininosuccinate lyase  MRPL28 - mitochondrial ribosomal protein l28  MRPS17 - mitochondrial ribosomal protein s17  SLC25A1 - solute carrier family 25 (mitochondrial carrier; citrate transporter), member 1  ACSF3 - acyl-coa synthetase family member 3  DRG2 - developmentally regulated gtp binding protein 2  RPL24 - ribosomal protein l24  RPL23A - ribosomal protein l23a  RPL21 - ribosomal protein l21  RPL19 - ribosomal protein l19  RPL18A - ribosomal protein l18a  CNDP2 - cndp dipeptidase 2 (metallopeptidase m20 family)  RPL18 - ribosomal protein l18  RPL17 - ribosomal protein l17  RPL13 - ribosomal protein l13  RPL12 - ribosomal protein l12  HBS1L - hbs1-like (s. cerevisiae)  RPL10 - ribosomal protein l10  RPL7A - ribosomal protein l7a  UGT8 - udp glycosyltransferase 8  RPL5 - ribosomal protein l5  RPL4 - ribosomal protein l4  RPS5 - ribosomal protein s5  RPS2 - ribosomal protein s2  RPLP2 - ribosomal protein, large, p2  MRPS12 - mitochondrial ribosomal protein s12  AGO2 - argonaute risc catalytic component 2  RPL36A - ribosomal protein l36a  PET112 - pet112 homolog (yeast)  MRPS2 - mitochondrial ribosomal protein s2  RPL37A - ribosomal protein l37a  RPL38 - ribosomal protein l38  RPL34 - ribosomal protein l34  RPL37 - ribosomal protein l37  RPL29 - ribosomal protein l29  RPL31 - ribosomal protein l31  B3GALT4 - udp-gal:betaglcnac beta 1,3-galactosyltransferase, polypeptide 4  RPL27 - ribosomal protein l27  RPL30 - ribosomal protein l30  RPL27A - ribosomal protein l27a  RPL28 - ribosomal protein l28  RPS24 - ribosomal protein s24  DLAT - dihydrolipoamide s-acetyltransferase  DLD - dihydrolipoamide dehydrogenase  RPS21 - ribosomal protein s21  B4GALNT1 - beta-1,4-n-acetyl-galactosaminyl transferase 1  RPS27 - ribosomal protein s27  RPS26 - ribosomal protein s26  PDHX - pyruvate dehydrogenase complex, component x  RPS25 - ribosomal protein s25  ASNSD1 - asparagine synthetase domain containing 1  RPS17 - ribosomal protein s17  RPS16 - ribosomal protein s16  RPS15A - ribosomal protein s15a  RPS20 - ribosomal protein s20  RPS19 - ribosomal protein s19  RPS18 - ribosomal protein s18  RPS11 - ribosomal protein s11  RPS8 - ribosomal protein s8  RPS9 - ribosomal protein s9  RPS14 - ribosomal protein s14  RPS13 - ribosomal protein s13  GUF1 - guf1 gtpase homolog (s. cerevisiae)  MRPS18B - mitochondrial ribosomal protein s18b  MRPS5 - mitochondrial ribosomal protein s5  MRPL41 - mitochondrial ribosomal protein l41  RPS7 - ribosomal protein s7  MRPL34 - mitochondrial ribosomal protein l34  PABPC4 - poly(a) binding protein, cytoplasmic 4 (inducible form)  PEMT - phosphatidylethanolamine n-methyltransferase  RPL10A - ribosomal protein l10a  DMD - dystrophin  RPL23 - ribosomal protein l23  IARS2 - isoleucyl-trna synthetase 2, mitochondrial  RPS28 - ribosomal protein s28  RPS29 - ribosomal protein s29  SPTSSA - serine palmitoyltransferase, small subunit a  FPGS - folylpolyglutamate synthase |
| GO:1901566 | organonitrogen compound biosynthetic process | 1.16E-11 | 1.24E-8 | 1.55 (10334,738,1776,197) | [+] Show genes  MRPL35 - mitochondrial ribosomal protein l35  SUCLA2 - succinate-coa ligase, adp-forming, beta subunit  STARD7 - star-related lipid transfer (start) domain containing 7  GFM2 - g elongation factor, mitochondrial 2  BPGM - 2,3-bisphosphoglycerate mutase  AASS - aminoadipate-semialdehyde synthase  FA2H - fatty acid 2-hydroxylase  LPCAT3 - lysophosphatidylcholine acyltransferase 3  COX10 - cytochrome c oxidase assembly homolog 10 (yeast)  GCH1 - gtp cyclohydrolase 1  BGN - biglycan  ACAT1 - acetyl-coa acetyltransferase 1  EIF4B - eukaryotic translation initiation factor 4b  EIF4EBP2 - eukaryotic translation initiation factor 4e binding protein 2  AKR1A1 - aldo-keto reductase family 1, member a1 (aldehyde reductase)  GMPS - guanine monphosphate synthase  MRPL27 - mitochondrial ribosomal protein l27  EIF4G1 - eukaryotic translation initiation factor 4 gamma, 1  MRPS36 - mitochondrial ribosomal protein s36  GGT5 - gamma-glutamyltransferase 5  GLUL - glutamate-ammonia ligase  CSGALNACT2 - chondroitin sulfate n-acetylgalactosaminyltransferase 2  ACSS1 - acyl-coa synthetase short-chain family member 1  TPI1 - triosephosphate isomerase 1  TECR - trans-2,3-enoyl-coa reductase  RWDD1 - rwd domain containing 1  ADCY2 - adenylate cyclase 2 (brain)  SMS - spermine synthase  RPSA - ribosomal protein sa  ADCY9 - adenylate cyclase 9  LCAT - lecithin-cholesterol acyltransferase  ADK - adenosine kinase  MRPS17 - mitochondrial ribosomal protein s17  HSPG2 - heparan sulfate proteoglycan 2  SLC25A1 - solute carrier family 25 (mitochondrial carrier; citrate transporter), member 1  GMPR - guanosine monophosphate reductase  ACSF3 - acyl-coa synthetase family member 3  NAMPT - nicotinamide phosphoribosyltransferase  RPL24 - ribosomal protein l24  MTR - 5-methyltetrahydrofolate-homocysteine methyltransferase  RPL23A - ribosomal protein l23a  RPL21 - ribosomal protein l21  ACCS - 1-aminocyclopropane-1-carboxylate synthase homolog (arabidopsis)(non-functional)  RPL19 - ribosomal protein l19  RPL18A - ribosomal protein l18a  CNDP2 - cndp dipeptidase 2 (metallopeptidase m20 family)  RPL18 - ribosomal protein l18  RPL17 - ribosomal protein l17  RPL13 - ribosomal protein l13  RPL12 - ribosomal protein l12  ADSL - adenylosuccinate lyase  FABP5 - fatty acid binding protein 5 (psoriasis-associated)  CTH - cystathionase (cystathionine gamma-lyase)  RPL10 - ribosomal protein l10  RPL7A - ribosomal protein l7a  SLC44A3 - solute carrier family 44, member 3  UGT8 - udp glycosyltransferase 8  MOCOS - molybdenum cofactor sulfurase  RPL5 - ribosomal protein l5  RPL4 - ribosomal protein l4  GPT2 - glutamic pyruvate transaminase (alanine aminotransferase) 2  AK1 - adenylate kinase 1  RPS5 - ribosomal protein s5  RPS2 - ribosomal protein s2  RPLP2 - ribosomal protein, large, p2  MRPS12 - mitochondrial ribosomal protein s12  RPL36A - ribosomal protein l36a  MRPS2 - mitochondrial ribosomal protein s2  PPCDC - phosphopantothenoylcysteine decarboxylase  RPL37A - ribosomal protein l37a  RPL38 - ribosomal protein l38  RPL34 - ribosomal protein l34  ALDH9A1 - aldehyde dehydrogenase 9 family, member a1  RPL37 - ribosomal protein l37  RPL29 - ribosomal protein l29  RPL31 - ribosomal protein l31  CYC1 - cytochrome c-1  RPL27 - ribosomal protein l27  B3GALT4 - udp-gal:betaglcnac beta 1,3-galactosyltransferase, polypeptide 4  RPL30 - ribosomal protein l30  RPL27A - ribosomal protein l27a  RPL28 - ribosomal protein l28  RPS24 - ribosomal protein s24  GALK1 - galactokinase 1  RPS21 - ribosomal protein s21  B4GALNT1 - beta-1,4-n-acetyl-galactosaminyl transferase 1  RPS27 - ribosomal protein s27  PDHX - pyruvate dehydrogenase complex, component x  RPS26 - ribosomal protein s26  RPS25 - ribosomal protein s25  RPS17 - ribosomal protein s17  RPS16 - ribosomal protein s16  RPS15A - ribosomal protein s15a  ALDOA - aldolase a, fructose-bisphosphate  RPS20 - ribosomal protein s20  RPS19 - ribosomal protein s19  RPS18 - ribosomal protein s18  RPS11 - ribosomal protein s11  RPS8 - ribosomal protein s8  RPS9 - ribosomal protein s9  RPS14 - ribosomal protein s14  GUF1 - guf1 gtpase homolog (s. cerevisiae)  RPS13 - ribosomal protein s13  UCKL1 - uridine-cytidine kinase 1-like 1  MRPS18B - mitochondrial ribosomal protein s18b  MRPS5 - mitochondrial ribosomal protein s5  MRPL41 - mitochondrial ribosomal protein l41  RPS7 - ribosomal protein s7  TYMS - thymidylate synthetase  MRPL34 - mitochondrial ribosomal protein l34  ANGPT1 - angiopoietin 1  NMNAT1 - nicotinamide nucleotide adenylyltransferase 1  GATM - glycine amidinotransferase (l-arginine:glycine amidinotransferase)  PABPC4 - poly(a) binding protein, cytoplasmic 4 (inducible form)  UGDH - udp-glucose 6-dehydrogenase  PEMT - phosphatidylethanolamine n-methyltransferase  AMPD3 - adenosine monophosphate deaminase 3  BCAN - brevican  RPL23 - ribosomal protein l23  GAPDH - glyceraldehyde-3-phosphate dehydrogenase  IARS2 - isoleucyl-trna synthetase 2, mitochondrial  PARP10 - poly (adp-ribose) polymerase family, member 10  RPS28 - ribosomal protein s28  RPS29 - ribosomal protein s29  AFMID - arylformamidase  CBS - cystathionine-beta-synthase  NCAN - neurocan  GSTM1 - glutathione s-transferase mu 1  GSTM4 - glutathione s-transferase mu 4  APOA1 - apolipoprotein a-i  SDSL - serine dehydratase-like  PPM1L - protein phosphatase, mg2+/mn2+ dependent, 1l  NAT8L - n-acetyltransferase 8-like (gcn5-related, putative)  CECR1 - cat eye syndrome chromosome region, candidate 1  RPL35 - ribosomal protein l35  NADSYN1 - nad synthetase 1  OGDH - oxoglutarate (alpha-ketoglutarate) dehydrogenase (lipoamide)  SDC1 - syndecan 1  COASY - coa synthase  APOE - apolipoprotein e  APRT - adenine phosphoribosyltransferase  RPL36 - ribosomal protein l36  TMLHE - trimethyllysine hydroxylase, epsilon  ATP5L - atp synthase, h+ transporting, mitochondrial fo complex, subunit g  MRPS18C - mitochondrial ribosomal protein s18c  PDHB - pyruvate dehydrogenase (lipoamide) beta  MRPL52 - mitochondrial ribosomal protein l52  PDHA1 - pyruvate dehydrogenase (lipoamide) alpha 1  VAPA - vamp (vesicle-associated membrane protein)-associated protein a, 33kda  CDO1 - cysteine dioxygenase type 1  FECH - ferrochelatase  ASL - argininosuccinate lyase  MRPL28 - mitochondrial ribosomal protein l28  NPPC - natriuretic peptide c  DAO - d-amino-acid oxidase  HEXA - hexosaminidase a (alpha polypeptide)  CHST8 - carbohydrate (n-acetylgalactosamine 4-0) sulfotransferase 8  MGST1 - microsomal glutathione s-transferase 1  DRG2 - developmentally regulated gtp binding protein 2  PGK1 - phosphoglycerate kinase 1  ATP5S - atp synthase, h+ transporting, mitochondrial fo complex, subunit s (factor b)  NME3 - nme/nm23 nucleoside diphosphate kinase 3  HBS1L - hbs1-like (s. cerevisiae)  AGPAT5 - 1-acylglycerol-3-phosphate o-acyltransferase 5  NMNAT3 - nicotinamide nucleotide adenylyltransferase 3  B3GNT4 - udp-glcnac:betagal beta-1,3-n-acetylglucosaminyltransferase 4  DPYD - dihydropyrimidine dehydrogenase  DUT - deoxyuridine triphosphatase  GLS2 - glutaminase 2 (liver, mitochondrial)  ATP5J - atp synthase, h+ transporting, mitochondrial fo complex, subunit f6  AGO2 - argonaute risc catalytic component 2  QPRT - quinolinate phosphoribosyltransferase  SLC1A3 - solute carrier family 1 (glial high affinity glutamate transporter), member 3  PET112 - pet112 homolog (yeast)  STARD10 - star-related lipid transfer (start) domain containing 10  ATP5C1 - atp synthase, h+ transporting, mitochondrial f1 complex, gamma polypeptide 1  ATP5F1 - atp synthase, h+ transporting, mitochondrial fo complex, subunit b1  ATP5G3 - atp synthase, h+ transporting, mitochondrial fo complex, subunit c3 (subunit 9)  CHDH - choline dehydrogenase  ATP5A1 - atp synthase, h+ transporting, mitochondrial f1 complex, alpha subunit 1, cardiac muscle  DTYMK - deoxythymidylate kinase (thymidylate kinase)  ATP5B - atp synthase, h+ transporting, mitochondrial f1 complex, beta polypeptide  IDH2 - isocitrate dehydrogenase 2 (nadp+), mitochondrial  DLAT - dihydrolipoamide s-acetyltransferase  DLD - dihydrolipoamide dehydrogenase  SLC25A39 - solute carrier family 25, member 39  ASNSD1 - asparagine synthetase domain containing 1  CSPG5 - chondroitin sulfate proteoglycan 5 (neuroglycan c)  SHMT2 - serine hydroxymethyltransferase 2 (mitochondrial)  NFE2L1 - nuclear factor (erythroid-derived 2)-like 1  ST3GAL1 - st3 beta-galactoside alpha-2,3-sialyltransferase 1  FLAD1 - flavin adenine dinucleotide synthetase 1  GPAM - glycerol-3-phosphate acyltransferase, mitochondrial  RPL10A - ribosomal protein l10a  DMD - dystrophin  SPTSSA - serine palmitoyltransferase, small subunit a  FPGS - folylpolyglutamate synthase |
| GO:0006518 | peptide metabolic process | 1.2E-11 | 1.2E-8 | 1.95 (10334,280,1776,94) | [+] Show genes  DRG2 - developmentally regulated gtp binding protein 2  GSTM1 - glutathione s-transferase mu 1  RPL24 - ribosomal protein l24  LHB - luteinizing hormone beta polypeptide  RPL23A - ribosomal protein l23a  AEBP1 - ae binding protein 1  GSTM4 - glutathione s-transferase mu 4  RPL21 - ribosomal protein l21  MRPL35 - mitochondrial ribosomal protein l35  RPL19 - ribosomal protein l19  RPL18A - ribosomal protein l18a  RPL18 - ribosomal protein l18  CNDP2 - cndp dipeptidase 2 (metallopeptidase m20 family)  RPL17 - ribosomal protein l17  RPL13 - ribosomal protein l13  GFM2 - g elongation factor, mitochondrial 2  RPL12 - ribosomal protein l12  HBS1L - hbs1-like (s. cerevisiae)  RPL10 - ribosomal protein l10  RPL7A - ribosomal protein l7a  RPL5 - ribosomal protein l5  RPL4 - ribosomal protein l4  RPL35 - ribosomal protein l35  CTSH - cathepsin h  RPS5 - ribosomal protein s5  EIF4B - eukaryotic translation initiation factor 4b  EIF4EBP2 - eukaryotic translation initiation factor 4e binding protein 2  RPS2 - ribosomal protein s2  RPLP2 - ribosomal protein, large, p2  CPM - carboxypeptidase m  CTSZ - cathepsin z  AGO2 - argonaute risc catalytic component 2  MRPS12 - mitochondrial ribosomal protein s12  RPL36A - ribosomal protein l36a  PET112 - pet112 homolog (yeast)  MRPS2 - mitochondrial ribosomal protein s2  PCSK6 - proprotein convertase subtilisin/kexin type 6  RPL37A - ribosomal protein l37a  RPL38 - ribosomal protein l38  RPL34 - ribosomal protein l34  SOD1 - superoxide dismutase 1, soluble  MRPL27 - mitochondrial ribosomal protein l27  RPL37 - ribosomal protein l37  RPL29 - ribosomal protein l29  EIF4G1 - eukaryotic translation initiation factor 4 gamma, 1  RPL31 - ribosomal protein l31  MRPS36 - mitochondrial ribosomal protein s36  CGA - glycoprotein hormones, alpha polypeptide  RPL36 - ribosomal protein l36  RPL27 - ribosomal protein l27  RPL30 - ribosomal protein l30  GGT5 - gamma-glutamyltransferase 5  RPL27A - ribosomal protein l27a  RPL28 - ribosomal protein l28  RPS24 - ribosomal protein s24  RPS21 - ribosomal protein s21  MRPS18C - mitochondrial ribosomal protein s18c  RPS27 - ribosomal protein s27  RPS26 - ribosomal protein s26  RPS25 - ribosomal protein s25  MRPL52 - mitochondrial ribosomal protein l52  RPS17 - ribosomal protein s17  RPS16 - ribosomal protein s16  RPS15A - ribosomal protein s15a  RPS20 - ribosomal protein s20  GLO1 - glyoxalase i  RPS19 - ribosomal protein s19  RPS18 - ribosomal protein s18  RPS11 - ribosomal protein s11  SYNPO2 - synaptopodin 2  RPS8 - ribosomal protein s8  RPS9 - ribosomal protein s9  RPS14 - ribosomal protein s14  SEC11A - sec11 homolog a (s. cerevisiae)  RWDD1 - rwd domain containing 1  NFE2L1 - nuclear factor (erythroid-derived 2)-like 1  GUF1 - guf1 gtpase homolog (s. cerevisiae)  RPS13 - ribosomal protein s13  MRPS18B - mitochondrial ribosomal protein s18b  MRPS5 - mitochondrial ribosomal protein s5  MRPL41 - mitochondrial ribosomal protein l41  RPS7 - ribosomal protein s7  MRPL34 - mitochondrial ribosomal protein l34  RPSA - ribosomal protein sa  MRPL28 - mitochondrial ribosomal protein l28  PABPC4 - poly(a) binding protein, cytoplasmic 4 (inducible form)  MRPS17 - mitochondrial ribosomal protein s17  DMD - dystrophin  RPL10A - ribosomal protein l10a  RPL23 - ribosomal protein l23  MGAT3 - mannosyl (beta-1,4-)-glycoprotein beta-1,4-n-acetylglucosaminyltransferase  IARS2 - isoleucyl-trna synthetase 2, mitochondrial  RPS28 - ribosomal protein s28  RPS29 - ribosomal protein s29 |
| GO:0043603 | cellular amide metabolic process | 1.95E-11 | 1.81E-8 | 1.70 (10334,471,1776,138) | [+] Show genes  GSTM1 - glutathione s-transferase mu 1  LHB - luteinizing hormone beta polypeptide  GSTM4 - glutathione s-transferase mu 4  HSD17B4 - hydroxysteroid (17-beta) dehydrogenase 4  MRPL35 - mitochondrial ribosomal protein l35  SUCLA2 - succinate-coa ligase, adp-forming, beta subunit  SUCLG2 - succinate-coa ligase, gdp-forming, beta subunit  GFM2 - g elongation factor, mitochondrial 2  BTD - biotinidase  AASS - aminoadipate-semialdehyde synthase  FA2H - fatty acid 2-hydroxylase  PC - pyruvate carboxylase  PCCA - propionyl coa carboxylase, alpha polypeptide  PCCB - propionyl coa carboxylase, beta polypeptide  GCH1 - gtp cyclohydrolase 1  RPL35 - ribosomal protein l35  ACAT1 - acetyl-coa acetyltransferase 1  EIF4B - eukaryotic translation initiation factor 4b  EIF4EBP2 - eukaryotic translation initiation factor 4e binding protein 2  CPM - carboxypeptidase m  PMVK - phosphomevalonate kinase  PCSK6 - proprotein convertase subtilisin/kexin type 6  OGDH - oxoglutarate (alpha-ketoglutarate) dehydrogenase (lipoamide)  MRPL27 - mitochondrial ribosomal protein l27  SOD1 - superoxide dismutase 1, soluble  EIF4G1 - eukaryotic translation initiation factor 4 gamma, 1  MRPS36 - mitochondrial ribosomal protein s36  RPL36 - ribosomal protein l36  GGT5 - gamma-glutamyltransferase 5  MRPS18C - mitochondrial ribosomal protein s18c  ACSS1 - acyl-coa synthetase short-chain family member 1  PDHB - pyruvate dehydrogenase (lipoamide) beta  MRPL52 - mitochondrial ribosomal protein l52  PDHA1 - pyruvate dehydrogenase (lipoamide) alpha 1  GLO1 - glyoxalase i  TECR - trans-2,3-enoyl-coa reductase  RWDD1 - rwd domain containing 1  RPSA - ribosomal protein sa  MRPL28 - mitochondrial ribosomal protein l28  ASL - argininosuccinate lyase  NEU3 - sialidase 3 (membrane sialidase)  NIT2 - nitrilase family, member 2  MRPS17 - mitochondrial ribosomal protein s17  MVD - mevalonate (diphospho) decarboxylase  HEXA - hexosaminidase a (alpha polypeptide)  SLC25A1 - solute carrier family 25 (mitochondrial carrier; citrate transporter), member 1  HEXB - hexosaminidase b (beta polypeptide)  MGAT3 - mannosyl (beta-1,4-)-glycoprotein beta-1,4-n-acetylglucosaminyltransferase  ACSF3 - acyl-coa synthetase family member 3  DRG2 - developmentally regulated gtp binding protein 2  RPL24 - ribosomal protein l24  AEBP1 - ae binding protein 1  RPL23A - ribosomal protein l23a  ACOT8 - acyl-coa thioesterase 8  RPL21 - ribosomal protein l21  RPL19 - ribosomal protein l19  RPL18A - ribosomal protein l18a  CNDP2 - cndp dipeptidase 2 (metallopeptidase m20 family)  RPL18 - ribosomal protein l18  RPL17 - ribosomal protein l17  RPL13 - ribosomal protein l13  UROC1 - urocanate hydratase 1  RPL12 - ribosomal protein l12  HBS1L - hbs1-like (s. cerevisiae)  RPL10 - ribosomal protein l10  RPL7A - ribosomal protein l7a  UGT8 - udp glycosyltransferase 8  RPL5 - ribosomal protein l5  RPL4 - ribosomal protein l4  CTSH - cathepsin h  RPS5 - ribosomal protein s5  RPS2 - ribosomal protein s2  RPLP2 - ribosomal protein, large, p2  CTSZ - cathepsin z  AGO2 - argonaute risc catalytic component 2  MRPS12 - mitochondrial ribosomal protein s12  RPL36A - ribosomal protein l36a  PET112 - pet112 homolog (yeast)  MRPS2 - mitochondrial ribosomal protein s2  RPL37A - ribosomal protein l37a  RPL38 - ribosomal protein l38  RPL34 - ribosomal protein l34  RPL37 - ribosomal protein l37  RPL29 - ribosomal protein l29  RPL31 - ribosomal protein l31  CGA - glycoprotein hormones, alpha polypeptide  RPL27 - ribosomal protein l27  B3GALT4 - udp-gal:betaglcnac beta 1,3-galactosyltransferase, polypeptide 4  RPL30 - ribosomal protein l30  RPL27A - ribosomal protein l27a  ABCD1 - atp-binding cassette, sub-family d (ald), member 1  RPL28 - ribosomal protein l28  RPS24 - ribosomal protein s24  DLAT - dihydrolipoamide s-acetyltransferase  DLD - dihydrolipoamide dehydrogenase  RPS21 - ribosomal protein s21  B4GALNT1 - beta-1,4-n-acetyl-galactosaminyl transferase 1  RPS27 - ribosomal protein s27  RPS26 - ribosomal protein s26  PDHX - pyruvate dehydrogenase complex, component x  RPS25 - ribosomal protein s25  ASNSD1 - asparagine synthetase domain containing 1  RPS17 - ribosomal protein s17  RPS16 - ribosomal protein s16  DLST - dihydrolipoamide s-succinyltransferase (e2 component of 2-oxo-glutarate complex)  RPS15A - ribosomal protein s15a  RPS20 - ribosomal protein s20  RPS19 - ribosomal protein s19  RPS18 - ribosomal protein s18  SHMT2 - serine hydroxymethyltransferase 2 (mitochondrial)  RPS11 - ribosomal protein s11  RPS8 - ribosomal protein s8  SYNPO2 - synaptopodin 2  RPS9 - ribosomal protein s9  RPS14 - ribosomal protein s14  SEC11A - sec11 homolog a (s. cerevisiae)  NFE2L1 - nuclear factor (erythroid-derived 2)-like 1  RPS13 - ribosomal protein s13  GUF1 - guf1 gtpase homolog (s. cerevisiae)  MRPS18B - mitochondrial ribosomal protein s18b  MRPS5 - mitochondrial ribosomal protein s5  MRPL41 - mitochondrial ribosomal protein l41  RPS7 - ribosomal protein s7  TYMS - thymidylate synthetase  MRPL34 - mitochondrial ribosomal protein l34  PABPC4 - poly(a) binding protein, cytoplasmic 4 (inducible form)  PEMT - phosphatidylethanolamine n-methyltransferase  GPAM - glycerol-3-phosphate acyltransferase, mitochondrial  RPL10A - ribosomal protein l10a  DMD - dystrophin  HMGCL - 3-hydroxymethyl-3-methylglutaryl-coa lyase  FOLR2 - folate receptor 2 (fetal)  RPL23 - ribosomal protein l23  IARS2 - isoleucyl-trna synthetase 2, mitochondrial  RPS28 - ribosomal protein s28  RPS29 - ribosomal protein s29  SPTSSA - serine palmitoyltransferase, small subunit a  FPGS - folylpolyglutamate synthase |
| GO:0044281 | small molecule metabolic process | 5.64E-11 | 4.91E-8 | 1.39 (10334,1275,1776,304) | [+] Show genes  LHB - luteinizing hormone beta polypeptide  HSD17B4 - hydroxysteroid (17-beta) dehydrogenase 4  SUCLA2 - succinate-coa ligase, adp-forming, beta subunit  SUCLG2 - succinate-coa ligase, gdp-forming, beta subunit  BPHL - biphenyl hydrolase-like (serine hydrolase)  BPGM - 2,3-bisphosphoglycerate mutase  BTD - biotinidase  CARD11 - caspase recruitment domain family, member 11  AASS - aminoadipate-semialdehyde synthase  LDHB - lactate dehydrogenase b  FA2H - fatty acid 2-hydroxylase  RPIA - ribose 5-phosphate isomerase a  BSG - basigin (ok blood group)  GCSH - glycine cleavage system protein h (aminomethyl carrier)  PMM2 - phosphomannomutase 2  GCH1 - gtp cyclohydrolase 1  DPM2 - dolichyl-phosphate mannosyltransferase polypeptide 2, regulatory subunit  RENBP - renin binding protein  COX7A1 - cytochrome c oxidase subunit viia polypeptide 1 (muscle)  RETSAT - retinol saturase (all-trans-retinol 13,14-reductase)  EPHA2 - eph receptor a2  ACAT1 - acetyl-coa acetyltransferase 1  BGN - biglycan  GK - glycerol kinase  DDAH1 - dimethylarginine dimethylaminohydrolase 1  PLP1 - proteolipid protein 1  ACO1 - aconitase 1, soluble  PTGR1 - prostaglandin reductase 1  ACO2 - aconitase 2, mitochondrial  BLMH - bleomycin hydrolase  AKR1A1 - aldo-keto reductase family 1, member a1 (aldehyde reductase)  LPL - lipoprotein lipase  GMPS - guanine monphosphate synthase  ACP5 - acid phosphatase 5, tartrate resistant  SORD - sorbitol dehydrogenase  CREM - camp responsive element modulator  TSTA3 - tissue specific transplantation antigen p35b  PDSS1 - prenyl (decaprenyl) diphosphate synthase, subunit 1  MRPS36 - mitochondrial ribosomal protein s36  CRAT - carnitine o-acetyltransferase  GGT5 - gamma-glutamyltransferase 5  FADS3 - fatty acid desaturase 3  HYI - hydroxypyruvate isomerase (putative)  GLUL - glutamate-ammonia ligase  CSGALNACT2 - chondroitin sulfate n-acetylgalactosaminyltransferase 2  NUDT17 - nudix (nucleoside diphosphate linked moiety x)-type motif 17  ACSS1 - acyl-coa synthetase short-chain family member 1  TPI1 - triosephosphate isomerase 1  DYSF - dysferlin, limb girdle muscular dystrophy 2b (autosomal recessive)  ACY1 - aminoacylase 1  GLO1 - glyoxalase i  CA4 - carbonic anhydrase iv  TECR - trans-2,3-enoyl-coa reductase  GLDC - glycine dehydrogenase (decarboxylating)  SHPK - sedoheptulokinase  LYVE1 - lymphatic vessel endothelial hyaluronan receptor 1  ADCY2 - adenylate cyclase 2 (brain)  SMS - spermine synthase  RORA - rar-related orphan receptor a  ADCY9 - adenylate cyclase 9  NIT2 - nitrilase family, member 2  LCAT - lecithin-cholesterol acyltransferase  ADK - adenosine kinase  MVD - mevalonate (diphospho) decarboxylase  POR - p450 (cytochrome) oxidoreductase  C3 - complement component 3  SLC25A1 - solute carrier family 25 (mitochondrial carrier; citrate transporter), member 1  LDHD - lactate dehydrogenase d  COQ10A - coenzyme q10 homolog a (s. cerevisiae)  CLPX - clpx caseinolytic peptidase x homolog (e. coli)  GMPR - guanosine monophosphate reductase  VKORC1 - vitamin k epoxide reductase complex, subunit 1  ACSF3 - acyl-coa synthetase family member 3  NAMPT - nicotinamide phosphoribosyltransferase  CYP46A1 - cytochrome p450, family 46, subfamily a, polypeptide 1  L2HGDH - l-2-hydroxyglutarate dehydrogenase  IDNK - idnk, gluconokinase homolog (e. coli)  UQCRC2 - ubiquinol-cytochrome c reductase core protein ii  PRKAA2 - protein kinase, amp-activated, alpha 2 catalytic subunit  SCPEP1 - serine carboxypeptidase 1  MTR - 5-methyltetrahydrofolate-homocysteine methyltransferase  UQCRB - ubiquinol-cytochrome c reductase binding protein  PRKAB2 - protein kinase, amp-activated, beta 2 non-catalytic subunit  QKI - qki, kh domain containing, rna binding  FAAH - fatty acid amide hydrolase  ACOT8 - acyl-coa thioesterase 8  FABP3 - fatty acid binding protein 3, muscle and heart (mammary-derived growth inhibitor)  ACCS - 1-aminocyclopropane-1-carboxylate synthase homolog (arabidopsis)(non-functional)  OMA1 - oma1 zinc metallopeptidase  ABCG2 - atp-binding cassette, sub-family g (white), member 2  FABP5 - fatty acid binding protein 5 (psoriasis-associated)  ADSL - adenylosuccinate lyase  CTH - cystathionase (cystathionine gamma-lyase)  KHK - ketohexokinase (fructokinase)  MOCOS - molybdenum cofactor sulfurase  GPT2 - glutamic pyruvate transaminase (alanine aminotransferase) 2  NUDT1 - nudix (nucleoside diphosphate linked moiety x)-type motif 1  AK1 - adenylate kinase 1  AKT2 - v-akt murine thymoma viral oncogene homolog 2  NUDT14 - nudix (nucleoside diphosphate linked moiety x)-type motif 14  VLDLR - very low density lipoprotein receptor  PPCDC - phosphopantothenoylcysteine decarboxylase  SULT1E1 - sulfotransferase family 1e, estrogen-preferring, member 1  VDAC1 - voltage-dependent anion channel 1  CYC1 - cytochrome c-1  ABCD1 - atp-binding cassette, sub-family d (ald), member 1  GALE - udp-galactose-4-epimerase  GALK1 - galactokinase 1  ALOX5AP - arachidonate 5-lipoxygenase-activating protein  PDHX - pyruvate dehydrogenase complex, component x  TEFM - transcription elongation factor, mitochondrial  STAT5B - signal transducer and activator of transcription 5b  ALDOA - aldolase a, fructose-bisphosphate  MFN1 - mitofusin 1  NT5C3A - 5'-nucleotidase, cytosolic iiia  ECHDC3 - enoyl coa hydratase domain containing 3  ZADH2 - zinc binding alcohol dehydrogenase domain containing 2  ACAD9 - acyl-coa dehydrogenase family, member 9  UCKL1 - uridine-cytidine kinase 1-like 1  GLRX5 - glutaredoxin 5  ABHD14B - abhydrolase domain containing 14b  PLAA - phospholipase a2-activating protein  TYMS - thymidylate synthetase  TXN - thioredoxin  CS - citrate synthase  ETFA - electron-transfer-flavoprotein, alpha polypeptide  MAPK14 - mitogen-activated protein kinase 14  GBAS - glioblastoma amplified sequence  ANGPT1 - angiopoietin 1  NMNAT1 - nicotinamide nucleotide adenylyltransferase 1  KCNJ11 - potassium inwardly-rectifying channel, subfamily j, member 11  GATM - glycine amidinotransferase (l-arginine:glycine amidinotransferase)  AMT - aminomethyltransferase  UGP2 - udp-glucose pyrophosphorylase 2  UGDH - udp-glucose 6-dehydrogenase  PEMT - phosphatidylethanolamine n-methyltransferase  AMPD3 - adenosine monophosphate deaminase 3  LYRM4 - lyr motif containing 4  BCAN - brevican  HMGCL - 3-hydroxymethyl-3-methylglutaryl-coa lyase  GAPDH - glyceraldehyde-3-phosphate dehydrogenase  EPHX1 - epoxide hydrolase 1, microsomal (xenobiotic)  EPHX2 - epoxide hydrolase 2, cytoplasmic  PARP10 - poly (adp-ribose) polymerase family, member 10  IARS2 - isoleucyl-trna synthetase 2, mitochondrial  MPP1 - membrane protein, palmitoylated 1, 55kda  SREBF1 - sterol regulatory element binding transcription factor 1  AIFM2 - apoptosis-inducing factor, mitochondrion-associated, 2  SLC27A4 - solute carrier family 27 (fatty acid transporter), member 4  CBS - cystathionine-beta-synthase  AFMID - arylformamidase  NCAN - neurocan  DCXR - dicarbonyl/l-xylulose reductase  FMO5 - flavin containing monooxygenase 5  GSTM1 - glutathione s-transferase mu 1  TCN2 - transcobalamin ii  KCNAB2 - potassium voltage-gated channel, shaker-related subfamily, beta member 2  GSTM4 - glutathione s-transferase mu 4  SDHD - succinate dehydrogenase complex, subunit d, integral membrane protein  SDHC - succinate dehydrogenase complex, subunit c, integral membrane protein, 15kda  APOA1 - apolipoprotein a-i  INPP5K - inositol polyphosphate-5-phosphatase k  SDHA - succinate dehydrogenase complex, subunit a, flavoprotein (fp)  SDSL - serine dehydratase-like  COQ3 - coenzyme q3 methyltransferase  DNPH1 - 2'-deoxynucleoside 5'-phosphate n-hydrolase 1  AOX1 - aldehyde oxidase 1  PC - pyruvate carboxylase  NAT8L - n-acetyltransferase 8-like (gcn5-related, putative)  ECI1 - enoyl-coa delta isomerase 1  ITIH5 - inter-alpha-trypsin inhibitor heavy chain family, member 5  PCCA - propionyl coa carboxylase, alpha polypeptide  OXCT1 - 3-oxoacid coa transferase 1  PCCB - propionyl coa carboxylase, beta polypeptide  CECR1 - cat eye syndrome chromosome region, candidate 1  CD38 - cd38 molecule  NADSYN1 - nad synthetase 1  P4HB - prolyl 4-hydroxylase, beta polypeptide  PMVK - phosphomevalonate kinase  AIMP1 - aminoacyl trna synthetase complex-interacting multifunctional protein 1  MRS2 - mrs2 magnesium transporter  ENTPD5 - ectonucleoside triphosphate diphosphohydrolase 5  OGDH - oxoglutarate (alpha-ketoglutarate) dehydrogenase (lipoamide)  HOGA1 - 4-hydroxy-2-oxoglutarate aldolase 1  AMDHD2 - amidohydrolase domain containing 2  COASY - coa synthase  APOE - apolipoprotein e  APRT - adenine phosphoribosyltransferase  DDO - d-aspartate oxidase  GUSB - glucuronidase, beta  PECR - peroxisomal trans-2-enoyl-coa reductase  ATP5L - atp synthase, h+ transporting, mitochondrial fo complex, subunit g  ADIPOR2 - adiponectin receptor 2  PDE8A - phosphodiesterase 8a  PDE7A - phosphodiesterase 7a  CYP11A1 - cytochrome p450, family 11, subfamily a, polypeptide 1  GNMT - glycine n-methyltransferase  PDHB - pyruvate dehydrogenase (lipoamide) beta  MDH2 - malate dehydrogenase 2, nad (mitochondrial)  CARS2 - cysteinyl-trna synthetase 2, mitochondrial (putative)  SC5D - sterol-c5-desaturase  PDHA1 - pyruvate dehydrogenase (lipoamide) alpha 1  CDO1 - cysteine dioxygenase type 1  PPM1K - protein phosphatase, mg2+/mn2+ dependent, 1k  PDK1 - pyruvate dehydrogenase kinase, isozyme 1  MMADHC - methylmalonic aciduria (cobalamin deficiency) cbld type, with homocystinuria  SULT1C4 - sulfotransferase family, cytosolic, 1c, member 4  PTGS1 - prostaglandin-endoperoxide synthase 1 (prostaglandin g/h synthase and cyclooxygenase)  COQ2 - coenzyme q2 4-hydroxybenzoate polyprenyltransferase  HADHA - hydroxyacyl-coa dehydrogenase/3-ketoacyl-coa thiolase/enoyl-coa hydratase (trifunctional protein), alpha subunit  FDX1 - ferredoxin 1  INSIG1 - insulin induced gene 1  DAK - dihydroxyacetone kinase 2 homolog (s. cerevisiae)  PCK2 - phosphoenolpyruvate carboxykinase 2 (mitochondrial)  ASL - argininosuccinate lyase  NUDT3 - nudix (nucleoside diphosphate linked moiety x)-type motif 3  NPPC - natriuretic peptide c  DAO - d-amino-acid oxidase  INPP5D - inositol polyphosphate-5-phosphatase, 145kda  ASMTL - acetylserotonin o-methyltransferase-like  PDE1A - phosphodiesterase 1a, calmodulin-dependent  HEXA - hexosaminidase a (alpha polypeptide)  FADS6 - fatty acid desaturase 6  HEXB - hexosaminidase b (beta polypeptide)  PDE4D - phosphodiesterase 4d, camp-specific  PDE4A - phosphodiesterase 4a, camp-specific  CYP27A1 - cytochrome p450, family 27, subfamily a, polypeptide 1  PDE4B - phosphodiesterase 4b, camp-specific  XDH - xanthine dehydrogenase  PGK1 - phosphoglycerate kinase 1  PGM5 - phosphoglucomutase 5  CROT - carnitine o-octanoyltransferase  FUOM - fucose mutarotase  NME3 - nme/nm23 nucleoside diphosphate kinase 3  RHOQ - ras homolog family member q  ATP5S - atp synthase, h+ transporting, mitochondrial fo complex, subunit s (factor b)  UROC1 - urocanate hydratase 1  AGPAT5 - 1-acylglycerol-3-phosphate o-acyltransferase 5  NMNAT3 - nicotinamide nucleotide adenylyltransferase 3  SLC16A1 - solute carrier family 16 (monocarboxylate transporter), member 1  AGMO - alkylglycerol monooxygenase  ABCD4 - atp-binding cassette, sub-family d (ald), member 4  SCLY - selenocysteine lyase  PTGR2 - prostaglandin reductase 2  ATF3 - activating transcription factor 3  B3GNT4 - udp-glcnac:betagal beta-1,3-n-acetylglucosaminyltransferase 4  RGN - regucalcin  MAN2B1 - mannosidase, alpha, class 2b, member 1  DPYD - dihydropyrimidine dehydrogenase  TM7SF2 - transmembrane 7 superfamily member 2  DUT - deoxyuridine triphosphatase  GLS2 - glutaminase 2 (liver, mitochondrial)  ATP5J - atp synthase, h+ transporting, mitochondrial fo complex, subunit f6  PEPD - peptidase d  NAAA - n-acylethanolamine acid amidase  QPRT - quinolinate phosphoribosyltransferase  SLC1A3 - solute carrier family 1 (glial high affinity glutamate transporter), member 3  PET112 - pet112 homolog (yeast)  PFKFB3 - 6-phosphofructo-2-kinase/fructose-2,6-biphosphatase 3  ATP5C1 - atp synthase, h+ transporting, mitochondrial f1 complex, gamma polypeptide 1  SLC39A8 - solute carrier family 39 (zinc transporter), member 8  PFKFB4 - 6-phosphofructo-2-kinase/fructose-2,6-biphosphatase 4  ATP5F1 - atp synthase, h+ transporting, mitochondrial fo complex, subunit b1  ATP5G3 - atp synthase, h+ transporting, mitochondrial fo complex, subunit c3 (subunit 9)  GALM - galactose mutarotase (aldose 1-epimerase)  NT5C - 5', 3'-nucleotidase, cytosolic  ATP5A1 - atp synthase, h+ transporting, mitochondrial f1 complex, alpha subunit 1, cardiac muscle  IDH3B - isocitrate dehydrogenase 3 (nad+) beta  DTYMK - deoxythymidylate kinase (thymidylate kinase)  ATP5B - atp synthase, h+ transporting, mitochondrial f1 complex, beta polypeptide  IDH2 - isocitrate dehydrogenase 2 (nadp+), mitochondrial  DLAT - dihydrolipoamide s-acetyltransferase  CYP4V2 - cytochrome p450, family 4, subfamily v, polypeptide 2  DLD - dihydrolipoamide dehydrogenase  EGLN3 - egl-9 family hypoxia-inducible factor 3  ASNSD1 - asparagine synthetase domain containing 1  CSPG5 - chondroitin sulfate proteoglycan 5 (neuroglycan c)  DLST - dihydrolipoamide s-succinyltransferase (e2 component of 2-oxo-glutarate complex)  LRP5 - low density lipoprotein receptor-related protein 5  EBP - emopamil binding protein (sterol isomerase)  SLC39A14 - solute carrier family 39 (zinc transporter), member 14  SHMT2 - serine hydroxymethyltransferase 2 (mitochondrial)  FARS2 - phenylalanyl-trna synthetase 2, mitochondrial  TGDS - tdp-glucose 4,6-dehydratase  NFE2L1 - nuclear factor (erythroid-derived 2)-like 1  ST3GAL1 - st3 beta-galactoside alpha-2,3-sialyltransferase 1  LHPP - phospholysine phosphohistidine inorganic pyrophosphate phosphatase  FLAD1 - flavin adenine dinucleotide synthetase 1  BDH1 - 3-hydroxybutyrate dehydrogenase, type 1  ACAD8 - acyl-coa dehydrogenase family, member 8  MGLL - monoglyceride lipase  HSD3B7 - hydroxy-delta-5-steroid dehydrogenase, 3 beta- and steroid delta-isomerase 7  NDUFAB1 - nadh dehydrogenase (ubiquinone) 1, alpha/beta subcomplex, 1, 8kda  CNBP - cchc-type zinc finger, nucleic acid binding protein  NDUFA9 - nadh dehydrogenase (ubiquinone) 1 alpha subcomplex, 9, 39kda  GPAM - glycerol-3-phosphate acyltransferase, mitochondrial  NNT - nicotinamide nucleotide transhydrogenase  SARDH - sarcosine dehydrogenase  RBP4 - retinol binding protein 4, plasma  FOLR2 - folate receptor 2 (fetal)  RDH5 - retinol dehydrogenase 5 (11-cis/9-cis)  NDUFS1 - nadh dehydrogenase (ubiquinone) fe-s protein 1, 75kda (nadh-coenzyme q reductase)  RBP1 - retinol binding protein 1, cellular  FPGS - folylpolyglutamate synthase |
| GO:0006612 | protein targeting to membrane | 8.1E-11 | 6.63E-8 | 2.34 (10334,139,1776,56) | [+] Show genes  RPL24 - ribosomal protein l24  RPL23A - ribosomal protein l23a  RPL21 - ribosomal protein l21  RPL19 - ribosomal protein l19  RPL18A - ribosomal protein l18a  RPL18 - ribosomal protein l18  RPL17 - ribosomal protein l17  RPL13 - ribosomal protein l13  RPL12 - ribosomal protein l12  RPL10 - ribosomal protein l10  RPL7A - ribosomal protein l7a  RPL5 - ribosomal protein l5  PEX19 - peroxisomal biogenesis factor 19  RPL4 - ribosomal protein l4  RPL35 - ribosomal protein l35  RPS5 - ribosomal protein s5  RPS2 - ribosomal protein s2  RPLP2 - ribosomal protein, large, p2  RPL36A - ribosomal protein l36a  RPL37A - ribosomal protein l37a  RPL38 - ribosomal protein l38  NCF1 - neutrophil cytosolic factor 1  RPL34 - ribosomal protein l34  RPL37 - ribosomal protein l37  RPL29 - ribosomal protein l29  RPL31 - ribosomal protein l31  RPL36 - ribosomal protein l36  RPL27 - ribosomal protein l27  ZDHHC18 - zinc finger, dhhc-type containing 18  RPL30 - ribosomal protein l30  RPL27A - ribosomal protein l27a  RPL28 - ribosomal protein l28  RPS24 - ribosomal protein s24  RPS21 - ribosomal protein s21  RPS27 - ribosomal protein s27  RPS26 - ribosomal protein s26  RPS25 - ribosomal protein s25  RPS17 - ribosomal protein s17  RPS16 - ribosomal protein s16  RPS15A - ribosomal protein s15a  RPS20 - ribosomal protein s20  RPS19 - ribosomal protein s19  RPS18 - ribosomal protein s18  RPS11 - ribosomal protein s11  RPS8 - ribosomal protein s8  RPS9 - ribosomal protein s9  RPS14 - ribosomal protein s14  RPS13 - ribosomal protein s13  RPS7 - ribosomal protein s7  RPSA - ribosomal protein sa  RPL10A - ribosomal protein l10a  PARD3 - par-3 partitioning defective 3 homolog (c. elegans)  RPL23 - ribosomal protein l23  ADORA1 - adenosine a1 receptor  RPS28 - ribosomal protein s28  RPS29 - ribosomal protein s29 |
| GO:0022900 | electron transport chain | 1.13E-10 | 8.72E-8 | 2.33 (10334,140,1776,56) | [+] Show genes  XDH - xanthine dehydrogenase  NQO2 - nad(p)h dehydrogenase, quinone 2  UQCRC2 - ubiquinol-cytochrome c reductase core protein ii  UQCRB - ubiquinol-cytochrome c reductase binding protein  COX6B1 - cytochrome c oxidase subunit vib polypeptide 1 (ubiquitous)  UQCRFS1 - ubiquinol-cytochrome c reductase, rieske iron-sulfur polypeptide 1  SDHD - succinate dehydrogenase complex, subunit d, integral membrane protein  SDHC - succinate dehydrogenase complex, subunit c, integral membrane protein, 15kda  COX4I1 - cytochrome c oxidase subunit iv isoform 1  NDUFA1 - nadh dehydrogenase (ubiquinone) 1 alpha subcomplex, 1, 7.5kda  SDHA - succinate dehydrogenase complex, subunit a, flavoprotein (fp)  NDUFA5 - nadh dehydrogenase (ubiquinone) 1 alpha subcomplex, 5  COX10 - cytochrome c oxidase assembly homolog 10 (yeast)  AOX1 - aldehyde oxidase 1  COX7C - cytochrome c oxidase subunit viic  COX1 - cytochrome c oxidase subunit i  COX7A1 - cytochrome c oxidase subunit viia polypeptide 1 (muscle)  COX6C - cytochrome c oxidase subunit vic  CYTB - cytochrome b  CYCS - cytochrome c, somatic  PINK1 - pten induced putative kinase 1  AKR1A1 - aldo-keto reductase family 1, member a1 (aldehyde reductase)  BID - bh3 interacting domain death agonist  NCF1 - neutrophil cytosolic factor 1  TSTA3 - tissue specific transplantation antigen p35b  NDUFA11 - nadh dehydrogenase (ubiquinone) 1 alpha subcomplex, 11, 14.7kda  CYC1 - cytochrome c-1  IDH3B - isocitrate dehydrogenase 3 (nad+) beta  CYBA - cytochrome b-245, alpha polypeptide  NDUFB11 - nadh dehydrogenase (ubiquinone) 1 beta subcomplex, 11, 17.3kda  DLD - dihydrolipoamide dehydrogenase  NDUFA12 - nadh dehydrogenase (ubiquinone) 1 alpha subcomplex, 12  COX5A - cytochrome c oxidase subunit va  GLDC - glycine dehydrogenase (decarboxylating)  GLRX5 - glutaredoxin 5  ETFA - electron-transfer-flavoprotein, alpha polypeptide  FDX1 - ferredoxin 1  NDUFC1 - nadh dehydrogenase (ubiquinone) 1, subcomplex unknown, 1, 6kda  NDUFB8 - nadh dehydrogenase (ubiquinone) 1 beta subcomplex, 8, 19kda  NDUFB9 - nadh dehydrogenase (ubiquinone) 1 beta subcomplex, 9, 22kda  NDUFB6 - nadh dehydrogenase (ubiquinone) 1 beta subcomplex, 6, 17kda  NDUFB4 - nadh dehydrogenase (ubiquinone) 1 beta subcomplex, 4, 15kda  NDUFB5 - nadh dehydrogenase (ubiquinone) 1 beta subcomplex, 5, 16kda  NDUFB3 - nadh dehydrogenase (ubiquinone) 1 beta subcomplex, 3, 12kda  NDUFAB1 - nadh dehydrogenase (ubiquinone) 1, alpha/beta subcomplex, 1, 8kda  NDUFB1 - nadh dehydrogenase (ubiquinone) 1 beta subcomplex, 1, 7kda  NDUFA9 - nadh dehydrogenase (ubiquinone) 1 alpha subcomplex, 9, 39kda  NDUFA10 - nadh dehydrogenase (ubiquinone) 1 alpha subcomplex, 10, 42kda  POR - p450 (cytochrome) oxidoreductase  TXNRD3 - thioredoxin reductase 3  NDUFV2 - nadh dehydrogenase (ubiquinone) flavoprotein 2, 24kda  NDUFS4 - nadh dehydrogenase (ubiquinone) fe-s protein 4, 18kda (nadh-coenzyme q reductase)  AIFM2 - apoptosis-inducing factor, mitochondrion-associated, 2  NDUFS3 - nadh dehydrogenase (ubiquinone) fe-s protein 3, 30kda (nadh-coenzyme q reductase)  NDUFS2 - nadh dehydrogenase (ubiquinone) fe-s protein 2, 49kda (nadh-coenzyme q reductase)  NDUFS1 - nadh dehydrogenase (ubiquinone) fe-s protein 1, 75kda (nadh-coenzyme q reductase) |
| GO:0022904 | respiratory electron transport chain | 1.19E-10 | 8.75E-8 | 2.69 (10334,91,1776,42) | [+] Show genes  UQCRC2 - ubiquinol-cytochrome c reductase core protein ii  NDUFB11 - nadh dehydrogenase (ubiquinone) 1 beta subcomplex, 11, 17.3kda  DLD - dihydrolipoamide dehydrogenase  UQCRB - ubiquinol-cytochrome c reductase binding protein  COX6B1 - cytochrome c oxidase subunit vib polypeptide 1 (ubiquitous)  UQCRFS1 - ubiquinol-cytochrome c reductase, rieske iron-sulfur polypeptide 1  SDHD - succinate dehydrogenase complex, subunit d, integral membrane protein  COX4I1 - cytochrome c oxidase subunit iv isoform 1  SDHC - succinate dehydrogenase complex, subunit c, integral membrane protein, 15kda  NDUFA1 - nadh dehydrogenase (ubiquinone) 1 alpha subcomplex, 1, 7.5kda  SDHA - succinate dehydrogenase complex, subunit a, flavoprotein (fp)  NDUFA12 - nadh dehydrogenase (ubiquinone) 1 alpha subcomplex, 12  NDUFA5 - nadh dehydrogenase (ubiquinone) 1 alpha subcomplex, 5  COX5A - cytochrome c oxidase subunit va  COX10 - cytochrome c oxidase assembly homolog 10 (yeast)  COX7C - cytochrome c oxidase subunit viic  COX1 - cytochrome c oxidase subunit i  COX6C - cytochrome c oxidase subunit vic  CYTB - cytochrome b  ETFA - electron-transfer-flavoprotein, alpha polypeptide  NDUFC1 - nadh dehydrogenase (ubiquinone) 1, subcomplex unknown, 1, 6kda  CYCS - cytochrome c, somatic  NDUFB8 - nadh dehydrogenase (ubiquinone) 1 beta subcomplex, 8, 19kda  NDUFB9 - nadh dehydrogenase (ubiquinone) 1 beta subcomplex, 9, 22kda  PINK1 - pten induced putative kinase 1  NDUFB6 - nadh dehydrogenase (ubiquinone) 1 beta subcomplex, 6, 17kda  NDUFB4 - nadh dehydrogenase (ubiquinone) 1 beta subcomplex, 4, 15kda  NDUFB5 - nadh dehydrogenase (ubiquinone) 1 beta subcomplex, 5, 16kda  NDUFB3 - nadh dehydrogenase (ubiquinone) 1 beta subcomplex, 3, 12kda  NDUFAB1 - nadh dehydrogenase (ubiquinone) 1, alpha/beta subcomplex, 1, 8kda  NDUFB1 - nadh dehydrogenase (ubiquinone) 1 beta subcomplex, 1, 7kda  NDUFA9 - nadh dehydrogenase (ubiquinone) 1 alpha subcomplex, 9, 39kda  NDUFA10 - nadh dehydrogenase (ubiquinone) 1 alpha subcomplex, 10, 42kda  BID - bh3 interacting domain death agonist  NDUFV2 - nadh dehydrogenase (ubiquinone) flavoprotein 2, 24kda  NDUFA11 - nadh dehydrogenase (ubiquinone) 1 alpha subcomplex, 11, 14.7kda  NDUFS4 - nadh dehydrogenase (ubiquinone) fe-s protein 4, 18kda (nadh-coenzyme q reductase)  AIFM2 - apoptosis-inducing factor, mitochondrion-associated, 2  NDUFS3 - nadh dehydrogenase (ubiquinone) fe-s protein 3, 30kda (nadh-coenzyme q reductase)  CYC1 - cytochrome c-1  NDUFS2 - nadh dehydrogenase (ubiquinone) fe-s protein 2, 49kda (nadh-coenzyme q reductase)  NDUFS1 - nadh dehydrogenase (ubiquinone) fe-s protein 1, 75kda (nadh-coenzyme q reductase) |
| GO:0055114 | oxidation-reduction process | 2.95E-10 | 2.05E-7 | 1.55 (10334,645,1776,172) | [+] Show genes  FMO5 - flavin containing monooxygenase 5  COX6B1 - cytochrome c oxidase subunit vib polypeptide 1 (ubiquitous)  KCNAB2 - potassium voltage-gated channel, shaker-related subfamily, beta member 2  HSD17B4 - hydroxysteroid (17-beta) dehydrogenase 4  SDHD - succinate dehydrogenase complex, subunit d, integral membrane protein  APOA1 - apolipoprotein a-i  SDHC - succinate dehydrogenase complex, subunit c, integral membrane protein, 15kda  COX4I1 - cytochrome c oxidase subunit iv isoform 1  NDUFA1 - nadh dehydrogenase (ubiquinone) 1 alpha subcomplex, 1, 7.5kda  SDHA - succinate dehydrogenase complex, subunit a, flavoprotein (fp)  NDUFA5 - nadh dehydrogenase (ubiquinone) 1 alpha subcomplex, 5  PDIA5 - protein disulfide isomerase family a, member 5  BPGM - 2,3-bisphosphoglycerate mutase  DHRS11 - dehydrogenase/reductase (sdr family) member 11  CP - ceruloplasmin (ferroxidase)  LDHB - lactate dehydrogenase b  AASS - aminoadipate-semialdehyde synthase  FA2H - fatty acid 2-hydroxylase  COX10 - cytochrome c oxidase assembly homolog 10 (yeast)  AOX1 - aldehyde oxidase 1  TXNRD2 - thioredoxin reductase 2  ECI1 - enoyl-coa delta isomerase 1  OXA1L - oxidase (cytochrome c) assembly 1-like  COX7C - cytochrome c oxidase subunit viic  COX7A1 - cytochrome c oxidase subunit viia polypeptide 1 (muscle)  COX6C - cytochrome c oxidase subunit vic  RETSAT - retinol saturase (all-trans-retinol 13,14-reductase)  CYCS - cytochrome c, somatic  ACAT1 - acetyl-coa acetyltransferase 1  NHLRC1 - nhl repeat containing 1  CYB561A3 - cytochrome b561 family, member a3  P4HB - prolyl 4-hydroxylase, beta polypeptide  CYB5R1 - cytochrome b5 reductase 1  PTGR1 - prostaglandin reductase 1  PIR - pirin (iron-binding nuclear protein)  AKR1A1 - aldo-keto reductase family 1, member a1 (aldehyde reductase)  BID - bh3 interacting domain death agonist  SORD - sorbitol dehydrogenase  GYS1 - glycogen synthase 1 (muscle)  SOD3 - superoxide dismutase 3, extracellular  SOD1 - superoxide dismutase 1, soluble  TSTA3 - tissue specific transplantation antigen p35b  DDO - d-aspartate oxidase  CRAT - carnitine o-acetyltransferase  PRDX3 - peroxiredoxin 3  GYG1 - glycogenin 1  PECR - peroxisomal trans-2-enoyl-coa reductase  NR1D1 - nuclear receptor subfamily 1, group d, member 1  FADS3 - fatty acid desaturase 3  TMLHE - trimethyllysine hydroxylase, epsilon  ADIPOR2 - adiponectin receptor 2  ACSS1 - acyl-coa synthetase short-chain family member 1  GNMT - glycine n-methyltransferase  CYP11A1 - cytochrome p450, family 11, subfamily a, polypeptide 1  TPI1 - triosephosphate isomerase 1  MDH2 - malate dehydrogenase 2, nad (mitochondrial)  SC5D - sterol-c5-desaturase  NDUFA12 - nadh dehydrogenase (ubiquinone) 1 alpha subcomplex, 12  CDO1 - cysteine dioxygenase type 1  TECR - trans-2,3-enoyl-coa reductase  GLDC - glycine dehydrogenase (decarboxylating)  PTGS1 - prostaglandin-endoperoxide synthase 1 (prostaglandin g/h synthase and cyclooxygenase)  HADHA - hydroxyacyl-coa dehydrogenase/3-ketoacyl-coa thiolase/enoyl-coa hydratase (trifunctional protein), alpha subunit  FDX1 - ferredoxin 1  HCCS - holocytochrome c synthase  GYG2 - glycogenin 2  QSOX1 - quiescin q6 sulfhydryl oxidase 1  DAO - d-amino-acid oxidase  POR - p450 (cytochrome) oxidoreductase  TXNRD3 - thioredoxin reductase 3  FADS6 - fatty acid desaturase 6  LDHD - lactate dehydrogenase d  COQ10A - coenzyme q10 homolog a (s. cerevisiae)  GMPR - guanosine monophosphate reductase  VKORC1 - vitamin k epoxide reductase complex, subunit 1  JHDM1D - jumonji c domain containing histone demethylase 1 homolog d (s. cerevisiae)  CYP46A1 - cytochrome p450, family 46, subfamily a, polypeptide 1  MGST1 - microsomal glutathione s-transferase 1  NXN - nucleoredoxin  L2HGDH - l-2-hydroxyglutarate dehydrogenase  CYP27A1 - cytochrome p450, family 27, subfamily a, polypeptide 1  NQO2 - nad(p)h dehydrogenase, quinone 2  UQCRC2 - ubiquinol-cytochrome c reductase core protein ii  HSD17B11 - hydroxysteroid (17-beta) dehydrogenase 11  XDH - xanthine dehydrogenase  UQCRB - ubiquinol-cytochrome c reductase binding protein  PGK1 - phosphoglycerate kinase 1  CROT - carnitine o-octanoyltransferase  ACOT8 - acyl-coa thioesterase 8  AIFM1 - apoptosis-inducing factor, mitochondrion-associated, 1  UQCRFS1 - ubiquinol-cytochrome c reductase, rieske iron-sulfur polypeptide 1  IFI30 - interferon, gamma-inducible protein 30  ADSL - adenylosuccinate lyase  TSTD1 - thiosulfate sulfurtransferase (rhodanese)-like domain containing 1  AGL - amylo-alpha-1, 6-glucosidase, 4-alpha-glucanotransferase  PHKA1 - phosphorylase kinase, alpha 1 (muscle)  PHKB - phosphorylase kinase, beta  AGMO - alkylglycerol monooxygenase  FTH1 - ferritin, heavy polypeptide 1  ABCD4 - atp-binding cassette, sub-family d (ald), member 4  COX1 - cytochrome c oxidase subunit i  PTGR2 - prostaglandin reductase 2  MTFR1L - mitochondrial fission regulator 1-like  DPYD - dihydropyrimidine dehydrogenase  CYTB - cytochrome b  DHRS1 - dehydrogenase/reductase (sdr family) member 1  TM7SF2 - transmembrane 7 superfamily member 2  PINK1 - pten induced putative kinase 1  AKT2 - v-akt murine thymoma viral oncogene homolog 2  LEPRE1 - leucine proline-enriched proteoglycan (leprecan) 1  PYGL - phosphorylase, glycogen, liver  PYGM - phosphorylase, glycogen, muscle  DUS1L - dihydrouridine synthase 1-like (s. cerevisiae)  NCF1 - neutrophil cytosolic factor 1  JMJD4 - jumonji domain containing 4  ALDH9A1 - aldehyde dehydrogenase 9 family, member a1  NDUFA11 - nadh dehydrogenase (ubiquinone) 1 alpha subcomplex, 11, 14.7kda  CHDH - choline dehydrogenase  CYC1 - cytochrome c-1  IDH3B - isocitrate dehydrogenase 3 (nad+) beta  CYBA - cytochrome b-245, alpha polypeptide  ABCD1 - atp-binding cassette, sub-family d (ald), member 1  NDUFB11 - nadh dehydrogenase (ubiquinone) 1 beta subcomplex, 11, 17.3kda  CYP4V2 - cytochrome p450, family 4, subfamily v, polypeptide 2  DLD - dihydrolipoamide dehydrogenase  EGLN3 - egl-9 family hypoxia-inducible factor 3  DHRS7 - dehydrogenase/reductase (sdr family) member 7  MICAL2 - microtubule associated monooxygenase, calponin and lim domain containing 2  ALDOA - aldolase a, fructose-bisphosphate  HSDL2 - hydroxysteroid dehydrogenase like 2  COX5A - cytochrome c oxidase subunit va  HIF1AN - hypoxia inducible factor 1, alpha subunit inhibitor  ZADH2 - zinc binding alcohol dehydrogenase domain containing 2  ALDH16A1 - aldehyde dehydrogenase 16 family, member a1  GPX7 - glutathione peroxidase 7  ACAD9 - acyl-coa dehydrogenase family, member 9  GLRX5 - glutaredoxin 5  STEAP3 - steap family member 3, metalloreductase  TXN - thioredoxin  ETFA - electron-transfer-flavoprotein, alpha polypeptide  MAPK14 - mitogen-activated protein kinase 14  NDUFC1 - nadh dehydrogenase (ubiquinone) 1, subcomplex unknown, 1, 6kda  ACAD8 - acyl-coa dehydrogenase family, member 8  BDH1 - 3-hydroxybutyrate dehydrogenase, type 1  NDUFB8 - nadh dehydrogenase (ubiquinone) 1 beta subcomplex, 8, 19kda  NDUFB9 - nadh dehydrogenase (ubiquinone) 1 beta subcomplex, 9, 22kda  NDUFB6 - nadh dehydrogenase (ubiquinone) 1 beta subcomplex, 6, 17kda  HSD3B7 - hydroxy-delta-5-steroid dehydrogenase, 3 beta- and steroid delta-isomerase 7  NDUFB4 - nadh dehydrogenase (ubiquinone) 1 beta subcomplex, 4, 15kda  NDUFB5 - nadh dehydrogenase (ubiquinone) 1 beta subcomplex, 5, 16kda  ALKBH3 - alkb, alkylation repair homolog 3 (e. coli)  NDUFB3 - nadh dehydrogenase (ubiquinone) 1 beta subcomplex, 3, 12kda  NDUFAB1 - nadh dehydrogenase (ubiquinone) 1, alpha/beta subcomplex, 1, 8kda  UGP2 - udp-glucose pyrophosphorylase 2  NDUFB1 - nadh dehydrogenase (ubiquinone) 1 beta subcomplex, 1, 7kda  UGDH - udp-glucose 6-dehydrogenase  NDUFA9 - nadh dehydrogenase (ubiquinone) 1 alpha subcomplex, 9, 39kda  NDUFA10 - nadh dehydrogenase (ubiquinone) 1 alpha subcomplex, 10, 42kda  NNT - nicotinamide nucleotide transhydrogenase  SARDH - sarcosine dehydrogenase  GAPDH - glyceraldehyde-3-phosphate dehydrogenase  RDH5 - retinol dehydrogenase 5 (11-cis/9-cis)  NDUFV2 - nadh dehydrogenase (ubiquinone) flavoprotein 2, 24kda  NDUFS4 - nadh dehydrogenase (ubiquinone) fe-s protein 4, 18kda (nadh-coenzyme q reductase)  CBS - cystathionine-beta-synthase  CCS - copper chaperone for superoxide dismutase  AIFM2 - apoptosis-inducing factor, mitochondrion-associated, 2  NDUFS3 - nadh dehydrogenase (ubiquinone) fe-s protein 3, 30kda (nadh-coenzyme q reductase)  NDUFS2 - nadh dehydrogenase (ubiquinone) fe-s protein 2, 49kda (nadh-coenzyme q reductase)  AIFM3 - apoptosis-inducing factor, mitochondrion-associated, 3  NDUFS1 - nadh dehydrogenase (ubiquinone) fe-s protein 1, 75kda (nadh-coenzyme q reductase)  DCXR - dicarbonyl/l-xylulose reductase |
| GO:0017144 | drug metabolic process | 1.27E-8 | 8.39E-6 | 1.63 (10334,413,1776,116) | [+] Show genes  LHB - luteinizing hormone beta polypeptide  FMO5 - flavin containing monooxygenase 5  TCN2 - transcobalamin ii  HSD17B4 - hydroxysteroid (17-beta) dehydrogenase 4  SDHD - succinate dehydrogenase complex, subunit d, integral membrane protein  SUCLA2 - succinate-coa ligase, adp-forming, beta subunit  SDHC - succinate dehydrogenase complex, subunit c, integral membrane protein, 15kda  SUCLG2 - succinate-coa ligase, gdp-forming, beta subunit  SDHA - succinate dehydrogenase complex, subunit a, flavoprotein (fp)  SDSL - serine dehydratase-like  BPGM - 2,3-bisphosphoglycerate mutase  BTD - biotinidase  AASS - aminoadipate-semialdehyde synthase  AOX1 - aldehyde oxidase 1  GCSH - glycine cleavage system protein h (aminomethyl carrier)  PC - pyruvate carboxylase  PCCA - propionyl coa carboxylase, alpha polypeptide  GCHFR - gtp cyclohydrolase i feedback regulator  PCCB - propionyl coa carboxylase, beta polypeptide  GCH1 - gtp cyclohydrolase 1  OXCT1 - 3-oxoacid coa transferase 1  CECR1 - cat eye syndrome chromosome region, candidate 1  RENBP - renin binding protein  COX7A1 - cytochrome c oxidase subunit viia polypeptide 1 (muscle)  ACAT1 - acetyl-coa acetyltransferase 1  BGN - biglycan  ACO1 - aconitase 1, soluble  ACO2 - aconitase 2, mitochondrial  AKR1A1 - aldo-keto reductase family 1, member a1 (aldehyde reductase)  ENTPD5 - ectonucleoside triphosphate diphosphohydrolase 5  ACP5 - acid phosphatase 5, tartrate resistant  OGDH - oxoglutarate (alpha-ketoglutarate) dehydrogenase (lipoamide)  AMDHD2 - amidohydrolase domain containing 2  SOD1 - superoxide dismutase 1, soluble  APRT - adenine phosphoribosyltransferase  LRTOMT - leucine rich transmembrane and o-methyltransferase domain containing  GGT5 - gamma-glutamyltransferase 5  PRDX3 - peroxiredoxin 3  ATP5L - atp synthase, h+ transporting, mitochondrial fo complex, subunit g  PECR - peroxisomal trans-2-enoyl-coa reductase  ACSS1 - acyl-coa synthetase short-chain family member 1  GNMT - glycine n-methyltransferase  CYP11A1 - cytochrome p450, family 11, subfamily a, polypeptide 1  TPI1 - triosephosphate isomerase 1  PDHB - pyruvate dehydrogenase (lipoamide) beta  MDH2 - malate dehydrogenase 2, nad (mitochondrial)  PDHA1 - pyruvate dehydrogenase (lipoamide) alpha 1  GLDC - glycine dehydrogenase (decarboxylating)  MMADHC - methylmalonic aciduria (cobalamin deficiency) cbld type, with homocystinuria  SULT1C4 - sulfotransferase family, cytosolic, 1c, member 4  SMS - spermine synthase  PCK2 - phosphoenolpyruvate carboxykinase 2 (mitochondrial)  RORA - rar-related orphan receptor a  ADK - adenosine kinase  DAO - d-amino-acid oxidase  POR - p450 (cytochrome) oxidoreductase  CLPX - clpx caseinolytic peptidase x homolog (e. coli)  VKORC1 - vitamin k epoxide reductase complex, subunit 1  CYP46A1 - cytochrome p450, family 46, subfamily a, polypeptide 1  CYP27A1 - cytochrome p450, family 27, subfamily a, polypeptide 1  UQCRC2 - ubiquinol-cytochrome c reductase core protein ii  MTR - 5-methyltetrahydrofolate-homocysteine methyltransferase  UQCRB - ubiquinol-cytochrome c reductase binding protein  PGK1 - phosphoglycerate kinase 1  ACOT8 - acyl-coa thioesterase 8  ATP5S - atp synthase, h+ transporting, mitochondrial fo complex, subunit s (factor b)  UROC1 - urocanate hydratase 1  ADSL - adenylosuccinate lyase  CTH - cystathionase (cystathionine gamma-lyase)  ABCD4 - atp-binding cassette, sub-family d (ald), member 4  DPYD - dihydropyrimidine dehydrogenase  AK1 - adenylate kinase 1  CTSH - cathepsin h  ATP5J - atp synthase, h+ transporting, mitochondrial fo complex, subunit f6  QPRT - quinolinate phosphoribosyltransferase  ATP5C1 - atp synthase, h+ transporting, mitochondrial f1 complex, gamma polypeptide 1  SULT1E1 - sulfotransferase family 1e, estrogen-preferring, member 1  ATP5F1 - atp synthase, h+ transporting, mitochondrial fo complex, subunit b1  ATP5G3 - atp synthase, h+ transporting, mitochondrial fo complex, subunit c3 (subunit 9)  CYC1 - cytochrome c-1  IDH3B - isocitrate dehydrogenase 3 (nad+) beta  ATP5A1 - atp synthase, h+ transporting, mitochondrial f1 complex, alpha subunit 1, cardiac muscle  CYBA - cytochrome b-245, alpha polypeptide  ATP5B - atp synthase, h+ transporting, mitochondrial f1 complex, beta polypeptide  IDH2 - isocitrate dehydrogenase 2 (nadp+), mitochondrial  ABCD1 - atp-binding cassette, sub-family d (ald), member 1  DLAT - dihydrolipoamide s-acetyltransferase  DLD - dihydrolipoamide dehydrogenase  GALK1 - galactokinase 1  TEFM - transcription elongation factor, mitochondrial  CSPG5 - chondroitin sulfate proteoglycan 5 (neuroglycan c)  DLST - dihydrolipoamide s-succinyltransferase (e2 component of 2-oxo-glutarate complex)  STAT5B - signal transducer and activator of transcription 5b  SAT2 - spermidine/spermine n1-acetyltransferase family member 2  ALDOA - aldolase a, fructose-bisphosphate  SHMT2 - serine hydroxymethyltransferase 2 (mitochondrial)  ST3GAL1 - st3 beta-galactoside alpha-2,3-sialyltransferase 1  FLAD1 - flavin adenine dinucleotide synthetase 1  TYMS - thymidylate synthetase  CS - citrate synthase  GBAS - glioblastoma amplified sequence  BDH1 - 3-hydroxybutyrate dehydrogenase, type 1  NMNAT1 - nicotinamide nucleotide adenylyltransferase 1  GATM - glycine amidinotransferase (l-arginine:glycine amidinotransferase)  AMT - aminomethyltransferase  PEMT - phosphatidylethanolamine n-methyltransferase  AMPD3 - adenosine monophosphate deaminase 3  NNT - nicotinamide nucleotide transhydrogenase  BCAN - brevican  HMGCL - 3-hydroxymethyl-3-methylglutaryl-coa lyase  FOLR2 - folate receptor 2 (fetal)  GAPDH - glyceraldehyde-3-phosphate dehydrogenase  CBS - cystathionine-beta-synthase  NCAN - neurocan  NDUFS1 - nadh dehydrogenase (ubiquinone) fe-s protein 1, 75kda (nadh-coenzyme q reductase)  FPGS - folylpolyglutamate synthase |
| GO:0019752 | carboxylic acid metabolic process | 4.49E-8 | 2.84E-5 | 1.47 (10334,653,1776,165) | [+] Show genes  GSTM1 - glutathione s-transferase mu 1  GSTM4 - glutathione s-transferase mu 4  HSD17B4 - hydroxysteroid (17-beta) dehydrogenase 4  SDHD - succinate dehydrogenase complex, subunit d, integral membrane protein  SUCLA2 - succinate-coa ligase, adp-forming, beta subunit  SDHC - succinate dehydrogenase complex, subunit c, integral membrane protein, 15kda  SUCLG2 - succinate-coa ligase, gdp-forming, beta subunit  SDHA - succinate dehydrogenase complex, subunit a, flavoprotein (fp)  BPHL - biphenyl hydrolase-like (serine hydrolase)  SDSL - serine dehydratase-like  BPGM - 2,3-bisphosphoglycerate mutase  BTD - biotinidase  AASS - aminoadipate-semialdehyde synthase  LDHB - lactate dehydrogenase b  FA2H - fatty acid 2-hydroxylase  BSG - basigin (ok blood group)  GCSH - glycine cleavage system protein h (aminomethyl carrier)  PC - pyruvate carboxylase  NAT8L - n-acetyltransferase 8-like (gcn5-related, putative)  ECI1 - enoyl-coa delta isomerase 1  ITIH5 - inter-alpha-trypsin inhibitor heavy chain family, member 5  PCCA - propionyl coa carboxylase, alpha polypeptide  GCH1 - gtp cyclohydrolase 1  PCCB - propionyl coa carboxylase, beta polypeptide  RENBP - renin binding protein  BGN - biglycan  ACAT1 - acetyl-coa acetyltransferase 1  DDAH1 - dimethylarginine dimethylaminohydrolase 1  PLP1 - proteolipid protein 1  ACO1 - aconitase 1, soluble  P4HB - prolyl 4-hydroxylase, beta polypeptide  PTGR1 - prostaglandin reductase 1  BLMH - bleomycin hydrolase  ACO2 - aconitase 2, mitochondrial  AKR1A1 - aldo-keto reductase family 1, member a1 (aldehyde reductase)  MRS2 - mrs2 magnesium transporter  AIMP1 - aminoacyl trna synthetase complex-interacting multifunctional protein 1  LPL - lipoprotein lipase  GMPS - guanine monphosphate synthase  OGDH - oxoglutarate (alpha-ketoglutarate) dehydrogenase (lipoamide)  HOGA1 - 4-hydroxy-2-oxoglutarate aldolase 1  SORD - sorbitol dehydrogenase  AMDHD2 - amidohydrolase domain containing 2  CREM - camp responsive element modulator  DDO - d-aspartate oxidase  MRPS36 - mitochondrial ribosomal protein s36  CRAT - carnitine o-acetyltransferase  GUSB - glucuronidase, beta  GGT5 - gamma-glutamyltransferase 5  PECR - peroxisomal trans-2-enoyl-coa reductase  FADS3 - fatty acid desaturase 3  HYI - hydroxypyruvate isomerase (putative)  ADIPOR2 - adiponectin receptor 2  GLUL - glutamate-ammonia ligase  ACSS1 - acyl-coa synthetase short-chain family member 1  GNMT - glycine n-methyltransferase  TPI1 - triosephosphate isomerase 1  PDHB - pyruvate dehydrogenase (lipoamide) beta  MDH2 - malate dehydrogenase 2, nad (mitochondrial)  CARS2 - cysteinyl-trna synthetase 2, mitochondrial (putative)  PDHA1 - pyruvate dehydrogenase (lipoamide) alpha 1  ACY1 - aminoacylase 1  GLO1 - glyoxalase i  CDO1 - cysteine dioxygenase type 1  TECR - trans-2,3-enoyl-coa reductase  GLDC - glycine dehydrogenase (decarboxylating)  PPM1K - protein phosphatase, mg2+/mn2+ dependent, 1k  PTGS1 - prostaglandin-endoperoxide synthase 1 (prostaglandin g/h synthase and cyclooxygenase)  HADHA - hydroxyacyl-coa dehydrogenase/3-ketoacyl-coa thiolase/enoyl-coa hydratase (trifunctional protein), alpha subunit  LYVE1 - lymphatic vessel endothelial hyaluronan receptor 1  SMS - spermine synthase  PCK2 - phosphoenolpyruvate carboxykinase 2 (mitochondrial)  ASL - argininosuccinate lyase  NIT2 - nitrilase family, member 2  DAO - d-amino-acid oxidase  POR - p450 (cytochrome) oxidoreductase  C3 - complement component 3  HEXA - hexosaminidase a (alpha polypeptide)  FADS6 - fatty acid desaturase 6  LDHD - lactate dehydrogenase d  HEXB - hexosaminidase b (beta polypeptide)  ACSF3 - acyl-coa synthetase family member 3  CYP46A1 - cytochrome p450, family 46, subfamily a, polypeptide 1  IDNK - idnk, gluconokinase homolog (e. coli)  L2HGDH - l-2-hydroxyglutarate dehydrogenase  CYP27A1 - cytochrome p450, family 27, subfamily a, polypeptide 1  PRKAA2 - protein kinase, amp-activated, alpha 2 catalytic subunit  SCPEP1 - serine carboxypeptidase 1  MTR - 5-methyltetrahydrofolate-homocysteine methyltransferase  PRKAB2 - protein kinase, amp-activated, beta 2 non-catalytic subunit  QKI - qki, kh domain containing, rna binding  PGK1 - phosphoglycerate kinase 1  FAAH - fatty acid amide hydrolase  CROT - carnitine o-octanoyltransferase  ACOT8 - acyl-coa thioesterase 8  FABP3 - fatty acid binding protein 3, muscle and heart (mammary-derived growth inhibitor)  ACCS - 1-aminocyclopropane-1-carboxylate synthase homolog (arabidopsis)(non-functional)  UROC1 - urocanate hydratase 1  CTH - cystathionase (cystathionine gamma-lyase)  SLC16A1 - solute carrier family 16 (monocarboxylate transporter), member 1  ABCD4 - atp-binding cassette, sub-family d (ald), member 4  GPT2 - glutamic pyruvate transaminase (alanine aminotransferase) 2  PTGR2 - prostaglandin reductase 2  SCLY - selenocysteine lyase  RGN - regucalcin  DPYD - dihydropyrimidine dehydrogenase  GLS2 - glutaminase 2 (liver, mitochondrial)  NAAA - n-acylethanolamine acid amidase  PEPD - peptidase d  QPRT - quinolinate phosphoribosyltransferase  SLC1A3 - solute carrier family 1 (glial high affinity glutamate transporter), member 3  PET112 - pet112 homolog (yeast)  SLC39A8 - solute carrier family 39 (zinc transporter), member 8  VDAC1 - voltage-dependent anion channel 1  IDH3B - isocitrate dehydrogenase 3 (nad+) beta  IDH2 - isocitrate dehydrogenase 2 (nadp+), mitochondrial  ABCD1 - atp-binding cassette, sub-family d (ald), member 1  CYP4V2 - cytochrome p450, family 4, subfamily v, polypeptide 2  DLAT - dihydrolipoamide s-acetyltransferase  DLD - dihydrolipoamide dehydrogenase  GALK1 - galactokinase 1  ALOX5AP - arachidonate 5-lipoxygenase-activating protein  EGLN3 - egl-9 family hypoxia-inducible factor 3  PDHX - pyruvate dehydrogenase complex, component x  ASNSD1 - asparagine synthetase domain containing 1  CSPG5 - chondroitin sulfate proteoglycan 5 (neuroglycan c)  DLST - dihydrolipoamide s-succinyltransferase (e2 component of 2-oxo-glutarate complex)  ALDOA - aldolase a, fructose-bisphosphate  SHMT2 - serine hydroxymethyltransferase 2 (mitochondrial)  FARS2 - phenylalanyl-trna synthetase 2, mitochondrial  ECHDC3 - enoyl coa hydratase domain containing 3  ZADH2 - zinc binding alcohol dehydrogenase domain containing 2  ACAD9 - acyl-coa dehydrogenase family, member 9  ST3GAL1 - st3 beta-galactoside alpha-2,3-sialyltransferase 1  PLAA - phospholipase a2-activating protein  TYMS - thymidylate synthetase  CS - citrate synthase  ETFA - electron-transfer-flavoprotein, alpha polypeptide  MAPK14 - mitogen-activated protein kinase 14  ACAD8 - acyl-coa dehydrogenase family, member 8  GATM - glycine amidinotransferase (l-arginine:glycine amidinotransferase)  MGLL - monoglyceride lipase  HSD3B7 - hydroxy-delta-5-steroid dehydrogenase, 3 beta- and steroid delta-isomerase 7  AMT - aminomethyltransferase  NDUFAB1 - nadh dehydrogenase (ubiquinone) 1, alpha/beta subcomplex, 1, 8kda  UGP2 - udp-glucose pyrophosphorylase 2  PEMT - phosphatidylethanolamine n-methyltransferase  UGDH - udp-glucose 6-dehydrogenase  GPAM - glycerol-3-phosphate acyltransferase, mitochondrial  NNT - nicotinamide nucleotide transhydrogenase  HMGCL - 3-hydroxymethyl-3-methylglutaryl-coa lyase  BCAN - brevican  SARDH - sarcosine dehydrogenase  FOLR2 - folate receptor 2 (fetal)  GAPDH - glyceraldehyde-3-phosphate dehydrogenase  EPHX1 - epoxide hydrolase 1, microsomal (xenobiotic)  EPHX2 - epoxide hydrolase 2, cytoplasmic  IARS2 - isoleucyl-trna synthetase 2, mitochondrial  CBS - cystathionine-beta-synthase  AFMID - arylformamidase  SLC27A4 - solute carrier family 27 (fatty acid transporter), member 4  NCAN - neurocan  RBP1 - retinol binding protein 1, cellular  FPGS - folylpolyglutamate synthase  DCXR - dicarbonyl/l-xylulose reductase |
| GO:0090150 | establishment of protein localization to membrane | 5.24E-8 | 3.17E-5 | 1.87 (10334,215,1776,69) | [+] Show genes  RPL24 - ribosomal protein l24  RPL23A - ribosomal protein l23a  RPL21 - ribosomal protein l21  RPL19 - ribosomal protein l19  RPL18A - ribosomal protein l18a  RPL18 - ribosomal protein l18  RPL17 - ribosomal protein l17  RPL13 - ribosomal protein l13  RPL12 - ribosomal protein l12  GOLGA4 - golgin a4  RPL10 - ribosomal protein l10  RPL7A - ribosomal protein l7a  RPL5 - ribosomal protein l5  PEX19 - peroxisomal biogenesis factor 19  RPL4 - ribosomal protein l4  OXA1L - oxidase (cytochrome c) assembly 1-like  RPL35 - ribosomal protein l35  RPS5 - ribosomal protein s5  RPS2 - ribosomal protein s2  RPLP2 - ribosomal protein, large, p2  RPL36A - ribosomal protein l36a  BID - bh3 interacting domain death agonist  RPL37A - ribosomal protein l37a  RPL38 - ribosomal protein l38  NCF1 - neutrophil cytosolic factor 1  SYS1 - sys1 golgi-localized integral membrane protein homolog (s. cerevisiae)  RPL34 - ribosomal protein l34  RPL37 - ribosomal protein l37  BAG6 - bcl2-associated athanogene 6  RPL29 - ribosomal protein l29  RPL31 - ribosomal protein l31  RPL36 - ribosomal protein l36  RPL27 - ribosomal protein l27  ZDHHC18 - zinc finger, dhhc-type containing 18  RPL30 - ribosomal protein l30  RPL27A - ribosomal protein l27a  RPL28 - ribosomal protein l28  RPS24 - ribosomal protein s24  RPS21 - ribosomal protein s21  SCRIB - scribbled planar cell polarity protein  RPS27 - ribosomal protein s27  RPS26 - ribosomal protein s26  RPS25 - ribosomal protein s25  RPS17 - ribosomal protein s17  RPS16 - ribosomal protein s16  RPS15A - ribosomal protein s15a  RPS20 - ribosomal protein s20  RPS19 - ribosomal protein s19  RPS18 - ribosomal protein s18  RPS11 - ribosomal protein s11  RPS8 - ribosomal protein s8  RPS9 - ribosomal protein s9  RPS14 - ribosomal protein s14  RPS13 - ribosomal protein s13  RPS7 - ribosomal protein s7  RAPSN - receptor-associated protein of the synapse  KRT18 - keratin 18  RPSA - ribosomal protein sa  RILPL1 - rab interacting lysosomal protein-like 1  RPL10A - ribosomal protein l10a  PARD3 - par-3 partitioning defective 3 homolog (c. elegans)  SAMM50 - samm50 sorting and assembly machinery component  ROMO1 - reactive oxygen species modulator 1  RPL23 - ribosomal protein l23  CLIP1 - cap-gly domain containing linker protein 1  ADORA1 - adenosine a1 receptor  RPS28 - ribosomal protein s28  RPS29 - ribosomal protein s29  OPTN - optineurin |
| GO:0072594 | establishment of protein localization to organelle | 1.22E-7 | 7.09E-5 | 1.65 (10334,342,1776,97) | [+] Show genes  SNF8 - snf8, escrt-ii complex subunit  HSD17B4 - hydroxysteroid (17-beta) dehydrogenase 4  TOMM70A - translocase of outer mitochondrial membrane 70 homolog a (s. cerevisiae)  CD68 - cd68 molecule  PMM2 - phosphomannomutase 2  OXA1L - oxidase (cytochrome c) assembly 1-like  ZFAND6 - zinc finger, an1-type domain 6  RPL35 - ribosomal protein l35  BID - bh3 interacting domain death agonist  TXNIP - thioredoxin interacting protein  CCDC101 - coiled-coil domain containing 101  DDO - d-aspartate oxidase  CRAT - carnitine o-acetyltransferase  RPL36 - ribosomal protein l36  PECR - peroxisomal trans-2-enoyl-coa reductase  VPS36 - vacuolar protein sorting 36 homolog (s. cerevisiae)  TP53 - tumor protein p53  RPSA - ribosomal protein sa  LAMP1 - lysosomal-associated membrane protein 1  CDKN1A - cyclin-dependent kinase inhibitor 1a (p21, cip1)  DAO - d-amino-acid oxidase  RPL24 - ribosomal protein l24  RPL23A - ribosomal protein l23a  ACOT8 - acyl-coa thioesterase 8  CROT - carnitine o-octanoyltransferase  AIFM1 - apoptosis-inducing factor, mitochondrion-associated, 1  RPL21 - ribosomal protein l21  RPL19 - ribosomal protein l19  RPL18A - ribosomal protein l18a  RPL18 - ribosomal protein l18  RPL17 - ribosomal protein l17  RPL13 - ribosomal protein l13  RPL12 - ribosomal protein l12  TIMM23 - translocase of inner mitochondrial membrane 23 homolog (yeast)  RPL10 - ribosomal protein l10  RPL7A - ribosomal protein l7a  TIMM44 - translocase of inner mitochondrial membrane 44 homolog (yeast)  AGT - angiotensinogen (serpin peptidase inhibitor, clade a, member 8)  RPL5 - ribosomal protein l5  PEX19 - peroxisomal biogenesis factor 19  RPL4 - ribosomal protein l4  RPS5 - ribosomal protein s5  PEX10 - peroxisomal biogenesis factor 10  PINK1 - pten induced putative kinase 1  IPO5 - importin 5  RPS2 - ribosomal protein s2  RPLP2 - ribosomal protein, large, p2  KPNA4 - karyopherin alpha 4 (importin alpha 3)  PIH1D1 - pih1 domain containing 1  KPNA3 - karyopherin alpha 3 (importin alpha 4)  RPL36A - ribosomal protein l36a  RPL37A - ribosomal protein l37a  RPL38 - ribosomal protein l38  RPL34 - ribosomal protein l34  RPL37 - ribosomal protein l37  C11orf73 - chromosome 11 open reading frame 73  RPL29 - ribosomal protein l29  RPL31 - ribosomal protein l31  RPL27 - ribosomal protein l27  RPL30 - ribosomal protein l30  RPL27A - ribosomal protein l27a  RPL28 - ribosomal protein l28  RPS24 - ribosomal protein s24  RPS21 - ribosomal protein s21  MFN2 - mitofusin 2  SCRIB - scribbled planar cell polarity protein  RPS27 - ribosomal protein s27  RPS26 - ribosomal protein s26  RPS25 - ribosomal protein s25  UBE2D1 - ubiquitin-conjugating enzyme e2d 1  RPS17 - ribosomal protein s17  RPS16 - ribosomal protein s16  RPS15A - ribosomal protein s15a  RPS20 - ribosomal protein s20  TSPAN17 - tetraspanin 17  RPS19 - ribosomal protein s19  RPS18 - ribosomal protein s18  RPS11 - ribosomal protein s11  RPS8 - ribosomal protein s8  RPS9 - ribosomal protein s9  RPS14 - ribosomal protein s14  RPS13 - ribosomal protein s13  SIX3 - six homeobox 3  RPS7 - ribosomal protein s7  SORT1 - sortilin 1  SIX2 - six homeobox 2  PHB2 - prohibitin 2  SAMM50 - samm50 sorting and assembly machinery component  ROMO1 - reactive oxygen species modulator 1  FAM53B - family with sequence similarity 53, member b  RPL10A - ribosomal protein l10a  HMGCL - 3-hydroxymethyl-3-methylglutaryl-coa lyase  RPL23 - ribosomal protein l23  EPHX2 - epoxide hydrolase 2, cytoplasmic  RPS28 - ribosomal protein s28  IPO13 - importin 13  RPS29 - ribosomal protein s29 |
| GO:0000956 | nuclear-transcribed mRNA catabolic process | 2.14E-7 | 1.19E-4 | 1.91 (10334,180,1776,59) | [+] Show genes  RPL24 - ribosomal protein l24  EXOSC5 - exosome component 5  RPL23A - ribosomal protein l23a  RPL21 - ribosomal protein l21  RPL19 - ribosomal protein l19  RPL18A - ribosomal protein l18a  RPL18 - ribosomal protein l18  RPL17 - ribosomal protein l17  RPL13 - ribosomal protein l13  RPL12 - ribosomal protein l12  HBS1L - hbs1-like (s. cerevisiae)  XRN1 - 5'-3' exoribonuclease 1  RPL10 - ribosomal protein l10  RPL7A - ribosomal protein l7a  RPL5 - ribosomal protein l5  RPL4 - ribosomal protein l4  RPL35 - ribosomal protein l35  RPS5 - ribosomal protein s5  RPS2 - ribosomal protein s2  RPLP2 - ribosomal protein, large, p2  RPL36A - ribosomal protein l36a  RPL37A - ribosomal protein l37a  RPL38 - ribosomal protein l38  RPL34 - ribosomal protein l34  RPL37 - ribosomal protein l37  RPL29 - ribosomal protein l29  EIF4G1 - eukaryotic translation initiation factor 4 gamma, 1  RPL31 - ribosomal protein l31  RPL36 - ribosomal protein l36  RPL27 - ribosomal protein l27  RPL30 - ribosomal protein l30  RPL27A - ribosomal protein l27a  RPL28 - ribosomal protein l28  RPS24 - ribosomal protein s24  RPS21 - ribosomal protein s21  RPS27 - ribosomal protein s27  RPS26 - ribosomal protein s26  RPS25 - ribosomal protein s25  RPS17 - ribosomal protein s17  RPS16 - ribosomal protein s16  RPS15A - ribosomal protein s15a  RPS20 - ribosomal protein s20  RPS19 - ribosomal protein s19  RPS18 - ribosomal protein s18  RPS11 - ribosomal protein s11  RPS8 - ribosomal protein s8  RPS9 - ribosomal protein s9  SAMD4A - sterile alpha motif domain containing 4a  RPS14 - ribosomal protein s14  RPS13 - ribosomal protein s13  CSDE1 - cold shock domain containing e1, rna-binding  RPS7 - ribosomal protein s7  RPSA - ribosomal protein sa  SMG5 - smg5 nonsense mediated mrna decay factor  LSM4 - lsm4 homolog, u6 small nuclear rna associated (s. cerevisiae)  RPL10A - ribosomal protein l10a  RPL23 - ribosomal protein l23  RPS28 - ribosomal protein s28  RPS29 - ribosomal protein s29 |
| GO:0055086 | nucleobase-containing small molecule metabolic process | 5.96E-7 | 3.19E-4 | 1.52 (10334,460,1776,120) | [+] Show genes  FMO5 - flavin containing monooxygenase 5  KCNAB2 - potassium voltage-gated channel, shaker-related subfamily, beta member 2  HSD17B4 - hydroxysteroid (17-beta) dehydrogenase 4  SUCLA2 - succinate-coa ligase, adp-forming, beta subunit  SUCLG2 - succinate-coa ligase, gdp-forming, beta subunit  BPGM - 2,3-bisphosphoglycerate mutase  CARD11 - caspase recruitment domain family, member 11  AASS - aminoadipate-semialdehyde synthase  DNPH1 - 2'-deoxynucleoside 5'-phosphate n-hydrolase 1  RPIA - ribose 5-phosphate isomerase a  AOX1 - aldehyde oxidase 1  PMM2 - phosphomannomutase 2  CECR1 - cat eye syndrome chromosome region, candidate 1  RENBP - renin binding protein  COX7A1 - cytochrome c oxidase subunit viia polypeptide 1 (muscle)  EPHA2 - eph receptor a2  ACAT1 - acetyl-coa acetyltransferase 1  CD38 - cd38 molecule  NADSYN1 - nad synthetase 1  PMVK - phosphomevalonate kinase  ENTPD5 - ectonucleoside triphosphate diphosphohydrolase 5  GMPS - guanine monphosphate synthase  OGDH - oxoglutarate (alpha-ketoglutarate) dehydrogenase (lipoamide)  AMDHD2 - amidohydrolase domain containing 2  COASY - coa synthase  TSTA3 - tissue specific transplantation antigen p35b  APRT - adenine phosphoribosyltransferase  ATP5L - atp synthase, h+ transporting, mitochondrial fo complex, subunit g  PDE8A - phosphodiesterase 8a  NUDT17 - nudix (nucleoside diphosphate linked moiety x)-type motif 17  PDE7A - phosphodiesterase 7a  ACSS1 - acyl-coa synthetase short-chain family member 1  GNMT - glycine n-methyltransferase  TPI1 - triosephosphate isomerase 1  PDHB - pyruvate dehydrogenase (lipoamide) beta  MDH2 - malate dehydrogenase 2, nad (mitochondrial)  PDHA1 - pyruvate dehydrogenase (lipoamide) alpha 1  TECR - trans-2,3-enoyl-coa reductase  SULT1C4 - sulfotransferase family, cytosolic, 1c, member 4  SHPK - sedoheptulokinase  ADCY2 - adenylate cyclase 2 (brain)  RORA - rar-related orphan receptor a  ADCY9 - adenylate cyclase 9  ADK - adenosine kinase  NUDT3 - nudix (nucleoside diphosphate linked moiety x)-type motif 3  NPPC - natriuretic peptide c  MVD - mevalonate (diphospho) decarboxylase  ASMTL - acetylserotonin o-methyltransferase-like  PDE1A - phosphodiesterase 1a, calmodulin-dependent  SLC25A1 - solute carrier family 25 (mitochondrial carrier; citrate transporter), member 1  GMPR - guanosine monophosphate reductase  CLPX - clpx caseinolytic peptidase x homolog (e. coli)  ACSF3 - acyl-coa synthetase family member 3  NAMPT - nicotinamide phosphoribosyltransferase  PDE4D - phosphodiesterase 4d, camp-specific  PDE4A - phosphodiesterase 4a, camp-specific  PDE4B - phosphodiesterase 4b, camp-specific  XDH - xanthine dehydrogenase  UQCRC2 - ubiquinol-cytochrome c reductase core protein ii  UQCRB - ubiquinol-cytochrome c reductase binding protein  PGK1 - phosphoglycerate kinase 1  CROT - carnitine o-octanoyltransferase  ACOT8 - acyl-coa thioesterase 8  ATP5S - atp synthase, h+ transporting, mitochondrial fo complex, subunit s (factor b)  RHOQ - ras homolog family member q  NME3 - nme/nm23 nucleoside diphosphate kinase 3  ADSL - adenylosuccinate lyase  AGPAT5 - 1-acylglycerol-3-phosphate o-acyltransferase 5  MOCOS - molybdenum cofactor sulfurase  NMNAT3 - nicotinamide nucleotide adenylyltransferase 3  NUDT1 - nudix (nucleoside diphosphate linked moiety x)-type motif 1  DPYD - dihydropyrimidine dehydrogenase  AK1 - adenylate kinase 1  DUT - deoxyuridine triphosphatase  ATP5J - atp synthase, h+ transporting, mitochondrial fo complex, subunit f6  QPRT - quinolinate phosphoribosyltransferase  NUDT14 - nudix (nucleoside diphosphate linked moiety x)-type motif 14  PPCDC - phosphopantothenoylcysteine decarboxylase  ATP5C1 - atp synthase, h+ transporting, mitochondrial f1 complex, gamma polypeptide 1  SULT1E1 - sulfotransferase family 1e, estrogen-preferring, member 1  ATP5F1 - atp synthase, h+ transporting, mitochondrial fo complex, subunit b1  ATP5G3 - atp synthase, h+ transporting, mitochondrial fo complex, subunit c3 (subunit 9)  NT5C - 5', 3'-nucleotidase, cytosolic  CYC1 - cytochrome c-1  ATP5A1 - atp synthase, h+ transporting, mitochondrial f1 complex, alpha subunit 1, cardiac muscle  ATP5B - atp synthase, h+ transporting, mitochondrial f1 complex, beta polypeptide  DTYMK - deoxythymidylate kinase (thymidylate kinase)  IDH2 - isocitrate dehydrogenase 2 (nadp+), mitochondrial  ABCD1 - atp-binding cassette, sub-family d (ald), member 1  DLAT - dihydrolipoamide s-acetyltransferase  DLD - dihydrolipoamide dehydrogenase  GALK1 - galactokinase 1  PDHX - pyruvate dehydrogenase complex, component x  TEFM - transcription elongation factor, mitochondrial  DLST - dihydrolipoamide s-succinyltransferase (e2 component of 2-oxo-glutarate complex)  ALDOA - aldolase a, fructose-bisphosphate  MFN1 - mitofusin 1  NT5C3A - 5'-nucleotidase, cytosolic iiia  SHMT2 - serine hydroxymethyltransferase 2 (mitochondrial)  TGDS - tdp-glucose 4,6-dehydratase  UCKL1 - uridine-cytidine kinase 1-like 1  LHPP - phospholysine phosphohistidine inorganic pyrophosphate phosphatase  ABHD14B - abhydrolase domain containing 14b  FLAD1 - flavin adenine dinucleotide synthetase 1  TYMS - thymidylate synthetase  GBAS - glioblastoma amplified sequence  NMNAT1 - nicotinamide nucleotide adenylyltransferase 1  UGP2 - udp-glucose pyrophosphorylase 2  PEMT - phosphatidylethanolamine n-methyltransferase  UGDH - udp-glucose 6-dehydrogenase  AMPD3 - adenosine monophosphate deaminase 3  GPAM - glycerol-3-phosphate acyltransferase, mitochondrial  NNT - nicotinamide nucleotide transhydrogenase  HMGCL - 3-hydroxymethyl-3-methylglutaryl-coa lyase  GAPDH - glyceraldehyde-3-phosphate dehydrogenase  PARP10 - poly (adp-ribose) polymerase family, member 10  MPP1 - membrane protein, palmitoylated 1, 55kda  AFMID - arylformamidase  NDUFS1 - nadh dehydrogenase (ubiquinone) fe-s protein 1, 75kda (nadh-coenzyme q reductase)  DCXR - dicarbonyl/l-xylulose reductase |
| GO:0006753 | nucleoside phosphate metabolic process | 5.96E-7 | 3.07E-4 | 1.56 (10334,399,1776,107) | [+] Show genes  FMO5 - flavin containing monooxygenase 5  KCNAB2 - potassium voltage-gated channel, shaker-related subfamily, beta member 2  HSD17B4 - hydroxysteroid (17-beta) dehydrogenase 4  SUCLA2 - succinate-coa ligase, adp-forming, beta subunit  SUCLG2 - succinate-coa ligase, gdp-forming, beta subunit  BPGM - 2,3-bisphosphoglycerate mutase  CARD11 - caspase recruitment domain family, member 11  AASS - aminoadipate-semialdehyde synthase  DNPH1 - 2'-deoxynucleoside 5'-phosphate n-hydrolase 1  RPIA - ribose 5-phosphate isomerase a  COX7A1 - cytochrome c oxidase subunit viia polypeptide 1 (muscle)  EPHA2 - eph receptor a2  ACAT1 - acetyl-coa acetyltransferase 1  CD38 - cd38 molecule  NADSYN1 - nad synthetase 1  PMVK - phosphomevalonate kinase  GMPS - guanine monphosphate synthase  ENTPD5 - ectonucleoside triphosphate diphosphohydrolase 5  OGDH - oxoglutarate (alpha-ketoglutarate) dehydrogenase (lipoamide)  COASY - coa synthase  APRT - adenine phosphoribosyltransferase  ATP5L - atp synthase, h+ transporting, mitochondrial fo complex, subunit g  PDE8A - phosphodiesterase 8a  NUDT17 - nudix (nucleoside diphosphate linked moiety x)-type motif 17  PDE7A - phosphodiesterase 7a  ACSS1 - acyl-coa synthetase short-chain family member 1  TPI1 - triosephosphate isomerase 1  PDHB - pyruvate dehydrogenase (lipoamide) beta  MDH2 - malate dehydrogenase 2, nad (mitochondrial)  PDHA1 - pyruvate dehydrogenase (lipoamide) alpha 1  TECR - trans-2,3-enoyl-coa reductase  SULT1C4 - sulfotransferase family, cytosolic, 1c, member 4  SHPK - sedoheptulokinase  ADCY2 - adenylate cyclase 2 (brain)  RORA - rar-related orphan receptor a  ADCY9 - adenylate cyclase 9  ADK - adenosine kinase  NUDT3 - nudix (nucleoside diphosphate linked moiety x)-type motif 3  MVD - mevalonate (diphospho) decarboxylase  NPPC - natriuretic peptide c  ASMTL - acetylserotonin o-methyltransferase-like  PDE1A - phosphodiesterase 1a, calmodulin-dependent  SLC25A1 - solute carrier family 25 (mitochondrial carrier; citrate transporter), member 1  GMPR - guanosine monophosphate reductase  CLPX - clpx caseinolytic peptidase x homolog (e. coli)  ACSF3 - acyl-coa synthetase family member 3  NAMPT - nicotinamide phosphoribosyltransferase  PDE4D - phosphodiesterase 4d, camp-specific  PDE4A - phosphodiesterase 4a, camp-specific  PDE4B - phosphodiesterase 4b, camp-specific  XDH - xanthine dehydrogenase  UQCRC2 - ubiquinol-cytochrome c reductase core protein ii  UQCRB - ubiquinol-cytochrome c reductase binding protein  PGK1 - phosphoglycerate kinase 1  ACOT8 - acyl-coa thioesterase 8  CROT - carnitine o-octanoyltransferase  NME3 - nme/nm23 nucleoside diphosphate kinase 3  RHOQ - ras homolog family member q  ATP5S - atp synthase, h+ transporting, mitochondrial fo complex, subunit s (factor b)  ADSL - adenylosuccinate lyase  AGPAT5 - 1-acylglycerol-3-phosphate o-acyltransferase 5  MOCOS - molybdenum cofactor sulfurase  NMNAT3 - nicotinamide nucleotide adenylyltransferase 3  NUDT1 - nudix (nucleoside diphosphate linked moiety x)-type motif 1  AK1 - adenylate kinase 1  DUT - deoxyuridine triphosphatase  ATP5J - atp synthase, h+ transporting, mitochondrial fo complex, subunit f6  QPRT - quinolinate phosphoribosyltransferase  NUDT14 - nudix (nucleoside diphosphate linked moiety x)-type motif 14  PPCDC - phosphopantothenoylcysteine decarboxylase  ATP5C1 - atp synthase, h+ transporting, mitochondrial f1 complex, gamma polypeptide 1  SULT1E1 - sulfotransferase family 1e, estrogen-preferring, member 1  ATP5F1 - atp synthase, h+ transporting, mitochondrial fo complex, subunit b1  ATP5G3 - atp synthase, h+ transporting, mitochondrial fo complex, subunit c3 (subunit 9)  NT5C - 5', 3'-nucleotidase, cytosolic  CYC1 - cytochrome c-1  ATP5A1 - atp synthase, h+ transporting, mitochondrial f1 complex, alpha subunit 1, cardiac muscle  ATP5B - atp synthase, h+ transporting, mitochondrial f1 complex, beta polypeptide  DTYMK - deoxythymidylate kinase (thymidylate kinase)  IDH2 - isocitrate dehydrogenase 2 (nadp+), mitochondrial  ABCD1 - atp-binding cassette, sub-family d (ald), member 1  DLAT - dihydrolipoamide s-acetyltransferase  DLD - dihydrolipoamide dehydrogenase  GALK1 - galactokinase 1  PDHX - pyruvate dehydrogenase complex, component x  TEFM - transcription elongation factor, mitochondrial  DLST - dihydrolipoamide s-succinyltransferase (e2 component of 2-oxo-glutarate complex)  ALDOA - aldolase a, fructose-bisphosphate  MFN1 - mitofusin 1  NT5C3A - 5'-nucleotidase, cytosolic iiia  UCKL1 - uridine-cytidine kinase 1-like 1  LHPP - phospholysine phosphohistidine inorganic pyrophosphate phosphatase  ABHD14B - abhydrolase domain containing 14b  FLAD1 - flavin adenine dinucleotide synthetase 1  TYMS - thymidylate synthetase  GBAS - glioblastoma amplified sequence  NMNAT1 - nicotinamide nucleotide adenylyltransferase 1  AMPD3 - adenosine monophosphate deaminase 3  GPAM - glycerol-3-phosphate acyltransferase, mitochondrial  NNT - nicotinamide nucleotide transhydrogenase  HMGCL - 3-hydroxymethyl-3-methylglutaryl-coa lyase  GAPDH - glyceraldehyde-3-phosphate dehydrogenase  PARP10 - poly (adp-ribose) polymerase family, member 10  MPP1 - membrane protein, palmitoylated 1, 55kda  AFMID - arylformamidase  NDUFS1 - nadh dehydrogenase (ubiquinone) fe-s protein 1, 75kda (nadh-coenzyme q reductase)  DCXR - dicarbonyl/l-xylulose reductase |
| GO:0006099 | tricarboxylic acid cycle | 6.44E-7 | 3.2E-4 | 3.27 (10334,32,1776,18) | [+] Show genes  DLAT - dihydrolipoamide s-acetyltransferase  DLD - dihydrolipoamide dehydrogenase  ACO1 - aconitase 1, soluble  SUCLA2 - succinate-coa ligase, adp-forming, beta subunit  PDHB - pyruvate dehydrogenase (lipoamide) beta  SDHD - succinate dehydrogenase complex, subunit d, integral membrane protein  MDH2 - malate dehydrogenase 2, nad (mitochondrial)  SDHC - succinate dehydrogenase complex, subunit c, integral membrane protein, 15kda  DLST - dihydrolipoamide s-succinyltransferase (e2 component of 2-oxo-glutarate complex)  PDHA1 - pyruvate dehydrogenase (lipoamide) alpha 1  SUCLG2 - succinate-coa ligase, gdp-forming, beta subunit  ACO2 - aconitase 2, mitochondrial  SDHA - succinate dehydrogenase complex, subunit a, flavoprotein (fp)  NNT - nicotinamide nucleotide transhydrogenase  OGDH - oxoglutarate (alpha-ketoglutarate) dehydrogenase (lipoamide)  IDH3B - isocitrate dehydrogenase 3 (nad+) beta  CS - citrate synthase  IDH2 - isocitrate dehydrogenase 2 (nadp+), mitochondrial |
| GO:0033108 | mitochondrial respiratory chain complex assembly | 7.04E-7 | 3.38E-4 | 2.30 (10334,86,1776,34) | [+] Show genes  NDUFB11 - nadh dehydrogenase (ubiquinone) 1 beta subcomplex, 11, 17.3kda  UQCRB - ubiquinol-cytochrome c reductase binding protein  AIFM1 - apoptosis-inducing factor, mitochondrion-associated, 1  COX14 - cytochrome c oxidase assembly homolog 14 (s. cerevisiae)  NDUFAF6 - nadh dehydrogenase (ubiquinone) complex i, assembly factor 6  NDUFA1 - nadh dehydrogenase (ubiquinone) 1 alpha subcomplex, 1, 7.5kda  NDUFA12 - nadh dehydrogenase (ubiquinone) 1 alpha subcomplex, 12  NDUFA5 - nadh dehydrogenase (ubiquinone) 1 alpha subcomplex, 5  OMA1 - oma1 zinc metallopeptidase  COX20 - cox20 cytochrome c oxidase assembly factor  ACAD9 - acyl-coa dehydrogenase family, member 9  OXA1L - oxidase (cytochrome c) assembly 1-like  TMEM126A - transmembrane protein 126a  NDUFC1 - nadh dehydrogenase (ubiquinone) 1, subcomplex unknown, 1, 6kda  NDUFAF3 - nadh dehydrogenase (ubiquinone) complex i, assembly factor 3  NDUFB8 - nadh dehydrogenase (ubiquinone) 1 beta subcomplex, 8, 19kda  NDUFB9 - nadh dehydrogenase (ubiquinone) 1 beta subcomplex, 9, 22kda  TACO1 - translational activator of mitochondrially encoded cytochrome c oxidase i  NDUFB6 - nadh dehydrogenase (ubiquinone) 1 beta subcomplex, 6, 17kda  NDUFB4 - nadh dehydrogenase (ubiquinone) 1 beta subcomplex, 4, 15kda  NDUFB5 - nadh dehydrogenase (ubiquinone) 1 beta subcomplex, 5, 16kda  NDUFB3 - nadh dehydrogenase (ubiquinone) 1 beta subcomplex, 3, 12kda  NDUFAB1 - nadh dehydrogenase (ubiquinone) 1, alpha/beta subcomplex, 1, 8kda  NDUFB1 - nadh dehydrogenase (ubiquinone) 1 beta subcomplex, 1, 7kda  NDUFA9 - nadh dehydrogenase (ubiquinone) 1 alpha subcomplex, 9, 39kda  NDUFA10 - nadh dehydrogenase (ubiquinone) 1 alpha subcomplex, 10, 42kda  SAMM50 - samm50 sorting and assembly machinery component  NDUFV2 - nadh dehydrogenase (ubiquinone) flavoprotein 2, 24kda  NDUFA11 - nadh dehydrogenase (ubiquinone) 1 alpha subcomplex, 11, 14.7kda  SMIM20 - small integral membrane protein 20  NDUFS4 - nadh dehydrogenase (ubiquinone) fe-s protein 4, 18kda (nadh-coenzyme q reductase)  NDUFS3 - nadh dehydrogenase (ubiquinone) fe-s protein 3, 30kda (nadh-coenzyme q reductase)  NDUFS2 - nadh dehydrogenase (ubiquinone) fe-s protein 2, 49kda (nadh-coenzyme q reductase)  NDUFS1 - nadh dehydrogenase (ubiquinone) fe-s protein 1, 75kda (nadh-coenzyme q reductase) |
| GO:0043436 | oxoacid metabolic process | 7.91E-7 | 3.67E-4 | 1.40 (10334,710,1776,171) | [+] Show genes  GSTM1 - glutathione s-transferase mu 1  HSD17B4 - hydroxysteroid (17-beta) dehydrogenase 4  GSTM4 - glutathione s-transferase mu 4  SUCLA2 - succinate-coa ligase, adp-forming, beta subunit  SDHD - succinate dehydrogenase complex, subunit d, integral membrane protein  SDHC - succinate dehydrogenase complex, subunit c, integral membrane protein, 15kda  SUCLG2 - succinate-coa ligase, gdp-forming, beta subunit  SDHA - succinate dehydrogenase complex, subunit a, flavoprotein (fp)  BPHL - biphenyl hydrolase-like (serine hydrolase)  SDSL - serine dehydratase-like  BPGM - 2,3-bisphosphoglycerate mutase  BTD - biotinidase  AASS - aminoadipate-semialdehyde synthase  LDHB - lactate dehydrogenase b  FA2H - fatty acid 2-hydroxylase  BSG - basigin (ok blood group)  GCSH - glycine cleavage system protein h (aminomethyl carrier)  PC - pyruvate carboxylase  NAT8L - n-acetyltransferase 8-like (gcn5-related, putative)  ECI1 - enoyl-coa delta isomerase 1  ITIH5 - inter-alpha-trypsin inhibitor heavy chain family, member 5  PCCA - propionyl coa carboxylase, alpha polypeptide  GCH1 - gtp cyclohydrolase 1  PCCB - propionyl coa carboxylase, beta polypeptide  RENBP - renin binding protein  BGN - biglycan  ACAT1 - acetyl-coa acetyltransferase 1  DDAH1 - dimethylarginine dimethylaminohydrolase 1  ACO1 - aconitase 1, soluble  PLP1 - proteolipid protein 1  P4HB - prolyl 4-hydroxylase, beta polypeptide  PTGR1 - prostaglandin reductase 1  BLMH - bleomycin hydrolase  ACO2 - aconitase 2, mitochondrial  AKR1A1 - aldo-keto reductase family 1, member a1 (aldehyde reductase)  MRS2 - mrs2 magnesium transporter  AIMP1 - aminoacyl trna synthetase complex-interacting multifunctional protein 1  LPL - lipoprotein lipase  GMPS - guanine monphosphate synthase  OGDH - oxoglutarate (alpha-ketoglutarate) dehydrogenase (lipoamide)  HOGA1 - 4-hydroxy-2-oxoglutarate aldolase 1  SORD - sorbitol dehydrogenase  AMDHD2 - amidohydrolase domain containing 2  CREM - camp responsive element modulator  DDO - d-aspartate oxidase  MRPS36 - mitochondrial ribosomal protein s36  CRAT - carnitine o-acetyltransferase  GUSB - glucuronidase, beta  GGT5 - gamma-glutamyltransferase 5  PECR - peroxisomal trans-2-enoyl-coa reductase  FADS3 - fatty acid desaturase 3  HYI - hydroxypyruvate isomerase (putative)  ADIPOR2 - adiponectin receptor 2  GLUL - glutamate-ammonia ligase  CSGALNACT2 - chondroitin sulfate n-acetylgalactosaminyltransferase 2  ACSS1 - acyl-coa synthetase short-chain family member 1  GNMT - glycine n-methyltransferase  TPI1 - triosephosphate isomerase 1  PDHB - pyruvate dehydrogenase (lipoamide) beta  MDH2 - malate dehydrogenase 2, nad (mitochondrial)  CARS2 - cysteinyl-trna synthetase 2, mitochondrial (putative)  PDHA1 - pyruvate dehydrogenase (lipoamide) alpha 1  ACY1 - aminoacylase 1  GLO1 - glyoxalase i  CDO1 - cysteine dioxygenase type 1  TECR - trans-2,3-enoyl-coa reductase  GLDC - glycine dehydrogenase (decarboxylating)  PPM1K - protein phosphatase, mg2+/mn2+ dependent, 1k  PTGS1 - prostaglandin-endoperoxide synthase 1 (prostaglandin g/h synthase and cyclooxygenase)  SULT1C4 - sulfotransferase family, cytosolic, 1c, member 4  HADHA - hydroxyacyl-coa dehydrogenase/3-ketoacyl-coa thiolase/enoyl-coa hydratase (trifunctional protein), alpha subunit  LYVE1 - lymphatic vessel endothelial hyaluronan receptor 1  SMS - spermine synthase  PCK2 - phosphoenolpyruvate carboxykinase 2 (mitochondrial)  ASL - argininosuccinate lyase  NIT2 - nitrilase family, member 2  DAO - d-amino-acid oxidase  POR - p450 (cytochrome) oxidoreductase  C3 - complement component 3  HEXA - hexosaminidase a (alpha polypeptide)  FADS6 - fatty acid desaturase 6  LDHD - lactate dehydrogenase d  HEXB - hexosaminidase b (beta polypeptide)  ACSF3 - acyl-coa synthetase family member 3  CYP46A1 - cytochrome p450, family 46, subfamily a, polypeptide 1  L2HGDH - l-2-hydroxyglutarate dehydrogenase  IDNK - idnk, gluconokinase homolog (e. coli)  CYP27A1 - cytochrome p450, family 27, subfamily a, polypeptide 1  SCPEP1 - serine carboxypeptidase 1  PRKAA2 - protein kinase, amp-activated, alpha 2 catalytic subunit  MTR - 5-methyltetrahydrofolate-homocysteine methyltransferase  QKI - qki, kh domain containing, rna binding  PRKAB2 - protein kinase, amp-activated, beta 2 non-catalytic subunit  PGK1 - phosphoglycerate kinase 1  FAAH - fatty acid amide hydrolase  CROT - carnitine o-octanoyltransferase  ACOT8 - acyl-coa thioesterase 8  FABP3 - fatty acid binding protein 3, muscle and heart (mammary-derived growth inhibitor)  ACCS - 1-aminocyclopropane-1-carboxylate synthase homolog (arabidopsis)(non-functional)  UROC1 - urocanate hydratase 1  CTH - cystathionase (cystathionine gamma-lyase)  SLC16A1 - solute carrier family 16 (monocarboxylate transporter), member 1  ABCD4 - atp-binding cassette, sub-family d (ald), member 4  GPT2 - glutamic pyruvate transaminase (alanine aminotransferase) 2  B3GNT4 - udp-glcnac:betagal beta-1,3-n-acetylglucosaminyltransferase 4  PTGR2 - prostaglandin reductase 2  SCLY - selenocysteine lyase  RGN - regucalcin  DPYD - dihydropyrimidine dehydrogenase  GLS2 - glutaminase 2 (liver, mitochondrial)  NAAA - n-acylethanolamine acid amidase  PEPD - peptidase d  QPRT - quinolinate phosphoribosyltransferase  SLC1A3 - solute carrier family 1 (glial high affinity glutamate transporter), member 3  PET112 - pet112 homolog (yeast)  SLC39A8 - solute carrier family 39 (zinc transporter), member 8  SULT1E1 - sulfotransferase family 1e, estrogen-preferring, member 1  VDAC1 - voltage-dependent anion channel 1  IDH3B - isocitrate dehydrogenase 3 (nad+) beta  IDH2 - isocitrate dehydrogenase 2 (nadp+), mitochondrial  ABCD1 - atp-binding cassette, sub-family d (ald), member 1  CYP4V2 - cytochrome p450, family 4, subfamily v, polypeptide 2  DLAT - dihydrolipoamide s-acetyltransferase  DLD - dihydrolipoamide dehydrogenase  GALK1 - galactokinase 1  ALOX5AP - arachidonate 5-lipoxygenase-activating protein  EGLN3 - egl-9 family hypoxia-inducible factor 3  PDHX - pyruvate dehydrogenase complex, component x  ASNSD1 - asparagine synthetase domain containing 1  CSPG5 - chondroitin sulfate proteoglycan 5 (neuroglycan c)  DLST - dihydrolipoamide s-succinyltransferase (e2 component of 2-oxo-glutarate complex)  ALDOA - aldolase a, fructose-bisphosphate  SHMT2 - serine hydroxymethyltransferase 2 (mitochondrial)  FARS2 - phenylalanyl-trna synthetase 2, mitochondrial  ECHDC3 - enoyl coa hydratase domain containing 3  ZADH2 - zinc binding alcohol dehydrogenase domain containing 2  ACAD9 - acyl-coa dehydrogenase family, member 9  ST3GAL1 - st3 beta-galactoside alpha-2,3-sialyltransferase 1  ABHD14B - abhydrolase domain containing 14b  PLAA - phospholipase a2-activating protein  TYMS - thymidylate synthetase  CS - citrate synthase  ETFA - electron-transfer-flavoprotein, alpha polypeptide  MAPK14 - mitogen-activated protein kinase 14  ANGPT1 - angiopoietin 1  ACAD8 - acyl-coa dehydrogenase family, member 8  GATM - glycine amidinotransferase (l-arginine:glycine amidinotransferase)  MGLL - monoglyceride lipase  HSD3B7 - hydroxy-delta-5-steroid dehydrogenase, 3 beta- and steroid delta-isomerase 7  AMT - aminomethyltransferase  NDUFAB1 - nadh dehydrogenase (ubiquinone) 1, alpha/beta subcomplex, 1, 8kda  UGP2 - udp-glucose pyrophosphorylase 2  UGDH - udp-glucose 6-dehydrogenase  PEMT - phosphatidylethanolamine n-methyltransferase  GPAM - glycerol-3-phosphate acyltransferase, mitochondrial  NNT - nicotinamide nucleotide transhydrogenase  HMGCL - 3-hydroxymethyl-3-methylglutaryl-coa lyase  BCAN - brevican  SARDH - sarcosine dehydrogenase  FOLR2 - folate receptor 2 (fetal)  GAPDH - glyceraldehyde-3-phosphate dehydrogenase  EPHX1 - epoxide hydrolase 1, microsomal (xenobiotic)  EPHX2 - epoxide hydrolase 2, cytoplasmic  IARS2 - isoleucyl-trna synthetase 2, mitochondrial  AFMID - arylformamidase  CBS - cystathionine-beta-synthase  SLC27A4 - solute carrier family 27 (fatty acid transporter), member 4  NCAN - neurocan  RBP1 - retinol binding protein 1, cellular  FPGS - folylpolyglutamate synthase  DCXR - dicarbonyl/l-xylulose reductase |
| GO:0009117 | nucleotide metabolic process | 8.08E-7 | 3.63E-4 | 1.56 (10334,392,1776,105) | [+] Show genes  FMO5 - flavin containing monooxygenase 5  KCNAB2 - potassium voltage-gated channel, shaker-related subfamily, beta member 2  HSD17B4 - hydroxysteroid (17-beta) dehydrogenase 4  SUCLA2 - succinate-coa ligase, adp-forming, beta subunit  SUCLG2 - succinate-coa ligase, gdp-forming, beta subunit  BPGM - 2,3-bisphosphoglycerate mutase  CARD11 - caspase recruitment domain family, member 11  AASS - aminoadipate-semialdehyde synthase  DNPH1 - 2'-deoxynucleoside 5'-phosphate n-hydrolase 1  RPIA - ribose 5-phosphate isomerase a  COX7A1 - cytochrome c oxidase subunit viia polypeptide 1 (muscle)  EPHA2 - eph receptor a2  ACAT1 - acetyl-coa acetyltransferase 1  CD38 - cd38 molecule  NADSYN1 - nad synthetase 1  PMVK - phosphomevalonate kinase  GMPS - guanine monphosphate synthase  ENTPD5 - ectonucleoside triphosphate diphosphohydrolase 5  OGDH - oxoglutarate (alpha-ketoglutarate) dehydrogenase (lipoamide)  COASY - coa synthase  APRT - adenine phosphoribosyltransferase  ATP5L - atp synthase, h+ transporting, mitochondrial fo complex, subunit g  PDE8A - phosphodiesterase 8a  NUDT17 - nudix (nucleoside diphosphate linked moiety x)-type motif 17  PDE7A - phosphodiesterase 7a  ACSS1 - acyl-coa synthetase short-chain family member 1  TPI1 - triosephosphate isomerase 1  PDHB - pyruvate dehydrogenase (lipoamide) beta  MDH2 - malate dehydrogenase 2, nad (mitochondrial)  PDHA1 - pyruvate dehydrogenase (lipoamide) alpha 1  TECR - trans-2,3-enoyl-coa reductase  SULT1C4 - sulfotransferase family, cytosolic, 1c, member 4  SHPK - sedoheptulokinase  ADCY2 - adenylate cyclase 2 (brain)  RORA - rar-related orphan receptor a  ADCY9 - adenylate cyclase 9  ADK - adenosine kinase  NUDT3 - nudix (nucleoside diphosphate linked moiety x)-type motif 3  MVD - mevalonate (diphospho) decarboxylase  NPPC - natriuretic peptide c  ASMTL - acetylserotonin o-methyltransferase-like  PDE1A - phosphodiesterase 1a, calmodulin-dependent  SLC25A1 - solute carrier family 25 (mitochondrial carrier; citrate transporter), member 1  CLPX - clpx caseinolytic peptidase x homolog (e. coli)  GMPR - guanosine monophosphate reductase  ACSF3 - acyl-coa synthetase family member 3  NAMPT - nicotinamide phosphoribosyltransferase  PDE4D - phosphodiesterase 4d, camp-specific  PDE4A - phosphodiesterase 4a, camp-specific  PDE4B - phosphodiesterase 4b, camp-specific  XDH - xanthine dehydrogenase  UQCRC2 - ubiquinol-cytochrome c reductase core protein ii  UQCRB - ubiquinol-cytochrome c reductase binding protein  PGK1 - phosphoglycerate kinase 1  ACOT8 - acyl-coa thioesterase 8  CROT - carnitine o-octanoyltransferase  RHOQ - ras homolog family member q  ATP5S - atp synthase, h+ transporting, mitochondrial fo complex, subunit s (factor b)  NME3 - nme/nm23 nucleoside diphosphate kinase 3  ADSL - adenylosuccinate lyase  AGPAT5 - 1-acylglycerol-3-phosphate o-acyltransferase 5  MOCOS - molybdenum cofactor sulfurase  NMNAT3 - nicotinamide nucleotide adenylyltransferase 3  NUDT1 - nudix (nucleoside diphosphate linked moiety x)-type motif 1  AK1 - adenylate kinase 1  DUT - deoxyuridine triphosphatase  ATP5J - atp synthase, h+ transporting, mitochondrial fo complex, subunit f6  QPRT - quinolinate phosphoribosyltransferase  PPCDC - phosphopantothenoylcysteine decarboxylase  ATP5C1 - atp synthase, h+ transporting, mitochondrial f1 complex, gamma polypeptide 1  SULT1E1 - sulfotransferase family 1e, estrogen-preferring, member 1  ATP5F1 - atp synthase, h+ transporting, mitochondrial fo complex, subunit b1  ATP5G3 - atp synthase, h+ transporting, mitochondrial fo complex, subunit c3 (subunit 9)  NT5C - 5', 3'-nucleotidase, cytosolic  CYC1 - cytochrome c-1  ATP5A1 - atp synthase, h+ transporting, mitochondrial f1 complex, alpha subunit 1, cardiac muscle  DTYMK - deoxythymidylate kinase (thymidylate kinase)  ATP5B - atp synthase, h+ transporting, mitochondrial f1 complex, beta polypeptide  IDH2 - isocitrate dehydrogenase 2 (nadp+), mitochondrial  ABCD1 - atp-binding cassette, sub-family d (ald), member 1  DLAT - dihydrolipoamide s-acetyltransferase  DLD - dihydrolipoamide dehydrogenase  GALK1 - galactokinase 1  PDHX - pyruvate dehydrogenase complex, component x  TEFM - transcription elongation factor, mitochondrial  DLST - dihydrolipoamide s-succinyltransferase (e2 component of 2-oxo-glutarate complex)  ALDOA - aldolase a, fructose-bisphosphate  MFN1 - mitofusin 1  NT5C3A - 5'-nucleotidase, cytosolic iiia  UCKL1 - uridine-cytidine kinase 1-like 1  ABHD14B - abhydrolase domain containing 14b  FLAD1 - flavin adenine dinucleotide synthetase 1  TYMS - thymidylate synthetase  GBAS - glioblastoma amplified sequence  NMNAT1 - nicotinamide nucleotide adenylyltransferase 1  AMPD3 - adenosine monophosphate deaminase 3  GPAM - glycerol-3-phosphate acyltransferase, mitochondrial  NNT - nicotinamide nucleotide transhydrogenase  HMGCL - 3-hydroxymethyl-3-methylglutaryl-coa lyase  GAPDH - glyceraldehyde-3-phosphate dehydrogenase  PARP10 - poly (adp-ribose) polymerase family, member 10  MPP1 - membrane protein, palmitoylated 1, 55kda  AFMID - arylformamidase  NDUFS1 - nadh dehydrogenase (ubiquinone) fe-s protein 1, 75kda (nadh-coenzyme q reductase)  DCXR - dicarbonyl/l-xylulose reductase |
| GO:0006082 | organic acid metabolic process | 8.77E-7 | 3.81E-4 | 1.40 (10334,721,1776,173) | [+] Show genes  GSTM1 - glutathione s-transferase mu 1  HSD17B4 - hydroxysteroid (17-beta) dehydrogenase 4  GSTM4 - glutathione s-transferase mu 4  SUCLA2 - succinate-coa ligase, adp-forming, beta subunit  SDHD - succinate dehydrogenase complex, subunit d, integral membrane protein  SDHC - succinate dehydrogenase complex, subunit c, integral membrane protein, 15kda  SUCLG2 - succinate-coa ligase, gdp-forming, beta subunit  SDHA - succinate dehydrogenase complex, subunit a, flavoprotein (fp)  BPHL - biphenyl hydrolase-like (serine hydrolase)  SDSL - serine dehydratase-like  BPGM - 2,3-bisphosphoglycerate mutase  BTD - biotinidase  AASS - aminoadipate-semialdehyde synthase  LDHB - lactate dehydrogenase b  FA2H - fatty acid 2-hydroxylase  BSG - basigin (ok blood group)  GCSH - glycine cleavage system protein h (aminomethyl carrier)  PC - pyruvate carboxylase  NAT8L - n-acetyltransferase 8-like (gcn5-related, putative)  ECI1 - enoyl-coa delta isomerase 1  ITIH5 - inter-alpha-trypsin inhibitor heavy chain family, member 5  PCCA - propionyl coa carboxylase, alpha polypeptide  GCH1 - gtp cyclohydrolase 1  PCCB - propionyl coa carboxylase, beta polypeptide  RENBP - renin binding protein  BGN - biglycan  ACAT1 - acetyl-coa acetyltransferase 1  DDAH1 - dimethylarginine dimethylaminohydrolase 1  PLP1 - proteolipid protein 1  ACO1 - aconitase 1, soluble  P4HB - prolyl 4-hydroxylase, beta polypeptide  PTGR1 - prostaglandin reductase 1  BLMH - bleomycin hydrolase  ACO2 - aconitase 2, mitochondrial  AKR1A1 - aldo-keto reductase family 1, member a1 (aldehyde reductase)  AIMP1 - aminoacyl trna synthetase complex-interacting multifunctional protein 1  MRS2 - mrs2 magnesium transporter  LPL - lipoprotein lipase  GMPS - guanine monphosphate synthase  OGDH - oxoglutarate (alpha-ketoglutarate) dehydrogenase (lipoamide)  HOGA1 - 4-hydroxy-2-oxoglutarate aldolase 1  SORD - sorbitol dehydrogenase  AMDHD2 - amidohydrolase domain containing 2  CREM - camp responsive element modulator  DDO - d-aspartate oxidase  MRPS36 - mitochondrial ribosomal protein s36  CRAT - carnitine o-acetyltransferase  GUSB - glucuronidase, beta  GGT5 - gamma-glutamyltransferase 5  PECR - peroxisomal trans-2-enoyl-coa reductase  FADS3 - fatty acid desaturase 3  HYI - hydroxypyruvate isomerase (putative)  ADIPOR2 - adiponectin receptor 2  GLUL - glutamate-ammonia ligase  CSGALNACT2 - chondroitin sulfate n-acetylgalactosaminyltransferase 2  ACSS1 - acyl-coa synthetase short-chain family member 1  GNMT - glycine n-methyltransferase  TPI1 - triosephosphate isomerase 1  PDHB - pyruvate dehydrogenase (lipoamide) beta  CARS2 - cysteinyl-trna synthetase 2, mitochondrial (putative)  MDH2 - malate dehydrogenase 2, nad (mitochondrial)  PDHA1 - pyruvate dehydrogenase (lipoamide) alpha 1  ACY1 - aminoacylase 1  GLO1 - glyoxalase i  CDO1 - cysteine dioxygenase type 1  TECR - trans-2,3-enoyl-coa reductase  GLDC - glycine dehydrogenase (decarboxylating)  PPM1K - protein phosphatase, mg2+/mn2+ dependent, 1k  PTGS1 - prostaglandin-endoperoxide synthase 1 (prostaglandin g/h synthase and cyclooxygenase)  SULT1C4 - sulfotransferase family, cytosolic, 1c, member 4  HADHA - hydroxyacyl-coa dehydrogenase/3-ketoacyl-coa thiolase/enoyl-coa hydratase (trifunctional protein), alpha subunit  LYVE1 - lymphatic vessel endothelial hyaluronan receptor 1  SMS - spermine synthase  PCK2 - phosphoenolpyruvate carboxykinase 2 (mitochondrial)  ASL - argininosuccinate lyase  NIT2 - nitrilase family, member 2  DAO - d-amino-acid oxidase  POR - p450 (cytochrome) oxidoreductase  C3 - complement component 3  HEXA - hexosaminidase a (alpha polypeptide)  FADS6 - fatty acid desaturase 6  LDHD - lactate dehydrogenase d  HEXB - hexosaminidase b (beta polypeptide)  ACSF3 - acyl-coa synthetase family member 3  CYP46A1 - cytochrome p450, family 46, subfamily a, polypeptide 1  IDNK - idnk, gluconokinase homolog (e. coli)  L2HGDH - l-2-hydroxyglutarate dehydrogenase  CYP27A1 - cytochrome p450, family 27, subfamily a, polypeptide 1  PRKAA2 - protein kinase, amp-activated, alpha 2 catalytic subunit  SCPEP1 - serine carboxypeptidase 1  MTR - 5-methyltetrahydrofolate-homocysteine methyltransferase  PRKAB2 - protein kinase, amp-activated, beta 2 non-catalytic subunit  QKI - qki, kh domain containing, rna binding  PGK1 - phosphoglycerate kinase 1  FAAH - fatty acid amide hydrolase  CROT - carnitine o-octanoyltransferase  ACOT8 - acyl-coa thioesterase 8  FABP3 - fatty acid binding protein 3, muscle and heart (mammary-derived growth inhibitor)  ACCS - 1-aminocyclopropane-1-carboxylate synthase homolog (arabidopsis)(non-functional)  UROC1 - urocanate hydratase 1  ABCG2 - atp-binding cassette, sub-family g (white), member 2  CTH - cystathionase (cystathionine gamma-lyase)  SLC16A1 - solute carrier family 16 (monocarboxylate transporter), member 1  ABCD4 - atp-binding cassette, sub-family d (ald), member 4  GPT2 - glutamic pyruvate transaminase (alanine aminotransferase) 2  SCLY - selenocysteine lyase  PTGR2 - prostaglandin reductase 2  B3GNT4 - udp-glcnac:betagal beta-1,3-n-acetylglucosaminyltransferase 4  RGN - regucalcin  DPYD - dihydropyrimidine dehydrogenase  GLS2 - glutaminase 2 (liver, mitochondrial)  NAAA - n-acylethanolamine acid amidase  PEPD - peptidase d  QPRT - quinolinate phosphoribosyltransferase  SLC1A3 - solute carrier family 1 (glial high affinity glutamate transporter), member 3  PET112 - pet112 homolog (yeast)  SLC39A8 - solute carrier family 39 (zinc transporter), member 8  SULT1E1 - sulfotransferase family 1e, estrogen-preferring, member 1  VDAC1 - voltage-dependent anion channel 1  IDH3B - isocitrate dehydrogenase 3 (nad+) beta  IDH2 - isocitrate dehydrogenase 2 (nadp+), mitochondrial  ABCD1 - atp-binding cassette, sub-family d (ald), member 1  DLAT - dihydrolipoamide s-acetyltransferase  CYP4V2 - cytochrome p450, family 4, subfamily v, polypeptide 2  DLD - dihydrolipoamide dehydrogenase  GALK1 - galactokinase 1  ALOX5AP - arachidonate 5-lipoxygenase-activating protein  EGLN3 - egl-9 family hypoxia-inducible factor 3  PDHX - pyruvate dehydrogenase complex, component x  ASNSD1 - asparagine synthetase domain containing 1  CSPG5 - chondroitin sulfate proteoglycan 5 (neuroglycan c)  DLST - dihydrolipoamide s-succinyltransferase (e2 component of 2-oxo-glutarate complex)  STAT5B - signal transducer and activator of transcription 5b  ALDOA - aldolase a, fructose-bisphosphate  SHMT2 - serine hydroxymethyltransferase 2 (mitochondrial)  FARS2 - phenylalanyl-trna synthetase 2, mitochondrial  ECHDC3 - enoyl coa hydratase domain containing 3  ZADH2 - zinc binding alcohol dehydrogenase domain containing 2  ACAD9 - acyl-coa dehydrogenase family, member 9  ST3GAL1 - st3 beta-galactoside alpha-2,3-sialyltransferase 1  ABHD14B - abhydrolase domain containing 14b  PLAA - phospholipase a2-activating protein  TYMS - thymidylate synthetase  CS - citrate synthase  ETFA - electron-transfer-flavoprotein, alpha polypeptide  MAPK14 - mitogen-activated protein kinase 14  ANGPT1 - angiopoietin 1  ACAD8 - acyl-coa dehydrogenase family, member 8  MGLL - monoglyceride lipase  GATM - glycine amidinotransferase (l-arginine:glycine amidinotransferase)  HSD3B7 - hydroxy-delta-5-steroid dehydrogenase, 3 beta- and steroid delta-isomerase 7  AMT - aminomethyltransferase  NDUFAB1 - nadh dehydrogenase (ubiquinone) 1, alpha/beta subcomplex, 1, 8kda  UGP2 - udp-glucose pyrophosphorylase 2  UGDH - udp-glucose 6-dehydrogenase  PEMT - phosphatidylethanolamine n-methyltransferase  GPAM - glycerol-3-phosphate acyltransferase, mitochondrial  NNT - nicotinamide nucleotide transhydrogenase  HMGCL - 3-hydroxymethyl-3-methylglutaryl-coa lyase  BCAN - brevican  SARDH - sarcosine dehydrogenase  FOLR2 - folate receptor 2 (fetal)  GAPDH - glyceraldehyde-3-phosphate dehydrogenase  EPHX1 - epoxide hydrolase 1, microsomal (xenobiotic)  EPHX2 - epoxide hydrolase 2, cytoplasmic  IARS2 - isoleucyl-trna synthetase 2, mitochondrial  AFMID - arylformamidase  CBS - cystathionine-beta-synthase  SLC27A4 - solute carrier family 27 (fatty acid transporter), member 4  NCAN - neurocan  RBP1 - retinol binding protein 1, cellular  FPGS - folylpolyglutamate synthase  DCXR - dicarbonyl/l-xylulose reductase |
| GO:0072521 | purine-containing compound metabolic process | 1.11E-6 | 4.69E-4 | 1.62 (10334,316,1776,88) | [+] Show genes  XDH - xanthine dehydrogenase  UQCRC2 - ubiquinol-cytochrome c reductase core protein ii  UQCRB - ubiquinol-cytochrome c reductase binding protein  PGK1 - phosphoglycerate kinase 1  CROT - carnitine o-octanoyltransferase  ACOT8 - acyl-coa thioesterase 8  HSD17B4 - hydroxysteroid (17-beta) dehydrogenase 4  SUCLA2 - succinate-coa ligase, adp-forming, beta subunit  SUCLG2 - succinate-coa ligase, gdp-forming, beta subunit  NME3 - nme/nm23 nucleoside diphosphate kinase 3  RHOQ - ras homolog family member q  ATP5S - atp synthase, h+ transporting, mitochondrial fo complex, subunit s (factor b)  ABCG2 - atp-binding cassette, sub-family g (white), member 2  BPGM - 2,3-bisphosphoglycerate mutase  ADSL - adenylosuccinate lyase  CARD11 - caspase recruitment domain family, member 11  AASS - aminoadipate-semialdehyde synthase  DNPH1 - 2'-deoxynucleoside 5'-phosphate n-hydrolase 1  MOCOS - molybdenum cofactor sulfurase  AOX1 - aldehyde oxidase 1  CECR1 - cat eye syndrome chromosome region, candidate 1  NUDT1 - nudix (nucleoside diphosphate linked moiety x)-type motif 1  DPYD - dihydropyrimidine dehydrogenase  COX7A1 - cytochrome c oxidase subunit viia polypeptide 1 (muscle)  EPHA2 - eph receptor a2  AK1 - adenylate kinase 1  ACAT1 - acetyl-coa acetyltransferase 1  ATP5J - atp synthase, h+ transporting, mitochondrial fo complex, subunit f6  PMVK - phosphomevalonate kinase  ENTPD5 - ectonucleoside triphosphate diphosphohydrolase 5  GMPS - guanine monphosphate synthase  PPCDC - phosphopantothenoylcysteine decarboxylase  ATP5C1 - atp synthase, h+ transporting, mitochondrial f1 complex, gamma polypeptide 1  OGDH - oxoglutarate (alpha-ketoglutarate) dehydrogenase (lipoamide)  ATP5F1 - atp synthase, h+ transporting, mitochondrial fo complex, subunit b1  COASY - coa synthase  SULT1E1 - sulfotransferase family 1e, estrogen-preferring, member 1  ATP5G3 - atp synthase, h+ transporting, mitochondrial fo complex, subunit c3 (subunit 9)  APRT - adenine phosphoribosyltransferase  NT5C - 5', 3'-nucleotidase, cytosolic  CYC1 - cytochrome c-1  ATP5A1 - atp synthase, h+ transporting, mitochondrial f1 complex, alpha subunit 1, cardiac muscle  ATP5B - atp synthase, h+ transporting, mitochondrial f1 complex, beta polypeptide  ATP5L - atp synthase, h+ transporting, mitochondrial fo complex, subunit g  ABCD1 - atp-binding cassette, sub-family d (ald), member 1  DLAT - dihydrolipoamide s-acetyltransferase  DLD - dihydrolipoamide dehydrogenase  GALK1 - galactokinase 1  PDE8A - phosphodiesterase 8a  PDE7A - phosphodiesterase 7a  ACSS1 - acyl-coa synthetase short-chain family member 1  GNMT - glycine n-methyltransferase  TPI1 - triosephosphate isomerase 1  PDHX - pyruvate dehydrogenase complex, component x  PDHB - pyruvate dehydrogenase (lipoamide) beta  TEFM - transcription elongation factor, mitochondrial  DLST - dihydrolipoamide s-succinyltransferase (e2 component of 2-oxo-glutarate complex)  PDHA1 - pyruvate dehydrogenase (lipoamide) alpha 1  ALDOA - aldolase a, fructose-bisphosphate  MFN1 - mitofusin 1  SHMT2 - serine hydroxymethyltransferase 2 (mitochondrial)  TECR - trans-2,3-enoyl-coa reductase  SULT1C4 - sulfotransferase family, cytosolic, 1c, member 4  ABHD14B - abhydrolase domain containing 14b  ADCY2 - adenylate cyclase 2 (brain)  GBAS - glioblastoma amplified sequence  NMNAT1 - nicotinamide nucleotide adenylyltransferase 1  RORA - rar-related orphan receptor a  ADCY9 - adenylate cyclase 9  ADK - adenosine kinase  NUDT3 - nudix (nucleoside diphosphate linked moiety x)-type motif 3  NPPC - natriuretic peptide c  MVD - mevalonate (diphospho) decarboxylase  PEMT - phosphatidylethanolamine n-methyltransferase  AMPD3 - adenosine monophosphate deaminase 3  GPAM - glycerol-3-phosphate acyltransferase, mitochondrial  PDE1A - phosphodiesterase 1a, calmodulin-dependent  SLC25A1 - solute carrier family 25 (mitochondrial carrier; citrate transporter), member 1  HMGCL - 3-hydroxymethyl-3-methylglutaryl-coa lyase  GAPDH - glyceraldehyde-3-phosphate dehydrogenase  GMPR - guanosine monophosphate reductase  CLPX - clpx caseinolytic peptidase x homolog (e. coli)  ACSF3 - acyl-coa synthetase family member 3  MPP1 - membrane protein, palmitoylated 1, 55kda  PDE4D - phosphodiesterase 4d, camp-specific  NDUFS1 - nadh dehydrogenase (ubiquinone) fe-s protein 1, 75kda (nadh-coenzyme q reductase)  PDE4A - phosphodiesterase 4a, camp-specific  PDE4B - phosphodiesterase 4b, camp-specific |
| GO:0002181 | cytoplasmic translation | 1.12E-6 | 4.58E-4 | 2.98 (10334,39,1776,20) | [+] Show genes  DRG2 - developmentally regulated gtp binding protein 2  RPL24 - ribosomal protein l24  RPSA - ribosomal protein sa  RPS21 - ribosomal protein s21  EIF4B - eukaryotic translation initiation factor 4b  RPS26 - ribosomal protein s26  RPL19 - ribosomal protein l19  RPL18A - ribosomal protein l18a  RPL18 - ribosomal protein l18  RPL17 - ribosomal protein l17  RPL36A - ribosomal protein l36a  RPL10A - ribosomal protein l10a  RPL38 - ribosomal protein l38  RWDD1 - rwd domain containing 1  RPL29 - ribosomal protein l29  RPS28 - ribosomal protein s28  RPL31 - ribosomal protein l31  RPS29 - ribosomal protein s29  RPL36 - ribosomal protein l36  RPL30 - ribosomal protein l30 |
| GO:0006101 | citrate metabolic process | 1.19E-6 | 4.73E-4 | 3.17 (10334,33,1776,18) | [+] Show genes  DLAT - dihydrolipoamide s-acetyltransferase  DLD - dihydrolipoamide dehydrogenase  ACO1 - aconitase 1, soluble  SDHD - succinate dehydrogenase complex, subunit d, integral membrane protein  PDHB - pyruvate dehydrogenase (lipoamide) beta  SUCLA2 - succinate-coa ligase, adp-forming, beta subunit  SDHC - succinate dehydrogenase complex, subunit c, integral membrane protein, 15kda  MDH2 - malate dehydrogenase 2, nad (mitochondrial)  PDHA1 - pyruvate dehydrogenase (lipoamide) alpha 1  DLST - dihydrolipoamide s-succinyltransferase (e2 component of 2-oxo-glutarate complex)  SUCLG2 - succinate-coa ligase, gdp-forming, beta subunit  ACO2 - aconitase 2, mitochondrial  SDHA - succinate dehydrogenase complex, subunit a, flavoprotein (fp)  NNT - nicotinamide nucleotide transhydrogenase  OGDH - oxoglutarate (alpha-ketoglutarate) dehydrogenase (lipoamide)  IDH3B - isocitrate dehydrogenase 3 (nad+) beta  CS - citrate synthase  IDH2 - isocitrate dehydrogenase 2 (nadp+), mitochondrial |
| GO:0006402 | mRNA catabolic process | 1.28E-6 | 4.96E-4 | 1.81 (10334,193,1776,60) | [+] Show genes  RPL24 - ribosomal protein l24  EXOSC5 - exosome component 5  RPL23A - ribosomal protein l23a  RPL21 - ribosomal protein l21  RPL19 - ribosomal protein l19  RPL18A - ribosomal protein l18a  RPL18 - ribosomal protein l18  RPL17 - ribosomal protein l17  RPL13 - ribosomal protein l13  RPL12 - ribosomal protein l12  HBS1L - hbs1-like (s. cerevisiae)  XRN1 - 5'-3' exoribonuclease 1  RPL10 - ribosomal protein l10  RPL7A - ribosomal protein l7a  AGO4 - argonaute risc catalytic component 4  RPL5 - ribosomal protein l5  RPL4 - ribosomal protein l4  RPL35 - ribosomal protein l35  RPS5 - ribosomal protein s5  RPS2 - ribosomal protein s2  RPLP2 - ribosomal protein, large, p2  RPL36A - ribosomal protein l36a  RPL37A - ribosomal protein l37a  RPL38 - ribosomal protein l38  RPL34 - ribosomal protein l34  RPL37 - ribosomal protein l37  RPL29 - ribosomal protein l29  EIF4G1 - eukaryotic translation initiation factor 4 gamma, 1  RPL31 - ribosomal protein l31  RPL36 - ribosomal protein l36  RPL27 - ribosomal protein l27  RPL30 - ribosomal protein l30  RPL27A - ribosomal protein l27a  RPL28 - ribosomal protein l28  RPS24 - ribosomal protein s24  RPS21 - ribosomal protein s21  RPS27 - ribosomal protein s27  RPS26 - ribosomal protein s26  RPS25 - ribosomal protein s25  RPS17 - ribosomal protein s17  RPS16 - ribosomal protein s16  RPS15A - ribosomal protein s15a  RPS20 - ribosomal protein s20  RPS19 - ribosomal protein s19  RPS18 - ribosomal protein s18  RPS11 - ribosomal protein s11  RPS8 - ribosomal protein s8  SAMD4A - sterile alpha motif domain containing 4a  RPS9 - ribosomal protein s9  RPS14 - ribosomal protein s14  RPS13 - ribosomal protein s13  CSDE1 - cold shock domain containing e1, rna-binding  RPS7 - ribosomal protein s7  RPSA - ribosomal protein sa  SMG5 - smg5 nonsense mediated mrna decay factor  LSM4 - lsm4 homolog, u6 small nuclear rna associated (s. cerevisiae)  RPL10A - ribosomal protein l10a  RPL23 - ribosomal protein l23  RPS28 - ribosomal protein s28  RPS29 - ribosomal protein s29 |
| GO:0006120 | mitochondrial electron transport, NADH to ubiquinone | 1.52E-6 | 5.7E-4 | 2.78 (10334,46,1776,22) | [+] Show genes  NDUFB11 - nadh dehydrogenase (ubiquinone) 1 beta subcomplex, 11, 17.3kda  DLD - dihydrolipoamide dehydrogenase  NDUFC1 - nadh dehydrogenase (ubiquinone) 1, subcomplex unknown, 1, 6kda  NDUFB8 - nadh dehydrogenase (ubiquinone) 1 beta subcomplex, 8, 19kda  NDUFB9 - nadh dehydrogenase (ubiquinone) 1 beta subcomplex, 9, 22kda  NDUFB6 - nadh dehydrogenase (ubiquinone) 1 beta subcomplex, 6, 17kda  NDUFB4 - nadh dehydrogenase (ubiquinone) 1 beta subcomplex, 4, 15kda  NDUFB5 - nadh dehydrogenase (ubiquinone) 1 beta subcomplex, 5, 16kda  NDUFB3 - nadh dehydrogenase (ubiquinone) 1 beta subcomplex, 3, 12kda  NDUFA1 - nadh dehydrogenase (ubiquinone) 1 alpha subcomplex, 1, 7.5kda  NDUFAB1 - nadh dehydrogenase (ubiquinone) 1, alpha/beta subcomplex, 1, 8kda  NDUFB1 - nadh dehydrogenase (ubiquinone) 1 beta subcomplex, 1, 7kda  NDUFA9 - nadh dehydrogenase (ubiquinone) 1 alpha subcomplex, 9, 39kda  NDUFA12 - nadh dehydrogenase (ubiquinone) 1 alpha subcomplex, 12  NDUFA10 - nadh dehydrogenase (ubiquinone) 1 alpha subcomplex, 10, 42kda  NDUFA5 - nadh dehydrogenase (ubiquinone) 1 alpha subcomplex, 5  NDUFV2 - nadh dehydrogenase (ubiquinone) flavoprotein 2, 24kda  NDUFA11 - nadh dehydrogenase (ubiquinone) 1 alpha subcomplex, 11, 14.7kda  NDUFS4 - nadh dehydrogenase (ubiquinone) fe-s protein 4, 18kda (nadh-coenzyme q reductase)  NDUFS3 - nadh dehydrogenase (ubiquinone) fe-s protein 3, 30kda (nadh-coenzyme q reductase)  NDUFS2 - nadh dehydrogenase (ubiquinone) fe-s protein 2, 49kda (nadh-coenzyme q reductase)  NDUFS1 - nadh dehydrogenase (ubiquinone) fe-s protein 1, 75kda (nadh-coenzyme q reductase) |
| GO:0010257 | NADH dehydrogenase complex assembly | 1.85E-6 | 6.78E-4 | 2.52 (10334,60,1776,26) | [+] Show genes  NDUFB11 - nadh dehydrogenase (ubiquinone) 1 beta subcomplex, 11, 17.3kda  NDUFAF6 - nadh dehydrogenase (ubiquinone) complex i, assembly factor 6  NDUFA1 - nadh dehydrogenase (ubiquinone) 1 alpha subcomplex, 1, 7.5kda  NDUFA12 - nadh dehydrogenase (ubiquinone) 1 alpha subcomplex, 12  NDUFA5 - nadh dehydrogenase (ubiquinone) 1 alpha subcomplex, 5  ACAD9 - acyl-coa dehydrogenase family, member 9  OXA1L - oxidase (cytochrome c) assembly 1-like  NDUFC1 - nadh dehydrogenase (ubiquinone) 1, subcomplex unknown, 1, 6kda  TMEM126A - transmembrane protein 126a  NDUFB8 - nadh dehydrogenase (ubiquinone) 1 beta subcomplex, 8, 19kda  NDUFAF3 - nadh dehydrogenase (ubiquinone) complex i, assembly factor 3  NDUFB9 - nadh dehydrogenase (ubiquinone) 1 beta subcomplex, 9, 22kda  NDUFB6 - nadh dehydrogenase (ubiquinone) 1 beta subcomplex, 6, 17kda  NDUFB4 - nadh dehydrogenase (ubiquinone) 1 beta subcomplex, 4, 15kda  NDUFB5 - nadh dehydrogenase (ubiquinone) 1 beta subcomplex, 5, 16kda  NDUFB3 - nadh dehydrogenase (ubiquinone) 1 beta subcomplex, 3, 12kda  NDUFAB1 - nadh dehydrogenase (ubiquinone) 1, alpha/beta subcomplex, 1, 8kda  NDUFB1 - nadh dehydrogenase (ubiquinone) 1 beta subcomplex, 1, 7kda  NDUFA9 - nadh dehydrogenase (ubiquinone) 1 alpha subcomplex, 9, 39kda  NDUFA10 - nadh dehydrogenase (ubiquinone) 1 alpha subcomplex, 10, 42kda  NDUFV2 - nadh dehydrogenase (ubiquinone) flavoprotein 2, 24kda  NDUFA11 - nadh dehydrogenase (ubiquinone) 1 alpha subcomplex, 11, 14.7kda  NDUFS4 - nadh dehydrogenase (ubiquinone) fe-s protein 4, 18kda (nadh-coenzyme q reductase)  NDUFS3 - nadh dehydrogenase (ubiquinone) fe-s protein 3, 30kda (nadh-coenzyme q reductase)  NDUFS2 - nadh dehydrogenase (ubiquinone) fe-s protein 2, 49kda (nadh-coenzyme q reductase)  NDUFS1 - nadh dehydrogenase (ubiquinone) fe-s protein 1, 75kda (nadh-coenzyme q reductase) |
| GO:0032981 | mitochondrial respiratory chain complex I assembly | 1.85E-6 | 6.6E-4 | 2.52 (10334,60,1776,26) | [+] Show genes  NDUFB11 - nadh dehydrogenase (ubiquinone) 1 beta subcomplex, 11, 17.3kda  NDUFAF6 - nadh dehydrogenase (ubiquinone) complex i, assembly factor 6  NDUFA1 - nadh dehydrogenase (ubiquinone) 1 alpha subcomplex, 1, 7.5kda  NDUFA12 - nadh dehydrogenase (ubiquinone) 1 alpha subcomplex, 12  NDUFA5 - nadh dehydrogenase (ubiquinone) 1 alpha subcomplex, 5  ACAD9 - acyl-coa dehydrogenase family, member 9  OXA1L - oxidase (cytochrome c) assembly 1-like  NDUFC1 - nadh dehydrogenase (ubiquinone) 1, subcomplex unknown, 1, 6kda  TMEM126A - transmembrane protein 126a  NDUFB8 - nadh dehydrogenase (ubiquinone) 1 beta subcomplex, 8, 19kda  NDUFAF3 - nadh dehydrogenase (ubiquinone) complex i, assembly factor 3  NDUFB9 - nadh dehydrogenase (ubiquinone) 1 beta subcomplex, 9, 22kda  NDUFB6 - nadh dehydrogenase (ubiquinone) 1 beta subcomplex, 6, 17kda  NDUFB4 - nadh dehydrogenase (ubiquinone) 1 beta subcomplex, 4, 15kda  NDUFB5 - nadh dehydrogenase (ubiquinone) 1 beta subcomplex, 5, 16kda  NDUFB3 - nadh dehydrogenase (ubiquinone) 1 beta subcomplex, 3, 12kda  NDUFAB1 - nadh dehydrogenase (ubiquinone) 1, alpha/beta subcomplex, 1, 8kda  NDUFB1 - nadh dehydrogenase (ubiquinone) 1 beta subcomplex, 1, 7kda  NDUFA9 - nadh dehydrogenase (ubiquinone) 1 alpha subcomplex, 9, 39kda  NDUFA10 - nadh dehydrogenase (ubiquinone) 1 alpha subcomplex, 10, 42kda  NDUFV2 - nadh dehydrogenase (ubiquinone) flavoprotein 2, 24kda  NDUFA11 - nadh dehydrogenase (ubiquinone) 1 alpha subcomplex, 11, 14.7kda  NDUFS4 - nadh dehydrogenase (ubiquinone) fe-s protein 4, 18kda (nadh-coenzyme q reductase)  NDUFS3 - nadh dehydrogenase (ubiquinone) fe-s protein 3, 30kda (nadh-coenzyme q reductase)  NDUFS2 - nadh dehydrogenase (ubiquinone) fe-s protein 2, 49kda (nadh-coenzyme q reductase)  NDUFS1 - nadh dehydrogenase (ubiquinone) fe-s protein 1, 75kda (nadh-coenzyme q reductase) |
| GO:0034655 | nucleobase-containing compound catabolic process | 2.33E-6 | 8.12E-4 | 1.58 (10334,335,1776,91) | [+] Show genes  XDH - xanthine dehydrogenase  RPL24 - ribosomal protein l24  PGK1 - phosphoglycerate kinase 1  EXOSC5 - exosome component 5  RPL23A - ribosomal protein l23a  RPL21 - ribosomal protein l21  RPL19 - ribosomal protein l19  RPL18A - ribosomal protein l18a  RPL18 - ribosomal protein l18  RPL17 - ribosomal protein l17  RPL13 - ribosomal protein l13  RPL12 - ribosomal protein l12  BPGM - 2,3-bisphosphoglycerate mutase  HBS1L - hbs1-like (s. cerevisiae)  XRN1 - 5'-3' exoribonuclease 1  RPL10 - ribosomal protein l10  DNPH1 - 2'-deoxynucleoside 5'-phosphate n-hydrolase 1  RPL7A - ribosomal protein l7a  AGO4 - argonaute risc catalytic component 4  RPL5 - ribosomal protein l5  RPL4 - ribosomal protein l4  CECR1 - cat eye syndrome chromosome region, candidate 1  NUDT1 - nudix (nucleoside diphosphate linked moiety x)-type motif 1  DPYD - dihydropyrimidine dehydrogenase  RPL35 - ribosomal protein l35  RPS5 - ribosomal protein s5  ACAT1 - acetyl-coa acetyltransferase 1  DUT - deoxyuridine triphosphatase  RPS2 - ribosomal protein s2  RPLP2 - ribosomal protein, large, p2  RPL36A - ribosomal protein l36a  ENTPD5 - ectonucleoside triphosphate diphosphohydrolase 5  RPL37A - ribosomal protein l37a  OGDH - oxoglutarate (alpha-ketoglutarate) dehydrogenase (lipoamide)  RPL38 - ribosomal protein l38  RPL34 - ribosomal protein l34  RPL37 - ribosomal protein l37  RPL29 - ribosomal protein l29  EIF4G1 - eukaryotic translation initiation factor 4 gamma, 1  RPL31 - ribosomal protein l31  NT5C - 5', 3'-nucleotidase, cytosolic  RPL36 - ribosomal protein l36  RPL27 - ribosomal protein l27  RPL30 - ribosomal protein l30  RPL27A - ribosomal protein l27a  ABCD1 - atp-binding cassette, sub-family d (ald), member 1  RPL28 - ribosomal protein l28  RPS24 - ribosomal protein s24  RPS21 - ribosomal protein s21  GALK1 - galactokinase 1  PDE8A - phosphodiesterase 8a  NUDT17 - nudix (nucleoside diphosphate linked moiety x)-type motif 17  PDE7A - phosphodiesterase 7a  RPS27 - ribosomal protein s27  TPI1 - triosephosphate isomerase 1  RPS26 - ribosomal protein s26  RPS25 - ribosomal protein s25  RPS17 - ribosomal protein s17  RPS16 - ribosomal protein s16  RPS15A - ribosomal protein s15a  H1F0 - h1 histone family, member 0  RPS20 - ribosomal protein s20  ALDOA - aldolase a, fructose-bisphosphate  RPS19 - ribosomal protein s19  NT5C3A - 5'-nucleotidase, cytosolic iiia  RPS18 - ribosomal protein s18  RPS11 - ribosomal protein s11  RPS8 - ribosomal protein s8  RPS9 - ribosomal protein s9  SAMD4A - sterile alpha motif domain containing 4a  RPS14 - ribosomal protein s14  RPS13 - ribosomal protein s13  CSDE1 - cold shock domain containing e1, rna-binding  RPS7 - ribosomal protein s7  DNASE1L3 - deoxyribonuclease i-like 3  RPSA - ribosomal protein sa  PABPC4 - poly(a) binding protein, cytoplasmic 4 (inducible form)  NUDT3 - nudix (nucleoside diphosphate linked moiety x)-type motif 3  SMG5 - smg5 nonsense mediated mrna decay factor  AMPD3 - adenosine monophosphate deaminase 3  LSM4 - lsm4 homolog, u6 small nuclear rna associated (s. cerevisiae)  RPL10A - ribosomal protein l10a  PDE1A - phosphodiesterase 1a, calmodulin-dependent  GAPDH - glyceraldehyde-3-phosphate dehydrogenase  RPL23 - ribosomal protein l23  RPS28 - ribosomal protein s28  PDE4D - phosphodiesterase 4d, camp-specific  RPS29 - ribosomal protein s29  PDE4A - phosphodiesterase 4a, camp-specific  DNASE1L1 - deoxyribonuclease i-like 1  PDE4B - phosphodiesterase 4b, camp-specific |
| GO:0006605 | protein targeting | 3.03E-6 | 1.03E-3 | 1.62 (10334,291,1776,81) | [+] Show genes  RPL24 - ribosomal protein l24  RHOD - ras homolog family member d  RPL23A - ribosomal protein l23a  CROT - carnitine o-octanoyltransferase  ACOT8 - acyl-coa thioesterase 8  TOMM70A - translocase of outer mitochondrial membrane 70 homolog a (s. cerevisiae)  HSD17B4 - hydroxysteroid (17-beta) dehydrogenase 4  RPL21 - ribosomal protein l21  RPL19 - ribosomal protein l19  RPL18A - ribosomal protein l18a  RPL18 - ribosomal protein l18  TRAK1 - trafficking protein, kinesin binding 1  RPL17 - ribosomal protein l17  AP4E1 - adaptor-related protein complex 4, epsilon 1 subunit  RPL13 - ribosomal protein l13  RPL12 - ribosomal protein l12  TIMM23 - translocase of inner mitochondrial membrane 23 homolog (yeast)  RPL10 - ribosomal protein l10  RPL7A - ribosomal protein l7a  TIMM44 - translocase of inner mitochondrial membrane 44 homolog (yeast)  HOMER3 - homer homolog 3 (drosophila)  RPL5 - ribosomal protein l5  PEX19 - peroxisomal biogenesis factor 19  PMM2 - phosphomannomutase 2  RPL4 - ribosomal protein l4  ZFAND6 - zinc finger, an1-type domain 6  RPL35 - ribosomal protein l35  PEX10 - peroxisomal biogenesis factor 10  RPS5 - ribosomal protein s5  RPS2 - ribosomal protein s2  RPLP2 - ribosomal protein, large, p2  SYNJ2BP - synaptojanin 2 binding protein  RPL36A - ribosomal protein l36a  BID - bh3 interacting domain death agonist  RPL37A - ribosomal protein l37a  NCF1 - neutrophil cytosolic factor 1  RPL38 - ribosomal protein l38  RPL34 - ribosomal protein l34  RPL37 - ribosomal protein l37  RPL29 - ribosomal protein l29  RPL31 - ribosomal protein l31  DDO - d-aspartate oxidase  CRAT - carnitine o-acetyltransferase  RPL36 - ribosomal protein l36  RPL27 - ribosomal protein l27  ZDHHC18 - zinc finger, dhhc-type containing 18  RPL30 - ribosomal protein l30  RPL27A - ribosomal protein l27a  PECR - peroxisomal trans-2-enoyl-coa reductase  RPL28 - ribosomal protein l28  RPS24 - ribosomal protein s24  MFN2 - mitofusin 2  RPS21 - ribosomal protein s21  RPS27 - ribosomal protein s27  RPS26 - ribosomal protein s26  RPS25 - ribosomal protein s25  UBE2D1 - ubiquitin-conjugating enzyme e2d 1  RPS17 - ribosomal protein s17  RPS16 - ribosomal protein s16  RPS15A - ribosomal protein s15a  RPS20 - ribosomal protein s20  RPS19 - ribosomal protein s19  RPS18 - ribosomal protein s18  RPS11 - ribosomal protein s11  RPS8 - ribosomal protein s8  RPS9 - ribosomal protein s9  RPS14 - ribosomal protein s14  RPS13 - ribosomal protein s13  RPS7 - ribosomal protein s7  RPSA - ribosomal protein sa  AKAP6 - a kinase (prka) anchor protein 6  SORT1 - sortilin 1  DAO - d-amino-acid oxidase  RPL10A - ribosomal protein l10a  PARD3 - par-3 partitioning defective 3 homolog (c. elegans)  HMGCL - 3-hydroxymethyl-3-methylglutaryl-coa lyase  RPL23 - ribosomal protein l23  EPHX2 - epoxide hydrolase 2, cytoplasmic  ADORA1 - adenosine a1 receptor  RPS28 - ribosomal protein s28  RPS29 - ribosomal protein s29 |
| GO:0006163 | purine nucleotide metabolic process | 4.04E-6 | 1.34E-3 | 1.61 (10334,293,1776,81) | [+] Show genes  XDH - xanthine dehydrogenase  UQCRC2 - ubiquinol-cytochrome c reductase core protein ii  UQCRB - ubiquinol-cytochrome c reductase binding protein  PGK1 - phosphoglycerate kinase 1  CROT - carnitine o-octanoyltransferase  ACOT8 - acyl-coa thioesterase 8  HSD17B4 - hydroxysteroid (17-beta) dehydrogenase 4  SUCLA2 - succinate-coa ligase, adp-forming, beta subunit  SUCLG2 - succinate-coa ligase, gdp-forming, beta subunit  RHOQ - ras homolog family member q  NME3 - nme/nm23 nucleoside diphosphate kinase 3  ATP5S - atp synthase, h+ transporting, mitochondrial fo complex, subunit s (factor b)  BPGM - 2,3-bisphosphoglycerate mutase  ADSL - adenylosuccinate lyase  CARD11 - caspase recruitment domain family, member 11  AASS - aminoadipate-semialdehyde synthase  DNPH1 - 2'-deoxynucleoside 5'-phosphate n-hydrolase 1  MOCOS - molybdenum cofactor sulfurase  NUDT1 - nudix (nucleoside diphosphate linked moiety x)-type motif 1  COX7A1 - cytochrome c oxidase subunit viia polypeptide 1 (muscle)  EPHA2 - eph receptor a2  AK1 - adenylate kinase 1  ACAT1 - acetyl-coa acetyltransferase 1  ATP5J - atp synthase, h+ transporting, mitochondrial fo complex, subunit f6  PMVK - phosphomevalonate kinase  GMPS - guanine monphosphate synthase  ENTPD5 - ectonucleoside triphosphate diphosphohydrolase 5  PPCDC - phosphopantothenoylcysteine decarboxylase  ATP5C1 - atp synthase, h+ transporting, mitochondrial f1 complex, gamma polypeptide 1  OGDH - oxoglutarate (alpha-ketoglutarate) dehydrogenase (lipoamide)  ATP5F1 - atp synthase, h+ transporting, mitochondrial fo complex, subunit b1  COASY - coa synthase  SULT1E1 - sulfotransferase family 1e, estrogen-preferring, member 1  ATP5G3 - atp synthase, h+ transporting, mitochondrial fo complex, subunit c3 (subunit 9)  APRT - adenine phosphoribosyltransferase  NT5C - 5', 3'-nucleotidase, cytosolic  CYC1 - cytochrome c-1  ATP5A1 - atp synthase, h+ transporting, mitochondrial f1 complex, alpha subunit 1, cardiac muscle  ATP5B - atp synthase, h+ transporting, mitochondrial f1 complex, beta polypeptide  ATP5L - atp synthase, h+ transporting, mitochondrial fo complex, subunit g  ABCD1 - atp-binding cassette, sub-family d (ald), member 1  DLAT - dihydrolipoamide s-acetyltransferase  DLD - dihydrolipoamide dehydrogenase  GALK1 - galactokinase 1  PDE8A - phosphodiesterase 8a  PDE7A - phosphodiesterase 7a  ACSS1 - acyl-coa synthetase short-chain family member 1  TPI1 - triosephosphate isomerase 1  PDHX - pyruvate dehydrogenase complex, component x  PDHB - pyruvate dehydrogenase (lipoamide) beta  TEFM - transcription elongation factor, mitochondrial  DLST - dihydrolipoamide s-succinyltransferase (e2 component of 2-oxo-glutarate complex)  PDHA1 - pyruvate dehydrogenase (lipoamide) alpha 1  ALDOA - aldolase a, fructose-bisphosphate  MFN1 - mitofusin 1  TECR - trans-2,3-enoyl-coa reductase  SULT1C4 - sulfotransferase family, cytosolic, 1c, member 4  ABHD14B - abhydrolase domain containing 14b  ADCY2 - adenylate cyclase 2 (brain)  GBAS - glioblastoma amplified sequence  NMNAT1 - nicotinamide nucleotide adenylyltransferase 1  RORA - rar-related orphan receptor a  ADCY9 - adenylate cyclase 9  ADK - adenosine kinase  NUDT3 - nudix (nucleoside diphosphate linked moiety x)-type motif 3  NPPC - natriuretic peptide c  MVD - mevalonate (diphospho) decarboxylase  AMPD3 - adenosine monophosphate deaminase 3  GPAM - glycerol-3-phosphate acyltransferase, mitochondrial  PDE1A - phosphodiesterase 1a, calmodulin-dependent  SLC25A1 - solute carrier family 25 (mitochondrial carrier; citrate transporter), member 1  HMGCL - 3-hydroxymethyl-3-methylglutaryl-coa lyase  GAPDH - glyceraldehyde-3-phosphate dehydrogenase  CLPX - clpx caseinolytic peptidase x homolog (e. coli)  GMPR - guanosine monophosphate reductase  ACSF3 - acyl-coa synthetase family member 3  MPP1 - membrane protein, palmitoylated 1, 55kda  PDE4D - phosphodiesterase 4d, camp-specific  NDUFS1 - nadh dehydrogenase (ubiquinone) fe-s protein 1, 75kda (nadh-coenzyme q reductase)  PDE4A - phosphodiesterase 4a, camp-specific  PDE4B - phosphodiesterase 4b, camp-specific |
| GO:0046700 | heterocycle catabolic process | 4.35E-6 | 1.41E-3 | 1.54 (10334,363,1776,96) | [+] Show genes  XDH - xanthine dehydrogenase  RPL24 - ribosomal protein l24  PGK1 - phosphoglycerate kinase 1  EXOSC5 - exosome component 5  RPL23A - ribosomal protein l23a  RPL21 - ribosomal protein l21  RPL19 - ribosomal protein l19  RPL18A - ribosomal protein l18a  RPL18 - ribosomal protein l18  RPL17 - ribosomal protein l17  RPL13 - ribosomal protein l13  UROC1 - urocanate hydratase 1  RPL12 - ribosomal protein l12  BPGM - 2,3-bisphosphoglycerate mutase  HBS1L - hbs1-like (s. cerevisiae)  XRN1 - 5'-3' exoribonuclease 1  RPL10 - ribosomal protein l10  DNPH1 - 2'-deoxynucleoside 5'-phosphate n-hydrolase 1  RPL7A - ribosomal protein l7a  AOX1 - aldehyde oxidase 1  AGO4 - argonaute risc catalytic component 4  RPL5 - ribosomal protein l5  RPL4 - ribosomal protein l4  CECR1 - cat eye syndrome chromosome region, candidate 1  NUDT1 - nudix (nucleoside diphosphate linked moiety x)-type motif 1  DPYD - dihydropyrimidine dehydrogenase  RPL35 - ribosomal protein l35  RPS5 - ribosomal protein s5  ACAT1 - acetyl-coa acetyltransferase 1  DUT - deoxyuridine triphosphatase  RPS2 - ribosomal protein s2  RPLP2 - ribosomal protein, large, p2  QPRT - quinolinate phosphoribosyltransferase  RPL36A - ribosomal protein l36a  ENTPD5 - ectonucleoside triphosphate diphosphohydrolase 5  RPL37A - ribosomal protein l37a  OGDH - oxoglutarate (alpha-ketoglutarate) dehydrogenase (lipoamide)  RPL38 - ribosomal protein l38  RPL34 - ribosomal protein l34  RPL37 - ribosomal protein l37  RPL29 - ribosomal protein l29  EIF4G1 - eukaryotic translation initiation factor 4 gamma, 1  RPL31 - ribosomal protein l31  NT5C - 5', 3'-nucleotidase, cytosolic  RPL36 - ribosomal protein l36  RPL27 - ribosomal protein l27  RPL30 - ribosomal protein l30  RPL27A - ribosomal protein l27a  ABCD1 - atp-binding cassette, sub-family d (ald), member 1  RPL28 - ribosomal protein l28  RPS24 - ribosomal protein s24  RPS21 - ribosomal protein s21  GALK1 - galactokinase 1  PDE8A - phosphodiesterase 8a  NUDT17 - nudix (nucleoside diphosphate linked moiety x)-type motif 17  PDE7A - phosphodiesterase 7a  RPS27 - ribosomal protein s27  TPI1 - triosephosphate isomerase 1  RPS26 - ribosomal protein s26  RPS25 - ribosomal protein s25  RPS17 - ribosomal protein s17  RPS16 - ribosomal protein s16  RPS15A - ribosomal protein s15a  H1F0 - h1 histone family, member 0  RPS20 - ribosomal protein s20  ALDOA - aldolase a, fructose-bisphosphate  RPS19 - ribosomal protein s19  RPS18 - ribosomal protein s18  NT5C3A - 5'-nucleotidase, cytosolic iiia  RPS11 - ribosomal protein s11  RPS8 - ribosomal protein s8  RPS9 - ribosomal protein s9  SAMD4A - sterile alpha motif domain containing 4a  RPS14 - ribosomal protein s14  RPS13 - ribosomal protein s13  CSDE1 - cold shock domain containing e1, rna-binding  RPS7 - ribosomal protein s7  DNASE1L3 - deoxyribonuclease i-like 3  RPSA - ribosomal protein sa  PABPC4 - poly(a) binding protein, cytoplasmic 4 (inducible form)  NUDT3 - nudix (nucleoside diphosphate linked moiety x)-type motif 3  SMG5 - smg5 nonsense mediated mrna decay factor  DAO - d-amino-acid oxidase  AMPD3 - adenosine monophosphate deaminase 3  LSM4 - lsm4 homolog, u6 small nuclear rna associated (s. cerevisiae)  RPL10A - ribosomal protein l10a  PDE1A - phosphodiesterase 1a, calmodulin-dependent  GAPDH - glyceraldehyde-3-phosphate dehydrogenase  RPL23 - ribosomal protein l23  RPS28 - ribosomal protein s28  PDE4D - phosphodiesterase 4d, camp-specific  RPS29 - ribosomal protein s29  AFMID - arylformamidase  PDE4A - phosphodiesterase 4a, camp-specific  DNASE1L1 - deoxyribonuclease i-like 1  PDE4B - phosphodiesterase 4b, camp-specific |
| GO:1901361 | organic cyclic compound catabolic process | 4.93E-6 | 1.56E-3 | 1.51 (10334,397,1776,103) | [+] Show genes  EXOSC5 - exosome component 5  BPGM - 2,3-bisphosphoglycerate mutase  XRN1 - 5'-3' exoribonuclease 1  DNPH1 - 2'-deoxynucleoside 5'-phosphate n-hydrolase 1  AOX1 - aldehyde oxidase 1  CECR1 - cat eye syndrome chromosome region, candidate 1  RPL35 - ribosomal protein l35  ACAT1 - acetyl-coa acetyltransferase 1  ENTPD5 - ectonucleoside triphosphate diphosphohydrolase 5  OGDH - oxoglutarate (alpha-ketoglutarate) dehydrogenase (lipoamide)  APOE - apolipoprotein e  EIF4G1 - eukaryotic translation initiation factor 4 gamma, 1  RPL36 - ribosomal protein l36  LRTOMT - leucine rich transmembrane and o-methyltransferase domain containing  NUDT17 - nudix (nucleoside diphosphate linked moiety x)-type motif 17  PDE8A - phosphodiesterase 8a  PDE7A - phosphodiesterase 7a  TPI1 - triosephosphate isomerase 1  H1F0 - h1 histone family, member 0  SAMD4A - sterile alpha motif domain containing 4a  CSDE1 - cold shock domain containing e1, rna-binding  RPSA - ribosomal protein sa  NUDT3 - nudix (nucleoside diphosphate linked moiety x)-type motif 3  SMG5 - smg5 nonsense mediated mrna decay factor  DAO - d-amino-acid oxidase  PDE1A - phosphodiesterase 1a, calmodulin-dependent  PDE4D - phosphodiesterase 4d, camp-specific  CYP46A1 - cytochrome p450, family 46, subfamily a, polypeptide 1  PDE4A - phosphodiesterase 4a, camp-specific  CYP27A1 - cytochrome p450, family 27, subfamily a, polypeptide 1  PDE4B - phosphodiesterase 4b, camp-specific  XDH - xanthine dehydrogenase  HSD17B11 - hydroxysteroid (17-beta) dehydrogenase 11  RPL24 - ribosomal protein l24  PGK1 - phosphoglycerate kinase 1  RPL23A - ribosomal protein l23a  RPL21 - ribosomal protein l21  RPL19 - ribosomal protein l19  RPL18A - ribosomal protein l18a  RPL18 - ribosomal protein l18  RPL17 - ribosomal protein l17  RPL13 - ribosomal protein l13  UROC1 - urocanate hydratase 1  RPL12 - ribosomal protein l12  HBS1L - hbs1-like (s. cerevisiae)  RPL10 - ribosomal protein l10  RPL7A - ribosomal protein l7a  AGO4 - argonaute risc catalytic component 4  RPL5 - ribosomal protein l5  RPL4 - ribosomal protein l4  NUDT1 - nudix (nucleoside diphosphate linked moiety x)-type motif 1  DPYD - dihydropyrimidine dehydrogenase  RPS5 - ribosomal protein s5  DUT - deoxyuridine triphosphatase  RPS2 - ribosomal protein s2  RPLP2 - ribosomal protein, large, p2  QPRT - quinolinate phosphoribosyltransferase  RPL36A - ribosomal protein l36a  RPL37A - ribosomal protein l37a  RPL38 - ribosomal protein l38  RPL34 - ribosomal protein l34  SULT1E1 - sulfotransferase family 1e, estrogen-preferring, member 1  RPL37 - ribosomal protein l37  RPL29 - ribosomal protein l29  RPL31 - ribosomal protein l31  NT5C - 5', 3'-nucleotidase, cytosolic  RPL27 - ribosomal protein l27  RPL30 - ribosomal protein l30  RPL27A - ribosomal protein l27a  ABCD1 - atp-binding cassette, sub-family d (ald), member 1  RPL28 - ribosomal protein l28  RPS24 - ribosomal protein s24  GALK1 - galactokinase 1  RPS21 - ribosomal protein s21  RPS27 - ribosomal protein s27  RPS26 - ribosomal protein s26  RPS25 - ribosomal protein s25  RPS17 - ribosomal protein s17  RPS16 - ribosomal protein s16  RPS15A - ribosomal protein s15a  ALDOA - aldolase a, fructose-bisphosphate  RPS20 - ribosomal protein s20  RPS19 - ribosomal protein s19  NT5C3A - 5'-nucleotidase, cytosolic iiia  RPS18 - ribosomal protein s18  RPS11 - ribosomal protein s11  RPS8 - ribosomal protein s8  RPS9 - ribosomal protein s9  RPS14 - ribosomal protein s14  RPS13 - ribosomal protein s13  RPS7 - ribosomal protein s7  DNASE1L3 - deoxyribonuclease i-like 3  PABPC4 - poly(a) binding protein, cytoplasmic 4 (inducible form)  AMPD3 - adenosine monophosphate deaminase 3  LSM4 - lsm4 homolog, u6 small nuclear rna associated (s. cerevisiae)  RPL10A - ribosomal protein l10a  GAPDH - glyceraldehyde-3-phosphate dehydrogenase  RPL23 - ribosomal protein l23  EPHX2 - epoxide hydrolase 2, cytoplasmic  RPS28 - ribosomal protein s28  RPS29 - ribosomal protein s29  AFMID - arylformamidase  DNASE1L1 - deoxyribonuclease i-like 1 |
| GO:0009150 | purine ribonucleotide metabolic process | 6.05E-6 | 1.87E-3 | 1.62 (10334,273,1776,76) | [+] Show genes  UQCRC2 - ubiquinol-cytochrome c reductase core protein ii  UQCRB - ubiquinol-cytochrome c reductase binding protein  PGK1 - phosphoglycerate kinase 1  CROT - carnitine o-octanoyltransferase  ACOT8 - acyl-coa thioesterase 8  HSD17B4 - hydroxysteroid (17-beta) dehydrogenase 4  SUCLA2 - succinate-coa ligase, adp-forming, beta subunit  SUCLG2 - succinate-coa ligase, gdp-forming, beta subunit  ATP5S - atp synthase, h+ transporting, mitochondrial fo complex, subunit s (factor b)  NME3 - nme/nm23 nucleoside diphosphate kinase 3  RHOQ - ras homolog family member q  ADSL - adenylosuccinate lyase  BPGM - 2,3-bisphosphoglycerate mutase  CARD11 - caspase recruitment domain family, member 11  AASS - aminoadipate-semialdehyde synthase  MOCOS - molybdenum cofactor sulfurase  COX7A1 - cytochrome c oxidase subunit viia polypeptide 1 (muscle)  EPHA2 - eph receptor a2  AK1 - adenylate kinase 1  ACAT1 - acetyl-coa acetyltransferase 1  ATP5J - atp synthase, h+ transporting, mitochondrial fo complex, subunit f6  PMVK - phosphomevalonate kinase  GMPS - guanine monphosphate synthase  ENTPD5 - ectonucleoside triphosphate diphosphohydrolase 5  PPCDC - phosphopantothenoylcysteine decarboxylase  ATP5C1 - atp synthase, h+ transporting, mitochondrial f1 complex, gamma polypeptide 1  OGDH - oxoglutarate (alpha-ketoglutarate) dehydrogenase (lipoamide)  ATP5F1 - atp synthase, h+ transporting, mitochondrial fo complex, subunit b1  SULT1E1 - sulfotransferase family 1e, estrogen-preferring, member 1  COASY - coa synthase  ATP5G3 - atp synthase, h+ transporting, mitochondrial fo complex, subunit c3 (subunit 9)  APRT - adenine phosphoribosyltransferase  CYC1 - cytochrome c-1  ATP5A1 - atp synthase, h+ transporting, mitochondrial f1 complex, alpha subunit 1, cardiac muscle  ATP5B - atp synthase, h+ transporting, mitochondrial f1 complex, beta polypeptide  ABCD1 - atp-binding cassette, sub-family d (ald), member 1  ATP5L - atp synthase, h+ transporting, mitochondrial fo complex, subunit g  DLAT - dihydrolipoamide s-acetyltransferase  DLD - dihydrolipoamide dehydrogenase  GALK1 - galactokinase 1  PDE8A - phosphodiesterase 8a  PDE7A - phosphodiesterase 7a  ACSS1 - acyl-coa synthetase short-chain family member 1  TPI1 - triosephosphate isomerase 1  PDHX - pyruvate dehydrogenase complex, component x  PDHB - pyruvate dehydrogenase (lipoamide) beta  TEFM - transcription elongation factor, mitochondrial  DLST - dihydrolipoamide s-succinyltransferase (e2 component of 2-oxo-glutarate complex)  PDHA1 - pyruvate dehydrogenase (lipoamide) alpha 1  ALDOA - aldolase a, fructose-bisphosphate  MFN1 - mitofusin 1  TECR - trans-2,3-enoyl-coa reductase  SULT1C4 - sulfotransferase family, cytosolic, 1c, member 4  ABHD14B - abhydrolase domain containing 14b  ADCY2 - adenylate cyclase 2 (brain)  GBAS - glioblastoma amplified sequence  NMNAT1 - nicotinamide nucleotide adenylyltransferase 1  RORA - rar-related orphan receptor a  ADCY9 - adenylate cyclase 9  ADK - adenosine kinase  NUDT3 - nudix (nucleoside diphosphate linked moiety x)-type motif 3  NPPC - natriuretic peptide c  MVD - mevalonate (diphospho) decarboxylase  AMPD3 - adenosine monophosphate deaminase 3  GPAM - glycerol-3-phosphate acyltransferase, mitochondrial  PDE1A - phosphodiesterase 1a, calmodulin-dependent  SLC25A1 - solute carrier family 25 (mitochondrial carrier; citrate transporter), member 1  HMGCL - 3-hydroxymethyl-3-methylglutaryl-coa lyase  GAPDH - glyceraldehyde-3-phosphate dehydrogenase  CLPX - clpx caseinolytic peptidase x homolog (e. coli)  ACSF3 - acyl-coa synthetase family member 3  MPP1 - membrane protein, palmitoylated 1, 55kda  PDE4D - phosphodiesterase 4d, camp-specific  NDUFS1 - nadh dehydrogenase (ubiquinone) fe-s protein 1, 75kda (nadh-coenzyme q reductase)  PDE4A - phosphodiesterase 4a, camp-specific  PDE4B - phosphodiesterase 4b, camp-specific |
| GO:0072350 | tricarboxylic acid metabolic process | 6.16E-6 | 1.86E-3 | 2.91 (10334,36,1776,18) | [+] Show genes  DLAT - dihydrolipoamide s-acetyltransferase  DLD - dihydrolipoamide dehydrogenase  ACO1 - aconitase 1, soluble  PDHB - pyruvate dehydrogenase (lipoamide) beta  SDHD - succinate dehydrogenase complex, subunit d, integral membrane protein  SUCLA2 - succinate-coa ligase, adp-forming, beta subunit  SDHC - succinate dehydrogenase complex, subunit c, integral membrane protein, 15kda  MDH2 - malate dehydrogenase 2, nad (mitochondrial)  PDHA1 - pyruvate dehydrogenase (lipoamide) alpha 1  DLST - dihydrolipoamide s-succinyltransferase (e2 component of 2-oxo-glutarate complex)  SUCLG2 - succinate-coa ligase, gdp-forming, beta subunit  ACO2 - aconitase 2, mitochondrial  SDHA - succinate dehydrogenase complex, subunit a, flavoprotein (fp)  NNT - nicotinamide nucleotide transhydrogenase  OGDH - oxoglutarate (alpha-ketoglutarate) dehydrogenase (lipoamide)  IDH3B - isocitrate dehydrogenase 3 (nad+) beta  CS - citrate synthase  IDH2 - isocitrate dehydrogenase 2 (nadp+), mitochondrial |
| GO:0044270 | cellular nitrogen compound catabolic process | 6.34E-6 | 1.88E-3 | 1.53 (10334,366,1776,96) | [+] Show genes  XDH - xanthine dehydrogenase  RPL24 - ribosomal protein l24  PGK1 - phosphoglycerate kinase 1  EXOSC5 - exosome component 5  RPL23A - ribosomal protein l23a  RPL21 - ribosomal protein l21  RPL19 - ribosomal protein l19  RPL18A - ribosomal protein l18a  RPL18 - ribosomal protein l18  RPL17 - ribosomal protein l17  RPL13 - ribosomal protein l13  UROC1 - urocanate hydratase 1  RPL12 - ribosomal protein l12  BPGM - 2,3-bisphosphoglycerate mutase  HBS1L - hbs1-like (s. cerevisiae)  XRN1 - 5'-3' exoribonuclease 1  RPL10 - ribosomal protein l10  DNPH1 - 2'-deoxynucleoside 5'-phosphate n-hydrolase 1  RPL7A - ribosomal protein l7a  AOX1 - aldehyde oxidase 1  AGO4 - argonaute risc catalytic component 4  RPL5 - ribosomal protein l5  RPL4 - ribosomal protein l4  CECR1 - cat eye syndrome chromosome region, candidate 1  NUDT1 - nudix (nucleoside diphosphate linked moiety x)-type motif 1  DPYD - dihydropyrimidine dehydrogenase  RPL35 - ribosomal protein l35  RPS5 - ribosomal protein s5  ACAT1 - acetyl-coa acetyltransferase 1  DUT - deoxyuridine triphosphatase  RPS2 - ribosomal protein s2  RPLP2 - ribosomal protein, large, p2  QPRT - quinolinate phosphoribosyltransferase  RPL36A - ribosomal protein l36a  ENTPD5 - ectonucleoside triphosphate diphosphohydrolase 5  RPL37A - ribosomal protein l37a  OGDH - oxoglutarate (alpha-ketoglutarate) dehydrogenase (lipoamide)  RPL38 - ribosomal protein l38  RPL34 - ribosomal protein l34  RPL37 - ribosomal protein l37  RPL29 - ribosomal protein l29  EIF4G1 - eukaryotic translation initiation factor 4 gamma, 1  RPL31 - ribosomal protein l31  NT5C - 5', 3'-nucleotidase, cytosolic  RPL36 - ribosomal protein l36  RPL27 - ribosomal protein l27  RPL30 - ribosomal protein l30  RPL27A - ribosomal protein l27a  RPL28 - ribosomal protein l28  ABCD1 - atp-binding cassette, sub-family d (ald), member 1  RPS24 - ribosomal protein s24  GALK1 - galactokinase 1  RPS21 - ribosomal protein s21  PDE8A - phosphodiesterase 8a  NUDT17 - nudix (nucleoside diphosphate linked moiety x)-type motif 17  PDE7A - phosphodiesterase 7a  RPS27 - ribosomal protein s27  TPI1 - triosephosphate isomerase 1  RPS26 - ribosomal protein s26  RPS25 - ribosomal protein s25  RPS17 - ribosomal protein s17  RPS16 - ribosomal protein s16  RPS15A - ribosomal protein s15a  H1F0 - h1 histone family, member 0  ALDOA - aldolase a, fructose-bisphosphate  RPS20 - ribosomal protein s20  RPS19 - ribosomal protein s19  NT5C3A - 5'-nucleotidase, cytosolic iiia  RPS18 - ribosomal protein s18  RPS11 - ribosomal protein s11  RPS8 - ribosomal protein s8  RPS9 - ribosomal protein s9  SAMD4A - sterile alpha motif domain containing 4a  RPS14 - ribosomal protein s14  RPS13 - ribosomal protein s13  CSDE1 - cold shock domain containing e1, rna-binding  RPS7 - ribosomal protein s7  DNASE1L3 - deoxyribonuclease i-like 3  RPSA - ribosomal protein sa  PABPC4 - poly(a) binding protein, cytoplasmic 4 (inducible form)  NUDT3 - nudix (nucleoside diphosphate linked moiety x)-type motif 3  SMG5 - smg5 nonsense mediated mrna decay factor  AMPD3 - adenosine monophosphate deaminase 3  LSM4 - lsm4 homolog, u6 small nuclear rna associated (s. cerevisiae)  POR - p450 (cytochrome) oxidoreductase  RPL10A - ribosomal protein l10a  PDE1A - phosphodiesterase 1a, calmodulin-dependent  RPL23 - ribosomal protein l23  GAPDH - glyceraldehyde-3-phosphate dehydrogenase  RPS28 - ribosomal protein s28  PDE4D - phosphodiesterase 4d, camp-specific  RPS29 - ribosomal protein s29  AFMID - arylformamidase  PDE4A - phosphodiesterase 4a, camp-specific  DNASE1L1 - deoxyribonuclease i-like 1  PDE4B - phosphodiesterase 4b, camp-specific |
| GO:1901576 | organic substance biosynthetic process | 7.74E-6 | 2.24E-3 | 1.21 (10334,1779,1776,370) | [+] Show genes  LHB - luteinizing hormone beta polypeptide  HSD17B4 - hydroxysteroid (17-beta) dehydrogenase 4  REV3L - rev3-like, polymerase (dna directed), zeta, catalytic subunit  PRRX1 - paired related homeobox 1  MRPL35 - mitochondrial ribosomal protein l35  HSF1 - heat shock transcription factor 1  POLR3D - polymerase (rna) iii (dna directed) polypeptide d, 44kda  SUCLA2 - succinate-coa ligase, adp-forming, beta subunit  ELOF1 - elongation factor 1 homolog (s. cerevisiae)  GFM2 - g elongation factor, mitochondrial 2  STARD7 - star-related lipid transfer (start) domain containing 7  BPGM - 2,3-bisphosphoglycerate mutase  DHRS11 - dehydrogenase/reductase (sdr family) member 11  AASS - aminoadipate-semialdehyde synthase  FA2H - fatty acid 2-hydroxylase  LPCAT3 - lysophosphatidylcholine acyltransferase 3  COX10 - cytochrome c oxidase assembly homolog 10 (yeast)  PMM2 - phosphomannomutase 2  DPM2 - dolichyl-phosphate mannosyltransferase polypeptide 2, regulatory subunit  GCH1 - gtp cyclohydrolase 1  RENBP - renin binding protein  BGN - biglycan  ACAT1 - acetyl-coa acetyltransferase 1  GK - glycerol kinase  EIF4B - eukaryotic translation initiation factor 4b  ABLIM2 - actin binding lim protein family, member 2  PLP1 - proteolipid protein 1  EIF4EBP2 - eukaryotic translation initiation factor 4e binding protein 2  SOX10 - sry (sex determining region y)-box 10  AKR1A1 - aldo-keto reductase family 1, member a1 (aldehyde reductase)  LPL - lipoprotein lipase  GMPS - guanine monphosphate synthase  MRPL33 - mitochondrial ribosomal protein l33  ALG12 - alg12, alpha-1,6-mannosyltransferase  ALG3 - alg3, alpha-1,3- mannosyltransferase  SORD - sorbitol dehydrogenase  AJUBA - ajuba lim protein  MRPL27 - mitochondrial ribosomal protein l27  TSTA3 - tissue specific transplantation antigen p35b  PDSS1 - prenyl (decaprenyl) diphosphate synthase, subunit 1  PLA2G12A - phospholipase a2, group xiia  EIF4G1 - eukaryotic translation initiation factor 4 gamma, 1  EIF5A - eukaryotic translation initiation factor 5a  MRPS36 - mitochondrial ribosomal protein s36  GGT5 - gamma-glutamyltransferase 5  NR1D1 - nuclear receptor subfamily 1, group d, member 1  FADS3 - fatty acid desaturase 3  GLUL - glutamate-ammonia ligase  NUDT17 - nudix (nucleoside diphosphate linked moiety x)-type motif 17  CSGALNACT2 - chondroitin sulfate n-acetylgalactosaminyltransferase 2  ACSS1 - acyl-coa synthetase short-chain family member 1  SNRPD3 - small nuclear ribonucleoprotein d3 polypeptide 18kda  EEF1G - eukaryotic translation elongation factor 1 gamma  TPI1 - triosephosphate isomerase 1  TP53 - tumor protein p53  TECR - trans-2,3-enoyl-coa reductase  RWDD1 - rwd domain containing 1  ADCY2 - adenylate cyclase 2 (brain)  SMS - spermine synthase  MRPL53 - mitochondrial ribosomal protein l53  RPSA - ribosomal protein sa  RORA - rar-related orphan receptor a  ADCY9 - adenylate cyclase 9  HIPK3 - homeodomain interacting protein kinase 3  LCAT - lecithin-cholesterol acyltransferase  GYG2 - glycogenin 2  ADK - adenosine kinase  MRPS17 - mitochondrial ribosomal protein s17  MVD - mevalonate (diphospho) decarboxylase  HSPG2 - heparan sulfate proteoglycan 2  MRPL54 - mitochondrial ribosomal protein l54  PIGL - phosphatidylinositol glycan anchor biosynthesis, class l  SLC25A1 - solute carrier family 25 (mitochondrial carrier; citrate transporter), member 1  COQ10A - coenzyme q10 homolog a (s. cerevisiae)  GMPR - guanosine monophosphate reductase  POLB - polymerase (dna directed), beta  ACSF3 - acyl-coa synthetase family member 3  NAMPT - nicotinamide phosphoribosyltransferase  POLR2H - polymerase (rna) ii (dna directed) polypeptide h  CYP46A1 - cytochrome p450, family 46, subfamily a, polypeptide 1  HSD17B11 - hydroxysteroid (17-beta) dehydrogenase 11  PRKAA2 - protein kinase, amp-activated, alpha 2 catalytic subunit  RPL24 - ribosomal protein l24  MTR - 5-methyltetrahydrofolate-homocysteine methyltransferase  QKI - qki, kh domain containing, rna binding  PRKAB2 - protein kinase, amp-activated, beta 2 non-catalytic subunit  RPL23A - ribosomal protein l23a  ACOT8 - acyl-coa thioesterase 8  RPL21 - ribosomal protein l21  RPL19 - ribosomal protein l19  ACCS - 1-aminocyclopropane-1-carboxylate synthase homolog (arabidopsis)(non-functional)  RPL18A - ribosomal protein l18a  HINT2 - histidine triad nucleotide binding protein 2  RPL18 - ribosomal protein l18  CNDP2 - cndp dipeptidase 2 (metallopeptidase m20 family)  RPL17 - ribosomal protein l17  RPL13 - ribosomal protein l13  RPL12 - ribosomal protein l12  FABP5 - fatty acid binding protein 5 (psoriasis-associated)  ADSL - adenylosuccinate lyase  RPL10 - ribosomal protein l10  AGL - amylo-alpha-1, 6-glucosidase, 4-alpha-glucanotransferase  CTH - cystathionase (cystathionine gamma-lyase)  RPL7A - ribosomal protein l7a  SLC44A3 - solute carrier family 44, member 3  UGT8 - udp glycosyltransferase 8  MOCOS - molybdenum cofactor sulfurase  RPL5 - ribosomal protein l5  RPL4 - ribosomal protein l4  GPT2 - glutamic pyruvate transaminase (alanine aminotransferase) 2  NUDT1 - nudix (nucleoside diphosphate linked moiety x)-type motif 1  AK1 - adenylate kinase 1  MRPS35 - mitochondrial ribosomal protein s35  RPS5 - ribosomal protein s5  MRPS28 - mitochondrial ribosomal protein s28  AKT2 - v-akt murine thymoma viral oncogene homolog 2  RPS2 - ribosomal protein s2  RPLP2 - ribosomal protein, large, p2  MRPS12 - mitochondrial ribosomal protein s12  MRPL12 - mitochondrial ribosomal protein l12  RPL36A - ribosomal protein l36a  MRPS2 - mitochondrial ribosomal protein s2  PPCDC - phosphopantothenoylcysteine decarboxylase  RPL37A - ribosomal protein l37a  RPL38 - ribosomal protein l38  RPL34 - ribosomal protein l34  DEK - dek oncogene  PTRF - polymerase i and transcript release factor  ALDH9A1 - aldehyde dehydrogenase 9 family, member a1  AURKAIP1 - aurora kinase a interacting protein 1  RPL37 - ribosomal protein l37  RPL29 - ribosomal protein l29  RPL31 - ribosomal protein l31  B3GALT4 - udp-gal:betaglcnac beta 1,3-galactosyltransferase, polypeptide 4  RPL27 - ribosomal protein l27  CYC1 - cytochrome c-1  RPL30 - ribosomal protein l30  RPL27A - ribosomal protein l27a  ABCD1 - atp-binding cassette, sub-family d (ald), member 1  RPL28 - ribosomal protein l28  CPNE6 - copine vi (neuronal)  RPS24 - ribosomal protein s24  GALK1 - galactokinase 1  RPS21 - ribosomal protein s21  B4GALNT1 - beta-1,4-n-acetyl-galactosaminyl transferase 1  ALOX5AP - arachidonate 5-lipoxygenase-activating protein  IRF9 - interferon regulatory factor 9  RPS27 - ribosomal protein s27  PDHX - pyruvate dehydrogenase complex, component x  RPS26 - ribosomal protein s26  ZNF768 - zinc finger protein 768  RPS25 - ribosomal protein s25  TEFM - transcription elongation factor, mitochondrial  RPS17 - ribosomal protein s17  RPS16 - ribosomal protein s16  RPS15A - ribosomal protein s15a  ALDOA - aldolase a, fructose-bisphosphate  RPS20 - ribosomal protein s20  RPS19 - ribosomal protein s19  RPS18 - ribosomal protein s18  MRPS26 - mitochondrial ribosomal protein s26  RPS11 - ribosomal protein s11  DOLPP1 - dolichyldiphosphatase 1  PRLR - prolactin receptor  RPS8 - ribosomal protein s8  RPS9 - ribosomal protein s9  RPS14 - ribosomal protein s14  ESR1 - estrogen receptor 1  UCKL1 - uridine-cytidine kinase 1-like 1  RPS13 - ribosomal protein s13  GUF1 - guf1 gtpase homolog (s. cerevisiae)  MRPS18B - mitochondrial ribosomal protein s18b  MRPS5 - mitochondrial ribosomal protein s5  MRPL41 - mitochondrial ribosomal protein l41  TYMS - thymidylate synthetase  RPS7 - ribosomal protein s7  MRPL34 - mitochondrial ribosomal protein l34  ANGPT1 - angiopoietin 1  ANG - angiogenin, ribonuclease, rnase a family, 5  NMNAT1 - nicotinamide nucleotide adenylyltransferase 1  GATM - glycine amidinotransferase (l-arginine:glycine amidinotransferase)  PABPC4 - poly(a) binding protein, cytoplasmic 4 (inducible form)  GNB2L1 - guanine nucleotide binding protein (g protein), beta polypeptide 2-like 1  UGP2 - udp-glucose pyrophosphorylase 2  UGDH - udp-glucose 6-dehydrogenase  PEMT - phosphatidylethanolamine n-methyltransferase  AMPD3 - adenosine monophosphate deaminase 3  KLF4 - kruppel-like factor 4 (gut)  ETNPPL - ethanolamine-phosphate phospho-lyase  HMGCL - 3-hydroxymethyl-3-methylglutaryl-coa lyase  BCAN - brevican  RPL23 - ribosomal protein l23  GAPDH - glyceraldehyde-3-phosphate dehydrogenase  EPHX2 - epoxide hydrolase 2, cytoplasmic  IARS2 - isoleucyl-trna synthetase 2, mitochondrial  PARP10 - poly (adp-ribose) polymerase family, member 10  RPS28 - ribosomal protein s28  SREBF1 - sterol regulatory element binding transcription factor 1  RPS29 - ribosomal protein s29  CBS - cystathionine-beta-synthase  AFMID - arylformamidase  NCAN - neurocan  RREB1 - ras responsive element binding protein 1  SRF - serum response factor (c-fos serum response element-binding transcription factor)  DCXR - dicarbonyl/l-xylulose reductase  GSTM1 - glutathione s-transferase mu 1  IL18 - interleukin 18 (interferon-gamma-inducing factor)  GSTM4 - glutathione s-transferase mu 4  TMEM38B - transmembrane protein 38b  DDT - d-dopachrome tautomerase  MITF - microphthalmia-associated transcription factor  APOA1 - apolipoprotein a-i  INPP5K - inositol polyphosphate-5-phosphatase k  PPM1L - protein phosphatase, mg2+/mn2+ dependent, 1l  SDSL - serine dehydratase-like  COQ3 - coenzyme q3 methyltransferase  DRAP1 - dr1-associated protein 1 (negative cofactor 2 alpha)  DNPH1 - 2'-deoxynucleoside 5'-phosphate n-hydrolase 1  MLF1 - myeloid leukemia factor 1  PC - pyruvate carboxylase  NAT8L - n-acetyltransferase 8-like (gcn5-related, putative)  OXA1L - oxidase (cytochrome c) assembly 1-like  PYURF - pigy upstream reading frame  CECR1 - cat eye syndrome chromosome region, candidate 1  RPL35 - ribosomal protein l35  STRA8 - stimulated by retinoic acid 8  NHLRC1 - nhl repeat containing 1  MRPL39 - mitochondrial ribosomal protein l39  NADSYN1 - nad synthetase 1  CYB5R1 - cytochrome b5 reductase 1  PMVK - phosphomevalonate kinase  PIR - pirin (iron-binding nuclear protein)  PMF1 - polyamine-modulated factor 1  INTS7 - integrator complex subunit 7  OGDH - oxoglutarate (alpha-ketoglutarate) dehydrogenase (lipoamide)  HOGA1 - 4-hydroxy-2-oxoglutarate aldolase 1  SDC1 - syndecan 1  GYS1 - glycogen synthase 1 (muscle)  AMDHD2 - amidohydrolase domain containing 2  TEAD1 - tea domain family member 1 (sv40 transcriptional enhancer factor)  IVNS1ABP - influenza virus ns1a binding protein  COASY - coa synthase  APOE - apolipoprotein e  POLR3C - polymerase (rna) iii (dna directed) polypeptide c (62kd)  APRT - adenine phosphoribosyltransferase  RPL36 - ribosomal protein l36  GYG1 - glycogenin 1  PECR - peroxisomal trans-2-enoyl-coa reductase  TMLHE - trimethyllysine hydroxylase, epsilon  ATP5L - atp synthase, h+ transporting, mitochondrial fo complex, subunit g  TAF11 - taf11 rna polymerase ii, tata box binding protein (tbp)-associated factor, 28kda  MRPS18C - mitochondrial ribosomal protein s18c  TAF10 - taf10 rna polymerase ii, tata box binding protein (tbp)-associated factor, 30kda  PDE8A - phosphodiesterase 8a  PDE7A - phosphodiesterase 7a  CYP11A1 - cytochrome p450, family 11, subfamily a, polypeptide 1  PDHB - pyruvate dehydrogenase (lipoamide) beta  MRPL52 - mitochondrial ribosomal protein l52  MDH2 - malate dehydrogenase 2, nad (mitochondrial)  PDHA1 - pyruvate dehydrogenase (lipoamide) alpha 1  SC5D - sterol-c5-desaturase  VAPA - vamp (vesicle-associated membrane protein)-associated protein a, 33kda  CDO1 - cysteine dioxygenase type 1  GTF2H5 - general transcription factor iih, polypeptide 5  PTGS1 - prostaglandin-endoperoxide synthase 1 (prostaglandin g/h synthase and cyclooxygenase)  COQ2 - coenzyme q2 4-hydroxybenzoate polyprenyltransferase  TAF12 - taf12 rna polymerase ii, tata box binding protein (tbp)-associated factor, 20kda  FECH - ferrochelatase  FDX1 - ferredoxin 1  INSIG1 - insulin induced gene 1  PCK2 - phosphoenolpyruvate carboxykinase 2 (mitochondrial)  ASL - argininosuccinate lyase  MRPL28 - mitochondrial ribosomal protein l28  NUDT3 - nudix (nucleoside diphosphate linked moiety x)-type motif 3  NPPC - natriuretic peptide c  CDKN1A - cyclin-dependent kinase inhibitor 1a (p21, cip1)  INPP5D - inositol polyphosphate-5-phosphatase, 145kda  DAO - d-amino-acid oxidase  PDE1A - phosphodiesterase 1a, calmodulin-dependent  HEXA - hexosaminidase a (alpha polypeptide)  CHST8 - carbohydrate (n-acetylgalactosamine 4-0) sulfotransferase 8  HEXB - hexosaminidase b (beta polypeptide)  FADS6 - fatty acid desaturase 6  TCEB2 - transcription elongation factor b (siii), polypeptide 2 (18kda, elongin b)  PDE4D - phosphodiesterase 4d, camp-specific  TCEB1 - transcription elongation factor b (siii), polypeptide 1 (15kda, elongin c)  PTPRC - protein tyrosine phosphatase, receptor type, c  MGST1 - microsomal glutathione s-transferase 1  PDE4A - phosphodiesterase 4a, camp-specific  PDE4B - phosphodiesterase 4b, camp-specific  CYP27A1 - cytochrome p450, family 27, subfamily a, polypeptide 1  XDH - xanthine dehydrogenase  DRG2 - developmentally regulated gtp binding protein 2  PGK1 - phosphoglycerate kinase 1  PI4K2B - phosphatidylinositol 4-kinase type 2 beta  NME3 - nme/nm23 nucleoside diphosphate kinase 3  ATP5S - atp synthase, h+ transporting, mitochondrial fo complex, subunit s (factor b)  HBS1L - hbs1-like (s. cerevisiae)  TFB2M - transcription factor b2, mitochondrial  AGPAT5 - 1-acylglycerol-3-phosphate o-acyltransferase 5  PCYT2 - phosphate cytidylyltransferase 2, ethanolamine  NMNAT3 - nicotinamide nucleotide adenylyltransferase 3  NMI - n-myc (and stat) interactor  AGMO - alkylglycerol monooxygenase  B3GNT4 - udp-glcnac:betagal beta-1,3-n-acetylglucosaminyltransferase 4  ATF3 - activating transcription factor 3  RGN - regucalcin  DPYD - dihydropyrimidine dehydrogenase  TM7SF2 - transmembrane 7 superfamily member 2  DUT - deoxyuridine triphosphatase  GLS2 - glutaminase 2 (liver, mitochondrial)  NFIX - nuclear factor i/x (ccaat-binding transcription factor)  AGO2 - argonaute risc catalytic component 2  ATP5J - atp synthase, h+ transporting, mitochondrial fo complex, subunit f6  QPRT - quinolinate phosphoribosyltransferase  SLC1A3 - solute carrier family 1 (glial high affinity glutamate transporter), member 3  PET112 - pet112 homolog (yeast)  PYGL - phosphorylase, glycogen, liver  STARD10 - star-related lipid transfer (start) domain containing 10  ATP5C1 - atp synthase, h+ transporting, mitochondrial f1 complex, gamma polypeptide 1  SLC39A8 - solute carrier family 39 (zinc transporter), member 8  ATP5F1 - atp synthase, h+ transporting, mitochondrial fo complex, subunit b1  ATP5G3 - atp synthase, h+ transporting, mitochondrial fo complex, subunit c3 (subunit 9)  SLC2A4 - solute carrier family 2 (facilitated glucose transporter), member 4  CHDH - choline dehydrogenase  NT5C - 5', 3'-nucleotidase, cytosolic  ATP5A1 - atp synthase, h+ transporting, mitochondrial f1 complex, alpha subunit 1, cardiac muscle  ATP5B - atp synthase, h+ transporting, mitochondrial f1 complex, beta polypeptide  DTYMK - deoxythymidylate kinase (thymidylate kinase)  IDH2 - isocitrate dehydrogenase 2 (nadp+), mitochondrial  DLAT - dihydrolipoamide s-acetyltransferase  DLD - dihydrolipoamide dehydrogenase  TEP1 - telomerase-associated protein 1  TEAD3 - tea domain family member 3  SLC25A39 - solute carrier family 25, member 39  ASNSD1 - asparagine synthetase domain containing 1  CSPG5 - chondroitin sulfate proteoglycan 5 (neuroglycan c)  ABTB1 - ankyrin repeat and btb (poz) domain containing 1  ETNK2 - ethanolamine kinase 2  MRPL50 - mitochondrial ribosomal protein l50  EBP - emopamil binding protein (sterol isomerase)  SLC39A14 - solute carrier family 39 (zinc transporter), member 14  SHMT2 - serine hydroxymethyltransferase 2 (mitochondrial)  NFIB - nuclear factor i/b  NFIL3 - nuclear factor, interleukin 3 regulated  RHNO1 - rad9-hus1-rad1 interacting nuclear orphan 1  DNAJC2 - dnaj (hsp40) homolog, subfamily c, member 2  ISG15 - isg15 ubiquitin-like modifier  NFE2L1 - nuclear factor (erythroid-derived 2)-like 1  EIF5A2 - eukaryotic translation initiation factor 5a2  ST3GAL1 - st3 beta-galactoside alpha-2,3-sialyltransferase 1  LHPP - phospholysine phosphohistidine inorganic pyrophosphate phosphatase  FLAD1 - flavin adenine dinucleotide synthetase 1  MTERFD3 - mterf domain containing 3  BDH1 - 3-hydroxybutyrate dehydrogenase, type 1  MGLL - monoglyceride lipase  HSD3B7 - hydroxy-delta-5-steroid dehydrogenase, 3 beta- and steroid delta-isomerase 7  NDUFAB1 - nadh dehydrogenase (ubiquinone) 1, alpha/beta subcomplex, 1, 8kda  CNBP - cchc-type zinc finger, nucleic acid binding protein  NDUFA9 - nadh dehydrogenase (ubiquinone) 1 alpha subcomplex, 9, 39kda  GPAM - glycerol-3-phosphate acyltransferase, mitochondrial  RPL10A - ribosomal protein l10a  DMD - dystrophin  BCL3 - b-cell cll/lymphoma 3  RBP4 - retinol binding protein 4, plasma  SPTSSA - serine palmitoyltransferase, small subunit a  PIGC - phosphatidylinositol glycan anchor biosynthesis, class c  TEAD2 - tea domain family member 2  RBP1 - retinol binding protein 1, cellular  FPGS - folylpolyglutamate synthase |
| GO:0072657 | protein localization to membrane | 1.13E-5 | 3.21E-3 | 1.50 (10334,385,1776,99) | [+] Show genes  EMP2 - epithelial membrane protein 2  F11R - f11 receptor  INPP5K - inositol polyphosphate-5-phosphatase k  BSG - basigin (ok blood group)  FLOT2 - flotillin 2  OXA1L - oxidase (cytochrome c) assembly 1-like  EPHA2 - eph receptor a2  RPL35 - ribosomal protein l35  P2RY1 - purinergic receptor p2y, g-protein coupled, 1  BID - bh3 interacting domain death agonist  IKBKB - inhibitor of kappa light polypeptide gene enhancer in b-cells, kinase beta  SYS1 - sys1 golgi-localized integral membrane protein homolog (s. cerevisiae)  APOE - apolipoprotein e  RPL36 - ribosomal protein l36  GLRB - glycine receptor, beta  ITGB7 - integrin, beta 7  ITGB2 - integrin, beta 2 (complement component 3 receptor 3 and 4 subunit)  KRT18 - keratin 18  RPSA - ribosomal protein sa  FCER1G - fc fragment of ige, high affinity i, receptor for; gamma polypeptide  ADORA1 - adenosine a1 receptor  CYP46A1 - cytochrome p450, family 46, subfamily a, polypeptide 1  OPTN - optineurin  RPL24 - ribosomal protein l24  RPL23A - ribosomal protein l23a  RPL21 - ribosomal protein l21  RPL19 - ribosomal protein l19  RPL18A - ribosomal protein l18a  RPL18 - ribosomal protein l18  RPL17 - ribosomal protein l17  RPL13 - ribosomal protein l13  RPL12 - ribosomal protein l12  GOLGA4 - golgin a4  RPL10 - ribosomal protein l10  RPL7A - ribosomal protein l7a  RPL5 - ribosomal protein l5  PEX19 - peroxisomal biogenesis factor 19  RPL4 - ribosomal protein l4  SLMAP - sarcolemma associated protein  RPS5 - ribosomal protein s5  AKT2 - v-akt murine thymoma viral oncogene homolog 2  RPS2 - ribosomal protein s2  RPLP2 - ribosomal protein, large, p2  SYNJ2BP - synaptojanin 2 binding protein  DVL1 - dishevelled segment polarity protein 1  RPL36A - ribosomal protein l36a  RPL37A - ribosomal protein l37a  NCF1 - neutrophil cytosolic factor 1  RPL38 - ribosomal protein l38  RPL34 - ribosomal protein l34  RPL37 - ribosomal protein l37  BAG6 - bcl2-associated athanogene 6  RPL29 - ribosomal protein l29  RPL31 - ribosomal protein l31  RPL27 - ribosomal protein l27  ZDHHC18 - zinc finger, dhhc-type containing 18  RPL30 - ribosomal protein l30  RPL27A - ribosomal protein l27a  RPL28 - ribosomal protein l28  RAB13 - rab13, member ras oncogene family  RPS24 - ribosomal protein s24  RPS21 - ribosomal protein s21  SCRIB - scribbled planar cell polarity protein  RPS27 - ribosomal protein s27  SLC9A3R1 - solute carrier family 9, subfamily a (nhe3, cation proton antiporter 3), member 3 regulator 1  RPS26 - ribosomal protein s26  RPS25 - ribosomal protein s25  SLC9A3R2 - solute carrier family 9, subfamily a (nhe3, cation proton antiporter 3), member 3 regulator 2  RPS17 - ribosomal protein s17  RPS16 - ribosomal protein s16  RPS15A - ribosomal protein s15a  TSPAN17 - tetraspanin 17  RPS20 - ribosomal protein s20  RPS19 - ribosomal protein s19  LRP4 - low density lipoprotein receptor-related protein 4  RPS18 - ribosomal protein s18  RPS11 - ribosomal protein s11  NRXN2 - neurexin 2  RPS8 - ribosomal protein s8  RPS9 - ribosomal protein s9  RPS14 - ribosomal protein s14  RPS13 - ribosomal protein s13  RPS7 - ribosomal protein s7  RAPSN - receptor-associated protein of the synapse  GRIN1 - glutamate receptor, ionotropic, n-methyl d-aspartate 1  PRKCZ - protein kinase c, zeta  FERMT2 - fermitin family member 2  RILPL1 - rab interacting lysosomal protein-like 1  RPL10A - ribosomal protein l10a  PARD3 - par-3 partitioning defective 3 homolog (c. elegans)  SAMM50 - samm50 sorting and assembly machinery component  ROMO1 - reactive oxygen species modulator 1  RPL23 - ribosomal protein l23  CLIP1 - cap-gly domain containing linker protein 1  RAB12 - rab12, member ras oncogene family  KCNB1 - potassium voltage-gated channel, shab-related subfamily, member 1  RPS28 - ribosomal protein s28  RPS29 - ribosomal protein s29  ANK1 - ankyrin 1, erythrocytic |
| GO:0019693 | ribose phosphate metabolic process | 1.22E-5 | 3.39E-3 | 1.57 (10334,301,1776,81) | [+] Show genes  UQCRC2 - ubiquinol-cytochrome c reductase core protein ii  UQCRB - ubiquinol-cytochrome c reductase binding protein  PGK1 - phosphoglycerate kinase 1  CROT - carnitine o-octanoyltransferase  ACOT8 - acyl-coa thioesterase 8  HSD17B4 - hydroxysteroid (17-beta) dehydrogenase 4  SUCLA2 - succinate-coa ligase, adp-forming, beta subunit  SUCLG2 - succinate-coa ligase, gdp-forming, beta subunit  ATP5S - atp synthase, h+ transporting, mitochondrial fo complex, subunit s (factor b)  NME3 - nme/nm23 nucleoside diphosphate kinase 3  RHOQ - ras homolog family member q  BPGM - 2,3-bisphosphoglycerate mutase  ADSL - adenylosuccinate lyase  CARD11 - caspase recruitment domain family, member 11  AASS - aminoadipate-semialdehyde synthase  RPIA - ribose 5-phosphate isomerase a  MOCOS - molybdenum cofactor sulfurase  COX7A1 - cytochrome c oxidase subunit viia polypeptide 1 (muscle)  EPHA2 - eph receptor a2  AK1 - adenylate kinase 1  ACAT1 - acetyl-coa acetyltransferase 1  ATP5J - atp synthase, h+ transporting, mitochondrial fo complex, subunit f6  PMVK - phosphomevalonate kinase  NUDT14 - nudix (nucleoside diphosphate linked moiety x)-type motif 14  PYGL - phosphorylase, glycogen, liver  GMPS - guanine monphosphate synthase  ENTPD5 - ectonucleoside triphosphate diphosphohydrolase 5  PPCDC - phosphopantothenoylcysteine decarboxylase  ATP5C1 - atp synthase, h+ transporting, mitochondrial f1 complex, gamma polypeptide 1  OGDH - oxoglutarate (alpha-ketoglutarate) dehydrogenase (lipoamide)  ATP5F1 - atp synthase, h+ transporting, mitochondrial fo complex, subunit b1  COASY - coa synthase  SULT1E1 - sulfotransferase family 1e, estrogen-preferring, member 1  ATP5G3 - atp synthase, h+ transporting, mitochondrial fo complex, subunit c3 (subunit 9)  APRT - adenine phosphoribosyltransferase  CYC1 - cytochrome c-1  ATP5A1 - atp synthase, h+ transporting, mitochondrial f1 complex, alpha subunit 1, cardiac muscle  ATP5B - atp synthase, h+ transporting, mitochondrial f1 complex, beta polypeptide  ATP5L - atp synthase, h+ transporting, mitochondrial fo complex, subunit g  ABCD1 - atp-binding cassette, sub-family d (ald), member 1  DLAT - dihydrolipoamide s-acetyltransferase  DLD - dihydrolipoamide dehydrogenase  GALK1 - galactokinase 1  PDE8A - phosphodiesterase 8a  PDE7A - phosphodiesterase 7a  ACSS1 - acyl-coa synthetase short-chain family member 1  TPI1 - triosephosphate isomerase 1  PDHX - pyruvate dehydrogenase complex, component x  PDHB - pyruvate dehydrogenase (lipoamide) beta  TEFM - transcription elongation factor, mitochondrial  DLST - dihydrolipoamide s-succinyltransferase (e2 component of 2-oxo-glutarate complex)  PDHA1 - pyruvate dehydrogenase (lipoamide) alpha 1  ALDOA - aldolase a, fructose-bisphosphate  MFN1 - mitofusin 1  TECR - trans-2,3-enoyl-coa reductase  UCKL1 - uridine-cytidine kinase 1-like 1  SULT1C4 - sulfotransferase family, cytosolic, 1c, member 4  ABHD14B - abhydrolase domain containing 14b  SHPK - sedoheptulokinase  ADCY2 - adenylate cyclase 2 (brain)  GBAS - glioblastoma amplified sequence  NMNAT1 - nicotinamide nucleotide adenylyltransferase 1  RORA - rar-related orphan receptor a  ADCY9 - adenylate cyclase 9  ADK - adenosine kinase  NUDT3 - nudix (nucleoside diphosphate linked moiety x)-type motif 3  NPPC - natriuretic peptide c  MVD - mevalonate (diphospho) decarboxylase  AMPD3 - adenosine monophosphate deaminase 3  GPAM - glycerol-3-phosphate acyltransferase, mitochondrial  PDE1A - phosphodiesterase 1a, calmodulin-dependent  SLC25A1 - solute carrier family 25 (mitochondrial carrier; citrate transporter), member 1  HMGCL - 3-hydroxymethyl-3-methylglutaryl-coa lyase  GAPDH - glyceraldehyde-3-phosphate dehydrogenase  CLPX - clpx caseinolytic peptidase x homolog (e. coli)  ACSF3 - acyl-coa synthetase family member 3  MPP1 - membrane protein, palmitoylated 1, 55kda  PDE4D - phosphodiesterase 4d, camp-specific  NDUFS1 - nadh dehydrogenase (ubiquinone) fe-s protein 1, 75kda (nadh-coenzyme q reductase)  PDE4A - phosphodiesterase 4a, camp-specific  PDE4B - phosphodiesterase 4b, camp-specific |
| GO:0019439 | aromatic compound catabolic process | 1.35E-5 | 3.68E-3 | 1.50 (10334,377,1776,97) | [+] Show genes  EXOSC5 - exosome component 5  BPGM - 2,3-bisphosphoglycerate mutase  XRN1 - 5'-3' exoribonuclease 1  DNPH1 - 2'-deoxynucleoside 5'-phosphate n-hydrolase 1  AOX1 - aldehyde oxidase 1  CECR1 - cat eye syndrome chromosome region, candidate 1  RPL35 - ribosomal protein l35  ACAT1 - acetyl-coa acetyltransferase 1  ENTPD5 - ectonucleoside triphosphate diphosphohydrolase 5  OGDH - oxoglutarate (alpha-ketoglutarate) dehydrogenase (lipoamide)  EIF4G1 - eukaryotic translation initiation factor 4 gamma, 1  RPL36 - ribosomal protein l36  LRTOMT - leucine rich transmembrane and o-methyltransferase domain containing  NUDT17 - nudix (nucleoside diphosphate linked moiety x)-type motif 17  PDE8A - phosphodiesterase 8a  PDE7A - phosphodiesterase 7a  TPI1 - triosephosphate isomerase 1  H1F0 - h1 histone family, member 0  SAMD4A - sterile alpha motif domain containing 4a  CSDE1 - cold shock domain containing e1, rna-binding  RPSA - ribosomal protein sa  NUDT3 - nudix (nucleoside diphosphate linked moiety x)-type motif 3  SMG5 - smg5 nonsense mediated mrna decay factor  PDE1A - phosphodiesterase 1a, calmodulin-dependent  PDE4D - phosphodiesterase 4d, camp-specific  PDE4A - phosphodiesterase 4a, camp-specific  PDE4B - phosphodiesterase 4b, camp-specific  XDH - xanthine dehydrogenase  RPL24 - ribosomal protein l24  PGK1 - phosphoglycerate kinase 1  RPL23A - ribosomal protein l23a  RPL21 - ribosomal protein l21  RPL19 - ribosomal protein l19  RPL18A - ribosomal protein l18a  RPL18 - ribosomal protein l18  RPL17 - ribosomal protein l17  RPL13 - ribosomal protein l13  RPL12 - ribosomal protein l12  UROC1 - urocanate hydratase 1  HBS1L - hbs1-like (s. cerevisiae)  RPL10 - ribosomal protein l10  RPL7A - ribosomal protein l7a  RPL5 - ribosomal protein l5  AGO4 - argonaute risc catalytic component 4  RPL4 - ribosomal protein l4  NUDT1 - nudix (nucleoside diphosphate linked moiety x)-type motif 1  DPYD - dihydropyrimidine dehydrogenase  RPS5 - ribosomal protein s5  DUT - deoxyuridine triphosphatase  RPS2 - ribosomal protein s2  RPLP2 - ribosomal protein, large, p2  RPL36A - ribosomal protein l36a  RPL37A - ribosomal protein l37a  RPL38 - ribosomal protein l38  RPL34 - ribosomal protein l34  RPL37 - ribosomal protein l37  RPL29 - ribosomal protein l29  RPL31 - ribosomal protein l31  NT5C - 5', 3'-nucleotidase, cytosolic  RPL27 - ribosomal protein l27  RPL30 - ribosomal protein l30  RPL27A - ribosomal protein l27a  RPL28 - ribosomal protein l28  ABCD1 - atp-binding cassette, sub-family d (ald), member 1  RPS24 - ribosomal protein s24  GALK1 - galactokinase 1  RPS21 - ribosomal protein s21  RPS27 - ribosomal protein s27  RPS26 - ribosomal protein s26  RPS25 - ribosomal protein s25  RPS17 - ribosomal protein s17  RPS16 - ribosomal protein s16  RPS15A - ribosomal protein s15a  ALDOA - aldolase a, fructose-bisphosphate  RPS20 - ribosomal protein s20  RPS19 - ribosomal protein s19  RPS18 - ribosomal protein s18  NT5C3A - 5'-nucleotidase, cytosolic iiia  RPS11 - ribosomal protein s11  RPS8 - ribosomal protein s8  RPS9 - ribosomal protein s9  RPS14 - ribosomal protein s14  RPS13 - ribosomal protein s13  RPS7 - ribosomal protein s7  DNASE1L3 - deoxyribonuclease i-like 3  PABPC4 - poly(a) binding protein, cytoplasmic 4 (inducible form)  AMPD3 - adenosine monophosphate deaminase 3  LSM4 - lsm4 homolog, u6 small nuclear rna associated (s. cerevisiae)  RPL10A - ribosomal protein l10a  RPL23 - ribosomal protein l23  GAPDH - glyceraldehyde-3-phosphate dehydrogenase  EPHX1 - epoxide hydrolase 1, microsomal (xenobiotic)  EPHX2 - epoxide hydrolase 2, cytoplasmic  RPS28 - ribosomal protein s28  RPS29 - ribosomal protein s29  AFMID - arylformamidase  DNASE1L1 - deoxyribonuclease i-like 1 |
| GO:0009058 | biosynthetic process | 1.45E-5 | 3.87E-3 | 1.20 (10334,1812,1776,374) | [+] Show genes  LHB - luteinizing hormone beta polypeptide  HSD17B4 - hydroxysteroid (17-beta) dehydrogenase 4  REV3L - rev3-like, polymerase (dna directed), zeta, catalytic subunit  PRRX1 - paired related homeobox 1  MRPL35 - mitochondrial ribosomal protein l35  HSF1 - heat shock transcription factor 1  POLR3D - polymerase (rna) iii (dna directed) polypeptide d, 44kda  SUCLA2 - succinate-coa ligase, adp-forming, beta subunit  ELOF1 - elongation factor 1 homolog (s. cerevisiae)  GFM2 - g elongation factor, mitochondrial 2  STARD7 - star-related lipid transfer (start) domain containing 7  BPGM - 2,3-bisphosphoglycerate mutase  DHRS11 - dehydrogenase/reductase (sdr family) member 11  AASS - aminoadipate-semialdehyde synthase  FA2H - fatty acid 2-hydroxylase  LPCAT3 - lysophosphatidylcholine acyltransferase 3  COX10 - cytochrome c oxidase assembly homolog 10 (yeast)  PMM2 - phosphomannomutase 2  GCHFR - gtp cyclohydrolase i feedback regulator  DPM2 - dolichyl-phosphate mannosyltransferase polypeptide 2, regulatory subunit  GCH1 - gtp cyclohydrolase 1  RENBP - renin binding protein  BGN - biglycan  ACAT1 - acetyl-coa acetyltransferase 1  GK - glycerol kinase  EIF4B - eukaryotic translation initiation factor 4b  ABLIM2 - actin binding lim protein family, member 2  PLP1 - proteolipid protein 1  EIF4EBP2 - eukaryotic translation initiation factor 4e binding protein 2  SOX10 - sry (sex determining region y)-box 10  AKR1A1 - aldo-keto reductase family 1, member a1 (aldehyde reductase)  LPL - lipoprotein lipase  GMPS - guanine monphosphate synthase  MRPL33 - mitochondrial ribosomal protein l33  ALG12 - alg12, alpha-1,6-mannosyltransferase  ALG3 - alg3, alpha-1,3- mannosyltransferase  SORD - sorbitol dehydrogenase  AJUBA - ajuba lim protein  MRPL27 - mitochondrial ribosomal protein l27  TSTA3 - tissue specific transplantation antigen p35b  SOD1 - superoxide dismutase 1, soluble  PDSS1 - prenyl (decaprenyl) diphosphate synthase, subunit 1  PLA2G12A - phospholipase a2, group xiia  EIF4G1 - eukaryotic translation initiation factor 4 gamma, 1  EIF5A - eukaryotic translation initiation factor 5a  MRPS36 - mitochondrial ribosomal protein s36  GGT5 - gamma-glutamyltransferase 5  NR1D1 - nuclear receptor subfamily 1, group d, member 1  FADS3 - fatty acid desaturase 3  GLUL - glutamate-ammonia ligase  NUDT17 - nudix (nucleoside diphosphate linked moiety x)-type motif 17  CSGALNACT2 - chondroitin sulfate n-acetylgalactosaminyltransferase 2  ACSS1 - acyl-coa synthetase short-chain family member 1  SNRPD3 - small nuclear ribonucleoprotein d3 polypeptide 18kda  EEF1G - eukaryotic translation elongation factor 1 gamma  TPI1 - triosephosphate isomerase 1  TP53 - tumor protein p53  COL1A1 - collagen, type i, alpha 1  TECR - trans-2,3-enoyl-coa reductase  RWDD1 - rwd domain containing 1  ADCY2 - adenylate cyclase 2 (brain)  SMS - spermine synthase  MRPL53 - mitochondrial ribosomal protein l53  RPSA - ribosomal protein sa  RORA - rar-related orphan receptor a  ADCY9 - adenylate cyclase 9  HIPK3 - homeodomain interacting protein kinase 3  LCAT - lecithin-cholesterol acyltransferase  GYG2 - glycogenin 2  ADK - adenosine kinase  MRPS17 - mitochondrial ribosomal protein s17  MVD - mevalonate (diphospho) decarboxylase  HSPG2 - heparan sulfate proteoglycan 2  MRPL54 - mitochondrial ribosomal protein l54  PIGL - phosphatidylinositol glycan anchor biosynthesis, class l  SLC25A1 - solute carrier family 25 (mitochondrial carrier; citrate transporter), member 1  COQ10A - coenzyme q10 homolog a (s. cerevisiae)  GMPR - guanosine monophosphate reductase  POLB - polymerase (dna directed), beta  ACSF3 - acyl-coa synthetase family member 3  NAMPT - nicotinamide phosphoribosyltransferase  POLR2H - polymerase (rna) ii (dna directed) polypeptide h  CYP46A1 - cytochrome p450, family 46, subfamily a, polypeptide 1  HSD17B11 - hydroxysteroid (17-beta) dehydrogenase 11  PRKAA2 - protein kinase, amp-activated, alpha 2 catalytic subunit  RPL24 - ribosomal protein l24  MTR - 5-methyltetrahydrofolate-homocysteine methyltransferase  QKI - qki, kh domain containing, rna binding  PRKAB2 - protein kinase, amp-activated, beta 2 non-catalytic subunit  RPL23A - ribosomal protein l23a  ACOT8 - acyl-coa thioesterase 8  RPL21 - ribosomal protein l21  RPL19 - ribosomal protein l19  ACCS - 1-aminocyclopropane-1-carboxylate synthase homolog (arabidopsis)(non-functional)  RPL18A - ribosomal protein l18a  HINT2 - histidine triad nucleotide binding protein 2  RPL18 - ribosomal protein l18  CNDP2 - cndp dipeptidase 2 (metallopeptidase m20 family)  RPL17 - ribosomal protein l17  RPL13 - ribosomal protein l13  RPL12 - ribosomal protein l12  FABP5 - fatty acid binding protein 5 (psoriasis-associated)  ADSL - adenylosuccinate lyase  RPL10 - ribosomal protein l10  AGL - amylo-alpha-1, 6-glucosidase, 4-alpha-glucanotransferase  CTH - cystathionase (cystathionine gamma-lyase)  SLC44A3 - solute carrier family 44, member 3  RPL7A - ribosomal protein l7a  UGT8 - udp glycosyltransferase 8  MOCOS - molybdenum cofactor sulfurase  RPL5 - ribosomal protein l5  RPL4 - ribosomal protein l4  GPT2 - glutamic pyruvate transaminase (alanine aminotransferase) 2  NUDT1 - nudix (nucleoside diphosphate linked moiety x)-type motif 1  AK1 - adenylate kinase 1  MRPS35 - mitochondrial ribosomal protein s35  RPS5 - ribosomal protein s5  MRPS28 - mitochondrial ribosomal protein s28  AKT2 - v-akt murine thymoma viral oncogene homolog 2  RPS2 - ribosomal protein s2  RPLP2 - ribosomal protein, large, p2  MRPS12 - mitochondrial ribosomal protein s12  MRPL12 - mitochondrial ribosomal protein l12  RPL36A - ribosomal protein l36a  MRPS2 - mitochondrial ribosomal protein s2  PPCDC - phosphopantothenoylcysteine decarboxylase  RPL37A - ribosomal protein l37a  RPL38 - ribosomal protein l38  RPL34 - ribosomal protein l34  DEK - dek oncogene  PTRF - polymerase i and transcript release factor  ALDH9A1 - aldehyde dehydrogenase 9 family, member a1  RPL37 - ribosomal protein l37  AURKAIP1 - aurora kinase a interacting protein 1  RPL29 - ribosomal protein l29  RPL31 - ribosomal protein l31  B3GALT4 - udp-gal:betaglcnac beta 1,3-galactosyltransferase, polypeptide 4  RPL27 - ribosomal protein l27  CYC1 - cytochrome c-1  RPL30 - ribosomal protein l30  RPL27A - ribosomal protein l27a  CYBA - cytochrome b-245, alpha polypeptide  ABCD1 - atp-binding cassette, sub-family d (ald), member 1  RPL28 - ribosomal protein l28  CPNE6 - copine vi (neuronal)  RPS24 - ribosomal protein s24  GALK1 - galactokinase 1  RPS21 - ribosomal protein s21  B4GALNT1 - beta-1,4-n-acetyl-galactosaminyl transferase 1  ALOX5AP - arachidonate 5-lipoxygenase-activating protein  RPS27 - ribosomal protein s27  IRF9 - interferon regulatory factor 9  PDHX - pyruvate dehydrogenase complex, component x  RPS26 - ribosomal protein s26  ZNF768 - zinc finger protein 768  RPS25 - ribosomal protein s25  TEFM - transcription elongation factor, mitochondrial  RPS17 - ribosomal protein s17  RPS16 - ribosomal protein s16  RPS15A - ribosomal protein s15a  ALDOA - aldolase a, fructose-bisphosphate  RPS20 - ribosomal protein s20  RPS19 - ribosomal protein s19  RPS18 - ribosomal protein s18  MRPS26 - mitochondrial ribosomal protein s26  RPS11 - ribosomal protein s11  DOLPP1 - dolichyldiphosphatase 1  PRLR - prolactin receptor  RPS8 - ribosomal protein s8  RPS9 - ribosomal protein s9  RPS14 - ribosomal protein s14  ESR1 - estrogen receptor 1  UCKL1 - uridine-cytidine kinase 1-like 1  RPS13 - ribosomal protein s13  GUF1 - guf1 gtpase homolog (s. cerevisiae)  MRPS18B - mitochondrial ribosomal protein s18b  MRPS5 - mitochondrial ribosomal protein s5  MRPL41 - mitochondrial ribosomal protein l41  TYMS - thymidylate synthetase  RPS7 - ribosomal protein s7  MRPL34 - mitochondrial ribosomal protein l34  ANGPT1 - angiopoietin 1  ANG - angiogenin, ribonuclease, rnase a family, 5  NMNAT1 - nicotinamide nucleotide adenylyltransferase 1  GATM - glycine amidinotransferase (l-arginine:glycine amidinotransferase)  PABPC4 - poly(a) binding protein, cytoplasmic 4 (inducible form)  GNB2L1 - guanine nucleotide binding protein (g protein), beta polypeptide 2-like 1  UGP2 - udp-glucose pyrophosphorylase 2  UGDH - udp-glucose 6-dehydrogenase  PEMT - phosphatidylethanolamine n-methyltransferase  AMPD3 - adenosine monophosphate deaminase 3  KLF4 - kruppel-like factor 4 (gut)  ETNPPL - ethanolamine-phosphate phospho-lyase  HMGCL - 3-hydroxymethyl-3-methylglutaryl-coa lyase  BCAN - brevican  RPL23 - ribosomal protein l23  GAPDH - glyceraldehyde-3-phosphate dehydrogenase  EPHX2 - epoxide hydrolase 2, cytoplasmic  IARS2 - isoleucyl-trna synthetase 2, mitochondrial  PARP10 - poly (adp-ribose) polymerase family, member 10  RPS28 - ribosomal protein s28  SREBF1 - sterol regulatory element binding transcription factor 1  RPS29 - ribosomal protein s29  CBS - cystathionine-beta-synthase  AFMID - arylformamidase  NCAN - neurocan  RREB1 - ras responsive element binding protein 1  SRF - serum response factor (c-fos serum response element-binding transcription factor)  DCXR - dicarbonyl/l-xylulose reductase  GSTM1 - glutathione s-transferase mu 1  IL18 - interleukin 18 (interferon-gamma-inducing factor)  GSTM4 - glutathione s-transferase mu 4  TMEM38B - transmembrane protein 38b  DDT - d-dopachrome tautomerase  MITF - microphthalmia-associated transcription factor  APOA1 - apolipoprotein a-i  INPP5K - inositol polyphosphate-5-phosphatase k  PPM1L - protein phosphatase, mg2+/mn2+ dependent, 1l  SDSL - serine dehydratase-like  COQ3 - coenzyme q3 methyltransferase  DRAP1 - dr1-associated protein 1 (negative cofactor 2 alpha)  DNPH1 - 2'-deoxynucleoside 5'-phosphate n-hydrolase 1  MLF1 - myeloid leukemia factor 1  PC - pyruvate carboxylase  NAT8L - n-acetyltransferase 8-like (gcn5-related, putative)  OXA1L - oxidase (cytochrome c) assembly 1-like  PYURF - pigy upstream reading frame  CECR1 - cat eye syndrome chromosome region, candidate 1  RPL35 - ribosomal protein l35  STRA8 - stimulated by retinoic acid 8  NHLRC1 - nhl repeat containing 1  MRPL39 - mitochondrial ribosomal protein l39  NADSYN1 - nad synthetase 1  CYB5R1 - cytochrome b5 reductase 1  PMVK - phosphomevalonate kinase  PIR - pirin (iron-binding nuclear protein)  PMF1 - polyamine-modulated factor 1  INTS7 - integrator complex subunit 7  OGDH - oxoglutarate (alpha-ketoglutarate) dehydrogenase (lipoamide)  HOGA1 - 4-hydroxy-2-oxoglutarate aldolase 1  SDC1 - syndecan 1  GYS1 - glycogen synthase 1 (muscle)  AMDHD2 - amidohydrolase domain containing 2  TEAD1 - tea domain family member 1 (sv40 transcriptional enhancer factor)  IVNS1ABP - influenza virus ns1a binding protein  COASY - coa synthase  APOE - apolipoprotein e  POLR3C - polymerase (rna) iii (dna directed) polypeptide c (62kd)  APRT - adenine phosphoribosyltransferase  RPL36 - ribosomal protein l36  GYG1 - glycogenin 1  PECR - peroxisomal trans-2-enoyl-coa reductase  TMLHE - trimethyllysine hydroxylase, epsilon  ATP5L - atp synthase, h+ transporting, mitochondrial fo complex, subunit g  TAF11 - taf11 rna polymerase ii, tata box binding protein (tbp)-associated factor, 28kda  MRPS18C - mitochondrial ribosomal protein s18c  TAF10 - taf10 rna polymerase ii, tata box binding protein (tbp)-associated factor, 30kda  PDE8A - phosphodiesterase 8a  PDE7A - phosphodiesterase 7a  CYP11A1 - cytochrome p450, family 11, subfamily a, polypeptide 1  MRPL52 - mitochondrial ribosomal protein l52  PDHB - pyruvate dehydrogenase (lipoamide) beta  MDH2 - malate dehydrogenase 2, nad (mitochondrial)  PDHA1 - pyruvate dehydrogenase (lipoamide) alpha 1  SC5D - sterol-c5-desaturase  VAPA - vamp (vesicle-associated membrane protein)-associated protein a, 33kda  CDO1 - cysteine dioxygenase type 1  GTF2H5 - general transcription factor iih, polypeptide 5  PTGS1 - prostaglandin-endoperoxide synthase 1 (prostaglandin g/h synthase and cyclooxygenase)  COQ2 - coenzyme q2 4-hydroxybenzoate polyprenyltransferase  TAF12 - taf12 rna polymerase ii, tata box binding protein (tbp)-associated factor, 20kda  FECH - ferrochelatase  FDX1 - ferredoxin 1  INSIG1 - insulin induced gene 1  PCK2 - phosphoenolpyruvate carboxykinase 2 (mitochondrial)  ASL - argininosuccinate lyase  MRPL28 - mitochondrial ribosomal protein l28  NUDT3 - nudix (nucleoside diphosphate linked moiety x)-type motif 3  NPPC - natriuretic peptide c  CDKN1A - cyclin-dependent kinase inhibitor 1a (p21, cip1)  INPP5D - inositol polyphosphate-5-phosphatase, 145kda  DAO - d-amino-acid oxidase  PDE1A - phosphodiesterase 1a, calmodulin-dependent  HEXA - hexosaminidase a (alpha polypeptide)  CHST8 - carbohydrate (n-acetylgalactosamine 4-0) sulfotransferase 8  HEXB - hexosaminidase b (beta polypeptide)  FADS6 - fatty acid desaturase 6  TCEB2 - transcription elongation factor b (siii), polypeptide 2 (18kda, elongin b)  PDE4D - phosphodiesterase 4d, camp-specific  TCEB1 - transcription elongation factor b (siii), polypeptide 1 (15kda, elongin c)  PTPRC - protein tyrosine phosphatase, receptor type, c  MGST1 - microsomal glutathione s-transferase 1  PDE4A - phosphodiesterase 4a, camp-specific  PDE4B - phosphodiesterase 4b, camp-specific  CYP27A1 - cytochrome p450, family 27, subfamily a, polypeptide 1  XDH - xanthine dehydrogenase  DRG2 - developmentally regulated gtp binding protein 2  PGK1 - phosphoglycerate kinase 1  PI4K2B - phosphatidylinositol 4-kinase type 2 beta  NME3 - nme/nm23 nucleoside diphosphate kinase 3  ATP5S - atp synthase, h+ transporting, mitochondrial fo complex, subunit s (factor b)  HBS1L - hbs1-like (s. cerevisiae)  TFB2M - transcription factor b2, mitochondrial  AGPAT5 - 1-acylglycerol-3-phosphate o-acyltransferase 5  PCYT2 - phosphate cytidylyltransferase 2, ethanolamine  NMNAT3 - nicotinamide nucleotide adenylyltransferase 3  NMI - n-myc (and stat) interactor  AGMO - alkylglycerol monooxygenase  B3GNT4 - udp-glcnac:betagal beta-1,3-n-acetylglucosaminyltransferase 4  ATF3 - activating transcription factor 3  RGN - regucalcin  DPYD - dihydropyrimidine dehydrogenase  TM7SF2 - transmembrane 7 superfamily member 2  DUT - deoxyuridine triphosphatase  GLS2 - glutaminase 2 (liver, mitochondrial)  NFIX - nuclear factor i/x (ccaat-binding transcription factor)  AGO2 - argonaute risc catalytic component 2  ATP5J - atp synthase, h+ transporting, mitochondrial fo complex, subunit f6  QPRT - quinolinate phosphoribosyltransferase  SLC1A3 - solute carrier family 1 (glial high affinity glutamate transporter), member 3  PET112 - pet112 homolog (yeast)  PYGL - phosphorylase, glycogen, liver  STARD10 - star-related lipid transfer (start) domain containing 10  ATP5C1 - atp synthase, h+ transporting, mitochondrial f1 complex, gamma polypeptide 1  SLC39A8 - solute carrier family 39 (zinc transporter), member 8  ATP5F1 - atp synthase, h+ transporting, mitochondrial fo complex, subunit b1  ATP5G3 - atp synthase, h+ transporting, mitochondrial fo complex, subunit c3 (subunit 9)  SLC2A4 - solute carrier family 2 (facilitated glucose transporter), member 4  CHDH - choline dehydrogenase  NT5C - 5', 3'-nucleotidase, cytosolic  ATP5A1 - atp synthase, h+ transporting, mitochondrial f1 complex, alpha subunit 1, cardiac muscle  ATP5B - atp synthase, h+ transporting, mitochondrial f1 complex, beta polypeptide  DTYMK - deoxythymidylate kinase (thymidylate kinase)  IDH2 - isocitrate dehydrogenase 2 (nadp+), mitochondrial  DLAT - dihydrolipoamide s-acetyltransferase  DLD - dihydrolipoamide dehydrogenase  TEP1 - telomerase-associated protein 1  TEAD3 - tea domain family member 3  SLC25A39 - solute carrier family 25, member 39  ASNSD1 - asparagine synthetase domain containing 1  CSPG5 - chondroitin sulfate proteoglycan 5 (neuroglycan c)  ABTB1 - ankyrin repeat and btb (poz) domain containing 1  ETNK2 - ethanolamine kinase 2  MRPL50 - mitochondrial ribosomal protein l50  EBP - emopamil binding protein (sterol isomerase)  SLC39A14 - solute carrier family 39 (zinc transporter), member 14  SHMT2 - serine hydroxymethyltransferase 2 (mitochondrial)  NFIB - nuclear factor i/b  NFIL3 - nuclear factor, interleukin 3 regulated  RHNO1 - rad9-hus1-rad1 interacting nuclear orphan 1  DNAJC2 - dnaj (hsp40) homolog, subfamily c, member 2  ISG15 - isg15 ubiquitin-like modifier  NFE2L1 - nuclear factor (erythroid-derived 2)-like 1  EIF5A2 - eukaryotic translation initiation factor 5a2  ST3GAL1 - st3 beta-galactoside alpha-2,3-sialyltransferase 1  LHPP - phospholysine phosphohistidine inorganic pyrophosphate phosphatase  FLAD1 - flavin adenine dinucleotide synthetase 1  MTERFD3 - mterf domain containing 3  BDH1 - 3-hydroxybutyrate dehydrogenase, type 1  MGLL - monoglyceride lipase  HSD3B7 - hydroxy-delta-5-steroid dehydrogenase, 3 beta- and steroid delta-isomerase 7  NDUFAB1 - nadh dehydrogenase (ubiquinone) 1, alpha/beta subcomplex, 1, 8kda  CNBP - cchc-type zinc finger, nucleic acid binding protein  NDUFA9 - nadh dehydrogenase (ubiquinone) 1 alpha subcomplex, 9, 39kda  GPAM - glycerol-3-phosphate acyltransferase, mitochondrial  RPL10A - ribosomal protein l10a  DMD - dystrophin  BCL3 - b-cell cll/lymphoma 3  RBP4 - retinol binding protein 4, plasma  SPTSSA - serine palmitoyltransferase, small subunit a  PIGC - phosphatidylinositol glycan anchor biosynthesis, class c  TEAD2 - tea domain family member 2  RBP1 - retinol binding protein 1, cellular  FPGS - folylpolyglutamate synthase |
| GO:0006401 | RNA catabolic process | 1.65E-5 | 4.34E-3 | 1.67 (10334,212,1776,61) | [+] Show genes  RPL24 - ribosomal protein l24  EXOSC5 - exosome component 5  RPL23A - ribosomal protein l23a  RPL21 - ribosomal protein l21  RPL19 - ribosomal protein l19  RPL18A - ribosomal protein l18a  RPL18 - ribosomal protein l18  RPL17 - ribosomal protein l17  RPL13 - ribosomal protein l13  RPL12 - ribosomal protein l12  HBS1L - hbs1-like (s. cerevisiae)  XRN1 - 5'-3' exoribonuclease 1  RPL10 - ribosomal protein l10  RPL7A - ribosomal protein l7a  AGO4 - argonaute risc catalytic component 4  RPL5 - ribosomal protein l5  RPL4 - ribosomal protein l4  RPL35 - ribosomal protein l35  RPS5 - ribosomal protein s5  RPS2 - ribosomal protein s2  RPLP2 - ribosomal protein, large, p2  RPL36A - ribosomal protein l36a  RPL37A - ribosomal protein l37a  RPL38 - ribosomal protein l38  RPL34 - ribosomal protein l34  RPL37 - ribosomal protein l37  RPL29 - ribosomal protein l29  EIF4G1 - eukaryotic translation initiation factor 4 gamma, 1  RPL31 - ribosomal protein l31  RPL36 - ribosomal protein l36  RPL27 - ribosomal protein l27  RPL30 - ribosomal protein l30  RPL27A - ribosomal protein l27a  RPL28 - ribosomal protein l28  RPS24 - ribosomal protein s24  RPS21 - ribosomal protein s21  RPS27 - ribosomal protein s27  RPS26 - ribosomal protein s26  RPS25 - ribosomal protein s25  RPS17 - ribosomal protein s17  RPS16 - ribosomal protein s16  RPS15A - ribosomal protein s15a  RPS20 - ribosomal protein s20  RPS19 - ribosomal protein s19  RPS18 - ribosomal protein s18  RPS11 - ribosomal protein s11  RPS8 - ribosomal protein s8  SAMD4A - sterile alpha motif domain containing 4a  RPS9 - ribosomal protein s9  RPS14 - ribosomal protein s14  RPS13 - ribosomal protein s13  CSDE1 - cold shock domain containing e1, rna-binding  RPS7 - ribosomal protein s7  RPSA - ribosomal protein sa  PABPC4 - poly(a) binding protein, cytoplasmic 4 (inducible form)  SMG5 - smg5 nonsense mediated mrna decay factor  LSM4 - lsm4 homolog, u6 small nuclear rna associated (s. cerevisiae)  RPL10A - ribosomal protein l10a  RPL23 - ribosomal protein l23  RPS28 - ribosomal protein s28  RPS29 - ribosomal protein s29 |
| GO:0009259 | ribonucleotide metabolic process | 1.71E-5 | 4.4E-3 | 1.57 (10334,285,1776,77) | [+] Show genes  UQCRC2 - ubiquinol-cytochrome c reductase core protein ii  UQCRB - ubiquinol-cytochrome c reductase binding protein  PGK1 - phosphoglycerate kinase 1  CROT - carnitine o-octanoyltransferase  ACOT8 - acyl-coa thioesterase 8  HSD17B4 - hydroxysteroid (17-beta) dehydrogenase 4  SUCLA2 - succinate-coa ligase, adp-forming, beta subunit  SUCLG2 - succinate-coa ligase, gdp-forming, beta subunit  RHOQ - ras homolog family member q  NME3 - nme/nm23 nucleoside diphosphate kinase 3  ATP5S - atp synthase, h+ transporting, mitochondrial fo complex, subunit s (factor b)  ADSL - adenylosuccinate lyase  BPGM - 2,3-bisphosphoglycerate mutase  CARD11 - caspase recruitment domain family, member 11  AASS - aminoadipate-semialdehyde synthase  MOCOS - molybdenum cofactor sulfurase  COX7A1 - cytochrome c oxidase subunit viia polypeptide 1 (muscle)  EPHA2 - eph receptor a2  AK1 - adenylate kinase 1  ACAT1 - acetyl-coa acetyltransferase 1  ATP5J - atp synthase, h+ transporting, mitochondrial fo complex, subunit f6  PMVK - phosphomevalonate kinase  GMPS - guanine monphosphate synthase  ENTPD5 - ectonucleoside triphosphate diphosphohydrolase 5  PPCDC - phosphopantothenoylcysteine decarboxylase  ATP5C1 - atp synthase, h+ transporting, mitochondrial f1 complex, gamma polypeptide 1  OGDH - oxoglutarate (alpha-ketoglutarate) dehydrogenase (lipoamide)  ATP5F1 - atp synthase, h+ transporting, mitochondrial fo complex, subunit b1  SULT1E1 - sulfotransferase family 1e, estrogen-preferring, member 1  COASY - coa synthase  ATP5G3 - atp synthase, h+ transporting, mitochondrial fo complex, subunit c3 (subunit 9)  APRT - adenine phosphoribosyltransferase  CYC1 - cytochrome c-1  ATP5A1 - atp synthase, h+ transporting, mitochondrial f1 complex, alpha subunit 1, cardiac muscle  ATP5B - atp synthase, h+ transporting, mitochondrial f1 complex, beta polypeptide  ATP5L - atp synthase, h+ transporting, mitochondrial fo complex, subunit g  ABCD1 - atp-binding cassette, sub-family d (ald), member 1  DLAT - dihydrolipoamide s-acetyltransferase  DLD - dihydrolipoamide dehydrogenase  GALK1 - galactokinase 1  PDE8A - phosphodiesterase 8a  PDE7A - phosphodiesterase 7a  ACSS1 - acyl-coa synthetase short-chain family member 1  TPI1 - triosephosphate isomerase 1  PDHX - pyruvate dehydrogenase complex, component x  PDHB - pyruvate dehydrogenase (lipoamide) beta  TEFM - transcription elongation factor, mitochondrial  DLST - dihydrolipoamide s-succinyltransferase (e2 component of 2-oxo-glutarate complex)  PDHA1 - pyruvate dehydrogenase (lipoamide) alpha 1  ALDOA - aldolase a, fructose-bisphosphate  MFN1 - mitofusin 1  TECR - trans-2,3-enoyl-coa reductase  UCKL1 - uridine-cytidine kinase 1-like 1  SULT1C4 - sulfotransferase family, cytosolic, 1c, member 4  ABHD14B - abhydrolase domain containing 14b  ADCY2 - adenylate cyclase 2 (brain)  GBAS - glioblastoma amplified sequence  NMNAT1 - nicotinamide nucleotide adenylyltransferase 1  RORA - rar-related orphan receptor a  ADCY9 - adenylate cyclase 9  ADK - adenosine kinase  NUDT3 - nudix (nucleoside diphosphate linked moiety x)-type motif 3  NPPC - natriuretic peptide c  MVD - mevalonate (diphospho) decarboxylase  AMPD3 - adenosine monophosphate deaminase 3  GPAM - glycerol-3-phosphate acyltransferase, mitochondrial  PDE1A - phosphodiesterase 1a, calmodulin-dependent  SLC25A1 - solute carrier family 25 (mitochondrial carrier; citrate transporter), member 1  HMGCL - 3-hydroxymethyl-3-methylglutaryl-coa lyase  GAPDH - glyceraldehyde-3-phosphate dehydrogenase  CLPX - clpx caseinolytic peptidase x homolog (e. coli)  ACSF3 - acyl-coa synthetase family member 3  MPP1 - membrane protein, palmitoylated 1, 55kda  PDE4D - phosphodiesterase 4d, camp-specific  NDUFS1 - nadh dehydrogenase (ubiquinone) fe-s protein 1, 75kda (nadh-coenzyme q reductase)  PDE4A - phosphodiesterase 4a, camp-specific  PDE4B - phosphodiesterase 4b, camp-specific |
| GO:0032787 | monocarboxylic acid metabolic process | 2.16E-5 | 5.46E-3 | 1.49 (10334,362,1776,93) | [+] Show genes  SCPEP1 - serine carboxypeptidase 1  PRKAA2 - protein kinase, amp-activated, alpha 2 catalytic subunit  GSTM1 - glutathione s-transferase mu 1  QKI - qki, kh domain containing, rna binding  PRKAB2 - protein kinase, amp-activated, beta 2 non-catalytic subunit  PGK1 - phosphoglycerate kinase 1  FAAH - fatty acid amide hydrolase  CROT - carnitine o-octanoyltransferase  ACOT8 - acyl-coa thioesterase 8  GSTM4 - glutathione s-transferase mu 4  HSD17B4 - hydroxysteroid (17-beta) dehydrogenase 4  ACCS - 1-aminocyclopropane-1-carboxylate synthase homolog (arabidopsis)(non-functional)  FABP3 - fatty acid binding protein 3, muscle and heart (mammary-derived growth inhibitor)  UROC1 - urocanate hydratase 1  BPGM - 2,3-bisphosphoglycerate mutase  BTD - biotinidase  LDHB - lactate dehydrogenase b  FA2H - fatty acid 2-hydroxylase  BSG - basigin (ok blood group)  PC - pyruvate carboxylase  SLC16A1 - solute carrier family 16 (monocarboxylate transporter), member 1  ECI1 - enoyl-coa delta isomerase 1  ABCD4 - atp-binding cassette, sub-family d (ald), member 4  PCCA - propionyl coa carboxylase, alpha polypeptide  PCCB - propionyl coa carboxylase, beta polypeptide  PTGR2 - prostaglandin reductase 2  ACAT1 - acetyl-coa acetyltransferase 1  BGN - biglycan  PLP1 - proteolipid protein 1  PTGR1 - prostaglandin reductase 1  NAAA - n-acylethanolamine acid amidase  MRS2 - mrs2 magnesium transporter  AKR1A1 - aldo-keto reductase family 1, member a1 (aldehyde reductase)  SLC1A3 - solute carrier family 1 (glial high affinity glutamate transporter), member 3  LPL - lipoprotein lipase  OGDH - oxoglutarate (alpha-ketoglutarate) dehydrogenase (lipoamide)  HOGA1 - 4-hydroxy-2-oxoglutarate aldolase 1  SORD - sorbitol dehydrogenase  CREM - camp responsive element modulator  CRAT - carnitine o-acetyltransferase  VDAC1 - voltage-dependent anion channel 1  GGT5 - gamma-glutamyltransferase 5  IDH2 - isocitrate dehydrogenase 2 (nadp+), mitochondrial  PECR - peroxisomal trans-2-enoyl-coa reductase  FADS3 - fatty acid desaturase 3  ABCD1 - atp-binding cassette, sub-family d (ald), member 1  CYP4V2 - cytochrome p450, family 4, subfamily v, polypeptide 2  DLAT - dihydrolipoamide s-acetyltransferase  ADIPOR2 - adiponectin receptor 2  HYI - hydroxypyruvate isomerase (putative)  DLD - dihydrolipoamide dehydrogenase  GALK1 - galactokinase 1  ACSS1 - acyl-coa synthetase short-chain family member 1  ALOX5AP - arachidonate 5-lipoxygenase-activating protein  TPI1 - triosephosphate isomerase 1  PDHX - pyruvate dehydrogenase complex, component x  ASNSD1 - asparagine synthetase domain containing 1  PDHB - pyruvate dehydrogenase (lipoamide) beta  CSPG5 - chondroitin sulfate proteoglycan 5 (neuroglycan c)  PDHA1 - pyruvate dehydrogenase (lipoamide) alpha 1  GLO1 - glyoxalase i  ALDOA - aldolase a, fructose-bisphosphate  TECR - trans-2,3-enoyl-coa reductase  ECHDC3 - enoyl coa hydratase domain containing 3  ZADH2 - zinc binding alcohol dehydrogenase domain containing 2  ACAD9 - acyl-coa dehydrogenase family, member 9  PTGS1 - prostaglandin-endoperoxide synthase 1 (prostaglandin g/h synthase and cyclooxygenase)  HADHA - hydroxyacyl-coa dehydrogenase/3-ketoacyl-coa thiolase/enoyl-coa hydratase (trifunctional protein), alpha subunit  PLAA - phospholipase a2-activating protein  MAPK14 - mitogen-activated protein kinase 14  ETFA - electron-transfer-flavoprotein, alpha polypeptide  MGLL - monoglyceride lipase  HSD3B7 - hydroxy-delta-5-steroid dehydrogenase, 3 beta- and steroid delta-isomerase 7  PCK2 - phosphoenolpyruvate carboxykinase 2 (mitochondrial)  NIT2 - nitrilase family, member 2  NDUFAB1 - nadh dehydrogenase (ubiquinone) 1, alpha/beta subcomplex, 1, 8kda  GPAM - glycerol-3-phosphate acyltransferase, mitochondrial  POR - p450 (cytochrome) oxidoreductase  C3 - complement component 3  BCAN - brevican  FADS6 - fatty acid desaturase 6  LDHD - lactate dehydrogenase d  GAPDH - glyceraldehyde-3-phosphate dehydrogenase  EPHX1 - epoxide hydrolase 1, microsomal (xenobiotic)  EPHX2 - epoxide hydrolase 2, cytoplasmic  ACSF3 - acyl-coa synthetase family member 3  SLC27A4 - solute carrier family 27 (fatty acid transporter), member 4  NCAN - neurocan  CYP46A1 - cytochrome p450, family 46, subfamily a, polypeptide 1  RBP1 - retinol binding protein 1, cellular  CYP27A1 - cytochrome p450, family 27, subfamily a, polypeptide 1  DCXR - dicarbonyl/l-xylulose reductase  IDNK - idnk, gluconokinase homolog (e. coli) |
| GO:0006732 | coenzyme metabolic process | 2.19E-5 | 5.43E-3 | 1.61 (10334,250,1776,69) | [+] Show genes  FMO5 - flavin containing monooxygenase 5  PGK1 - phosphoglycerate kinase 1  CROT - carnitine o-octanoyltransferase  ACOT8 - acyl-coa thioesterase 8  KCNAB2 - potassium voltage-gated channel, shaker-related subfamily, beta member 2  HSD17B4 - hydroxysteroid (17-beta) dehydrogenase 4  SUCLA2 - succinate-coa ligase, adp-forming, beta subunit  SUCLG2 - succinate-coa ligase, gdp-forming, beta subunit  BPGM - 2,3-bisphosphoglycerate mutase  BTD - biotinidase  COQ3 - coenzyme q3 methyltransferase  AASS - aminoadipate-semialdehyde synthase  RPIA - ribose 5-phosphate isomerase a  MOCOS - molybdenum cofactor sulfurase  NMNAT3 - nicotinamide nucleotide adenylyltransferase 3  PC - pyruvate carboxylase  PCCA - propionyl coa carboxylase, alpha polypeptide  GCH1 - gtp cyclohydrolase 1  PCCB - propionyl coa carboxylase, beta polypeptide  RGN - regucalcin  ACAT1 - acetyl-coa acetyltransferase 1  CD38 - cd38 molecule  NADSYN1 - nad synthetase 1  PMVK - phosphomevalonate kinase  QPRT - quinolinate phosphoribosyltransferase  AKR1A1 - aldo-keto reductase family 1, member a1 (aldehyde reductase)  PPCDC - phosphopantothenoylcysteine decarboxylase  OGDH - oxoglutarate (alpha-ketoglutarate) dehydrogenase (lipoamide)  COASY - coa synthase  PDSS1 - prenyl (decaprenyl) diphosphate synthase, subunit 1  IDH2 - isocitrate dehydrogenase 2 (nadp+), mitochondrial  ABCD1 - atp-binding cassette, sub-family d (ald), member 1  DLAT - dihydrolipoamide s-acetyltransferase  DLD - dihydrolipoamide dehydrogenase  GALK1 - galactokinase 1  NUDT17 - nudix (nucleoside diphosphate linked moiety x)-type motif 17  ACSS1 - acyl-coa synthetase short-chain family member 1  TPI1 - triosephosphate isomerase 1  PDHX - pyruvate dehydrogenase complex, component x  GNMT - glycine n-methyltransferase  PDHB - pyruvate dehydrogenase (lipoamide) beta  MDH2 - malate dehydrogenase 2, nad (mitochondrial)  DLST - dihydrolipoamide s-succinyltransferase (e2 component of 2-oxo-glutarate complex)  PDHA1 - pyruvate dehydrogenase (lipoamide) alpha 1  ALDOA - aldolase a, fructose-bisphosphate  SHMT2 - serine hydroxymethyltransferase 2 (mitochondrial)  TECR - trans-2,3-enoyl-coa reductase  COQ2 - coenzyme q2 4-hydroxybenzoate polyprenyltransferase  SHPK - sedoheptulokinase  FLAD1 - flavin adenine dinucleotide synthetase 1  TYMS - thymidylate synthetase  NMNAT1 - nicotinamide nucleotide adenylyltransferase 1  MVD - mevalonate (diphospho) decarboxylase  PEMT - phosphatidylethanolamine n-methyltransferase  NDUFA9 - nadh dehydrogenase (ubiquinone) 1 alpha subcomplex, 9, 39kda  GPAM - glycerol-3-phosphate acyltransferase, mitochondrial  NNT - nicotinamide nucleotide transhydrogenase  SLC25A1 - solute carrier family 25 (mitochondrial carrier; citrate transporter), member 1  HMGCL - 3-hydroxymethyl-3-methylglutaryl-coa lyase  FOLR2 - folate receptor 2 (fetal)  GAPDH - glyceraldehyde-3-phosphate dehydrogenase  COQ10A - coenzyme q10 homolog a (s. cerevisiae)  ACSF3 - acyl-coa synthetase family member 3  PARP10 - poly (adp-ribose) polymerase family, member 10  NAMPT - nicotinamide phosphoribosyltransferase  AFMID - arylformamidase  AIFM2 - apoptosis-inducing factor, mitochondrion-associated, 2  FPGS - folylpolyglutamate synthase  DCXR - dicarbonyl/l-xylulose reductase |
| GO:0006733 | oxidoreduction coenzyme metabolic process | 3.33E-5 | 8.12E-3 | 1.98 (10334,100,1776,34) | [+] Show genes  FMO5 - flavin containing monooxygenase 5  DLD - dihydrolipoamide dehydrogenase  GALK1 - galactokinase 1  NUDT17 - nudix (nucleoside diphosphate linked moiety x)-type motif 17  PGK1 - phosphoglycerate kinase 1  KCNAB2 - potassium voltage-gated channel, shaker-related subfamily, beta member 2  TPI1 - triosephosphate isomerase 1  PDHX - pyruvate dehydrogenase complex, component x  PDHB - pyruvate dehydrogenase (lipoamide) beta  MDH2 - malate dehydrogenase 2, nad (mitochondrial)  PDHA1 - pyruvate dehydrogenase (lipoamide) alpha 1  ALDOA - aldolase a, fructose-bisphosphate  BPGM - 2,3-bisphosphoglycerate mutase  COQ3 - coenzyme q3 methyltransferase  RPIA - ribose 5-phosphate isomerase a  NMNAT3 - nicotinamide nucleotide adenylyltransferase 3  COQ2 - coenzyme q2 4-hydroxybenzoate polyprenyltransferase  SHPK - sedoheptulokinase  NMNAT1 - nicotinamide nucleotide adenylyltransferase 1  CD38 - cd38 molecule  NADSYN1 - nad synthetase 1  NDUFA9 - nadh dehydrogenase (ubiquinone) 1 alpha subcomplex, 9, 39kda  QPRT - quinolinate phosphoribosyltransferase  NNT - nicotinamide nucleotide transhydrogenase  OGDH - oxoglutarate (alpha-ketoglutarate) dehydrogenase (lipoamide)  GAPDH - glyceraldehyde-3-phosphate dehydrogenase  COQ10A - coenzyme q10 homolog a (s. cerevisiae)  PARP10 - poly (adp-ribose) polymerase family, member 10  PDSS1 - prenyl (decaprenyl) diphosphate synthase, subunit 1  NAMPT - nicotinamide phosphoribosyltransferase  AFMID - arylformamidase  AIFM2 - apoptosis-inducing factor, mitochondrion-associated, 2  DCXR - dicarbonyl/l-xylulose reductase  IDH2 - isocitrate dehydrogenase 2 (nadp+), mitochondrial |
| GO:0044249 | cellular biosynthetic process | 4.02E-5 | 9.63E-3 | 1.20 (10334,1696,1776,349) | [+] Show genes  LHB - luteinizing hormone beta polypeptide  HSD17B4 - hydroxysteroid (17-beta) dehydrogenase 4  REV3L - rev3-like, polymerase (dna directed), zeta, catalytic subunit  PRRX1 - paired related homeobox 1  MRPL35 - mitochondrial ribosomal protein l35  HSF1 - heat shock transcription factor 1  POLR3D - polymerase (rna) iii (dna directed) polypeptide d, 44kda  SUCLA2 - succinate-coa ligase, adp-forming, beta subunit  ELOF1 - elongation factor 1 homolog (s. cerevisiae)  GFM2 - g elongation factor, mitochondrial 2  STARD7 - star-related lipid transfer (start) domain containing 7  BPGM - 2,3-bisphosphoglycerate mutase  DHRS11 - dehydrogenase/reductase (sdr family) member 11  AASS - aminoadipate-semialdehyde synthase  FA2H - fatty acid 2-hydroxylase  LPCAT3 - lysophosphatidylcholine acyltransferase 3  COX10 - cytochrome c oxidase assembly homolog 10 (yeast)  PMM2 - phosphomannomutase 2  GCHFR - gtp cyclohydrolase i feedback regulator  DPM2 - dolichyl-phosphate mannosyltransferase polypeptide 2, regulatory subunit  GCH1 - gtp cyclohydrolase 1  RENBP - renin binding protein  BGN - biglycan  ACAT1 - acetyl-coa acetyltransferase 1  GK - glycerol kinase  EIF4B - eukaryotic translation initiation factor 4b  ABLIM2 - actin binding lim protein family, member 2  PLP1 - proteolipid protein 1  SOX10 - sry (sex determining region y)-box 10  EIF4EBP2 - eukaryotic translation initiation factor 4e binding protein 2  AKR1A1 - aldo-keto reductase family 1, member a1 (aldehyde reductase)  LPL - lipoprotein lipase  GMPS - guanine monphosphate synthase  MRPL33 - mitochondrial ribosomal protein l33  AJUBA - ajuba lim protein  MRPL27 - mitochondrial ribosomal protein l27  SOD1 - superoxide dismutase 1, soluble  PDSS1 - prenyl (decaprenyl) diphosphate synthase, subunit 1  TSTA3 - tissue specific transplantation antigen p35b  PLA2G12A - phospholipase a2, group xiia  EIF4G1 - eukaryotic translation initiation factor 4 gamma, 1  EIF5A - eukaryotic translation initiation factor 5a  MRPS36 - mitochondrial ribosomal protein s36  GGT5 - gamma-glutamyltransferase 5  NR1D1 - nuclear receptor subfamily 1, group d, member 1  FADS3 - fatty acid desaturase 3  GLUL - glutamate-ammonia ligase  NUDT17 - nudix (nucleoside diphosphate linked moiety x)-type motif 17  CSGALNACT2 - chondroitin sulfate n-acetylgalactosaminyltransferase 2  ACSS1 - acyl-coa synthetase short-chain family member 1  SNRPD3 - small nuclear ribonucleoprotein d3 polypeptide 18kda  EEF1G - eukaryotic translation elongation factor 1 gamma  TPI1 - triosephosphate isomerase 1  TP53 - tumor protein p53  TECR - trans-2,3-enoyl-coa reductase  RWDD1 - rwd domain containing 1  ADCY2 - adenylate cyclase 2 (brain)  SMS - spermine synthase  MRPL53 - mitochondrial ribosomal protein l53  RPSA - ribosomal protein sa  RORA - rar-related orphan receptor a  ADCY9 - adenylate cyclase 9  HIPK3 - homeodomain interacting protein kinase 3  LCAT - lecithin-cholesterol acyltransferase  GYG2 - glycogenin 2  ADK - adenosine kinase  MRPS17 - mitochondrial ribosomal protein s17  MVD - mevalonate (diphospho) decarboxylase  MRPL54 - mitochondrial ribosomal protein l54  PIGL - phosphatidylinositol glycan anchor biosynthesis, class l  SLC25A1 - solute carrier family 25 (mitochondrial carrier; citrate transporter), member 1  COQ10A - coenzyme q10 homolog a (s. cerevisiae)  GMPR - guanosine monophosphate reductase  POLB - polymerase (dna directed), beta  ACSF3 - acyl-coa synthetase family member 3  NAMPT - nicotinamide phosphoribosyltransferase  POLR2H - polymerase (rna) ii (dna directed) polypeptide h  CYP46A1 - cytochrome p450, family 46, subfamily a, polypeptide 1  HSD17B11 - hydroxysteroid (17-beta) dehydrogenase 11  PRKAA2 - protein kinase, amp-activated, alpha 2 catalytic subunit  RPL24 - ribosomal protein l24  MTR - 5-methyltetrahydrofolate-homocysteine methyltransferase  QKI - qki, kh domain containing, rna binding  PRKAB2 - protein kinase, amp-activated, beta 2 non-catalytic subunit  RPL23A - ribosomal protein l23a  ACOT8 - acyl-coa thioesterase 8  RPL21 - ribosomal protein l21  RPL19 - ribosomal protein l19  ACCS - 1-aminocyclopropane-1-carboxylate synthase homolog (arabidopsis)(non-functional)  RPL18A - ribosomal protein l18a  RPL18 - ribosomal protein l18  CNDP2 - cndp dipeptidase 2 (metallopeptidase m20 family)  RPL17 - ribosomal protein l17  RPL13 - ribosomal protein l13  RPL12 - ribosomal protein l12  FABP5 - fatty acid binding protein 5 (psoriasis-associated)  ADSL - adenylosuccinate lyase  RPL10 - ribosomal protein l10  AGL - amylo-alpha-1, 6-glucosidase, 4-alpha-glucanotransferase  CTH - cystathionase (cystathionine gamma-lyase)  SLC44A3 - solute carrier family 44, member 3  RPL7A - ribosomal protein l7a  UGT8 - udp glycosyltransferase 8  MOCOS - molybdenum cofactor sulfurase  RPL5 - ribosomal protein l5  RPL4 - ribosomal protein l4  GPT2 - glutamic pyruvate transaminase (alanine aminotransferase) 2  NUDT1 - nudix (nucleoside diphosphate linked moiety x)-type motif 1  AK1 - adenylate kinase 1  MRPS35 - mitochondrial ribosomal protein s35  RPS5 - ribosomal protein s5  MRPS28 - mitochondrial ribosomal protein s28  AKT2 - v-akt murine thymoma viral oncogene homolog 2  RPS2 - ribosomal protein s2  RPLP2 - ribosomal protein, large, p2  MRPS12 - mitochondrial ribosomal protein s12  MRPL12 - mitochondrial ribosomal protein l12  RPL36A - ribosomal protein l36a  MRPS2 - mitochondrial ribosomal protein s2  PPCDC - phosphopantothenoylcysteine decarboxylase  RPL37A - ribosomal protein l37a  RPL38 - ribosomal protein l38  RPL34 - ribosomal protein l34  DEK - dek oncogene  PTRF - polymerase i and transcript release factor  AURKAIP1 - aurora kinase a interacting protein 1  RPL37 - ribosomal protein l37  ALDH9A1 - aldehyde dehydrogenase 9 family, member a1  RPL29 - ribosomal protein l29  RPL31 - ribosomal protein l31  B3GALT4 - udp-gal:betaglcnac beta 1,3-galactosyltransferase, polypeptide 4  RPL27 - ribosomal protein l27  CYC1 - cytochrome c-1  RPL30 - ribosomal protein l30  RPL27A - ribosomal protein l27a  CYBA - cytochrome b-245, alpha polypeptide  ABCD1 - atp-binding cassette, sub-family d (ald), member 1  RPL28 - ribosomal protein l28  CPNE6 - copine vi (neuronal)  RPS24 - ribosomal protein s24  GALK1 - galactokinase 1  RPS21 - ribosomal protein s21  B4GALNT1 - beta-1,4-n-acetyl-galactosaminyl transferase 1  ALOX5AP - arachidonate 5-lipoxygenase-activating protein  IRF9 - interferon regulatory factor 9  RPS27 - ribosomal protein s27  PDHX - pyruvate dehydrogenase complex, component x  RPS26 - ribosomal protein s26  ZNF768 - zinc finger protein 768  RPS25 - ribosomal protein s25  TEFM - transcription elongation factor, mitochondrial  RPS17 - ribosomal protein s17  RPS16 - ribosomal protein s16  RPS15A - ribosomal protein s15a  ALDOA - aldolase a, fructose-bisphosphate  RPS20 - ribosomal protein s20  RPS19 - ribosomal protein s19  RPS18 - ribosomal protein s18  MRPS26 - mitochondrial ribosomal protein s26  RPS11 - ribosomal protein s11  DOLPP1 - dolichyldiphosphatase 1  RPS8 - ribosomal protein s8  RPS9 - ribosomal protein s9  RPS14 - ribosomal protein s14  ESR1 - estrogen receptor 1  RPS13 - ribosomal protein s13  UCKL1 - uridine-cytidine kinase 1-like 1  GUF1 - guf1 gtpase homolog (s. cerevisiae)  MRPS18B - mitochondrial ribosomal protein s18b  MRPS5 - mitochondrial ribosomal protein s5  MRPL41 - mitochondrial ribosomal protein l41  TYMS - thymidylate synthetase  RPS7 - ribosomal protein s7  MRPL34 - mitochondrial ribosomal protein l34  ANGPT1 - angiopoietin 1  ANG - angiogenin, ribonuclease, rnase a family, 5  NMNAT1 - nicotinamide nucleotide adenylyltransferase 1  GATM - glycine amidinotransferase (l-arginine:glycine amidinotransferase)  PABPC4 - poly(a) binding protein, cytoplasmic 4 (inducible form)  GNB2L1 - guanine nucleotide binding protein (g protein), beta polypeptide 2-like 1  UGP2 - udp-glucose pyrophosphorylase 2  UGDH - udp-glucose 6-dehydrogenase  PEMT - phosphatidylethanolamine n-methyltransferase  AMPD3 - adenosine monophosphate deaminase 3  KLF4 - kruppel-like factor 4 (gut)  ETNPPL - ethanolamine-phosphate phospho-lyase  HMGCL - 3-hydroxymethyl-3-methylglutaryl-coa lyase  BCAN - brevican  RPL23 - ribosomal protein l23  GAPDH - glyceraldehyde-3-phosphate dehydrogenase  EPHX2 - epoxide hydrolase 2, cytoplasmic  IARS2 - isoleucyl-trna synthetase 2, mitochondrial  PARP10 - poly (adp-ribose) polymerase family, member 10  RPS28 - ribosomal protein s28  SREBF1 - sterol regulatory element binding transcription factor 1  RPS29 - ribosomal protein s29  CBS - cystathionine-beta-synthase  AFMID - arylformamidase  NCAN - neurocan  RREB1 - ras responsive element binding protein 1  SRF - serum response factor (c-fos serum response element-binding transcription factor)  GSTM1 - glutathione s-transferase mu 1  GSTM4 - glutathione s-transferase mu 4  TMEM38B - transmembrane protein 38b  DDT - d-dopachrome tautomerase  MITF - microphthalmia-associated transcription factor  APOA1 - apolipoprotein a-i  INPP5K - inositol polyphosphate-5-phosphatase k  PPM1L - protein phosphatase, mg2+/mn2+ dependent, 1l  SDSL - serine dehydratase-like  COQ3 - coenzyme q3 methyltransferase  DRAP1 - dr1-associated protein 1 (negative cofactor 2 alpha)  DNPH1 - 2'-deoxynucleoside 5'-phosphate n-hydrolase 1  MLF1 - myeloid leukemia factor 1  NAT8L - n-acetyltransferase 8-like (gcn5-related, putative)  OXA1L - oxidase (cytochrome c) assembly 1-like  PYURF - pigy upstream reading frame  CECR1 - cat eye syndrome chromosome region, candidate 1  RPL35 - ribosomal protein l35  STRA8 - stimulated by retinoic acid 8  NHLRC1 - nhl repeat containing 1  MRPL39 - mitochondrial ribosomal protein l39  NADSYN1 - nad synthetase 1  PMVK - phosphomevalonate kinase  PIR - pirin (iron-binding nuclear protein)  PMF1 - polyamine-modulated factor 1  INTS7 - integrator complex subunit 7  OGDH - oxoglutarate (alpha-ketoglutarate) dehydrogenase (lipoamide)  HOGA1 - 4-hydroxy-2-oxoglutarate aldolase 1  GYS1 - glycogen synthase 1 (muscle)  AMDHD2 - amidohydrolase domain containing 2  TEAD1 - tea domain family member 1 (sv40 transcriptional enhancer factor)  IVNS1ABP - influenza virus ns1a binding protein  COASY - coa synthase  APOE - apolipoprotein e  POLR3C - polymerase (rna) iii (dna directed) polypeptide c (62kd)  APRT - adenine phosphoribosyltransferase  RPL36 - ribosomal protein l36  GYG1 - glycogenin 1  PECR - peroxisomal trans-2-enoyl-coa reductase  TMLHE - trimethyllysine hydroxylase, epsilon  ATP5L - atp synthase, h+ transporting, mitochondrial fo complex, subunit g  TAF11 - taf11 rna polymerase ii, tata box binding protein (tbp)-associated factor, 28kda  MRPS18C - mitochondrial ribosomal protein s18c  TAF10 - taf10 rna polymerase ii, tata box binding protein (tbp)-associated factor, 30kda  PDE8A - phosphodiesterase 8a  PDE7A - phosphodiesterase 7a  CYP11A1 - cytochrome p450, family 11, subfamily a, polypeptide 1  PDHB - pyruvate dehydrogenase (lipoamide) beta  MRPL52 - mitochondrial ribosomal protein l52  PDHA1 - pyruvate dehydrogenase (lipoamide) alpha 1  VAPA - vamp (vesicle-associated membrane protein)-associated protein a, 33kda  CDO1 - cysteine dioxygenase type 1  GTF2H5 - general transcription factor iih, polypeptide 5  PTGS1 - prostaglandin-endoperoxide synthase 1 (prostaglandin g/h synthase and cyclooxygenase)  COQ2 - coenzyme q2 4-hydroxybenzoate polyprenyltransferase  TAF12 - taf12 rna polymerase ii, tata box binding protein (tbp)-associated factor, 20kda  FECH - ferrochelatase  FDX1 - ferredoxin 1  PCK2 - phosphoenolpyruvate carboxykinase 2 (mitochondrial)  ASL - argininosuccinate lyase  MRPL28 - mitochondrial ribosomal protein l28  NUDT3 - nudix (nucleoside diphosphate linked moiety x)-type motif 3  NPPC - natriuretic peptide c  CDKN1A - cyclin-dependent kinase inhibitor 1a (p21, cip1)  INPP5D - inositol polyphosphate-5-phosphatase, 145kda  DAO - d-amino-acid oxidase  PDE1A - phosphodiesterase 1a, calmodulin-dependent  CHST8 - carbohydrate (n-acetylgalactosamine 4-0) sulfotransferase 8  HEXB - hexosaminidase b (beta polypeptide)  FADS6 - fatty acid desaturase 6  TCEB2 - transcription elongation factor b (siii), polypeptide 2 (18kda, elongin b)  PDE4D - phosphodiesterase 4d, camp-specific  TCEB1 - transcription elongation factor b (siii), polypeptide 1 (15kda, elongin c)  MGST1 - microsomal glutathione s-transferase 1  PDE4A - phosphodiesterase 4a, camp-specific  PDE4B - phosphodiesterase 4b, camp-specific  CYP27A1 - cytochrome p450, family 27, subfamily a, polypeptide 1  XDH - xanthine dehydrogenase  DRG2 - developmentally regulated gtp binding protein 2  PGK1 - phosphoglycerate kinase 1  PI4K2B - phosphatidylinositol 4-kinase type 2 beta  NME3 - nme/nm23 nucleoside diphosphate kinase 3  ATP5S - atp synthase, h+ transporting, mitochondrial fo complex, subunit s (factor b)  HBS1L - hbs1-like (s. cerevisiae)  TFB2M - transcription factor b2, mitochondrial  AGPAT5 - 1-acylglycerol-3-phosphate o-acyltransferase 5  PCYT2 - phosphate cytidylyltransferase 2, ethanolamine  NMNAT3 - nicotinamide nucleotide adenylyltransferase 3  NMI - n-myc (and stat) interactor  AGMO - alkylglycerol monooxygenase  B3GNT4 - udp-glcnac:betagal beta-1,3-n-acetylglucosaminyltransferase 4  RGN - regucalcin  DPYD - dihydropyrimidine dehydrogenase  DUT - deoxyuridine triphosphatase  GLS2 - glutaminase 2 (liver, mitochondrial)  NFIX - nuclear factor i/x (ccaat-binding transcription factor)  AGO2 - argonaute risc catalytic component 2  ATP5J - atp synthase, h+ transporting, mitochondrial fo complex, subunit f6  QPRT - quinolinate phosphoribosyltransferase  SLC1A3 - solute carrier family 1 (glial high affinity glutamate transporter), member 3  PET112 - pet112 homolog (yeast)  STARD10 - star-related lipid transfer (start) domain containing 10  ATP5C1 - atp synthase, h+ transporting, mitochondrial f1 complex, gamma polypeptide 1  SLC39A8 - solute carrier family 39 (zinc transporter), member 8  ATP5F1 - atp synthase, h+ transporting, mitochondrial fo complex, subunit b1  ATP5G3 - atp synthase, h+ transporting, mitochondrial fo complex, subunit c3 (subunit 9)  CHDH - choline dehydrogenase  NT5C - 5', 3'-nucleotidase, cytosolic  ATP5A1 - atp synthase, h+ transporting, mitochondrial f1 complex, alpha subunit 1, cardiac muscle  ATP5B - atp synthase, h+ transporting, mitochondrial f1 complex, beta polypeptide  DTYMK - deoxythymidylate kinase (thymidylate kinase)  IDH2 - isocitrate dehydrogenase 2 (nadp+), mitochondrial  DLAT - dihydrolipoamide s-acetyltransferase  DLD - dihydrolipoamide dehydrogenase  TEP1 - telomerase-associated protein 1  TEAD3 - tea domain family member 3  SLC25A39 - solute carrier family 25, member 39  ASNSD1 - asparagine synthetase domain containing 1  CSPG5 - chondroitin sulfate proteoglycan 5 (neuroglycan c)  ABTB1 - ankyrin repeat and btb (poz) domain containing 1  ETNK2 - ethanolamine kinase 2  MRPL50 - mitochondrial ribosomal protein l50  SHMT2 - serine hydroxymethyltransferase 2 (mitochondrial)  NFIB - nuclear factor i/b  NFIL3 - nuclear factor, interleukin 3 regulated  RHNO1 - rad9-hus1-rad1 interacting nuclear orphan 1  DNAJC2 - dnaj (hsp40) homolog, subfamily c, member 2  ISG15 - isg15 ubiquitin-like modifier  NFE2L1 - nuclear factor (erythroid-derived 2)-like 1  EIF5A2 - eukaryotic translation initiation factor 5a2  ST3GAL1 - st3 beta-galactoside alpha-2,3-sialyltransferase 1  LHPP - phospholysine phosphohistidine inorganic pyrophosphate phosphatase  FLAD1 - flavin adenine dinucleotide synthetase 1  MTERFD3 - mterf domain containing 3  BDH1 - 3-hydroxybutyrate dehydrogenase, type 1  MGLL - monoglyceride lipase  HSD3B7 - hydroxy-delta-5-steroid dehydrogenase, 3 beta- and steroid delta-isomerase 7  NDUFAB1 - nadh dehydrogenase (ubiquinone) 1, alpha/beta subcomplex, 1, 8kda  NDUFA9 - nadh dehydrogenase (ubiquinone) 1 alpha subcomplex, 9, 39kda  GPAM - glycerol-3-phosphate acyltransferase, mitochondrial  RPL10A - ribosomal protein l10a  DMD - dystrophin  BCL3 - b-cell cll/lymphoma 3  SPTSSA - serine palmitoyltransferase, small subunit a  PIGC - phosphatidylinositol glycan anchor biosynthesis, class c  TEAD2 - tea domain family member 2  RBP1 - retinol binding protein 1, cellular  FPGS - folylpolyglutamate synthase |
| GO:0044283 | small molecule biosynthetic process | 4.88E-5 | 1.15E-2 | 1.42 (10334,442,1776,108) | [+] Show genes  GSTM1 - glutathione s-transferase mu 1  LHB - luteinizing hormone beta polypeptide  GSTM4 - glutathione s-transferase mu 4  HSD17B4 - hydroxysteroid (17-beta) dehydrogenase 4  APOA1 - apolipoprotein a-i  SDSL - serine dehydratase-like  BPGM - 2,3-bisphosphoglycerate mutase  COQ3 - coenzyme q3 methyltransferase  AASS - aminoadipate-semialdehyde synthase  FA2H - fatty acid 2-hydroxylase  DNPH1 - 2'-deoxynucleoside 5'-phosphate n-hydrolase 1  PC - pyruvate carboxylase  NAT8L - n-acetyltransferase 8-like (gcn5-related, putative)  GCH1 - gtp cyclohydrolase 1  CECR1 - cat eye syndrome chromosome region, candidate 1  ACAT1 - acetyl-coa acetyltransferase 1  BGN - biglycan  PLP1 - proteolipid protein 1  PMVK - phosphomevalonate kinase  AKR1A1 - aldo-keto reductase family 1, member a1 (aldehyde reductase)  LPL - lipoprotein lipase  GMPS - guanine monphosphate synthase  OGDH - oxoglutarate (alpha-ketoglutarate) dehydrogenase (lipoamide)  SORD - sorbitol dehydrogenase  HOGA1 - 4-hydroxy-2-oxoglutarate aldolase 1  PDSS1 - prenyl (decaprenyl) diphosphate synthase, subunit 1  APRT - adenine phosphoribosyltransferase  GGT5 - gamma-glutamyltransferase 5  FADS3 - fatty acid desaturase 3  PECR - peroxisomal trans-2-enoyl-coa reductase  GLUL - glutamate-ammonia ligase  PDE8A - phosphodiesterase 8a  NUDT17 - nudix (nucleoside diphosphate linked moiety x)-type motif 17  PDE7A - phosphodiesterase 7a  ACSS1 - acyl-coa synthetase short-chain family member 1  TPI1 - triosephosphate isomerase 1  MDH2 - malate dehydrogenase 2, nad (mitochondrial)  SC5D - sterol-c5-desaturase  CDO1 - cysteine dioxygenase type 1  TECR - trans-2,3-enoyl-coa reductase  PTGS1 - prostaglandin-endoperoxide synthase 1 (prostaglandin g/h synthase and cyclooxygenase)  COQ2 - coenzyme q2 4-hydroxybenzoate polyprenyltransferase  INSIG1 - insulin induced gene 1  PCK2 - phosphoenolpyruvate carboxykinase 2 (mitochondrial)  ASL - argininosuccinate lyase  ADK - adenosine kinase  NUDT3 - nudix (nucleoside diphosphate linked moiety x)-type motif 3  MVD - mevalonate (diphospho) decarboxylase  PDE1A - phosphodiesterase 1a, calmodulin-dependent  SLC25A1 - solute carrier family 25 (mitochondrial carrier; citrate transporter), member 1  FADS6 - fatty acid desaturase 6  COQ10A - coenzyme q10 homolog a (s. cerevisiae)  ACSF3 - acyl-coa synthetase family member 3  PDE4D - phosphodiesterase 4d, camp-specific  CYP46A1 - cytochrome p450, family 46, subfamily a, polypeptide 1  PDE4A - phosphodiesterase 4a, camp-specific  CYP27A1 - cytochrome p450, family 27, subfamily a, polypeptide 1  PDE4B - phosphodiesterase 4b, camp-specific  PRKAA2 - protein kinase, amp-activated, alpha 2 catalytic subunit  XDH - xanthine dehydrogenase  PRKAB2 - protein kinase, amp-activated, beta 2 non-catalytic subunit  QKI - qki, kh domain containing, rna binding  MTR - 5-methyltetrahydrofolate-homocysteine methyltransferase  PGK1 - phosphoglycerate kinase 1  ACOT8 - acyl-coa thioesterase 8  ACCS - 1-aminocyclopropane-1-carboxylate synthase homolog (arabidopsis)(non-functional)  NME3 - nme/nm23 nucleoside diphosphate kinase 3  CTH - cystathionase (cystathionine gamma-lyase)  GPT2 - glutamic pyruvate transaminase (alanine aminotransferase) 2  ATF3 - activating transcription factor 3  RGN - regucalcin  NUDT1 - nudix (nucleoside diphosphate linked moiety x)-type motif 1  DPYD - dihydropyrimidine dehydrogenase  DUT - deoxyuridine triphosphatase  TM7SF2 - transmembrane 7 superfamily member 2  GLS2 - glutaminase 2 (liver, mitochondrial)  SLC1A3 - solute carrier family 1 (glial high affinity glutamate transporter), member 3  NT5C - 5', 3'-nucleotidase, cytosolic  DTYMK - deoxythymidylate kinase (thymidylate kinase)  ABCD1 - atp-binding cassette, sub-family d (ald), member 1  GALK1 - galactokinase 1  ALOX5AP - arachidonate 5-lipoxygenase-activating protein  ASNSD1 - asparagine synthetase domain containing 1  CSPG5 - chondroitin sulfate proteoglycan 5 (neuroglycan c)  EBP - emopamil binding protein (sterol isomerase)  ALDOA - aldolase a, fructose-bisphosphate  SLC39A14 - solute carrier family 39 (zinc transporter), member 14  SHMT2 - serine hydroxymethyltransferase 2 (mitochondrial)  UCKL1 - uridine-cytidine kinase 1-like 1  BDH1 - 3-hydroxybutyrate dehydrogenase, type 1  GATM - glycine amidinotransferase (l-arginine:glycine amidinotransferase)  MGLL - monoglyceride lipase  HSD3B7 - hydroxy-delta-5-steroid dehydrogenase, 3 beta- and steroid delta-isomerase 7  NDUFAB1 - nadh dehydrogenase (ubiquinone) 1, alpha/beta subcomplex, 1, 8kda  UGP2 - udp-glucose pyrophosphorylase 2  CNBP - cchc-type zinc finger, nucleic acid binding protein  UGDH - udp-glucose 6-dehydrogenase  NDUFA9 - nadh dehydrogenase (ubiquinone) 1 alpha subcomplex, 9, 39kda  AMPD3 - adenosine monophosphate deaminase 3  BCAN - brevican  HMGCL - 3-hydroxymethyl-3-methylglutaryl-coa lyase  RBP4 - retinol binding protein 4, plasma  GAPDH - glyceraldehyde-3-phosphate dehydrogenase  EPHX2 - epoxide hydrolase 2, cytoplasmic  CBS - cystathionine-beta-synthase  NCAN - neurocan  RBP1 - retinol binding protein 1, cellular  FPGS - folylpolyglutamate synthase |
| GO:0098662 | inorganic cation transmembrane transport | 5.45E-5 | 1.26E-2 | 1.52 (10334,294,1776,77) | [+] Show genes  SLC6A11 - solute carrier family 6 (neurotransmitter transporter), member 11  SLC4A10 - solute carrier family 4, sodium bicarbonate transporter, member 10  COX6B1 - cytochrome c oxidase subunit vib polypeptide 1 (ubiquitous)  SLC6A9 - solute carrier family 6 (neurotransmitter transporter, glycine), member 9  ATP2B2 - atpase, ca++ transporting, plasma membrane 2  KCNAB2 - potassium voltage-gated channel, shaker-related subfamily, beta member 2  ATP2A2 - atpase, ca++ transporting, cardiac muscle, slow twitch 2  TMEM38B - transmembrane protein 38b  SLC8A3 - solute carrier family 8 (sodium/calcium exchanger), member 3  COX4I1 - cytochrome c oxidase subunit iv isoform 1  MFSD3 - major facilitator superfamily domain containing 3  ATP5S - atp synthase, h+ transporting, mitochondrial fo complex, subunit s (factor b)  NIPA1 - non imprinted in prader-willi/angelman syndrome 1  SLC9A5 - solute carrier family 9, subfamily a (nhe5, cation proton antiporter 5), member 5  ATOX1 - antioxidant 1 copper chaperone  COX10 - cytochrome c oxidase assembly homolog 10 (yeast)  SLC9A2 - solute carrier family 9, subfamily a (nhe2, cation proton antiporter 2), member 2  SLC25A37 - solute carrier family 25 (mitochondrial iron transporter), member 37  COX7C - cytochrome c oxidase subunit viic  SLC4A4 - solute carrier family 4 (sodium bicarbonate cotransporter), member 4  COX1 - cytochrome c oxidase subunit i  TCIRG1 - t-cell, immune regulator 1, atpase, h+ transporting, lysosomal v0 subunit a3  SLC25A3 - solute carrier family 25 (mitochondrial carrier; phosphate carrier), member 3  COX7A1 - cytochrome c oxidase subunit viia polypeptide 1 (muscle)  CYTB - cytochrome b  COX6C - cytochrome c oxidase subunit vic  ASIC1 - acid-sensing (proton-gated) ion channel 1  P2RX4 - purinergic receptor p2x, ligand-gated ion channel, 4  ATP6V1G2 - atpase, h+ transporting, lysosomal 13kda, v1 subunit g2  CCL19 - chemokine (c-c motif) ligand 19  SLC8B1 - solute carrier family 8 (sodium/lithium/calcium exchanger), member b1  CACNB1 - calcium channel, voltage-dependent, beta 1 subunit  CACNA2D1 - calcium channel, voltage-dependent, alpha 2/delta subunit 1  ATP5J - atp synthase, h+ transporting, mitochondrial fo complex, subunit f6  MRS2 - mrs2 magnesium transporter  SLC1A3 - solute carrier family 1 (glial high affinity glutamate transporter), member 3  ATP5C1 - atp synthase, h+ transporting, mitochondrial f1 complex, gamma polypeptide 1  SLC39A8 - solute carrier family 39 (zinc transporter), member 8  ATP5F1 - atp synthase, h+ transporting, mitochondrial fo complex, subunit b1  ATP5G3 - atp synthase, h+ transporting, mitochondrial fo complex, subunit c3 (subunit 9)  ANO6 - anoctamin 6  SLC6A1 - solute carrier family 6 (neurotransmitter transporter), member 1  VDAC1 - voltage-dependent anion channel 1  CYC1 - cytochrome c-1  ATP5A1 - atp synthase, h+ transporting, mitochondrial f1 complex, alpha subunit 1, cardiac muscle  ATP5B - atp synthase, h+ transporting, mitochondrial f1 complex, beta polypeptide  CCL21 - chemokine (c-c motif) ligand 21  ATP5L - atp synthase, h+ transporting, mitochondrial fo complex, subunit g  SLC24A3 - solute carrier family 24 (sodium/potassium/calcium exchanger), member 3  CUL5 - cullin 5  MCU - mitochondrial calcium uniporter  SCN1B - sodium channel, voltage-gated, type i, beta subunit  SLC12A5 - solute carrier family 12 (potassium/chloride transporter), member 5  SLC25A28 - solute carrier family 25 (mitochondrial iron transporter), member 28  SLC39A14 - solute carrier family 39 (zinc transporter), member 14  COX5A - cytochrome c oxidase subunit va  TMEM37 - transmembrane protein 37  TMEM38A - transmembrane protein 38a  TRPV2 - transient receptor potential cation channel, subfamily v, member 2  ANXA9 - annexin a9  KCNK12 - potassium channel, subfamily k, member 12  SCN4B - sodium channel, voltage-gated, type iv, beta subunit  GRIN1 - glutamate receptor, ionotropic, n-methyl d-aspartate 1  KCNJ11 - potassium inwardly-rectifying channel, subfamily j, member 11  SLC39A12 - solute carrier family 39 (zinc transporter), member 12  NOL3 - nucleolar protein 3 (apoptosis repressor with card domain)  MICU2 - mitochondrial calcium uptake 2  KCNJ10 - potassium inwardly-rectifying channel, subfamily j, member 10  UCP2 - uncoupling protein 2 (mitochondrial, proton carrier)  PHB2 - prohibitin 2  NNT - nicotinamide nucleotide transhydrogenase  TPCN1 - two pore segment channel 1  SLC20A2 - solute carrier family 20 (phosphate transporter), member 2  SLC31A1 - solute carrier family 31 (copper transporter), member 1  ORAI1 - orai calcium release-activated calcium modulator 1  KCNB1 - potassium voltage-gated channel, shab-related subfamily, member 1  PTPRC - protein tyrosine phosphatase, receptor type, c |
| GO:0016999 | antibiotic metabolic process | 7.05E-5 | 1.61E-2 | 2.01 (10334,87,1776,30) | [+] Show genes  DLAT - dihydrolipoamide s-acetyltransferase  DLD - dihydrolipoamide dehydrogenase  ACSS1 - acyl-coa synthetase short-chain family member 1  SDHD - succinate dehydrogenase complex, subunit d, integral membrane protein  SUCLA2 - succinate-coa ligase, adp-forming, beta subunit  PDHB - pyruvate dehydrogenase (lipoamide) beta  SDHC - succinate dehydrogenase complex, subunit c, integral membrane protein, 15kda  MDH2 - malate dehydrogenase 2, nad (mitochondrial)  SUCLG2 - succinate-coa ligase, gdp-forming, beta subunit  DLST - dihydrolipoamide s-succinyltransferase (e2 component of 2-oxo-glutarate complex)  PDHA1 - pyruvate dehydrogenase (lipoamide) alpha 1  SDHA - succinate dehydrogenase complex, subunit a, flavoprotein (fp)  UROC1 - urocanate hydratase 1  ST3GAL1 - st3 beta-galactoside alpha-2,3-sialyltransferase 1  SULT1C4 - sulfotransferase family, cytosolic, 1c, member 4  RENBP - renin binding protein  CS - citrate synthase  PCK2 - phosphoenolpyruvate carboxykinase 2 (mitochondrial)  ACO1 - aconitase 1, soluble  ACO2 - aconitase 2, mitochondrial  AKR1A1 - aldo-keto reductase family 1, member a1 (aldehyde reductase)  NNT - nicotinamide nucleotide transhydrogenase  OGDH - oxoglutarate (alpha-ketoglutarate) dehydrogenase (lipoamide)  AMDHD2 - amidohydrolase domain containing 2  SULT1E1 - sulfotransferase family 1e, estrogen-preferring, member 1  SOD1 - superoxide dismutase 1, soluble  IDH3B - isocitrate dehydrogenase 3 (nad+) beta  CYBA - cytochrome b-245, alpha polypeptide  PRDX3 - peroxiredoxin 3  IDH2 - isocitrate dehydrogenase 2 (nadp+), mitochondrial |
| GO:0051704 | multi-organism process | 7.54E-5 | 1.69E-2 | 1.26 (10334,982,1776,213) | [+] Show genes  LGALS9 - lectin, galactoside-binding, soluble, 9  BNIP3 - bcl2/adenovirus e1b 19kda interacting protein 3  DHX58 - dexh (asp-glu-x-his) box polypeptide 58  EXOSC5 - exosome component 5  PLAC8 - placenta-specific 8  POLR3D - polymerase (rna) iii (dna directed) polypeptide d, 44kda  HSF1 - heat shock transcription factor 1  UNC93B1 - unc-93 homolog b1 (c. elegans)  RAB11FIP4 - rab11 family interacting protein 4 (class ii)  PYCARD - pyd and card domain containing  EPHA2 - eph receptor a2  CXCL14 - chemokine (c-x-c motif) ligand 14  EIF4EBP2 - eukaryotic translation initiation factor 4e binding protein 2  LPL - lipoprotein lipase  ACP5 - acid phosphatase 5, tartrate resistant  CRHR1 - corticotropin releasing hormone receptor 1  FAM46C - family with sequence similarity 46, member c  EIF4G1 - eukaryotic translation initiation factor 4 gamma, 1  CCDC86 - coiled-coil domain containing 86  ICAM1 - intercellular adhesion molecule 1  KRT19 - keratin 19  TRDC - t cell receptor delta constant  EEF1G - eukaryotic translation elongation factor 1 gamma  TP53 - tumor protein p53  KRT8 - keratin 8  NUP37 - nucleoporin 37kda  RNF5 - ring finger protein 5, e3 ubiquitin protein ligase  KRT18 - keratin 18  RPSA - ribosomal protein sa  LAMP1 - lysosomal-associated membrane protein 1  KIRREL3 - kin of irre like 3 (drosophila)  C1QA - complement component 1, q subcomponent, a chain  RBM38 - rna binding motif protein 38  C3 - complement component 3  SLC20A2 - solute carrier family 20 (phosphate transporter), member 2  FAM20A - family with sequence similarity 20, member a  ADM - adrenomedullin  NAMPT - nicotinamide phosphoribosyltransferase  OPTN - optineurin  BATF3 - basic leucine zipper transcription factor, atf-like 3  RPL24 - ribosomal protein l24  RPL23A - ribosomal protein l23a  ACOT8 - acyl-coa thioesterase 8  RPL21 - ribosomal protein l21  RPL19 - ribosomal protein l19  RPL18A - ribosomal protein l18a  RPL18 - ribosomal protein l18  USF2 - upstream transcription factor 2, c-fos interacting  RPL17 - ribosomal protein l17  RPL13 - ribosomal protein l13  RPL12 - ribosomal protein l12  RPL10 - ribosomal protein l10  RPL7A - ribosomal protein l7a  AGT - angiotensinogen (serpin peptidase inhibitor, clade a, member 8)  RPL5 - ribosomal protein l5  SUV39H1 - suppressor of variegation 3-9 homolog 1 (drosophila)  VAMP8 - vesicle-associated membrane protein 8  RPL4 - ribosomal protein l4  RPS5 - ribosomal protein s5  IPO5 - importin 5  RPS2 - ribosomal protein s2  NMT2 - n-myristoyltransferase 2  RPLP2 - ribosomal protein, large, p2  KPNA4 - karyopherin alpha 4 (importin alpha 3)  KPNA3 - karyopherin alpha 3 (importin alpha 4)  APLNR - apelin receptor  RPL36A - ribosomal protein l36a  MAFF - v-maf avian musculoaponeurotic fibrosarcoma oncogene homolog f  RPL37A - ribosomal protein l37a  RPL38 - ribosomal protein l38  ATXN1L - ataxin 1-like  RPL34 - ribosomal protein l34  CARM1 - coactivator-associated arginine methyltransferase 1  DEK - dek oncogene  CX3CR1 - chemokine (c-x3-c motif) receptor 1  RPL37 - ribosomal protein l37  RPL29 - ribosomal protein l29  RPL31 - ribosomal protein l31  RPL27 - ribosomal protein l27  VDAC1 - voltage-dependent anion channel 1  RPL30 - ribosomal protein l30  RPL27A - ribosomal protein l27a  RPL28 - ribosomal protein l28  IFI6 - interferon, alpha-inducible protein 6  RPS24 - ribosomal protein s24  RPS21 - ribosomal protein s21  CUL5 - cullin 5  RPS27 - ribosomal protein s27  IRF9 - interferon regulatory factor 9  HIPK2 - homeodomain interacting protein kinase 2  RPS26 - ribosomal protein s26  RPS25 - ribosomal protein s25  RPS17 - ribosomal protein s17  RPS16 - ribosomal protein s16  RPS15A - ribosomal protein s15a  STAT5B - signal transducer and activator of transcription 5b  RPS20 - ribosomal protein s20  RPS19 - ribosomal protein s19  MCTS1 - malignant t cell amplified sequence 1  RPS18 - ribosomal protein s18  NT5C3A - 5'-nucleotidase, cytosolic iiia  RPS11 - ribosomal protein s11  PRL - prolactin  IL17RC - interleukin 17 receptor c  RPS8 - ribosomal protein s8  PRLR - prolactin receptor  NRXN2 - neurexin 2  RPS9 - ribosomal protein s9  RPS14 - ribosomal protein s14  RPS13 - ribosomal protein s13  UCKL1 - uridine-cytidine kinase 1-like 1  RPS7 - ribosomal protein s7  MAPK14 - mitogen-activated protein kinase 14  ANG - angiogenin, ribonuclease, rnase a family, 5  IFITM3 - interferon induced transmembrane protein 3  GNB2L1 - guanine nucleotide binding protein (g protein), beta polypeptide 2-like 1  TAPT1 - transmembrane anterior posterior transformation 1  BIN1 - bridging integrator 1  WFDC2 - wap four-disulfide core domain 2  ROMO1 - reactive oxygen species modulator 1  RIOK3 - rio kinase 3  GSDMD - gasdermin d  NEXN - nexilin (f actin binding protein)  GAPDH - glyceraldehyde-3-phosphate dehydrogenase  RPL23 - ribosomal protein l23  RPS28 - ribosomal protein s28  UBE3A - ubiquitin protein ligase e3a  RPS29 - ribosomal protein s29  PRSS2 - protease, serine, 2 (trypsin 2)  F11R - f11 receptor  CXCL9 - chemokine (c-x-c motif) ligand 9  INPP5K - inositol polyphosphate-5-phosphatase k  INHBB - inhibin, beta b  PCBP1 - poly(rc) binding protein 1  PC - pyruvate carboxylase  SELPLG - selectin p ligand  RPL35 - ribosomal protein l35  CCL19 - chemokine (c-c motif) ligand 19  CD38 - cd38 molecule  PSMA4 - proteasome (prosome, macropain) subunit, alpha type, 4  IL2RG - interleukin 2 receptor, gamma  PSMB9 - proteasome (prosome, macropain) subunit, beta type, 9  CCL4 - chemokine (c-c motif) ligand 4  PSMB8 - proteasome (prosome, macropain) subunit, beta type, 8  AIMP1 - aminoacyl trna synthetase complex-interacting multifunctional protein 1  TREM2 - triggering receptor expressed on myeloid cells 2  PSMB5 - proteasome (prosome, macropain) subunit, beta type, 5  IKBKB - inhibitor of kappa light polypeptide gene enhancer in b-cells, kinase beta  IVNS1ABP - influenza virus ns1a binding protein  NUCKS1 - nuclear casein kinase and cyclin-dependent kinase substrate 1  APOE - apolipoprotein e  POLR3C - polymerase (rna) iii (dna directed) polypeptide c (62kd)  MOG - myelin oligodendrocyte glycoprotein  DDO - d-aspartate oxidase  FGR - feline gardner-rasheed sarcoma viral oncogene homolog  NLGN3 - neuroligin 3  RPL36 - ribosomal protein l36  IL6R - interleukin 6 receptor  ADIPOR2 - adiponectin receptor 2  TAF11 - taf11 rna polymerase ii, tata box binding protein (tbp)-associated factor, 28kda  BAIAP2L1 - bai1-associated protein 2-like 1  ATXN1 - ataxin 1  VAPA - vamp (vesicle-associated membrane protein)-associated protein a, 33kda  CHMP2A - charged multivesicular body protein 2a  ITGB6 - integrin, beta 6  ITGB7 - integrin, beta 7  HCK - hemopoietic cell kinase  SIVA1 - siva1, apoptosis-inducing factor  MET - met proto-oncogene  CXCL10 - chemokine (c-x-c motif) ligand 10  S100A9 - s100 calcium binding protein a9  HEXB - hexosaminidase b (beta polypeptide)  IRF1 - interferon regulatory factor 1  CLIC5 - chloride intracellular channel 5  SYNGR2 - synaptogyrin 2  FCER1G - fc fragment of ige, high affinity i, receptor for; gamma polypeptide  TCEB2 - transcription elongation factor b (siii), polypeptide 2 (18kda, elongin b)  TCEB1 - transcription elongation factor b (siii), polypeptide 1 (15kda, elongin c)  PTPRC - protein tyrosine phosphatase, receptor type, c  HIST2H2BE - histone cluster 2, h2be  DKKL1 - dickkopf-like 1  ULBP3 - ul16 binding protein 3  COTL1 - coactosin-like 1 (dictyostelium)  IFNAR2 - interferon (alpha, beta and omega) receptor 2  IRF8 - interferon regulatory factor 8  RCC1 - regulator of chromosome condensation 1  DVL1 - dishevelled segment polarity protein 1  PYGL - phosphorylase, glycogen, liver  TBKBP1 - tbk1 binding protein 1  CARD9 - caspase recruitment domain family, member 9  CFP - complement factor properdin  UBR3 - ubiquitin protein ligase e3 component n-recognin 3 (putative)  DDIT4 - dna-damage-inducible transcript 4  SCRIB - scribbled planar cell polarity protein  TEAD3 - tea domain family member 3  TF - transferrin  AKAP8L - a kinase (prka) anchor protein 8-like  NFIB - nuclear factor i/b  ISG15 - isg15 ubiquitin-like modifier  AVPR1A - arginine vasopressin receptor 1a  RARRES2 - retinoic acid receptor responder (tazarotene induced) 2  TFRC - transferrin receptor (p90, cd71)  LTBR - lymphotoxin beta receptor (tnfr superfamily, member 3)  GRIN1 - glutamate receptor, ionotropic, n-methyl d-aspartate 1  FCHSD1 - fch and double sh3 domains 1  AP2S1 - adaptor-related protein complex 2, sigma 1 subunit  IGFBP2 - insulin-like growth factor binding protein 2, 36kda  IGFBP5 - insulin-like growth factor binding protein 5  GPAM - glycerol-3-phosphate acyltransferase, mitochondrial  PHB2 - prohibitin 2  RPL10A - ribosomal protein l10a  BCL3 - b-cell cll/lymphoma 3  CUL4A - cullin 4a |
| GO:0030316 | osteoclast differentiation | 7.55E-5 | 1.67E-2 | 3.17 (10334,22,1776,12) | [+] Show genes  TRAF6 - tnf receptor-associated factor 6, e3 ubiquitin protein ligase  EPHA2 - eph receptor a2  GAB2 - grb2-associated binding protein 2  FCER1G - fc fragment of ige, high affinity i, receptor for; gamma polypeptide  TYROBP - tyro protein tyrosine kinase binding protein  TFRC - transferrin receptor (p90, cd71)  TF - transferrin  MITF - microphthalmia-associated transcription factor  TCIRG1 - t-cell, immune regulator 1, atpase, h+ transporting, lysosomal v0 subunit a3  GLO1 - glyoxalase i  TREM2 - triggering receptor expressed on myeloid cells 2  MAPK14 - mitogen-activated protein kinase 14 |

Species used: Homo sapiens

The system has recognized 12276 genes out of 13663 gene terms entered by the user.  
 12276 genes were recognized by gene symbol and 0 genes by other gene IDs .  
1847 duplicate genes were removed (keeping the highest ranking instance of each gene) leaving a total of 10429 genes.  
Only 10334 of these genes are associated with a GO term.

The GOrilla database is periodically updated using the GO database and other sources.  
The GOrilla database was last updated on Aug 29, 2020

This results page will be available on this site for one month from now (until
Oct 1, 2020
). You can bookmark this page and come back to it later.

  
**'P-value'** is the enrichment p-value computed according to the mHG or HG model. This p-value is not corrected for multiple testing of 13915 GO terms.  
  
**'FDR q-value'** is the correction of the above p-value for multiple testing using the Benjamini and Hochberg (1995) method.   
Namely, for the ith term (ranked according to p-value) the FDR q-value is (p-value \* number of GO terms) / i.   
  
**Enrichment (N, B, n, b)** is defined as follows:  
N - is the total number of genes  
B - is the total number of genes associated with a specific GO term  
n - is the number of genes in the top of the user's input list or in the target set when appropriate  
b - is the number of genes in the intersection  
Enrichment = (b/n) / (B/N)  
  
**Genes:** For each GO term you can see the list of associated genes that appear in the optimal top of the list.  
Each gene name is specified by gene symbol followed by a short description of the gene   

Back to the GOrilla main page
