# Supplementary material for: Genome-Wide Co-Expression Distributions as a Metric to Prioritize Genes of Functional Importance
Source: Genes (Basel). 2020 Oct 20;11(10):1231. doi: 10.3390/genes11101231 (PMC7593939; doi:10.3390/genes11101231)
Supplement: Supplementary file 1 [file genes-11-01231-s001.zip › SupplementaryFile3_FE_shape2.html]

Results


*P-value color scale*

|  |  |  |  |  |
| --- | --- | --- | --- | --- |
| > 10-3 | 10-3 to 10-5 | 10-5 to 10-7 | 10-7 to 10-9 | < 10-9 |


|  |  |  |  |  |  |
| --- | --- | --- | --- | --- | --- |
| **GO term** | **Description** | **P-value** | **FDR q-value** | **Enrichment (N, B, n, b)** | **Genes** |
| GO:1901566 | organonitrogen compound biosynthetic process | 4.16E-7 | 5.78E-3 | 1.70 (10334,738,716,87) | [+] Show genes  RPL26 - ribosomal protein l26  GSTM1 - glutathione s-transferase mu 1  ELOVL2 - elovl fatty acid elongase 2  UROS - uroporphyrinogen iii synthase  UROD - uroporphyrinogen decarboxylase  PNMT - phenylethanolamine n-methyltransferase  TMEM14C - transmembrane protein 14c  GSTA3 - glutathione s-transferase alpha 3  ABAT - 4-aminobutyrate aminotransferase  GOT1 - glutamic-oxaloacetic transaminase 1, soluble  DDC - dopa decarboxylase (aromatic l-amino acid decarboxylase)  SDC4 - syndecan 4  MRPS18A - mitochondrial ribosomal protein s18a  RPL8 - ribosomal protein l8  RPL6 - ribosomal protein l6  GPC1 - glypican 1  DCN - decorin  SLC25A15 - solute carrier family 25 (mitochondrial carrier; ornithine transporter) member 15  CHKB - choline kinase beta  RSAD1 - radical s-adenosyl methionine domain containing 1  CARNS1 - carnosine synthase 1  ACADM - acyl-coa dehydrogenase, c-4 to c-12 straight chain  GSTO1 - glutathione s-transferase omega 1  OPLAH - 5-oxoprolinase (atp-hydrolysing)  RPS3A - ribosomal protein s3a  MRPL55 - mitochondrial ribosomal protein l55  SPTSSB - serine palmitoyltransferase, small subunit b  ATPIF1 - atpase inhibitory factor 1  MRPL9 - mitochondrial ribosomal protein l9  GPC3 - glypican 3  ATP8 - atp synthase f0 subunit 8  RPS3 - ribosomal protein s3  NMRAL1 - nmra-like family domain containing 1  ATP5O - atp synthase, h+ transporting, mitochondrial f1 complex, o subunit  ATP6 - atp synthase f0 subunit 6  AK4 - adenylate kinase 4  RPLP0 - ribosomal protein, large, p0  FURIN - furin (paired basic amino acid cleaving enzyme)  AGPAT6 - 1-acylglycerol-3-phosphate o-acyltransferase 6  RPL39 - ribosomal protein l39  RPL35A - ribosomal protein l35a  STOML2 - stomatin (epb72)-like 2  NME6 - nme/nm23 nucleoside diphosphate kinase 6  ATP5G2 - atp synthase, h+ transporting, mitochondrial fo complex, subunit c2 (subunit 9)  B4GALT1 - udp-gal:betaglcnac beta 1,4- galactosyltransferase, polypeptide 1  GUK1 - guanylate kinase 1  ALAS1 - aminolevulinate, delta-, synthase 1  ALDH7A1 - aldehyde dehydrogenase 7 family, member a1  MPC2 - mitochondrial pyruvate carrier 2  MOCS2 - molybdenum cofactor synthesis 2  GPR37 - g protein-coupled receptor 37 (endothelin receptor type b-like)  MRPS16 - mitochondrial ribosomal protein s16  SLC35D1 - solute carrier family 35 (udp-glca/udp-galnac transporter), member d1  MRPL37 - mitochondrial ribosomal protein l37  ST8SIA5 - st8 alpha-n-acetyl-neuraminide alpha-2,8-sialyltransferase 5  SGPL1 - sphingosine-1-phosphate lyase 1  MRPL13 - mitochondrial ribosomal protein l13  RPS27A - ribosomal protein s27a  GLUD1 - glutamate dehydrogenase 1  PAPSS2 - 3'-phosphoadenosine 5'-phosphosulfate synthase 2  RPS10 - ribosomal protein s10  ESD - esterase d  MRPL42 - mitochondrial ribosomal protein l42  ST3GAL4 - st3 beta-galactoside alpha-2,3-sialyltransferase 4  ADCY7 - adenylate cyclase 7  SMPD2 - sphingomyelin phosphodiesterase 2, neutral membrane (neutral sphingomyelinase)  MRPL11 - mitochondrial ribosomal protein l11  PRKD2 - protein kinase d2  BBOX1 - butyrobetaine (gamma), 2-oxoglutarate dioxygenase (gamma-butyrobetaine hydroxylase) 1  NDST1 - n-deacetylase/n-sulfotransferase (heparan glucosaminyl) 1  MRPL16 - mitochondrial ribosomal protein l16  DBH - dopamine beta-hydroxylase (dopamine beta-monooxygenase)  MRPS7 - mitochondrial ribosomal protein s7  NPR1 - natriuretic peptide receptor a/guanylate cyclase a (atrionatriuretic peptide receptor a)  PANK1 - pantothenate kinase 1  CBR4 - carbonyl reductase 4  ST3GAL6 - st3 beta-galactoside alpha-2,3-sialyltransferase 6  APIP - apaf1 interacting protein  SLC11A2 - solute carrier family 11 (proton-coupled divalent metal ion transporter), member 2  MRPS14 - mitochondrial ribosomal protein s14  CKMT2 - creatine kinase, mitochondrial 2 (sarcomeric)  MFSD2A - major facilitator superfamily domain containing 2a  DHTKD1 - dehydrogenase e1 and transketolase domain containing 1  AK3 - adenylate kinase 3  ILVBL - ilvb (bacterial acetolactate synthase)-like  MGST2 - microsomal glutathione s-transferase 2  MGST3 - microsomal glutathione s-transferase 3 |
| GO:0070125 | mitochondrial translational elongation | 1.17E-6 | 8.15E-3 | 3.36 (10334,86,716,20) | [+] Show genes  MRPS16 - mitochondrial ribosomal protein s16  MRPL37 - mitochondrial ribosomal protein l37  MRPL22 - mitochondrial ribosomal protein l22  MRPL55 - mitochondrial ribosomal protein l55  MRPL13 - mitochondrial ribosomal protein l13  MRPL9 - mitochondrial ribosomal protein l9  MRPL16 - mitochondrial ribosomal protein l16  TUFM - tu translation elongation factor, mitochondrial  MRPS7 - mitochondrial ribosomal protein s7  MRPL19 - mitochondrial ribosomal protein l19  MRPL4 - mitochondrial ribosomal protein l4  MRPS18A - mitochondrial ribosomal protein s18a  MRPS14 - mitochondrial ribosomal protein s14  MRPS9 - mitochondrial ribosomal protein s9  MRPL42 - mitochondrial ribosomal protein l42  MRPL38 - mitochondrial ribosomal protein l38  MRPL46 - mitochondrial ribosomal protein l46  MRPL21 - mitochondrial ribosomal protein l21  MRPL11 - mitochondrial ribosomal protein l11  MRPS31 - mitochondrial ribosomal protein s31 |
| GO:0006415 | translational termination | 3.6E-6 | 1.67E-2 | 3.14 (10334,92,716,20) | [+] Show genes  APEH - acylaminoacyl-peptide hydrolase  MRPS16 - mitochondrial ribosomal protein s16  MRPL37 - mitochondrial ribosomal protein l37  MRPL22 - mitochondrial ribosomal protein l22  MRPL55 - mitochondrial ribosomal protein l55  MRPL13 - mitochondrial ribosomal protein l13  MRPL9 - mitochondrial ribosomal protein l9  MRPL16 - mitochondrial ribosomal protein l16  MRPS7 - mitochondrial ribosomal protein s7  MRPL19 - mitochondrial ribosomal protein l19  MRPL4 - mitochondrial ribosomal protein l4  MRPS18A - mitochondrial ribosomal protein s18a  MRPS14 - mitochondrial ribosomal protein s14  MRPL42 - mitochondrial ribosomal protein l42  MRPS9 - mitochondrial ribosomal protein s9  MRPL38 - mitochondrial ribosomal protein l38  MRPL46 - mitochondrial ribosomal protein l46  MRPL21 - mitochondrial ribosomal protein l21  MRPL11 - mitochondrial ribosomal protein l11  MRPS31 - mitochondrial ribosomal protein s31 |
| GO:0055085 | transmembrane transport | 4E-6 | 1.39E-2 | 1.65 (10334,710,716,81) | [+] Show genes  ND5 - nadh dehydrogenase, subunit 5 (complex i)  CHRNA3 - cholinergic receptor, nicotinic, alpha 3 (neuronal)  DDIT3 - dna-damage-inducible transcript 3  ND4 - nadh dehydrogenase, subunit 4 (complex i)  SFXN5 - sideroflexin 5  SLC7A2 - solute carrier family 7 (cationic amino acid transporter, y+ system), member 2  SLC31A2 - solute carrier family 31 (copper transporter), member 2  SLC16A3 - solute carrier family 16 (monocarboxylate transporter), member 3  TTYH1 - tweety homolog 1 (drosophila)  TIMM13 - translocase of inner mitochondrial membrane 13 homolog (yeast)  TIMM10 - translocase of inner mitochondrial membrane 10 homolog (yeast)  SLC16A6 - solute carrier family 16, member 6  ANXA6 - annexin a6  PEX5 - peroxisomal biogenesis factor 5  SLC25A15 - solute carrier family 25 (mitochondrial carrier; ornithine transporter) member 15  COX3 - cytochrome c oxidase iii  COX7B - cytochrome c oxidase subunit viib  CTNS - cystinosin, lysosomal cystine transporter  SLC46A3 - solute carrier family 46, member 3  PHB - prohibitin  PSMB3 - proteasome (prosome, macropain) subunit, beta type, 3  PSMB2 - proteasome (prosome, macropain) subunit, beta type, 2  PSMA7 - proteasome (prosome, macropain) subunit, alpha type, 7  SLC1A1 - solute carrier family 1 (neuronal/epithelial high affinity glutamate transporter, system xag), member 1  SLCO4A1 - solute carrier organic anion transporter family, member 4a1  SCARB1 - scavenger receptor class b, member 1  ATP5O - atp synthase, h+ transporting, mitochondrial f1 complex, o subunit  ATP8 - atp synthase f0 subunit 8  ATP6 - atp synthase f0 subunit 6  SLC25A11 - solute carrier family 25 (mitochondrial carrier; oxoglutarate carrier), member 11  GJA4 - gap junction protein, alpha 4, 37kda  CLDN4 - claudin 4  PSMB7 - proteasome (prosome, macropain) subunit, beta type, 7  SLC1A2 - solute carrier family 1 (glial high affinity glutamate transporter), member 2  PSMB6 - proteasome (prosome, macropain) subunit, beta type, 6  PSMB4 - proteasome (prosome, macropain) subunit, beta type, 4  CYB5A - cytochrome b5 type a (microsomal)  TMEM63B - transmembrane protein 63b  PSMC3 - proteasome (prosome, macropain) 26s subunit, atpase, 3  TST - thiosulfate sulfurtransferase (rhodanese)  SLC35A3 - solute carrier family 35 (udp-n-acetylglucosamine (udp-glcnac) transporter), member a3  SLC35C2 - solute carrier family 35 (gdp-fucose transporter), member c2  ABCC9 - atp-binding cassette, sub-family c (cftr/mrp), member 9  STOML2 - stomatin (epb72)-like 2  PSMC1 - proteasome (prosome, macropain) 26s subunit, atpase, 1  ZFAND2B - zinc finger, an1-type domain 2b  ATP5G2 - atp synthase, h+ transporting, mitochondrial fo complex, subunit c2 (subunit 9)  PSMD4 - proteasome (prosome, macropain) 26s subunit, non-atpase, 4  MPC2 - mitochondrial pyruvate carrier 2  AQP1 - aquaporin 1 (colton blood group)  SLC35D1 - solute carrier family 35 (udp-glca/udp-galnac transporter), member d1  PSME1 - proteasome (prosome, macropain) activator subunit 1 (pa28 alpha)  SFXN4 - sideroflexin 4  RPS27A - ribosomal protein s27a  PMPCB - peptidase (mitochondrial processing) beta  TOMM22 - translocase of outer mitochondrial membrane 22 homolog (yeast)  SLC22A23 - solute carrier family 22, member 23  SLC25A25 - solute carrier family 25 (mitochondrial carrier; phosphate carrier), member 25  SLC16A7 - solute carrier family 16 (monocarboxylate transporter), member 7  TAP1 - transporter 1, atp-binding cassette, sub-family b (mdr/tap)  PDZK1 - pdz domain containing 1  SLC38A3 - solute carrier family 38, member 3  TRAM2 - translocation associated membrane protein 2  PMPCA - peptidase (mitochondrial processing) alpha  TIMM17B - translocase of inner mitochondrial membrane 17 homolog b (yeast)  GRPEL1 - grpe-like 1, mitochondrial (e. coli)  KCNJ12 - potassium inwardly-rectifying channel, subfamily j, member 12  SPNS2 - spinster homolog 2 (drosophila)  CLIC3 - chloride intracellular channel 3  SLC25A42 - solute carrier family 25, member 42  NDUFA13 - nadh dehydrogenase (ubiquinone) 1 alpha subcomplex, 13  TMEM120A - transmembrane protein 120a  SLC11A2 - solute carrier family 11 (proton-coupled divalent metal ion transporter), member 2  ABCC1 - atp-binding cassette, sub-family c (cftr/mrp), member 1  MPV17 - mpv17 mitochondrial inner membrane protein  MFSD2A - major facilitator superfamily domain containing 2a  TMC8 - transmembrane channel-like 8  SLC41A2 - solute carrier family 41 (magnesium transporter), member 2  ATP6V0E1 - atpase, h+ transporting, lysosomal 9kda, v0 subunit e1  LONP2 - lon peptidase 2, peroxisomal  FXYD7 - fxyd domain containing ion transport regulator 7 |
| GO:0070126 | mitochondrial translational termination | 4.88E-6 | 1.36E-2 | 3.19 (10334,86,716,19) | [+] Show genes  MRPS16 - mitochondrial ribosomal protein s16  MRPL37 - mitochondrial ribosomal protein l37  MRPL22 - mitochondrial ribosomal protein l22  MRPL55 - mitochondrial ribosomal protein l55  MRPL13 - mitochondrial ribosomal protein l13  MRPL9 - mitochondrial ribosomal protein l9  MRPL16 - mitochondrial ribosomal protein l16  MRPS7 - mitochondrial ribosomal protein s7  MRPL19 - mitochondrial ribosomal protein l19  MRPL4 - mitochondrial ribosomal protein l4  MRPS18A - mitochondrial ribosomal protein s18a  MRPS14 - mitochondrial ribosomal protein s14  MRPS9 - mitochondrial ribosomal protein s9  MRPL42 - mitochondrial ribosomal protein l42  MRPL38 - mitochondrial ribosomal protein l38  MRPL46 - mitochondrial ribosomal protein l46  MRPL21 - mitochondrial ribosomal protein l21  MRPL11 - mitochondrial ribosomal protein l11  MRPS31 - mitochondrial ribosomal protein s31 |
| GO:0006518 | peptide metabolic process | 2.07E-5 | 4.81E-2 | 2.01 (10334,280,716,39) | [+] Show genes  GLRX2 - glutaredoxin 2  RPL26 - ribosomal protein l26  GSTA5 - glutathione s-transferase alpha 5  MRPS16 - mitochondrial ribosomal protein s16  MRPL37 - mitochondrial ribosomal protein l37  GSTM1 - glutathione s-transferase mu 1  APEH - acylaminoacyl-peptide hydrolase  ERAP1 - endoplasmic reticulum aminopeptidase 1  MRPL13 - mitochondrial ribosomal protein l13  RPS27A - ribosomal protein s27a  GSTA3 - glutathione s-transferase alpha 3  MRPS18A - mitochondrial ribosomal protein s18a  RPS10 - ribosomal protein s10  RPL8 - ribosomal protein l8  FSHB - follicle stimulating hormone, beta polypeptide  RPL6 - ribosomal protein l6  MRPL42 - mitochondrial ribosomal protein l42  CARNS1 - carnosine synthase 1  CTNS - cystinosin, lysosomal cystine transporter  MRPL11 - mitochondrial ribosomal protein l11  OPLAH - 5-oxoprolinase (atp-hydrolysing)  RPS3A - ribosomal protein s3a  MRPL55 - mitochondrial ribosomal protein l55  MRPL9 - mitochondrial ribosomal protein l9  MME - membrane metallo-endopeptidase  RPS3 - ribosomal protein s3  MRPL16 - mitochondrial ribosomal protein l16  MRPS7 - mitochondrial ribosomal protein s7  DNPEP - aspartyl aminopeptidase  FURIN - furin (paired basic amino acid cleaving enzyme)  RPLP0 - ribosomal protein, large, p0  IDH1 - isocitrate dehydrogenase 1 (nadp+), soluble  MRPS14 - mitochondrial ribosomal protein s14  IDE - insulin-degrading enzyme  RPL39 - ribosomal protein l39  RPL35A - ribosomal protein l35a  CPXM1 - carboxypeptidase x (m14 family), member 1  TSHB - thyroid stimulating hormone, beta  MGST2 - microsomal glutathione s-transferase 2 |
| GO:0033238 | regulation of cellular amine metabolic process | 3.38E-5 | 6.72E-2 | 3.28 (10334,66,716,15) | [+] Show genes  PSMB3 - proteasome (prosome, macropain) subunit, beta type, 3  PSMB2 - proteasome (prosome, macropain) subunit, beta type, 2  PSME1 - proteasome (prosome, macropain) activator subunit 1 (pa28 alpha)  PSMA7 - proteasome (prosome, macropain) subunit, alpha type, 7  ABAT - 4-aminobutyrate aminotransferase  PSMB7 - proteasome (prosome, macropain) subunit, beta type, 7  PSMB6 - proteasome (prosome, macropain) subunit, beta type, 6  PSMB4 - proteasome (prosome, macropain) subunit, beta type, 4  CLN3 - ceroid-lipofuscinosis, neuronal 3  PSMC3 - proteasome (prosome, macropain) 26s subunit, atpase, 3  PSMC1 - proteasome (prosome, macropain) 26s subunit, atpase, 1  MAOB - monoamine oxidase b  SIRT4 - sirtuin 4  PSMD4 - proteasome (prosome, macropain) 26s subunit, non-atpase, 4  GPR37 - g protein-coupled receptor 37 (endothelin receptor type b-like) |
| GO:0006414 | translational elongation | 4.41E-5 | 7.67E-2 | 2.67 (10334,108,716,20) | [+] Show genes  MRPS16 - mitochondrial ribosomal protein s16  MRPL37 - mitochondrial ribosomal protein l37  MRPL22 - mitochondrial ribosomal protein l22  MRPL55 - mitochondrial ribosomal protein l55  MRPL13 - mitochondrial ribosomal protein l13  MRPL9 - mitochondrial ribosomal protein l9  MRPL16 - mitochondrial ribosomal protein l16  TUFM - tu translation elongation factor, mitochondrial  MRPS7 - mitochondrial ribosomal protein s7  MRPL19 - mitochondrial ribosomal protein l19  MRPL4 - mitochondrial ribosomal protein l4  MRPS18A - mitochondrial ribosomal protein s18a  MRPS14 - mitochondrial ribosomal protein s14  MRPL42 - mitochondrial ribosomal protein l42  MRPS9 - mitochondrial ribosomal protein s9  MRPL38 - mitochondrial ribosomal protein l38  MRPL21 - mitochondrial ribosomal protein l21  MRPL46 - mitochondrial ribosomal protein l46  MRPL11 - mitochondrial ribosomal protein l11  MRPS31 - mitochondrial ribosomal protein s31 |
| GO:0002479 | antigen processing and presentation of exogenous peptide antigen via MHC class I, TAP-dependent | 5.48E-5 | 8.47E-2 | 3.31 (10334,61,716,14) | [+] Show genes  PSMB3 - proteasome (prosome, macropain) subunit, beta type, 3  PSMB2 - proteasome (prosome, macropain) subunit, beta type, 2  PSME1 - proteasome (prosome, macropain) activator subunit 1 (pa28 alpha)  PSMA7 - proteasome (prosome, macropain) subunit, alpha type, 7  IKBKG - inhibitor of kappa light polypeptide gene enhancer in b-cells, kinase gamma  PSMB7 - proteasome (prosome, macropain) subunit, beta type, 7  PSMB6 - proteasome (prosome, macropain) subunit, beta type, 6  PSMB4 - proteasome (prosome, macropain) subunit, beta type, 4  B2M - beta-2-microglobulin  FCGR1A - fc fragment of igg, high affinity ia, receptor (cd64)  PSMC3 - proteasome (prosome, macropain) 26s subunit, atpase, 3  PSMC1 - proteasome (prosome, macropain) 26s subunit, atpase, 1  TAP1 - transporter 1, atp-binding cassette, sub-family b (mdr/tap)  PSMD4 - proteasome (prosome, macropain) 26s subunit, non-atpase, 4 |

Species used: Homo sapiens

The system has recognized 11204 genes out of 12485 gene terms entered by the user.  
 11204 genes were recognized by gene symbol and 0 genes by other gene IDs .  
775 duplicate genes were removed (keeping the highest ranking instance of each gene) leaving a total of 10429 genes.  
Only 10334 of these genes are associated with a GO term.

The GOrilla database is periodically updated using the GO database and other sources.  
The GOrilla database was last updated on Aug 29, 2020

This results page will be available on this site for one month from now (until
Oct 1, 2020
). You can bookmark this page and come back to it later.

  
**'P-value'** is the enrichment p-value computed according to the mHG or HG model. This p-value is not corrected for multiple testing of 13915 GO terms.  
  
**'FDR q-value'** is the correction of the above p-value for multiple testing using the Benjamini and Hochberg (1995) method.   
Namely, for the ith term (ranked according to p-value) the FDR q-value is (p-value \* number of GO terms) / i.   
  
**Enrichment (N, B, n, b)** is defined as follows:  
N - is the total number of genes  
B - is the total number of genes associated with a specific GO term  
n - is the number of genes in the top of the user's input list or in the target set when appropriate  
b - is the number of genes in the intersection  
Enrichment = (b/n) / (B/N)  
  
**Genes:** For each GO term you can see the list of associated genes that appear in the optimal top of the list.  
Each gene name is specified by gene symbol followed by a short description of the gene   

Back to the GOrilla main page
