# Supplementary material for: Genome-Wide Co-Expression Distributions as a Metric to Prioritize Genes of Functional Importance
Source: Genes (Basel). 2020 Oct 20;11(10):1231. doi: 10.3390/genes11101231 (PMC7593939; doi:10.3390/genes11101231)
Supplement: Supplementary file 1 [file genes-11-01231-s001.zip › SupplementaryFile4_FE_shape3.html]

Results


*P-value color scale*

|  |  |  |  |  |
| --- | --- | --- | --- | --- |
| > 10-3 | 10-3 to 10-5 | 10-5 to 10-7 | 10-7 to 10-9 | < 10-9 |


|  |  |  |  |  |  |
| --- | --- | --- | --- | --- | --- |
| **GO term** | **Description** | **P-value** | **FDR q-value** | **Enrichment (N, B, n, b)** | **Genes** |
| GO:0044281 | small molecule metabolic process | 2.09E-34 | 2.91E-30 | 2.38 (10334,1275,667,196) | [+] Show genes  COX5B - cytochrome c oxidase subunit vb  SUCLG1 - succinate-coa ligase, alpha subunit  AMACR - alpha-methylacyl-coa racemase  BDH2 - 3-hydroxybutyrate dehydrogenase, type 2  ENO3 - enolase 3 (beta, muscle)  CES2 - carboxylesterase 2  CA14 - carbonic anhydrase xiv  LDHA - lactate dehydrogenase a  ACAA1 - acetyl-coa acyltransferase 1  SLC25A13 - solute carrier family 25 (aspartate/glutamate carrier), member 13  ACADL - acyl-coa dehydrogenase, long chain  ACADS - acyl-coa dehydrogenase, c-2 to c-3 short chain  ACADVL - acyl-coa dehydrogenase, very long chain  CPT2 - carnitine palmitoyltransferase 2  ATP5J2 - atp synthase, h+ transporting, mitochondrial fo complex, subunit f2  TTR - transthyretin  DHRS4 - dehydrogenase/reductase (sdr family) member 4  CPT1B - carnitine palmitoyltransferase 1b (muscle)  CPS1 - carbamoyl-phosphate synthase 1, mitochondrial  EHHADH - enoyl-coa, hydratase/3-hydroxyacyl coa dehydrogenase  BHMT - betaine--homocysteine s-methyltransferase  HPD - 4-hydroxyphenylpyruvate dioxygenase  TTPA - tocopherol (alpha) transfer protein  MMAB - methylmalonic aciduria (cobalamin deficiency) cblb type  GHR - growth hormone receptor  PIPOX - pipecolic acid oxidase  LIPC - lipase, hepatic  ADHFE1 - alcohol dehydrogenase, iron containing, 1  SMPDL3A - sphingomyelin phosphodiesterase, acid-like 3a  APOBEC2 - apolipoprotein b mrna editing enzyme, catalytic polypeptide-like 2  GGT1 - gamma-glutamyltransferase 1  ACOX2 - acyl-coa oxidase 2, branched chain  FAHD1 - fumarylacetoacetate hydrolase domain containing 1  VNN1 - vanin 1  CA3 - carbonic anhydrase iii, muscle specific  PPARGC1A - peroxisome proliferator-activated receptor gamma, coactivator 1 alpha  ADA - adenosine deaminase  PM20D1 - peptidase m20 domain containing 1  PPARA - peroxisome proliferator-activated receptor alpha  PON1 - paraoxonase 1  APOA5 - apolipoprotein a-v  GLYAT - glycine-n-acyltransferase  FTCD - formimidoyltransferase cyclodeaminase  ALDH1L1 - aldehyde dehydrogenase 1 family, member l1  ADH6 - alcohol dehydrogenase 6 (class v)  ADH4 - alcohol dehydrogenase 4 (class ii), pi polypeptide  DGAT2 - diacylglycerol o-acyltransferase 2  PRODH2 - proline dehydrogenase (oxidase) 2  ECHS1 - enoyl coa hydratase, short chain, 1, mitochondrial  SLC17A1 - solute carrier family 17 (organic anion transporter), member 1  ECH1 - enoyl coa hydratase 1, peroxisomal  ECI2 - enoyl-coa delta isomerase 2  AKR1C3 - aldo-keto reductase family 1, member c3  HSD17B8 - hydroxysteroid (17-beta) dehydrogenase 8  UQCRC1 - ubiquinol-cytochrome c reductase core protein i  ACAA2 - acetyl-coa acyltransferase 2  AGMAT - agmatine ureohydrolase (agmatinase)  MCEE - methylmalonyl coa epimerase  MTHFR - methylenetetrahydrofolate reductase (nad(p)h)  ACSL1 - acyl-coa synthetase long-chain family member 1  MTHFD1 - methylenetetrahydrofolate dehydrogenase (nadp+ dependent) 1, methenyltetrahydrofolate cyclohydrolase, formyltetrahydrofolate synthetase  ALDH4A1 - aldehyde dehydrogenase 4 family, member a1  CHCHD10 - coiled-coil-helix-coiled-coil-helix domain containing 10  GK5 - glycerol kinase 5 (putative)  FAH - fumarylacetoacetate hydrolase (fumarylacetoacetase)  ASPG - asparaginase homolog (s. cerevisiae)  ABHD1 - abhydrolase domain containing 1  PNPLA2 - patatin-like phospholipase domain containing 2  AK2 - adenylate kinase 2  SLC37A4 - solute carrier family 37 (glucose-6-phosphate transporter), member 4  CHST13 - carbohydrate (chondroitin 4) sulfotransferase 13  AHCY - adenosylhomocysteinase  SULT2A1 - sulfotransferase family, cytosolic, 2a, dehydroepiandrosterone (dhea)-preferring, member 1  ATP5H - atp synthase, h+ transporting, mitochondrial fo complex, subunit d  AGXT - alanine-glyoxylate aminotransferase  COQ4 - coenzyme q4 homolog (s. cerevisiae)  HGD - homogentisate 1,2-dioxygenase  MSRA - methionine sulfoxide reductase a  COQ9 - coenzyme q9 homolog (s. cerevisiae)  G6PC - glucose-6-phosphatase, catalytic subunit  ALDH2 - aldehyde dehydrogenase 2 family (mitochondrial)  ACSM5 - acyl-coa synthetase medium-chain family member 5  AGXT2 - alanine--glyoxylate aminotransferase 2  MACROD1 - macro domain containing 1  GAMT - guanidinoacetate n-methyltransferase  PLCD4 - phospholipase c, delta 4  LPIN1 - lipin 1  ALKBH7 - alkb, alkylation repair homolog 7 (e. coli)  ALDOB - aldolase b, fructose-bisphosphate  GRHPR - glyoxylate reductase/hydroxypyruvate reductase  ADTRP - androgen-dependent tfpi-regulating protein  ETFDH - electron-transferring-flavoprotein dehydrogenase  GCDH - glutaryl-coa dehydrogenase  CAT - catalase  GC - group-specific component (vitamin d binding protein)  RXRA - retinoid x receptor, alpha  UCP3 - uncoupling protein 3 (mitochondrial, proton carrier)  AMPD1 - adenosine monophosphate deaminase 1  HMGCS2 - 3-hydroxy-3-methylglutaryl-coa synthase 2 (mitochondrial)  CBR1 - carbonyl reductase 1  SLC27A2 - solute carrier family 27 (fatty acid transporter), member 2  FBP2 - fructose-1,6-bisphosphatase 2  MPST - mercaptopyruvate sulfurtransferase  SLC27A5 - solute carrier family 27 (fatty acid transporter), member 5  URAD - ureidoimidazoline (2-oxo-4-hydroxy-4-carboxy-5-) decarboxylase  CCBL1 - cysteine conjugate-beta lyase, cytoplasmic  HAO2 - hydroxyacid oxidase 2 (long chain)  ANGPTL3 - angiopoietin-like 3  LGMN - legumain  GADL1 - glutamate decarboxylase-like 1  APOA2 - apolipoprotein a-ii  SDHB - succinate dehydrogenase complex, subunit b, iron sulfur (ip)  ENTPD8 - ectonucleoside triphosphate diphosphohydrolase 8  FMO1 - flavin containing monooxygenase 1  APOA4 - apolipoprotein a-iv  OTC - ornithine carbamoyltransferase  IMPA2 - inositol(myo)-1(or 4)-monophosphatase 2  APOF - apolipoprotein f  GSTZ1 - glutathione s-transferase zeta 1  ACSM2A - acyl-coa synthetase medium-chain family member 2a  DHRS3 - dehydrogenase/reductase (sdr family) member 3  ASPDH - aspartate dehydrogenase domain containing  CD36 - cd36 molecule (thrombospondin receptor)  TDO2 - tryptophan 2,3-dioxygenase  PAH - phenylalanine hydroxylase  FH - fumarate hydratase  MOCS1 - molybdenum cofactor synthesis 1  UPB1 - ureidopropionase, beta  CYP4A11 - cytochrome p450, family 4, subfamily a, polypeptide 11  NMRK2 - nicotinamide riboside kinase 2  CYP8B1 - cytochrome p450, family 8, subfamily b, polypeptide 1  CES1 - carboxylesterase 1  ALDH8A1 - aldehyde dehydrogenase 8 family, member a1  CYP2E1 - cytochrome p450, family 2, subfamily e, polypeptide 1  CYP3A5 - cytochrome p450, family 3, subfamily a, polypeptide 5  SCARF1 - scavenger receptor class f, member 1  ADSSL1 - adenylosuccinate synthase like 1  CYP2C19 - cytochrome p450, family 2, subfamily c, polypeptide 19  ENPP1 - ectonucleotide pyrophosphatase/phosphodiesterase 1  TAT - tyrosine aminotransferase  ITIH1 - inter-alpha-trypsin inhibitor heavy chain 1  PDK4 - pyruvate dehydrogenase kinase, isozyme 4  ARG1 - arginase 1  ITIH2 - inter-alpha-trypsin inhibitor heavy chain 2  HADH - hydroxyacyl-coa dehydrogenase  ITIH3 - inter-alpha-trypsin inhibitor heavy chain 3  SCP2 - sterol carrier protein 2  PDK2 - pyruvate dehydrogenase kinase, isozyme 2  ITIH4 - inter-alpha-trypsin inhibitor heavy chain family, member 4  HAGH - hydroxyacylglutathione hydrolase  CYP1A2 - cytochrome p450, family 1, subfamily a, polypeptide 2  PTPLA - protein tyrosine phosphatase-like (proline instead of catalytic arginine), member a  HSD17B10 - hydroxysteroid (17-beta) dehydrogenase 10  IYD - iodotyrosine deiodinase  PCK1 - phosphoenolpyruvate carboxykinase 1 (soluble)  ASS1 - argininosuccinate synthase 1  GIMAP7 - gtpase, imap family member 7  TMEM86B - transmembrane protein 86b  GLYCTK - glycerate kinase  RDH16 - retinol dehydrogenase 16 (all-trans)  CRYL1 - crystallin, lambda 1  AGPAT2 - 1-acylglycerol-3-phosphate o-acyltransferase 2  ACSM3 - acyl-coa synthetase medium-chain family member 3  MMACHC - methylmalonic aciduria (cobalamin deficiency) cblc type, with homocystinuria  SEPSECS - sep (o-phosphoserine) trna:sec (selenocysteine) trna synthase  MLYCD - malonyl-coa decarboxylase  PGM1 - phosphoglucomutase 1  PGAM2 - phosphoglycerate mutase 2 (muscle)  ATF4 - activating transcription factor 4  GOT2 - glutamic-oxaloacetic transaminase 2, mitochondrial  GPD1 - glycerol-3-phosphate dehydrogenase 1 (soluble)  DPYS - dihydropyrimidinase  AKR1C4 - aldo-keto reductase family 1, member c4  GCAT - glycine c-acetyltransferase  UGT1A6 - udp glucuronosyltransferase 1 family, polypeptide a6  ATP5I - atp synthase, h+ transporting, mitochondrial fo complex, subunit e  MAT1A - methionine adenosyltransferase i, alpha  ATP5E - atp synthase, h+ transporting, mitochondrial f1 complex, epsilon subunit  HACL1 - 2-hydroxyacyl-coa lyase 1  ATP5D - atp synthase, h+ transporting, mitochondrial f1 complex, delta subunit  ATP5G1 - atp synthase, h+ transporting, mitochondrial fo complex, subunit c1 (subunit 9)  IDH3G - isocitrate dehydrogenase 3 (nad+) gamma  DECR2 - 2,4-dienoyl coa reductase 2, peroxisomal  PFKFB1 - 6-phosphofructo-2-kinase/fructose-2,6-biphosphatase 1  SHMT1 - serine hydroxymethyltransferase 1 (soluble)  ST3GAL3 - st3 beta-galactoside alpha-2,3-sialyltransferase 3  MPC1 - mitochondrial pyruvate carrier 1  PKLR - pyruvate kinase, liver and rbc  HAAO - 3-hydroxyanthranilate 3,4-dioxygenase  ECHDC2 - enoyl coa hydratase domain containing 2  IGF1 - insulin-like growth factor 1 (somatomedin c)  ADI1 - acireductone dioxygenase 1  PHYH - phytanoyl-coa 2-hydroxylase  CKM - creatine kinase, muscle  NDUFS6 - nadh dehydrogenase (ubiquinone) fe-s protein 6, 13kda (nadh-coenzyme q reductase)  FGGY - fggy carbohydrate kinase domain containing |
| GO:0006082 | organic acid metabolic process | 3.96E-34 | 2.76E-30 | 2.99 (10334,721,667,139) | [+] Show genes  ANGPTL3 - angiopoietin-like 3  GADL1 - glutamate decarboxylase-like 1  SDHB - succinate dehydrogenase complex, subunit b, iron sulfur (ip)  SUCLG1 - succinate-coa ligase, alpha subunit  FMO1 - flavin containing monooxygenase 1  AMACR - alpha-methylacyl-coa racemase  BDH2 - 3-hydroxybutyrate dehydrogenase, type 2  OTC - ornithine carbamoyltransferase  ENO3 - enolase 3 (beta, muscle)  CES2 - carboxylesterase 2  LDHA - lactate dehydrogenase a  GSTZ1 - glutathione s-transferase zeta 1  ACAA1 - acetyl-coa acyltransferase 1  ACADL - acyl-coa dehydrogenase, long chain  ACADS - acyl-coa dehydrogenase, c-2 to c-3 short chain  ACSM2A - acyl-coa synthetase medium-chain family member 2a  ACADVL - acyl-coa dehydrogenase, very long chain  CPT2 - carnitine palmitoyltransferase 2  CPT1B - carnitine palmitoyltransferase 1b (muscle)  CPS1 - carbamoyl-phosphate synthase 1, mitochondrial  CD36 - cd36 molecule (thrombospondin receptor)  EHHADH - enoyl-coa, hydratase/3-hydroxyacyl coa dehydrogenase  BHMT - betaine--homocysteine s-methyltransferase  HPD - 4-hydroxyphenylpyruvate dioxygenase  TDO2 - tryptophan 2,3-dioxygenase  LIPC - lipase, hepatic  GHR - growth hormone receptor  PIPOX - pipecolic acid oxidase  ADHFE1 - alcohol dehydrogenase, iron containing, 1  PAH - phenylalanine hydroxylase  FH - fumarate hydratase  GGT1 - gamma-glutamyltransferase 1  UPB1 - ureidopropionase, beta  ACOX2 - acyl-coa oxidase 2, branched chain  FAHD1 - fumarylacetoacetate hydrolase domain containing 1  CYP4A11 - cytochrome p450, family 4, subfamily a, polypeptide 11  CYP8B1 - cytochrome p450, family 8, subfamily b, polypeptide 1  ALDH8A1 - aldehyde dehydrogenase 8 family, member a1  CES1 - carboxylesterase 1  CYP2E1 - cytochrome p450, family 2, subfamily e, polypeptide 1  CYP3A5 - cytochrome p450, family 3, subfamily a, polypeptide 5  ADSSL1 - adenylosuccinate synthase like 1  VNN1 - vanin 1  CYP2C19 - cytochrome p450, family 2, subfamily c, polypeptide 19  ENPP1 - ectonucleotide pyrophosphatase/phosphodiesterase 1  TAT - tyrosine aminotransferase  PPARGC1A - peroxisome proliferator-activated receptor gamma, coactivator 1 alpha  ITIH1 - inter-alpha-trypsin inhibitor heavy chain 1  ITIH2 - inter-alpha-trypsin inhibitor heavy chain 2  ARG1 - arginase 1  ITIH3 - inter-alpha-trypsin inhibitor heavy chain 3  SCP2 - sterol carrier protein 2  HADH - hydroxyacyl-coa dehydrogenase  ITIH4 - inter-alpha-trypsin inhibitor heavy chain family, member 4  PM20D1 - peptidase m20 domain containing 1  HAGH - hydroxyacylglutathione hydrolase  CYP1A2 - cytochrome p450, family 1, subfamily a, polypeptide 2  PPARA - peroxisome proliferator-activated receptor alpha  PTPLA - protein tyrosine phosphatase-like (proline instead of catalytic arginine), member a  HSD17B10 - hydroxysteroid (17-beta) dehydrogenase 10  PON1 - paraoxonase 1  IYD - iodotyrosine deiodinase  PCK1 - phosphoenolpyruvate carboxykinase 1 (soluble)  GLYAT - glycine-n-acyltransferase  FTCD - formimidoyltransferase cyclodeaminase  ADH6 - alcohol dehydrogenase 6 (class v)  ALDH1L1 - aldehyde dehydrogenase 1 family, member l1  ASS1 - argininosuccinate synthase 1  ADH4 - alcohol dehydrogenase 4 (class ii), pi polypeptide  PRODH2 - proline dehydrogenase (oxidase) 2  CRYL1 - crystallin, lambda 1  ECHS1 - enoyl coa hydratase, short chain, 1, mitochondrial  SLC17A1 - solute carrier family 17 (organic anion transporter), member 1  ACSM3 - acyl-coa synthetase medium-chain family member 3  SEPSECS - sep (o-phosphoserine) trna:sec (selenocysteine) trna synthase  ECH1 - enoyl coa hydratase 1, peroxisomal  ECI2 - enoyl-coa delta isomerase 2  HSD17B8 - hydroxysteroid (17-beta) dehydrogenase 8  AKR1C3 - aldo-keto reductase family 1, member c3  MLYCD - malonyl-coa decarboxylase  ACAA2 - acetyl-coa acyltransferase 2  AGMAT - agmatine ureohydrolase (agmatinase)  PGM1 - phosphoglucomutase 1  PGAM2 - phosphoglycerate mutase 2 (muscle)  MTHFR - methylenetetrahydrofolate reductase (nad(p)h)  MCEE - methylmalonyl coa epimerase  ACSL1 - acyl-coa synthetase long-chain family member 1  MTHFD1 - methylenetetrahydrofolate dehydrogenase (nadp+ dependent) 1, methenyltetrahydrofolate cyclohydrolase, formyltetrahydrofolate synthetase  ATF4 - activating transcription factor 4  GOT2 - glutamic-oxaloacetic transaminase 2, mitochondrial  FAH - fumarylacetoacetate hydrolase (fumarylacetoacetase)  ALDH4A1 - aldehyde dehydrogenase 4 family, member a1  ASPG - asparaginase homolog (s. cerevisiae)  ABHD1 - abhydrolase domain containing 1  DPYS - dihydropyrimidinase  AKR1C4 - aldo-keto reductase family 1, member c4  GCAT - glycine c-acetyltransferase  CHST13 - carbohydrate (chondroitin 4) sulfotransferase 13  UGT1A6 - udp glucuronosyltransferase 1 family, polypeptide a6  AHCY - adenosylhomocysteinase  SULT2A1 - sulfotransferase family, cytosolic, 2a, dehydroepiandrosterone (dhea)-preferring, member 1  AGXT - alanine-glyoxylate aminotransferase  MAT1A - methionine adenosyltransferase i, alpha  HGD - homogentisate 1,2-dioxygenase  MSRA - methionine sulfoxide reductase a  HACL1 - 2-hydroxyacyl-coa lyase 1  IDH3G - isocitrate dehydrogenase 3 (nad+) gamma  G6PC - glucose-6-phosphatase, catalytic subunit  DECR2 - 2,4-dienoyl coa reductase 2, peroxisomal  PFKFB1 - 6-phosphofructo-2-kinase/fructose-2,6-biphosphatase 1  ACSM5 - acyl-coa synthetase medium-chain family member 5  AGXT2 - alanine--glyoxylate aminotransferase 2  SHMT1 - serine hydroxymethyltransferase 1 (soluble)  GAMT - guanidinoacetate n-methyltransferase  LPIN1 - lipin 1  ALKBH7 - alkb, alkylation repair homolog 7 (e. coli)  ALDOB - aldolase b, fructose-bisphosphate  GRHPR - glyoxylate reductase/hydroxypyruvate reductase  ADTRP - androgen-dependent tfpi-regulating protein  ST3GAL3 - st3 beta-galactoside alpha-2,3-sialyltransferase 3  ETFDH - electron-transferring-flavoprotein dehydrogenase  MPC1 - mitochondrial pyruvate carrier 1  PKLR - pyruvate kinase, liver and rbc  HAAO - 3-hydroxyanthranilate 3,4-dioxygenase  ECHDC2 - enoyl coa hydratase domain containing 2  IGF1 - insulin-like growth factor 1 (somatomedin c)  GCDH - glutaryl-coa dehydrogenase  UCP3 - uncoupling protein 3 (mitochondrial, proton carrier)  ADI1 - acireductone dioxygenase 1  PHYH - phytanoyl-coa 2-hydroxylase  CKM - creatine kinase, muscle  NDUFS6 - nadh dehydrogenase (ubiquinone) fe-s protein 6, 13kda (nadh-coenzyme q reductase)  CBR1 - carbonyl reductase 1  SLC27A2 - solute carrier family 27 (fatty acid transporter), member 2  MPST - mercaptopyruvate sulfurtransferase  SLC27A5 - solute carrier family 27 (fatty acid transporter), member 5  URAD - ureidoimidazoline (2-oxo-4-hydroxy-4-carboxy-5-) decarboxylase  CCBL1 - cysteine conjugate-beta lyase, cytoplasmic  HAO2 - hydroxyacid oxidase 2 (long chain) |
| GO:0019752 | carboxylic acid metabolic process | 6.23E-34 | 2.89E-30 | 3.11 (10334,653,667,131) | [+] Show genes  ANGPTL3 - angiopoietin-like 3  GADL1 - glutamate decarboxylase-like 1  SDHB - succinate dehydrogenase complex, subunit b, iron sulfur (ip)  SUCLG1 - succinate-coa ligase, alpha subunit  AMACR - alpha-methylacyl-coa racemase  BDH2 - 3-hydroxybutyrate dehydrogenase, type 2  OTC - ornithine carbamoyltransferase  ENO3 - enolase 3 (beta, muscle)  CES2 - carboxylesterase 2  LDHA - lactate dehydrogenase a  GSTZ1 - glutathione s-transferase zeta 1  ACAA1 - acetyl-coa acyltransferase 1  ACADL - acyl-coa dehydrogenase, long chain  ACADS - acyl-coa dehydrogenase, c-2 to c-3 short chain  ACSM2A - acyl-coa synthetase medium-chain family member 2a  ACADVL - acyl-coa dehydrogenase, very long chain  CPT2 - carnitine palmitoyltransferase 2  CPT1B - carnitine palmitoyltransferase 1b (muscle)  CPS1 - carbamoyl-phosphate synthase 1, mitochondrial  CD36 - cd36 molecule (thrombospondin receptor)  EHHADH - enoyl-coa, hydratase/3-hydroxyacyl coa dehydrogenase  BHMT - betaine--homocysteine s-methyltransferase  HPD - 4-hydroxyphenylpyruvate dioxygenase  TDO2 - tryptophan 2,3-dioxygenase  PIPOX - pipecolic acid oxidase  LIPC - lipase, hepatic  ADHFE1 - alcohol dehydrogenase, iron containing, 1  PAH - phenylalanine hydroxylase  FH - fumarate hydratase  GGT1 - gamma-glutamyltransferase 1  UPB1 - ureidopropionase, beta  ACOX2 - acyl-coa oxidase 2, branched chain  FAHD1 - fumarylacetoacetate hydrolase domain containing 1  CYP4A11 - cytochrome p450, family 4, subfamily a, polypeptide 11  CYP8B1 - cytochrome p450, family 8, subfamily b, polypeptide 1  ALDH8A1 - aldehyde dehydrogenase 8 family, member a1  CES1 - carboxylesterase 1  CYP2E1 - cytochrome p450, family 2, subfamily e, polypeptide 1  CYP3A5 - cytochrome p450, family 3, subfamily a, polypeptide 5  ADSSL1 - adenylosuccinate synthase like 1  VNN1 - vanin 1  CYP2C19 - cytochrome p450, family 2, subfamily c, polypeptide 19  TAT - tyrosine aminotransferase  PPARGC1A - peroxisome proliferator-activated receptor gamma, coactivator 1 alpha  ITIH1 - inter-alpha-trypsin inhibitor heavy chain 1  ITIH2 - inter-alpha-trypsin inhibitor heavy chain 2  ARG1 - arginase 1  ITIH3 - inter-alpha-trypsin inhibitor heavy chain 3  HADH - hydroxyacyl-coa dehydrogenase  SCP2 - sterol carrier protein 2  ITIH4 - inter-alpha-trypsin inhibitor heavy chain family, member 4  PM20D1 - peptidase m20 domain containing 1  HAGH - hydroxyacylglutathione hydrolase  CYP1A2 - cytochrome p450, family 1, subfamily a, polypeptide 2  PPARA - peroxisome proliferator-activated receptor alpha  PTPLA - protein tyrosine phosphatase-like (proline instead of catalytic arginine), member a  HSD17B10 - hydroxysteroid (17-beta) dehydrogenase 10  PON1 - paraoxonase 1  IYD - iodotyrosine deiodinase  PCK1 - phosphoenolpyruvate carboxykinase 1 (soluble)  GLYAT - glycine-n-acyltransferase  FTCD - formimidoyltransferase cyclodeaminase  ADH6 - alcohol dehydrogenase 6 (class v)  ALDH1L1 - aldehyde dehydrogenase 1 family, member l1  ASS1 - argininosuccinate synthase 1  ADH4 - alcohol dehydrogenase 4 (class ii), pi polypeptide  PRODH2 - proline dehydrogenase (oxidase) 2  CRYL1 - crystallin, lambda 1  ECHS1 - enoyl coa hydratase, short chain, 1, mitochondrial  ACSM3 - acyl-coa synthetase medium-chain family member 3  SEPSECS - sep (o-phosphoserine) trna:sec (selenocysteine) trna synthase  ECH1 - enoyl coa hydratase 1, peroxisomal  ECI2 - enoyl-coa delta isomerase 2  HSD17B8 - hydroxysteroid (17-beta) dehydrogenase 8  AKR1C3 - aldo-keto reductase family 1, member c3  MLYCD - malonyl-coa decarboxylase  ACAA2 - acetyl-coa acyltransferase 2  AGMAT - agmatine ureohydrolase (agmatinase)  PGM1 - phosphoglucomutase 1  PGAM2 - phosphoglycerate mutase 2 (muscle)  MCEE - methylmalonyl coa epimerase  MTHFR - methylenetetrahydrofolate reductase (nad(p)h)  ACSL1 - acyl-coa synthetase long-chain family member 1  MTHFD1 - methylenetetrahydrofolate dehydrogenase (nadp+ dependent) 1, methenyltetrahydrofolate cyclohydrolase, formyltetrahydrofolate synthetase  ATF4 - activating transcription factor 4  GOT2 - glutamic-oxaloacetic transaminase 2, mitochondrial  ALDH4A1 - aldehyde dehydrogenase 4 family, member a1  FAH - fumarylacetoacetate hydrolase (fumarylacetoacetase)  ASPG - asparaginase homolog (s. cerevisiae)  ABHD1 - abhydrolase domain containing 1  DPYS - dihydropyrimidinase  AKR1C4 - aldo-keto reductase family 1, member c4  GCAT - glycine c-acetyltransferase  UGT1A6 - udp glucuronosyltransferase 1 family, polypeptide a6  SULT2A1 - sulfotransferase family, cytosolic, 2a, dehydroepiandrosterone (dhea)-preferring, member 1  AHCY - adenosylhomocysteinase  AGXT - alanine-glyoxylate aminotransferase  MAT1A - methionine adenosyltransferase i, alpha  HGD - homogentisate 1,2-dioxygenase  MSRA - methionine sulfoxide reductase a  HACL1 - 2-hydroxyacyl-coa lyase 1  IDH3G - isocitrate dehydrogenase 3 (nad+) gamma  DECR2 - 2,4-dienoyl coa reductase 2, peroxisomal  PFKFB1 - 6-phosphofructo-2-kinase/fructose-2,6-biphosphatase 1  ACSM5 - acyl-coa synthetase medium-chain family member 5  AGXT2 - alanine--glyoxylate aminotransferase 2  SHMT1 - serine hydroxymethyltransferase 1 (soluble)  GAMT - guanidinoacetate n-methyltransferase  LPIN1 - lipin 1  ALKBH7 - alkb, alkylation repair homolog 7 (e. coli)  ALDOB - aldolase b, fructose-bisphosphate  GRHPR - glyoxylate reductase/hydroxypyruvate reductase  ADTRP - androgen-dependent tfpi-regulating protein  ETFDH - electron-transferring-flavoprotein dehydrogenase  MPC1 - mitochondrial pyruvate carrier 1  PKLR - pyruvate kinase, liver and rbc  HAAO - 3-hydroxyanthranilate 3,4-dioxygenase  ECHDC2 - enoyl coa hydratase domain containing 2  IGF1 - insulin-like growth factor 1 (somatomedin c)  GCDH - glutaryl-coa dehydrogenase  UCP3 - uncoupling protein 3 (mitochondrial, proton carrier)  ADI1 - acireductone dioxygenase 1  PHYH - phytanoyl-coa 2-hydroxylase  CKM - creatine kinase, muscle  NDUFS6 - nadh dehydrogenase (ubiquinone) fe-s protein 6, 13kda (nadh-coenzyme q reductase)  CBR1 - carbonyl reductase 1  SLC27A2 - solute carrier family 27 (fatty acid transporter), member 2  MPST - mercaptopyruvate sulfurtransferase  SLC27A5 - solute carrier family 27 (fatty acid transporter), member 5  CCBL1 - cysteine conjugate-beta lyase, cytoplasmic  HAO2 - hydroxyacid oxidase 2 (long chain) |
| GO:0043436 | oxoacid metabolic process | 7.92E-32 | 2.75E-28 | 2.92 (10334,710,667,134) | [+] Show genes  ANGPTL3 - angiopoietin-like 3  GADL1 - glutamate decarboxylase-like 1  SDHB - succinate dehydrogenase complex, subunit b, iron sulfur (ip)  SUCLG1 - succinate-coa ligase, alpha subunit  AMACR - alpha-methylacyl-coa racemase  BDH2 - 3-hydroxybutyrate dehydrogenase, type 2  OTC - ornithine carbamoyltransferase  ENO3 - enolase 3 (beta, muscle)  CES2 - carboxylesterase 2  LDHA - lactate dehydrogenase a  GSTZ1 - glutathione s-transferase zeta 1  ACAA1 - acetyl-coa acyltransferase 1  ACADL - acyl-coa dehydrogenase, long chain  ACADS - acyl-coa dehydrogenase, c-2 to c-3 short chain  ACSM2A - acyl-coa synthetase medium-chain family member 2a  ACADVL - acyl-coa dehydrogenase, very long chain  CPT2 - carnitine palmitoyltransferase 2  CPT1B - carnitine palmitoyltransferase 1b (muscle)  CPS1 - carbamoyl-phosphate synthase 1, mitochondrial  CD36 - cd36 molecule (thrombospondin receptor)  EHHADH - enoyl-coa, hydratase/3-hydroxyacyl coa dehydrogenase  BHMT - betaine--homocysteine s-methyltransferase  HPD - 4-hydroxyphenylpyruvate dioxygenase  TDO2 - tryptophan 2,3-dioxygenase  LIPC - lipase, hepatic  PIPOX - pipecolic acid oxidase  ADHFE1 - alcohol dehydrogenase, iron containing, 1  PAH - phenylalanine hydroxylase  FH - fumarate hydratase  GGT1 - gamma-glutamyltransferase 1  UPB1 - ureidopropionase, beta  ACOX2 - acyl-coa oxidase 2, branched chain  FAHD1 - fumarylacetoacetate hydrolase domain containing 1  CYP4A11 - cytochrome p450, family 4, subfamily a, polypeptide 11  CYP8B1 - cytochrome p450, family 8, subfamily b, polypeptide 1  CES1 - carboxylesterase 1  ALDH8A1 - aldehyde dehydrogenase 8 family, member a1  CYP2E1 - cytochrome p450, family 2, subfamily e, polypeptide 1  CYP3A5 - cytochrome p450, family 3, subfamily a, polypeptide 5  ADSSL1 - adenylosuccinate synthase like 1  VNN1 - vanin 1  CYP2C19 - cytochrome p450, family 2, subfamily c, polypeptide 19  ENPP1 - ectonucleotide pyrophosphatase/phosphodiesterase 1  TAT - tyrosine aminotransferase  PPARGC1A - peroxisome proliferator-activated receptor gamma, coactivator 1 alpha  ITIH1 - inter-alpha-trypsin inhibitor heavy chain 1  ITIH2 - inter-alpha-trypsin inhibitor heavy chain 2  ARG1 - arginase 1  ITIH3 - inter-alpha-trypsin inhibitor heavy chain 3  HADH - hydroxyacyl-coa dehydrogenase  SCP2 - sterol carrier protein 2  ITIH4 - inter-alpha-trypsin inhibitor heavy chain family, member 4  PM20D1 - peptidase m20 domain containing 1  HAGH - hydroxyacylglutathione hydrolase  CYP1A2 - cytochrome p450, family 1, subfamily a, polypeptide 2  PPARA - peroxisome proliferator-activated receptor alpha  PTPLA - protein tyrosine phosphatase-like (proline instead of catalytic arginine), member a  HSD17B10 - hydroxysteroid (17-beta) dehydrogenase 10  PON1 - paraoxonase 1  IYD - iodotyrosine deiodinase  PCK1 - phosphoenolpyruvate carboxykinase 1 (soluble)  GLYAT - glycine-n-acyltransferase  FTCD - formimidoyltransferase cyclodeaminase  ALDH1L1 - aldehyde dehydrogenase 1 family, member l1  ADH6 - alcohol dehydrogenase 6 (class v)  ASS1 - argininosuccinate synthase 1  ADH4 - alcohol dehydrogenase 4 (class ii), pi polypeptide  PRODH2 - proline dehydrogenase (oxidase) 2  CRYL1 - crystallin, lambda 1  ECHS1 - enoyl coa hydratase, short chain, 1, mitochondrial  ACSM3 - acyl-coa synthetase medium-chain family member 3  SEPSECS - sep (o-phosphoserine) trna:sec (selenocysteine) trna synthase  ECH1 - enoyl coa hydratase 1, peroxisomal  ECI2 - enoyl-coa delta isomerase 2  HSD17B8 - hydroxysteroid (17-beta) dehydrogenase 8  AKR1C3 - aldo-keto reductase family 1, member c3  MLYCD - malonyl-coa decarboxylase  ACAA2 - acetyl-coa acyltransferase 2  AGMAT - agmatine ureohydrolase (agmatinase)  PGM1 - phosphoglucomutase 1  PGAM2 - phosphoglycerate mutase 2 (muscle)  MCEE - methylmalonyl coa epimerase  MTHFR - methylenetetrahydrofolate reductase (nad(p)h)  ACSL1 - acyl-coa synthetase long-chain family member 1  MTHFD1 - methylenetetrahydrofolate dehydrogenase (nadp+ dependent) 1, methenyltetrahydrofolate cyclohydrolase, formyltetrahydrofolate synthetase  ATF4 - activating transcription factor 4  GOT2 - glutamic-oxaloacetic transaminase 2, mitochondrial  ALDH4A1 - aldehyde dehydrogenase 4 family, member a1  FAH - fumarylacetoacetate hydrolase (fumarylacetoacetase)  ASPG - asparaginase homolog (s. cerevisiae)  ABHD1 - abhydrolase domain containing 1  DPYS - dihydropyrimidinase  AKR1C4 - aldo-keto reductase family 1, member c4  GCAT - glycine c-acetyltransferase  CHST13 - carbohydrate (chondroitin 4) sulfotransferase 13  UGT1A6 - udp glucuronosyltransferase 1 family, polypeptide a6  AHCY - adenosylhomocysteinase  SULT2A1 - sulfotransferase family, cytosolic, 2a, dehydroepiandrosterone (dhea)-preferring, member 1  AGXT - alanine-glyoxylate aminotransferase  MAT1A - methionine adenosyltransferase i, alpha  HGD - homogentisate 1,2-dioxygenase  MSRA - methionine sulfoxide reductase a  HACL1 - 2-hydroxyacyl-coa lyase 1  IDH3G - isocitrate dehydrogenase 3 (nad+) gamma  DECR2 - 2,4-dienoyl coa reductase 2, peroxisomal  PFKFB1 - 6-phosphofructo-2-kinase/fructose-2,6-biphosphatase 1  ACSM5 - acyl-coa synthetase medium-chain family member 5  AGXT2 - alanine--glyoxylate aminotransferase 2  SHMT1 - serine hydroxymethyltransferase 1 (soluble)  GAMT - guanidinoacetate n-methyltransferase  LPIN1 - lipin 1  ALKBH7 - alkb, alkylation repair homolog 7 (e. coli)  ALDOB - aldolase b, fructose-bisphosphate  GRHPR - glyoxylate reductase/hydroxypyruvate reductase  ADTRP - androgen-dependent tfpi-regulating protein  ST3GAL3 - st3 beta-galactoside alpha-2,3-sialyltransferase 3  ETFDH - electron-transferring-flavoprotein dehydrogenase  MPC1 - mitochondrial pyruvate carrier 1  PKLR - pyruvate kinase, liver and rbc  HAAO - 3-hydroxyanthranilate 3,4-dioxygenase  ECHDC2 - enoyl coa hydratase domain containing 2  IGF1 - insulin-like growth factor 1 (somatomedin c)  GCDH - glutaryl-coa dehydrogenase  UCP3 - uncoupling protein 3 (mitochondrial, proton carrier)  ADI1 - acireductone dioxygenase 1  PHYH - phytanoyl-coa 2-hydroxylase  CKM - creatine kinase, muscle  NDUFS6 - nadh dehydrogenase (ubiquinone) fe-s protein 6, 13kda (nadh-coenzyme q reductase)  CBR1 - carbonyl reductase 1  SLC27A2 - solute carrier family 27 (fatty acid transporter), member 2  MPST - mercaptopyruvate sulfurtransferase  SLC27A5 - solute carrier family 27 (fatty acid transporter), member 5  CCBL1 - cysteine conjugate-beta lyase, cytoplasmic  HAO2 - hydroxyacid oxidase 2 (long chain) |
| GO:0055114 | oxidation-reduction process | 5.93E-29 | 1.65E-25 | 2.93 (10334,645,667,122) | [+] Show genes  COX6A2 - cytochrome c oxidase subunit via polypeptide 2  APOA2 - apolipoprotein a-ii  NDUFA3 - nadh dehydrogenase (ubiquinone) 1 alpha subcomplex, 3, 9kda  NDUFA4 - nadh dehydrogenase (ubiquinone) 1 alpha subcomplex, 4, 9kda  SDHB - succinate dehydrogenase complex, subunit b, iron sulfur (ip)  COX5B - cytochrome c oxidase subunit vb  NDUFA2 - nadh dehydrogenase (ubiquinone) 1 alpha subcomplex, 2, 8kda  FMO1 - flavin containing monooxygenase 1  AMACR - alpha-methylacyl-coa racemase  NDUFA7 - nadh dehydrogenase (ubiquinone) 1 alpha subcomplex, 7, 14.5kda  HSD11B1 - hydroxysteroid (11-beta) dehydrogenase 1  FMO3 - flavin containing monooxygenase 3  DHRS7C - dehydrogenase/reductase (sdr family) member 7c  BDH2 - 3-hydroxybutyrate dehydrogenase, type 2  ENO3 - enolase 3 (beta, muscle)  AOC1 - amine oxidase, copper containing 1  FOXRED1 - fad-dependent oxidoreductase domain containing 1  PPP1R3B - protein phosphatase 1, regulatory subunit 3b  LDHA - lactate dehydrogenase a  ACAA1 - acetyl-coa acyltransferase 1  SLC25A13 - solute carrier family 25 (aspartate/glutamate carrier), member 13  PHYHD1 - phytanoyl-coa dioxygenase domain containing 1  COX7C - cytochrome c oxidase subunit viic  ACADL - acyl-coa dehydrogenase, long chain  ACADS - acyl-coa dehydrogenase, c-2 to c-3 short chain  ACADVL - acyl-coa dehydrogenase, very long chain  BLOC1S1 - biogenesis of lysosomal organelles complex-1, subunit 1  DHRS3 - dehydrogenase/reductase (sdr family) member 3  CPT2 - carnitine palmitoyltransferase 2  ASPDH - aspartate dehydrogenase domain containing  DHRS4 - dehydrogenase/reductase (sdr family) member 4  CPT1B - carnitine palmitoyltransferase 1b (muscle)  UQCR10 - ubiquinol-cytochrome c reductase, complex iii subunit x  HSD17B13 - hydroxysteroid (17-beta) dehydrogenase 13  EHHADH - enoyl-coa, hydratase/3-hydroxyacyl coa dehydrogenase  HPD - 4-hydroxyphenylpyruvate dioxygenase  TDO2 - tryptophan 2,3-dioxygenase  IMMP2L - imp2 inner mitochondrial membrane peptidase-like (s. cerevisiae)  PIPOX - pipecolic acid oxidase  GYS2 - glycogen synthase 2 (liver)  ADHFE1 - alcohol dehydrogenase, iron containing, 1  PAH - phenylalanine hydroxylase  BLVRB - biliverdin reductase b (flavin reductase (nadph))  MSRB3 - methionine sulfoxide reductase b3  ACOX2 - acyl-coa oxidase 2, branched chain  CYP4A11 - cytochrome p450, family 4, subfamily a, polypeptide 11  CYP8B1 - cytochrome p450, family 8, subfamily b, polypeptide 1  ALDH8A1 - aldehyde dehydrogenase 8 family, member a1  CYP2E1 - cytochrome p450, family 2, subfamily e, polypeptide 1  CYP3A5 - cytochrome p450, family 3, subfamily a, polypeptide 5  CYP2C19 - cytochrome p450, family 2, subfamily c, polypeptide 19  PPARGC1A - peroxisome proliferator-activated receptor gamma, coactivator 1 alpha  HADH - hydroxyacyl-coa dehydrogenase  SCP2 - sterol carrier protein 2  CYP1A2 - cytochrome p450, family 1, subfamily a, polypeptide 2  HSD17B10 - hydroxysteroid (17-beta) dehydrogenase 10  IYD - iodotyrosine deiodinase  HSD17B6 - hydroxysteroid (17-beta) dehydrogenase 6  ALDH1L1 - aldehyde dehydrogenase 1 family, member l1  ADH6 - alcohol dehydrogenase 6 (class v)  ADH4 - alcohol dehydrogenase 4 (class ii), pi polypeptide  PRODH2 - proline dehydrogenase (oxidase) 2  RDH16 - retinol dehydrogenase 16 (all-trans)  DHRS7B - dehydrogenase/reductase (sdr family) member 7b  CRYL1 - crystallin, lambda 1  ECHS1 - enoyl coa hydratase, short chain, 1, mitochondrial  MMACHC - methylmalonic aciduria (cobalamin deficiency) cblc type, with homocystinuria  ECH1 - enoyl coa hydratase 1, peroxisomal  ECI2 - enoyl-coa delta isomerase 2  HSD17B8 - hydroxysteroid (17-beta) dehydrogenase 8  AKR1C3 - aldo-keto reductase family 1, member c3  UQCRC1 - ubiquinol-cytochrome c reductase core protein i  MLYCD - malonyl-coa decarboxylase  HSD17B14 - hydroxysteroid (17-beta) dehydrogenase 14  AOC3 - amine oxidase, copper containing 3  ACAA2 - acetyl-coa acyltransferase 2  PGM1 - phosphoglucomutase 1  NDUFS7 - nadh dehydrogenase (ubiquinone) fe-s protein 7, 20kda (nadh-coenzyme q reductase)  PGAM2 - phosphoglycerate mutase 2 (muscle)  MTHFR - methylenetetrahydrofolate reductase (nad(p)h)  MTHFD1 - methylenetetrahydrofolate dehydrogenase (nadp+ dependent) 1, methenyltetrahydrofolate cyclohydrolase, formyltetrahydrofolate synthetase  ALDH4A1 - aldehyde dehydrogenase 4 family, member a1  GPD1 - glycerol-3-phosphate dehydrogenase 1 (soluble)  AKR1C4 - aldo-keto reductase family 1, member c4  ETHE1 - ethylmalonic encephalopathy 1  HGD - homogentisate 1,2-dioxygenase  MSRA - methionine sulfoxide reductase a  PPP1R3C - protein phosphatase 1, regulatory subunit 3c  HACL1 - 2-hydroxyacyl-coa lyase 1  ATP5D - atp synthase, h+ transporting, mitochondrial f1 complex, delta subunit  COQ9 - coenzyme q9 homolog (s. cerevisiae)  G6PC - glucose-6-phosphatase, catalytic subunit  DECR2 - 2,4-dienoyl coa reductase 2, peroxisomal  ALDH2 - aldehyde dehydrogenase 2 family (mitochondrial)  PPP1R3A - protein phosphatase 1, regulatory subunit 3a  PYROXD2 - pyridine nucleotide-disulphide oxidoreductase domain 2  ALKBH7 - alkb, alkylation repair homolog 7 (e. coli)  ALDOB - aldolase b, fructose-bisphosphate  GRHPR - glyoxylate reductase/hydroxypyruvate reductase  DHRS12 - dehydrogenase/reductase (sdr family) member 12  ETFDH - electron-transferring-flavoprotein dehydrogenase  PKLR - pyruvate kinase, liver and rbc  HAAO - 3-hydroxyanthranilate 3,4-dioxygenase  NDUFB10 - nadh dehydrogenase (ubiquinone) 1 beta subcomplex, 10, 22kda  NDUFB7 - nadh dehydrogenase (ubiquinone) 1 beta subcomplex, 7, 18kda  ECHDC2 - enoyl coa hydratase domain containing 2  GCDH - glutaryl-coa dehydrogenase  CAT - catalase  ADI1 - acireductone dioxygenase 1  UQCR11 - ubiquinol-cytochrome c reductase, complex iii subunit xi  PHYH - phytanoyl-coa 2-hydroxylase  STBD1 - starch binding domain 1  NDUFS8 - nadh dehydrogenase (ubiquinone) fe-s protein 8, 23kda (nadh-coenzyme q reductase)  NDUFS6 - nadh dehydrogenase (ubiquinone) fe-s protein 6, 13kda (nadh-coenzyme q reductase)  CBR1 - carbonyl reductase 1  SLC27A2 - solute carrier family 27 (fatty acid transporter), member 2  NDUFS5 - nadh dehydrogenase (ubiquinone) fe-s protein 5, 15kda (nadh-coenzyme q reductase)  HMOX1 - heme oxygenase (decycling) 1  NDUFV1 - nadh dehydrogenase (ubiquinone) flavoprotein 1, 51kda  SLC27A5 - solute carrier family 27 (fatty acid transporter), member 5  HAO2 - hydroxyacid oxidase 2 (long chain)  UQCRQ - ubiquinol-cytochrome c reductase, complex iii subunit vii, 9.5kda |
| GO:0032787 | monocarboxylic acid metabolic process | 1.59E-27 | 3.68E-24 | 3.68 (10334,362,667,86) | [+] Show genes  ECI2 - enoyl-coa delta isomerase 2  HSD17B8 - hydroxysteroid (17-beta) dehydrogenase 8  AKR1C3 - aldo-keto reductase family 1, member c3  MLYCD - malonyl-coa decarboxylase  ANGPTL3 - angiopoietin-like 3  ACAA2 - acetyl-coa acyltransferase 2  PGM1 - phosphoglucomutase 1  AMACR - alpha-methylacyl-coa racemase  PGAM2 - phosphoglycerate mutase 2 (muscle)  MCEE - methylmalonyl coa epimerase  BDH2 - 3-hydroxybutyrate dehydrogenase, type 2  ACSL1 - acyl-coa synthetase long-chain family member 1  GOT2 - glutamic-oxaloacetic transaminase 2, mitochondrial  ATF4 - activating transcription factor 4  ENO3 - enolase 3 (beta, muscle)  ALDH4A1 - aldehyde dehydrogenase 4 family, member a1  FAH - fumarylacetoacetate hydrolase (fumarylacetoacetase)  CES2 - carboxylesterase 2  LDHA - lactate dehydrogenase a  ACAA1 - acetyl-coa acyltransferase 1  ACADL - acyl-coa dehydrogenase, long chain  ACADS - acyl-coa dehydrogenase, c-2 to c-3 short chain  ACSM2A - acyl-coa synthetase medium-chain family member 2a  ABHD1 - abhydrolase domain containing 1  ACADVL - acyl-coa dehydrogenase, very long chain  CPT2 - carnitine palmitoyltransferase 2  AKR1C4 - aldo-keto reductase family 1, member c4  CPT1B - carnitine palmitoyltransferase 1b (muscle)  CD36 - cd36 molecule (thrombospondin receptor)  UGT1A6 - udp glucuronosyltransferase 1 family, polypeptide a6  SULT2A1 - sulfotransferase family, cytosolic, 2a, dehydroepiandrosterone (dhea)-preferring, member 1  AGXT - alanine-glyoxylate aminotransferase  EHHADH - enoyl-coa, hydratase/3-hydroxyacyl coa dehydrogenase  HACL1 - 2-hydroxyacyl-coa lyase 1  LIPC - lipase, hepatic  GGT1 - gamma-glutamyltransferase 1  DECR2 - 2,4-dienoyl coa reductase 2, peroxisomal  PFKFB1 - 6-phosphofructo-2-kinase/fructose-2,6-biphosphatase 1  ACSM5 - acyl-coa synthetase medium-chain family member 5  ACOX2 - acyl-coa oxidase 2, branched chain  CYP4A11 - cytochrome p450, family 4, subfamily a, polypeptide 11  AGXT2 - alanine--glyoxylate aminotransferase 2  CYP8B1 - cytochrome p450, family 8, subfamily b, polypeptide 1  CES1 - carboxylesterase 1  ALDH8A1 - aldehyde dehydrogenase 8 family, member a1  CYP2E1 - cytochrome p450, family 2, subfamily e, polypeptide 1  LPIN1 - lipin 1  CYP3A5 - cytochrome p450, family 3, subfamily a, polypeptide 5  ALKBH7 - alkb, alkylation repair homolog 7 (e. coli)  VNN1 - vanin 1  ALDOB - aldolase b, fructose-bisphosphate  CYP2C19 - cytochrome p450, family 2, subfamily c, polypeptide 19  PPARGC1A - peroxisome proliferator-activated receptor gamma, coactivator 1 alpha  GRHPR - glyoxylate reductase/hydroxypyruvate reductase  ADTRP - androgen-dependent tfpi-regulating protein  HADH - hydroxyacyl-coa dehydrogenase  SCP2 - sterol carrier protein 2  CYP1A2 - cytochrome p450, family 1, subfamily a, polypeptide 2  HAGH - hydroxyacylglutathione hydrolase  PTPLA - protein tyrosine phosphatase-like (proline instead of catalytic arginine), member a  PPARA - peroxisome proliferator-activated receptor alpha  MPC1 - mitochondrial pyruvate carrier 1  ETFDH - electron-transferring-flavoprotein dehydrogenase  PKLR - pyruvate kinase, liver and rbc  HAAO - 3-hydroxyanthranilate 3,4-dioxygenase  PON1 - paraoxonase 1  ECHDC2 - enoyl coa hydratase domain containing 2  PCK1 - phosphoenolpyruvate carboxykinase 1 (soluble)  GLYAT - glycine-n-acyltransferase  IGF1 - insulin-like growth factor 1 (somatomedin c)  FTCD - formimidoyltransferase cyclodeaminase  GCDH - glutaryl-coa dehydrogenase  ADH6 - alcohol dehydrogenase 6 (class v)  UCP3 - uncoupling protein 3 (mitochondrial, proton carrier)  ADH4 - alcohol dehydrogenase 4 (class ii), pi polypeptide  PHYH - phytanoyl-coa 2-hydroxylase  PRODH2 - proline dehydrogenase (oxidase) 2  CRYL1 - crystallin, lambda 1  NDUFS6 - nadh dehydrogenase (ubiquinone) fe-s protein 6, 13kda (nadh-coenzyme q reductase)  CBR1 - carbonyl reductase 1  SLC27A2 - solute carrier family 27 (fatty acid transporter), member 2  ECHS1 - enoyl coa hydratase, short chain, 1, mitochondrial  ACSM3 - acyl-coa synthetase medium-chain family member 3  SLC27A5 - solute carrier family 27 (fatty acid transporter), member 5  HAO2 - hydroxyacid oxidase 2 (long chain)  ECH1 - enoyl coa hydratase 1, peroxisomal |
| GO:0044282 | small molecule catabolic process | 2.98E-26 | 5.93E-23 | 3.76 (10334,330,667,80) | [+] Show genes  ECI2 - enoyl-coa delta isomerase 2  AKR1C3 - aldo-keto reductase family 1, member c3  ACAA2 - acetyl-coa acyltransferase 2  GADL1 - glutamate decarboxylase-like 1  PGM1 - phosphoglucomutase 1  ENTPD8 - ectonucleoside triphosphate diphosphohydrolase 8  AMACR - alpha-methylacyl-coa racemase  PGAM2 - phosphoglycerate mutase 2 (muscle)  MCEE - methylmalonyl coa epimerase  BDH2 - 3-hydroxybutyrate dehydrogenase, type 2  GOT2 - glutamic-oxaloacetic transaminase 2, mitochondrial  OTC - ornithine carbamoyltransferase  ENO3 - enolase 3 (beta, muscle)  GK5 - glycerol kinase 5 (putative)  ALDH4A1 - aldehyde dehydrogenase 4 family, member a1  FAH - fumarylacetoacetate hydrolase (fumarylacetoacetase)  IMPA2 - inositol(myo)-1(or 4)-monophosphatase 2  GSTZ1 - glutathione s-transferase zeta 1  ACAA1 - acetyl-coa acyltransferase 1  ACADL - acyl-coa dehydrogenase, long chain  ACADS - acyl-coa dehydrogenase, c-2 to c-3 short chain  DPYS - dihydropyrimidinase  ABHD1 - abhydrolase domain containing 1  ACADVL - acyl-coa dehydrogenase, very long chain  CPT2 - carnitine palmitoyltransferase 2  CPT1B - carnitine palmitoyltransferase 1b (muscle)  GCAT - glycine c-acetyltransferase  SULT2A1 - sulfotransferase family, cytosolic, 2a, dehydroepiandrosterone (dhea)-preferring, member 1  AHCY - adenosylhomocysteinase  AGXT - alanine-glyoxylate aminotransferase  EHHADH - enoyl-coa, hydratase/3-hydroxyacyl coa dehydrogenase  MAT1A - methionine adenosyltransferase i, alpha  HPD - 4-hydroxyphenylpyruvate dioxygenase  HGD - homogentisate 1,2-dioxygenase  TDO2 - tryptophan 2,3-dioxygenase  HACL1 - 2-hydroxyacyl-coa lyase 1  PIPOX - pipecolic acid oxidase  ADHFE1 - alcohol dehydrogenase, iron containing, 1  PAH - phenylalanine hydroxylase  APOBEC2 - apolipoprotein b mrna editing enzyme, catalytic polypeptide-like 2  DECR2 - 2,4-dienoyl coa reductase 2, peroxisomal  ALDH2 - aldehyde dehydrogenase 2 family (mitochondrial)  UPB1 - ureidopropionase, beta  ACOX2 - acyl-coa oxidase 2, branched chain  CYP4A11 - cytochrome p450, family 4, subfamily a, polypeptide 11  AGXT2 - alanine--glyoxylate aminotransferase 2  ALDH8A1 - aldehyde dehydrogenase 8 family, member a1  SHMT1 - serine hydroxymethyltransferase 1 (soluble)  LPIN1 - lipin 1  SCARF1 - scavenger receptor class f, member 1  ALDOB - aldolase b, fructose-bisphosphate  TAT - tyrosine aminotransferase  ADTRP - androgen-dependent tfpi-regulating protein  ARG1 - arginase 1  HADH - hydroxyacyl-coa dehydrogenase  SCP2 - sterol carrier protein 2  ADA - adenosine deaminase  HAGH - hydroxyacylglutathione hydrolase  HSD17B10 - hydroxysteroid (17-beta) dehydrogenase 10  ETFDH - electron-transferring-flavoprotein dehydrogenase  PKLR - pyruvate kinase, liver and rbc  HAAO - 3-hydroxyanthranilate 3,4-dioxygenase  PON1 - paraoxonase 1  ECHDC2 - enoyl coa hydratase domain containing 2  PCK1 - phosphoenolpyruvate carboxykinase 1 (soluble)  FTCD - formimidoyltransferase cyclodeaminase  GCDH - glutaryl-coa dehydrogenase  ALDH1L1 - aldehyde dehydrogenase 1 family, member l1  ADH4 - alcohol dehydrogenase 4 (class ii), pi polypeptide  PHYH - phytanoyl-coa 2-hydroxylase  PRODH2 - proline dehydrogenase (oxidase) 2  GLYCTK - glycerate kinase  CRYL1 - crystallin, lambda 1  SLC27A2 - solute carrier family 27 (fatty acid transporter), member 2  ECHS1 - enoyl coa hydratase, short chain, 1, mitochondrial  MPST - mercaptopyruvate sulfurtransferase  ECH1 - enoyl coa hydratase 1, peroxisomal  URAD - ureidoimidazoline (2-oxo-4-hydroxy-4-carboxy-5-) decarboxylase  HAO2 - hydroxyacid oxidase 2 (long chain)  CCBL1 - cysteine conjugate-beta lyase, cytoplasmic |
| GO:0016054 | organic acid catabolic process | 6.52E-24 | 1.13E-20 | 4.38 (10334,216,667,61) | [+] Show genes  ECI2 - enoyl-coa delta isomerase 2  ACAA2 - acetyl-coa acyltransferase 2  GADL1 - glutamate decarboxylase-like 1  AMACR - alpha-methylacyl-coa racemase  MCEE - methylmalonyl coa epimerase  BDH2 - 3-hydroxybutyrate dehydrogenase, type 2  GOT2 - glutamic-oxaloacetic transaminase 2, mitochondrial  OTC - ornithine carbamoyltransferase  FAH - fumarylacetoacetate hydrolase (fumarylacetoacetase)  ALDH4A1 - aldehyde dehydrogenase 4 family, member a1  ACAA1 - acetyl-coa acyltransferase 1  GSTZ1 - glutathione s-transferase zeta 1  ACADL - acyl-coa dehydrogenase, long chain  ACADS - acyl-coa dehydrogenase, c-2 to c-3 short chain  ABHD1 - abhydrolase domain containing 1  ACADVL - acyl-coa dehydrogenase, very long chain  CPT2 - carnitine palmitoyltransferase 2  CPT1B - carnitine palmitoyltransferase 1b (muscle)  GCAT - glycine c-acetyltransferase  SULT2A1 - sulfotransferase family, cytosolic, 2a, dehydroepiandrosterone (dhea)-preferring, member 1  AHCY - adenosylhomocysteinase  AGXT - alanine-glyoxylate aminotransferase  EHHADH - enoyl-coa, hydratase/3-hydroxyacyl coa dehydrogenase  MAT1A - methionine adenosyltransferase i, alpha  HPD - 4-hydroxyphenylpyruvate dioxygenase  HGD - homogentisate 1,2-dioxygenase  TDO2 - tryptophan 2,3-dioxygenase  HACL1 - 2-hydroxyacyl-coa lyase 1  PIPOX - pipecolic acid oxidase  ADHFE1 - alcohol dehydrogenase, iron containing, 1  PAH - phenylalanine hydroxylase  DECR2 - 2,4-dienoyl coa reductase 2, peroxisomal  ACOX2 - acyl-coa oxidase 2, branched chain  CYP4A11 - cytochrome p450, family 4, subfamily a, polypeptide 11  AGXT2 - alanine--glyoxylate aminotransferase 2  ALDH8A1 - aldehyde dehydrogenase 8 family, member a1  SHMT1 - serine hydroxymethyltransferase 1 (soluble)  LPIN1 - lipin 1  TAT - tyrosine aminotransferase  ADTRP - androgen-dependent tfpi-regulating protein  ARG1 - arginase 1  HADH - hydroxyacyl-coa dehydrogenase  SCP2 - sterol carrier protein 2  HSD17B10 - hydroxysteroid (17-beta) dehydrogenase 10  ETFDH - electron-transferring-flavoprotein dehydrogenase  HAAO - 3-hydroxyanthranilate 3,4-dioxygenase  PON1 - paraoxonase 1  ECHDC2 - enoyl coa hydratase domain containing 2  PCK1 - phosphoenolpyruvate carboxykinase 1 (soluble)  FTCD - formimidoyltransferase cyclodeaminase  GCDH - glutaryl-coa dehydrogenase  ALDH1L1 - aldehyde dehydrogenase 1 family, member l1  PHYH - phytanoyl-coa 2-hydroxylase  PRODH2 - proline dehydrogenase (oxidase) 2  CRYL1 - crystallin, lambda 1  SLC27A2 - solute carrier family 27 (fatty acid transporter), member 2  ECHS1 - enoyl coa hydratase, short chain, 1, mitochondrial  MPST - mercaptopyruvate sulfurtransferase  ECH1 - enoyl coa hydratase 1, peroxisomal  HAO2 - hydroxyacid oxidase 2 (long chain)  CCBL1 - cysteine conjugate-beta lyase, cytoplasmic |
| GO:0046395 | carboxylic acid catabolic process | 6.52E-24 | 1.01E-20 | 4.38 (10334,216,667,61) | [+] Show genes  ECI2 - enoyl-coa delta isomerase 2  ACAA2 - acetyl-coa acyltransferase 2  GADL1 - glutamate decarboxylase-like 1  AMACR - alpha-methylacyl-coa racemase  MCEE - methylmalonyl coa epimerase  BDH2 - 3-hydroxybutyrate dehydrogenase, type 2  GOT2 - glutamic-oxaloacetic transaminase 2, mitochondrial  OTC - ornithine carbamoyltransferase  ALDH4A1 - aldehyde dehydrogenase 4 family, member a1  FAH - fumarylacetoacetate hydrolase (fumarylacetoacetase)  ACAA1 - acetyl-coa acyltransferase 1  GSTZ1 - glutathione s-transferase zeta 1  ACADL - acyl-coa dehydrogenase, long chain  ACADS - acyl-coa dehydrogenase, c-2 to c-3 short chain  ABHD1 - abhydrolase domain containing 1  ACADVL - acyl-coa dehydrogenase, very long chain  CPT2 - carnitine palmitoyltransferase 2  CPT1B - carnitine palmitoyltransferase 1b (muscle)  GCAT - glycine c-acetyltransferase  AHCY - adenosylhomocysteinase  SULT2A1 - sulfotransferase family, cytosolic, 2a, dehydroepiandrosterone (dhea)-preferring, member 1  AGXT - alanine-glyoxylate aminotransferase  EHHADH - enoyl-coa, hydratase/3-hydroxyacyl coa dehydrogenase  MAT1A - methionine adenosyltransferase i, alpha  HPD - 4-hydroxyphenylpyruvate dioxygenase  HGD - homogentisate 1,2-dioxygenase  TDO2 - tryptophan 2,3-dioxygenase  HACL1 - 2-hydroxyacyl-coa lyase 1  PIPOX - pipecolic acid oxidase  ADHFE1 - alcohol dehydrogenase, iron containing, 1  PAH - phenylalanine hydroxylase  DECR2 - 2,4-dienoyl coa reductase 2, peroxisomal  ACOX2 - acyl-coa oxidase 2, branched chain  CYP4A11 - cytochrome p450, family 4, subfamily a, polypeptide 11  AGXT2 - alanine--glyoxylate aminotransferase 2  ALDH8A1 - aldehyde dehydrogenase 8 family, member a1  SHMT1 - serine hydroxymethyltransferase 1 (soluble)  LPIN1 - lipin 1  TAT - tyrosine aminotransferase  ADTRP - androgen-dependent tfpi-regulating protein  ARG1 - arginase 1  HADH - hydroxyacyl-coa dehydrogenase  SCP2 - sterol carrier protein 2  HSD17B10 - hydroxysteroid (17-beta) dehydrogenase 10  ETFDH - electron-transferring-flavoprotein dehydrogenase  HAAO - 3-hydroxyanthranilate 3,4-dioxygenase  PON1 - paraoxonase 1  ECHDC2 - enoyl coa hydratase domain containing 2  PCK1 - phosphoenolpyruvate carboxykinase 1 (soluble)  FTCD - formimidoyltransferase cyclodeaminase  GCDH - glutaryl-coa dehydrogenase  ALDH1L1 - aldehyde dehydrogenase 1 family, member l1  PHYH - phytanoyl-coa 2-hydroxylase  PRODH2 - proline dehydrogenase (oxidase) 2  CRYL1 - crystallin, lambda 1  SLC27A2 - solute carrier family 27 (fatty acid transporter), member 2  ECHS1 - enoyl coa hydratase, short chain, 1, mitochondrial  MPST - mercaptopyruvate sulfurtransferase  ECH1 - enoyl coa hydratase 1, peroxisomal  HAO2 - hydroxyacid oxidase 2 (long chain)  CCBL1 - cysteine conjugate-beta lyase, cytoplasmic |
| GO:0017144 | drug metabolic process | 6.53E-24 | 9.08E-21 | 3.26 (10334,413,667,87) | [+] Show genes  UQCRC1 - ubiquinol-cytochrome c reductase core protein i  AKR1C3 - aldo-keto reductase family 1, member c3  PGM1 - phosphoglucomutase 1  SDHB - succinate dehydrogenase complex, subunit b, iron sulfur (ip)  COX5B - cytochrome c oxidase subunit vb  SUCLG1 - succinate-coa ligase, alpha subunit  FMO1 - flavin containing monooxygenase 1  PGAM2 - phosphoglycerate mutase 2 (muscle)  APOA4 - apolipoprotein a-iv  MTHFR - methylenetetrahydrofolate reductase (nad(p)h)  ACSL1 - acyl-coa synthetase long-chain family member 1  BDH2 - 3-hydroxybutyrate dehydrogenase, type 2  MTHFD1 - methylenetetrahydrofolate dehydrogenase (nadp+ dependent) 1, methenyltetrahydrofolate cyclohydrolase, formyltetrahydrofolate synthetase  ENO3 - enolase 3 (beta, muscle)  ATF4 - activating transcription factor 4  CHCHD10 - coiled-coil-helix-coiled-coil-helix domain containing 10  FAH - fumarylacetoacetate hydrolase (fumarylacetoacetase)  LDHA - lactate dehydrogenase a  SLC25A13 - solute carrier family 25 (aspartate/glutamate carrier), member 13  GSTZ1 - glutathione s-transferase zeta 1  ACAA1 - acetyl-coa acyltransferase 1  DPYS - dihydropyrimidinase  AK2 - adenylate kinase 2  ATP5J2 - atp synthase, h+ transporting, mitochondrial fo complex, subunit f2  AKR1C4 - aldo-keto reductase family 1, member c4  GCAT - glycine c-acetyltransferase  CPS1 - carbamoyl-phosphate synthase 1, mitochondrial  AHCY - adenosylhomocysteinase  SULT2A1 - sulfotransferase family, cytosolic, 2a, dehydroepiandrosterone (dhea)-preferring, member 1  ATP5H - atp synthase, h+ transporting, mitochondrial fo complex, subunit d  ATP5I - atp synthase, h+ transporting, mitochondrial fo complex, subunit e  AGXT - alanine-glyoxylate aminotransferase  CHIA - chitinase, acidic  BHMT - betaine--homocysteine s-methyltransferase  MAT1A - methionine adenosyltransferase i, alpha  ETHE1 - ethylmalonic encephalopathy 1  HPD - 4-hydroxyphenylpyruvate dioxygenase  HGD - homogentisate 1,2-dioxygenase  ATP5E - atp synthase, h+ transporting, mitochondrial f1 complex, epsilon subunit  PIPOX - pipecolic acid oxidase  MMAB - methylmalonic aciduria (cobalamin deficiency) cblb type  ATP5D - atp synthase, h+ transporting, mitochondrial f1 complex, delta subunit  ATP5G1 - atp synthase, h+ transporting, mitochondrial fo complex, subunit c1 (subunit 9)  PAH - phenylalanine hydroxylase  FH - fumarate hydratase  NR1I2 - nuclear receptor subfamily 1, group i, member 2  GGT1 - gamma-glutamyltransferase 1  IDH3G - isocitrate dehydrogenase 3 (nad+) gamma  ALDH2 - aldehyde dehydrogenase 2 family (mitochondrial)  PFKFB1 - 6-phosphofructo-2-kinase/fructose-2,6-biphosphatase 1  FAHD1 - fumarylacetoacetate hydrolase domain containing 1  CYP4A11 - cytochrome p450, family 4, subfamily a, polypeptide 11  AGXT2 - alanine--glyoxylate aminotransferase 2  ALDH8A1 - aldehyde dehydrogenase 8 family, member a1  CHPT1 - choline phosphotransferase 1  CYP2E1 - cytochrome p450, family 2, subfamily e, polypeptide 1  SHMT1 - serine hydroxymethyltransferase 1 (soluble)  GAMT - guanidinoacetate n-methyltransferase  CYP3A5 - cytochrome p450, family 3, subfamily a, polypeptide 5  ALDOB - aldolase b, fructose-bisphosphate  ADSSL1 - adenylosuccinate synthase like 1  CYP2C19 - cytochrome p450, family 2, subfamily c, polypeptide 19  ENPP1 - ectonucleotide pyrophosphatase/phosphodiesterase 1  TAT - tyrosine aminotransferase  SCP2 - sterol carrier protein 2  ADA - adenosine deaminase  CYP1A2 - cytochrome p450, family 1, subfamily a, polypeptide 2  PKLR - pyruvate kinase, liver and rbc  HAAO - 3-hydroxyanthranilate 3,4-dioxygenase  GLYAT - glycine-n-acyltransferase  PCK1 - phosphoenolpyruvate carboxykinase 1 (soluble)  IGF1 - insulin-like growth factor 1 (somatomedin c)  FTCD - formimidoyltransferase cyclodeaminase  CAT - catalase  ADH6 - alcohol dehydrogenase 6 (class v)  ALDH1L1 - aldehyde dehydrogenase 1 family, member l1  ADH4 - alcohol dehydrogenase 4 (class ii), pi polypeptide  ADI1 - acireductone dioxygenase 1  AMPD1 - adenosine monophosphate deaminase 1  CKM - creatine kinase, muscle  HMGCS2 - 3-hydroxy-3-methylglutaryl-coa synthase 2 (mitochondrial)  CBR1 - carbonyl reductase 1  MPST - mercaptopyruvate sulfurtransferase  SLC27A5 - solute carrier family 27 (fatty acid transporter), member 5  URAD - ureidoimidazoline (2-oxo-4-hydroxy-4-carboxy-5-) decarboxylase  CCBL1 - cysteine conjugate-beta lyase, cytoplasmic  MMACHC - methylmalonic aciduria (cobalamin deficiency) cblc type, with homocystinuria |
| GO:0003012 | muscle system process | 1.25E-21 | 1.58E-18 | 4.60 (10334,175,667,52) | [+] Show genes  ATP2A1 - atpase, ca++ transporting, cardiac muscle, fast twitch 1  CHRNE - cholinergic receptor, nicotinic, epsilon (muscle)  TRIM63 - tripartite motif containing 63, e3 ubiquitin protein ligase  PGAM2 - phosphoglycerate mutase 2 (muscle)  CHRNA1 - cholinergic receptor, nicotinic, alpha 1 (muscle)  CASQ2 - calsequestrin 2 (cardiac muscle)  HRC - histidine rich calcium binding protein  LMOD3 - leiomodin 3 (fetal)  MYLK2 - myosin light chain kinase 2  FXYD1 - fxyd domain containing ion transport regulator 1  KLHL41 - kelch-like family member 41  MB - myoglobin  PLN - phospholamban  TTN - titin  LMOD2 - leiomodin 2 (cardiac)  CACNA1S - calcium channel, voltage-dependent, l type, alpha 1s subunit  MYL3 - myosin, light chain 3, alkali; ventricular, skeletal, slow  DES - desmin  MYL2 - myosin, light chain 2, regulatory, cardiac, slow  TNNC2 - troponin c type 2 (fast)  TMOD4 - tropomodulin 4 (muscle)  ACTA1 - actin, alpha 1, skeletal muscle  RCSD1 - rcsd domain containing 1  TNNI2 - troponin i type 2 (skeletal, fast)  TNNI1 - troponin i type 1 (skeletal, slow)  TNNC1 - troponin c type 1 (slow)  TNNT3 - troponin t type 3 (skeletal, fast)  SMTN - smoothelin  ANKRD2 - ankyrin repeat domain 2 (stretch responsive muscle)  MYH1 - myosin, heavy chain 1, skeletal muscle, adult  MYH2 - myosin, heavy chain 2, skeletal muscle, adult  KLF15 - kruppel-like factor 15  TPM2 - tropomyosin 2 (beta)  CSRP3 - cysteine and glycine-rich protein 3 (cardiac lim protein)  MYL1 - myosin, light chain 1, alkali; skeletal, fast  GAMT - guanidinoacetate n-methyltransferase  ACTN3 - actinin, alpha 3  PPARGC1A - peroxisome proliferator-activated receptor gamma, coactivator 1 alpha  TCAP - titin-cap  MYLPF - myosin light chain, phosphorylatable, fast skeletal muscle  STAC3 - sh3 and cysteine rich domain 3  IGF1 - insulin-like growth factor 1 (somatomedin c)  MYOM2 - myomesin 2  LMOD1 - leiomodin 1 (smooth muscle)  TRIM72 - tripartite motif containing 72  CAV3 - caveolin 3  RYR1 - ryanodine receptor 1 (skeletal)  SMPX - small muscle protein, x-linked  MYOM3 - myomesin 3  NDUFS6 - nadh dehydrogenase (ubiquinone) fe-s protein 6, 13kda (nadh-coenzyme q reductase)  MYOT - myotilin  HMOX1 - heme oxygenase (decycling) 1 |
| GO:0072376 | protein activation cascade | 3.04E-21 | 3.53E-18 | 8.48 (10334,53,667,29) | [+] Show genes  F11 - coagulation factor xi  F10 - coagulation factor x  F9 - coagulation factor ix  C9 - complement component 9  FGG - fibrinogen gamma chain  MASP1 - mannan-binding lectin serine peptidase 1 (c4/c2 activating component of ra-reactive factor)  APCS - amyloid p component, serum  CRP - c-reactive protein, pentraxin-related  FN1 - fibronectin 1  F12 - coagulation factor xii (hageman factor)  FGA - fibrinogen alpha chain  FGB - fibrinogen beta chain  MASP2 - mannan-binding lectin serine peptidase 2  MBL2 - mannose-binding lectin (protein c) 2, soluble  CFB - complement factor b  C1R - complement component 1, r subcomponent  C2 - complement component 2  KNG1 - kininogen 1  C4BPA - complement component 4 binding protein, alpha  APOH - apolipoprotein h (beta-2-glycoprotein i)  C4BPB - complement component 4 binding protein, beta  F2 - coagulation factor ii (thrombin)  C5 - complement component 5  C6 - complement component 6  KLKB1 - kallikrein b, plasma (fletcher factor) 1  C8B - complement component 8, beta polypeptide  C8A - complement component 8, alpha polypeptide  F7 - coagulation factor vii (serum prothrombin conversion accelerator)  C8G - complement component 8, gamma polypeptide |
| GO:0016042 | lipid catabolic process | 3.71E-21 | 3.97E-18 | 4.28 (10334,199,667,55) | [+] Show genes  ECI2 - enoyl-coa delta isomerase 2  AKR1C3 - aldo-keto reductase family 1, member c3  HSD17B14 - hydroxysteroid (17-beta) dehydrogenase 14  ANGPTL3 - angiopoietin-like 3  ACAA2 - acetyl-coa acyltransferase 2  APOA2 - apolipoprotein a-ii  FABP1 - fatty acid binding protein 1, liver  AMACR - alpha-methylacyl-coa racemase  HSD11B1 - hydroxysteroid (11-beta) dehydrogenase 1  APOA4 - apolipoprotein a-iv  MCEE - methylmalonyl coa epimerase  BDH2 - 3-hydroxybutyrate dehydrogenase, type 2  CES2 - carboxylesterase 2  ASPG - asparaginase homolog (s. cerevisiae)  ACAA1 - acetyl-coa acyltransferase 1  ACADL - acyl-coa dehydrogenase, long chain  ACADS - acyl-coa dehydrogenase, c-2 to c-3 short chain  PNPLA2 - patatin-like phospholipase domain containing 2  ABHD1 - abhydrolase domain containing 1  ACADVL - acyl-coa dehydrogenase, very long chain  CPT2 - carnitine palmitoyltransferase 2  CPT1B - carnitine palmitoyltransferase 1b (muscle)  CPS1 - carbamoyl-phosphate synthase 1, mitochondrial  EHHADH - enoyl-coa, hydratase/3-hydroxyacyl coa dehydrogenase  HACL1 - 2-hydroxyacyl-coa lyase 1  APOC2 - apolipoprotein c-ii  LIPC - lipase, hepatic  APOC3 - apolipoprotein c-iii  PAFAH2 - platelet-activating factor acetylhydrolase 2, 40kda  DECR2 - 2,4-dienoyl coa reductase 2, peroxisomal  ACOX2 - acyl-coa oxidase 2, branched chain  CYP4A11 - cytochrome p450, family 4, subfamily a, polypeptide 11  CES1 - carboxylesterase 1  PLBD1 - phospholipase b domain containing 1  STS - steroid sulfatase (microsomal), isozyme s  LPIN1 - lipin 1  PLCD4 - phospholipase c, delta 4  SCARF1 - scavenger receptor class f, member 1  CES3 - carboxylesterase 3  ADTRP - androgen-dependent tfpi-regulating protein  HADH - hydroxyacyl-coa dehydrogenase  SCP2 - sterol carrier protein 2  CYP1A2 - cytochrome p450, family 1, subfamily a, polypeptide 2  ETFDH - electron-transferring-flavoprotein dehydrogenase  HSD17B6 - hydroxysteroid (17-beta) dehydrogenase 6  APOA5 - apolipoprotein a-v  ECHDC2 - enoyl coa hydratase domain containing 2  PCK1 - phosphoenolpyruvate carboxykinase 1 (soluble)  GCDH - glutaryl-coa dehydrogenase  PHYH - phytanoyl-coa 2-hydroxylase  PLA2G12B - phospholipase a2, group xiib  SLC27A2 - solute carrier family 27 (fatty acid transporter), member 2  ECHS1 - enoyl coa hydratase, short chain, 1, mitochondrial  ECH1 - enoyl coa hydratase 1, peroxisomal  HAO2 - hydroxyacid oxidase 2 (long chain) |
| GO:0006631 | fatty acid metabolic process | 7.87E-21 | 7.82E-18 | 4.03 (10334,223,667,58) | [+] Show genes  ECI2 - enoyl-coa delta isomerase 2  HSD17B8 - hydroxysteroid (17-beta) dehydrogenase 8  AKR1C3 - aldo-keto reductase family 1, member c3  MLYCD - malonyl-coa decarboxylase  ANGPTL3 - angiopoietin-like 3  ACAA2 - acetyl-coa acyltransferase 2  AMACR - alpha-methylacyl-coa racemase  MCEE - methylmalonyl coa epimerase  ACSL1 - acyl-coa synthetase long-chain family member 1  BDH2 - 3-hydroxybutyrate dehydrogenase, type 2  CES2 - carboxylesterase 2  ACAA1 - acetyl-coa acyltransferase 1  ACADL - acyl-coa dehydrogenase, long chain  ACADS - acyl-coa dehydrogenase, c-2 to c-3 short chain  ABHD1 - abhydrolase domain containing 1  ACSM2A - acyl-coa synthetase medium-chain family member 2a  ACADVL - acyl-coa dehydrogenase, very long chain  CPT2 - carnitine palmitoyltransferase 2  AKR1C4 - aldo-keto reductase family 1, member c4  CPT1B - carnitine palmitoyltransferase 1b (muscle)  CD36 - cd36 molecule (thrombospondin receptor)  EHHADH - enoyl-coa, hydratase/3-hydroxyacyl coa dehydrogenase  HACL1 - 2-hydroxyacyl-coa lyase 1  LIPC - lipase, hepatic  GGT1 - gamma-glutamyltransferase 1  DECR2 - 2,4-dienoyl coa reductase 2, peroxisomal  ACSM5 - acyl-coa synthetase medium-chain family member 5  ACOX2 - acyl-coa oxidase 2, branched chain  CYP4A11 - cytochrome p450, family 4, subfamily a, polypeptide 11  CES1 - carboxylesterase 1  CYP2E1 - cytochrome p450, family 2, subfamily e, polypeptide 1  LPIN1 - lipin 1  ALKBH7 - alkb, alkylation repair homolog 7 (e. coli)  CYP2C19 - cytochrome p450, family 2, subfamily c, polypeptide 19  PPARGC1A - peroxisome proliferator-activated receptor gamma, coactivator 1 alpha  ADTRP - androgen-dependent tfpi-regulating protein  SCP2 - sterol carrier protein 2  HADH - hydroxyacyl-coa dehydrogenase  CYP1A2 - cytochrome p450, family 1, subfamily a, polypeptide 2  PPARA - peroxisome proliferator-activated receptor alpha  PTPLA - protein tyrosine phosphatase-like (proline instead of catalytic arginine), member a  ETFDH - electron-transferring-flavoprotein dehydrogenase  PON1 - paraoxonase 1  ECHDC2 - enoyl coa hydratase domain containing 2  PCK1 - phosphoenolpyruvate carboxykinase 1 (soluble)  GCDH - glutaryl-coa dehydrogenase  UCP3 - uncoupling protein 3 (mitochondrial, proton carrier)  ADH4 - alcohol dehydrogenase 4 (class ii), pi polypeptide  PHYH - phytanoyl-coa 2-hydroxylase  CRYL1 - crystallin, lambda 1  NDUFS6 - nadh dehydrogenase (ubiquinone) fe-s protein 6, 13kda (nadh-coenzyme q reductase)  CBR1 - carbonyl reductase 1  SLC27A2 - solute carrier family 27 (fatty acid transporter), member 2  ECHS1 - enoyl coa hydratase, short chain, 1, mitochondrial  ACSM3 - acyl-coa synthetase medium-chain family member 3  SLC27A5 - solute carrier family 27 (fatty acid transporter), member 5  ECH1 - enoyl coa hydratase 1, peroxisomal  HAO2 - hydroxyacid oxidase 2 (long chain) |
| GO:0006629 | lipid metabolic process | 4.79E-20 | 4.45E-17 | 2.36 (10334,796,667,121) | [+] Show genes  LGMN - legumain  ANGPTL3 - angiopoietin-like 3  APOA2 - apolipoprotein a-ii  HSD11B1 - hydroxysteroid (11-beta) dehydrogenase 1  AMACR - alpha-methylacyl-coa racemase  APOA4 - apolipoprotein a-iv  BDH2 - 3-hydroxybutyrate dehydrogenase, type 2  CES2 - carboxylesterase 2  APOF - apolipoprotein f  IMPA2 - inositol(myo)-1(or 4)-monophosphatase 2  ACAA1 - acetyl-coa acyltransferase 1  ACADL - acyl-coa dehydrogenase, long chain  ACADS - acyl-coa dehydrogenase, c-2 to c-3 short chain  ACSM2A - acyl-coa synthetase medium-chain family member 2a  ACADVL - acyl-coa dehydrogenase, very long chain  CPT2 - carnitine palmitoyltransferase 2  DHRS3 - dehydrogenase/reductase (sdr family) member 3  TTR - transthyretin  DHRS4 - dehydrogenase/reductase (sdr family) member 4  CPT1B - carnitine palmitoyltransferase 1b (muscle)  CPS1 - carbamoyl-phosphate synthase 1, mitochondrial  CD36 - cd36 molecule (thrombospondin receptor)  IL1RN - interleukin 1 receptor antagonist  EHHADH - enoyl-coa, hydratase/3-hydroxyacyl coa dehydrogenase  TTPA - tocopherol (alpha) transfer protein  FITM1 - fat storage-inducing transmembrane protein 1  APOC2 - apolipoprotein c-ii  APOC3 - apolipoprotein c-iii  LIPC - lipase, hepatic  SDC2 - syndecan 2  APOC4 - apolipoprotein c-iv  PAFAH2 - platelet-activating factor acetylhydrolase 2, 40kda  APOH - apolipoprotein h (beta-2-glycoprotein i)  NR1I2 - nuclear receptor subfamily 1, group i, member 2  GGT1 - gamma-glutamyltransferase 1  PLEK - pleckstrin  ACOX2 - acyl-coa oxidase 2, branched chain  CYP4A11 - cytochrome p450, family 4, subfamily a, polypeptide 11  CYP8B1 - cytochrome p450, family 8, subfamily b, polypeptide 1  CHPT1 - choline phosphotransferase 1  ALDH8A1 - aldehyde dehydrogenase 8 family, member a1  CES1 - carboxylesterase 1  CYP2E1 - cytochrome p450, family 2, subfamily e, polypeptide 1  STS - steroid sulfatase (microsomal), isozyme s  PLBD1 - phospholipase b domain containing 1  CYP3A5 - cytochrome p450, family 3, subfamily a, polypeptide 5  SCARF1 - scavenger receptor class f, member 1  PCTP - phosphatidylcholine transfer protein  CYP2C19 - cytochrome p450, family 2, subfamily c, polypeptide 19  PPARGC1A - peroxisome proliferator-activated receptor gamma, coactivator 1 alpha  HADH - hydroxyacyl-coa dehydrogenase  SCP2 - sterol carrier protein 2  PM20D1 - peptidase m20 domain containing 1  CYP1A2 - cytochrome p450, family 1, subfamily a, polypeptide 2  PPARA - peroxisome proliferator-activated receptor alpha  PTPLA - protein tyrosine phosphatase-like (proline instead of catalytic arginine), member a  HSD17B10 - hydroxysteroid (17-beta) dehydrogenase 10  PON1 - paraoxonase 1  C19orf80 - chromosome 19 open reading frame 80  HSD17B6 - hydroxysteroid (17-beta) dehydrogenase 6  APOA5 - apolipoprotein a-v  TM6SF2 - transmembrane 6 superfamily member 2  PCK1 - phosphoenolpyruvate carboxykinase 1 (soluble)  ADH6 - alcohol dehydrogenase 6 (class v)  ADH4 - alcohol dehydrogenase 4 (class ii), pi polypeptide  DGAT2 - diacylglycerol o-acyltransferase 2  TMEM86B - transmembrane protein 86b  RDH16 - retinol dehydrogenase 16 (all-trans)  PLA2G12B - phospholipase a2, group xiib  APOM - apolipoprotein m  CRYL1 - crystallin, lambda 1  AGPAT2 - 1-acylglycerol-3-phosphate o-acyltransferase 2  ECHS1 - enoyl coa hydratase, short chain, 1, mitochondrial  ACSM3 - acyl-coa synthetase medium-chain family member 3  ECH1 - enoyl coa hydratase 1, peroxisomal  ECI2 - enoyl-coa delta isomerase 2  AKR1C3 - aldo-keto reductase family 1, member c3  HSD17B8 - hydroxysteroid (17-beta) dehydrogenase 8  MLYCD - malonyl-coa decarboxylase  HSD17B14 - hydroxysteroid (17-beta) dehydrogenase 14  ACAA2 - acetyl-coa acyltransferase 2  FABP1 - fatty acid binding protein 1, liver  ORMDL3 - orm1-like 3 (s. cerevisiae)  MCEE - methylmalonyl coa epimerase  ACSL1 - acyl-coa synthetase long-chain family member 1  GK5 - glycerol kinase 5 (putative)  ASPG - asparaginase homolog (s. cerevisiae)  GPD1 - glycerol-3-phosphate dehydrogenase 1 (soluble)  PNPLA2 - patatin-like phospholipase domain containing 2  ABHD1 - abhydrolase domain containing 1  AKR1C4 - aldo-keto reductase family 1, member c4  ANGPTL4 - angiopoietin-like 4  SULT2A1 - sulfotransferase family, cytosolic, 2a, dehydroepiandrosterone (dhea)-preferring, member 1  HACL1 - 2-hydroxyacyl-coa lyase 1  DECR2 - 2,4-dienoyl coa reductase 2, peroxisomal  G6PC - glucose-6-phosphatase, catalytic subunit  ACSM5 - acyl-coa synthetase medium-chain family member 5  PHOSPHO1 - phosphatase, orphan 1  LPIN1 - lipin 1  PLCD4 - phospholipase c, delta 4  ALKBH7 - alkb, alkylation repair homolog 7 (e. coli)  CES3 - carboxylesterase 3  ADTRP - androgen-dependent tfpi-regulating protein  ETFDH - electron-transferring-flavoprotein dehydrogenase  SERPINA6 - serpin peptidase inhibitor, clade a (alpha-1 antiproteinase, antitrypsin), member 6  ECHDC2 - enoyl coa hydratase domain containing 2  GCDH - glutaryl-coa dehydrogenase  CAT - catalase  GC - group-specific component (vitamin d binding protein)  RXRA - retinoid x receptor, alpha  UCP3 - uncoupling protein 3 (mitochondrial, proton carrier)  CAV3 - caveolin 3  PHYH - phytanoyl-coa 2-hydroxylase  HMGCS2 - 3-hydroxy-3-methylglutaryl-coa synthase 2 (mitochondrial)  NDUFS6 - nadh dehydrogenase (ubiquinone) fe-s protein 6, 13kda (nadh-coenzyme q reductase)  SLC27A2 - solute carrier family 27 (fatty acid transporter), member 2  CBR1 - carbonyl reductase 1  THRSP - thyroid hormone responsive  SLC27A5 - solute carrier family 27 (fatty acid transporter), member 5  HAO2 - hydroxyacid oxidase 2 (long chain)  NR1I3 - nuclear receptor subfamily 1, group i, member 3 |
| GO:0006936 | muscle contraction | 1.96E-19 | 1.71E-16 | 4.74 (10334,147,667,45) | [+] Show genes  ANKRD2 - ankyrin repeat domain 2 (stretch responsive muscle)  MYH1 - myosin, heavy chain 1, skeletal muscle, adult  CHRNE - cholinergic receptor, nicotinic, epsilon (muscle)  MYH2 - myosin, heavy chain 2, skeletal muscle, adult  TPM2 - tropomyosin 2 (beta)  CSRP3 - cysteine and glycine-rich protein 3 (cardiac lim protein)  MYL1 - myosin, light chain 1, alkali; skeletal, fast  GAMT - guanidinoacetate n-methyltransferase  TRIM63 - tripartite motif containing 63, e3 ubiquitin protein ligase  PGAM2 - phosphoglycerate mutase 2 (muscle)  CHRNA1 - cholinergic receptor, nicotinic, alpha 1 (muscle)  CASQ2 - calsequestrin 2 (cardiac muscle)  HRC - histidine rich calcium binding protein  TCAP - titin-cap  MYLPF - myosin light chain, phosphorylatable, fast skeletal muscle  LMOD3 - leiomodin 3 (fetal)  MYLK2 - myosin light chain kinase 2  FXYD1 - fxyd domain containing ion transport regulator 1  KLHL41 - kelch-like family member 41  STAC3 - sh3 and cysteine rich domain 3  MB - myoglobin  MYOM2 - myomesin 2  LMOD1 - leiomodin 1 (smooth muscle)  TRIM72 - tripartite motif containing 72  CAV3 - caveolin 3  RYR1 - ryanodine receptor 1 (skeletal)  TTN - titin  LMOD2 - leiomodin 2 (cardiac)  CACNA1S - calcium channel, voltage-dependent, l type, alpha 1s subunit  MYL3 - myosin, light chain 3, alkali; ventricular, skeletal, slow  DES - desmin  TNNC2 - troponin c type 2 (fast)  MYL2 - myosin, light chain 2, regulatory, cardiac, slow  TMOD4 - tropomodulin 4 (muscle)  ACTA1 - actin, alpha 1, skeletal muscle  SMPX - small muscle protein, x-linked  MYOM3 - myomesin 3  NDUFS6 - nadh dehydrogenase (ubiquinone) fe-s protein 6, 13kda (nadh-coenzyme q reductase)  TNNI2 - troponin i type 2 (skeletal, fast)  RCSD1 - rcsd domain containing 1  MYOT - myotilin  TNNI1 - troponin i type 1 (skeletal, slow)  TNNC1 - troponin c type 1 (slow)  TNNT3 - troponin t type 3 (skeletal, fast)  SMTN - smoothelin |
| GO:0006091 | generation of precursor metabolites and energy | 1.61E-18 | 1.32E-15 | 3.37 (10334,294,667,64) | [+] Show genes  UQCRC1 - ubiquinol-cytochrome c reductase core protein i  COX6A2 - cytochrome c oxidase subunit via polypeptide 2  PGM1 - phosphoglucomutase 1  NDUFA3 - nadh dehydrogenase (ubiquinone) 1 alpha subcomplex, 3, 9kda  NDUFA4 - nadh dehydrogenase (ubiquinone) 1 alpha subcomplex, 4, 9kda  NDUFS7 - nadh dehydrogenase (ubiquinone) fe-s protein 7, 20kda (nadh-coenzyme q reductase)  COX5B - cytochrome c oxidase subunit vb  SDHB - succinate dehydrogenase complex, subunit b, iron sulfur (ip)  NDUFA2 - nadh dehydrogenase (ubiquinone) 1 alpha subcomplex, 2, 8kda  NDUFA7 - nadh dehydrogenase (ubiquinone) 1 alpha subcomplex, 7, 14.5kda  PGAM2 - phosphoglycerate mutase 2 (muscle)  BDH2 - 3-hydroxybutyrate dehydrogenase, type 2  ENO3 - enolase 3 (beta, muscle)  CHCHD10 - coiled-coil-helix-coiled-coil-helix domain containing 10  ALDH4A1 - aldehyde dehydrogenase 4 family, member a1  LDHA - lactate dehydrogenase a  PPP1R3B - protein phosphatase 1, regulatory subunit 3b  GCGR - glucagon receptor  SLC25A13 - solute carrier family 25 (aspartate/glutamate carrier), member 13  COX7C - cytochrome c oxidase subunit viic  ACADVL - acyl-coa dehydrogenase, very long chain  BLOC1S1 - biogenesis of lysosomal organelles complex-1, subunit 1  DHRS3 - dehydrogenase/reductase (sdr family) member 3  ATP5J2 - atp synthase, h+ transporting, mitochondrial fo complex, subunit f2  UQCR10 - ubiquinol-cytochrome c reductase, complex iii subunit x  AKR1C4 - aldo-keto reductase family 1, member c4  ATP5I - atp synthase, h+ transporting, mitochondrial fo complex, subunit e  ATP5H - atp synthase, h+ transporting, mitochondrial fo complex, subunit d  ATP5E - atp synthase, h+ transporting, mitochondrial f1 complex, epsilon subunit  PPP1R3C - protein phosphatase 1, regulatory subunit 3c  ATP5D - atp synthase, h+ transporting, mitochondrial f1 complex, delta subunit  IMMP2L - imp2 inner mitochondrial membrane peptidase-like (s. cerevisiae)  ATP5G1 - atp synthase, h+ transporting, mitochondrial fo complex, subunit c1 (subunit 9)  GYS2 - glycogen synthase 2 (liver)  COQ9 - coenzyme q9 homolog (s. cerevisiae)  G6PC - glucose-6-phosphatase, catalytic subunit  ALDH2 - aldehyde dehydrogenase 2 family (mitochondrial)  PPP1R3A - protein phosphatase 1, regulatory subunit 3a  PFKFB1 - 6-phosphofructo-2-kinase/fructose-2,6-biphosphatase 1  COX17 - cox17 cytochrome c oxidase copper chaperone  ALDOB - aldolase b, fructose-bisphosphate  ENPP1 - ectonucleotide pyrophosphatase/phosphodiesterase 1  PPARGC1A - peroxisome proliferator-activated receptor gamma, coactivator 1 alpha  CYP1A2 - cytochrome p450, family 1, subfamily a, polypeptide 2  ETFDH - electron-transferring-flavoprotein dehydrogenase  PKLR - pyruvate kinase, liver and rbc  HAAO - 3-hydroxyanthranilate 3,4-dioxygenase  NDUFB10 - nadh dehydrogenase (ubiquinone) 1 beta subcomplex, 10, 22kda  HSD17B6 - hydroxysteroid (17-beta) dehydrogenase 6  NDUFB7 - nadh dehydrogenase (ubiquinone) 1 beta subcomplex, 7, 18kda  CAT - catalase  ADH6 - alcohol dehydrogenase 6 (class v)  ADH4 - alcohol dehydrogenase 4 (class ii), pi polypeptide  UQCR11 - ubiquinol-cytochrome c reductase, complex iii subunit xi  STBD1 - starch binding domain 1  RDH16 - retinol dehydrogenase 16 (all-trans)  HMGCS2 - 3-hydroxy-3-methylglutaryl-coa synthase 2 (mitochondrial)  NDUFS8 - nadh dehydrogenase (ubiquinone) fe-s protein 8, 23kda (nadh-coenzyme q reductase)  NDUFS6 - nadh dehydrogenase (ubiquinone) fe-s protein 6, 13kda (nadh-coenzyme q reductase)  SLC25A4 - solute carrier family 25 (mitochondrial carrier; adenine nucleotide translocator), member 4  NDUFS5 - nadh dehydrogenase (ubiquinone) fe-s protein 5, 15kda (nadh-coenzyme q reductase)  NDUFV1 - nadh dehydrogenase (ubiquinone) flavoprotein 1, 51kda  SLC27A5 - solute carrier family 27 (fatty acid transporter), member 5  UQCRQ - ubiquinol-cytochrome c reductase, complex iii subunit vii, 9.5kda |
| GO:0072329 | monocarboxylic acid catabolic process | 3.89E-17 | 3.01E-14 | 5.32 (10334,102,667,35) | [+] Show genes  ECI2 - enoyl-coa delta isomerase 2  ACOX2 - acyl-coa oxidase 2, branched chain  CYP4A11 - cytochrome p450, family 4, subfamily a, polypeptide 11  ACAA2 - acetyl-coa acyltransferase 2  AGXT2 - alanine--glyoxylate aminotransferase 2  LPIN1 - lipin 1  AMACR - alpha-methylacyl-coa racemase  MCEE - methylmalonyl coa epimerase  BDH2 - 3-hydroxybutyrate dehydrogenase, type 2  ADTRP - androgen-dependent tfpi-regulating protein  FAH - fumarylacetoacetate hydrolase (fumarylacetoacetase)  SCP2 - sterol carrier protein 2  HADH - hydroxyacyl-coa dehydrogenase  ACAA1 - acetyl-coa acyltransferase 1  ACADL - acyl-coa dehydrogenase, long chain  ACADS - acyl-coa dehydrogenase, c-2 to c-3 short chain  ETFDH - electron-transferring-flavoprotein dehydrogenase  ABHD1 - abhydrolase domain containing 1  ACADVL - acyl-coa dehydrogenase, very long chain  CPT2 - carnitine palmitoyltransferase 2  CPT1B - carnitine palmitoyltransferase 1b (muscle)  ECHDC2 - enoyl coa hydratase domain containing 2  PCK1 - phosphoenolpyruvate carboxykinase 1 (soluble)  GCDH - glutaryl-coa dehydrogenase  SULT2A1 - sulfotransferase family, cytosolic, 2a, dehydroepiandrosterone (dhea)-preferring, member 1  AGXT - alanine-glyoxylate aminotransferase  EHHADH - enoyl-coa, hydratase/3-hydroxyacyl coa dehydrogenase  PHYH - phytanoyl-coa 2-hydroxylase  HACL1 - 2-hydroxyacyl-coa lyase 1  CRYL1 - crystallin, lambda 1  SLC27A2 - solute carrier family 27 (fatty acid transporter), member 2  ECHS1 - enoyl coa hydratase, short chain, 1, mitochondrial  DECR2 - 2,4-dienoyl coa reductase 2, peroxisomal  HAO2 - hydroxyacid oxidase 2 (long chain)  ECH1 - enoyl coa hydratase 1, peroxisomal |
| GO:0030049 | muscle filament sliding | 2.07E-16 | 1.52E-13 | 10.15 (10334,29,667,19) | [+] Show genes  MYH2 - myosin, heavy chain 2, skeletal muscle, adult  TPM2 - tropomyosin 2 (beta)  MYL1 - myosin, light chain 1, alkali; skeletal, fast  ACTN2 - actinin, alpha 2  ACTN3 - actinin, alpha 3  TTN - titin  NEB - nebulin  MYL3 - myosin, light chain 3, alkali; ventricular, skeletal, slow  DES - desmin  TNNC2 - troponin c type 2 (fast)  MYBPC1 - myosin binding protein c, slow type  MYL2 - myosin, light chain 2, regulatory, cardiac, slow  ACTA1 - actin, alpha 1, skeletal muscle  TNNI2 - troponin i type 2 (skeletal, fast)  TNNI1 - troponin i type 1 (skeletal, slow)  TCAP - titin-cap  TNNC1 - troponin c type 1 (slow)  TNNT3 - troponin t type 3 (skeletal, fast)  MYBPC2 - myosin binding protein c, fast type |
| GO:0033275 | actin-myosin filament sliding | 2.07E-16 | 1.44E-13 | 10.15 (10334,29,667,19) | [+] Show genes  MYH2 - myosin, heavy chain 2, skeletal muscle, adult  TPM2 - tropomyosin 2 (beta)  MYL1 - myosin, light chain 1, alkali; skeletal, fast  ACTN2 - actinin, alpha 2  ACTN3 - actinin, alpha 3  TTN - titin  NEB - nebulin  MYL3 - myosin, light chain 3, alkali; ventricular, skeletal, slow  DES - desmin  TNNC2 - troponin c type 2 (fast)  MYBPC1 - myosin binding protein c, slow type  MYL2 - myosin, light chain 2, regulatory, cardiac, slow  ACTA1 - actin, alpha 1, skeletal muscle  TNNI2 - troponin i type 2 (skeletal, fast)  TNNI1 - troponin i type 1 (skeletal, slow)  TCAP - titin-cap  TNNC1 - troponin c type 1 (slow)  TNNT3 - troponin t type 3 (skeletal, fast)  MYBPC2 - myosin binding protein c, fast type |
| GO:0002526 | acute inflammatory response | 3.1E-16 | 2.05E-13 | 9.39 (10334,33,667,20) | [+] Show genes  APCS - amyloid p component, serum  CRP - c-reactive protein, pentraxin-related  FN1 - fibronectin 1  F12 - coagulation factor xii (hageman factor)  MBL2 - mannose-binding lectin (protein c) 2, soluble  SAA1 - serum amyloid a1  APOA2 - apolipoprotein a-ii  LBP - lipopolysaccharide binding protein  ASS1 - argininosuccinate synthase 1  HP - haptoglobin  SERPINA1 - serpin peptidase inhibitor, clade a (alpha-1 antiproteinase, antitrypsin), member 1  VNN1 - vanin 1  HAMP - hepcidin antimicrobial peptide  ITIH4 - inter-alpha-trypsin inhibitor heavy chain family, member 4  F2 - coagulation factor ii (thrombin)  SERPINC1 - serpin peptidase inhibitor, clade c (antithrombin), member 1  SAA4 - serum amyloid a4, constitutive  TFR2 - transferrin receptor 2  NUPR1 - nuclear protein, transcriptional regulator, 1  SERPINF2 - serpin peptidase inhibitor, clade f (alpha-2 antiplasmin, pigment epithelium derived factor), member 2 |
| GO:0044255 | cellular lipid metabolic process | 9.21E-16 | 5.82E-13 | 2.33 (10334,645,667,97) | [+] Show genes  ANGPTL3 - angiopoietin-like 3  APOA2 - apolipoprotein a-ii  AMACR - alpha-methylacyl-coa racemase  APOA4 - apolipoprotein a-iv  BDH2 - 3-hydroxybutyrate dehydrogenase, type 2  CES2 - carboxylesterase 2  IMPA2 - inositol(myo)-1(or 4)-monophosphatase 2  ACAA1 - acetyl-coa acyltransferase 1  ACADL - acyl-coa dehydrogenase, long chain  ACADS - acyl-coa dehydrogenase, c-2 to c-3 short chain  ACSM2A - acyl-coa synthetase medium-chain family member 2a  ACADVL - acyl-coa dehydrogenase, very long chain  DHRS3 - dehydrogenase/reductase (sdr family) member 3  CPT2 - carnitine palmitoyltransferase 2  TTR - transthyretin  CPT1B - carnitine palmitoyltransferase 1b (muscle)  CPS1 - carbamoyl-phosphate synthase 1, mitochondrial  CD36 - cd36 molecule (thrombospondin receptor)  EHHADH - enoyl-coa, hydratase/3-hydroxyacyl coa dehydrogenase  FITM1 - fat storage-inducing transmembrane protein 1  APOC2 - apolipoprotein c-ii  APOC3 - apolipoprotein c-iii  SDC2 - syndecan 2  LIPC - lipase, hepatic  APOH - apolipoprotein h (beta-2-glycoprotein i)  GGT1 - gamma-glutamyltransferase 1  PLEK - pleckstrin  ACOX2 - acyl-coa oxidase 2, branched chain  CYP4A11 - cytochrome p450, family 4, subfamily a, polypeptide 11  CHPT1 - choline phosphotransferase 1  CES1 - carboxylesterase 1  ALDH8A1 - aldehyde dehydrogenase 8 family, member a1  CYP2E1 - cytochrome p450, family 2, subfamily e, polypeptide 1  STS - steroid sulfatase (microsomal), isozyme s  PLBD1 - phospholipase b domain containing 1  CYP3A5 - cytochrome p450, family 3, subfamily a, polypeptide 5  PCTP - phosphatidylcholine transfer protein  CYP2C19 - cytochrome p450, family 2, subfamily c, polypeptide 19  PPARGC1A - peroxisome proliferator-activated receptor gamma, coactivator 1 alpha  HADH - hydroxyacyl-coa dehydrogenase  SCP2 - sterol carrier protein 2  PM20D1 - peptidase m20 domain containing 1  CYP1A2 - cytochrome p450, family 1, subfamily a, polypeptide 2  PPARA - peroxisome proliferator-activated receptor alpha  PTPLA - protein tyrosine phosphatase-like (proline instead of catalytic arginine), member a  PON1 - paraoxonase 1  C19orf80 - chromosome 19 open reading frame 80  APOA5 - apolipoprotein a-v  PCK1 - phosphoenolpyruvate carboxykinase 1 (soluble)  ADH6 - alcohol dehydrogenase 6 (class v)  ADH4 - alcohol dehydrogenase 4 (class ii), pi polypeptide  DGAT2 - diacylglycerol o-acyltransferase 2  TMEM86B - transmembrane protein 86b  PLA2G12B - phospholipase a2, group xiib  RDH16 - retinol dehydrogenase 16 (all-trans)  CRYL1 - crystallin, lambda 1  APOM - apolipoprotein m  AGPAT2 - 1-acylglycerol-3-phosphate o-acyltransferase 2  ECHS1 - enoyl coa hydratase, short chain, 1, mitochondrial  ACSM3 - acyl-coa synthetase medium-chain family member 3  ECH1 - enoyl coa hydratase 1, peroxisomal  ECI2 - enoyl-coa delta isomerase 2  HSD17B8 - hydroxysteroid (17-beta) dehydrogenase 8  AKR1C3 - aldo-keto reductase family 1, member c3  MLYCD - malonyl-coa decarboxylase  ACAA2 - acetyl-coa acyltransferase 2  FABP1 - fatty acid binding protein 1, liver  ORMDL3 - orm1-like 3 (s. cerevisiae)  MCEE - methylmalonyl coa epimerase  ACSL1 - acyl-coa synthetase long-chain family member 1  GK5 - glycerol kinase 5 (putative)  GPD1 - glycerol-3-phosphate dehydrogenase 1 (soluble)  ABHD1 - abhydrolase domain containing 1  PNPLA2 - patatin-like phospholipase domain containing 2  AKR1C4 - aldo-keto reductase family 1, member c4  HACL1 - 2-hydroxyacyl-coa lyase 1  G6PC - glucose-6-phosphatase, catalytic subunit  DECR2 - 2,4-dienoyl coa reductase 2, peroxisomal  ACSM5 - acyl-coa synthetase medium-chain family member 5  PHOSPHO1 - phosphatase, orphan 1  PLCD4 - phospholipase c, delta 4  LPIN1 - lipin 1  ALKBH7 - alkb, alkylation repair homolog 7 (e. coli)  ADTRP - androgen-dependent tfpi-regulating protein  ETFDH - electron-transferring-flavoprotein dehydrogenase  ECHDC2 - enoyl coa hydratase domain containing 2  GCDH - glutaryl-coa dehydrogenase  CAT - catalase  UCP3 - uncoupling protein 3 (mitochondrial, proton carrier)  CAV3 - caveolin 3  PHYH - phytanoyl-coa 2-hydroxylase  HMGCS2 - 3-hydroxy-3-methylglutaryl-coa synthase 2 (mitochondrial)  SLC27A2 - solute carrier family 27 (fatty acid transporter), member 2  CBR1 - carbonyl reductase 1  NDUFS6 - nadh dehydrogenase (ubiquinone) fe-s protein 6, 13kda (nadh-coenzyme q reductase)  SLC27A5 - solute carrier family 27 (fatty acid transporter), member 5  HAO2 - hydroxyacid oxidase 2 (long chain) |
| GO:0009062 | fatty acid catabolic process | 2.98E-15 | 1.8E-12 | 5.47 (10334,85,667,30) | [+] Show genes  ECI2 - enoyl-coa delta isomerase 2  ACOX2 - acyl-coa oxidase 2, branched chain  CYP4A11 - cytochrome p450, family 4, subfamily a, polypeptide 11  ACAA2 - acetyl-coa acyltransferase 2  LPIN1 - lipin 1  AMACR - alpha-methylacyl-coa racemase  MCEE - methylmalonyl coa epimerase  BDH2 - 3-hydroxybutyrate dehydrogenase, type 2  ADTRP - androgen-dependent tfpi-regulating protein  HADH - hydroxyacyl-coa dehydrogenase  SCP2 - sterol carrier protein 2  ACAA1 - acetyl-coa acyltransferase 1  ACADL - acyl-coa dehydrogenase, long chain  ACADS - acyl-coa dehydrogenase, c-2 to c-3 short chain  ABHD1 - abhydrolase domain containing 1  ETFDH - electron-transferring-flavoprotein dehydrogenase  ACADVL - acyl-coa dehydrogenase, very long chain  CPT2 - carnitine palmitoyltransferase 2  CPT1B - carnitine palmitoyltransferase 1b (muscle)  ECHDC2 - enoyl coa hydratase domain containing 2  PCK1 - phosphoenolpyruvate carboxykinase 1 (soluble)  GCDH - glutaryl-coa dehydrogenase  EHHADH - enoyl-coa, hydratase/3-hydroxyacyl coa dehydrogenase  PHYH - phytanoyl-coa 2-hydroxylase  HACL1 - 2-hydroxyacyl-coa lyase 1  SLC27A2 - solute carrier family 27 (fatty acid transporter), member 2  ECHS1 - enoyl coa hydratase, short chain, 1, mitochondrial  DECR2 - 2,4-dienoyl coa reductase 2, peroxisomal  ECH1 - enoyl coa hydratase 1, peroxisomal  HAO2 - hydroxyacid oxidase 2 (long chain) |
| GO:0044242 | cellular lipid catabolic process | 3.24E-15 | 1.88E-12 | 4.16 (10334,149,667,40) | [+] Show genes  ECI2 - enoyl-coa delta isomerase 2  ACOX2 - acyl-coa oxidase 2, branched chain  AKR1C3 - aldo-keto reductase family 1, member c3  CYP4A11 - cytochrome p450, family 4, subfamily a, polypeptide 11  ANGPTL3 - angiopoietin-like 3  ACAA2 - acetyl-coa acyltransferase 2  APOA2 - apolipoprotein a-ii  FABP1 - fatty acid binding protein 1, liver  PLBD1 - phospholipase b domain containing 1  LPIN1 - lipin 1  AMACR - alpha-methylacyl-coa racemase  MCEE - methylmalonyl coa epimerase  BDH2 - 3-hydroxybutyrate dehydrogenase, type 2  ADTRP - androgen-dependent tfpi-regulating protein  SCP2 - sterol carrier protein 2  HADH - hydroxyacyl-coa dehydrogenase  ACAA1 - acetyl-coa acyltransferase 1  ACADL - acyl-coa dehydrogenase, long chain  ACADS - acyl-coa dehydrogenase, c-2 to c-3 short chain  ETFDH - electron-transferring-flavoprotein dehydrogenase  ABHD1 - abhydrolase domain containing 1  PNPLA2 - patatin-like phospholipase domain containing 2  ACADVL - acyl-coa dehydrogenase, very long chain  CPT2 - carnitine palmitoyltransferase 2  CPT1B - carnitine palmitoyltransferase 1b (muscle)  APOA5 - apolipoprotein a-v  ECHDC2 - enoyl coa hydratase domain containing 2  CPS1 - carbamoyl-phosphate synthase 1, mitochondrial  PCK1 - phosphoenolpyruvate carboxykinase 1 (soluble)  GCDH - glutaryl-coa dehydrogenase  EHHADH - enoyl-coa, hydratase/3-hydroxyacyl coa dehydrogenase  PHYH - phytanoyl-coa 2-hydroxylase  HACL1 - 2-hydroxyacyl-coa lyase 1  LIPC - lipase, hepatic  APOC3 - apolipoprotein c-iii  SLC27A2 - solute carrier family 27 (fatty acid transporter), member 2  ECHS1 - enoyl coa hydratase, short chain, 1, mitochondrial  DECR2 - 2,4-dienoyl coa reductase 2, peroxisomal  HAO2 - hydroxyacid oxidase 2 (long chain)  ECH1 - enoyl coa hydratase 1, peroxisomal |
| GO:0019395 | fatty acid oxidation | 1.3E-14 | 7.25E-12 | 5.81 (10334,72,667,27) | [+] Show genes  ECI2 - enoyl-coa delta isomerase 2  ACOX2 - acyl-coa oxidase 2, branched chain  MLYCD - malonyl-coa decarboxylase  ACAA2 - acetyl-coa acyltransferase 2  AMACR - alpha-methylacyl-coa racemase  BDH2 - 3-hydroxybutyrate dehydrogenase, type 2  PPARGC1A - peroxisome proliferator-activated receptor gamma, coactivator 1 alpha  SCP2 - sterol carrier protein 2  HADH - hydroxyacyl-coa dehydrogenase  ACAA1 - acetyl-coa acyltransferase 1  ACADL - acyl-coa dehydrogenase, long chain  ACADS - acyl-coa dehydrogenase, c-2 to c-3 short chain  ETFDH - electron-transferring-flavoprotein dehydrogenase  ACADVL - acyl-coa dehydrogenase, very long chain  CPT2 - carnitine palmitoyltransferase 2  CPT1B - carnitine palmitoyltransferase 1b (muscle)  ECHDC2 - enoyl coa hydratase domain containing 2  GCDH - glutaryl-coa dehydrogenase  EHHADH - enoyl-coa, hydratase/3-hydroxyacyl coa dehydrogenase  ADH4 - alcohol dehydrogenase 4 (class ii), pi polypeptide  PHYH - phytanoyl-coa 2-hydroxylase  HACL1 - 2-hydroxyacyl-coa lyase 1  SLC27A2 - solute carrier family 27 (fatty acid transporter), member 2  ECHS1 - enoyl coa hydratase, short chain, 1, mitochondrial  DECR2 - 2,4-dienoyl coa reductase 2, peroxisomal  ECH1 - enoyl coa hydratase 1, peroxisomal  HAO2 - hydroxyacid oxidase 2 (long chain) |
| GO:1901605 | alpha-amino acid metabolic process | 1.56E-14 | 8.35E-12 | 3.83 (10334,170,667,42) | [+] Show genes  AGXT2 - alanine--glyoxylate aminotransferase 2  AGMAT - agmatine ureohydrolase (agmatinase)  ALDH8A1 - aldehyde dehydrogenase 8 family, member a1  SHMT1 - serine hydroxymethyltransferase 1 (soluble)  GAMT - guanidinoacetate n-methyltransferase  ADSSL1 - adenylosuccinate synthase like 1  MTHFR - methylenetetrahydrofolate reductase (nad(p)h)  MTHFD1 - methylenetetrahydrofolate dehydrogenase (nadp+ dependent) 1, methenyltetrahydrofolate cyclohydrolase, formyltetrahydrofolate synthetase  TAT - tyrosine aminotransferase  OTC - ornithine carbamoyltransferase  ATF4 - activating transcription factor 4  GOT2 - glutamic-oxaloacetic transaminase 2, mitochondrial  ALDH4A1 - aldehyde dehydrogenase 4 family, member a1  FAH - fumarylacetoacetate hydrolase (fumarylacetoacetase)  ARG1 - arginase 1  GSTZ1 - glutathione s-transferase zeta 1  HAAO - 3-hydroxyanthranilate 3,4-dioxygenase  IYD - iodotyrosine deiodinase  GCAT - glycine c-acetyltransferase  CPS1 - carbamoyl-phosphate synthase 1, mitochondrial  GLYAT - glycine-n-acyltransferase  GCDH - glutaryl-coa dehydrogenase  AHCY - adenosylhomocysteinase  FTCD - formimidoyltransferase cyclodeaminase  AGXT - alanine-glyoxylate aminotransferase  BHMT - betaine--homocysteine s-methyltransferase  ASS1 - argininosuccinate synthase 1  ADI1 - acireductone dioxygenase 1  MAT1A - methionine adenosyltransferase i, alpha  HPD - 4-hydroxyphenylpyruvate dioxygenase  HGD - homogentisate 1,2-dioxygenase  TDO2 - tryptophan 2,3-dioxygenase  PRODH2 - proline dehydrogenase (oxidase) 2  MSRA - methionine sulfoxide reductase a  CKM - creatine kinase, muscle  PIPOX - pipecolic acid oxidase  ADHFE1 - alcohol dehydrogenase, iron containing, 1  PAH - phenylalanine hydroxylase  MPST - mercaptopyruvate sulfurtransferase  GGT1 - gamma-glutamyltransferase 1  CCBL1 - cysteine conjugate-beta lyase, cytoplasmic  SEPSECS - sep (o-phosphoserine) trna:sec (selenocysteine) trna synthase |
| GO:0034440 | lipid oxidation | 1.94E-14 | 1E-11 | 5.73 (10334,73,667,27) | [+] Show genes  ECI2 - enoyl-coa delta isomerase 2  ACOX2 - acyl-coa oxidase 2, branched chain  MLYCD - malonyl-coa decarboxylase  ACAA2 - acetyl-coa acyltransferase 2  AMACR - alpha-methylacyl-coa racemase  BDH2 - 3-hydroxybutyrate dehydrogenase, type 2  PPARGC1A - peroxisome proliferator-activated receptor gamma, coactivator 1 alpha  HADH - hydroxyacyl-coa dehydrogenase  SCP2 - sterol carrier protein 2  ACAA1 - acetyl-coa acyltransferase 1  ACADL - acyl-coa dehydrogenase, long chain  ACADS - acyl-coa dehydrogenase, c-2 to c-3 short chain  ETFDH - electron-transferring-flavoprotein dehydrogenase  ACADVL - acyl-coa dehydrogenase, very long chain  CPT2 - carnitine palmitoyltransferase 2  CPT1B - carnitine palmitoyltransferase 1b (muscle)  ECHDC2 - enoyl coa hydratase domain containing 2  GCDH - glutaryl-coa dehydrogenase  EHHADH - enoyl-coa, hydratase/3-hydroxyacyl coa dehydrogenase  ADH4 - alcohol dehydrogenase 4 (class ii), pi polypeptide  PHYH - phytanoyl-coa 2-hydroxylase  HACL1 - 2-hydroxyacyl-coa lyase 1  SLC27A2 - solute carrier family 27 (fatty acid transporter), member 2  ECHS1 - enoyl coa hydratase, short chain, 1, mitochondrial  DECR2 - 2,4-dienoyl coa reductase 2, peroxisomal  HAO2 - hydroxyacid oxidase 2 (long chain)  ECH1 - enoyl coa hydratase 1, peroxisomal |
| GO:0006953 | acute-phase response | 4.56E-14 | 2.27E-11 | 11.07 (10334,21,667,15) | [+] Show genes  APCS - amyloid p component, serum  CRP - c-reactive protein, pentraxin-related  FN1 - fibronectin 1  SAA1 - serum amyloid a1  MBL2 - mannose-binding lectin (protein c) 2, soluble  LBP - lipopolysaccharide binding protein  ASS1 - argininosuccinate synthase 1  HP - haptoglobin  SERPINA1 - serpin peptidase inhibitor, clade a (alpha-1 antiproteinase, antitrypsin), member 1  HAMP - hepcidin antimicrobial peptide  ITIH4 - inter-alpha-trypsin inhibitor heavy chain family, member 4  F2 - coagulation factor ii (thrombin)  SAA4 - serum amyloid a4, constitutive  TFR2 - transferrin receptor 2  SERPINF2 - serpin peptidase inhibitor, clade f (alpha-2 antiplasmin, pigment epithelium derived factor), member 2 |
| GO:0008202 | steroid metabolic process | 1.32E-13 | 6.33E-11 | 3.76 (10334,165,667,40) | [+] Show genes  AKR1C3 - aldo-keto reductase family 1, member c3  HSD17B8 - hydroxysteroid (17-beta) dehydrogenase 8  ACOX2 - acyl-coa oxidase 2, branched chain  HSD17B14 - hydroxysteroid (17-beta) dehydrogenase 14  ANGPTL3 - angiopoietin-like 3  LGMN - legumain  ACAA2 - acetyl-coa acyltransferase 2  CYP8B1 - cytochrome p450, family 8, subfamily b, polypeptide 1  APOA2 - apolipoprotein a-ii  CES1 - carboxylesterase 1  CYP2E1 - cytochrome p450, family 2, subfamily e, polypeptide 1  STS - steroid sulfatase (microsomal), isozyme s  CYP3A5 - cytochrome p450, family 3, subfamily a, polypeptide 5  HSD11B1 - hydroxysteroid (11-beta) dehydrogenase 1  AMACR - alpha-methylacyl-coa racemase  SCARF1 - scavenger receptor class f, member 1  APOA4 - apolipoprotein a-iv  CYP2C19 - cytochrome p450, family 2, subfamily c, polypeptide 19  PPARGC1A - peroxisome proliferator-activated receptor gamma, coactivator 1 alpha  SCP2 - sterol carrier protein 2  APOF - apolipoprotein f  ACAA1 - acetyl-coa acyltransferase 1  CYP1A2 - cytochrome p450, family 1, subfamily a, polypeptide 2  PON1 - paraoxonase 1  SERPINA6 - serpin peptidase inhibitor, clade a (alpha-1 antiproteinase, antitrypsin), member 6  DHRS4 - dehydrogenase/reductase (sdr family) member 4  HSD17B6 - hydroxysteroid (17-beta) dehydrogenase 6  AKR1C4 - aldo-keto reductase family 1, member c4  APOA5 - apolipoprotein a-v  SULT2A1 - sulfotransferase family, cytosolic, 2a, dehydroepiandrosterone (dhea)-preferring, member 1  CAT - catalase  GC - group-specific component (vitamin d binding protein)  RXRA - retinoid x receptor, alpha  RDH16 - retinol dehydrogenase 16 (all-trans)  LIPC - lipase, hepatic  HMGCS2 - 3-hydroxy-3-methylglutaryl-coa synthase 2 (mitochondrial)  SLC27A2 - solute carrier family 27 (fatty acid transporter), member 2  NR1I2 - nuclear receptor subfamily 1, group i, member 2  G6PC - glucose-6-phosphatase, catalytic subunit  SLC27A5 - solute carrier family 27 (fatty acid transporter), member 5 |
| GO:0070252 | actin-mediated cell contraction | 3.86E-13 | 1.79E-10 | 7.55 (10334,39,667,19) | [+] Show genes  MYH2 - myosin, heavy chain 2, skeletal muscle, adult  TPM2 - tropomyosin 2 (beta)  MYL1 - myosin, light chain 1, alkali; skeletal, fast  ACTN2 - actinin, alpha 2  ACTN3 - actinin, alpha 3  TTN - titin  NEB - nebulin  MYL3 - myosin, light chain 3, alkali; ventricular, skeletal, slow  DES - desmin  TNNC2 - troponin c type 2 (fast)  MYBPC1 - myosin binding protein c, slow type  MYL2 - myosin, light chain 2, regulatory, cardiac, slow  ACTA1 - actin, alpha 1, skeletal muscle  TNNI2 - troponin i type 2 (skeletal, fast)  TNNI1 - troponin i type 1 (skeletal, slow)  TCAP - titin-cap  TNNC1 - troponin c type 1 (slow)  TNNT3 - troponin t type 3 (skeletal, fast)  MYBPC2 - myosin binding protein c, fast type |
| GO:0051186 | cofactor metabolic process | 1.61E-12 | 7.21E-10 | 2.61 (10334,368,667,62) | [+] Show genes  HSD17B8 - hydroxysteroid (17-beta) dehydrogenase 8  AKR1C3 - aldo-keto reductase family 1, member c3  MLYCD - malonyl-coa decarboxylase  PGM1 - phosphoglucomutase 1  HMBS - hydroxymethylbilane synthase  FMO1 - flavin containing monooxygenase 1  PGAM2 - phosphoglycerate mutase 2 (muscle)  APOA4 - apolipoprotein a-iv  MCEE - methylmalonyl coa epimerase  MTHFR - methylenetetrahydrofolate reductase (nad(p)h)  ACSL1 - acyl-coa synthetase long-chain family member 1  BDH2 - 3-hydroxybutyrate dehydrogenase, type 2  MTHFD1 - methylenetetrahydrofolate dehydrogenase (nadp+ dependent) 1, methenyltetrahydrofolate cyclohydrolase, formyltetrahydrofolate synthetase  GOT2 - glutamic-oxaloacetic transaminase 2, mitochondrial  ENO3 - enolase 3 (beta, muscle)  LDHA - lactate dehydrogenase a  GSTZ1 - glutathione s-transferase zeta 1  GPD1 - glycerol-3-phosphate dehydrogenase 1 (soluble)  GSTT1 - glutathione s-transferase theta 1  HPX - hemopexin  ACSM2A - acyl-coa synthetase medium-chain family member 2a  ASPDH - aspartate dehydrogenase domain containing  AKR1C4 - aldo-keto reductase family 1, member c4  AHCY - adenosylhomocysteinase  MAT1A - methionine adenosyltransferase i, alpha  COQ4 - coenzyme q4 homolog (s. cerevisiae)  ETHE1 - ethylmalonic encephalopathy 1  TDO2 - tryptophan 2,3-dioxygenase  ABCB6 - atp-binding cassette, sub-family b (mdr/tap), member 6  MMAB - methylmalonic aciduria (cobalamin deficiency) cblb type  PIPOX - pipecolic acid oxidase  ADHFE1 - alcohol dehydrogenase, iron containing, 1  BLVRB - biliverdin reductase b (flavin reductase (nadph))  COQ9 - coenzyme q9 homolog (s. cerevisiae)  GGT1 - gamma-glutamyltransferase 1  PFKFB1 - 6-phosphofructo-2-kinase/fructose-2,6-biphosphatase 1  MOCS1 - molybdenum cofactor synthesis 1  ACSM5 - acyl-coa synthetase medium-chain family member 5  NMRK2 - nicotinamide riboside kinase 2  SHMT1 - serine hydroxymethyltransferase 1 (soluble)  VNN1 - vanin 1  ALDOB - aldolase b, fructose-bisphosphate  TAT - tyrosine aminotransferase  AMBP - alpha-1-microglobulin/bikunin precursor  HAGH - hydroxyacylglutathione hydrolase  CYP1A2 - cytochrome p450, family 1, subfamily a, polypeptide 2  PTPLA - protein tyrosine phosphatase-like (proline instead of catalytic arginine), member a  MPC1 - mitochondrial pyruvate carrier 1  PKLR - pyruvate kinase, liver and rbc  HAAO - 3-hydroxyanthranilate 3,4-dioxygenase  GLYAT - glycine-n-acyltransferase  FTCD - formimidoyltransferase cyclodeaminase  GCDH - glutaryl-coa dehydrogenase  CAT - catalase  ALDH1L1 - aldehyde dehydrogenase 1 family, member l1  ADH4 - alcohol dehydrogenase 4 (class ii), pi polypeptide  PHYH - phytanoyl-coa 2-hydroxylase  DGAT2 - diacylglycerol o-acyltransferase 2  HMGCS2 - 3-hydroxy-3-methylglutaryl-coa synthase 2 (mitochondrial)  HMOX1 - heme oxygenase (decycling) 1  ACSM3 - acyl-coa synthetase medium-chain family member 3  MMACHC - methylmalonic aciduria (cobalamin deficiency) cblc type, with homocystinuria |
| GO:0006635 | fatty acid beta-oxidation | 3.34E-12 | 1.45E-9 | 6.14 (10334,53,667,21) | [+] Show genes  ECI2 - enoyl-coa delta isomerase 2  CPT2 - carnitine palmitoyltransferase 2  ACOX2 - acyl-coa oxidase 2, branched chain  CPT1B - carnitine palmitoyltransferase 1b (muscle)  ECHDC2 - enoyl coa hydratase domain containing 2  ACAA2 - acetyl-coa acyltransferase 2  GCDH - glutaryl-coa dehydrogenase  EHHADH - enoyl-coa, hydratase/3-hydroxyacyl coa dehydrogenase  AMACR - alpha-methylacyl-coa racemase  BDH2 - 3-hydroxybutyrate dehydrogenase, type 2  HADH - hydroxyacyl-coa dehydrogenase  SCP2 - sterol carrier protein 2  SLC27A2 - solute carrier family 27 (fatty acid transporter), member 2  ECHS1 - enoyl coa hydratase, short chain, 1, mitochondrial  ACAA1 - acetyl-coa acyltransferase 1  ACADL - acyl-coa dehydrogenase, long chain  ACADS - acyl-coa dehydrogenase, c-2 to c-3 short chain  DECR2 - 2,4-dienoyl coa reductase 2, peroxisomal  ETFDH - electron-transferring-flavoprotein dehydrogenase  ECH1 - enoyl coa hydratase 1, peroxisomal  ACADVL - acyl-coa dehydrogenase, very long chain |
| GO:0006520 | cellular amino acid metabolic process | 4.43E-12 | 1.87E-9 | 2.99 (10334,249,667,48) | [+] Show genes  GADL1 - glutamate decarboxylase-like 1  AGMAT - agmatine ureohydrolase (agmatinase)  AGXT2 - alanine--glyoxylate aminotransferase 2  ALDH8A1 - aldehyde dehydrogenase 8 family, member a1  SHMT1 - serine hydroxymethyltransferase 1 (soluble)  GAMT - guanidinoacetate n-methyltransferase  ADSSL1 - adenylosuccinate synthase like 1  MTHFR - methylenetetrahydrofolate reductase (nad(p)h)  MTHFD1 - methylenetetrahydrofolate dehydrogenase (nadp+ dependent) 1, methenyltetrahydrofolate cyclohydrolase, formyltetrahydrofolate synthetase  GOT2 - glutamic-oxaloacetic transaminase 2, mitochondrial  ATF4 - activating transcription factor 4  TAT - tyrosine aminotransferase  OTC - ornithine carbamoyltransferase  FAH - fumarylacetoacetate hydrolase (fumarylacetoacetase)  ALDH4A1 - aldehyde dehydrogenase 4 family, member a1  ARG1 - arginase 1  ASPG - asparaginase homolog (s. cerevisiae)  GSTZ1 - glutathione s-transferase zeta 1  PM20D1 - peptidase m20 domain containing 1  DPYS - dihydropyrimidinase  HSD17B10 - hydroxysteroid (17-beta) dehydrogenase 10  HAAO - 3-hydroxyanthranilate 3,4-dioxygenase  IYD - iodotyrosine deiodinase  GCAT - glycine c-acetyltransferase  GLYAT - glycine-n-acyltransferase  CPS1 - carbamoyl-phosphate synthase 1, mitochondrial  FTCD - formimidoyltransferase cyclodeaminase  AHCY - adenosylhomocysteinase  GCDH - glutaryl-coa dehydrogenase  AGXT - alanine-glyoxylate aminotransferase  ASS1 - argininosuccinate synthase 1  BHMT - betaine--homocysteine s-methyltransferase  ADI1 - acireductone dioxygenase 1  MAT1A - methionine adenosyltransferase i, alpha  HPD - 4-hydroxyphenylpyruvate dioxygenase  HGD - homogentisate 1,2-dioxygenase  TDO2 - tryptophan 2,3-dioxygenase  PRODH2 - proline dehydrogenase (oxidase) 2  MSRA - methionine sulfoxide reductase a  CKM - creatine kinase, muscle  PIPOX - pipecolic acid oxidase  ADHFE1 - alcohol dehydrogenase, iron containing, 1  PAH - phenylalanine hydroxylase  GGT1 - gamma-glutamyltransferase 1  MPST - mercaptopyruvate sulfurtransferase  SEPSECS - sep (o-phosphoserine) trna:sec (selenocysteine) trna synthase  CCBL1 - cysteine conjugate-beta lyase, cytoplasmic  UPB1 - ureidopropionase, beta |
| GO:0046394 | carboxylic acid biosynthetic process | 9.83E-12 | 4.02E-9 | 3.01 (10334,237,667,46) | [+] Show genes  HSD17B8 - hydroxysteroid (17-beta) dehydrogenase 8  ACOX2 - acyl-coa oxidase 2, branched chain  MLYCD - malonyl-coa decarboxylase  CYP4A11 - cytochrome p450, family 4, subfamily a, polypeptide 11  AGXT2 - alanine--glyoxylate aminotransferase 2  GADL1 - glutamate decarboxylase-like 1  PGM1 - phosphoglucomutase 1  CYP8B1 - cytochrome p450, family 8, subfamily b, polypeptide 1  ALDH8A1 - aldehyde dehydrogenase 8 family, member a1  SHMT1 - serine hydroxymethyltransferase 1 (soluble)  CYP2E1 - cytochrome p450, family 2, subfamily e, polypeptide 1  GAMT - guanidinoacetate n-methyltransferase  AMACR - alpha-methylacyl-coa racemase  PGAM2 - phosphoglycerate mutase 2 (muscle)  ALDOB - aldolase b, fructose-bisphosphate  MTHFR - methylenetetrahydrofolate reductase (nad(p)h)  MTHFD1 - methylenetetrahydrofolate dehydrogenase (nadp+ dependent) 1, methenyltetrahydrofolate cyclohydrolase, formyltetrahydrofolate synthetase  GOT2 - glutamic-oxaloacetic transaminase 2, mitochondrial  ENO3 - enolase 3 (beta, muscle)  OTC - ornithine carbamoyltransferase  ASPG - asparaginase homolog (s. cerevisiae)  SCP2 - sterol carrier protein 2  LDHA - lactate dehydrogenase a  CYP1A2 - cytochrome p450, family 1, subfamily a, polypeptide 2  PTPLA - protein tyrosine phosphatase-like (proline instead of catalytic arginine), member a  ABHD1 - abhydrolase domain containing 1  ACSM2A - acyl-coa synthetase medium-chain family member 2a  PKLR - pyruvate kinase, liver and rbc  HAAO - 3-hydroxyanthranilate 3,4-dioxygenase  AKR1C4 - aldo-keto reductase family 1, member c4  CPS1 - carbamoyl-phosphate synthase 1, mitochondrial  AGXT - alanine-glyoxylate aminotransferase  ASS1 - argininosuccinate synthase 1  BHMT - betaine--homocysteine s-methyltransferase  ADI1 - acireductone dioxygenase 1  LIPC - lipase, hepatic  PAH - phenylalanine hydroxylase  SLC27A2 - solute carrier family 27 (fatty acid transporter), member 2  GGT1 - gamma-glutamyltransferase 1  DECR2 - 2,4-dienoyl coa reductase 2, peroxisomal  ACSM3 - acyl-coa synthetase medium-chain family member 3  SLC27A5 - solute carrier family 27 (fatty acid transporter), member 5  PFKFB1 - 6-phosphofructo-2-kinase/fructose-2,6-biphosphatase 1  CCBL1 - cysteine conjugate-beta lyase, cytoplasmic  UPB1 - ureidopropionase, beta  ACSM5 - acyl-coa synthetase medium-chain family member 5 |
| GO:0016053 | organic acid biosynthetic process | 1.15E-11 | 4.55E-9 | 2.99 (10334,238,667,46) | [+] Show genes  HSD17B8 - hydroxysteroid (17-beta) dehydrogenase 8  ACOX2 - acyl-coa oxidase 2, branched chain  MLYCD - malonyl-coa decarboxylase  CYP4A11 - cytochrome p450, family 4, subfamily a, polypeptide 11  AGXT2 - alanine--glyoxylate aminotransferase 2  GADL1 - glutamate decarboxylase-like 1  PGM1 - phosphoglucomutase 1  CYP8B1 - cytochrome p450, family 8, subfamily b, polypeptide 1  ALDH8A1 - aldehyde dehydrogenase 8 family, member a1  SHMT1 - serine hydroxymethyltransferase 1 (soluble)  CYP2E1 - cytochrome p450, family 2, subfamily e, polypeptide 1  GAMT - guanidinoacetate n-methyltransferase  AMACR - alpha-methylacyl-coa racemase  PGAM2 - phosphoglycerate mutase 2 (muscle)  ALDOB - aldolase b, fructose-bisphosphate  MTHFR - methylenetetrahydrofolate reductase (nad(p)h)  MTHFD1 - methylenetetrahydrofolate dehydrogenase (nadp+ dependent) 1, methenyltetrahydrofolate cyclohydrolase, formyltetrahydrofolate synthetase  GOT2 - glutamic-oxaloacetic transaminase 2, mitochondrial  ENO3 - enolase 3 (beta, muscle)  OTC - ornithine carbamoyltransferase  ASPG - asparaginase homolog (s. cerevisiae)  SCP2 - sterol carrier protein 2  LDHA - lactate dehydrogenase a  CYP1A2 - cytochrome p450, family 1, subfamily a, polypeptide 2  PTPLA - protein tyrosine phosphatase-like (proline instead of catalytic arginine), member a  ABHD1 - abhydrolase domain containing 1  ACSM2A - acyl-coa synthetase medium-chain family member 2a  PKLR - pyruvate kinase, liver and rbc  HAAO - 3-hydroxyanthranilate 3,4-dioxygenase  AKR1C4 - aldo-keto reductase family 1, member c4  CPS1 - carbamoyl-phosphate synthase 1, mitochondrial  AGXT - alanine-glyoxylate aminotransferase  ASS1 - argininosuccinate synthase 1  BHMT - betaine--homocysteine s-methyltransferase  ADI1 - acireductone dioxygenase 1  LIPC - lipase, hepatic  PAH - phenylalanine hydroxylase  SLC27A2 - solute carrier family 27 (fatty acid transporter), member 2  GGT1 - gamma-glutamyltransferase 1  DECR2 - 2,4-dienoyl coa reductase 2, peroxisomal  ACSM3 - acyl-coa synthetase medium-chain family member 3  SLC27A5 - solute carrier family 27 (fatty acid transporter), member 5  PFKFB1 - 6-phosphofructo-2-kinase/fructose-2,6-biphosphatase 1  CCBL1 - cysteine conjugate-beta lyase, cytoplasmic  UPB1 - ureidopropionase, beta  ACSM5 - acyl-coa synthetase medium-chain family member 5 |
| GO:0002673 | regulation of acute inflammatory response | 1.75E-11 | 6.77E-9 | 5.71 (10334,57,667,21) | [+] Show genes  C9 - complement component 9  APCS - amyloid p component, serum  F12 - coagulation factor xii (hageman factor)  CPN2 - carboxypeptidase n, polypeptide 2  CFB - complement factor b  C1R - complement component 1, r subcomponent  C2 - complement component 2  CPB2 - carboxypeptidase b2 (plasma)  FCGR2B - fc fragment of igg, low affinity iib, receptor (cd32)  C4BPA - complement component 4 binding protein, alpha  C4BPB - complement component 4 binding protein, beta  F2 - coagulation factor ii (thrombin)  C5 - complement component 5  VTN - vitronectin  C6 - complement component 6  KLKB1 - kallikrein b, plasma (fletcher factor) 1  C8B - complement component 8, beta polypeptide  C8A - complement component 8, alpha polypeptide  CD5L - cd5 molecule-like  C8G - complement component 8, gamma polypeptide  CREB3L3 - camp responsive element binding protein 3-like 3 |
| GO:0009063 | cellular amino acid catabolic process | 2.11E-11 | 7.94E-9 | 4.30 (10334,101,667,28) | [+] Show genes  GADL1 - glutamate decarboxylase-like 1  AGXT2 - alanine--glyoxylate aminotransferase 2  ALDH8A1 - aldehyde dehydrogenase 8 family, member a1  SHMT1 - serine hydroxymethyltransferase 1 (soluble)  TAT - tyrosine aminotransferase  OTC - ornithine carbamoyltransferase  GOT2 - glutamic-oxaloacetic transaminase 2, mitochondrial  ALDH4A1 - aldehyde dehydrogenase 4 family, member a1  FAH - fumarylacetoacetate hydrolase (fumarylacetoacetase)  ARG1 - arginase 1  GSTZ1 - glutathione s-transferase zeta 1  HSD17B10 - hydroxysteroid (17-beta) dehydrogenase 10  HAAO - 3-hydroxyanthranilate 3,4-dioxygenase  GCAT - glycine c-acetyltransferase  GCDH - glutaryl-coa dehydrogenase  FTCD - formimidoyltransferase cyclodeaminase  AHCY - adenosylhomocysteinase  AGXT - alanine-glyoxylate aminotransferase  MAT1A - methionine adenosyltransferase i, alpha  HPD - 4-hydroxyphenylpyruvate dioxygenase  HGD - homogentisate 1,2-dioxygenase  TDO2 - tryptophan 2,3-dioxygenase  PRODH2 - proline dehydrogenase (oxidase) 2  PIPOX - pipecolic acid oxidase  ADHFE1 - alcohol dehydrogenase, iron containing, 1  PAH - phenylalanine hydroxylase  MPST - mercaptopyruvate sulfurtransferase  CCBL1 - cysteine conjugate-beta lyase, cytoplasmic |
| GO:1901606 | alpha-amino acid catabolic process | 2.58E-11 | 9.45E-9 | 4.72 (10334,82,667,25) | [+] Show genes  AGXT2 - alanine--glyoxylate aminotransferase 2  ALDH8A1 - aldehyde dehydrogenase 8 family, member a1  SHMT1 - serine hydroxymethyltransferase 1 (soluble)  GOT2 - glutamic-oxaloacetic transaminase 2, mitochondrial  OTC - ornithine carbamoyltransferase  TAT - tyrosine aminotransferase  ALDH4A1 - aldehyde dehydrogenase 4 family, member a1  FAH - fumarylacetoacetate hydrolase (fumarylacetoacetase)  ARG1 - arginase 1  GSTZ1 - glutathione s-transferase zeta 1  HAAO - 3-hydroxyanthranilate 3,4-dioxygenase  GCAT - glycine c-acetyltransferase  AHCY - adenosylhomocysteinase  FTCD - formimidoyltransferase cyclodeaminase  GCDH - glutaryl-coa dehydrogenase  AGXT - alanine-glyoxylate aminotransferase  MAT1A - methionine adenosyltransferase i, alpha  HPD - 4-hydroxyphenylpyruvate dioxygenase  HGD - homogentisate 1,2-dioxygenase  TDO2 - tryptophan 2,3-dioxygenase  PRODH2 - proline dehydrogenase (oxidase) 2  PIPOX - pipecolic acid oxidase  ADHFE1 - alcohol dehydrogenase, iron containing, 1  PAH - phenylalanine hydroxylase  CCBL1 - cysteine conjugate-beta lyase, cytoplasmic |
| GO:0044283 | small molecule biosynthetic process | 2.8E-11 | 1E-8 | 2.35 (10334,442,667,67) | [+] Show genes  AKR1C3 - aldo-keto reductase family 1, member c3  HSD17B8 - hydroxysteroid (17-beta) dehydrogenase 8  MLYCD - malonyl-coa decarboxylase  ACAA2 - acetyl-coa acyltransferase 2  GADL1 - glutamate decarboxylase-like 1  PGM1 - phosphoglucomutase 1  AMACR - alpha-methylacyl-coa racemase  PGAM2 - phosphoglycerate mutase 2 (muscle)  APOA4 - apolipoprotein a-iv  MTHFR - methylenetetrahydrofolate reductase (nad(p)h)  BDH2 - 3-hydroxybutyrate dehydrogenase, type 2  MTHFD1 - methylenetetrahydrofolate dehydrogenase (nadp+ dependent) 1, methenyltetrahydrofolate cyclohydrolase, formyltetrahydrofolate synthetase  OTC - ornithine carbamoyltransferase  ATF4 - activating transcription factor 4  GOT2 - glutamic-oxaloacetic transaminase 2, mitochondrial  ENO3 - enolase 3 (beta, muscle)  ASPG - asparaginase homolog (s. cerevisiae)  LDHA - lactate dehydrogenase a  IMPA2 - inositol(myo)-1(or 4)-monophosphatase 2  SLC25A13 - solute carrier family 25 (aspartate/glutamate carrier), member 13  GPD1 - glycerol-3-phosphate dehydrogenase 1 (soluble)  ACSM2A - acyl-coa synthetase medium-chain family member 2a  ABHD1 - abhydrolase domain containing 1  SLC37A4 - solute carrier family 37 (glucose-6-phosphate transporter), member 4  AKR1C4 - aldo-keto reductase family 1, member c4  CPS1 - carbamoyl-phosphate synthase 1, mitochondrial  AGXT - alanine-glyoxylate aminotransferase  BHMT - betaine--homocysteine s-methyltransferase  COQ4 - coenzyme q4 homolog (s. cerevisiae)  LIPC - lipase, hepatic  SMPDL3A - sphingomyelin phosphodiesterase, acid-like 3a  COQ9 - coenzyme q9 homolog (s. cerevisiae)  PAH - phenylalanine hydroxylase  GGT1 - gamma-glutamyltransferase 1  G6PC - glucose-6-phosphatase, catalytic subunit  DECR2 - 2,4-dienoyl coa reductase 2, peroxisomal  PFKFB1 - 6-phosphofructo-2-kinase/fructose-2,6-biphosphatase 1  ACSM5 - acyl-coa synthetase medium-chain family member 5  UPB1 - ureidopropionase, beta  ACOX2 - acyl-coa oxidase 2, branched chain  CYP4A11 - cytochrome p450, family 4, subfamily a, polypeptide 11  AGXT2 - alanine--glyoxylate aminotransferase 2  CYP8B1 - cytochrome p450, family 8, subfamily b, polypeptide 1  ALDH8A1 - aldehyde dehydrogenase 8 family, member a1  CES1 - carboxylesterase 1  CYP2E1 - cytochrome p450, family 2, subfamily e, polypeptide 1  SHMT1 - serine hydroxymethyltransferase 1 (soluble)  GAMT - guanidinoacetate n-methyltransferase  ALDOB - aldolase b, fructose-bisphosphate  ENPP1 - ectonucleotide pyrophosphatase/phosphodiesterase 1  PPARGC1A - peroxisome proliferator-activated receptor gamma, coactivator 1 alpha  SCP2 - sterol carrier protein 2  ADA - adenosine deaminase  CYP1A2 - cytochrome p450, family 1, subfamily a, polypeptide 2  PTPLA - protein tyrosine phosphatase-like (proline instead of catalytic arginine), member a  PKLR - pyruvate kinase, liver and rbc  HAAO - 3-hydroxyanthranilate 3,4-dioxygenase  APOA5 - apolipoprotein a-v  PCK1 - phosphoenolpyruvate carboxykinase 1 (soluble)  ASS1 - argininosuccinate synthase 1  ADI1 - acireductone dioxygenase 1  HMGCS2 - 3-hydroxy-3-methylglutaryl-coa synthase 2 (mitochondrial)  SLC27A2 - solute carrier family 27 (fatty acid transporter), member 2  FBP2 - fructose-1,6-bisphosphatase 2  SLC27A5 - solute carrier family 27 (fatty acid transporter), member 5  ACSM3 - acyl-coa synthetase medium-chain family member 3  CCBL1 - cysteine conjugate-beta lyase, cytoplasmic |
| GO:2000257 | regulation of protein activation cascade | 3.84E-11 | 1.34E-8 | 6.93 (10334,38,667,17) | [+] Show genes  C9 - complement component 9  CPN2 - carboxypeptidase n, polypeptide 2  CFB - complement factor b  C1R - complement component 1, r subcomponent  C2 - complement component 2  CPB2 - carboxypeptidase b2 (plasma)  C4BPA - complement component 4 binding protein, alpha  C4BPB - complement component 4 binding protein, beta  SERPINC1 - serpin peptidase inhibitor, clade c (antithrombin), member 1  F2 - coagulation factor ii (thrombin)  VTN - vitronectin  C5 - complement component 5  C6 - complement component 6  C8B - complement component 8, beta polypeptide  C8A - complement component 8, alpha polypeptide  CD5L - cd5 molecule-like  C8G - complement component 8, gamma polypeptide |
| GO:0002920 | regulation of humoral immune response | 3.99E-11 | 1.35E-8 | 6.49 (10334,43,667,18) | [+] Show genes  C9 - complement component 9  CFB - complement factor b  CPN2 - carboxypeptidase n, polypeptide 2  C1R - complement component 1, r subcomponent  C2 - complement component 2  CPB2 - carboxypeptidase b2 (plasma)  FCGR2B - fc fragment of igg, low affinity iib, receptor (cd32)  C4BPA - complement component 4 binding protein, alpha  C4BPB - complement component 4 binding protein, beta  F2 - coagulation factor ii (thrombin)  C5 - complement component 5  VTN - vitronectin  C6 - complement component 6  C8B - complement component 8, beta polypeptide  C8A - complement component 8, alpha polypeptide  HPX - hemopexin  CD5L - cd5 molecule-like  C8G - complement component 8, gamma polypeptide |
| GO:0006941 | striated muscle contraction | 5.45E-11 | 1.81E-8 | 5.42 (10334,60,667,21) | [+] Show genes  KLHL41 - kelch-like family member 41  STAC3 - sh3 and cysteine rich domain 3  MB - myoglobin  CSRP3 - cysteine and glycine-rich protein 3 (cardiac lim protein)  MYL1 - myosin, light chain 1, alkali; skeletal, fast  PGAM2 - phosphoglycerate mutase 2 (muscle)  TTN - titin  CHRNA1 - cholinergic receptor, nicotinic, alpha 1 (muscle)  CASQ2 - calsequestrin 2 (cardiac muscle)  MYL3 - myosin, light chain 3, alkali; ventricular, skeletal, slow  MYL2 - myosin, light chain 2, regulatory, cardiac, slow  TNNC2 - troponin c type 2 (fast)  SMPX - small muscle protein, x-linked  TNNI2 - troponin i type 2 (skeletal, fast)  RCSD1 - rcsd domain containing 1  TCAP - titin-cap  TNNI1 - troponin i type 1 (skeletal, slow)  TNNC1 - troponin c type 1 (slow)  TNNT3 - troponin t type 3 (skeletal, fast)  LMOD3 - leiomodin 3 (fetal)  MYLK2 - myosin light chain kinase 2 |
| GO:0045214 | sarcomere organization | 7.53E-11 | 2.44E-8 | 7.75 (10334,30,667,15) | [+] Show genes  LDB3 - lim domain binding 3  KLHL41 - kelch-like family member 41  CAPN3 - calpain 3, (p94)  CSRP3 - cysteine and glycine-rich protein 3 (cardiac lim protein)  SYNPO2L - synaptopodin 2-like  ACTN2 - actinin, alpha 2  MYOZ1 - myozenin 1  TTN - titin  LMOD2 - leiomodin 2 (cardiac)  ANKRD1 - ankyrin repeat domain 1 (cardiac muscle)  CASQ2 - calsequestrin 2 (cardiac muscle)  MYOZ2 - myozenin 2  TCAP - titin-cap  TNNT3 - troponin t type 3 (skeletal, fast)  MYPN - myopalladin |
| GO:0006956 | complement activation | 9.73E-11 | 3.08E-8 | 7.08 (10334,35,667,16) | [+] Show genes  C9 - complement component 9  MASP1 - mannan-binding lectin serine peptidase 1 (c4/c2 activating component of ra-reactive factor)  APCS - amyloid p component, serum  CRP - c-reactive protein, pentraxin-related  MASP2 - mannan-binding lectin serine peptidase 2  CFB - complement factor b  MBL2 - mannose-binding lectin (protein c) 2, soluble  C1R - complement component 1, r subcomponent  C2 - complement component 2  C4BPA - complement component 4 binding protein, alpha  C4BPB - complement component 4 binding protein, beta  C5 - complement component 5  C6 - complement component 6  C8B - complement component 8, beta polypeptide  C8A - complement component 8, alpha polypeptide  C8G - complement component 8, gamma polypeptide |
| GO:1901615 | organic hydroxy compound metabolic process | 1.11E-10 | 3.43E-8 | 2.67 (10334,290,667,50) | [+] Show genes  AKR1C3 - aldo-keto reductase family 1, member c3  LGMN - legumain  ANGPTL3 - angiopoietin-like 3  ACAA2 - acetyl-coa acyltransferase 2  APOA2 - apolipoprotein a-ii  AMACR - alpha-methylacyl-coa racemase  APOA4 - apolipoprotein a-iv  GK5 - glycerol kinase 5 (putative)  FAH - fumarylacetoacetate hydrolase (fumarylacetoacetase)  LDHA - lactate dehydrogenase a  APOF - apolipoprotein f  IMPA2 - inositol(myo)-1(or 4)-monophosphatase 2  ACAA1 - acetyl-coa acyltransferase 1  GPD1 - glycerol-3-phosphate dehydrogenase 1 (soluble)  PNPLA2 - patatin-like phospholipase domain containing 2  DHRS3 - dehydrogenase/reductase (sdr family) member 3  TTR - transthyretin  DHRS4 - dehydrogenase/reductase (sdr family) member 4  AKR1C4 - aldo-keto reductase family 1, member c4  SULT2A1 - sulfotransferase family, cytosolic, 2a, dehydroepiandrosterone (dhea)-preferring, member 1  TTPA - tocopherol (alpha) transfer protein  LIPC - lipase, hepatic  PAH - phenylalanine hydroxylase  ALDH2 - aldehyde dehydrogenase 2 family (mitochondrial)  ACOX2 - acyl-coa oxidase 2, branched chain  CYP4A11 - cytochrome p450, family 4, subfamily a, polypeptide 11  CYP8B1 - cytochrome p450, family 8, subfamily b, polypeptide 1  CES1 - carboxylesterase 1  CYP2E1 - cytochrome p450, family 2, subfamily e, polypeptide 1  PLCD4 - phospholipase c, delta 4  CYP3A5 - cytochrome p450, family 3, subfamily a, polypeptide 5  SCARF1 - scavenger receptor class f, member 1  SCP2 - sterol carrier protein 2  HAGH - hydroxyacylglutathione hydrolase  CYP1A2 - cytochrome p450, family 1, subfamily a, polypeptide 2  PON1 - paraoxonase 1  IYD - iodotyrosine deiodinase  APOA5 - apolipoprotein a-v  PCK1 - phosphoenolpyruvate carboxykinase 1 (soluble)  IGF1 - insulin-like growth factor 1 (somatomedin c)  CAT - catalase  GC - group-specific component (vitamin d binding protein)  RXRA - retinoid x receptor, alpha  ADH6 - alcohol dehydrogenase 6 (class v)  ADH4 - alcohol dehydrogenase 4 (class ii), pi polypeptide  DGAT2 - diacylglycerol o-acyltransferase 2  RDH16 - retinol dehydrogenase 16 (all-trans)  HMGCS2 - 3-hydroxy-3-methylglutaryl-coa synthase 2 (mitochondrial)  SLC27A2 - solute carrier family 27 (fatty acid transporter), member 2  SLC27A5 - solute carrier family 27 (fatty acid transporter), member 5 |
| GO:1901575 | organic substance catabolic process | 1.27E-10 | 3.85E-8 | 1.69 (10334,1268,667,138) | [+] Show genes  LGMN - legumain  ANGPTL3 - angiopoietin-like 3  GADL1 - glutamate decarboxylase-like 1  APOA2 - apolipoprotein a-ii  ENTPD8 - ectonucleoside triphosphate diphosphohydrolase 8  HSD11B1 - hydroxysteroid (11-beta) dehydrogenase 1  AMACR - alpha-methylacyl-coa racemase  CDC34 - cell division cycle 34  APOA4 - apolipoprotein a-iv  BDH2 - 3-hydroxybutyrate dehydrogenase, type 2  OTC - ornithine carbamoyltransferase  ENO3 - enolase 3 (beta, muscle)  CES2 - carboxylesterase 2  FBXW5 - f-box and wd repeat domain containing 5  LDHA - lactate dehydrogenase a  ENDOG - endonuclease g  IMPA2 - inositol(myo)-1(or 4)-monophosphatase 2  GSTZ1 - glutathione s-transferase zeta 1  ACAA1 - acetyl-coa acyltransferase 1  ACADL - acyl-coa dehydrogenase, long chain  ACADS - acyl-coa dehydrogenase, c-2 to c-3 short chain  ACADVL - acyl-coa dehydrogenase, very long chain  CPT2 - carnitine palmitoyltransferase 2  CPT1B - carnitine palmitoyltransferase 1b (muscle)  CPS1 - carbamoyl-phosphate synthase 1, mitochondrial  UBE2L6 - ubiquitin-conjugating enzyme e2l 6  EHHADH - enoyl-coa, hydratase/3-hydroxyacyl coa dehydrogenase  BHMT - betaine--homocysteine s-methyltransferase  HPD - 4-hydroxyphenylpyruvate dioxygenase  TDO2 - tryptophan 2,3-dioxygenase  APOC2 - apolipoprotein c-ii  SDC2 - syndecan 2  APOC3 - apolipoprotein c-iii  PSMC5 - proteasome (prosome, macropain) 26s subunit, atpase, 5  PIPOX - pipecolic acid oxidase  LIPC - lipase, hepatic  PAFAH2 - platelet-activating factor acetylhydrolase 2, 40kda  ADHFE1 - alcohol dehydrogenase, iron containing, 1  DCAF11 - ddb1 and cul4 associated factor 11  SMPDL3A - sphingomyelin phosphodiesterase, acid-like 3a  PAH - phenylalanine hydroxylase  BLVRB - biliverdin reductase b (flavin reductase (nadph))  APOBEC2 - apolipoprotein b mrna editing enzyme, catalytic polypeptide-like 2  GGT1 - gamma-glutamyltransferase 1  UPB1 - ureidopropionase, beta  ACOX2 - acyl-coa oxidase 2, branched chain  CYP4A11 - cytochrome p450, family 4, subfamily a, polypeptide 11  ALDH8A1 - aldehyde dehydrogenase 8 family, member a1  CES1 - carboxylesterase 1  ABHD17A - abhydrolase domain containing 17a  STS - steroid sulfatase (microsomal), isozyme s  PLBD1 - phospholipase b domain containing 1  CYP3A5 - cytochrome p450, family 3, subfamily a, polypeptide 5  SCARF1 - scavenger receptor class f, member 1  ENPP1 - ectonucleotide pyrophosphatase/phosphodiesterase 1  TAT - tyrosine aminotransferase  UBXN1 - ubx domain protein 1  ARG1 - arginase 1  SCP2 - sterol carrier protein 2  HADH - hydroxyacyl-coa dehydrogenase  BAG3 - bcl2-associated athanogene 3  ADA - adenosine deaminase  CYP1A2 - cytochrome p450, family 1, subfamily a, polypeptide 2  HAGH - hydroxyacylglutathione hydrolase  UPF2 - upf2 regulator of nonsense transcripts homolog (yeast)  HSD17B10 - hydroxysteroid (17-beta) dehydrogenase 10  PON1 - paraoxonase 1  HSD17B6 - hydroxysteroid (17-beta) dehydrogenase 6  APOA5 - apolipoprotein a-v  PCK1 - phosphoenolpyruvate carboxykinase 1 (soluble)  FEM1A - fem-1 homolog a (c. elegans)  FTCD - formimidoyltransferase cyclodeaminase  ALDH1L1 - aldehyde dehydrogenase 1 family, member l1  ADH4 - alcohol dehydrogenase 4 (class ii), pi polypeptide  PRODH2 - proline dehydrogenase (oxidase) 2  GLYCTK - glycerate kinase  PLA2G12B - phospholipase a2, group xiib  CRYL1 - crystallin, lambda 1  ECHS1 - enoyl coa hydratase, short chain, 1, mitochondrial  KEAP1 - kelch-like ech-associated protein 1  ECH1 - enoyl coa hydratase 1, peroxisomal  ECI2 - enoyl-coa delta isomerase 2  AKR1C3 - aldo-keto reductase family 1, member c3  MLYCD - malonyl-coa decarboxylase  FBXO17 - f-box protein 17  HSD17B14 - hydroxysteroid (17-beta) dehydrogenase 14  ACAA2 - acetyl-coa acyltransferase 2  CAPN3 - calpain 3, (p94)  PGM1 - phosphoglucomutase 1  FABP1 - fatty acid binding protein 1, liver  PGAM2 - phosphoglycerate mutase 2 (muscle)  MCEE - methylmalonyl coa epimerase  GOT2 - glutamic-oxaloacetic transaminase 2, mitochondrial  FAH - fumarylacetoacetate hydrolase (fumarylacetoacetase)  ALDH4A1 - aldehyde dehydrogenase 4 family, member a1  GK5 - glycerol kinase 5 (putative)  ASPG - asparaginase homolog (s. cerevisiae)  GPD1 - glycerol-3-phosphate dehydrogenase 1 (soluble)  PNPLA2 - patatin-like phospholipase domain containing 2  DPYS - dihydropyrimidinase  ABHD1 - abhydrolase domain containing 1  GCAT - glycine c-acetyltransferase  AHCY - adenosylhomocysteinase  SULT2A1 - sulfotransferase family, cytosolic, 2a, dehydroepiandrosterone (dhea)-preferring, member 1  AGXT - alanine-glyoxylate aminotransferase  CHIA - chitinase, acidic  MAT1A - methionine adenosyltransferase i, alpha  WWP1 - ww domain containing e3 ubiquitin protein ligase 1  HGD - homogentisate 1,2-dioxygenase  HACL1 - 2-hydroxyacyl-coa lyase 1  DECR2 - 2,4-dienoyl coa reductase 2, peroxisomal  G6PC - glucose-6-phosphatase, catalytic subunit  ALDH2 - aldehyde dehydrogenase 2 family (mitochondrial)  PFKFB1 - 6-phosphofructo-2-kinase/fructose-2,6-biphosphatase 1  UBA52 - ubiquitin a-52 residue ribosomal protein fusion product 1  AGXT2 - alanine--glyoxylate aminotransferase 2  SHMT1 - serine hydroxymethyltransferase 1 (soluble)  LPIN1 - lipin 1  PLCD4 - phospholipase c, delta 4  ALDOB - aldolase b, fructose-bisphosphate  CES3 - carboxylesterase 3  ASB2 - ankyrin repeat and socs box containing 2  AMBP - alpha-1-microglobulin/bikunin precursor  ADTRP - androgen-dependent tfpi-regulating protein  ETFDH - electron-transferring-flavoprotein dehydrogenase  PKLR - pyruvate kinase, liver and rbc  HAAO - 3-hydroxyanthranilate 3,4-dioxygenase  ECHDC2 - enoyl coa hydratase domain containing 2  GCDH - glutaryl-coa dehydrogenase  TRIM72 - tripartite motif containing 72  PHYH - phytanoyl-coa 2-hydroxylase  STBD1 - starch binding domain 1  SLC27A2 - solute carrier family 27 (fatty acid transporter), member 2  HMOX1 - heme oxygenase (decycling) 1  MPST - mercaptopyruvate sulfurtransferase  CCBL1 - cysteine conjugate-beta lyase, cytoplasmic  URAD - ureidoimidazoline (2-oxo-4-hydroxy-4-carboxy-5-) decarboxylase  HAO2 - hydroxyacid oxidase 2 (long chain) |
| GO:1900046 | regulation of hemostasis | 1.49E-10 | 4.42E-8 | 5.44 (10334,57,667,20) | [+] Show genes  F11 - coagulation factor xi  FGG - fibrinogen gamma chain  F12 - coagulation factor xii (hageman factor)  CD36 - cd36 molecule (thrombospondin receptor)  PROC - protein c (inactivator of coagulation factors va and viiia)  CPB2 - carboxypeptidase b2 (plasma)  ADTRP - androgen-dependent tfpi-regulating protein  FGA - fibrinogen alpha chain  HRG - histidine-rich glycoprotein  KNG1 - kininogen 1  FGB - fibrinogen beta chain  APOH - apolipoprotein h (beta-2-glycoprotein i)  SERPINC1 - serpin peptidase inhibitor, clade c (antithrombin), member 1  PLG - plasminogen  F2 - coagulation factor ii (thrombin)  VTN - vitronectin  KLKB1 - kallikrein b, plasma (fletcher factor) 1  SERPINF2 - serpin peptidase inhibitor, clade f (alpha-2 antiplasmin, pigment epithelium derived factor), member 2  F7 - coagulation factor vii (serum prothrombin conversion accelerator)  PLEK - pleckstrin |
| GO:0030193 | regulation of blood coagulation | 1.49E-10 | 4.33E-8 | 5.44 (10334,57,667,20) | [+] Show genes  F11 - coagulation factor xi  FGG - fibrinogen gamma chain  F12 - coagulation factor xii (hageman factor)  CD36 - cd36 molecule (thrombospondin receptor)  PROC - protein c (inactivator of coagulation factors va and viiia)  CPB2 - carboxypeptidase b2 (plasma)  ADTRP - androgen-dependent tfpi-regulating protein  FGA - fibrinogen alpha chain  HRG - histidine-rich glycoprotein  KNG1 - kininogen 1  FGB - fibrinogen beta chain  APOH - apolipoprotein h (beta-2-glycoprotein i)  SERPINC1 - serpin peptidase inhibitor, clade c (antithrombin), member 1  PLG - plasminogen  F2 - coagulation factor ii (thrombin)  VTN - vitronectin  KLKB1 - kallikrein b, plasma (fletcher factor) 1  SERPINF2 - serpin peptidase inhibitor, clade f (alpha-2 antiplasmin, pigment epithelium derived factor), member 2  F7 - coagulation factor vii (serum prothrombin conversion accelerator)  PLEK - pleckstrin |
| GO:0006958 | complement activation, classical pathway | 1.74E-10 | 4.95E-8 | 8.03 (10334,27,667,14) | [+] Show genes  C9 - complement component 9  APCS - amyloid p component, serum  CRP - c-reactive protein, pentraxin-related  MASP2 - mannan-binding lectin serine peptidase 2  MBL2 - mannose-binding lectin (protein c) 2, soluble  C1R - complement component 1, r subcomponent  C2 - complement component 2  C4BPA - complement component 4 binding protein, alpha  C4BPB - complement component 4 binding protein, beta  C5 - complement component 5  C6 - complement component 6  C8B - complement component 8, beta polypeptide  C8A - complement component 8, alpha polypeptide  C8G - complement component 8, gamma polypeptide |
| GO:0006869 | lipid transport | 2.47E-10 | 6.88E-8 | 3.22 (10334,173,667,36) | [+] Show genes  APOA2 - apolipoprotein a-ii  CES1 - carboxylesterase 1  FABP1 - fatty acid binding protein 1, liver  APOA4 - apolipoprotein a-iv  PCTP - phosphatidylcholine transfer protein  ACSL1 - acyl-coa synthetase long-chain family member 1  SFTPA1 - surfactant protein a1  SLC10A1 - solute carrier family 10 (sodium/bile acid cotransporter), member 1  GOT2 - glutamic-oxaloacetic transaminase 2, mitochondrial  SCP2 - sterol carrier protein 2  APOF - apolipoprotein f  PPARA - peroxisome proliferator-activated receptor alpha  SERPINA5 - serpin peptidase inhibitor, clade a (alpha-1 antiproteinase, antitrypsin), member 5  CPT2 - carnitine palmitoyltransferase 2  SLCO1B3 - solute carrier organic anion transporter family, member 1b3  AKR1C4 - aldo-keto reductase family 1, member c4  CPT1B - carnitine palmitoyltransferase 1b (muscle)  APOA5 - apolipoprotein a-v  AQP9 - aquaporin 9  SLC25A20 - solute carrier family 25 (carnitine/acylcarnitine translocase), member 20  CD36 - cd36 molecule (thrombospondin receptor)  SLC51B - solute carrier family 51, beta subunit  LBP - lipopolysaccharide binding protein  RXRA - retinoid x receptor, alpha  TTPA - tocopherol (alpha) transfer protein  APOC2 - apolipoprotein c-ii  LIPC - lipase, hepatic  APOC3 - apolipoprotein c-iii  PLA2G12B - phospholipase a2, group xiib  APOC4 - apolipoprotein c-iv  APOM - apolipoprotein m  APOH - apolipoprotein h (beta-2-glycoprotein i)  SLC27A2 - solute carrier family 27 (fatty acid transporter), member 2  ATP8B3 - atpase, aminophospholipid transporter, class i, type 8b, member 3  THRSP - thyroid hormone responsive  SLC27A5 - solute carrier family 27 (fatty acid transporter), member 5 |
| GO:1900047 | negative regulation of hemostasis | 2.73E-10 | 7.45E-8 | 6.70 (10334,37,667,16) | [+] Show genes  F11 - coagulation factor xi  FGG - fibrinogen gamma chain  F12 - coagulation factor xii (hageman factor)  PROC - protein c (inactivator of coagulation factors va and viiia)  CPB2 - carboxypeptidase b2 (plasma)  ADTRP - androgen-dependent tfpi-regulating protein  HRG - histidine-rich glycoprotein  FGA - fibrinogen alpha chain  KNG1 - kininogen 1  FGB - fibrinogen beta chain  APOH - apolipoprotein h (beta-2-glycoprotein i)  F2 - coagulation factor ii (thrombin)  PLG - plasminogen  VTN - vitronectin  KLKB1 - kallikrein b, plasma (fletcher factor) 1  SERPINF2 - serpin peptidase inhibitor, clade f (alpha-2 antiplasmin, pigment epithelium derived factor), member 2 |
| GO:0030449 | regulation of complement activation | 2.73E-10 | 7.31E-8 | 6.70 (10334,37,667,16) | [+] Show genes  C9 - complement component 9  CPN2 - carboxypeptidase n, polypeptide 2  CFB - complement factor b  C1R - complement component 1, r subcomponent  CPB2 - carboxypeptidase b2 (plasma)  C2 - complement component 2  C4BPA - complement component 4 binding protein, alpha  C4BPB - complement component 4 binding protein, beta  F2 - coagulation factor ii (thrombin)  C5 - complement component 5  VTN - vitronectin  C6 - complement component 6  C8B - complement component 8, beta polypeptide  C8A - complement component 8, alpha polypeptide  CD5L - cd5 molecule-like  C8G - complement component 8, gamma polypeptide |
| GO:0030195 | negative regulation of blood coagulation | 2.73E-10 | 7.17E-8 | 6.70 (10334,37,667,16) | [+] Show genes  F11 - coagulation factor xi  FGG - fibrinogen gamma chain  F12 - coagulation factor xii (hageman factor)  PROC - protein c (inactivator of coagulation factors va and viiia)  CPB2 - carboxypeptidase b2 (plasma)  ADTRP - androgen-dependent tfpi-regulating protein  HRG - histidine-rich glycoprotein  FGA - fibrinogen alpha chain  KNG1 - kininogen 1  FGB - fibrinogen beta chain  APOH - apolipoprotein h (beta-2-glycoprotein i)  PLG - plasminogen  F2 - coagulation factor ii (thrombin)  VTN - vitronectin  KLKB1 - kallikrein b, plasma (fletcher factor) 1  SERPINF2 - serpin peptidase inhibitor, clade f (alpha-2 antiplasmin, pigment epithelium derived factor), member 2 |
| GO:0003008 | system process | 3.02E-10 | 7.79E-8 | 1.99 (10334,670,667,86) | [+] Show genes  AKR1C3 - aldo-keto reductase family 1, member c3  ATP2A1 - atpase, ca++ transporting, cardiac muscle, fast twitch 1  CHRNE - cholinergic receptor, nicotinic, epsilon (muscle)  LGMN - legumain  FABP1 - fatty acid binding protein 1, liver  ADRB2 - adrenoceptor beta 2, surface  SLC22A18 - solute carrier family 22, member 18  TRIM63 - tripartite motif containing 63, e3 ubiquitin protein ligase  PGAM2 - phosphoglycerate mutase 2 (muscle)  CHRNA1 - cholinergic receptor, nicotinic, alpha 1 (muscle)  CASQ2 - calsequestrin 2 (cardiac muscle)  BTC - betacellulin  HOMER2 - homer homolog 2 (drosophila)  HRC - histidine rich calcium binding protein  LMOD3 - leiomodin 3 (fetal)  DNAJC19 - dnaj (hsp40) homolog, subfamily c, member 19  MYLK2 - myosin light chain kinase 2  TIMM8B - translocase of inner mitochondrial membrane 8 homolog b (yeast)  DHRS3 - dehydrogenase/reductase (sdr family) member 3  FXYD1 - fxyd domain containing ion transport regulator 1  KLHL41 - kelch-like family member 41  AQP9 - aquaporin 9  P2RY2 - purinergic receptor p2y, g-protein coupled, 2  CPS1 - carbamoyl-phosphate synthase 1, mitochondrial  MB - myoglobin  ABCC6 - atp-binding cassette, sub-family c (cftr/mrp), member 6  PLN - phospholamban  CD36 - cd36 molecule (thrombospondin receptor)  HPN - hepsin  TTN - titin  LMOD2 - leiomodin 2 (cardiac)  CACNA1S - calcium channel, voltage-dependent, l type, alpha 1s subunit  DES - desmin  MYL3 - myosin, light chain 3, alkali; ventricular, skeletal, slow  TNNC2 - troponin c type 2 (fast)  MYL2 - myosin, light chain 2, regulatory, cardiac, slow  TMOD4 - tropomodulin 4 (muscle)  ACTA1 - actin, alpha 1, skeletal muscle  IMMP2L - imp2 inner mitochondrial membrane peptidase-like (s. cerevisiae)  KNG1 - kininogen 1  TNNI2 - troponin i type 2 (skeletal, fast)  RCSD1 - rcsd domain containing 1  TNNI1 - troponin i type 1 (skeletal, slow)  TNNC1 - troponin c type 1 (slow)  RGS14 - regulator of g-protein signaling 14  TNNT3 - troponin t type 3 (skeletal, fast)  SERPINF2 - serpin peptidase inhibitor, clade f (alpha-2 antiplasmin, pigment epithelium derived factor), member 2  RGS16 - regulator of g-protein signaling 16  SMTN - smoothelin  ANKRD2 - ankyrin repeat domain 2 (stretch responsive muscle)  FGG - fibrinogen gamma chain  CYP4A11 - cytochrome p450, family 4, subfamily a, polypeptide 11  MYH1 - myosin, heavy chain 1, skeletal muscle, adult  KLF15 - kruppel-like factor 15  MYH2 - myosin, heavy chain 2, skeletal muscle, adult  TPM2 - tropomyosin 2 (beta)  CRP - c-reactive protein, pentraxin-related  CSRP3 - cysteine and glycine-rich protein 3 (cardiac lim protein)  MYL1 - myosin, light chain 1, alkali; skeletal, fast  GAMT - guanidinoacetate n-methyltransferase  ACTN3 - actinin, alpha 3  ATXN7 - ataxin 7  GRHPR - glyoxylate reductase/hydroxypyruvate reductase  PPARGC1A - peroxisome proliferator-activated receptor gamma, coactivator 1 alpha  FGA - fibrinogen alpha chain  FGB - fibrinogen beta chain  TCAP - titin-cap  MYLPF - myosin light chain, phosphorylatable, fast skeletal muscle  SERPINF1 - serpin peptidase inhibitor, clade f (alpha-2 antiplasmin, pigment epithelium derived factor), member 1  PTP4A3 - protein tyrosine phosphatase type iva, member 3  STAC3 - sh3 and cysteine rich domain 3  IGF1 - insulin-like growth factor 1 (somatomedin c)  RP9 - retinitis pigmentosa 9 (autosomal dominant)  MYOM2 - myomesin 2  LMOD1 - leiomodin 1 (smooth muscle)  TRIM72 - tripartite motif containing 72  CAV3 - caveolin 3  RYR1 - ryanodine receptor 1 (skeletal)  PKN1 - protein kinase n1  SMPX - small muscle protein, x-linked  MYOM3 - myomesin 3  SGCG - sarcoglycan, gamma (35kda dystrophin-associated glycoprotein)  NDUFS6 - nadh dehydrogenase (ubiquinone) fe-s protein 6, 13kda (nadh-coenzyme q reductase)  MYOT - myotilin  HMOX1 - heme oxygenase (decycling) 1  SMTNL1 - smoothelin-like 1 |
| GO:0006959 | humoral immune response | 5E-10 | 1.26E-7 | 4.32 (10334,86,667,24) | [+] Show genes  S100A12 - s100 calcium binding protein a12  C9 - complement component 9  MASP1 - mannan-binding lectin serine peptidase 1 (c4/c2 activating component of ra-reactive factor)  APCS - amyloid p component, serum  CRP - c-reactive protein, pentraxin-related  MASP2 - mannan-binding lectin serine peptidase 2  MBL2 - mannose-binding lectin (protein c) 2, soluble  CFB - complement factor b  C1R - complement component 1, r subcomponent  C2 - complement component 2  LEAP2 - liver expressed antimicrobial peptide 2  C4BPA - complement component 4 binding protein, alpha  HRG - histidine-rich glycoprotein  FGA - fibrinogen alpha chain  FGB - fibrinogen beta chain  C4BPB - complement component 4 binding protein, beta  SPON2 - spondin 2, extracellular matrix protein  F2 - coagulation factor ii (thrombin)  C5 - complement component 5  C6 - complement component 6  C8B - complement component 8, beta polypeptide  C8A - complement component 8, alpha polypeptide  CXCL3 - chemokine (c-x-c motif) ligand 3  C8G - complement component 8, gamma polypeptide |
| GO:0050818 | regulation of coagulation | 6.02E-10 | 1.5E-7 | 5.08 (10334,61,667,20) | [+] Show genes  F11 - coagulation factor xi  FGG - fibrinogen gamma chain  F12 - coagulation factor xii (hageman factor)  CD36 - cd36 molecule (thrombospondin receptor)  PROC - protein c (inactivator of coagulation factors va and viiia)  CPB2 - carboxypeptidase b2 (plasma)  ADTRP - androgen-dependent tfpi-regulating protein  FGA - fibrinogen alpha chain  HRG - histidine-rich glycoprotein  KNG1 - kininogen 1  FGB - fibrinogen beta chain  APOH - apolipoprotein h (beta-2-glycoprotein i)  SERPINC1 - serpin peptidase inhibitor, clade c (antithrombin), member 1  F2 - coagulation factor ii (thrombin)  PLG - plasminogen  VTN - vitronectin  KLKB1 - kallikrein b, plasma (fletcher factor) 1  SERPINF2 - serpin peptidase inhibitor, clade f (alpha-2 antiplasmin, pigment epithelium derived factor), member 2  F7 - coagulation factor vii (serum prothrombin conversion accelerator)  PLEK - pleckstrin |
| GO:0006721 | terpenoid metabolic process | 7.84E-10 | 1.91E-7 | 4.78 (10334,68,667,21) | [+] Show genes  DHRS3 - dehydrogenase/reductase (sdr family) member 3  AKR1C3 - aldo-keto reductase family 1, member c3  TTR - transthyretin  AKR1C4 - aldo-keto reductase family 1, member c4  APOA2 - apolipoprotein a-ii  ALDH8A1 - aldehyde dehydrogenase 8 family, member a1  CYP2E1 - cytochrome p450, family 2, subfamily e, polypeptide 1  ADH6 - alcohol dehydrogenase 6 (class v)  CYP3A5 - cytochrome p450, family 3, subfamily a, polypeptide 5  ADH4 - alcohol dehydrogenase 4 (class ii), pi polypeptide  APOA4 - apolipoprotein a-iv  CYP2C19 - cytochrome p450, family 2, subfamily c, polypeptide 19  DGAT2 - diacylglycerol o-acyltransferase 2  APOC2 - apolipoprotein c-ii  RDH16 - retinol dehydrogenase 16 (all-trans)  SDC2 - syndecan 2  APOC3 - apolipoprotein c-iii  APOM - apolipoprotein m  HMGCS2 - 3-hydroxy-3-methylglutaryl-coa synthase 2 (mitochondrial)  CYP1A2 - cytochrome p450, family 1, subfamily a, polypeptide 2  PNPLA2 - patatin-like phospholipase domain containing 2 |
| GO:0006805 | xenobiotic metabolic process | 8.36E-10 | 2.01E-7 | 5.00 (10334,62,667,20) | [+] Show genes  S100A12 - s100 calcium binding protein a12  GLYAT - glycine-n-acyltransferase  AOC3 - amine oxidase, copper containing 3  RORC - rar-related orphan receptor c  UGT1A6 - udp glucuronosyltransferase 1 family, polypeptide a6  CES1 - carboxylesterase 1  CYP2E1 - cytochrome p450, family 2, subfamily e, polypeptide 1  CYP3A5 - cytochrome p450, family 3, subfamily a, polypeptide 5  FMO1 - flavin containing monooxygenase 1  FMO3 - flavin containing monooxygenase 3  CYP2C19 - cytochrome p450, family 2, subfamily c, polypeptide 19  ACSL1 - acyl-coa synthetase long-chain family member 1  CES3 - carboxylesterase 3  GHR - growth hormone receptor  CES2 - carboxylesterase 2  AOC1 - amine oxidase, copper containing 1  ACAA1 - acetyl-coa acyltransferase 1  CYP1A2 - cytochrome p450, family 1, subfamily a, polypeptide 2  GGT1 - gamma-glutamyltransferase 1  NR1I2 - nuclear receptor subfamily 1, group i, member 2 |
| GO:0022900 | electron transport chain | 8.91E-10 | 2.1E-7 | 3.43 (10334,140,667,31) | [+] Show genes  UQCRC1 - ubiquinol-cytochrome c reductase core protein i  COX6A2 - cytochrome c oxidase subunit via polypeptide 2  NDUFA3 - nadh dehydrogenase (ubiquinone) 1 alpha subcomplex, 3, 9kda  NDUFA4 - nadh dehydrogenase (ubiquinone) 1 alpha subcomplex, 4, 9kda  COX5B - cytochrome c oxidase subunit vb  SDHB - succinate dehydrogenase complex, subunit b, iron sulfur (ip)  NDUFS7 - nadh dehydrogenase (ubiquinone) fe-s protein 7, 20kda (nadh-coenzyme q reductase)  NDUFA2 - nadh dehydrogenase (ubiquinone) 1 alpha subcomplex, 2, 8kda  NDUFA7 - nadh dehydrogenase (ubiquinone) 1 alpha subcomplex, 7, 14.5kda  PPARGC1A - peroxisome proliferator-activated receptor gamma, coactivator 1 alpha  ALDH4A1 - aldehyde dehydrogenase 4 family, member a1  CYP1A2 - cytochrome p450, family 1, subfamily a, polypeptide 2  COX7C - cytochrome c oxidase subunit viic  ETFDH - electron-transferring-flavoprotein dehydrogenase  HAAO - 3-hydroxyanthranilate 3,4-dioxygenase  DHRS3 - dehydrogenase/reductase (sdr family) member 3  NDUFB10 - nadh dehydrogenase (ubiquinone) 1 beta subcomplex, 10, 22kda  HSD17B6 - hydroxysteroid (17-beta) dehydrogenase 6  UQCR10 - ubiquinol-cytochrome c reductase, complex iii subunit x  AKR1C4 - aldo-keto reductase family 1, member c4  NDUFB7 - nadh dehydrogenase (ubiquinone) 1 beta subcomplex, 7, 18kda  UQCR11 - ubiquinol-cytochrome c reductase, complex iii subunit xi  IMMP2L - imp2 inner mitochondrial membrane peptidase-like (s. cerevisiae)  RDH16 - retinol dehydrogenase 16 (all-trans)  NDUFS8 - nadh dehydrogenase (ubiquinone) fe-s protein 8, 23kda (nadh-coenzyme q reductase)  COQ9 - coenzyme q9 homolog (s. cerevisiae)  NDUFS6 - nadh dehydrogenase (ubiquinone) fe-s protein 6, 13kda (nadh-coenzyme q reductase)  NDUFS5 - nadh dehydrogenase (ubiquinone) fe-s protein 5, 15kda (nadh-coenzyme q reductase)  NDUFV1 - nadh dehydrogenase (ubiquinone) flavoprotein 1, 51kda  ALDH2 - aldehyde dehydrogenase 2 family (mitochondrial)  UQCRQ - ubiquinol-cytochrome c reductase, complex iii subunit vii, 9.5kda |
| GO:0050819 | negative regulation of coagulation | 1.11E-9 | 2.57E-7 | 6.20 (10334,40,667,16) | [+] Show genes  F11 - coagulation factor xi  FGG - fibrinogen gamma chain  F12 - coagulation factor xii (hageman factor)  PROC - protein c (inactivator of coagulation factors va and viiia)  CPB2 - carboxypeptidase b2 (plasma)  ADTRP - androgen-dependent tfpi-regulating protein  HRG - histidine-rich glycoprotein  FGA - fibrinogen alpha chain  KNG1 - kininogen 1  FGB - fibrinogen beta chain  APOH - apolipoprotein h (beta-2-glycoprotein i)  F2 - coagulation factor ii (thrombin)  PLG - plasminogen  VTN - vitronectin  KLKB1 - kallikrein b, plasma (fletcher factor) 1  SERPINF2 - serpin peptidase inhibitor, clade f (alpha-2 antiplasmin, pigment epithelium derived factor), member 2 |
| GO:0032501 | multicellular organismal process | 1.29E-9 | 2.95E-7 | 1.53 (10334,1734,667,171) | [+] Show genes  LGMN - legumain  APOA2 - apolipoprotein a-ii  COX5B - cytochrome c oxidase subunit vb  NDUFA2 - nadh dehydrogenase (ubiquinone) 1 alpha subcomplex, 2, 8kda  SLC22A18 - solute carrier family 22, member 18  SPP2 - secreted phosphoprotein 2, 24kda  APOA4 - apolipoprotein a-iv  SLC29A1 - solute carrier family 29 (equilibrative nucleoside transporter), member 1  BTC - betacellulin  HRC - histidine rich calcium binding protein  PPDPF - pancreatic progenitor cell differentiation and proliferation factor  ENDOG - endonuclease g  ACADL - acyl-coa dehydrogenase, long chain  SERPINA5 - serpin peptidase inhibitor, clade a (alpha-1 antiproteinase, antitrypsin), member 5  COBL - cordon-bleu wh2 repeat protein  ACADVL - acyl-coa dehydrogenase, very long chain  DHRS3 - dehydrogenase/reductase (sdr family) member 3  FXYD1 - fxyd domain containing ion transport regulator 1  CPT2 - carnitine palmitoyltransferase 2  KLHL41 - kelch-like family member 41  AQP9 - aquaporin 9  P2RY2 - purinergic receptor p2y, g-protein coupled, 2  CPS1 - carbamoyl-phosphate synthase 1, mitochondrial  ABCC6 - atp-binding cassette, sub-family c (cftr/mrp), member 6  PLN - phospholamban  CD36 - cd36 molecule (thrombospondin receptor)  GADD45G - growth arrest and dna-damage-inducible, gamma  HPN - hepsin  TTN - titin  CPB2 - carboxypeptidase b2 (plasma)  MYL3 - myosin, light chain 3, alkali; ventricular, skeletal, slow  DES - desmin  MYL2 - myosin, light chain 2, regulatory, cardiac, slow  HAMP - hepcidin antimicrobial peptide  TMOD4 - tropomodulin 4 (muscle)  APOC2 - apolipoprotein c-ii  IMMP2L - imp2 inner mitochondrial membrane peptidase-like (s. cerevisiae)  LIPC - lipase, hepatic  APOC3 - apolipoprotein c-iii  ACTA1 - actin, alpha 1, skeletal muscle  APOC4 - apolipoprotein c-iv  PROZ - protein z, vitamin k-dependent plasma glycoprotein  PAFAH2 - platelet-activating factor acetylhydrolase 2, 40kda  PLG - plasminogen  RCSD1 - rcsd domain containing 1  YBX3 - y box binding protein 3  RGS14 - regulator of g-protein signaling 14  SERPINF2 - serpin peptidase inhibitor, clade f (alpha-2 antiplasmin, pigment epithelium derived factor), member 2  RGS16 - regulator of g-protein signaling 16  MYH1 - myosin, heavy chain 1, skeletal muscle, adult  FGG - fibrinogen gamma chain  CYP4A11 - cytochrome p450, family 4, subfamily a, polypeptide 11  MYH2 - myosin, heavy chain 2, skeletal muscle, adult  TPM2 - tropomyosin 2 (beta)  MYF6 - myogenic factor 6 (herculin)  EPN1 - epsin 1  MYL1 - myosin, light chain 1, alkali; skeletal, fast  STS - steroid sulfatase (microsomal), isozyme s  PAM16 - presequence translocase-associated motor 16 homolog (s. cerevisiae)  ACTN3 - actinin, alpha 3  TBATA - thymus, brain and testes associated  ATXN7 - ataxin 7  PPARGC1A - peroxisome proliferator-activated receptor gamma, coactivator 1 alpha  FGA - fibrinogen alpha chain  ARG1 - arginase 1  FGB - fibrinogen beta chain  PM20D1 - peptidase m20 domain containing 1  TCAP - titin-cap  MYLPF - myosin light chain, phosphorylatable, fast skeletal muscle  CYP1A2 - cytochrome p450, family 1, subfamily a, polypeptide 2  SERPINF1 - serpin peptidase inhibitor, clade f (alpha-2 antiplasmin, pigment epithelium derived factor), member 1  PTPLA - protein tyrosine phosphatase-like (proline instead of catalytic arginine), member a  PPARA - peroxisome proliferator-activated receptor alpha  SERPIND1 - serpin peptidase inhibitor, clade d (heparin cofactor), member 1  PTP4A3 - protein tyrosine phosphatase type iva, member 3  APOA5 - apolipoprotein a-v  RORC - rar-related orphan receptor c  POLR2I - polymerase (rna) ii (dna directed) polypeptide i, 14.5kda  OSGIN1 - oxidative stress induced growth inhibitor 1  POLR2J - polymerase (rna) ii (dna directed) polypeptide j, 13.3kda  MYOM2 - myomesin 2  RP9 - retinitis pigmentosa 9 (autosomal dominant)  DGAT2 - diacylglycerol o-acyltransferase 2  ARVCF - armadillo repeat gene deleted in velocardiofacial syndrome  SMPX - small muscle protein, x-linked  APOM - apolipoprotein m  MYOM3 - myomesin 3  MLXIPL - mlx interacting protein-like  C4BPB - complement component 4 binding protein, beta  MYOT - myotilin  YBX1 - y box binding protein 1  SMTNL1 - smoothelin-like 1  C6 - complement component 6  KEAP1 - kelch-like ech-associated protein 1  F11 - coagulation factor xi  AKR1C3 - aldo-keto reductase family 1, member c3  ATP2A1 - atpase, ca++ transporting, cardiac muscle, fast twitch 1  F10 - coagulation factor x  CHRNE - cholinergic receptor, nicotinic, epsilon (muscle)  F9 - coagulation factor ix  F13B - coagulation factor xiii, b polypeptide  F12 - coagulation factor xii (hageman factor)  ZNF358 - zinc finger protein 358  FABP1 - fatty acid binding protein 1, liver  ADRB2 - adrenoceptor beta 2, surface  TRIM63 - tripartite motif containing 63, e3 ubiquitin protein ligase  SERPINA10 - serpin peptidase inhibitor, clade a (alpha-1 antiproteinase, antitrypsin), member 10  PGAM2 - phosphoglycerate mutase 2 (muscle)  CHRNA1 - cholinergic receptor, nicotinic, alpha 1 (muscle)  CASQ2 - calsequestrin 2 (cardiac muscle)  SFTPA1 - surfactant protein a1  GOT2 - glutamic-oxaloacetic transaminase 2, mitochondrial  ATF4 - activating transcription factor 4  HOMER2 - homer homolog 2 (drosophila)  SERPINC1 - serpin peptidase inhibitor, clade c (antithrombin), member 1  LMOD3 - leiomodin 3 (fetal)  DNAJC19 - dnaj (hsp40) homolog, subfamily c, member 19  CACNG6 - calcium channel, voltage-dependent, gamma subunit 6  MYLK2 - myosin light chain kinase 2  MYO18B - myosin xviiib  TIMM8B - translocase of inner mitochondrial membrane 8 homolog b (yeast)  CACNG1 - calcium channel, voltage-dependent, gamma subunit 1  MB - myoglobin  SLC25A20 - solute carrier family 25 (carnitine/acylcarnitine translocase), member 20  SLC40A1 - solute carrier family 40 (iron-regulated transporter), member 1  CHIA - chitinase, acidic  MARCO - macrophage receptor with collagenous structure  CACNA1S - calcium channel, voltage-dependent, l type, alpha 1s subunit  LMOD2 - leiomodin 2 (cardiac)  TNNC2 - troponin c type 2 (fast)  KNG1 - kininogen 1  TNNI2 - troponin i type 2 (skeletal, fast)  F2 - coagulation factor ii (thrombin)  TNNI1 - troponin i type 1 (skeletal, slow)  ALB - albumin  TNNC1 - troponin c type 1 (slow)  G6PC - glucose-6-phosphatase, catalytic subunit  TNNT3 - troponin t type 3 (skeletal, fast)  F7 - coagulation factor vii (serum prothrombin conversion accelerator)  SMTN - smoothelin  ANKRD2 - ankyrin repeat domain 2 (stretch responsive muscle)  KLF15 - kruppel-like factor 15  PHOSPHO1 - phosphatase, orphan 1  CRP - c-reactive protein, pentraxin-related  CSRP3 - cysteine and glycine-rich protein 3 (cardiac lim protein)  GAMT - guanidinoacetate n-methyltransferase  PROC - protein c (inactivator of coagulation factors va and viiia)  GRHPR - glyoxylate reductase/hydroxypyruvate reductase  CES3 - carboxylesterase 3  AMBP - alpha-1-microglobulin/bikunin precursor  MAFB - v-maf avian musculoaponeurotic fibrosarcoma oncogene homolog b  FOXA3 - forkhead box a3  STAC3 - sh3 and cysteine rich domain 3  EPHA1 - eph receptor a1  IGF1 - insulin-like growth factor 1 (somatomedin c)  TRIM54 - tripartite motif containing 54  LMOD1 - leiomodin 1 (smooth muscle)  SCGB1A1 - secretoglobin, family 1a, member 1 (uteroglobin)  TRIM72 - tripartite motif containing 72  RXRA - retinoid x receptor, alpha  UCP3 - uncoupling protein 3 (mitochondrial, proton carrier)  CAV3 - caveolin 3  JPH2 - junctophilin 2  RYR1 - ryanodine receptor 1 (skeletal)  SERPINA1 - serpin peptidase inhibitor, clade a (alpha-1 antiproteinase, antitrypsin), member 1  PKN1 - protein kinase n1  SGCG - sarcoglycan, gamma (35kda dystrophin-associated glycoprotein)  NDUFS6 - nadh dehydrogenase (ubiquinone) fe-s protein 6, 13kda (nadh-coenzyme q reductase)  HMOX1 - heme oxygenase (decycling) 1  MAF - v-maf avian musculoaponeurotic fibrosarcoma oncogene homolog  ERBB2 - v-erb-b2 avian erythroblastic leukemia viral oncogene homolog 2 |
| GO:0009072 | aromatic amino acid family metabolic process | 1.43E-9 | 3.21E-7 | 7.75 (10334,26,667,13) | [+] Show genes  IYD - iodotyrosine deiodinase  GCDH - glutaryl-coa dehydrogenase  FTCD - formimidoyltransferase cyclodeaminase  HPD - 4-hydroxyphenylpyruvate dioxygenase  HGD - homogentisate 1,2-dioxygenase  TDO2 - tryptophan 2,3-dioxygenase  MTHFD1 - methylenetetrahydrofolate dehydrogenase (nadp+ dependent) 1, methenyltetrahydrofolate cyclohydrolase, formyltetrahydrofolate synthetase  TAT - tyrosine aminotransferase  FAH - fumarylacetoacetate hydrolase (fumarylacetoacetase)  PAH - phenylalanine hydroxylase  GSTZ1 - glutathione s-transferase zeta 1  CCBL1 - cysteine conjugate-beta lyase, cytoplasmic  HAAO - 3-hydroxyanthranilate 3,4-dioxygenase |
| GO:0006641 | triglyceride metabolic process | 1.43E-9 | 3.16E-7 | 5.73 (10334,46,667,17) | [+] Show genes  APOA5 - apolipoprotein a-v  CPS1 - carbamoyl-phosphate synthase 1, mitochondrial  APOA2 - apolipoprotein a-ii  CYP2E1 - cytochrome p450, family 2, subfamily e, polypeptide 1  CAT - catalase  FABP1 - fatty acid binding protein 1, liver  LPIN1 - lipin 1  CAV3 - caveolin 3  ACSL1 - acyl-coa synthetase long-chain family member 1  DGAT2 - diacylglycerol o-acyltransferase 2  APOC3 - apolipoprotein c-iii  LIPC - lipase, hepatic  GK5 - glycerol kinase 5 (putative)  APOH - apolipoprotein h (beta-2-glycoprotein i)  G6PC - glucose-6-phosphatase, catalytic subunit  SLC27A5 - solute carrier family 27 (fatty acid transporter), member 5  PNPLA2 - patatin-like phospholipase domain containing 2 |
| GO:0031638 | zymogen activation | 1.81E-9 | 3.93E-7 | 7.00 (10334,31,667,14) | [+] Show genes  F11 - coagulation factor xi  FGG - fibrinogen gamma chain  F9 - coagulation factor ix  LGMN - legumain  F12 - coagulation factor xii (hageman factor)  C1R - complement component 1, r subcomponent  HP - haptoglobin  FGA - fibrinogen alpha chain  FGB - fibrinogen beta chain  APOH - apolipoprotein h (beta-2-glycoprotein i)  KLKB1 - kallikrein b, plasma (fletcher factor) 1  CIDEB - cell death-inducing dffa-like effector b  GGT1 - gamma-glutamyltransferase 1  CD5L - cd5 molecule-like |
| GO:0042737 | drug catabolic process | 2.12E-9 | 4.55E-7 | 4.37 (10334,78,667,22) | [+] Show genes  GCAT - glycine c-acetyltransferase  PCK1 - phosphoenolpyruvate carboxykinase 1 (soluble)  SULT2A1 - sulfotransferase family, cytosolic, 2a, dehydroepiandrosterone (dhea)-preferring, member 1  CAT - catalase  CYP2E1 - cytochrome p450, family 2, subfamily e, polypeptide 1  CYP3A5 - cytochrome p450, family 3, subfamily a, polypeptide 5  CHIA - chitinase, acidic  APOA4 - apolipoprotein a-iv  HPD - 4-hydroxyphenylpyruvate dioxygenase  CYP2C19 - cytochrome p450, family 2, subfamily c, polypeptide 19  HGD - homogentisate 1,2-dioxygenase  TAT - tyrosine aminotransferase  PIPOX - pipecolic acid oxidase  FAH - fumarylacetoacetate hydrolase (fumarylacetoacetase)  ADA - adenosine deaminase  PAH - phenylalanine hydroxylase  GSTZ1 - glutathione s-transferase zeta 1  CYP1A2 - cytochrome p450, family 1, subfamily a, polypeptide 2  NR1I2 - nuclear receptor subfamily 1, group i, member 2  DPYS - dihydropyrimidinase  ALDH2 - aldehyde dehydrogenase 2 family (mitochondrial)  CCBL1 - cysteine conjugate-beta lyase, cytoplasmic |
| GO:0055088 | lipid homeostasis | 2.53E-9 | 5.34E-7 | 3.76 (10334,107,667,26) | [+] Show genes  ACOX2 - acyl-coa oxidase 2, branched chain  ANGPTL3 - angiopoietin-like 3  CES1 - carboxylesterase 1  APOA2 - apolipoprotein a-ii  ORMDL3 - orm1-like 3 (s. cerevisiae)  APOA4 - apolipoprotein a-iv  ACSM2A - acyl-coa synthetase medium-chain family member 2a  PNPLA2 - patatin-like phospholipase domain containing 2  C19orf80 - chromosome 19 open reading frame 80  APOA5 - apolipoprotein a-v  ANGPTL4 - angiopoietin-like 4  TM6SF2 - transmembrane 6 superfamily member 2  CAV3 - caveolin 3  DGAT2 - diacylglycerol o-acyltransferase 2  APOC2 - apolipoprotein c-ii  APOC3 - apolipoprotein c-iii  LIPC - lipase, hepatic  PLA2G12B - phospholipase a2, group xiib  APOC4 - apolipoprotein c-iv  APOM - apolipoprotein m  MLXIPL - mlx interacting protein-like  ASGR2 - asialoglycoprotein receptor 2  NR1I2 - nuclear receptor subfamily 1, group i, member 2  G6PC - glucose-6-phosphatase, catalytic subunit  ACSM3 - acyl-coa synthetase medium-chain family member 3  NR1I3 - nuclear receptor subfamily 1, group i, member 3 |
| GO:0061045 | negative regulation of wound healing | 3.24E-9 | 6.73E-7 | 5.16 (10334,54,667,18) | [+] Show genes  F11 - coagulation factor xi  FGG - fibrinogen gamma chain  APCS - amyloid p component, serum  F12 - coagulation factor xii (hageman factor)  MYOZ1 - myozenin 1  PROC - protein c (inactivator of coagulation factors va and viiia)  CPB2 - carboxypeptidase b2 (plasma)  ADTRP - androgen-dependent tfpi-regulating protein  FGA - fibrinogen alpha chain  HRG - histidine-rich glycoprotein  KNG1 - kininogen 1  FGB - fibrinogen beta chain  APOH - apolipoprotein h (beta-2-glycoprotein i)  PLG - plasminogen  F2 - coagulation factor ii (thrombin)  VTN - vitronectin  KLKB1 - kallikrein b, plasma (fletcher factor) 1  SERPINF2 - serpin peptidase inhibitor, clade f (alpha-2 antiplasmin, pigment epithelium derived factor), member 2 |
| GO:0009056 | catabolic process | 3.9E-9 | 7.98E-7 | 1.57 (10334,1460,667,148) | [+] Show genes  ANGPTL3 - angiopoietin-like 3  LGMN - legumain  GADL1 - glutamate decarboxylase-like 1  APOA2 - apolipoprotein a-ii  MMP23B - matrix metallopeptidase 23b  ENTPD8 - ectonucleoside triphosphate diphosphohydrolase 8  HSD11B1 - hydroxysteroid (11-beta) dehydrogenase 1  AMACR - alpha-methylacyl-coa racemase  CDC34 - cell division cycle 34  APOA4 - apolipoprotein a-iv  BDH2 - 3-hydroxybutyrate dehydrogenase, type 2  OTC - ornithine carbamoyltransferase  ENO3 - enolase 3 (beta, muscle)  CES2 - carboxylesterase 2  FBXW5 - f-box and wd repeat domain containing 5  LDHA - lactate dehydrogenase a  ENDOG - endonuclease g  IMPA2 - inositol(myo)-1(or 4)-monophosphatase 2  GSTZ1 - glutathione s-transferase zeta 1  ACAA1 - acetyl-coa acyltransferase 1  ACADL - acyl-coa dehydrogenase, long chain  ACADS - acyl-coa dehydrogenase, c-2 to c-3 short chain  ACADVL - acyl-coa dehydrogenase, very long chain  CPT2 - carnitine palmitoyltransferase 2  CPT1B - carnitine palmitoyltransferase 1b (muscle)  CPS1 - carbamoyl-phosphate synthase 1, mitochondrial  UBE2L6 - ubiquitin-conjugating enzyme e2l 6  CD36 - cd36 molecule (thrombospondin receptor)  EHHADH - enoyl-coa, hydratase/3-hydroxyacyl coa dehydrogenase  BHMT - betaine--homocysteine s-methyltransferase  HPD - 4-hydroxyphenylpyruvate dioxygenase  TDO2 - tryptophan 2,3-dioxygenase  APOC2 - apolipoprotein c-ii  SDC2 - syndecan 2  APOC3 - apolipoprotein c-iii  PSMC5 - proteasome (prosome, macropain) 26s subunit, atpase, 5  LIPC - lipase, hepatic  PIPOX - pipecolic acid oxidase  PAFAH2 - platelet-activating factor acetylhydrolase 2, 40kda  ADHFE1 - alcohol dehydrogenase, iron containing, 1  DCAF11 - ddb1 and cul4 associated factor 11  SMPDL3A - sphingomyelin phosphodiesterase, acid-like 3a  PAH - phenylalanine hydroxylase  BLVRB - biliverdin reductase b (flavin reductase (nadph))  APOBEC2 - apolipoprotein b mrna editing enzyme, catalytic polypeptide-like 2  GGT1 - gamma-glutamyltransferase 1  NR1I2 - nuclear receptor subfamily 1, group i, member 2  UPB1 - ureidopropionase, beta  ACOX2 - acyl-coa oxidase 2, branched chain  CYP4A11 - cytochrome p450, family 4, subfamily a, polypeptide 11  CES1 - carboxylesterase 1  ALDH8A1 - aldehyde dehydrogenase 8 family, member a1  CYP2E1 - cytochrome p450, family 2, subfamily e, polypeptide 1  ABHD17A - abhydrolase domain containing 17a  STS - steroid sulfatase (microsomal), isozyme s  PLBD1 - phospholipase b domain containing 1  CYP3A5 - cytochrome p450, family 3, subfamily a, polypeptide 5  SCARF1 - scavenger receptor class f, member 1  CYP2C19 - cytochrome p450, family 2, subfamily c, polypeptide 19  ENPP1 - ectonucleotide pyrophosphatase/phosphodiesterase 1  TAT - tyrosine aminotransferase  PPARGC1A - peroxisome proliferator-activated receptor gamma, coactivator 1 alpha  UBXN1 - ubx domain protein 1  ARG1 - arginase 1  SCP2 - sterol carrier protein 2  HADH - hydroxyacyl-coa dehydrogenase  BAG3 - bcl2-associated athanogene 3  ADA - adenosine deaminase  PM20D1 - peptidase m20 domain containing 1  CYP1A2 - cytochrome p450, family 1, subfamily a, polypeptide 2  HAGH - hydroxyacylglutathione hydrolase  UPF2 - upf2 regulator of nonsense transcripts homolog (yeast)  HSD17B10 - hydroxysteroid (17-beta) dehydrogenase 10  PON1 - paraoxonase 1  HSD17B6 - hydroxysteroid (17-beta) dehydrogenase 6  APOA5 - apolipoprotein a-v  PCK1 - phosphoenolpyruvate carboxykinase 1 (soluble)  FEM1A - fem-1 homolog a (c. elegans)  FTCD - formimidoyltransferase cyclodeaminase  ALDH1L1 - aldehyde dehydrogenase 1 family, member l1  ADH4 - alcohol dehydrogenase 4 (class ii), pi polypeptide  PRODH2 - proline dehydrogenase (oxidase) 2  GLYCTK - glycerate kinase  PLA2G12B - phospholipase a2, group xiib  CRYL1 - crystallin, lambda 1  ECHS1 - enoyl coa hydratase, short chain, 1, mitochondrial  KEAP1 - kelch-like ech-associated protein 1  ECH1 - enoyl coa hydratase 1, peroxisomal  ECI2 - enoyl-coa delta isomerase 2  AKR1C3 - aldo-keto reductase family 1, member c3  MLYCD - malonyl-coa decarboxylase  FBXO17 - f-box protein 17  HSD17B14 - hydroxysteroid (17-beta) dehydrogenase 14  CAPN3 - calpain 3, (p94)  ACAA2 - acetyl-coa acyltransferase 2  PGM1 - phosphoglucomutase 1  FABP1 - fatty acid binding protein 1, liver  PGAM2 - phosphoglycerate mutase 2 (muscle)  TMPRSS6 - transmembrane protease, serine 6  MCEE - methylmalonyl coa epimerase  ACSL1 - acyl-coa synthetase long-chain family member 1  GOT2 - glutamic-oxaloacetic transaminase 2, mitochondrial  FAH - fumarylacetoacetate hydrolase (fumarylacetoacetase)  ALDH4A1 - aldehyde dehydrogenase 4 family, member a1  GK5 - glycerol kinase 5 (putative)  ASPG - asparaginase homolog (s. cerevisiae)  GPD1 - glycerol-3-phosphate dehydrogenase 1 (soluble)  PNPLA2 - patatin-like phospholipase domain containing 2  DPYS - dihydropyrimidinase  ABHD1 - abhydrolase domain containing 1  GCAT - glycine c-acetyltransferase  SULT2A1 - sulfotransferase family, cytosolic, 2a, dehydroepiandrosterone (dhea)-preferring, member 1  AHCY - adenosylhomocysteinase  AGXT - alanine-glyoxylate aminotransferase  CHIA - chitinase, acidic  MAT1A - methionine adenosyltransferase i, alpha  WWP1 - ww domain containing e3 ubiquitin protein ligase 1  HGD - homogentisate 1,2-dioxygenase  HACL1 - 2-hydroxyacyl-coa lyase 1  DECR2 - 2,4-dienoyl coa reductase 2, peroxisomal  G6PC - glucose-6-phosphatase, catalytic subunit  ALDH2 - aldehyde dehydrogenase 2 family (mitochondrial)  PFKFB1 - 6-phosphofructo-2-kinase/fructose-2,6-biphosphatase 1  UBA52 - ubiquitin a-52 residue ribosomal protein fusion product 1  AGXT2 - alanine--glyoxylate aminotransferase 2  SHMT1 - serine hydroxymethyltransferase 1 (soluble)  LPIN1 - lipin 1  PLCD4 - phospholipase c, delta 4  ALDOB - aldolase b, fructose-bisphosphate  CES3 - carboxylesterase 3  ASB2 - ankyrin repeat and socs box containing 2  ADTRP - androgen-dependent tfpi-regulating protein  AMBP - alpha-1-microglobulin/bikunin precursor  ETFDH - electron-transferring-flavoprotein dehydrogenase  PKLR - pyruvate kinase, liver and rbc  HAAO - 3-hydroxyanthranilate 3,4-dioxygenase  ECHDC2 - enoyl coa hydratase domain containing 2  GCDH - glutaryl-coa dehydrogenase  CAT - catalase  TRIM72 - tripartite motif containing 72  PHYH - phytanoyl-coa 2-hydroxylase  STBD1 - starch binding domain 1  SLC27A2 - solute carrier family 27 (fatty acid transporter), member 2  HMOX1 - heme oxygenase (decycling) 1  MPST - mercaptopyruvate sulfurtransferase  HAO2 - hydroxyacid oxidase 2 (long chain)  URAD - ureidoimidazoline (2-oxo-4-hydroxy-4-carboxy-5-) decarboxylase  CCBL1 - cysteine conjugate-beta lyase, cytoplasmic |
| GO:0030048 | actin filament-based movement | 4.31E-9 | 8.69E-7 | 4.83 (10334,61,667,19) | [+] Show genes  MYH2 - myosin, heavy chain 2, skeletal muscle, adult  TPM2 - tropomyosin 2 (beta)  MYL1 - myosin, light chain 1, alkali; skeletal, fast  ACTN2 - actinin, alpha 2  ACTN3 - actinin, alpha 3  TTN - titin  NEB - nebulin  MYL3 - myosin, light chain 3, alkali; ventricular, skeletal, slow  DES - desmin  MYBPC1 - myosin binding protein c, slow type  MYL2 - myosin, light chain 2, regulatory, cardiac, slow  TNNC2 - troponin c type 2 (fast)  ACTA1 - actin, alpha 1, skeletal muscle  TNNI2 - troponin i type 2 (skeletal, fast)  TCAP - titin-cap  TNNI1 - troponin i type 1 (skeletal, slow)  TNNC1 - troponin c type 1 (slow)  TNNT3 - troponin t type 3 (skeletal, fast)  MYBPC2 - myosin binding protein c, fast type |
| GO:0001523 | retinoid metabolic process | 4.53E-9 | 9.01E-7 | 5.07 (10334,55,667,18) | [+] Show genes  DHRS3 - dehydrogenase/reductase (sdr family) member 3  AKR1C3 - aldo-keto reductase family 1, member c3  TTR - transthyretin  AKR1C4 - aldo-keto reductase family 1, member c4  APOA2 - apolipoprotein a-ii  ALDH8A1 - aldehyde dehydrogenase 8 family, member a1  ADH6 - alcohol dehydrogenase 6 (class v)  CYP3A5 - cytochrome p450, family 3, subfamily a, polypeptide 5  ADH4 - alcohol dehydrogenase 4 (class ii), pi polypeptide  APOA4 - apolipoprotein a-iv  DGAT2 - diacylglycerol o-acyltransferase 2  APOC2 - apolipoprotein c-ii  RDH16 - retinol dehydrogenase 16 (all-trans)  SDC2 - syndecan 2  APOC3 - apolipoprotein c-iii  APOM - apolipoprotein m  CYP1A2 - cytochrome p450, family 1, subfamily a, polypeptide 2  PNPLA2 - patatin-like phospholipase domain containing 2 |
| GO:0006720 | isoprenoid metabolic process | 5.96E-9 | 1.17E-6 | 4.16 (10334,82,667,22) | [+] Show genes  DHRS3 - dehydrogenase/reductase (sdr family) member 3  AKR1C3 - aldo-keto reductase family 1, member c3  TTR - transthyretin  AKR1C4 - aldo-keto reductase family 1, member c4  APOA2 - apolipoprotein a-ii  ALDH8A1 - aldehyde dehydrogenase 8 family, member a1  CYP2E1 - cytochrome p450, family 2, subfamily e, polypeptide 1  ADH6 - alcohol dehydrogenase 6 (class v)  CYP3A5 - cytochrome p450, family 3, subfamily a, polypeptide 5  ADH4 - alcohol dehydrogenase 4 (class ii), pi polypeptide  PHYH - phytanoyl-coa 2-hydroxylase  APOA4 - apolipoprotein a-iv  CYP2C19 - cytochrome p450, family 2, subfamily c, polypeptide 19  DGAT2 - diacylglycerol o-acyltransferase 2  APOC2 - apolipoprotein c-ii  RDH16 - retinol dehydrogenase 16 (all-trans)  SDC2 - syndecan 2  APOC3 - apolipoprotein c-iii  APOM - apolipoprotein m  HMGCS2 - 3-hydroxy-3-methylglutaryl-coa synthase 2 (mitochondrial)  CYP1A2 - cytochrome p450, family 1, subfamily a, polypeptide 2  PNPLA2 - patatin-like phospholipase domain containing 2 |
| GO:0055090 | acylglycerol homeostasis | 7.79E-9 | 1.5E-6 | 6.95 (10334,29,667,13) | [+] Show genes  C19orf80 - chromosome 19 open reading frame 80  APOA5 - apolipoprotein a-v  ANGPTL4 - angiopoietin-like 4  ANGPTL3 - angiopoietin-like 3  APOA4 - apolipoprotein a-iv  DGAT2 - diacylglycerol o-acyltransferase 2  APOC2 - apolipoprotein c-ii  PLA2G12B - phospholipase a2, group xiib  APOC3 - apolipoprotein c-iii  LIPC - lipase, hepatic  APOC4 - apolipoprotein c-iv  MLXIPL - mlx interacting protein-like  ACSM2A - acyl-coa synthetase medium-chain family member 2a |
| GO:0070328 | triglyceride homeostasis | 7.79E-9 | 1.48E-6 | 6.95 (10334,29,667,13) | [+] Show genes  C19orf80 - chromosome 19 open reading frame 80  ANGPTL4 - angiopoietin-like 4  APOA5 - apolipoprotein a-v  ANGPTL3 - angiopoietin-like 3  APOA4 - apolipoprotein a-iv  DGAT2 - diacylglycerol o-acyltransferase 2  APOC2 - apolipoprotein c-ii  PLA2G12B - phospholipase a2, group xiib  APOC3 - apolipoprotein c-iii  LIPC - lipase, hepatic  APOC4 - apolipoprotein c-iv  MLXIPL - mlx interacting protein-like  ACSM2A - acyl-coa synthetase medium-chain family member 2a |
| GO:0015711 | organic anion transport | 8E-9 | 1.5E-6 | 2.60 (10334,250,667,42) | [+] Show genes  SLC16A13 - solute carrier family 16, member 13  APOA2 - apolipoprotein a-ii  FABP1 - fatty acid binding protein 1, liver  APOA4 - apolipoprotein a-iv  PCTP - phosphatidylcholine transfer protein  SLC16A5 - solute carrier family 16 (monocarboxylate transporter), member 5  ACSL1 - acyl-coa synthetase long-chain family member 1  CA3 - carbonic anhydrase iii, muscle specific  SLC10A1 - solute carrier family 10 (sodium/bile acid cotransporter), member 1  GOT2 - glutamic-oxaloacetic transaminase 2, mitochondrial  SLC38A4 - solute carrier family 38, member 4  SCP2 - sterol carrier protein 2  CA14 - carbonic anhydrase xiv  SLC25A13 - solute carrier family 25 (aspartate/glutamate carrier), member 13  PPARA - peroxisome proliferator-activated receptor alpha  MPC1 - mitochondrial pyruvate carrier 1  CPT2 - carnitine palmitoyltransferase 2  SLCO1B3 - solute carrier organic anion transporter family, member 1b3  SLC17A2 - solute carrier family 17, member 2  SLC37A4 - solute carrier family 37 (glucose-6-phosphate transporter), member 4  AKR1C4 - aldo-keto reductase family 1, member c4  SLC25A47 - solute carrier family 25, member 47  APOA5 - apolipoprotein a-v  CPT1B - carnitine palmitoyltransferase 1b (muscle)  AQP9 - aquaporin 9  CD36 - cd36 molecule (thrombospondin receptor)  SLC25A20 - solute carrier family 25 (carnitine/acylcarnitine translocase), member 20  SLC51B - solute carrier family 51, beta subunit  AGXT - alanine-glyoxylate aminotransferase  RXRA - retinoid x receptor, alpha  SLC25A30 - solute carrier family 25, member 30  APOC2 - apolipoprotein c-ii  APOC3 - apolipoprotein c-iii  PLA2G12B - phospholipase a2, group xiib  SLC27A2 - solute carrier family 27 (fatty acid transporter), member 2  SLC25A4 - solute carrier family 25 (mitochondrial carrier; adenine nucleotide translocator), member 4  ATP8B3 - atpase, aminophospholipid transporter, class i, type 8b, member 3  SLC22A7 - solute carrier family 22 (organic anion transporter), member 7  SLC17A1 - solute carrier family 17 (organic anion transporter), member 1  THRSP - thyroid hormone responsive  G6PC - glucose-6-phosphatase, catalytic subunit  SLC27A5 - solute carrier family 27 (fatty acid transporter), member 5 |
| GO:0022904 | respiratory electron transport chain | 9.42E-9 | 1.75E-6 | 3.92 (10334,91,667,23) | [+] Show genes  NDUFB10 - nadh dehydrogenase (ubiquinone) 1 beta subcomplex, 10, 22kda  UQCRC1 - ubiquinol-cytochrome c reductase core protein i  COX6A2 - cytochrome c oxidase subunit via polypeptide 2  UQCR10 - ubiquinol-cytochrome c reductase, complex iii subunit x  NDUFB7 - nadh dehydrogenase (ubiquinone) 1 beta subcomplex, 7, 18kda  NDUFA3 - nadh dehydrogenase (ubiquinone) 1 alpha subcomplex, 3, 9kda  NDUFA4 - nadh dehydrogenase (ubiquinone) 1 alpha subcomplex, 4, 9kda  SDHB - succinate dehydrogenase complex, subunit b, iron sulfur (ip)  COX5B - cytochrome c oxidase subunit vb  NDUFS7 - nadh dehydrogenase (ubiquinone) fe-s protein 7, 20kda (nadh-coenzyme q reductase)  NDUFA2 - nadh dehydrogenase (ubiquinone) 1 alpha subcomplex, 2, 8kda  NDUFA7 - nadh dehydrogenase (ubiquinone) 1 alpha subcomplex, 7, 14.5kda  UQCR11 - ubiquinol-cytochrome c reductase, complex iii subunit xi  PPARGC1A - peroxisome proliferator-activated receptor gamma, coactivator 1 alpha  IMMP2L - imp2 inner mitochondrial membrane peptidase-like (s. cerevisiae)  NDUFS8 - nadh dehydrogenase (ubiquinone) fe-s protein 8, 23kda (nadh-coenzyme q reductase)  COQ9 - coenzyme q9 homolog (s. cerevisiae)  NDUFS6 - nadh dehydrogenase (ubiquinone) fe-s protein 6, 13kda (nadh-coenzyme q reductase)  NDUFS5 - nadh dehydrogenase (ubiquinone) fe-s protein 5, 15kda (nadh-coenzyme q reductase)  COX7C - cytochrome c oxidase subunit viic  NDUFV1 - nadh dehydrogenase (ubiquinone) flavoprotein 1, 51kda  ETFDH - electron-transferring-flavoprotein dehydrogenase  UQCRQ - ubiquinol-cytochrome c reductase, complex iii subunit vii, 9.5kda |
| GO:0051917 | regulation of fibrinolysis | 1.04E-8 | 1.91E-6 | 10.73 (10334,13,667,9) | [+] Show genes  F11 - coagulation factor xi  HRG - histidine-rich glycoprotein  APOH - apolipoprotein h (beta-2-glycoprotein i)  F12 - coagulation factor xii (hageman factor)  F2 - coagulation factor ii (thrombin)  PLG - plasminogen  KLKB1 - kallikrein b, plasma (fletcher factor) 1  SERPINF2 - serpin peptidase inhibitor, clade f (alpha-2 antiplasmin, pigment epithelium derived factor), member 2  CPB2 - carboxypeptidase b2 (plasma) |
| GO:0042730 | fibrinolysis | 1.51E-8 | 2.73E-6 | 9.11 (10334,17,667,10) | [+] Show genes  FGG - fibrinogen gamma chain  HRG - histidine-rich glycoprotein  FGA - fibrinogen alpha chain  FGB - fibrinogen beta chain  F12 - coagulation factor xii (hageman factor)  F2 - coagulation factor ii (thrombin)  PLG - plasminogen  KLKB1 - kallikrein b, plasma (fletcher factor) 1  SERPINF2 - serpin peptidase inhibitor, clade f (alpha-2 antiplasmin, pigment epithelium derived factor), member 2  CPB2 - carboxypeptidase b2 (plasma) |
| GO:1903317 | regulation of protein maturation | 1.56E-8 | 2.78E-6 | 3.96 (10334,86,667,22) | [+] Show genes  C19orf80 - chromosome 19 open reading frame 80  C9 - complement component 9  F12 - coagulation factor xii (hageman factor)  CFB - complement factor b  CPN2 - carboxypeptidase n, polypeptide 2  HPN - hepsin  C1R - complement component 1, r subcomponent  C2 - complement component 2  CPB2 - carboxypeptidase b2 (plasma)  C4BPA - complement component 4 binding protein, alpha  C4BPB - complement component 4 binding protein, beta  F2 - coagulation factor ii (thrombin)  C5 - complement component 5  VTN - vitronectin  TFR2 - transferrin receptor 2  C6 - complement component 6  KLKB1 - kallikrein b, plasma (fletcher factor) 1  C8B - complement component 8, beta polypeptide  SERPINF2 - serpin peptidase inhibitor, clade f (alpha-2 antiplasmin, pigment epithelium derived factor), member 2  C8A - complement component 8, alpha polypeptide  CD5L - cd5 molecule-like  C8G - complement component 8, gamma polypeptide |
| GO:0061041 | regulation of wound healing | 1.67E-8 | 2.95E-6 | 3.68 (10334,101,667,24) | [+] Show genes  F11 - coagulation factor xi  FGG - fibrinogen gamma chain  APCS - amyloid p component, serum  CAPN3 - calpain 3, (p94)  F12 - coagulation factor xii (hageman factor)  CD36 - cd36 molecule (thrombospondin receptor)  MYOZ1 - myozenin 1  PROC - protein c (inactivator of coagulation factors va and viiia)  CPB2 - carboxypeptidase b2 (plasma)  ADTRP - androgen-dependent tfpi-regulating protein  KNG1 - kininogen 1  HRG - histidine-rich glycoprotein  FGA - fibrinogen alpha chain  FGB - fibrinogen beta chain  APOH - apolipoprotein h (beta-2-glycoprotein i)  F2 - coagulation factor ii (thrombin)  SERPINC1 - serpin peptidase inhibitor, clade c (antithrombin), member 1  PLG - plasminogen  VTN - vitronectin  KLKB1 - kallikrein b, plasma (fletcher factor) 1  HRAS - harvey rat sarcoma viral oncogene homolog  SERPINF2 - serpin peptidase inhibitor, clade f (alpha-2 antiplasmin, pigment epithelium derived factor), member 2  F7 - coagulation factor vii (serum prothrombin conversion accelerator)  PLEK - pleckstrin |
| GO:0072521 | purine-containing compound metabolic process | 1.96E-8 | 3.42E-6 | 2.35 (10334,316,667,48) | [+] Show genes  HSD17B8 - hydroxysteroid (17-beta) dehydrogenase 8  UQCRC1 - ubiquinol-cytochrome c reductase core protein i  MLYCD - malonyl-coa decarboxylase  PGM1 - phosphoglucomutase 1  MACROD1 - macro domain containing 1  SHMT1 - serine hydroxymethyltransferase 1 (soluble)  COX5B - cytochrome c oxidase subunit vb  PGAM2 - phosphoglycerate mutase 2 (muscle)  ADSSL1 - adenylosuccinate synthase like 1  ALDOB - aldolase b, fructose-bisphosphate  MCEE - methylmalonyl coa epimerase  ACSL1 - acyl-coa synthetase long-chain family member 1  ENPP1 - ectonucleotide pyrophosphatase/phosphodiesterase 1  MTHFD1 - methylenetetrahydrofolate dehydrogenase (nadp+ dependent) 1, methenyltetrahydrofolate cyclohydrolase, formyltetrahydrofolate synthetase  ENO3 - enolase 3 (beta, muscle)  CHCHD10 - coiled-coil-helix-coiled-coil-helix domain containing 10  ADA - adenosine deaminase  LDHA - lactate dehydrogenase a  SLC25A13 - solute carrier family 25 (aspartate/glutamate carrier), member 13  PTPLA - protein tyrosine phosphatase-like (proline instead of catalytic arginine), member a  MPC1 - mitochondrial pyruvate carrier 1  ACSM2A - acyl-coa synthetase medium-chain family member 2a  PKLR - pyruvate kinase, liver and rbc  AK2 - adenylate kinase 2  TTR - transthyretin  ATP5J2 - atp synthase, h+ transporting, mitochondrial fo complex, subunit f2  GLYAT - glycine-n-acyltransferase  GCDH - glutaryl-coa dehydrogenase  SULT2A1 - sulfotransferase family, cytosolic, 2a, dehydroepiandrosterone (dhea)-preferring, member 1  AHCY - adenosylhomocysteinase  ATP5I - atp synthase, h+ transporting, mitochondrial fo complex, subunit e  ATP5H - atp synthase, h+ transporting, mitochondrial fo complex, subunit d  GIMAP7 - gtpase, imap family member 7  AMPD1 - adenosine monophosphate deaminase 1  TDO2 - tryptophan 2,3-dioxygenase  DGAT2 - diacylglycerol o-acyltransferase 2  ATP5E - atp synthase, h+ transporting, mitochondrial f1 complex, epsilon subunit  ATP5D - atp synthase, h+ transporting, mitochondrial f1 complex, delta subunit  PIPOX - pipecolic acid oxidase  ATP5G1 - atp synthase, h+ transporting, mitochondrial fo complex, subunit c1 (subunit 9)  HMGCS2 - 3-hydroxy-3-methylglutaryl-coa synthase 2 (mitochondrial)  SLC17A1 - solute carrier family 17 (organic anion transporter), member 1  G6PC - glucose-6-phosphatase, catalytic subunit  ACSM3 - acyl-coa synthetase medium-chain family member 3  PFKFB1 - 6-phosphofructo-2-kinase/fructose-2,6-biphosphatase 1  MOCS1 - molybdenum cofactor synthesis 1  URAD - ureidoimidazoline (2-oxo-4-hydroxy-4-carboxy-5-) decarboxylase  ACSM5 - acyl-coa synthetase medium-chain family member 5 |
| GO:0015718 | monocarboxylic acid transport | 2.02E-8 | 3.47E-6 | 4.07 (10334,80,667,21) | [+] Show genes  CPT2 - carnitine palmitoyltransferase 2  SLCO1B3 - solute carrier organic anion transporter family, member 1b3  AKR1C4 - aldo-keto reductase family 1, member c4  CPT1B - carnitine palmitoyltransferase 1b (muscle)  AQP9 - aquaporin 9  CD36 - cd36 molecule (thrombospondin receptor)  SLC25A20 - solute carrier family 25 (carnitine/acylcarnitine translocase), member 20  SLC16A13 - solute carrier family 16, member 13  SLC51B - solute carrier family 51, beta subunit  FABP1 - fatty acid binding protein 1, liver  RXRA - retinoid x receptor, alpha  SLC16A5 - solute carrier family 16 (monocarboxylate transporter), member 5  ACSL1 - acyl-coa synthetase long-chain family member 1  PLA2G12B - phospholipase a2, group xiib  SLC10A1 - solute carrier family 10 (sodium/bile acid cotransporter), member 1  GOT2 - glutamic-oxaloacetic transaminase 2, mitochondrial  SLC27A2 - solute carrier family 27 (fatty acid transporter), member 2  THRSP - thyroid hormone responsive  PPARA - peroxisome proliferator-activated receptor alpha  SLC27A5 - solute carrier family 27 (fatty acid transporter), member 5  MPC1 - mitochondrial pyruvate carrier 1 |
| GO:0016101 | diterpenoid metabolic process | 2.14E-8 | 3.64E-6 | 4.65 (10334,60,667,18) | [+] Show genes  DHRS3 - dehydrogenase/reductase (sdr family) member 3  AKR1C3 - aldo-keto reductase family 1, member c3  TTR - transthyretin  AKR1C4 - aldo-keto reductase family 1, member c4  APOA2 - apolipoprotein a-ii  ALDH8A1 - aldehyde dehydrogenase 8 family, member a1  ADH6 - alcohol dehydrogenase 6 (class v)  CYP3A5 - cytochrome p450, family 3, subfamily a, polypeptide 5  ADH4 - alcohol dehydrogenase 4 (class ii), pi polypeptide  APOA4 - apolipoprotein a-iv  DGAT2 - diacylglycerol o-acyltransferase 2  APOC2 - apolipoprotein c-ii  RDH16 - retinol dehydrogenase 16 (all-trans)  SDC2 - syndecan 2  APOC3 - apolipoprotein c-iii  APOM - apolipoprotein m  CYP1A2 - cytochrome p450, family 1, subfamily a, polypeptide 2  PNPLA2 - patatin-like phospholipase domain containing 2 |
| GO:0006625 | protein targeting to peroxisome | 2.86E-8 | 4.79E-6 | 4.57 (10334,61,667,18) | [+] Show genes  ECI2 - enoyl-coa delta isomerase 2  ACOX2 - acyl-coa oxidase 2, branched chain  MLYCD - malonyl-coa decarboxylase  DHRS4 - dehydrogenase/reductase (sdr family) member 4  UBA52 - ubiquitin a-52 residue ribosomal protein fusion product 1  CAT - catalase  AGXT - alanine-glyoxylate aminotransferase  EHHADH - enoyl-coa, hydratase/3-hydroxyacyl coa dehydrogenase  AMACR - alpha-methylacyl-coa racemase  PHYH - phytanoyl-coa 2-hydroxylase  HACL1 - 2-hydroxyacyl-coa lyase 1  PIPOX - pipecolic acid oxidase  SCP2 - sterol carrier protein 2  SLC27A2 - solute carrier family 27 (fatty acid transporter), member 2  ACAA1 - acetyl-coa acyltransferase 1  DECR2 - 2,4-dienoyl coa reductase 2, peroxisomal  ECH1 - enoyl coa hydratase 1, peroxisomal  HAO2 - hydroxyacid oxidase 2 (long chain) |
| GO:0072663 | establishment of protein localization to peroxisome | 2.86E-8 | 4.74E-6 | 4.57 (10334,61,667,18) | [+] Show genes  ECI2 - enoyl-coa delta isomerase 2  ACOX2 - acyl-coa oxidase 2, branched chain  MLYCD - malonyl-coa decarboxylase  DHRS4 - dehydrogenase/reductase (sdr family) member 4  UBA52 - ubiquitin a-52 residue ribosomal protein fusion product 1  CAT - catalase  AGXT - alanine-glyoxylate aminotransferase  EHHADH - enoyl-coa, hydratase/3-hydroxyacyl coa dehydrogenase  AMACR - alpha-methylacyl-coa racemase  PHYH - phytanoyl-coa 2-hydroxylase  HACL1 - 2-hydroxyacyl-coa lyase 1  PIPOX - pipecolic acid oxidase  SCP2 - sterol carrier protein 2  SLC27A2 - solute carrier family 27 (fatty acid transporter), member 2  ACAA1 - acetyl-coa acyltransferase 1  DECR2 - 2,4-dienoyl coa reductase 2, peroxisomal  HAO2 - hydroxyacid oxidase 2 (long chain)  ECH1 - enoyl coa hydratase 1, peroxisomal |
| GO:0072662 | protein localization to peroxisome | 2.86E-8 | 4.68E-6 | 4.57 (10334,61,667,18) | [+] Show genes  ECI2 - enoyl-coa delta isomerase 2  ACOX2 - acyl-coa oxidase 2, branched chain  MLYCD - malonyl-coa decarboxylase  DHRS4 - dehydrogenase/reductase (sdr family) member 4  UBA52 - ubiquitin a-52 residue ribosomal protein fusion product 1  CAT - catalase  AGXT - alanine-glyoxylate aminotransferase  EHHADH - enoyl-coa, hydratase/3-hydroxyacyl coa dehydrogenase  AMACR - alpha-methylacyl-coa racemase  PHYH - phytanoyl-coa 2-hydroxylase  HACL1 - 2-hydroxyacyl-coa lyase 1  PIPOX - pipecolic acid oxidase  SCP2 - sterol carrier protein 2  SLC27A2 - solute carrier family 27 (fatty acid transporter), member 2  ACAA1 - acetyl-coa acyltransferase 1  DECR2 - 2,4-dienoyl coa reductase 2, peroxisomal  ECH1 - enoyl coa hydratase 1, peroxisomal  HAO2 - hydroxyacid oxidase 2 (long chain) |
| GO:0043574 | peroxisomal transport | 3.79E-8 | 6.13E-6 | 4.50 (10334,62,667,18) | [+] Show genes  ECI2 - enoyl-coa delta isomerase 2  ACOX2 - acyl-coa oxidase 2, branched chain  MLYCD - malonyl-coa decarboxylase  DHRS4 - dehydrogenase/reductase (sdr family) member 4  UBA52 - ubiquitin a-52 residue ribosomal protein fusion product 1  CAT - catalase  AGXT - alanine-glyoxylate aminotransferase  EHHADH - enoyl-coa, hydratase/3-hydroxyacyl coa dehydrogenase  AMACR - alpha-methylacyl-coa racemase  PHYH - phytanoyl-coa 2-hydroxylase  HACL1 - 2-hydroxyacyl-coa lyase 1  PIPOX - pipecolic acid oxidase  SCP2 - sterol carrier protein 2  SLC27A2 - solute carrier family 27 (fatty acid transporter), member 2  ACAA1 - acetyl-coa acyltransferase 1  DECR2 - 2,4-dienoyl coa reductase 2, peroxisomal  HAO2 - hydroxyacid oxidase 2 (long chain)  ECH1 - enoyl coa hydratase 1, peroxisomal |
| GO:1903035 | negative regulation of response to wounding | 4.99E-8 | 7.98E-6 | 4.43 (10334,63,667,18) | [+] Show genes  F11 - coagulation factor xi  FGG - fibrinogen gamma chain  APCS - amyloid p component, serum  F12 - coagulation factor xii (hageman factor)  MYOZ1 - myozenin 1  PROC - protein c (inactivator of coagulation factors va and viiia)  CPB2 - carboxypeptidase b2 (plasma)  ADTRP - androgen-dependent tfpi-regulating protein  FGA - fibrinogen alpha chain  HRG - histidine-rich glycoprotein  KNG1 - kininogen 1  FGB - fibrinogen beta chain  APOH - apolipoprotein h (beta-2-glycoprotein i)  PLG - plasminogen  F2 - coagulation factor ii (thrombin)  VTN - vitronectin  KLKB1 - kallikrein b, plasma (fletcher factor) 1  SERPINF2 - serpin peptidase inhibitor, clade f (alpha-2 antiplasmin, pigment epithelium derived factor), member 2 |
| GO:0070613 | regulation of protein processing | 5.11E-8 | 8.08E-6 | 3.87 (10334,84,667,21) | [+] Show genes  C19orf80 - chromosome 19 open reading frame 80  C9 - complement component 9  F12 - coagulation factor xii (hageman factor)  CFB - complement factor b  CPN2 - carboxypeptidase n, polypeptide 2  HPN - hepsin  C1R - complement component 1, r subcomponent  C2 - complement component 2  CPB2 - carboxypeptidase b2 (plasma)  C4BPA - complement component 4 binding protein, alpha  C4BPB - complement component 4 binding protein, beta  F2 - coagulation factor ii (thrombin)  C5 - complement component 5  VTN - vitronectin  C6 - complement component 6  KLKB1 - kallikrein b, plasma (fletcher factor) 1  C8B - complement component 8, beta polypeptide  SERPINF2 - serpin peptidase inhibitor, clade f (alpha-2 antiplasmin, pigment epithelium derived factor), member 2  C8A - complement component 8, alpha polypeptide  CD5L - cd5 molecule-like  C8G - complement component 8, gamma polypeptide |
| GO:0009074 | aromatic amino acid family catabolic process | 6.37E-8 | 9.96E-6 | 8.15 (10334,19,667,10) | [+] Show genes  HGD - homogentisate 1,2-dioxygenase  TDO2 - tryptophan 2,3-dioxygenase  TAT - tyrosine aminotransferase  FAH - fumarylacetoacetate hydrolase (fumarylacetoacetase)  PAH - phenylalanine hydroxylase  FTCD - formimidoyltransferase cyclodeaminase  GSTZ1 - glutathione s-transferase zeta 1  CCBL1 - cysteine conjugate-beta lyase, cytoplasmic  HAAO - 3-hydroxyanthranilate 3,4-dioxygenase  HPD - 4-hydroxyphenylpyruvate dioxygenase |
| GO:0015980 | energy derivation by oxidation of organic compounds | 6.38E-8 | 9.87E-6 | 3.56 (10334,100,667,23) | [+] Show genes  UQCRC1 - ubiquinol-cytochrome c reductase core protein i  COX6A2 - cytochrome c oxidase subunit via polypeptide 2  UQCR10 - ubiquinol-cytochrome c reductase, complex iii subunit x  PGM1 - phosphoglucomutase 1  CAT - catalase  NDUFA4 - nadh dehydrogenase (ubiquinone) 1 alpha subcomplex, 4, 9kda  NDUFS7 - nadh dehydrogenase (ubiquinone) fe-s protein 7, 20kda (nadh-coenzyme q reductase)  COX5B - cytochrome c oxidase subunit vb  SDHB - succinate dehydrogenase complex, subunit b, iron sulfur (ip)  PPP1R3C - protein phosphatase 1, regulatory subunit 3c  STBD1 - starch binding domain 1  ATP5D - atp synthase, h+ transporting, mitochondrial f1 complex, delta subunit  PPARGC1A - peroxisome proliferator-activated receptor gamma, coactivator 1 alpha  GYS2 - glycogen synthase 2 (liver)  NDUFS8 - nadh dehydrogenase (ubiquinone) fe-s protein 8, 23kda (nadh-coenzyme q reductase)  PPP1R3B - protein phosphatase 1, regulatory subunit 3b  SLC25A13 - solute carrier family 25 (aspartate/glutamate carrier), member 13  COX7C - cytochrome c oxidase subunit viic  CYP1A2 - cytochrome p450, family 1, subfamily a, polypeptide 2  G6PC - glucose-6-phosphatase, catalytic subunit  PPP1R3A - protein phosphatase 1, regulatory subunit 3a  ACADVL - acyl-coa dehydrogenase, very long chain  BLOC1S1 - biogenesis of lysosomal organelles complex-1, subunit 1 |
| GO:0030258 | lipid modification | 8.24E-8 | 1.26E-5 | 2.92 (10334,159,667,30) | [+] Show genes  ECI2 - enoyl-coa delta isomerase 2  ACOX2 - acyl-coa oxidase 2, branched chain  MLYCD - malonyl-coa decarboxylase  ACAA2 - acetyl-coa acyltransferase 2  CYP2E1 - cytochrome p450, family 2, subfamily e, polypeptide 1  CYP3A5 - cytochrome p450, family 3, subfamily a, polypeptide 5  AMACR - alpha-methylacyl-coa racemase  BDH2 - 3-hydroxybutyrate dehydrogenase, type 2  PPARGC1A - peroxisome proliferator-activated receptor gamma, coactivator 1 alpha  HADH - hydroxyacyl-coa dehydrogenase  SCP2 - sterol carrier protein 2  IMPA2 - inositol(myo)-1(or 4)-monophosphatase 2  ACAA1 - acetyl-coa acyltransferase 1  ACADL - acyl-coa dehydrogenase, long chain  ACADS - acyl-coa dehydrogenase, c-2 to c-3 short chain  ETFDH - electron-transferring-flavoprotein dehydrogenase  ACADVL - acyl-coa dehydrogenase, very long chain  CPT2 - carnitine palmitoyltransferase 2  CPT1B - carnitine palmitoyltransferase 1b (muscle)  ECHDC2 - enoyl coa hydratase domain containing 2  GCDH - glutaryl-coa dehydrogenase  EHHADH - enoyl-coa, hydratase/3-hydroxyacyl coa dehydrogenase  ADH4 - alcohol dehydrogenase 4 (class ii), pi polypeptide  PHYH - phytanoyl-coa 2-hydroxylase  HACL1 - 2-hydroxyacyl-coa lyase 1  SLC27A2 - solute carrier family 27 (fatty acid transporter), member 2  ECHS1 - enoyl coa hydratase, short chain, 1, mitochondrial  DECR2 - 2,4-dienoyl coa reductase 2, peroxisomal  HAO2 - hydroxyacid oxidase 2 (long chain)  ECH1 - enoyl coa hydratase 1, peroxisomal |
| GO:0006094 | gluconeogenesis | 9.17E-8 | 1.39E-5 | 5.42 (10334,40,667,14) | [+] Show genes  SLC37A4 - solute carrier family 37 (glucose-6-phosphate transporter), member 4  PCK1 - phosphoenolpyruvate carboxykinase 1 (soluble)  PGM1 - phosphoglucomutase 1  PGAM2 - phosphoglycerate mutase 2 (muscle)  ALDOB - aldolase b, fructose-bisphosphate  PPARGC1A - peroxisome proliferator-activated receptor gamma, coactivator 1 alpha  ENO3 - enolase 3 (beta, muscle)  ATF4 - activating transcription factor 4  GOT2 - glutamic-oxaloacetic transaminase 2, mitochondrial  SLC25A13 - solute carrier family 25 (aspartate/glutamate carrier), member 13  FBP2 - fructose-1,6-bisphosphatase 2  GPD1 - glycerol-3-phosphate dehydrogenase 1 (soluble)  G6PC - glucose-6-phosphatase, catalytic subunit  PFKFB1 - 6-phosphofructo-2-kinase/fructose-2,6-biphosphatase 1 |
| GO:0043648 | dicarboxylic acid metabolic process | 1.11E-7 | 1.66E-5 | 4.03 (10334,73,667,19) | [+] Show genes  PCK1 - phosphoenolpyruvate carboxykinase 1 (soluble)  FTCD - formimidoyltransferase cyclodeaminase  SHMT1 - serine hydroxymethyltransferase 1 (soluble)  SDHB - succinate dehydrogenase complex, subunit b, iron sulfur (ip)  ALDH1L1 - aldehyde dehydrogenase 1 family, member l1  ASS1 - argininosuccinate synthase 1  PHYH - phytanoyl-coa 2-hydroxylase  ADSSL1 - adenylosuccinate synthase like 1  MTHFR - methylenetetrahydrofolate reductase (nad(p)h)  PRODH2 - proline dehydrogenase (oxidase) 2  MTHFD1 - methylenetetrahydrofolate dehydrogenase (nadp+ dependent) 1, methenyltetrahydrofolate cyclohydrolase, formyltetrahydrofolate synthetase  GOT2 - glutamic-oxaloacetic transaminase 2, mitochondrial  GRHPR - glyoxylate reductase/hydroxypyruvate reductase  TAT - tyrosine aminotransferase  ALDH4A1 - aldehyde dehydrogenase 4 family, member a1  ADHFE1 - alcohol dehydrogenase, iron containing, 1  FH - fumarate hydratase  GGT1 - gamma-glutamyltransferase 1  HAAO - 3-hydroxyanthranilate 3,4-dioxygenase |
| GO:0019835 | cytolysis | 1.13E-7 | 1.68E-5 | 10.33 (10334,12,667,8) | [+] Show genes  C9 - complement component 9  HRG - histidine-rich glycoprotein  F2 - coagulation factor ii (thrombin)  C5 - complement component 5  C6 - complement component 6  C8B - complement component 8, beta polypeptide  C8A - complement component 8, alpha polypeptide  C8G - complement component 8, gamma polypeptide |
| GO:0030239 | myofibril assembly | 1.44E-7 | 2.12E-5 | 6.82 (10334,25,667,11) | [+] Show genes  MYL2 - myosin, light chain 2, regulatory, cardiac, slow  KLHL41 - kelch-like family member 41  TMOD4 - tropomodulin 4 (muscle)  CAPN3 - calpain 3, (p94)  CSRP3 - cysteine and glycine-rich protein 3 (cardiac lim protein)  LMOD1 - leiomodin 1 (smooth muscle)  TCAP - titin-cap  MYOZ1 - myozenin 1  LMOD3 - leiomodin 3 (fetal)  TTN - titin  LMOD2 - leiomodin 2 (cardiac) |
| GO:0006570 | tyrosine metabolic process | 1.45E-7 | 2.11E-5 | 12.05 (10334,9,667,7) | [+] Show genes  HGD - homogentisate 1,2-dioxygenase  IYD - iodotyrosine deiodinase  TAT - tyrosine aminotransferase  FAH - fumarylacetoacetate hydrolase (fumarylacetoacetase)  PAH - phenylalanine hydroxylase  GSTZ1 - glutathione s-transferase zeta 1  HPD - 4-hydroxyphenylpyruvate dioxygenase |
| GO:1903034 | regulation of response to wounding | 1.62E-7 | 2.33E-5 | 3.20 (10334,121,667,25) | [+] Show genes  F11 - coagulation factor xi  FGG - fibrinogen gamma chain  APCS - amyloid p component, serum  CAPN3 - calpain 3, (p94)  F12 - coagulation factor xii (hageman factor)  SCARF1 - scavenger receptor class f, member 1  PROC - protein c (inactivator of coagulation factors va and viiia)  ADTRP - androgen-dependent tfpi-regulating protein  FGA - fibrinogen alpha chain  HRG - histidine-rich glycoprotein  FGB - fibrinogen beta chain  SERPINC1 - serpin peptidase inhibitor, clade c (antithrombin), member 1  HRAS - harvey rat sarcoma viral oncogene homolog  CD36 - cd36 molecule (thrombospondin receptor)  MYOZ1 - myozenin 1  CPB2 - carboxypeptidase b2 (plasma)  KNG1 - kininogen 1  APOH - apolipoprotein h (beta-2-glycoprotein i)  PLG - plasminogen  F2 - coagulation factor ii (thrombin)  VTN - vitronectin  KLKB1 - kallikrein b, plasma (fletcher factor) 1  SERPINF2 - serpin peptidase inhibitor, clade f (alpha-2 antiplasmin, pigment epithelium derived factor), member 2  F7 - coagulation factor vii (serum prothrombin conversion accelerator)  PLEK - pleckstrin |
| GO:0034308 | primary alcohol metabolic process | 1.78E-7 | 2.52E-5 | 4.84 (10334,48,667,15) | [+] Show genes  DHRS3 - dehydrogenase/reductase (sdr family) member 3  AKR1C3 - aldo-keto reductase family 1, member c3  TTR - transthyretin  AKR1C4 - aldo-keto reductase family 1, member c4  IGF1 - insulin-like growth factor 1 (somatomedin c)  SULT2A1 - sulfotransferase family, cytosolic, 2a, dehydroepiandrosterone (dhea)-preferring, member 1  ADH6 - alcohol dehydrogenase 6 (class v)  CYP3A5 - cytochrome p450, family 3, subfamily a, polypeptide 5  ADH4 - alcohol dehydrogenase 4 (class ii), pi polypeptide  DGAT2 - diacylglycerol o-acyltransferase 2  RDH16 - retinol dehydrogenase 16 (all-trans)  CYP1A2 - cytochrome p450, family 1, subfamily a, polypeptide 2  GPD1 - glycerol-3-phosphate dehydrogenase 1 (soluble)  ALDH2 - aldehyde dehydrogenase 2 family (mitochondrial)  PNPLA2 - patatin-like phospholipase domain containing 2 |
| GO:0007031 | peroxisome organization | 1.81E-7 | 2.54E-5 | 4.10 (10334,68,667,18) | [+] Show genes  ECI2 - enoyl-coa delta isomerase 2  ACOX2 - acyl-coa oxidase 2, branched chain  MLYCD - malonyl-coa decarboxylase  DHRS4 - dehydrogenase/reductase (sdr family) member 4  UBA52 - ubiquitin a-52 residue ribosomal protein fusion product 1  CAT - catalase  AGXT - alanine-glyoxylate aminotransferase  EHHADH - enoyl-coa, hydratase/3-hydroxyacyl coa dehydrogenase  AMACR - alpha-methylacyl-coa racemase  PHYH - phytanoyl-coa 2-hydroxylase  HACL1 - 2-hydroxyacyl-coa lyase 1  PIPOX - pipecolic acid oxidase  SCP2 - sterol carrier protein 2  SLC27A2 - solute carrier family 27 (fatty acid transporter), member 2  ACAA1 - acetyl-coa acyltransferase 1  DECR2 - 2,4-dienoyl coa reductase 2, peroxisomal  HAO2 - hydroxyacid oxidase 2 (long chain)  ECH1 - enoyl coa hydratase 1, peroxisomal |
| GO:0050878 | regulation of body fluid levels | 2.12E-7 | 2.95E-5 | 2.52 (10334,221,667,36) | [+] Show genes  F11 - coagulation factor xi  F10 - coagulation factor x  CYP4A11 - cytochrome p450, family 4, subfamily a, polypeptide 11  FGG - fibrinogen gamma chain  F9 - coagulation factor ix  F13B - coagulation factor xiii, b polypeptide  F12 - coagulation factor xii (hageman factor)  SERPINA10 - serpin peptidase inhibitor, clade a (alpha-1 antiproteinase, antitrypsin), member 10  PROC - protein c (inactivator of coagulation factors va and viiia)  SLC29A1 - solute carrier family 29 (equilibrative nucleoside transporter), member 1  BTC - betacellulin  GOT2 - glutamic-oxaloacetic transaminase 2, mitochondrial  ADTRP - androgen-dependent tfpi-regulating protein  FGA - fibrinogen alpha chain  HRG - histidine-rich glycoprotein  FGB - fibrinogen beta chain  ADA - adenosine deaminase  SERPINC1 - serpin peptidase inhibitor, clade c (antithrombin), member 1  SERPINA5 - serpin peptidase inhibitor, clade a (alpha-1 antiproteinase, antitrypsin), member 5  SERPIND1 - serpin peptidase inhibitor, clade d (heparin cofactor), member 1  P2RY2 - purinergic receptor p2y, g-protein coupled, 2  CD36 - cd36 molecule (thrombospondin receptor)  SERPINA1 - serpin peptidase inhibitor, clade a (alpha-1 antiproteinase, antitrypsin), member 1  CPB2 - carboxypeptidase b2 (plasma)  PAFAH2 - platelet-activating factor acetylhydrolase 2, 40kda  PROZ - protein z, vitamin k-dependent plasma glycoprotein  KNG1 - kininogen 1  APOH - apolipoprotein h (beta-2-glycoprotein i)  C4BPB - complement component 4 binding protein, beta  PLG - plasminogen  F2 - coagulation factor ii (thrombin)  VTN - vitronectin  KLKB1 - kallikrein b, plasma (fletcher factor) 1  SERPINF2 - serpin peptidase inhibitor, clade f (alpha-2 antiplasmin, pigment epithelium derived factor), member 2  F7 - coagulation factor vii (serum prothrombin conversion accelerator)  PLEK - pleckstrin |
| GO:0006638 | neutral lipid metabolic process | 2.28E-7 | 3.14E-5 | 4.25 (10334,62,667,17) | [+] Show genes  APOA5 - apolipoprotein a-v  CPS1 - carbamoyl-phosphate synthase 1, mitochondrial  APOA2 - apolipoprotein a-ii  CAT - catalase  CYP2E1 - cytochrome p450, family 2, subfamily e, polypeptide 1  FABP1 - fatty acid binding protein 1, liver  LPIN1 - lipin 1  CAV3 - caveolin 3  DGAT2 - diacylglycerol o-acyltransferase 2  ACSL1 - acyl-coa synthetase long-chain family member 1  APOC3 - apolipoprotein c-iii  LIPC - lipase, hepatic  GK5 - glycerol kinase 5 (putative)  APOH - apolipoprotein h (beta-2-glycoprotein i)  G6PC - glucose-6-phosphatase, catalytic subunit  SLC27A5 - solute carrier family 27 (fatty acid transporter), member 5  PNPLA2 - patatin-like phospholipase domain containing 2 |
| GO:0006639 | acylglycerol metabolic process | 2.28E-7 | 3.11E-5 | 4.25 (10334,62,667,17) | [+] Show genes  APOA5 - apolipoprotein a-v  CPS1 - carbamoyl-phosphate synthase 1, mitochondrial  APOA2 - apolipoprotein a-ii  CAT - catalase  CYP2E1 - cytochrome p450, family 2, subfamily e, polypeptide 1  FABP1 - fatty acid binding protein 1, liver  LPIN1 - lipin 1  CAV3 - caveolin 3  DGAT2 - diacylglycerol o-acyltransferase 2  ACSL1 - acyl-coa synthetase long-chain family member 1  APOC3 - apolipoprotein c-iii  LIPC - lipase, hepatic  GK5 - glycerol kinase 5 (putative)  APOH - apolipoprotein h (beta-2-glycoprotein i)  G6PC - glucose-6-phosphatase, catalytic subunit  SLC27A5 - solute carrier family 27 (fatty acid transporter), member 5  PNPLA2 - patatin-like phospholipase domain containing 2 |
| GO:0008652 | cellular amino acid biosynthetic process | 2.3E-7 | 3.11E-5 | 4.04 (10334,69,667,18) | [+] Show genes  CPS1 - carbamoyl-phosphate synthase 1, mitochondrial  GADL1 - glutamate decarboxylase-like 1  AGXT2 - alanine--glyoxylate aminotransferase 2  SHMT1 - serine hydroxymethyltransferase 1 (soluble)  GAMT - guanidinoacetate n-methyltransferase  AGXT - alanine-glyoxylate aminotransferase  ASS1 - argininosuccinate synthase 1  BHMT - betaine--homocysteine s-methyltransferase  ADI1 - acireductone dioxygenase 1  MTHFR - methylenetetrahydrofolate reductase (nad(p)h)  MTHFD1 - methylenetetrahydrofolate dehydrogenase (nadp+ dependent) 1, methenyltetrahydrofolate cyclohydrolase, formyltetrahydrofolate synthetase  GOT2 - glutamic-oxaloacetic transaminase 2, mitochondrial  OTC - ornithine carbamoyltransferase  ASPG - asparaginase homolog (s. cerevisiae)  PAH - phenylalanine hydroxylase  GGT1 - gamma-glutamyltransferase 1  CCBL1 - cysteine conjugate-beta lyase, cytoplasmic  UPB1 - ureidopropionase, beta |
| GO:0019319 | hexose biosynthetic process | 2.58E-7 | 3.46E-5 | 5.04 (10334,43,667,14) | [+] Show genes  SLC37A4 - solute carrier family 37 (glucose-6-phosphate transporter), member 4  PCK1 - phosphoenolpyruvate carboxykinase 1 (soluble)  PGM1 - phosphoglucomutase 1  PGAM2 - phosphoglycerate mutase 2 (muscle)  ALDOB - aldolase b, fructose-bisphosphate  PPARGC1A - peroxisome proliferator-activated receptor gamma, coactivator 1 alpha  ENO3 - enolase 3 (beta, muscle)  ATF4 - activating transcription factor 4  GOT2 - glutamic-oxaloacetic transaminase 2, mitochondrial  SLC25A13 - solute carrier family 25 (aspartate/glutamate carrier), member 13  FBP2 - fructose-1,6-bisphosphatase 2  GPD1 - glycerol-3-phosphate dehydrogenase 1 (soluble)  G6PC - glucose-6-phosphatase, catalytic subunit  PFKFB1 - 6-phosphofructo-2-kinase/fructose-2,6-biphosphatase 1 |
| GO:0055002 | striated muscle cell development | 3.24E-7 | 4.29E-5 | 4.65 (10334,50,667,15) | [+] Show genes  KLHL41 - kelch-like family member 41  STAC3 - sh3 and cysteine rich domain 3  IGF1 - insulin-like growth factor 1 (somatomedin c)  ACTN2 - actinin, alpha 2  CAV3 - caveolin 3  TTN - titin  RYR1 - ryanodine receptor 1 (skeletal)  NEB - nebulin  NRAP - nebulin-related anchoring protein  MYL2 - myosin, light chain 2, regulatory, cardiac, slow  ACTA1 - actin, alpha 1, skeletal muscle  TCAP - titin-cap  KLHL40 - kelch-like family member 40  LMOD3 - leiomodin 3 (fetal)  MYO18B - myosin xviiib |
| GO:0050817 | coagulation | 3.42E-7 | 4.49E-5 | 3.37 (10334,101,667,22) | [+] Show genes  F11 - coagulation factor xi  SERPIND1 - serpin peptidase inhibitor, clade d (heparin cofactor), member 1  F10 - coagulation factor x  F9 - coagulation factor ix  FGG - fibrinogen gamma chain  F13B - coagulation factor xiii, b polypeptide  F12 - coagulation factor xii (hageman factor)  CD36 - cd36 molecule (thrombospondin receptor)  PROC - protein c (inactivator of coagulation factors va and viiia)  SERPINA10 - serpin peptidase inhibitor, clade a (alpha-1 antiproteinase, antitrypsin), member 10  SERPINA1 - serpin peptidase inhibitor, clade a (alpha-1 antiproteinase, antitrypsin), member 1  CPB2 - carboxypeptidase b2 (plasma)  PAFAH2 - platelet-activating factor acetylhydrolase 2, 40kda  PROZ - protein z, vitamin k-dependent plasma glycoprotein  FGA - fibrinogen alpha chain  FGB - fibrinogen beta chain  C4BPB - complement component 4 binding protein, beta  F2 - coagulation factor ii (thrombin)  SERPINC1 - serpin peptidase inhibitor, clade c (antithrombin), member 1  PLG - plasminogen  SERPINA5 - serpin peptidase inhibitor, clade a (alpha-1 antiproteinase, antitrypsin), member 5  F7 - coagulation factor vii (serum prothrombin conversion accelerator) |
| GO:0007596 | blood coagulation | 3.42E-7 | 4.44E-5 | 3.37 (10334,101,667,22) | [+] Show genes  F11 - coagulation factor xi  SERPIND1 - serpin peptidase inhibitor, clade d (heparin cofactor), member 1  F10 - coagulation factor x  F9 - coagulation factor ix  FGG - fibrinogen gamma chain  F13B - coagulation factor xiii, b polypeptide  F12 - coagulation factor xii (hageman factor)  CD36 - cd36 molecule (thrombospondin receptor)  PROC - protein c (inactivator of coagulation factors va and viiia)  SERPINA10 - serpin peptidase inhibitor, clade a (alpha-1 antiproteinase, antitrypsin), member 10  SERPINA1 - serpin peptidase inhibitor, clade a (alpha-1 antiproteinase, antitrypsin), member 1  CPB2 - carboxypeptidase b2 (plasma)  PAFAH2 - platelet-activating factor acetylhydrolase 2, 40kda  PROZ - protein z, vitamin k-dependent plasma glycoprotein  FGA - fibrinogen alpha chain  FGB - fibrinogen beta chain  C4BPB - complement component 4 binding protein, beta  F2 - coagulation factor ii (thrombin)  SERPINC1 - serpin peptidase inhibitor, clade c (antithrombin), member 1  PLG - plasminogen  SERPINA5 - serpin peptidase inhibitor, clade a (alpha-1 antiproteinase, antitrypsin), member 5  F7 - coagulation factor vii (serum prothrombin conversion accelerator) |
| GO:1900048 | positive regulation of hemostasis | 3.73E-7 | 4.81E-5 | 7.04 (10334,22,667,10) | [+] Show genes  HRG - histidine-rich glycoprotein  APOH - apolipoprotein h (beta-2-glycoprotein i)  F12 - coagulation factor xii (hageman factor)  CD36 - cd36 molecule (thrombospondin receptor)  PLG - plasminogen  F2 - coagulation factor ii (thrombin)  SERPINF2 - serpin peptidase inhibitor, clade f (alpha-2 antiplasmin, pigment epithelium derived factor), member 2  F7 - coagulation factor vii (serum prothrombin conversion accelerator)  PLEK - pleckstrin  CPB2 - carboxypeptidase b2 (plasma) |
| GO:0030194 | positive regulation of blood coagulation | 3.73E-7 | 4.76E-5 | 7.04 (10334,22,667,10) | [+] Show genes  HRG - histidine-rich glycoprotein  APOH - apolipoprotein h (beta-2-glycoprotein i)  F12 - coagulation factor xii (hageman factor)  CD36 - cd36 molecule (thrombospondin receptor)  F2 - coagulation factor ii (thrombin)  PLG - plasminogen  SERPINF2 - serpin peptidase inhibitor, clade f (alpha-2 antiplasmin, pigment epithelium derived factor), member 2  F7 - coagulation factor vii (serum prothrombin conversion accelerator)  PLEK - pleckstrin  CPB2 - carboxypeptidase b2 (plasma) |
| GO:0006558 | L-phenylalanine metabolic process | 4.58E-7 | 5.79E-5 | 10.85 (10334,10,667,7) | [+] Show genes  HGD - homogentisate 1,2-dioxygenase  TAT - tyrosine aminotransferase  FAH - fumarylacetoacetate hydrolase (fumarylacetoacetase)  PAH - phenylalanine hydroxylase  GSTZ1 - glutathione s-transferase zeta 1  CCBL1 - cysteine conjugate-beta lyase, cytoplasmic  HPD - 4-hydroxyphenylpyruvate dioxygenase |
| GO:0006559 | L-phenylalanine catabolic process | 4.58E-7 | 5.74E-5 | 10.85 (10334,10,667,7) | [+] Show genes  HGD - homogentisate 1,2-dioxygenase  TAT - tyrosine aminotransferase  FAH - fumarylacetoacetate hydrolase (fumarylacetoacetase)  PAH - phenylalanine hydroxylase  GSTZ1 - glutathione s-transferase zeta 1  CCBL1 - cysteine conjugate-beta lyase, cytoplasmic  HPD - 4-hydroxyphenylpyruvate dioxygenase |
| GO:1902221 | erythrose 4-phosphate/phosphoenolpyruvate family amino acid metabolic process | 4.58E-7 | 5.68E-5 | 10.85 (10334,10,667,7) | [+] Show genes  HGD - homogentisate 1,2-dioxygenase  TAT - tyrosine aminotransferase  FAH - fumarylacetoacetate hydrolase (fumarylacetoacetase)  PAH - phenylalanine hydroxylase  GSTZ1 - glutathione s-transferase zeta 1  CCBL1 - cysteine conjugate-beta lyase, cytoplasmic  HPD - 4-hydroxyphenylpyruvate dioxygenase |
| GO:1902222 | erythrose 4-phosphate/phosphoenolpyruvate family amino acid catabolic process | 4.58E-7 | 5.63E-5 | 10.85 (10334,10,667,7) | [+] Show genes  HGD - homogentisate 1,2-dioxygenase  TAT - tyrosine aminotransferase  FAH - fumarylacetoacetate hydrolase (fumarylacetoacetase)  PAH - phenylalanine hydroxylase  GSTZ1 - glutathione s-transferase zeta 1  CCBL1 - cysteine conjugate-beta lyase, cytoplasmic  HPD - 4-hydroxyphenylpyruvate dioxygenase |
| GO:0055086 | nucleobase-containing small molecule metabolic process | 5.46E-7 | 6.67E-5 | 1.95 (10334,460,667,58) | [+] Show genes  HSD17B8 - hydroxysteroid (17-beta) dehydrogenase 8  UQCRC1 - ubiquinol-cytochrome c reductase core protein i  MLYCD - malonyl-coa decarboxylase  PGM1 - phosphoglucomutase 1  COX5B - cytochrome c oxidase subunit vb  ENTPD8 - ectonucleoside triphosphate diphosphohydrolase 8  FMO1 - flavin containing monooxygenase 1  PGAM2 - phosphoglycerate mutase 2 (muscle)  MCEE - methylmalonyl coa epimerase  ACSL1 - acyl-coa synthetase long-chain family member 1  MTHFD1 - methylenetetrahydrofolate dehydrogenase (nadp+ dependent) 1, methenyltetrahydrofolate cyclohydrolase, formyltetrahydrofolate synthetase  ENO3 - enolase 3 (beta, muscle)  CHCHD10 - coiled-coil-helix-coiled-coil-helix domain containing 10  LDHA - lactate dehydrogenase a  SLC25A13 - solute carrier family 25 (aspartate/glutamate carrier), member 13  GPD1 - glycerol-3-phosphate dehydrogenase 1 (soluble)  ACSM2A - acyl-coa synthetase medium-chain family member 2a  DPYS - dihydropyrimidinase  AK2 - adenylate kinase 2  ASPDH - aspartate dehydrogenase domain containing  ATP5J2 - atp synthase, h+ transporting, mitochondrial fo complex, subunit f2  TTR - transthyretin  CPS1 - carbamoyl-phosphate synthase 1, mitochondrial  SULT2A1 - sulfotransferase family, cytosolic, 2a, dehydroepiandrosterone (dhea)-preferring, member 1  AHCY - adenosylhomocysteinase  ATP5I - atp synthase, h+ transporting, mitochondrial fo complex, subunit e  ATP5H - atp synthase, h+ transporting, mitochondrial fo complex, subunit d  TDO2 - tryptophan 2,3-dioxygenase  ATP5E - atp synthase, h+ transporting, mitochondrial f1 complex, epsilon subunit  PIPOX - pipecolic acid oxidase  ATP5D - atp synthase, h+ transporting, mitochondrial f1 complex, delta subunit  ATP5G1 - atp synthase, h+ transporting, mitochondrial fo complex, subunit c1 (subunit 9)  SMPDL3A - sphingomyelin phosphodiesterase, acid-like 3a  APOBEC2 - apolipoprotein b mrna editing enzyme, catalytic polypeptide-like 2  MOCS1 - molybdenum cofactor synthesis 1  PFKFB1 - 6-phosphofructo-2-kinase/fructose-2,6-biphosphatase 1  UPB1 - ureidopropionase, beta  ACSM5 - acyl-coa synthetase medium-chain family member 5  NMRK2 - nicotinamide riboside kinase 2  MACROD1 - macro domain containing 1  SHMT1 - serine hydroxymethyltransferase 1 (soluble)  ADSSL1 - adenylosuccinate synthase like 1  ALDOB - aldolase b, fructose-bisphosphate  ENPP1 - ectonucleotide pyrophosphatase/phosphodiesterase 1  ADA - adenosine deaminase  PTPLA - protein tyrosine phosphatase-like (proline instead of catalytic arginine), member a  MPC1 - mitochondrial pyruvate carrier 1  PKLR - pyruvate kinase, liver and rbc  HAAO - 3-hydroxyanthranilate 3,4-dioxygenase  GLYAT - glycine-n-acyltransferase  GCDH - glutaryl-coa dehydrogenase  GIMAP7 - gtpase, imap family member 7  AMPD1 - adenosine monophosphate deaminase 1  DGAT2 - diacylglycerol o-acyltransferase 2  HMGCS2 - 3-hydroxy-3-methylglutaryl-coa synthase 2 (mitochondrial)  AGPAT2 - 1-acylglycerol-3-phosphate o-acyltransferase 2  ACSM3 - acyl-coa synthetase medium-chain family member 3  URAD - ureidoimidazoline (2-oxo-4-hydroxy-4-carboxy-5-) decarboxylase |
| GO:0006820 | anion transport | 5.57E-7 | 6.74E-5 | 2.20 (10334,310,667,44) | [+] Show genes  APOA2 - apolipoprotein a-ii  SLC16A13 - solute carrier family 16, member 13  FABP1 - fatty acid binding protein 1, liver  APOA4 - apolipoprotein a-iv  PCTP - phosphatidylcholine transfer protein  SLC16A5 - solute carrier family 16 (monocarboxylate transporter), member 5  ACSL1 - acyl-coa synthetase long-chain family member 1  ENPP1 - ectonucleotide pyrophosphatase/phosphodiesterase 1  CA3 - carbonic anhydrase iii, muscle specific  GOT2 - glutamic-oxaloacetic transaminase 2, mitochondrial  SLC10A1 - solute carrier family 10 (sodium/bile acid cotransporter), member 1  SLC38A4 - solute carrier family 38, member 4  SCP2 - sterol carrier protein 2  CA14 - carbonic anhydrase xiv  SLC25A13 - solute carrier family 25 (aspartate/glutamate carrier), member 13  PPARA - peroxisome proliferator-activated receptor alpha  MPC1 - mitochondrial pyruvate carrier 1  CPT2 - carnitine palmitoyltransferase 2  FXYD1 - fxyd domain containing ion transport regulator 1  SLCO1B3 - solute carrier organic anion transporter family, member 1b3  SLC17A2 - solute carrier family 17, member 2  SLC37A4 - solute carrier family 37 (glucose-6-phosphate transporter), member 4  AKR1C4 - aldo-keto reductase family 1, member c4  SLC25A47 - solute carrier family 25, member 47  APOA5 - apolipoprotein a-v  CPT1B - carnitine palmitoyltransferase 1b (muscle)  AQP9 - aquaporin 9  CD36 - cd36 molecule (thrombospondin receptor)  SLC25A20 - solute carrier family 25 (carnitine/acylcarnitine translocase), member 20  SLC51B - solute carrier family 51, beta subunit  AGXT - alanine-glyoxylate aminotransferase  RXRA - retinoid x receptor, alpha  SLC25A30 - solute carrier family 25, member 30  APOC2 - apolipoprotein c-ii  APOC3 - apolipoprotein c-iii  PLA2G12B - phospholipase a2, group xiib  SLC27A2 - solute carrier family 27 (fatty acid transporter), member 2  SLC25A4 - solute carrier family 25 (mitochondrial carrier; adenine nucleotide translocator), member 4  SLC22A7 - solute carrier family 22 (organic anion transporter), member 7  ATP8B3 - atpase, aminophospholipid transporter, class i, type 8b, member 3  SLC17A1 - solute carrier family 17 (organic anion transporter), member 1  THRSP - thyroid hormone responsive  G6PC - glucose-6-phosphatase, catalytic subunit  SLC27A5 - solute carrier family 27 (fatty acid transporter), member 5 |
| GO:0072330 | monocarboxylic acid biosynthetic process | 5.68E-7 | 6.81E-5 | 2.80 (10334,155,667,28) | [+] Show genes  ACOX2 - acyl-coa oxidase 2, branched chain  HSD17B8 - hydroxysteroid (17-beta) dehydrogenase 8  CYP4A11 - cytochrome p450, family 4, subfamily a, polypeptide 11  MLYCD - malonyl-coa decarboxylase  CYP8B1 - cytochrome p450, family 8, subfamily b, polypeptide 1  PGM1 - phosphoglucomutase 1  ALDH8A1 - aldehyde dehydrogenase 8 family, member a1  CYP2E1 - cytochrome p450, family 2, subfamily e, polypeptide 1  AMACR - alpha-methylacyl-coa racemase  PGAM2 - phosphoglycerate mutase 2 (muscle)  ALDOB - aldolase b, fructose-bisphosphate  ENO3 - enolase 3 (beta, muscle)  SCP2 - sterol carrier protein 2  LDHA - lactate dehydrogenase a  CYP1A2 - cytochrome p450, family 1, subfamily a, polypeptide 2  PTPLA - protein tyrosine phosphatase-like (proline instead of catalytic arginine), member a  ACSM2A - acyl-coa synthetase medium-chain family member 2a  ABHD1 - abhydrolase domain containing 1  PKLR - pyruvate kinase, liver and rbc  AKR1C4 - aldo-keto reductase family 1, member c4  AGXT - alanine-glyoxylate aminotransferase  LIPC - lipase, hepatic  SLC27A2 - solute carrier family 27 (fatty acid transporter), member 2  DECR2 - 2,4-dienoyl coa reductase 2, peroxisomal  SLC27A5 - solute carrier family 27 (fatty acid transporter), member 5  ACSM3 - acyl-coa synthetase medium-chain family member 3  PFKFB1 - 6-phosphofructo-2-kinase/fructose-2,6-biphosphatase 1  ACSM5 - acyl-coa synthetase medium-chain family member 5 |
| GO:0006732 | coenzyme metabolic process | 6E-7 | 7.14E-5 | 2.35 (10334,250,667,38) | [+] Show genes  HSD17B8 - hydroxysteroid (17-beta) dehydrogenase 8  MLYCD - malonyl-coa decarboxylase  PGM1 - phosphoglucomutase 1  NMRK2 - nicotinamide riboside kinase 2  SHMT1 - serine hydroxymethyltransferase 1 (soluble)  FMO1 - flavin containing monooxygenase 1  PGAM2 - phosphoglycerate mutase 2 (muscle)  VNN1 - vanin 1  ALDOB - aldolase b, fructose-bisphosphate  MCEE - methylmalonyl coa epimerase  MTHFR - methylenetetrahydrofolate reductase (nad(p)h)  ACSL1 - acyl-coa synthetase long-chain family member 1  MTHFD1 - methylenetetrahydrofolate dehydrogenase (nadp+ dependent) 1, methenyltetrahydrofolate cyclohydrolase, formyltetrahydrofolate synthetase  ENO3 - enolase 3 (beta, muscle)  LDHA - lactate dehydrogenase a  GPD1 - glycerol-3-phosphate dehydrogenase 1 (soluble)  PTPLA - protein tyrosine phosphatase-like (proline instead of catalytic arginine), member a  MPC1 - mitochondrial pyruvate carrier 1  ACSM2A - acyl-coa synthetase medium-chain family member 2a  PKLR - pyruvate kinase, liver and rbc  HAAO - 3-hydroxyanthranilate 3,4-dioxygenase  ASPDH - aspartate dehydrogenase domain containing  GLYAT - glycine-n-acyltransferase  FTCD - formimidoyltransferase cyclodeaminase  AHCY - adenosylhomocysteinase  GCDH - glutaryl-coa dehydrogenase  ALDH1L1 - aldehyde dehydrogenase 1 family, member l1  MAT1A - methionine adenosyltransferase i, alpha  COQ4 - coenzyme q4 homolog (s. cerevisiae)  DGAT2 - diacylglycerol o-acyltransferase 2  TDO2 - tryptophan 2,3-dioxygenase  PIPOX - pipecolic acid oxidase  HMGCS2 - 3-hydroxy-3-methylglutaryl-coa synthase 2 (mitochondrial)  COQ9 - coenzyme q9 homolog (s. cerevisiae)  ACSM3 - acyl-coa synthetase medium-chain family member 3  PFKFB1 - 6-phosphofructo-2-kinase/fructose-2,6-biphosphatase 1  MOCS1 - molybdenum cofactor synthesis 1  ACSM5 - acyl-coa synthetase medium-chain family member 5 |
| GO:0042572 | retinol metabolic process | 6.22E-7 | 7.34E-5 | 6.74 (10334,23,667,10) | [+] Show genes  DHRS3 - dehydrogenase/reductase (sdr family) member 3  DGAT2 - diacylglycerol o-acyltransferase 2  AKR1C3 - aldo-keto reductase family 1, member c3  TTR - transthyretin  RDH16 - retinol dehydrogenase 16 (all-trans)  CYP1A2 - cytochrome p450, family 1, subfamily a, polypeptide 2  ADH6 - alcohol dehydrogenase 6 (class v)  CYP3A5 - cytochrome p450, family 3, subfamily a, polypeptide 5  PNPLA2 - patatin-like phospholipase domain containing 2  ADH4 - alcohol dehydrogenase 4 (class ii), pi polypeptide |
| GO:0050820 | positive regulation of coagulation | 6.22E-7 | 7.28E-5 | 6.74 (10334,23,667,10) | [+] Show genes  HRG - histidine-rich glycoprotein  APOH - apolipoprotein h (beta-2-glycoprotein i)  F12 - coagulation factor xii (hageman factor)  CD36 - cd36 molecule (thrombospondin receptor)  PLG - plasminogen  F2 - coagulation factor ii (thrombin)  SERPINF2 - serpin peptidase inhibitor, clade f (alpha-2 antiplasmin, pigment epithelium derived factor), member 2  F7 - coagulation factor vii (serum prothrombin conversion accelerator)  PLEK - pleckstrin  CPB2 - carboxypeptidase b2 (plasma) |
| GO:0009066 | aspartate family amino acid metabolic process | 6.59E-7 | 7.64E-5 | 4.72 (10334,46,667,14) | [+] Show genes  GCAT - glycine c-acetyltransferase  AHCY - adenosylhomocysteinase  GCDH - glutaryl-coa dehydrogenase  ASS1 - argininosuccinate synthase 1  BHMT - betaine--homocysteine s-methyltransferase  ADI1 - acireductone dioxygenase 1  MAT1A - methionine adenosyltransferase i, alpha  ADSSL1 - adenylosuccinate synthase like 1  MTHFR - methylenetetrahydrofolate reductase (nad(p)h)  MTHFD1 - methylenetetrahydrofolate dehydrogenase (nadp+ dependent) 1, methenyltetrahydrofolate cyclohydrolase, formyltetrahydrofolate synthetase  MSRA - methionine sulfoxide reductase a  PIPOX - pipecolic acid oxidase  ATF4 - activating transcription factor 4  GOT2 - glutamic-oxaloacetic transaminase 2, mitochondrial |
| GO:0031032 | actomyosin structure organization | 6.87E-7 | 7.9E-5 | 3.48 (10334,89,667,20) | [+] Show genes  LDB3 - lim domain binding 3  KLHL41 - kelch-like family member 41  CAPN3 - calpain 3, (p94)  SYNPO2L - synaptopodin 2-like  CSRP3 - cysteine and glycine-rich protein 3 (cardiac lim protein)  LMOD1 - leiomodin 1 (smooth muscle)  FRMD3 - ferm domain containing 3  MYOZ1 - myozenin 1  ACTN2 - actinin, alpha 2  TTN - titin  ANKRD1 - ankyrin repeat domain 1 (cardiac muscle)  LMOD2 - leiomodin 2 (cardiac)  CASQ2 - calsequestrin 2 (cardiac muscle)  MYL2 - myosin, light chain 2, regulatory, cardiac, slow  TMOD4 - tropomodulin 4 (muscle)  MYOZ2 - myozenin 2  TCAP - titin-cap  LMOD3 - leiomodin 3 (fetal)  TNNT3 - troponin t type 3 (skeletal, fast)  MYPN - myopalladin |
| GO:0045333 | cellular respiration | 7.5E-7 | 8.55E-5 | 4.38 (10334,53,667,15) | [+] Show genes  UQCRC1 - ubiquinol-cytochrome c reductase core protein i  COX6A2 - cytochrome c oxidase subunit via polypeptide 2  UQCR10 - ubiquinol-cytochrome c reductase, complex iii subunit x  CAT - catalase  NDUFA4 - nadh dehydrogenase (ubiquinone) 1 alpha subcomplex, 4, 9kda  NDUFS7 - nadh dehydrogenase (ubiquinone) fe-s protein 7, 20kda (nadh-coenzyme q reductase)  COX5B - cytochrome c oxidase subunit vb  SDHB - succinate dehydrogenase complex, subunit b, iron sulfur (ip)  ATP5D - atp synthase, h+ transporting, mitochondrial f1 complex, delta subunit  PPARGC1A - peroxisome proliferator-activated receptor gamma, coactivator 1 alpha  NDUFS8 - nadh dehydrogenase (ubiquinone) fe-s protein 8, 23kda (nadh-coenzyme q reductase)  SLC25A13 - solute carrier family 25 (aspartate/glutamate carrier), member 13  COX7C - cytochrome c oxidase subunit viic  CYP1A2 - cytochrome p450, family 1, subfamily a, polypeptide 2  BLOC1S1 - biogenesis of lysosomal organelles complex-1, subunit 1 |
| GO:0009168 | purine ribonucleoside monophosphate biosynthetic process | 7.91E-7 | 8.94E-5 | 3.59 (10334,82,667,19) | [+] Show genes  AK2 - adenylate kinase 2  ATP5J2 - atp synthase, h+ transporting, mitochondrial fo complex, subunit f2  PGM1 - phosphoglucomutase 1  ATP5H - atp synthase, h+ transporting, mitochondrial fo complex, subunit d  ATP5I - atp synthase, h+ transporting, mitochondrial fo complex, subunit e  COX5B - cytochrome c oxidase subunit vb  AMPD1 - adenosine monophosphate deaminase 1  PGAM2 - phosphoglycerate mutase 2 (muscle)  ALDOB - aldolase b, fructose-bisphosphate  ADSSL1 - adenylosuccinate synthase like 1  ATP5E - atp synthase, h+ transporting, mitochondrial f1 complex, epsilon subunit  ENO3 - enolase 3 (beta, muscle)  ATP5D - atp synthase, h+ transporting, mitochondrial f1 complex, delta subunit  ATP5G1 - atp synthase, h+ transporting, mitochondrial fo complex, subunit c1 (subunit 9)  ADA - adenosine deaminase  LDHA - lactate dehydrogenase a  SLC25A13 - solute carrier family 25 (aspartate/glutamate carrier), member 13  PKLR - pyruvate kinase, liver and rbc  PFKFB1 - 6-phosphofructo-2-kinase/fructose-2,6-biphosphatase 1 |
| GO:0007599 | hemostasis | 8.25E-7 | 9.26E-5 | 3.22 (10334,106,667,22) | [+] Show genes  F11 - coagulation factor xi  SERPIND1 - serpin peptidase inhibitor, clade d (heparin cofactor), member 1  F10 - coagulation factor x  F9 - coagulation factor ix  FGG - fibrinogen gamma chain  F13B - coagulation factor xiii, b polypeptide  F12 - coagulation factor xii (hageman factor)  CD36 - cd36 molecule (thrombospondin receptor)  PROC - protein c (inactivator of coagulation factors va and viiia)  SERPINA10 - serpin peptidase inhibitor, clade a (alpha-1 antiproteinase, antitrypsin), member 10  SERPINA1 - serpin peptidase inhibitor, clade a (alpha-1 antiproteinase, antitrypsin), member 1  CPB2 - carboxypeptidase b2 (plasma)  PAFAH2 - platelet-activating factor acetylhydrolase 2, 40kda  PROZ - protein z, vitamin k-dependent plasma glycoprotein  FGA - fibrinogen alpha chain  FGB - fibrinogen beta chain  C4BPB - complement component 4 binding protein, beta  F2 - coagulation factor ii (thrombin)  SERPINC1 - serpin peptidase inhibitor, clade c (antithrombin), member 1  PLG - plasminogen  SERPINA5 - serpin peptidase inhibitor, clade a (alpha-1 antiproteinase, antitrypsin), member 5  F7 - coagulation factor vii (serum prothrombin conversion accelerator) |
| GO:0050727 | regulation of inflammatory response | 8.67E-7 | 9.65E-5 | 2.42 (10334,224,667,35) | [+] Show genes  FAM132A - family with sequence similarity 132, member a  C9 - complement component 9  APCS - amyloid p component, serum  F12 - coagulation factor xii (hageman factor)  PROC - protein c (inactivator of coagulation factors va and viiia)  ADA - adenosine deaminase  SERPINF1 - serpin peptidase inhibitor, clade f (alpha-2 antiplasmin, pigment epithelium derived factor), member 1  PPARA - peroxisome proliferator-activated receptor alpha  CD5L - cd5 molecule-like  CREB3L3 - camp responsive element binding protein 3-like 3  S100A12 - s100 calcium binding protein a12  TNIP1 - tnfaip3 interacting protein 1  FEM1A - fem-1 homolog a (c. elegans)  IGF1 - insulin-like growth factor 1 (somatomedin c)  SAA1 - serum amyloid a1  CFB - complement factor b  CPN2 - carboxypeptidase n, polypeptide 2  SCGB1A1 - secretoglobin, family 1a, member 1 (uteroglobin)  LBP - lipopolysaccharide binding protein  C1R - complement component 1, r subcomponent  FCGR2B - fc fragment of igg, low affinity iib, receptor (cd32)  C2 - complement component 2  CPB2 - carboxypeptidase b2 (plasma)  C4BPA - complement component 4 binding protein, alpha  C4BPB - complement component 4 binding protein, beta  F2 - coagulation factor ii (thrombin)  VTN - vitronectin  C5 - complement component 5  NUPR1 - nuclear protein, transcriptional regulator, 1  GGT1 - gamma-glutamyltransferase 1  C6 - complement component 6  KLKB1 - kallikrein b, plasma (fletcher factor) 1  C8B - complement component 8, beta polypeptide  C8A - complement component 8, alpha polypeptide  C8G - complement component 8, gamma polypeptide |
| GO:0008152 | metabolic process | 8.97E-7 | 9.9E-5 | 1.16 (10334,5680,667,426) | [+] Show genes  COX6A2 - cytochrome c oxidase subunit via polypeptide 2  DAPK2 - death-associated protein kinase 2  COX5B - cytochrome c oxidase subunit vb  SPP2 - secreted phosphoprotein 2, 24kda  AMACR - alpha-methylacyl-coa racemase  AOC1 - amine oxidase, copper containing 1  CA14 - carbonic anhydrase xiv  FOXRED1 - fad-dependent oxidoreductase domain containing 1  LDHA - lactate dehydrogenase a  GCGR - glucagon receptor  ACAA1 - acetyl-coa acyltransferase 1  COX7C - cytochrome c oxidase subunit viic  ACADL - acyl-coa dehydrogenase, long chain  ACADS - acyl-coa dehydrogenase, c-2 to c-3 short chain  ACADVL - acyl-coa dehydrogenase, very long chain  BLOC1S1 - biogenesis of lysosomal organelles complex-1, subunit 1  CPT2 - carnitine palmitoyltransferase 2  ATP5J2 - atp synthase, h+ transporting, mitochondrial fo complex, subunit f2  DHRS4 - dehydrogenase/reductase (sdr family) member 4  CPT1B - carnitine palmitoyltransferase 1b (muscle)  CPS1 - carbamoyl-phosphate synthase 1, mitochondrial  FITM1 - fat storage-inducing transmembrane protein 1  CPB2 - carboxypeptidase b2 (plasma)  GHR - growth hormone receptor  PIPOX - pipecolic acid oxidase  LIPC - lipase, hepatic  MMAB - methylmalonic aciduria (cobalamin deficiency) cblb type  POLD4 - polymerase (dna-directed), delta 4, accessory subunit  ADHFE1 - alcohol dehydrogenase, iron containing, 1  SMPDL3A - sphingomyelin phosphodiesterase, acid-like 3a  PLG - plasminogen  APOBEC2 - apolipoprotein b mrna editing enzyme, catalytic polypeptide-like 2  PPM1J - protein phosphatase, mg2+/mn2+ dependent, 1j  GGT1 - gamma-glutamyltransferase 1  PLEK - pleckstrin  ACOX2 - acyl-coa oxidase 2, branched chain  LSM10 - lsm10, u7 small nuclear rna associated  PLBD1 - phospholipase b domain containing 1  ACTN2 - actinin, alpha 2  PCTP - phosphatidylcholine transfer protein  PPARGC1A - peroxisome proliferator-activated receptor gamma, coactivator 1 alpha  BAG3 - bcl2-associated athanogene 3  ADA - adenosine deaminase  PPARA - peroxisome proliferator-activated receptor alpha  PON1 - paraoxonase 1  APOA5 - apolipoprotein a-v  FEM1A - fem-1 homolog a (c. elegans)  POLR2I - polymerase (rna) ii (dna directed) polypeptide i, 14.5kda  POLR2J - polymerase (rna) ii (dna directed) polypeptide j, 13.3kda  FTCD - formimidoyltransferase cyclodeaminase  ALDH1L1 - aldehyde dehydrogenase 1 family, member l1  ADH6 - alcohol dehydrogenase 6 (class v)  ADH4 - alcohol dehydrogenase 4 (class ii), pi polypeptide  DGAT2 - diacylglycerol o-acyltransferase 2  PRODH2 - proline dehydrogenase (oxidase) 2  PLA2G12B - phospholipase a2, group xiib  SLC17A1 - solute carrier family 17 (organic anion transporter), member 1  HSD17B8 - hydroxysteroid (17-beta) dehydrogenase 8  FBXO17 - f-box protein 17  HSD17B14 - hydroxysteroid (17-beta) dehydrogenase 14  AGMAT - agmatine ureohydrolase (agmatinase)  NDUFS7 - nadh dehydrogenase (ubiquinone) fe-s protein 7, 20kda (nadh-coenzyme q reductase)  ADRB2 - adrenoceptor beta 2, surface  TRIM63 - tripartite motif containing 63, e3 ubiquitin protein ligase  SERPINA10 - serpin peptidase inhibitor, clade a (alpha-1 antiproteinase, antitrypsin), member 10  TMPRSS6 - transmembrane protease, serine 6  MCEE - methylmalonyl coa epimerase  ASB11 - ankyrin repeat and socs box containing 11  SFTPA1 - surfactant protein a1  ASB5 - ankyrin repeat and socs box containing 5  GK5 - glycerol kinase 5 (putative)  ASPG - asparaginase homolog (s. cerevisiae)  ABHD1 - abhydrolase domain containing 1  NHEJ1 - nonhomologous end-joining factor 1  AK2 - adenylate kinase 2  SLC37A4 - solute carrier family 37 (glucose-6-phosphate transporter), member 4  ANGPTL4 - angiopoietin-like 4  CHST13 - carbohydrate (chondroitin 4) sulfotransferase 13  AHCY - adenosylhomocysteinase  SULT2A1 - sulfotransferase family, cytosolic, 2a, dehydroepiandrosterone (dhea)-preferring, member 1  AGXT - alanine-glyoxylate aminotransferase  WWP1 - ww domain containing e3 ubiquitin protein ligase 1  COQ4 - coenzyme q4 homolog (s. cerevisiae)  PPP1R3C - protein phosphatase 1, regulatory subunit 3c  KNG1 - kininogen 1  ALB - albumin  KLKB1 - kallikrein b, plasma (fletcher factor) 1  G6PC - glucose-6-phosphatase, catalytic subunit  ALDH2 - aldehyde dehydrogenase 2 family (mitochondrial)  PPP1R3A - protein phosphatase 1, regulatory subunit 3a  PYROXD2 - pyridine nucleotide-disulphide oxidoreductase domain 2  PHOSPHO1 - phosphatase, orphan 1  CRP - c-reactive protein, pentraxin-related  GAMT - guanidinoacetate n-methyltransferase  PLCD4 - phospholipase c, delta 4  METTL7A - methyltransferase like 7a  ALKBH7 - alkb, alkylation repair homolog 7 (e. coli)  PROC - protein c (inactivator of coagulation factors va and viiia)  ALDOB - aldolase b, fructose-bisphosphate  GRHPR - glyoxylate reductase/hydroxypyruvate reductase  ADTRP - androgen-dependent tfpi-regulating protein  AMBP - alpha-1-microglobulin/bikunin precursor  DHRS12 - dehydrogenase/reductase (sdr family) member 12  DUSP13 - dual specificity phosphatase 13  AMY2B - amylase, alpha 2b (pancreatic)  GCDH - glutaryl-coa dehydrogenase  GC - group-specific component (vitamin d binding protein)  TRIM72 - tripartite motif containing 72  AMPD1 - adenosine monophosphate deaminase 1  UQCR11 - ubiquinol-cytochrome c reductase, complex iii subunit xi  PKN1 - protein kinase n1  SLC27A2 - solute carrier family 27 (fatty acid transporter), member 2  SLC25A4 - solute carrier family 25 (mitochondrial carrier; adenine nucleotide translocator), member 4  SLC27A5 - solute carrier family 27 (fatty acid transporter), member 5  HAO2 - hydroxyacid oxidase 2 (long chain)  APCS - amyloid p component, serum  APOA2 - apolipoprotein a-ii  SDHB - succinate dehydrogenase complex, subunit b, iron sulfur (ip)  APOA4 - apolipoprotein a-iv  PPP1R3B - protein phosphatase 1, regulatory subunit 3b  APOF - apolipoprotein f  GSTZ1 - glutathione s-transferase zeta 1  PHYHD1 - phytanoyl-coa dioxygenase domain containing 1  GSTT1 - glutathione s-transferase theta 1  ASB12 - ankyrin repeat and socs box containing 12  ACSM2A - acyl-coa synthetase medium-chain family member 2a  DHRS3 - dehydrogenase/reductase (sdr family) member 3  ASPDH - aspartate dehydrogenase domain containing  UBE2L6 - ubiquitin-conjugating enzyme e2l 6  APOC2 - apolipoprotein c-ii  FBXO40 - f-box protein 40  IMMP2L - imp2 inner mitochondrial membrane peptidase-like (s. cerevisiae)  APOC3 - apolipoprotein c-iii  SDC2 - syndecan 2  GYS2 - glycogen synthase 2 (liver)  APOC4 - apolipoprotein c-iv  PAFAH2 - platelet-activating factor acetylhydrolase 2, 40kda  APOH - apolipoprotein h (beta-2-glycoprotein i)  PAH - phenylalanine hydroxylase  TBRG4 - transforming growth factor beta regulator 4  MSRB3 - methionine sulfoxide reductase b3  MOCS1 - molybdenum cofactor synthesis 1  UPB1 - ureidopropionase, beta  CYP4A11 - cytochrome p450, family 4, subfamily a, polypeptide 11  INMT - indolethylamine n-methyltransferase  CYP8B1 - cytochrome p450, family 8, subfamily b, polypeptide 1  GADD45GIP1 - growth arrest and dna-damage-inducible, gamma interacting protein 1  CYP2E1 - cytochrome p450, family 2, subfamily e, polypeptide 1  STS - steroid sulfatase (microsomal), isozyme s  CYP3A5 - cytochrome p450, family 3, subfamily a, polypeptide 5  ADSSL1 - adenylosuccinate synthase like 1  ATXN7 - ataxin 7  CYP2C19 - cytochrome p450, family 2, subfamily c, polypeptide 19  ENPP1 - ectonucleotide pyrophosphatase/phosphodiesterase 1  ARG1 - arginase 1  PDK4 - pyruvate dehydrogenase kinase, isozyme 4  HADH - hydroxyacyl-coa dehydrogenase  SCP2 - sterol carrier protein 2  PDK2 - pyruvate dehydrogenase kinase, isozyme 2  HAGH - hydroxyacylglutathione hydrolase  CYP1A2 - cytochrome p450, family 1, subfamily a, polypeptide 2  PTPLA - protein tyrosine phosphatase-like (proline instead of catalytic arginine), member a  HABP2 - hyaluronan binding protein 2  UPF2 - upf2 regulator of nonsense transcripts homolog (yeast)  HSD17B10 - hydroxysteroid (17-beta) dehydrogenase 10  S100A12 - s100 calcium binding protein a12  IYD - iodotyrosine deiodinase  SERPIND1 - serpin peptidase inhibitor, clade d (heparin cofactor), member 1  PTP4A3 - protein tyrosine phosphatase type iva, member 3  TM6SF2 - transmembrane 6 superfamily member 2  PCK1 - phosphoenolpyruvate carboxykinase 1 (soluble)  SAA1 - serum amyloid a1  ASS1 - argininosuccinate synthase 1  GIMAP7 - gtpase, imap family member 7  ART3 - adp-ribosyltransferase 3  MAPK12 - mitogen-activated protein kinase 12  TMEM86B - transmembrane protein 86b  DHRS7B - dehydrogenase/reductase (sdr family) member 7b  TCEANC - transcription elongation factor a (sii) n-terminal and central domain containing  SNORA17 - small nucleolar rna, h/aca box 17  ASGR1 - asialoglycoprotein receptor 1  ASGR2 - asialoglycoprotein receptor 2  ZDHHC4 - zinc finger, dhhc-type containing 4  ACSM3 - acyl-coa synthetase medium-chain family member 3  MMACHC - methylmalonic aciduria (cobalamin deficiency) cblc type, with homocystinuria  PGM1 - phosphoglucomutase 1  LRRC2 - leucine rich repeat containing 2  PGAM2 - phosphoglycerate mutase 2 (muscle)  ATF4 - activating transcription factor 4  GOT2 - glutamic-oxaloacetic transaminase 2, mitochondrial  ABLIM3 - actin binding lim protein family, member 3  CINP - cyclin-dependent kinase 2 interacting protein  SERPINC1 - serpin peptidase inhibitor, clade c (antithrombin), member 1  GPD1 - glycerol-3-phosphate dehydrogenase 1 (soluble)  DPYS - dihydropyrimidinase  MYLK2 - myosin light chain kinase 2  MBL2 - mannose-binding lectin (protein c) 2, soluble  ATP5I - atp synthase, h+ transporting, mitochondrial fo complex, subunit e  METTL7B - methyltransferase like 7b  MAT1A - methionine adenosyltransferase i, alpha  HACL1 - 2-hydroxyacyl-coa lyase 1  ATP5E - atp synthase, h+ transporting, mitochondrial f1 complex, epsilon subunit  ATP5D - atp synthase, h+ transporting, mitochondrial f1 complex, delta subunit  ATP5G1 - atp synthase, h+ transporting, mitochondrial fo complex, subunit c1 (subunit 9)  POLDIP2 - polymerase (dna-directed), delta interacting protein 2  DECR2 - 2,4-dienoyl coa reductase 2, peroxisomal  PFKFB1 - 6-phosphofructo-2-kinase/fructose-2,6-biphosphatase 1  PIN4 - protein (peptidylprolyl cis/trans isomerase) nima-interacting, 4 (parvulin)  SHMT1 - serine hydroxymethyltransferase 1 (soluble)  AZGP1 - alpha-2-glycoprotein 1, zinc-binding  TRPT1 - trna phosphotransferase 1  ASB2 - ankyrin repeat and socs box containing 2  ST3GAL3 - st3 beta-galactoside alpha-2,3-sialyltransferase 3  MPC1 - mitochondrial pyruvate carrier 1  PKLR - pyruvate kinase, liver and rbc  STYXL1 - serine/threonine/tyrosine interacting-like 1  ECHDC2 - enoyl coa hydratase domain containing 2  ADI1 - acireductone dioxygenase 1  PHYH - phytanoyl-coa 2-hydroxylase  SERPINA1 - serpin peptidase inhibitor, clade a (alpha-1 antiproteinase, antitrypsin), member 1  STBD1 - starch binding domain 1  FGGY - fggy carbohydrate kinase domain containing  MAF - v-maf avian musculoaponeurotic fibrosarcoma oncogene homolog  NDUFA3 - nadh dehydrogenase (ubiquinone) 1 alpha subcomplex, 3, 9kda  NDUFA4 - nadh dehydrogenase (ubiquinone) 1 alpha subcomplex, 4, 9kda  NDUFA2 - nadh dehydrogenase (ubiquinone) 1 alpha subcomplex, 2, 8kda  SUCLG1 - succinate-coa ligase, alpha subunit  HSD11B1 - hydroxysteroid (11-beta) dehydrogenase 1  NDUFA7 - nadh dehydrogenase (ubiquinone) 1 alpha subcomplex, 7, 14.5kda  SLC29A1 - solute carrier family 29 (equilibrative nucleoside transporter), member 1  DHRS7C - dehydrogenase/reductase (sdr family) member 7c  BTC - betacellulin  BDH2 - 3-hydroxybutyrate dehydrogenase, type 2  ENO3 - enolase 3 (beta, muscle)  CES2 - carboxylesterase 2  HRC - histidine rich calcium binding protein  ENDOG - endonuclease g  SLC25A13 - solute carrier family 25 (aspartate/glutamate carrier), member 13  TRIM7 - tripartite motif containing 7  HRAS - harvey rat sarcoma viral oncogene homolog  HPX - hemopexin  RCL1 - rna terminal phosphate cyclase-like 1  KLHL41 - kelch-like family member 41  TTR - transthyretin  UQCR10 - ubiquinol-cytochrome c reductase, complex iii subunit x  TNIP1 - tnfaip3 interacting protein 1  HSD17B13 - hydroxysteroid (17-beta) dehydrogenase 13  EIF4EBP1 - eukaryotic translation initiation factor 4e binding protein 1  DUSP27 - dual specificity phosphatase 27 (putative)  CFB - complement factor b  EHHADH - enoyl-coa, hydratase/3-hydroxyacyl coa dehydrogenase  HPN - hepsin  BHMT - betaine--homocysteine s-methyltransferase  HP - haptoglobin  TTN - titin  HPD - 4-hydroxyphenylpyruvate dioxygenase  TTPA - tocopherol (alpha) transfer protein  PROZ - protein z, vitamin k-dependent plasma glycoprotein  BLVRB - biliverdin reductase b (flavin reductase (nadph))  NR1I2 - nuclear receptor subfamily 1, group i, member 2  FAHD1 - fumarylacetoacetate hydrolase domain containing 1  C9 - complement component 9  EEF1D - eukaryotic translation elongation factor 1 delta (guanine nucleotide exchange protein)  COX17 - cox17 cytochrome c oxidase copper chaperone  TRIB1 - tribbles homolog 1 (drosophila)  CHPT1 - choline phosphotransferase 1  ABHD17A - abhydrolase domain containing 17a  VNN1 - vanin 1  CA3 - carbonic anhydrase iii, muscle specific  PM20D1 - peptidase m20 domain containing 1  HFE2 - hemochromatosis type 2 (juvenile)  SLC17A2 - solute carrier family 17, member 2  GLYAT - glycine-n-acyltransferase  RORC - rar-related orphan receptor c  RP9 - retinitis pigmentosa 9 (autosomal dominant)  MRPS34 - mitochondrial ribosomal protein s34  C1R - complement component 1, r subcomponent  C2 - complement component 2  C4BPA - complement component 4 binding protein, alpha  SNORA70 - small nucleolar rna, h/aca box 70  C4BPB - complement component 4 binding protein, beta  ECHS1 - enoyl coa hydratase, short chain, 1, mitochondrial  C5 - complement component 5  C6 - complement component 6  C8B - complement component 8, beta polypeptide  C8A - complement component 8, alpha polypeptide  ECH1 - enoyl coa hydratase 1, peroxisomal  LRRC39 - leucine rich repeat containing 39  C8G - complement component 8, gamma polypeptide  F11 - coagulation factor xi  ECI2 - enoyl-coa delta isomerase 2  F10 - coagulation factor x  UQCRC1 - ubiquinol-cytochrome c reductase core protein i  AKR1C3 - aldo-keto reductase family 1, member c3  F9 - coagulation factor ix  MRPL23 - mitochondrial ribosomal protein l23  CAPN3 - calpain 3, (p94)  ACAA2 - acetyl-coa acyltransferase 2  AOC3 - amine oxidase, copper containing 3  F12 - coagulation factor xii (hageman factor)  FABP1 - fatty acid binding protein 1, liver  HMBS - hydroxymethylbilane synthase  MTHFR - methylenetetrahydrofolate reductase (nad(p)h)  ACSL1 - acyl-coa synthetase long-chain family member 1  MTHFD1 - methylenetetrahydrofolate dehydrogenase (nadp+ dependent) 1, methenyltetrahydrofolate cyclohydrolase, formyltetrahydrofolate synthetase  CHCHD10 - coiled-coil-helix-coiled-coil-helix domain containing 10  ALDH4A1 - aldehyde dehydrogenase 4 family, member a1  FAH - fumarylacetoacetate hydrolase (fumarylacetoacetase)  RPL3L - ribosomal protein l3-like  PNPLA2 - patatin-like phospholipase domain containing 2  ATP5H - atp synthase, h+ transporting, mitochondrial fo complex, subunit d  HGD - homogentisate 1,2-dioxygenase  MSRA - methionine sulfoxide reductase a  ABCB6 - atp-binding cassette, sub-family b (mdr/tap), member 6  MST1 - macrophage stimulating 1 (hepatocyte growth factor-like)  COQ9 - coenzyme q9 homolog (s. cerevisiae)  F2 - coagulation factor ii (thrombin)  F7 - coagulation factor vii (serum prothrombin conversion accelerator)  ACSM5 - acyl-coa synthetase medium-chain family member 5  UBA52 - ubiquitin a-52 residue ribosomal protein fusion product 1  AGXT2 - alanine--glyoxylate aminotransferase 2  MACROD1 - macro domain containing 1  LPIN1 - lipin 1  MRPL14 - mitochondrial ribosomal protein l14  ESRRA - estrogen-related receptor alpha  MRPS15 - mitochondrial ribosomal protein s15  MRPL40 - mitochondrial ribosomal protein l40  ETFDH - electron-transferring-flavoprotein dehydrogenase  CD5L - cd5 molecule-like  MRPL36 - mitochondrial ribosomal protein l36  SMYD1 - set and mynd domain containing 1  SERPINA6 - serpin peptidase inhibitor, clade a (alpha-1 antiproteinase, antitrypsin), member 6  EPHA1 - eph receptor a1  CAT - catalase  TRIM54 - tripartite motif containing 54  PHPT1 - phosphohistidine phosphatase 1  RXRA - retinoid x receptor, alpha  UCP3 - uncoupling protein 3 (mitochondrial, proton carrier)  CAV3 - caveolin 3  RXRG - retinoid x receptor, gamma  HMGCS2 - 3-hydroxy-3-methylglutaryl-coa synthase 2 (mitochondrial)  CBR1 - carbonyl reductase 1  RNPEPL1 - arginyl aminopeptidase (aminopeptidase b)-like 1  FBP2 - fructose-1,6-bisphosphatase 2  HMOX1 - heme oxygenase (decycling) 1  NUPR1 - nuclear protein, transcriptional regulator, 1  MPST - mercaptopyruvate sulfurtransferase  NR2F6 - nuclear receptor subfamily 2, group f, member 6  CCBL1 - cysteine conjugate-beta lyase, cytoplasmic  URAD - ureidoimidazoline (2-oxo-4-hydroxy-4-carboxy-5-) decarboxylase  NR1I3 - nuclear receptor subfamily 1, group i, member 3  ERBB2 - v-erb-b2 avian erythroblastic leukemia viral oncogene homolog 2  MASP1 - mannan-binding lectin serine peptidase 1 (c4/c2 activating component of ra-reactive factor)  ANGPTL3 - angiopoietin-like 3  LGMN - legumain  FN1 - fibronectin 1  GADL1 - glutamate decarboxylase-like 1  MMP23B - matrix metallopeptidase 23b  ENTPD8 - ectonucleoside triphosphate diphosphohydrolase 8  ADPRHL1 - adp-ribosylhydrolase like 1  FMO1 - flavin containing monooxygenase 1  EEPD1 - endonuclease/exonuclease/phosphatase family domain containing 1  CDC34 - cell division cycle 34  FMO3 - flavin containing monooxygenase 3  OTC - ornithine carbamoyltransferase  FBXW5 - f-box and wd repeat domain containing 5  IMPA2 - inositol(myo)-1(or 4)-monophosphatase 2  CD36 - cd36 molecule (thrombospondin receptor)  IL1RN - interleukin 1 receptor antagonist  TDO2 - tryptophan 2,3-dioxygenase  PSMC5 - proteasome (prosome, macropain) 26s subunit, atpase, 5  DCAF11 - ddb1 and cul4 associated factor 11  FH - fumarate hydratase  FGG - fibrinogen gamma chain  NMRK2 - nicotinamide riboside kinase 2  ALDH8A1 - aldehyde dehydrogenase 8 family, member a1  CES1 - carboxylesterase 1  SCARF1 - scavenger receptor class f, member 1  MRPS25 - mitochondrial ribosomal protein s25  TAT - tyrosine aminotransferase  ITIH1 - inter-alpha-trypsin inhibitor heavy chain 1  UBXN1 - ubx domain protein 1  FGA - fibrinogen alpha chain  ITIH2 - inter-alpha-trypsin inhibitor heavy chain 2  FGB - fibrinogen beta chain  ITIH3 - inter-alpha-trypsin inhibitor heavy chain 3  ITIH4 - inter-alpha-trypsin inhibitor heavy chain family, member 4  C19orf80 - chromosome 19 open reading frame 80  HSD17B6 - hydroxysteroid (17-beta) dehydrogenase 6  MRPL2 - mitochondrial ribosomal protein l2  GLYCTK - glycerate kinase  RDH16 - retinol dehydrogenase 16 (all-trans)  CRYL1 - crystallin, lambda 1  APOM - apolipoprotein m  AGPAT2 - 1-acylglycerol-3-phosphate o-acyltransferase 2  YBX1 - y box binding protein 1  TCEA3 - transcription elongation factor a (sii), 3  SMTNL1 - smoothelin-like 1  KEAP1 - kelch-like ech-associated protein 1  SEPSECS - sep (o-phosphoserine) trna:sec (selenocysteine) trna synthase  MLYCD - malonyl-coa decarboxylase  ORMDL3 - orm1-like 3 (s. cerevisiae)  CIDEB - cell death-inducing dffa-like effector b  AKR1C4 - aldo-keto reductase family 1, member c4  GCAT - glycine c-acetyltransferase  UGT1A6 - udp glucuronosyltransferase 1 family, polypeptide a6  CHIA - chitinase, acidic  ETHE1 - ethylmalonic encephalopathy 1  IDH3G - isocitrate dehydrogenase 3 (nad+) gamma  CES3 - carboxylesterase 3  NFIC - nuclear factor i/c (ccaat-binding transcription factor)  HAAO - 3-hydroxyanthranilate 3,4-dioxygenase  NDUFB10 - nadh dehydrogenase (ubiquinone) 1 beta subcomplex, 10, 22kda  MASP2 - mannan-binding lectin serine peptidase 2  NDUFB7 - nadh dehydrogenase (ubiquinone) 1 beta subcomplex, 7, 18kda  IGF1 - insulin-like growth factor 1 (somatomedin c)  IGFBP1 - insulin-like growth factor binding protein 1  IGFALS - insulin-like growth factor binding protein, acid labile subunit  THOP1 - thimet oligopeptidase 1  CKM - creatine kinase, muscle  NDUFS8 - nadh dehydrogenase (ubiquinone) fe-s protein 8, 23kda (nadh-coenzyme q reductase)  NDUFS6 - nadh dehydrogenase (ubiquinone) fe-s protein 6, 13kda (nadh-coenzyme q reductase)  NDUFS5 - nadh dehydrogenase (ubiquinone) fe-s protein 5, 15kda (nadh-coenzyme q reductase)  NDUFV1 - nadh dehydrogenase (ubiquinone) flavoprotein 1, 51kda  THRSP - thyroid hormone responsive  UQCRQ - ubiquinol-cytochrome c reductase, complex iii subunit vii, 9.5kda |
| GO:0051004 | regulation of lipoprotein lipase activity | 9.47E-7 | 1.04E-4 | 7.34 (10334,19,667,9) | [+] Show genes  C19orf80 - chromosome 19 open reading frame 80  APOC2 - apolipoprotein c-ii  APOC3 - apolipoprotein c-iii  LIPC - lipase, hepatic  APOA5 - apolipoprotein a-v  ANGPTL4 - angiopoietin-like 4  ANGPTL3 - angiopoietin-like 3  APOH - apolipoprotein h (beta-2-glycoprotein i)  APOA4 - apolipoprotein a-iv |
| GO:0048747 | muscle fiber development | 9.96E-7 | 1.08E-4 | 5.31 (10334,35,667,12) | [+] Show genes  MYL2 - myosin, light chain 2, regulatory, cardiac, slow  NRAP - nebulin-related anchoring protein  KLHL41 - kelch-like family member 41  ACTA1 - actin, alpha 1, skeletal muscle  STAC3 - sh3 and cysteine rich domain 3  TCAP - titin-cap  KLHL40 - kelch-like family member 40  LMOD3 - leiomodin 3 (fetal)  TTN - titin  RYR1 - ryanodine receptor 1 (skeletal)  MYO18B - myosin xviiib  NEB - nebulin |
| GO:0000096 | sulfur amino acid metabolic process | 9.96E-7 | 1.07E-4 | 5.31 (10334,35,667,12) | [+] Show genes  MTHFR - methylenetetrahydrofolate reductase (nad(p)h)  MSRA - methionine sulfoxide reductase a  MTHFD1 - methylenetetrahydrofolate dehydrogenase (nadp+ dependent) 1, methenyltetrahydrofolate cyclohydrolase, formyltetrahydrofolate synthetase  CPS1 - carbamoyl-phosphate synthase 1, mitochondrial  GADL1 - glutamate decarboxylase-like 1  AHCY - adenosylhomocysteinase  AGXT - alanine-glyoxylate aminotransferase  GGT1 - gamma-glutamyltransferase 1  MPST - mercaptopyruvate sulfurtransferase  BHMT - betaine--homocysteine s-methyltransferase  MAT1A - methionine adenosyltransferase i, alpha  ADI1 - acireductone dioxygenase 1 |
| GO:0006572 | tyrosine catabolic process | 1.1E-6 | 1.18E-4 | 15.49 (10334,5,667,5) | [+] Show genes  HGD - homogentisate 1,2-dioxygenase  TAT - tyrosine aminotransferase  FAH - fumarylacetoacetate hydrolase (fumarylacetoacetase)  GSTZ1 - glutathione s-transferase zeta 1  HPD - 4-hydroxyphenylpyruvate dioxygenase |
| GO:0009127 | purine nucleoside monophosphate biosynthetic process | 1.17E-6 | 1.25E-4 | 3.50 (10334,84,667,19) | [+] Show genes  AK2 - adenylate kinase 2  ATP5J2 - atp synthase, h+ transporting, mitochondrial fo complex, subunit f2  PGM1 - phosphoglucomutase 1  ATP5H - atp synthase, h+ transporting, mitochondrial fo complex, subunit d  ATP5I - atp synthase, h+ transporting, mitochondrial fo complex, subunit e  COX5B - cytochrome c oxidase subunit vb  AMPD1 - adenosine monophosphate deaminase 1  PGAM2 - phosphoglycerate mutase 2 (muscle)  ADSSL1 - adenylosuccinate synthase like 1  ALDOB - aldolase b, fructose-bisphosphate  ATP5E - atp synthase, h+ transporting, mitochondrial f1 complex, epsilon subunit  ENO3 - enolase 3 (beta, muscle)  ATP5D - atp synthase, h+ transporting, mitochondrial f1 complex, delta subunit  ATP5G1 - atp synthase, h+ transporting, mitochondrial fo complex, subunit c1 (subunit 9)  ADA - adenosine deaminase  LDHA - lactate dehydrogenase a  SLC25A13 - solute carrier family 25 (aspartate/glutamate carrier), member 13  PKLR - pyruvate kinase, liver and rbc  PFKFB1 - 6-phosphofructo-2-kinase/fructose-2,6-biphosphatase 1 |
| GO:0009124 | nucleoside monophosphate biosynthetic process | 1.2E-6 | 1.26E-4 | 3.25 (10334,100,667,21) | [+] Show genes  AK2 - adenylate kinase 2  ATP5J2 - atp synthase, h+ transporting, mitochondrial fo complex, subunit f2  PGM1 - phosphoglucomutase 1  SHMT1 - serine hydroxymethyltransferase 1 (soluble)  ATP5I - atp synthase, h+ transporting, mitochondrial fo complex, subunit e  ATP5H - atp synthase, h+ transporting, mitochondrial fo complex, subunit d  COX5B - cytochrome c oxidase subunit vb  ENTPD8 - ectonucleoside triphosphate diphosphohydrolase 8  AMPD1 - adenosine monophosphate deaminase 1  PGAM2 - phosphoglycerate mutase 2 (muscle)  ALDOB - aldolase b, fructose-bisphosphate  ADSSL1 - adenylosuccinate synthase like 1  ATP5E - atp synthase, h+ transporting, mitochondrial f1 complex, epsilon subunit  ATP5D - atp synthase, h+ transporting, mitochondrial f1 complex, delta subunit  ENO3 - enolase 3 (beta, muscle)  ATP5G1 - atp synthase, h+ transporting, mitochondrial fo complex, subunit c1 (subunit 9)  LDHA - lactate dehydrogenase a  ADA - adenosine deaminase  SLC25A13 - solute carrier family 25 (aspartate/glutamate carrier), member 13  PKLR - pyruvate kinase, liver and rbc  PFKFB1 - 6-phosphofructo-2-kinase/fructose-2,6-biphosphatase 1 |
| GO:0044248 | cellular catabolic process | 1.24E-6 | 1.29E-4 | 1.49 (10334,1319,667,127) | [+] Show genes  ANGPTL3 - angiopoietin-like 3  LGMN - legumain  GADL1 - glutamate decarboxylase-like 1  APOA2 - apolipoprotein a-ii  ENTPD8 - ectonucleoside triphosphate diphosphohydrolase 8  AMACR - alpha-methylacyl-coa racemase  CDC34 - cell division cycle 34  APOA4 - apolipoprotein a-iv  BDH2 - 3-hydroxybutyrate dehydrogenase, type 2  OTC - ornithine carbamoyltransferase  ENO3 - enolase 3 (beta, muscle)  FBXW5 - f-box and wd repeat domain containing 5  LDHA - lactate dehydrogenase a  ENDOG - endonuclease g  GSTZ1 - glutathione s-transferase zeta 1  ACAA1 - acetyl-coa acyltransferase 1  ACADL - acyl-coa dehydrogenase, long chain  ACADS - acyl-coa dehydrogenase, c-2 to c-3 short chain  ACADVL - acyl-coa dehydrogenase, very long chain  CPT2 - carnitine palmitoyltransferase 2  CPT1B - carnitine palmitoyltransferase 1b (muscle)  CPS1 - carbamoyl-phosphate synthase 1, mitochondrial  CD36 - cd36 molecule (thrombospondin receptor)  UBE2L6 - ubiquitin-conjugating enzyme e2l 6  EHHADH - enoyl-coa, hydratase/3-hydroxyacyl coa dehydrogenase  BHMT - betaine--homocysteine s-methyltransferase  HPD - 4-hydroxyphenylpyruvate dioxygenase  TDO2 - tryptophan 2,3-dioxygenase  APOC3 - apolipoprotein c-iii  PSMC5 - proteasome (prosome, macropain) 26s subunit, atpase, 5  LIPC - lipase, hepatic  PIPOX - pipecolic acid oxidase  ADHFE1 - alcohol dehydrogenase, iron containing, 1  DCAF11 - ddb1 and cul4 associated factor 11  SMPDL3A - sphingomyelin phosphodiesterase, acid-like 3a  BLVRB - biliverdin reductase b (flavin reductase (nadph))  PAH - phenylalanine hydroxylase  APOBEC2 - apolipoprotein b mrna editing enzyme, catalytic polypeptide-like 2  GGT1 - gamma-glutamyltransferase 1  NR1I2 - nuclear receptor subfamily 1, group i, member 2  UPB1 - ureidopropionase, beta  ACOX2 - acyl-coa oxidase 2, branched chain  CYP4A11 - cytochrome p450, family 4, subfamily a, polypeptide 11  ALDH8A1 - aldehyde dehydrogenase 8 family, member a1  CYP2E1 - cytochrome p450, family 2, subfamily e, polypeptide 1  PLBD1 - phospholipase b domain containing 1  CYP3A5 - cytochrome p450, family 3, subfamily a, polypeptide 5  CYP2C19 - cytochrome p450, family 2, subfamily c, polypeptide 19  ENPP1 - ectonucleotide pyrophosphatase/phosphodiesterase 1  TAT - tyrosine aminotransferase  PPARGC1A - peroxisome proliferator-activated receptor gamma, coactivator 1 alpha  UBXN1 - ubx domain protein 1  ARG1 - arginase 1  HADH - hydroxyacyl-coa dehydrogenase  BAG3 - bcl2-associated athanogene 3  SCP2 - sterol carrier protein 2  ADA - adenosine deaminase  PM20D1 - peptidase m20 domain containing 1  HAGH - hydroxyacylglutathione hydrolase  CYP1A2 - cytochrome p450, family 1, subfamily a, polypeptide 2  UPF2 - upf2 regulator of nonsense transcripts homolog (yeast)  HSD17B10 - hydroxysteroid (17-beta) dehydrogenase 10  PON1 - paraoxonase 1  APOA5 - apolipoprotein a-v  PCK1 - phosphoenolpyruvate carboxykinase 1 (soluble)  FEM1A - fem-1 homolog a (c. elegans)  FTCD - formimidoyltransferase cyclodeaminase  ALDH1L1 - aldehyde dehydrogenase 1 family, member l1  ADH4 - alcohol dehydrogenase 4 (class ii), pi polypeptide  PRODH2 - proline dehydrogenase (oxidase) 2  CRYL1 - crystallin, lambda 1  ECHS1 - enoyl coa hydratase, short chain, 1, mitochondrial  KEAP1 - kelch-like ech-associated protein 1  ECH1 - enoyl coa hydratase 1, peroxisomal  ECI2 - enoyl-coa delta isomerase 2  AKR1C3 - aldo-keto reductase family 1, member c3  MLYCD - malonyl-coa decarboxylase  FBXO17 - f-box protein 17  ACAA2 - acetyl-coa acyltransferase 2  PGM1 - phosphoglucomutase 1  FABP1 - fatty acid binding protein 1, liver  PGAM2 - phosphoglycerate mutase 2 (muscle)  MCEE - methylmalonyl coa epimerase  ACSL1 - acyl-coa synthetase long-chain family member 1  GOT2 - glutamic-oxaloacetic transaminase 2, mitochondrial  FAH - fumarylacetoacetate hydrolase (fumarylacetoacetase)  ALDH4A1 - aldehyde dehydrogenase 4 family, member a1  GK5 - glycerol kinase 5 (putative)  PNPLA2 - patatin-like phospholipase domain containing 2  ABHD1 - abhydrolase domain containing 1  DPYS - dihydropyrimidinase  GCAT - glycine c-acetyltransferase  AHCY - adenosylhomocysteinase  SULT2A1 - sulfotransferase family, cytosolic, 2a, dehydroepiandrosterone (dhea)-preferring, member 1  AGXT - alanine-glyoxylate aminotransferase  CHIA - chitinase, acidic  WWP1 - ww domain containing e3 ubiquitin protein ligase 1  MAT1A - methionine adenosyltransferase i, alpha  HGD - homogentisate 1,2-dioxygenase  HACL1 - 2-hydroxyacyl-coa lyase 1  DECR2 - 2,4-dienoyl coa reductase 2, peroxisomal  G6PC - glucose-6-phosphatase, catalytic subunit  ALDH2 - aldehyde dehydrogenase 2 family (mitochondrial)  PFKFB1 - 6-phosphofructo-2-kinase/fructose-2,6-biphosphatase 1  UBA52 - ubiquitin a-52 residue ribosomal protein fusion product 1  AGXT2 - alanine--glyoxylate aminotransferase 2  SHMT1 - serine hydroxymethyltransferase 1 (soluble)  LPIN1 - lipin 1  ALDOB - aldolase b, fructose-bisphosphate  ASB2 - ankyrin repeat and socs box containing 2  AMBP - alpha-1-microglobulin/bikunin precursor  ADTRP - androgen-dependent tfpi-regulating protein  ETFDH - electron-transferring-flavoprotein dehydrogenase  PKLR - pyruvate kinase, liver and rbc  HAAO - 3-hydroxyanthranilate 3,4-dioxygenase  ECHDC2 - enoyl coa hydratase domain containing 2  GCDH - glutaryl-coa dehydrogenase  CAT - catalase  TRIM72 - tripartite motif containing 72  PHYH - phytanoyl-coa 2-hydroxylase  STBD1 - starch binding domain 1  SLC27A2 - solute carrier family 27 (fatty acid transporter), member 2  HMOX1 - heme oxygenase (decycling) 1  MPST - mercaptopyruvate sulfurtransferase  CCBL1 - cysteine conjugate-beta lyase, cytoplasmic  URAD - ureidoimidazoline (2-oxo-4-hydroxy-4-carboxy-5-) decarboxylase  HAO2 - hydroxyacid oxidase 2 (long chain) |
| GO:0065008 | regulation of biological quality | 1.25E-6 | 1.29E-4 | 1.34 (10334,2378,667,205) | [+] Show genes  SYPL2 - synaptophysin-like 2  APOPT1 - apoptogenic 1, mitochondrial  AKT1S1 - akt1 substrate 1 (proline-rich)  PLAC8 - placenta-specific 8  HSD11B1 - hydroxysteroid (11-beta) dehydrogenase 1  SLC29A1 - solute carrier family 29 (equilibrative nucleoside transporter), member 1  DHRS7C - dehydrogenase/reductase (sdr family) member 7c  BDH2 - 3-hydroxybutyrate dehydrogenase, type 2  BTC - betacellulin  HRG - histidine-rich glycoprotein  HRC - histidine rich calcium binding protein  ENDOG - endonuclease g  GCGR - glucagon receptor  ACAA1 - acetyl-coa acyltransferase 1  ACADL - acyl-coa dehydrogenase, long chain  HRAS - harvey rat sarcoma viral oncogene homolog  HPX - hemopexin  ACADVL - acyl-coa dehydrogenase, very long chain  CPT2 - carnitine palmitoyltransferase 2  FXYD1 - fxyd domain containing ion transport regulator 1  TTR - transthyretin  CPS1 - carbamoyl-phosphate synthase 1, mitochondrial  PLN - phospholamban  CPN2 - carboxypeptidase n, polypeptide 2  GADD45G - growth arrest and dna-damage-inducible, gamma  PALMD - palmdelphin  ECSIT - ecsit signalling integrator  HPN - hepsin  BHMT - betaine--homocysteine s-methyltransferase  CPB2 - carboxypeptidase b2 (plasma)  TTPA - tocopherol (alpha) transfer protein  FITM1 - fat storage-inducing transmembrane protein 1  MYL3 - myosin, light chain 3, alkali; ventricular, skeletal, slow  HAMP - hepcidin antimicrobial peptide  MYL2 - myosin, light chain 2, regulatory, cardiac, slow  TMOD4 - tropomodulin 4 (muscle)  POLD4 - polymerase (dna-directed), delta 4, accessory subunit  GHR - growth hormone receptor  LIPC - lipase, hepatic  PROZ - protein z, vitamin k-dependent plasma glycoprotein  PLG - plasminogen  NR1I2 - nuclear receptor subfamily 1, group i, member 2  SERPINF2 - serpin peptidase inhibitor, clade f (alpha-2 antiplasmin, pigment epithelium derived factor), member 2  PLEK - pleckstrin  ACOX2 - acyl-coa oxidase 2, branched chain  ACTN2 - actinin, alpha 2  ACTN3 - actinin, alpha 3  PCTP - phosphatidylcholine transfer protein  PPARGC1A - peroxisome proliferator-activated receptor gamma, coactivator 1 alpha  BAG3 - bcl2-associated athanogene 3  ADA - adenosine deaminase  PM20D1 - peptidase m20 domain containing 1  PPARA - peroxisome proliferator-activated receptor alpha  HFE2 - hemochromatosis type 2 (juvenile)  APOA5 - apolipoprotein a-v  GLYAT - glycine-n-acyltransferase  ADH6 - alcohol dehydrogenase 6 (class v)  ADH4 - alcohol dehydrogenase 4 (class ii), pi polypeptide  DGAT2 - diacylglycerol o-acyltransferase 2  PLA2G12B - phospholipase a2, group xiib  SLC22A1 - solute carrier family 22 (organic cation transporter), member 1  C4BPB - complement component 4 binding protein, beta  ATP8B3 - atpase, aminophospholipid transporter, class i, type 8b, member 3  TPT1 - tumor protein, translationally-controlled 1  FAM132A - family with sequence similarity 132, member a  F11 - coagulation factor xi  F10 - coagulation factor x  HSD17B8 - hydroxysteroid (17-beta) dehydrogenase 8  AKR1C3 - aldo-keto reductase family 1, member c3  F9 - coagulation factor ix  HSD17B14 - hydroxysteroid (17-beta) dehydrogenase 14  CAPN3 - calpain 3, (p94)  ACAA2 - acetyl-coa acyltransferase 2  F13B - coagulation factor xiii, b polypeptide  F12 - coagulation factor xii (hageman factor)  ADRB2 - adrenoceptor beta 2, surface  SERPINA10 - serpin peptidase inhibitor, clade a (alpha-1 antiproteinase, antitrypsin), member 10  TMPRSS6 - transmembrane protease, serine 6  CASQ2 - calsequestrin 2 (cardiac muscle)  ACSL1 - acyl-coa synthetase long-chain family member 1  CHCHD10 - coiled-coil-helix-coiled-coil-helix domain containing 10  HOMER2 - homer homolog 2 (drosophila)  AFM - afamin  PNPLA2 - patatin-like phospholipase domain containing 2  ANGPTL4 - angiopoietin-like 4  AGXT - alanine-glyoxylate aminotransferase  ABCB6 - atp-binding cassette, sub-family b (mdr/tap), member 6  KNG1 - kininogen 1  F2 - coagulation factor ii (thrombin)  ALB - albumin  KLKB1 - kallikrein b, plasma (fletcher factor) 1  G6PC - glucose-6-phosphatase, catalytic subunit  F7 - coagulation factor vii (serum prothrombin conversion accelerator)  UBA52 - ubiquitin a-52 residue ribosomal protein fusion product 1  KLF15 - kruppel-like factor 15  CRP - c-reactive protein, pentraxin-related  AGXT2 - alanine--glyoxylate aminotransferase 2  CSRP3 - cysteine and glycine-rich protein 3 (cardiac lim protein)  LPIN1 - lipin 1  PROC - protein c (inactivator of coagulation factors va and viiia)  ALKBH7 - alkb, alkylation repair homolog 7 (e. coli)  ADTRP - androgen-dependent tfpi-regulating protein  MAFB - v-maf avian musculoaponeurotic fibrosarcoma oncogene homolog b  FOXA3 - forkhead box a3  SERPINA6 - serpin peptidase inhibitor, clade a (alpha-1 antiproteinase, antitrypsin), member 6  ISOC2 - isochorismatase domain containing 2  LMOD1 - leiomodin 1 (smooth muscle)  SCGB1A1 - secretoglobin, family 1a, member 1 (uteroglobin)  PHPT1 - phosphohistidine phosphatase 1  CAV3 - caveolin 3  RYR1 - ryanodine receptor 1 (skeletal)  JPH2 - junctophilin 2  PKN1 - protein kinase n1  SLC25A4 - solute carrier family 25 (mitochondrial carrier; adenine nucleotide translocator), member 4  HMOX1 - heme oxygenase (decycling) 1  NR1I3 - nuclear receptor subfamily 1, group i, member 3  LGMN - legumain  ANGPTL3 - angiopoietin-like 3  FN1 - fibronectin 1  APOA2 - apolipoprotein a-ii  APOA4 - apolipoprotein a-iv  OTC - ornithine carbamoyltransferase  SERPINA5 - serpin peptidase inhibitor, clade a (alpha-1 antiproteinase, antitrypsin), member 5  ACSM2A - acyl-coa synthetase medium-chain family member 2a  DHRS3 - dehydrogenase/reductase (sdr family) member 3  AQP9 - aquaporin 9  P2RY2 - purinergic receptor p2y, g-protein coupled, 2  CCL14 - chemokine (c-c motif) ligand 14  CD36 - cd36 molecule (thrombospondin receptor)  IL1RN - interleukin 1 receptor antagonist  APOC2 - apolipoprotein c-ii  PSMC5 - proteasome (prosome, macropain) 26s subunit, atpase, 5  APOC3 - apolipoprotein c-iii  APOC4 - apolipoprotein c-iv  PAFAH2 - platelet-activating factor acetylhydrolase 2, 40kda  APOH - apolipoprotein h (beta-2-glycoprotein i)  PAH - phenylalanine hydroxylase  TBRG4 - transforming growth factor beta regulator 4  FH - fumarate hydratase  YBX3 - y box binding protein 3  RGS14 - regulator of g-protein signaling 14  UPB1 - ureidopropionase, beta  CYP4A11 - cytochrome p450, family 4, subfamily a, polypeptide 11  FGG - fibrinogen gamma chain  CES1 - carboxylesterase 1  ALDH8A1 - aldehyde dehydrogenase 8 family, member a1  CYP3A5 - cytochrome p450, family 3, subfamily a, polypeptide 5  ENPP1 - ectonucleotide pyrophosphatase/phosphodiesterase 1  PDK4 - pyruvate dehydrogenase kinase, isozyme 4  FGA - fibrinogen alpha chain  FGB - fibrinogen beta chain  HADH - hydroxyacyl-coa dehydrogenase  PDK2 - pyruvate dehydrogenase kinase, isozyme 2  CYP1A2 - cytochrome p450, family 1, subfamily a, polypeptide 2  SERPINF1 - serpin peptidase inhibitor, clade f (alpha-2 antiplasmin, pigment epithelium derived factor), member 1  IYD - iodotyrosine deiodinase  C19orf80 - chromosome 19 open reading frame 80  SERPIND1 - serpin peptidase inhibitor, clade d (heparin cofactor), member 1  HSD17B6 - hydroxysteroid (17-beta) dehydrogenase 6  PTP4A3 - protein tyrosine phosphatase type iva, member 3  TM6SF2 - transmembrane 6 superfamily member 2  PCK1 - phosphoenolpyruvate carboxykinase 1 (soluble)  SAA1 - serum amyloid a1  SERPINA7 - serpin peptidase inhibitor, clade a (alpha-1 antiproteinase, antitrypsin), member 7  SLC51B - solute carrier family 51, beta subunit  ASS1 - argininosuccinate synthase 1  RDH16 - retinol dehydrogenase 16 (all-trans)  APOM - apolipoprotein m  MLXIPL - mlx interacting protein-like  YBX1 - y box binding protein 1  ASGR2 - asialoglycoprotein receptor 2  SMTNL1 - smoothelin-like 1  KEAP1 - kelch-like ech-associated protein 1  MPV17L - mpv17 mitochondrial membrane protein-like  ACSM3 - acyl-coa synthetase medium-chain family member 3  ATP2A1 - atpase, ca++ transporting, cardiac muscle, fast twitch 1  CHRNE - cholinergic receptor, nicotinic, epsilon (muscle)  RILP - rab interacting lysosomal protein  ORMDL3 - orm1-like 3 (s. cerevisiae)  CHRNA1 - cholinergic receptor, nicotinic, alpha 1 (muscle)  ATF4 - activating transcription factor 4  GOT2 - glutamic-oxaloacetic transaminase 2, mitochondrial  POPDC3 - popeye domain containing 3  SERPINC1 - serpin peptidase inhibitor, clade c (antithrombin), member 1  LMOD3 - leiomodin 3 (fetal)  DPYS - dihydropyrimidinase  AKR1C4 - aldo-keto reductase family 1, member c4  GCAT - glycine c-acetyltransferase  PLIN5 - perilipin 5  SLC40A1 - solute carrier family 40 (iron-regulated transporter), member 1  LMOD2 - leiomodin 2 (cardiac)  VTN - vitronectin  TNNC1 - troponin c type 1 (slow)  SHMT1 - serine hydroxymethyltransferase 1 (soluble)  AZGP1 - alpha-2-glycoprotein 1, zinc-binding  GRB10 - growth factor receptor-bound protein 10  TFR2 - transferrin receptor 2  HAAO - 3-hydroxyanthranilate 3,4-dioxygenase  SLC39A5 - solute carrier family 39 (zinc transporter), member 5  IGF1 - insulin-like growth factor 1 (somatomedin c)  NEB - nebulin  SERPINA1 - serpin peptidase inhibitor, clade a (alpha-1 antiproteinase, antitrypsin), member 1  GET4 - golgi to er traffic protein 4 homolog (s. cerevisiae)  PIGR - polymeric immunoglobulin receptor  FGGY - fggy carbohydrate kinase domain containing |
| GO:0042632 | cholesterol homeostasis | 1.26E-6 | 1.3E-4 | 4.00 (10334,62,667,16) | [+] Show genes  APOA5 - apolipoprotein a-v  ANGPTL3 - angiopoietin-like 3  APOA2 - apolipoprotein a-ii  CES1 - carboxylesterase 1  CAV3 - caveolin 3  APOA4 - apolipoprotein a-iv  DGAT2 - diacylglycerol o-acyltransferase 2  APOC2 - apolipoprotein c-ii  LIPC - lipase, hepatic  APOC3 - apolipoprotein c-iii  PLA2G12B - phospholipase a2, group xiib  APOM - apolipoprotein m  NR1I2 - nuclear receptor subfamily 1, group i, member 2  G6PC - glucose-6-phosphatase, catalytic subunit  ACSM3 - acyl-coa synthetase medium-chain family member 3  NR1I3 - nuclear receptor subfamily 1, group i, member 3 |
| GO:0046364 | monosaccharide biosynthetic process | 1.55E-6 | 1.58E-4 | 4.43 (10334,49,667,14) | [+] Show genes  SLC37A4 - solute carrier family 37 (glucose-6-phosphate transporter), member 4  PCK1 - phosphoenolpyruvate carboxykinase 1 (soluble)  PGM1 - phosphoglucomutase 1  PGAM2 - phosphoglycerate mutase 2 (muscle)  ALDOB - aldolase b, fructose-bisphosphate  GOT2 - glutamic-oxaloacetic transaminase 2, mitochondrial  ATF4 - activating transcription factor 4  ENO3 - enolase 3 (beta, muscle)  PPARGC1A - peroxisome proliferator-activated receptor gamma, coactivator 1 alpha  FBP2 - fructose-1,6-bisphosphatase 2  SLC25A13 - solute carrier family 25 (aspartate/glutamate carrier), member 13  GPD1 - glycerol-3-phosphate dehydrogenase 1 (soluble)  G6PC - glucose-6-phosphatase, catalytic subunit  PFKFB1 - 6-phosphofructo-2-kinase/fructose-2,6-biphosphatase 1 |
| GO:0006066 | alcohol metabolic process | 1.56E-6 | 1.59E-4 | 2.43 (10334,210,667,33) | [+] Show genes  AKR1C3 - aldo-keto reductase family 1, member c3  ANGPTL3 - angiopoietin-like 3  ACAA2 - acetyl-coa acyltransferase 2  APOA2 - apolipoprotein a-ii  CES1 - carboxylesterase 1  PLCD4 - phospholipase c, delta 4  CYP3A5 - cytochrome p450, family 3, subfamily a, polypeptide 5  SCARF1 - scavenger receptor class f, member 1  APOA4 - apolipoprotein a-iv  GK5 - glycerol kinase 5 (putative)  IMPA2 - inositol(myo)-1(or 4)-monophosphatase 2  APOF - apolipoprotein f  CYP1A2 - cytochrome p450, family 1, subfamily a, polypeptide 2  GPD1 - glycerol-3-phosphate dehydrogenase 1 (soluble)  PNPLA2 - patatin-like phospholipase domain containing 2  PON1 - paraoxonase 1  DHRS3 - dehydrogenase/reductase (sdr family) member 3  TTR - transthyretin  DHRS4 - dehydrogenase/reductase (sdr family) member 4  AKR1C4 - aldo-keto reductase family 1, member c4  APOA5 - apolipoprotein a-v  PCK1 - phosphoenolpyruvate carboxykinase 1 (soluble)  IGF1 - insulin-like growth factor 1 (somatomedin c)  SULT2A1 - sulfotransferase family, cytosolic, 2a, dehydroepiandrosterone (dhea)-preferring, member 1  CAT - catalase  ADH6 - alcohol dehydrogenase 6 (class v)  RXRA - retinoid x receptor, alpha  ADH4 - alcohol dehydrogenase 4 (class ii), pi polypeptide  DGAT2 - diacylglycerol o-acyltransferase 2  RDH16 - retinol dehydrogenase 16 (all-trans)  LIPC - lipase, hepatic  HMGCS2 - 3-hydroxy-3-methylglutaryl-coa synthase 2 (mitochondrial)  ALDH2 - aldehyde dehydrogenase 2 family (mitochondrial) |
| GO:0003009 | skeletal muscle contraction | 1.58E-6 | 1.59E-4 | 6.20 (10334,25,667,10) | [+] Show genes  TNNC2 - troponin c type 2 (fast)  STAC3 - sh3 and cysteine rich domain 3  MB - myoglobin  TNNI2 - troponin i type 2 (skeletal, fast)  RCSD1 - rcsd domain containing 1  TNNI1 - troponin i type 1 (skeletal, slow)  TCAP - titin-cap  TNNC1 - troponin c type 1 (slow)  TNNT3 - troponin t type 3 (skeletal, fast)  CHRNA1 - cholinergic receptor, nicotinic, alpha 1 (muscle) |
| GO:0055092 | sterol homeostasis | 1.6E-6 | 1.6E-4 | 3.93 (10334,63,667,16) | [+] Show genes  APOA5 - apolipoprotein a-v  ANGPTL3 - angiopoietin-like 3  APOA2 - apolipoprotein a-ii  CES1 - carboxylesterase 1  CAV3 - caveolin 3  APOA4 - apolipoprotein a-iv  DGAT2 - diacylglycerol o-acyltransferase 2  APOC2 - apolipoprotein c-ii  LIPC - lipase, hepatic  APOC3 - apolipoprotein c-iii  PLA2G12B - phospholipase a2, group xiib  APOM - apolipoprotein m  NR1I2 - nuclear receptor subfamily 1, group i, member 2  G6PC - glucose-6-phosphatase, catalytic subunit  ACSM3 - acyl-coa synthetase medium-chain family member 3  NR1I3 - nuclear receptor subfamily 1, group i, member 3 |
| GO:0042445 | hormone metabolic process | 1.6E-6 | 1.59E-4 | 3.10 (10334,110,667,22) | [+] Show genes  DHRS3 - dehydrogenase/reductase (sdr family) member 3  IYD - iodotyrosine deiodinase  HSD17B8 - hydroxysteroid (17-beta) dehydrogenase 8  AKR1C3 - aldo-keto reductase family 1, member c3  TTR - transthyretin  SERPINA6 - serpin peptidase inhibitor, clade a (alpha-1 antiproteinase, antitrypsin), member 6  HSD17B6 - hydroxysteroid (17-beta) dehydrogenase 6  AKR1C4 - aldo-keto reductase family 1, member c4  HSD17B14 - hydroxysteroid (17-beta) dehydrogenase 14  ALDH8A1 - aldehyde dehydrogenase 8 family, member a1  CES1 - carboxylesterase 1  ADH6 - alcohol dehydrogenase 6 (class v)  CYP3A5 - cytochrome p450, family 3, subfamily a, polypeptide 5  ADH4 - alcohol dehydrogenase 4 (class ii), pi polypeptide  HSD11B1 - hydroxysteroid (11-beta) dehydrogenase 1  DGAT2 - diacylglycerol o-acyltransferase 2  RDH16 - retinol dehydrogenase 16 (all-trans)  GHR - growth hormone receptor  PPARGC1A - peroxisome proliferator-activated receptor gamma, coactivator 1 alpha  ACAA1 - acetyl-coa acyltransferase 1  CYP1A2 - cytochrome p450, family 1, subfamily a, polypeptide 2  PNPLA2 - patatin-like phospholipase domain containing 2 |
| GO:0050996 | positive regulation of lipid catabolic process | 1.62E-6 | 1.6E-4 | 6.97 (10334,20,667,9) | [+] Show genes  APOC2 - apolipoprotein c-ii  APOA5 - apolipoprotein a-v  ANGPTL3 - angiopoietin-like 3  PLIN5 - perilipin 5  APOA2 - apolipoprotein a-ii  FABP1 - fatty acid binding protein 1, liver  PPARA - peroxisome proliferator-activated receptor alpha  PNPLA2 - patatin-like phospholipase domain containing 2  APOA4 - apolipoprotein a-iv |
| GO:0006163 | purine nucleotide metabolic process | 1.98E-6 | 1.94E-4 | 2.17 (10334,293,667,41) | [+] Show genes  UQCRC1 - ubiquinol-cytochrome c reductase core protein i  HSD17B8 - hydroxysteroid (17-beta) dehydrogenase 8  MLYCD - malonyl-coa decarboxylase  PGM1 - phosphoglucomutase 1  COX5B - cytochrome c oxidase subunit vb  PGAM2 - phosphoglycerate mutase 2 (muscle)  ADSSL1 - adenylosuccinate synthase like 1  ALDOB - aldolase b, fructose-bisphosphate  MCEE - methylmalonyl coa epimerase  ACSL1 - acyl-coa synthetase long-chain family member 1  ENPP1 - ectonucleotide pyrophosphatase/phosphodiesterase 1  MTHFD1 - methylenetetrahydrofolate dehydrogenase (nadp+ dependent) 1, methenyltetrahydrofolate cyclohydrolase, formyltetrahydrofolate synthetase  ENO3 - enolase 3 (beta, muscle)  CHCHD10 - coiled-coil-helix-coiled-coil-helix domain containing 10  ADA - adenosine deaminase  LDHA - lactate dehydrogenase a  SLC25A13 - solute carrier family 25 (aspartate/glutamate carrier), member 13  PTPLA - protein tyrosine phosphatase-like (proline instead of catalytic arginine), member a  MPC1 - mitochondrial pyruvate carrier 1  ACSM2A - acyl-coa synthetase medium-chain family member 2a  PKLR - pyruvate kinase, liver and rbc  AK2 - adenylate kinase 2  ATP5J2 - atp synthase, h+ transporting, mitochondrial fo complex, subunit f2  GLYAT - glycine-n-acyltransferase  SULT2A1 - sulfotransferase family, cytosolic, 2a, dehydroepiandrosterone (dhea)-preferring, member 1  GCDH - glutaryl-coa dehydrogenase  ATP5I - atp synthase, h+ transporting, mitochondrial fo complex, subunit e  ATP5H - atp synthase, h+ transporting, mitochondrial fo complex, subunit d  GIMAP7 - gtpase, imap family member 7  AMPD1 - adenosine monophosphate deaminase 1  DGAT2 - diacylglycerol o-acyltransferase 2  TDO2 - tryptophan 2,3-dioxygenase  ATP5E - atp synthase, h+ transporting, mitochondrial f1 complex, epsilon subunit  ATP5D - atp synthase, h+ transporting, mitochondrial f1 complex, delta subunit  PIPOX - pipecolic acid oxidase  ATP5G1 - atp synthase, h+ transporting, mitochondrial fo complex, subunit c1 (subunit 9)  HMGCS2 - 3-hydroxy-3-methylglutaryl-coa synthase 2 (mitochondrial)  ACSM3 - acyl-coa synthetase medium-chain family member 3  PFKFB1 - 6-phosphofructo-2-kinase/fructose-2,6-biphosphatase 1  MOCS1 - molybdenum cofactor synthesis 1  ACSM5 - acyl-coa synthetase medium-chain family member 5 |
| GO:0055001 | muscle cell development | 2.08E-6 | 2.02E-4 | 4.08 (10334,57,667,15) | [+] Show genes  KLHL41 - kelch-like family member 41  STAC3 - sh3 and cysteine rich domain 3  IGF1 - insulin-like growth factor 1 (somatomedin c)  ACTN2 - actinin, alpha 2  CAV3 - caveolin 3  TTN - titin  RYR1 - ryanodine receptor 1 (skeletal)  NEB - nebulin  NRAP - nebulin-related anchoring protein  MYL2 - myosin, light chain 2, regulatory, cardiac, slow  ACTA1 - actin, alpha 1, skeletal muscle  TCAP - titin-cap  KLHL40 - kelch-like family member 40  LMOD3 - leiomodin 3 (fetal)  MYO18B - myosin xviiib |
| GO:0046942 | carboxylic acid transport | 2.09E-6 | 2.02E-4 | 2.63 (10334,165,667,28) | [+] Show genes  SLC16A13 - solute carrier family 16, member 13  FABP1 - fatty acid binding protein 1, liver  ACSL1 - acyl-coa synthetase long-chain family member 1  SLC16A5 - solute carrier family 16 (monocarboxylate transporter), member 5  GOT2 - glutamic-oxaloacetic transaminase 2, mitochondrial  SLC10A1 - solute carrier family 10 (sodium/bile acid cotransporter), member 1  SLC38A4 - solute carrier family 38, member 4  SLC25A13 - solute carrier family 25 (aspartate/glutamate carrier), member 13  PPARA - peroxisome proliferator-activated receptor alpha  MPC1 - mitochondrial pyruvate carrier 1  CPT2 - carnitine palmitoyltransferase 2  SLC17A2 - solute carrier family 17, member 2  SLCO1B3 - solute carrier organic anion transporter family, member 1b3  SLC25A47 - solute carrier family 25, member 47  CPT1B - carnitine palmitoyltransferase 1b (muscle)  AKR1C4 - aldo-keto reductase family 1, member c4  AQP9 - aquaporin 9  CD36 - cd36 molecule (thrombospondin receptor)  SLC25A20 - solute carrier family 25 (carnitine/acylcarnitine translocase), member 20  SLC51B - solute carrier family 51, beta subunit  AGXT - alanine-glyoxylate aminotransferase  RXRA - retinoid x receptor, alpha  SLC25A30 - solute carrier family 25, member 30  PLA2G12B - phospholipase a2, group xiib  SLC27A2 - solute carrier family 27 (fatty acid transporter), member 2  SLC17A1 - solute carrier family 17 (organic anion transporter), member 1  THRSP - thyroid hormone responsive  SLC27A5 - solute carrier family 27 (fatty acid transporter), member 5 |
| GO:0009150 | purine ribonucleotide metabolic process | 2.1E-6 | 2.02E-4 | 2.21 (10334,273,667,39) | [+] Show genes  HSD17B8 - hydroxysteroid (17-beta) dehydrogenase 8  UQCRC1 - ubiquinol-cytochrome c reductase core protein i  MLYCD - malonyl-coa decarboxylase  PGM1 - phosphoglucomutase 1  COX5B - cytochrome c oxidase subunit vb  PGAM2 - phosphoglycerate mutase 2 (muscle)  ALDOB - aldolase b, fructose-bisphosphate  ADSSL1 - adenylosuccinate synthase like 1  MCEE - methylmalonyl coa epimerase  ACSL1 - acyl-coa synthetase long-chain family member 1  ENPP1 - ectonucleotide pyrophosphatase/phosphodiesterase 1  ENO3 - enolase 3 (beta, muscle)  CHCHD10 - coiled-coil-helix-coiled-coil-helix domain containing 10  LDHA - lactate dehydrogenase a  SLC25A13 - solute carrier family 25 (aspartate/glutamate carrier), member 13  PTPLA - protein tyrosine phosphatase-like (proline instead of catalytic arginine), member a  MPC1 - mitochondrial pyruvate carrier 1  ACSM2A - acyl-coa synthetase medium-chain family member 2a  PKLR - pyruvate kinase, liver and rbc  AK2 - adenylate kinase 2  ATP5J2 - atp synthase, h+ transporting, mitochondrial fo complex, subunit f2  GLYAT - glycine-n-acyltransferase  SULT2A1 - sulfotransferase family, cytosolic, 2a, dehydroepiandrosterone (dhea)-preferring, member 1  GCDH - glutaryl-coa dehydrogenase  ATP5I - atp synthase, h+ transporting, mitochondrial fo complex, subunit e  ATP5H - atp synthase, h+ transporting, mitochondrial fo complex, subunit d  GIMAP7 - gtpase, imap family member 7  AMPD1 - adenosine monophosphate deaminase 1  DGAT2 - diacylglycerol o-acyltransferase 2  TDO2 - tryptophan 2,3-dioxygenase  ATP5E - atp synthase, h+ transporting, mitochondrial f1 complex, epsilon subunit  ATP5D - atp synthase, h+ transporting, mitochondrial f1 complex, delta subunit  PIPOX - pipecolic acid oxidase  ATP5G1 - atp synthase, h+ transporting, mitochondrial fo complex, subunit c1 (subunit 9)  HMGCS2 - 3-hydroxy-3-methylglutaryl-coa synthase 2 (mitochondrial)  ACSM3 - acyl-coa synthetase medium-chain family member 3  PFKFB1 - 6-phosphofructo-2-kinase/fructose-2,6-biphosphatase 1  MOCS1 - molybdenum cofactor synthesis 1  ACSM5 - acyl-coa synthetase medium-chain family member 5 |
| GO:0006575 | cellular modified amino acid metabolic process | 2.12E-6 | 2.02E-4 | 2.88 (10334,129,667,24) | [+] Show genes  IYD - iodotyrosine deiodinase  CPT1B - carnitine palmitoyltransferase 1b (muscle)  AHCY - adenosylhomocysteinase  FTCD - formimidoyltransferase cyclodeaminase  SHMT1 - serine hydroxymethyltransferase 1 (soluble)  GAMT - guanidinoacetate n-methyltransferase  ALDH1L1 - aldehyde dehydrogenase 1 family, member l1  BHMT - betaine--homocysteine s-methyltransferase  ASS1 - argininosuccinate synthase 1  ETHE1 - ethylmalonic encephalopathy 1  VNN1 - vanin 1  MTHFR - methylenetetrahydrofolate reductase (nad(p)h)  MTHFD1 - methylenetetrahydrofolate dehydrogenase (nadp+ dependent) 1, methenyltetrahydrofolate cyclohydrolase, formyltetrahydrofolate synthetase  CKM - creatine kinase, muscle  GOT2 - glutamic-oxaloacetic transaminase 2, mitochondrial  PIPOX - pipecolic acid oxidase  ALDH4A1 - aldehyde dehydrogenase 4 family, member a1  GSTZ1 - glutathione s-transferase zeta 1  ACADL - acyl-coa dehydrogenase, long chain  HAGH - hydroxyacylglutathione hydrolase  GSTT1 - glutathione s-transferase theta 1  GGT1 - gamma-glutamyltransferase 1  CCBL1 - cysteine conjugate-beta lyase, cytoplasmic  MMACHC - methylmalonic aciduria (cobalamin deficiency) cblc type, with homocystinuria |
| GO:0015849 | organic acid transport | 2.36E-6 | 2.23E-4 | 2.61 (10334,166,667,28) | [+] Show genes  SLC16A13 - solute carrier family 16, member 13  FABP1 - fatty acid binding protein 1, liver  SLC16A5 - solute carrier family 16 (monocarboxylate transporter), member 5  ACSL1 - acyl-coa synthetase long-chain family member 1  SLC10A1 - solute carrier family 10 (sodium/bile acid cotransporter), member 1  GOT2 - glutamic-oxaloacetic transaminase 2, mitochondrial  SLC38A4 - solute carrier family 38, member 4  SLC25A13 - solute carrier family 25 (aspartate/glutamate carrier), member 13  PPARA - peroxisome proliferator-activated receptor alpha  MPC1 - mitochondrial pyruvate carrier 1  CPT2 - carnitine palmitoyltransferase 2  SLC17A2 - solute carrier family 17, member 2  SLCO1B3 - solute carrier organic anion transporter family, member 1b3  SLC25A47 - solute carrier family 25, member 47  CPT1B - carnitine palmitoyltransferase 1b (muscle)  AKR1C4 - aldo-keto reductase family 1, member c4  AQP9 - aquaporin 9  CD36 - cd36 molecule (thrombospondin receptor)  SLC25A20 - solute carrier family 25 (carnitine/acylcarnitine translocase), member 20  SLC51B - solute carrier family 51, beta subunit  AGXT - alanine-glyoxylate aminotransferase  RXRA - retinoid x receptor, alpha  SLC25A30 - solute carrier family 25, member 30  PLA2G12B - phospholipase a2, group xiib  SLC27A2 - solute carrier family 27 (fatty acid transporter), member 2  SLC17A1 - solute carrier family 17 (organic anion transporter), member 1  THRSP - thyroid hormone responsive  SLC27A5 - solute carrier family 27 (fatty acid transporter), member 5 |
| GO:0002576 | platelet degranulation | 2.44E-6 | 2.3E-4 | 3.23 (10334,96,667,20) | [+] Show genes  A1BG - alpha-1-b glycoprotein  FGG - fibrinogen gamma chain  FN1 - fibronectin 1  IGF1 - insulin-like growth factor 1 (somatomedin c)  CD36 - cd36 molecule (thrombospondin receptor)  ACTN2 - actinin, alpha 2  SPP2 - secreted phosphoprotein 2, 24kda  TTN - titin  SERPINA1 - serpin peptidase inhibitor, clade a (alpha-1 antiproteinase, antitrypsin), member 1  FGA - fibrinogen alpha chain  HRG - histidine-rich glycoprotein  KNG1 - kininogen 1  ITIH3 - inter-alpha-trypsin inhibitor heavy chain 3  FGB - fibrinogen beta chain  ITIH4 - inter-alpha-trypsin inhibitor heavy chain family, member 4  APOH - apolipoprotein h (beta-2-glycoprotein i)  PLG - plasminogen  ALB - albumin  SERPINF2 - serpin peptidase inhibitor, clade f (alpha-2 antiplasmin, pigment epithelium derived factor), member 2  PLEK - pleckstrin |
| GO:0006754 | ATP biosynthetic process | 2.64E-6 | 2.46E-4 | 4.01 (10334,58,667,15) | [+] Show genes  ATP5J2 - atp synthase, h+ transporting, mitochondrial fo complex, subunit f2  PGM1 - phosphoglucomutase 1  ATP5H - atp synthase, h+ transporting, mitochondrial fo complex, subunit d  ATP5I - atp synthase, h+ transporting, mitochondrial fo complex, subunit e  COX5B - cytochrome c oxidase subunit vb  PGAM2 - phosphoglycerate mutase 2 (muscle)  ALDOB - aldolase b, fructose-bisphosphate  ATP5E - atp synthase, h+ transporting, mitochondrial f1 complex, epsilon subunit  ENO3 - enolase 3 (beta, muscle)  ATP5D - atp synthase, h+ transporting, mitochondrial f1 complex, delta subunit  ATP5G1 - atp synthase, h+ transporting, mitochondrial fo complex, subunit c1 (subunit 9)  LDHA - lactate dehydrogenase a  SLC25A13 - solute carrier family 25 (aspartate/glutamate carrier), member 13  PKLR - pyruvate kinase, liver and rbc  PFKFB1 - 6-phosphofructo-2-kinase/fructose-2,6-biphosphatase 1 |
| GO:0007597 | blood coagulation, intrinsic pathway | 2.69E-6 | 2.5E-4 | 9.04 (10334,12,667,7) | [+] Show genes  F11 - coagulation factor xi  F9 - coagulation factor ix  KNG1 - kininogen 1  APOH - apolipoprotein h (beta-2-glycoprotein i)  F12 - coagulation factor xii (hageman factor)  F2 - coagulation factor ii (thrombin)  KLKB1 - kallikrein b, plasma (fletcher factor) 1 |
| GO:0090303 | positive regulation of wound healing | 3.19E-6 | 2.94E-4 | 4.48 (10334,45,667,13) | [+] Show genes  CAPN3 - calpain 3, (p94)  F12 - coagulation factor xii (hageman factor)  CD36 - cd36 molecule (thrombospondin receptor)  CPB2 - carboxypeptidase b2 (plasma)  HRG - histidine-rich glycoprotein  APOH - apolipoprotein h (beta-2-glycoprotein i)  PLG - plasminogen  F2 - coagulation factor ii (thrombin)  VTN - vitronectin  HRAS - harvey rat sarcoma viral oncogene homolog  SERPINF2 - serpin peptidase inhibitor, clade f (alpha-2 antiplasmin, pigment epithelium derived factor), member 2  F7 - coagulation factor vii (serum prothrombin conversion accelerator)  PLEK - pleckstrin |
| GO:0006811 | ion transport | 3.32E-6 | 3.04E-4 | 1.68 (10334,710,667,77) | [+] Show genes  ATP2A1 - atpase, ca++ transporting, cardiac muscle, fast twitch 1  CHRNE - cholinergic receptor, nicotinic, epsilon (muscle)  COX6A2 - cytochrome c oxidase subunit via polypeptide 2  CLDN15 - claudin 15  APOA2 - apolipoprotein a-ii  SLC16A13 - solute carrier family 16, member 13  NDUFA4 - nadh dehydrogenase (ubiquinone) 1 alpha subcomplex, 4, 9kda  FABP1 - fatty acid binding protein 1, liver  NDUFS7 - nadh dehydrogenase (ubiquinone) fe-s protein 7, 20kda (nadh-coenzyme q reductase)  COX5B - cytochrome c oxidase subunit vb  SLC22A18 - solute carrier family 22, member 18  CHRNA1 - cholinergic receptor, nicotinic, alpha 1 (muscle)  APOA4 - apolipoprotein a-iv  CASQ2 - calsequestrin 2 (cardiac muscle)  ACSL1 - acyl-coa synthetase long-chain family member 1  SLC16A5 - solute carrier family 16 (monocarboxylate transporter), member 5  GOT2 - glutamic-oxaloacetic transaminase 2, mitochondrial  SLC10A1 - solute carrier family 10 (sodium/bile acid cotransporter), member 1  CA14 - carbonic anhydrase xiv  SLC25A13 - solute carrier family 25 (aspartate/glutamate carrier), member 13  COX7C - cytochrome c oxidase subunit viic  CACNG6 - calcium channel, voltage-dependent, gamma subunit 6  CPT2 - carnitine palmitoyltransferase 2  FXYD1 - fxyd domain containing ion transport regulator 1  ATP5J2 - atp synthase, h+ transporting, mitochondrial fo complex, subunit f2  SLCO1B3 - solute carrier organic anion transporter family, member 1b3  SLC37A4 - solute carrier family 37 (glucose-6-phosphate transporter), member 4  AKR1C4 - aldo-keto reductase family 1, member c4  SLC25A47 - solute carrier family 25, member 47  CPT1B - carnitine palmitoyltransferase 1b (muscle)  CACNG1 - calcium channel, voltage-dependent, gamma subunit 1  AQP9 - aquaporin 9  PLN - phospholamban  CD36 - cd36 molecule (thrombospondin receptor)  SLC25A20 - solute carrier family 25 (carnitine/acylcarnitine translocase), member 20  ATP5H - atp synthase, h+ transporting, mitochondrial fo complex, subunit d  ATP5I - atp synthase, h+ transporting, mitochondrial fo complex, subunit e  AGXT - alanine-glyoxylate aminotransferase  SLC40A1 - solute carrier family 40 (iron-regulated transporter), member 1  HPN - hepsin  WWP1 - ww domain containing e3 ubiquitin protein ligase 1  SLC25A30 - solute carrier family 25, member 30  CACNA1S - calcium channel, voltage-dependent, l type, alpha 1s subunit  APOC2 - apolipoprotein c-ii  ATP5E - atp synthase, h+ transporting, mitochondrial f1 complex, epsilon subunit  APOC3 - apolipoprotein c-iii  ATP5D - atp synthase, h+ transporting, mitochondrial f1 complex, delta subunit  ATP5G1 - atp synthase, h+ transporting, mitochondrial fo complex, subunit c1 (subunit 9)  G6PC - glucose-6-phosphatase, catalytic subunit  COX17 - cox17 cytochrome c oxidase copper chaperone  PCTP - phosphatidylcholine transfer protein  CA3 - carbonic anhydrase iii, muscle specific  ENPP1 - ectonucleotide pyrophosphatase/phosphodiesterase 1  SLC38A4 - solute carrier family 38, member 4  SCP2 - sterol carrier protein 2  ADA - adenosine deaminase  TFR2 - transferrin receptor 2  PPARA - peroxisome proliferator-activated receptor alpha  MPC1 - mitochondrial pyruvate carrier 1  SLC39A5 - solute carrier family 39 (zinc transporter), member 5  SLC17A2 - solute carrier family 17, member 2  APOA5 - apolipoprotein a-v  SLC51B - solute carrier family 51, beta subunit  RXRA - retinoid x receptor, alpha  UCP3 - uncoupling protein 3 (mitochondrial, proton carrier)  JPH2 - junctophilin 2  RYR1 - ryanodine receptor 1 (skeletal)  PLA2G12B - phospholipase a2, group xiib  SLC22A1 - solute carrier family 22 (organic cation transporter), member 1  SLC25A4 - solute carrier family 25 (mitochondrial carrier; adenine nucleotide translocator), member 4  SLC27A2 - solute carrier family 27 (fatty acid transporter), member 2  ATP8B3 - atpase, aminophospholipid transporter, class i, type 8b, member 3  SLC22A7 - solute carrier family 22 (organic anion transporter), member 7  SLC17A1 - solute carrier family 17 (organic anion transporter), member 1  THRSP - thyroid hormone responsive  SLC27A5 - solute carrier family 27 (fatty acid transporter), member 5  TPT1 - tumor protein, translationally-controlled 1 |
| GO:1903036 | positive regulation of response to wounding | 3.38E-6 | 3.08E-4 | 4.17 (10334,52,667,14) | [+] Show genes  CAPN3 - calpain 3, (p94)  F12 - coagulation factor xii (hageman factor)  CD36 - cd36 molecule (thrombospondin receptor)  SCARF1 - scavenger receptor class f, member 1  CPB2 - carboxypeptidase b2 (plasma)  HRG - histidine-rich glycoprotein  APOH - apolipoprotein h (beta-2-glycoprotein i)  PLG - plasminogen  F2 - coagulation factor ii (thrombin)  VTN - vitronectin  HRAS - harvey rat sarcoma viral oncogene homolog  SERPINF2 - serpin peptidase inhibitor, clade f (alpha-2 antiplasmin, pigment epithelium derived factor), member 2  F7 - coagulation factor vii (serum prothrombin conversion accelerator)  PLEK - pleckstrin |
| GO:0015850 | organic hydroxy compound transport | 3.54E-6 | 3.2E-4 | 3.40 (10334,82,667,18) | [+] Show genes  SLCO1B3 - solute carrier organic anion transporter family, member 1b3  AKR1C4 - aldo-keto reductase family 1, member c4  APOA5 - apolipoprotein a-v  AQP9 - aquaporin 9  AQP7 - aquaporin 7  CD36 - cd36 molecule (thrombospondin receptor)  APOA2 - apolipoprotein a-ii  CES1 - carboxylesterase 1  SLC51B - solute carrier family 51, beta subunit  RXRA - retinoid x receptor, alpha  APOA4 - apolipoprotein a-iv  APOC2 - apolipoprotein c-ii  SLC10A1 - solute carrier family 10 (sodium/bile acid cotransporter), member 1  APOC3 - apolipoprotein c-iii  LIPC - lipase, hepatic  APOM - apolipoprotein m  SLC22A1 - solute carrier family 22 (organic cation transporter), member 1  SLC27A5 - solute carrier family 27 (fatty acid transporter), member 5 |
| GO:0006954 | inflammatory response | 3.63E-6 | 3.26E-4 | 2.35 (10334,218,667,33) | [+] Show genes  APCS - amyloid p component, serum  CRP - c-reactive protein, pentraxin-related  AOC3 - amine oxidase, copper containing 3  FN1 - fibronectin 1  F12 - coagulation factor xii (hageman factor)  CSRP3 - cysteine and glycine-rich protein 3 (cardiac lim protein)  APOA2 - apolipoprotein a-ii  VNN1 - vanin 1  ITIH4 - inter-alpha-trypsin inhibitor heavy chain family, member 4  SERPINC1 - serpin peptidase inhibitor, clade c (antithrombin), member 1  TFR2 - transferrin receptor 2  CD5L - cd5 molecule-like  S100A12 - s100 calcium binding protein a12  CCL16 - chemokine (c-c motif) ligand 16  TNIP1 - tnfaip3 interacting protein 1  MBL2 - mannose-binding lectin (protein c) 2, soluble  CCL14 - chemokine (c-c motif) ligand 14  SAA1 - serum amyloid a1  AHCY - adenosylhomocysteinase  LBP - lipopolysaccharide binding protein  IL1RN - interleukin 1 receptor antagonist  ASS1 - argininosuccinate synthase 1  HP - haptoglobin  SERPINA1 - serpin peptidase inhibitor, clade a (alpha-1 antiproteinase, antitrypsin), member 1  FCGR2B - fc fragment of igg, low affinity iib, receptor (cd32)  HAMP - hepcidin antimicrobial peptide  KNG1 - kininogen 1  F2 - coagulation factor ii (thrombin)  SAA4 - serum amyloid a4, constitutive  C5 - complement component 5  NUPR1 - nuclear protein, transcriptional regulator, 1  SERPINF2 - serpin peptidase inhibitor, clade f (alpha-2 antiplasmin, pigment epithelium derived factor), member 2  CXCL3 - chemokine (c-x-c motif) ligand 3 |
| GO:0034754 | cellular hormone metabolic process | 3.84E-6 | 3.42E-4 | 3.70 (10334,67,667,16) | [+] Show genes  DHRS3 - dehydrogenase/reductase (sdr family) member 3  AKR1C3 - aldo-keto reductase family 1, member c3  HSD17B8 - hydroxysteroid (17-beta) dehydrogenase 8  TTR - transthyretin  HSD17B6 - hydroxysteroid (17-beta) dehydrogenase 6  HSD17B14 - hydroxysteroid (17-beta) dehydrogenase 14  AKR1C4 - aldo-keto reductase family 1, member c4  ALDH8A1 - aldehyde dehydrogenase 8 family, member a1  ADH6 - alcohol dehydrogenase 6 (class v)  CYP3A5 - cytochrome p450, family 3, subfamily a, polypeptide 5  ADH4 - alcohol dehydrogenase 4 (class ii), pi polypeptide  DGAT2 - diacylglycerol o-acyltransferase 2  RDH16 - retinol dehydrogenase 16 (all-trans)  PPARGC1A - peroxisome proliferator-activated receptor gamma, coactivator 1 alpha  CYP1A2 - cytochrome p450, family 1, subfamily a, polypeptide 2  PNPLA2 - patatin-like phospholipase domain containing 2 |
| GO:0060048 | cardiac muscle contraction | 3.89E-6 | 3.45E-4 | 5.16 (10334,33,667,11) | [+] Show genes  MYL3 - myosin, light chain 3, alkali; ventricular, skeletal, slow  CASQ2 - calsequestrin 2 (cardiac muscle)  MYL2 - myosin, light chain 2, regulatory, cardiac, slow  CSRP3 - cysteine and glycine-rich protein 3 (cardiac lim protein)  TNNI2 - troponin i type 2 (skeletal, fast)  TNNI1 - troponin i type 1 (skeletal, slow)  TCAP - titin-cap  MYL1 - myosin, light chain 1, alkali; skeletal, fast  TNNC1 - troponin c type 1 (slow)  MYLK2 - myosin light chain kinase 2  TTN - titin |
| GO:0006753 | nucleoside phosphate metabolic process | 4.07E-6 | 3.59E-4 | 1.94 (10334,399,667,50) | [+] Show genes  HSD17B8 - hydroxysteroid (17-beta) dehydrogenase 8  UQCRC1 - ubiquinol-cytochrome c reductase core protein i  MLYCD - malonyl-coa decarboxylase  PGM1 - phosphoglucomutase 1  COX5B - cytochrome c oxidase subunit vb  ENTPD8 - ectonucleoside triphosphate diphosphohydrolase 8  FMO1 - flavin containing monooxygenase 1  PGAM2 - phosphoglycerate mutase 2 (muscle)  MCEE - methylmalonyl coa epimerase  ACSL1 - acyl-coa synthetase long-chain family member 1  MTHFD1 - methylenetetrahydrofolate dehydrogenase (nadp+ dependent) 1, methenyltetrahydrofolate cyclohydrolase, formyltetrahydrofolate synthetase  ENO3 - enolase 3 (beta, muscle)  CHCHD10 - coiled-coil-helix-coiled-coil-helix domain containing 10  LDHA - lactate dehydrogenase a  SLC25A13 - solute carrier family 25 (aspartate/glutamate carrier), member 13  GPD1 - glycerol-3-phosphate dehydrogenase 1 (soluble)  ACSM2A - acyl-coa synthetase medium-chain family member 2a  AK2 - adenylate kinase 2  ASPDH - aspartate dehydrogenase domain containing  ATP5J2 - atp synthase, h+ transporting, mitochondrial fo complex, subunit f2  SULT2A1 - sulfotransferase family, cytosolic, 2a, dehydroepiandrosterone (dhea)-preferring, member 1  ATP5I - atp synthase, h+ transporting, mitochondrial fo complex, subunit e  ATP5H - atp synthase, h+ transporting, mitochondrial fo complex, subunit d  TDO2 - tryptophan 2,3-dioxygenase  ATP5E - atp synthase, h+ transporting, mitochondrial f1 complex, epsilon subunit  PIPOX - pipecolic acid oxidase  ATP5D - atp synthase, h+ transporting, mitochondrial f1 complex, delta subunit  ATP5G1 - atp synthase, h+ transporting, mitochondrial fo complex, subunit c1 (subunit 9)  SMPDL3A - sphingomyelin phosphodiesterase, acid-like 3a  MOCS1 - molybdenum cofactor synthesis 1  PFKFB1 - 6-phosphofructo-2-kinase/fructose-2,6-biphosphatase 1  ACSM5 - acyl-coa synthetase medium-chain family member 5  NMRK2 - nicotinamide riboside kinase 2  SHMT1 - serine hydroxymethyltransferase 1 (soluble)  ADSSL1 - adenylosuccinate synthase like 1  ALDOB - aldolase b, fructose-bisphosphate  ENPP1 - ectonucleotide pyrophosphatase/phosphodiesterase 1  ADA - adenosine deaminase  PTPLA - protein tyrosine phosphatase-like (proline instead of catalytic arginine), member a  MPC1 - mitochondrial pyruvate carrier 1  PKLR - pyruvate kinase, liver and rbc  HAAO - 3-hydroxyanthranilate 3,4-dioxygenase  GLYAT - glycine-n-acyltransferase  GCDH - glutaryl-coa dehydrogenase  GIMAP7 - gtpase, imap family member 7  AMPD1 - adenosine monophosphate deaminase 1  DGAT2 - diacylglycerol o-acyltransferase 2  HMGCS2 - 3-hydroxy-3-methylglutaryl-coa synthase 2 (mitochondrial)  AGPAT2 - 1-acylglycerol-3-phosphate o-acyltransferase 2  ACSM3 - acyl-coa synthetase medium-chain family member 3 |
| GO:0032101 | regulation of response to external stimulus | 4.12E-6 | 3.61E-4 | 1.82 (10334,501,667,59) | [+] Show genes  F11 - coagulation factor xi  FAM132A - family with sequence similarity 132, member a  APCS - amyloid p component, serum  LGMN - legumain  CAPN3 - calpain 3, (p94)  F12 - coagulation factor xii (hageman factor)  DAPK2 - death-associated protein kinase 2  HRG - histidine-rich glycoprotein  SERPINC1 - serpin peptidase inhibitor, clade c (antithrombin), member 1  CREB3L3 - camp responsive element binding protein 3-like 3  TNIP1 - tnfaip3 interacting protein 1  CD36 - cd36 molecule (thrombospondin receptor)  CPN2 - carboxypeptidase n, polypeptide 2  CFB - complement factor b  NFKBIL1 - nuclear factor of kappa light polypeptide gene enhancer in b-cells inhibitor-like 1  CPB2 - carboxypeptidase b2 (plasma)  MST1 - macrophage stimulating 1 (hepatocyte growth factor-like)  KNG1 - kininogen 1  APOH - apolipoprotein h (beta-2-glycoprotein i)  PLG - plasminogen  F2 - coagulation factor ii (thrombin)  VTN - vitronectin  KLKB1 - kallikrein b, plasma (fletcher factor) 1  GGT1 - gamma-glutamyltransferase 1  SERPINF2 - serpin peptidase inhibitor, clade f (alpha-2 antiplasmin, pigment epithelium derived factor), member 2  F7 - coagulation factor vii (serum prothrombin conversion accelerator)  PLEK - pleckstrin  FGG - fibrinogen gamma chain  C9 - complement component 9  TRIB1 - tribbles homolog 1 (drosophila)  SCARF1 - scavenger receptor class f, member 1  PROC - protein c (inactivator of coagulation factors va and viiia)  ADTRP - androgen-dependent tfpi-regulating protein  FGA - fibrinogen alpha chain  ARG1 - arginase 1  FGB - fibrinogen beta chain  ADA - adenosine deaminase  SERPINF1 - serpin peptidase inhibitor, clade f (alpha-2 antiplasmin, pigment epithelium derived factor), member 1  PPARA - peroxisome proliferator-activated receptor alpha  CD5L - cd5 molecule-like  S100A12 - s100 calcium binding protein a12  MTUS1 - microtubule associated tumor suppressor 1  FEM1A - fem-1 homolog a (c. elegans)  IGF1 - insulin-like growth factor 1 (somatomedin c)  SAA1 - serum amyloid a1  SCGB1A1 - secretoglobin, family 1a, member 1 (uteroglobin)  LBP - lipopolysaccharide binding protein  MYOZ1 - myozenin 1  C1R - complement component 1, r subcomponent  FCGR2B - fc fragment of igg, low affinity iib, receptor (cd32)  C2 - complement component 2  C4BPA - complement component 4 binding protein, alpha  C4BPB - complement component 4 binding protein, beta  C5 - complement component 5  NUPR1 - nuclear protein, transcriptional regulator, 1  C6 - complement component 6  C8B - complement component 8, beta polypeptide  C8A - complement component 8, alpha polypeptide  C8G - complement component 8, gamma polypeptide |
| GO:0009156 | ribonucleoside monophosphate biosynthetic process | 4.98E-6 | 4.33E-4 | 3.20 (10334,92,667,19) | [+] Show genes  AK2 - adenylate kinase 2  ATP5J2 - atp synthase, h+ transporting, mitochondrial fo complex, subunit f2  PGM1 - phosphoglucomutase 1  ATP5H - atp synthase, h+ transporting, mitochondrial fo complex, subunit d  ATP5I - atp synthase, h+ transporting, mitochondrial fo complex, subunit e  COX5B - cytochrome c oxidase subunit vb  AMPD1 - adenosine monophosphate deaminase 1  PGAM2 - phosphoglycerate mutase 2 (muscle)  ADSSL1 - adenylosuccinate synthase like 1  ALDOB - aldolase b, fructose-bisphosphate  ATP5E - atp synthase, h+ transporting, mitochondrial f1 complex, epsilon subunit  ENO3 - enolase 3 (beta, muscle)  ATP5D - atp synthase, h+ transporting, mitochondrial f1 complex, delta subunit  ATP5G1 - atp synthase, h+ transporting, mitochondrial fo complex, subunit c1 (subunit 9)  ADA - adenosine deaminase  LDHA - lactate dehydrogenase a  SLC25A13 - solute carrier family 25 (aspartate/glutamate carrier), member 13  PKLR - pyruvate kinase, liver and rbc  PFKFB1 - 6-phosphofructo-2-kinase/fructose-2,6-biphosphatase 1 |
| GO:0009064 | glutamine family amino acid metabolic process | 5.5E-6 | 4.76E-4 | 4.02 (10334,54,667,14) | [+] Show genes  CPS1 - carbamoyl-phosphate synthase 1, mitochondrial  AGMAT - agmatine ureohydrolase (agmatinase)  FTCD - formimidoyltransferase cyclodeaminase  ASS1 - argininosuccinate synthase 1  ADSSL1 - adenylosuccinate synthase like 1  PRODH2 - proline dehydrogenase (oxidase) 2  GOT2 - glutamic-oxaloacetic transaminase 2, mitochondrial  OTC - ornithine carbamoyltransferase  TAT - tyrosine aminotransferase  FAH - fumarylacetoacetate hydrolase (fumarylacetoacetase)  ALDH4A1 - aldehyde dehydrogenase 4 family, member a1  ADHFE1 - alcohol dehydrogenase, iron containing, 1  ARG1 - arginase 1  GGT1 - gamma-glutamyltransferase 1 |
| GO:0019637 | organophosphate metabolic process | 5.72E-6 | 4.92E-4 | 1.68 (10334,684,667,74) | [+] Show genes  UQCRC1 - ubiquinol-cytochrome c reductase core protein i  HSD17B8 - hydroxysteroid (17-beta) dehydrogenase 8  MLYCD - malonyl-coa decarboxylase  ANGPTL3 - angiopoietin-like 3  PGM1 - phosphoglucomutase 1  APOA2 - apolipoprotein a-ii  COX5B - cytochrome c oxidase subunit vb  ENTPD8 - ectonucleoside triphosphate diphosphohydrolase 8  FMO1 - flavin containing monooxygenase 1  PGAM2 - phosphoglycerate mutase 2 (muscle)  APOA4 - apolipoprotein a-iv  MCEE - methylmalonyl coa epimerase  ACSL1 - acyl-coa synthetase long-chain family member 1  MTHFD1 - methylenetetrahydrofolate dehydrogenase (nadp+ dependent) 1, methenyltetrahydrofolate cyclohydrolase, formyltetrahydrofolate synthetase  ENO3 - enolase 3 (beta, muscle)  CHCHD10 - coiled-coil-helix-coiled-coil-helix domain containing 10  GK5 - glycerol kinase 5 (putative)  LDHA - lactate dehydrogenase a  IMPA2 - inositol(myo)-1(or 4)-monophosphatase 2  SLC25A13 - solute carrier family 25 (aspartate/glutamate carrier), member 13  GPD1 - glycerol-3-phosphate dehydrogenase 1 (soluble)  ACSM2A - acyl-coa synthetase medium-chain family member 2a  AK2 - adenylate kinase 2  ATP5J2 - atp synthase, h+ transporting, mitochondrial fo complex, subunit f2  ASPDH - aspartate dehydrogenase domain containing  CPS1 - carbamoyl-phosphate synthase 1, mitochondrial  SULT2A1 - sulfotransferase family, cytosolic, 2a, dehydroepiandrosterone (dhea)-preferring, member 1  ATP5H - atp synthase, h+ transporting, mitochondrial fo complex, subunit d  ATP5I - atp synthase, h+ transporting, mitochondrial fo complex, subunit e  FITM1 - fat storage-inducing transmembrane protein 1  TDO2 - tryptophan 2,3-dioxygenase  ATP5E - atp synthase, h+ transporting, mitochondrial f1 complex, epsilon subunit  PIPOX - pipecolic acid oxidase  LIPC - lipase, hepatic  ATP5D - atp synthase, h+ transporting, mitochondrial f1 complex, delta subunit  ATP5G1 - atp synthase, h+ transporting, mitochondrial fo complex, subunit c1 (subunit 9)  SMPDL3A - sphingomyelin phosphodiesterase, acid-like 3a  G6PC - glucose-6-phosphatase, catalytic subunit  MOCS1 - molybdenum cofactor synthesis 1  PFKFB1 - 6-phosphofructo-2-kinase/fructose-2,6-biphosphatase 1  ACSM5 - acyl-coa synthetase medium-chain family member 5  PLEK - pleckstrin  PHOSPHO1 - phosphatase, orphan 1  NMRK2 - nicotinamide riboside kinase 2  CHPT1 - choline phosphotransferase 1  SHMT1 - serine hydroxymethyltransferase 1 (soluble)  PLBD1 - phospholipase b domain containing 1  LPIN1 - lipin 1  PLCD4 - phospholipase c, delta 4  ALDOB - aldolase b, fructose-bisphosphate  PCTP - phosphatidylcholine transfer protein  ADSSL1 - adenylosuccinate synthase like 1  ENPP1 - ectonucleotide pyrophosphatase/phosphodiesterase 1  ADA - adenosine deaminase  PTPLA - protein tyrosine phosphatase-like (proline instead of catalytic arginine), member a  MPC1 - mitochondrial pyruvate carrier 1  PKLR - pyruvate kinase, liver and rbc  HAAO - 3-hydroxyanthranilate 3,4-dioxygenase  PON1 - paraoxonase 1  APOA5 - apolipoprotein a-v  GLYAT - glycine-n-acyltransferase  GCDH - glutaryl-coa dehydrogenase  GIMAP7 - gtpase, imap family member 7  AMPD1 - adenosine monophosphate deaminase 1  DGAT2 - diacylglycerol o-acyltransferase 2  TMEM86B - transmembrane protein 86b  GLYCTK - glycerate kinase  CKM - creatine kinase, muscle  PLA2G12B - phospholipase a2, group xiib  CRYL1 - crystallin, lambda 1  HMGCS2 - 3-hydroxy-3-methylglutaryl-coa synthase 2 (mitochondrial)  AGPAT2 - 1-acylglycerol-3-phosphate o-acyltransferase 2  FBP2 - fructose-1,6-bisphosphatase 2  ACSM3 - acyl-coa synthetase medium-chain family member 3 |
| GO:0019217 | regulation of fatty acid metabolic process | 5.79E-6 | 4.94E-4 | 3.59 (10334,69,667,16) | [+] Show genes  MLYCD - malonyl-coa decarboxylase  APOA5 - apolipoprotein a-v  PLIN5 - perilipin 5  FABP1 - fatty acid binding protein 1, liver  APOA4 - apolipoprotein a-iv  DGAT2 - diacylglycerol o-acyltransferase 2  APOC2 - apolipoprotein c-ii  APOC3 - apolipoprotein c-iii  PPARGC1A - peroxisome proliferator-activated receptor gamma, coactivator 1 alpha  MLXIPL - mlx interacting protein-like  PDK4 - pyruvate dehydrogenase kinase, isozyme 4  PDK2 - pyruvate dehydrogenase kinase, isozyme 2  GSTZ1 - glutathione s-transferase zeta 1  ACADL - acyl-coa dehydrogenase, long chain  PPARA - peroxisome proliferator-activated receptor alpha  ACADVL - acyl-coa dehydrogenase, very long chain |
| GO:0009259 | ribonucleotide metabolic process | 6.09E-6 | 5.16E-4 | 2.12 (10334,285,667,39) | [+] Show genes  UQCRC1 - ubiquinol-cytochrome c reductase core protein i  HSD17B8 - hydroxysteroid (17-beta) dehydrogenase 8  MLYCD - malonyl-coa decarboxylase  PGM1 - phosphoglucomutase 1  COX5B - cytochrome c oxidase subunit vb  PGAM2 - phosphoglycerate mutase 2 (muscle)  ALDOB - aldolase b, fructose-bisphosphate  ADSSL1 - adenylosuccinate synthase like 1  MCEE - methylmalonyl coa epimerase  ACSL1 - acyl-coa synthetase long-chain family member 1  ENPP1 - ectonucleotide pyrophosphatase/phosphodiesterase 1  ENO3 - enolase 3 (beta, muscle)  CHCHD10 - coiled-coil-helix-coiled-coil-helix domain containing 10  LDHA - lactate dehydrogenase a  SLC25A13 - solute carrier family 25 (aspartate/glutamate carrier), member 13  PTPLA - protein tyrosine phosphatase-like (proline instead of catalytic arginine), member a  MPC1 - mitochondrial pyruvate carrier 1  ACSM2A - acyl-coa synthetase medium-chain family member 2a  PKLR - pyruvate kinase, liver and rbc  AK2 - adenylate kinase 2  ATP5J2 - atp synthase, h+ transporting, mitochondrial fo complex, subunit f2  GLYAT - glycine-n-acyltransferase  SULT2A1 - sulfotransferase family, cytosolic, 2a, dehydroepiandrosterone (dhea)-preferring, member 1  GCDH - glutaryl-coa dehydrogenase  ATP5I - atp synthase, h+ transporting, mitochondrial fo complex, subunit e  ATP5H - atp synthase, h+ transporting, mitochondrial fo complex, subunit d  GIMAP7 - gtpase, imap family member 7  AMPD1 - adenosine monophosphate deaminase 1  DGAT2 - diacylglycerol o-acyltransferase 2  TDO2 - tryptophan 2,3-dioxygenase  ATP5E - atp synthase, h+ transporting, mitochondrial f1 complex, epsilon subunit  ATP5D - atp synthase, h+ transporting, mitochondrial f1 complex, delta subunit  PIPOX - pipecolic acid oxidase  ATP5G1 - atp synthase, h+ transporting, mitochondrial fo complex, subunit c1 (subunit 9)  HMGCS2 - 3-hydroxy-3-methylglutaryl-coa synthase 2 (mitochondrial)  ACSM3 - acyl-coa synthetase medium-chain family member 3  PFKFB1 - 6-phosphofructo-2-kinase/fructose-2,6-biphosphatase 1  MOCS1 - molybdenum cofactor synthesis 1  ACSM5 - acyl-coa synthetase medium-chain family member 5 |
| GO:0008228 | opsonization | 6.27E-6 | 5.29E-4 | 12.91 (10334,6,667,5) | [+] Show genes  SFTPA1 - surfactant protein a1  CRP - c-reactive protein, pentraxin-related  MBL2 - mannose-binding lectin (protein c) 2, soluble  SPON2 - spondin 2, extracellular matrix protein  LBP - lipopolysaccharide binding protein |
| GO:0009060 | aerobic respiration | 6.59E-6 | 5.52E-4 | 4.53 (10334,41,667,12) | [+] Show genes  UQCRC1 - ubiquinol-cytochrome c reductase core protein i  COX6A2 - cytochrome c oxidase subunit via polypeptide 2  ATP5D - atp synthase, h+ transporting, mitochondrial f1 complex, delta subunit  UQCR10 - ubiquinol-cytochrome c reductase, complex iii subunit x  NDUFS8 - nadh dehydrogenase (ubiquinone) fe-s protein 8, 23kda (nadh-coenzyme q reductase)  CAT - catalase  NDUFA4 - nadh dehydrogenase (ubiquinone) 1 alpha subcomplex, 4, 9kda  COX7C - cytochrome c oxidase subunit viic  NDUFS7 - nadh dehydrogenase (ubiquinone) fe-s protein 7, 20kda (nadh-coenzyme q reductase)  COX5B - cytochrome c oxidase subunit vb  SDHB - succinate dehydrogenase complex, subunit b, iron sulfur (ip)  BLOC1S1 - biogenesis of lysosomal organelles complex-1, subunit 1 |
| GO:0072522 | purine-containing compound biosynthetic process | 7.22E-6 | 6.02E-4 | 2.52 (10334,166,667,27) | [+] Show genes  HSD17B8 - hydroxysteroid (17-beta) dehydrogenase 8  MLYCD - malonyl-coa decarboxylase  PGM1 - phosphoglucomutase 1  SHMT1 - serine hydroxymethyltransferase 1 (soluble)  COX5B - cytochrome c oxidase subunit vb  PGAM2 - phosphoglycerate mutase 2 (muscle)  ADSSL1 - adenylosuccinate synthase like 1  ALDOB - aldolase b, fructose-bisphosphate  ACSL1 - acyl-coa synthetase long-chain family member 1  MTHFD1 - methylenetetrahydrofolate dehydrogenase (nadp+ dependent) 1, methenyltetrahydrofolate cyclohydrolase, formyltetrahydrofolate synthetase  ENO3 - enolase 3 (beta, muscle)  ADA - adenosine deaminase  LDHA - lactate dehydrogenase a  SLC25A13 - solute carrier family 25 (aspartate/glutamate carrier), member 13  PTPLA - protein tyrosine phosphatase-like (proline instead of catalytic arginine), member a  MPC1 - mitochondrial pyruvate carrier 1  PKLR - pyruvate kinase, liver and rbc  AK2 - adenylate kinase 2  ATP5J2 - atp synthase, h+ transporting, mitochondrial fo complex, subunit f2  GCDH - glutaryl-coa dehydrogenase  ATP5I - atp synthase, h+ transporting, mitochondrial fo complex, subunit e  ATP5H - atp synthase, h+ transporting, mitochondrial fo complex, subunit d  AMPD1 - adenosine monophosphate deaminase 1  ATP5E - atp synthase, h+ transporting, mitochondrial f1 complex, epsilon subunit  ATP5D - atp synthase, h+ transporting, mitochondrial f1 complex, delta subunit  ATP5G1 - atp synthase, h+ transporting, mitochondrial fo complex, subunit c1 (subunit 9)  PFKFB1 - 6-phosphofructo-2-kinase/fructose-2,6-biphosphatase 1 |
| GO:0006164 | purine nucleotide biosynthetic process | 8.58E-6 | 7.1E-4 | 2.55 (10334,158,667,26) | [+] Show genes  HSD17B8 - hydroxysteroid (17-beta) dehydrogenase 8  MLYCD - malonyl-coa decarboxylase  PGM1 - phosphoglucomutase 1  COX5B - cytochrome c oxidase subunit vb  PGAM2 - phosphoglycerate mutase 2 (muscle)  ADSSL1 - adenylosuccinate synthase like 1  ALDOB - aldolase b, fructose-bisphosphate  ACSL1 - acyl-coa synthetase long-chain family member 1  MTHFD1 - methylenetetrahydrofolate dehydrogenase (nadp+ dependent) 1, methenyltetrahydrofolate cyclohydrolase, formyltetrahydrofolate synthetase  ENO3 - enolase 3 (beta, muscle)  ADA - adenosine deaminase  LDHA - lactate dehydrogenase a  SLC25A13 - solute carrier family 25 (aspartate/glutamate carrier), member 13  PTPLA - protein tyrosine phosphatase-like (proline instead of catalytic arginine), member a  MPC1 - mitochondrial pyruvate carrier 1  PKLR - pyruvate kinase, liver and rbc  AK2 - adenylate kinase 2  ATP5J2 - atp synthase, h+ transporting, mitochondrial fo complex, subunit f2  GCDH - glutaryl-coa dehydrogenase  ATP5H - atp synthase, h+ transporting, mitochondrial fo complex, subunit d  ATP5I - atp synthase, h+ transporting, mitochondrial fo complex, subunit e  AMPD1 - adenosine monophosphate deaminase 1  ATP5E - atp synthase, h+ transporting, mitochondrial f1 complex, epsilon subunit  ATP5D - atp synthase, h+ transporting, mitochondrial f1 complex, delta subunit  ATP5G1 - atp synthase, h+ transporting, mitochondrial fo complex, subunit c1 (subunit 9)  PFKFB1 - 6-phosphofructo-2-kinase/fructose-2,6-biphosphatase 1 |
| GO:1901607 | alpha-amino acid biosynthetic process | 8.73E-6 | 7.18E-4 | 3.87 (10334,56,667,14) | [+] Show genes  CPS1 - carbamoyl-phosphate synthase 1, mitochondrial  AGXT2 - alanine--glyoxylate aminotransferase 2  SHMT1 - serine hydroxymethyltransferase 1 (soluble)  AGXT - alanine-glyoxylate aminotransferase  GAMT - guanidinoacetate n-methyltransferase  BHMT - betaine--homocysteine s-methyltransferase  ASS1 - argininosuccinate synthase 1  ADI1 - acireductone dioxygenase 1  MTHFR - methylenetetrahydrofolate reductase (nad(p)h)  MTHFD1 - methylenetetrahydrofolate dehydrogenase (nadp+ dependent) 1, methenyltetrahydrofolate cyclohydrolase, formyltetrahydrofolate synthetase  GOT2 - glutamic-oxaloacetic transaminase 2, mitochondrial  OTC - ornithine carbamoyltransferase  PAH - phenylalanine hydroxylase  GGT1 - gamma-glutamyltransferase 1 |
| GO:1901568 | fatty acid derivative metabolic process | 9.58E-6 | 7.84E-4 | 3.07 (10334,96,667,19) | [+] Show genes  PON1 - paraoxonase 1  AKR1C3 - aldo-keto reductase family 1, member c3  HSD17B8 - hydroxysteroid (17-beta) dehydrogenase 8  CYP4A11 - cytochrome p450, family 4, subfamily a, polypeptide 11  AKR1C4 - aldo-keto reductase family 1, member c4  GCDH - glutaryl-coa dehydrogenase  CYP2E1 - cytochrome p450, family 2, subfamily e, polypeptide 1  CYP2C19 - cytochrome p450, family 2, subfamily c, polypeptide 19  DGAT2 - diacylglycerol o-acyltransferase 2  ACSL1 - acyl-coa synthetase long-chain family member 1  BDH2 - 3-hydroxybutyrate dehydrogenase, type 2  HMGCS2 - 3-hydroxy-3-methylglutaryl-coa synthase 2 (mitochondrial)  CES2 - carboxylesterase 2  CBR1 - carbonyl reductase 1  CYP1A2 - cytochrome p450, family 1, subfamily a, polypeptide 2  GGT1 - gamma-glutamyltransferase 1  PTPLA - protein tyrosine phosphatase-like (proline instead of catalytic arginine), member a  SLC27A5 - solute carrier family 27 (fatty acid transporter), member 5  ACSM2A - acyl-coa synthetase medium-chain family member 2a |
| GO:0046034 | ATP metabolic process | 9.58E-6 | 7.79E-4 | 3.07 (10334,96,667,19) | [+] Show genes  AK2 - adenylate kinase 2  UQCRC1 - ubiquinol-cytochrome c reductase core protein i  ATP5J2 - atp synthase, h+ transporting, mitochondrial fo complex, subunit f2  PGM1 - phosphoglucomutase 1  ATP5H - atp synthase, h+ transporting, mitochondrial fo complex, subunit d  ATP5I - atp synthase, h+ transporting, mitochondrial fo complex, subunit e  COX5B - cytochrome c oxidase subunit vb  PGAM2 - phosphoglycerate mutase 2 (muscle)  ALDOB - aldolase b, fructose-bisphosphate  ENPP1 - ectonucleotide pyrophosphatase/phosphodiesterase 1  ATP5E - atp synthase, h+ transporting, mitochondrial f1 complex, epsilon subunit  ATP5D - atp synthase, h+ transporting, mitochondrial f1 complex, delta subunit  ENO3 - enolase 3 (beta, muscle)  ATP5G1 - atp synthase, h+ transporting, mitochondrial fo complex, subunit c1 (subunit 9)  CHCHD10 - coiled-coil-helix-coiled-coil-helix domain containing 10  LDHA - lactate dehydrogenase a  SLC25A13 - solute carrier family 25 (aspartate/glutamate carrier), member 13  PKLR - pyruvate kinase, liver and rbc  PFKFB1 - 6-phosphofructo-2-kinase/fructose-2,6-biphosphatase 1 |
| GO:0034375 | high-density lipoprotein particle remodeling | 1.04E-5 | 8.42E-4 | 7.75 (10334,14,667,7) | [+] Show genes  APOC2 - apolipoprotein c-ii  LIPC - lipase, hepatic  APOC3 - apolipoprotein c-iii  APOM - apolipoprotein m  APOA2 - apolipoprotein a-ii  ALB - albumin  APOA4 - apolipoprotein a-iv |
| GO:0015721 | bile acid and bile salt transport | 1.04E-5 | 8.37E-4 | 7.75 (10334,14,667,7) | [+] Show genes  SLCO1B3 - solute carrier organic anion transporter family, member 1b3  SLC10A1 - solute carrier family 10 (sodium/bile acid cotransporter), member 1  AKR1C4 - aldo-keto reductase family 1, member c4  AQP9 - aquaporin 9  SLC51B - solute carrier family 51, beta subunit  RXRA - retinoid x receptor, alpha  SLC27A5 - solute carrier family 27 (fatty acid transporter), member 5 |
| GO:0033540 | fatty acid beta-oxidation using acyl-CoA oxidase | 1.04E-5 | 8.32E-4 | 7.75 (10334,14,667,7) | [+] Show genes  ECI2 - enoyl-coa delta isomerase 2  ACOX2 - acyl-coa oxidase 2, branched chain  SCP2 - sterol carrier protein 2  ACAA1 - acetyl-coa acyltransferase 1  EHHADH - enoyl-coa, hydratase/3-hydroxyacyl coa dehydrogenase  DECR2 - 2,4-dienoyl coa reductase 2, peroxisomal  AMACR - alpha-methylacyl-coa racemase |
| GO:0048878 | chemical homeostasis | 1.04E-5 | 8.3E-4 | 1.68 (10334,647,667,70) | [+] Show genes  ATP2A1 - atpase, ca++ transporting, cardiac muscle, fast twitch 1  SYPL2 - synaptophysin-like 2  ANGPTL3 - angiopoietin-like 3  CAPN3 - calpain 3, (p94)  APOA2 - apolipoprotein a-ii  ORMDL3 - orm1-like 3 (s. cerevisiae)  APOA4 - apolipoprotein a-iv  TMPRSS6 - transmembrane protease, serine 6  SLC29A1 - solute carrier family 29 (equilibrative nucleoside transporter), member 1  BDH2 - 3-hydroxybutyrate dehydrogenase, type 2  OTC - ornithine carbamoyltransferase  ATF4 - activating transcription factor 4  HOMER2 - homer homolog 2 (drosophila)  HRC - histidine rich calcium binding protein  GCGR - glucagon receptor  ENDOG - endonuclease g  PNPLA2 - patatin-like phospholipase domain containing 2  ACSM2A - acyl-coa synthetase medium-chain family member 2a  HPX - hemopexin  ANGPTL4 - angiopoietin-like 4  AQP9 - aquaporin 9  P2RY2 - purinergic receptor p2y, g-protein coupled, 2  CPS1 - carbamoyl-phosphate synthase 1, mitochondrial  PLN - phospholamban  CD36 - cd36 molecule (thrombospondin receptor)  CCL14 - chemokine (c-c motif) ligand 14  SLC40A1 - solute carrier family 40 (iron-regulated transporter), member 1  CPB2 - carboxypeptidase b2 (plasma)  TTPA - tocopherol (alpha) transfer protein  HAMP - hepcidin antimicrobial peptide  APOC2 - apolipoprotein c-ii  ABCB6 - atp-binding cassette, sub-family b (mdr/tap), member 6  APOC3 - apolipoprotein c-iii  LIPC - lipase, hepatic  APOC4 - apolipoprotein c-iv  KNG1 - kininogen 1  F2 - coagulation factor ii (thrombin)  NR1I2 - nuclear receptor subfamily 1, group i, member 2  G6PC - glucose-6-phosphatase, catalytic subunit  ACOX2 - acyl-coa oxidase 2, branched chain  CYP4A11 - cytochrome p450, family 4, subfamily a, polypeptide 11  KLF15 - kruppel-like factor 15  CSRP3 - cysteine and glycine-rich protein 3 (cardiac lim protein)  CES1 - carboxylesterase 1  ENPP1 - ectonucleotide pyrophosphatase/phosphodiesterase 1  PPARGC1A - peroxisome proliferator-activated receptor gamma, coactivator 1 alpha  PDK4 - pyruvate dehydrogenase kinase, isozyme 4  PDK2 - pyruvate dehydrogenase kinase, isozyme 2  TFR2 - transferrin receptor 2  SERPINF1 - serpin peptidase inhibitor, clade f (alpha-2 antiplasmin, pigment epithelium derived factor), member 1  HFE2 - hemochromatosis type 2 (juvenile)  SLC39A5 - solute carrier family 39 (zinc transporter), member 5  C19orf80 - chromosome 19 open reading frame 80  FOXA3 - forkhead box a3  APOA5 - apolipoprotein a-v  TM6SF2 - transmembrane 6 superfamily member 2  PCK1 - phosphoenolpyruvate carboxykinase 1 (soluble)  SAA1 - serum amyloid a1  CAV3 - caveolin 3  JPH2 - junctophilin 2  RYR1 - ryanodine receptor 1 (skeletal)  DGAT2 - diacylglycerol o-acyltransferase 2  PLA2G12B - phospholipase a2, group xiib  APOM - apolipoprotein m  MLXIPL - mlx interacting protein-like  HMOX1 - heme oxygenase (decycling) 1  ASGR2 - asialoglycoprotein receptor 2  ACSM3 - acyl-coa synthetase medium-chain family member 3  NR1I3 - nuclear receptor subfamily 1, group i, member 3  TPT1 - tumor protein, translationally-controlled 1 |
| GO:0006790 | sulfur compound metabolic process | 1.11E-5 | 8.77E-4 | 2.12 (10334,271,667,37) | [+] Show genes  HSD17B8 - hydroxysteroid (17-beta) dehydrogenase 8  MLYCD - malonyl-coa decarboxylase  GADL1 - glutamate decarboxylase-like 1  MCEE - methylmalonyl coa epimerase  MTHFR - methylenetetrahydrofolate reductase (nad(p)h)  ACSL1 - acyl-coa synthetase long-chain family member 1  ENPP1 - ectonucleotide pyrophosphatase/phosphodiesterase 1  MTHFD1 - methylenetetrahydrofolate dehydrogenase (nadp+ dependent) 1, methenyltetrahydrofolate cyclohydrolase, formyltetrahydrofolate synthetase  ST3GAL3 - st3 beta-galactoside alpha-2,3-sialyltransferase 3  GSTZ1 - glutathione s-transferase zeta 1  HAGH - hydroxyacylglutathione hydrolase  GSTT1 - glutathione s-transferase theta 1  PTPLA - protein tyrosine phosphatase-like (proline instead of catalytic arginine), member a  MPC1 - mitochondrial pyruvate carrier 1  ACSM2A - acyl-coa synthetase medium-chain family member 2a  CHST13 - carbohydrate (chondroitin 4) sulfotransferase 13  GLYAT - glycine-n-acyltransferase  CPS1 - carbamoyl-phosphate synthase 1, mitochondrial  SULT2A1 - sulfotransferase family, cytosolic, 2a, dehydroepiandrosterone (dhea)-preferring, member 1  AHCY - adenosylhomocysteinase  GCDH - glutaryl-coa dehydrogenase  AGXT - alanine-glyoxylate aminotransferase  BHMT - betaine--homocysteine s-methyltransferase  ADI1 - acireductone dioxygenase 1  MAT1A - methionine adenosyltransferase i, alpha  ETHE1 - ethylmalonic encephalopathy 1  TDO2 - tryptophan 2,3-dioxygenase  DGAT2 - diacylglycerol o-acyltransferase 2  MSRA - methionine sulfoxide reductase a  PIPOX - pipecolic acid oxidase  GHR - growth hormone receptor  HMGCS2 - 3-hydroxy-3-methylglutaryl-coa synthase 2 (mitochondrial)  GGT1 - gamma-glutamyltransferase 1  MPST - mercaptopyruvate sulfurtransferase  ACSM3 - acyl-coa synthetase medium-chain family member 3  MMACHC - methylmalonic aciduria (cobalamin deficiency) cblc type, with homocystinuria  ACSM5 - acyl-coa synthetase medium-chain family member 5 |
| GO:0034368 | protein-lipid complex remodeling | 1.15E-5 | 9.07E-4 | 6.52 (10334,19,667,8) | [+] Show genes  APOC2 - apolipoprotein c-ii  LIPC - lipase, hepatic  APOC3 - apolipoprotein c-iii  APOA5 - apolipoprotein a-v  APOM - apolipoprotein m  APOA2 - apolipoprotein a-ii  ALB - albumin  APOA4 - apolipoprotein a-iv |
| GO:0034369 | plasma lipoprotein particle remodeling | 1.15E-5 | 9.02E-4 | 6.52 (10334,19,667,8) | [+] Show genes  APOC2 - apolipoprotein c-ii  LIPC - lipase, hepatic  APOC3 - apolipoprotein c-iii  APOA5 - apolipoprotein a-v  APOM - apolipoprotein m  APOA2 - apolipoprotein a-ii  ALB - albumin  APOA4 - apolipoprotein a-iv |
| GO:0051918 | negative regulation of fibrinolysis | 1.19E-5 | 9.23E-4 | 9.30 (10334,10,667,6) | [+] Show genes  HRG - histidine-rich glycoprotein  APOH - apolipoprotein h (beta-2-glycoprotein i)  F2 - coagulation factor ii (thrombin)  PLG - plasminogen  SERPINF2 - serpin peptidase inhibitor, clade f (alpha-2 antiplasmin, pigment epithelium derived factor), member 2  CPB2 - carboxypeptidase b2 (plasma) |
| GO:0034370 | triglyceride-rich lipoprotein particle remodeling | 1.19E-5 | 9.17E-4 | 9.30 (10334,10,667,6) | [+] Show genes  APOC2 - apolipoprotein c-ii  APOA5 - apolipoprotein a-v  LIPC - lipase, hepatic  APOC3 - apolipoprotein c-iii  APOA2 - apolipoprotein a-ii  APOA4 - apolipoprotein a-iv |
| GO:0046487 | glyoxylate metabolic process | 1.19E-5 | 9.12E-4 | 9.30 (10334,10,667,6) | [+] Show genes  PRODH2 - proline dehydrogenase (oxidase) 2  GRHPR - glyoxylate reductase/hydroxypyruvate reductase  GOT2 - glutamic-oxaloacetic transaminase 2, mitochondrial  ALDH4A1 - aldehyde dehydrogenase 4 family, member a1  AGXT2 - alanine--glyoxylate aminotransferase 2  AGXT - alanine-glyoxylate aminotransferase |
| GO:0031639 | plasminogen activation | 1.19E-5 | 9.07E-4 | 9.30 (10334,10,667,6) | [+] Show genes  F11 - coagulation factor xi  FGG - fibrinogen gamma chain  FGA - fibrinogen alpha chain  FGB - fibrinogen beta chain  APOH - apolipoprotein h (beta-2-glycoprotein i)  KLKB1 - kallikrein b, plasma (fletcher factor) 1 |
| GO:1901293 | nucleoside phosphate biosynthetic process | 1.19E-5 | 9.05E-4 | 2.25 (10334,220,667,32) | [+] Show genes  HSD17B8 - hydroxysteroid (17-beta) dehydrogenase 8  MLYCD - malonyl-coa decarboxylase  NMRK2 - nicotinamide riboside kinase 2  PGM1 - phosphoglucomutase 1  SHMT1 - serine hydroxymethyltransferase 1 (soluble)  COX5B - cytochrome c oxidase subunit vb  ENTPD8 - ectonucleoside triphosphate diphosphohydrolase 8  PGAM2 - phosphoglycerate mutase 2 (muscle)  ALDOB - aldolase b, fructose-bisphosphate  ADSSL1 - adenylosuccinate synthase like 1  ACSL1 - acyl-coa synthetase long-chain family member 1  MTHFD1 - methylenetetrahydrofolate dehydrogenase (nadp+ dependent) 1, methenyltetrahydrofolate cyclohydrolase, formyltetrahydrofolate synthetase  ENO3 - enolase 3 (beta, muscle)  ADA - adenosine deaminase  LDHA - lactate dehydrogenase a  SLC25A13 - solute carrier family 25 (aspartate/glutamate carrier), member 13  PTPLA - protein tyrosine phosphatase-like (proline instead of catalytic arginine), member a  MPC1 - mitochondrial pyruvate carrier 1  PKLR - pyruvate kinase, liver and rbc  HAAO - 3-hydroxyanthranilate 3,4-dioxygenase  AK2 - adenylate kinase 2  ATP5J2 - atp synthase, h+ transporting, mitochondrial fo complex, subunit f2  ASPDH - aspartate dehydrogenase domain containing  GCDH - glutaryl-coa dehydrogenase  ATP5H - atp synthase, h+ transporting, mitochondrial fo complex, subunit d  ATP5I - atp synthase, h+ transporting, mitochondrial fo complex, subunit e  AMPD1 - adenosine monophosphate deaminase 1  ATP5E - atp synthase, h+ transporting, mitochondrial f1 complex, epsilon subunit  ATP5D - atp synthase, h+ transporting, mitochondrial f1 complex, delta subunit  ATP5G1 - atp synthase, h+ transporting, mitochondrial fo complex, subunit c1 (subunit 9)  AGPAT2 - 1-acylglycerol-3-phosphate o-acyltransferase 2  PFKFB1 - 6-phosphofructo-2-kinase/fructose-2,6-biphosphatase 1 |
| GO:0009117 | nucleotide metabolic process | 1.2E-5 | 9.05E-4 | 1.90 (10334,392,667,48) | [+] Show genes  UQCRC1 - ubiquinol-cytochrome c reductase core protein i  HSD17B8 - hydroxysteroid (17-beta) dehydrogenase 8  MLYCD - malonyl-coa decarboxylase  NMRK2 - nicotinamide riboside kinase 2  PGM1 - phosphoglucomutase 1  SHMT1 - serine hydroxymethyltransferase 1 (soluble)  COX5B - cytochrome c oxidase subunit vb  FMO1 - flavin containing monooxygenase 1  PGAM2 - phosphoglycerate mutase 2 (muscle)  ALDOB - aldolase b, fructose-bisphosphate  ADSSL1 - adenylosuccinate synthase like 1  MCEE - methylmalonyl coa epimerase  ACSL1 - acyl-coa synthetase long-chain family member 1  MTHFD1 - methylenetetrahydrofolate dehydrogenase (nadp+ dependent) 1, methenyltetrahydrofolate cyclohydrolase, formyltetrahydrofolate synthetase  ENPP1 - ectonucleotide pyrophosphatase/phosphodiesterase 1  ENO3 - enolase 3 (beta, muscle)  CHCHD10 - coiled-coil-helix-coiled-coil-helix domain containing 10  ADA - adenosine deaminase  LDHA - lactate dehydrogenase a  SLC25A13 - solute carrier family 25 (aspartate/glutamate carrier), member 13  GPD1 - glycerol-3-phosphate dehydrogenase 1 (soluble)  PTPLA - protein tyrosine phosphatase-like (proline instead of catalytic arginine), member a  MPC1 - mitochondrial pyruvate carrier 1  ACSM2A - acyl-coa synthetase medium-chain family member 2a  PKLR - pyruvate kinase, liver and rbc  HAAO - 3-hydroxyanthranilate 3,4-dioxygenase  AK2 - adenylate kinase 2  ATP5J2 - atp synthase, h+ transporting, mitochondrial fo complex, subunit f2  ASPDH - aspartate dehydrogenase domain containing  GLYAT - glycine-n-acyltransferase  GCDH - glutaryl-coa dehydrogenase  SULT2A1 - sulfotransferase family, cytosolic, 2a, dehydroepiandrosterone (dhea)-preferring, member 1  ATP5H - atp synthase, h+ transporting, mitochondrial fo complex, subunit d  ATP5I - atp synthase, h+ transporting, mitochondrial fo complex, subunit e  GIMAP7 - gtpase, imap family member 7  AMPD1 - adenosine monophosphate deaminase 1  TDO2 - tryptophan 2,3-dioxygenase  DGAT2 - diacylglycerol o-acyltransferase 2  ATP5E - atp synthase, h+ transporting, mitochondrial f1 complex, epsilon subunit  ATP5D - atp synthase, h+ transporting, mitochondrial f1 complex, delta subunit  PIPOX - pipecolic acid oxidase  ATP5G1 - atp synthase, h+ transporting, mitochondrial fo complex, subunit c1 (subunit 9)  HMGCS2 - 3-hydroxy-3-methylglutaryl-coa synthase 2 (mitochondrial)  AGPAT2 - 1-acylglycerol-3-phosphate o-acyltransferase 2  ACSM3 - acyl-coa synthetase medium-chain family member 3  PFKFB1 - 6-phosphofructo-2-kinase/fructose-2,6-biphosphatase 1  MOCS1 - molybdenum cofactor synthesis 1  ACSM5 - acyl-coa synthetase medium-chain family member 5 |
| GO:0010466 | negative regulation of peptidase activity | 1.23E-5 | 9.22E-4 | 2.68 (10334,133,667,23) | [+] Show genes  SERPIND1 - serpin peptidase inhibitor, clade d (heparin cofactor), member 1  SERPINA6 - serpin peptidase inhibitor, clade a (alpha-1 antiproteinase, antitrypsin), member 6  SERPINA7 - serpin peptidase inhibitor, clade a (alpha-1 antiproteinase, antitrypsin), member 7  FABP1 - fatty acid binding protein 1, liver  SPP2 - secreted phosphoprotein 2, 24kda  SERPINA10 - serpin peptidase inhibitor, clade a (alpha-1 antiproteinase, antitrypsin), member 10  SERPINA1 - serpin peptidase inhibitor, clade a (alpha-1 antiproteinase, antitrypsin), member 1  FETUB - fetuin b  SERPINA11 - serpin peptidase inhibitor, clade a (alpha-1 antiproteinase, antitrypsin), member 11  AMBP - alpha-1-microglobulin/bikunin precursor  UBXN1 - ubx domain protein 1  ITIH1 - inter-alpha-trypsin inhibitor heavy chain 1  KNG1 - kininogen 1  HRG - histidine-rich glycoprotein  ITIH2 - inter-alpha-trypsin inhibitor heavy chain 2  ITIH3 - inter-alpha-trypsin inhibitor heavy chain 3  ITIH4 - inter-alpha-trypsin inhibitor heavy chain family, member 4  SERPINC1 - serpin peptidase inhibitor, clade c (antithrombin), member 1  VTN - vitronectin  C5 - complement component 5  SERPINF1 - serpin peptidase inhibitor, clade f (alpha-2 antiplasmin, pigment epithelium derived factor), member 1  SERPINF2 - serpin peptidase inhibitor, clade f (alpha-2 antiplasmin, pigment epithelium derived factor), member 2  SERPINA5 - serpin peptidase inhibitor, clade a (alpha-1 antiproteinase, antitrypsin), member 5 |
| GO:0010038 | response to metal ion | 1.25E-5 | 9.32E-4 | 2.19 (10334,241,667,34) | [+] Show genes  AKR1C3 - aldo-keto reductase family 1, member c3  FGG - fibrinogen gamma chain  LGMN - legumain  CAPN3 - calpain 3, (p94)  PGAM2 - phosphoglycerate mutase 2 (muscle)  CASQ2 - calsequestrin 2 (cardiac muscle)  ATF4 - activating transcription factor 4  PPARGC1A - peroxisome proliferator-activated receptor gamma, coactivator 1 alpha  OTC - ornithine carbamoyltransferase  TAT - tyrosine aminotransferase  AOC1 - amine oxidase, copper containing 1  FGA - fibrinogen alpha chain  ARG1 - arginase 1  FGB - fibrinogen beta chain  ENDOG - endonuclease g  TFR2 - transferrin receptor 2  SLC25A13 - solute carrier family 25 (aspartate/glutamate carrier), member 13  CYP1A2 - cytochrome p450, family 1, subfamily a, polypeptide 2  SERPINF1 - serpin peptidase inhibitor, clade f (alpha-2 antiplasmin, pigment epithelium derived factor), member 1  PKLR - pyruvate kinase, liver and rbc  HAAO - 3-hydroxyanthranilate 3,4-dioxygenase  AQP9 - aquaporin 9  CPS1 - carbamoyl-phosphate synthase 1, mitochondrial  PLN - phospholamban  CAT - catalase  ASS1 - argininosuccinate synthase 1  CHP2 - calcineurin-like ef-hand protein 2  TTN - titin  RYR1 - ryanodine receptor 1 (skeletal)  HAMP - hepcidin antimicrobial peptide  ATP5D - atp synthase, h+ transporting, mitochondrial f1 complex, delta subunit  ACTA1 - actin, alpha 1, skeletal muscle  HMOX1 - heme oxygenase (decycling) 1  TNNC1 - troponin c type 1 (slow) |
| GO:0009206 | purine ribonucleoside triphosphate biosynthetic process | 1.47E-5 | 1.09E-3 | 3.52 (10334,66,667,15) | [+] Show genes  ATP5J2 - atp synthase, h+ transporting, mitochondrial fo complex, subunit f2  PGM1 - phosphoglucomutase 1  ATP5H - atp synthase, h+ transporting, mitochondrial fo complex, subunit d  ATP5I - atp synthase, h+ transporting, mitochondrial fo complex, subunit e  COX5B - cytochrome c oxidase subunit vb  PGAM2 - phosphoglycerate mutase 2 (muscle)  ALDOB - aldolase b, fructose-bisphosphate  ATP5E - atp synthase, h+ transporting, mitochondrial f1 complex, epsilon subunit  ENO3 - enolase 3 (beta, muscle)  ATP5D - atp synthase, h+ transporting, mitochondrial f1 complex, delta subunit  ATP5G1 - atp synthase, h+ transporting, mitochondrial fo complex, subunit c1 (subunit 9)  LDHA - lactate dehydrogenase a  SLC25A13 - solute carrier family 25 (aspartate/glutamate carrier), member 13  PKLR - pyruvate kinase, liver and rbc  PFKFB1 - 6-phosphofructo-2-kinase/fructose-2,6-biphosphatase 1 |
| GO:0015908 | fatty acid transport | 1.47E-5 | 1.09E-3 | 4.23 (10334,44,667,12) | [+] Show genes  CPT2 - carnitine palmitoyltransferase 2  ACSL1 - acyl-coa synthetase long-chain family member 1  PLA2G12B - phospholipase a2, group xiib  CPT1B - carnitine palmitoyltransferase 1b (muscle)  GOT2 - glutamic-oxaloacetic transaminase 2, mitochondrial  SLC25A20 - solute carrier family 25 (carnitine/acylcarnitine translocase), member 20  CD36 - cd36 molecule (thrombospondin receptor)  SLC27A2 - solute carrier family 27 (fatty acid transporter), member 2  FABP1 - fatty acid binding protein 1, liver  THRSP - thyroid hormone responsive  PPARA - peroxisome proliferator-activated receptor alpha  SLC27A5 - solute carrier family 27 (fatty acid transporter), member 5 |
| GO:0046320 | regulation of fatty acid oxidation | 1.47E-5 | 1.08E-3 | 5.58 (10334,25,667,9) | [+] Show genes  DGAT2 - diacylglycerol o-acyltransferase 2  MLYCD - malonyl-coa decarboxylase  PPARGC1A - peroxisome proliferator-activated receptor gamma, coactivator 1 alpha  PDK4 - pyruvate dehydrogenase kinase, isozyme 4  PLIN5 - perilipin 5  ACADL - acyl-coa dehydrogenase, long chain  FABP1 - fatty acid binding protein 1, liver  PPARA - peroxisome proliferator-activated receptor alpha  ACADVL - acyl-coa dehydrogenase, very long chain |
| GO:0034381 | plasma lipoprotein particle clearance | 1.5E-5 | 1.1E-3 | 5.00 (10334,31,667,10) | [+] Show genes  DGAT2 - diacylglycerol o-acyltransferase 2  APOC2 - apolipoprotein c-ii  APOC3 - apolipoprotein c-iii  LIPC - lipase, hepatic  CES3 - carboxylesterase 3  APOM - apolipoprotein m  APOC4 - apolipoprotein c-iv  CD36 - cd36 molecule (thrombospondin receptor)  APOA2 - apolipoprotein a-ii  HMOX1 - heme oxygenase (decycling) 1 |
| GO:0009167 | purine ribonucleoside monophosphate metabolic process | 1.61E-5 | 1.18E-3 | 2.71 (10334,126,667,22) | [+] Show genes  AK2 - adenylate kinase 2  ATP5J2 - atp synthase, h+ transporting, mitochondrial fo complex, subunit f2  UQCRC1 - ubiquinol-cytochrome c reductase core protein i  PGM1 - phosphoglucomutase 1  ATP5I - atp synthase, h+ transporting, mitochondrial fo complex, subunit e  ATP5H - atp synthase, h+ transporting, mitochondrial fo complex, subunit d  COX5B - cytochrome c oxidase subunit vb  AMPD1 - adenosine monophosphate deaminase 1  PGAM2 - phosphoglycerate mutase 2 (muscle)  ALDOB - aldolase b, fructose-bisphosphate  ADSSL1 - adenylosuccinate synthase like 1  ATP5E - atp synthase, h+ transporting, mitochondrial f1 complex, epsilon subunit  ENPP1 - ectonucleotide pyrophosphatase/phosphodiesterase 1  ATP5D - atp synthase, h+ transporting, mitochondrial f1 complex, delta subunit  ENO3 - enolase 3 (beta, muscle)  ATP5G1 - atp synthase, h+ transporting, mitochondrial fo complex, subunit c1 (subunit 9)  CHCHD10 - coiled-coil-helix-coiled-coil-helix domain containing 10  LDHA - lactate dehydrogenase a  ADA - adenosine deaminase  SLC25A13 - solute carrier family 25 (aspartate/glutamate carrier), member 13  PKLR - pyruvate kinase, liver and rbc  PFKFB1 - 6-phosphofructo-2-kinase/fructose-2,6-biphosphatase 1 |
| GO:0051919 | positive regulation of fibrinolysis | 1.72E-5 | 1.25E-3 | 15.49 (10334,4,667,4) | [+] Show genes  F11 - coagulation factor xi  F12 - coagulation factor xii (hageman factor)  PLG - plasminogen  KLKB1 - kallikrein b, plasma (fletcher factor) 1 |
| GO:0035634 | response to stilbenoid | 1.72E-5 | 1.24E-3 | 15.49 (10334,4,667,4) | [+] Show genes  PPARGC1A - peroxisome proliferator-activated receptor gamma, coactivator 1 alpha  CD36 - cd36 molecule (thrombospondin receptor)  G6PC - glucose-6-phosphatase, catalytic subunit  APOA4 - apolipoprotein a-iv |
| GO:0035995 | detection of muscle stretch | 1.72E-5 | 1.23E-3 | 15.49 (10334,4,667,4) | [+] Show genes  CSRP3 - cysteine and glycine-rich protein 3 (cardiac lim protein)  TCAP - titin-cap  CAV3 - caveolin 3  TTN - titin |
| GO:0009145 | purine nucleoside triphosphate biosynthetic process | 1.78E-5 | 1.27E-3 | 3.47 (10334,67,667,15) | [+] Show genes  ATP5J2 - atp synthase, h+ transporting, mitochondrial fo complex, subunit f2  PGM1 - phosphoglucomutase 1  ATP5H - atp synthase, h+ transporting, mitochondrial fo complex, subunit d  ATP5I - atp synthase, h+ transporting, mitochondrial fo complex, subunit e  COX5B - cytochrome c oxidase subunit vb  PGAM2 - phosphoglycerate mutase 2 (muscle)  ALDOB - aldolase b, fructose-bisphosphate  ATP5E - atp synthase, h+ transporting, mitochondrial f1 complex, epsilon subunit  ENO3 - enolase 3 (beta, muscle)  ATP5D - atp synthase, h+ transporting, mitochondrial f1 complex, delta subunit  ATP5G1 - atp synthase, h+ transporting, mitochondrial fo complex, subunit c1 (subunit 9)  LDHA - lactate dehydrogenase a  SLC25A13 - solute carrier family 25 (aspartate/glutamate carrier), member 13  PKLR - pyruvate kinase, liver and rbc  PFKFB1 - 6-phosphofructo-2-kinase/fructose-2,6-biphosphatase 1 |
| GO:0034367 | protein-containing complex remodeling | 1.81E-5 | 1.29E-3 | 6.20 (10334,20,667,8) | [+] Show genes  APOC2 - apolipoprotein c-ii  LIPC - lipase, hepatic  APOC3 - apolipoprotein c-iii  APOA5 - apolipoprotein a-v  APOM - apolipoprotein m  APOA2 - apolipoprotein a-ii  ALB - albumin  APOA4 - apolipoprotein a-iv |
| GO:0043691 | reverse cholesterol transport | 1.84E-5 | 1.3E-3 | 7.23 (10334,15,667,7) | [+] Show genes  APOC2 - apolipoprotein c-ii  LIPC - lipase, hepatic  APOC3 - apolipoprotein c-iii  APOM - apolipoprotein m  CES1 - carboxylesterase 1  APOA2 - apolipoprotein a-ii  APOA4 - apolipoprotein a-iv |
| GO:0042537 | benzene-containing compound metabolic process | 1.84E-5 | 1.29E-3 | 7.23 (10334,15,667,7) | [+] Show genes  TDO2 - tryptophan 2,3-dioxygenase  GOT2 - glutamic-oxaloacetic transaminase 2, mitochondrial  ALDH8A1 - aldehyde dehydrogenase 8 family, member a1  ACAA1 - acetyl-coa acyltransferase 1  CYP2E1 - cytochrome p450, family 2, subfamily e, polypeptide 1  CCBL1 - cysteine conjugate-beta lyase, cytoplasmic  HAAO - 3-hydroxyanthranilate 3,4-dioxygenase |
| GO:0019216 | regulation of lipid metabolic process | 1.92E-5 | 1.34E-3 | 2.00 (10334,310,667,40) | [+] Show genes  AKR1C3 - aldo-keto reductase family 1, member c3  MLYCD - malonyl-coa decarboxylase  CYP4A11 - cytochrome p450, family 4, subfamily a, polypeptide 11  ANGPTL3 - angiopoietin-like 3  CES1 - carboxylesterase 1  APOA2 - apolipoprotein a-ii  FABP1 - fatty acid binding protein 1, liver  ORMDL3 - orm1-like 3 (s. cerevisiae)  APOA4 - apolipoprotein a-iv  ACSL1 - acyl-coa synthetase long-chain family member 1  PPARGC1A - peroxisome proliferator-activated receptor gamma, coactivator 1 alpha  PDK4 - pyruvate dehydrogenase kinase, isozyme 4  PDK2 - pyruvate dehydrogenase kinase, isozyme 2  GSTZ1 - glutathione s-transferase zeta 1  ACADL - acyl-coa dehydrogenase, long chain  DNAJC19 - dnaj (hsp40) homolog, subfamily c, member 19  PPARA - peroxisome proliferator-activated receptor alpha  PNPLA2 - patatin-like phospholipase domain containing 2  ACADVL - acyl-coa dehydrogenase, very long chain  CPT2 - carnitine palmitoyltransferase 2  NFYB - nuclear transcription factor y, beta  C19orf80 - chromosome 19 open reading frame 80  APOA5 - apolipoprotein a-v  ANGPTL4 - angiopoietin-like 4  TM6SF2 - transmembrane 6 superfamily member 2  HSD17B13 - hydroxysteroid (17-beta) dehydrogenase 13  PCK1 - phosphoenolpyruvate carboxykinase 1 (soluble)  PLIN5 - perilipin 5  RORC - rar-related orphan receptor c  CD36 - cd36 molecule (thrombospondin receptor)  SULT2A1 - sulfotransferase family, cytosolic, 2a, dehydroepiandrosterone (dhea)-preferring, member 1  RXRA - retinoid x receptor, alpha  ANKRD1 - ankyrin repeat domain 1 (cardiac muscle)  DGAT2 - diacylglycerol o-acyltransferase 2  APOC2 - apolipoprotein c-ii  APOC3 - apolipoprotein c-iii  MLXIPL - mlx interacting protein-like  HMGCS2 - 3-hydroxy-3-methylglutaryl-coa synthase 2 (mitochondrial)  F2 - coagulation factor ii (thrombin)  THRSP - thyroid hormone responsive |
| GO:0010951 | negative regulation of endopeptidase activity | 2.08E-5 | 1.45E-3 | 2.66 (10334,128,667,22) | [+] Show genes  SERPIND1 - serpin peptidase inhibitor, clade d (heparin cofactor), member 1  SERPINA6 - serpin peptidase inhibitor, clade a (alpha-1 antiproteinase, antitrypsin), member 6  SERPINA7 - serpin peptidase inhibitor, clade a (alpha-1 antiproteinase, antitrypsin), member 7  FABP1 - fatty acid binding protein 1, liver  SPP2 - secreted phosphoprotein 2, 24kda  SERPINA10 - serpin peptidase inhibitor, clade a (alpha-1 antiproteinase, antitrypsin), member 10  SERPINA1 - serpin peptidase inhibitor, clade a (alpha-1 antiproteinase, antitrypsin), member 1  FETUB - fetuin b  SERPINA11 - serpin peptidase inhibitor, clade a (alpha-1 antiproteinase, antitrypsin), member 11  AMBP - alpha-1-microglobulin/bikunin precursor  ITIH1 - inter-alpha-trypsin inhibitor heavy chain 1  KNG1 - kininogen 1  HRG - histidine-rich glycoprotein  ITIH2 - inter-alpha-trypsin inhibitor heavy chain 2  ITIH3 - inter-alpha-trypsin inhibitor heavy chain 3  ITIH4 - inter-alpha-trypsin inhibitor heavy chain family, member 4  SERPINC1 - serpin peptidase inhibitor, clade c (antithrombin), member 1  VTN - vitronectin  C5 - complement component 5  SERPINF1 - serpin peptidase inhibitor, clade f (alpha-2 antiplasmin, pigment epithelium derived factor), member 1  SERPINF2 - serpin peptidase inhibitor, clade f (alpha-2 antiplasmin, pigment epithelium derived factor), member 2  SERPINA5 - serpin peptidase inhibitor, clade a (alpha-1 antiproteinase, antitrypsin), member 5 |
| GO:0009126 | purine nucleoside monophosphate metabolic process | 2.08E-5 | 1.44E-3 | 2.66 (10334,128,667,22) | [+] Show genes  AK2 - adenylate kinase 2  ATP5J2 - atp synthase, h+ transporting, mitochondrial fo complex, subunit f2  UQCRC1 - ubiquinol-cytochrome c reductase core protein i  PGM1 - phosphoglucomutase 1  ATP5I - atp synthase, h+ transporting, mitochondrial fo complex, subunit e  ATP5H - atp synthase, h+ transporting, mitochondrial fo complex, subunit d  COX5B - cytochrome c oxidase subunit vb  AMPD1 - adenosine monophosphate deaminase 1  PGAM2 - phosphoglycerate mutase 2 (muscle)  ALDOB - aldolase b, fructose-bisphosphate  ADSSL1 - adenylosuccinate synthase like 1  ATP5E - atp synthase, h+ transporting, mitochondrial f1 complex, epsilon subunit  ENPP1 - ectonucleotide pyrophosphatase/phosphodiesterase 1  ATP5D - atp synthase, h+ transporting, mitochondrial f1 complex, delta subunit  ENO3 - enolase 3 (beta, muscle)  ATP5G1 - atp synthase, h+ transporting, mitochondrial fo complex, subunit c1 (subunit 9)  CHCHD10 - coiled-coil-helix-coiled-coil-helix domain containing 10  LDHA - lactate dehydrogenase a  ADA - adenosine deaminase  SLC25A13 - solute carrier family 25 (aspartate/glutamate carrier), member 13  PKLR - pyruvate kinase, liver and rbc  PFKFB1 - 6-phosphofructo-2-kinase/fructose-2,6-biphosphatase 1 |
| GO:0009144 | purine nucleoside triphosphate metabolic process | 2.08E-5 | 1.44E-3 | 2.66 (10334,128,667,22) | [+] Show genes  AK2 - adenylate kinase 2  ATP5J2 - atp synthase, h+ transporting, mitochondrial fo complex, subunit f2  UQCRC1 - ubiquinol-cytochrome c reductase core protein i  PGM1 - phosphoglucomutase 1  ATP5H - atp synthase, h+ transporting, mitochondrial fo complex, subunit d  ATP5I - atp synthase, h+ transporting, mitochondrial fo complex, subunit e  COX5B - cytochrome c oxidase subunit vb  GIMAP7 - gtpase, imap family member 7  PGAM2 - phosphoglycerate mutase 2 (muscle)  ALDOB - aldolase b, fructose-bisphosphate  ATP5E - atp synthase, h+ transporting, mitochondrial f1 complex, epsilon subunit  ENPP1 - ectonucleotide pyrophosphatase/phosphodiesterase 1  ATP5D - atp synthase, h+ transporting, mitochondrial f1 complex, delta subunit  ENO3 - enolase 3 (beta, muscle)  ATP5G1 - atp synthase, h+ transporting, mitochondrial fo complex, subunit c1 (subunit 9)  CHCHD10 - coiled-coil-helix-coiled-coil-helix domain containing 10  LDHA - lactate dehydrogenase a  ADA - adenosine deaminase  SLC25A13 - solute carrier family 25 (aspartate/glutamate carrier), member 13  PKLR - pyruvate kinase, liver and rbc  PFKFB1 - 6-phosphofructo-2-kinase/fructose-2,6-biphosphatase 1  MOCS1 - molybdenum cofactor synthesis 1 |
| GO:0045923 | positive regulation of fatty acid metabolic process | 2.13E-5 | 1.46E-3 | 5.36 (10334,26,667,9) | [+] Show genes  APOC2 - apolipoprotein c-ii  MLYCD - malonyl-coa decarboxylase  APOA5 - apolipoprotein a-v  PPARGC1A - peroxisome proliferator-activated receptor gamma, coactivator 1 alpha  MLXIPL - mlx interacting protein-like  PLIN5 - perilipin 5  FABP1 - fatty acid binding protein 1, liver  PPARA - peroxisome proliferator-activated receptor alpha  APOA4 - apolipoprotein a-iv |
| GO:0019693 | ribose phosphate metabolic process | 2.22E-5 | 1.51E-3 | 2.01 (10334,301,667,39) | [+] Show genes  HSD17B8 - hydroxysteroid (17-beta) dehydrogenase 8  UQCRC1 - ubiquinol-cytochrome c reductase core protein i  MLYCD - malonyl-coa decarboxylase  PGM1 - phosphoglucomutase 1  COX5B - cytochrome c oxidase subunit vb  PGAM2 - phosphoglycerate mutase 2 (muscle)  ALDOB - aldolase b, fructose-bisphosphate  ADSSL1 - adenylosuccinate synthase like 1  MCEE - methylmalonyl coa epimerase  ACSL1 - acyl-coa synthetase long-chain family member 1  ENPP1 - ectonucleotide pyrophosphatase/phosphodiesterase 1  ENO3 - enolase 3 (beta, muscle)  CHCHD10 - coiled-coil-helix-coiled-coil-helix domain containing 10  LDHA - lactate dehydrogenase a  SLC25A13 - solute carrier family 25 (aspartate/glutamate carrier), member 13  PTPLA - protein tyrosine phosphatase-like (proline instead of catalytic arginine), member a  MPC1 - mitochondrial pyruvate carrier 1  ACSM2A - acyl-coa synthetase medium-chain family member 2a  PKLR - pyruvate kinase, liver and rbc  AK2 - adenylate kinase 2  ATP5J2 - atp synthase, h+ transporting, mitochondrial fo complex, subunit f2  GLYAT - glycine-n-acyltransferase  SULT2A1 - sulfotransferase family, cytosolic, 2a, dehydroepiandrosterone (dhea)-preferring, member 1  GCDH - glutaryl-coa dehydrogenase  ATP5I - atp synthase, h+ transporting, mitochondrial fo complex, subunit e  ATP5H - atp synthase, h+ transporting, mitochondrial fo complex, subunit d  GIMAP7 - gtpase, imap family member 7  AMPD1 - adenosine monophosphate deaminase 1  DGAT2 - diacylglycerol o-acyltransferase 2  TDO2 - tryptophan 2,3-dioxygenase  ATP5E - atp synthase, h+ transporting, mitochondrial f1 complex, epsilon subunit  ATP5D - atp synthase, h+ transporting, mitochondrial f1 complex, delta subunit  PIPOX - pipecolic acid oxidase  ATP5G1 - atp synthase, h+ transporting, mitochondrial fo complex, subunit c1 (subunit 9)  HMGCS2 - 3-hydroxy-3-methylglutaryl-coa synthase 2 (mitochondrial)  ACSM3 - acyl-coa synthetase medium-chain family member 3  PFKFB1 - 6-phosphofructo-2-kinase/fructose-2,6-biphosphatase 1  MOCS1 - molybdenum cofactor synthesis 1  ACSM5 - acyl-coa synthetase medium-chain family member 5 |
| GO:0071704 | organic substance metabolic process | 2.32E-5 | 1.57E-3 | 1.15 (10334,5311,667,394) | [+] Show genes  DAPK2 - death-associated protein kinase 2  COX5B - cytochrome c oxidase subunit vb  SPP2 - secreted phosphoprotein 2, 24kda  AMACR - alpha-methylacyl-coa racemase  AOC1 - amine oxidase, copper containing 1  LDHA - lactate dehydrogenase a  ACAA1 - acetyl-coa acyltransferase 1  ACADL - acyl-coa dehydrogenase, long chain  ACADS - acyl-coa dehydrogenase, c-2 to c-3 short chain  ACADVL - acyl-coa dehydrogenase, very long chain  BLOC1S1 - biogenesis of lysosomal organelles complex-1, subunit 1  CPT2 - carnitine palmitoyltransferase 2  ATP5J2 - atp synthase, h+ transporting, mitochondrial fo complex, subunit f2  DHRS4 - dehydrogenase/reductase (sdr family) member 4  CPT1B - carnitine palmitoyltransferase 1b (muscle)  CPS1 - carbamoyl-phosphate synthase 1, mitochondrial  FITM1 - fat storage-inducing transmembrane protein 1  CPB2 - carboxypeptidase b2 (plasma)  GHR - growth hormone receptor  PIPOX - pipecolic acid oxidase  LIPC - lipase, hepatic  MMAB - methylmalonic aciduria (cobalamin deficiency) cblb type  POLD4 - polymerase (dna-directed), delta 4, accessory subunit  ADHFE1 - alcohol dehydrogenase, iron containing, 1  SMPDL3A - sphingomyelin phosphodiesterase, acid-like 3a  PLG - plasminogen  APOBEC2 - apolipoprotein b mrna editing enzyme, catalytic polypeptide-like 2  PPM1J - protein phosphatase, mg2+/mn2+ dependent, 1j  GGT1 - gamma-glutamyltransferase 1  PLEK - pleckstrin  ACOX2 - acyl-coa oxidase 2, branched chain  LSM10 - lsm10, u7 small nuclear rna associated  PLBD1 - phospholipase b domain containing 1  ACTN2 - actinin, alpha 2  PCTP - phosphatidylcholine transfer protein  PPARGC1A - peroxisome proliferator-activated receptor gamma, coactivator 1 alpha  BAG3 - bcl2-associated athanogene 3  ADA - adenosine deaminase  PPARA - peroxisome proliferator-activated receptor alpha  PON1 - paraoxonase 1  APOA5 - apolipoprotein a-v  POLR2I - polymerase (rna) ii (dna directed) polypeptide i, 14.5kda  FEM1A - fem-1 homolog a (c. elegans)  POLR2J - polymerase (rna) ii (dna directed) polypeptide j, 13.3kda  FTCD - formimidoyltransferase cyclodeaminase  ADH6 - alcohol dehydrogenase 6 (class v)  ALDH1L1 - aldehyde dehydrogenase 1 family, member l1  ADH4 - alcohol dehydrogenase 4 (class ii), pi polypeptide  PRODH2 - proline dehydrogenase (oxidase) 2  DGAT2 - diacylglycerol o-acyltransferase 2  PLA2G12B - phospholipase a2, group xiib  SLC17A1 - solute carrier family 17 (organic anion transporter), member 1  HSD17B8 - hydroxysteroid (17-beta) dehydrogenase 8  FBXO17 - f-box protein 17  HSD17B14 - hydroxysteroid (17-beta) dehydrogenase 14  AGMAT - agmatine ureohydrolase (agmatinase)  ADRB2 - adrenoceptor beta 2, surface  TRIM63 - tripartite motif containing 63, e3 ubiquitin protein ligase  SERPINA10 - serpin peptidase inhibitor, clade a (alpha-1 antiproteinase, antitrypsin), member 10  TMPRSS6 - transmembrane protease, serine 6  MCEE - methylmalonyl coa epimerase  ASB11 - ankyrin repeat and socs box containing 11  SFTPA1 - surfactant protein a1  ASB5 - ankyrin repeat and socs box containing 5  GK5 - glycerol kinase 5 (putative)  ASPG - asparaginase homolog (s. cerevisiae)  ABHD1 - abhydrolase domain containing 1  NHEJ1 - nonhomologous end-joining factor 1  AK2 - adenylate kinase 2  SLC37A4 - solute carrier family 37 (glucose-6-phosphate transporter), member 4  ANGPTL4 - angiopoietin-like 4  CHST13 - carbohydrate (chondroitin 4) sulfotransferase 13  AHCY - adenosylhomocysteinase  SULT2A1 - sulfotransferase family, cytosolic, 2a, dehydroepiandrosterone (dhea)-preferring, member 1  AGXT - alanine-glyoxylate aminotransferase  WWP1 - ww domain containing e3 ubiquitin protein ligase 1  COQ4 - coenzyme q4 homolog (s. cerevisiae)  PPP1R3C - protein phosphatase 1, regulatory subunit 3c  KNG1 - kininogen 1  ALB - albumin  KLKB1 - kallikrein b, plasma (fletcher factor) 1  G6PC - glucose-6-phosphatase, catalytic subunit  ALDH2 - aldehyde dehydrogenase 2 family (mitochondrial)  PPP1R3A - protein phosphatase 1, regulatory subunit 3a  PHOSPHO1 - phosphatase, orphan 1  CRP - c-reactive protein, pentraxin-related  GAMT - guanidinoacetate n-methyltransferase  PLCD4 - phospholipase c, delta 4  PROC - protein c (inactivator of coagulation factors va and viiia)  ALKBH7 - alkb, alkylation repair homolog 7 (e. coli)  ALDOB - aldolase b, fructose-bisphosphate  GRHPR - glyoxylate reductase/hydroxypyruvate reductase  ADTRP - androgen-dependent tfpi-regulating protein  AMBP - alpha-1-microglobulin/bikunin precursor  DUSP13 - dual specificity phosphatase 13  AMY2B - amylase, alpha 2b (pancreatic)  GCDH - glutaryl-coa dehydrogenase  GC - group-specific component (vitamin d binding protein)  TRIM72 - tripartite motif containing 72  AMPD1 - adenosine monophosphate deaminase 1  PKN1 - protein kinase n1  SLC27A2 - solute carrier family 27 (fatty acid transporter), member 2  SLC27A5 - solute carrier family 27 (fatty acid transporter), member 5  HAO2 - hydroxyacid oxidase 2 (long chain)  APCS - amyloid p component, serum  APOA2 - apolipoprotein a-ii  SDHB - succinate dehydrogenase complex, subunit b, iron sulfur (ip)  APOA4 - apolipoprotein a-iv  PPP1R3B - protein phosphatase 1, regulatory subunit 3b  APOF - apolipoprotein f  GSTZ1 - glutathione s-transferase zeta 1  GSTT1 - glutathione s-transferase theta 1  ASB12 - ankyrin repeat and socs box containing 12  ACSM2A - acyl-coa synthetase medium-chain family member 2a  DHRS3 - dehydrogenase/reductase (sdr family) member 3  ASPDH - aspartate dehydrogenase domain containing  UBE2L6 - ubiquitin-conjugating enzyme e2l 6  APOC2 - apolipoprotein c-ii  FBXO40 - f-box protein 40  APOC3 - apolipoprotein c-iii  SDC2 - syndecan 2  IMMP2L - imp2 inner mitochondrial membrane peptidase-like (s. cerevisiae)  GYS2 - glycogen synthase 2 (liver)  APOC4 - apolipoprotein c-iv  PAFAH2 - platelet-activating factor acetylhydrolase 2, 40kda  APOH - apolipoprotein h (beta-2-glycoprotein i)  PAH - phenylalanine hydroxylase  TBRG4 - transforming growth factor beta regulator 4  MSRB3 - methionine sulfoxide reductase b3  MOCS1 - molybdenum cofactor synthesis 1  UPB1 - ureidopropionase, beta  CYP4A11 - cytochrome p450, family 4, subfamily a, polypeptide 11  INMT - indolethylamine n-methyltransferase  CYP8B1 - cytochrome p450, family 8, subfamily b, polypeptide 1  CYP2E1 - cytochrome p450, family 2, subfamily e, polypeptide 1  GADD45GIP1 - growth arrest and dna-damage-inducible, gamma interacting protein 1  STS - steroid sulfatase (microsomal), isozyme s  CYP3A5 - cytochrome p450, family 3, subfamily a, polypeptide 5  ADSSL1 - adenylosuccinate synthase like 1  ATXN7 - ataxin 7  CYP2C19 - cytochrome p450, family 2, subfamily c, polypeptide 19  ENPP1 - ectonucleotide pyrophosphatase/phosphodiesterase 1  ARG1 - arginase 1  PDK4 - pyruvate dehydrogenase kinase, isozyme 4  HADH - hydroxyacyl-coa dehydrogenase  SCP2 - sterol carrier protein 2  PDK2 - pyruvate dehydrogenase kinase, isozyme 2  HAGH - hydroxyacylglutathione hydrolase  CYP1A2 - cytochrome p450, family 1, subfamily a, polypeptide 2  HABP2 - hyaluronan binding protein 2  UPF2 - upf2 regulator of nonsense transcripts homolog (yeast)  PTPLA - protein tyrosine phosphatase-like (proline instead of catalytic arginine), member a  HSD17B10 - hydroxysteroid (17-beta) dehydrogenase 10  IYD - iodotyrosine deiodinase  SERPIND1 - serpin peptidase inhibitor, clade d (heparin cofactor), member 1  PTP4A3 - protein tyrosine phosphatase type iva, member 3  TM6SF2 - transmembrane 6 superfamily member 2  PCK1 - phosphoenolpyruvate carboxykinase 1 (soluble)  SAA1 - serum amyloid a1  GIMAP7 - gtpase, imap family member 7  ASS1 - argininosuccinate synthase 1  ART3 - adp-ribosyltransferase 3  TMEM86B - transmembrane protein 86b  MAPK12 - mitogen-activated protein kinase 12  TCEANC - transcription elongation factor a (sii) n-terminal and central domain containing  SNORA17 - small nucleolar rna, h/aca box 17  ASGR1 - asialoglycoprotein receptor 1  ASGR2 - asialoglycoprotein receptor 2  ZDHHC4 - zinc finger, dhhc-type containing 4  ACSM3 - acyl-coa synthetase medium-chain family member 3  MMACHC - methylmalonic aciduria (cobalamin deficiency) cblc type, with homocystinuria  PGM1 - phosphoglucomutase 1  LRRC2 - leucine rich repeat containing 2  PGAM2 - phosphoglycerate mutase 2 (muscle)  GOT2 - glutamic-oxaloacetic transaminase 2, mitochondrial  ATF4 - activating transcription factor 4  ABLIM3 - actin binding lim protein family, member 3  CINP - cyclin-dependent kinase 2 interacting protein  SERPINC1 - serpin peptidase inhibitor, clade c (antithrombin), member 1  GPD1 - glycerol-3-phosphate dehydrogenase 1 (soluble)  DPYS - dihydropyrimidinase  MYLK2 - myosin light chain kinase 2  MBL2 - mannose-binding lectin (protein c) 2, soluble  ATP5I - atp synthase, h+ transporting, mitochondrial fo complex, subunit e  MAT1A - methionine adenosyltransferase i, alpha  HACL1 - 2-hydroxyacyl-coa lyase 1  ATP5E - atp synthase, h+ transporting, mitochondrial f1 complex, epsilon subunit  ATP5D - atp synthase, h+ transporting, mitochondrial f1 complex, delta subunit  ATP5G1 - atp synthase, h+ transporting, mitochondrial fo complex, subunit c1 (subunit 9)  POLDIP2 - polymerase (dna-directed), delta interacting protein 2  DECR2 - 2,4-dienoyl coa reductase 2, peroxisomal  PFKFB1 - 6-phosphofructo-2-kinase/fructose-2,6-biphosphatase 1  PIN4 - protein (peptidylprolyl cis/trans isomerase) nima-interacting, 4 (parvulin)  SHMT1 - serine hydroxymethyltransferase 1 (soluble)  AZGP1 - alpha-2-glycoprotein 1, zinc-binding  ASB2 - ankyrin repeat and socs box containing 2  ST3GAL3 - st3 beta-galactoside alpha-2,3-sialyltransferase 3  TRPT1 - trna phosphotransferase 1  MPC1 - mitochondrial pyruvate carrier 1  PKLR - pyruvate kinase, liver and rbc  STYXL1 - serine/threonine/tyrosine interacting-like 1  ECHDC2 - enoyl coa hydratase domain containing 2  ADI1 - acireductone dioxygenase 1  PHYH - phytanoyl-coa 2-hydroxylase  SERPINA1 - serpin peptidase inhibitor, clade a (alpha-1 antiproteinase, antitrypsin), member 1  STBD1 - starch binding domain 1  FGGY - fggy carbohydrate kinase domain containing  MAF - v-maf avian musculoaponeurotic fibrosarcoma oncogene homolog  SUCLG1 - succinate-coa ligase, alpha subunit  HSD11B1 - hydroxysteroid (11-beta) dehydrogenase 1  NDUFA7 - nadh dehydrogenase (ubiquinone) 1 alpha subcomplex, 7, 14.5kda  SLC29A1 - solute carrier family 29 (equilibrative nucleoside transporter), member 1  BTC - betacellulin  BDH2 - 3-hydroxybutyrate dehydrogenase, type 2  ENO3 - enolase 3 (beta, muscle)  CES2 - carboxylesterase 2  HRC - histidine rich calcium binding protein  ENDOG - endonuclease g  SLC25A13 - solute carrier family 25 (aspartate/glutamate carrier), member 13  HRAS - harvey rat sarcoma viral oncogene homolog  TRIM7 - tripartite motif containing 7  HPX - hemopexin  RCL1 - rna terminal phosphate cyclase-like 1  KLHL41 - kelch-like family member 41  TTR - transthyretin  TNIP1 - tnfaip3 interacting protein 1  DUSP27 - dual specificity phosphatase 27 (putative)  CFB - complement factor b  HPN - hepsin  EHHADH - enoyl-coa, hydratase/3-hydroxyacyl coa dehydrogenase  BHMT - betaine--homocysteine s-methyltransferase  HP - haptoglobin  TTN - titin  HPD - 4-hydroxyphenylpyruvate dioxygenase  TTPA - tocopherol (alpha) transfer protein  PROZ - protein z, vitamin k-dependent plasma glycoprotein  BLVRB - biliverdin reductase b (flavin reductase (nadph))  NR1I2 - nuclear receptor subfamily 1, group i, member 2  FAHD1 - fumarylacetoacetate hydrolase domain containing 1  C9 - complement component 9  EEF1D - eukaryotic translation elongation factor 1 delta (guanine nucleotide exchange protein)  TRIB1 - tribbles homolog 1 (drosophila)  CHPT1 - choline phosphotransferase 1  ABHD17A - abhydrolase domain containing 17a  VNN1 - vanin 1  PM20D1 - peptidase m20 domain containing 1  HFE2 - hemochromatosis type 2 (juvenile)  GLYAT - glycine-n-acyltransferase  RORC - rar-related orphan receptor c  RP9 - retinitis pigmentosa 9 (autosomal dominant)  MRPS34 - mitochondrial ribosomal protein s34  C1R - complement component 1, r subcomponent  C2 - complement component 2  SNORA70 - small nucleolar rna, h/aca box 70  C4BPA - complement component 4 binding protein, alpha  C4BPB - complement component 4 binding protein, beta  ECHS1 - enoyl coa hydratase, short chain, 1, mitochondrial  C5 - complement component 5  C6 - complement component 6  C8B - complement component 8, beta polypeptide  C8A - complement component 8, alpha polypeptide  ECH1 - enoyl coa hydratase 1, peroxisomal  LRRC39 - leucine rich repeat containing 39  C8G - complement component 8, gamma polypeptide  F11 - coagulation factor xi  ECI2 - enoyl-coa delta isomerase 2  F10 - coagulation factor x  UQCRC1 - ubiquinol-cytochrome c reductase core protein i  AKR1C3 - aldo-keto reductase family 1, member c3  F9 - coagulation factor ix  MRPL23 - mitochondrial ribosomal protein l23  ACAA2 - acetyl-coa acyltransferase 2  CAPN3 - calpain 3, (p94)  AOC3 - amine oxidase, copper containing 3  F12 - coagulation factor xii (hageman factor)  FABP1 - fatty acid binding protein 1, liver  HMBS - hydroxymethylbilane synthase  MTHFR - methylenetetrahydrofolate reductase (nad(p)h)  ACSL1 - acyl-coa synthetase long-chain family member 1  MTHFD1 - methylenetetrahydrofolate dehydrogenase (nadp+ dependent) 1, methenyltetrahydrofolate cyclohydrolase, formyltetrahydrofolate synthetase  FAH - fumarylacetoacetate hydrolase (fumarylacetoacetase)  ALDH4A1 - aldehyde dehydrogenase 4 family, member a1  CHCHD10 - coiled-coil-helix-coiled-coil-helix domain containing 10  RPL3L - ribosomal protein l3-like  PNPLA2 - patatin-like phospholipase domain containing 2  ATP5H - atp synthase, h+ transporting, mitochondrial fo complex, subunit d  HGD - homogentisate 1,2-dioxygenase  MSRA - methionine sulfoxide reductase a  ABCB6 - atp-binding cassette, sub-family b (mdr/tap), member 6  MST1 - macrophage stimulating 1 (hepatocyte growth factor-like)  COQ9 - coenzyme q9 homolog (s. cerevisiae)  F2 - coagulation factor ii (thrombin)  F7 - coagulation factor vii (serum prothrombin conversion accelerator)  ACSM5 - acyl-coa synthetase medium-chain family member 5  UBA52 - ubiquitin a-52 residue ribosomal protein fusion product 1  AGXT2 - alanine--glyoxylate aminotransferase 2  MACROD1 - macro domain containing 1  LPIN1 - lipin 1  MRPL14 - mitochondrial ribosomal protein l14  ESRRA - estrogen-related receptor alpha  MRPS15 - mitochondrial ribosomal protein s15  MRPL40 - mitochondrial ribosomal protein l40  ETFDH - electron-transferring-flavoprotein dehydrogenase  CD5L - cd5 molecule-like  SMYD1 - set and mynd domain containing 1  MRPL36 - mitochondrial ribosomal protein l36  SERPINA6 - serpin peptidase inhibitor, clade a (alpha-1 antiproteinase, antitrypsin), member 6  EPHA1 - eph receptor a1  CAT - catalase  TRIM54 - tripartite motif containing 54  PHPT1 - phosphohistidine phosphatase 1  RXRA - retinoid x receptor, alpha  UCP3 - uncoupling protein 3 (mitochondrial, proton carrier)  CAV3 - caveolin 3  RXRG - retinoid x receptor, gamma  HMGCS2 - 3-hydroxy-3-methylglutaryl-coa synthase 2 (mitochondrial)  CBR1 - carbonyl reductase 1  RNPEPL1 - arginyl aminopeptidase (aminopeptidase b)-like 1  HMOX1 - heme oxygenase (decycling) 1  FBP2 - fructose-1,6-bisphosphatase 2  NUPR1 - nuclear protein, transcriptional regulator, 1  MPST - mercaptopyruvate sulfurtransferase  NR2F6 - nuclear receptor subfamily 2, group f, member 6  CCBL1 - cysteine conjugate-beta lyase, cytoplasmic  URAD - ureidoimidazoline (2-oxo-4-hydroxy-4-carboxy-5-) decarboxylase  NR1I3 - nuclear receptor subfamily 1, group i, member 3  ERBB2 - v-erb-b2 avian erythroblastic leukemia viral oncogene homolog 2  MASP1 - mannan-binding lectin serine peptidase 1 (c4/c2 activating component of ra-reactive factor)  ANGPTL3 - angiopoietin-like 3  LGMN - legumain  FN1 - fibronectin 1  GADL1 - glutamate decarboxylase-like 1  MMP23B - matrix metallopeptidase 23b  ENTPD8 - ectonucleoside triphosphate diphosphohydrolase 8  ADPRHL1 - adp-ribosylhydrolase like 1  FMO1 - flavin containing monooxygenase 1  EEPD1 - endonuclease/exonuclease/phosphatase family domain containing 1  CDC34 - cell division cycle 34  OTC - ornithine carbamoyltransferase  FBXW5 - f-box and wd repeat domain containing 5  IMPA2 - inositol(myo)-1(or 4)-monophosphatase 2  CD36 - cd36 molecule (thrombospondin receptor)  IL1RN - interleukin 1 receptor antagonist  TDO2 - tryptophan 2,3-dioxygenase  PSMC5 - proteasome (prosome, macropain) 26s subunit, atpase, 5  DCAF11 - ddb1 and cul4 associated factor 11  FH - fumarate hydratase  FGG - fibrinogen gamma chain  NMRK2 - nicotinamide riboside kinase 2  CES1 - carboxylesterase 1  ALDH8A1 - aldehyde dehydrogenase 8 family, member a1  SCARF1 - scavenger receptor class f, member 1  MRPS25 - mitochondrial ribosomal protein s25  TAT - tyrosine aminotransferase  ITIH1 - inter-alpha-trypsin inhibitor heavy chain 1  UBXN1 - ubx domain protein 1  FGA - fibrinogen alpha chain  ITIH2 - inter-alpha-trypsin inhibitor heavy chain 2  FGB - fibrinogen beta chain  ITIH3 - inter-alpha-trypsin inhibitor heavy chain 3  ITIH4 - inter-alpha-trypsin inhibitor heavy chain family, member 4  C19orf80 - chromosome 19 open reading frame 80  HSD17B6 - hydroxysteroid (17-beta) dehydrogenase 6  MRPL2 - mitochondrial ribosomal protein l2  GLYCTK - glycerate kinase  RDH16 - retinol dehydrogenase 16 (all-trans)  CRYL1 - crystallin, lambda 1  APOM - apolipoprotein m  AGPAT2 - 1-acylglycerol-3-phosphate o-acyltransferase 2  YBX1 - y box binding protein 1  TCEA3 - transcription elongation factor a (sii), 3  SMTNL1 - smoothelin-like 1  KEAP1 - kelch-like ech-associated protein 1  SEPSECS - sep (o-phosphoserine) trna:sec (selenocysteine) trna synthase  MLYCD - malonyl-coa decarboxylase  ORMDL3 - orm1-like 3 (s. cerevisiae)  CIDEB - cell death-inducing dffa-like effector b  AKR1C4 - aldo-keto reductase family 1, member c4  GCAT - glycine c-acetyltransferase  UGT1A6 - udp glucuronosyltransferase 1 family, polypeptide a6  CHIA - chitinase, acidic  ETHE1 - ethylmalonic encephalopathy 1  IDH3G - isocitrate dehydrogenase 3 (nad+) gamma  CES3 - carboxylesterase 3  NFIC - nuclear factor i/c (ccaat-binding transcription factor)  HAAO - 3-hydroxyanthranilate 3,4-dioxygenase  MASP2 - mannan-binding lectin serine peptidase 2  IGF1 - insulin-like growth factor 1 (somatomedin c)  IGFBP1 - insulin-like growth factor binding protein 1  IGFALS - insulin-like growth factor binding protein, acid labile subunit  THOP1 - thimet oligopeptidase 1  CKM - creatine kinase, muscle  NDUFS6 - nadh dehydrogenase (ubiquinone) fe-s protein 6, 13kda (nadh-coenzyme q reductase)  THRSP - thyroid hormone responsive |
| GO:0006952 | defense response | 2.33E-5 | 1.57E-3 | 1.72 (10334,530,667,59) | [+] Show genes  APCS - amyloid p component, serum  MASP1 - mannan-binding lectin serine peptidase 1 (c4/c2 activating component of ra-reactive factor)  PLAC8 - placenta-specific 8  AOC3 - amine oxidase, copper containing 3  FN1 - fibronectin 1  F12 - coagulation factor xii (hageman factor)  APOA2 - apolipoprotein a-ii  APOA4 - apolipoprotein a-iv  GSDMB - gasdermin b  HRG - histidine-rich glycoprotein  SERPINC1 - serpin peptidase inhibitor, clade c (antithrombin), member 1  HRAS - harvey rat sarcoma viral oncogene homolog  CCL16 - chemokine (c-c motif) ligand 16  TNIP1 - tnfaip3 interacting protein 1  CD36 - cd36 molecule (thrombospondin receptor)  MBL2 - mannose-binding lectin (protein c) 2, soluble  CCL14 - chemokine (c-c motif) ligand 14  CFB - complement factor b  AHCY - adenosylhomocysteinase  IL1RN - interleukin 1 receptor antagonist  ECSIT - ecsit signalling integrator  HP - haptoglobin  MARCO - macrophage receptor with collagenous structure  HAMP - hepcidin antimicrobial peptide  KNG1 - kininogen 1  F2 - coagulation factor ii (thrombin)  CD209 - cd209 molecule  SERPINF2 - serpin peptidase inhibitor, clade f (alpha-2 antiplasmin, pigment epithelium derived factor), member 2  C9 - complement component 9  CRP - c-reactive protein, pentraxin-related  CSRP3 - cysteine and glycine-rich protein 3 (cardiac lim protein)  VNN1 - vanin 1  FGA - fibrinogen alpha chain  ARG1 - arginase 1  FGB - fibrinogen beta chain  ITIH4 - inter-alpha-trypsin inhibitor heavy chain family, member 4  TFR2 - transferrin receptor 2  CD5L - cd5 molecule-like  S100A12 - s100 calcium binding protein a12  MASP2 - mannan-binding lectin serine peptidase 2  SAA1 - serum amyloid a1  LBP - lipopolysaccharide binding protein  ASS1 - argininosuccinate synthase 1  C1R - complement component 1, r subcomponent  FCGR2B - fc fragment of igg, low affinity iib, receptor (cd32)  SERPINA1 - serpin peptidase inhibitor, clade a (alpha-1 antiproteinase, antitrypsin), member 1  C2 - complement component 2  LEAP2 - liver expressed antimicrobial peptide 2  C4BPA - complement component 4 binding protein, alpha  C4BPB - complement component 4 binding protein, beta  SPON2 - spondin 2, extracellular matrix protein  SAA4 - serum amyloid a4, constitutive  C5 - complement component 5  C6 - complement component 6  NUPR1 - nuclear protein, transcriptional regulator, 1  C8B - complement component 8, beta polypeptide  CXCL3 - chemokine (c-x-c motif) ligand 3  C8A - complement component 8, alpha polypeptide  C8G - complement component 8, gamma polypeptide |
| GO:0009165 | nucleotide biosynthetic process | 2.34E-5 | 1.57E-3 | 2.21 (10334,217,667,31) | [+] Show genes  HSD17B8 - hydroxysteroid (17-beta) dehydrogenase 8  MLYCD - malonyl-coa decarboxylase  NMRK2 - nicotinamide riboside kinase 2  PGM1 - phosphoglucomutase 1  SHMT1 - serine hydroxymethyltransferase 1 (soluble)  COX5B - cytochrome c oxidase subunit vb  PGAM2 - phosphoglycerate mutase 2 (muscle)  ALDOB - aldolase b, fructose-bisphosphate  ADSSL1 - adenylosuccinate synthase like 1  ACSL1 - acyl-coa synthetase long-chain family member 1  MTHFD1 - methylenetetrahydrofolate dehydrogenase (nadp+ dependent) 1, methenyltetrahydrofolate cyclohydrolase, formyltetrahydrofolate synthetase  ENO3 - enolase 3 (beta, muscle)  ADA - adenosine deaminase  LDHA - lactate dehydrogenase a  SLC25A13 - solute carrier family 25 (aspartate/glutamate carrier), member 13  PTPLA - protein tyrosine phosphatase-like (proline instead of catalytic arginine), member a  MPC1 - mitochondrial pyruvate carrier 1  PKLR - pyruvate kinase, liver and rbc  HAAO - 3-hydroxyanthranilate 3,4-dioxygenase  AK2 - adenylate kinase 2  ATP5J2 - atp synthase, h+ transporting, mitochondrial fo complex, subunit f2  ASPDH - aspartate dehydrogenase domain containing  GCDH - glutaryl-coa dehydrogenase  ATP5H - atp synthase, h+ transporting, mitochondrial fo complex, subunit d  ATP5I - atp synthase, h+ transporting, mitochondrial fo complex, subunit e  AMPD1 - adenosine monophosphate deaminase 1  ATP5E - atp synthase, h+ transporting, mitochondrial f1 complex, epsilon subunit  ATP5D - atp synthase, h+ transporting, mitochondrial f1 complex, delta subunit  ATP5G1 - atp synthase, h+ transporting, mitochondrial fo complex, subunit c1 (subunit 9)  AGPAT2 - 1-acylglycerol-3-phosphate o-acyltransferase 2  PFKFB1 - 6-phosphofructo-2-kinase/fructose-2,6-biphosphatase 1 |
| GO:0006120 | mitochondrial electron transport, NADH to ubiquinone | 2.41E-5 | 1.61E-3 | 4.04 (10334,46,667,12) | [+] Show genes  NDUFB10 - nadh dehydrogenase (ubiquinone) 1 beta subcomplex, 10, 22kda  NDUFB7 - nadh dehydrogenase (ubiquinone) 1 beta subcomplex, 7, 18kda  NDUFS8 - nadh dehydrogenase (ubiquinone) fe-s protein 8, 23kda (nadh-coenzyme q reductase)  COQ9 - coenzyme q9 homolog (s. cerevisiae)  NDUFS6 - nadh dehydrogenase (ubiquinone) fe-s protein 6, 13kda (nadh-coenzyme q reductase)  NDUFA3 - nadh dehydrogenase (ubiquinone) 1 alpha subcomplex, 3, 9kda  NDUFS5 - nadh dehydrogenase (ubiquinone) fe-s protein 5, 15kda (nadh-coenzyme q reductase)  NDUFA4 - nadh dehydrogenase (ubiquinone) 1 alpha subcomplex, 4, 9kda  NDUFS7 - nadh dehydrogenase (ubiquinone) fe-s protein 7, 20kda (nadh-coenzyme q reductase)  NDUFV1 - nadh dehydrogenase (ubiquinone) flavoprotein 1, 51kda  NDUFA2 - nadh dehydrogenase (ubiquinone) 1 alpha subcomplex, 2, 8kda  NDUFA7 - nadh dehydrogenase (ubiquinone) 1 alpha subcomplex, 7, 14.5kda |
| GO:0010906 | regulation of glucose metabolic process | 2.43E-5 | 1.62E-3 | 3.10 (10334,85,667,17) | [+] Show genes  FAM132A - family with sequence similarity 132, member a  MLYCD - malonyl-coa decarboxylase  RORC - rar-related orphan receptor c  IGF1 - insulin-like growth factor 1 (somatomedin c)  ACTN3 - actinin, alpha 3  DGAT2 - diacylglycerol o-acyltransferase 2  PPP1R3C - protein phosphatase 1, regulatory subunit 3c  ENPP1 - ectonucleotide pyrophosphatase/phosphodiesterase 1  PPARGC1A - peroxisome proliferator-activated receptor gamma, coactivator 1 alpha  MST1 - macrophage stimulating 1 (hepatocyte growth factor-like)  PDK4 - pyruvate dehydrogenase kinase, isozyme 4  PPP1R3B - protein phosphatase 1, regulatory subunit 3b  PDK2 - pyruvate dehydrogenase kinase, isozyme 2  GCGR - glucagon receptor  PPARA - peroxisome proliferator-activated receptor alpha  PPP1R3A - protein phosphatase 1, regulatory subunit 3a  PFKFB1 - 6-phosphofructo-2-kinase/fructose-2,6-biphosphatase 1 |
| GO:0010896 | regulation of triglyceride catabolic process | 2.47E-5 | 1.64E-3 | 8.45 (10334,11,667,6) | [+] Show genes  APOC2 - apolipoprotein c-ii  APOA5 - apolipoprotein a-v  APOC3 - apolipoprotein c-iii  PLIN5 - perilipin 5  PNPLA2 - patatin-like phospholipase domain containing 2  APOA4 - apolipoprotein a-iv |
| GO:0006957 | complement activation, alternative pathway | 2.47E-5 | 1.63E-3 | 8.45 (10334,11,667,6) | [+] Show genes  C9 - complement component 9  CFB - complement factor b  C5 - complement component 5  C8B - complement component 8, beta polypeptide  C8A - complement component 8, alpha polypeptide  C8G - complement component 8, gamma polypeptide |
| GO:0009152 | purine ribonucleotide biosynthetic process | 2.73E-5 | 1.79E-3 | 2.50 (10334,149,667,24) | [+] Show genes  AK2 - adenylate kinase 2  ATP5J2 - atp synthase, h+ transporting, mitochondrial fo complex, subunit f2  HSD17B8 - hydroxysteroid (17-beta) dehydrogenase 8  MLYCD - malonyl-coa decarboxylase  PGM1 - phosphoglucomutase 1  GCDH - glutaryl-coa dehydrogenase  ATP5H - atp synthase, h+ transporting, mitochondrial fo complex, subunit d  ATP5I - atp synthase, h+ transporting, mitochondrial fo complex, subunit e  COX5B - cytochrome c oxidase subunit vb  AMPD1 - adenosine monophosphate deaminase 1  PGAM2 - phosphoglycerate mutase 2 (muscle)  ALDOB - aldolase b, fructose-bisphosphate  ADSSL1 - adenylosuccinate synthase like 1  ACSL1 - acyl-coa synthetase long-chain family member 1  ATP5E - atp synthase, h+ transporting, mitochondrial f1 complex, epsilon subunit  ATP5D - atp synthase, h+ transporting, mitochondrial f1 complex, delta subunit  ENO3 - enolase 3 (beta, muscle)  ATP5G1 - atp synthase, h+ transporting, mitochondrial fo complex, subunit c1 (subunit 9)  LDHA - lactate dehydrogenase a  SLC25A13 - solute carrier family 25 (aspartate/glutamate carrier), member 13  PTPLA - protein tyrosine phosphatase-like (proline instead of catalytic arginine), member a  MPC1 - mitochondrial pyruvate carrier 1  PKLR - pyruvate kinase, liver and rbc  PFKFB1 - 6-phosphofructo-2-kinase/fructose-2,6-biphosphatase 1 |
| GO:0010675 | regulation of cellular carbohydrate metabolic process | 2.73E-5 | 1.79E-3 | 2.86 (10334,103,667,19) | [+] Show genes  FAM132A - family with sequence similarity 132, member a  MLYCD - malonyl-coa decarboxylase  RORC - rar-related orphan receptor c  IGF1 - insulin-like growth factor 1 (somatomedin c)  ACTN3 - actinin, alpha 3  DGAT2 - diacylglycerol o-acyltransferase 2  PPP1R3C - protein phosphatase 1, regulatory subunit 3c  ENPP1 - ectonucleotide pyrophosphatase/phosphodiesterase 1  PPARGC1A - peroxisome proliferator-activated receptor gamma, coactivator 1 alpha  MST1 - macrophage stimulating 1 (hepatocyte growth factor-like)  PDK4 - pyruvate dehydrogenase kinase, isozyme 4  PPP1R3B - protein phosphatase 1, regulatory subunit 3b  PDK2 - pyruvate dehydrogenase kinase, isozyme 2  GCGR - glucagon receptor  NUPR1 - nuclear protein, transcriptional regulator, 1  PPARA - peroxisome proliferator-activated receptor alpha  PPP1R3A - protein phosphatase 1, regulatory subunit 3a  PFKFB1 - 6-phosphofructo-2-kinase/fructose-2,6-biphosphatase 1  PLEK - pleckstrin |
| GO:0009205 | purine ribonucleoside triphosphate metabolic process | 2.75E-5 | 1.79E-3 | 2.69 (10334,121,667,21) | [+] Show genes  AK2 - adenylate kinase 2  ATP5J2 - atp synthase, h+ transporting, mitochondrial fo complex, subunit f2  UQCRC1 - ubiquinol-cytochrome c reductase core protein i  PGM1 - phosphoglucomutase 1  ATP5H - atp synthase, h+ transporting, mitochondrial fo complex, subunit d  ATP5I - atp synthase, h+ transporting, mitochondrial fo complex, subunit e  COX5B - cytochrome c oxidase subunit vb  GIMAP7 - gtpase, imap family member 7  PGAM2 - phosphoglycerate mutase 2 (muscle)  ALDOB - aldolase b, fructose-bisphosphate  ATP5E - atp synthase, h+ transporting, mitochondrial f1 complex, epsilon subunit  ENPP1 - ectonucleotide pyrophosphatase/phosphodiesterase 1  ATP5D - atp synthase, h+ transporting, mitochondrial f1 complex, delta subunit  ENO3 - enolase 3 (beta, muscle)  ATP5G1 - atp synthase, h+ transporting, mitochondrial fo complex, subunit c1 (subunit 9)  CHCHD10 - coiled-coil-helix-coiled-coil-helix domain containing 10  LDHA - lactate dehydrogenase a  SLC25A13 - solute carrier family 25 (aspartate/glutamate carrier), member 13  PKLR - pyruvate kinase, liver and rbc  PFKFB1 - 6-phosphofructo-2-kinase/fructose-2,6-biphosphatase 1  MOCS1 - molybdenum cofactor synthesis 1 |
| GO:0033108 | mitochondrial respiratory chain complex assembly | 2.85E-5 | 1.84E-3 | 3.06 (10334,86,667,17) | [+] Show genes  NDUFB10 - nadh dehydrogenase (ubiquinone) 1 beta subcomplex, 10, 22kda  APOPT1 - apoptogenic 1, mitochondrial  UQCR10 - ubiquinol-cytochrome c reductase, complex iii subunit x  BCS1L - bc1 (ubiquinol-cytochrome c reductase) synthesis-like  NDUFB7 - nadh dehydrogenase (ubiquinone) 1 beta subcomplex, 7, 18kda  COX17 - cox17 cytochrome c oxidase copper chaperone  NDUFA3 - nadh dehydrogenase (ubiquinone) 1 alpha subcomplex, 3, 9kda  NDUFS7 - nadh dehydrogenase (ubiquinone) fe-s protein 7, 20kda (nadh-coenzyme q reductase)  ECSIT - ecsit signalling integrator  NDUFA2 - nadh dehydrogenase (ubiquinone) 1 alpha subcomplex, 2, 8kda  NDUFA7 - nadh dehydrogenase (ubiquinone) 1 alpha subcomplex, 7, 14.5kda  IMMP2L - imp2 inner mitochondrial membrane peptidase-like (s. cerevisiae)  FOXRED1 - fad-dependent oxidoreductase domain containing 1  NDUFS8 - nadh dehydrogenase (ubiquinone) fe-s protein 8, 23kda (nadh-coenzyme q reductase)  NDUFS6 - nadh dehydrogenase (ubiquinone) fe-s protein 6, 13kda (nadh-coenzyme q reductase)  NDUFS5 - nadh dehydrogenase (ubiquinone) fe-s protein 5, 15kda (nadh-coenzyme q reductase)  NDUFV1 - nadh dehydrogenase (ubiquinone) flavoprotein 1, 51kda |
| GO:0008206 | bile acid metabolic process | 3.01E-5 | 1.94E-3 | 5.16 (10334,27,667,9) | [+] Show genes  ACOX2 - acyl-coa oxidase 2, branched chain  AKR1C4 - aldo-keto reductase family 1, member c4  SCP2 - sterol carrier protein 2  CYP8B1 - cytochrome p450, family 8, subfamily b, polypeptide 1  SLC27A2 - solute carrier family 27 (fatty acid transporter), member 2  SULT2A1 - sulfotransferase family, cytosolic, 2a, dehydroepiandrosterone (dhea)-preferring, member 1  ACAA1 - acetyl-coa acyltransferase 1  SLC27A5 - solute carrier family 27 (fatty acid transporter), member 5  AMACR - alpha-methylacyl-coa racemase |
| GO:0050994 | regulation of lipid catabolic process | 3.07E-5 | 1.97E-3 | 4.26 (10334,40,667,11) | [+] Show genes  APOC2 - apolipoprotein c-ii  MLYCD - malonyl-coa decarboxylase  APOC3 - apolipoprotein c-iii  APOA5 - apolipoprotein a-v  ANGPTL3 - angiopoietin-like 3  PLIN5 - perilipin 5  APOA2 - apolipoprotein a-ii  FABP1 - fatty acid binding protein 1, liver  PPARA - peroxisome proliferator-activated receptor alpha  PNPLA2 - patatin-like phospholipase domain containing 2  APOA4 - apolipoprotein a-iv |
| GO:0060415 | muscle tissue morphogenesis | 3.07E-5 | 1.96E-3 | 4.26 (10334,40,667,11) | [+] Show genes  MYL3 - myosin, light chain 3, alkali; ventricular, skeletal, slow  MYL2 - myosin, light chain 2, regulatory, cardiac, slow  MYF6 - myogenic factor 6 (herculin)  TNNI1 - troponin i type 1 (skeletal, slow)  TCAP - titin-cap  TNNC1 - troponin c type 1 (slow)  RXRA - retinoid x receptor, alpha  XIRP2 - xin actin-binding repeat containing 2  MYLK2 - myosin light chain kinase 2  TTN - titin  ANKRD1 - ankyrin repeat domain 1 (cardiac muscle) |
| GO:0016999 | antibiotic metabolic process | 3.33E-5 | 2.12E-3 | 3.03 (10334,87,667,17) | [+] Show genes  AKR1C3 - aldo-keto reductase family 1, member c3  FAHD1 - fumarylacetoacetate hydrolase domain containing 1  CYP4A11 - cytochrome p450, family 4, subfamily a, polypeptide 11  AKR1C4 - aldo-keto reductase family 1, member c4  PCK1 - phosphoenolpyruvate carboxykinase 1 (soluble)  FTCD - formimidoyltransferase cyclodeaminase  SULT2A1 - sulfotransferase family, cytosolic, 2a, dehydroepiandrosterone (dhea)-preferring, member 1  CAT - catalase  SDHB - succinate dehydrogenase complex, subunit b, iron sulfur (ip)  ADH6 - alcohol dehydrogenase 6 (class v)  SUCLG1 - succinate-coa ligase, alpha subunit  ADH4 - alcohol dehydrogenase 4 (class ii), pi polypeptide  APOA4 - apolipoprotein a-iv  FH - fumarate hydratase  CYP1A2 - cytochrome p450, family 1, subfamily a, polypeptide 2  IDH3G - isocitrate dehydrogenase 3 (nad+) gamma  ALDH2 - aldehyde dehydrogenase 2 family (mitochondrial) |
| GO:0009123 | nucleoside monophosphate metabolic process | 3.41E-5 | 2.16E-3 | 2.46 (10334,151,667,24) | [+] Show genes  AK2 - adenylate kinase 2  ATP5J2 - atp synthase, h+ transporting, mitochondrial fo complex, subunit f2  UQCRC1 - ubiquinol-cytochrome c reductase core protein i  PGM1 - phosphoglucomutase 1  SHMT1 - serine hydroxymethyltransferase 1 (soluble)  ATP5I - atp synthase, h+ transporting, mitochondrial fo complex, subunit e  ATP5H - atp synthase, h+ transporting, mitochondrial fo complex, subunit d  COX5B - cytochrome c oxidase subunit vb  ENTPD8 - ectonucleoside triphosphate diphosphohydrolase 8  AMPD1 - adenosine monophosphate deaminase 1  PGAM2 - phosphoglycerate mutase 2 (muscle)  ALDOB - aldolase b, fructose-bisphosphate  ADSSL1 - adenylosuccinate synthase like 1  ATP5E - atp synthase, h+ transporting, mitochondrial f1 complex, epsilon subunit  ENPP1 - ectonucleotide pyrophosphatase/phosphodiesterase 1  ATP5D - atp synthase, h+ transporting, mitochondrial f1 complex, delta subunit  ENO3 - enolase 3 (beta, muscle)  ATP5G1 - atp synthase, h+ transporting, mitochondrial fo complex, subunit c1 (subunit 9)  CHCHD10 - coiled-coil-helix-coiled-coil-helix domain containing 10  LDHA - lactate dehydrogenase a  ADA - adenosine deaminase  SLC25A13 - solute carrier family 25 (aspartate/glutamate carrier), member 13  PKLR - pyruvate kinase, liver and rbc  PFKFB1 - 6-phosphofructo-2-kinase/fructose-2,6-biphosphatase 1 |
| GO:1905952 | regulation of lipid localization | 3.53E-5 | 2.22E-3 | 2.90 (10334,96,667,18) | [+] Show genes  PON1 - paraoxonase 1  CYP4A11 - cytochrome p450, family 4, subfamily a, polypeptide 11  APOA5 - apolipoprotein a-v  CRP - c-reactive protein, pentraxin-related  PLIN5 - perilipin 5  CD36 - cd36 molecule (thrombospondin receptor)  APOA2 - apolipoprotein a-ii  CES1 - carboxylesterase 1  ALKBH7 - alkb, alkylation repair homolog 7 (e. coli)  EEPD1 - endonuclease/exonuclease/phosphatase family domain containing 1  APOA4 - apolipoprotein a-iv  FITM1 - fat storage-inducing transmembrane protein 1  APOC2 - apolipoprotein c-ii  APOC3 - apolipoprotein c-iii  APOC4 - apolipoprotein c-iv  SCP2 - sterol carrier protein 2  PPARA - peroxisome proliferator-activated receptor alpha  PNPLA2 - patatin-like phospholipase domain containing 2 |
| GO:0001676 | long-chain fatty acid metabolic process | 3.69E-5 | 2.31E-3 | 3.44 (10334,63,667,14) | [+] Show genes  AKR1C3 - aldo-keto reductase family 1, member c3  CYP4A11 - cytochrome p450, family 4, subfamily a, polypeptide 11  CYP2E1 - cytochrome p450, family 2, subfamily e, polypeptide 1  CYP2C19 - cytochrome p450, family 2, subfamily c, polypeptide 19  ACSL1 - acyl-coa synthetase long-chain family member 1  HACL1 - 2-hydroxyacyl-coa lyase 1  ADTRP - androgen-dependent tfpi-regulating protein  SCP2 - sterol carrier protein 2  SLC27A2 - solute carrier family 27 (fatty acid transporter), member 2  CBR1 - carbonyl reductase 1  ACAA1 - acetyl-coa acyltransferase 1  ACADL - acyl-coa dehydrogenase, long chain  CYP1A2 - cytochrome p450, family 1, subfamily a, polypeptide 2  SLC27A5 - solute carrier family 27 (fatty acid transporter), member 5 |
| GO:0062014 | negative regulation of small molecule metabolic process | 3.7E-5 | 2.31E-3 | 3.27 (10334,71,667,15) | [+] Show genes  FAM132A - family with sequence similarity 132, member a  AKR1C3 - aldo-keto reductase family 1, member c3  PLIN5 - perilipin 5  ACTN3 - actinin, alpha 3  DGAT2 - diacylglycerol o-acyltransferase 2  APOC3 - apolipoprotein c-iii  PPARGC1A - peroxisome proliferator-activated receptor gamma, coactivator 1 alpha  MST1 - macrophage stimulating 1 (hepatocyte growth factor-like)  MLXIPL - mlx interacting protein-like  ACADL - acyl-coa dehydrogenase, long chain  NUPR1 - nuclear protein, transcriptional regulator, 1  PPARA - peroxisome proliferator-activated receptor alpha  ACADVL - acyl-coa dehydrogenase, very long chain  PFKFB1 - 6-phosphofructo-2-kinase/fructose-2,6-biphosphatase 1  PLEK - pleckstrin |
| GO:0055008 | cardiac muscle tissue morphogenesis | 3.72E-5 | 2.31E-3 | 4.56 (10334,34,667,10) | [+] Show genes  MYL3 - myosin, light chain 3, alkali; ventricular, skeletal, slow  MYL2 - myosin, light chain 2, regulatory, cardiac, slow  TNNI1 - troponin i type 1 (skeletal, slow)  TCAP - titin-cap  TNNC1 - troponin c type 1 (slow)  RXRA - retinoid x receptor, alpha  XIRP2 - xin actin-binding repeat containing 2  MYLK2 - myosin light chain kinase 2  TTN - titin  ANKRD1 - ankyrin repeat domain 1 (cardiac muscle) |
| GO:0009201 | ribonucleoside triphosphate biosynthetic process | 4.39E-5 | 2.72E-3 | 3.23 (10334,72,667,15) | [+] Show genes  ATP5J2 - atp synthase, h+ transporting, mitochondrial fo complex, subunit f2  PGM1 - phosphoglucomutase 1  ATP5H - atp synthase, h+ transporting, mitochondrial fo complex, subunit d  ATP5I - atp synthase, h+ transporting, mitochondrial fo complex, subunit e  COX5B - cytochrome c oxidase subunit vb  PGAM2 - phosphoglycerate mutase 2 (muscle)  ALDOB - aldolase b, fructose-bisphosphate  ATP5E - atp synthase, h+ transporting, mitochondrial f1 complex, epsilon subunit  ENO3 - enolase 3 (beta, muscle)  ATP5D - atp synthase, h+ transporting, mitochondrial f1 complex, delta subunit  ATP5G1 - atp synthase, h+ transporting, mitochondrial fo complex, subunit c1 (subunit 9)  LDHA - lactate dehydrogenase a  SLC25A13 - solute carrier family 25 (aspartate/glutamate carrier), member 13  PKLR - pyruvate kinase, liver and rbc  PFKFB1 - 6-phosphofructo-2-kinase/fructose-2,6-biphosphatase 1 |
| GO:1901566 | organonitrogen compound biosynthetic process | 4.42E-5 | 2.72E-3 | 1.57 (10334,738,667,75) | [+] Show genes  HSD17B8 - hydroxysteroid (17-beta) dehydrogenase 8  MLYCD - malonyl-coa decarboxylase  MRPL23 - mitochondrial ribosomal protein l23  GADL1 - glutamate decarboxylase-like 1  AGMAT - agmatine ureohydrolase (agmatinase)  PGM1 - phosphoglucomutase 1  APOA2 - apolipoprotein a-ii  COX5B - cytochrome c oxidase subunit vb  HMBS - hydroxymethylbilane synthase  NDUFA7 - nadh dehydrogenase (ubiquinone) 1 alpha subcomplex, 7, 14.5kda  PGAM2 - phosphoglycerate mutase 2 (muscle)  MTHFR - methylenetetrahydrofolate reductase (nad(p)h)  ACSL1 - acyl-coa synthetase long-chain family member 1  BDH2 - 3-hydroxybutyrate dehydrogenase, type 2  MTHFD1 - methylenetetrahydrofolate dehydrogenase (nadp+ dependent) 1, methenyltetrahydrofolate cyclohydrolase, formyltetrahydrofolate synthetase  OTC - ornithine carbamoyltransferase  GOT2 - glutamic-oxaloacetic transaminase 2, mitochondrial  ENO3 - enolase 3 (beta, muscle)  ASPG - asparaginase homolog (s. cerevisiae)  LDHA - lactate dehydrogenase a  RPL3L - ribosomal protein l3-like  GSTZ1 - glutathione s-transferase zeta 1  SLC25A13 - solute carrier family 25 (aspartate/glutamate carrier), member 13  GSTT1 - glutathione s-transferase theta 1  AK2 - adenylate kinase 2  ASPDH - aspartate dehydrogenase domain containing  ATP5J2 - atp synthase, h+ transporting, mitochondrial fo complex, subunit f2  CHST13 - carbohydrate (chondroitin 4) sulfotransferase 13  TNIP1 - tnfaip3 interacting protein 1  CPS1 - carbamoyl-phosphate synthase 1, mitochondrial  ATP5H - atp synthase, h+ transporting, mitochondrial fo complex, subunit d  ATP5I - atp synthase, h+ transporting, mitochondrial fo complex, subunit e  AGXT - alanine-glyoxylate aminotransferase  BHMT - betaine--homocysteine s-methyltransferase  ATP5E - atp synthase, h+ transporting, mitochondrial f1 complex, epsilon subunit  ABCB6 - atp-binding cassette, sub-family b (mdr/tap), member 6  SDC2 - syndecan 2  ATP5D - atp synthase, h+ transporting, mitochondrial f1 complex, delta subunit  ATP5G1 - atp synthase, h+ transporting, mitochondrial fo complex, subunit c1 (subunit 9)  PAH - phenylalanine hydroxylase  GGT1 - gamma-glutamyltransferase 1  MOCS1 - molybdenum cofactor synthesis 1  PFKFB1 - 6-phosphofructo-2-kinase/fructose-2,6-biphosphatase 1  UPB1 - ureidopropionase, beta  PHOSPHO1 - phosphatase, orphan 1  AGXT2 - alanine--glyoxylate aminotransferase 2  NMRK2 - nicotinamide riboside kinase 2  CHPT1 - choline phosphotransferase 1  SHMT1 - serine hydroxymethyltransferase 1 (soluble)  GAMT - guanidinoacetate n-methyltransferase  LPIN1 - lipin 1  ALDOB - aldolase b, fructose-bisphosphate  PCTP - phosphatidylcholine transfer protein  ADSSL1 - adenylosuccinate synthase like 1  ST3GAL3 - st3 beta-galactoside alpha-2,3-sialyltransferase 3  ARG1 - arginase 1  MRPS15 - mitochondrial ribosomal protein s15  ADA - adenosine deaminase  HAGH - hydroxyacylglutathione hydrolase  PTPLA - protein tyrosine phosphatase-like (proline instead of catalytic arginine), member a  MPC1 - mitochondrial pyruvate carrier 1  MRPL36 - mitochondrial ribosomal protein l36  PKLR - pyruvate kinase, liver and rbc  HAAO - 3-hydroxyanthranilate 3,4-dioxygenase  MRPL2 - mitochondrial ribosomal protein l2  IGF1 - insulin-like growth factor 1 (somatomedin c)  GCDH - glutaryl-coa dehydrogenase  MRPS34 - mitochondrial ribosomal protein s34  ASS1 - argininosuccinate synthase 1  ADI1 - acireductone dioxygenase 1  AMPD1 - adenosine monophosphate deaminase 1  CKM - creatine kinase, muscle  AGPAT2 - 1-acylglycerol-3-phosphate o-acyltransferase 2  CCBL1 - cysteine conjugate-beta lyase, cytoplasmic  URAD - ureidoimidazoline (2-oxo-4-hydroxy-4-carboxy-5-) decarboxylase |
| GO:0006937 | regulation of muscle contraction | 4.52E-5 | 2.77E-3 | 2.96 (10334,89,667,17) | [+] Show genes  ATP2A1 - atpase, ca++ transporting, cardiac muscle, fast twitch 1  PLN - phospholamban  ADRB2 - adrenoceptor beta 2, surface  CAV3 - caveolin 3  ACTN3 - actinin, alpha 3  CASQ2 - calsequestrin 2 (cardiac muscle)  MYL3 - myosin, light chain 3, alkali; ventricular, skeletal, slow  TNNC2 - troponin c type 2 (fast)  MYL2 - myosin, light chain 2, regulatory, cardiac, slow  HSPB6 - heat shock protein, alpha-crystallin-related, b6  ADA - adenosine deaminase  HRC - histidine rich calcium binding protein  TNNI2 - troponin i type 2 (skeletal, fast)  TNNI1 - troponin i type 1 (skeletal, slow)  TNNC1 - troponin c type 1 (slow)  TNNT3 - troponin t type 3 (skeletal, fast)  MYLK2 - myosin light chain kinase 2 |
| GO:0016051 | carbohydrate biosynthetic process | 4.7E-5 | 2.87E-3 | 2.85 (10334,98,667,18) | [+] Show genes  SLC37A4 - solute carrier family 37 (glucose-6-phosphate transporter), member 4  CHST13 - carbohydrate (chondroitin 4) sulfotransferase 13  PCK1 - phosphoenolpyruvate carboxykinase 1 (soluble)  PGM1 - phosphoglucomutase 1  PGAM2 - phosphoglycerate mutase 2 (muscle)  ALDOB - aldolase b, fructose-bisphosphate  PPP1R3C - protein phosphatase 1, regulatory subunit 3c  ENO3 - enolase 3 (beta, muscle)  PPARGC1A - peroxisome proliferator-activated receptor gamma, coactivator 1 alpha  GOT2 - glutamic-oxaloacetic transaminase 2, mitochondrial  ATF4 - activating transcription factor 4  GYS2 - glycogen synthase 2 (liver)  IMPA2 - inositol(myo)-1(or 4)-monophosphatase 2  FBP2 - fructose-1,6-bisphosphatase 2  SLC25A13 - solute carrier family 25 (aspartate/glutamate carrier), member 13  GPD1 - glycerol-3-phosphate dehydrogenase 1 (soluble)  G6PC - glucose-6-phosphatase, catalytic subunit  PFKFB1 - 6-phosphofructo-2-kinase/fructose-2,6-biphosphatase 1 |
| GO:0051188 | cofactor biosynthetic process | 4.84E-5 | 2.94E-3 | 2.27 (10334,184,667,27) | [+] Show genes  HSD17B8 - hydroxysteroid (17-beta) dehydrogenase 8  MLYCD - malonyl-coa decarboxylase  PGM1 - phosphoglucomutase 1  NMRK2 - nicotinamide riboside kinase 2  HMBS - hydroxymethylbilane synthase  PGAM2 - phosphoglycerate mutase 2 (muscle)  ALDOB - aldolase b, fructose-bisphosphate  BDH2 - 3-hydroxybutyrate dehydrogenase, type 2  ACSL1 - acyl-coa synthetase long-chain family member 1  MTHFD1 - methylenetetrahydrofolate dehydrogenase (nadp+ dependent) 1, methenyltetrahydrofolate cyclohydrolase, formyltetrahydrofolate synthetase  ENO3 - enolase 3 (beta, muscle)  LDHA - lactate dehydrogenase a  CYP1A2 - cytochrome p450, family 1, subfamily a, polypeptide 2  HAGH - hydroxyacylglutathione hydrolase  PTPLA - protein tyrosine phosphatase-like (proline instead of catalytic arginine), member a  MPC1 - mitochondrial pyruvate carrier 1  PKLR - pyruvate kinase, liver and rbc  HAAO - 3-hydroxyanthranilate 3,4-dioxygenase  ASPDH - aspartate dehydrogenase domain containing  GCDH - glutaryl-coa dehydrogenase  MAT1A - methionine adenosyltransferase i, alpha  COQ4 - coenzyme q4 homolog (s. cerevisiae)  ABCB6 - atp-binding cassette, sub-family b (mdr/tap), member 6  COQ9 - coenzyme q9 homolog (s. cerevisiae)  GGT1 - gamma-glutamyltransferase 1  MOCS1 - molybdenum cofactor synthesis 1  PFKFB1 - 6-phosphofructo-2-kinase/fructose-2,6-biphosphatase 1 |
| GO:0014904 | myotube cell development | 4.97E-5 | 3.01E-3 | 6.38 (10334,17,667,7) | [+] Show genes  KLHL41 - kelch-like family member 41  ACTA1 - actin, alpha 1, skeletal muscle  STAC3 - sh3 and cysteine rich domain 3  IGF1 - insulin-like growth factor 1 (somatomedin c)  KLHL40 - kelch-like family member 40  LMOD3 - leiomodin 3 (fetal)  RYR1 - ryanodine receptor 1 (skeletal) |
| GO:0006555 | methionine metabolic process | 4.97E-5 | 2.99E-3 | 6.38 (10334,17,667,7) | [+] Show genes  MTHFR - methylenetetrahydrofolate reductase (nad(p)h)  MTHFD1 - methylenetetrahydrofolate dehydrogenase (nadp+ dependent) 1, methenyltetrahydrofolate cyclohydrolase, formyltetrahydrofolate synthetase  MSRA - methionine sulfoxide reductase a  AHCY - adenosylhomocysteinase  BHMT - betaine--homocysteine s-methyltransferase  ADI1 - acireductone dioxygenase 1  MAT1A - methionine adenosyltransferase i, alpha |
| GO:0010035 | response to inorganic substance | 5.07E-5 | 3.04E-3 | 1.87 (10334,357,667,43) | [+] Show genes  AKR1C3 - aldo-keto reductase family 1, member c3  FGG - fibrinogen gamma chain  LGMN - legumain  CAPN3 - calpain 3, (p94)  CYP2E1 - cytochrome p450, family 2, subfamily e, polypeptide 1  FABP1 - fatty acid binding protein 1, liver  PGAM2 - phosphoglycerate mutase 2 (muscle)  CASQ2 - calsequestrin 2 (cardiac muscle)  ATF4 - activating transcription factor 4  TAT - tyrosine aminotransferase  PPARGC1A - peroxisome proliferator-activated receptor gamma, coactivator 1 alpha  OTC - ornithine carbamoyltransferase  FGA - fibrinogen alpha chain  ARG1 - arginase 1  AOC1 - amine oxidase, copper containing 1  FGB - fibrinogen beta chain  ADA - adenosine deaminase  LDHA - lactate dehydrogenase a  ENDOG - endonuclease g  TFR2 - transferrin receptor 2  SLC25A13 - solute carrier family 25 (aspartate/glutamate carrier), member 13  CYP1A2 - cytochrome p450, family 1, subfamily a, polypeptide 2  SERPINF1 - serpin peptidase inhibitor, clade f (alpha-2 antiplasmin, pigment epithelium derived factor), member 1  PKLR - pyruvate kinase, liver and rbc  HAAO - 3-hydroxyanthranilate 3,4-dioxygenase  PON1 - paraoxonase 1  AQP9 - aquaporin 9  MB - myoglobin  CPS1 - carbamoyl-phosphate synthase 1, mitochondrial  PLN - phospholamban  SCGB1A1 - secretoglobin, family 1a, member 1 (uteroglobin)  CAT - catalase  ASS1 - argininosuccinate synthase 1  UCP3 - uncoupling protein 3 (mitochondrial, proton carrier)  CHP2 - calcineurin-like ef-hand protein 2  HP - haptoglobin  TTN - titin  RYR1 - ryanodine receptor 1 (skeletal)  HAMP - hepcidin antimicrobial peptide  ATP5D - atp synthase, h+ transporting, mitochondrial f1 complex, delta subunit  ACTA1 - actin, alpha 1, skeletal muscle  HMOX1 - heme oxygenase (decycling) 1  TNNC1 - troponin c type 1 (slow) |
| GO:0009141 | nucleoside triphosphate metabolic process | 5.11E-5 | 3.05E-3 | 2.46 (10334,145,667,23) | [+] Show genes  AK2 - adenylate kinase 2  ATP5J2 - atp synthase, h+ transporting, mitochondrial fo complex, subunit f2  UQCRC1 - ubiquinol-cytochrome c reductase core protein i  PGM1 - phosphoglucomutase 1  ATP5H - atp synthase, h+ transporting, mitochondrial fo complex, subunit d  ATP5I - atp synthase, h+ transporting, mitochondrial fo complex, subunit e  COX5B - cytochrome c oxidase subunit vb  GIMAP7 - gtpase, imap family member 7  PGAM2 - phosphoglycerate mutase 2 (muscle)  ALDOB - aldolase b, fructose-bisphosphate  ATP5E - atp synthase, h+ transporting, mitochondrial f1 complex, epsilon subunit  ENPP1 - ectonucleotide pyrophosphatase/phosphodiesterase 1  ATP5D - atp synthase, h+ transporting, mitochondrial f1 complex, delta subunit  ENO3 - enolase 3 (beta, muscle)  ATP5G1 - atp synthase, h+ transporting, mitochondrial fo complex, subunit c1 (subunit 9)  CHCHD10 - coiled-coil-helix-coiled-coil-helix domain containing 10  SMPDL3A - sphingomyelin phosphodiesterase, acid-like 3a  LDHA - lactate dehydrogenase a  ADA - adenosine deaminase  SLC25A13 - solute carrier family 25 (aspartate/glutamate carrier), member 13  PKLR - pyruvate kinase, liver and rbc  PFKFB1 - 6-phosphofructo-2-kinase/fructose-2,6-biphosphatase 1  MOCS1 - molybdenum cofactor synthesis 1 |
| GO:0006694 | steroid biosynthetic process | 5.24E-5 | 3.12E-3 | 2.93 (10334,90,667,17) | [+] Show genes  AKR1C3 - aldo-keto reductase family 1, member c3  HSD17B8 - hydroxysteroid (17-beta) dehydrogenase 8  ACOX2 - acyl-coa oxidase 2, branched chain  HSD17B6 - hydroxysteroid (17-beta) dehydrogenase 6  APOA5 - apolipoprotein a-v  HSD17B14 - hydroxysteroid (17-beta) dehydrogenase 14  AKR1C4 - aldo-keto reductase family 1, member c4  ACAA2 - acetyl-coa acyltransferase 2  CYP8B1 - cytochrome p450, family 8, subfamily b, polypeptide 1  CES1 - carboxylesterase 1  AMACR - alpha-methylacyl-coa racemase  HSD11B1 - hydroxysteroid (11-beta) dehydrogenase 1  APOA4 - apolipoprotein a-iv  HMGCS2 - 3-hydroxy-3-methylglutaryl-coa synthase 2 (mitochondrial)  SCP2 - sterol carrier protein 2  SLC27A2 - solute carrier family 27 (fatty acid transporter), member 2  SLC27A5 - solute carrier family 27 (fatty acid transporter), member 5 |
| GO:0030029 | actin filament-based process | 5.26E-5 | 3.11E-3 | 1.90 (10334,335,667,41) | [+] Show genes  MYH2 - myosin, heavy chain 2, skeletal muscle, adult  TPM2 - tropomyosin 2 (beta)  CAPN3 - calpain 3, (p94)  CSRP3 - cysteine and glycine-rich protein 3 (cardiac lim protein)  MYL1 - myosin, light chain 1, alkali; skeletal, fast  FRMD3 - ferm domain containing 3  ACTN2 - actinin, alpha 2  ACTN3 - actinin, alpha 3  CASQ2 - calsequestrin 2 (cardiac muscle)  MYBPC1 - myosin binding protein c, slow type  MYOZ2 - myozenin 2  ABLIM3 - actin binding lim protein family, member 3  MOB2 - mob kinase activator 2  TCAP - titin-cap  LMOD3 - leiomodin 3 (fetal)  MYBPC2 - myosin binding protein c, fast type  LDB3 - lim domain binding 3  KLHL41 - kelch-like family member 41  SYNPO2L - synaptopodin 2-like  LMOD1 - leiomodin 1 (smooth muscle)  MYOZ1 - myozenin 1  XIRP2 - xin actin-binding repeat containing 2  TTN - titin  ANKRD1 - ankyrin repeat domain 1 (cardiac muscle)  NEB - nebulin  LMOD2 - leiomodin 2 (cardiac)  MYL3 - myosin, light chain 3, alkali; ventricular, skeletal, slow  DES - desmin  TNNC2 - troponin c type 2 (fast)  MYL2 - myosin, light chain 2, regulatory, cardiac, slow  TMOD4 - tropomodulin 4 (muscle)  ACTA1 - actin, alpha 1, skeletal muscle  AUTS2 - autism susceptibility candidate 2  TNNI2 - troponin i type 2 (skeletal, fast)  TNNI1 - troponin i type 1 (skeletal, slow)  TNNC1 - troponin c type 1 (slow)  SMTNL1 - smoothelin-like 1  TNNT3 - troponin t type 3 (skeletal, fast)  MYPN - myopalladin  SMTN - smoothelin  PLEK - pleckstrin |
| GO:0009161 | ribonucleoside monophosphate metabolic process | 5.46E-5 | 3.22E-3 | 2.51 (10334,136,667,22) | [+] Show genes  AK2 - adenylate kinase 2  ATP5J2 - atp synthase, h+ transporting, mitochondrial fo complex, subunit f2  UQCRC1 - ubiquinol-cytochrome c reductase core protein i  PGM1 - phosphoglucomutase 1  ATP5I - atp synthase, h+ transporting, mitochondrial fo complex, subunit e  ATP5H - atp synthase, h+ transporting, mitochondrial fo complex, subunit d  COX5B - cytochrome c oxidase subunit vb  AMPD1 - adenosine monophosphate deaminase 1  PGAM2 - phosphoglycerate mutase 2 (muscle)  ALDOB - aldolase b, fructose-bisphosphate  ADSSL1 - adenylosuccinate synthase like 1  ATP5E - atp synthase, h+ transporting, mitochondrial f1 complex, epsilon subunit  ENPP1 - ectonucleotide pyrophosphatase/phosphodiesterase 1  ATP5D - atp synthase, h+ transporting, mitochondrial f1 complex, delta subunit  ENO3 - enolase 3 (beta, muscle)  ATP5G1 - atp synthase, h+ transporting, mitochondrial fo complex, subunit c1 (subunit 9)  CHCHD10 - coiled-coil-helix-coiled-coil-helix domain containing 10  LDHA - lactate dehydrogenase a  ADA - adenosine deaminase  SLC25A13 - solute carrier family 25 (aspartate/glutamate carrier), member 13  PKLR - pyruvate kinase, liver and rbc  PFKFB1 - 6-phosphofructo-2-kinase/fructose-2,6-biphosphatase 1 |
| GO:0010677 | negative regulation of cellular carbohydrate metabolic process | 5.72E-5 | 3.36E-3 | 4.81 (10334,29,667,9) | [+] Show genes  FAM132A - family with sequence similarity 132, member a  ENPP1 - ectonucleotide pyrophosphatase/phosphodiesterase 1  PPARGC1A - peroxisome proliferator-activated receptor gamma, coactivator 1 alpha  MST1 - macrophage stimulating 1 (hepatocyte growth factor-like)  NUPR1 - nuclear protein, transcriptional regulator, 1  PPARA - peroxisome proliferator-activated receptor alpha  ACTN3 - actinin, alpha 3  PFKFB1 - 6-phosphofructo-2-kinase/fructose-2,6-biphosphatase 1  PLEK - pleckstrin |
| GO:0009199 | ribonucleoside triphosphate metabolic process | 5.77E-5 | 3.38E-3 | 2.56 (10334,127,667,21) | [+] Show genes  AK2 - adenylate kinase 2  ATP5J2 - atp synthase, h+ transporting, mitochondrial fo complex, subunit f2  UQCRC1 - ubiquinol-cytochrome c reductase core protein i  PGM1 - phosphoglucomutase 1  ATP5H - atp synthase, h+ transporting, mitochondrial fo complex, subunit d  ATP5I - atp synthase, h+ transporting, mitochondrial fo complex, subunit e  COX5B - cytochrome c oxidase subunit vb  GIMAP7 - gtpase, imap family member 7  PGAM2 - phosphoglycerate mutase 2 (muscle)  ALDOB - aldolase b, fructose-bisphosphate  ATP5E - atp synthase, h+ transporting, mitochondrial f1 complex, epsilon subunit  ENPP1 - ectonucleotide pyrophosphatase/phosphodiesterase 1  ATP5D - atp synthase, h+ transporting, mitochondrial f1 complex, delta subunit  ENO3 - enolase 3 (beta, muscle)  ATP5G1 - atp synthase, h+ transporting, mitochondrial fo complex, subunit c1 (subunit 9)  CHCHD10 - coiled-coil-helix-coiled-coil-helix domain containing 10  LDHA - lactate dehydrogenase a  SLC25A13 - solute carrier family 25 (aspartate/glutamate carrier), member 13  PKLR - pyruvate kinase, liver and rbc  PFKFB1 - 6-phosphofructo-2-kinase/fructose-2,6-biphosphatase 1  MOCS1 - molybdenum cofactor synthesis 1 |
| GO:0006081 | cellular aldehyde metabolic process | 5.92E-5 | 3.44E-3 | 3.72 (10334,50,667,12) | [+] Show genes  PRODH2 - proline dehydrogenase (oxidase) 2  AKR1C3 - aldo-keto reductase family 1, member c3  GLYCTK - glycerate kinase  GRHPR - glyoxylate reductase/hydroxypyruvate reductase  GOT2 - glutamic-oxaloacetic transaminase 2, mitochondrial  ALDH4A1 - aldehyde dehydrogenase 4 family, member a1  AGXT2 - alanine--glyoxylate aminotransferase 2  ALDH8A1 - aldehyde dehydrogenase 8 family, member a1  HAGH - hydroxyacylglutathione hydrolase  AGXT - alanine-glyoxylate aminotransferase  ADH4 - alcohol dehydrogenase 4 (class ii), pi polypeptide  ALDOB - aldolase b, fructose-bisphosphate |
| GO:0050848 | regulation of calcium-mediated signaling | 6.38E-5 | 3.7E-3 | 3.29 (10334,66,667,14) | [+] Show genes  PLN - phospholamban  IGF1 - insulin-like growth factor 1 (somatomedin c)  CHP2 - calcineurin-like ef-hand protein 2  MYOZ1 - myozenin 1  ACTN3 - actinin, alpha 3  JPH2 - junctophilin 2  CASQ2 - calsequestrin 2 (cardiac muscle)  MYOZ2 - myozenin 2  HOMER2 - homer homolog 2 (drosophila)  HRC - histidine rich calcium binding protein  PDK2 - pyruvate dehydrogenase kinase, isozyme 2  ADA - adenosine deaminase  C10orf71 - chromosome 10 open reading frame 71  PLEK - pleckstrin |
| GO:0090257 | regulation of muscle system process | 6.85E-5 | 3.96E-3 | 2.47 (10334,138,667,22) | [+] Show genes  MLIP - muscular lmna-interacting protein  ATP2A1 - atpase, ca++ transporting, cardiac muscle, fast twitch 1  PLN - phospholamban  IGF1 - insulin-like growth factor 1 (somatomedin c)  ADRB2 - adrenoceptor beta 2, surface  TRIM63 - tripartite motif containing 63, e3 ubiquitin protein ligase  CAV3 - caveolin 3  ACTN3 - actinin, alpha 3  MYL3 - myosin, light chain 3, alkali; ventricular, skeletal, slow  CASQ2 - calsequestrin 2 (cardiac muscle)  TNNC2 - troponin c type 2 (fast)  HAMP - hepcidin antimicrobial peptide  MYL2 - myosin, light chain 2, regulatory, cardiac, slow  HSPB6 - heat shock protein, alpha-crystallin-related, b6  HRC - histidine rich calcium binding protein  ADA - adenosine deaminase  TNNI2 - troponin i type 2 (skeletal, fast)  TNNI1 - troponin i type 1 (skeletal, slow)  TNNC1 - troponin c type 1 (slow)  TNNT3 - troponin t type 3 (skeletal, fast)  PPARA - peroxisome proliferator-activated receptor alpha  MYLK2 - myosin light chain kinase 2 |
| GO:0006006 | glucose metabolic process | 7.01E-5 | 4.03E-3 | 2.86 (10334,92,667,17) | [+] Show genes  SLC37A4 - solute carrier family 37 (glucose-6-phosphate transporter), member 4  PCK1 - phosphoenolpyruvate carboxykinase 1 (soluble)  PGM1 - phosphoglucomutase 1  PGAM2 - phosphoglycerate mutase 2 (muscle)  ALDOB - aldolase b, fructose-bisphosphate  GOT2 - glutamic-oxaloacetic transaminase 2, mitochondrial  ATF4 - activating transcription factor 4  ENO3 - enolase 3 (beta, muscle)  PPARGC1A - peroxisome proliferator-activated receptor gamma, coactivator 1 alpha  PDK4 - pyruvate dehydrogenase kinase, isozyme 4  PDK2 - pyruvate dehydrogenase kinase, isozyme 2  FBP2 - fructose-1,6-bisphosphatase 2  SLC25A13 - solute carrier family 25 (aspartate/glutamate carrier), member 13  GPD1 - glycerol-3-phosphate dehydrogenase 1 (soluble)  G6PC - glucose-6-phosphatase, catalytic subunit  PKLR - pyruvate kinase, liver and rbc  PFKFB1 - 6-phosphofructo-2-kinase/fructose-2,6-biphosphatase 1 |
| GO:0042180 | cellular ketone metabolic process | 7.29E-5 | 4.17E-3 | 3.65 (10334,51,667,12) | [+] Show genes  TDO2 - tryptophan 2,3-dioxygenase  AKR1C3 - aldo-keto reductase family 1, member c3  DHRS4 - dehydrogenase/reductase (sdr family) member 4  AKR1C4 - aldo-keto reductase family 1, member c4  GOT2 - glutamic-oxaloacetic transaminase 2, mitochondrial  COQ9 - coenzyme q9 homolog (s. cerevisiae)  ALDH8A1 - aldehyde dehydrogenase 8 family, member a1  HAGH - hydroxyacylglutathione hydrolase  GPD1 - glycerol-3-phosphate dehydrogenase 1 (soluble)  ADH4 - alcohol dehydrogenase 4 (class ii), pi polypeptide  CCBL1 - cysteine conjugate-beta lyase, cytoplasmic  COQ4 - coenzyme q4 homolog (s. cerevisiae) |
| GO:0032102 | negative regulation of response to external stimulus | 7.3E-5 | 4.17E-3 | 2.15 (10334,209,667,29) | [+] Show genes  FAM132A - family with sequence similarity 132, member a  F11 - coagulation factor xi  FGG - fibrinogen gamma chain  APCS - amyloid p component, serum  F12 - coagulation factor xii (hageman factor)  TRIB1 - tribbles homolog 1 (drosophila)  PROC - protein c (inactivator of coagulation factors va and viiia)  ADTRP - androgen-dependent tfpi-regulating protein  HRG - histidine-rich glycoprotein  FGA - fibrinogen alpha chain  FGB - fibrinogen beta chain  ADA - adenosine deaminase  SERPINF1 - serpin peptidase inhibitor, clade f (alpha-2 antiplasmin, pigment epithelium derived factor), member 1  PPARA - peroxisome proliferator-activated receptor alpha  FEM1A - fem-1 homolog a (c. elegans)  IGF1 - insulin-like growth factor 1 (somatomedin c)  SAA1 - serum amyloid a1  NFKBIL1 - nuclear factor of kappa light polypeptide gene enhancer in b-cells inhibitor-like 1  MYOZ1 - myozenin 1  FCGR2B - fc fragment of igg, low affinity iib, receptor (cd32)  CPB2 - carboxypeptidase b2 (plasma)  KNG1 - kininogen 1  APOH - apolipoprotein h (beta-2-glycoprotein i)  PLG - plasminogen  F2 - coagulation factor ii (thrombin)  VTN - vitronectin  C5 - complement component 5  KLKB1 - kallikrein b, plasma (fletcher factor) 1  SERPINF2 - serpin peptidase inhibitor, clade f (alpha-2 antiplasmin, pigment epithelium derived factor), member 2 |
| GO:0009260 | ribonucleotide biosynthetic process | 7.99E-5 | 4.54E-3 | 2.34 (10334,159,667,24) | [+] Show genes  AK2 - adenylate kinase 2  ATP5J2 - atp synthase, h+ transporting, mitochondrial fo complex, subunit f2  HSD17B8 - hydroxysteroid (17-beta) dehydrogenase 8  MLYCD - malonyl-coa decarboxylase  PGM1 - phosphoglucomutase 1  GCDH - glutaryl-coa dehydrogenase  ATP5I - atp synthase, h+ transporting, mitochondrial fo complex, subunit e  ATP5H - atp synthase, h+ transporting, mitochondrial fo complex, subunit d  COX5B - cytochrome c oxidase subunit vb  AMPD1 - adenosine monophosphate deaminase 1  PGAM2 - phosphoglycerate mutase 2 (muscle)  ALDOB - aldolase b, fructose-bisphosphate  ADSSL1 - adenylosuccinate synthase like 1  ACSL1 - acyl-coa synthetase long-chain family member 1  ATP5E - atp synthase, h+ transporting, mitochondrial f1 complex, epsilon subunit  ATP5D - atp synthase, h+ transporting, mitochondrial f1 complex, delta subunit  ENO3 - enolase 3 (beta, muscle)  ATP5G1 - atp synthase, h+ transporting, mitochondrial fo complex, subunit c1 (subunit 9)  LDHA - lactate dehydrogenase a  SLC25A13 - solute carrier family 25 (aspartate/glutamate carrier), member 13  PTPLA - protein tyrosine phosphatase-like (proline instead of catalytic arginine), member a  MPC1 - mitochondrial pyruvate carrier 1  PKLR - pyruvate kinase, liver and rbc  PFKFB1 - 6-phosphofructo-2-kinase/fructose-2,6-biphosphatase 1 |
| GO:0010901 | regulation of very-low-density lipoprotein particle remodeling | 8.16E-5 | 4.62E-3 | 12.39 (10334,5,667,4) | [+] Show genes  APOC2 - apolipoprotein c-ii  APOA5 - apolipoprotein a-v  APOC3 - apolipoprotein c-iii  APOA2 - apolipoprotein a-ii |
| GO:0030240 | skeletal muscle thin filament assembly | 8.16E-5 | 4.6E-3 | 12.39 (10334,5,667,4) | [+] Show genes  ACTA1 - actin, alpha 1, skeletal muscle  TCAP - titin-cap  LMOD3 - leiomodin 3 (fetal)  TTN - titin |
| GO:0072378 | blood coagulation, fibrin clot formation | 8.16E-5 | 4.58E-3 | 12.39 (10334,5,667,4) | [+] Show genes  FGG - fibrinogen gamma chain  FGA - fibrinogen alpha chain  FN1 - fibronectin 1  FGB - fibrinogen beta chain |
| GO:0015909 | long-chain fatty acid transport | 8.29E-5 | 4.63E-3 | 4.19 (10334,37,667,10) | [+] Show genes  CPT2 - carnitine palmitoyltransferase 2  ACSL1 - acyl-coa synthetase long-chain family member 1  PLA2G12B - phospholipase a2, group xiib  CPT1B - carnitine palmitoyltransferase 1b (muscle)  SLC25A20 - solute carrier family 25 (carnitine/acylcarnitine translocase), member 20  CD36 - cd36 molecule (thrombospondin receptor)  SLC27A2 - solute carrier family 27 (fatty acid transporter), member 2  FABP1 - fatty acid binding protein 1, liver  THRSP - thyroid hormone responsive  SLC27A5 - solute carrier family 27 (fatty acid transporter), member 5 |
| GO:0051346 | negative regulation of hydrolase activity | 8.4E-5 | 4.68E-3 | 1.97 (10334,275,667,35) | [+] Show genes  APCS - amyloid p component, serum  ANGPTL3 - angiopoietin-like 3  APOA2 - apolipoprotein a-ii  FABP1 - fatty acid binding protein 1, liver  SPP2 - secreted phosphoprotein 2, 24kda  PAM16 - presequence translocase-associated motor 16 homolog (s. cerevisiae)  SERPINA10 - serpin peptidase inhibitor, clade a (alpha-1 antiproteinase, antitrypsin), member 10  FETUB - fetuin b  SERPINA11 - serpin peptidase inhibitor, clade a (alpha-1 antiproteinase, antitrypsin), member 11  AMBP - alpha-1-microglobulin/bikunin precursor  UBXN1 - ubx domain protein 1  ITIH1 - inter-alpha-trypsin inhibitor heavy chain 1  HRG - histidine-rich glycoprotein  ITIH2 - inter-alpha-trypsin inhibitor heavy chain 2  ITIH3 - inter-alpha-trypsin inhibitor heavy chain 3  ITIH4 - inter-alpha-trypsin inhibitor heavy chain family, member 4  SERPINC1 - serpin peptidase inhibitor, clade c (antithrombin), member 1  SERPINF1 - serpin peptidase inhibitor, clade f (alpha-2 antiplasmin, pigment epithelium derived factor), member 1  HRAS - harvey rat sarcoma viral oncogene homolog  SERPINA5 - serpin peptidase inhibitor, clade a (alpha-1 antiproteinase, antitrypsin), member 5  STYXL1 - serine/threonine/tyrosine interacting-like 1  SERPIND1 - serpin peptidase inhibitor, clade d (heparin cofactor), member 1  SERPINA6 - serpin peptidase inhibitor, clade a (alpha-1 antiproteinase, antitrypsin), member 6  ANGPTL4 - angiopoietin-like 4  PLIN5 - perilipin 5  PLN - phospholamban  SERPINA7 - serpin peptidase inhibitor, clade a (alpha-1 antiproteinase, antitrypsin), member 7  MYOZ1 - myozenin 1  PPP1R27 - protein phosphatase 1, regulatory subunit 27  SERPINA1 - serpin peptidase inhibitor, clade a (alpha-1 antiproteinase, antitrypsin), member 1  APOC3 - apolipoprotein c-iii  KNG1 - kininogen 1  VTN - vitronectin  C5 - complement component 5  SERPINF2 - serpin peptidase inhibitor, clade f (alpha-2 antiplasmin, pigment epithelium derived factor), member 2 |
| GO:0010257 | NADH dehydrogenase complex assembly | 9.2E-5 | 5.1E-3 | 3.36 (10334,60,667,13) | [+] Show genes  NDUFB10 - nadh dehydrogenase (ubiquinone) 1 beta subcomplex, 10, 22kda  NDUFB7 - nadh dehydrogenase (ubiquinone) 1 beta subcomplex, 7, 18kda  BCS1L - bc1 (ubiquinol-cytochrome c reductase) synthesis-like  NDUFA3 - nadh dehydrogenase (ubiquinone) 1 alpha subcomplex, 3, 9kda  NDUFS7 - nadh dehydrogenase (ubiquinone) fe-s protein 7, 20kda (nadh-coenzyme q reductase)  ECSIT - ecsit signalling integrator  NDUFA2 - nadh dehydrogenase (ubiquinone) 1 alpha subcomplex, 2, 8kda  NDUFA7 - nadh dehydrogenase (ubiquinone) 1 alpha subcomplex, 7, 14.5kda  FOXRED1 - fad-dependent oxidoreductase domain containing 1  NDUFS8 - nadh dehydrogenase (ubiquinone) fe-s protein 8, 23kda (nadh-coenzyme q reductase)  NDUFS6 - nadh dehydrogenase (ubiquinone) fe-s protein 6, 13kda (nadh-coenzyme q reductase)  NDUFS5 - nadh dehydrogenase (ubiquinone) fe-s protein 5, 15kda (nadh-coenzyme q reductase)  NDUFV1 - nadh dehydrogenase (ubiquinone) flavoprotein 1, 51kda |
| GO:0032981 | mitochondrial respiratory chain complex I assembly | 9.2E-5 | 5.08E-3 | 3.36 (10334,60,667,13) | [+] Show genes  NDUFB10 - nadh dehydrogenase (ubiquinone) 1 beta subcomplex, 10, 22kda  NDUFB7 - nadh dehydrogenase (ubiquinone) 1 beta subcomplex, 7, 18kda  BCS1L - bc1 (ubiquinol-cytochrome c reductase) synthesis-like  NDUFA3 - nadh dehydrogenase (ubiquinone) 1 alpha subcomplex, 3, 9kda  NDUFS7 - nadh dehydrogenase (ubiquinone) fe-s protein 7, 20kda (nadh-coenzyme q reductase)  ECSIT - ecsit signalling integrator  NDUFA2 - nadh dehydrogenase (ubiquinone) 1 alpha subcomplex, 2, 8kda  NDUFA7 - nadh dehydrogenase (ubiquinone) 1 alpha subcomplex, 7, 14.5kda  FOXRED1 - fad-dependent oxidoreductase domain containing 1  NDUFS8 - nadh dehydrogenase (ubiquinone) fe-s protein 8, 23kda (nadh-coenzyme q reductase)  NDUFS6 - nadh dehydrogenase (ubiquinone) fe-s protein 6, 13kda (nadh-coenzyme q reductase)  NDUFS5 - nadh dehydrogenase (ubiquinone) fe-s protein 5, 15kda (nadh-coenzyme q reductase)  NDUFV1 - nadh dehydrogenase (ubiquinone) flavoprotein 1, 51kda |
| GO:0009058 | biosynthetic process | 9.64E-5 | 5.3E-3 | 1.32 (10334,1812,667,154) | [+] Show genes  GADL1 - glutamate decarboxylase-like 1  APOA2 - apolipoprotein a-ii  COX5B - cytochrome c oxidase subunit vb  ENTPD8 - ectonucleoside triphosphate diphosphohydrolase 8  NDUFA7 - nadh dehydrogenase (ubiquinone) 1 alpha subcomplex, 7, 14.5kda  HSD11B1 - hydroxysteroid (11-beta) dehydrogenase 1  AMACR - alpha-methylacyl-coa racemase  APOA4 - apolipoprotein a-iv  BDH2 - 3-hydroxybutyrate dehydrogenase, type 2  OTC - ornithine carbamoyltransferase  ENO3 - enolase 3 (beta, muscle)  LDHA - lactate dehydrogenase a  IMPA2 - inositol(myo)-1(or 4)-monophosphatase 2  SLC25A13 - solute carrier family 25 (aspartate/glutamate carrier), member 13  GSTZ1 - glutathione s-transferase zeta 1  GSTT1 - glutathione s-transferase theta 1  ACSM2A - acyl-coa synthetase medium-chain family member 2a  ATP5J2 - atp synthase, h+ transporting, mitochondrial fo complex, subunit f2  ASPDH - aspartate dehydrogenase domain containing  TNIP1 - tnfaip3 interacting protein 1  CPS1 - carbamoyl-phosphate synthase 1, mitochondrial  UBE2L6 - ubiquitin-conjugating enzyme e2l 6  BHMT - betaine--homocysteine s-methyltransferase  FITM1 - fat storage-inducing transmembrane protein 1  LIPC - lipase, hepatic  POLD4 - polymerase (dna-directed), delta 4, accessory subunit  SDC2 - syndecan 2  GYS2 - glycogen synthase 2 (liver)  SMPDL3A - sphingomyelin phosphodiesterase, acid-like 3a  PAH - phenylalanine hydroxylase  NR1I2 - nuclear receptor subfamily 1, group i, member 2  GGT1 - gamma-glutamyltransferase 1  MOCS1 - molybdenum cofactor synthesis 1  UPB1 - ureidopropionase, beta  ACOX2 - acyl-coa oxidase 2, branched chain  CYP4A11 - cytochrome p450, family 4, subfamily a, polypeptide 11  EEF1D - eukaryotic translation elongation factor 1 delta (guanine nucleotide exchange protein)  NMRK2 - nicotinamide riboside kinase 2  CYP8B1 - cytochrome p450, family 8, subfamily b, polypeptide 1  ALDH8A1 - aldehyde dehydrogenase 8 family, member a1  CES1 - carboxylesterase 1  CHPT1 - choline phosphotransferase 1  LSM10 - lsm10, u7 small nuclear rna associated  CYP2E1 - cytochrome p450, family 2, subfamily e, polypeptide 1  GADD45GIP1 - growth arrest and dna-damage-inducible, gamma interacting protein 1  ADSSL1 - adenylosuccinate synthase like 1  PCTP - phosphatidylcholine transfer protein  MRPS25 - mitochondrial ribosomal protein s25  ENPP1 - ectonucleotide pyrophosphatase/phosphodiesterase 1  PPARGC1A - peroxisome proliferator-activated receptor gamma, coactivator 1 alpha  TAT - tyrosine aminotransferase  ARG1 - arginase 1  SCP2 - sterol carrier protein 2  ADA - adenosine deaminase  PM20D1 - peptidase m20 domain containing 1  HAGH - hydroxyacylglutathione hydrolase  CYP1A2 - cytochrome p450, family 1, subfamily a, polypeptide 2  PTPLA - protein tyrosine phosphatase-like (proline instead of catalytic arginine), member a  PPARA - peroxisome proliferator-activated receptor alpha  HSD17B6 - hydroxysteroid (17-beta) dehydrogenase 6  MRPL2 - mitochondrial ribosomal protein l2  APOA5 - apolipoprotein a-v  PCK1 - phosphoenolpyruvate carboxykinase 1 (soluble)  RORC - rar-related orphan receptor c  POLR2I - polymerase (rna) ii (dna directed) polypeptide i, 14.5kda  POLR2J - polymerase (rna) ii (dna directed) polypeptide j, 13.3kda  MRPS34 - mitochondrial ribosomal protein s34  ALDH1L1 - aldehyde dehydrogenase 1 family, member l1  ASS1 - argininosuccinate synthase 1  DGAT2 - diacylglycerol o-acyltransferase 2  CRYL1 - crystallin, lambda 1  TCEANC - transcription elongation factor a (sii) n-terminal and central domain containing  AGPAT2 - 1-acylglycerol-3-phosphate o-acyltransferase 2  TCEA3 - transcription elongation factor a (sii), 3  ACSM3 - acyl-coa synthetase medium-chain family member 3  SEPSECS - sep (o-phosphoserine) trna:sec (selenocysteine) trna synthase  AKR1C3 - aldo-keto reductase family 1, member c3  HSD17B8 - hydroxysteroid (17-beta) dehydrogenase 8  MRPL23 - mitochondrial ribosomal protein l23  MLYCD - malonyl-coa decarboxylase  HSD17B14 - hydroxysteroid (17-beta) dehydrogenase 14  ACAA2 - acetyl-coa acyltransferase 2  AGMAT - agmatine ureohydrolase (agmatinase)  PGM1 - phosphoglucomutase 1  HMBS - hydroxymethylbilane synthase  PGAM2 - phosphoglycerate mutase 2 (muscle)  MTHFR - methylenetetrahydrofolate reductase (nad(p)h)  ACSL1 - acyl-coa synthetase long-chain family member 1  MTHFD1 - methylenetetrahydrofolate dehydrogenase (nadp+ dependent) 1, methenyltetrahydrofolate cyclohydrolase, formyltetrahydrofolate synthetase  GOT2 - glutamic-oxaloacetic transaminase 2, mitochondrial  ATF4 - activating transcription factor 4  GK5 - glycerol kinase 5 (putative)  ASPG - asparaginase homolog (s. cerevisiae)  ABLIM3 - actin binding lim protein family, member 3  CINP - cyclin-dependent kinase 2 interacting protein  RPL3L - ribosomal protein l3-like  GPD1 - glycerol-3-phosphate dehydrogenase 1 (soluble)  ABHD1 - abhydrolase domain containing 1  PNPLA2 - patatin-like phospholipase domain containing 2  AK2 - adenylate kinase 2  SLC37A4 - solute carrier family 37 (glucose-6-phosphate transporter), member 4  AKR1C4 - aldo-keto reductase family 1, member c4  GCAT - glycine c-acetyltransferase  CHST13 - carbohydrate (chondroitin 4) sulfotransferase 13  ATP5H - atp synthase, h+ transporting, mitochondrial fo complex, subunit d  ATP5I - atp synthase, h+ transporting, mitochondrial fo complex, subunit e  AGXT - alanine-glyoxylate aminotransferase  MAT1A - methionine adenosyltransferase i, alpha  COQ4 - coenzyme q4 homolog (s. cerevisiae)  ATP5E - atp synthase, h+ transporting, mitochondrial f1 complex, epsilon subunit  PPP1R3C - protein phosphatase 1, regulatory subunit 3c  ABCB6 - atp-binding cassette, sub-family b (mdr/tap), member 6  ATP5D - atp synthase, h+ transporting, mitochondrial f1 complex, delta subunit  ATP5G1 - atp synthase, h+ transporting, mitochondrial fo complex, subunit c1 (subunit 9)  COQ9 - coenzyme q9 homolog (s. cerevisiae)  POLDIP2 - polymerase (dna-directed), delta interacting protein 2  DECR2 - 2,4-dienoyl coa reductase 2, peroxisomal  G6PC - glucose-6-phosphatase, catalytic subunit  PFKFB1 - 6-phosphofructo-2-kinase/fructose-2,6-biphosphatase 1  ACSM5 - acyl-coa synthetase medium-chain family member 5  UBA52 - ubiquitin a-52 residue ribosomal protein fusion product 1  PHOSPHO1 - phosphatase, orphan 1  AGXT2 - alanine--glyoxylate aminotransferase 2  SHMT1 - serine hydroxymethyltransferase 1 (soluble)  GAMT - guanidinoacetate n-methyltransferase  LPIN1 - lipin 1  MRPL14 - mitochondrial ribosomal protein l14  ALDOB - aldolase b, fructose-bisphosphate  NFIC - nuclear factor i/c (ccaat-binding transcription factor)  ESRRA - estrogen-related receptor alpha  ST3GAL3 - st3 beta-galactoside alpha-2,3-sialyltransferase 3  MRPS15 - mitochondrial ribosomal protein s15  MRPL40 - mitochondrial ribosomal protein l40  MPC1 - mitochondrial pyruvate carrier 1  MRPL36 - mitochondrial ribosomal protein l36  PKLR - pyruvate kinase, liver and rbc  HAAO - 3-hydroxyanthranilate 3,4-dioxygenase  IGF1 - insulin-like growth factor 1 (somatomedin c)  GCDH - glutaryl-coa dehydrogenase  RXRA - retinoid x receptor, alpha  RXRG - retinoid x receptor, gamma  ADI1 - acireductone dioxygenase 1  AMPD1 - adenosine monophosphate deaminase 1  CKM - creatine kinase, muscle  HMGCS2 - 3-hydroxy-3-methylglutaryl-coa synthase 2 (mitochondrial)  SLC27A2 - solute carrier family 27 (fatty acid transporter), member 2  FBP2 - fructose-1,6-bisphosphatase 2  MPST - mercaptopyruvate sulfurtransferase  SLC27A5 - solute carrier family 27 (fatty acid transporter), member 5  URAD - ureidoimidazoline (2-oxo-4-hydroxy-4-carboxy-5-) decarboxylase  CCBL1 - cysteine conjugate-beta lyase, cytoplasmic  NR2F6 - nuclear receptor subfamily 2, group f, member 6  NR1I3 - nuclear receptor subfamily 1, group i, member 3  MAF - v-maf avian musculoaponeurotic fibrosarcoma oncogene homolog |

Species used: Homo sapiens

The system has recognized 11159 genes out of 12440 gene terms entered by the user.  
 11159 genes were recognized by gene symbol and 0 genes by other gene IDs .  
730 duplicate genes were removed (keeping the highest ranking instance of each gene) leaving a total of 10429 genes.  
Only 10334 of these genes are associated with a GO term.

The GOrilla database is periodically updated using the GO database and other sources.  
The GOrilla database was last updated on Aug 29, 2020

This results page will be available on this site for one month from now (until
Oct 1, 2020
). You can bookmark this page and come back to it later.

  
**'P-value'** is the enrichment p-value computed according to the mHG or HG model. This p-value is not corrected for multiple testing of 13915 GO terms.  
  
**'FDR q-value'** is the correction of the above p-value for multiple testing using the Benjamini and Hochberg (1995) method.   
Namely, for the i^th^ term (ranked according to p-value) the FDR q-value is (p-value \* number of GO terms) / i.   
  
**Enrichment (N, B, n, b)** is defined as follows:  
N - is the total number of genes  
B - is the total number of genes associated with a specific GO term  
n - is the number of genes in the top of the user's input list or in the target set when appropriate  
b - is the number of genes in the intersection  
Enrichment = (b/n) / (B/N)  
  
**Genes:** For each GO term you can see the list of associated genes that appear in the optimal top of the list.  
Each gene name is specified by gene symbol followed by a short description of the gene   

Back to the GOrilla main page
