# Supplementary material for: Genome-Wide Co-Expression Distributions as a Metric to Prioritize Genes of Functional Importance
Source: Genes (Basel). 2020 Oct 20;11(10):1231. doi: 10.3390/genes11101231 (PMC7593939; doi:10.3390/genes11101231)
Supplement: Supplementary file 1 [file genes-11-01231-s001.zip › SupplementaryFile5_FE_shape4.html]

Results


*P-value color scale*

|  |  |  |  |  |
| --- | --- | --- | --- | --- |
| > 10-3 | 10-3 to 10-5 | 10-5 to 10-7 | 10-7 to 10-9 | < 10-9 |


|  |  |  |  |  |  |
| --- | --- | --- | --- | --- | --- |
| **GO term** | **Description** | **P-value** | **FDR q-value** | **Enrichment (N, B, n, b)** | **Genes** |
| GO:0090304 | nucleic acid metabolic process | 9.61E-23 | 1.34E-18 | 1.15 (10334,1649,6996,1282) | [+] Show genes  ELL - elongation factor rna polymerase ii  EXOSC9 - exosome component 9  EXOSC10 - exosome component 10  PMS2 - pms2 postmeiotic segregation increased 2 (s. cerevisiae)  ZCCHC8 - zinc finger, cchc domain containing 8  RNPC3 - rna-binding region (rnp1, rrm) containing 3  SYMPK - symplekin  SF3A3 - splicing factor 3a, subunit 3, 60kda  HNRNPA0 - heterogeneous nuclear ribonucleoprotein a0  AARS - alanyl-trna synthetase  PNN - pinin, desmosome associated protein  SF3A2 - splicing factor 3a, subunit 2, 66kda  ABL1 - c-abl oncogene 1, non-receptor tyrosine kinase  CNOT11 - ccr4-not transcription complex, subunit 11  OBFC1 - oligonucleotide/oligosaccharide-binding fold containing 1  EBNA1BP2 - ebna1 binding protein 2  PMS1 - pms1 postmeiotic segregation increased 1 (s. cerevisiae)  ASCC3 - activating signal cointegrator 1 complex subunit 3  FTSJ3 - ftsj homolog 3 (e. coli)  PNRC2 - proline-rich nuclear receptor coactivator 2  TXNL4A - thioredoxin-like 4a  NOP9 - nop9 nucleolar protein  CHD7 - chromodomain helicase dna binding protein 7  PLRG1 - pleiotropic regulator 1  LYAR - ly1 antibody reactive  PAPOLA - poly(a) polymerase alpha  TCERG1 - transcription elongation regulator 1  PABPN1 - poly(a) binding protein, nuclear 1  TAF15 - taf15 rna polymerase ii, tata box binding protein (tbp)-associated factor, 68kda  RNPS1 - rna binding protein s1, serine-rich domain  OTUB1 - otu domain, ubiquitin aldehyde binding 1  EHMT2 - euchromatic histone-lysine n-methyltransferase 2  SPIN1 - spindlin 1  MORF4L1 - mortality factor 4 like 1  THUMPD1 - thump domain containing 1  ACTL6A - actin-like 6a  TRRAP - transformation/transcription domain-associated protein  PPIA - peptidylprolyl isomerase a (cyclophilin a)  MCMBP - minichromosome maintenance complex binding protein  TRMT10B - trna methyltransferase 10 homolog b (s. cerevisiae)  ADAR - adenosine deaminase, rna-specific  ADARB1 - adenosine deaminase, rna-specific, b1  WDR3 - wd repeat domain 3  ACTR5 - arp5 actin-related protein 5 homolog (yeast)  CPSF4 - cleavage and polyadenylation specific factor 4, 30kda  MED1 - mediator complex subunit 1  CDC45 - cell division cycle 45  PPA1 - pyrophosphatase (inorganic) 1  AXIN2 - axin 2  CDC7 - cell division cycle 7  PPARD - peroxisome proliferator-activated receptor delta  SMC1A - structural maintenance of chromosomes 1a  POLRMT - polymerase (rna) mitochondrial (dna directed)  MAML3 - mastermind-like 3 (drosophila)  RBM10 - rna binding motif protein 10  POLR2K - polymerase (rna) ii (dna directed) polypeptide k, 7.0kda  TSEN15 - tsen15 trna splicing endonuclease subunit  GNAS - gnas complex locus  PARP1 - poly (adp-ribose) polymerase 1  POLH - polymerase (dna directed), eta  PPP1R13L - protein phosphatase 1, regulatory subunit 13 like  NOP10 - nop10 ribonucleoprotein  POLD2 - polymerase (dna directed), delta 2, accessory subunit  POLD1 - polymerase (dna directed), delta 1, catalytic subunit  POLR2G - polymerase (rna) ii (dna directed) polypeptide g  NOL10 - nucleolar protein 10  POLR2F - polymerase (rna) ii (dna directed) polypeptide f  ELAC1 - elac ribonuclease z 1  POLR2E - polymerase (rna) ii (dna directed) polypeptide e, 25kda  SMPD3 - sphingomyelin phosphodiesterase 3, neutral membrane (neutral sphingomyelinase ii)  POLR2D - polymerase (rna) ii (dna directed) polypeptide d  POLR2C - polymerase (rna) ii (dna directed) polypeptide c, 33kda  POLR2B - polymerase (rna) ii (dna directed) polypeptide b, 140kda  RUVBL2 - ruvb-like 2 (e. coli)  PARP4 - poly (adp-ribose) polymerase family, member 4  POLR2A - polymerase (rna) ii (dna directed) polypeptide a, 220kda  HNRNPUL1 - heterogeneous nuclear ribonucleoprotein u-like 1  INTS9 - integrator complex subunit 9  RPP14 - ribonuclease p/mrp 14kda subunit  KRR1 - krr1, small subunit (ssu) processome component, homolog (yeast)  PRKACA - protein kinase, camp-dependent, catalytic, alpha  NELFE - negative elongation factor complex member e  WDR12 - wd repeat domain 12  EHMT1 - euchromatic histone-lysine n-methyltransferase 1  NR5A1 - nuclear receptor subfamily 5, group a, member 1  PRIM2 - primase, dna, polypeptide 2 (58kda)  PRIM1 - primase, dna, polypeptide 1 (49kda)  FUS - fused in sarcoma  FRG1 - fshd region gene 1  METTL8 - methyltransferase like 8  PRCC - papillary renal cell carcinoma (translocation-associated)  POLR3A - polymerase (rna) iii (dna directed) polypeptide a, 155kda  CLASRP - clk4-associating serine/arginine rich protein  GEMIN6 - gem (nuclear organelle) associated protein 6  PRDM4 - pr domain containing 4  N4BP2 - nedd4 binding protein 2  ATF7IP - activating transcription factor 7 interacting protein  PPP5C - protein phosphatase 5, catalytic subunit  UHRF2 - ubiquitin-like with phd and ring finger domains 2, e3 ubiquitin protein ligase  SDE2 - sde2 telomere maintenance homolog (s. pombe)  PCID2 - pci domain containing 2  SNRNP35 - small nuclear ribonucleoprotein 35kda (u11/u12)  USP7 - ubiquitin specific peptidase 7 (herpes virus-associated)  XRCC6 - x-ray repair complementing defective repair in chinese hamster cells 6  RNF168 - ring finger protein 168, e3 ubiquitin protein ligase  PPP2R2A - protein phosphatase 2, regulatory subunit b, alpha  CMTR2 - cap methyltransferase 2  RIOK2 - rio kinase 2  PPP2R1A - protein phosphatase 2, regulatory subunit a, alpha  CPSF6 - cleavage and polyadenylation specific factor 6, 68kda  SLBP - stem-loop binding protein  NUDT21 - nudix (nucleoside diphosphate linked moiety x)-type motif 21  DIS3L - dis3 mitotic control homolog (s. cerevisiae)-like  DDX52 - dead (asp-glu-ala-asp) box polypeptide 52  DDX51 - dead (asp-glu-ala-asp) box polypeptide 51  PPP1R8 - protein phosphatase 1, regulatory subunit 8  RBFA - ribosome binding factor a (putative)  PPP2CA - protein phosphatase 2, catalytic subunit, alpha isozyme  CPSF7 - cleavage and polyadenylation specific factor 7, 59kda  DIDO1 - death inducer-obliterator 1  CBLL1 - cbl proto-oncogene-like 1, e3 ubiquitin protein ligase  TOPBP1 - topoisomerase (dna) ii binding protein 1  RMI2 - recq mediated genome instability 2  RPAP2 - rna polymerase ii associated protein 2  HSF2BP - heat shock transcription factor 2 binding protein  DDX39B - dead (asp-glu-ala-asp) box polypeptide 39b  TDRKH - tudor and kh domain containing  IWS1 - iws1 homolog (s. cerevisiae)  NARS2 - asparaginyl-trna synthetase 2, mitochondrial (putative)  NPLOC4 - nuclear protein localization 4 homolog (s. cerevisiae)  PRPF40A - prp40 pre-mrna processing factor 40 homolog a (s. cerevisiae)  DDX27 - dead (asp-glu-ala-asp) box polypeptide 27  PAPD7 - pap associated domain containing 7  INTS8 - integrator complex subunit 8  CDK2AP1 - cyclin-dependent kinase 2 associated protein 1  SNORA8 - small nucleolar rna, h/aca box 8  SNIP1 - smad nuclear interacting protein 1  TSR1 - tsr1, 20s rrna accumulation, homolog (s. cerevisiae)  POLR3E - polymerase (rna) iii (dna directed) polypeptide e (80kd)  MAPK3 - mitogen-activated protein kinase 3  GEMIN7 - gem (nuclear organelle) associated protein 7  COPS6 - cop9 signalosome subunit 6  CLP1 - cleavage and polyadenylation factor i subunit 1  CCDC88A - coiled-coil domain containing 88a  NT5C3B - 5'-nucleotidase, cytosolic iiib  CCDC94 - coiled-coil domain containing 94  POLR3B - polymerase (rna) iii (dna directed) polypeptide b  NR4A3 - nuclear receptor subfamily 4, group a, member 3  RBM22 - rna binding motif protein 22  NSUN5 - nop2/sun domain family, member 5  SF3B2 - splicing factor 3b, subunit 2, 145kda  DEDD2 - death effector domain containing 2  GARS - glycyl-trna synthetase  APEX1 - apex nuclease (multifunctional dna repair enzyme) 1  FASTKD1 - fast kinase domains 1  MTPAP - mitochondrial poly(a) polymerase  DHX36 - deah (asp-glu-ala-his) box polypeptide 36  RBM23 - rna binding motif protein 23  DALRD3 - dalr anticodon binding domain containing 3  SMC6 - structural maintenance of chromosomes 6  PARN - poly(a)-specific ribonuclease  GSPT1 - g1 to s phase transition 1  RFWD3 - ring finger and wd repeat domain 3  TREX1 - three prime repair exonuclease 1  DARS2 - aspartyl-trna synthetase 2, mitochondrial  ZCCHC6 - zinc finger, cchc domain containing 6  RNF113A - ring finger protein 113a  GTF2F1 - general transcription factor iif, polypeptide 1, 74kda  GTF2F2 - general transcription factor iif, polypeptide 2, 30kda  PRMT6 - protein arginine methyltransferase 6  GTF2E1 - general transcription factor iie, polypeptide 1, alpha 56kda  GTF2E2 - general transcription factor iie, polypeptide 2, beta 34kda  NEIL1 - nei endonuclease viii-like 1 (e. coli)  GTF2H2 - general transcription factor iih, polypeptide 2, 44kda  GTF2H3 - general transcription factor iih, polypeptide 3, 34kda  PCBP2 - poly(rc) binding protein 2  INTS10 - integrator complex subunit 10  GTF2H1 - general transcription factor iih, polypeptide 1, 62kda  GTF2A2 - general transcription factor iia, 2, 12kda  SMG8 - smg8 nonsense mediated mrna decay factor  GTF2B - general transcription factor iib  MSH6 - muts homolog 6 (e. coli)  GTF2A1 - general transcription factor iia, 1, 19/37kda  USB1 - u6 snrna biogenesis 1  APBB1 - amyloid beta (a4) precursor protein-binding, family b, member 1 (fe65)  GTF3C2 - general transcription factor iiic, polypeptide 2, beta 110kda  RIF1 - rap1 interacting factor homolog (yeast)  POLG2 - polymerase (dna directed), gamma 2, accessory subunit  GTF2I - general transcription factor iii  GTF2H4 - general transcription factor iih, polypeptide 4, 52kda  GTF3A - general transcription factor iiia  PRKRIP1 - prkr interacting protein 1 (il11 inducible)  PA2G4 - proliferation-associated 2g4, 38kda  BRF1 - brf1, rna polymerase iii transcription initiation factor 90 kda subunit  NOL9 - nucleolar protein 9  RPRD1A - regulation of nuclear pre-mrna domain containing 1a  CNTD1 - cyclin n-terminal domain containing 1  GTF3C1 - general transcription factor iiic, polypeptide 1, alpha 220kda  MAP1S - microtubule-associated protein 1s  POLI - polymerase (dna directed) iota  APP - amyloid beta (a4) precursor protein  DDX20 - dead (asp-glu-ala-asp) box polypeptide 20  CELF3 - cugbp, elav-like family member 3  H2AFX - h2a histone family, member x  DCP2 - decapping mrna 2  SNORA43 - small nucleolar rna, h/aca box 43  PAPD4 - pap associated domain containing 4  PAX8 - paired box 8  NSMCE2 - non-smc element 2, mms21 homolog (s. cerevisiae)  SUPT16H - suppressor of ty 16 homolog (s. cerevisiae)  ARRB2 - arrestin, beta 2  ARRB1 - arrestin, beta 1  CDC73 - cell division cycle 73  GPATCH1 - g patch domain containing 1  SAMD4B - sterile alpha motif domain containing 4b  HARS - histidyl-trna synthetase  BAZ2A - bromodomain adjacent to zinc finger domain, 2a  PCNA - proliferating cell nuclear antigen  LSM6 - lsm6 homolog, u6 small nuclear rna associated (s. cerevisiae)  MAGOHB - mago-nashi homolog b (drosophila)  MTFMT - mitochondrial methionyl-trna formyltransferase  HEATR1 - heat repeat containing 1  TARSL2 - threonyl-trna synthetase-like 2  LEO1 - leo1, paf1/rna polymerase ii complex component, homolog (s. cerevisiae)  RPL7L1 - ribosomal protein l7-like 1  FANCL - fanconi anemia, complementation group l  PRPF38B - prp38 pre-mrna processing factor 38 (yeast) domain containing b  PWP1 - pwp1 homolog (s. cerevisiae)  WRAP53 - wd repeat containing, antisense to tp53  SNRNP25 - small nuclear ribonucleoprotein 25kda (u11/u12)  RBM28 - rna binding motif protein 28  THAP1 - thap domain containing, apoptosis associated protein 1  HDAC1 - histone deacetylase 1  PGBD5 - piggybac transposable element derived 5  SNORA11 - small nucleolar rna, h/aca box 11  ELP3 - elongator acetyltransferase complex subunit 3  KAT7 - k(lysine) acetyltransferase 7  XPA - xeroderma pigmentosum, complementation group a  ZBTB1 - zinc finger and btb domain containing 1  XPC - xeroderma pigmentosum, complementation group c  ZNF365 - zinc finger protein 365  XRCC1 - x-ray repair complementing defective repair in chinese hamster cells 1  PAGR1 - paxip1 associated glutamate-rich protein 1  DIS3 - dis3 mitotic control homolog (s. cerevisiae)  SNORD86 - small nucleolar rna, c/d box 86  ZHX2 - zinc fingers and homeoboxes 2  AP5S1 - adaptor-related protein complex 5, sigma 1 subunit  XBP1 - x-box binding protein 1  LARP6 - la ribonucleoprotein domain family, member 6  WHSC1 - wolf-hirschhorn syndrome candidate 1  NELFA - negative elongation factor complex member a  ZFC3H1 - zinc finger, c3h1-type containing  SNORD12 - small nucleolar rna, c/d box 12  ALKBH2 - alkb, alkylation repair homolog 2 (e. coli)  WDR33 - wd repeat domain 33  DCP1B - decapping mrna 1b  WARS - tryptophanyl-trna synthetase  BAX - bcl2-associated x protein  SCAF8 - sr-related ctd-associated factor 8  SMU1 - smu-1 suppressor of mec-8 and unc-52 homolog (c. elegans)  PUF60 - poly-u binding splicing factor 60kda  THOC6 - tho complex 6 homolog (drosophila)  RAVER2 - ribonucleoprotein, ptb-binding 2  NAT10 - n-acetyltransferase 10 (gcn5-related)  BACH1 - btb and cnc homology 1, basic leucine zipper transcription factor 1  ZNF45 - zinc finger protein 45  DPH3 - diphthamide biosynthesis 3  BRCC3 - brca1/brca2-containing complex, subunit 3  EXD2 - exonuclease 3'-5' domain containing 2  TYW1 - trna-yw synthesizing protein 1 homolog (s. cerevisiae)  ASXL2 - additional sex combs like 2 (drosophila)  ELP2 - elongator acetyltransferase complex subunit 2  XRN2 - 5'-3' exoribonuclease 2  ASXL1 - additional sex combs like 1 (drosophila)  ATRX - alpha thalassemia/mental retardation syndrome x-linked  PKNOX1 - pbx/knotted 1 homeobox 1  CASC3 - cancer susceptibility candidate 3  PSTK - phosphoseryl-trna kinase  IMP3 - imp3, u3 small nucleolar ribonucleoprotein, homolog (yeast)  PIH1D2 - pih1 domain containing 2  EXOSC8 - exosome component 8  SF1 - splicing factor 1  U2AF2 - u2 small nuclear rna auxiliary factor 2  PSPC1 - paraspeckle component 1  NUDT15 - nudix (nucleoside diphosphate linked moiety x)-type motif 15  RPUSD3 - rna pseudouridylate synthase domain containing 3  BRF2 - brf2, rna polymerase iii transcription initiation factor 50 kda subunit  DDX42 - dead (asp-glu-ala-asp) box helicase 42  UBE2W - ubiquitin-conjugating enzyme e2w (putative)  XRCC5 - x-ray repair complementing defective repair in chinese hamster cells 5 (double-strand-break rejoining)  XRCC4 - x-ray repair complementing defective repair in chinese hamster cells 4  ECD - ecdysoneless homolog (drosophila)  POLR3H - polymerase (rna) iii (dna directed) polypeptide h (22.9kd)  GRSF1 - g-rich rna sequence binding factor 1  CWF19L1 - cwf19-like 1, cell cycle control (s. pombe)  CCND1 - cyclin d1  PARK7 - parkinson protein 7  PNKP - polynucleotide kinase 3'-phosphatase  QRSL1 - glutaminyl-trna synthase (glutamine-hydrolyzing)-like 1  CCNK - cyclin k  CDKAL1 - cdk5 regulatory subunit associated protein 1-like 1  SMNDC1 - survival motor neuron domain containing 1  RFC5 - replication factor c (activator 1) 5, 36.5kda  BCAS2 - breast carcinoma amplified sequence 2  RFC2 - replication factor c (activator 1) 2, 40kda  CWC27 - cwc27 spliceosome-associated protein homolog (s. cerevisiae)  RFC3 - replication factor c (activator 1) 3, 38kda  SAP18 - sin3a-associated protein, 18kda  TRIM24 - tripartite motif containing 24  TAF1D - tata box binding protein (tbp)-associated factor, rna polymerase i, d, 41kda  SNORD24 - small nucleolar rna, c/d box 24  SF3A1 - splicing factor 3a, subunit 1, 120kda  CWC25 - cwc25 spliceosome-associated protein homolog (s. cerevisiae)  RELA - v-rel avian reticuloendotheliosis viral oncogene homolog a  TEX10 - testis expressed 10  SNAPC5 - small nuclear rna activating complex, polypeptide 5, 19kda  SNORD55 - small nucleolar rna, c/d box 55  RECQL - recq protein-like (dna helicase q1-like)  ZFP36L2 - zfp36 ring finger protein-like 2  ZFP36L1 - zfp36 ring finger protein-like 1  ALKBH5 - alkb, alkylation repair homolog 5 (e. coli)  UPF1 - upf1 regulator of nonsense transcripts homolog (yeast)  NSUN2 - nop2/sun rna methyltransferase family, member 2  METTL16 - methyltransferase like 16  ALKBH1 - alkb, alkylation repair homolog 1 (e. coli)  FTO - fat mass and obesity associated  RLF - rearranged l-myc fusion  ELP6 - elongator acetyltransferase complex subunit 6  NOC4L - nucleolar complex associated 4 homolog (s. cerevisiae)  FBLL1 - fibrillarin-like 1  TATDN3 - tatd dnase domain containing 3  LDB1 - lim domain binding 1  DSCC1 - dna replication and sister chromatid cohesion 1  WARS2 - tryptophanyl trna synthetase 2, mitochondrial  KAT2B - k(lysine) acetyltransferase 2b  TSC22D1 - tsc22 domain family, member 1  WDR55 - wd repeat domain 55  PSMD14 - proteasome (prosome, macropain) 26s subunit, non-atpase, 14  ZNF259 - zinc finger protein 259  GIN1 - gypsy retrotransposon integrase 1  C1orf86 - chromosome 1 open reading frame 86  SCNM1 - sodium channel modifier 1  IARS - isoleucyl-trna synthetase  PRPF4B - prp4 pre-mrna processing factor 4 homolog b (yeast)  TRIT1 - trna isopentenyltransferase 1  NABP2 - nucleic acid binding protein 2  HUS1 - hus1 checkpoint homolog (s. pombe)  TSEN34 - tsen34 trna splicing endonuclease subunit  RNASE6 - ribonuclease, rnase a family, k6  U2AF1L4 - u2 small nuclear rna auxiliary factor 1-like 4  HNRNPR - heterogeneous nuclear ribonucleoprotein r  ERI3 - eri1 exoribonuclease family member 3  BUD31 - bud31 homolog (s. cerevisiae)  GATAD2A - gata zinc finger domain containing 2a  HERC2 - hect and rld domain containing e3 ubiquitin protein ligase 2  RNF111 - ring finger protein 111  BYSL - bystin-like  SRRM1 - serine/arginine repetitive matrix 1  TET2 - tet methylcytosine dioxygenase 2  HSPD1 - heat shock 60kda protein 1 (chaperonin)  NSMCE1 - non-smc element 1 homolog (s. cerevisiae)  MED18 - mediator complex subunit 18  TYW3 - trna-yw synthesizing protein 3 homolog (s. cerevisiae)  FUBP3 - far upstream element (fuse) binding protein 3  SF3B4 - splicing factor 3b, subunit 4, 49kda  HSP90AA1 - heat shock protein 90kda alpha (cytosolic), class a member 1  C7orf49 - chromosome 7 open reading frame 49  ZMPSTE24 - zinc metallopeptidase ste24  MBD4 - methyl-cpg binding domain protein 4  STUB1 - stip1 homology and u-box containing protein 1, e3 ubiquitin protein ligase  C5AR1 - complement component 5a receptor 1  TCEANC2 - transcription elongation factor a (sii) n-terminal and central domain containing 2  HSPA8 - heat shock 70kda protein 8  PPIE - peptidylprolyl isomerase e (cyclophilin e)  MCRS1 - microspherule protein 1  HMGN1 - high mobility group nucleosome binding domain 1  HMGB3 - high mobility group box 3  HMGB2 - high mobility group box 2  RPL17 - ribosomal protein l17  HMGB1 - high mobility group box 1  RPL15 - ribosomal protein l15  C1D - c1d nuclear receptor corepressor  TRMT12 - trna methyltransferase 12 homolog (s. cerevisiae)  RPL11 - ribosomal protein l11  RPL9 - ribosomal protein l9  NOL8 - nucleolar protein 8  RPL7 - ribosomal protein l7  INTS6 - integrator complex subunit 6  PPIH - peptidylprolyl isomerase h (cyclophilin h)  SLC30A9 - solute carrier family 30 (zinc transporter), member 9  CASP3 - caspase 3, apoptosis-related cysteine peptidase  RPL3 - ribosomal protein l3  AGO1 - argonaute risc catalytic component 1  RPA3 - replication protein a3, 14kda  CARS - cysteinyl-trna synthetase  MAD2L2 - mad2 mitotic arrest deficient-like 2 (yeast)  RPA1 - replication protein a1, 70kda  RPA2 - replication protein a2, 32kda  RPS4X - ribosomal protein s4, x-linked  ZBTB48 - zinc finger and btb domain containing 48  SRSF9 - serine/arginine-rich splicing factor 9  RPS4Y1 - ribosomal protein s4, y-linked 1  MRPL1 - mitochondrial ribosomal protein l1  ACD - adrenocortical dysplasia homolog (mouse)  GPBP1 - gc-rich promoter binding protein 1  NOL6 - nucleolar protein 6 (rna-associated)  KRI1 - kri1 homolog (s. cerevisiae)  MRPL44 - mitochondrial ribosomal protein l44  NR2C2AP - nuclear receptor 2c2-associated protein  SYNCRIP - synaptotagmin binding, cytoplasmic rna interacting protein  INTS3 - integrator complex subunit 3  RPL32 - ribosomal protein l32  ELP4 - elongator acetyltransferase complex subunit 4  UPF3B - upf3 regulator of nonsense transcripts homolog b (yeast)  UPF3A - upf3 regulator of nonsense transcripts homolog a (yeast)  RPS23 - ribosomal protein s23  PAPOLG - poly(a) polymerase gamma  RAVER1 - ribonucleoprotein, ptb-binding 1  CCNT2 - cyclin t2  PINX1 - pin2/terf1 interacting, telomerase inhibitor 1  NPM3 - nucleophosmin/nucleoplasmin 3  INO80C - ino80 complex subunit c  CCNT1 - cyclin t1  THG1L - trna-histidine guanylyltransferase 1-like (s. cerevisiae)  RNGTT - rna guanylyltransferase and 5'-phosphatase  CCNH - cyclin h  RNMT - rna (guanine-7-) methyltransferase  HNRNPU - heterogeneous nuclear ribonucleoprotein u (scaffold attachment factor a)  SWSAP1 - swim-type zinc finger 7 associated protein 1  HNRNPK - heterogeneous nuclear ribonucleoprotein k  HNRNPL - heterogeneous nuclear ribonucleoprotein l  RPS15 - ribosomal protein s15  RPS12 - ribosomal protein s12  TIPIN - timeless interacting protein  MRPS11 - mitochondrial ribosomal protein s11  FBXO6 - f-box protein 6  TINF2 - terf1 (trf1)-interacting nuclear factor 2  CBFA2T3 - core-binding factor, runt domain, alpha subunit 2; translocated to, 3  CBFB - core-binding factor, beta subunit  NSA2 - nsa2 ribosome biogenesis homolog (s. cerevisiae)  HNRNPA2B1 - heterogeneous nuclear ribonucleoprotein a2/b1  HNRNPA1 - heterogeneous nuclear ribonucleoprotein a1  HNRNPC - heterogeneous nuclear ribonucleoprotein c (c1/c2)  HNRNPF - heterogeneous nuclear ribonucleoprotein f  RXRB - retinoid x receptor, beta  HNRNPD - heterogeneous nuclear ribonucleoprotein d (au-rich element rna binding protein 1, 37kda)  HNRNPH1 - heterogeneous nuclear ribonucleoprotein h1 (h)  ADPRHL2 - adp-ribosylhydrolase like 2  HNRNPH3 - heterogeneous nuclear ribonucleoprotein h3 (2h9)  HNRNPH2 - heterogeneous nuclear ribonucleoprotein h2 (h')  SARS2 - seryl-trna synthetase 2, mitochondrial  ZSWIM7 - zinc finger, swim-type containing 7  TUT1 - terminal uridylyl transferase 1, u6 snrna-specific  CCNC - cyclin c  HMGA1 - high mobility group at-hook 1  TRMT10C - trna methyltransferase 10 homolog c (s. cerevisiae)  PRMT5 - protein arginine methyltransferase 5  NABP1 - nucleic acid binding protein 1  NR4A1 - nuclear receptor subfamily 4, group a, member 1  PDE12 - phosphodiesterase 12  CD2BP2 - cd2 (cytoplasmic tail) binding protein 2  CHTF8 - ctf8, chromosome transmission fidelity factor 8 homolog (s. cerevisiae)  RRM1 - ribonucleotide reductase m1  RBM27 - rna binding motif protein 27  IKBKAP - inhibitor of kappa light polypeptide gene enhancer in b-cells, kinase complex-associated protein  HTATSF1 - hiv-1 tat specific factor 1  ZNF638 - zinc finger protein 638  CDC5L - cell division cycle 5-like  TBL3 - transducin (beta)-like 3  RBM15 - rna binding motif protein 15  POLR3F - polymerase (rna) iii (dna directed) polypeptide f, 39 kda  RBMX - rna binding motif protein, x-linked  HNRNPA3 - heterogeneous nuclear ribonucleoprotein a3  PPM1D - protein phosphatase, mg2+/mn2+ dependent, 1d  POLL - polymerase (dna directed), lambda  SRSF12 - serine/arginine-rich splicing factor 12  PRPF8 - pre-mrna processing factor 8  PRPF19 - pre-mrna processing factor 19  UTP20 - utp20, small subunit (ssu) processome component, homolog (yeast)  CDC14B - cell division cycle 14b  WDR36 - wd repeat domain 36  KHDRBS3 - kh domain containing, rna binding, signal transduction associated 3  PATL1 - protein associated with topoisomerase ii homolog 1 (yeast)  ADAT2 - adenosine deaminase, trna-specific 2  LMO4 - lim domain only 4  TAF6L - taf6-like rna polymerase ii, p300/cbp-associated factor (pcaf)-associated factor, 65kda  COPS7B - cop9 signalosome subunit 7b  POLR3G - polymerase (rna) iii (dna directed) polypeptide g (32kd)  COPS3 - cop9 signalosome subunit 3  GAR1 - gar1 ribonucleoprotein  TERF2IP - telomeric repeat binding factor 2, interacting protein  ORAOV1 - oral cancer overexpressed 1  RNASEH2A - ribonuclease h2, subunit a  PRKRA - protein kinase, interferon-inducible double stranded rna dependent activator  PTBP1 - polypyrimidine tract binding protein 1  PTEN - phosphatase and tensin homolog  CETN2 - centrin, ef-hand protein, 2  JMY - junction mediating and regulatory protein, p53 cofactor  YARS - tyrosyl-trna synthetase  ZBTB8OS - zinc finger and btb domain containing 8 opposite strand  RRP1 - ribosomal rna processing 1  KHSRP - kh-type splicing regulatory protein  CHRAC1 - chromatin accessibility complex 1  CIB1 - calcium and integrin binding 1 (calmyrin)  DDX17 - dead (asp-glu-ala-asp) box helicase 17  POLR1E - polymerase (rna) i polypeptide e, 53kda  CHERP - calcium homeostasis endoplasmic reticulum protein  PRPF18 - pre-mrna processing factor 18  KAT5 - k(lysine) acetyltransferase 5  GPKOW - g patch domain and kow motifs  EIF6 - eukaryotic translation initiation factor 6  CEBPG - ccaat/enhancer binding protein (c/ebp), gamma  NOP56 - nop56 ribonucleoprotein  SLU7 - slu7 splicing factor homolog (s. cerevisiae)  PTMA - prothymosin, alpha  CDK4 - cyclin-dependent kinase 4  CDK9 - cyclin-dependent kinase 9  RNASET2 - ribonuclease t2  CDK8 - cyclin-dependent kinase 8  RTCA - rna 3'-terminal phosphate cyclase  CDK7 - cyclin-dependent kinase 7  CDK13 - cyclin-dependent kinase 13  LSM3 - lsm3 homolog, u6 small nuclear rna associated (s. cerevisiae)  PCBP3 - poly(rc) binding protein 3  CDKN2D - cyclin-dependent kinase inhibitor 2d (p19, inhibits cdk4)  CSDC2 - cold shock domain containing c2, rna binding  APEX2 - apex nuclease (apurinic/apyrimidinic endonuclease) 2  RUVBL1 - ruvb-like 1 (e. coli)  RPP38 - ribonuclease p/mrp 38kda subunit  MOCS3 - molybdenum cofactor synthesis 3  DIMT1 - dim1 dimethyladenosine transferase 1 homolog (s. cerevisiae)  CRCP - cgrp receptor component  CDK2 - cyclin-dependent kinase 2  NOP14 - nop14 nucleolar protein  RAD1 - rad1 homolog (s. pombe)  WDR4 - wd repeat domain 4  RRN3 - rrn3 rna polymerase i transcription factor homolog (s. cerevisiae)  PURA - purine-rich element binding protein a  GTPBP2 - gtp binding protein 2  LRWD1 - leucine-rich repeats and wd repeat domain containing 1  METTL6 - methyltransferase like 6  ZNHIT6 - zinc finger, hit-type containing 6  PAPD5 - pap associated domain containing 5  NUDT16 - nudix (nucleoside diphosphate linked moiety x)-type motif 16  POP4 - processing of precursor 4, ribonuclease p/mrp subunit (s. cerevisiae)  SRSF10 - serine/arginine-rich splicing factor 10  AFF4 - af4/fmr2 family, member 4  AHCYL1 - adenosylhomocysteinase-like 1  MMS19 - mms19 nucleotide excision repair homolog (s. cerevisiae)  MTERFD2 - mterf domain containing 2  OSGEPL1 - o-sialoglycoprotein endopeptidase-like 1  METTL2A - methyltransferase like 2a  QARS - glutaminyl-trna synthetase  RRAGC - ras-related gtp binding c  RAD21 - rad21 homolog (s. pombe)  RAD17 - rad17 homolog (s. pombe)  RPP40 - ribonuclease p/mrp 40kda subunit  PAF1 - paf1, rna polymerase ii associated factor, homolog (s. cerevisiae)  INO80 - ino80 complex subunit  DDX56 - dead (asp-glu-ala-asp) box helicase 56  IGHMBP2 - immunoglobulin mu binding protein 2  RC3H2 - ring finger and ccch-type domains 2  RBPJ - recombination signal binding protein for immunoglobulin kappa j region  PABPC1 - poly(a) binding protein, cytoplasmic 1  TRUB2 - trub pseudouridine (psi) synthase homolog 2 (e. coli)  FAM35A - family with sequence similarity 35, member a  DUSP11 - dual specificity phosphatase 11 (rna/rnp complex 1-interacting)  RARG - retinoic acid receptor, gamma  RBM26 - rna binding motif protein 26  RARB - retinoic acid receptor, beta  RARS - arginyl-trna synthetase  CELF1 - cugbp, elav-like family member 1  KHDRBS1 - kh domain containing, rna binding, signal transduction associated 1  TAF8 - taf8 rna polymerase ii, tata box binding protein (tbp)-associated factor, 43kda  CELF2 - cugbp, elav-like family member 2  IK - ik cytokine, down-regulator of hla ii  MLH3 - mutl homolog 3 (e. coli)  DDX49 - dead (asp-glu-ala-asp) box polypeptide 49  CTCF - ccctc-binding factor (zinc finger protein)  PRMT7 - protein arginine methyltransferase 7  RBBP7 - retinoblastoma binding protein 7  SMARCA5 - swi/snf related, matrix associated, actin dependent regulator of chromatin, subfamily a, member 5  RBBP8 - retinoblastoma binding protein 8  RBMS1 - rna binding motif, single stranded interacting protein 1  DIS3L2 - dis3 mitotic control homolog (s. cerevisiae)-like 2  RBM3 - rna binding motif (rnp1, rrm) protein 3  RBM4 - rna binding motif protein 4  DIEXF - digestive organ expansion factor homolog (zebrafish)  PELP1 - proline, glutamate and leucine rich protein 1  GEMIN2 - gem (nuclear organelle) associated protein 2  TYW5 - trna-yw synthesizing protein 5  RBBP6 - retinoblastoma binding protein 6  SND1 - staphylococcal nuclease and tudor domain containing 1  DGCR8 - dgcr8 microprocessor complex subunit  RBBP4 - retinoblastoma binding protein 4  CUL4B - cullin 4b  PUS7 - pseudouridylate synthase 7 homolog (s. cerevisiae)  ERCC8 - excision repair cross-complementing rodent repair deficiency, complementation group 8  DHX16 - deah (asp-glu-ala-his) box polypeptide 16  EXOSC4 - exosome component 4  ANKRD16 - ankyrin repeat domain 16  PCIF1 - pdx1 c-terminal inhibiting factor 1  PTGES3 - prostaglandin e synthase 3 (cytosolic)  NFAT5 - nuclear factor of activated t-cells 5, tonicity-responsive  POLD3 - polymerase (dna-directed), delta 3, accessory subunit  USP39 - ubiquitin specific peptidase 39  SUPT3H - suppressor of ty 3 homolog (s. cerevisiae)  RSRC1 - arginine/serine-rich coiled-coil 1  WTAP - wilms tumor 1 associated protein  RBM39 - rna binding motif protein 39  LIG4 - ligase iv, dna, atp-dependent  TBRG1 - transforming growth factor beta regulator 1  MKI67IP - mki67 (fha domain) interacting nucleolar phosphoprotein  FBXO18 - f-box protein, helicase, 18  METTL14 - methyltransferase like 14  LIG1 - ligase i, dna, atp-dependent  LIG3 - ligase iii, dna, atp-dependent  CIRH1A - cirrhosis, autosomal recessive 1a (cirhin)  DOT1L - dot1-like histone h3k79 methyltransferase  CWC22 - cwc22 spliceosome-associated protein homolog (s. cerevisiae)  SP1 - sp1 transcription factor  CIR1 - corepressor interacting with rbpj, 1  MUM1 - melanoma associated antigen (mutated) 1  RPP25L - ribonuclease p/mrp 25kda subunit-like  SLX4 - slx4 structure-specific endonuclease subunit  CHD1L - chromodomain helicase dna binding protein 1-like  GTPBP4 - gtp binding protein 4  LSM2 - lsm2 homolog, u6 small nuclear rna associated (s. cerevisiae)  GTF2IRD1 - gtf2i repeat domain containing 1  ORC3 - origin recognition complex, subunit 3  SON - son dna binding protein  ORC6 - origin recognition complex, subunit 6  CREBL2 - camp responsive element binding protein-like 2  CREBBP - creb binding protein  ZC3H8 - zinc finger ccch-type containing 8  SUGP1 - surp and g patch domain containing 1  CREB1 - camp responsive element binding protein 1  PRPF38A - prp38 pre-mrna processing factor 38 (yeast) domain containing a  SMUG1 - single-strand-selective monofunctional uracil-dna glycosylase 1  SNRPN - small nuclear ribonucleoprotein polypeptide n  SNRPB2 - small nuclear ribonucleoprotein polypeptide b  POLK - polymerase (dna directed) kappa  RPRD1B - regulation of nuclear pre-mrna domain containing 1b  SNRPD1 - small nuclear ribonucleoprotein d1 polypeptide 16kda  DDX41 - dead (asp-glu-ala-asp) box polypeptide 41  INIP - ints3 and nabp interacting protein  SNRPF - small nuclear ribonucleoprotein polypeptide f  SNAPC4 - small nuclear rna activating complex, polypeptide 4, 190kda  TBPL1 - tbp-like 1  YTHDF2 - yth domain family, member 2  SNRNP70 - small nuclear ribonucleoprotein 70kda (u1)  MAK16 - mak16 homolog (s. cerevisiae)  SNRPA - small nuclear ribonucleoprotein polypeptide a  RNF138 - ring finger protein 138, e3 ubiquitin protein ligase  EEF1E1 - eukaryotic translation elongation factor 1 epsilon 1  SNRPA1 - small nuclear ribonucleoprotein polypeptide a'  SNRPB - small nuclear ribonucleoprotein polypeptides b and b1  SNAPC2 - small nuclear rna activating complex, polypeptide 2, 45kda  SNAPC1 - small nuclear rna activating complex, polypeptide 1, 43kda  SNAPC3 - small nuclear rna activating complex, polypeptide 3, 50kda  POLR1C - polymerase (rna) i polypeptide c, 30kda  SMN1 - survival of motor neuron 1, telomeric  RBM17 - rna binding motif protein 17  HEMK1 - hemk methyltransferase family member 1  SMARCB1 - swi/snf related, matrix associated, actin dependent regulator of chromatin, subfamily b, member 1  UBR5 - ubiquitin protein ligase e3 component n-recognin 5  LSM5 - lsm5 homolog, u6 small nuclear rna associated (s. cerevisiae)  SMARCD3 - swi/snf related, matrix associated, actin dependent regulator of chromatin, subfamily d, member 3  UCHL5 - ubiquitin carboxyl-terminal hydrolase l5  MED20 - mediator complex subunit 20  RBM25 - rna binding motif protein 25  COL4A2 - collagen, type iv, alpha 2  SMARCA1 - swi/snf related, matrix associated, actin dependent regulator of chromatin, subfamily a, member 1  PLD3 - phospholipase d family, member 3  EDC4 - enhancer of mrna decapping 4  CACTIN - cactin, spliceosome c complex subunit  TAOK3 - tao kinase 3  FZR1 - fizzy/cell division cycle 20 related 1 (drosophila)  ZBTB7A - zinc finger and btb domain containing 7a  CRNKL1 - crooked neck pre-mrna splicing factor-like 1 (drosophila)  CDC40 - cell division cycle 40  SCAF1 - sr-related ctd-associated factor 1  MED27 - mediator complex subunit 27  MED26 - mediator complex subunit 26  TFIP11 - tuftelin interacting protein 11  CSTF1 - cleavage stimulation factor, 3' pre-rna, subunit 1, 50kda  POLR1B - polymerase (rna) i polypeptide b, 128kda  FTSJ1 - ftsj rna methyltransferase homolog 1 (e. coli)  DBR1 - debranching rna lariats 1  MED17 - mediator complex subunit 17  MED23 - mediator complex subunit 23  PRPF40B - prp40 pre-mrna processing factor 40 homolog b (s. cerevisiae)  MRTO4 - mrna turnover 4 homolog (s. cerevisiae)  CSTF3 - cleavage stimulation factor, 3' pre-rna, subunit 3, 77kda  RPF2 - ribosome production factor 2 homolog (s. cerevisiae)  CSTF2 - cleavage stimulation factor, 3' pre-rna, subunit 2, 64kda  PRPF6 - pre-mrna processing factor 6  SUPT6H - suppressor of ty 6 homolog (s. cerevisiae)  CTGF - connective tissue growth factor  SUPV3L1 - suppressor of var1, 3-like 1 (s. cerevisiae)  PICK1 - protein interacting with prkca 1  SUPT4H1 - suppressor of ty 4 homolog 1 (s. cerevisiae)  ING4 - inhibitor of growth family, member 4  SUPT5H - suppressor of ty 5 homolog (s. cerevisiae)  GTPBP3 - gtp binding protein 3 (mitochondrial)  UBL5 - ubiquitin-like 5  TRMT11 - trna methyltransferase 11 homolog (s. cerevisiae)  RECQL5 - recq protein-like 5  KPNA2 - karyopherin alpha 2 (rag cohort 1, importin alpha 1)  SBDS - shwachman-bodian-diamond syndrome  KPNB1 - karyopherin (importin) beta 1  KPNA1 - karyopherin alpha 1 (importin alpha 5)  SUV420H1 - suppressor of variegation 4-20 homolog 1 (drosophila)  LACTB2 - lactamase, beta 2  DDX23 - dead (asp-glu-ala-asp) box polypeptide 23  GPBP1L1 - gc-rich promoter binding protein 1-like 1  TFB1M - transcription factor b1, mitochondrial  MED21 - mediator complex subunit 21  MED10 - mediator complex subunit 10  PAN3 - pan3 poly(a) specific ribonuclease subunit homolog (s. cerevisiae)  SUV420H2 - suppressor of variegation 4-20 homolog 2 (drosophila)  SNRNP40 - small nuclear ribonucleoprotein 40kda (u5)  ZRANB2 - zinc finger, ran-binding domain containing 2  RPAIN - rpa interacting protein  LONP1 - lon peptidase 1, mitochondrial  POLDIP3 - polymerase (dna-directed), delta interacting protein 3  NIPBL - nipped-b homolog (drosophila)  BUD13 - bud13 homolog (s. cerevisiae)  STAT3 - signal transducer and activator of transcription 3 (acute-phase response factor)  ASF1A - anti-silencing function 1a histone chaperone  PPIG - peptidylprolyl isomerase g (cyclophilin g)  POLR3GL - polymerase (rna) iii (dna directed) polypeptide g (32kd)-like  SSRP1 - structure specific recognition protein 1  ING5 - inhibitor of growth family, member 5  SLC25A33 - solute carrier family 25 (pyrimidine nucleotide carrier), member 33  BOD1L1 - biorientation of chromosomes in cell division 1-like 1  DHX35 - deah (asp-glu-ala-his) box polypeptide 35  PHF5A - phd finger protein 5a  TRIP12 - thyroid hormone receptor interactor 12  GTF3C5 - general transcription factor iiic, polypeptide 5, 63kda  NUDT16L1 - nudix (nucleoside diphosphate linked moiety x)-type motif 16-like 1  ZNHIT3 - zinc finger, hit-type containing 3  SRPK2 - srsf protein kinase 2  SRPK1 - srsf protein kinase 1  UTP23 - utp23, small subunit (ssu) processome component, homolog (yeast)  TROVE2 - trove domain family, member 2  COPS2 - cop9 signalosome subunit 2  DDX47 - dead (asp-glu-ala-asp) box polypeptide 47  SSBP1 - single-stranded dna binding protein 1, mitochondrial  SSB - sjogren syndrome antigen b (autoantigen la)  EFTUD2 - elongation factor tu gtp binding domain containing 2  THOC3 - tho complex 3  SARNP - sap domain containing ribonucleoprotein  LEF1 - lymphoid enhancer-binding factor 1  GTF3C4 - general transcription factor iiic, polypeptide 4, 90kda  THADA - thyroid adenoma associated  GTF3C3 - general transcription factor iiic, polypeptide 3, 102kda  MTO1 - mitochondrial trna translation optimization 1  CNOT8 - ccr4-not transcription complex, subunit 8  NSUN3 - nop2/sun domain family, member 3  SHPRH - snf2 histone linker phd ring helicase, e3 ubiquitin protein ligase  CDK12 - cyclin-dependent kinase 12  SNORD31 - small nucleolar rna, c/d box 31  DHX9 - deah (asp-glu-ala-his) box helicase 9  SNORD29 - small nucleolar rna, c/d box 29  DHX8 - deah (asp-glu-ala-his) box polypeptide 8  TSR2 - tsr2, 20s rrna accumulation, homolog (s. cerevisiae)  SRSF11 - serine/arginine-rich splicing factor 11  DDX6 - dead (asp-glu-ala-asp) box helicase 6  DDX5 - dead (asp-glu-ala-asp) box helicase 5  LUC7L3 - luc7-like 3 (s. cerevisiae)  DDX3X - dead (asp-glu-ala-asp) box helicase 3, x-linked  DDX1 - dead (asp-glu-ala-asp) box helicase 1  SYF2 - syf2 pre-mrna-splicing factor  SPRTN - sprt-like n-terminal domain  SNORD22 - small nucleolar rna, c/d box 22  SIN3A - sin3 transcription regulator family member a  SNORD30 - small nucleolar rna, c/d box 30  SAMHD1 - sam domain and hd domain 1  WWTR1 - ww domain containing transcription regulator 1  SET - set nuclear oncogene  DDB1 - damage-specific dna binding protein 1, 127kda  SETMAR - set domain and mariner transposase fusion gene  MLH1 - mutl homolog 1, colon cancer, nonpolyposis type 2 (e. coli)  RSF1 - remodeling and spacing factor 1  MCM9 - minichromosome maintenance complex component 9  GEMIN5 - gem (nuclear organelle) associated protein 5  NOL11 - nucleolar protein 11  MLLT3 - myeloid/lymphoid or mixed-lineage leukemia (trithorax homolog, drosophila); translocated to, 3  MLLT1 - myeloid/lymphoid or mixed-lineage leukemia (trithorax homolog, drosophila); translocated to, 1  MED14 - mediator complex subunit 14  WDR46 - wd repeat domain 46  THUMPD3 - thump domain containing 3  PHAX - phosphorylated adaptor for rna export  NELFB - negative elongation factor complex member b  CPSF3 - cleavage and polyadenylation specific factor 3, 73kda  POT1 - protection of telomeres 1  NAA38 - n(alpha)-acetyltransferase 38, natc auxiliary subunit  LSM7 - lsm7 homolog, u6 small nuclear rna associated (s. cerevisiae)  CNOT10 - ccr4-not transcription complex, subunit 10  RCHY1 - ring finger and chy zinc finger domain containing 1, e3 ubiquitin protein ligase  TATDN1 - tatd dnase domain containing 1  UIMC1 - ubiquitin interaction motif containing 1  MNAT1 - mnat cdk-activating kinase assembly factor 1  DFFA - dna fragmentation factor, 45kda, alpha polypeptide  MTA2 - metastasis associated 1 family, member 2  POLR1A - polymerase (rna) i polypeptide a, 194kda  NOLC1 - nucleolar and coiled-body phosphoprotein 1  ZNF473 - zinc finger protein 473  KCTD13 - potassium channel tetramerization domain containing 13  REXO1 - rex1, rna exonuclease 1 homolog (s. cerevisiae)  DCAF13 - ddb1 and cul4 associated factor 13  MNT - mnt, max dimerization protein  POLR3K - polymerase (rna) iii (dna directed) polypeptide k, 12.3 kda  DDX10 - dead (asp-glu-ala-asp) box polypeptide 10  WBP11 - ww domain binding protein 11  WDR18 - wd repeat domain 18  DHX15 - deah (asp-glu-ala-his) box helicase 15  CNOT6 - ccr4-not transcription complex, subunit 6  MCM7 - minichromosome maintenance complex component 7  NSRP1 - nuclear speckle splicing regulatory protein 1  CWF19L2 - cwf19-like 2, cell cycle control (s. pombe)  ATXN2 - ataxin 2  ERI1 - exoribonuclease 1  SCAF4 - sr-related ctd-associated factor 4  MECP2 - methyl cpg binding protein 2 (rett syndrome)  PELO - pelota homolog (drosophila)  MEF2A - myocyte enhancer factor 2a  MED30 - mediator complex subunit 30  DDX21 - dead (asp-glu-ala-asp) box helicase 21  BCDIN3D - bcdin3 domain containing  PPIL3 - peptidylprolyl isomerase (cyclophilin)-like 3  RPAP1 - rna polymerase ii associated protein 1  CPSF2 - cleavage and polyadenylation specific factor 2, 100kda  MEN1 - multiple endocrine neoplasia i  DARS - aspartyl-trna synthetase  WDR75 - wd repeat domain 75  INTS2 - integrator complex subunit 2  ANGEL2 - angel homolog 2 (drosophila)  XPO5 - exportin 5  SCAF11 - sr-related ctd-associated factor 11  UTP15 - utp15, u3 small nucleolar ribonucleoprotein, homolog (s. cerevisiae)  METTL1 - methyltransferase like 1  HINFP - histone h4 transcription factor  MFAP1 - microfibrillar-associated protein 1  REXO2 - rna exonuclease 2  AARS2 - alanyl-trna synthetase 2, mitochondrial  RNASEH2C - ribonuclease h2, subunit c  PPP1R1B - protein phosphatase 1, regulatory (inhibitor) subunit 1b  SARS - seryl-trna synthetase  NGDN - neuroguidin, eif4e binding protein  MBD3 - methyl-cpg binding domain protein 3  NYNRIN - nyn domain and retroviral integrase containing  ATRIP - atr interacting protein  CBFA2T2 - core-binding factor, runt domain, alpha subunit 2; translocated to, 2  RRP9 - ribosomal rna processing 9, small subunit (ssu) processome component, homolog (yeast)  MAGOH - mago-nashi homolog, proliferation-associated (drosophila)  EPC2 - enhancer of polycomb homolog 2 (drosophila)  PRPF3 - pre-mrna processing factor 3  LARS - leucyl-trna synthetase  PRPF4 - prp4 pre-mrna processing factor 4 homolog (yeast)  RQCD1 - rcd1 required for cell differentiation1 homolog (s. pombe)  CNOT6L - ccr4-not transcription complex, subunit 6-like  EAF1 - ell associated factor 1  SMC3 - structural maintenance of chromosomes 3  TAOK1 - tao kinase 1  PUS3 - pseudouridylate synthase 3  MARS - methionyl-trna synthetase  MAPT - microtubule-associated protein tau  RNASEH1 - ribonuclease h1  DOM3Z - dom-3 homolog z (c. elegans)  MTA1 - metastasis associated 1  SIRT6 - sirtuin 6  PRPF31 - pre-mrna processing factor 31  DR1 - down-regulator of transcription 1, tbp-binding (negative cofactor 2)  TRMT5 - trna methyltransferase 5  TNKS1BP1 - tankyrase 1 binding protein 1, 182kda  USP10 - ubiquitin specific peptidase 10  SKI - v-ski avian sarcoma viral oncogene homolog  MBD1 - methyl-cpg binding domain protein 1  SKIV2L - superkiller viralicidic activity 2-like (s. cerevisiae)  REV1 - rev1, polymerase (dna directed)  SART1 - squamous cell carcinoma antigen recognized by t cells  CHTOP - chromatin target of prmt1  ZCRB1 - zinc finger cchc-type and rna binding motif 1  WDR48 - wd repeat domain 48  MCM3 - minichromosome maintenance complex component 3  TRMT112 - trna methyltransferase 11-2 homolog (s. cerevisiae)  MCM6 - minichromosome maintenance complex component 6  MCM5 - minichromosome maintenance complex component 5  RTFDC1 - replication termination factor 2 domain containing 1  NELFCD - negative elongation factor complex member c/d  MCM2 - minichromosome maintenance complex component 2  CWC15 - cwc15 spliceosome-associated protein homolog (s. cerevisiae)  RTCB - rna 2',3'-cyclic phosphate and 5'-oh ligase  NUDT12 - nudix (nucleoside diphosphate linked moiety x)-type motif 12  DTX1 - deltex homolog 1 (drosophila)  PPIL1 - peptidylprolyl isomerase (cyclophilin)-like 1  DKC1 - dyskeratosis congenita 1, dyskerin  PNPT1 - polyribonucleotide nucleotidyltransferase 1  RPL14 - ribosomal protein l14  RIOK1 - rio kinase 1  ZNF445 - zinc finger protein 445  RNASEK - ribonuclease, rnase k  NEIL2 - nei endonuclease viii-like 2 (e. coli)  RBFOX3 - rna binding protein, fox-1 homolog (c. elegans) 3  SESN2 - sestrin 2  PARP9 - poly (adp-ribose) polymerase family, member 9  CDK5RAP1 - cdk5 regulatory subunit associated protein 1  RNF8 - ring finger protein 8, e3 ubiquitin protein ligase  DHX37 - deah (asp-glu-ala-his) box polypeptide 37  UVSSA - uv-stimulated scaffold protein a  ESF1 - esf1, nucleolar pre-rrna processing protein, homolog (s. cerevisiae)  TAF1B - tata box binding protein (tbp)-associated factor, rna polymerase i, b, 63kda  DNASE2 - deoxyribonuclease ii, lysosomal  SFPQ - splicing factor proline/glutamine-rich  PHRF1 - phd and ring finger domains 1  PCF11 - pcf11 cleavage and polyadenylation factor subunit  MED15 - mediator complex subunit 15  PIAS4 - protein inhibitor of activated stat, 4  SRSF1 - serine/arginine-rich splicing factor 1  SMAD2 - smad family member 2  SRRT - serrate rna effector molecule homolog (arabidopsis)  SRSF2 - serine/arginine-rich splicing factor 2  GMEB2 - glucocorticoid modulatory element binding protein 2  SRSF3 - serine/arginine-rich splicing factor 3  DNMT1 - dna (cytosine-5-)-methyltransferase 1  SRSF4 - serine/arginine-rich splicing factor 4  TRDMT1 - trna aspartic acid methyltransferase 1  SRSF5 - serine/arginine-rich splicing factor 5  DNMT3A - dna (cytosine-5-)-methyltransferase 3 alpha  TMTC1 - transmembrane and tetratricopeptide repeat containing 1  TDP2 - tyrosyl-dna phosphodiesterase 2  SRSF6 - serine/arginine-rich splicing factor 6  SRSF7 - serine/arginine-rich splicing factor 7  SFSWAP - splicing factor, suppressor of white-apricot homolog (drosophila)  TRA2B - transformer 2 beta homolog (drosophila)  LARP7 - la ribonucleoprotein domain family, member 7  TAF1C - tata box binding protein (tbp)-associated factor, rna polymerase i, c, 110kda  TAF9B - taf9b rna polymerase ii, tata box binding protein (tbp)-associated factor, 31kda  RSL1D1 - ribosomal l1 domain containing 1  SMAD4 - smad family member 4  NBAS - neuroblastoma amplified sequence  EME1 - essential meiotic structure-specific endonuclease 1  TRMT6 - trna methyltransferase 6 homolog (s. cerevisiae)  INTS1 - integrator complex subunit 1  RBM4B - rna binding motif protein 4b  SENP3 - sumo1/sentrin/smt3 specific peptidase 3  SMARCAD1 - swi/snf-related, matrix-associated actin-dependent regulator of chromatin, subfamily a, containing dead/h box 1  NCBP2 - nuclear cap binding protein subunit 2, 20kda  NCBP1 - nuclear cap binding protein subunit 1, 80kda  TSN - translin  G3BP1 - gtpase activating protein (sh3 domain) binding protein 1  MBNL2 - muscleblind-like splicing regulator 2  FAN1 - fancd2/fanci-associated nuclease 1  SUGP2 - surp and g patch domain containing 2  NAF1 - nuclear assembly factor 1 ribonucleoprotein  TRMT1L - trna methyltransferase 1 homolog (s. cerevisiae)-like  SNW1 - snw domain containing 1  HNRNPM - heterogeneous nuclear ribonucleoprotein m  MGME1 - mitochondrial genome maintenance exonuclease 1  ELL2 - elongation factor, rna polymerase ii, 2  WRNIP1 - werner helicase interacting protein 1  TRIM28 - tripartite motif containing 28  DTD1 - d-tyrosyl-trna deacylase 1  DROSHA - drosha, ribonuclease type iii  KIN - kin, antigenic determinant of reca protein homolog (mouse)  EP300 - e1a binding protein p300  NAP1L1 - nucleosome assembly protein 1-like 1  NASP - nuclear autoantigenic sperm protein (histone-binding)  NARS - asparaginyl-trna synthetase  HABP4 - hyaluronan binding protein 4  NBN - nibrin  UBQLN4 - ubiquilin 4  CELF4 - cugbp, elav-like family member 4  RBM7 - rna binding motif protein 7  RBM6 - rna binding motif protein 6  RBM5 - rna binding motif protein 5  EGR2 - early growth response 2  TTF1 - transcription termination factor, rna polymerase i  ALYREF - aly/ref export factor  INTS4 - integrator complex subunit 4  TFPT - tcf3 (e2a) fusion partner (in childhood leukemia)  KDM2A - lysine (k)-specific demethylase 2a  ELAVL1 - elav (embryonic lethal, abnormal vision, drosophila)-like 1 (hu antigen r)  MPHOSPH6 - m-phase phosphoprotein 6  CCDC130 - coiled-coil domain containing 130  ZNF326 - zinc finger protein 326  MPHOSPH10 - m-phase phosphoprotein 10 (u3 small nucleolar ribonucleoprotein)  ELAVL4 - elav (embryonic lethal, abnormal vision, drosophila)-like 4  CDADC1 - cytidine and dcmp deaminase domain containing 1  URM1 - ubiquitin related modifier 1  USPL1 - ubiquitin specific peptidase like 1  PDCD11 - programmed cell death 11  ABT1 - activator of basal transcription 1  TOPORS - topoisomerase i binding, arginine/serine-rich, e3 ubiquitin protein ligase  TSNAX - translin-associated factor x  FIP1L1 - factor interacting with papola and cpsf1  ACIN1 - apoptotic chromatin condensation inducer 1  CNOT1 - ccr4-not transcription complex, subunit 1  LAS1L - las1-like (s. cerevisiae)  EXOSC7 - exosome component 7  SPEN - spen homolog, transcriptional regulator (drosophila)  ISG20L2 - interferon stimulated exonuclease gene 20kda-like 2  PDCD7 - programmed cell death 7  TP53BP1 - tumor protein p53 binding protein 1  HUWE1 - hect, uba and wwe domain containing 1, e3 ubiquitin protein ligase  TRMT10A - trna methyltransferase 10 homolog a (s. cerevisiae)  TOP3A - topoisomerase (dna) iii alpha  CNOT7 - ccr4-not transcription complex, subunit 7  TOP2B - topoisomerase (dna) ii beta 180kda  PQBP1 - polyglutamine binding protein 1  TOP1 - topoisomerase (dna) i  TRA2A - transformer 2 alpha homolog (drosophila)  KDM1A - lysine (k)-specific demethylase 1a  INO80B - ino80 complex subunit b  SF3B5 - splicing factor 3b, subunit 5, 10kda  CPSF1 - cleavage and polyadenylation specific factor 1, 160kda  RBM15B - rna binding motif protein 15b  SNRNP200 - small nuclear ribonucleoprotein 200kda (u5)  HNRNPLL - heterogeneous nuclear ribonucleoprotein l-like  NCOA6 - nuclear receptor coactivator 6  IMP4 - imp4, u3 small nucleolar ribonucleoprotein, homolog (yeast)  SMG1 - smg1 phosphatidylinositol 3-kinase-related kinase  E4F1 - e4f transcription factor 1  NT5M - 5',3'-nucleotidase, mitochondrial  ENDOD1 - endonuclease domain containing 1  NRBP1 - nuclear receptor binding protein 1  MUTYH - muty homolog (e. coli)  XAB2 - xpa binding protein 2  PDS5B - pds5, regulator of cohesion maintenance, homolog b (s. cerevisiae)  ENY2 - enhancer of yellow 2 homolog (drosophila)  RAD50 - rad50 homolog (s. cerevisiae)  C11orf30 - chromosome 11 open reading frame 30  RRP1B - ribosomal rna processing 1b  CMTR1 - cap methyltransferase 1  NR2C1 - nuclear receptor subfamily 2, group c, member 1  SETX - senataxin  DUS3L - dihydrouridine synthase 3-like (s. cerevisiae)  NR2C2 - nuclear receptor subfamily 2, group c, member 2  MARS2 - methionyl-trna synthetase 2, mitochondrial  MED25 - mediator complex subunit 25  MED6 - mediator complex subunit 6  NAT14 - n-acetyltransferase 14 (gcn5-related, putative)  MTRR - 5-methyltetrahydrofolate-homocysteine methyltransferase reductase  REXO4 - rex4, rna exonuclease 4 homolog (s. cerevisiae)  PTBP3 - polypyrimidine tract binding protein 3  INTS12 - integrator complex subunit 12  ZC3H13 - zinc finger ccch-type containing 13  USP1 - ubiquitin specific peptidase 1  FANCE - fanconi anemia, complementation group e  SNORD107 - small nucleolar rna, c/d box 107  UNG - uracil-dna glycosylase  MTF1 - metal-regulatory transcription factor 1  FARSA - phenylalanyl-trna synthetase, alpha subunit  NR1H2 - nuclear receptor subfamily 1, group h, member 2  C19orf40 - chromosome 19 open reading frame 40  PARP2 - poly (adp-ribose) polymerase 2  DCPS - decapping enzyme, scavenger  UTP3 - utp3, small subunit (ssu) processome component, homolog (s. cerevisiae)  MED16 - mediator complex subunit 16  SMC5 - structural maintenance of chromosomes 5  CHAF1A - chromatin assembly factor 1, subunit a (p150)  DDX24 - dead (asp-glu-ala-asp) box helicase 24  FARSB - phenylalanyl-trna synthetase, beta subunit  ZC3H3 - zinc finger ccch-type containing 3  UVRAG - uv radiation resistance associated  VARS - valyl-trna synthetase  EYA3 - eyes absent homolog 3 (drosophila)  CCNL1 - cyclin l1  ENDOV - endonuclease v  VCP - valosin containing protein  WDR43 - wd repeat domain 43  RARS2 - arginyl-trna synthetase 2, mitochondrial  MSH3 - muts homolog 3 (e. coli)  MSH2 - muts homolog 2, colon cancer, nonpolyposis type 1 (e. coli)  ERCC5 - excision repair cross-complementing rodent repair deficiency, complementation group 5  ERCC4 - excision repair cross-complementing rodent repair deficiency, complementation group 4  PAN2 - pan2 poly(a) specific ribonuclease subunit homolog (s. cerevisiae)  ERCC3 - excision repair cross-complementing rodent repair deficiency, complementation group 3  LIN52 - lin-52 homolog (c. elegans)  YTHDC1 - yth domain containing 1  THOC2 - tho complex 2  ASTE1 - asteroid homolog 1 (drosophila)  UBE2A - ubiquitin-conjugating enzyme e2a  NOB1 - nin1/rpn12 binding protein 1 homolog (s. cerevisiae)  ERN1 - endoplasmic reticulum to nucleus signaling 1  UBB - ubiquitin b  VARS2 - valyl-trna synthetase 2, mitochondrial  HIRA - histone cell cycle regulator  COMMD1 - copper metabolism (murr1) domain containing 1  TTC5 - tetratricopeptide repeat domain 5  SNORA3 - small nucleolar rna, h/aca box 3  RBM8A - rna binding motif protein 8a  ETV1 - ets variant 1  MDN1 - mdn1, midasin homolog (yeast)  POLR2M - polymerase (rna) ii (dna directed) polypeptide m  ETF1 - eukaryotic translation termination factor 1  USP3 - ubiquitin specific peptidase 3  BABAM1 - brisc and brca1 a complex member 1  SUMO1 - small ubiquitin-like modifier 1  UBE2T - ubiquitin-conjugating enzyme e2t (putative)  UBTF - upstream binding transcription factor, rna polymerase i  ZNF830 - zinc finger protein 830  METTL5 - methyltransferase like 5  RRS1 - rrs1 ribosome biogenesis regulator homolog (s. cerevisiae)  MED4 - mediator complex subunit 4  JMJD6 - jumonji domain containing 6  THOC1 - tho complex 1  SETD2 - set domain containing 2  EPRS - glutamyl-prolyl-trna synthetase  RBX1 - ring-box 1, e3 ubiquitin protein ligase  ZC3H7A - zinc finger ccch-type containing 7a  MRE11A - mre11 meiotic recombination 11 homolog a (s. cerevisiae)  UBE2V2 - ubiquitin-conjugating enzyme e2 variant 2  ERCC2 - excision repair cross-complementing rodent repair deficiency, complementation group 2  THRAP3 - thyroid hormone receptor associated protein 3  MED12 - mediator complex subunit 12  MED13 - mediator complex subunit 13  UBE2N - ubiquitin-conjugating enzyme e2n  UBE2V1 - ubiquitin-conjugating enzyme e2 variant 1  ZC3H7B - zinc finger ccch-type containing 7b  MED24 - mediator complex subunit 24  FMR1 - fragile x mental retardation 1  TCOF1 - treacher collins-franceschetti syndrome 1  HENMT1 - hen1 methyltransferase homolog 1 (arabidopsis)  DTX3L - deltex 3-like (drosophila)  ORC4 - origin recognition complex, subunit 4  INTS5 - integrator complex subunit 5  ORC2 - origin recognition complex, subunit 2  ORC5 - origin recognition complex, subunit 5  PDS5A - pds5, regulator of cohesion maintenance, homolog a (s. cerevisiae)  BOP1 - block of proliferation 1  THUMPD2 - thump domain containing 2  ERCC6L2 - excision repair cross-complementing rodent repair deficiency, complementation group 6-like 2  FLNA - filamin a, alpha  RPRD2 - regulation of nuclear pre-mrna domain containing 2  PPP4R2 - protein phosphatase 4, regulatory subunit 2  FOXO3 - forkhead box o3  RBM19 - rna binding motif protein 19  UTP6 - utp6, small subunit (ssu) processome component, homolog (yeast)  DCP1A - decapping mrna 1a  AP5Z1 - adaptor-related protein complex 5, zeta 1 subunit  RPUSD1 - rna pseudouridylate synthase domain containing 1  MED8 - mediator complex subunit 8  DDX46 - dead (asp-glu-ala-asp) box polypeptide 46  TDG - thymine-dna glycosylase  SMG6 - smg6 nonsense mediated mrna decay factor  OGG1 - 8-oxoguanine dna glycosylase  EXOSC3 - exosome component 3  WDR61 - wd repeat domain 61  SMG7 - smg7 nonsense mediated mrna decay factor  CSTF2T - cleavage stimulation factor, 3' pre-rna, subunit 2, 64kda, tau variant  EXOSC1 - exosome component 1  APITD1 - apoptosis-inducing, taf9-like domain 1  TAF9 - taf9 rna polymerase ii, tata box binding protein (tbp)-associated factor, 32kda  BMS1 - bms1 ribosome biogenesis factor  TAF7 - taf7 rna polymerase ii, tata box binding protein (tbp)-associated factor, 55kda  FAM98B - family with sequence similarity 98, member b  RRP15 - ribosomal rna processing 15 homolog (s. cerevisiae)  KHNYN - kh and nyn domain containing  TAF6 - taf6 rna polymerase ii, tata box binding protein (tbp)-associated factor, 80kda  U2SURP - u2 snrnp-associated surp domain containing  SMCHD1 - structural maintenance of chromosomes flexible hinge domain containing 1  TAF4 - taf4 rna polymerase ii, tata box binding protein (tbp)-associated factor, 135kda  TAF2 - taf2 rna polymerase ii, tata box binding protein (tbp)-associated factor, 150kda  NTHL1 - nth endonuclease iii-like 1 (e. coli)  EIF4A3 - eukaryotic translation initiation factor 4a3  TARS - threonyl-trna synthetase  NR4A2 - nuclear receptor subfamily 4, group a, member 2  ZBTB7B - zinc finger and btb domain containing 7b  TARBP1 - tar (hiv-1) rna binding protein 1  FEN1 - flap structure-specific endonuclease 1  DHX38 - deah (asp-glu-ala-his) box polypeptide 38  NVL - nuclear vcp-like  SMG9 - smg9 nonsense mediated mrna decay factor  TAF13 - taf13 rna polymerase ii, tata box binding protein (tbp)-associated factor, 18kda  ZCCHC11 - zinc finger, cchc domain containing 11  LARS2 - leucyl-trna synthetase 2, mitochondrial  INO80E - ino80 complex subunit e  NPM1 - nucleophosmin (nucleolar phosphoprotein b23, numatrin)  KMT2E - lysine (k)-specific methyltransferase 2e  FCF1 - fcf1 rrna-processing protein  GINS1 - gins complex subunit 1 (psf1 homolog)  TSEN54 - tsen54 trna splicing endonuclease subunit  CTIF - cbp80/20-dependent translation initiation factor  TCF3 - transcription factor 3  PUM2 - pumilio homolog 2 (drosophila)  POLR1D - polymerase (rna) i polypeptide d, 16kda  DMAP1 - dna methyltransferase 1 associated protein 1  TCEB3 - transcription elongation factor b (siii), polypeptide 3 (110kda, elongin a)  TRNT1 - trna nucleotidyl transferase, cca-adding, 1  UTP18 - utp18 small subunit (ssu) processome component homolog (yeast)  RRNAD1 - ribosomal rna adenine dimethylase domain containing 1  TOE1 - target of egr1, member 1 (nuclear)  TCEA2 - transcription elongation factor a (sii), 2  ANGEL1 - angel homolog 1 (drosophila)  SIDT2 - sid1 transmembrane family, member 2  TCEA1 - transcription elongation factor a (sii), 1  DONSON - downstream neighbor of son  NRBF2 - nuclear receptor binding factor 2  NOP2 - nop2 nucleolar protein  NONO - non-pou domain containing, octamer-binding  VPRBP - vpr (hiv-1) binding protein  TK1 - thymidine kinase 1, soluble  TK2 - thymidine kinase 2, mitochondrial  MUS81 - mus81 structure-specific endonuclease subunit  NME1 - nme/nm23 nucleoside diphosphate kinase 1  SNORD118 - small nucleolar rna, c/d box 118  PPWD1 - peptidylprolyl isomerase domain and wd repeat containing 1  NOTCH4 - notch 4  NDNL2 - necdin-like 2  NOVA2 - neuro-oncological ventral antigen 2  NOVA1 - neuro-oncological ventral antigen 1  AQR - aquarius homolog (mouse)  EXOSC2 - exosome component 2  CNOT2 - ccr4-not transcription complex, subunit 2  PUM1 - pumilio homolog 1 (drosophila)  CNOT4 - ccr4-not transcription complex, subunit 4  CNOT3 - ccr4-not transcription complex, subunit 3  NOTCH1 - notch 1  SIRT1 - sirtuin 1  NOTCH3 - notch 3  CTC1 - cts telomere maintenance complex component 1  NOTCH2 - notch 2  DHX34 - deah (asp-glu-ala-his) box polypeptide 34  NFYA - nuclear transcription factor y, alpha  NFRKB - nuclear factor related to kappab binding protein  THOC7 - tho complex 7 homolog (drosophila)  EDC3 - enhancer of mrna decapping 3  ACTR8 - arp8 actin-related protein 8 homolog (yeast)  ZMAT2 - zinc finger, matrin-type 2  PHF3 - phd finger protein 3  SRFBP1 - serum response factor binding protein 1  NFKB1 - nuclear factor of kappa light polypeptide gene enhancer in b-cells 1  NFKBIB - nuclear factor of kappa light polypeptide gene enhancer in b-cells inhibitor, beta  TNFAIP1 - tumor necrosis factor, alpha-induced protein 1 (endothelial)  HARS2 - histidyl-trna synthetase 2, mitochondrial  TARDBP - tar dna binding protein  SNRNP48 - small nuclear ribonucleoprotein 48kda (u11/u12)  NHP2L1 - nhp2 non-histone chromosome protein 2-like 1 (s. cerevisiae)  ZNRD1 - zinc ribbon domain containing 1  SART3 - squamous cell carcinoma antigen recognized by t cells 3  SF3B1 - splicing factor 3b, subunit 1, 155kda  SF3B3 - splicing factor 3b, subunit 3, 130kda  MDC1 - mediator of dna-damage checkpoint 1  TTC37 - tetratricopeptide repeat domain 37  RRM2B - ribonucleotide reductase m2 b (tp53 inducible)  TFAM - transcription factor a, mitochondrial  SMARCAL1 - swi/snf related, matrix associated, actin dependent regulator of chromatin, subfamily a-like 1  HARBI1 - harbinger transposase derived 1  TERF1 - telomeric repeat binding factor (nima-interacting) 1  TERF2 - telomeric repeat binding factor 2  CTR9 - ctr9, paf1/rna polymerase ii complex component  GTF3C6 - general transcription factor iiic, polypeptide 6, alpha 35kda  SKIV2L2 - superkiller viralicidic activity 2-like 2 (s. cerevisiae)  TFDP1 - transcription factor dp-1  EPC1 - enhancer of polycomb homolog 1 (drosophila)  MORF4L2 - mortality factor 4 like 2  BCCIP - brca2 and cdkn1a interacting protein  NR2F1 - nuclear receptor subfamily 2, group f, member 1  PES1 - pescadillo ribosomal biogenesis factor 1  TFCP2 - transcription factor cp2  ZFYVE26 - zinc finger, fyve domain containing 26  TGS1 - trimethylguanosine synthase 1  NFIA - nuclear factor i/a  POLE4 - polymerase (dna-directed), epsilon 4, accessory subunit  KIAA0391 - kiaa0391  RBFOX2 - rna binding protein, fox-1 homolog (c. elegans) 2  CTNNBL1 - catenin, beta like 1  MEPCE - methylphosphate capping enzyme  GEMIN4 - gem (nuclear organelle) associated protein 4  FOSB - fbj murine osteosarcoma viral oncogene homolog b  FOS - fbj murine osteosarcoma viral oncogene homolog  DRG1 - developmentally regulated gtp binding protein 1  SRRM2 - serine/arginine repetitive matrix 2  COPS7A - cop9 signalosome subunit 7a  GATC - glutamyl-trna(gln) amidotransferase, subunit c  TARS2 - threonyl-trna synthetase 2, mitochondrial (putative)  METTL3 - methyltransferase like 3  KIAA0430 - kiaa0430  THRA - thyroid hormone receptor, alpha |
| GO:0006397 | mRNA processing | 1.03E-22 | 7.13E-19 | 1.31 (10334,383,6996,340) | [+] Show genes  RSRC1 - arginine/serine-rich coiled-coil 1  NCBP2 - nuclear cap binding protein subunit 2, 20kda  SMNDC1 - survival motor neuron domain containing 1  WTAP - wilms tumor 1 associated protein  NCBP1 - nuclear cap binding protein subunit 1, 80kda  BCAS2 - breast carcinoma amplified sequence 2  CWC27 - cwc27 spliceosome-associated protein homolog (s. cerevisiae)  SAP18 - sin3a-associated protein, 18kda  ZCCHC8 - zinc finger, cchc domain containing 8  RBM39 - rna binding motif protein 39  RNPC3 - rna-binding region (rnp1, rrm) containing 3  SYMPK - symplekin  SF3A3 - splicing factor 3a, subunit 3, 60kda  MBNL2 - muscleblind-like splicing regulator 2  HNRNPA0 - heterogeneous nuclear ribonucleoprotein a0  SUGP2 - surp and g patch domain containing 2  PNN - pinin, desmosome associated protein  SF3A1 - splicing factor 3a, subunit 1, 120kda  METTL14 - methyltransferase like 14  SF3A2 - splicing factor 3a, subunit 2, 66kda  CWC25 - cwc25 spliceosome-associated protein homolog (s. cerevisiae)  SNW1 - snw domain containing 1  HNRNPM - heterogeneous nuclear ribonucleoprotein m  KIN - kin, antigenic determinant of reca protein homolog (mouse)  ZFP36L1 - zfp36 ring finger protein-like 1  ALKBH5 - alkb, alkylation repair homolog 5 (e. coli)  HABP4 - hyaluronan binding protein 4  CWC22 - cwc22 spliceosome-associated protein homolog (s. cerevisiae)  CELF4 - cugbp, elav-like family member 4  TXNL4A - thioredoxin-like 4a  CIR1 - corepressor interacting with rbpj, 1  RBM6 - rna binding motif protein 6  RBM5 - rna binding motif protein 5  PLRG1 - pleiotropic regulator 1  ALYREF - aly/ref export factor  PAPOLA - poly(a) polymerase alpha  LSM2 - lsm2 homolog, u6 small nuclear rna associated (s. cerevisiae)  TCERG1 - transcription elongation regulator 1  PABPN1 - poly(a) binding protein, nuclear 1  RNPS1 - rna binding protein s1, serine-rich domain  SON - son dna binding protein  ELAVL1 - elav (embryonic lethal, abnormal vision, drosophila)-like 1 (hu antigen r)  ZNF326 - zinc finger protein 326  ELAVL4 - elav (embryonic lethal, abnormal vision, drosophila)-like 4  SUGP1 - surp and g patch domain containing 1  PDCD11 - programmed cell death 11  PRPF38A - prp38 pre-mrna processing factor 38 (yeast) domain containing a  FIP1L1 - factor interacting with papola and cpsf1  ACIN1 - apoptotic chromatin condensation inducer 1  SNRPN - small nuclear ribonucleoprotein polypeptide n  SNRPB2 - small nuclear ribonucleoprotein polypeptide b  RPRD1B - regulation of nuclear pre-mrna domain containing 1b  ZNF259 - zinc finger protein 259  SNRPD1 - small nuclear ribonucleoprotein d1 polypeptide 16kda  DDX41 - dead (asp-glu-ala-asp) box polypeptide 41  SPEN - spen homolog, transcriptional regulator (drosophila)  SNRPF - small nuclear ribonucleoprotein polypeptide f  PDCD7 - programmed cell death 7  SCNM1 - sodium channel modifier 1  SNRNP70 - small nuclear ribonucleoprotein 70kda (u1)  SNRPA - small nuclear ribonucleoprotein polypeptide a  SNRPA1 - small nuclear ribonucleoprotein polypeptide a'  SNRPB - small nuclear ribonucleoprotein polypeptides b and b1  ADAR - adenosine deaminase, rna-specific  PRPF4B - prp4 pre-mrna processing factor 4 homolog b (yeast)  ADARB1 - adenosine deaminase, rna-specific, b1  TSEN34 - tsen34 trna splicing endonuclease subunit  PQBP1 - polyglutamine binding protein 1  CPSF4 - cleavage and polyadenylation specific factor 4, 30kda  TRA2A - transformer 2 alpha homolog (drosophila)  U2AF1L4 - u2 small nuclear rna auxiliary factor 1-like 4  KDM1A - lysine (k)-specific demethylase 1a  RBM17 - rna binding motif protein 17  CPSF1 - cleavage and polyadenylation specific factor 1, 160kda  HNRNPR - heterogeneous nuclear ribonucleoprotein r  SF3B5 - splicing factor 3b, subunit 5, 10kda  BUD31 - bud31 homolog (s. cerevisiae)  RBM15B - rna binding motif protein 15b  SNRNP200 - small nuclear ribonucleoprotein 200kda (u5)  HNRNPLL - heterogeneous nuclear ribonucleoprotein l-like  RBM10 - rna binding motif protein 10  LSM5 - lsm5 homolog, u6 small nuclear rna associated (s. cerevisiae)  POLR2K - polymerase (rna) ii (dna directed) polypeptide k, 7.0kda  SRRM1 - serine/arginine repetitive matrix 1  TSEN15 - tsen15 trna splicing endonuclease subunit  XAB2 - xpa binding protein 2  RBM25 - rna binding motif protein 25  RRP1B - ribosomal rna processing 1b  SF3B4 - splicing factor 3b, subunit 4, 49kda  CACTIN - cactin, spliceosome c complex subunit  CMTR1 - cap methyltransferase 1  CRNKL1 - crooked neck pre-mrna splicing factor-like 1 (drosophila)  POLR2G - polymerase (rna) ii (dna directed) polypeptide g  POLR2F - polymerase (rna) ii (dna directed) polypeptide f  POLR2E - polymerase (rna) ii (dna directed) polypeptide e, 25kda  CDC40 - cell division cycle 40  SCAF1 - sr-related ctd-associated factor 1  POLR2D - polymerase (rna) ii (dna directed) polypeptide d  POLR2C - polymerase (rna) ii (dna directed) polypeptide c, 33kda  POLR2B - polymerase (rna) ii (dna directed) polypeptide b, 140kda  POLR2A - polymerase (rna) ii (dna directed) polypeptide a, 220kda  HSPA8 - heat shock 70kda protein 8  HNRNPUL1 - heterogeneous nuclear ribonucleoprotein u-like 1  TFIP11 - tuftelin interacting protein 11  CSTF1 - cleavage stimulation factor, 3' pre-rna, subunit 1, 50kda  PPIE - peptidylprolyl isomerase e (cyclophilin e)  PRKACA - protein kinase, camp-dependent, catalytic, alpha  DBR1 - debranching rna lariats 1  PTBP3 - polypyrimidine tract binding protein 3  PRPF40B - prp40 pre-mrna processing factor 40 homolog b (s. cerevisiae)  ZC3H13 - zinc finger ccch-type containing 13  CSTF3 - cleavage stimulation factor, 3' pre-rna, subunit 3, 77kda  PRPF6 - pre-mrna processing factor 6  CSTF2 - cleavage stimulation factor, 3' pre-rna, subunit 2, 64kda  FUS - fused in sarcoma  SUPT6H - suppressor of ty 6 homolog (s. cerevisiae)  FRG1 - fshd region gene 1  PRCC - papillary renal cell carcinoma (translocation-associated)  CLASRP - clk4-associating serine/arginine rich protein  SUPT4H1 - suppressor of ty 4 homolog 1 (s. cerevisiae)  SUPT5H - suppressor of ty 5 homolog (s. cerevisiae)  GEMIN6 - gem (nuclear organelle) associated protein 6  PPIH - peptidylprolyl isomerase h (cyclophilin h)  UBL5 - ubiquitin-like 5  SRSF9 - serine/arginine-rich splicing factor 9  DCPS - decapping enzyme, scavenger  SNRNP35 - small nuclear ribonucleoprotein 35kda (u11/u12)  CMTR2 - cap methyltransferase 2  CPSF6 - cleavage and polyadenylation specific factor 6, 68kda  SLBP - stem-loop binding protein  NUDT21 - nudix (nucleoside diphosphate linked moiety x)-type motif 21  PPP1R8 - protein phosphatase 1, regulatory subunit 8  ZC3H3 - zinc finger ccch-type containing 3  CPSF7 - cleavage and polyadenylation specific factor 7, 59kda  DDX23 - dead (asp-glu-ala-asp) box polypeptide 23  SYNCRIP - synaptotagmin binding, cytoplasmic rna interacting protein  PAN3 - pan3 poly(a) specific ribonuclease subunit homolog (s. cerevisiae)  UPF3B - upf3 regulator of nonsense transcripts homolog b (yeast)  SNRNP40 - small nuclear ribonucleoprotein 40kda (u5)  ZRANB2 - zinc finger, ran-binding domain containing 2  DDX39B - dead (asp-glu-ala-asp) box polypeptide 39b  PAPOLG - poly(a) polymerase gamma  RAVER1 - ribonucleoprotein, ptb-binding 1  IWS1 - iws1 homolog (s. cerevisiae)  PAN2 - pan2 poly(a) specific ribonuclease subunit homolog (s. cerevisiae)  ERCC3 - excision repair cross-complementing rodent repair deficiency, complementation group 3  POLDIP3 - polymerase (dna-directed), delta interacting protein 3  YTHDC1 - yth domain containing 1  THOC2 - tho complex 2  BUD13 - bud13 homolog (s. cerevisiae)  CCNH - cyclin h  RNGTT - rna guanylyltransferase and 5'-phosphatase  ERN1 - endoplasmic reticulum to nucleus signaling 1  RNMT - rna (guanine-7-) methyltransferase  HNRNPU - heterogeneous nuclear ribonucleoprotein u (scaffold attachment factor a)  HNRNPK - heterogeneous nuclear ribonucleoprotein k  HNRNPL - heterogeneous nuclear ribonucleoprotein l  PRPF40A - prp40 pre-mrna processing factor 40 homolog a (s. cerevisiae)  PAPD7 - pap associated domain containing 7  RBM8A - rna binding motif protein 8a  DHX35 - deah (asp-glu-ala-his) box polypeptide 35  PHF5A - phd finger protein 5a  SNIP1 - smad nuclear interacting protein 1  HNRNPA2B1 - heterogeneous nuclear ribonucleoprotein a2/b1  GEMIN7 - gem (nuclear organelle) associated protein 7  HNRNPA1 - heterogeneous nuclear ribonucleoprotein a1  ZNF830 - zinc finger protein 830  HNRNPC - heterogeneous nuclear ribonucleoprotein c (c1/c2)  HNRNPF - heterogeneous nuclear ribonucleoprotein f  HNRNPD - heterogeneous nuclear ribonucleoprotein d (au-rich element rna binding protein 1, 37kda)  HNRNPH1 - heterogeneous nuclear ribonucleoprotein h1 (h)  DDX47 - dead (asp-glu-ala-asp) box polypeptide 47  CLP1 - cleavage and polyadenylation factor i subunit 1  HNRNPH3 - heterogeneous nuclear ribonucleoprotein h3 (2h9)  JMJD6 - jumonji domain containing 6  HNRNPH2 - heterogeneous nuclear ribonucleoprotein h2 (h')  THOC1 - tho complex 1  THOC3 - tho complex 3  EFTUD2 - elongation factor tu gtp binding domain containing 2  CCDC94 - coiled-coil domain containing 94  TUT1 - terminal uridylyl transferase 1, u6 snrna-specific  SARNP - sap domain containing ribonucleoprotein  RBM22 - rna binding motif protein 22  ERCC2 - excision repair cross-complementing rodent repair deficiency, complementation group 2  THRAP3 - thyroid hormone receptor associated protein 3  PDE12 - phosphodiesterase 12  CD2BP2 - cd2 (cytoplasmic tail) binding protein 2  SF3B2 - splicing factor 3b, subunit 2, 145kda  RBM27 - rna binding motif protein 27  CDK12 - cyclin-dependent kinase 12  DHX9 - deah (asp-glu-ala-his) box helicase 9  MTPAP - mitochondrial poly(a) polymerase  DHX8 - deah (asp-glu-ala-his) box polypeptide 8  HTATSF1 - hiv-1 tat specific factor 1  SRSF11 - serine/arginine-rich splicing factor 11  FMR1 - fragile x mental retardation 1  RBM23 - rna binding motif protein 23  DDX5 - dead (asp-glu-ala-asp) box helicase 5  CDC5L - cell division cycle 5-like  SYF2 - syf2 pre-mrna-splicing factor  RBM15 - rna binding motif protein 15  ZCCHC6 - zinc finger, cchc domain containing 6  RNF113A - ring finger protein 113a  RBMX - rna binding motif protein, x-linked  GTF2F1 - general transcription factor iif, polypeptide 1, 74kda  GTF2F2 - general transcription factor iif, polypeptide 2, 30kda  HNRNPA3 - heterogeneous nuclear ribonucleoprotein a3  RPRD2 - regulation of nuclear pre-mrna domain containing 2  GTF2H2 - general transcription factor iih, polypeptide 2, 44kda  PCBP2 - poly(rc) binding protein 2  GTF2H3 - general transcription factor iih, polypeptide 3, 34kda  GEMIN5 - gem (nuclear organelle) associated protein 5  GTF2H1 - general transcription factor iih, polypeptide 1, 62kda  SRSF12 - serine/arginine-rich splicing factor 12  PPP4R2 - protein phosphatase 4, regulatory subunit 2  PRPF8 - pre-mrna processing factor 8  PRPF19 - pre-mrna processing factor 19  CPSF3 - cleavage and polyadenylation specific factor 3, 73kda  NAA38 - n(alpha)-acetyltransferase 38, natc auxiliary subunit  RBM19 - rna binding motif protein 19  LSM7 - lsm7 homolog, u6 small nuclear rna associated (s. cerevisiae)  KHDRBS3 - kh domain containing, rna binding, signal transduction associated 3  GTF2H4 - general transcription factor iih, polypeptide 4, 52kda  PRKRIP1 - prkr interacting protein 1 (il11 inducible)  RPRD1A - regulation of nuclear pre-mrna domain containing 1a  MNAT1 - mnat cdk-activating kinase assembly factor 1  DDX46 - dead (asp-glu-ala-asp) box polypeptide 46  ZNF473 - zinc finger protein 473  APP - amyloid beta (a4) precursor protein  CSTF2T - cleavage stimulation factor, 3' pre-rna, subunit 2, 64kda, tau variant  WBP11 - ww domain binding protein 11  DHX15 - deah (asp-glu-ala-his) box helicase 15  NSRP1 - nuclear speckle splicing regulatory protein 1  CWF19L2 - cwf19-like 2, cell cycle control (s. pombe)  U2SURP - u2 snrnp-associated surp domain containing  PAPD4 - pap associated domain containing 4  PTBP1 - polypyrimidine tract binding protein 1  SCAF4 - sr-related ctd-associated factor 4  CDC73 - cell division cycle 73  GPATCH1 - g patch domain containing 1  EIF4A3 - eukaryotic translation initiation factor 4a3  KHSRP - kh-type splicing regulatory protein  PPIL3 - peptidylprolyl isomerase (cyclophilin)-like 3  DDX17 - dead (asp-glu-ala-asp) box helicase 17  CHERP - calcium homeostasis endoplasmic reticulum protein  DHX38 - deah (asp-glu-ala-his) box polypeptide 38  CPSF2 - cleavage and polyadenylation specific factor 2, 100kda  PRPF18 - pre-mrna processing factor 18  GPKOW - g patch domain and kow motifs  ZCCHC11 - zinc finger, cchc domain containing 11  SLU7 - slu7 splicing factor homolog (s. cerevisiae)  LSM6 - lsm6 homolog, u6 small nuclear rna associated (s. cerevisiae)  SCAF11 - sr-related ctd-associated factor 11  MAGOHB - mago-nashi homolog b (drosophila)  CDK7 - cyclin-dependent kinase 7  CDK13 - cyclin-dependent kinase 13  MFAP1 - microfibrillar-associated protein 1  LSM3 - lsm3 homolog, u6 small nuclear rna associated (s. cerevisiae)  LEO1 - leo1, paf1/rna polymerase ii complex component, homolog (s. cerevisiae)  CSDC2 - cold shock domain containing c2, rna binding  PRPF38B - prp38 pre-mrna processing factor 38 (yeast) domain containing b  TSEN54 - tsen54 trna splicing endonuclease subunit  SNRNP25 - small nuclear ribonucleoprotein 25kda (u11/u12)  RBM28 - rna binding motif protein 28  MAGOH - mago-nashi homolog, proliferation-associated (drosophila)  NONO - non-pou domain containing, octamer-binding  PAPD5 - pap associated domain containing 5  PRPF3 - pre-mrna processing factor 3  PRPF4 - prp4 pre-mrna processing factor 4 homolog (yeast)  CNOT6L - ccr4-not transcription complex, subunit 6-like  SRSF10 - serine/arginine-rich splicing factor 10  PPWD1 - peptidylprolyl isomerase domain and wd repeat containing 1  NOVA2 - neuro-oncological ventral antigen 2  NOVA1 - neuro-oncological ventral antigen 1  AHCYL1 - adenosylhomocysteinase-like 1  AQR - aquarius homolog (mouse)  PRPF31 - pre-mrna processing factor 31  THOC7 - tho complex 7 homolog (drosophila)  ZMAT2 - zinc finger, matrin-type 2  SART1 - squamous cell carcinoma antigen recognized by t cells  CHTOP - chromatin target of prmt1  WDR33 - wd repeat domain 33  ZCRB1 - zinc finger cchc-type and rna binding motif 1  TARDBP - tar dna binding protein  SNRNP48 - small nuclear ribonucleoprotein 48kda (u11/u12)  CWC15 - cwc15 spliceosome-associated protein homolog (s. cerevisiae)  PAF1 - paf1, rna polymerase ii associated factor, homolog (s. cerevisiae)  NHP2L1 - nhp2 non-histone chromosome protein 2-like 1 (s. cerevisiae)  SART3 - squamous cell carcinoma antigen recognized by t cells 3  SF3B1 - splicing factor 3b, subunit 1, 155kda  SF3B3 - splicing factor 3b, subunit 3, 130kda  SMU1 - smu-1 suppressor of mec-8 and unc-52 homolog (c. elegans)  SCAF8 - sr-related ctd-associated factor 8  PPIL1 - peptidylprolyl isomerase (cyclophilin)-like 1  PUF60 - poly-u binding splicing factor 60kda  PNPT1 - polyribonucleotide nucleotidyltransferase 1  RAVER2 - ribonucleoprotein, ptb-binding 2  THOC6 - tho complex 6 homolog (drosophila)  PABPC1 - poly(a) binding protein, cytoplasmic 1  TRUB2 - trub pseudouridine (psi) synthase homolog 2 (e. coli)  SKIV2L2 - superkiller viralicidic activity 2-like 2 (s. cerevisiae)  RBM26 - rna binding motif protein 26  XRN2 - 5'-3' exoribonuclease 2  RBFOX3 - rna binding protein, fox-1 homolog (c. elegans) 3  CELF1 - cugbp, elav-like family member 1  KHDRBS1 - kh domain containing, rna binding, signal transduction associated 1  CASC3 - cancer susceptibility candidate 3  IK - ik cytokine, down-regulator of hla ii  SFPQ - splicing factor proline/glutamine-rich  PHRF1 - phd and ring finger domains 1  PCF11 - pcf11 cleavage and polyadenylation factor subunit  SF1 - splicing factor 1  RBFOX2 - rna binding protein, fox-1 homolog (c. elegans) 2  PSPC1 - paraspeckle component 1  U2AF2 - u2 small nuclear rna auxiliary factor 2  SRSF1 - serine/arginine-rich splicing factor 1  SRRT - serrate rna effector molecule homolog (arabidopsis)  SRSF2 - serine/arginine-rich splicing factor 2  RBM4 - rna binding motif protein 4  SRSF3 - serine/arginine-rich splicing factor 3  SRSF4 - serine/arginine-rich splicing factor 4  CTNNBL1 - catenin, beta like 1  SRSF5 - serine/arginine-rich splicing factor 5  SRSF6 - serine/arginine-rich splicing factor 6  SRSF7 - serine/arginine-rich splicing factor 7  GEMIN2 - gem (nuclear organelle) associated protein 2  SFSWAP - splicing factor, suppressor of white-apricot homolog (drosophila)  RBBP6 - retinoblastoma binding protein 6  TRA2B - transformer 2 beta homolog (drosophila)  LARP7 - la ribonucleoprotein domain family, member 7  DHX16 - deah (asp-glu-ala-his) box polypeptide 16  RPUSD3 - rna pseudouridylate synthase domain containing 3  DDX42 - dead (asp-glu-ala-asp) box helicase 42  SRRM2 - serine/arginine repetitive matrix 2  ECD - ecdysoneless homolog (drosophila)  CWF19L1 - cwf19-like 1, cell cycle control (s. pombe)  USP39 - ubiquitin specific peptidase 39  GRSF1 - g-rich rna sequence binding factor 1  METTL3 - methyltransferase like 3  RBM4B - rna binding motif protein 4b |
| GO:0000377 | RNA splicing, via transesterification reactions with bulged adenosine as nucleophile | 9.78E-19 | 4.54E-15 | 1.35 (10334,242,6996,221) | [+] Show genes  RSRC1 - arginine/serine-rich coiled-coil 1  NCBP2 - nuclear cap binding protein subunit 2, 20kda  SMNDC1 - survival motor neuron domain containing 1  NCBP1 - nuclear cap binding protein subunit 1, 80kda  BCAS2 - breast carcinoma amplified sequence 2  CWC27 - cwc27 spliceosome-associated protein homolog (s. cerevisiae)  ZCCHC8 - zinc finger, cchc domain containing 8  RNPC3 - rna-binding region (rnp1, rrm) containing 3  SYMPK - symplekin  SF3A3 - splicing factor 3a, subunit 3, 60kda  HNRNPA0 - heterogeneous nuclear ribonucleoprotein a0  PNN - pinin, desmosome associated protein  SF3A1 - splicing factor 3a, subunit 1, 120kda  METTL14 - methyltransferase like 14  SF3A2 - splicing factor 3a, subunit 2, 66kda  CWC25 - cwc25 spliceosome-associated protein homolog (s. cerevisiae)  SNW1 - snw domain containing 1  HNRNPM - heterogeneous nuclear ribonucleoprotein m  CWC22 - cwc22 spliceosome-associated protein homolog (s. cerevisiae)  TXNL4A - thioredoxin-like 4a  CELF4 - cugbp, elav-like family member 4  RBM6 - rna binding motif protein 6  RBM5 - rna binding motif protein 5  PLRG1 - pleiotropic regulator 1  ALYREF - aly/ref export factor  PAPOLA - poly(a) polymerase alpha  LSM2 - lsm2 homolog, u6 small nuclear rna associated (s. cerevisiae)  PABPN1 - poly(a) binding protein, nuclear 1  RNPS1 - rna binding protein s1, serine-rich domain  ELAVL1 - elav (embryonic lethal, abnormal vision, drosophila)-like 1 (hu antigen r)  SUGP1 - surp and g patch domain containing 1  PRPF38A - prp38 pre-mrna processing factor 38 (yeast) domain containing a  SNRPN - small nuclear ribonucleoprotein polypeptide n  FIP1L1 - factor interacting with papola and cpsf1  SNRPB2 - small nuclear ribonucleoprotein polypeptide b  SNRPD1 - small nuclear ribonucleoprotein d1 polypeptide 16kda  DDX41 - dead (asp-glu-ala-asp) box polypeptide 41  SPEN - spen homolog, transcriptional regulator (drosophila)  SNRPF - small nuclear ribonucleoprotein polypeptide f  PDCD7 - programmed cell death 7  SCNM1 - sodium channel modifier 1  SNRNP70 - small nuclear ribonucleoprotein 70kda (u1)  SNRPA - small nuclear ribonucleoprotein polypeptide a  SNRPA1 - small nuclear ribonucleoprotein polypeptide a'  SNRPB - small nuclear ribonucleoprotein polypeptides b and b1  PRPF4B - prp4 pre-mrna processing factor 4 homolog b (yeast)  PQBP1 - polyglutamine binding protein 1  CPSF4 - cleavage and polyadenylation specific factor 4, 30kda  TRA2A - transformer 2 alpha homolog (drosophila)  U2AF1L4 - u2 small nuclear rna auxiliary factor 1-like 4  KDM1A - lysine (k)-specific demethylase 1a  RBM17 - rna binding motif protein 17  CPSF1 - cleavage and polyadenylation specific factor 1, 160kda  HNRNPR - heterogeneous nuclear ribonucleoprotein r  SF3B5 - splicing factor 3b, subunit 5, 10kda  BUD31 - bud31 homolog (s. cerevisiae)  RBM15B - rna binding motif protein 15b  SNRNP200 - small nuclear ribonucleoprotein 200kda (u5)  RBM10 - rna binding motif protein 10  LSM5 - lsm5 homolog, u6 small nuclear rna associated (s. cerevisiae)  POLR2K - polymerase (rna) ii (dna directed) polypeptide k, 7.0kda  SRRM1 - serine/arginine repetitive matrix 1  XAB2 - xpa binding protein 2  SF3B4 - splicing factor 3b, subunit 4, 49kda  CACTIN - cactin, spliceosome c complex subunit  CRNKL1 - crooked neck pre-mrna splicing factor-like 1 (drosophila)  POLR2G - polymerase (rna) ii (dna directed) polypeptide g  POLR2F - polymerase (rna) ii (dna directed) polypeptide f  POLR2E - polymerase (rna) ii (dna directed) polypeptide e, 25kda  POLR2D - polymerase (rna) ii (dna directed) polypeptide d  CDC40 - cell division cycle 40  POLR2C - polymerase (rna) ii (dna directed) polypeptide c, 33kda  POLR2B - polymerase (rna) ii (dna directed) polypeptide b, 140kda  HSPA8 - heat shock 70kda protein 8  POLR2A - polymerase (rna) ii (dna directed) polypeptide a, 220kda  HNRNPUL1 - heterogeneous nuclear ribonucleoprotein u-like 1  TFIP11 - tuftelin interacting protein 11  CSTF1 - cleavage stimulation factor, 3' pre-rna, subunit 1, 50kda  PPIE - peptidylprolyl isomerase e (cyclophilin e)  DBR1 - debranching rna lariats 1  PRPF40B - prp40 pre-mrna processing factor 40 homolog b (s. cerevisiae)  CSTF3 - cleavage stimulation factor, 3' pre-rna, subunit 3, 77kda  CSTF2 - cleavage stimulation factor, 3' pre-rna, subunit 2, 64kda  PRPF6 - pre-mrna processing factor 6  FUS - fused in sarcoma  FRG1 - fshd region gene 1  PRCC - papillary renal cell carcinoma (translocation-associated)  PPIH - peptidylprolyl isomerase h (cyclophilin h)  GEMIN6 - gem (nuclear organelle) associated protein 6  UBL5 - ubiquitin-like 5  SRSF9 - serine/arginine-rich splicing factor 9  DCPS - decapping enzyme, scavenger  SNRNP35 - small nuclear ribonucleoprotein 35kda (u11/u12)  NUDT21 - nudix (nucleoside diphosphate linked moiety x)-type motif 21  CPSF7 - cleavage and polyadenylation specific factor 7, 59kda  DDX23 - dead (asp-glu-ala-asp) box polypeptide 23  SYNCRIP - synaptotagmin binding, cytoplasmic rna interacting protein  UPF3B - upf3 regulator of nonsense transcripts homolog b (yeast)  SNRNP40 - small nuclear ribonucleoprotein 40kda (u5)  DDX39B - dead (asp-glu-ala-asp) box polypeptide 39b  RAVER1 - ribonucleoprotein, ptb-binding 1  YTHDC1 - yth domain containing 1  BUD13 - bud13 homolog (s. cerevisiae)  HNRNPU - heterogeneous nuclear ribonucleoprotein u (scaffold attachment factor a)  HNRNPK - heterogeneous nuclear ribonucleoprotein k  HNRNPL - heterogeneous nuclear ribonucleoprotein l  PRPF40A - prp40 pre-mrna processing factor 40 homolog a (s. cerevisiae)  RBM8A - rna binding motif protein 8a  DHX35 - deah (asp-glu-ala-his) box polypeptide 35  PHF5A - phd finger protein 5a  SNIP1 - smad nuclear interacting protein 1  HNRNPA2B1 - heterogeneous nuclear ribonucleoprotein a2/b1  GEMIN7 - gem (nuclear organelle) associated protein 7  HNRNPA1 - heterogeneous nuclear ribonucleoprotein a1  HNRNPC - heterogeneous nuclear ribonucleoprotein c (c1/c2)  HNRNPF - heterogeneous nuclear ribonucleoprotein f  HNRNPD - heterogeneous nuclear ribonucleoprotein d (au-rich element rna binding protein 1, 37kda)  HNRNPH1 - heterogeneous nuclear ribonucleoprotein h1 (h)  CLP1 - cleavage and polyadenylation factor i subunit 1  HNRNPH3 - heterogeneous nuclear ribonucleoprotein h3 (2h9)  HNRNPH2 - heterogeneous nuclear ribonucleoprotein h2 (h')  EFTUD2 - elongation factor tu gtp binding domain containing 2  RBM22 - rna binding motif protein 22  CD2BP2 - cd2 (cytoplasmic tail) binding protein 2  SF3B2 - splicing factor 3b, subunit 2, 145kda  DHX9 - deah (asp-glu-ala-his) box helicase 9  DHX8 - deah (asp-glu-ala-his) box polypeptide 8  HTATSF1 - hiv-1 tat specific factor 1  SRSF11 - serine/arginine-rich splicing factor 11  DDX5 - dead (asp-glu-ala-asp) box helicase 5  CDC5L - cell division cycle 5-like  SYF2 - syf2 pre-mrna-splicing factor  RBM15 - rna binding motif protein 15  RNF113A - ring finger protein 113a  RBMX - rna binding motif protein, x-linked  GTF2F1 - general transcription factor iif, polypeptide 1, 74kda  HNRNPA3 - heterogeneous nuclear ribonucleoprotein a3  GTF2F2 - general transcription factor iif, polypeptide 2, 30kda  PCBP2 - poly(rc) binding protein 2  GEMIN5 - gem (nuclear organelle) associated protein 5  SRSF12 - serine/arginine-rich splicing factor 12  PRPF8 - pre-mrna processing factor 8  PRPF19 - pre-mrna processing factor 19  CPSF3 - cleavage and polyadenylation specific factor 3, 73kda  NAA38 - n(alpha)-acetyltransferase 38, natc auxiliary subunit  RBM19 - rna binding motif protein 19  LSM7 - lsm7 homolog, u6 small nuclear rna associated (s. cerevisiae)  DDX46 - dead (asp-glu-ala-asp) box polypeptide 46  CSTF2T - cleavage stimulation factor, 3' pre-rna, subunit 2, 64kda, tau variant  WBP11 - ww domain binding protein 11  DHX15 - deah (asp-glu-ala-his) box helicase 15  CWF19L2 - cwf19-like 2, cell cycle control (s. pombe)  U2SURP - u2 snrnp-associated surp domain containing  PTBP1 - polypyrimidine tract binding protein 1  GPATCH1 - g patch domain containing 1  EIF4A3 - eukaryotic translation initiation factor 4a3  PPIL3 - peptidylprolyl isomerase (cyclophilin)-like 3  DDX17 - dead (asp-glu-ala-asp) box helicase 17  CHERP - calcium homeostasis endoplasmic reticulum protein  CPSF2 - cleavage and polyadenylation specific factor 2, 100kda  DHX38 - deah (asp-glu-ala-his) box polypeptide 38  GPKOW - g patch domain and kow motifs  SLU7 - slu7 splicing factor homolog (s. cerevisiae)  LSM6 - lsm6 homolog, u6 small nuclear rna associated (s. cerevisiae)  MAGOHB - mago-nashi homolog b (drosophila)  CDK13 - cyclin-dependent kinase 13  MFAP1 - microfibrillar-associated protein 1  LSM3 - lsm3 homolog, u6 small nuclear rna associated (s. cerevisiae)  SNRNP25 - small nuclear ribonucleoprotein 25kda (u11/u12)  NONO - non-pou domain containing, octamer-binding  MAGOH - mago-nashi homolog, proliferation-associated (drosophila)  PRPF3 - pre-mrna processing factor 3  PRPF4 - prp4 pre-mrna processing factor 4 homolog (yeast)  SRSF10 - serine/arginine-rich splicing factor 10  PPWD1 - peptidylprolyl isomerase domain and wd repeat containing 1  NOVA2 - neuro-oncological ventral antigen 2  NOVA1 - neuro-oncological ventral antigen 1  AQR - aquarius homolog (mouse)  PRPF31 - pre-mrna processing factor 31  ZMAT2 - zinc finger, matrin-type 2  SART1 - squamous cell carcinoma antigen recognized by t cells  WDR33 - wd repeat domain 33  ZCRB1 - zinc finger cchc-type and rna binding motif 1  CWC15 - cwc15 spliceosome-associated protein homolog (s. cerevisiae)  SNRNP48 - small nuclear ribonucleoprotein 48kda (u11/u12)  NHP2L1 - nhp2 non-histone chromosome protein 2-like 1 (s. cerevisiae)  SART3 - squamous cell carcinoma antigen recognized by t cells 3  SF3B1 - splicing factor 3b, subunit 1, 155kda  SF3B3 - splicing factor 3b, subunit 3, 130kda  SMU1 - smu-1 suppressor of mec-8 and unc-52 homolog (c. elegans)  PPIL1 - peptidylprolyl isomerase (cyclophilin)-like 1  PUF60 - poly-u binding splicing factor 60kda  RAVER2 - ribonucleoprotein, ptb-binding 2  PABPC1 - poly(a) binding protein, cytoplasmic 1  SKIV2L2 - superkiller viralicidic activity 2-like 2 (s. cerevisiae)  CASC3 - cancer susceptibility candidate 3  IK - ik cytokine, down-regulator of hla ii  SFPQ - splicing factor proline/glutamine-rich  PCF11 - pcf11 cleavage and polyadenylation factor subunit  SF1 - splicing factor 1  PSPC1 - paraspeckle component 1  U2AF2 - u2 small nuclear rna auxiliary factor 2  SRSF1 - serine/arginine-rich splicing factor 1  SRRT - serrate rna effector molecule homolog (arabidopsis)  SRSF2 - serine/arginine-rich splicing factor 2  RBM4 - rna binding motif protein 4  SRSF3 - serine/arginine-rich splicing factor 3  SRSF4 - serine/arginine-rich splicing factor 4  CTNNBL1 - catenin, beta like 1  SRSF5 - serine/arginine-rich splicing factor 5  SRSF6 - serine/arginine-rich splicing factor 6  SRSF7 - serine/arginine-rich splicing factor 7  SFSWAP - splicing factor, suppressor of white-apricot homolog (drosophila)  TRA2B - transformer 2 beta homolog (drosophila)  DHX16 - deah (asp-glu-ala-his) box polypeptide 16  DDX42 - dead (asp-glu-ala-asp) box helicase 42  SRRM2 - serine/arginine repetitive matrix 2  CWF19L1 - cwf19-like 1, cell cycle control (s. pombe)  USP39 - ubiquitin specific peptidase 39  METTL3 - methyltransferase like 3  RBM4B - rna binding motif protein 4b |
| GO:0000398 | mRNA splicing, via spliceosome | 9.78E-19 | 3.4E-15 | 1.35 (10334,242,6996,221) | [+] Show genes  RSRC1 - arginine/serine-rich coiled-coil 1  NCBP2 - nuclear cap binding protein subunit 2, 20kda  SMNDC1 - survival motor neuron domain containing 1  NCBP1 - nuclear cap binding protein subunit 1, 80kda  BCAS2 - breast carcinoma amplified sequence 2  CWC27 - cwc27 spliceosome-associated protein homolog (s. cerevisiae)  ZCCHC8 - zinc finger, cchc domain containing 8  RNPC3 - rna-binding region (rnp1, rrm) containing 3  SYMPK - symplekin  SF3A3 - splicing factor 3a, subunit 3, 60kda  HNRNPA0 - heterogeneous nuclear ribonucleoprotein a0  PNN - pinin, desmosome associated protein  SF3A1 - splicing factor 3a, subunit 1, 120kda  METTL14 - methyltransferase like 14  SF3A2 - splicing factor 3a, subunit 2, 66kda  SNW1 - snw domain containing 1  CWC25 - cwc25 spliceosome-associated protein homolog (s. cerevisiae)  HNRNPM - heterogeneous nuclear ribonucleoprotein m  CWC22 - cwc22 spliceosome-associated protein homolog (s. cerevisiae)  CELF4 - cugbp, elav-like family member 4  TXNL4A - thioredoxin-like 4a  RBM6 - rna binding motif protein 6  RBM5 - rna binding motif protein 5  PLRG1 - pleiotropic regulator 1  ALYREF - aly/ref export factor  PAPOLA - poly(a) polymerase alpha  LSM2 - lsm2 homolog, u6 small nuclear rna associated (s. cerevisiae)  PABPN1 - poly(a) binding protein, nuclear 1  RNPS1 - rna binding protein s1, serine-rich domain  ELAVL1 - elav (embryonic lethal, abnormal vision, drosophila)-like 1 (hu antigen r)  SUGP1 - surp and g patch domain containing 1  PRPF38A - prp38 pre-mrna processing factor 38 (yeast) domain containing a  SNRPN - small nuclear ribonucleoprotein polypeptide n  FIP1L1 - factor interacting with papola and cpsf1  SNRPB2 - small nuclear ribonucleoprotein polypeptide b  SNRPD1 - small nuclear ribonucleoprotein d1 polypeptide 16kda  DDX41 - dead (asp-glu-ala-asp) box polypeptide 41  SPEN - spen homolog, transcriptional regulator (drosophila)  SNRPF - small nuclear ribonucleoprotein polypeptide f  PDCD7 - programmed cell death 7  SCNM1 - sodium channel modifier 1  SNRNP70 - small nuclear ribonucleoprotein 70kda (u1)  SNRPA - small nuclear ribonucleoprotein polypeptide a  SNRPA1 - small nuclear ribonucleoprotein polypeptide a'  SNRPB - small nuclear ribonucleoprotein polypeptides b and b1  PRPF4B - prp4 pre-mrna processing factor 4 homolog b (yeast)  PQBP1 - polyglutamine binding protein 1  CPSF4 - cleavage and polyadenylation specific factor 4, 30kda  RBM17 - rna binding motif protein 17  KDM1A - lysine (k)-specific demethylase 1a  U2AF1L4 - u2 small nuclear rna auxiliary factor 1-like 4  TRA2A - transformer 2 alpha homolog (drosophila)  SF3B5 - splicing factor 3b, subunit 5, 10kda  HNRNPR - heterogeneous nuclear ribonucleoprotein r  CPSF1 - cleavage and polyadenylation specific factor 1, 160kda  BUD31 - bud31 homolog (s. cerevisiae)  RBM15B - rna binding motif protein 15b  SNRNP200 - small nuclear ribonucleoprotein 200kda (u5)  RBM10 - rna binding motif protein 10  POLR2K - polymerase (rna) ii (dna directed) polypeptide k, 7.0kda  LSM5 - lsm5 homolog, u6 small nuclear rna associated (s. cerevisiae)  SRRM1 - serine/arginine repetitive matrix 1  XAB2 - xpa binding protein 2  SF3B4 - splicing factor 3b, subunit 4, 49kda  CACTIN - cactin, spliceosome c complex subunit  CRNKL1 - crooked neck pre-mrna splicing factor-like 1 (drosophila)  POLR2G - polymerase (rna) ii (dna directed) polypeptide g  POLR2F - polymerase (rna) ii (dna directed) polypeptide f  POLR2E - polymerase (rna) ii (dna directed) polypeptide e, 25kda  CDC40 - cell division cycle 40  POLR2D - polymerase (rna) ii (dna directed) polypeptide d  POLR2C - polymerase (rna) ii (dna directed) polypeptide c, 33kda  POLR2B - polymerase (rna) ii (dna directed) polypeptide b, 140kda  POLR2A - polymerase (rna) ii (dna directed) polypeptide a, 220kda  HSPA8 - heat shock 70kda protein 8  HNRNPUL1 - heterogeneous nuclear ribonucleoprotein u-like 1  TFIP11 - tuftelin interacting protein 11  CSTF1 - cleavage stimulation factor, 3' pre-rna, subunit 1, 50kda  PPIE - peptidylprolyl isomerase e (cyclophilin e)  DBR1 - debranching rna lariats 1  PRPF40B - prp40 pre-mrna processing factor 40 homolog b (s. cerevisiae)  CSTF3 - cleavage stimulation factor, 3' pre-rna, subunit 3, 77kda  PRPF6 - pre-mrna processing factor 6  CSTF2 - cleavage stimulation factor, 3' pre-rna, subunit 2, 64kda  FUS - fused in sarcoma  FRG1 - fshd region gene 1  PRCC - papillary renal cell carcinoma (translocation-associated)  PPIH - peptidylprolyl isomerase h (cyclophilin h)  GEMIN6 - gem (nuclear organelle) associated protein 6  UBL5 - ubiquitin-like 5  SRSF9 - serine/arginine-rich splicing factor 9  DCPS - decapping enzyme, scavenger  SNRNP35 - small nuclear ribonucleoprotein 35kda (u11/u12)  NUDT21 - nudix (nucleoside diphosphate linked moiety x)-type motif 21  CPSF7 - cleavage and polyadenylation specific factor 7, 59kda  DDX23 - dead (asp-glu-ala-asp) box polypeptide 23  SYNCRIP - synaptotagmin binding, cytoplasmic rna interacting protein  UPF3B - upf3 regulator of nonsense transcripts homolog b (yeast)  SNRNP40 - small nuclear ribonucleoprotein 40kda (u5)  DDX39B - dead (asp-glu-ala-asp) box polypeptide 39b  RAVER1 - ribonucleoprotein, ptb-binding 1  YTHDC1 - yth domain containing 1  BUD13 - bud13 homolog (s. cerevisiae)  HNRNPU - heterogeneous nuclear ribonucleoprotein u (scaffold attachment factor a)  HNRNPK - heterogeneous nuclear ribonucleoprotein k  HNRNPL - heterogeneous nuclear ribonucleoprotein l  PRPF40A - prp40 pre-mrna processing factor 40 homolog a (s. cerevisiae)  RBM8A - rna binding motif protein 8a  DHX35 - deah (asp-glu-ala-his) box polypeptide 35  PHF5A - phd finger protein 5a  SNIP1 - smad nuclear interacting protein 1  HNRNPA2B1 - heterogeneous nuclear ribonucleoprotein a2/b1  GEMIN7 - gem (nuclear organelle) associated protein 7  HNRNPA1 - heterogeneous nuclear ribonucleoprotein a1  HNRNPC - heterogeneous nuclear ribonucleoprotein c (c1/c2)  HNRNPF - heterogeneous nuclear ribonucleoprotein f  HNRNPD - heterogeneous nuclear ribonucleoprotein d (au-rich element rna binding protein 1, 37kda)  HNRNPH1 - heterogeneous nuclear ribonucleoprotein h1 (h)  CLP1 - cleavage and polyadenylation factor i subunit 1  HNRNPH3 - heterogeneous nuclear ribonucleoprotein h3 (2h9)  HNRNPH2 - heterogeneous nuclear ribonucleoprotein h2 (h')  EFTUD2 - elongation factor tu gtp binding domain containing 2  RBM22 - rna binding motif protein 22  CD2BP2 - cd2 (cytoplasmic tail) binding protein 2  SF3B2 - splicing factor 3b, subunit 2, 145kda  DHX9 - deah (asp-glu-ala-his) box helicase 9  DHX8 - deah (asp-glu-ala-his) box polypeptide 8  HTATSF1 - hiv-1 tat specific factor 1  SRSF11 - serine/arginine-rich splicing factor 11  DDX5 - dead (asp-glu-ala-asp) box helicase 5  CDC5L - cell division cycle 5-like  SYF2 - syf2 pre-mrna-splicing factor  RBM15 - rna binding motif protein 15  RNF113A - ring finger protein 113a  RBMX - rna binding motif protein, x-linked  GTF2F1 - general transcription factor iif, polypeptide 1, 74kda  HNRNPA3 - heterogeneous nuclear ribonucleoprotein a3  GTF2F2 - general transcription factor iif, polypeptide 2, 30kda  PCBP2 - poly(rc) binding protein 2  GEMIN5 - gem (nuclear organelle) associated protein 5  SRSF12 - serine/arginine-rich splicing factor 12  PRPF8 - pre-mrna processing factor 8  PRPF19 - pre-mrna processing factor 19  CPSF3 - cleavage and polyadenylation specific factor 3, 73kda  NAA38 - n(alpha)-acetyltransferase 38, natc auxiliary subunit  RBM19 - rna binding motif protein 19  LSM7 - lsm7 homolog, u6 small nuclear rna associated (s. cerevisiae)  DDX46 - dead (asp-glu-ala-asp) box polypeptide 46  CSTF2T - cleavage stimulation factor, 3' pre-rna, subunit 2, 64kda, tau variant  WBP11 - ww domain binding protein 11  DHX15 - deah (asp-glu-ala-his) box helicase 15  CWF19L2 - cwf19-like 2, cell cycle control (s. pombe)  U2SURP - u2 snrnp-associated surp domain containing  PTBP1 - polypyrimidine tract binding protein 1  GPATCH1 - g patch domain containing 1  EIF4A3 - eukaryotic translation initiation factor 4a3  PPIL3 - peptidylprolyl isomerase (cyclophilin)-like 3  DDX17 - dead (asp-glu-ala-asp) box helicase 17  CHERP - calcium homeostasis endoplasmic reticulum protein  CPSF2 - cleavage and polyadenylation specific factor 2, 100kda  DHX38 - deah (asp-glu-ala-his) box polypeptide 38  GPKOW - g patch domain and kow motifs  SLU7 - slu7 splicing factor homolog (s. cerevisiae)  LSM6 - lsm6 homolog, u6 small nuclear rna associated (s. cerevisiae)  MAGOHB - mago-nashi homolog b (drosophila)  CDK13 - cyclin-dependent kinase 13  MFAP1 - microfibrillar-associated protein 1  LSM3 - lsm3 homolog, u6 small nuclear rna associated (s. cerevisiae)  SNRNP25 - small nuclear ribonucleoprotein 25kda (u11/u12)  NONO - non-pou domain containing, octamer-binding  MAGOH - mago-nashi homolog, proliferation-associated (drosophila)  PRPF3 - pre-mrna processing factor 3  PRPF4 - prp4 pre-mrna processing factor 4 homolog (yeast)  SRSF10 - serine/arginine-rich splicing factor 10  PPWD1 - peptidylprolyl isomerase domain and wd repeat containing 1  NOVA2 - neuro-oncological ventral antigen 2  NOVA1 - neuro-oncological ventral antigen 1  AQR - aquarius homolog (mouse)  PRPF31 - pre-mrna processing factor 31  ZMAT2 - zinc finger, matrin-type 2  SART1 - squamous cell carcinoma antigen recognized by t cells  WDR33 - wd repeat domain 33  ZCRB1 - zinc finger cchc-type and rna binding motif 1  SNRNP48 - small nuclear ribonucleoprotein 48kda (u11/u12)  CWC15 - cwc15 spliceosome-associated protein homolog (s. cerevisiae)  NHP2L1 - nhp2 non-histone chromosome protein 2-like 1 (s. cerevisiae)  SART3 - squamous cell carcinoma antigen recognized by t cells 3  SF3B1 - splicing factor 3b, subunit 1, 155kda  SF3B3 - splicing factor 3b, subunit 3, 130kda  SMU1 - smu-1 suppressor of mec-8 and unc-52 homolog (c. elegans)  PPIL1 - peptidylprolyl isomerase (cyclophilin)-like 1  PUF60 - poly-u binding splicing factor 60kda  RAVER2 - ribonucleoprotein, ptb-binding 2  PABPC1 - poly(a) binding protein, cytoplasmic 1  SKIV2L2 - superkiller viralicidic activity 2-like 2 (s. cerevisiae)  CASC3 - cancer susceptibility candidate 3  IK - ik cytokine, down-regulator of hla ii  SFPQ - splicing factor proline/glutamine-rich  PCF11 - pcf11 cleavage and polyadenylation factor subunit  SF1 - splicing factor 1  SRSF1 - serine/arginine-rich splicing factor 1  U2AF2 - u2 small nuclear rna auxiliary factor 2  PSPC1 - paraspeckle component 1  RBM4 - rna binding motif protein 4  SRSF2 - serine/arginine-rich splicing factor 2  SRRT - serrate rna effector molecule homolog (arabidopsis)  SRSF3 - serine/arginine-rich splicing factor 3  SRSF4 - serine/arginine-rich splicing factor 4  SRSF5 - serine/arginine-rich splicing factor 5  CTNNBL1 - catenin, beta like 1  SRSF6 - serine/arginine-rich splicing factor 6  SRSF7 - serine/arginine-rich splicing factor 7  SFSWAP - splicing factor, suppressor of white-apricot homolog (drosophila)  TRA2B - transformer 2 beta homolog (drosophila)  DHX16 - deah (asp-glu-ala-his) box polypeptide 16  DDX42 - dead (asp-glu-ala-asp) box helicase 42  SRRM2 - serine/arginine repetitive matrix 2  USP39 - ubiquitin specific peptidase 39  CWF19L1 - cwf19-like 1, cell cycle control (s. pombe)  METTL3 - methyltransferase like 3  RBM4B - rna binding motif protein 4b |
| GO:0000375 | RNA splicing, via transesterification reactions | 1.06E-18 | 2.94E-15 | 1.35 (10334,247,6996,225) | [+] Show genes  RSRC1 - arginine/serine-rich coiled-coil 1  NCBP2 - nuclear cap binding protein subunit 2, 20kda  SMNDC1 - survival motor neuron domain containing 1  NCBP1 - nuclear cap binding protein subunit 1, 80kda  BCAS2 - breast carcinoma amplified sequence 2  CWC27 - cwc27 spliceosome-associated protein homolog (s. cerevisiae)  ZCCHC8 - zinc finger, cchc domain containing 8  RNPC3 - rna-binding region (rnp1, rrm) containing 3  SYMPK - symplekin  SF3A3 - splicing factor 3a, subunit 3, 60kda  HNRNPA0 - heterogeneous nuclear ribonucleoprotein a0  PNN - pinin, desmosome associated protein  SF3A1 - splicing factor 3a, subunit 1, 120kda  METTL14 - methyltransferase like 14  SF3A2 - splicing factor 3a, subunit 2, 66kda  SNW1 - snw domain containing 1  CWC25 - cwc25 spliceosome-associated protein homolog (s. cerevisiae)  HNRNPM - heterogeneous nuclear ribonucleoprotein m  CWC22 - cwc22 spliceosome-associated protein homolog (s. cerevisiae)  CELF4 - cugbp, elav-like family member 4  TXNL4A - thioredoxin-like 4a  RBM6 - rna binding motif protein 6  RBM5 - rna binding motif protein 5  PLRG1 - pleiotropic regulator 1  ALYREF - aly/ref export factor  PAPOLA - poly(a) polymerase alpha  LSM2 - lsm2 homolog, u6 small nuclear rna associated (s. cerevisiae)  PABPN1 - poly(a) binding protein, nuclear 1  RNPS1 - rna binding protein s1, serine-rich domain  ELAVL1 - elav (embryonic lethal, abnormal vision, drosophila)-like 1 (hu antigen r)  MPHOSPH10 - m-phase phosphoprotein 10 (u3 small nucleolar ribonucleoprotein)  SUGP1 - surp and g patch domain containing 1  PRPF38A - prp38 pre-mrna processing factor 38 (yeast) domain containing a  SNRPN - small nuclear ribonucleoprotein polypeptide n  FIP1L1 - factor interacting with papola and cpsf1  SNRPB2 - small nuclear ribonucleoprotein polypeptide b  SNRPD1 - small nuclear ribonucleoprotein d1 polypeptide 16kda  DDX41 - dead (asp-glu-ala-asp) box polypeptide 41  SPEN - spen homolog, transcriptional regulator (drosophila)  SNRPF - small nuclear ribonucleoprotein polypeptide f  PDCD7 - programmed cell death 7  SCNM1 - sodium channel modifier 1  SNRNP70 - small nuclear ribonucleoprotein 70kda (u1)  SNRPA - small nuclear ribonucleoprotein polypeptide a  SNRPA1 - small nuclear ribonucleoprotein polypeptide a'  SNRPB - small nuclear ribonucleoprotein polypeptides b and b1  PRPF4B - prp4 pre-mrna processing factor 4 homolog b (yeast)  PQBP1 - polyglutamine binding protein 1  CPSF4 - cleavage and polyadenylation specific factor 4, 30kda  RBM17 - rna binding motif protein 17  KDM1A - lysine (k)-specific demethylase 1a  U2AF1L4 - u2 small nuclear rna auxiliary factor 1-like 4  TRA2A - transformer 2 alpha homolog (drosophila)  SF3B5 - splicing factor 3b, subunit 5, 10kda  HNRNPR - heterogeneous nuclear ribonucleoprotein r  CPSF1 - cleavage and polyadenylation specific factor 1, 160kda  BUD31 - bud31 homolog (s. cerevisiae)  RBM15B - rna binding motif protein 15b  SNRNP200 - small nuclear ribonucleoprotein 200kda (u5)  RBM10 - rna binding motif protein 10  POLR2K - polymerase (rna) ii (dna directed) polypeptide k, 7.0kda  LSM5 - lsm5 homolog, u6 small nuclear rna associated (s. cerevisiae)  SRRM1 - serine/arginine repetitive matrix 1  XAB2 - xpa binding protein 2  SF3B4 - splicing factor 3b, subunit 4, 49kda  CACTIN - cactin, spliceosome c complex subunit  CRNKL1 - crooked neck pre-mrna splicing factor-like 1 (drosophila)  POLR2G - polymerase (rna) ii (dna directed) polypeptide g  POLR2F - polymerase (rna) ii (dna directed) polypeptide f  POLR2E - polymerase (rna) ii (dna directed) polypeptide e, 25kda  CDC40 - cell division cycle 40  POLR2D - polymerase (rna) ii (dna directed) polypeptide d  POLR2C - polymerase (rna) ii (dna directed) polypeptide c, 33kda  POLR2B - polymerase (rna) ii (dna directed) polypeptide b, 140kda  POLR2A - polymerase (rna) ii (dna directed) polypeptide a, 220kda  HSPA8 - heat shock 70kda protein 8  HNRNPUL1 - heterogeneous nuclear ribonucleoprotein u-like 1  TFIP11 - tuftelin interacting protein 11  CSTF1 - cleavage stimulation factor, 3' pre-rna, subunit 1, 50kda  PPIE - peptidylprolyl isomerase e (cyclophilin e)  DBR1 - debranching rna lariats 1  PRPF40B - prp40 pre-mrna processing factor 40 homolog b (s. cerevisiae)  CSTF3 - cleavage stimulation factor, 3' pre-rna, subunit 3, 77kda  PRPF6 - pre-mrna processing factor 6  CSTF2 - cleavage stimulation factor, 3' pre-rna, subunit 2, 64kda  FUS - fused in sarcoma  FRG1 - fshd region gene 1  PRCC - papillary renal cell carcinoma (translocation-associated)  PPIH - peptidylprolyl isomerase h (cyclophilin h)  GEMIN6 - gem (nuclear organelle) associated protein 6  UBL5 - ubiquitin-like 5  SRSF9 - serine/arginine-rich splicing factor 9  DCPS - decapping enzyme, scavenger  SNRNP35 - small nuclear ribonucleoprotein 35kda (u11/u12)  NUDT21 - nudix (nucleoside diphosphate linked moiety x)-type motif 21  CPSF7 - cleavage and polyadenylation specific factor 7, 59kda  DDX23 - dead (asp-glu-ala-asp) box polypeptide 23  SYNCRIP - synaptotagmin binding, cytoplasmic rna interacting protein  UPF3B - upf3 regulator of nonsense transcripts homolog b (yeast)  SNRNP40 - small nuclear ribonucleoprotein 40kda (u5)  DDX39B - dead (asp-glu-ala-asp) box polypeptide 39b  RAVER1 - ribonucleoprotein, ptb-binding 1  YTHDC1 - yth domain containing 1  BUD13 - bud13 homolog (s. cerevisiae)  HNRNPU - heterogeneous nuclear ribonucleoprotein u (scaffold attachment factor a)  HNRNPK - heterogeneous nuclear ribonucleoprotein k  HNRNPL - heterogeneous nuclear ribonucleoprotein l  PRPF40A - prp40 pre-mrna processing factor 40 homolog a (s. cerevisiae)  RBM8A - rna binding motif protein 8a  DHX35 - deah (asp-glu-ala-his) box polypeptide 35  PHF5A - phd finger protein 5a  SNIP1 - smad nuclear interacting protein 1  HNRNPA2B1 - heterogeneous nuclear ribonucleoprotein a2/b1  GEMIN7 - gem (nuclear organelle) associated protein 7  HNRNPA1 - heterogeneous nuclear ribonucleoprotein a1  HNRNPC - heterogeneous nuclear ribonucleoprotein c (c1/c2)  HNRNPF - heterogeneous nuclear ribonucleoprotein f  HNRNPD - heterogeneous nuclear ribonucleoprotein d (au-rich element rna binding protein 1, 37kda)  HNRNPH1 - heterogeneous nuclear ribonucleoprotein h1 (h)  CLP1 - cleavage and polyadenylation factor i subunit 1  HNRNPH3 - heterogeneous nuclear ribonucleoprotein h3 (2h9)  HNRNPH2 - heterogeneous nuclear ribonucleoprotein h2 (h')  EFTUD2 - elongation factor tu gtp binding domain containing 2  RBM22 - rna binding motif protein 22  CD2BP2 - cd2 (cytoplasmic tail) binding protein 2  SF3B2 - splicing factor 3b, subunit 2, 145kda  DHX9 - deah (asp-glu-ala-his) box helicase 9  DHX8 - deah (asp-glu-ala-his) box polypeptide 8  HTATSF1 - hiv-1 tat specific factor 1  SRSF11 - serine/arginine-rich splicing factor 11  DDX5 - dead (asp-glu-ala-asp) box helicase 5  CDC5L - cell division cycle 5-like  SYF2 - syf2 pre-mrna-splicing factor  RBM15 - rna binding motif protein 15  RNF113A - ring finger protein 113a  RBMX - rna binding motif protein, x-linked  GTF2F1 - general transcription factor iif, polypeptide 1, 74kda  HNRNPA3 - heterogeneous nuclear ribonucleoprotein a3  GTF2F2 - general transcription factor iif, polypeptide 2, 30kda  PCBP2 - poly(rc) binding protein 2  GEMIN5 - gem (nuclear organelle) associated protein 5  SRSF12 - serine/arginine-rich splicing factor 12  PRPF8 - pre-mrna processing factor 8  PRPF19 - pre-mrna processing factor 19  CPSF3 - cleavage and polyadenylation specific factor 3, 73kda  NAA38 - n(alpha)-acetyltransferase 38, natc auxiliary subunit  RBM19 - rna binding motif protein 19  LSM7 - lsm7 homolog, u6 small nuclear rna associated (s. cerevisiae)  DDX46 - dead (asp-glu-ala-asp) box polypeptide 46  CSTF2T - cleavage stimulation factor, 3' pre-rna, subunit 2, 64kda, tau variant  WBP11 - ww domain binding protein 11  DHX15 - deah (asp-glu-ala-his) box helicase 15  CWF19L2 - cwf19-like 2, cell cycle control (s. pombe)  U2SURP - u2 snrnp-associated surp domain containing  PTBP1 - polypyrimidine tract binding protein 1  GPATCH1 - g patch domain containing 1  EIF4A3 - eukaryotic translation initiation factor 4a3  KHSRP - kh-type splicing regulatory protein  PPIL3 - peptidylprolyl isomerase (cyclophilin)-like 3  DDX17 - dead (asp-glu-ala-asp) box helicase 17  CHERP - calcium homeostasis endoplasmic reticulum protein  CPSF2 - cleavage and polyadenylation specific factor 2, 100kda  DHX38 - deah (asp-glu-ala-his) box polypeptide 38  GPKOW - g patch domain and kow motifs  SLU7 - slu7 splicing factor homolog (s. cerevisiae)  LSM6 - lsm6 homolog, u6 small nuclear rna associated (s. cerevisiae)  SCAF11 - sr-related ctd-associated factor 11  MAGOHB - mago-nashi homolog b (drosophila)  CDK13 - cyclin-dependent kinase 13  MFAP1 - microfibrillar-associated protein 1  LSM3 - lsm3 homolog, u6 small nuclear rna associated (s. cerevisiae)  SNRNP25 - small nuclear ribonucleoprotein 25kda (u11/u12)  NONO - non-pou domain containing, octamer-binding  MAGOH - mago-nashi homolog, proliferation-associated (drosophila)  PRPF3 - pre-mrna processing factor 3  PRPF4 - prp4 pre-mrna processing factor 4 homolog (yeast)  SRSF10 - serine/arginine-rich splicing factor 10  PPWD1 - peptidylprolyl isomerase domain and wd repeat containing 1  NOVA2 - neuro-oncological ventral antigen 2  NOVA1 - neuro-oncological ventral antigen 1  AQR - aquarius homolog (mouse)  PRPF31 - pre-mrna processing factor 31  ZMAT2 - zinc finger, matrin-type 2  SART1 - squamous cell carcinoma antigen recognized by t cells  WDR33 - wd repeat domain 33  ZCRB1 - zinc finger cchc-type and rna binding motif 1  SNRNP48 - small nuclear ribonucleoprotein 48kda (u11/u12)  CWC15 - cwc15 spliceosome-associated protein homolog (s. cerevisiae)  NHP2L1 - nhp2 non-histone chromosome protein 2-like 1 (s. cerevisiae)  SART3 - squamous cell carcinoma antigen recognized by t cells 3  SF3B1 - splicing factor 3b, subunit 1, 155kda  SF3B3 - splicing factor 3b, subunit 3, 130kda  SMU1 - smu-1 suppressor of mec-8 and unc-52 homolog (c. elegans)  PPIL1 - peptidylprolyl isomerase (cyclophilin)-like 1  PUF60 - poly-u binding splicing factor 60kda  RAVER2 - ribonucleoprotein, ptb-binding 2  PABPC1 - poly(a) binding protein, cytoplasmic 1  SKIV2L2 - superkiller viralicidic activity 2-like 2 (s. cerevisiae)  CASC3 - cancer susceptibility candidate 3  IK - ik cytokine, down-regulator of hla ii  SFPQ - splicing factor proline/glutamine-rich  PCF11 - pcf11 cleavage and polyadenylation factor subunit  SF1 - splicing factor 1  SRSF1 - serine/arginine-rich splicing factor 1  U2AF2 - u2 small nuclear rna auxiliary factor 2  PSPC1 - paraspeckle component 1  RBM4 - rna binding motif protein 4  SRSF2 - serine/arginine-rich splicing factor 2  SRRT - serrate rna effector molecule homolog (arabidopsis)  SRSF3 - serine/arginine-rich splicing factor 3  SRSF4 - serine/arginine-rich splicing factor 4  SRSF5 - serine/arginine-rich splicing factor 5  CTNNBL1 - catenin, beta like 1  SRSF6 - serine/arginine-rich splicing factor 6  SRSF7 - serine/arginine-rich splicing factor 7  GEMIN2 - gem (nuclear organelle) associated protein 2  SFSWAP - splicing factor, suppressor of white-apricot homolog (drosophila)  TRA2B - transformer 2 beta homolog (drosophila)  DHX16 - deah (asp-glu-ala-his) box polypeptide 16  DDX42 - dead (asp-glu-ala-asp) box helicase 42  SRRM2 - serine/arginine repetitive matrix 2  USP39 - ubiquitin specific peptidase 39  CWF19L1 - cwf19-like 1, cell cycle control (s. pombe)  METTL3 - methyltransferase like 3  RBM4B - rna binding motif protein 4b |
| GO:0006325 | chromatin organization | 1.28E-18 | 2.98E-15 | 1.25 (10334,482,6996,409) | [+] Show genes  PCGF5 - polycomb group ring finger 5  WAC - ww domain containing adaptor with coiled-coil  MSL3 - male-specific lethal 3 homolog (drosophila)  PHF21A - phd finger protein 21a  TADA1 - transcriptional adaptor 1  NCOR1 - nuclear receptor corepressor 1  DOT1L - dot1-like histone h3k79 methyltransferase  MIER1 - mesoderm induction early response 1, transcriptional regulator  KAT2A - k(lysine) acetyltransferase 2a  NCOA3 - nuclear receptor coactivator 3  L3MBTL3 - l(3)mbt-like 3 (drosophila)  BRD8 - bromodomain containing 8  MUM1 - melanoma associated antigen (mutated) 1  CHD7 - chromodomain helicase dna binding protein 7  CHD1L - chromodomain helicase dna binding protein 1-like  H2AFY - h2a histone family, member y  ACTB - actin, beta  EHMT2 - euchromatic histone-lysine n-methyltransferase 2  SPIN1 - spindlin 1  GATAD1 - gata zinc finger domain containing 1  MORF4L1 - mortality factor 4 like 1  CREBBP - creb binding protein  ATF2 - activating transcription factor 2  CLOCK - clock circadian regulator  ACTL6A - actin-like 6a  TRRAP - transformation/transcription domain-associated protein  HDAC11 - histone deacetylase 11  ARID1A - at rich interactive domain 1a (swi-like)  SETD6 - set domain containing 6  ACTR5 - arp5 actin-related protein 5 homolog (yeast)  SMARCE1 - swi/snf related, matrix associated, actin dependent regulator of chromatin, subfamily e, member 1  NAA60 - n(alpha)-acetyltransferase 60, natf catalytic subunit  BAP1 - brca1 associated protein-1 (ubiquitin carboxy-terminal hydrolase)  ACTL6B - actin-like 6b  SMARCB1 - swi/snf related, matrix associated, actin dependent regulator of chromatin, subfamily b, member 1  KDM5C - lysine (k)-specific demethylase 5c  SMARCC1 - swi/snf related, matrix associated, actin dependent regulator of chromatin, subfamily c, member 1  HLTF - helicase-like transcription factor  SMARCA4 - swi/snf related, matrix associated, actin dependent regulator of chromatin, subfamily a, member 4  SMARCD2 - swi/snf related, matrix associated, actin dependent regulator of chromatin, subfamily d, member 2  SMARCD3 - swi/snf related, matrix associated, actin dependent regulator of chromatin, subfamily d, member 3  SMARCC2 - swi/snf related, matrix associated, actin dependent regulator of chromatin, subfamily c, member 2  SMARCD1 - swi/snf related, matrix associated, actin dependent regulator of chromatin, subfamily d, member 1  SMARCA1 - swi/snf related, matrix associated, actin dependent regulator of chromatin, subfamily a, member 1  SMARCA2 - swi/snf related, matrix associated, actin dependent regulator of chromatin, subfamily a, member 2  H2AFY2 - h2a histone family, member y2  PHF17 - phd finger protein 17  KMT2C - lysine (k)-specific methyltransferase 2c  ZBTB7A - zinc finger and btb domain containing 7a  DPY30 - dpy-30 homolog (c. elegans)  ZNF462 - zinc finger protein 462  NAP1L5 - nucleosome assembly protein 1-like 5  RUVBL2 - ruvb-like 2 (e. coli)  CHD6 - chromodomain helicase dna binding protein 6  RCOR3 - rest corepressor 3  EHMT1 - euchromatic histone-lysine n-methyltransferase 1  KDM2B - lysine (k)-specific demethylase 2b  SUPT6H - suppressor of ty 6 homolog (s. cerevisiae)  SUPV3L1 - suppressor of var1, 3-like 1 (s. cerevisiae)  NAA40 - n(alpha)-acetyltransferase 40, natd catalytic subunit, homolog (s. cerevisiae)  CTBP1 - c-terminal binding protein 1  SUPT4H1 - suppressor of ty 4 homolog 1 (s. cerevisiae)  ING4 - inhibitor of growth family, member 4  PRDM4 - pr domain containing 4  PPP5C - protein phosphatase 5, catalytic subunit  CTNNB1 - catenin (cadherin-associated protein), beta 1, 88kda  USP7 - ubiquitin specific peptidase 7 (herpes virus-associated)  RNF168 - ring finger protein 168, e3 ubiquitin protein ligase  SUV420H1 - suppressor of variegation 4-20 homolog 1 (drosophila)  BRD1 - bromodomain containing 1  PCGF1 - polycomb group ring finger 1  AURKC - aurora kinase c  PHF20L1 - phd finger protein 20-like 1  SUV420H2 - suppressor of variegation 4-20 homolog 2 (drosophila)  KANSL3 - kat8 regulatory nsl complex subunit 3  NIPBL - nipped-b homolog (drosophila)  ASF1A - anti-silencing function 1a histone chaperone  KMT2D - lysine (k)-specific methyltransferase 2d  JAK2 - janus kinase 2  ING5 - inhibitor of growth family, member 5  BRMS1 - breast cancer metastasis suppressor 1  YEATS4 - yeats domain containing 4  PHF20 - phd finger protein 20  ZFPM1 - zinc finger protein, fog family member 1  SRPK1 - srsf protein kinase 1  HMGN3 - high mobility group nucleosomal binding domain 3  KAT6A - k(lysine) acetyltransferase 6a  ZNF335 - zinc finger protein 335  TLK2 - tousled-like kinase 2  PRKCB - protein kinase c, beta  BRD3 - bromodomain containing 3  PRKCD - protein kinase c, delta  BRMS1L - breast cancer metastasis-suppressor 1-like  LEF1 - lymphoid enhancer-binding factor 1  PRKCA - protein kinase c, alpha  GTF3C4 - general transcription factor iiic, polypeptide 4, 90kda  YEATS2 - yeats domain containing 2  SHPRH - snf2 histone linker phd ring helicase, e3 ubiquitin protein ligase  ARID4B - at rich interactive domain 4b (rbp1-like)  SIN3A - sin3 transcription regulator family member a  SET - set nuclear oncogene  DDB1 - damage-specific dna binding protein 1, 127kda  SETMAR - set domain and mariner transposase fusion gene  PRMT6 - protein arginine methyltransferase 6  MSL2 - male-specific lethal 2 homolog (drosophila)  ZNF304 - zinc finger protein 304  KDM3B - lysine (k)-specific demethylase 3b  RSF1 - remodeling and spacing factor 1  CSRP2BP - csrp2 binding protein  GTF2B - general transcription factor iib  PCGF2 - polycomb group ring finger 2  KMT2A - lysine (k)-specific methyltransferase 2a  APBB1 - amyloid beta (a4) precursor protein-binding, family b, member 1 (fe65)  RIF1 - rap1 interacting factor homolog (yeast)  RPS6KA5 - ribosomal protein s6 kinase, 90kda, polypeptide 5  TBL1XR1 - transducin (beta)-like 1 x-linked receptor 1  PBRM1 - polybromo 1  IPO4 - importin 4  UIMC1 - ubiquitin interaction motif containing 1  GATAD2B - gata zinc finger domain containing 2b  MTA2 - metastasis associated 1 family, member 2  SETD5 - set domain containing 5  MLF1IP - mlf1 interacting protein  RCBTB1 - regulator of chromosome condensation (rcc1) and btb (poz) domain containing protein 1  H3F3A - h3 histone, family 3a  HIST1H2BB - histone cluster 1, h2bb  BRPF1 - bromodomain and phd finger containing, 1  H2AFZ - h2a histone family, member z  H2AFX - h2a histone family, member x  SUPT16H - suppressor of ty 16 homolog (s. cerevisiae)  SPTY2D1 - spt2, suppressor of ty, domain containing 1 (s. cerevisiae)  ARRB1 - arrestin, beta 1  SS18L1 - synovial sarcoma translocation gene on chromosome 18-like 1  CDC73 - cell division cycle 73  CHD5 - chromodomain helicase dna binding protein 5  MECP2 - methyl cpg binding protein 2 (rett syndrome)  ARID1B - at rich interactive domain 1b (swi1-like)  BRWD1 - bromodomain and wd repeat domain containing 1  BAZ2A - bromodomain adjacent to zinc finger domain, 2a  HCFC1 - host cell factor c1 (vp16-accessory protein)  MEN1 - multiple endocrine neoplasia i  DAPK3 - death-associated protein kinase 3  DAXX - death-domain associated protein  MAP3K12 - mitogen-activated protein kinase kinase kinase 12  ZZZ3 - zinc finger, zz-type containing 3  KAT8 - k(lysine) acetyltransferase 8  LEO1 - leo1, paf1/rna polymerase ii complex component, homolog (s. cerevisiae)  MTA3 - metastasis associated 1 family, member 3  PWP1 - pwp1 homolog (s. cerevisiae)  HDAC2 - histone deacetylase 2  SATB1 - satb homeobox 1  MBD3 - methyl-cpg binding domain protein 3  HDAC1 - histone deacetylase 1  SAFB - scaffold attachment factor b  KAT7 - k(lysine) acetyltransferase 7  EPC2 - enhancer of polycomb homolog 2 (drosophila)  ZBTB1 - zinc finger and btb domain containing 1  PHF19 - phd finger protein 19  FAM50A - family with sequence similarity 50, member a  BAHD1 - bromo adjacent homology domain containing 1  PAGR1 - paxip1 associated glutamate-rich protein 1  MORC2 - morc family cw-type zinc finger 2  PHF2 - phd finger protein 2  PHF1 - phd finger protein 1  RERE - arginine-glutamic acid dipeptide (re) repeats  MTA1 - metastasis associated 1  SIRT6 - sirtuin 6  DR1 - down-regulator of transcription 1, tbp-binding (negative cofactor 2)  SKP1 - s-phase kinase-associated protein 1  WHSC1 - wolf-hirschhorn syndrome candidate 1  SFMBT1 - scm-like with four mbt domains 1  AEBP2 - ae binding protein 2  ZMIZ2 - zinc finger, miz-type containing 2  TRMT112 - trna methyltransferase 11-2 homolog (s. cerevisiae)  USP36 - ubiquitin specific peptidase 36  VRK1 - vaccinia related kinase 1  CDAN1 - codanin 1  MCM2 - minichromosome maintenance complex component 2  ASH2L - ash2 (absent, small, or homeotic)-like (drosophila)  ARID2 - at rich interactive domain 2 (arid, rfx-like)  COPRS - coordinator of prmt5, differentiation stimulator  MTF2 - metal response element binding transcription factor 2  L3MBTL2 - l(3)mbt-like 2 (drosophila)  BRCC3 - brca1/brca2-containing complex, subunit 3  EP400 - e1a binding protein p400  ASXL1 - additional sex combs like 1 (drosophila)  CENPO - centromere protein o  ATRX - alpha thalassemia/mental retardation syndrome x-linked  RNF8 - ring finger protein 8, e3 ubiquitin protein ligase  BAZ1B - bromodomain adjacent to zinc finger domain, 1b  SFPQ - splicing factor proline/glutamine-rich  PHF10 - phd finger protein 10  DNMT1 - dna (cytosine-5-)-methyltransferase 1  ZNF711 - zinc finger protein 711  MBIP - map3k12 binding inhibitory protein 1  HDAC7 - histone deacetylase 7  DNMT3A - dna (cytosine-5-)-methyltransferase 3 alpha  MRGBP - mrg/morf4l binding protein  CHD8 - chromodomain helicase dna binding protein 8  RPS6KA4 - ribosomal protein s6 kinase, 90kda, polypeptide 4  NOC2L - nucleolar complex associated 2 homolog (s. cerevisiae)  H1FX - h1 histone family, member x  PARK7 - parkinson protein 7  SMARCAD1 - swi/snf-related, matrix-associated actin-dependent regulator of chromatin, subfamily a, containing dead/h box 1  ANP32E - acidic (leucine-rich) nuclear phosphoprotein 32 family, member e  WHSC1L1 - wolf-hirschhorn syndrome candidate 1-like 1  ELK4 - elk4, ets-domain protein (srf accessory protein 1)  CENPV - centromere protein v  RELA - v-rel avian reticuloendotheliosis viral oncogene homolog a  PRMT1 - protein arginine methyltransferase 1  TRIM28 - tripartite motif containing 28  BCOR - bcl6 corepressor  PRMT2 - protein arginine methyltransferase 2  EP300 - e1a binding protein p300  NAP1L1 - nucleosome assembly protein 1-like 1  NASP - nuclear autoantigenic sperm protein (histone-binding)  DPF2 - d4, zinc and double phd fingers family 2  NAP1L4 - nucleosome assembly protein 1-like 4  TDRD3 - tudor domain containing 3  RLF - rearranged l-myc fusion  RING1 - ring finger protein 1  HIST3H2BB - histone cluster 3, h2bb  SPHK2 - sphingosine kinase 2  FBLL1 - fibrillarin-like 1  TSPYL1 - tspy-like 1  BMI1 - bmi1 polycomb ring finger oncogene  KDM2A - lysine (k)-specific demethylase 2a  PCGF3 - polycomb group ring finger 3  LDB1 - lim domain binding 1  KAT2B - k(lysine) acetyltransferase 2b  CRTC2 - creb regulated transcription coactivator 2  CENPM - centromere protein m  PHC1 - polyhomeotic homolog 1 (drosophila)  HUWE1 - hect, uba and wwe domain containing 1, e3 ubiquitin protein ligase  HCFC2 - host cell factor c2  UBN1 - ubinuclein 1  KDM4B - lysine (k)-specific demethylase 4b  TOP1 - topoisomerase (dna) i  BRD2 - bromodomain containing 2  KDM1A - lysine (k)-specific demethylase 1a  RNF2 - ring finger protein 2  ATXN7L3 - ataxin 7-like 3  INO80B - ino80 complex subunit b  MCM3AP - minichromosome maintenance complex component 3 associated protein  TET2 - tet methylcytosine dioxygenase 2  PHF13 - phd finger protein 13  ENY2 - enhancer of yellow 2 homolog (drosophila)  MBTD1 - mbt domain containing 1  C11orf30 - chromosome 11 open reading frame 30  TRIM37 - tripartite motif containing 37  ZMPSTE24 - zinc metallopeptidase ste24  SETD1B - set domain containing 1b  KDM4C - lysine (k)-specific demethylase 4c  MCRS1 - microspherule protein 1  HMGN1 - high mobility group nucleosome binding domain 1  HMGB3 - high mobility group box 3  NRDE2 - nrde-2, necessary for rna interference, domain containing  HMGB2 - high mobility group box 2  HMGB1 - high mobility group box 1  HLCS - holocarboxylase synthetase (biotin-(proprionyl-coa-carboxylase (atp-hydrolysing)) ligase)  BRD9 - bromodomain containing 9  BPTF - bromodomain phd finger transcription factor  KANSL1 - kat8 regulatory nsl complex subunit 1  HDAC5 - histone deacetylase 5  HDAC6 - histone deacetylase 6  MT3 - metallothionein 3  UTP3 - utp3, small subunit (ssu) processome component, homolog (s. cerevisiae)  KDM6B - lysine (k)-specific demethylase 6b  PHF8 - phd finger protein 8  CHAF1A - chromatin assembly factor 1, subunit a (p150)  TADA3 - transcriptional adaptor 3  KDM6A - lysine (k)-specific demethylase 6a  EYA3 - eyes absent homolog 3 (drosophila)  EZH1 - enhancer of zeste homolog 1 (drosophila)  TTLL12 - tubulin tyrosine ligase-like family, member 12  KANSL1L - kat8 regulatory nsl complex subunit 1-like  ELMSAN1 - elm2 and myb/sant-like domain containing 1  NPM3 - nucleophosmin/nucleoplasmin 3  EED - embryonic ectoderm development  UBE2A - ubiquitin-conjugating enzyme e2a  INO80C - ino80 complex subunit c  HMG20A - high mobility group 20a  BANP - btg3 associated nuclear protein  SUPT7L - suppressor of ty 7 (s. cerevisiae)-like  HNRNPU - heterogeneous nuclear ribonucleoprotein u (scaffold attachment factor a)  RCOR1 - rest corepressor 1  HIRA - histone cell cycle regulator  ZNFX1 - zinc finger, nfx1-type containing 1  ZMIZ1 - zinc finger, miz-type containing 1  C17orf49 - chromosome 17 open reading frame 49  USP3 - ubiquitin specific peptidase 3  BABAM1 - brisc and brca1 a complex member 1  HNRNPC - heterogeneous nuclear ribonucleoprotein c (c1/c2)  USP15 - ubiquitin specific peptidase 15  KANSL2 - kat8 regulatory nsl complex subunit 2  JMJD6 - jumonji domain containing 6  SETD2 - set domain containing 2  RBM14 - rna binding motif protein 14  HMGA1 - high mobility group at-hook 1  PRMT5 - protein arginine methyltransferase 5  UBE2N - ubiquitin-conjugating enzyme e2n  VPS72 - vacuolar protein sorting 72 homolog (s. cerevisiae)  HAT1 - histone acetyltransferase 1  DTX3L - deltex 3-like (drosophila)  TSPYL4 - tspy-like 4  SETDB1 - set domain, bifurcated 1  TLK1 - tousled-like kinase 1  MEAF6 - myst/esa1-associated factor 6  ING2 - inhibitor of growth family, member 2  PPM1D - protein phosphatase, mg2+/mn2+ dependent, 1d  USP16 - ubiquitin specific peptidase 16  HDAC8 - histone deacetylase 8  ASH1L - ash1 (absent, small, or homeotic)-like (drosophila)  SIN3B - sin3 transcription regulator family member b  SMYD3 - set and mynd domain containing 3  HR - hair growth associated  TAF6L - taf6-like rna polymerase ii, p300/cbp-associated factor (pcaf)-associated factor, 65kda  TDG - thymine-dna glycosylase  CBX4 - chromobox homolog 4  KDM3A - lysine (k)-specific demethylase 3a  WDR61 - wd repeat domain 61  APITD1 - apoptosis-inducing, taf9-like domain 1  TAF9 - taf9 rna polymerase ii, tata box binding protein (tbp)-associated factor, 32kda  CENPC - centromere protein c  TAF6 - taf6 rna polymerase ii, tata box binding protein (tbp)-associated factor, 80kda  TADA2A - transcriptional adaptor 2a  SMCHD1 - structural maintenance of chromosomes flexible hinge domain containing 1  ANP32B - acidic (leucine-rich) nuclear phosphoprotein 32 family, member b  PHF15 - phd finger protein 15  USP22 - ubiquitin specific peptidase 22  CHRAC1 - chromatin accessibility complex 1  SUDS3 - suppressor of defective silencing 3 homolog (s. cerevisiae)  KAT5 - k(lysine) acetyltransferase 5  MAP3K7 - mitogen-activated protein kinase kinase kinase 7  ACTR6 - arp6 actin-related protein 6 homolog (yeast)  CDK5 - cyclin-dependent kinase 5  INO80E - ino80 complex subunit e  PTMA - prothymosin, alpha  NPM1 - nucleophosmin (nucleolar phosphoprotein b23, numatrin)  KMT2E - lysine (k)-specific methyltransferase 2e  UFL1 - ufm1-specific ligase 1  RUVBL1 - ruvb-like 1 (e. coli)  RNF40 - ring finger protein 40, e3 ubiquitin protein ligase  DMAP1 - dna methyltransferase 1 associated protein 1  CDK2 - cyclin-dependent kinase 2  CHD9 - chromodomain helicase dna binding protein 9  VPRBP - vpr (hiv-1) binding protein  ITGB3BP - integrin beta 3 binding protein (beta3-endonexin)  LRWD1 - leucine-rich repeats and wd repeat domain containing 1  NSD1 - nuclear receptor binding set domain protein 1  RSBN1 - round spermatid basic protein 1  RYBP - ring1 and yy1 binding protein  ZMYND11 - zinc finger, mynd-type containing 11  KDM5B - lysine (k)-specific demethylase 5b  SIRT1 - sirtuin 1  BAZ2B - bromodomain adjacent to zinc finger domain, 2b  BRPF3 - bromodomain and phd finger containing, 3  HDAC4 - histone deacetylase 4  CHD4 - chromodomain helicase dna binding protein 4  TOX - thymocyte selection-associated high mobility group box  CHD2 - chromodomain helicase dna binding protein 2  CBX6 - chromobox homolog 6  CHD3 - chromodomain helicase dna binding protein 3  ACTR8 - arp8 actin-related protein 8 homolog (yeast)  CHD1 - chromodomain helicase dna binding protein 1  CXXC1 - cxxc finger protein 1  CENPT - centromere protein t  KMT2B - lysine (k)-specific methyltransferase 2b  BRD4 - bromodomain containing 4  PAF1 - paf1, rna polymerase ii associated factor, homolog (s. cerevisiae)  MSL1 - male-specific lethal 1 homolog (drosophila)  HDAC9 - histone deacetylase 9  SART3 - squamous cell carcinoma antigen recognized by t cells 3  INO80 - ino80 complex subunit  SETD1A - set domain containing 1a  SUZ12 - suz12 polycomb repressive complex 2 subunit  RCOR2 - rest corepressor 2  MIER2 - mesoderm induction early response 1, family member 2  TFAM - transcription factor a, mitochondrial  CTR9 - ctr9, paf1/rna polymerase ii complex component  PPM1F - protein phosphatase, mg2+/mn2+ dependent, 1f  EPC1 - enhancer of polycomb homolog 1 (drosophila)  CBX7 - chromobox homolog 7  MORF4L2 - mortality factor 4 like 2  USP21 - ubiquitin specific peptidase 21  ING3 - inhibitor of growth family, member 3  CTCF - ccctc-binding factor (zinc finger protein)  PRMT7 - protein arginine methyltransferase 7  JMJD1C - jumonji domain containing 1c  RBL2 - retinoblastoma-like 2 (p130)  RBBP7 - retinoblastoma binding protein 7  SMARCA5 - swi/snf related, matrix associated, actin dependent regulator of chromatin, subfamily a, member 5  OGT - o-linked n-acetylglucosamine (glcnac) transferase  RB1 - retinoblastoma 1  ARID4A - at rich interactive domain 4a (rbp1-like)  HIRIP3 - hira interacting protein 3  H2AFV - h2a histone family, member v  RBBP5 - retinoblastoma binding protein 5  TSPYL2 - tspy-like 2  RNF20 - ring finger protein 20, e3 ubiquitin protein ligase  KDM5A - lysine (k)-specific demethylase 5a  RBBP4 - retinoblastoma binding protein 4  KDM4A - lysine (k)-specific demethylase 4a  CUL4B - cullin 4b  PHF14 - phd finger protein 14  CABIN1 - calcineurin binding protein 1  KAT6B - k(lysine) acetyltransferase 6b  HP1BP3 - heterochromatin protein 1, binding protein 3  SUPT3H - suppressor of ty 3 homolog (s. cerevisiae) |
| GO:0008380 | RNA splicing | 7.9E-18 | 1.57E-14 | 1.30 (10334,333,6996,292) | [+] Show genes  RSRC1 - arginine/serine-rich coiled-coil 1  SMNDC1 - survival motor neuron domain containing 1  NCBP2 - nuclear cap binding protein subunit 2, 20kda  WTAP - wilms tumor 1 associated protein  BCAS2 - breast carcinoma amplified sequence 2  NCBP1 - nuclear cap binding protein subunit 1, 80kda  CWC27 - cwc27 spliceosome-associated protein homolog (s. cerevisiae)  SAP18 - sin3a-associated protein, 18kda  ZCCHC8 - zinc finger, cchc domain containing 8  RBM39 - rna binding motif protein 39  SYMPK - symplekin  RNPC3 - rna-binding region (rnp1, rrm) containing 3  SF3A3 - splicing factor 3a, subunit 3, 60kda  MBNL2 - muscleblind-like splicing regulator 2  HNRNPA0 - heterogeneous nuclear ribonucleoprotein a0  SUGP2 - surp and g patch domain containing 2  PNN - pinin, desmosome associated protein  SF3A1 - splicing factor 3a, subunit 1, 120kda  METTL14 - methyltransferase like 14  SF3A2 - splicing factor 3a, subunit 2, 66kda  SNW1 - snw domain containing 1  CWC25 - cwc25 spliceosome-associated protein homolog (s. cerevisiae)  HNRNPM - heterogeneous nuclear ribonucleoprotein m  HABP4 - hyaluronan binding protein 4  CWC22 - cwc22 spliceosome-associated protein homolog (s. cerevisiae)  TXNL4A - thioredoxin-like 4a  CELF4 - cugbp, elav-like family member 4  CIR1 - corepressor interacting with rbpj, 1  RBM6 - rna binding motif protein 6  RBM5 - rna binding motif protein 5  PLRG1 - pleiotropic regulator 1  ALYREF - aly/ref export factor  PAPOLA - poly(a) polymerase alpha  LSM2 - lsm2 homolog, u6 small nuclear rna associated (s. cerevisiae)  TCERG1 - transcription elongation regulator 1  PABPN1 - poly(a) binding protein, nuclear 1  TAF15 - taf15 rna polymerase ii, tata box binding protein (tbp)-associated factor, 68kda  RNPS1 - rna binding protein s1, serine-rich domain  SON - son dna binding protein  ELAVL1 - elav (embryonic lethal, abnormal vision, drosophila)-like 1 (hu antigen r)  CCDC130 - coiled-coil domain containing 130  ZNF326 - zinc finger protein 326  ELAVL4 - elav (embryonic lethal, abnormal vision, drosophila)-like 4  MPHOSPH10 - m-phase phosphoprotein 10 (u3 small nucleolar ribonucleoprotein)  SUGP1 - surp and g patch domain containing 1  PRPF38A - prp38 pre-mrna processing factor 38 (yeast) domain containing a  ACIN1 - apoptotic chromatin condensation inducer 1  FIP1L1 - factor interacting with papola and cpsf1  SNRPN - small nuclear ribonucleoprotein polypeptide n  SNRPB2 - small nuclear ribonucleoprotein polypeptide b  ZNF259 - zinc finger protein 259  SNRPD1 - small nuclear ribonucleoprotein d1 polypeptide 16kda  DDX41 - dead (asp-glu-ala-asp) box polypeptide 41  SPEN - spen homolog, transcriptional regulator (drosophila)  SNRPF - small nuclear ribonucleoprotein polypeptide f  PDCD7 - programmed cell death 7  SCNM1 - sodium channel modifier 1  SNRNP70 - small nuclear ribonucleoprotein 70kda (u1)  SNRPA - small nuclear ribonucleoprotein polypeptide a  SNRPA1 - small nuclear ribonucleoprotein polypeptide a'  SNRPB - small nuclear ribonucleoprotein polypeptides b and b1  PRPF4B - prp4 pre-mrna processing factor 4 homolog b (yeast)  TSEN34 - tsen34 trna splicing endonuclease subunit  PQBP1 - polyglutamine binding protein 1  CPSF4 - cleavage and polyadenylation specific factor 4, 30kda  TRA2A - transformer 2 alpha homolog (drosophila)  U2AF1L4 - u2 small nuclear rna auxiliary factor 1-like 4  KDM1A - lysine (k)-specific demethylase 1a  RBM17 - rna binding motif protein 17  CPSF1 - cleavage and polyadenylation specific factor 1, 160kda  HNRNPR - heterogeneous nuclear ribonucleoprotein r  SF3B5 - splicing factor 3b, subunit 5, 10kda  BUD31 - bud31 homolog (s. cerevisiae)  RBM15B - rna binding motif protein 15b  SNRNP200 - small nuclear ribonucleoprotein 200kda (u5)  RBM10 - rna binding motif protein 10  LSM5 - lsm5 homolog, u6 small nuclear rna associated (s. cerevisiae)  POLR2K - polymerase (rna) ii (dna directed) polypeptide k, 7.0kda  SRRM1 - serine/arginine repetitive matrix 1  TSEN15 - tsen15 trna splicing endonuclease subunit  XAB2 - xpa binding protein 2  RBM25 - rna binding motif protein 25  RRP1B - ribosomal rna processing 1b  SF3B4 - splicing factor 3b, subunit 4, 49kda  CACTIN - cactin, spliceosome c complex subunit  CRNKL1 - crooked neck pre-mrna splicing factor-like 1 (drosophila)  POLR2G - polymerase (rna) ii (dna directed) polypeptide g  POLR2F - polymerase (rna) ii (dna directed) polypeptide f  POLR2E - polymerase (rna) ii (dna directed) polypeptide e, 25kda  POLR2D - polymerase (rna) ii (dna directed) polypeptide d  CDC40 - cell division cycle 40  SCAF1 - sr-related ctd-associated factor 1  POLR2C - polymerase (rna) ii (dna directed) polypeptide c, 33kda  POLR2B - polymerase (rna) ii (dna directed) polypeptide b, 140kda  POLR2A - polymerase (rna) ii (dna directed) polypeptide a, 220kda  HSPA8 - heat shock 70kda protein 8  HNRNPUL1 - heterogeneous nuclear ribonucleoprotein u-like 1  TFIP11 - tuftelin interacting protein 11  CSTF1 - cleavage stimulation factor, 3' pre-rna, subunit 1, 50kda  PPIE - peptidylprolyl isomerase e (cyclophilin e)  DBR1 - debranching rna lariats 1  PTBP3 - polypyrimidine tract binding protein 3  PRPF40B - prp40 pre-mrna processing factor 40 homolog b (s. cerevisiae)  ZC3H13 - zinc finger ccch-type containing 13  CSTF3 - cleavage stimulation factor, 3' pre-rna, subunit 3, 77kda  CSTF2 - cleavage stimulation factor, 3' pre-rna, subunit 2, 64kda  PRPF6 - pre-mrna processing factor 6  FUS - fused in sarcoma  SUPT6H - suppressor of ty 6 homolog (s. cerevisiae)  FRG1 - fshd region gene 1  PRCC - papillary renal cell carcinoma (translocation-associated)  CLASRP - clk4-associating serine/arginine rich protein  GEMIN6 - gem (nuclear organelle) associated protein 6  PPIH - peptidylprolyl isomerase h (cyclophilin h)  UBL5 - ubiquitin-like 5  SRSF9 - serine/arginine-rich splicing factor 9  DCPS - decapping enzyme, scavenger  SNRNP35 - small nuclear ribonucleoprotein 35kda (u11/u12)  PPP2R1A - protein phosphatase 2, regulatory subunit a, alpha  NUDT21 - nudix (nucleoside diphosphate linked moiety x)-type motif 21  PPP1R8 - protein phosphatase 1, regulatory subunit 8  PPP2CA - protein phosphatase 2, catalytic subunit, alpha isozyme  CPSF7 - cleavage and polyadenylation specific factor 7, 59kda  DDX23 - dead (asp-glu-ala-asp) box polypeptide 23  SYNCRIP - synaptotagmin binding, cytoplasmic rna interacting protein  UPF3B - upf3 regulator of nonsense transcripts homolog b (yeast)  SNRNP40 - small nuclear ribonucleoprotein 40kda (u5)  ZRANB2 - zinc finger, ran-binding domain containing 2  DDX39B - dead (asp-glu-ala-asp) box polypeptide 39b  RAVER1 - ribonucleoprotein, ptb-binding 1  IWS1 - iws1 homolog (s. cerevisiae)  YTHDC1 - yth domain containing 1  THOC2 - tho complex 2  BUD13 - bud13 homolog (s. cerevisiae)  ERN1 - endoplasmic reticulum to nucleus signaling 1  PPIG - peptidylprolyl isomerase g (cyclophilin g)  HNRNPU - heterogeneous nuclear ribonucleoprotein u (scaffold attachment factor a)  HNRNPK - heterogeneous nuclear ribonucleoprotein k  HNRNPL - heterogeneous nuclear ribonucleoprotein l  PRPF40A - prp40 pre-mrna processing factor 40 homolog a (s. cerevisiae)  RBM8A - rna binding motif protein 8a  DHX35 - deah (asp-glu-ala-his) box polypeptide 35  PHF5A - phd finger protein 5a  SNIP1 - smad nuclear interacting protein 1  SRPK2 - srsf protein kinase 2  SRPK1 - srsf protein kinase 1  HNRNPA2B1 - heterogeneous nuclear ribonucleoprotein a2/b1  GEMIN7 - gem (nuclear organelle) associated protein 7  HNRNPA1 - heterogeneous nuclear ribonucleoprotein a1  ZNF830 - zinc finger protein 830  HNRNPC - heterogeneous nuclear ribonucleoprotein c (c1/c2)  HNRNPF - heterogeneous nuclear ribonucleoprotein f  HNRNPD - heterogeneous nuclear ribonucleoprotein d (au-rich element rna binding protein 1, 37kda)  HNRNPH1 - heterogeneous nuclear ribonucleoprotein h1 (h)  DDX47 - dead (asp-glu-ala-asp) box polypeptide 47  CLP1 - cleavage and polyadenylation factor i subunit 1  HNRNPH3 - heterogeneous nuclear ribonucleoprotein h3 (2h9)  JMJD6 - jumonji domain containing 6  HNRNPH2 - heterogeneous nuclear ribonucleoprotein h2 (h')  THOC1 - tho complex 1  THOC3 - tho complex 3  EFTUD2 - elongation factor tu gtp binding domain containing 2  CCDC94 - coiled-coil domain containing 94  RBM22 - rna binding motif protein 22  THRAP3 - thyroid hormone receptor associated protein 3  CD2BP2 - cd2 (cytoplasmic tail) binding protein 2  SF3B2 - splicing factor 3b, subunit 2, 145kda  CDK12 - cyclin-dependent kinase 12  DHX9 - deah (asp-glu-ala-his) box helicase 9  DHX8 - deah (asp-glu-ala-his) box polypeptide 8  HTATSF1 - hiv-1 tat specific factor 1  FMR1 - fragile x mental retardation 1  RBM23 - rna binding motif protein 23  SRSF11 - serine/arginine-rich splicing factor 11  DDX5 - dead (asp-glu-ala-asp) box helicase 5  LUC7L3 - luc7-like 3 (s. cerevisiae)  ZNF638 - zinc finger protein 638  DDX1 - dead (asp-glu-ala-asp) box helicase 1  CDC5L - cell division cycle 5-like  SYF2 - syf2 pre-mrna-splicing factor  RBM15 - rna binding motif protein 15  RNF113A - ring finger protein 113a  RBMX - rna binding motif protein, x-linked  GTF2F1 - general transcription factor iif, polypeptide 1, 74kda  HNRNPA3 - heterogeneous nuclear ribonucleoprotein a3  GTF2F2 - general transcription factor iif, polypeptide 2, 30kda  PCBP2 - poly(rc) binding protein 2  GEMIN5 - gem (nuclear organelle) associated protein 5  PPP4R2 - protein phosphatase 4, regulatory subunit 2  SRSF12 - serine/arginine-rich splicing factor 12  PRPF8 - pre-mrna processing factor 8  PRPF19 - pre-mrna processing factor 19  USB1 - u6 snrna biogenesis 1  CPSF3 - cleavage and polyadenylation specific factor 3, 73kda  NAA38 - n(alpha)-acetyltransferase 38, natc auxiliary subunit  RBM19 - rna binding motif protein 19  LSM7 - lsm7 homolog, u6 small nuclear rna associated (s. cerevisiae)  PRKRIP1 - prkr interacting protein 1 (il11 inducible)  DDX46 - dead (asp-glu-ala-asp) box polypeptide 46  CSTF2T - cleavage stimulation factor, 3' pre-rna, subunit 2, 64kda, tau variant  WBP11 - ww domain binding protein 11  DHX15 - deah (asp-glu-ala-his) box helicase 15  CELF3 - cugbp, elav-like family member 3  FAM98B - family with sequence similarity 98, member b  NSRP1 - nuclear speckle splicing regulatory protein 1  CWF19L2 - cwf19-like 2, cell cycle control (s. pombe)  U2SURP - u2 snrnp-associated surp domain containing  PTBP1 - polypyrimidine tract binding protein 1  GPATCH1 - g patch domain containing 1  EIF4A3 - eukaryotic translation initiation factor 4a3  ZBTB8OS - zinc finger and btb domain containing 8 opposite strand  KHSRP - kh-type splicing regulatory protein  PPIL3 - peptidylprolyl isomerase (cyclophilin)-like 3  DDX17 - dead (asp-glu-ala-asp) box helicase 17  CHERP - calcium homeostasis endoplasmic reticulum protein  DHX38 - deah (asp-glu-ala-his) box polypeptide 38  CPSF2 - cleavage and polyadenylation specific factor 2, 100kda  PRPF18 - pre-mrna processing factor 18  GPKOW - g patch domain and kow motifs  SLU7 - slu7 splicing factor homolog (s. cerevisiae)  LSM6 - lsm6 homolog, u6 small nuclear rna associated (s. cerevisiae)  SCAF11 - sr-related ctd-associated factor 11  MAGOHB - mago-nashi homolog b (drosophila)  CDK13 - cyclin-dependent kinase 13  MFAP1 - microfibrillar-associated protein 1  LSM3 - lsm3 homolog, u6 small nuclear rna associated (s. cerevisiae)  PRPF38B - prp38 pre-mrna processing factor 38 (yeast) domain containing b  TSEN54 - tsen54 trna splicing endonuclease subunit  SNRNP25 - small nuclear ribonucleoprotein 25kda (u11/u12)  RBM28 - rna binding motif protein 28  NONO - non-pou domain containing, octamer-binding  MAGOH - mago-nashi homolog, proliferation-associated (drosophila)  PRPF3 - pre-mrna processing factor 3  PRPF4 - prp4 pre-mrna processing factor 4 homolog (yeast)  SRSF10 - serine/arginine-rich splicing factor 10  PPWD1 - peptidylprolyl isomerase domain and wd repeat containing 1  NOVA2 - neuro-oncological ventral antigen 2  NOVA1 - neuro-oncological ventral antigen 1  AQR - aquarius homolog (mouse)  PRPF31 - pre-mrna processing factor 31  THOC7 - tho complex 7 homolog (drosophila)  ZMAT2 - zinc finger, matrin-type 2  SART1 - squamous cell carcinoma antigen recognized by t cells  WDR33 - wd repeat domain 33  ZCRB1 - zinc finger cchc-type and rna binding motif 1  RRAGC - ras-related gtp binding c  TARDBP - tar dna binding protein  SNRNP48 - small nuclear ribonucleoprotein 48kda (u11/u12)  CWC15 - cwc15 spliceosome-associated protein homolog (s. cerevisiae)  RTCB - rna 2',3'-cyclic phosphate and 5'-oh ligase  NHP2L1 - nhp2 non-histone chromosome protein 2-like 1 (s. cerevisiae)  SART3 - squamous cell carcinoma antigen recognized by t cells 3  SF3B1 - splicing factor 3b, subunit 1, 155kda  SF3B3 - splicing factor 3b, subunit 3, 130kda  SMU1 - smu-1 suppressor of mec-8 and unc-52 homolog (c. elegans)  PPIL1 - peptidylprolyl isomerase (cyclophilin)-like 1  PUF60 - poly-u binding splicing factor 60kda  RAVER2 - ribonucleoprotein, ptb-binding 2  THOC6 - tho complex 6 homolog (drosophila)  PABPC1 - poly(a) binding protein, cytoplasmic 1  SKIV2L2 - superkiller viralicidic activity 2-like 2 (s. cerevisiae)  RBFOX3 - rna binding protein, fox-1 homolog (c. elegans) 3  CASC3 - cancer susceptibility candidate 3  IK - ik cytokine, down-regulator of hla ii  SFPQ - splicing factor proline/glutamine-rich  PCF11 - pcf11 cleavage and polyadenylation factor subunit  SF1 - splicing factor 1  RBFOX2 - rna binding protein, fox-1 homolog (c. elegans) 2  PSPC1 - paraspeckle component 1  U2AF2 - u2 small nuclear rna auxiliary factor 2  SRSF1 - serine/arginine-rich splicing factor 1  SRRT - serrate rna effector molecule homolog (arabidopsis)  SRSF2 - serine/arginine-rich splicing factor 2  RBM4 - rna binding motif protein 4  SRSF3 - serine/arginine-rich splicing factor 3  SRSF4 - serine/arginine-rich splicing factor 4  CTNNBL1 - catenin, beta like 1  SRSF5 - serine/arginine-rich splicing factor 5  SRSF6 - serine/arginine-rich splicing factor 6  GEMIN2 - gem (nuclear organelle) associated protein 2  SRSF7 - serine/arginine-rich splicing factor 7  SFSWAP - splicing factor, suppressor of white-apricot homolog (drosophila)  TRA2B - transformer 2 beta homolog (drosophila)  LARP7 - la ribonucleoprotein domain family, member 7  DHX16 - deah (asp-glu-ala-his) box polypeptide 16  DDX42 - dead (asp-glu-ala-asp) box helicase 42  SRRM2 - serine/arginine repetitive matrix 2  ECD - ecdysoneless homolog (drosophila)  CWF19L1 - cwf19-like 1, cell cycle control (s. pombe)  USP39 - ubiquitin specific peptidase 39  METTL3 - methyltransferase like 3  RBM4B - rna binding motif protein 4b |
| GO:0006396 | RNA processing | 1.16E-17 | 2.02E-14 | 1.20 (10334,724,6996,589) | [+] Show genes  RSRC1 - arginine/serine-rich coiled-coil 1  WTAP - wilms tumor 1 associated protein  EXOSC9 - exosome component 9  EXOSC10 - exosome component 10  ZCCHC8 - zinc finger, cchc domain containing 8  RBM39 - rna binding motif protein 39  SYMPK - symplekin  RNPC3 - rna-binding region (rnp1, rrm) containing 3  SF3A3 - splicing factor 3a, subunit 3, 60kda  HNRNPA0 - heterogeneous nuclear ribonucleoprotein a0  AARS - alanyl-trna synthetase  MKI67IP - mki67 (fha domain) interacting nucleolar phosphoprotein  PNN - pinin, desmosome associated protein  METTL14 - methyltransferase like 14  SF3A2 - splicing factor 3a, subunit 2, 66kda  CIRH1A - cirrhosis, autosomal recessive 1a (cirhin)  EBNA1BP2 - ebna1 binding protein 2  CWC22 - cwc22 spliceosome-associated protein homolog (s. cerevisiae)  FTSJ3 - ftsj homolog 3 (e. coli)  TXNL4A - thioredoxin-like 4a  CIR1 - corepressor interacting with rbpj, 1  NOP9 - nop9 nucleolar protein  RPP25L - ribonuclease p/mrp 25kda subunit-like  CHD7 - chromodomain helicase dna binding protein 7  PLRG1 - pleiotropic regulator 1  LYAR - ly1 antibody reactive  GTPBP4 - gtp binding protein 4  PAPOLA - poly(a) polymerase alpha  LSM2 - lsm2 homolog, u6 small nuclear rna associated (s. cerevisiae)  TCERG1 - transcription elongation regulator 1  PABPN1 - poly(a) binding protein, nuclear 1  TAF15 - taf15 rna polymerase ii, tata box binding protein (tbp)-associated factor, 68kda  RNPS1 - rna binding protein s1, serine-rich domain  SON - son dna binding protein  SUGP1 - surp and g patch domain containing 1  PRPF38A - prp38 pre-mrna processing factor 38 (yeast) domain containing a  SNRPN - small nuclear ribonucleoprotein polypeptide n  THUMPD1 - thump domain containing 1  SNRPB2 - small nuclear ribonucleoprotein polypeptide b  RPRD1B - regulation of nuclear pre-mrna domain containing 1b  SNRPD1 - small nuclear ribonucleoprotein d1 polypeptide 16kda  DDX41 - dead (asp-glu-ala-asp) box polypeptide 41  SNRPF - small nuclear ribonucleoprotein polypeptide f  SNRNP70 - small nuclear ribonucleoprotein 70kda (u1)  SNRPA - small nuclear ribonucleoprotein polypeptide a  MAK16 - mak16 homolog (s. cerevisiae)  SNRPA1 - small nuclear ribonucleoprotein polypeptide a'  SNRPB - small nuclear ribonucleoprotein polypeptides b and b1  TRMT10B - trna methyltransferase 10 homolog b (s. cerevisiae)  ADAR - adenosine deaminase, rna-specific  ADARB1 - adenosine deaminase, rna-specific, b1  WDR3 - wd repeat domain 3  CPSF4 - cleavage and polyadenylation specific factor 4, 30kda  RBM17 - rna binding motif protein 17  RBM10 - rna binding motif protein 10  POLR2K - polymerase (rna) ii (dna directed) polypeptide k, 7.0kda  LSM5 - lsm5 homolog, u6 small nuclear rna associated (s. cerevisiae)  TSEN15 - tsen15 trna splicing endonuclease subunit  RBM25 - rna binding motif protein 25  CACTIN - cactin, spliceosome c complex subunit  NOP10 - nop10 ribonucleoprotein  CRNKL1 - crooked neck pre-mrna splicing factor-like 1 (drosophila)  NOL10 - nucleolar protein 10  POLR2G - polymerase (rna) ii (dna directed) polypeptide g  POLR2F - polymerase (rna) ii (dna directed) polypeptide f  POLR2E - polymerase (rna) ii (dna directed) polypeptide e, 25kda  POLR2D - polymerase (rna) ii (dna directed) polypeptide d  CDC40 - cell division cycle 40  SCAF1 - sr-related ctd-associated factor 1  POLR2C - polymerase (rna) ii (dna directed) polypeptide c, 33kda  POLR2B - polymerase (rna) ii (dna directed) polypeptide b, 140kda  POLR2A - polymerase (rna) ii (dna directed) polypeptide a, 220kda  HNRNPUL1 - heterogeneous nuclear ribonucleoprotein u-like 1  TFIP11 - tuftelin interacting protein 11  INTS9 - integrator complex subunit 9  CSTF1 - cleavage stimulation factor, 3' pre-rna, subunit 1, 50kda  RPP14 - ribonuclease p/mrp 14kda subunit  FTSJ1 - ftsj rna methyltransferase homolog 1 (e. coli)  KRR1 - krr1, small subunit (ssu) processome component, homolog (yeast)  DBR1 - debranching rna lariats 1  PRKACA - protein kinase, camp-dependent, catalytic, alpha  WDR12 - wd repeat domain 12  PRPF40B - prp40 pre-mrna processing factor 40 homolog b (s. cerevisiae)  MRTO4 - mrna turnover 4 homolog (s. cerevisiae)  CSTF3 - cleavage stimulation factor, 3' pre-rna, subunit 3, 77kda  RPF2 - ribosome production factor 2 homolog (s. cerevisiae)  PRPF6 - pre-mrna processing factor 6  CSTF2 - cleavage stimulation factor, 3' pre-rna, subunit 2, 64kda  FUS - fused in sarcoma  SUPT6H - suppressor of ty 6 homolog (s. cerevisiae)  SUPV3L1 - suppressor of var1, 3-like 1 (s. cerevisiae)  FRG1 - fshd region gene 1  METTL8 - methyltransferase like 8  PRCC - papillary renal cell carcinoma (translocation-associated)  CLASRP - clk4-associating serine/arginine rich protein  SUPT4H1 - suppressor of ty 4 homolog 1 (s. cerevisiae)  SUPT5H - suppressor of ty 5 homolog (s. cerevisiae)  GEMIN6 - gem (nuclear organelle) associated protein 6  GTPBP3 - gtp binding protein 3 (mitochondrial)  UBL5 - ubiquitin-like 5  TRMT11 - trna methyltransferase 11 homolog (s. cerevisiae)  SNRNP35 - small nuclear ribonucleoprotein 35kda (u11/u12)  CMTR2 - cap methyltransferase 2  SLBP - stem-loop binding protein  CPSF6 - cleavage and polyadenylation specific factor 6, 68kda  PPP2R1A - protein phosphatase 2, regulatory subunit a, alpha  RIOK2 - rio kinase 2  NUDT21 - nudix (nucleoside diphosphate linked moiety x)-type motif 21  SBDS - shwachman-bodian-diamond syndrome  DDX52 - dead (asp-glu-ala-asp) box polypeptide 52  DDX51 - dead (asp-glu-ala-asp) box polypeptide 51  PPP1R8 - protein phosphatase 1, regulatory subunit 8  RBFA - ribosome binding factor a (putative)  PPP2CA - protein phosphatase 2, catalytic subunit, alpha isozyme  CPSF7 - cleavage and polyadenylation specific factor 7, 59kda  DDX23 - dead (asp-glu-ala-asp) box polypeptide 23  TFB1M - transcription factor b1, mitochondrial  PAN3 - pan3 poly(a) specific ribonuclease subunit homolog (s. cerevisiae)  SNRNP40 - small nuclear ribonucleoprotein 40kda (u5)  ZRANB2 - zinc finger, ran-binding domain containing 2  DDX39B - dead (asp-glu-ala-asp) box polypeptide 39b  IWS1 - iws1 homolog (s. cerevisiae)  POLDIP3 - polymerase (dna-directed), delta interacting protein 3  BUD13 - bud13 homolog (s. cerevisiae)  PPIG - peptidylprolyl isomerase g (cyclophilin g)  PRPF40A - prp40 pre-mrna processing factor 40 homolog a (s. cerevisiae)  PAPD7 - pap associated domain containing 7  DDX27 - dead (asp-glu-ala-asp) box polypeptide 27  INTS8 - integrator complex subunit 8  DHX35 - deah (asp-glu-ala-his) box polypeptide 35  SNORA8 - small nucleolar rna, h/aca box 8  PHF5A - phd finger protein 5a  SNIP1 - smad nuclear interacting protein 1  TSR1 - tsr1, 20s rrna accumulation, homolog (s. cerevisiae)  SRPK2 - srsf protein kinase 2  ZNHIT3 - zinc finger, hit-type containing 3  SRPK1 - srsf protein kinase 1  GEMIN7 - gem (nuclear organelle) associated protein 7  DDX47 - dead (asp-glu-ala-asp) box polypeptide 47  CLP1 - cleavage and polyadenylation factor i subunit 1  SSB - sjogren syndrome antigen b (autoantigen la)  THOC3 - tho complex 3  EFTUD2 - elongation factor tu gtp binding domain containing 2  CCDC94 - coiled-coil domain containing 94  SARNP - sap domain containing ribonucleoprotein  RBM22 - rna binding motif protein 22  THADA - thyroid adenoma associated  MTO1 - mitochondrial trna translation optimization 1  NSUN5 - nop2/sun domain family, member 5  NSUN3 - nop2/sun domain family, member 3  SF3B2 - splicing factor 3b, subunit 2, 145kda  DEDD2 - death effector domain containing 2  CDK12 - cyclin-dependent kinase 12  DHX9 - deah (asp-glu-ala-his) box helicase 9  SNORD31 - small nucleolar rna, c/d box 31  DHX8 - deah (asp-glu-ala-his) box polypeptide 8  MTPAP - mitochondrial poly(a) polymerase  TSR2 - tsr2, 20s rrna accumulation, homolog (s. cerevisiae)  SNORD29 - small nucleolar rna, c/d box 29  SRSF11 - serine/arginine-rich splicing factor 11  RBM23 - rna binding motif protein 23  DDX5 - dead (asp-glu-ala-asp) box helicase 5  LUC7L3 - luc7-like 3 (s. cerevisiae)  DDX1 - dead (asp-glu-ala-asp) box helicase 1  SYF2 - syf2 pre-mrna-splicing factor  PARN - poly(a)-specific ribonuclease  SNORD22 - small nucleolar rna, c/d box 22  ZCCHC6 - zinc finger, cchc domain containing 6  RNF113A - ring finger protein 113a  SNORD30 - small nucleolar rna, c/d box 30  GTF2F1 - general transcription factor iif, polypeptide 1, 74kda  GTF2F2 - general transcription factor iif, polypeptide 2, 30kda  GTF2H2 - general transcription factor iih, polypeptide 2, 44kda  PCBP2 - poly(rc) binding protein 2  GTF2H3 - general transcription factor iih, polypeptide 3, 34kda  INTS10 - integrator complex subunit 10  GEMIN5 - gem (nuclear organelle) associated protein 5  GTF2H1 - general transcription factor iih, polypeptide 1, 62kda  NOL11 - nucleolar protein 11  THUMPD3 - thump domain containing 3  WDR46 - wd repeat domain 46  USB1 - u6 snrna biogenesis 1  CPSF3 - cleavage and polyadenylation specific factor 3, 73kda  NAA38 - n(alpha)-acetyltransferase 38, natc auxiliary subunit  LSM7 - lsm7 homolog, u6 small nuclear rna associated (s. cerevisiae)  GTF2H4 - general transcription factor iih, polypeptide 4, 52kda  PRKRIP1 - prkr interacting protein 1 (il11 inducible)  PA2G4 - proliferation-associated 2g4, 38kda  RPRD1A - regulation of nuclear pre-mrna domain containing 1a  NOL9 - nucleolar protein 9  MNAT1 - mnat cdk-activating kinase assembly factor 1  ZNF473 - zinc finger protein 473  NOLC1 - nucleolar and coiled-body phosphoprotein 1  APP - amyloid beta (a4) precursor protein  DCAF13 - ddb1 and cul4 associated factor 13  DDX20 - dead (asp-glu-ala-asp) box polypeptide 20  DDX10 - dead (asp-glu-ala-asp) box polypeptide 10  WDR18 - wd repeat domain 18  WBP11 - ww domain binding protein 11  DHX15 - deah (asp-glu-ala-his) box helicase 15  CELF3 - cugbp, elav-like family member 3  NSRP1 - nuclear speckle splicing regulatory protein 1  CWF19L2 - cwf19-like 2, cell cycle control (s. pombe)  SNORA43 - small nucleolar rna, h/aca box 43  PAPD4 - pap associated domain containing 4  ERI1 - exoribonuclease 1  SCAF4 - sr-related ctd-associated factor 4  CDC73 - cell division cycle 73  GPATCH1 - g patch domain containing 1  DDX21 - dead (asp-glu-ala-asp) box helicase 21  BCDIN3D - bcdin3 domain containing  PPIL3 - peptidylprolyl isomerase (cyclophilin)-like 3  CPSF2 - cleavage and polyadenylation specific factor 2, 100kda  WDR75 - wd repeat domain 75  INTS2 - integrator complex subunit 2  LSM6 - lsm6 homolog, u6 small nuclear rna associated (s. cerevisiae)  SCAF11 - sr-related ctd-associated factor 11  UTP15 - utp15, u3 small nucleolar ribonucleoprotein, homolog (s. cerevisiae)  MAGOHB - mago-nashi homolog b (drosophila)  MTFMT - mitochondrial methionyl-trna formyltransferase  METTL1 - methyltransferase like 1  HEATR1 - heat repeat containing 1  MFAP1 - microfibrillar-associated protein 1  AARS2 - alanyl-trna synthetase 2, mitochondrial  LEO1 - leo1, paf1/rna polymerase ii complex component, homolog (s. cerevisiae)  RPL7L1 - ribosomal protein l7-like 1  PRPF38B - prp38 pre-mrna processing factor 38 (yeast) domain containing b  SARS - seryl-trna synthetase  NGDN - neuroguidin, eif4e binding protein  RBM28 - rna binding motif protein 28  SNRNP25 - small nuclear ribonucleoprotein 25kda (u11/u12)  SNORA11 - small nucleolar rna, h/aca box 11  ELP3 - elongator acetyltransferase complex subunit 3  RRP9 - ribosomal rna processing 9, small subunit (ssu) processome component, homolog (yeast)  MAGOH - mago-nashi homolog, proliferation-associated (drosophila)  PRPF3 - pre-mrna processing factor 3  PRPF4 - prp4 pre-mrna processing factor 4 homolog (yeast)  CNOT6L - ccr4-not transcription complex, subunit 6-like  DIS3 - dis3 mitotic control homolog (s. cerevisiae)  SNORD86 - small nucleolar rna, c/d box 86  PUS3 - pseudouridylate synthase 3  PRPF31 - pre-mrna processing factor 31  LARP6 - la ribonucleoprotein domain family, member 6  TRMT5 - trna methyltransferase 5  CHTOP - chromatin target of prmt1  SART1 - squamous cell carcinoma antigen recognized by t cells  ZFC3H1 - zinc finger, c3h1-type containing  SNORD12 - small nucleolar rna, c/d box 12  WDR33 - wd repeat domain 33  ZCRB1 - zinc finger cchc-type and rna binding motif 1  TRMT112 - trna methyltransferase 11-2 homolog (s. cerevisiae)  CWC15 - cwc15 spliceosome-associated protein homolog (s. cerevisiae)  RTCB - rna 2',3'-cyclic phosphate and 5'-oh ligase  SCAF8 - sr-related ctd-associated factor 8  SMU1 - smu-1 suppressor of mec-8 and unc-52 homolog (c. elegans)  PPIL1 - peptidylprolyl isomerase (cyclophilin)-like 1  DKC1 - dyskeratosis congenita 1, dyskerin  PUF60 - poly-u binding splicing factor 60kda  PNPT1 - polyribonucleotide nucleotidyltransferase 1  THOC6 - tho complex 6 homolog (drosophila)  RAVER2 - ribonucleoprotein, ptb-binding 2  NAT10 - n-acetyltransferase 10 (gcn5-related)  RPL14 - ribosomal protein l14  RIOK1 - rio kinase 1  DPH3 - diphthamide biosynthesis 3  TYW1 - trna-yw synthesizing protein 1 homolog (s. cerevisiae)  ELP2 - elongator acetyltransferase complex subunit 2  XRN2 - 5'-3' exoribonuclease 2  RBFOX3 - rna binding protein, fox-1 homolog (c. elegans) 3  CDK5RAP1 - cdk5 regulatory subunit associated protein 1  DHX37 - deah (asp-glu-ala-his) box polypeptide 37  CASC3 - cancer susceptibility candidate 3  PIH1D2 - pih1 domain containing 2  IMP3 - imp3, u3 small nucleolar ribonucleoprotein, homolog (yeast)  ESF1 - esf1, nucleolar pre-rrna processing protein, homolog (s. cerevisiae)  EXOSC8 - exosome component 8  SFPQ - splicing factor proline/glutamine-rich  PHRF1 - phd and ring finger domains 1  PCF11 - pcf11 cleavage and polyadenylation factor subunit  SF1 - splicing factor 1  SRSF1 - serine/arginine-rich splicing factor 1  U2AF2 - u2 small nuclear rna auxiliary factor 2  PSPC1 - paraspeckle component 1  SMAD2 - smad family member 2  SRSF2 - serine/arginine-rich splicing factor 2  SRRT - serrate rna effector molecule homolog (arabidopsis)  SRSF3 - serine/arginine-rich splicing factor 3  TRDMT1 - trna aspartic acid methyltransferase 1  SRSF4 - serine/arginine-rich splicing factor 4  SRSF5 - serine/arginine-rich splicing factor 5  TMTC1 - transmembrane and tetratricopeptide repeat containing 1  SRSF6 - serine/arginine-rich splicing factor 6  SRSF7 - serine/arginine-rich splicing factor 7  SFSWAP - splicing factor, suppressor of white-apricot homolog (drosophila)  TRA2B - transformer 2 beta homolog (drosophila)  LARP7 - la ribonucleoprotein domain family, member 7  RPUSD3 - rna pseudouridylate synthase domain containing 3  DDX42 - dead (asp-glu-ala-asp) box helicase 42  RSL1D1 - ribosomal l1 domain containing 1  ECD - ecdysoneless homolog (drosophila)  GRSF1 - g-rich rna sequence binding factor 1  CWF19L1 - cwf19-like 1, cell cycle control (s. pombe)  TRMT6 - trna methyltransferase 6 homolog (s. cerevisiae)  INTS1 - integrator complex subunit 1  SENP3 - sumo1/sentrin/smt3 specific peptidase 3  RBM4B - rna binding motif protein 4b  CDKAL1 - cdk5 regulatory subunit associated protein 1-like 1  NCBP2 - nuclear cap binding protein subunit 2, 20kda  SMNDC1 - survival motor neuron domain containing 1  NCBP1 - nuclear cap binding protein subunit 1, 80kda  BCAS2 - breast carcinoma amplified sequence 2  CWC27 - cwc27 spliceosome-associated protein homolog (s. cerevisiae)  TSN - translin  SAP18 - sin3a-associated protein, 18kda  MBNL2 - muscleblind-like splicing regulator 2  SNORD24 - small nucleolar rna, c/d box 24  NAF1 - nuclear assembly factor 1 ribonucleoprotein  SUGP2 - surp and g patch domain containing 2  TRMT1L - trna methyltransferase 1 homolog (s. cerevisiae)-like  SF3A1 - splicing factor 3a, subunit 1, 120kda  CWC25 - cwc25 spliceosome-associated protein homolog (s. cerevisiae)  SNW1 - snw domain containing 1  HNRNPM - heterogeneous nuclear ribonucleoprotein m  TEX10 - testis expressed 10  SNORD55 - small nucleolar rna, c/d box 55  DROSHA - drosha, ribonuclease type iii  KIN - kin, antigenic determinant of reca protein homolog (mouse)  ZFP36L1 - zfp36 ring finger protein-like 1  HABP4 - hyaluronan binding protein 4  ALKBH5 - alkb, alkylation repair homolog 5 (e. coli)  NSUN2 - nop2/sun rna methyltransferase family, member 2  METTL16 - methyltransferase like 16  ALKBH1 - alkb, alkylation repair homolog 1 (e. coli)  CELF4 - cugbp, elav-like family member 4  ELP6 - elongator acetyltransferase complex subunit 6  RBM6 - rna binding motif protein 6  RBM5 - rna binding motif protein 5  ALYREF - aly/ref export factor  INTS4 - integrator complex subunit 4  NOC4L - nucleolar complex associated 4 homolog (s. cerevisiae)  FBLL1 - fibrillarin-like 1  ELAVL1 - elav (embryonic lethal, abnormal vision, drosophila)-like 1 (hu antigen r)  MPHOSPH6 - m-phase phosphoprotein 6  ZNF326 - zinc finger protein 326  CCDC130 - coiled-coil domain containing 130  MPHOSPH10 - m-phase phosphoprotein 10 (u3 small nucleolar ribonucleoprotein)  ELAVL4 - elav (embryonic lethal, abnormal vision, drosophila)-like 4  URM1 - ubiquitin related modifier 1  PDCD11 - programmed cell death 11  ABT1 - activator of basal transcription 1  WDR55 - wd repeat domain 55  TSNAX - translin-associated factor x  ACIN1 - apoptotic chromatin condensation inducer 1  FIP1L1 - factor interacting with papola and cpsf1  ZNF259 - zinc finger protein 259  LAS1L - las1-like (s. cerevisiae)  EXOSC7 - exosome component 7  SPEN - spen homolog, transcriptional regulator (drosophila)  ISG20L2 - interferon stimulated exonuclease gene 20kda-like 2  PDCD7 - programmed cell death 7  SCNM1 - sodium channel modifier 1  TRMT10A - trna methyltransferase 10 homolog a (s. cerevisiae)  TRIT1 - trna isopentenyltransferase 1  PRPF4B - prp4 pre-mrna processing factor 4 homolog b (yeast)  TSEN34 - tsen34 trna splicing endonuclease subunit  PQBP1 - polyglutamine binding protein 1  KDM1A - lysine (k)-specific demethylase 1a  U2AF1L4 - u2 small nuclear rna auxiliary factor 1-like 4  TRA2A - transformer 2 alpha homolog (drosophila)  SF3B5 - splicing factor 3b, subunit 5, 10kda  HNRNPR - heterogeneous nuclear ribonucleoprotein r  CPSF1 - cleavage and polyadenylation specific factor 1, 160kda  ERI3 - eri1 exoribonuclease family member 3  BUD31 - bud31 homolog (s. cerevisiae)  RBM15B - rna binding motif protein 15b  SNRNP200 - small nuclear ribonucleoprotein 200kda (u5)  HNRNPLL - heterogeneous nuclear ribonucleoprotein l-like  IMP4 - imp4, u3 small nucleolar ribonucleoprotein, homolog (yeast)  BYSL - bystin-like  SRRM1 - serine/arginine repetitive matrix 1  XAB2 - xpa binding protein 2  TYW3 - trna-yw synthesizing protein 3 homolog (s. cerevisiae)  RRP1B - ribosomal rna processing 1b  SF3B4 - splicing factor 3b, subunit 4, 49kda  CMTR1 - cap methyltransferase 1  SETX - senataxin  DUS3L - dihydrouridine synthase 3-like (s. cerevisiae)  HSPA8 - heat shock 70kda protein 8  PPIE - peptidylprolyl isomerase e (cyclophilin e)  REXO4 - rex4, rna exonuclease 4 homolog (s. cerevisiae)  PTBP3 - polypyrimidine tract binding protein 3  INTS12 - integrator complex subunit 12  ZC3H13 - zinc finger ccch-type containing 13  C1D - c1d nuclear receptor corepressor  TRMT12 - trna methyltransferase 12 homolog (s. cerevisiae)  RPL11 - ribosomal protein l11  NOL8 - nucleolar protein 8  RPL7 - ribosomal protein l7  PPIH - peptidylprolyl isomerase h (cyclophilin h)  INTS6 - integrator complex subunit 6  SNORD107 - small nucleolar rna, c/d box 107  AGO1 - argonaute risc catalytic component 1  SRSF9 - serine/arginine-rich splicing factor 9  DCPS - decapping enzyme, scavenger  MRPL1 - mitochondrial ribosomal protein l1  UTP3 - utp3, small subunit (ssu) processome component, homolog (s. cerevisiae)  NOL6 - nucleolar protein 6 (rna-associated)  ZC3H3 - zinc finger ccch-type containing 3  MRPL44 - mitochondrial ribosomal protein l44  SYNCRIP - synaptotagmin binding, cytoplasmic rna interacting protein  CCNL1 - cyclin l1  INTS3 - integrator complex subunit 3  ELP4 - elongator acetyltransferase complex subunit 4  UPF3B - upf3 regulator of nonsense transcripts homolog b (yeast)  WDR43 - wd repeat domain 43  RAVER1 - ribonucleoprotein, ptb-binding 1  PAPOLG - poly(a) polymerase gamma  PAN2 - pan2 poly(a) specific ribonuclease subunit homolog (s. cerevisiae)  ERCC3 - excision repair cross-complementing rodent repair deficiency, complementation group 3  YTHDC1 - yth domain containing 1  THOC2 - tho complex 2  NPM3 - nucleophosmin/nucleoplasmin 3  THG1L - trna-histidine guanylyltransferase 1-like (s. cerevisiae)  ERN1 - endoplasmic reticulum to nucleus signaling 1  CCNH - cyclin h  RNGTT - rna guanylyltransferase and 5'-phosphatase  NOB1 - nin1/rpn12 binding protein 1 homolog (s. cerevisiae)  RNMT - rna (guanine-7-) methyltransferase  HNRNPU - heterogeneous nuclear ribonucleoprotein u (scaffold attachment factor a)  HNRNPK - heterogeneous nuclear ribonucleoprotein k  HNRNPL - heterogeneous nuclear ribonucleoprotein l  RPS15 - ribosomal protein s15  MRPS11 - mitochondrial ribosomal protein s11  SNORA3 - small nucleolar rna, h/aca box 3  RBM8A - rna binding motif protein 8a  MDN1 - mdn1, midasin homolog (yeast)  NSA2 - nsa2 ribosome biogenesis homolog (s. cerevisiae)  HNRNPA2B1 - heterogeneous nuclear ribonucleoprotein a2/b1  HNRNPA1 - heterogeneous nuclear ribonucleoprotein a1  ZNF830 - zinc finger protein 830  HNRNPC - heterogeneous nuclear ribonucleoprotein c (c1/c2)  METTL5 - methyltransferase like 5  HNRNPF - heterogeneous nuclear ribonucleoprotein f  HNRNPD - heterogeneous nuclear ribonucleoprotein d (au-rich element rna binding protein 1, 37kda)  HNRNPH1 - heterogeneous nuclear ribonucleoprotein h1 (h)  HNRNPH3 - heterogeneous nuclear ribonucleoprotein h3 (2h9)  JMJD6 - jumonji domain containing 6  HNRNPH2 - heterogeneous nuclear ribonucleoprotein h2 (h')  THOC1 - tho complex 1  TUT1 - terminal uridylyl transferase 1, u6 snrna-specific  ZC3H7A - zinc finger ccch-type containing 7a  TRMT10C - trna methyltransferase 10 homolog c (s. cerevisiae)  ERCC2 - excision repair cross-complementing rodent repair deficiency, complementation group 2  THRAP3 - thyroid hormone receptor associated protein 3  CD2BP2 - cd2 (cytoplasmic tail) binding protein 2  PDE12 - phosphodiesterase 12  ZC3H7B - zinc finger ccch-type containing 7b  RBM27 - rna binding motif protein 27  IKBKAP - inhibitor of kappa light polypeptide gene enhancer in b-cells, kinase complex-associated protein  HTATSF1 - hiv-1 tat specific factor 1  FMR1 - fragile x mental retardation 1  HENMT1 - hen1 methyltransferase homolog 1 (arabidopsis)  CDC5L - cell division cycle 5-like  ZNF638 - zinc finger protein 638  TBL3 - transducin (beta)-like 3  RBM15 - rna binding motif protein 15  INTS5 - integrator complex subunit 5  RBMX - rna binding motif protein, x-linked  HNRNPA3 - heterogeneous nuclear ribonucleoprotein a3  BOP1 - block of proliferation 1  THUMPD2 - thump domain containing 2  RPRD2 - regulation of nuclear pre-mrna domain containing 2  SRSF12 - serine/arginine-rich splicing factor 12  PPP4R2 - protein phosphatase 4, regulatory subunit 2  PRPF8 - pre-mrna processing factor 8  PRPF19 - pre-mrna processing factor 19  UTP20 - utp20, small subunit (ssu) processome component, homolog (yeast)  RBM19 - rna binding motif protein 19  WDR36 - wd repeat domain 36  UTP6 - utp6, small subunit (ssu) processome component, homolog (yeast)  KHDRBS3 - kh domain containing, rna binding, signal transduction associated 3  ADAT2 - adenosine deaminase, trna-specific 2  RPUSD1 - rna pseudouridylate synthase domain containing 1  DDX46 - dead (asp-glu-ala-asp) box polypeptide 46  EXOSC3 - exosome component 3  GAR1 - gar1 ribonucleoprotein  CSTF2T - cleavage stimulation factor, 3' pre-rna, subunit 2, 64kda, tau variant  EXOSC1 - exosome component 1  BMS1 - bms1 ribosome biogenesis factor  FAM98B - family with sequence similarity 98, member b  RRP15 - ribosomal rna processing 15 homolog (s. cerevisiae)  U2SURP - u2 snrnp-associated surp domain containing  PRKRA - protein kinase, interferon-inducible double stranded rna dependent activator  PTBP1 - polypyrimidine tract binding protein 1  EIF4A3 - eukaryotic translation initiation factor 4a3  ZBTB8OS - zinc finger and btb domain containing 8 opposite strand  RRP1 - ribosomal rna processing 1  KHSRP - kh-type splicing regulatory protein  TARBP1 - tar (hiv-1) rna binding protein 1  DDX17 - dead (asp-glu-ala-asp) box helicase 17  CHERP - calcium homeostasis endoplasmic reticulum protein  DHX38 - deah (asp-glu-ala-his) box polypeptide 38  PRPF18 - pre-mrna processing factor 18  GPKOW - g patch domain and kow motifs  NVL - nuclear vcp-like  ZCCHC11 - zinc finger, cchc domain containing 11  EIF6 - eukaryotic translation initiation factor 6  NOP56 - nop56 ribonucleoprotein  SLU7 - slu7 splicing factor homolog (s. cerevisiae)  RTCA - rna 3'-terminal phosphate cyclase  CDK7 - cyclin-dependent kinase 7  CDK13 - cyclin-dependent kinase 13  FCF1 - fcf1 rrna-processing protein  LSM3 - lsm3 homolog, u6 small nuclear rna associated (s. cerevisiae)  CSDC2 - cold shock domain containing c2, rna binding  TSEN54 - tsen54 trna splicing endonuclease subunit  PUM2 - pumilio homolog 2 (drosophila)  RPP38 - ribonuclease p/mrp 38kda subunit  MOCS3 - molybdenum cofactor synthesis 3  DIMT1 - dim1 dimethyladenosine transferase 1 homolog (s. cerevisiae)  TRNT1 - trna nucleotidyl transferase, cca-adding, 1  UTP18 - utp18 small subunit (ssu) processome component homolog (yeast)  TOE1 - target of egr1, member 1 (nuclear)  RRNAD1 - ribosomal rna adenine dimethylase domain containing 1  NOP14 - nop14 nucleolar protein  WDR4 - wd repeat domain 4  NOP2 - nop2 nucleolar protein  NONO - non-pou domain containing, octamer-binding  METTL6 - methyltransferase like 6  ZNHIT6 - zinc finger, hit-type containing 6  PAPD5 - pap associated domain containing 5  POP4 - processing of precursor 4, ribonuclease p/mrp subunit (s. cerevisiae)  SRSF10 - serine/arginine-rich splicing factor 10  SNORD118 - small nucleolar rna, c/d box 118  PPWD1 - peptidylprolyl isomerase domain and wd repeat containing 1  NOVA2 - neuro-oncological ventral antigen 2  NOVA1 - neuro-oncological ventral antigen 1  AHCYL1 - adenosylhomocysteinase-like 1  AQR - aquarius homolog (mouse)  EXOSC2 - exosome component 2  PUM1 - pumilio homolog 1 (drosophila)  THOC7 - tho complex 7 homolog (drosophila)  MTERFD2 - mterf domain containing 2  ZMAT2 - zinc finger, matrin-type 2  SRFBP1 - serum response factor binding protein 1  OSGEPL1 - o-sialoglycoprotein endopeptidase-like 1  METTL2A - methyltransferase like 2a  RRAGC - ras-related gtp binding c  TARDBP - tar dna binding protein  SNRNP48 - small nuclear ribonucleoprotein 48kda (u11/u12)  RPP40 - ribonuclease p/mrp 40kda subunit  PAF1 - paf1, rna polymerase ii associated factor, homolog (s. cerevisiae)  NHP2L1 - nhp2 non-histone chromosome protein 2-like 1 (s. cerevisiae)  SART3 - squamous cell carcinoma antigen recognized by t cells 3  DDX56 - dead (asp-glu-ala-asp) box helicase 56  SF3B1 - splicing factor 3b, subunit 1, 155kda  SF3B3 - splicing factor 3b, subunit 3, 130kda  PABPC1 - poly(a) binding protein, cytoplasmic 1  TRUB2 - trub pseudouridine (psi) synthase homolog 2 (e. coli)  SKIV2L2 - superkiller viralicidic activity 2-like 2 (s. cerevisiae)  DUSP11 - dual specificity phosphatase 11 (rna/rnp complex 1-interacting)  RBM26 - rna binding motif protein 26  PES1 - pescadillo ribosomal biogenesis factor 1  CELF1 - cugbp, elav-like family member 1  KHDRBS1 - kh domain containing, rna binding, signal transduction associated 1  TGS1 - trimethylguanosine synthase 1  CELF2 - cugbp, elav-like family member 2  IK - ik cytokine, down-regulator of hla ii  DDX49 - dead (asp-glu-ala-asp) box polypeptide 49  KIAA0391 - kiaa0391  RBFOX2 - rna binding protein, fox-1 homolog (c. elegans) 2  RBMS1 - rna binding motif, single stranded interacting protein 1  RBM3 - rna binding motif (rnp1, rrm) protein 3  RBM4 - rna binding motif protein 4  DIEXF - digestive organ expansion factor homolog (zebrafish)  PELP1 - proline, glutamate and leucine rich protein 1  CTNNBL1 - catenin, beta like 1  GEMIN2 - gem (nuclear organelle) associated protein 2  TYW5 - trna-yw synthesizing protein 5  RBBP6 - retinoblastoma binding protein 6  DGCR8 - dgcr8 microprocessor complex subunit  GEMIN4 - gem (nuclear organelle) associated protein 4  PUS7 - pseudouridylate synthase 7 homolog (s. cerevisiae)  DHX16 - deah (asp-glu-ala-his) box polypeptide 16  EXOSC4 - exosome component 4  ANKRD16 - ankyrin repeat domain 16  SRRM2 - serine/arginine repetitive matrix 2  USP39 - ubiquitin specific peptidase 39  METTL3 - methyltransferase like 3 |
| GO:0019219 | regulation of nucleobase-containing compound metabolic process | 5.83E-17 | 9.01E-14 | 1.10 (10334,2424,6996,1806) | [+] Show genes  EXOSC9 - exosome component 9  EXOSC10 - exosome component 10  MSL3 - male-specific lethal 3 homolog (drosophila)  TADA1 - transcriptional adaptor 1  HNRNPA0 - heterogeneous nuclear ribonucleoprotein a0  CBX1 - chromobox homolog 1  ABCA2 - atp-binding cassette, sub-family a (abc1), member 2  ABL1 - c-abl oncogene 1, non-receptor tyrosine kinase  ZNF821 - zinc finger protein 821  PML - promyelocytic leukemia  MLLT11 - myeloid/lymphoid or mixed-lineage leukemia (trithorax homolog, drosophila); translocated to, 11  SUPT20H - suppressor of ty 20 homolog (s. cerevisiae)  YWHAQ - tyrosine 3-monooxygenase/tryptophan 5-monooxygenase activation protein, theta polypeptide  BRD8 - bromodomain containing 8  CHD7 - chromodomain helicase dna binding protein 7  LYAR - ly1 antibody reactive  PAPOLA - poly(a) polymerase alpha  ZNF354B - zinc finger protein 354b  TCERG1 - transcription elongation regulator 1  RNPS1 - rna binding protein s1, serine-rich domain  OTUB1 - otu domain, ubiquitin aldehyde binding 1  EHMT2 - euchromatic histone-lysine n-methyltransferase 2  SPIN1 - spindlin 1  TASP1 - taspase, threonine aspartase, 1  SUB1 - sub1 homolog (s. cerevisiae)  MORF4L1 - mortality factor 4 like 1  ZNF511 - zinc finger protein 511  PPID - peptidylprolyl isomerase d  ACTL6A - actin-like 6a  ACTN1 - actinin, alpha 1  ACTN4 - actinin, alpha 4  ACVR2A - activin a receptor, type iia  ACVRL1 - activin a receptor type ii-like 1  ACVR1 - activin a receptor, type i  ACVR1B - activin a receptor, type ib  ZNF331 - zinc finger protein 331  CPSF4 - cleavage and polyadenylation specific factor 4, 30kda  MED1 - mediator complex subunit 1  ADCY1 - adenylate cyclase 1 (brain)  PPARD - peroxisome proliferator-activated receptor delta  MAML3 - mastermind-like 3 (drosophila)  POLR2K - polymerase (rna) ii (dna directed) polypeptide k, 7.0kda  PARP1 - poly (adp-ribose) polymerase 1  POLH - polymerase (dna directed), eta  H2AFY2 - h2a histone family, member y2  PPP1R13L - protein phosphatase 1, regulatory subunit 13 like  HES6 - hairy and enhancer of split 6 (drosophila)  POLR2G - polymerase (rna) ii (dna directed) polypeptide g  SMPD3 - sphingomyelin phosphodiesterase 3, neutral membrane (neutral sphingomyelinase ii)  PARP4 - poly (adp-ribose) polymerase family, member 4  RUVBL2 - ruvb-like 2 (e. coli)  POLR2A - polymerase (rna) ii (dna directed) polypeptide a, 220kda  CDK5RAP2 - cdk5 regulatory subunit associated protein 2  RCOR3 - rest corepressor 3  AES - amino-terminal enhancer of split  NUP133 - nucleoporin 133kda  NUPL2 - nucleoporin like 2  FAM46B - family with sequence similarity 46, member b  JAG1 - jagged 1  PRDM4 - pr domain containing 4  PPP3R1 - protein phosphatase 3, regulatory subunit b, alpha  ATF7IP - activating transcription factor 7 interacting protein  ZNF689 - zinc finger protein 689  LRIF1 - ligand dependent nuclear receptor interacting factor 1  PPP2R5B - protein phosphatase 2, regulatory subunit b', beta  PPP4C - protein phosphatase 4, catalytic subunit  ZNF653 - zinc finger protein 653  AKT1 - v-akt murine thymoma viral oncogene homolog 1  PCID2 - pci domain containing 2  CPSF6 - cleavage and polyadenylation specific factor 6, 68kda  PPP2R1A - protein phosphatase 2, regulatory subunit a, alpha  RIOK2 - rio kinase 2  NUDT21 - nudix (nucleoside diphosphate linked moiety x)-type motif 21  WWP2 - ww domain containing e3 ubiquitin protein ligase 2  AHR - aryl hydrocarbon receptor  ZNF526 - zinc finger protein 526  FOXP4 - forkhead box p4  PPP2CA - protein phosphatase 2, catalytic subunit, alpha isozyme  PPP1R10 - protein phosphatase 1, regulatory subunit 10  RMI2 - recq mediated genome instability 2  ZNF83 - zinc finger protein 83  ESCO1 - establishment of sister chromatid cohesion n-acetyltransferase 1  KANSL3 - kat8 regulatory nsl complex subunit 3  IWS1 - iws1 homolog (s. cerevisiae)  PRNP - prion protein  ATF7 - activating transcription factor 7  AMFR - autocrine motility factor receptor, e3 ubiquitin protein ligase  PRKRIR - protein-kinase, interferon-inducible double stranded rna dependent inhibitor, repressor of (p58 repressor)  PAPD7 - pap associated domain containing 7  MAP2K5 - mitogen-activated protein kinase kinase 5  MAP2K7 - mitogen-activated protein kinase kinase 7  ZNF692 - zinc finger protein 692  EIF2AK2 - eukaryotic translation initiation factor 2-alpha kinase 2  PIM2 - pim-2 oncogene  MAP2K1 - mitogen-activated protein kinase kinase 1  MAP2K2 - mitogen-activated protein kinase kinase 2  MAPK10 - mitogen-activated protein kinase 10  MAPK9 - mitogen-activated protein kinase 9  MAPK11 - mitogen-activated protein kinase 11  MAPK8 - mitogen-activated protein kinase 8  MAPK7 - mitogen-activated protein kinase 7  GCN1L1 - gcn1 general control of amino-acid synthesis 1-like 1 (yeast)  MAPK3 - mitogen-activated protein kinase 3  MAPK1 - mitogen-activated protein kinase 1  ADIRF - adipogenesis regulatory factor  PRKD1 - protein kinase d1  NDC1 - ndc1 transmembrane nucleoporin  ANXA3 - annexin a3  CCDC88A - coiled-coil domain containing 88a  PRKCH - protein kinase c, eta  PRKCI - protein kinase c, iota  CCDC85B - coiled-coil domain containing 85b  PRKCB - protein kinase c, beta  PRKCD - protein kinase c, delta  RBM22 - rna binding motif protein 22  PRKCA - protein kinase c, alpha  PRKAR1A - protein kinase, camp-dependent, regulatory, type i, alpha  YEATS2 - yeats domain containing 2  PKIB - protein kinase (camp-dependent, catalytic) inhibitor beta  PRKAG1 - protein kinase, amp-activated, gamma 1 non-catalytic subunit  APEX1 - apex nuclease (multifunctional dna repair enzyme) 1  RBM23 - rna binding motif protein 23  ZNF605 - zinc finger protein 605  XIAP - x-linked inhibitor of apoptosis  BIRC2 - baculoviral iap repeat containing 2  PARN - poly(a)-specific ribonuclease  KLF12 - kruppel-like factor 12  TREX1 - three prime repair exonuclease 1  SHQ1 - shq1, h/aca ribonucleoprotein assembly factor  PBX2 - pre-b-cell leukemia homeobox 2  PBX3 - pre-b-cell leukemia homeobox 3  PRMT6 - protein arginine methyltransferase 6  PBX1 - pre-b-cell leukemia homeobox 1  PCBP2 - poly(rc) binding protein 2  PCBD1 - pterin-4 alpha-carbinolamine dehydratase/dimerization cofactor of hepatocyte nuclear factor 1 alpha  APBB2 - amyloid beta (a4) precursor protein-binding, family b, member 2  CHP1 - calcineurin-like ef-hand protein 1  APBB1 - amyloid beta (a4) precursor protein-binding, family b, member 1 (fe65)  RIF1 - rap1 interacting factor homolog (yeast)  POLG2 - polymerase (dna directed), gamma 2, accessory subunit  KIAA1551 - kiaa1551  PA2G4 - proliferation-associated 2g4, 38kda  RPRD1A - regulation of nuclear pre-mrna domain containing 1a  ARF4 - adp-ribosylation factor 4  ZHX1 - zinc fingers and homeoboxes 1  SBNO1 - strawberry notch homolog 1 (drosophila)  IRAK3 - interleukin-1 receptor-associated kinase 3  SETD5 - set domain containing 5  APP - amyloid beta (a4) precursor protein  BBS7 - bardet-biedl syndrome 7  DDX20 - dead (asp-glu-ala-asp) box polypeptide 20  PAK3 - p21 protein (cdc42/rac)-activated kinase 3  PAM - peptidylglycine alpha-amidating monooxygenase  ARNTL - aryl hydrocarbon receptor nuclear translocator-like  ARNT - aryl hydrocarbon receptor nuclear translocator  CELF3 - cugbp, elav-like family member 3  PDPR - pyruvate dehydrogenase phosphatase regulatory subunit  SUPT16H - suppressor of ty 16 homolog (s. cerevisiae)  PDGFRB - platelet-derived growth factor receptor, beta polypeptide  ARRB2 - arrestin, beta 2  ARRB1 - arrestin, beta 1  PDGFB - platelet-derived growth factor beta polypeptide  SAMD4B - sterile alpha motif domain containing 4b  RHOA - ras homolog family member a  STRAP - serine/threonine kinase receptor associated protein  PSIP1 - pc4 and sfrs1 interacting protein 1  PDK3 - pyruvate dehydrogenase kinase, isozyme 3  MED9 - mediator complex subunit 9  FSTL1 - follistatin-like 1  SLC2A6 - solute carrier family 2 (facilitated glucose transporter), member 6  BAZ2A - bromodomain adjacent to zinc finger domain, 2a  WDR70 - wd repeat domain 70  PCNA - proliferating cell nuclear antigen  CHMP1A - charged multivesicular body protein 1a  JDP2 - jun dimerization protein 2  CDK16 - cyclin-dependent kinase 16  AKIRIN2 - akirin 2  HEATR1 - heat repeat containing 1  CDK17 - cyclin-dependent kinase 17  LEO1 - leo1, paf1/rna polymerase ii complex component, homolog (s. cerevisiae)  ERLIN2 - er lipid raft associated 2  PWP1 - pwp1 homolog (s. cerevisiae)  WRAP53 - wd repeat containing, antisense to tp53  PDE2A - phosphodiesterase 2a, cgmp-stimulated  THAP1 - thap domain containing, apoptosis associated protein 1  PKIG - protein kinase (camp-dependent, catalytic) inhibitor gamma  ELP3 - elongator acetyltransferase complex subunit 3  ASCL1 - achaete-scute complex homolog 1 (drosophila)  KAT7 - k(lysine) acetyltransferase 7  RGMB - rgm domain family, member b  SBNO2 - strawberry notch homolog 2 (drosophila)  ZNF444 - zinc finger protein 444  ZBTB1 - zinc finger and btb domain containing 1  ZNF365 - zinc finger protein 365  PGAM1 - phosphoglycerate mutase 1 (brain)  FBXW7 - f-box and wd repeat domain containing 7, e3 ubiquitin protein ligase  BAHD1 - bromo adjacent homology domain containing 1  DIS3 - dis3 mitotic control homolog (s. cerevisiae)  ZHX2 - zinc fingers and homeoboxes 2  MORC2 - morc family cw-type zinc finger 2  PHF2 - phd finger protein 2  PHF1 - phd finger protein 1  FOXJ3 - forkhead box j3  RERE - arginine-glutamic acid dipeptide (re) repeats  ZFHX3 - zinc finger homeobox 3  ATP7A - atpase, cu++ transporting, alpha polypeptide  PEX14 - peroxisomal biogenesis factor 14  ADNP2 - adnp homeobox 2  AEBP2 - ae binding protein 2  IMPACT - impact rwd domain protein  ZNF507 - zinc finger protein 507  LGR4 - leucine-rich repeat containing g protein-coupled receptor 4  ATP2B4 - atpase, ca++ transporting, plasma membrane 4  ZNF652 - zinc finger protein 652  BAX - bcl2-associated x protein  SCAF8 - sr-related ctd-associated factor 8  SMU1 - smu-1 suppressor of mec-8 and unc-52 homolog (c. elegans)  PKD1 - polycystic kidney disease 1 (autosomal dominant)  PKD2 - polycystic kidney disease 2 (autosomal dominant)  PUF60 - poly-u binding splicing factor 60kda  MTF2 - metal response element binding transcription factor 2  NAT10 - n-acetyltransferase 10 (gcn5-related)  BACH1 - btb and cnc homology 1, basic leucine zipper transcription factor 1  PIN1 - peptidylprolyl cis/trans isomerase, nima-interacting 1  PIK3R2 - phosphoinositide-3-kinase, regulatory subunit 2 (beta)  PIK3R1 - phosphoinositide-3-kinase, regulatory subunit 1 (alpha)  ASXL2 - additional sex combs like 2 (drosophila)  PLAGL1 - pleiomorphic adenoma gene-like 1  ELP2 - elongator acetyltransferase complex subunit 2  ATRX - alpha thalassemia/mental retardation syndrome x-linked  PKNOX1 - pbx/knotted 1 homeobox 1  EXOSC8 - exosome component 8  TMEM100 - transmembrane protein 100  PHF10 - phd finger protein 10  U2AF2 - u2 small nuclear rna auxiliary factor 2  PSPC1 - paraspeckle component 1  BCL7A - b-cell cll/lymphoma 7a  MRGBP - mrg/morf4l binding protein  BCL9 - b-cell cll/lymphoma 9  PIK3CA - phosphatidylinositol-4,5-bisphosphate 3-kinase, catalytic subunit alpha  BRF2 - brf2, rna polymerase iii transcription initiation factor 50 kda subunit  PIM1 - pim-1 oncogene  ECD - ecdysoneless homolog (drosophila)  CCND1 - cyclin d1  PARK7 - parkinson protein 7  PNKP - polynucleotide kinase 3'-phosphatase  C3orf33 - chromosome 3 open reading frame 33  TRIM27 - tripartite motif containing 27  RFC5 - replication factor c (activator 1) 5, 36.5kda  RFC2 - replication factor c (activator 1) 2, 40kda  RFC3 - replication factor c (activator 1) 3, 38kda  SAP18 - sin3a-associated protein, 18kda  TSHZ2 - teashirt zinc finger homeobox 2  RFXAP - regulatory factor x-associated protein  RFX5 - regulatory factor x, 5 (influences hla class ii expression)  WHSC1L1 - wolf-hirschhorn syndrome candidate 1-like 1  RFX1 - regulatory factor x, 1 (influences hla class ii expression)  RELA - v-rel avian reticuloendotheliosis viral oncogene homolog a  APBB3 - amyloid beta (a4) precursor protein-binding, family b, member 3  BCOR - bcl6 corepressor  ZFP36L2 - zfp36 ring finger protein-like 2  ZFP36L1 - zfp36 ring finger protein-like 1  RET - ret proto-oncogene  DPF2 - d4, zinc and double phd fingers family 2  ALKBH5 - alkb, alkylation repair homolog 5 (e. coli)  NSUN2 - nop2/sun rna methyltransferase family, member 2  UPF1 - upf1 regulator of nonsense transcripts homolog (yeast)  CC2D1A - coiled-coil and c2 domain containing 1a  RLF - rearranged l-myc fusion  RING1 - ring finger protein 1  BMI1 - bmi1 polycomb ring finger oncogene  PCGF3 - polycomb group ring finger 3  BMPR1A - bone morphogenetic protein receptor, type ia  BMPR2 - bone morphogenetic protein receptor, type ii (serine/threonine kinase)  GON4L - gon-4-like (c. elegans)  BMPR1B - bone morphogenetic protein receptor, type ib  BMP7 - bone morphogenetic protein 7  PSMD14 - proteasome (prosome, macropain) 26s subunit, non-atpase, 14  FLOT1 - flotillin 1  BCAS3 - breast carcinoma amplified sequence 3  ABCE1 - atp-binding cassette, sub-family e (oabp), member 1  AHI1 - abelson helper integration site 1  RNF4 - ring finger protein 4  BRD2 - bromodomain containing 2  RNF2 - ring finger protein 2  HNRNPR - heterogeneous nuclear ribonucleoprotein r  ZNF280D - zinc finger protein 280d  GATAD2A - gata zinc finger domain containing 2a  RNF6 - ring finger protein (c3h2c3 type) 6  ROCK1 - rho-associated, coiled-coil containing protein kinase 1  RNF111 - ring finger protein 111  TSPO - translocator protein (18kda)  TET2 - tet methylcytosine dioxygenase 2  MED18 - mediator complex subunit 18  DENND4A - denn/madd domain containing 4a  MBTD1 - mbt domain containing 1  SF3B4 - splicing factor 3b, subunit 4, 49kda  ZMPSTE24 - zinc metallopeptidase ste24  TRIM44 - tripartite motif containing 44  TAB1 - tgf-beta activated kinase 1/map3k7 binding protein 1  PPIE - peptidylprolyl isomerase e (cyclophilin e)  MCRS1 - microspherule protein 1  NRDE2 - nrde-2, necessary for rna interference, domain containing  C1D - c1d nuclear receptor corepressor  SLC30A9 - solute carrier family 30 (zinc transporter), member 9  MAD2L2 - mad2 mitotic arrest deficient-like 2 (yeast)  PID1 - phosphotyrosine interaction domain containing 1  RPA2 - replication protein a2, 32kda  PHIP - pleckstrin homology domain interacting protein  NRARP - notch-regulated ankyrin repeat protein  ZNF787 - zinc finger protein 787  TRIM38 - tripartite motif containing 38  TADA3 - transcriptional adaptor 3  CALR - calreticulin  ZNF792 - zinc finger protein 792  WTIP - wilms tumor 1 interacting protein  NCOA2 - nuclear receptor coactivator 2  SYNCRIP - synaptotagmin binding, cytoplasmic rna interacting protein  CREB3 - camp responsive element binding protein 3  CITED2 - cbp/p300-interacting transactivator, with glu/asp-rich carboxy-terminal domain, 2  CCT6A - chaperonin containing tcp1, subunit 6a (zeta 1)  CCNT2 - cyclin t2  PINX1 - pin2/terf1 interacting, telomerase inhibitor 1  CCND3 - cyclin d3  CCNT1 - cyclin t1  HMG20A - high mobility group 20a  CCNH - cyclin h  BANP - btg3 associated nuclear protein  TIPIN - timeless interacting protein  RPS6KA1 - ribosomal protein s6 kinase, 90kda, polypeptide 1  CBFA2T3 - core-binding factor, runt domain, alpha subunit 2; translocated to, 3  CBFB - core-binding factor, beta subunit  PIAS3 - protein inhibitor of activated stat, 3  RXRB - retinoid x receptor, beta  CAV1 - caveolin 1, caveolae protein, 22kda  CCNC - cyclin c  RBM14 - rna binding motif protein 14  PRMT5 - protein arginine methyltransferase 5  CHTF8 - ctf8, chromosome transmission fidelity factor 8 homolog (s. cerevisiae)  CDK11B - cyclin-dependent kinase 11b  MXD4 - max dimerization protein 4  CDC5L - cell division cycle 5-like  HTRA1 - htra serine peptidase 1  POLR3F - polymerase (rna) iii (dna directed) polypeptide f, 39 kda  HEXIM1 - hexamethylene bis-acetamide inducible 1  ERLIN1 - er lipid raft associated 1  SPIN2A - spindlin family, member 2a  NCOA7 - nuclear receptor coactivator 7  PSEN1 - presenilin 1  USP16 - ubiquitin specific peptidase 16  SRSF12 - serine/arginine-rich splicing factor 12  PSMA1 - proteasome (prosome, macropain) subunit, alpha type, 1  PSMA2 - proteasome (prosome, macropain) subunit, alpha type, 2  CD81 - cd81 molecule  CAMKK2 - calcium/calmodulin-dependent protein kinase kinase 2, beta  PSMB1 - proteasome (prosome, macropain) subunit, beta type, 1  PSMA5 - proteasome (prosome, macropain) subunit, alpha type, 5  PSMA3 - proteasome (prosome, macropain) subunit, alpha type, 3  RIPPLY2 - ripply transcriptional repressor 2  PSMB10 - proteasome (prosome, macropain) subunit, beta type, 10  KHDRBS3 - kh domain containing, rna binding, signal transduction associated 3  FAM173B - family with sequence similarity 173, member b  PSMC6 - proteasome (prosome, macropain) 26s subunit, atpase, 6  TAF6L - taf6-like rna polymerase ii, p300/cbp-associated factor (pcaf)-associated factor, 65kda  PSMD1 - proteasome (prosome, macropain) 26s subunit, non-atpase, 1  PSMC4 - proteasome (prosome, macropain) 26s subunit, atpase, 4  SOX18 - sry (sex determining region y)-box 18  POLR3G - polymerase (rna) iii (dna directed) polypeptide g (32kd)  PSMC2 - proteasome (prosome, macropain) 26s subunit, atpase, 2  PSMD9 - proteasome (prosome, macropain) 26s subunit, non-atpase, 9  PSMD10 - proteasome (prosome, macropain) 26s subunit, non-atpase, 10  PSMD7 - proteasome (prosome, macropain) 26s subunit, non-atpase, 7  PSMD8 - proteasome (prosome, macropain) 26s subunit, non-atpase, 8  PSMD5 - proteasome (prosome, macropain) 26s subunit, non-atpase, 5  TERF2IP - telomeric repeat binding factor 2, interacting protein  PSMD2 - proteasome (prosome, macropain) 26s subunit, non-atpase, 2  SLC38A2 - solute carrier family 38, member 2  PSMD12 - proteasome (prosome, macropain) 26s subunit, non-atpase, 12  ATG7 - autophagy related 7  PSMD13 - proteasome (prosome, macropain) 26s subunit, non-atpase, 13  PTBP1 - polypyrimidine tract binding protein 1  PTEN - phosphatase and tensin homolog  LAMTOR5 - late endosomal/lysosomal adaptor, mapk and mtor activator 5  JMY - junction mediating and regulatory protein, p53 cofactor  MYBBP1A - myb binding protein (p160) 1a  FBLN5 - fibulin 5  CIB1 - calcium and integrin binding 1 (calmyrin)  PTGIS - prostaglandin i2 (prostacyclin) synthase  DDX17 - dead (asp-glu-ala-asp) box helicase 17  KAT5 - k(lysine) acetyltransferase 5  ZFP69 - zfp69 zinc finger protein  CEBPG - ccaat/enhancer binding protein (c/ebp), gamma  CDK5 - cyclin-dependent kinase 5  CDK9 - cyclin-dependent kinase 9  CCT7 - chaperonin containing tcp1, subunit 7 (eta)  CDK8 - cyclin-dependent kinase 8  LIN54 - lin-54 homolog (c. elegans)  CDK7 - cyclin-dependent kinase 7  CDKN1C - cyclin-dependent kinase inhibitor 1c (p57, kip2)  CDKN1B - cyclin-dependent kinase inhibitor 1b (p27, kip1)  CCT2 - chaperonin containing tcp1, subunit 2 (beta)  CCT4 - chaperonin containing tcp1, subunit 4 (delta)  PCBP3 - poly(rc) binding protein 3  PTPN2 - protein tyrosine phosphatase, non-receptor type 2  HTATIP2 - hiv-1 tat interactive protein 2, 30kda  CDK2 - cyclin-dependent kinase 2  RRN3 - rrn3 rna polymerase i transcription factor homolog (s. cerevisiae)  PURA - purine-rich element binding protein a  PURB - purine-rich element binding protein b  PDP1 - pyruvate dehyrogenase phosphatase catalytic subunit 1  NUDT16 - nudix (nucleoside diphosphate linked moiety x)-type motif 16  ZMYND11 - zinc finger, mynd-type containing 11  SRSF10 - serine/arginine-rich splicing factor 10  PTPRK - protein tyrosine phosphatase, receptor type, k  PTPRN - protein tyrosine phosphatase, receptor type, n  JMJD8 - jumonji domain containing 8  AHCYL1 - adenosylhomocysteinase-like 1  PEX2 - peroxisomal biogenesis factor 2  NUP50 - nucleoporin 50kda  KDM5B - lysine (k)-specific demethylase 5b  MPHOSPH8 - m-phase phosphoprotein 8  PXN - paxillin  CHD4 - chromodomain helicase dna binding protein 4  CHD2 - chromodomain helicase dna binding protein 2  CHD3 - chromodomain helicase dna binding protein 3  CHD1 - chromodomain helicase dna binding protein 1  MTERFD2 - mterf domain containing 2  QARS - glutaminyl-trna synthetase  MYPOP - myb-related transcription factor, partner of profilin  RAD21 - rad21 homolog (s. pombe)  RAD17 - rad17 homolog (s. pombe)  PARP14 - poly (adp-ribose) polymerase family, member 14  ZNF181 - zinc finger protein 181  PAF1 - paf1, rna polymerase ii associated factor, homolog (s. cerevisiae)  RAC1 - ras-related c3 botulinum toxin substrate 1 (rho family, small gtp binding protein rac1)  ZNF268 - zinc finger protein 268  INO80 - ino80 complex subunit  ZNF513 - zinc finger protein 513  GMEB1 - glucocorticoid modulatory element binding protein 1  RC3H2 - ring finger and ccch-type domains 2  RANBP2 - ran binding protein 2  CCT8 - chaperonin containing tcp1, subunit 8 (theta)  MIER2 - mesoderm induction early response 1, family member 2  FAM35A - family with sequence similarity 35, member a  RARG - retinoic acid receptor, gamma  RARB - retinoic acid receptor, beta  DLL4 - delta-like 4 (drosophila)  RASA1 - ras p21 protein activator (gtpase activating protein) 1  CELF1 - cugbp, elav-like family member 1  CLU - clusterin  KHDRBS1 - kh domain containing, rna binding, signal transduction associated 1  TAF8 - taf8 rna polymerase ii, tata box binding protein (tbp)-associated factor, 43kda  CLK1 - cdc-like kinase 1  GNL3L - guanine nucleotide binding protein-like 3 (nucleolar)-like  CELF2 - cugbp, elav-like family member 2  MAGEL2 - mage-like 2  CLK2 - cdc-like kinase 2  DDX49 - dead (asp-glu-ala-asp) box polypeptide 49  CLK3 - cdc-like kinase 3  CTCF - ccctc-binding factor (zinc finger protein)  RAI1 - retinoic acid induced 1  RBL2 - retinoblastoma-like 2 (p130)  RBBP7 - retinoblastoma binding protein 7  RBBP8 - retinoblastoma binding protein 8  MAP3K2 - mitogen-activated protein kinase kinase kinase 2  RBM3 - rna binding motif (rnp1, rrm) protein 3  RBM4 - rna binding motif protein 4  RB1 - retinoblastoma 1  ARID4A - at rich interactive domain 4a (rbp1-like)  RBBP6 - retinoblastoma binding protein 6  KDM5A - lysine (k)-specific demethylase 5a  NUP35 - nucleoporin 35kda  ERCC8 - excision repair cross-complementing rodent repair deficiency, complementation group 8  EXOSC4 - exosome component 4  PTGES3 - prostaglandin e synthase 3 (cytosolic)  NFAT5 - nuclear factor of activated t-cells 5, tonicity-responsive  CKS1B - cdc28 protein kinase regulatory subunit 1b  CIRBP - cold inducible rna binding protein  CHUK - conserved helix-loop-helix ubiquitous kinase  CERS1 - ceramide synthase 1  ZMYND8 - zinc finger, mynd-type containing 8  ANKRA2 - ankyrin repeat, family a (rfxank-like), 2  RBAK - rb-associated krab zinc finger  NFATC2IP - nuclear factor of activated t-cells, cytoplasmic, calcineurin-dependent 2 interacting protein  FBXO18 - f-box protein, helicase, 18  PATZ1 - poz (btb) and at hook containing zinc finger 1  METTL14 - methyltransferase like 14  SPP1 - secreted phosphoprotein 1  SSBP2 - single-stranded dna binding protein 2  KPNA6 - karyopherin alpha 6 (importin alpha 7)  CIRH1A - cirrhosis, autosomal recessive 1a (cirhin)  COX7A2 - cytochrome c oxidase subunit viia polypeptide 2 (liver)  MIER1 - mesoderm induction early response 1, transcriptional regulator  CWC22 - cwc22 spliceosome-associated protein homolog (s. cerevisiae)  ZNF382 - zinc finger protein 382  ZNF705A - zinc finger protein 705a  SP3 - sp3 transcription factor  SP4 - sp4 transcription factor  SP1 - sp1 transcription factor  ZNF410 - zinc finger protein 410  SP2 - sp2 transcription factor  ARL2BP - adp-ribosylation factor-like 2 binding protein  SOX4 - sry (sex determining region y)-box 4  GTPBP4 - gtp binding protein 4  WBP2 - ww domain binding protein 2  ORC3 - origin recognition complex, subunit 3  SON - son dna binding protein  HES4 - hairy and enhancer of split 4 (drosophila)  LEMD3 - lem domain containing 3  CREBL2 - camp responsive element binding protein-like 2  GATAD1 - gata zinc finger domain containing 1  ATF6B - activating transcription factor 6 beta  CREBBP - creb binding protein  ATF2 - activating transcription factor 2  CREB1 - camp responsive element binding protein 1  DDX58 - dead (asp-glu-ala-asp) box polypeptide 58  CRABP2 - cellular retinoic acid binding protein 2  RPRD1B - regulation of nuclear pre-mrna domain containing 1b  PLK3 - polo-like kinase 3  SNAPC4 - small nuclear rna activating complex, polypeptide 4, 190kda  SNCA - synuclein, alpha (non a4 component of amyloid precursor)  SNRNP70 - small nuclear ribonucleoprotein 70kda (u1)  ZBED5 - zinc finger, bed-type containing 5  SNRPA - small nuclear ribonucleoprotein polypeptide a  CREBZF - creb/atf bzip transcription factor  EID1 - ep300 interacting inhibitor of differentiation 1  TP53INP2 - tumor protein p53 inducible nuclear protein 2  PTBP2 - polypyrimidine tract binding protein 2  RBM17 - rna binding motif protein 17  SMARCE1 - swi/snf related, matrix associated, actin dependent regulator of chromatin, subfamily e, member 1  SMO - smoothened, frizzled family receptor  SMARCB1 - swi/snf related, matrix associated, actin dependent regulator of chromatin, subfamily b, member 1  SMARCC1 - swi/snf related, matrix associated, actin dependent regulator of chromatin, subfamily c, member 1  HLTF - helicase-like transcription factor  SSBP3 - single stranded dna binding protein 3  SMARCA4 - swi/snf related, matrix associated, actin dependent regulator of chromatin, subfamily a, member 4  SNX6 - sorting nexin 6  SMARCD2 - swi/snf related, matrix associated, actin dependent regulator of chromatin, subfamily d, member 2  SMARCD3 - swi/snf related, matrix associated, actin dependent regulator of chromatin, subfamily d, member 3  SMARCC2 - swi/snf related, matrix associated, actin dependent regulator of chromatin, subfamily c, member 2  SMARCD1 - swi/snf related, matrix associated, actin dependent regulator of chromatin, subfamily d, member 1  WIZ - widely interspaced zinc finger motifs  RBM25 - rna binding motif protein 25  SMARCA1 - swi/snf related, matrix associated, actin dependent regulator of chromatin, subfamily a, member 1  SMARCA2 - swi/snf related, matrix associated, actin dependent regulator of chromatin, subfamily a, member 2  SGK3 - serum/glucocorticoid regulated kinase family, member 3  CACTIN - cactin, spliceosome c complex subunit  KMT2C - lysine (k)-specific methyltransferase 2c  ZNF462 - zinc finger protein 462  PPIL4 - peptidylprolyl isomerase (cyclophilin)-like 4  TFIP11 - tuftelin interacting protein 11  SENP2 - sumo1/sentrin/smt3 specific peptidase 2  CST3 - cystatin c  ZNF318 - zinc finger protein 318  PRPF6 - pre-mrna processing factor 6  KDM2B - lysine (k)-specific demethylase 2b  SUPT6H - suppressor of ty 6 homolog (s. cerevisiae)  SUPV3L1 - suppressor of var1, 3-like 1 (s. cerevisiae)  CBY1 - chibby homolog 1 (drosophila)  CACNG7 - calcium channel, voltage-dependent, gamma subunit 7  CTBP1 - c-terminal binding protein 1  CTBP2 - c-terminal binding protein 2  SUPT4H1 - suppressor of ty 4 homolog 1 (s. cerevisiae)  SUPT5H - suppressor of ty 5 homolog (s. cerevisiae)  CTNNB1 - catenin (cadherin-associated protein), beta 1, 88kda  SAV1 - salvador homolog 1 (drosophila)  CUX1 - cut-like homeobox 1  NIF3L1 - nif3 ngg1 interacting factor 3-like 1 (s. cerevisiae)  NEK4 - nima-related kinase 4  STK3 - serine/threonine kinase 3  PCGF1 - polycomb group ring finger 1  NPNT - nephronectin  FKBP8 - fk506 binding protein 8, 38kda  GPBP1L1 - gc-rich promoter binding protein 1-like 1  CYLD - cylindromatosis (turban tumor syndrome)  SUV420H2 - suppressor of variegation 4-20 homolog 2 (drosophila)  TGIF2 - tgfb-induced factor homeobox 2  BCL6B - b-cell cll/lymphoma 6, member b  CRK - v-crk avian sarcoma virus ct10 oncogene homolog  CELF6 - cugbp, elav-like family member 6  NIPBL - nipped-b homolog (drosophila)  STAT2 - signal transducer and activator of transcription 2, 113kda  CRY1 - cryptochrome 1 (photolyase-like)  STAT1 - signal transducer and activator of transcription 1, 91kda  STAT3 - signal transducer and activator of transcription 3 (acute-phase response factor)  CRY2 - cryptochrome 2 (photolyase-like)  STAT5A - signal transducer and activator of transcription 5a  ASF1A - anti-silencing function 1a histone chaperone  STAT6 - signal transducer and activator of transcription 6, interleukin-4 induced  PARM1 - prostate androgen-regulated mucin-like protein 1  BRMS1 - breast cancer metastasis suppressor 1  ZNF496 - zinc finger protein 496  CRYM - crystallin, mu  SS18 - synovial sarcoma translocation, chromosome 18  PHF5A - phd finger protein 5a  GPATCH3 - g patch domain containing 3  SRPK2 - srsf protein kinase 2  SRPK1 - srsf protein kinase 1  TAB3 - tgf-beta activated kinase 1/map3k7 binding protein 3  ZNF335 - zinc finger protein 335  BAMBI - bmp and activin membrane-bound inhibitor  HAVCR2 - hepatitis a virus cellular receptor 2  ZC3H10 - zinc finger ccch-type containing 10  SRI - sorcin  SRC - v-src avian sarcoma (schmidt-ruppin a-2) viral oncogene homolog  DNAJB5 - dnaj (hsp40) homolog, subfamily b, member 5  SREBF2 - sterol regulatory element binding transcription factor 2  UBE2O - ubiquitin-conjugating enzyme e2o  PRDX5 - peroxiredoxin 5  RNF169 - ring finger protein 169  DHX9 - deah (asp-glu-ala-his) box helicase 9  TRAPPC2 - trafficking protein particle complex 2  KANK2 - kn motif and ankyrin repeat domains 2  DDX5 - dead (asp-glu-ala-asp) box helicase 5  DDX3X - dead (asp-glu-ala-asp) box helicase 3, x-linked  SEC13 - sec13 homolog (s. cerevisiae)  DDX1 - dead (asp-glu-ala-asp) box helicase 1  SIN3A - sin3 transcription regulator family member a  SET - set nuclear oncogene  WWTR1 - ww domain containing transcription regulator 1  SETMAR - set domain and mariner transposase fusion gene  ZNF304 - zinc finger protein 304  MAP2K4 - mitogen-activated protein kinase kinase 4  ZNF286A - zinc finger protein 286a  NOL11 - nucleolar protein 11  PBXIP1 - pre-b-cell leukemia homeobox interacting protein 1  DBP - d site of albumin promoter (albumin d-box) binding protein  NELFB - negative elongation factor complex member b  POT1 - protection of telomeres 1  AHCTF1 - at hook containing transcription factor 1  CLK4 - cdc-like kinase 4  ZBTB38 - zinc finger and btb domain containing 38  ZNF551 - zinc finger protein 551  DFFA - dna fragmentation factor, 45kda, alpha polypeptide  GATAD2B - gata zinc finger domain containing 2b  ZNF473 - zinc finger protein 473  KCTD13 - potassium channel tetramerization domain containing 13  YTHDF3 - yth domain family, member 3  WDR18 - wd repeat domain 18  CNOT6 - ccr4-not transcription complex, subunit 6  ZNF451 - zinc finger protein 451  SS18L1 - synovial sarcoma translocation gene on chromosome 18-like 1  SCAF4 - sr-related ctd-associated factor 4  CHD5 - chromodomain helicase dna binding protein 5  ARID1B - at rich interactive domain 1b (swi1-like)  MED30 - mediator complex subunit 30  MKL2 - mkl/myocardin-like 2  DAPK3 - death-associated protein kinase 3  ANGEL2 - angel homolog 2 (drosophila)  DAXX - death-domain associated protein  ZZZ3 - zinc finger, zz-type containing 3  HINFP - histone h4 transcription factor  DGKQ - diacylglycerol kinase, theta 110kda  MTA3 - metastasis associated 1 family, member 3  ZNF608 - zinc finger protein 608  MAVS - mitochondrial antiviral signaling protein  DAP - death-associated protein  SARS - seryl-trna synthetase  SATB1 - satb homeobox 1  RPTOR - regulatory associated protein of mtor, complex 1  SAFB - scaffold attachment factor b  SH2B1 - sh2b adaptor protein 1  SALL2 - sal-like 2 (drosophila)  SERBP1 - serpine1 mrna binding protein 1  DRD2 - dopamine receptor d2  EPC2 - enhancer of polycomb homolog 2 (drosophila)  PHF19 - phd finger protein 19  SLC9A1 - solute carrier family 9, subfamily a (nhe1, cation proton antiporter 1), member 1  IRF2BP1 - interferon regulatory factor 2 binding protein 1  CNOT6L - ccr4-not transcription complex, subunit 6-like  EAF1 - ell associated factor 1  PDP2 - pyruvate dehyrogenase phosphatase catalytic subunit 2  ATN1 - atrophin 1  DOM3Z - dom-3 homolog z (c. elegans)  C10orf137 - chromosome 10 open reading frame 137  DR1 - down-regulator of transcription 1, tbp-binding (negative cofactor 2)  MAPK15 - mitogen-activated protein kinase 15  SKI - v-ski avian sarcoma viral oncogene homolog  SKIL - ski-like oncogene  DYRK1A - dual-specificity tyrosine-(y)-phosphorylation regulated kinase 1a  MKL1 - megakaryoblastic leukemia (translocation) 1  DVL2 - dishevelled segment polarity protein 2  DVL3 - dishevelled segment polarity protein 3  GIGYF2 - grb10 interacting gyf protein 2  USP36 - ubiquitin specific peptidase 36  FNIP2 - folliculin interacting protein 2  HOMEZ - homeobox and leucine zipper encoding  EBF4 - early b-cell factor 4  ANKRD17 - ankyrin repeat domain 17  ZFHX2 - zinc finger homeobox 2  DTX1 - deltex homolog 1 (drosophila)  DKC1 - dyskeratosis congenita 1, dyskerin  PNPT1 - polyribonucleotide nucleotidyltransferase 1  DLG1 - discs, large homolog 1 (drosophila)  SHOX2 - short stature homeobox 2  SIAH2 - siah e3 ubiquitin protein ligase 2  POGK - pogo transposable element with krab domain  DHFR - dihydrofolate reductase  PHF12 - phd finger protein 12  CALCOCO1 - calcium binding and coiled-coil domain 1  SFPQ - splicing factor proline/glutamine-rich  ZBTB4 - zinc finger and btb domain containing 4  SFRP1 - secreted frizzled-related protein 1  SFRP2 - secreted frizzled-related protein 2  SFRP4 - secreted frizzled-related protein 4  SRSF1 - serine/arginine-rich splicing factor 1  GMEB2 - glucocorticoid modulatory element binding protein 2  DNM2 - dynamin 2  SRSF2 - serine/arginine-rich splicing factor 2  SPAG8 - sperm associated antigen 8  SRSF3 - serine/arginine-rich splicing factor 3  DNMT1 - dna (cytosine-5-)-methyltransferase 1  TRDMT1 - trna aspartic acid methyltransferase 1  SRSF4 - serine/arginine-rich splicing factor 4  SRSF5 - serine/arginine-rich splicing factor 5  DNMT3A - dna (cytosine-5-)-methyltransferase 3 alpha  SRSF6 - serine/arginine-rich splicing factor 6  EIF2AK4 - eukaryotic translation initiation factor 2 alpha kinase 4  SRSF7 - serine/arginine-rich splicing factor 7  SFSWAP - splicing factor, suppressor of white-apricot homolog (drosophila)  TRA2B - transformer 2 beta homolog (drosophila)  CHD8 - chromodomain helicase dna binding protein 8  TNRC6C - trinucleotide repeat containing 6c  SGK1 - serum/glucocorticoid regulated kinase 1  NOC2L - nucleolar complex associated 2 homolog (s. cerevisiae)  ZNF317 - zinc finger protein 317  USP37 - ubiquitin specific peptidase 37  SMARCAD1 - swi/snf-related, matrix-associated actin-dependent regulator of chromatin, subfamily a, containing dead/h box 1  MEIS3 - meis homeobox 3  NCBP2 - nuclear cap binding protein subunit 2, 20kda  MAPRE3 - microtubule-associated protein, rp/eb family, member 3  ELK1 - elk1, member of ets oncogene family  SPIRE1 - spire actin nucleation factor 1  NAF1 - nuclear assembly factor 1 ribonucleoprotein  ELK3 - elk3, ets-domain protein (srf accessory protein 2)  ELK4 - elk4, ets-domain protein (srf accessory protein 1)  SNW1 - snw domain containing 1  SCAP - srebf chaperone  WRNIP1 - werner helicase interacting protein 1  EPAS1 - endothelial pas domain protein 1  EP300 - e1a binding protein p300  HABP4 - hyaluronan binding protein 4  ATF6 - activating transcription factor 6  UBQLN4 - ubiquilin 4  ENO1 - enolase 1, (alpha)  EIF4A2 - eukaryotic translation initiation factor 4a2  CELF4 - cugbp, elav-like family member 4  EGR2 - early growth response 2  EGR3 - early growth response 3  CCT5 - chaperonin containing tcp1, subunit 5 (epsilon)  ZC3HAV1 - zinc finger ccch-type, antiviral 1  TTF1 - transcription termination factor, rna polymerase i  TRIM32 - tripartite motif containing 32  SPHK2 - sphingosine kinase 2  KDM2A - lysine (k)-specific demethylase 2a  ELAVL1 - elav (embryonic lethal, abnormal vision, drosophila)-like 1 (hu antigen r)  ELF1 - e74-like factor 1 (ets domain transcription factor)  ZNF326 - zinc finger protein 326  ELAVL4 - elav (embryonic lethal, abnormal vision, drosophila)-like 4  ELF2 - e74-like factor 2 (ets domain transcription factor)  TSG101 - tumor susceptibility gene 101  TCF25 - transcription factor 25 (basic helix-loop-helix)  EFNA1 - ephrin-a1  CNOT1 - ccr4-not transcription complex, subunit 1  EXOSC7 - exosome component 7  CTNNBIP1 - catenin, beta interacting protein 1  SPEN - spen homolog, transcriptional regulator (drosophila)  BBX - bobby sox homolog (drosophila)  PHC1 - polyhomeotic homolog 1 (drosophila)  TP53BP1 - tumor protein p53 binding protein 1  PHC2 - polyhomeotic homolog 2 (drosophila)  EGR1 - early growth response 1  ZNF292 - zinc finger protein 292  EGFR - epidermal growth factor receptor  MEGF8 - multiple egf-like-domains 8  CELSR2 - cadherin, egf lag seven-pass g-type receptor 2  KDM1A - lysine (k)-specific demethylase 1a  ATXN7L3 - ataxin 7-like 3  HNRNPLL - heterogeneous nuclear ribonucleoprotein l-like  NCOA6 - nuclear receptor coactivator 6  CCT3 - chaperonin containing tcp1, subunit 3 (gamma)  E2F6 - e2f transcription factor 6  SMG1 - smg1 phosphatidylinositol 3-kinase-related kinase  E4F1 - e4f transcription factor 1  ZHX3 - zinc fingers and homeoboxes 3  E2F4 - e2f transcription factor 4, p107/p130-binding  E2F5 - e2f transcription factor 5, p130-binding  ENY2 - enhancer of yellow 2 homolog (drosophila)  C11orf30 - chromosome 11 open reading frame 30  RRP1B - ribosomal rna processing 1b  EDNRB - endothelin receptor type b  TRAF1 - tnf receptor-associated factor 1  S1PR1 - sphingosine-1-phosphate receptor 1  TRAF3 - tnf receptor-associated factor 3  TRAF2 - tnf receptor-associated factor 2  NR2C1 - nuclear receptor subfamily 2, group c, member 1  ZBTB47 - zinc finger and btb domain containing 47  SETX - senataxin  NR2C2 - nuclear receptor subfamily 2, group c, member 2  ZNF609 - zinc finger protein 609  TPR - translocated promoter region, nuclear basket protein  WAPAL - wings apart-like homolog (drosophila)  ERC1 - elks/rab6-interacting/cast family member 1  PPRC1 - peroxisome proliferator-activated receptor gamma, coactivator-related 1  NAT14 - n-acetyltransferase 14 (gcn5-related, putative)  KDM4C - lysine (k)-specific demethylase 4c  ZNF423 - zinc finger protein 423  REXO4 - rex4, rna exonuclease 4 homolog (s. cerevisiae)  CAMK1D - calcium/calmodulin-dependent protein kinase id  USF1 - upstream transcription factor 1  NUP107 - nucleoporin 107kda  USP1 - ubiquitin specific peptidase 1  ZBTB43 - zinc finger and btb domain containing 43  PTK2B - protein tyrosine kinase 2 beta  PITHD1 - pith (c-terminal proteasome-interacting domain of thioredoxin-like) domain containing 1  TNRC6B - trinucleotide repeat containing 6b  BPTF - bromodomain phd finger transcription factor  TAB2 - tgf-beta activated kinase 1/map3k7 binding protein 2  NR1H2 - nuclear receptor subfamily 1, group h, member 2  CAMTA2 - calmodulin binding transcription activator 2  ETV3 - ets variant 3  ETV5 - ets variant 5  POGZ - pogo transposable element with znf domain  TWSG1 - twisted gastrulation homolog 1 (drosophila)  PLXND1 - plexin d1  ETV6 - ets variant 6  VHL - von hippel-lindau tumor suppressor, e3 ubiquitin protein ligase  VIM - vimentin  KDM6B - lysine (k)-specific demethylase 6b  PHF8 - phd finger protein 8  EZR - ezrin  EWSR1 - ews rna-binding protein 1  TRIM41 - tripartite motif containing 41  TBX20 - t-box 20  PCBP4 - poly(rc) binding protein 4  KCTD1 - potassium channel tetramerization domain containing 1  CREB3L1 - camp responsive element binding protein 3-like 1  ZC3H3 - zinc finger ccch-type containing 3  EYA3 - eyes absent homolog 3 (drosophila)  EZH1 - enhancer of zeste homolog 1 (drosophila)  CCNL1 - cyclin l1  CIC - capicua transcriptional repressor  F2R - coagulation factor ii (thrombin) receptor  VCP - valosin containing protein  WDR43 - wd repeat domain 43  ZNF575 - zinc finger protein 575  ERF - ets2 repressor factor  ERCC4 - excision repair cross-complementing rodent repair deficiency, complementation group 4  NUP205 - nucleoporin 205kda  YTHDC1 - yth domain containing 1  ELMSAN1 - elm2 and myb/sant-like domain containing 1  THAP11 - thap domain containing 11  ERN1 - endoplasmic reticulum to nucleus signaling 1  ZNF248 - zinc finger protein 248  UBB - ubiquitin b  RCOR1 - rest corepressor 1  TULP3 - tubby like protein 3  HIRA - histone cell cycle regulator  ZNFX1 - zinc finger, nfx1-type containing 1  TTC5 - tetratricopeptide repeat domain 5  ETV1 - ets variant 1  ETS1 - v-ets avian erythroblastosis virus e26 oncogene homolog 1  ZMIZ1 - zinc finger, miz-type containing 1  UBP1 - upstream binding protein 1 (lbp-1a)  SUMO1 - small ubiquitin-like modifier 1  FAM168A - family with sequence similarity 168, member a  UBTF - upstream binding transcription factor, rna polymerase i  ZNF830 - zinc finger protein 830  PELI1 - pellino e3 ubiquitin protein ligase 1  CHURC1 - churchill domain containing 1  SMURF1 - smad specific e3 ubiquitin protein ligase 1  ZC3H4 - zinc finger ccch-type containing 4  JMJD6 - jumonji domain containing 6  UBE2I - ubiquitin-conjugating enzyme e2i  UBE2V2 - ubiquitin-conjugating enzyme e2 variant 2  ERCC2 - excision repair cross-complementing rodent repair deficiency, complementation group 2  NUP210 - nucleoporin 210kda  UBE2L3 - ubiquitin-conjugating enzyme e2l 3  UBE2N - ubiquitin-conjugating enzyme e2n  UBE2V1 - ubiquitin-conjugating enzyme e2 variant 1  VPS72 - vacuolar protein sorting 72 homolog (s. cerevisiae)  FMR1 - fragile x mental retardation 1  MGA - mga, max dimerization protein  TCP1 - t-complex 1  WWC3 - wwc family member 3  ZEB1 - zinc finger e-box binding homeobox 1  TCF12 - transcription factor 12  ZNF354A - zinc finger protein 354a  TCF20 - transcription factor 20 (ar1)  CAMTA1 - calmodulin binding transcription activator 1  PDS5A - pds5, regulator of cohesion maintenance, homolog a (s. cerevisiae)  FLI1 - fli-1 proto-oncogene, ets transcription factor  FLNA - filamin a, alpha  PLCB1 - phospholipase c, beta 1 (phosphoinositide-specific)  FOXS1 - forkhead box s1  FOXO3 - forkhead box o3  HDAC8 - histone deacetylase 8  ASH1L - ash1 (absent, small, or homeotic)-like (drosophila)  SIN3B - sin3 transcription regulator family member b  DCP1A - decapping mrna 1a  NACC1 - nucleus accumbens associated 1, ben and btb (poz) domain containing  HR - hair growth associated  MED8 - mediator complex subunit 8  CAND1 - cullin-associated and neddylation-dissociated 1  TDG - thymine-dna glycosylase  SMG6 - smg6 nonsense mediated mrna decay factor  PRDX2 - peroxiredoxin 2  EAPP - e2f-associated phosphoprotein  FBXW11 - f-box and wd repeat domain containing 11  KDM3A - lysine (k)-specific demethylase 3a  FAM208A - family with sequence similarity 208, member a  FHL2 - four and a half lim domains 2  NUP160 - nucleoporin 160kda  TAF9 - taf9 rna polymerase ii, tata box binding protein (tbp)-associated factor, 32kda  FGFR2 - fibroblast growth factor receptor 2  TAF7 - taf7 rna polymerase ii, tata box binding protein (tbp)-associated factor, 55kda  TAF6 - taf6 rna polymerase ii, tata box binding protein (tbp)-associated factor, 80kda  FGFR1 - fibroblast growth factor receptor 1  TADA2A - transcriptional adaptor 2a  FGF9 - fibroblast growth factor 9  SMCHD1 - structural maintenance of chromosomes flexible hinge domain containing 1  TACC1 - transforming, acidic coiled-coil containing protein 1  FGF1 - fibroblast growth factor 1 (acidic)  TIRAP - toll-interleukin 1 receptor (tir) domain containing adaptor protein  FGF2 - fibroblast growth factor 2 (basic)  USP22 - ubiquitin specific peptidase 22  TARBP1 - tar (hiv-1) rna binding protein 1  FER - fer (fps/fes related) tyrosine kinase  MAP3K7 - mitogen-activated protein kinase kinase kinase 7  ADNP - activity-dependent neuroprotector homeobox  KMT2E - lysine (k)-specific methyltransferase 2e  MED13L - mediator complex subunit 13-like  ZNF395 - zinc finger protein 395  TAZ - tafazzin  MYNN - myoneurin  CRTC1 - creb regulated transcription coactivator 1  UFL1 - ufm1-specific ligase 1  TCF3 - transcription factor 3  PUM2 - pumilio homolog 2 (drosophila)  TCEB3 - transcription elongation factor b (siii), polypeptide 3 (110kda, elongin a)  DMAP1 - dna methyltransferase 1 associated protein 1  TCF4 - transcription factor 4  LARP1 - la ribonucleoprotein domain family, member 1  EFEMP1 - egf containing fibulin-like extracellular matrix protein 1  NKRF - nfkb repressing factor  ZNF629 - zinc finger protein 629  FBN1 - fibrillin 1  LANCL2 - lanc lantibiotic synthetase component c-like 2 (bacterial)  TCEA2 - transcription elongation factor a (sii), 2  TCEA1 - transcription elongation factor a (sii), 1  ERBB2IP - erbb2 interacting protein  ITGB3BP - integrin beta 3 binding protein (beta3-endonexin)  RYBP - ring1 and yy1 binding protein  MTOR - mechanistic target of rapamycin (serine/threonine kinase)  PAXBP1 - pax3 and pax7 binding protein 1  TLE1 - transducin-like enhancer of split 1 (e(sp1) homolog, drosophila)  TLE3 - transducin-like enhancer of split 3 (e(sp1) homolog, drosophila)  TLE4 - transducin-like enhancer of split 4 (e(sp1) homolog, drosophila)  TLR3 - toll-like receptor 3  EXOSC2 - exosome component 2  SIRT1 - sirtuin 1  MKX - mohawk homeobox  TMF1 - tata element modulatory factor 1  ACTR8 - arp8 actin-related protein 8 homolog (yeast)  TMSB4X - thymosin beta 4, x-linked  CBX6 - chromobox homolog 6  HEY1 - hairy/enhancer-of-split related with yrpw motif 1  TMPO - thymopoietin  CBX5 - chromobox homolog 5  BRD4 - bromodomain containing 4  TNFAIP1 - tumor necrosis factor, alpha-induced protein 1 (endothelial)  BCL9L - b-cell cll/lymphoma 9-like  TARDBP - tar dna binding protein  TNFRSF1A - tumor necrosis factor receptor superfamily, member 1a  TNFRSF1B - tumor necrosis factor receptor superfamily, member 1b  TMBIM6 - transmembrane bax inhibitor motif containing 6  NUP188 - nucleoporin 188kda  SUZ12 - suz12 polycomb repressive complex 2 subunit  TEF - thyrotrophic embryonic factor  POFUT1 - protein o-fucosyltransferase 1  RCOR2 - rest corepressor 2  TFAM - transcription factor a, mitochondrial  TERF1 - telomeric repeat binding factor (nima-interacting) 1  TERF2 - telomeric repeat binding factor 2  TFDP1 - transcription factor dp-1  NR2F2 - nuclear receptor subfamily 2, group f, member 2  TFE3 - transcription factor binding to ighm enhancer 3  CBX7 - chromobox homolog 7  ISCU - iron-sulfur cluster assembly enzyme  NR2F1 - nuclear receptor subfamily 2, group f, member 1  TFCP2 - transcription factor cp2  ZNF740 - zinc finger protein 740  TGFA - transforming growth factor, alpha  HEY2 - hairy/enhancer-of-split related with yrpw motif 2  FNIP1 - folliculin interacting protein 1  YLPM1 - ylp motif containing 1  TGFBR1 - transforming growth factor, beta receptor 1  TGFB2 - transforming growth factor, beta 2  RBFOX2 - rna binding protein, fox-1 homolog (c. elegans) 2  TGFB3 - transforming growth factor, beta 3  TGFB1 - transforming growth factor, beta 1  TGFB1I1 - transforming growth factor beta 1 induced transcript 1  TP53INP1 - tumor protein p53 inducible nuclear protein 1  H2AFV - h2a histone family, member v  MEPCE - methylphosphate capping enzyme  RNF20 - ring finger protein 20, e3 ubiquitin protein ligase  TGFBR3 - transforming growth factor, beta receptor iii  FOSL2 - fos-like antigen 2  FOSB - fbj murine osteosarcoma viral oncogene homolog b  FOS - fbj murine osteosarcoma viral oncogene homolog  KAT6B - k(lysine) acetyltransferase 6b  TIA1 - tia1 cytotoxic granule-associated rna binding protein  METTL3 - methyltransferase like 3  THRA - thyroid hormone receptor, alpha  ELL - elongation factor rna polymerase ii  ZNF239 - zinc finger protein 239  PHC3 - polyhomeotic homolog 3 (drosophila)  OBFC1 - oligonucleotide/oligosaccharide-binding fold containing 1  KAT2A - k(lysine) acetyltransferase 2a  NRIP1 - nuclear receptor interacting protein 1  NCOA3 - nuclear receptor coactivator 3  ANP32A - acidic (leucine-rich) nuclear phosphoprotein 32 family, member a  WWC2 - ww and c2 domain containing 2  PABPN1 - poly(a) binding protein, nuclear 1  TAF15 - taf15 rna polymerase ii, tata box binding protein (tbp)-associated factor, 68kda  GJA1 - gap junction protein, alpha 1, 43kda  AXIN1 - axin 1  TRRAP - transformation/transcription domain-associated protein  PICALM - phosphatidylinositol binding clathrin assembly protein  ZC3H14 - zinc finger ccch-type containing 14  DLL1 - delta-like 1 (drosophila)  ARID1A - at rich interactive domain 1a (swi-like)  SETD6 - set domain containing 6  FZD6 - frizzled family receptor 6  ACTR5 - arp5 actin-related protein 5 homolog (yeast)  CDC45 - cell division cycle 45  FZD4 - frizzled family receptor 4  GCLC - glutamate-cysteine ligase, catalytic subunit  FZD1 - frizzled family receptor 1  BAP1 - brca1 associated protein-1 (ubiquitin carboxy-terminal hydrolase)  ZNF613 - zinc finger protein 613  AXIN2 - axin 2  CDC7 - cell division cycle 7  NUP85 - nucleoporin 85kda  KDM5C - lysine (k)-specific demethylase 5c  USP9X - ubiquitin specific peptidase 9, x-linked  RBM10 - rna binding motif protein 10  PHF17 - phd finger protein 17  TTC21B - tetratricopeptide repeat domain 21b  SLTM - safb-like, transcription modulator  NELFE - negative elongation factor complex member e  EHMT1 - euchromatic histone-lysine n-methyltransferase 1  NR5A1 - nuclear receptor subfamily 5, group a, member 1  FUS - fused in sarcoma  NAA40 - n(alpha)-acetyltransferase 40, natd catalytic subunit, homolog (s. cerevisiae)  USP7 - ubiquitin specific peptidase 7 (herpes virus-associated)  XRCC6 - x-ray repair complementing defective repair in chinese hamster cells 6  RNF168 - ring finger protein 168, e3 ubiquitin protein ligase  GABPB1 - ga binding protein transcription factor, beta subunit 1  GABPA - ga binding protein transcription factor, alpha subunit 60kda  CPSF7 - cleavage and polyadenylation specific factor 7, 59kda  RPAP2 - rna polymerase ii associated protein 2  DDX39B - dead (asp-glu-ala-asp) box polypeptide 39b  KMT2D - lysine (k)-specific methyltransferase 2d  AAAS - achalasia, adrenocortical insufficiency, alacrimia  ZNF419 - zinc finger protein 419  YEATS4 - yeats domain containing 4  SNIP1 - smad nuclear interacting protein 1  ZFPM1 - zinc finger protein, fog family member 1  ZNF212 - zinc finger protein 212  EID2 - ep300 interacting inhibitor of differentiation 2  ZNF383 - zinc finger protein 383  GATA2 - gata binding protein 2  KAT6A - k(lysine) acetyltransferase 6a  NUP214 - nucleoporin 214kda  NR4A3 - nuclear receptor subfamily 4, group a, member 3  BRD3 - bromodomain containing 3  GAS6 - growth arrest-specific 6  NCOA4 - nuclear receptor coactivator 4  CARF - calcium responsive transcription factor  MLLT10 - myeloid/lymphoid or mixed-lineage leukemia (trithorax homolog, drosophila); translocated to, 10  ZNF408 - zinc finger protein 408  DEDD2 - death effector domain containing 2  FASTKD1 - fast kinase domains 1  DHX36 - deah (asp-glu-ala-his) box polypeptide 36  ZNF189 - zinc finger protein 189  ZSCAN26 - zinc finger and scan domain containing 26  ZNF177 - zinc finger protein 177  ZNF175 - zinc finger protein 175  ZNF174 - zinc finger protein 174  ZNF184 - zinc finger protein 184  SSBP4 - single stranded dna binding protein 4  GTF2F1 - general transcription factor iif, polypeptide 1, 74kda  ZNF148 - zinc finger protein 148  ZBTB17 - zinc finger and btb domain containing 17  BEND5 - ben domain containing 5  GTF2H2 - general transcription factor iih, polypeptide 2, 44kda  NEIL1 - nei endonuclease viii-like 1 (e. coli)  TRIM26 - tripartite motif containing 26  GTF2H3 - general transcription factor iih, polypeptide 3, 34kda  GTF2H1 - general transcription factor iih, polypeptide 1, 62kda  VEZF1 - vascular endothelial zinc finger 1  ZNF142 - zinc finger protein 142  MCPH1 - microcephalin 1  ZNF140 - zinc finger protein 140  GTF2B - general transcription factor iib  PCGF2 - polycomb group ring finger 2  MSH6 - muts homolog 6 (e. coli)  ZNF132 - zinc finger protein 132  ZNF131 - zinc finger protein 131  ZNF134 - zinc finger protein 134  ZNF133 - zinc finger protein 133  TBL1XR1 - transducin (beta)-like 1 x-linked receptor 1  GTF2I - general transcription factor iii  GTF2H4 - general transcription factor iih, polypeptide 4, 52kda  BRF1 - brf1, rna polymerase iii transcription initiation factor 90 kda subunit  ZNF79 - zinc finger protein 79  ZNF70 - zinc finger protein 70  RBM12B - rna binding motif protein 12b  ZNF75D - zinc finger protein 75d  ZNF76 - zinc finger protein 76  H3F3A - h3 histone, family 3a  BRPF1 - bromodomain and phd finger containing, 1  H2AFZ - h2a histone family, member z  FZD5 - frizzled family receptor 5  SAP130 - sin3a-associated protein, 130kda  H2AFX - h2a histone family, member x  DCP2 - decapping mrna 2  PAPD4 - pap associated domain containing 4  PAX8 - paired box 8  MIER3 - mesoderm induction early response 1, family member 3  CDC73 - cell division cycle 73  NKAP - nfkb activating protein  THAP5 - thap domain containing 5  LRP8 - low density lipoprotein receptor-related protein 8, apolipoprotein e receptor  HCFC1 - host cell factor c1 (vp16-accessory protein)  PRDM2 - pr domain containing 2, with znf domain  MAP3K12 - mitogen-activated protein kinase kinase kinase 12  ZNF226 - zinc finger protein 226  HDAC2 - histone deacetylase 2  HMBOX1 - homeobox containing 1  ZCCHC12 - zinc finger, cchc domain containing 12  HDGF - hepatoma-derived growth factor  ZNF213 - zinc finger protein 213  HDAC1 - histone deacetylase 1  HCLS1 - hematopoietic cell-specific lyn substrate 1  ZNF205 - zinc finger protein 205  ZNF207 - zinc finger protein 207  XPA - xeroderma pigmentosum, complementation group a  XPC - xeroderma pigmentosum, complementation group c  GOLGB1 - golgin b1  XPO1 - exportin 1 (crm1 homolog, yeast)  XRCC1 - x-ray repair complementing defective repair in chinese hamster cells 1  PAGR1 - paxip1 associated glutamate-rich protein 1  ZXDC - zxd family zinc finger c  AAED1 - ahpc/tsa antioxidant enzyme domain containing 1  XBP1 - x-box binding protein 1  ZNF367 - zinc finger protein 367  BHLHE41 - basic helix-loop-helix family, member e41  WFS1 - wolfram syndrome 1 (wolframin)  WHSC1 - wolf-hirschhorn syndrome candidate 1  NELFA - negative elongation factor complex member a  ARID2 - at rich interactive domain 2 (arid, rfx-like)  ZNF35 - zinc finger protein 35  ZKSCAN1 - zinc finger with krab and scan domains 1  FBXL15 - f-box and leucine-rich repeat protein 15  ZNF576 - zinc finger protein 576  ZNF33B - zinc finger protein 33b  ZNF32 - zinc finger protein 32  ZNF45 - zinc finger protein 45  BRCC3 - brca1/brca2-containing complex, subunit 3  MZF1 - myeloid zinc finger 1  ZNF18 - zinc finger protein 18  ZNF16 - zinc finger protein 16  ASXL1 - additional sex combs like 1 (drosophila)  ZNF384 - zinc finger protein 384  TNIP2 - tnfaip3 interacting protein 2  RBM42 - rna binding motif protein 42  ZNF24 - zinc finger protein 24  ZFPL1 - zinc finger protein-like 1  ZFX - zinc finger protein, x-linked  SF1 - splicing factor 1  ZBTB14 - zinc finger and btb domain containing 14  ZNF711 - zinc finger protein 711  ZNF7 - zinc finger protein 7  ZNF8 - zinc finger protein 8  ZNF2 - zinc finger protein 2  ZNF3 - zinc finger protein 3  YES1 - v-yes-1 yamaguchi sarcoma viral oncogene homolog 1  XRCC5 - x-ray repair complementing defective repair in chinese hamster cells 5 (double-strand-break rejoining)  GRSF1 - g-rich rna sequence binding factor 1  YWHAZ - tyrosine 3-monooxygenase/tryptophan 5-monooxygenase activation protein, zeta polypeptide  GSK3A - glycogen synthase kinase 3 alpha  YWHAB - tyrosine 3-monooxygenase/tryptophan 5-monooxygenase activation protein, beta polypeptide  CCNK - cyclin k  HSF2 - heat shock transcription factor 2  TRIM24 - tripartite motif containing 24  TAF1D - tata box binding protein (tbp)-associated factor, rna polymerase i, d, 41kda  CREG1 - cellular repressor of e1a-stimulated genes 1  HSBP1 - heat shock factor binding protein 1  FLCN - folliculin  PRMT2 - protein arginine methyltransferase 2  HESX1 - hesx homeobox 1  SAP30 - sin3a-associated protein, 30kda  METTL16 - methyltransferase like 16  FTO - fat mass and obesity associated  CFLAR - casp8 and fadd-like apoptosis regulator  SECISBP2 - secis binding protein 2  MED11 - mediator complex subunit 11  WDR77 - wd repeat domain 77  LDB1 - lim domain binding 1  DSCC1 - dna replication and sister chromatid cohesion 1  KAT2B - k(lysine) acetyltransferase 2b  TSC22D1 - tsc22 domain family, member 1  CRTC2 - creb regulated transcription coactivator 2  ZNF259 - zinc finger protein 259  TAX1BP1 - tax1 (human t-cell leukemia virus type i) binding protein 1  CC2D1B - coiled-coil and c2 domain containing 1b  NABP2 - nucleic acid binding protein 2  HUS1 - hus1 checkpoint homolog (s. pombe)  THAP8 - thap domain containing 8  BUD31 - bud31 homolog (s. cerevisiae)  ZNF655 - zinc finger protein 655  TRAK2 - trafficking protein, kinesin binding 2  HSPD1 - heat shock 60kda protein 1 (chaperonin)  DNAJB1 - dnaj (hsp40) homolog, subfamily b, member 1  ZNF48 - zinc finger protein 48  BCL10 - b-cell cll/lymphoma 10  FUBP3 - far upstream element (fuse) binding protein 3  HSP90AA1 - heat shock protein 90kda alpha (cytosolic), class a member 1  C7orf49 - chromosome 7 open reading frame 49  BTRC - beta-transducin repeat containing e3 ubiquitin protein ligase  HSPA8 - heat shock 70kda protein 8  HMGN1 - high mobility group nucleosome binding domain 1  HMGB3 - high mobility group box 3  HMGB2 - high mobility group box 2  HMGB1 - high mobility group box 1  BRD9 - bromodomain containing 9  TNKS - tankyrase, trf1-interacting ankyrin-related adp-ribose polymerase  DAZAP1 - daz associated protein 1  ZBTB10 - zinc finger and btb domain containing 10  AGO1 - argonaute risc catalytic component 1  CAPRIN2 - caprin family member 2  ZBTB48 - zinc finger and btb domain containing 48  SRSF9 - serine/arginine-rich splicing factor 9  HYAL2 - hyaluronoglucosaminidase 2  ACD - adrenocortical dysplasia homolog (mouse)  GPBP1 - gc-rich promoter binding protein 1  HFE - hemochromatosis  HINT1 - histidine triad nucleotide binding protein 1  ELP4 - elongator acetyltransferase complex subunit 4  HIVEP1 - human immunodeficiency virus type i enhancer binding protein 1  HIF1A - hypoxia inducible factor 1, alpha subunit (basic helix-loop-helix transcription factor)  LPIN3 - lipin 3  TRADD - tnfrsf1a-associated via death domain  MYCBP - myc binding protein  EED - embryonic ectoderm development  URI1 - uri1, prefoldin-like chaperone  RIPK1 - receptor (tnfrsf)-interacting serine-threonine kinase 1  HNRNPU - heterogeneous nuclear ribonucleoprotein u (scaffold attachment factor a)  HNRNPK - heterogeneous nuclear ribonucleoprotein k  HNRNPL - heterogeneous nuclear ribonucleoprotein l  TNFSF13 - tumor necrosis factor (ligand) superfamily, member 13  FBXO4 - f-box protein 4  TINF2 - terf1 (trf1)-interacting nuclear factor 2  HNRNPA2B1 - heterogeneous nuclear ribonucleoprotein a2/b1  HNRNPA1 - heterogeneous nuclear ribonucleoprotein a1  HNRNPC - heterogeneous nuclear ribonucleoprotein c (c1/c2)  RIPK2 - receptor-interacting serine-threonine kinase 2  HNRNPAB - heterogeneous nuclear ribonucleoprotein a/b  HNRNPF - heterogeneous nuclear ribonucleoprotein f  HNRNPD - heterogeneous nuclear ribonucleoprotein d (au-rich element rna binding protein 1, 37kda)  HNRNPH1 - heterogeneous nuclear ribonucleoprotein h1 (h)  HNRNPH3 - heterogeneous nuclear ribonucleoprotein h3 (2h9)  HNRNPH2 - heterogeneous nuclear ribonucleoprotein h2 (h')  ZNF584 - zinc finger protein 584  HMGA1 - high mobility group at-hook 1  FAM129B - family with sequence similarity 129, member b  NR4A1 - nuclear receptor subfamily 4, group a, member 1  PDE12 - phosphodiesterase 12  RFX7 - regulatory factor x, 7  HTATSF1 - hiv-1 tat specific factor 1  FOXK2 - forkhead box k2  HAT1 - histone acetyltransferase 1  CRTC3 - creb regulated transcription coactivator 3  RBM15 - rna binding motif protein 15  TNRC6A - trinucleotide repeat containing 6a  RBMX - rna binding motif protein, x-linked  PARG - poly (adp-ribose) glycohydrolase  ING2 - inhibitor of growth family, member 2  ZNF574 - zinc finger protein 574  PPM1D - protein phosphatase, mg2+/mn2+ dependent, 1d  CREB3L2 - camp responsive element binding protein 3-like 2  IPPK - inositol 1,3,4,5,6-pentakisphosphate 2-kinase  ILF2 - interleukin enhancer binding factor 2  PRPF19 - pre-mrna processing factor 19  ILF3 - interleukin enhancer binding factor 3, 90kda  ILK - integrin-linked kinase  PIAS1 - protein inhibitor of activated stat, 1  SMYD3 - set and mynd domain containing 3  SLCO3A1 - solute carrier organic anion transporter family, member 3a1  MAPKAPK5 - mitogen-activated protein kinase-activated protein kinase 5  SMURF2 - smad specific e3 ubiquitin protein ligase 2  CGGBP1 - cgg triplet repeat binding protein 1  LMO4 - lim domain only 4  CAMK1 - calcium/calmodulin-dependent protein kinase i  CBX4 - chromobox homolog 4  CSRNP1 - cysteine-serine-rich nuclear protein 1  ZNF25 - zinc finger protein 25  STK16 - serine/threonine kinase 16  ITGA3 - integrin, alpha 3 (antigen cd49c, alpha 3 subunit of vla-3 receptor)  KHSRP - kh-type splicing regulatory protein  CASK - calcium/calmodulin-dependent serine protein kinase (maguk family)  SUDS3 - suppressor of defective silencing 3 homolog (s. cerevisiae)  EIF6 - eukaryotic translation initiation factor 6  SKAP1 - src kinase associated phosphoprotein 1  GZF1 - gdnf-inducible zinc finger protein 1  INSR - insulin receptor  INSM1 - insulinoma-associated 1  CDK13 - cyclin-dependent kinase 13  PDCD4 - programmed cell death 4 (neoplastic transformation inhibitor)  RFXANK - regulatory factor x-associated ankyrin-containing protein  CSDC2 - cold shock domain containing c2, rna binding  KLF7 - kruppel-like factor 7 (ubiquitous)  IRF2 - interferon regulatory factor 2  IRF3 - interferon regulatory factor 3  RUVBL1 - ruvb-like 1 (e. coli)  IKZF5 - ikaros family zinc finger 5 (pegasus)  DIMT1 - dim1 dimethyladenosine transferase 1 homolog (s. cerevisiae)  MED19 - mediator complex subunit 19  ITGA6 - integrin, alpha 6  IRAK2 - interleukin-1 receptor-associated kinase 2  IRAK1 - interleukin-1 receptor-associated kinase 1  CACYBP - calcyclin binding protein  ZSCAN31 - zinc finger and scan domain containing 31  KIAA1324L - kiaa1324-like  PAPD5 - pap associated domain containing 5  NSD1 - nuclear receptor binding set domain protein 1  ZBTB11 - zinc finger and btb domain containing 11  RNF25 - ring finger protein 25  MMS19 - mms19 nucleotide excision repair homolog (s. cerevisiae)  DKK3 - dickkopf wnt signaling pathway inhibitor 3  SNX5 - sorting nexin 5  ZNF777 - zinc finger protein 777  ID2 - inhibitor of dna binding 2, dominant negative helix-loop-helix protein  BRPF3 - bromodomain and phd finger containing, 3  ID3 - inhibitor of dna binding 3, dominant negative helix-loop-helix protein  STK36 - serine/threonine kinase 36  IRF2BPL - interferon regulatory factor 2 binding protein-like  JAZF1 - jazf zinc finger 1  FOXK1 - forkhead box k1  MAGEF1 - melanoma antigen family f, 1  ZNF282 - zinc finger protein 282  IGHMBP2 - immunoglobulin mu binding protein 2  RBPJ - recombination signal binding protein for immunoglobulin kappa j region  NCK2 - nck adaptor protein 2  PABPC1 - poly(a) binding protein, cytoplasmic 1  USP21 - ubiquitin specific peptidase 21  ZBTB9 - zinc finger and btb domain containing 9  ZBTB12 - zinc finger and btb domain containing 12  BCAR3 - breast cancer anti-estrogen resistance 3  IK - ik cytokine, down-regulator of hla ii  JMJD1C - jumonji domain containing 1c  IGBP1 - immunoglobulin (cd79a) binding protein 1  SMARCA5 - swi/snf related, matrix associated, actin dependent regulator of chromatin, subfamily a, member 5  OGT - o-linked n-acetylglucosamine (glcnac) transferase  PELP1 - proline, glutamate and leucine rich protein 1  RAE1 - rae1 rna export 1 homolog (s. pombe)  TSPYL2 - tspy-like 2  SND1 - staphylococcal nuclease and tudor domain containing 1  CUL2 - cullin 2  ZNF667 - zinc finger protein 667  CUL3 - cullin 3  FOXP1 - forkhead box p1  SUPT3H - suppressor of ty 3 homolog (s. cerevisiae)  BEND6 - ben domain containing 6  ZNF572 - zinc finger protein 572  PCGF5 - polycomb group ring finger 5  WAC - ww domain containing adaptor with coiled-coil  WTAP - wilms tumor 1 associated protein  RBM39 - rna binding motif protein 39  ZNF414 - zinc finger protein 414  PHF21A - phd finger protein 21a  ZNF771 - zinc finger protein 771  IER2 - immediate early response 2  LIG3 - ligase iii, dna, atp-dependent  NCOR1 - nuclear receptor corepressor 1  MTPN - myotrophin  NCOR2 - nuclear receptor corepressor 2  DOT1L - dot1-like histone h3k79 methyltransferase  EFCAB7 - ef-hand calcium binding domain 7  L3MBTL3 - l(3)mbt-like 3 (drosophila)  ZNF333 - zinc finger protein 333  SCAND1 - scan domain containing 1  CIR1 - corepressor interacting with rbpj, 1  SLX4 - slx4 structure-specific endonuclease subunit  H2AFY - h2a histone family, member y  FADS1 - fatty acid desaturase 1  GTF2IRD1 - gtf2i repeat domain containing 1  GTPBP1 - gtp binding protein 1  ZNF527 - zinc finger protein 527  SPIRE2 - spire actin nucleation factor 2  HOPX - hop homeobox  ZNF316 - zinc finger protein 316  ZC3H8 - zinc finger ccch-type containing 8  SOX13 - sry (sex determining region y)-box 13  LMO1 - lim domain only 1 (rhombotin 1)  IER5 - immediate early response 5  NACC2 - nacc family member 2, ben and btb (poz) domain containing  CLOCK - clock circadian regulator  KLF3 - kruppel-like factor 3 (basic)  PRKAG2 - protein kinase, amp-activated, gamma 2 non-catalytic subunit  DDX41 - dead (asp-glu-ala-asp) box polypeptide 41  FXR2 - fragile x mental retardation, autosomal homolog 2  LITAF - lipopolysaccharide-induced tnf factor  TBPL1 - tbp-like 1  YTHDF2 - yth domain family, member 2  ZFP62 - zfp62 zinc finger protein  ZBTB41 - zinc finger and btb domain containing 41  ZGPAT - zinc finger, ccch-type with g patch domain  ACTL6B - actin-like 6b  UBR5 - ubiquitin protein ligase e3 component n-recognin 5  IL27RA - interleukin 27 receptor, alpha  MED20 - mediator complex subunit 20  AFAP1L2 - actin filament associated protein 1-like 2  ROCK2 - rho-associated, coiled-coil containing protein kinase 2  CRLF3 - cytokine receptor-like factor 3  MAPK8IP1 - mitogen-activated protein kinase 8 interacting protein 1  GLYR1 - glyoxylate reductase 1 homolog (arabidopsis)  ZBTB7A - zinc finger and btb domain containing 7a  DPY30 - dpy-30 homolog (c. elegans)  MBTPS2 - membrane-bound transcription factor peptidase, site 2  MAGED1 - melanoma antigen family d, 1  GLIS2 - glis family zinc finger 2  IRF2BP2 - interferon regulatory factor 2 binding protein 2  MED27 - mediator complex subunit 27  MED26 - mediator complex subunit 26  CHD6 - chromodomain helicase dna binding protein 6  MED17 - mediator complex subunit 17  MED23 - mediator complex subunit 23  ZNF580 - zinc finger protein 580  CDYL - chromodomain protein, y-like  GTF2IRD2 - gtf2i repeat domain containing 2  ZNF710 - zinc finger protein 710  ING4 - inhibitor of growth family, member 4  EIF2AK3 - eukaryotic translation initiation factor 2-alpha kinase 3  INSIG2 - insulin induced gene 2  RLIM - ring finger protein, lim domain interacting  KRAS - kirsten rat sarcoma viral oncogene homolog  RECQL5 - recq protein-like 5  TNPO1 - transportin 1  TGFBRAP1 - transforming growth factor, beta receptor associated protein 1  ZNF706 - zinc finger protein 706  CIAO1 - cytosolic iron-sulfur protein assembly 1  KPNA2 - karyopherin alpha 2 (rag cohort 1, importin alpha 1)  KPNA1 - karyopherin alpha 1 (importin alpha 5)  SUV420H1 - suppressor of variegation 4-20 homolog 1 (drosophila)  PHF20L1 - phd finger protein 20-like 1  MED21 - mediator complex subunit 21  MED10 - mediator complex subunit 10  KIT - v-kit hardy-zuckerman 4 feline sarcoma viral oncogene homolog  ZFP28 - zfp28 zinc finger protein  JUP - junction plakoglobin  JAK2 - janus kinase 2  ING5 - inhibitor of growth family, member 5  SLC25A33 - solute carrier family 25 (pyrimidine nucleotide carrier), member 33  ZNF219 - zinc finger protein 219  DNAJC30 - dnaj (hsp40) homolog, subfamily c, member 30  PHF20 - phd finger protein 20  TRIP12 - thyroid hormone receptor interactor 12  NUDT16L1 - nudix (nucleoside diphosphate linked moiety x)-type motif 16-like 1  KCNK2 - potassium channel, subfamily k, member 2  ZNHIT3 - zinc finger, hit-type containing 3  ZNF397 - zinc finger protein 397  TRIP4 - thyroid hormone receptor interactor 4  HMGN3 - high mobility group nucleosomal binding domain 3  PHF6 - phd finger protein 6  ZNF235 - zinc finger protein 235  COPS2 - cop9 signalosome subunit 2  ZNF639 - zinc finger protein 639  SARNP - sap domain containing ribonucleoprotein  LEF1 - lymphoid enhancer-binding factor 1  BRMS1L - breast cancer metastasis-suppressor 1-like  CNOT8 - ccr4-not transcription complex, subunit 8  CDK12 - cyclin-dependent kinase 12  ZNF280B - zinc finger protein 280b  ARID4B - at rich interactive domain 4b (rbp1-like)  MAP3K10 - mitogen-activated protein kinase kinase kinase 10  MLH1 - mutl homolog 1, colon cancer, nonpolyposis type 2 (e. coli)  ITGB1BP1 - integrin beta 1 binding protein 1  KDM3B - lysine (k)-specific demethylase 3b  RSF1 - remodeling and spacing factor 1  MLLT6 - myeloid/lymphoid or mixed-lineage leukemia (trithorax homolog, drosophila); translocated to, 6  MLLT3 - myeloid/lymphoid or mixed-lineage leukemia (trithorax homolog, drosophila); translocated to, 3  MED14 - mediator complex subunit 14  MLLT1 - myeloid/lymphoid or mixed-lineage leukemia (trithorax homolog, drosophila); translocated to, 1  ZBTB22 - zinc finger and btb domain containing 22  KMT2A - lysine (k)-specific methyltransferase 2a  RPS6KA5 - ribosomal protein s6 kinase, 90kda, polypeptide 5  SUFU - suppressor of fused homolog (drosophila)  NLK - nemo-like kinase  MNAT1 - mnat cdk-activating kinase assembly factor 1  UIMC1 - ubiquitin interaction motif containing 1  MTA2 - metastasis associated 1 family, member 2  NOLC1 - nucleolar and coiled-body phosphoprotein 1  EIF2A - eukaryotic translation initiation factor 2a, 65kda  CD200 - cd200 molecule  WWOX - ww domain containing oxidoreductase  MNT - mnt, max dimerization protein  CCPG1 - cell cycle progression 1  NSRP1 - nuclear speckle splicing regulatory protein 1  ME1 - malic enzyme 1, nadp(+)-dependent, cytosolic  SPTY2D1 - spt2, suppressor of ty, domain containing 1 (s. cerevisiae)  PELO - pelota homolog (drosophila)  MECP2 - methyl cpg binding protein 2 (rett syndrome)  MEF2A - myocyte enhancer factor 2a  ME2 - malic enzyme 2, nad(+)-dependent, mitochondrial  PRICKLE1 - prickle homolog 1 (drosophila)  ZBED1 - zinc finger, bed-type containing 1  BCDIN3D - bcdin3 domain containing  MEIS2 - meis homeobox 2  BRWD1 - bromodomain and wd repeat domain containing 1  ZNF664 - zinc finger protein 664  MEIS1 - meis homeobox 1  MAP3K4 - mitogen-activated protein kinase kinase kinase 4  MAP3K5 - mitogen-activated protein kinase kinase kinase 5  SLC33A1 - solute carrier family 33 (acetyl-coa transporter), member 1  MEN1 - multiple endocrine neoplasia i  WDR75 - wd repeat domain 75  COX7A2L - cytochrome c oxidase subunit viia polypeptide 2 like  UTP15 - utp15, u3 small nucleolar ribonucleoprotein, homolog (s. cerevisiae)  KAT8 - k(lysine) acetyltransferase 8  ZNF644 - zinc finger protein 644  ARHGEF2 - rho/rac guanine nucleotide exchange factor (gef) 2  LACC1 - laccase (multicopper oxidoreductase) domain containing 1  MBD3 - methyl-cpg binding domain protein 3  PTOV1 - prostate tumor overexpressed 1  ZNF394 - zinc finger protein 394  CBFA2T2 - core-binding factor, runt domain, alpha subunit 2; translocated to, 2  MAGOH - mago-nashi homolog, proliferation-associated (drosophila)  CXXC5 - cxxc finger protein 5  RQCD1 - rcd1 required for cell differentiation1 homolog (s. pombe)  SMC3 - structural maintenance of chromosomes 3  IL23A - interleukin 23, alpha subunit p19  MARS - methionyl-trna synthetase  SIRT6 - sirtuin 6  MTA1 - metastasis associated 1  PPHLN1 - periphilin 1  NUP43 - nucleoporin 43kda  MBD1 - methyl-cpg binding domain protein 1  SFMBT1 - scm-like with four mbt domains 1  MAX - myc associated factor x  ZMIZ2 - zinc finger, miz-type containing 2  LDB2 - lim domain binding 2  TRMT112 - trna methyltransferase 11-2 homolog (s. cerevisiae)  ZFP90 - zfp90 zinc finger protein  CDAN1 - codanin 1  NELFCD - negative elongation factor complex member c/d  ASH2L - ash2 (absent, small, or homeotic)-like (drosophila)  CTDSPL2 - ctd (carboxy-terminal domain, rna polymerase ii, polypeptide a) small phosphatase like 2  ZSCAN29 - zinc finger and scan domain containing 29  THAP2 - thap domain containing, apoptosis associated protein 2  ITCH - itchy e3 ubiquitin protein ligase  ZNF484 - zinc finger protein 484  L3MBTL2 - l(3)mbt-like 2 (drosophila)  RIOK1 - rio kinase 1  LRP6 - low density lipoprotein receptor-related protein 6  ZNF445 - zinc finger protein 445  AIP - aryl hydrocarbon receptor interacting protein  TRAPPC9 - trafficking protein particle complex 9  LUM - lumican  BTAF1 - btaf1 rna polymerase ii, b-tfiid transcription factor-associated, 170kda  UBA3 - ubiquitin-like modifier activating enzyme 3  RBFOX3 - rna binding protein, fox-1 homolog (c. elegans) 3  SESN2 - sestrin 2  PARP9 - poly (adp-ribose) polymerase family, member 9  RNF8 - ring finger protein 8, e3 ubiquitin protein ligase  BAZ1B - bromodomain adjacent to zinc finger domain, 1b  MXD1 - max dimerization protein 1  MED15 - mediator complex subunit 15  PIAS4 - protein inhibitor of activated stat, 4  TRIM33 - tripartite motif containing 33  SMAD2 - smad family member 2  SRRT - serrate rna effector molecule homolog (arabidopsis)  HDAC7 - histone deacetylase 7  KLF16 - kruppel-like factor 16  ARMCX3 - armadillo repeat containing, x-linked 3  TDP2 - tyrosyl-dna phosphodiesterase 2  LARP7 - la ribonucleoprotein domain family, member 7  MAFG - v-maf avian musculoaponeurotic fibrosarcoma oncogene homolog g  RPS6KA4 - ribosomal protein s6 kinase, 90kda, polypeptide 4  TAF9B - taf9b rna polymerase ii, tata box binding protein (tbp)-associated factor, 31kda  SMAD4 - smad family member 4  NBAS - neuroblastoma amplified sequence  H1FX - h1 histone family, member x  SMAD5 - smad family member 5  SMAD7 - smad family member 7  WASL - wiskott-aldrich syndrome-like  RBM4B - rna binding motif protein 4b  ZNF565 - zinc finger protein 565  SAP30BP - sap30 binding protein  YAF2 - yy1 associated factor 2  TAGLN3 - transgelin 3  SIX5 - six homeobox 5  NCBP1 - nuclear cap binding protein subunit 1, 80kda  NDN - necdin, melanoma antigen (mage) family member  ZNF582 - zinc finger protein 582  NCK1 - nck adaptor protein 1  BRD7 - bromodomain containing 7  NCL - nucleolin  MBNL2 - muscleblind-like splicing regulator 2  CEBPZ - ccaat/enhancer binding protein (c/ebp), zeta  TICAM1 - toll-like receptor adaptor molecule 1  CCNL2 - cyclin l2  HNRNPM - heterogeneous nuclear ribonucleoprotein m  TRIM28 - tripartite motif containing 28  DROSHA - drosha, ribonuclease type iii  DNAJC15 - dnaj (hsp40) homolog, subfamily c, member 15  TBK1 - tank-binding kinase 1  NBN - nibrin  ZNF197 - zinc finger protein 197  TDRD3 - tudor domain containing 3  RBM7 - rna binding motif protein 7  RBM5 - rna binding motif protein 5  TRIM11 - tripartite motif containing 11  PPP1R12A - protein phosphatase 1, regulatory subunit 12a  MYT1 - myelin transcription factor 1  ALYREF - aly/ref export factor  NAB1 - ngfi-a binding protein 1 (egr1 binding protein 1)  CSRNP2 - cysteine-serine-rich nuclear protein 2  SENP1 - sumo1/sentrin specific peptidase 1  NAB2 - ngfi-a binding protein 2 (egr1 binding protein 2)  RNF41 - ring finger protein 41  TSHZ1 - teashirt zinc finger homeobox 1  PSME3 - proteasome (prosome, macropain) activator subunit 3 (pa28 gamma; ki)  TRIM13 - tripartite motif containing 13  TRIM8 - tripartite motif containing 8  MYO6 - myosin vi  LBH - limb bud and heart development  ABT1 - activator of basal transcription 1  TOPORS - topoisomerase i binding, arginine/serine-rich, e3 ubiquitin protein ligase  MYD88 - myeloid differentiation primary response 88  SEH1L - seh1-like (s. cerevisiae)  ZNF362 - zinc finger protein 362  HCFC2 - host cell factor c2  CNOT7 - ccr4-not transcription complex, subunit 7  MXI1 - max interactor 1, dimerization protein  PQBP1 - polyglutamine binding protein 1  TRA2A - transformer 2 alpha homolog (drosophila)  ACTR2 - arp2 actin-related protein 2 homolog (yeast)  ACTR3 - arp3 actin-related protein 3 homolog (yeast)  RBM15B - rna binding motif protein 15b  TSFM - ts translation elongation factor, mitochondrial  RAD50 - rad50 homolog (s. cerevisiae)  TRIM37 - tripartite motif containing 37  ZNF558 - zinc finger protein 558  LRPPRC - leucine-rich pentatricopeptide repeat containing  ZNF263 - zinc finger protein 263  PURG - purine-rich element binding protein g  RBM12 - rna binding motif protein 12  MED25 - mediator complex subunit 25  MED6 - mediator complex subunit 6  PTBP3 - polypyrimidine tract binding protein 3  DMTF1 - cyclin d binding myb-like transcription factor 1  ZBTB33 - zinc finger and btb domain containing 33  HDAC5 - histone deacetylase 5  MTF1 - metal-regulatory transcription factor 1  SRA1 - steroid receptor rna activator 1  HDAC6 - histone deacetylase 6  RNF187 - ring finger protein 187  MT3 - metallothionein 3  MED16 - mediator complex subunit 16  CCDC22 - coiled-coil domain containing 22  DNM1L - dynamin 1-like  DNAJB6 - dnaj (hsp40) homolog, subfamily b, member 6  MSH3 - muts homolog 3 (e. coli)  MSH2 - muts homolog 2, colon cancer, nonpolyposis type 1 (e. coli)  CITED1 - cbp/p300-interacting transactivator, with glu/asp-rich carboxy-terminal domain, 1  ZBTB5 - zinc finger and btb domain containing 5  ZBTB40 - zinc finger and btb domain containing 40  SUPT7L - suppressor of ty 7 (s. cerevisiae)-like  RGCC - regulator of cell cycle  COMMD1 - copper metabolism (murr1) domain containing 1  RBM8A - rna binding motif protein 8a  USP3 - ubiquitin specific peptidase 3  BABAM1 - brisc and brca1 a complex member 1  USP15 - ubiquitin specific peptidase 15  MED4 - mediator complex subunit 4  THOC1 - tho complex 1  SETD2 - set domain containing 2  RBX1 - ring-box 1, e3 ubiquitin protein ligase  MRE11A - mre11 meiotic recombination 11 homolog a (s. cerevisiae)  NUP153 - nucleoporin 153kda  THRAP3 - thyroid hormone receptor associated protein 3  MED12 - mediator complex subunit 12  MED13 - mediator complex subunit 13  PSMD6 - proteasome (prosome, macropain) 26s subunit, non-atpase, 6  MED24 - mediator complex subunit 24  ZC3H6 - zinc finger ccch-type containing 6  DTX3L - deltex 3-like (drosophila)  ZNF34 - zinc finger protein 34  SETDB1 - set domain, bifurcated 1  ORC2 - origin recognition complex, subunit 2  ORC5 - origin recognition complex, subunit 5  TOX4 - tox high mobility group box family member 4  ZNF436 - zinc finger protein 436  RNF141 - ring finger protein 141  AGRN - agrin  PPP4R2 - protein phosphatase 4, regulatory subunit 2  NDFIP1 - nedd4 family interacting protein 1  RBM19 - rna binding motif protein 19  G3BP2 - gtpase activating protein (sh3 domain) binding protein 2  OGG1 - 8-oxoguanine dna glycosylase  ZSCAN16 - zinc finger and scan domain containing 16  TNKS2 - tankyrase, trf1-interacting ankyrin-related adp-ribose polymerase 2  EXOSC3 - exosome component 3  EXOSC1 - exosome component 1  DDR2 - discoidin domain receptor tyrosine kinase 2  BCLAF1 - bcl2-associated transcription factor 1  DAB2IP - dab2 interacting protein  NUMA1 - nuclear mitotic apparatus protein 1  NUP88 - nucleoporin 88kda  GMNN - geminin, dna replication inhibitor  NUP98 - nucleoporin 98kda  NR4A2 - nuclear receptor subfamily 4, group a, member 2  ZNF593 - zinc finger protein 593  ZBTB7B - zinc finger and btb domain containing 7b  NVL - nuclear vcp-like  ZNF691 - zinc finger protein 691  CREBRF - creb3 regulatory factor  ZNF623 - zinc finger protein 623  NMD3 - nmd3 ribosome export adaptor  NPM1 - nucleophosmin (nucleolar phosphoprotein b23, numatrin)  ARHGEF11 - rho guanine nucleotide exchange factor (gef) 11  ZBTB24 - zinc finger and btb domain containing 24  ZEB2 - zinc finger e-box binding homeobox 2  THAP4 - thap domain containing 4  RNF40 - ring finger protein 40, e3 ubiquitin protein ligase  TSC22D2 - tsc22 domain family, member 2  NFXL1 - nuclear transcription factor, x-box binding-like 1  NRF1 - nuclear respiratory factor 1  DONSON - downstream neighbor of son  NOP2 - nop2 nucleolar protein  NONO - non-pou domain containing, octamer-binding  VPRBP - vpr (hiv-1) binding protein  MDFIC - myod family inhibitor domain containing  NOTCH4 - notch 4  IFT74 - intraflagellar transport 74 homolog (chlamydomonas)  NOVA2 - neuro-oncological ventral antigen 2  NOVA1 - neuro-oncological ventral antigen 1  NPAT - nuclear protein, ataxia-telangiectasia locus  CEP290 - centrosomal protein 290kda  CNOT2 - ccr4-not transcription complex, subunit 2  NAA15 - n(alpha)-acetyltransferase 15, nata auxiliary subunit  PUM1 - pumilio homolog 1 (drosophila)  NOS3 - nitric oxide synthase 3 (endothelial cell)  CNOT3 - ccr4-not transcription complex, subunit 3  NOTCH1 - notch 1  BAZ2B - bromodomain adjacent to zinc finger domain, 2b  NOTCH3 - notch 3  CTC1 - cts telomere maintenance complex component 1  ST18 - suppression of tumorigenicity 18 (breast carcinoma) (zinc finger protein)  DHX34 - deah (asp-glu-ala-his) box polypeptide 34  NOTCH2 - notch 2  NFYA - nuclear transcription factor y, alpha  HDAC4 - histone deacetylase 4  TOX - thymocyte selection-associated high mobility group box  NFYC - nuclear transcription factor y, gamma  ZFYVE16 - zinc finger, fyve domain containing 16  CXXC1 - cxxc finger protein 1  NFKBIA - nuclear factor of kappa light polypeptide gene enhancer in b-cells inhibitor, alpha  NFKB1 - nuclear factor of kappa light polypeptide gene enhancer in b-cells 1  KMT2B - lysine (k)-specific methyltransferase 2b  NFKBIB - nuclear factor of kappa light polypeptide gene enhancer in b-cells inhibitor, beta  NFKBIE - nuclear factor of kappa light polypeptide gene enhancer in b-cells inhibitor, epsilon  NKX2-2 - nk2 homeobox 2  HDAC9 - histone deacetylase 9  ZNF606 - zinc finger protein 606  SART3 - squamous cell carcinoma antigen recognized by t cells 3  ZNF614 - zinc finger protein 614  ZFP2 - zfp2 zinc finger protein  NEO1 - neogenin 1  SMARCAL1 - swi/snf related, matrix associated, actin dependent regulator of chromatin, subfamily a-like 1  NEDD8 - neural precursor cell expressed, developmentally down-regulated 8  CTR9 - ctr9, paf1/rna polymerase ii complex component  PPM1F - protein phosphatase, mg2+/mn2+ dependent, 1f  EPC1 - enhancer of polycomb homolog 1 (drosophila)  NFE2L2 - nuclear factor (erythroid-derived 2)-like 2  MORF4L2 - mortality factor 4 like 2  RASL11A - ras-like, family 11, member a  NFATC4 - nuclear factor of activated t-cells, cytoplasmic, calcineurin-dependent 4  KLHL15 - kelch-like family member 15  NUP155 - nucleoporin 155kda  NFIA - nuclear factor i/a  CDK5RAP3 - cdk5 regulatory subunit associated protein 3  NUP93 - nucleoporin 93kda  ZNF746 - zinc finger protein 746  VGLL4 - vestigial like 4 (drosophila)  LRRC14 - leucine rich repeat containing 14  KDM4A - lysine (k)-specific demethylase 4a  PHF14 - phd finger protein 14  HP1BP3 - heterochromatin protein 1, binding protein 3  ZNF432 - zinc finger protein 432  SAFB2 - scaffold attachment factor b2  MYEF2 - myelin expression factor 2  LPIN2 - lipin 2 |
| GO:0043170 | macromolecule metabolic process | 1.4E-15 | 1.95E-12 | 1.07 (10334,4157,6996,2998) | [+] Show genes  AFG3L2 - afg3 atpase family member 3-like 2 (s. cerevisiae)  EXOSC9 - exosome component 9  MSL3 - male-specific lethal 3 homolog (drosophila)  EXOSC10 - exosome component 10  OTUD5 - otu domain containing 5  AGAP3 - arfgap with gtpase domain, ankyrin repeat and ph domain 3  ZCCHC8 - zinc finger, cchc domain containing 8  PMS2 - pms2 postmeiotic segregation increased 2 (s. cerevisiae)  RNPC3 - rna-binding region (rnp1, rrm) containing 3  TADA1 - transcriptional adaptor 1  SF3A3 - splicing factor 3a, subunit 3, 60kda  HNRNPA0 - heterogeneous nuclear ribonucleoprotein a0  AARS - alanyl-trna synthetase  PNN - pinin, desmosome associated protein  ABCA1 - atp-binding cassette, sub-family a (abc1), member 1  SEC61B - sec61 beta subunit  ABCF1 - atp-binding cassette, sub-family f (gcn20), member 1  OS9 - osteosarcoma amplified 9, endoplasmic reticulum lectin  ABL1 - c-abl oncogene 1, non-receptor tyrosine kinase  GALNT10 - udp-n-acetyl-alpha-d-galactosamine:polypeptide n-acetylgalactosaminyltransferase 10 (galnac-t10)  PML - promyelocytic leukemia  CNOT11 - ccr4-not transcription complex, subunit 11  ACACA - acetyl-coa carboxylase alpha  PMM1 - phosphomannomutase 1  EBNA1BP2 - ebna1 binding protein 2  RAB40B - rab40b, member ras oncogene family  CKAP4 - cytoskeleton-associated protein 4  PMS1 - pms1 postmeiotic segregation increased 1 (s. cerevisiae)  ASCC3 - activating signal cointegrator 1 complex subunit 3  FTSJ3 - ftsj homolog 3 (e. coli)  UBE2Q1 - ubiquitin-conjugating enzyme e2q family member 1  PNRC2 - proline-rich nuclear receptor coactivator 2  BRD8 - bromodomain containing 8  TXNL4A - thioredoxin-like 4a  PLOD2 - procollagen-lysine, 2-oxoglutarate 5-dioxygenase 2  ACHE - acetylcholinesterase  MAN1A2 - mannosidase, alpha, class 1a, member 2  LRRC40 - leucine rich repeat containing 40  PLOD1 - procollagen-lysine, 2-oxoglutarate 5-dioxygenase 1  RFFL - ring finger and fyve-like domain containing e3 ubiquitin protein ligase  CHD7 - chromodomain helicase dna binding protein 7  PLRG1 - pleiotropic regulator 1  LYAR - ly1 antibody reactive  PAPOLA - poly(a) polymerase alpha  TCERG1 - transcription elongation regulator 1  ACTB - actin, beta  RNPS1 - rna binding protein s1, serine-rich domain  OTUB1 - otu domain, ubiquitin aldehyde binding 1  EHMT2 - euchromatic histone-lysine n-methyltransferase 2  SPIN1 - spindlin 1  TASP1 - taspase, threonine aspartase, 1  KIF16B - kinesin family member 16b  MORF4L1 - mortality factor 4 like 1  ZDHHC7 - zinc finger, dhhc-type containing 7  POMGNT1 - protein o-linked mannose n-acetylglucosaminyltransferase 1 (beta 1,2-)  CHCHD1 - coiled-coil-helix-coiled-coil-helix domain containing 1  THUMPD1 - thump domain containing 1  PPIC - peptidylprolyl isomerase c (cyclophilin c)  PPID - peptidylprolyl isomerase d  ACTL6A - actin-like 6a  PPEF1 - protein phosphatase, ef-hand calcium binding domain 1  USP20 - ubiquitin specific peptidase 20  PPIA - peptidylprolyl isomerase a (cyclophilin a)  USP19 - ubiquitin specific peptidase 19  PPIB - peptidylprolyl isomerase b (cyclophilin b)  ACVR2A - activin a receptor, type iia  ACVRL1 - activin a receptor type ii-like 1  PARL - presenilin associated, rhomboid-like  FGL2 - fibrinogen-like 2  ACVR1 - activin a receptor, type i  ACVR1B - activin a receptor, type ib  NDUFAF7 - nadh dehydrogenase (ubiquinone) complex i, assembly factor 7  ADAR - adenosine deaminase, rna-specific  ADAM10 - adam metallopeptidase domain 10  ADARB1 - adenosine deaminase, rna-specific, b1  WDR3 - wd repeat domain 3  MRPS30 - mitochondrial ribosomal protein s30  CPSF4 - cleavage and polyadenylation specific factor 4, 30kda  MED1 - mediator complex subunit 1  MMP24 - matrix metallopeptidase 24 (membrane-inserted)  PPA1 - pyrophosphatase (inorganic) 1  PPARD - peroxisome proliferator-activated receptor delta  POLRMT - polymerase (rna) mitochondrial (dna directed)  MAML3 - mastermind-like 3 (drosophila)  POLR2K - polymerase (rna) ii (dna directed) polypeptide k, 7.0kda  ADD1 - adducin 1 (alpha)  TSEN15 - tsen15 trna splicing endonuclease subunit  ADH5 - alcohol dehydrogenase 5 (class iii), chi polypeptide  PARP1 - poly (adp-ribose) polymerase 1  POLH - polymerase (dna directed), eta  ADPRH - adp-ribosylarginine hydrolase  PPP1R13L - protein phosphatase 1, regulatory subunit 13 like  NOP10 - nop10 ribonucleoprotein  TNFRSF19 - tumor necrosis factor receptor superfamily, member 19  POLD2 - polymerase (dna directed), delta 2, accessory subunit  POLD1 - polymerase (dna directed), delta 1, catalytic subunit  CHST12 - carbohydrate (chondroitin 4) sulfotransferase 12  POLR2G - polymerase (rna) ii (dna directed) polypeptide g  ELAC1 - elac ribonuclease z 1  POLR2F - polymerase (rna) ii (dna directed) polypeptide f  POLR2E - polymerase (rna) ii (dna directed) polypeptide e, 25kda  SMPD3 - sphingomyelin phosphodiesterase 3, neutral membrane (neutral sphingomyelinase ii)  POLR2D - polymerase (rna) ii (dna directed) polypeptide d  POLR2C - polymerase (rna) ii (dna directed) polypeptide c, 33kda  POLR2B - polymerase (rna) ii (dna directed) polypeptide b, 140kda  RUVBL2 - ruvb-like 2 (e. coli)  PARP4 - poly (adp-ribose) polymerase family, member 4  POLR2A - polymerase (rna) ii (dna directed) polypeptide a, 220kda  HNRNPUL1 - heterogeneous nuclear ribonucleoprotein u-like 1  AP2A2 - adaptor-related protein complex 2, alpha 2 subunit  AP2A1 - adaptor-related protein complex 2, alpha 1 subunit  RPP14 - ribonuclease p/mrp 14kda subunit  AP2B1 - adaptor-related protein complex 2, beta 1 subunit  INTS9 - integrator complex subunit 9  PRKAB1 - protein kinase, amp-activated, beta 1 non-catalytic subunit  ATE1 - arginyltransferase 1  PRKACB - protein kinase, camp-dependent, catalytic, beta  RCOR3 - rest corepressor 3  PRKACA - protein kinase, camp-dependent, catalytic, alpha  KRR1 - krr1, small subunit (ssu) processome component, homolog (yeast)  WDR12 - wd repeat domain 12  NUP133 - nucleoporin 133kda  SRGN - serglycin  ADRBK2 - adrenergic, beta, receptor kinase 2  ADRBK1 - adrenergic, beta, receptor kinase 1  PRIM2 - primase, dna, polypeptide 2 (58kda)  PRIM1 - primase, dna, polypeptide 1 (49kda)  NUPL2 - nucleoporin like 2  EDEM2 - er degradation enhancer, mannosidase alpha-like 2  PRCC - papillary renal cell carcinoma (translocation-associated)  PRCP - prolylcarboxypeptidase (angiotensinase c)  POLR3A - polymerase (rna) iii (dna directed) polypeptide a, 155kda  CHFR - checkpoint with forkhead and ring finger domains, e3 ubiquitin protein ligase  CLASRP - clk4-associating serine/arginine rich protein  PRELP - proline/arginine-rich end leucine-rich repeat protein  PRDM4 - pr domain containing 4  PPP3R1 - protein phosphatase 3, regulatory subunit b, alpha  N4BP2 - nedd4 binding protein 2  ATF7IP - activating transcription factor 7 interacting protein  CIT - citron (rho-interacting, serine/threonine kinase 21)  PPP5C - protein phosphatase 5, catalytic subunit  PPP6C - protein phosphatase 6, catalytic subunit  PPT1 - palmitoyl-protein thioesterase 1  UHRF2 - ubiquitin-like with phd and ring finger domains 2, e3 ubiquitin protein ligase  AGA - aspartylglucosaminidase  PPP2R5D - protein phosphatase 2, regulatory subunit b', delta  PPP2R5C - protein phosphatase 2, regulatory subunit b', gamma  CSGALNACT1 - chondroitin sulfate n-acetylgalactosaminyltransferase 1  PPP2R5B - protein phosphatase 2, regulatory subunit b', beta  PPP4C - protein phosphatase 4, catalytic subunit  AKT1 - v-akt murine thymoma viral oncogene homolog 1  PCID2 - pci domain containing 2  SNRNP35 - small nuclear ribonucleoprotein 35kda (u11/u12)  PPP2R5E - protein phosphatase 2, regulatory subunit b', epsilon isoform  PPP2R2A - protein phosphatase 2, regulatory subunit b, alpha  PPP2R1B - protein phosphatase 2, regulatory subunit a, beta  CMTR2 - cap methyltransferase 2  PPP2R1A - protein phosphatase 2, regulatory subunit a, alpha  RIOK2 - rio kinase 2  CPSF6 - cleavage and polyadenylation specific factor 6, 68kda  NUDT21 - nudix (nucleoside diphosphate linked moiety x)-type motif 21  WWP2 - ww domain containing e3 ubiquitin protein ligase 2  PPP2R2C - protein phosphatase 2, regulatory subunit b, gamma  DIS3L - dis3 mitotic control homolog (s. cerevisiae)-like  PPP2R2B - protein phosphatase 2, regulatory subunit b, beta  DDX52 - dead (asp-glu-ala-asp) box polypeptide 52  DDX51 - dead (asp-glu-ala-asp) box polypeptide 51  PPP1R8 - protein phosphatase 1, regulatory subunit 8  TRIOBP - trio and f-actin binding protein  PPP2CA - protein phosphatase 2, catalytic subunit, alpha isozyme  PPP2CB - protein phosphatase 2, catalytic subunit, beta isozyme  MBD5 - methyl-cpg binding domain protein 5  DIDO1 - death inducer-obliterator 1  DUSP14 - dual specificity phosphatase 14  TOPBP1 - topoisomerase (dna) ii binding protein 1  PPM1G - protein phosphatase, mg2+/mn2+ dependent, 1g  RMI2 - recq mediated genome instability 2  PPP1R2 - protein phosphatase 1, regulatory (inhibitor) subunit 2  HSF2BP - heat shock transcription factor 2 binding protein  PPP1CC - protein phosphatase 1, catalytic subunit, gamma isozyme  ESCO1 - establishment of sister chromatid cohesion n-acetyltransferase 1  KANSL3 - kat8 regulatory nsl complex subunit 3  IWS1 - iws1 homolog (s. cerevisiae)  TDRKH - tudor and kh domain containing  SCYL2 - scy1-like 2 (s. cerevisiae)  LIAS - lipoic acid synthetase  NPLOC4 - nuclear protein localization 4 homolog (s. cerevisiae)  AMFR - autocrine motility factor receptor, e3 ubiquitin protein ligase  RNF126 - ring finger protein 126  DNAJC3 - dnaj (hsp40) homolog, subfamily c, member 3  PRPF40A - prp40 pre-mrna processing factor 40 homolog a (s. cerevisiae)  DDX27 - dead (asp-glu-ala-asp) box polypeptide 27  PAPD7 - pap associated domain containing 7  MAP2K5 - mitogen-activated protein kinase kinase 5  INTS8 - integrator complex subunit 8  MAP2K7 - mitogen-activated protein kinase kinase 7  EIF2AK2 - eukaryotic translation initiation factor 2-alpha kinase 2  PIM2 - pim-2 oncogene  PIGV - phosphatidylinositol glycan anchor biosynthesis, class v  MAPK13 - mitogen-activated protein kinase 13  GALNT13 - udp-n-acetyl-alpha-d-galactosamine:polypeptide n-acetylgalactosaminyltransferase 13 (galnac-t13)  RAB31 - rab31, member ras oncogene family  MAP2K1 - mitogen-activated protein kinase kinase 1  RNF157 - ring finger protein 157  MAP2K2 - mitogen-activated protein kinase kinase 2  MAPK10 - mitogen-activated protein kinase 10  MAPK9 - mitogen-activated protein kinase 9  TRIM59 - tripartite motif containing 59  MAPK11 - mitogen-activated protein kinase 11  MAPK8 - mitogen-activated protein kinase 8  METAP2 - methionyl aminopeptidase 2  MAPK7 - mitogen-activated protein kinase 7  TSR1 - tsr1, 20s rrna accumulation, homolog (s. cerevisiae)  POLR3E - polymerase (rna) iii (dna directed) polypeptide e (80kd)  GCN1L1 - gcn1 general control of amino-acid synthesis 1-like 1 (yeast)  MAPK3 - mitogen-activated protein kinase 3  MAPK1 - mitogen-activated protein kinase 1  COPS6 - cop9 signalosome subunit 6  PRKCSH - protein kinase c substrate 80k-h  CLP1 - cleavage and polyadenylation factor i subunit 1  NDC1 - ndc1 transmembrane nucleoporin  PRKD1 - protein kinase d1  CCDC88A - coiled-coil domain containing 88a  PKN2 - protein kinase n2  PRKCH - protein kinase c, eta  CCDC94 - coiled-coil domain containing 94  NT5C3B - 5'-nucleotidase, cytosolic iiib  POLR3B - polymerase (rna) iii (dna directed) polypeptide b  PRKCI - protein kinase c, iota  TLK2 - tousled-like kinase 2  PRKCE - protein kinase c, epsilon  ANXA2 - annexin a2  PRKCB - protein kinase c, beta  PRKCD - protein kinase c, delta  RBM22 - rna binding motif protein 22  PRKCA - protein kinase c, alpha  PRKAR1B - protein kinase, camp-dependent, regulatory, type i, beta  NSUN5 - nop2/sun domain family, member 5  YEATS2 - yeats domain containing 2  SF3B2 - splicing factor 3b, subunit 2, 145kda  PRKAG1 - protein kinase, amp-activated, gamma 1 non-catalytic subunit  APEX1 - apex nuclease (multifunctional dna repair enzyme) 1  MTPAP - mitochondrial poly(a) polymerase  UBR7 - ubiquitin protein ligase e3 component n-recognin 7 (putative)  RBM23 - rna binding motif protein 23  DUSP12 - dual specificity phosphatase 12  KLHL2 - kelch-like family member 2  XIAP - x-linked inhibitor of apoptosis  DALRD3 - dalr anticodon binding domain containing 3  BIRC3 - baculoviral iap repeat containing 3  PARN - poly(a)-specific ribonuclease  BIRC2 - baculoviral iap repeat containing 2  RFWD3 - ring finger and wd repeat domain 3  DARS2 - aspartyl-trna synthetase 2, mitochondrial  TREX1 - three prime repair exonuclease 1  APLP2 - amyloid beta (a4) precursor-like protein 2  MGAT4B - mannosyl (alpha-1,3-)-glycoprotein beta-1,4-n-acetylglucosaminyltransferase, isozyme b  PRMT6 - protein arginine methyltransferase 6  MSL2 - male-specific lethal 2 homolog (drosophila)  MRPS10 - mitochondrial ribosomal protein s10  MAN1B1 - mannosidase, alpha, class 1b, member 1  INTS10 - integrator complex subunit 10  PCBP2 - poly(rc) binding protein 2  SMG8 - smg8 nonsense mediated mrna decay factor  DCUN1D3 - dcn1, defective in cullin neddylation 1, domain containing 3  RNF220 - ring finger protein 220  APC - adenomatous polyposis coli  APBB1 - amyloid beta (a4) precursor protein-binding, family b, member 1 (fe65)  CHP1 - calcineurin-like ef-hand protein 1  GALNT6 - udp-n-acetyl-alpha-d-galactosamine:polypeptide n-acetylgalactosaminyltransferase 6 (galnac-t6)  RIF1 - rap1 interacting factor homolog (yeast)  POLG2 - polymerase (dna directed), gamma 2, accessory subunit  DPY19L4 - dpy-19-like 4 (c. elegans)  RNF139 - ring finger protein 139  P4HA1 - prolyl 4-hydroxylase, alpha polypeptide i  PEBP1 - phosphatidylethanolamine binding protein 1  PA2G4 - proliferation-associated 2g4, 38kda  TRIM23 - tripartite motif containing 23  RPRD1A - regulation of nuclear pre-mrna domain containing 1a  CNTD1 - cyclin n-terminal domain containing 1  ARF4 - adp-ribosylation factor 4  MAP1S - microtubule-associated protein 1s  POLI - polymerase (dna directed) iota  IRAK3 - interleukin-1 receptor-associated kinase 3  SETD5 - set domain containing 5  AKAP13 - a kinase (prka) anchor protein 13  APP - amyloid beta (a4) precursor protein  PAK2 - p21 protein (cdc42/rac)-activated kinase 2  DDX20 - dead (asp-glu-ala-asp) box polypeptide 20  PAK3 - p21 protein (cdc42/rac)-activated kinase 3  LEPREL1 - leprecan-like 1  PAM - peptidylglycine alpha-amidating monooxygenase  MRPL3 - mitochondrial ribosomal protein l3  CANT1 - calcium activated nucleotidase 1  PDGFA - platelet-derived growth factor alpha polypeptide  ARNTL - aryl hydrocarbon receptor nuclear translocator-like  CELF3 - cugbp, elav-like family member 3  MAP4K5 - mitogen-activated protein kinase kinase kinase kinase 5  NSMCE2 - non-smc element 2, mms21 homolog (s. cerevisiae)  SUPT16H - suppressor of ty 16 homolog (s. cerevisiae)  PDGFRB - platelet-derived growth factor receptor, beta polypeptide  ARSB - arylsulfatase b  ARRB2 - arrestin, beta 2  PDGFRA - platelet-derived growth factor receptor, alpha polypeptide  ARRB1 - arrestin, beta 1  PDGFB - platelet-derived growth factor beta polypeptide  GPATCH1 - g patch domain containing 1  PDPK1 - 3-phosphoinositide dependent protein kinase-1  SAMD4B - sterile alpha motif domain containing 4b  WDYHV1 - wdyhv motif containing 1  RHOA - ras homolog family member a  PDK3 - pyruvate dehydrogenase kinase, isozyme 3  FSTL1 - follistatin-like 1  BAZ2A - bromodomain adjacent to zinc finger domain, 2a  PCNA - proliferating cell nuclear antigen  CHMP1A - charged multivesicular body protein 1a  LSM6 - lsm6 homolog, u6 small nuclear rna associated (s. cerevisiae)  ISCA2 - iron-sulfur cluster assembly 2  PLEKHJ1 - pleckstrin homology domain containing, family j member 1  PCMT1 - protein-l-isoaspartate (d-aspartate) o-methyltransferase  FICD - fic domain containing  MAGOHB - mago-nashi homolog b (drosophila)  CDK16 - cyclin-dependent kinase 16  MTFMT - mitochondrial methionyl-trna formyltransferase  PCSK2 - proprotein convertase subtilisin/kexin type 2  HEATR1 - heat repeat containing 1  TARSL2 - threonyl-trna synthetase-like 2  CDK17 - cyclin-dependent kinase 17  LEO1 - leo1, paf1/rna polymerase ii complex component, homolog (s. cerevisiae)  ASPH - aspartate beta-hydroxylase  FANCL - fanconi anemia, complementation group l  RPL7L1 - ribosomal protein l7-like 1  PCSK1 - proprotein convertase subtilisin/kexin type 1  PRPF38B - prp38 pre-mrna processing factor 38 (yeast) domain containing b  ERLIN2 - er lipid raft associated 2  SRBD1 - s1 rna binding domain 1  PWP1 - pwp1 homolog (s. cerevisiae)  WRAP53 - wd repeat containing, antisense to tp53  SOCS4 - suppressor of cytokine signaling 4  CAPN10 - calpain 10  RBM28 - rna binding motif protein 28  THAP1 - thap domain containing, apoptosis associated protein 1  ANKZF1 - ankyrin repeat and zinc finger domain containing 1  ELP3 - elongator acetyltransferase complex subunit 3  KAT7 - k(lysine) acetyltransferase 7  PGGT1B - protein geranylgeranyltransferase type i, beta subunit  DENND3 - denn/madd domain containing 3  CPPED1 - calcineurin-like phosphoesterase domain containing 1  PGM3 - phosphoglucomutase 3  ZBTB1 - zinc finger and btb domain containing 1  ZNF365 - zinc finger protein 365  UEVLD - uev and lactate/malate dehyrogenase domains  RNF180 - ring finger protein 180  UBOX5 - u-box domain containing 5  FBXW7 - f-box and wd repeat domain containing 7, e3 ubiquitin protein ligase  RNF121 - ring finger protein 121  DIS3 - dis3 mitotic control homolog (s. cerevisiae)  ZHX2 - zinc fingers and homeoboxes 2  PHF2 - phd finger protein 2  UFSP2 - ufm1-specific peptidase 2  LRRK2 - leucine-rich repeat kinase 2  DNAJC24 - dnaj (hsp40) homolog, subfamily c, member 24  AP5S1 - adaptor-related protein complex 5, sigma 1 subunit  LARP6 - la ribonucleoprotein domain family, member 6  SPPL3 - signal peptide peptidase like 3  SLC35C1 - solute carrier family 35 (gdp-fucose transporter), member c1  PEX12 - peroxisomal biogenesis factor 12  PEX13 - peroxisomal biogenesis factor 13  PEX14 - peroxisomal biogenesis factor 14  ATP7A - atpase, cu++ transporting, alpha polypeptide  PENK - proenkephalin  LMTK2 - lemur tyrosine kinase 2  CHSY1 - chondroitin sulfate synthase 1  ICK - intestinal cell (mak-like) kinase  ALKBH2 - alkb, alkylation repair homolog 2 (e. coli)  WDR33 - wd repeat domain 33  PPM1E - protein phosphatase, mg2+/mn2+ dependent, 1e  VASH1 - vasohibin 1  AAK1 - ap2 associated kinase 1  COPRS - coordinator of prmt5, differentiation stimulator  PKD1 - polycystic kidney disease 1 (autosomal dominant)  SMU1 - smu-1 suppressor of mec-8 and unc-52 homolog (c. elegans)  SCAF8 - sr-related ctd-associated factor 8  BAX - bcl2-associated x protein  UBA6 - ubiquitin-like modifier activating enzyme 6  CPXM2 - carboxypeptidase x (m14 family), member 2  PUF60 - poly-u binding splicing factor 60kda  USP40 - ubiquitin specific peptidase 40  BAK1 - bcl2-antagonist/killer 1  RASA3 - ras p21 protein activator 3  PHLDA1 - pleckstrin homology-like domain, family a, member 1  RAVER2 - ribonucleoprotein, ptb-binding 2  NAT10 - n-acetyltransferase 10 (gcn5-related)  BACH1 - btb and cnc homology 1, basic leucine zipper transcription factor 1  PIN1 - peptidylprolyl cis/trans isomerase, nima-interacting 1  BAD - bcl2-associated agonist of cell death  DPH3 - diphthamide biosynthesis 3  EXD2 - exonuclease 3'-5' domain containing 2  PIK3R1 - phosphoinositide-3-kinase, regulatory subunit 1 (alpha)  PLAT - plasminogen activator, tissue  TYW1 - trna-yw synthesizing protein 1 homolog (s. cerevisiae)  ASXL2 - additional sex combs like 2 (drosophila)  AXL - axl receptor tyrosine kinase  PCMTD2 - protein-l-isoaspartate (d-aspartate) o-methyltransferase domain containing 2  ELP2 - elongator acetyltransferase complex subunit 2  XRN2 - 5'-3' exoribonuclease 2  PEF1 - penta-ef-hand domain containing 1  ATRX - alpha thalassemia/mental retardation syndrome x-linked  PKNOX1 - pbx/knotted 1 homeobox 1  CASC3 - cancer susceptibility candidate 3  OGFOD1 - 2-oxoglutarate and iron-dependent oxygenase domain containing 1  PSTK - phosphoseryl-trna kinase  IMP3 - imp3, u3 small nucleolar ribonucleoprotein, homolog (yeast)  EXOSC8 - exosome component 8  PIH1D2 - pih1 domain containing 2  PHF10 - phd finger protein 10  RNF13 - ring finger protein 13  PSPC1 - paraspeckle component 1  U2AF2 - u2 small nuclear rna auxiliary factor 2  NUDT15 - nudix (nucleoside diphosphate linked moiety x)-type motif 15  BCR - breakpoint cluster region  PHKG2 - phosphorylase kinase, gamma 2 (testis)  FBXW12 - f-box and wd repeat domain containing 12  FKBP9 - fk506 binding protein 9, 63 kda  STK38 - serine/threonine kinase 38  MRGBP - mrg/morf4l binding protein  SUMF1 - sulfatase modifying factor 1  PIK3CA - phosphatidylinositol-4,5-bisphosphate 3-kinase, catalytic subunit alpha  RHOT1 - ras homolog family member t1  RPUSD3 - rna pseudouridylate synthase domain containing 3  PIM1 - pim-1 oncogene  BRF2 - brf2, rna polymerase iii transcription initiation factor 50 kda subunit  DDX42 - dead (asp-glu-ala-asp) box helicase 42  UBE2W - ubiquitin-conjugating enzyme e2w (putative)  BCL2 - b-cell cll/lymphoma 2  PIK3C3 - phosphatidylinositol 3-kinase, catalytic subunit type 3  MGAT4A - mannosyl (alpha-1,3-)-glycoprotein beta-1,4-n-acetylglucosaminyltransferase, isozyme a  ECD - ecdysoneless homolog (drosophila)  PIGH - phosphatidylinositol glycan anchor biosynthesis, class h  CWF19L1 - cwf19-like 1, cell cycle control (s. pombe)  LYPLA2 - lysophospholipase ii  CCND1 - cyclin d1  PARK7 - parkinson protein 7  B4GALT7 - xylosylprotein beta 1,4-galactosyltransferase, polypeptide 7  PGM2 - phosphoglucomutase 2  PNKP - polynucleotide kinase 3'-phosphatase  PIGF - phosphatidylinositol glycan anchor biosynthesis, class f  QRSL1 - glutaminyl-trna synthase (glutamine-hydrolyzing)-like 1  TRIM27 - tripartite motif containing 27  CDKAL1 - cdk5 regulatory subunit associated protein 1-like 1  SMNDC1 - survival motor neuron domain containing 1  RFC5 - replication factor c (activator 1) 5, 36.5kda  BCAS2 - breast carcinoma amplified sequence 2  BNIP3L - bcl2/adenovirus e1b 19kda interacting protein 3-like  RFC2 - replication factor c (activator 1) 2, 40kda  CWC27 - cwc27 spliceosome-associated protein homolog (s. cerevisiae)  RFC3 - replication factor c (activator 1) 3, 38kda  SAP18 - sin3a-associated protein, 18kda  MAEA - macrophage erythroblast attacher  BRAF - v-raf murine sarcoma viral oncogene homolog b  BCKDK - branched chain ketoacid dehydrogenase kinase  WHSC1L1 - wolf-hirschhorn syndrome candidate 1-like 1  SF3A1 - splicing factor 3a, subunit 1, 120kda  RELA - v-rel avian reticuloendotheliosis viral oncogene homolog a  CDKL4 - cyclin-dependent kinase-like 4  CWC25 - cwc25 spliceosome-associated protein homolog (s. cerevisiae)  TEX10 - testis expressed 10  BCOR - bcl6 corepressor  SNAPC5 - small nuclear rna activating complex, polypeptide 5, 19kda  RECQL - recq protein-like (dna helicase q1-like)  DPP8 - dipeptidyl-peptidase 8  ZFP36L2 - zfp36 ring finger protein-like 2  DCAF16 - ddb1 and cul4 associated factor 16  ZFP36L1 - zfp36 ring finger protein-like 1  PAK4 - p21 protein (cdc42/rac)-activated kinase 4  RET - ret proto-oncogene  ALKBH5 - alkb, alkylation repair homolog 5 (e. coli)  DPF2 - d4, zinc and double phd fingers family 2  SH3RF3 - sh3 domain containing ring finger 3  DUSP15 - dual specificity phosphatase 15  UPF1 - upf1 regulator of nonsense transcripts homolog (yeast)  NSUN2 - nop2/sun rna methyltransferase family, member 2  GALNTL6 - udp-n-acetyl-alpha-d-galactosamine:polypeptide n-acetylgalactosaminyltransferase-like 6  RLF - rearranged l-myc fusion  ELP6 - elongator acetyltransferase complex subunit 6  SNRK - snf related kinase  RING1 - ring finger protein 1  PIGG - phosphatidylinositol glycan anchor biosynthesis, class g  TMEM5 - transmembrane protein 5  FBLL1 - fibrillarin-like 1  TATDN3 - tatd dnase domain containing 3  BMP1 - bone morphogenetic protein 1  BMI1 - bmi1 polycomb ring finger oncogene  PCGF3 - polycomb group ring finger 3  BMPR1A - bone morphogenetic protein receptor, type ia  BMPR2 - bone morphogenetic protein receptor, type ii (serine/threonine kinase)  WARS2 - tryptophanyl trna synthetase 2, mitochondrial  BMPR1B - bone morphogenetic protein receptor, type ib  FBXL12 - f-box and leucine-rich repeat protein 12  WDR55 - wd repeat domain 55  ABCA7 - atp-binding cassette, sub-family a (abc1), member 7  TRPM7 - transient receptor potential cation channel, subfamily m, member 7  PSMD14 - proteasome (prosome, macropain) 26s subunit, non-atpase, 14  MRPL49 - mitochondrial ribosomal protein l49  RNF19B - ring finger protein 19b  CTDSPL - ctd (carboxy-terminal domain, rna polymerase ii, polypeptide a) small phosphatase-like  TMEM258 - transmembrane protein 258  GIN1 - gypsy retrotransposon integrase 1  TRIT1 - trna isopentenyltransferase 1  UHMK1 - u2af homology motif (uhm) kinase 1  RNASE6 - ribonuclease, rnase a family, k6  MSLN - mesothelin  RNF4 - ring finger protein 4  DCAF7 - ddb1 and cul4 associated factor 7  RNF2 - ring finger protein 2  HNRNPR - heterogeneous nuclear ribonucleoprotein r  RNPEP - arginyl aminopeptidase (aminopeptidase b)  GPHN - gephyrin  GATAD2A - gata zinc finger domain containing 2a  RNF6 - ring finger protein (c3h2c3 type) 6  QPCTL - glutaminyl-peptide cyclotransferase-like  ROCK1 - rho-associated, coiled-coil containing protein kinase 1  LYPLAL1 - lysophospholipase-like 1  RNF111 - ring finger protein 111  BYSL - bystin-like  SRRM1 - serine/arginine repetitive matrix 1  DNAJB12 - dnaj (hsp40) homolog, subfamily b, member 12  TET2 - tet methylcytosine dioxygenase 2  STAM2 - signal transducing adaptor molecule (sh3 domain and itam motif) 2  TRPM4 - transient receptor potential cation channel, subfamily m, member 4  MED18 - mediator complex subunit 18  TYW3 - trna-yw synthesizing protein 3 homolog (s. cerevisiae)  SF3B4 - splicing factor 3b, subunit 4, 49kda  RAMP2 - receptor (g protein-coupled) activity modifying protein 2  RAMP3 - receptor (g protein-coupled) activity modifying protein 3  RAMP1 - receptor (g protein-coupled) activity modifying protein 1  CAPN5 - calpain 5  ZMPSTE24 - zinc metallopeptidase ste24  STUB1 - stip1 homology and u-box containing protein 1, e3 ubiquitin protein ligase  C5AR1 - complement component 5a receptor 1  TCEANC2 - transcription elongation factor a (sii) n-terminal and central domain containing 2  UBE4B - ubiquitination factor e4b  TRIM44 - tripartite motif containing 44  TAB1 - tgf-beta activated kinase 1/map3k7 binding protein 1  CAPNS1 - calpain, small subunit 1  PPIE - peptidylprolyl isomerase e (cyclophilin e)  CAPN2 - calpain 2, (m/ii) large subunit  CAPN1 - calpain 1, (mu/i) large subunit  MCRS1 - microspherule protein 1  MRPL20 - mitochondrial ribosomal protein l20  ZER1 - zyg-11 related, cell cycle regulator  RPL17 - ribosomal protein l17  CAMLG - calcium modulating ligand  RPL15 - ribosomal protein l15  VPS37C - vacuolar protein sorting 37 homolog c (s. cerevisiae)  C1D - c1d nuclear receptor corepressor  CAMK2B - calcium/calmodulin-dependent protein kinase ii beta  RPL11 - ribosomal protein l11  TRMT12 - trna methyltransferase 12 homolog (s. cerevisiae)  PTCD3 - pentatricopeptide repeat domain 3  RPL9 - ribosomal protein l9  NOL8 - nucleolar protein 8  FKBP14 - fk506 binding protein 14, 22 kda  CASP8 - caspase 8, apoptosis-related cysteine peptidase  RPL7 - ribosomal protein l7  CASP9 - caspase 9, apoptosis-related cysteine peptidase  PIBF1 - progesterone immunomodulatory binding factor 1  PPIH - peptidylprolyl isomerase h (cyclophilin h)  SLC30A9 - solute carrier family 30 (zinc transporter), member 9  CASP2 - caspase 2, apoptosis-related cysteine peptidase  RPL3 - ribosomal protein l3  CASP3 - caspase 3, apoptosis-related cysteine peptidase  RPA3 - replication protein a3, 14kda  CARS - cysteinyl-trna synthetase  B3GALT6 - udp-gal:betagal beta 1,3-galactosyltransferase polypeptide 6  MAD2L2 - mad2 mitotic arrest deficient-like 2 (yeast)  RPA1 - replication protein a1, 70kda  RPA2 - replication protein a2, 32kda  RPS4X - ribosomal protein s4, x-linked  CAD - carbamoyl-phosphate synthetase 2, aspartate transcarbamylase, and dihydroorotase  RPS4Y1 - ribosomal protein s4, y-linked 1  RPN2 - ribophorin ii  RPN1 - ribophorin i  UBE2E3 - ubiquitin-conjugating enzyme e2e 3  HERC6 - hect and rld domain containing e3 ubiquitin protein ligase family member 6  RMND1 - required for meiotic nuclear division 1 homolog (s. cerevisiae)  TRIM38 - tripartite motif containing 38  DDR1 - discoidin domain receptor tyrosine kinase 1  TADA3 - transcriptional adaptor 3  CALR - calreticulin  CALU - calumenin  NR2C2AP - nuclear receptor 2c2-associated protein  SYNCRIP - synaptotagmin binding, cytoplasmic rna interacting protein  RPL32 - ribosomal protein l32  STK25 - serine/threonine kinase 25  GID8 - gid complex subunit 8  LRRC41 - leucine rich repeat containing 41  CRTAP - cartilage associated protein  RPS23 - ribosomal protein s23  RAVER1 - ribonucleoprotein, ptb-binding 1  CCNT2 - cyclin t2  PINX1 - pin2/terf1 interacting, telomerase inhibitor 1  CLN6 - ceroid-lipofuscinosis, neuronal 6, late infantile, variant  NPM3 - nucleophosmin/nucleoplasmin 3  INO80C - ino80 complex subunit c  THG1L - trna-histidine guanylyltransferase 1-like (s. cerevisiae)  CCNT1 - cyclin t1  CCNH - cyclin h  TMEM132A - transmembrane protein 132a  SSH3 - slingshot protein phosphatase 3  SWSAP1 - swim-type zinc finger 7 associated protein 1  ANAPC10 - anaphase promoting complex subunit 10  RPS15 - ribosomal protein s15  TIPIN - timeless interacting protein  RPS12 - ribosomal protein s12  CD9 - cd9 molecule  TRNAU1AP - trna selenocysteine 1 associated protein 1  RPS6KA2 - ribosomal protein s6 kinase, 90kda, polypeptide 2  NRBP2 - nuclear receptor binding protein 2  RPS6KA1 - ribosomal protein s6 kinase, 90kda, polypeptide 1  PARP16 - poly (adp-ribose) polymerase family, member 16  RPS6KB1 - ribosomal protein s6 kinase, 70kda, polypeptide 1  RPS6KB2 - ribosomal protein s6 kinase, 70kda, polypeptide 2  RNF125 - ring finger protein 125, e3 ubiquitin protein ligase  CBFA2T3 - core-binding factor, runt domain, alpha subunit 2; translocated to, 3  CBFB - core-binding factor, beta subunit  NSA2 - nsa2 ribosome biogenesis homolog (s. cerevisiae)  CBL - cbl proto-oncogene, e3 ubiquitin protein ligase  SERPINH1 - serpin peptidase inhibitor, clade h (heat shock protein 47), member 1, (collagen binding protein 1)  MRPL10 - mitochondrial ribosomal protein l10  RHBDL2 - rhomboid, veinlet-like 2 (drosophila)  RXRB - retinoid x receptor, beta  PIAS3 - protein inhibitor of activated stat, 3  KANSL2 - kat8 regulatory nsl complex subunit 2  CAV1 - caveolin 1, caveolae protein, 22kda  ADPRHL2 - adp-ribosylhydrolase like 2  SARS2 - seryl-trna synthetase 2, mitochondrial  CPQ - carboxypeptidase q  ZSWIM7 - zinc finger, swim-type containing 7  UBE2R2 - ubiquitin-conjugating enzyme e2r 2  IMPAD1 - inositol monophosphatase domain containing 1  CCNC - cyclin c  RBM14 - rna binding motif protein 14  TRMT10C - trna methyltransferase 10 homolog c (s. cerevisiae)  PRMT5 - protein arginine methyltransferase 5  CD2BP2 - cd2 (cytoplasmic tail) binding protein 2  RRM1 - ribonucleotide reductase m1  CHTF8 - ctf8, chromosome transmission fidelity factor 8 homolog (s. cerevisiae)  RBM27 - rna binding motif protein 27  TRIM3 - tripartite motif containing 3  CDK11B - cyclin-dependent kinase 11b  CDC5L - cell division cycle 5-like  TBL3 - transducin (beta)-like 3  TGOLN2 - trans-golgi network protein 2  HTRA1 - htra serine peptidase 1  STAMBP - stam binding protein  PSAP - prosaposin  POLR3F - polymerase (rna) iii (dna directed) polypeptide f, 39 kda  CDH2 - cadherin 2, type 1, n-cadherin (neuronal)  KCTD5 - potassium channel tetramerization domain containing 5  ERLIN1 - er lipid raft associated 1  CDC27 - cell division cycle 27  ANKIB1 - ankyrin repeat and ibr domain containing 1  MRPS21 - mitochondrial ribosomal protein s21  POMT1 - protein-o-mannosyltransferase 1  PSEN1 - presenilin 1  PSEN2 - presenilin 2 (alzheimer disease 4)  TOLLIP - toll interacting protein  PSKH1 - protein serine kinase h1  USP16 - ubiquitin specific peptidase 16  PSMA1 - proteasome (prosome, macropain) subunit, alpha type, 1  SRSF12 - serine/arginine-rich splicing factor 12  PSMA2 - proteasome (prosome, macropain) subunit, alpha type, 2  RNF216 - ring finger protein 216  PRPF8 - pre-mrna processing factor 8  CD81 - cd81 molecule  CAMKK2 - calcium/calmodulin-dependent protein kinase kinase 2, beta  PSMB1 - proteasome (prosome, macropain) subunit, beta type, 1  PSMA5 - proteasome (prosome, macropain) subunit, alpha type, 5  WDR36 - wd repeat domain 36  NPRL2 - nitrogen permease regulator-like 2 (s. cerevisiae)  PSMA3 - proteasome (prosome, macropain) subunit, alpha type, 3  PSMB10 - proteasome (prosome, macropain) subunit, beta type, 10  KHDRBS3 - kh domain containing, rna binding, signal transduction associated 3  ADAT2 - adenosine deaminase, trna-specific 2  CD44 - cd44 molecule (indian blood group)  UBLCP1 - ubiquitin-like domain containing ctd phosphatase 1  PSMC6 - proteasome (prosome, macropain) 26s subunit, atpase, 6  TAF6L - taf6-like rna polymerase ii, p300/cbp-associated factor (pcaf)-associated factor, 65kda  FAM173B - family with sequence similarity 173, member b  PSMD1 - proteasome (prosome, macropain) 26s subunit, non-atpase, 1  PSMC4 - proteasome (prosome, macropain) 26s subunit, atpase, 4  POLR3G - polymerase (rna) iii (dna directed) polypeptide g (32kd)  DPM3 - dolichyl-phosphate mannosyltransferase polypeptide 3  PSMC2 - proteasome (prosome, macropain) 26s subunit, atpase, 2  PSMD9 - proteasome (prosome, macropain) 26s subunit, non-atpase, 9  PSMD10 - proteasome (prosome, macropain) 26s subunit, non-atpase, 10  GAR1 - gar1 ribonucleoprotein  PSMD7 - proteasome (prosome, macropain) 26s subunit, non-atpase, 7  TSPAN33 - tetraspanin 33  PSMD8 - proteasome (prosome, macropain) 26s subunit, non-atpase, 8  DNAJC10 - dnaj (hsp40) homolog, subfamily c, member 10  PSMD5 - proteasome (prosome, macropain) 26s subunit, non-atpase, 5  PSMD2 - proteasome (prosome, macropain) 26s subunit, non-atpase, 2  TERF2IP - telomeric repeat binding factor 2, interacting protein  PSMD12 - proteasome (prosome, macropain) 26s subunit, non-atpase, 12  PITRM1 - pitrilysin metallopeptidase 1  PSMD13 - proteasome (prosome, macropain) 26s subunit, non-atpase, 13  ATG7 - autophagy related 7  LEPREL2 - leprecan-like 2  RNASEH2A - ribonuclease h2, subunit a  KLHL17 - kelch-like family member 17  PTBP1 - polypyrimidine tract binding protein 1  GLRX3 - glutaredoxin 3  PTEN - phosphatase and tensin homolog  CETN2 - centrin, ef-hand protein, 2  JMY - junction mediating and regulatory protein, p53 cofactor  SETD4 - set domain containing 4  PRDX4 - peroxiredoxin 4  CFL1 - cofilin 1 (non-muscle)  ZBTB8OS - zinc finger and btb domain containing 8 opposite strand  CHRAC1 - chromatin accessibility complex 1  CIB1 - calcium and integrin binding 1 (calmyrin)  DDX17 - dead (asp-glu-ala-asp) box helicase 17  CHERP - calcium homeostasis endoplasmic reticulum protein  KAT5 - k(lysine) acetyltransferase 5  PTK2 - protein tyrosine kinase 2  PTK7 - protein tyrosine kinase 7  CEBPG - ccaat/enhancer binding protein (c/ebp), gamma  NOP56 - nop56 ribonucleoprotein  SLU7 - slu7 splicing factor homolog (s. cerevisiae)  CDK5 - cyclin-dependent kinase 5  PTMA - prothymosin, alpha  CDK4 - cyclin-dependent kinase 4  TWF1 - twinfilin actin-binding protein 1  CDK9 - cyclin-dependent kinase 9  CDK8 - cyclin-dependent kinase 8  CDK7 - cyclin-dependent kinase 7  SPOPL - speckle-type poz protein-like  PCBP3 - poly(rc) binding protein 3  PTPN3 - protein tyrosine phosphatase, non-receptor type 3  CDKN2D - cyclin-dependent kinase inhibitor 2d (p19, inhibits cdk4)  PTPN2 - protein tyrosine phosphatase, non-receptor type 2  PTPN1 - protein tyrosine phosphatase, non-receptor type 1  PTPN9 - protein tyrosine phosphatase, non-receptor type 9  PTPN11 - protein tyrosine phosphatase, non-receptor type 11  HTATIP2 - hiv-1 tat interactive protein 2, 30kda  PPM1M - protein phosphatase, mg2+/mn2+ dependent, 1m  PTPRA - protein tyrosine phosphatase, receptor type, a  RPP38 - ribonuclease p/mrp 38kda subunit  PTPN12 - protein tyrosine phosphatase, non-receptor type 12  PTPN13 - protein tyrosine phosphatase, non-receptor type 13 (apo-1/cd95 (fas)-associated phosphatase)  PTPRD - protein tyrosine phosphatase, receptor type, d  PTPRB - protein tyrosine phosphatase, receptor type, b  SLC35A1 - solute carrier family 35 (cmp-sialic acid transporter), member a1  PTPRG - protein tyrosine phosphatase, receptor type, g  ARFGEF2 - adp-ribosylation factor guanine nucleotide-exchange factor 2 (brefeldin a-inhibited)  CDK2 - cyclin-dependent kinase 2  PTPRE - protein tyrosine phosphatase, receptor type, e  PTPRF - protein tyrosine phosphatase, receptor type, f  RAD1 - rad1 homolog (s. pombe)  WDR4 - wd repeat domain 4  PURA - purine-rich element binding protein a  RRN3 - rrn3 rna polymerase i transcription factor homolog (s. cerevisiae)  PDP1 - pyruvate dehyrogenase phosphatase catalytic subunit 1  GTPBP2 - gtp binding protein 2  ZNHIT6 - zinc finger, hit-type containing 6  METTL6 - methyltransferase like 6  P4HTM - prolyl 4-hydroxylase, transmembrane (endoplasmic reticulum)  POP4 - processing of precursor 4, ribonuclease p/mrp subunit (s. cerevisiae)  NUDT16 - nudix (nucleoside diphosphate linked moiety x)-type motif 16  PTPRR - protein tyrosine phosphatase, receptor type, r  PTPRS - protein tyrosine phosphatase, receptor type, s  PTPRK - protein tyrosine phosphatase, receptor type, k  SRSF10 - serine/arginine-rich splicing factor 10  PTPRM - protein tyrosine phosphatase, receptor type, m  PTPRN - protein tyrosine phosphatase, receptor type, n  PYGB - phosphorylase, glycogen; brain  PLK2 - polo-like kinase 2  AHCYL1 - adenosylhomocysteinase-like 1  PEX2 - peroxisomal biogenesis factor 2  NUP50 - nucleoporin 50kda  CHML - choroideremia-like (rab escort protein 2)  CHM - choroideremia (rab escort protein 1)  KDM5B - lysine (k)-specific demethylase 5b  CHCHD4 - coiled-coil-helix-coiled-coil-helix domain containing 4  CHGB - chromogranin b (secretogranin 1)  MXRA8 - matrix-remodelling associated 8  RAB6A - rab6a, member ras oncogene family  MAP4K2 - mitogen-activated protein kinase kinase kinase kinase 2  RAB5A - rab5a, member ras oncogene family  RAB3A - rab3a, member ras oncogene family  RAB3B - rab3b, member ras oncogene family  CHD1 - chromodomain helicase dna binding protein 1  MTERFD2 - mterf domain containing 2  FRS2 - fibroblast growth factor receptor substrate 2  RAB2A - rab2a, member ras oncogene family  RAB1A - rab1a, member ras oncogene family  METTL2A - methyltransferase like 2a  QARS - glutaminyl-trna synthetase  RAD21 - rad21 homolog (s. pombe)  RAD17 - rad17 homolog (s. pombe)  PARP14 - poly (adp-ribose) polymerase family, member 14  PAF1 - paf1, rna polymerase ii associated factor, homolog (s. cerevisiae)  RPP40 - ribonuclease p/mrp 40kda subunit  MSL1 - male-specific lethal 1 homolog (drosophila)  FBXL19 - f-box and leucine-rich repeat protein 19  INO80 - ino80 complex subunit  DDX56 - dead (asp-glu-ala-asp) box helicase 56  NDFIP2 - nedd4 family interacting protein 2  RC3H2 - ring finger and ccch-type domains 2  RANBP2 - ran binding protein 2  RANGAP1 - ran gtpase activating protein 1  CLTC - clathrin, heavy chain (hc)  MIER2 - mesoderm induction early response 1, family member 2  MOK - mok protein kinase  USP53 - ubiquitin specific peptidase 53  B3GNT2 - udp-glcnac:betagal beta-1,3-n-acetylglucosaminyltransferase 2  FAM35A - family with sequence similarity 35, member a  RARG - retinoic acid receptor, gamma  CLN5 - ceroid-lipofuscinosis, neuronal 5  SGTB - small glutamine-rich tetratricopeptide repeat (tpr)-containing, beta  RARB - retinoic acid receptor, beta  RARS - arginyl-trna synthetase  RRAGA - ras-related gtp binding a  RASA1 - ras p21 protein activator (gtpase activating protein) 1  CLTA - clathrin, light chain a  CELF1 - cugbp, elav-like family member 1  KHDRBS1 - kh domain containing, rna binding, signal transduction associated 1  CLU - clusterin  TAF8 - taf8 rna polymerase ii, tata box binding protein (tbp)-associated factor, 43kda  CLK1 - cdc-like kinase 1  MAGEL2 - mage-like 2  CELF2 - cugbp, elav-like family member 2  CLK2 - cdc-like kinase 2  ING3 - inhibitor of growth family, member 3  TPP1 - tripeptidyl peptidase i  DDX49 - dead (asp-glu-ala-asp) box polypeptide 49  CLK3 - cdc-like kinase 3  CTCF - ccctc-binding factor (zinc finger protein)  PRMT7 - protein arginine methyltransferase 7  RBBP7 - retinoblastoma binding protein 7  TMX3 - thioredoxin-related transmembrane protein 3  RBBP8 - retinoblastoma binding protein 8  RBMS1 - rna binding motif, single stranded interacting protein 1  MAP3K2 - mitogen-activated protein kinase kinase kinase 2  RBM3 - rna binding motif (rnp1, rrm) protein 3  DIS3L2 - dis3 mitotic control homolog (s. cerevisiae)-like 2  RBM4 - rna binding motif protein 4  CHPF2 - chondroitin polymerizing factor 2  ARID4A - at rich interactive domain 4a (rbp1-like)  AP2M1 - adaptor-related protein complex 2, mu 1 subunit  RBBP5 - retinoblastoma binding protein 5  TYW5 - trna-yw synthesizing protein 5  RBBP6 - retinoblastoma binding protein 6  KDM5A - lysine (k)-specific demethylase 5a  DGCR8 - dgcr8 microprocessor complex subunit  RBBP4 - retinoblastoma binding protein 4  NUP35 - nucleoporin 35kda  PUS7 - pseudouridylate synthase 7 homolog (s. cerevisiae)  MGEA5 - meningioma expressed antigen 5 (hyaluronidase)  ERCC8 - excision repair cross-complementing rodent repair deficiency, complementation group 8  RCN1 - reticulocalbin 1, ef-hand calcium binding domain  EXOSC4 - exosome component 4  ANKRD16 - ankyrin repeat domain 16  YME1L1 - yme1-like 1 atpase  PTGES3 - prostaglandin e synthase 3 (cytosolic)  NFAT5 - nuclear factor of activated t-cells 5, tonicity-responsive  POLD3 - polymerase (dna-directed), delta 3, accessory subunit  CHMP4B - charged multivesicular body protein 4b  PIGU - phosphatidylinositol glycan anchor biosynthesis, class u  USP39 - ubiquitin specific peptidase 39  ZDHHC13 - zinc finger, dhhc-type containing 13  CHUK - conserved helix-loop-helix ubiquitous kinase  SPTAN1 - spectrin, alpha, non-erythrocytic 1  MKRN1 - makorin ring finger protein 1  MKRN2 - makorin ring finger protein 2  TBRG1 - transforming growth factor beta regulator 1  TMTC4 - transmembrane and tetratricopeptide repeat containing 4  SPTBN4 - spectrin, beta, non-erythrocytic 4  CD2AP - cd2-associated protein  FBXO18 - f-box protein, helicase, 18  METTL14 - methyltransferase like 14  SPP1 - secreted phosphoprotein 1  ANO8 - anoctamin 8  SPG7 - spastic paraplegia 7 (pure and complicated autosomal recessive)  ALG10 - alg10, alpha-1,2-glucosyltransferase  CIRH1A - cirrhosis, autosomal recessive 1a (cirhin)  MIER1 - mesoderm induction early response 1, transcriptional regulator  PPP1R15B - protein phosphatase 1, regulatory subunit 15b  CWC22 - cwc22 spliceosome-associated protein homolog (s. cerevisiae)  BACE1 - beta-site app-cleaving enzyme 1  ABHD13 - abhydrolase domain containing 13  SP1 - sp1 transcription factor  METTL12 - methyltransferase like 12  MUM1 - melanoma associated antigen (mutated) 1  ATG4C - autophagy related 4c, cysteine peptidase  CPD - carboxypeptidase d  CPE - carboxypeptidase e  GTPBP4 - gtp binding protein 4  LSM2 - lsm2 homolog, u6 small nuclear rna associated (s. cerevisiae)  SOS1 - son of sevenless homolog 1 (drosophila)  FBXL20 - f-box and leucine-rich repeat protein 20  ORC3 - origin recognition complex, subunit 3  SON - son dna binding protein  ORC6 - origin recognition complex, subunit 6  CAPN15 - calpain 15  RAB40C - rab40c, member ras oncogene family  CREBL2 - camp responsive element binding protein-like 2  CREBBP - creb binding protein  SUGP1 - surp and g patch domain containing 1  ATF2 - activating transcription factor 2  CREB1 - camp responsive element binding protein 1  DDX58 - dead (asp-glu-ala-asp) box polypeptide 58  SNX1 - sorting nexin 1  PRPF38A - prp38 pre-mrna processing factor 38 (yeast) domain containing a  SMUG1 - single-strand-selective monofunctional uracil-dna glycosylase 1  MARK4 - map/microtubule affinity-regulating kinase 4  SNRPN - small nuclear ribonucleoprotein polypeptide n  SNRPB2 - small nuclear ribonucleoprotein polypeptide b  RPRD1B - regulation of nuclear pre-mrna domain containing 1b  SNRPD1 - small nuclear ribonucleoprotein d1 polypeptide 16kda  INIP - ints3 and nabp interacting protein  SNRPF - small nuclear ribonucleoprotein polypeptide f  PLK3 - polo-like kinase 3  SNAPC4 - small nuclear rna activating complex, polypeptide 4, 190kda  SNCA - synuclein, alpha (non a4 component of amyloid precursor)  SNRNP70 - small nuclear ribonucleoprotein 70kda (u1)  SNRPA - small nuclear ribonucleoprotein polypeptide a  SNRPA1 - small nuclear ribonucleoprotein polypeptide a'  SNRPB - small nuclear ribonucleoprotein polypeptides b and b1  SUMO2 - small ubiquitin-like modifier 2  SNAP25 - synaptosomal-associated protein, 25kda  SNAPC2 - small nuclear rna activating complex, polypeptide 2, 45kda  SNAPC1 - small nuclear rna activating complex, polypeptide 1, 43kda  SNAPC3 - small nuclear rna activating complex, polypeptide 3, 50kda  TP53INP2 - tumor protein p53 inducible nuclear protein 2  SMN1 - survival of motor neuron 1, telomeric  RBM17 - rna binding motif protein 17  SUMO3 - small ubiquitin-like modifier 3  SMARCB1 - swi/snf related, matrix associated, actin dependent regulator of chromatin, subfamily b, member 1  HLTF - helicase-like transcription factor  LSM5 - lsm5 homolog, u6 small nuclear rna associated (s. cerevisiae)  SMARCD3 - swi/snf related, matrix associated, actin dependent regulator of chromatin, subfamily d, member 3  RBM25 - rna binding motif protein 25  COL4A2 - collagen, type iv, alpha 2  SMARCA1 - swi/snf related, matrix associated, actin dependent regulator of chromatin, subfamily a, member 1  PLD3 - phospholipase d family, member 3  EDC4 - enhancer of mrna decapping 4  SGK3 - serum/glucocorticoid regulated kinase family, member 3  CACTIN - cactin, spliceosome c complex subunit  KMT2C - lysine (k)-specific methyltransferase 2c  PRKD3 - protein kinase d3  RAB38 - rab38, member ras oncogene family  PPIL4 - peptidylprolyl isomerase (cyclophilin)-like 4  SCAF1 - sr-related ctd-associated factor 1  OSTC - oligosaccharyltransferase complex subunit (non-catalytic)  TMEM2 - transmembrane protein 2  TFIP11 - tuftelin interacting protein 11  CSTF1 - cleavage stimulation factor, 3' pre-rna, subunit 1, 50kda  SENP2 - sumo1/sentrin/smt3 specific peptidase 2  CST3 - cystatin c  FTSJ1 - ftsj rna methyltransferase homolog 1 (e. coli)  NAT6 - n-acetyltransferase 6 (gcn5-related)  USP32 - ubiquitin specific peptidase 32  SHC2 - shc (src homology 2 domain containing) transforming protein 2  PRPF40B - prp40 pre-mrna processing factor 40 homolog b (s. cerevisiae)  CSTF3 - cleavage stimulation factor, 3' pre-rna, subunit 3, 77kda  CSTF2 - cleavage stimulation factor, 3' pre-rna, subunit 2, 64kda  PRPF6 - pre-mrna processing factor 6  KLHL12 - kelch-like family member 12  KDM2B - lysine (k)-specific demethylase 2b  SUPT6H - suppressor of ty 6 homolog (s. cerevisiae)  CTGF - connective tissue growth factor  SUPV3L1 - suppressor of var1, 3-like 1 (s. cerevisiae)  CACNG7 - calcium channel, voltage-dependent, gamma subunit 7  DSTYK - dual serine/threonine and tyrosine protein kinase  CTBP1 - c-terminal binding protein 1  SUPT4H1 - suppressor of ty 4 homolog 1 (s. cerevisiae)  SUPT5H - suppressor of ty 5 homolog (s. cerevisiae)  GTPBP3 - gtp binding protein 3 (mitochondrial)  RASGRP3 - ras guanyl releasing protein 3 (calcium and dag-regulated)  UBL5 - ubiquitin-like 5  CTNNB1 - catenin (cadherin-associated protein), beta 1, 88kda  PPIL2 - peptidylprolyl isomerase (cyclophilin)-like 2  TRMT11 - trna methyltransferase 11 homolog (s. cerevisiae)  PIGO - phosphatidylinositol glycan anchor biosynthesis, class o  STX1A - syntaxin 1a (brain)  CTSD - cathepsin d  LNX1 - ligand of numb-protein x 1, e3 ubiquitin protein ligase  CTSB - cathepsin b  USP30 - ubiquitin specific peptidase 30  FUT10 - fucosyltransferase 10 (alpha (1,3) fucosyltransferase)  CTSS - cathepsin s  CTSO - cathepsin o  CTSV - cathepsin v  PISD - phosphatidylserine decarboxylase  NEK4 - nima-related kinase 4  STK3 - serine/threonine kinase 3  BRD1 - bromodomain containing 1  PCGF1 - polycomb group ring finger 1  FKBP8 - fk506 binding protein 8, 38kda  GPBP1L1 - gc-rich promoter binding protein 1-like 1  AURKC - aurora kinase c  STK10 - serine/threonine kinase 10  PAN3 - pan3 poly(a) specific ribonuclease subunit homolog (s. cerevisiae)  ISPD - isoprenoid synthase domain containing  CYLD - cylindromatosis (turban tumor syndrome)  SUV420H2 - suppressor of variegation 4-20 homolog 2 (drosophila)  CDKL5 - cyclin-dependent kinase-like 5  STK4 - serine/threonine kinase 4  CRKL - v-crk avian sarcoma virus ct10 oncogene homolog-like  HECTD1 - hect domain containing e3 ubiquitin protein ligase 1  EEFSEC - eukaryotic elongation factor, selenocysteine-trna-specific  FKBP10 - fk506 binding protein 10, 65 kda  NIPBL - nipped-b homolog (drosophila)  CRY1 - cryptochrome 1 (photolyase-like)  BUD13 - bud13 homolog (s. cerevisiae)  STAT3 - signal transducer and activator of transcription 3 (acute-phase response factor)  CRY2 - cryptochrome 2 (photolyase-like)  ASF1A - anti-silencing function 1a histone chaperone  ANAPC13 - anaphase promoting complex subunit 13  AGBL5 - atp/gtp binding protein-like 5  SSRP1 - structure specific recognition protein 1  ARMC8 - armadillo repeat containing 8  DCAF12 - ddb1 and cul4 associated factor 12  BRMS1 - breast cancer metastasis suppressor 1  SPCS3 - signal peptidase complex subunit 3 homolog (s. cerevisiae)  BOD1L1 - biorientation of chromosomes in cell division 1-like 1  NAA35 - n(alpha)-acetyltransferase 35, natc auxiliary subunit  DHX35 - deah (asp-glu-ala-his) box polypeptide 35  SUMF2 - sulfatase modifying factor 2  PHF5A - phd finger protein 5a  CSF1R - colony stimulating factor 1 receptor  CSF1 - colony stimulating factor 1 (macrophage)  FBXO7 - f-box protein 7  SRPK2 - srsf protein kinase 2  SRPK1 - srsf protein kinase 1  QPCT - glutaminyl-peptide cyclotransferase  TAB3 - tgf-beta activated kinase 1/map3k7 binding protein 3  TROVE2 - trove domain family, member 2  KLHL22 - kelch-like family member 22  GALNT11 - udp-n-acetyl-alpha-d-galactosamine:polypeptide n-acetylgalactosaminyltransferase 11 (galnac-t11)  ZNF335 - zinc finger protein 335  SSBP1 - single-stranded dna binding protein 1, mitochondrial  PTPN5 - protein tyrosine phosphatase, non-receptor type 5 (striatum-enriched)  SSB - sjogren syndrome antigen b (autoantigen la)  CSK - c-src tyrosine kinase  CSNK1G2 - casein kinase 1, gamma 2  TTLL1 - tubulin tyrosine ligase-like family, member 1  CSNK1A1 - casein kinase 1, alpha 1  SPTBN1 - spectrin, beta, non-erythrocytic 1  CSNK1D - casein kinase 1, delta  RNF214 - ring finger protein 214  CSNK2A2 - casein kinase 2, alpha prime polypeptide  SRC - v-src avian sarcoma (schmidt-ruppin a-2) viral oncogene homolog  MRPL17 - mitochondrial ribosomal protein l17  CSNK1G3 - casein kinase 1, gamma 3  CSNK2A1 - casein kinase 2, alpha 1 polypeptide  VIPAS39 - vps33b interacting protein, apical-basolateral polarity regulator, spe-39 homolog  ZDHHC12 - zinc finger, dhhc-type containing 12  THADA - thyroid adenoma associated  ARIH1 - ariadne homolog, ubiquitin-conjugating enzyme e2 binding protein, 1 (drosophila)  UBE2O - ubiquitin-conjugating enzyme e2o  MTO1 - mitochondrial trna translation optimization 1  NSUN3 - nop2/sun domain family, member 3  FBXL2 - f-box and leucine-rich repeat protein 2  SHPRH - snf2 histone linker phd ring helicase, e3 ubiquitin protein ligase  BACE2 - beta-site app-cleaving enzyme 2  RNF169 - ring finger protein 169  DHX9 - deah (asp-glu-ala-his) box helicase 9  SEL1L - sel-1 suppressor of lin-12-like (c. elegans)  DHX8 - deah (asp-glu-ala-his) box polypeptide 8  TSR2 - tsr2, 20s rrna accumulation, homolog (s. cerevisiae)  DDX6 - dead (asp-glu-ala-asp) box helicase 6  DDX5 - dead (asp-glu-ala-asp) box helicase 5  FBXO33 - f-box protein 33  DDX3X - dead (asp-glu-ala-asp) box helicase 3, x-linked  DDX1 - dead (asp-glu-ala-asp) box helicase 1  SYF2 - syf2 pre-mrna-splicing factor  DDOST - dolichyl-diphosphooligosaccharide--protein glycosyltransferase subunit (non-catalytic)  KBTBD2 - kelch repeat and btb (poz) domain containing 2  SDF2 - stromal cell-derived factor 2  DPH6 - diphthamine biosynthesis 6  SIN3A - sin3 transcription regulator family member a  TPGS2 - tubulin polyglutamylase complex subunit 2  FAM98A - family with sequence similarity 98, member a  RSPRY1 - ring finger and spry domain containing 1  SAMHD1 - sam domain and hd domain 1  SET - set nuclear oncogene  WWTR1 - ww domain containing transcription regulator 1  SETMAR - set domain and mariner transposase fusion gene  DDB1 - damage-specific dna binding protein 1, 127kda  MAP2K4 - mitogen-activated protein kinase kinase 4  PTPN23 - protein tyrosine phosphatase, non-receptor type 23  SENP7 - sumo1/sentrin specific peptidase 7  MCM9 - minichromosome maintenance complex component 9  RCN3 - reticulocalbin 3, ef-hand calcium binding domain  ZDHHC24 - zinc finger, dhhc-type containing 24  GEMIN5 - gem (nuclear organelle) associated protein 5  KBTBD6 - kelch repeat and btb (poz) domain containing 6  NOL11 - nucleolar protein 11  CSRP2BP - csrp2 binding protein  ZDHHC5 - zinc finger, dhhc-type containing 5  THUMPD3 - thump domain containing 3  NELFB - negative elongation factor complex member b  POT1 - protection of telomeres 1  SCYL1 - scy1-like 1 (s. cerevisiae)  CLK4 - cdc-like kinase 4  KLHL13 - kelch-like family member 13  CNOT10 - ccr4-not transcription complex, subunit 10  RNF19A - ring finger protein 19a, e3 ubiquitin protein ligase  RCHY1 - ring finger and chy zinc finger domain containing 1, e3 ubiquitin protein ligase  ASPHD1 - aspartate beta-hydroxylase domain containing 1  DFFA - dna fragmentation factor, 45kda, alpha polypeptide  PPM1H - protein phosphatase, mg2+/mn2+ dependent, 1h  POLR1A - polymerase (rna) i polypeptide a, 194kda  GALNT16 - udp-n-acetyl-alpha-d-galactosamine:polypeptide n-acetylgalactosaminyltransferase 16  KCTD13 - potassium channel tetramerization domain containing 13  ZNF473 - zinc finger protein 473  REXO1 - rex1, rna exonuclease 1 homolog (s. cerevisiae)  DCAF13 - ddb1 and cul4 associated factor 13  BIRC6 - baculoviral iap repeat containing 6  RHBDD2 - rhomboid domain containing 2  DDX10 - dead (asp-glu-ala-asp) box polypeptide 10  WDR18 - wd repeat domain 18  ZDHHC20 - zinc finger, dhhc-type containing 20  BTBD6 - btb (poz) domain containing 6  DHX15 - deah (asp-glu-ala-his) box helicase 15  CNOT6 - ccr4-not transcription complex, subunit 6  LTN1 - listerin e3 ubiquitin protein ligase 1  DNM3 - dynamin 3  ATXN2 - ataxin 2  ZNF451 - zinc finger protein 451  GLCE - glucuronic acid epimerase  ERI1 - exoribonuclease 1  UBXN7 - ubx domain protein 7  SCAF4 - sr-related ctd-associated factor 4  CHD5 - chromodomain helicase dna binding protein 5  MCFD2 - multiple coagulation factor deficiency 2  MED30 - mediator complex subunit 30  RIMKLB - ribosomal modification protein rimk-like family member b  RPAP1 - rna polymerase ii associated protein 1  USP31 - ubiquitin specific peptidase 31  NLN - neurolysin (metallopeptidase m3 family)  DCAF15 - ddb1 and cul4 associated factor 15  DARS - aspartyl-trna synthetase  INTS2 - integrator complex subunit 2  DAPK3 - death-associated protein kinase 3  XPO5 - exportin 5  ANGEL2 - angel homolog 2 (drosophila)  C2CD3 - c2 calcium-dependent domain containing 3  ZZZ3 - zinc finger, zz-type containing 3  HINFP - histone h4 transcription factor  DAG1 - dystroglycan 1 (dystrophin-associated glycoprotein 1)  LRSAM1 - leucine rich repeat and sterile alpha motif containing 1  ULK3 - unc-51 like kinase 3  AARS2 - alanyl-trna synthetase 2, mitochondrial  SEC11C - sec11 homolog c (s. cerevisiae)  REXO2 - rna exonuclease 2  MTA3 - metastasis associated 1 family, member 3  DGKQ - diacylglycerol kinase, theta 110kda  CLIP3 - cap-gly domain containing linker protein 3  DAPK1 - death-associated protein kinase 1  TIPARP - tcdd-inducible poly(adp-ribose) polymerase  HACE1 - hect domain and ankyrin repeat containing e3 ubiquitin protein ligase 1  SARS - seryl-trna synthetase  SATB1 - satb homeobox 1  MIB1 - mindbomb e3 ubiquitin protein ligase 1  SBF1 - set binding factor 1  DAD1 - defender against cell death 1  NGDN - neuroguidin, eif4e binding protein  NYNRIN - nyn domain and retroviral integrase containing  KLHL42 - kelch-like family member 42  TRPC4AP - transient receptor potential cation channel, subfamily c, member 4 associated protein  EPC2 - enhancer of polycomb homolog 2 (drosophila)  DRD2 - dopamine receptor d2  CDC26 - cell division cycle 26  SLC9A1 - solute carrier family 9, subfamily a (nhe1, cation proton antiporter 1), member 1  IRF2BP1 - interferon regulatory factor 2 binding protein 1  CNOT6L - ccr4-not transcription complex, subunit 6-like  EAF1 - ell associated factor 1  TAOK1 - tao kinase 1  PDP2 - pyruvate dehyrogenase phosphatase catalytic subunit 2  KLHL8 - kelch-like family member 8  DPH2 - dph2 homolog (s. cerevisiae)  SHANK3 - sh3 and multiple ankyrin repeat domains 3  DPH1 - diphthamide biosynthesis 1  RNASEH1 - ribonuclease h1  DPAGT1 - dolichyl-phosphate (udp-n-acetylglucosamine) n-acetylglucosaminephosphotransferase 1 (glcnac-1-p transferase)  WIPI2 - wd repeat domain, phosphoinositide interacting 2  USP35 - ubiquitin specific peptidase 35  DOM3Z - dom-3 homolog z (c. elegans)  PRPF31 - pre-mrna processing factor 31  DR1 - down-regulator of transcription 1, tbp-binding (negative cofactor 2)  WSB1 - wd repeat and socs box containing 1  TRMT5 - trna methyltransferase 5  ALG2 - alg2, alpha-1,3/1,6-mannosyltransferase  ZNRF2 - zinc and ring finger 2  DUSP8 - dual specificity phosphatase 8  TNKS1BP1 - tankyrase 1 binding protein 1, 182kda  MAPK15 - mitogen-activated protein kinase 15  SKI - v-ski avian sarcoma viral oncogene homolog  DUSP5 - dual specificity phosphatase 5  SKIV2L - superkiller viralicidic activity 2-like (s. cerevisiae)  DUSP6 - dual specificity phosphatase 6  SKP1 - s-phase kinase-associated protein 1  DUSP7 - dual specificity phosphatase 7  DYRK1A - dual-specificity tyrosine-(y)-phosphorylation regulated kinase 1a  TOR1A - torsin family 1, member a (torsin a)  GFM1 - g elongation factor, mitochondrial 1  CHTOP - chromatin target of prmt1  GGA1 - golgi-associated, gamma adaptin ear containing, arf binding protein 1  ABHD12 - abhydrolase domain containing 12  HERC4 - hect and rld domain containing e3 ubiquitin protein ligase 4  WDR48 - wd repeat domain 48  ZCRB1 - zinc finger cchc-type and rna binding motif 1  GIGYF2 - grb10 interacting gyf protein 2  USP36 - ubiquitin specific peptidase 36  FNIP2 - folliculin interacting protein 2  SENP6 - sumo1/sentrin specific peptidase 6  HBEGF - heparin-binding egf-like growth factor  DTX1 - deltex homolog 1 (drosophila)  DKC1 - dyskeratosis congenita 1, dyskerin  DLG3 - discs, large homolog 3 (drosophila)  DLG4 - discs, large homolog 4 (drosophila)  PNPT1 - polyribonucleotide nucleotidyltransferase 1  DLG1 - discs, large homolog 1 (drosophila)  RNASEK - ribonuclease, rnase k  NEIL2 - nei endonuclease viii-like 2 (e. coli)  FBXW8 - f-box and wd repeat domain containing 8  SH3RF1 - sh3 domain containing ring finger 1  FBXO25 - f-box protein 25  ST6GAL1 - st6 beta-galactosamide alpha-2,6-sialyltranferase 1  DHCR24 - 24-dehydrocholesterol reductase  SIAH2 - siah e3 ubiquitin protein ligase 2  SIAH1 - siah e3 ubiquitin protein ligase 1  DGUOK - deoxyguanosine kinase  EP400 - e1a binding protein p400  FBXL3 - f-box and leucine-rich repeat protein 3  DHPS - deoxyhypusine synthase  ST3GAL2 - st3 beta-galactoside alpha-2,3-sialyltransferase 2  B3GAT3 - beta-1,3-glucuronyltransferase 3 (glucuronosyltransferase i)  RICTOR - rptor independent companion of mtor, complex 2  FBXL5 - f-box and leucine-rich repeat protein 5  DHX37 - deah (asp-glu-ala-his) box polypeptide 37  FBXO2 - f-box protein 2  UVSSA - uv-stimulated scaffold protein a  SFPQ - splicing factor proline/glutamine-rich  DNASE2 - deoxyribonuclease ii, lysosomal  PELI3 - pellino e3 ubiquitin protein ligase family member 3  PHRF1 - phd and ring finger domains 1  SFRP1 - secreted frizzled-related protein 1  FBXW2 - f-box and wd repeat domain containing 2  SRSF1 - serine/arginine-rich splicing factor 1  SRSF2 - serine/arginine-rich splicing factor 2  GMEB2 - glucocorticoid modulatory element binding protein 2  DNM2 - dynamin 2  SRSF3 - serine/arginine-rich splicing factor 3  DNMT1 - dna (cytosine-5-)-methyltransferase 1  SRSF4 - serine/arginine-rich splicing factor 4  TRDMT1 - trna aspartic acid methyltransferase 1  SRSF5 - serine/arginine-rich splicing factor 5  DNMT3A - dna (cytosine-5-)-methyltransferase 3 alpha  SRSF6 - serine/arginine-rich splicing factor 6  SRSF7 - serine/arginine-rich splicing factor 7  EIF2AK4 - eukaryotic translation initiation factor 2 alpha kinase 4  SFSWAP - splicing factor, suppressor of white-apricot homolog (drosophila)  ALS2 - amyotrophic lateral sclerosis 2 (juvenile)  TRA2B - transformer 2 beta homolog (drosophila)  NAT9 - n-acetyltransferase 9 (gcn5-related, putative)  SCG5 - secretogranin v (7b2 protein)  ALG11 - alg11, alpha-1,2-mannosyltransferase  RSL1D1 - ribosomal l1 domain containing 1  SGK1 - serum/glucocorticoid regulated kinase 1  SGTA - small glutamine-rich tetratricopeptide repeat (tpr)-containing, alpha  GAL3ST3 - galactose-3-o-sulfotransferase 3  IFT172 - intraflagellar transport 172 homolog (chlamydomonas)  USP37 - ubiquitin specific peptidase 37  INTS1 - integrator complex subunit 1  SENP3 - sumo1/sentrin/smt3 specific peptidase 3  SMARCAD1 - swi/snf-related, matrix-associated actin-dependent regulator of chromatin, subfamily a, containing dead/h box 1  NCBP2 - nuclear cap binding protein subunit 2, 20kda  MARK2 - map/microtubule affinity-regulating kinase 2  CHMP4C - charged multivesicular body protein 4c  TSN - translin  FAN1 - fancd2/fanci-associated nuclease 1  NAF1 - nuclear assembly factor 1 ribonucleoprotein  C1GALT1 - core 1 synthase, glycoprotein-n-acetylgalactosamine 3-beta-galactosyltransferase, 1  ELK4 - elk4, ets-domain protein (srf accessory protein 1)  SNW1 - snw domain containing 1  WRNIP1 - werner helicase interacting protein 1  MGME1 - mitochondrial genome maintenance exonuclease 1  ELL2 - elongation factor, rna polymerase ii, 2  DTD1 - d-tyrosyl-trna deacylase 1  EPAS1 - endothelial pas domain protein 1  DPH7 - diphthamide biosynthesis 7  EP300 - e1a binding protein p300  KIN - kin, antigenic determinant of reca protein homolog (mouse)  SEPHS1 - selenophosphate synthetase 1  HABP4 - hyaluronan binding protein 4  UBQLN4 - ubiquilin 4  TIMM50 - translocase of inner mitochondrial membrane 50 homolog (s. cerevisiae)  CELF4 - cugbp, elav-like family member 4  UGGT1 - udp-glucose glycoprotein glucosyltransferase 1  EGR2 - early growth response 2  TTC3 - tetratricopeptide repeat domain 3  TTF1 - transcription termination factor, rna polymerase i  EIF2S1 - eukaryotic translation initiation factor 2, subunit 1 alpha, 35kda  INTS4 - integrator complex subunit 4  TRIM32 - tripartite motif containing 32  SPHK2 - sphingosine kinase 2  DSEL - dermatan sulfate epimerase-like  KDM2A - lysine (k)-specific demethylase 2a  TMEM129 - transmembrane protein 129  ELAVL1 - elav (embryonic lethal, abnormal vision, drosophila)-like 1 (hu antigen r)  ZNF326 - zinc finger protein 326  ELAVL4 - elav (embryonic lethal, abnormal vision, drosophila)-like 4  METTL18 - methyltransferase like 18  TSG101 - tumor susceptibility gene 101  EIF4G2 - eukaryotic translation initiation factor 4 gamma, 2  PDCD11 - programmed cell death 11  MAST1 - microtubule associated serine/threonine kinase 1  TSNAX - translin-associated factor x  NRIP3 - nuclear receptor interacting protein 3  ACIN1 - apoptotic chromatin condensation inducer 1  YAE1D1 - yae1 domain containing 1  CCDC47 - coiled-coil domain containing 47  MVB12A - multivesicular body subunit 12a  CNOT1 - ccr4-not transcription complex, subunit 1  EEF2 - eukaryotic translation elongation factor 2  EXOSC7 - exosome component 7  SPEN - spen homolog, transcriptional regulator (drosophila)  FBXO21 - f-box protein 21  PGAP3 - post-gpi attachment to proteins 3  RNF149 - ring finger protein 149  STK38L - serine/threonine kinase 38 like  TULP4 - tubby like protein 4  PIGM - phosphatidylinositol glycan anchor biosynthesis, class m  EEF1A1 - eukaryotic translation elongation factor 1 alpha 1  EEF1A2 - eukaryotic translation elongation factor 1 alpha 2  TP53BP1 - tumor protein p53 binding protein 1  PHC1 - polyhomeotic homolog 1 (drosophila)  POGLUT1 - protein o-glucosyltransferase 1  TOP3A - topoisomerase (dna) iii alpha  TRMT10A - trna methyltransferase 10 homolog a (s. cerevisiae)  TOP2B - topoisomerase (dna) ii beta 180kda  PHLPP2 - ph domain and leucine rich repeat protein phosphatase 2  EGFR - epidermal growth factor receptor  USP33 - ubiquitin specific peptidase 33  MAST3 - microtubule associated serine/threonine kinase 3  FAM20C - family with sequence similarity 20, member c  TOP1 - topoisomerase (dna) i  KDM4B - lysine (k)-specific demethylase 4b  KDM1A - lysine (k)-specific demethylase 1a  GOLGA7B - golgin a7 family, member b  ATXN7L3 - ataxin 7-like 3  PARP6 - poly (adp-ribose) polymerase family, member 6  SNRNP200 - small nuclear ribonucleoprotein 200kda (u5)  EOGT - egf domain-specific o-linked n-acetylglucosamine (glcnac) transferase  HNRNPLL - heterogeneous nuclear ribonucleoprotein l-like  NCOA6 - nuclear receptor coactivator 6  IMP4 - imp4, u3 small nucleolar ribonucleoprotein, homolog (yeast)  UBE2Q2 - ubiquitin-conjugating enzyme e2q family member 2  TRIO - trio rho guanine nucleotide exchange factor  E4F1 - e4f transcription factor 1  SMG1 - smg1 phosphatidylinositol 3-kinase-related kinase  NT5M - 5',3'-nucleotidase, mitochondrial  ENDOD1 - endonuclease domain containing 1  TNIK - traf2 and nck interacting kinase  XAB2 - xpa binding protein 2  PDS5B - pds5, regulator of cohesion maintenance, homolog b (s. cerevisiae)  ENY2 - enhancer of yellow 2 homolog (drosophila)  DNER - delta/notch-like egf repeat containing  MRPS22 - mitochondrial ribosomal protein s22  C11orf30 - chromosome 11 open reading frame 30  RRP1B - ribosomal rna processing 1b  MYCBP2 - myc binding protein 2, e3 ubiquitin protein ligase  TRAF1 - tnf receptor-associated factor 1  ERP44 - endoplasmic reticulum protein 44  HSP90B1 - heat shock protein 90kda beta (grp94), member 1  CMTR1 - cap methyltransferase 1  TRAF3 - tnf receptor-associated factor 3  TRAF2 - tnf receptor-associated factor 2  SPPL2B - signal peptide peptidase like 2b  NR2C1 - nuclear receptor subfamily 2, group c, member 1  SETX - senataxin  DUS3L - dihydrouridine synthase 3-like (s. cerevisiae)  SETD1B - set domain containing 1b  FEM1C - fem-1 homolog c (c. elegans)  NR2C2 - nuclear receptor subfamily 2, group c, member 2  ECE1 - endothelin converting enzyme 1  MARS2 - methionyl-trna synthetase 2, mitochondrial  TPR - translocated promoter region, nuclear basket protein  GGA2 - golgi-associated, gamma adaptin ear containing, arf binding protein 2  ERC1 - elks/rab6-interacting/cast family member 1  NAT14 - n-acetyltransferase 14 (gcn5-related, putative)  KDM4C - lysine (k)-specific demethylase 4c  LMF2 - lipase maturation factor 2  TRIM35 - tripartite motif containing 35  REXO4 - rex4, rna exonuclease 4 homolog (s. cerevisiae)  PIGW - phosphatidylinositol glycan anchor biosynthesis, class w  CAMK1D - calcium/calmodulin-dependent protein kinase id  INTS12 - integrator complex subunit 12  TTLL5 - tubulin tyrosine ligase-like family, member 5  ZC3H13 - zinc finger ccch-type containing 13  NUP107 - nucleoporin 107kda  CDK19 - cyclin-dependent kinase 19  DERL3 - derlin 3  USP1 - ubiquitin specific peptidase 1  FANCE - fanconi anemia, complementation group e  PTK2B - protein tyrosine kinase 2 beta  MRPS27 - mitochondrial ribosomal protein s27  PCNP - pest proteolytic signal containing nuclear protein  SNORD107 - small nucleolar rna, c/d box 107  PARP11 - poly (adp-ribose) polymerase family, member 11  UNG - uracil-dna glycosylase  CUL9 - cullin 9  KANSL1 - kat8 regulatory nsl complex subunit 1  FARSA - phenylalanyl-trna synthetase, alpha subunit  USP4 - ubiquitin specific peptidase 4 (proto-oncogene)  RNF185 - ring finger protein 185  NR1H2 - nuclear receptor subfamily 1, group h, member 2  TAB2 - tgf-beta activated kinase 1/map3k7 binding protein 2  C19orf40 - chromosome 19 open reading frame 40  FBLN1 - fibulin 1  ZNF598 - zinc finger protein 598  VGF - vgf nerve growth factor inducible  LAMA1 - laminin, alpha 1  SPSB3 - spla/ryanodine receptor domain and socs box containing 3  VHL - von hippel-lindau tumor suppressor, e3 ubiquitin protein ligase  UTP3 - utp3, small subunit (ssu) processome component, homolog (s. cerevisiae)  KDM6B - lysine (k)-specific demethylase 6b  EZR - ezrin  PHF8 - phd finger protein 8  TRIM41 - tripartite motif containing 41  SMC5 - structural maintenance of chromosomes 5  EXT2 - exostosin glycosyltransferase 2  EXT1 - exostosin glycosyltransferase 1  DDX24 - dead (asp-glu-ala-asp) box helicase 24  KDM6A - lysine (k)-specific demethylase 6a  DCUN1D4 - dcn1, defective in cullin neddylation 1, domain containing 4  EXTL2 - exostosin-like glycosyltransferase 2  EXTL3 - exostosin-like glycosyltransferase 3  UVRAG - uv radiation resistance associated  ACKR3 - atypical chemokine receptor 3  ZC3H3 - zinc finger ccch-type containing 3  VARS - valyl-trna synthetase  EYA3 - eyes absent homolog 3 (drosophila)  EZH1 - enhancer of zeste homolog 1 (drosophila)  CCNL1 - cyclin l1  ENDOV - endonuclease v  TRIM47 - tripartite motif containing 47  PDXP - pyridoxal (pyridoxine, vitamin b6) phosphatase  VCP - valosin containing protein  F3 - coagulation factor iii (thromboplastin, tissue factor)  WDR43 - wd repeat domain 43  RARS2 - arginyl-trna synthetase 2, mitochondrial  TTLL12 - tubulin tyrosine ligase-like family, member 12  ERCC5 - excision repair cross-complementing rodent repair deficiency, complementation group 5  NEK9 - nima-related kinase 9  ERCC4 - excision repair cross-complementing rodent repair deficiency, complementation group 4  LIN52 - lin-52 homolog (c. elegans)  ERCC3 - excision repair cross-complementing rodent repair deficiency, complementation group 3  NUP205 - nucleoporin 205kda  YTHDC1 - yth domain containing 1  THOC2 - tho complex 2  GGA3 - golgi-associated, gamma adaptin ear containing, arf binding protein 3  ELMSAN1 - elm2 and myb/sant-like domain containing 1  UBE2A - ubiquitin-conjugating enzyme e2a  WDR20 - wd repeat domain 20  UBA1 - ubiquitin-like modifier activating enzyme 1  ERN1 - endoplasmic reticulum to nucleus signaling 1  UBB - ubiquitin b  VARS2 - valyl-trna synthetase 2, mitochondrial  ARRDC4 - arrestin domain containing 4  RCOR1 - rest corepressor 1  ASPHD2 - aspartate beta-hydroxylase domain containing 2  HIRA - histone cell cycle regulator  TTC5 - tetratricopeptide repeat domain 5  TYRO3 - tyro3 protein tyrosine kinase  FAF2 - fas associated factor family member 2  ETV1 - ets variant 1  TPGS1 - tubulin polyglutamylase complex subunit 1  MDN1 - mdn1, midasin homolog (yeast)  TYK2 - tyrosine kinase 2  ATG4B - autophagy related 4b, cysteine peptidase  GANAB - glucosidase, alpha; neutral ab  UBXN4 - ubx domain protein 4  ETF1 - eukaryotic translation termination factor 1  COG7 - component of oligomeric golgi complex 7  UCHL3 - ubiquitin carboxyl-terminal esterase l3 (ubiquitin thiolesterase)  UBXN11 - ubx domain protein 11  SCYL3 - scy1-like 3 (s. cerevisiae)  PKDCC - protein kinase domain containing, cytoplasmic  SUMO1 - small ubiquitin-like modifier 1  UCHL1 - ubiquitin carboxyl-terminal esterase l1 (ubiquitin thiolesterase)  UBTF - upstream binding transcription factor, rna polymerase i  UGCG - udp-glucose ceramide glucosyltransferase  ZNF830 - zinc finger protein 830  RRS1 - rrs1 ribosome biogenesis regulator homolog (s. cerevisiae)  PELI1 - pellino e3 ubiquitin protein ligase 1  SMURF1 - smad specific e3 ubiquitin protein ligase 1  JMJD6 - jumonji domain containing 6  MRPL47 - mitochondrial ribosomal protein l47  UBE2H - ubiquitin-conjugating enzyme e2h  UBE2I - ubiquitin-conjugating enzyme e2i  EPRS - glutamyl-prolyl-trna synthetase  UBE2D2 - ubiquitin-conjugating enzyme e2d 2  EPHB6 - eph receptor b6  FBXO28 - f-box protein 28  UBE2V2 - ubiquitin-conjugating enzyme e2 variant 2  LONRF1 - lon peptidase n-terminal domain and ring finger 1  ADCK1 - aarf domain containing kinase 1  ERCC2 - excision repair cross-complementing rodent repair deficiency, complementation group 2  UBE2L3 - ubiquitin-conjugating enzyme e2l 3  MAN1C1 - mannosidase, alpha, class 1c, member 1  NUP210 - nucleoporin 210kda  EPS15 - epidermal growth factor receptor pathway substrate 15  UBE2N - ubiquitin-conjugating enzyme e2n  UBE2V1 - ubiquitin-conjugating enzyme e2 variant 1  ZC3H7B - zinc finger ccch-type containing 7b  FMOD - fibromodulin  PSENEN - presenilin enhancer 2 homolog (c. elegans)  ZC3H15 - zinc finger ccch-type containing 15  FMR1 - fragile x mental retardation 1  PIK3IP1 - phosphoinositide-3-kinase interacting protein 1  TCOF1 - treacher collins-franceschetti syndrome 1  HENMT1 - hen1 methyltransferase homolog 1 (arabidopsis)  TMEM165 - transmembrane protein 165  FNTA - farnesyltransferase, caax box, alpha  FLT1 - fms-related tyrosine kinase 1  USE1 - unconventional snare in the er 1 homolog (s. cerevisiae)  PDS5A - pds5, regulator of cohesion maintenance, homolog a (s. cerevisiae)  TMEM106A - transmembrane protein 106a  BOP1 - block of proliferation 1  WSB2 - wd repeat and socs box containing 2  FLNA - filamin a, alpha  RPRD2 - regulation of nuclear pre-mrna domain containing 2  CHST14 - carbohydrate (n-acetylgalactosamine 4-0) sulfotransferase 14  SIK2 - salt-inducible kinase 2  PHLPP1 - ph domain and leucine rich repeat protein phosphatase 1  FOXO3 - forkhead box o3  HDAC8 - histone deacetylase 8  LARP4 - la ribonucleoprotein domain family, member 4  ASH1L - ash1 (absent, small, or homeotic)-like (drosophila)  SIN3B - sin3 transcription regulator family member b  FKBP15 - fk506 binding protein 15, 133kda  UTP6 - utp6, small subunit (ssu) processome component, homolog (yeast)  DCP1A - decapping mrna 1a  FKBP4 - fk506 binding protein 4, 59kda  ZDHHC22 - zinc finger, dhhc-type containing 22  FKBP2 - fk506 binding protein 2, 13kda  MGRN1 - mahogunin ring finger 1, e3 ubiquitin protein ligase  RPUSD1 - rna pseudouridylate synthase domain containing 1  HR - hair growth associated  MED8 - mediator complex subunit 8  FKBP5 - fk506 binding protein 5  CAND1 - cullin-associated and neddylation-dissociated 1  FKBP1A - fk506 binding protein 1a, 12kda  TDG - thymine-dna glycosylase  SMG6 - smg6 nonsense mediated mrna decay factor  FKBP1B - fk506 binding protein 1b, 12.6 kda  FBXW11 - f-box and wd repeat domain containing 11  POFUT2 - protein o-fucosyltransferase 2  KLHL18 - kelch-like family member 18  KDM3A - lysine (k)-specific demethylase 3a  RNF130 - ring finger protein 130  CSTF2T - cleavage stimulation factor, 3' pre-rna, subunit 2, 64kda, tau variant  PPP1R11 - protein phosphatase 1, regulatory (inhibitor) subunit 11  NUP160 - nucleoporin 160kda  TAF9 - taf9 rna polymerase ii, tata box binding protein (tbp)-associated factor, 32kda  FGFR2 - fibroblast growth factor receptor 2  TAF7 - taf7 rna polymerase ii, tata box binding protein (tbp)-associated factor, 55kda  GPC5 - glypican 5  UBR4 - ubiquitin protein ligase e3 component n-recognin 4  FAM98B - family with sequence similarity 98, member b  MBD6 - methyl-cpg binding domain protein 6  KLHL7 - kelch-like family member 7  FGF13 - fibroblast growth factor 13  TAF6 - taf6 rna polymerase ii, tata box binding protein (tbp)-associated factor, 80kda  KHNYN - kh and nyn domain containing  U2SURP - u2 snrnp-associated surp domain containing  FGFR1 - fibroblast growth factor receptor 1  TADA2A - transcriptional adaptor 2a  FGF9 - fibroblast growth factor 9  TAF4 - taf4 rna polymerase ii, tata box binding protein (tbp)-associated factor, 135kda  SMCHD1 - structural maintenance of chromosomes flexible hinge domain containing 1  TAF2 - taf2 rna polymerase ii, tata box binding protein (tbp)-associated factor, 150kda  ADAM17 - adam metallopeptidase domain 17  NSFL1C - nsfl1 (p97) cofactor (p47)  PHF15 - phd finger protein 15  FGF1 - fibroblast growth factor 1 (acidic)  FGF2 - fibroblast growth factor 2 (basic)  TARS - threonyl-trna synthetase  DPY19L1 - dpy-19-like 1 (c. elegans)  USP22 - ubiquitin specific peptidase 22  SASH1 - sam and sh3 domain containing 1  TARBP1 - tar (hiv-1) rna binding protein 1  TRIM2 - tripartite motif containing 2  FEN1 - flap structure-specific endonuclease 1  GPC4 - glypican 4  FER - fer (fps/fes related) tyrosine kinase  SMG9 - smg9 nonsense mediated mrna decay factor  TAF13 - taf13 rna polymerase ii, tata box binding protein (tbp)-associated factor, 18kda  MAP3K7 - mitogen-activated protein kinase kinase kinase 7  ALG1 - alg1, chitobiosyldiphosphodolichol beta-mannosyltransferase  ZCCHC11 - zinc finger, cchc domain containing 11  LARS2 - leucyl-trna synthetase 2, mitochondrial  INO80E - ino80 complex subunit e  RNF114 - ring finger protein 114  ZDHHC17 - zinc finger, dhhc-type containing 17  KMT2E - lysine (k)-specific methyltransferase 2e  SIK3 - sik family kinase 3  PGP - phosphoglycolate phosphatase  NCSTN - nicastrin  FKTN - fukutin  SULF2 - sulfatase 2  TSEN54 - tsen54 trna splicing endonuclease subunit  UFL1 - ufm1-specific ligase 1  KLHL9 - kelch-like family member 9  TCF3 - transcription factor 3  TRIM9 - tripartite motif containing 9  TENC1 - tensin like c1 domain containing phosphatase (tensin 2)  PUM2 - pumilio homolog 2 (drosophila)  DMAP1 - dna methyltransferase 1 associated protein 1  TCEB3 - transcription elongation factor b (siii), polypeptide 3 (110kda, elongin a)  OBSL1 - obscurin-like 1  EFEMP1 - egf containing fibulin-like extracellular matrix protein 1  CTTNBP2NL - cttnbp2 n-terminal like  FBN1 - fibrillin 1  TOE1 - target of egr1, member 1 (nuclear)  TCEA2 - transcription elongation factor a (sii), 2  ANGEL1 - angel homolog 1 (drosophila)  RNF208 - ring finger protein 208  TCEA1 - transcription elongation factor a (sii), 1  USP24 - ubiquitin specific peptidase 24  TIE1 - tyrosine kinase with immunoglobulin-like and egf-like domains 1  TIMP1 - timp metallopeptidase inhibitor 1  TK1 - thymidine kinase 1, soluble  TK2 - thymidine kinase 2, mitochondrial  RYBP - ring1 and yy1 binding protein  MTOR - mechanistic target of rapamycin (serine/threonine kinase)  SNORD118 - small nucleolar rna, c/d box 118  STK32C - serine/threonine kinase 32c  PPWD1 - peptidylprolyl isomerase domain and wd repeat containing 1  NDNL2 - necdin-like 2  ATP13A2 - atpase type 13a2  TLR3 - toll-like receptor 3  PIGS - phosphatidylinositol glycan anchor biosynthesis, class s  EXOSC2 - exosome component 2  SIRT1 - sirtuin 1  ACTR8 - arp8 actin-related protein 8 homolog (yeast)  ICMT - isoprenylcysteine carboxyl methyltransferase  PHF3 - phd finger protein 3  BRD4 - bromodomain containing 4  CAPN7 - calpain 7  TNFAIP1 - tumor necrosis factor, alpha-induced protein 1 (endothelial)  FBXO44 - f-box protein 44  HARS2 - histidyl-trna synthetase 2, mitochondrial  TARDBP - tar dna binding protein  SF3B1 - splicing factor 3b, subunit 1, 155kda  SF3B3 - splicing factor 3b, subunit 3, 130kda  KCTD21 - potassium channel tetramerization domain containing 21  KCTD2 - potassium channel tetramerization domain containing 2  TEK - tek tyrosine kinase, endothelial  NUP188 - nucleoporin 188kda  SUZ12 - suz12 polycomb repressive complex 2 subunit  PGM2L1 - phosphoglucomutase 2-like 1  P4HA3 - prolyl 4-hydroxylase, alpha polypeptide iii  POFUT1 - protein o-fucosyltransferase 1  RCOR2 - rest corepressor 2  TFAM - transcription factor a, mitochondrial  HARBI1 - harbinger transposase derived 1  TERF1 - telomeric repeat binding factor (nima-interacting) 1  TERF2 - telomeric repeat binding factor 2  MORC3 - morc family cw-type zinc finger 3  TESK1 - testis-specific kinase 1  GTF3C6 - general transcription factor iiic, polypeptide 6, alpha 35kda  SKIV2L2 - superkiller viralicidic activity 2-like 2 (s. cerevisiae)  TFDP1 - transcription factor dp-1  BCCIP - brca2 and cdkn1a interacting protein  NR2F1 - nuclear receptor subfamily 2, group f, member 1  PES1 - pescadillo ribosomal biogenesis factor 1  TFCP2 - transcription factor cp2  FXN - frataxin  ZFYVE26 - zinc finger, fyve domain containing 26  TGS1 - trimethylguanosine synthase 1  TGFA - transforming growth factor, alpha  CEP41 - centrosomal protein 41kda  POLE4 - polymerase (dna-directed), epsilon 4, accessory subunit  TGFBR1 - transforming growth factor, beta receptor 1  TGFB2 - transforming growth factor, beta 2  RBFOX2 - rna binding protein, fox-1 homolog (c. elegans) 2  TGFB1 - transforming growth factor, beta 1  TGFB1I1 - transforming growth factor beta 1 induced transcript 1  MAPK8IP2 - mitogen-activated protein kinase 8 interacting protein 2  TSPAN15 - tetraspanin 15  PIGN - phosphatidylinositol glycan anchor biosynthesis, class n  CTNNBL1 - catenin, beta like 1  MEPCE - methylphosphate capping enzyme  CDK20 - cyclin-dependent kinase 20  RNF20 - ring finger protein 20, e3 ubiquitin protein ligase  TGFBR2 - transforming growth factor, beta receptor ii (70/80kda)  TGFBR3 - transforming growth factor, beta receptor iii  FOSB - fbj murine osteosarcoma viral oncogene homolog b  FOS - fbj murine osteosarcoma viral oncogene homolog  WDR45B - wd repeat domain 45b  SRRM2 - serine/arginine repetitive matrix 2  THBS1 - thrombospondin 1  KAT6B - k(lysine) acetyltransferase 6b  GATC - glutamyl-trna(gln) amidotransferase, subunit c  THY1 - thy-1 cell surface antigen  METTL3 - methyltransferase like 3  THRA - thyroid hormone receptor, alpha  MMD - monocyte to macrophage differentiation-associated  ELL - elongation factor rna polymerase ii  DNAJB14 - dnaj (hsp40) homolog, subfamily b, member 14  GFPT1 - glutamine--fructose-6-phosphate transaminase 1  GFRA1 - gdnf family receptor alpha 1  TMTC2 - transmembrane and tetratricopeptide repeat containing 2  GFRA2 - gdnf family receptor alpha 2  SYMPK - symplekin  USP54 - ubiquitin specific peptidase 54  ATAT1 - alpha tubulin acetyltransferase 1  SF3A2 - splicing factor 3a, subunit 2, 66kda  GCNT1 - glucosaminyl (n-acetyl) transferase 1, core 2  LZTR1 - leucine-zipper-like transcription regulator 1  OBFC1 - oligonucleotide/oligosaccharide-binding fold containing 1  KAT2A - k(lysine) acetyltransferase 2a  NCOA3 - nuclear receptor coactivator 3  FBXL18 - f-box and leucine-rich repeat protein 18  NOP9 - nop9 nucleolar protein  NAA25 - n(alpha)-acetyltransferase 25, natb auxiliary subunit  PABPN1 - poly(a) binding protein, nuclear 1  TAF15 - taf15 rna polymerase ii, tata box binding protein (tbp)-associated factor, 68kda  GGTA1P - glycoprotein, alpha-galactosyltransferase 1 pseudogene  TMTC3 - transmembrane and tetratricopeptide repeat containing 3  GGT7 - gamma-glutamyltransferase 7  TRRAP - transformation/transcription domain-associated protein  MCMBP - minichromosome maintenance complex binding protein  PICALM - phosphatidylinositol binding clathrin assembly protein  HDAC11 - histone deacetylase 11  SERF1A - small edrk-rich factor 1a (telomeric)  TRMT10B - trna methyltransferase 10 homolog b (s. cerevisiae)  SETD6 - set domain containing 6  ACTR5 - arp5 actin-related protein 5 homolog (yeast)  NAA60 - n(alpha)-acetyltransferase 60, natf catalytic subunit  CDC45 - cell division cycle 45  BAP1 - brca1 associated protein-1 (ubiquitin carboxy-terminal hydrolase)  AXIN2 - axin 2  GLB1 - galactosidase, beta 1  NUP85 - nucleoporin 85kda  CDC7 - cell division cycle 7  BRAP - brca1 associated protein  KDM5C - lysine (k)-specific demethylase 5c  SMC1A - structural maintenance of chromosomes 1a  USP9X - ubiquitin specific peptidase 9, x-linked  RBM10 - rna binding motif protein 10  USP11 - ubiquitin specific peptidase 11  GNAS - gnas complex locus  PHF17 - phd finger protein 17  ERMP1 - endoplasmic reticulum metallopeptidase 1  GMFB - glia maturation factor, beta  NOL10 - nucleolar protein 10  NELFE - negative elongation factor complex member e  EHMT1 - euchromatic histone-lysine n-methyltransferase 1  NR5A1 - nuclear receptor subfamily 5, group a, member 1  FUCA1 - fucosidase, alpha-l- 1, tissue  FUCA2 - fucosidase, alpha-l- 2, plasma  CAMKMT - calmodulin-lysine n-methyltransferase  FUS - fused in sarcoma  FRG1 - fshd region gene 1  METTL8 - methyltransferase like 8  NAA40 - n(alpha)-acetyltransferase 40, natd catalytic subunit, homolog (s. cerevisiae)  GEMIN6 - gem (nuclear organelle) associated protein 6  SDE2 - sde2 telomere maintenance homolog (s. pombe)  RAB7A - rab7a, member ras oncogene family  USP7 - ubiquitin specific peptidase 7 (herpes virus-associated)  GAA - glucosidase, alpha; acid  RNF168 - ring finger protein 168, e3 ubiquitin protein ligase  XRCC6 - x-ray repair complementing defective repair in chinese hamster cells 6  SLBP - stem-loop binding protein  FUT4 - fucosyltransferase 4 (alpha (1,3) fucosyltransferase, myeloid-specific)  ST8SIA4 - st8 alpha-n-acetyl-neuraminide alpha-2,8-sialyltransferase 4  RBFA - ribosome binding factor a (putative)  CPSF7 - cleavage and polyadenylation specific factor 7, 59kda  FUT8 - fucosyltransferase 8 (alpha (1,6) fucosyltransferase)  CBLL1 - cbl proto-oncogene-like 1, e3 ubiquitin protein ligase  FYN - fyn oncogene related to src, fgr, yes  RPAP2 - rna polymerase ii associated protein 2  UBA5 - ubiquitin-like modifier activating enzyme 5  DDX39B - dead (asp-glu-ala-asp) box polypeptide 39b  ABHD16A - abhydrolase domain containing 16a  GAK - cyclin g associated kinase  GALNT2 - udp-n-acetyl-alpha-d-galactosamine:polypeptide n-acetylgalactosaminyltransferase 2 (galnac-t2)  TTLL7 - tubulin tyrosine ligase-like family, member 7  USP5 - ubiquitin specific peptidase 5 (isopeptidase t)  NARS2 - asparaginyl-trna synthetase 2, mitochondrial (putative)  KCTD17 - potassium channel tetramerization domain containing 17  GALNT1 - udp-n-acetyl-alpha-d-galactosamine:polypeptide n-acetylgalactosaminyltransferase 1 (galnac-t1)  GALNS - galactosamine (n-acetyl)-6-sulfate sulfatase  KMT2D - lysine (k)-specific methyltransferase 2d  SPRED1 - sprouty-related, evh1 domain containing 1  AAAS - achalasia, adrenocortical insufficiency, alacrimia  CDK2AP1 - cyclin-dependent kinase 2 associated protein 1  YEATS4 - yeats domain containing 4  SNORA8 - small nucleolar rna, h/aca box 8  SNIP1 - smad nuclear interacting protein 1  GEMIN7 - gem (nuclear organelle) associated protein 7  KAT6A - k(lysine) acetyltransferase 6a  UBXN8 - ubx domain protein 8  TUSC3 - tumor suppressor candidate 3  GBE1 - glucan (1,4-alpha-), branching enzyme 1  STAM - signal transducing adaptor molecule (sh3 domain and itam motif) 1  NUP214 - nucleoporin 214kda  NR4A3 - nuclear receptor subfamily 4, group a, member 3  KLHL36 - kelch-like family member 36  GAS6 - growth arrest-specific 6  DEDD2 - death effector domain containing 2  GARS - glycyl-trna synthetase  FASTKD1 - fast kinase domains 1  DHX36 - deah (asp-glu-ala-his) box polypeptide 36  FN3KRP - fructosamine 3 kinase related protein  SMC6 - structural maintenance of chromosomes 6  RNF112 - ring finger protein 112  GSPT1 - g1 to s phase transition 1  GSN - gelsolin  ZCCHC6 - zinc finger, cchc domain containing 6  RNF113A - ring finger protein 113a  PARP8 - poly (adp-ribose) polymerase family, member 8  GTF2F1 - general transcription factor iif, polypeptide 1, 74kda  GTF2F2 - general transcription factor iif, polypeptide 2, 30kda  HECTD3 - hect domain containing e3 ubiquitin protein ligase 3  GTF2E1 - general transcription factor iie, polypeptide 1, alpha 56kda  GTF2E2 - general transcription factor iie, polypeptide 2, beta 34kda  NEIL1 - nei endonuclease viii-like 1 (e. coli)  GTF2H2 - general transcription factor iih, polypeptide 2, 44kda  TRIM26 - tripartite motif containing 26  GTF2H3 - general transcription factor iih, polypeptide 3, 34kda  GTF2H1 - general transcription factor iih, polypeptide 1, 62kda  USP27X - ubiquitin specific peptidase 27, x-linked  GTF2A2 - general transcription factor iia, 2, 12kda  GTF2B - general transcription factor iib  PCGF2 - polycomb group ring finger 2  MSH6 - muts homolog 6 (e. coli)  USB1 - u6 snrna biogenesis 1  GTF2A1 - general transcription factor iia, 1, 19/37kda  GTF3C2 - general transcription factor iiic, polypeptide 2, beta 110kda  TBL1XR1 - transducin (beta)-like 1 x-linked receptor 1  GTF2I - general transcription factor iii  GTF2H4 - general transcription factor iih, polypeptide 4, 52kda  PRKRIP1 - prkr interacting protein 1 (il11 inducible)  GTF3A - general transcription factor iiia  NOL9 - nucleolar protein 9  BRF1 - brf1, rna polymerase iii transcription initiation factor 90 kda subunit  GTF3C1 - general transcription factor iiic, polypeptide 1, alpha 220kda  ZYG11B - zyg-11 family member b, cell cycle regulator  ZDHHC14 - zinc finger, dhhc-type containing 14  H3F3A - h3 histone, family 3a  HIST1H2BB - histone cluster 1, h2bb  BRPF1 - bromodomain and phd finger containing, 1  SCG2 - secretogranin ii  H2AFX - h2a histone family, member x  DCP2 - decapping mrna 2  MUL1 - mitochondrial e3 ubiquitin protein ligase 1  SNORA43 - small nucleolar rna, h/aca box 43  PAPD4 - pap associated domain containing 4  PAX8 - paired box 8  CHPF - chondroitin polymerizing factor  RNF103 - ring finger protein 103  MOGS - mannosyl-oligosaccharide glucosidase  CDC73 - cell division cycle 73  HARS - histidyl-trna synthetase  DAP3 - death associated protein 3  LRP8 - low density lipoprotein receptor-related protein 8, apolipoprotein e receptor  PTP4A1 - protein tyrosine phosphatase type iva, member 1  HCFC1 - host cell factor c1 (vp16-accessory protein)  MAP3K12 - mitogen-activated protein kinase kinase kinase 12  SCRN3 - secernin 3  NDNF - neuron-derived neurotrophic factor  FUT11 - fucosyltransferase 11 (alpha (1,3) fucosyltransferase)  HDAC2 - histone deacetylase 2  SNRNP25 - small nuclear ribonucleoprotein 25kda (u11/u12)  HDAC1 - histone deacetylase 1  PGBD5 - piggybac transposable element derived 5  SNORA11 - small nucleolar rna, h/aca box 11  XPA - xeroderma pigmentosum, complementation group a  XPC - xeroderma pigmentosum, complementation group c  GOLGA2 - golgin a2  GNS - glucosamine (n-acetyl)-6-sulfatase  XRCC1 - x-ray repair complementing defective repair in chinese hamster cells 1  XPNPEP1 - x-prolyl aminopeptidase (aminopeptidase p) 1, soluble  PAGR1 - paxip1 associated glutamate-rich protein 1  SNORD86 - small nucleolar rna, c/d box 86  XBP1 - x-box binding protein 1  EIF4H - eukaryotic translation initiation factor 4h  WEE1 - wee1 homolog (s. pombe)  IMMP1L - imp1 inner mitochondrial membrane peptidase-like (s. cerevisiae)  WFS1 - wolfram syndrome 1 (wolframin)  WHSC1 - wolf-hirschhorn syndrome candidate 1  AMN1 - antagonist of mitotic exit network 1 homolog (s. cerevisiae)  NELFA - negative elongation factor complex member a  DTX3 - deltex homolog 3 (drosophila)  ZFC3H1 - zinc finger, c3h1-type containing  SNORD12 - small nucleolar rna, c/d box 12  VRK1 - vaccinia related kinase 1  VRK2 - vaccinia related kinase 2  DCP1B - decapping mrna 1b  VWF - von willebrand factor  WARS - tryptophanyl-trna synthetase  GRK5 - g protein-coupled receptor kinase 5  GRK6 - g protein-coupled receptor kinase 6  FBXL15 - f-box and leucine-rich repeat protein 15  THOC6 - tho complex 6 homolog (drosophila)  ZNF45 - zinc finger protein 45  BRCC3 - brca1/brca2-containing complex, subunit 3  GRB2 - growth factor receptor-bound protein 2  GNPTAB - n-acetylglucosamine-1-phosphate transferase, alpha and beta subunits  ASXL1 - additional sex combs like 1 (drosophila)  TNIP2 - tnfaip3 interacting protein 2  SF1 - splicing factor 1  ZNF711 - zinc finger protein 711  YES1 - v-yes-1 yamaguchi sarcoma viral oncogene homolog 1  DCAF10 - ddb1 and cul4 associated factor 10  XRCC5 - x-ray repair complementing defective repair in chinese hamster cells 5 (double-strand-break rejoining)  XRCC4 - x-ray repair complementing defective repair in chinese hamster cells 4  POLR3H - polymerase (rna) iii (dna directed) polypeptide h (22.9kd)  GRSF1 - g-rich rna sequence binding factor 1  GSK3B - glycogen synthase kinase 3 beta  YWHAZ - tyrosine 3-monooxygenase/tryptophan 5-monooxygenase activation protein, zeta polypeptide  PGAM5 - phosphoglycerate mutase family member 5  GSK3A - glycogen synthase kinase 3 alpha  YWHAE - tyrosine 3-monooxygenase/tryptophan 5-monooxygenase activation protein, epsilon polypeptide  YWHAB - tyrosine 3-monooxygenase/tryptophan 5-monooxygenase activation protein, beta polypeptide  PDIA3 - protein disulfide isomerase family a, member 3  NDUFAF5 - nadh dehydrogenase (ubiquinone) complex i, assembly factor 5  CCNK - cyclin k  DPM1 - dolichyl-phosphate mannosyltransferase polypeptide 1, catalytic subunit  DNAJB2 - dnaj (hsp40) homolog, subfamily b, member 2  RNF26 - ring finger protein 26  TRIM24 - tripartite motif containing 24  TAF1D - tata box binding protein (tbp)-associated factor, rna polymerase i, d, 41kda  SNORD24 - small nucleolar rna, c/d box 24  METTL22 - methyltransferase like 22  PRMT1 - protein arginine methyltransferase 1  PRMT2 - protein arginine methyltransferase 2  SNORD55 - small nucleolar rna, c/d box 55  FKRP - fukutin related protein  FBXO45 - f-box protein 45  RPL22L1 - ribosomal protein l22-like 1  DERL1 - derlin 1  DCAF5 - ddb1 and cul4 associated factor 5  KCTD6 - potassium channel tetramerization domain containing 6  METTL16 - methyltransferase like 16  FTO - fat mass and obesity associated  ALKBH1 - alkb, alkylation repair homolog 1 (e. coli)  KDELC1 - kdel (lys-asp-glu-leu) containing 1  ALG8 - alg8, alpha-1,3-glucosyltransferase  GGH - gamma-glutamyl hydrolase (conjugase, folylpolygammaglutamyl hydrolase)  CFLAR - casp8 and fadd-like apoptosis regulator  SYNGAP1 - synaptic ras gtpase activating protein 1  SECISBP2 - secis binding protein 2  NOC4L - nucleolar complex associated 4 homolog (s. cerevisiae)  MED11 - mediator complex subunit 11  ST3GAL5 - st3 beta-galactoside alpha-2,3-sialyltransferase 5  TMUB2 - transmembrane and ubiquitin-like domain containing 2  STK19 - serine/threonine kinase 19  RNF215 - ring finger protein 215  LDB1 - lim domain binding 1  DSCC1 - dna replication and sister chromatid cohesion 1  KAT2B - k(lysine) acetyltransferase 2b  TSC22D1 - tsc22 domain family, member 1  CRTC2 - creb regulated transcription coactivator 2  CDC16 - cell division cycle 16  DDA1 - det1 and ddb1 associated 1  ZNF259 - zinc finger protein 259  GID4 - gid complex subunit 4  NAE1 - nedd8 activating enzyme e1 subunit 1  RPS6KC1 - ribosomal protein s6 kinase, 52kda, polypeptide 1  SCNM1 - sodium channel modifier 1  C1orf86 - chromosome 1 open reading frame 86  IARS - isoleucyl-trna synthetase  PRPF4B - prp4 pre-mrna processing factor 4 homolog b (yeast)  MTMR2 - myotubularin related protein 2  NABP2 - nucleic acid binding protein 2  MTMR3 - myotubularin related protein 3  TSEN34 - tsen34 trna splicing endonuclease subunit  HUS1 - hus1 checkpoint homolog (s. pombe)  CPNE1 - copine i  U2AF1L4 - u2 small nuclear rna auxiliary factor 1-like 4  MCM3AP - minichromosome maintenance complex component 3 associated protein  ERI3 - eri1 exoribonuclease family member 3  BUD31 - bud31 homolog (s. cerevisiae)  CPNE3 - copine iii  HERC3 - hect and rld domain containing e3 ubiquitin protein ligase 3  HERC2 - hect and rld domain containing e3 ubiquitin protein ligase 2  HERC1 - hect and rld domain containing e3 ubiquitin protein ligase family member 1  TRAK2 - trafficking protein, kinesin binding 2  HSPD1 - heat shock 60kda protein 1 (chaperonin)  NSMCE1 - non-smc element 1 homolog (s. cerevisiae)  BCL10 - b-cell cll/lymphoma 10  FUBP3 - far upstream element (fuse) binding protein 3  HSP90AA1 - heat shock protein 90kda alpha (cytosolic), class a member 1  C7orf49 - chromosome 7 open reading frame 49  BTRC - beta-transducin repeat containing e3 ubiquitin protein ligase  MBD4 - methyl-cpg binding domain protein 4  PCYOX1L - prenylcysteine oxidase 1 like  HSPA8 - heat shock 70kda protein 8  UBR1 - ubiquitin protein ligase e3 component n-recognin 1  USP12 - ubiquitin specific peptidase 12  WNK1 - wnk lysine deficient protein kinase 1  HMGN1 - high mobility group nucleosome binding domain 1  UBE2Z - ubiquitin-conjugating enzyme e2z  HMGB3 - high mobility group box 3  HMGB2 - high mobility group box 2  LAMTOR3 - late endosomal/lysosomal adaptor, mapk and mtor activator 3  HMGB1 - high mobility group box 1  MPPE1 - metallophosphoesterase 1  HLCS - holocarboxylase synthetase (biotin-(proprionyl-coa-carboxylase (atp-hydrolysing)) ligase)  SENP5 - sumo1/sentrin specific peptidase 5  IRS2 - insulin receptor substrate 2  STK33 - serine/threonine kinase 33  TNKS - tankyrase, trf1-interacting ankyrin-related adp-ribose polymerase  INTS6 - integrator complex subunit 6  AGO1 - argonaute risc catalytic component 1  LATS2 - large tumor suppressor kinase 2  EIF3F - eukaryotic translation initiation factor 3, subunit f  BECN1 - beclin 1, autophagy related  ZBTB48 - zinc finger and btb domain containing 48  SRSF9 - serine/arginine-rich splicing factor 9  PEA15 - phosphoprotein enriched in astrocytes 15  MRPL1 - mitochondrial ribosomal protein l1  HIPK1 - homeodomain interacting protein kinase 1  HYAL2 - hyaluronoglucosaminidase 2  ACD - adrenocortical dysplasia homolog (mouse)  GPBP1 - gc-rich promoter binding protein 1  NOL6 - nucleolar protein 6 (rna-associated)  B4GALT4 - udp-gal:betaglcnac beta 1,4- galactosyltransferase, polypeptide 4  B4GALT3 - udp-gal:betaglcnac beta 1,4- galactosyltransferase, polypeptide 3  B4GALT2 - udp-gal:betaglcnac beta 1,4- galactosyltransferase, polypeptide 2  KRI1 - kri1 homolog (s. cerevisiae)  CDC23 - cell division cycle 23  MRPL44 - mitochondrial ribosomal protein l44  UBE2K - ubiquitin-conjugating enzyme e2k  INTS3 - integrator complex subunit 3  ELP4 - elongator acetyltransferase complex subunit 4  B3GALNT1 - beta-1,3-n-acetylgalactosaminyltransferase 1 (globoside blood group)  UPF3B - upf3 regulator of nonsense transcripts homolog b (yeast)  B3GALT2 - udp-gal:betaglcnac beta 1,3-galactosyltransferase, polypeptide 2  UPF3A - upf3 regulator of nonsense transcripts homolog a (yeast)  HIF1A - hypoxia inducible factor 1, alpha subunit (basic helix-loop-helix transcription factor)  MBTPS1 - membrane-bound transcription factor peptidase, site 1  PAPOLG - poly(a) polymerase gamma  ERAL1 - era-like 12s mitochondrial rrna chaperone 1  SNX3 - sorting nexin 3  CTSF - cathepsin f  GBGT1 - globoside alpha-1,3-n-acetylgalactosaminyltransferase 1  EED - embryonic ectoderm development  RNGTT - rna guanylyltransferase and 5'-phosphatase  RNMT - rna (guanine-7-) methyltransferase  RIPK1 - receptor (tnfrsf)-interacting serine-threonine kinase 1  HNRNPU - heterogeneous nuclear ribonucleoprotein u (scaffold attachment factor a)  FBXO10 - f-box protein 10  MRPS24 - mitochondrial ribosomal protein s24  HNRNPK - heterogeneous nuclear ribonucleoprotein k  FBXO22 - f-box protein 22  HNRNPL - heterogeneous nuclear ribonucleoprotein l  MRPS6 - mitochondrial ribosomal protein s6  FBXO4 - f-box protein 4  FBXO8 - f-box protein 8  MRPS11 - mitochondrial ribosomal protein s11  FBXO6 - f-box protein 6  ADAM23 - adam metallopeptidase domain 23  VPS33B - vacuolar protein sorting 33 homolog b (yeast)  TINF2 - terf1 (trf1)-interacting nuclear factor 2  FBXO3 - f-box protein 3  MRPL32 - mitochondrial ribosomal protein l32  ADAM15 - adam metallopeptidase domain 15  ADAM9 - adam metallopeptidase domain 9  RAB11A - rab11a, member ras oncogene family  HNRNPA2B1 - heterogeneous nuclear ribonucleoprotein a2/b1  HNRNPA1 - heterogeneous nuclear ribonucleoprotein a1  HNRNPC - heterogeneous nuclear ribonucleoprotein c (c1/c2)  RIPK2 - receptor-interacting serine-threonine kinase 2  PORCN - porcupine homolog (drosophila)  HNRNPF - heterogeneous nuclear ribonucleoprotein f  HNRNPD - heterogeneous nuclear ribonucleoprotein d (au-rich element rna binding protein 1, 37kda)  HNRNPH1 - heterogeneous nuclear ribonucleoprotein h1 (h)  HNRNPH3 - heterogeneous nuclear ribonucleoprotein h3 (2h9)  DNAJC18 - dnaj (hsp40) homolog, subfamily c, member 18  MTMR1 - myotubularin related protein 1  HNRNPH2 - heterogeneous nuclear ribonucleoprotein h2 (h')  FBXO15 - f-box protein 15  TUT1 - terminal uridylyl transferase 1, u6 snrna-specific  USP46 - ubiquitin specific peptidase 46  HMGA1 - high mobility group at-hook 1  VWA1 - von willebrand factor a domain containing 1  HMMR - hyaluronan-mediated motility receptor (rhamm)  PTPLB - protein tyrosine phosphatase-like (proline instead of catalytic arginine), member b  PTPN18 - protein tyrosine phosphatase, non-receptor type 18 (brain-derived)  NABP1 - nucleic acid binding protein 1  NR4A1 - nuclear receptor subfamily 4, group a, member 1  STT3B - stt3b, subunit of the oligosaccharyltransferase complex (catalytic)  PDE12 - phosphodiesterase 12  IKBKAP - inhibitor of kappa light polypeptide gene enhancer in b-cells, kinase complex-associated protein  HTATSF1 - hiv-1 tat specific factor 1  FOXK2 - forkhead box k2  HAT1 - histone acetyltransferase 1  ZNF638 - zinc finger protein 638  RPS6KA6 - ribosomal protein s6 kinase, 90kda, polypeptide 6  RBM15 - rna binding motif protein 15  ENC1 - ectodermal-neural cortex 1 (with btb domain)  PGAP2 - post-gpi attachment to proteins 2  NDST2 - n-deacetylase/n-sulfotransferase (heparan glucosaminyl) 2  RBMX - rna binding motif protein, x-linked  INHA - inhibin, alpha  HNRNPA3 - heterogeneous nuclear ribonucleoprotein a3  DESI1 - desumoylating isopeptidase 1  RMND5B - required for meiotic nuclear division 5 homolog b (s. cerevisiae)  PARG - poly (adp-ribose) glycohydrolase  HTRA2 - htra serine peptidase 2  MEAF6 - myst/esa1-associated factor 6  STK39 - serine threonine kinase 39  ENGASE - endo-beta-n-acetylglucosaminidase  PPM1D - protein phosphatase, mg2+/mn2+ dependent, 1d  POLL - polymerase (dna directed), lambda  PCSK1N - proprotein convertase subtilisin/kexin type 1 inhibitor  UBE2S - ubiquitin-conjugating enzyme e2s  ILF3 - interleukin enhancer binding factor 3, 90kda  PRPF19 - pre-mrna processing factor 19  MAP4K3 - mitogen-activated protein kinase kinase kinase kinase 3  UTP20 - utp20, small subunit (ssu) processome component, homolog (yeast)  ILK - integrin-linked kinase  CDC14B - cell division cycle 14b  PIAS1 - protein inhibitor of activated stat, 1  SMYD3 - set and mynd domain containing 3  MAPKAPK5 - mitogen-activated protein kinase-activated protein kinase 5  SMURF2 - smad specific e3 ubiquitin protein ligase 2  METTL17 - methyltransferase like 17  PATL1 - protein associated with topoisomerase ii homolog 1 (yeast)  LMO4 - lim domain only 4  CAMK1 - calcium/calmodulin-dependent protein kinase i  COPS7B - cop9 signalosome subunit 7b  CHST1 - carbohydrate (keratan sulfate gal-6) sulfotransferase 1  CBX4 - chromobox homolog 4  COPS3 - cop9 signalosome subunit 3  GORASP1 - golgi reassembly stacking protein 1, 65kda  ANAPC1 - anaphase promoting complex subunit 1  IL6ST - interleukin 6 signal transducer (gp130, oncostatin m receptor)  ORAOV1 - oral cancer overexpressed 1  SERP1 - stress-associated endoplasmic reticulum protein 1  PRKRA - protein kinase, interferon-inducible double stranded rna dependent activator  STK16 - serine/threonine kinase 16  YARS - tyrosyl-trna synthetase  RRP1 - ribosomal rna processing 1  RNF115 - ring finger protein 115  KHSRP - kh-type splicing regulatory protein  NFU1 - nfu1 iron-sulfur cluster scaffold homolog (s. cerevisiae)  ERLEC1 - endoplasmic reticulum lectin 1  CASK - calcium/calmodulin-dependent serine protein kinase (maguk family)  ITGAV - integrin, alpha v  POLR1E - polymerase (rna) i polypeptide e, 53kda  CDK10 - cyclin-dependent kinase 10  SUDS3 - suppressor of defective silencing 3 homolog (s. cerevisiae)  PRPF18 - pre-mrna processing factor 18  ITGB1 - integrin, beta 1 (fibronectin receptor, beta polypeptide, antigen cd29 includes mdf2, msk12)  GPKOW - g patch domain and kow motifs  ZDHHC6 - zinc finger, dhhc-type containing 6  EIF6 - eukaryotic translation initiation factor 6  ATG3 - autophagy related 3  RNASET2 - ribonuclease t2  RTCA - rna 3'-terminal phosphate cyclase  INSR - insulin receptor  CDK13 - cyclin-dependent kinase 13  AKTIP - akt interacting protein  KLHL20 - kelch-like family member 20  MPP5 - membrane protein, palmitoylated 5 (maguk p55 subfamily member 5)  LSM3 - lsm3 homolog, u6 small nuclear rna associated (s. cerevisiae)  CSDC2 - cold shock domain containing c2, rna binding  APEX2 - apex nuclease (apurinic/apyrimidinic endonuclease) 2  RUVBL1 - ruvb-like 1 (e. coli)  MOCS3 - molybdenum cofactor synthesis 3  DIMT1 - dim1 dimethyladenosine transferase 1 homolog (s. cerevisiae)  IRAK2 - interleukin-1 receptor-associated kinase 2  CRCP - cgrp receptor component  NOP14 - nop14 nucleolar protein  FOLH1B - folate hydrolase 1b  IRAK1 - interleukin-1 receptor-associated kinase 1  EIF2AK1 - eukaryotic translation initiation factor 2-alpha kinase 1  LRWD1 - leucine-rich repeats and wd repeat domain containing 1  PAPD5 - pap associated domain containing 5  NSD1 - nuclear receptor binding set domain protein 1  HS3ST5 - heparan sulfate (glucosamine) 3-o-sulfotransferase 5  RFWD2 - ring finger and wd repeat domain 2, e3 ubiquitin protein ligase  RNF25 - ring finger protein 25  AFF4 - af4/fmr2 family, member 4  MMS19 - mms19 nucleotide excision repair homolog (s. cerevisiae)  PALD1 - phosphatase domain containing, paladin 1  SNX5 - sorting nexin 5  ERAP2 - endoplasmic reticulum aminopeptidase 2  BRPF3 - bromodomain and phd finger containing, 3  PDF - peptide deformylase (mitochondrial)  STK36 - serine/threonine kinase 36  IRF2BPL - interferon regulatory factor 2 binding protein-like  OSGEPL1 - o-sialoglycoprotein endopeptidase-like 1  RRAGC - ras-related gtp binding c  FOXK1 - forkhead box k1  MAGEF1 - melanoma antigen family f, 1  IDUA - iduronidase, alpha-l-  XYLT2 - xylosyltransferase ii  IDS - iduronate 2-sulfatase  VPS4A - vacuolar protein sorting 4 homolog a (s. cerevisiae)  FN3K - fructosamine 3 kinase  IGHMBP2 - immunoglobulin mu binding protein 2  STK24 - serine/threonine kinase 24  GFRA4 - gdnf family receptor alpha 4  RBPJ - recombination signal binding protein for immunoglobulin kappa j region  RASAL1 - ras protein activator like 1 (gap1 like)  OARD1 - o-acyl-adp-ribose deacylase 1  PABPC1 - poly(a) binding protein, cytoplasmic 1  DYRK3 - dual-specificity tyrosine-(y)-phosphorylation regulated kinase 3  RNF11 - ring finger protein 11  TRUB2 - trub pseudouridine (psi) synthase homolog 2 (e. coli)  DUSP11 - dual specificity phosphatase 11 (rna/rnp complex 1-interacting)  SPOP - speckle-type poz protein  CYFIP2 - cytoplasmic fmr1 interacting protein 2  SPARCL1 - sparc-like 1 (hevin)  RBM26 - rna binding motif protein 26  USP21 - ubiquitin specific peptidase 21  ULK1 - unc-51 like autophagy activating kinase 1  GOLPH3 - golgi phosphoprotein 3 (coat-protein)  IK - ik cytokine, down-regulator of hla ii  MLH3 - mutl homolog 3 (e. coli)  JMJD1C - jumonji domain containing 1c  SMARCA5 - swi/snf related, matrix associated, actin dependent regulator of chromatin, subfamily a, member 5  CDC42BPA - cdc42 binding protein kinase alpha (dmpk-like)  IGF1R - insulin-like growth factor 1 receptor  OGT - o-linked n-acetylglucosamine (glcnac) transferase  DIEXF - digestive organ expansion factor homolog (zebrafish)  PELP1 - proline, glutamate and leucine rich protein 1  RAE1 - rae1 rna export 1 homolog (s. pombe)  LAT - linker for activation of t cells  GEMIN2 - gem (nuclear organelle) associated protein 2  NSG1 - neuron specific gene family member 1  IGFBP6 - insulin-like growth factor binding protein 6  SND1 - staphylococcal nuclease and tudor domain containing 1  CUL4B - cullin 4b  DHX16 - deah (asp-glu-ala-his) box polypeptide 16  XPNPEP3 - x-prolyl aminopeptidase (aminopeptidase p) 3, putative  CUL1 - cullin 1  CUL2 - cullin 2  PCIF1 - pdx1 c-terminal inhibiting factor 1  CUL3 - cullin 3  TPST2 - tyrosylprotein sulfotransferase 2  B3GAT1 - beta-1,3-glucuronyltransferase 1 (glucuronosyltransferase p)  SUPT3H - suppressor of ty 3 homolog (s. cerevisiae)  CACNG4 - calcium channel, voltage-dependent, gamma subunit 4  ZUFSP - zinc finger with ufm1-specific peptidase domain  TPST1 - tyrosylprotein sulfotransferase 1  RSRC1 - arginine/serine-rich coiled-coil 1  PCGF5 - polycomb group ring finger 5  MEX3C - mex-3 rna binding family member c  AMZ2 - archaelysin family metallopeptidase 2  WTAP - wilms tumor 1 associated protein  WAC - ww domain containing adaptor with coiled-coil  RBM39 - rna binding motif protein 39  LIG4 - ligase iv, dna, atp-dependent  MKI67IP - mki67 (fha domain) interacting nucleolar phosphoprotein  LIMK1 - lim domain kinase 1  LIG1 - ligase i, dna, atp-dependent  LIG3 - ligase iii, dna, atp-dependent  VPS37A - vacuolar protein sorting 37 homolog a (s. cerevisiae)  ZDHHC3 - zinc finger, dhhc-type containing 3  LDLR - low density lipoprotein receptor  AATK - apoptosis-associated tyrosine kinase  DOT1L - dot1-like histone h3k79 methyltransferase  ABCG1 - atp-binding cassette, sub-family g (white), member 1  LOXL1 - lysyl oxidase-like 1  RAB3D - rab3d, member ras oncogene family  LNPEP - leucyl/cystinyl aminopeptidase  BRSK1 - br serine/threonine kinase 1  CIR1 - corepressor interacting with rbpj, 1  NRG2 - neuregulin 2  SYVN1 - synovial apoptosis inhibitor 1, synoviolin  BFAR - bifunctional apoptosis regulator  RPP25L - ribonuclease p/mrp 25kda subunit-like  SLX4 - slx4 structure-specific endonuclease subunit  CHD1L - chromodomain helicase dna binding protein 1-like  PTPDC1 - protein tyrosine phosphatase domain containing 1  NEURL4 - neuralized homolog 4 (drosophila)  GTF2IRD1 - gtf2i repeat domain containing 1  UBAP1 - ubiquitin associated protein 1  LIPE - lipase, hormone-sensitive  UBXN2B - ubx domain protein 2b  GTPBP1 - gtp binding protein 1  MINPP1 - multiple inositol-polyphosphate phosphatase 1  LIMK2 - lim domain kinase 2  PREPL - prolyl endopeptidase-like  ZC3H8 - zinc finger ccch-type containing 8  GOLM1 - golgi membrane protein 1  CDC42BPB - cdc42 binding protein kinase beta (dmpk-like)  CLOCK - clock circadian regulator  HGSNAT - heparan-alpha-glucosaminide n-acetyltransferase  LMAN1 - lectin, mannose-binding, 1  PRKAG2 - protein kinase, amp-activated, gamma 2 non-catalytic subunit  ADAMTS4 - adam metallopeptidase with thrombospondin type 1 motif, 4  POLK - polymerase (dna directed) kappa  DDX41 - dead (asp-glu-ala-asp) box polypeptide 41  ADAMTS1 - adam metallopeptidase with thrombospondin type 1 motif, 1  ANAPC5 - anaphase promoting complex subunit 5  ANAPC7 - anaphase promoting complex subunit 7  MRPL43 - mitochondrial ribosomal protein l43  TBPL1 - tbp-like 1  YTHDF2 - yth domain family, member 2  NPEPPS - aminopeptidase puromycin sensitive  MAK16 - mak16 homolog (s. cerevisiae)  EEF1E1 - eukaryotic translation elongation factor 1 epsilon 1  KTN1 - kinectin 1 (kinesin receptor)  RNF138 - ring finger protein 138, e3 ubiquitin protein ligase  VPS4B - vacuolar protein sorting 4 homolog b (s. cerevisiae)  TMEM59 - transmembrane protein 59  KRT10 - keratin 10  POLR1C - polymerase (rna) i polypeptide c, 30kda  PPME1 - protein phosphatase methylesterase 1  HEMK1 - hemk methyltransferase family member 1  ACTL6B - actin-like 6b  LAMP2 - lysosomal-associated membrane protein 2  UBR5 - ubiquitin protein ligase e3 component n-recognin 5  CHST15 - carbohydrate (n-acetylgalactosamine 4-sulfate 6-o) sulfotransferase 15  TMA7 - translation machinery associated 7 homolog (s. cerevisiae)  MED20 - mediator complex subunit 20  UCHL5 - ubiquitin carboxyl-terminal hydrolase l5  ATG5 - autophagy related 5  ROCK2 - rho-associated, coiled-coil containing protein kinase 2  MAPK8IP1 - mitogen-activated protein kinase 8 interacting protein 1  PIGB - phosphatidylinositol glycan anchor biosynthesis, class b  CHST10 - carbohydrate sulfotransferase 10  TAOK3 - tao kinase 3  FZR1 - fizzy/cell division cycle 20 related 1 (drosophila)  ZBTB7A - zinc finger and btb domain containing 7a  CRNKL1 - crooked neck pre-mrna splicing factor-like 1 (drosophila)  DPY30 - dpy-30 homolog (c. elegans)  CDC40 - cell division cycle 40  LAMC1 - laminin, gamma 1 (formerly lamb2)  MBTPS2 - membrane-bound transcription factor peptidase, site 2  IRF2BP2 - interferon regulatory factor 2 binding protein 2  MED27 - mediator complex subunit 27  MED26 - mediator complex subunit 26  NAGPA - n-acetylglucosamine-1-phosphodiester alpha-n-acetylglucosaminidase  POLR1B - polymerase (rna) i polypeptide b, 128kda  DBR1 - debranching rna lariats 1  GALNT18 - udp-n-acetyl-alpha-d-galactosamine:polypeptide n-acetylgalactosaminyltransferase 18  MED17 - mediator complex subunit 17  MED23 - mediator complex subunit 23  VPS28 - vacuolar protein sorting 28 homolog (s. cerevisiae)  CHST2 - carbohydrate (n-acetylglucosamine-6-o) sulfotransferase 2  ASB7 - ankyrin repeat and socs box containing 7  MRTO4 - mrna turnover 4 homolog (s. cerevisiae)  RPF2 - ribosome production factor 2 homolog (s. cerevisiae)  ASB8 - ankyrin repeat and socs box containing 8  ARHGEF6 - rac/cdc42 guanine nucleotide exchange factor (gef) 6  KDR - kinase insert domain receptor (a type iii receptor tyrosine kinase)  PICK1 - protein interacting with prkca 1  ASB6 - ankyrin repeat and socs box containing 6  ING4 - inhibitor of growth family, member 4  EIF2AK3 - eukaryotic translation initiation factor 2-alpha kinase 3  USP48 - ubiquitin specific peptidase 48  ITM2B - integral membrane protein 2b  IRAK4 - interleukin-1 receptor-associated kinase 4  MAP4K4 - mitogen-activated protein kinase kinase kinase kinase 4  RHBDD1 - rhomboid domain containing 1  RLIM - ring finger protein, lim domain interacting  ASB3 - ankyrin repeat and socs box containing 3  TRAF7 - tnf receptor-associated factor 7, e3 ubiquitin protein ligase  RECQL5 - recq protein-like 5  KRAS - kirsten rat sarcoma viral oncogene homolog  NAA20 - n(alpha)-acetyltransferase 20, natb catalytic subunit  GOLGA7 - golgin a7  HS6ST1 - heparan sulfate 6-o-sulfotransferase 1  CIAO1 - cytosolic iron-sulfur protein assembly 1  RPL26L1 - ribosomal protein l26-like 1  KPNA2 - karyopherin alpha 2 (rag cohort 1, importin alpha 1)  SBDS - shwachman-bodian-diamond syndrome  KPNB1 - karyopherin (importin) beta 1  KPNA1 - karyopherin alpha 1 (importin alpha 5)  ZDHHC9 - zinc finger, dhhc-type containing 9  CAMKK1 - calcium/calmodulin-dependent protein kinase kinase 1, alpha  SUV420H1 - suppressor of variegation 4-20 homolog 1 (drosophila)  TRAPPC12 - trafficking protein particle complex 12  LACTB2 - lactamase, beta 2  APH1A - aph1a gamma secretase subunit  DDX23 - dead (asp-glu-ala-asp) box polypeptide 23  PHF20L1 - phd finger protein 20-like 1  TFB1M - transcription factor b1, mitochondrial  MED10 - mediator complex subunit 10  MED21 - mediator complex subunit 21  ABHD17B - abhydrolase domain containing 17b  KIT - v-kit hardy-zuckerman 4 feline sarcoma viral oncogene homolog  SNRNP40 - small nuclear ribonucleoprotein 40kda (u5)  ZRANB2 - zinc finger, ran-binding domain containing 2  SH3GLB1 - sh3-domain grb2-like endophilin b1  RPAIN - rpa interacting protein  LONP1 - lon peptidase 1, mitochondrial  POLDIP3 - polymerase (dna-directed), delta interacting protein 3  MRPL51 - mitochondrial ribosomal protein l51  MRPL30 - mitochondrial ribosomal protein l30  NOA1 - nitric oxide associated 1  SHISA5 - shisa family member 5  DCUN1D5 - dcn1, defective in cullin neddylation 1, domain containing 5  UBE4A - ubiquitination factor e4a  FBXW9 - f-box and wd repeat domain containing 9  PPIG - peptidylprolyl isomerase g (cyclophilin g)  POLR3GL - polymerase (rna) iii (dna directed) polypeptide g (32kd)-like  ZDHHC16 - zinc finger, dhhc-type containing 16  JAK1 - janus kinase 1  JAK2 - janus kinase 2  ING5 - inhibitor of growth family, member 5  JAK3 - janus kinase 3  ANKRD39 - ankyrin repeat domain 39  SLC25A33 - solute carrier family 25 (pyrimidine nucleotide carrier), member 33  STT3A - stt3a, subunit of the oligosaccharyltransferase complex (catalytic)  MAST4 - microtubule associated serine/threonine kinase family member 4  PPT2 - palmitoyl-protein thioesterase 2  PIGP - phosphatidylinositol glycan anchor biosynthesis, class p  PHF20 - phd finger protein 20  TMEM246 - transmembrane protein 246  TRIP12 - thyroid hormone receptor interactor 12  GTF3C5 - general transcription factor iiic, polypeptide 5, 63kda  NUDT16L1 - nudix (nucleoside diphosphate linked moiety x)-type motif 16-like 1  ZNHIT3 - zinc finger, hit-type containing 3  UTP23 - utp23, small subunit (ssu) processome component, homolog (yeast)  COPS2 - cop9 signalosome subunit 2  DDX47 - dead (asp-glu-ala-asp) box polypeptide 47  ZDHHC2 - zinc finger, dhhc-type containing 2  RSL24D1 - ribosomal l24 domain containing 1  THOC3 - tho complex 3  EFTUD2 - elongation factor tu gtp binding domain containing 2  CRBN - cereblon  MIA3 - melanoma inhibitory activity family, member 3  TAOK2 - tao kinase 2  SARNP - sap domain containing ribonucleoprotein  B4GALT6 - udp-gal:betaglcnac beta 1,4- galactosyltransferase, polypeptide 6  BRMS1L - breast cancer metastasis-suppressor 1-like  LEF1 - lymphoid enhancer-binding factor 1  GTF3C4 - general transcription factor iiic, polypeptide 4, 90kda  MRPL45 - mitochondrial ribosomal protein l45  GTF3C3 - general transcription factor iiic, polypeptide 3, 102kda  CNOT8 - ccr4-not transcription complex, subunit 8  CCDC115 - coiled-coil domain containing 115  B4GALT5 - udp-gal:betaglcnac beta 1,4- galactosyltransferase, polypeptide 5  SNORD31 - small nucleolar rna, c/d box 31  CDK12 - cyclin-dependent kinase 12  SNORD29 - small nucleolar rna, c/d box 29  SRSF11 - serine/arginine-rich splicing factor 11  LUC7L3 - luc7-like 3 (s. cerevisiae)  KCTD10 - potassium channel tetramerization domain containing 10  SNX25 - sorting nexin 25  ARID4B - at rich interactive domain 4b (rbp1-like)  SOCS6 - suppressor of cytokine signaling 6  SPRTN - sprt-like n-terminal domain  MIPEP - mitochondrial intermediate peptidase  SNORD22 - small nucleolar rna, c/d box 22  STK35 - serine/threonine kinase 35  SNORD30 - small nucleolar rna, c/d box 30  MAP3K10 - mitogen-activated protein kinase kinase kinase 10  DUSP19 - dual specificity phosphatase 19  MIB2 - mindbomb e3 ubiquitin protein ligase 2  MLH1 - mutl homolog 1, colon cancer, nonpolyposis type 2 (e. coli)  KDM3B - lysine (k)-specific demethylase 3b  RSF1 - remodeling and spacing factor 1  MLLT3 - myeloid/lymphoid or mixed-lineage leukemia (trithorax homolog, drosophila); translocated to, 3  MED14 - mediator complex subunit 14  MLLT1 - myeloid/lymphoid or mixed-lineage leukemia (trithorax homolog, drosophila); translocated to, 1  WDR46 - wd repeat domain 46  MAP3K11 - mitogen-activated protein kinase kinase kinase 11  KMT2A - lysine (k)-specific methyltransferase 2a  PHAX - phosphorylated adaptor for rna export  CPSF3 - cleavage and polyadenylation specific factor 3, 73kda  MMP2 - matrix metallopeptidase 2 (gelatinase a, 72kda gelatinase, 72kda type iv collagenase)  NAA38 - n(alpha)-acetyltransferase 38, natc auxiliary subunit  NUMBL - numb homolog (drosophila)-like  RPS6KA5 - ribosomal protein s6 kinase, 90kda, polypeptide 5  CCDC8 - coiled-coil domain containing 8  SUFU - suppressor of fused homolog (drosophila)  LSM7 - lsm7 homolog, u6 small nuclear rna associated (s. cerevisiae)  STK17A - serine/threonine kinase 17a  MMP14 - matrix metallopeptidase 14 (membrane-inserted)  NLK - nemo-like kinase  TATDN1 - tatd dnase domain containing 1  UIMC1 - ubiquitin interaction motif containing 1  MNAT1 - mnat cdk-activating kinase assembly factor 1  RAB11B - rab11b, member ras oncogene family  DNAJB11 - dnaj (hsp40) homolog, subfamily b, member 11  MTA2 - metastasis associated 1 family, member 2  NOLC1 - nucleolar and coiled-body phosphoprotein 1  EIF2A - eukaryotic translation initiation factor 2a, 65kda  MNT - mnt, max dimerization protein  POLR3K - polymerase (rna) iii (dna directed) polypeptide k, 12.3 kda  WBP11 - ww domain binding protein 11  CD46 - cd46 molecule, complement regulatory protein  MCM7 - minichromosome maintenance complex component 7  NSRP1 - nuclear speckle splicing regulatory protein 1  CWF19L2 - cwf19-like 2, cell cycle control (s. pombe)  KBTBD7 - kelch repeat and btb (poz) domain containing 7  KDELC2 - kdel (lys-asp-glu-leu) containing 2  DNAJB9 - dnaj (hsp40) homolog, subfamily b, member 9  BUB3 - bub3 mitotic checkpoint protein  MECP2 - methyl cpg binding protein 2 (rett syndrome)  PELO - pelota homolog (drosophila)  MAGT1 - magnesium transporter 1  MEF2A - myocyte enhancer factor 2a  DDX21 - dead (asp-glu-ala-asp) box helicase 21  ZBED1 - zinc finger, bed-type containing 1  PPIL3 - peptidylprolyl isomerase (cyclophilin)-like 3  BCDIN3D - bcdin3 domain containing  CPSF2 - cleavage and polyadenylation specific factor 2, 100kda  MAP3K4 - mitogen-activated protein kinase kinase kinase 4  DCLK1 - doublecortin-like kinase 1  MAP3K5 - mitogen-activated protein kinase kinase kinase 5  MAP3K1 - mitogen-activated protein kinase kinase kinase 1, e3 ubiquitin protein ligase  MAP3K3 - mitogen-activated protein kinase kinase kinase 3  PCSK7 - proprotein convertase subtilisin/kexin type 7  MEN1 - multiple endocrine neoplasia i  WDR75 - wd repeat domain 75  RAB8A - rab8a, member ras oncogene family  USP42 - ubiquitin specific peptidase 42  SCAF11 - sr-related ctd-associated factor 11  UTP15 - utp15, u3 small nucleolar ribonucleoprotein, homolog (s. cerevisiae)  METTL1 - methyltransferase like 1  MFAP1 - microfibrillar-associated protein 1  KAT8 - k(lysine) acetyltransferase 8  RNASEH2C - ribonuclease h2, subunit c  MFGE8 - milk fat globule-egf factor 8 protein  FBXL14 - f-box and leucine-rich repeat protein 14  PPP1R1B - protein phosphatase 1, regulatory (inhibitor) subunit 1b  MFAP4 - microfibrillar-associated protein 4  ATG12 - autophagy related 12  MGAT1 - mannosyl (alpha-1,3-)-glycoprotein beta-1,2-n-acetylglucosaminyltransferase  MGAT2 - mannosyl (alpha-1,6-)-glycoprotein beta-1,2-n-acetylglucosaminyltransferase  HGS - hepatocyte growth factor-regulated tyrosine kinase substrate  MGAT5 - mannosyl (alpha-1,6-)-glycoprotein beta-1,6-n-acetyl-glucosaminyltransferase  NEMF - nuclear export mediator factor  MBD3 - methyl-cpg binding domain protein 3  KITLG - kit ligand  B3GALTL - beta 1,3-galactosyltransferase-like  ATRIP - atr interacting protein  MAN1A1 - mannosidase, alpha, class 1a, member 1  CBFA2T2 - core-binding factor, runt domain, alpha subunit 2; translocated to, 2  MAN2A2 - mannosidase, alpha, class 2a, member 2  ZC3HC1 - zinc finger, c3hc-type containing 1  COG3 - component of oligomeric golgi complex 3  RRP9 - ribosomal rna processing 9, small subunit (ssu) processome component, homolog (yeast)  JKAMP - jnk1/mapk8-associated membrane protein  MAGOH - mago-nashi homolog, proliferation-associated (drosophila)  CHST9 - carbohydrate (n-acetylgalactosamine 4-0) sulfotransferase 9  PRPF3 - pre-mrna processing factor 3  LARS - leucyl-trna synthetase  PRPF4 - prp4 pre-mrna processing factor 4 homolog (yeast)  RQCD1 - rcd1 required for cell differentiation1 homolog (s. pombe)  SMC3 - structural maintenance of chromosomes 3  PUS3 - pseudouridylate synthase 3  MARS - methionyl-trna synthetase  MARK3 - map/microtubule affinity-regulating kinase 3  MARK1 - map/microtubule affinity-regulating kinase 1  DOHH - deoxyhypusine hydroxylase/monooxygenase  MAPT - microtubule-associated protein tau  APH1B - aph1b gamma secretase subunit  MDP1 - magnesium-dependent phosphatase 1  LATS1 - large tumor suppressor kinase 1  MTA1 - metastasis associated 1  SIRT6 - sirtuin 6  MTMR4 - myotubularin related protein 4  MTMR7 - myotubularin related protein 7  MTMR6 - myotubularin related protein 6  MANBA - mannosidase, beta a, lysosomal  MAN2A1 - mannosidase, alpha, class 2a, member 1  USP8 - ubiquitin specific peptidase 8  USP14 - ubiquitin specific peptidase 14 (trna-guanine transglycosylase)  USP10 - ubiquitin specific peptidase 10  UBE2J1 - ubiquitin-conjugating enzyme e2, j1  NUP43 - nucleoporin 43kda  MBD1 - methyl-cpg binding domain protein 1  REV1 - rev1, polymerase (dna directed)  PKMYT1 - protein kinase, membrane associated tyrosine/threonine 1  SART1 - squamous cell carcinoma antigen recognized by t cells  MATK - megakaryocyte-associated tyrosine kinase  UFC1 - ubiquitin-fold modifier conjugating enzyme 1  TRMT112 - trna methyltransferase 11-2 homolog (s. cerevisiae)  MCM3 - minichromosome maintenance complex component 3  MCM6 - minichromosome maintenance complex component 6  RTFDC1 - replication termination factor 2 domain containing 1  MCM5 - minichromosome maintenance complex component 5  TTBK2 - tau tubulin kinase 2  NELFCD - negative elongation factor complex member c/d  CWC15 - cwc15 spliceosome-associated protein homolog (s. cerevisiae)  MCM2 - minichromosome maintenance complex component 2  ASH2L - ash2 (absent, small, or homeotic)-like (drosophila)  RTCB - rna 2',3'-cyclic phosphate and 5'-oh ligase  CTDSPL2 - ctd (carboxy-terminal domain, rna polymerase ii, polypeptide a) small phosphatase like 2  NUDT12 - nudix (nucleoside diphosphate linked moiety x)-type motif 12  PPIL1 - peptidylprolyl isomerase (cyclophilin)-like 1  ITCH - itchy e3 ubiquitin protein ligase  NFS1 - nfs1 cysteine desulfurase  FBXL16 - f-box and leucine-rich repeat protein 16  MRPL48 - mitochondrial ribosomal protein l48  MRPS23 - mitochondrial ribosomal protein s23  MRPS33 - mitochondrial ribosomal protein s33  LTA4H - leukotriene a4 hydrolase  FAM96B - family with sequence similarity 96, member b  LRP1 - low density lipoprotein receptor-related protein 1  RPL14 - ribosomal protein l14  RIOK1 - rio kinase 1  ZNF445 - zinc finger protein 445  AIP - aryl hydrocarbon receptor interacting protein  OTUD6B - otu domain containing 6b  NUB1 - negative regulator of ubiquitin-like proteins 1  ASB1 - ankyrin repeat and socs box containing 1  LUM - lumican  LYN - v-yes-1 yamaguchi sarcoma viral related oncogene homolog  UBE2M - ubiquitin-conjugating enzyme e2m  UBA3 - ubiquitin-like modifier activating enzyme 3  RBFOX3 - rna binding protein, fox-1 homolog (c. elegans) 3  SESN2 - sestrin 2  PARP9 - poly (adp-ribose) polymerase family, member 9  CDK5RAP1 - cdk5 regulatory subunit associated protein 1  RNF8 - ring finger protein 8, e3 ubiquitin protein ligase  LTBP1 - latent transforming growth factor beta binding protein 1  RPS6KL1 - ribosomal protein s6 kinase-like 1  FKBP7 - fk506 binding protein 7  BAZ1B - bromodomain adjacent to zinc finger domain, 1b  RHBDL1 - rhomboid, veinlet-like 1 (drosophila)  TAF1B - tata box binding protein (tbp)-associated factor, rna polymerase i, b, 63kda  ESF1 - esf1, nucleolar pre-rrna processing protein, homolog (s. cerevisiae)  PCF11 - pcf11 cleavage and polyadenylation factor subunit  MED15 - mediator complex subunit 15  PIAS4 - protein inhibitor of activated stat, 4  TRIM33 - tripartite motif containing 33  SMAD2 - smad family member 2  SRRT - serrate rna effector molecule homolog (arabidopsis)  MBIP - map3k12 binding inhibitory protein 1  HDAC7 - histone deacetylase 7  TMTC1 - transmembrane and tetratricopeptide repeat containing 1  TDP2 - tyrosyl-dna phosphodiesterase 2  CDKL2 - cyclin-dependent kinase-like 2 (cdc2-related kinase)  TAF1C - tata box binding protein (tbp)-associated factor, rna polymerase i, c, 110kda  LARP7 - la ribonucleoprotein domain family, member 7  RPS6KA4 - ribosomal protein s6 kinase, 90kda, polypeptide 4  KCTD11 - potassium channel tetramerization domain containing 11  PLOD3 - procollagen-lysine, 2-oxoglutarate 5-dioxygenase 3  DPH5 - diphthamide biosynthesis 5  TAF9B - taf9b rna polymerase ii, tata box binding protein (tbp)-associated factor, 31kda  SMAD4 - smad family member 4  NBAS - neuroblastoma amplified sequence  SMAD5 - smad family member 5  EME1 - essential meiotic structure-specific endonuclease 1  P4HA2 - prolyl 4-hydroxylase, alpha polypeptide ii  SMAD7 - smad family member 7  TMEM199 - transmembrane protein 199  TRMT6 - trna methyltransferase 6 homolog (s. cerevisiae)  RBM4B - rna binding motif protein 4b  PIGT - phosphatidylinositol glycan anchor biosynthesis, class t  MYLIP - myosin regulatory light chain interacting protein  NCBP1 - nuclear cap binding protein subunit 1, 80kda  TSPAN14 - tetraspanin 14  G3BP1 - gtpase activating protein (sh3 domain) binding protein 1  NCK1 - nck adaptor protein 1  MBNL2 - muscleblind-like splicing regulator 2  DPY19L3 - dpy-19-like 3 (c. elegans)  SUGP2 - surp and g patch domain containing 2  TRMT1L - trna methyltransferase 1 homolog (s. cerevisiae)-like  ABI2 - abl-interactor 2  HNRNPM - heterogeneous nuclear ribonucleoprotein m  NAGLU - n-acetylglucosaminidase, alpha  TRIM28 - tripartite motif containing 28  DROSHA - drosha, ribonuclease type iii  ISCA1 - iron-sulfur cluster assembly 1  TMEM208 - transmembrane protein 208  NAP1L1 - nucleosome assembly protein 1-like 1  ATP6AP2 - atpase, h+ transporting, lysosomal accessory protein 2  SCG3 - secretogranin iii  NASP - nuclear autoantigenic sperm protein (histone-binding)  NARS - asparaginyl-trna synthetase  NCAM1 - neural cell adhesion molecule 1  TBK1 - tank-binding kinase 1  NBN - nibrin  RBM7 - rna binding motif protein 7  NHLRC3 - nhl repeat containing 3  RBM6 - rna binding motif protein 6  RBM5 - rna binding motif protein 5  ZDHHC1 - zinc finger, dhhc-type containing 1  ZDHHC8 - zinc finger, dhhc-type containing 8  TRIM11 - tripartite motif containing 11  PPP1R12A - protein phosphatase 1, regulatory subunit 12a  TNK2 - tyrosine kinase, non-receptor, 2  ALYREF - aly/ref export factor  NDEL1 - nude neurodevelopment protein 1-like 1  SENP1 - sumo1/sentrin specific peptidase 1  RNF41 - ring finger protein 41  TFPT - tcf3 (e2a) fusion partner (in childhood leukemia)  PRMT3 - protein arginine methyltransferase 3  PSME3 - proteasome (prosome, macropain) activator subunit 3 (pa28 gamma; ki)  CCDC130 - coiled-coil domain containing 130  MPHOSPH6 - m-phase phosphoprotein 6  MYLK - myosin light chain kinase  APOLD1 - apolipoprotein l domain containing 1  MPHOSPH10 - m-phase phosphoprotein 10 (u3 small nucleolar ribonucleoprotein)  CDADC1 - cytidine and dcmp deaminase domain containing 1  CALCRL - calcitonin receptor-like  TRIM13 - tripartite motif containing 13  URM1 - ubiquitin related modifier 1  TRIM8 - tripartite motif containing 8  USPL1 - ubiquitin specific peptidase like 1  ABT1 - activator of basal transcription 1  TOPORS - topoisomerase i binding, arginine/serine-rich, e3 ubiquitin protein ligase  FIP1L1 - factor interacting with papola and cpsf1  DPP3 - dipeptidyl-peptidase 3  RAB1B - rab1b, member ras oncogene family  LAS1L - las1-like (s. cerevisiae)  ISG20L2 - interferon stimulated exonuclease gene 20kda-like 2  SEH1L - seh1-like (s. cerevisiae)  PDCD7 - programmed cell death 7  PTPRU - protein tyrosine phosphatase, receptor type, u  MYH9 - myosin, heavy chain 9, non-muscle  HUWE1 - hect, uba and wwe domain containing 1, e3 ubiquitin protein ligase  CNOT7 - ccr4-not transcription complex, subunit 7  ALG5 - alg5, dolichyl-phosphate beta-glucosyltransferase  PQBP1 - polyglutamine binding protein 1  MX1 - myxovirus (influenza virus) resistance 1, interferon-inducible protein p78 (mouse)  TRA2A - transformer 2 alpha homolog (drosophila)  INO80B - ino80 complex subunit b  TSPAN5 - tetraspanin 5  SF3B5 - splicing factor 3b, subunit 5, 10kda  CPSF1 - cleavage and polyadenylation specific factor 1, 160kda  B3GALNT2 - beta-1,3-n-acetylgalactosaminyltransferase 2  RBM15B - rna binding motif protein 15b  CTDSP2 - ctd (carboxy-terminal domain, rna polymerase ii, polypeptide a) small phosphatase 2  ANAPC4 - anaphase promoting complex subunit 4  TSFM - ts translation elongation factor, mitochondrial  RNF170 - ring finger protein 170  FEM1B - fem-1 homolog b (c. elegans)  NRBP1 - nuclear receptor binding protein 1  MUTYH - muty homolog (e. coli)  RAD50 - rad50 homolog (s. cerevisiae)  TRIM37 - tripartite motif containing 37  CDC42EP5 - cdc42 effector protein (rho gtpase binding) 5  SHARPIN - shank-associated rh domain interactor  PDIA6 - protein disulfide isomerase family a, member 6  MED25 - mediator complex subunit 25  MED6 - mediator complex subunit 6  AKT3 - v-akt murine thymoma viral oncogene homolog 3  ABI1 - abl-interactor 1  MTRR - 5-methyltetrahydrofolate-homocysteine methyltransferase reductase  PTBP3 - polypyrimidine tract binding protein 3  PPP4R1 - protein phosphatase 4, regulatory subunit 1  PDCD6 - programmed cell death 6  MTM1 - myotubularin 1  TANK - traf family member-associated nfkb activator  HDAC5 - histone deacetylase 5  MTF1 - metal-regulatory transcription factor 1  PDCD6IP - programmed cell death 6 interacting protein  HDAC6 - histone deacetylase 6  PARP2 - poly (adp-ribose) polymerase 2  RNF187 - ring finger protein 187  SH2D3C - sh2 domain containing 3c  DCPS - decapping enzyme, scavenger  MT3 - metallothionein 3  MED16 - mediator complex subunit 16  PIGK - phosphatidylinositol glycan anchor biosynthesis, class k  CHAF1A - chromatin assembly factor 1, subunit a (p150)  CCDC22 - coiled-coil domain containing 22  SAE1 - sumo1 activating enzyme subunit 1  FARSB - phenylalanyl-trna synthetase, beta subunit  ABCC5 - atp-binding cassette, sub-family c (cftr/mrp), member 5  PDIK1L - pdlim1 interacting kinase 1 like  RANBP9 - ran binding protein 9  UBA2 - ubiquitin-like modifier activating enzyme 2  JOSD1 - josephin domain containing 1  KANSL1L - kat8 regulatory nsl complex subunit 1-like  MSH3 - muts homolog 3 (e. coli)  MSH2 - muts homolog 2, colon cancer, nonpolyposis type 1 (e. coli)  PAN2 - pan2 poly(a) specific ribonuclease subunit homolog (s. cerevisiae)  ASTE1 - asteroid homolog 1 (drosophila)  FAM20B - family with sequence similarity 20, member b  NOB1 - nin1/rpn12 binding protein 1 homolog (s. cerevisiae)  SUPT7L - suppressor of ty 7 (s. cerevisiae)-like  FBXO38 - f-box protein 38  RGCC - regulator of cell cycle  GFPT2 - glutamine-fructose-6-phosphate transaminase 2  OXSR1 - oxidative stress responsive 1  COMMD1 - copper metabolism (murr1) domain containing 1  SNORA3 - small nucleolar rna, h/aca box 3  SPCS1 - signal peptidase complex subunit 1 homolog (s. cerevisiae)  RBM8A - rna binding motif protein 8a  HM13 - histocompatibility (minor) 13  POLR2M - polymerase (rna) ii (dna directed) polypeptide m  USP3 - ubiquitin specific peptidase 3  BABAM1 - brisc and brca1 a complex member 1  UBE2T - ubiquitin-conjugating enzyme e2t (putative)  MRPL15 - mitochondrial ribosomal protein l15  METTL5 - methyltransferase like 5  USP15 - ubiquitin specific peptidase 15  HS3ST1 - heparan sulfate (glucosamine) 3-o-sulfotransferase 1  TTL - tubulin tyrosine ligase  CCDC59 - coiled-coil domain containing 59  MED4 - mediator complex subunit 4  THOC1 - tho complex 1  C1GALT1C1 - c1galt1-specific chaperone 1  RCE1 - rce1 homolog, prenyl protein protease (s. cerevisiae)  SETD2 - set domain containing 2  MRPL18 - mitochondrial ribosomal protein l18  RBX1 - ring-box 1, e3 ubiquitin protein ligase  DUSP18 - dual specificity phosphatase 18  ZC3H7A - zinc finger ccch-type containing 7a  MRE11A - mre11 meiotic recombination 11 homolog a (s. cerevisiae)  HSCB - hscb iron-sulfur cluster co-chaperone homolog (e. coli)  NUP153 - nucleoporin 153kda  THRAP3 - thyroid hormone receptor associated protein 3  MED12 - mediator complex subunit 12  MPI - mannose phosphate isomerase  MED13 - mediator complex subunit 13  PSMD6 - proteasome (prosome, macropain) 26s subunit, non-atpase, 6  WDSUB1 - wd repeat, sterile alpha motif and u-box domain containing 1  MED24 - mediator complex subunit 24  DTX3L - deltex 3-like (drosophila)  PJA2 - praja ring finger 2, e3 ubiquitin protein ligase  AREL1 - apoptosis resistant e3 ubiquitin protein ligase 1  SETDB1 - set domain, bifurcated 1  ORC4 - origin recognition complex, subunit 4  INTS5 - integrator complex subunit 5  ORC2 - origin recognition complex, subunit 2  TLK1 - tousled-like kinase 1  ORC5 - origin recognition complex, subunit 5  RNF141 - ring finger protein 141  THUMPD2 - thump domain containing 2  ERCC6L2 - excision repair cross-complementing rodent repair deficiency, complementation group 6-like 2  PTAR1 - protein prenyltransferase alpha subunit repeat containing 1  AGRN - agrin  PPP4R2 - protein phosphatase 4, regulatory subunit 2  NDFIP1 - nedd4 family interacting protein 1  RNF38 - ring finger protein 38  XXYLT1 - xyloside xylosyltransferase 1  RBM19 - rna binding motif protein 19  KLHL21 - kelch-like family member 21  METTL21A - methyltransferase like 21a  AP5Z1 - adaptor-related protein complex 5, zeta 1 subunit  TMCC2 - transmembrane and coiled-coil domain family 2  DDX46 - dead (asp-glu-ala-asp) box polypeptide 46  OGG1 - 8-oxoguanine dna glycosylase  OGN - osteoglycin  TNKS2 - tankyrase, trf1-interacting ankyrin-related adp-ribose polymerase 2  EXOSC3 - exosome component 3  SMG7 - smg7 nonsense mediated mrna decay factor  WDR61 - wd repeat domain 61  UBXN6 - ubx domain protein 6  APITD1 - apoptosis-inducing, taf9-like domain 1  EXOSC1 - exosome component 1  BMS1 - bms1 ribosome biogenesis factor  RRP15 - ribosomal rna processing 15 homolog (s. cerevisiae)  SPCS2 - signal peptidase complex subunit 2 homolog (s. cerevisiae)  SCRN1 - secernin 1  DDR2 - discoidin domain receptor tyrosine kinase 2  TOMM20 - translocase of outer mitochondrial membrane 20 homolog (yeast)  NTHL1 - nth endonuclease iii-like 1 (e. coli)  DAB2IP - dab2 interacting protein  EIF4A3 - eukaryotic translation initiation factor 4a3  NUP88 - nucleoporin 88kda  NUP98 - nucleoporin 98kda  NR4A2 - nuclear receptor subfamily 4, group a, member 2  ZBTB7B - zinc finger and btb domain containing 7b  NUCB1 - nucleobindin 1  ST8SIA3 - st8 alpha-n-acetyl-neuraminide alpha-2,8-sialyltransferase 3  DHX38 - deah (asp-glu-ala-his) box polypeptide 38  NVL - nuclear vcp-like  LAP3 - leucine aminopeptidase 3  RNF144A - ring finger protein 144a  ILKAP - integrin-linked kinase-associated serine/threonine phosphatase  DNAJC6 - dnaj (hsp40) homolog, subfamily c, member 6  NPM1 - nucleophosmin (nucleolar phosphoprotein b23, numatrin)  SPATA2 - spermatogenesis associated 2  GINS1 - gins complex subunit 1 (psf1 homolog)  FCF1 - fcf1 rrna-processing protein  KLHL5 - kelch-like family member 5  CTIF - cbp80/20-dependent translation initiation factor  DUSP16 - dual specificity phosphatase 16  RNF40 - ring finger protein 40, e3 ubiquitin protein ligase  SPOCK2 - sparc/osteonectin, cwcv and kazal-like domains proteoglycan (testican) 2  POLR1D - polymerase (rna) i polypeptide d, 16kda  RB1CC1 - rb1-inducible coiled-coil 1  TRNT1 - trna nucleotidyl transferase, cca-adding, 1  CUL7 - cullin 7  UTP18 - utp18 small subunit (ssu) processome component homolog (yeast)  PAQR3 - progestin and adipoq receptor family member iii  RRNAD1 - ribosomal rna adenine dimethylase domain containing 1  NRAS - neuroblastoma ras viral (v-ras) oncogene homolog  SIDT2 - sid1 transmembrane family, member 2  UBQLN1 - ubiquilin 1  DONSON - downstream neighbor of son  NMT1 - n-myristoyltransferase 1  NRBF2 - nuclear receptor binding factor 2  NOP2 - nop2 nucleolar protein  VPRBP - vpr (hiv-1) binding protein  NONO - non-pou domain containing, octamer-binding  ECE2 - endothelin converting enzyme 2  RNF34 - ring finger protein 34, e3 ubiquitin protein ligase  MUS81 - mus81 structure-specific endonuclease subunit  NME1 - nme/nm23 nucleoside diphosphate kinase 1  FBXO11 - f-box protein 11  UBQLN2 - ubiquilin 2  ULK2 - unc-51 like autophagy activating kinase 2  NOTCH4 - notch 4  NOVA2 - neuro-oncological ventral antigen 2  HERPUD1 - homocysteine-inducible, endoplasmic reticulum stress-inducible, ubiquitin-like domain member 1  SPSB1 - spla/ryanodine receptor domain and socs box containing 1  NOVA1 - neuro-oncological ventral antigen 1  ERO1L - ero1-like (s. cerevisiae)  KCTD7 - potassium channel tetramerization domain containing 7  AQR - aquarius homolog (mouse)  CNOT2 - ccr4-not transcription complex, subunit 2  NAA15 - n(alpha)-acetyltransferase 15, nata auxiliary subunit  PUM1 - pumilio homolog 1 (drosophila)  CNOT4 - ccr4-not transcription complex, subunit 4  CNOT3 - ccr4-not transcription complex, subunit 3  NOTCH1 - notch 1  CTC1 - cts telomere maintenance complex component 1  NOTCH3 - notch 3  DHX34 - deah (asp-glu-ala-his) box polypeptide 34  NOTCH2 - notch 2  NFYA - nuclear transcription factor y, alpha  HDAC4 - histone deacetylase 4  THOC7 - tho complex 7 homolog (drosophila)  NFRKB - nuclear factor related to kappab binding protein  EDC3 - enhancer of mrna decapping 3  ASRGL1 - asparaginase like 1  CXXC1 - cxxc finger protein 1  ZMAT2 - zinc finger, matrin-type 2  NFKBIA - nuclear factor of kappa light polypeptide gene enhancer in b-cells inhibitor, alpha  SRFBP1 - serum response factor binding protein 1  NFKB1 - nuclear factor of kappa light polypeptide gene enhancer in b-cells 1  KMT2B - lysine (k)-specific methyltransferase 2b  ST6GALNAC6 - st6 (alpha-n-acetyl-neuraminyl-2,3-beta-galactosyl-1,3)-n-acetylgalactosaminide alpha-2,6-sialyltransferase 6  NFKBIB - nuclear factor of kappa light polypeptide gene enhancer in b-cells inhibitor, beta  PIK3R4 - phosphoinositide-3-kinase, regulatory subunit 4  SLK - ste20-like kinase  SNRNP48 - small nuclear ribonucleoprotein 48kda (u11/u12)  VCPIP1 - valosin containing protein (p97)/p47 complex interacting protein 1  NKTR - natural killer-tumor recognition sequence  USP34 - ubiquitin specific peptidase 34  NHP2L1 - nhp2 non-histone chromosome protein 2-like 1 (s. cerevisiae)  HDAC9 - histone deacetylase 9  ZNRD1 - zinc ribbon domain containing 1  SART3 - squamous cell carcinoma antigen recognized by t cells 3  RNF145 - ring finger protein 145  SETD1A - set domain containing 1a  EHD3 - eh-domain containing 3  CCP110 - centriolar coiled coil protein 110kda  SOCS7 - suppressor of cytokine signaling 7  MDC1 - mediator of dna-damage checkpoint 1  TTC37 - tetratricopeptide repeat domain 37  HS2ST1 - heparan sulfate 2-o-sulfotransferase 1  NEK3 - nima-related kinase 3  MINK1 - misshapen-like kinase 1  TTLL4 - tubulin tyrosine ligase-like family, member 4  SOCS5 - suppressor of cytokine signaling 5  RRM2B - ribonucleotide reductase m2 b (tp53 inducible)  NEFL - neurofilament, light polypeptide  SMARCAL1 - swi/snf related, matrix associated, actin dependent regulator of chromatin, subfamily a-like 1  NEK1 - nima-related kinase 1  NEDD8 - neural precursor cell expressed, developmentally down-regulated 8  CTR9 - ctr9, paf1/rna polymerase ii complex component  PPM1F - protein phosphatase, mg2+/mn2+ dependent, 1f  CPEB4 - cytoplasmic polyadenylation element binding protein 4  EPC1 - enhancer of polycomb homolog 1 (drosophila)  NFE2L2 - nuclear factor (erythroid-derived 2)-like 2  MORF4L2 - mortality factor 4 like 2  KLHL15 - kelch-like family member 15  NUP155 - nucleoporin 155kda  NFIA - nuclear factor i/a  NF1 - neurofibromin 1  CHST11 - carbohydrate (chondroitin 4) sulfotransferase 11  CDK5RAP3 - cdk5 regulatory subunit associated protein 3  DCAF8 - ddb1 and cul4 associated factor 8  VPS37D - vacuolar protein sorting 37 homolog d (s. cerevisiae)  EDEM1 - er degradation enhancer, mannosidase alpha-like 1  KIAA0391 - kiaa0391  RAPGEF2 - rap guanine nucleotide exchange factor (gef) 2  EDEM3 - er degradation enhancer, mannosidase alpha-like 3  NUP93 - nucleoporin 93kda  CALY - calcyon neuron-specific vesicular protein  GEMIN4 - gem (nuclear organelle) associated protein 4  KDM4A - lysine (k)-specific demethylase 4a  PHF14 - phd finger protein 14  DRG1 - developmentally regulated gtp binding protein 1  SDC3 - syndecan 3  COPS7A - cop9 signalosome subunit 7a  TARS2 - threonyl-trna synthetase 2, mitochondrial (putative)  DZIP3 - daz interacting zinc finger protein 3  KIAA0430 - kiaa0430 |
| GO:0043412 | macromolecule modification | 2.26E-15 | 2.86E-12 | 1.10 (10334,2281,6996,1697) | [+] Show genes  OTUD5 - otu domain containing 5  MSL3 - male-specific lethal 3 homolog (drosophila)  TADA1 - transcriptional adaptor 1  AARS - alanyl-trna synthetase  ABCA1 - atp-binding cassette, sub-family a (abc1), member 1  OS9 - osteosarcoma amplified 9, endoplasmic reticulum lectin  ABL1 - c-abl oncogene 1, non-receptor tyrosine kinase  GALNT10 - udp-n-acetyl-alpha-d-galactosamine:polypeptide n-acetylgalactosaminyltransferase 10 (galnac-t10)  PML - promyelocytic leukemia  PMM1 - phosphomannomutase 1  RAB40B - rab40b, member ras oncogene family  CKAP4 - cytoskeleton-associated protein 4  ASCC3 - activating signal cointegrator 1 complex subunit 3  FTSJ3 - ftsj homolog 3 (e. coli)  UBE2Q1 - ubiquitin-conjugating enzyme e2q family member 1  BRD8 - bromodomain containing 8  PLOD2 - procollagen-lysine, 2-oxoglutarate 5-dioxygenase 2  MAN1A2 - mannosidase, alpha, class 1a, member 2  LRRC40 - leucine rich repeat containing 40  PLOD1 - procollagen-lysine, 2-oxoglutarate 5-dioxygenase 1  RFFL - ring finger and fyve-like domain containing e3 ubiquitin protein ligase  ACTB - actin, beta  OTUB1 - otu domain, ubiquitin aldehyde binding 1  EHMT2 - euchromatic histone-lysine n-methyltransferase 2  MORF4L1 - mortality factor 4 like 1  ZDHHC7 - zinc finger, dhhc-type containing 7  POMGNT1 - protein o-linked mannose n-acetylglucosaminyltransferase 1 (beta 1,2-)  THUMPD1 - thump domain containing 1  PPIC - peptidylprolyl isomerase c (cyclophilin c)  PPID - peptidylprolyl isomerase d  ACTL6A - actin-like 6a  PPEF1 - protein phosphatase, ef-hand calcium binding domain 1  USP20 - ubiquitin specific peptidase 20  PPIA - peptidylprolyl isomerase a (cyclophilin a)  USP19 - ubiquitin specific peptidase 19  PPIB - peptidylprolyl isomerase b (cyclophilin b)  ACVR2A - activin a receptor, type iia  ACVRL1 - activin a receptor type ii-like 1  ACVR1 - activin a receptor, type i  NDUFAF7 - nadh dehydrogenase (ubiquinone) complex i, assembly factor 7  ACVR1B - activin a receptor, type ib  ADAR - adenosine deaminase, rna-specific  ADAM10 - adam metallopeptidase domain 10  ADARB1 - adenosine deaminase, rna-specific, b1  MED1 - mediator complex subunit 1  ADH5 - alcohol dehydrogenase 5 (class iii), chi polypeptide  PARP1 - poly (adp-ribose) polymerase 1  ADPRH - adp-ribosylarginine hydrolase  NOP10 - nop10 ribonucleoprotein  TNFRSF19 - tumor necrosis factor receptor superfamily, member 19  PARP4 - poly (adp-ribose) polymerase family, member 4  RUVBL2 - ruvb-like 2 (e. coli)  ATE1 - arginyltransferase 1  PRKAB1 - protein kinase, amp-activated, beta 1 non-catalytic subunit  PRKACB - protein kinase, camp-dependent, catalytic, beta  RCOR3 - rest corepressor 3  PRKACA - protein kinase, camp-dependent, catalytic, alpha  NUP133 - nucleoporin 133kda  ADRBK2 - adrenergic, beta, receptor kinase 2  ADRBK1 - adrenergic, beta, receptor kinase 1  NUPL2 - nucleoporin like 2  EDEM2 - er degradation enhancer, mannosidase alpha-like 2  CHFR - checkpoint with forkhead and ring finger domains, e3 ubiquitin protein ligase  PRDM4 - pr domain containing 4  PPP3R1 - protein phosphatase 3, regulatory subunit b, alpha  CIT - citron (rho-interacting, serine/threonine kinase 21)  ATF7IP - activating transcription factor 7 interacting protein  PPP5C - protein phosphatase 5, catalytic subunit  PPP6C - protein phosphatase 6, catalytic subunit  UHRF2 - ubiquitin-like with phd and ring finger domains 2, e3 ubiquitin protein ligase  PPT1 - palmitoyl-protein thioesterase 1  AGA - aspartylglucosaminidase  PPP2R5D - protein phosphatase 2, regulatory subunit b', delta  PPP2R5C - protein phosphatase 2, regulatory subunit b', gamma  PPP2R5B - protein phosphatase 2, regulatory subunit b', beta  AKT1 - v-akt murine thymoma viral oncogene homolog 1  PPP4C - protein phosphatase 4, catalytic subunit  PPP2R5E - protein phosphatase 2, regulatory subunit b', epsilon isoform  PPP2R2A - protein phosphatase 2, regulatory subunit b, alpha  PPP2R1B - protein phosphatase 2, regulatory subunit a, beta  CMTR2 - cap methyltransferase 2  RIOK2 - rio kinase 2  PPP2R1A - protein phosphatase 2, regulatory subunit a, alpha  WWP2 - ww domain containing e3 ubiquitin protein ligase 2  PPP2R2C - protein phosphatase 2, regulatory subunit b, gamma  PPP2R2B - protein phosphatase 2, regulatory subunit b, beta  TRIOBP - trio and f-actin binding protein  PPP2CA - protein phosphatase 2, catalytic subunit, alpha isozyme  PPP2CB - protein phosphatase 2, catalytic subunit, beta isozyme  MBD5 - methyl-cpg binding domain protein 5  DUSP14 - dual specificity phosphatase 14  PPM1G - protein phosphatase, mg2+/mn2+ dependent, 1g  PPP1CC - protein phosphatase 1, catalytic subunit, gamma isozyme  ESCO1 - establishment of sister chromatid cohesion n-acetyltransferase 1  KANSL3 - kat8 regulatory nsl complex subunit 3  TDRKH - tudor and kh domain containing  SCYL2 - scy1-like 2 (s. cerevisiae)  LIAS - lipoic acid synthetase  AMFR - autocrine motility factor receptor, e3 ubiquitin protein ligase  RNF126 - ring finger protein 126  DNAJC3 - dnaj (hsp40) homolog, subfamily c, member 3  MAP2K5 - mitogen-activated protein kinase kinase 5  MAP2K7 - mitogen-activated protein kinase kinase 7  EIF2AK2 - eukaryotic translation initiation factor 2-alpha kinase 2  PIM2 - pim-2 oncogene  GALNT13 - udp-n-acetyl-alpha-d-galactosamine:polypeptide n-acetylgalactosaminyltransferase 13 (galnac-t13)  PIGV - phosphatidylinositol glycan anchor biosynthesis, class v  MAPK13 - mitogen-activated protein kinase 13  RNF157 - ring finger protein 157  MAP2K1 - mitogen-activated protein kinase kinase 1  MAP2K2 - mitogen-activated protein kinase kinase 2  MAPK10 - mitogen-activated protein kinase 10  MAPK9 - mitogen-activated protein kinase 9  TRIM59 - tripartite motif containing 59  MAPK11 - mitogen-activated protein kinase 11  METAP2 - methionyl aminopeptidase 2  MAPK8 - mitogen-activated protein kinase 8  MAPK7 - mitogen-activated protein kinase 7  MAPK3 - mitogen-activated protein kinase 3  MAPK1 - mitogen-activated protein kinase 1  COPS6 - cop9 signalosome subunit 6  PRKCSH - protein kinase c substrate 80k-h  NDC1 - ndc1 transmembrane nucleoporin  PRKD1 - protein kinase d1  PKN2 - protein kinase n2  PRKCH - protein kinase c, eta  PRKCI - protein kinase c, iota  TLK2 - tousled-like kinase 2  PRKCE - protein kinase c, epsilon  PRKCB - protein kinase c, beta  PRKCD - protein kinase c, delta  PRKCA - protein kinase c, alpha  PRKAR1B - protein kinase, camp-dependent, regulatory, type i, beta  NSUN5 - nop2/sun domain family, member 5  YEATS2 - yeats domain containing 2  PRKAG1 - protein kinase, amp-activated, gamma 1 non-catalytic subunit  APEX1 - apex nuclease (multifunctional dna repair enzyme) 1  UBR7 - ubiquitin protein ligase e3 component n-recognin 7 (putative)  DUSP12 - dual specificity phosphatase 12  KLHL2 - kelch-like family member 2  XIAP - x-linked inhibitor of apoptosis  BIRC3 - baculoviral iap repeat containing 3  PARN - poly(a)-specific ribonuclease  BIRC2 - baculoviral iap repeat containing 2  RFWD3 - ring finger and wd repeat domain 3  TREX1 - three prime repair exonuclease 1  APLP2 - amyloid beta (a4) precursor-like protein 2  MGAT4B - mannosyl (alpha-1,3-)-glycoprotein beta-1,4-n-acetylglucosaminyltransferase, isozyme b  PRMT6 - protein arginine methyltransferase 6  MSL2 - male-specific lethal 2 homolog (drosophila)  MAN1B1 - mannosidase, alpha, class 1b, member 1  DCUN1D3 - dcn1, defective in cullin neddylation 1, domain containing 3  APC - adenomatous polyposis coli  RNF220 - ring finger protein 220  APBB1 - amyloid beta (a4) precursor protein-binding, family b, member 1 (fe65)  GALNT6 - udp-n-acetyl-alpha-d-galactosamine:polypeptide n-acetylgalactosaminyltransferase 6 (galnac-t6)  DPY19L4 - dpy-19-like 4 (c. elegans)  RNF139 - ring finger protein 139  P4HA1 - prolyl 4-hydroxylase, alpha polypeptide i  PEBP1 - phosphatidylethanolamine binding protein 1  TRIM23 - tripartite motif containing 23  RPRD1A - regulation of nuclear pre-mrna domain containing 1a  ARF4 - adp-ribosylation factor 4  IRAK3 - interleukin-1 receptor-associated kinase 3  AKAP13 - a kinase (prka) anchor protein 13  SETD5 - set domain containing 5  APP - amyloid beta (a4) precursor protein  PAK2 - p21 protein (cdc42/rac)-activated kinase 2  PAK3 - p21 protein (cdc42/rac)-activated kinase 3  LEPREL1 - leprecan-like 1  PAM - peptidylglycine alpha-amidating monooxygenase  PDGFA - platelet-derived growth factor alpha polypeptide  MAP4K5 - mitogen-activated protein kinase kinase kinase kinase 5  NSMCE2 - non-smc element 2, mms21 homolog (s. cerevisiae)  PDGFRB - platelet-derived growth factor receptor, beta polypeptide  ARRB2 - arrestin, beta 2  PDGFRA - platelet-derived growth factor receptor, alpha polypeptide  ARRB1 - arrestin, beta 1  PDGFB - platelet-derived growth factor beta polypeptide  PDPK1 - 3-phosphoinositide dependent protein kinase-1  WDYHV1 - wdyhv motif containing 1  RHOA - ras homolog family member a  PDK3 - pyruvate dehydrogenase kinase, isozyme 3  FSTL1 - follistatin-like 1  BAZ2A - bromodomain adjacent to zinc finger domain, 2a  PCNA - proliferating cell nuclear antigen  FICD - fic domain containing  PCMT1 - protein-l-isoaspartate (d-aspartate) o-methyltransferase  MTFMT - mitochondrial methionyl-trna formyltransferase  CDK16 - cyclin-dependent kinase 16  CDK17 - cyclin-dependent kinase 17  ASPH - aspartate beta-hydroxylase  LEO1 - leo1, paf1/rna polymerase ii complex component, homolog (s. cerevisiae)  FANCL - fanconi anemia, complementation group l  PWP1 - pwp1 homolog (s. cerevisiae)  SOCS4 - suppressor of cytokine signaling 4  KAT7 - k(lysine) acetyltransferase 7  ELP3 - elongator acetyltransferase complex subunit 3  PGGT1B - protein geranylgeranyltransferase type i, beta subunit  CPPED1 - calcineurin-like phosphoesterase domain containing 1  PGM3 - phosphoglucomutase 3  UBOX5 - u-box domain containing 5  RNF180 - ring finger protein 180  UEVLD - uev and lactate/malate dehyrogenase domains  FBXW7 - f-box and wd repeat domain containing 7, e3 ubiquitin protein ligase  RNF121 - ring finger protein 121  PHF2 - phd finger protein 2  LRRK2 - leucine-rich repeat kinase 2  DNAJC24 - dnaj (hsp40) homolog, subfamily c, member 24  PEX12 - peroxisomal biogenesis factor 12  SLC35C1 - solute carrier family 35 (gdp-fucose transporter), member c1  PEX13 - peroxisomal biogenesis factor 13  PEX14 - peroxisomal biogenesis factor 14  ATP7A - atpase, cu++ transporting, alpha polypeptide  PENK - proenkephalin  LMTK2 - lemur tyrosine kinase 2  ICK - intestinal cell (mak-like) kinase  ALKBH2 - alkb, alkylation repair homolog 2 (e. coli)  PPM1E - protein phosphatase, mg2+/mn2+ dependent, 1e  AAK1 - ap2 associated kinase 1  COPRS - coordinator of prmt5, differentiation stimulator  PKD1 - polycystic kidney disease 1 (autosomal dominant)  UBA6 - ubiquitin-like modifier activating enzyme 6  USP40 - ubiquitin specific peptidase 40  RASA3 - ras p21 protein activator 3  NAT10 - n-acetyltransferase 10 (gcn5-related)  PIN1 - peptidylprolyl cis/trans isomerase, nima-interacting 1  DPH3 - diphthamide biosynthesis 3  PIK3R1 - phosphoinositide-3-kinase, regulatory subunit 1 (alpha)  PLAT - plasminogen activator, tissue  TYW1 - trna-yw synthesizing protein 1 homolog (s. cerevisiae)  ASXL2 - additional sex combs like 2 (drosophila)  AXL - axl receptor tyrosine kinase  PCMTD2 - protein-l-isoaspartate (d-aspartate) o-methyltransferase domain containing 2  ELP2 - elongator acetyltransferase complex subunit 2  PEF1 - penta-ef-hand domain containing 1  ATRX - alpha thalassemia/mental retardation syndrome x-linked  OGFOD1 - 2-oxoglutarate and iron-dependent oxygenase domain containing 1  RNF13 - ring finger protein 13  PHF10 - phd finger protein 10  BCR - breakpoint cluster region  FBXW12 - f-box and wd repeat domain containing 12  PHKG2 - phosphorylase kinase, gamma 2 (testis)  FKBP9 - fk506 binding protein 9, 63 kda  STK38 - serine/threonine kinase 38  MRGBP - mrg/morf4l binding protein  SUMF1 - sulfatase modifying factor 1  RHOT1 - ras homolog family member t1  PIK3CA - phosphatidylinositol-4,5-bisphosphate 3-kinase, catalytic subunit alpha  RPUSD3 - rna pseudouridylate synthase domain containing 3  PIM1 - pim-1 oncogene  UBE2W - ubiquitin-conjugating enzyme e2w (putative)  BCL2 - b-cell cll/lymphoma 2  PIK3C3 - phosphatidylinositol 3-kinase, catalytic subunit type 3  MGAT4A - mannosyl (alpha-1,3-)-glycoprotein beta-1,4-n-acetylglucosaminyltransferase, isozyme a  PIGH - phosphatidylinositol glycan anchor biosynthesis, class h  LYPLA2 - lysophospholipase ii  CCND1 - cyclin d1  PARK7 - parkinson protein 7  B4GALT7 - xylosylprotein beta 1,4-galactosyltransferase, polypeptide 7  PIGF - phosphatidylinositol glycan anchor biosynthesis, class f  TRIM27 - tripartite motif containing 27  CDKAL1 - cdk5 regulatory subunit associated protein 1-like 1  CWC27 - cwc27 spliceosome-associated protein homolog (s. cerevisiae)  BCKDK - branched chain ketoacid dehydrogenase kinase  BRAF - v-raf murine sarcoma viral oncogene homolog b  WHSC1L1 - wolf-hirschhorn syndrome candidate 1-like 1  CDKL4 - cyclin-dependent kinase-like 4  BCOR - bcl6 corepressor  ZFP36L2 - zfp36 ring finger protein-like 2  DCAF16 - ddb1 and cul4 associated factor 16  PAK4 - p21 protein (cdc42/rac)-activated kinase 4  ZFP36L1 - zfp36 ring finger protein-like 1  RET - ret proto-oncogene  SH3RF3 - sh3 domain containing ring finger 3  DPF2 - d4, zinc and double phd fingers family 2  DUSP15 - dual specificity phosphatase 15  ALKBH5 - alkb, alkylation repair homolog 5 (e. coli)  NSUN2 - nop2/sun rna methyltransferase family, member 2  GALNTL6 - udp-n-acetyl-alpha-d-galactosamine:polypeptide n-acetylgalactosaminyltransferase-like 6  RLF - rearranged l-myc fusion  ELP6 - elongator acetyltransferase complex subunit 6  RING1 - ring finger protein 1  SNRK - snf related kinase  PIGG - phosphatidylinositol glycan anchor biosynthesis, class g  TMEM5 - transmembrane protein 5  FBLL1 - fibrillarin-like 1  BMI1 - bmi1 polycomb ring finger oncogene  PCGF3 - polycomb group ring finger 3  BMPR1A - bone morphogenetic protein receptor, type ia  BMPR2 - bone morphogenetic protein receptor, type ii (serine/threonine kinase)  BMPR1B - bone morphogenetic protein receptor, type ib  FBXL12 - f-box and leucine-rich repeat protein 12  ABCA7 - atp-binding cassette, sub-family a (abc1), member 7  PSMD14 - proteasome (prosome, macropain) 26s subunit, non-atpase, 14  TRPM7 - transient receptor potential cation channel, subfamily m, member 7  RNF19B - ring finger protein 19b  CTDSPL - ctd (carboxy-terminal domain, rna polymerase ii, polypeptide a) small phosphatase-like  TMEM258 - transmembrane protein 258  TRIT1 - trna isopentenyltransferase 1  UHMK1 - u2af homology motif (uhm) kinase 1  MSLN - mesothelin  RNF4 - ring finger protein 4  DCAF7 - ddb1 and cul4 associated factor 7  RNF2 - ring finger protein 2  GPHN - gephyrin  RNF6 - ring finger protein (c3h2c3 type) 6  GATAD2A - gata zinc finger domain containing 2a  QPCTL - glutaminyl-peptide cyclotransferase-like  ROCK1 - rho-associated, coiled-coil containing protein kinase 1  LYPLAL1 - lysophospholipase-like 1  RNF111 - ring finger protein 111  TET2 - tet methylcytosine dioxygenase 2  STAM2 - signal transducing adaptor molecule (sh3 domain and itam motif) 2  TRPM4 - transient receptor potential cation channel, subfamily m, member 4  TYW3 - trna-yw synthesizing protein 3 homolog (s. cerevisiae)  ZMPSTE24 - zinc metallopeptidase ste24  STUB1 - stip1 homology and u-box containing protein 1, e3 ubiquitin protein ligase  UBE4B - ubiquitination factor e4b  TRIM44 - tripartite motif containing 44  TAB1 - tgf-beta activated kinase 1/map3k7 binding protein 1  PPIE - peptidylprolyl isomerase e (cyclophilin e)  MCRS1 - microspherule protein 1  CAMK2B - calcium/calmodulin-dependent protein kinase ii beta  TRMT12 - trna methyltransferase 12 homolog (s. cerevisiae)  FKBP14 - fk506 binding protein 14, 22 kda  PIBF1 - progesterone immunomodulatory binding factor 1  PPIH - peptidylprolyl isomerase h (cyclophilin h)  B3GALT6 - udp-gal:betagal beta 1,3-galactosyltransferase polypeptide 6  CAD - carbamoyl-phosphate synthetase 2, aspartate transcarbamylase, and dihydroorotase  RPN2 - ribophorin ii  RPN1 - ribophorin i  UBE2E3 - ubiquitin-conjugating enzyme e2e 3  HERC6 - hect and rld domain containing e3 ubiquitin protein ligase family member 6  DDR1 - discoidin domain receptor tyrosine kinase 1  TRIM38 - tripartite motif containing 38  TADA3 - transcriptional adaptor 3  CALU - calumenin  STK25 - serine/threonine kinase 25  LRRC41 - leucine rich repeat containing 41  CRTAP - cartilage associated protein  INO80C - ino80 complex subunit c  CCNT1 - cyclin t1  THG1L - trna-histidine guanylyltransferase 1-like (s. cerevisiae)  CCNH - cyclin h  TMEM132A - transmembrane protein 132a  SSH3 - slingshot protein phosphatase 3  ANAPC10 - anaphase promoting complex subunit 10  RPS6KA2 - ribosomal protein s6 kinase, 90kda, polypeptide 2  NRBP2 - nuclear receptor binding protein 2  RPS6KA1 - ribosomal protein s6 kinase, 90kda, polypeptide 1  PARP16 - poly (adp-ribose) polymerase family, member 16  RPS6KB1 - ribosomal protein s6 kinase, 70kda, polypeptide 1  RPS6KB2 - ribosomal protein s6 kinase, 70kda, polypeptide 2  RNF125 - ring finger protein 125, e3 ubiquitin protein ligase  CBFB - core-binding factor, beta subunit  CBL - cbl proto-oncogene, e3 ubiquitin protein ligase  PIAS3 - protein inhibitor of activated stat, 3  KANSL2 - kat8 regulatory nsl complex subunit 2  UBE2R2 - ubiquitin-conjugating enzyme e2r 2  RBM14 - rna binding motif protein 14  TRMT10C - trna methyltransferase 10 homolog c (s. cerevisiae)  PRMT5 - protein arginine methyltransferase 5  TRIM3 - tripartite motif containing 3  CDK11B - cyclin-dependent kinase 11b  TGOLN2 - trans-golgi network protein 2  STAMBP - stam binding protein  CDH2 - cadherin 2, type 1, n-cadherin (neuronal)  CDC27 - cell division cycle 27  ANKIB1 - ankyrin repeat and ibr domain containing 1  POMT1 - protein-o-mannosyltransferase 1  PSEN1 - presenilin 1  USP16 - ubiquitin specific peptidase 16  PSKH1 - protein serine kinase h1  PSMA1 - proteasome (prosome, macropain) subunit, alpha type, 1  RNF216 - ring finger protein 216  PSMA2 - proteasome (prosome, macropain) subunit, alpha type, 2  CAMKK2 - calcium/calmodulin-dependent protein kinase kinase 2, beta  PSMB1 - proteasome (prosome, macropain) subunit, beta type, 1  PSMA5 - proteasome (prosome, macropain) subunit, alpha type, 5  NPRL2 - nitrogen permease regulator-like 2 (s. cerevisiae)  PSMA3 - proteasome (prosome, macropain) subunit, alpha type, 3  PSMB10 - proteasome (prosome, macropain) subunit, beta type, 10  ADAT2 - adenosine deaminase, trna-specific 2  UBLCP1 - ubiquitin-like domain containing ctd phosphatase 1  TAF6L - taf6-like rna polymerase ii, p300/cbp-associated factor (pcaf)-associated factor, 65kda  PSMC6 - proteasome (prosome, macropain) 26s subunit, atpase, 6  FAM173B - family with sequence similarity 173, member b  PSMD1 - proteasome (prosome, macropain) 26s subunit, non-atpase, 1  PSMC4 - proteasome (prosome, macropain) 26s subunit, atpase, 4  PSMC2 - proteasome (prosome, macropain) 26s subunit, atpase, 2  DPM3 - dolichyl-phosphate mannosyltransferase polypeptide 3  PSMD9 - proteasome (prosome, macropain) 26s subunit, non-atpase, 9  PSMD10 - proteasome (prosome, macropain) 26s subunit, non-atpase, 10  GAR1 - gar1 ribonucleoprotein  PSMD7 - proteasome (prosome, macropain) 26s subunit, non-atpase, 7  PSMD8 - proteasome (prosome, macropain) 26s subunit, non-atpase, 8  PSMD5 - proteasome (prosome, macropain) 26s subunit, non-atpase, 5  PSMD2 - proteasome (prosome, macropain) 26s subunit, non-atpase, 2  PSMD12 - proteasome (prosome, macropain) 26s subunit, non-atpase, 12  PSMD13 - proteasome (prosome, macropain) 26s subunit, non-atpase, 13  ATG7 - autophagy related 7  LEPREL2 - leprecan-like 2  KLHL17 - kelch-like family member 17  PTEN - phosphatase and tensin homolog  SETD4 - set domain containing 4  PRDX4 - peroxiredoxin 4  CFL1 - cofilin 1 (non-muscle)  KAT5 - k(lysine) acetyltransferase 5  PTK2 - protein tyrosine kinase 2  PTK7 - protein tyrosine kinase 7  CDK5 - cyclin-dependent kinase 5  CDK4 - cyclin-dependent kinase 4  TWF1 - twinfilin actin-binding protein 1  CDK9 - cyclin-dependent kinase 9  CDK8 - cyclin-dependent kinase 8  CDK7 - cyclin-dependent kinase 7  SPOPL - speckle-type poz protein-like  PTPN3 - protein tyrosine phosphatase, non-receptor type 3  PTPN2 - protein tyrosine phosphatase, non-receptor type 2  PTPN1 - protein tyrosine phosphatase, non-receptor type 1  PTPN9 - protein tyrosine phosphatase, non-receptor type 9  HTATIP2 - hiv-1 tat interactive protein 2, 30kda  PTPN11 - protein tyrosine phosphatase, non-receptor type 11  PPM1M - protein phosphatase, mg2+/mn2+ dependent, 1m  PTPRA - protein tyrosine phosphatase, receptor type, a  PTPN12 - protein tyrosine phosphatase, non-receptor type 12  PTPN13 - protein tyrosine phosphatase, non-receptor type 13 (apo-1/cd95 (fas)-associated phosphatase)  PTPRD - protein tyrosine phosphatase, receptor type, d  PTPRB - protein tyrosine phosphatase, receptor type, b  SLC35A1 - solute carrier family 35 (cmp-sialic acid transporter), member a1  PTPRG - protein tyrosine phosphatase, receptor type, g  CDK2 - cyclin-dependent kinase 2  PTPRE - protein tyrosine phosphatase, receptor type, e  PTPRF - protein tyrosine phosphatase, receptor type, f  WDR4 - wd repeat domain 4  PDP1 - pyruvate dehyrogenase phosphatase catalytic subunit 1  METTL6 - methyltransferase like 6  P4HTM - prolyl 4-hydroxylase, transmembrane (endoplasmic reticulum)  NUDT16 - nudix (nucleoside diphosphate linked moiety x)-type motif 16  PTPRR - protein tyrosine phosphatase, receptor type, r  PTPRS - protein tyrosine phosphatase, receptor type, s  PTPRK - protein tyrosine phosphatase, receptor type, k  PTPRM - protein tyrosine phosphatase, receptor type, m  PTPRN - protein tyrosine phosphatase, receptor type, n  PLK2 - polo-like kinase 2  CHML - choroideremia-like (rab escort protein 2)  NUP50 - nucleoporin 50kda  PEX2 - peroxisomal biogenesis factor 2  CHM - choroideremia (rab escort protein 1)  CHCHD4 - coiled-coil-helix-coiled-coil-helix domain containing 4  KDM5B - lysine (k)-specific demethylase 5b  CHGB - chromogranin b (secretogranin 1)  MXRA8 - matrix-remodelling associated 8  RAB6A - rab6a, member ras oncogene family  MAP4K2 - mitogen-activated protein kinase kinase kinase kinase 2  RAB5A - rab5a, member ras oncogene family  RAB3A - rab3a, member ras oncogene family  FRS2 - fibroblast growth factor receptor substrate 2  RAB3B - rab3b, member ras oncogene family  MTERFD2 - mterf domain containing 2  CHD1 - chromodomain helicase dna binding protein 1  RAB2A - rab2a, member ras oncogene family  RAB1A - rab1a, member ras oncogene family  METTL2A - methyltransferase like 2a  PARP14 - poly (adp-ribose) polymerase family, member 14  PAF1 - paf1, rna polymerase ii associated factor, homolog (s. cerevisiae)  MSL1 - male-specific lethal 1 homolog (drosophila)  FBXL19 - f-box and leucine-rich repeat protein 19  INO80 - ino80 complex subunit  RC3H2 - ring finger and ccch-type domains 2  RANBP2 - ran binding protein 2  RANGAP1 - ran gtpase activating protein 1  MIER2 - mesoderm induction early response 1, family member 2  MOK - mok protein kinase  USP53 - ubiquitin specific peptidase 53  B3GNT2 - udp-glcnac:betagal beta-1,3-n-acetylglucosaminyltransferase 2  RRAGA - ras-related gtp binding a  RASA1 - ras p21 protein activator (gtpase activating protein) 1  CLK1 - cdc-like kinase 1  MAGEL2 - mage-like 2  CLK2 - cdc-like kinase 2  ING3 - inhibitor of growth family, member 3  CLK3 - cdc-like kinase 3  CTCF - ccctc-binding factor (zinc finger protein)  PRMT7 - protein arginine methyltransferase 7  RBBP7 - retinoblastoma binding protein 7  TMX3 - thioredoxin-related transmembrane protein 3  MAP3K2 - mitogen-activated protein kinase kinase kinase 2  ARID4A - at rich interactive domain 4a (rbp1-like)  RBBP5 - retinoblastoma binding protein 5  TYW5 - trna-yw synthesizing protein 5  RBBP6 - retinoblastoma binding protein 6  KDM5A - lysine (k)-specific demethylase 5a  NUP35 - nucleoporin 35kda  ERCC8 - excision repair cross-complementing rodent repair deficiency, complementation group 8  PUS7 - pseudouridylate synthase 7 homolog (s. cerevisiae)  MGEA5 - meningioma expressed antigen 5 (hyaluronidase)  RCN1 - reticulocalbin 1, ef-hand calcium binding domain  EXOSC4 - exosome component 4  ANKRD16 - ankyrin repeat domain 16  PIGU - phosphatidylinositol glycan anchor biosynthesis, class u  USP39 - ubiquitin specific peptidase 39  CHUK - conserved helix-loop-helix ubiquitous kinase  ZDHHC13 - zinc finger, dhhc-type containing 13  SPTAN1 - spectrin, alpha, non-erythrocytic 1  MKRN1 - makorin ring finger protein 1  MKRN2 - makorin ring finger protein 2  TMTC4 - transmembrane and tetratricopeptide repeat containing 4  SPTBN4 - spectrin, beta, non-erythrocytic 4  FBXO18 - f-box protein, helicase, 18  METTL14 - methyltransferase like 14  SPP1 - secreted phosphoprotein 1  ANO8 - anoctamin 8  ALG10 - alg10, alpha-1,2-glucosyltransferase  PPP1R15B - protein phosphatase 1, regulatory subunit 15b  MIER1 - mesoderm induction early response 1, transcriptional regulator  ABHD13 - abhydrolase domain containing 13  METTL12 - methyltransferase like 12  CPE - carboxypeptidase e  SOS1 - son of sevenless homolog 1 (drosophila)  FBXL20 - f-box and leucine-rich repeat protein 20  RAB40C - rab40c, member ras oncogene family  CREBBP - creb binding protein  ATF2 - activating transcription factor 2  CREB1 - camp responsive element binding protein 1  DDX58 - dead (asp-glu-ala-asp) box polypeptide 58  SMUG1 - single-strand-selective monofunctional uracil-dna glycosylase 1  MARK4 - map/microtubule affinity-regulating kinase 4  RPRD1B - regulation of nuclear pre-mrna domain containing 1b  PLK3 - polo-like kinase 3  SNRPB - small nuclear ribonucleoprotein polypeptides b and b1  SUMO2 - small ubiquitin-like modifier 2  SUMO3 - small ubiquitin-like modifier 3  HLTF - helicase-like transcription factor  SGK3 - serum/glucocorticoid regulated kinase family, member 3  KMT2C - lysine (k)-specific methyltransferase 2c  PRKD3 - protein kinase d3  RAB38 - rab38, member ras oncogene family  PPIL4 - peptidylprolyl isomerase (cyclophilin)-like 4  OSTC - oligosaccharyltransferase complex subunit (non-catalytic)  SENP2 - sumo1/sentrin/smt3 specific peptidase 2  CST3 - cystatin c  FTSJ1 - ftsj rna methyltransferase homolog 1 (e. coli)  NAT6 - n-acetyltransferase 6 (gcn5-related)  USP32 - ubiquitin specific peptidase 32  SHC2 - shc (src homology 2 domain containing) transforming protein 2  KLHL12 - kelch-like family member 12  KDM2B - lysine (k)-specific demethylase 2b  DSTYK - dual serine/threonine and tyrosine protein kinase  CTBP1 - c-terminal binding protein 1  GTPBP3 - gtp binding protein 3 (mitochondrial)  RASGRP3 - ras guanyl releasing protein 3 (calcium and dag-regulated)  UBL5 - ubiquitin-like 5  CTNNB1 - catenin (cadherin-associated protein), beta 1, 88kda  PPIL2 - peptidylprolyl isomerase (cyclophilin)-like 2  PIGO - phosphatidylinositol glycan anchor biosynthesis, class o  TRMT11 - trna methyltransferase 11 homolog (s. cerevisiae)  STX1A - syntaxin 1a (brain)  LNX1 - ligand of numb-protein x 1, e3 ubiquitin protein ligase  USP30 - ubiquitin specific peptidase 30  FUT10 - fucosyltransferase 10 (alpha (1,3) fucosyltransferase)  NEK4 - nima-related kinase 4  STK3 - serine/threonine kinase 3  BRD1 - bromodomain containing 1  PCGF1 - polycomb group ring finger 1  FKBP8 - fk506 binding protein 8, 38kda  AURKC - aurora kinase c  STK10 - serine/threonine kinase 10  PAN3 - pan3 poly(a) specific ribonuclease subunit homolog (s. cerevisiae)  CYLD - cylindromatosis (turban tumor syndrome)  SUV420H2 - suppressor of variegation 4-20 homolog 2 (drosophila)  ISPD - isoprenoid synthase domain containing  CDKL5 - cyclin-dependent kinase-like 5  STK4 - serine/threonine kinase 4  CRKL - v-crk avian sarcoma virus ct10 oncogene homolog-like  HECTD1 - hect domain containing e3 ubiquitin protein ligase 1  FKBP10 - fk506 binding protein 10, 65 kda  CRY1 - cryptochrome 1 (photolyase-like)  CRY2 - cryptochrome 2 (photolyase-like)  ANAPC13 - anaphase promoting complex subunit 13  AGBL5 - atp/gtp binding protein-like 5  DCAF12 - ddb1 and cul4 associated factor 12  BRMS1 - breast cancer metastasis suppressor 1  NAA35 - n(alpha)-acetyltransferase 35, natc auxiliary subunit  SUMF2 - sulfatase modifying factor 2  CSF1R - colony stimulating factor 1 receptor  CSF1 - colony stimulating factor 1 (macrophage)  FBXO7 - f-box protein 7  SRPK2 - srsf protein kinase 2  QPCT - glutaminyl-peptide cyclotransferase  SRPK1 - srsf protein kinase 1  TAB3 - tgf-beta activated kinase 1/map3k7 binding protein 3  KLHL22 - kelch-like family member 22  GALNT11 - udp-n-acetyl-alpha-d-galactosamine:polypeptide n-acetylgalactosaminyltransferase 11 (galnac-t11)  PTPN5 - protein tyrosine phosphatase, non-receptor type 5 (striatum-enriched)  ZNF335 - zinc finger protein 335  CSK - c-src tyrosine kinase  SSB - sjogren syndrome antigen b (autoantigen la)  CSNK1G2 - casein kinase 1, gamma 2  TTLL1 - tubulin tyrosine ligase-like family, member 1  CSNK1A1 - casein kinase 1, alpha 1  SPTBN1 - spectrin, beta, non-erythrocytic 1  CSNK1D - casein kinase 1, delta  CSNK2A2 - casein kinase 2, alpha prime polypeptide  RNF214 - ring finger protein 214  SRC - v-src avian sarcoma (schmidt-ruppin a-2) viral oncogene homolog  CSNK1G3 - casein kinase 1, gamma 3  CSNK2A1 - casein kinase 2, alpha 1 polypeptide  VIPAS39 - vps33b interacting protein, apical-basolateral polarity regulator, spe-39 homolog  ZDHHC12 - zinc finger, dhhc-type containing 12  ARIH1 - ariadne homolog, ubiquitin-conjugating enzyme e2 binding protein, 1 (drosophila)  THADA - thyroid adenoma associated  UBE2O - ubiquitin-conjugating enzyme e2o  MTO1 - mitochondrial trna translation optimization 1  NSUN3 - nop2/sun domain family, member 3  FBXL2 - f-box and leucine-rich repeat protein 2  SHPRH - snf2 histone linker phd ring helicase, e3 ubiquitin protein ligase  RNF169 - ring finger protein 169  SEL1L - sel-1 suppressor of lin-12-like (c. elegans)  FBXO33 - f-box protein 33  DDOST - dolichyl-diphosphooligosaccharide--protein glycosyltransferase subunit (non-catalytic)  DPH6 - diphthamine biosynthesis 6  SIN3A - sin3 transcription regulator family member a  SDF2 - stromal cell-derived factor 2  TPGS2 - tubulin polyglutamylase complex subunit 2  FAM98A - family with sequence similarity 98, member a  RSPRY1 - ring finger and spry domain containing 1  WWTR1 - ww domain containing transcription regulator 1  SETMAR - set domain and mariner transposase fusion gene  DDB1 - damage-specific dna binding protein 1, 127kda  MAP2K4 - mitogen-activated protein kinase kinase 4  PTPN23 - protein tyrosine phosphatase, non-receptor type 23  SENP7 - sumo1/sentrin specific peptidase 7  ZDHHC24 - zinc finger, dhhc-type containing 24  KBTBD6 - kelch repeat and btb (poz) domain containing 6  CSRP2BP - csrp2 binding protein  ZDHHC5 - zinc finger, dhhc-type containing 5  THUMPD3 - thump domain containing 3  SCYL1 - scy1-like 1 (s. cerevisiae)  CLK4 - cdc-like kinase 4  KLHL13 - kelch-like family member 13  RNF19A - ring finger protein 19a, e3 ubiquitin protein ligase  RCHY1 - ring finger and chy zinc finger domain containing 1, e3 ubiquitin protein ligase  ASPHD1 - aspartate beta-hydroxylase domain containing 1  PPM1H - protein phosphatase, mg2+/mn2+ dependent, 1h  GALNT16 - udp-n-acetyl-alpha-d-galactosamine:polypeptide n-acetylgalactosaminyltransferase 16  KCTD13 - potassium channel tetramerization domain containing 13  DCAF13 - ddb1 and cul4 associated factor 13  BIRC6 - baculoviral iap repeat containing 6  BTBD6 - btb (poz) domain containing 6  ZDHHC20 - zinc finger, dhhc-type containing 20  LTN1 - listerin e3 ubiquitin protein ligase 1  ZNF451 - zinc finger protein 451  UBXN7 - ubx domain protein 7  CHD5 - chromodomain helicase dna binding protein 5  MCFD2 - multiple coagulation factor deficiency 2  MED30 - mediator complex subunit 30  RIMKLB - ribosomal modification protein rimk-like family member b  USP31 - ubiquitin specific peptidase 31  DCAF15 - ddb1 and cul4 associated factor 15  DAPK3 - death-associated protein kinase 3  ZZZ3 - zinc finger, zz-type containing 3  LRSAM1 - leucine rich repeat and sterile alpha motif containing 1  ULK3 - unc-51 like kinase 3  AARS2 - alanyl-trna synthetase 2, mitochondrial  DGKQ - diacylglycerol kinase, theta 110kda  MTA3 - metastasis associated 1 family, member 3  CLIP3 - cap-gly domain containing linker protein 3  DAPK1 - death-associated protein kinase 1  TIPARP - tcdd-inducible poly(adp-ribose) polymerase  HACE1 - hect domain and ankyrin repeat containing e3 ubiquitin protein ligase 1  SATB1 - satb homeobox 1  MIB1 - mindbomb e3 ubiquitin protein ligase 1  SBF1 - set binding factor 1  DAD1 - defender against cell death 1  TRPC4AP - transient receptor potential cation channel, subfamily c, member 4 associated protein  KLHL42 - kelch-like family member 42  EPC2 - enhancer of polycomb homolog 2 (drosophila)  CDC26 - cell division cycle 26  IRF2BP1 - interferon regulatory factor 2 binding protein 1  TAOK1 - tao kinase 1  PDP2 - pyruvate dehyrogenase phosphatase catalytic subunit 2  KLHL8 - kelch-like family member 8  DPH2 - dph2 homolog (s. cerevisiae)  SHANK3 - sh3 and multiple ankyrin repeat domains 3  DPH1 - diphthamide biosynthesis 1  WIPI2 - wd repeat domain, phosphoinositide interacting 2  DPAGT1 - dolichyl-phosphate (udp-n-acetylglucosamine) n-acetylglucosaminephosphotransferase 1 (glcnac-1-p transferase)  USP35 - ubiquitin specific peptidase 35  DR1 - down-regulator of transcription 1, tbp-binding (negative cofactor 2)  WSB1 - wd repeat and socs box containing 1  TRMT5 - trna methyltransferase 5  ALG2 - alg2, alpha-1,3/1,6-mannosyltransferase  ZNRF2 - zinc and ring finger 2  DUSP8 - dual specificity phosphatase 8  MAPK15 - mitogen-activated protein kinase 15  DUSP5 - dual specificity phosphatase 5  DUSP6 - dual specificity phosphatase 6  SKP1 - s-phase kinase-associated protein 1  DUSP7 - dual specificity phosphatase 7  DYRK1A - dual-specificity tyrosine-(y)-phosphorylation regulated kinase 1a  TOR1A - torsin family 1, member a (torsin a)  ABHD12 - abhydrolase domain containing 12  HERC4 - hect and rld domain containing e3 ubiquitin protein ligase 4  WDR48 - wd repeat domain 48  USP36 - ubiquitin specific peptidase 36  FNIP2 - folliculin interacting protein 2  SENP6 - sumo1/sentrin specific peptidase 6  HBEGF - heparin-binding egf-like growth factor  DTX1 - deltex homolog 1 (drosophila)  DKC1 - dyskeratosis congenita 1, dyskerin  DLG3 - discs, large homolog 3 (drosophila)  DLG4 - discs, large homolog 4 (drosophila)  DLG1 - discs, large homolog 1 (drosophila)  FBXW8 - f-box and wd repeat domain containing 8  NEIL2 - nei endonuclease viii-like 2 (e. coli)  SH3RF1 - sh3 domain containing ring finger 1  FBXO25 - f-box protein 25  ST6GAL1 - st6 beta-galactosamide alpha-2,6-sialyltranferase 1  SIAH2 - siah e3 ubiquitin protein ligase 2  SIAH1 - siah e3 ubiquitin protein ligase 1  DGUOK - deoxyguanosine kinase  FBXL3 - f-box and leucine-rich repeat protein 3  EP400 - e1a binding protein p400  DHPS - deoxyhypusine synthase  ST3GAL2 - st3 beta-galactoside alpha-2,3-sialyltransferase 2  B3GAT3 - beta-1,3-glucuronyltransferase 3 (glucuronosyltransferase i)  FBXL5 - f-box and leucine-rich repeat protein 5  RICTOR - rptor independent companion of mtor, complex 2  FBXO2 - f-box protein 2  UVSSA - uv-stimulated scaffold protein a  SFPQ - splicing factor proline/glutamine-rich  PELI3 - pellino e3 ubiquitin protein ligase family member 3  FBXW2 - f-box and wd repeat domain containing 2  DNMT1 - dna (cytosine-5-)-methyltransferase 1  TRDMT1 - trna aspartic acid methyltransferase 1  DNMT3A - dna (cytosine-5-)-methyltransferase 3 alpha  EIF2AK4 - eukaryotic translation initiation factor 2 alpha kinase 4  NAT9 - n-acetyltransferase 9 (gcn5-related, putative)  ALG11 - alg11, alpha-1,2-mannosyltransferase  SGK1 - serum/glucocorticoid regulated kinase 1  USP37 - ubiquitin specific peptidase 37  SENP3 - sumo1/sentrin/smt3 specific peptidase 3  SMARCAD1 - swi/snf-related, matrix-associated actin-dependent regulator of chromatin, subfamily a, containing dead/h box 1  MARK2 - map/microtubule affinity-regulating kinase 2  NAF1 - nuclear assembly factor 1 ribonucleoprotein  C1GALT1 - core 1 synthase, glycoprotein-n-acetylgalactosamine 3-beta-galactosyltransferase, 1  ELK4 - elk4, ets-domain protein (srf accessory protein 1)  EPAS1 - endothelial pas domain protein 1  EP300 - e1a binding protein p300  DPH7 - diphthamide biosynthesis 7  SEPHS1 - selenophosphate synthetase 1  TIMM50 - translocase of inner mitochondrial membrane 50 homolog (s. cerevisiae)  UGGT1 - udp-glucose glycoprotein glucosyltransferase 1  EGR2 - early growth response 2  TTC3 - tetratricopeptide repeat domain 3  EIF2S1 - eukaryotic translation initiation factor 2, subunit 1 alpha, 35kda  TRIM32 - tripartite motif containing 32  SPHK2 - sphingosine kinase 2  KDM2A - lysine (k)-specific demethylase 2a  TMEM129 - transmembrane protein 129  METTL18 - methyltransferase like 18  TSG101 - tumor susceptibility gene 101  MAST1 - microtubule associated serine/threonine kinase 1  FBXO21 - f-box protein 21  PGAP3 - post-gpi attachment to proteins 3  RNF149 - ring finger protein 149  STK38L - serine/threonine kinase 38 like  PIGM - phosphatidylinositol glycan anchor biosynthesis, class m  TULP4 - tubby like protein 4  PHC1 - polyhomeotic homolog 1 (drosophila)  POGLUT1 - protein o-glucosyltransferase 1  TRMT10A - trna methyltransferase 10 homolog a (s. cerevisiae)  EGFR - epidermal growth factor receptor  PHLPP2 - ph domain and leucine rich repeat protein phosphatase 2  USP33 - ubiquitin specific peptidase 33  MAST3 - microtubule associated serine/threonine kinase 3  TOP1 - topoisomerase (dna) i  FAM20C - family with sequence similarity 20, member c  KDM4B - lysine (k)-specific demethylase 4b  GOLGA7B - golgin a7 family, member b  KDM1A - lysine (k)-specific demethylase 1a  ATXN7L3 - ataxin 7-like 3  PARP6 - poly (adp-ribose) polymerase family, member 6  EOGT - egf domain-specific o-linked n-acetylglucosamine (glcnac) transferase  UBE2Q2 - ubiquitin-conjugating enzyme e2q family member 2  SMG1 - smg1 phosphatidylinositol 3-kinase-related kinase  TRIO - trio rho guanine nucleotide exchange factor  E4F1 - e4f transcription factor 1  TNIK - traf2 and nck interacting kinase  ENY2 - enhancer of yellow 2 homolog (drosophila)  MYCBP2 - myc binding protein 2, e3 ubiquitin protein ligase  TRAF1 - tnf receptor-associated factor 1  CMTR1 - cap methyltransferase 1  HSP90B1 - heat shock protein 90kda beta (grp94), member 1  TRAF3 - tnf receptor-associated factor 3  TRAF2 - tnf receptor-associated factor 2  SETX - senataxin  DUS3L - dihydrouridine synthase 3-like (s. cerevisiae)  SETD1B - set domain containing 1b  FEM1C - fem-1 homolog c (c. elegans)  TPR - translocated promoter region, nuclear basket protein  ERC1 - elks/rab6-interacting/cast family member 1  KDM4C - lysine (k)-specific demethylase 4c  TRIM35 - tripartite motif containing 35  CAMK1D - calcium/calmodulin-dependent protein kinase id  PIGW - phosphatidylinositol glycan anchor biosynthesis, class w  TTLL5 - tubulin tyrosine ligase-like family, member 5  ZC3H13 - zinc finger ccch-type containing 13  NUP107 - nucleoporin 107kda  CDK19 - cyclin-dependent kinase 19  DERL3 - derlin 3  USP1 - ubiquitin specific peptidase 1  PTK2B - protein tyrosine kinase 2 beta  PCNP - pest proteolytic signal containing nuclear protein  PARP11 - poly (adp-ribose) polymerase family, member 11  UNG - uracil-dna glycosylase  CUL9 - cullin 9  KANSL1 - kat8 regulatory nsl complex subunit 1  USP4 - ubiquitin specific peptidase 4 (proto-oncogene)  RNF185 - ring finger protein 185  TAB2 - tgf-beta activated kinase 1/map3k7 binding protein 2  ZNF598 - zinc finger protein 598  LAMA1 - laminin, alpha 1  VGF - vgf nerve growth factor inducible  SPSB3 - spla/ryanodine receptor domain and socs box containing 3  VHL - von hippel-lindau tumor suppressor, e3 ubiquitin protein ligase  KDM6B - lysine (k)-specific demethylase 6b  PHF8 - phd finger protein 8  TRIM41 - tripartite motif containing 41  EXT2 - exostosin glycosyltransferase 2  EXT1 - exostosin glycosyltransferase 1  EXTL2 - exostosin-like glycosyltransferase 2  DCUN1D4 - dcn1, defective in cullin neddylation 1, domain containing 4  KDM6A - lysine (k)-specific demethylase 6a  EXTL3 - exostosin-like glycosyltransferase 3  EYA3 - eyes absent homolog 3 (drosophila)  EZH1 - enhancer of zeste homolog 1 (drosophila)  TRIM47 - tripartite motif containing 47  PDXP - pyridoxal (pyridoxine, vitamin b6) phosphatase  VCP - valosin containing protein  TTLL12 - tubulin tyrosine ligase-like family, member 12  NEK9 - nima-related kinase 9  ERCC3 - excision repair cross-complementing rodent repair deficiency, complementation group 3  NUP205 - nucleoporin 205kda  ELMSAN1 - elm2 and myb/sant-like domain containing 1  WDR20 - wd repeat domain 20  UBE2A - ubiquitin-conjugating enzyme e2a  UBA1 - ubiquitin-like modifier activating enzyme 1  ERN1 - endoplasmic reticulum to nucleus signaling 1  UBB - ubiquitin b  ARRDC4 - arrestin domain containing 4  RCOR1 - rest corepressor 1  ASPHD2 - aspartate beta-hydroxylase domain containing 2  TYRO3 - tyro3 protein tyrosine kinase  TPGS1 - tubulin polyglutamylase complex subunit 1  TYK2 - tyrosine kinase 2  ATG4B - autophagy related 4b, cysteine peptidase  ETF1 - eukaryotic translation termination factor 1  COG7 - component of oligomeric golgi complex 7  UCHL3 - ubiquitin carboxyl-terminal esterase l3 (ubiquitin thiolesterase)  PKDCC - protein kinase domain containing, cytoplasmic  SCYL3 - scy1-like 3 (s. cerevisiae)  SUMO1 - small ubiquitin-like modifier 1  UCHL1 - ubiquitin carboxyl-terminal esterase l1 (ubiquitin thiolesterase)  UGCG - udp-glucose ceramide glucosyltransferase  PELI1 - pellino e3 ubiquitin protein ligase 1  SMURF1 - smad specific e3 ubiquitin protein ligase 1  JMJD6 - jumonji domain containing 6  UBE2H - ubiquitin-conjugating enzyme e2h  UBE2I - ubiquitin-conjugating enzyme e2i  EPHB6 - eph receptor b6  UBE2D2 - ubiquitin-conjugating enzyme e2d 2  FBXO28 - f-box protein 28  UBE2V2 - ubiquitin-conjugating enzyme e2 variant 2  LONRF1 - lon peptidase n-terminal domain and ring finger 1  ADCK1 - aarf domain containing kinase 1  ERCC2 - excision repair cross-complementing rodent repair deficiency, complementation group 2  MAN1C1 - mannosidase, alpha, class 1c, member 1  NUP210 - nucleoporin 210kda  UBE2L3 - ubiquitin-conjugating enzyme e2l 3  UBE2N - ubiquitin-conjugating enzyme e2n  UBE2V1 - ubiquitin-conjugating enzyme e2 variant 1  HENMT1 - hen1 methyltransferase homolog 1 (arabidopsis)  TMEM165 - transmembrane protein 165  FNTA - farnesyltransferase, caax box, alpha  FLT1 - fms-related tyrosine kinase 1  FLNA - filamin a, alpha  WSB2 - wd repeat and socs box containing 2  SIK2 - salt-inducible kinase 2  PHLPP1 - ph domain and leucine rich repeat protein phosphatase 1  HDAC8 - histone deacetylase 8  ASH1L - ash1 (absent, small, or homeotic)-like (drosophila)  SIN3B - sin3 transcription regulator family member b  FKBP15 - fk506 binding protein 15, 133kda  FKBP4 - fk506 binding protein 4, 59kda  FKBP2 - fk506 binding protein 2, 13kda  ZDHHC22 - zinc finger, dhhc-type containing 22  MGRN1 - mahogunin ring finger 1, e3 ubiquitin protein ligase  RPUSD1 - rna pseudouridylate synthase domain containing 1  HR - hair growth associated  MED8 - mediator complex subunit 8  FKBP5 - fk506 binding protein 5  CAND1 - cullin-associated and neddylation-dissociated 1  FKBP1A - fk506 binding protein 1a, 12kda  TDG - thymine-dna glycosylase  FKBP1B - fk506 binding protein 1b, 12.6 kda  FBXW11 - f-box and wd repeat domain containing 11  POFUT2 - protein o-fucosyltransferase 2  KLHL18 - kelch-like family member 18  KDM3A - lysine (k)-specific demethylase 3a  RNF130 - ring finger protein 130  NUP160 - nucleoporin 160kda  PPP1R11 - protein phosphatase 1, regulatory (inhibitor) subunit 11  FGFR2 - fibroblast growth factor receptor 2  TAF9 - taf9 rna polymerase ii, tata box binding protein (tbp)-associated factor, 32kda  UBR4 - ubiquitin protein ligase e3 component n-recognin 4  FAM98B - family with sequence similarity 98, member b  MBD6 - methyl-cpg binding domain protein 6  KLHL7 - kelch-like family member 7  FGF13 - fibroblast growth factor 13  TAF6 - taf6 rna polymerase ii, tata box binding protein (tbp)-associated factor, 80kda  FGFR1 - fibroblast growth factor receptor 1  FGF9 - fibroblast growth factor 9  TADA2A - transcriptional adaptor 2a  PHF15 - phd finger protein 15  FGF1 - fibroblast growth factor 1 (acidic)  FGF2 - fibroblast growth factor 2 (basic)  DPY19L1 - dpy-19-like 1 (c. elegans)  USP22 - ubiquitin specific peptidase 22  SASH1 - sam and sh3 domain containing 1  TARBP1 - tar (hiv-1) rna binding protein 1  TRIM2 - tripartite motif containing 2  FER - fer (fps/fes related) tyrosine kinase  MAP3K7 - mitogen-activated protein kinase kinase kinase 7  ALG1 - alg1, chitobiosyldiphosphodolichol beta-mannosyltransferase  INO80E - ino80 complex subunit e  ZDHHC17 - zinc finger, dhhc-type containing 17  RNF114 - ring finger protein 114  KMT2E - lysine (k)-specific methyltransferase 2e  SIK3 - sik family kinase 3  PGP - phosphoglycolate phosphatase  FKTN - fukutin  UFL1 - ufm1-specific ligase 1  TENC1 - tensin like c1 domain containing phosphatase (tensin 2)  KLHL9 - kelch-like family member 9  TRIM9 - tripartite motif containing 9  DMAP1 - dna methyltransferase 1 associated protein 1  EFEMP1 - egf containing fibulin-like extracellular matrix protein 1  OBSL1 - obscurin-like 1  CTTNBP2NL - cttnbp2 n-terminal like  FBN1 - fibrillin 1  USP24 - ubiquitin specific peptidase 24  RNF208 - ring finger protein 208  TIE1 - tyrosine kinase with immunoglobulin-like and egf-like domains 1  TIMP1 - timp metallopeptidase inhibitor 1  RYBP - ring1 and yy1 binding protein  MTOR - mechanistic target of rapamycin (serine/threonine kinase)  STK32C - serine/threonine kinase 32c  PPWD1 - peptidylprolyl isomerase domain and wd repeat containing 1  ATP13A2 - atpase type 13a2  TLR3 - toll-like receptor 3  PIGS - phosphatidylinositol glycan anchor biosynthesis, class s  SIRT1 - sirtuin 1  ACTR8 - arp8 actin-related protein 8 homolog (yeast)  ICMT - isoprenylcysteine carboxyl methyltransferase  BRD4 - bromodomain containing 4  FBXO44 - f-box protein 44  TNFAIP1 - tumor necrosis factor, alpha-induced protein 1 (endothelial)  KCTD21 - potassium channel tetramerization domain containing 21  TEK - tek tyrosine kinase, endothelial  NUP188 - nucleoporin 188kda  SUZ12 - suz12 polycomb repressive complex 2 subunit  P4HA3 - prolyl 4-hydroxylase, alpha polypeptide iii  POFUT1 - protein o-fucosyltransferase 1  RCOR2 - rest corepressor 2  MORC3 - morc family cw-type zinc finger 3  TESK1 - testis-specific kinase 1  FXN - frataxin  TGS1 - trimethylguanosine synthase 1  TGFA - transforming growth factor, alpha  CEP41 - centrosomal protein 41kda  TGFBR1 - transforming growth factor, beta receptor 1  TGFB2 - transforming growth factor, beta 2  TGFB1 - transforming growth factor, beta 1  MAPK8IP2 - mitogen-activated protein kinase 8 interacting protein 2  PIGN - phosphatidylinositol glycan anchor biosynthesis, class n  MEPCE - methylphosphate capping enzyme  CDK20 - cyclin-dependent kinase 20  TGFBR2 - transforming growth factor, beta receptor ii (70/80kda)  RNF20 - ring finger protein 20, e3 ubiquitin protein ligase  TGFBR3 - transforming growth factor, beta receptor iii  FOS - fbj murine osteosarcoma viral oncogene homolog  WDR45B - wd repeat domain 45b  THBS1 - thrombospondin 1  KAT6B - k(lysine) acetyltransferase 6b  METTL3 - methyltransferase like 3  THY1 - thy-1 cell surface antigen  MMD - monocyte to macrophage differentiation-associated  GFPT1 - glutamine--fructose-6-phosphate transaminase 1  GFRA1 - gdnf family receptor alpha 1  TMTC2 - transmembrane and tetratricopeptide repeat containing 2  GFRA2 - gdnf family receptor alpha 2  USP54 - ubiquitin specific peptidase 54  ATAT1 - alpha tubulin acetyltransferase 1  GCNT1 - glucosaminyl (n-acetyl) transferase 1, core 2  LZTR1 - leucine-zipper-like transcription regulator 1  KAT2A - k(lysine) acetyltransferase 2a  NCOA3 - nuclear receptor coactivator 3  FBXL18 - f-box and leucine-rich repeat protein 18  NAA25 - n(alpha)-acetyltransferase 25, natb auxiliary subunit  PABPN1 - poly(a) binding protein, nuclear 1  GGTA1P - glycoprotein, alpha-galactosyltransferase 1 pseudogene  TMTC3 - transmembrane and tetratricopeptide repeat containing 3  TRRAP - transformation/transcription domain-associated protein  HDAC11 - histone deacetylase 11  TRMT10B - trna methyltransferase 10 homolog b (s. cerevisiae)  SETD6 - set domain containing 6  ACTR5 - arp5 actin-related protein 5 homolog (yeast)  NAA60 - n(alpha)-acetyltransferase 60, natf catalytic subunit  BAP1 - brca1 associated protein-1 (ubiquitin carboxy-terminal hydrolase)  CDC7 - cell division cycle 7  NUP85 - nucleoporin 85kda  BRAP - brca1 associated protein  KDM5C - lysine (k)-specific demethylase 5c  USP9X - ubiquitin specific peptidase 9, x-linked  USP11 - ubiquitin specific peptidase 11  GNAS - gnas complex locus  PHF17 - phd finger protein 17  GMFB - glia maturation factor, beta  EHMT1 - euchromatic histone-lysine n-methyltransferase 1  FUCA2 - fucosidase, alpha-l- 2, plasma  CAMKMT - calmodulin-lysine n-methyltransferase  METTL8 - methyltransferase like 8  NAA40 - n(alpha)-acetyltransferase 40, natd catalytic subunit, homolog (s. cerevisiae)  SDE2 - sde2 telomere maintenance homolog (s. pombe)  USP7 - ubiquitin specific peptidase 7 (herpes virus-associated)  RNF168 - ring finger protein 168, e3 ubiquitin protein ligase  FUT4 - fucosyltransferase 4 (alpha (1,3) fucosyltransferase, myeloid-specific)  ST8SIA4 - st8 alpha-n-acetyl-neuraminide alpha-2,8-sialyltransferase 4  FUT8 - fucosyltransferase 8 (alpha (1,6) fucosyltransferase)  CBLL1 - cbl proto-oncogene-like 1, e3 ubiquitin protein ligase  FYN - fyn oncogene related to src, fgr, yes  RPAP2 - rna polymerase ii associated protein 2  UBA5 - ubiquitin-like modifier activating enzyme 5  ABHD16A - abhydrolase domain containing 16a  GAK - cyclin g associated kinase  GALNT2 - udp-n-acetyl-alpha-d-galactosamine:polypeptide n-acetylgalactosaminyltransferase 2 (galnac-t2)  TTLL7 - tubulin tyrosine ligase-like family, member 7  USP5 - ubiquitin specific peptidase 5 (isopeptidase t)  GALNT1 - udp-n-acetyl-alpha-d-galactosamine:polypeptide n-acetylgalactosaminyltransferase 1 (galnac-t1)  KMT2D - lysine (k)-specific methyltransferase 2d  SPRED1 - sprouty-related, evh1 domain containing 1  AAAS - achalasia, adrenocortical insufficiency, alacrimia  YEATS4 - yeats domain containing 4  KAT6A - k(lysine) acetyltransferase 6a  TUSC3 - tumor suppressor candidate 3  STAM - signal transducing adaptor molecule (sh3 domain and itam motif) 1  NUP214 - nucleoporin 214kda  KLHL36 - kelch-like family member 36  GAS6 - growth arrest-specific 6  FN3KRP - fructosamine 3 kinase related protein  RNF112 - ring finger protein 112  GSPT1 - g1 to s phase transition 1  RNF113A - ring finger protein 113a  PARP8 - poly (adp-ribose) polymerase family, member 8  HECTD3 - hect domain containing e3 ubiquitin protein ligase 3  NEIL1 - nei endonuclease viii-like 1 (e. coli)  TRIM26 - tripartite motif containing 26  GTF2H3 - general transcription factor iih, polypeptide 3, 34kda  GTF2H1 - general transcription factor iih, polypeptide 1, 62kda  USP27X - ubiquitin specific peptidase 27, x-linked  GTF2B - general transcription factor iib  PCGF2 - polycomb group ring finger 2  TBL1XR1 - transducin (beta)-like 1 x-linked receptor 1  GTF2H4 - general transcription factor iih, polypeptide 4, 52kda  ZDHHC14 - zinc finger, dhhc-type containing 14  HIST1H2BB - histone cluster 1, h2bb  BRPF1 - bromodomain and phd finger containing, 1  SCG2 - secretogranin ii  MUL1 - mitochondrial e3 ubiquitin protein ligase 1  RNF103 - ring finger protein 103  MOGS - mannosyl-oligosaccharide glucosidase  CDC73 - cell division cycle 73  PTP4A1 - protein tyrosine phosphatase type iva, member 1  HCFC1 - host cell factor c1 (vp16-accessory protein)  MAP3K12 - mitogen-activated protein kinase kinase kinase 12  NDNF - neuron-derived neurotrophic factor  FUT11 - fucosyltransferase 11 (alpha (1,3) fucosyltransferase)  HDAC2 - histone deacetylase 2  HDAC1 - histone deacetylase 1  GOLGA2 - golgin a2  PAGR1 - paxip1 associated glutamate-rich protein 1  WEE1 - wee1 homolog (s. pombe)  WFS1 - wolfram syndrome 1 (wolframin)  WHSC1 - wolf-hirschhorn syndrome candidate 1  DTX3 - deltex homolog 3 (drosophila)  VRK1 - vaccinia related kinase 1  VRK2 - vaccinia related kinase 2  GRK5 - g protein-coupled receptor kinase 5  GRK6 - g protein-coupled receptor kinase 6  FBXL15 - f-box and leucine-rich repeat protein 15  BRCC3 - brca1/brca2-containing complex, subunit 3  GRB2 - growth factor receptor-bound protein 2  ASXL1 - additional sex combs like 1 (drosophila)  TNIP2 - tnfaip3 interacting protein 2  ZNF711 - zinc finger protein 711  YES1 - v-yes-1 yamaguchi sarcoma viral oncogene homolog 1  DCAF10 - ddb1 and cul4 associated factor 10  GSK3B - glycogen synthase kinase 3 beta  PGAM5 - phosphoglycerate mutase family member 5  GSK3A - glycogen synthase kinase 3 alpha  YWHAZ - tyrosine 3-monooxygenase/tryptophan 5-monooxygenase activation protein, zeta polypeptide  YWHAE - tyrosine 3-monooxygenase/tryptophan 5-monooxygenase activation protein, epsilon polypeptide  YWHAB - tyrosine 3-monooxygenase/tryptophan 5-monooxygenase activation protein, beta polypeptide  CCNK - cyclin k  NDUFAF5 - nadh dehydrogenase (ubiquinone) complex i, assembly factor 5  DPM1 - dolichyl-phosphate mannosyltransferase polypeptide 1, catalytic subunit  RNF26 - ring finger protein 26  TRIM24 - tripartite motif containing 24  METTL22 - methyltransferase like 22  PRMT1 - protein arginine methyltransferase 1  PRMT2 - protein arginine methyltransferase 2  FKRP - fukutin related protein  FBXO45 - f-box protein 45  DERL1 - derlin 1  DCAF5 - ddb1 and cul4 associated factor 5  KCTD6 - potassium channel tetramerization domain containing 6  METTL16 - methyltransferase like 16  FTO - fat mass and obesity associated  ALKBH1 - alkb, alkylation repair homolog 1 (e. coli)  KDELC1 - kdel (lys-asp-glu-leu) containing 1  ALG8 - alg8, alpha-1,3-glucosyltransferase  SYNGAP1 - synaptic ras gtpase activating protein 1  MED11 - mediator complex subunit 11  ST3GAL5 - st3 beta-galactoside alpha-2,3-sialyltransferase 5  RNF215 - ring finger protein 215  STK19 - serine/threonine kinase 19  LDB1 - lim domain binding 1  DSCC1 - dna replication and sister chromatid cohesion 1  KAT2B - k(lysine) acetyltransferase 2b  CRTC2 - creb regulated transcription coactivator 2  CDC16 - cell division cycle 16  DDA1 - det1 and ddb1 associated 1  GID4 - gid complex subunit 4  NAE1 - nedd8 activating enzyme e1 subunit 1  RPS6KC1 - ribosomal protein s6 kinase, 52kda, polypeptide 1  PRPF4B - prp4 pre-mrna processing factor 4 homolog b (yeast)  MTMR2 - myotubularin related protein 2  MTMR3 - myotubularin related protein 3  HUS1 - hus1 checkpoint homolog (s. pombe)  MCM3AP - minichromosome maintenance complex component 3 associated protein  CPNE3 - copine iii  HERC3 - hect and rld domain containing e3 ubiquitin protein ligase 3  HERC2 - hect and rld domain containing e3 ubiquitin protein ligase 2  HERC1 - hect and rld domain containing e3 ubiquitin protein ligase family member 1  TRAK2 - trafficking protein, kinesin binding 2  NSMCE1 - non-smc element 1 homolog (s. cerevisiae)  BCL10 - b-cell cll/lymphoma 10  BTRC - beta-transducin repeat containing e3 ubiquitin protein ligase  MBD4 - methyl-cpg binding domain protein 4  UBR1 - ubiquitin protein ligase e3 component n-recognin 1  USP12 - ubiquitin specific peptidase 12  WNK1 - wnk lysine deficient protein kinase 1  UBE2Z - ubiquitin-conjugating enzyme e2z  LAMTOR3 - late endosomal/lysosomal adaptor, mapk and mtor activator 3  MPPE1 - metallophosphoesterase 1  HLCS - holocarboxylase synthetase (biotin-(proprionyl-coa-carboxylase (atp-hydrolysing)) ligase)  SENP5 - sumo1/sentrin specific peptidase 5  IRS2 - insulin receptor substrate 2  STK33 - serine/threonine kinase 33  TNKS - tankyrase, trf1-interacting ankyrin-related adp-ribose polymerase  LATS2 - large tumor suppressor kinase 2  EIF3F - eukaryotic translation initiation factor 3, subunit f  BECN1 - beclin 1, autophagy related  PEA15 - phosphoprotein enriched in astrocytes 15  HIPK1 - homeodomain interacting protein kinase 1  B4GALT4 - udp-gal:betaglcnac beta 1,4- galactosyltransferase, polypeptide 4  B4GALT3 - udp-gal:betaglcnac beta 1,4- galactosyltransferase, polypeptide 3  B4GALT2 - udp-gal:betaglcnac beta 1,4- galactosyltransferase, polypeptide 2  CDC23 - cell division cycle 23  UBE2K - ubiquitin-conjugating enzyme e2k  ELP4 - elongator acetyltransferase complex subunit 4  B3GALNT1 - beta-1,3-n-acetylgalactosaminyltransferase 1 (globoside blood group)  B3GALT2 - udp-gal:betaglcnac beta 1,3-galactosyltransferase, polypeptide 2  HIF1A - hypoxia inducible factor 1, alpha subunit (basic helix-loop-helix transcription factor)  MBTPS1 - membrane-bound transcription factor peptidase, site 1  SNX3 - sorting nexin 3  GBGT1 - globoside alpha-1,3-n-acetylgalactosaminyltransferase 1  EED - embryonic ectoderm development  RNGTT - rna guanylyltransferase and 5'-phosphatase  RNMT - rna (guanine-7-) methyltransferase  RIPK1 - receptor (tnfrsf)-interacting serine-threonine kinase 1  FBXO10 - f-box protein 10  FBXO22 - f-box protein 22  FBXO4 - f-box protein 4  FBXO6 - f-box protein 6  VPS33B - vacuolar protein sorting 33 homolog b (yeast)  FBXO3 - f-box protein 3  RAB11A - rab11a, member ras oncogene family  RIPK2 - receptor-interacting serine-threonine kinase 2  PORCN - porcupine homolog (drosophila)  MTMR1 - myotubularin related protein 1  FBXO15 - f-box protein 15  USP46 - ubiquitin specific peptidase 46  PTPLB - protein tyrosine phosphatase-like (proline instead of catalytic arginine), member b  VWA1 - von willebrand factor a domain containing 1  PTPN18 - protein tyrosine phosphatase, non-receptor type 18 (brain-derived)  STT3B - stt3b, subunit of the oligosaccharyltransferase complex (catalytic)  IKBKAP - inhibitor of kappa light polypeptide gene enhancer in b-cells, kinase complex-associated protein  FOXK2 - forkhead box k2  HAT1 - histone acetyltransferase 1  RPS6KA6 - ribosomal protein s6 kinase, 90kda, polypeptide 6  RBM15 - rna binding motif protein 15  ENC1 - ectodermal-neural cortex 1 (with btb domain)  PGAP2 - post-gpi attachment to proteins 2  DESI1 - desumoylating isopeptidase 1  MEAF6 - myst/esa1-associated factor 6  STK39 - serine threonine kinase 39  ENGASE - endo-beta-n-acetylglucosaminidase  PPM1D - protein phosphatase, mg2+/mn2+ dependent, 1d  UBE2S - ubiquitin-conjugating enzyme e2s  ILF3 - interleukin enhancer binding factor 3, 90kda  PRPF19 - pre-mrna processing factor 19  MAP4K3 - mitogen-activated protein kinase kinase kinase kinase 3  ILK - integrin-linked kinase  CDC14B - cell division cycle 14b  PIAS1 - protein inhibitor of activated stat, 1  SMYD3 - set and mynd domain containing 3  MAPKAPK5 - mitogen-activated protein kinase-activated protein kinase 5  SMURF2 - smad specific e3 ubiquitin protein ligase 2  CAMK1 - calcium/calmodulin-dependent protein kinase i  COPS7B - cop9 signalosome subunit 7b  CBX4 - chromobox homolog 4  COPS3 - cop9 signalosome subunit 3  GORASP1 - golgi reassembly stacking protein 1, 65kda  ANAPC1 - anaphase promoting complex subunit 1  SERP1 - stress-associated endoplasmic reticulum protein 1  PRKRA - protein kinase, interferon-inducible double stranded rna dependent activator  STK16 - serine/threonine kinase 16  RNF115 - ring finger protein 115  CASK - calcium/calmodulin-dependent serine protein kinase (maguk family)  ITGAV - integrin, alpha v  CDK10 - cyclin-dependent kinase 10  SUDS3 - suppressor of defective silencing 3 homolog (s. cerevisiae)  ZDHHC6 - zinc finger, dhhc-type containing 6  ATG3 - autophagy related 3  INSR - insulin receptor  CDK13 - cyclin-dependent kinase 13  KLHL20 - kelch-like family member 20  AKTIP - akt interacting protein  RUVBL1 - ruvb-like 1 (e. coli)  MOCS3 - molybdenum cofactor synthesis 3  DIMT1 - dim1 dimethyladenosine transferase 1 homolog (s. cerevisiae)  IRAK2 - interleukin-1 receptor-associated kinase 2  FOLH1B - folate hydrolase 1b  IRAK1 - interleukin-1 receptor-associated kinase 1  EIF2AK1 - eukaryotic translation initiation factor 2-alpha kinase 1  NSD1 - nuclear receptor binding set domain protein 1  HS3ST5 - heparan sulfate (glucosamine) 3-o-sulfotransferase 5  RFWD2 - ring finger and wd repeat domain 2, e3 ubiquitin protein ligase  RNF25 - ring finger protein 25  PALD1 - phosphatase domain containing, paladin 1  BRPF3 - bromodomain and phd finger containing, 3  PDF - peptide deformylase (mitochondrial)  STK36 - serine/threonine kinase 36  IRF2BPL - interferon regulatory factor 2 binding protein-like  OSGEPL1 - o-sialoglycoprotein endopeptidase-like 1  FOXK1 - forkhead box k1  MAGEF1 - melanoma antigen family f, 1  FN3K - fructosamine 3 kinase  STK24 - serine/threonine kinase 24  GFRA4 - gdnf family receptor alpha 4  RASAL1 - ras protein activator like 1 (gap1 like)  OARD1 - o-acyl-adp-ribose deacylase 1  DYRK3 - dual-specificity tyrosine-(y)-phosphorylation regulated kinase 3  RNF11 - ring finger protein 11  TRUB2 - trub pseudouridine (psi) synthase homolog 2 (e. coli)  DUSP11 - dual specificity phosphatase 11 (rna/rnp complex 1-interacting)  SPOP - speckle-type poz protein  SPARCL1 - sparc-like 1 (hevin)  USP21 - ubiquitin specific peptidase 21  ULK1 - unc-51 like autophagy activating kinase 1  JMJD1C - jumonji domain containing 1c  IGF1R - insulin-like growth factor 1 receptor  CDC42BPA - cdc42 binding protein kinase alpha (dmpk-like)  OGT - o-linked n-acetylglucosamine (glcnac) transferase  LAT - linker for activation of t cells  RAE1 - rae1 rna export 1 homolog (s. pombe)  CUL4B - cullin 4b  CUL1 - cullin 1  CUL2 - cullin 2  PCIF1 - pdx1 c-terminal inhibiting factor 1  CUL3 - cullin 3  TPST2 - tyrosylprotein sulfotransferase 2  B3GAT1 - beta-1,3-glucuronyltransferase 1 (glucuronosyltransferase p)  SUPT3H - suppressor of ty 3 homolog (s. cerevisiae)  TPST1 - tyrosylprotein sulfotransferase 1  RSRC1 - arginine/serine-rich coiled-coil 1  MEX3C - mex-3 rna binding family member c  PCGF5 - polycomb group ring finger 5  WAC - ww domain containing adaptor with coiled-coil  WTAP - wilms tumor 1 associated protein  LIMK1 - lim domain kinase 1  ZDHHC3 - zinc finger, dhhc-type containing 3  AATK - apoptosis-associated tyrosine kinase  DOT1L - dot1-like histone h3k79 methyltransferase  LOXL1 - lysyl oxidase-like 1  LNPEP - leucyl/cystinyl aminopeptidase  RAB3D - rab3d, member ras oncogene family  BRSK1 - br serine/threonine kinase 1  NRG2 - neuregulin 2  SYVN1 - synovial apoptosis inhibitor 1, synoviolin  BFAR - bifunctional apoptosis regulator  PTPDC1 - protein tyrosine phosphatase domain containing 1  NEURL4 - neuralized homolog 4 (drosophila)  LIPE - lipase, hormone-sensitive  LIMK2 - lim domain kinase 2  MINPP1 - multiple inositol-polyphosphate phosphatase 1  GOLM1 - golgi membrane protein 1  CDC42BPB - cdc42 binding protein kinase beta (dmpk-like)  CLOCK - clock circadian regulator  LMAN1 - lectin, mannose-binding, 1  PRKAG2 - protein kinase, amp-activated, gamma 2 non-catalytic subunit  ANAPC5 - anaphase promoting complex subunit 5  ANAPC7 - anaphase promoting complex subunit 7  NPEPPS - aminopeptidase puromycin sensitive  KTN1 - kinectin 1 (kinesin receptor)  RNF138 - ring finger protein 138, e3 ubiquitin protein ligase  KRT10 - keratin 10  PPME1 - protein phosphatase methylesterase 1  HEMK1 - hemk methyltransferase family member 1  ACTL6B - actin-like 6b  UBR5 - ubiquitin protein ligase e3 component n-recognin 5  UCHL5 - ubiquitin carboxyl-terminal hydrolase l5  MED20 - mediator complex subunit 20  ATG5 - autophagy related 5  ROCK2 - rho-associated, coiled-coil containing protein kinase 2  MAPK8IP1 - mitogen-activated protein kinase 8 interacting protein 1  PIGB - phosphatidylinositol glycan anchor biosynthesis, class b  TAOK3 - tao kinase 3  FZR1 - fizzy/cell division cycle 20 related 1 (drosophila)  DPY30 - dpy-30 homolog (c. elegans)  LAMC1 - laminin, gamma 1 (formerly lamb2)  IRF2BP2 - interferon regulatory factor 2 binding protein 2  MED27 - mediator complex subunit 27  NAGPA - n-acetylglucosamine-1-phosphodiester alpha-n-acetylglucosaminidase  GALNT18 - udp-n-acetyl-alpha-d-galactosamine:polypeptide n-acetylgalactosaminyltransferase 18  ASB7 - ankyrin repeat and socs box containing 7  ASB8 - ankyrin repeat and socs box containing 8  ARHGEF6 - rac/cdc42 guanine nucleotide exchange factor (gef) 6  KDR - kinase insert domain receptor (a type iii receptor tyrosine kinase)  ASB6 - ankyrin repeat and socs box containing 6  PICK1 - protein interacting with prkca 1  ING4 - inhibitor of growth family, member 4  EIF2AK3 - eukaryotic translation initiation factor 2-alpha kinase 3  USP48 - ubiquitin specific peptidase 48  IRAK4 - interleukin-1 receptor-associated kinase 4  MAP4K4 - mitogen-activated protein kinase kinase kinase kinase 4  RHBDD1 - rhomboid domain containing 1  RLIM - ring finger protein, lim domain interacting  ASB3 - ankyrin repeat and socs box containing 3  TRAF7 - tnf receptor-associated factor 7, e3 ubiquitin protein ligase  KRAS - kirsten rat sarcoma viral oncogene homolog  NAA20 - n(alpha)-acetyltransferase 20, natb catalytic subunit  GOLGA7 - golgin a7  ZDHHC9 - zinc finger, dhhc-type containing 9  CAMKK1 - calcium/calmodulin-dependent protein kinase kinase 1, alpha  SUV420H1 - suppressor of variegation 4-20 homolog 1 (drosophila)  PHF20L1 - phd finger protein 20-like 1  TFB1M - transcription factor b1, mitochondrial  MED10 - mediator complex subunit 10  MED21 - mediator complex subunit 21  ABHD17B - abhydrolase domain containing 17b  KIT - v-kit hardy-zuckerman 4 feline sarcoma viral oncogene homolog  SHISA5 - shisa family member 5  DCUN1D5 - dcn1, defective in cullin neddylation 1, domain containing 5  UBE4A - ubiquitination factor e4a  FBXW9 - f-box and wd repeat domain containing 9  PPIG - peptidylprolyl isomerase g (cyclophilin g)  JAK1 - janus kinase 1  ZDHHC16 - zinc finger, dhhc-type containing 16  JAK2 - janus kinase 2  JAK3 - janus kinase 3  ING5 - inhibitor of growth family, member 5  ANKRD39 - ankyrin repeat domain 39  STT3A - stt3a, subunit of the oligosaccharyltransferase complex (catalytic)  MAST4 - microtubule associated serine/threonine kinase family member 4  PIGP - phosphatidylinositol glycan anchor biosynthesis, class p  PPT2 - palmitoyl-protein thioesterase 2  PHF20 - phd finger protein 20  TMEM246 - transmembrane protein 246  TRIP12 - thyroid hormone receptor interactor 12  COPS2 - cop9 signalosome subunit 2  ZDHHC2 - zinc finger, dhhc-type containing 2  CRBN - cereblon  MIA3 - melanoma inhibitory activity family, member 3  TAOK2 - tao kinase 2  LEF1 - lymphoid enhancer-binding factor 1  B4GALT6 - udp-gal:betaglcnac beta 1,4- galactosyltransferase, polypeptide 6  BRMS1L - breast cancer metastasis-suppressor 1-like  GTF3C4 - general transcription factor iiic, polypeptide 4, 90kda  B4GALT5 - udp-gal:betaglcnac beta 1,4- galactosyltransferase, polypeptide 5  CDK12 - cyclin-dependent kinase 12  KCTD10 - potassium channel tetramerization domain containing 10  ARID4B - at rich interactive domain 4b (rbp1-like)  SOCS6 - suppressor of cytokine signaling 6  STK35 - serine/threonine kinase 35  MAP3K10 - mitogen-activated protein kinase kinase kinase 10  DUSP19 - dual specificity phosphatase 19  MIB2 - mindbomb e3 ubiquitin protein ligase 2  KDM3B - lysine (k)-specific demethylase 3b  RSF1 - remodeling and spacing factor 1  MAP3K11 - mitogen-activated protein kinase kinase kinase 11  KMT2A - lysine (k)-specific methyltransferase 2a  RPS6KA5 - ribosomal protein s6 kinase, 90kda, polypeptide 5  CCDC8 - coiled-coil domain containing 8  STK17A - serine/threonine kinase 17a  NLK - nemo-like kinase  UIMC1 - ubiquitin interaction motif containing 1  RAB11B - rab11b, member ras oncogene family  MTA2 - metastasis associated 1 family, member 2  EIF2A - eukaryotic translation initiation factor 2a, 65kda  KBTBD7 - kelch repeat and btb (poz) domain containing 7  KDELC2 - kdel (lys-asp-glu-leu) containing 2  MECP2 - methyl cpg binding protein 2 (rett syndrome)  MEF2A - myocyte enhancer factor 2a  MAGT1 - magnesium transporter 1  ZBED1 - zinc finger, bed-type containing 1  PPIL3 - peptidylprolyl isomerase (cyclophilin)-like 3  BCDIN3D - bcdin3 domain containing  MAP3K4 - mitogen-activated protein kinase kinase kinase 4  MAP3K5 - mitogen-activated protein kinase kinase kinase 5  DCLK1 - doublecortin-like kinase 1  MAP3K1 - mitogen-activated protein kinase kinase kinase 1, e3 ubiquitin protein ligase  MAP3K3 - mitogen-activated protein kinase kinase kinase 3  MEN1 - multiple endocrine neoplasia i  RAB8A - rab8a, member ras oncogene family  USP42 - ubiquitin specific peptidase 42  METTL1 - methyltransferase like 1  KAT8 - k(lysine) acetyltransferase 8  MFGE8 - milk fat globule-egf factor 8 protein  FBXL14 - f-box and leucine-rich repeat protein 14  ATG12 - autophagy related 12  MGAT1 - mannosyl (alpha-1,3-)-glycoprotein beta-1,2-n-acetylglucosaminyltransferase  MGAT2 - mannosyl (alpha-1,6-)-glycoprotein beta-1,2-n-acetylglucosaminyltransferase  HGS - hepatocyte growth factor-regulated tyrosine kinase substrate  MGAT5 - mannosyl (alpha-1,6-)-glycoprotein beta-1,6-n-acetyl-glucosaminyltransferase  MBD3 - methyl-cpg binding domain protein 3  KITLG - kit ligand  B3GALTL - beta 1,3-galactosyltransferase-like  MAN1A1 - mannosidase, alpha, class 1a, member 1  MAN2A2 - mannosidase, alpha, class 2a, member 2  ZC3HC1 - zinc finger, c3hc-type containing 1  COG3 - component of oligomeric golgi complex 3  PUS3 - pseudouridylate synthase 3  MARK3 - map/microtubule affinity-regulating kinase 3  MARK1 - map/microtubule affinity-regulating kinase 1  DOHH - deoxyhypusine hydroxylase/monooxygenase  MAPT - microtubule-associated protein tau  MDP1 - magnesium-dependent phosphatase 1  LATS1 - large tumor suppressor kinase 1  MTA1 - metastasis associated 1  SIRT6 - sirtuin 6  MTMR4 - myotubularin related protein 4  MTMR7 - myotubularin related protein 7  MANBA - mannosidase, beta a, lysosomal  MTMR6 - myotubularin related protein 6  MAN2A1 - mannosidase, alpha, class 2a, member 1  USP8 - ubiquitin specific peptidase 8  USP14 - ubiquitin specific peptidase 14 (trna-guanine transglycosylase)  USP10 - ubiquitin specific peptidase 10  UBE2J1 - ubiquitin-conjugating enzyme e2, j1  NUP43 - nucleoporin 43kda  PKMYT1 - protein kinase, membrane associated tyrosine/threonine 1  MATK - megakaryocyte-associated tyrosine kinase  UFC1 - ubiquitin-fold modifier conjugating enzyme 1  TRMT112 - trna methyltransferase 11-2 homolog (s. cerevisiae)  TTBK2 - tau tubulin kinase 2  ASH2L - ash2 (absent, small, or homeotic)-like (drosophila)  CTDSPL2 - ctd (carboxy-terminal domain, rna polymerase ii, polypeptide a) small phosphatase like 2  PPIL1 - peptidylprolyl isomerase (cyclophilin)-like 1  NFS1 - nfs1 cysteine desulfurase  FBXL16 - f-box and leucine-rich repeat protein 16  ITCH - itchy e3 ubiquitin protein ligase  RIOK1 - rio kinase 1  AIP - aryl hydrocarbon receptor interacting protein  OTUD6B - otu domain containing 6b  NUB1 - negative regulator of ubiquitin-like proteins 1  ASB1 - ankyrin repeat and socs box containing 1  LYN - v-yes-1 yamaguchi sarcoma viral related oncogene homolog  UBE2M - ubiquitin-conjugating enzyme e2m  UBA3 - ubiquitin-like modifier activating enzyme 3  PARP9 - poly (adp-ribose) polymerase family, member 9  CDK5RAP1 - cdk5 regulatory subunit associated protein 1  RNF8 - ring finger protein 8, e3 ubiquitin protein ligase  LTBP1 - latent transforming growth factor beta binding protein 1  FKBP7 - fk506 binding protein 7  RPS6KL1 - ribosomal protein s6 kinase-like 1  BAZ1B - bromodomain adjacent to zinc finger domain, 1b  PIAS4 - protein inhibitor of activated stat, 4  TRIM33 - tripartite motif containing 33  SMAD2 - smad family member 2  MBIP - map3k12 binding inhibitory protein 1  HDAC7 - histone deacetylase 7  TMTC1 - transmembrane and tetratricopeptide repeat containing 1  CDKL2 - cyclin-dependent kinase-like 2 (cdc2-related kinase)  LARP7 - la ribonucleoprotein domain family, member 7  RPS6KA4 - ribosomal protein s6 kinase, 90kda, polypeptide 4  KCTD11 - potassium channel tetramerization domain containing 11  PLOD3 - procollagen-lysine, 2-oxoglutarate 5-dioxygenase 3  DPH5 - diphthamide biosynthesis 5  TAF9B - taf9b rna polymerase ii, tata box binding protein (tbp)-associated factor, 31kda  SMAD4 - smad family member 4  SMAD5 - smad family member 5  P4HA2 - prolyl 4-hydroxylase, alpha polypeptide ii  SMAD7 - smad family member 7  TRMT6 - trna methyltransferase 6 homolog (s. cerevisiae)  PIGT - phosphatidylinositol glycan anchor biosynthesis, class t  MYLIP - myosin regulatory light chain interacting protein  NCK1 - nck adaptor protein 1  DPY19L3 - dpy-19-like 3 (c. elegans)  TRMT1L - trna methyltransferase 1 homolog (s. cerevisiae)-like  ABI2 - abl-interactor 2  TRIM28 - tripartite motif containing 28  SCG3 - secretogranin iii  NCAM1 - neural cell adhesion molecule 1  TBK1 - tank-binding kinase 1  NHLRC3 - nhl repeat containing 3  ZDHHC1 - zinc finger, dhhc-type containing 1  ZDHHC8 - zinc finger, dhhc-type containing 8  PPP1R12A - protein phosphatase 1, regulatory subunit 12a  TRIM11 - tripartite motif containing 11  TNK2 - tyrosine kinase, non-receptor, 2  SENP1 - sumo1/sentrin specific peptidase 1  RNF41 - ring finger protein 41  TFPT - tcf3 (e2a) fusion partner (in childhood leukemia)  PRMT3 - protein arginine methyltransferase 3  PSME3 - proteasome (prosome, macropain) activator subunit 3 (pa28 gamma; ki)  MYLK - myosin light chain kinase  CDADC1 - cytidine and dcmp deaminase domain containing 1  URM1 - ubiquitin related modifier 1  TRIM13 - tripartite motif containing 13  TRIM8 - tripartite motif containing 8  USPL1 - ubiquitin specific peptidase like 1  TOPORS - topoisomerase i binding, arginine/serine-rich, e3 ubiquitin protein ligase  RAB1B - rab1b, member ras oncogene family  SEH1L - seh1-like (s. cerevisiae)  PTPRU - protein tyrosine phosphatase, receptor type, u  HUWE1 - hect, uba and wwe domain containing 1, e3 ubiquitin protein ligase  ALG5 - alg5, dolichyl-phosphate beta-glucosyltransferase  INO80B - ino80 complex subunit b  B3GALNT2 - beta-1,3-n-acetylgalactosaminyltransferase 2  RBM15B - rna binding motif protein 15b  CTDSP2 - ctd (carboxy-terminal domain, rna polymerase ii, polypeptide a) small phosphatase 2  ANAPC4 - anaphase promoting complex subunit 4  RNF170 - ring finger protein 170  FEM1B - fem-1 homolog b (c. elegans)  MUTYH - muty homolog (e. coli)  TRIM37 - tripartite motif containing 37  CDC42EP5 - cdc42 effector protein (rho gtpase binding) 5  SHARPIN - shank-associated rh domain interactor  PDIA6 - protein disulfide isomerase family a, member 6  AKT3 - v-akt murine thymoma viral oncogene homolog 3  ABI1 - abl-interactor 1  MTRR - 5-methyltetrahydrofolate-homocysteine methyltransferase reductase  PPP4R1 - protein phosphatase 4, regulatory subunit 1  PDCD6 - programmed cell death 6  MTM1 - myotubularin 1  HDAC5 - histone deacetylase 5  HDAC6 - histone deacetylase 6  PARP2 - poly (adp-ribose) polymerase 2  RNF187 - ring finger protein 187  SH2D3C - sh2 domain containing 3c  MT3 - metallothionein 3  PIGK - phosphatidylinositol glycan anchor biosynthesis, class k  CCDC22 - coiled-coil domain containing 22  SAE1 - sumo1 activating enzyme subunit 1  PDIK1L - pdlim1 interacting kinase 1 like  RANBP9 - ran binding protein 9  UBA2 - ubiquitin-like modifier activating enzyme 2  JOSD1 - josephin domain containing 1  KANSL1L - kat8 regulatory nsl complex subunit 1-like  SUPT7L - suppressor of ty 7 (s. cerevisiae)-like  FBXO38 - f-box protein 38  GFPT2 - glutamine-fructose-6-phosphate transaminase 2  OXSR1 - oxidative stress responsive 1  COMMD1 - copper metabolism (murr1) domain containing 1  USP3 - ubiquitin specific peptidase 3  BABAM1 - brisc and brca1 a complex member 1  UBE2T - ubiquitin-conjugating enzyme e2t (putative)  METTL5 - methyltransferase like 5  USP15 - ubiquitin specific peptidase 15  TTL - tubulin tyrosine ligase  C1GALT1C1 - c1galt1-specific chaperone 1  SETD2 - set domain containing 2  RCE1 - rce1 homolog, prenyl protein protease (s. cerevisiae)  DUSP18 - dual specificity phosphatase 18  RBX1 - ring-box 1, e3 ubiquitin protein ligase  NUP153 - nucleoporin 153kda  MPI - mannose phosphate isomerase  MED12 - mediator complex subunit 12  WDSUB1 - wd repeat, sterile alpha motif and u-box domain containing 1  PSMD6 - proteasome (prosome, macropain) 26s subunit, non-atpase, 6  DTX3L - deltex 3-like (drosophila)  PJA2 - praja ring finger 2, e3 ubiquitin protein ligase  AREL1 - apoptosis resistant e3 ubiquitin protein ligase 1  SETDB1 - set domain, bifurcated 1  TLK1 - tousled-like kinase 1  RNF141 - ring finger protein 141  THUMPD2 - thump domain containing 2  PTAR1 - protein prenyltransferase alpha subunit repeat containing 1  PPP4R2 - protein phosphatase 4, regulatory subunit 2  RNF38 - ring finger protein 38  XXYLT1 - xyloside xylosyltransferase 1  METTL21A - methyltransferase like 21a  KLHL21 - kelch-like family member 21  OGG1 - 8-oxoguanine dna glycosylase  EXOSC3 - exosome component 3  TNKS2 - tankyrase, trf1-interacting ankyrin-related adp-ribose polymerase 2  WDR61 - wd repeat domain 61  DDR2 - discoidin domain receptor tyrosine kinase 2  TOMM20 - translocase of outer mitochondrial membrane 20 homolog (yeast)  NTHL1 - nth endonuclease iii-like 1 (e. coli)  DAB2IP - dab2 interacting protein  NUP88 - nucleoporin 88kda  NUP98 - nucleoporin 98kda  NUCB1 - nucleobindin 1  ST8SIA3 - st8 alpha-n-acetyl-neuraminide alpha-2,8-sialyltransferase 3  RNF144A - ring finger protein 144a  ILKAP - integrin-linked kinase-associated serine/threonine phosphatase  DNAJC6 - dnaj (hsp40) homolog, subfamily c, member 6  SPATA2 - spermatogenesis associated 2  KLHL5 - kelch-like family member 5  DUSP16 - dual specificity phosphatase 16  RNF40 - ring finger protein 40, e3 ubiquitin protein ligase  SPOCK2 - sparc/osteonectin, cwcv and kazal-like domains proteoglycan (testican) 2  CUL7 - cullin 7  PAQR3 - progestin and adipoq receptor family member iii  RRNAD1 - ribosomal rna adenine dimethylase domain containing 1  NRAS - neuroblastoma ras viral (v-ras) oncogene homolog  NMT1 - n-myristoyltransferase 1  NOP2 - nop2 nucleolar protein  VPRBP - vpr (hiv-1) binding protein  RNF34 - ring finger protein 34, e3 ubiquitin protein ligase  FBXO11 - f-box protein 11  ULK2 - unc-51 like autophagy activating kinase 2  HERPUD1 - homocysteine-inducible, endoplasmic reticulum stress-inducible, ubiquitin-like domain member 1  SPSB1 - spla/ryanodine receptor domain and socs box containing 1  ERO1L - ero1-like (s. cerevisiae)  KCTD7 - potassium channel tetramerization domain containing 7  NAA15 - n(alpha)-acetyltransferase 15, nata auxiliary subunit  CNOT4 - ccr4-not transcription complex, subunit 4  HDAC4 - histone deacetylase 4  NFRKB - nuclear factor related to kappab binding protein  CXXC1 - cxxc finger protein 1  NFKBIA - nuclear factor of kappa light polypeptide gene enhancer in b-cells inhibitor, alpha  NFKB1 - nuclear factor of kappa light polypeptide gene enhancer in b-cells 1  KMT2B - lysine (k)-specific methyltransferase 2b  ST6GALNAC6 - st6 (alpha-n-acetyl-neuraminyl-2,3-beta-galactosyl-1,3)-n-acetylgalactosaminide alpha-2,6-sialyltransferase 6  PIK3R4 - phosphoinositide-3-kinase, regulatory subunit 4  SLK - ste20-like kinase  VCPIP1 - valosin containing protein (p97)/p47 complex interacting protein 1  NKTR - natural killer-tumor recognition sequence  USP34 - ubiquitin specific peptidase 34  HDAC9 - histone deacetylase 9  RNF145 - ring finger protein 145  SETD1A - set domain containing 1a  CCP110 - centriolar coiled coil protein 110kda  SOCS7 - suppressor of cytokine signaling 7  NEK3 - nima-related kinase 3  MINK1 - misshapen-like kinase 1  TTLL4 - tubulin tyrosine ligase-like family, member 4  SOCS5 - suppressor of cytokine signaling 5  NEFL - neurofilament, light polypeptide  NEK1 - nima-related kinase 1  NEDD8 - neural precursor cell expressed, developmentally down-regulated 8  CTR9 - ctr9, paf1/rna polymerase ii complex component  PPM1F - protein phosphatase, mg2+/mn2+ dependent, 1f  NFE2L2 - nuclear factor (erythroid-derived 2)-like 2  EPC1 - enhancer of polycomb homolog 1 (drosophila)  MORF4L2 - mortality factor 4 like 2  KLHL15 - kelch-like family member 15  NUP155 - nucleoporin 155kda  NF1 - neurofibromin 1  CDK5RAP3 - cdk5 regulatory subunit associated protein 3  DCAF8 - ddb1 and cul4 associated factor 8  EDEM1 - er degradation enhancer, mannosidase alpha-like 1  KIAA0391 - kiaa0391  RAPGEF2 - rap guanine nucleotide exchange factor (gef) 2  EDEM3 - er degradation enhancer, mannosidase alpha-like 3  NUP93 - nucleoporin 93kda  KDM4A - lysine (k)-specific demethylase 4a  PHF14 - phd finger protein 14  COPS7A - cop9 signalosome subunit 7a  DZIP3 - daz interacting zinc finger protein 3 |
| GO:0016070 | RNA metabolic process | 2.81E-15 | 3.26E-12 | 1.14 (10334,1231,6996,951) | [+] Show genes  ELL - elongation factor rna polymerase ii  EXOSC9 - exosome component 9  EXOSC10 - exosome component 10  ZCCHC8 - zinc finger, cchc domain containing 8  RNPC3 - rna-binding region (rnp1, rrm) containing 3  SYMPK - symplekin  SF3A3 - splicing factor 3a, subunit 3, 60kda  HNRNPA0 - heterogeneous nuclear ribonucleoprotein a0  AARS - alanyl-trna synthetase  PNN - pinin, desmosome associated protein  SF3A2 - splicing factor 3a, subunit 2, 66kda  CNOT11 - ccr4-not transcription complex, subunit 11  EBNA1BP2 - ebna1 binding protein 2  FTSJ3 - ftsj homolog 3 (e. coli)  PNRC2 - proline-rich nuclear receptor coactivator 2  TXNL4A - thioredoxin-like 4a  NOP9 - nop9 nucleolar protein  CHD7 - chromodomain helicase dna binding protein 7  PLRG1 - pleiotropic regulator 1  LYAR - ly1 antibody reactive  PAPOLA - poly(a) polymerase alpha  PABPN1 - poly(a) binding protein, nuclear 1  TCERG1 - transcription elongation regulator 1  TAF15 - taf15 rna polymerase ii, tata box binding protein (tbp)-associated factor, 68kda  RNPS1 - rna binding protein s1, serine-rich domain  SPIN1 - spindlin 1  THUMPD1 - thump domain containing 1  TRMT10B - trna methyltransferase 10 homolog b (s. cerevisiae)  ADAR - adenosine deaminase, rna-specific  ADARB1 - adenosine deaminase, rna-specific, b1  WDR3 - wd repeat domain 3  CPSF4 - cleavage and polyadenylation specific factor 4, 30kda  MED1 - mediator complex subunit 1  PPA1 - pyrophosphatase (inorganic) 1  PPARD - peroxisome proliferator-activated receptor delta  POLRMT - polymerase (rna) mitochondrial (dna directed)  RBM10 - rna binding motif protein 10  MAML3 - mastermind-like 3 (drosophila)  POLR2K - polymerase (rna) ii (dna directed) polypeptide k, 7.0kda  TSEN15 - tsen15 trna splicing endonuclease subunit  PARP1 - poly (adp-ribose) polymerase 1  PPP1R13L - protein phosphatase 1, regulatory subunit 13 like  NOP10 - nop10 ribonucleoprotein  POLR2G - polymerase (rna) ii (dna directed) polypeptide g  NOL10 - nucleolar protein 10  ELAC1 - elac ribonuclease z 1  POLR2F - polymerase (rna) ii (dna directed) polypeptide f  POLR2E - polymerase (rna) ii (dna directed) polypeptide e, 25kda  POLR2D - polymerase (rna) ii (dna directed) polypeptide d  POLR2C - polymerase (rna) ii (dna directed) polypeptide c, 33kda  POLR2B - polymerase (rna) ii (dna directed) polypeptide b, 140kda  POLR2A - polymerase (rna) ii (dna directed) polypeptide a, 220kda  HNRNPUL1 - heterogeneous nuclear ribonucleoprotein u-like 1  RPP14 - ribonuclease p/mrp 14kda subunit  INTS9 - integrator complex subunit 9  KRR1 - krr1, small subunit (ssu) processome component, homolog (yeast)  PRKACA - protein kinase, camp-dependent, catalytic, alpha  NELFE - negative elongation factor complex member e  WDR12 - wd repeat domain 12  NR5A1 - nuclear receptor subfamily 5, group a, member 1  PRIM2 - primase, dna, polypeptide 2 (58kda)  PRIM1 - primase, dna, polypeptide 1 (49kda)  FUS - fused in sarcoma  FRG1 - fshd region gene 1  METTL8 - methyltransferase like 8  PRCC - papillary renal cell carcinoma (translocation-associated)  POLR3A - polymerase (rna) iii (dna directed) polypeptide a, 155kda  CLASRP - clk4-associating serine/arginine rich protein  GEMIN6 - gem (nuclear organelle) associated protein 6  PRDM4 - pr domain containing 4  PPP5C - protein phosphatase 5, catalytic subunit  PCID2 - pci domain containing 2  SNRNP35 - small nuclear ribonucleoprotein 35kda (u11/u12)  PPP2R2A - protein phosphatase 2, regulatory subunit b, alpha  CMTR2 - cap methyltransferase 2  RIOK2 - rio kinase 2  SLBP - stem-loop binding protein  PPP2R1A - protein phosphatase 2, regulatory subunit a, alpha  CPSF6 - cleavage and polyadenylation specific factor 6, 68kda  NUDT21 - nudix (nucleoside diphosphate linked moiety x)-type motif 21  DIS3L - dis3 mitotic control homolog (s. cerevisiae)-like  DDX52 - dead (asp-glu-ala-asp) box polypeptide 52  DDX51 - dead (asp-glu-ala-asp) box polypeptide 51  PPP1R8 - protein phosphatase 1, regulatory subunit 8  RBFA - ribosome binding factor a (putative)  PPP2CA - protein phosphatase 2, catalytic subunit, alpha isozyme  CPSF7 - cleavage and polyadenylation specific factor 7, 59kda  DIDO1 - death inducer-obliterator 1  CBLL1 - cbl proto-oncogene-like 1, e3 ubiquitin protein ligase  RPAP2 - rna polymerase ii associated protein 2  HSF2BP - heat shock transcription factor 2 binding protein  DDX39B - dead (asp-glu-ala-asp) box polypeptide 39b  TDRKH - tudor and kh domain containing  IWS1 - iws1 homolog (s. cerevisiae)  NARS2 - asparaginyl-trna synthetase 2, mitochondrial (putative)  PRPF40A - prp40 pre-mrna processing factor 40 homolog a (s. cerevisiae)  PAPD7 - pap associated domain containing 7  DDX27 - dead (asp-glu-ala-asp) box polypeptide 27  INTS8 - integrator complex subunit 8  SNORA8 - small nucleolar rna, h/aca box 8  SNIP1 - smad nuclear interacting protein 1  TSR1 - tsr1, 20s rrna accumulation, homolog (s. cerevisiae)  POLR3E - polymerase (rna) iii (dna directed) polypeptide e (80kd)  GEMIN7 - gem (nuclear organelle) associated protein 7  MAPK3 - mitogen-activated protein kinase 3  CLP1 - cleavage and polyadenylation factor i subunit 1  NT5C3B - 5'-nucleotidase, cytosolic iiib  CCDC94 - coiled-coil domain containing 94  POLR3B - polymerase (rna) iii (dna directed) polypeptide b  NR4A3 - nuclear receptor subfamily 4, group a, member 3  RBM22 - rna binding motif protein 22  NSUN5 - nop2/sun domain family, member 5  SF3B2 - splicing factor 3b, subunit 2, 145kda  DEDD2 - death effector domain containing 2  GARS - glycyl-trna synthetase  APEX1 - apex nuclease (multifunctional dna repair enzyme) 1  FASTKD1 - fast kinase domains 1  DHX36 - deah (asp-glu-ala-his) box polypeptide 36  MTPAP - mitochondrial poly(a) polymerase  RBM23 - rna binding motif protein 23  DALRD3 - dalr anticodon binding domain containing 3  PARN - poly(a)-specific ribonuclease  GSPT1 - g1 to s phase transition 1  DARS2 - aspartyl-trna synthetase 2, mitochondrial  ZCCHC6 - zinc finger, cchc domain containing 6  RNF113A - ring finger protein 113a  GTF2F1 - general transcription factor iif, polypeptide 1, 74kda  GTF2F2 - general transcription factor iif, polypeptide 2, 30kda  GTF2E1 - general transcription factor iie, polypeptide 1, alpha 56kda  GTF2E2 - general transcription factor iie, polypeptide 2, beta 34kda  GTF2H2 - general transcription factor iih, polypeptide 2, 44kda  PCBP2 - poly(rc) binding protein 2  GTF2H3 - general transcription factor iih, polypeptide 3, 34kda  INTS10 - integrator complex subunit 10  GTF2H1 - general transcription factor iih, polypeptide 1, 62kda  SMG8 - smg8 nonsense mediated mrna decay factor  GTF2A2 - general transcription factor iia, 2, 12kda  GTF2B - general transcription factor iib  GTF2A1 - general transcription factor iia, 1, 19/37kda  USB1 - u6 snrna biogenesis 1  GTF3C2 - general transcription factor iiic, polypeptide 2, beta 110kda  GTF2I - general transcription factor iii  GTF2H4 - general transcription factor iih, polypeptide 4, 52kda  PRKRIP1 - prkr interacting protein 1 (il11 inducible)  GTF3A - general transcription factor iiia  PA2G4 - proliferation-associated 2g4, 38kda  BRF1 - brf1, rna polymerase iii transcription initiation factor 90 kda subunit  RPRD1A - regulation of nuclear pre-mrna domain containing 1a  NOL9 - nucleolar protein 9  GTF3C1 - general transcription factor iiic, polypeptide 1, alpha 220kda  APP - amyloid beta (a4) precursor protein  DDX20 - dead (asp-glu-ala-asp) box polypeptide 20  CELF3 - cugbp, elav-like family member 3  DCP2 - decapping mrna 2  PAPD4 - pap associated domain containing 4  SNORA43 - small nucleolar rna, h/aca box 43  PAX8 - paired box 8  SUPT16H - suppressor of ty 16 homolog (s. cerevisiae)  ARRB2 - arrestin, beta 2  ARRB1 - arrestin, beta 1  CDC73 - cell division cycle 73  GPATCH1 - g patch domain containing 1  SAMD4B - sterile alpha motif domain containing 4b  HARS - histidyl-trna synthetase  BAZ2A - bromodomain adjacent to zinc finger domain, 2a  PCNA - proliferating cell nuclear antigen  LSM6 - lsm6 homolog, u6 small nuclear rna associated (s. cerevisiae)  MAGOHB - mago-nashi homolog b (drosophila)  MTFMT - mitochondrial methionyl-trna formyltransferase  TARSL2 - threonyl-trna synthetase-like 2  HEATR1 - heat repeat containing 1  LEO1 - leo1, paf1/rna polymerase ii complex component, homolog (s. cerevisiae)  RPL7L1 - ribosomal protein l7-like 1  PRPF38B - prp38 pre-mrna processing factor 38 (yeast) domain containing b  PWP1 - pwp1 homolog (s. cerevisiae)  SNRNP25 - small nuclear ribonucleoprotein 25kda (u11/u12)  RBM28 - rna binding motif protein 28  THAP1 - thap domain containing, apoptosis associated protein 1  SNORA11 - small nucleolar rna, h/aca box 11  ELP3 - elongator acetyltransferase complex subunit 3  ZBTB1 - zinc finger and btb domain containing 1  DIS3 - dis3 mitotic control homolog (s. cerevisiae)  ZHX2 - zinc fingers and homeoboxes 2  SNORD86 - small nucleolar rna, c/d box 86  LARP6 - la ribonucleoprotein domain family, member 6  XBP1 - x-box binding protein 1  NELFA - negative elongation factor complex member a  ZFC3H1 - zinc finger, c3h1-type containing  SNORD12 - small nucleolar rna, c/d box 12  WDR33 - wd repeat domain 33  DCP1B - decapping mrna 1b  WARS - tryptophanyl-trna synthetase  BAX - bcl2-associated x protein  SCAF8 - sr-related ctd-associated factor 8  SMU1 - smu-1 suppressor of mec-8 and unc-52 homolog (c. elegans)  PUF60 - poly-u binding splicing factor 60kda  THOC6 - tho complex 6 homolog (drosophila)  RAVER2 - ribonucleoprotein, ptb-binding 2  NAT10 - n-acetyltransferase 10 (gcn5-related)  DPH3 - diphthamide biosynthesis 3  ZNF45 - zinc finger protein 45  EXD2 - exonuclease 3'-5' domain containing 2  TYW1 - trna-yw synthesizing protein 1 homolog (s. cerevisiae)  ASXL2 - additional sex combs like 2 (drosophila)  XRN2 - 5'-3' exoribonuclease 2  ELP2 - elongator acetyltransferase complex subunit 2  ASXL1 - additional sex combs like 1 (drosophila)  PKNOX1 - pbx/knotted 1 homeobox 1  CASC3 - cancer susceptibility candidate 3  PSTK - phosphoseryl-trna kinase  EXOSC8 - exosome component 8  IMP3 - imp3, u3 small nucleolar ribonucleoprotein, homolog (yeast)  PIH1D2 - pih1 domain containing 2  SF1 - splicing factor 1  U2AF2 - u2 small nuclear rna auxiliary factor 2  PSPC1 - paraspeckle component 1  RPUSD3 - rna pseudouridylate synthase domain containing 3  BRF2 - brf2, rna polymerase iii transcription initiation factor 50 kda subunit  DDX42 - dead (asp-glu-ala-asp) box helicase 42  ECD - ecdysoneless homolog (drosophila)  POLR3H - polymerase (rna) iii (dna directed) polypeptide h (22.9kd)  GRSF1 - g-rich rna sequence binding factor 1  CWF19L1 - cwf19-like 1, cell cycle control (s. pombe)  CCND1 - cyclin d1  QRSL1 - glutaminyl-trna synthase (glutamine-hydrolyzing)-like 1  CCNK - cyclin k  CDKAL1 - cdk5 regulatory subunit associated protein 1-like 1  SMNDC1 - survival motor neuron domain containing 1  BCAS2 - breast carcinoma amplified sequence 2  CWC27 - cwc27 spliceosome-associated protein homolog (s. cerevisiae)  SAP18 - sin3a-associated protein, 18kda  TRIM24 - tripartite motif containing 24  TAF1D - tata box binding protein (tbp)-associated factor, rna polymerase i, d, 41kda  SNORD24 - small nucleolar rna, c/d box 24  SF3A1 - splicing factor 3a, subunit 1, 120kda  RELA - v-rel avian reticuloendotheliosis viral oncogene homolog a  CWC25 - cwc25 spliceosome-associated protein homolog (s. cerevisiae)  TEX10 - testis expressed 10  SNAPC5 - small nuclear rna activating complex, polypeptide 5, 19kda  SNORD55 - small nucleolar rna, c/d box 55  ZFP36L2 - zfp36 ring finger protein-like 2  ZFP36L1 - zfp36 ring finger protein-like 1  ALKBH5 - alkb, alkylation repair homolog 5 (e. coli)  NSUN2 - nop2/sun rna methyltransferase family, member 2  UPF1 - upf1 regulator of nonsense transcripts homolog (yeast)  METTL16 - methyltransferase like 16  FTO - fat mass and obesity associated  ALKBH1 - alkb, alkylation repair homolog 1 (e. coli)  ELP6 - elongator acetyltransferase complex subunit 6  NOC4L - nucleolar complex associated 4 homolog (s. cerevisiae)  FBLL1 - fibrillarin-like 1  LDB1 - lim domain binding 1  WARS2 - tryptophanyl trna synthetase 2, mitochondrial  KAT2B - k(lysine) acetyltransferase 2b  TSC22D1 - tsc22 domain family, member 1  WDR55 - wd repeat domain 55  ZNF259 - zinc finger protein 259  SCNM1 - sodium channel modifier 1  IARS - isoleucyl-trna synthetase  TRIT1 - trna isopentenyltransferase 1  PRPF4B - prp4 pre-mrna processing factor 4 homolog b (yeast)  NABP2 - nucleic acid binding protein 2  TSEN34 - tsen34 trna splicing endonuclease subunit  RNASE6 - ribonuclease, rnase a family, k6  U2AF1L4 - u2 small nuclear rna auxiliary factor 1-like 4  HNRNPR - heterogeneous nuclear ribonucleoprotein r  ERI3 - eri1 exoribonuclease family member 3  BUD31 - bud31 homolog (s. cerevisiae)  BYSL - bystin-like  SRRM1 - serine/arginine repetitive matrix 1  MED18 - mediator complex subunit 18  TYW3 - trna-yw synthesizing protein 3 homolog (s. cerevisiae)  FUBP3 - far upstream element (fuse) binding protein 3  SF3B4 - splicing factor 3b, subunit 4, 49kda  C5AR1 - complement component 5a receptor 1  TCEANC2 - transcription elongation factor a (sii) n-terminal and central domain containing 2  HSPA8 - heat shock 70kda protein 8  PPIE - peptidylprolyl isomerase e (cyclophilin e)  RPL17 - ribosomal protein l17  RPL15 - ribosomal protein l15  C1D - c1d nuclear receptor corepressor  RPL11 - ribosomal protein l11  TRMT12 - trna methyltransferase 12 homolog (s. cerevisiae)  RPL9 - ribosomal protein l9  NOL8 - nucleolar protein 8  RPL7 - ribosomal protein l7  INTS6 - integrator complex subunit 6  PPIH - peptidylprolyl isomerase h (cyclophilin h)  RPL3 - ribosomal protein l3  AGO1 - argonaute risc catalytic component 1  CARS - cysteinyl-trna synthetase  RPS4X - ribosomal protein s4, x-linked  SRSF9 - serine/arginine-rich splicing factor 9  RPS4Y1 - ribosomal protein s4, y-linked 1  MRPL1 - mitochondrial ribosomal protein l1  GPBP1 - gc-rich promoter binding protein 1  NOL6 - nucleolar protein 6 (rna-associated)  KRI1 - kri1 homolog (s. cerevisiae)  MRPL44 - mitochondrial ribosomal protein l44  NR2C2AP - nuclear receptor 2c2-associated protein  SYNCRIP - synaptotagmin binding, cytoplasmic rna interacting protein  INTS3 - integrator complex subunit 3  RPL32 - ribosomal protein l32  ELP4 - elongator acetyltransferase complex subunit 4  UPF3B - upf3 regulator of nonsense transcripts homolog b (yeast)  UPF3A - upf3 regulator of nonsense transcripts homolog a (yeast)  RPS23 - ribosomal protein s23  PAPOLG - poly(a) polymerase gamma  RAVER1 - ribonucleoprotein, ptb-binding 1  CCNT2 - cyclin t2  NPM3 - nucleophosmin/nucleoplasmin 3  THG1L - trna-histidine guanylyltransferase 1-like (s. cerevisiae)  CCNT1 - cyclin t1  CCNH - cyclin h  RNGTT - rna guanylyltransferase and 5'-phosphatase  RNMT - rna (guanine-7-) methyltransferase  HNRNPU - heterogeneous nuclear ribonucleoprotein u (scaffold attachment factor a)  HNRNPK - heterogeneous nuclear ribonucleoprotein k  HNRNPL - heterogeneous nuclear ribonucleoprotein l  RPS15 - ribosomal protein s15  RPS12 - ribosomal protein s12  MRPS11 - mitochondrial ribosomal protein s11  CBFA2T3 - core-binding factor, runt domain, alpha subunit 2; translocated to, 3  CBFB - core-binding factor, beta subunit  NSA2 - nsa2 ribosome biogenesis homolog (s. cerevisiae)  HNRNPA2B1 - heterogeneous nuclear ribonucleoprotein a2/b1  HNRNPA1 - heterogeneous nuclear ribonucleoprotein a1  HNRNPC - heterogeneous nuclear ribonucleoprotein c (c1/c2)  RXRB - retinoid x receptor, beta  HNRNPF - heterogeneous nuclear ribonucleoprotein f  HNRNPD - heterogeneous nuclear ribonucleoprotein d (au-rich element rna binding protein 1, 37kda)  HNRNPH1 - heterogeneous nuclear ribonucleoprotein h1 (h)  HNRNPH3 - heterogeneous nuclear ribonucleoprotein h3 (2h9)  SARS2 - seryl-trna synthetase 2, mitochondrial  HNRNPH2 - heterogeneous nuclear ribonucleoprotein h2 (h')  TUT1 - terminal uridylyl transferase 1, u6 snrna-specific  CCNC - cyclin c  TRMT10C - trna methyltransferase 10 homolog c (s. cerevisiae)  PRMT5 - protein arginine methyltransferase 5  NR4A1 - nuclear receptor subfamily 4, group a, member 1  NABP1 - nucleic acid binding protein 1  PDE12 - phosphodiesterase 12  CD2BP2 - cd2 (cytoplasmic tail) binding protein 2  RBM27 - rna binding motif protein 27  IKBKAP - inhibitor of kappa light polypeptide gene enhancer in b-cells, kinase complex-associated protein  HTATSF1 - hiv-1 tat specific factor 1  ZNF638 - zinc finger protein 638  CDC5L - cell division cycle 5-like  TBL3 - transducin (beta)-like 3  RBM15 - rna binding motif protein 15  POLR3F - polymerase (rna) iii (dna directed) polypeptide f, 39 kda  RBMX - rna binding motif protein, x-linked  HNRNPA3 - heterogeneous nuclear ribonucleoprotein a3  PPM1D - protein phosphatase, mg2+/mn2+ dependent, 1d  SRSF12 - serine/arginine-rich splicing factor 12  PRPF8 - pre-mrna processing factor 8  PRPF19 - pre-mrna processing factor 19  UTP20 - utp20, small subunit (ssu) processome component, homolog (yeast)  WDR36 - wd repeat domain 36  KHDRBS3 - kh domain containing, rna binding, signal transduction associated 3  PATL1 - protein associated with topoisomerase ii homolog 1 (yeast)  ADAT2 - adenosine deaminase, trna-specific 2  LMO4 - lim domain only 4  TAF6L - taf6-like rna polymerase ii, p300/cbp-associated factor (pcaf)-associated factor, 65kda  POLR3G - polymerase (rna) iii (dna directed) polypeptide g (32kd)  GAR1 - gar1 ribonucleoprotein  RNASEH2A - ribonuclease h2, subunit a  PRKRA - protein kinase, interferon-inducible double stranded rna dependent activator  PTBP1 - polypyrimidine tract binding protein 1  PTEN - phosphatase and tensin homolog  YARS - tyrosyl-trna synthetase  ZBTB8OS - zinc finger and btb domain containing 8 opposite strand  RRP1 - ribosomal rna processing 1  KHSRP - kh-type splicing regulatory protein  DDX17 - dead (asp-glu-ala-asp) box helicase 17  POLR1E - polymerase (rna) i polypeptide e, 53kda  CHERP - calcium homeostasis endoplasmic reticulum protein  PRPF18 - pre-mrna processing factor 18  GPKOW - g patch domain and kow motifs  NOP56 - nop56 ribonucleoprotein  EIF6 - eukaryotic translation initiation factor 6  CEBPG - ccaat/enhancer binding protein (c/ebp), gamma  SLU7 - slu7 splicing factor homolog (s. cerevisiae)  CDK4 - cyclin-dependent kinase 4  PTMA - prothymosin, alpha  CDK9 - cyclin-dependent kinase 9  RNASET2 - ribonuclease t2  CDK8 - cyclin-dependent kinase 8  RTCA - rna 3'-terminal phosphate cyclase  CDK7 - cyclin-dependent kinase 7  CDK13 - cyclin-dependent kinase 13  PCBP3 - poly(rc) binding protein 3  LSM3 - lsm3 homolog, u6 small nuclear rna associated (s. cerevisiae)  CSDC2 - cold shock domain containing c2, rna binding  RPP38 - ribonuclease p/mrp 38kda subunit  MOCS3 - molybdenum cofactor synthesis 3  DIMT1 - dim1 dimethyladenosine transferase 1 homolog (s. cerevisiae)  CRCP - cgrp receptor component  NOP14 - nop14 nucleolar protein  WDR4 - wd repeat domain 4  RRN3 - rrn3 rna polymerase i transcription factor homolog (s. cerevisiae)  GTPBP2 - gtp binding protein 2  METTL6 - methyltransferase like 6  ZNHIT6 - zinc finger, hit-type containing 6  PAPD5 - pap associated domain containing 5  NUDT16 - nudix (nucleoside diphosphate linked moiety x)-type motif 16  POP4 - processing of precursor 4, ribonuclease p/mrp subunit (s. cerevisiae)  SRSF10 - serine/arginine-rich splicing factor 10  AFF4 - af4/fmr2 family, member 4  AHCYL1 - adenosylhomocysteinase-like 1  MMS19 - mms19 nucleotide excision repair homolog (s. cerevisiae)  MTERFD2 - mterf domain containing 2  OSGEPL1 - o-sialoglycoprotein endopeptidase-like 1  METTL2A - methyltransferase like 2a  QARS - glutaminyl-trna synthetase  RRAGC - ras-related gtp binding c  PAF1 - paf1, rna polymerase ii associated factor, homolog (s. cerevisiae)  RPP40 - ribonuclease p/mrp 40kda subunit  INO80 - ino80 complex subunit  DDX56 - dead (asp-glu-ala-asp) box helicase 56  RC3H2 - ring finger and ccch-type domains 2  RBPJ - recombination signal binding protein for immunoglobulin kappa j region  PABPC1 - poly(a) binding protein, cytoplasmic 1  TRUB2 - trub pseudouridine (psi) synthase homolog 2 (e. coli)  DUSP11 - dual specificity phosphatase 11 (rna/rnp complex 1-interacting)  RARG - retinoic acid receptor, gamma  RBM26 - rna binding motif protein 26  RARB - retinoic acid receptor, beta  RARS - arginyl-trna synthetase  CELF1 - cugbp, elav-like family member 1  KHDRBS1 - kh domain containing, rna binding, signal transduction associated 1  TAF8 - taf8 rna polymerase ii, tata box binding protein (tbp)-associated factor, 43kda  CELF2 - cugbp, elav-like family member 2  IK - ik cytokine, down-regulator of hla ii  DDX49 - dead (asp-glu-ala-asp) box polypeptide 49  SMARCA5 - swi/snf related, matrix associated, actin dependent regulator of chromatin, subfamily a, member 5  RBMS1 - rna binding motif, single stranded interacting protein 1  RBM3 - rna binding motif (rnp1, rrm) protein 3  DIS3L2 - dis3 mitotic control homolog (s. cerevisiae)-like 2  RBM4 - rna binding motif protein 4  DIEXF - digestive organ expansion factor homolog (zebrafish)  PELP1 - proline, glutamate and leucine rich protein 1  GEMIN2 - gem (nuclear organelle) associated protein 2  TYW5 - trna-yw synthesizing protein 5  RBBP6 - retinoblastoma binding protein 6  SND1 - staphylococcal nuclease and tudor domain containing 1  DGCR8 - dgcr8 microprocessor complex subunit  PUS7 - pseudouridylate synthase 7 homolog (s. cerevisiae)  DHX16 - deah (asp-glu-ala-his) box polypeptide 16  EXOSC4 - exosome component 4  ANKRD16 - ankyrin repeat domain 16  PCIF1 - pdx1 c-terminal inhibiting factor 1  NFAT5 - nuclear factor of activated t-cells 5, tonicity-responsive  USP39 - ubiquitin specific peptidase 39  SUPT3H - suppressor of ty 3 homolog (s. cerevisiae)  RSRC1 - arginine/serine-rich coiled-coil 1  WTAP - wilms tumor 1 associated protein  RBM39 - rna binding motif protein 39  MKI67IP - mki67 (fha domain) interacting nucleolar phosphoprotein  METTL14 - methyltransferase like 14  LIG1 - ligase i, dna, atp-dependent  LIG3 - ligase iii, dna, atp-dependent  CIRH1A - cirrhosis, autosomal recessive 1a (cirhin)  CWC22 - cwc22 spliceosome-associated protein homolog (s. cerevisiae)  SP1 - sp1 transcription factor  CIR1 - corepressor interacting with rbpj, 1  RPP25L - ribonuclease p/mrp 25kda subunit-like  GTPBP4 - gtp binding protein 4  LSM2 - lsm2 homolog, u6 small nuclear rna associated (s. cerevisiae)  GTF2IRD1 - gtf2i repeat domain containing 1  SON - son dna binding protein  CREBL2 - camp responsive element binding protein-like 2  CREBBP - creb binding protein  SUGP1 - surp and g patch domain containing 1  ZC3H8 - zinc finger ccch-type containing 8  CREB1 - camp responsive element binding protein 1  PRPF38A - prp38 pre-mrna processing factor 38 (yeast) domain containing a  SNRPN - small nuclear ribonucleoprotein polypeptide n  SNRPB2 - small nuclear ribonucleoprotein polypeptide b  RPRD1B - regulation of nuclear pre-mrna domain containing 1b  SNRPD1 - small nuclear ribonucleoprotein d1 polypeptide 16kda  DDX41 - dead (asp-glu-ala-asp) box polypeptide 41  SNRPF - small nuclear ribonucleoprotein polypeptide f  SNAPC4 - small nuclear rna activating complex, polypeptide 4, 190kda  YTHDF2 - yth domain family, member 2  SNRNP70 - small nuclear ribonucleoprotein 70kda (u1)  TBPL1 - tbp-like 1  SNRPA - small nuclear ribonucleoprotein polypeptide a  MAK16 - mak16 homolog (s. cerevisiae)  SNRPA1 - small nuclear ribonucleoprotein polypeptide a'  EEF1E1 - eukaryotic translation elongation factor 1 epsilon 1  SNRPB - small nuclear ribonucleoprotein polypeptides b and b1  SNAPC2 - small nuclear rna activating complex, polypeptide 2, 45kda  SNAPC1 - small nuclear rna activating complex, polypeptide 1, 43kda  SNAPC3 - small nuclear rna activating complex, polypeptide 3, 50kda  SMN1 - survival of motor neuron 1, telomeric  POLR1C - polymerase (rna) i polypeptide c, 30kda  RBM17 - rna binding motif protein 17  LSM5 - lsm5 homolog, u6 small nuclear rna associated (s. cerevisiae)  SMARCD3 - swi/snf related, matrix associated, actin dependent regulator of chromatin, subfamily d, member 3  MED20 - mediator complex subunit 20  RBM25 - rna binding motif protein 25  COL4A2 - collagen, type iv, alpha 2  EDC4 - enhancer of mrna decapping 4  CACTIN - cactin, spliceosome c complex subunit  ZBTB7A - zinc finger and btb domain containing 7a  CRNKL1 - crooked neck pre-mrna splicing factor-like 1 (drosophila)  CDC40 - cell division cycle 40  SCAF1 - sr-related ctd-associated factor 1  MED27 - mediator complex subunit 27  TFIP11 - tuftelin interacting protein 11  MED26 - mediator complex subunit 26  CSTF1 - cleavage stimulation factor, 3' pre-rna, subunit 1, 50kda  POLR1B - polymerase (rna) i polypeptide b, 128kda  FTSJ1 - ftsj rna methyltransferase homolog 1 (e. coli)  DBR1 - debranching rna lariats 1  MED17 - mediator complex subunit 17  MED23 - mediator complex subunit 23  PRPF40B - prp40 pre-mrna processing factor 40 homolog b (s. cerevisiae)  CSTF3 - cleavage stimulation factor, 3' pre-rna, subunit 3, 77kda  MRTO4 - mrna turnover 4 homolog (s. cerevisiae)  PRPF6 - pre-mrna processing factor 6  CSTF2 - cleavage stimulation factor, 3' pre-rna, subunit 2, 64kda  RPF2 - ribosome production factor 2 homolog (s. cerevisiae)  SUPT6H - suppressor of ty 6 homolog (s. cerevisiae)  CTGF - connective tissue growth factor  SUPV3L1 - suppressor of var1, 3-like 1 (s. cerevisiae)  SUPT4H1 - suppressor of ty 4 homolog 1 (s. cerevisiae)  SUPT5H - suppressor of ty 5 homolog (s. cerevisiae)  GTPBP3 - gtp binding protein 3 (mitochondrial)  UBL5 - ubiquitin-like 5  TRMT11 - trna methyltransferase 11 homolog (s. cerevisiae)  SBDS - shwachman-bodian-diamond syndrome  LACTB2 - lactamase, beta 2  DDX23 - dead (asp-glu-ala-asp) box polypeptide 23  GPBP1L1 - gc-rich promoter binding protein 1-like 1  TFB1M - transcription factor b1, mitochondrial  PAN3 - pan3 poly(a) specific ribonuclease subunit homolog (s. cerevisiae)  MED10 - mediator complex subunit 10  MED21 - mediator complex subunit 21  SNRNP40 - small nuclear ribonucleoprotein 40kda (u5)  ZRANB2 - zinc finger, ran-binding domain containing 2  POLDIP3 - polymerase (dna-directed), delta interacting protein 3  BUD13 - bud13 homolog (s. cerevisiae)  STAT3 - signal transducer and activator of transcription 3 (acute-phase response factor)  PPIG - peptidylprolyl isomerase g (cyclophilin g)  POLR3GL - polymerase (rna) iii (dna directed) polypeptide g (32kd)-like  SSRP1 - structure specific recognition protein 1  SLC25A33 - solute carrier family 25 (pyrimidine nucleotide carrier), member 33  DHX35 - deah (asp-glu-ala-his) box polypeptide 35  PHF5A - phd finger protein 5a  NUDT16L1 - nudix (nucleoside diphosphate linked moiety x)-type motif 16-like 1  GTF3C5 - general transcription factor iiic, polypeptide 5, 63kda  SRPK2 - srsf protein kinase 2  ZNHIT3 - zinc finger, hit-type containing 3  SRPK1 - srsf protein kinase 1  UTP23 - utp23, small subunit (ssu) processome component, homolog (yeast)  TROVE2 - trove domain family, member 2  COPS2 - cop9 signalosome subunit 2  DDX47 - dead (asp-glu-ala-asp) box polypeptide 47  SSB - sjogren syndrome antigen b (autoantigen la)  THOC3 - tho complex 3  EFTUD2 - elongation factor tu gtp binding domain containing 2  SARNP - sap domain containing ribonucleoprotein  GTF3C4 - general transcription factor iiic, polypeptide 4, 90kda  THADA - thyroid adenoma associated  GTF3C3 - general transcription factor iiic, polypeptide 3, 102kda  MTO1 - mitochondrial trna translation optimization 1  CNOT8 - ccr4-not transcription complex, subunit 8  NSUN3 - nop2/sun domain family, member 3  SNORD31 - small nucleolar rna, c/d box 31  CDK12 - cyclin-dependent kinase 12  DHX9 - deah (asp-glu-ala-his) box helicase 9  DHX8 - deah (asp-glu-ala-his) box polypeptide 8  SNORD29 - small nucleolar rna, c/d box 29  TSR2 - tsr2, 20s rrna accumulation, homolog (s. cerevisiae)  DDX6 - dead (asp-glu-ala-asp) box helicase 6  SRSF11 - serine/arginine-rich splicing factor 11  DDX5 - dead (asp-glu-ala-asp) box helicase 5  DDX3X - dead (asp-glu-ala-asp) box helicase 3, x-linked  LUC7L3 - luc7-like 3 (s. cerevisiae)  DDX1 - dead (asp-glu-ala-asp) box helicase 1  SYF2 - syf2 pre-mrna-splicing factor  SNORD22 - small nucleolar rna, c/d box 22  SAMHD1 - sam domain and hd domain 1  SNORD30 - small nucleolar rna, c/d box 30  WWTR1 - ww domain containing transcription regulator 1  MLH1 - mutl homolog 1, colon cancer, nonpolyposis type 2 (e. coli)  RSF1 - remodeling and spacing factor 1  GEMIN5 - gem (nuclear organelle) associated protein 5  NOL11 - nucleolar protein 11  MLLT3 - myeloid/lymphoid or mixed-lineage leukemia (trithorax homolog, drosophila); translocated to, 3  MED14 - mediator complex subunit 14  MLLT1 - myeloid/lymphoid or mixed-lineage leukemia (trithorax homolog, drosophila); translocated to, 1  THUMPD3 - thump domain containing 3  WDR46 - wd repeat domain 46  PHAX - phosphorylated adaptor for rna export  NELFB - negative elongation factor complex member b  CPSF3 - cleavage and polyadenylation specific factor 3, 73kda  NAA38 - n(alpha)-acetyltransferase 38, natc auxiliary subunit  LSM7 - lsm7 homolog, u6 small nuclear rna associated (s. cerevisiae)  CNOT10 - ccr4-not transcription complex, subunit 10  MNAT1 - mnat cdk-activating kinase assembly factor 1  POLR1A - polymerase (rna) i polypeptide a, 194kda  ZNF473 - zinc finger protein 473  NOLC1 - nucleolar and coiled-body phosphoprotein 1  DCAF13 - ddb1 and cul4 associated factor 13  MNT - mnt, max dimerization protein  POLR3K - polymerase (rna) iii (dna directed) polypeptide k, 12.3 kda  DDX10 - dead (asp-glu-ala-asp) box polypeptide 10  WDR18 - wd repeat domain 18  WBP11 - ww domain binding protein 11  DHX15 - deah (asp-glu-ala-his) box helicase 15  CNOT6 - ccr4-not transcription complex, subunit 6  NSRP1 - nuclear speckle splicing regulatory protein 1  CWF19L2 - cwf19-like 2, cell cycle control (s. pombe)  ATXN2 - ataxin 2  ERI1 - exoribonuclease 1  SCAF4 - sr-related ctd-associated factor 4  MECP2 - methyl cpg binding protein 2 (rett syndrome)  PELO - pelota homolog (drosophila)  MEF2A - myocyte enhancer factor 2a  MED30 - mediator complex subunit 30  DDX21 - dead (asp-glu-ala-asp) box helicase 21  PPIL3 - peptidylprolyl isomerase (cyclophilin)-like 3  BCDIN3D - bcdin3 domain containing  RPAP1 - rna polymerase ii associated protein 1  CPSF2 - cleavage and polyadenylation specific factor 2, 100kda  DARS - aspartyl-trna synthetase  INTS2 - integrator complex subunit 2  WDR75 - wd repeat domain 75  ANGEL2 - angel homolog 2 (drosophila)  XPO5 - exportin 5  SCAF11 - sr-related ctd-associated factor 11  UTP15 - utp15, u3 small nucleolar ribonucleoprotein, homolog (s. cerevisiae)  HINFP - histone h4 transcription factor  METTL1 - methyltransferase like 1  MFAP1 - microfibrillar-associated protein 1  REXO2 - rna exonuclease 2  AARS2 - alanyl-trna synthetase 2, mitochondrial  RNASEH2C - ribonuclease h2, subunit c  PPP1R1B - protein phosphatase 1, regulatory (inhibitor) subunit 1b  SARS - seryl-trna synthetase  NGDN - neuroguidin, eif4e binding protein  NYNRIN - nyn domain and retroviral integrase containing  CBFA2T2 - core-binding factor, runt domain, alpha subunit 2; translocated to, 2  RRP9 - ribosomal rna processing 9, small subunit (ssu) processome component, homolog (yeast)  MAGOH - mago-nashi homolog, proliferation-associated (drosophila)  PRPF3 - pre-mrna processing factor 3  LARS - leucyl-trna synthetase  PRPF4 - prp4 pre-mrna processing factor 4 homolog (yeast)  RQCD1 - rcd1 required for cell differentiation1 homolog (s. pombe)  CNOT6L - ccr4-not transcription complex, subunit 6-like  EAF1 - ell associated factor 1  MARS - methionyl-trna synthetase  PUS3 - pseudouridylate synthase 3  MAPT - microtubule-associated protein tau  RNASEH1 - ribonuclease h1  DOM3Z - dom-3 homolog z (c. elegans)  PRPF31 - pre-mrna processing factor 31  DR1 - down-regulator of transcription 1, tbp-binding (negative cofactor 2)  TRMT5 - trna methyltransferase 5  TNKS1BP1 - tankyrase 1 binding protein 1, 182kda  SKI - v-ski avian sarcoma viral oncogene homolog  SKIV2L - superkiller viralicidic activity 2-like (s. cerevisiae)  MBD1 - methyl-cpg binding domain protein 1  CHTOP - chromatin target of prmt1  SART1 - squamous cell carcinoma antigen recognized by t cells  ZCRB1 - zinc finger cchc-type and rna binding motif 1  TRMT112 - trna methyltransferase 11-2 homolog (s. cerevisiae)  NELFCD - negative elongation factor complex member c/d  CWC15 - cwc15 spliceosome-associated protein homolog (s. cerevisiae)  RTCB - rna 2',3'-cyclic phosphate and 5'-oh ligase  NUDT12 - nudix (nucleoside diphosphate linked moiety x)-type motif 12  DTX1 - deltex homolog 1 (drosophila)  PPIL1 - peptidylprolyl isomerase (cyclophilin)-like 1  DKC1 - dyskeratosis congenita 1, dyskerin  PNPT1 - polyribonucleotide nucleotidyltransferase 1  RPL14 - ribosomal protein l14  RIOK1 - rio kinase 1  RNASEK - ribonuclease, rnase k  RBFOX3 - rna binding protein, fox-1 homolog (c. elegans) 3  CDK5RAP1 - cdk5 regulatory subunit associated protein 1  DHX37 - deah (asp-glu-ala-his) box polypeptide 37  TAF1B - tata box binding protein (tbp)-associated factor, rna polymerase i, b, 63kda  ESF1 - esf1, nucleolar pre-rrna processing protein, homolog (s. cerevisiae)  SFPQ - splicing factor proline/glutamine-rich  PHRF1 - phd and ring finger domains 1  PCF11 - pcf11 cleavage and polyadenylation factor subunit  MED15 - mediator complex subunit 15  SRSF1 - serine/arginine-rich splicing factor 1  SRSF2 - serine/arginine-rich splicing factor 2  GMEB2 - glucocorticoid modulatory element binding protein 2  SRRT - serrate rna effector molecule homolog (arabidopsis)  SMAD2 - smad family member 2  SRSF3 - serine/arginine-rich splicing factor 3  SRSF4 - serine/arginine-rich splicing factor 4  TRDMT1 - trna aspartic acid methyltransferase 1  SRSF5 - serine/arginine-rich splicing factor 5  SRSF6 - serine/arginine-rich splicing factor 6  TMTC1 - transmembrane and tetratricopeptide repeat containing 1  SRSF7 - serine/arginine-rich splicing factor 7  SFSWAP - splicing factor, suppressor of white-apricot homolog (drosophila)  TRA2B - transformer 2 beta homolog (drosophila)  TAF1C - tata box binding protein (tbp)-associated factor, rna polymerase i, c, 110kda  LARP7 - la ribonucleoprotein domain family, member 7  TAF9B - taf9b rna polymerase ii, tata box binding protein (tbp)-associated factor, 31kda  RSL1D1 - ribosomal l1 domain containing 1  SMAD4 - smad family member 4  NBAS - neuroblastoma amplified sequence  INTS1 - integrator complex subunit 1  TRMT6 - trna methyltransferase 6 homolog (s. cerevisiae)  RBM4B - rna binding motif protein 4b  SENP3 - sumo1/sentrin/smt3 specific peptidase 3  NCBP2 - nuclear cap binding protein subunit 2, 20kda  NCBP1 - nuclear cap binding protein subunit 1, 80kda  TSN - translin  MBNL2 - muscleblind-like splicing regulator 2  SUGP2 - surp and g patch domain containing 2  NAF1 - nuclear assembly factor 1 ribonucleoprotein  TRMT1L - trna methyltransferase 1 homolog (s. cerevisiae)-like  SNW1 - snw domain containing 1  HNRNPM - heterogeneous nuclear ribonucleoprotein m  ELL2 - elongation factor, rna polymerase ii, 2  TRIM28 - tripartite motif containing 28  DTD1 - d-tyrosyl-trna deacylase 1  DROSHA - drosha, ribonuclease type iii  KIN - kin, antigenic determinant of reca protein homolog (mouse)  HABP4 - hyaluronan binding protein 4  NARS - asparaginyl-trna synthetase  CELF4 - cugbp, elav-like family member 4  RBM7 - rna binding motif protein 7  RBM6 - rna binding motif protein 6  RBM5 - rna binding motif protein 5  EGR2 - early growth response 2  TTF1 - transcription termination factor, rna polymerase i  ALYREF - aly/ref export factor  INTS4 - integrator complex subunit 4  ELAVL1 - elav (embryonic lethal, abnormal vision, drosophila)-like 1 (hu antigen r)  CCDC130 - coiled-coil domain containing 130  ZNF326 - zinc finger protein 326  MPHOSPH6 - m-phase phosphoprotein 6  ELAVL4 - elav (embryonic lethal, abnormal vision, drosophila)-like 4  MPHOSPH10 - m-phase phosphoprotein 10 (u3 small nucleolar ribonucleoprotein)  URM1 - ubiquitin related modifier 1  USPL1 - ubiquitin specific peptidase like 1  PDCD11 - programmed cell death 11  ABT1 - activator of basal transcription 1  TSNAX - translin-associated factor x  TOPORS - topoisomerase i binding, arginine/serine-rich, e3 ubiquitin protein ligase  ACIN1 - apoptotic chromatin condensation inducer 1  FIP1L1 - factor interacting with papola and cpsf1  CNOT1 - ccr4-not transcription complex, subunit 1  EXOSC7 - exosome component 7  LAS1L - las1-like (s. cerevisiae)  SPEN - spen homolog, transcriptional regulator (drosophila)  ISG20L2 - interferon stimulated exonuclease gene 20kda-like 2  PDCD7 - programmed cell death 7  TRMT10A - trna methyltransferase 10 homolog a (s. cerevisiae)  CNOT7 - ccr4-not transcription complex, subunit 7  PQBP1 - polyglutamine binding protein 1  TRA2A - transformer 2 alpha homolog (drosophila)  KDM1A - lysine (k)-specific demethylase 1a  SF3B5 - splicing factor 3b, subunit 5, 10kda  CPSF1 - cleavage and polyadenylation specific factor 1, 160kda  RBM15B - rna binding motif protein 15b  SNRNP200 - small nuclear ribonucleoprotein 200kda (u5)  HNRNPLL - heterogeneous nuclear ribonucleoprotein l-like  IMP4 - imp4, u3 small nucleolar ribonucleoprotein, homolog (yeast)  NCOA6 - nuclear receptor coactivator 6  SMG1 - smg1 phosphatidylinositol 3-kinase-related kinase  NRBP1 - nuclear receptor binding protein 1  XAB2 - xpa binding protein 2  ENY2 - enhancer of yellow 2 homolog (drosophila)  RRP1B - ribosomal rna processing 1b  CMTR1 - cap methyltransferase 1  NR2C1 - nuclear receptor subfamily 2, group c, member 1  SETX - senataxin  DUS3L - dihydrouridine synthase 3-like (s. cerevisiae)  NR2C2 - nuclear receptor subfamily 2, group c, member 2  MARS2 - methionyl-trna synthetase 2, mitochondrial  MED25 - mediator complex subunit 25  MED6 - mediator complex subunit 6  NAT14 - n-acetyltransferase 14 (gcn5-related, putative)  REXO4 - rex4, rna exonuclease 4 homolog (s. cerevisiae)  PTBP3 - polypyrimidine tract binding protein 3  INTS12 - integrator complex subunit 12  ZC3H13 - zinc finger ccch-type containing 13  SNORD107 - small nucleolar rna, c/d box 107  FARSA - phenylalanyl-trna synthetase, alpha subunit  MTF1 - metal-regulatory transcription factor 1  NR1H2 - nuclear receptor subfamily 1, group h, member 2  DCPS - decapping enzyme, scavenger  UTP3 - utp3, small subunit (ssu) processome component, homolog (s. cerevisiae)  MED16 - mediator complex subunit 16  DDX24 - dead (asp-glu-ala-asp) box helicase 24  FARSB - phenylalanyl-trna synthetase, beta subunit  ZC3H3 - zinc finger ccch-type containing 3  VARS - valyl-trna synthetase  CCNL1 - cyclin l1  ENDOV - endonuclease v  WDR43 - wd repeat domain 43  RARS2 - arginyl-trna synthetase 2, mitochondrial  ERCC3 - excision repair cross-complementing rodent repair deficiency, complementation group 3  LIN52 - lin-52 homolog (c. elegans)  PAN2 - pan2 poly(a) specific ribonuclease subunit homolog (s. cerevisiae)  YTHDC1 - yth domain containing 1  THOC2 - tho complex 2  NOB1 - nin1/rpn12 binding protein 1 homolog (s. cerevisiae)  ERN1 - endoplasmic reticulum to nucleus signaling 1  VARS2 - valyl-trna synthetase 2, mitochondrial  HIRA - histone cell cycle regulator  SNORA3 - small nucleolar rna, h/aca box 3  ETV1 - ets variant 1  RBM8A - rna binding motif protein 8a  MDN1 - mdn1, midasin homolog (yeast)  POLR2M - polymerase (rna) ii (dna directed) polypeptide m  ETF1 - eukaryotic translation termination factor 1  UBTF - upstream binding transcription factor, rna polymerase i  ZNF830 - zinc finger protein 830  METTL5 - methyltransferase like 5  RRS1 - rrs1 ribosome biogenesis regulator homolog (s. cerevisiae)  MED4 - mediator complex subunit 4  JMJD6 - jumonji domain containing 6  THOC1 - tho complex 1  SETD2 - set domain containing 2  EPRS - glutamyl-prolyl-trna synthetase  ZC3H7A - zinc finger ccch-type containing 7a  ERCC2 - excision repair cross-complementing rodent repair deficiency, complementation group 2  THRAP3 - thyroid hormone receptor associated protein 3  MED12 - mediator complex subunit 12  MED13 - mediator complex subunit 13  ZC3H7B - zinc finger ccch-type containing 7b  MED24 - mediator complex subunit 24  FMR1 - fragile x mental retardation 1  HENMT1 - hen1 methyltransferase homolog 1 (arabidopsis)  TCOF1 - treacher collins-franceschetti syndrome 1  INTS5 - integrator complex subunit 5  BOP1 - block of proliferation 1  THUMPD2 - thump domain containing 2  RPRD2 - regulation of nuclear pre-mrna domain containing 2  FLNA - filamin a, alpha  PPP4R2 - protein phosphatase 4, regulatory subunit 2  FOXO3 - forkhead box o3  RBM19 - rna binding motif protein 19  UTP6 - utp6, small subunit (ssu) processome component, homolog (yeast)  DCP1A - decapping mrna 1a  RPUSD1 - rna pseudouridylate synthase domain containing 1  MED8 - mediator complex subunit 8  DDX46 - dead (asp-glu-ala-asp) box polypeptide 46  SMG6 - smg6 nonsense mediated mrna decay factor  EXOSC3 - exosome component 3  WDR61 - wd repeat domain 61  SMG7 - smg7 nonsense mediated mrna decay factor  CSTF2T - cleavage stimulation factor, 3' pre-rna, subunit 2, 64kda, tau variant  EXOSC1 - exosome component 1  TAF9 - taf9 rna polymerase ii, tata box binding protein (tbp)-associated factor, 32kda  BMS1 - bms1 ribosome biogenesis factor  TAF7 - taf7 rna polymerase ii, tata box binding protein (tbp)-associated factor, 55kda  FAM98B - family with sequence similarity 98, member b  RRP15 - ribosomal rna processing 15 homolog (s. cerevisiae)  TAF6 - taf6 rna polymerase ii, tata box binding protein (tbp)-associated factor, 80kda  KHNYN - kh and nyn domain containing  U2SURP - u2 snrnp-associated surp domain containing  TAF4 - taf4 rna polymerase ii, tata box binding protein (tbp)-associated factor, 135kda  TAF2 - taf2 rna polymerase ii, tata box binding protein (tbp)-associated factor, 150kda  EIF4A3 - eukaryotic translation initiation factor 4a3  TARS - threonyl-trna synthetase  NR4A2 - nuclear receptor subfamily 4, group a, member 2  ZBTB7B - zinc finger and btb domain containing 7b  TARBP1 - tar (hiv-1) rna binding protein 1  FEN1 - flap structure-specific endonuclease 1  DHX38 - deah (asp-glu-ala-his) box polypeptide 38  NVL - nuclear vcp-like  SMG9 - smg9 nonsense mediated mrna decay factor  TAF13 - taf13 rna polymerase ii, tata box binding protein (tbp)-associated factor, 18kda  ZCCHC11 - zinc finger, cchc domain containing 11  LARS2 - leucyl-trna synthetase 2, mitochondrial  FCF1 - fcf1 rrna-processing protein  TSEN54 - tsen54 trna splicing endonuclease subunit  CTIF - cbp80/20-dependent translation initiation factor  PUM2 - pumilio homolog 2 (drosophila)  TCEB3 - transcription elongation factor b (siii), polypeptide 3 (110kda, elongin a)  POLR1D - polymerase (rna) i polypeptide d, 16kda  TRNT1 - trna nucleotidyl transferase, cca-adding, 1  UTP18 - utp18 small subunit (ssu) processome component homolog (yeast)  RRNAD1 - ribosomal rna adenine dimethylase domain containing 1  TOE1 - target of egr1, member 1 (nuclear)  TCEA2 - transcription elongation factor a (sii), 2  ANGEL1 - angel homolog 1 (drosophila)  TCEA1 - transcription elongation factor a (sii), 1  SIDT2 - sid1 transmembrane family, member 2  NRBF2 - nuclear receptor binding factor 2  NOP2 - nop2 nucleolar protein  NONO - non-pou domain containing, octamer-binding  SNORD118 - small nucleolar rna, c/d box 118  PPWD1 - peptidylprolyl isomerase domain and wd repeat containing 1  NOTCH4 - notch 4  NOVA2 - neuro-oncological ventral antigen 2  NOVA1 - neuro-oncological ventral antigen 1  AQR - aquarius homolog (mouse)  EXOSC2 - exosome component 2  CNOT2 - ccr4-not transcription complex, subunit 2  PUM1 - pumilio homolog 1 (drosophila)  CNOT4 - ccr4-not transcription complex, subunit 4  CNOT3 - ccr4-not transcription complex, subunit 3  NOTCH1 - notch 1  NOTCH3 - notch 3  NOTCH2 - notch 2  DHX34 - deah (asp-glu-ala-his) box polypeptide 34  NFYA - nuclear transcription factor y, alpha  NFRKB - nuclear factor related to kappab binding protein  THOC7 - tho complex 7 homolog (drosophila)  EDC3 - enhancer of mrna decapping 3  PHF3 - phd finger protein 3  ZMAT2 - zinc finger, matrin-type 2  SRFBP1 - serum response factor binding protein 1  NFKB1 - nuclear factor of kappa light polypeptide gene enhancer in b-cells 1  NFKBIB - nuclear factor of kappa light polypeptide gene enhancer in b-cells inhibitor, beta  HARS2 - histidyl-trna synthetase 2, mitochondrial  TARDBP - tar dna binding protein  SNRNP48 - small nuclear ribonucleoprotein 48kda (u11/u12)  NHP2L1 - nhp2 non-histone chromosome protein 2-like 1 (s. cerevisiae)  ZNRD1 - zinc ribbon domain containing 1  SART3 - squamous cell carcinoma antigen recognized by t cells 3  SF3B1 - splicing factor 3b, subunit 1, 155kda  SF3B3 - splicing factor 3b, subunit 3, 130kda  TTC37 - tetratricopeptide repeat domain 37  TFAM - transcription factor a, mitochondrial  CTR9 - ctr9, paf1/rna polymerase ii complex component  SKIV2L2 - superkiller viralicidic activity 2-like 2 (s. cerevisiae)  GTF3C6 - general transcription factor iiic, polypeptide 6, alpha 35kda  TFDP1 - transcription factor dp-1  EPC1 - enhancer of polycomb homolog 1 (drosophila)  PES1 - pescadillo ribosomal biogenesis factor 1  NR2F1 - nuclear receptor subfamily 2, group f, member 1  TFCP2 - transcription factor cp2  TGS1 - trimethylguanosine synthase 1  KIAA0391 - kiaa0391  RBFOX2 - rna binding protein, fox-1 homolog (c. elegans) 2  CTNNBL1 - catenin, beta like 1  MEPCE - methylphosphate capping enzyme  GEMIN4 - gem (nuclear organelle) associated protein 4  FOSB - fbj murine osteosarcoma viral oncogene homolog b  FOS - fbj murine osteosarcoma viral oncogene homolog  DRG1 - developmentally regulated gtp binding protein 1  SRRM2 - serine/arginine repetitive matrix 2  GATC - glutamyl-trna(gln) amidotransferase, subunit c  TARS2 - threonyl-trna synthetase 2, mitochondrial (putative)  METTL3 - methyltransferase like 3  THRA - thyroid hormone receptor, alpha |
| GO:0051640 | organelle localization | 9.56E-15 | 1.02E-11 | 1.25 (10334,389,6996,329) | [+] Show genes  MAPRE1 - microtubule-associated protein, rp/eb family, member 1  CHMP4C - charged multivesicular body protein 4c  MARK2 - map/microtubule affinity-regulating kinase 2  KIFAP3 - kinesin-associated protein 3  AKAP9 - a kinase (prka) anchor protein (yotiao) 9  CEP89 - centrosomal protein 89kda  AP3M2 - adaptor-related protein complex 3, mu 2 subunit  SPIRE1 - spire actin nucleation factor 1  FLCN - folliculin  GEM - gtp binding protein overexpressed in skeletal muscle  MKS1 - meckel syndrome, type 1  HAUS7 - haus augmin-like complex, subunit 7  RAB3IP - rab3a interacting protein  LIN7A - lin-7 homolog a (c. elegans)  HOOK3 - hook microtubule-tethering protein 3  KATNB1 - katanin p80 (wd repeat containing) subunit b 1  SPAST - spastin  MKKS - mckusick-kaufman syndrome  KAT2A - k(lysine) acetyltransferase 2a  NUBP1 - nucleotide binding protein 1  LTV1 - ltv1 homolog (s. cerevisiae)  RAB3D - rab3d, member ras oncogene family  TUBG1 - tubulin, gamma 1  AP3B2 - adaptor-related protein complex 3, beta 2 subunit  NOP9 - nop9 nucleolar protein  SYBU - syntabulin (syntaxin-interacting)  FNBP1L - formin binding protein 1-like  NDEL1 - nude neurodevelopment protein 1-like 1  PLXNA2 - plexin a2  UBXN2B - ubx domain protein 2b  DEF8 - differentially expressed in fdcp 8 homolog (mouse)  SYNJ1 - synaptojanin 1  GJA1 - gap junction protein, alpha 1, 43kda  AZI1 - 5-azacytidine induced 1  BTBD8 - btb (poz) domain containing 8  SPIRE2 - spire actin nucleation factor 2  MYO1D - myosin id  MYO5A - myosin va (heavy chain 12, myoxin)  MYO1E - myosin ie  MYO6 - myosin vi  LLGL1 - lethal giant larvae homolog 1 (drosophila)  MARK4 - map/microtubule affinity-regulating kinase 4  NDE1 - nude neurodevelopment protein 1  ACTN4 - actinin, alpha 4  ABCE1 - atp-binding cassette, sub-family e (oabp), member 1  SEH1L - seh1-like (s. cerevisiae)  SNCA - synuclein, alpha (non a4 component of amyloid precursor)  MYH10 - myosin, heavy chain 10, non-muscle  MYH9 - myosin, heavy chain 9, non-muscle  RAB10 - rab10, member ras oncogene family  VPS4B - vacuolar protein sorting 4 homolog b (s. cerevisiae)  KXD1 - kxdl motif containing 1  SNAP25 - synaptosomal-associated protein, 25kda  HAUS6 - haus augmin-like complex, subunit 6  STX6 - syntaxin 6  TMEM201 - transmembrane protein 201  AHI1 - abelson helper integration site 1  AP3S2 - adaptor-related protein complex 3, sigma 2 subunit  GPSM2 - g-protein signaling modulator 2  ACTR2 - arp2 actin-related protein 2 homolog (yeast)  UNC13A - unc-13 homolog a (c. elegans)  ACTR3 - arp3 actin-related protein 3 homolog (yeast)  RABEPK - rab9 effector protein with kelch motifs  ATCAY - ataxia, cerebellar, cayman type  TRAK2 - trafficking protein, kinesin binding 2  SPRY1 - sprouty homolog 1, antagonist of fgf signaling (drosophila)  SPRY2 - sprouty homolog 2 (drosophila)  HSP90AA1 - heat shock protein 90kda alpha (cytosolic), class a member 1  LRPPRC - leucine-rich pentatricopeptide repeat containing  STX8 - syntaxin 8  ACTR1A - arp1 actin-related protein 1 homolog a, centractin alpha (yeast)  CEP76 - centrosomal protein 76kda  CPLX3 - complexin 3  AP3D1 - adaptor-related protein complex 3, delta 1 subunit  FBF1 - fas (tnfrsf6) binding factor 1  STX12 - syntaxin 12  WASF1 - was protein family, member 1  BLOC1S3 - biogenesis of lysosomal organelles complex-1, subunit 3  SYN1 - synapsin i  CDK5RAP2 - cdk5 regulatory subunit associated protein 2  SYN2 - synapsin ii  PRKACA - protein kinase, camp-dependent, catalytic, alpha  BOD1 - biorientation of chromosomes in cell division 1  KIF5C - kinesin family member 5c  KIF3C - kinesin family member 3c  SYT1 - synaptotagmin i  KIF5B - kinesin family member 5b  SYT5 - synaptotagmin v  KIF1B - kinesin family member 1b  SYT4 - synaptotagmin iv  KIF5A - kinesin family member 5a  SUN2 - sad1 and unc84 domain containing 2  KIF3A - kinesin family member 3a  DYNLL1 - dynein, light chain, lc8-type 1  PIBF1 - progesterone immunomodulatory binding factor 1  CTBP2 - c-terminal binding protein 2  MTM1 - myotubularin 1  VAMP2 - vesicle-associated membrane protein 2 (synaptobrevin 2)  FGFR1OP - fgfr1 oncogene partner  CCDC41 - coiled-coil domain containing 41  HDAC6 - histone deacetylase 6  CTNNB1 - catenin (cadherin-associated protein), beta 1, 88kda  RAB3C - rab3c, member ras oncogene family  CNTRL - centriolin  BECN1 - beclin 1, autophagy related  CLASP2 - cytoplasmic linker associated protein 2  STXBP1 - syntaxin binding protein 1  STX5 - syntaxin 5  STX4 - syntaxin 4  STX3 - syntaxin 3  STX1A - syntaxin 1a (brain)  RAB7A - rab7a, member ras oncogene family  STX17 - syntaxin 17  NDRG4 - ndrg family member 4  MGARP - mitochondria-localized glutamic acid-rich protein  EZR - ezrin  PPP2R1A - protein phosphatase 2, regulatory subunit a, alpha  KPNB1 - karyopherin (importin) beta 1  LAMTOR1 - late endosomal/lysosomal adaptor, mapk and mtor activator 1  STXBP3 - syntaxin binding protein 3  SYT3 - synaptotagmin iii  UVRAG - uv radiation resistance associated  TRAPPC12 - trafficking protein particle complex 12  UNC13B - unc-13 homolog b (c. elegans)  DNM1L - dynamin 1-like  CDC23 - cell division cycle 23  VPS33A - vacuolar protein sorting 33 homolog a (s. cerevisiae)  TCTN2 - tectonic family member 2  STK25 - serine/threonine kinase 25  EXOC4 - exocyst complex component 4  KIT - v-kit hardy-zuckerman 4 feline sarcoma viral oncogene homolog  VTI1B - vesicle transport through interaction with t-snares 1b  HIF1A - hypoxia inducible factor 1, alpha subunit (basic helix-loop-helix transcription factor)  PINX1 - pin2/terf1 interacting, telomerase inhibitor 1  TMEM216 - transmembrane protein 216  TSNARE1 - t-snare domain containing 1  TUBB - tubulin, beta class i  CHMP7 - charged multivesicular body protein 7  UBB - ubiquitin b  RPS15 - ribosomal protein s15  TUBB4B - tubulin, beta 4b class ivb  VPS33B - vacuolar protein sorting 33 homolog b (yeast)  KIF3B - kinesin family member 3b  TPGS1 - tubulin polyglutamylase complex subunit 1  POLR2M - polymerase (rna) ii (dna directed) polypeptide m  CBL - cbl proto-oncogene, e3 ubiquitin protein ligase  BLOC1S5 - biogenesis of lysosomal organelles complex-1, subunit 5, muted  RAB11A - rab11a, member ras oncogene family  UCHL1 - ubiquitin carboxyl-terminal esterase l1 (ubiquitin thiolesterase)  CHMP4A - charged multivesicular body protein 4a  RRS1 - rrs1 ribosome biogenesis regulator homolog (s. cerevisiae)  CAV2 - caveolin 2  SYT11 - synaptotagmin xi  HAUS1 - haus augmin-like complex, subunit 1  SNAP29 - synaptosomal-associated protein, 29kda  CSNK1D - casein kinase 1, delta  VAMP3 - vesicle-associated membrane protein 3  NR4A3 - nuclear receptor subfamily 4, group a, member 3  STX2 - syntaxin 2  PRKAR2B - protein kinase, camp-dependent, regulatory, type ii, beta  EXOC6B - exocyst complex component 6b  TMEM230 - transmembrane protein 230  MSTO1 - misato 1, mitochondrial distribution and morphology regulator  SDAD1 - sda1 domain containing 1  B9D2 - b9 protein domain 2  SCFD1 - sec1 family domain containing 1  RAB8B - rab8b, member ras oncogene family  FAM98A - family with sequence similarity 98, member a  SYT17 - synaptotagmin xvii  PLEKHM1 - pleckstrin homology domain containing, family m (with run domain) member 1  MLH1 - mutl homolog 1, colon cancer, nonpolyposis type 2 (e. coli)  RHOT2 - ras homolog family member t2  DCTN1 - dynactin 1  PPFIA2 - protein tyrosine phosphatase, receptor type, f polypeptide (ptprf), interacting protein (liprin), alpha 2  TBCCD1 - tbcc domain containing 1  PSEN2 - presenilin 2 (alzheimer disease 4)  DCTN3 - dynactin 3 (p22)  ACTR10 - actin-related protein 10 homolog (s. cerevisiae)  MCPH1 - microcephalin 1  PACS2 - phosphofurin acidic cluster sorting protein 2  RAB15 - rab15, member ras oncogene family  ARCN1 - archain 1  AP3B1 - adaptor-related protein complex 3, beta 1 subunit  PDCD10 - programmed cell death 10  BICD2 - bicaudal d homolog 2 (drosophila)  YKT6 - ykt6 v-snare homolog (s. cerevisiae)  PPFIA3 - protein tyrosine phosphatase, receptor type, f polypeptide (ptprf), interacting protein (liprin), alpha 3  ODF2 - outer dense fiber of sperm tails 2  MAP1S - microtubule-associated protein 1s  PAFAH1B1 - platelet-activating factor acetylhydrolase 1b, regulatory subunit 1 (45kda)  RAB11B - rab11b, member ras oncogene family  SYT9 - synaptotagmin ix  ARL8B - adp-ribosylation factor-like 8b  FBXW11 - f-box and wd repeat domain containing 11  VPS11 - vacuolar protein sorting 11 homolog (s. cerevisiae)  BBS7 - bardet-biedl syndrome 7  OPA1 - optic atrophy 1 (autosomal dominant)  DYNLT1 - dynein, light chain, tctex-type 1  STX1B - syntaxin 1b  CEP250 - centrosomal protein 250kda  TCTN1 - tectonic family member 1  CKAP5 - cytoskeleton associated protein 5  HAUS5 - haus augmin-like complex, subunit 5  NSF - n-ethylmaleimide-sensitive factor  MUL1 - mitochondrial e3 ubiquitin protein ligase 1  ESYT1 - extended synaptotagmin-like protein 1  DCTN2 - dynactin 2 (p50)  TUBA1A - tubulin, alpha 1a  CETN2 - centrin, ef-hand protein, 2  VAPB - vamp (vesicle-associated membrane protein)-associated protein b and c  ALMS1 - alstrom syndrome 1  NSFL1C - nsfl1 (p97) cofactor (p47)  NUMA1 - nuclear mitotic apparatus protein 1  CLASP1 - cytoplasmic linker associated protein 1  NUP88 - nucleoporin 88kda  MEF2A - myocyte enhancer factor 2a  ESYT2 - extended synaptotagmin-like protein 2  ZW10 - zw10 kinetochore protein  RIMS3 - regulating synaptic membrane exocytosis 3  ITGB1 - integrin, beta 1 (fibronectin receptor, beta polypeptide, antigen cd29 includes mdf2, msk12)  PTK2 - protein tyrosine kinase 2  CEP120 - centrosomal protein 120kda  EIF6 - eukaryotic translation initiation factor 6  PIP5K1C - phosphatidylinositol-4-phosphate 5-kinase, type i, gamma  CDK5 - cyclin-dependent kinase 5  RAB8A - rab8a, member ras oncogene family  CHMP1A - charged multivesicular body protein 1a  CEP78 - centrosomal protein 78kda  NMD3 - nmd3 ribosome export adaptor  NPM1 - nucleophosmin (nucleolar phosphoprotein b23, numatrin)  C2CD3 - c2 calcium-dependent domain containing 3  NPHP1 - nephronophthisis 1 (juvenile)  PCM1 - pericentriolar material 1  CADPS - ca++-dependent secretion activator  SPICE1 - spindle and centriole associated protein 1  ARHGEF2 - rho/rac guanine nucleotide exchange factor (gef) 2  SFI1 - sfi1 homolog, spindle assembly associated (yeast)  CHMP2B - charged multivesicular body protein 2b  TRIM9 - tripartite motif containing 9  ARL6 - adp-ribosylation factor-like 6  USO1 - uso1 vesicle transport factor  SCLT1 - sodium channel and clathrin linker 1  HTT - huntingtin  HAUS2 - haus augmin-like complex, subunit 2  SPG11 - spastic paraplegia 11 (autosomal recessive)  TCTN3 - tectonic family member 3  TMEM106B - transmembrane protein 106b  HAUS3 - haus augmin-like complex, subunit 3  MTOR - mechanistic target of rapamycin (serine/threonine kinase)  XPO1 - exportin 1 (crm1 homolog, yeast)  BLOC1S2 - biogenesis of lysosomal organelles complex-1, subunit 2  MARK1 - map/microtubule affinity-regulating kinase 1  MAPT - microtubule-associated protein tau  MYO19 - myosin xix  MAP6 - microtubule-associated protein 6  LRRK2 - leucine-rich repeat kinase 2  BLOC1S4 - biogenesis of lysosomal organelles complex-1, subunit 4, cappuccino  CEP290 - centrosomal protein 290kda  MAP1B - microtubule-associated protein 1b  RIMS2 - regulating synaptic membrane exocytosis 2  CHGA - chromogranin a (parathyroid secretory protein 1)  TANC2 - tetratricopeptide repeat, ankyrin repeat and coiled-coil containing 2  CEP57 - centrosomal protein 57kda  PVRL2 - poliovirus receptor-related 2 (herpesvirus entry mediator b)  RAB6A - rab6a, member ras oncogene family  LSG1 - large 60s subunit nuclear export gtpase 1  ATG14 - autophagy related 14  ATP6AP1 - atpase, h+ transporting, lysosomal accessory protein 1  PEX13 - peroxisomal biogenesis factor 13  PEX14 - peroxisomal biogenesis factor 14  RAB3A - rab3a, member ras oncogene family  RAB3B - rab3b, member ras oncogene family  TOR1A - torsin family 1, member a (torsin a)  SNPH - syntaphilin  CPLX2 - complexin 2  RAB1A - rab1a, member ras oncogene family  ARHGAP21 - rho gtpase activating protein 21  PEX1 - peroxisomal biogenesis factor 1  CHMP5 - charged multivesicular body protein 5  DOCK7 - dedicator of cytokinesis 7  TTBK2 - tau tubulin kinase 2  BRAT1 - brca1-associated atm activator 1  VPS4A - vacuolar protein sorting 4 homolog a (s. cerevisiae)  CCP110 - centriolar coiled coil protein 110kda  RAB27A - rab27a, member ras oncogene family  SYT7 - synaptotagmin vii  COPG2 - coatomer protein complex, subunit gamma 2  IQCB1 - iq motif containing b1  RANBP2 - ran binding protein 2  DLG1 - discs, large homolog 1 (drosophila)  VPS18 - vacuolar protein sorting 18 homolog (s. cerevisiae)  DNAJC5 - dnaj (hsp40) homolog, subfamily c, member 5  NEFL - neurofilament, light polypeptide  COPG1 - coatomer protein complex, subunit gamma 1  BLOC1S6 - biogenesis of lysosomal organelles complex-1, subunit 6, pallidin  AP3M1 - adaptor-related protein complex 3, mu 1 subunit  TERF1 - telomeric repeat binding factor (nima-interacting) 1  EXOC6 - exocyst complex component 6  CEP70 - centrosomal protein 70kda  PDZD8 - pdz domain containing 8  BCCIP - brca2 and cdkn1a interacting protein  BBS5 - bardet-biedl syndrome 5  SPAG9 - sperm associated antigen 9  FEZ1 - fasciculation and elongation protein zeta 1 (zygin i)  KIF1A - kinesin family member 1a  STX7 - syntaxin 7  CEP41 - centrosomal protein 41kda  CROCC - ciliary rootlet coiled-coil, rootletin  DYNC1H1 - dynein, cytoplasmic 1, heavy chain 1  DYNC1I1 - dynein, cytoplasmic 1, intermediate chain 1  DYNC1I2 - dynein, cytoplasmic 1, intermediate chain 2  DYNC1LI2 - dynein, cytoplasmic 1, light intermediate chain 2  RAB34 - rab34, member ras oncogene family  DNM2 - dynamin 2  LAT - linker for activation of t cells  CEP63 - centrosomal protein 63kda  RHOT1 - ras homolog family member t1  DOC2B - double c2-like domains, beta  B9D1 - b9 protein domain 1  CUL3 - cullin 3  SYTL4 - synaptotagmin-like 4  CHMP4B - charged multivesicular body protein 4b  YWHAG - tyrosine 3-monooxygenase/tryptophan 5-monooxygenase activation protein, gamma polypeptide  YWHAZ - tyrosine 3-monooxygenase/tryptophan 5-monooxygenase activation protein, zeta polypeptide  BBS4 - bardet-biedl syndrome 4  BBS2 - bardet-biedl syndrome 2  WASL - wiskott-aldrich syndrome-like  VPS45 - vacuolar protein sorting 45 homolog (s. cerevisiae)  YWHAE - tyrosine 3-monooxygenase/tryptophan 5-monooxygenase activation protein, epsilon polypeptide |
| GO:0051252 | regulation of RNA metabolic process | 1.45E-13 | 1.44E-10 | 1.09 (10334,2193,6996,1625) | [+] Show genes  EXOSC9 - exosome component 9  MSL3 - male-specific lethal 3 homolog (drosophila)  TADA1 - transcriptional adaptor 1  HNRNPA0 - heterogeneous nuclear ribonucleoprotein a0  CBX1 - chromobox homolog 1  ABCA2 - atp-binding cassette, sub-family a (abc1), member 2  ABL1 - c-abl oncogene 1, non-receptor tyrosine kinase  ZNF821 - zinc finger protein 821  PML - promyelocytic leukemia  MLLT11 - myeloid/lymphoid or mixed-lineage leukemia (trithorax homolog, drosophila); translocated to, 11  SUPT20H - suppressor of ty 20 homolog (s. cerevisiae)  YWHAQ - tyrosine 3-monooxygenase/tryptophan 5-monooxygenase activation protein, theta polypeptide  BRD8 - bromodomain containing 8  CHD7 - chromodomain helicase dna binding protein 7  LYAR - ly1 antibody reactive  PAPOLA - poly(a) polymerase alpha  ZNF354B - zinc finger protein 354b  TCERG1 - transcription elongation regulator 1  RNPS1 - rna binding protein s1, serine-rich domain  EHMT2 - euchromatic histone-lysine n-methyltransferase 2  SPIN1 - spindlin 1  TASP1 - taspase, threonine aspartase, 1  SUB1 - sub1 homolog (s. cerevisiae)  MORF4L1 - mortality factor 4 like 1  ZNF511 - zinc finger protein 511  PPID - peptidylprolyl isomerase d  ACTL6A - actin-like 6a  ACTN1 - actinin, alpha 1  ACTN4 - actinin, alpha 4  ACVR2A - activin a receptor, type iia  ACVRL1 - activin a receptor type ii-like 1  ACVR1 - activin a receptor, type i  ACVR1B - activin a receptor, type ib  ZNF331 - zinc finger protein 331  CPSF4 - cleavage and polyadenylation specific factor 4, 30kda  MED1 - mediator complex subunit 1  ADCY1 - adenylate cyclase 1 (brain)  PPARD - peroxisome proliferator-activated receptor delta  MAML3 - mastermind-like 3 (drosophila)  POLR2K - polymerase (rna) ii (dna directed) polypeptide k, 7.0kda  PARP1 - poly (adp-ribose) polymerase 1  H2AFY2 - h2a histone family, member y2  PPP1R13L - protein phosphatase 1, regulatory subunit 13 like  HES6 - hairy and enhancer of split 6 (drosophila)  POLR2G - polymerase (rna) ii (dna directed) polypeptide g  SMPD3 - sphingomyelin phosphodiesterase 3, neutral membrane (neutral sphingomyelinase ii)  RUVBL2 - ruvb-like 2 (e. coli)  POLR2A - polymerase (rna) ii (dna directed) polypeptide a, 220kda  CDK5RAP2 - cdk5 regulatory subunit associated protein 2  RCOR3 - rest corepressor 3  AES - amino-terminal enhancer of split  NUP133 - nucleoporin 133kda  FAM46B - family with sequence similarity 46, member b  JAG1 - jagged 1  PRDM4 - pr domain containing 4  PPP3R1 - protein phosphatase 3, regulatory subunit b, alpha  ATF7IP - activating transcription factor 7 interacting protein  ZNF689 - zinc finger protein 689  LRIF1 - ligand dependent nuclear receptor interacting factor 1  PPP2R5B - protein phosphatase 2, regulatory subunit b', beta  AKT1 - v-akt murine thymoma viral oncogene homolog 1  ZNF653 - zinc finger protein 653  PCID2 - pci domain containing 2  CPSF6 - cleavage and polyadenylation specific factor 6, 68kda  PPP2R1A - protein phosphatase 2, regulatory subunit a, alpha  RIOK2 - rio kinase 2  NUDT21 - nudix (nucleoside diphosphate linked moiety x)-type motif 21  WWP2 - ww domain containing e3 ubiquitin protein ligase 2  AHR - aryl hydrocarbon receptor  ZNF526 - zinc finger protein 526  FOXP4 - forkhead box p4  PPP2CA - protein phosphatase 2, catalytic subunit, alpha isozyme  ZNF83 - zinc finger protein 83  KANSL3 - kat8 regulatory nsl complex subunit 3  IWS1 - iws1 homolog (s. cerevisiae)  PRNP - prion protein  ATF7 - activating transcription factor 7  AMFR - autocrine motility factor receptor, e3 ubiquitin protein ligase  PRKRIR - protein-kinase, interferon-inducible double stranded rna dependent inhibitor, repressor of (p58 repressor)  PAPD7 - pap associated domain containing 7  MAP2K5 - mitogen-activated protein kinase kinase 5  MAP2K7 - mitogen-activated protein kinase kinase 7  EIF2AK2 - eukaryotic translation initiation factor 2-alpha kinase 2  ZNF692 - zinc finger protein 692  PIM2 - pim-2 oncogene  MAP2K1 - mitogen-activated protein kinase kinase 1  MAP2K2 - mitogen-activated protein kinase kinase 2  MAPK10 - mitogen-activated protein kinase 10  MAPK9 - mitogen-activated protein kinase 9  MAPK11 - mitogen-activated protein kinase 11  MAPK8 - mitogen-activated protein kinase 8  MAPK7 - mitogen-activated protein kinase 7  GCN1L1 - gcn1 general control of amino-acid synthesis 1-like 1 (yeast)  MAPK3 - mitogen-activated protein kinase 3  MAPK1 - mitogen-activated protein kinase 1  PRKD1 - protein kinase d1  ADIRF - adipogenesis regulatory factor  ANXA3 - annexin a3  PRKCH - protein kinase c, eta  PRKCI - protein kinase c, iota  CCDC85B - coiled-coil domain containing 85b  PRKCB - protein kinase c, beta  PRKCD - protein kinase c, delta  RBM22 - rna binding motif protein 22  PRKCA - protein kinase c, alpha  PRKAR1A - protein kinase, camp-dependent, regulatory, type i, alpha  YEATS2 - yeats domain containing 2  APEX1 - apex nuclease (multifunctional dna repair enzyme) 1  RBM23 - rna binding motif protein 23  ZNF605 - zinc finger protein 605  XIAP - x-linked inhibitor of apoptosis  BIRC2 - baculoviral iap repeat containing 2  PARN - poly(a)-specific ribonuclease  KLF12 - kruppel-like factor 12  SHQ1 - shq1, h/aca ribonucleoprotein assembly factor  PBX2 - pre-b-cell leukemia homeobox 2  PBX3 - pre-b-cell leukemia homeobox 3  PRMT6 - protein arginine methyltransferase 6  PBX1 - pre-b-cell leukemia homeobox 1  PCBP2 - poly(rc) binding protein 2  PCBD1 - pterin-4 alpha-carbinolamine dehydratase/dimerization cofactor of hepatocyte nuclear factor 1 alpha  APBB2 - amyloid beta (a4) precursor protein-binding, family b, member 2  APBB1 - amyloid beta (a4) precursor protein-binding, family b, member 1 (fe65)  CHP1 - calcineurin-like ef-hand protein 1  RIF1 - rap1 interacting factor homolog (yeast)  KIAA1551 - kiaa1551  PA2G4 - proliferation-associated 2g4, 38kda  RPRD1A - regulation of nuclear pre-mrna domain containing 1a  ARF4 - adp-ribosylation factor 4  ZHX1 - zinc fingers and homeoboxes 1  SBNO1 - strawberry notch homolog 1 (drosophila)  IRAK3 - interleukin-1 receptor-associated kinase 3  SETD5 - set domain containing 5  APP - amyloid beta (a4) precursor protein  BBS7 - bardet-biedl syndrome 7  DDX20 - dead (asp-glu-ala-asp) box polypeptide 20  PAM - peptidylglycine alpha-amidating monooxygenase  ARNTL - aryl hydrocarbon receptor nuclear translocator-like  ARNT - aryl hydrocarbon receptor nuclear translocator  CELF3 - cugbp, elav-like family member 3  SUPT16H - suppressor of ty 16 homolog (s. cerevisiae)  ARRB2 - arrestin, beta 2  ARRB1 - arrestin, beta 1  PDGFB - platelet-derived growth factor beta polypeptide  SAMD4B - sterile alpha motif domain containing 4b  STRAP - serine/threonine kinase receptor associated protein  RHOA - ras homolog family member a  PSIP1 - pc4 and sfrs1 interacting protein 1  MED9 - mediator complex subunit 9  FSTL1 - follistatin-like 1  BAZ2A - bromodomain adjacent to zinc finger domain, 2a  PCNA - proliferating cell nuclear antigen  CHMP1A - charged multivesicular body protein 1a  JDP2 - jun dimerization protein 2  CDK16 - cyclin-dependent kinase 16  AKIRIN2 - akirin 2  HEATR1 - heat repeat containing 1  CDK17 - cyclin-dependent kinase 17  LEO1 - leo1, paf1/rna polymerase ii complex component, homolog (s. cerevisiae)  ERLIN2 - er lipid raft associated 2  PWP1 - pwp1 homolog (s. cerevisiae)  PDE2A - phosphodiesterase 2a, cgmp-stimulated  THAP1 - thap domain containing, apoptosis associated protein 1  PKIG - protein kinase (camp-dependent, catalytic) inhibitor gamma  ASCL1 - achaete-scute complex homolog 1 (drosophila)  KAT7 - k(lysine) acetyltransferase 7  ELP3 - elongator acetyltransferase complex subunit 3  RGMB - rgm domain family, member b  SBNO2 - strawberry notch homolog 2 (drosophila)  ZNF444 - zinc finger protein 444  ZBTB1 - zinc finger and btb domain containing 1  FBXW7 - f-box and wd repeat domain containing 7, e3 ubiquitin protein ligase  BAHD1 - bromo adjacent homology domain containing 1  DIS3 - dis3 mitotic control homolog (s. cerevisiae)  ZHX2 - zinc fingers and homeoboxes 2  PHF2 - phd finger protein 2  MORC2 - morc family cw-type zinc finger 2  PHF1 - phd finger protein 1  FOXJ3 - forkhead box j3  RERE - arginine-glutamic acid dipeptide (re) repeats  ZFHX3 - zinc finger homeobox 3  PEX14 - peroxisomal biogenesis factor 14  ADNP2 - adnp homeobox 2  AEBP2 - ae binding protein 2  ZNF507 - zinc finger protein 507  IMPACT - impact rwd domain protein  LGR4 - leucine-rich repeat containing g protein-coupled receptor 4  ATP2B4 - atpase, ca++ transporting, plasma membrane 4  ZNF652 - zinc finger protein 652  PKD1 - polycystic kidney disease 1 (autosomal dominant)  SMU1 - smu-1 suppressor of mec-8 and unc-52 homolog (c. elegans)  SCAF8 - sr-related ctd-associated factor 8  PKD2 - polycystic kidney disease 2 (autosomal dominant)  PUF60 - poly-u binding splicing factor 60kda  MTF2 - metal response element binding transcription factor 2  BACH1 - btb and cnc homology 1, basic leucine zipper transcription factor 1  PIN1 - peptidylprolyl cis/trans isomerase, nima-interacting 1  PIK3R2 - phosphoinositide-3-kinase, regulatory subunit 2 (beta)  PIK3R1 - phosphoinositide-3-kinase, regulatory subunit 1 (alpha)  ASXL2 - additional sex combs like 2 (drosophila)  PLAGL1 - pleiomorphic adenoma gene-like 1  ELP2 - elongator acetyltransferase complex subunit 2  ATRX - alpha thalassemia/mental retardation syndrome x-linked  PKNOX1 - pbx/knotted 1 homeobox 1  EXOSC8 - exosome component 8  TMEM100 - transmembrane protein 100  PHF10 - phd finger protein 10  PSPC1 - paraspeckle component 1  U2AF2 - u2 small nuclear rna auxiliary factor 2  BCL7A - b-cell cll/lymphoma 7a  MRGBP - mrg/morf4l binding protein  BCL9 - b-cell cll/lymphoma 9  BRF2 - brf2, rna polymerase iii transcription initiation factor 50 kda subunit  PIM1 - pim-1 oncogene  ECD - ecdysoneless homolog (drosophila)  CCND1 - cyclin d1  PARK7 - parkinson protein 7  C3orf33 - chromosome 3 open reading frame 33  TRIM27 - tripartite motif containing 27  SAP18 - sin3a-associated protein, 18kda  TSHZ2 - teashirt zinc finger homeobox 2  RFXAP - regulatory factor x-associated protein  RFX5 - regulatory factor x, 5 (influences hla class ii expression)  WHSC1L1 - wolf-hirschhorn syndrome candidate 1-like 1  RFX1 - regulatory factor x, 1 (influences hla class ii expression)  APBB3 - amyloid beta (a4) precursor protein-binding, family b, member 3  RELA - v-rel avian reticuloendotheliosis viral oncogene homolog a  BCOR - bcl6 corepressor  ZFP36L2 - zfp36 ring finger protein-like 2  ZFP36L1 - zfp36 ring finger protein-like 1  RET - ret proto-oncogene  DPF2 - d4, zinc and double phd fingers family 2  ALKBH5 - alkb, alkylation repair homolog 5 (e. coli)  UPF1 - upf1 regulator of nonsense transcripts homolog (yeast)  NSUN2 - nop2/sun rna methyltransferase family, member 2  CC2D1A - coiled-coil and c2 domain containing 1a  RLF - rearranged l-myc fusion  RING1 - ring finger protein 1  BMI1 - bmi1 polycomb ring finger oncogene  PCGF3 - polycomb group ring finger 3  BMPR1A - bone morphogenetic protein receptor, type ia  BMPR2 - bone morphogenetic protein receptor, type ii (serine/threonine kinase)  GON4L - gon-4-like (c. elegans)  BMPR1B - bone morphogenetic protein receptor, type ib  BMP7 - bone morphogenetic protein 7  PSMD14 - proteasome (prosome, macropain) 26s subunit, non-atpase, 14  FLOT1 - flotillin 1  BCAS3 - breast carcinoma amplified sequence 3  ABCE1 - atp-binding cassette, sub-family e (oabp), member 1  AHI1 - abelson helper integration site 1  RNF4 - ring finger protein 4  BRD2 - bromodomain containing 2  RNF2 - ring finger protein 2  HNRNPR - heterogeneous nuclear ribonucleoprotein r  ZNF280D - zinc finger protein 280d  GATAD2A - gata zinc finger domain containing 2a  RNF6 - ring finger protein (c3h2c3 type) 6  ROCK1 - rho-associated, coiled-coil containing protein kinase 1  RNF111 - ring finger protein 111  TET2 - tet methylcytosine dioxygenase 2  MED18 - mediator complex subunit 18  DENND4A - denn/madd domain containing 4a  MBTD1 - mbt domain containing 1  SF3B4 - splicing factor 3b, subunit 4, 49kda  ZMPSTE24 - zinc metallopeptidase ste24  TRIM44 - tripartite motif containing 44  TAB1 - tgf-beta activated kinase 1/map3k7 binding protein 1  PPIE - peptidylprolyl isomerase e (cyclophilin e)  MCRS1 - microspherule protein 1  NRDE2 - nrde-2, necessary for rna interference, domain containing  C1D - c1d nuclear receptor corepressor  SLC30A9 - solute carrier family 30 (zinc transporter), member 9  MAD2L2 - mad2 mitotic arrest deficient-like 2 (yeast)  PID1 - phosphotyrosine interaction domain containing 1  PHIP - pleckstrin homology domain interacting protein  ZNF787 - zinc finger protein 787  NRARP - notch-regulated ankyrin repeat protein  TRIM38 - tripartite motif containing 38  TADA3 - transcriptional adaptor 3  CALR - calreticulin  ZNF792 - zinc finger protein 792  WTIP - wilms tumor 1 interacting protein  NCOA2 - nuclear receptor coactivator 2  SYNCRIP - synaptotagmin binding, cytoplasmic rna interacting protein  CREB3 - camp responsive element binding protein 3  CITED2 - cbp/p300-interacting transactivator, with glu/asp-rich carboxy-terminal domain, 2  CCNT2 - cyclin t2  CCND3 - cyclin d3  CCNT1 - cyclin t1  CCNH - cyclin h  HMG20A - high mobility group 20a  BANP - btg3 associated nuclear protein  RPS6KA1 - ribosomal protein s6 kinase, 90kda, polypeptide 1  CBFA2T3 - core-binding factor, runt domain, alpha subunit 2; translocated to, 3  CBFB - core-binding factor, beta subunit  PIAS3 - protein inhibitor of activated stat, 3  RXRB - retinoid x receptor, beta  CAV1 - caveolin 1, caveolae protein, 22kda  CCNC - cyclin c  RBM14 - rna binding motif protein 14  PRMT5 - protein arginine methyltransferase 5  CDK11B - cyclin-dependent kinase 11b  MXD4 - max dimerization protein 4  CDC5L - cell division cycle 5-like  HTRA1 - htra serine peptidase 1  POLR3F - polymerase (rna) iii (dna directed) polypeptide f, 39 kda  HEXIM1 - hexamethylene bis-acetamide inducible 1  ERLIN1 - er lipid raft associated 1  SPIN2A - spindlin family, member 2a  NCOA7 - nuclear receptor coactivator 7  PSEN1 - presenilin 1  USP16 - ubiquitin specific peptidase 16  PSMA1 - proteasome (prosome, macropain) subunit, alpha type, 1  SRSF12 - serine/arginine-rich splicing factor 12  PSMA2 - proteasome (prosome, macropain) subunit, alpha type, 2  CD81 - cd81 molecule  CAMKK2 - calcium/calmodulin-dependent protein kinase kinase 2, beta  PSMB1 - proteasome (prosome, macropain) subunit, beta type, 1  PSMA5 - proteasome (prosome, macropain) subunit, alpha type, 5  PSMA3 - proteasome (prosome, macropain) subunit, alpha type, 3  RIPPLY2 - ripply transcriptional repressor 2  PSMB10 - proteasome (prosome, macropain) subunit, beta type, 10  KHDRBS3 - kh domain containing, rna binding, signal transduction associated 3  PSMC6 - proteasome (prosome, macropain) 26s subunit, atpase, 6  TAF6L - taf6-like rna polymerase ii, p300/cbp-associated factor (pcaf)-associated factor, 65kda  PSMD1 - proteasome (prosome, macropain) 26s subunit, non-atpase, 1  PSMC4 - proteasome (prosome, macropain) 26s subunit, atpase, 4  SOX18 - sry (sex determining region y)-box 18  POLR3G - polymerase (rna) iii (dna directed) polypeptide g (32kd)  PSMC2 - proteasome (prosome, macropain) 26s subunit, atpase, 2  PSMD9 - proteasome (prosome, macropain) 26s subunit, non-atpase, 9  PSMD10 - proteasome (prosome, macropain) 26s subunit, non-atpase, 10  PSMD7 - proteasome (prosome, macropain) 26s subunit, non-atpase, 7  PSMD8 - proteasome (prosome, macropain) 26s subunit, non-atpase, 8  PSMD5 - proteasome (prosome, macropain) 26s subunit, non-atpase, 5  TERF2IP - telomeric repeat binding factor 2, interacting protein  PSMD2 - proteasome (prosome, macropain) 26s subunit, non-atpase, 2  SLC38A2 - solute carrier family 38, member 2  PSMD12 - proteasome (prosome, macropain) 26s subunit, non-atpase, 12  PSMD13 - proteasome (prosome, macropain) 26s subunit, non-atpase, 13  PTBP1 - polypyrimidine tract binding protein 1  PTEN - phosphatase and tensin homolog  LAMTOR5 - late endosomal/lysosomal adaptor, mapk and mtor activator 5  JMY - junction mediating and regulatory protein, p53 cofactor  MYBBP1A - myb binding protein (p160) 1a  FBLN5 - fibulin 5  PTGIS - prostaglandin i2 (prostacyclin) synthase  CIB1 - calcium and integrin binding 1 (calmyrin)  DDX17 - dead (asp-glu-ala-asp) box helicase 17  KAT5 - k(lysine) acetyltransferase 5  ZFP69 - zfp69 zinc finger protein  CEBPG - ccaat/enhancer binding protein (c/ebp), gamma  CDK5 - cyclin-dependent kinase 5  CDK9 - cyclin-dependent kinase 9  CDK8 - cyclin-dependent kinase 8  LIN54 - lin-54 homolog (c. elegans)  CDK7 - cyclin-dependent kinase 7  CDKN1C - cyclin-dependent kinase inhibitor 1c (p57, kip2)  CDKN1B - cyclin-dependent kinase inhibitor 1b (p27, kip1)  PCBP3 - poly(rc) binding protein 3  PTPN2 - protein tyrosine phosphatase, non-receptor type 2  HTATIP2 - hiv-1 tat interactive protein 2, 30kda  CDK2 - cyclin-dependent kinase 2  PURA - purine-rich element binding protein a  RRN3 - rrn3 rna polymerase i transcription factor homolog (s. cerevisiae)  PURB - purine-rich element binding protein b  NUDT16 - nudix (nucleoside diphosphate linked moiety x)-type motif 16  ZMYND11 - zinc finger, mynd-type containing 11  PTPRK - protein tyrosine phosphatase, receptor type, k  SRSF10 - serine/arginine-rich splicing factor 10  PTPRN - protein tyrosine phosphatase, receptor type, n  AHCYL1 - adenosylhomocysteinase-like 1  PEX2 - peroxisomal biogenesis factor 2  MPHOSPH8 - m-phase phosphoprotein 8  KDM5B - lysine (k)-specific demethylase 5b  PXN - paxillin  CHD4 - chromodomain helicase dna binding protein 4  CHD2 - chromodomain helicase dna binding protein 2  CHD3 - chromodomain helicase dna binding protein 3  MTERFD2 - mterf domain containing 2  CHD1 - chromodomain helicase dna binding protein 1  QARS - glutaminyl-trna synthetase  MYPOP - myb-related transcription factor, partner of profilin  RAD21 - rad21 homolog (s. pombe)  PARP14 - poly (adp-ribose) polymerase family, member 14  PAF1 - paf1, rna polymerase ii associated factor, homolog (s. cerevisiae)  ZNF181 - zinc finger protein 181  ZNF268 - zinc finger protein 268  INO80 - ino80 complex subunit  ZNF513 - zinc finger protein 513  GMEB1 - glucocorticoid modulatory element binding protein 1  RC3H2 - ring finger and ccch-type domains 2  MIER2 - mesoderm induction early response 1, family member 2  RARG - retinoic acid receptor, gamma  RARB - retinoic acid receptor, beta  DLL4 - delta-like 4 (drosophila)  RASA1 - ras p21 protein activator (gtpase activating protein) 1  CELF1 - cugbp, elav-like family member 1  KHDRBS1 - kh domain containing, rna binding, signal transduction associated 1  CLU - clusterin  TAF8 - taf8 rna polymerase ii, tata box binding protein (tbp)-associated factor, 43kda  CLK1 - cdc-like kinase 1  CELF2 - cugbp, elav-like family member 2  MAGEL2 - mage-like 2  CLK2 - cdc-like kinase 2  DDX49 - dead (asp-glu-ala-asp) box polypeptide 49  CLK3 - cdc-like kinase 3  CTCF - ccctc-binding factor (zinc finger protein)  RAI1 - retinoic acid induced 1  RBL2 - retinoblastoma-like 2 (p130)  RBBP7 - retinoblastoma binding protein 7  RBBP8 - retinoblastoma binding protein 8  MAP3K2 - mitogen-activated protein kinase kinase kinase 2  RBM3 - rna binding motif (rnp1, rrm) protein 3  RBM4 - rna binding motif protein 4  RB1 - retinoblastoma 1  ARID4A - at rich interactive domain 4a (rbp1-like)  KDM5A - lysine (k)-specific demethylase 5a  NUP35 - nucleoporin 35kda  EXOSC4 - exosome component 4  NFAT5 - nuclear factor of activated t-cells 5, tonicity-responsive  CKS1B - cdc28 protein kinase regulatory subunit 1b  CIRBP - cold inducible rna binding protein  CHUK - conserved helix-loop-helix ubiquitous kinase  ZMYND8 - zinc finger, mynd-type containing 8  ANKRA2 - ankyrin repeat, family a (rfxank-like), 2  RBAK - rb-associated krab zinc finger  NFATC2IP - nuclear factor of activated t-cells, cytoplasmic, calcineurin-dependent 2 interacting protein  PATZ1 - poz (btb) and at hook containing zinc finger 1  METTL14 - methyltransferase like 14  SPP1 - secreted phosphoprotein 1  SSBP2 - single-stranded dna binding protein 2  KPNA6 - karyopherin alpha 6 (importin alpha 7)  CIRH1A - cirrhosis, autosomal recessive 1a (cirhin)  MIER1 - mesoderm induction early response 1, transcriptional regulator  CWC22 - cwc22 spliceosome-associated protein homolog (s. cerevisiae)  ZNF382 - zinc finger protein 382  ZNF705A - zinc finger protein 705a  SP3 - sp3 transcription factor  SP4 - sp4 transcription factor  SP1 - sp1 transcription factor  SP2 - sp2 transcription factor  ZNF410 - zinc finger protein 410  ARL2BP - adp-ribosylation factor-like 2 binding protein  SOX4 - sry (sex determining region y)-box 4  WBP2 - ww domain binding protein 2  SON - son dna binding protein  HES4 - hairy and enhancer of split 4 (drosophila)  LEMD3 - lem domain containing 3  CREBL2 - camp responsive element binding protein-like 2  GATAD1 - gata zinc finger domain containing 1  ATF6B - activating transcription factor 6 beta  CREBBP - creb binding protein  ATF2 - activating transcription factor 2  CREB1 - camp responsive element binding protein 1  DDX58 - dead (asp-glu-ala-asp) box polypeptide 58  CRABP2 - cellular retinoic acid binding protein 2  RPRD1B - regulation of nuclear pre-mrna domain containing 1b  PLK3 - polo-like kinase 3  SNAPC4 - small nuclear rna activating complex, polypeptide 4, 190kda  SNCA - synuclein, alpha (non a4 component of amyloid precursor)  SNRNP70 - small nuclear ribonucleoprotein 70kda (u1)  ZBED5 - zinc finger, bed-type containing 5  SNRPA - small nuclear ribonucleoprotein polypeptide a  CREBZF - creb/atf bzip transcription factor  EID1 - ep300 interacting inhibitor of differentiation 1  TP53INP2 - tumor protein p53 inducible nuclear protein 2  PTBP2 - polypyrimidine tract binding protein 2  RBM17 - rna binding motif protein 17  SMARCE1 - swi/snf related, matrix associated, actin dependent regulator of chromatin, subfamily e, member 1  SMO - smoothened, frizzled family receptor  SMARCB1 - swi/snf related, matrix associated, actin dependent regulator of chromatin, subfamily b, member 1  SMARCC1 - swi/snf related, matrix associated, actin dependent regulator of chromatin, subfamily c, member 1  HLTF - helicase-like transcription factor  SSBP3 - single stranded dna binding protein 3  SMARCA4 - swi/snf related, matrix associated, actin dependent regulator of chromatin, subfamily a, member 4  SNX6 - sorting nexin 6  SMARCD2 - swi/snf related, matrix associated, actin dependent regulator of chromatin, subfamily d, member 2  SMARCD3 - swi/snf related, matrix associated, actin dependent regulator of chromatin, subfamily d, member 3  SMARCC2 - swi/snf related, matrix associated, actin dependent regulator of chromatin, subfamily c, member 2  SMARCD1 - swi/snf related, matrix associated, actin dependent regulator of chromatin, subfamily d, member 1  WIZ - widely interspaced zinc finger motifs  RBM25 - rna binding motif protein 25  SMARCA1 - swi/snf related, matrix associated, actin dependent regulator of chromatin, subfamily a, member 1  SMARCA2 - swi/snf related, matrix associated, actin dependent regulator of chromatin, subfamily a, member 2  SGK3 - serum/glucocorticoid regulated kinase family, member 3  CACTIN - cactin, spliceosome c complex subunit  KMT2C - lysine (k)-specific methyltransferase 2c  ZNF462 - zinc finger protein 462  PPIL4 - peptidylprolyl isomerase (cyclophilin)-like 4  SENP2 - sumo1/sentrin/smt3 specific peptidase 2  ZNF318 - zinc finger protein 318  PRPF6 - pre-mrna processing factor 6  KDM2B - lysine (k)-specific demethylase 2b  SUPT6H - suppressor of ty 6 homolog (s. cerevisiae)  SUPV3L1 - suppressor of var1, 3-like 1 (s. cerevisiae)  CBY1 - chibby homolog 1 (drosophila)  CACNG7 - calcium channel, voltage-dependent, gamma subunit 7  CTBP1 - c-terminal binding protein 1  CTBP2 - c-terminal binding protein 2  SUPT4H1 - suppressor of ty 4 homolog 1 (s. cerevisiae)  SUPT5H - suppressor of ty 5 homolog (s. cerevisiae)  CTNNB1 - catenin (cadherin-associated protein), beta 1, 88kda  SAV1 - salvador homolog 1 (drosophila)  CUX1 - cut-like homeobox 1  NIF3L1 - nif3 ngg1 interacting factor 3-like 1 (s. cerevisiae)  NEK4 - nima-related kinase 4  STK3 - serine/threonine kinase 3  PCGF1 - polycomb group ring finger 1  NPNT - nephronectin  FKBP8 - fk506 binding protein 8, 38kda  GPBP1L1 - gc-rich promoter binding protein 1-like 1  CYLD - cylindromatosis (turban tumor syndrome)  TGIF2 - tgfb-induced factor homeobox 2  BCL6B - b-cell cll/lymphoma 6, member b  CRK - v-crk avian sarcoma virus ct10 oncogene homolog  CELF6 - cugbp, elav-like family member 6  NIPBL - nipped-b homolog (drosophila)  STAT2 - signal transducer and activator of transcription 2, 113kda  CRY1 - cryptochrome 1 (photolyase-like)  STAT1 - signal transducer and activator of transcription 1, 91kda  STAT3 - signal transducer and activator of transcription 3 (acute-phase response factor)  CRY2 - cryptochrome 2 (photolyase-like)  STAT5A - signal transducer and activator of transcription 5a  ASF1A - anti-silencing function 1a histone chaperone  STAT6 - signal transducer and activator of transcription 6, interleukin-4 induced  BRMS1 - breast cancer metastasis suppressor 1  ZNF496 - zinc finger protein 496  CRYM - crystallin, mu  SS18 - synovial sarcoma translocation, chromosome 18  PHF5A - phd finger protein 5a  GPATCH3 - g patch domain containing 3  SRPK2 - srsf protein kinase 2  SRPK1 - srsf protein kinase 1  TAB3 - tgf-beta activated kinase 1/map3k7 binding protein 3  ZNF335 - zinc finger protein 335  BAMBI - bmp and activin membrane-bound inhibitor  HAVCR2 - hepatitis a virus cellular receptor 2  ZC3H10 - zinc finger ccch-type containing 10  SRI - sorcin  SRC - v-src avian sarcoma (schmidt-ruppin a-2) viral oncogene homolog  DNAJB5 - dnaj (hsp40) homolog, subfamily b, member 5  SREBF2 - sterol regulatory element binding transcription factor 2  UBE2O - ubiquitin-conjugating enzyme e2o  PRDX5 - peroxiredoxin 5  DHX9 - deah (asp-glu-ala-his) box helicase 9  KANK2 - kn motif and ankyrin repeat domains 2  TRAPPC2 - trafficking protein particle complex 2  DDX5 - dead (asp-glu-ala-asp) box helicase 5  DDX3X - dead (asp-glu-ala-asp) box helicase 3, x-linked  DDX1 - dead (asp-glu-ala-asp) box helicase 1  SIN3A - sin3 transcription regulator family member a  SET - set nuclear oncogene  WWTR1 - ww domain containing transcription regulator 1  ZNF304 - zinc finger protein 304  ZNF286A - zinc finger protein 286a  NOL11 - nucleolar protein 11  PBXIP1 - pre-b-cell leukemia homeobox interacting protein 1  DBP - d site of albumin promoter (albumin d-box) binding protein  NELFB - negative elongation factor complex member b  AHCTF1 - at hook containing transcription factor 1  CLK4 - cdc-like kinase 4  ZBTB38 - zinc finger and btb domain containing 38  GATAD2B - gata zinc finger domain containing 2b  ZNF551 - zinc finger protein 551  ZNF473 - zinc finger protein 473  YTHDF3 - yth domain family, member 3  CNOT6 - ccr4-not transcription complex, subunit 6  ZNF451 - zinc finger protein 451  SS18L1 - synovial sarcoma translocation gene on chromosome 18-like 1  SCAF4 - sr-related ctd-associated factor 4  CHD5 - chromodomain helicase dna binding protein 5  ARID1B - at rich interactive domain 1b (swi1-like)  MED30 - mediator complex subunit 30  MKL2 - mkl/myocardin-like 2  DAPK3 - death-associated protein kinase 3  DAXX - death-domain associated protein  ANGEL2 - angel homolog 2 (drosophila)  ZZZ3 - zinc finger, zz-type containing 3  HINFP - histone h4 transcription factor  MTA3 - metastasis associated 1 family, member 3  DGKQ - diacylglycerol kinase, theta 110kda  ZNF608 - zinc finger protein 608  MAVS - mitochondrial antiviral signaling protein  DAP - death-associated protein  SARS - seryl-trna synthetase  SATB1 - satb homeobox 1  RPTOR - regulatory associated protein of mtor, complex 1  SAFB - scaffold attachment factor b  SALL2 - sal-like 2 (drosophila)  SERBP1 - serpine1 mrna binding protein 1  DRD2 - dopamine receptor d2  EPC2 - enhancer of polycomb homolog 2 (drosophila)  PHF19 - phd finger protein 19  SLC9A1 - solute carrier family 9, subfamily a (nhe1, cation proton antiporter 1), member 1  IRF2BP1 - interferon regulatory factor 2 binding protein 1  CNOT6L - ccr4-not transcription complex, subunit 6-like  EAF1 - ell associated factor 1  ATN1 - atrophin 1  DOM3Z - dom-3 homolog z (c. elegans)  C10orf137 - chromosome 10 open reading frame 137  DR1 - down-regulator of transcription 1, tbp-binding (negative cofactor 2)  SKI - v-ski avian sarcoma viral oncogene homolog  SKIL - ski-like oncogene  DYRK1A - dual-specificity tyrosine-(y)-phosphorylation regulated kinase 1a  MKL1 - megakaryoblastic leukemia (translocation) 1  DVL2 - dishevelled segment polarity protein 2  DVL3 - dishevelled segment polarity protein 3  GIGYF2 - grb10 interacting gyf protein 2  USP36 - ubiquitin specific peptidase 36  FNIP2 - folliculin interacting protein 2  HOMEZ - homeobox and leucine zipper encoding  EBF4 - early b-cell factor 4  ZFHX2 - zinc finger homeobox 2  DTX1 - deltex homolog 1 (drosophila)  DKC1 - dyskeratosis congenita 1, dyskerin  PNPT1 - polyribonucleotide nucleotidyltransferase 1  DLG1 - discs, large homolog 1 (drosophila)  SHOX2 - short stature homeobox 2  SIAH2 - siah e3 ubiquitin protein ligase 2  POGK - pogo transposable element with krab domain  DHFR - dihydrofolate reductase  PHF12 - phd finger protein 12  CALCOCO1 - calcium binding and coiled-coil domain 1  ZBTB4 - zinc finger and btb domain containing 4  SFPQ - splicing factor proline/glutamine-rich  SFRP1 - secreted frizzled-related protein 1  SFRP2 - secreted frizzled-related protein 2  SFRP4 - secreted frizzled-related protein 4  SRSF1 - serine/arginine-rich splicing factor 1  GMEB2 - glucocorticoid modulatory element binding protein 2  DNM2 - dynamin 2  SRSF2 - serine/arginine-rich splicing factor 2  SPAG8 - sperm associated antigen 8  DNMT1 - dna (cytosine-5-)-methyltransferase 1  SRSF3 - serine/arginine-rich splicing factor 3  TRDMT1 - trna aspartic acid methyltransferase 1  SRSF4 - serine/arginine-rich splicing factor 4  DNMT3A - dna (cytosine-5-)-methyltransferase 3 alpha  SRSF5 - serine/arginine-rich splicing factor 5  SRSF6 - serine/arginine-rich splicing factor 6  SRSF7 - serine/arginine-rich splicing factor 7  EIF2AK4 - eukaryotic translation initiation factor 2 alpha kinase 4  SFSWAP - splicing factor, suppressor of white-apricot homolog (drosophila)  TRA2B - transformer 2 beta homolog (drosophila)  CHD8 - chromodomain helicase dna binding protein 8  TNRC6C - trinucleotide repeat containing 6c  NOC2L - nucleolar complex associated 2 homolog (s. cerevisiae)  SGK1 - serum/glucocorticoid regulated kinase 1  ZNF317 - zinc finger protein 317  MEIS3 - meis homeobox 3  NCBP2 - nuclear cap binding protein subunit 2, 20kda  MAPRE3 - microtubule-associated protein, rp/eb family, member 3  ELK1 - elk1, member of ets oncogene family  NAF1 - nuclear assembly factor 1 ribonucleoprotein  ELK3 - elk3, ets-domain protein (srf accessory protein 2)  ELK4 - elk4, ets-domain protein (srf accessory protein 1)  SNW1 - snw domain containing 1  SCAP - srebf chaperone  EPAS1 - endothelial pas domain protein 1  EP300 - e1a binding protein p300  HABP4 - hyaluronan binding protein 4  ATF6 - activating transcription factor 6  ENO1 - enolase 1, (alpha)  EIF4A2 - eukaryotic translation initiation factor 4a2  CELF4 - cugbp, elav-like family member 4  EGR2 - early growth response 2  EGR3 - early growth response 3  ZC3HAV1 - zinc finger ccch-type, antiviral 1  TRIM32 - tripartite motif containing 32  KDM2A - lysine (k)-specific demethylase 2a  ELAVL1 - elav (embryonic lethal, abnormal vision, drosophila)-like 1 (hu antigen r)  ELF1 - e74-like factor 1 (ets domain transcription factor)  ZNF326 - zinc finger protein 326  ELAVL4 - elav (embryonic lethal, abnormal vision, drosophila)-like 4  ELF2 - e74-like factor 2 (ets domain transcription factor)  TCF25 - transcription factor 25 (basic helix-loop-helix)  TSG101 - tumor susceptibility gene 101  CNOT1 - ccr4-not transcription complex, subunit 1  EFNA1 - ephrin-a1  EXOSC7 - exosome component 7  SPEN - spen homolog, transcriptional regulator (drosophila)  CTNNBIP1 - catenin, beta interacting protein 1  BBX - bobby sox homolog (drosophila)  PHC1 - polyhomeotic homolog 1 (drosophila)  TP53BP1 - tumor protein p53 binding protein 1  PHC2 - polyhomeotic homolog 2 (drosophila)  EGR1 - early growth response 1  ZNF292 - zinc finger protein 292  EGFR - epidermal growth factor receptor  MEGF8 - multiple egf-like-domains 8  CELSR2 - cadherin, egf lag seven-pass g-type receptor 2  KDM1A - lysine (k)-specific demethylase 1a  ATXN7L3 - ataxin 7-like 3  HNRNPLL - heterogeneous nuclear ribonucleoprotein l-like  NCOA6 - nuclear receptor coactivator 6  E2F6 - e2f transcription factor 6  E4F1 - e4f transcription factor 1  ZHX3 - zinc fingers and homeoboxes 3  E2F4 - e2f transcription factor 4, p107/p130-binding  E2F5 - e2f transcription factor 5, p130-binding  ENY2 - enhancer of yellow 2 homolog (drosophila)  C11orf30 - chromosome 11 open reading frame 30  RRP1B - ribosomal rna processing 1b  EDNRB - endothelin receptor type b  TRAF1 - tnf receptor-associated factor 1  S1PR1 - sphingosine-1-phosphate receptor 1  TRAF3 - tnf receptor-associated factor 3  TRAF2 - tnf receptor-associated factor 2  NR2C1 - nuclear receptor subfamily 2, group c, member 1  SETX - senataxin  ZBTB47 - zinc finger and btb domain containing 47  NR2C2 - nuclear receptor subfamily 2, group c, member 2  TPR - translocated promoter region, nuclear basket protein  ZNF609 - zinc finger protein 609  ERC1 - elks/rab6-interacting/cast family member 1  PPRC1 - peroxisome proliferator-activated receptor gamma, coactivator-related 1  NAT14 - n-acetyltransferase 14 (gcn5-related, putative)  KDM4C - lysine (k)-specific demethylase 4c  ZNF423 - zinc finger protein 423  REXO4 - rex4, rna exonuclease 4 homolog (s. cerevisiae)  CAMK1D - calcium/calmodulin-dependent protein kinase id  USF1 - upstream transcription factor 1  NUP107 - nucleoporin 107kda  ZBTB43 - zinc finger and btb domain containing 43  BPTF - bromodomain phd finger transcription factor  PITHD1 - pith (c-terminal proteasome-interacting domain of thioredoxin-like) domain containing 1  TNRC6B - trinucleotide repeat containing 6b  NR1H2 - nuclear receptor subfamily 1, group h, member 2  TAB2 - tgf-beta activated kinase 1/map3k7 binding protein 2  ETV3 - ets variant 3  CAMTA2 - calmodulin binding transcription activator 2  ETV5 - ets variant 5  POGZ - pogo transposable element with znf domain  TWSG1 - twisted gastrulation homolog 1 (drosophila)  ETV6 - ets variant 6  PLXND1 - plexin d1  VHL - von hippel-lindau tumor suppressor, e3 ubiquitin protein ligase  VIM - vimentin  KDM6B - lysine (k)-specific demethylase 6b  EZR - ezrin  PHF8 - phd finger protein 8  TBX20 - t-box 20  EWSR1 - ews rna-binding protein 1  TRIM41 - tripartite motif containing 41  KCTD1 - potassium channel tetramerization domain containing 1  PCBP4 - poly(rc) binding protein 4  CREB3L1 - camp responsive element binding protein 3-like 1  ZC3H3 - zinc finger ccch-type containing 3  EZH1 - enhancer of zeste homolog 1 (drosophila)  CIC - capicua transcriptional repressor  CCNL1 - cyclin l1  F2R - coagulation factor ii (thrombin) receptor  WDR43 - wd repeat domain 43  ERF - ets2 repressor factor  ZNF575 - zinc finger protein 575  YTHDC1 - yth domain containing 1  ELMSAN1 - elm2 and myb/sant-like domain containing 1  THAP11 - thap domain containing 11  ERN1 - endoplasmic reticulum to nucleus signaling 1  ZNF248 - zinc finger protein 248  UBB - ubiquitin b  RCOR1 - rest corepressor 1  TULP3 - tubby like protein 3  ZNFX1 - zinc finger, nfx1-type containing 1  HIRA - histone cell cycle regulator  TTC5 - tetratricopeptide repeat domain 5  ETV1 - ets variant 1  ETS1 - v-ets avian erythroblastosis virus e26 oncogene homolog 1  ZMIZ1 - zinc finger, miz-type containing 1  UBP1 - upstream binding protein 1 (lbp-1a)  SUMO1 - small ubiquitin-like modifier 1  UBTF - upstream binding transcription factor, rna polymerase i  PELI1 - pellino e3 ubiquitin protein ligase 1  CHURC1 - churchill domain containing 1  SMURF1 - smad specific e3 ubiquitin protein ligase 1  ZC3H4 - zinc finger ccch-type containing 4  JMJD6 - jumonji domain containing 6  UBE2I - ubiquitin-conjugating enzyme e2i  UBE2L3 - ubiquitin-conjugating enzyme e2l 3  UBE2N - ubiquitin-conjugating enzyme e2n  UBE2V1 - ubiquitin-conjugating enzyme e2 variant 1  VPS72 - vacuolar protein sorting 72 homolog (s. cerevisiae)  FMR1 - fragile x mental retardation 1  MGA - mga, max dimerization protein  WWC3 - wwc family member 3  ZEB1 - zinc finger e-box binding homeobox 1  TCF12 - transcription factor 12  ZNF354A - zinc finger protein 354a  CAMTA1 - calmodulin binding transcription activator 1  TCF20 - transcription factor 20 (ar1)  FLI1 - fli-1 proto-oncogene, ets transcription factor  FLNA - filamin a, alpha  PLCB1 - phospholipase c, beta 1 (phosphoinositide-specific)  FOXS1 - forkhead box s1  FOXO3 - forkhead box o3  HDAC8 - histone deacetylase 8  ASH1L - ash1 (absent, small, or homeotic)-like (drosophila)  SIN3B - sin3 transcription regulator family member b  DCP1A - decapping mrna 1a  NACC1 - nucleus accumbens associated 1, ben and btb (poz) domain containing  HR - hair growth associated  MED8 - mediator complex subunit 8  CAND1 - cullin-associated and neddylation-dissociated 1  TDG - thymine-dna glycosylase  PRDX2 - peroxiredoxin 2  FBXW11 - f-box and wd repeat domain containing 11  EAPP - e2f-associated phosphoprotein  FAM208A - family with sequence similarity 208, member a  KDM3A - lysine (k)-specific demethylase 3a  FHL2 - four and a half lim domains 2  TAF9 - taf9 rna polymerase ii, tata box binding protein (tbp)-associated factor, 32kda  FGFR2 - fibroblast growth factor receptor 2  TAF7 - taf7 rna polymerase ii, tata box binding protein (tbp)-associated factor, 55kda  TAF6 - taf6 rna polymerase ii, tata box binding protein (tbp)-associated factor, 80kda  FGFR1 - fibroblast growth factor receptor 1  TADA2A - transcriptional adaptor 2a  FGF9 - fibroblast growth factor 9  TACC1 - transforming, acidic coiled-coil containing protein 1  FGF1 - fibroblast growth factor 1 (acidic)  TIRAP - toll-interleukin 1 receptor (tir) domain containing adaptor protein  FGF2 - fibroblast growth factor 2 (basic)  USP22 - ubiquitin specific peptidase 22  TARBP1 - tar (hiv-1) rna binding protein 1  FER - fer (fps/fes related) tyrosine kinase  MAP3K7 - mitogen-activated protein kinase kinase kinase 7  ADNP - activity-dependent neuroprotector homeobox  MED13L - mediator complex subunit 13-like  KMT2E - lysine (k)-specific methyltransferase 2e  ZNF395 - zinc finger protein 395  MYNN - myoneurin  CRTC1 - creb regulated transcription coactivator 1  UFL1 - ufm1-specific ligase 1  TCF3 - transcription factor 3  PUM2 - pumilio homolog 2 (drosophila)  TCEB3 - transcription elongation factor b (siii), polypeptide 3 (110kda, elongin a)  DMAP1 - dna methyltransferase 1 associated protein 1  TCF4 - transcription factor 4  LARP1 - la ribonucleoprotein domain family, member 1  EFEMP1 - egf containing fibulin-like extracellular matrix protein 1  NKRF - nfkb repressing factor  FBN1 - fibrillin 1  ZNF629 - zinc finger protein 629  LANCL2 - lanc lantibiotic synthetase component c-like 2 (bacterial)  TCEA2 - transcription elongation factor a (sii), 2  ERBB2IP - erbb2 interacting protein  TCEA1 - transcription elongation factor a (sii), 1  ITGB3BP - integrin beta 3 binding protein (beta3-endonexin)  RYBP - ring1 and yy1 binding protein  MTOR - mechanistic target of rapamycin (serine/threonine kinase)  PAXBP1 - pax3 and pax7 binding protein 1  TLE1 - transducin-like enhancer of split 1 (e(sp1) homolog, drosophila)  TLE3 - transducin-like enhancer of split 3 (e(sp1) homolog, drosophila)  TLE4 - transducin-like enhancer of split 4 (e(sp1) homolog, drosophila)  TLR3 - toll-like receptor 3  EXOSC2 - exosome component 2  SIRT1 - sirtuin 1  MKX - mohawk homeobox  TMF1 - tata element modulatory factor 1  TMSB4X - thymosin beta 4, x-linked  ACTR8 - arp8 actin-related protein 8 homolog (yeast)  CBX6 - chromobox homolog 6  HEY1 - hairy/enhancer-of-split related with yrpw motif 1  TMPO - thymopoietin  CBX5 - chromobox homolog 5  BRD4 - bromodomain containing 4  BCL9L - b-cell cll/lymphoma 9-like  TARDBP - tar dna binding protein  TNFRSF1A - tumor necrosis factor receptor superfamily, member 1a  TNFRSF1B - tumor necrosis factor receptor superfamily, member 1b  TMBIM6 - transmembrane bax inhibitor motif containing 6  SUZ12 - suz12 polycomb repressive complex 2 subunit  POFUT1 - protein o-fucosyltransferase 1  TEF - thyrotrophic embryonic factor  RCOR2 - rest corepressor 2  TFAM - transcription factor a, mitochondrial  TFDP1 - transcription factor dp-1  NR2F2 - nuclear receptor subfamily 2, group f, member 2  TFE3 - transcription factor binding to ighm enhancer 3  CBX7 - chromobox homolog 7  NR2F1 - nuclear receptor subfamily 2, group f, member 1  TFCP2 - transcription factor cp2  TGFA - transforming growth factor, alpha  ZNF740 - zinc finger protein 740  FNIP1 - folliculin interacting protein 1  HEY2 - hairy/enhancer-of-split related with yrpw motif 2  TGFBR1 - transforming growth factor, beta receptor 1  TGFB2 - transforming growth factor, beta 2  RBFOX2 - rna binding protein, fox-1 homolog (c. elegans) 2  TGFB3 - transforming growth factor, beta 3  TGFB1 - transforming growth factor, beta 1  TGFB1I1 - transforming growth factor beta 1 induced transcript 1  TP53INP1 - tumor protein p53 inducible nuclear protein 1  H2AFV - h2a histone family, member v  MEPCE - methylphosphate capping enzyme  RNF20 - ring finger protein 20, e3 ubiquitin protein ligase  TGFBR3 - transforming growth factor, beta receptor iii  FOSL2 - fos-like antigen 2  FOSB - fbj murine osteosarcoma viral oncogene homolog b  FOS - fbj murine osteosarcoma viral oncogene homolog  KAT6B - k(lysine) acetyltransferase 6b  TIA1 - tia1 cytotoxic granule-associated rna binding protein  METTL3 - methyltransferase like 3  THRA - thyroid hormone receptor, alpha  ELL - elongation factor rna polymerase ii  ZNF239 - zinc finger protein 239  PHC3 - polyhomeotic homolog 3 (drosophila)  NRIP1 - nuclear receptor interacting protein 1  KAT2A - k(lysine) acetyltransferase 2a  NCOA3 - nuclear receptor coactivator 3  ANP32A - acidic (leucine-rich) nuclear phosphoprotein 32 family, member a  WWC2 - ww and c2 domain containing 2  PABPN1 - poly(a) binding protein, nuclear 1  TAF15 - taf15 rna polymerase ii, tata box binding protein (tbp)-associated factor, 68kda  AXIN1 - axin 1  TRRAP - transformation/transcription domain-associated protein  PICALM - phosphatidylinositol binding clathrin assembly protein  ZC3H14 - zinc finger ccch-type containing 14  ARID1A - at rich interactive domain 1a (swi-like)  DLL1 - delta-like 1 (drosophila)  SETD6 - set domain containing 6  FZD6 - frizzled family receptor 6  ACTR5 - arp5 actin-related protein 5 homolog (yeast)  CDC45 - cell division cycle 45  GCLC - glutamate-cysteine ligase, catalytic subunit  FZD4 - frizzled family receptor 4  FZD1 - frizzled family receptor 1  BAP1 - brca1 associated protein-1 (ubiquitin carboxy-terminal hydrolase)  ZNF613 - zinc finger protein 613  AXIN2 - axin 2  NUP85 - nucleoporin 85kda  KDM5C - lysine (k)-specific demethylase 5c  USP9X - ubiquitin specific peptidase 9, x-linked  RBM10 - rna binding motif protein 10  PHF17 - phd finger protein 17  TTC21B - tetratricopeptide repeat domain 21b  SLTM - safb-like, transcription modulator  NELFE - negative elongation factor complex member e  EHMT1 - euchromatic histone-lysine n-methyltransferase 1  NR5A1 - nuclear receptor subfamily 5, group a, member 1  FUS - fused in sarcoma  NAA40 - n(alpha)-acetyltransferase 40, natd catalytic subunit, homolog (s. cerevisiae)  USP7 - ubiquitin specific peptidase 7 (herpes virus-associated)  RNF168 - ring finger protein 168, e3 ubiquitin protein ligase  XRCC6 - x-ray repair complementing defective repair in chinese hamster cells 6  GABPB1 - ga binding protein transcription factor, beta subunit 1  GABPA - ga binding protein transcription factor, alpha subunit 60kda  CPSF7 - cleavage and polyadenylation specific factor 7, 59kda  RPAP2 - rna polymerase ii associated protein 2  DDX39B - dead (asp-glu-ala-asp) box polypeptide 39b  KMT2D - lysine (k)-specific methyltransferase 2d  ZNF419 - zinc finger protein 419  YEATS4 - yeats domain containing 4  SNIP1 - smad nuclear interacting protein 1  ZFPM1 - zinc finger protein, fog family member 1  ZNF212 - zinc finger protein 212  EID2 - ep300 interacting inhibitor of differentiation 2  ZNF383 - zinc finger protein 383  GATA2 - gata binding protein 2  KAT6A - k(lysine) acetyltransferase 6a  NUP214 - nucleoporin 214kda  NR4A3 - nuclear receptor subfamily 4, group a, member 3  BRD3 - bromodomain containing 3  GAS6 - growth arrest-specific 6  CARF - calcium responsive transcription factor  NCOA4 - nuclear receptor coactivator 4  ZNF408 - zinc finger protein 408  DEDD2 - death effector domain containing 2  MLLT10 - myeloid/lymphoid or mixed-lineage leukemia (trithorax homolog, drosophila); translocated to, 10  FASTKD1 - fast kinase domains 1  DHX36 - deah (asp-glu-ala-his) box polypeptide 36  ZNF189 - zinc finger protein 189  ZSCAN26 - zinc finger and scan domain containing 26  ZNF177 - zinc finger protein 177  ZNF175 - zinc finger protein 175  ZNF174 - zinc finger protein 174  ZNF184 - zinc finger protein 184  SSBP4 - single stranded dna binding protein 4  GTF2F1 - general transcription factor iif, polypeptide 1, 74kda  ZNF148 - zinc finger protein 148  BEND5 - ben domain containing 5  ZBTB17 - zinc finger and btb domain containing 17  GTF2H2 - general transcription factor iih, polypeptide 2, 44kda  GTF2H3 - general transcription factor iih, polypeptide 3, 34kda  TRIM26 - tripartite motif containing 26  VEZF1 - vascular endothelial zinc finger 1  GTF2H1 - general transcription factor iih, polypeptide 1, 62kda  ZNF142 - zinc finger protein 142  MCPH1 - microcephalin 1  ZNF140 - zinc finger protein 140  GTF2B - general transcription factor iib  PCGF2 - polycomb group ring finger 2  ZNF132 - zinc finger protein 132  ZNF131 - zinc finger protein 131  ZNF134 - zinc finger protein 134  ZNF133 - zinc finger protein 133  TBL1XR1 - transducin (beta)-like 1 x-linked receptor 1  GTF2I - general transcription factor iii  GTF2H4 - general transcription factor iih, polypeptide 4, 52kda  BRF1 - brf1, rna polymerase iii transcription initiation factor 90 kda subunit  ZNF79 - zinc finger protein 79  ZNF70 - zinc finger protein 70  RBM12B - rna binding motif protein 12b  ZNF75D - zinc finger protein 75d  ZNF76 - zinc finger protein 76  H3F3A - h3 histone, family 3a  BRPF1 - bromodomain and phd finger containing, 1  H2AFZ - h2a histone family, member z  SAP130 - sin3a-associated protein, 130kda  H2AFX - h2a histone family, member x  DCP2 - decapping mrna 2  FZD5 - frizzled family receptor 5  PAPD4 - pap associated domain containing 4  PAX8 - paired box 8  MIER3 - mesoderm induction early response 1, family member 3  CDC73 - cell division cycle 73  NKAP - nfkb activating protein  THAP5 - thap domain containing 5  LRP8 - low density lipoprotein receptor-related protein 8, apolipoprotein e receptor  HCFC1 - host cell factor c1 (vp16-accessory protein)  PRDM2 - pr domain containing 2, with znf domain  MAP3K12 - mitogen-activated protein kinase kinase kinase 12  ZNF226 - zinc finger protein 226  HDAC2 - histone deacetylase 2  HMBOX1 - homeobox containing 1  HDGF - hepatoma-derived growth factor  ZCCHC12 - zinc finger, cchc domain containing 12  ZNF213 - zinc finger protein 213  HDAC1 - histone deacetylase 1  HCLS1 - hematopoietic cell-specific lyn substrate 1  ZNF205 - zinc finger protein 205  ZNF207 - zinc finger protein 207  XPA - xeroderma pigmentosum, complementation group a  XPC - xeroderma pigmentosum, complementation group c  GOLGB1 - golgin b1  XPO1 - exportin 1 (crm1 homolog, yeast)  PAGR1 - paxip1 associated glutamate-rich protein 1  ZXDC - zxd family zinc finger c  XBP1 - x-box binding protein 1  ZNF367 - zinc finger protein 367  BHLHE41 - basic helix-loop-helix family, member e41  WFS1 - wolfram syndrome 1 (wolframin)  WHSC1 - wolf-hirschhorn syndrome candidate 1  NELFA - negative elongation factor complex member a  ARID2 - at rich interactive domain 2 (arid, rfx-like)  ZNF35 - zinc finger protein 35  ZKSCAN1 - zinc finger with krab and scan domains 1  FBXL15 - f-box and leucine-rich repeat protein 15  ZNF33B - zinc finger protein 33b  ZNF576 - zinc finger protein 576  ZNF32 - zinc finger protein 32  ZNF45 - zinc finger protein 45  MZF1 - myeloid zinc finger 1  ZNF18 - zinc finger protein 18  ZNF16 - zinc finger protein 16  ASXL1 - additional sex combs like 1 (drosophila)  ZNF384 - zinc finger protein 384  TNIP2 - tnfaip3 interacting protein 2  RBM42 - rna binding motif protein 42  ZNF24 - zinc finger protein 24  ZFPL1 - zinc finger protein-like 1  ZFX - zinc finger protein, x-linked  SF1 - splicing factor 1  ZBTB14 - zinc finger and btb domain containing 14  ZNF711 - zinc finger protein 711  ZNF7 - zinc finger protein 7  ZNF8 - zinc finger protein 8  ZNF2 - zinc finger protein 2  ZNF3 - zinc finger protein 3  YES1 - v-yes-1 yamaguchi sarcoma viral oncogene homolog 1  XRCC5 - x-ray repair complementing defective repair in chinese hamster cells 5 (double-strand-break rejoining)  GRSF1 - g-rich rna sequence binding factor 1  YWHAZ - tyrosine 3-monooxygenase/tryptophan 5-monooxygenase activation protein, zeta polypeptide  GSK3A - glycogen synthase kinase 3 alpha  YWHAB - tyrosine 3-monooxygenase/tryptophan 5-monooxygenase activation protein, beta polypeptide  CCNK - cyclin k  HSF2 - heat shock transcription factor 2  TRIM24 - tripartite motif containing 24  TAF1D - tata box binding protein (tbp)-associated factor, rna polymerase i, d, 41kda  CREG1 - cellular repressor of e1a-stimulated genes 1  HSBP1 - heat shock factor binding protein 1  FLCN - folliculin  PRMT2 - protein arginine methyltransferase 2  HESX1 - hesx homeobox 1  SAP30 - sin3a-associated protein, 30kda  METTL16 - methyltransferase like 16  FTO - fat mass and obesity associated  CFLAR - casp8 and fadd-like apoptosis regulator  SECISBP2 - secis binding protein 2  MED11 - mediator complex subunit 11  LDB1 - lim domain binding 1  WDR77 - wd repeat domain 77  KAT2B - k(lysine) acetyltransferase 2b  TSC22D1 - tsc22 domain family, member 1  CRTC2 - creb regulated transcription coactivator 2  ZNF259 - zinc finger protein 259  TAX1BP1 - tax1 (human t-cell leukemia virus type i) binding protein 1  CC2D1B - coiled-coil and c2 domain containing 1b  THAP8 - thap domain containing 8  BUD31 - bud31 homolog (s. cerevisiae)  ZNF655 - zinc finger protein 655  TRAK2 - trafficking protein, kinesin binding 2  HSPD1 - heat shock 60kda protein 1 (chaperonin)  DNAJB1 - dnaj (hsp40) homolog, subfamily b, member 1  ZNF48 - zinc finger protein 48  BCL10 - b-cell cll/lymphoma 10  FUBP3 - far upstream element (fuse) binding protein 3  BTRC - beta-transducin repeat containing e3 ubiquitin protein ligase  HSPA8 - heat shock 70kda protein 8  HMGN1 - high mobility group nucleosome binding domain 1  HMGB3 - high mobility group box 3  HMGB2 - high mobility group box 2  HMGB1 - high mobility group box 1  BRD9 - bromodomain containing 9  TNKS - tankyrase, trf1-interacting ankyrin-related adp-ribose polymerase  DAZAP1 - daz associated protein 1  ZBTB10 - zinc finger and btb domain containing 10  AGO1 - argonaute risc catalytic component 1  CAPRIN2 - caprin family member 2  ZBTB48 - zinc finger and btb domain containing 48  SRSF9 - serine/arginine-rich splicing factor 9  HYAL2 - hyaluronoglucosaminidase 2  GPBP1 - gc-rich promoter binding protein 1  HFE - hemochromatosis  HINT1 - histidine triad nucleotide binding protein 1  ELP4 - elongator acetyltransferase complex subunit 4  HIVEP1 - human immunodeficiency virus type i enhancer binding protein 1  HIF1A - hypoxia inducible factor 1, alpha subunit (basic helix-loop-helix transcription factor)  LPIN3 - lipin 3  TRADD - tnfrsf1a-associated via death domain  MYCBP - myc binding protein  EED - embryonic ectoderm development  URI1 - uri1, prefoldin-like chaperone  HNRNPU - heterogeneous nuclear ribonucleoprotein u (scaffold attachment factor a)  RIPK1 - receptor (tnfrsf)-interacting serine-threonine kinase 1  HNRNPK - heterogeneous nuclear ribonucleoprotein k  HNRNPL - heterogeneous nuclear ribonucleoprotein l  TNFSF13 - tumor necrosis factor (ligand) superfamily, member 13  HNRNPA2B1 - heterogeneous nuclear ribonucleoprotein a2/b1  HNRNPA1 - heterogeneous nuclear ribonucleoprotein a1  HNRNPC - heterogeneous nuclear ribonucleoprotein c (c1/c2)  HNRNPAB - heterogeneous nuclear ribonucleoprotein a/b  RIPK2 - receptor-interacting serine-threonine kinase 2  HNRNPF - heterogeneous nuclear ribonucleoprotein f  HNRNPD - heterogeneous nuclear ribonucleoprotein d (au-rich element rna binding protein 1, 37kda)  HNRNPH1 - heterogeneous nuclear ribonucleoprotein h1 (h)  HNRNPH3 - heterogeneous nuclear ribonucleoprotein h3 (2h9)  HNRNPH2 - heterogeneous nuclear ribonucleoprotein h2 (h')  ZNF584 - zinc finger protein 584  HMGA1 - high mobility group at-hook 1  FAM129B - family with sequence similarity 129, member b  NR4A1 - nuclear receptor subfamily 4, group a, member 1  PDE12 - phosphodiesterase 12  RFX7 - regulatory factor x, 7  HTATSF1 - hiv-1 tat specific factor 1  FOXK2 - forkhead box k2  HAT1 - histone acetyltransferase 1  CRTC3 - creb regulated transcription coactivator 3  RBM15 - rna binding motif protein 15  TNRC6A - trinucleotide repeat containing 6a  RBMX - rna binding motif protein, x-linked  ING2 - inhibitor of growth family, member 2  ZNF574 - zinc finger protein 574  PPM1D - protein phosphatase, mg2+/mn2+ dependent, 1d  CREB3L2 - camp responsive element binding protein 3-like 2  IPPK - inositol 1,3,4,5,6-pentakisphosphate 2-kinase  ILF2 - interleukin enhancer binding factor 2  ILF3 - interleukin enhancer binding factor 3, 90kda  PRPF19 - pre-mrna processing factor 19  ILK - integrin-linked kinase  PIAS1 - protein inhibitor of activated stat, 1  SLCO3A1 - solute carrier organic anion transporter family, member 3a1  SMYD3 - set and mynd domain containing 3  SMURF2 - smad specific e3 ubiquitin protein ligase 2  CGGBP1 - cgg triplet repeat binding protein 1  LMO4 - lim domain only 4  CAMK1 - calcium/calmodulin-dependent protein kinase i  CBX4 - chromobox homolog 4  CSRNP1 - cysteine-serine-rich nuclear protein 1  ZNF25 - zinc finger protein 25  STK16 - serine/threonine kinase 16  ITGA3 - integrin, alpha 3 (antigen cd49c, alpha 3 subunit of vla-3 receptor)  KHSRP - kh-type splicing regulatory protein  CASK - calcium/calmodulin-dependent serine protein kinase (maguk family)  SUDS3 - suppressor of defective silencing 3 homolog (s. cerevisiae)  SKAP1 - src kinase associated phosphoprotein 1  GZF1 - gdnf-inducible zinc finger protein 1  INSR - insulin receptor  INSM1 - insulinoma-associated 1  CDK13 - cyclin-dependent kinase 13  PDCD4 - programmed cell death 4 (neoplastic transformation inhibitor)  RFXANK - regulatory factor x-associated ankyrin-containing protein  CSDC2 - cold shock domain containing c2, rna binding  KLF7 - kruppel-like factor 7 (ubiquitous)  IRF2 - interferon regulatory factor 2  IRF3 - interferon regulatory factor 3  IKZF5 - ikaros family zinc finger 5 (pegasus)  RUVBL1 - ruvb-like 1 (e. coli)  DIMT1 - dim1 dimethyladenosine transferase 1 homolog (s. cerevisiae)  MED19 - mediator complex subunit 19  ITGA6 - integrin, alpha 6  IRAK2 - interleukin-1 receptor-associated kinase 2  IRAK1 - interleukin-1 receptor-associated kinase 1  ZSCAN31 - zinc finger and scan domain containing 31  KIAA1324L - kiaa1324-like  PAPD5 - pap associated domain containing 5  NSD1 - nuclear receptor binding set domain protein 1  ZBTB11 - zinc finger and btb domain containing 11  RNF25 - ring finger protein 25  DKK3 - dickkopf wnt signaling pathway inhibitor 3  MMS19 - mms19 nucleotide excision repair homolog (s. cerevisiae)  SNX5 - sorting nexin 5  ZNF777 - zinc finger protein 777  ID2 - inhibitor of dna binding 2, dominant negative helix-loop-helix protein  ID3 - inhibitor of dna binding 3, dominant negative helix-loop-helix protein  STK36 - serine/threonine kinase 36  IRF2BPL - interferon regulatory factor 2 binding protein-like  JAZF1 - jazf zinc finger 1  FOXK1 - forkhead box k1  ZNF282 - zinc finger protein 282  IGHMBP2 - immunoglobulin mu binding protein 2  RBPJ - recombination signal binding protein for immunoglobulin kappa j region  NCK2 - nck adaptor protein 2  PABPC1 - poly(a) binding protein, cytoplasmic 1  ZBTB9 - zinc finger and btb domain containing 9  USP21 - ubiquitin specific peptidase 21  ZBTB12 - zinc finger and btb domain containing 12  IK - ik cytokine, down-regulator of hla ii  JMJD1C - jumonji domain containing 1c  IGBP1 - immunoglobulin (cd79a) binding protein 1  SMARCA5 - swi/snf related, matrix associated, actin dependent regulator of chromatin, subfamily a, member 5  OGT - o-linked n-acetylglucosamine (glcnac) transferase  PELP1 - proline, glutamate and leucine rich protein 1  SND1 - staphylococcal nuclease and tudor domain containing 1  CUL2 - cullin 2  ZNF667 - zinc finger protein 667  CUL3 - cullin 3  FOXP1 - forkhead box p1  SUPT3H - suppressor of ty 3 homolog (s. cerevisiae)  BEND6 - ben domain containing 6  ZNF572 - zinc finger protein 572  PCGF5 - polycomb group ring finger 5  WTAP - wilms tumor 1 associated protein  WAC - ww domain containing adaptor with coiled-coil  ZNF414 - zinc finger protein 414  PHF21A - phd finger protein 21a  RBM39 - rna binding motif protein 39  ZNF771 - zinc finger protein 771  IER2 - immediate early response 2  NCOR1 - nuclear receptor corepressor 1  MTPN - myotrophin  NCOR2 - nuclear receptor corepressor 2  DOT1L - dot1-like histone h3k79 methyltransferase  EFCAB7 - ef-hand calcium binding domain 7  L3MBTL3 - l(3)mbt-like 3 (drosophila)  ZNF333 - zinc finger protein 333  CIR1 - corepressor interacting with rbpj, 1  SCAND1 - scan domain containing 1  H2AFY - h2a histone family, member y  FADS1 - fatty acid desaturase 1  GTF2IRD1 - gtf2i repeat domain containing 1  GTPBP1 - gtp binding protein 1  ZNF527 - zinc finger protein 527  HOPX - hop homeobox  ZNF316 - zinc finger protein 316  ZC3H8 - zinc finger ccch-type containing 8  SOX13 - sry (sex determining region y)-box 13  LMO1 - lim domain only 1 (rhombotin 1)  NACC2 - nacc family member 2, ben and btb (poz) domain containing  IER5 - immediate early response 5  CLOCK - clock circadian regulator  KLF3 - kruppel-like factor 3 (basic)  DDX41 - dead (asp-glu-ala-asp) box polypeptide 41  FXR2 - fragile x mental retardation, autosomal homolog 2  LITAF - lipopolysaccharide-induced tnf factor  YTHDF2 - yth domain family, member 2  TBPL1 - tbp-like 1  ZBTB41 - zinc finger and btb domain containing 41  ZFP62 - zfp62 zinc finger protein  ZGPAT - zinc finger, ccch-type with g patch domain  ACTL6B - actin-like 6b  MED20 - mediator complex subunit 20  AFAP1L2 - actin filament associated protein 1-like 2  ROCK2 - rho-associated, coiled-coil containing protein kinase 2  MAPK8IP1 - mitogen-activated protein kinase 8 interacting protein 1  CRLF3 - cytokine receptor-like factor 3  GLYR1 - glyoxylate reductase 1 homolog (arabidopsis)  ZBTB7A - zinc finger and btb domain containing 7a  DPY30 - dpy-30 homolog (c. elegans)  MBTPS2 - membrane-bound transcription factor peptidase, site 2  MAGED1 - melanoma antigen family d, 1  GLIS2 - glis family zinc finger 2  IRF2BP2 - interferon regulatory factor 2 binding protein 2  MED27 - mediator complex subunit 27  MED26 - mediator complex subunit 26  CHD6 - chromodomain helicase dna binding protein 6  MED17 - mediator complex subunit 17  MED23 - mediator complex subunit 23  ZNF580 - zinc finger protein 580  CDYL - chromodomain protein, y-like  GTF2IRD2 - gtf2i repeat domain containing 2  ZNF710 - zinc finger protein 710  ING4 - inhibitor of growth family, member 4  INSIG2 - insulin induced gene 2  EIF2AK3 - eukaryotic translation initiation factor 2-alpha kinase 3  RLIM - ring finger protein, lim domain interacting  KRAS - kirsten rat sarcoma viral oncogene homolog  RECQL5 - recq protein-like 5  TNPO1 - transportin 1  TGFBRAP1 - transforming growth factor, beta receptor associated protein 1  ZNF706 - zinc finger protein 706  CIAO1 - cytosolic iron-sulfur protein assembly 1  PHF20L1 - phd finger protein 20-like 1  MED10 - mediator complex subunit 10  MED21 - mediator complex subunit 21  ZFP28 - zfp28 zinc finger protein  KIT - v-kit hardy-zuckerman 4 feline sarcoma viral oncogene homolog  JUP - junction plakoglobin  JAK2 - janus kinase 2  ING5 - inhibitor of growth family, member 5  ZNF219 - zinc finger protein 219  PHF20 - phd finger protein 20  ZNF397 - zinc finger protein 397  ZNHIT3 - zinc finger, hit-type containing 3  TRIP4 - thyroid hormone receptor interactor 4  HMGN3 - high mobility group nucleosomal binding domain 3  PHF6 - phd finger protein 6  ZNF235 - zinc finger protein 235  COPS2 - cop9 signalosome subunit 2  ZNF639 - zinc finger protein 639  SARNP - sap domain containing ribonucleoprotein  LEF1 - lymphoid enhancer-binding factor 1  BRMS1L - breast cancer metastasis-suppressor 1-like  CNOT8 - ccr4-not transcription complex, subunit 8  CDK12 - cyclin-dependent kinase 12  ZNF280B - zinc finger protein 280b  ARID4B - at rich interactive domain 4b (rbp1-like)  MAP3K10 - mitogen-activated protein kinase kinase kinase 10  KDM3B - lysine (k)-specific demethylase 3b  ITGB1BP1 - integrin beta 1 binding protein 1  RSF1 - remodeling and spacing factor 1  MLLT6 - myeloid/lymphoid or mixed-lineage leukemia (trithorax homolog, drosophila); translocated to, 6  MLLT3 - myeloid/lymphoid or mixed-lineage leukemia (trithorax homolog, drosophila); translocated to, 3  MLLT1 - myeloid/lymphoid or mixed-lineage leukemia (trithorax homolog, drosophila); translocated to, 1  MED14 - mediator complex subunit 14  ZBTB22 - zinc finger and btb domain containing 22  KMT2A - lysine (k)-specific methyltransferase 2a  RPS6KA5 - ribosomal protein s6 kinase, 90kda, polypeptide 5  SUFU - suppressor of fused homolog (drosophila)  NLK - nemo-like kinase  MNAT1 - mnat cdk-activating kinase assembly factor 1  UIMC1 - ubiquitin interaction motif containing 1  MTA2 - metastasis associated 1 family, member 2  NOLC1 - nucleolar and coiled-body phosphoprotein 1  EIF2A - eukaryotic translation initiation factor 2a, 65kda  CD200 - cd200 molecule  WWOX - ww domain containing oxidoreductase  MNT - mnt, max dimerization protein  CCPG1 - cell cycle progression 1  NSRP1 - nuclear speckle splicing regulatory protein 1  SPTY2D1 - spt2, suppressor of ty, domain containing 1 (s. cerevisiae)  PELO - pelota homolog (drosophila)  MECP2 - methyl cpg binding protein 2 (rett syndrome)  MEF2A - myocyte enhancer factor 2a  PRICKLE1 - prickle homolog 1 (drosophila)  ZBED1 - zinc finger, bed-type containing 1  BCDIN3D - bcdin3 domain containing  MEIS2 - meis homeobox 2  BRWD1 - bromodomain and wd repeat domain containing 1  ZNF664 - zinc finger protein 664  MEIS1 - meis homeobox 1  MAP3K5 - mitogen-activated protein kinase kinase kinase 5  SLC33A1 - solute carrier family 33 (acetyl-coa transporter), member 1  MEN1 - multiple endocrine neoplasia i  WDR75 - wd repeat domain 75  UTP15 - utp15, u3 small nucleolar ribonucleoprotein, homolog (s. cerevisiae)  KAT8 - k(lysine) acetyltransferase 8  ZNF644 - zinc finger protein 644  ARHGEF2 - rho/rac guanine nucleotide exchange factor (gef) 2  MBD3 - methyl-cpg binding domain protein 3  PTOV1 - prostate tumor overexpressed 1  ZNF394 - zinc finger protein 394  CBFA2T2 - core-binding factor, runt domain, alpha subunit 2; translocated to, 2  MAGOH - mago-nashi homolog, proliferation-associated (drosophila)  CXXC5 - cxxc finger protein 5  RQCD1 - rcd1 required for cell differentiation1 homolog (s. pombe)  IL23A - interleukin 23, alpha subunit p19  MARS - methionyl-trna synthetase  MTA1 - metastasis associated 1  SIRT6 - sirtuin 6  PPHLN1 - periphilin 1  MBD1 - methyl-cpg binding domain protein 1  SFMBT1 - scm-like with four mbt domains 1  MAX - myc associated factor x  ZMIZ2 - zinc finger, miz-type containing 2  TRMT112 - trna methyltransferase 11-2 homolog (s. cerevisiae)  LDB2 - lim domain binding 2  ZFP90 - zfp90 zinc finger protein  NELFCD - negative elongation factor complex member c/d  ASH2L - ash2 (absent, small, or homeotic)-like (drosophila)  CTDSPL2 - ctd (carboxy-terminal domain, rna polymerase ii, polypeptide a) small phosphatase like 2  ZSCAN29 - zinc finger and scan domain containing 29  THAP2 - thap domain containing, apoptosis associated protein 2  ITCH - itchy e3 ubiquitin protein ligase  ZNF484 - zinc finger protein 484  L3MBTL2 - l(3)mbt-like 2 (drosophila)  RIOK1 - rio kinase 1  LRP6 - low density lipoprotein receptor-related protein 6  ZNF445 - zinc finger protein 445  AIP - aryl hydrocarbon receptor interacting protein  TRAPPC9 - trafficking protein particle complex 9  LUM - lumican  BTAF1 - btaf1 rna polymerase ii, b-tfiid transcription factor-associated, 170kda  RBFOX3 - rna binding protein, fox-1 homolog (c. elegans) 3  UBA3 - ubiquitin-like modifier activating enzyme 3  SESN2 - sestrin 2  PARP9 - poly (adp-ribose) polymerase family, member 9  RNF8 - ring finger protein 8, e3 ubiquitin protein ligase  BAZ1B - bromodomain adjacent to zinc finger domain, 1b  MED15 - mediator complex subunit 15  MXD1 - max dimerization protein 1  PIAS4 - protein inhibitor of activated stat, 4  TRIM33 - tripartite motif containing 33  SRRT - serrate rna effector molecule homolog (arabidopsis)  SMAD2 - smad family member 2  HDAC7 - histone deacetylase 7  KLF16 - kruppel-like factor 16  ARMCX3 - armadillo repeat containing, x-linked 3  TDP2 - tyrosyl-dna phosphodiesterase 2  LARP7 - la ribonucleoprotein domain family, member 7  MAFG - v-maf avian musculoaponeurotic fibrosarcoma oncogene homolog g  RPS6KA4 - ribosomal protein s6 kinase, 90kda, polypeptide 4  TAF9B - taf9b rna polymerase ii, tata box binding protein (tbp)-associated factor, 31kda  SMAD4 - smad family member 4  NBAS - neuroblastoma amplified sequence  SMAD5 - smad family member 5  H1FX - h1 histone family, member x  SMAD7 - smad family member 7  WASL - wiskott-aldrich syndrome-like  RBM4B - rna binding motif protein 4b  ZNF565 - zinc finger protein 565  SAP30BP - sap30 binding protein  YAF2 - yy1 associated factor 2  NCBP1 - nuclear cap binding protein subunit 1, 80kda  TAGLN3 - transgelin 3  SIX5 - six homeobox 5  NDN - necdin, melanoma antigen (mage) family member  ZNF582 - zinc finger protein 582  NCK1 - nck adaptor protein 1  BRD7 - bromodomain containing 7  NCL - nucleolin  MBNL2 - muscleblind-like splicing regulator 2  CEBPZ - ccaat/enhancer binding protein (c/ebp), zeta  TICAM1 - toll-like receptor adaptor molecule 1  CCNL2 - cyclin l2  HNRNPM - heterogeneous nuclear ribonucleoprotein m  TRIM28 - tripartite motif containing 28  DROSHA - drosha, ribonuclease type iii  TBK1 - tank-binding kinase 1  ZNF197 - zinc finger protein 197  TDRD3 - tudor domain containing 3  RBM7 - rna binding motif protein 7  RBM5 - rna binding motif protein 5  TRIM11 - tripartite motif containing 11  PPP1R12A - protein phosphatase 1, regulatory subunit 12a  MYT1 - myelin transcription factor 1  ALYREF - aly/ref export factor  NAB1 - ngfi-a binding protein 1 (egr1 binding protein 1)  CSRNP2 - cysteine-serine-rich nuclear protein 2  SENP1 - sumo1/sentrin specific peptidase 1  RNF41 - ring finger protein 41  NAB2 - ngfi-a binding protein 2 (egr1 binding protein 2)  TSHZ1 - teashirt zinc finger homeobox 1  PSME3 - proteasome (prosome, macropain) activator subunit 3 (pa28 gamma; ki)  TRIM13 - tripartite motif containing 13  TRIM8 - tripartite motif containing 8  MYO6 - myosin vi  ABT1 - activator of basal transcription 1  LBH - limb bud and heart development  TOPORS - topoisomerase i binding, arginine/serine-rich, e3 ubiquitin protein ligase  MYD88 - myeloid differentiation primary response 88  ZNF362 - zinc finger protein 362  HCFC2 - host cell factor c2  CNOT7 - ccr4-not transcription complex, subunit 7  MXI1 - max interactor 1, dimerization protein  PQBP1 - polyglutamine binding protein 1  TRA2A - transformer 2 alpha homolog (drosophila)  ACTR2 - arp2 actin-related protein 2 homolog (yeast)  ACTR3 - arp3 actin-related protein 3 homolog (yeast)  RBM15B - rna binding motif protein 15b  TSFM - ts translation elongation factor, mitochondrial  TRIM37 - tripartite motif containing 37  ZNF558 - zinc finger protein 558  LRPPRC - leucine-rich pentatricopeptide repeat containing  ZNF263 - zinc finger protein 263  PURG - purine-rich element binding protein g  RBM12 - rna binding motif protein 12  MED25 - mediator complex subunit 25  MED6 - mediator complex subunit 6  PTBP3 - polypyrimidine tract binding protein 3  DMTF1 - cyclin d binding myb-like transcription factor 1  ZBTB33 - zinc finger and btb domain containing 33  MTF1 - metal-regulatory transcription factor 1  HDAC5 - histone deacetylase 5  SRA1 - steroid receptor rna activator 1  HDAC6 - histone deacetylase 6  RNF187 - ring finger protein 187  MT3 - metallothionein 3  MED16 - mediator complex subunit 16  CCDC22 - coiled-coil domain containing 22  DNAJB6 - dnaj (hsp40) homolog, subfamily b, member 6  CITED1 - cbp/p300-interacting transactivator, with glu/asp-rich carboxy-terminal domain, 1  ZBTB5 - zinc finger and btb domain containing 5  ZBTB40 - zinc finger and btb domain containing 40  SUPT7L - suppressor of ty 7 (s. cerevisiae)-like  RGCC - regulator of cell cycle  COMMD1 - copper metabolism (murr1) domain containing 1  RBM8A - rna binding motif protein 8a  USP3 - ubiquitin specific peptidase 3  USP15 - ubiquitin specific peptidase 15  MED4 - mediator complex subunit 4  THOC1 - tho complex 1  SETD2 - set domain containing 2  RBX1 - ring-box 1, e3 ubiquitin protein ligase  THRAP3 - thyroid hormone receptor associated protein 3  MED12 - mediator complex subunit 12  MED13 - mediator complex subunit 13  PSMD6 - proteasome (prosome, macropain) 26s subunit, non-atpase, 6  MED24 - mediator complex subunit 24  ZC3H6 - zinc finger ccch-type containing 6  DTX3L - deltex 3-like (drosophila)  ZNF34 - zinc finger protein 34  SETDB1 - set domain, bifurcated 1  ORC2 - origin recognition complex, subunit 2  TOX4 - tox high mobility group box family member 4  ZNF436 - zinc finger protein 436  RNF141 - ring finger protein 141  AGRN - agrin  RBM19 - rna binding motif protein 19  G3BP2 - gtpase activating protein (sh3 domain) binding protein 2  OGG1 - 8-oxoguanine dna glycosylase  ZSCAN16 - zinc finger and scan domain containing 16  EXOSC3 - exosome component 3  EXOSC1 - exosome component 1  DDR2 - discoidin domain receptor tyrosine kinase 2  DAB2IP - dab2 interacting protein  BCLAF1 - bcl2-associated transcription factor 1  NUMA1 - nuclear mitotic apparatus protein 1  GMNN - geminin, dna replication inhibitor  NUP98 - nucleoporin 98kda  NR4A2 - nuclear receptor subfamily 4, group a, member 2  ZNF593 - zinc finger protein 593  ZBTB7B - zinc finger and btb domain containing 7b  ZNF691 - zinc finger protein 691  CREBRF - creb3 regulatory factor  ZNF623 - zinc finger protein 623  NPM1 - nucleophosmin (nucleolar phosphoprotein b23, numatrin)  NMD3 - nmd3 ribosome export adaptor  ARHGEF11 - rho guanine nucleotide exchange factor (gef) 11  ZBTB24 - zinc finger and btb domain containing 24  ZEB2 - zinc finger e-box binding homeobox 2  THAP4 - thap domain containing 4  RNF40 - ring finger protein 40, e3 ubiquitin protein ligase  TSC22D2 - tsc22 domain family, member 2  NRF1 - nuclear respiratory factor 1  NFXL1 - nuclear transcription factor, x-box binding-like 1  NOP2 - nop2 nucleolar protein  VPRBP - vpr (hiv-1) binding protein  NONO - non-pou domain containing, octamer-binding  MDFIC - myod family inhibitor domain containing  NOTCH4 - notch 4  IFT74 - intraflagellar transport 74 homolog (chlamydomonas)  NOVA2 - neuro-oncological ventral antigen 2  NOVA1 - neuro-oncological ventral antigen 1  NPAT - nuclear protein, ataxia-telangiectasia locus  CEP290 - centrosomal protein 290kda  CNOT2 - ccr4-not transcription complex, subunit 2  PUM1 - pumilio homolog 1 (drosophila)  NAA15 - n(alpha)-acetyltransferase 15, nata auxiliary subunit  CNOT3 - ccr4-not transcription complex, subunit 3  NOTCH1 - notch 1  BAZ2B - bromodomain adjacent to zinc finger domain, 2b  NOTCH3 - notch 3  ST18 - suppression of tumorigenicity 18 (breast carcinoma) (zinc finger protein)  NOTCH2 - notch 2  DHX34 - deah (asp-glu-ala-his) box polypeptide 34  NFYA - nuclear transcription factor y, alpha  HDAC4 - histone deacetylase 4  TOX - thymocyte selection-associated high mobility group box  NFYC - nuclear transcription factor y, gamma  CXXC1 - cxxc finger protein 1  ZFYVE16 - zinc finger, fyve domain containing 16  NFKBIA - nuclear factor of kappa light polypeptide gene enhancer in b-cells inhibitor, alpha  NFKB1 - nuclear factor of kappa light polypeptide gene enhancer in b-cells 1  KMT2B - lysine (k)-specific methyltransferase 2b  NFKBIB - nuclear factor of kappa light polypeptide gene enhancer in b-cells inhibitor, beta  NFKBIE - nuclear factor of kappa light polypeptide gene enhancer in b-cells inhibitor, epsilon  NKX2-2 - nk2 homeobox 2  HDAC9 - histone deacetylase 9  ZNF606 - zinc finger protein 606  SART3 - squamous cell carcinoma antigen recognized by t cells 3  ZNF614 - zinc finger protein 614  ZFP2 - zfp2 zinc finger protein  NEO1 - neogenin 1  SMARCAL1 - swi/snf related, matrix associated, actin dependent regulator of chromatin, subfamily a-like 1  NEDD8 - neural precursor cell expressed, developmentally down-regulated 8  CTR9 - ctr9, paf1/rna polymerase ii complex component  PPM1F - protein phosphatase, mg2+/mn2+ dependent, 1f  EPC1 - enhancer of polycomb homolog 1 (drosophila)  NFE2L2 - nuclear factor (erythroid-derived 2)-like 2  MORF4L2 - mortality factor 4 like 2  RASL11A - ras-like, family 11, member a  NFATC4 - nuclear factor of activated t-cells, cytoplasmic, calcineurin-dependent 4  NFIA - nuclear factor i/a  CDK5RAP3 - cdk5 regulatory subunit associated protein 3  ZNF746 - zinc finger protein 746  VGLL4 - vestigial like 4 (drosophila)  LRRC14 - leucine rich repeat containing 14  KDM4A - lysine (k)-specific demethylase 4a  PHF14 - phd finger protein 14  HP1BP3 - heterochromatin protein 1, binding protein 3  ZNF432 - zinc finger protein 432  SAFB2 - scaffold attachment factor b2  MYEF2 - myelin expression factor 2  LPIN2 - lipin 2 |
| GO:0006464 | cellular protein modification process | 4.21E-13 | 3.91E-10 | 1.09 (10334,2137,6996,1583) | [+] Show genes  OTUD5 - otu domain containing 5  MSL3 - male-specific lethal 3 homolog (drosophila)  TADA1 - transcriptional adaptor 1  ABCA1 - atp-binding cassette, sub-family a (abc1), member 1  OS9 - osteosarcoma amplified 9, endoplasmic reticulum lectin  ABL1 - c-abl oncogene 1, non-receptor tyrosine kinase  GALNT10 - udp-n-acetyl-alpha-d-galactosamine:polypeptide n-acetylgalactosaminyltransferase 10 (galnac-t10)  PML - promyelocytic leukemia  PMM1 - phosphomannomutase 1  RAB40B - rab40b, member ras oncogene family  CKAP4 - cytoskeleton-associated protein 4  UBE2Q1 - ubiquitin-conjugating enzyme e2q family member 1  BRD8 - bromodomain containing 8  PLOD2 - procollagen-lysine, 2-oxoglutarate 5-dioxygenase 2  LRRC40 - leucine rich repeat containing 40  MAN1A2 - mannosidase, alpha, class 1a, member 2  PLOD1 - procollagen-lysine, 2-oxoglutarate 5-dioxygenase 1  RFFL - ring finger and fyve-like domain containing e3 ubiquitin protein ligase  ACTB - actin, beta  OTUB1 - otu domain, ubiquitin aldehyde binding 1  EHMT2 - euchromatic histone-lysine n-methyltransferase 2  MORF4L1 - mortality factor 4 like 1  ZDHHC7 - zinc finger, dhhc-type containing 7  POMGNT1 - protein o-linked mannose n-acetylglucosaminyltransferase 1 (beta 1,2-)  PPIC - peptidylprolyl isomerase c (cyclophilin c)  PPID - peptidylprolyl isomerase d  ACTL6A - actin-like 6a  PPEF1 - protein phosphatase, ef-hand calcium binding domain 1  USP20 - ubiquitin specific peptidase 20  PPIA - peptidylprolyl isomerase a (cyclophilin a)  USP19 - ubiquitin specific peptidase 19  PPIB - peptidylprolyl isomerase b (cyclophilin b)  ACVR2A - activin a receptor, type iia  ACVRL1 - activin a receptor type ii-like 1  ACVR1 - activin a receptor, type i  NDUFAF7 - nadh dehydrogenase (ubiquinone) complex i, assembly factor 7  ACVR1B - activin a receptor, type ib  ADAM10 - adam metallopeptidase domain 10  MED1 - mediator complex subunit 1  ADH5 - alcohol dehydrogenase 5 (class iii), chi polypeptide  PARP1 - poly (adp-ribose) polymerase 1  ADPRH - adp-ribosylarginine hydrolase  TNFRSF19 - tumor necrosis factor receptor superfamily, member 19  PARP4 - poly (adp-ribose) polymerase family, member 4  RUVBL2 - ruvb-like 2 (e. coli)  ATE1 - arginyltransferase 1  PRKAB1 - protein kinase, amp-activated, beta 1 non-catalytic subunit  RCOR3 - rest corepressor 3  PRKACB - protein kinase, camp-dependent, catalytic, beta  PRKACA - protein kinase, camp-dependent, catalytic, alpha  NUP133 - nucleoporin 133kda  ADRBK2 - adrenergic, beta, receptor kinase 2  ADRBK1 - adrenergic, beta, receptor kinase 1  NUPL2 - nucleoporin like 2  EDEM2 - er degradation enhancer, mannosidase alpha-like 2  CHFR - checkpoint with forkhead and ring finger domains, e3 ubiquitin protein ligase  PRDM4 - pr domain containing 4  PPP3R1 - protein phosphatase 3, regulatory subunit b, alpha  CIT - citron (rho-interacting, serine/threonine kinase 21)  PPP5C - protein phosphatase 5, catalytic subunit  PPP6C - protein phosphatase 6, catalytic subunit  PPT1 - palmitoyl-protein thioesterase 1  UHRF2 - ubiquitin-like with phd and ring finger domains 2, e3 ubiquitin protein ligase  AGA - aspartylglucosaminidase  PPP2R5D - protein phosphatase 2, regulatory subunit b', delta  PPP2R5C - protein phosphatase 2, regulatory subunit b', gamma  PPP2R5B - protein phosphatase 2, regulatory subunit b', beta  PPP4C - protein phosphatase 4, catalytic subunit  AKT1 - v-akt murine thymoma viral oncogene homolog 1  PPP2R5E - protein phosphatase 2, regulatory subunit b', epsilon isoform  PPP2R2A - protein phosphatase 2, regulatory subunit b, alpha  PPP2R1B - protein phosphatase 2, regulatory subunit a, beta  RIOK2 - rio kinase 2  PPP2R1A - protein phosphatase 2, regulatory subunit a, alpha  WWP2 - ww domain containing e3 ubiquitin protein ligase 2  PPP2R2C - protein phosphatase 2, regulatory subunit b, gamma  PPP2R2B - protein phosphatase 2, regulatory subunit b, beta  TRIOBP - trio and f-actin binding protein  PPP2CA - protein phosphatase 2, catalytic subunit, alpha isozyme  PPP2CB - protein phosphatase 2, catalytic subunit, beta isozyme  MBD5 - methyl-cpg binding domain protein 5  DUSP14 - dual specificity phosphatase 14  PPM1G - protein phosphatase, mg2+/mn2+ dependent, 1g  PPP1CC - protein phosphatase 1, catalytic subunit, gamma isozyme  ESCO1 - establishment of sister chromatid cohesion n-acetyltransferase 1  KANSL3 - kat8 regulatory nsl complex subunit 3  SCYL2 - scy1-like 2 (s. cerevisiae)  LIAS - lipoic acid synthetase  AMFR - autocrine motility factor receptor, e3 ubiquitin protein ligase  DNAJC3 - dnaj (hsp40) homolog, subfamily c, member 3  RNF126 - ring finger protein 126  MAP2K5 - mitogen-activated protein kinase kinase 5  MAP2K7 - mitogen-activated protein kinase kinase 7  PIM2 - pim-2 oncogene  EIF2AK2 - eukaryotic translation initiation factor 2-alpha kinase 2  GALNT13 - udp-n-acetyl-alpha-d-galactosamine:polypeptide n-acetylgalactosaminyltransferase 13 (galnac-t13)  MAPK13 - mitogen-activated protein kinase 13  PIGV - phosphatidylinositol glycan anchor biosynthesis, class v  RNF157 - ring finger protein 157  MAP2K1 - mitogen-activated protein kinase kinase 1  MAP2K2 - mitogen-activated protein kinase kinase 2  MAPK10 - mitogen-activated protein kinase 10  MAPK9 - mitogen-activated protein kinase 9  TRIM59 - tripartite motif containing 59  MAPK11 - mitogen-activated protein kinase 11  METAP2 - methionyl aminopeptidase 2  MAPK8 - mitogen-activated protein kinase 8  MAPK7 - mitogen-activated protein kinase 7  MAPK3 - mitogen-activated protein kinase 3  MAPK1 - mitogen-activated protein kinase 1  COPS6 - cop9 signalosome subunit 6  PRKCSH - protein kinase c substrate 80k-h  NDC1 - ndc1 transmembrane nucleoporin  PRKD1 - protein kinase d1  PKN2 - protein kinase n2  PRKCH - protein kinase c, eta  PRKCI - protein kinase c, iota  TLK2 - tousled-like kinase 2  PRKCE - protein kinase c, epsilon  PRKCB - protein kinase c, beta  PRKCD - protein kinase c, delta  PRKCA - protein kinase c, alpha  PRKAR1B - protein kinase, camp-dependent, regulatory, type i, beta  YEATS2 - yeats domain containing 2  PRKAG1 - protein kinase, amp-activated, gamma 1 non-catalytic subunit  UBR7 - ubiquitin protein ligase e3 component n-recognin 7 (putative)  DUSP12 - dual specificity phosphatase 12  KLHL2 - kelch-like family member 2  XIAP - x-linked inhibitor of apoptosis  BIRC3 - baculoviral iap repeat containing 3  BIRC2 - baculoviral iap repeat containing 2  RFWD3 - ring finger and wd repeat domain 3  APLP2 - amyloid beta (a4) precursor-like protein 2  MGAT4B - mannosyl (alpha-1,3-)-glycoprotein beta-1,4-n-acetylglucosaminyltransferase, isozyme b  PRMT6 - protein arginine methyltransferase 6  MSL2 - male-specific lethal 2 homolog (drosophila)  MAN1B1 - mannosidase, alpha, class 1b, member 1  DCUN1D3 - dcn1, defective in cullin neddylation 1, domain containing 3  RNF220 - ring finger protein 220  APC - adenomatous polyposis coli  APBB1 - amyloid beta (a4) precursor protein-binding, family b, member 1 (fe65)  GALNT6 - udp-n-acetyl-alpha-d-galactosamine:polypeptide n-acetylgalactosaminyltransferase 6 (galnac-t6)  RNF139 - ring finger protein 139  DPY19L4 - dpy-19-like 4 (c. elegans)  P4HA1 - prolyl 4-hydroxylase, alpha polypeptide i  PEBP1 - phosphatidylethanolamine binding protein 1  TRIM23 - tripartite motif containing 23  RPRD1A - regulation of nuclear pre-mrna domain containing 1a  ARF4 - adp-ribosylation factor 4  IRAK3 - interleukin-1 receptor-associated kinase 3  SETD5 - set domain containing 5  AKAP13 - a kinase (prka) anchor protein 13  APP - amyloid beta (a4) precursor protein  PAK2 - p21 protein (cdc42/rac)-activated kinase 2  LEPREL1 - leprecan-like 1  PAK3 - p21 protein (cdc42/rac)-activated kinase 3  PAM - peptidylglycine alpha-amidating monooxygenase  PDGFA - platelet-derived growth factor alpha polypeptide  MAP4K5 - mitogen-activated protein kinase kinase kinase kinase 5  NSMCE2 - non-smc element 2, mms21 homolog (s. cerevisiae)  PDGFRB - platelet-derived growth factor receptor, beta polypeptide  ARRB2 - arrestin, beta 2  ARRB1 - arrestin, beta 1  PDGFRA - platelet-derived growth factor receptor, alpha polypeptide  PDGFB - platelet-derived growth factor beta polypeptide  PDPK1 - 3-phosphoinositide dependent protein kinase-1  RHOA - ras homolog family member a  WDYHV1 - wdyhv motif containing 1  PDK3 - pyruvate dehydrogenase kinase, isozyme 3  FSTL1 - follistatin-like 1  BAZ2A - bromodomain adjacent to zinc finger domain, 2a  PCNA - proliferating cell nuclear antigen  PCMT1 - protein-l-isoaspartate (d-aspartate) o-methyltransferase  FICD - fic domain containing  MTFMT - mitochondrial methionyl-trna formyltransferase  CDK16 - cyclin-dependent kinase 16  CDK17 - cyclin-dependent kinase 17  LEO1 - leo1, paf1/rna polymerase ii complex component, homolog (s. cerevisiae)  ASPH - aspartate beta-hydroxylase  FANCL - fanconi anemia, complementation group l  PWP1 - pwp1 homolog (s. cerevisiae)  SOCS4 - suppressor of cytokine signaling 4  KAT7 - k(lysine) acetyltransferase 7  PGGT1B - protein geranylgeranyltransferase type i, beta subunit  PGM3 - phosphoglucomutase 3  CPPED1 - calcineurin-like phosphoesterase domain containing 1  UBOX5 - u-box domain containing 5  RNF180 - ring finger protein 180  UEVLD - uev and lactate/malate dehyrogenase domains  FBXW7 - f-box and wd repeat domain containing 7, e3 ubiquitin protein ligase  RNF121 - ring finger protein 121  PHF2 - phd finger protein 2  LRRK2 - leucine-rich repeat kinase 2  DNAJC24 - dnaj (hsp40) homolog, subfamily c, member 24  SLC35C1 - solute carrier family 35 (gdp-fucose transporter), member c1  PEX12 - peroxisomal biogenesis factor 12  PEX13 - peroxisomal biogenesis factor 13  ATP7A - atpase, cu++ transporting, alpha polypeptide  PEX14 - peroxisomal biogenesis factor 14  PENK - proenkephalin  LMTK2 - lemur tyrosine kinase 2  ICK - intestinal cell (mak-like) kinase  PPM1E - protein phosphatase, mg2+/mn2+ dependent, 1e  AAK1 - ap2 associated kinase 1  COPRS - coordinator of prmt5, differentiation stimulator  PKD1 - polycystic kidney disease 1 (autosomal dominant)  UBA6 - ubiquitin-like modifier activating enzyme 6  USP40 - ubiquitin specific peptidase 40  RASA3 - ras p21 protein activator 3  PIN1 - peptidylprolyl cis/trans isomerase, nima-interacting 1  DPH3 - diphthamide biosynthesis 3  PIK3R1 - phosphoinositide-3-kinase, regulatory subunit 1 (alpha)  PLAT - plasminogen activator, tissue  AXL - axl receptor tyrosine kinase  ASXL2 - additional sex combs like 2 (drosophila)  PCMTD2 - protein-l-isoaspartate (d-aspartate) o-methyltransferase domain containing 2  PEF1 - penta-ef-hand domain containing 1  OGFOD1 - 2-oxoglutarate and iron-dependent oxygenase domain containing 1  PHF10 - phd finger protein 10  RNF13 - ring finger protein 13  BCR - breakpoint cluster region  FKBP9 - fk506 binding protein 9, 63 kda  PHKG2 - phosphorylase kinase, gamma 2 (testis)  FBXW12 - f-box and wd repeat domain containing 12  STK38 - serine/threonine kinase 38  MRGBP - mrg/morf4l binding protein  SUMF1 - sulfatase modifying factor 1  PIK3CA - phosphatidylinositol-4,5-bisphosphate 3-kinase, catalytic subunit alpha  RHOT1 - ras homolog family member t1  PIM1 - pim-1 oncogene  BCL2 - b-cell cll/lymphoma 2  UBE2W - ubiquitin-conjugating enzyme e2w (putative)  PIK3C3 - phosphatidylinositol 3-kinase, catalytic subunit type 3  MGAT4A - mannosyl (alpha-1,3-)-glycoprotein beta-1,4-n-acetylglucosaminyltransferase, isozyme a  PIGH - phosphatidylinositol glycan anchor biosynthesis, class h  LYPLA2 - lysophospholipase ii  CCND1 - cyclin d1  PARK7 - parkinson protein 7  B4GALT7 - xylosylprotein beta 1,4-galactosyltransferase, polypeptide 7  PIGF - phosphatidylinositol glycan anchor biosynthesis, class f  TRIM27 - tripartite motif containing 27  CWC27 - cwc27 spliceosome-associated protein homolog (s. cerevisiae)  BRAF - v-raf murine sarcoma viral oncogene homolog b  BCKDK - branched chain ketoacid dehydrogenase kinase  WHSC1L1 - wolf-hirschhorn syndrome candidate 1-like 1  CDKL4 - cyclin-dependent kinase-like 4  BCOR - bcl6 corepressor  ZFP36L2 - zfp36 ring finger protein-like 2  ZFP36L1 - zfp36 ring finger protein-like 1  PAK4 - p21 protein (cdc42/rac)-activated kinase 4  DCAF16 - ddb1 and cul4 associated factor 16  RET - ret proto-oncogene  DUSP15 - dual specificity phosphatase 15  DPF2 - d4, zinc and double phd fingers family 2  SH3RF3 - sh3 domain containing ring finger 3  GALNTL6 - udp-n-acetyl-alpha-d-galactosamine:polypeptide n-acetylgalactosaminyltransferase-like 6  RLF - rearranged l-myc fusion  SNRK - snf related kinase  RING1 - ring finger protein 1  PIGG - phosphatidylinositol glycan anchor biosynthesis, class g  TMEM5 - transmembrane protein 5  FBLL1 - fibrillarin-like 1  BMI1 - bmi1 polycomb ring finger oncogene  PCGF3 - polycomb group ring finger 3  BMPR1A - bone morphogenetic protein receptor, type ia  BMPR2 - bone morphogenetic protein receptor, type ii (serine/threonine kinase)  BMPR1B - bone morphogenetic protein receptor, type ib  FBXL12 - f-box and leucine-rich repeat protein 12  ABCA7 - atp-binding cassette, sub-family a (abc1), member 7  TRPM7 - transient receptor potential cation channel, subfamily m, member 7  PSMD14 - proteasome (prosome, macropain) 26s subunit, non-atpase, 14  RNF19B - ring finger protein 19b  CTDSPL - ctd (carboxy-terminal domain, rna polymerase ii, polypeptide a) small phosphatase-like  TMEM258 - transmembrane protein 258  UHMK1 - u2af homology motif (uhm) kinase 1  MSLN - mesothelin  RNF4 - ring finger protein 4  DCAF7 - ddb1 and cul4 associated factor 7  RNF2 - ring finger protein 2  GPHN - gephyrin  RNF6 - ring finger protein (c3h2c3 type) 6  QPCTL - glutaminyl-peptide cyclotransferase-like  ROCK1 - rho-associated, coiled-coil containing protein kinase 1  LYPLAL1 - lysophospholipase-like 1  RNF111 - ring finger protein 111  TET2 - tet methylcytosine dioxygenase 2  STAM2 - signal transducing adaptor molecule (sh3 domain and itam motif) 2  TRPM4 - transient receptor potential cation channel, subfamily m, member 4  ZMPSTE24 - zinc metallopeptidase ste24  STUB1 - stip1 homology and u-box containing protein 1, e3 ubiquitin protein ligase  UBE4B - ubiquitination factor e4b  TRIM44 - tripartite motif containing 44  TAB1 - tgf-beta activated kinase 1/map3k7 binding protein 1  PPIE - peptidylprolyl isomerase e (cyclophilin e)  MCRS1 - microspherule protein 1  CAMK2B - calcium/calmodulin-dependent protein kinase ii beta  FKBP14 - fk506 binding protein 14, 22 kda  PIBF1 - progesterone immunomodulatory binding factor 1  PPIH - peptidylprolyl isomerase h (cyclophilin h)  B3GALT6 - udp-gal:betagal beta 1,3-galactosyltransferase polypeptide 6  CAD - carbamoyl-phosphate synthetase 2, aspartate transcarbamylase, and dihydroorotase  RPN2 - ribophorin ii  RPN1 - ribophorin i  UBE2E3 - ubiquitin-conjugating enzyme e2e 3  HERC6 - hect and rld domain containing e3 ubiquitin protein ligase family member 6  DDR1 - discoidin domain receptor tyrosine kinase 1  TRIM38 - tripartite motif containing 38  TADA3 - transcriptional adaptor 3  CALU - calumenin  STK25 - serine/threonine kinase 25  LRRC41 - leucine rich repeat containing 41  CRTAP - cartilage associated protein  INO80C - ino80 complex subunit c  CCNT1 - cyclin t1  CCNH - cyclin h  TMEM132A - transmembrane protein 132a  SSH3 - slingshot protein phosphatase 3  ANAPC10 - anaphase promoting complex subunit 10  RPS6KA2 - ribosomal protein s6 kinase, 90kda, polypeptide 2  NRBP2 - nuclear receptor binding protein 2  RPS6KA1 - ribosomal protein s6 kinase, 90kda, polypeptide 1  PARP16 - poly (adp-ribose) polymerase family, member 16  RPS6KB1 - ribosomal protein s6 kinase, 70kda, polypeptide 1  RPS6KB2 - ribosomal protein s6 kinase, 70kda, polypeptide 2  RNF125 - ring finger protein 125, e3 ubiquitin protein ligase  CBFB - core-binding factor, beta subunit  CBL - cbl proto-oncogene, e3 ubiquitin protein ligase  PIAS3 - protein inhibitor of activated stat, 3  KANSL2 - kat8 regulatory nsl complex subunit 2  UBE2R2 - ubiquitin-conjugating enzyme e2r 2  RBM14 - rna binding motif protein 14  PRMT5 - protein arginine methyltransferase 5  TRIM3 - tripartite motif containing 3  CDK11B - cyclin-dependent kinase 11b  TGOLN2 - trans-golgi network protein 2  STAMBP - stam binding protein  CDH2 - cadherin 2, type 1, n-cadherin (neuronal)  CDC27 - cell division cycle 27  ANKIB1 - ankyrin repeat and ibr domain containing 1  POMT1 - protein-o-mannosyltransferase 1  PSEN1 - presenilin 1  PSKH1 - protein serine kinase h1  USP16 - ubiquitin specific peptidase 16  PSMA1 - proteasome (prosome, macropain) subunit, alpha type, 1  PSMA2 - proteasome (prosome, macropain) subunit, alpha type, 2  RNF216 - ring finger protein 216  CAMKK2 - calcium/calmodulin-dependent protein kinase kinase 2, beta  PSMB1 - proteasome (prosome, macropain) subunit, beta type, 1  NPRL2 - nitrogen permease regulator-like 2 (s. cerevisiae)  PSMA5 - proteasome (prosome, macropain) subunit, alpha type, 5  PSMA3 - proteasome (prosome, macropain) subunit, alpha type, 3  PSMB10 - proteasome (prosome, macropain) subunit, beta type, 10  UBLCP1 - ubiquitin-like domain containing ctd phosphatase 1  TAF6L - taf6-like rna polymerase ii, p300/cbp-associated factor (pcaf)-associated factor, 65kda  PSMC6 - proteasome (prosome, macropain) 26s subunit, atpase, 6  FAM173B - family with sequence similarity 173, member b  PSMD1 - proteasome (prosome, macropain) 26s subunit, non-atpase, 1  PSMC4 - proteasome (prosome, macropain) 26s subunit, atpase, 4  DPM3 - dolichyl-phosphate mannosyltransferase polypeptide 3  PSMC2 - proteasome (prosome, macropain) 26s subunit, atpase, 2  PSMD9 - proteasome (prosome, macropain) 26s subunit, non-atpase, 9  PSMD10 - proteasome (prosome, macropain) 26s subunit, non-atpase, 10  PSMD7 - proteasome (prosome, macropain) 26s subunit, non-atpase, 7  PSMD8 - proteasome (prosome, macropain) 26s subunit, non-atpase, 8  PSMD5 - proteasome (prosome, macropain) 26s subunit, non-atpase, 5  PSMD2 - proteasome (prosome, macropain) 26s subunit, non-atpase, 2  PSMD12 - proteasome (prosome, macropain) 26s subunit, non-atpase, 12  PSMD13 - proteasome (prosome, macropain) 26s subunit, non-atpase, 13  ATG7 - autophagy related 7  LEPREL2 - leprecan-like 2  KLHL17 - kelch-like family member 17  PTEN - phosphatase and tensin homolog  SETD4 - set domain containing 4  PRDX4 - peroxiredoxin 4  CFL1 - cofilin 1 (non-muscle)  KAT5 - k(lysine) acetyltransferase 5  PTK2 - protein tyrosine kinase 2  PTK7 - protein tyrosine kinase 7  CDK5 - cyclin-dependent kinase 5  CDK4 - cyclin-dependent kinase 4  TWF1 - twinfilin actin-binding protein 1  CDK9 - cyclin-dependent kinase 9  CDK8 - cyclin-dependent kinase 8  CDK7 - cyclin-dependent kinase 7  SPOPL - speckle-type poz protein-like  PTPN3 - protein tyrosine phosphatase, non-receptor type 3  PTPN2 - protein tyrosine phosphatase, non-receptor type 2  PTPN1 - protein tyrosine phosphatase, non-receptor type 1  PTPN9 - protein tyrosine phosphatase, non-receptor type 9  PPM1M - protein phosphatase, mg2+/mn2+ dependent, 1m  PTPN11 - protein tyrosine phosphatase, non-receptor type 11  HTATIP2 - hiv-1 tat interactive protein 2, 30kda  PTPRA - protein tyrosine phosphatase, receptor type, a  PTPN12 - protein tyrosine phosphatase, non-receptor type 12  PTPN13 - protein tyrosine phosphatase, non-receptor type 13 (apo-1/cd95 (fas)-associated phosphatase)  PTPRD - protein tyrosine phosphatase, receptor type, d  PTPRB - protein tyrosine phosphatase, receptor type, b  SLC35A1 - solute carrier family 35 (cmp-sialic acid transporter), member a1  PTPRG - protein tyrosine phosphatase, receptor type, g  CDK2 - cyclin-dependent kinase 2  PTPRE - protein tyrosine phosphatase, receptor type, e  PTPRF - protein tyrosine phosphatase, receptor type, f  PDP1 - pyruvate dehyrogenase phosphatase catalytic subunit 1  P4HTM - prolyl 4-hydroxylase, transmembrane (endoplasmic reticulum)  PTPRR - protein tyrosine phosphatase, receptor type, r  PTPRS - protein tyrosine phosphatase, receptor type, s  PTPRK - protein tyrosine phosphatase, receptor type, k  PTPRM - protein tyrosine phosphatase, receptor type, m  PTPRN - protein tyrosine phosphatase, receptor type, n  PLK2 - polo-like kinase 2  PEX2 - peroxisomal biogenesis factor 2  NUP50 - nucleoporin 50kda  CHML - choroideremia-like (rab escort protein 2)  CHM - choroideremia (rab escort protein 1)  KDM5B - lysine (k)-specific demethylase 5b  CHCHD4 - coiled-coil-helix-coiled-coil-helix domain containing 4  CHGB - chromogranin b (secretogranin 1)  RAB6A - rab6a, member ras oncogene family  MXRA8 - matrix-remodelling associated 8  MAP4K2 - mitogen-activated protein kinase kinase kinase kinase 2  RAB5A - rab5a, member ras oncogene family  RAB3A - rab3a, member ras oncogene family  RAB3B - rab3b, member ras oncogene family  FRS2 - fibroblast growth factor receptor substrate 2  RAB2A - rab2a, member ras oncogene family  RAB1A - rab1a, member ras oncogene family  PARP14 - poly (adp-ribose) polymerase family, member 14  PAF1 - paf1, rna polymerase ii associated factor, homolog (s. cerevisiae)  FBXL19 - f-box and leucine-rich repeat protein 19  MSL1 - male-specific lethal 1 homolog (drosophila)  INO80 - ino80 complex subunit  RC3H2 - ring finger and ccch-type domains 2  RANBP2 - ran binding protein 2  RANGAP1 - ran gtpase activating protein 1  MIER2 - mesoderm induction early response 1, family member 2  USP53 - ubiquitin specific peptidase 53  MOK - mok protein kinase  B3GNT2 - udp-glcnac:betagal beta-1,3-n-acetylglucosaminyltransferase 2  RRAGA - ras-related gtp binding a  RASA1 - ras p21 protein activator (gtpase activating protein) 1  CLK1 - cdc-like kinase 1  MAGEL2 - mage-like 2  CLK2 - cdc-like kinase 2  ING3 - inhibitor of growth family, member 3  CLK3 - cdc-like kinase 3  PRMT7 - protein arginine methyltransferase 7  RBBP7 - retinoblastoma binding protein 7  TMX3 - thioredoxin-related transmembrane protein 3  MAP3K2 - mitogen-activated protein kinase kinase kinase 2  ARID4A - at rich interactive domain 4a (rbp1-like)  RBBP5 - retinoblastoma binding protein 5  RBBP6 - retinoblastoma binding protein 6  KDM5A - lysine (k)-specific demethylase 5a  NUP35 - nucleoporin 35kda  MGEA5 - meningioma expressed antigen 5 (hyaluronidase)  ERCC8 - excision repair cross-complementing rodent repair deficiency, complementation group 8  RCN1 - reticulocalbin 1, ef-hand calcium binding domain  USP39 - ubiquitin specific peptidase 39  PIGU - phosphatidylinositol glycan anchor biosynthesis, class u  ZDHHC13 - zinc finger, dhhc-type containing 13  CHUK - conserved helix-loop-helix ubiquitous kinase  SPTAN1 - spectrin, alpha, non-erythrocytic 1  MKRN1 - makorin ring finger protein 1  MKRN2 - makorin ring finger protein 2  TMTC4 - transmembrane and tetratricopeptide repeat containing 4  SPTBN4 - spectrin, beta, non-erythrocytic 4  FBXO18 - f-box protein, helicase, 18  SPP1 - secreted phosphoprotein 1  ANO8 - anoctamin 8  ALG10 - alg10, alpha-1,2-glucosyltransferase  MIER1 - mesoderm induction early response 1, transcriptional regulator  PPP1R15B - protein phosphatase 1, regulatory subunit 15b  ABHD13 - abhydrolase domain containing 13  METTL12 - methyltransferase like 12  CPE - carboxypeptidase e  SOS1 - son of sevenless homolog 1 (drosophila)  FBXL20 - f-box and leucine-rich repeat protein 20  RAB40C - rab40c, member ras oncogene family  CREBBP - creb binding protein  ATF2 - activating transcription factor 2  CREB1 - camp responsive element binding protein 1  DDX58 - dead (asp-glu-ala-asp) box polypeptide 58  MARK4 - map/microtubule affinity-regulating kinase 4  RPRD1B - regulation of nuclear pre-mrna domain containing 1b  PLK3 - polo-like kinase 3  SNRPB - small nuclear ribonucleoprotein polypeptides b and b1  SUMO2 - small ubiquitin-like modifier 2  SUMO3 - small ubiquitin-like modifier 3  HLTF - helicase-like transcription factor  SGK3 - serum/glucocorticoid regulated kinase family, member 3  KMT2C - lysine (k)-specific methyltransferase 2c  PRKD3 - protein kinase d3  RAB38 - rab38, member ras oncogene family  PPIL4 - peptidylprolyl isomerase (cyclophilin)-like 4  OSTC - oligosaccharyltransferase complex subunit (non-catalytic)  SENP2 - sumo1/sentrin/smt3 specific peptidase 2  CST3 - cystatin c  USP32 - ubiquitin specific peptidase 32  NAT6 - n-acetyltransferase 6 (gcn5-related)  SHC2 - shc (src homology 2 domain containing) transforming protein 2  KDM2B - lysine (k)-specific demethylase 2b  KLHL12 - kelch-like family member 12  DSTYK - dual serine/threonine and tyrosine protein kinase  CTBP1 - c-terminal binding protein 1  RASGRP3 - ras guanyl releasing protein 3 (calcium and dag-regulated)  UBL5 - ubiquitin-like 5  CTNNB1 - catenin (cadherin-associated protein), beta 1, 88kda  PPIL2 - peptidylprolyl isomerase (cyclophilin)-like 2  PIGO - phosphatidylinositol glycan anchor biosynthesis, class o  STX1A - syntaxin 1a (brain)  LNX1 - ligand of numb-protein x 1, e3 ubiquitin protein ligase  USP30 - ubiquitin specific peptidase 30  FUT10 - fucosyltransferase 10 (alpha (1,3) fucosyltransferase)  NEK4 - nima-related kinase 4  STK3 - serine/threonine kinase 3  PCGF1 - polycomb group ring finger 1  BRD1 - bromodomain containing 1  FKBP8 - fk506 binding protein 8, 38kda  AURKC - aurora kinase c  STK10 - serine/threonine kinase 10  SUV420H2 - suppressor of variegation 4-20 homolog 2 (drosophila)  CYLD - cylindromatosis (turban tumor syndrome)  ISPD - isoprenoid synthase domain containing  PAN3 - pan3 poly(a) specific ribonuclease subunit homolog (s. cerevisiae)  CDKL5 - cyclin-dependent kinase-like 5  STK4 - serine/threonine kinase 4  HECTD1 - hect domain containing e3 ubiquitin protein ligase 1  CRKL - v-crk avian sarcoma virus ct10 oncogene homolog-like  FKBP10 - fk506 binding protein 10, 65 kda  CRY1 - cryptochrome 1 (photolyase-like)  CRY2 - cryptochrome 2 (photolyase-like)  ANAPC13 - anaphase promoting complex subunit 13  AGBL5 - atp/gtp binding protein-like 5  DCAF12 - ddb1 and cul4 associated factor 12  BRMS1 - breast cancer metastasis suppressor 1  NAA35 - n(alpha)-acetyltransferase 35, natc auxiliary subunit  SUMF2 - sulfatase modifying factor 2  CSF1R - colony stimulating factor 1 receptor  FBXO7 - f-box protein 7  CSF1 - colony stimulating factor 1 (macrophage)  SRPK2 - srsf protein kinase 2  SRPK1 - srsf protein kinase 1  QPCT - glutaminyl-peptide cyclotransferase  TAB3 - tgf-beta activated kinase 1/map3k7 binding protein 3  KLHL22 - kelch-like family member 22  GALNT11 - udp-n-acetyl-alpha-d-galactosamine:polypeptide n-acetylgalactosaminyltransferase 11 (galnac-t11)  ZNF335 - zinc finger protein 335  PTPN5 - protein tyrosine phosphatase, non-receptor type 5 (striatum-enriched)  CSK - c-src tyrosine kinase  CSNK1G2 - casein kinase 1, gamma 2  CSNK1A1 - casein kinase 1, alpha 1  TTLL1 - tubulin tyrosine ligase-like family, member 1  CSNK1D - casein kinase 1, delta  SPTBN1 - spectrin, beta, non-erythrocytic 1  RNF214 - ring finger protein 214  CSNK2A2 - casein kinase 2, alpha prime polypeptide  CSNK1G3 - casein kinase 1, gamma 3  SRC - v-src avian sarcoma (schmidt-ruppin a-2) viral oncogene homolog  CSNK2A1 - casein kinase 2, alpha 1 polypeptide  VIPAS39 - vps33b interacting protein, apical-basolateral polarity regulator, spe-39 homolog  ZDHHC12 - zinc finger, dhhc-type containing 12  ARIH1 - ariadne homolog, ubiquitin-conjugating enzyme e2 binding protein, 1 (drosophila)  UBE2O - ubiquitin-conjugating enzyme e2o  FBXL2 - f-box and leucine-rich repeat protein 2  SHPRH - snf2 histone linker phd ring helicase, e3 ubiquitin protein ligase  RNF169 - ring finger protein 169  SEL1L - sel-1 suppressor of lin-12-like (c. elegans)  FBXO33 - f-box protein 33  DDOST - dolichyl-diphosphooligosaccharide--protein glycosyltransferase subunit (non-catalytic)  SIN3A - sin3 transcription regulator family member a  SDF2 - stromal cell-derived factor 2  DPH6 - diphthamine biosynthesis 6  TPGS2 - tubulin polyglutamylase complex subunit 2  FAM98A - family with sequence similarity 98, member a  RSPRY1 - ring finger and spry domain containing 1  WWTR1 - ww domain containing transcription regulator 1  SETMAR - set domain and mariner transposase fusion gene  DDB1 - damage-specific dna binding protein 1, 127kda  MAP2K4 - mitogen-activated protein kinase kinase 4  PTPN23 - protein tyrosine phosphatase, non-receptor type 23  SENP7 - sumo1/sentrin specific peptidase 7  ZDHHC24 - zinc finger, dhhc-type containing 24  KBTBD6 - kelch repeat and btb (poz) domain containing 6  CSRP2BP - csrp2 binding protein  ZDHHC5 - zinc finger, dhhc-type containing 5  SCYL1 - scy1-like 1 (s. cerevisiae)  CLK4 - cdc-like kinase 4  KLHL13 - kelch-like family member 13  RNF19A - ring finger protein 19a, e3 ubiquitin protein ligase  RCHY1 - ring finger and chy zinc finger domain containing 1, e3 ubiquitin protein ligase  ASPHD1 - aspartate beta-hydroxylase domain containing 1  PPM1H - protein phosphatase, mg2+/mn2+ dependent, 1h  GALNT16 - udp-n-acetyl-alpha-d-galactosamine:polypeptide n-acetylgalactosaminyltransferase 16  KCTD13 - potassium channel tetramerization domain containing 13  DCAF13 - ddb1 and cul4 associated factor 13  BIRC6 - baculoviral iap repeat containing 6  BTBD6 - btb (poz) domain containing 6  ZDHHC20 - zinc finger, dhhc-type containing 20  LTN1 - listerin e3 ubiquitin protein ligase 1  ZNF451 - zinc finger protein 451  UBXN7 - ubx domain protein 7  CHD5 - chromodomain helicase dna binding protein 5  MCFD2 - multiple coagulation factor deficiency 2  MED30 - mediator complex subunit 30  RIMKLB - ribosomal modification protein rimk-like family member b  USP31 - ubiquitin specific peptidase 31  DCAF15 - ddb1 and cul4 associated factor 15  DAPK3 - death-associated protein kinase 3  ZZZ3 - zinc finger, zz-type containing 3  LRSAM1 - leucine rich repeat and sterile alpha motif containing 1  ULK3 - unc-51 like kinase 3  MTA3 - metastasis associated 1 family, member 3  DGKQ - diacylglycerol kinase, theta 110kda  CLIP3 - cap-gly domain containing linker protein 3  DAPK1 - death-associated protein kinase 1  TIPARP - tcdd-inducible poly(adp-ribose) polymerase  HACE1 - hect domain and ankyrin repeat containing e3 ubiquitin protein ligase 1  SATB1 - satb homeobox 1  MIB1 - mindbomb e3 ubiquitin protein ligase 1  DAD1 - defender against cell death 1  SBF1 - set binding factor 1  TRPC4AP - transient receptor potential cation channel, subfamily c, member 4 associated protein  KLHL42 - kelch-like family member 42  EPC2 - enhancer of polycomb homolog 2 (drosophila)  CDC26 - cell division cycle 26  IRF2BP1 - interferon regulatory factor 2 binding protein 1  TAOK1 - tao kinase 1  PDP2 - pyruvate dehyrogenase phosphatase catalytic subunit 2  KLHL8 - kelch-like family member 8  DPH2 - dph2 homolog (s. cerevisiae)  SHANK3 - sh3 and multiple ankyrin repeat domains 3  DPH1 - diphthamide biosynthesis 1  DPAGT1 - dolichyl-phosphate (udp-n-acetylglucosamine) n-acetylglucosaminephosphotransferase 1 (glcnac-1-p transferase)  WIPI2 - wd repeat domain, phosphoinositide interacting 2  USP35 - ubiquitin specific peptidase 35  DR1 - down-regulator of transcription 1, tbp-binding (negative cofactor 2)  WSB1 - wd repeat and socs box containing 1  ALG2 - alg2, alpha-1,3/1,6-mannosyltransferase  ZNRF2 - zinc and ring finger 2  DUSP8 - dual specificity phosphatase 8  MAPK15 - mitogen-activated protein kinase 15  DUSP5 - dual specificity phosphatase 5  DUSP6 - dual specificity phosphatase 6  SKP1 - s-phase kinase-associated protein 1  DUSP7 - dual specificity phosphatase 7  DYRK1A - dual-specificity tyrosine-(y)-phosphorylation regulated kinase 1a  TOR1A - torsin family 1, member a (torsin a)  ABHD12 - abhydrolase domain containing 12  HERC4 - hect and rld domain containing e3 ubiquitin protein ligase 4  WDR48 - wd repeat domain 48  USP36 - ubiquitin specific peptidase 36  FNIP2 - folliculin interacting protein 2  SENP6 - sumo1/sentrin specific peptidase 6  HBEGF - heparin-binding egf-like growth factor  DTX1 - deltex homolog 1 (drosophila)  DLG3 - discs, large homolog 3 (drosophila)  DLG4 - discs, large homolog 4 (drosophila)  DLG1 - discs, large homolog 1 (drosophila)  FBXW8 - f-box and wd repeat domain containing 8  SH3RF1 - sh3 domain containing ring finger 1  FBXO25 - f-box protein 25  ST6GAL1 - st6 beta-galactosamide alpha-2,6-sialyltranferase 1  SIAH2 - siah e3 ubiquitin protein ligase 2  SIAH1 - siah e3 ubiquitin protein ligase 1  DGUOK - deoxyguanosine kinase  FBXL3 - f-box and leucine-rich repeat protein 3  EP400 - e1a binding protein p400  DHPS - deoxyhypusine synthase  B3GAT3 - beta-1,3-glucuronyltransferase 3 (glucuronosyltransferase i)  ST3GAL2 - st3 beta-galactoside alpha-2,3-sialyltransferase 2  FBXL5 - f-box and leucine-rich repeat protein 5  RICTOR - rptor independent companion of mtor, complex 2  FBXO2 - f-box protein 2  UVSSA - uv-stimulated scaffold protein a  SFPQ - splicing factor proline/glutamine-rich  PELI3 - pellino e3 ubiquitin protein ligase family member 3  FBXW2 - f-box and wd repeat domain containing 2  EIF2AK4 - eukaryotic translation initiation factor 2 alpha kinase 4  NAT9 - n-acetyltransferase 9 (gcn5-related, putative)  ALG11 - alg11, alpha-1,2-mannosyltransferase  SGK1 - serum/glucocorticoid regulated kinase 1  USP37 - ubiquitin specific peptidase 37  SENP3 - sumo1/sentrin/smt3 specific peptidase 3  SMARCAD1 - swi/snf-related, matrix-associated actin-dependent regulator of chromatin, subfamily a, containing dead/h box 1  MARK2 - map/microtubule affinity-regulating kinase 2  C1GALT1 - core 1 synthase, glycoprotein-n-acetylgalactosamine 3-beta-galactosyltransferase, 1  ELK4 - elk4, ets-domain protein (srf accessory protein 1)  EPAS1 - endothelial pas domain protein 1  EP300 - e1a binding protein p300  DPH7 - diphthamide biosynthesis 7  SEPHS1 - selenophosphate synthetase 1  TIMM50 - translocase of inner mitochondrial membrane 50 homolog (s. cerevisiae)  UGGT1 - udp-glucose glycoprotein glucosyltransferase 1  EGR2 - early growth response 2  TTC3 - tetratricopeptide repeat domain 3  EIF2S1 - eukaryotic translation initiation factor 2, subunit 1 alpha, 35kda  TRIM32 - tripartite motif containing 32  SPHK2 - sphingosine kinase 2  KDM2A - lysine (k)-specific demethylase 2a  TMEM129 - transmembrane protein 129  METTL18 - methyltransferase like 18  TSG101 - tumor susceptibility gene 101  MAST1 - microtubule associated serine/threonine kinase 1  FBXO21 - f-box protein 21  PGAP3 - post-gpi attachment to proteins 3  RNF149 - ring finger protein 149  STK38L - serine/threonine kinase 38 like  TULP4 - tubby like protein 4  PIGM - phosphatidylinositol glycan anchor biosynthesis, class m  PHC1 - polyhomeotic homolog 1 (drosophila)  POGLUT1 - protein o-glucosyltransferase 1  PHLPP2 - ph domain and leucine rich repeat protein phosphatase 2  EGFR - epidermal growth factor receptor  USP33 - ubiquitin specific peptidase 33  MAST3 - microtubule associated serine/threonine kinase 3  KDM4B - lysine (k)-specific demethylase 4b  FAM20C - family with sequence similarity 20, member c  TOP1 - topoisomerase (dna) i  KDM1A - lysine (k)-specific demethylase 1a  GOLGA7B - golgin a7 family, member b  ATXN7L3 - ataxin 7-like 3  PARP6 - poly (adp-ribose) polymerase family, member 6  EOGT - egf domain-specific o-linked n-acetylglucosamine (glcnac) transferase  UBE2Q2 - ubiquitin-conjugating enzyme e2q family member 2  E4F1 - e4f transcription factor 1  TRIO - trio rho guanine nucleotide exchange factor  SMG1 - smg1 phosphatidylinositol 3-kinase-related kinase  TNIK - traf2 and nck interacting kinase  ENY2 - enhancer of yellow 2 homolog (drosophila)  MYCBP2 - myc binding protein 2, e3 ubiquitin protein ligase  TRAF1 - tnf receptor-associated factor 1  HSP90B1 - heat shock protein 90kda beta (grp94), member 1  TRAF3 - tnf receptor-associated factor 3  TRAF2 - tnf receptor-associated factor 2  SETX - senataxin  SETD1B - set domain containing 1b  FEM1C - fem-1 homolog c (c. elegans)  TPR - translocated promoter region, nuclear basket protein  ERC1 - elks/rab6-interacting/cast family member 1  KDM4C - lysine (k)-specific demethylase 4c  TRIM35 - tripartite motif containing 35  PIGW - phosphatidylinositol glycan anchor biosynthesis, class w  CAMK1D - calcium/calmodulin-dependent protein kinase id  TTLL5 - tubulin tyrosine ligase-like family, member 5  NUP107 - nucleoporin 107kda  CDK19 - cyclin-dependent kinase 19  DERL3 - derlin 3  USP1 - ubiquitin specific peptidase 1  PTK2B - protein tyrosine kinase 2 beta  PCNP - pest proteolytic signal containing nuclear protein  PARP11 - poly (adp-ribose) polymerase family, member 11  CUL9 - cullin 9  KANSL1 - kat8 regulatory nsl complex subunit 1  RNF185 - ring finger protein 185  USP4 - ubiquitin specific peptidase 4 (proto-oncogene)  TAB2 - tgf-beta activated kinase 1/map3k7 binding protein 2  ZNF598 - zinc finger protein 598  VGF - vgf nerve growth factor inducible  LAMA1 - laminin, alpha 1  SPSB3 - spla/ryanodine receptor domain and socs box containing 3  VHL - von hippel-lindau tumor suppressor, e3 ubiquitin protein ligase  KDM6B - lysine (k)-specific demethylase 6b  PHF8 - phd finger protein 8  TRIM41 - tripartite motif containing 41  EXT2 - exostosin glycosyltransferase 2  EXT1 - exostosin glycosyltransferase 1  KDM6A - lysine (k)-specific demethylase 6a  DCUN1D4 - dcn1, defective in cullin neddylation 1, domain containing 4  EXTL2 - exostosin-like glycosyltransferase 2  EXTL3 - exostosin-like glycosyltransferase 3  EYA3 - eyes absent homolog 3 (drosophila)  EZH1 - enhancer of zeste homolog 1 (drosophila)  TRIM47 - tripartite motif containing 47  PDXP - pyridoxal (pyridoxine, vitamin b6) phosphatase  VCP - valosin containing protein  TTLL12 - tubulin tyrosine ligase-like family, member 12  NEK9 - nima-related kinase 9  ERCC3 - excision repair cross-complementing rodent repair deficiency, complementation group 3  NUP205 - nucleoporin 205kda  ELMSAN1 - elm2 and myb/sant-like domain containing 1  WDR20 - wd repeat domain 20  UBE2A - ubiquitin-conjugating enzyme e2a  UBA1 - ubiquitin-like modifier activating enzyme 1  ERN1 - endoplasmic reticulum to nucleus signaling 1  UBB - ubiquitin b  ARRDC4 - arrestin domain containing 4  RCOR1 - rest corepressor 1  ASPHD2 - aspartate beta-hydroxylase domain containing 2  TYRO3 - tyro3 protein tyrosine kinase  TPGS1 - tubulin polyglutamylase complex subunit 1  TYK2 - tyrosine kinase 2  ATG4B - autophagy related 4b, cysteine peptidase  ETF1 - eukaryotic translation termination factor 1  COG7 - component of oligomeric golgi complex 7  UCHL3 - ubiquitin carboxyl-terminal esterase l3 (ubiquitin thiolesterase)  PKDCC - protein kinase domain containing, cytoplasmic  SCYL3 - scy1-like 3 (s. cerevisiae)  SUMO1 - small ubiquitin-like modifier 1  UCHL1 - ubiquitin carboxyl-terminal esterase l1 (ubiquitin thiolesterase)  UGCG - udp-glucose ceramide glucosyltransferase  PELI1 - pellino e3 ubiquitin protein ligase 1  SMURF1 - smad specific e3 ubiquitin protein ligase 1  JMJD6 - jumonji domain containing 6  UBE2H - ubiquitin-conjugating enzyme e2h  UBE2I - ubiquitin-conjugating enzyme e2i  EPHB6 - eph receptor b6  UBE2D2 - ubiquitin-conjugating enzyme e2d 2  FBXO28 - f-box protein 28  UBE2V2 - ubiquitin-conjugating enzyme e2 variant 2  LONRF1 - lon peptidase n-terminal domain and ring finger 1  ADCK1 - aarf domain containing kinase 1  ERCC2 - excision repair cross-complementing rodent repair deficiency, complementation group 2  MAN1C1 - mannosidase, alpha, class 1c, member 1  NUP210 - nucleoporin 210kda  UBE2L3 - ubiquitin-conjugating enzyme e2l 3  UBE2N - ubiquitin-conjugating enzyme e2n  UBE2V1 - ubiquitin-conjugating enzyme e2 variant 1  TMEM165 - transmembrane protein 165  FNTA - farnesyltransferase, caax box, alpha  FLT1 - fms-related tyrosine kinase 1  WSB2 - wd repeat and socs box containing 2  FLNA - filamin a, alpha  SIK2 - salt-inducible kinase 2  PHLPP1 - ph domain and leucine rich repeat protein phosphatase 1  HDAC8 - histone deacetylase 8  ASH1L - ash1 (absent, small, or homeotic)-like (drosophila)  SIN3B - sin3 transcription regulator family member b  FKBP15 - fk506 binding protein 15, 133kda  FKBP4 - fk506 binding protein 4, 59kda  FKBP2 - fk506 binding protein 2, 13kda  ZDHHC22 - zinc finger, dhhc-type containing 22  MGRN1 - mahogunin ring finger 1, e3 ubiquitin protein ligase  HR - hair growth associated  MED8 - mediator complex subunit 8  FKBP5 - fk506 binding protein 5  CAND1 - cullin-associated and neddylation-dissociated 1  FKBP1A - fk506 binding protein 1a, 12kda  FKBP1B - fk506 binding protein 1b, 12.6 kda  FBXW11 - f-box and wd repeat domain containing 11  POFUT2 - protein o-fucosyltransferase 2  KLHL18 - kelch-like family member 18  KDM3A - lysine (k)-specific demethylase 3a  RNF130 - ring finger protein 130  NUP160 - nucleoporin 160kda  PPP1R11 - protein phosphatase 1, regulatory (inhibitor) subunit 11  FGFR2 - fibroblast growth factor receptor 2  TAF9 - taf9 rna polymerase ii, tata box binding protein (tbp)-associated factor, 32kda  UBR4 - ubiquitin protein ligase e3 component n-recognin 4  FAM98B - family with sequence similarity 98, member b  MBD6 - methyl-cpg binding domain protein 6  KLHL7 - kelch-like family member 7  FGF13 - fibroblast growth factor 13  TAF6 - taf6 rna polymerase ii, tata box binding protein (tbp)-associated factor, 80kda  FGFR1 - fibroblast growth factor receptor 1  FGF9 - fibroblast growth factor 9  TADA2A - transcriptional adaptor 2a  PHF15 - phd finger protein 15  FGF1 - fibroblast growth factor 1 (acidic)  FGF2 - fibroblast growth factor 2 (basic)  DPY19L1 - dpy-19-like 1 (c. elegans)  USP22 - ubiquitin specific peptidase 22  SASH1 - sam and sh3 domain containing 1  TRIM2 - tripartite motif containing 2  FER - fer (fps/fes related) tyrosine kinase  MAP3K7 - mitogen-activated protein kinase kinase kinase 7  ALG1 - alg1, chitobiosyldiphosphodolichol beta-mannosyltransferase  INO80E - ino80 complex subunit e  ZDHHC17 - zinc finger, dhhc-type containing 17  RNF114 - ring finger protein 114  KMT2E - lysine (k)-specific methyltransferase 2e  SIK3 - sik family kinase 3  PGP - phosphoglycolate phosphatase  FKTN - fukutin  UFL1 - ufm1-specific ligase 1  TENC1 - tensin like c1 domain containing phosphatase (tensin 2)  KLHL9 - kelch-like family member 9  TRIM9 - tripartite motif containing 9  DMAP1 - dna methyltransferase 1 associated protein 1  EFEMP1 - egf containing fibulin-like extracellular matrix protein 1  OBSL1 - obscurin-like 1  CTTNBP2NL - cttnbp2 n-terminal like  FBN1 - fibrillin 1  USP24 - ubiquitin specific peptidase 24  RNF208 - ring finger protein 208  TIE1 - tyrosine kinase with immunoglobulin-like and egf-like domains 1  TIMP1 - timp metallopeptidase inhibitor 1  RYBP - ring1 and yy1 binding protein  MTOR - mechanistic target of rapamycin (serine/threonine kinase)  STK32C - serine/threonine kinase 32c  PPWD1 - peptidylprolyl isomerase domain and wd repeat containing 1  ATP13A2 - atpase type 13a2  TLR3 - toll-like receptor 3  PIGS - phosphatidylinositol glycan anchor biosynthesis, class s  SIRT1 - sirtuin 1  ACTR8 - arp8 actin-related protein 8 homolog (yeast)  ICMT - isoprenylcysteine carboxyl methyltransferase  BRD4 - bromodomain containing 4  FBXO44 - f-box protein 44  TNFAIP1 - tumor necrosis factor, alpha-induced protein 1 (endothelial)  KCTD21 - potassium channel tetramerization domain containing 21  TEK - tek tyrosine kinase, endothelial  NUP188 - nucleoporin 188kda  SUZ12 - suz12 polycomb repressive complex 2 subunit  P4HA3 - prolyl 4-hydroxylase, alpha polypeptide iii  POFUT1 - protein o-fucosyltransferase 1  RCOR2 - rest corepressor 2  MORC3 - morc family cw-type zinc finger 3  TESK1 - testis-specific kinase 1  FXN - frataxin  TGFA - transforming growth factor, alpha  CEP41 - centrosomal protein 41kda  TGFBR1 - transforming growth factor, beta receptor 1  TGFB2 - transforming growth factor, beta 2  TGFB1 - transforming growth factor, beta 1  MAPK8IP2 - mitogen-activated protein kinase 8 interacting protein 2  PIGN - phosphatidylinositol glycan anchor biosynthesis, class n  CDK20 - cyclin-dependent kinase 20  TGFBR2 - transforming growth factor, beta receptor ii (70/80kda)  RNF20 - ring finger protein 20, e3 ubiquitin protein ligase  TGFBR3 - transforming growth factor, beta receptor iii  WDR45B - wd repeat domain 45b  THBS1 - thrombospondin 1  KAT6B - k(lysine) acetyltransferase 6b  THY1 - thy-1 cell surface antigen  MMD - monocyte to macrophage differentiation-associated  GFPT1 - glutamine--fructose-6-phosphate transaminase 1  GFRA1 - gdnf family receptor alpha 1  GFRA2 - gdnf family receptor alpha 2  TMTC2 - transmembrane and tetratricopeptide repeat containing 2  USP54 - ubiquitin specific peptidase 54  ATAT1 - alpha tubulin acetyltransferase 1  GCNT1 - glucosaminyl (n-acetyl) transferase 1, core 2  LZTR1 - leucine-zipper-like transcription regulator 1  KAT2A - k(lysine) acetyltransferase 2a  NCOA3 - nuclear receptor coactivator 3  FBXL18 - f-box and leucine-rich repeat protein 18  NAA25 - n(alpha)-acetyltransferase 25, natb auxiliary subunit  PABPN1 - poly(a) binding protein, nuclear 1  GGTA1P - glycoprotein, alpha-galactosyltransferase 1 pseudogene  TMTC3 - transmembrane and tetratricopeptide repeat containing 3  TRRAP - transformation/transcription domain-associated protein  HDAC11 - histone deacetylase 11  SETD6 - set domain containing 6  ACTR5 - arp5 actin-related protein 5 homolog (yeast)  NAA60 - n(alpha)-acetyltransferase 60, natf catalytic subunit  BAP1 - brca1 associated protein-1 (ubiquitin carboxy-terminal hydrolase)  CDC7 - cell division cycle 7  NUP85 - nucleoporin 85kda  BRAP - brca1 associated protein  KDM5C - lysine (k)-specific demethylase 5c  USP9X - ubiquitin specific peptidase 9, x-linked  USP11 - ubiquitin specific peptidase 11  PHF17 - phd finger protein 17  GMFB - glia maturation factor, beta  EHMT1 - euchromatic histone-lysine n-methyltransferase 1  FUCA2 - fucosidase, alpha-l- 2, plasma  CAMKMT - calmodulin-lysine n-methyltransferase  NAA40 - n(alpha)-acetyltransferase 40, natd catalytic subunit, homolog (s. cerevisiae)  SDE2 - sde2 telomere maintenance homolog (s. pombe)  USP7 - ubiquitin specific peptidase 7 (herpes virus-associated)  RNF168 - ring finger protein 168, e3 ubiquitin protein ligase  FUT4 - fucosyltransferase 4 (alpha (1,3) fucosyltransferase, myeloid-specific)  ST8SIA4 - st8 alpha-n-acetyl-neuraminide alpha-2,8-sialyltransferase 4  FUT8 - fucosyltransferase 8 (alpha (1,6) fucosyltransferase)  CBLL1 - cbl proto-oncogene-like 1, e3 ubiquitin protein ligase  FYN - fyn oncogene related to src, fgr, yes  RPAP2 - rna polymerase ii associated protein 2  UBA5 - ubiquitin-like modifier activating enzyme 5  GAK - cyclin g associated kinase  GALNT2 - udp-n-acetyl-alpha-d-galactosamine:polypeptide n-acetylgalactosaminyltransferase 2 (galnac-t2)  TTLL7 - tubulin tyrosine ligase-like family, member 7  USP5 - ubiquitin specific peptidase 5 (isopeptidase t)  GALNT1 - udp-n-acetyl-alpha-d-galactosamine:polypeptide n-acetylgalactosaminyltransferase 1 (galnac-t1)  SPRED1 - sprouty-related, evh1 domain containing 1  KMT2D - lysine (k)-specific methyltransferase 2d  AAAS - achalasia, adrenocortical insufficiency, alacrimia  YEATS4 - yeats domain containing 4  KAT6A - k(lysine) acetyltransferase 6a  TUSC3 - tumor suppressor candidate 3  STAM - signal transducing adaptor molecule (sh3 domain and itam motif) 1  NUP214 - nucleoporin 214kda  KLHL36 - kelch-like family member 36  GAS6 - growth arrest-specific 6  FN3KRP - fructosamine 3 kinase related protein  RNF112 - ring finger protein 112  GSPT1 - g1 to s phase transition 1  RNF113A - ring finger protein 113a  PARP8 - poly (adp-ribose) polymerase family, member 8  HECTD3 - hect domain containing e3 ubiquitin protein ligase 3  GTF2H3 - general transcription factor iih, polypeptide 3, 34kda  TRIM26 - tripartite motif containing 26  GTF2H1 - general transcription factor iih, polypeptide 1, 62kda  USP27X - ubiquitin specific peptidase 27, x-linked  GTF2B - general transcription factor iib  PCGF2 - polycomb group ring finger 2  TBL1XR1 - transducin (beta)-like 1 x-linked receptor 1  GTF2H4 - general transcription factor iih, polypeptide 4, 52kda  ZDHHC14 - zinc finger, dhhc-type containing 14  HIST1H2BB - histone cluster 1, h2bb  BRPF1 - bromodomain and phd finger containing, 1  SCG2 - secretogranin ii  MUL1 - mitochondrial e3 ubiquitin protein ligase 1  RNF103 - ring finger protein 103  MOGS - mannosyl-oligosaccharide glucosidase  CDC73 - cell division cycle 73  PTP4A1 - protein tyrosine phosphatase type iva, member 1  HCFC1 - host cell factor c1 (vp16-accessory protein)  MAP3K12 - mitogen-activated protein kinase kinase kinase 12  FUT11 - fucosyltransferase 11 (alpha (1,3) fucosyltransferase)  NDNF - neuron-derived neurotrophic factor  HDAC2 - histone deacetylase 2  HDAC1 - histone deacetylase 1  GOLGA2 - golgin a2  PAGR1 - paxip1 associated glutamate-rich protein 1  WEE1 - wee1 homolog (s. pombe)  WFS1 - wolfram syndrome 1 (wolframin)  WHSC1 - wolf-hirschhorn syndrome candidate 1  DTX3 - deltex homolog 3 (drosophila)  VRK1 - vaccinia related kinase 1  VRK2 - vaccinia related kinase 2  GRK5 - g protein-coupled receptor kinase 5  GRK6 - g protein-coupled receptor kinase 6  FBXL15 - f-box and leucine-rich repeat protein 15  BRCC3 - brca1/brca2-containing complex, subunit 3  GRB2 - growth factor receptor-bound protein 2  ASXL1 - additional sex combs like 1 (drosophila)  TNIP2 - tnfaip3 interacting protein 2  ZNF711 - zinc finger protein 711  YES1 - v-yes-1 yamaguchi sarcoma viral oncogene homolog 1  DCAF10 - ddb1 and cul4 associated factor 10  GSK3B - glycogen synthase kinase 3 beta  YWHAZ - tyrosine 3-monooxygenase/tryptophan 5-monooxygenase activation protein, zeta polypeptide  GSK3A - glycogen synthase kinase 3 alpha  PGAM5 - phosphoglycerate mutase family member 5  YWHAE - tyrosine 3-monooxygenase/tryptophan 5-monooxygenase activation protein, epsilon polypeptide  YWHAB - tyrosine 3-monooxygenase/tryptophan 5-monooxygenase activation protein, beta polypeptide  NDUFAF5 - nadh dehydrogenase (ubiquinone) complex i, assembly factor 5  CCNK - cyclin k  DPM1 - dolichyl-phosphate mannosyltransferase polypeptide 1, catalytic subunit  RNF26 - ring finger protein 26  TRIM24 - tripartite motif containing 24  METTL22 - methyltransferase like 22  PRMT1 - protein arginine methyltransferase 1  PRMT2 - protein arginine methyltransferase 2  FKRP - fukutin related protein  FBXO45 - f-box protein 45  DERL1 - derlin 1  DCAF5 - ddb1 and cul4 associated factor 5  KCTD6 - potassium channel tetramerization domain containing 6  KDELC1 - kdel (lys-asp-glu-leu) containing 1  ALG8 - alg8, alpha-1,3-glucosyltransferase  SYNGAP1 - synaptic ras gtpase activating protein 1  MED11 - mediator complex subunit 11  ST3GAL5 - st3 beta-galactoside alpha-2,3-sialyltransferase 5  STK19 - serine/threonine kinase 19  RNF215 - ring finger protein 215  LDB1 - lim domain binding 1  DSCC1 - dna replication and sister chromatid cohesion 1  KAT2B - k(lysine) acetyltransferase 2b  CRTC2 - creb regulated transcription coactivator 2  DDA1 - det1 and ddb1 associated 1  CDC16 - cell division cycle 16  RPS6KC1 - ribosomal protein s6 kinase, 52kda, polypeptide 1  NAE1 - nedd8 activating enzyme e1 subunit 1  GID4 - gid complex subunit 4  PRPF4B - prp4 pre-mrna processing factor 4 homolog b (yeast)  MTMR2 - myotubularin related protein 2  MTMR3 - myotubularin related protein 3  HUS1 - hus1 checkpoint homolog (s. pombe)  MCM3AP - minichromosome maintenance complex component 3 associated protein  CPNE3 - copine iii  HERC3 - hect and rld domain containing e3 ubiquitin protein ligase 3  HERC2 - hect and rld domain containing e3 ubiquitin protein ligase 2  HERC1 - hect and rld domain containing e3 ubiquitin protein ligase family member 1  TRAK2 - trafficking protein, kinesin binding 2  NSMCE1 - non-smc element 1 homolog (s. cerevisiae)  BCL10 - b-cell cll/lymphoma 10  BTRC - beta-transducin repeat containing e3 ubiquitin protein ligase  UBR1 - ubiquitin protein ligase e3 component n-recognin 1  USP12 - ubiquitin specific peptidase 12  WNK1 - wnk lysine deficient protein kinase 1  UBE2Z - ubiquitin-conjugating enzyme e2z  LAMTOR3 - late endosomal/lysosomal adaptor, mapk and mtor activator 3  MPPE1 - metallophosphoesterase 1  HLCS - holocarboxylase synthetase (biotin-(proprionyl-coa-carboxylase (atp-hydrolysing)) ligase)  SENP5 - sumo1/sentrin specific peptidase 5  STK33 - serine/threonine kinase 33  IRS2 - insulin receptor substrate 2  TNKS - tankyrase, trf1-interacting ankyrin-related adp-ribose polymerase  LATS2 - large tumor suppressor kinase 2  EIF3F - eukaryotic translation initiation factor 3, subunit f  BECN1 - beclin 1, autophagy related  PEA15 - phosphoprotein enriched in astrocytes 15  HIPK1 - homeodomain interacting protein kinase 1  B4GALT4 - udp-gal:betaglcnac beta 1,4- galactosyltransferase, polypeptide 4  B4GALT3 - udp-gal:betaglcnac beta 1,4- galactosyltransferase, polypeptide 3  B4GALT2 - udp-gal:betaglcnac beta 1,4- galactosyltransferase, polypeptide 2  CDC23 - cell division cycle 23  UBE2K - ubiquitin-conjugating enzyme e2k  B3GALNT1 - beta-1,3-n-acetylgalactosaminyltransferase 1 (globoside blood group)  B3GALT2 - udp-gal:betaglcnac beta 1,3-galactosyltransferase, polypeptide 2  HIF1A - hypoxia inducible factor 1, alpha subunit (basic helix-loop-helix transcription factor)  MBTPS1 - membrane-bound transcription factor peptidase, site 1  SNX3 - sorting nexin 3  GBGT1 - globoside alpha-1,3-n-acetylgalactosaminyltransferase 1  EED - embryonic ectoderm development  RNGTT - rna guanylyltransferase and 5'-phosphatase  FBXO10 - f-box protein 10  RIPK1 - receptor (tnfrsf)-interacting serine-threonine kinase 1  FBXO22 - f-box protein 22  FBXO4 - f-box protein 4  FBXO6 - f-box protein 6  VPS33B - vacuolar protein sorting 33 homolog b (yeast)  FBXO3 - f-box protein 3  RAB11A - rab11a, member ras oncogene family  RIPK2 - receptor-interacting serine-threonine kinase 2  PORCN - porcupine homolog (drosophila)  MTMR1 - myotubularin related protein 1  FBXO15 - f-box protein 15  USP46 - ubiquitin specific peptidase 46  VWA1 - von willebrand factor a domain containing 1  PTPLB - protein tyrosine phosphatase-like (proline instead of catalytic arginine), member b  PTPN18 - protein tyrosine phosphatase, non-receptor type 18 (brain-derived)  STT3B - stt3b, subunit of the oligosaccharyltransferase complex (catalytic)  FOXK2 - forkhead box k2  HAT1 - histone acetyltransferase 1  RPS6KA6 - ribosomal protein s6 kinase, 90kda, polypeptide 6  PGAP2 - post-gpi attachment to proteins 2  ENC1 - ectodermal-neural cortex 1 (with btb domain)  DESI1 - desumoylating isopeptidase 1  MEAF6 - myst/esa1-associated factor 6  STK39 - serine threonine kinase 39  ENGASE - endo-beta-n-acetylglucosaminidase  PPM1D - protein phosphatase, mg2+/mn2+ dependent, 1d  UBE2S - ubiquitin-conjugating enzyme e2s  ILF3 - interleukin enhancer binding factor 3, 90kda  PRPF19 - pre-mrna processing factor 19  MAP4K3 - mitogen-activated protein kinase kinase kinase kinase 3  ILK - integrin-linked kinase  CDC14B - cell division cycle 14b  PIAS1 - protein inhibitor of activated stat, 1  SMYD3 - set and mynd domain containing 3  MAPKAPK5 - mitogen-activated protein kinase-activated protein kinase 5  SMURF2 - smad specific e3 ubiquitin protein ligase 2  CAMK1 - calcium/calmodulin-dependent protein kinase i  COPS7B - cop9 signalosome subunit 7b  CBX4 - chromobox homolog 4  COPS3 - cop9 signalosome subunit 3  GORASP1 - golgi reassembly stacking protein 1, 65kda  ANAPC1 - anaphase promoting complex subunit 1  SERP1 - stress-associated endoplasmic reticulum protein 1  PRKRA - protein kinase, interferon-inducible double stranded rna dependent activator  STK16 - serine/threonine kinase 16  RNF115 - ring finger protein 115  CASK - calcium/calmodulin-dependent serine protein kinase (maguk family)  ITGAV - integrin, alpha v  CDK10 - cyclin-dependent kinase 10  SUDS3 - suppressor of defective silencing 3 homolog (s. cerevisiae)  ZDHHC6 - zinc finger, dhhc-type containing 6  ATG3 - autophagy related 3  INSR - insulin receptor  CDK13 - cyclin-dependent kinase 13  KLHL20 - kelch-like family member 20  AKTIP - akt interacting protein  RUVBL1 - ruvb-like 1 (e. coli)  MOCS3 - molybdenum cofactor synthesis 3  IRAK2 - interleukin-1 receptor-associated kinase 2  FOLH1B - folate hydrolase 1b  IRAK1 - interleukin-1 receptor-associated kinase 1  EIF2AK1 - eukaryotic translation initiation factor 2-alpha kinase 1  NSD1 - nuclear receptor binding set domain protein 1  HS3ST5 - heparan sulfate (glucosamine) 3-o-sulfotransferase 5  RFWD2 - ring finger and wd repeat domain 2, e3 ubiquitin protein ligase  RNF25 - ring finger protein 25  PALD1 - phosphatase domain containing, paladin 1  BRPF3 - bromodomain and phd finger containing, 3  PDF - peptide deformylase (mitochondrial)  STK36 - serine/threonine kinase 36  IRF2BPL - interferon regulatory factor 2 binding protein-like  FOXK1 - forkhead box k1  MAGEF1 - melanoma antigen family f, 1  FN3K - fructosamine 3 kinase  STK24 - serine/threonine kinase 24  GFRA4 - gdnf family receptor alpha 4  RASAL1 - ras protein activator like 1 (gap1 like)  OARD1 - o-acyl-adp-ribose deacylase 1  DYRK3 - dual-specificity tyrosine-(y)-phosphorylation regulated kinase 3  RNF11 - ring finger protein 11  DUSP11 - dual specificity phosphatase 11 (rna/rnp complex 1-interacting)  SPOP - speckle-type poz protein  SPARCL1 - sparc-like 1 (hevin)  USP21 - ubiquitin specific peptidase 21  ULK1 - unc-51 like autophagy activating kinase 1  JMJD1C - jumonji domain containing 1c  IGF1R - insulin-like growth factor 1 receptor  CDC42BPA - cdc42 binding protein kinase alpha (dmpk-like)  OGT - o-linked n-acetylglucosamine (glcnac) transferase  LAT - linker for activation of t cells  RAE1 - rae1 rna export 1 homolog (s. pombe)  CUL4B - cullin 4b  CUL1 - cullin 1  CUL2 - cullin 2  CUL3 - cullin 3  TPST2 - tyrosylprotein sulfotransferase 2  B3GAT1 - beta-1,3-glucuronyltransferase 1 (glucuronosyltransferase p)  SUPT3H - suppressor of ty 3 homolog (s. cerevisiae)  TPST1 - tyrosylprotein sulfotransferase 1  RSRC1 - arginine/serine-rich coiled-coil 1  MEX3C - mex-3 rna binding family member c  PCGF5 - polycomb group ring finger 5  WAC - ww domain containing adaptor with coiled-coil  LIMK1 - lim domain kinase 1  ZDHHC3 - zinc finger, dhhc-type containing 3  AATK - apoptosis-associated tyrosine kinase  DOT1L - dot1-like histone h3k79 methyltransferase  LOXL1 - lysyl oxidase-like 1  LNPEP - leucyl/cystinyl aminopeptidase  RAB3D - rab3d, member ras oncogene family  BRSK1 - br serine/threonine kinase 1  NRG2 - neuregulin 2  SYVN1 - synovial apoptosis inhibitor 1, synoviolin  BFAR - bifunctional apoptosis regulator  PTPDC1 - protein tyrosine phosphatase domain containing 1  NEURL4 - neuralized homolog 4 (drosophila)  LIPE - lipase, hormone-sensitive  LIMK2 - lim domain kinase 2  MINPP1 - multiple inositol-polyphosphate phosphatase 1  GOLM1 - golgi membrane protein 1  CDC42BPB - cdc42 binding protein kinase beta (dmpk-like)  CLOCK - clock circadian regulator  LMAN1 - lectin, mannose-binding, 1  PRKAG2 - protein kinase, amp-activated, gamma 2 non-catalytic subunit  ANAPC5 - anaphase promoting complex subunit 5  ANAPC7 - anaphase promoting complex subunit 7  NPEPPS - aminopeptidase puromycin sensitive  KTN1 - kinectin 1 (kinesin receptor)  RNF138 - ring finger protein 138, e3 ubiquitin protein ligase  KRT10 - keratin 10  PPME1 - protein phosphatase methylesterase 1  HEMK1 - hemk methyltransferase family member 1  ACTL6B - actin-like 6b  UBR5 - ubiquitin protein ligase e3 component n-recognin 5  UCHL5 - ubiquitin carboxyl-terminal hydrolase l5  MED20 - mediator complex subunit 20  ATG5 - autophagy related 5  ROCK2 - rho-associated, coiled-coil containing protein kinase 2  MAPK8IP1 - mitogen-activated protein kinase 8 interacting protein 1  PIGB - phosphatidylinositol glycan anchor biosynthesis, class b  TAOK3 - tao kinase 3  FZR1 - fizzy/cell division cycle 20 related 1 (drosophila)  DPY30 - dpy-30 homolog (c. elegans)  LAMC1 - laminin, gamma 1 (formerly lamb2)  IRF2BP2 - interferon regulatory factor 2 binding protein 2  MED27 - mediator complex subunit 27  NAGPA - n-acetylglucosamine-1-phosphodiester alpha-n-acetylglucosaminidase  GALNT18 - udp-n-acetyl-alpha-d-galactosamine:polypeptide n-acetylgalactosaminyltransferase 18  ASB7 - ankyrin repeat and socs box containing 7  ASB8 - ankyrin repeat and socs box containing 8  ARHGEF6 - rac/cdc42 guanine nucleotide exchange factor (gef) 6  KDR - kinase insert domain receptor (a type iii receptor tyrosine kinase)  PICK1 - protein interacting with prkca 1  ASB6 - ankyrin repeat and socs box containing 6  ING4 - inhibitor of growth family, member 4  EIF2AK3 - eukaryotic translation initiation factor 2-alpha kinase 3  USP48 - ubiquitin specific peptidase 48  IRAK4 - interleukin-1 receptor-associated kinase 4  MAP4K4 - mitogen-activated protein kinase kinase kinase kinase 4  RHBDD1 - rhomboid domain containing 1  RLIM - ring finger protein, lim domain interacting  ASB3 - ankyrin repeat and socs box containing 3  TRAF7 - tnf receptor-associated factor 7, e3 ubiquitin protein ligase  KRAS - kirsten rat sarcoma viral oncogene homolog  NAA20 - n(alpha)-acetyltransferase 20, natb catalytic subunit  GOLGA7 - golgin a7  ZDHHC9 - zinc finger, dhhc-type containing 9  CAMKK1 - calcium/calmodulin-dependent protein kinase kinase 1, alpha  SUV420H1 - suppressor of variegation 4-20 homolog 1 (drosophila)  PHF20L1 - phd finger protein 20-like 1  ABHD17B - abhydrolase domain containing 17b  MED10 - mediator complex subunit 10  MED21 - mediator complex subunit 21  KIT - v-kit hardy-zuckerman 4 feline sarcoma viral oncogene homolog  SHISA5 - shisa family member 5  DCUN1D5 - dcn1, defective in cullin neddylation 1, domain containing 5  UBE4A - ubiquitination factor e4a  FBXW9 - f-box and wd repeat domain containing 9  PPIG - peptidylprolyl isomerase g (cyclophilin g)  JAK1 - janus kinase 1  ZDHHC16 - zinc finger, dhhc-type containing 16  JAK2 - janus kinase 2  JAK3 - janus kinase 3  ING5 - inhibitor of growth family, member 5  ANKRD39 - ankyrin repeat domain 39  STT3A - stt3a, subunit of the oligosaccharyltransferase complex (catalytic)  MAST4 - microtubule associated serine/threonine kinase family member 4  PIGP - phosphatidylinositol glycan anchor biosynthesis, class p  PHF20 - phd finger protein 20  TMEM246 - transmembrane protein 246  TRIP12 - thyroid hormone receptor interactor 12  COPS2 - cop9 signalosome subunit 2  ZDHHC2 - zinc finger, dhhc-type containing 2  CRBN - cereblon  MIA3 - melanoma inhibitory activity family, member 3  TAOK2 - tao kinase 2  LEF1 - lymphoid enhancer-binding factor 1  B4GALT6 - udp-gal:betaglcnac beta 1,4- galactosyltransferase, polypeptide 6  BRMS1L - breast cancer metastasis-suppressor 1-like  GTF3C4 - general transcription factor iiic, polypeptide 4, 90kda  B4GALT5 - udp-gal:betaglcnac beta 1,4- galactosyltransferase, polypeptide 5  CDK12 - cyclin-dependent kinase 12  KCTD10 - potassium channel tetramerization domain containing 10  ARID4B - at rich interactive domain 4b (rbp1-like)  SOCS6 - suppressor of cytokine signaling 6  STK35 - serine/threonine kinase 35  MAP3K10 - mitogen-activated protein kinase kinase kinase 10  DUSP19 - dual specificity phosphatase 19  MIB2 - mindbomb e3 ubiquitin protein ligase 2  KDM3B - lysine (k)-specific demethylase 3b  RSF1 - remodeling and spacing factor 1  MAP3K11 - mitogen-activated protein kinase kinase kinase 11  KMT2A - lysine (k)-specific methyltransferase 2a  RPS6KA5 - ribosomal protein s6 kinase, 90kda, polypeptide 5  CCDC8 - coiled-coil domain containing 8  STK17A - serine/threonine kinase 17a  NLK - nemo-like kinase  UIMC1 - ubiquitin interaction motif containing 1  RAB11B - rab11b, member ras oncogene family  MTA2 - metastasis associated 1 family, member 2  EIF2A - eukaryotic translation initiation factor 2a, 65kda  KDELC2 - kdel (lys-asp-glu-leu) containing 2  KBTBD7 - kelch repeat and btb (poz) domain containing 7  MECP2 - methyl cpg binding protein 2 (rett syndrome)  MEF2A - myocyte enhancer factor 2a  MAGT1 - magnesium transporter 1  ZBED1 - zinc finger, bed-type containing 1  PPIL3 - peptidylprolyl isomerase (cyclophilin)-like 3  MAP3K4 - mitogen-activated protein kinase kinase kinase 4  MAP3K5 - mitogen-activated protein kinase kinase kinase 5  DCLK1 - doublecortin-like kinase 1  MAP3K1 - mitogen-activated protein kinase kinase kinase 1, e3 ubiquitin protein ligase  MAP3K3 - mitogen-activated protein kinase kinase kinase 3  MEN1 - multiple endocrine neoplasia i  RAB8A - rab8a, member ras oncogene family  USP42 - ubiquitin specific peptidase 42  KAT8 - k(lysine) acetyltransferase 8  MFGE8 - milk fat globule-egf factor 8 protein  FBXL14 - f-box and leucine-rich repeat protein 14  ATG12 - autophagy related 12  MGAT1 - mannosyl (alpha-1,3-)-glycoprotein beta-1,2-n-acetylglucosaminyltransferase  MGAT2 - mannosyl (alpha-1,6-)-glycoprotein beta-1,2-n-acetylglucosaminyltransferase  MGAT5 - mannosyl (alpha-1,6-)-glycoprotein beta-1,6-n-acetyl-glucosaminyltransferase  HGS - hepatocyte growth factor-regulated tyrosine kinase substrate  MBD3 - methyl-cpg binding domain protein 3  KITLG - kit ligand  B3GALTL - beta 1,3-galactosyltransferase-like  MAN1A1 - mannosidase, alpha, class 1a, member 1  MAN2A2 - mannosidase, alpha, class 2a, member 2  ZC3HC1 - zinc finger, c3hc-type containing 1  COG3 - component of oligomeric golgi complex 3  MARK3 - map/microtubule affinity-regulating kinase 3  MARK1 - map/microtubule affinity-regulating kinase 1  DOHH - deoxyhypusine hydroxylase/monooxygenase  MAPT - microtubule-associated protein tau  MDP1 - magnesium-dependent phosphatase 1  LATS1 - large tumor suppressor kinase 1  SIRT6 - sirtuin 6  MTA1 - metastasis associated 1  MTMR4 - myotubularin related protein 4  MTMR7 - myotubularin related protein 7  MANBA - mannosidase, beta a, lysosomal  MTMR6 - myotubularin related protein 6  MAN2A1 - mannosidase, alpha, class 2a, member 1  USP8 - ubiquitin specific peptidase 8  USP14 - ubiquitin specific peptidase 14 (trna-guanine transglycosylase)  USP10 - ubiquitin specific peptidase 10  UBE2J1 - ubiquitin-conjugating enzyme e2, j1  NUP43 - nucleoporin 43kda  PKMYT1 - protein kinase, membrane associated tyrosine/threonine 1  MATK - megakaryocyte-associated tyrosine kinase  UFC1 - ubiquitin-fold modifier conjugating enzyme 1  TRMT112 - trna methyltransferase 11-2 homolog (s. cerevisiae)  TTBK2 - tau tubulin kinase 2  ASH2L - ash2 (absent, small, or homeotic)-like (drosophila)  CTDSPL2 - ctd (carboxy-terminal domain, rna polymerase ii, polypeptide a) small phosphatase like 2  PPIL1 - peptidylprolyl isomerase (cyclophilin)-like 1  NFS1 - nfs1 cysteine desulfurase  FBXL16 - f-box and leucine-rich repeat protein 16  ITCH - itchy e3 ubiquitin protein ligase  RIOK1 - rio kinase 1  AIP - aryl hydrocarbon receptor interacting protein  OTUD6B - otu domain containing 6b  NUB1 - negative regulator of ubiquitin-like proteins 1  ASB1 - ankyrin repeat and socs box containing 1  LYN - v-yes-1 yamaguchi sarcoma viral related oncogene homolog  UBE2M - ubiquitin-conjugating enzyme e2m  UBA3 - ubiquitin-like modifier activating enzyme 3  PARP9 - poly (adp-ribose) polymerase family, member 9  RNF8 - ring finger protein 8, e3 ubiquitin protein ligase  LTBP1 - latent transforming growth factor beta binding protein 1  FKBP7 - fk506 binding protein 7  RPS6KL1 - ribosomal protein s6 kinase-like 1  BAZ1B - bromodomain adjacent to zinc finger domain, 1b  PIAS4 - protein inhibitor of activated stat, 4  TRIM33 - tripartite motif containing 33  SMAD2 - smad family member 2  MBIP - map3k12 binding inhibitory protein 1  HDAC7 - histone deacetylase 7  TMTC1 - transmembrane and tetratricopeptide repeat containing 1  CDKL2 - cyclin-dependent kinase-like 2 (cdc2-related kinase)  RPS6KA4 - ribosomal protein s6 kinase, 90kda, polypeptide 4  KCTD11 - potassium channel tetramerization domain containing 11  PLOD3 - procollagen-lysine, 2-oxoglutarate 5-dioxygenase 3  DPH5 - diphthamide biosynthesis 5  TAF9B - taf9b rna polymerase ii, tata box binding protein (tbp)-associated factor, 31kda  SMAD4 - smad family member 4  SMAD5 - smad family member 5  P4HA2 - prolyl 4-hydroxylase, alpha polypeptide ii  SMAD7 - smad family member 7  PIGT - phosphatidylinositol glycan anchor biosynthesis, class t  MYLIP - myosin regulatory light chain interacting protein  NCK1 - nck adaptor protein 1  DPY19L3 - dpy-19-like 3 (c. elegans)  ABI2 - abl-interactor 2  TRIM28 - tripartite motif containing 28  SCG3 - secretogranin iii  NCAM1 - neural cell adhesion molecule 1  TBK1 - tank-binding kinase 1  NHLRC3 - nhl repeat containing 3  ZDHHC1 - zinc finger, dhhc-type containing 1  ZDHHC8 - zinc finger, dhhc-type containing 8  PPP1R12A - protein phosphatase 1, regulatory subunit 12a  TRIM11 - tripartite motif containing 11  TNK2 - tyrosine kinase, non-receptor, 2  SENP1 - sumo1/sentrin specific peptidase 1  TFPT - tcf3 (e2a) fusion partner (in childhood leukemia)  RNF41 - ring finger protein 41  PRMT3 - protein arginine methyltransferase 3  PSME3 - proteasome (prosome, macropain) activator subunit 3 (pa28 gamma; ki)  MYLK - myosin light chain kinase  URM1 - ubiquitin related modifier 1  TRIM13 - tripartite motif containing 13  TRIM8 - tripartite motif containing 8  USPL1 - ubiquitin specific peptidase like 1  TOPORS - topoisomerase i binding, arginine/serine-rich, e3 ubiquitin protein ligase  RAB1B - rab1b, member ras oncogene family  SEH1L - seh1-like (s. cerevisiae)  PTPRU - protein tyrosine phosphatase, receptor type, u  HUWE1 - hect, uba and wwe domain containing 1, e3 ubiquitin protein ligase  ALG5 - alg5, dolichyl-phosphate beta-glucosyltransferase  INO80B - ino80 complex subunit b  B3GALNT2 - beta-1,3-n-acetylgalactosaminyltransferase 2  CTDSP2 - ctd (carboxy-terminal domain, rna polymerase ii, polypeptide a) small phosphatase 2  ANAPC4 - anaphase promoting complex subunit 4  RNF170 - ring finger protein 170  FEM1B - fem-1 homolog b (c. elegans)  TRIM37 - tripartite motif containing 37  CDC42EP5 - cdc42 effector protein (rho gtpase binding) 5  PDIA6 - protein disulfide isomerase family a, member 6  SHARPIN - shank-associated rh domain interactor  AKT3 - v-akt murine thymoma viral oncogene homolog 3  ABI1 - abl-interactor 1  PPP4R1 - protein phosphatase 4, regulatory subunit 1  PDCD6 - programmed cell death 6  MTM1 - myotubularin 1  HDAC5 - histone deacetylase 5  HDAC6 - histone deacetylase 6  RNF187 - ring finger protein 187  PARP2 - poly (adp-ribose) polymerase 2  SH2D3C - sh2 domain containing 3c  MT3 - metallothionein 3  PIGK - phosphatidylinositol glycan anchor biosynthesis, class k  CCDC22 - coiled-coil domain containing 22  SAE1 - sumo1 activating enzyme subunit 1  PDIK1L - pdlim1 interacting kinase 1 like  RANBP9 - ran binding protein 9  UBA2 - ubiquitin-like modifier activating enzyme 2  KANSL1L - kat8 regulatory nsl complex subunit 1-like  JOSD1 - josephin domain containing 1  SUPT7L - suppressor of ty 7 (s. cerevisiae)-like  FBXO38 - f-box protein 38  GFPT2 - glutamine-fructose-6-phosphate transaminase 2  OXSR1 - oxidative stress responsive 1  COMMD1 - copper metabolism (murr1) domain containing 1  USP3 - ubiquitin specific peptidase 3  BABAM1 - brisc and brca1 a complex member 1  UBE2T - ubiquitin-conjugating enzyme e2t (putative)  USP15 - ubiquitin specific peptidase 15  TTL - tubulin tyrosine ligase  C1GALT1C1 - c1galt1-specific chaperone 1  RCE1 - rce1 homolog, prenyl protein protease (s. cerevisiae)  SETD2 - set domain containing 2  RBX1 - ring-box 1, e3 ubiquitin protein ligase  DUSP18 - dual specificity phosphatase 18  NUP153 - nucleoporin 153kda  MPI - mannose phosphate isomerase  MED12 - mediator complex subunit 12  PSMD6 - proteasome (prosome, macropain) 26s subunit, non-atpase, 6  WDSUB1 - wd repeat, sterile alpha motif and u-box domain containing 1  DTX3L - deltex 3-like (drosophila)  PJA2 - praja ring finger 2, e3 ubiquitin protein ligase  AREL1 - apoptosis resistant e3 ubiquitin protein ligase 1  SETDB1 - set domain, bifurcated 1  TLK1 - tousled-like kinase 1  RNF141 - ring finger protein 141  PTAR1 - protein prenyltransferase alpha subunit repeat containing 1  PPP4R2 - protein phosphatase 4, regulatory subunit 2  RNF38 - ring finger protein 38  XXYLT1 - xyloside xylosyltransferase 1  KLHL21 - kelch-like family member 21  METTL21A - methyltransferase like 21a  TNKS2 - tankyrase, trf1-interacting ankyrin-related adp-ribose polymerase 2  WDR61 - wd repeat domain 61  DDR2 - discoidin domain receptor tyrosine kinase 2  TOMM20 - translocase of outer mitochondrial membrane 20 homolog (yeast)  DAB2IP - dab2 interacting protein  NUP88 - nucleoporin 88kda  NUP98 - nucleoporin 98kda  NUCB1 - nucleobindin 1  ST8SIA3 - st8 alpha-n-acetyl-neuraminide alpha-2,8-sialyltransferase 3  RNF144A - ring finger protein 144a  DNAJC6 - dnaj (hsp40) homolog, subfamily c, member 6  ILKAP - integrin-linked kinase-associated serine/threonine phosphatase  SPATA2 - spermatogenesis associated 2  KLHL5 - kelch-like family member 5  DUSP16 - dual specificity phosphatase 16  RNF40 - ring finger protein 40, e3 ubiquitin protein ligase  SPOCK2 - sparc/osteonectin, cwcv and kazal-like domains proteoglycan (testican) 2  PAQR3 - progestin and adipoq receptor family member iii  CUL7 - cullin 7  NRAS - neuroblastoma ras viral (v-ras) oncogene homolog  NMT1 - n-myristoyltransferase 1  VPRBP - vpr (hiv-1) binding protein  RNF34 - ring finger protein 34, e3 ubiquitin protein ligase  FBXO11 - f-box protein 11  ULK2 - unc-51 like autophagy activating kinase 2  HERPUD1 - homocysteine-inducible, endoplasmic reticulum stress-inducible, ubiquitin-like domain member 1  ERO1L - ero1-like (s. cerevisiae)  SPSB1 - spla/ryanodine receptor domain and socs box containing 1  KCTD7 - potassium channel tetramerization domain containing 7  NAA15 - n(alpha)-acetyltransferase 15, nata auxiliary subunit  CNOT4 - ccr4-not transcription complex, subunit 4  NFRKB - nuclear factor related to kappab binding protein  HDAC4 - histone deacetylase 4  CXXC1 - cxxc finger protein 1  NFKBIA - nuclear factor of kappa light polypeptide gene enhancer in b-cells inhibitor, alpha  NFKB1 - nuclear factor of kappa light polypeptide gene enhancer in b-cells 1  KMT2B - lysine (k)-specific methyltransferase 2b  ST6GALNAC6 - st6 (alpha-n-acetyl-neuraminyl-2,3-beta-galactosyl-1,3)-n-acetylgalactosaminide alpha-2,6-sialyltransferase 6  PIK3R4 - phosphoinositide-3-kinase, regulatory subunit 4  SLK - ste20-like kinase  VCPIP1 - valosin containing protein (p97)/p47 complex interacting protein 1  NKTR - natural killer-tumor recognition sequence  USP34 - ubiquitin specific peptidase 34  HDAC9 - histone deacetylase 9  RNF145 - ring finger protein 145  SETD1A - set domain containing 1a  CCP110 - centriolar coiled coil protein 110kda  SOCS7 - suppressor of cytokine signaling 7  NEK3 - nima-related kinase 3  MINK1 - misshapen-like kinase 1  TTLL4 - tubulin tyrosine ligase-like family, member 4  SOCS5 - suppressor of cytokine signaling 5  NEFL - neurofilament, light polypeptide  NEK1 - nima-related kinase 1  NEDD8 - neural precursor cell expressed, developmentally down-regulated 8  CTR9 - ctr9, paf1/rna polymerase ii complex component  PPM1F - protein phosphatase, mg2+/mn2+ dependent, 1f  NFE2L2 - nuclear factor (erythroid-derived 2)-like 2  EPC1 - enhancer of polycomb homolog 1 (drosophila)  MORF4L2 - mortality factor 4 like 2  KLHL15 - kelch-like family member 15  NUP155 - nucleoporin 155kda  NF1 - neurofibromin 1  CDK5RAP3 - cdk5 regulatory subunit associated protein 3  DCAF8 - ddb1 and cul4 associated factor 8  EDEM1 - er degradation enhancer, mannosidase alpha-like 1  RAPGEF2 - rap guanine nucleotide exchange factor (gef) 2  EDEM3 - er degradation enhancer, mannosidase alpha-like 3  NUP93 - nucleoporin 93kda  KDM4A - lysine (k)-specific demethylase 4a  PHF14 - phd finger protein 14  COPS7A - cop9 signalosome subunit 7a  DZIP3 - daz interacting zinc finger protein 3 |
| GO:0036211 | protein modification process | 4.21E-13 | 3.66E-10 | 1.09 (10334,2137,6996,1583) | [+] Show genes  MSL3 - male-specific lethal 3 homolog (drosophila)  OTUD5 - otu domain containing 5  TADA1 - transcriptional adaptor 1  ABCA1 - atp-binding cassette, sub-family a (abc1), member 1  OS9 - osteosarcoma amplified 9, endoplasmic reticulum lectin  ABL1 - c-abl oncogene 1, non-receptor tyrosine kinase  GALNT10 - udp-n-acetyl-alpha-d-galactosamine:polypeptide n-acetylgalactosaminyltransferase 10 (galnac-t10)  PML - promyelocytic leukemia  PMM1 - phosphomannomutase 1  RAB40B - rab40b, member ras oncogene family  CKAP4 - cytoskeleton-associated protein 4  UBE2Q1 - ubiquitin-conjugating enzyme e2q family member 1  BRD8 - bromodomain containing 8  PLOD2 - procollagen-lysine, 2-oxoglutarate 5-dioxygenase 2  LRRC40 - leucine rich repeat containing 40  MAN1A2 - mannosidase, alpha, class 1a, member 2  PLOD1 - procollagen-lysine, 2-oxoglutarate 5-dioxygenase 1  RFFL - ring finger and fyve-like domain containing e3 ubiquitin protein ligase  ACTB - actin, beta  OTUB1 - otu domain, ubiquitin aldehyde binding 1  EHMT2 - euchromatic histone-lysine n-methyltransferase 2  MORF4L1 - mortality factor 4 like 1  ZDHHC7 - zinc finger, dhhc-type containing 7  POMGNT1 - protein o-linked mannose n-acetylglucosaminyltransferase 1 (beta 1,2-)  PPIC - peptidylprolyl isomerase c (cyclophilin c)  PPID - peptidylprolyl isomerase d  ACTL6A - actin-like 6a  PPEF1 - protein phosphatase, ef-hand calcium binding domain 1  PPIA - peptidylprolyl isomerase a (cyclophilin a)  USP20 - ubiquitin specific peptidase 20  PPIB - peptidylprolyl isomerase b (cyclophilin b)  USP19 - ubiquitin specific peptidase 19  ACVR2A - activin a receptor, type iia  ACVRL1 - activin a receptor type ii-like 1  ACVR1 - activin a receptor, type i  ACVR1B - activin a receptor, type ib  NDUFAF7 - nadh dehydrogenase (ubiquinone) complex i, assembly factor 7  ADAM10 - adam metallopeptidase domain 10  MED1 - mediator complex subunit 1  ADH5 - alcohol dehydrogenase 5 (class iii), chi polypeptide  PARP1 - poly (adp-ribose) polymerase 1  ADPRH - adp-ribosylarginine hydrolase  TNFRSF19 - tumor necrosis factor receptor superfamily, member 19  PARP4 - poly (adp-ribose) polymerase family, member 4  RUVBL2 - ruvb-like 2 (e. coli)  ATE1 - arginyltransferase 1  PRKAB1 - protein kinase, amp-activated, beta 1 non-catalytic subunit  RCOR3 - rest corepressor 3  PRKACB - protein kinase, camp-dependent, catalytic, beta  PRKACA - protein kinase, camp-dependent, catalytic, alpha  NUP133 - nucleoporin 133kda  ADRBK2 - adrenergic, beta, receptor kinase 2  ADRBK1 - adrenergic, beta, receptor kinase 1  NUPL2 - nucleoporin like 2  EDEM2 - er degradation enhancer, mannosidase alpha-like 2  CHFR - checkpoint with forkhead and ring finger domains, e3 ubiquitin protein ligase  PRDM4 - pr domain containing 4  PPP3R1 - protein phosphatase 3, regulatory subunit b, alpha  CIT - citron (rho-interacting, serine/threonine kinase 21)  PPP5C - protein phosphatase 5, catalytic subunit  PPP6C - protein phosphatase 6, catalytic subunit  PPT1 - palmitoyl-protein thioesterase 1  UHRF2 - ubiquitin-like with phd and ring finger domains 2, e3 ubiquitin protein ligase  AGA - aspartylglucosaminidase  PPP2R5D - protein phosphatase 2, regulatory subunit b', delta  PPP2R5C - protein phosphatase 2, regulatory subunit b', gamma  PPP2R5B - protein phosphatase 2, regulatory subunit b', beta  PPP4C - protein phosphatase 4, catalytic subunit  AKT1 - v-akt murine thymoma viral oncogene homolog 1  PPP2R5E - protein phosphatase 2, regulatory subunit b', epsilon isoform  PPP2R2A - protein phosphatase 2, regulatory subunit b, alpha  PPP2R1B - protein phosphatase 2, regulatory subunit a, beta  PPP2R1A - protein phosphatase 2, regulatory subunit a, alpha  RIOK2 - rio kinase 2  WWP2 - ww domain containing e3 ubiquitin protein ligase 2  PPP2R2C - protein phosphatase 2, regulatory subunit b, gamma  PPP2R2B - protein phosphatase 2, regulatory subunit b, beta  TRIOBP - trio and f-actin binding protein  PPP2CA - protein phosphatase 2, catalytic subunit, alpha isozyme  PPP2CB - protein phosphatase 2, catalytic subunit, beta isozyme  MBD5 - methyl-cpg binding domain protein 5  DUSP14 - dual specificity phosphatase 14  PPM1G - protein phosphatase, mg2+/mn2+ dependent, 1g  PPP1CC - protein phosphatase 1, catalytic subunit, gamma isozyme  ESCO1 - establishment of sister chromatid cohesion n-acetyltransferase 1  KANSL3 - kat8 regulatory nsl complex subunit 3  SCYL2 - scy1-like 2 (s. cerevisiae)  LIAS - lipoic acid synthetase  AMFR - autocrine motility factor receptor, e3 ubiquitin protein ligase  RNF126 - ring finger protein 126  DNAJC3 - dnaj (hsp40) homolog, subfamily c, member 3  MAP2K5 - mitogen-activated protein kinase kinase 5  MAP2K7 - mitogen-activated protein kinase kinase 7  EIF2AK2 - eukaryotic translation initiation factor 2-alpha kinase 2  PIM2 - pim-2 oncogene  MAPK13 - mitogen-activated protein kinase 13  GALNT13 - udp-n-acetyl-alpha-d-galactosamine:polypeptide n-acetylgalactosaminyltransferase 13 (galnac-t13)  PIGV - phosphatidylinositol glycan anchor biosynthesis, class v  MAP2K1 - mitogen-activated protein kinase kinase 1  RNF157 - ring finger protein 157  MAP2K2 - mitogen-activated protein kinase kinase 2  MAPK10 - mitogen-activated protein kinase 10  MAPK9 - mitogen-activated protein kinase 9  MAPK11 - mitogen-activated protein kinase 11  TRIM59 - tripartite motif containing 59  MAPK8 - mitogen-activated protein kinase 8  METAP2 - methionyl aminopeptidase 2  MAPK7 - mitogen-activated protein kinase 7  MAPK3 - mitogen-activated protein kinase 3  MAPK1 - mitogen-activated protein kinase 1  COPS6 - cop9 signalosome subunit 6  PRKCSH - protein kinase c substrate 80k-h  PRKD1 - protein kinase d1  NDC1 - ndc1 transmembrane nucleoporin  PKN2 - protein kinase n2  PRKCH - protein kinase c, eta  TLK2 - tousled-like kinase 2  PRKCI - protein kinase c, iota  PRKCE - protein kinase c, epsilon  PRKCB - protein kinase c, beta  PRKCD - protein kinase c, delta  PRKCA - protein kinase c, alpha  PRKAR1B - protein kinase, camp-dependent, regulatory, type i, beta  YEATS2 - yeats domain containing 2  PRKAG1 - protein kinase, amp-activated, gamma 1 non-catalytic subunit  UBR7 - ubiquitin protein ligase e3 component n-recognin 7 (putative)  DUSP12 - dual specificity phosphatase 12  KLHL2 - kelch-like family member 2  XIAP - x-linked inhibitor of apoptosis  BIRC3 - baculoviral iap repeat containing 3  BIRC2 - baculoviral iap repeat containing 2  RFWD3 - ring finger and wd repeat domain 3  APLP2 - amyloid beta (a4) precursor-like protein 2  MGAT4B - mannosyl (alpha-1,3-)-glycoprotein beta-1,4-n-acetylglucosaminyltransferase, isozyme b  PRMT6 - protein arginine methyltransferase 6  MSL2 - male-specific lethal 2 homolog (drosophila)  MAN1B1 - mannosidase, alpha, class 1b, member 1  DCUN1D3 - dcn1, defective in cullin neddylation 1, domain containing 3  RNF220 - ring finger protein 220  APC - adenomatous polyposis coli  APBB1 - amyloid beta (a4) precursor protein-binding, family b, member 1 (fe65)  GALNT6 - udp-n-acetyl-alpha-d-galactosamine:polypeptide n-acetylgalactosaminyltransferase 6 (galnac-t6)  RNF139 - ring finger protein 139  DPY19L4 - dpy-19-like 4 (c. elegans)  P4HA1 - prolyl 4-hydroxylase, alpha polypeptide i  PEBP1 - phosphatidylethanolamine binding protein 1  TRIM23 - tripartite motif containing 23  RPRD1A - regulation of nuclear pre-mrna domain containing 1a  ARF4 - adp-ribosylation factor 4  IRAK3 - interleukin-1 receptor-associated kinase 3  SETD5 - set domain containing 5  AKAP13 - a kinase (prka) anchor protein 13  APP - amyloid beta (a4) precursor protein  PAK2 - p21 protein (cdc42/rac)-activated kinase 2  LEPREL1 - leprecan-like 1  PAK3 - p21 protein (cdc42/rac)-activated kinase 3  PAM - peptidylglycine alpha-amidating monooxygenase  PDGFA - platelet-derived growth factor alpha polypeptide  MAP4K5 - mitogen-activated protein kinase kinase kinase kinase 5  NSMCE2 - non-smc element 2, mms21 homolog (s. cerevisiae)  PDGFRB - platelet-derived growth factor receptor, beta polypeptide  ARRB2 - arrestin, beta 2  ARRB1 - arrestin, beta 1  PDGFRA - platelet-derived growth factor receptor, alpha polypeptide  PDGFB - platelet-derived growth factor beta polypeptide  PDPK1 - 3-phosphoinositide dependent protein kinase-1  RHOA - ras homolog family member a  WDYHV1 - wdyhv motif containing 1  PDK3 - pyruvate dehydrogenase kinase, isozyme 3  FSTL1 - follistatin-like 1  BAZ2A - bromodomain adjacent to zinc finger domain, 2a  PCNA - proliferating cell nuclear antigen  PCMT1 - protein-l-isoaspartate (d-aspartate) o-methyltransferase  FICD - fic domain containing  MTFMT - mitochondrial methionyl-trna formyltransferase  CDK16 - cyclin-dependent kinase 16  CDK17 - cyclin-dependent kinase 17  LEO1 - leo1, paf1/rna polymerase ii complex component, homolog (s. cerevisiae)  ASPH - aspartate beta-hydroxylase  FANCL - fanconi anemia, complementation group l  PWP1 - pwp1 homolog (s. cerevisiae)  SOCS4 - suppressor of cytokine signaling 4  KAT7 - k(lysine) acetyltransferase 7  PGGT1B - protein geranylgeranyltransferase type i, beta subunit  PGM3 - phosphoglucomutase 3  CPPED1 - calcineurin-like phosphoesterase domain containing 1  UBOX5 - u-box domain containing 5  RNF180 - ring finger protein 180  UEVLD - uev and lactate/malate dehyrogenase domains  FBXW7 - f-box and wd repeat domain containing 7, e3 ubiquitin protein ligase  RNF121 - ring finger protein 121  PHF2 - phd finger protein 2  LRRK2 - leucine-rich repeat kinase 2  DNAJC24 - dnaj (hsp40) homolog, subfamily c, member 24  SLC35C1 - solute carrier family 35 (gdp-fucose transporter), member c1  PEX12 - peroxisomal biogenesis factor 12  PEX13 - peroxisomal biogenesis factor 13  ATP7A - atpase, cu++ transporting, alpha polypeptide  PEX14 - peroxisomal biogenesis factor 14  PENK - proenkephalin  LMTK2 - lemur tyrosine kinase 2  ICK - intestinal cell (mak-like) kinase  PPM1E - protein phosphatase, mg2+/mn2+ dependent, 1e  AAK1 - ap2 associated kinase 1  COPRS - coordinator of prmt5, differentiation stimulator  PKD1 - polycystic kidney disease 1 (autosomal dominant)  UBA6 - ubiquitin-like modifier activating enzyme 6  USP40 - ubiquitin specific peptidase 40  RASA3 - ras p21 protein activator 3  PIN1 - peptidylprolyl cis/trans isomerase, nima-interacting 1  DPH3 - diphthamide biosynthesis 3  PIK3R1 - phosphoinositide-3-kinase, regulatory subunit 1 (alpha)  PLAT - plasminogen activator, tissue  AXL - axl receptor tyrosine kinase  ASXL2 - additional sex combs like 2 (drosophila)  PCMTD2 - protein-l-isoaspartate (d-aspartate) o-methyltransferase domain containing 2  PEF1 - penta-ef-hand domain containing 1  OGFOD1 - 2-oxoglutarate and iron-dependent oxygenase domain containing 1  PHF10 - phd finger protein 10  RNF13 - ring finger protein 13  BCR - breakpoint cluster region  FKBP9 - fk506 binding protein 9, 63 kda  PHKG2 - phosphorylase kinase, gamma 2 (testis)  FBXW12 - f-box and wd repeat domain containing 12  STK38 - serine/threonine kinase 38  MRGBP - mrg/morf4l binding protein  SUMF1 - sulfatase modifying factor 1  PIK3CA - phosphatidylinositol-4,5-bisphosphate 3-kinase, catalytic subunit alpha  RHOT1 - ras homolog family member t1  PIM1 - pim-1 oncogene  BCL2 - b-cell cll/lymphoma 2  UBE2W - ubiquitin-conjugating enzyme e2w (putative)  PIK3C3 - phosphatidylinositol 3-kinase, catalytic subunit type 3  MGAT4A - mannosyl (alpha-1,3-)-glycoprotein beta-1,4-n-acetylglucosaminyltransferase, isozyme a  PIGH - phosphatidylinositol glycan anchor biosynthesis, class h  LYPLA2 - lysophospholipase ii  CCND1 - cyclin d1  PARK7 - parkinson protein 7  B4GALT7 - xylosylprotein beta 1,4-galactosyltransferase, polypeptide 7  PIGF - phosphatidylinositol glycan anchor biosynthesis, class f  TRIM27 - tripartite motif containing 27  CWC27 - cwc27 spliceosome-associated protein homolog (s. cerevisiae)  BRAF - v-raf murine sarcoma viral oncogene homolog b  BCKDK - branched chain ketoacid dehydrogenase kinase  WHSC1L1 - wolf-hirschhorn syndrome candidate 1-like 1  CDKL4 - cyclin-dependent kinase-like 4  BCOR - bcl6 corepressor  ZFP36L2 - zfp36 ring finger protein-like 2  ZFP36L1 - zfp36 ring finger protein-like 1  PAK4 - p21 protein (cdc42/rac)-activated kinase 4  DCAF16 - ddb1 and cul4 associated factor 16  RET - ret proto-oncogene  DUSP15 - dual specificity phosphatase 15  DPF2 - d4, zinc and double phd fingers family 2  SH3RF3 - sh3 domain containing ring finger 3  GALNTL6 - udp-n-acetyl-alpha-d-galactosamine:polypeptide n-acetylgalactosaminyltransferase-like 6  RLF - rearranged l-myc fusion  SNRK - snf related kinase  RING1 - ring finger protein 1  PIGG - phosphatidylinositol glycan anchor biosynthesis, class g  TMEM5 - transmembrane protein 5  FBLL1 - fibrillarin-like 1  BMI1 - bmi1 polycomb ring finger oncogene  PCGF3 - polycomb group ring finger 3  BMPR1A - bone morphogenetic protein receptor, type ia  BMPR2 - bone morphogenetic protein receptor, type ii (serine/threonine kinase)  BMPR1B - bone morphogenetic protein receptor, type ib  FBXL12 - f-box and leucine-rich repeat protein 12  ABCA7 - atp-binding cassette, sub-family a (abc1), member 7  TRPM7 - transient receptor potential cation channel, subfamily m, member 7  PSMD14 - proteasome (prosome, macropain) 26s subunit, non-atpase, 14  RNF19B - ring finger protein 19b  CTDSPL - ctd (carboxy-terminal domain, rna polymerase ii, polypeptide a) small phosphatase-like  TMEM258 - transmembrane protein 258  UHMK1 - u2af homology motif (uhm) kinase 1  MSLN - mesothelin  RNF4 - ring finger protein 4  DCAF7 - ddb1 and cul4 associated factor 7  RNF2 - ring finger protein 2  GPHN - gephyrin  RNF6 - ring finger protein (c3h2c3 type) 6  QPCTL - glutaminyl-peptide cyclotransferase-like  ROCK1 - rho-associated, coiled-coil containing protein kinase 1  LYPLAL1 - lysophospholipase-like 1  RNF111 - ring finger protein 111  TET2 - tet methylcytosine dioxygenase 2  STAM2 - signal transducing adaptor molecule (sh3 domain and itam motif) 2  TRPM4 - transient receptor potential cation channel, subfamily m, member 4  ZMPSTE24 - zinc metallopeptidase ste24  STUB1 - stip1 homology and u-box containing protein 1, e3 ubiquitin protein ligase  UBE4B - ubiquitination factor e4b  TRIM44 - tripartite motif containing 44  TAB1 - tgf-beta activated kinase 1/map3k7 binding protein 1  PPIE - peptidylprolyl isomerase e (cyclophilin e)  MCRS1 - microspherule protein 1  CAMK2B - calcium/calmodulin-dependent protein kinase ii beta  FKBP14 - fk506 binding protein 14, 22 kda  PIBF1 - progesterone immunomodulatory binding factor 1  PPIH - peptidylprolyl isomerase h (cyclophilin h)  B3GALT6 - udp-gal:betagal beta 1,3-galactosyltransferase polypeptide 6  CAD - carbamoyl-phosphate synthetase 2, aspartate transcarbamylase, and dihydroorotase  RPN2 - ribophorin ii  RPN1 - ribophorin i  UBE2E3 - ubiquitin-conjugating enzyme e2e 3  HERC6 - hect and rld domain containing e3 ubiquitin protein ligase family member 6  TRIM38 - tripartite motif containing 38  DDR1 - discoidin domain receptor tyrosine kinase 1  TADA3 - transcriptional adaptor 3  CALU - calumenin  STK25 - serine/threonine kinase 25  LRRC41 - leucine rich repeat containing 41  CRTAP - cartilage associated protein  INO80C - ino80 complex subunit c  CCNT1 - cyclin t1  CCNH - cyclin h  TMEM132A - transmembrane protein 132a  ANAPC10 - anaphase promoting complex subunit 10  SSH3 - slingshot protein phosphatase 3  RPS6KA2 - ribosomal protein s6 kinase, 90kda, polypeptide 2  NRBP2 - nuclear receptor binding protein 2  RPS6KA1 - ribosomal protein s6 kinase, 90kda, polypeptide 1  PARP16 - poly (adp-ribose) polymerase family, member 16  RPS6KB1 - ribosomal protein s6 kinase, 70kda, polypeptide 1  RPS6KB2 - ribosomal protein s6 kinase, 70kda, polypeptide 2  RNF125 - ring finger protein 125, e3 ubiquitin protein ligase  CBFB - core-binding factor, beta subunit  CBL - cbl proto-oncogene, e3 ubiquitin protein ligase  PIAS3 - protein inhibitor of activated stat, 3  KANSL2 - kat8 regulatory nsl complex subunit 2  UBE2R2 - ubiquitin-conjugating enzyme e2r 2  RBM14 - rna binding motif protein 14  PRMT5 - protein arginine methyltransferase 5  TRIM3 - tripartite motif containing 3  CDK11B - cyclin-dependent kinase 11b  TGOLN2 - trans-golgi network protein 2  STAMBP - stam binding protein  CDH2 - cadherin 2, type 1, n-cadherin (neuronal)  CDC27 - cell division cycle 27  ANKIB1 - ankyrin repeat and ibr domain containing 1  POMT1 - protein-o-mannosyltransferase 1  PSEN1 - presenilin 1  USP16 - ubiquitin specific peptidase 16  PSKH1 - protein serine kinase h1  PSMA1 - proteasome (prosome, macropain) subunit, alpha type, 1  RNF216 - ring finger protein 216  PSMA2 - proteasome (prosome, macropain) subunit, alpha type, 2  CAMKK2 - calcium/calmodulin-dependent protein kinase kinase 2, beta  PSMB1 - proteasome (prosome, macropain) subunit, beta type, 1  PSMA5 - proteasome (prosome, macropain) subunit, alpha type, 5  NPRL2 - nitrogen permease regulator-like 2 (s. cerevisiae)  PSMA3 - proteasome (prosome, macropain) subunit, alpha type, 3  PSMB10 - proteasome (prosome, macropain) subunit, beta type, 10  UBLCP1 - ubiquitin-like domain containing ctd phosphatase 1  FAM173B - family with sequence similarity 173, member b  PSMC6 - proteasome (prosome, macropain) 26s subunit, atpase, 6  TAF6L - taf6-like rna polymerase ii, p300/cbp-associated factor (pcaf)-associated factor, 65kda  PSMD1 - proteasome (prosome, macropain) 26s subunit, non-atpase, 1  PSMC4 - proteasome (prosome, macropain) 26s subunit, atpase, 4  PSMC2 - proteasome (prosome, macropain) 26s subunit, atpase, 2  DPM3 - dolichyl-phosphate mannosyltransferase polypeptide 3  PSMD9 - proteasome (prosome, macropain) 26s subunit, non-atpase, 9  PSMD10 - proteasome (prosome, macropain) 26s subunit, non-atpase, 10  PSMD7 - proteasome (prosome, macropain) 26s subunit, non-atpase, 7  PSMD8 - proteasome (prosome, macropain) 26s subunit, non-atpase, 8  PSMD5 - proteasome (prosome, macropain) 26s subunit, non-atpase, 5  PSMD2 - proteasome (prosome, macropain) 26s subunit, non-atpase, 2  PSMD12 - proteasome (prosome, macropain) 26s subunit, non-atpase, 12  ATG7 - autophagy related 7  PSMD13 - proteasome (prosome, macropain) 26s subunit, non-atpase, 13  LEPREL2 - leprecan-like 2  KLHL17 - kelch-like family member 17  PTEN - phosphatase and tensin homolog  SETD4 - set domain containing 4  PRDX4 - peroxiredoxin 4  CFL1 - cofilin 1 (non-muscle)  KAT5 - k(lysine) acetyltransferase 5  PTK2 - protein tyrosine kinase 2  PTK7 - protein tyrosine kinase 7  CDK5 - cyclin-dependent kinase 5  CDK4 - cyclin-dependent kinase 4  TWF1 - twinfilin actin-binding protein 1  CDK9 - cyclin-dependent kinase 9  CDK8 - cyclin-dependent kinase 8  CDK7 - cyclin-dependent kinase 7  SPOPL - speckle-type poz protein-like  PTPN3 - protein tyrosine phosphatase, non-receptor type 3  PTPN2 - protein tyrosine phosphatase, non-receptor type 2  PTPN1 - protein tyrosine phosphatase, non-receptor type 1  PTPN9 - protein tyrosine phosphatase, non-receptor type 9  HTATIP2 - hiv-1 tat interactive protein 2, 30kda  PTPN11 - protein tyrosine phosphatase, non-receptor type 11  PPM1M - protein phosphatase, mg2+/mn2+ dependent, 1m  PTPRA - protein tyrosine phosphatase, receptor type, a  PTPN12 - protein tyrosine phosphatase, non-receptor type 12  PTPN13 - protein tyrosine phosphatase, non-receptor type 13 (apo-1/cd95 (fas)-associated phosphatase)  PTPRD - protein tyrosine phosphatase, receptor type, d  PTPRB - protein tyrosine phosphatase, receptor type, b  SLC35A1 - solute carrier family 35 (cmp-sialic acid transporter), member a1  PTPRG - protein tyrosine phosphatase, receptor type, g  CDK2 - cyclin-dependent kinase 2  PTPRE - protein tyrosine phosphatase, receptor type, e  PTPRF - protein tyrosine phosphatase, receptor type, f  PDP1 - pyruvate dehyrogenase phosphatase catalytic subunit 1  P4HTM - prolyl 4-hydroxylase, transmembrane (endoplasmic reticulum)  PTPRR - protein tyrosine phosphatase, receptor type, r  PTPRS - protein tyrosine phosphatase, receptor type, s  PTPRK - protein tyrosine phosphatase, receptor type, k  PTPRM - protein tyrosine phosphatase, receptor type, m  PTPRN - protein tyrosine phosphatase, receptor type, n  PLK2 - polo-like kinase 2  CHML - choroideremia-like (rab escort protein 2)  NUP50 - nucleoporin 50kda  PEX2 - peroxisomal biogenesis factor 2  CHM - choroideremia (rab escort protein 1)  CHCHD4 - coiled-coil-helix-coiled-coil-helix domain containing 4  KDM5B - lysine (k)-specific demethylase 5b  CHGB - chromogranin b (secretogranin 1)  MXRA8 - matrix-remodelling associated 8  RAB6A - rab6a, member ras oncogene family  MAP4K2 - mitogen-activated protein kinase kinase kinase kinase 2  RAB5A - rab5a, member ras oncogene family  RAB3A - rab3a, member ras oncogene family  FRS2 - fibroblast growth factor receptor substrate 2  RAB3B - rab3b, member ras oncogene family  RAB2A - rab2a, member ras oncogene family  RAB1A - rab1a, member ras oncogene family  PARP14 - poly (adp-ribose) polymerase family, member 14  PAF1 - paf1, rna polymerase ii associated factor, homolog (s. cerevisiae)  MSL1 - male-specific lethal 1 homolog (drosophila)  FBXL19 - f-box and leucine-rich repeat protein 19  INO80 - ino80 complex subunit  RC3H2 - ring finger and ccch-type domains 2  RANBP2 - ran binding protein 2  RANGAP1 - ran gtpase activating protein 1  MIER2 - mesoderm induction early response 1, family member 2  MOK - mok protein kinase  USP53 - ubiquitin specific peptidase 53  B3GNT2 - udp-glcnac:betagal beta-1,3-n-acetylglucosaminyltransferase 2  RRAGA - ras-related gtp binding a  RASA1 - ras p21 protein activator (gtpase activating protein) 1  CLK1 - cdc-like kinase 1  MAGEL2 - mage-like 2  CLK2 - cdc-like kinase 2  ING3 - inhibitor of growth family, member 3  CLK3 - cdc-like kinase 3  PRMT7 - protein arginine methyltransferase 7  RBBP7 - retinoblastoma binding protein 7  TMX3 - thioredoxin-related transmembrane protein 3  MAP3K2 - mitogen-activated protein kinase kinase kinase 2  ARID4A - at rich interactive domain 4a (rbp1-like)  RBBP5 - retinoblastoma binding protein 5  RBBP6 - retinoblastoma binding protein 6  KDM5A - lysine (k)-specific demethylase 5a  NUP35 - nucleoporin 35kda  ERCC8 - excision repair cross-complementing rodent repair deficiency, complementation group 8  MGEA5 - meningioma expressed antigen 5 (hyaluronidase)  RCN1 - reticulocalbin 1, ef-hand calcium binding domain  PIGU - phosphatidylinositol glycan anchor biosynthesis, class u  USP39 - ubiquitin specific peptidase 39  CHUK - conserved helix-loop-helix ubiquitous kinase  ZDHHC13 - zinc finger, dhhc-type containing 13  SPTAN1 - spectrin, alpha, non-erythrocytic 1  MKRN1 - makorin ring finger protein 1  MKRN2 - makorin ring finger protein 2  TMTC4 - transmembrane and tetratricopeptide repeat containing 4  SPTBN4 - spectrin, beta, non-erythrocytic 4  FBXO18 - f-box protein, helicase, 18  SPP1 - secreted phosphoprotein 1  ANO8 - anoctamin 8  ALG10 - alg10, alpha-1,2-glucosyltransferase  PPP1R15B - protein phosphatase 1, regulatory subunit 15b  MIER1 - mesoderm induction early response 1, transcriptional regulator  ABHD13 - abhydrolase domain containing 13  METTL12 - methyltransferase like 12  CPE - carboxypeptidase e  SOS1 - son of sevenless homolog 1 (drosophila)  FBXL20 - f-box and leucine-rich repeat protein 20  RAB40C - rab40c, member ras oncogene family  CREBBP - creb binding protein  ATF2 - activating transcription factor 2  CREB1 - camp responsive element binding protein 1  DDX58 - dead (asp-glu-ala-asp) box polypeptide 58  MARK4 - map/microtubule affinity-regulating kinase 4  RPRD1B - regulation of nuclear pre-mrna domain containing 1b  PLK3 - polo-like kinase 3  SNRPB - small nuclear ribonucleoprotein polypeptides b and b1  SUMO2 - small ubiquitin-like modifier 2  SUMO3 - small ubiquitin-like modifier 3  HLTF - helicase-like transcription factor  SGK3 - serum/glucocorticoid regulated kinase family, member 3  KMT2C - lysine (k)-specific methyltransferase 2c  PRKD3 - protein kinase d3  RAB38 - rab38, member ras oncogene family  PPIL4 - peptidylprolyl isomerase (cyclophilin)-like 4  OSTC - oligosaccharyltransferase complex subunit (non-catalytic)  SENP2 - sumo1/sentrin/smt3 specific peptidase 2  CST3 - cystatin c  NAT6 - n-acetyltransferase 6 (gcn5-related)  USP32 - ubiquitin specific peptidase 32  SHC2 - shc (src homology 2 domain containing) transforming protein 2  KLHL12 - kelch-like family member 12  KDM2B - lysine (k)-specific demethylase 2b  DSTYK - dual serine/threonine and tyrosine protein kinase  CTBP1 - c-terminal binding protein 1  RASGRP3 - ras guanyl releasing protein 3 (calcium and dag-regulated)  UBL5 - ubiquitin-like 5  CTNNB1 - catenin (cadherin-associated protein), beta 1, 88kda  PPIL2 - peptidylprolyl isomerase (cyclophilin)-like 2  PIGO - phosphatidylinositol glycan anchor biosynthesis, class o  STX1A - syntaxin 1a (brain)  LNX1 - ligand of numb-protein x 1, e3 ubiquitin protein ligase  USP30 - ubiquitin specific peptidase 30  FUT10 - fucosyltransferase 10 (alpha (1,3) fucosyltransferase)  NEK4 - nima-related kinase 4  STK3 - serine/threonine kinase 3  BRD1 - bromodomain containing 1  PCGF1 - polycomb group ring finger 1  FKBP8 - fk506 binding protein 8, 38kda  AURKC - aurora kinase c  STK10 - serine/threonine kinase 10  PAN3 - pan3 poly(a) specific ribonuclease subunit homolog (s. cerevisiae)  ISPD - isoprenoid synthase domain containing  CYLD - cylindromatosis (turban tumor syndrome)  SUV420H2 - suppressor of variegation 4-20 homolog 2 (drosophila)  CDKL5 - cyclin-dependent kinase-like 5  STK4 - serine/threonine kinase 4  CRKL - v-crk avian sarcoma virus ct10 oncogene homolog-like  HECTD1 - hect domain containing e3 ubiquitin protein ligase 1  FKBP10 - fk506 binding protein 10, 65 kda  CRY1 - cryptochrome 1 (photolyase-like)  CRY2 - cryptochrome 2 (photolyase-like)  ANAPC13 - anaphase promoting complex subunit 13  AGBL5 - atp/gtp binding protein-like 5  DCAF12 - ddb1 and cul4 associated factor 12  BRMS1 - breast cancer metastasis suppressor 1  NAA35 - n(alpha)-acetyltransferase 35, natc auxiliary subunit  SUMF2 - sulfatase modifying factor 2  CSF1R - colony stimulating factor 1 receptor  CSF1 - colony stimulating factor 1 (macrophage)  FBXO7 - f-box protein 7  SRPK2 - srsf protein kinase 2  QPCT - glutaminyl-peptide cyclotransferase  SRPK1 - srsf protein kinase 1  TAB3 - tgf-beta activated kinase 1/map3k7 binding protein 3  KLHL22 - kelch-like family member 22  GALNT11 - udp-n-acetyl-alpha-d-galactosamine:polypeptide n-acetylgalactosaminyltransferase 11 (galnac-t11)  PTPN5 - protein tyrosine phosphatase, non-receptor type 5 (striatum-enriched)  ZNF335 - zinc finger protein 335  CSK - c-src tyrosine kinase  CSNK1G2 - casein kinase 1, gamma 2  TTLL1 - tubulin tyrosine ligase-like family, member 1  CSNK1A1 - casein kinase 1, alpha 1  SPTBN1 - spectrin, beta, non-erythrocytic 1  CSNK1D - casein kinase 1, delta  CSNK2A2 - casein kinase 2, alpha prime polypeptide  RNF214 - ring finger protein 214  SRC - v-src avian sarcoma (schmidt-ruppin a-2) viral oncogene homolog  CSNK1G3 - casein kinase 1, gamma 3  CSNK2A1 - casein kinase 2, alpha 1 polypeptide  VIPAS39 - vps33b interacting protein, apical-basolateral polarity regulator, spe-39 homolog  ZDHHC12 - zinc finger, dhhc-type containing 12  ARIH1 - ariadne homolog, ubiquitin-conjugating enzyme e2 binding protein, 1 (drosophila)  UBE2O - ubiquitin-conjugating enzyme e2o  FBXL2 - f-box and leucine-rich repeat protein 2  SHPRH - snf2 histone linker phd ring helicase, e3 ubiquitin protein ligase  RNF169 - ring finger protein 169  SEL1L - sel-1 suppressor of lin-12-like (c. elegans)  FBXO33 - f-box protein 33  DDOST - dolichyl-diphosphooligosaccharide--protein glycosyltransferase subunit (non-catalytic)  SIN3A - sin3 transcription regulator family member a  SDF2 - stromal cell-derived factor 2  DPH6 - diphthamine biosynthesis 6  TPGS2 - tubulin polyglutamylase complex subunit 2  FAM98A - family with sequence similarity 98, member a  RSPRY1 - ring finger and spry domain containing 1  WWTR1 - ww domain containing transcription regulator 1  SETMAR - set domain and mariner transposase fusion gene  DDB1 - damage-specific dna binding protein 1, 127kda  MAP2K4 - mitogen-activated protein kinase kinase 4  PTPN23 - protein tyrosine phosphatase, non-receptor type 23  SENP7 - sumo1/sentrin specific peptidase 7  ZDHHC24 - zinc finger, dhhc-type containing 24  KBTBD6 - kelch repeat and btb (poz) domain containing 6  CSRP2BP - csrp2 binding protein  ZDHHC5 - zinc finger, dhhc-type containing 5  SCYL1 - scy1-like 1 (s. cerevisiae)  CLK4 - cdc-like kinase 4  KLHL13 - kelch-like family member 13  RNF19A - ring finger protein 19a, e3 ubiquitin protein ligase  RCHY1 - ring finger and chy zinc finger domain containing 1, e3 ubiquitin protein ligase  ASPHD1 - aspartate beta-hydroxylase domain containing 1  PPM1H - protein phosphatase, mg2+/mn2+ dependent, 1h  GALNT16 - udp-n-acetyl-alpha-d-galactosamine:polypeptide n-acetylgalactosaminyltransferase 16  KCTD13 - potassium channel tetramerization domain containing 13  DCAF13 - ddb1 and cul4 associated factor 13  BIRC6 - baculoviral iap repeat containing 6  BTBD6 - btb (poz) domain containing 6  ZDHHC20 - zinc finger, dhhc-type containing 20  LTN1 - listerin e3 ubiquitin protein ligase 1  ZNF451 - zinc finger protein 451  UBXN7 - ubx domain protein 7  CHD5 - chromodomain helicase dna binding protein 5  MCFD2 - multiple coagulation factor deficiency 2  MED30 - mediator complex subunit 30  RIMKLB - ribosomal modification protein rimk-like family member b  USP31 - ubiquitin specific peptidase 31  DCAF15 - ddb1 and cul4 associated factor 15  DAPK3 - death-associated protein kinase 3  ZZZ3 - zinc finger, zz-type containing 3  LRSAM1 - leucine rich repeat and sterile alpha motif containing 1  ULK3 - unc-51 like kinase 3  MTA3 - metastasis associated 1 family, member 3  DGKQ - diacylglycerol kinase, theta 110kda  CLIP3 - cap-gly domain containing linker protein 3  DAPK1 - death-associated protein kinase 1  TIPARP - tcdd-inducible poly(adp-ribose) polymerase  HACE1 - hect domain and ankyrin repeat containing e3 ubiquitin protein ligase 1  SATB1 - satb homeobox 1  MIB1 - mindbomb e3 ubiquitin protein ligase 1  DAD1 - defender against cell death 1  SBF1 - set binding factor 1  TRPC4AP - transient receptor potential cation channel, subfamily c, member 4 associated protein  KLHL42 - kelch-like family member 42  EPC2 - enhancer of polycomb homolog 2 (drosophila)  CDC26 - cell division cycle 26  IRF2BP1 - interferon regulatory factor 2 binding protein 1  TAOK1 - tao kinase 1  PDP2 - pyruvate dehyrogenase phosphatase catalytic subunit 2  KLHL8 - kelch-like family member 8  DPH2 - dph2 homolog (s. cerevisiae)  SHANK3 - sh3 and multiple ankyrin repeat domains 3  DPH1 - diphthamide biosynthesis 1  DPAGT1 - dolichyl-phosphate (udp-n-acetylglucosamine) n-acetylglucosaminephosphotransferase 1 (glcnac-1-p transferase)  WIPI2 - wd repeat domain, phosphoinositide interacting 2  USP35 - ubiquitin specific peptidase 35  DR1 - down-regulator of transcription 1, tbp-binding (negative cofactor 2)  WSB1 - wd repeat and socs box containing 1  ALG2 - alg2, alpha-1,3/1,6-mannosyltransferase  ZNRF2 - zinc and ring finger 2  DUSP8 - dual specificity phosphatase 8  MAPK15 - mitogen-activated protein kinase 15  DUSP5 - dual specificity phosphatase 5  DUSP6 - dual specificity phosphatase 6  SKP1 - s-phase kinase-associated protein 1  DUSP7 - dual specificity phosphatase 7  DYRK1A - dual-specificity tyrosine-(y)-phosphorylation regulated kinase 1a  TOR1A - torsin family 1, member a (torsin a)  ABHD12 - abhydrolase domain containing 12  HERC4 - hect and rld domain containing e3 ubiquitin protein ligase 4  WDR48 - wd repeat domain 48  USP36 - ubiquitin specific peptidase 36  FNIP2 - folliculin interacting protein 2  SENP6 - sumo1/sentrin specific peptidase 6  HBEGF - heparin-binding egf-like growth factor  DTX1 - deltex homolog 1 (drosophila)  DLG3 - discs, large homolog 3 (drosophila)  DLG4 - discs, large homolog 4 (drosophila)  DLG1 - discs, large homolog 1 (drosophila)  FBXW8 - f-box and wd repeat domain containing 8  SH3RF1 - sh3 domain containing ring finger 1  FBXO25 - f-box protein 25  ST6GAL1 - st6 beta-galactosamide alpha-2,6-sialyltranferase 1  SIAH2 - siah e3 ubiquitin protein ligase 2  SIAH1 - siah e3 ubiquitin protein ligase 1  DGUOK - deoxyguanosine kinase  FBXL3 - f-box and leucine-rich repeat protein 3  EP400 - e1a binding protein p400  DHPS - deoxyhypusine synthase  B3GAT3 - beta-1,3-glucuronyltransferase 3 (glucuronosyltransferase i)  ST3GAL2 - st3 beta-galactoside alpha-2,3-sialyltransferase 2  FBXL5 - f-box and leucine-rich repeat protein 5  RICTOR - rptor independent companion of mtor, complex 2  FBXO2 - f-box protein 2  UVSSA - uv-stimulated scaffold protein a  SFPQ - splicing factor proline/glutamine-rich  PELI3 - pellino e3 ubiquitin protein ligase family member 3  FBXW2 - f-box and wd repeat domain containing 2  EIF2AK4 - eukaryotic translation initiation factor 2 alpha kinase 4  NAT9 - n-acetyltransferase 9 (gcn5-related, putative)  ALG11 - alg11, alpha-1,2-mannosyltransferase  SGK1 - serum/glucocorticoid regulated kinase 1  USP37 - ubiquitin specific peptidase 37  SENP3 - sumo1/sentrin/smt3 specific peptidase 3  SMARCAD1 - swi/snf-related, matrix-associated actin-dependent regulator of chromatin, subfamily a, containing dead/h box 1  MARK2 - map/microtubule affinity-regulating kinase 2  C1GALT1 - core 1 synthase, glycoprotein-n-acetylgalactosamine 3-beta-galactosyltransferase, 1  ELK4 - elk4, ets-domain protein (srf accessory protein 1)  EPAS1 - endothelial pas domain protein 1  EP300 - e1a binding protein p300  DPH7 - diphthamide biosynthesis 7  SEPHS1 - selenophosphate synthetase 1  TIMM50 - translocase of inner mitochondrial membrane 50 homolog (s. cerevisiae)  UGGT1 - udp-glucose glycoprotein glucosyltransferase 1  EGR2 - early growth response 2  TTC3 - tetratricopeptide repeat domain 3  EIF2S1 - eukaryotic translation initiation factor 2, subunit 1 alpha, 35kda  TRIM32 - tripartite motif containing 32  SPHK2 - sphingosine kinase 2  KDM2A - lysine (k)-specific demethylase 2a  TMEM129 - transmembrane protein 129  METTL18 - methyltransferase like 18  TSG101 - tumor susceptibility gene 101  MAST1 - microtubule associated serine/threonine kinase 1  FBXO21 - f-box protein 21  PGAP3 - post-gpi attachment to proteins 3  RNF149 - ring finger protein 149  STK38L - serine/threonine kinase 38 like  TULP4 - tubby like protein 4  PIGM - phosphatidylinositol glycan anchor biosynthesis, class m  PHC1 - polyhomeotic homolog 1 (drosophila)  POGLUT1 - protein o-glucosyltransferase 1  PHLPP2 - ph domain and leucine rich repeat protein phosphatase 2  EGFR - epidermal growth factor receptor  USP33 - ubiquitin specific peptidase 33  MAST3 - microtubule associated serine/threonine kinase 3  KDM4B - lysine (k)-specific demethylase 4b  FAM20C - family with sequence similarity 20, member c  TOP1 - topoisomerase (dna) i  KDM1A - lysine (k)-specific demethylase 1a  GOLGA7B - golgin a7 family, member b  ATXN7L3 - ataxin 7-like 3  PARP6 - poly (adp-ribose) polymerase family, member 6  EOGT - egf domain-specific o-linked n-acetylglucosamine (glcnac) transferase  UBE2Q2 - ubiquitin-conjugating enzyme e2q family member 2  E4F1 - e4f transcription factor 1  TRIO - trio rho guanine nucleotide exchange factor  SMG1 - smg1 phosphatidylinositol 3-kinase-related kinase  TNIK - traf2 and nck interacting kinase  ENY2 - enhancer of yellow 2 homolog (drosophila)  MYCBP2 - myc binding protein 2, e3 ubiquitin protein ligase  TRAF1 - tnf receptor-associated factor 1  HSP90B1 - heat shock protein 90kda beta (grp94), member 1  TRAF3 - tnf receptor-associated factor 3  TRAF2 - tnf receptor-associated factor 2  SETX - senataxin  SETD1B - set domain containing 1b  FEM1C - fem-1 homolog c (c. elegans)  TPR - translocated promoter region, nuclear basket protein  ERC1 - elks/rab6-interacting/cast family member 1  KDM4C - lysine (k)-specific demethylase 4c  TRIM35 - tripartite motif containing 35  PIGW - phosphatidylinositol glycan anchor biosynthesis, class w  CAMK1D - calcium/calmodulin-dependent protein kinase id  TTLL5 - tubulin tyrosine ligase-like family, member 5  NUP107 - nucleoporin 107kda  CDK19 - cyclin-dependent kinase 19  DERL3 - derlin 3  USP1 - ubiquitin specific peptidase 1  PTK2B - protein tyrosine kinase 2 beta  PCNP - pest proteolytic signal containing nuclear protein  PARP11 - poly (adp-ribose) polymerase family, member 11  CUL9 - cullin 9  KANSL1 - kat8 regulatory nsl complex subunit 1  RNF185 - ring finger protein 185  USP4 - ubiquitin specific peptidase 4 (proto-oncogene)  TAB2 - tgf-beta activated kinase 1/map3k7 binding protein 2  ZNF598 - zinc finger protein 598  VGF - vgf nerve growth factor inducible  LAMA1 - laminin, alpha 1  SPSB3 - spla/ryanodine receptor domain and socs box containing 3  VHL - von hippel-lindau tumor suppressor, e3 ubiquitin protein ligase  KDM6B - lysine (k)-specific demethylase 6b  PHF8 - phd finger protein 8  TRIM41 - tripartite motif containing 41  EXT2 - exostosin glycosyltransferase 2  EXT1 - exostosin glycosyltransferase 1  KDM6A - lysine (k)-specific demethylase 6a  DCUN1D4 - dcn1, defective in cullin neddylation 1, domain containing 4  EXTL2 - exostosin-like glycosyltransferase 2  EXTL3 - exostosin-like glycosyltransferase 3  EYA3 - eyes absent homolog 3 (drosophila)  EZH1 - enhancer of zeste homolog 1 (drosophila)  TRIM47 - tripartite motif containing 47  PDXP - pyridoxal (pyridoxine, vitamin b6) phosphatase  VCP - valosin containing protein  TTLL12 - tubulin tyrosine ligase-like family, member 12  NEK9 - nima-related kinase 9  ERCC3 - excision repair cross-complementing rodent repair deficiency, complementation group 3  NUP205 - nucleoporin 205kda  ELMSAN1 - elm2 and myb/sant-like domain containing 1  WDR20 - wd repeat domain 20  UBE2A - ubiquitin-conjugating enzyme e2a  UBA1 - ubiquitin-like modifier activating enzyme 1  ERN1 - endoplasmic reticulum to nucleus signaling 1  UBB - ubiquitin b  ARRDC4 - arrestin domain containing 4  RCOR1 - rest corepressor 1  ASPHD2 - aspartate beta-hydroxylase domain containing 2  TYRO3 - tyro3 protein tyrosine kinase  TPGS1 - tubulin polyglutamylase complex subunit 1  TYK2 - tyrosine kinase 2  ATG4B - autophagy related 4b, cysteine peptidase  ETF1 - eukaryotic translation termination factor 1  COG7 - component of oligomeric golgi complex 7  UCHL3 - ubiquitin carboxyl-terminal esterase l3 (ubiquitin thiolesterase)  PKDCC - protein kinase domain containing, cytoplasmic  SCYL3 - scy1-like 3 (s. cerevisiae)  SUMO1 - small ubiquitin-like modifier 1  UCHL1 - ubiquitin carboxyl-terminal esterase l1 (ubiquitin thiolesterase)  UGCG - udp-glucose ceramide glucosyltransferase  PELI1 - pellino e3 ubiquitin protein ligase 1  SMURF1 - smad specific e3 ubiquitin protein ligase 1  JMJD6 - jumonji domain containing 6  UBE2H - ubiquitin-conjugating enzyme e2h  UBE2I - ubiquitin-conjugating enzyme e2i  EPHB6 - eph receptor b6  UBE2D2 - ubiquitin-conjugating enzyme e2d 2  FBXO28 - f-box protein 28  UBE2V2 - ubiquitin-conjugating enzyme e2 variant 2  LONRF1 - lon peptidase n-terminal domain and ring finger 1  ADCK1 - aarf domain containing kinase 1  ERCC2 - excision repair cross-complementing rodent repair deficiency, complementation group 2  MAN1C1 - mannosidase, alpha, class 1c, member 1  NUP210 - nucleoporin 210kda  UBE2L3 - ubiquitin-conjugating enzyme e2l 3  UBE2N - ubiquitin-conjugating enzyme e2n  UBE2V1 - ubiquitin-conjugating enzyme e2 variant 1  TMEM165 - transmembrane protein 165  FNTA - farnesyltransferase, caax box, alpha  FLT1 - fms-related tyrosine kinase 1  WSB2 - wd repeat and socs box containing 2  FLNA - filamin a, alpha  SIK2 - salt-inducible kinase 2  PHLPP1 - ph domain and leucine rich repeat protein phosphatase 1  HDAC8 - histone deacetylase 8  ASH1L - ash1 (absent, small, or homeotic)-like (drosophila)  SIN3B - sin3 transcription regulator family member b  FKBP15 - fk506 binding protein 15, 133kda  FKBP4 - fk506 binding protein 4, 59kda  FKBP2 - fk506 binding protein 2, 13kda  ZDHHC22 - zinc finger, dhhc-type containing 22  MGRN1 - mahogunin ring finger 1, e3 ubiquitin protein ligase  HR - hair growth associated  MED8 - mediator complex subunit 8  FKBP5 - fk506 binding protein 5  CAND1 - cullin-associated and neddylation-dissociated 1  FKBP1A - fk506 binding protein 1a, 12kda  FKBP1B - fk506 binding protein 1b, 12.6 kda  FBXW11 - f-box and wd repeat domain containing 11  POFUT2 - protein o-fucosyltransferase 2  KLHL18 - kelch-like family member 18  KDM3A - lysine (k)-specific demethylase 3a  RNF130 - ring finger protein 130  NUP160 - nucleoporin 160kda  PPP1R11 - protein phosphatase 1, regulatory (inhibitor) subunit 11  FGFR2 - fibroblast growth factor receptor 2  TAF9 - taf9 rna polymerase ii, tata box binding protein (tbp)-associated factor, 32kda  UBR4 - ubiquitin protein ligase e3 component n-recognin 4  FAM98B - family with sequence similarity 98, member b  MBD6 - methyl-cpg binding domain protein 6  KLHL7 - kelch-like family member 7  FGF13 - fibroblast growth factor 13  TAF6 - taf6 rna polymerase ii, tata box binding protein (tbp)-associated factor, 80kda  FGFR1 - fibroblast growth factor receptor 1  FGF9 - fibroblast growth factor 9  TADA2A - transcriptional adaptor 2a  PHF15 - phd finger protein 15  FGF1 - fibroblast growth factor 1 (acidic)  FGF2 - fibroblast growth factor 2 (basic)  DPY19L1 - dpy-19-like 1 (c. elegans)  USP22 - ubiquitin specific peptidase 22  SASH1 - sam and sh3 domain containing 1  TRIM2 - tripartite motif containing 2  FER - fer (fps/fes related) tyrosine kinase  MAP3K7 - mitogen-activated protein kinase kinase kinase 7  ALG1 - alg1, chitobiosyldiphosphodolichol beta-mannosyltransferase  INO80E - ino80 complex subunit e  ZDHHC17 - zinc finger, dhhc-type containing 17  RNF114 - ring finger protein 114  KMT2E - lysine (k)-specific methyltransferase 2e  SIK3 - sik family kinase 3  PGP - phosphoglycolate phosphatase  FKTN - fukutin  UFL1 - ufm1-specific ligase 1  TENC1 - tensin like c1 domain containing phosphatase (tensin 2)  KLHL9 - kelch-like family member 9  TRIM9 - tripartite motif containing 9  DMAP1 - dna methyltransferase 1 associated protein 1  EFEMP1 - egf containing fibulin-like extracellular matrix protein 1  OBSL1 - obscurin-like 1  CTTNBP2NL - cttnbp2 n-terminal like  FBN1 - fibrillin 1  USP24 - ubiquitin specific peptidase 24  RNF208 - ring finger protein 208  TIE1 - tyrosine kinase with immunoglobulin-like and egf-like domains 1  TIMP1 - timp metallopeptidase inhibitor 1  RYBP - ring1 and yy1 binding protein  MTOR - mechanistic target of rapamycin (serine/threonine kinase)  STK32C - serine/threonine kinase 32c  PPWD1 - peptidylprolyl isomerase domain and wd repeat containing 1  ATP13A2 - atpase type 13a2  TLR3 - toll-like receptor 3  PIGS - phosphatidylinositol glycan anchor biosynthesis, class s  SIRT1 - sirtuin 1  ACTR8 - arp8 actin-related protein 8 homolog (yeast)  ICMT - isoprenylcysteine carboxyl methyltransferase  BRD4 - bromodomain containing 4  FBXO44 - f-box protein 44  TNFAIP1 - tumor necrosis factor, alpha-induced protein 1 (endothelial)  KCTD21 - potassium channel tetramerization domain containing 21  TEK - tek tyrosine kinase, endothelial  NUP188 - nucleoporin 188kda  SUZ12 - suz12 polycomb repressive complex 2 subunit  P4HA3 - prolyl 4-hydroxylase, alpha polypeptide iii  POFUT1 - protein o-fucosyltransferase 1  RCOR2 - rest corepressor 2  MORC3 - morc family cw-type zinc finger 3  TESK1 - testis-specific kinase 1  FXN - frataxin  TGFA - transforming growth factor, alpha  CEP41 - centrosomal protein 41kda  TGFBR1 - transforming growth factor, beta receptor 1  TGFB2 - transforming growth factor, beta 2  TGFB1 - transforming growth factor, beta 1  MAPK8IP2 - mitogen-activated protein kinase 8 interacting protein 2  PIGN - phosphatidylinositol glycan anchor biosynthesis, class n  CDK20 - cyclin-dependent kinase 20  TGFBR2 - transforming growth factor, beta receptor ii (70/80kda)  RNF20 - ring finger protein 20, e3 ubiquitin protein ligase  TGFBR3 - transforming growth factor, beta receptor iii  WDR45B - wd repeat domain 45b  THBS1 - thrombospondin 1  KAT6B - k(lysine) acetyltransferase 6b  THY1 - thy-1 cell surface antigen  MMD - monocyte to macrophage differentiation-associated  GFPT1 - glutamine--fructose-6-phosphate transaminase 1  GFRA1 - gdnf family receptor alpha 1  GFRA2 - gdnf family receptor alpha 2  TMTC2 - transmembrane and tetratricopeptide repeat containing 2  USP54 - ubiquitin specific peptidase 54  ATAT1 - alpha tubulin acetyltransferase 1  GCNT1 - glucosaminyl (n-acetyl) transferase 1, core 2  LZTR1 - leucine-zipper-like transcription regulator 1  KAT2A - k(lysine) acetyltransferase 2a  NCOA3 - nuclear receptor coactivator 3  FBXL18 - f-box and leucine-rich repeat protein 18  NAA25 - n(alpha)-acetyltransferase 25, natb auxiliary subunit  PABPN1 - poly(a) binding protein, nuclear 1  GGTA1P - glycoprotein, alpha-galactosyltransferase 1 pseudogene  TMTC3 - transmembrane and tetratricopeptide repeat containing 3  TRRAP - transformation/transcription domain-associated protein  HDAC11 - histone deacetylase 11  SETD6 - set domain containing 6  ACTR5 - arp5 actin-related protein 5 homolog (yeast)  NAA60 - n(alpha)-acetyltransferase 60, natf catalytic subunit  BAP1 - brca1 associated protein-1 (ubiquitin carboxy-terminal hydrolase)  CDC7 - cell division cycle 7  NUP85 - nucleoporin 85kda  BRAP - brca1 associated protein  KDM5C - lysine (k)-specific demethylase 5c  USP9X - ubiquitin specific peptidase 9, x-linked  USP11 - ubiquitin specific peptidase 11  PHF17 - phd finger protein 17  GMFB - glia maturation factor, beta  EHMT1 - euchromatic histone-lysine n-methyltransferase 1  FUCA2 - fucosidase, alpha-l- 2, plasma  CAMKMT - calmodulin-lysine n-methyltransferase  NAA40 - n(alpha)-acetyltransferase 40, natd catalytic subunit, homolog (s. cerevisiae)  SDE2 - sde2 telomere maintenance homolog (s. pombe)  USP7 - ubiquitin specific peptidase 7 (herpes virus-associated)  RNF168 - ring finger protein 168, e3 ubiquitin protein ligase  FUT4 - fucosyltransferase 4 (alpha (1,3) fucosyltransferase, myeloid-specific)  ST8SIA4 - st8 alpha-n-acetyl-neuraminide alpha-2,8-sialyltransferase 4  FUT8 - fucosyltransferase 8 (alpha (1,6) fucosyltransferase)  CBLL1 - cbl proto-oncogene-like 1, e3 ubiquitin protein ligase  FYN - fyn oncogene related to src, fgr, yes  RPAP2 - rna polymerase ii associated protein 2  UBA5 - ubiquitin-like modifier activating enzyme 5  GAK - cyclin g associated kinase  GALNT2 - udp-n-acetyl-alpha-d-galactosamine:polypeptide n-acetylgalactosaminyltransferase 2 (galnac-t2)  TTLL7 - tubulin tyrosine ligase-like family, member 7  USP5 - ubiquitin specific peptidase 5 (isopeptidase t)  GALNT1 - udp-n-acetyl-alpha-d-galactosamine:polypeptide n-acetylgalactosaminyltransferase 1 (galnac-t1)  SPRED1 - sprouty-related, evh1 domain containing 1  KMT2D - lysine (k)-specific methyltransferase 2d  AAAS - achalasia, adrenocortical insufficiency, alacrimia  YEATS4 - yeats domain containing 4  KAT6A - k(lysine) acetyltransferase 6a  TUSC3 - tumor suppressor candidate 3  STAM - signal transducing adaptor molecule (sh3 domain and itam motif) 1  NUP214 - nucleoporin 214kda  KLHL36 - kelch-like family member 36  GAS6 - growth arrest-specific 6  FN3KRP - fructosamine 3 kinase related protein  RNF112 - ring finger protein 112  GSPT1 - g1 to s phase transition 1  RNF113A - ring finger protein 113a  PARP8 - poly (adp-ribose) polymerase family, member 8  HECTD3 - hect domain containing e3 ubiquitin protein ligase 3  GTF2H3 - general transcription factor iih, polypeptide 3, 34kda  TRIM26 - tripartite motif containing 26  GTF2H1 - general transcription factor iih, polypeptide 1, 62kda  USP27X - ubiquitin specific peptidase 27, x-linked  GTF2B - general transcription factor iib  PCGF2 - polycomb group ring finger 2  TBL1XR1 - transducin (beta)-like 1 x-linked receptor 1  GTF2H4 - general transcription factor iih, polypeptide 4, 52kda  ZDHHC14 - zinc finger, dhhc-type containing 14  HIST1H2BB - histone cluster 1, h2bb  BRPF1 - bromodomain and phd finger containing, 1  SCG2 - secretogranin ii  MUL1 - mitochondrial e3 ubiquitin protein ligase 1  RNF103 - ring finger protein 103  MOGS - mannosyl-oligosaccharide glucosidase  CDC73 - cell division cycle 73  PTP4A1 - protein tyrosine phosphatase type iva, member 1  HCFC1 - host cell factor c1 (vp16-accessory protein)  MAP3K12 - mitogen-activated protein kinase kinase kinase 12  FUT11 - fucosyltransferase 11 (alpha (1,3) fucosyltransferase)  NDNF - neuron-derived neurotrophic factor  HDAC2 - histone deacetylase 2  HDAC1 - histone deacetylase 1  GOLGA2 - golgin a2  PAGR1 - paxip1 associated glutamate-rich protein 1  WEE1 - wee1 homolog (s. pombe)  WFS1 - wolfram syndrome 1 (wolframin)  WHSC1 - wolf-hirschhorn syndrome candidate 1  DTX3 - deltex homolog 3 (drosophila)  VRK1 - vaccinia related kinase 1  VRK2 - vaccinia related kinase 2  GRK5 - g protein-coupled receptor kinase 5  GRK6 - g protein-coupled receptor kinase 6  FBXL15 - f-box and leucine-rich repeat protein 15  BRCC3 - brca1/brca2-containing complex, subunit 3  GRB2 - growth factor receptor-bound protein 2  ASXL1 - additional sex combs like 1 (drosophila)  TNIP2 - tnfaip3 interacting protein 2  ZNF711 - zinc finger protein 711  YES1 - v-yes-1 yamaguchi sarcoma viral oncogene homolog 1  DCAF10 - ddb1 and cul4 associated factor 10  GSK3B - glycogen synthase kinase 3 beta  YWHAZ - tyrosine 3-monooxygenase/tryptophan 5-monooxygenase activation protein, zeta polypeptide  GSK3A - glycogen synthase kinase 3 alpha  PGAM5 - phosphoglycerate mutase family member 5  YWHAE - tyrosine 3-monooxygenase/tryptophan 5-monooxygenase activation protein, epsilon polypeptide  YWHAB - tyrosine 3-monooxygenase/tryptophan 5-monooxygenase activation protein, beta polypeptide  NDUFAF5 - nadh dehydrogenase (ubiquinone) complex i, assembly factor 5  CCNK - cyclin k  DPM1 - dolichyl-phosphate mannosyltransferase polypeptide 1, catalytic subunit  RNF26 - ring finger protein 26  TRIM24 - tripartite motif containing 24  METTL22 - methyltransferase like 22  PRMT1 - protein arginine methyltransferase 1  PRMT2 - protein arginine methyltransferase 2  FKRP - fukutin related protein  FBXO45 - f-box protein 45  DERL1 - derlin 1  DCAF5 - ddb1 and cul4 associated factor 5  KCTD6 - potassium channel tetramerization domain containing 6  KDELC1 - kdel (lys-asp-glu-leu) containing 1  ALG8 - alg8, alpha-1,3-glucosyltransferase  SYNGAP1 - synaptic ras gtpase activating protein 1  MED11 - mediator complex subunit 11  ST3GAL5 - st3 beta-galactoside alpha-2,3-sialyltransferase 5  STK19 - serine/threonine kinase 19  RNF215 - ring finger protein 215  LDB1 - lim domain binding 1  DSCC1 - dna replication and sister chromatid cohesion 1  KAT2B - k(lysine) acetyltransferase 2b  CRTC2 - creb regulated transcription coactivator 2  DDA1 - det1 and ddb1 associated 1  CDC16 - cell division cycle 16  RPS6KC1 - ribosomal protein s6 kinase, 52kda, polypeptide 1  NAE1 - nedd8 activating enzyme e1 subunit 1  GID4 - gid complex subunit 4  PRPF4B - prp4 pre-mrna processing factor 4 homolog b (yeast)  MTMR2 - myotubularin related protein 2  MTMR3 - myotubularin related protein 3  HUS1 - hus1 checkpoint homolog (s. pombe)  MCM3AP - minichromosome maintenance complex component 3 associated protein  CPNE3 - copine iii  HERC3 - hect and rld domain containing e3 ubiquitin protein ligase 3  HERC2 - hect and rld domain containing e3 ubiquitin protein ligase 2  HERC1 - hect and rld domain containing e3 ubiquitin protein ligase family member 1  TRAK2 - trafficking protein, kinesin binding 2  NSMCE1 - non-smc element 1 homolog (s. cerevisiae)  BCL10 - b-cell cll/lymphoma 10  BTRC - beta-transducin repeat containing e3 ubiquitin protein ligase  UBR1 - ubiquitin protein ligase e3 component n-recognin 1  USP12 - ubiquitin specific peptidase 12  WNK1 - wnk lysine deficient protein kinase 1  UBE2Z - ubiquitin-conjugating enzyme e2z  LAMTOR3 - late endosomal/lysosomal adaptor, mapk and mtor activator 3  MPPE1 - metallophosphoesterase 1  HLCS - holocarboxylase synthetase (biotin-(proprionyl-coa-carboxylase (atp-hydrolysing)) ligase)  SENP5 - sumo1/sentrin specific peptidase 5  STK33 - serine/threonine kinase 33  IRS2 - insulin receptor substrate 2  TNKS - tankyrase, trf1-interacting ankyrin-related adp-ribose polymerase  LATS2 - large tumor suppressor kinase 2  EIF3F - eukaryotic translation initiation factor 3, subunit f  BECN1 - beclin 1, autophagy related  PEA15 - phosphoprotein enriched in astrocytes 15  HIPK1 - homeodomain interacting protein kinase 1  B4GALT4 - udp-gal:betaglcnac beta 1,4- galactosyltransferase, polypeptide 4  B4GALT3 - udp-gal:betaglcnac beta 1,4- galactosyltransferase, polypeptide 3  B4GALT2 - udp-gal:betaglcnac beta 1,4- galactosyltransferase, polypeptide 2  CDC23 - cell division cycle 23  UBE2K - ubiquitin-conjugating enzyme e2k  B3GALNT1 - beta-1,3-n-acetylgalactosaminyltransferase 1 (globoside blood group)  B3GALT2 - udp-gal:betaglcnac beta 1,3-galactosyltransferase, polypeptide 2  HIF1A - hypoxia inducible factor 1, alpha subunit (basic helix-loop-helix transcription factor)  MBTPS1 - membrane-bound transcription factor peptidase, site 1  SNX3 - sorting nexin 3  GBGT1 - globoside alpha-1,3-n-acetylgalactosaminyltransferase 1  EED - embryonic ectoderm development  RNGTT - rna guanylyltransferase and 5'-phosphatase  FBXO10 - f-box protein 10  RIPK1 - receptor (tnfrsf)-interacting serine-threonine kinase 1  FBXO22 - f-box protein 22  FBXO4 - f-box protein 4  FBXO6 - f-box protein 6  VPS33B - vacuolar protein sorting 33 homolog b (yeast)  FBXO3 - f-box protein 3  RAB11A - rab11a, member ras oncogene family  RIPK2 - receptor-interacting serine-threonine kinase 2  PORCN - porcupine homolog (drosophila)  MTMR1 - myotubularin related protein 1  FBXO15 - f-box protein 15  USP46 - ubiquitin specific peptidase 46  VWA1 - von willebrand factor a domain containing 1  PTPLB - protein tyrosine phosphatase-like (proline instead of catalytic arginine), member b  PTPN18 - protein tyrosine phosphatase, non-receptor type 18 (brain-derived)  STT3B - stt3b, subunit of the oligosaccharyltransferase complex (catalytic)  FOXK2 - forkhead box k2  HAT1 - histone acetyltransferase 1  RPS6KA6 - ribosomal protein s6 kinase, 90kda, polypeptide 6  PGAP2 - post-gpi attachment to proteins 2  ENC1 - ectodermal-neural cortex 1 (with btb domain)  DESI1 - desumoylating isopeptidase 1  MEAF6 - myst/esa1-associated factor 6  STK39 - serine threonine kinase 39  ENGASE - endo-beta-n-acetylglucosaminidase  PPM1D - protein phosphatase, mg2+/mn2+ dependent, 1d  UBE2S - ubiquitin-conjugating enzyme e2s  PRPF19 - pre-mrna processing factor 19  ILF3 - interleukin enhancer binding factor 3, 90kda  ILK - integrin-linked kinase  MAP4K3 - mitogen-activated protein kinase kinase kinase kinase 3  CDC14B - cell division cycle 14b  PIAS1 - protein inhibitor of activated stat, 1  SMYD3 - set and mynd domain containing 3  SMURF2 - smad specific e3 ubiquitin protein ligase 2  MAPKAPK5 - mitogen-activated protein kinase-activated protein kinase 5  CAMK1 - calcium/calmodulin-dependent protein kinase i  COPS7B - cop9 signalosome subunit 7b  CBX4 - chromobox homolog 4  COPS3 - cop9 signalosome subunit 3  GORASP1 - golgi reassembly stacking protein 1, 65kda  ANAPC1 - anaphase promoting complex subunit 1  SERP1 - stress-associated endoplasmic reticulum protein 1  PRKRA - protein kinase, interferon-inducible double stranded rna dependent activator  STK16 - serine/threonine kinase 16  RNF115 - ring finger protein 115  CASK - calcium/calmodulin-dependent serine protein kinase (maguk family)  ITGAV - integrin, alpha v  SUDS3 - suppressor of defective silencing 3 homolog (s. cerevisiae)  CDK10 - cyclin-dependent kinase 10  ZDHHC6 - zinc finger, dhhc-type containing 6  ATG3 - autophagy related 3  INSR - insulin receptor  AKTIP - akt interacting protein  KLHL20 - kelch-like family member 20  CDK13 - cyclin-dependent kinase 13  RUVBL1 - ruvb-like 1 (e. coli)  MOCS3 - molybdenum cofactor synthesis 3  IRAK2 - interleukin-1 receptor-associated kinase 2  FOLH1B - folate hydrolase 1b  IRAK1 - interleukin-1 receptor-associated kinase 1  EIF2AK1 - eukaryotic translation initiation factor 2-alpha kinase 1  HS3ST5 - heparan sulfate (glucosamine) 3-o-sulfotransferase 5  NSD1 - nuclear receptor binding set domain protein 1  RFWD2 - ring finger and wd repeat domain 2, e3 ubiquitin protein ligase  RNF25 - ring finger protein 25  PALD1 - phosphatase domain containing, paladin 1  BRPF3 - bromodomain and phd finger containing, 3  PDF - peptide deformylase (mitochondrial)  STK36 - serine/threonine kinase 36  IRF2BPL - interferon regulatory factor 2 binding protein-like  FOXK1 - forkhead box k1  MAGEF1 - melanoma antigen family f, 1  FN3K - fructosamine 3 kinase  STK24 - serine/threonine kinase 24  GFRA4 - gdnf family receptor alpha 4  RASAL1 - ras protein activator like 1 (gap1 like)  OARD1 - o-acyl-adp-ribose deacylase 1  RNF11 - ring finger protein 11  DYRK3 - dual-specificity tyrosine-(y)-phosphorylation regulated kinase 3  DUSP11 - dual specificity phosphatase 11 (rna/rnp complex 1-interacting)  SPOP - speckle-type poz protein  SPARCL1 - sparc-like 1 (hevin)  USP21 - ubiquitin specific peptidase 21  ULK1 - unc-51 like autophagy activating kinase 1  JMJD1C - jumonji domain containing 1c  CDC42BPA - cdc42 binding protein kinase alpha (dmpk-like)  IGF1R - insulin-like growth factor 1 receptor  OGT - o-linked n-acetylglucosamine (glcnac) transferase  RAE1 - rae1 rna export 1 homolog (s. pombe)  LAT - linker for activation of t cells  CUL4B - cullin 4b  CUL1 - cullin 1  CUL2 - cullin 2  CUL3 - cullin 3  B3GAT1 - beta-1,3-glucuronyltransferase 1 (glucuronosyltransferase p)  TPST2 - tyrosylprotein sulfotransferase 2  SUPT3H - suppressor of ty 3 homolog (s. cerevisiae)  TPST1 - tyrosylprotein sulfotransferase 1  RSRC1 - arginine/serine-rich coiled-coil 1  PCGF5 - polycomb group ring finger 5  MEX3C - mex-3 rna binding family member c  WAC - ww domain containing adaptor with coiled-coil  LIMK1 - lim domain kinase 1  ZDHHC3 - zinc finger, dhhc-type containing 3  AATK - apoptosis-associated tyrosine kinase  DOT1L - dot1-like histone h3k79 methyltransferase  LOXL1 - lysyl oxidase-like 1  RAB3D - rab3d, member ras oncogene family  LNPEP - leucyl/cystinyl aminopeptidase  BRSK1 - br serine/threonine kinase 1  BFAR - bifunctional apoptosis regulator  SYVN1 - synovial apoptosis inhibitor 1, synoviolin  NRG2 - neuregulin 2  PTPDC1 - protein tyrosine phosphatase domain containing 1  NEURL4 - neuralized homolog 4 (drosophila)  LIPE - lipase, hormone-sensitive  MINPP1 - multiple inositol-polyphosphate phosphatase 1  LIMK2 - lim domain kinase 2  GOLM1 - golgi membrane protein 1  CDC42BPB - cdc42 binding protein kinase beta (dmpk-like)  LMAN1 - lectin, mannose-binding, 1  CLOCK - clock circadian regulator  PRKAG2 - protein kinase, amp-activated, gamma 2 non-catalytic subunit  ANAPC5 - anaphase promoting complex subunit 5  ANAPC7 - anaphase promoting complex subunit 7  NPEPPS - aminopeptidase puromycin sensitive  RNF138 - ring finger protein 138, e3 ubiquitin protein ligase  KTN1 - kinectin 1 (kinesin receptor)  KRT10 - keratin 10  PPME1 - protein phosphatase methylesterase 1  HEMK1 - hemk methyltransferase family member 1  ACTL6B - actin-like 6b  UBR5 - ubiquitin protein ligase e3 component n-recognin 5  MED20 - mediator complex subunit 20  UCHL5 - ubiquitin carboxyl-terminal hydrolase l5  ATG5 - autophagy related 5  ROCK2 - rho-associated, coiled-coil containing protein kinase 2  MAPK8IP1 - mitogen-activated protein kinase 8 interacting protein 1  PIGB - phosphatidylinositol glycan anchor biosynthesis, class b  TAOK3 - tao kinase 3  FZR1 - fizzy/cell division cycle 20 related 1 (drosophila)  DPY30 - dpy-30 homolog (c. elegans)  LAMC1 - laminin, gamma 1 (formerly lamb2)  IRF2BP2 - interferon regulatory factor 2 binding protein 2  MED27 - mediator complex subunit 27  NAGPA - n-acetylglucosamine-1-phosphodiester alpha-n-acetylglucosaminidase  GALNT18 - udp-n-acetyl-alpha-d-galactosamine:polypeptide n-acetylgalactosaminyltransferase 18  ASB7 - ankyrin repeat and socs box containing 7  ASB8 - ankyrin repeat and socs box containing 8  ARHGEF6 - rac/cdc42 guanine nucleotide exchange factor (gef) 6  KDR - kinase insert domain receptor (a type iii receptor tyrosine kinase)  ASB6 - ankyrin repeat and socs box containing 6  PICK1 - protein interacting with prkca 1  ING4 - inhibitor of growth family, member 4  EIF2AK3 - eukaryotic translation initiation factor 2-alpha kinase 3  USP48 - ubiquitin specific peptidase 48  IRAK4 - interleukin-1 receptor-associated kinase 4  MAP4K4 - mitogen-activated protein kinase kinase kinase kinase 4  RLIM - ring finger protein, lim domain interacting  RHBDD1 - rhomboid domain containing 1  ASB3 - ankyrin repeat and socs box containing 3  TRAF7 - tnf receptor-associated factor 7, e3 ubiquitin protein ligase  KRAS - kirsten rat sarcoma viral oncogene homolog  NAA20 - n(alpha)-acetyltransferase 20, natb catalytic subunit  GOLGA7 - golgin a7  ZDHHC9 - zinc finger, dhhc-type containing 9  SUV420H1 - suppressor of variegation 4-20 homolog 1 (drosophila)  CAMKK1 - calcium/calmodulin-dependent protein kinase kinase 1, alpha  PHF20L1 - phd finger protein 20-like 1  MED21 - mediator complex subunit 21  MED10 - mediator complex subunit 10  ABHD17B - abhydrolase domain containing 17b  KIT - v-kit hardy-zuckerman 4 feline sarcoma viral oncogene homolog  SHISA5 - shisa family member 5  DCUN1D5 - dcn1, defective in cullin neddylation 1, domain containing 5  UBE4A - ubiquitination factor e4a  FBXW9 - f-box and wd repeat domain containing 9  PPIG - peptidylprolyl isomerase g (cyclophilin g)  ZDHHC16 - zinc finger, dhhc-type containing 16  JAK1 - janus kinase 1  JAK2 - janus kinase 2  ANKRD39 - ankyrin repeat domain 39  ING5 - inhibitor of growth family, member 5  JAK3 - janus kinase 3  STT3A - stt3a, subunit of the oligosaccharyltransferase complex (catalytic)  MAST4 - microtubule associated serine/threonine kinase family member 4  PIGP - phosphatidylinositol glycan anchor biosynthesis, class p  PHF20 - phd finger protein 20  TRIP12 - thyroid hormone receptor interactor 12  TMEM246 - transmembrane protein 246  COPS2 - cop9 signalosome subunit 2  ZDHHC2 - zinc finger, dhhc-type containing 2  CRBN - cereblon  MIA3 - melanoma inhibitory activity family, member 3  TAOK2 - tao kinase 2  BRMS1L - breast cancer metastasis-suppressor 1-like  B4GALT6 - udp-gal:betaglcnac beta 1,4- galactosyltransferase, polypeptide 6  LEF1 - lymphoid enhancer-binding factor 1  GTF3C4 - general transcription factor iiic, polypeptide 4, 90kda  B4GALT5 - udp-gal:betaglcnac beta 1,4- galactosyltransferase, polypeptide 5  CDK12 - cyclin-dependent kinase 12  KCTD10 - potassium channel tetramerization domain containing 10  ARID4B - at rich interactive domain 4b (rbp1-like)  SOCS6 - suppressor of cytokine signaling 6  STK35 - serine/threonine kinase 35  MAP3K10 - mitogen-activated protein kinase kinase kinase 10  DUSP19 - dual specificity phosphatase 19  MIB2 - mindbomb e3 ubiquitin protein ligase 2  KDM3B - lysine (k)-specific demethylase 3b  RSF1 - remodeling and spacing factor 1  MAP3K11 - mitogen-activated protein kinase kinase kinase 11  KMT2A - lysine (k)-specific methyltransferase 2a  RPS6KA5 - ribosomal protein s6 kinase, 90kda, polypeptide 5  CCDC8 - coiled-coil domain containing 8  STK17A - serine/threonine kinase 17a  NLK - nemo-like kinase  UIMC1 - ubiquitin interaction motif containing 1  RAB11B - rab11b, member ras oncogene family  MTA2 - metastasis associated 1 family, member 2  EIF2A - eukaryotic translation initiation factor 2a, 65kda  KBTBD7 - kelch repeat and btb (poz) domain containing 7  KDELC2 - kdel (lys-asp-glu-leu) containing 2  MECP2 - methyl cpg binding protein 2 (rett syndrome)  MAGT1 - magnesium transporter 1  MEF2A - myocyte enhancer factor 2a  ZBED1 - zinc finger, bed-type containing 1  PPIL3 - peptidylprolyl isomerase (cyclophilin)-like 3  MAP3K4 - mitogen-activated protein kinase kinase kinase 4  DCLK1 - doublecortin-like kinase 1  MAP3K5 - mitogen-activated protein kinase kinase kinase 5  MAP3K1 - mitogen-activated protein kinase kinase kinase 1, e3 ubiquitin protein ligase  MAP3K3 - mitogen-activated protein kinase kinase kinase 3  MEN1 - multiple endocrine neoplasia i  RAB8A - rab8a, member ras oncogene family  USP42 - ubiquitin specific peptidase 42  KAT8 - k(lysine) acetyltransferase 8  FBXL14 - f-box and leucine-rich repeat protein 14  MFGE8 - milk fat globule-egf factor 8 protein  ATG12 - autophagy related 12  MGAT1 - mannosyl (alpha-1,3-)-glycoprotein beta-1,2-n-acetylglucosaminyltransferase  MGAT2 - mannosyl (alpha-1,6-)-glycoprotein beta-1,2-n-acetylglucosaminyltransferase  HGS - hepatocyte growth factor-regulated tyrosine kinase substrate  MGAT5 - mannosyl (alpha-1,6-)-glycoprotein beta-1,6-n-acetyl-glucosaminyltransferase  MBD3 - methyl-cpg binding domain protein 3  KITLG - kit ligand  B3GALTL - beta 1,3-galactosyltransferase-like  MAN1A1 - mannosidase, alpha, class 1a, member 1  MAN2A2 - mannosidase, alpha, class 2a, member 2  ZC3HC1 - zinc finger, c3hc-type containing 1  COG3 - component of oligomeric golgi complex 3  MARK3 - map/microtubule affinity-regulating kinase 3  MARK1 - map/microtubule affinity-regulating kinase 1  MAPT - microtubule-associated protein tau  DOHH - deoxyhypusine hydroxylase/monooxygenase  MDP1 - magnesium-dependent phosphatase 1  LATS1 - large tumor suppressor kinase 1  MTA1 - metastasis associated 1  SIRT6 - sirtuin 6  MTMR4 - myotubularin related protein 4  MTMR7 - myotubularin related protein 7  MTMR6 - myotubularin related protein 6  MANBA - mannosidase, beta a, lysosomal  MAN2A1 - mannosidase, alpha, class 2a, member 1  USP8 - ubiquitin specific peptidase 8  USP14 - ubiquitin specific peptidase 14 (trna-guanine transglycosylase)  USP10 - ubiquitin specific peptidase 10  UBE2J1 - ubiquitin-conjugating enzyme e2, j1  NUP43 - nucleoporin 43kda  PKMYT1 - protein kinase, membrane associated tyrosine/threonine 1  MATK - megakaryocyte-associated tyrosine kinase  UFC1 - ubiquitin-fold modifier conjugating enzyme 1  TRMT112 - trna methyltransferase 11-2 homolog (s. cerevisiae)  TTBK2 - tau tubulin kinase 2  ASH2L - ash2 (absent, small, or homeotic)-like (drosophila)  CTDSPL2 - ctd (carboxy-terminal domain, rna polymerase ii, polypeptide a) small phosphatase like 2  PPIL1 - peptidylprolyl isomerase (cyclophilin)-like 1  ITCH - itchy e3 ubiquitin protein ligase  FBXL16 - f-box and leucine-rich repeat protein 16  NFS1 - nfs1 cysteine desulfurase  RIOK1 - rio kinase 1  OTUD6B - otu domain containing 6b  AIP - aryl hydrocarbon receptor interacting protein  NUB1 - negative regulator of ubiquitin-like proteins 1  ASB1 - ankyrin repeat and socs box containing 1  LYN - v-yes-1 yamaguchi sarcoma viral related oncogene homolog  UBE2M - ubiquitin-conjugating enzyme e2m  UBA3 - ubiquitin-like modifier activating enzyme 3  PARP9 - poly (adp-ribose) polymerase family, member 9  RNF8 - ring finger protein 8, e3 ubiquitin protein ligase  LTBP1 - latent transforming growth factor beta binding protein 1  RPS6KL1 - ribosomal protein s6 kinase-like 1  FKBP7 - fk506 binding protein 7  BAZ1B - bromodomain adjacent to zinc finger domain, 1b  PIAS4 - protein inhibitor of activated stat, 4  TRIM33 - tripartite motif containing 33  SMAD2 - smad family member 2  MBIP - map3k12 binding inhibitory protein 1  HDAC7 - histone deacetylase 7  TMTC1 - transmembrane and tetratricopeptide repeat containing 1  CDKL2 - cyclin-dependent kinase-like 2 (cdc2-related kinase)  KCTD11 - potassium channel tetramerization domain containing 11  RPS6KA4 - ribosomal protein s6 kinase, 90kda, polypeptide 4  PLOD3 - procollagen-lysine, 2-oxoglutarate 5-dioxygenase 3  DPH5 - diphthamide biosynthesis 5  TAF9B - taf9b rna polymerase ii, tata box binding protein (tbp)-associated factor, 31kda  SMAD4 - smad family member 4  SMAD5 - smad family member 5  P4HA2 - prolyl 4-hydroxylase, alpha polypeptide ii  SMAD7 - smad family member 7  PIGT - phosphatidylinositol glycan anchor biosynthesis, class t  MYLIP - myosin regulatory light chain interacting protein  NCK1 - nck adaptor protein 1  DPY19L3 - dpy-19-like 3 (c. elegans)  ABI2 - abl-interactor 2  TRIM28 - tripartite motif containing 28  SCG3 - secretogranin iii  TBK1 - tank-binding kinase 1  NCAM1 - neural cell adhesion molecule 1  NHLRC3 - nhl repeat containing 3  ZDHHC1 - zinc finger, dhhc-type containing 1  ZDHHC8 - zinc finger, dhhc-type containing 8  TRIM11 - tripartite motif containing 11  PPP1R12A - protein phosphatase 1, regulatory subunit 12a  TNK2 - tyrosine kinase, non-receptor, 2  SENP1 - sumo1/sentrin specific peptidase 1  RNF41 - ring finger protein 41  TFPT - tcf3 (e2a) fusion partner (in childhood leukemia)  PRMT3 - protein arginine methyltransferase 3  PSME3 - proteasome (prosome, macropain) activator subunit 3 (pa28 gamma; ki)  MYLK - myosin light chain kinase  TRIM13 - tripartite motif containing 13  URM1 - ubiquitin related modifier 1  TRIM8 - tripartite motif containing 8  USPL1 - ubiquitin specific peptidase like 1  TOPORS - topoisomerase i binding, arginine/serine-rich, e3 ubiquitin protein ligase  RAB1B - rab1b, member ras oncogene family  SEH1L - seh1-like (s. cerevisiae)  PTPRU - protein tyrosine phosphatase, receptor type, u  HUWE1 - hect, uba and wwe domain containing 1, e3 ubiquitin protein ligase  ALG5 - alg5, dolichyl-phosphate beta-glucosyltransferase  INO80B - ino80 complex subunit b  B3GALNT2 - beta-1,3-n-acetylgalactosaminyltransferase 2  CTDSP2 - ctd (carboxy-terminal domain, rna polymerase ii, polypeptide a) small phosphatase 2  ANAPC4 - anaphase promoting complex subunit 4  RNF170 - ring finger protein 170  FEM1B - fem-1 homolog b (c. elegans)  TRIM37 - tripartite motif containing 37  CDC42EP5 - cdc42 effector protein (rho gtpase binding) 5  SHARPIN - shank-associated rh domain interactor  PDIA6 - protein disulfide isomerase family a, member 6  AKT3 - v-akt murine thymoma viral oncogene homolog 3  ABI1 - abl-interactor 1  PPP4R1 - protein phosphatase 4, regulatory subunit 1  PDCD6 - programmed cell death 6  MTM1 - myotubularin 1  HDAC5 - histone deacetylase 5  HDAC6 - histone deacetylase 6  PARP2 - poly (adp-ribose) polymerase 2  RNF187 - ring finger protein 187  SH2D3C - sh2 domain containing 3c  MT3 - metallothionein 3  PIGK - phosphatidylinositol glycan anchor biosynthesis, class k  CCDC22 - coiled-coil domain containing 22  SAE1 - sumo1 activating enzyme subunit 1  PDIK1L - pdlim1 interacting kinase 1 like  RANBP9 - ran binding protein 9  UBA2 - ubiquitin-like modifier activating enzyme 2  JOSD1 - josephin domain containing 1  KANSL1L - kat8 regulatory nsl complex subunit 1-like  SUPT7L - suppressor of ty 7 (s. cerevisiae)-like  FBXO38 - f-box protein 38  GFPT2 - glutamine-fructose-6-phosphate transaminase 2  OXSR1 - oxidative stress responsive 1  COMMD1 - copper metabolism (murr1) domain containing 1  USP3 - ubiquitin specific peptidase 3  BABAM1 - brisc and brca1 a complex member 1  UBE2T - ubiquitin-conjugating enzyme e2t (putative)  USP15 - ubiquitin specific peptidase 15  TTL - tubulin tyrosine ligase  C1GALT1C1 - c1galt1-specific chaperone 1  SETD2 - set domain containing 2  RCE1 - rce1 homolog, prenyl protein protease (s. cerevisiae)  DUSP18 - dual specificity phosphatase 18  RBX1 - ring-box 1, e3 ubiquitin protein ligase  NUP153 - nucleoporin 153kda  MED12 - mediator complex subunit 12  MPI - mannose phosphate isomerase  WDSUB1 - wd repeat, sterile alpha motif and u-box domain containing 1  PSMD6 - proteasome (prosome, macropain) 26s subunit, non-atpase, 6  DTX3L - deltex 3-like (drosophila)  PJA2 - praja ring finger 2, e3 ubiquitin protein ligase  AREL1 - apoptosis resistant e3 ubiquitin protein ligase 1  SETDB1 - set domain, bifurcated 1  TLK1 - tousled-like kinase 1  RNF141 - ring finger protein 141  PTAR1 - protein prenyltransferase alpha subunit repeat containing 1  PPP4R2 - protein phosphatase 4, regulatory subunit 2  RNF38 - ring finger protein 38  XXYLT1 - xyloside xylosyltransferase 1  METTL21A - methyltransferase like 21a  KLHL21 - kelch-like family member 21  TNKS2 - tankyrase, trf1-interacting ankyrin-related adp-ribose polymerase 2  WDR61 - wd repeat domain 61  DDR2 - discoidin domain receptor tyrosine kinase 2  TOMM20 - translocase of outer mitochondrial membrane 20 homolog (yeast)  DAB2IP - dab2 interacting protein  NUP88 - nucleoporin 88kda  NUP98 - nucleoporin 98kda  NUCB1 - nucleobindin 1  ST8SIA3 - st8 alpha-n-acetyl-neuraminide alpha-2,8-sialyltransferase 3  RNF144A - ring finger protein 144a  ILKAP - integrin-linked kinase-associated serine/threonine phosphatase  DNAJC6 - dnaj (hsp40) homolog, subfamily c, member 6  SPATA2 - spermatogenesis associated 2  KLHL5 - kelch-like family member 5  DUSP16 - dual specificity phosphatase 16  RNF40 - ring finger protein 40, e3 ubiquitin protein ligase  SPOCK2 - sparc/osteonectin, cwcv and kazal-like domains proteoglycan (testican) 2  CUL7 - cullin 7  PAQR3 - progestin and adipoq receptor family member iii  NRAS - neuroblastoma ras viral (v-ras) oncogene homolog  NMT1 - n-myristoyltransferase 1  VPRBP - vpr (hiv-1) binding protein  RNF34 - ring finger protein 34, e3 ubiquitin protein ligase  FBXO11 - f-box protein 11  ULK2 - unc-51 like autophagy activating kinase 2  ERO1L - ero1-like (s. cerevisiae)  HERPUD1 - homocysteine-inducible, endoplasmic reticulum stress-inducible, ubiquitin-like domain member 1  SPSB1 - spla/ryanodine receptor domain and socs box containing 1  KCTD7 - potassium channel tetramerization domain containing 7  NAA15 - n(alpha)-acetyltransferase 15, nata auxiliary subunit  CNOT4 - ccr4-not transcription complex, subunit 4  HDAC4 - histone deacetylase 4  NFRKB - nuclear factor related to kappab binding protein  CXXC1 - cxxc finger protein 1  NFKBIA - nuclear factor of kappa light polypeptide gene enhancer in b-cells inhibitor, alpha  NFKB1 - nuclear factor of kappa light polypeptide gene enhancer in b-cells 1  KMT2B - lysine (k)-specific methyltransferase 2b  ST6GALNAC6 - st6 (alpha-n-acetyl-neuraminyl-2,3-beta-galactosyl-1,3)-n-acetylgalactosaminide alpha-2,6-sialyltransferase 6  PIK3R4 - phosphoinositide-3-kinase, regulatory subunit 4  SLK - ste20-like kinase  NKTR - natural killer-tumor recognition sequence  VCPIP1 - valosin containing protein (p97)/p47 complex interacting protein 1  USP34 - ubiquitin specific peptidase 34  HDAC9 - histone deacetylase 9  RNF145 - ring finger protein 145  SETD1A - set domain containing 1a  CCP110 - centriolar coiled coil protein 110kda  SOCS7 - suppressor of cytokine signaling 7  MINK1 - misshapen-like kinase 1  NEK3 - nima-related kinase 3  TTLL4 - tubulin tyrosine ligase-like family, member 4  SOCS5 - suppressor of cytokine signaling 5  NEFL - neurofilament, light polypeptide  NEK1 - nima-related kinase 1  NEDD8 - neural precursor cell expressed, developmentally down-regulated 8  CTR9 - ctr9, paf1/rna polymerase ii complex component  PPM1F - protein phosphatase, mg2+/mn2+ dependent, 1f  EPC1 - enhancer of polycomb homolog 1 (drosophila)  NFE2L2 - nuclear factor (erythroid-derived 2)-like 2  MORF4L2 - mortality factor 4 like 2  KLHL15 - kelch-like family member 15  NUP155 - nucleoporin 155kda  NF1 - neurofibromin 1  CDK5RAP3 - cdk5 regulatory subunit associated protein 3  DCAF8 - ddb1 and cul4 associated factor 8  EDEM1 - er degradation enhancer, mannosidase alpha-like 1  RAPGEF2 - rap guanine nucleotide exchange factor (gef) 2  EDEM3 - er degradation enhancer, mannosidase alpha-like 3  NUP93 - nucleoporin 93kda  KDM4A - lysine (k)-specific demethylase 4a  PHF14 - phd finger protein 14  COPS7A - cop9 signalosome subunit 7a  DZIP3 - daz interacting zinc finger protein 3 |
| GO:0044260 | cellular macromolecule metabolic process | 2.02E-12 | 1.65E-9 | 1.07 (10334,3294,6996,2383) | [+] Show genes  AFG3L2 - afg3 atpase family member 3-like 2 (s. cerevisiae)  EXOSC9 - exosome component 9  MSL3 - male-specific lethal 3 homolog (drosophila)  EXOSC10 - exosome component 10  OTUD5 - otu domain containing 5  AGAP3 - arfgap with gtpase domain, ankyrin repeat and ph domain 3  ZCCHC8 - zinc finger, cchc domain containing 8  PMS2 - pms2 postmeiotic segregation increased 2 (s. cerevisiae)  TADA1 - transcriptional adaptor 1  ABCA1 - atp-binding cassette, sub-family a (abc1), member 1  SEC61B - sec61 beta subunit  ABCF1 - atp-binding cassette, sub-family f (gcn20), member 1  OS9 - osteosarcoma amplified 9, endoplasmic reticulum lectin  ABL1 - c-abl oncogene 1, non-receptor tyrosine kinase  GALNT10 - udp-n-acetyl-alpha-d-galactosamine:polypeptide n-acetylgalactosaminyltransferase 10 (galnac-t10)  PML - promyelocytic leukemia  CNOT11 - ccr4-not transcription complex, subunit 11  PMM1 - phosphomannomutase 1  RAB40B - rab40b, member ras oncogene family  CKAP4 - cytoskeleton-associated protein 4  PMS1 - pms1 postmeiotic segregation increased 1 (s. cerevisiae)  ASCC3 - activating signal cointegrator 1 complex subunit 3  FTSJ3 - ftsj homolog 3 (e. coli)  UBE2Q1 - ubiquitin-conjugating enzyme e2q family member 1  PNRC2 - proline-rich nuclear receptor coactivator 2  BRD8 - bromodomain containing 8  PLOD2 - procollagen-lysine, 2-oxoglutarate 5-dioxygenase 2  MAN1A2 - mannosidase, alpha, class 1a, member 2  ACHE - acetylcholinesterase  LRRC40 - leucine rich repeat containing 40  PLOD1 - procollagen-lysine, 2-oxoglutarate 5-dioxygenase 1  RFFL - ring finger and fyve-like domain containing e3 ubiquitin protein ligase  ACTB - actin, beta  RNPS1 - rna binding protein s1, serine-rich domain  OTUB1 - otu domain, ubiquitin aldehyde binding 1  EHMT2 - euchromatic histone-lysine n-methyltransferase 2  SPIN1 - spindlin 1  KIF16B - kinesin family member 16b  MORF4L1 - mortality factor 4 like 1  ZDHHC7 - zinc finger, dhhc-type containing 7  POMGNT1 - protein o-linked mannose n-acetylglucosaminyltransferase 1 (beta 1,2-)  CHCHD1 - coiled-coil-helix-coiled-coil-helix domain containing 1  PPIC - peptidylprolyl isomerase c (cyclophilin c)  PPID - peptidylprolyl isomerase d  ACTL6A - actin-like 6a  PPEF1 - protein phosphatase, ef-hand calcium binding domain 1  USP20 - ubiquitin specific peptidase 20  PPIA - peptidylprolyl isomerase a (cyclophilin a)  USP19 - ubiquitin specific peptidase 19  PPIB - peptidylprolyl isomerase b (cyclophilin b)  ACVR2A - activin a receptor, type iia  ACVRL1 - activin a receptor type ii-like 1  ACVR1 - activin a receptor, type i  ACVR1B - activin a receptor, type ib  NDUFAF7 - nadh dehydrogenase (ubiquinone) complex i, assembly factor 7  ADAM10 - adam metallopeptidase domain 10  MRPS30 - mitochondrial ribosomal protein s30  MED1 - mediator complex subunit 1  PPARD - peroxisome proliferator-activated receptor delta  POLRMT - polymerase (rna) mitochondrial (dna directed)  POLR2K - polymerase (rna) ii (dna directed) polypeptide k, 7.0kda  ADD1 - adducin 1 (alpha)  ADH5 - alcohol dehydrogenase 5 (class iii), chi polypeptide  PARP1 - poly (adp-ribose) polymerase 1  POLH - polymerase (dna directed), eta  ADPRH - adp-ribosylarginine hydrolase  PPP1R13L - protein phosphatase 1, regulatory subunit 13 like  NOP10 - nop10 ribonucleoprotein  TNFRSF19 - tumor necrosis factor receptor superfamily, member 19  POLD2 - polymerase (dna directed), delta 2, accessory subunit  POLD1 - polymerase (dna directed), delta 1, catalytic subunit  CHST12 - carbohydrate (chondroitin 4) sulfotransferase 12  POLR2G - polymerase (rna) ii (dna directed) polypeptide g  POLR2F - polymerase (rna) ii (dna directed) polypeptide f  POLR2E - polymerase (rna) ii (dna directed) polypeptide e, 25kda  SMPD3 - sphingomyelin phosphodiesterase 3, neutral membrane (neutral sphingomyelinase ii)  POLR2D - polymerase (rna) ii (dna directed) polypeptide d  POLR2C - polymerase (rna) ii (dna directed) polypeptide c, 33kda  POLR2B - polymerase (rna) ii (dna directed) polypeptide b, 140kda  RUVBL2 - ruvb-like 2 (e. coli)  PARP4 - poly (adp-ribose) polymerase family, member 4  POLR2A - polymerase (rna) ii (dna directed) polypeptide a, 220kda  AP2A2 - adaptor-related protein complex 2, alpha 2 subunit  AP2A1 - adaptor-related protein complex 2, alpha 1 subunit  AP2B1 - adaptor-related protein complex 2, beta 1 subunit  INTS9 - integrator complex subunit 9  ATE1 - arginyltransferase 1  PRKAB1 - protein kinase, amp-activated, beta 1 non-catalytic subunit  RCOR3 - rest corepressor 3  PRKACB - protein kinase, camp-dependent, catalytic, beta  PRKACA - protein kinase, camp-dependent, catalytic, alpha  NUP133 - nucleoporin 133kda  ADRBK2 - adrenergic, beta, receptor kinase 2  ADRBK1 - adrenergic, beta, receptor kinase 1  PRIM2 - primase, dna, polypeptide 2 (58kda)  NUPL2 - nucleoporin like 2  PRIM1 - primase, dna, polypeptide 1 (49kda)  EDEM2 - er degradation enhancer, mannosidase alpha-like 2  POLR3A - polymerase (rna) iii (dna directed) polypeptide a, 155kda  CHFR - checkpoint with forkhead and ring finger domains, e3 ubiquitin protein ligase  PRDM4 - pr domain containing 4  PPP3R1 - protein phosphatase 3, regulatory subunit b, alpha  CIT - citron (rho-interacting, serine/threonine kinase 21)  ATF7IP - activating transcription factor 7 interacting protein  PPP5C - protein phosphatase 5, catalytic subunit  PPP6C - protein phosphatase 6, catalytic subunit  UHRF2 - ubiquitin-like with phd and ring finger domains 2, e3 ubiquitin protein ligase  PPT1 - palmitoyl-protein thioesterase 1  AGA - aspartylglucosaminidase  PPP2R5D - protein phosphatase 2, regulatory subunit b', delta  PPP2R5C - protein phosphatase 2, regulatory subunit b', gamma  PPP2R5B - protein phosphatase 2, regulatory subunit b', beta  CSGALNACT1 - chondroitin sulfate n-acetylgalactosaminyltransferase 1  AKT1 - v-akt murine thymoma viral oncogene homolog 1  PPP4C - protein phosphatase 4, catalytic subunit  PPP2R5E - protein phosphatase 2, regulatory subunit b', epsilon isoform  PPP2R2A - protein phosphatase 2, regulatory subunit b, alpha  CMTR2 - cap methyltransferase 2  PPP2R1B - protein phosphatase 2, regulatory subunit a, beta  RIOK2 - rio kinase 2  PPP2R1A - protein phosphatase 2, regulatory subunit a, alpha  WWP2 - ww domain containing e3 ubiquitin protein ligase 2  DIS3L - dis3 mitotic control homolog (s. cerevisiae)-like  PPP2R2C - protein phosphatase 2, regulatory subunit b, gamma  PPP2R2B - protein phosphatase 2, regulatory subunit b, beta  PPP1R8 - protein phosphatase 1, regulatory subunit 8  TRIOBP - trio and f-actin binding protein  PPP2CA - protein phosphatase 2, catalytic subunit, alpha isozyme  PPP2CB - protein phosphatase 2, catalytic subunit, beta isozyme  DIDO1 - death inducer-obliterator 1  MBD5 - methyl-cpg binding domain protein 5  DUSP14 - dual specificity phosphatase 14  TOPBP1 - topoisomerase (dna) ii binding protein 1  RMI2 - recq mediated genome instability 2  PPM1G - protein phosphatase, mg2+/mn2+ dependent, 1g  PPP1R2 - protein phosphatase 1, regulatory (inhibitor) subunit 2  HSF2BP - heat shock transcription factor 2 binding protein  PPP1CC - protein phosphatase 1, catalytic subunit, gamma isozyme  ESCO1 - establishment of sister chromatid cohesion n-acetyltransferase 1  KANSL3 - kat8 regulatory nsl complex subunit 3  TDRKH - tudor and kh domain containing  IWS1 - iws1 homolog (s. cerevisiae)  SCYL2 - scy1-like 2 (s. cerevisiae)  LIAS - lipoic acid synthetase  NPLOC4 - nuclear protein localization 4 homolog (s. cerevisiae)  AMFR - autocrine motility factor receptor, e3 ubiquitin protein ligase  DNAJC3 - dnaj (hsp40) homolog, subfamily c, member 3  RNF126 - ring finger protein 126  PAPD7 - pap associated domain containing 7  MAP2K5 - mitogen-activated protein kinase kinase 5  MAP2K7 - mitogen-activated protein kinase kinase 7  INTS8 - integrator complex subunit 8  PIM2 - pim-2 oncogene  EIF2AK2 - eukaryotic translation initiation factor 2-alpha kinase 2  GALNT13 - udp-n-acetyl-alpha-d-galactosamine:polypeptide n-acetylgalactosaminyltransferase 13 (galnac-t13)  RAB31 - rab31, member ras oncogene family  MAPK13 - mitogen-activated protein kinase 13  PIGV - phosphatidylinositol glycan anchor biosynthesis, class v  RNF157 - ring finger protein 157  MAP2K1 - mitogen-activated protein kinase kinase 1  MAP2K2 - mitogen-activated protein kinase kinase 2  MAPK10 - mitogen-activated protein kinase 10  MAPK9 - mitogen-activated protein kinase 9  MAPK11 - mitogen-activated protein kinase 11  TRIM59 - tripartite motif containing 59  METAP2 - methionyl aminopeptidase 2  MAPK8 - mitogen-activated protein kinase 8  MAPK7 - mitogen-activated protein kinase 7  GCN1L1 - gcn1 general control of amino-acid synthesis 1-like 1 (yeast)  POLR3E - polymerase (rna) iii (dna directed) polypeptide e (80kd)  MAPK3 - mitogen-activated protein kinase 3  MAPK1 - mitogen-activated protein kinase 1  COPS6 - cop9 signalosome subunit 6  PRKCSH - protein kinase c substrate 80k-h  PRKD1 - protein kinase d1  NDC1 - ndc1 transmembrane nucleoporin  CCDC88A - coiled-coil domain containing 88a  PKN2 - protein kinase n2  NT5C3B - 5'-nucleotidase, cytosolic iiib  PRKCH - protein kinase c, eta  TLK2 - tousled-like kinase 2  PRKCI - protein kinase c, iota  POLR3B - polymerase (rna) iii (dna directed) polypeptide b  PRKCE - protein kinase c, epsilon  PRKCB - protein kinase c, beta  PRKCD - protein kinase c, delta  PRKCA - protein kinase c, alpha  PRKAR1B - protein kinase, camp-dependent, regulatory, type i, beta  NSUN5 - nop2/sun domain family, member 5  YEATS2 - yeats domain containing 2  PRKAG1 - protein kinase, amp-activated, gamma 1 non-catalytic subunit  APEX1 - apex nuclease (multifunctional dna repair enzyme) 1  MTPAP - mitochondrial poly(a) polymerase  UBR7 - ubiquitin protein ligase e3 component n-recognin 7 (putative)  DUSP12 - dual specificity phosphatase 12  KLHL2 - kelch-like family member 2  XIAP - x-linked inhibitor of apoptosis  BIRC3 - baculoviral iap repeat containing 3  BIRC2 - baculoviral iap repeat containing 2  PARN - poly(a)-specific ribonuclease  RFWD3 - ring finger and wd repeat domain 3  APLP2 - amyloid beta (a4) precursor-like protein 2  TREX1 - three prime repair exonuclease 1  MGAT4B - mannosyl (alpha-1,3-)-glycoprotein beta-1,4-n-acetylglucosaminyltransferase, isozyme b  PRMT6 - protein arginine methyltransferase 6  MSL2 - male-specific lethal 2 homolog (drosophila)  MAN1B1 - mannosidase, alpha, class 1b, member 1  MRPS10 - mitochondrial ribosomal protein s10  PCBP2 - poly(rc) binding protein 2  INTS10 - integrator complex subunit 10  DCUN1D3 - dcn1, defective in cullin neddylation 1, domain containing 3  SMG8 - smg8 nonsense mediated mrna decay factor  APC - adenomatous polyposis coli  RNF220 - ring finger protein 220  APBB1 - amyloid beta (a4) precursor protein-binding, family b, member 1 (fe65)  GALNT6 - udp-n-acetyl-alpha-d-galactosamine:polypeptide n-acetylgalactosaminyltransferase 6 (galnac-t6)  RIF1 - rap1 interacting factor homolog (yeast)  POLG2 - polymerase (dna directed), gamma 2, accessory subunit  RNF139 - ring finger protein 139  DPY19L4 - dpy-19-like 4 (c. elegans)  P4HA1 - prolyl 4-hydroxylase, alpha polypeptide i  PEBP1 - phosphatidylethanolamine binding protein 1  TRIM23 - tripartite motif containing 23  CNTD1 - cyclin n-terminal domain containing 1  RPRD1A - regulation of nuclear pre-mrna domain containing 1a  ARF4 - adp-ribosylation factor 4  MAP1S - microtubule-associated protein 1s  POLI - polymerase (dna directed) iota  IRAK3 - interleukin-1 receptor-associated kinase 3  AKAP13 - a kinase (prka) anchor protein 13  SETD5 - set domain containing 5  APP - amyloid beta (a4) precursor protein  PAK2 - p21 protein (cdc42/rac)-activated kinase 2  LEPREL1 - leprecan-like 1  PAK3 - p21 protein (cdc42/rac)-activated kinase 3  MRPL3 - mitochondrial ribosomal protein l3  PAM - peptidylglycine alpha-amidating monooxygenase  CANT1 - calcium activated nucleotidase 1  ARNTL - aryl hydrocarbon receptor nuclear translocator-like  PDGFA - platelet-derived growth factor alpha polypeptide  CELF3 - cugbp, elav-like family member 3  MAP4K5 - mitogen-activated protein kinase kinase kinase kinase 5  NSMCE2 - non-smc element 2, mms21 homolog (s. cerevisiae)  SUPT16H - suppressor of ty 16 homolog (s. cerevisiae)  PDGFRB - platelet-derived growth factor receptor, beta polypeptide  ARRB2 - arrestin, beta 2  ARRB1 - arrestin, beta 1  PDGFRA - platelet-derived growth factor receptor, alpha polypeptide  PDGFB - platelet-derived growth factor beta polypeptide  SAMD4B - sterile alpha motif domain containing 4b  PDPK1 - 3-phosphoinositide dependent protein kinase-1  RHOA - ras homolog family member a  WDYHV1 - wdyhv motif containing 1  PDK3 - pyruvate dehydrogenase kinase, isozyme 3  FSTL1 - follistatin-like 1  BAZ2A - bromodomain adjacent to zinc finger domain, 2a  PCNA - proliferating cell nuclear antigen  LSM6 - lsm6 homolog, u6 small nuclear rna associated (s. cerevisiae)  FICD - fic domain containing  PCMT1 - protein-l-isoaspartate (d-aspartate) o-methyltransferase  PLEKHJ1 - pleckstrin homology domain containing, family j member 1  MAGOHB - mago-nashi homolog b (drosophila)  MTFMT - mitochondrial methionyl-trna formyltransferase  CDK16 - cyclin-dependent kinase 16  CDK17 - cyclin-dependent kinase 17  LEO1 - leo1, paf1/rna polymerase ii complex component, homolog (s. cerevisiae)  ASPH - aspartate beta-hydroxylase  FANCL - fanconi anemia, complementation group l  ERLIN2 - er lipid raft associated 2  PWP1 - pwp1 homolog (s. cerevisiae)  SRBD1 - s1 rna binding domain 1  WRAP53 - wd repeat containing, antisense to tp53  SOCS4 - suppressor of cytokine signaling 4  THAP1 - thap domain containing, apoptosis associated protein 1  ANKZF1 - ankyrin repeat and zinc finger domain containing 1  KAT7 - k(lysine) acetyltransferase 7  DENND3 - denn/madd domain containing 3  PGGT1B - protein geranylgeranyltransferase type i, beta subunit  PGM3 - phosphoglucomutase 3  CPPED1 - calcineurin-like phosphoesterase domain containing 1  ZBTB1 - zinc finger and btb domain containing 1  ZNF365 - zinc finger protein 365  UBOX5 - u-box domain containing 5  RNF180 - ring finger protein 180  UEVLD - uev and lactate/malate dehyrogenase domains  FBXW7 - f-box and wd repeat domain containing 7, e3 ubiquitin protein ligase  DIS3 - dis3 mitotic control homolog (s. cerevisiae)  RNF121 - ring finger protein 121  ZHX2 - zinc fingers and homeoboxes 2  PHF2 - phd finger protein 2  LRRK2 - leucine-rich repeat kinase 2  DNAJC24 - dnaj (hsp40) homolog, subfamily c, member 24  AP5S1 - adaptor-related protein complex 5, sigma 1 subunit  SPPL3 - signal peptide peptidase like 3  PEX12 - peroxisomal biogenesis factor 12  SLC35C1 - solute carrier family 35 (gdp-fucose transporter), member c1  PEX13 - peroxisomal biogenesis factor 13  ATP7A - atpase, cu++ transporting, alpha polypeptide  PEX14 - peroxisomal biogenesis factor 14  PENK - proenkephalin  LMTK2 - lemur tyrosine kinase 2  ICK - intestinal cell (mak-like) kinase  ALKBH2 - alkb, alkylation repair homolog 2 (e. coli)  WDR33 - wd repeat domain 33  PPM1E - protein phosphatase, mg2+/mn2+ dependent, 1e  AAK1 - ap2 associated kinase 1  COPRS - coordinator of prmt5, differentiation stimulator  PKD1 - polycystic kidney disease 1 (autosomal dominant)  UBA6 - ubiquitin-like modifier activating enzyme 6  USP40 - ubiquitin specific peptidase 40  RASA3 - ras p21 protein activator 3  PHLDA1 - pleckstrin homology-like domain, family a, member 1  BACH1 - btb and cnc homology 1, basic leucine zipper transcription factor 1  PIN1 - peptidylprolyl cis/trans isomerase, nima-interacting 1  DPH3 - diphthamide biosynthesis 3  EXD2 - exonuclease 3'-5' domain containing 2  PIK3R1 - phosphoinositide-3-kinase, regulatory subunit 1 (alpha)  PLAT - plasminogen activator, tissue  AXL - axl receptor tyrosine kinase  ASXL2 - additional sex combs like 2 (drosophila)  PCMTD2 - protein-l-isoaspartate (d-aspartate) o-methyltransferase domain containing 2  XRN2 - 5'-3' exoribonuclease 2  ATRX - alpha thalassemia/mental retardation syndrome x-linked  PEF1 - penta-ef-hand domain containing 1  PKNOX1 - pbx/knotted 1 homeobox 1  CASC3 - cancer susceptibility candidate 3  PSTK - phosphoseryl-trna kinase  OGFOD1 - 2-oxoglutarate and iron-dependent oxygenase domain containing 1  EXOSC8 - exosome component 8  RNF13 - ring finger protein 13  PHF10 - phd finger protein 10  NUDT15 - nudix (nucleoside diphosphate linked moiety x)-type motif 15  BCR - breakpoint cluster region  FKBP9 - fk506 binding protein 9, 63 kda  FBXW12 - f-box and wd repeat domain containing 12  PHKG2 - phosphorylase kinase, gamma 2 (testis)  STK38 - serine/threonine kinase 38  MRGBP - mrg/morf4l binding protein  SUMF1 - sulfatase modifying factor 1  RHOT1 - ras homolog family member t1  PIK3CA - phosphatidylinositol-4,5-bisphosphate 3-kinase, catalytic subunit alpha  PIM1 - pim-1 oncogene  BCL2 - b-cell cll/lymphoma 2  UBE2W - ubiquitin-conjugating enzyme e2w (putative)  MGAT4A - mannosyl (alpha-1,3-)-glycoprotein beta-1,4-n-acetylglucosaminyltransferase, isozyme a  PIK3C3 - phosphatidylinositol 3-kinase, catalytic subunit type 3  PIGH - phosphatidylinositol glycan anchor biosynthesis, class h  LYPLA2 - lysophospholipase ii  CCND1 - cyclin d1  PARK7 - parkinson protein 7  B4GALT7 - xylosylprotein beta 1,4-galactosyltransferase, polypeptide 7  PNKP - polynucleotide kinase 3'-phosphatase  PGM2 - phosphoglucomutase 2  PIGF - phosphatidylinositol glycan anchor biosynthesis, class f  QRSL1 - glutaminyl-trna synthase (glutamine-hydrolyzing)-like 1  TRIM27 - tripartite motif containing 27  BNIP3L - bcl2/adenovirus e1b 19kda interacting protein 3-like  RFC5 - replication factor c (activator 1) 5, 36.5kda  CWC27 - cwc27 spliceosome-associated protein homolog (s. cerevisiae)  RFC2 - replication factor c (activator 1) 2, 40kda  RFC3 - replication factor c (activator 1) 3, 38kda  MAEA - macrophage erythroblast attacher  BRAF - v-raf murine sarcoma viral oncogene homolog b  BCKDK - branched chain ketoacid dehydrogenase kinase  WHSC1L1 - wolf-hirschhorn syndrome candidate 1-like 1  CDKL4 - cyclin-dependent kinase-like 4  RELA - v-rel avian reticuloendotheliosis viral oncogene homolog a  SNAPC5 - small nuclear rna activating complex, polypeptide 5, 19kda  BCOR - bcl6 corepressor  RECQL - recq protein-like (dna helicase q1-like)  ZFP36L2 - zfp36 ring finger protein-like 2  ZFP36L1 - zfp36 ring finger protein-like 1  PAK4 - p21 protein (cdc42/rac)-activated kinase 4  DCAF16 - ddb1 and cul4 associated factor 16  RET - ret proto-oncogene  DUSP15 - dual specificity phosphatase 15  SH3RF3 - sh3 domain containing ring finger 3  DPF2 - d4, zinc and double phd fingers family 2  ALKBH5 - alkb, alkylation repair homolog 5 (e. coli)  NSUN2 - nop2/sun rna methyltransferase family, member 2  UPF1 - upf1 regulator of nonsense transcripts homolog (yeast)  GALNTL6 - udp-n-acetyl-alpha-d-galactosamine:polypeptide n-acetylgalactosaminyltransferase-like 6  RLF - rearranged l-myc fusion  RING1 - ring finger protein 1  SNRK - snf related kinase  PIGG - phosphatidylinositol glycan anchor biosynthesis, class g  TMEM5 - transmembrane protein 5  FBLL1 - fibrillarin-like 1  BMI1 - bmi1 polycomb ring finger oncogene  PCGF3 - polycomb group ring finger 3  BMPR1A - bone morphogenetic protein receptor, type ia  BMPR2 - bone morphogenetic protein receptor, type ii (serine/threonine kinase)  BMPR1B - bone morphogenetic protein receptor, type ib  FBXL12 - f-box and leucine-rich repeat protein 12  ABCA7 - atp-binding cassette, sub-family a (abc1), member 7  PSMD14 - proteasome (prosome, macropain) 26s subunit, non-atpase, 14  MRPL49 - mitochondrial ribosomal protein l49  TRPM7 - transient receptor potential cation channel, subfamily m, member 7  RNF19B - ring finger protein 19b  CTDSPL - ctd (carboxy-terminal domain, rna polymerase ii, polypeptide a) small phosphatase-like  TMEM258 - transmembrane protein 258  GIN1 - gypsy retrotransposon integrase 1  UHMK1 - u2af homology motif (uhm) kinase 1  RNASE6 - ribonuclease, rnase a family, k6  MSLN - mesothelin  RNF4 - ring finger protein 4  DCAF7 - ddb1 and cul4 associated factor 7  RNF2 - ring finger protein 2  GPHN - gephyrin  RNF6 - ring finger protein (c3h2c3 type) 6  GATAD2A - gata zinc finger domain containing 2a  QPCTL - glutaminyl-peptide cyclotransferase-like  ROCK1 - rho-associated, coiled-coil containing protein kinase 1  LYPLAL1 - lysophospholipase-like 1  RNF111 - ring finger protein 111  DNAJB12 - dnaj (hsp40) homolog, subfamily b, member 12  TET2 - tet methylcytosine dioxygenase 2  STAM2 - signal transducing adaptor molecule (sh3 domain and itam motif) 2  TRPM4 - transient receptor potential cation channel, subfamily m, member 4  TYW3 - trna-yw synthesizing protein 3 homolog (s. cerevisiae)  RAMP2 - receptor (g protein-coupled) activity modifying protein 2  RAMP3 - receptor (g protein-coupled) activity modifying protein 3  RAMP1 - receptor (g protein-coupled) activity modifying protein 1  ZMPSTE24 - zinc metallopeptidase ste24  STUB1 - stip1 homology and u-box containing protein 1, e3 ubiquitin protein ligase  C5AR1 - complement component 5a receptor 1  TCEANC2 - transcription elongation factor a (sii) n-terminal and central domain containing 2  UBE4B - ubiquitination factor e4b  TRIM44 - tripartite motif containing 44  TAB1 - tgf-beta activated kinase 1/map3k7 binding protein 1  PPIE - peptidylprolyl isomerase e (cyclophilin e)  CAPN1 - calpain 1, (mu/i) large subunit  MCRS1 - microspherule protein 1  MRPL20 - mitochondrial ribosomal protein l20  CAMLG - calcium modulating ligand  RPL17 - ribosomal protein l17  VPS37C - vacuolar protein sorting 37 homolog c (s. cerevisiae)  RPL15 - ribosomal protein l15  CAMK2B - calcium/calmodulin-dependent protein kinase ii beta  TRMT12 - trna methyltransferase 12 homolog (s. cerevisiae)  RPL11 - ribosomal protein l11  RPL9 - ribosomal protein l9  PTCD3 - pentatricopeptide repeat domain 3  FKBP14 - fk506 binding protein 14, 22 kda  RPL7 - ribosomal protein l7  PIBF1 - progesterone immunomodulatory binding factor 1  PPIH - peptidylprolyl isomerase h (cyclophilin h)  SLC30A9 - solute carrier family 30 (zinc transporter), member 9  CASP3 - caspase 3, apoptosis-related cysteine peptidase  RPL3 - ribosomal protein l3  B3GALT6 - udp-gal:betagal beta 1,3-galactosyltransferase polypeptide 6  RPA3 - replication protein a3, 14kda  MAD2L2 - mad2 mitotic arrest deficient-like 2 (yeast)  RPA1 - replication protein a1, 70kda  RPA2 - replication protein a2, 32kda  RPS4X - ribosomal protein s4, x-linked  CAD - carbamoyl-phosphate synthetase 2, aspartate transcarbamylase, and dihydroorotase  RPS4Y1 - ribosomal protein s4, y-linked 1  RPN2 - ribophorin ii  RPN1 - ribophorin i  UBE2E3 - ubiquitin-conjugating enzyme e2e 3  HERC6 - hect and rld domain containing e3 ubiquitin protein ligase family member 6  RMND1 - required for meiotic nuclear division 1 homolog (s. cerevisiae)  DDR1 - discoidin domain receptor tyrosine kinase 1  TRIM38 - tripartite motif containing 38  TADA3 - transcriptional adaptor 3  CALU - calumenin  STK25 - serine/threonine kinase 25  RPL32 - ribosomal protein l32  GID8 - gid complex subunit 8  LRRC41 - leucine rich repeat containing 41  CRTAP - cartilage associated protein  RPS23 - ribosomal protein s23  CCNT2 - cyclin t2  PINX1 - pin2/terf1 interacting, telomerase inhibitor 1  CLN6 - ceroid-lipofuscinosis, neuronal 6, late infantile, variant  NPM3 - nucleophosmin/nucleoplasmin 3  INO80C - ino80 complex subunit c  CCNT1 - cyclin t1  CCNH - cyclin h  TMEM132A - transmembrane protein 132a  ANAPC10 - anaphase promoting complex subunit 10  SWSAP1 - swim-type zinc finger 7 associated protein 1  SSH3 - slingshot protein phosphatase 3  RPS15 - ribosomal protein s15  RPS12 - ribosomal protein s12  TIPIN - timeless interacting protein  CD9 - cd9 molecule  RPS6KA2 - ribosomal protein s6 kinase, 90kda, polypeptide 2  TRNAU1AP - trna selenocysteine 1 associated protein 1  NRBP2 - nuclear receptor binding protein 2  RPS6KA1 - ribosomal protein s6 kinase, 90kda, polypeptide 1  PARP16 - poly (adp-ribose) polymerase family, member 16  RPS6KB1 - ribosomal protein s6 kinase, 70kda, polypeptide 1  RPS6KB2 - ribosomal protein s6 kinase, 70kda, polypeptide 2  RNF125 - ring finger protein 125, e3 ubiquitin protein ligase  CBFA2T3 - core-binding factor, runt domain, alpha subunit 2; translocated to, 3  CBFB - core-binding factor, beta subunit  CBL - cbl proto-oncogene, e3 ubiquitin protein ligase  MRPL10 - mitochondrial ribosomal protein l10  PIAS3 - protein inhibitor of activated stat, 3  CAV1 - caveolin 1, caveolae protein, 22kda  KANSL2 - kat8 regulatory nsl complex subunit 2  ADPRHL2 - adp-ribosylhydrolase like 2  ZSWIM7 - zinc finger, swim-type containing 7  UBE2R2 - ubiquitin-conjugating enzyme e2r 2  RBM14 - rna binding motif protein 14  TRMT10C - trna methyltransferase 10 homolog c (s. cerevisiae)  PRMT5 - protein arginine methyltransferase 5  CHTF8 - ctf8, chromosome transmission fidelity factor 8 homolog (s. cerevisiae)  RRM1 - ribonucleotide reductase m1  TRIM3 - tripartite motif containing 3  CDK11B - cyclin-dependent kinase 11b  CDC5L - cell division cycle 5-like  TGOLN2 - trans-golgi network protein 2  STAMBP - stam binding protein  POLR3F - polymerase (rna) iii (dna directed) polypeptide f, 39 kda  PSAP - prosaposin  CDH2 - cadherin 2, type 1, n-cadherin (neuronal)  ERLIN1 - er lipid raft associated 1  KCTD5 - potassium channel tetramerization domain containing 5  CDC27 - cell division cycle 27  ANKIB1 - ankyrin repeat and ibr domain containing 1  POMT1 - protein-o-mannosyltransferase 1  MRPS21 - mitochondrial ribosomal protein s21  PSEN1 - presenilin 1  PSEN2 - presenilin 2 (alzheimer disease 4)  TOLLIP - toll interacting protein  USP16 - ubiquitin specific peptidase 16  PSKH1 - protein serine kinase h1  PSMA1 - proteasome (prosome, macropain) subunit, alpha type, 1  RNF216 - ring finger protein 216  PSMA2 - proteasome (prosome, macropain) subunit, alpha type, 2  CD81 - cd81 molecule  CAMKK2 - calcium/calmodulin-dependent protein kinase kinase 2, beta  PSMB1 - proteasome (prosome, macropain) subunit, beta type, 1  NPRL2 - nitrogen permease regulator-like 2 (s. cerevisiae)  PSMA5 - proteasome (prosome, macropain) subunit, alpha type, 5  PSMA3 - proteasome (prosome, macropain) subunit, alpha type, 3  PSMB10 - proteasome (prosome, macropain) subunit, beta type, 10  UBLCP1 - ubiquitin-like domain containing ctd phosphatase 1  FAM173B - family with sequence similarity 173, member b  TAF6L - taf6-like rna polymerase ii, p300/cbp-associated factor (pcaf)-associated factor, 65kda  PSMC6 - proteasome (prosome, macropain) 26s subunit, atpase, 6  PSMD1 - proteasome (prosome, macropain) 26s subunit, non-atpase, 1  PSMC4 - proteasome (prosome, macropain) 26s subunit, atpase, 4  POLR3G - polymerase (rna) iii (dna directed) polypeptide g (32kd)  PSMC2 - proteasome (prosome, macropain) 26s subunit, atpase, 2  DPM3 - dolichyl-phosphate mannosyltransferase polypeptide 3  PSMD9 - proteasome (prosome, macropain) 26s subunit, non-atpase, 9  GAR1 - gar1 ribonucleoprotein  PSMD10 - proteasome (prosome, macropain) 26s subunit, non-atpase, 10  TSPAN33 - tetraspanin 33  PSMD7 - proteasome (prosome, macropain) 26s subunit, non-atpase, 7  DNAJC10 - dnaj (hsp40) homolog, subfamily c, member 10  PSMD8 - proteasome (prosome, macropain) 26s subunit, non-atpase, 8  PSMD5 - proteasome (prosome, macropain) 26s subunit, non-atpase, 5  TERF2IP - telomeric repeat binding factor 2, interacting protein  PSMD2 - proteasome (prosome, macropain) 26s subunit, non-atpase, 2  PSMD12 - proteasome (prosome, macropain) 26s subunit, non-atpase, 12  ATG7 - autophagy related 7  PSMD13 - proteasome (prosome, macropain) 26s subunit, non-atpase, 13  LEPREL2 - leprecan-like 2  RNASEH2A - ribonuclease h2, subunit a  KLHL17 - kelch-like family member 17  CETN2 - centrin, ef-hand protein, 2  PTEN - phosphatase and tensin homolog  JMY - junction mediating and regulatory protein, p53 cofactor  SETD4 - set domain containing 4  PRDX4 - peroxiredoxin 4  CFL1 - cofilin 1 (non-muscle)  CIB1 - calcium and integrin binding 1 (calmyrin)  CHRAC1 - chromatin accessibility complex 1  KAT5 - k(lysine) acetyltransferase 5  PTK2 - protein tyrosine kinase 2  PTK7 - protein tyrosine kinase 7  CDK5 - cyclin-dependent kinase 5  CDK4 - cyclin-dependent kinase 4  PTMA - prothymosin, alpha  TWF1 - twinfilin actin-binding protein 1  CDK9 - cyclin-dependent kinase 9  CDK8 - cyclin-dependent kinase 8  CDK7 - cyclin-dependent kinase 7  SPOPL - speckle-type poz protein-like  CDKN2D - cyclin-dependent kinase inhibitor 2d (p19, inhibits cdk4)  PTPN3 - protein tyrosine phosphatase, non-receptor type 3  PTPN2 - protein tyrosine phosphatase, non-receptor type 2  PTPN1 - protein tyrosine phosphatase, non-receptor type 1  PTPN9 - protein tyrosine phosphatase, non-receptor type 9  PPM1M - protein phosphatase, mg2+/mn2+ dependent, 1m  HTATIP2 - hiv-1 tat interactive protein 2, 30kda  PTPN11 - protein tyrosine phosphatase, non-receptor type 11  PTPRA - protein tyrosine phosphatase, receptor type, a  PTPN12 - protein tyrosine phosphatase, non-receptor type 12  PTPN13 - protein tyrosine phosphatase, non-receptor type 13 (apo-1/cd95 (fas)-associated phosphatase)  PTPRD - protein tyrosine phosphatase, receptor type, d  PTPRB - protein tyrosine phosphatase, receptor type, b  SLC35A1 - solute carrier family 35 (cmp-sialic acid transporter), member a1  ARFGEF2 - adp-ribosylation factor guanine nucleotide-exchange factor 2 (brefeldin a-inhibited)  PTPRG - protein tyrosine phosphatase, receptor type, g  CDK2 - cyclin-dependent kinase 2  PTPRE - protein tyrosine phosphatase, receptor type, e  PTPRF - protein tyrosine phosphatase, receptor type, f  WDR4 - wd repeat domain 4  RAD1 - rad1 homolog (s. pombe)  PURA - purine-rich element binding protein a  PDP1 - pyruvate dehyrogenase phosphatase catalytic subunit 1  GTPBP2 - gtp binding protein 2  METTL6 - methyltransferase like 6  P4HTM - prolyl 4-hydroxylase, transmembrane (endoplasmic reticulum)  NUDT16 - nudix (nucleoside diphosphate linked moiety x)-type motif 16  PTPRR - protein tyrosine phosphatase, receptor type, r  PTPRS - protein tyrosine phosphatase, receptor type, s  PTPRK - protein tyrosine phosphatase, receptor type, k  PTPRM - protein tyrosine phosphatase, receptor type, m  PTPRN - protein tyrosine phosphatase, receptor type, n  PLK2 - polo-like kinase 2  PYGB - phosphorylase, glycogen; brain  NUP50 - nucleoporin 50kda  CHML - choroideremia-like (rab escort protein 2)  PEX2 - peroxisomal biogenesis factor 2  CHM - choroideremia (rab escort protein 1)  KDM5B - lysine (k)-specific demethylase 5b  CHCHD4 - coiled-coil-helix-coiled-coil-helix domain containing 4  CHGB - chromogranin b (secretogranin 1)  RAB6A - rab6a, member ras oncogene family  MXRA8 - matrix-remodelling associated 8  MAP4K2 - mitogen-activated protein kinase kinase kinase kinase 2  RAB5A - rab5a, member ras oncogene family  RAB3A - rab3a, member ras oncogene family  MTERFD2 - mterf domain containing 2  FRS2 - fibroblast growth factor receptor substrate 2  RAB3B - rab3b, member ras oncogene family  RAB2A - rab2a, member ras oncogene family  RAB1A - rab1a, member ras oncogene family  METTL2A - methyltransferase like 2a  RAD21 - rad21 homolog (s. pombe)  RAD17 - rad17 homolog (s. pombe)  PARP14 - poly (adp-ribose) polymerase family, member 14  PAF1 - paf1, rna polymerase ii associated factor, homolog (s. cerevisiae)  FBXL19 - f-box and leucine-rich repeat protein 19  MSL1 - male-specific lethal 1 homolog (drosophila)  INO80 - ino80 complex subunit  NDFIP2 - nedd4 family interacting protein 2  RC3H2 - ring finger and ccch-type domains 2  RANBP2 - ran binding protein 2  RANGAP1 - ran gtpase activating protein 1  CLTC - clathrin, heavy chain (hc)  MIER2 - mesoderm induction early response 1, family member 2  MOK - mok protein kinase  USP53 - ubiquitin specific peptidase 53  B3GNT2 - udp-glcnac:betagal beta-1,3-n-acetylglucosaminyltransferase 2  FAM35A - family with sequence similarity 35, member a  CLN5 - ceroid-lipofuscinosis, neuronal 5  SGTB - small glutamine-rich tetratricopeptide repeat (tpr)-containing, beta  RRAGA - ras-related gtp binding a  RASA1 - ras p21 protein activator (gtpase activating protein) 1  CLTA - clathrin, light chain a  TAF8 - taf8 rna polymerase ii, tata box binding protein (tbp)-associated factor, 43kda  CLK1 - cdc-like kinase 1  MAGEL2 - mage-like 2  CLK2 - cdc-like kinase 2  ING3 - inhibitor of growth family, member 3  CTCF - ccctc-binding factor (zinc finger protein)  CLK3 - cdc-like kinase 3  PRMT7 - protein arginine methyltransferase 7  RBBP7 - retinoblastoma binding protein 7  TMX3 - thioredoxin-related transmembrane protein 3  RBBP8 - retinoblastoma binding protein 8  RBMS1 - rna binding motif, single stranded interacting protein 1  MAP3K2 - mitogen-activated protein kinase kinase kinase 2  DIS3L2 - dis3 mitotic control homolog (s. cerevisiae)-like 2  ARID4A - at rich interactive domain 4a (rbp1-like)  AP2M1 - adaptor-related protein complex 2, mu 1 subunit  RBBP5 - retinoblastoma binding protein 5  RBBP6 - retinoblastoma binding protein 6  KDM5A - lysine (k)-specific demethylase 5a  RBBP4 - retinoblastoma binding protein 4  NUP35 - nucleoporin 35kda  ERCC8 - excision repair cross-complementing rodent repair deficiency, complementation group 8  MGEA5 - meningioma expressed antigen 5 (hyaluronidase)  RCN1 - reticulocalbin 1, ef-hand calcium binding domain  EXOSC4 - exosome component 4  YME1L1 - yme1-like 1 atpase  PTGES3 - prostaglandin e synthase 3 (cytosolic)  NFAT5 - nuclear factor of activated t-cells 5, tonicity-responsive  POLD3 - polymerase (dna-directed), delta 3, accessory subunit  USP39 - ubiquitin specific peptidase 39  PIGU - phosphatidylinositol glycan anchor biosynthesis, class u  ZDHHC13 - zinc finger, dhhc-type containing 13  CHUK - conserved helix-loop-helix ubiquitous kinase  SPTAN1 - spectrin, alpha, non-erythrocytic 1  MKRN1 - makorin ring finger protein 1  MKRN2 - makorin ring finger protein 2  TBRG1 - transforming growth factor beta regulator 1  TMTC4 - transmembrane and tetratricopeptide repeat containing 4  SPTBN4 - spectrin, beta, non-erythrocytic 4  CD2AP - cd2-associated protein  FBXO18 - f-box protein, helicase, 18  METTL14 - methyltransferase like 14  SPP1 - secreted phosphoprotein 1  ANO8 - anoctamin 8  SPG7 - spastic paraplegia 7 (pure and complicated autosomal recessive)  ALG10 - alg10, alpha-1,2-glucosyltransferase  MIER1 - mesoderm induction early response 1, transcriptional regulator  PPP1R15B - protein phosphatase 1, regulatory subunit 15b  BACE1 - beta-site app-cleaving enzyme 1  ABHD13 - abhydrolase domain containing 13  SP1 - sp1 transcription factor  METTL12 - methyltransferase like 12  MUM1 - melanoma associated antigen (mutated) 1  CPE - carboxypeptidase e  LSM2 - lsm2 homolog, u6 small nuclear rna associated (s. cerevisiae)  SOS1 - son of sevenless homolog 1 (drosophila)  ORC3 - origin recognition complex, subunit 3  FBXL20 - f-box and leucine-rich repeat protein 20  ORC6 - origin recognition complex, subunit 6  RAB40C - rab40c, member ras oncogene family  CREBL2 - camp responsive element binding protein-like 2  CREBBP - creb binding protein  ATF2 - activating transcription factor 2  CREB1 - camp responsive element binding protein 1  DDX58 - dead (asp-glu-ala-asp) box polypeptide 58  SNX1 - sorting nexin 1  SMUG1 - single-strand-selective monofunctional uracil-dna glycosylase 1  MARK4 - map/microtubule affinity-regulating kinase 4  RPRD1B - regulation of nuclear pre-mrna domain containing 1b  INIP - ints3 and nabp interacting protein  PLK3 - polo-like kinase 3  SNAPC4 - small nuclear rna activating complex, polypeptide 4, 190kda  SNCA - synuclein, alpha (non a4 component of amyloid precursor)  SNRPB - small nuclear ribonucleoprotein polypeptides b and b1  SUMO2 - small ubiquitin-like modifier 2  SNAP25 - synaptosomal-associated protein, 25kda  SNAPC2 - small nuclear rna activating complex, polypeptide 2, 45kda  SNAPC1 - small nuclear rna activating complex, polypeptide 1, 43kda  SNAPC3 - small nuclear rna activating complex, polypeptide 3, 50kda  TP53INP2 - tumor protein p53 inducible nuclear protein 2  RBM17 - rna binding motif protein 17  SUMO3 - small ubiquitin-like modifier 3  SMARCB1 - swi/snf related, matrix associated, actin dependent regulator of chromatin, subfamily b, member 1  HLTF - helicase-like transcription factor  LSM5 - lsm5 homolog, u6 small nuclear rna associated (s. cerevisiae)  SMARCD3 - swi/snf related, matrix associated, actin dependent regulator of chromatin, subfamily d, member 3  COL4A2 - collagen, type iv, alpha 2  SMARCA1 - swi/snf related, matrix associated, actin dependent regulator of chromatin, subfamily a, member 1  PLD3 - phospholipase d family, member 3  EDC4 - enhancer of mrna decapping 4  SGK3 - serum/glucocorticoid regulated kinase family, member 3  KMT2C - lysine (k)-specific methyltransferase 2c  PRKD3 - protein kinase d3  RAB38 - rab38, member ras oncogene family  PPIL4 - peptidylprolyl isomerase (cyclophilin)-like 4  SCAF1 - sr-related ctd-associated factor 1  OSTC - oligosaccharyltransferase complex subunit (non-catalytic)  TFIP11 - tuftelin interacting protein 11  SENP2 - sumo1/sentrin/smt3 specific peptidase 2  FTSJ1 - ftsj rna methyltransferase homolog 1 (e. coli)  CST3 - cystatin c  USP32 - ubiquitin specific peptidase 32  NAT6 - n-acetyltransferase 6 (gcn5-related)  SHC2 - shc (src homology 2 domain containing) transforming protein 2  KLHL12 - kelch-like family member 12  KDM2B - lysine (k)-specific demethylase 2b  SUPT6H - suppressor of ty 6 homolog (s. cerevisiae)  CTGF - connective tissue growth factor  SUPV3L1 - suppressor of var1, 3-like 1 (s. cerevisiae)  CACNG7 - calcium channel, voltage-dependent, gamma subunit 7  DSTYK - dual serine/threonine and tyrosine protein kinase  CTBP1 - c-terminal binding protein 1  SUPT4H1 - suppressor of ty 4 homolog 1 (s. cerevisiae)  SUPT5H - suppressor of ty 5 homolog (s. cerevisiae)  GTPBP3 - gtp binding protein 3 (mitochondrial)  RASGRP3 - ras guanyl releasing protein 3 (calcium and dag-regulated)  UBL5 - ubiquitin-like 5  CTNNB1 - catenin (cadherin-associated protein), beta 1, 88kda  PPIL2 - peptidylprolyl isomerase (cyclophilin)-like 2  TRMT11 - trna methyltransferase 11 homolog (s. cerevisiae)  PIGO - phosphatidylinositol glycan anchor biosynthesis, class o  STX1A - syntaxin 1a (brain)  LNX1 - ligand of numb-protein x 1, e3 ubiquitin protein ligase  USP30 - ubiquitin specific peptidase 30  FUT10 - fucosyltransferase 10 (alpha (1,3) fucosyltransferase)  NEK4 - nima-related kinase 4  STK3 - serine/threonine kinase 3  PCGF1 - polycomb group ring finger 1  BRD1 - bromodomain containing 1  FKBP8 - fk506 binding protein 8, 38kda  GPBP1L1 - gc-rich promoter binding protein 1-like 1  AURKC - aurora kinase c  STK10 - serine/threonine kinase 10  PAN3 - pan3 poly(a) specific ribonuclease subunit homolog (s. cerevisiae)  ISPD - isoprenoid synthase domain containing  SUV420H2 - suppressor of variegation 4-20 homolog 2 (drosophila)  CYLD - cylindromatosis (turban tumor syndrome)  CDKL5 - cyclin-dependent kinase-like 5  STK4 - serine/threonine kinase 4  HECTD1 - hect domain containing e3 ubiquitin protein ligase 1  CRKL - v-crk avian sarcoma virus ct10 oncogene homolog-like  EEFSEC - eukaryotic elongation factor, selenocysteine-trna-specific  FKBP10 - fk506 binding protein 10, 65 kda  NIPBL - nipped-b homolog (drosophila)  CRY1 - cryptochrome 1 (photolyase-like)  STAT3 - signal transducer and activator of transcription 3 (acute-phase response factor)  CRY2 - cryptochrome 2 (photolyase-like)  ASF1A - anti-silencing function 1a histone chaperone  ANAPC13 - anaphase promoting complex subunit 13  AGBL5 - atp/gtp binding protein-like 5  SSRP1 - structure specific recognition protein 1  ARMC8 - armadillo repeat containing 8  DCAF12 - ddb1 and cul4 associated factor 12  BRMS1 - breast cancer metastasis suppressor 1  SPCS3 - signal peptidase complex subunit 3 homolog (s. cerevisiae)  BOD1L1 - biorientation of chromosomes in cell division 1-like 1  NAA35 - n(alpha)-acetyltransferase 35, natc auxiliary subunit  SUMF2 - sulfatase modifying factor 2  CSF1R - colony stimulating factor 1 receptor  FBXO7 - f-box protein 7  CSF1 - colony stimulating factor 1 (macrophage)  SRPK2 - srsf protein kinase 2  SRPK1 - srsf protein kinase 1  QPCT - glutaminyl-peptide cyclotransferase  TAB3 - tgf-beta activated kinase 1/map3k7 binding protein 3  TROVE2 - trove domain family, member 2  KLHL22 - kelch-like family member 22  GALNT11 - udp-n-acetyl-alpha-d-galactosamine:polypeptide n-acetylgalactosaminyltransferase 11 (galnac-t11)  ZNF335 - zinc finger protein 335  SSBP1 - single-stranded dna binding protein 1, mitochondrial  PTPN5 - protein tyrosine phosphatase, non-receptor type 5 (striatum-enriched)  SSB - sjogren syndrome antigen b (autoantigen la)  CSK - c-src tyrosine kinase  CSNK1G2 - casein kinase 1, gamma 2  CSNK1A1 - casein kinase 1, alpha 1  TTLL1 - tubulin tyrosine ligase-like family, member 1  SPTBN1 - spectrin, beta, non-erythrocytic 1  CSNK1D - casein kinase 1, delta  RNF214 - ring finger protein 214  CSNK2A2 - casein kinase 2, alpha prime polypeptide  SRC - v-src avian sarcoma (schmidt-ruppin a-2) viral oncogene homolog  MRPL17 - mitochondrial ribosomal protein l17  CSNK1G3 - casein kinase 1, gamma 3  CSNK2A1 - casein kinase 2, alpha 1 polypeptide  VIPAS39 - vps33b interacting protein, apical-basolateral polarity regulator, spe-39 homolog  ZDHHC12 - zinc finger, dhhc-type containing 12  THADA - thyroid adenoma associated  ARIH1 - ariadne homolog, ubiquitin-conjugating enzyme e2 binding protein, 1 (drosophila)  UBE2O - ubiquitin-conjugating enzyme e2o  MTO1 - mitochondrial trna translation optimization 1  NSUN3 - nop2/sun domain family, member 3  FBXL2 - f-box and leucine-rich repeat protein 2  SHPRH - snf2 histone linker phd ring helicase, e3 ubiquitin protein ligase  RNF169 - ring finger protein 169  DHX9 - deah (asp-glu-ala-his) box helicase 9  SEL1L - sel-1 suppressor of lin-12-like (c. elegans)  DDX6 - dead (asp-glu-ala-asp) box helicase 6  DDX5 - dead (asp-glu-ala-asp) box helicase 5  FBXO33 - f-box protein 33  DDX1 - dead (asp-glu-ala-asp) box helicase 1  DDOST - dolichyl-diphosphooligosaccharide--protein glycosyltransferase subunit (non-catalytic)  SDF2 - stromal cell-derived factor 2  DPH6 - diphthamine biosynthesis 6  SIN3A - sin3 transcription regulator family member a  TPGS2 - tubulin polyglutamylase complex subunit 2  FAM98A - family with sequence similarity 98, member a  RSPRY1 - ring finger and spry domain containing 1  SAMHD1 - sam domain and hd domain 1  SET - set nuclear oncogene  WWTR1 - ww domain containing transcription regulator 1  SETMAR - set domain and mariner transposase fusion gene  DDB1 - damage-specific dna binding protein 1, 127kda  MAP2K4 - mitogen-activated protein kinase kinase 4  PTPN23 - protein tyrosine phosphatase, non-receptor type 23  SENP7 - sumo1/sentrin specific peptidase 7  MCM9 - minichromosome maintenance complex component 9  ZDHHC24 - zinc finger, dhhc-type containing 24  GEMIN5 - gem (nuclear organelle) associated protein 5  KBTBD6 - kelch repeat and btb (poz) domain containing 6  CSRP2BP - csrp2 binding protein  ZDHHC5 - zinc finger, dhhc-type containing 5  THUMPD3 - thump domain containing 3  NELFB - negative elongation factor complex member b  POT1 - protection of telomeres 1  SCYL1 - scy1-like 1 (s. cerevisiae)  CLK4 - cdc-like kinase 4  KLHL13 - kelch-like family member 13  CNOT10 - ccr4-not transcription complex, subunit 10  RNF19A - ring finger protein 19a, e3 ubiquitin protein ligase  RCHY1 - ring finger and chy zinc finger domain containing 1, e3 ubiquitin protein ligase  ASPHD1 - aspartate beta-hydroxylase domain containing 1  DFFA - dna fragmentation factor, 45kda, alpha polypeptide  PPM1H - protein phosphatase, mg2+/mn2+ dependent, 1h  GALNT16 - udp-n-acetyl-alpha-d-galactosamine:polypeptide n-acetylgalactosaminyltransferase 16  KCTD13 - potassium channel tetramerization domain containing 13  DCAF13 - ddb1 and cul4 associated factor 13  BIRC6 - baculoviral iap repeat containing 6  RHBDD2 - rhomboid domain containing 2  ZDHHC20 - zinc finger, dhhc-type containing 20  BTBD6 - btb (poz) domain containing 6  CNOT6 - ccr4-not transcription complex, subunit 6  LTN1 - listerin e3 ubiquitin protein ligase 1  DNM3 - dynamin 3  ZNF451 - zinc finger protein 451  GLCE - glucuronic acid epimerase  ERI1 - exoribonuclease 1  UBXN7 - ubx domain protein 7  CHD5 - chromodomain helicase dna binding protein 5  MCFD2 - multiple coagulation factor deficiency 2  MED30 - mediator complex subunit 30  RIMKLB - ribosomal modification protein rimk-like family member b  RPAP1 - rna polymerase ii associated protein 1  USP31 - ubiquitin specific peptidase 31  DCAF15 - ddb1 and cul4 associated factor 15  DARS - aspartyl-trna synthetase  INTS2 - integrator complex subunit 2  DAPK3 - death-associated protein kinase 3  ZZZ3 - zinc finger, zz-type containing 3  HINFP - histone h4 transcription factor  LRSAM1 - leucine rich repeat and sterile alpha motif containing 1  ULK3 - unc-51 like kinase 3  SEC11C - sec11 homolog c (s. cerevisiae)  MTA3 - metastasis associated 1 family, member 3  DGKQ - diacylglycerol kinase, theta 110kda  CLIP3 - cap-gly domain containing linker protein 3  DAPK1 - death-associated protein kinase 1  TIPARP - tcdd-inducible poly(adp-ribose) polymerase  HACE1 - hect domain and ankyrin repeat containing e3 ubiquitin protein ligase 1  SARS - seryl-trna synthetase  SATB1 - satb homeobox 1  MIB1 - mindbomb e3 ubiquitin protein ligase 1  SBF1 - set binding factor 1  DAD1 - defender against cell death 1  NYNRIN - nyn domain and retroviral integrase containing  KLHL42 - kelch-like family member 42  TRPC4AP - transient receptor potential cation channel, subfamily c, member 4 associated protein  EPC2 - enhancer of polycomb homolog 2 (drosophila)  DRD2 - dopamine receptor d2  CDC26 - cell division cycle 26  IRF2BP1 - interferon regulatory factor 2 binding protein 1  CNOT6L - ccr4-not transcription complex, subunit 6-like  EAF1 - ell associated factor 1  TAOK1 - tao kinase 1  PDP2 - pyruvate dehyrogenase phosphatase catalytic subunit 2  KLHL8 - kelch-like family member 8  DPH2 - dph2 homolog (s. cerevisiae)  SHANK3 - sh3 and multiple ankyrin repeat domains 3  DPH1 - diphthamide biosynthesis 1  RNASEH1 - ribonuclease h1  DPAGT1 - dolichyl-phosphate (udp-n-acetylglucosamine) n-acetylglucosaminephosphotransferase 1 (glcnac-1-p transferase)  WIPI2 - wd repeat domain, phosphoinositide interacting 2  USP35 - ubiquitin specific peptidase 35  DOM3Z - dom-3 homolog z (c. elegans)  DR1 - down-regulator of transcription 1, tbp-binding (negative cofactor 2)  WSB1 - wd repeat and socs box containing 1  TRMT5 - trna methyltransferase 5  ALG2 - alg2, alpha-1,3/1,6-mannosyltransferase  ZNRF2 - zinc and ring finger 2  DUSP8 - dual specificity phosphatase 8  TNKS1BP1 - tankyrase 1 binding protein 1, 182kda  MAPK15 - mitogen-activated protein kinase 15  SKI - v-ski avian sarcoma viral oncogene homolog  DUSP5 - dual specificity phosphatase 5  SKIV2L - superkiller viralicidic activity 2-like (s. cerevisiae)  DUSP6 - dual specificity phosphatase 6  SKP1 - s-phase kinase-associated protein 1  DUSP7 - dual specificity phosphatase 7  DYRK1A - dual-specificity tyrosine-(y)-phosphorylation regulated kinase 1a  TOR1A - torsin family 1, member a (torsin a)  GFM1 - g elongation factor, mitochondrial 1  GGA1 - golgi-associated, gamma adaptin ear containing, arf binding protein 1  ABHD12 - abhydrolase domain containing 12  HERC4 - hect and rld domain containing e3 ubiquitin protein ligase 4  WDR48 - wd repeat domain 48  GIGYF2 - grb10 interacting gyf protein 2  USP36 - ubiquitin specific peptidase 36  FNIP2 - folliculin interacting protein 2  SENP6 - sumo1/sentrin specific peptidase 6  HBEGF - heparin-binding egf-like growth factor  DTX1 - deltex homolog 1 (drosophila)  DKC1 - dyskeratosis congenita 1, dyskerin  DLG3 - discs, large homolog 3 (drosophila)  DLG4 - discs, large homolog 4 (drosophila)  PNPT1 - polyribonucleotide nucleotidyltransferase 1  DLG1 - discs, large homolog 1 (drosophila)  NEIL2 - nei endonuclease viii-like 2 (e. coli)  FBXW8 - f-box and wd repeat domain containing 8  SH3RF1 - sh3 domain containing ring finger 1  FBXO25 - f-box protein 25  ST6GAL1 - st6 beta-galactosamide alpha-2,6-sialyltranferase 1  SIAH2 - siah e3 ubiquitin protein ligase 2  SIAH1 - siah e3 ubiquitin protein ligase 1  DGUOK - deoxyguanosine kinase  EP400 - e1a binding protein p400  FBXL3 - f-box and leucine-rich repeat protein 3  DHPS - deoxyhypusine synthase  ST3GAL2 - st3 beta-galactoside alpha-2,3-sialyltransferase 2  B3GAT3 - beta-1,3-glucuronyltransferase 3 (glucuronosyltransferase i)  RICTOR - rptor independent companion of mtor, complex 2  FBXL5 - f-box and leucine-rich repeat protein 5  FBXO2 - f-box protein 2  UVSSA - uv-stimulated scaffold protein a  SFPQ - splicing factor proline/glutamine-rich  DNASE2 - deoxyribonuclease ii, lysosomal  PELI3 - pellino e3 ubiquitin protein ligase family member 3  PHRF1 - phd and ring finger domains 1  FBXW2 - f-box and wd repeat domain containing 2  GMEB2 - glucocorticoid modulatory element binding protein 2  DNM2 - dynamin 2  DNMT1 - dna (cytosine-5-)-methyltransferase 1  TRDMT1 - trna aspartic acid methyltransferase 1  DNMT3A - dna (cytosine-5-)-methyltransferase 3 alpha  EIF2AK4 - eukaryotic translation initiation factor 2 alpha kinase 4  ALS2 - amyotrophic lateral sclerosis 2 (juvenile)  NAT9 - n-acetyltransferase 9 (gcn5-related, putative)  ALG11 - alg11, alpha-1,2-mannosyltransferase  SGK1 - serum/glucocorticoid regulated kinase 1  SGTA - small glutamine-rich tetratricopeptide repeat (tpr)-containing, alpha  GAL3ST3 - galactose-3-o-sulfotransferase 3  USP37 - ubiquitin specific peptidase 37  INTS1 - integrator complex subunit 1  SENP3 - sumo1/sentrin/smt3 specific peptidase 3  SMARCAD1 - swi/snf-related, matrix-associated actin-dependent regulator of chromatin, subfamily a, containing dead/h box 1  NCBP2 - nuclear cap binding protein subunit 2, 20kda  MARK2 - map/microtubule affinity-regulating kinase 2  TSN - translin  FAN1 - fancd2/fanci-associated nuclease 1  C1GALT1 - core 1 synthase, glycoprotein-n-acetylgalactosamine 3-beta-galactosyltransferase, 1  ELK4 - elk4, ets-domain protein (srf accessory protein 1)  WRNIP1 - werner helicase interacting protein 1  MGME1 - mitochondrial genome maintenance exonuclease 1  ELL2 - elongation factor, rna polymerase ii, 2  DTD1 - d-tyrosyl-trna deacylase 1  EPAS1 - endothelial pas domain protein 1  DPH7 - diphthamide biosynthesis 7  EP300 - e1a binding protein p300  KIN - kin, antigenic determinant of reca protein homolog (mouse)  SEPHS1 - selenophosphate synthetase 1  UBQLN4 - ubiquilin 4  TIMM50 - translocase of inner mitochondrial membrane 50 homolog (s. cerevisiae)  UGGT1 - udp-glucose glycoprotein glucosyltransferase 1  EGR2 - early growth response 2  TTC3 - tetratricopeptide repeat domain 3  TTF1 - transcription termination factor, rna polymerase i  EIF2S1 - eukaryotic translation initiation factor 2, subunit 1 alpha, 35kda  INTS4 - integrator complex subunit 4  TRIM32 - tripartite motif containing 32  SPHK2 - sphingosine kinase 2  KDM2A - lysine (k)-specific demethylase 2a  TMEM129 - transmembrane protein 129  METTL18 - methyltransferase like 18  TSG101 - tumor susceptibility gene 101  EIF4G2 - eukaryotic translation initiation factor 4 gamma, 2  MAST1 - microtubule associated serine/threonine kinase 1  CCDC47 - coiled-coil domain containing 47  MVB12A - multivesicular body subunit 12a  CNOT1 - ccr4-not transcription complex, subunit 1  EEF2 - eukaryotic translation elongation factor 2  EXOSC7 - exosome component 7  FBXO21 - f-box protein 21  PGAP3 - post-gpi attachment to proteins 3  RNF149 - ring finger protein 149  STK38L - serine/threonine kinase 38 like  TULP4 - tubby like protein 4  PIGM - phosphatidylinositol glycan anchor biosynthesis, class m  EEF1A1 - eukaryotic translation elongation factor 1 alpha 1  EEF1A2 - eukaryotic translation elongation factor 1 alpha 2  TP53BP1 - tumor protein p53 binding protein 1  PHC1 - polyhomeotic homolog 1 (drosophila)  POGLUT1 - protein o-glucosyltransferase 1  TOP3A - topoisomerase (dna) iii alpha  TRMT10A - trna methyltransferase 10 homolog a (s. cerevisiae)  TOP2B - topoisomerase (dna) ii beta 180kda  PHLPP2 - ph domain and leucine rich repeat protein phosphatase 2  EGFR - epidermal growth factor receptor  USP33 - ubiquitin specific peptidase 33  MAST3 - microtubule associated serine/threonine kinase 3  FAM20C - family with sequence similarity 20, member c  TOP1 - topoisomerase (dna) i  KDM4B - lysine (k)-specific demethylase 4b  KDM1A - lysine (k)-specific demethylase 1a  GOLGA7B - golgin a7 family, member b  ATXN7L3 - ataxin 7-like 3  PARP6 - poly (adp-ribose) polymerase family, member 6  EOGT - egf domain-specific o-linked n-acetylglucosamine (glcnac) transferase  UBE2Q2 - ubiquitin-conjugating enzyme e2q family member 2  TRIO - trio rho guanine nucleotide exchange factor  E4F1 - e4f transcription factor 1  SMG1 - smg1 phosphatidylinositol 3-kinase-related kinase  NT5M - 5',3'-nucleotidase, mitochondrial  TNIK - traf2 and nck interacting kinase  XAB2 - xpa binding protein 2  PDS5B - pds5, regulator of cohesion maintenance, homolog b (s. cerevisiae)  ENY2 - enhancer of yellow 2 homolog (drosophila)  DNER - delta/notch-like egf repeat containing  MRPS22 - mitochondrial ribosomal protein s22  C11orf30 - chromosome 11 open reading frame 30  MYCBP2 - myc binding protein 2, e3 ubiquitin protein ligase  TRAF1 - tnf receptor-associated factor 1  ERP44 - endoplasmic reticulum protein 44  HSP90B1 - heat shock protein 90kda beta (grp94), member 1  CMTR1 - cap methyltransferase 1  TRAF3 - tnf receptor-associated factor 3  TRAF2 - tnf receptor-associated factor 2  SETX - senataxin  SETD1B - set domain containing 1b  FEM1C - fem-1 homolog c (c. elegans)  TPR - translocated promoter region, nuclear basket protein  GGA2 - golgi-associated, gamma adaptin ear containing, arf binding protein 2  ERC1 - elks/rab6-interacting/cast family member 1  KDM4C - lysine (k)-specific demethylase 4c  TRIM35 - tripartite motif containing 35  REXO4 - rex4, rna exonuclease 4 homolog (s. cerevisiae)  PIGW - phosphatidylinositol glycan anchor biosynthesis, class w  CAMK1D - calcium/calmodulin-dependent protein kinase id  INTS12 - integrator complex subunit 12  TTLL5 - tubulin tyrosine ligase-like family, member 5  ZC3H13 - zinc finger ccch-type containing 13  NUP107 - nucleoporin 107kda  CDK19 - cyclin-dependent kinase 19  DERL3 - derlin 3  USP1 - ubiquitin specific peptidase 1  FANCE - fanconi anemia, complementation group e  PTK2B - protein tyrosine kinase 2 beta  MRPS27 - mitochondrial ribosomal protein s27  PCNP - pest proteolytic signal containing nuclear protein  PARP11 - poly (adp-ribose) polymerase family, member 11  UNG - uracil-dna glycosylase  CUL9 - cullin 9  KANSL1 - kat8 regulatory nsl complex subunit 1  USP4 - ubiquitin specific peptidase 4 (proto-oncogene)  RNF185 - ring finger protein 185  TAB2 - tgf-beta activated kinase 1/map3k7 binding protein 2  C19orf40 - chromosome 19 open reading frame 40  ZNF598 - zinc finger protein 598  VGF - vgf nerve growth factor inducible  LAMA1 - laminin, alpha 1  SPSB3 - spla/ryanodine receptor domain and socs box containing 3  VHL - von hippel-lindau tumor suppressor, e3 ubiquitin protein ligase  KDM6B - lysine (k)-specific demethylase 6b  EZR - ezrin  PHF8 - phd finger protein 8  TRIM41 - tripartite motif containing 41  SMC5 - structural maintenance of chromosomes 5  EXT2 - exostosin glycosyltransferase 2  EXT1 - exostosin glycosyltransferase 1  KDM6A - lysine (k)-specific demethylase 6a  DCUN1D4 - dcn1, defective in cullin neddylation 1, domain containing 4  EXTL2 - exostosin-like glycosyltransferase 2  EXTL3 - exostosin-like glycosyltransferase 3  UVRAG - uv radiation resistance associated  ACKR3 - atypical chemokine receptor 3  EYA3 - eyes absent homolog 3 (drosophila)  EZH1 - enhancer of zeste homolog 1 (drosophila)  ENDOV - endonuclease v  TRIM47 - tripartite motif containing 47  PDXP - pyridoxal (pyridoxine, vitamin b6) phosphatase  VCP - valosin containing protein  RARS2 - arginyl-trna synthetase 2, mitochondrial  TTLL12 - tubulin tyrosine ligase-like family, member 12  NEK9 - nima-related kinase 9  ERCC5 - excision repair cross-complementing rodent repair deficiency, complementation group 5  ERCC4 - excision repair cross-complementing rodent repair deficiency, complementation group 4  NUP205 - nucleoporin 205kda  ERCC3 - excision repair cross-complementing rodent repair deficiency, complementation group 3  LIN52 - lin-52 homolog (c. elegans)  ELMSAN1 - elm2 and myb/sant-like domain containing 1  GGA3 - golgi-associated, gamma adaptin ear containing, arf binding protein 3  UBE2A - ubiquitin-conjugating enzyme e2a  WDR20 - wd repeat domain 20  UBA1 - ubiquitin-like modifier activating enzyme 1  ERN1 - endoplasmic reticulum to nucleus signaling 1  UBB - ubiquitin b  ARRDC4 - arrestin domain containing 4  RCOR1 - rest corepressor 1  ASPHD2 - aspartate beta-hydroxylase domain containing 2  HIRA - histone cell cycle regulator  TTC5 - tetratricopeptide repeat domain 5  TYRO3 - tyro3 protein tyrosine kinase  ETV1 - ets variant 1  FAF2 - fas associated factor family member 2  TPGS1 - tubulin polyglutamylase complex subunit 1  TYK2 - tyrosine kinase 2  ATG4B - autophagy related 4b, cysteine peptidase  GANAB - glucosidase, alpha; neutral ab  ETF1 - eukaryotic translation termination factor 1  UBXN4 - ubx domain protein 4  COG7 - component of oligomeric golgi complex 7  UCHL3 - ubiquitin carboxyl-terminal esterase l3 (ubiquitin thiolesterase)  UBXN11 - ubx domain protein 11  SCYL3 - scy1-like 3 (s. cerevisiae)  PKDCC - protein kinase domain containing, cytoplasmic  SUMO1 - small ubiquitin-like modifier 1  UCHL1 - ubiquitin carboxyl-terminal esterase l1 (ubiquitin thiolesterase)  UBTF - upstream binding transcription factor, rna polymerase i  UGCG - udp-glucose ceramide glucosyltransferase  ZNF830 - zinc finger protein 830  PELI1 - pellino e3 ubiquitin protein ligase 1  SMURF1 - smad specific e3 ubiquitin protein ligase 1  JMJD6 - jumonji domain containing 6  MRPL47 - mitochondrial ribosomal protein l47  UBE2H - ubiquitin-conjugating enzyme e2h  UBE2I - ubiquitin-conjugating enzyme e2i  UBE2D2 - ubiquitin-conjugating enzyme e2d 2  EPHB6 - eph receptor b6  FBXO28 - f-box protein 28  UBE2V2 - ubiquitin-conjugating enzyme e2 variant 2  LONRF1 - lon peptidase n-terminal domain and ring finger 1  ADCK1 - aarf domain containing kinase 1  ERCC2 - excision repair cross-complementing rodent repair deficiency, complementation group 2  UBE2L3 - ubiquitin-conjugating enzyme e2l 3  MAN1C1 - mannosidase, alpha, class 1c, member 1  NUP210 - nucleoporin 210kda  EPS15 - epidermal growth factor receptor pathway substrate 15  UBE2N - ubiquitin-conjugating enzyme e2n  UBE2V1 - ubiquitin-conjugating enzyme e2 variant 1  PSENEN - presenilin enhancer 2 homolog (c. elegans)  ZC3H15 - zinc finger ccch-type containing 15  TCOF1 - treacher collins-franceschetti syndrome 1  HENMT1 - hen1 methyltransferase homolog 1 (arabidopsis)  TMEM165 - transmembrane protein 165  FNTA - farnesyltransferase, caax box, alpha  FLT1 - fms-related tyrosine kinase 1  PDS5A - pds5, regulator of cohesion maintenance, homolog a (s. cerevisiae)  TMEM106A - transmembrane protein 106a  WSB2 - wd repeat and socs box containing 2  FLNA - filamin a, alpha  RPRD2 - regulation of nuclear pre-mrna domain containing 2  CHST14 - carbohydrate (n-acetylgalactosamine 4-0) sulfotransferase 14  SIK2 - salt-inducible kinase 2  PHLPP1 - ph domain and leucine rich repeat protein phosphatase 1  FOXO3 - forkhead box o3  HDAC8 - histone deacetylase 8  LARP4 - la ribonucleoprotein domain family, member 4  ASH1L - ash1 (absent, small, or homeotic)-like (drosophila)  SIN3B - sin3 transcription regulator family member b  FKBP15 - fk506 binding protein 15, 133kda  DCP1A - decapping mrna 1a  FKBP4 - fk506 binding protein 4, 59kda  ZDHHC22 - zinc finger, dhhc-type containing 22  FKBP2 - fk506 binding protein 2, 13kda  MGRN1 - mahogunin ring finger 1, e3 ubiquitin protein ligase  HR - hair growth associated  MED8 - mediator complex subunit 8  FKBP5 - fk506 binding protein 5  CAND1 - cullin-associated and neddylation-dissociated 1  FKBP1A - fk506 binding protein 1a, 12kda  TDG - thymine-dna glycosylase  SMG6 - smg6 nonsense mediated mrna decay factor  FKBP1B - fk506 binding protein 1b, 12.6 kda  FBXW11 - f-box and wd repeat domain containing 11  POFUT2 - protein o-fucosyltransferase 2  KLHL18 - kelch-like family member 18  KDM3A - lysine (k)-specific demethylase 3a  RNF130 - ring finger protein 130  PPP1R11 - protein phosphatase 1, regulatory (inhibitor) subunit 11  NUP160 - nucleoporin 160kda  TAF9 - taf9 rna polymerase ii, tata box binding protein (tbp)-associated factor, 32kda  FGFR2 - fibroblast growth factor receptor 2  TAF7 - taf7 rna polymerase ii, tata box binding protein (tbp)-associated factor, 55kda  UBR4 - ubiquitin protein ligase e3 component n-recognin 4  FAM98B - family with sequence similarity 98, member b  MBD6 - methyl-cpg binding domain protein 6  KLHL7 - kelch-like family member 7  FGF13 - fibroblast growth factor 13  TAF6 - taf6 rna polymerase ii, tata box binding protein (tbp)-associated factor, 80kda  FGFR1 - fibroblast growth factor receptor 1  TADA2A - transcriptional adaptor 2a  FGF9 - fibroblast growth factor 9  TAF4 - taf4 rna polymerase ii, tata box binding protein (tbp)-associated factor, 135kda  SMCHD1 - structural maintenance of chromosomes flexible hinge domain containing 1  TAF2 - taf2 rna polymerase ii, tata box binding protein (tbp)-associated factor, 150kda  ADAM17 - adam metallopeptidase domain 17  NSFL1C - nsfl1 (p97) cofactor (p47)  PHF15 - phd finger protein 15  FGF1 - fibroblast growth factor 1 (acidic)  FGF2 - fibroblast growth factor 2 (basic)  DPY19L1 - dpy-19-like 1 (c. elegans)  USP22 - ubiquitin specific peptidase 22  SASH1 - sam and sh3 domain containing 1  TARBP1 - tar (hiv-1) rna binding protein 1  TRIM2 - tripartite motif containing 2  FEN1 - flap structure-specific endonuclease 1  FER - fer (fps/fes related) tyrosine kinase  SMG9 - smg9 nonsense mediated mrna decay factor  TAF13 - taf13 rna polymerase ii, tata box binding protein (tbp)-associated factor, 18kda  MAP3K7 - mitogen-activated protein kinase kinase kinase 7  ALG1 - alg1, chitobiosyldiphosphodolichol beta-mannosyltransferase  ZCCHC11 - zinc finger, cchc domain containing 11  LARS2 - leucyl-trna synthetase 2, mitochondrial  INO80E - ino80 complex subunit e  RNF114 - ring finger protein 114  ZDHHC17 - zinc finger, dhhc-type containing 17  KMT2E - lysine (k)-specific methyltransferase 2e  SIK3 - sik family kinase 3  PGP - phosphoglycolate phosphatase  NCSTN - nicastrin  FKTN - fukutin  SULF2 - sulfatase 2  UFL1 - ufm1-specific ligase 1  KLHL9 - kelch-like family member 9  TCF3 - transcription factor 3  TRIM9 - tripartite motif containing 9  TENC1 - tensin like c1 domain containing phosphatase (tensin 2)  DMAP1 - dna methyltransferase 1 associated protein 1  TCEB3 - transcription elongation factor b (siii), polypeptide 3 (110kda, elongin a)  OBSL1 - obscurin-like 1  EFEMP1 - egf containing fibulin-like extracellular matrix protein 1  CTTNBP2NL - cttnbp2 n-terminal like  FBN1 - fibrillin 1  RNF208 - ring finger protein 208  TCEA1 - transcription elongation factor a (sii), 1  USP24 - ubiquitin specific peptidase 24  TIE1 - tyrosine kinase with immunoglobulin-like and egf-like domains 1  TIMP1 - timp metallopeptidase inhibitor 1  TK1 - thymidine kinase 1, soluble  TK2 - thymidine kinase 2, mitochondrial  RYBP - ring1 and yy1 binding protein  MTOR - mechanistic target of rapamycin (serine/threonine kinase)  STK32C - serine/threonine kinase 32c  PPWD1 - peptidylprolyl isomerase domain and wd repeat containing 1  NDNL2 - necdin-like 2  ATP13A2 - atpase type 13a2  TLR3 - toll-like receptor 3  PIGS - phosphatidylinositol glycan anchor biosynthesis, class s  EXOSC2 - exosome component 2  SIRT1 - sirtuin 1  ACTR8 - arp8 actin-related protein 8 homolog (yeast)  ICMT - isoprenylcysteine carboxyl methyltransferase  PHF3 - phd finger protein 3  BRD4 - bromodomain containing 4  TNFAIP1 - tumor necrosis factor, alpha-induced protein 1 (endothelial)  FBXO44 - f-box protein 44  HARS2 - histidyl-trna synthetase 2, mitochondrial  TARDBP - tar dna binding protein  KCTD21 - potassium channel tetramerization domain containing 21  KCTD2 - potassium channel tetramerization domain containing 2  TEK - tek tyrosine kinase, endothelial  NUP188 - nucleoporin 188kda  SUZ12 - suz12 polycomb repressive complex 2 subunit  PGM2L1 - phosphoglucomutase 2-like 1  P4HA3 - prolyl 4-hydroxylase, alpha polypeptide iii  POFUT1 - protein o-fucosyltransferase 1  RCOR2 - rest corepressor 2  TFAM - transcription factor a, mitochondrial  TERF1 - telomeric repeat binding factor (nima-interacting) 1  TERF2 - telomeric repeat binding factor 2  MORC3 - morc family cw-type zinc finger 3  TESK1 - testis-specific kinase 1  GTF3C6 - general transcription factor iiic, polypeptide 6, alpha 35kda  SKIV2L2 - superkiller viralicidic activity 2-like 2 (s. cerevisiae)  TFDP1 - transcription factor dp-1  BCCIP - brca2 and cdkn1a interacting protein  TFCP2 - transcription factor cp2  FXN - frataxin  ZFYVE26 - zinc finger, fyve domain containing 26  TGS1 - trimethylguanosine synthase 1  TGFA - transforming growth factor, alpha  CEP41 - centrosomal protein 41kda  POLE4 - polymerase (dna-directed), epsilon 4, accessory subunit  TGFBR1 - transforming growth factor, beta receptor 1  TGFB2 - transforming growth factor, beta 2  TGFB1 - transforming growth factor, beta 1  TGFB1I1 - transforming growth factor beta 1 induced transcript 1  MAPK8IP2 - mitogen-activated protein kinase 8 interacting protein 2  TSPAN15 - tetraspanin 15  PIGN - phosphatidylinositol glycan anchor biosynthesis, class n  MEPCE - methylphosphate capping enzyme  CDK20 - cyclin-dependent kinase 20  RNF20 - ring finger protein 20, e3 ubiquitin protein ligase  TGFBR2 - transforming growth factor, beta receptor ii (70/80kda)  TGFBR3 - transforming growth factor, beta receptor iii  FOSB - fbj murine osteosarcoma viral oncogene homolog b  FOS - fbj murine osteosarcoma viral oncogene homolog  WDR45B - wd repeat domain 45b  THBS1 - thrombospondin 1  KAT6B - k(lysine) acetyltransferase 6b  GATC - glutamyl-trna(gln) amidotransferase, subunit c  THY1 - thy-1 cell surface antigen  METTL3 - methyltransferase like 3  THRA - thyroid hormone receptor, alpha  MMD - monocyte to macrophage differentiation-associated  ELL - elongation factor rna polymerase ii  DNAJB14 - dnaj (hsp40) homolog, subfamily b, member 14  GFPT1 - glutamine--fructose-6-phosphate transaminase 1  GFRA1 - gdnf family receptor alpha 1  TMTC2 - transmembrane and tetratricopeptide repeat containing 2  GFRA2 - gdnf family receptor alpha 2  USP54 - ubiquitin specific peptidase 54  ATAT1 - alpha tubulin acetyltransferase 1  GCNT1 - glucosaminyl (n-acetyl) transferase 1, core 2  LZTR1 - leucine-zipper-like transcription regulator 1  OBFC1 - oligonucleotide/oligosaccharide-binding fold containing 1  KAT2A - k(lysine) acetyltransferase 2a  NCOA3 - nuclear receptor coactivator 3  FBXL18 - f-box and leucine-rich repeat protein 18  NAA25 - n(alpha)-acetyltransferase 25, natb auxiliary subunit  PABPN1 - poly(a) binding protein, nuclear 1  TAF15 - taf15 rna polymerase ii, tata box binding protein (tbp)-associated factor, 68kda  GGTA1P - glycoprotein, alpha-galactosyltransferase 1 pseudogene  TMTC3 - transmembrane and tetratricopeptide repeat containing 3  GGT7 - gamma-glutamyltransferase 7  TRRAP - transformation/transcription domain-associated protein  MCMBP - minichromosome maintenance complex binding protein  PICALM - phosphatidylinositol binding clathrin assembly protein  HDAC11 - histone deacetylase 11  TRMT10B - trna methyltransferase 10 homolog b (s. cerevisiae)  SETD6 - set domain containing 6  ACTR5 - arp5 actin-related protein 5 homolog (yeast)  NAA60 - n(alpha)-acetyltransferase 60, natf catalytic subunit  CDC45 - cell division cycle 45  BAP1 - brca1 associated protein-1 (ubiquitin carboxy-terminal hydrolase)  AXIN2 - axin 2  NUP85 - nucleoporin 85kda  CDC7 - cell division cycle 7  BRAP - brca1 associated protein  KDM5C - lysine (k)-specific demethylase 5c  SMC1A - structural maintenance of chromosomes 1a  USP9X - ubiquitin specific peptidase 9, x-linked  USP11 - ubiquitin specific peptidase 11  GNAS - gnas complex locus  PHF17 - phd finger protein 17  GMFB - glia maturation factor, beta  NELFE - negative elongation factor complex member e  EHMT1 - euchromatic histone-lysine n-methyltransferase 1  FUCA2 - fucosidase, alpha-l- 2, plasma  CAMKMT - calmodulin-lysine n-methyltransferase  METTL8 - methyltransferase like 8  NAA40 - n(alpha)-acetyltransferase 40, natd catalytic subunit, homolog (s. cerevisiae)  SDE2 - sde2 telomere maintenance homolog (s. pombe)  RAB7A - rab7a, member ras oncogene family  USP7 - ubiquitin specific peptidase 7 (herpes virus-associated)  GAA - glucosidase, alpha; acid  RNF168 - ring finger protein 168, e3 ubiquitin protein ligase  XRCC6 - x-ray repair complementing defective repair in chinese hamster cells 6  FUT4 - fucosyltransferase 4 (alpha (1,3) fucosyltransferase, myeloid-specific)  ST8SIA4 - st8 alpha-n-acetyl-neuraminide alpha-2,8-sialyltransferase 4  FUT8 - fucosyltransferase 8 (alpha (1,6) fucosyltransferase)  CBLL1 - cbl proto-oncogene-like 1, e3 ubiquitin protein ligase  FYN - fyn oncogene related to src, fgr, yes  RPAP2 - rna polymerase ii associated protein 2  UBA5 - ubiquitin-like modifier activating enzyme 5  GAK - cyclin g associated kinase  GALNT2 - udp-n-acetyl-alpha-d-galactosamine:polypeptide n-acetylgalactosaminyltransferase 2 (galnac-t2)  TTLL7 - tubulin tyrosine ligase-like family, member 7  USP5 - ubiquitin specific peptidase 5 (isopeptidase t)  KCTD17 - potassium channel tetramerization domain containing 17  GALNT1 - udp-n-acetyl-alpha-d-galactosamine:polypeptide n-acetylgalactosaminyltransferase 1 (galnac-t1)  KMT2D - lysine (k)-specific methyltransferase 2d  SPRED1 - sprouty-related, evh1 domain containing 1  AAAS - achalasia, adrenocortical insufficiency, alacrimia  CDK2AP1 - cyclin-dependent kinase 2 associated protein 1  YEATS4 - yeats domain containing 4  KAT6A - k(lysine) acetyltransferase 6a  UBXN8 - ubx domain protein 8  TUSC3 - tumor suppressor candidate 3  GBE1 - glucan (1,4-alpha-), branching enzyme 1  STAM - signal transducing adaptor molecule (sh3 domain and itam motif) 1  NUP214 - nucleoporin 214kda  KLHL36 - kelch-like family member 36  GAS6 - growth arrest-specific 6  DEDD2 - death effector domain containing 2  FN3KRP - fructosamine 3 kinase related protein  SMC6 - structural maintenance of chromosomes 6  RNF112 - ring finger protein 112  GSPT1 - g1 to s phase transition 1  GSN - gelsolin  ZCCHC6 - zinc finger, cchc domain containing 6  RNF113A - ring finger protein 113a  PARP8 - poly (adp-ribose) polymerase family, member 8  GTF2F1 - general transcription factor iif, polypeptide 1, 74kda  GTF2F2 - general transcription factor iif, polypeptide 2, 30kda  HECTD3 - hect domain containing e3 ubiquitin protein ligase 3  GTF2E1 - general transcription factor iie, polypeptide 1, alpha 56kda  GTF2E2 - general transcription factor iie, polypeptide 2, beta 34kda  NEIL1 - nei endonuclease viii-like 1 (e. coli)  GTF2H2 - general transcription factor iih, polypeptide 2, 44kda  TRIM26 - tripartite motif containing 26  GTF2H3 - general transcription factor iih, polypeptide 3, 34kda  GTF2H1 - general transcription factor iih, polypeptide 1, 62kda  USP27X - ubiquitin specific peptidase 27, x-linked  GTF2A2 - general transcription factor iia, 2, 12kda  GTF2B - general transcription factor iib  PCGF2 - polycomb group ring finger 2  MSH6 - muts homolog 6 (e. coli)  GTF2A1 - general transcription factor iia, 1, 19/37kda  GTF3C2 - general transcription factor iiic, polypeptide 2, beta 110kda  TBL1XR1 - transducin (beta)-like 1 x-linked receptor 1  GTF2I - general transcription factor iii  GTF2H4 - general transcription factor iih, polypeptide 4, 52kda  GTF3A - general transcription factor iiia  BRF1 - brf1, rna polymerase iii transcription initiation factor 90 kda subunit  GTF3C1 - general transcription factor iiic, polypeptide 1, alpha 220kda  ZDHHC14 - zinc finger, dhhc-type containing 14  H3F3A - h3 histone, family 3a  HIST1H2BB - histone cluster 1, h2bb  BRPF1 - bromodomain and phd finger containing, 1  SCG2 - secretogranin ii  H2AFX - h2a histone family, member x  DCP2 - decapping mrna 2  MUL1 - mitochondrial e3 ubiquitin protein ligase 1  PAPD4 - pap associated domain containing 4  PAX8 - paired box 8  RNF103 - ring finger protein 103  MOGS - mannosyl-oligosaccharide glucosidase  CDC73 - cell division cycle 73  HARS - histidyl-trna synthetase  DAP3 - death associated protein 3  PTP4A1 - protein tyrosine phosphatase type iva, member 1  HCFC1 - host cell factor c1 (vp16-accessory protein)  MAP3K12 - mitogen-activated protein kinase kinase kinase 12  NDNF - neuron-derived neurotrophic factor  FUT11 - fucosyltransferase 11 (alpha (1,3) fucosyltransferase)  HDAC2 - histone deacetylase 2  HDAC1 - histone deacetylase 1  PGBD5 - piggybac transposable element derived 5  XPA - xeroderma pigmentosum, complementation group a  XPC - xeroderma pigmentosum, complementation group c  GOLGA2 - golgin a2  XRCC1 - x-ray repair complementing defective repair in chinese hamster cells 1  PAGR1 - paxip1 associated glutamate-rich protein 1  XBP1 - x-box binding protein 1  EIF4H - eukaryotic translation initiation factor 4h  WEE1 - wee1 homolog (s. pombe)  IMMP1L - imp1 inner mitochondrial membrane peptidase-like (s. cerevisiae)  WFS1 - wolfram syndrome 1 (wolframin)  WHSC1 - wolf-hirschhorn syndrome candidate 1  AMN1 - antagonist of mitotic exit network 1 homolog (s. cerevisiae)  NELFA - negative elongation factor complex member a  DTX3 - deltex homolog 3 (drosophila)  VRK1 - vaccinia related kinase 1  VRK2 - vaccinia related kinase 2  DCP1B - decapping mrna 1b  WARS - tryptophanyl-trna synthetase  GRK5 - g protein-coupled receptor kinase 5  GRK6 - g protein-coupled receptor kinase 6  FBXL15 - f-box and leucine-rich repeat protein 15  BRCC3 - brca1/brca2-containing complex, subunit 3  GRB2 - growth factor receptor-bound protein 2  GNPTAB - n-acetylglucosamine-1-phosphate transferase, alpha and beta subunits  ASXL1 - additional sex combs like 1 (drosophila)  TNIP2 - tnfaip3 interacting protein 2  ZNF711 - zinc finger protein 711  YES1 - v-yes-1 yamaguchi sarcoma viral oncogene homolog 1  DCAF10 - ddb1 and cul4 associated factor 10  XRCC5 - x-ray repair complementing defective repair in chinese hamster cells 5 (double-strand-break rejoining)  XRCC4 - x-ray repair complementing defective repair in chinese hamster cells 4  POLR3H - polymerase (rna) iii (dna directed) polypeptide h (22.9kd)  GSK3B - glycogen synthase kinase 3 beta  YWHAZ - tyrosine 3-monooxygenase/tryptophan 5-monooxygenase activation protein, zeta polypeptide  PGAM5 - phosphoglycerate mutase family member 5  GSK3A - glycogen synthase kinase 3 alpha  YWHAE - tyrosine 3-monooxygenase/tryptophan 5-monooxygenase activation protein, epsilon polypeptide  YWHAB - tyrosine 3-monooxygenase/tryptophan 5-monooxygenase activation protein, beta polypeptide  NDUFAF5 - nadh dehydrogenase (ubiquinone) complex i, assembly factor 5  CCNK - cyclin k  DPM1 - dolichyl-phosphate mannosyltransferase polypeptide 1, catalytic subunit  DNAJB2 - dnaj (hsp40) homolog, subfamily b, member 2  RNF26 - ring finger protein 26  TRIM24 - tripartite motif containing 24  METTL22 - methyltransferase like 22  PRMT1 - protein arginine methyltransferase 1  PRMT2 - protein arginine methyltransferase 2  FKRP - fukutin related protein  FBXO45 - f-box protein 45  RPL22L1 - ribosomal protein l22-like 1  DERL1 - derlin 1  DCAF5 - ddb1 and cul4 associated factor 5  KCTD6 - potassium channel tetramerization domain containing 6  METTL16 - methyltransferase like 16  FTO - fat mass and obesity associated  ALKBH1 - alkb, alkylation repair homolog 1 (e. coli)  KDELC1 - kdel (lys-asp-glu-leu) containing 1  ALG8 - alg8, alpha-1,3-glucosyltransferase  SYNGAP1 - synaptic ras gtpase activating protein 1  SECISBP2 - secis binding protein 2  MED11 - mediator complex subunit 11  ST3GAL5 - st3 beta-galactoside alpha-2,3-sialyltransferase 5  TMUB2 - transmembrane and ubiquitin-like domain containing 2  STK19 - serine/threonine kinase 19  RNF215 - ring finger protein 215  LDB1 - lim domain binding 1  DSCC1 - dna replication and sister chromatid cohesion 1  KAT2B - k(lysine) acetyltransferase 2b  TSC22D1 - tsc22 domain family, member 1  CRTC2 - creb regulated transcription coactivator 2  CDC16 - cell division cycle 16  DDA1 - det1 and ddb1 associated 1  ZNF259 - zinc finger protein 259  GID4 - gid complex subunit 4  NAE1 - nedd8 activating enzyme e1 subunit 1  RPS6KC1 - ribosomal protein s6 kinase, 52kda, polypeptide 1  C1orf86 - chromosome 1 open reading frame 86  PRPF4B - prp4 pre-mrna processing factor 4 homolog b (yeast)  MTMR2 - myotubularin related protein 2  NABP2 - nucleic acid binding protein 2  MTMR3 - myotubularin related protein 3  HUS1 - hus1 checkpoint homolog (s. pombe)  MCM3AP - minichromosome maintenance complex component 3 associated protein  ERI3 - eri1 exoribonuclease family member 3  CPNE3 - copine iii  HERC3 - hect and rld domain containing e3 ubiquitin protein ligase 3  HERC2 - hect and rld domain containing e3 ubiquitin protein ligase 2  HERC1 - hect and rld domain containing e3 ubiquitin protein ligase family member 1  TRAK2 - trafficking protein, kinesin binding 2  HSPD1 - heat shock 60kda protein 1 (chaperonin)  NSMCE1 - non-smc element 1 homolog (s. cerevisiae)  BCL10 - b-cell cll/lymphoma 10  FUBP3 - far upstream element (fuse) binding protein 3  HSP90AA1 - heat shock protein 90kda alpha (cytosolic), class a member 1  C7orf49 - chromosome 7 open reading frame 49  BTRC - beta-transducin repeat containing e3 ubiquitin protein ligase  MBD4 - methyl-cpg binding domain protein 4  PCYOX1L - prenylcysteine oxidase 1 like  UBR1 - ubiquitin protein ligase e3 component n-recognin 1  USP12 - ubiquitin specific peptidase 12  WNK1 - wnk lysine deficient protein kinase 1  HMGN1 - high mobility group nucleosome binding domain 1  UBE2Z - ubiquitin-conjugating enzyme e2z  HMGB3 - high mobility group box 3  HMGB2 - high mobility group box 2  LAMTOR3 - late endosomal/lysosomal adaptor, mapk and mtor activator 3  HMGB1 - high mobility group box 1  MPPE1 - metallophosphoesterase 1  HLCS - holocarboxylase synthetase (biotin-(proprionyl-coa-carboxylase (atp-hydrolysing)) ligase)  SENP5 - sumo1/sentrin specific peptidase 5  IRS2 - insulin receptor substrate 2  STK33 - serine/threonine kinase 33  TNKS - tankyrase, trf1-interacting ankyrin-related adp-ribose polymerase  INTS6 - integrator complex subunit 6  AGO1 - argonaute risc catalytic component 1  LATS2 - large tumor suppressor kinase 2  EIF3F - eukaryotic translation initiation factor 3, subunit f  BECN1 - beclin 1, autophagy related  ZBTB48 - zinc finger and btb domain containing 48  PEA15 - phosphoprotein enriched in astrocytes 15  MRPL1 - mitochondrial ribosomal protein l1  HIPK1 - homeodomain interacting protein kinase 1  ACD - adrenocortical dysplasia homolog (mouse)  GPBP1 - gc-rich promoter binding protein 1  B4GALT4 - udp-gal:betaglcnac beta 1,4- galactosyltransferase, polypeptide 4  B4GALT3 - udp-gal:betaglcnac beta 1,4- galactosyltransferase, polypeptide 3  B4GALT2 - udp-gal:betaglcnac beta 1,4- galactosyltransferase, polypeptide 2  CDC23 - cell division cycle 23  MRPL44 - mitochondrial ribosomal protein l44  UBE2K - ubiquitin-conjugating enzyme e2k  INTS3 - integrator complex subunit 3  B3GALNT1 - beta-1,3-n-acetylgalactosaminyltransferase 1 (globoside blood group)  UPF3B - upf3 regulator of nonsense transcripts homolog b (yeast)  B3GALT2 - udp-gal:betaglcnac beta 1,3-galactosyltransferase, polypeptide 2  UPF3A - upf3 regulator of nonsense transcripts homolog a (yeast)  HIF1A - hypoxia inducible factor 1, alpha subunit (basic helix-loop-helix transcription factor)  MBTPS1 - membrane-bound transcription factor peptidase, site 1  ERAL1 - era-like 12s mitochondrial rrna chaperone 1  SNX3 - sorting nexin 3  GBGT1 - globoside alpha-1,3-n-acetylgalactosaminyltransferase 1  EED - embryonic ectoderm development  RNGTT - rna guanylyltransferase and 5'-phosphatase  RNMT - rna (guanine-7-) methyltransferase  RIPK1 - receptor (tnfrsf)-interacting serine-threonine kinase 1  FBXO10 - f-box protein 10  MRPS24 - mitochondrial ribosomal protein s24  FBXO22 - f-box protein 22  MRPS6 - mitochondrial ribosomal protein s6  FBXO4 - f-box protein 4  FBXO8 - f-box protein 8  MRPS11 - mitochondrial ribosomal protein s11  FBXO6 - f-box protein 6  VPS33B - vacuolar protein sorting 33 homolog b (yeast)  TINF2 - terf1 (trf1)-interacting nuclear factor 2  FBXO3 - f-box protein 3  MRPL32 - mitochondrial ribosomal protein l32  RAB11A - rab11a, member ras oncogene family  RIPK2 - receptor-interacting serine-threonine kinase 2  PORCN - porcupine homolog (drosophila)  HNRNPD - heterogeneous nuclear ribonucleoprotein d (au-rich element rna binding protein 1, 37kda)  DNAJC18 - dnaj (hsp40) homolog, subfamily c, member 18  MTMR1 - myotubularin related protein 1  FBXO15 - f-box protein 15  USP46 - ubiquitin specific peptidase 46  HMGA1 - high mobility group at-hook 1  VWA1 - von willebrand factor a domain containing 1  PTPLB - protein tyrosine phosphatase-like (proline instead of catalytic arginine), member b  PTPN18 - protein tyrosine phosphatase, non-receptor type 18 (brain-derived)  NABP1 - nucleic acid binding protein 1  STT3B - stt3b, subunit of the oligosaccharyltransferase complex (catalytic)  PDE12 - phosphodiesterase 12  FOXK2 - forkhead box k2  HAT1 - histone acetyltransferase 1  RPS6KA6 - ribosomal protein s6 kinase, 90kda, polypeptide 6  RBM15 - rna binding motif protein 15  ENC1 - ectodermal-neural cortex 1 (with btb domain)  PGAP2 - post-gpi attachment to proteins 2  NDST2 - n-deacetylase/n-sulfotransferase (heparan glucosaminyl) 2  RBMX - rna binding motif protein, x-linked  INHA - inhibin, alpha  DESI1 - desumoylating isopeptidase 1  RMND5B - required for meiotic nuclear division 5 homolog b (s. cerevisiae)  HTRA2 - htra serine peptidase 2  MEAF6 - myst/esa1-associated factor 6  STK39 - serine threonine kinase 39  ENGASE - endo-beta-n-acetylglucosaminidase  PPM1D - protein phosphatase, mg2+/mn2+ dependent, 1d  POLL - polymerase (dna directed), lambda  UBE2S - ubiquitin-conjugating enzyme e2s  PRPF19 - pre-mrna processing factor 19  ILF3 - interleukin enhancer binding factor 3, 90kda  MAP4K3 - mitogen-activated protein kinase kinase kinase kinase 3  ILK - integrin-linked kinase  CDC14B - cell division cycle 14b  PIAS1 - protein inhibitor of activated stat, 1  SMYD3 - set and mynd domain containing 3  MAPKAPK5 - mitogen-activated protein kinase-activated protein kinase 5  SMURF2 - smad specific e3 ubiquitin protein ligase 2  METTL17 - methyltransferase like 17  PATL1 - protein associated with topoisomerase ii homolog 1 (yeast)  LMO4 - lim domain only 4  CAMK1 - calcium/calmodulin-dependent protein kinase i  COPS7B - cop9 signalosome subunit 7b  CBX4 - chromobox homolog 4  COPS3 - cop9 signalosome subunit 3  GORASP1 - golgi reassembly stacking protein 1, 65kda  ANAPC1 - anaphase promoting complex subunit 1  IL6ST - interleukin 6 signal transducer (gp130, oncostatin m receptor)  ORAOV1 - oral cancer overexpressed 1  SERP1 - stress-associated endoplasmic reticulum protein 1  PRKRA - protein kinase, interferon-inducible double stranded rna dependent activator  STK16 - serine/threonine kinase 16  RNF115 - ring finger protein 115  KHSRP - kh-type splicing regulatory protein  CASK - calcium/calmodulin-dependent serine protein kinase (maguk family)  ITGAV - integrin, alpha v  POLR1E - polymerase (rna) i polypeptide e, 53kda  CDK10 - cyclin-dependent kinase 10  SUDS3 - suppressor of defective silencing 3 homolog (s. cerevisiae)  ITGB1 - integrin, beta 1 (fibronectin receptor, beta polypeptide, antigen cd29 includes mdf2, msk12)  ZDHHC6 - zinc finger, dhhc-type containing 6  ATG3 - autophagy related 3  RNASET2 - ribonuclease t2  INSR - insulin receptor  CDK13 - cyclin-dependent kinase 13  AKTIP - akt interacting protein  KLHL20 - kelch-like family member 20  LSM3 - lsm3 homolog, u6 small nuclear rna associated (s. cerevisiae)  APEX2 - apex nuclease (apurinic/apyrimidinic endonuclease) 2  RUVBL1 - ruvb-like 1 (e. coli)  MOCS3 - molybdenum cofactor synthesis 3  DIMT1 - dim1 dimethyladenosine transferase 1 homolog (s. cerevisiae)  CRCP - cgrp receptor component  IRAK2 - interleukin-1 receptor-associated kinase 2  FOLH1B - folate hydrolase 1b  IRAK1 - interleukin-1 receptor-associated kinase 1  EIF2AK1 - eukaryotic translation initiation factor 2-alpha kinase 1  LRWD1 - leucine-rich repeats and wd repeat domain containing 1  PAPD5 - pap associated domain containing 5  NSD1 - nuclear receptor binding set domain protein 1  HS3ST5 - heparan sulfate (glucosamine) 3-o-sulfotransferase 5  RFWD2 - ring finger and wd repeat domain 2, e3 ubiquitin protein ligase  RNF25 - ring finger protein 25  AFF4 - af4/fmr2 family, member 4  MMS19 - mms19 nucleotide excision repair homolog (s. cerevisiae)  PALD1 - phosphatase domain containing, paladin 1  SNX5 - sorting nexin 5  BRPF3 - bromodomain and phd finger containing, 3  PDF - peptide deformylase (mitochondrial)  STK36 - serine/threonine kinase 36  IRF2BPL - interferon regulatory factor 2 binding protein-like  RRAGC - ras-related gtp binding c  FOXK1 - forkhead box k1  MAGEF1 - melanoma antigen family f, 1  XYLT2 - xylosyltransferase ii  VPS4A - vacuolar protein sorting 4 homolog a (s. cerevisiae)  FN3K - fructosamine 3 kinase  IGHMBP2 - immunoglobulin mu binding protein 2  STK24 - serine/threonine kinase 24  GFRA4 - gdnf family receptor alpha 4  RASAL1 - ras protein activator like 1 (gap1 like)  OARD1 - o-acyl-adp-ribose deacylase 1  PABPC1 - poly(a) binding protein, cytoplasmic 1  DYRK3 - dual-specificity tyrosine-(y)-phosphorylation regulated kinase 3  RNF11 - ring finger protein 11  DUSP11 - dual specificity phosphatase 11 (rna/rnp complex 1-interacting)  SPOP - speckle-type poz protein  SPARCL1 - sparc-like 1 (hevin)  USP21 - ubiquitin specific peptidase 21  ULK1 - unc-51 like autophagy activating kinase 1  GOLPH3 - golgi phosphoprotein 3 (coat-protein)  MLH3 - mutl homolog 3 (e. coli)  JMJD1C - jumonji domain containing 1c  CDC42BPA - cdc42 binding protein kinase alpha (dmpk-like)  IGF1R - insulin-like growth factor 1 receptor  OGT - o-linked n-acetylglucosamine (glcnac) transferase  RAE1 - rae1 rna export 1 homolog (s. pombe)  LAT - linker for activation of t cells  NSG1 - neuron specific gene family member 1  IGFBP6 - insulin-like growth factor binding protein 6  SND1 - staphylococcal nuclease and tudor domain containing 1  CUL4B - cullin 4b  CUL1 - cullin 1  CUL2 - cullin 2  PCIF1 - pdx1 c-terminal inhibiting factor 1  CUL3 - cullin 3  TPST2 - tyrosylprotein sulfotransferase 2  B3GAT1 - beta-1,3-glucuronyltransferase 1 (glucuronosyltransferase p)  SUPT3H - suppressor of ty 3 homolog (s. cerevisiae)  CACNG4 - calcium channel, voltage-dependent, gamma subunit 4  TPST1 - tyrosylprotein sulfotransferase 1  RSRC1 - arginine/serine-rich coiled-coil 1  PCGF5 - polycomb group ring finger 5  MEX3C - mex-3 rna binding family member c  WTAP - wilms tumor 1 associated protein  WAC - ww domain containing adaptor with coiled-coil  LIG4 - ligase iv, dna, atp-dependent  MKI67IP - mki67 (fha domain) interacting nucleolar phosphoprotein  LIMK1 - lim domain kinase 1  LIG1 - ligase i, dna, atp-dependent  VPS37A - vacuolar protein sorting 37 homolog a (s. cerevisiae)  LIG3 - ligase iii, dna, atp-dependent  ZDHHC3 - zinc finger, dhhc-type containing 3  AATK - apoptosis-associated tyrosine kinase  DOT1L - dot1-like histone h3k79 methyltransferase  LOXL1 - lysyl oxidase-like 1  RAB3D - rab3d, member ras oncogene family  LNPEP - leucyl/cystinyl aminopeptidase  BRSK1 - br serine/threonine kinase 1  NRG2 - neuregulin 2  SYVN1 - synovial apoptosis inhibitor 1, synoviolin  BFAR - bifunctional apoptosis regulator  SLX4 - slx4 structure-specific endonuclease subunit  CHD1L - chromodomain helicase dna binding protein 1-like  PTPDC1 - protein tyrosine phosphatase domain containing 1  NEURL4 - neuralized homolog 4 (drosophila)  GTF2IRD1 - gtf2i repeat domain containing 1  UBAP1 - ubiquitin associated protein 1  UBXN2B - ubx domain protein 2b  LIPE - lipase, hormone-sensitive  GTPBP1 - gtp binding protein 1  MINPP1 - multiple inositol-polyphosphate phosphatase 1  LIMK2 - lim domain kinase 2  ZC3H8 - zinc finger ccch-type containing 8  GOLM1 - golgi membrane protein 1  CDC42BPB - cdc42 binding protein kinase beta (dmpk-like)  CLOCK - clock circadian regulator  LMAN1 - lectin, mannose-binding, 1  PRKAG2 - protein kinase, amp-activated, gamma 2 non-catalytic subunit  POLK - polymerase (dna directed) kappa  ANAPC5 - anaphase promoting complex subunit 5  ANAPC7 - anaphase promoting complex subunit 7  MRPL43 - mitochondrial ribosomal protein l43  TBPL1 - tbp-like 1  YTHDF2 - yth domain family, member 2  NPEPPS - aminopeptidase puromycin sensitive  RNF138 - ring finger protein 138, e3 ubiquitin protein ligase  KTN1 - kinectin 1 (kinesin receptor)  VPS4B - vacuolar protein sorting 4 homolog b (s. cerevisiae)  KRT10 - keratin 10  POLR1C - polymerase (rna) i polypeptide c, 30kda  PPME1 - protein phosphatase methylesterase 1  HEMK1 - hemk methyltransferase family member 1  ACTL6B - actin-like 6b  LAMP2 - lysosomal-associated membrane protein 2  UBR5 - ubiquitin protein ligase e3 component n-recognin 5  TMA7 - translation machinery associated 7 homolog (s. cerevisiae)  MED20 - mediator complex subunit 20  UCHL5 - ubiquitin carboxyl-terminal hydrolase l5  ATG5 - autophagy related 5  ROCK2 - rho-associated, coiled-coil containing protein kinase 2  MAPK8IP1 - mitogen-activated protein kinase 8 interacting protein 1  PIGB - phosphatidylinositol glycan anchor biosynthesis, class b  CHST10 - carbohydrate sulfotransferase 10  TAOK3 - tao kinase 3  FZR1 - fizzy/cell division cycle 20 related 1 (drosophila)  ZBTB7A - zinc finger and btb domain containing 7a  DPY30 - dpy-30 homolog (c. elegans)  LAMC1 - laminin, gamma 1 (formerly lamb2)  IRF2BP2 - interferon regulatory factor 2 binding protein 2  MED27 - mediator complex subunit 27  NAGPA - n-acetylglucosamine-1-phosphodiester alpha-n-acetylglucosaminidase  POLR1B - polymerase (rna) i polypeptide b, 128kda  GALNT18 - udp-n-acetyl-alpha-d-galactosamine:polypeptide n-acetylgalactosaminyltransferase 18  VPS28 - vacuolar protein sorting 28 homolog (s. cerevisiae)  MRTO4 - mrna turnover 4 homolog (s. cerevisiae)  ASB7 - ankyrin repeat and socs box containing 7  ASB8 - ankyrin repeat and socs box containing 8  ARHGEF6 - rac/cdc42 guanine nucleotide exchange factor (gef) 6  KDR - kinase insert domain receptor (a type iii receptor tyrosine kinase)  PICK1 - protein interacting with prkca 1  ASB6 - ankyrin repeat and socs box containing 6  ING4 - inhibitor of growth family, member 4  EIF2AK3 - eukaryotic translation initiation factor 2-alpha kinase 3  USP48 - ubiquitin specific peptidase 48  ITM2B - integral membrane protein 2b  IRAK4 - interleukin-1 receptor-associated kinase 4  MAP4K4 - mitogen-activated protein kinase kinase kinase kinase 4  RHBDD1 - rhomboid domain containing 1  RLIM - ring finger protein, lim domain interacting  ASB3 - ankyrin repeat and socs box containing 3  TRAF7 - tnf receptor-associated factor 7, e3 ubiquitin protein ligase  RECQL5 - recq protein-like 5  KRAS - kirsten rat sarcoma viral oncogene homolog  NAA20 - n(alpha)-acetyltransferase 20, natb catalytic subunit  GOLGA7 - golgin a7  HS6ST1 - heparan sulfate 6-o-sulfotransferase 1  KPNA2 - karyopherin alpha 2 (rag cohort 1, importin alpha 1)  RPL26L1 - ribosomal protein l26-like 1  KPNB1 - karyopherin (importin) beta 1  KPNA1 - karyopherin alpha 1 (importin alpha 5)  ZDHHC9 - zinc finger, dhhc-type containing 9  CAMKK1 - calcium/calmodulin-dependent protein kinase kinase 1, alpha  SUV420H1 - suppressor of variegation 4-20 homolog 1 (drosophila)  APH1A - aph1a gamma secretase subunit  PHF20L1 - phd finger protein 20-like 1  TFB1M - transcription factor b1, mitochondrial  MED10 - mediator complex subunit 10  MED21 - mediator complex subunit 21  ABHD17B - abhydrolase domain containing 17b  KIT - v-kit hardy-zuckerman 4 feline sarcoma viral oncogene homolog  SH3GLB1 - sh3-domain grb2-like endophilin b1  RPAIN - rpa interacting protein  LONP1 - lon peptidase 1, mitochondrial  MRPL51 - mitochondrial ribosomal protein l51  MRPL30 - mitochondrial ribosomal protein l30  NOA1 - nitric oxide associated 1  SHISA5 - shisa family member 5  DCUN1D5 - dcn1, defective in cullin neddylation 1, domain containing 5  UBE4A - ubiquitination factor e4a  FBXW9 - f-box and wd repeat domain containing 9  PPIG - peptidylprolyl isomerase g (cyclophilin g)  POLR3GL - polymerase (rna) iii (dna directed) polypeptide g (32kd)-like  ZDHHC16 - zinc finger, dhhc-type containing 16  JAK1 - janus kinase 1  JAK2 - janus kinase 2  ING5 - inhibitor of growth family, member 5  ANKRD39 - ankyrin repeat domain 39  JAK3 - janus kinase 3  SLC25A33 - solute carrier family 25 (pyrimidine nucleotide carrier), member 33  STT3A - stt3a, subunit of the oligosaccharyltransferase complex (catalytic)  MAST4 - microtubule associated serine/threonine kinase family member 4  PIGP - phosphatidylinositol glycan anchor biosynthesis, class p  PHF20 - phd finger protein 20  TMEM246 - transmembrane protein 246  TRIP12 - thyroid hormone receptor interactor 12  GTF3C5 - general transcription factor iiic, polypeptide 5, 63kda  COPS2 - cop9 signalosome subunit 2  ZDHHC2 - zinc finger, dhhc-type containing 2  RSL24D1 - ribosomal l24 domain containing 1  CRBN - cereblon  MIA3 - melanoma inhibitory activity family, member 3  TAOK2 - tao kinase 2  B4GALT6 - udp-gal:betaglcnac beta 1,4- galactosyltransferase, polypeptide 6  BRMS1L - breast cancer metastasis-suppressor 1-like  LEF1 - lymphoid enhancer-binding factor 1  GTF3C4 - general transcription factor iiic, polypeptide 4, 90kda  MRPL45 - mitochondrial ribosomal protein l45  GTF3C3 - general transcription factor iiic, polypeptide 3, 102kda  CNOT8 - ccr4-not transcription complex, subunit 8  CCDC115 - coiled-coil domain containing 115  B4GALT5 - udp-gal:betaglcnac beta 1,4- galactosyltransferase, polypeptide 5  CDK12 - cyclin-dependent kinase 12  KCTD10 - potassium channel tetramerization domain containing 10  SNX25 - sorting nexin 25  ARID4B - at rich interactive domain 4b (rbp1-like)  SOCS6 - suppressor of cytokine signaling 6  SPRTN - sprt-like n-terminal domain  MIPEP - mitochondrial intermediate peptidase  STK35 - serine/threonine kinase 35  MAP3K10 - mitogen-activated protein kinase kinase kinase 10  DUSP19 - dual specificity phosphatase 19  MLH1 - mutl homolog 1, colon cancer, nonpolyposis type 2 (e. coli)  MIB2 - mindbomb e3 ubiquitin protein ligase 2  KDM3B - lysine (k)-specific demethylase 3b  RSF1 - remodeling and spacing factor 1  MLLT3 - myeloid/lymphoid or mixed-lineage leukemia (trithorax homolog, drosophila); translocated to, 3  MLLT1 - myeloid/lymphoid or mixed-lineage leukemia (trithorax homolog, drosophila); translocated to, 1  MAP3K11 - mitogen-activated protein kinase kinase kinase 11  PHAX - phosphorylated adaptor for rna export  KMT2A - lysine (k)-specific methyltransferase 2a  MMP2 - matrix metallopeptidase 2 (gelatinase a, 72kda gelatinase, 72kda type iv collagenase)  RPS6KA5 - ribosomal protein s6 kinase, 90kda, polypeptide 5  CCDC8 - coiled-coil domain containing 8  LSM7 - lsm7 homolog, u6 small nuclear rna associated (s. cerevisiae)  STK17A - serine/threonine kinase 17a  NLK - nemo-like kinase  TATDN1 - tatd dnase domain containing 1  MNAT1 - mnat cdk-activating kinase assembly factor 1  UIMC1 - ubiquitin interaction motif containing 1  RAB11B - rab11b, member ras oncogene family  MTA2 - metastasis associated 1 family, member 2  EIF2A - eukaryotic translation initiation factor 2a, 65kda  MNT - mnt, max dimerization protein  POLR3K - polymerase (rna) iii (dna directed) polypeptide k, 12.3 kda  MCM7 - minichromosome maintenance complex component 7  KBTBD7 - kelch repeat and btb (poz) domain containing 7  KDELC2 - kdel (lys-asp-glu-leu) containing 2  DNAJB9 - dnaj (hsp40) homolog, subfamily b, member 9  BUB3 - bub3 mitotic checkpoint protein  PELO - pelota homolog (drosophila)  MECP2 - methyl cpg binding protein 2 (rett syndrome)  MAGT1 - magnesium transporter 1  MEF2A - myocyte enhancer factor 2a  DDX21 - dead (asp-glu-ala-asp) box helicase 21  ZBED1 - zinc finger, bed-type containing 1  BCDIN3D - bcdin3 domain containing  PPIL3 - peptidylprolyl isomerase (cyclophilin)-like 3  MAP3K4 - mitogen-activated protein kinase kinase kinase 4  DCLK1 - doublecortin-like kinase 1  MAP3K5 - mitogen-activated protein kinase kinase kinase 5  MAP3K1 - mitogen-activated protein kinase kinase kinase 1, e3 ubiquitin protein ligase  MAP3K3 - mitogen-activated protein kinase kinase kinase 3  MEN1 - multiple endocrine neoplasia i  RAB8A - rab8a, member ras oncogene family  USP42 - ubiquitin specific peptidase 42  METTL1 - methyltransferase like 1  KAT8 - k(lysine) acetyltransferase 8  RNASEH2C - ribonuclease h2, subunit c  FBXL14 - f-box and leucine-rich repeat protein 14  MFGE8 - milk fat globule-egf factor 8 protein  PPP1R1B - protein phosphatase 1, regulatory (inhibitor) subunit 1b  ATG12 - autophagy related 12  MGAT1 - mannosyl (alpha-1,3-)-glycoprotein beta-1,2-n-acetylglucosaminyltransferase  MGAT2 - mannosyl (alpha-1,6-)-glycoprotein beta-1,2-n-acetylglucosaminyltransferase  HGS - hepatocyte growth factor-regulated tyrosine kinase substrate  MGAT5 - mannosyl (alpha-1,6-)-glycoprotein beta-1,6-n-acetyl-glucosaminyltransferase  NEMF - nuclear export mediator factor  MBD3 - methyl-cpg binding domain protein 3  KITLG - kit ligand  B3GALTL - beta 1,3-galactosyltransferase-like  ATRIP - atr interacting protein  MAN1A1 - mannosidase, alpha, class 1a, member 1  CBFA2T2 - core-binding factor, runt domain, alpha subunit 2; translocated to, 2  MAN2A2 - mannosidase, alpha, class 2a, member 2  ZC3HC1 - zinc finger, c3hc-type containing 1  COG3 - component of oligomeric golgi complex 3  JKAMP - jnk1/mapk8-associated membrane protein  MAGOH - mago-nashi homolog, proliferation-associated (drosophila)  CHST9 - carbohydrate (n-acetylgalactosamine 4-0) sulfotransferase 9  RQCD1 - rcd1 required for cell differentiation1 homolog (s. pombe)  SMC3 - structural maintenance of chromosomes 3  MARS - methionyl-trna synthetase  MARK3 - map/microtubule affinity-regulating kinase 3  MARK1 - map/microtubule affinity-regulating kinase 1  DOHH - deoxyhypusine hydroxylase/monooxygenase  MAPT - microtubule-associated protein tau  APH1B - aph1b gamma secretase subunit  MDP1 - magnesium-dependent phosphatase 1  LATS1 - large tumor suppressor kinase 1  MTA1 - metastasis associated 1  SIRT6 - sirtuin 6  MTMR4 - myotubularin related protein 4  MTMR7 - myotubularin related protein 7  MTMR6 - myotubularin related protein 6  MANBA - mannosidase, beta a, lysosomal  MAN2A1 - mannosidase, alpha, class 2a, member 1  USP8 - ubiquitin specific peptidase 8  USP14 - ubiquitin specific peptidase 14 (trna-guanine transglycosylase)  USP10 - ubiquitin specific peptidase 10  UBE2J1 - ubiquitin-conjugating enzyme e2, j1  NUP43 - nucleoporin 43kda  MBD1 - methyl-cpg binding domain protein 1  REV1 - rev1, polymerase (dna directed)  PKMYT1 - protein kinase, membrane associated tyrosine/threonine 1  MATK - megakaryocyte-associated tyrosine kinase  UFC1 - ubiquitin-fold modifier conjugating enzyme 1  MCM3 - minichromosome maintenance complex component 3  TRMT112 - trna methyltransferase 11-2 homolog (s. cerevisiae)  MCM6 - minichromosome maintenance complex component 6  MCM5 - minichromosome maintenance complex component 5  RTFDC1 - replication termination factor 2 domain containing 1  TTBK2 - tau tubulin kinase 2  NELFCD - negative elongation factor complex member c/d  MCM2 - minichromosome maintenance complex component 2  ASH2L - ash2 (absent, small, or homeotic)-like (drosophila)  CTDSPL2 - ctd (carboxy-terminal domain, rna polymerase ii, polypeptide a) small phosphatase like 2  NUDT12 - nudix (nucleoside diphosphate linked moiety x)-type motif 12  PPIL1 - peptidylprolyl isomerase (cyclophilin)-like 1  NFS1 - nfs1 cysteine desulfurase  ITCH - itchy e3 ubiquitin protein ligase  MRPL48 - mitochondrial ribosomal protein l48  FBXL16 - f-box and leucine-rich repeat protein 16  MRPS23 - mitochondrial ribosomal protein s23  MRPS33 - mitochondrial ribosomal protein s33  LTA4H - leukotriene a4 hydrolase  LRP1 - low density lipoprotein receptor-related protein 1  RPL14 - ribosomal protein l14  RIOK1 - rio kinase 1  ZNF445 - zinc finger protein 445  AIP - aryl hydrocarbon receptor interacting protein  OTUD6B - otu domain containing 6b  NUB1 - negative regulator of ubiquitin-like proteins 1  ASB1 - ankyrin repeat and socs box containing 1  LYN - v-yes-1 yamaguchi sarcoma viral related oncogene homolog  UBE2M - ubiquitin-conjugating enzyme e2m  UBA3 - ubiquitin-like modifier activating enzyme 3  SESN2 - sestrin 2  PARP9 - poly (adp-ribose) polymerase family, member 9  RNF8 - ring finger protein 8, e3 ubiquitin protein ligase  LTBP1 - latent transforming growth factor beta binding protein 1  RPS6KL1 - ribosomal protein s6 kinase-like 1  FKBP7 - fk506 binding protein 7  BAZ1B - bromodomain adjacent to zinc finger domain, 1b  TAF1B - tata box binding protein (tbp)-associated factor, rna polymerase i, b, 63kda  PIAS4 - protein inhibitor of activated stat, 4  TRIM33 - tripartite motif containing 33  SRRT - serrate rna effector molecule homolog (arabidopsis)  SMAD2 - smad family member 2  MBIP - map3k12 binding inhibitory protein 1  HDAC7 - histone deacetylase 7  TMTC1 - transmembrane and tetratricopeptide repeat containing 1  TDP2 - tyrosyl-dna phosphodiesterase 2  CDKL2 - cyclin-dependent kinase-like 2 (cdc2-related kinase)  TAF1C - tata box binding protein (tbp)-associated factor, rna polymerase i, c, 110kda  LARP7 - la ribonucleoprotein domain family, member 7  RPS6KA4 - ribosomal protein s6 kinase, 90kda, polypeptide 4  KCTD11 - potassium channel tetramerization domain containing 11  PLOD3 - procollagen-lysine, 2-oxoglutarate 5-dioxygenase 3  DPH5 - diphthamide biosynthesis 5  TAF9B - taf9b rna polymerase ii, tata box binding protein (tbp)-associated factor, 31kda  SMAD4 - smad family member 4  NBAS - neuroblastoma amplified sequence  EME1 - essential meiotic structure-specific endonuclease 1  SMAD5 - smad family member 5  P4HA2 - prolyl 4-hydroxylase, alpha polypeptide ii  TMEM199 - transmembrane protein 199  SMAD7 - smad family member 7  TRMT6 - trna methyltransferase 6 homolog (s. cerevisiae)  PIGT - phosphatidylinositol glycan anchor biosynthesis, class t  MYLIP - myosin regulatory light chain interacting protein  NCBP1 - nuclear cap binding protein subunit 1, 80kda  TSPAN14 - tetraspanin 14  NCK1 - nck adaptor protein 1  DPY19L3 - dpy-19-like 3 (c. elegans)  TRMT1L - trna methyltransferase 1 homolog (s. cerevisiae)-like  ABI2 - abl-interactor 2  TRIM28 - tripartite motif containing 28  DROSHA - drosha, ribonuclease type iii  NAP1L1 - nucleosome assembly protein 1-like 1  TMEM208 - transmembrane protein 208  SCG3 - secretogranin iii  NASP - nuclear autoantigenic sperm protein (histone-binding)  TBK1 - tank-binding kinase 1  NCAM1 - neural cell adhesion molecule 1  NBN - nibrin  NHLRC3 - nhl repeat containing 3  RBM7 - rna binding motif protein 7  ZDHHC1 - zinc finger, dhhc-type containing 1  ZDHHC8 - zinc finger, dhhc-type containing 8  TRIM11 - tripartite motif containing 11  PPP1R12A - protein phosphatase 1, regulatory subunit 12a  TNK2 - tyrosine kinase, non-receptor, 2  ALYREF - aly/ref export factor  SENP1 - sumo1/sentrin specific peptidase 1  RNF41 - ring finger protein 41  TFPT - tcf3 (e2a) fusion partner (in childhood leukemia)  PRMT3 - protein arginine methyltransferase 3  PSME3 - proteasome (prosome, macropain) activator subunit 3 (pa28 gamma; ki)  MYLK - myosin light chain kinase  CDADC1 - cytidine and dcmp deaminase domain containing 1  CALCRL - calcitonin receptor-like  URM1 - ubiquitin related modifier 1  TRIM13 - tripartite motif containing 13  TRIM8 - tripartite motif containing 8  USPL1 - ubiquitin specific peptidase like 1  ABT1 - activator of basal transcription 1  TOPORS - topoisomerase i binding, arginine/serine-rich, e3 ubiquitin protein ligase  RAB1B - rab1b, member ras oncogene family  SEH1L - seh1-like (s. cerevisiae)  PTPRU - protein tyrosine phosphatase, receptor type, u  HUWE1 - hect, uba and wwe domain containing 1, e3 ubiquitin protein ligase  CNOT7 - ccr4-not transcription complex, subunit 7  ALG5 - alg5, dolichyl-phosphate beta-glucosyltransferase  MX1 - myxovirus (influenza virus) resistance 1, interferon-inducible protein p78 (mouse)  TSPAN5 - tetraspanin 5  INO80B - ino80 complex subunit b  B3GALNT2 - beta-1,3-n-acetylgalactosaminyltransferase 2  RBM15B - rna binding motif protein 15b  CTDSP2 - ctd (carboxy-terminal domain, rna polymerase ii, polypeptide a) small phosphatase 2  ANAPC4 - anaphase promoting complex subunit 4  TSFM - ts translation elongation factor, mitochondrial  RNF170 - ring finger protein 170  FEM1B - fem-1 homolog b (c. elegans)  MUTYH - muty homolog (e. coli)  RAD50 - rad50 homolog (s. cerevisiae)  TRIM37 - tripartite motif containing 37  CDC42EP5 - cdc42 effector protein (rho gtpase binding) 5  PDIA6 - protein disulfide isomerase family a, member 6  SHARPIN - shank-associated rh domain interactor  AKT3 - v-akt murine thymoma viral oncogene homolog 3  ABI1 - abl-interactor 1  MTRR - 5-methyltetrahydrofolate-homocysteine methyltransferase reductase  PPP4R1 - protein phosphatase 4, regulatory subunit 1  PDCD6 - programmed cell death 6  MTM1 - myotubularin 1  HDAC5 - histone deacetylase 5  MTF1 - metal-regulatory transcription factor 1  HDAC6 - histone deacetylase 6  PARP2 - poly (adp-ribose) polymerase 2  RNF187 - ring finger protein 187  SH2D3C - sh2 domain containing 3c  DCPS - decapping enzyme, scavenger  MT3 - metallothionein 3  MED16 - mediator complex subunit 16  PIGK - phosphatidylinositol glycan anchor biosynthesis, class k  CHAF1A - chromatin assembly factor 1, subunit a (p150)  CCDC22 - coiled-coil domain containing 22  SAE1 - sumo1 activating enzyme subunit 1  FARSB - phenylalanyl-trna synthetase, beta subunit  PDIK1L - pdlim1 interacting kinase 1 like  RANBP9 - ran binding protein 9  UBA2 - ubiquitin-like modifier activating enzyme 2  JOSD1 - josephin domain containing 1  KANSL1L - kat8 regulatory nsl complex subunit 1-like  MSH3 - muts homolog 3 (e. coli)  MSH2 - muts homolog 2, colon cancer, nonpolyposis type 1 (e. coli)  PAN2 - pan2 poly(a) specific ribonuclease subunit homolog (s. cerevisiae)  FAM20B - family with sequence similarity 20, member b  SUPT7L - suppressor of ty 7 (s. cerevisiae)-like  FBXO38 - f-box protein 38  GFPT2 - glutamine-fructose-6-phosphate transaminase 2  OXSR1 - oxidative stress responsive 1  COMMD1 - copper metabolism (murr1) domain containing 1  SPCS1 - signal peptidase complex subunit 1 homolog (s. cerevisiae)  HM13 - histocompatibility (minor) 13  RBM8A - rna binding motif protein 8a  USP3 - ubiquitin specific peptidase 3  BABAM1 - brisc and brca1 a complex member 1  UBE2T - ubiquitin-conjugating enzyme e2t (putative)  MRPL15 - mitochondrial ribosomal protein l15  METTL5 - methyltransferase like 5  USP15 - ubiquitin specific peptidase 15  TTL - tubulin tyrosine ligase  CCDC59 - coiled-coil domain containing 59  MED4 - mediator complex subunit 4  THOC1 - tho complex 1  C1GALT1C1 - c1galt1-specific chaperone 1  RCE1 - rce1 homolog, prenyl protein protease (s. cerevisiae)  SETD2 - set domain containing 2  MRPL18 - mitochondrial ribosomal protein l18  RBX1 - ring-box 1, e3 ubiquitin protein ligase  DUSP18 - dual specificity phosphatase 18  MRE11A - mre11 meiotic recombination 11 homolog a (s. cerevisiae)  NUP153 - nucleoporin 153kda  THRAP3 - thyroid hormone receptor associated protein 3  MED12 - mediator complex subunit 12  MPI - mannose phosphate isomerase  PSMD6 - proteasome (prosome, macropain) 26s subunit, non-atpase, 6  WDSUB1 - wd repeat, sterile alpha motif and u-box domain containing 1  DTX3L - deltex 3-like (drosophila)  PJA2 - praja ring finger 2, e3 ubiquitin protein ligase  AREL1 - apoptosis resistant e3 ubiquitin protein ligase 1  SETDB1 - set domain, bifurcated 1  ORC4 - origin recognition complex, subunit 4  INTS5 - integrator complex subunit 5  ORC2 - origin recognition complex, subunit 2  TLK1 - tousled-like kinase 1  ORC5 - origin recognition complex, subunit 5  RNF141 - ring finger protein 141  THUMPD2 - thump domain containing 2  ERCC6L2 - excision repair cross-complementing rodent repair deficiency, complementation group 6-like 2  PTAR1 - protein prenyltransferase alpha subunit repeat containing 1  PPP4R2 - protein phosphatase 4, regulatory subunit 2  NDFIP1 - nedd4 family interacting protein 1  RNF38 - ring finger protein 38  XXYLT1 - xyloside xylosyltransferase 1  KLHL21 - kelch-like family member 21  METTL21A - methyltransferase like 21a  AP5Z1 - adaptor-related protein complex 5, zeta 1 subunit  OGG1 - 8-oxoguanine dna glycosylase  TNKS2 - tankyrase, trf1-interacting ankyrin-related adp-ribose polymerase 2  EXOSC3 - exosome component 3  WDR61 - wd repeat domain 61  SMG7 - smg7 nonsense mediated mrna decay factor  APITD1 - apoptosis-inducing, taf9-like domain 1  EXOSC1 - exosome component 1  SPCS2 - signal peptidase complex subunit 2 homolog (s. cerevisiae)  DDR2 - discoidin domain receptor tyrosine kinase 2  TOMM20 - translocase of outer mitochondrial membrane 20 homolog (yeast)  NTHL1 - nth endonuclease iii-like 1 (e. coli)  DAB2IP - dab2 interacting protein  EIF4A3 - eukaryotic translation initiation factor 4a3  NUP88 - nucleoporin 88kda  NUP98 - nucleoporin 98kda  NR4A2 - nuclear receptor subfamily 4, group a, member 2  ZBTB7B - zinc finger and btb domain containing 7b  NUCB1 - nucleobindin 1  ST8SIA3 - st8 alpha-n-acetyl-neuraminide alpha-2,8-sialyltransferase 3  RNF144A - ring finger protein 144a  DNAJC6 - dnaj (hsp40) homolog, subfamily c, member 6  ILKAP - integrin-linked kinase-associated serine/threonine phosphatase  NPM1 - nucleophosmin (nucleolar phosphoprotein b23, numatrin)  SPATA2 - spermatogenesis associated 2  GINS1 - gins complex subunit 1 (psf1 homolog)  KLHL5 - kelch-like family member 5  CTIF - cbp80/20-dependent translation initiation factor  DUSP16 - dual specificity phosphatase 16  RNF40 - ring finger protein 40, e3 ubiquitin protein ligase  SPOCK2 - sparc/osteonectin, cwcv and kazal-like domains proteoglycan (testican) 2  POLR1D - polymerase (rna) i polypeptide d, 16kda  RB1CC1 - rb1-inducible coiled-coil 1  CUL7 - cullin 7  PAQR3 - progestin and adipoq receptor family member iii  RRNAD1 - ribosomal rna adenine dimethylase domain containing 1  NRAS - neuroblastoma ras viral (v-ras) oncogene homolog  SIDT2 - sid1 transmembrane family, member 2  UBQLN1 - ubiquilin 1  DONSON - downstream neighbor of son  NMT1 - n-myristoyltransferase 1  NOP2 - nop2 nucleolar protein  VPRBP - vpr (hiv-1) binding protein  NONO - non-pou domain containing, octamer-binding  RNF34 - ring finger protein 34, e3 ubiquitin protein ligase  MUS81 - mus81 structure-specific endonuclease subunit  NME1 - nme/nm23 nucleoside diphosphate kinase 1  FBXO11 - f-box protein 11  UBQLN2 - ubiquilin 2  ULK2 - unc-51 like autophagy activating kinase 2  SPSB1 - spla/ryanodine receptor domain and socs box containing 1  HERPUD1 - homocysteine-inducible, endoplasmic reticulum stress-inducible, ubiquitin-like domain member 1  ERO1L - ero1-like (s. cerevisiae)  KCTD7 - potassium channel tetramerization domain containing 7  AQR - aquarius homolog (mouse)  CNOT2 - ccr4-not transcription complex, subunit 2  NAA15 - n(alpha)-acetyltransferase 15, nata auxiliary subunit  CNOT4 - ccr4-not transcription complex, subunit 4  CNOT3 - ccr4-not transcription complex, subunit 3  CTC1 - cts telomere maintenance complex component 1  DHX34 - deah (asp-glu-ala-his) box polypeptide 34  NFYA - nuclear transcription factor y, alpha  HDAC4 - histone deacetylase 4  NFRKB - nuclear factor related to kappab binding protein  EDC3 - enhancer of mrna decapping 3  CXXC1 - cxxc finger protein 1  NFKBIA - nuclear factor of kappa light polypeptide gene enhancer in b-cells inhibitor, alpha  NFKB1 - nuclear factor of kappa light polypeptide gene enhancer in b-cells 1  KMT2B - lysine (k)-specific methyltransferase 2b  ST6GALNAC6 - st6 (alpha-n-acetyl-neuraminyl-2,3-beta-galactosyl-1,3)-n-acetylgalactosaminide alpha-2,6-sialyltransferase 6  NFKBIB - nuclear factor of kappa light polypeptide gene enhancer in b-cells inhibitor, beta  PIK3R4 - phosphoinositide-3-kinase, regulatory subunit 4  SLK - ste20-like kinase  VCPIP1 - valosin containing protein (p97)/p47 complex interacting protein 1  NKTR - natural killer-tumor recognition sequence  USP34 - ubiquitin specific peptidase 34  HDAC9 - histone deacetylase 9  RNF145 - ring finger protein 145  SETD1A - set domain containing 1a  EHD3 - eh-domain containing 3  CCP110 - centriolar coiled coil protein 110kda  SOCS7 - suppressor of cytokine signaling 7  MDC1 - mediator of dna-damage checkpoint 1  TTC37 - tetratricopeptide repeat domain 37  HS2ST1 - heparan sulfate 2-o-sulfotransferase 1  NEK3 - nima-related kinase 3  MINK1 - misshapen-like kinase 1  TTLL4 - tubulin tyrosine ligase-like family, member 4  SOCS5 - suppressor of cytokine signaling 5  RRM2B - ribonucleotide reductase m2 b (tp53 inducible)  NEFL - neurofilament, light polypeptide  SMARCAL1 - swi/snf related, matrix associated, actin dependent regulator of chromatin, subfamily a-like 1  NEK1 - nima-related kinase 1  NEDD8 - neural precursor cell expressed, developmentally down-regulated 8  CTR9 - ctr9, paf1/rna polymerase ii complex component  PPM1F - protein phosphatase, mg2+/mn2+ dependent, 1f  CPEB4 - cytoplasmic polyadenylation element binding protein 4  EPC1 - enhancer of polycomb homolog 1 (drosophila)  NFE2L2 - nuclear factor (erythroid-derived 2)-like 2  MORF4L2 - mortality factor 4 like 2  KLHL15 - kelch-like family member 15  NUP155 - nucleoporin 155kda  NFIA - nuclear factor i/a  NF1 - neurofibromin 1  CHST11 - carbohydrate (chondroitin 4) sulfotransferase 11  CDK5RAP3 - cdk5 regulatory subunit associated protein 3  DCAF8 - ddb1 and cul4 associated factor 8  VPS37D - vacuolar protein sorting 37 homolog d (s. cerevisiae)  EDEM1 - er degradation enhancer, mannosidase alpha-like 1  KIAA0391 - kiaa0391  RAPGEF2 - rap guanine nucleotide exchange factor (gef) 2  EDEM3 - er degradation enhancer, mannosidase alpha-like 3  NUP93 - nucleoporin 93kda  CALY - calcyon neuron-specific vesicular protein  KDM4A - lysine (k)-specific demethylase 4a  PHF14 - phd finger protein 14  DRG1 - developmentally regulated gtp binding protein 1  COPS7A - cop9 signalosome subunit 7a  DZIP3 - daz interacting zinc finger protein 3  KIAA0430 - kiaa0430 |
| GO:2000112 | regulation of cellular macromolecule biosynthetic process | 1.14E-11 | 8.84E-9 | 1.08 (10334,2294,6996,1684) | [+] Show genes  EXOSC9 - exosome component 9  MSL3 - male-specific lethal 3 homolog (drosophila)  EXOSC10 - exosome component 10  TADA1 - transcriptional adaptor 1  AARS - alanyl-trna synthetase  CBX1 - chromobox homolog 1  ABCA2 - atp-binding cassette, sub-family a (abc1), member 2  ZNF821 - zinc finger protein 821  ABL1 - c-abl oncogene 1, non-receptor tyrosine kinase  PML - promyelocytic leukemia  MLLT11 - myeloid/lymphoid or mixed-lineage leukemia (trithorax homolog, drosophila); translocated to, 11  CNOT11 - ccr4-not transcription complex, subunit 11  SUPT20H - suppressor of ty 20 homolog (s. cerevisiae)  YWHAQ - tyrosine 3-monooxygenase/tryptophan 5-monooxygenase activation protein, theta polypeptide  BRD8 - bromodomain containing 8  CHD7 - chromodomain helicase dna binding protein 7  LYAR - ly1 antibody reactive  TCERG1 - transcription elongation regulator 1  ZNF354B - zinc finger protein 354b  EHMT2 - euchromatic histone-lysine n-methyltransferase 2  TASP1 - taspase, threonine aspartase, 1  SPIN1 - spindlin 1  SUB1 - sub1 homolog (s. cerevisiae)  MORF4L1 - mortality factor 4 like 1  ZNF511 - zinc finger protein 511  PPID - peptidylprolyl isomerase d  ACTL6A - actin-like 6a  ACVR2A - activin a receptor, type iia  ACVRL1 - activin a receptor type ii-like 1  ACVR1 - activin a receptor, type i  ACVR1B - activin a receptor, type ib  ZNF331 - zinc finger protein 331  MED1 - mediator complex subunit 1  ADCY1 - adenylate cyclase 1 (brain)  PPARD - peroxisome proliferator-activated receptor delta  MAML3 - mastermind-like 3 (drosophila)  POLR2K - polymerase (rna) ii (dna directed) polypeptide k, 7.0kda  FAM129A - family with sequence similarity 129, member a  PARP1 - poly (adp-ribose) polymerase 1  H2AFY2 - h2a histone family, member y2  PPP1R13L - protein phosphatase 1, regulatory subunit 13 like  HES6 - hairy and enhancer of split 6 (drosophila)  POLR2G - polymerase (rna) ii (dna directed) polypeptide g  SMPD3 - sphingomyelin phosphodiesterase 3, neutral membrane (neutral sphingomyelinase ii)  POLR2D - polymerase (rna) ii (dna directed) polypeptide d  RUVBL2 - ruvb-like 2 (e. coli)  POLR2A - polymerase (rna) ii (dna directed) polypeptide a, 220kda  PARP4 - poly (adp-ribose) polymerase family, member 4  CDK5RAP2 - cdk5 regulatory subunit associated protein 2  RCOR3 - rest corepressor 3  AES - amino-terminal enhancer of split  NUP133 - nucleoporin 133kda  JAG1 - jagged 1  PRDM4 - pr domain containing 4  PPP3R1 - protein phosphatase 3, regulatory subunit b, alpha  ATF7IP - activating transcription factor 7 interacting protein  ZNF689 - zinc finger protein 689  LRIF1 - ligand dependent nuclear receptor interacting factor 1  PPP2R5B - protein phosphatase 2, regulatory subunit b', beta  ZNF653 - zinc finger protein 653  AKT1 - v-akt murine thymoma viral oncogene homolog 1  PCID2 - pci domain containing 2  PPP2R1A - protein phosphatase 2, regulatory subunit a, alpha  WWP2 - ww domain containing e3 ubiquitin protein ligase 2  AHR - aryl hydrocarbon receptor  ZNF526 - zinc finger protein 526  FOXP4 - forkhead box p4  PPP2CA - protein phosphatase 2, catalytic subunit, alpha isozyme  ZNF83 - zinc finger protein 83  ESCO1 - establishment of sister chromatid cohesion n-acetyltransferase 1  KANSL3 - kat8 regulatory nsl complex subunit 3  PRNP - prion protein  ATF7 - activating transcription factor 7  AMFR - autocrine motility factor receptor, e3 ubiquitin protein ligase  DNAJC3 - dnaj (hsp40) homolog, subfamily c, member 3  PRKRIR - protein-kinase, interferon-inducible double stranded rna dependent inhibitor, repressor of (p58 repressor)  MAP2K5 - mitogen-activated protein kinase kinase 5  MAP2K7 - mitogen-activated protein kinase kinase 7  ZNF692 - zinc finger protein 692  PIM2 - pim-2 oncogene  EIF2AK2 - eukaryotic translation initiation factor 2-alpha kinase 2  MAP2K1 - mitogen-activated protein kinase kinase 1  EIF4E3 - eukaryotic translation initiation factor 4e family member 3  MAP2K2 - mitogen-activated protein kinase kinase 2  MAPK10 - mitogen-activated protein kinase 10  MAPK9 - mitogen-activated protein kinase 9  MAPK11 - mitogen-activated protein kinase 11  MAPK8 - mitogen-activated protein kinase 8  MAPK7 - mitogen-activated protein kinase 7  GCN1L1 - gcn1 general control of amino-acid synthesis 1-like 1 (yeast)  MAPK3 - mitogen-activated protein kinase 3  MAPK1 - mitogen-activated protein kinase 1  ADIRF - adipogenesis regulatory factor  PRKD1 - protein kinase d1  CCDC88A - coiled-coil domain containing 88a  ANXA3 - annexin a3  PRKCH - protein kinase c, eta  PRKCI - protein kinase c, iota  CCDC85B - coiled-coil domain containing 85b  PRKCB - protein kinase c, beta  NSUN5 - nop2/sun domain family, member 5  PRKAR1A - protein kinase, camp-dependent, regulatory, type i, alpha  YEATS2 - yeats domain containing 2  PKIB - protein kinase (camp-dependent, catalytic) inhibitor beta  APEX1 - apex nuclease (multifunctional dna repair enzyme) 1  RBM23 - rna binding motif protein 23  ZNF605 - zinc finger protein 605  XIAP - x-linked inhibitor of apoptosis  PARN - poly(a)-specific ribonuclease  KLF12 - kruppel-like factor 12  PBX2 - pre-b-cell leukemia homeobox 2  PRMT6 - protein arginine methyltransferase 6  PBX3 - pre-b-cell leukemia homeobox 3  PBX1 - pre-b-cell leukemia homeobox 1  PCBD1 - pterin-4 alpha-carbinolamine dehydratase/dimerization cofactor of hepatocyte nuclear factor 1 alpha  APBB2 - amyloid beta (a4) precursor protein-binding, family b, member 2  CHP1 - calcineurin-like ef-hand protein 1  APBB1 - amyloid beta (a4) precursor protein-binding, family b, member 1 (fe65)  RIF1 - rap1 interacting factor homolog (yeast)  POLG2 - polymerase (dna directed), gamma 2, accessory subunit  RNF139 - ring finger protein 139  KIAA1551 - kiaa1551  PA2G4 - proliferation-associated 2g4, 38kda  RPRD1A - regulation of nuclear pre-mrna domain containing 1a  ZHX1 - zinc fingers and homeoboxes 1  ARF4 - adp-ribosylation factor 4  SBNO1 - strawberry notch homolog 1 (drosophila)  IRAK3 - interleukin-1 receptor-associated kinase 3  SETD5 - set domain containing 5  APP - amyloid beta (a4) precursor protein  BBS7 - bardet-biedl syndrome 7  DDX20 - dead (asp-glu-ala-asp) box polypeptide 20  PAK3 - p21 protein (cdc42/rac)-activated kinase 3  PAM - peptidylglycine alpha-amidating monooxygenase  ARNTL - aryl hydrocarbon receptor nuclear translocator-like  ARNT - aryl hydrocarbon receptor nuclear translocator  SUPT16H - suppressor of ty 16 homolog (s. cerevisiae)  PDGFRB - platelet-derived growth factor receptor, beta polypeptide  ARRB2 - arrestin, beta 2  ARRB1 - arrestin, beta 1  PDGFB - platelet-derived growth factor beta polypeptide  SAMD4B - sterile alpha motif domain containing 4b  STRAP - serine/threonine kinase receptor associated protein  RHOA - ras homolog family member a  PSIP1 - pc4 and sfrs1 interacting protein 1  MED9 - mediator complex subunit 9  FSTL1 - follistatin-like 1  BAZ2A - bromodomain adjacent to zinc finger domain, 2a  PCNA - proliferating cell nuclear antigen  CHMP1A - charged multivesicular body protein 1a  JDP2 - jun dimerization protein 2  CDK16 - cyclin-dependent kinase 16  AKIRIN2 - akirin 2  HEATR1 - heat repeat containing 1  CDK17 - cyclin-dependent kinase 17  LEO1 - leo1, paf1/rna polymerase ii complex component, homolog (s. cerevisiae)  ERLIN2 - er lipid raft associated 2  PWP1 - pwp1 homolog (s. cerevisiae)  WRAP53 - wd repeat containing, antisense to tp53  PDE2A - phosphodiesterase 2a, cgmp-stimulated  THAP1 - thap domain containing, apoptosis associated protein 1  PKIG - protein kinase (camp-dependent, catalytic) inhibitor gamma  ELP3 - elongator acetyltransferase complex subunit 3  ASCL1 - achaete-scute complex homolog 1 (drosophila)  KAT7 - k(lysine) acetyltransferase 7  RGMB - rgm domain family, member b  SBNO2 - strawberry notch homolog 2 (drosophila)  ZNF444 - zinc finger protein 444  ZBTB1 - zinc finger and btb domain containing 1  ZNF365 - zinc finger protein 365  FBXW7 - f-box and wd repeat domain containing 7, e3 ubiquitin protein ligase  BAHD1 - bromo adjacent homology domain containing 1  ZHX2 - zinc fingers and homeoboxes 2  PHF2 - phd finger protein 2  MORC2 - morc family cw-type zinc finger 2  PHF1 - phd finger protein 1  FOXJ3 - forkhead box j3  RERE - arginine-glutamic acid dipeptide (re) repeats  DNAJC24 - dnaj (hsp40) homolog, subfamily c, member 24  LARP6 - la ribonucleoprotein domain family, member 6  ZFHX3 - zinc finger homeobox 3  PEX14 - peroxisomal biogenesis factor 14  ADNP2 - adnp homeobox 2  AEBP2 - ae binding protein 2  ZNF507 - zinc finger protein 507  IMPACT - impact rwd domain protein  LGR4 - leucine-rich repeat containing g protein-coupled receptor 4  ATP2B4 - atpase, ca++ transporting, plasma membrane 4  ZNF652 - zinc finger protein 652  PKD1 - polycystic kidney disease 1 (autosomal dominant)  SCAF8 - sr-related ctd-associated factor 8  PKD2 - polycystic kidney disease 2 (autosomal dominant)  MTF2 - metal response element binding transcription factor 2  NAT10 - n-acetyltransferase 10 (gcn5-related)  BACH1 - btb and cnc homology 1, basic leucine zipper transcription factor 1  PIN1 - peptidylprolyl cis/trans isomerase, nima-interacting 1  PIK3R2 - phosphoinositide-3-kinase, regulatory subunit 2 (beta)  DPH3 - diphthamide biosynthesis 3  PIK3R1 - phosphoinositide-3-kinase, regulatory subunit 1 (alpha)  ASXL2 - additional sex combs like 2 (drosophila)  PLAGL1 - pleiomorphic adenoma gene-like 1  ELP2 - elongator acetyltransferase complex subunit 2  ATRX - alpha thalassemia/mental retardation syndrome x-linked  PKNOX1 - pbx/knotted 1 homeobox 1  PKM - pyruvate kinase, muscle  CASC3 - cancer susceptibility candidate 3  OGFOD1 - 2-oxoglutarate and iron-dependent oxygenase domain containing 1  TMEM100 - transmembrane protein 100  PHF10 - phd finger protein 10  RABL3 - rab, member of ras oncogene family-like 3  PSPC1 - paraspeckle component 1  BCL7A - b-cell cll/lymphoma 7a  MRGBP - mrg/morf4l binding protein  BCL9 - b-cell cll/lymphoma 9  RPUSD3 - rna pseudouridylate synthase domain containing 3  BRF2 - brf2, rna polymerase iii transcription initiation factor 50 kda subunit  PIM1 - pim-1 oncogene  BCL2 - b-cell cll/lymphoma 2  ECD - ecdysoneless homolog (drosophila)  CCND1 - cyclin d1  PARK7 - parkinson protein 7  PNKP - polynucleotide kinase 3'-phosphatase  C3orf33 - chromosome 3 open reading frame 33  TRIM27 - tripartite motif containing 27  RFC5 - replication factor c (activator 1) 5, 36.5kda  RFC2 - replication factor c (activator 1) 2, 40kda  RFC3 - replication factor c (activator 1) 3, 38kda  SAP18 - sin3a-associated protein, 18kda  TSHZ2 - teashirt zinc finger homeobox 2  RFXAP - regulatory factor x-associated protein  RFX5 - regulatory factor x, 5 (influences hla class ii expression)  WHSC1L1 - wolf-hirschhorn syndrome candidate 1-like 1  EIF1B - eukaryotic translation initiation factor 1b  RFX1 - regulatory factor x, 1 (influences hla class ii expression)  APBB3 - amyloid beta (a4) precursor protein-binding, family b, member 3  RELA - v-rel avian reticuloendotheliosis viral oncogene homolog a  BCOR - bcl6 corepressor  ZFP36L2 - zfp36 ring finger protein-like 2  ZFP36L1 - zfp36 ring finger protein-like 1  RET - ret proto-oncogene  DPF2 - d4, zinc and double phd fingers family 2  UPF1 - upf1 regulator of nonsense transcripts homolog (yeast)  CC2D1A - coiled-coil and c2 domain containing 1a  RLF - rearranged l-myc fusion  RING1 - ring finger protein 1  BMI1 - bmi1 polycomb ring finger oncogene  PCGF3 - polycomb group ring finger 3  BMPR1A - bone morphogenetic protein receptor, type ia  BMPR2 - bone morphogenetic protein receptor, type ii (serine/threonine kinase)  GON4L - gon-4-like (c. elegans)  BMPR1B - bone morphogenetic protein receptor, type ib  BMP7 - bone morphogenetic protein 7  ABCA7 - atp-binding cassette, sub-family a (abc1), member 7  PSMD14 - proteasome (prosome, macropain) 26s subunit, non-atpase, 14  FLOT1 - flotillin 1  BCAS3 - breast carcinoma amplified sequence 3  UHMK1 - u2af homology motif (uhm) kinase 1  AHI1 - abelson helper integration site 1  RNF4 - ring finger protein 4  BRD2 - bromodomain containing 2  RNF2 - ring finger protein 2  HNRNPR - heterogeneous nuclear ribonucleoprotein r  ZNF280D - zinc finger protein 280d  RNF6 - ring finger protein (c3h2c3 type) 6  GATAD2A - gata zinc finger domain containing 2a  ROCK1 - rho-associated, coiled-coil containing protein kinase 1  RNF111 - ring finger protein 111  TET2 - tet methylcytosine dioxygenase 2  MED18 - mediator complex subunit 18  DENND4A - denn/madd domain containing 4a  MBTD1 - mbt domain containing 1  RAMP1 - receptor (g protein-coupled) activity modifying protein 1  ZMPSTE24 - zinc metallopeptidase ste24  TRIM44 - tripartite motif containing 44  TAB1 - tgf-beta activated kinase 1/map3k7 binding protein 1  PPIE - peptidylprolyl isomerase e (cyclophilin e)  MCRS1 - microspherule protein 1  NRDE2 - nrde-2, necessary for rna interference, domain containing  C1D - c1d nuclear receptor corepressor  PTCD3 - pentatricopeptide repeat domain 3  SLC30A9 - solute carrier family 30 (zinc transporter), member 9  MAD2L2 - mad2 mitotic arrest deficient-like 2 (yeast)  PID1 - phosphotyrosine interaction domain containing 1  PHIP - pleckstrin homology domain interacting protein  RPS4X - ribosomal protein s4, x-linked  NRARP - notch-regulated ankyrin repeat protein  ZNF787 - zinc finger protein 787  RMND1 - required for meiotic nuclear division 1 homolog (s. cerevisiae)  TRIM38 - tripartite motif containing 38  TADA3 - transcriptional adaptor 3  CALR - calreticulin  ZNF792 - zinc finger protein 792  WTIP - wilms tumor 1 interacting protein  NCOA2 - nuclear receptor coactivator 2  SYNCRIP - synaptotagmin binding, cytoplasmic rna interacting protein  CREB3 - camp responsive element binding protein 3  CITED2 - cbp/p300-interacting transactivator, with glu/asp-rich carboxy-terminal domain, 2  CCT6A - chaperonin containing tcp1, subunit 6a (zeta 1)  CCNT2 - cyclin t2  PINX1 - pin2/terf1 interacting, telomerase inhibitor 1  CCND3 - cyclin d3  CCNT1 - cyclin t1  HMG20A - high mobility group 20a  CCNH - cyclin h  BANP - btg3 associated nuclear protein  TIPIN - timeless interacting protein  ACER2 - alkaline ceramidase 2  TRNAU1AP - trna selenocysteine 1 associated protein 1  RPS6KA1 - ribosomal protein s6 kinase, 90kda, polypeptide 1  RPS6KB1 - ribosomal protein s6 kinase, 70kda, polypeptide 1  RPS6KB2 - ribosomal protein s6 kinase, 70kda, polypeptide 2  CBFA2T3 - core-binding factor, runt domain, alpha subunit 2; translocated to, 3  CBFB - core-binding factor, beta subunit  PIAS3 - protein inhibitor of activated stat, 3  RXRB - retinoid x receptor, beta  CAV1 - caveolin 1, caveolae protein, 22kda  RBM14 - rna binding motif protein 14  CCNC - cyclin c  TRMT10C - trna methyltransferase 10 homolog c (s. cerevisiae)  PRMT5 - protein arginine methyltransferase 5  CHTF8 - ctf8, chromosome transmission fidelity factor 8 homolog (s. cerevisiae)  CDK11B - cyclin-dependent kinase 11b  CDC5L - cell division cycle 5-like  MXD4 - max dimerization protein 4  HTRA1 - htra serine peptidase 1  POLR3F - polymerase (rna) iii (dna directed) polypeptide f, 39 kda  HEXIM1 - hexamethylene bis-acetamide inducible 1  ERLIN1 - er lipid raft associated 1  SPIN2A - spindlin family, member 2a  NCOA7 - nuclear receptor coactivator 7  POMT1 - protein-o-mannosyltransferase 1  PSEN1 - presenilin 1  USP16 - ubiquitin specific peptidase 16  PSMA1 - proteasome (prosome, macropain) subunit, alpha type, 1  PSMA2 - proteasome (prosome, macropain) subunit, alpha type, 2  CD81 - cd81 molecule  CAMKK2 - calcium/calmodulin-dependent protein kinase kinase 2, beta  PSMB1 - proteasome (prosome, macropain) subunit, beta type, 1  PSMA5 - proteasome (prosome, macropain) subunit, alpha type, 5  PSMA3 - proteasome (prosome, macropain) subunit, alpha type, 3  PSMB10 - proteasome (prosome, macropain) subunit, beta type, 10  RIPPLY2 - ripply transcriptional repressor 2  TAF6L - taf6-like rna polymerase ii, p300/cbp-associated factor (pcaf)-associated factor, 65kda  PSMC6 - proteasome (prosome, macropain) 26s subunit, atpase, 6  PSMD1 - proteasome (prosome, macropain) 26s subunit, non-atpase, 1  PSMC4 - proteasome (prosome, macropain) 26s subunit, atpase, 4  SOX18 - sry (sex determining region y)-box 18  POLR3G - polymerase (rna) iii (dna directed) polypeptide g (32kd)  PSMC2 - proteasome (prosome, macropain) 26s subunit, atpase, 2  PSMD9 - proteasome (prosome, macropain) 26s subunit, non-atpase, 9  PSMD10 - proteasome (prosome, macropain) 26s subunit, non-atpase, 10  PSMD7 - proteasome (prosome, macropain) 26s subunit, non-atpase, 7  PSMD8 - proteasome (prosome, macropain) 26s subunit, non-atpase, 8  PSMD5 - proteasome (prosome, macropain) 26s subunit, non-atpase, 5  PSMD2 - proteasome (prosome, macropain) 26s subunit, non-atpase, 2  TERF2IP - telomeric repeat binding factor 2, interacting protein  PSMD12 - proteasome (prosome, macropain) 26s subunit, non-atpase, 12  ATG7 - autophagy related 7  PSMD13 - proteasome (prosome, macropain) 26s subunit, non-atpase, 13  PTBP1 - polypyrimidine tract binding protein 1  LAMTOR5 - late endosomal/lysosomal adaptor, mapk and mtor activator 5  PTEN - phosphatase and tensin homolog  JMY - junction mediating and regulatory protein, p53 cofactor  MYBBP1A - myb binding protein (p160) 1a  FBLN5 - fibulin 5  CIB1 - calcium and integrin binding 1 (calmyrin)  PTGIS - prostaglandin i2 (prostacyclin) synthase  DDX17 - dead (asp-glu-ala-asp) box helicase 17  KAT5 - k(lysine) acetyltransferase 5  ZFP69 - zfp69 zinc finger protein  CEBPG - ccaat/enhancer binding protein (c/ebp), gamma  CDK5 - cyclin-dependent kinase 5  CDK4 - cyclin-dependent kinase 4  CCT7 - chaperonin containing tcp1, subunit 7 (eta)  CDK9 - cyclin-dependent kinase 9  CDK8 - cyclin-dependent kinase 8  LIN54 - lin-54 homolog (c. elegans)  CDK7 - cyclin-dependent kinase 7  CDKN1C - cyclin-dependent kinase inhibitor 1c (p57, kip2)  CCT2 - chaperonin containing tcp1, subunit 2 (beta)  CDKN1B - cyclin-dependent kinase inhibitor 1b (p27, kip1)  CCT4 - chaperonin containing tcp1, subunit 4 (delta)  PCBP3 - poly(rc) binding protein 3  PTPN2 - protein tyrosine phosphatase, non-receptor type 2  HTATIP2 - hiv-1 tat interactive protein 2, 30kda  CDK2 - cyclin-dependent kinase 2  ARFGEF1 - adp-ribosylation factor guanine nucleotide-exchange factor 1 (brefeldin a-inhibited)  PURA - purine-rich element binding protein a  RRN3 - rrn3 rna polymerase i transcription factor homolog (s. cerevisiae)  PURB - purine-rich element binding protein b  GTPBP2 - gtp binding protein 2  ZMYND11 - zinc finger, mynd-type containing 11  SRSF10 - serine/arginine-rich splicing factor 10  PTPRK - protein tyrosine phosphatase, receptor type, k  PTPRN - protein tyrosine phosphatase, receptor type, n  PEX2 - peroxisomal biogenesis factor 2  MPHOSPH8 - m-phase phosphoprotein 8  KDM5B - lysine (k)-specific demethylase 5b  CHD4 - chromodomain helicase dna binding protein 4  CHD2 - chromodomain helicase dna binding protein 2  CHD3 - chromodomain helicase dna binding protein 3  MTERFD2 - mterf domain containing 2  CHD1 - chromodomain helicase dna binding protein 1  QARS - glutaminyl-trna synthetase  MYPOP - myb-related transcription factor, partner of profilin  RAD21 - rad21 homolog (s. pombe)  RAD17 - rad17 homolog (s. pombe)  PAF1 - paf1, rna polymerase ii associated factor, homolog (s. cerevisiae)  ZNF181 - zinc finger protein 181  RAC1 - ras-related c3 botulinum toxin substrate 1 (rho family, small gtp binding protein rac1)  ZNF268 - zinc finger protein 268  INO80 - ino80 complex subunit  ZNF513 - zinc finger protein 513  GMEB1 - glucocorticoid modulatory element binding protein 1  CCT8 - chaperonin containing tcp1, subunit 8 (theta)  MIER2 - mesoderm induction early response 1, family member 2  RARG - retinoic acid receptor, gamma  RARB - retinoic acid receptor, beta  DLL4 - delta-like 4 (drosophila)  CELF1 - cugbp, elav-like family member 1  NECAB2 - n-terminal ef-hand calcium binding protein 2  CLU - clusterin  KHDRBS1 - kh domain containing, rna binding, signal transduction associated 1  TAF8 - taf8 rna polymerase ii, tata box binding protein (tbp)-associated factor, 43kda  GNL3L - guanine nucleotide binding protein-like 3 (nucleolar)-like  MAGEL2 - mage-like 2  CTCF - ccctc-binding factor (zinc finger protein)  RBL2 - retinoblastoma-like 2 (p130)  RAI1 - retinoic acid induced 1  RBBP7 - retinoblastoma binding protein 7  RBBP8 - retinoblastoma binding protein 8  MAP3K2 - mitogen-activated protein kinase kinase kinase 2  RBM3 - rna binding motif (rnp1, rrm) protein 3  RBM4 - rna binding motif protein 4  RB1 - retinoblastoma 1  ARID4A - at rich interactive domain 4a (rbp1-like)  RBBP6 - retinoblastoma binding protein 6  KDM5A - lysine (k)-specific demethylase 5a  NUP35 - nucleoporin 35kda  PUS7 - pseudouridylate synthase 7 homolog (s. cerevisiae)  PTGES3 - prostaglandin e synthase 3 (cytosolic)  CKS1B - cdc28 protein kinase regulatory subunit 1b  NFAT5 - nuclear factor of activated t-cells 5, tonicity-responsive  CIRBP - cold inducible rna binding protein  DHX29 - deah (asp-glu-ala-his) box polypeptide 29  CHUK - conserved helix-loop-helix ubiquitous kinase  CERS1 - ceramide synthase 1  ZMYND8 - zinc finger, mynd-type containing 8  ANKRA2 - ankyrin repeat, family a (rfxank-like), 2  RBAK - rb-associated krab zinc finger  NFATC2IP - nuclear factor of activated t-cells, cytoplasmic, calcineurin-dependent 2 interacting protein  PATZ1 - poz (btb) and at hook containing zinc finger 1  FBXO18 - f-box protein, helicase, 18  METTL14 - methyltransferase like 14  SPP1 - secreted phosphoprotein 1  SSBP2 - single-stranded dna binding protein 2  KPNA6 - karyopherin alpha 6 (importin alpha 7)  CIRH1A - cirrhosis, autosomal recessive 1a (cirhin)  MIER1 - mesoderm induction early response 1, transcriptional regulator  PPP1R15B - protein phosphatase 1, regulatory subunit 15b  ZNF382 - zinc finger protein 382  ZNF705A - zinc finger protein 705a  SP3 - sp3 transcription factor  SP4 - sp4 transcription factor  SP1 - sp1 transcription factor  SP2 - sp2 transcription factor  ZNF410 - zinc finger protein 410  SOX4 - sry (sex determining region y)-box 4  GTPBP4 - gtp binding protein 4  WBP2 - ww domain binding protein 2  ORC3 - origin recognition complex, subunit 3  HES4 - hairy and enhancer of split 4 (drosophila)  LEMD3 - lem domain containing 3  GATAD1 - gata zinc finger domain containing 1  CREBL2 - camp responsive element binding protein-like 2  ATF6B - activating transcription factor 6 beta  CREBBP - creb binding protein  SOAT1 - sterol o-acyltransferase 1  ATF2 - activating transcription factor 2  CREB1 - camp responsive element binding protein 1  DDX58 - dead (asp-glu-ala-asp) box polypeptide 58  CRABP2 - cellular retinoic acid binding protein 2  RPRD1B - regulation of nuclear pre-mrna domain containing 1b  PLK3 - polo-like kinase 3  SNAPC4 - small nuclear rna activating complex, polypeptide 4, 190kda  SNCA - synuclein, alpha (non a4 component of amyloid precursor)  ZBED5 - zinc finger, bed-type containing 5  CREBZF - creb/atf bzip transcription factor  EID1 - ep300 interacting inhibitor of differentiation 1  TP53INP2 - tumor protein p53 inducible nuclear protein 2  PTBP2 - polypyrimidine tract binding protein 2  SMARCE1 - swi/snf related, matrix associated, actin dependent regulator of chromatin, subfamily e, member 1  SMO - smoothened, frizzled family receptor  SMARCB1 - swi/snf related, matrix associated, actin dependent regulator of chromatin, subfamily b, member 1  SMARCC1 - swi/snf related, matrix associated, actin dependent regulator of chromatin, subfamily c, member 1  HLTF - helicase-like transcription factor  SMARCA4 - swi/snf related, matrix associated, actin dependent regulator of chromatin, subfamily a, member 4  SNX6 - sorting nexin 6  SSBP3 - single stranded dna binding protein 3  SMARCD2 - swi/snf related, matrix associated, actin dependent regulator of chromatin, subfamily d, member 2  SMARCD3 - swi/snf related, matrix associated, actin dependent regulator of chromatin, subfamily d, member 3  SMARCC2 - swi/snf related, matrix associated, actin dependent regulator of chromatin, subfamily c, member 2  SMARCD1 - swi/snf related, matrix associated, actin dependent regulator of chromatin, subfamily d, member 1  WIZ - widely interspaced zinc finger motifs  SMARCA1 - swi/snf related, matrix associated, actin dependent regulator of chromatin, subfamily a, member 1  SMARCA2 - swi/snf related, matrix associated, actin dependent regulator of chromatin, subfamily a, member 2  SGK3 - serum/glucocorticoid regulated kinase family, member 3  CACTIN - cactin, spliceosome c complex subunit  KMT2C - lysine (k)-specific methyltransferase 2c  ZNF462 - zinc finger protein 462  PPIL4 - peptidylprolyl isomerase (cyclophilin)-like 4  SENP2 - sumo1/sentrin/smt3 specific peptidase 2  CST3 - cystatin c  ZNF318 - zinc finger protein 318  PRPF6 - pre-mrna processing factor 6  KDM2B - lysine (k)-specific demethylase 2b  SUPT6H - suppressor of ty 6 homolog (s. cerevisiae)  CBY1 - chibby homolog 1 (drosophila)  CTBP1 - c-terminal binding protein 1  SUPT4H1 - suppressor of ty 4 homolog 1 (s. cerevisiae)  CTBP2 - c-terminal binding protein 2  SUPT5H - suppressor of ty 5 homolog (s. cerevisiae)  RAB3GAP2 - rab3 gtpase activating protein subunit 2 (non-catalytic)  CTNNB1 - catenin (cadherin-associated protein), beta 1, 88kda  SAV1 - salvador homolog 1 (drosophila)  CUX1 - cut-like homeobox 1  NIF3L1 - nif3 ngg1 interacting factor 3-like 1 (s. cerevisiae)  NEK4 - nima-related kinase 4  STK3 - serine/threonine kinase 3  MPV17L2 - mpv17 mitochondrial membrane protein-like 2  PCGF1 - polycomb group ring finger 1  NPNT - nephronectin  FKBP8 - fk506 binding protein 8, 38kda  GPBP1L1 - gc-rich promoter binding protein 1-like 1  CYLD - cylindromatosis (turban tumor syndrome)  TGIF2 - tgfb-induced factor homeobox 2  BCL6B - b-cell cll/lymphoma 6, member b  CRK - v-crk avian sarcoma virus ct10 oncogene homolog  EEFSEC - eukaryotic elongation factor, selenocysteine-trna-specific  NIPBL - nipped-b homolog (drosophila)  CRY1 - cryptochrome 1 (photolyase-like)  STAT2 - signal transducer and activator of transcription 2, 113kda  STAT1 - signal transducer and activator of transcription 1, 91kda  STAT3 - signal transducer and activator of transcription 3 (acute-phase response factor)  CRY2 - cryptochrome 2 (photolyase-like)  ASF1A - anti-silencing function 1a histone chaperone  STAT5A - signal transducer and activator of transcription 5a  STAT6 - signal transducer and activator of transcription 6, interleukin-4 induced  PARM1 - prostate androgen-regulated mucin-like protein 1  BRMS1 - breast cancer metastasis suppressor 1  CRYM - crystallin, mu  ZNF496 - zinc finger protein 496  SS18 - synovial sarcoma translocation, chromosome 18  PHF5A - phd finger protein 5a  GPATCH3 - g patch domain containing 3  TAB3 - tgf-beta activated kinase 1/map3k7 binding protein 3  BAMBI - bmp and activin membrane-bound inhibitor  ZNF335 - zinc finger protein 335  HAVCR2 - hepatitis a virus cellular receptor 2  SRI - sorcin  SRC - v-src avian sarcoma (schmidt-ruppin a-2) viral oncogene homolog  DNAJB5 - dnaj (hsp40) homolog, subfamily b, member 5  SREBF2 - sterol regulatory element binding transcription factor 2  UBE2O - ubiquitin-conjugating enzyme e2o  NSUN3 - nop2/sun domain family, member 3  SRP9 - signal recognition particle 9kda  PRDX5 - peroxiredoxin 5  BACE2 - beta-site app-cleaving enzyme 2  DHX9 - deah (asp-glu-ala-his) box helicase 9  TRAPPC2 - trafficking protein particle complex 2  KANK2 - kn motif and ankyrin repeat domains 2  DDX5 - dead (asp-glu-ala-asp) box helicase 5  DDX3X - dead (asp-glu-ala-asp) box helicase 3, x-linked  DDX1 - dead (asp-glu-ala-asp) box helicase 1  SIN3A - sin3 transcription regulator family member a  DPH6 - diphthamine biosynthesis 6  SET - set nuclear oncogene  WWTR1 - ww domain containing transcription regulator 1  ZNF304 - zinc finger protein 304  MAP2K4 - mitogen-activated protein kinase kinase 4  ZNF286A - zinc finger protein 286a  GEMIN5 - gem (nuclear organelle) associated protein 5  NOL11 - nucleolar protein 11  PBXIP1 - pre-b-cell leukemia homeobox interacting protein 1  DBP - d site of albumin promoter (albumin d-box) binding protein  NELFB - negative elongation factor complex member b  POT1 - protection of telomeres 1  AHCTF1 - at hook containing transcription factor 1  CNOT10 - ccr4-not transcription complex, subunit 10  ZBTB38 - zinc finger and btb domain containing 38  GATAD2B - gata zinc finger domain containing 2b  ZNF551 - zinc finger protein 551  ZNF473 - zinc finger protein 473  KCTD13 - potassium channel tetramerization domain containing 13  YTHDF3 - yth domain family, member 3  WDR18 - wd repeat domain 18  CNOT6 - ccr4-not transcription complex, subunit 6  LTN1 - listerin e3 ubiquitin protein ligase 1  ATXN2 - ataxin 2  ZNF451 - zinc finger protein 451  SCAF4 - sr-related ctd-associated factor 4  SS18L1 - synovial sarcoma translocation gene on chromosome 18-like 1  CHD5 - chromodomain helicase dna binding protein 5  ARID1B - at rich interactive domain 1b (swi1-like)  MED30 - mediator complex subunit 30  MKL2 - mkl/myocardin-like 2  DAPK3 - death-associated protein kinase 3  DAXX - death-domain associated protein  ZZZ3 - zinc finger, zz-type containing 3  HINFP - histone h4 transcription factor  MTA3 - metastasis associated 1 family, member 3  DGKQ - diacylglycerol kinase, theta 110kda  ZNF608 - zinc finger protein 608  DAPK1 - death-associated protein kinase 1  DAP - death-associated protein  MAVS - mitochondrial antiviral signaling protein  SARS - seryl-trna synthetase  SATB1 - satb homeobox 1  NGDN - neuroguidin, eif4e binding protein  RPTOR - regulatory associated protein of mtor, complex 1  SAFB - scaffold attachment factor b  SH2B1 - sh2b adaptor protein 1  SALL2 - sal-like 2 (drosophila)  EPC2 - enhancer of polycomb homolog 2 (drosophila)  DRD2 - dopamine receptor d2  PHF19 - phd finger protein 19  SLC9A1 - solute carrier family 9, subfamily a (nhe1, cation proton antiporter 1), member 1  IRF2BP1 - interferon regulatory factor 2 binding protein 1  EAF1 - ell associated factor 1  CNOT6L - ccr4-not transcription complex, subunit 6-like  ATN1 - atrophin 1  DPH2 - dph2 homolog (s. cerevisiae)  DPH1 - diphthamide biosynthesis 1  C10orf137 - chromosome 10 open reading frame 137  DR1 - down-regulator of transcription 1, tbp-binding (negative cofactor 2)  MAPK15 - mitogen-activated protein kinase 15  SKI - v-ski avian sarcoma viral oncogene homolog  SKIL - ski-like oncogene  UNK - unkempt homolog (drosophila)  DYRK1A - dual-specificity tyrosine-(y)-phosphorylation regulated kinase 1a  MKL1 - megakaryoblastic leukemia (translocation) 1  DVL2 - dishevelled segment polarity protein 2  DVL3 - dishevelled segment polarity protein 3  GIGYF2 - grb10 interacting gyf protein 2  FNIP2 - folliculin interacting protein 2  HOMEZ - homeobox and leucine zipper encoding  EBF4 - early b-cell factor 4  ANKRD17 - ankyrin repeat domain 17  ZFHX2 - zinc finger homeobox 2  HBEGF - heparin-binding egf-like growth factor  LSM14A - lsm14a, scd6 homolog a (s. cerevisiae)  DKC1 - dyskeratosis congenita 1, dyskerin  DLG1 - discs, large homolog 1 (drosophila)  SHOX2 - short stature homeobox 2  POGK - pogo transposable element with krab domain  DHFR - dihydrofolate reductase  PHF12 - phd finger protein 12  CALCOCO1 - calcium binding and coiled-coil domain 1  ZBTB4 - zinc finger and btb domain containing 4  SFPQ - splicing factor proline/glutamine-rich  SFRP1 - secreted frizzled-related protein 1  SFRP2 - secreted frizzled-related protein 2  SFRP4 - secreted frizzled-related protein 4  DNM2 - dynamin 2  GMEB2 - glucocorticoid modulatory element binding protein 2  DNMT1 - dna (cytosine-5-)-methyltransferase 1  SPAG8 - sperm associated antigen 8  DNMT3A - dna (cytosine-5-)-methyltransferase 3 alpha  EIF2AK4 - eukaryotic translation initiation factor 2 alpha kinase 4  CHD8 - chromodomain helicase dna binding protein 8  SGK1 - serum/glucocorticoid regulated kinase 1  NOC2L - nucleolar complex associated 2 homolog (s. cerevisiae)  TNRC6C - trinucleotide repeat containing 6c  ZNF317 - zinc finger protein 317  USP37 - ubiquitin specific peptidase 37  PPP1R3F - protein phosphatase 1, regulatory subunit 3f  MEIS3 - meis homeobox 3  NCBP2 - nuclear cap binding protein subunit 2, 20kda  MAPRE3 - microtubule-associated protein, rp/eb family, member 3  TSC1 - tuberous sclerosis 1  ELK1 - elk1, member of ets oncogene family  NAF1 - nuclear assembly factor 1 ribonucleoprotein  ELK3 - elk3, ets-domain protein (srf accessory protein 2)  ELK4 - elk4, ets-domain protein (srf accessory protein 1)  SNW1 - snw domain containing 1  SCAP - srebf chaperone  WRNIP1 - werner helicase interacting protein 1  EPAS1 - endothelial pas domain protein 1  EP300 - e1a binding protein p300  DPH7 - diphthamide biosynthesis 7  HABP4 - hyaluronan binding protein 4  ATF6 - activating transcription factor 6  ENO1 - enolase 1, (alpha)  RAB3GAP1 - rab3 gtpase activating protein subunit 1 (catalytic)  EIF4A2 - eukaryotic translation initiation factor 4a2  EIF4E - eukaryotic translation initiation factor 4e  CELF4 - cugbp, elav-like family member 4  EGR2 - early growth response 2  EGR3 - early growth response 3  CCT5 - chaperonin containing tcp1, subunit 5 (epsilon)  TTF1 - transcription termination factor, rna polymerase i  EIF2S1 - eukaryotic translation initiation factor 2, subunit 1 alpha, 35kda  TRIM32 - tripartite motif containing 32  EIF2B1 - eukaryotic translation initiation factor 2b, subunit 1 alpha, 26kda  EIF2S3 - eukaryotic translation initiation factor 2, subunit 3 gamma, 52kda  KDM2A - lysine (k)-specific demethylase 2a  ELAVL1 - elav (embryonic lethal, abnormal vision, drosophila)-like 1 (hu antigen r)  ELF1 - e74-like factor 1 (ets domain transcription factor)  ZNF326 - zinc finger protein 326  ELF2 - e74-like factor 2 (ets domain transcription factor)  TSG101 - tumor susceptibility gene 101  TCF25 - transcription factor 25 (basic helix-loop-helix)  EIF4G2 - eukaryotic translation initiation factor 4 gamma, 2  EFNA1 - ephrin-a1  CNOT1 - ccr4-not transcription complex, subunit 1  EEF2 - eukaryotic translation elongation factor 2  CTNNBIP1 - catenin, beta interacting protein 1  SPEN - spen homolog, transcriptional regulator (drosophila)  BBX - bobby sox homolog (drosophila)  TP53BP1 - tumor protein p53 binding protein 1  PHC1 - polyhomeotic homolog 1 (drosophila)  PHC2 - polyhomeotic homolog 2 (drosophila)  EGR1 - early growth response 1  ZNF292 - zinc finger protein 292  EGFR - epidermal growth factor receptor  MEGF8 - multiple egf-like-domains 8  CELSR2 - cadherin, egf lag seven-pass g-type receptor 2  KDM1A - lysine (k)-specific demethylase 1a  ATXN7L3 - ataxin 7-like 3  HNRNPLL - heterogeneous nuclear ribonucleoprotein l-like  NCOA6 - nuclear receptor coactivator 6  E2F6 - e2f transcription factor 6  CCT3 - chaperonin containing tcp1, subunit 3 (gamma)  E4F1 - e4f transcription factor 1  ZHX3 - zinc fingers and homeoboxes 3  E2F4 - e2f transcription factor 4, p107/p130-binding  E2F5 - e2f transcription factor 5, p130-binding  ENY2 - enhancer of yellow 2 homolog (drosophila)  C11orf30 - chromosome 11 open reading frame 30  RRP1B - ribosomal rna processing 1b  EDNRB - endothelin receptor type b  TRAF1 - tnf receptor-associated factor 1  S1PR1 - sphingosine-1-phosphate receptor 1  TRAF3 - tnf receptor-associated factor 3  TRAF2 - tnf receptor-associated factor 2  NR2C1 - nuclear receptor subfamily 2, group c, member 1  ZBTB47 - zinc finger and btb domain containing 47  SETX - senataxin  NR2C2 - nuclear receptor subfamily 2, group c, member 2  ZNF609 - zinc finger protein 609  TPR - translocated promoter region, nuclear basket protein  WAPAL - wings apart-like homolog (drosophila)  ERC1 - elks/rab6-interacting/cast family member 1  PPRC1 - peroxisome proliferator-activated receptor gamma, coactivator-related 1  KDM4C - lysine (k)-specific demethylase 4c  NAT14 - n-acetyltransferase 14 (gcn5-related, putative)  ZNF423 - zinc finger protein 423  REXO4 - rex4, rna exonuclease 4 homolog (s. cerevisiae)  CAMK1D - calcium/calmodulin-dependent protein kinase id  USF1 - upstream transcription factor 1  NUP107 - nucleoporin 107kda  ZBTB43 - zinc finger and btb domain containing 43  PTK2B - protein tyrosine kinase 2 beta  MRPS27 - mitochondrial ribosomal protein s27  BPTF - bromodomain phd finger transcription factor  PITHD1 - pith (c-terminal proteasome-interacting domain of thioredoxin-like) domain containing 1  TNRC6B - trinucleotide repeat containing 6b  NR1H2 - nuclear receptor subfamily 1, group h, member 2  TAB2 - tgf-beta activated kinase 1/map3k7 binding protein 2  ETV3 - ets variant 3  CAMTA2 - calmodulin binding transcription activator 2  ZNF598 - zinc finger protein 598  ETV5 - ets variant 5  POGZ - pogo transposable element with znf domain  TWSG1 - twisted gastrulation homolog 1 (drosophila)  ETV6 - ets variant 6  PLXND1 - plexin d1  VHL - von hippel-lindau tumor suppressor, e3 ubiquitin protein ligase  VIM - vimentin  KDM6B - lysine (k)-specific demethylase 6b  PHF8 - phd finger protein 8  EZR - ezrin  EWSR1 - ews rna-binding protein 1  TRIM41 - tripartite motif containing 41  TBX20 - t-box 20  KCTD1 - potassium channel tetramerization domain containing 1  CREB3L1 - camp responsive element binding protein 3-like 1  EZH1 - enhancer of zeste homolog 1 (drosophila)  CIC - capicua transcriptional repressor  CCNL1 - cyclin l1  F2R - coagulation factor ii (thrombin) receptor  WDR43 - wd repeat domain 43  ERF - ets2 repressor factor  ZNF575 - zinc finger protein 575  ELMSAN1 - elm2 and myb/sant-like domain containing 1  THAP11 - thap domain containing 11  ZNF248 - zinc finger protein 248  UBB - ubiquitin b  LARP4B - la ribonucleoprotein domain family, member 4b  RCOR1 - rest corepressor 1  TULP3 - tubby like protein 3  ZNFX1 - zinc finger, nfx1-type containing 1  HIRA - histone cell cycle regulator  TTC5 - tetratricopeptide repeat domain 5  ETV1 - ets variant 1  ETS1 - v-ets avian erythroblastosis virus e26 oncogene homolog 1  ETF1 - eukaryotic translation termination factor 1  ZMIZ1 - zinc finger, miz-type containing 1  CYFIP1 - cytoplasmic fmr1 interacting protein 1  UBP1 - upstream binding protein 1 (lbp-1a)  SUMO1 - small ubiquitin-like modifier 1  UBTF - upstream binding transcription factor, rna polymerase i  ZNF830 - zinc finger protein 830  PELI1 - pellino e3 ubiquitin protein ligase 1  CHURC1 - churchill domain containing 1  SMURF1 - smad specific e3 ubiquitin protein ligase 1  ZC3H4 - zinc finger ccch-type containing 4  JMJD6 - jumonji domain containing 6  EPRS - glutamyl-prolyl-trna synthetase  UBE2I - ubiquitin-conjugating enzyme e2i  UBE2L3 - ubiquitin-conjugating enzyme e2l 3  UBE2N - ubiquitin-conjugating enzyme e2n  UBE2V1 - ubiquitin-conjugating enzyme e2 variant 1  VPS72 - vacuolar protein sorting 72 homolog (s. cerevisiae)  FMR1 - fragile x mental retardation 1  TCOF1 - treacher collins-franceschetti syndrome 1  MGA - mga, max dimerization protein  TCP1 - t-complex 1  ZEB1 - zinc finger e-box binding homeobox 1  WWC3 - wwc family member 3  TCF12 - transcription factor 12  ZNF354A - zinc finger protein 354a  CAMTA1 - calmodulin binding transcription activator 1  TCF20 - transcription factor 20 (ar1)  PDS5A - pds5, regulator of cohesion maintenance, homolog a (s. cerevisiae)  FLI1 - fli-1 proto-oncogene, ets transcription factor  FLNA - filamin a, alpha  PLCB1 - phospholipase c, beta 1 (phosphoinositide-specific)  FOXS1 - forkhead box s1  FOXO3 - forkhead box o3  LARP4 - la ribonucleoprotein domain family, member 4  HDAC8 - histone deacetylase 8  ASH1L - ash1 (absent, small, or homeotic)-like (drosophila)  SIN3B - sin3 transcription regulator family member b  NACC1 - nucleus accumbens associated 1, ben and btb (poz) domain containing  HR - hair growth associated  MED8 - mediator complex subunit 8  CAND1 - cullin-associated and neddylation-dissociated 1  TDG - thymine-dna glycosylase  SMG6 - smg6 nonsense mediated mrna decay factor  PRDX2 - peroxiredoxin 2  EAPP - e2f-associated phosphoprotein  FBXW11 - f-box and wd repeat domain containing 11  FAM208A - family with sequence similarity 208, member a  KDM3A - lysine (k)-specific demethylase 3a  FHL2 - four and a half lim domains 2  FGFR2 - fibroblast growth factor receptor 2  TAF9 - taf9 rna polymerase ii, tata box binding protein (tbp)-associated factor, 32kda  TAF7 - taf7 rna polymerase ii, tata box binding protein (tbp)-associated factor, 55kda  TAF6 - taf6 rna polymerase ii, tata box binding protein (tbp)-associated factor, 80kda  FGFR1 - fibroblast growth factor receptor 1  FGF9 - fibroblast growth factor 9  TADA2A - transcriptional adaptor 2a  TIRAP - toll-interleukin 1 receptor (tir) domain containing adaptor protein  FGF1 - fibroblast growth factor 1 (acidic)  FGF2 - fibroblast growth factor 2 (basic)  USP22 - ubiquitin specific peptidase 22  TARBP1 - tar (hiv-1) rna binding protein 1  FER - fer (fps/fes related) tyrosine kinase  MAP3K7 - mitogen-activated protein kinase kinase kinase 7  ADNP - activity-dependent neuroprotector homeobox  KMT2E - lysine (k)-specific methyltransferase 2e  MED13L - mediator complex subunit 13-like  NCSTN - nicastrin  FKTN - fukutin  ZNF395 - zinc finger protein 395  MYNN - myoneurin  CRTC1 - creb regulated transcription coactivator 1  UFL1 - ufm1-specific ligase 1  TCF3 - transcription factor 3  PUM2 - pumilio homolog 2 (drosophila)  DMAP1 - dna methyltransferase 1 associated protein 1  TCEB3 - transcription elongation factor b (siii), polypeptide 3 (110kda, elongin a)  LARP1 - la ribonucleoprotein domain family, member 1  TCF4 - transcription factor 4  EFEMP1 - egf containing fibulin-like extracellular matrix protein 1  NKRF - nfkb repressing factor  ZNF629 - zinc finger protein 629  FBN1 - fibrillin 1  LANCL2 - lanc lantibiotic synthetase component c-like 2 (bacterial)  TCEA2 - transcription elongation factor a (sii), 2  ERBB2IP - erbb2 interacting protein  TCEA1 - transcription elongation factor a (sii), 1  ITGB3BP - integrin beta 3 binding protein (beta3-endonexin)  RYBP - ring1 and yy1 binding protein  MTOR - mechanistic target of rapamycin (serine/threonine kinase)  PAXBP1 - pax3 and pax7 binding protein 1  TLE1 - transducin-like enhancer of split 1 (e(sp1) homolog, drosophila)  TLE3 - transducin-like enhancer of split 3 (e(sp1) homolog, drosophila)  TLE4 - transducin-like enhancer of split 4 (e(sp1) homolog, drosophila)  TLR3 - toll-like receptor 3  SIRT1 - sirtuin 1  MKX - mohawk homeobox  TMF1 - tata element modulatory factor 1  ACTR8 - arp8 actin-related protein 8 homolog (yeast)  TMSB4X - thymosin beta 4, x-linked  CBX6 - chromobox homolog 6  HEY1 - hairy/enhancer-of-split related with yrpw motif 1  TMPO - thymopoietin  CBX5 - chromobox homolog 5  BRD4 - bromodomain containing 4  TNFAIP1 - tumor necrosis factor, alpha-induced protein 1 (endothelial)  TARDBP - tar dna binding protein  BCL9L - b-cell cll/lymphoma 9-like  TNFRSF1A - tumor necrosis factor receptor superfamily, member 1a  TMBIM6 - transmembrane bax inhibitor motif containing 6  EIF4ENIF1 - eukaryotic translation initiation factor 4e nuclear import factor 1  SUZ12 - suz12 polycomb repressive complex 2 subunit  POFUT1 - protein o-fucosyltransferase 1  TEF - thyrotrophic embryonic factor  RCOR2 - rest corepressor 2  TFAM - transcription factor a, mitochondrial  INPP5E - inositol polyphosphate-5-phosphatase, 72 kda  TERF1 - telomeric repeat binding factor (nima-interacting) 1  TERF2 - telomeric repeat binding factor 2  TFDP1 - transcription factor dp-1  NR2F2 - nuclear receptor subfamily 2, group f, member 2  TFE3 - transcription factor binding to ighm enhancer 3  CBX7 - chromobox homolog 7  NR2F1 - nuclear receptor subfamily 2, group f, member 1  TFCP2 - transcription factor cp2  ZNF740 - zinc finger protein 740  TGFA - transforming growth factor, alpha  FNIP1 - folliculin interacting protein 1  HEY2 - hairy/enhancer-of-split related with yrpw motif 2  TGFBR1 - transforming growth factor, beta receptor 1  TGFB2 - transforming growth factor, beta 2  RBFOX2 - rna binding protein, fox-1 homolog (c. elegans) 2  TGFB3 - transforming growth factor, beta 3  TGFB1 - transforming growth factor, beta 1  TGFB1I1 - transforming growth factor beta 1 induced transcript 1  TP53INP1 - tumor protein p53 inducible nuclear protein 1  H2AFV - h2a histone family, member v  MEPCE - methylphosphate capping enzyme  RNF20 - ring finger protein 20, e3 ubiquitin protein ligase  TGFBR3 - transforming growth factor, beta receptor iii  FOSL2 - fos-like antigen 2  FOSB - fbj murine osteosarcoma viral oncogene homolog b  FOS - fbj murine osteosarcoma viral oncogene homolog  THBS1 - thrombospondin 1  KAT6B - k(lysine) acetyltransferase 6b  TIA1 - tia1 cytotoxic granule-associated rna binding protein  METTL3 - methyltransferase like 3  THRA - thyroid hormone receptor, alpha  ELL - elongation factor rna polymerase ii  ZNF239 - zinc finger protein 239  PHC3 - polyhomeotic homolog 3 (drosophila)  OBFC1 - oligonucleotide/oligosaccharide-binding fold containing 1  NRIP1 - nuclear receptor interacting protein 1  KAT2A - k(lysine) acetyltransferase 2a  NCOA3 - nuclear receptor coactivator 3  WWC2 - ww and c2 domain containing 2  TAF15 - taf15 rna polymerase ii, tata box binding protein (tbp)-associated factor, 68kda  GJA1 - gap junction protein, alpha 1, 43kda  AXIN1 - axin 1  TRRAP - transformation/transcription domain-associated protein  PICALM - phosphatidylinositol binding clathrin assembly protein  ARID1A - at rich interactive domain 1a (swi-like)  DLL1 - delta-like 1 (drosophila)  SETD6 - set domain containing 6  FZD6 - frizzled family receptor 6  GLE1 - gle1 rna export mediator  ACTR5 - arp5 actin-related protein 5 homolog (yeast)  CDC45 - cell division cycle 45  FZD4 - frizzled family receptor 4  GCLC - glutamate-cysteine ligase, catalytic subunit  FZD1 - frizzled family receptor 1  BAP1 - brca1 associated protein-1 (ubiquitin carboxy-terminal hydrolase)  ZNF613 - zinc finger protein 613  CDC7 - cell division cycle 7  NUP85 - nucleoporin 85kda  KDM5C - lysine (k)-specific demethylase 5c  USP9X - ubiquitin specific peptidase 9, x-linked  RBM10 - rna binding motif protein 10  TTC21B - tetratricopeptide repeat domain 21b  SLTM - safb-like, transcription modulator  NELFE - negative elongation factor complex member e  EHMT1 - euchromatic histone-lysine n-methyltransferase 1  NR5A1 - nuclear receptor subfamily 5, group a, member 1  FUS - fused in sarcoma  NAA40 - n(alpha)-acetyltransferase 40, natd catalytic subunit, homolog (s. cerevisiae)  USP7 - ubiquitin specific peptidase 7 (herpes virus-associated)  RNF168 - ring finger protein 168, e3 ubiquitin protein ligase  XRCC6 - x-ray repair complementing defective repair in chinese hamster cells 6  GABPB1 - ga binding protein transcription factor, beta subunit 1  GABPA - ga binding protein transcription factor, alpha subunit 60kda  RPAP2 - rna polymerase ii associated protein 2  DDX39B - dead (asp-glu-ala-asp) box polypeptide 39b  KMT2D - lysine (k)-specific methyltransferase 2d  ZNF419 - zinc finger protein 419  YEATS4 - yeats domain containing 4  ZFPM1 - zinc finger protein, fog family member 1  SNIP1 - smad nuclear interacting protein 1  ZNF212 - zinc finger protein 212  EID2 - ep300 interacting inhibitor of differentiation 2  GBA - glucosidase, beta, acid  ZNF383 - zinc finger protein 383  GATA2 - gata binding protein 2  KAT6A - k(lysine) acetyltransferase 6a  NR4A3 - nuclear receptor subfamily 4, group a, member 3  BRD3 - bromodomain containing 3  GAS6 - growth arrest-specific 6  CARF - calcium responsive transcription factor  DEDD2 - death effector domain containing 2  ZNF408 - zinc finger protein 408  MLLT10 - myeloid/lymphoid or mixed-lineage leukemia (trithorax homolog, drosophila); translocated to, 10  DHX36 - deah (asp-glu-ala-his) box polypeptide 36  ZNF189 - zinc finger protein 189  ZSCAN26 - zinc finger and scan domain containing 26  GSPT1 - g1 to s phase transition 1  ZNF177 - zinc finger protein 177  ZNF175 - zinc finger protein 175  ZNF174 - zinc finger protein 174  ZNF184 - zinc finger protein 184  SSBP4 - single stranded dna binding protein 4  GTF2F1 - general transcription factor iif, polypeptide 1, 74kda  ZNF148 - zinc finger protein 148  BEND5 - ben domain containing 5  ZBTB17 - zinc finger and btb domain containing 17  GTF2H2 - general transcription factor iih, polypeptide 2, 44kda  TRIM26 - tripartite motif containing 26  GTF2H3 - general transcription factor iih, polypeptide 3, 34kda  VEZF1 - vascular endothelial zinc finger 1  GTF2H1 - general transcription factor iih, polypeptide 1, 62kda  ZNF142 - zinc finger protein 142  MCPH1 - microcephalin 1  ZNF140 - zinc finger protein 140  GTF2B - general transcription factor iib  PCGF2 - polycomb group ring finger 2  ZNF132 - zinc finger protein 132  ZNF131 - zinc finger protein 131  ZNF134 - zinc finger protein 134  ZNF133 - zinc finger protein 133  TBL1XR1 - transducin (beta)-like 1 x-linked receptor 1  GTF2I - general transcription factor iii  GTF2H4 - general transcription factor iih, polypeptide 4, 52kda  BRF1 - brf1, rna polymerase iii transcription initiation factor 90 kda subunit  ZNF79 - zinc finger protein 79  ZNF70 - zinc finger protein 70  ZNF75D - zinc finger protein 75d  ZNF76 - zinc finger protein 76  H3F3A - h3 histone, family 3a  BRPF1 - bromodomain and phd finger containing, 1  H2AFZ - h2a histone family, member z  DCP2 - decapping mrna 2  H2AFX - h2a histone family, member x  SAP130 - sin3a-associated protein, 130kda  FZD5 - frizzled family receptor 5  PAX8 - paired box 8  MIER3 - mesoderm induction early response 1, family member 3  CDC73 - cell division cycle 73  NKAP - nfkb activating protein  THAP5 - thap domain containing 5  LRP8 - low density lipoprotein receptor-related protein 8, apolipoprotein e receptor  HCFC1 - host cell factor c1 (vp16-accessory protein)  PRDM2 - pr domain containing 2, with znf domain  MAP3K12 - mitogen-activated protein kinase kinase kinase 12  ZNF226 - zinc finger protein 226  HDAC2 - histone deacetylase 2  HMBOX1 - homeobox containing 1  HDGF - hepatoma-derived growth factor  ZNF213 - zinc finger protein 213  HDAC1 - histone deacetylase 1  HCLS1 - hematopoietic cell-specific lyn substrate 1  ZNF205 - zinc finger protein 205  ZNF207 - zinc finger protein 207  XPA - xeroderma pigmentosum, complementation group a  GOLGA2 - golgin a2  XPC - xeroderma pigmentosum, complementation group c  GOLGB1 - golgin b1  XPO1 - exportin 1 (crm1 homolog, yeast)  PAGR1 - paxip1 associated glutamate-rich protein 1  ZXDC - zxd family zinc finger c  XBP1 - x-box binding protein 1  ZNF367 - zinc finger protein 367  BHLHE41 - basic helix-loop-helix family, member e41  EIF4H - eukaryotic translation initiation factor 4h  WFS1 - wolfram syndrome 1 (wolframin)  WHSC1 - wolf-hirschhorn syndrome candidate 1  NELFA - negative elongation factor complex member a  ARID2 - at rich interactive domain 2 (arid, rfx-like)  ZNF35 - zinc finger protein 35  ZKSCAN1 - zinc finger with krab and scan domains 1  FBXL15 - f-box and leucine-rich repeat protein 15  ZNF33B - zinc finger protein 33b  ZNF576 - zinc finger protein 576  ZNF32 - zinc finger protein 32  ZNF45 - zinc finger protein 45  MZF1 - myeloid zinc finger 1  ZNF18 - zinc finger protein 18  ZNF16 - zinc finger protein 16  ASXL1 - additional sex combs like 1 (drosophila)  ZNF384 - zinc finger protein 384  TNIP2 - tnfaip3 interacting protein 2  ZNF24 - zinc finger protein 24  ZFPL1 - zinc finger protein-like 1  ZFX - zinc finger protein, x-linked  ZBTB14 - zinc finger and btb domain containing 14  ZNF711 - zinc finger protein 711  ZNF7 - zinc finger protein 7  ZNF8 - zinc finger protein 8  ZNF2 - zinc finger protein 2  ZNF3 - zinc finger protein 3  YES1 - v-yes-1 yamaguchi sarcoma viral oncogene homolog 1  XRCC5 - x-ray repair complementing defective repair in chinese hamster cells 5 (double-strand-break rejoining)  GSK3B - glycogen synthase kinase 3 beta  GSK3A - glycogen synthase kinase 3 alpha  YWHAB - tyrosine 3-monooxygenase/tryptophan 5-monooxygenase activation protein, beta polypeptide  CCNK - cyclin k  HSF2 - heat shock transcription factor 2  TRIM24 - tripartite motif containing 24  TAF1D - tata box binding protein (tbp)-associated factor, rna polymerase i, d, 41kda  CREG1 - cellular repressor of e1a-stimulated genes 1  HSBP1 - heat shock factor binding protein 1  FLCN - folliculin  PRMT1 - protein arginine methyltransferase 1  PRMT2 - protein arginine methyltransferase 2  HESX1 - hesx homeobox 1  SAP30 - sin3a-associated protein, 30kda  METTL16 - methyltransferase like 16  ALKBH1 - alkb, alkylation repair homolog 1 (e. coli)  FTO - fat mass and obesity associated  CFLAR - casp8 and fadd-like apoptosis regulator  SECISBP2 - secis binding protein 2  MED11 - mediator complex subunit 11  LDB1 - lim domain binding 1  WDR77 - wd repeat domain 77  DSCC1 - dna replication and sister chromatid cohesion 1  KAT2B - k(lysine) acetyltransferase 2b  TSC22D1 - tsc22 domain family, member 1  CRTC2 - creb regulated transcription coactivator 2  ZNF259 - zinc finger protein 259  TAX1BP1 - tax1 (human t-cell leukemia virus type i) binding protein 1  CDC123 - cell division cycle 123  CC2D1B - coiled-coil and c2 domain containing 1b  HUS1 - hus1 checkpoint homolog (s. pombe)  EIF2B2 - eukaryotic translation initiation factor 2b, subunit 2 beta, 39kda  THAP8 - thap domain containing 8  BUD31 - bud31 homolog (s. cerevisiae)  EIF2B5 - eukaryotic translation initiation factor 2b, subunit 5 epsilon, 82kda  ZNF655 - zinc finger protein 655  TRAK2 - trafficking protein, kinesin binding 2  HSPD1 - heat shock 60kda protein 1 (chaperonin)  DNAJB1 - dnaj (hsp40) homolog, subfamily b, member 1  ZNF48 - zinc finger protein 48  BCL10 - b-cell cll/lymphoma 10  FUBP3 - far upstream element (fuse) binding protein 3  HSP90AA1 - heat shock protein 90kda alpha (cytosolic), class a member 1  BTRC - beta-transducin repeat containing e3 ubiquitin protein ligase  HSPA8 - heat shock 70kda protein 8  EIF4EBP3 - eukaryotic translation initiation factor 4e binding protein 3  HMGN1 - high mobility group nucleosome binding domain 1  HMGB3 - high mobility group box 3  HMGB2 - high mobility group box 2  HMGB1 - high mobility group box 1  EIF3C - eukaryotic translation initiation factor 3, subunit c  EIF3D - eukaryotic translation initiation factor 3, subunit d  BRD9 - bromodomain containing 9  EIF3B - eukaryotic translation initiation factor 3, subunit b  IRS2 - insulin receptor substrate 2  TNKS - tankyrase, trf1-interacting ankyrin-related adp-ribose polymerase  EIF4G3 - eukaryotic translation initiation factor 4 gamma, 3  ZBTB10 - zinc finger and btb domain containing 10  AGO1 - argonaute risc catalytic component 1  CAPRIN2 - caprin family member 2  ZBTB48 - zinc finger and btb domain containing 48  HYAL2 - hyaluronoglucosaminidase 2  ACD - adrenocortical dysplasia homolog (mouse)  GPBP1 - gc-rich promoter binding protein 1  HFE - hemochromatosis  HINT1 - histidine triad nucleotide binding protein 1  HIVEP1 - human immunodeficiency virus type i enhancer binding protein 1  ELP4 - elongator acetyltransferase complex subunit 4  UPF3B - upf3 regulator of nonsense transcripts homolog b (yeast)  UPF3A - upf3 regulator of nonsense transcripts homolog a (yeast)  HIF1A - hypoxia inducible factor 1, alpha subunit (basic helix-loop-helix transcription factor)  LPIN3 - lipin 3  TRADD - tnfrsf1a-associated via death domain  MYCBP - myc binding protein  EED - embryonic ectoderm development  URI1 - uri1, prefoldin-like chaperone  HNRNPU - heterogeneous nuclear ribonucleoprotein u (scaffold attachment factor a)  RIPK1 - receptor (tnfrsf)-interacting serine-threonine kinase 1  HNRNPK - heterogeneous nuclear ribonucleoprotein k  HNRNPL - heterogeneous nuclear ribonucleoprotein l  FBXO4 - f-box protein 4  TINF2 - terf1 (trf1)-interacting nuclear factor 2  HNRNPA2B1 - heterogeneous nuclear ribonucleoprotein a2/b1  HNRNPA1 - heterogeneous nuclear ribonucleoprotein a1  HNRNPC - heterogeneous nuclear ribonucleoprotein c (c1/c2)  HNRNPAB - heterogeneous nuclear ribonucleoprotein a/b  RIPK2 - receptor-interacting serine-threonine kinase 2  HNRNPD - heterogeneous nuclear ribonucleoprotein d (au-rich element rna binding protein 1, 37kda)  HMGA1 - high mobility group at-hook 1  ZNF584 - zinc finger protein 584  FAM129B - family with sequence similarity 129, member b  NR4A1 - nuclear receptor subfamily 4, group a, member 1  RFX7 - regulatory factor x, 7  FOXK2 - forkhead box k2  HTATSF1 - hiv-1 tat specific factor 1  EIF3K - eukaryotic translation initiation factor 3, subunit k  HAT1 - histone acetyltransferase 1  CRTC3 - creb regulated transcription coactivator 3  RBM15 - rna binding motif protein 15  TNRC6A - trinucleotide repeat containing 6a  ENC1 - ectodermal-neural cortex 1 (with btb domain)  RBMX - rna binding motif protein, x-linked  ING2 - inhibitor of growth family, member 2  PPM1D - protein phosphatase, mg2+/mn2+ dependent, 1d  ZNF574 - zinc finger protein 574  CREB3L2 - camp responsive element binding protein 3-like 2  IPPK - inositol 1,3,4,5,6-pentakisphosphate 2-kinase  ILF2 - interleukin enhancer binding factor 2  ILF3 - interleukin enhancer binding factor 3, 90kda  ILK - integrin-linked kinase  PIAS1 - protein inhibitor of activated stat, 1  SLCO3A1 - solute carrier organic anion transporter family, member 3a1  SMYD3 - set and mynd domain containing 3  MAPKAPK5 - mitogen-activated protein kinase-activated protein kinase 5  SMURF2 - smad specific e3 ubiquitin protein ligase 2  CGGBP1 - cgg triplet repeat binding protein 1  LMO4 - lim domain only 4  CAMK1 - calcium/calmodulin-dependent protein kinase i  CBX4 - chromobox homolog 4  CSRNP1 - cysteine-serine-rich nuclear protein 1  SERP1 - stress-associated endoplasmic reticulum protein 1  ZNF25 - zinc finger protein 25  STK16 - serine/threonine kinase 16  ITGA3 - integrin, alpha 3 (antigen cd49c, alpha 3 subunit of vla-3 receptor)  KHSRP - kh-type splicing regulatory protein  CASK - calcium/calmodulin-dependent serine protein kinase (maguk family)  SUDS3 - suppressor of defective silencing 3 homolog (s. cerevisiae)  EIF6 - eukaryotic translation initiation factor 6  SKAP1 - src kinase associated phosphoprotein 1  GZF1 - gdnf-inducible zinc finger protein 1  INSR - insulin receptor  INSM1 - insulinoma-associated 1  CDK13 - cyclin-dependent kinase 13  PDCD4 - programmed cell death 4 (neoplastic transformation inhibitor)  RFXANK - regulatory factor x-associated ankyrin-containing protein  KLF7 - kruppel-like factor 7 (ubiquitous)  IRF2 - interferon regulatory factor 2  IRF3 - interferon regulatory factor 3  IREB2 - iron-responsive element binding protein 2  IKZF5 - ikaros family zinc finger 5 (pegasus)  RUVBL1 - ruvb-like 1 (e. coli)  MED19 - mediator complex subunit 19  ITGA6 - integrin, alpha 6  IRAK2 - interleukin-1 receptor-associated kinase 2  IRAK1 - interleukin-1 receptor-associated kinase 1  CACYBP - calcyclin binding protein  EIF2AK1 - eukaryotic translation initiation factor 2-alpha kinase 1  ZSCAN31 - zinc finger and scan domain containing 31  PAPD5 - pap associated domain containing 5  KIAA1324L - kiaa1324-like  NSD1 - nuclear receptor binding set domain protein 1  ZBTB11 - zinc finger and btb domain containing 11  RNF25 - ring finger protein 25  MMS19 - mms19 nucleotide excision repair homolog (s. cerevisiae)  DKK3 - dickkopf wnt signaling pathway inhibitor 3  SNX5 - sorting nexin 5  ID2 - inhibitor of dna binding 2, dominant negative helix-loop-helix protein  ZNF777 - zinc finger protein 777  BRPF3 - bromodomain and phd finger containing, 3  ID3 - inhibitor of dna binding 3, dominant negative helix-loop-helix protein  STK36 - serine/threonine kinase 36  IRF2BPL - interferon regulatory factor 2 binding protein-like  JAZF1 - jazf zinc finger 1  FOXK1 - forkhead box k1  ZNF282 - zinc finger protein 282  IGHMBP2 - immunoglobulin mu binding protein 2  RBPJ - recombination signal binding protein for immunoglobulin kappa j region  PABPC1 - poly(a) binding protein, cytoplasmic 1  NCK2 - nck adaptor protein 2  TRUB2 - trub pseudouridine (psi) synthase homolog 2 (e. coli)  USP21 - ubiquitin specific peptidase 21  ZBTB9 - zinc finger and btb domain containing 9  ZBTB12 - zinc finger and btb domain containing 12  BCAR3 - breast cancer anti-estrogen resistance 3  IGBP1 - immunoglobulin (cd79a) binding protein 1  JMJD1C - jumonji domain containing 1c  SMARCA5 - swi/snf related, matrix associated, actin dependent regulator of chromatin, subfamily a, member 5  OGT - o-linked n-acetylglucosamine (glcnac) transferase  PELP1 - proline, glutamate and leucine rich protein 1  TSPYL2 - tspy-like 2  PCIF1 - pdx1 c-terminal inhibiting factor 1  CUL2 - cullin 2  CUL3 - cullin 3  ZNF667 - zinc finger protein 667  FOXP1 - forkhead box p1  SUPT3H - suppressor of ty 3 homolog (s. cerevisiae)  ZNF572 - zinc finger protein 572  PCGF5 - polycomb group ring finger 5  WAC - ww domain containing adaptor with coiled-coil  PHF21A - phd finger protein 21a  ZNF414 - zinc finger protein 414  ZNF771 - zinc finger protein 771  IER2 - immediate early response 2  LIG3 - ligase iii, dna, atp-dependent  NCOR1 - nuclear receptor corepressor 1  MTPN - myotrophin  NCOR2 - nuclear receptor corepressor 2  DOT1L - dot1-like histone h3k79 methyltransferase  EFCAB7 - ef-hand calcium binding domain 7  L3MBTL3 - l(3)mbt-like 3 (drosophila)  ZNF333 - zinc finger protein 333  CIR1 - corepressor interacting with rbpj, 1  H2AFY - h2a histone family, member y  FADS1 - fatty acid desaturase 1  GTF2IRD1 - gtf2i repeat domain containing 1  ZNF527 - zinc finger protein 527  HOPX - hop homeobox  SOX13 - sry (sex determining region y)-box 13  ZC3H8 - zinc finger ccch-type containing 8  ZNF316 - zinc finger protein 316  LMO1 - lim domain only 1 (rhombotin 1)  NACC2 - nacc family member 2, ben and btb (poz) domain containing  IER5 - immediate early response 5  CLOCK - clock circadian regulator  KLF3 - kruppel-like factor 3 (basic)  DDX41 - dead (asp-glu-ala-asp) box polypeptide 41  FXR2 - fragile x mental retardation, autosomal homolog 2  LITAF - lipopolysaccharide-induced tnf factor  YTHDF2 - yth domain family, member 2  TBPL1 - tbp-like 1  ZBTB41 - zinc finger and btb domain containing 41  ZFP62 - zfp62 zinc finger protein  TMEM59 - transmembrane protein 59  ZGPAT - zinc finger, ccch-type with g patch domain  ACTL6B - actin-like 6b  EIF4E2 - eukaryotic translation initiation factor 4e family member 2  MED20 - mediator complex subunit 20  AFAP1L2 - actin filament associated protein 1-like 2  ROCK2 - rho-associated, coiled-coil containing protein kinase 2  MAPK8IP1 - mitogen-activated protein kinase 8 interacting protein 1  CRLF3 - cytokine receptor-like factor 3  NGRN - neugrin, neurite outgrowth associated  GLYR1 - glyoxylate reductase 1 homolog (arabidopsis)  ZBTB7A - zinc finger and btb domain containing 7a  DPY30 - dpy-30 homolog (c. elegans)  MBTPS2 - membrane-bound transcription factor peptidase, site 2  IRF2BP2 - interferon regulatory factor 2 binding protein 2  GLIS2 - glis family zinc finger 2  MAGED1 - melanoma antigen family d, 1  MED27 - mediator complex subunit 27  MED26 - mediator complex subunit 26  CHD6 - chromodomain helicase dna binding protein 6  MED17 - mediator complex subunit 17  MED23 - mediator complex subunit 23  ZNF580 - zinc finger protein 580  GTF2IRD2 - gtf2i repeat domain containing 2  ZNF710 - zinc finger protein 710  ING4 - inhibitor of growth family, member 4  INSIG2 - insulin induced gene 2  EIF2AK3 - eukaryotic translation initiation factor 2-alpha kinase 3  ITM2B - integral membrane protein 2b  RLIM - ring finger protein, lim domain interacting  RECQL5 - recq protein-like 5  KRAS - kirsten rat sarcoma viral oncogene homolog  ZNF706 - zinc finger protein 706  TGFBRAP1 - transforming growth factor, beta receptor associated protein 1  CIAO1 - cytosolic iron-sulfur protein assembly 1  PHF20L1 - phd finger protein 20-like 1  MED10 - mediator complex subunit 10  MED21 - mediator complex subunit 21  ZFP28 - zfp28 zinc finger protein  KIT - v-kit hardy-zuckerman 4 feline sarcoma viral oncogene homolog  POLDIP3 - polymerase (dna-directed), delta interacting protein 3  PAIP2 - poly(a) binding protein interacting protein 2  JUP - junction plakoglobin  JAK2 - janus kinase 2  JAK3 - janus kinase 3  ING5 - inhibitor of growth family, member 5  ZNF219 - zinc finger protein 219  PHF20 - phd finger protein 20  KCNK2 - potassium channel, subfamily k, member 2  ZNF397 - zinc finger protein 397  ZNHIT3 - zinc finger, hit-type containing 3  TRIP4 - thyroid hormone receptor interactor 4  HMGN3 - high mobility group nucleosomal binding domain 3  PHF6 - phd finger protein 6  ZNF235 - zinc finger protein 235  COPS2 - cop9 signalosome subunit 2  ZNF639 - zinc finger protein 639  SARNP - sap domain containing ribonucleoprotein  LEF1 - lymphoid enhancer-binding factor 1  BRMS1L - breast cancer metastasis-suppressor 1-like  CNOT8 - ccr4-not transcription complex, subunit 8  CDK12 - cyclin-dependent kinase 12  ZNF280B - zinc finger protein 280b  ARID4B - at rich interactive domain 4b (rbp1-like)  MAP3K10 - mitogen-activated protein kinase kinase kinase 10  ITGB1BP1 - integrin beta 1 binding protein 1  KDM3B - lysine (k)-specific demethylase 3b  RSF1 - remodeling and spacing factor 1  MLLT6 - myeloid/lymphoid or mixed-lineage leukemia (trithorax homolog, drosophila); translocated to, 6  MLLT3 - myeloid/lymphoid or mixed-lineage leukemia (trithorax homolog, drosophila); translocated to, 3  MLLT1 - myeloid/lymphoid or mixed-lineage leukemia (trithorax homolog, drosophila); translocated to, 1  MED14 - mediator complex subunit 14  KMT2A - lysine (k)-specific methyltransferase 2a  ZBTB22 - zinc finger and btb domain containing 22  RPS6KA5 - ribosomal protein s6 kinase, 90kda, polypeptide 5  SUFU - suppressor of fused homolog (drosophila)  NLK - nemo-like kinase  MNAT1 - mnat cdk-activating kinase assembly factor 1  UIMC1 - ubiquitin interaction motif containing 1  MTA2 - metastasis associated 1 family, member 2  NOLC1 - nucleolar and coiled-body phosphoprotein 1  EIF2A - eukaryotic translation initiation factor 2a, 65kda  WWOX - ww domain containing oxidoreductase  CD200 - cd200 molecule  MNT - mnt, max dimerization protein  CCPG1 - cell cycle progression 1  SPTY2D1 - spt2, suppressor of ty, domain containing 1 (s. cerevisiae)  PELO - pelota homolog (drosophila)  MECP2 - methyl cpg binding protein 2 (rett syndrome)  MEF2A - myocyte enhancer factor 2a  PRICKLE1 - prickle homolog 1 (drosophila)  ZBED1 - zinc finger, bed-type containing 1  MEIS2 - meis homeobox 2  BRWD1 - bromodomain and wd repeat domain containing 1  ZNF664 - zinc finger protein 664  MEIS1 - meis homeobox 1  MAP3K4 - mitogen-activated protein kinase kinase kinase 4  MAP3K5 - mitogen-activated protein kinase kinase kinase 5  SLC33A1 - solute carrier family 33 (acetyl-coa transporter), member 1  MEN1 - multiple endocrine neoplasia i  WDR75 - wd repeat domain 75  UTP15 - utp15, u3 small nucleolar ribonucleoprotein, homolog (s. cerevisiae)  KAT8 - k(lysine) acetyltransferase 8  ZNF644 - zinc finger protein 644  ARHGEF2 - rho/rac guanine nucleotide exchange factor (gef) 2  NEMF - nuclear export mediator factor  MBD3 - methyl-cpg binding domain protein 3  PTOV1 - prostate tumor overexpressed 1  ZNF394 - zinc finger protein 394  CBFA2T2 - core-binding factor, runt domain, alpha subunit 2; translocated to, 2  MAGOH - mago-nashi homolog, proliferation-associated (drosophila)  CXXC5 - cxxc finger protein 5  RQCD1 - rcd1 required for cell differentiation1 homolog (s. pombe)  SMC3 - structural maintenance of chromosomes 3  IL23A - interleukin 23, alpha subunit p19  MARS - methionyl-trna synthetase  MTA1 - metastasis associated 1  SIRT6 - sirtuin 6  PPHLN1 - periphilin 1  MBD1 - methyl-cpg binding domain protein 1  SFMBT1 - scm-like with four mbt domains 1  MAX - myc associated factor x  ZMIZ2 - zinc finger, miz-type containing 2  LDB2 - lim domain binding 2  ZFP90 - zfp90 zinc finger protein  CDAN1 - codanin 1  NELFCD - negative elongation factor complex member c/d  ASH2L - ash2 (absent, small, or homeotic)-like (drosophila)  CTDSPL2 - ctd (carboxy-terminal domain, rna polymerase ii, polypeptide a) small phosphatase like 2  ZSCAN29 - zinc finger and scan domain containing 29  THAP2 - thap domain containing, apoptosis associated protein 2  ITCH - itchy e3 ubiquitin protein ligase  ZNF484 - zinc finger protein 484  L3MBTL2 - l(3)mbt-like 2 (drosophila)  LRP6 - low density lipoprotein receptor-related protein 6  ZNF445 - zinc finger protein 445  TRAPPC9 - trafficking protein particle complex 9  LUM - lumican  BTAF1 - btaf1 rna polymerase ii, b-tfiid transcription factor-associated, 170kda  UBA3 - ubiquitin-like modifier activating enzyme 3  SESN2 - sestrin 2  CDK5RAP1 - cdk5 regulatory subunit associated protein 1  PARP9 - poly (adp-ribose) polymerase family, member 9  RNF8 - ring finger protein 8, e3 ubiquitin protein ligase  BAZ1B - bromodomain adjacent to zinc finger domain, 1b  MED15 - mediator complex subunit 15  MXD1 - max dimerization protein 1  PIAS4 - protein inhibitor of activated stat, 4  TRIM33 - tripartite motif containing 33  SMAD2 - smad family member 2  SRRT - serrate rna effector molecule homolog (arabidopsis)  KLF16 - kruppel-like factor 16  HDAC7 - histone deacetylase 7  ARMCX3 - armadillo repeat containing, x-linked 3  CAPRIN1 - cell cycle associated protein 1  LARP7 - la ribonucleoprotein domain family, member 7  MAFG - v-maf avian musculoaponeurotic fibrosarcoma oncogene homolog g  RPS6KA4 - ribosomal protein s6 kinase, 90kda, polypeptide 4  DPH5 - diphthamide biosynthesis 5  TAF9B - taf9b rna polymerase ii, tata box binding protein (tbp)-associated factor, 31kda  SMAD4 - smad family member 4  SMAD5 - smad family member 5  H1FX - h1 histone family, member x  SMAD7 - smad family member 7  WASL - wiskott-aldrich syndrome-like  RBM4B - rna binding motif protein 4b  ZNF565 - zinc finger protein 565  SAP30BP - sap30 binding protein  YAF2 - yy1 associated factor 2  NCBP1 - nuclear cap binding protein subunit 1, 80kda  SIX5 - six homeobox 5  TAGLN3 - transgelin 3  ITM2C - integral membrane protein 2c  NDN - necdin, melanoma antigen (mage) family member  ZNF582 - zinc finger protein 582  BRD7 - bromodomain containing 7  NCK1 - nck adaptor protein 1  DDX25 - dead (asp-glu-ala-asp) box helicase 25  NCL - nucleolin  CEBPZ - ccaat/enhancer binding protein (c/ebp), zeta  TICAM1 - toll-like receptor adaptor molecule 1  CCNL2 - cyclin l2  TRIM28 - tripartite motif containing 28  TBK1 - tank-binding kinase 1  NBN - nibrin  ZNF197 - zinc finger protein 197  PPP1R12A - protein phosphatase 1, regulatory subunit 12a  TRIM11 - tripartite motif containing 11  MYT1 - myelin transcription factor 1  ALYREF - aly/ref export factor  SENP1 - sumo1/sentrin specific peptidase 1  CSRNP2 - cysteine-serine-rich nuclear protein 2  NAB1 - ngfi-a binding protein 1 (egr1 binding protein 1)  NAB2 - ngfi-a binding protein 2 (egr1 binding protein 2)  RNF41 - ring finger protein 41  TSHZ1 - teashirt zinc finger homeobox 1  PSME3 - proteasome (prosome, macropain) activator subunit 3 (pa28 gamma; ki)  TRIM13 - tripartite motif containing 13  TRIM8 - tripartite motif containing 8  MYO6 - myosin vi  LBH - limb bud and heart development  ABT1 - activator of basal transcription 1  TOPORS - topoisomerase i binding, arginine/serine-rich, e3 ubiquitin protein ligase  MYD88 - myeloid differentiation primary response 88  ZNF362 - zinc finger protein 362  HCFC2 - host cell factor c2  CNOT7 - ccr4-not transcription complex, subunit 7  MXI1 - max interactor 1, dimerization protein  PQBP1 - polyglutamine binding protein 1  ACTR2 - arp2 actin-related protein 2 homolog (yeast)  ACTR3 - arp3 actin-related protein 3 homolog (yeast)  TSFM - ts translation elongation factor, mitochondrial  TRIM37 - tripartite motif containing 37  ZNF558 - zinc finger protein 558  LRPPRC - leucine-rich pentatricopeptide repeat containing  ZNF263 - zinc finger protein 263  PURG - purine-rich element binding protein g  MED25 - mediator complex subunit 25  TRAP1 - tnf receptor-associated protein 1  MED6 - mediator complex subunit 6  PTBP3 - polypyrimidine tract binding protein 3  DMTF1 - cyclin d binding myb-like transcription factor 1  MTIF2 - mitochondrial translational initiation factor 2  ZBTB33 - zinc finger and btb domain containing 33  HDAC5 - histone deacetylase 5  MTF1 - metal-regulatory transcription factor 1  HDAC6 - histone deacetylase 6  RNF187 - ring finger protein 187  MT3 - metallothionein 3  MED16 - mediator complex subunit 16  CCDC22 - coiled-coil domain containing 22  DNAJB6 - dnaj (hsp40) homolog, subfamily b, member 6  MSH3 - muts homolog 3 (e. coli)  CITED1 - cbp/p300-interacting transactivator, with glu/asp-rich carboxy-terminal domain, 1  ZBTB5 - zinc finger and btb domain containing 5  ZBTB40 - zinc finger and btb domain containing 40  RGCC - regulator of cell cycle  COMMD1 - copper metabolism (murr1) domain containing 1  RBM8A - rna binding motif protein 8a  USP3 - ubiquitin specific peptidase 3  METTL5 - methyltransferase like 5  USP15 - ubiquitin specific peptidase 15  MED4 - mediator complex subunit 4  THOC1 - tho complex 1  SETD2 - set domain containing 2  RBX1 - ring-box 1, e3 ubiquitin protein ligase  THRAP3 - thyroid hormone receptor associated protein 3  MED12 - mediator complex subunit 12  MED13 - mediator complex subunit 13  PSMD6 - proteasome (prosome, macropain) 26s subunit, non-atpase, 6  MED24 - mediator complex subunit 24  ZC3H6 - zinc finger ccch-type containing 6  DTX3L - deltex 3-like (drosophila)  SETDB1 - set domain, bifurcated 1  ZNF34 - zinc finger protein 34  ORC2 - origin recognition complex, subunit 2  ORC5 - origin recognition complex, subunit 5  TOX4 - tox high mobility group box family member 4  ZNF436 - zinc finger protein 436  RNF141 - ring finger protein 141  AGRN - agrin  EPM2AIP1 - epm2a (laforin) interacting protein 1  G3BP2 - gtpase activating protein (sh3 domain) binding protein 2  OGG1 - 8-oxoguanine dna glycosylase  ZSCAN16 - zinc finger and scan domain containing 16  TNKS2 - tankyrase, trf1-interacting ankyrin-related adp-ribose polymerase 2  DDR2 - discoidin domain receptor tyrosine kinase 2  DAB2IP - dab2 interacting protein  BCLAF1 - bcl2-associated transcription factor 1  NUMA1 - nuclear mitotic apparatus protein 1  EIF4A3 - eukaryotic translation initiation factor 4a3  GMNN - geminin, dna replication inhibitor  NR4A2 - nuclear receptor subfamily 4, group a, member 2  ZNF593 - zinc finger protein 593  ZBTB7B - zinc finger and btb domain containing 7b  MATR3 - matrin 3  NVL - nuclear vcp-like  ZNF691 - zinc finger protein 691  CREBRF - creb3 regulatory factor  ZNF623 - zinc finger protein 623  NPM1 - nucleophosmin (nucleolar phosphoprotein b23, numatrin)  ARHGEF11 - rho guanine nucleotide exchange factor (gef) 11  ZBTB24 - zinc finger and btb domain containing 24  ZEB2 - zinc finger e-box binding homeobox 2  THAP4 - thap domain containing 4  CTIF - cbp80/20-dependent translation initiation factor  TSC22D2 - tsc22 domain family, member 2  NFXL1 - nuclear transcription factor, x-box binding-like 1  NRF1 - nuclear respiratory factor 1  DONSON - downstream neighbor of son  NOP2 - nop2 nucleolar protein  VPRBP - vpr (hiv-1) binding protein  NONO - non-pou domain containing, octamer-binding  MDFIC - myod family inhibitor domain containing  NOTCH4 - notch 4  IFT74 - intraflagellar transport 74 homolog (chlamydomonas)  CEP290 - centrosomal protein 290kda  NPAT - nuclear protein, ataxia-telangiectasia locus  CNOT2 - ccr4-not transcription complex, subunit 2  NAA15 - n(alpha)-acetyltransferase 15, nata auxiliary subunit  PUM1 - pumilio homolog 1 (drosophila)  CNOT3 - ccr4-not transcription complex, subunit 3  NOTCH1 - notch 1  BAZ2B - bromodomain adjacent to zinc finger domain, 2b  NOTCH3 - notch 3  ST18 - suppression of tumorigenicity 18 (breast carcinoma) (zinc finger protein)  CTC1 - cts telomere maintenance complex component 1  NOTCH2 - notch 2  NFYA - nuclear transcription factor y, alpha  HDAC4 - histone deacetylase 4  TOX - thymocyte selection-associated high mobility group box  NFYC - nuclear transcription factor y, gamma  CXXC1 - cxxc finger protein 1  ZFYVE16 - zinc finger, fyve domain containing 16  NFKBIA - nuclear factor of kappa light polypeptide gene enhancer in b-cells inhibitor, alpha  NFKB1 - nuclear factor of kappa light polypeptide gene enhancer in b-cells 1  KMT2B - lysine (k)-specific methyltransferase 2b  NFKBIB - nuclear factor of kappa light polypeptide gene enhancer in b-cells inhibitor, beta  NFKBIE - nuclear factor of kappa light polypeptide gene enhancer in b-cells inhibitor, epsilon  NKX2-2 - nk2 homeobox 2  ZNF606 - zinc finger protein 606  HDAC9 - histone deacetylase 9  ZNF614 - zinc finger protein 614  ZFP2 - zfp2 zinc finger protein  NEO1 - neogenin 1  SMARCAL1 - swi/snf related, matrix associated, actin dependent regulator of chromatin, subfamily a-like 1  NEDD8 - neural precursor cell expressed, developmentally down-regulated 8  CTR9 - ctr9, paf1/rna polymerase ii complex component  PPM1F - protein phosphatase, mg2+/mn2+ dependent, 1f  CPEB4 - cytoplasmic polyadenylation element binding protein 4  NFE2L2 - nuclear factor (erythroid-derived 2)-like 2  EPC1 - enhancer of polycomb homolog 1 (drosophila)  RASL11A - ras-like, family 11, member a  MORF4L2 - mortality factor 4 like 2  NFATC4 - nuclear factor of activated t-cells, cytoplasmic, calcineurin-dependent 4  NFIA - nuclear factor i/a  CDK5RAP3 - cdk5 regulatory subunit associated protein 3  ZNF746 - zinc finger protein 746  VGLL4 - vestigial like 4 (drosophila)  LRRC14 - leucine rich repeat containing 14  KDM4A - lysine (k)-specific demethylase 4a  PHF14 - phd finger protein 14  HP1BP3 - heterochromatin protein 1, binding protein 3  ZNF432 - zinc finger protein 432  SAFB2 - scaffold attachment factor b2  MYEF2 - myelin expression factor 2  LPIN2 - lipin 2 |
| GO:1903047 | mitotic cell cycle process | 1.26E-11 | 9.22E-9 | 1.21 (10334,426,6996,349) | [+] Show genes  MAPRE1 - microtubule-associated protein, rp/eb family, member 1  EMD - emerin  CHMP4C - charged multivesicular body protein 4c  AKAP9 - a kinase (prka) anchor protein (yotiao) 9  EML1 - echinoderm microtubule associated protein like 1  LIG1 - ligase i, dna, atp-dependent  TTC19 - tetratricopeptide repeat domain 19  RACGAP1 - rac gtpase activating protein 1  HAUS7 - haus augmin-like complex, subunit 7  ENSA - endosulfine alpha  PRMT1 - protein arginine methyltransferase 1  KLHDC8B - kelch domain containing 8b  EP300 - e1a binding protein p300  KATNB1 - katanin p80 (wd repeat containing) subunit b 1  PML - promyelocytic leukemia  CNOT11 - ccr4-not transcription complex, subunit 11  NASP - nuclear autoantigenic sperm protein (histone-binding)  SPAST - spastin  LZTS2 - leucine zipper, putative tumor suppressor 2  NBN - nibrin  NCAPH2 - non-smc condensin ii complex, subunit h2  EIF4E - eukaryotic translation initiation factor 4e  TUBG1 - tubulin, gamma 1  BRSK1 - br serine/threonine kinase 1  ZFYVE19 - zinc finger, fyve domain containing 19  PPP1R12A - protein phosphatase 1, regulatory subunit 12a  SOX4 - sry (sex determining region y)-box 4  NDEL1 - nude neurodevelopment protein 1-like 1  ORC3 - origin recognition complex, subunit 3  UBXN2B - ubx domain protein 2b  ORC6 - origin recognition complex, subunit 6  SON - son dna binding protein  GJA1 - gap junction protein, alpha 1, 43kda  AZI1 - 5-azacytidine induced 1  ATF2 - activating transcription factor 2  DSCC1 - dna replication and sister chromatid cohesion 1  LMNA - lamin a/c  FBXL12 - f-box and leucine-rich repeat protein 12  CDC16 - cell division cycle 16  CNOT1 - ccr4-not transcription complex, subunit 1  ZNF259 - zinc finger protein 259  NDE1 - nude neurodevelopment protein 1  PLK3 - polo-like kinase 3  NAE1 - nedd8 activating enzyme e1 subunit 1  ANAPC7 - anaphase promoting complex subunit 7  SEH1L - seh1-like (s. cerevisiae)  RHOU - ras homolog family member u  MYH10 - myosin, heavy chain 10, non-muscle  MIS12 - mis12 kinetochore complex component  ACVR1 - activin a receptor, type i  MAP9 - microtubule-associated protein 9  ACVR1B - activin a receptor, type ib  VPS4B - vacuolar protein sorting 4 homolog b (s. cerevisiae)  CNOT7 - ccr4-not transcription complex, subunit 7  HAUS6 - haus augmin-like complex, subunit 6  NABP2 - nucleic acid binding protein 2  TOP2B - topoisomerase (dna) ii beta 180kda  HUS1 - hus1 checkpoint homolog (s. pombe)  GPSM2 - g-protein signaling modulator 2  CDC45 - cell division cycle 45  PPAT - phosphoribosyl pyrophosphate amidotransferase  PPME1 - protein phosphatase methylesterase 1  CDC7 - cell division cycle 7  SMC1A - structural maintenance of chromosomes 1a  ROCK1 - rho-associated, coiled-coil containing protein kinase 1  E2F6 - e2f transcription factor 6  STMN1 - stathmin 1  SPRY1 - sprouty homolog 1, antagonist of fgf signaling (drosophila)  SPRY2 - sprouty homolog 2 (drosophila)  E2F4 - e2f transcription factor 4, p107/p130-binding  PHF13 - phd finger protein 13  PDS5B - pds5, regulator of cohesion maintenance, homolog b (s. cerevisiae)  ROCK2 - rho-associated, coiled-coil containing protein kinase 2  CRLF3 - cytokine receptor-like factor 3  HSP90AA1 - heat shock protein 90kda alpha (cytosolic), class a member 1  ACTR1A - arp1 actin-related protein 1 homolog a, centractin alpha (yeast)  CEP76 - centrosomal protein 76kda  CNTROB - centrobin, centrosomal brca2 interacting protein  TAOK3 - tao kinase 3  AKAP8 - a kinase (prka) anchor protein 8  BTRC - beta-transducin repeat containing e3 ubiquitin protein ligase  STAG1 - stromal antigen 1  SMPD3 - sphingomyelin phosphodiesterase 3, neutral membrane (neutral sphingomyelinase ii)  TPR - translocated promoter region, nuclear basket protein  CDK5RAP2 - cdk5 regulatory subunit associated protein 2  PRKACA - protein kinase, camp-dependent, catalytic, alpha  ZWILCH - zwilch kinetochore protein  BOD1 - biorientation of chromosomes in cell division 1  KIF2A - kinesin heavy chain member 2a  PRIM2 - primase, dna, polypeptide 2 (58kda)  PRIM1 - primase, dna, polypeptide 1 (49kda)  SUN2 - sad1 and unc84 domain containing 2  PRCC - papillary renal cell carcinoma (translocation-associated)  STK33 - serine/threonine kinase 33  PIBF1 - progesterone immunomodulatory binding factor 1  DYNLL1 - dynein, light chain, lc8-type 1  CHFR - checkpoint with forkhead and ring finger domains, e3 ubiquitin protein ligase  TNKS - tankyrase, trf1-interacting ankyrin-related adp-ribose polymerase  ZWINT - zw10 interacting kinetochore protein  CASP2 - caspase 2, apoptosis-related cysteine peptidase  CIT - citron (rho-interacting, serine/threonine kinase 21)  FGFR1OP - fgfr1 oncogene partner  RPA3 - replication protein a3, 14kda  PPP6C - protein phosphatase 6, catalytic subunit  MAD2L2 - mad2 mitotic arrest deficient-like 2 (yeast)  LATS2 - large tumor suppressor kinase 2  PDCD6IP - programmed cell death 6 interacting protein  RPA1 - replication protein a1, 70kda  RPA2 - replication protein a2, 32kda  CNTRL - centriolin  CLASP2 - cytoplasmic linker associated protein 2  BECN1 - beclin 1, autophagy related  POGZ - pogo transposable element with znf domain  PPP2R2A - protein phosphatase 2, regulatory subunit b, alpha  PHF8 - phd finger protein 8  PPP2R1A - protein phosphatase 2, regulatory subunit a, alpha  SBDS - shwachman-bodian-diamond syndrome  KPNB1 - karyopherin (importin) beta 1  PCBP4 - poly(rc) binding protein 4  ANKLE2 - ankyrin repeat and lem domain containing 2  KIFC2 - kinesin family member c2  RMDN1 - regulator of microtubule dynamics 1  PPP2CA - protein phosphatase 2, catalytic subunit, alpha isozyme  CDC23 - cell division cycle 23  AURKC - aurora kinase c  TOPBP1 - topoisomerase (dna) ii binding protein 1  INTS3 - integrator complex subunit 3  VCP - valosin containing protein  SMC4 - structural maintenance of chromosomes 4  CNEP1R1 - ctd nuclear envelope phosphatase 1 regulatory subunit 1  MSH2 - muts homolog 2, colon cancer, nonpolyposis type 1 (e. coli)  NEK9 - nima-related kinase 9  PINX1 - pin2/terf1 interacting, telomerase inhibitor 1  NIPBL - nipped-b homolog (drosophila)  TUBB - tubulin, beta class i  RAB35 - rab35, member ras oncogene family  CCND3 - cyclin d3  NCAPD2 - non-smc condensin i complex, subunit d2  CCNH - cyclin h  CCNG2 - cyclin g2  CHMP7 - charged multivesicular body protein 7  RGCC - regulator of cell cycle  AAAS - achalasia, adrenocortical insufficiency, alacrimia  HIRA - histone cell cycle regulator  TIPIN - timeless interacting protein  PAPD7 - pap associated domain containing 7  TUBB4B - tubulin, beta 4b class ivb  KIF3B - kinesin family member 3b  PIM2 - pim-2 oncogene  RPS6KB1 - ribosomal protein s6 kinase, 70kda, polypeptide 1  SNX33 - sorting nexin 33  RAB11A - rab11a, member ras oncogene family  ZNF830 - zinc finger protein 830  CHMP4A - charged multivesicular body protein 4a  CCNI - cyclin i  RRS1 - rrs1 ribosome biogenesis regulator homolog (s. cerevisiae)  KLHL22 - kelch-like family member 22  HAUS1 - haus augmin-like complex, subunit 1  SETD2 - set domain containing 2  CSNK1D - casein kinase 1, delta  SPTBN1 - spectrin, beta, non-erythrocytic 1  CCND2 - cyclin d2  TAOK2 - tao kinase 2  PRKCB - protein kinase c, beta  MRE11A - mre11 meiotic recombination 11 homolog a (s. cerevisiae)  PRKAR2B - protein kinase, camp-dependent, regulatory, type ii, beta  PRKCA - protein kinase c, alpha  NABP1 - nucleic acid binding protein 1  EPS8 - epidermal growth factor receptor pathway substrate 8  CNOT8 - ccr4-not transcription complex, subunit 8  CHTF8 - ctf8, chromosome transmission fidelity factor 8 homolog (s. cerevisiae)  MSTO1 - misato 1, mitochondrial distribution and morphology regulator  CHAMP1 - chromosome alignment maintaining phosphoprotein 1  XIAP - x-linked inhibitor of apoptosis  SYF2 - syf2 pre-mrna-splicing factor  STAMBP - stam binding protein  RFWD3 - ring finger and wd repeat domain 3  GSPT1 - g1 to s phase transition 1  ORC4 - origin recognition complex, subunit 4  ORC2 - origin recognition complex, subunit 2  ORC5 - origin recognition complex, subunit 5  CCNI2 - cyclin i family, member 2  CDC27 - cell division cycle 27  PDS5A - pds5, regulator of cohesion maintenance, homolog a (s. cerevisiae)  SETMAR - set domain and mariner transposase fusion gene  DCTN1 - dynactin 1  FLNA - filamin a, alpha  PPM1D - protein phosphatase, mg2+/mn2+ dependent, 1d  DCTN3 - dynactin 3 (p22)  USP16 - ubiquitin specific peptidase 16  PLCB1 - phospholipase c, beta 1 (phosphoinositide-specific)  MCPH1 - microcephalin 1  CSRP2BP - csrp2 binding protein  UBE2S - ubiquitin-conjugating enzyme e2s  APC - adenomatous polyposis coli  PIAS1 - protein inhibitor of activated stat, 1  NCAPD3 - non-smc condensin ii complex, subunit d3  CNOT10 - ccr4-not transcription complex, subunit 10  ARF1 - adp-ribosylation factor 1  ODF2 - outer dense fiber of sperm tails 2  MNAT1 - mnat cdk-activating kinase assembly factor 1  PAFAH1B1 - platelet-activating factor acetylhydrolase 1b, regulatory subunit 1 (45kda)  REEP4 - receptor accessory protein 4  FBXW11 - f-box and wd repeat domain containing 11  ANAPC1 - anaphase promoting complex subunit 1  DYNLT1 - dynein, light chain, tctex-type 1  CNOT6 - ccr4-not transcription complex, subunit 6  CEP250 - centrosomal protein 250kda  CKAP5 - cytoskeleton associated protein 5  ARL3 - adp-ribosylation factor-like 3  HAUS5 - haus augmin-like complex, subunit 5  MCM7 - minichromosome maintenance complex component 7  CENPC - centromere protein c  DCTN2 - dynactin 2 (p50)  TUBA1A - tubulin, alpha 1a  CETN2 - centrin, ef-hand protein, 2  TAF2 - taf2 rna polymerase ii, tata box binding protein (tbp)-associated factor, 150kda  TACC1 - transforming, acidic coiled-coil containing protein 1  CCNY - cyclin y  ALMS1 - alstrom syndrome 1  NSFL1C - nsfl1 (p97) cofactor (p47)  CFL1 - cofilin 1 (non-muscle)  NUMA1 - nuclear mitotic apparatus protein 1  RHOB - ras homolog family member b  MECP2 - methyl cpg binding protein 2 (rett syndrome)  BUB3 - bub3 mitotic checkpoint protein  CLASP1 - cytoplasmic linker associated protein 1  RHOC - ras homolog family member c  GMNN - geminin, dna replication inhibitor  RHOA - ras homolog family member a  ZW10 - zw10 kinetochore protein  KNSTRN - kinetochore-localized astrin/spag5 binding protein  ITGB1 - integrin, beta 1 (fibronectin receptor, beta polypeptide, antigen cd29 includes mdf2, msk12)  EFHC1 - ef-hand domain (c-terminal) containing 1  CDK5 - cyclin-dependent kinase 5  PCNA - proliferating cell nuclear antigen  CDK4 - cyclin-dependent kinase 4  CHMP1A - charged multivesicular body protein 1a  CEP78 - centrosomal protein 78kda  CDK7 - cyclin-dependent kinase 7  PCM1 - pericentriolar material 1  CDK16 - cyclin-dependent kinase 16  HINFP - histone h4 transcription factor  TBCE - tubulin folding cofactor e  SCC4 - sister chromatid cohesion 4  CDKN1B - cyclin-dependent kinase inhibitor 1b (p27, kip1)  GINS1 - gins complex subunit 1 (psf1 homolog)  CDK17 - cyclin-dependent kinase 17  SPICE1 - spindle and centriole associated protein 1  CDKN2D - cyclin-dependent kinase inhibitor 2d (p19, inhibits cdk4)  ARHGEF2 - rho/rac guanine nucleotide exchange factor (gef) 2  SFI1 - sfi1 homolog, spindle assembly associated (yeast)  CHMP2B - charged multivesicular body protein 2b  HTT - huntingtin  CUL7 - cullin 7  CDK2 - cyclin-dependent kinase 2  ZNF207 - zinc finger protein 207  HAUS2 - haus augmin-like complex, subunit 2  DONSON - downstream neighbor of son  GOLGA2 - golgin a2  XPC - xeroderma pigmentosum, complementation group c  HAUS3 - haus augmin-like complex, subunit 3  MUS81 - mus81 structure-specific endonuclease subunit  RQCD1 - rcd1 required for cell differentiation1 homolog (s. pombe)  CNOT6L - ccr4-not transcription complex, subunit 6-like  SMC3 - structural maintenance of chromosomes 3  TAOK1 - tao kinase 1  PLK2 - polo-like kinase 2  NPAT - nuclear protein, ataxia-telangiectasia locus  CEP290 - centrosomal protein 290kda  LATS1 - large tumor suppressor kinase 1  CNOT2 - ccr4-not transcription complex, subunit 2  CNOT4 - ccr4-not transcription complex, subunit 4  CNOT3 - ccr4-not transcription complex, subunit 3  CEP57 - centrosomal protein 57kda  USP8 - ubiquitin specific peptidase 8  ID2 - inhibitor of dna binding 2, dominant negative helix-loop-helix protein  TNKS1BP1 - tankyrase 1 binding protein 1, 182kda  UNC119 - unc-119 homolog (c. elegans)  WEE1 - wee1 homolog (s. pombe)  SKP1 - s-phase kinase-associated protein 1  PKMYT1 - protein kinase, membrane associated tyrosine/threonine 1  MAX - myc associated factor x  BRD4 - bromodomain containing 4  MCM3 - minichromosome maintenance complex component 3  GIGYF2 - grb10 interacting gyf protein 2  TUBG2 - tubulin, gamma 2  VRK1 - vaccinia related kinase 1  CHMP5 - charged multivesicular body protein 5  MCM6 - minichromosome maintenance complex component 6  RAD21 - rad21 homolog (s. pombe)  MCM5 - minichromosome maintenance complex component 5  RTFDC1 - replication termination factor 2 domain containing 1  RAD17 - rad17 homolog (s. pombe)  MCM2 - minichromosome maintenance complex component 2  MAD1L1 - mad1 mitotic arrest deficient-like 1 (yeast)  INO80 - ino80 complex subunit  VPS4A - vacuolar protein sorting 4 homolog a (s. cerevisiae)  CCP110 - centriolar coiled coil protein 110kda  MDC1 - mediator of dna-damage checkpoint 1  BAX - bcl2-associated x protein  FBXL15 - f-box and leucine-rich repeat protein 15  DLG1 - discs, large homolog 1 (drosophila)  PHLDA1 - pleckstrin homology-like domain, family a, member 1  BACH1 - btb and cnc homology 1, basic leucine zipper transcription factor 1  WRAP73 - wd repeat containing, antisense to tp73  SNX18 - sorting nexin 18  CEP70 - centrosomal protein 70kda  TFDP1 - transcription factor dp-1  ARHGEF10 - rho guanine nucleotide exchange factor (gef) 10  PLAGL1 - pleiomorphic adenoma gene-like 1  FBXL3 - f-box and leucine-rich repeat protein 3  BCCIP - brca2 and cdkn1a interacting protein  DCTN6 - dynactin 6  RASA1 - ras p21 protein activator (gtpase activating protein) 1  DHFR - dihydrofolate reductase  ZFYVE26 - zinc finger, fyve domain containing 26  KHDRBS1 - kh domain containing, rna binding, signal transduction associated 1  CHMP3 - charged multivesicular body protein 3  CEP41 - centrosomal protein 41kda  POLE4 - polymerase (dna-directed), epsilon 4, accessory subunit  IK - ik cytokine, down-regulator of hla ii  CDK5RAP3 - cdk5 regulatory subunit associated protein 3  MITD1 - mit, microtubule interacting and transport, domain containing 1  RBL2 - retinoblastoma-like 2 (p130)  REEP3 - receptor accessory protein 3  DYNC1H1 - dynein, cytoplasmic 1, heavy chain 1  RBBP8 - retinoblastoma binding protein 8  DYNC1I2 - dynein, cytoplasmic 1, intermediate chain 2  TGFB1 - transforming growth factor, beta 1  DIS3L2 - dis3 mitotic control homolog (s. cerevisiae)-like 2  DNM2 - dynamin 2  RB1 - retinoblastoma 1  STAG2 - stromal antigen 2  CEP63 - centrosomal protein 63kda  CUL4B - cullin 4b  CUL1 - cullin 1  CUL2 - cullin 2  CUL3 - cullin 3  CKS1B - cdc28 protein kinase regulatory subunit 1b  BCL2L1 - bcl2-like 1  CHMP4B - charged multivesicular body protein 4b  MZT1 - mitotic spindle organizing protein 1  YWHAG - tyrosine 3-monooxygenase/tryptophan 5-monooxygenase activation protein, gamma polypeptide  CCND1 - cyclin d1  USP37 - ubiquitin specific peptidase 37  EME1 - essential meiotic structure-specific endonuclease 1  BBS4 - bardet-biedl syndrome 4  YWHAE - tyrosine 3-monooxygenase/tryptophan 5-monooxygenase activation protein, epsilon polypeptide |
| GO:0016569 | covalent chromatin modification | 3.29E-11 | 2.29E-8 | 1.25 (10334,286,6996,242) | [+] Show genes  SMARCAD1 - swi/snf-related, matrix-associated actin-dependent regulator of chromatin, subfamily a, containing dead/h box 1  PCGF5 - polycomb group ring finger 5  WAC - ww domain containing adaptor with coiled-coil  MSL3 - male-specific lethal 3 homolog (drosophila)  TADA1 - transcriptional adaptor 1  WHSC1L1 - wolf-hirschhorn syndrome candidate 1-like 1  ELK4 - elk4, ets-domain protein (srf accessory protein 1)  PRMT1 - protein arginine methyltransferase 1  BCOR - bcl6 corepressor  PRMT2 - protein arginine methyltransferase 2  EP300 - e1a binding protein p300  DPF2 - d4, zinc and double phd fingers family 2  DOT1L - dot1-like histone h3k79 methyltransferase  MIER1 - mesoderm induction early response 1, transcriptional regulator  KAT2A - k(lysine) acetyltransferase 2a  NCOA3 - nuclear receptor coactivator 3  RLF - rearranged l-myc fusion  BRD8 - bromodomain containing 8  RING1 - ring finger protein 1  SPHK2 - sphingosine kinase 2  FBLL1 - fibrillarin-like 1  BMI1 - bmi1 polycomb ring finger oncogene  KDM2A - lysine (k)-specific demethylase 2a  EHMT2 - euchromatic histone-lysine n-methyltransferase 2  PCGF3 - polycomb group ring finger 3  LDB1 - lim domain binding 1  CREBBP - creb binding protein  MORF4L1 - mortality factor 4 like 1  ATF2 - activating transcription factor 2  KAT2B - k(lysine) acetyltransferase 2b  CLOCK - clock circadian regulator  CRTC2 - creb regulated transcription coactivator 2  ACTL6A - actin-like 6a  TRRAP - transformation/transcription domain-associated protein  HDAC11 - histone deacetylase 11  PHC1 - polyhomeotic homolog 1 (drosophila)  HUWE1 - hect, uba and wwe domain containing 1, e3 ubiquitin protein ligase  SETD6 - set domain containing 6  KDM4B - lysine (k)-specific demethylase 4b  NAA60 - n(alpha)-acetyltransferase 60, natf catalytic subunit  KDM1A - lysine (k)-specific demethylase 1a  RNF2 - ring finger protein 2  ATXN7L3 - ataxin 7-like 3  MCM3AP - minichromosome maintenance complex component 3 associated protein  BAP1 - brca1 associated protein-1 (ubiquitin carboxy-terminal hydrolase)  ACTL6B - actin-like 6b  KDM5C - lysine (k)-specific demethylase 5c  TET2 - tet methylcytosine dioxygenase 2  ENY2 - enhancer of yellow 2 homolog (drosophila)  TRIM37 - tripartite motif containing 37  PHF17 - phd finger protein 17  KMT2C - lysine (k)-specific methyltransferase 2c  ZMPSTE24 - zinc metallopeptidase ste24  DPY30 - dpy-30 homolog (c. elegans)  SETD1B - set domain containing 1b  RUVBL2 - ruvb-like 2 (e. coli)  KDM4C - lysine (k)-specific demethylase 4c  RCOR3 - rest corepressor 3  EHMT1 - euchromatic histone-lysine n-methyltransferase 1  MCRS1 - microspherule protein 1  KDM2B - lysine (k)-specific demethylase 2b  HLCS - holocarboxylase synthetase (biotin-(proprionyl-coa-carboxylase (atp-hydrolysing)) ligase)  NAA40 - n(alpha)-acetyltransferase 40, natd catalytic subunit, homolog (s. cerevisiae)  ING4 - inhibitor of growth family, member 4  PRDM4 - pr domain containing 4  PPP5C - protein phosphatase 5, catalytic subunit  HDAC5 - histone deacetylase 5  KANSL1 - kat8 regulatory nsl complex subunit 1  HDAC6 - histone deacetylase 6  MT3 - metallothionein 3  USP7 - ubiquitin specific peptidase 7 (herpes virus-associated)  RNF168 - ring finger protein 168, e3 ubiquitin protein ligase  KDM6B - lysine (k)-specific demethylase 6b  PHF8 - phd finger protein 8  TADA3 - transcriptional adaptor 3  KDM6A - lysine (k)-specific demethylase 6a  SUV420H1 - suppressor of variegation 4-20 homolog 1 (drosophila)  EYA3 - eyes absent homolog 3 (drosophila)  PCGF1 - polycomb group ring finger 1  BRD1 - bromodomain containing 1  EZH1 - enhancer of zeste homolog 1 (drosophila)  AURKC - aurora kinase c  PHF20L1 - phd finger protein 20-like 1  SUV420H2 - suppressor of variegation 4-20 homolog 2 (drosophila)  TTLL12 - tubulin tyrosine ligase-like family, member 12  KANSL1L - kat8 regulatory nsl complex subunit 1-like  KANSL3 - kat8 regulatory nsl complex subunit 3  ELMSAN1 - elm2 and myb/sant-like domain containing 1  EED - embryonic ectoderm development  UBE2A - ubiquitin-conjugating enzyme e2a  SUPT7L - suppressor of ty 7 (s. cerevisiae)-like  KMT2D - lysine (k)-specific methyltransferase 2d  RCOR1 - rest corepressor 1  JAK2 - janus kinase 2  ING5 - inhibitor of growth family, member 5  BRMS1 - breast cancer metastasis suppressor 1  YEATS4 - yeats domain containing 4  PHF20 - phd finger protein 20  USP3 - ubiquitin specific peptidase 3  USP15 - ubiquitin specific peptidase 15  KANSL2 - kat8 regulatory nsl complex subunit 2  KAT6A - k(lysine) acetyltransferase 6a  ZNF335 - zinc finger protein 335  JMJD6 - jumonji domain containing 6  SETD2 - set domain containing 2  RBM14 - rna binding motif protein 14  PRKCB - protein kinase c, beta  PRKCD - protein kinase c, delta  LEF1 - lymphoid enhancer-binding factor 1  BRMS1L - breast cancer metastasis-suppressor 1-like  PRKCA - protein kinase c, alpha  GTF3C4 - general transcription factor iiic, polypeptide 4, 90kda  PRMT5 - protein arginine methyltransferase 5  UBE2N - ubiquitin-conjugating enzyme e2n  YEATS2 - yeats domain containing 2  HAT1 - histone acetyltransferase 1  DTX3L - deltex 3-like (drosophila)  ARID4B - at rich interactive domain 4b (rbp1-like)  SETDB1 - set domain, bifurcated 1  SIN3A - sin3 transcription regulator family member a  DDB1 - damage-specific dna binding protein 1, 127kda  SETMAR - set domain and mariner transposase fusion gene  PRMT6 - protein arginine methyltransferase 6  MSL2 - male-specific lethal 2 homolog (drosophila)  MEAF6 - myst/esa1-associated factor 6  KDM3B - lysine (k)-specific demethylase 3b  RSF1 - remodeling and spacing factor 1  USP16 - ubiquitin specific peptidase 16  CSRP2BP - csrp2 binding protein  GTF2B - general transcription factor iib  HDAC8 - histone deacetylase 8  PCGF2 - polycomb group ring finger 2  ASH1L - ash1 (absent, small, or homeotic)-like (drosophila)  KMT2A - lysine (k)-specific methyltransferase 2a  APBB1 - amyloid beta (a4) precursor protein-binding, family b, member 1 (fe65)  SIN3B - sin3 transcription regulator family member b  RPS6KA5 - ribosomal protein s6 kinase, 90kda, polypeptide 5  SMYD3 - set and mynd domain containing 3  TBL1XR1 - transducin (beta)-like 1 x-linked receptor 1  HR - hair growth associated  TAF6L - taf6-like rna polymerase ii, p300/cbp-associated factor (pcaf)-associated factor, 65kda  UIMC1 - ubiquitin interaction motif containing 1  MTA2 - metastasis associated 1 family, member 2  SETD5 - set domain containing 5  WDR61 - wd repeat domain 61  KDM3A - lysine (k)-specific demethylase 3a  TAF9 - taf9 rna polymerase ii, tata box binding protein (tbp)-associated factor, 32kda  BRPF1 - bromodomain and phd finger containing, 1  TAF6 - taf6 rna polymerase ii, tata box binding protein (tbp)-associated factor, 80kda  TADA2A - transcriptional adaptor 2a  ARRB1 - arrestin, beta 1  CDC73 - cell division cycle 73  PHF15 - phd finger protein 15  CHD5 - chromodomain helicase dna binding protein 5  MECP2 - methyl cpg binding protein 2 (rett syndrome)  USP22 - ubiquitin specific peptidase 22  SUDS3 - suppressor of defective silencing 3 homolog (s. cerevisiae)  KAT5 - k(lysine) acetyltransferase 5  MAP3K7 - mitogen-activated protein kinase kinase kinase 7  BAZ2A - bromodomain adjacent to zinc finger domain, 2a  MEN1 - multiple endocrine neoplasia i  HCFC1 - host cell factor c1 (vp16-accessory protein)  CDK5 - cyclin-dependent kinase 5  MAP3K12 - mitogen-activated protein kinase kinase kinase 12  KMT2E - lysine (k)-specific methyltransferase 2e  ZZZ3 - zinc finger, zz-type containing 3  KAT8 - k(lysine) acetyltransferase 8  MTA3 - metastasis associated 1 family, member 3  LEO1 - leo1, paf1/rna polymerase ii complex component, homolog (s. cerevisiae)  PWP1 - pwp1 homolog (s. cerevisiae)  UFL1 - ufm1-specific ligase 1  RUVBL1 - ruvb-like 1 (e. coli)  HDAC2 - histone deacetylase 2  SATB1 - satb homeobox 1  RNF40 - ring finger protein 40, e3 ubiquitin protein ligase  DMAP1 - dna methyltransferase 1 associated protein 1  MBD3 - methyl-cpg binding domain protein 3  HDAC1 - histone deacetylase 1  CDK2 - cyclin-dependent kinase 2  KAT7 - k(lysine) acetyltransferase 7  VPRBP - vpr (hiv-1) binding protein  EPC2 - enhancer of polycomb homolog 2 (drosophila)  NSD1 - nuclear receptor binding set domain protein 1  RYBP - ring1 and yy1 binding protein  PAGR1 - paxip1 associated glutamate-rich protein 1  PHF2 - phd finger protein 2  KDM5B - lysine (k)-specific demethylase 5b  SIRT6 - sirtuin 6  MTA1 - metastasis associated 1  DR1 - down-regulator of transcription 1, tbp-binding (negative cofactor 2)  SIRT1 - sirtuin 1  BRPF3 - bromodomain and phd finger containing, 3  HDAC4 - histone deacetylase 4  SKP1 - s-phase kinase-associated protein 1  CXXC1 - cxxc finger protein 1  CHD1 - chromodomain helicase dna binding protein 1  WHSC1 - wolf-hirschhorn syndrome candidate 1  KMT2B - lysine (k)-specific methyltransferase 2b  TRMT112 - trna methyltransferase 11-2 homolog (s. cerevisiae)  USP36 - ubiquitin specific peptidase 36  VRK1 - vaccinia related kinase 1  PAF1 - paf1, rna polymerase ii associated factor, homolog (s. cerevisiae)  ASH2L - ash2 (absent, small, or homeotic)-like (drosophila)  COPRS - coordinator of prmt5, differentiation stimulator  HDAC9 - histone deacetylase 9  MSL1 - male-specific lethal 1 homolog (drosophila)  SETD1A - set domain containing 1a  SUZ12 - suz12 polycomb repressive complex 2 subunit  RCOR2 - rest corepressor 2  MIER2 - mesoderm induction early response 1, family member 2  BRCC3 - brca1/brca2-containing complex, subunit 3  CTR9 - ctr9, paf1/rna polymerase ii complex component  PPM1F - protein phosphatase, mg2+/mn2+ dependent, 1f  EPC1 - enhancer of polycomb homolog 1 (drosophila)  MORF4L2 - mortality factor 4 like 2  EP400 - e1a binding protein p400  USP21 - ubiquitin specific peptidase 21  ASXL1 - additional sex combs like 1 (drosophila)  RNF8 - ring finger protein 8, e3 ubiquitin protein ligase  BAZ1B - bromodomain adjacent to zinc finger domain, 1b  ING3 - inhibitor of growth family, member 3  PRMT7 - protein arginine methyltransferase 7  JMJD1C - jumonji domain containing 1c  SFPQ - splicing factor proline/glutamine-rich  PHF10 - phd finger protein 10  OGT - o-linked n-acetylglucosamine (glcnac) transferase  MBIP - map3k12 binding inhibitory protein 1  ZNF711 - zinc finger protein 711  ARID4A - at rich interactive domain 4a (rbp1-like)  HDAC7 - histone deacetylase 7  DNMT3A - dna (cytosine-5-)-methyltransferase 3 alpha  RBBP5 - retinoblastoma binding protein 5  RNF20 - ring finger protein 20, e3 ubiquitin protein ligase  MRGBP - mrg/morf4l binding protein  KDM5A - lysine (k)-specific demethylase 5a  KDM4A - lysine (k)-specific demethylase 4a  CUL4B - cullin 4b  RPS6KA4 - ribosomal protein s6 kinase, 90kda, polypeptide 4  PHF14 - phd finger protein 14  KAT6B - k(lysine) acetyltransferase 6b  PARK7 - parkinson protein 7  SUPT3H - suppressor of ty 3 homolog (s. cerevisiae) |
| GO:0016570 | histone modification | 5.24E-11 | 3.47E-8 | 1.25 (10334,284,6996,240) | [+] Show genes  SMARCAD1 - swi/snf-related, matrix-associated actin-dependent regulator of chromatin, subfamily a, containing dead/h box 1  PCGF5 - polycomb group ring finger 5  WAC - ww domain containing adaptor with coiled-coil  MSL3 - male-specific lethal 3 homolog (drosophila)  TADA1 - transcriptional adaptor 1  WHSC1L1 - wolf-hirschhorn syndrome candidate 1-like 1  ELK4 - elk4, ets-domain protein (srf accessory protein 1)  PRMT1 - protein arginine methyltransferase 1  BCOR - bcl6 corepressor  PRMT2 - protein arginine methyltransferase 2  EP300 - e1a binding protein p300  DPF2 - d4, zinc and double phd fingers family 2  MIER1 - mesoderm induction early response 1, transcriptional regulator  DOT1L - dot1-like histone h3k79 methyltransferase  KAT2A - k(lysine) acetyltransferase 2a  NCOA3 - nuclear receptor coactivator 3  RLF - rearranged l-myc fusion  BRD8 - bromodomain containing 8  RING1 - ring finger protein 1  SPHK2 - sphingosine kinase 2  FBLL1 - fibrillarin-like 1  BMI1 - bmi1 polycomb ring finger oncogene  KDM2A - lysine (k)-specific demethylase 2a  EHMT2 - euchromatic histone-lysine n-methyltransferase 2  PCGF3 - polycomb group ring finger 3  LDB1 - lim domain binding 1  CREBBP - creb binding protein  MORF4L1 - mortality factor 4 like 1  ATF2 - activating transcription factor 2  KAT2B - k(lysine) acetyltransferase 2b  CLOCK - clock circadian regulator  CRTC2 - creb regulated transcription coactivator 2  ACTL6A - actin-like 6a  TRRAP - transformation/transcription domain-associated protein  HDAC11 - histone deacetylase 11  PHC1 - polyhomeotic homolog 1 (drosophila)  HUWE1 - hect, uba and wwe domain containing 1, e3 ubiquitin protein ligase  SETD6 - set domain containing 6  KDM4B - lysine (k)-specific demethylase 4b  NAA60 - n(alpha)-acetyltransferase 60, natf catalytic subunit  KDM1A - lysine (k)-specific demethylase 1a  RNF2 - ring finger protein 2  ATXN7L3 - ataxin 7-like 3  MCM3AP - minichromosome maintenance complex component 3 associated protein  BAP1 - brca1 associated protein-1 (ubiquitin carboxy-terminal hydrolase)  ACTL6B - actin-like 6b  KDM5C - lysine (k)-specific demethylase 5c  TET2 - tet methylcytosine dioxygenase 2  ENY2 - enhancer of yellow 2 homolog (drosophila)  TRIM37 - tripartite motif containing 37  KMT2C - lysine (k)-specific methyltransferase 2c  PHF17 - phd finger protein 17  ZMPSTE24 - zinc metallopeptidase ste24  DPY30 - dpy-30 homolog (c. elegans)  SETD1B - set domain containing 1b  RUVBL2 - ruvb-like 2 (e. coli)  KDM4C - lysine (k)-specific demethylase 4c  RCOR3 - rest corepressor 3  EHMT1 - euchromatic histone-lysine n-methyltransferase 1  MCRS1 - microspherule protein 1  KDM2B - lysine (k)-specific demethylase 2b  HLCS - holocarboxylase synthetase (biotin-(proprionyl-coa-carboxylase (atp-hydrolysing)) ligase)  NAA40 - n(alpha)-acetyltransferase 40, natd catalytic subunit, homolog (s. cerevisiae)  ING4 - inhibitor of growth family, member 4  PRDM4 - pr domain containing 4  PPP5C - protein phosphatase 5, catalytic subunit  KANSL1 - kat8 regulatory nsl complex subunit 1  HDAC5 - histone deacetylase 5  HDAC6 - histone deacetylase 6  MT3 - metallothionein 3  USP7 - ubiquitin specific peptidase 7 (herpes virus-associated)  RNF168 - ring finger protein 168, e3 ubiquitin protein ligase  KDM6B - lysine (k)-specific demethylase 6b  PHF8 - phd finger protein 8  TADA3 - transcriptional adaptor 3  KDM6A - lysine (k)-specific demethylase 6a  SUV420H1 - suppressor of variegation 4-20 homolog 1 (drosophila)  EYA3 - eyes absent homolog 3 (drosophila)  BRD1 - bromodomain containing 1  PCGF1 - polycomb group ring finger 1  EZH1 - enhancer of zeste homolog 1 (drosophila)  PHF20L1 - phd finger protein 20-like 1  AURKC - aurora kinase c  SUV420H2 - suppressor of variegation 4-20 homolog 2 (drosophila)  TTLL12 - tubulin tyrosine ligase-like family, member 12  KANSL1L - kat8 regulatory nsl complex subunit 1-like  KANSL3 - kat8 regulatory nsl complex subunit 3  ELMSAN1 - elm2 and myb/sant-like domain containing 1  EED - embryonic ectoderm development  UBE2A - ubiquitin-conjugating enzyme e2a  SUPT7L - suppressor of ty 7 (s. cerevisiae)-like  KMT2D - lysine (k)-specific methyltransferase 2d  RCOR1 - rest corepressor 1  JAK2 - janus kinase 2  ING5 - inhibitor of growth family, member 5  BRMS1 - breast cancer metastasis suppressor 1  YEATS4 - yeats domain containing 4  PHF20 - phd finger protein 20  USP3 - ubiquitin specific peptidase 3  USP15 - ubiquitin specific peptidase 15  KANSL2 - kat8 regulatory nsl complex subunit 2  KAT6A - k(lysine) acetyltransferase 6a  ZNF335 - zinc finger protein 335  JMJD6 - jumonji domain containing 6  SETD2 - set domain containing 2  RBM14 - rna binding motif protein 14  PRKCB - protein kinase c, beta  PRKCD - protein kinase c, delta  LEF1 - lymphoid enhancer-binding factor 1  BRMS1L - breast cancer metastasis-suppressor 1-like  PRKCA - protein kinase c, alpha  GTF3C4 - general transcription factor iiic, polypeptide 4, 90kda  PRMT5 - protein arginine methyltransferase 5  UBE2N - ubiquitin-conjugating enzyme e2n  YEATS2 - yeats domain containing 2  HAT1 - histone acetyltransferase 1  DTX3L - deltex 3-like (drosophila)  ARID4B - at rich interactive domain 4b (rbp1-like)  SETDB1 - set domain, bifurcated 1  SIN3A - sin3 transcription regulator family member a  DDB1 - damage-specific dna binding protein 1, 127kda  SETMAR - set domain and mariner transposase fusion gene  PRMT6 - protein arginine methyltransferase 6  MSL2 - male-specific lethal 2 homolog (drosophila)  MEAF6 - myst/esa1-associated factor 6  KDM3B - lysine (k)-specific demethylase 3b  RSF1 - remodeling and spacing factor 1  USP16 - ubiquitin specific peptidase 16  CSRP2BP - csrp2 binding protein  GTF2B - general transcription factor iib  HDAC8 - histone deacetylase 8  PCGF2 - polycomb group ring finger 2  ASH1L - ash1 (absent, small, or homeotic)-like (drosophila)  KMT2A - lysine (k)-specific methyltransferase 2a  APBB1 - amyloid beta (a4) precursor protein-binding, family b, member 1 (fe65)  SIN3B - sin3 transcription regulator family member b  RPS6KA5 - ribosomal protein s6 kinase, 90kda, polypeptide 5  SMYD3 - set and mynd domain containing 3  TBL1XR1 - transducin (beta)-like 1 x-linked receptor 1  HR - hair growth associated  TAF6L - taf6-like rna polymerase ii, p300/cbp-associated factor (pcaf)-associated factor, 65kda  UIMC1 - ubiquitin interaction motif containing 1  MTA2 - metastasis associated 1 family, member 2  SETD5 - set domain containing 5  WDR61 - wd repeat domain 61  KDM3A - lysine (k)-specific demethylase 3a  TAF9 - taf9 rna polymerase ii, tata box binding protein (tbp)-associated factor, 32kda  BRPF1 - bromodomain and phd finger containing, 1  TAF6 - taf6 rna polymerase ii, tata box binding protein (tbp)-associated factor, 80kda  TADA2A - transcriptional adaptor 2a  ARRB1 - arrestin, beta 1  CDC73 - cell division cycle 73  PHF15 - phd finger protein 15  CHD5 - chromodomain helicase dna binding protein 5  MECP2 - methyl cpg binding protein 2 (rett syndrome)  USP22 - ubiquitin specific peptidase 22  SUDS3 - suppressor of defective silencing 3 homolog (s. cerevisiae)  KAT5 - k(lysine) acetyltransferase 5  MAP3K7 - mitogen-activated protein kinase kinase kinase 7  BAZ2A - bromodomain adjacent to zinc finger domain, 2a  HCFC1 - host cell factor c1 (vp16-accessory protein)  MEN1 - multiple endocrine neoplasia i  CDK5 - cyclin-dependent kinase 5  MAP3K12 - mitogen-activated protein kinase kinase kinase 12  KMT2E - lysine (k)-specific methyltransferase 2e  ZZZ3 - zinc finger, zz-type containing 3  KAT8 - k(lysine) acetyltransferase 8  LEO1 - leo1, paf1/rna polymerase ii complex component, homolog (s. cerevisiae)  MTA3 - metastasis associated 1 family, member 3  UFL1 - ufm1-specific ligase 1  PWP1 - pwp1 homolog (s. cerevisiae)  RUVBL1 - ruvb-like 1 (e. coli)  HDAC2 - histone deacetylase 2  RNF40 - ring finger protein 40, e3 ubiquitin protein ligase  SATB1 - satb homeobox 1  DMAP1 - dna methyltransferase 1 associated protein 1  MBD3 - methyl-cpg binding domain protein 3  HDAC1 - histone deacetylase 1  CDK2 - cyclin-dependent kinase 2  KAT7 - k(lysine) acetyltransferase 7  VPRBP - vpr (hiv-1) binding protein  EPC2 - enhancer of polycomb homolog 2 (drosophila)  NSD1 - nuclear receptor binding set domain protein 1  RYBP - ring1 and yy1 binding protein  PAGR1 - paxip1 associated glutamate-rich protein 1  PHF2 - phd finger protein 2  KDM5B - lysine (k)-specific demethylase 5b  MTA1 - metastasis associated 1  SIRT6 - sirtuin 6  DR1 - down-regulator of transcription 1, tbp-binding (negative cofactor 2)  SIRT1 - sirtuin 1  BRPF3 - bromodomain and phd finger containing, 3  HDAC4 - histone deacetylase 4  CXXC1 - cxxc finger protein 1  SKP1 - s-phase kinase-associated protein 1  WHSC1 - wolf-hirschhorn syndrome candidate 1  KMT2B - lysine (k)-specific methyltransferase 2b  TRMT112 - trna methyltransferase 11-2 homolog (s. cerevisiae)  VRK1 - vaccinia related kinase 1  USP36 - ubiquitin specific peptidase 36  PAF1 - paf1, rna polymerase ii associated factor, homolog (s. cerevisiae)  ASH2L - ash2 (absent, small, or homeotic)-like (drosophila)  MSL1 - male-specific lethal 1 homolog (drosophila)  HDAC9 - histone deacetylase 9  COPRS - coordinator of prmt5, differentiation stimulator  SETD1A - set domain containing 1a  SUZ12 - suz12 polycomb repressive complex 2 subunit  RCOR2 - rest corepressor 2  MIER2 - mesoderm induction early response 1, family member 2  CTR9 - ctr9, paf1/rna polymerase ii complex component  BRCC3 - brca1/brca2-containing complex, subunit 3  PPM1F - protein phosphatase, mg2+/mn2+ dependent, 1f  EPC1 - enhancer of polycomb homolog 1 (drosophila)  MORF4L2 - mortality factor 4 like 2  EP400 - e1a binding protein p400  USP21 - ubiquitin specific peptidase 21  ASXL1 - additional sex combs like 1 (drosophila)  RNF8 - ring finger protein 8, e3 ubiquitin protein ligase  BAZ1B - bromodomain adjacent to zinc finger domain, 1b  ING3 - inhibitor of growth family, member 3  PRMT7 - protein arginine methyltransferase 7  SFPQ - splicing factor proline/glutamine-rich  JMJD1C - jumonji domain containing 1c  PHF10 - phd finger protein 10  OGT - o-linked n-acetylglucosamine (glcnac) transferase  ZNF711 - zinc finger protein 711  MBIP - map3k12 binding inhibitory protein 1  HDAC7 - histone deacetylase 7  ARID4A - at rich interactive domain 4a (rbp1-like)  RBBP5 - retinoblastoma binding protein 5  KDM5A - lysine (k)-specific demethylase 5a  MRGBP - mrg/morf4l binding protein  RNF20 - ring finger protein 20, e3 ubiquitin protein ligase  KDM4A - lysine (k)-specific demethylase 4a  RPS6KA4 - ribosomal protein s6 kinase, 90kda, polypeptide 4  CUL4B - cullin 4b  PHF14 - phd finger protein 14  KAT6B - k(lysine) acetyltransferase 6b  PARK7 - parkinson protein 7  SUPT3H - suppressor of ty 3 homolog (s. cerevisiae) |
| GO:0010556 | regulation of macromolecule biosynthetic process | 5.82E-11 | 3.68E-8 | 1.08 (10334,2371,6996,1733) | [+] Show genes  EXOSC9 - exosome component 9  EXOSC10 - exosome component 10  MSL3 - male-specific lethal 3 homolog (drosophila)  TADA1 - transcriptional adaptor 1  AARS - alanyl-trna synthetase  CBX1 - chromobox homolog 1  ABCA2 - atp-binding cassette, sub-family a (abc1), member 2  ZNF821 - zinc finger protein 821  ABL1 - c-abl oncogene 1, non-receptor tyrosine kinase  PML - promyelocytic leukemia  MLLT11 - myeloid/lymphoid or mixed-lineage leukemia (trithorax homolog, drosophila); translocated to, 11  CNOT11 - ccr4-not transcription complex, subunit 11  SUPT20H - suppressor of ty 20 homolog (s. cerevisiae)  YWHAQ - tyrosine 3-monooxygenase/tryptophan 5-monooxygenase activation protein, theta polypeptide  BRD8 - bromodomain containing 8  CHD7 - chromodomain helicase dna binding protein 7  LYAR - ly1 antibody reactive  TCERG1 - transcription elongation regulator 1  ZNF354B - zinc finger protein 354b  EHMT2 - euchromatic histone-lysine n-methyltransferase 2  TASP1 - taspase, threonine aspartase, 1  SPIN1 - spindlin 1  SUB1 - sub1 homolog (s. cerevisiae)  MORF4L1 - mortality factor 4 like 1  ZNF511 - zinc finger protein 511  PPID - peptidylprolyl isomerase d  ACTL6A - actin-like 6a  ACTN1 - actinin, alpha 1  ACTN4 - actinin, alpha 4  ACVR2A - activin a receptor, type iia  ACVRL1 - activin a receptor type ii-like 1  ACVR1 - activin a receptor, type i  ACVR1B - activin a receptor, type ib  ZNF331 - zinc finger protein 331  MED1 - mediator complex subunit 1  ADCY1 - adenylate cyclase 1 (brain)  PPARD - peroxisome proliferator-activated receptor delta  MAML3 - mastermind-like 3 (drosophila)  POLR2K - polymerase (rna) ii (dna directed) polypeptide k, 7.0kda  FAM129A - family with sequence similarity 129, member a  PARP1 - poly (adp-ribose) polymerase 1  H2AFY2 - h2a histone family, member y2  PPP1R13L - protein phosphatase 1, regulatory subunit 13 like  HES6 - hairy and enhancer of split 6 (drosophila)  POLR2G - polymerase (rna) ii (dna directed) polypeptide g  POLR2D - polymerase (rna) ii (dna directed) polypeptide d  SMPD3 - sphingomyelin phosphodiesterase 3, neutral membrane (neutral sphingomyelinase ii)  PARP4 - poly (adp-ribose) polymerase family, member 4  RUVBL2 - ruvb-like 2 (e. coli)  POLR2A - polymerase (rna) ii (dna directed) polypeptide a, 220kda  AP2A1 - adaptor-related protein complex 2, alpha 1 subunit  CDK5RAP2 - cdk5 regulatory subunit associated protein 2  RCOR3 - rest corepressor 3  AES - amino-terminal enhancer of split  NUP133 - nucleoporin 133kda  JAG1 - jagged 1  PRDM4 - pr domain containing 4  PPP3R1 - protein phosphatase 3, regulatory subunit b, alpha  ATF7IP - activating transcription factor 7 interacting protein  ZNF689 - zinc finger protein 689  LRIF1 - ligand dependent nuclear receptor interacting factor 1  PPP2R5B - protein phosphatase 2, regulatory subunit b', beta  ZNF653 - zinc finger protein 653  AKT1 - v-akt murine thymoma viral oncogene homolog 1  PCID2 - pci domain containing 2  PPP2R1A - protein phosphatase 2, regulatory subunit a, alpha  WWP2 - ww domain containing e3 ubiquitin protein ligase 2  AHR - aryl hydrocarbon receptor  ZNF526 - zinc finger protein 526  FOXP4 - forkhead box p4  PPP2CA - protein phosphatase 2, catalytic subunit, alpha isozyme  ZNF83 - zinc finger protein 83  ESCO1 - establishment of sister chromatid cohesion n-acetyltransferase 1  KANSL3 - kat8 regulatory nsl complex subunit 3  PRNP - prion protein  ATF7 - activating transcription factor 7  AMFR - autocrine motility factor receptor, e3 ubiquitin protein ligase  DNAJC3 - dnaj (hsp40) homolog, subfamily c, member 3  PRKRIR - protein-kinase, interferon-inducible double stranded rna dependent inhibitor, repressor of (p58 repressor)  MAP2K5 - mitogen-activated protein kinase kinase 5  MAP2K7 - mitogen-activated protein kinase kinase 7  EIF2AK2 - eukaryotic translation initiation factor 2-alpha kinase 2  ZNF692 - zinc finger protein 692  PIM2 - pim-2 oncogene  MAP2K1 - mitogen-activated protein kinase kinase 1  EIF4E3 - eukaryotic translation initiation factor 4e family member 3  MAP2K2 - mitogen-activated protein kinase kinase 2  MAPK10 - mitogen-activated protein kinase 10  MAPK9 - mitogen-activated protein kinase 9  MAPK11 - mitogen-activated protein kinase 11  MAPK8 - mitogen-activated protein kinase 8  MAPK7 - mitogen-activated protein kinase 7  GCN1L1 - gcn1 general control of amino-acid synthesis 1-like 1 (yeast)  MAPK3 - mitogen-activated protein kinase 3  MAPK1 - mitogen-activated protein kinase 1  ADIRF - adipogenesis regulatory factor  PRKD1 - protein kinase d1  ANXA3 - annexin a3  CCDC88A - coiled-coil domain containing 88a  PRKCH - protein kinase c, eta  PRKCI - protein kinase c, iota  CCDC85B - coiled-coil domain containing 85b  PRKCB - protein kinase c, beta  PRKCD - protein kinase c, delta  NSUN5 - nop2/sun domain family, member 5  PRKAR1A - protein kinase, camp-dependent, regulatory, type i, alpha  PKIB - protein kinase (camp-dependent, catalytic) inhibitor beta  YEATS2 - yeats domain containing 2  APEX1 - apex nuclease (multifunctional dna repair enzyme) 1  RBM23 - rna binding motif protein 23  ZNF605 - zinc finger protein 605  XIAP - x-linked inhibitor of apoptosis  PARN - poly(a)-specific ribonuclease  BIRC2 - baculoviral iap repeat containing 2  KLF12 - kruppel-like factor 12  PBX2 - pre-b-cell leukemia homeobox 2  PBX3 - pre-b-cell leukemia homeobox 3  PRMT6 - protein arginine methyltransferase 6  PBX1 - pre-b-cell leukemia homeobox 1  PCBD1 - pterin-4 alpha-carbinolamine dehydratase/dimerization cofactor of hepatocyte nuclear factor 1 alpha  APBB2 - amyloid beta (a4) precursor protein-binding, family b, member 2  CHP1 - calcineurin-like ef-hand protein 1  APBB1 - amyloid beta (a4) precursor protein-binding, family b, member 1 (fe65)  RIF1 - rap1 interacting factor homolog (yeast)  POLG2 - polymerase (dna directed), gamma 2, accessory subunit  RNF139 - ring finger protein 139  KIAA1551 - kiaa1551  PA2G4 - proliferation-associated 2g4, 38kda  RPRD1A - regulation of nuclear pre-mrna domain containing 1a  ARF4 - adp-ribosylation factor 4  ZHX1 - zinc fingers and homeoboxes 1  SBNO1 - strawberry notch homolog 1 (drosophila)  IRAK3 - interleukin-1 receptor-associated kinase 3  SETD5 - set domain containing 5  APP - amyloid beta (a4) precursor protein  BBS7 - bardet-biedl syndrome 7  DDX20 - dead (asp-glu-ala-asp) box polypeptide 20  PAK3 - p21 protein (cdc42/rac)-activated kinase 3  PAM - peptidylglycine alpha-amidating monooxygenase  ARNTL - aryl hydrocarbon receptor nuclear translocator-like  ARNT - aryl hydrocarbon receptor nuclear translocator  SUPT16H - suppressor of ty 16 homolog (s. cerevisiae)  PDGFRB - platelet-derived growth factor receptor, beta polypeptide  ARRB2 - arrestin, beta 2  ARRB1 - arrestin, beta 1  PDGFB - platelet-derived growth factor beta polypeptide  SAMD4B - sterile alpha motif domain containing 4b  RHOA - ras homolog family member a  STRAP - serine/threonine kinase receptor associated protein  PSIP1 - pc4 and sfrs1 interacting protein 1  MED9 - mediator complex subunit 9  FSTL1 - follistatin-like 1  BAZ2A - bromodomain adjacent to zinc finger domain, 2a  PCNA - proliferating cell nuclear antigen  CHMP1A - charged multivesicular body protein 1a  JDP2 - jun dimerization protein 2  CDK16 - cyclin-dependent kinase 16  AKIRIN2 - akirin 2  HEATR1 - heat repeat containing 1  CDK17 - cyclin-dependent kinase 17  LEO1 - leo1, paf1/rna polymerase ii complex component, homolog (s. cerevisiae)  ERLIN2 - er lipid raft associated 2  PWP1 - pwp1 homolog (s. cerevisiae)  WRAP53 - wd repeat containing, antisense to tp53  PDE2A - phosphodiesterase 2a, cgmp-stimulated  THAP1 - thap domain containing, apoptosis associated protein 1  PKIG - protein kinase (camp-dependent, catalytic) inhibitor gamma  ELP3 - elongator acetyltransferase complex subunit 3  ASCL1 - achaete-scute complex homolog 1 (drosophila)  KAT7 - k(lysine) acetyltransferase 7  RGMB - rgm domain family, member b  PGGT1B - protein geranylgeranyltransferase type i, beta subunit  SBNO2 - strawberry notch homolog 2 (drosophila)  ZNF444 - zinc finger protein 444  ZBTB1 - zinc finger and btb domain containing 1  ZNF365 - zinc finger protein 365  FBXW7 - f-box and wd repeat domain containing 7, e3 ubiquitin protein ligase  BAHD1 - bromo adjacent homology domain containing 1  ZHX2 - zinc fingers and homeoboxes 2  PHF2 - phd finger protein 2  MORC2 - morc family cw-type zinc finger 2  PHF1 - phd finger protein 1  FOXJ3 - forkhead box j3  LRRK2 - leucine-rich repeat kinase 2  RERE - arginine-glutamic acid dipeptide (re) repeats  DNAJC24 - dnaj (hsp40) homolog, subfamily c, member 24  LARP6 - la ribonucleoprotein domain family, member 6  ZFHX3 - zinc finger homeobox 3  PEX14 - peroxisomal biogenesis factor 14  ADNP2 - adnp homeobox 2  AEBP2 - ae binding protein 2  ZNF507 - zinc finger protein 507  IMPACT - impact rwd domain protein  LGR4 - leucine-rich repeat containing g protein-coupled receptor 4  ATP2B4 - atpase, ca++ transporting, plasma membrane 4  ZNF652 - zinc finger protein 652  SCAF8 - sr-related ctd-associated factor 8  PKD1 - polycystic kidney disease 1 (autosomal dominant)  PKD2 - polycystic kidney disease 2 (autosomal dominant)  MTF2 - metal response element binding transcription factor 2  NAT10 - n-acetyltransferase 10 (gcn5-related)  BACH1 - btb and cnc homology 1, basic leucine zipper transcription factor 1  PIN1 - peptidylprolyl cis/trans isomerase, nima-interacting 1  PIK3R2 - phosphoinositide-3-kinase, regulatory subunit 2 (beta)  DPH3 - diphthamide biosynthesis 3  PIK3R1 - phosphoinositide-3-kinase, regulatory subunit 1 (alpha)  ASXL2 - additional sex combs like 2 (drosophila)  PLAGL1 - pleiomorphic adenoma gene-like 1  ELP2 - elongator acetyltransferase complex subunit 2  ATRX - alpha thalassemia/mental retardation syndrome x-linked  PKNOX1 - pbx/knotted 1 homeobox 1  PKM - pyruvate kinase, muscle  CASC3 - cancer susceptibility candidate 3  OGFOD1 - 2-oxoglutarate and iron-dependent oxygenase domain containing 1  TMEM100 - transmembrane protein 100  PHF10 - phd finger protein 10  RABL3 - rab, member of ras oncogene family-like 3  PSPC1 - paraspeckle component 1  BCL7A - b-cell cll/lymphoma 7a  MRGBP - mrg/morf4l binding protein  BCL9 - b-cell cll/lymphoma 9  RPUSD3 - rna pseudouridylate synthase domain containing 3  BRF2 - brf2, rna polymerase iii transcription initiation factor 50 kda subunit  PIM1 - pim-1 oncogene  BCL2 - b-cell cll/lymphoma 2  ECD - ecdysoneless homolog (drosophila)  CCND1 - cyclin d1  PARK7 - parkinson protein 7  PNKP - polynucleotide kinase 3'-phosphatase  C3orf33 - chromosome 3 open reading frame 33  TRIM27 - tripartite motif containing 27  RFC5 - replication factor c (activator 1) 5, 36.5kda  RFC2 - replication factor c (activator 1) 2, 40kda  SAP18 - sin3a-associated protein, 18kda  RFC3 - replication factor c (activator 1) 3, 38kda  TSHZ2 - teashirt zinc finger homeobox 2  RFXAP - regulatory factor x-associated protein  RFX5 - regulatory factor x, 5 (influences hla class ii expression)  WHSC1L1 - wolf-hirschhorn syndrome candidate 1-like 1  EIF1B - eukaryotic translation initiation factor 1b  RFX1 - regulatory factor x, 1 (influences hla class ii expression)  RELA - v-rel avian reticuloendotheliosis viral oncogene homolog a  APBB3 - amyloid beta (a4) precursor protein-binding, family b, member 3  BCOR - bcl6 corepressor  ZFP36L2 - zfp36 ring finger protein-like 2  ZFP36L1 - zfp36 ring finger protein-like 1  RET - ret proto-oncogene  DPF2 - d4, zinc and double phd fingers family 2  UPF1 - upf1 regulator of nonsense transcripts homolog (yeast)  CC2D1A - coiled-coil and c2 domain containing 1a  RLF - rearranged l-myc fusion  RING1 - ring finger protein 1  CNPY2 - canopy fgf signaling regulator 2  BMI1 - bmi1 polycomb ring finger oncogene  PCGF3 - polycomb group ring finger 3  BMPR1A - bone morphogenetic protein receptor, type ia  BMPR2 - bone morphogenetic protein receptor, type ii (serine/threonine kinase)  GON4L - gon-4-like (c. elegans)  BMPR1B - bone morphogenetic protein receptor, type ib  BMP7 - bone morphogenetic protein 7  ABCA7 - atp-binding cassette, sub-family a (abc1), member 7  PSMD14 - proteasome (prosome, macropain) 26s subunit, non-atpase, 14  FLOT1 - flotillin 1  BCAS3 - breast carcinoma amplified sequence 3  UHMK1 - u2af homology motif (uhm) kinase 1  AHI1 - abelson helper integration site 1  RNF4 - ring finger protein 4  BRD2 - bromodomain containing 2  RNF2 - ring finger protein 2  HNRNPR - heterogeneous nuclear ribonucleoprotein r  ZNF280D - zinc finger protein 280d  GATAD2A - gata zinc finger domain containing 2a  RNF6 - ring finger protein (c3h2c3 type) 6  ROCK1 - rho-associated, coiled-coil containing protein kinase 1  RNF111 - ring finger protein 111  TET2 - tet methylcytosine dioxygenase 2  MED18 - mediator complex subunit 18  DENND4A - denn/madd domain containing 4a  MBTD1 - mbt domain containing 1  RAMP1 - receptor (g protein-coupled) activity modifying protein 1  ZMPSTE24 - zinc metallopeptidase ste24  TRIM44 - tripartite motif containing 44  TAB1 - tgf-beta activated kinase 1/map3k7 binding protein 1  PPIE - peptidylprolyl isomerase e (cyclophilin e)  MCRS1 - microspherule protein 1  NRDE2 - nrde-2, necessary for rna interference, domain containing  C1D - c1d nuclear receptor corepressor  PTCD3 - pentatricopeptide repeat domain 3  SLC30A9 - solute carrier family 30 (zinc transporter), member 9  MAD2L2 - mad2 mitotic arrest deficient-like 2 (yeast)  PID1 - phosphotyrosine interaction domain containing 1  PHIP - pleckstrin homology domain interacting protein  RPS4X - ribosomal protein s4, x-linked  NRARP - notch-regulated ankyrin repeat protein  ZNF787 - zinc finger protein 787  RMND1 - required for meiotic nuclear division 1 homolog (s. cerevisiae)  TRIM38 - tripartite motif containing 38  TADA3 - transcriptional adaptor 3  CALR - calreticulin  ZNF792 - zinc finger protein 792  WTIP - wilms tumor 1 interacting protein  NCOA2 - nuclear receptor coactivator 2  SYNCRIP - synaptotagmin binding, cytoplasmic rna interacting protein  CREB3 - camp responsive element binding protein 3  CITED2 - cbp/p300-interacting transactivator, with glu/asp-rich carboxy-terminal domain, 2  CCT6A - chaperonin containing tcp1, subunit 6a (zeta 1)  CCNT2 - cyclin t2  PINX1 - pin2/terf1 interacting, telomerase inhibitor 1  CCND3 - cyclin d3  CCNT1 - cyclin t1  HMG20A - high mobility group 20a  CCNH - cyclin h  BANP - btg3 associated nuclear protein  TIPIN - timeless interacting protein  ACER2 - alkaline ceramidase 2  TRNAU1AP - trna selenocysteine 1 associated protein 1  RPS6KA1 - ribosomal protein s6 kinase, 90kda, polypeptide 1  RPS6KB1 - ribosomal protein s6 kinase, 70kda, polypeptide 1  RPS6KB2 - ribosomal protein s6 kinase, 70kda, polypeptide 2  CBFA2T3 - core-binding factor, runt domain, alpha subunit 2; translocated to, 3  CBFB - core-binding factor, beta subunit  PIAS3 - protein inhibitor of activated stat, 3  RXRB - retinoid x receptor, beta  CAV1 - caveolin 1, caveolae protein, 22kda  RBM14 - rna binding motif protein 14  CCNC - cyclin c  TRMT10C - trna methyltransferase 10 homolog c (s. cerevisiae)  PRMT5 - protein arginine methyltransferase 5  CHTF8 - ctf8, chromosome transmission fidelity factor 8 homolog (s. cerevisiae)  CDK11B - cyclin-dependent kinase 11b  MXD4 - max dimerization protein 4  CDC5L - cell division cycle 5-like  HTRA1 - htra serine peptidase 1  POLR3F - polymerase (rna) iii (dna directed) polypeptide f, 39 kda  HEXIM1 - hexamethylene bis-acetamide inducible 1  ERLIN1 - er lipid raft associated 1  SPIN2A - spindlin family, member 2a  NCOA7 - nuclear receptor coactivator 7  POMT1 - protein-o-mannosyltransferase 1  PSEN1 - presenilin 1  USP16 - ubiquitin specific peptidase 16  PSMA1 - proteasome (prosome, macropain) subunit, alpha type, 1  PSMA2 - proteasome (prosome, macropain) subunit, alpha type, 2  CD81 - cd81 molecule  CAMKK2 - calcium/calmodulin-dependent protein kinase kinase 2, beta  PSMB1 - proteasome (prosome, macropain) subunit, beta type, 1  PSMA5 - proteasome (prosome, macropain) subunit, alpha type, 5  PSMA3 - proteasome (prosome, macropain) subunit, alpha type, 3  RIPPLY2 - ripply transcriptional repressor 2  PSMB10 - proteasome (prosome, macropain) subunit, beta type, 10  TAF6L - taf6-like rna polymerase ii, p300/cbp-associated factor (pcaf)-associated factor, 65kda  PSMC6 - proteasome (prosome, macropain) 26s subunit, atpase, 6  PSMD1 - proteasome (prosome, macropain) 26s subunit, non-atpase, 1  PSMC4 - proteasome (prosome, macropain) 26s subunit, atpase, 4  SOX18 - sry (sex determining region y)-box 18  POLR3G - polymerase (rna) iii (dna directed) polypeptide g (32kd)  PSMC2 - proteasome (prosome, macropain) 26s subunit, atpase, 2  PSMD9 - proteasome (prosome, macropain) 26s subunit, non-atpase, 9  PSMD10 - proteasome (prosome, macropain) 26s subunit, non-atpase, 10  PSMD7 - proteasome (prosome, macropain) 26s subunit, non-atpase, 7  PSMD8 - proteasome (prosome, macropain) 26s subunit, non-atpase, 8  PSMD5 - proteasome (prosome, macropain) 26s subunit, non-atpase, 5  PSMD2 - proteasome (prosome, macropain) 26s subunit, non-atpase, 2  TERF2IP - telomeric repeat binding factor 2, interacting protein  PSMD12 - proteasome (prosome, macropain) 26s subunit, non-atpase, 12  ATG7 - autophagy related 7  PSMD13 - proteasome (prosome, macropain) 26s subunit, non-atpase, 13  PTBP1 - polypyrimidine tract binding protein 1  LAMTOR5 - late endosomal/lysosomal adaptor, mapk and mtor activator 5  PTEN - phosphatase and tensin homolog  JMY - junction mediating and regulatory protein, p53 cofactor  MYBBP1A - myb binding protein (p160) 1a  FBLN5 - fibulin 5  CIB1 - calcium and integrin binding 1 (calmyrin)  PTGIS - prostaglandin i2 (prostacyclin) synthase  DDX17 - dead (asp-glu-ala-asp) box helicase 17  KAT5 - k(lysine) acetyltransferase 5  ZFP69 - zfp69 zinc finger protein  CEBPG - ccaat/enhancer binding protein (c/ebp), gamma  CDK5 - cyclin-dependent kinase 5  CDK4 - cyclin-dependent kinase 4  CCT7 - chaperonin containing tcp1, subunit 7 (eta)  CDK9 - cyclin-dependent kinase 9  CDK8 - cyclin-dependent kinase 8  LIN54 - lin-54 homolog (c. elegans)  CDK7 - cyclin-dependent kinase 7  CDKN1C - cyclin-dependent kinase inhibitor 1c (p57, kip2)  CCT2 - chaperonin containing tcp1, subunit 2 (beta)  CDKN1B - cyclin-dependent kinase inhibitor 1b (p27, kip1)  CCT4 - chaperonin containing tcp1, subunit 4 (delta)  PCBP3 - poly(rc) binding protein 3  PTPN2 - protein tyrosine phosphatase, non-receptor type 2  HTATIP2 - hiv-1 tat interactive protein 2, 30kda  ARFGEF1 - adp-ribosylation factor guanine nucleotide-exchange factor 1 (brefeldin a-inhibited)  CDK2 - cyclin-dependent kinase 2  RRN3 - rrn3 rna polymerase i transcription factor homolog (s. cerevisiae)  PURA - purine-rich element binding protein a  PURB - purine-rich element binding protein b  GTPBP2 - gtp binding protein 2  ZMYND11 - zinc finger, mynd-type containing 11  SRSF10 - serine/arginine-rich splicing factor 10  PTPRK - protein tyrosine phosphatase, receptor type, k  PTPRN - protein tyrosine phosphatase, receptor type, n  PEX2 - peroxisomal biogenesis factor 2  MPHOSPH8 - m-phase phosphoprotein 8  KDM5B - lysine (k)-specific demethylase 5b  PXN - paxillin  CHD4 - chromodomain helicase dna binding protein 4  CHD2 - chromodomain helicase dna binding protein 2  CHD3 - chromodomain helicase dna binding protein 3  MTERFD2 - mterf domain containing 2  CHD1 - chromodomain helicase dna binding protein 1  QARS - glutaminyl-trna synthetase  HSPH1 - heat shock 105kda/110kda protein 1  MYPOP - myb-related transcription factor, partner of profilin  RAD21 - rad21 homolog (s. pombe)  RAD17 - rad17 homolog (s. pombe)  SEC24A - sec24 family, member a (s. cerevisiae)  PARP14 - poly (adp-ribose) polymerase family, member 14  ZNF181 - zinc finger protein 181  PAF1 - paf1, rna polymerase ii associated factor, homolog (s. cerevisiae)  RAC1 - ras-related c3 botulinum toxin substrate 1 (rho family, small gtp binding protein rac1)  ZNF268 - zinc finger protein 268  INO80 - ino80 complex subunit  ZNF513 - zinc finger protein 513  GMEB1 - glucocorticoid modulatory element binding protein 1  CCT8 - chaperonin containing tcp1, subunit 8 (theta)  CLTC - clathrin, heavy chain (hc)  MIER2 - mesoderm induction early response 1, family member 2  RARG - retinoic acid receptor, gamma  RARB - retinoic acid receptor, beta  DLL4 - delta-like 4 (drosophila)  NECAB2 - n-terminal ef-hand calcium binding protein 2  CELF1 - cugbp, elav-like family member 1  CLU - clusterin  KHDRBS1 - kh domain containing, rna binding, signal transduction associated 1  TAF8 - taf8 rna polymerase ii, tata box binding protein (tbp)-associated factor, 43kda  GNL3L - guanine nucleotide binding protein-like 3 (nucleolar)-like  MAGEL2 - mage-like 2  CTCF - ccctc-binding factor (zinc finger protein)  RBL2 - retinoblastoma-like 2 (p130)  RAI1 - retinoic acid induced 1  RBBP7 - retinoblastoma binding protein 7  RBBP8 - retinoblastoma binding protein 8  MAP3K2 - mitogen-activated protein kinase kinase kinase 2  RBM3 - rna binding motif (rnp1, rrm) protein 3  RBM4 - rna binding motif protein 4  RB1 - retinoblastoma 1  ARID4A - at rich interactive domain 4a (rbp1-like)  RBBP6 - retinoblastoma binding protein 6  KDM5A - lysine (k)-specific demethylase 5a  NUP35 - nucleoporin 35kda  PUS7 - pseudouridylate synthase 7 homolog (s. cerevisiae)  PTGES3 - prostaglandin e synthase 3 (cytosolic)  CKS1B - cdc28 protein kinase regulatory subunit 1b  NFAT5 - nuclear factor of activated t-cells 5, tonicity-responsive  CIRBP - cold inducible rna binding protein  DHX29 - deah (asp-glu-ala-his) box polypeptide 29  CHUK - conserved helix-loop-helix ubiquitous kinase  CERS1 - ceramide synthase 1  ZMYND8 - zinc finger, mynd-type containing 8  ANKRA2 - ankyrin repeat, family a (rfxank-like), 2  RBAK - rb-associated krab zinc finger  NFATC2IP - nuclear factor of activated t-cells, cytoplasmic, calcineurin-dependent 2 interacting protein  FBXO18 - f-box protein, helicase, 18  PATZ1 - poz (btb) and at hook containing zinc finger 1  METTL14 - methyltransferase like 14  SPP1 - secreted phosphoprotein 1  SSBP2 - single-stranded dna binding protein 2  KPNA6 - karyopherin alpha 6 (importin alpha 7)  CIRH1A - cirrhosis, autosomal recessive 1a (cirhin)  PPP1R15B - protein phosphatase 1, regulatory subunit 15b  MIER1 - mesoderm induction early response 1, transcriptional regulator  ZNF382 - zinc finger protein 382  ZNF705A - zinc finger protein 705a  SP3 - sp3 transcription factor  SP4 - sp4 transcription factor  SP1 - sp1 transcription factor  ZNF410 - zinc finger protein 410  SP2 - sp2 transcription factor  ARL2BP - adp-ribosylation factor-like 2 binding protein  SOX4 - sry (sex determining region y)-box 4  GTPBP4 - gtp binding protein 4  WBP2 - ww domain binding protein 2  ORC3 - origin recognition complex, subunit 3  HES4 - hairy and enhancer of split 4 (drosophila)  LEMD3 - lem domain containing 3  GATAD1 - gata zinc finger domain containing 1  CREBL2 - camp responsive element binding protein-like 2  ATF6B - activating transcription factor 6 beta  SOAT1 - sterol o-acyltransferase 1  CREBBP - creb binding protein  ATF2 - activating transcription factor 2  CREB1 - camp responsive element binding protein 1  DDX58 - dead (asp-glu-ala-asp) box polypeptide 58  CRABP2 - cellular retinoic acid binding protein 2  RPRD1B - regulation of nuclear pre-mrna domain containing 1b  PLK3 - polo-like kinase 3  SNAPC4 - small nuclear rna activating complex, polypeptide 4, 190kda  SNCA - synuclein, alpha (non a4 component of amyloid precursor)  ZBED5 - zinc finger, bed-type containing 5  CREBZF - creb/atf bzip transcription factor  EID1 - ep300 interacting inhibitor of differentiation 1  TP53INP2 - tumor protein p53 inducible nuclear protein 2  PTBP2 - polypyrimidine tract binding protein 2  SMARCE1 - swi/snf related, matrix associated, actin dependent regulator of chromatin, subfamily e, member 1  SMO - smoothened, frizzled family receptor  SMARCB1 - swi/snf related, matrix associated, actin dependent regulator of chromatin, subfamily b, member 1  SMARCC1 - swi/snf related, matrix associated, actin dependent regulator of chromatin, subfamily c, member 1  HLTF - helicase-like transcription factor  SSBP3 - single stranded dna binding protein 3  SMARCA4 - swi/snf related, matrix associated, actin dependent regulator of chromatin, subfamily a, member 4  SNX6 - sorting nexin 6  SMARCD2 - swi/snf related, matrix associated, actin dependent regulator of chromatin, subfamily d, member 2  SMARCD3 - swi/snf related, matrix associated, actin dependent regulator of chromatin, subfamily d, member 3  SMARCC2 - swi/snf related, matrix associated, actin dependent regulator of chromatin, subfamily c, member 2  SMARCD1 - swi/snf related, matrix associated, actin dependent regulator of chromatin, subfamily d, member 1  WIZ - widely interspaced zinc finger motifs  SMARCA1 - swi/snf related, matrix associated, actin dependent regulator of chromatin, subfamily a, member 1  SMARCA2 - swi/snf related, matrix associated, actin dependent regulator of chromatin, subfamily a, member 2  SGK3 - serum/glucocorticoid regulated kinase family, member 3  CACTIN - cactin, spliceosome c complex subunit  KMT2C - lysine (k)-specific methyltransferase 2c  ZNF462 - zinc finger protein 462  PPIL4 - peptidylprolyl isomerase (cyclophilin)-like 4  TMEFF2 - transmembrane protein with egf-like and two follistatin-like domains 2  SENP2 - sumo1/sentrin/smt3 specific peptidase 2  CST3 - cystatin c  ZNF318 - zinc finger protein 318  PRPF6 - pre-mrna processing factor 6  KDM2B - lysine (k)-specific demethylase 2b  SUPT6H - suppressor of ty 6 homolog (s. cerevisiae)  CBY1 - chibby homolog 1 (drosophila)  CTBP1 - c-terminal binding protein 1  CTBP2 - c-terminal binding protein 2  SUPT4H1 - suppressor of ty 4 homolog 1 (s. cerevisiae)  SUPT5H - suppressor of ty 5 homolog (s. cerevisiae)  RAB3GAP2 - rab3 gtpase activating protein subunit 2 (non-catalytic)  CTNNB1 - catenin (cadherin-associated protein), beta 1, 88kda  SAV1 - salvador homolog 1 (drosophila)  CUX1 - cut-like homeobox 1  NIF3L1 - nif3 ngg1 interacting factor 3-like 1 (s. cerevisiae)  NEK4 - nima-related kinase 4  STK3 - serine/threonine kinase 3  MPV17L2 - mpv17 mitochondrial membrane protein-like 2  PCGF1 - polycomb group ring finger 1  NPNT - nephronectin  FKBP8 - fk506 binding protein 8, 38kda  GPBP1L1 - gc-rich promoter binding protein 1-like 1  CYLD - cylindromatosis (turban tumor syndrome)  TGIF2 - tgfb-induced factor homeobox 2  BCL6B - b-cell cll/lymphoma 6, member b  CRK - v-crk avian sarcoma virus ct10 oncogene homolog  EEFSEC - eukaryotic elongation factor, selenocysteine-trna-specific  NIPBL - nipped-b homolog (drosophila)  STAT2 - signal transducer and activator of transcription 2, 113kda  CRY1 - cryptochrome 1 (photolyase-like)  STAT1 - signal transducer and activator of transcription 1, 91kda  CRY2 - cryptochrome 2 (photolyase-like)  STAT3 - signal transducer and activator of transcription 3 (acute-phase response factor)  ASF1A - anti-silencing function 1a histone chaperone  STAT5A - signal transducer and activator of transcription 5a  STAT6 - signal transducer and activator of transcription 6, interleukin-4 induced  PARM1 - prostate androgen-regulated mucin-like protein 1  BRMS1 - breast cancer metastasis suppressor 1  ZNF496 - zinc finger protein 496  CRYM - crystallin, mu  SS18 - synovial sarcoma translocation, chromosome 18  PHF5A - phd finger protein 5a  GPATCH3 - g patch domain containing 3  TAB3 - tgf-beta activated kinase 1/map3k7 binding protein 3  BAMBI - bmp and activin membrane-bound inhibitor  ZNF335 - zinc finger protein 335  HAVCR2 - hepatitis a virus cellular receptor 2  SRI - sorcin  SRC - v-src avian sarcoma (schmidt-ruppin a-2) viral oncogene homolog  DNAJB5 - dnaj (hsp40) homolog, subfamily b, member 5  SREBF2 - sterol regulatory element binding transcription factor 2  UBE2O - ubiquitin-conjugating enzyme e2o  NSUN3 - nop2/sun domain family, member 3  SRP9 - signal recognition particle 9kda  PRDX5 - peroxiredoxin 5  BACE2 - beta-site app-cleaving enzyme 2  DHX9 - deah (asp-glu-ala-his) box helicase 9  TRAPPC2 - trafficking protein particle complex 2  KANK2 - kn motif and ankyrin repeat domains 2  DDX5 - dead (asp-glu-ala-asp) box helicase 5  DDX3X - dead (asp-glu-ala-asp) box helicase 3, x-linked  DDX1 - dead (asp-glu-ala-asp) box helicase 1  DPH6 - diphthamine biosynthesis 6  SIN3A - sin3 transcription regulator family member a  SET - set nuclear oncogene  WWTR1 - ww domain containing transcription regulator 1  ZNF304 - zinc finger protein 304  MAP2K4 - mitogen-activated protein kinase kinase 4  ZNF286A - zinc finger protein 286a  GEMIN5 - gem (nuclear organelle) associated protein 5  NOL11 - nucleolar protein 11  PBXIP1 - pre-b-cell leukemia homeobox interacting protein 1  DBP - d site of albumin promoter (albumin d-box) binding protein  NELFB - negative elongation factor complex member b  POT1 - protection of telomeres 1  AHCTF1 - at hook containing transcription factor 1  CNOT10 - ccr4-not transcription complex, subunit 10  ZBTB38 - zinc finger and btb domain containing 38  ZNF551 - zinc finger protein 551  GATAD2B - gata zinc finger domain containing 2b  ZNF473 - zinc finger protein 473  KCTD13 - potassium channel tetramerization domain containing 13  YTHDF3 - yth domain family, member 3  WDR18 - wd repeat domain 18  CNOT6 - ccr4-not transcription complex, subunit 6  LTN1 - listerin e3 ubiquitin protein ligase 1  ATXN2 - ataxin 2  ZNF451 - zinc finger protein 451  SS18L1 - synovial sarcoma translocation gene on chromosome 18-like 1  SCAF4 - sr-related ctd-associated factor 4  CHD5 - chromodomain helicase dna binding protein 5  ARID1B - at rich interactive domain 1b (swi1-like)  MED30 - mediator complex subunit 30  MKL2 - mkl/myocardin-like 2  DAPK3 - death-associated protein kinase 3  DAXX - death-domain associated protein  ZZZ3 - zinc finger, zz-type containing 3  HINFP - histone h4 transcription factor  DGKQ - diacylglycerol kinase, theta 110kda  MTA3 - metastasis associated 1 family, member 3  DAPK1 - death-associated protein kinase 1  ZNF608 - zinc finger protein 608  MAVS - mitochondrial antiviral signaling protein  DAP - death-associated protein  SARS - seryl-trna synthetase  SATB1 - satb homeobox 1  NGDN - neuroguidin, eif4e binding protein  RPTOR - regulatory associated protein of mtor, complex 1  SAFB - scaffold attachment factor b  SH2B1 - sh2b adaptor protein 1  SALL2 - sal-like 2 (drosophila)  EPC2 - enhancer of polycomb homolog 2 (drosophila)  DRD2 - dopamine receptor d2  PHF19 - phd finger protein 19  SLC9A1 - solute carrier family 9, subfamily a (nhe1, cation proton antiporter 1), member 1  IRF2BP1 - interferon regulatory factor 2 binding protein 1  EAF1 - ell associated factor 1  CNOT6L - ccr4-not transcription complex, subunit 6-like  ATN1 - atrophin 1  DPH2 - dph2 homolog (s. cerevisiae)  DPH1 - diphthamide biosynthesis 1  C10orf137 - chromosome 10 open reading frame 137  DR1 - down-regulator of transcription 1, tbp-binding (negative cofactor 2)  MAPK15 - mitogen-activated protein kinase 15  SKI - v-ski avian sarcoma viral oncogene homolog  UNK - unkempt homolog (drosophila)  SKIL - ski-like oncogene  DYRK1A - dual-specificity tyrosine-(y)-phosphorylation regulated kinase 1a  MKL1 - megakaryoblastic leukemia (translocation) 1  DVL2 - dishevelled segment polarity protein 2  DVL3 - dishevelled segment polarity protein 3  GIGYF2 - grb10 interacting gyf protein 2  FNIP2 - folliculin interacting protein 2  HOMEZ - homeobox and leucine zipper encoding  EBF4 - early b-cell factor 4  ANKRD17 - ankyrin repeat domain 17  ZFHX2 - zinc finger homeobox 2  HBEGF - heparin-binding egf-like growth factor  LSM14A - lsm14a, scd6 homolog a (s. cerevisiae)  DTX1 - deltex homolog 1 (drosophila)  DKC1 - dyskeratosis congenita 1, dyskerin  DLG1 - discs, large homolog 1 (drosophila)  SHOX2 - short stature homeobox 2  SIAH2 - siah e3 ubiquitin protein ligase 2  POGK - pogo transposable element with krab domain  DHFR - dihydrofolate reductase  PHF12 - phd finger protein 12  CALCOCO1 - calcium binding and coiled-coil domain 1  SFPQ - splicing factor proline/glutamine-rich  ZBTB4 - zinc finger and btb domain containing 4  SFRP1 - secreted frizzled-related protein 1  SFRP2 - secreted frizzled-related protein 2  SFRP4 - secreted frizzled-related protein 4  DNM2 - dynamin 2  GMEB2 - glucocorticoid modulatory element binding protein 2  SRSF2 - serine/arginine-rich splicing factor 2  SPAG8 - sperm associated antigen 8  DNMT1 - dna (cytosine-5-)-methyltransferase 1  DNMT3A - dna (cytosine-5-)-methyltransferase 3 alpha  EIF2AK4 - eukaryotic translation initiation factor 2 alpha kinase 4  CHD8 - chromodomain helicase dna binding protein 8  SGK1 - serum/glucocorticoid regulated kinase 1  TNRC6C - trinucleotide repeat containing 6c  NOC2L - nucleolar complex associated 2 homolog (s. cerevisiae)  ZNF317 - zinc finger protein 317  USP37 - ubiquitin specific peptidase 37  PPP1R3F - protein phosphatase 1, regulatory subunit 3f  MEIS3 - meis homeobox 3  NCBP2 - nuclear cap binding protein subunit 2, 20kda  MAPRE3 - microtubule-associated protein, rp/eb family, member 3  TSC1 - tuberous sclerosis 1  ELK1 - elk1, member of ets oncogene family  NAF1 - nuclear assembly factor 1 ribonucleoprotein  ELK3 - elk3, ets-domain protein (srf accessory protein 2)  ELK4 - elk4, ets-domain protein (srf accessory protein 1)  SNW1 - snw domain containing 1  SCAP - srebf chaperone  WRNIP1 - werner helicase interacting protein 1  EPAS1 - endothelial pas domain protein 1  DPH7 - diphthamide biosynthesis 7  EP300 - e1a binding protein p300  HABP4 - hyaluronan binding protein 4  ATF6 - activating transcription factor 6  ENO1 - enolase 1, (alpha)  RAB3GAP1 - rab3 gtpase activating protein subunit 1 (catalytic)  EIF4A2 - eukaryotic translation initiation factor 4a2  CELF4 - cugbp, elav-like family member 4  EIF4E - eukaryotic translation initiation factor 4e  EGR2 - early growth response 2  CCT5 - chaperonin containing tcp1, subunit 5 (epsilon)  EGR3 - early growth response 3  TTF1 - transcription termination factor, rna polymerase i  EIF2S1 - eukaryotic translation initiation factor 2, subunit 1 alpha, 35kda  EIF2B1 - eukaryotic translation initiation factor 2b, subunit 1 alpha, 26kda  TRIM32 - tripartite motif containing 32  EIF2S3 - eukaryotic translation initiation factor 2, subunit 3 gamma, 52kda  SPHK2 - sphingosine kinase 2  KDM2A - lysine (k)-specific demethylase 2a  ELAVL1 - elav (embryonic lethal, abnormal vision, drosophila)-like 1 (hu antigen r)  ZNF326 - zinc finger protein 326  ELF1 - e74-like factor 1 (ets domain transcription factor)  ELF2 - e74-like factor 2 (ets domain transcription factor)  TSG101 - tumor susceptibility gene 101  TCF25 - transcription factor 25 (basic helix-loop-helix)  EIF4G2 - eukaryotic translation initiation factor 4 gamma, 2  EFNA1 - ephrin-a1  CNOT1 - ccr4-not transcription complex, subunit 1  EEF2 - eukaryotic translation elongation factor 2  CTNNBIP1 - catenin, beta interacting protein 1  SPEN - spen homolog, transcriptional regulator (drosophila)  BBX - bobby sox homolog (drosophila)  PHC1 - polyhomeotic homolog 1 (drosophila)  TP53BP1 - tumor protein p53 binding protein 1  PHC2 - polyhomeotic homolog 2 (drosophila)  EGR1 - early growth response 1  ZNF292 - zinc finger protein 292  EGFR - epidermal growth factor receptor  MEGF8 - multiple egf-like-domains 8  CELSR2 - cadherin, egf lag seven-pass g-type receptor 2  KDM1A - lysine (k)-specific demethylase 1a  ATXN7L3 - ataxin 7-like 3  HNRNPLL - heterogeneous nuclear ribonucleoprotein l-like  NCOA6 - nuclear receptor coactivator 6  E2F6 - e2f transcription factor 6  CCT3 - chaperonin containing tcp1, subunit 3 (gamma)  E4F1 - e4f transcription factor 1  ZHX3 - zinc fingers and homeoboxes 3  E2F4 - e2f transcription factor 4, p107/p130-binding  E2F5 - e2f transcription factor 5, p130-binding  ENY2 - enhancer of yellow 2 homolog (drosophila)  C11orf30 - chromosome 11 open reading frame 30  RRP1B - ribosomal rna processing 1b  EDNRB - endothelin receptor type b  TRAF1 - tnf receptor-associated factor 1  S1PR1 - sphingosine-1-phosphate receptor 1  TRAF3 - tnf receptor-associated factor 3  TRAF2 - tnf receptor-associated factor 2  NR2C1 - nuclear receptor subfamily 2, group c, member 1  SETX - senataxin  ZBTB47 - zinc finger and btb domain containing 47  NR2C2 - nuclear receptor subfamily 2, group c, member 2  ZNF609 - zinc finger protein 609  TPR - translocated promoter region, nuclear basket protein  WAPAL - wings apart-like homolog (drosophila)  ERC1 - elks/rab6-interacting/cast family member 1  PPRC1 - peroxisome proliferator-activated receptor gamma, coactivator-related 1  KDM4C - lysine (k)-specific demethylase 4c  NAT14 - n-acetyltransferase 14 (gcn5-related, putative)  ZNF423 - zinc finger protein 423  REXO4 - rex4, rna exonuclease 4 homolog (s. cerevisiae)  CAMK1D - calcium/calmodulin-dependent protein kinase id  USF1 - upstream transcription factor 1  NUP107 - nucleoporin 107kda  ZBTB43 - zinc finger and btb domain containing 43  PTK2B - protein tyrosine kinase 2 beta  MRPS27 - mitochondrial ribosomal protein s27  PITHD1 - pith (c-terminal proteasome-interacting domain of thioredoxin-like) domain containing 1  BPTF - bromodomain phd finger transcription factor  TNRC6B - trinucleotide repeat containing 6b  TAB2 - tgf-beta activated kinase 1/map3k7 binding protein 2  NR1H2 - nuclear receptor subfamily 1, group h, member 2  CAMTA2 - calmodulin binding transcription activator 2  ETV3 - ets variant 3  ETV5 - ets variant 5  ZNF598 - zinc finger protein 598  TWSG1 - twisted gastrulation homolog 1 (drosophila)  POGZ - pogo transposable element with znf domain  PLXND1 - plexin d1  ETV6 - ets variant 6  VHL - von hippel-lindau tumor suppressor, e3 ubiquitin protein ligase  KDM6B - lysine (k)-specific demethylase 6b  VIM - vimentin  PHF8 - phd finger protein 8  EZR - ezrin  TRIM41 - tripartite motif containing 41  EWSR1 - ews rna-binding protein 1  TBX20 - t-box 20  KCTD1 - potassium channel tetramerization domain containing 1  CREB3L1 - camp responsive element binding protein 3-like 1  EZH1 - enhancer of zeste homolog 1 (drosophila)  CCNL1 - cyclin l1  CIC - capicua transcriptional repressor  F2R - coagulation factor ii (thrombin) receptor  WDR43 - wd repeat domain 43  ZNF575 - zinc finger protein 575  ERF - ets2 repressor factor  ELMSAN1 - elm2 and myb/sant-like domain containing 1  THAP11 - thap domain containing 11  ZNF248 - zinc finger protein 248  UBB - ubiquitin b  LARP4B - la ribonucleoprotein domain family, member 4b  RCOR1 - rest corepressor 1  TULP3 - tubby like protein 3  HIRA - histone cell cycle regulator  ZNFX1 - zinc finger, nfx1-type containing 1  TTC5 - tetratricopeptide repeat domain 5  ETV1 - ets variant 1  ETS1 - v-ets avian erythroblastosis virus e26 oncogene homolog 1  ZMIZ1 - zinc finger, miz-type containing 1  ETF1 - eukaryotic translation termination factor 1  CYFIP1 - cytoplasmic fmr1 interacting protein 1  UBP1 - upstream binding protein 1 (lbp-1a)  SUMO1 - small ubiquitin-like modifier 1  UBTF - upstream binding transcription factor, rna polymerase i  ZNF830 - zinc finger protein 830  PELI1 - pellino e3 ubiquitin protein ligase 1  CHURC1 - churchill domain containing 1  SMURF1 - smad specific e3 ubiquitin protein ligase 1  ZC3H4 - zinc finger ccch-type containing 4  JMJD6 - jumonji domain containing 6  EPRS - glutamyl-prolyl-trna synthetase  UBE2I - ubiquitin-conjugating enzyme e2i  UBE2L3 - ubiquitin-conjugating enzyme e2l 3  UBE2N - ubiquitin-conjugating enzyme e2n  UBE2V1 - ubiquitin-conjugating enzyme e2 variant 1  VPS72 - vacuolar protein sorting 72 homolog (s. cerevisiae)  FMR1 - fragile x mental retardation 1  TCOF1 - treacher collins-franceschetti syndrome 1  MGA - mga, max dimerization protein  TCP1 - t-complex 1  WWC3 - wwc family member 3  ZEB1 - zinc finger e-box binding homeobox 1  TCF12 - transcription factor 12  ZNF354A - zinc finger protein 354a  TCF20 - transcription factor 20 (ar1)  CAMTA1 - calmodulin binding transcription activator 1  TMEM106A - transmembrane protein 106a  PDS5A - pds5, regulator of cohesion maintenance, homolog a (s. cerevisiae)  FLI1 - fli-1 proto-oncogene, ets transcription factor  FLNA - filamin a, alpha  PLCB1 - phospholipase c, beta 1 (phosphoinositide-specific)  FOXS1 - forkhead box s1  FOXO3 - forkhead box o3  LARP4 - la ribonucleoprotein domain family, member 4  HDAC8 - histone deacetylase 8  ASH1L - ash1 (absent, small, or homeotic)-like (drosophila)  SIN3B - sin3 transcription regulator family member b  NACC1 - nucleus accumbens associated 1, ben and btb (poz) domain containing  HR - hair growth associated  MED8 - mediator complex subunit 8  CAND1 - cullin-associated and neddylation-dissociated 1  TDG - thymine-dna glycosylase  SMG6 - smg6 nonsense mediated mrna decay factor  PRDX2 - peroxiredoxin 2  FBXW11 - f-box and wd repeat domain containing 11  EAPP - e2f-associated phosphoprotein  KDM3A - lysine (k)-specific demethylase 3a  FAM208A - family with sequence similarity 208, member a  FHL2 - four and a half lim domains 2  TAF9 - taf9 rna polymerase ii, tata box binding protein (tbp)-associated factor, 32kda  FGFR2 - fibroblast growth factor receptor 2  TAF7 - taf7 rna polymerase ii, tata box binding protein (tbp)-associated factor, 55kda  TAF6 - taf6 rna polymerase ii, tata box binding protein (tbp)-associated factor, 80kda  FGFR1 - fibroblast growth factor receptor 1  TADA2A - transcriptional adaptor 2a  FGF9 - fibroblast growth factor 9  TACC1 - transforming, acidic coiled-coil containing protein 1  FGF1 - fibroblast growth factor 1 (acidic)  TIRAP - toll-interleukin 1 receptor (tir) domain containing adaptor protein  FGF2 - fibroblast growth factor 2 (basic)  USP22 - ubiquitin specific peptidase 22  TARBP1 - tar (hiv-1) rna binding protein 1  FER - fer (fps/fes related) tyrosine kinase  MAP3K7 - mitogen-activated protein kinase kinase kinase 7  ADNP - activity-dependent neuroprotector homeobox  MED13L - mediator complex subunit 13-like  KMT2E - lysine (k)-specific methyltransferase 2e  NCSTN - nicastrin  FKTN - fukutin  ZNF395 - zinc finger protein 395  MYNN - myoneurin  CRTC1 - creb regulated transcription coactivator 1  UFL1 - ufm1-specific ligase 1  TCF3 - transcription factor 3  PUM2 - pumilio homolog 2 (drosophila)  TCEB3 - transcription elongation factor b (siii), polypeptide 3 (110kda, elongin a)  DMAP1 - dna methyltransferase 1 associated protein 1  TCF4 - transcription factor 4  LARP1 - la ribonucleoprotein domain family, member 1  EFEMP1 - egf containing fibulin-like extracellular matrix protein 1  NKRF - nfkb repressing factor  FBN1 - fibrillin 1  ZNF629 - zinc finger protein 629  LANCL2 - lanc lantibiotic synthetase component c-like 2 (bacterial)  TCEA2 - transcription elongation factor a (sii), 2  TCEA1 - transcription elongation factor a (sii), 1  ERBB2IP - erbb2 interacting protein  ITGB3BP - integrin beta 3 binding protein (beta3-endonexin)  ORMDL1 - orm1-like 1 (s. cerevisiae)  RYBP - ring1 and yy1 binding protein  MTOR - mechanistic target of rapamycin (serine/threonine kinase)  PAXBP1 - pax3 and pax7 binding protein 1  TLE1 - transducin-like enhancer of split 1 (e(sp1) homolog, drosophila)  TLE3 - transducin-like enhancer of split 3 (e(sp1) homolog, drosophila)  TLE4 - transducin-like enhancer of split 4 (e(sp1) homolog, drosophila)  TLR3 - toll-like receptor 3  SIRT1 - sirtuin 1  MKX - mohawk homeobox  TMF1 - tata element modulatory factor 1  TMSB4X - thymosin beta 4, x-linked  ACTR8 - arp8 actin-related protein 8 homolog (yeast)  CBX6 - chromobox homolog 6  HEY1 - hairy/enhancer-of-split related with yrpw motif 1  TMPO - thymopoietin  CBX5 - chromobox homolog 5  BRD4 - bromodomain containing 4  TNFAIP1 - tumor necrosis factor, alpha-induced protein 1 (endothelial)  BCL9L - b-cell cll/lymphoma 9-like  TARDBP - tar dna binding protein  TNFRSF1A - tumor necrosis factor receptor superfamily, member 1a  TNFRSF1B - tumor necrosis factor receptor superfamily, member 1b  TMBIM6 - transmembrane bax inhibitor motif containing 6  EIF4ENIF1 - eukaryotic translation initiation factor 4e nuclear import factor 1  SUZ12 - suz12 polycomb repressive complex 2 subunit  TEF - thyrotrophic embryonic factor  POFUT1 - protein o-fucosyltransferase 1  RCOR2 - rest corepressor 2  TFAM - transcription factor a, mitochondrial  INPP5E - inositol polyphosphate-5-phosphatase, 72 kda  TERF1 - telomeric repeat binding factor (nima-interacting) 1  TERF2 - telomeric repeat binding factor 2  TFDP1 - transcription factor dp-1  NR2F2 - nuclear receptor subfamily 2, group f, member 2  TFE3 - transcription factor binding to ighm enhancer 3  CBX7 - chromobox homolog 7  NR2F1 - nuclear receptor subfamily 2, group f, member 1  TFCP2 - transcription factor cp2  TGFA - transforming growth factor, alpha  ZNF740 - zinc finger protein 740  FNIP1 - folliculin interacting protein 1  HEY2 - hairy/enhancer-of-split related with yrpw motif 2  TGFBR1 - transforming growth factor, beta receptor 1  TGFB2 - transforming growth factor, beta 2  RBFOX2 - rna binding protein, fox-1 homolog (c. elegans) 2  TGFB3 - transforming growth factor, beta 3  TGFB1 - transforming growth factor, beta 1  TGFB1I1 - transforming growth factor beta 1 induced transcript 1  TP53INP1 - tumor protein p53 inducible nuclear protein 1  H2AFV - h2a histone family, member v  MEPCE - methylphosphate capping enzyme  RNF20 - ring finger protein 20, e3 ubiquitin protein ligase  TGFBR3 - transforming growth factor, beta receptor iii  FOSL2 - fos-like antigen 2  FOSB - fbj murine osteosarcoma viral oncogene homolog b  FOS - fbj murine osteosarcoma viral oncogene homolog  THBS1 - thrombospondin 1  KAT6B - k(lysine) acetyltransferase 6b  TIA1 - tia1 cytotoxic granule-associated rna binding protein  METTL3 - methyltransferase like 3  THRA - thyroid hormone receptor, alpha  ELL - elongation factor rna polymerase ii  ZNF239 - zinc finger protein 239  PHC3 - polyhomeotic homolog 3 (drosophila)  OBFC1 - oligonucleotide/oligosaccharide-binding fold containing 1  KAT2A - k(lysine) acetyltransferase 2a  NRIP1 - nuclear receptor interacting protein 1  NCOA3 - nuclear receptor coactivator 3  WWC2 - ww and c2 domain containing 2  TAF15 - taf15 rna polymerase ii, tata box binding protein (tbp)-associated factor, 68kda  GJA1 - gap junction protein, alpha 1, 43kda  AXIN1 - axin 1  TRRAP - transformation/transcription domain-associated protein  PICALM - phosphatidylinositol binding clathrin assembly protein  DLL1 - delta-like 1 (drosophila)  ARID1A - at rich interactive domain 1a (swi-like)  SETD6 - set domain containing 6  FZD6 - frizzled family receptor 6  GLE1 - gle1 rna export mediator  ACTR5 - arp5 actin-related protein 5 homolog (yeast)  CDC45 - cell division cycle 45  FZD4 - frizzled family receptor 4  GCLC - glutamate-cysteine ligase, catalytic subunit  FZD1 - frizzled family receptor 1  BAP1 - brca1 associated protein-1 (ubiquitin carboxy-terminal hydrolase)  ZNF613 - zinc finger protein 613  NUP85 - nucleoporin 85kda  CDC7 - cell division cycle 7  KDM5C - lysine (k)-specific demethylase 5c  USP9X - ubiquitin specific peptidase 9, x-linked  RBM10 - rna binding motif protein 10  PHF17 - phd finger protein 17  TTC21B - tetratricopeptide repeat domain 21b  SLTM - safb-like, transcription modulator  NELFE - negative elongation factor complex member e  EHMT1 - euchromatic histone-lysine n-methyltransferase 1  NR5A1 - nuclear receptor subfamily 5, group a, member 1  FUS - fused in sarcoma  NAA40 - n(alpha)-acetyltransferase 40, natd catalytic subunit, homolog (s. cerevisiae)  USP7 - ubiquitin specific peptidase 7 (herpes virus-associated)  XRCC6 - x-ray repair complementing defective repair in chinese hamster cells 6  RNF168 - ring finger protein 168, e3 ubiquitin protein ligase  GABPB1 - ga binding protein transcription factor, beta subunit 1  GABPA - ga binding protein transcription factor, alpha subunit 60kda  RPAP2 - rna polymerase ii associated protein 2  DDX39B - dead (asp-glu-ala-asp) box polypeptide 39b  KMT2D - lysine (k)-specific methyltransferase 2d  ZNF419 - zinc finger protein 419  YEATS4 - yeats domain containing 4  SNIP1 - smad nuclear interacting protein 1  ZFPM1 - zinc finger protein, fog family member 1  ZNF212 - zinc finger protein 212  EID2 - ep300 interacting inhibitor of differentiation 2  ZNF383 - zinc finger protein 383  GBA - glucosidase, beta, acid  GATA2 - gata binding protein 2  KAT6A - k(lysine) acetyltransferase 6a  NR4A3 - nuclear receptor subfamily 4, group a, member 3  BRD3 - bromodomain containing 3  GAS6 - growth arrest-specific 6  NCOA4 - nuclear receptor coactivator 4  CARF - calcium responsive transcription factor  MLLT10 - myeloid/lymphoid or mixed-lineage leukemia (trithorax homolog, drosophila); translocated to, 10  ZNF408 - zinc finger protein 408  DEDD2 - death effector domain containing 2  DHX36 - deah (asp-glu-ala-his) box polypeptide 36  ZNF189 - zinc finger protein 189  ZSCAN26 - zinc finger and scan domain containing 26  GSTP1 - glutathione s-transferase pi 1  GSPT1 - g1 to s phase transition 1  ZNF177 - zinc finger protein 177  ZNF175 - zinc finger protein 175  ZNF174 - zinc finger protein 174  ZNF184 - zinc finger protein 184  SSBP4 - single stranded dna binding protein 4  GTF2F1 - general transcription factor iif, polypeptide 1, 74kda  ZNF148 - zinc finger protein 148  ZBTB17 - zinc finger and btb domain containing 17  BEND5 - ben domain containing 5  GTF2H2 - general transcription factor iih, polypeptide 2, 44kda  GTF2H3 - general transcription factor iih, polypeptide 3, 34kda  TRIM26 - tripartite motif containing 26  GTF2H1 - general transcription factor iih, polypeptide 1, 62kda  VEZF1 - vascular endothelial zinc finger 1  ZNF142 - zinc finger protein 142  MCPH1 - microcephalin 1  ZNF140 - zinc finger protein 140  GTF2B - general transcription factor iib  PCGF2 - polycomb group ring finger 2  ZNF132 - zinc finger protein 132  ZNF131 - zinc finger protein 131  ZNF134 - zinc finger protein 134  ZNF133 - zinc finger protein 133  TBL1XR1 - transducin (beta)-like 1 x-linked receptor 1  GTF2I - general transcription factor iii  GTF2H4 - general transcription factor iih, polypeptide 4, 52kda  BRF1 - brf1, rna polymerase iii transcription initiation factor 90 kda subunit  ZNF79 - zinc finger protein 79  ZNF70 - zinc finger protein 70  ZNF75D - zinc finger protein 75d  ZNF76 - zinc finger protein 76  H3F3A - h3 histone, family 3a  BRPF1 - bromodomain and phd finger containing, 1  H2AFZ - h2a histone family, member z  FZD5 - frizzled family receptor 5  H2AFX - h2a histone family, member x  SAP130 - sin3a-associated protein, 130kda  DCP2 - decapping mrna 2  PAX8 - paired box 8  MIER3 - mesoderm induction early response 1, family member 3  CDC73 - cell division cycle 73  NKAP - nfkb activating protein  THAP5 - thap domain containing 5  LRP8 - low density lipoprotein receptor-related protein 8, apolipoprotein e receptor  HCFC1 - host cell factor c1 (vp16-accessory protein)  PRDM2 - pr domain containing 2, with znf domain  MAP3K12 - mitogen-activated protein kinase kinase kinase 12  ZNF226 - zinc finger protein 226  HDAC2 - histone deacetylase 2  HMBOX1 - homeobox containing 1  ZCCHC12 - zinc finger, cchc domain containing 12  HDGF - hepatoma-derived growth factor  ZNF213 - zinc finger protein 213  HDAC1 - histone deacetylase 1  HCLS1 - hematopoietic cell-specific lyn substrate 1  ZNF205 - zinc finger protein 205  ZNF207 - zinc finger protein 207  XPA - xeroderma pigmentosum, complementation group a  XPC - xeroderma pigmentosum, complementation group c  GOLGA2 - golgin a2  GOLGB1 - golgin b1  XPO1 - exportin 1 (crm1 homolog, yeast)  PAGR1 - paxip1 associated glutamate-rich protein 1  ZXDC - zxd family zinc finger c  XBP1 - x-box binding protein 1  ZNF367 - zinc finger protein 367  BHLHE41 - basic helix-loop-helix family, member e41  EIF4H - eukaryotic translation initiation factor 4h  WFS1 - wolfram syndrome 1 (wolframin)  WHSC1 - wolf-hirschhorn syndrome candidate 1  NELFA - negative elongation factor complex member a  ARID2 - at rich interactive domain 2 (arid, rfx-like)  ZNF35 - zinc finger protein 35  ZKSCAN1 - zinc finger with krab and scan domains 1  FBXL15 - f-box and leucine-rich repeat protein 15  ZNF576 - zinc finger protein 576  ZNF33B - zinc finger protein 33b  ZNF32 - zinc finger protein 32  ZNF45 - zinc finger protein 45  MZF1 - myeloid zinc finger 1  ZNF18 - zinc finger protein 18  ZNF16 - zinc finger protein 16  ASXL1 - additional sex combs like 1 (drosophila)  ZNF384 - zinc finger protein 384  TNIP2 - tnfaip3 interacting protein 2  ZNF24 - zinc finger protein 24  ZFPL1 - zinc finger protein-like 1  ZFX - zinc finger protein, x-linked  SF1 - splicing factor 1  ZBTB14 - zinc finger and btb domain containing 14  ZNF711 - zinc finger protein 711  ZNF7 - zinc finger protein 7  ZNF8 - zinc finger protein 8  ZNF2 - zinc finger protein 2  ZNF3 - zinc finger protein 3  YES1 - v-yes-1 yamaguchi sarcoma viral oncogene homolog 1  XRCC5 - x-ray repair complementing defective repair in chinese hamster cells 5 (double-strand-break rejoining)  GSK3B - glycogen synthase kinase 3 beta  GSK3A - glycogen synthase kinase 3 alpha  YWHAB - tyrosine 3-monooxygenase/tryptophan 5-monooxygenase activation protein, beta polypeptide  CCNK - cyclin k  HSF2 - heat shock transcription factor 2  TRIM24 - tripartite motif containing 24  TAF1D - tata box binding protein (tbp)-associated factor, rna polymerase i, d, 41kda  CREG1 - cellular repressor of e1a-stimulated genes 1  FLCN - folliculin  HSBP1 - heat shock factor binding protein 1  PRMT1 - protein arginine methyltransferase 1  PRMT2 - protein arginine methyltransferase 2  HESX1 - hesx homeobox 1  SAP30 - sin3a-associated protein, 30kda  METTL16 - methyltransferase like 16  ALKBH1 - alkb, alkylation repair homolog 1 (e. coli)  FTO - fat mass and obesity associated  CFLAR - casp8 and fadd-like apoptosis regulator  SECISBP2 - secis binding protein 2  MED11 - mediator complex subunit 11  WDR77 - wd repeat domain 77  LDB1 - lim domain binding 1  DSCC1 - dna replication and sister chromatid cohesion 1  KAT2B - k(lysine) acetyltransferase 2b  TSC22D1 - tsc22 domain family, member 1  CRTC2 - creb regulated transcription coactivator 2  ZNF259 - zinc finger protein 259  TAX1BP1 - tax1 (human t-cell leukemia virus type i) binding protein 1  CDC123 - cell division cycle 123  CC2D1B - coiled-coil and c2 domain containing 1b  HUS1 - hus1 checkpoint homolog (s. pombe)  THAP8 - thap domain containing 8  EIF2B2 - eukaryotic translation initiation factor 2b, subunit 2 beta, 39kda  BUD31 - bud31 homolog (s. cerevisiae)  ZNF655 - zinc finger protein 655  EIF2B5 - eukaryotic translation initiation factor 2b, subunit 5 epsilon, 82kda  TRAK2 - trafficking protein, kinesin binding 2  HSPD1 - heat shock 60kda protein 1 (chaperonin)  DNAJB1 - dnaj (hsp40) homolog, subfamily b, member 1  ZNF48 - zinc finger protein 48  BCL10 - b-cell cll/lymphoma 10  FUBP3 - far upstream element (fuse) binding protein 3  HSP90AA1 - heat shock protein 90kda alpha (cytosolic), class a member 1  BTRC - beta-transducin repeat containing e3 ubiquitin protein ligase  HSPA8 - heat shock 70kda protein 8  EIF4EBP3 - eukaryotic translation initiation factor 4e binding protein 3  HMGN1 - high mobility group nucleosome binding domain 1  HMGB3 - high mobility group box 3  HMGB2 - high mobility group box 2  HMGB1 - high mobility group box 1  EIF3C - eukaryotic translation initiation factor 3, subunit c  BRD9 - bromodomain containing 9  EIF3D - eukaryotic translation initiation factor 3, subunit d  EIF3B - eukaryotic translation initiation factor 3, subunit b  IRS2 - insulin receptor substrate 2  TNKS - tankyrase, trf1-interacting ankyrin-related adp-ribose polymerase  EIF4G3 - eukaryotic translation initiation factor 4 gamma, 3  ZBTB10 - zinc finger and btb domain containing 10  AGO1 - argonaute risc catalytic component 1  CAPRIN2 - caprin family member 2  ZBTB48 - zinc finger and btb domain containing 48  HYAL2 - hyaluronoglucosaminidase 2  ACD - adrenocortical dysplasia homolog (mouse)  GPBP1 - gc-rich promoter binding protein 1  HFE - hemochromatosis  HINT1 - histidine triad nucleotide binding protein 1  ELP4 - elongator acetyltransferase complex subunit 4  HIVEP1 - human immunodeficiency virus type i enhancer binding protein 1  UPF3B - upf3 regulator of nonsense transcripts homolog b (yeast)  UPF3A - upf3 regulator of nonsense transcripts homolog a (yeast)  HIF1A - hypoxia inducible factor 1, alpha subunit (basic helix-loop-helix transcription factor)  LPIN3 - lipin 3  TRADD - tnfrsf1a-associated via death domain  MYCBP - myc binding protein  EED - embryonic ectoderm development  URI1 - uri1, prefoldin-like chaperone  RIPK1 - receptor (tnfrsf)-interacting serine-threonine kinase 1  HNRNPU - heterogeneous nuclear ribonucleoprotein u (scaffold attachment factor a)  HNRNPK - heterogeneous nuclear ribonucleoprotein k  HNRNPL - heterogeneous nuclear ribonucleoprotein l  FBXO4 - f-box protein 4  TINF2 - terf1 (trf1)-interacting nuclear factor 2  HNRNPA2B1 - heterogeneous nuclear ribonucleoprotein a2/b1  HNRNPA1 - heterogeneous nuclear ribonucleoprotein a1  HNRNPC - heterogeneous nuclear ribonucleoprotein c (c1/c2)  RIPK2 - receptor-interacting serine-threonine kinase 2  HNRNPAB - heterogeneous nuclear ribonucleoprotein a/b  HNRNPD - heterogeneous nuclear ribonucleoprotein d (au-rich element rna binding protein 1, 37kda)  ZNF584 - zinc finger protein 584  HMGA1 - high mobility group at-hook 1  FAM129B - family with sequence similarity 129, member b  NR4A1 - nuclear receptor subfamily 4, group a, member 1  RFX7 - regulatory factor x, 7  HTATSF1 - hiv-1 tat specific factor 1  FOXK2 - forkhead box k2  EIF3K - eukaryotic translation initiation factor 3, subunit k  HAT1 - histone acetyltransferase 1  CRTC3 - creb regulated transcription coactivator 3  RBM15 - rna binding motif protein 15  TNRC6A - trinucleotide repeat containing 6a  ENC1 - ectodermal-neural cortex 1 (with btb domain)  RBMX - rna binding motif protein, x-linked  ING2 - inhibitor of growth family, member 2  ZNF574 - zinc finger protein 574  PPM1D - protein phosphatase, mg2+/mn2+ dependent, 1d  CREB3L2 - camp responsive element binding protein 3-like 2  IPPK - inositol 1,3,4,5,6-pentakisphosphate 2-kinase  ILF2 - interleukin enhancer binding factor 2  ILF3 - interleukin enhancer binding factor 3, 90kda  ILK - integrin-linked kinase  PIAS1 - protein inhibitor of activated stat, 1  SMYD3 - set and mynd domain containing 3  SLCO3A1 - solute carrier organic anion transporter family, member 3a1  MAPKAPK5 - mitogen-activated protein kinase-activated protein kinase 5  SMURF2 - smad specific e3 ubiquitin protein ligase 2  CGGBP1 - cgg triplet repeat binding protein 1  LMO4 - lim domain only 4  CAMK1 - calcium/calmodulin-dependent protein kinase i  CBX4 - chromobox homolog 4  CSRNP1 - cysteine-serine-rich nuclear protein 1  SERP1 - stress-associated endoplasmic reticulum protein 1  ZNF25 - zinc finger protein 25  STK16 - serine/threonine kinase 16  ITGA3 - integrin, alpha 3 (antigen cd49c, alpha 3 subunit of vla-3 receptor)  KHSRP - kh-type splicing regulatory protein  CASK - calcium/calmodulin-dependent serine protein kinase (maguk family)  ITGAV - integrin, alpha v  SUDS3 - suppressor of defective silencing 3 homolog (s. cerevisiae)  EIF6 - eukaryotic translation initiation factor 6  SKAP1 - src kinase associated phosphoprotein 1  GZF1 - gdnf-inducible zinc finger protein 1  INSR - insulin receptor  INSM1 - insulinoma-associated 1  CDK13 - cyclin-dependent kinase 13  PDCD4 - programmed cell death 4 (neoplastic transformation inhibitor)  RFXANK - regulatory factor x-associated ankyrin-containing protein  IRF2 - interferon regulatory factor 2  KLF7 - kruppel-like factor 7 (ubiquitous)  IRF3 - interferon regulatory factor 3  RUVBL1 - ruvb-like 1 (e. coli)  IREB2 - iron-responsive element binding protein 2  IKZF5 - ikaros family zinc finger 5 (pegasus)  MED19 - mediator complex subunit 19  ITGA6 - integrin, alpha 6  IRAK2 - interleukin-1 receptor-associated kinase 2  IRAK1 - interleukin-1 receptor-associated kinase 1  CACYBP - calcyclin binding protein  ZSCAN31 - zinc finger and scan domain containing 31  EIF2AK1 - eukaryotic translation initiation factor 2-alpha kinase 1  KIAA1324L - kiaa1324-like  PAPD5 - pap associated domain containing 5  NSD1 - nuclear receptor binding set domain protein 1  ZBTB11 - zinc finger and btb domain containing 11  RNF25 - ring finger protein 25  MMS19 - mms19 nucleotide excision repair homolog (s. cerevisiae)  DKK3 - dickkopf wnt signaling pathway inhibitor 3  SNX5 - sorting nexin 5  ZNF777 - zinc finger protein 777  ID2 - inhibitor of dna binding 2, dominant negative helix-loop-helix protein  BRPF3 - bromodomain and phd finger containing, 3  ID3 - inhibitor of dna binding 3, dominant negative helix-loop-helix protein  STK36 - serine/threonine kinase 36  IRF2BPL - interferon regulatory factor 2 binding protein-like  JAZF1 - jazf zinc finger 1  PLA2G6 - phospholipase a2, group vi (cytosolic, calcium-independent)  FOXK1 - forkhead box k1  ZNF282 - zinc finger protein 282  IGHMBP2 - immunoglobulin mu binding protein 2  RBPJ - recombination signal binding protein for immunoglobulin kappa j region  NSMAF - neutral sphingomyelinase (n-smase) activation associated factor  PABPC1 - poly(a) binding protein, cytoplasmic 1  NCK2 - nck adaptor protein 2  TRUB2 - trub pseudouridine (psi) synthase homolog 2 (e. coli)  ZBTB9 - zinc finger and btb domain containing 9  USP21 - ubiquitin specific peptidase 21  ZBTB12 - zinc finger and btb domain containing 12  BCAR3 - breast cancer anti-estrogen resistance 3  JMJD1C - jumonji domain containing 1c  IGBP1 - immunoglobulin (cd79a) binding protein 1  SMARCA5 - swi/snf related, matrix associated, actin dependent regulator of chromatin, subfamily a, member 5  OGT - o-linked n-acetylglucosamine (glcnac) transferase  PELP1 - proline, glutamate and leucine rich protein 1  TSPYL2 - tspy-like 2  SND1 - staphylococcal nuclease and tudor domain containing 1  CUL2 - cullin 2  PCIF1 - pdx1 c-terminal inhibiting factor 1  ZNF667 - zinc finger protein 667  CUL3 - cullin 3  FOXP1 - forkhead box p1  SUPT3H - suppressor of ty 3 homolog (s. cerevisiae)  BEND6 - ben domain containing 6  ZNF572 - zinc finger protein 572  PCGF5 - polycomb group ring finger 5  WAC - ww domain containing adaptor with coiled-coil  ZNF414 - zinc finger protein 414  PHF21A - phd finger protein 21a  ZNF771 - zinc finger protein 771  AKAP12 - a kinase (prka) anchor protein 12  IER2 - immediate early response 2  LIG3 - ligase iii, dna, atp-dependent  NCOR1 - nuclear receptor corepressor 1  MTPN - myotrophin  NCOR2 - nuclear receptor corepressor 2  DOT1L - dot1-like histone h3k79 methyltransferase  EFCAB7 - ef-hand calcium binding domain 7  L3MBTL3 - l(3)mbt-like 3 (drosophila)  ZNF333 - zinc finger protein 333  SCAND1 - scan domain containing 1  CIR1 - corepressor interacting with rbpj, 1  H2AFY - h2a histone family, member y  FADS1 - fatty acid desaturase 1  GTF2IRD1 - gtf2i repeat domain containing 1  ZNF527 - zinc finger protein 527  HOPX - hop homeobox  ZNF316 - zinc finger protein 316  ZC3H8 - zinc finger ccch-type containing 8  SOX13 - sry (sex determining region y)-box 13  LMO1 - lim domain only 1 (rhombotin 1)  IER5 - immediate early response 5  NACC2 - nacc family member 2, ben and btb (poz) domain containing  CLOCK - clock circadian regulator  KLF3 - kruppel-like factor 3 (basic)  DDX41 - dead (asp-glu-ala-asp) box polypeptide 41  FXR2 - fragile x mental retardation, autosomal homolog 2  LITAF - lipopolysaccharide-induced tnf factor  TBPL1 - tbp-like 1  YTHDF2 - yth domain family, member 2  ZFP62 - zfp62 zinc finger protein  ZBTB41 - zinc finger and btb domain containing 41  TMEM59 - transmembrane protein 59  ZGPAT - zinc finger, ccch-type with g patch domain  ACTL6B - actin-like 6b  EIF4E2 - eukaryotic translation initiation factor 4e family member 2  MED20 - mediator complex subunit 20  AFAP1L2 - actin filament associated protein 1-like 2  ROCK2 - rho-associated, coiled-coil containing protein kinase 2  CRLF3 - cytokine receptor-like factor 3  MAPK8IP1 - mitogen-activated protein kinase 8 interacting protein 1  NGRN - neugrin, neurite outgrowth associated  GLYR1 - glyoxylate reductase 1 homolog (arabidopsis)  ZBTB7A - zinc finger and btb domain containing 7a  DPY30 - dpy-30 homolog (c. elegans)  MBTPS2 - membrane-bound transcription factor peptidase, site 2  MAGED1 - melanoma antigen family d, 1  GLIS2 - glis family zinc finger 2  IRF2BP2 - interferon regulatory factor 2 binding protein 2  MED27 - mediator complex subunit 27  MED26 - mediator complex subunit 26  CHD6 - chromodomain helicase dna binding protein 6  MED17 - mediator complex subunit 17  MED23 - mediator complex subunit 23  ZNF580 - zinc finger protein 580  CDYL - chromodomain protein, y-like  GTF2IRD2 - gtf2i repeat domain containing 2  KDR - kinase insert domain receptor (a type iii receptor tyrosine kinase)  ZNF710 - zinc finger protein 710  ING4 - inhibitor of growth family, member 4  INSIG2 - insulin induced gene 2  EIF2AK3 - eukaryotic translation initiation factor 2-alpha kinase 3  ITM2B - integral membrane protein 2b  RLIM - ring finger protein, lim domain interacting  KRAS - kirsten rat sarcoma viral oncogene homolog  RECQL5 - recq protein-like 5  TGFBRAP1 - transforming growth factor, beta receptor associated protein 1  ZNF706 - zinc finger protein 706  CIAO1 - cytosolic iron-sulfur protein assembly 1  PHF20L1 - phd finger protein 20-like 1  MED21 - mediator complex subunit 21  MED10 - mediator complex subunit 10  KIT - v-kit hardy-zuckerman 4 feline sarcoma viral oncogene homolog  ZFP28 - zfp28 zinc finger protein  POLDIP3 - polymerase (dna-directed), delta interacting protein 3  PAIP2 - poly(a) binding protein interacting protein 2  JUP - junction plakoglobin  JAK2 - janus kinase 2  ING5 - inhibitor of growth family, member 5  JAK3 - janus kinase 3  ZNF219 - zinc finger protein 219  PHF20 - phd finger protein 20  KCNK2 - potassium channel, subfamily k, member 2  ZNHIT3 - zinc finger, hit-type containing 3  ZNF397 - zinc finger protein 397  TRIP4 - thyroid hormone receptor interactor 4  HMGN3 - high mobility group nucleosomal binding domain 3  PHF6 - phd finger protein 6  ZNF235 - zinc finger protein 235  COPS2 - cop9 signalosome subunit 2  ZNF639 - zinc finger protein 639  SARNP - sap domain containing ribonucleoprotein  LEF1 - lymphoid enhancer-binding factor 1  BRMS1L - breast cancer metastasis-suppressor 1-like  CNOT8 - ccr4-not transcription complex, subunit 8  CDK12 - cyclin-dependent kinase 12  ZNF280B - zinc finger protein 280b  ARID4B - at rich interactive domain 4b (rbp1-like)  MAP3K10 - mitogen-activated protein kinase kinase kinase 10  KDM3B - lysine (k)-specific demethylase 3b  ITGB1BP1 - integrin beta 1 binding protein 1  RSF1 - remodeling and spacing factor 1  MLLT6 - myeloid/lymphoid or mixed-lineage leukemia (trithorax homolog, drosophila); translocated to, 6  SAMD8 - sterile alpha motif domain containing 8  MLLT3 - myeloid/lymphoid or mixed-lineage leukemia (trithorax homolog, drosophila); translocated to, 3  MED14 - mediator complex subunit 14  MLLT1 - myeloid/lymphoid or mixed-lineage leukemia (trithorax homolog, drosophila); translocated to, 1  ZBTB22 - zinc finger and btb domain containing 22  KMT2A - lysine (k)-specific methyltransferase 2a  RPS6KA5 - ribosomal protein s6 kinase, 90kda, polypeptide 5  SUFU - suppressor of fused homolog (drosophila)  NLK - nemo-like kinase  MNAT1 - mnat cdk-activating kinase assembly factor 1  UIMC1 - ubiquitin interaction motif containing 1  MTA2 - metastasis associated 1 family, member 2  NOLC1 - nucleolar and coiled-body phosphoprotein 1  EIF2A - eukaryotic translation initiation factor 2a, 65kda  CD200 - cd200 molecule  WWOX - ww domain containing oxidoreductase  MNT - mnt, max dimerization protein  CCPG1 - cell cycle progression 1  SPTY2D1 - spt2, suppressor of ty, domain containing 1 (s. cerevisiae)  PELO - pelota homolog (drosophila)  MECP2 - methyl cpg binding protein 2 (rett syndrome)  MEF2A - myocyte enhancer factor 2a  PRICKLE1 - prickle homolog 1 (drosophila)  ZBED1 - zinc finger, bed-type containing 1  MEIS2 - meis homeobox 2  BRWD1 - bromodomain and wd repeat domain containing 1  ZNF664 - zinc finger protein 664  MEIS1 - meis homeobox 1  MAP3K4 - mitogen-activated protein kinase kinase kinase 4  MAP3K5 - mitogen-activated protein kinase kinase kinase 5  SLC33A1 - solute carrier family 33 (acetyl-coa transporter), member 1  MEN1 - multiple endocrine neoplasia i  WDR75 - wd repeat domain 75  UTP15 - utp15, u3 small nucleolar ribonucleoprotein, homolog (s. cerevisiae)  KAT8 - k(lysine) acetyltransferase 8  ZNF644 - zinc finger protein 644  ARHGEF2 - rho/rac guanine nucleotide exchange factor (gef) 2  NEMF - nuclear export mediator factor  MBD3 - methyl-cpg binding domain protein 3  PTOV1 - prostate tumor overexpressed 1  ZNF394 - zinc finger protein 394  CBFA2T2 - core-binding factor, runt domain, alpha subunit 2; translocated to, 2  MAGOH - mago-nashi homolog, proliferation-associated (drosophila)  CXXC5 - cxxc finger protein 5  RQCD1 - rcd1 required for cell differentiation1 homolog (s. pombe)  SMC3 - structural maintenance of chromosomes 3  MARS - methionyl-trna synthetase  IL23A - interleukin 23, alpha subunit p19  SIRT6 - sirtuin 6  MTA1 - metastasis associated 1  PPHLN1 - periphilin 1  UBE2J1 - ubiquitin-conjugating enzyme e2, j1  MBD1 - methyl-cpg binding domain protein 1  SFMBT1 - scm-like with four mbt domains 1  MAX - myc associated factor x  ZMIZ2 - zinc finger, miz-type containing 2  LDB2 - lim domain binding 2  ZFP90 - zfp90 zinc finger protein  NELFCD - negative elongation factor complex member c/d  CDAN1 - codanin 1  ASH2L - ash2 (absent, small, or homeotic)-like (drosophila)  ZSCAN29 - zinc finger and scan domain containing 29  CTDSPL2 - ctd (carboxy-terminal domain, rna polymerase ii, polypeptide a) small phosphatase like 2  THAP2 - thap domain containing, apoptosis associated protein 2  ITCH - itchy e3 ubiquitin protein ligase  ZNF484 - zinc finger protein 484  L3MBTL2 - l(3)mbt-like 2 (drosophila)  LRP6 - low density lipoprotein receptor-related protein 6  ZNF445 - zinc finger protein 445  AIP - aryl hydrocarbon receptor interacting protein  TRAPPC9 - trafficking protein particle complex 9  ASB1 - ankyrin repeat and socs box containing 1  LUM - lumican  BTAF1 - btaf1 rna polymerase ii, b-tfiid transcription factor-associated, 170kda  UBA3 - ubiquitin-like modifier activating enzyme 3  SESN2 - sestrin 2  CDK5RAP1 - cdk5 regulatory subunit associated protein 1  PARP9 - poly (adp-ribose) polymerase family, member 9  RNF8 - ring finger protein 8, e3 ubiquitin protein ligase  BAZ1B - bromodomain adjacent to zinc finger domain, 1b  MED15 - mediator complex subunit 15  MXD1 - max dimerization protein 1  PIAS4 - protein inhibitor of activated stat, 4  TRIM33 - tripartite motif containing 33  SRRT - serrate rna effector molecule homolog (arabidopsis)  SMAD2 - smad family member 2  KLF16 - kruppel-like factor 16  HDAC7 - histone deacetylase 7  ARMCX3 - armadillo repeat containing, x-linked 3  TDP2 - tyrosyl-dna phosphodiesterase 2  CAPRIN1 - cell cycle associated protein 1  LARP7 - la ribonucleoprotein domain family, member 7  RPS6KA4 - ribosomal protein s6 kinase, 90kda, polypeptide 4  MAFG - v-maf avian musculoaponeurotic fibrosarcoma oncogene homolog g  DPH5 - diphthamide biosynthesis 5  TAF9B - taf9b rna polymerase ii, tata box binding protein (tbp)-associated factor, 31kda  SMAD4 - smad family member 4  SMAD5 - smad family member 5  H1FX - h1 histone family, member x  SMAD7 - smad family member 7  WASL - wiskott-aldrich syndrome-like  RBM4B - rna binding motif protein 4b  ZNF565 - zinc finger protein 565  SAP30BP - sap30 binding protein  YAF2 - yy1 associated factor 2  TAGLN3 - transgelin 3  SIX5 - six homeobox 5  NCBP1 - nuclear cap binding protein subunit 1, 80kda  ITM2C - integral membrane protein 2c  NDN - necdin, melanoma antigen (mage) family member  ZNF582 - zinc finger protein 582  NCK1 - nck adaptor protein 1  BRD7 - bromodomain containing 7  NCL - nucleolin  DDX25 - dead (asp-glu-ala-asp) box helicase 25  CEBPZ - ccaat/enhancer binding protein (c/ebp), zeta  TICAM1 - toll-like receptor adaptor molecule 1  CCNL2 - cyclin l2  ORMDL2 - orm1-like 2 (s. cerevisiae)  TRIM28 - tripartite motif containing 28  TBK1 - tank-binding kinase 1  NBN - nibrin  ZNF197 - zinc finger protein 197  TDRD3 - tudor domain containing 3  TRIM11 - tripartite motif containing 11  PPP1R12A - protein phosphatase 1, regulatory subunit 12a  MYT1 - myelin transcription factor 1  ALYREF - aly/ref export factor  NAB1 - ngfi-a binding protein 1 (egr1 binding protein 1)  SENP1 - sumo1/sentrin specific peptidase 1  CSRNP2 - cysteine-serine-rich nuclear protein 2  NAB2 - ngfi-a binding protein 2 (egr1 binding protein 2)  RNF41 - ring finger protein 41  TSHZ1 - teashirt zinc finger homeobox 1  ANKRD13C - ankyrin repeat domain 13c  PSME3 - proteasome (prosome, macropain) activator subunit 3 (pa28 gamma; ki)  TRIM13 - tripartite motif containing 13  TRIM8 - tripartite motif containing 8  MYO6 - myosin vi  LBH - limb bud and heart development  ABT1 - activator of basal transcription 1  TOPORS - topoisomerase i binding, arginine/serine-rich, e3 ubiquitin protein ligase  MYD88 - myeloid differentiation primary response 88  ZNF362 - zinc finger protein 362  HCFC2 - host cell factor c2  CNOT7 - ccr4-not transcription complex, subunit 7  MXI1 - max interactor 1, dimerization protein  PQBP1 - polyglutamine binding protein 1  ACTR2 - arp2 actin-related protein 2 homolog (yeast)  ACTR3 - arp3 actin-related protein 3 homolog (yeast)  TSFM - ts translation elongation factor, mitochondrial  TRIM37 - tripartite motif containing 37  ZNF558 - zinc finger protein 558  LRPPRC - leucine-rich pentatricopeptide repeat containing  ZNF263 - zinc finger protein 263  PURG - purine-rich element binding protein g  MED25 - mediator complex subunit 25  TRAP1 - tnf receptor-associated protein 1  MED6 - mediator complex subunit 6  PTBP3 - polypyrimidine tract binding protein 3  DMTF1 - cyclin d binding myb-like transcription factor 1  MTIF2 - mitochondrial translational initiation factor 2  ZBTB33 - zinc finger and btb domain containing 33  HDAC5 - histone deacetylase 5  MTF1 - metal-regulatory transcription factor 1  SRA1 - steroid receptor rna activator 1  HDAC6 - histone deacetylase 6  RNF187 - ring finger protein 187  MT3 - metallothionein 3  MED16 - mediator complex subunit 16  CCDC22 - coiled-coil domain containing 22  DNAJB6 - dnaj (hsp40) homolog, subfamily b, member 6  MSH3 - muts homolog 3 (e. coli)  CITED1 - cbp/p300-interacting transactivator, with glu/asp-rich carboxy-terminal domain, 1  ZBTB5 - zinc finger and btb domain containing 5  ZBTB40 - zinc finger and btb domain containing 40  SUPT7L - suppressor of ty 7 (s. cerevisiae)-like  RGCC - regulator of cell cycle  COMMD1 - copper metabolism (murr1) domain containing 1  RBM8A - rna binding motif protein 8a  USP3 - ubiquitin specific peptidase 3  METTL5 - methyltransferase like 5  USP15 - ubiquitin specific peptidase 15  MED4 - mediator complex subunit 4  THOC1 - tho complex 1  SETD2 - set domain containing 2  RBX1 - ring-box 1, e3 ubiquitin protein ligase  THRAP3 - thyroid hormone receptor associated protein 3  MED12 - mediator complex subunit 12  MED13 - mediator complex subunit 13  PSMD6 - proteasome (prosome, macropain) 26s subunit, non-atpase, 6  MED24 - mediator complex subunit 24  ZC3H6 - zinc finger ccch-type containing 6  DTX3L - deltex 3-like (drosophila)  ZNF34 - zinc finger protein 34  SETDB1 - set domain, bifurcated 1  ORC2 - origin recognition complex, subunit 2  ORC5 - origin recognition complex, subunit 5  TOX4 - tox high mobility group box family member 4  ZNF436 - zinc finger protein 436  RNF141 - ring finger protein 141  EPM2AIP1 - epm2a (laforin) interacting protein 1  AGRN - agrin  G3BP2 - gtpase activating protein (sh3 domain) binding protein 2  OGG1 - 8-oxoguanine dna glycosylase  ZSCAN16 - zinc finger and scan domain containing 16  TNKS2 - tankyrase, trf1-interacting ankyrin-related adp-ribose polymerase 2  DDR2 - discoidin domain receptor tyrosine kinase 2  DAB2IP - dab2 interacting protein  BCLAF1 - bcl2-associated transcription factor 1  NUMA1 - nuclear mitotic apparatus protein 1  EIF4A3 - eukaryotic translation initiation factor 4a3  GMNN - geminin, dna replication inhibitor  NUP98 - nucleoporin 98kda  NR4A2 - nuclear receptor subfamily 4, group a, member 2  ZNF593 - zinc finger protein 593  ZBTB7B - zinc finger and btb domain containing 7b  MATR3 - matrin 3  NVL - nuclear vcp-like  ZNF691 - zinc finger protein 691  CREBRF - creb3 regulatory factor  ZNF623 - zinc finger protein 623  NPM1 - nucleophosmin (nucleolar phosphoprotein b23, numatrin)  NMD3 - nmd3 ribosome export adaptor  ARHGEF11 - rho guanine nucleotide exchange factor (gef) 11  ZBTB24 - zinc finger and btb domain containing 24  ZEB2 - zinc finger e-box binding homeobox 2  THAP4 - thap domain containing 4  CTIF - cbp80/20-dependent translation initiation factor  TSC22D2 - tsc22 domain family, member 2  NRF1 - nuclear respiratory factor 1  NFXL1 - nuclear transcription factor, x-box binding-like 1  DONSON - downstream neighbor of son  NOP2 - nop2 nucleolar protein  VPRBP - vpr (hiv-1) binding protein  NONO - non-pou domain containing, octamer-binding  MDFIC - myod family inhibitor domain containing  NOTCH4 - notch 4  IFT74 - intraflagellar transport 74 homolog (chlamydomonas)  NPAT - nuclear protein, ataxia-telangiectasia locus  CEP290 - centrosomal protein 290kda  CNOT2 - ccr4-not transcription complex, subunit 2  PUM1 - pumilio homolog 1 (drosophila)  NAA15 - n(alpha)-acetyltransferase 15, nata auxiliary subunit  CNOT3 - ccr4-not transcription complex, subunit 3  NOTCH1 - notch 1  BAZ2B - bromodomain adjacent to zinc finger domain, 2b  NOTCH3 - notch 3  CTC1 - cts telomere maintenance complex component 1  ST18 - suppression of tumorigenicity 18 (breast carcinoma) (zinc finger protein)  NOTCH2 - notch 2  NFYA - nuclear transcription factor y, alpha  HDAC4 - histone deacetylase 4  TOX - thymocyte selection-associated high mobility group box  NFYC - nuclear transcription factor y, gamma  ZFYVE16 - zinc finger, fyve domain containing 16  CXXC1 - cxxc finger protein 1  NFKBIA - nuclear factor of kappa light polypeptide gene enhancer in b-cells inhibitor, alpha  NFKB1 - nuclear factor of kappa light polypeptide gene enhancer in b-cells 1  KMT2B - lysine (k)-specific methyltransferase 2b  NFKBIB - nuclear factor of kappa light polypeptide gene enhancer in b-cells inhibitor, beta  NFKBIE - nuclear factor of kappa light polypeptide gene enhancer in b-cells inhibitor, epsilon  NKX2-2 - nk2 homeobox 2  HDAC9 - histone deacetylase 9  ZNF606 - zinc finger protein 606  ZNF614 - zinc finger protein 614  ZFP2 - zfp2 zinc finger protein  NEO1 - neogenin 1  SMARCAL1 - swi/snf related, matrix associated, actin dependent regulator of chromatin, subfamily a-like 1  NEDD8 - neural precursor cell expressed, developmentally down-regulated 8  CTR9 - ctr9, paf1/rna polymerase ii complex component  PPM1F - protein phosphatase, mg2+/mn2+ dependent, 1f  CPEB4 - cytoplasmic polyadenylation element binding protein 4  EPC1 - enhancer of polycomb homolog 1 (drosophila)  NFE2L2 - nuclear factor (erythroid-derived 2)-like 2  MORF4L2 - mortality factor 4 like 2  RASL11A - ras-like, family 11, member a  NFATC4 - nuclear factor of activated t-cells, cytoplasmic, calcineurin-dependent 4  NFIA - nuclear factor i/a  CDK5RAP3 - cdk5 regulatory subunit associated protein 3  ZNF746 - zinc finger protein 746  VGLL4 - vestigial like 4 (drosophila)  LRRC14 - leucine rich repeat containing 14  KDM4A - lysine (k)-specific demethylase 4a  PHF14 - phd finger protein 14  HP1BP3 - heterochromatin protein 1, binding protein 3  ZNF432 - zinc finger protein 432  SAFB2 - scaffold attachment factor b2  MYEF2 - myelin expression factor 2  LPIN2 - lipin 2 |
| GO:0018193 | peptidyl-amino acid modification | 6.66E-11 | 4.03E-8 | 1.16 (10334,647,6996,510) | [+] Show genes  MSL3 - male-specific lethal 3 homolog (drosophila)  TADA1 - transcriptional adaptor 1  ATAT1 - alpha tubulin acetyltransferase 1  ZDHHC3 - zinc finger, dhhc-type containing 3  ABL1 - c-abl oncogene 1, non-receptor tyrosine kinase  AATK - apoptosis-associated tyrosine kinase  DOT1L - dot1-like histone h3k79 methyltransferase  KAT2A - k(lysine) acetyltransferase 2a  NCOA3 - nuclear receptor coactivator 3  LOXL1 - lysyl oxidase-like 1  BRD8 - bromodomain containing 8  METTL12 - methyltransferase like 12  PLOD2 - procollagen-lysine, 2-oxoglutarate 5-dioxygenase 2  RAB3D - rab3d, member ras oncogene family  BRSK1 - br serine/threonine kinase 1  PLOD1 - procollagen-lysine, 2-oxoglutarate 5-dioxygenase 1  NAA25 - n(alpha)-acetyltransferase 25, natb auxiliary subunit  EHMT2 - euchromatic histone-lysine n-methyltransferase 2  MORF4L1 - mortality factor 4 like 1  CREBBP - creb binding protein  ATF2 - activating transcription factor 2  CDC42BPB - cdc42 binding protein kinase beta (dmpk-like)  LMAN1 - lectin, mannose-binding, 1  ZDHHC7 - zinc finger, dhhc-type containing 7  CLOCK - clock circadian regulator  PPIC - peptidylprolyl isomerase c (cyclophilin c)  PPID - peptidylprolyl isomerase d  ACTL6A - actin-like 6a  TRRAP - transformation/transcription domain-associated protein  PPIA - peptidylprolyl isomerase a (cyclophilin a)  PPIB - peptidylprolyl isomerase b (cyclophilin b)  ACVR1 - activin a receptor, type i  ACVR1B - activin a receptor, type ib  NDUFAF7 - nadh dehydrogenase (ubiquinone) complex i, assembly factor 7  SETD6 - set domain containing 6  SUMO2 - small ubiquitin-like modifier 2  NAA60 - n(alpha)-acetyltransferase 60, natf catalytic subunit  NUP85 - nucleoporin 85kda  SUMO3 - small ubiquitin-like modifier 3  CDC7 - cell division cycle 7  ACTL6B - actin-like 6b  ADH5 - alcohol dehydrogenase 5 (class iii), chi polypeptide  ROCK2 - rho-associated, coiled-coil containing protein kinase 2  PARP1 - poly (adp-ribose) polymerase 1  SGK3 - serum/glucocorticoid regulated kinase family, member 3  KMT2C - lysine (k)-specific methyltransferase 2c  PHF17 - phd finger protein 17  DPY30 - dpy-30 homolog (c. elegans)  PPIL4 - peptidylprolyl isomerase (cyclophilin)-like 4  OSTC - oligosaccharyltransferase complex subunit (non-catalytic)  RUVBL2 - ruvb-like 2 (e. coli)  SENP2 - sumo1/sentrin/smt3 specific peptidase 2  PRKACA - protein kinase, camp-dependent, catalytic, alpha  NAT6 - n-acetyltransferase 6 (gcn5-related)  EHMT1 - euchromatic histone-lysine n-methyltransferase 1  NUP133 - nucleoporin 133kda  ADRBK1 - adrenergic, beta, receptor kinase 1  CAMKMT - calmodulin-lysine n-methyltransferase  NUPL2 - nucleoporin like 2  KDR - kinase insert domain receptor (a type iii receptor tyrosine kinase)  DSTYK - dual serine/threonine and tyrosine protein kinase  NAA40 - n(alpha)-acetyltransferase 40, natd catalytic subunit, homolog (s. cerevisiae)  ING4 - inhibitor of growth family, member 4  PRDM4 - pr domain containing 4  EIF2AK3 - eukaryotic translation initiation factor 2-alpha kinase 3  CIT - citron (rho-interacting, serine/threonine kinase 21)  PPIL2 - peptidylprolyl isomerase (cyclophilin)-like 2  AKT1 - v-akt murine thymoma viral oncogene homolog 1  STX1A - syntaxin 1a (brain)  NAA20 - n(alpha)-acetyltransferase 20, natb catalytic subunit  GOLGA7 - golgin a7  ZDHHC9 - zinc finger, dhhc-type containing 9  SUV420H1 - suppressor of variegation 4-20 homolog 1 (drosophila)  FUT8 - fucosyltransferase 8 (alpha (1,6) fucosyltransferase)  BRD1 - bromodomain containing 1  FKBP8 - fk506 binding protein 8, 38kda  AURKC - aurora kinase c  PHF20L1 - phd finger protein 20-like 1  FYN - fyn oncogene related to src, fgr, yes  SUV420H2 - suppressor of variegation 4-20 homolog 2 (drosophila)  KIT - v-kit hardy-zuckerman 4 feline sarcoma viral oncogene homolog  STK4 - serine/threonine kinase 4  ESCO1 - establishment of sister chromatid cohesion n-acetyltransferase 1  KANSL3 - kat8 regulatory nsl complex subunit 3  FKBP10 - fk506 binding protein 10, 65 kda  LIAS - lipoic acid synthetase  GALNT2 - udp-n-acetyl-alpha-d-galactosamine:polypeptide n-acetylgalactosaminyltransferase 2 (galnac-t2)  TTLL7 - tubulin tyrosine ligase-like family, member 7  PPIG - peptidylprolyl isomerase g (cyclophilin g)  GALNT1 - udp-n-acetyl-alpha-d-galactosamine:polypeptide n-acetylgalactosaminyltransferase 1 (galnac-t1)  KMT2D - lysine (k)-specific methyltransferase 2d  AAAS - achalasia, adrenocortical insufficiency, alacrimia  AGBL5 - atp/gtp binding protein-like 5  JAK1 - janus kinase 1  JAK2 - janus kinase 2  JAK3 - janus kinase 3  ING5 - inhibitor of growth family, member 5  MAP2K5 - mitogen-activated protein kinase kinase 5  NAA35 - n(alpha)-acetyltransferase 35, natc auxiliary subunit  STT3A - stt3a, subunit of the oligosaccharyltransferase complex (catalytic)  MAP2K7 - mitogen-activated protein kinase kinase 7  EIF2AK2 - eukaryotic translation initiation factor 2-alpha kinase 2  YEATS4 - yeats domain containing 4  GALNT13 - udp-n-acetyl-alpha-d-galactosamine:polypeptide n-acetylgalactosaminyltransferase 13 (galnac-t13)  MAST4 - microtubule associated serine/threonine kinase family member 4  MAPK13 - mitogen-activated protein kinase 13  MAP2K1 - mitogen-activated protein kinase kinase 1  MAP2K2 - mitogen-activated protein kinase kinase 2  PHF20 - phd finger protein 20  CSF1R - colony stimulating factor 1 receptor  MAPK9 - mitogen-activated protein kinase 9  MAPK8 - mitogen-activated protein kinase 8  METAP2 - methionyl aminopeptidase 2  MAPK7 - mitogen-activated protein kinase 7  SRPK2 - srsf protein kinase 2  QPCT - glutaminyl-peptide cyclotransferase  MAPK3 - mitogen-activated protein kinase 3  MAPK1 - mitogen-activated protein kinase 1  GALNT11 - udp-n-acetyl-alpha-d-galactosamine:polypeptide n-acetylgalactosaminyltransferase 11 (galnac-t11)  KAT6A - k(lysine) acetyltransferase 6a  ZNF335 - zinc finger protein 335  ZDHHC2 - zinc finger, dhhc-type containing 2  TUSC3 - tumor suppressor candidate 3  NDC1 - ndc1 transmembrane nucleoporin  PRKD1 - protein kinase d1  CSK - c-src tyrosine kinase  PKN2 - protein kinase n2  CSNK1G2 - casein kinase 1, gamma 2  TTLL1 - tubulin tyrosine ligase-like family, member 1  PRKCH - protein kinase c, eta  CSNK1A1 - casein kinase 1, alpha 1  PRKCI - protein kinase c, iota  CSNK1D - casein kinase 1, delta  TLK2 - tousled-like kinase 2  NUP214 - nucleoporin 214kda  PRKCE - protein kinase c, epsilon  CSNK2A2 - casein kinase 2, alpha prime polypeptide  PRKCB - protein kinase c, beta  CSNK1G3 - casein kinase 1, gamma 3  SRC - v-src avian sarcoma (schmidt-ruppin a-2) viral oncogene homolog  PRKCD - protein kinase c, delta  CSNK2A1 - casein kinase 2, alpha 1 polypeptide  VIPAS39 - vps33b interacting protein, apical-basolateral polarity regulator, spe-39 homolog  LEF1 - lymphoid enhancer-binding factor 1  PRKCA - protein kinase c, alpha  ZDHHC12 - zinc finger, dhhc-type containing 12  GTF3C4 - general transcription factor iiic, polypeptide 4, 90kda  GAS6 - growth arrest-specific 6  YEATS2 - yeats domain containing 2  ARID4B - at rich interactive domain 4b (rbp1-like)  DDOST - dolichyl-diphosphooligosaccharide--protein glycosyltransferase subunit (non-catalytic)  DPH6 - diphthamine biosynthesis 6  TPGS2 - tubulin polyglutamylase complex subunit 2  RNF113A - ring finger protein 113a  MAP3K10 - mitogen-activated protein kinase kinase kinase 10  SETMAR - set domain and mariner transposase fusion gene  PRMT6 - protein arginine methyltransferase 6  MSL2 - male-specific lethal 2 homolog (drosophila)  MAP2K4 - mitogen-activated protein kinase kinase 4  SENP7 - sumo1/sentrin specific peptidase 7  RSF1 - remodeling and spacing factor 1  ZDHHC24 - zinc finger, dhhc-type containing 24  CSRP2BP - csrp2 binding protein  GTF2B - general transcription factor iib  PCGF2 - polycomb group ring finger 2  APBB1 - amyloid beta (a4) precursor protein-binding, family b, member 1 (fe65)  KMT2A - lysine (k)-specific methyltransferase 2a  RPS6KA5 - ribosomal protein s6 kinase, 90kda, polypeptide 5  SCYL1 - scy1-like 1 (s. cerevisiae)  DPY19L4 - dpy-19-like 4 (c. elegans)  P4HA1 - prolyl 4-hydroxylase, alpha polypeptide i  CLK4 - cdc-like kinase 4  NLK - nemo-like kinase  ASPHD1 - aspartate beta-hydroxylase domain containing 1  GALNT16 - udp-n-acetyl-alpha-d-galactosamine:polypeptide n-acetylgalactosaminyltransferase 16  SETD5 - set domain containing 5  ZDHHC14 - zinc finger, dhhc-type containing 14  PAK2 - p21 protein (cdc42/rac)-activated kinase 2  LEPREL1 - leprecan-like 1  ZDHHC20 - zinc finger, dhhc-type containing 20  BRPF1 - bromodomain and phd finger containing, 1  MUL1 - mitochondrial e3 ubiquitin protein ligase 1  KDELC2 - kdel (lys-asp-glu-leu) containing 2  NSMCE2 - non-smc element 2, mms21 homolog (s. cerevisiae)  ZNF451 - zinc finger protein 451  PDGFRB - platelet-derived growth factor receptor, beta polypeptide  ARRB1 - arrestin, beta 1  PDGFRA - platelet-derived growth factor receptor, alpha polypeptide  CHD5 - chromodomain helicase dna binding protein 5  PDGFB - platelet-derived growth factor beta polypeptide  MECP2 - methyl cpg binding protein 2 (rett syndrome)  MCFD2 - multiple coagulation factor deficiency 2  MAGT1 - magnesium transporter 1  PDPK1 - 3-phosphoinositide dependent protein kinase-1  PDK3 - pyruvate dehydrogenase kinase, isozyme 3  ZBED1 - zinc finger, bed-type containing 1  PPIL3 - peptidylprolyl isomerase (cyclophilin)-like 3  DCLK1 - doublecortin-like kinase 1  MEN1 - multiple endocrine neoplasia i  HCFC1 - host cell factor c1 (vp16-accessory protein)  MAP3K12 - mitogen-activated protein kinase kinase kinase 12  ZZZ3 - zinc finger, zz-type containing 3  KAT8 - k(lysine) acetyltransferase 8  ASPH - aspartate beta-hydroxylase  DGKQ - diacylglycerol kinase, theta 110kda  CLIP3 - cap-gly domain containing linker protein 3  PWP1 - pwp1 homolog (s. cerevisiae)  MGAT1 - mannosyl (alpha-1,3-)-glycoprotein beta-1,2-n-acetylglucosaminyltransferase  NDNF - neuron-derived neurotrophic factor  MGAT2 - mannosyl (alpha-1,6-)-glycoprotein beta-1,2-n-acetylglucosaminyltransferase  DAD1 - defender against cell death 1  MGAT5 - mannosyl (alpha-1,6-)-glycoprotein beta-1,6-n-acetyl-glucosaminyltransferase  MBD3 - methyl-cpg binding domain protein 3  KAT7 - k(lysine) acetyltransferase 7  EPC2 - enhancer of polycomb homolog 2 (drosophila)  PAGR1 - paxip1 associated glutamate-rich protein 1  MARK3 - map/microtubule affinity-regulating kinase 3  DPH2 - dph2 homolog (s. cerevisiae)  MARK1 - map/microtubule affinity-regulating kinase 1  DPH1 - diphthamide biosynthesis 1  DOHH - deoxyhypusine hydroxylase/monooxygenase  LRRK2 - leucine-rich repeat kinase 2  LATS1 - large tumor suppressor kinase 1  DNAJC24 - dnaj (hsp40) homolog, subfamily c, member 24  DR1 - down-regulator of transcription 1, tbp-binding (negative cofactor 2)  UBE2J1 - ubiquitin-conjugating enzyme e2, j1  WEE1 - wee1 homolog (s. pombe)  NUP43 - nucleoporin 43kda  ATP7A - atpase, cu++ transporting, alpha polypeptide  WHSC1 - wolf-hirschhorn syndrome candidate 1  DYRK1A - dual-specificity tyrosine-(y)-phosphorylation regulated kinase 1a  LMTK2 - lemur tyrosine kinase 2  MATK - megakaryocyte-associated tyrosine kinase  TRMT112 - trna methyltransferase 11-2 homolog (s. cerevisiae)  VRK1 - vaccinia related kinase 1  SENP6 - sumo1/sentrin specific peptidase 6  TTBK2 - tau tubulin kinase 2  VRK2 - vaccinia related kinase 2  ASH2L - ash2 (absent, small, or homeotic)-like (drosophila)  COPRS - coordinator of prmt5, differentiation stimulator  PKD1 - polycystic kidney disease 1 (autosomal dominant)  PPIL1 - peptidylprolyl isomerase (cyclophilin)-like 1  PIN1 - peptidylprolyl cis/trans isomerase, nima-interacting 1  DPH3 - diphthamide biosynthesis 3  AIP - aryl hydrocarbon receptor interacting protein  ST6GAL1 - st6 beta-galactosamide alpha-2,6-sialyltranferase 1  AXL - axl receptor tyrosine kinase  EP400 - e1a binding protein p400  LYN - v-yes-1 yamaguchi sarcoma viral related oncogene homolog  DHPS - deoxyhypusine synthase  RICTOR - rptor independent companion of mtor, complex 2  FKBP7 - fk506 binding protein 7  BAZ1B - bromodomain adjacent to zinc finger domain, 1b  OGFOD1 - 2-oxoglutarate and iron-dependent oxygenase domain containing 1  PHF10 - phd finger protein 10  PIAS4 - protein inhibitor of activated stat, 4  ZNF711 - zinc finger protein 711  MBIP - map3k12 binding inhibitory protein 1  FKBP9 - fk506 binding protein 9, 63 kda  STK38 - serine/threonine kinase 38  MRGBP - mrg/morf4l binding protein  RPS6KA4 - ribosomal protein s6 kinase, 90kda, polypeptide 4  PLOD3 - procollagen-lysine, 2-oxoglutarate 5-dioxygenase 3  YES1 - v-yes-1 yamaguchi sarcoma viral oncogene homolog 1  DPH5 - diphthamide biosynthesis 5  BCL2 - b-cell cll/lymphoma 2  SGK1 - serum/glucocorticoid regulated kinase 1  GSK3B - glycogen synthase kinase 3 beta  PARK7 - parkinson protein 7  GSK3A - glycogen synthase kinase 3 alpha  P4HA2 - prolyl 4-hydroxylase, alpha polypeptide ii  SENP3 - sumo1/sentrin/smt3 specific peptidase 3  NDUFAF5 - nadh dehydrogenase (ubiquinone) complex i, assembly factor 5  DPM1 - dolichyl-phosphate mannosyltransferase polypeptide 1, catalytic subunit  MARK2 - map/microtubule affinity-regulating kinase 2  CWC27 - cwc27 spliceosome-associated protein homolog (s. cerevisiae)  DPY19L3 - dpy-19-like 3 (c. elegans)  WHSC1L1 - wolf-hirschhorn syndrome candidate 1-like 1  ABI2 - abl-interactor 2  METTL22 - methyltransferase like 22  PRMT1 - protein arginine methyltransferase 1  PRMT2 - protein arginine methyltransferase 2  TRIM28 - tripartite motif containing 28  DPH7 - diphthamide biosynthesis 7  EP300 - e1a binding protein p300  RET - ret proto-oncogene  DPF2 - d4, zinc and double phd fingers family 2  TBK1 - tank-binding kinase 1  GALNTL6 - udp-n-acetyl-alpha-d-galactosamine:polypeptide n-acetylgalactosaminyltransferase-like 6  RLF - rearranged l-myc fusion  KDELC1 - kdel (lys-asp-glu-leu) containing 1  UGGT1 - udp-glucose glycoprotein glucosyltransferase 1  ZDHHC1 - zinc finger, dhhc-type containing 1  ZDHHC8 - zinc finger, dhhc-type containing 8  EGR2 - early growth response 2  ALG8 - alg8, alpha-1,3-glucosyltransferase  TNK2 - tyrosine kinase, non-receptor, 2  SENP1 - sumo1/sentrin specific peptidase 1  SPHK2 - sphingosine kinase 2  PRMT3 - protein arginine methyltransferase 3  METTL18 - methyltransferase like 18  LDB1 - lim domain binding 1  USPL1 - ubiquitin specific peptidase like 1  KAT2B - k(lysine) acetyltransferase 2b  MAST1 - microtubule associated serine/threonine kinase 1  TOPORS - topoisomerase i binding, arginine/serine-rich, e3 ubiquitin protein ligase  CRTC2 - creb regulated transcription coactivator 2  STK38L - serine/threonine kinase 38 like  SEH1L - seh1-like (s. cerevisiae)  POGLUT1 - protein o-glucosyltransferase 1  EGFR - epidermal growth factor receptor  ALG5 - alg5, dolichyl-phosphate beta-glucosyltransferase  UHMK1 - u2af homology motif (uhm) kinase 1  MAST3 - microtubule associated serine/threonine kinase 3  TOP1 - topoisomerase (dna) i  GOLGA7B - golgin a7 family, member b  MCM3AP - minichromosome maintenance complex component 3 associated protein  QPCTL - glutaminyl-peptide cyclotransferase-like  EOGT - egf domain-specific o-linked n-acetylglucosamine (glcnac) transferase  ROCK1 - rho-associated, coiled-coil containing protein kinase 1  SMG1 - smg1 phosphatidylinositol 3-kinase-related kinase  TET2 - tet methylcytosine dioxygenase 2  TRPM4 - transient receptor potential cation channel, subfamily m, member 4  ZMPSTE24 - zinc metallopeptidase ste24  SETD1B - set domain containing 1b  TPR - translocated promoter region, nuclear basket protein  AKT3 - v-akt murine thymoma viral oncogene homolog 3  ABI1 - abl-interactor 1  PPIE - peptidylprolyl isomerase e (cyclophilin e)  WNK1 - wnk lysine deficient protein kinase 1  CAMK1D - calcium/calmodulin-dependent protein kinase id  MCRS1 - microspherule protein 1  TTLL5 - tubulin tyrosine ligase-like family, member 5  NUP107 - nucleoporin 107kda  DERL3 - derlin 3  SENP5 - sumo1/sentrin specific peptidase 5  FKBP14 - fk506 binding protein 14, 22 kda  PTK2B - protein tyrosine kinase 2 beta  PIBF1 - progesterone immunomodulatory binding factor 1  PPIH - peptidylprolyl isomerase h (cyclophilin h)  TNKS - tankyrase, trf1-interacting ankyrin-related adp-ribose polymerase  KANSL1 - kat8 regulatory nsl complex subunit 1  LATS2 - large tumor suppressor kinase 2  HDAC6 - histone deacetylase 6  CAD - carbamoyl-phosphate synthetase 2, aspartate transcarbamylase, and dihydroorotase  PARP2 - poly (adp-ribose) polymerase 2  RPN2 - ribophorin ii  RPN1 - ribophorin i  HIPK1 - homeodomain interacting protein kinase 1  DDR1 - discoidin domain receptor tyrosine kinase 1  TADA3 - transcriptional adaptor 3  KDM6A - lysine (k)-specific demethylase 6a  SAE1 - sumo1 activating enzyme subunit 1  EZH1 - enhancer of zeste homolog 1 (drosophila)  CRTAP - cartilage associated protein  UBA2 - ubiquitin-like modifier activating enzyme 2  KANSL1L - kat8 regulatory nsl complex subunit 1-like  TTLL12 - tubulin tyrosine ligase-like family, member 12  NUP205 - nucleoporin 205kda  EED - embryonic ectoderm development  ERN1 - endoplasmic reticulum to nucleus signaling 1  SUPT7L - suppressor of ty 7 (s. cerevisiae)-like  RIPK1 - receptor (tnfrsf)-interacting serine-threonine kinase 1  ASPHD2 - aspartate beta-hydroxylase domain containing 2  OXSR1 - oxidative stress responsive 1  TYRO3 - tyro3 protein tyrosine kinase  VPS33B - vacuolar protein sorting 33 homolog b (yeast)  TPGS1 - tubulin polyglutamylase complex subunit 1  TYK2 - tyrosine kinase 2  RPS6KB1 - ribosomal protein s6 kinase, 70kda, polypeptide 1  PKDCC - protein kinase domain containing, cytoplasmic  SUMO1 - small ubiquitin-like modifier 1  RIPK2 - receptor-interacting serine-threonine kinase 2  PIAS3 - protein inhibitor of activated stat, 3  TTL - tubulin tyrosine ligase  KANSL2 - kat8 regulatory nsl complex subunit 2  JMJD6 - jumonji domain containing 6  SETD2 - set domain containing 2  UBE2I - ubiquitin-conjugating enzyme e2i  EPHB6 - eph receptor b6  NUP153 - nucleoporin 153kda  PRMT5 - protein arginine methyltransferase 5  STT3B - stt3b, subunit of the oligosaccharyltransferase complex (catalytic)  NUP210 - nucleoporin 210kda  HAT1 - histone acetyltransferase 1  SETDB1 - set domain, bifurcated 1  FLT1 - fms-related tyrosine kinase 1  TLK1 - tousled-like kinase 1  MEAF6 - myst/esa1-associated factor 6  STK39 - serine threonine kinase 39  ILK - integrin-linked kinase  ASH1L - ash1 (absent, small, or homeotic)-like (drosophila)  PIAS1 - protein inhibitor of activated stat, 1  CAMKK2 - calcium/calmodulin-dependent protein kinase kinase 2, beta  FKBP15 - fk506 binding protein 15, 133kda  SMYD3 - set and mynd domain containing 3  METTL21A - methyltransferase like 21a  MAPKAPK5 - mitogen-activated protein kinase-activated protein kinase 5  FKBP4 - fk506 binding protein 4, 59kda  FKBP2 - fk506 binding protein 2, 13kda  ZDHHC22 - zinc finger, dhhc-type containing 22  FKBP5 - fk506 binding protein 5  TAF6L - taf6-like rna polymerase ii, p300/cbp-associated factor (pcaf)-associated factor, 65kda  FAM173B - family with sequence similarity 173, member b  FKBP1A - fk506 binding protein 1a, 12kda  CAMK1 - calcium/calmodulin-dependent protein kinase i  CBX4 - chromobox homolog 4  FKBP1B - fk506 binding protein 1b, 12.6 kda  DPM3 - dolichyl-phosphate mannosyltransferase polypeptide 3  WDR61 - wd repeat domain 61  KDM3A - lysine (k)-specific demethylase 3a  NUP160 - nucleoporin 160kda  TAF9 - taf9 rna polymerase ii, tata box binding protein (tbp)-associated factor, 32kda  FGFR2 - fibroblast growth factor receptor 2  LEPREL2 - leprecan-like 2  TAF6 - taf6 rna polymerase ii, tata box binding protein (tbp)-associated factor, 80kda  FGFR1 - fibroblast growth factor receptor 1  TADA2A - transcriptional adaptor 2a  DDR2 - discoidin domain receptor tyrosine kinase 2  STK16 - serine/threonine kinase 16  SETD4 - set domain containing 4  PRDX4 - peroxiredoxin 4  PHF15 - phd finger protein 15  NUP88 - nucleoporin 88kda  NUP98 - nucleoporin 98kda  DPY19L1 - dpy-19-like 1 (c. elegans)  USP22 - ubiquitin specific peptidase 22  CDK10 - cyclin-dependent kinase 10  KAT5 - k(lysine) acetyltransferase 5  FER - fer (fps/fes related) tyrosine kinase  PTK2 - protein tyrosine kinase 2  ZDHHC6 - zinc finger, dhhc-type containing 6  MAP3K7 - mitogen-activated protein kinase kinase kinase 7  CDK5 - cyclin-dependent kinase 5  TWF1 - twinfilin actin-binding protein 1  KMT2E - lysine (k)-specific methyltransferase 2e  INSR - insulin receptor  RUVBL1 - ruvb-like 1 (e. coli)  SPOCK2 - sparc/osteonectin, cwcv and kazal-like domains proteoglycan (testican) 2  DMAP1 - dna methyltransferase 1 associated protein 1  MOCS3 - molybdenum cofactor synthesis 3  EFEMP1 - egf containing fibulin-like extracellular matrix protein 1  CDK2 - cyclin-dependent kinase 2  FOLH1B - folate hydrolase 1b  TIE1 - tyrosine kinase with immunoglobulin-like and egf-like domains 1  NMT1 - n-myristoyltransferase 1  VPRBP - vpr (hiv-1) binding protein  P4HTM - prolyl 4-hydroxylase, transmembrane (endoplasmic reticulum)  NSD1 - nuclear receptor binding set domain protein 1  MTOR - mechanistic target of rapamycin (serine/threonine kinase)  STK32C - serine/threonine kinase 32c  FBXO11 - f-box protein 11  PPWD1 - peptidylprolyl isomerase domain and wd repeat containing 1  PLK2 - polo-like kinase 2  ATP13A2 - atpase type 13a2  ERO1L - ero1-like (s. cerevisiae)  NUP50 - nucleoporin 50kda  CHCHD4 - coiled-coil-helix-coiled-coil-helix domain containing 4  NAA15 - n(alpha)-acetyltransferase 15, nata auxiliary subunit  SIRT1 - sirtuin 1  RAB6A - rab6a, member ras oncogene family  BRPF3 - bromodomain and phd finger containing, 3  HDAC4 - histone deacetylase 4  PDF - peptide deformylase (mitochondrial)  CXXC1 - cxxc finger protein 1  RAB3B - rab3b, member ras oncogene family  KMT2B - lysine (k)-specific methyltransferase 2b  NKTR - natural killer-tumor recognition sequence  MSL1 - male-specific lethal 1 homolog (drosophila)  HDAC9 - histone deacetylase 9  SETD1A - set domain containing 1a  NUP188 - nucleoporin 188kda  TEK - tek tyrosine kinase, endothelial  SUZ12 - suz12 polycomb repressive complex 2 subunit  P4HA3 - prolyl 4-hydroxylase, alpha polypeptide iii  RANBP2 - ran binding protein 2  TTLL4 - tubulin tyrosine ligase-like family, member 4  RANGAP1 - ran gtpase activating protein 1  NEK1 - nima-related kinase 1  DYRK3 - dual-specificity tyrosine-(y)-phosphorylation regulated kinase 3  MORC3 - morc family cw-type zinc finger 3  CTR9 - ctr9, paf1/rna polymerase ii complex component  TESK1 - testis-specific kinase 1  EPC1 - enhancer of polycomb homolog 1 (drosophila)  MORF4L2 - mortality factor 4 like 2  ULK1 - unc-51 like autophagy activating kinase 1  NUP155 - nucleoporin 155kda  CLK1 - cdc-like kinase 1  CEP41 - centrosomal protein 41kda  CLK2 - cdc-like kinase 2  ING3 - inhibitor of growth family, member 3  CLK3 - cdc-like kinase 3  TGFBR1 - transforming growth factor, beta receptor 1  PRMT7 - protein arginine methyltransferase 7  TMX3 - thioredoxin-related transmembrane protein 3  IGF1R - insulin-like growth factor 1 receptor  CDC42BPA - cdc42 binding protein kinase alpha (dmpk-like)  OGT - o-linked n-acetylglucosamine (glcnac) transferase  ARID4A - at rich interactive domain 4a (rbp1-like)  NUP93 - nucleoporin 93kda  RAE1 - rae1 rna export 1 homolog (s. pombe)  RBBP5 - retinoblastoma binding protein 5  TGFBR2 - transforming growth factor, beta receptor ii (70/80kda)  NUP35 - nucleoporin 35kda  PHF14 - phd finger protein 14  KAT6B - k(lysine) acetyltransferase 6b  TPST2 - tyrosylprotein sulfotransferase 2  CHUK - conserved helix-loop-helix ubiquitous kinase  SUPT3H - suppressor of ty 3 homolog (s. cerevisiae)  TPST1 - tyrosylprotein sulfotransferase 1 |
| GO:0007017 | microtubule-based process | 9.55E-11 | 5.54E-8 | 1.21 (10334,400,6996,327) | [+] Show genes  MAPRE1 - microtubule-associated protein, rp/eb family, member 1  DST - dystonin  MARK2 - map/microtubule affinity-regulating kinase 2  MAPRE3 - microtubule-associated protein, rp/eb family, member 3  AKAP9 - a kinase (prka) anchor protein (yotiao) 9  KIFAP3 - kinesin-associated protein 3  AP3M2 - adaptor-related protein complex 3, mu 2 subunit  WDR19 - wd repeat domain 19  SPIRE1 - spire actin nucleation factor 1  KIF21A - kinesin family member 21a  IFT46 - intraflagellar transport 46 homolog (chlamydomonas)  EML1 - echinoderm microtubule associated protein like 1  WDR47 - wd repeat domain 47  RACGAP1 - rac gtpase activating protein 1  HAUS7 - haus augmin-like complex, subunit 7  NCOR1 - nuclear receptor corepressor 1  SSX2IP - synovial sarcoma, x breakpoint 2 interacting protein  SPG7 - spastic paraplegia 7 (pure and complicated autosomal recessive)  HOOK3 - hook microtubule-tethering protein 3  KATNB1 - katanin p80 (wd repeat containing) subunit b 1  LZTS2 - leucine zipper, putative tumor suppressor 2  SPAST - spastin  NCKAP5L - nck-associated protein 5-like  KIF27 - kinesin family member 27  SRRD - srr1 domain containing  TUB - tubby bipartite transcription factor  WDR73 - wd repeat domain 73  TUBB2A - tubulin, beta 2a class iia  TUBG1 - tubulin, gamma 1  AP3B2 - adaptor-related protein complex 3, beta 2 subunit  BRSK1 - br serine/threonine kinase 1  SYBU - syntabulin (syntaxin-interacting)  PPP1R12A - protein phosphatase 1, regulatory subunit 12a  TTC30A - tetratricopeptide repeat domain 30a  NDEL1 - nude neurodevelopment protein 1-like 1  UBXN2B - ubx domain protein 2b  SON - son dna binding protein  GJA1 - gap junction protein, alpha 1, 43kda  AZI1 - 5-azacytidine induced 1  SPIRE2 - spire actin nucleation factor 2  KIF16B - kinesin family member 16b  LIMK2 - lim domain kinase 2  LMNA - lamin a/c  LLGL1 - lethal giant larvae homolog 1 (drosophila)  MARK4 - map/microtubule affinity-regulating kinase 4  ZNF259 - zinc finger protein 259  CEP19 - centrosomal protein 19kda  NDE1 - nude neurodevelopment protein 1  BCAS3 - breast carcinoma amplified sequence 3  PLK3 - polo-like kinase 3  RAB21 - rab21, member ras oncogene family  MYH9 - myosin, heavy chain 9, non-muscle  KTN1 - kinectin 1 (kinesin receptor)  MAP9 - microtubule-associated protein 9  VPS4B - vacuolar protein sorting 4 homolog b (s. cerevisiae)  ARL8A - adp-ribosylation factor-like 8a  HAUS6 - haus augmin-like complex, subunit 6  TMEM201 - transmembrane protein 201  CELSR2 - cadherin, egf lag seven-pass g-type receptor 2  AP3S2 - adaptor-related protein complex 3, sigma 2 subunit  GPSM2 - g-protein signaling modulator 2  ACTR2 - arp2 actin-related protein 2 homolog (yeast)  CNP - 2',3'-cyclic nucleotide 3' phosphodiesterase  ACTR3 - arp3 actin-related protein 3 homolog (yeast)  TMEM108 - transmembrane protein 108  SMC1A - structural maintenance of chromosomes 1a  CLUAP1 - clusterin associated protein 1  STMN1 - stathmin 1  TRAK2 - trafficking protein, kinesin binding 2  SPRY1 - sprouty homolog 1, antagonist of fgf signaling (drosophila)  SPRY2 - sprouty homolog 2 (drosophila)  E2F4 - e2f transcription factor 4, p107/p130-binding  LRPPRC - leucine-rich pentatricopeptide repeat containing  CNTROB - centrobin, centrosomal brca2 interacting protein  AP3D1 - adaptor-related protein complex 3, delta 1 subunit  TUBGCP2 - tubulin, gamma complex associated protein 2  STAG1 - stromal antigen 1  BLOC1S3 - biogenesis of lysosomal organelles complex-1, subunit 3  HSPA8 - heat shock 70kda protein 8  WASF1 - was protein family, member 1  CCSAP - centriole, cilia and spindle-associated protein  CDK5RAP2 - cdk5 regulatory subunit associated protein 2  TTC21B - tetratricopeptide repeat domain 21b  KATNA1 - katanin p60 (atpase containing) subunit a 1  EML2 - echinoderm microtubule associated protein like 2  KIFC3 - kinesin family member c3  TTLL5 - tubulin tyrosine ligase-like family, member 5  KIF5C - kinesin family member 5c  KIF3C - kinesin family member 3c  KIF2A - kinesin heavy chain member 2a  KIF5B - kinesin family member 5b  KIF1B - kinesin family member 1b  KIF5A - kinesin family member 5a  SUN2 - sad1 and unc84 domain containing 2  KIF3A - kinesin family member 3a  DYNLL1 - dynein, light chain, lc8-type 1  PIBF1 - progesterone immunomodulatory binding factor 1  TNKS - tankyrase, trf1-interacting ankyrin-related adp-ribose polymerase  EML3 - echinoderm microtubule associated protein like 3  CUL9 - cullin 9  DYNC1LI1 - dynein, cytoplasmic 1, light intermediate chain 1  FGFR1OP - fgfr1 oncogene partner  CLASP2 - cytoplasmic linker associated protein 2  PPP2R3C - protein phosphatase 2, regulatory subunit b'', gamma  VAMP4 - vesicle-associated membrane protein 4  MGARP - mitochondria-localized glutamic acid-rich protein  TNPO1 - transportin 1  BBS12 - bardet-biedl syndrome 12  EZR - ezrin  PPP2R1A - protein phosphatase 2, regulatory subunit a, alpha  KPNB1 - karyopherin (importin) beta 1  SBDS - shwachman-bodian-diamond syndrome  KIFC2 - kinesin family member c2  RMDN1 - regulator of microtubule dynamics 1  UVRAG - uv radiation resistance associated  KLC1 - kinesin light chain 1  CALM2 - calmodulin 2 (phosphorylase kinase, delta)  CRIPT - cysteine-rich pdz-binding protein  VBP1 - von hippel-lindau binding protein 1  AURKC - aurora kinase c  RANBP9 - ran binding protein 9  IFT122 - intraflagellar transport 122 homolog (chlamydomonas)  TUBA1C - tubulin, alpha 1c  VCP - valosin containing protein  HIF1A - hypoxia inducible factor 1, alpha subunit (basic helix-loop-helix transcription factor)  MAPK8IP3 - mitogen-activated protein kinase 8 interacting protein 3  STMN2 - stathmin-like 2  CRMP1 - collapsin response mediator protein 1  TUBA1B - tubulin, alpha 1b  TUBB - tubulin, beta class i  CEP68 - centrosomal protein 68kda  TUBGCP5 - tubulin, gamma complex associated protein 5  TTLL7 - tubulin tyrosine ligase-like family, member 7  IFT27 - intraflagellar transport 27 homolog (chlamydomonas)  UBB - ubiquitin b  HNRNPU - heterogeneous nuclear ribonucleoprotein u (scaffold attachment factor a)  IFT81 - intraflagellar transport 81 homolog (chlamydomonas)  AAAS - achalasia, adrenocortical insufficiency, alacrimia  TUBB4B - tubulin, beta 4b class ivb  TUBB3 - tubulin, beta 3 class iii  KIF3B - kinesin family member 3b  TPGS1 - tubulin polyglutamylase complex subunit 1  IFT88 - intraflagellar transport 88 homolog (chlamydomonas)  SS18 - synovial sarcoma translocation, chromosome 18  BLOC1S5 - biogenesis of lysosomal organelles complex-1, subunit 5, muted  RAB11A - rab11a, member ras oncogene family  UCHL1 - ubiquitin carboxyl-terminal esterase l1 (ubiquitin thiolesterase)  KLC2 - kinesin light chain 2  MAPRE2 - microtubule-associated protein, rp/eb family, member 2  TTL - tubulin tyrosine ligase  HAUS1 - haus augmin-like complex, subunit 1  CCDC88A - coiled-coil domain containing 88a  TUBGCP3 - tubulin, gamma complex associated protein 3  SETD2 - set domain containing 2  TTLL1 - tubulin tyrosine ligase-like family, member 1  CSNK1D - casein kinase 1, delta  RBM14 - rna binding motif protein 14  TUBE1 - tubulin, epsilon 1  CCDC78 - coiled-coil domain containing 78  MSTO1 - misato 1, mitochondrial distribution and morphology regulator  XIAP - x-linked inhibitor of apoptosis  CAMSAP2 - calmodulin regulated spectrin-associated protein family, member 2  PLK1S1 - polo-like kinase 1 substrate 1  CCDC120 - coiled-coil domain containing 120  PARD6A - par-6 partitioning defective 6 homolog alpha (c. elegans)  STMN3 - stathmin-like 3  RHOT2 - ras homolog family member t2  MLH1 - mutl homolog 1, colon cancer, nonpolyposis type 2 (e. coli)  DCTN1 - dynactin 1  FLNA - filamin a, alpha  CCSER2 - coiled-coil serine-rich protein 2  WDR34 - wd repeat domain 34  FOPNL - fgfr1op n-terminal like  DCTN3 - dynactin 3 (p22)  ACTR10 - actin-related protein 10 homolog (s. cerevisiae)  APBA1 - amyloid beta (a4) precursor protein-binding, family a, member 1  MCPH1 - microcephalin 1  CEP350 - centrosomal protein 350kda  MAP7D3 - map7 domain containing 3  MAP3K11 - mitogen-activated protein kinase kinase kinase 11  CHP1 - calcineurin-like ef-hand protein 1  SPATA7 - spermatogenesis associated 7  CDC14B - cell division cycle 14b  CCDC8 - coiled-coil domain containing 8  DPCD - deleted in primary ciliary dyskinesia homolog (mouse)  AP3B1 - adaptor-related protein complex 3, beta 1 subunit  BICD2 - bicaudal d homolog 2 (drosophila)  KATNAL1 - katanin p60 subunit a-like 1  EML4 - echinoderm microtubule associated protein like 4  RNF19A - ring finger protein 19a, e3 ubiquitin protein ligase  KIF13B - kinesin family member 13b  MAP1S - microtubule-associated protein 1s  PAFAH1B1 - platelet-activating factor acetylhydrolase 1b, regulatory subunit 1 (45kda)  ARL8B - adp-ribosylation factor-like 8b  FBXW11 - f-box and wd repeat domain containing 11  APP - amyloid beta (a4) precursor protein  OPA1 - optic atrophy 1 (autosomal dominant)  DYNLT1 - dynein, light chain, tctex-type 1  GAS2L1 - growth arrest-specific 2 like 1  IFT43 - intraflagellar transport 43 homolog (chlamydomonas)  CKAP5 - cytoskeleton associated protein 5  ARL3 - adp-ribosylation factor-like 3  HAUS5 - haus augmin-like complex, subunit 5  ARL2 - adp-ribosylation factor-like 2  FGF13 - fibroblast growth factor 13  TUBGCP4 - tubulin, gamma complex associated protein 4  DCTN2 - dynactin 2 (p50)  TUBA1A - tubulin, alpha 1a  CETN2 - centrin, ef-hand protein, 2  IFT57 - intraflagellar transport 57 homolog (chlamydomonas)  TACC1 - transforming, acidic coiled-coil containing protein 1  CETN3 - centrin, ef-hand protein, 3  NSFL1C - nsfl1 (p97) cofactor (p47)  NUMA1 - nuclear mitotic apparatus protein 1  MECP2 - methyl cpg binding protein 2 (rett syndrome)  CLASP1 - cytoplasmic linker associated protein 1  ZW10 - zw10 kinetochore protein  RHOA - ras homolog family member a  CIB1 - calcium and integrin binding 1 (calmyrin)  KNSTRN - kinetochore-localized astrin/spag5 binding protein  TBCEL - tubulin folding cofactor e-like  ITGB1 - integrin, beta 1 (fibronectin receptor, beta polypeptide, antigen cd29 includes mdf2, msk12)  FER - fer (fps/fes related) tyrosine kinase  PTK2 - protein tyrosine kinase 2  EFHC1 - ef-hand domain (c-terminal) containing 1  CEP120 - centrosomal protein 120kda  TEKT2 - tektin 2 (testicular)  CDK5 - cyclin-dependent kinase 5  SPA17 - sperm autoantigenic protein 17  WDR60 - wd repeat domain 60  NPM1 - nucleophosmin (nucleolar phosphoprotein b23, numatrin)  SIK3 - sik family kinase 3  PCM1 - pericentriolar material 1  TBCE - tubulin folding cofactor e  DAG1 - dystroglycan 1 (dystrophin-associated glycoprotein 1)  TBCD - tubulin folding cofactor d  SPICE1 - spindle and centriole associated protein 1  RABL2B - rab, member of ras oncogene family-like 2b  ARHGEF2 - rho/rac guanine nucleotide exchange factor (gef) 2  CLIP3 - cap-gly domain containing linker protein 3  TUBB2B - tubulin, beta 2b class iib  OBSL1 - obscurin-like 1  IFT52 - intraflagellar transport 52 homolog (chlamydomonas)  HTT - huntingtin  CUL7 - cullin 7  CDK2 - cyclin-dependent kinase 2  ZNF207 - zinc finger protein 207  HAUS2 - haus augmin-like complex, subunit 2  PURA - purine-rich element binding protein a  SPG11 - spastic paraplegia 11 (autosomal recessive)  TUBGCP6 - tubulin, gamma complex associated protein 6  GOLGA2 - golgin a2  HAUS3 - haus augmin-like complex, subunit 3  TRAF3IP1 - tnf receptor-associated factor 3 interacting protein 1  TAOK1 - tao kinase 1  SMC3 - structural maintenance of chromosomes 3  BLOC1S2 - biogenesis of lysosomal organelles complex-1, subunit 2  IFT74 - intraflagellar transport 74 homolog (chlamydomonas)  MARK3 - map/microtubule affinity-regulating kinase 3  PLK2 - polo-like kinase 2  MARK1 - map/microtubule affinity-regulating kinase 1  MAPT - microtubule-associated protein tau  MAP6 - microtubule-associated protein 6  BLOC1S4 - biogenesis of lysosomal organelles complex-1, subunit 4, cappuccino  MAP2 - microtubule-associated protein 2  MAP1B - microtubule-associated protein 1b  MAP1A - microtubule-associated protein 1a  CEP57 - centrosomal protein 57kda  RAB6A - rab6a, member ras oncogene family  DYNLRB2 - dynein, light chain, roadblock-type 2  CLIP2 - cap-gly domain containing linker protein 2  DYNLRB1 - dynein, light chain, roadblock-type 1  PEX13 - peroxisomal biogenesis factor 13  STK36 - serine/threonine kinase 36  CHD3 - chromodomain helicase dna binding protein 3  WEE1 - wee1 homolog (s. pombe)  PEX14 - peroxisomal biogenesis factor 14  RAB1A - rab1a, member ras oncogene family  ARHGAP21 - rho gtpase activating protein 21  ICK - intestinal cell (mak-like) kinase  PEX1 - peroxisomal biogenesis factor 1  TUBG2 - tubulin, gamma 2  IFT140 - intraflagellar transport 140 homolog (chlamydomonas)  DOCK7 - dedicator of cytokinesis 7  TTBK2 - tau tubulin kinase 2  SLK - ste20-like kinase  NME5 - nme/nm23 family member 5  INO80 - ino80 complex subunit  CCP110 - centriolar coiled coil protein 110kda  COPG2 - coatomer protein complex, subunit gamma 2  MAP7 - microtubule-associated protein 7  RSPH9 - radial spoke head 9 homolog (chlamydomonas)  TTLL4 - tubulin tyrosine ligase-like family, member 4  DLG1 - discs, large homolog 1 (drosophila)  GCC2 - grip and coiled-coil domain containing 2  NEFL - neurofilament, light polypeptide  COPG1 - coatomer protein complex, subunit gamma 1  BLOC1S6 - biogenesis of lysosomal organelles complex-1, subunit 6, pallidin  AP3M1 - adaptor-related protein complex 3, mu 1 subunit  DYNC2LI1 - dynein, cytoplasmic 2, light intermediate chain 1  RSPH1 - radial spoke head 1 homolog (chlamydomonas)  TERF2 - telomeric repeat binding factor 2  WRAP73 - wd repeat containing, antisense to tp73  FSD1 - fibronectin type iii and spry domain containing 1  ARHGEF10 - rho guanine nucleotide exchange factor (gef) 10  DCTN6 - dynactin 6  BCCIP - brca2 and cdkn1a interacting protein  TPPP3 - tubulin polymerization-promoting protein family member 3  CAMSAP1 - calmodulin regulated spectrin-associated protein 1  KIF1A - kinesin family member 1a  ATRX - alpha thalassemia/mental retardation syndrome x-linked  CROCC - ciliary rootlet coiled-coil, rootletin  SFPQ - splicing factor proline/glutamine-rich  DYNC1H1 - dynein, cytoplasmic 1, heavy chain 1  DYNC1I1 - dynein, cytoplasmic 1, intermediate chain 1  DYNC1I2 - dynein, cytoplasmic 1, intermediate chain 2  GABARAP - gaba(a) receptor-associated protein  DYNC1LI2 - dynein, cytoplasmic 1, light intermediate chain 2  STAG2 - stromal antigen 2  CEP63 - centrosomal protein 63kda  RHOT1 - ras homolog family member t1  MZT1 - mitotic spindle organizing protein 1  IFT172 - intraflagellar transport 172 homolog (chlamydomonas)  CCDC66 - coiled-coil domain containing 66  NAV1 - neuron navigator 1  BBS4 - bardet-biedl syndrome 4  BBS2 - bardet-biedl syndrome 2 |
| GO:0006259 | DNA metabolic process | 2.78E-10 | 1.55E-7 | 1.18 (10334,526,6996,419) | [+] Show genes  PMS2 - pms2 postmeiotic segregation increased 2 (s. cerevisiae)  TBRG1 - transforming growth factor beta regulator 1  LIG4 - ligase iv, dna, atp-dependent  FBXO18 - f-box protein, helicase, 18  LIG1 - ligase i, dna, atp-dependent  LIG3 - ligase iii, dna, atp-dependent  ABL1 - c-abl oncogene 1, non-receptor tyrosine kinase  OBFC1 - oligonucleotide/oligosaccharide-binding fold containing 1  DOT1L - dot1-like histone h3k79 methyltransferase  PMS1 - pms1 postmeiotic segregation increased 1 (s. cerevisiae)  ASCC3 - activating signal cointegrator 1 complex subunit 3  MUM1 - melanoma associated antigen (mutated) 1  SLX4 - slx4 structure-specific endonuclease subunit  CHD1L - chromodomain helicase dna binding protein 1-like  OTUB1 - otu domain, ubiquitin aldehyde binding 1  ORC3 - origin recognition complex, subunit 3  EHMT2 - euchromatic histone-lysine n-methyltransferase 2  ORC6 - origin recognition complex, subunit 6  MORF4L1 - mortality factor 4 like 1  SMUG1 - single-strand-selective monofunctional uracil-dna glycosylase 1  POLK - polymerase (dna directed) kappa  ACTL6A - actin-like 6a  INIP - ints3 and nabp interacting protein  TRRAP - transformation/transcription domain-associated protein  MCMBP - minichromosome maintenance complex binding protein  PPIA - peptidylprolyl isomerase a (cyclophilin a)  RNF138 - ring finger protein 138, e3 ubiquitin protein ligase  ACTR5 - arp5 actin-related protein 5 homolog (yeast)  CDC45 - cell division cycle 45  RBM17 - rna binding motif protein 17  AXIN2 - axin 2  HEMK1 - hemk methyltransferase family member 1  CDC7 - cell division cycle 7  SMARCB1 - swi/snf related, matrix associated, actin dependent regulator of chromatin, subfamily b, member 1  SMC1A - structural maintenance of chromosomes 1a  UBR5 - ubiquitin protein ligase e3 component n-recognin 5  POLR2K - polymerase (rna) ii (dna directed) polypeptide k, 7.0kda  UCHL5 - ubiquitin carboxyl-terminal hydrolase l5  GNAS - gnas complex locus  SMARCA1 - swi/snf related, matrix associated, actin dependent regulator of chromatin, subfamily a, member 1  PLD3 - phospholipase d family, member 3  PARP1 - poly (adp-ribose) polymerase 1  POLH - polymerase (dna directed), eta  NOP10 - nop10 ribonucleoprotein  TAOK3 - tao kinase 3  POLD2 - polymerase (dna directed), delta 2, accessory subunit  FZR1 - fizzy/cell division cycle 20 related 1 (drosophila)  POLD1 - polymerase (dna directed), delta 1, catalytic subunit  ZBTB7A - zinc finger and btb domain containing 7a  POLR2G - polymerase (rna) ii (dna directed) polypeptide g  POLR2F - polymerase (rna) ii (dna directed) polypeptide f  POLR2E - polymerase (rna) ii (dna directed) polypeptide e, 25kda  SMPD3 - sphingomyelin phosphodiesterase 3, neutral membrane (neutral sphingomyelinase ii)  POLR2D - polymerase (rna) ii (dna directed) polypeptide d  POLR2C - polymerase (rna) ii (dna directed) polypeptide c, 33kda  POLR2B - polymerase (rna) ii (dna directed) polypeptide b, 140kda  POLR2A - polymerase (rna) ii (dna directed) polypeptide a, 220kda  PARP4 - poly (adp-ribose) polymerase family, member 4  RUVBL2 - ruvb-like 2 (e. coli)  TFIP11 - tuftelin interacting protein 11  EHMT1 - euchromatic histone-lysine n-methyltransferase 1  PRIM2 - primase, dna, polypeptide 2 (58kda)  PRIM1 - primase, dna, polypeptide 1 (49kda)  CTGF - connective tissue growth factor  SUPV3L1 - suppressor of var1, 3-like 1 (s. cerevisiae)  PICK1 - protein interacting with prkca 1  ING4 - inhibitor of growth family, member 4  ATF7IP - activating transcription factor 7 interacting protein  PPP5C - protein phosphatase 5, catalytic subunit  UHRF2 - ubiquitin-like with phd and ring finger domains 2, e3 ubiquitin protein ligase  SDE2 - sde2 telomere maintenance homolog (s. pombe)  RECQL5 - recq protein-like 5  USP7 - ubiquitin specific peptidase 7 (herpes virus-associated)  XRCC6 - x-ray repair complementing defective repair in chinese hamster cells 6  RNF168 - ring finger protein 168, e3 ubiquitin protein ligase  KPNA2 - karyopherin alpha 2 (rag cohort 1, importin alpha 1)  KPNB1 - karyopherin (importin) beta 1  KPNA1 - karyopherin alpha 1 (importin alpha 5)  SUV420H1 - suppressor of variegation 4-20 homolog 1 (drosophila)  TOPBP1 - topoisomerase (dna) ii binding protein 1  RMI2 - recq mediated genome instability 2  SUV420H2 - suppressor of variegation 4-20 homolog 2 (drosophila)  RPAIN - rpa interacting protein  LONP1 - lon peptidase 1, mitochondrial  TDRKH - tudor and kh domain containing  NIPBL - nipped-b homolog (drosophila)  ASF1A - anti-silencing function 1a histone chaperone  NPLOC4 - nuclear protein localization 4 homolog (s. cerevisiae)  SSRP1 - structure specific recognition protein 1  ING5 - inhibitor of growth family, member 5  PAPD7 - pap associated domain containing 7  BOD1L1 - biorientation of chromosomes in cell division 1-like 1  CDK2AP1 - cyclin-dependent kinase 2 associated protein 1  TRIP12 - thyroid hormone receptor interactor 12  COPS6 - cop9 signalosome subunit 6  COPS2 - cop9 signalosome subunit 2  SSBP1 - single-stranded dna binding protein 1, mitochondrial  CCDC88A - coiled-coil domain containing 88a  LEF1 - lymphoid enhancer-binding factor 1  SHPRH - snf2 histone linker phd ring helicase, e3 ubiquitin protein ligase  DHX9 - deah (asp-glu-ala-his) box helicase 9  APEX1 - apex nuclease (multifunctional dna repair enzyme) 1  DDX1 - dead (asp-glu-ala-asp) box helicase 1  SMC6 - structural maintenance of chromosomes 6  SPRTN - sprt-like n-terminal domain  RFWD3 - ring finger and wd repeat domain 3  TREX1 - three prime repair exonuclease 1  SIN3A - sin3 transcription regulator family member a  RNF113A - ring finger protein 113a  SAMHD1 - sam domain and hd domain 1  SET - set nuclear oncogene  DDB1 - damage-specific dna binding protein 1, 127kda  SETMAR - set domain and mariner transposase fusion gene  PRMT6 - protein arginine methyltransferase 6  MLH1 - mutl homolog 1, colon cancer, nonpolyposis type 2 (e. coli)  GTF2H2 - general transcription factor iih, polypeptide 2, 44kda  NEIL1 - nei endonuclease viii-like 1 (e. coli)  GTF2H3 - general transcription factor iih, polypeptide 3, 34kda  MCM9 - minichromosome maintenance complex component 9  GTF2H1 - general transcription factor iih, polypeptide 1, 62kda  MSH6 - muts homolog 6 (e. coli)  APBB1 - amyloid beta (a4) precursor protein-binding, family b, member 1 (fe65)  POT1 - protection of telomeres 1  RIF1 - rap1 interacting factor homolog (yeast)  POLG2 - polymerase (dna directed), gamma 2, accessory subunit  GTF2H4 - general transcription factor iih, polypeptide 4, 52kda  CNTD1 - cyclin n-terminal domain containing 1  RCHY1 - ring finger and chy zinc finger domain containing 1, e3 ubiquitin protein ligase  TATDN1 - tatd dnase domain containing 1  MAP1S - microtubule-associated protein 1s  MNAT1 - mnat cdk-activating kinase assembly factor 1  UIMC1 - ubiquitin interaction motif containing 1  POLI - polymerase (dna directed) iota  DFFA - dna fragmentation factor, 45kda, alpha polypeptide  MTA2 - metastasis associated 1 family, member 2  KCTD13 - potassium channel tetramerization domain containing 13  MCM7 - minichromosome maintenance complex component 7  H2AFX - h2a histone family, member x  NSMCE2 - non-smc element 2, mms21 homolog (s. cerevisiae)  SUPT16H - suppressor of ty 16 homolog (s. cerevisiae)  ERI1 - exoribonuclease 1  BAZ2A - bromodomain adjacent to zinc finger domain, 2a  MEN1 - multiple endocrine neoplasia i  PCNA - proliferating cell nuclear antigen  HINFP - histone h4 transcription factor  FANCL - fanconi anemia, complementation group l  WRAP53 - wd repeat containing, antisense to tp53  MBD3 - methyl-cpg binding domain protein 3  HDAC1 - histone deacetylase 1  NYNRIN - nyn domain and retroviral integrase containing  PGBD5 - piggybac transposable element derived 5  KAT7 - k(lysine) acetyltransferase 7  ATRIP - atr interacting protein  EPC2 - enhancer of polycomb homolog 2 (drosophila)  XPA - xeroderma pigmentosum, complementation group a  ZBTB1 - zinc finger and btb domain containing 1  XPC - xeroderma pigmentosum, complementation group c  ZNF365 - zinc finger protein 365  TAOK1 - tao kinase 1  SMC3 - structural maintenance of chromosomes 3  XRCC1 - x-ray repair complementing defective repair in chinese hamster cells 1  PAGR1 - paxip1 associated glutamate-rich protein 1  RNASEH1 - ribonuclease h1  MTA1 - metastasis associated 1  SIRT6 - sirtuin 6  AP5S1 - adaptor-related protein complex 5, sigma 1 subunit  TNKS1BP1 - tankyrase 1 binding protein 1, 182kda  USP10 - ubiquitin specific peptidase 10  WHSC1 - wolf-hirschhorn syndrome candidate 1  REV1 - rev1, polymerase (dna directed)  ALKBH2 - alkb, alkylation repair homolog 2 (e. coli)  WDR33 - wd repeat domain 33  WDR48 - wd repeat domain 48  MCM3 - minichromosome maintenance complex component 3  MCM6 - minichromosome maintenance complex component 6  MCM5 - minichromosome maintenance complex component 5  RTFDC1 - replication termination factor 2 domain containing 1  MCM2 - minichromosome maintenance complex component 2  DKC1 - dyskeratosis congenita 1, dyskerin  BACH1 - btb and cnc homology 1, basic leucine zipper transcription factor 1  ZNF445 - zinc finger protein 445  NEIL2 - nei endonuclease viii-like 2 (e. coli)  BRCC3 - brca1/brca2-containing complex, subunit 3  EXD2 - exonuclease 3'-5' domain containing 2  XRN2 - 5'-3' exoribonuclease 2  SESN2 - sestrin 2  PARP9 - poly (adp-ribose) polymerase family, member 9  ATRX - alpha thalassemia/mental retardation syndrome x-linked  RNF8 - ring finger protein 8, e3 ubiquitin protein ligase  UVSSA - uv-stimulated scaffold protein a  SFPQ - splicing factor proline/glutamine-rich  DNASE2 - deoxyribonuclease ii, lysosomal  PIAS4 - protein inhibitor of activated stat, 4  NUDT15 - nudix (nucleoside diphosphate linked moiety x)-type motif 15  DNMT1 - dna (cytosine-5-)-methyltransferase 1  DNMT3A - dna (cytosine-5-)-methyltransferase 3 alpha  TDP2 - tyrosyl-dna phosphodiesterase 2  UBE2W - ubiquitin-conjugating enzyme e2w (putative)  XRCC5 - x-ray repair complementing defective repair in chinese hamster cells 5 (double-strand-break rejoining)  XRCC4 - x-ray repair complementing defective repair in chinese hamster cells 4  PARK7 - parkinson protein 7  EME1 - essential meiotic structure-specific endonuclease 1  PNKP - polynucleotide kinase 3'-phosphatase  SMARCAD1 - swi/snf-related, matrix-associated actin-dependent regulator of chromatin, subfamily a, containing dead/h box 1  RFC5 - replication factor c (activator 1) 5, 36.5kda  RFC2 - replication factor c (activator 1) 2, 40kda  TSN - translin  RFC3 - replication factor c (activator 1) 3, 38kda  FAN1 - fancd2/fanci-associated nuclease 1  MGME1 - mitochondrial genome maintenance exonuclease 1  WRNIP1 - werner helicase interacting protein 1  TRIM28 - tripartite motif containing 28  DTD1 - d-tyrosyl-trna deacylase 1  EP300 - e1a binding protein p300  KIN - kin, antigenic determinant of reca protein homolog (mouse)  RECQL - recq protein-like (dna helicase q1-like)  NAP1L1 - nucleosome assembly protein 1-like 1  NASP - nuclear autoantigenic sperm protein (histone-binding)  ALKBH5 - alkb, alkylation repair homolog 5 (e. coli)  UPF1 - upf1 regulator of nonsense transcripts homolog (yeast)  NBN - nibrin  UBQLN4 - ubiquilin 4  FTO - fat mass and obesity associated  RLF - rearranged l-myc fusion  ALKBH1 - alkb, alkylation repair homolog 1 (e. coli)  ALYREF - aly/ref export factor  TFPT - tcf3 (e2a) fusion partner (in childhood leukemia)  KDM2A - lysine (k)-specific demethylase 2a  CDADC1 - cytidine and dcmp deaminase domain containing 1  DSCC1 - dna replication and sister chromatid cohesion 1  PSMD14 - proteasome (prosome, macropain) 26s subunit, non-atpase, 14  ZNF259 - zinc finger protein 259  GIN1 - gypsy retrotransposon integrase 1  C1orf86 - chromosome 1 open reading frame 86  TP53BP1 - tumor protein p53 binding protein 1  HUWE1 - hect, uba and wwe domain containing 1, e3 ubiquitin protein ligase  TOP3A - topoisomerase (dna) iii alpha  TOP2B - topoisomerase (dna) ii beta 180kda  NABP2 - nucleic acid binding protein 2  HUS1 - hus1 checkpoint homolog (s. pombe)  TOP1 - topoisomerase (dna) i  INO80B - ino80 complex subunit b  ERI3 - eri1 exoribonuclease family member 3  GATAD2A - gata zinc finger domain containing 2a  HERC2 - hect and rld domain containing e3 ubiquitin protein ligase 2  RNF111 - ring finger protein 111  E4F1 - e4f transcription factor 1  SMG1 - smg1 phosphatidylinositol 3-kinase-related kinase  NT5M - 5',3'-nucleotidase, mitochondrial  TET2 - tet methylcytosine dioxygenase 2  XAB2 - xpa binding protein 2  MUTYH - muty homolog (e. coli)  HSPD1 - heat shock 60kda protein 1 (chaperonin)  PDS5B - pds5, regulator of cohesion maintenance, homolog b (s. cerevisiae)  NSMCE1 - non-smc element 1 homolog (s. cerevisiae)  RAD50 - rad50 homolog (s. cerevisiae)  C11orf30 - chromosome 11 open reading frame 30  HSP90AA1 - heat shock protein 90kda alpha (cytosolic), class a member 1  C7orf49 - chromosome 7 open reading frame 49  ZMPSTE24 - zinc metallopeptidase ste24  MBD4 - methyl-cpg binding domain protein 4  STUB1 - stip1 homology and u-box containing protein 1, e3 ubiquitin protein ligase  SETX - senataxin  PPIE - peptidylprolyl isomerase e (cyclophilin e)  MTRR - 5-methyltetrahydrofolate-homocysteine methyltransferase reductase  REXO4 - rex4, rna exonuclease 4 homolog (s. cerevisiae)  MCRS1 - microspherule protein 1  HMGN1 - high mobility group nucleosome binding domain 1  HMGB3 - high mobility group box 3  HMGB2 - high mobility group box 2  HMGB1 - high mobility group box 1  USP1 - ubiquitin specific peptidase 1  FANCE - fanconi anemia, complementation group e  SLC30A9 - solute carrier family 30 (zinc transporter), member 9  UNG - uracil-dna glycosylase  CASP3 - caspase 3, apoptosis-related cysteine peptidase  RPA3 - replication protein a3, 14kda  MAD2L2 - mad2 mitotic arrest deficient-like 2 (yeast)  RPA1 - replication protein a1, 70kda  RPA2 - replication protein a2, 32kda  C19orf40 - chromosome 19 open reading frame 40  ZBTB48 - zinc finger and btb domain containing 48  PARP2 - poly (adp-ribose) polymerase 2  SMC5 - structural maintenance of chromosomes 5  ACD - adrenocortical dysplasia homolog (mouse)  CHAF1A - chromatin assembly factor 1, subunit a (p150)  UVRAG - uv radiation resistance associated  EYA3 - eyes absent homolog 3 (drosophila)  ENDOV - endonuclease v  INTS3 - integrator complex subunit 3  VCP - valosin containing protein  MSH3 - muts homolog 3 (e. coli)  MSH2 - muts homolog 2, colon cancer, nonpolyposis type 1 (e. coli)  ERCC5 - excision repair cross-complementing rodent repair deficiency, complementation group 5  PINX1 - pin2/terf1 interacting, telomerase inhibitor 1  ERCC4 - excision repair cross-complementing rodent repair deficiency, complementation group 4  ERCC3 - excision repair cross-complementing rodent repair deficiency, complementation group 3  UBE2A - ubiquitin-conjugating enzyme e2a  INO80C - ino80 complex subunit c  CCNH - cyclin h  UBB - ubiquitin b  SWSAP1 - swim-type zinc finger 7 associated protein 1  TIPIN - timeless interacting protein  TTC5 - tetratricopeptide repeat domain 5  COMMD1 - copper metabolism (murr1) domain containing 1  FBXO6 - f-box protein 6  TINF2 - terf1 (trf1)-interacting nuclear factor 2  USP3 - ubiquitin specific peptidase 3  SUMO1 - small ubiquitin-like modifier 1  BABAM1 - brisc and brca1 a complex member 1  UBE2T - ubiquitin-conjugating enzyme e2t (putative)  ZNF830 - zinc finger protein 830  ADPRHL2 - adp-ribosylhydrolase like 2  THOC1 - tho complex 1  ZSWIM7 - zinc finger, swim-type containing 7  SETD2 - set domain containing 2  RBX1 - ring-box 1, e3 ubiquitin protein ligase  HMGA1 - high mobility group at-hook 1  MRE11A - mre11 meiotic recombination 11 homolog a (s. cerevisiae)  UBE2V2 - ubiquitin-conjugating enzyme e2 variant 2  NABP1 - nucleic acid binding protein 1  ERCC2 - excision repair cross-complementing rodent repair deficiency, complementation group 2  CHTF8 - ctf8, chromosome transmission fidelity factor 8 homolog (s. cerevisiae)  RRM1 - ribonucleotide reductase m1  UBE2N - ubiquitin-conjugating enzyme e2n  UBE2V1 - ubiquitin-conjugating enzyme e2 variant 1  CDC5L - cell division cycle 5-like  DTX3L - deltex 3-like (drosophila)  ORC4 - origin recognition complex, subunit 4  ORC2 - origin recognition complex, subunit 2  ORC5 - origin recognition complex, subunit 5  PDS5A - pds5, regulator of cohesion maintenance, homolog a (s. cerevisiae)  ERCC6L2 - excision repair cross-complementing rodent repair deficiency, complementation group 6-like 2  PPM1D - protein phosphatase, mg2+/mn2+ dependent, 1d  POLL - polymerase (dna directed), lambda  PRPF19 - pre-mrna processing factor 19  CDC14B - cell division cycle 14b  AP5Z1 - adaptor-related protein complex 5, zeta 1 subunit  TDG - thymine-dna glycosylase  COPS7B - cop9 signalosome subunit 7b  OGG1 - 8-oxoguanine dna glycosylase  COPS3 - cop9 signalosome subunit 3  EXOSC3 - exosome component 3  GAR1 - gar1 ribonucleoprotein  TERF2IP - telomeric repeat binding factor 2, interacting protein  ORAOV1 - oral cancer overexpressed 1  APITD1 - apoptosis-inducing, taf9-like domain 1  RNASEH2A - ribonuclease h2, subunit a  SMCHD1 - structural maintenance of chromosomes flexible hinge domain containing 1  CETN2 - centrin, ef-hand protein, 2  JMY - junction mediating and regulatory protein, p53 cofactor  NTHL1 - nth endonuclease iii-like 1 (e. coli)  CHRAC1 - chromatin accessibility complex 1  CIB1 - calcium and integrin binding 1 (calmyrin)  FEN1 - flap structure-specific endonuclease 1  KAT5 - k(lysine) acetyltransferase 5  INO80E - ino80 complex subunit e  CDK9 - cyclin-dependent kinase 9  NPM1 - nucleophosmin (nucleolar phosphoprotein b23, numatrin)  KMT2E - lysine (k)-specific methyltransferase 2e  CDK7 - cyclin-dependent kinase 7  GINS1 - gins complex subunit 1 (psf1 homolog)  CDKN2D - cyclin-dependent kinase inhibitor 2d (p19, inhibits cdk4)  APEX2 - apex nuclease (apurinic/apyrimidinic endonuclease) 2  RUVBL1 - ruvb-like 1 (e. coli)  TCF3 - transcription factor 3  DMAP1 - dna methyltransferase 1 associated protein 1  CDK2 - cyclin-dependent kinase 2  TCEA1 - transcription elongation factor a (sii), 1  RAD1 - rad1 homolog (s. pombe)  PURA - purine-rich element binding protein a  DONSON - downstream neighbor of son  VPRBP - vpr (hiv-1) binding protein  NONO - non-pou domain containing, octamer-binding  LRWD1 - leucine-rich repeats and wd repeat domain containing 1  PAPD5 - pap associated domain containing 5  TK1 - thymidine kinase 1, soluble  TK2 - thymidine kinase 2, mitochondrial  NME1 - nme/nm23 nucleoside diphosphate kinase 1  MUS81 - mus81 structure-specific endonuclease subunit  NDNL2 - necdin-like 2  MMS19 - mms19 nucleotide excision repair homolog (s. cerevisiae)  AQR - aquarius homolog (mouse)  SIRT1 - sirtuin 1  CTC1 - cts telomere maintenance complex component 1  NFRKB - nuclear factor related to kappab binding protein  ACTR8 - arp8 actin-related protein 8 homolog (yeast)  TNFAIP1 - tumor necrosis factor, alpha-induced protein 1 (endothelial)  RAD21 - rad21 homolog (s. pombe)  RAD17 - rad17 homolog (s. pombe)  INO80 - ino80 complex subunit  MDC1 - mediator of dna-damage checkpoint 1  IGHMBP2 - immunoglobulin mu binding protein 2  RRM2B - ribonucleotide reductase m2 b (tp53 inducible)  SMARCAL1 - swi/snf related, matrix associated, actin dependent regulator of chromatin, subfamily a-like 1  TERF1 - telomeric repeat binding factor (nima-interacting) 1  TERF2 - telomeric repeat binding factor 2  FAM35A - family with sequence similarity 35, member a  MORF4L2 - mortality factor 4 like 2  BCCIP - brca2 and cdkn1a interacting protein  ZFYVE26 - zinc finger, fyve domain containing 26  NFIA - nuclear factor i/a  POLE4 - polymerase (dna-directed), epsilon 4, accessory subunit  MLH3 - mutl homolog 3 (e. coli)  CTCF - ccctc-binding factor (zinc finger protein)  PRMT7 - protein arginine methyltransferase 7  RBBP7 - retinoblastoma binding protein 7  RBBP8 - retinoblastoma binding protein 8  RBMS1 - rna binding motif, single stranded interacting protein 1  RBBP6 - retinoblastoma binding protein 6  RBBP4 - retinoblastoma binding protein 4  CUL4B - cullin 4b  ERCC8 - excision repair cross-complementing rodent repair deficiency, complementation group 8  FOS - fbj murine osteosarcoma viral oncogene homolog  EXOSC4 - exosome component 4  PTGES3 - prostaglandin e synthase 3 (cytosolic)  COPS7A - cop9 signalosome subunit 7a  POLD3 - polymerase (dna-directed), delta 3, accessory subunit  KIAA0430 - kiaa0430 |
| GO:0044782 | cilium organization | 3.06E-10 | 1.64E-7 | 1.33 (10334,141,6996,127) | [+] Show genes  KIFAP3 - kinesin-associated protein 3  B9D2 - b9 protein domain 2  CEP89 - centrosomal protein 89kda  WDR19 - wd repeat domain 19  ATAT1 - alpha tubulin acetyltransferase 1  GSN - gelsolin  ABLIM1 - actin binding lim protein 1  GORAB - golgin, rab6-interacting  IFT46 - intraflagellar transport 46 homolog (chlamydomonas)  MKS1 - meckel syndrome, type 1  WWTR1 - ww domain containing transcription regulator 1  SSX2IP - synovial sarcoma, x breakpoint 2 interacting protein  DCTN1 - dynactin 1  FLNA - filamin a, alpha  PTPN23 - protein tyrosine phosphatase, non-receptor type 23  WDR34 - wd repeat domain 34  FOPNL - fgfr1op n-terminal like  ARL13B - adp-ribosylation factor-like 13b  CCDC28B - coiled-coil domain containing 28b  MKKS - mckusick-kaufman syndrome  KIF27 - kinesin family member 27  CDC14B - cell division cycle 14b  EXOC5 - exocyst complex component 5  TMEM17 - transmembrane protein 17  OCRL - oculocerebrorenal syndrome of lowe  FNBP1L - formin binding protein 1-like  PTPDC1 - protein tyrosine phosphatase domain containing 1  ODF2 - outer dense fiber of sperm tails 2  AZI1 - 5-azacytidine induced 1  RAB23 - rab23, member ras oncogene family  BBS7 - bardet-biedl syndrome 7  EHD1 - eh-domain containing 1  AKAP4 - a kinase (prka) anchor protein 4  IFT43 - intraflagellar transport 43 homolog (chlamydomonas)  MARK4 - map/microtubule affinity-regulating kinase 4  CEP250 - centrosomal protein 250kda  TCTN1 - tectonic family member 1  ARL3 - adp-ribosylation factor-like 3  CEP19 - centrosomal protein 19kda  KIAA0586 - kiaa0586  IFT57 - intraflagellar transport 57 homolog (chlamydomonas)  TMEM231 - transmembrane protein 231  SNX10 - sorting nexin 10  CELSR2 - cadherin, egf lag seven-pass g-type receptor 2  AHI1 - abelson helper integration site 1  ACTR2 - arp2 actin-related protein 2 homolog (yeast)  ACTR3 - arp3 actin-related protein 3 homolog (yeast)  BBS9 - bardet-biedl syndrome 9  TEKT2 - tektin 2 (testicular)  RAB8A - rab8a, member ras oncogene family  WDR60 - wd repeat domain 60  CEP78 - centrosomal protein 78kda  CLUAP1 - clusterin associated protein 1  C2CD3 - c2 calcium-dependent domain containing 3  TTC8 - tetratricopeptide repeat domain 8  PCM1 - pericentriolar material 1  NUDCD3 - nudc domain containing 3  FAM161A - family with sequence similarity 161, member a  E2F4 - e2f transcription factor 4, p107/p130-binding  RABL2B - rab, member of ras oncogene family-like 2b  ATP6V1D - atpase, h+ transporting, lysosomal 34kda, v1 subunit d  ARL6 - adp-ribosylation factor-like 6  SCLT1 - sodium channel and clathrin linker 1  LAMA5 - laminin, alpha 5  IFT52 - intraflagellar transport 52 homolog (chlamydomonas)  FBF1 - fas (tnfrsf6) binding factor 1  ARMC9 - armadillo repeat containing 9  POC1B - poc1 centriolar protein b  TCTN3 - tectonic family member 3  TMEM138 - transmembrane protein 138  TRAF3IP1 - tnf receptor-associated factor 3 interacting protein 1  IFT74 - intraflagellar transport 74 homolog (chlamydomonas)  KIF3A - kinesin family member 3a  PARVA - parvin, alpha  MNS1 - meiosis-specific nuclear structural 1  PIBF1 - progesterone immunomodulatory binding factor 1  DYNLL1 - dynein, light chain, lc8-type 1  ATP6V0D1 - atpase, h+ transporting, lysosomal 38kda, v0 subunit d1  CEP290 - centrosomal protein 290kda  RSG1 - rem2 and rab-like small gtpase 1  DZIP1 - daz interacting zinc finger protein 1  FAM161B - family with sequence similarity 161, member b  CCDC41 - coiled-coil domain containing 41  NOTCH1 - notch 1  PVRL2 - poliovirus receptor-related 2 (herpesvirus entry mediator b)  HDAC6 - histone deacetylase 6  STK36 - serine/threonine kinase 36  RILPL2 - rab interacting lysosomal protein-like 2  TMEM237 - transmembrane protein 237  UNC119B - unc-119 homolog b (c. elegans)  ICK - intestinal cell (mak-like) kinase  IFT140 - intraflagellar transport 140 homolog (chlamydomonas)  TTBK2 - tau tubulin kinase 2  TCTEX1D2 - tctex1 domain containing 2  TCTN2 - tectonic family member 2  IFT122 - intraflagellar transport 122 homolog (chlamydomonas)  NME5 - nme/nm23 family member 5  TTC17 - tetratricopeptide repeat domain 17  EHD2 - eh-domain containing 2  EHD3 - eh-domain containing 3  CCP110 - centriolar coiled coil protein 110kda  EHD4 - eh-domain containing 4  IQCB1 - iq motif containing b1  PKD2 - polycystic kidney disease 2 (autosomal dominant)  TMEM216 - transmembrane protein 216  RSPH9 - radial spoke head 9 homolog (chlamydomonas)  NEK1 - nima-related kinase 1  TMEM80 - transmembrane protein 80  IFT81 - intraflagellar transport 81 homolog (chlamydomonas)  BBS5 - bardet-biedl syndrome 5  IFT88 - intraflagellar transport 88 homolog (chlamydomonas)  CEP41 - centrosomal protein 41kda  CROCC - ciliary rootlet coiled-coil, rootletin  TXNDC15 - thioredoxin domain containing 15  WDR11 - wd repeat domain 11  TROVE2 - trove domain family, member 2  GALNT11 - udp-n-acetyl-alpha-d-galactosamine:polypeptide n-acetylgalactosaminyltransferase 11 (galnac-t11)  SNAP29 - synaptosomal-associated protein, 29kda  ATXN10 - ataxin 10  CSNK1D - casein kinase 1, delta  B9D1 - b9 protein domain 1  IFT172 - intraflagellar transport 172 homolog (chlamydomonas)  CCDC66 - coiled-coil domain containing 66  ASAP1 - arfgap with sh3 domain, ankyrin repeat and ph domain 1  BBS4 - bardet-biedl syndrome 4  BBS2 - bardet-biedl syndrome 2  TMEM107 - transmembrane protein 107 |
| GO:0051301 | cell division | 4.43E-10 | 2.28E-7 | 1.25 (10334,262,6996,221) | [+] Show genes  CCNK - cyclin k  MAPRE1 - microtubule-associated protein, rp/eb family, member 1  MAPRE3 - microtubule-associated protein, rp/eb family, member 3  LIG4 - ligase iv, dna, atp-dependent  MAEA - macrophage erythroblast attacher  CD2AP - cd2-associated protein  LIG1 - ligase i, dna, atp-dependent  CENPV - centromere protein v  LIG3 - ligase iii, dna, atp-dependent  HAUS7 - haus augmin-like complex, subunit 7  ENSA - endosulfine alpha  KATNB1 - katanin p80 (wd repeat containing) subunit b 1  ZFP36L2 - zfp36 ring finger protein-like 2  MPLKIP - m-phase specific plk1 interacting protein  NSUN2 - nop2/sun rna methyltransferase family, member 2  TXNL4A - thioredoxin-like 4a  ZFYVE19 - zinc finger, fyve domain containing 19  BCAR1 - breast cancer anti-estrogen resistance 1  TSG101 - tumor susceptibility gene 101  MARK4 - map/microtubule affinity-regulating kinase 4  CDC16 - cell division cycle 16  NDE1 - nude neurodevelopment protein 1  ANAPC5 - anaphase promoting complex subunit 5  MCMBP - minichromosome maintenance complex binding protein  ANAPC7 - anaphase promoting complex subunit 7  CDC123 - cell division cycle 123  SEH1L - seh1-like (s. cerevisiae)  MIS12 - mis12 kinetochore complex component  CABLES2 - cdk5 and abl enzyme substrate 2  ARL8A - adp-ribosylation factor-like 8a  HAUS6 - haus augmin-like complex, subunit 6  TOP1 - topoisomerase (dna) i  GPSM2 - g-protein signaling modulator 2  ACTR2 - arp2 actin-related protein 2 homolog (yeast)  ACTR3 - arp3 actin-related protein 3 homolog (yeast)  CDC7 - cell division cycle 7  SMC1A - structural maintenance of chromosomes 1a  USP9X - ubiquitin specific peptidase 9, x-linked  ANAPC4 - anaphase promoting complex subunit 4  E4F1 - e4f transcription factor 1  PHF13 - phd finger protein 13  PDS5B - pds5, regulator of cohesion maintenance, homolog b (s. cerevisiae)  GNAI3 - guanine nucleotide binding protein (g protein), alpha inhibiting activity polypeptide 3  GNAI2 - guanine nucleotide binding protein (g protein), alpha inhibiting activity polypeptide 2  GNAI1 - guanine nucleotide binding protein (g protein), alpha inhibiting activity polypeptide 1  FZR1 - fizzy/cell division cycle 20 related 1 (drosophila)  STAG1 - stromal antigen 1  TPR - translocated promoter region, nuclear basket protein  WAPAL - wings apart-like homolog (drosophila)  CCSAP - centriole, cilia and spindle-associated protein  KATNA1 - katanin p60 (atpase containing) subunit a 1  BOD1 - biorientation of chromosomes in cell division 1  ZWILCH - zwilch kinetochore protein  NUMB - numb homolog (drosophila)  KIF2A - kinesin heavy chain member 2a  SENP5 - sumo1/sentrin specific peptidase 5  CHFR - checkpoint with forkhead and ring finger domains, e3 ubiquitin protein ligase  TNKS - tankyrase, trf1-interacting ankyrin-related adp-ribose polymerase  ZWINT - zw10 interacting kinetochore protein  DYNC1LI1 - dynein, cytoplasmic 1, light intermediate chain 1  MAD2L2 - mad2 mitotic arrest deficient-like 2 (yeast)  LATS2 - large tumor suppressor kinase 2  SDE2 - sde2 telomere maintenance homolog (s. pombe)  CNTRL - centriolin  CLASP2 - cytoplasmic linker associated protein 2  BECN1 - beclin 1, autophagy related  ETV5 - ets variant 5  POGZ - pogo transposable element with znf domain  RECQL5 - recq protein-like 5  SMC5 - structural maintenance of chromosomes 5  ANKLE2 - ankyrin repeat and lem domain containing 2  NEK4 - nima-related kinase 4  TRIOBP - trio and f-actin binding protein  CDC23 - cell division cycle 23  AURKC - aurora kinase c  TUBA1C - tubulin, alpha 1c  SMC4 - structural maintenance of chromosomes 4  KIT - v-kit hardy-zuckerman 4 feline sarcoma viral oncogene homolog  PPP1CC - protein phosphatase 1, catalytic subunit, gamma isozyme  CCNT2 - cyclin t2  NEK9 - nima-related kinase 9  TUBA1B - tubulin, alpha 1b  TUBB - tubulin, beta class i  THOC2 - tho complex 2  NCAPD2 - non-smc condensin i complex, subunit d2  CCND3 - cyclin d3  CCNT1 - cyclin t1  CCNG2 - cyclin g2  ANAPC13 - anaphase promoting complex subunit 13  HNRNPU - heterogeneous nuclear ribonucleoprotein u (scaffold attachment factor a)  ANAPC10 - anaphase promoting complex subunit 10  PRPF40A - prp40 pre-mrna processing factor 40 homolog a (s. cerevisiae)  TIPIN - timeless interacting protein  BABAM1 - brisc and brca1 a complex member 1  EPB41 - erythrocyte membrane protein band 4.1 (elliptocytosis 1, rh-linked)  EPB41L2 - erythrocyte membrane protein band 4.1-like 2  ZNF830 - zinc finger protein 830  MAPRE2 - microtubule-associated protein, rp/eb family, member 2  KLHL22 - kelch-like family member 22  HAUS1 - haus augmin-like complex, subunit 1  PKN2 - protein kinase n2  CSNK1A1 - casein kinase 1, alpha 1  UBE2I - ubiquitin-conjugating enzyme e2i  PRKCE - protein kinase c, epsilon  CCND2 - cyclin d2  LEF1 - lymphoid enhancer-binding factor 1  ERCC2 - excision repair cross-complementing rodent repair deficiency, complementation group 2  PARD6A - par-6 partitioning defective 6 homolog alpha (c. elegans)  CDC27 - cell division cycle 27  WWTR1 - ww domain containing transcription regulator 1  PDS5A - pds5, regulator of cohesion maintenance, homolog a (s. cerevisiae)  DCTN1 - dynactin 1  ING2 - inhibitor of growth family, member 2  USP16 - ubiquitin specific peptidase 16  UBE2S - ubiquitin-conjugating enzyme e2s  NUMBL - numb homolog (drosophila)-like  KLHL21 - kelch-like family member 21  NCAPD3 - non-smc condensin ii complex, subunit d3  AHCTF1 - at hook containing transcription factor 1  KLHL13 - kelch-like family member 13  PAFAH1B1 - platelet-activating factor acetylhydrolase 1b, regulatory subunit 1 (45kda)  REEP4 - receptor accessory protein 4  ARL8B - adp-ribosylation factor-like 8b  DYNLT3 - dynein, light chain, tctex-type 3  KLHL18 - kelch-like family member 18  BIRC6 - baculoviral iap repeat containing 6  ANAPC1 - anaphase promoting complex subunit 1  DYNLT1 - dynein, light chain, tctex-type 1  APITD1 - apoptosis-inducing, taf9-like domain 1  FGFR2 - fibroblast growth factor receptor 2  CKAP5 - cytoskeleton associated protein 5  SSSCA1 - sjogren syndrome/scleroderma autoantigen 1  HAUS5 - haus augmin-like complex, subunit 5  CENPC - centromere protein c  FGFR1 - fibroblast growth factor receptor 1  NSMCE2 - non-smc element 2, mms21 homolog (s. cerevisiae)  TUBA1A - tubulin, alpha 1a  CETN2 - centrin, ef-hand protein, 2  CCNY - cyclin y  TACC1 - transforming, acidic coiled-coil containing protein 1  CETN3 - centrin, ef-hand protein, 3  IST1 - increased sodium tolerance 1 homolog (yeast)  SKA2 - spindle and kinetochore associated complex subunit 2  BUB3 - bub3 mitotic checkpoint protein  NUMA1 - nuclear mitotic apparatus protein 1  PELO - pelota homolog (drosophila)  CLASP1 - cytoplasmic linker associated protein 1  ZW10 - zw10 kinetochore protein  ARF6 - adp-ribosylation factor 6  KNSTRN - kinetochore-localized astrin/spag5 binding protein  CIB1 - calcium and integrin binding 1 (calmyrin)  EVI5 - ecotropic viral integration site 5  CDK5 - cyclin-dependent kinase 5  CDK4 - cyclin-dependent kinase 4  CHMP1A - charged multivesicular body protein 1a  CDK7 - cyclin-dependent kinase 7  SPECC1L - sperm antigen with calponin homology and coiled-coil domains 1-like  SCC4 - sister chromatid cohesion 4  SPICE1 - spindle and centriole associated protein 1  ARHGEF2 - rho/rac guanine nucleotide exchange factor (gef) 2  RUVBL1 - ruvb-like 1 (e. coli)  KLHL9 - kelch-like family member 9  RCC2 - regulator of chromosome condensation 2  CDK2 - cyclin-dependent kinase 2  ZNF207 - zinc finger protein 207  HAUS2 - haus augmin-like complex, subunit 2  ZC3HC1 - zinc finger, c3hc-type containing 1  RAB11FIP3 - rab11 family interacting protein 3 (class ii)  KLHL42 - kelch-like family member 42  ITGB3BP - integrin beta 3 binding protein (beta3-endonexin)  PAPD5 - pap associated domain containing 5  CDC26 - cell division cycle 26  GOLGA2 - golgin a2  HAUS3 - haus augmin-like complex, subunit 3  MICAL3 - microtubule associated monooxygenase, calponin and lim domain containing 3  SMC3 - structural maintenance of chromosomes 3  LATS1 - large tumor suppressor kinase 1  NOTCH1 - notch 1  DIXDC1 - dix domain containing 1  WEE1 - wee1 homolog (s. pombe)  ACTR8 - arp8 actin-related protein 8 homolog (yeast)  NUP43 - nucleoporin 43kda  CENPT - centromere protein t  SDCCAG3 - serologically defined colon cancer antigen 3  VRK1 - vaccinia related kinase 1  RAD21 - rad21 homolog (s. pombe)  MAD1L1 - mad1 mitotic arrest deficient-like 1 (yeast)  INO80 - ino80 complex subunit  VPS4A - vacuolar protein sorting 4 homolog a (s. cerevisiae)  LRRCC1 - leucine rich repeat and coiled-coil centrosomal protein 1  RALA - v-ral simian leukemia viral oncogene homolog a (ras related)  RALB - v-ral simian leukemia viral oncogene homolog b  NEK3 - nima-related kinase 3  CLTC - clathrin, heavy chain (hc)  NEK1 - nima-related kinase 1  TERF1 - telomeric repeat binding factor (nima-interacting) 1  DYRK3 - dual-specificity tyrosine-(y)-phosphorylation regulated kinase 3  NEDD9 - neural precursor cell expressed, developmentally down-regulated 9  BRCC3 - brca1/brca2-containing complex, subunit 3  FSD1 - fibronectin type iii and spry domain containing 1  ZNF16 - zinc finger protein 16  CLTA - clathrin, light chain a  RNF8 - ring finger protein 8, e3 ubiquitin protein ligase  REEP3 - receptor accessory protein 3  DYNC1H1 - dynein, cytoplasmic 1, heavy chain 1  RBBP8 - retinoblastoma binding protein 8  TGFB2 - transforming growth factor, beta 2  DIS3L2 - dis3 mitotic control homolog (s. cerevisiae)-like 2  RB1 - retinoblastoma 1  STAG2 - stromal antigen 2  RAE1 - rae1 rna export 1 homolog (s. pombe)  CEP63 - centrosomal protein 63kda  CDK20 - cyclin-dependent kinase 20  CUL3 - cullin 3  PIK3C3 - phosphatidylinositol 3-kinase, catalytic subunit type 3  NUDC - nudc nuclear distribution protein  CKS1B - cdc28 protein kinase regulatory subunit 1b  USP39 - ubiquitin specific peptidase 39  CCND1 - cyclin d1  USP37 - ubiquitin specific peptidase 37  WASL - wiskott-aldrich syndrome-like |
| GO:0051656 | establishment of organelle localization | 5.44E-10 | 2.7E-7 | 1.27 (10334,218,6996,187) | [+] Show genes  CHMP4C - charged multivesicular body protein 4c  SDAD1 - sda1 domain containing 1  KIFAP3 - kinesin-associated protein 3  AP3M2 - adaptor-related protein complex 3, mu 2 subunit  SPIRE1 - spire actin nucleation factor 1  SYT17 - synaptotagmin xvii  GEM - gtp binding protein overexpressed in skeletal muscle  MLH1 - mutl homolog 1, colon cancer, nonpolyposis type 2 (e. coli)  RHOT2 - ras homolog family member t2  DCTN1 - dynactin 1  LIN7A - lin-7 homolog a (c. elegans)  PPFIA2 - protein tyrosine phosphatase, receptor type, f polypeptide (ptprf), interacting protein (liprin), alpha 2  HOOK3 - hook microtubule-tethering protein 3  KATNB1 - katanin p80 (wd repeat containing) subunit b 1  ACTR10 - actin-related protein 10 homolog (s. cerevisiae)  MCPH1 - microcephalin 1  SPAST - spastin  MKKS - mckusick-kaufman syndrome  LTV1 - ltv1 homolog (s. cerevisiae)  AP3B2 - adaptor-related protein complex 3, beta 2 subunit  NOP9 - nop9 nucleolar protein  SYBU - syntabulin (syntaxin-interacting)  AP3B1 - adaptor-related protein complex 3, beta 1 subunit  PDCD10 - programmed cell death 10  BICD2 - bicaudal d homolog 2 (drosophila)  FNBP1L - formin binding protein 1-like  NDEL1 - nude neurodevelopment protein 1-like 1  MAP1S - microtubule-associated protein 1s  PAFAH1B1 - platelet-activating factor acetylhydrolase 1b, regulatory subunit 1 (45kda)  UBXN2B - ubx domain protein 2b  RAB11B - rab11b, member ras oncogene family  SYNJ1 - synaptojanin 1  SYT9 - synaptotagmin ix  GJA1 - gap junction protein, alpha 1, 43kda  BTBD8 - btb (poz) domain containing 8  SPIRE2 - spire actin nucleation factor 2  FBXW11 - f-box and wd repeat domain containing 11  MYO1D - myosin id  BBS7 - bardet-biedl syndrome 7  MYO5A - myosin va (heavy chain 12, myoxin)  OPA1 - optic atrophy 1 (autosomal dominant)  MYO1E - myosin ie  DYNLT1 - dynein, light chain, tctex-type 1  MYO6 - myosin vi  STX1B - syntaxin 1b  LLGL1 - lethal giant larvae homolog 1 (drosophila)  NDE1 - nude neurodevelopment protein 1  ACTN4 - actinin, alpha 4  ABCE1 - atp-binding cassette, sub-family e (oabp), member 1  DCTN2 - dynactin 2 (p50)  SEH1L - seh1-like (s. cerevisiae)  SNCA - synuclein, alpha (non a4 component of amyloid precursor)  MYH10 - myosin, heavy chain 10, non-muscle  MYH9 - myosin, heavy chain 9, non-muscle  NSFL1C - nsfl1 (p97) cofactor (p47)  NUMA1 - nuclear mitotic apparatus protein 1  VPS4B - vacuolar protein sorting 4 homolog b (s. cerevisiae)  NUP88 - nucleoporin 88kda  CLASP1 - cytoplasmic linker associated protein 1  SNAP25 - synaptosomal-associated protein, 25kda  ZW10 - zw10 kinetochore protein  TMEM201 - transmembrane protein 201  AP3S2 - adaptor-related protein complex 3, sigma 2 subunit  GPSM2 - g-protein signaling modulator 2  RIMS3 - regulating synaptic membrane exocytosis 3  ITGB1 - integrin, beta 1 (fibronectin receptor, beta polypeptide, antigen cd29 includes mdf2, msk12)  ACTR2 - arp2 actin-related protein 2 homolog (yeast)  ACTR3 - arp3 actin-related protein 3 homolog (yeast)  PTK2 - protein tyrosine kinase 2  CEP120 - centrosomal protein 120kda  EIF6 - eukaryotic translation initiation factor 6  PIP5K1C - phosphatidylinositol-4-phosphate 5-kinase, type i, gamma  CDK5 - cyclin-dependent kinase 5  CHMP1A - charged multivesicular body protein 1a  NPM1 - nucleophosmin (nucleolar phosphoprotein b23, numatrin)  NMD3 - nmd3 ribosome export adaptor  TRAK2 - trafficking protein, kinesin binding 2  PCM1 - pericentriolar material 1  SPRY1 - sprouty homolog 1, antagonist of fgf signaling (drosophila)  SPRY2 - sprouty homolog 2 (drosophila)  CADPS - ca++-dependent secretion activator  SPICE1 - spindle and centriole associated protein 1  ARHGEF2 - rho/rac guanine nucleotide exchange factor (gef) 2  CHMP2B - charged multivesicular body protein 2b  LRPPRC - leucine-rich pentatricopeptide repeat containing  TRIM9 - tripartite motif containing 9  ARL6 - adp-ribosylation factor-like 6  CPLX3 - complexin 3  AP3D1 - adaptor-related protein complex 3, delta 1 subunit  HTT - huntingtin  WASF1 - was protein family, member 1  BLOC1S3 - biogenesis of lysosomal organelles complex-1, subunit 3  SPG11 - spastic paraplegia 11 (autosomal recessive)  CDK5RAP2 - cdk5 regulatory subunit associated protein 2  BOD1 - biorientation of chromosomes in cell division 1  KIF5C - kinesin family member 5c  XPO1 - exportin 1 (crm1 homolog, yeast)  KIF3C - kinesin family member 3c  SYT1 - synaptotagmin i  KIF5B - kinesin family member 5b  SYT5 - synaptotagmin v  SYT4 - synaptotagmin iv  KIF1B - kinesin family member 1b  KIF5A - kinesin family member 5a  BLOC1S2 - biogenesis of lysosomal organelles complex-1, subunit 2  MARK1 - map/microtubule affinity-regulating kinase 1  SUN2 - sad1 and unc84 domain containing 2  MAPT - microtubule-associated protein tau  MYO19 - myosin xix  KIF3A - kinesin family member 3a  BLOC1S4 - biogenesis of lysosomal organelles complex-1, subunit 4, cappuccino  PIBF1 - progesterone immunomodulatory binding factor 1  MAP1B - microtubule-associated protein 1b  RIMS2 - regulating synaptic membrane exocytosis 2  VAMP2 - vesicle-associated membrane protein 2 (synaptobrevin 2)  CHGA - chromogranin a (parathyroid secretory protein 1)  TANC2 - tetratricopeptide repeat, ankyrin repeat and coiled-coil containing 2  CCDC41 - coiled-coil domain containing 41  PVRL2 - poliovirus receptor-related 2 (herpesvirus entry mediator b)  CTNNB1 - catenin (cadherin-associated protein), beta 1, 88kda  RAB6A - rab6a, member ras oncogene family  LSG1 - large 60s subunit nuclear export gtpase 1  CLASP2 - cytoplasmic linker associated protein 2  BECN1 - beclin 1, autophagy related  ATP6AP1 - atpase, h+ transporting, lysosomal accessory protein 1  PEX14 - peroxisomal biogenesis factor 14  RAB7A - rab7a, member ras oncogene family  STX1A - syntaxin 1a (brain)  RAB3A - rab3a, member ras oncogene family  MGARP - mitochondria-localized glutamic acid-rich protein  TOR1A - torsin family 1, member a (torsin a)  EZR - ezrin  RAB1A - rab1a, member ras oncogene family  CPLX2 - complexin 2  ARHGAP21 - rho gtpase activating protein 21  KPNB1 - karyopherin (importin) beta 1  SYT3 - synaptotagmin iii  CHMP5 - charged multivesicular body protein 5  DOCK7 - dedicator of cytokinesis 7  TRAPPC12 - trafficking protein particle complex 12  DNM1L - dynamin 1-like  CDC23 - cell division cycle 23  STK25 - serine/threonine kinase 25  VPS4A - vacuolar protein sorting 4 homolog a (s. cerevisiae)  KIT - v-kit hardy-zuckerman 4 feline sarcoma viral oncogene homolog  RAB27A - rab27a, member ras oncogene family  SYT7 - synaptotagmin vii  HIF1A - hypoxia inducible factor 1, alpha subunit (basic helix-loop-helix transcription factor)  COPG2 - coatomer protein complex, subunit gamma 2  PINX1 - pin2/terf1 interacting, telomerase inhibitor 1  DLG1 - discs, large homolog 1 (drosophila)  DNAJC5 - dnaj (hsp40) homolog, subfamily c, member 5  BLOC1S6 - biogenesis of lysosomal organelles complex-1, subunit 6, pallidin  NEFL - neurofilament, light polypeptide  COPG1 - coatomer protein complex, subunit gamma 1  AP3M1 - adaptor-related protein complex 3, mu 1 subunit  TERF1 - telomeric repeat binding factor (nima-interacting) 1  CHMP7 - charged multivesicular body protein 7  UBB - ubiquitin b  BCCIP - brca2 and cdkn1a interacting protein  BBS5 - bardet-biedl syndrome 5  RPS15 - ribosomal protein s15  FEZ1 - fasciculation and elongation protein zeta 1 (zygin i)  KIF1A - kinesin family member 1a  KIF3B - kinesin family member 3b  CROCC - ciliary rootlet coiled-coil, rootletin  DYNC1H1 - dynein, cytoplasmic 1, heavy chain 1  DYNC1I1 - dynein, cytoplasmic 1, intermediate chain 1  CBL - cbl proto-oncogene, e3 ubiquitin protein ligase  BLOC1S5 - biogenesis of lysosomal organelles complex-1, subunit 5, muted  RAB11A - rab11a, member ras oncogene family  UCHL1 - ubiquitin carboxyl-terminal esterase l1 (ubiquitin thiolesterase)  DNM2 - dynamin 2  CHMP4A - charged multivesicular body protein 4a  RRS1 - rrs1 ribosome biogenesis regulator homolog (s. cerevisiae)  LAT - linker for activation of t cells  SYT11 - synaptotagmin xi  RHOT1 - ras homolog family member t1  DOC2B - double c2-like domains, beta  CUL3 - cullin 3  NR4A3 - nuclear receptor subfamily 4, group a, member 3  CHMP4B - charged multivesicular body protein 4b  TMEM230 - transmembrane protein 230  YWHAZ - tyrosine 3-monooxygenase/tryptophan 5-monooxygenase activation protein, zeta polypeptide  BBS4 - bardet-biedl syndrome 4  BBS2 - bardet-biedl syndrome 2  WASL - wiskott-aldrich syndrome-like |
| GO:0031124 | mRNA 3'-end processing | 1E-9 | 4.8E-7 | 1.42 (10334,76,6996,73) | [+] Show genes  MTPAP - mitochondrial poly(a) polymerase  CSTF1 - cleavage stimulation factor, 3' pre-rna, subunit 1, 50kda  NCBP2 - nuclear cap binding protein subunit 2, 20kda  SRSF11 - serine/arginine-rich splicing factor 11  NCBP1 - nuclear cap binding protein subunit 1, 80kda  MAGOH - mago-nashi homolog, proliferation-associated (drosophila)  SYMPK - symplekin  PAPD5 - pap associated domain containing 5  CSTF3 - cleavage stimulation factor, 3' pre-rna, subunit 3, 77kda  ZCCHC6 - zinc finger, cchc domain containing 6  CSTF2 - cleavage stimulation factor, 3' pre-rna, subunit 2, 64kda  AHCYL1 - adenosylhomocysteinase-like 1  RPRD2 - regulation of nuclear pre-mrna domain containing 2  CPSF3 - cleavage and polyadenylation specific factor 3, 73kda  SRSF9 - serine/arginine-rich splicing factor 9  THOC7 - tho complex 7 homolog (drosophila)  SLBP - stem-loop binding protein  CHTOP - chromatin target of prmt1  NUDT21 - nudix (nucleoside diphosphate linked moiety x)-type motif 21  ALYREF - aly/ref export factor  RPRD1A - regulation of nuclear pre-mrna domain containing 1a  PAPOLA - poly(a) polymerase alpha  PABPN1 - poly(a) binding protein, nuclear 1  WDR33 - wd repeat domain 33  RNPS1 - rna binding protein s1, serine-rich domain  ZC3H3 - zinc finger ccch-type containing 3  CPSF7 - cleavage and polyadenylation specific factor 7, 59kda  ZNF473 - zinc finger protein 473  PAF1 - paf1, rna polymerase ii associated factor, homolog (s. cerevisiae)  APP - amyloid beta (a4) precursor protein  UPF3B - upf3 regulator of nonsense transcripts homolog b (yeast)  CSTF2T - cleavage stimulation factor, 3' pre-rna, subunit 2, 64kda, tau variant  DDX39B - dead (asp-glu-ala-asp) box polypeptide 39b  FIP1L1 - factor interacting with papola and cpsf1  SCAF8 - sr-related ctd-associated factor 8  RPRD1B - regulation of nuclear pre-mrna domain containing 1b  PAPOLG - poly(a) polymerase gamma  POLDIP3 - polymerase (dna-directed), delta interacting protein 3  PNPT1 - polyribonucleotide nucleotidyltransferase 1  THOC2 - tho complex 2  PAPD4 - pap associated domain containing 4  THOC6 - tho complex 6 homolog (drosophila)  PABPC1 - poly(a) binding protein, cytoplasmic 1  CDC73 - cell division cycle 73  EIF4A3 - eukaryotic translation initiation factor 4a3  CPSF4 - cleavage and polyadenylation specific factor 4, 30kda  RBM8A - rna binding motif protein 8a  U2AF1L4 - u2 small nuclear rna auxiliary factor 1-like 4  CPSF1 - cleavage and polyadenylation specific factor 1, 160kda  CPSF2 - cleavage and polyadenylation specific factor 2, 100kda  DHX38 - deah (asp-glu-ala-his) box polypeptide 38  CASC3 - cancer susceptibility candidate 3  ZCCHC11 - zinc finger, cchc domain containing 11  SLU7 - slu7 splicing factor homolog (s. cerevisiae)  PCF11 - pcf11 cleavage and polyadenylation factor subunit  SRSF1 - serine/arginine-rich splicing factor 1  SRRM1 - serine/arginine repetitive matrix 1  U2AF2 - u2 small nuclear rna auxiliary factor 2  SRSF2 - serine/arginine-rich splicing factor 2  MAGOHB - mago-nashi homolog b (drosophila)  SRSF3 - serine/arginine-rich splicing factor 3  SRSF4 - serine/arginine-rich splicing factor 4  SRSF5 - serine/arginine-rich splicing factor 5  SRSF6 - serine/arginine-rich splicing factor 6  SRSF7 - serine/arginine-rich splicing factor 7  LEO1 - leo1, paf1/rna polymerase ii complex component, homolog (s. cerevisiae)  CLP1 - cleavage and polyadenylation factor i subunit 1  THOC1 - tho complex 1  THOC3 - tho complex 3  TUT1 - terminal uridylyl transferase 1, u6 snrna-specific  SARNP - sap domain containing ribonucleoprotein  GRSF1 - g-rich rna sequence binding factor 1  CDC40 - cell division cycle 40 |
| GO:0022402 | cell cycle process | 1.04E-9 | 4.8E-7 | 1.16 (10334,619,6996,485) | [+] Show genes  TBRG1 - transforming growth factor beta regulator 1  LIG1 - ligase i, dna, atp-dependent  HAUS7 - haus augmin-like complex, subunit 7  NCOR1 - nuclear receptor corepressor 1  ABL1 - c-abl oncogene 1, non-receptor tyrosine kinase  SSX2IP - synovial sarcoma, x breakpoint 2 interacting protein  PML - promyelocytic leukemia  CNOT11 - ccr4-not transcription complex, subunit 11  SPAST - spastin  LZTS2 - leucine zipper, putative tumor suppressor 2  SYCE1L - synaptonemal complex central element protein 1-like  BRSK1 - br serine/threonine kinase 1  ZFYVE19 - zinc finger, fyve domain containing 19  SLX4 - slx4 structure-specific endonuclease subunit  SOX4 - sry (sex determining region y)-box 4  UBXN2B - ubx domain protein 2b  ORC3 - origin recognition complex, subunit 3  SON - son dna binding protein  ORC6 - origin recognition complex, subunit 6  GJA1 - gap junction protein, alpha 1, 43kda  SPIRE2 - spire actin nucleation factor 2  LIMK2 - lim domain kinase 2  ATF2 - activating transcription factor 2  LMNA - lamin a/c  LLGL1 - lethal giant larvae homolog 1 (drosophila)  PRKAG2 - protein kinase, amp-activated, gamma 2 non-catalytic subunit  MCMBP - minichromosome maintenance complex binding protein  PLK3 - polo-like kinase 3  ANAPC7 - anaphase promoting complex subunit 7  RHOU - ras homolog family member u  ACVR1 - activin a receptor, type i  MAP9 - microtubule-associated protein 9  ACVR1B - activin a receptor, type ib  VPS4B - vacuolar protein sorting 4 homolog b (s. cerevisiae)  CDC45 - cell division cycle 45  PPME1 - protein phosphatase methylesterase 1  PPAT - phosphoribosyl pyrophosphate amidotransferase  CDC7 - cell division cycle 7  SMC1A - structural maintenance of chromosomes 1a  STMN1 - stathmin 1  ROCK2 - rho-associated, coiled-coil containing protein kinase 2  CRLF3 - cytokine receptor-like factor 3  TAOK3 - tao kinase 3  CNTROB - centrobin, centrosomal brca2 interacting protein  CEP76 - centrosomal protein 76kda  POLD2 - polymerase (dna directed), delta 2, accessory subunit  POLD1 - polymerase (dna directed), delta 1, catalytic subunit  TUBGCP2 - tubulin, gamma complex associated protein 2  SMPD3 - sphingomyelin phosphodiesterase 3, neutral membrane (neutral sphingomyelinase ii)  PRKAB1 - protein kinase, amp-activated, beta 1 non-catalytic subunit  CDK5RAP2 - cdk5 regulatory subunit associated protein 2  PRKACA - protein kinase, camp-dependent, catalytic, alpha  KIF2A - kinesin heavy chain member 2a  PRIM2 - primase, dna, polypeptide 2 (58kda)  PRIM1 - primase, dna, polypeptide 1 (49kda)  SUN2 - sad1 and unc84 domain containing 2  KIF3A - kinesin family member 3a  PRCC - papillary renal cell carcinoma (translocation-associated)  CHFR - checkpoint with forkhead and ring finger domains, e3 ubiquitin protein ligase  ING4 - inhibitor of growth family, member 4  ZWINT - zw10 interacting kinetochore protein  CIT - citron (rho-interacting, serine/threonine kinase 21)  FGFR1OP - fgfr1 oncogene partner  PPP6C - protein phosphatase 6, catalytic subunit  CNTRL - centriolin  RECQL5 - recq protein-like 5  PPP2R2A - protein phosphatase 2, regulatory subunit b, alpha  PPP2R1A - protein phosphatase 2, regulatory subunit a, alpha  KPNB1 - karyopherin (importin) beta 1  SBDS - shwachman-bodian-diamond syndrome  RMDN1 - regulator of microtubule dynamics 1  PPP2CA - protein phosphatase 2, catalytic subunit, alpha isozyme  AURKC - aurora kinase c  TOPBP1 - topoisomerase (dna) ii binding protein 1  PPM1G - protein phosphatase, mg2+/mn2+ dependent, 1g  CNEP1R1 - ctd nuclear envelope phosphatase 1 regulatory subunit 1  ESCO1 - establishment of sister chromatid cohesion n-acetyltransferase 1  TDRKH - tudor and kh domain containing  NIPBL - nipped-b homolog (drosophila)  TUBGCP5 - tubulin, gamma complex associated protein 5  RAB35 - rab35, member ras oncogene family  PRNP - prion protein  AAAS - achalasia, adrenocortical insufficiency, alacrimia  PAPD7 - pap associated domain containing 7  KIF3B - kinesin family member 3b  PIM2 - pim-2 oncogene  MAP2K1 - mitogen-activated protein kinase kinase 1  SNX33 - sorting nexin 33  CCNI - cyclin i  MAPRE2 - microtubule-associated protein, rp/eb family, member 2  KLHL22 - kelch-like family member 22  NDC1 - ndc1 transmembrane nucleoporin  HAUS1 - haus augmin-like complex, subunit 1  SPTBN1 - spectrin, beta, non-erythrocytic 1  CSNK1D - casein kinase 1, delta  PRKCB - protein kinase c, beta  TAOK2 - tao kinase 2  PRKAR2B - protein kinase, camp-dependent, regulatory, type ii, beta  PRKCA - protein kinase c, alpha  TUBE1 - tubulin, epsilon 1  CNOT8 - ccr4-not transcription complex, subunit 8  PRKAR1A - protein kinase, camp-dependent, regulatory, type i, alpha  PRKAG1 - protein kinase, amp-activated, gamma 1 non-catalytic subunit  MSTO1 - misato 1, mitochondrial distribution and morphology regulator  XIAP - x-linked inhibitor of apoptosis  SYF2 - syf2 pre-mrna-splicing factor  RNF112 - ring finger protein 112  RFWD3 - ring finger and wd repeat domain 3  GSPT1 - g1 to s phase transition 1  CCNI2 - cyclin i family, member 2  SETMAR - set domain and mariner transposase fusion gene  ANXA11 - annexin a11  MLH1 - mutl homolog 1, colon cancer, nonpolyposis type 2 (e. coli)  DCTN1 - dynactin 1  DCTN3 - dynactin 3 (p22)  MCPH1 - microcephalin 1  CSRP2BP - csrp2 binding protein  APBB2 - amyloid beta (a4) precursor protein-binding, family b, member 2  APC - adenomatous polyposis coli  MSH6 - muts homolog 6 (e. coli)  MAP3K11 - mitogen-activated protein kinase kinase kinase 11  APBB1 - amyloid beta (a4) precursor protein-binding, family b, member 1 (fe65)  CNOT10 - ccr4-not transcription complex, subunit 10  ARF1 - adp-ribosylation factor 1  CNTD1 - cyclin n-terminal domain containing 1  MNAT1 - mnat cdk-activating kinase assembly factor 1  PAFAH1B1 - platelet-activating factor acetylhydrolase 1b, regulatory subunit 1 (45kda)  CNOT6 - ccr4-not transcription complex, subunit 6  CEP250 - centrosomal protein 250kda  ARL3 - adp-ribosylation factor-like 3  MCM7 - minichromosome maintenance complex component 7  ARL2 - adp-ribosylation factor-like 2  TUBA1A - tubulin, alpha 1a  ALMS1 - alstrom syndrome 1  MECP2 - methyl cpg binding protein 2 (rett syndrome)  RHOB - ras homolog family member b  BUB3 - bub3 mitotic checkpoint protein  RHOC - ras homolog family member c  ZW10 - zw10 kinetochore protein  RHOA - ras homolog family member a  KNSTRN - kinetochore-localized astrin/spag5 binding protein  WDR6 - wd repeat domain 6  PCNA - proliferating cell nuclear antigen  CHMP1A - charged multivesicular body protein 1a  CEP78 - centrosomal protein 78kda  BRINP1 - bone morphogenetic protein/retinoic acid inducible neural-specific 1  C2CD3 - c2 calcium-dependent domain containing 3  PCM1 - pericentriolar material 1  HINFP - histone h4 transcription factor  CDK16 - cyclin-dependent kinase 16  CDK17 - cyclin-dependent kinase 17  ARHGEF2 - rho/rac guanine nucleotide exchange factor (gef) 2  CHMP2B - charged multivesicular body protein 2b  HTT - huntingtin  RPTOR - regulatory associated protein of mtor, complex 1  ZNF207 - zinc finger protein 207  HAUS2 - haus augmin-like complex, subunit 2  TUBGCP6 - tubulin, gamma complex associated protein 6  XPC - xeroderma pigmentosum, complementation group c  GOLGA2 - golgin a2  HAUS3 - haus augmin-like complex, subunit 3  FBXW7 - f-box and wd repeat domain containing 7, e3 ubiquitin protein ligase  CNOT6L - ccr4-not transcription complex, subunit 6-like  RQCD1 - rcd1 required for cell differentiation1 homolog (s. pombe)  TAOK1 - tao kinase 1  SMC3 - structural maintenance of chromosomes 3  LATS1 - large tumor suppressor kinase 1  USP8 - ubiquitin specific peptidase 8  ZFHX3 - zinc finger homeobox 3  TNKS1BP1 - tankyrase 1 binding protein 1, 182kda  WEE1 - wee1 homolog (s. pombe)  UNC119 - unc-119 homolog (c. elegans)  SKIL - ski-like oncogene  SKP1 - s-phase kinase-associated protein 1  PKMYT1 - protein kinase, membrane associated tyrosine/threonine 1  MAX - myc associated factor x  GIGYF2 - grb10 interacting gyf protein 2  MCM3 - minichromosome maintenance complex component 3  MCM6 - minichromosome maintenance complex component 6  CHMP5 - charged multivesicular body protein 5  VRK1 - vaccinia related kinase 1  RTFDC1 - replication termination factor 2 domain containing 1  MCM5 - minichromosome maintenance complex component 5  VASH1 - vasohibin 1  MCM2 - minichromosome maintenance complex component 2  BAX - bcl2-associated x protein  PKD1 - polycystic kidney disease 1 (autosomal dominant)  PKD2 - polycystic kidney disease 2 (autosomal dominant)  FBXL15 - f-box and leucine-rich repeat protein 15  PNPT1 - polyribonucleotide nucleotidyltransferase 1  DLG1 - discs, large homolog 1 (drosophila)  PHLDA1 - pleckstrin homology-like domain, family a, member 1  BACH1 - btb and cnc homology 1, basic leucine zipper transcription factor 1  PHGDH - phosphoglycerate dehydrogenase  DHCR24 - 24-dehydrocholesterol reductase  PLAGL1 - pleiomorphic adenoma gene-like 1  FBXL3 - f-box and leucine-rich repeat protein 3  DHFR - dihydrofolate reductase  ATRX - alpha thalassemia/mental retardation syndrome x-linked  CHMP3 - charged multivesicular body protein 3  DYNC1H1 - dynein, cytoplasmic 1, heavy chain 1  DYNC1I2 - dynein, cytoplasmic 1, intermediate chain 2  DNM2 - dynamin 2  EIF2AK4 - eukaryotic translation initiation factor 2 alpha kinase 4  BCL2L1 - bcl2-like 1  MZT1 - mitotic spindle organizing protein 1  YWHAG - tyrosine 3-monooxygenase/tryptophan 5-monooxygenase activation protein, gamma polypeptide  CCND1 - cyclin d1  USP37 - ubiquitin specific peptidase 37  EME1 - essential meiotic structure-specific endonuclease 1  BBS4 - bardet-biedl syndrome 4  WASL - wiskott-aldrich syndrome-like  YWHAE - tyrosine 3-monooxygenase/tryptophan 5-monooxygenase activation protein, epsilon polypeptide  SMARCAD1 - swi/snf-related, matrix-associated actin-dependent regulator of chromatin, subfamily a, containing dead/h box 1  MAPRE1 - microtubule-associated protein, rp/eb family, member 1  EMD - emerin  RFC5 - replication factor c (activator 1) 5, 36.5kda  CHMP4C - charged multivesicular body protein 4c  MAPRE3 - microtubule-associated protein, rp/eb family, member 3  RFC2 - replication factor c (activator 1) 2, 40kda  RFC3 - replication factor c (activator 1) 3, 38kda  AKAP9 - a kinase (prka) anchor protein (yotiao) 9  CAB39L - calcium binding protein 39-like  SPIRE1 - spire actin nucleation factor 1  EML1 - echinoderm microtubule associated protein like 1  TTC19 - tetratricopeptide repeat domain 19  RACGAP1 - rac gtpase activating protein 1  ENSA - endosulfine alpha  PRMT1 - protein arginine methyltransferase 1  KLHDC8B - kelch domain containing 8b  KATNB1 - katanin p80 (wd repeat containing) subunit b 1  EP300 - e1a binding protein p300  NASP - nuclear autoantigenic sperm protein (histone-binding)  NSUN2 - nop2/sun rna methyltransferase family, member 2  UPF1 - upf1 regulator of nonsense transcripts homolog (yeast)  NBN - nibrin  NCAPH2 - non-smc condensin ii complex, subunit h2  RRAGB - ras-related gtp binding b  EIF4E - eukaryotic translation initiation factor 4e  TUBG1 - tubulin, gamma 1  PPP1R12A - protein phosphatase 1, regulatory subunit 12a  NDEL1 - nude neurodevelopment protein 1-like 1  AZI1 - 5-azacytidine induced 1  STRADA - ste20-related kinase adaptor alpha  RHEB - ras homolog enriched in brain  EIF4G2 - eukaryotic translation initiation factor 4 gamma, 2  TSG101 - tumor susceptibility gene 101  DSCC1 - dna replication and sister chromatid cohesion 1  KAT2B - k(lysine) acetyltransferase 2b  FBXL12 - f-box and leucine-rich repeat protein 12  CDC16 - cell division cycle 16  CNOT1 - ccr4-not transcription complex, subunit 1  ZNF259 - zinc finger protein 259  NDE1 - nude neurodevelopment protein 1  NAE1 - nedd8 activating enzyme e1 subunit 1  SEH1L - seh1-like (s. cerevisiae)  CDC123 - cell division cycle 123  MYH10 - myosin, heavy chain 10, non-muscle  MIS12 - mis12 kinetochore complex component  MYH9 - myosin, heavy chain 9, non-muscle  TOP3A - topoisomerase (dna) iii alpha  CNOT7 - ccr4-not transcription complex, subunit 7  HAUS6 - haus augmin-like complex, subunit 6  NABP2 - nucleic acid binding protein 2  TOP2B - topoisomerase (dna) ii beta 180kda  HUS1 - hus1 checkpoint homolog (s. pombe)  USP33 - ubiquitin specific peptidase 33  UHMK1 - u2af homology motif (uhm) kinase 1  GPSM2 - g-protein signaling modulator 2  ACTR2 - arp2 actin-related protein 2 homolog (yeast)  ACTR3 - arp3 actin-related protein 3 homolog (yeast)  ROCK1 - rho-associated, coiled-coil containing protein kinase 1  E2F6 - e2f transcription factor 6  E4F1 - e4f transcription factor 1  SPRY1 - sprouty homolog 1, antagonist of fgf signaling (drosophila)  SPRY2 - sprouty homolog 2 (drosophila)  PHF13 - phd finger protein 13  E2F4 - e2f transcription factor 4, p107/p130-binding  PDS5B - pds5, regulator of cohesion maintenance, homolog b (s. cerevisiae)  RAD50 - rad50 homolog (s. cerevisiae)  HSP90AA1 - heat shock protein 90kda alpha (cytosolic), class a member 1  ACTR1A - arp1 actin-related protein 1 homolog a, centractin alpha (yeast)  AKAP8 - a kinase (prka) anchor protein 8  BTRC - beta-transducin repeat containing e3 ubiquitin protein ligase  STAG1 - stromal antigen 1  TPR - translocated promoter region, nuclear basket protein  WAPAL - wings apart-like homolog (drosophila)  ZWILCH - zwilch kinetochore protein  BOD1 - biorientation of chromosomes in cell division 1  LAMTOR3 - late endosomal/lysosomal adaptor, mapk and mtor activator 3  STK33 - serine/threonine kinase 33  PIBF1 - progesterone immunomodulatory binding factor 1  DYNLL1 - dynein, light chain, lc8-type 1  TNKS - tankyrase, trf1-interacting ankyrin-related adp-ribose polymerase  CASP2 - caspase 2, apoptosis-related cysteine peptidase  RPA3 - replication protein a3, 14kda  MAD2L2 - mad2 mitotic arrest deficient-like 2 (yeast)  LATS2 - large tumor suppressor kinase 2  PDCD6IP - programmed cell death 6 interacting protein  RPA1 - replication protein a1, 70kda  RPA2 - replication protein a2, 32kda  CLASP2 - cytoplasmic linker associated protein 2  BECN1 - beclin 1, autophagy related  POGZ - pogo transposable element with znf domain  EZR - ezrin  PHF8 - phd finger protein 8  SMC5 - structural maintenance of chromosomes 5  PCBP4 - poly(rc) binding protein 4  LAMTOR1 - late endosomal/lysosomal adaptor, mapk and mtor activator 1  ANKLE2 - ankyrin repeat and lem domain containing 2  KIFC2 - kinesin family member c2  UVRAG - uv radiation resistance associated  CDC23 - cell division cycle 23  INTS3 - integrator complex subunit 3  VCP - valosin containing protein  SMC4 - structural maintenance of chromosomes 4  MSH3 - muts homolog 3 (e. coli)  MSH2 - muts homolog 2, colon cancer, nonpolyposis type 1 (e. coli)  NEK9 - nima-related kinase 9  ERCC4 - excision repair cross-complementing rodent repair deficiency, complementation group 4  PINX1 - pin2/terf1 interacting, telomerase inhibitor 1  TUBB - tubulin, beta class i  CEP68 - centrosomal protein 68kda  CCND3 - cyclin d3  NCAPD2 - non-smc condensin i complex, subunit d2  CCNH - cyclin h  ERN1 - endoplasmic reticulum to nucleus signaling 1  CCNG2 - cyclin g2  CHMP7 - charged multivesicular body protein 7  UBB - ubiquitin b  RGCC - regulator of cell cycle  HIRA - histone cell cycle regulator  TIPIN - timeless interacting protein  TUBB4B - tubulin, beta 4b class ivb  RPS6KB1 - ribosomal protein s6 kinase, 70kda, polypeptide 1  RAB11A - rab11a, member ras oncogene family  ZNF830 - zinc finger protein 830  CHMP4A - charged multivesicular body protein 4a  RRS1 - rrs1 ribosome biogenesis regulator homolog (s. cerevisiae)  YTHDC2 - yth domain containing 2  TUBGCP3 - tubulin, gamma complex associated protein 3  SETD2 - set domain containing 2  CCND2 - cyclin d2  MRE11A - mre11 meiotic recombination 11 homolog a (s. cerevisiae)  NABP1 - nucleic acid binding protein 1  UBE2L3 - ubiquitin-conjugating enzyme e2l 3  EPS8 - epidermal growth factor receptor pathway substrate 8  CHTF8 - ctf8, chromosome transmission fidelity factor 8 homolog (s. cerevisiae)  CHAMP1 - chromosome alignment maintaining phosphoprotein 1  PLK1S1 - polo-like kinase 1 substrate 1  STAMBP - stam binding protein  ORC4 - origin recognition complex, subunit 4  ORC2 - origin recognition complex, subunit 2  PARD6A - par-6 partitioning defective 6 homolog alpha (c. elegans)  ORC5 - origin recognition complex, subunit 5  CDC27 - cell division cycle 27  PDS5A - pds5, regulator of cohesion maintenance, homolog a (s. cerevisiae)  INHA - inhibin, alpha  SGSM3 - small g protein signaling modulator 3  FLNA - filamin a, alpha  ING2 - inhibitor of growth family, member 2  PPM1D - protein phosphatase, mg2+/mn2+ dependent, 1d  USP16 - ubiquitin specific peptidase 16  PLCB1 - phospholipase c, beta 1 (phosphoinositide-specific)  UBE2S - ubiquitin-conjugating enzyme e2s  HDAC8 - histone deacetylase 8  ILK - integrin-linked kinase  CDC14B - cell division cycle 14b  PIAS1 - protein inhibitor of activated stat, 1  NCAPD3 - non-smc condensin ii complex, subunit d3  ODF2 - outer dense fiber of sperm tails 2  REEP4 - receptor accessory protein 4  FBXW11 - f-box and wd repeat domain containing 11  ANAPC1 - anaphase promoting complex subunit 1  DYNLT1 - dynein, light chain, tctex-type 1  GAS2L1 - growth arrest-specific 2 like 1  APITD1 - apoptosis-inducing, taf9-like domain 1  CKAP5 - cytoskeleton associated protein 5  PSMD13 - proteasome (prosome, macropain) 26s subunit, non-atpase, 13  HAUS5 - haus augmin-like complex, subunit 5  CENPC - centromere protein c  TUBGCP4 - tubulin, gamma complex associated protein 4  DCTN2 - dynactin 2 (p50)  CETN2 - centrin, ef-hand protein, 2  LAMTOR5 - late endosomal/lysosomal adaptor, mapk and mtor activator 5  JMY - junction mediating and regulatory protein, p53 cofactor  TAF2 - taf2 rna polymerase ii, tata box binding protein (tbp)-associated factor, 150kda  CCNY - cyclin y  TACC1 - transforming, acidic coiled-coil containing protein 1  CETN3 - centrin, ef-hand protein, 3  IST1 - increased sodium tolerance 1 homolog (yeast)  CFL1 - cofilin 1 (non-muscle)  NSFL1C - nsfl1 (p97) cofactor (p47)  NUMA1 - nuclear mitotic apparatus protein 1  CLASP1 - cytoplasmic linker associated protein 1  GMNN - geminin, dna replication inhibitor  SLC38A9 - solute carrier family 38, member 9  FEN1 - flap structure-specific endonuclease 1  ITGB1 - integrin, beta 1 (fibronectin receptor, beta polypeptide, antigen cd29 includes mdf2, msk12)  EFHC1 - ef-hand domain (c-terminal) containing 1  CEP120 - centrosomal protein 120kda  CDK5 - cyclin-dependent kinase 5  CDK4 - cyclin-dependent kinase 4  NPM1 - nucleophosmin (nucleolar phosphoprotein b23, numatrin)  KMT2E - lysine (k)-specific methyltransferase 2e  CDK7 - cyclin-dependent kinase 7  CDKN1C - cyclin-dependent kinase inhibitor 1c (p57, kip2)  TBCE - tubulin folding cofactor e  CDKN1B - cyclin-dependent kinase inhibitor 1b (p27, kip1)  SCC4 - sister chromatid cohesion 4  GINS1 - gins complex subunit 1 (psf1 homolog)  CDKN2D - cyclin-dependent kinase inhibitor 2d (p19, inhibits cdk4)  SPICE1 - spindle and centriole associated protein 1  SFI1 - sfi1 homolog, spindle assembly associated (yeast)  CUL7 - cullin 7  CDK2 - cyclin-dependent kinase 2  RAD1 - rad1 homolog (s. pombe)  DONSON - downstream neighbor of son  SLC2A8 - solute carrier family 2 (facilitated glucose transporter), member 8  MTOR - mechanistic target of rapamycin (serine/threonine kinase)  MUS81 - mus81 structure-specific endonuclease subunit  PLK2 - polo-like kinase 2  CEP290 - centrosomal protein 290kda  NPAT - nuclear protein, ataxia-telangiectasia locus  CNOT2 - ccr4-not transcription complex, subunit 2  CNOT4 - ccr4-not transcription complex, subunit 4  CNOT3 - ccr4-not transcription complex, subunit 3  NOTCH1 - notch 1  CEP57 - centrosomal protein 57kda  NOTCH2 - notch 2  ID2 - inhibitor of dna binding 2, dominant negative helix-loop-helix protein  CHD3 - chromodomain helicase dna binding protein 3  BRD4 - bromodomain containing 4  RRAGC - ras-related gtp binding c  TUBG2 - tubulin, gamma 2  RAD21 - rad21 homolog (s. pombe)  RAD17 - rad17 homolog (s. pombe)  MAD1L1 - mad1 mitotic arrest deficient-like 1 (yeast)  VPS4A - vacuolar protein sorting 4 homolog a (s. cerevisiae)  INO80 - ino80 complex subunit  CCP110 - centriolar coiled coil protein 110kda  MDC1 - mediator of dna-damage checkpoint 1  CHORDC1 - cysteine and histidine-rich domain (chord) containing 1  RPRM - reprimo, tp53 dependent g2 arrest mediator candidate  TERF1 - telomeric repeat binding factor (nima-interacting) 1  SNX18 - sorting nexin 18  WRAP73 - wd repeat containing, antisense to tp73  CEP70 - centrosomal protein 70kda  TFDP1 - transcription factor dp-1  ARHGEF10 - rho guanine nucleotide exchange factor (gef) 10  CGREF1 - cell growth regulator with ef-hand domain 1  CGRRF1 - cell growth regulator with ring finger domain 1  DCTN6 - dynactin 6  BCCIP - brca2 and cdkn1a interacting protein  RRAGA - ras-related gtp binding a  RASA1 - ras p21 protein activator (gtpase activating protein) 1  ZFYVE26 - zinc finger, fyve domain containing 26  KHDRBS1 - kh domain containing, rna binding, signal transduction associated 1  CEP41 - centrosomal protein 41kda  POLE4 - polymerase (dna-directed), epsilon 4, accessory subunit  IK - ik cytokine, down-regulator of hla ii  MLH3 - mutl homolog 3 (e. coli)  TGFBR1 - transforming growth factor, beta receptor 1  MITD1 - mit, microtubule interacting and transport, domain containing 1  CDK5RAP3 - cdk5 regulatory subunit associated protein 3  CROCC - ciliary rootlet coiled-coil, rootletin  RBL2 - retinoblastoma-like 2 (p130)  REEP3 - receptor accessory protein 3  RBBP8 - retinoblastoma binding protein 8  TGFB2 - transforming growth factor, beta 2  TGFB1 - transforming growth factor, beta 1  DIS3L2 - dis3 mitotic control homolog (s. cerevisiae)-like 2  RB1 - retinoblastoma 1  STAG2 - stromal antigen 2  TP53INP1 - tumor protein p53 inducible nuclear protein 1  CEP63 - centrosomal protein 63kda  CUL4B - cullin 4b  CUL1 - cullin 1  CUL2 - cullin 2  THBS1 - thrombospondin 1  CUL3 - cullin 3  CKS1B - cdc28 protein kinase regulatory subunit 1b  POLD3 - polymerase (dna-directed), delta 3, accessory subunit  CHMP4B - charged multivesicular body protein 4b  KIAA0430 - kiaa0430 |
| GO:0007049 | cell cycle | 1.54E-9 | 6.92E-7 | 1.22 (10334,302,6996,250) | [+] Show genes  CCNK - cyclin k  WTAP - wilms tumor 1 associated protein  BRD7 - bromodomain containing 7  LIG4 - ligase iv, dna, atp-dependent  MAEA - macrophage erythroblast attacher  CD2AP - cd2-associated protein  GEM - gtp binding protein overexpressed in skeletal muscle  CENPV - centromere protein v  LIG3 - ligase iii, dna, atp-dependent  ENSA - endosulfine alpha  ABL1 - c-abl oncogene 1, non-receptor tyrosine kinase  RABGAP1 - rab gtpase activating protein 1  PAK4 - p21 protein (cdc42/rac)-activated kinase 4  MPLKIP - m-phase specific plk1 interacting protein  NBN - nibrin  BLCAP - bladder cancer associated protein  TXNL4A - thioredoxin-like 4a  TUBB2A - tubulin, beta 2a class iia  RBM7 - rna binding motif protein 7  TUBG1 - tubulin, gamma 1  PPP1R12A - protein phosphatase 1, regulatory subunit 12a  SUGT1 - sgt1, suppressor of g2 allele of skp1 (s. cerevisiae)  PSME3 - proteasome (prosome, macropain) activator subunit 3 (pa28 gamma; ki)  SPIN1 - spindlin 1  PRR5 - proline rich 5 (renal)  CREBL2 - camp responsive element binding protein-like 2  MARK4 - map/microtubule affinity-regulating kinase 4  CCNDBP1 - cyclin d-type binding-protein 1  PLK3 - polo-like kinase 3  ANAPC5 - anaphase promoting complex subunit 5  CABLES2 - cdk5 and abl enzyme substrate 2  TP53BP2 - tumor protein p53 binding protein, 2  TOP3A - topoisomerase (dna) iii alpha  ARL8A - adp-ribosylation factor-like 8a  EID1 - ep300 interacting inhibitor of differentiation 1  RNF2 - ring finger protein 2  SMARCB1 - swi/snf related, matrix associated, actin dependent regulator of chromatin, subfamily b, member 1  SMC1A - structural maintenance of chromosomes 1a  USP9X - ubiquitin specific peptidase 9, x-linked  ANAPC4 - anaphase promoting complex subunit 4  TET2 - tet methylcytosine dioxygenase 2  PHF13 - phd finger protein 13  GNAI3 - guanine nucleotide binding protein (g protein), alpha inhibiting activity polypeptide 3  GNAI2 - guanine nucleotide binding protein (g protein), alpha inhibiting activity polypeptide 2  GNAI1 - guanine nucleotide binding protein (g protein), alpha inhibiting activity polypeptide 1  FZR1 - fizzy/cell division cycle 20 related 1 (drosophila)  AKAP8 - a kinase (prka) anchor protein 8  TUBGCP2 - tubulin, gamma complex associated protein 2  OSGIN2 - oxidative stress induced growth inhibitor family member 2  WAPAL - wings apart-like homolog (drosophila)  CCSAP - centriole, cilia and spindle-associated protein  KATNA1 - katanin p60 (atpase containing) subunit a 1  DMTF1 - cyclin d binding myb-like transcription factor 1  ZNF318 - zinc finger protein 318  SUN2 - sad1 and unc84 domain containing 2  SENP5 - sumo1/sentrin specific peptidase 5  PCNP - pest proteolytic signal containing nuclear protein  CIT - citron (rho-interacting, serine/threonine kinase 21)  BEX2 - brain expressed x-linked 2  PPP5C - protein phosphatase 5, catalytic subunit  DYNC1LI1 - dynein, cytoplasmic 1, light intermediate chain 1  UHRF2 - ubiquitin-like with phd and ring finger domains 2, e3 ubiquitin protein ligase  RPA1 - replication protein a1, 70kda  SDE2 - sde2 telomere maintenance homolog (s. pombe)  RECQL5 - recq protein-like 5  RIOK2 - rio kinase 2  TXLNG - taxilin gamma  AHR - aryl hydrocarbon receptor  CHAF1A - chromatin assembly factor 1, subunit a (p150)  TADA3 - transcriptional adaptor 3  NEK4 - nima-related kinase 4  TRIOBP - trio and f-actin binding protein  PPP2CA - protein phosphatase 2, catalytic subunit, alpha isozyme  CDC23 - cell division cycle 23  PDIK1L - pdlim1 interacting kinase 1 like  AURKC - aurora kinase c  TUBA1C - tubulin, alpha 1c  STK10 - serine/threonine kinase 10  CYLD - cylindromatosis (turban tumor syndrome)  PPP1CC - protein phosphatase 1, catalytic subunit, gamma isozyme  CCNT2 - cyclin t2  TUBA1B - tubulin, alpha 1b  GAK - cyclin g associated kinase  TUBB - tubulin, beta class i  TUBGCP5 - tubulin, gamma complex associated protein 5  CCNT1 - cyclin t1  ERH - enhancer of rudimentary homolog (drosophila)  BANP - btg3 associated nuclear protein  ANAPC13 - anaphase promoting complex subunit 13  HNRNPU - heterogeneous nuclear ribonucleoprotein u (scaffold attachment factor a)  ANAPC10 - anaphase promoting complex subunit 10  URGCP - upregulator of cell proliferation  PRPF40A - prp40 pre-mrna processing factor 40 homolog a (s. cerevisiae)  TUBB4B - tubulin, beta 4b class ivb  TUBB3 - tubulin, beta 3 class iii  RINT1 - rad50 interactor 1  CDK2AP1 - cyclin-dependent kinase 2 associated protein 1  RPS6KA1 - ribosomal protein s6 kinase, 90kda, polypeptide 1  MAPK13 - mitogen-activated protein kinase 13  YEATS4 - yeats domain containing 4  MAPK7 - mitogen-activated protein kinase 7  USP3 - ubiquitin specific peptidase 3  EPB41 - erythrocyte membrane protein band 4.1 (elliptocytosis 1, rh-linked)  BABAM1 - brisc and brca1 a complex member 1  PDCD2L - programmed cell death 2-like  EPB41L2 - erythrocyte membrane protein band 4.1-like 2  MAPK3 - mitogen-activated protein kinase 3  MAPK1 - mitogen-activated protein kinase 1  ZNF830 - zinc finger protein 830  YTHDC2 - yth domain containing 2  PKN2 - protein kinase n2  TUBGCP3 - tubulin, gamma complex associated protein 3  CSNK1A1 - casein kinase 1, alpha 1  TLK2 - tousled-like kinase 2  UBE2I - ubiquitin-conjugating enzyme e2i  CSNK2A2 - casein kinase 2, alpha prime polypeptide  PRKCE - protein kinase c, epsilon  NUP214 - nucleoporin 214kda  SRC - v-src avian sarcoma (schmidt-ruppin a-2) viral oncogene homolog  CSNK2A1 - casein kinase 2, alpha 1 polypeptide  PRKCD - protein kinase c, delta  TUBE1 - tubulin, epsilon 1  RRM1 - ribonucleotide reductase m1  CDK11B - cyclin-dependent kinase 11b  CDC5L - cell division cycle 5-like  STK35 - serine/threonine kinase 35  TLK1 - tousled-like kinase 1  SPIN2A - spindlin family, member 2a  DCTN1 - dynactin 1  DCTN3 - dynactin 3 (p22)  MCPH1 - microcephalin 1  USP16 - ubiquitin specific peptidase 16  CDC14B - cell division cycle 14b  RIF1 - rap1 interacting factor homolog (yeast)  KLHL21 - kelch-like family member 21  POC5 - poc5 centriolar protein  AHCTF1 - at hook containing transcription factor 1  PBRM1 - polybromo 1  KLHL13 - kelch-like family member 13  EML4 - echinoderm microtubule associated protein like 4  APPL2 - adaptor protein, phosphotyrosine interaction, ph domain and leucine zipper containing 2  CAMK1 - calcium/calmodulin-dependent protein kinase i  RASSF4 - ras association (ralgds/af-6) domain family member 4  ARL8B - adp-ribosylation factor-like 8b  NOLC1 - nucleolar and coiled-body phosphoprotein 1  KLHL18 - kelch-like family member 18  DYNLT3 - dynein, light chain, tctex-type 3  BIRC6 - baculoviral iap repeat containing 6  RCBTB1 - regulator of chromosome condensation (rcc1) and btb (poz) domain containing protein 1  CCPG1 - cell cycle progression 1  CEP250 - centrosomal protein 250kda  SSSCA1 - sjogren syndrome/scleroderma autoantigen 1  H2AFX - h2a histone family, member x  CENPC - centromere protein c  TUBGCP4 - tubulin, gamma complex associated protein 4  NSMCE2 - non-smc element 2, mms21 homolog (s. cerevisiae)  TUBA1A - tubulin, alpha 1a  CETN2 - centrin, ef-hand protein, 2  SKA2 - spindle and kinetochore associated complex subunit 2  CDC73 - cell division cycle 73  BUB3 - bub3 mitotic checkpoint protein  NUMA1 - nuclear mitotic apparatus protein 1  DAB2IP - dab2 interacting protein  PELO - pelota homolog (drosophila)  NUP88 - nucleoporin 88kda  FAM107A - family with sequence similarity 107, member a  ZW10 - zw10 kinetochore protein  THAP5 - thap domain containing 5  USP22 - ubiquitin specific peptidase 22  ARF6 - adp-ribosylation factor 6  CIB1 - calcium and integrin binding 1 (calmyrin)  EVI5 - ecotropic viral integration site 5  FAM32A - family with sequence similarity 32, member a  PTP4A1 - protein tyrosine phosphatase type iva, member 1  MEN1 - multiple endocrine neoplasia i  HCFC1 - host cell factor c1 (vp16-accessory protein)  LIN54 - lin-54 homolog (c. elegans)  INSM1 - insulinoma-associated 1  SPECC1L - sperm antigen with calponin homology and coiled-coil domains 1-like  TBCD - tubulin folding cofactor d  TUBB2B - tubulin, beta 2b class iib  APEX2 - apex nuclease (apurinic/apyrimidinic endonuclease) 2  PTPN11 - protein tyrosine phosphatase, non-receptor type 11  HACE1 - hect domain and ankyrin repeat containing e3 ubiquitin protein ligase 1  RUVBL1 - ruvb-like 1 (e. coli)  KLHL9 - kelch-like family member 9  RB1CC1 - rb1-inducible coiled-coil 1  AZI2 - 5-azacytidine induced 2  THAP1 - thap domain containing, apoptosis associated protein 1  RCC2 - regulator of chromosome condensation 2  CDK2 - cyclin-dependent kinase 2  RAB11FIP3 - rab11 family interacting protein 3 (class ii)  ZC3HC1 - zinc finger, c3hc-type containing 1  KLHL42 - kelch-like family member 42  TUBGCP6 - tubulin, gamma complex associated protein 6  ITGB3BP - integrin beta 3 binding protein (beta3-endonexin)  PAPD5 - pap associated domain containing 5  CDC26 - cell division cycle 26  MICAL3 - microtubule associated monooxygenase, calponin and lim domain containing 3  ZMYND11 - zinc finger, mynd-type containing 11  SMC3 - structural maintenance of chromosomes 3  PLK2 - polo-like kinase 2  MNS1 - meiosis-specific nuclear structural 1  NUP43 - nucleoporin 43kda  ACTR8 - arp8 actin-related protein 8 homolog (yeast)  CENPT - centromere protein t  PKMYT1 - protein kinase, membrane associated tyrosine/threonine 1  SDCCAG3 - serologically defined colon cancer antigen 3  TUBG2 - tubulin, gamma 2  APPL1 - adaptor protein, phosphotyrosine interaction, ph domain and leucine zipper containing 1  VCPIP1 - valosin containing protein (p97)/p47 complex interacting protein 1  LRRCC1 - leucine rich repeat and coiled-coil centrosomal protein 1  RALA - v-ral simian leukemia viral oncogene homolog a (ras related)  RALB - v-ral simian leukemia viral oncogene homolog b  NEK3 - nima-related kinase 3  MARVELD1 - marvel domain containing 1  CLTC - clathrin, heavy chain (hc)  PIN1 - peptidylprolyl cis/trans isomerase, nima-interacting 1  NEK1 - nima-related kinase 1  RSPH1 - radial spoke head 1 homolog (chlamydomonas)  NEDD9 - neural precursor cell expressed, developmentally down-regulated 9  TERF2 - telomeric repeat binding factor 2  DYRK3 - dual-specificity tyrosine-(y)-phosphorylation regulated kinase 3  BRCC3 - brca1/brca2-containing complex, subunit 3  FSD1 - fibronectin type iii and spry domain containing 1  SIAH2 - siah e3 ubiquitin protein ligase 2  SIAH1 - siah e3 ubiquitin protein ligase 1  ZNF16 - zinc finger protein 16  UBA3 - ubiquitin-like modifier activating enzyme 3  CLTA - clathrin, light chain a  RNF8 - ring finger protein 8, e3 ubiquitin protein ligase  IK - ik cytokine, down-regulator of hla ii  DYNC1H1 - dynein, cytoplasmic 1, heavy chain 1  RBBP8 - retinoblastoma binding protein 8  DIS3L2 - dis3 mitotic control homolog (s. cerevisiae)-like 2  SRSF2 - serine/arginine-rich splicing factor 2  NUDT15 - nudix (nucleoside diphosphate linked moiety x)-type motif 15  SPAG8 - sperm associated antigen 8  STAG2 - stromal antigen 2  DNMT3A - dna (cytosine-5-)-methyltransferase 3 alpha  TUSC2 - tumor suppressor candidate 2  RAE1 - rae1 rna export 1 homolog (s. pombe)  TSPYL2 - tspy-like 2  CDK20 - cyclin-dependent kinase 20  RBBP4 - retinoblastoma binding protein 4  KCTD11 - potassium channel tetramerization domain containing 11  PIM1 - pim-1 oncogene  PIK3C3 - phosphatidylinositol 3-kinase, catalytic subunit type 3  NUDC - nudc nuclear distribution protein  USP39 - ubiquitin specific peptidase 39 |
| GO:0050684 | regulation of mRNA processing | 1.76E-9 | 7.64E-7 | 1.33 (10334,130,6996,117) | [+] Show genes  DHX9 - deah (asp-glu-ala-his) box helicase 9  DHX36 - deah (asp-glu-ala-his) box polypeptide 36  NCBP2 - nuclear cap binding protein subunit 2, 20kda  RBM23 - rna binding motif protein 23  NCBP1 - nuclear cap binding protein subunit 1, 80kda  FMR1 - fragile x mental retardation 1  WTAP - wilms tumor 1 associated protein  DDX5 - dead (asp-glu-ala-asp) box helicase 5  CDK11B - cyclin-dependent kinase 11b  SAP18 - sin3a-associated protein, 18kda  RBM39 - rna binding motif protein 39  NCL - nucleolin  MBNL2 - muscleblind-like splicing regulator 2  RBM15 - rna binding motif protein 15  RBMX - rna binding motif protein, x-linked  SNW1 - snw domain containing 1  ZFP36L1 - zfp36 ring finger protein-like 1  SRSF12 - serine/arginine-rich splicing factor 12  CWC22 - cwc22 spliceosome-associated protein homolog (s. cerevisiae)  PRPF19 - pre-mrna processing factor 19  METTL16 - methyltransferase like 16  CELF4 - cugbp, elav-like family member 4  RBM19 - rna binding motif protein 19  RBM7 - rna binding motif protein 7  RBM5 - rna binding motif protein 5  KHDRBS3 - kh domain containing, rna binding, signal transduction associated 3  PAPOLA - poly(a) polymerase alpha  PABPN1 - poly(a) binding protein, nuclear 1  RNPS1 - rna binding protein s1, serine-rich domain  SON - son dna binding protein  CELF3 - cugbp, elav-like family member 3  NSRP1 - nuclear speckle splicing regulatory protein 1  FXR2 - fragile x mental retardation, autosomal homolog 2  PTBP1 - polypyrimidine tract binding protein 1  SNRNP70 - small nuclear ribonucleoprotein 70kda (u1)  ZC3H14 - zinc finger ccch-type containing 14  SNRPA - small nuclear ribonucleoprotein polypeptide a  CDC73 - cell division cycle 73  NUP98 - nucleoporin 98kda  DDX17 - dead (asp-glu-ala-asp) box helicase 17  CPSF4 - cleavage and polyadenylation specific factor 4, 30kda  TRA2A - transformer 2 alpha homolog (drosophila)  RBM17 - rna binding motif protein 17  RBM15B - rna binding motif protein 15b  RBM10 - rna binding motif protein 10  CDK9 - cyclin-dependent kinase 9  RBM25 - rna binding motif protein 25  LEO1 - leo1, paf1/rna polymerase ii complex component, homolog (s. cerevisiae)  SF3B4 - splicing factor 3b, subunit 4, 49kda  RNF40 - ring finger protein 40, e3 ubiquitin protein ligase  ZBTB7A - zinc finger and btb domain containing 7a  SAFB - scaffold attachment factor b  HSPA8 - heat shock 70kda protein 8  SLTM - safb-like, transcription modulator  MAGOH - mago-nashi homolog, proliferation-associated (drosophila)  NELFE - negative elongation factor complex member e  PTBP3 - polypyrimidine tract binding protein 3  SRSF10 - serine/arginine-rich splicing factor 10  SUPT6H - suppressor of ty 6 homolog (s. cerevisiae)  NOVA2 - neuro-oncological ventral antigen 2  AHCYL1 - adenosylhomocysteinase-like 1  NOVA1 - neuro-oncological ventral antigen 1  SUPT5H - suppressor of ty 5 homolog (s. cerevisiae)  DAZAP1 - daz associated protein 1  SRSF9 - serine/arginine-rich splicing factor 9  DYRK1A - dual-specificity tyrosine-(y)-phosphorylation regulated kinase 1a  CPSF6 - cleavage and polyadenylation specific factor 6, 68kda  NUDT21 - nudix (nucleoside diphosphate linked moiety x)-type motif 21  PCBP4 - poly(rc) binding protein 4  ZC3H3 - zinc finger ccch-type containing 3  CPSF7 - cleavage and polyadenylation specific factor 7, 59kda  PAF1 - paf1, rna polymerase ii associated factor, homolog (s. cerevisiae)  SART3 - squamous cell carcinoma antigen recognized by t cells 3  SMU1 - smu-1 suppressor of mec-8 and unc-52 homolog (c. elegans)  PUF60 - poly-u binding splicing factor 60kda  IWS1 - iws1 homolog (s. cerevisiae)  CELF6 - cugbp, elav-like family member 6  YTHDC1 - yth domain containing 1  CCNT1 - cyclin t1  CTR9 - ctr9, paf1/rna polymerase ii complex component  HNRNPU - heterogeneous nuclear ribonucleoprotein u (scaffold attachment factor a)  HNRNPK - heterogeneous nuclear ribonucleoprotein k  HNRNPL - heterogeneous nuclear ribonucleoprotein l  RBFOX3 - rna binding protein, fox-1 homolog (c. elegans) 3  CELF1 - cugbp, elav-like family member 1  KHDRBS1 - kh domain containing, rna binding, signal transduction associated 1  RBM8A - rna binding motif protein 8a  RBM42 - rna binding motif protein 42  CELF2 - cugbp, elav-like family member 2  IK - ik cytokine, down-regulator of hla ii  RBFOX2 - rna binding protein, fox-1 homolog (c. elegans) 2  SF1 - splicing factor 1  SRPK2 - srsf protein kinase 2  RBM3 - rna binding motif (rnp1, rrm) protein 3  HNRNPA2B1 - heterogeneous nuclear ribonucleoprotein a2/b1  U2AF2 - u2 small nuclear rna auxiliary factor 2  SRPK1 - srsf protein kinase 1  SRSF1 - serine/arginine-rich splicing factor 1  SRSF2 - serine/arginine-rich splicing factor 2  HNRNPA1 - heterogeneous nuclear ribonucleoprotein a1  RBM4 - rna binding motif protein 4  SRSF3 - serine/arginine-rich splicing factor 3  SRSF4 - serine/arginine-rich splicing factor 4  SRSF5 - serine/arginine-rich splicing factor 5  SRSF6 - serine/arginine-rich splicing factor 6  SRSF7 - serine/arginine-rich splicing factor 7  SFSWAP - splicing factor, suppressor of white-apricot homolog (drosophila)  TRA2B - transformer 2 beta homolog (drosophila)  RNF20 - ring finger protein 20, e3 ubiquitin protein ligase  JMJD6 - jumonji domain containing 6  LARP7 - la ribonucleoprotein domain family, member 7  ZC3H10 - zinc finger ccch-type containing 10  CIRBP - cold inducible rna binding protein  TIA1 - tia1 cytotoxic granule-associated rna binding protein  SAFB2 - scaffold attachment factor b2  THRAP3 - thyroid hormone receptor associated protein 3  RBM4B - rna binding motif protein 4b |
| GO:0051168 | nuclear export | 2.26E-9 | 9.54E-7 | 1.32 (10334,139,6996,124) | [+] Show genes  DDX19B - dead (asp-glu-ala-asp) box polypeptide 19b  NCBP2 - nuclear cap binding protein subunit 2, 20kda  SRSF11 - serine/arginine-rich splicing factor 11  NCBP1 - nuclear cap binding protein subunit 1, 80kda  SDAD1 - sda1 domain containing 1  TSC1 - tuberous sclerosis 1  SEC13 - sec13 homolog (s. cerevisiae)  SYMPK - symplekin  DDX25 - dead (asp-glu-ala-asp) box helicase 25  DESI1 - desumoylating isopeptidase 1  RANBP3 - ran binding protein 3  ALKBH5 - alkb, alkylation repair homolog 5 (e. coli)  LZTS2 - leucine zipper, putative tumor suppressor 2  AGFG1 - arfgap with fg repeats 1  UPF1 - upf1 regulator of nonsense transcripts homolog (yeast)  XPOT - exportin, trna  CHP1 - calcineurin-like ef-hand protein 1  PHAX - phosphorylated adaptor for rna export  LTV1 - ltv1 homolog (s. cerevisiae)  CPSF3 - cleavage and polyadenylation specific factor 3, 73kda  EIF4E - eukaryotic translation initiation factor 4e  NOP9 - nop9 nucleolar protein  EGR2 - early growth response 2  ALYREF - aly/ref export factor  PABPN1 - poly(a) binding protein, nuclear 1  RNPS1 - rna binding protein s1, serine-rich domain  SMG6 - smg6 nonsense mediated mrna decay factor  STRADA - ste20-related kinase adaptor alpha  AKAP13 - a kinase (prka) anchor protein 13  NUTF2 - nuclear transport factor 2  SMG7 - smg7 nonsense mediated mrna decay factor  NUP160 - nucleoporin 160kda  FIP1L1 - factor interacting with papola and cpsf1  ABCE1 - atp-binding cassette, sub-family e (oabp), member 1  SEH1L - seh1-like (s. cerevisiae)  NUP88 - nucleoporin 88kda  EIF4A3 - eukaryotic translation initiation factor 4a3  NUP98 - nucleoporin 98kda  GLE1 - gle1 rna export mediator  CPSF4 - cleavage and polyadenylation specific factor 4, 30kda  U2AF1L4 - u2 small nuclear rna auxiliary factor 1-like 4  MCM3AP - minichromosome maintenance complex component 3 associated protein  CPSF2 - cleavage and polyadenylation specific factor 2, 100kda  CPSF1 - cleavage and polyadenylation specific factor 1, 160kda  DHX38 - deah (asp-glu-ala-his) box polypeptide 38  RBM15B - rna binding motif protein 15b  NUP85 - nucleoporin 85kda  EIF6 - eukaryotic translation initiation factor 6  SLU7 - slu7 splicing factor homolog (s. cerevisiae)  XPO5 - exportin 5  NMD3 - nmd3 ribosome export adaptor  NPM1 - nucleophosmin (nucleolar phosphoprotein b23, numatrin)  SMG1 - smg1 phosphatidylinositol 3-kinase-related kinase  SRRM1 - serine/arginine repetitive matrix 1  MAGOHB - mago-nashi homolog b (drosophila)  ENY2 - enhancer of yellow 2 homolog (drosophila)  XPO7 - exportin 7  DUSP16 - dual specificity phosphatase 16  NEMF - nuclear export mediator factor  POLR2D - polymerase (rna) ii (dna directed) polypeptide d  CDC40 - cell division cycle 40  TPR - translocated promoter region, nuclear basket protein  HSPA9 - heat shock 70kda protein 9 (mortalin)  DDX19A - dead (asp-glu-ala-asp) box polypeptide 19a  MAGOH - mago-nashi homolog, proliferation-associated (drosophila)  NUP133 - nucleoporin 133kda  XPO1 - exportin 1 (crm1 homolog, yeast)  NUP107 - nucleoporin 107kda  NUPL2 - nucleoporin like 2  AHCYL1 - adenosylhomocysteinase-like 1  NUP50 - nucleoporin 50kda  LSG1 - large 60s subunit nuclear export gtpase 1  SRSF9 - serine/arginine-rich splicing factor 9  THOC7 - tho complex 7 homolog (drosophila)  NUP43 - nucleoporin 43kda  PCID2 - pci domain containing 2  SLBP - stem-loop binding protein  CHTOP - chromatin target of prmt1  WDR33 - wd repeat domain 33  NOL6 - nucleolar protein 6 (rna-associated)  CALR - calreticulin  FYTTD1 - forty-two-three domain containing 1  UPF3B - upf3 regulator of nonsense transcripts homolog b (yeast)  DDX39B - dead (asp-glu-ala-asp) box polypeptide 39b  PKD1 - polycystic kidney disease 1 (autosomal dominant)  EIF4ENIF1 - eukaryotic translation initiation factor 4e nuclear import factor 1  NUP188 - nucleoporin 188kda  NUP205 - nucleoporin 205kda  RANBP2 - ran binding protein 2  POLDIP3 - polymerase (dna-directed), delta interacting protein 3  YTHDC1 - yth domain containing 1  THOC2 - tho complex 2  THOC6 - tho complex 6 homolog (drosophila)  AAAS - achalasia, adrenocortical insufficiency, alacrimia  RPS15 - ribosomal protein s15  NUP155 - nucleoporin 155kda  RBM8A - rna binding motif protein 8a  CASC3 - cancer susceptibility candidate 3  CSE1L - cse1 chromosome segregation 1-like (yeast)  U2AF2 - u2 small nuclear rna auxiliary factor 2  SRSF1 - serine/arginine-rich splicing factor 1  HNRNPA2B1 - heterogeneous nuclear ribonucleoprotein a2/b1  TGFB1 - transforming growth factor, beta 1  HNRNPA1 - heterogeneous nuclear ribonucleoprotein a1  SRSF2 - serine/arginine-rich splicing factor 2  SRSF3 - serine/arginine-rich splicing factor 3  SRSF4 - serine/arginine-rich splicing factor 4  RRS1 - rrs1 ribosome biogenesis regulator homolog (s. cerevisiae)  NUP93 - nucleoporin 93kda  SRSF5 - serine/arginine-rich splicing factor 5  SRSF6 - serine/arginine-rich splicing factor 6  RAE1 - rae1 rna export 1 homolog (s. pombe)  XPO6 - exportin 6  SRSF7 - serine/arginine-rich splicing factor 7  SMURF1 - smad specific e3 ubiquitin protein ligase 1  NUP35 - nucleoporin 35kda  SSB - sjogren syndrome antigen b (autoantigen la)  NDC1 - ndc1 transmembrane nucleoporin  THOC1 - tho complex 1  THOC3 - tho complex 3  NUP214 - nucleoporin 214kda  SARNP - sap domain containing ribonucleoprotein  NUP153 - nucleoporin 153kda  NUP210 - nucleoporin 210kda |
| GO:0030030 | cell projection organization | 2.33E-9 | 9.53E-7 | 1.16 (10334,551,6996,434) | [+] Show genes  AFG3L2 - afg3 atpase family member 3-like 2 (s. cerevisiae)  CEP89 - centrosomal protein 89kda  WDR19 - wd repeat domain 19  ABLIM1 - actin binding lim protein 1  ATAT1 - alpha tubulin acetyltransferase 1  CD2AP - cd2-associated protein  LRRC16A - leucine rich repeat containing 16a  SPTBN4 - spectrin, beta, non-erythrocytic 4  PPP1R9A - protein phosphatase 1, regulatory subunit 9a  PLXNB1 - plexin b1  ABL1 - c-abl oncogene 1, non-receptor tyrosine kinase  SSX2IP - synovial sarcoma, x breakpoint 2 interacting protein  SPAST - spastin  PMP22 - peripheral myelin protein 22  MKKS - mckusick-kaufman syndrome  KIF27 - kinesin family member 27  BRSK1 - br serine/threonine kinase 1  PTPDC1 - protein tyrosine phosphatase domain containing 1  ACTB - actin, beta  GJA1 - gap junction protein, alpha 1, 43kda  CREB1 - camp responsive element binding protein 1  DOCK10 - dedicator of cytokinesis 10  SNX2 - sorting nexin 2  EHD1 - eh-domain containing 1  SNX1 - sorting nexin 1  LLGL1 - lethal giant larvae homolog 1 (drosophila)  MARK4 - map/microtubule affinity-regulating kinase 4  CEP19 - centrosomal protein 19kda  PICALM - phosphatidylinositol binding clathrin assembly protein  RHOU - ras homolog family member u  FSCN1 - fascin homolog 1, actin-bundling protein (strongylocentrotus purpuratus)  RAB10 - rab10, member ras oncogene family  ADARB1 - adenosine deaminase, rna-specific, b1  BAG4 - bcl2-associated athanogene 4  TRAPPC4 - trafficking protein particle complex 4  CNR1 - cannabinoid receptor 1 (brain)  CNP - 2',3'-cyclic nucleotide 3' phosphodiesterase  ADCY1 - adenylate cyclase 1 (brain)  CNTN1 - contactin 1  NUP85 - nucleoporin 85kda  ACTL6B - actin-like 6b  ARFIP2 - adp-ribosylation factor interacting protein 2  ATCAY - ataxia, cerebellar, cayman type  STMN1 - stathmin 1  ATP6V1D - atpase, h+ transporting, lysosomal 34kda, v1 subunit d  L1CAM - l1 cell adhesion molecule  TAOK3 - tao kinase 3  LAMA5 - laminin, alpha 5  FBF1 - fas (tnfrsf6) binding factor 1  PLEKHA1 - pleckstrin homology domain containing, family a (phosphoinositide binding specific) member 1  ARHGEF6 - rac/cdc42 guanine nucleotide exchange factor (gef) 6  CBY1 - chibby homolog 1 (drosophila)  PICK1 - protein interacting with prkca 1  CTNNA1 - catenin (cadherin-associated protein), alpha 1, 102kda  KIF3A - kinesin family member 3a  PARVA - parvin, alpha  CTNND2 - catenin (cadherin-associated protein), delta 2  CTNNA2 - catenin (cadherin-associated protein), alpha 2  CCDC41 - coiled-coil domain containing 41  MAP4K4 - mitogen-activated protein kinase kinase kinase kinase 4  STX3 - syntaxin 3  AKT1 - v-akt murine thymoma viral oncogene homolog 1  MANF - mesencephalic astrocyte-derived neurotrophic factor  STRN - striatin, calmodulin binding protein  UNC119B - unc-119 homolog b (c. elegans)  TBC1D23 - tbc1 domain family, member 23  TRIOBP - trio and f-actin binding protein  TCTEX1D2 - tctex1 domain containing 2  TCTN2 - tectonic family member 2  FYN - fyn oncogene related to src, fgr, yes  IFT122 - intraflagellar transport 122 homolog (chlamydomonas)  TTC17 - tetratricopeptide repeat domain 17  KIT - v-kit hardy-zuckerman 4 feline sarcoma viral oncogene homolog  STMN2 - stathmin-like 2  CRKL - v-crk avian sarcoma virus ct10 oncogene homolog-like  CRK - v-crk avian sarcoma virus ct10 oncogene homolog  TMEM216 - transmembrane protein 216  TCHP - trichoplein, keratin filament binding  RAB35 - rab35, member ras oncogene family  IFT27 - intraflagellar transport 27 homolog (chlamydomonas)  PRNP - prion protein  GPRIN1 - g protein regulated inducer of neurite outgrowth 1  KCTD17 - potassium channel tetramerization domain containing 17  JAK2 - janus kinase 2  DFNB31 - deafness, autosomal recessive 31  IFT88 - intraflagellar transport 88 homolog (chlamydomonas)  CSF1R - colony stimulating factor 1 receptor  TXNDC15 - thioredoxin domain containing 15  BLOC1S5 - biogenesis of lysosomal organelles complex-1, subunit 5, muted  WDR11 - wd repeat domain 11  TROVE2 - trove domain family, member 2  GALNT11 - udp-n-acetyl-alpha-d-galactosamine:polypeptide n-acetylgalactosaminyltransferase 11 (galnac-t11)  MICALL2 - mical-like 2  ZNF335 - zinc finger protein 335  CCDC88A - coiled-coil domain containing 88a  SNAP29 - synaptosomal-associated protein, 29kda  ATXN10 - ataxin 10  PKN2 - protein kinase n2  CSNK1D - casein kinase 1, delta  TAOK2 - tao kinase 2  B4GALT6 - udp-gal:betaglcnac beta 1,4- galactosyltransferase, polypeptide 6  PLEKHO1 - pleckstrin homology domain containing, family o member 1  TMEM107 - transmembrane protein 107  B4GALT5 - udp-gal:betaglcnac beta 1,4- galactosyltransferase, polypeptide 5  S1PR2 - sphingosine-1-phosphate receptor 2  CCDC78 - coiled-coil domain containing 78  GSN - gelsolin  ATP8A2 - atpase, aminophospholipid transporter, class i, type 8a, member 2  SDCBP - syndecan binding protein (syntenin)  PDCL - phosducin-like  WWTR1 - ww domain containing transcription regulator 1  DCTN1 - dynactin 1  PTPN23 - protein tyrosine phosphatase, non-receptor type 23  WDR34 - wd repeat domain 34  PACSIN2 - protein kinase c and casein kinase substrate in neurons 2  FOPNL - fgfr1op n-terminal like  DBN1 - drebrin 1  APBB1 - amyloid beta (a4) precursor protein-binding, family b, member 1 (fe65)  NUMBL - numb homolog (drosophila)-like  UNC5A - unc-5 homolog a (c. elegans)  ARF1 - adp-ribosylation factor 1  MAP1S - microtubule-associated protein 1s  PAFAH1B1 - platelet-activating factor acetylhydrolase 1b, regulatory subunit 1 (45kda)  APOD - apolipoprotein d  RAB23 - rab23, member ras oncogene family  APP - amyloid beta (a4) precursor protein  BBS7 - bardet-biedl syndrome 7  PAK3 - p21 protein (cdc42/rac)-activated kinase 3  CEP250 - centrosomal protein 250kda  PDGFA - platelet-derived growth factor alpha polypeptide  TCTN1 - tectonic family member 1  ARL3 - adp-ribosylation factor-like 3  DNM3 - dynamin 3  ANKRD27 - ankyrin repeat domain 27 (vps9 domain)  AMIGO1 - adhesion molecule with ig-like domain 1  PDGFRB - platelet-derived growth factor receptor, beta polypeptide  IFT57 - intraflagellar transport 57 homolog (chlamydomonas)  TMEM231 - transmembrane protein 231  SS18L1 - synovial sarcoma translocation gene on chromosome 18-like 1  TBC1D24 - tbc1 domain family, member 24  MECP2 - methyl cpg binding protein 2 (rett syndrome)  ARID1B - at rich interactive domain 1b (swi1-like)  MEF2A - myocyte enhancer factor 2a  RHOA - ras homolog family member a  ODF2L - outer dense fiber of sperm tails 2-like  ARF6 - adp-ribosylation factor 6  DCLK1 - doublecortin-like kinase 1  RAB8A - rab8a, member ras oncogene family  WDR60 - wd repeat domain 60  S100B - s100 calcium binding protein b  CEP78 - centrosomal protein 78kda  C2CD3 - c2 calcium-dependent domain containing 3  TTC8 - tetratricopeptide repeat domain 8  PCM1 - pericentriolar material 1  CDK16 - cyclin-dependent kinase 16  KIDINS220 - kinase d-interacting substrate, 220kda  DAG1 - dystroglycan 1 (dystrophin-associated glycoprotein 1)  FAM161A - family with sequence similarity 161, member a  RABL2B - rab, member of ras oncogene family-like 2b  HDAC2 - histone deacetylase 2  ARL6 - adp-ribosylation factor-like 6  SH2B1 - sh2b adaptor protein 1  MICALL1 - mical-like 1  DRD2 - dopamine receptor d2  TCTN3 - tectonic family member 3  BTBD3 - btb (poz) domain containing 3  ATP1A3 - atpase, na+/k+ transporting, alpha 3 polypeptide  ZNF365 - zinc finger protein 365  TMEM138 - transmembrane protein 138  TRAF3IP1 - tnf receptor-associated factor 3 interacting protein 1  ATP1B2 - atpase, na+/k+ transporting, beta 2 polypeptide  TAOK1 - tao kinase 1  MAPT - microtubule-associated protein tau  SHANK3 - sh3 and multiple ankyrin repeat domains 3  MAP6 - microtubule-associated protein 6  LRRK2 - leucine-rich repeat kinase 2  BLOC1S4 - biogenesis of lysosomal organelles complex-1, subunit 4, cappuccino  MAP2 - microtubule-associated protein 2  MNS1 - meiosis-specific nuclear structural 1  ATP6V0D1 - atpase, h+ transporting, lysosomal 38kda, v0 subunit d1  RERE - arginine-glutamic acid dipeptide (re) repeats  MAP1B - microtubule-associated protein 1b  MAP1A - microtubule-associated protein 1a  RSG1 - rem2 and rab-like small gtpase 1  DZIP1 - daz interacting zinc finger protein 1  FAM161B - family with sequence similarity 161, member b  GPM6B - glycoprotein m6b  LIMA1 - lim domain and actin binding 1  NYAP1 - neuronal tyrosine-phosphorylated phosphoinositide-3-kinase adaptor 1  WEE1 - wee1 homolog (s. pombe)  ATP7A - atpase, cu++ transporting, alpha polypeptide  RILPL2 - rab interacting lysosomal protein-like 2  FRYL - fry-like  MATN2 - matrilin 2  TOR1A - torsin family 1, member a (torsin a)  LMTK2 - lemur tyrosine kinase 2  TANC1 - tetratricopeptide repeat, ankyrin repeat and coiled-coil containing 1  ICK - intestinal cell (mak-like) kinase  DOCK7 - dedicator of cytokinesis 7  TTBK2 - tau tubulin kinase 2  MCF2 - mcf.2 cell line derived transforming sequence  PKD2 - polycystic kidney disease 2 (autosomal dominant)  PITPNA - phosphatidylinositol transfer protein, alpha  DLG4 - discs, large homolog 4 (drosophila)  BLOC1S6 - biogenesis of lysosomal organelles complex-1, subunit 6, pallidin  PHGDH - phosphoglycerate dehydrogenase  SEMA5A - sema domain, seven thrombospondin repeats (type 1 and type 1-like), transmembrane domain (tm) and short cytoplasmic domain, (semaphorin) 5a  SCIN - scinderin  LYN - v-yes-1 yamaguchi sarcoma viral related oncogene homolog  JAM3 - junctional adhesion molecule 3  DHFR - dihydrofolate reductase  ZFYVE27 - zinc finger, fyve domain containing 27  TWF2 - twinfilin actin-binding protein 2  RAB34 - rab34, member ras oncogene family  DNM2 - dynamin 2  RTN4RL1 - reticulon 4 receptor-like 1  ARHGAP35 - rho gtpase activating protein 35  ALS2 - amyotrophic lateral sclerosis 2 (juvenile)  ZSWIM6 - zinc finger, swim-type containing 6  BCL2 - b-cell cll/lymphoma 2  SGK1 - serum/glucocorticoid regulated kinase 1  IFT172 - intraflagellar transport 172 homolog (chlamydomonas)  CCDC66 - coiled-coil domain containing 66  GSK3B - glycogen synthase kinase 3 beta  BBS4 - bardet-biedl syndrome 4  BBS2 - bardet-biedl syndrome 2  WASL - wiskott-aldrich syndrome-like  EMP1 - epithelial membrane protein 1  MARK2 - map/microtubule affinity-regulating kinase 2  NDN - necdin, melanoma antigen (mage) family member  TSC1 - tuberous sclerosis 1  CTTN - cortactin  NCK1 - nck adaptor protein 1  KIFAP3 - kinesin-associated protein 3  SAMD14 - sterile alpha motif domain containing 14  GORAB - golgin, rab6-interacting  IFT46 - intraflagellar transport 46 homolog (chlamydomonas)  MKS1 - meckel syndrome, type 1  NRP1 - neuropilin 1  PRMT1 - protein arginine methyltransferase 1  FBXO45 - f-box protein 45  RDX - radixin  ARL13B - adp-ribosylation factor-like 13b  WASF2 - was protein family, member 2  CCDC28B - coiled-coil domain containing 28b  NUBP1 - nucleotide binding protein 1  MTSS1L - metastasis suppressor 1-like  ALKBH1 - alkb, alkylation repair homolog 1 (e. coli)  HPRT1 - hypoxanthine phosphoribosyltransferase 1  TMEM17 - transmembrane protein 17  FNBP1L - formin binding protein 1-like  NDEL1 - nude neurodevelopment protein 1-like 1  SYNGAP1 - synaptic ras gtpase activating protein 1  TRIM32 - tripartite motif containing 32  MYLK - myosin light chain kinase  AZI1 - 5-azacytidine induced 1  BTBD8 - btb (poz) domain containing 8  BMP7 - bone morphogenetic protein 7  AKAP4 - a kinase (prka) anchor protein 4  ZNF259 - zinc finger protein 259  FLOT1 - flotillin 1  ARHGEF7 - rho guanine nucleotide exchange factor (gef) 7  MYH10 - myosin, heavy chain 10, non-muscle  MYH9 - myosin, heavy chain 9, non-muscle  ARPC5 - actin related protein 2/3 complex, subunit 5, 16kda  SNX10 - sorting nexin 10  TOP2B - topoisomerase (dna) ii beta 180kda  EGFR - epidermal growth factor receptor  MTMR2 - myotubularin related protein 2  MEGF8 - multiple egf-like-domains 8  UHMK1 - u2af homology motif (uhm) kinase 1  PQBP1 - polyglutamine binding protein 1  CELSR2 - cadherin, egf lag seven-pass g-type receptor 2  AHI1 - abelson helper integration site 1  TSPAN2 - tetraspanin 2  ACTR2 - arp2 actin-related protein 2 homolog (yeast)  ACTR3 - arp3 actin-related protein 3 homolog (yeast)  TMEM108 - transmembrane protein 108  ROCK1 - rho-associated, coiled-coil containing protein kinase 1  CLUAP1 - clusterin associated protein 1  NUBP2 - nucleotide binding protein 2  HERC1 - hect and rld domain containing e3 ubiquitin protein ligase family member 1  TRAK2 - trafficking protein, kinesin binding 2  TSPO - translocator protein (18kda)  TNIK - traf2 and nck interacting kinase  E2F4 - e2f transcription factor 4, p107/p130-binding  E2F5 - e2f transcription factor 5, p130-binding  HSP90AA1 - heat shock protein 90kda alpha (cytosolic), class a member 1  MYCBP2 - myc binding protein 2, e3 ubiquitin protein ligase  LPAR1 - lysophosphatidic acid receptor 1  S1PR1 - sphingosine-1-phosphate receptor 1  UBE4B - ubiquitination factor e4b  BLOC1S3 - biogenesis of lysosomal organelles complex-1, subunit 3  WASF1 - was protein family, member 1  VAV3 - vav 3 guanine nucleotide exchange factor  ABI1 - abl-interactor 1  NUMB - numb homolog (drosophila)  HMGB1 - high mobility group box 1  PTK2B - protein tyrosine kinase 2 beta  PIBF1 - progesterone immunomodulatory binding factor 1  DYNLL1 - dynein, light chain, lc8-type 1  CASP3 - caspase 3, apoptosis-related cysteine peptidase  BAIAP2 - bai1-associated protein 2  HDAC6 - histone deacetylase 6  EZR - ezrin  TMEM237 - transmembrane protein 237  SLC9A6 - solute carrier family 9, subfamily a (nhe6, cation proton antiporter 6), member 6  EPB41L3 - erythrocyte membrane protein band 4.1-like 3  DDR1 - discoidin domain receptor tyrosine kinase 1  WTIP - wilms tumor 1 interacting protein  VAV2 - vav 2 guanine nucleotide exchange factor  NCDN - neurochondrin  VCL - vinculin  SLC25A46 - solute carrier family 25, member 46  GLDN - gliomedin  MAPK8IP3 - mitogen-activated protein kinase 8 interacting protein 3  DBNL - drebrin-like  UBB - ubiquitin b  CORO1B - coronin, actin binding protein, 1b  IFT81 - intraflagellar transport 81 homolog (chlamydomonas)  CYFIP1 - cytoplasmic fmr1 interacting protein 1  DMTN - dematin actin binding protein  FAM206A - family with sequence similarity 206, member a  P2RY12 - purinergic receptor p2y, g-protein coupled, 12  RAB11A - rab11a, member ras oncogene family  SEC24B - sec24 family, member b (s. cerevisiae)  EPHB6 - eph receptor b6  CAMSAP2 - calmodulin regulated spectrin-associated protein family, member 2  B9D2 - b9 protein domain 2  STMN3 - stathmin-like 3  FLNA - filamin a, alpha  PSEN1 - presenilin 1  ARC - activity-regulated cytoskeleton-associated protein  SHANK1 - sh3 and multiple ankyrin repeat domains 1  ILK - integrin-linked kinase  CDC14B - cell division cycle 14b  EXOC5 - exocyst complex component 5  OCRL - oculocerebrorenal syndrome of lowe  ODF2 - outer dense fiber of sperm tails 2  DYNLT1 - dynein, light chain, tctex-type 1  GAS7 - growth arrest-specific 7  IFT43 - intraflagellar transport 43 homolog (chlamydomonas)  FGFR2 - fibroblast growth factor receptor 2  KIAA0586 - kiaa0586  PTEN - phosphatase and tensin homolog  ITGA1 - integrin, alpha 1  ITGA3 - integrin, alpha 3 (antigen cd49c, alpha 3 subunit of vla-3 receptor)  DAB2IP - dab2 interacting protein  SZT2 - seizure threshold 2 homolog (mouse)  NR4A2 - nuclear receptor subfamily 4, group a, member 2  FGD1 - fyve, rhogef and ph domain containing 1  CDK10 - cyclin-dependent kinase 10  ATL1 - atlastin gtpase 1  ITGB1 - integrin, beta 1 (fibronectin receptor, beta polypeptide, antigen cd29 includes mdf2, msk12)  PTK2 - protein tyrosine kinase 2  BBS9 - bardet-biedl syndrome 9  TEKT2 - tektin 2 (testicular)  CDK5 - cyclin-dependent kinase 5  INPPL1 - inositol polyphosphate phosphatase-like 1  PTN - pleiotrophin  NPHP1 - nephronophthisis 1 (juvenile)  INSR - insulin receptor  NUDCD3 - nudc domain containing 3  ZEB2 - zinc finger e-box binding homeobox 2  KLF7 - kruppel-like factor 7 (ubiquitous)  SLC11A2 - solute carrier family 11 (proton-coupled divalent metal ion transporter), member 2  PTPN11 - protein tyrosine phosphatase, non-receptor type 11  CNTN4 - contactin 4  CDH11 - cadherin 11, type 2, ob-cadherin (osteoblast)  NPTX1 - neuronal pentraxin i  FGD5 - fyve, rhogef and ph domain containing 5  SCLT1 - sodium channel and clathrin linker 1  IFT52 - intraflagellar transport 52 homolog (chlamydomonas)  ITGA6 - integrin, alpha 6  ARFGEF1 - adp-ribosylation factor guanine nucleotide-exchange factor 1 (brefeldin a-inhibited)  PTPRF - protein tyrosine phosphatase, receptor type, f  NRCAM - neuronal cell adhesion molecule  NCKAP1 - nck-associated protein 1  ARMC9 - armadillo repeat containing 9  CHRNB2 - cholinergic receptor, nicotinic, beta 2 (neuronal)  POC1B - poc1 centriolar protein b  TMEM106B - transmembrane protein 106b  LRP12 - low density lipoprotein receptor-related protein 12  MTOR - mechanistic target of rapamycin (serine/threonine kinase)  PTPRK - protein tyrosine phosphatase, receptor type, k  PTPRM - protein tyrosine phosphatase, receptor type, m  BLOC1S2 - biogenesis of lysosomal organelles complex-1, subunit 2  IFT74 - intraflagellar transport 74 homolog (chlamydomonas)  CEP290 - centrosomal protein 290kda  PACSIN1 - protein kinase c and casein kinase substrate in neurons 1  NPY - neuropeptide y  NOTCH1 - notch 1  PVRL2 - poliovirus receptor-related 2 (herpesvirus entry mediator b)  PIP5K1A - phosphatidylinositol-4-phosphate 5-kinase, type i, alpha  STK36 - serine/threonine kinase 36  RAB3A - rab3a, member ras oncogene family  WASF3 - was protein family, member 3  SDCCAG3 - serologically defined colon cancer antigen 3  IFT140 - intraflagellar transport 140 homolog (chlamydomonas)  RAC3 - ras-related c3 botulinum toxin substrate 3 (rho family, small gtp binding protein rac3)  RAC1 - ras-related c3 botulinum toxin substrate 1 (rho family, small gtp binding protein rac1)  NME5 - nme/nm23 family member 5  EHD2 - eh-domain containing 2  EHD3 - eh-domain containing 3  CCP110 - centriolar coiled coil protein 110kda  EHD4 - eh-domain containing 4  IQCB1 - iq motif containing b1  NEK3 - nima-related kinase 3  MINK1 - misshapen-like kinase 1  RSPH9 - radial spoke head 9 homolog (chlamydomonas)  NEFL - neurofilament, light polypeptide  NCK2 - nck adaptor protein 2  NEK1 - nima-related kinase 1  TMEM80 - transmembrane protein 80  WRAP73 - wd repeat containing, antisense to tp73  USP21 - ubiquitin specific peptidase 21  BBS5 - bardet-biedl syndrome 5  ULK1 - unc-51 like autophagy activating kinase 1  CAMSAP1 - calmodulin regulated spectrin-associated protein 1  GOLPH3 - golgi phosphoprotein 3 (coat-protein)  CEP41 - centrosomal protein 41kda  RAP2A - rap2a, member of ras oncogene family  PHACTR1 - phosphatase and actin regulator 1  CROCC - ciliary rootlet coiled-coil, rootletin  IGF1R - insulin-like growth factor 1 receptor  RBFOX2 - rna binding protein, fox-1 homolog (c. elegans) 2  RAPGEF2 - rap guanine nucleotide exchange factor (gef) 2  ARHGEF4 - rho guanine nucleotide exchange factor (gef) 4  MAPK8IP2 - mitogen-activated protein kinase 8 interacting protein 2  RB1 - retinoblastoma 1  CUL4B - cullin 4b  B9D1 - b9 protein domain 1  CUL3 - cullin 3  ASAP1 - arfgap with sh3 domain, ankyrin repeat and ph domain 1 |
| GO:0060271 | cilium assembly | 2.35E-9 | 9.35E-7 | 1.33 (10334,129,6996,116) | [+] Show genes  CEP89 - centrosomal protein 89kda  B9D2 - b9 protein domain 2  WDR19 - wd repeat domain 19  ABLIM1 - actin binding lim protein 1  GSN - gelsolin  ATAT1 - alpha tubulin acetyltransferase 1  GORAB - golgin, rab6-interacting  IFT46 - intraflagellar transport 46 homolog (chlamydomonas)  MKS1 - meckel syndrome, type 1  WWTR1 - ww domain containing transcription regulator 1  SSX2IP - synovial sarcoma, x breakpoint 2 interacting protein  DCTN1 - dynactin 1  FLNA - filamin a, alpha  PTPN23 - protein tyrosine phosphatase, non-receptor type 23  WDR34 - wd repeat domain 34  FOPNL - fgfr1op n-terminal like  ARL13B - adp-ribosylation factor-like 13b  CCDC28B - coiled-coil domain containing 28b  MKKS - mckusick-kaufman syndrome  KIF27 - kinesin family member 27  CDC14B - cell division cycle 14b  EXOC5 - exocyst complex component 5  TMEM17 - transmembrane protein 17  OCRL - oculocerebrorenal syndrome of lowe  FNBP1L - formin binding protein 1-like  PTPDC1 - protein tyrosine phosphatase domain containing 1  AZI1 - 5-azacytidine induced 1  RAB23 - rab23, member ras oncogene family  BBS7 - bardet-biedl syndrome 7  EHD1 - eh-domain containing 1  AKAP4 - a kinase (prka) anchor protein 4  IFT43 - intraflagellar transport 43 homolog (chlamydomonas)  CEP250 - centrosomal protein 250kda  TCTN1 - tectonic family member 1  CEP19 - centrosomal protein 19kda  ARL3 - adp-ribosylation factor-like 3  KIAA0586 - kiaa0586  IFT57 - intraflagellar transport 57 homolog (chlamydomonas)  TMEM231 - transmembrane protein 231  SNX10 - sorting nexin 10  CELSR2 - cadherin, egf lag seven-pass g-type receptor 2  AHI1 - abelson helper integration site 1  ACTR2 - arp2 actin-related protein 2 homolog (yeast)  ACTR3 - arp3 actin-related protein 3 homolog (yeast)  BBS9 - bardet-biedl syndrome 9  TEKT2 - tektin 2 (testicular)  WDR60 - wd repeat domain 60  RAB8A - rab8a, member ras oncogene family  CLUAP1 - clusterin associated protein 1  C2CD3 - c2 calcium-dependent domain containing 3  TTC8 - tetratricopeptide repeat domain 8  PCM1 - pericentriolar material 1  NUDCD3 - nudc domain containing 3  FAM161A - family with sequence similarity 161, member a  E2F4 - e2f transcription factor 4, p107/p130-binding  RABL2B - rab, member of ras oncogene family-like 2b  ATP6V1D - atpase, h+ transporting, lysosomal 34kda, v1 subunit d  ARL6 - adp-ribosylation factor-like 6  SCLT1 - sodium channel and clathrin linker 1  LAMA5 - laminin, alpha 5  IFT52 - intraflagellar transport 52 homolog (chlamydomonas)  FBF1 - fas (tnfrsf6) binding factor 1  ARMC9 - armadillo repeat containing 9  POC1B - poc1 centriolar protein b  TCTN3 - tectonic family member 3  TMEM138 - transmembrane protein 138  TRAF3IP1 - tnf receptor-associated factor 3 interacting protein 1  IFT74 - intraflagellar transport 74 homolog (chlamydomonas)  KIF3A - kinesin family member 3a  PARVA - parvin, alpha  PIBF1 - progesterone immunomodulatory binding factor 1  DYNLL1 - dynein, light chain, lc8-type 1  ATP6V0D1 - atpase, h+ transporting, lysosomal 38kda, v0 subunit d1  CEP290 - centrosomal protein 290kda  RSG1 - rem2 and rab-like small gtpase 1  DZIP1 - daz interacting zinc finger protein 1  CCDC41 - coiled-coil domain containing 41  NOTCH1 - notch 1  HDAC6 - histone deacetylase 6  STK36 - serine/threonine kinase 36  RILPL2 - rab interacting lysosomal protein-like 2  TMEM237 - transmembrane protein 237  UNC119B - unc-119 homolog b (c. elegans)  ICK - intestinal cell (mak-like) kinase  IFT140 - intraflagellar transport 140 homolog (chlamydomonas)  TTBK2 - tau tubulin kinase 2  TCTEX1D2 - tctex1 domain containing 2  TCTN2 - tectonic family member 2  IFT122 - intraflagellar transport 122 homolog (chlamydomonas)  NME5 - nme/nm23 family member 5  EHD2 - eh-domain containing 2  EHD3 - eh-domain containing 3  EHD4 - eh-domain containing 4  IQCB1 - iq motif containing b1  TMEM216 - transmembrane protein 216  RSPH9 - radial spoke head 9 homolog (chlamydomonas)  NEK1 - nima-related kinase 1  TMEM80 - transmembrane protein 80  IFT81 - intraflagellar transport 81 homolog (chlamydomonas)  BBS5 - bardet-biedl syndrome 5  IFT88 - intraflagellar transport 88 homolog (chlamydomonas)  CEP41 - centrosomal protein 41kda  TXNDC15 - thioredoxin domain containing 15  WDR11 - wd repeat domain 11  TROVE2 - trove domain family, member 2  GALNT11 - udp-n-acetyl-alpha-d-galactosamine:polypeptide n-acetylgalactosaminyltransferase 11 (galnac-t11)  SNAP29 - synaptosomal-associated protein, 29kda  ATXN10 - ataxin 10  CSNK1D - casein kinase 1, delta  B9D1 - b9 protein domain 1  IFT172 - intraflagellar transport 172 homolog (chlamydomonas)  CCDC66 - coiled-coil domain containing 66  ASAP1 - arfgap with sh3 domain, ankyrin repeat and ph domain 1  BBS4 - bardet-biedl syndrome 4  BBS2 - bardet-biedl syndrome 2  TMEM107 - transmembrane protein 107 |
| GO:0031123 | RNA 3'-end processing | 2.67E-9 | 1.03E-6 | 1.35 (10334,108,6996,99) | [+] Show genes  MTPAP - mitochondrial poly(a) polymerase  NCBP2 - nuclear cap binding protein subunit 2, 20kda  EXOSC9 - exosome component 9  SRSF11 - serine/arginine-rich splicing factor 11  NCBP1 - nuclear cap binding protein subunit 1, 80kda  EXOSC10 - exosome component 10  SYMPK - symplekin  PARN - poly(a)-specific ribonuclease  INTS5 - integrator complex subunit 5  ZCCHC6 - zinc finger, cchc domain containing 6  RPRD2 - regulation of nuclear pre-mrna domain containing 2  USB1 - u6 snrna biogenesis 1  CPSF3 - cleavage and polyadenylation specific factor 3, 73kda  ALYREF - aly/ref export factor  RPRD1A - regulation of nuclear pre-mrna domain containing 1a  PAPOLA - poly(a) polymerase alpha  FBLL1 - fibrillarin-like 1  PABPN1 - poly(a) binding protein, nuclear 1  RNPS1 - rna binding protein s1, serine-rich domain  ZNF473 - zinc finger protein 473  EXOSC3 - exosome component 3  APP - amyloid beta (a4) precursor protein  CSTF2T - cleavage stimulation factor, 3' pre-rna, subunit 2, 64kda, tau variant  FIP1L1 - factor interacting with papola and cpsf1  RPRD1B - regulation of nuclear pre-mrna domain containing 1b  EXOSC7 - exosome component 7  PAPD4 - pap associated domain containing 4  ERI1 - exoribonuclease 1  CDC73 - cell division cycle 73  EIF4A3 - eukaryotic translation initiation factor 4a3  CPSF4 - cleavage and polyadenylation specific factor 4, 30kda  U2AF1L4 - u2 small nuclear rna auxiliary factor 1-like 4  CPSF1 - cleavage and polyadenylation specific factor 1, 160kda  CPSF2 - cleavage and polyadenylation specific factor 2, 100kda  DHX38 - deah (asp-glu-ala-his) box polypeptide 38  ERI3 - eri1 exoribonuclease family member 3  ZCCHC11 - zinc finger, cchc domain containing 11  SLU7 - slu7 splicing factor homolog (s. cerevisiae)  INTS2 - integrator complex subunit 2  SRRM1 - serine/arginine repetitive matrix 1  MAGOHB - mago-nashi homolog b (drosophila)  LEO1 - leo1, paf1/rna polymerase ii complex component, homolog (s. cerevisiae)  TRNT1 - trna nucleotidyl transferase, cca-adding, 1  TOE1 - target of egr1, member 1 (nuclear)  CDC40 - cell division cycle 40  CSTF1 - cleavage stimulation factor, 3' pre-rna, subunit 1, 50kda  MAGOH - mago-nashi homolog, proliferation-associated (drosophila)  PAPD5 - pap associated domain containing 5  INTS12 - integrator complex subunit 12  CSTF3 - cleavage stimulation factor, 3' pre-rna, subunit 3, 77kda  CSTF2 - cleavage stimulation factor, 3' pre-rna, subunit 2, 64kda  SUPV3L1 - suppressor of var1, 3-like 1 (s. cerevisiae)  AHCYL1 - adenosylhomocysteinase-like 1  EXOSC2 - exosome component 2  INTS6 - integrator complex subunit 6  SRSF9 - serine/arginine-rich splicing factor 9  THOC7 - tho complex 7 homolog (drosophila)  SLBP - stem-loop binding protein  NUDT21 - nudix (nucleoside diphosphate linked moiety x)-type motif 21  CHTOP - chromatin target of prmt1  WDR33 - wd repeat domain 33  ZC3H3 - zinc finger ccch-type containing 3  CPSF7 - cleavage and polyadenylation specific factor 7, 59kda  PAF1 - paf1, rna polymerase ii associated factor, homolog (s. cerevisiae)  UPF3B - upf3 regulator of nonsense transcripts homolog b (yeast)  DDX39B - dead (asp-glu-ala-asp) box polypeptide 39b  SCAF8 - sr-related ctd-associated factor 8  PAPOLG - poly(a) polymerase gamma  DKC1 - dyskeratosis congenita 1, dyskerin  POLDIP3 - polymerase (dna-directed), delta interacting protein 3  PNPT1 - polyribonucleotide nucleotidyltransferase 1  THOC2 - tho complex 2  THOC6 - tho complex 6 homolog (drosophila)  PABPC1 - poly(a) binding protein, cytoplasmic 1  PAPD7 - pap associated domain containing 7  RBM8A - rna binding motif protein 8a  INTS8 - integrator complex subunit 8  CASC3 - cancer susceptibility candidate 3  EXOSC8 - exosome component 8  PCF11 - pcf11 cleavage and polyadenylation factor subunit  U2AF2 - u2 small nuclear rna auxiliary factor 2  SRSF1 - serine/arginine-rich splicing factor 1  SRSF2 - serine/arginine-rich splicing factor 2  SRSF3 - serine/arginine-rich splicing factor 3  SRSF4 - serine/arginine-rich splicing factor 4  SRSF5 - serine/arginine-rich splicing factor 5  SRSF6 - serine/arginine-rich splicing factor 6  SRSF7 - serine/arginine-rich splicing factor 7  CLP1 - cleavage and polyadenylation factor i subunit 1  SSB - sjogren syndrome antigen b (autoantigen la)  LARP7 - la ribonucleoprotein domain family, member 7  THOC1 - tho complex 1  THOC3 - tho complex 3  EXOSC4 - exosome component 4  TUT1 - terminal uridylyl transferase 1, u6 snrna-specific  SARNP - sap domain containing ribonucleoprotein  TRMT10C - trna methyltransferase 10 homolog c (s. cerevisiae)  GRSF1 - g-rich rna sequence binding factor 1  INTS1 - integrator complex subunit 1 |
| GO:0050794 | regulation of cellular process | 2.73E-9 | 1.03E-6 | 1.03 (10334,6081,6996,4254) | [+] Show genes  AGAP2 - arfgap with gtpase domain, ankyrin repeat and ph domain 2  MSL3 - male-specific lethal 3 homolog (drosophila)  AGAP3 - arfgap with gtpase domain, ankyrin repeat and ph domain 3  TADA1 - transcriptional adaptor 1  AAMP - angio-associated, migratory cell protein  AARS - alanyl-trna synthetase  HNRNPA0 - heterogeneous nuclear ribonucleoprotein a0  BTG3 - btg family, member 3  CBX1 - chromobox homolog 1  ABCA1 - atp-binding cassette, sub-family a (abc1), member 1  ABCA2 - atp-binding cassette, sub-family a (abc1), member 2  OS9 - osteosarcoma amplified 9, endoplasmic reticulum lectin  SERINC3 - serine incorporator 3  ABL1 - c-abl oncogene 1, non-receptor tyrosine kinase  SSX2IP - synovial sarcoma, x breakpoint 2 interacting protein  ERP29 - endoplasmic reticulum protein 29  ABR - active bcr-related  MLLT11 - myeloid/lymphoid or mixed-lineage leukemia (trithorax homolog, drosophila); translocated to, 11  ACACA - acetyl-coa carboxylase alpha  RAB40B - rab40b, member ras oncogene family  YWHAQ - tyrosine 3-monooxygenase/tryptophan 5-monooxygenase activation protein, theta polypeptide  LURAP1 - leucine rich adaptor protein 1  TMED10 - transmembrane emp24-like trafficking protein 10 (yeast)  BRD8 - bromodomain containing 8  ACLY - atp citrate lyase  ACHE - acetylcholinesterase  RFFL - ring finger and fyve-like domain containing e3 ubiquitin protein ligase  PAPOLA - poly(a) polymerase alpha  ZNF354B - zinc finger protein 354b  TCERG1 - transcription elongation regulator 1  ACTB - actin, beta  RNPS1 - rna binding protein s1, serine-rich domain  EHMT2 - euchromatic histone-lysine n-methyltransferase 2  SPIN1 - spindlin 1  SUB1 - sub1 homolog (s. cerevisiae)  ACTG1 - actin, gamma 1  MORF4L1 - mortality factor 4 like 1  RALBP1 - rala binding protein 1  EHD1 - eh-domain containing 1  ZNF511 - zinc finger protein 511  ACTL6A - actin-like 6a  ACTN1 - actinin, alpha 1  USP20 - ubiquitin specific peptidase 20  USP19 - ubiquitin specific peptidase 19  ACTN4 - actinin, alpha 4  ACVR2A - activin a receptor, type iia  ACVRL1 - activin a receptor type ii-like 1  FGL2 - fibrinogen-like 2  FAM122A - family with sequence similarity 122a  ACVR1 - activin a receptor, type i  ACVR1B - activin a receptor, type ib  ADAR - adenosine deaminase, rna-specific  RAB10 - rab10, member ras oncogene family  ADAM10 - adam metallopeptidase domain 10  LYPD1 - ly6/plaur domain containing 1  ADARB1 - adenosine deaminase, rna-specific, b1  ADCY6 - adenylate cyclase 6  CPSF4 - cleavage and polyadenylation specific factor 4, 30kda  ADCY5 - adenylate cyclase 5  RUNDC3A - run domain containing 3a  ADCY1 - adenylate cyclase 1 (brain)  ADCY3 - adenylate cyclase 3  RAB39B - rab39b, member ras oncogene family  ADD2 - adducin 2 (beta)  ADD3 - adducin 3 (gamma)  ADCYAP1R1 - adenylate cyclase activating polypeptide 1 (pituitary) receptor type i  ADD1 - adducin 1 (alpha)  FAM129A - family with sequence similarity 129, member a  PARP1 - poly (adp-ribose) polymerase 1  ADPRH - adp-ribosylarginine hydrolase  PPP1R13L - protein phosphatase 1, regulatory subunit 13 like  CNTROB - centrobin, centrosomal brca2 interacting protein  SNAP47 - synaptosomal-associated protein, 47kda  ACAP3 - arfgap with coiled-coil, ankyrin repeat and ph domains 3  RUVBL2 - ruvb-like 2 (e. coli)  PARP4 - poly (adp-ribose) polymerase family, member 4  AP2A2 - adaptor-related protein complex 2, alpha 2 subunit  AP2A1 - adaptor-related protein complex 2, alpha 1 subunit  AP2B1 - adaptor-related protein complex 2, beta 1 subunit  AP1G1 - adaptor-related protein complex 1, gamma 1 subunit  AES - amino-terminal enhancer of split  ADRBK2 - adrenergic, beta, receptor kinase 2  ADRBK1 - adrenergic, beta, receptor kinase 1  NUPL2 - nucleoporin like 2  FAM46B - family with sequence similarity 46, member b  JAG1 - jagged 1  ZWINT - zw10 interacting kinetochore protein  PRDM4 - pr domain containing 4  CIT - citron (rho-interacting, serine/threonine kinase 21)  FGFR1OP - fgfr1 oncogene partner  ZNF689 - zinc finger protein 689  UHRF2 - ubiquitin-like with phd and ring finger domains 2, e3 ubiquitin protein ligase  ARHGEF25 - rho guanine nucleotide exchange factor (gef) 25  RAB3C - rab3c, member ras oncogene family  CNTRL - centriolin  WDFY2 - wd repeat and fyve domain containing 2  AKT1 - v-akt murine thymoma viral oncogene homolog 1  ZNF653 - zinc finger protein 653  OGFR - opioid growth factor receptor  CPSF6 - cleavage and polyadenylation specific factor 6, 68kda  EVI5L - ecotropic viral integration site 5-like  NUDT21 - nudix (nucleoside diphosphate linked moiety x)-type motif 21  WWP2 - ww domain containing e3 ubiquitin protein ligase 2  AHR - aryl hydrocarbon receptor  ABHD2 - abhydrolase domain containing 2  ZNF526 - zinc finger protein 526  TRIOBP - trio and f-actin binding protein  FOXP4 - forkhead box p4  DIDO1 - death inducer-obliterator 1  DUSP14 - dual specificity phosphatase 14  TOPBP1 - topoisomerase (dna) ii binding protein 1  RAPGEF4 - rap guanine nucleotide exchange factor (gef) 4  RMI2 - recq mediated genome instability 2  TMEM115 - transmembrane protein 115  ALCAM - activated leukocyte cell adhesion molecule  STMN2 - stathmin-like 2  ESCO1 - establishment of sister chromatid cohesion n-acetyltransferase 1  AKR1B1 - aldo-keto reductase family 1, member b1 (aldose reductase)  RAB35 - rab35, member ras oncogene family  IFT27 - intraflagellar transport 27 homolog (chlamydomonas)  FMNL2 - formin-like 2  ATF7 - activating transcription factor 7  AMFR - autocrine motility factor receptor, e3 ubiquitin protein ligase  RHPN1 - rhophilin, rho gtpase binding protein 1  PAPD7 - pap associated domain containing 7  PIM2 - pim-2 oncogene  RAB31 - rab31, member ras oncogene family  RNF157 - ring finger protein 157  DSTN - destrin (actin depolymerizing factor)  METAP2 - methionyl aminopeptidase 2  GCN1L1 - gcn1 general control of amino-acid synthesis 1-like 1 (yeast)  CISD2 - cdgsh iron sulfur domain 2  CCNI - cyclin i  MAPRE2 - microtubule-associated protein, rp/eb family, member 2  OSBPL7 - oxysterol binding protein-like 7  OSBPL8 - oxysterol binding protein-like 8  ADIRF - adipogenesis regulatory factor  HAUS1 - haus augmin-like complex, subunit 1  ANXA3 - annexin a3  TLK2 - tousled-like kinase 2  ANXA2 - annexin a2  CCDC85B - coiled-coil domain containing 85b  VASN - vasorin  SLC25A5 - solute carrier family 25 (mitochondrial carrier; adenine nucleotide translocator), member 5  APEX1 - apex nuclease (multifunctional dna repair enzyme) 1  ZNF605 - zinc finger protein 605  DUSP12 - dual specificity phosphatase 12  CMTM3 - cklf-like marvel transmembrane domain containing 3  XIAP - x-linked inhibitor of apoptosis  ATXN2L - ataxin 2-like  BIRC3 - baculoviral iap repeat containing 3  NOXO1 - nadph oxidase organizer 1  BIRC2 - baculoviral iap repeat containing 2  KLF12 - kruppel-like factor 12  TREX1 - three prime repair exonuclease 1  APLP2 - amyloid beta (a4) precursor-like protein 2  APLP1 - amyloid beta (a4) precursor-like protein 1  NXPH4 - neurexophilin 4  ANXA5 - annexin a5  PACSIN2 - protein kinase c and casein kinase substrate in neurons 2  DCTN3 - dynactin 3 (p22)  APBA1 - amyloid beta (a4) precursor protein-binding, family a, member 1  DCUN1D3 - dcn1, defective in cullin neddylation 1, domain containing 3  APBB2 - amyloid beta (a4) precursor protein-binding, family b, member 2  APC - adenomatous polyposis coli  APBB1 - amyloid beta (a4) precursor protein-binding, family b, member 1 (fe65)  CHP1 - calcineurin-like ef-hand protein 1  POLG2 - polymerase (dna directed), gamma 2, accessory subunit  RNF139 - ring finger protein 139  PDCD10 - programmed cell death 10  ARF1 - adp-ribosylation factor 1  GPR176 - g protein-coupled receptor 176  ARF4 - adp-ribosylation factor 4  ZHX1 - zinc fingers and homeoboxes 1  APOD - apolipoprotein d  IRAK3 - interleukin-1 receptor-associated kinase 3  AKAP13 - a kinase (prka) anchor protein 13  AKAP11 - a kinase (prka) anchor protein 11  APP - amyloid beta (a4) precursor protein  AKAP10 - a kinase (prka) anchor protein 10  DDX20 - dead (asp-glu-ala-asp) box polypeptide 20  CANT1 - calcium activated nucleotidase 1  CEP250 - centrosomal protein 250kda  ARNTL - aryl hydrocarbon receptor nuclear translocator-like  CELF3 - cugbp, elav-like family member 3  ARNT - aryl hydrocarbon receptor nuclear translocator  ARL3 - adp-ribosylation factor-like 3  NISCH - nischarin  ARL2 - adp-ribosylation factor-like 2  MAP4K5 - mitogen-activated protein kinase kinase kinase kinase 5  ARHGDIG - rho gdp dissociation inhibitor (gdi) gamma  SUPT16H - suppressor of ty 16 homolog (s. cerevisiae)  ARSB - arylsulfatase b  ARRB2 - arrestin, beta 2  ARRB1 - arrestin, beta 1  RHOB - ras homolog family member b  RHOC - ras homolog family member c  ARG2 - arginase 2  FAM107A - family with sequence similarity 107, member a  STRAP - serine/threonine kinase receptor associated protein  RHOA - ras homolog family member a  ARF6 - adp-ribosylation factor 6  PSIP1 - pc4 and sfrs1 interacting protein 1  FSTL1 - follistatin-like 1  ARHGDIA - rho gdp dissociation inhibitor (gdi) alpha  SLC2A6 - solute carrier family 2 (facilitated glucose transporter), member 6  ZNF277 - zinc finger protein 277  ARHGAP5 - rho gtpase activating protein 5  WDR6 - wd repeat domain 6  ARHGAP1 - rho gtpase activating protein 1  RND3 - rho family gtpase 3  BAZ2A - bromodomain adjacent to zinc finger domain, 2a  ASNS - asparagine synthetase (glutamine-hydrolyzing)  ASNA1 - arsa arsenite transporter, atp-binding, homolog 1 (bacterial)  JDP2 - jun dimerization protein 2  FICD - fic domain containing  NUDT4 - nudix (nucleoside diphosphate linked moiety x)-type motif 4  LEO1 - leo1, paf1/rna polymerase ii complex component, homolog (s. cerevisiae)  ASPH - aspartate beta-hydroxylase  ERLIN2 - er lipid raft associated 2  FRMD6 - ferm domain containing 6  PWP1 - pwp1 homolog (s. cerevisiae)  TBC1D8 - tbc1 domain family, member 8 (with gram domain)  SOCS4 - suppressor of cytokine signaling 4  CDC37 - cell division cycle 37  CAPN10 - calpain 10  KPTN - kaptin (actin binding protein)  PLA2G16 - phospholipase a2, group xvi  GLMN - glomulin, fkbp associated protein  PKIG - protein kinase (camp-dependent, catalytic) inhibitor gamma  KAT7 - k(lysine) acetyltransferase 7  ASCL1 - achaete-scute complex homolog 1 (drosophila)  DENND3 - denn/madd domain containing 3  SBNO2 - strawberry notch homolog 2 (drosophila)  ATP2B1 - atpase, ca++ transporting, plasma membrane 1  EPN2 - epsin 2  RUFY3 - run and fyve domain containing 3  ATP2A3 - atpase, ca++ transporting, ubiquitous  ZBTB1 - zinc finger and btb domain containing 1  ZNF365 - zinc finger protein 365  ATP1A1 - atpase, na+/k+ transporting, alpha 1 polypeptide  ATP1B2 - atpase, na+/k+ transporting, beta 2 polypeptide  ATP1B3 - atpase, na+/k+ transporting, beta 3 polypeptide  BAHD1 - bromo adjacent homology domain containing 1  ATP1B1 - atpase, na+/k+ transporting, beta 1 polypeptide  DIS3 - dis3 mitotic control homolog (s. cerevisiae)  ZHX2 - zinc fingers and homeoboxes 2  ANKRD6 - ankyrin repeat domain 6  MORC2 - morc family cw-type zinc finger 2  LRRK2 - leucine-rich repeat kinase 2  FOXJ3 - forkhead box j3  RERE - arginine-glutamic acid dipeptide (re) repeats  CLSTN1 - calsyntenin 1  DNAJC24 - dnaj (hsp40) homolog, subfamily c, member 24  DZIP1 - daz interacting zinc finger protein 1  SEC31A - sec31 homolog a (s. cerevisiae)  ZFHX3 - zinc finger homeobox 3  SPPL3 - signal peptide peptidase like 3  ATP6V1E1 - atpase, h+ transporting, lysosomal 31kda, v1 subunit e1  ATG14 - autophagy related 14  ATP6V0B - atpase, h+ transporting, lysosomal 21kda, v0 subunit b  ATP6V0A1 - atpase, h+ transporting, lysosomal v0 subunit a1  ATP6AP1 - atpase, h+ transporting, lysosomal accessory protein 1  CNKSR2 - connector enhancer of kinase suppressor of ras 2  ATP7A - atpase, cu++ transporting, alpha polypeptide  PPP6R1 - protein phosphatase 6, regulatory subunit 1  ADNP2 - adnp homeobox 2  LMTK2 - lemur tyrosine kinase 2  AEBP2 - ae binding protein 2  ATP6V1A - atpase, h+ transporting, lysosomal 70kda, v1 subunit a  CHSY1 - chondroitin sulfate synthase 1  ATP6V1B2 - atpase, h+ transporting, lysosomal 56/58kda, v1 subunit b2  ICK - intestinal cell (mak-like) kinase  ATP6V0C - atpase, h+ transporting, lysosomal 16kda, v0 subunit c  ATP6V1C1 - atpase, h+ transporting, lysosomal 42kda, v1 subunit c1  PPM1E - protein phosphatase, mg2+/mn2+ dependent, 1e  RAB11FIP2 - rab11 family interacting protein 2 (class i)  ZNF507 - zinc finger protein 507  VASH1 - vasohibin 1  AAK1 - ap2 associated kinase 1  ATP2B4 - atpase, ca++ transporting, plasma membrane 4  ZNF652 - zinc finger protein 652  DLGAP4 - discs, large (drosophila) homolog-associated protein 4  SCAF8 - sr-related ctd-associated factor 8  BAX - bcl2-associated x protein  PUF60 - poly-u binding splicing factor 60kda  MTF2 - metal response element binding transcription factor 2  BAK1 - bcl2-antagonist/killer 1  RASA3 - ras p21 protein activator 3  PHLDA1 - pleckstrin homology-like domain, family a, member 1  BAG1 - bcl2-associated athanogene  BACH1 - btb and cnc homology 1, basic leucine zipper transcription factor 1  BAD - bcl2-associated agonist of cell death  PDZD8 - pdz domain containing 8  MRAS - muscle ras oncogene homolog  AXL - axl receptor tyrosine kinase  ZFYVE27 - zinc finger, fyve domain containing 27  KIF1A - kinesin family member 1a  ATRX - alpha thalassemia/mental retardation syndrome x-linked  PEF1 - penta-ef-hand domain containing 1  CASC3 - cancer susceptibility candidate 3  GABARAPL2 - gaba(a) receptor-associated protein-like 2  TWF2 - twinfilin actin-binding protein 2  EXOSC8 - exosome component 8  RNF13 - ring finger protein 13  GABARAP - gaba(a) receptor-associated protein  U2AF2 - u2 small nuclear rna auxiliary factor 2  PDAP1 - pdgfa associated protein 1  TUSC2 - tumor suppressor candidate 2  BCR - breakpoint cluster region  STK38 - serine/threonine kinase 38  BCL7A - b-cell cll/lymphoma 7a  BCL9 - b-cell cll/lymphoma 9  DDX42 - dead (asp-glu-ala-asp) box helicase 42  BCL2 - b-cell cll/lymphoma 2  BCL2L2 - bcl2-like 2  BCL2L1 - bcl2-like 1  ECD - ecdysoneless homolog (drosophila)  BCHE - butyrylcholinesterase  CCND1 - cyclin d1  PARK7 - parkinson protein 7  B4GALT7 - xylosylprotein beta 1,4-galactosyltransferase, polypeptide 7  BBS4 - bardet-biedl syndrome 4  PNKP - polynucleotide kinase 3'-phosphatase  BBS2 - bardet-biedl syndrome 2  RGS19 - regulator of g-protein signaling 19  TBC1D20 - tbc1 domain family, member 20  DST - dystonin  BNIP3L - bcl2/adenovirus e1b 19kda interacting protein 3-like  BNIP1 - bcl2/adenovirus e1b 19kda interacting protein 1  SAP18 - sin3a-associated protein, 18kda  BNIP2 - bcl2/adenovirus e1b 19kda interacting protein 2  TSHZ2 - teashirt zinc finger homeobox 2  MAEA - macrophage erythroblast attacher  DNAJA2 - dnaj (hsp40) homolog, subfamily a, member 2  BRAF - v-raf murine sarcoma viral oncogene homolog b  EIF1B - eukaryotic translation initiation factor 1b  APBB3 - amyloid beta (a4) precursor protein-binding, family b, member 3  KATNB1 - katanin p80 (wd repeat containing) subunit b 1  ZFP36L2 - zfp36 ring finger protein-like 2  PAK4 - p21 protein (cdc42/rac)-activated kinase 4  ZFP36L1 - zfp36 ring finger protein-like 1  LANCL1 - lanc lantibiotic synthetase component c-like 1 (bacterial)  DUSP15 - dual specificity phosphatase 15  RRAGB - ras-related gtp binding b  CNPY2 - canopy fgf signaling regulator 2  DRAM2 - dna-damage regulated autophagy modulator 2  TFG - trk-fused gene  BMP1 - bone morphogenetic protein 1  BMI1 - bmi1 polycomb ring finger oncogene  VSTM2L - v-set and transmembrane domain containing 2 like  PCGF3 - polycomb group ring finger 3  BMPR1A - bone morphogenetic protein receptor, type ia  BMPR2 - bone morphogenetic protein receptor, type ii (serine/threonine kinase)  BMPR1B - bone morphogenetic protein receptor, type ib  BMP7 - bone morphogenetic protein 7  ABCA7 - atp-binding cassette, sub-family a (abc1), member 7  FITM2 - fat storage-inducing transmembrane protein 2  PSMD14 - proteasome (prosome, macropain) 26s subunit, non-atpase, 14  FLOT1 - flotillin 1  RNF19B - ring finger protein 19b  CTDSPL - ctd (carboxy-terminal domain, rna polymerase ii, polypeptide a) small phosphatase-like  DAGLA - diacylglycerol lipase, alpha  LDLRAD4 - low density lipoprotein receptor class a domain containing 4  PTTG1IP - pituitary tumor-transforming 1 interacting protein  FMNL1 - formin-like 1  COQ7 - coenzyme q7 homolog, ubiquinone (yeast)  CA2 - carbonic anhydrase ii  CA8 - carbonic anhydrase viii  UHMK1 - u2af homology motif (uhm) kinase 1  HNRNPR - heterogeneous nuclear ribonucleoprotein r  CACNA1C - calcium channel, voltage-dependent, l type, alpha 1c subunit  CALCOCO2 - calcium binding and coiled-coil domain 2  TSPO - translocator protein (18kda)  SPRY1 - sprouty homolog 1, antagonist of fgf signaling (drosophila)  SPRY2 - sprouty homolog 2 (drosophila)  STAM2 - signal transducing adaptor molecule (sh3 domain and itam motif) 2  DENND4A - denn/madd domain containing 4a  CDK2AP2 - cyclin-dependent kinase 2 associated protein 2  SF3B4 - splicing factor 3b, subunit 4, 49kda  RAMP2 - receptor (g protein-coupled) activity modifying protein 2  TPRG1L - tumor protein p63 regulated 1-like  RAMP3 - receptor (g protein-coupled) activity modifying protein 3  RAMP1 - receptor (g protein-coupled) activity modifying protein 1  CAPN5 - calpain 5  AKAP8 - a kinase (prka) anchor protein 8  ZMPSTE24 - zinc metallopeptidase ste24  STUB1 - stip1 homology and u-box containing protein 1, e3 ubiquitin protein ligase  C5AR1 - complement component 5a receptor 1  EFS - embryonal fyn-associated substrate  UBE4B - ubiquitination factor e4b  OSGIN2 - oxidative stress induced growth inhibitor family member 2  CCSAP - centriole, cilia and spindle-associated protein  CAPZA1 - capping protein (actin filament) muscle z-line, alpha 1  TAB1 - tgf-beta activated kinase 1/map3k7 binding protein 1  CAPS - calcyphosine  LRRC24 - leucine rich repeat containing 24  VAV3 - vav 3 guanine nucleotide exchange factor  PPIE - peptidylprolyl isomerase e (cyclophilin e)  CAPNS1 - calpain, small subunit 1  CAPN2 - calpain 2, (m/ii) large subunit  FAM3C - family with sequence similarity 3, member c  LRRN2 - leucine rich repeat neuronal 2  CAPN1 - calpain 1, (mu/i) large subunit  MCRS1 - microspherule protein 1  CANX - calnexin  ZER1 - zyg-11 related, cell cycle regulator  TIMM17A - translocase of inner mitochondrial membrane 17 homolog a (yeast)  CAMLG - calcium modulating ligand  OLFM1 - olfactomedin 1  C1D - c1d nuclear receptor corepressor  CAMK2B - calcium/calmodulin-dependent protein kinase ii beta  CASP8 - caspase 8, apoptosis-related cysteine peptidase  CASP9 - caspase 9, apoptosis-related cysteine peptidase  PIBF1 - progesterone immunomodulatory binding factor 1  SLC30A9 - solute carrier family 30 (zinc transporter), member 9  CASP2 - caspase 2, apoptosis-related cysteine peptidase  CASP3 - caspase 3, apoptosis-related cysteine peptidase  BAIAP2 - bai1-associated protein 2  MAD2L2 - mad2 mitotic arrest deficient-like 2 (yeast)  CAPZB - capping protein (actin filament) muscle z-line, beta  CAP1 - cap, adenylate cyclase-associated protein 1 (yeast)  ZNF787 - zinc finger protein 787  NRARP - notch-regulated ankyrin repeat protein  CACNB3 - calcium channel, voltage-dependent, beta 3 subunit  SLC9A6 - solute carrier family 9, subfamily a (nhe6, cation proton antiporter 6), member 6  CACNB2 - calcium channel, voltage-dependent, beta 2 subunit  TRIM38 - tripartite motif containing 38  DDR1 - discoidin domain receptor tyrosine kinase 1  TADA3 - transcriptional adaptor 3  SEMA6B - sema domain, transmembrane domain (tm), and cytoplasmic domain, (semaphorin) 6b  CALR - calreticulin  SEMA4B - sema domain, immunoglobulin domain (ig), transmembrane domain (tm) and short cytoplasmic domain, (semaphorin) 4b  ZNF792 - zinc finger protein 792  UNC13B - unc-13 homolog b (c. elegans)  CALM2 - calmodulin 2 (phosphorylase kinase, delta)  WTIP - wilms tumor 1 interacting protein  NCOA2 - nuclear receptor coactivator 2  SYNCRIP - synaptotagmin binding, cytoplasmic rna interacting protein  VAT1 - vesicle amine transport protein 1 homolog (t. californica)  STK25 - serine/threonine kinase 25  CREB3 - camp responsive element binding protein 3  CRTAP - cartilage associated protein  MFSD12 - major facilitator superfamily domain containing 12  CITED2 - cbp/p300-interacting transactivator, with glu/asp-rich carboxy-terminal domain, 2  CCT6A - chaperonin containing tcp1, subunit 6a (zeta 1)  CCNT2 - cyclin t2  MICU1 - mitochondrial calcium uptake 1  CCND3 - cyclin d3  CCNT1 - cyclin t1  CCNH - cyclin h  HMG20A - high mobility group 20a  CCNG2 - cyclin g2  ANAPC10 - anaphase promoting complex subunit 10  CORO2B - coronin, actin binding protein, 2b  ATP8A1 - atpase, aminophospholipid transporter (aplt), class i, type 8a, member 1  NDRG1 - n-myc downstream regulated 1  DLC1 - deleted in liver cancer 1  CD9 - cd9 molecule  TUBB4B - tubulin, beta 4b class ivb  TUBB3 - tubulin, beta 3 class iii  CD247 - cd247 molecule  CYB5D2 - cytochrome b5 domain containing 2  CBFA2T3 - core-binding factor, runt domain, alpha subunit 2; translocated to, 3  CBFB - core-binding factor, beta subunit  RAPGEF3 - rap guanine nucleotide exchange factor (gef) 3  CBL - cbl proto-oncogene, e3 ubiquitin protein ligase  SERPINH1 - serpin peptidase inhibitor, clade h (heat shock protein 47), member 1, (collagen binding protein 1)  MYL9 - myosin, light chain 9, regulatory  CAV2 - caveolin 2  PIAS3 - protein inhibitor of activated stat, 3  CAV1 - caveolin 1, caveolae protein, 22kda  SEZ6 - seizure related 6 homolog (mouse)  ANKRD13B - ankyrin repeat domain 13b  SEC24B - sec24 family, member b (s. cerevisiae)  KRIT1 - krit1, ankyrin repeat containing  CFDP1 - craniofacial development protein 1  CCNC - cyclin c  RBM14 - rna binding motif protein 14  CCND2 - cyclin d2  SPON1 - spondin 1, extracellular matrix protein  PRMT5 - protein arginine methyltransferase 5  CD2BP2 - cd2 (cytoplasmic tail) binding protein 2  CDK11B - cyclin-dependent kinase 11b  CDC5L - cell division cycle 5-like  MXD4 - max dimerization protein 4  STAMBP - stam binding protein  POLR3F - polymerase (rna) iii (dna directed) polypeptide f, 39 kda  CDH3 - cadherin 3, type 1, p-cadherin (placental)  CDH2 - cadherin 2, type 1, n-cadherin (neuronal)  HEXIM1 - hexamethylene bis-acetamide inducible 1  ERLIN1 - er lipid raft associated 1  CDC27 - cell division cycle 27  STXBP5 - syntaxin binding protein 5 (tomosyn)  NCOA7 - nuclear receptor coactivator 7  POMT1 - protein-o-mannosyltransferase 1  CD151 - cd151 molecule (raph blood group)  USP16 - ubiquitin specific peptidase 16  CDC42EP3 - cdc42 effector protein (rho gtpase binding) 3  SRSF12 - serine/arginine-rich splicing factor 12  CD81 - cd81 molecule  CAMKK2 - calcium/calmodulin-dependent protein kinase kinase 2, beta  STARD4 - star-related lipid transfer (start) domain containing 4  WDR36 - wd repeat domain 36  SCARB2 - scavenger receptor class b, member 2  NPRL2 - nitrogen permease regulator-like 2 (s. cerevisiae)  KHDRBS3 - kh domain containing, rna binding, signal transduction associated 3  CD53 - cd53 molecule  RIPPLY2 - ripply transcriptional repressor 2  IRAK1BP1 - interleukin-1 receptor-associated kinase 1 binding protein 1  CD47 - cd47 molecule  SPINT2 - serine peptidase inhibitor, kunitz type, 2  CD44 - cd44 molecule (indian blood group)  FAM173B - family with sequence similarity 173, member b  TAF6L - taf6-like rna polymerase ii, p300/cbp-associated factor (pcaf)-associated factor, 65kda  POLR3G - polymerase (rna) iii (dna directed) polypeptide g (32kd)  CD34 - cd34 molecule  GAS2L1 - growth arrest-specific 2 like 1  ATG7 - autophagy related 7  LEPREL2 - leprecan-like 2  PRRC1 - proline-rich coiled-coil 1  DCTN2 - dynactin 2 (p50)  CETN2 - centrin, ef-hand protein, 2  LAMTOR5 - late endosomal/lysosomal adaptor, mapk and mtor activator 5  JMY - junction mediating and regulatory protein, p53 cofactor  ANP32B - acidic (leucine-rich) nuclear phosphoprotein 32 family, member b  PRDX4 - peroxiredoxin 4  CFL1 - cofilin 1 (non-muscle)  MYBBP1A - myb binding protein (p160) 1a  FBLN5 - fibulin 5  CIB1 - calcium and integrin binding 1 (calmyrin)  DDX17 - dead (asp-glu-ala-asp) box helicase 17  CLGN - calmegin  CHERP - calcium homeostasis endoplasmic reticulum protein  KAT5 - k(lysine) acetyltransferase 5  HYOU1 - hypoxia up-regulated 1  IPO8 - importin 8  IPO7 - importin 7  CEBPG - ccaat/enhancer binding protein (c/ebp), gamma  CDK5 - cyclin-dependent kinase 5  CDK4 - cyclin-dependent kinase 4  CCT7 - chaperonin containing tcp1, subunit 7 (eta)  CDK9 - cyclin-dependent kinase 9  CDK8 - cyclin-dependent kinase 8  LIN54 - lin-54 homolog (c. elegans)  CDK7 - cyclin-dependent kinase 7  NPC2 - niemann-pick disease, type c2  CDKN1C - cyclin-dependent kinase inhibitor 1c (p57, kip2)  CCT2 - chaperonin containing tcp1, subunit 2 (beta)  CDKN1B - cyclin-dependent kinase inhibitor 1b (p27, kip1)  CCT4 - chaperonin containing tcp1, subunit 4 (delta)  IFITM2 - interferon induced transmembrane protein 2  CDKN2D - cyclin-dependent kinase inhibitor 2d (p19, inhibits cdk4)  CDH6 - cadherin 6, type 2, k-cadherin (fetal kidney)  ARPC1A - actin related protein 2/3 complex, subunit 1a, 41kda  HTATIP2 - hiv-1 tat interactive protein 2, 30kda  ARL6IP5 - adp-ribosylation-like factor 6 interacting protein 5  CDH11 - cadherin 11, type 2, ob-cadherin (osteoblast)  CDH8 - cadherin 8, type 2  AGPAT1 - 1-acylglycerol-3-phosphate o-acyltransferase 1  SPTLC1 - serine palmitoyltransferase, long chain base subunit 1  ARFGEF2 - adp-ribosylation factor guanine nucleotide-exchange factor 2 (brefeldin a-inhibited)  ARFGEF1 - adp-ribosylation factor guanine nucleotide-exchange factor 1 (brefeldin a-inhibited)  CDK2 - cyclin-dependent kinase 2  NCKAP1 - nck-associated protein 1  CHRNB2 - cholinergic receptor, nicotinic, beta 2 (neuronal)  NUDT16 - nudix (nucleoside diphosphate linked moiety x)-type motif 16  ZMYND11 - zinc finger, mynd-type containing 11  SRSF10 - serine/arginine-rich splicing factor 10  DCBLD2 - discoidin, cub and lccl domain containing 2  PLK2 - polo-like kinase 2  AHCYL1 - adenosylhomocysteinase-like 1  CHML - choroideremia-like (rab escort protein 2)  NUP50 - nucleoporin 50kda  CHM - choroideremia (rab escort protein 1)  KDM5B - lysine (k)-specific demethylase 5b  CHN1 - chimerin 1  CHL1 - cell adhesion molecule l1-like  GIPC1 - gipc pdz domain containing family, member 1  CHGA - chromogranin a (parathyroid secretory protein 1)  CHGB - chromogranin b (secretogranin 1)  CHD4 - chromodomain helicase dna binding protein 4  CHD2 - chromodomain helicase dna binding protein 2  CHD3 - chromodomain helicase dna binding protein 3  CHD1 - chromodomain helicase dna binding protein 1  MTERFD2 - mterf domain containing 2  FRS2 - fibroblast growth factor receptor substrate 2  CPLX2 - complexin 2  WASF3 - was protein family, member 3  NOXA1 - nadph oxidase activator 1  HSPH1 - heat shock 105kda/110kda protein 1  SDCCAG3 - serologically defined colon cancer antigen 3  SEC24A - sec24 family, member a (s. cerevisiae)  TMEM198 - transmembrane protein 198  ZNF268 - zinc finger protein 268  ZNF513 - zinc finger protein 513  PNMA2 - paraneoplastic ma antigen 2  GMEB1 - glucocorticoid modulatory element binding protein 1  CCT8 - chaperonin containing tcp1, subunit 8 (theta)  CNPY3 - canopy fgf signaling regulator 3  CLTC - clathrin, heavy chain (hc)  GNB5 - guanine nucleotide binding protein (g protein), beta 5  CGREF1 - cell growth regulator with ef-hand domain 1  CGRRF1 - cell growth regulator with ring finger domain 1  CLPTM1 - cleft lip and palate associated transmembrane protein 1  RRAGA - ras-related gtp binding a  GNA13 - guanine nucleotide binding protein (g protein), alpha 13  CLIC1 - chloride intracellular channel 1  CELF1 - cugbp, elav-like family member 1  CLU - clusterin  KHDRBS1 - kh domain containing, rna binding, signal transduction associated 1  CLK1 - cdc-like kinase 1  TAF8 - taf8 rna polymerase ii, tata box binding protein (tbp)-associated factor, 43kda  CELF2 - cugbp, elav-like family member 2  CLK2 - cdc-like kinase 2  TPP1 - tripeptidyl peptidase i  CLK3 - cdc-like kinase 3  CTCF - ccctc-binding factor (zinc finger protein)  RAI1 - retinoic acid induced 1  RBBP9 - retinoblastoma binding protein 9  MAP3K2 - mitogen-activated protein kinase kinase kinase 2  CLCN6 - chloride channel, voltage-sensitive 6  DIS3L2 - dis3 mitotic control homolog (s. cerevisiae)-like 2  TMEM150A - transmembrane protein 150a  AP2M1 - adaptor-related protein complex 2, mu 1 subunit  ANKRD54 - ankyrin repeat domain 54  NUP35 - nucleoporin 35kda  ERCC8 - excision repair cross-complementing rodent repair deficiency, complementation group 8  YME1L1 - yme1-like 1 atpase  PTGES3 - prostaglandin e synthase 3 (cytosolic)  CKS1B - cdc28 protein kinase regulatory subunit 1b  NFAT5 - nuclear factor of activated t-cells 5, tonicity-responsive  CHMP4B - charged multivesicular body protein 4b  CIRBP - cold inducible rna binding protein  PIGU - phosphatidylinositol glycan anchor biosynthesis, class u  CHUK - conserved helix-loop-helix ubiquitous kinase  CERS1 - ceramide synthase 1  ZMYND8 - zinc finger, mynd-type containing 8  NFATC2IP - nuclear factor of activated t-cells, cytoplasmic, calcineurin-dependent 2 interacting protein  TBRG1 - transforming growth factor beta regulator 1  ATAD1 - atpase family, aaa domain containing 1  CD2AP - cd2-associated protein  FBXO18 - f-box protein, helicase, 18  PATZ1 - poz (btb) and at hook containing zinc finger 1  CORO1C - coronin, actin binding protein, 1c  SSBP2 - single-stranded dna binding protein 2  SPRYD3 - spry domain containing 3  KPNA6 - karyopherin alpha 6 (importin alpha 7)  FIZ1 - flt3-interacting zinc finger 1  COX11 - cytochrome c oxidase assembly homolog 11 (yeast)  CIRH1A - cirrhosis, autosomal recessive 1a (cirhin)  COX7A2 - cytochrome c oxidase subunit viia polypeptide 2 (liver)  FAM89B - family with sequence similarity 89, member b  PPP1R15B - protein phosphatase 1, regulatory subunit 15b  ZNF382 - zinc finger protein 382  IQSEC3 - iq motif and sec7 domain 3  BACE1 - beta-site app-cleaving enzyme 1  ZNF705A - zinc finger protein 705a  CPT1A - carnitine palmitoyltransferase 1a (liver)  WDR73 - wd repeat domain 73  ZNF346 - zinc finger protein 346  ARL2BP - adp-ribosylation factor-like 2 binding protein  DDAH2 - dimethylarginine dimethylaminohydrolase 2  ZFYVE19 - zinc finger, fyve domain containing 19  CPE - carboxypeptidase e  RAB2B - rab2b, member ras oncogene family  GTPBP4 - gtp binding protein 4  WBP2 - ww domain binding protein 2  ORC3 - origin recognition complex, subunit 3  FBXL20 - f-box and leucine-rich repeat protein 20  ORC6 - origin recognition complex, subunit 6  HEBP2 - heme binding protein 2  LEMD3 - lem domain containing 3  CREBL2 - camp responsive element binding protein-like 2  ATF6B - activating transcription factor 6 beta  CREBBP - creb binding protein  ATF2 - activating transcription factor 2  CGNL1 - cingulin-like 1  CREB1 - camp responsive element binding protein 1  DDX58 - dead (asp-glu-ala-asp) box polypeptide 58  CRABP2 - cellular retinoic acid binding protein 2  CRABP1 - cellular retinoic acid binding protein 1  CCNDBP1 - cyclin d-type binding-protein 1  CADM1 - cell adhesion molecule 1  PPP1R16A - protein phosphatase 1, regulatory subunit 16a  PLK3 - polo-like kinase 3  CNN1 - calponin 1, basic, smooth muscle  KCNE4 - potassium voltage-gated channel, isk-related family, member 4  RERG - ras-like, estrogen-regulated, growth inhibitor  EID1 - ep300 interacting inhibitor of differentiation 1  RBM17 - rna binding motif protein 17  CNN2 - calponin 2  CNR1 - cannabinoid receptor 1 (brain)  FRRS1L - ferric-chelate reductase 1-like  CNTN1 - contactin 1  CNTFR - ciliary neurotrophic factor receptor  ARFIP2 - adp-ribosylation factor interacting protein 2  SSBP3 - single stranded dna binding protein 3  ATCAY - ataxia, cerebellar, cayman type  COL4A2 - collagen, type iv, alpha 2  HSPBP1 - hspa (heat shock 70kda) binding protein, cytoplasmic cochaperone 1  COL5A2 - collagen, type v, alpha 2  SGK3 - serum/glucocorticoid regulated kinase family, member 3  SH3BP4 - sh3-domain binding protein 4  PRKD3 - protein kinase d3  COPA - coatomer protein complex, subunit alpha  RAB38 - rab38, member ras oncogene family  PPIL4 - peptidylprolyl isomerase (cyclophilin)-like 4  COL16A1 - collagen, type xvi, alpha 1  TMEFF2 - transmembrane protein with egf-like and two follistatin-like domains 2  PANX1 - pannexin 1  TFIP11 - tuftelin interacting protein 11  CSTB - cystatin b (stefin b)  CST3 - cystatin c  EML2 - echinoderm microtubule associated protein like 2  NAT6 - n-acetyltransferase 6 (gcn5-related)  SHC2 - shc (src homology 2 domain containing) transforming protein 2  HYPK - huntingtin interacting protein k  ZNF318 - zinc finger protein 318  PRPF6 - pre-mrna processing factor 6  KDM2B - lysine (k)-specific demethylase 2b  SLC9A7 - solute carrier family 9, subfamily a (nhe7, cation proton antiporter 7), member 7  CTGF - connective tissue growth factor  CBY1 - chibby homolog 1 (drosophila)  SUN2 - sad1 and unc84 domain containing 2  DSTYK - dual serine/threonine and tyrosine protein kinase  CTNNA1 - catenin (cadherin-associated protein), alpha 1, 102kda  SLC24A2 - solute carrier family 24 (sodium/potassium/calcium exchanger), member 2  CTBP1 - c-terminal binding protein 1  CTBP2 - c-terminal binding protein 2  CTNND1 - catenin (cadherin-associated protein), delta 1  CTNND2 - catenin (cadherin-associated protein), delta 2  BEX2 - brain expressed x-linked 2  RASGRP3 - ras guanyl releasing protein 3 (calcium and dag-regulated)  CTNNA2 - catenin (cadherin-associated protein), alpha 2  RAB3GAP2 - rab3 gtpase activating protein subunit 2 (non-catalytic)  CTNNB1 - catenin (cadherin-associated protein), beta 1, 88kda  CTSD - cathepsin d  CTSB - cathepsin b  MGARP - mitochondria-localized glutamic acid-rich protein  CUX1 - cut-like homeobox 1  USP30 - ubiquitin specific peptidase 30  FLRT2 - fibronectin leucine rich transmembrane protein 2  CTSS - cathepsin s  IL17RA - interleukin 17 receptor a  CTSV - cathepsin v  MPV17L2 - mpv17 mitochondrial membrane protein-like 2  BRD1 - bromodomain containing 1  PCGF1 - polycomb group ring finger 1  FKBP8 - fk506 binding protein 8, 38kda  CXADR - coxsackie virus and adenovirus receptor  IFIT5 - interferon-induced protein with tetratricopeptide repeats 5  MTCH1 - mitochondrial carrier 1  SUV420H2 - suppressor of variegation 4-20 homolog 2 (drosophila)  CYLD - cylindromatosis (turban tumor syndrome)  TXN2 - thioredoxin 2  CRKL - v-crk avian sarcoma virus ct10 oncogene homolog-like  CRK - v-crk avian sarcoma virus ct10 oncogene homolog  AGPAT9 - 1-acylglycerol-3-phosphate o-acyltransferase 9  CRMP1 - collapsin response mediator protein 1  NIPBL - nipped-b homolog (drosophila)  CRY1 - cryptochrome 1 (photolyase-like)  RAB26 - rab26, member ras oncogene family  CRY2 - cryptochrome 2 (photolyase-like)  ASF1A - anti-silencing function 1a histone chaperone  PARM1 - prostate androgen-regulated mucin-like protein 1  BRMS1 - breast cancer metastasis suppressor 1  ZNF496 - zinc finger protein 496  CRYM - crystallin, mu  PHF5A - phd finger protein 5a  CSF1R - colony stimulating factor 1 receptor  CSF1 - colony stimulating factor 1 (macrophage)  FBXO7 - f-box protein 7  KLHL22 - kelch-like family member 22  BAMBI - bmp and activin membrane-bound inhibitor  CSK - c-src tyrosine kinase  HAVCR2 - hepatitis a virus cellular receptor 2  CSNK1G2 - casein kinase 1, gamma 2  ZC3H10 - zinc finger ccch-type containing 10  CSNK1A1 - casein kinase 1, alpha 1  CSNK1D - casein kinase 1, delta  CSNK2A2 - casein kinase 2, alpha prime polypeptide  TNFAIP8 - tumor necrosis factor, alpha-induced protein 8  CSNK1G3 - casein kinase 1, gamma 3  CSNK2A1 - casein kinase 2, alpha 1 polypeptide  DNAJB5 - dnaj (hsp40) homolog, subfamily b, member 5  ARIH1 - ariadne homolog, ubiquitin-conjugating enzyme e2 binding protein, 1 (drosophila)  FBXL2 - f-box and leucine-rich repeat protein 2  PRDX5 - peroxiredoxin 5  BACE2 - beta-site app-cleaving enzyme 2  DHX9 - deah (asp-glu-ala-his) box helicase 9  KANK2 - kn motif and ankyrin repeat domains 2  DDX6 - dead (asp-glu-ala-asp) box helicase 6  DDX5 - dead (asp-glu-ala-asp) box helicase 5  DDX3X - dead (asp-glu-ala-asp) box helicase 3, x-linked  PNKD - paroxysmal nonkinesigenic dyskinesia  DDX1 - dead (asp-glu-ala-asp) box helicase 1  SYF2 - syf2 pre-mrna-splicing factor  KBTBD2 - kelch repeat and btb (poz) domain containing 2  SIN3A - sin3 transcription regulator family member a  DPH6 - diphthamine biosynthesis 6  MIDN - midnolin  FAM98A - family with sequence similarity 98, member a  SAMHD1 - sam domain and hd domain 1  WWTR1 - ww domain containing transcription regulator 1  DDB1 - damage-specific dna binding protein 1, 127kda  RHOT2 - ras homolog family member t2  DCTN1 - dynactin 1  PTPN23 - protein tyrosine phosphatase, non-receptor type 23  CLIC4 - chloride intracellular channel 4  GEMIN5 - gem (nuclear organelle) associated protein 5  NOL11 - nucleolar protein 11  CNRIP1 - cannabinoid receptor interacting protein 1  MYRIP - myosin viia and rab interacting protein  DBN1 - drebrin 1  ATL3 - atlastin gtpase 3  DBP - d site of albumin promoter (albumin d-box) binding protein  NELFB - negative elongation factor complex member b  POT1 - protection of telomeres 1  AHCTF1 - at hook containing transcription factor 1  CNOT10 - ccr4-not transcription complex, subunit 10  KLHL13 - kelch-like family member 13  COCH - cochlin  RNF19A - ring finger protein 19a, e3 ubiquitin protein ligase  UNC5A - unc-5 homolog a (c. elegans)  RCHY1 - ring finger and chy zinc finger domain containing 1, e3 ubiquitin protein ligase  DFFA - dna fragmentation factor, 45kda, alpha polypeptide  ZNF551 - zinc finger protein 551  ZNF473 - zinc finger protein 473  LTN1 - listerin e3 ubiquitin protein ligase 1  DNM3 - dynamin 3  SIPA1L1 - signal-induced proliferation-associated 1 like 1  ZNF451 - zinc finger protein 451  ZNF622 - zinc finger protein 622  TMEM150C - transmembrane protein 150c  SS18L1 - synovial sarcoma translocation gene on chromosome 18-like 1  CHD5 - chromodomain helicase dna binding protein 5  TMEM98 - transmembrane protein 98  MED30 - mediator complex subunit 30  KNSTRN - kinetochore-localized astrin/spag5 binding protein  NSMF - nmda receptor synaptonuclear signaling and neuronal migration factor  DCAF15 - ddb1 and cul4 associated factor 15  PYGO2 - pygopus homolog 2 (drosophila)  DAPK3 - death-associated protein kinase 3  ANGEL2 - angel homolog 2 (drosophila)  DAXX - death-domain associated protein  BRINP1 - bone morphogenetic protein/retinoic acid inducible neural-specific 1  C2CD3 - c2 calcium-dependent domain containing 3  ZZZ3 - zinc finger, zz-type containing 3  HINFP - histone h4 transcription factor  DGKA - diacylglycerol kinase, alpha 80kda  DAG1 - dystroglycan 1 (dystrophin-associated glycoprotein 1)  LRSAM1 - leucine rich repeat and sterile alpha motif containing 1  ULK3 - unc-51 like kinase 3  DGKB - diacylglycerol kinase, beta 90kda  DGKQ - diacylglycerol kinase, theta 110kda  DAPK1 - death-associated protein kinase 1  CLIP3 - cap-gly domain containing linker protein 3  DAP - death-associated protein  IBTK - inhibitor of bruton agammaglobulinemia tyrosine kinase  TIPARP - tcdd-inducible poly(adp-ribose) polymerase  CYP51A1 - cytochrome p450, family 51, subfamily a, polypeptide 1  STARD13 - star-related lipid transfer (start) domain containing 13  CHMP2B - charged multivesicular body protein 2b  DAD1 - defender against cell death 1  NGDN - neuroguidin, eif4e binding protein  SH2B1 - sh2b adaptor protein 1  SERBP1 - serpine1 mrna binding protein 1  GAPVD1 - gtpase activating protein and vps9 domains 1  DRD2 - dopamine receptor d2  EPC2 - enhancer of polycomb homolog 2 (drosophila)  TCTN3 - tectonic family member 3  PHF19 - phd finger protein 19  NKD2 - naked cuticle homolog 2 (drosophila)  IRF2BP1 - interferon regulatory factor 2 binding protein 1  TRAF3IP1 - tnf receptor-associated factor 3 interacting protein 1  EAF1 - ell associated factor 1  ATN1 - atrophin 1  SYDE1 - synapse defective 1, rho gtpase, homolog 1 (c. elegans)  DPH2 - dph2 homolog (s. cerevisiae)  SHANK3 - sh3 and multiple ankyrin repeat domains 3  DPH1 - diphthamide biosynthesis 1  DOM3Z - dom-3 homolog z (c. elegans)  DOK1 - docking protein 1, 62kda (downstream of tyrosine kinase 1)  C10orf137 - chromosome 10 open reading frame 137  STRIP1 - striatin interacting protein 1  DR1 - down-regulator of transcription 1, tbp-binding (negative cofactor 2)  DPYSL3 - dihydropyrimidinase-like 3  WSB1 - wd repeat and socs box containing 1  TANC2 - tetratricopeptide repeat, ankyrin repeat and coiled-coil containing 2  DPYSL2 - dihydropyrimidinase-like 2  ZCCHC3 - zinc finger, cchc domain containing 3  DUSP8 - dual specificity phosphatase 8  TNKS1BP1 - tankyrase 1 binding protein 1, 182kda  DIXDC1 - dix domain containing 1  GPSM1 - g-protein signaling modulator 1  DUSP5 - dual specificity phosphatase 5  UNK - unkempt homolog (drosophila)  DUSP6 - dual specificity phosphatase 6  DUSP7 - dual specificity phosphatase 7  DYRK1A - dual-specificity tyrosine-(y)-phosphorylation regulated kinase 1a  TOR1A - torsin family 1, member a (torsin a)  CHTOP - chromatin target of prmt1  DVL2 - dishevelled segment polarity protein 2  HERC4 - hect and rld domain containing e3 ubiquitin protein ligase 4  DVL3 - dishevelled segment polarity protein 3  GIGYF2 - grb10 interacting gyf protein 2  APPL1 - adaptor protein, phosphotyrosine interaction, ph domain and leucine zipper containing 1  DOCK7 - dedicator of cytokinesis 7  SENP6 - sumo1/sentrin specific peptidase 6  RHPN2 - rhophilin, rho gtpase binding protein 2  ANKRD17 - ankyrin repeat domain 17  ZFHX2 - zinc finger homeobox 2  HBEGF - heparin-binding egf-like growth factor  LSM14A - lsm14a, scd6 homolog a (s. cerevisiae)  DTX1 - deltex homolog 1 (drosophila)  DKC1 - dyskeratosis congenita 1, dyskerin  DLG3 - discs, large homolog 3 (drosophila)  DLG4 - discs, large homolog 4 (drosophila)  PNPT1 - polyribonucleotide nucleotidyltransferase 1  DLG1 - discs, large homolog 1 (drosophila)  BLOC1S6 - biogenesis of lysosomal organelles complex-1, subunit 6, pallidin  ANKRD13A - ankyrin repeat domain 13a  FBXW8 - f-box and wd repeat domain containing 8  DHCR24 - 24-dehydrocholesterol reductase  DHCR7 - 7-dehydrocholesterol reductase  DGUOK - deoxyguanosine kinase  SCIN - scinderin  FBXL3 - f-box and leucine-rich repeat protein 3  DHPS - deoxyhypusine synthase  B3GAT3 - beta-1,3-glucuronyltransferase 3 (glucuronosyltransferase i)  DHFR - dihydrofolate reductase  FBXL5 - f-box and leucine-rich repeat protein 5  NQO1 - nad(p)h dehydrogenase, quinone 1  FBXO2 - f-box protein 2  DYNC1H1 - dynein, cytoplasmic 1, heavy chain 1  DYNC1I2 - dynein, cytoplasmic 1, intermediate chain 2  DNM2 - dynamin 2  GMEB2 - glucocorticoid modulatory element binding protein 2  DNMT1 - dna (cytosine-5-)-methyltransferase 1  SPAG8 - sperm associated antigen 8  TRDMT1 - trna aspartic acid methyltransferase 1  DNMT3A - dna (cytosine-5-)-methyltransferase 3 alpha  EIF2AK4 - eukaryotic translation initiation factor 2 alpha kinase 4  DOCK1 - dedicator of cytokinesis 1  DOCK3 - dedicator of cytokinesis 3  HPS4 - hermansky-pudlak syndrome 4  RSL1D1 - ribosomal l1 domain containing 1  NOC2L - nucleolar complex associated 2 homolog (s. cerevisiae)  IFT172 - intraflagellar transport 172 homolog (chlamydomonas)  PPP1R3F - protein phosphatase 1, regulatory subunit 3f  MAPRE1 - microtubule-associated protein, rp/eb family, member 1  EMD - emerin  NCBP2 - nuclear cap binding protein subunit 2, 20kda  CHMP4C - charged multivesicular body protein 4c  MARK2 - map/microtubule affinity-regulating kinase 2  MAPRE3 - microtubule-associated protein, rp/eb family, member 3  PLA2R1 - phospholipase a2 receptor 1, 180kda  CTTN - cortactin  KIFAP3 - kinesin-associated protein 3  ELK1 - elk1, member of ets oncogene family  NAF1 - nuclear assembly factor 1 ribonucleoprotein  GORAB - golgin, rab6-interacting  ELN - elastin  ELK3 - elk3, ets-domain protein (srf accessory protein 2)  ELK4 - elk4, ets-domain protein (srf accessory protein 1)  SNW1 - snw domain containing 1  ENSA - endosulfine alpha  SCAP - srebf chaperone  EPAS1 - endothelial pas domain protein 1  EP300 - e1a binding protein p300  DPH7 - diphthamide biosynthesis 7  HABP4 - hyaluronan binding protein 4  ATF6 - activating transcription factor 6  ENO2 - enolase 2 (gamma, neuronal)  SRRD - srr1 domain containing  ENO1 - enolase 1, (alpha)  MOB1B - mob kinase activator 1b  RAB18 - rab18, member ras oncogene family  RAB3GAP1 - rab3 gtpase activating protein subunit 1 (catalytic)  EIF4A2 - eukaryotic translation initiation factor 4a2  EIF4E - eukaryotic translation initiation factor 4e  DAPL1 - death associated protein-like 1  EGR2 - early growth response 2  EGR3 - early growth response 3  CCT5 - chaperonin containing tcp1, subunit 5 (epsilon)  EIF2S1 - eukaryotic translation initiation factor 2, subunit 1 alpha, 35kda  TRIM32 - tripartite motif containing 32  EIF2B1 - eukaryotic translation initiation factor 2b, subunit 1 alpha, 26kda  EIF2S3 - eukaryotic translation initiation factor 2, subunit 3 gamma, 52kda  SERPINB1 - serpin peptidase inhibitor, clade b (ovalbumin), member 1  KDM2A - lysine (k)-specific demethylase 2a  ELAVL1 - elav (embryonic lethal, abnormal vision, drosophila)-like 1 (hu antigen r)  ELF1 - e74-like factor 1 (ets domain transcription factor)  AZI1 - 5-azacytidine induced 1  STRADA - ste20-related kinase adaptor alpha  ELAVL4 - elav (embryonic lethal, abnormal vision, drosophila)-like 4  ELF2 - e74-like factor 2 (ets domain transcription factor)  EIF4G2 - eukaryotic translation initiation factor 4 gamma, 2  TCF25 - transcription factor 25 (basic helix-loop-helix)  LMBRD2 - lmbr1 domain containing 2  MAST1 - microtubule associated serine/threonine kinase 1  ACIN1 - apoptotic chromatin condensation inducer 1  FAIM2 - fas apoptotic inhibitory molecule 2  CNOT1 - ccr4-not transcription complex, subunit 1  EFNA1 - ephrin-a1  MVB12A - multivesicular body subunit 12a  EEF2 - eukaryotic translation elongation factor 2  EXOSC7 - exosome component 7  SPEN - spen homolog, transcriptional regulator (drosophila)  RAB21 - rab21, member ras oncogene family  STK38L - serine/threonine kinase 38 like  KLHDC10 - kelch domain containing 10  DAAM1 - dishevelled associated activator of morphogenesis 1  EEF1A1 - eukaryotic translation elongation factor 1 alpha 1  MAPKBP1 - mitogen-activated protein kinase binding protein 1  EEF1A2 - eukaryotic translation elongation factor 1 alpha 2  PHC1 - polyhomeotic homolog 1 (drosophila)  PHC2 - polyhomeotic homolog 2 (drosophila)  EGR1 - early growth response 1  ZNF292 - zinc finger protein 292  PHLPP2 - ph domain and leucine rich repeat protein phosphatase 2  EGFR - epidermal growth factor receptor  MEGF8 - multiple egf-like-domains 8  USP33 - ubiquitin specific peptidase 33  MAST3 - microtubule associated serine/threonine kinase 3  CELSR2 - cadherin, egf lag seven-pass g-type receptor 2  KDM1A - lysine (k)-specific demethylase 1a  EFNB2 - ephrin-b2  EFNB1 - ephrin-b1  UNC13A - unc-13 homolog a (c. elegans)  EFNA5 - ephrin-a5  HNRNPLL - heterogeneous nuclear ribonucleoprotein l-like  ZSWIM8 - zinc finger, swim-type containing 8  NCOA6 - nuclear receptor coactivator 6  E2F6 - e2f transcription factor 6  E4F1 - e4f transcription factor 1  SMG1 - smg1 phosphatidylinositol 3-kinase-related kinase  ZHX3 - zinc fingers and homeoboxes 3  TNIK - traf2 and nck interacting kinase  E2F4 - e2f transcription factor 4, p107/p130-binding  SHKBP1 - sh3kbp1 binding protein 1  E2F5 - e2f transcription factor 5, p130-binding  PDS5B - pds5, regulator of cohesion maintenance, homolog b (s. cerevisiae)  DNER - delta/notch-like egf repeat containing  RRP1B - ribosomal rna processing 1b  EDNRB - endothelin receptor type b  MYCBP2 - myc binding protein 2, e3 ubiquitin protein ligase  LPAR1 - lysophosphatidic acid receptor 1  ERP44 - endoplasmic reticulum protein 44  S1PR1 - sphingosine-1-phosphate receptor 1  SETX - senataxin  ZBTB47 - zinc finger and btb domain containing 47  ECE1 - endothelin converting enzyme 1  ZNF609 - zinc finger protein 609  WAPAL - wings apart-like homolog (drosophila)  ERC1 - elks/rab6-interacting/cast family member 1  PPRC1 - peroxisome proliferator-activated receptor gamma, coactivator-related 1  KDM4C - lysine (k)-specific demethylase 4c  ZNF423 - zinc finger protein 423  PEG10 - paternally expressed 10  TRIM35 - tripartite motif containing 35  BOD1 - biorientation of chromosomes in cell division 1  TMEM259 - transmembrane protein 259  SIPA1L3 - signal-induced proliferation-associated 1 like 3  ARHGAP26 - rho gtpase activating protein 26  SARM1 - sterile alpha and tir motif containing 1  CDK19 - cyclin-dependent kinase 19  DERL3 - derlin 3  IQSEC2 - iq motif and sec7 domain 2  FSTL4 - follistatin-like 4  ZBTB43 - zinc finger and btb domain containing 43  PTK2B - protein tyrosine kinase 2 beta  ACSL3 - acyl-coa synthetase long-chain family member 3  MRPS27 - mitochondrial ribosomal protein s27  RAP1GAP2 - rap1 gtpase activating protein 2  ACSL4 - acyl-coa synthetase long-chain family member 4  TNRC6B - trinucleotide repeat containing 6b  BPTF - bromodomain phd finger transcription factor  CUL9 - cullin 9  FASN - fatty acid synthase  RNF185 - ring finger protein 185  TAB2 - tgf-beta activated kinase 1/map3k7 binding protein 2  FBLN1 - fibulin 1  ETV3 - ets variant 3  CAMTA2 - calmodulin binding transcription activator 2  CLASP2 - cytoplasmic linker associated protein 2  ETV5 - ets variant 5  ZNF598 - zinc finger protein 598  POGZ - pogo transposable element with znf domain  EVC - ellis van creveld syndrome  PLXND1 - plexin d1  ETV6 - ets variant 6  KDM6B - lysine (k)-specific demethylase 6b  PHF8 - phd finger protein 8  SMC5 - structural maintenance of chromosomes 5  EWSR1 - ews rna-binding protein 1  TRIM41 - tripartite motif containing 41  ESAM - endothelial cell adhesion molecule  EPB41L3 - erythrocyte membrane protein band 4.1-like 3  EXT2 - exostosin glycosyltransferase 2  EXT1 - exostosin glycosyltransferase 1  ANKLE2 - ankyrin repeat and lem domain containing 2  DCUN1D4 - dcn1, defective in cullin neddylation 1, domain containing 4  FMNL3 - formin-like 3  EXTL2 - exostosin-like glycosyltransferase 2  LRCH1 - leucine-rich repeats and calponin homology (ch) domain containing 1  CREB3L1 - camp responsive element binding protein 3-like 1  EXTL3 - exostosin-like glycosyltransferase 3  ZC3H3 - zinc finger ccch-type containing 3  EYA3 - eyes absent homolog 3 (drosophila)  EZH1 - enhancer of zeste homolog 1 (drosophila)  GRAMD4 - gram domain containing 4  CIC - capicua transcriptional repressor  F2R - coagulation factor ii (thrombin) receptor  NCDN - neurochondrin  F3 - coagulation factor iii (thromboplastin, tissue factor)  WDR43 - wd repeat domain 43  MAPK8IP3 - mitogen-activated protein kinase 8 interacting protein 3  ERF - ets2 repressor factor  TTLL12 - tubulin tyrosine ligase-like family, member 12  ERCC5 - excision repair cross-complementing rodent repair deficiency, complementation group 5  ERCC4 - excision repair cross-complementing rodent repair deficiency, complementation group 4  NUP205 - nucleoporin 205kda  ERCC3 - excision repair cross-complementing rodent repair deficiency, complementation group 3  LIN52 - lin-52 homolog (c. elegans)  YTHDC1 - yth domain containing 1  GGA3 - golgi-associated, gamma adaptin ear containing, arf binding protein 3  ELMSAN1 - elm2 and myb/sant-like domain containing 1  ERN1 - endoplasmic reticulum to nucleus signaling 1  GPD1L - glycerol-3-phosphate dehydrogenase 1-like  PHLDB1 - pleckstrin homology-like domain, family b, member 1  KANK1 - kn motif and ankyrin repeat domains 1  ARRDC4 - arrestin domain containing 4  NDUFAF2 - nadh dehydrogenase (ubiquinone) complex i, assembly factor 2  LARP4B - la ribonucleoprotein domain family, member 4b  RCOR1 - rest corepressor 1  DIP2A - dip2 disco-interacting protein 2 homolog a (drosophila)  MESDC2 - mesoderm development candidate 2  TTC5 - tetratricopeptide repeat domain 5  RGL1 - ral guanine nucleotide dissociation stimulator-like 1  ETV1 - ets variant 1  ETS1 - v-ets avian erythroblastosis virus e26 oncogene homolog 1  ETF1 - eukaryotic translation termination factor 1  CYFIP1 - cytoplasmic fmr1 interacting protein 1  ARL6IP1 - adp-ribosylation factor-like 6 interacting protein 1  DMTN - dematin actin binding protein  RGS7BP - regulator of g-protein signaling 7 binding protein  PKDCC - protein kinase domain containing, cytoplasmic  EPB41 - erythrocyte membrane protein band 4.1 (elliptocytosis 1, rh-linked)  FAM168A - family with sequence similarity 168, member a  ZNF830 - zinc finger protein 830  RRS1 - rrs1 ribosome biogenesis regulator homolog (s. cerevisiae)  RASL10B - ras-like, family 10, member b  CHURC1 - churchill domain containing 1  SYT11 - synaptotagmin xi  ZC3H4 - zinc finger ccch-type containing 4  JMJD6 - jumonji domain containing 6  MYADM - myeloid-associated differentiation marker  EPRS - glutamyl-prolyl-trna synthetase  EPHB6 - eph receptor b6  STX2 - syntaxin 2  TBC1D12 - tbc1 domain family, member 12  ERCC2 - excision repair cross-complementing rodent repair deficiency, complementation group 2  NUP210 - nucleoporin 210kda  EPS8 - epidermal growth factor receptor pathway substrate 8  EPS15 - epidermal growth factor receptor pathway substrate 15  ARHGEF9 - cdc42 guanine nucleotide exchange factor (gef) 9  MCF2L - mcf.2 cell line derived transforming sequence-like  PIK3IP1 - phosphoinositide-3-kinase interacting protein 1  FMR1 - fragile x mental retardation 1  EXOC7 - exocyst complex component 7  MGA - mga, max dimerization protein  DNMBP - dynamin binding protein  CAMSAP2 - calmodulin regulated spectrin-associated protein family, member 2  FNTA - farnesyltransferase, caax box, alpha  FLT1 - fms-related tyrosine kinase 1  SCFD1 - sec1 family domain containing 1  DDHD2 - ddhd domain containing 2  DENND5A - denn/madd domain containing 5a  CAMTA1 - calmodulin binding transcription activator 1  PDS5A - pds5, regulator of cohesion maintenance, homolog a (s. cerevisiae)  TMEM106A - transmembrane protein 106a  FLI1 - fli-1 proto-oncogene, ets transcription factor  FLII - flightless i homolog (drosophila)  BOP1 - block of proliferation 1  FLNA - filamin a, alpha  FLNB - filamin b, beta  SIK2 - salt-inducible kinase 2  PLCB1 - phospholipase c, beta 1 (phosphoinositide-specific)  ARC - activity-regulated cytoskeleton-associated protein  FOXS1 - forkhead box s1  PHLPP1 - ph domain and leucine rich repeat protein phosphatase 1  KIAA0922 - kiaa0922  FOXO3 - forkhead box o3  LARP4 - la ribonucleoprotein domain family, member 4  SIN3B - sin3 transcription regulator family member b  FKBP15 - fk506 binding protein 15, 133kda  NACC1 - nucleus accumbens associated 1, ben and btb (poz) domain containing  FKBP4 - fk506 binding protein 4, 59kda  BICD2 - bicaudal d homolog 2 (drosophila)  VPS26B - vacuolar protein sorting 26 homolog b (s. pombe)  MGRN1 - mahogunin ring finger 1, e3 ubiquitin protein ligase  PLCD3 - phospholipase c, delta 3  KIF13B - kinesin family member 13b  MED8 - mediator complex subunit 8  FKBP1A - fk506 binding protein 1a, 12kda  SMG6 - smg6 nonsense mediated mrna decay factor  ANKS1A - ankyrin repeat and sterile alpha motif domain containing 1a  FKBP1B - fk506 binding protein 1b, 12.6 kda  FBXW11 - f-box and wd repeat domain containing 11  POFUT2 - protein o-fucosyltransferase 2  KLHL18 - kelch-like family member 18  FAM208A - family with sequence similarity 208, member a  CLEC16A - c-type lectin domain family 16, member a  FHL2 - four and a half lim domains 2  STX1B - syntaxin 1b  FHIT - fragile histidine triad  NUP160 - nucleoporin 160kda  FGFR2 - fibroblast growth factor receptor 2  BTBD9 - btb (poz) domain containing 9  GPC5 - glypican 5  HAUS5 - haus augmin-like complex, subunit 5  FGF13 - fibroblast growth factor 13  DOCK9 - dedicator of cytokinesis 9  FGFR1 - fibroblast growth factor receptor 1  CYGB - cytoglobin  FGF9 - fibroblast growth factor 9  FGF12 - fibroblast growth factor 12  SMCHD1 - structural maintenance of chromosomes flexible hinge domain containing 1  FGF1 - fibroblast growth factor 1 (acidic)  TIRAP - toll-interleukin 1 receptor (tir) domain containing adaptor protein  FGF2 - fibroblast growth factor 2 (basic)  CLASP1 - cytoplasmic linker associated protein 1  SZT2 - seizure threshold 2 homolog (mouse)  USP22 - ubiquitin specific peptidase 22  SASH1 - sam and sh3 domain containing 1  TBC1D30 - tbc1 domain family, member 30  FGD1 - fyve, rhogef and ph domain containing 1  TRIM2 - tripartite motif containing 2  FEN1 - flap structure-specific endonuclease 1  GPC4 - glypican 4  FER - fer (fps/fes related) tyrosine kinase  FAM45A - family with sequence similarity 45, member a  EFHC1 - ef-hand domain (c-terminal) containing 1  DNAJC13 - dnaj (hsp40) homolog, subfamily c, member 13  ZCCHC11 - zinc finger, cchc domain containing 11  PIP5K1C - phosphatidylinositol-4-phosphate 5-kinase, type i, gamma  ADNP - activity-dependent neuroprotector homeobox  ZDHHC17 - zinc finger, dhhc-type containing 17  MED13L - mediator complex subunit 13-like  SIK3 - sik family kinase 3  NCSTN - nicastrin  FKTN - fukutin  CRTC1 - creb regulated transcription coactivator 1  UFL1 - ufm1-specific ligase 1  TRIM9 - tripartite motif containing 9  TENC1 - tensin like c1 domain containing phosphatase (tensin 2)  PPP1R13B - protein phosphatase 1, regulatory subunit 13b  PUM2 - pumilio homolog 2 (drosophila)  LARP1 - la ribonucleoprotein domain family, member 1  EFEMP1 - egf containing fibulin-like extracellular matrix protein 1  OBSL1 - obscurin-like 1  ARHGEF12 - rho guanine nucleotide exchange factor (gef) 12  FBN1 - fibrillin 1  ZNF629 - zinc finger protein 629  LRRC4B - leucine rich repeat containing 4b  CAMK2N2 - calcium/calmodulin-dependent protein kinase ii inhibitor 2  ITGB3BP - integrin beta 3 binding protein (beta3-endonexin)  SYAP1 - synapse associated protein 1  RYBP - ring1 and yy1 binding protein  ORMDL1 - orm1-like 1 (s. cerevisiae)  MTOR - mechanistic target of rapamycin (serine/threonine kinase)  PAXBP1 - pax3 and pax7 binding protein 1  GPR161 - g protein-coupled receptor 161  ATP13A2 - atpase type 13a2  EXOSC2 - exosome component 2  SIRT1 - sirtuin 1  NCS1 - neuronal calcium sensor 1  ABCA5 - atp-binding cassette, sub-family a (abc1), member 5  CADPS2 - ca++-dependent secretion activator 2  CBX6 - chromobox homolog 6  ACTR8 - arp8 actin-related protein 8 homolog (yeast)  HEY1 - hairy/enhancer-of-split related with yrpw motif 1  NPTXR - neuronal pentraxin receptor  CBX5 - chromobox homolog 5  BRD4 - bromodomain containing 4  CAPN7 - calpain 7  TARDBP - tar dna binding protein  TBCK - tbc1 domain containing kinase  ARHGAP18 - rho gtpase activating protein 18  PRKCDBP - protein kinase c, delta binding protein  NUP188 - nucleoporin 188kda  SUZ12 - suz12 polycomb repressive complex 2 subunit  MYL12B - myosin, light chain 12b, regulatory  POFUT1 - protein o-fucosyltransferase 1  MRAP2 - melanocortin 2 receptor accessory protein 2  CMTM7 - cklf-like marvel transmembrane domain containing 7  MORC3 - morc family cw-type zinc finger 3  CBX7 - chromobox homolog 7  ISCU - iron-sulfur cluster assembly enzyme  PES1 - pescadillo ribosomal biogenesis factor 1  FXN - frataxin  ZFYVE26 - zinc finger, fyve domain containing 26  RIMBP2 - rims binding protein 2  CEP41 - centrosomal protein 41kda  FNIP1 - folliculin interacting protein 1  HEY2 - hairy/enhancer-of-split related with yrpw motif 2  MACF1 - microtubule-actin crosslinking factor 1  ATP6V0A2 - atpase, h+ transporting, lysosomal v0 subunit a2  RBFOX2 - rna binding protein, fox-1 homolog (c. elegans) 2  MAPK8IP2 - mitogen-activated protein kinase 8 interacting protein 2  TSPAN15 - tetraspanin 15  TP53INP1 - tumor protein p53 inducible nuclear protein 1  TSPAN12 - tetraspanin 12  H2AFV - h2a histone family, member v  CDK20 - cyclin-dependent kinase 20  FOSL2 - fos-like antigen 2  FOSB - fbj murine osteosarcoma viral oncogene homolog b  FOS - fbj murine osteosarcoma viral oncogene homolog  ARHGAP12 - rho gtpase activating protein 12  CABIN1 - calcineurin binding protein 1  SYTL4 - synaptotagmin-like 4  KAT6B - k(lysine) acetyltransferase 6b  MMD - monocyte to macrophage differentiation-associated  GFER - growth factor, augmenter of liver regeneration  GFPT1 - glutamine--fructose-6-phosphate transaminase 1  GFRA1 - gdnf family receptor alpha 1  GFRA2 - gdnf family receptor alpha 2  GDI1 - gdp dissociation inhibitor 1  GDI2 - gdp dissociation inhibitor 2  GEM - gtp binding protein overexpressed in skeletal muscle  KAT2A - k(lysine) acetyltransferase 2a  GLA - galactosidase, alpha  GJA5 - gap junction protein, alpha 5, 40kda  GJA1 - gap junction protein, alpha 1, 43kda  GPR180 - g protein-coupled receptor 180  TMTC3 - transmembrane and tetratricopeptide repeat containing 3  GRASP - grp1 (general receptor for phosphoinositides 1)-associated scaffold protein  NKIRAS1 - nfkb inhibitor interacting ras-like 1  DLL1 - delta-like 1 (drosophila)  GLG1 - golgi glycoprotein 1  GCLM - glutamate-cysteine ligase, modifier subunit  GLE1 - gle1 rna export mediator  GCLC - glutamate-cysteine ligase, catalytic subunit  GNG7 - guanine nucleotide binding protein (g protein), gamma 7  GNG3 - guanine nucleotide binding protein (g protein), gamma 3  GNG4 - guanine nucleotide binding protein (g protein), gamma 4  GNB2 - guanine nucleotide binding protein (g protein), beta polypeptide 2  GNAZ - guanine nucleotide binding protein (g protein), alpha z polypeptide  GNB1 - guanine nucleotide binding protein (g protein), beta polypeptide 1  MOSPD2 - motile sperm domain containing 2  GNAS - gnas complex locus  GNAO1 - guanine nucleotide binding protein (g protein), alpha activating activity polypeptide o  GNAQ - guanine nucleotide binding protein (g protein), q polypeptide  GNAI3 - guanine nucleotide binding protein (g protein), alpha inhibiting activity polypeptide 3  GNAI2 - guanine nucleotide binding protein (g protein), alpha inhibiting activity polypeptide 2  GNAI1 - guanine nucleotide binding protein (g protein), alpha inhibiting activity polypeptide 1  GNA12 - guanine nucleotide binding protein (g protein) alpha 12  GNA11 - guanine nucleotide binding protein (g protein), alpha 11 (gq class)  GMFB - glia maturation factor, beta  CNST - consortin, connexin sorting protein  NR5A1 - nuclear receptor subfamily 5, group a, member 1  FUS - fused in sarcoma  GPR89A - g protein-coupled receptor 89a  SDE2 - sde2 telomere maintenance homolog (s. pombe)  GABBR1 - gamma-aminobutyric acid (gaba) b receptor, 1  GAB1 - grb2-associated binding protein 1  RNF168 - ring finger protein 168, e3 ubiquitin protein ligase  XRCC6 - x-ray repair complementing defective repair in chinese hamster cells 6  BBS12 - bardet-biedl syndrome 12  GABPB1 - ga binding protein transcription factor, beta subunit 1  GABPA - ga binding protein transcription factor, alpha subunit 60kda  FUT4 - fucosyltransferase 4 (alpha (1,3) fucosyltransferase, myeloid-specific)  FUT8 - fucosyltransferase 8 (alpha (1,6) fucosyltransferase)  FYN - fyn oncogene related to src, fgr, yes  DARC - duffy blood group, atypical chemokine receptor  G6PD - glucose-6-phosphate dehydrogenase  GAK - cyclin g associated kinase  SPRED1 - sprouty-related, evh1 domain containing 1  GABRG2 - gamma-aminobutyric acid (gaba) a receptor, gamma 2  GABRB3 - gamma-aminobutyric acid (gaba) a receptor, beta 3  GABRD - gamma-aminobutyric acid (gaba) a receptor, delta  ZFPM1 - zinc finger protein, fog family member 1  EID2 - ep300 interacting inhibitor of differentiation 2  GBA - glucosidase, beta, acid  ZNF383 - zinc finger protein 383  GATA2 - gata binding protein 2  GAS6 - growth arrest-specific 6  DEDD2 - death effector domain containing 2  DHX36 - deah (asp-glu-ala-his) box polypeptide 36  GSTP1 - glutathione s-transferase pi 1  GSR - glutathione reductase  GSPT1 - g1 to s phase transition 1  GSN - gelsolin  SSBP4 - single stranded dna binding protein 4  GTF2F1 - general transcription factor iif, polypeptide 1, 74kda  GTF2F2 - general transcription factor iif, polypeptide 2, 30kda  GTF2H2 - general transcription factor iih, polypeptide 2, 44kda  GTF2H3 - general transcription factor iih, polypeptide 3, 34kda  GTF2H1 - general transcription factor iih, polypeptide 1, 62kda  GTF2B - general transcription factor iib  MSH6 - muts homolog 6 (e. coli)  GUCY1B3 - guanylate cyclase 1, soluble, beta 3  GUCY1A3 - guanylate cyclase 1, soluble, alpha 3  GTF2I - general transcription factor iii  GTF2H4 - general transcription factor iih, polypeptide 4, 52kda  BRF1 - brf1, rna polymerase iii transcription initiation factor 90 kda subunit  H3F3A - h3 histone, family 3a  H2AFZ - h2a histone family, member z  DCP2 - decapping mrna 2  H2AFX - h2a histone family, member x  PAPD4 - pap associated domain containing 4  MIER3 - mesoderm induction early response 1, family member 3  THAP5 - thap domain containing 5  HCFC1 - host cell factor c1 (vp16-accessory protein)  TMEM64 - transmembrane protein 64  HDAC2 - histone deacetylase 2  HDGF - hepatoma-derived growth factor  ZCCHC12 - zinc finger, cchc domain containing 12  HTT - huntingtin  HDAC1 - histone deacetylase 1  HCLS1 - hematopoietic cell-specific lyn substrate 1  GNL1 - guanine nucleotide binding protein-like 1  GOLGA2 - golgin a2  GOLGB1 - golgin b1  HIGD2A - hig1 hypoxia inducible domain family, member 2a  AAED1 - ahpc/tsa antioxidant enzyme domain containing 1  SCAMP5 - secretory carrier membrane protein 5  GPM6B - glycoprotein m6b  ZNF367 - zinc finger protein 367  GPR4 - g protein-coupled receptor 4  DTX3 - deltex homolog 3 (drosophila)  GPR19 - g protein-coupled receptor 19  ARID2 - at rich interactive domain 2 (arid, rfx-like)  PAOX - polyamine oxidase (exo-n4-amino)  MSMP - microseminoprotein, prostate associated  GRK5 - g protein-coupled receptor kinase 5  GRK6 - g protein-coupled receptor kinase 6  GRB2 - growth factor receptor-bound protein 2  ASXL1 - additional sex combs like 1 (drosophila)  ZNF384 - zinc finger protein 384  GRID1 - glutamate receptor, ionotropic, delta 1  GRIA4 - glutamate receptor, ionotropic, ampa 4  GRIK5 - glutamate receptor, ionotropic, kainate 5  GRIK2 - glutamate receptor, ionotropic, kainate 2  ARHGAP35 - rho gtpase activating protein 35  GRINA - glutamate receptor, ionotropic, n-methyl d-aspartate-associated protein 1 (glutamate binding)  GRSF1 - g-rich rna sequence binding factor 1  GSK3B - glycogen synthase kinase 3 beta  GSK3A - glycogen synthase kinase 3 alpha  PDIA3 - protein disulfide isomerase family a, member 3  TRIM65 - tripartite motif containing 65  DNAJB2 - dnaj (hsp40) homolog, subfamily b, member 2  DNAJA1 - dnaj (hsp40) homolog, subfamily a, member 1  HSF2 - heat shock transcription factor 2  SAMD14 - sterile alpha motif domain containing 14  ARHGAP27 - rho gtpase activating protein 27  HSBP1 - heat shock factor binding protein 1  FLCN - folliculin  CENPV - centromere protein v  PRMT1 - protein arginine methyltransferase 1  PRMT2 - protein arginine methyltransferase 2  ARL13B - adp-ribosylation factor-like 13b  KCTD6 - potassium channel tetramerization domain containing 6  HPRT1 - hypoxanthine phosphoribosyltransferase 1  SPRED2 - sprouty-related, evh1 domain containing 2  TMEM17 - transmembrane protein 17  CRTC2 - creb regulated transcription coactivator 2  ICA1 - islet cell autoantigen 1, 69kda  TXLNA - taxilin alpha  RPS6KC1 - ribosomal protein s6 kinase, 52kda, polypeptide 1  CC2D1B - coiled-coil and c2 domain containing 1b  HUS1 - hus1 checkpoint homolog (s. pombe)  THAP8 - thap domain containing 8  CADM4 - cell adhesion molecule 4  HTR1F - 5-hydroxytryptamine (serotonin) receptor 1f, g protein-coupled  HSPD1 - heat shock 60kda protein 1 (chaperonin)  NSMCE1 - non-smc element 1 homolog (s. cerevisiae)  DNAJB1 - dnaj (hsp40) homolog, subfamily b, member 1  ZNF48 - zinc finger protein 48  HSP90AA1 - heat shock protein 90kda alpha (cytosolic), class a member 1  HSPA9 - heat shock 70kda protein 9 (mortalin)  UBR1 - ubiquitin protein ligase e3 component n-recognin 1  HSPA8 - heat shock 70kda protein 8  OSTF1 - osteoclast stimulating factor 1  HMGN1 - high mobility group nucleosome binding domain 1  HMGB3 - high mobility group box 3  HMGB2 - high mobility group box 2  HMGB1 - high mobility group box 1  DAZAP1 - daz associated protein 1  AGO1 - argonaute risc catalytic component 1  LATS2 - large tumor suppressor kinase 2  ZBTB48 - zinc finger and btb domain containing 48  HIPK1 - homeodomain interacting protein kinase 1  NAIF1 - nuclear apoptosis inducing factor 1  HFE - hemochromatosis  HIP1 - huntingtin interacting protein 1  UBE2K - ubiquitin-conjugating enzyme e2k  TBL2 - transducin (beta)-like 2  HINT1 - histidine triad nucleotide binding protein 1  HIVEP1 - human immunodeficiency virus type i enhancer binding protein 1  ELP4 - elongator acetyltransferase complex subunit 4  HIF1A - hypoxia inducible factor 1, alpha subunit (basic helix-loop-helix transcription factor)  MYCBP - myc binding protein  TUBB - tubulin, beta class i  SERGEF - secretion regulating guanine nucleotide exchange factor  HNRNPU - heterogeneous nuclear ribonucleoprotein u (scaffold attachment factor a)  FBXO10 - f-box protein 10  HNRNPK - heterogeneous nuclear ribonucleoprotein k  FBXO22 - f-box protein 22  HNRNPL - heterogeneous nuclear ribonucleoprotein l  FBXO4 - f-box protein 4  FBXO8 - f-box protein 8  FBXO6 - f-box protein 6  TINF2 - terf1 (trf1)-interacting nuclear factor 2  HNRNPA2B1 - heterogeneous nuclear ribonucleoprotein a2/b1  HNRNPA1 - heterogeneous nuclear ribonucleoprotein a1  HNRNPC - heterogeneous nuclear ribonucleoprotein c (c1/c2)  HNRNPAB - heterogeneous nuclear ribonucleoprotein a/b  HNRNPF - heterogeneous nuclear ribonucleoprotein f  HNRNPD - heterogeneous nuclear ribonucleoprotein d (au-rich element rna binding protein 1, 37kda)  HNRNPH1 - heterogeneous nuclear ribonucleoprotein h1 (h)  HNRNPH3 - heterogeneous nuclear ribonucleoprotein h3 (2h9)  HNRNPH2 - heterogeneous nuclear ribonucleoprotein h2 (h')  UNC13D - unc-13 homolog d (c. elegans)  HMGCR - 3-hydroxy-3-methylglutaryl-coa reductase  HMGA1 - high mobility group at-hook 1  ZNF584 - zinc finger protein 584  HMMR - hyaluronan-mediated motility receptor (rhamm)  PTPN18 - protein tyrosine phosphatase, non-receptor type 18 (brain-derived)  NR4A1 - nuclear receptor subfamily 4, group a, member 1  PDE12 - phosphodiesterase 12  FOXK2 - forkhead box k2  HTATSF1 - hiv-1 tat specific factor 1  EIF3K - eukaryotic translation initiation factor 3, subunit k  RPS6KA6 - ribosomal protein s6 kinase, 90kda, polypeptide 6  TNRC6A - trinucleotide repeat containing 6a  IL13RA1 - interleukin 13 receptor, alpha 1  RAB30 - rab30, member ras oncogene family  IL11RA - interleukin 11 receptor, alpha  RBMX - rna binding motif protein, x-linked  INHA - inhibin, alpha  DESI1 - desumoylating isopeptidase 1  SGSM3 - small g protein signaling modulator 3  HTRA2 - htra serine peptidase 2  STK39 - serine threonine kinase 39  ING2 - inhibitor of growth family, member 2  PCSK1N - proprotein convertase subtilisin/kexin type 1 inhibitor  KCNMB4 - potassium large conductance calcium-activated channel, subfamily m, beta member 4  ILF2 - interleukin enhancer binding factor 2  UBE2S - ubiquitin-conjugating enzyme e2s  PRPF19 - pre-mrna processing factor 19  ILF3 - interleukin enhancer binding factor 3, 90kda  ILK - integrin-linked kinase  UTP20 - utp20, small subunit (ssu) processome component, homolog (yeast)  IMPA1 - inositol(myo)-1(or 4)-monophosphatase 1  SLCO3A1 - solute carrier organic anion transporter family, member 3a1  IL10RA - interleukin 10 receptor, alpha  IL6ST - interleukin 6 signal transducer (gp130, oncostatin m receptor)  C5AR2 - complement component 5a receptor 2  ITGAE - integrin, alpha e (antigen cd103, human mucosal lymphocyte antigen 1; alpha polypeptide)  SERP1 - stress-associated endoplasmic reticulum protein 1  ZNF25 - zinc finger protein 25  CCNY - cyclin y  ITGA1 - integrin, alpha 1  ITGA3 - integrin, alpha 3 (antigen cd49c, alpha 3 subunit of vla-3 receptor)  ITGB5 - integrin, beta 5  RNF115 - ring finger protein 115  ERLEC1 - endoplasmic reticulum lectin 1  ITGAV - integrin, alpha v  ARFIP1 - adp-ribosylation factor interacting protein 1  ITGB1 - integrin, beta 1 (fibronectin receptor, beta polypeptide, antigen cd29 includes mdf2, msk12)  GPR162 - g protein-coupled receptor 162  ITGB4 - integrin, beta 4  EIF6 - eukaryotic translation initiation factor 6  TPCN2 - two pore segment channel 2  INPPL1 - inositol polyphosphate phosphatase-like 1  RND1 - rho family gtpase 1  INSR - insulin receptor  INSM1 - insulinoma-associated 1  PCDH17 - protocadherin 17  KLHL20 - kelch-like family member 20  INPP1 - inositol polyphosphate-1-phosphatase  PDCD4 - programmed cell death 4 (neoplastic transformation inhibitor)  INPP5B - inositol polyphosphate-5-phosphatase, 75kda  INPP5A - inositol polyphosphate-5-phosphatase, 40kda  CSDC2 - cold shock domain containing c2, rna binding  INPP4A - inositol polyphosphate-4-phosphatase, type i, 107kda  IRF2 - interferon regulatory factor 2  IRF3 - interferon regulatory factor 3  IREB2 - iron-responsive element binding protein 2  UNC5B - unc-5 homolog b (c. elegans)  DIMT1 - dim1 dimethyladenosine transferase 1 homolog (s. cerevisiae)  MED19 - mediator complex subunit 19  ITGA6 - integrin, alpha 6  CRCP - cgrp receptor component  IRAK2 - interleukin-1 receptor-associated kinase 2  IRAK1 - interleukin-1 receptor-associated kinase 1  CACYBP - calcyclin binding protein  NAPEPLD - n-acyl phosphatidylethanolamine phospholipase d  EIF2AK1 - eukaryotic translation initiation factor 2-alpha kinase 1  KIAA1324L - kiaa1324-like  PDE7B - phosphodiesterase 7b  ZBTB11 - zinc finger and btb domain containing 11  KCTD20 - potassium channel tetramerization domain containing 20  IFNAR1 - interferon (alpha, beta and omega) receptor 1  DKK3 - dickkopf wnt signaling pathway inhibitor 3  SNX5 - sorting nexin 5  INVS - inversin  IFNGR1 - interferon gamma receptor 1  ZNF777 - zinc finger protein 777  ID2 - inhibitor of dna binding 2, dominant negative helix-loop-helix protein  BRPF3 - bromodomain and phd finger containing, 3  ID3 - inhibitor of dna binding 3, dominant negative helix-loop-helix protein  STK36 - serine/threonine kinase 36  JAZF1 - jazf zinc finger 1  FOXK1 - forkhead box k1  BRAT1 - brca1-associated atm activator 1  TMED4 - transmembrane emp24 protein transport domain containing 4  VPS4A - vacuolar protein sorting 4 homolog a (s. cerevisiae)  IGHMBP2 - immunoglobulin mu binding protein 2  CHORDC1 - cysteine and histidine-rich domain (chord) containing 1  RBPJ - recombination signal binding protein for immunoglobulin kappa j region  PABPC1 - poly(a) binding protein, cytoplasmic 1  TRUB2 - trub pseudouridine (psi) synthase homolog 2 (e. coli)  C6orf89 - chromosome 6 open reading frame 89  CYFIP2 - cytoplasmic fmr1 interacting protein 2  ZBTB9 - zinc finger and btb domain containing 9  USP21 - ubiquitin specific peptidase 21  ZBTB12 - zinc finger and btb domain containing 12  NGFRAP1 - nerve growth factor receptor (tnfrsf16) associated protein 1  PHACTR1 - phosphatase and actin regulator 1  NPTN - neuroplastin  IK - ik cytokine, down-regulator of hla ii  JMJD1C - jumonji domain containing 1c  IGBP1 - immunoglobulin (cd79a) binding protein 1  ATP2C1 - atpase, ca++ transporting, type 2c, member 1  IGF1R - insulin-like growth factor 1 receptor  FAM171A1 - family with sequence similarity 171, member a1  PELP1 - proline, glutamate and leucine rich protein 1  IGF2R - insulin-like growth factor 2 receptor  LAT - linker for activation of t cells  NSG1 - neuron specific gene family member 1  IGFBP6 - insulin-like growth factor binding protein 6  SND1 - staphylococcal nuclease and tudor domain containing 1  B9D1 - b9 protein domain 1  FOXP1 - forkhead box p1  CACNG4 - calcium channel, voltage-dependent, gamma subunit 4  BEND6 - ben domain containing 6  ZNF572 - zinc finger protein 572  MEX3C - mex-3 rna binding family member c  WAC - ww domain containing adaptor with coiled-coil  PHF21A - phd finger protein 21a  LIG4 - ligase iv, dna, atp-dependent  ZNF771 - zinc finger protein 771  LIMK1 - lim domain kinase 1  SPG21 - spastic paraplegia 21 (autosomal recessive, mast syndrome)  LIFR - leukemia inhibitory factor receptor alpha  LIG3 - ligase iii, dna, atp-dependent  ZDHHC3 - zinc finger, dhhc-type containing 3  FAM13B - family with sequence similarity 13, member b  LDLR - low density lipoprotein receptor  MTPN - myotrophin  LFNG - lfng o-fucosylpeptide 3-beta-n-acetylglucosaminyltransferase  LETM1 - leucine zipper-ef-hand containing transmembrane protein 1  LNPEP - leucyl/cystinyl aminopeptidase  SCAND1 - scan domain containing 1  BFAR - bifunctional apoptosis regulator  CD320 - cd320 molecule  BET1L - bet1 golgi vesicular membrane trafficking protein-like  FADS1 - fatty acid desaturase 1  UBXN2B - ubx domain protein 2b  LMO1 - lim domain only 1 (rhombotin 1)  LMNB1 - lamin b1  IER5 - immediate early response 5  NACC2 - nacc family member 2, ben and btb (poz) domain containing  DNAJC27 - dnaj (hsp40) homolog, subfamily c, member 27  LMNA - lamin a/c  LMAN1 - lectin, mannose-binding, 1  LLGL1 - lethal giant larvae homolog 1 (drosophila)  KLF3 - kruppel-like factor 3 (basic)  PRKAG2 - protein kinase, amp-activated, gamma 2 non-catalytic subunit  DDX41 - dead (asp-glu-ala-asp) box polypeptide 41  SUCO - sun domain containing ossification factor  ANAPC5 - anaphase promoting complex subunit 5  ANAPC7 - anaphase promoting complex subunit 7  YTHDF2 - yth domain family, member 2  RNF138 - ring finger protein 138, e3 ubiquitin protein ligase  IP6K2 - inositol hexakisphosphate kinase 2  ZFP62 - zfp62 zinc finger protein  PPME1 - protein phosphatase methylesterase 1  ACTL6B - actin-like 6b  UBR5 - ubiquitin protein ligase e3 component n-recognin 5  STMN1 - stathmin 1  UCHL5 - ubiquitin carboxyl-terminal hydrolase l5  ATRAID - all-trans retinoic acid-induced differentiation factor  ATP6V1D - atpase, h+ transporting, lysosomal 34kda, v1 subunit d  LCP1 - lymphocyte cytosolic protein 1 (l-plastin)  ANGPT4 - angiopoietin 4  CRLF3 - cytokine receptor-like factor 3  NGRN - neugrin, neurite outgrowth associated  L1CAM - l1 cell adhesion molecule  TAOK3 - tao kinase 3  FZR1 - fizzy/cell division cycle 20 related 1 (drosophila)  ZBTB7A - zinc finger and btb domain containing 7a  LAMA5 - laminin, alpha 5  LAMA4 - laminin, alpha 4  LAMC1 - laminin, gamma 1 (formerly lamb2)  MBTPS2 - membrane-bound transcription factor peptidase, site 2  VPS28 - vacuolar protein sorting 28 homolog (s. cerevisiae)  ZNF580 - zinc finger protein 580  ASB7 - ankyrin repeat and socs box containing 7  ASB8 - ankyrin repeat and socs box containing 8  KIF2A - kinesin heavy chain member 2a  KIF5B - kinesin family member 5b  KDR - kinase insert domain receptor (a type iii receptor tyrosine kinase)  ASB6 - ankyrin repeat and socs box containing 6  ING4 - inhibitor of growth family, member 4  KCNN2 - potassium intermediate/small conductance calcium-activated channel, subfamily n, member 2  INSIG2 - insulin induced gene 2  DYNC1LI1 - dynein, cytoplasmic 1, light intermediate chain 1  KCNMA1 - potassium large conductance calcium-activated channel, subfamily m, alpha member 1  IRAK4 - interleukin-1 receptor-associated kinase 4  RLIM - ring finger protein, lim domain interacting  ASB3 - ankyrin repeat and socs box containing 3  SOGA1 - suppressor of glucose, autophagy associated 1  KRAS - kirsten rat sarcoma viral oncogene homolog  TNPO1 - transportin 1  IER3IP1 - immediate early response 3 interacting protein 1  ZNF706 - zinc finger protein 706  KPNA2 - karyopherin alpha 2 (rag cohort 1, importin alpha 1)  KPNB1 - karyopherin (importin) beta 1  KPNA1 - karyopherin alpha 1 (importin alpha 5)  WFDC3 - wap four-disulfide core domain 3  SUV420H1 - suppressor of variegation 4-20 homolog 1 (drosophila)  TRAPPC12 - trafficking protein particle complex 12  APH1A - aph1a gamma secretase subunit  PHF20L1 - phd finger protein 20-like 1  ABHD17B - abhydrolase domain containing 17b  ZFP28 - zfp28 zinc finger protein  KIT - v-kit hardy-zuckerman 4 feline sarcoma viral oncogene homolog  ABHD5 - abhydrolase domain containing 5  SH3GLB1 - sh3-domain grb2-like endophilin b1  KCNA5 - potassium voltage-gated channel, shaker-related subfamily, member 5  SHISA5 - shisa family member 5  PAIP2 - poly(a) binding protein interacting protein 2  JUP - junction plakoglobin  CRIM1 - cysteine rich transmembrane bmp regulator 1 (chordin-like)  JAG2 - jagged 2  JAK1 - janus kinase 1  JAK2 - janus kinase 2  JAK3 - janus kinase 3  ZNF219 - zinc finger protein 219  ITPK1 - inositol-tetrakisphosphate 1-kinase  RIMS4 - regulating synaptic membrane exocytosis 4  ITPR1 - inositol 1,4,5-trisphosphate receptor, type 1  PHF20 - phd finger protein 20  ITPR2 - inositol 1,4,5-trisphosphate receptor, type 2  KCNK2 - potassium channel, subfamily k, member 2  DDX47 - dead (asp-glu-ala-asp) box polypeptide 47  ZDHHC2 - zinc finger, dhhc-type containing 2  CRBN - cereblon  KCNH2 - potassium voltage-gated channel, subfamily h (eag-related), member 2  ZNF639 - zinc finger protein 639  LEF1 - lymphoid enhancer-binding factor 1  PLEKHO1 - pleckstrin homology domain containing, family o member 1  SMCR8 - smith-magenis syndrome chromosome region, candidate 8  KCNC4 - potassium voltage-gated channel, shaw-related subfamily, member 4  CDK12 - cyclin-dependent kinase 12  SIRPA - signal-regulatory protein alpha  TMEM8B - transmembrane protein 8b  ZNF280B - zinc finger protein 280b  ARID4B - at rich interactive domain 4b (rbp1-like)  TM7SF3 - transmembrane 7 superfamily member 3  RAB8B - rab8b, member ras oncogene family  MIF - macrophage migration inhibitory factor (glycosylation-inhibiting factor)  ATP8A2 - atpase, aminophospholipid transporter, class i, type 8a, member 2  CCNI2 - cyclin i family, member 2  SYT17 - synaptotagmin xvii  MAP3K10 - mitogen-activated protein kinase kinase kinase 10  DUSP19 - dual specificity phosphatase 19  MIB2 - mindbomb e3 ubiquitin protein ligase 2  MLH1 - mutl homolog 1, colon cancer, nonpolyposis type 2 (e. coli)  MKLN1 - muskelin 1, intracellular mediator containing kelch motifs  KDM3B - lysine (k)-specific demethylase 3b  RSF1 - remodeling and spacing factor 1  MLLT6 - myeloid/lymphoid or mixed-lineage leukemia (trithorax homolog, drosophila); translocated to, 6  SAMD8 - sterile alpha motif domain containing 8  MLLT3 - myeloid/lymphoid or mixed-lineage leukemia (trithorax homolog, drosophila); translocated to, 3  MLLT1 - myeloid/lymphoid or mixed-lineage leukemia (trithorax homolog, drosophila); translocated to, 1  MAP3K11 - mitogen-activated protein kinase kinase kinase 11  KMT2A - lysine (k)-specific methyltransferase 2a  MMP2 - matrix metallopeptidase 2 (gelatinase a, 72kda gelatinase, 72kda type iv collagenase)  HECA - headcase homolog (drosophila)  SUFU - suppressor of fused homolog (drosophila)  GPRC5B - g protein-coupled receptor, family c, group 5, member b  MMP14 - matrix metallopeptidase 14 (membrane-inserted)  VPS29 - vacuolar protein sorting 29 homolog (s. cerevisiae)  NLK - nemo-like kinase  MNAT1 - mnat cdk-activating kinase assembly factor 1  UIMC1 - ubiquitin interaction motif containing 1  DNAJB11 - dnaj (hsp40) homolog, subfamily b, member 11  SYT9 - synaptotagmin ix  RAB23 - rab23, member ras oncogene family  WWOX - ww domain containing oxidoreductase  CD200 - cd200 molecule  MNT - mnt, max dimerization protein  CD46 - cd46 molecule, complement regulatory protein  SESN3 - sestrin 3  MCM7 - minichromosome maintenance complex component 7  DNAJB9 - dnaj (hsp40) homolog, subfamily b, member 9  ME1 - malic enzyme 1, nadp(+)-dependent, cytosolic  SPTY2D1 - spt2, suppressor of ty, domain containing 1 (s. cerevisiae)  MECP2 - methyl cpg binding protein 2 (rett syndrome)  PELO - pelota homolog (drosophila)  MEF2A - myocyte enhancer factor 2a  ME2 - malic enzyme 2, nad(+)-dependent, mitochondrial  PRICKLE1 - prickle homolog 1 (drosophila)  BCDIN3D - bcdin3 domain containing  MEIS2 - meis homeobox 2  BRWD1 - bromodomain and wd repeat domain containing 1  ZNF664 - zinc finger protein 664  MEIS1 - meis homeobox 1  MAP3K4 - mitogen-activated protein kinase kinase kinase 4  MAP3K5 - mitogen-activated protein kinase kinase kinase 5  MAP3K1 - mitogen-activated protein kinase kinase kinase 1, e3 ubiquitin protein ligase  MAP3K3 - mitogen-activated protein kinase kinase kinase 3  MEN1 - multiple endocrine neoplasia i  RAB8A - rab8a, member ras oncogene family  IL17D - interleukin 17d  ZFYVE1 - zinc finger, fyve domain containing 1  MFGE8 - milk fat globule-egf factor 8 protein  STX18 - syntaxin 18  MGAT5 - mannosyl (alpha-1,6-)-glycoprotein beta-1,6-n-acetyl-glucosaminyltransferase  LACC1 - laccase (multicopper oxidoreductase) domain containing 1  MBD3 - methyl-cpg binding domain protein 3  KITLG - kit ligand  PTOV1 - prostate tumor overexpressed 1  ZC3HC1 - zinc finger, c3hc-type containing 1  MAL - mal, t-cell differentiation protein  MAGOH - mago-nashi homolog, proliferation-associated (drosophila)  GSKIP - gsk3b interacting protein  CXXC5 - cxxc finger protein 5  LARS - leucyl-trna synthetase  NCKIPSD - nck interacting protein with sh3 domain  MARS - methionyl-trna synthetase  IL23A - interleukin 23, alpha subunit p19  MARK3 - map/microtubule affinity-regulating kinase 3  MARK1 - map/microtubule affinity-regulating kinase 1  MAPT - microtubule-associated protein tau  MAP6 - microtubule-associated protein 6  RAB14 - rab14, member ras oncogene family  MAP2 - microtubule-associated protein 2  MAP1B - microtubule-associated protein 1b  MAP1A - microtubule-associated protein 1a  SIRT6 - sirtuin 6  MAOA - monoamine oxidase a  MAN2A1 - mannosidase, alpha, class 2a, member 1  PPHLN1 - periphilin 1  LIMA1 - lim domain and actin binding 1  UBE2J1 - ubiquitin-conjugating enzyme e2, j1  EVL - enah/vasp-like  MBD1 - methyl-cpg binding domain protein 1  SFMBT1 - scm-like with four mbt domains 1  MAX - myc associated factor x  MATK - megakaryocyte-associated tyrosine kinase  TRMT112 - trna methyltransferase 11-2 homolog (s. cerevisiae)  ZFP90 - zfp90 zinc finger protein  CHMP5 - charged multivesicular body protein 5  TTBK2 - tau tubulin kinase 2  MCF2 - mcf.2 cell line derived transforming sequence  CDAN1 - codanin 1  NELFCD - negative elongation factor complex member c/d  MCM2 - minichromosome maintenance complex component 2  MCC - mutated in colorectal cancers  MCAM - melanoma cell adhesion molecule  ZSCAN29 - zinc finger and scan domain containing 29  CTDSPL2 - ctd (carboxy-terminal domain, rna polymerase ii, polypeptide a) small phosphatase like 2  PTPLAD1 - protein tyrosine phosphatase-like a domain containing 1  ANKFY1 - ankyrin repeat and fyve domain containing 1  LRPAP1 - low density lipoprotein receptor-related protein associated protein 1  IL34 - interleukin 34  LRP1 - low density lipoprotein receptor-related protein 1  DYNC2LI1 - dynein, cytoplasmic 2, light intermediate chain 1  LRP6 - low density lipoprotein receptor-related protein 6  LRP3 - low density lipoprotein receptor-related protein 3  NUB1 - negative regulator of ubiquitin-like proteins 1  TOM1L2 - target of myb1-like 2 (chicken)  LY6H - lymphocyte antigen 6 complex, locus h  LY6E - lymphocyte antigen 6 complex, locus e  ASB1 - ankyrin repeat and socs box containing 1  LUM - lumican  LYN - v-yes-1 yamaguchi sarcoma viral related oncogene homolog  TPPP3 - tubulin polymerization-promoting protein family member 3  RBFOX3 - rna binding protein, fox-1 homolog (c. elegans) 3  CDK5RAP1 - cdk5 regulatory subunit associated protein 1  LTBP3 - latent transforming growth factor beta binding protein 3  CHMP3 - charged multivesicular body protein 3  LTBP1 - latent transforming growth factor beta binding protein 1  AZIN1 - antizyme inhibitor 1  MED15 - mediator complex subunit 15  MXD1 - max dimerization protein 1  PIAS4 - protein inhibitor of activated stat, 4  TRIM33 - tripartite motif containing 33  SRRT - serrate rna effector molecule homolog (arabidopsis)  SMAD2 - smad family member 2  MBIP - map3k12 binding inhibitory protein 1  RTN4RL1 - reticulon 4 receptor-like 1  HDAC7 - histone deacetylase 7  ARMCX3 - armadillo repeat containing, x-linked 3  TDP2 - tyrosyl-dna phosphodiesterase 2  FAM49B - family with sequence similarity 49, member b  CAPRIN1 - cell cycle associated protein 1  LARP7 - la ribonucleoprotein domain family, member 7  NBR1 - neighbor of brca1 gene 1  KCTD11 - potassium channel tetramerization domain containing 11  MAFG - v-maf avian musculoaponeurotic fibrosarcoma oncogene homolog g  ATP6V1H - atpase, h+ transporting, lysosomal 50/57kda, v1 subunit h  DPH5 - diphthamide biosynthesis 5  TAF9B - taf9b rna polymerase ii, tata box binding protein (tbp)-associated factor, 31kda  SMAD4 - smad family member 4  NBAS - neuroblastoma amplified sequence  EME1 - essential meiotic structure-specific endonuclease 1  SMAD5 - smad family member 5  SMAD7 - smad family member 7  ZNF565 - zinc finger protein 565  SAP30BP - sap30 binding protein  MYLIP - myosin regulatory light chain interacting protein  NCBP1 - nuclear cap binding protein subunit 1, 80kda  TAGLN3 - transgelin 3  SIX5 - six homeobox 5  NDN - necdin, melanoma antigen (mage) family member  ZNF582 - zinc finger protein 582  BRD7 - bromodomain containing 7  NCK1 - nck adaptor protein 1  DDX25 - dead (asp-glu-ala-asp) box helicase 25  NCL - nucleolin  TICAM1 - toll-like receptor adaptor molecule 1  RACGAP1 - rac gtpase activating protein 1  HNRNPM - heterogeneous nuclear ribonucleoprotein m  ORMDL2 - orm1-like 2 (s. cerevisiae)  DROSHA - drosha, ribonuclease type iii  NAP1L1 - nucleosome assembly protein 1-like 1  DNAJC15 - dnaj (hsp40) homolog, subfamily c, member 15  NCAM1 - neural cell adhesion molecule 1  TBK1 - tank-binding kinase 1  NBN - nibrin  NUBP1 - nucleotide binding protein 1  MYO9B - myosin ixb  APCDD1 - adenomatosis polyposis coli down-regulated 1  MYO10 - myosin x  ZDHHC1 - zinc finger, dhhc-type containing 1  PPP1R12A - protein phosphatase 1, regulatory subunit 12a  MYT1 - myelin transcription factor 1  SENP1 - sumo1/sentrin specific peptidase 1  NAB1 - ngfi-a binding protein 1 (egr1 binding protein 1)  TFPT - tcf3 (e2a) fusion partner (in childhood leukemia)  NAB2 - ngfi-a binding protein 2 (egr1 binding protein 2)  WIPF2 - was/wasl interacting protein family, member 2  MYLK - myosin light chain kinase  TMOD2 - tropomodulin 2 (neuronal)  MYO1D - myosin id  MYO5A - myosin va (heavy chain 12, myoxin)  MYO1E - myosin ie  MYO6 - myosin vi  ABT1 - activator of basal transcription 1  MYD88 - myeloid differentiation primary response 88  ZNF362 - zinc finger protein 362  MYH10 - myosin, heavy chain 10, non-muscle  MYH9 - myosin, heavy chain 9, non-muscle  HCFC2 - host cell factor c2  CNOT7 - ccr4-not transcription complex, subunit 7  MXI1 - max interactor 1, dimerization protein  MX1 - myxovirus (influenza virus) resistance 1, interferon-inducible protein p78 (mouse)  GPSM2 - g-protein signaling modulator 2  TRA2A - transformer 2 alpha homolog (drosophila)  RBM15B - rna binding motif protein 15b  ANAPC4 - anaphase promoting complex subunit 4  NRBP1 - nuclear receptor binding protein 1  CERS2 - ceramide synthase 2  SLC25A24 - solute carrier family 25 (mitochondrial carrier; phosphate carrier), member 24  TRIM37 - tripartite motif containing 37  ZNF558 - zinc finger protein 558  CDC42EP5 - cdc42 effector protein (rho gtpase binding) 5  CPLX3 - complexin 3  PURG - purine-rich element binding protein g  DIRAS1 - diras family, gtp-binding ras-like 1  SNX12 - sorting nexin 12  MTIF2 - mitochondrial translational initiation factor 2  MTM1 - myotubularin 1  MTF1 - metal-regulatory transcription factor 1  RNF187 - ring finger protein 187  DCPS - decapping enzyme, scavenger  MT3 - metallothionein 3  GIT1 - g protein-coupled receptor kinase interacting arfgap 1  CCDC22 - coiled-coil domain containing 22  EXOC8 - exocyst complex component 8  MSN - moesin  MSH3 - muts homolog 3 (e. coli)  MSH2 - muts homolog 2, colon cancer, nonpolyposis type 1 (e. coli)  CITED1 - cbp/p300-interacting transactivator, with glu/asp-rich carboxy-terminal domain, 1  DBNL - drebrin-like  RGCC - regulator of cell cycle  IFT81 - intraflagellar transport 81 homolog (chlamydomonas)  TMEM14A - transmembrane protein 14a  COMMD1 - copper metabolism (murr1) domain containing 1  STXBP6 - syntaxin binding protein 6 (amisyn)  BABAM1 - brisc and brca1 a complex member 1  CHMP4A - charged multivesicular body protein 4a  METTL5 - methyltransferase like 5  TTL - tubulin tyrosine ligase  MED4 - mediator complex subunit 4  SETD2 - set domain containing 2  DUSP18 - dual specificity phosphatase 18  MRE11A - mre11 meiotic recombination 11 homolog a (s. cerevisiae)  MPP2 - membrane protein, palmitoylated 2 (maguk p55 subfamily member 2)  WDR91 - wd repeat domain 91  GPR155 - g protein-coupled receptor 155  DTX3L - deltex 3-like (drosophila)  ORC2 - origin recognition complex, subunit 2  PARD6A - par-6 partitioning defective 6 homolog alpha (c. elegans)  ORC5 - origin recognition complex, subunit 5  STMN3 - stathmin-like 3  RNF141 - ring finger protein 141  PPP4R2 - protein phosphatase 4, regulatory subunit 2  SHANK1 - sh3 and multiple ankyrin repeat domains 1  GLIPR2 - gli pathogenesis-related 2  TBC1D25 - tbc1 domain family, member 25  OAZ2 - ornithine decarboxylase antizyme 2  OAZ1 - ornithine decarboxylase antizyme 1  ODC1 - ornithine decarboxylase 1  OCRL - oculocerebrorenal syndrome of lowe  CCNYL1 - cyclin y-like 1  ODF2 - outer dense fiber of sperm tails 2  OGG1 - 8-oxoguanine dna glycosylase  OGN - osteoglycin  EXOSC3 - exosome component 3  OPA1 - optic atrophy 1 (autosomal dominant)  EXOSC1 - exosome component 1  FIS1 - fission 1 (mitochondrial outer membrane) homolog (s. cerevisiae)  NSF - n-ethylmaleimide-sensitive factor  DDR2 - discoidin domain receptor tyrosine kinase 2  GOLT1B - golgi transport 1b  NUMA1 - nuclear mitotic apparatus protein 1  DAB2IP - dab2 interacting protein  NUP88 - nucleoporin 88kda  GMNN - geminin, dna replication inhibitor  NUP98 - nucleoporin 98kda  SLC38A9 - solute carrier family 38, member 9  NR4A2 - nuclear receptor subfamily 4, group a, member 2  NTS - neurotensin  ZNF593 - zinc finger protein 593  NUCB1 - nucleobindin 1  ZBTB7B - zinc finger and btb domain containing 7b  NUCB2 - nucleobindin 2  TXNDC12 - thioredoxin domain containing 12 (endoplasmic reticulum)  NVL - nuclear vcp-like  ZNF691 - zinc finger protein 691  CEP120 - centrosomal protein 120kda  CREBRF - creb3 regulatory factor  NMD3 - nmd3 ribosome export adaptor  NPM1 - nucleophosmin (nucleolar phosphoprotein b23, numatrin)  NPHP1 - nephronophthisis 1 (juvenile)  THAP4 - thap domain containing 4  SPICE1 - spindle and centriole associated protein 1  IGSF11 - immunoglobulin superfamily, member 11  CNTN4 - contactin 4  NPY1R - neuropeptide y receptor y1  NPTX1 - neuronal pentraxin i  FGD5 - fyve, rhogef and ph domain containing 5  NPTX2 - neuronal pentraxin ii  IFT52 - intraflagellar transport 52 homolog (chlamydomonas)  NRGN - neurogranin (protein kinase c substrate, rc3)  PAQR3 - progestin and adipoq receptor family member iii  NFXL1 - nuclear transcription factor, x-box binding-like 1  NRF1 - nuclear respiratory factor 1  ADIPOR1 - adiponectin receptor 1  SH3D19 - sh3 domain containing 19  NRAS - neuroblastoma ras viral (v-ras) oncogene homolog  SIDT2 - sid1 transmembrane family, member 2  NRCAM - neuronal cell adhesion molecule  UBQLN1 - ubiquilin 1  NMT1 - n-myristoyltransferase 1  DONSON - downstream neighbor of son  NRBF2 - nuclear receptor binding factor 2  NOP2 - nop2 nucleolar protein  NONO - non-pou domain containing, octamer-binding  SLC2A8 - solute carrier family 2 (facilitated glucose transporter), member 8  CDIP1 - cell death-inducing p53 target 1  LRP12 - low density lipoprotein receptor-related protein 12  NMB - neuromedin b  NME1 - nme/nm23 nucleoside diphosphate kinase 1  MDFIC - myod family inhibitor domain containing  UBQLN2 - ubiquilin 2  NOTCH4 - notch 4  NOVA2 - neuro-oncological ventral antigen 2  NOVA1 - neuro-oncological ventral antigen 1  ERO1L - ero1-like (s. cerevisiae)  NPAT - nuclear protein, ataxia-telangiectasia locus  CNOT2 - ccr4-not transcription complex, subunit 2  NOS3 - nitric oxide synthase 3 (endothelial cell)  CNOT4 - ccr4-not transcription complex, subunit 4  PACSIN1 - protein kinase c and casein kinase substrate in neurons 1  CNOT3 - ccr4-not transcription complex, subunit 3  NPY - neuropeptide y  BAZ2B - bromodomain adjacent to zinc finger domain, 2b  NOTCH1 - notch 1  NOTCH3 - notch 3  AMOTL1 - angiomotin like 1  NOTCH2 - notch 2  NFYA - nuclear transcription factor y, alpha  KCNIP2 - kv channel interacting protein 2  KCNIP1 - kv channel interacting protein 1  NFYC - nuclear transcription factor y, gamma  CXXC1 - cxxc finger protein 1  NFKBIA - nuclear factor of kappa light polypeptide gene enhancer in b-cells inhibitor, alpha  NFKB1 - nuclear factor of kappa light polypeptide gene enhancer in b-cells 1  NFKBIB - nuclear factor of kappa light polypeptide gene enhancer in b-cells inhibitor, beta  NFKBIE - nuclear factor of kappa light polypeptide gene enhancer in b-cells inhibitor, epsilon  PIK3R4 - phosphoinositide-3-kinase, regulatory subunit 4  NKX2-2 - nk2 homeobox 2  CNKSR3 - cnksr family member 3  TAX1BP3 - tax1 (human t-cell leukemia virus type i) binding protein 3  EHD2 - eh-domain containing 2  EHD3 - eh-domain containing 3  EHD4 - eh-domain containing 4  SOCS7 - suppressor of cytokine signaling 7  NEK3 - nima-related kinase 3  MINK1 - misshapen-like kinase 1  NEO1 - neogenin 1  RRM2B - ribonucleotide reductase m2 b (tp53 inducible)  NEFL - neurofilament, light polypeptide  SMARCAL1 - swi/snf related, matrix associated, actin dependent regulator of chromatin, subfamily a-like 1  NEDD8 - neural precursor cell expressed, developmentally down-regulated 8  NEDD9 - neural precursor cell expressed, developmentally down-regulated 9  WRAP73 - wd repeat containing, antisense to tp73  ITSN2 - intersectin 2  NFE2L2 - nuclear factor (erythroid-derived 2)-like 2  NFATC4 - nuclear factor of activated t-cells, cytoplasmic, calcineurin-dependent 4  CAMSAP1 - calmodulin regulated spectrin-associated protein 1  NFIA - nuclear factor i/a  NF1 - neurofibromin 1  CHST11 - carbohydrate (chondroitin 4) sulfotransferase 11  ARHGEF4 - rho guanine nucleotide exchange factor (gef) 4  ARHGEF3 - rho guanine nucleotide exchange factor (gef) 3  ATP6V0E2 - atpase, h+ transporting v0 subunit e2  ZNF746 - zinc finger protein 746  CALY - calcyon neuron-specific vesicular protein  RDH10 - retinol dehydrogenase 10 (all-trans)  DRG1 - developmentally regulated gtp binding protein 1  NSDHL - nad(p) dependent steroid dehydrogenase-like  HP1BP3 - heterochromatin protein 1, binding protein 3  ASAP1 - arfgap with sh3 domain, ankyrin repeat and ph domain 1  MYEF2 - myelin expression factor 2  EXOSC9 - exosome component 9  EXOSC10 - exosome component 10  LRRC16A - leucine rich repeat containing 16a  PPP1R9A - protein phosphatase 1, regulatory subunit 9a  HAUS7 - haus augmin-like complex, subunit 7  PLXNB1 - plexin b1  PLXNA3 - plexin a3  ZNF821 - zinc finger protein 821  PML - promyelocytic leukemia  CNOT11 - ccr4-not transcription complex, subunit 11  SUPT20H - suppressor of ty 20 homolog (s. cerevisiae)  PMP22 - peripheral myelin protein 22  LRRC40 - leucine rich repeat containing 40  SYBU - syntabulin (syntaxin-interacting)  PLP2 - proteolipid protein 2 (colonic epithelium-enriched)  PLRG1 - pleiotropic regulator 1  CHD7 - chromodomain helicase dna binding protein 7  LYAR - ly1 antibody reactive  PLXNA1 - plexin a1  PLXNA2 - plexin a2  PLCB4 - phospholipase c, beta 4  PLCB3 - phospholipase c, beta 3 (phosphatidylinositol-specific)  OTUB1 - otu domain, ubiquitin aldehyde binding 1  PLAUR - plasminogen activator, urokinase receptor  TASP1 - taspase, threonine aspartase, 1  ASAP3 - arfgap with sh3 domain, ankyrin repeat and ph domain 3  PLCG1 - phospholipase c, gamma 1  PLCL1 - phospholipase c-like 1  PRR5 - proline rich 5 (renal)  KIF16B - kinesin family member 16b  PLD2 - phospholipase d2  DOCK10 - dedicator of cytokinesis 10  AMBRA1 - autophagy/beclin-1 regulator 1  ZDHHC7 - zinc finger, dhhc-type containing 7  CAMK2N1 - calcium/calmodulin-dependent protein kinase ii inhibitor 1  PPID - peptidylprolyl isomerase d  PPEF1 - protein phosphatase, ef-hand calcium binding domain 1  AP1AR - adaptor-related protein complex 1 associated regulatory protein  PPIA - peptidylprolyl isomerase a (cyclophilin a)  PARL - presenilin associated, rhomboid-like  DNAJA4 - dnaj (hsp40) homolog, subfamily a, member 4  LRRC59 - leucine rich repeat containing 59  ZNF331 - zinc finger protein 331  MED1 - mediator complex subunit 1  PPARD - peroxisome proliferator-activated receptor delta  MAML3 - mastermind-like 3 (drosophila)  POLR2K - polymerase (rna) ii (dna directed) polypeptide k, 7.0kda  POLH - polymerase (dna directed), eta  H2AFY2 - h2a histone family, member y2  TNFRSF19 - tumor necrosis factor receptor superfamily, member 19  HES6 - hairy and enhancer of split 6 (drosophila)  POLR2G - polymerase (rna) ii (dna directed) polypeptide g  POLR2F - polymerase (rna) ii (dna directed) polypeptide f  POLR2E - polymerase (rna) ii (dna directed) polypeptide e, 25kda  POLR2D - polymerase (rna) ii (dna directed) polypeptide d  SMPD3 - sphingomyelin phosphodiesterase 3, neutral membrane (neutral sphingomyelinase ii)  POLR2C - polymerase (rna) ii (dna directed) polypeptide c, 33kda  POLR2B - polymerase (rna) ii (dna directed) polypeptide b, 140kda  POLR2A - polymerase (rna) ii (dna directed) polypeptide a, 220kda  TMEM30A - transmembrane protein 30a  CDK5RAP2 - cdk5 regulatory subunit associated protein 2  PRKAB1 - protein kinase, amp-activated, beta 1 non-catalytic subunit  RCOR3 - rest corepressor 3  PRKACB - protein kinase, camp-dependent, catalytic, beta  PRKACA - protein kinase, camp-dependent, catalytic, alpha  WDR12 - wd repeat domain 12  NUP133 - nucleoporin 133kda  SRGN - serglycin  VPS35 - vacuolar protein sorting 35 homolog (s. cerevisiae)  EDEM2 - er degradation enhancer, mannosidase alpha-like 2  PRCC - papillary renal cell carcinoma (translocation-associated)  PRCP - prolylcarboxypeptidase (angiotensinase c)  PARVA - parvin, alpha  CHFR - checkpoint with forkhead and ring finger domains, e3 ubiquitin protein ligase  PPP3R1 - protein phosphatase 3, regulatory subunit b, alpha  ATF7IP - activating transcription factor 7 interacting protein  PPP5C - protein phosphatase 5, catalytic subunit  PPT1 - palmitoyl-protein thioesterase 1  PPP2R5D - protein phosphatase 2, regulatory subunit b', delta  LRIF1 - ligand dependent nuclear receptor interacting factor 1  PPP2R5C - protein phosphatase 2, regulatory subunit b', gamma  PPP2R5B - protein phosphatase 2, regulatory subunit b', beta  PPP4C - protein phosphatase 4, catalytic subunit  PCID2 - pci domain containing 2  PPP2R5E - protein phosphatase 2, regulatory subunit b', epsilon isoform  PPP2R2A - protein phosphatase 2, regulatory subunit b, alpha  PPP2R1B - protein phosphatase 2, regulatory subunit a, beta  RIOK2 - rio kinase 2  PPP2R1A - protein phosphatase 2, regulatory subunit a, alpha  TXLNG - taxilin gamma  PPP2R2C - protein phosphatase 2, regulatory subunit b, gamma  PPP2R2B - protein phosphatase 2, regulatory subunit b, beta  PPP1R7 - protein phosphatase 1, regulatory subunit 7  PPP1R8 - protein phosphatase 1, regulatory subunit 8  PPP2CA - protein phosphatase 2, catalytic subunit, alpha isozyme  PPP2CB - protein phosphatase 2, catalytic subunit, beta isozyme  MBD5 - methyl-cpg binding domain protein 5  PPP1R10 - protein phosphatase 1, regulatory subunit 10  EXOC1 - exocyst complex component 1  IFT122 - intraflagellar transport 122 homolog (chlamydomonas)  PPM1G - protein phosphatase, mg2+/mn2+ dependent, 1g  PPP1R2 - protein phosphatase 1, regulatory (inhibitor) subunit 2  ZNF83 - zinc finger protein 83  PPP1CC - protein phosphatase 1, catalytic subunit, gamma isozyme  KANSL3 - kat8 regulatory nsl complex subunit 3  IWS1 - iws1 homolog (s. cerevisiae)  SCYL2 - scy1-like 2 (s. cerevisiae)  RUFY2 - run and fyve domain containing 2  DENND4C - denn/madd domain containing 4c  PRNP - prion protein  NPLOC4 - nuclear protein localization 4 homolog (s. cerevisiae)  RNF126 - ring finger protein 126  DNAJC3 - dnaj (hsp40) homolog, subfamily c, member 3  PRKRIR - protein-kinase, interferon-inducible double stranded rna dependent inhibitor, repressor of (p58 repressor)  PRPF40A - prp40 pre-mrna processing factor 40 homolog a (s. cerevisiae)  TMEM127 - transmembrane protein 127  MAP2K5 - mitogen-activated protein kinase kinase 5  MAP2K7 - mitogen-activated protein kinase kinase 7  EIF2AK2 - eukaryotic translation initiation factor 2-alpha kinase 2  ZNF692 - zinc finger protein 692  MAPK13 - mitogen-activated protein kinase 13  MAP2K1 - mitogen-activated protein kinase kinase 1  EIF4E3 - eukaryotic translation initiation factor 4e family member 3  MAP2K2 - mitogen-activated protein kinase kinase 2  MAPK10 - mitogen-activated protein kinase 10  MAPK9 - mitogen-activated protein kinase 9  MAPK11 - mitogen-activated protein kinase 11  TRIM59 - tripartite motif containing 59  MAPK8 - mitogen-activated protein kinase 8  MAPK7 - mitogen-activated protein kinase 7  WDR11 - wd repeat domain 11  MAPK3 - mitogen-activated protein kinase 3  MAPK1 - mitogen-activated protein kinase 1  LMBR1L - limb development membrane protein 1-like  TENM3 - teneurin transmembrane protein 3  PRKCSH - protein kinase c substrate 80k-h  PRKD1 - protein kinase d1  NDC1 - ndc1 transmembrane nucleoporin  CCDC88A - coiled-coil domain containing 88a  PKN2 - protein kinase n2  PRKCH - protein kinase c, eta  CCDC94 - coiled-coil domain containing 94  PRKCI - protein kinase c, iota  PRKCE - protein kinase c, epsilon  RADIL - ras association and dil domains  PRKCB - protein kinase c, beta  PRKCD - protein kinase c, delta  RBM22 - rna binding motif protein 22  PRKAR2B - protein kinase, camp-dependent, regulatory, type ii, beta  VAC14 - vac14 homolog (s. cerevisiae)  PRKCA - protein kinase c, alpha  PRKAR1B - protein kinase, camp-dependent, regulatory, type i, beta  NSUN5 - nop2/sun domain family, member 5  PRKAR1A - protein kinase, camp-dependent, regulatory, type i, alpha  FRMD4A - ferm domain containing 4a  PKIB - protein kinase (camp-dependent, catalytic) inhibitor beta  YEATS2 - yeats domain containing 2  PRKAG1 - protein kinase, amp-activated, gamma 1 non-catalytic subunit  RBM23 - rna binding motif protein 23  PARN - poly(a)-specific ribonuclease  RFWD3 - ring finger and wd repeat domain 3  SHQ1 - shq1, h/aca ribonucleoprotein assembly factor  PDCL - phosducin-like  TMEM33 - transmembrane protein 33  PBX2 - pre-b-cell leukemia homeobox 2  PBX3 - pre-b-cell leukemia homeobox 3  PRMT6 - protein arginine methyltransferase 6  PBX1 - pre-b-cell leukemia homeobox 1  PCBP2 - poly(rc) binding protein 2  TBCCD1 - tbcc domain containing 1  PCBD1 - pterin-4 alpha-carbinolamine dehydratase/dimerization cofactor of hepatocyte nuclear factor 1 alpha  SMG8 - smg8 nonsense mediated mrna decay factor  RNF220 - ring finger protein 220  FAIM - fas apoptotic inhibitory molecule  RIF1 - rap1 interacting factor homolog (yeast)  VPS13D - vacuolar protein sorting 13 homolog d (s. cerevisiae)  RIC8B - ric8 guanine nucleotide exchange factor b  PBRM1 - polybromo 1  KIAA1551 - kiaa1551  PEBP1 - phosphatidylethanolamine binding protein 1  PA2G4 - proliferation-associated 2g4, 38kda  APPL2 - adaptor protein, phosphotyrosine interaction, ph domain and leucine zipper containing 2  RPRD1A - regulation of nuclear pre-mrna domain containing 1a  MAP1S - microtubule-associated protein 1s  GOLPH3L - golgi phosphoprotein 3-like  PAFAH1B1 - platelet-activating factor acetylhydrolase 1b, regulatory subunit 1 (45kda)  PAFAH1B2 - platelet-activating factor acetylhydrolase 1b, catalytic subunit 2 (30kda)  PPP1R42 - protein phosphatase 1, regulatory subunit 42  SBNO1 - strawberry notch homolog 1 (drosophila)  PRDX1 - peroxiredoxin 1  SETD5 - set domain containing 5  BBS7 - bardet-biedl syndrome 7  PAK2 - p21 protein (cdc42/rac)-activated kinase 2  PAK3 - p21 protein (cdc42/rac)-activated kinase 3  LEPREL1 - leprecan-like 1  PALM - paralemmin  PAM - peptidylglycine alpha-amidating monooxygenase  PDGFA - platelet-derived growth factor alpha polypeptide  OXR1 - oxidation resistance 1  PDE1B - phosphodiesterase 1b, calmodulin-dependent  PDE9A - phosphodiesterase 9a  PDPR - pyruvate dehydrogenase phosphatase regulatory subunit  NSMCE2 - non-smc element 2, mms21 homolog (s. cerevisiae)  IFT57 - intraflagellar transport 57 homolog (chlamydomonas)  PDGFRB - platelet-derived growth factor receptor, beta polypeptide  PDGFRA - platelet-derived growth factor receptor, alpha polypeptide  PDGFB - platelet-derived growth factor beta polypeptide  FAM83H - family with sequence similarity 83, member h  PDPK1 - 3-phosphoinositide dependent protein kinase-1  SAMD4B - sterile alpha motif domain containing 4b  PDK3 - pyruvate dehydrogenase kinase, isozyme 3  MED9 - mediator complex subunit 9  PECAM1 - platelet/endothelial cell adhesion molecule 1  WDR70 - wd repeat domain 70  PCNA - proliferating cell nuclear antigen  CHMP1A - charged multivesicular body protein 1a  ARHGAP17 - rho gtpase activating protein 17  AGGF1 - angiogenic factor with g patch and fha domains 1  PCM1 - pericentriolar material 1  CDK16 - cyclin-dependent kinase 16  AKIRIN2 - akirin 2  HEATR1 - heat repeat containing 1  CDK17 - cyclin-dependent kinase 17  FANCL - fanconi anemia, complementation group l  PDCD2 - programmed cell death 2  WRAP53 - wd repeat containing, antisense to tp53  PDE2A - phosphodiesterase 2a, cgmp-stimulated  THAP1 - thap domain containing, apoptosis associated protein 1  PDE3B - phosphodiesterase 3b, cgmp-inhibited  ELP3 - elongator acetyltransferase complex subunit 3  HAUS2 - haus augmin-like complex, subunit 2  RGMB - rgm domain family, member b  PGGT1B - protein geranylgeranyltransferase type i, beta subunit  ZNF444 - zinc finger protein 444  PGAM1 - phosphoglycerate mutase 1 (brain)  RNF180 - ring finger protein 180  PFN2 - profilin 2  FBXW7 - f-box and wd repeat domain containing 7, e3 ubiquitin protein ligase  PGF - placental growth factor  RNF121 - ring finger protein 121  UFSP2 - ufm1-specific peptidase 2  PHF2 - phd finger protein 2  PHF1 - phd finger protein 1  ACER3 - alkaline ceramidase 3  MNS1 - meiosis-specific nuclear structural 1  LARP6 - la ribonucleoprotein domain family, member 6  SH3PXD2B - sh3 and px domains 2b  SLC35C1 - solute carrier family 35 (gdp-fucose transporter), member c1  PEX14 - peroxisomal biogenesis factor 14  TCP11L1 - t-complex 11, testis-specific-like 1  PENK - proenkephalin  PFKL - phosphofructokinase, liver  IMPACT - impact rwd domain protein  LGR4 - leucine-rich repeat containing g protein-coupled receptor 4  PFDN2 - prefoldin subunit 2  RELL2 - relt-like 2  SMU1 - smu-1 suppressor of mec-8 and unc-52 homolog (c. elegans)  PKD1 - polycystic kidney disease 1 (autosomal dominant)  PKD2 - polycystic kidney disease 2 (autosomal dominant)  MOB1A - mob kinase activator 1a  PITPNA - phosphatidylinositol transfer protein, alpha  PIP4K2A - phosphatidylinositol-5-phosphate 4-kinase, type ii, alpha  NAT10 - n-acetyltransferase 10 (gcn5-related)  PI4KB - phosphatidylinositol 4-kinase, catalytic, beta  PIN1 - peptidylprolyl cis/trans isomerase, nima-interacting 1  PIK3R2 - phosphoinositide-3-kinase, regulatory subunit 2 (beta)  PI4KA - phosphatidylinositol 4-kinase, catalytic, alpha  DPH3 - diphthamide biosynthesis 3  TMEM57 - transmembrane protein 57  PIK3R1 - phosphoinositide-3-kinase, regulatory subunit 1 (alpha)  TMEM39A - transmembrane protein 39a  PLAT - plasminogen activator, tissue  ASXL2 - additional sex combs like 2 (drosophila)  PLAGL1 - pleiomorphic adenoma gene-like 1  ELP2 - elongator acetyltransferase complex subunit 2  PKP2 - plakophilin 2  PKNOX1 - pbx/knotted 1 homeobox 1  PKM - pyruvate kinase, muscle  OGFOD1 - 2-oxoglutarate and iron-dependent oxygenase domain containing 1  SERPINI1 - serpin peptidase inhibitor, clade i (neuroserpin), member 1  TMEM100 - transmembrane protein 100  PHF10 - phd finger protein 10  RABL3 - rab, member of ras oncogene family-like 3  SERPINE2 - serpin peptidase inhibitor, clade e (nexin, plasminogen activator inhibitor type 1), member 2  SERPINB8 - serpin peptidase inhibitor, clade b (ovalbumin), member 8  PSPC1 - paraspeckle component 1  NUDT15 - nudix (nucleoside diphosphate linked moiety x)-type motif 15  C7orf43 - chromosome 7 open reading frame 43  WDR41 - wd repeat domain 41  PHKG2 - phosphorylase kinase, gamma 2 (testis)  MRGBP - mrg/morf4l binding protein  PIK3CB - phosphatidylinositol-4,5-bisphosphate 3-kinase, catalytic subunit beta  RPUSD3 - rna pseudouridylate synthase domain containing 3  RHOT1 - ras homolog family member t1  PIK3CA - phosphatidylinositol-4,5-bisphosphate 3-kinase, catalytic subunit alpha  PPP6R3 - protein phosphatase 6, regulatory subunit 3  BRF2 - brf2, rna polymerase iii transcription initiation factor 50 kda subunit  PIM1 - pim-1 oncogene  PIK3C2B - phosphatidylinositol-4-phosphate 3-kinase, catalytic subunit type 2 beta  PIK3C3 - phosphatidylinositol 3-kinase, catalytic subunit type 3  C3orf33 - chromosome 3 open reading frame 33  TRIM27 - tripartite motif containing 27  PXK - px domain containing serine/threonine kinase  RFC5 - replication factor c (activator 1) 5, 36.5kda  RFC2 - replication factor c (activator 1) 2, 40kda  RFC3 - replication factor c (activator 1) 3, 38kda  RFXAP - regulatory factor x-associated protein  SEMA4C - sema domain, immunoglobulin domain (ig), transmembrane domain (tm) and short cytoplasmic domain, (semaphorin) 4c  RFX5 - regulatory factor x, 5 (influences hla class ii expression)  WHSC1L1 - wolf-hirschhorn syndrome candidate 1-like 1  RFX1 - regulatory factor x, 1 (influences hla class ii expression)  MKS1 - meckel syndrome, type 1  CDKL4 - cyclin-dependent kinase-like 4  RELA - v-rel avian reticuloendotheliosis viral oncogene homolog a  BCOR - bcl6 corepressor  RDX - radixin  RET - ret proto-oncogene  ALKBH5 - alkb, alkylation repair homolog 5 (e. coli)  SH3RF3 - sh3 domain containing ring finger 3  DPF2 - d4, zinc and double phd fingers family 2  UPF1 - upf1 regulator of nonsense transcripts homolog (yeast)  NSUN2 - nop2/sun rna methyltransferase family, member 2  RIT1 - ras-like without caax 1  CC2D1A - coiled-coil and c2 domain containing 1a  RLF - rearranged l-myc fusion  ELP6 - elongator acetyltransferase complex subunit 6  SNRK - snf related kinase  RING1 - ring finger protein 1  FNBP1L - formin binding protein 1-like  RGS10 - regulator of g-protein signaling 10  RGS7 - regulator of g-protein signaling 7  DEF8 - differentially expressed in fdcp 8 homolog (mouse)  RGS1 - regulator of g-protein signaling 1  RGS4 - regulator of g-protein signaling 4  RGS3 - regulator of g-protein signaling 3  RHEB - ras homolog enriched in brain  GON4L - gon-4-like (c. elegans)  FBXL12 - f-box and leucine-rich repeat protein 12  NDE1 - nude neurodevelopment protein 1  BCAS3 - breast carcinoma amplified sequence 3  ABCE1 - atp-binding cassette, sub-family e (oabp), member 1  VPS13C - vacuolar protein sorting 13 homolog c (s. cerevisiae)  HAUS6 - haus augmin-like complex, subunit 6  AHI1 - abelson helper integration site 1  RNF4 - ring finger protein 4  BRD2 - bromodomain containing 2  RNF2 - ring finger protein 2  ZNF280D - zinc finger protein 280d  GATAD2A - gata zinc finger domain containing 2a  RNF6 - ring finger protein (c3h2c3 type) 6  ROBO1 - roundabout, axon guidance receptor, homolog 1 (drosophila)  ROBO2 - roundabout, axon guidance receptor, homolog 2 (drosophila)  PPP1R12C - protein phosphatase 1, regulatory subunit 12c  ROCK1 - rho-associated, coiled-coil containing protein kinase 1  RNF111 - ring finger protein 111  TET2 - tet methylcytosine dioxygenase 2  TRPM4 - transient receptor potential cation channel, subfamily m, member 4  MED18 - mediator complex subunit 18  MBTD1 - mbt domain containing 1  FBLIM1 - filamin binding lim protein 1  TRIM44 - tripartite motif containing 44  ZWILCH - zwilch kinetochore protein  NRDE2 - nrde-2, necessary for rna interference, domain containing  PLEKHB2 - pleckstrin homology domain containing, family b (evectins) member 2  RPL11 - ribosomal protein l11  PTCD3 - pentatricopeptide repeat domain 3  FKBP14 - fk506 binding protein 14, 22 kda  RPA3 - replication protein a3, 14kda  PID1 - phosphotyrosine interaction domain containing 1  RPA1 - replication protein a1, 70kda  PHIP - pleckstrin homology domain interacting protein  RPA2 - replication protein a2, 32kda  RPS4X - ribosomal protein s4, x-linked  PPP2R3C - protein phosphatase 2, regulatory subunit b'', gamma  STAC2 - sh3 and cysteine rich domain 2  RMND1 - required for meiotic nuclear division 1 homolog (s. cerevisiae)  LAMTOR1 - late endosomal/lysosomal adaptor, mapk and mtor activator 1  PAK1IP1 - pak1 interacting protein 1  GID8 - gid complex subunit 8  SLC35F6 - solute carrier family 35, member f6  PINX1 - pin2/terf1 interacting, telomerase inhibitor 1  CLN6 - ceroid-lipofuscinosis, neuronal 6, late infantile, variant  BEX5 - brain expressed, x-linked 5  BANP - btg3 associated nuclear protein  SSH3 - slingshot protein phosphatase 3  TIPIN - timeless interacting protein  ACER2 - alkaline ceramidase 2  TRNAU1AP - trna selenocysteine 1 associated protein 1  RPS6KA2 - ribosomal protein s6 kinase, 90kda, polypeptide 2  NRBP2 - nuclear receptor binding protein 2  RPS6KA1 - ribosomal protein s6 kinase, 90kda, polypeptide 1  PARP16 - poly (adp-ribose) polymerase family, member 16  RPS6KB1 - ribosomal protein s6 kinase, 70kda, polypeptide 1  RPS6KB2 - ribosomal protein s6 kinase, 70kda, polypeptide 2  RNF125 - ring finger protein 125, e3 ubiquitin protein ligase  OCIAD1 - ocia domain containing 1  FAM206A - family with sequence similarity 206, member a  RXRB - retinoid x receptor, beta  RSU1 - ras suppressor protein 1  TRMT10C - trna methyltransferase 10 homolog c (s. cerevisiae)  RRAS - related ras viral (r-ras) oncogene homolog  CHTF8 - ctf8, chromosome transmission fidelity factor 8 homolog (s. cerevisiae)  HTRA1 - htra serine peptidase 1  PSAP - prosaposin  RIN2 - ras and rab interactor 2  SPIN2A - spindlin family, member 2a  ANKIB1 - ankyrin repeat and ibr domain containing 1  PSEN1 - presenilin 1  PSEN2 - presenilin 2 (alzheimer disease 4)  TOLLIP - toll interacting protein  PSMA1 - proteasome (prosome, macropain) subunit, alpha type, 1  NLE1 - notchless homolog 1 (drosophila)  RNF216 - ring finger protein 216  PSMA2 - proteasome (prosome, macropain) subunit, alpha type, 2  SMCR7L - smith-magenis syndrome chromosome region, candidate 7-like  PSMB1 - proteasome (prosome, macropain) subunit, beta type, 1  PSMA5 - proteasome (prosome, macropain) subunit, alpha type, 5  PSMA3 - proteasome (prosome, macropain) subunit, alpha type, 3  GNG2 - guanine nucleotide binding protein (g protein), gamma 2  PSMB10 - proteasome (prosome, macropain) subunit, beta type, 10  GPR173 - g protein-coupled receptor 173  PSMC6 - proteasome (prosome, macropain) 26s subunit, atpase, 6  PSMD1 - proteasome (prosome, macropain) 26s subunit, non-atpase, 1  PSMC4 - proteasome (prosome, macropain) 26s subunit, atpase, 4  SOX18 - sry (sex determining region y)-box 18  PSMC2 - proteasome (prosome, macropain) 26s subunit, atpase, 2  PSMD9 - proteasome (prosome, macropain) 26s subunit, non-atpase, 9  PSMD10 - proteasome (prosome, macropain) 26s subunit, non-atpase, 10  PSMD7 - proteasome (prosome, macropain) 26s subunit, non-atpase, 7  DNAJC10 - dnaj (hsp40) homolog, subfamily c, member 10  PSMD8 - proteasome (prosome, macropain) 26s subunit, non-atpase, 8  PSMD5 - proteasome (prosome, macropain) 26s subunit, non-atpase, 5  TERF2IP - telomeric repeat binding factor 2, interacting protein  PSMD2 - proteasome (prosome, macropain) 26s subunit, non-atpase, 2  SLC38A2 - solute carrier family 38, member 2  PSMD12 - proteasome (prosome, macropain) 26s subunit, non-atpase, 12  PSMD13 - proteasome (prosome, macropain) 26s subunit, non-atpase, 13  PTBP1 - polypyrimidine tract binding protein 1  PTEN - phosphatase and tensin homolog  PTGIS - prostaglandin i2 (prostacyclin) synthase  PTK2 - protein tyrosine kinase 2  ZFP69 - zfp69 zinc finger protein  PTK7 - protein tyrosine kinase 7  PTMA - prothymosin, alpha  TWF1 - twinfilin actin-binding protein 1  PTN - pleiotrophin  SPOPL - speckle-type poz protein-like  PCBP3 - poly(rc) binding protein 3  PTPN3 - protein tyrosine phosphatase, non-receptor type 3  PTPN2 - protein tyrosine phosphatase, non-receptor type 2  PTPN1 - protein tyrosine phosphatase, non-receptor type 1  PTPN9 - protein tyrosine phosphatase, non-receptor type 9  PTPN11 - protein tyrosine phosphatase, non-receptor type 11  PTPRA - protein tyrosine phosphatase, receptor type, a  PTPN12 - protein tyrosine phosphatase, non-receptor type 12  PTPN13 - protein tyrosine phosphatase, non-receptor type 13 (apo-1/cd95 (fas)-associated phosphatase)  PTPRD - protein tyrosine phosphatase, receptor type, d  PTPRG - protein tyrosine phosphatase, receptor type, g  PTPRE - protein tyrosine phosphatase, receptor type, e  PTPRF - protein tyrosine phosphatase, receptor type, f  RAD1 - rad1 homolog (s. pombe)  PURA - purine-rich element binding protein a  RRN3 - rrn3 rna polymerase i transcription factor homolog (s. cerevisiae)  PDP1 - pyruvate dehyrogenase phosphatase catalytic subunit 1  PURB - purine-rich element binding protein b  GTPBP2 - gtp binding protein 2  P4HTM - prolyl 4-hydroxylase, transmembrane (endoplasmic reticulum)  TMEM106B - transmembrane protein 106b  LRRN3 - leucine rich repeat neuronal 3  PTPRR - protein tyrosine phosphatase, receptor type, r  PTPRS - protein tyrosine phosphatase, receptor type, s  PTPRK - protein tyrosine phosphatase, receptor type, k  PTPRM - protein tyrosine phosphatase, receptor type, m  PTPRN - protein tyrosine phosphatase, receptor type, n  PYCR1 - pyrroline-5-carboxylate reductase 1  RELL1 - relt-like 1  JMJD8 - jumonji domain containing 8  LEPROT - leptin receptor overlapping transcript  PEX2 - peroxisomal biogenesis factor 2  MPHOSPH8 - m-phase phosphoprotein 8  PXN - paxillin  PVRL1 - poliovirus receptor-related 1 (herpesvirus entry mediator c)  PVRL2 - poliovirus receptor-related 2 (herpesvirus entry mediator b)  MAP4K2 - mitogen-activated protein kinase kinase kinase kinase 2  RAB5A - rab5a, member ras oncogene family  RAB5B - rab5b, member ras oncogene family  GNB1L - guanine nucleotide binding protein (g protein), beta polypeptide 1-like  RAB4A - rab4a, member ras oncogene family  RAB3A - rab3a, member ras oncogene family  RAB3B - rab3b, member ras oncogene family  RAB1A - rab1a, member ras oncogene family  QARS - glutaminyl-trna synthetase  MYPOP - myb-related transcription factor, partner of profilin  RAD21 - rad21 homolog (s. pombe)  RAD17 - rad17 homolog (s. pombe)  RAC3 - ras-related c3 botulinum toxin substrate 3 (rho family, small gtp binding protein rac3)  PARP14 - poly (adp-ribose) polymerase family, member 14  ZNF181 - zinc finger protein 181  PAF1 - paf1, rna polymerase ii associated factor, homolog (s. cerevisiae)  RAC1 - ras-related c3 botulinum toxin substrate 1 (rho family, small gtp binding protein rac1)  RAB5C - rab5c, member ras oncogene family  RABIF - rab interacting factor  INO80 - ino80 complex subunit  DDX56 - dead (asp-glu-ala-asp) box helicase 56  RAB27A - rab27a, member ras oncogene family  NDFIP2 - nedd4 family interacting protein 2  RALA - v-ral simian leukemia viral oncogene homolog a (ras related)  RALB - v-ral simian leukemia viral oncogene homolog b  RALGDS - ral guanine nucleotide dissociation stimulator  RC3H2 - ring finger and ccch-type domains 2  TOMM7 - translocase of outer mitochondrial membrane 7 homolog (yeast)  RANBP2 - ran binding protein 2  RANGAP1 - ran gtpase activating protein 1  RAP1A - rap1a, member of ras oncogene family  MIER2 - mesoderm induction early response 1, family member 2  MOK - mok protein kinase  FAM35A - family with sequence similarity 35, member a  ROBO4 - roundabout, axon guidance receptor, homolog 4 (drosophila)  RARG - retinoic acid receptor, gamma  SGTB - small glutamine-rich tetratricopeptide repeat (tpr)-containing, beta  RARB - retinoic acid receptor, beta  DLL4 - delta-like 4 (drosophila)  RASA1 - ras p21 protein activator (gtpase activating protein) 1  RAP1B - rap1b, member of ras oncogene family  NECAB2 - n-terminal ef-hand calcium binding protein 2  GNL3L - guanine nucleotide binding protein-like 3 (nucleolar)-like  MAGEL2 - mage-like 2  RAP1GAP - rap1 gtpase activating protein  RAP2B - rap2b, member of ras oncogene family  RAP2A - rap2a, member of ras oncogene family  ING3 - inhibitor of growth family, member 3  DDX49 - dead (asp-glu-ala-asp) box polypeptide 49  RBL2 - retinoblastoma-like 2 (p130)  RBBP7 - retinoblastoma binding protein 7  TMX3 - thioredoxin-related transmembrane protein 3  RBBP8 - retinoblastoma binding protein 8  RBM3 - rna binding motif (rnp1, rrm) protein 3  TMCO1 - transmembrane and coiled-coil domains 1  RBM4 - rna binding motif protein 4  RB1 - retinoblastoma 1  ARID4A - at rich interactive domain 4a (rbp1-like)  RBBP5 - retinoblastoma binding protein 5  RBBP6 - retinoblastoma binding protein 6  KDM5A - lysine (k)-specific demethylase 5a  RBBP4 - retinoblastoma binding protein 4  PUS7 - pseudouridylate synthase 7 homolog (s. cerevisiae)  EXOSC4 - exosome component 4  ANKRD13D - ankyrin repeat domain 13 family, member d  DHX29 - deah (asp-glu-ala-his) box polypeptide 29  ZDHHC13 - zinc finger, dhhc-type containing 13  ANKRA2 - ankyrin repeat, family a (rfxank-like), 2  SPTAN1 - spectrin, alpha, non-erythrocytic 1  RBAK - rb-associated krab zinc finger  SCUBE2 - signal peptide, cub domain, egf-like 2  NCOA5 - nuclear receptor coactivator 5  WDR19 - wd repeat domain 19  SPTBN4 - spectrin, beta, non-erythrocytic 4  METTL14 - methyltransferase like 14  SPOCK1 - sparc/osteonectin, cwcv and kazal-like domains proteoglycan (testican) 1  SPP1 - secreted phosphoprotein 1  EPG5 - ectopic p-granules autophagy protein 5 homolog (c. elegans)  TLDC1 - tbc/lysm-associated domain containing 1  SPARC - secreted protein, acidic, cysteine-rich (osteonectin)  DENND1A - denn/madd domain containing 1a  SPAST - spastin  MIER1 - mesoderm induction early response 1, transcriptional regulator  CWC22 - cwc22 spliceosome-associated protein homolog (s. cerevisiae)  GBA2 - glucosidase, beta (bile acid) 2  SP3 - sp3 transcription factor  SP4 - sp4 transcription factor  SP1 - sp1 transcription factor  ZNF410 - zinc finger protein 410  SP2 - sp2 transcription factor  SOX4 - sry (sex determining region y)-box 4  TIPRL - tip41, tor signaling pathway regulator-like (s. cerevisiae)  SOS2 - son of sevenless homolog 2 (drosophila)  SOS1 - son of sevenless homolog 1 (drosophila)  MAGI3 - membrane associated guanylate kinase, ww and pdz domain containing 3  SON - son dna binding protein  HES4 - hairy and enhancer of split 4 (drosophila)  RAB40C - rab40c, member ras oncogene family  GATAD1 - gata zinc finger domain containing 1  SOAT1 - sterol o-acyltransferase 1  MARK4 - map/microtubule affinity-regulating kinase 4  RPRD1B - regulation of nuclear pre-mrna domain containing 1b  C16orf52 - chromosome 16 open reading frame 52  SNAPC4 - small nuclear rna activating complex, polypeptide 4, 190kda  RHOU - ras homolog family member u  SNCA - synuclein, alpha (non a4 component of amyloid precursor)  SNCG - synuclein, gamma (breast cancer-specific protein 1)  FSCN1 - fascin homolog 1, actin-bundling protein (strongylocentrotus purpuratus)  SNRNP70 - small nuclear ribonucleoprotein 70kda (u1)  ZBED5 - zinc finger, bed-type containing 5  SNRPA - small nuclear ribonucleoprotein polypeptide a  SNRPA1 - small nuclear ribonucleoprotein polypeptide a'  CREBZF - creb/atf bzip transcription factor  SUMO2 - small ubiquitin-like modifier 2  SNAP25 - synaptosomal-associated protein, 25kda  PLEKHB1 - pleckstrin homology domain containing, family b (evectins) member 1  SRPRB - signal recognition particle receptor, b subunit  SNCB - synuclein, beta  TP53INP2 - tumor protein p53 inducible nuclear protein 2  PTBP2 - polypyrimidine tract binding protein 2  SMARCE1 - swi/snf related, matrix associated, actin dependent regulator of chromatin, subfamily e, member 1  SMO - smoothened, frizzled family receptor  SMPD1 - sphingomyelin phosphodiesterase 1, acid lysosomal  SMARCB1 - swi/snf related, matrix associated, actin dependent regulator of chromatin, subfamily b, member 1  SMARCC1 - swi/snf related, matrix associated, actin dependent regulator of chromatin, subfamily c, member 1  HLTF - helicase-like transcription factor  SNX6 - sorting nexin 6  SMARCA4 - swi/snf related, matrix associated, actin dependent regulator of chromatin, subfamily a, member 4  SMARCD2 - swi/snf related, matrix associated, actin dependent regulator of chromatin, subfamily d, member 2  SMARCD3 - swi/snf related, matrix associated, actin dependent regulator of chromatin, subfamily d, member 3  SMARCC2 - swi/snf related, matrix associated, actin dependent regulator of chromatin, subfamily c, member 2  SMARCD1 - swi/snf related, matrix associated, actin dependent regulator of chromatin, subfamily d, member 1  WIZ - widely interspaced zinc finger motifs  SLIT3 - slit homolog 3 (drosophila)  MID1IP1 - mid1 interacting protein 1  RBM25 - rna binding motif protein 25  SMARCA1 - swi/snf related, matrix associated, actin dependent regulator of chromatin, subfamily a, member 1  SMARCA2 - swi/snf related, matrix associated, actin dependent regulator of chromatin, subfamily a, member 2  ABRACL - abra c-terminal like  CACTIN - cactin, spliceosome c complex subunit  KMT2C - lysine (k)-specific methyltransferase 2c  EPS15L1 - epidermal growth factor receptor pathway substrate 15-like 1  ZNF462 - zinc finger protein 462  SLC16A2 - solute carrier family 16, member 2 (thyroid hormone transporter)  SLC20A1 - solute carrier family 20 (phosphate transporter), member 1  ARHGAP22 - rho gtpase activating protein 22  SLC18A2 - solute carrier family 18 (vesicular monoamine transporter), member 2  SYN1 - synapsin i  SYP - synaptophysin  SENP2 - sumo1/sentrin/smt3 specific peptidase 2  PLEKHA1 - pleckstrin homology domain containing, family a (phosphoinositide binding specific) member 1  TAC1 - tachykinin, precursor 1  SYT1 - synaptotagmin i  GNB4 - guanine nucleotide binding protein (g protein), beta polypeptide 4  SYT5 - synaptotagmin v  KLHL12 - kelch-like family member 12  SYT4 - synaptotagmin iv  SUPT6H - suppressor of ty 6 homolog (s. cerevisiae)  SUPV3L1 - suppressor of var1, 3-like 1 (s. cerevisiae)  ABCC8 - atp-binding cassette, sub-family c (cftr/mrp), member 8  CACNG7 - calcium channel, voltage-dependent, gamma subunit 7  SUPT4H1 - suppressor of ty 4 homolog 1 (s. cerevisiae)  SUPT5H - suppressor of ty 5 homolog (s. cerevisiae)  VAMP2 - vesicle-associated membrane protein 2 (synaptobrevin 2)  MFSD8 - major facilitator superfamily domain containing 8  UBL5 - ubiquitin-like 5  STXBP1 - syntaxin binding protein 1  STX5 - syntaxin 5  SAV1 - salvador homolog 1 (drosophila)  STX4 - syntaxin 4  STX3 - syntaxin 3  STX1A - syntaxin 1a (brain)  ELOVL5 - elovl fatty acid elongase 5  STRN - striatin, calmodulin binding protein  NIF3L1 - nif3 ngg1 interacting factor 3-like 1 (s. cerevisiae)  STXBP3 - syntaxin binding protein 3  NEK4 - nima-related kinase 4  STK3 - serine/threonine kinase 3  TCTEX1D2 - tctex1 domain containing 2  FAM3A - family with sequence similarity 3, member a  STAU1 - staufen double-stranded rna binding protein 1  NPNT - nephronectin  GPBP1L1 - gc-rich promoter binding protein 1-like 1  STC1 - stanniocalcin 1  AURKC - aurora kinase c  STK10 - serine/threonine kinase 10  PAN3 - pan3 poly(a) specific ribonuclease subunit homolog (s. cerevisiae)  EXOC4 - exocyst complex component 4  CDKL5 - cyclin-dependent kinase-like 5  TGIF2 - tgfb-induced factor homeobox 2  CNEP1R1 - ctd nuclear envelope phosphatase 1 regulatory subunit 1  STK4 - serine/threonine kinase 4  BCL6B - b-cell cll/lymphoma 6, member b  ST5 - suppression of tumorigenicity 5  C12orf44 - chromosome 12 open reading frame 44  ST13 - suppression of tumorigenicity 13 (colon carcinoma) (hsp70 interacting protein)  EEFSEC - eukaryotic elongation factor, selenocysteine-trna-specific  CELF6 - cugbp, elav-like family member 6  STAT2 - signal transducer and activator of transcription 2, 113kda  SMAP1 - small arfgap 1  STAT1 - signal transducer and activator of transcription 1, 91kda  STAT3 - signal transducer and activator of transcription 3 (acute-phase response factor)  STAT5A - signal transducer and activator of transcription 5a  STAT6 - signal transducer and activator of transcription 6, interleukin-4 induced  SGMS1 - sphingomyelin synthase 1  SSRP1 - structure specific recognition protein 1  SSTR2 - somatostatin receptor 2  BOD1L1 - biorientation of chromosomes in cell division 1-like 1  NAA35 - n(alpha)-acetyltransferase 35, natc auxiliary subunit  RINT1 - rad50 interactor 1  SCOC - short coiled-coil protein  RIC8A - ric8 guanine nucleotide exchange factor a  SS18 - synovial sarcoma translocation, chromosome 18  MIIP - migration and invasion inhibitory protein  NAPB - n-ethylmaleimide-sensitive factor attachment protein, beta  GPATCH3 - g patch domain containing 3  SNX33 - sorting nexin 33  ELMO2 - engulfment and cell motility 2  BLOC1S5 - biogenesis of lysosomal organelles complex-1, subunit 5, muted  SRPK2 - srsf protein kinase 2  SRPK1 - srsf protein kinase 1  TAB3 - tgf-beta activated kinase 1/map3k7 binding protein 3  TROVE2 - trove domain family, member 2  GALNT11 - udp-n-acetyl-alpha-d-galactosamine:polypeptide n-acetylgalactosaminyltransferase 11 (galnac-t11)  SSR1 - signal sequence receptor, alpha  SSBP1 - single-stranded dna binding protein 1, mitochondrial  ZNF335 - zinc finger protein 335  SQLE - squalene epoxidase  SPTBN1 - spectrin, beta, non-erythrocytic 1  SRI - sorcin  SRC - v-src avian sarcoma (schmidt-ruppin a-2) viral oncogene homolog  SREBF2 - sterol regulatory element binding transcription factor 2  UBE2O - ubiquitin-conjugating enzyme e2o  NSUN3 - nop2/sun domain family, member 3  SRP9 - signal recognition particle 9kda  RNF169 - ring finger protein 169  SEL1L - sel-1 suppressor of lin-12-like (c. elegans)  TRAPPC2 - trafficking protein particle complex 2  SEC14L1 - sec14-like 1 (s. cerevisiae)  SEC13 - sec13 homolog (s. cerevisiae)  KIAA1244 - kiaa1244  SDCBP - syndecan binding protein (syntenin)  SET - set nuclear oncogene  SETMAR - set domain and mariner transposase fusion gene  JPH3 - junctophilin 3  ZNF304 - zinc finger protein 304  MAP2K4 - mitogen-activated protein kinase kinase 4  RCN3 - reticulocalbin 3, ef-hand calcium binding domain  ZNF286A - zinc finger protein 286a  PBXIP1 - pre-b-cell leukemia homeobox interacting protein 1  SEMA3F - sema domain, immunoglobulin domain (ig), short basic domain, secreted, (semaphorin) 3f  ABHD6 - abhydrolase domain containing 6  CLK4 - cdc-like kinase 4  ZBTB38 - zinc finger and btb domain containing 38  GATAD2B - gata zinc finger domain containing 2b  RALGAPA1 - ral gtpase activating protein, alpha subunit 1 (catalytic)  KCTD13 - potassium channel tetramerization domain containing 13  NDRG3 - ndrg family member 3  BIRC6 - baculoviral iap repeat containing 6  YTHDF3 - yth domain family, member 3  PLEKHG5 - pleckstrin homology domain containing, family g (with rhogef domain) member 5  RHBDD2 - rhomboid domain containing 2  WDR18 - wd repeat domain 18  CNOT6 - ccr4-not transcription complex, subunit 6  SCN2B - sodium channel, voltage-gated, type ii, beta subunit  ATXN2 - ataxin 2  AMIGO1 - adhesion molecule with ig-like domain 1  SCAF4 - sr-related ctd-associated factor 4  ARID1B - at rich interactive domain 1b (swi1-like)  SCO1 - sco1 cytochrome c oxidase assembly protein  ODF2L - outer dense fiber of sperm tails 2-like  MKL2 - mkl/myocardin-like 2  HEG1 - heart development protein with egf-like domains 1  TCP11L2 - t-complex 11, testis-specific-like 2  NLN - neurolysin (metallopeptidase m3 family)  S100B - s100 calcium binding protein b  XPO5 - exportin 5  ARHGAP31 - rho gtpase activating protein 31  KIDINS220 - kinase d-interacting substrate, 220kda  MTA3 - metastasis associated 1 family, member 3  S100A10 - s100 calcium binding protein a10  ZNF608 - zinc finger protein 608  MAVS - mitochondrial antiviral signaling protein  HACE1 - hect domain and ankyrin repeat containing e3 ubiquitin protein ligase 1  SARS - seryl-trna synthetase  TSPAN31 - tetraspanin 31  SAT1 - spermidine/spermine n1-acetyltransferase 1  TBC1D14 - tbc1 domain family, member 14  SATB1 - satb homeobox 1  MIB1 - mindbomb e3 ubiquitin protein ligase 1  RPTOR - regulatory associated protein of mtor, complex 1  SAFB - scaffold attachment factor b  SALL2 - sal-like 2 (drosophila)  KLHL42 - kelch-like family member 42  CDC26 - cell division cycle 26  NLGN2 - neuroligin 2  SLC9A1 - solute carrier family 9, subfamily a (nhe1, cation proton antiporter 1), member 1  SLC8A1 - solute carrier family 8 (sodium/calcium exchanger), member 1  CNOT6L - ccr4-not transcription complex, subunit 6-like  SLC8A2 - solute carrier family 8 (sodium/calcium exchanger), member 2  TAOK1 - tao kinase 1  PDP2 - pyruvate dehyrogenase phosphatase catalytic subunit 2  SLC7A1 - solute carrier family 7 (cationic amino acid transporter, y+ system), member 1  ARRDC3 - arrestin domain containing 3  SEMA6A - sema domain, transmembrane domain (tm), and cytoplasmic domain, (semaphorin) 6a  DOCK6 - dedicator of cytokinesis 6  SIPA1L2 - signal-induced proliferation-associated 1 like 2  SLC12A2 - solute carrier family 12 (sodium/potassium/chloride transporter), member 2  PREX1 - phosphatidylinositol-3,4,5-trisphosphate-dependent rac exchange factor 1  MAPK15 - mitogen-activated protein kinase 15  NYAP1 - neuronal tyrosine-phosphorylated phosphoinositide-3-kinase adaptor 1  SKI - v-ski avian sarcoma viral oncogene homolog  SKIL - ski-like oncogene  SKP1 - s-phase kinase-associated protein 1  WDFY1 - wd repeat and fyve domain containing 1  MKL1 - megakaryoblastic leukemia (translocation) 1  SLC2A1 - solute carrier family 2 (facilitated glucose transporter), member 1  ARHGAP21 - rho gtpase activating protein 21  SYT13 - synaptotagmin xiii  WDR48 - wd repeat domain 48  USP36 - ubiquitin specific peptidase 36  FNIP2 - folliculin interacting protein 2  HOMEZ - homeobox and leucine zipper encoding  EBF4 - early b-cell factor 4  RANBP10 - ran binding protein 10  KIAA1462 - kiaa1462  SH3GL3 - sh3-domain grb2-like 3  VPS18 - vacuolar protein sorting 18 homolog (s. cerevisiae)  STXBP4 - syntaxin binding protein 4  SHOX2 - short stature homeobox 2  SH3RF1 - sh3 domain containing ring finger 1  SIAH2 - siah e3 ubiquitin protein ligase 2  SIAH1 - siah e3 ubiquitin protein ligase 1  POGK - pogo transposable element with krab domain  ZSWIM5 - zinc finger, swim-type containing 5  RICTOR - rptor independent companion of mtor, complex 2  PHF12 - phd finger protein 12  SIPA1 - signal-induced proliferation-associated 1  CALCOCO1 - calcium binding and coiled-coil domain 1  SFPQ - splicing factor proline/glutamine-rich  ZBTB4 - zinc finger and btb domain containing 4  SFRP1 - secreted frizzled-related protein 1  PELI3 - pellino e3 ubiquitin protein ligase family member 3  SFRP2 - secreted frizzled-related protein 2  SFRP4 - secreted frizzled-related protein 4  SRSF1 - serine/arginine-rich splicing factor 1  SRSF2 - serine/arginine-rich splicing factor 2  SRSF3 - serine/arginine-rich splicing factor 3  EPB41L5 - erythrocyte membrane protein band 4.1 like 5  SRSF4 - serine/arginine-rich splicing factor 4  SRSF5 - serine/arginine-rich splicing factor 5  SRSF6 - serine/arginine-rich splicing factor 6  SRSF7 - serine/arginine-rich splicing factor 7  SFSWAP - splicing factor, suppressor of white-apricot homolog (drosophila)  TRA2B - transformer 2 beta homolog (drosophila)  ALS2 - amyotrophic lateral sclerosis 2 (juvenile)  CHD8 - chromodomain helicase dna binding protein 8  LRRC4C - leucine rich repeat containing 4c  ZSWIM6 - zinc finger, swim-type containing 6  SCG5 - secretogranin v (7b2 protein)  SGK1 - serum/glucocorticoid regulated kinase 1  TNRC6C - trinucleotide repeat containing 6c  ZNF317 - zinc finger protein 317  SGTA - small glutamine-rich tetratricopeptide repeat (tpr)-containing, alpha  USP37 - ubiquitin specific peptidase 37  ITSN1 - intersectin 1 (sh3 domain protein)  SH3GL1 - sh3-domain grb2-like 1  SMARCAD1 - swi/snf-related, matrix-associated actin-dependent regulator of chromatin, subfamily a, containing dead/h box 1  MEIS3 - meis homeobox 3  TSC1 - tuberous sclerosis 1  SPIRE1 - spire actin nucleation factor 1  IFT46 - intraflagellar transport 46 homolog (chlamydomonas)  WRNIP1 - werner helicase interacting protein 1  TRPC1 - transient receptor potential cation channel, subfamily c, member 1  UBQLN4 - ubiquilin 4  TUB - tubby bipartite transcription factor  GRIPAP1 - grip1 associated protein 1  CELF4 - cugbp, elav-like family member 4  CDC42SE1 - cdc42 small effector 1  TUBG1 - tubulin, gamma 1  TUFT1 - tuftelin 1  DNAJC7 - dnaj (hsp40) homolog, subfamily c, member 7  ZC3HAV1 - zinc finger ccch-type, antiviral 1  TTF1 - transcription termination factor, rna polymerase i  GPR137 - g protein-coupled receptor 137  SPHK2 - sphingosine kinase 2  SAR1A - sar1 homolog a (s. cerevisiae)  ZNF326 - zinc finger protein 326  TSG101 - tumor susceptibility gene 101  TSC2 - tuberous sclerosis 2  AKIP1 - a kinase (prka) interacting protein 1  TMEM9B - tmem9 domain family, member b  CCDC47 - coiled-coil domain containing 47  CTNNBIP1 - catenin, beta interacting protein 1  RNF149 - ring finger protein 149  BBX - bobby sox homolog (drosophila)  CDC42SE2 - cdc42 small effector 2  TP53BP1 - tumor protein p53 binding protein 1  TP53BP2 - tumor protein p53 binding protein, 2  TOP3A - topoisomerase (dna) iii alpha  POGLUT1 - protein o-glucosyltransferase 1  TOP2B - topoisomerase (dna) ii beta 180kda  FAM20C - family with sequence similarity 20, member c  ATXN7L3 - ataxin 7-like 3  PARP6 - poly (adp-ribose) polymerase family, member 6  CCT3 - chaperonin containing tcp1, subunit 3 (gamma)  TRIO - trio rho guanine nucleotide exchange factor  TRIP6 - thyroid hormone receptor interactor 6  MFF - mitochondrial fission factor  ENY2 - enhancer of yellow 2 homolog (drosophila)  C11orf30 - chromosome 11 open reading frame 30  TRAF1 - tnf receptor-associated factor 1  HSP90B1 - heat shock protein 90kda beta (grp94), member 1  PMEPA1 - prostate transmembrane protein, androgen induced 1  TRAF3 - tnf receptor-associated factor 3  TRAF2 - tnf receptor-associated factor 2  SPPL2B - signal peptide peptidase like 2b  NR2C1 - nuclear receptor subfamily 2, group c, member 1  NR2C2 - nuclear receptor subfamily 2, group c, member 2  TPR - translocated promoter region, nuclear basket protein  NCLN - nicalin  LXN - latexin  NAT14 - n-acetyltransferase 14 (gcn5-related, putative)  REXO4 - rex4, rna exonuclease 4 homolog (s. cerevisiae)  CAMK1D - calcium/calmodulin-dependent protein kinase id  USF1 - upstream transcription factor 1  NUP107 - nucleoporin 107kda  GOPC - golgi-associated pdz and coiled-coil motif containing  USP1 - ubiquitin specific peptidase 1  UNG - uracil-dna glycosylase  AVEN - apoptosis, caspase activation inhibitor  PITHD1 - pith (c-terminal proteasome-interacting domain of thioredoxin-like) domain containing 1  USP4 - ubiquitin specific peptidase 4 (proto-oncogene)  NR1H2 - nuclear receptor subfamily 1, group h, member 2  TWSG1 - twisted gastrulation homolog 1 (drosophila)  PLSCR3 - phospholipid scramblase 3  VGF - vgf nerve growth factor inducible  LAMA1 - laminin, alpha 1  VHL - von hippel-lindau tumor suppressor, e3 ubiquitin protein ligase  VIM - vimentin  EZR - ezrin  TBX20 - t-box 20  PCBP4 - poly(rc) binding protein 4  KCTD1 - potassium channel tetramerization domain containing 1  KDM6A - lysine (k)-specific demethylase 6a  ACKR3 - atypical chemokine receptor 3  UVRAG - uv radiation resistance associated  VASP - vasodilator-stimulated phosphoprotein  VAV2 - vav 2 guanine nucleotide exchange factor  VCAM1 - vascular cell adhesion molecule 1  CCNL1 - cyclin l1  CIAPIN1 - cytokine induced apoptosis inhibitor 1  VCL - vinculin  VCP - valosin containing protein  PDXP - pyridoxal (pyridoxine, vitamin b6) phosphatase  ZNF575 - zinc finger protein 575  ATP10A - atpase, class v, type 10a  MCOLN1 - mucolipin 1  TMEM145 - transmembrane protein 145  THOC2 - tho complex 2  THAP11 - thap domain containing 11  EMC10 - er membrane protein complex subunit 10  UBE2A - ubiquitin-conjugating enzyme e2a  ZNF248 - zinc finger protein 248  PPP1R37 - protein phosphatase 1, regulatory subunit 37  UBB - ubiquitin b  CORO1B - coronin, actin binding protein, 1b  TULP3 - tubby like protein 3  HIRA - histone cell cycle regulator  ZNFX1 - zinc finger, nfx1-type containing 1  TYRO3 - tyro3 protein tyrosine kinase  TYK2 - tyrosine kinase 2  SLC39A10 - solute carrier family 39 (zinc transporter), member 10  ZMIZ1 - zinc finger, miz-type containing 1  TXNRD1 - thioredoxin reductase 1  SLC44A2 - solute carrier family 44 (choline transporter), member 2  UBP1 - upstream binding protein 1 (lbp-1a)  SUMO1 - small ubiquitin-like modifier 1  UCHL1 - ubiquitin carboxyl-terminal esterase l1 (ubiquitin thiolesterase)  UBTF - upstream binding transcription factor, rna polymerase i  RALGAPB - ral gtpase activating protein, beta subunit (non-catalytic)  UGCG - udp-glucose ceramide glucosyltransferase  PELI1 - pellino e3 ubiquitin protein ligase 1  SMURF1 - smad specific e3 ubiquitin protein ligase 1  UBE2I - ubiquitin-conjugating enzyme e2i  UBE2D2 - ubiquitin-conjugating enzyme e2d 2  UBE2V2 - ubiquitin-conjugating enzyme e2 variant 2  ADCK1 - aarf domain containing kinase 1  UBE2L3 - ubiquitin-conjugating enzyme e2l 3  UBE2N - ubiquitin-conjugating enzyme e2n  UBE2V1 - ubiquitin-conjugating enzyme e2 variant 1  TEX2 - testis expressed 2  VPS72 - vacuolar protein sorting 72 homolog (s. cerevisiae)  PSENEN - presenilin enhancer 2 homolog (c. elegans)  ZC3H15 - zinc finger ccch-type containing 15  TCOF1 - treacher collins-franceschetti syndrome 1  TCP1 - t-complex 1  WWC3 - wwc family member 3  ZEB1 - zinc finger e-box binding homeobox 1  BRK1 - brick1, scar/wave actin-nucleating complex subunit  TCF12 - transcription factor 12  ITFG2 - integrin alpha fg-gap repeat containing 2  ZNF354A - zinc finger protein 354a  TCF20 - transcription factor 20 (ar1)  WSB2 - wd repeat and socs box containing 2  HDAC8 - histone deacetylase 8  ASH1L - ash1 (absent, small, or homeotic)-like (drosophila)  DCP1A - decapping mrna 1a  SCN3B - sodium channel, voltage-gated, type iii, beta subunit  HR - hair growth associated  CAND1 - cullin-associated and neddylation-dissociated 1  TDG - thymine-dna glycosylase  PRDX2 - peroxiredoxin 2  EAPP - e2f-associated phosphoprotein  DYNLT3 - dynein, light chain, tctex-type 3  VPS11 - vacuolar protein sorting 11 homolog (s. cerevisiae)  KDM3A - lysine (k)-specific demethylase 3a  DYNLT1 - dynein, light chain, tctex-type 1  PPP1R11 - protein phosphatase 1, regulatory (inhibitor) subunit 11  SLC24A5 - solute carrier family 24 (sodium/potassium/calcium exchanger), member 5  TAF9 - taf9 rna polymerase ii, tata box binding protein (tbp)-associated factor, 32kda  TAF7 - taf7 rna polymerase ii, tata box binding protein (tbp)-associated factor, 55kda  FAM98B - family with sequence similarity 98, member b  TAF6 - taf6 rna polymerase ii, tata box binding protein (tbp)-associated factor, 80kda  TADA2A - transcriptional adaptor 2a  GNG12 - guanine nucleotide binding protein (g protein), gamma 12  TAF4 - taf4 rna polymerase ii, tata box binding protein (tbp)-associated factor, 135kda  TAF2 - taf2 rna polymerase ii, tata box binding protein (tbp)-associated factor, 150kda  ADAM17 - adam metallopeptidase domain 17  TACC1 - transforming, acidic coiled-coil containing protein 1  NSFL1C - nsfl1 (p97) cofactor (p47)  TARBP1 - tar (hiv-1) rna binding protein 1  TALDO1 - transaldolase 1  TAF13 - taf13 rna polymerase ii, tata box binding protein (tbp)-associated factor, 18kda  MAP3K7 - mitogen-activated protein kinase kinase kinase 7  PDGFC - platelet derived growth factor c  ZC4H2 - zinc finger, c4h2 domain containing  KMT2E - lysine (k)-specific methyltransferase 2e  PGP - phosphoglycolate phosphatase  TBCD - tubulin folding cofactor d  ZNF395 - zinc finger protein 395  TAZ - tafazzin  MYNN - myoneurin  SULF2 - sulfatase 2  TCF3 - transcription factor 3  KLHL9 - kelch-like family member 9  DMAP1 - dna methyltransferase 1 associated protein 1  TCEB3 - transcription elongation factor b (siii), polypeptide 3 (110kda, elongin a)  TCF4 - transcription factor 4  NKRF - nfkb repressing factor  RCC2 - regulator of chromosome condensation 2  LANCL2 - lanc lantibiotic synthetase component c-like 2 (bacterial)  TCEA2 - transcription elongation factor a (sii), 2  ERBB2IP - erbb2 interacting protein  TCEA1 - transcription elongation factor a (sii), 1  TIE1 - tyrosine kinase with immunoglobulin-like and egf-like domains 1  TIMP1 - timp metallopeptidase inhibitor 1  TIMP2 - timp metallopeptidase inhibitor 2  TIMP3 - timp metallopeptidase inhibitor 3  TIMP4 - timp metallopeptidase inhibitor 4  TJP1 - tight junction protein 1  TLE1 - transducin-like enhancer of split 1 (e(sp1) homolog, drosophila)  TLE3 - transducin-like enhancer of split 3 (e(sp1) homolog, drosophila)  STK32C - serine/threonine kinase 32c  BLOC1S2 - biogenesis of lysosomal organelles complex-1, subunit 2  TLE4 - transducin-like enhancer of split 4 (e(sp1) homolog, drosophila)  NDNL2 - necdin-like 2  TLN1 - talin 1  TLR3 - toll-like receptor 3  MKX - mohawk homeobox  TSPAN6 - tetraspanin 6  TMF1 - tata element modulatory factor 1  GPR137B - g protein-coupled receptor 137b  TMSB4X - thymosin beta 4, x-linked  TMPO - thymopoietin  TNFAIP1 - tumor necrosis factor, alpha-induced protein 1 (endothelial)  BCL9L - b-cell cll/lymphoma 9-like  TNFRSF1A - tumor necrosis factor receptor superfamily, member 1a  TNFRSF1B - tumor necrosis factor receptor superfamily, member 1b  KCTD21 - potassium channel tetramerization domain containing 21  TMBIM6 - transmembrane bax inhibitor motif containing 6  EIF4ENIF1 - eukaryotic translation initiation factor 4e nuclear import factor 1  TEK - tek tyrosine kinase, endothelial  RPRM - reprimo, tp53 dependent g2 arrest mediator candidate  TEF - thyrotrophic embryonic factor  RCOR2 - rest corepressor 2  TFAM - transcription factor a, mitochondrial  DIABLO - diablo, iap-binding mitochondrial protein  INPP5E - inositol polyphosphate-5-phosphatase, 72 kda  TERF1 - telomeric repeat binding factor (nima-interacting) 1  TERF2 - telomeric repeat binding factor 2  TESK1 - testis-specific kinase 1  TFDP1 - transcription factor dp-1  NR2F2 - nuclear receptor subfamily 2, group f, member 2  TFE3 - transcription factor binding to ighm enhancer 3  BCCIP - brca2 and cdkn1a interacting protein  NR2F1 - nuclear receptor subfamily 2, group f, member 1  TFCP2 - transcription factor cp2  TGFA - transforming growth factor, alpha  ZNF740 - zinc finger protein 740  TGFBR1 - transforming growth factor, beta receptor 1  YLPM1 - ylp motif containing 1  TGFB2 - transforming growth factor, beta 2  TGFB3 - transforming growth factor, beta 3  TGFB1 - transforming growth factor, beta 1  TGFB1I1 - transforming growth factor beta 1 induced transcript 1  RASSF3 - ras association (ralgds/af-6) domain family member 3  CTNNBL1 - catenin, beta like 1  MEPCE - methylphosphate capping enzyme  RNF20 - ring finger protein 20, e3 ubiquitin protein ligase  TGFBR2 - transforming growth factor, beta receptor ii (70/80kda)  TGFBR3 - transforming growth factor, beta receptor iii  TMX4 - thioredoxin-related transmembrane protein 4  THBS1 - thrombospondin 1  THBD - thrombomodulin  TIA1 - tia1 cytotoxic granule-associated rna binding protein  THY1 - thy-1 cell surface antigen  METTL3 - methyltransferase like 3  THRA - thyroid hormone receptor, alpha  ELL - elongation factor rna polymerase ii  ZNF239 - zinc finger protein 239  SYMPK - symplekin  ATAT1 - alpha tubulin acetyltransferase 1  WLS - wntless homolog (drosophila)  SF3A2 - splicing factor 3a, subunit 2, 66kda  PHC3 - polyhomeotic homolog 3 (drosophila)  LZTR1 - leucine-zipper-like transcription regulator 1  FAM192A - family with sequence similarity 192, member a  OBFC1 - oligonucleotide/oligosaccharide-binding fold containing 1  PLEKHH3 - pleckstrin homology domain containing, family h (with myth4 domain) member 3  MKKS - mckusick-kaufman syndrome  NRIP1 - nuclear receptor interacting protein 1  NCOA3 - nuclear receptor coactivator 3  PANK2 - pantothenate kinase 2  ANP32A - acidic (leucine-rich) nuclear phosphoprotein 32 family, member a  SEMA6D - sema domain, transmembrane domain (tm), and cytoplasmic domain, (semaphorin) 6d  WWC2 - ww and c2 domain containing 2  PABPN1 - poly(a) binding protein, nuclear 1  TAF15 - taf15 rna polymerase ii, tata box binding protein (tbp)-associated factor, 68kda  RND2 - rho family gtpase 2  NPRL3 - nitrogen permease regulator-like 3 (s. cerevisiae)  AXIN1 - axin 1  TRRAP - transformation/transcription domain-associated protein  PICALM - phosphatidylinositol binding clathrin assembly protein  GGNBP2 - gametogenetin binding protein 2  CAAP1 - caspase activity and apoptosis inhibitor 1  LPCAT1 - lysophosphatidylcholine acyltransferase 1  ZC3H14 - zinc finger ccch-type containing 14  ARID1A - at rich interactive domain 1a (swi-like)  MAP9 - microtubule-associated protein 9  SETD6 - set domain containing 6  FZD6 - frizzled family receptor 6  FZD8 - frizzled family receptor 8  ACTR5 - arp5 actin-related protein 5 homolog (yeast)  CDC45 - cell division cycle 45  FZD4 - frizzled family receptor 4  FZD1 - frizzled family receptor 1  BAP1 - brca1 associated protein-1 (ubiquitin carboxy-terminal hydrolase)  AXIN2 - axin 2  ZNF613 - zinc finger protein 613  NUP85 - nucleoporin 85kda  CDC7 - cell division cycle 7  BRAP - brca1 associated protein  KDM5C - lysine (k)-specific demethylase 5c  USP9X - ubiquitin specific peptidase 9, x-linked  RBM10 - rna binding motif protein 10  PHF17 - phd finger protein 17  CEP76 - centrosomal protein 76kda  TTC21B - tetratricopeptide repeat domain 21b  MMRN2 - multimerin 2  SLTM - safb-like, transcription modulator  NELFE - negative elongation factor complex member e  EHMT1 - euchromatic histone-lysine n-methyltransferase 1  CAMKMT - calmodulin-lysine n-methyltransferase  NAA40 - n(alpha)-acetyltransferase 40, natd catalytic subunit, homolog (s. cerevisiae)  PIP4K2C - phosphatidylinositol-5-phosphate 4-kinase, type ii, gamma  FZD3 - frizzled family receptor 3  SEMA3B - sema domain, immunoglobulin domain (ig), short basic domain, secreted, (semaphorin) 3b  RAB7A - rab7a, member ras oncogene family  USP7 - ubiquitin specific peptidase 7 (herpes virus-associated)  CSPP1 - centrosome and spindle pole associated protein 1  MANF - mesencephalic astrocyte-derived neurotrophic factor  CPSF7 - cleavage and polyadenylation specific factor 7, 59kda  TCTN2 - tectonic family member 2  CBLL1 - cbl proto-oncogene-like 1, e3 ubiquitin protein ligase  RPAP2 - rna polymerase ii associated protein 2  UBA5 - ubiquitin-like modifier activating enzyme 5  DDX39B - dead (asp-glu-ala-asp) box polypeptide 39b  WDR59 - wd repeat domain 59  USP5 - ubiquitin specific peptidase 5 (isopeptidase t)  KCTD17 - potassium channel tetramerization domain containing 17  KMT2D - lysine (k)-specific methyltransferase 2d  AAAS - achalasia, adrenocortical insufficiency, alacrimia  ZNF419 - zinc finger protein 419  CDK2AP1 - cyclin-dependent kinase 2 associated protein 1  IFT88 - intraflagellar transport 88 homolog (chlamydomonas)  YEATS4 - yeats domain containing 4  SNIP1 - smad nuclear interacting protein 1  ZNF212 - zinc finger protein 212  TXNDC15 - thioredoxin domain containing 15  KATNBL1 - katanin p80 subunit b-like 1  ST7 - suppression of tumorigenicity 7  KAT6A - k(lysine) acetyltransferase 6a  MICALL2 - mical-like 2  STAM - signal transducing adaptor molecule (sh3 domain and itam motif) 1  NUP214 - nucleoporin 214kda  NR4A3 - nuclear receptor subfamily 4, group a, member 3  BRD3 - bromodomain containing 3  SHOC2 - soc-2 suppressor of clear homolog (c. elegans)  CARF - calcium responsive transcription factor  NCOA4 - nuclear receptor coactivator 4  MLLT10 - myeloid/lymphoid or mixed-lineage leukemia (trithorax homolog, drosophila); translocated to, 10  ZNF408 - zinc finger protein 408  FASTKD1 - fast kinase domains 1  ZNF189 - zinc finger protein 189  ZSCAN26 - zinc finger and scan domain containing 26  SMC6 - structural maintenance of chromosomes 6  RNF112 - ring finger protein 112  ZNF177 - zinc finger protein 177  ZNF175 - zinc finger protein 175  ZNF174 - zinc finger protein 174  ZCCHC6 - zinc finger, cchc domain containing 6  ZNF184 - zinc finger protein 184  RNF113A - ring finger protein 113a  ZNF148 - zinc finger protein 148  BEND5 - ben domain containing 5  ZBTB17 - zinc finger and btb domain containing 17  NEIL1 - nei endonuclease viii-like 1 (e. coli)  TRIM26 - tripartite motif containing 26  VEZF1 - vascular endothelial zinc finger 1  AKIRIN1 - akirin 1  USP27X - ubiquitin specific peptidase 27, x-linked  ZNF142 - zinc finger protein 142  MCPH1 - microcephalin 1  ZNF140 - zinc finger protein 140  TMEM204 - transmembrane protein 204  PCGF2 - polycomb group ring finger 2  ZNF132 - zinc finger protein 132  ZNF131 - zinc finger protein 131  ZNF134 - zinc finger protein 134  ZNF133 - zinc finger protein 133  TBL1XR1 - transducin (beta)-like 1 x-linked receptor 1  PRKRIP1 - prkr interacting protein 1 (il11 inducible)  ZNF79 - zinc finger protein 79  ZYG11B - zyg-11 family member b, cell cycle regulator  ZNF70 - zinc finger protein 70  RBM12B - rna binding motif protein 12b  ZNF75D - zinc finger protein 75d  ZNF76 - zinc finger protein 76  TCTN1 - tectonic family member 1  BRPF1 - bromodomain and phd finger containing, 1  SCG2 - secretogranin ii  SAP130 - sin3a-associated protein, 130kda  FZD5 - frizzled family receptor 5  MUL1 - mitochondrial e3 ubiquitin protein ligase 1  CXCR4 - chemokine (c-x-c motif) receptor 4  C10orf76 - chromosome 10 open reading frame 76  PAX8 - paired box 8  TUBA1A - tubulin, alpha 1a  TMEM231 - transmembrane protein 231  ALMS1 - alstrom syndrome 1  CDC73 - cell division cycle 73  NKAP - nfkb activating protein  DAP3 - death associated protein 3  LRP8 - low density lipoprotein receptor-related protein 8, apolipoprotein e receptor  PTP4A1 - protein tyrosine phosphatase type iva, member 1  ROGDI - rogdi homolog (drosophila)  PRDM2 - pr domain containing 2, with znf domain  MAP3K12 - mitogen-activated protein kinase kinase kinase 12  ZYX - zyxin  SLC30A4 - solute carrier family 30 (zinc transporter), member 4  OGFRL1 - opioid growth factor receptor-like 1  SLC30A1 - solute carrier family 30 (zinc transporter), member 1  ZNF226 - zinc finger protein 226  NDNF - neuron-derived neurotrophic factor  HMBOX1 - homeobox containing 1  ZNF213 - zinc finger protein 213  ZNF205 - zinc finger protein 205  ZNF207 - zinc finger protein 207  LRFN3 - leucine rich repeat and fibronectin type iii domain containing 3  XPA - xeroderma pigmentosum, complementation group a  XPC - xeroderma pigmentosum, complementation group c  HAUS3 - haus augmin-like complex, subunit 3  XPO1 - exportin 1 (crm1 homolog, yeast)  XRCC1 - x-ray repair complementing defective repair in chinese hamster cells 1  PAGR1 - paxip1 associated glutamate-rich protein 1  ZXDC - zxd family zinc finger c  RSG1 - rem2 and rab-like small gtpase 1  XBP1 - x-box binding protein 1  BHLHE41 - basic helix-loop-helix family, member e41  EIF4H - eukaryotic translation initiation factor 4h  WEE1 - wee1 homolog (s. pombe)  WFS1 - wolfram syndrome 1 (wolframin)  WHSC1 - wolf-hirschhorn syndrome candidate 1  NELFA - negative elongation factor complex member a  VRK2 - vaccinia related kinase 2  VSNL1 - visinin-like 1  WARS - tryptophanyl-trna synthetase  ZNF35 - zinc finger protein 35  ZKSCAN1 - zinc finger with krab and scan domains 1  FBXL15 - f-box and leucine-rich repeat protein 15  ZNF576 - zinc finger protein 576  ZNF33B - zinc finger protein 33b  ZNF32 - zinc finger protein 32  THOC6 - tho complex 6 homolog (drosophila)  ZNF45 - zinc finger protein 45  BRCC3 - brca1/brca2-containing complex, subunit 3  MZF1 - myeloid zinc finger 1  FSD1 - fibronectin type iii and spry domain containing 1  ZNF18 - zinc finger protein 18  ZNF16 - zinc finger protein 16  TNIP2 - tnfaip3 interacting protein 2  RBM42 - rna binding motif protein 42  ZNF24 - zinc finger protein 24  ZFPL1 - zinc finger protein-like 1  ZFX - zinc finger protein, x-linked  SF1 - splicing factor 1  ZBTB14 - zinc finger and btb domain containing 14  ZNF711 - zinc finger protein 711  ZNF7 - zinc finger protein 7  ZNF8 - zinc finger protein 8  ZNF2 - zinc finger protein 2  ZNF3 - zinc finger protein 3  YES1 - v-yes-1 yamaguchi sarcoma viral oncogene homolog 1  XRCC5 - x-ray repair complementing defective repair in chinese hamster cells 5 (double-strand-break rejoining)  YWHAG - tyrosine 3-monooxygenase/tryptophan 5-monooxygenase activation protein, gamma polypeptide  YWHAZ - tyrosine 3-monooxygenase/tryptophan 5-monooxygenase activation protein, zeta polypeptide  YWHAE - tyrosine 3-monooxygenase/tryptophan 5-monooxygenase activation protein, epsilon polypeptide  YWHAB - tyrosine 3-monooxygenase/tryptophan 5-monooxygenase activation protein, beta polypeptide  CCNK - cyclin k  MAPKAP1 - mitogen-activated protein kinase associated protein 1  TRIM24 - tripartite motif containing 24  TAF1D - tata box binding protein (tbp)-associated factor, rna polymerase i, d, 41kda  CREG1 - cellular repressor of e1a-stimulated genes 1  PEX11B - peroxisomal biogenesis factor 11 beta  CHAC1 - chac, cation transport regulator homolog 1 (e. coli)  PEX11A - peroxisomal biogenesis factor 11 alpha  NRP1 - neuropilin 1  NRP2 - neuropilin 2  IQGAP1 - iq motif containing gtpase activating protein 1  HESX1 - hesx homeobox 1  PHF23 - phd finger protein 23  SAP30 - sin3a-associated protein, 30kda  DERL1 - derlin 1  METTL16 - methyltransferase like 16  ALKBH1 - alkb, alkylation repair homolog 1 (e. coli)  FTO - fat mass and obesity associated  CFLAR - casp8 and fadd-like apoptosis regulator  SYNGAP1 - synaptic ras gtpase activating protein 1  SECISBP2 - secis binding protein 2  MED11 - mediator complex subunit 11  SYNJ1 - synaptojanin 1  STK19 - serine/threonine kinase 19  LDB1 - lim domain binding 1  WDR77 - wd repeat domain 77  ALDH1A2 - aldehyde dehydrogenase 1 family, member a2  DSCC1 - dna replication and sister chromatid cohesion 1  KAT2B - k(lysine) acetyltransferase 2b  TSC22D1 - tsc22 domain family, member 1  AKAP4 - a kinase (prka) anchor protein 4  TMEM109 - transmembrane protein 109  CDC16 - cell division cycle 16  DDA1 - det1 and ddb1 associated 1  ZNF259 - zinc finger protein 259  TAX1BP1 - tax1 (human t-cell leukemia virus type i) binding protein 1  NAE1 - nedd8 activating enzyme e1 subunit 1  CDC123 - cell division cycle 123  ARHGEF7 - rho guanine nucleotide exchange factor (gef) 7  METRN - meteorin, glial cell differentiation regulator  DBNDD1 - dysbindin (dystrobrevin binding protein 1) domain containing 1  NABP2 - nucleic acid binding protein 2  MTMR2 - myotubularin related protein 2  MTMR3 - myotubularin related protein 3  CPNE1 - copine i  EIF2B2 - eukaryotic translation initiation factor 2b, subunit 2 beta, 39kda  EIF2B3 - eukaryotic translation initiation factor 2b, subunit 3 gamma, 58kda  BUD31 - bud31 homolog (s. cerevisiae)  CPNE3 - copine iii  ZNF655 - zinc finger protein 655  EIF2B5 - eukaryotic translation initiation factor 2b, subunit 5 epsilon, 82kda  TMEM108 - transmembrane protein 108  HERC1 - hect and rld domain containing e3 ubiquitin protein ligase family member 1  TRAK2 - trafficking protein, kinesin binding 2  MTMR9 - myotubularin related protein 9  CACNA1H - calcium channel, voltage-dependent, t type, alpha 1h subunit  RASL11B - ras-like, family 11, member b  BCL10 - b-cell cll/lymphoma 10  FUBP3 - far upstream element (fuse) binding protein 3  C7orf49 - chromosome 7 open reading frame 49  CDK5R2 - cyclin-dependent kinase 5, regulatory subunit 2 (p39)  AP3D1 - adaptor-related protein complex 3, delta 1 subunit  BTRC - beta-transducin repeat containing e3 ubiquitin protein ligase  SKAP2 - src kinase associated phosphoprotein 2  BAIAP3 - bai1-associated protein 3  WASF1 - was protein family, member 1  NADK - nad kinase  WNK1 - wnk lysine deficient protein kinase 1  EIF4EBP3 - eukaryotic translation initiation factor 4e binding protein 3  UBE2Z - ubiquitin-conjugating enzyme e2z  NUMB - numb homolog (drosophila)  LAMTOR3 - late endosomal/lysosomal adaptor, mapk and mtor activator 3  PHACTR4 - phosphatase and actin regulator 4  EIF3C - eukaryotic translation initiation factor 3, subunit c  EIF3D - eukaryotic translation initiation factor 3, subunit d  BRD9 - bromodomain containing 9  EIF3A - eukaryotic translation initiation factor 3, subunit a  EIF3B - eukaryotic translation initiation factor 3, subunit b  STK33 - serine/threonine kinase 33  IRS2 - insulin receptor substrate 2  DYNLL1 - dynein, light chain, lc8-type 1  TNKS - tankyrase, trf1-interacting ankyrin-related adp-ribose polymerase  EIF4G3 - eukaryotic translation initiation factor 4 gamma, 3  ZBTB10 - zinc finger and btb domain containing 10  AACS - acetoacetyl-coa synthetase  CAPRIN2 - caprin family member 2  BECN1 - beclin 1, autophagy related  SRSF9 - serine/arginine-rich splicing factor 9  PEA15 - phosphoprotein enriched in astrocytes 15  VAMP4 - vesicle-associated membrane protein 4  NDRG4 - ndrg family member 4  TMEM237 - transmembrane protein 237  HYAL2 - hyaluronoglucosaminidase 2  ACD - adrenocortical dysplasia homolog (mouse)  GPBP1 - gc-rich promoter binding protein 1  RAPH1 - ras association (ralgds/af-6) and pleckstrin homology domains 1  TMEM135 - transmembrane protein 135  CDC23 - cell division cycle 23  INTS3 - integrator complex subunit 3  MARCKSL1 - marcks-like 1  UPF3B - upf3 regulator of nonsense transcripts homolog b (yeast)  UPF3A - upf3 regulator of nonsense transcripts homolog a (yeast)  LPIN3 - lipin 3  MBTPS1 - membrane-bound transcription factor peptidase, site 1  TRADD - tnfrsf1a-associated via death domain  SNX3 - sorting nexin 3  SNX4 - sorting nexin 4  CTNNAL1 - catenin (cadherin-associated protein), alpha-like 1  EED - embryonic ectoderm development  URI1 - uri1, prefoldin-like chaperone  GBF1 - golgi brefeldin a resistant guanine nucleotide exchange factor 1  RIPK1 - receptor (tnfrsf)-interacting serine-threonine kinase 1  TNFSF13 - tumor necrosis factor (ligand) superfamily, member 13  TNFSF12 - tumor necrosis factor (ligand) superfamily, member 12  ADAM15 - adam metallopeptidase domain 15  ADAM9 - adam metallopeptidase domain 9  CD164 - cd164 molecule, sialomucin  P2RY12 - purinergic receptor p2y, g-protein coupled, 12  RAB11A - rab11a, member ras oncogene family  TNFRSF6B - tumor necrosis factor receptor superfamily, member 6b, decoy  RIPK2 - receptor-interacting serine-threonine kinase 2  PORCN - porcupine homolog (drosophila)  NAPA - n-ethylmaleimide-sensitive factor attachment protein, alpha  MTMR1 - myotubularin related protein 1  YTHDC2 - yth domain containing 2  AIDA - axin interactor, dorsalization associated  RGS11 - regulator of g-protein signaling 11  USP46 - ubiquitin specific peptidase 46  FAM129B - family with sequence similarity 129, member b  RGS9 - regulator of g-protein signaling 9  DLK1 - delta-like 1 homolog (drosophila)  NABP1 - nucleic acid binding protein 1  RFX7 - regulatory factor x, 7  DEPTOR - dep domain containing mtor-interacting protein  HAT1 - histone acetyltransferase 1  EPS8L2 - eps8-like 2  CRTC3 - creb regulated transcription coactivator 3  RBM15 - rna binding motif protein 15  ENC1 - ectodermal-neural cortex 1 (with btb domain)  PKP4 - plakophilin 4  PIK3R3 - phosphoinositide-3-kinase, regulatory subunit 3 (gamma)  PARG - poly (adp-ribose) glycohydrolase  MEAF6 - myst/esa1-associated factor 6  PPFIA2 - protein tyrosine phosphatase, receptor type, f polypeptide (ptprf), interacting protein (liprin), alpha 2  PPFIA1 - protein tyrosine phosphatase, receptor type, f polypeptide (ptprf), interacting protein (liprin), alpha 1  PPM1D - protein phosphatase, mg2+/mn2+ dependent, 1d  ZNF574 - zinc finger protein 574  CREB3L2 - camp responsive element binding protein 3-like 2  IPPK - inositol 1,3,4,5,6-pentakisphosphate 2-kinase  TNS3 - tensin 3  RGS5 - regulator of g-protein signaling 5  MAP4K3 - mitogen-activated protein kinase kinase kinase kinase 3  CDC14B - cell division cycle 14b  PIAS1 - protein inhibitor of activated stat, 1  SMYD3 - set and mynd domain containing 3  SMURF2 - smad specific e3 ubiquitin protein ligase 2  MAPKAPK5 - mitogen-activated protein kinase-activated protein kinase 5  AP3B1 - adaptor-related protein complex 3, beta 1 subunit  CGGBP1 - cgg triplet repeat binding protein 1  WDR13 - wd repeat domain 13  LMO4 - lim domain only 4  PPFIA3 - protein tyrosine phosphatase, receptor type, f polypeptide (ptprf), interacting protein (liprin), alpha 3  API5 - apoptosis inhibitor 5  CAMK1 - calcium/calmodulin-dependent protein kinase i  CBX4 - chromobox homolog 4  COPS3 - cop9 signalosome subunit 3  GORASP1 - golgi reassembly stacking protein 1, 65kda  ANAPC1 - anaphase promoting complex subunit 1  DGKE - diacylglycerol kinase, epsilon 64kda  CSRNP1 - cysteine-serine-rich nuclear protein 1  DGKZ - diacylglycerol kinase, zeta  GIGYF1 - grb10 interacting gyf protein 1  VPS16 - vacuolar protein sorting 16 homolog (s. cerevisiae)  PRKRA - protein kinase, interferon-inducible double stranded rna dependent activator  STK16 - serine/threonine kinase 16  MADD - map-kinase activating death domain  KHSRP - kh-type splicing regulatory protein  CASK - calcium/calmodulin-dependent serine protein kinase (maguk family)  CDK10 - cyclin-dependent kinase 10  SUDS3 - suppressor of defective silencing 3 homolog (s. cerevisiae)  ZDHHC6 - zinc finger, dhhc-type containing 6  SKAP1 - src kinase associated phosphoprotein 1  GZF1 - gdnf-inducible zinc finger protein 1  ARAP3 - arfgap with rhogap domain, ankyrin repeat and ph domain 3  ATG3 - autophagy related 3  UNC5C - unc-5 homolog c (c. elegans)  PDE8B - phosphodiesterase 8b  CDK13 - cyclin-dependent kinase 13  AKTIP - akt interacting protein  CADPS - ca++-dependent secretion activator  RFXANK - regulatory factor x-associated ankyrin-containing protein  KLF7 - kruppel-like factor 7 (ubiquitous)  IKZF5 - ikaros family zinc finger 5 (pegasus)  RUVBL1 - ruvb-like 1 (e. coli)  USO1 - uso1 vesicle transport factor  ZMAT3 - zinc finger, matrin-type 3  AZI2 - 5-azacytidine induced 2  ARHGEF28 - rho guanine nucleotide exchange factor (gef) 28  ZSCAN31 - zinc finger and scan domain containing 31  PAPD5 - pap associated domain containing 5  NSD1 - nuclear receptor binding set domain protein 1  RFWD2 - ring finger and wd repeat domain 2, e3 ubiquitin protein ligase  LMBR1 - limb development membrane protein 1  RNF25 - ring finger protein 25  MMS19 - mms19 nucleotide excision repair homolog (s. cerevisiae)  HERPUD2 - herpud family member 2  PIP5K1A - phosphatidylinositol-4-phosphate 5-kinase, type i, alpha  ERAP2 - endoplasmic reticulum aminopeptidase 2  PDF - peptide deformylase (mitochondrial)  IRF2BPL - interferon regulatory factor 2 binding protein-like  PLA2G6 - phospholipase a2, group vi (cytosolic, calcium-independent)  PIP5K1B - phosphatidylinositol-4-phosphate 5-kinase, type i, beta  RRAGC - ras-related gtp binding c  TMBIM1 - transmembrane bax inhibitor motif containing 1  MAGEF1 - melanoma antigen family f, 1  NME5 - nme/nm23 family member 5  MAD1L1 - mad1 mitotic arrest deficient-like 1 (yeast)  LTBP4 - latent transforming growth factor beta binding protein 4  ZNF282 - zinc finger protein 282  STK24 - serine/threonine kinase 24  GFRA4 - gdnf family receptor alpha 4  RASAL1 - ras protein activator like 1 (gap1 like)  NSMAF - neutral sphingomyelinase (n-smase) activation associated factor  LRRC4 - leucine rich repeat containing 4  NCK2 - nck adaptor protein 2  DYRK3 - dual-specificity tyrosine-(y)-phosphorylation regulated kinase 3  RBM26 - rna binding motif protein 26  SPARCL1 - sparc-like 1 (hevin)  ULK1 - unc-51 like autophagy activating kinase 1  BCAR3 - breast cancer anti-estrogen resistance 3  GOLPH3 - golgi phosphoprotein 3 (coat-protein)  SORBS2 - sorbin and sh3 domain containing 2  SMARCA5 - swi/snf related, matrix associated, actin dependent regulator of chromatin, subfamily a, member 5  CDC42BPA - cdc42 binding protein kinase alpha (dmpk-like)  OGT - o-linked n-acetylglucosamine (glcnac) transferase  RAE1 - rae1 rna export 1 homolog (s. pombe)  TSPYL2 - tspy-like 2  CUL4B - cullin 4b  DOC2B - double c2-like domains, beta  CUL1 - cullin 1  PCIF1 - pdx1 c-terminal inhibiting factor 1  CUL2 - cullin 2  CUL3 - cullin 3  ZNF667 - zinc finger protein 667  SUPT3H - suppressor of ty 3 homolog (s. cerevisiae)  PCGF5 - polycomb group ring finger 5  WTAP - wilms tumor 1 associated protein  HVCN1 - hydrogen voltage-gated channel 1  RBM39 - rna binding motif protein 39  ZNF414 - zinc finger protein 414  PITPNM1 - phosphatidylinositol transfer protein, membrane-associated 1  PDIA4 - protein disulfide isomerase family a, member 4  AKAP12 - a kinase (prka) anchor protein 12  IER2 - immediate early response 2  NCOR1 - nuclear receptor corepressor 1  NCOR2 - nuclear receptor corepressor 2  HOOK3 - hook microtubule-tethering protein 3  CARTPT - cart prepropeptide  LZTS2 - leucine zipper, putative tumor suppressor 2  DOT1L - dot1-like histone h3k79 methyltransferase  ABCG1 - atp-binding cassette, sub-family g (white), member 1  EFCAB7 - ef-hand calcium binding domain 7  ATP6V1G1 - atpase, h+ transporting, lysosomal 13kda, v1 subunit g1  L3MBTL3 - l(3)mbt-like 3 (drosophila)  TICAM2 - toll-like receptor adaptor molecule 2  RAB3D - rab3d, member ras oncogene family  ZNF333 - zinc finger protein 333  CIR1 - corepressor interacting with rbpj, 1  BRSK1 - br serine/threonine kinase 1  SYVN1 - synovial apoptosis inhibitor 1, synoviolin  NRG2 - neuregulin 2  VPS26A - vacuolar protein sorting 26 homolog a (s. pombe)  SLX4 - slx4 structure-specific endonuclease subunit  CHD1L - chromodomain helicase dna binding protein 1-like  SEC22B - sec22 vesicle trafficking protein homolog b (s. cerevisiae) (gene/pseudogene)  H2AFY - h2a histone family, member y  GOSR2 - golgi snap receptor complex member 2  GTF2IRD1 - gtf2i repeat domain containing 1  GTPBP1 - gtp binding protein 1  ZNF527 - zinc finger protein 527  JPH4 - junctophilin 4  BCAR1 - breast cancer anti-estrogen resistance 1  SPIRE2 - spire actin nucleation factor 2  H6PD - hexose-6-phosphate dehydrogenase (glucose 1-dehydrogenase)  FAM120B - family with sequence similarity 120b  HOPX - hop homeobox  PREPL - prolyl endopeptidase-like  SOX13 - sry (sex determining region y)-box 13  ZC3H8 - zinc finger ccch-type containing 8  ZNF316 - zinc finger protein 316  JAGN1 - jagunal homolog 1 (drosophila)  CDC42BPB - cdc42 binding protein kinase beta (dmpk-like)  CLOCK - clock circadian regulator  ADAMTS1 - adam metallopeptidase with thrombospondin type 1 motif, 1  FXR2 - fragile x mental retardation, autosomal homolog 2  LITAF - lipopolysaccharide-induced tnf factor  SPTLC2 - serine palmitoyltransferase, long chain base subunit 2  TBPL1 - tbp-like 1  NPEPPS - aminopeptidase puromycin sensitive  EEF1E1 - eukaryotic translation elongation factor 1 epsilon 1  VPS4B - vacuolar protein sorting 4 homolog b (s. cerevisiae)  ZBTB41 - zinc finger and btb domain containing 41  GOSR1 - golgi snap receptor complex member 1  BAG5 - bcl2-associated athanogene 5  TMEM59 - transmembrane protein 59  BAG4 - bcl2-associated athanogene 4  ZGPAT - zinc finger, ccch-type with g patch domain  TP53I3 - tumor protein p53 inducible protein 3  EI24 - etoposide induced 2.4  AKAP7 - a kinase (prka) anchor protein 7  IL27RA - interleukin 27 receptor, alpha  EIF4E2 - eukaryotic translation initiation factor 4e family member 2  MED20 - mediator complex subunit 20  ATG5 - autophagy related 5  AFAP1L2 - actin filament associated protein 1-like 2  ROCK2 - rho-associated, coiled-coil containing protein kinase 2  SLC25A27 - solute carrier family 25, member 27  MAPK8IP1 - mitogen-activated protein kinase 8 interacting protein 1  GLYR1 - glyoxylate reductase 1 homolog (arabidopsis)  DPY30 - dpy-30 homolog (c. elegans)  SLC4A8 - solute carrier family 4, sodium bicarbonate cotransporter, member 8  RPH3AL - rabphilin 3a-like (without c2 domains)  MAGED1 - melanoma antigen family d, 1  GLIS2 - glis family zinc finger 2  IRF2BP2 - interferon regulatory factor 2 binding protein 2  MED27 - mediator complex subunit 27  MED26 - mediator complex subunit 26  CHD6 - chromodomain helicase dna binding protein 6  MED17 - mediator complex subunit 17  MED23 - mediator complex subunit 23  ANTXR1 - anthrax toxin receptor 1  CDYL - chromodomain protein, y-like  RPF2 - ribosome production factor 2 homolog (s. cerevisiae)  GTF2IRD2 - gtf2i repeat domain containing 2  ARHGEF6 - rac/cdc42 guanine nucleotide exchange factor (gef) 6  ZNF710 - zinc finger protein 710  PICK1 - protein interacting with prkca 1  TMEM117 - transmembrane protein 117  EIF2AK3 - eukaryotic translation initiation factor 2-alpha kinase 3  ITM2B - integral membrane protein 2b  MAP4K4 - mitogen-activated protein kinase kinase kinase kinase 4  RHBDD1 - rhomboid domain containing 1  TRAF7 - tnf receptor-associated factor 7, e3 ubiquitin protein ligase  RECQL5 - recq protein-like 5  TGFBRAP1 - transforming growth factor, beta receptor associated protein 1  CIAO1 - cytosolic iron-sulfur protein assembly 1  WDR24 - wd repeat domain 24  SYT3 - synaptotagmin iii  CAMKK1 - calcium/calmodulin-dependent protein kinase kinase 1, alpha  CRIPT - cysteine-rich pdz-binding protein  CYP7B1 - cytochrome p450, family 7, subfamily b, polypeptide 1  GARNL3 - gtpase activating rap/rangap domain-like 3  TJP2 - tight junction protein 2  ARHGAP29 - rho gtpase activating protein 29  MED21 - mediator complex subunit 21  MED10 - mediator complex subunit 10  POLDIP3 - polymerase (dna-directed), delta interacting protein 3  LHFPL4 - lipoma hmgic fusion partner-like 4  RAB9A - rab9a, member ras oncogene family  NOA1 - nitric oxide associated 1  TCHP - trichoplein, keratin filament binding  TXNL1 - thioredoxin-like 1  DCUN1D5 - dcn1, defective in cullin neddylation 1, domain containing 5  SLIT2 - slit homolog 2 (drosophila)  ITGBL1 - integrin, beta-like 1 (with egf-like repeat domains)  ING5 - inhibitor of growth family, member 5  SLC25A33 - solute carrier family 25 (pyrimidine nucleotide carrier), member 33  TMEM110 - transmembrane protein 110  KIF3B - kinesin family member 3b  DNAJC30 - dnaj (hsp40) homolog, subfamily c, member 30  ZFYVE9 - zinc finger, fyve domain containing 9  MAST4 - microtubule associated serine/threonine kinase family member 4  BTBD10 - btb (poz) domain containing 10  TRIP12 - thyroid hormone receptor interactor 12  NUDT16L1 - nudix (nucleoside diphosphate linked moiety x)-type motif 16-like 1  ZNHIT3 - zinc finger, hit-type containing 3  ZNF397 - zinc finger protein 397  TRIP4 - thyroid hormone receptor interactor 4  HMGN3 - high mobility group nucleosomal binding domain 3  PHF6 - phd finger protein 6  FAM213A - family with sequence similarity 213, member a  ZNF235 - zinc finger protein 235  COPS2 - cop9 signalosome subunit 2  MIEN1 - migration and invasion enhancer 1  VAMP3 - vesicle-associated membrane protein 3  MIA3 - melanoma inhibitory activity family, member 3  TAOK2 - tao kinase 2  PEAR1 - platelet endothelial aggregation receptor 1  SARNP - sap domain containing ribonucleoprotein  BRMS1L - breast cancer metastasis-suppressor 1-like  VPS25 - vacuolar protein sorting 25 homolog (s. cerevisiae)  CNOT8 - ccr4-not transcription complex, subunit 8  ATP6V1F - atpase, h+ transporting, lysosomal 14kda, v1 subunit f  S1PR2 - sphingosine-1-phosphate receptor 2  KCTD10 - potassium channel tetramerization domain containing 10  SNX25 - sorting nexin 25  CD83 - cd83 molecule  SOCS6 - suppressor of cytokine signaling 6  SPRTN - sprt-like n-terminal domain  BCL7B - b-cell cll/lymphoma 7b  CYTH1 - cytohesin 1  ITGB1BP1 - integrin beta 1 binding protein 1  CYTH3 - cytohesin 3  CYTH2 - cytohesin 2  MED14 - mediator complex subunit 14  ZBTB22 - zinc finger and btb domain containing 22  CTTNBP2 - cortactin binding protein 2  NUMBL - numb homolog (drosophila)-like  RPS6KA5 - ribosomal protein s6 kinase, 90kda, polypeptide 5  CCDC8 - coiled-coil domain containing 8  NCALD - neurocalcin delta  STK17A - serine/threonine kinase 17a  CACNA2D2 - calcium channel, voltage-dependent, alpha 2/delta subunit 2  MFHAS1 - malignant fibrous histiocytoma amplified sequence 1  TM2D1 - tm2 domain containing 1  RAB11B - rab11b, member ras oncogene family  MTA2 - metastasis associated 1 family, member 2  RASSF4 - ras association (ralgds/af-6) domain family member 4  NOLC1 - nucleolar and coiled-body phosphoprotein 1  EIF2A - eukaryotic translation initiation factor 2a, 65kda  MAGI1 - membrane associated guanylate kinase, ww and pdz domain containing 1  PNMA1 - paraneoplastic ma antigen 1  IFI27L2 - interferon, alpha-inducible protein 27-like 2  DLG5 - discs, large homolog 5 (drosophila)  CCPG1 - cell cycle progression 1  ZMYM3 - zinc finger, mym-type 3  ZMYM4 - zinc finger, mym-type 4  NSRP1 - nuclear speckle splicing regulatory protein 1  ANKRD27 - ankyrin repeat domain 27 (vps9 domain)  KBTBD7 - kelch repeat and btb (poz) domain containing 7  XPR1 - xenotropic and polytropic retrovirus receptor 1  VAPB - vamp (vesicle-associated membrane protein)-associated protein b and c  SKA2 - spindle and kinetochore associated complex subunit 2  BUB3 - bub3 mitotic checkpoint protein  REPS2 - ralbp1 associated eps domain containing 2  WDR54 - wd repeat domain 54  ZW10 - zw10 kinetochore protein  SLC35B2 - solute carrier family 35 (adenosine 3'-phospho 5'-phosphosulfate transporter), member b2  DDX21 - dead (asp-glu-ala-asp) box helicase 21  ZBED1 - zinc finger, bed-type containing 1  DEDD - death effector domain containing  DCLK1 - doublecortin-like kinase 1  SLC33A1 - solute carrier family 33 (acetyl-coa transporter), member 1  AMIGO2 - adhesion molecule with ig-like domain 2  WDR75 - wd repeat domain 75  USP42 - ubiquitin specific peptidase 42  CEP78 - centrosomal protein 78kda  COX7A2L - cytochrome c oxidase subunit viia polypeptide 2 like  EBAG9 - estrogen receptor binding site associated, antigen, 9  UTP15 - utp15, u3 small nucleolar ribonucleoprotein, homolog (s. cerevisiae)  FAM161A - family with sequence similarity 161, member a  KAT8 - k(lysine) acetyltransferase 8  ZNF644 - zinc finger protein 644  ARHGEF2 - rho/rac guanine nucleotide exchange factor (gef) 2  OSMR - oncostatin m receptor  PPP1R1B - protein phosphatase 1, regulatory (inhibitor) subunit 1b  TUBB2B - tubulin, beta 2b class iib  ARL6 - adp-ribosylation factor-like 6  SYNGR1 - synaptogyrin 1  HGS - hepatocyte growth factor-regulated tyrosine kinase substrate  NEMF - nuclear export mediator factor  ZNF394 - zinc finger protein 394  ATRIP - atr interacting protein  ARHGEF1 - rho guanine nucleotide exchange factor (gef) 1  CBFA2T2 - core-binding factor, runt domain, alpha subunit 2; translocated to, 2  RABEP1 - rabaptin, rab gtpase binding effector protein 1  RQCD1 - rcd1 required for cell differentiation1 homolog (s. pombe)  SMC3 - structural maintenance of chromosomes 3  APH1B - aph1b gamma secretase subunit  ATP6V0D1 - atpase, h+ transporting, lysosomal 38kda, v0 subunit d1  LATS1 - large tumor suppressor kinase 1  MTA1 - metastasis associated 1  MTMR4 - myotubularin related protein 4  RAB33B - rab33b, member ras oncogene family  USP8 - ubiquitin specific peptidase 8  USP14 - ubiquitin specific peptidase 14 (trna-guanine transglycosylase)  USP10 - ubiquitin specific peptidase 10  NUP43 - nucleoporin 43kda  UNC119 - unc-119 homolog (c. elegans)  PKMYT1 - protein kinase, membrane associated tyrosine/threonine 1  SART1 - squamous cell carcinoma antigen recognized by t cells  ZMIZ2 - zinc finger, miz-type containing 2  LDB2 - lim domain binding 2  DIRAS3 - diras family, gtp-binding ras-like 3  ASH2L - ash2 (absent, small, or homeotic)-like (drosophila)  THAP2 - thap domain containing, apoptosis associated protein 2  SYT7 - synaptotagmin vii  ITCH - itchy e3 ubiquitin protein ligase  ZNF484 - zinc finger protein 484  L3MBTL2 - l(3)mbt-like 2 (drosophila)  RIOK1 - rio kinase 1  ZNF445 - zinc finger protein 445  FAM57B - family with sequence similarity 57, member b  AIP - aryl hydrocarbon receptor interacting protein  SEMA5A - sema domain, seven thrombospondin repeats (type 1 and type 1-like), transmembrane domain (tm) and short cytoplasmic domain, (semaphorin) 5a  TRAPPC9 - trafficking protein particle complex 9  BTAF1 - btaf1 rna polymerase ii, b-tfiid transcription factor-associated, 170kda  SPAG9 - sperm associated antigen 9  JAM3 - junctional adhesion molecule 3  UBE2M - ubiquitin-conjugating enzyme e2m  UBA3 - ubiquitin-like modifier activating enzyme 3  SESN2 - sestrin 2  PARP9 - poly (adp-ribose) polymerase family, member 9  HIP1R - huntingtin interacting protein 1 related  RNF8 - ring finger protein 8, e3 ubiquitin protein ligase  BAZ1B - bromodomain adjacent to zinc finger domain, 1b  RHBDL1 - rhomboid, veinlet-like 1 (drosophila)  MPZL1 - myelin protein zero-like 1  RAB34 - rab34, member ras oncogene family  KLF16 - kruppel-like factor 16  CDKL2 - cyclin-dependent kinase-like 2 (cdc2-related kinase)  RPS6KA4 - ribosomal protein s6 kinase, 90kda, polypeptide 4  H1FX - h1 histone family, member x  WASL - wiskott-aldrich syndrome-like  FRMD8 - ferm domain containing 8  RBM4B - rna binding motif protein 4b  YAF2 - yy1 associated factor 2  ANP32E - acidic (leucine-rich) nuclear phosphoprotein 32 family, member e  ITM2C - integral membrane protein 2c  FAM13A - family with sequence similarity 13, member a  G3BP1 - gtpase activating protein (sh3 domain) binding protein 1  TSPAN14 - tetraspanin 14  AKAP9 - a kinase (prka) anchor protein (yotiao) 9  CAB39L - calcium binding protein 39-like  MBNL2 - muscleblind-like splicing regulator 2  CEBPZ - ccaat/enhancer binding protein (c/ebp), zeta  ABI2 - abl-interactor 2  CCNL2 - cyclin l2  TRIM28 - tripartite motif containing 28  ATP6AP2 - atpase, h+ transporting, lysosomal accessory protein 2  WASF2 - was protein family, member 2  ZNF197 - zinc finger protein 197  TDRD3 - tudor domain containing 3  FAM49A - family with sequence similarity 49, member a  CNIH - cornichon homolog (drosophila)  YIPF5 - yip1 domain family, member 5  RBM7 - rna binding motif protein 7  RBM5 - rna binding motif protein 5  TRIM11 - tripartite motif containing 11  TNK2 - tyrosine kinase, non-receptor, 2  NDEL1 - nude neurodevelopment protein 1-like 1  ALYREF - aly/ref export factor  CSRNP2 - cysteine-serine-rich nuclear protein 2  RNF41 - ring finger protein 41  TSHZ1 - teashirt zinc finger homeobox 1  PRMT3 - protein arginine methyltransferase 3  CLPB - clpb caseinolytic peptidase b homolog (e. coli)  ANKRD13C - ankyrin repeat domain 13c  PSME3 - proteasome (prosome, macropain) activator subunit 3 (pa28 gamma; ki)  MPHOSPH10 - m-phase phosphoprotein 10 (u3 small nucleolar ribonucleoprotein)  APOLD1 - apolipoprotein l domain containing 1  TNFAIP8L3 - tumor necrosis factor, alpha-induced protein 8-like 3  CALCRL - calcitonin receptor-like  TRIM13 - tripartite motif containing 13  TRIM8 - tripartite motif containing 8  LBH - limb bud and heart development  INADL - inad-like (drosophila)  TOPORS - topoisomerase i binding, arginine/serine-rich, e3 ubiquitin protein ligase  SNX27 - sorting nexin family member 27  RAB1B - rab1b, member ras oncogene family  ARPC5L - actin related protein 2/3 complex, subunit 5-like  SEH1L - seh1-like (s. cerevisiae)  CABLES2 - cdk5 and abl enzyme substrate 2  PTPRU - protein tyrosine phosphatase, receptor type, u  HUWE1 - hect, uba and wwe domain containing 1, e3 ubiquitin protein ligase  ARPC5 - actin related protein 2/3 complex, subunit 5, 16kda  COL4A3BP - collagen, type iv, alpha 3 (goodpasture antigen) binding protein  PQBP1 - polyglutamine binding protein 1  TSPAN5 - tetraspanin 5  ACTR2 - arp2 actin-related protein 2 homolog (yeast)  ACTR3 - arp3 actin-related protein 3 homolog (yeast)  ARPC3 - actin related protein 2/3 complex, subunit 3, 21kda  ARPC4 - actin related protein 2/3 complex, subunit 4, 20kda  CTDSP2 - ctd (carboxy-terminal domain, rna polymerase ii, polypeptide a) small phosphatase 2  ARPC2 - actin related protein 2/3 complex, subunit 2, 34kda  TSFM - ts translation elongation factor, mitochondrial  FEM1B - fem-1 homolog b (c. elegans)  RAD50 - rad50 homolog (s. cerevisiae)  LRPPRC - leucine-rich pentatricopeptide repeat containing  ZNF263 - zinc finger protein 263  ACTR1A - arp1 actin-related protein 1 homolog a, centractin alpha (yeast)  SPRY4 - sprouty homolog 4 (drosophila)  BCAP31 - b-cell receptor-associated protein 31  RBM12 - rna binding motif protein 12  SHARPIN - shank-associated rh domain interactor  PDIA6 - protein disulfide isomerase family a, member 6  MED25 - mediator complex subunit 25  TRAP1 - tnf receptor-associated protein 1  BLOC1S3 - biogenesis of lysosomal organelles complex-1, subunit 3  MED6 - mediator complex subunit 6  AKT3 - v-akt murine thymoma viral oncogene homolog 3  ABI1 - abl-interactor 1  RNF207 - ring finger protein 207  PTBP3 - polypyrimidine tract binding protein 3  PPP4R1 - protein phosphatase 4, regulatory subunit 1  DMTF1 - cyclin d binding myb-like transcription factor 1  SH2B3 - sh2b adaptor protein 3  PDCD6 - programmed cell death 6  ZBTB33 - zinc finger and btb domain containing 33  TANK - traf family member-associated nfkb activator  HDAC5 - histone deacetylase 5  PDCD6IP - programmed cell death 6 interacting protein  SRA1 - steroid receptor rna activator 1  OCLN - occludin  HDAC6 - histone deacetylase 6  PARP2 - poly (adp-ribose) polymerase 2  HMGXB4 - hmg box domain containing 4  SH2D3C - sh2 domain containing 3c  TOM1 - target of myb1 (chicken)  MED16 - mediator complex subunit 16  SAE1 - sumo1 activating enzyme subunit 1  DNM1L - dynamin 1-like  RANBP9 - ran binding protein 9  DNAJB6 - dnaj (hsp40) homolog, subfamily b, member 6  UBA2 - ubiquitin-like modifier activating enzyme 2  LPGAT1 - lysophosphatidylglycerol acyltransferase 1  ZBTB5 - zinc finger and btb domain containing 5  PAN2 - pan2 poly(a) specific ribonuclease subunit homolog (s. cerevisiae)  ZBTB40 - zinc finger and btb domain containing 40  SUPT7L - suppressor of ty 7 (s. cerevisiae)-like  WDR1 - wd repeat domain 1  FBXO38 - f-box protein 38  TMX1 - thioredoxin-related transmembrane protein 1  SGPP1 - sphingosine-1-phosphate phosphatase 1  OXSR1 - oxidative stress responsive 1  RBM8A - rna binding motif protein 8a  POLR2M - polymerase (rna) ii (dna directed) polypeptide m  USP3 - ubiquitin specific peptidase 3  USP15 - ubiquitin specific peptidase 15  THOC1 - tho complex 1  HNRNPDL - heterogeneous nuclear ribonucleoprotein d-like  RBX1 - ring-box 1, e3 ubiquitin protein ligase  NUP153 - nucleoporin 153kda  THRAP3 - thyroid hormone receptor associated protein 3  MED12 - mediator complex subunit 12  MED13 - mediator complex subunit 13  PSMD6 - proteasome (prosome, macropain) 26s subunit, non-atpase, 6  LRIG2 - leucine-rich repeats and immunoglobulin-like domains 2  GLTPD1 - glycolipid transfer protein domain containing 1  MED24 - mediator complex subunit 24  ZC3H6 - zinc finger ccch-type containing 6  TRIL - tlr4 interactor with leucine-rich repeats  PJA2 - praja ring finger 2, e3 ubiquitin protein ligase  AREL1 - apoptosis resistant e3 ubiquitin protein ligase 1  SETDB1 - set domain, bifurcated 1  ZNF34 - zinc finger protein 34  FCHSD2 - fch and double sh3 domains 2  TLK1 - tousled-like kinase 1  TOX4 - tox high mobility group box family member 4  ZNF436 - zinc finger protein 436  PLEKHM1 - pleckstrin homology domain containing, family m (with run domain) member 1  ELMO1 - engulfment and cell motility 1  ENHO - energy homeostasis associated  C2CD5 - c2 calcium-dependent domain containing 5  PRR7 - proline rich 7 (synaptic)  AGRN - agrin  EPM2AIP1 - epm2a (laforin) interacting protein 1  C2CD2L - c2cd2-like  NDFIP1 - nedd4 family interacting protein 1  KIAA0319 - kiaa0319  PPP1R26 - protein phosphatase 1, regulatory subunit 26  RAB15 - rab15, member ras oncogene family  RBM19 - rna binding motif protein 19  KLHL21 - kelch-like family member 21  SRGAP3 - slit-robo rho gtpase activating protein 3  G3BP2 - gtpase activating protein (sh3 domain) binding protein 2  ARHGAP39 - rho gtpase activating protein 39  ARHGAP44 - rho gtpase activating protein 44  ZSCAN16 - zinc finger and scan domain containing 16  TNKS2 - tankyrase, trf1-interacting ankyrin-related adp-ribose polymerase 2  WDR61 - wd repeat domain 61  SMG7 - smg7 nonsense mediated mrna decay factor  FIG4 - fig4 homolog, sac1 lipid phosphatase domain containing (s. cerevisiae)  SNAP91 - synaptosomal-associated protein, 91kda  CD276 - cd276 molecule  APITD1 - apoptosis-inducing, taf9-like domain 1  CKAP5 - cytoskeleton associated protein 5  KIAA0586 - kiaa0586  DAZAP2 - daz associated protein 2  IST1 - increased sodium tolerance 1 homolog (yeast)  BCLAF1 - bcl2-associated transcription factor 1  EIF4A3 - eukaryotic translation initiation factor 4a3  ATG13 - autophagy related 13  MATR3 - matrin 3  RIMS3 - regulating synaptic membrane exocytosis 3  SNX17 - sorting nexin 17  TBC1D5 - tbc1 domain family, member 5  PIEZO1 - piezo-type mechanosensitive ion channel component 1  RNF144A - ring finger protein 144a  ZNF623 - zinc finger protein 623  DNAJC6 - dnaj (hsp40) homolog, subfamily c, member 6  ARHGEF17 - rho guanine nucleotide exchange factor (gef) 17  RGP1 - rgp1 retrograde golgi transport homolog (s. cerevisiae)  ARHGEF11 - rho guanine nucleotide exchange factor (gef) 11  SPATA2 - spermatogenesis associated 2  COLEC12 - collectin sub-family member 12  AMIGO3 - adhesion molecule with ig-like domain 3  ZBTB24 - zinc finger and btb domain containing 24  ZEB2 - zinc finger e-box binding homeobox 2  SFI1 - sfi1 homolog, spindle assembly associated (yeast)  CTIF - cbp80/20-dependent translation initiation factor  DUSP16 - dual specificity phosphatase 16  RNF40 - ring finger protein 40, e3 ubiquitin protein ligase  SPOCK2 - sparc/osteonectin, cwcv and kazal-like domains proteoglycan (testican) 2  DDHD1 - ddhd domain containing 1  RB1CC1 - rb1-inducible coiled-coil 1  TSC22D2 - tsc22 domain family, member 2  CUL7 - cullin 7  SH3BP5L - sh3-binding domain protein 5-like  GRIP2 - glutamate receptor interacting protein 2  GIT2 - g protein-coupled receptor kinase interacting arfgap 2  RAB11FIP3 - rab11 family interacting protein 3 (class ii)  VPRBP - vpr (hiv-1) binding protein  ARMC9 - armadillo repeat containing 9  RNF34 - ring finger protein 34, e3 ubiquitin protein ligase  MUS81 - mus81 structure-specific endonuclease subunit  FBXO11 - f-box protein 11  IFT74 - intraflagellar transport 74 homolog (chlamydomonas)  ULK2 - unc-51 like autophagy activating kinase 2  KIAA0355 - kiaa0355  HERPUD1 - homocysteine-inducible, endoplasmic reticulum stress-inducible, ubiquitin-like domain member 1  MYO19 - myosin xix  USP6NL - usp6 n-terminal like  CEP290 - centrosomal protein 290kda  RIMS2 - regulating synaptic membrane exocytosis 2  NAA15 - n(alpha)-acetyltransferase 15, nata auxiliary subunit  PUM1 - pumilio homolog 1 (drosophila)  PPP6R2 - protein phosphatase 6, regulatory subunit 2  CEP57 - centrosomal protein 57kda  ST18 - suppression of tumorigenicity 18 (breast carcinoma) (zinc finger protein)  CTC1 - cts telomere maintenance complex component 1  DHX34 - deah (asp-glu-ala-his) box polypeptide 34  LZTS3 - leucine zipper, putative tumor suppressor family member 3  HDAC4 - histone deacetylase 4  TOX - thymocyte selection-associated high mobility group box  ZFYVE16 - zinc finger, fyve domain containing 16  KMT2B - lysine (k)-specific methyltransferase 2b  IFT140 - intraflagellar transport 140 homolog (chlamydomonas)  PHACTR2 - phosphatase and actin regulator 2  SLK - ste20-like kinase  CLSTN3 - calsyntenin 3  USP34 - ubiquitin specific peptidase 34  HDAC9 - histone deacetylase 9  ZNF606 - zinc finger protein 606  SART3 - squamous cell carcinoma antigen recognized by t cells 3  SETD1A - set domain containing 1a  CCP110 - centriolar coiled coil protein 110kda  ZNF614 - zinc finger protein 614  ZFP2 - zfp2 zinc finger protein  MDC1 - mediator of dna-damage checkpoint 1  IQCB1 - iq motif containing b1  KCNIP4 - kv channel interacting protein 4  SOCS5 - suppressor of cytokine signaling 5  DNAJC5 - dnaj (hsp40) homolog, subfamily c, member 5  RALGPS1 - ral gef with ph domain and sh3 binding motif 1  GPR153 - g protein-coupled receptor 153  CTR9 - ctr9, paf1/rna polymerase ii complex component  PPM1F - protein phosphatase, mg2+/mn2+ dependent, 1f  CEP70 - centrosomal protein 70kda  CPEB4 - cytoplasmic polyadenylation element binding protein 4  ARHGEF10 - rho guanine nucleotide exchange factor (gef) 10  EPC1 - enhancer of polycomb homolog 1 (drosophila)  MORF4L2 - mortality factor 4 like 2  RASL11A - ras-like, family 11, member a  KLHL15 - kelch-like family member 15  PDGFD - platelet derived growth factor d  FEZ1 - fasciculation and elongation protein zeta 1 (zygin i)  FEZ2 - fasciculation and elongation protein zeta 2 (zygin ii)  NUP155 - nucleoporin 155kda  MED28 - mediator complex subunit 28  GNA14 - guanine nucleotide binding protein (g protein), alpha 14  CDK5RAP3 - cdk5 regulatory subunit associated protein 3  CROCC - ciliary rootlet coiled-coil, rootletin  EDEM1 - er degradation enhancer, mannosidase alpha-like 1  RAPGEF2 - rap guanine nucleotide exchange factor (gef) 2  NUP93 - nucleoporin 93kda  VGLL4 - vestigial like 4 (drosophila)  CEP63 - centrosomal protein 63kda  LRRC14 - leucine rich repeat containing 14  DEPDC5 - dep domain containing 5  KDM4A - lysine (k)-specific demethylase 4a  PHF14 - phd finger protein 14  TTI1 - telo2 interacting protein 1  ZNF432 - zinc finger protein 432  SAFB2 - scaffold attachment factor b2  KIAA0430 - kiaa0430  LPIN2 - lipin 2 |
| GO:1903506 | regulation of nucleic acid-templated transcription | 4.22E-9 | 1.54E-6 | 1.08 (10334,1978,6996,1446) | [+] Show genes  ELL - elongation factor rna polymerase ii  EXOSC9 - exosome component 9  ZNF239 - zinc finger protein 239  MSL3 - male-specific lethal 3 homolog (drosophila)  TADA1 - transcriptional adaptor 1  CBX1 - chromobox homolog 1  ABCA2 - atp-binding cassette, sub-family a (abc1), member 2  ABL1 - c-abl oncogene 1, non-receptor tyrosine kinase  ZNF821 - zinc finger protein 821  PHC3 - polyhomeotic homolog 3 (drosophila)  PML - promyelocytic leukemia  MLLT11 - myeloid/lymphoid or mixed-lineage leukemia (trithorax homolog, drosophila); translocated to, 11  SUPT20H - suppressor of ty 20 homolog (s. cerevisiae)  YWHAQ - tyrosine 3-monooxygenase/tryptophan 5-monooxygenase activation protein, theta polypeptide  KAT2A - k(lysine) acetyltransferase 2a  NRIP1 - nuclear receptor interacting protein 1  NCOA3 - nuclear receptor coactivator 3  BRD8 - bromodomain containing 8  WWC2 - ww and c2 domain containing 2  CHD7 - chromodomain helicase dna binding protein 7  LYAR - ly1 antibody reactive  ZNF354B - zinc finger protein 354b  TCERG1 - transcription elongation regulator 1  TAF15 - taf15 rna polymerase ii, tata box binding protein (tbp)-associated factor, 68kda  EHMT2 - euchromatic histone-lysine n-methyltransferase 2  SPIN1 - spindlin 1  TASP1 - taspase, threonine aspartase, 1  SUB1 - sub1 homolog (s. cerevisiae)  MORF4L1 - mortality factor 4 like 1  ZNF511 - zinc finger protein 511  PPID - peptidylprolyl isomerase d  ACTL6A - actin-like 6a  AXIN1 - axin 1  ACTN1 - actinin, alpha 1  TRRAP - transformation/transcription domain-associated protein  PICALM - phosphatidylinositol binding clathrin assembly protein  ACTN4 - actinin, alpha 4  ACVR2A - activin a receptor, type iia  ACVRL1 - activin a receptor type ii-like 1  ACVR1 - activin a receptor, type i  DLL1 - delta-like 1 (drosophila)  ARID1A - at rich interactive domain 1a (swi-like)  ACVR1B - activin a receptor, type ib  SETD6 - set domain containing 6  FZD6 - frizzled family receptor 6  ACTR5 - arp5 actin-related protein 5 homolog (yeast)  ZNF331 - zinc finger protein 331  CDC45 - cell division cycle 45  MED1 - mediator complex subunit 1  GCLC - glutamate-cysteine ligase, catalytic subunit  FZD4 - frizzled family receptor 4  FZD1 - frizzled family receptor 1  BAP1 - brca1 associated protein-1 (ubiquitin carboxy-terminal hydrolase)  ADCY1 - adenylate cyclase 1 (brain)  ZNF613 - zinc finger protein 613  NUP85 - nucleoporin 85kda  PPARD - peroxisome proliferator-activated receptor delta  KDM5C - lysine (k)-specific demethylase 5c  USP9X - ubiquitin specific peptidase 9, x-linked  RBM10 - rna binding motif protein 10  MAML3 - mastermind-like 3 (drosophila)  POLR2K - polymerase (rna) ii (dna directed) polypeptide k, 7.0kda  PARP1 - poly (adp-ribose) polymerase 1  H2AFY2 - h2a histone family, member y2  PHF17 - phd finger protein 17  PPP1R13L - protein phosphatase 1, regulatory subunit 13 like  HES6 - hairy and enhancer of split 6 (drosophila)  SMPD3 - sphingomyelin phosphodiesterase 3, neutral membrane (neutral sphingomyelinase ii)  POLR2A - polymerase (rna) ii (dna directed) polypeptide a, 220kda  RUVBL2 - ruvb-like 2 (e. coli)  TTC21B - tetratricopeptide repeat domain 21b  CDK5RAP2 - cdk5 regulatory subunit associated protein 2  RCOR3 - rest corepressor 3  SLTM - safb-like, transcription modulator  NELFE - negative elongation factor complex member e  EHMT1 - euchromatic histone-lysine n-methyltransferase 1  AES - amino-terminal enhancer of split  NR5A1 - nuclear receptor subfamily 5, group a, member 1  NUP133 - nucleoporin 133kda  FUS - fused in sarcoma  NAA40 - n(alpha)-acetyltransferase 40, natd catalytic subunit, homolog (s. cerevisiae)  JAG1 - jagged 1  PRDM4 - pr domain containing 4  PPP3R1 - protein phosphatase 3, regulatory subunit b, alpha  ATF7IP - activating transcription factor 7 interacting protein  ZNF689 - zinc finger protein 689  LRIF1 - ligand dependent nuclear receptor interacting factor 1  PPP2R5B - protein phosphatase 2, regulatory subunit b', beta  AKT1 - v-akt murine thymoma viral oncogene homolog 1  ZNF653 - zinc finger protein 653  USP7 - ubiquitin specific peptidase 7 (herpes virus-associated)  PCID2 - pci domain containing 2  XRCC6 - x-ray repair complementing defective repair in chinese hamster cells 6  RNF168 - ring finger protein 168, e3 ubiquitin protein ligase  GABPB1 - ga binding protein transcription factor, beta subunit 1  PPP2R1A - protein phosphatase 2, regulatory subunit a, alpha  GABPA - ga binding protein transcription factor, alpha subunit 60kda  WWP2 - ww domain containing e3 ubiquitin protein ligase 2  AHR - aryl hydrocarbon receptor  ZNF526 - zinc finger protein 526  FOXP4 - forkhead box p4  PPP2CA - protein phosphatase 2, catalytic subunit, alpha isozyme  RPAP2 - rna polymerase ii associated protein 2  ZNF83 - zinc finger protein 83  DDX39B - dead (asp-glu-ala-asp) box polypeptide 39b  KANSL3 - kat8 regulatory nsl complex subunit 3  PRNP - prion protein  ATF7 - activating transcription factor 7  KMT2D - lysine (k)-specific methyltransferase 2d  AMFR - autocrine motility factor receptor, e3 ubiquitin protein ligase  PRKRIR - protein-kinase, interferon-inducible double stranded rna dependent inhibitor, repressor of (p58 repressor)  ZNF419 - zinc finger protein 419  MAP2K5 - mitogen-activated protein kinase kinase 5  MAP2K7 - mitogen-activated protein kinase kinase 7  EIF2AK2 - eukaryotic translation initiation factor 2-alpha kinase 2  PIM2 - pim-2 oncogene  ZNF692 - zinc finger protein 692  YEATS4 - yeats domain containing 4  MAP2K1 - mitogen-activated protein kinase kinase 1  MAP2K2 - mitogen-activated protein kinase kinase 2  SNIP1 - smad nuclear interacting protein 1  ZFPM1 - zinc finger protein, fog family member 1  ZNF212 - zinc finger protein 212  MAPK10 - mitogen-activated protein kinase 10  EID2 - ep300 interacting inhibitor of differentiation 2  MAPK9 - mitogen-activated protein kinase 9  ZNF383 - zinc finger protein 383  MAPK11 - mitogen-activated protein kinase 11  MAPK8 - mitogen-activated protein kinase 8  MAPK7 - mitogen-activated protein kinase 7  GCN1L1 - gcn1 general control of amino-acid synthesis 1-like 1 (yeast)  GATA2 - gata binding protein 2  MAPK3 - mitogen-activated protein kinase 3  MAPK1 - mitogen-activated protein kinase 1  KAT6A - k(lysine) acetyltransferase 6a  PRKD1 - protein kinase d1  ADIRF - adipogenesis regulatory factor  ANXA3 - annexin a3  PRKCH - protein kinase c, eta  PRKCI - protein kinase c, iota  CCDC85B - coiled-coil domain containing 85b  NR4A3 - nuclear receptor subfamily 4, group a, member 3  PRKCB - protein kinase c, beta  BRD3 - bromodomain containing 3  GAS6 - growth arrest-specific 6  PRKAR1A - protein kinase, camp-dependent, regulatory, type i, alpha  CARF - calcium responsive transcription factor  NCOA4 - nuclear receptor coactivator 4  MLLT10 - myeloid/lymphoid or mixed-lineage leukemia (trithorax homolog, drosophila); translocated to, 10  ZNF408 - zinc finger protein 408  DEDD2 - death effector domain containing 2  YEATS2 - yeats domain containing 2  APEX1 - apex nuclease (multifunctional dna repair enzyme) 1  DHX36 - deah (asp-glu-ala-his) box polypeptide 36  ZNF189 - zinc finger protein 189  ZSCAN26 - zinc finger and scan domain containing 26  RBM23 - rna binding motif protein 23  ZNF605 - zinc finger protein 605  XIAP - x-linked inhibitor of apoptosis  BIRC2 - baculoviral iap repeat containing 2  ZNF177 - zinc finger protein 177  KLF12 - kruppel-like factor 12  ZNF175 - zinc finger protein 175  ZNF174 - zinc finger protein 174  ZNF184 - zinc finger protein 184  SSBP4 - single stranded dna binding protein 4  GTF2F1 - general transcription factor iif, polypeptide 1, 74kda  PBX2 - pre-b-cell leukemia homeobox 2  PBX3 - pre-b-cell leukemia homeobox 3  PRMT6 - protein arginine methyltransferase 6  ZNF148 - zinc finger protein 148  PBX1 - pre-b-cell leukemia homeobox 1  ZBTB17 - zinc finger and btb domain containing 17  BEND5 - ben domain containing 5  GTF2H2 - general transcription factor iih, polypeptide 2, 44kda  GTF2H3 - general transcription factor iih, polypeptide 3, 34kda  TRIM26 - tripartite motif containing 26  GTF2H1 - general transcription factor iih, polypeptide 1, 62kda  VEZF1 - vascular endothelial zinc finger 1  PCBD1 - pterin-4 alpha-carbinolamine dehydratase/dimerization cofactor of hepatocyte nuclear factor 1 alpha  ZNF142 - zinc finger protein 142  MCPH1 - microcephalin 1  ZNF140 - zinc finger protein 140  APBB2 - amyloid beta (a4) precursor protein-binding, family b, member 2  GTF2B - general transcription factor iib  PCGF2 - polycomb group ring finger 2  APBB1 - amyloid beta (a4) precursor protein-binding, family b, member 1 (fe65)  CHP1 - calcineurin-like ef-hand protein 1  ZNF132 - zinc finger protein 132  ZNF131 - zinc finger protein 131  RIF1 - rap1 interacting factor homolog (yeast)  ZNF134 - zinc finger protein 134  ZNF133 - zinc finger protein 133  TBL1XR1 - transducin (beta)-like 1 x-linked receptor 1  GTF2I - general transcription factor iii  GTF2H4 - general transcription factor iih, polypeptide 4, 52kda  KIAA1551 - kiaa1551  PA2G4 - proliferation-associated 2g4, 38kda  BRF1 - brf1, rna polymerase iii transcription initiation factor 90 kda subunit  RPRD1A - regulation of nuclear pre-mrna domain containing 1a  ARF4 - adp-ribosylation factor 4  ZHX1 - zinc fingers and homeoboxes 1  ZNF79 - zinc finger protein 79  SBNO1 - strawberry notch homolog 1 (drosophila)  IRAK3 - interleukin-1 receptor-associated kinase 3  SETD5 - set domain containing 5  APP - amyloid beta (a4) precursor protein  ZNF70 - zinc finger protein 70  BBS7 - bardet-biedl syndrome 7  DDX20 - dead (asp-glu-ala-asp) box polypeptide 20  ZNF75D - zinc finger protein 75d  PAM - peptidylglycine alpha-amidating monooxygenase  ZNF76 - zinc finger protein 76  H3F3A - h3 histone, family 3a  ARNTL - aryl hydrocarbon receptor nuclear translocator-like  ARNT - aryl hydrocarbon receptor nuclear translocator  BRPF1 - bromodomain and phd finger containing, 1  H2AFZ - h2a histone family, member z  FZD5 - frizzled family receptor 5  SAP130 - sin3a-associated protein, 130kda  H2AFX - h2a histone family, member x  PAX8 - paired box 8  SUPT16H - suppressor of ty 16 homolog (s. cerevisiae)  MIER3 - mesoderm induction early response 1, family member 3  ARRB2 - arrestin, beta 2  ARRB1 - arrestin, beta 1  CDC73 - cell division cycle 73  PDGFB - platelet-derived growth factor beta polypeptide  NKAP - nfkb activating protein  THAP5 - thap domain containing 5  RHOA - ras homolog family member a  STRAP - serine/threonine kinase receptor associated protein  PSIP1 - pc4 and sfrs1 interacting protein 1  MED9 - mediator complex subunit 9  FSTL1 - follistatin-like 1  LRP8 - low density lipoprotein receptor-related protein 8, apolipoprotein e receptor  BAZ2A - bromodomain adjacent to zinc finger domain, 2a  HCFC1 - host cell factor c1 (vp16-accessory protein)  PCNA - proliferating cell nuclear antigen  PRDM2 - pr domain containing 2, with znf domain  CHMP1A - charged multivesicular body protein 1a  MAP3K12 - mitogen-activated protein kinase kinase kinase 12  JDP2 - jun dimerization protein 2  CDK16 - cyclin-dependent kinase 16  AKIRIN2 - akirin 2  HEATR1 - heat repeat containing 1  CDK17 - cyclin-dependent kinase 17  LEO1 - leo1, paf1/rna polymerase ii complex component, homolog (s. cerevisiae)  ERLIN2 - er lipid raft associated 2  ZNF226 - zinc finger protein 226  PWP1 - pwp1 homolog (s. cerevisiae)  PDE2A - phosphodiesterase 2a, cgmp-stimulated  HDAC2 - histone deacetylase 2  HMBOX1 - homeobox containing 1  HDGF - hepatoma-derived growth factor  ZCCHC12 - zinc finger, cchc domain containing 12  ZNF213 - zinc finger protein 213  THAP1 - thap domain containing, apoptosis associated protein 1  HDAC1 - histone deacetylase 1  ZNF205 - zinc finger protein 205  HCLS1 - hematopoietic cell-specific lyn substrate 1  PKIG - protein kinase (camp-dependent, catalytic) inhibitor gamma  ZNF207 - zinc finger protein 207  KAT7 - k(lysine) acetyltransferase 7  ASCL1 - achaete-scute complex homolog 1 (drosophila)  ELP3 - elongator acetyltransferase complex subunit 3  RGMB - rgm domain family, member b  SBNO2 - strawberry notch homolog 2 (drosophila)  ZNF444 - zinc finger protein 444  XPA - xeroderma pigmentosum, complementation group a  ZBTB1 - zinc finger and btb domain containing 1  XPC - xeroderma pigmentosum, complementation group c  FBXW7 - f-box and wd repeat domain containing 7, e3 ubiquitin protein ligase  XPO1 - exportin 1 (crm1 homolog, yeast)  GOLGB1 - golgin b1  BAHD1 - bromo adjacent homology domain containing 1  PAGR1 - paxip1 associated glutamate-rich protein 1  ZHX2 - zinc fingers and homeoboxes 2  MORC2 - morc family cw-type zinc finger 2  PHF2 - phd finger protein 2  PHF1 - phd finger protein 1  FOXJ3 - forkhead box j3  RERE - arginine-glutamic acid dipeptide (re) repeats  ZXDC - zxd family zinc finger c  XBP1 - x-box binding protein 1  ZNF367 - zinc finger protein 367  BHLHE41 - basic helix-loop-helix family, member e41  ZFHX3 - zinc finger homeobox 3  PEX14 - peroxisomal biogenesis factor 14  WFS1 - wolfram syndrome 1 (wolframin)  WHSC1 - wolf-hirschhorn syndrome candidate 1  ADNP2 - adnp homeobox 2  NELFA - negative elongation factor complex member a  AEBP2 - ae binding protein 2  IMPACT - impact rwd domain protein  ZNF507 - zinc finger protein 507  LGR4 - leucine-rich repeat containing g protein-coupled receptor 4  ARID2 - at rich interactive domain 2 (arid, rfx-like)  ATP2B4 - atpase, ca++ transporting, plasma membrane 4  ZNF652 - zinc finger protein 652  SCAF8 - sr-related ctd-associated factor 8  PKD1 - polycystic kidney disease 1 (autosomal dominant)  PKD2 - polycystic kidney disease 2 (autosomal dominant)  ZNF35 - zinc finger protein 35  ZKSCAN1 - zinc finger with krab and scan domains 1  FBXL15 - f-box and leucine-rich repeat protein 15  MTF2 - metal response element binding transcription factor 2  ZNF576 - zinc finger protein 576  ZNF33B - zinc finger protein 33b  ZNF32 - zinc finger protein 32  BACH1 - btb and cnc homology 1, basic leucine zipper transcription factor 1  PIN1 - peptidylprolyl cis/trans isomerase, nima-interacting 1  PIK3R2 - phosphoinositide-3-kinase, regulatory subunit 2 (beta)  ZNF45 - zinc finger protein 45  MZF1 - myeloid zinc finger 1  PIK3R1 - phosphoinositide-3-kinase, regulatory subunit 1 (alpha)  ZNF18 - zinc finger protein 18  ASXL2 - additional sex combs like 2 (drosophila)  ZNF16 - zinc finger protein 16  PLAGL1 - pleiomorphic adenoma gene-like 1  ELP2 - elongator acetyltransferase complex subunit 2  ASXL1 - additional sex combs like 1 (drosophila)  ZNF384 - zinc finger protein 384  TNIP2 - tnfaip3 interacting protein 2  ATRX - alpha thalassemia/mental retardation syndrome x-linked  ZNF24 - zinc finger protein 24  PKNOX1 - pbx/knotted 1 homeobox 1  ZFPL1 - zinc finger protein-like 1  ZFX - zinc finger protein, x-linked  TMEM100 - transmembrane protein 100  PHF10 - phd finger protein 10  SF1 - splicing factor 1  PSPC1 - paraspeckle component 1  ZBTB14 - zinc finger and btb domain containing 14  ZNF711 - zinc finger protein 711  ZNF7 - zinc finger protein 7  ZNF8 - zinc finger protein 8  BCL7A - b-cell cll/lymphoma 7a  ZNF2 - zinc finger protein 2  MRGBP - mrg/morf4l binding protein  ZNF3 - zinc finger protein 3  BCL9 - b-cell cll/lymphoma 9  YES1 - v-yes-1 yamaguchi sarcoma viral oncogene homolog 1  BRF2 - brf2, rna polymerase iii transcription initiation factor 50 kda subunit  PIM1 - pim-1 oncogene  XRCC5 - x-ray repair complementing defective repair in chinese hamster cells 5 (double-strand-break rejoining)  ECD - ecdysoneless homolog (drosophila)  CCND1 - cyclin d1  GSK3A - glycogen synthase kinase 3 alpha  PARK7 - parkinson protein 7  C3orf33 - chromosome 3 open reading frame 33  YWHAB - tyrosine 3-monooxygenase/tryptophan 5-monooxygenase activation protein, beta polypeptide  CCNK - cyclin k  TRIM27 - tripartite motif containing 27  HSF2 - heat shock transcription factor 2  SAP18 - sin3a-associated protein, 18kda  TRIM24 - tripartite motif containing 24  TAF1D - tata box binding protein (tbp)-associated factor, rna polymerase i, d, 41kda  TSHZ2 - teashirt zinc finger homeobox 2  RFXAP - regulatory factor x-associated protein  CREG1 - cellular repressor of e1a-stimulated genes 1  RFX5 - regulatory factor x, 5 (influences hla class ii expression)  WHSC1L1 - wolf-hirschhorn syndrome candidate 1-like 1  FLCN - folliculin  HSBP1 - heat shock factor binding protein 1  RFX1 - regulatory factor x, 1 (influences hla class ii expression)  RELA - v-rel avian reticuloendotheliosis viral oncogene homolog a  APBB3 - amyloid beta (a4) precursor protein-binding, family b, member 3  PRMT2 - protein arginine methyltransferase 2  BCOR - bcl6 corepressor  ZFP36L2 - zfp36 ring finger protein-like 2  ZFP36L1 - zfp36 ring finger protein-like 1  RET - ret proto-oncogene  HESX1 - hesx homeobox 1  SAP30 - sin3a-associated protein, 30kda  DPF2 - d4, zinc and double phd fingers family 2  CC2D1A - coiled-coil and c2 domain containing 1a  RLF - rearranged l-myc fusion  RING1 - ring finger protein 1  CFLAR - casp8 and fadd-like apoptosis regulator  MED11 - mediator complex subunit 11  BMI1 - bmi1 polycomb ring finger oncogene  PCGF3 - polycomb group ring finger 3  WDR77 - wd repeat domain 77  LDB1 - lim domain binding 1  BMPR1A - bone morphogenetic protein receptor, type ia  BMPR2 - bone morphogenetic protein receptor, type ii (serine/threonine kinase)  BMPR1B - bone morphogenetic protein receptor, type ib  GON4L - gon-4-like (c. elegans)  KAT2B - k(lysine) acetyltransferase 2b  TSC22D1 - tsc22 domain family, member 1  CRTC2 - creb regulated transcription coactivator 2  BMP7 - bone morphogenetic protein 7  PSMD14 - proteasome (prosome, macropain) 26s subunit, non-atpase, 14  ZNF259 - zinc finger protein 259  FLOT1 - flotillin 1  TAX1BP1 - tax1 (human t-cell leukemia virus type i) binding protein 1  BCAS3 - breast carcinoma amplified sequence 3  CC2D1B - coiled-coil and c2 domain containing 1b  AHI1 - abelson helper integration site 1  THAP8 - thap domain containing 8  RNF4 - ring finger protein 4  BRD2 - bromodomain containing 2  RNF2 - ring finger protein 2  BUD31 - bud31 homolog (s. cerevisiae)  ZNF280D - zinc finger protein 280d  GATAD2A - gata zinc finger domain containing 2a  RNF6 - ring finger protein (c3h2c3 type) 6  ZNF655 - zinc finger protein 655  RNF111 - ring finger protein 111  TRAK2 - trafficking protein, kinesin binding 2  TET2 - tet methylcytosine dioxygenase 2  HSPD1 - heat shock 60kda protein 1 (chaperonin)  DNAJB1 - dnaj (hsp40) homolog, subfamily b, member 1  MED18 - mediator complex subunit 18  ZNF48 - zinc finger protein 48  DENND4A - denn/madd domain containing 4a  BCL10 - b-cell cll/lymphoma 10  MBTD1 - mbt domain containing 1  FUBP3 - far upstream element (fuse) binding protein 3  BTRC - beta-transducin repeat containing e3 ubiquitin protein ligase  ZMPSTE24 - zinc metallopeptidase ste24  HSPA8 - heat shock 70kda protein 8  TRIM44 - tripartite motif containing 44  TAB1 - tgf-beta activated kinase 1/map3k7 binding protein 1  PPIE - peptidylprolyl isomerase e (cyclophilin e)  MCRS1 - microspherule protein 1  HMGN1 - high mobility group nucleosome binding domain 1  HMGB3 - high mobility group box 3  NRDE2 - nrde-2, necessary for rna interference, domain containing  HMGB2 - high mobility group box 2  HMGB1 - high mobility group box 1  C1D - c1d nuclear receptor corepressor  BRD9 - bromodomain containing 9  TNKS - tankyrase, trf1-interacting ankyrin-related adp-ribose polymerase  SLC30A9 - solute carrier family 30 (zinc transporter), member 9  ZBTB10 - zinc finger and btb domain containing 10  AGO1 - argonaute risc catalytic component 1  MAD2L2 - mad2 mitotic arrest deficient-like 2 (yeast)  CAPRIN2 - caprin family member 2  PID1 - phosphotyrosine interaction domain containing 1  PHIP - pleckstrin homology domain interacting protein  ZBTB48 - zinc finger and btb domain containing 48  ZNF787 - zinc finger protein 787  NRARP - notch-regulated ankyrin repeat protein  HYAL2 - hyaluronoglucosaminidase 2  GPBP1 - gc-rich promoter binding protein 1  TRIM38 - tripartite motif containing 38  TADA3 - transcriptional adaptor 3  CALR - calreticulin  ZNF792 - zinc finger protein 792  HFE - hemochromatosis  WTIP - wilms tumor 1 interacting protein  NCOA2 - nuclear receptor coactivator 2  HINT1 - histidine triad nucleotide binding protein 1  ELP4 - elongator acetyltransferase complex subunit 4  HIVEP1 - human immunodeficiency virus type i enhancer binding protein 1  CREB3 - camp responsive element binding protein 3  HIF1A - hypoxia inducible factor 1, alpha subunit (basic helix-loop-helix transcription factor)  LPIN3 - lipin 3  CITED2 - cbp/p300-interacting transactivator, with glu/asp-rich carboxy-terminal domain, 2  TRADD - tnfrsf1a-associated via death domain  CCNT2 - cyclin t2  MYCBP - myc binding protein  EED - embryonic ectoderm development  URI1 - uri1, prefoldin-like chaperone  CCND3 - cyclin d3  CCNT1 - cyclin t1  CCNH - cyclin h  HMG20A - high mobility group 20a  BANP - btg3 associated nuclear protein  RIPK1 - receptor (tnfrsf)-interacting serine-threonine kinase 1  HNRNPU - heterogeneous nuclear ribonucleoprotein u (scaffold attachment factor a)  HNRNPK - heterogeneous nuclear ribonucleoprotein k  RPS6KA1 - ribosomal protein s6 kinase, 90kda, polypeptide 1  CBFA2T3 - core-binding factor, runt domain, alpha subunit 2; translocated to, 3  CBFB - core-binding factor, beta subunit  HNRNPA2B1 - heterogeneous nuclear ribonucleoprotein a2/b1  RIPK2 - receptor-interacting serine-threonine kinase 2  HNRNPAB - heterogeneous nuclear ribonucleoprotein a/b  RXRB - retinoid x receptor, beta  PIAS3 - protein inhibitor of activated stat, 3  HNRNPD - heterogeneous nuclear ribonucleoprotein d (au-rich element rna binding protein 1, 37kda)  CAV1 - caveolin 1, caveolae protein, 22kda  CCNC - cyclin c  RBM14 - rna binding motif protein 14  ZNF584 - zinc finger protein 584  HMGA1 - high mobility group at-hook 1  FAM129B - family with sequence similarity 129, member b  PRMT5 - protein arginine methyltransferase 5  NR4A1 - nuclear receptor subfamily 4, group a, member 1  RFX7 - regulatory factor x, 7  HTATSF1 - hiv-1 tat specific factor 1  FOXK2 - forkhead box k2  HAT1 - histone acetyltransferase 1  CDK11B - cyclin-dependent kinase 11b  MXD4 - max dimerization protein 4  CDC5L - cell division cycle 5-like  CRTC3 - creb regulated transcription coactivator 3  RBM15 - rna binding motif protein 15  HTRA1 - htra serine peptidase 1  POLR3F - polymerase (rna) iii (dna directed) polypeptide f, 39 kda  HEXIM1 - hexamethylene bis-acetamide inducible 1  ERLIN1 - er lipid raft associated 1  RBMX - rna binding motif protein, x-linked  SPIN2A - spindlin family, member 2a  NCOA7 - nuclear receptor coactivator 7  PSEN1 - presenilin 1  ING2 - inhibitor of growth family, member 2  ZNF574 - zinc finger protein 574  PPM1D - protein phosphatase, mg2+/mn2+ dependent, 1d  CREB3L2 - camp responsive element binding protein 3-like 2  USP16 - ubiquitin specific peptidase 16  PSMA1 - proteasome (prosome, macropain) subunit, alpha type, 1  IPPK - inositol 1,3,4,5,6-pentakisphosphate 2-kinase  PSMA2 - proteasome (prosome, macropain) subunit, alpha type, 2  ILF2 - interleukin enhancer binding factor 2  ILF3 - interleukin enhancer binding factor 3, 90kda  ILK - integrin-linked kinase  CD81 - cd81 molecule  PIAS1 - protein inhibitor of activated stat, 1  CAMKK2 - calcium/calmodulin-dependent protein kinase kinase 2, beta  PSMB1 - proteasome (prosome, macropain) subunit, beta type, 1  SMYD3 - set and mynd domain containing 3  SLCO3A1 - solute carrier organic anion transporter family, member 3a1  SMURF2 - smad specific e3 ubiquitin protein ligase 2  PSMA5 - proteasome (prosome, macropain) subunit, alpha type, 5  PSMA3 - proteasome (prosome, macropain) subunit, alpha type, 3  RIPPLY2 - ripply transcriptional repressor 2  PSMB10 - proteasome (prosome, macropain) subunit, beta type, 10  CGGBP1 - cgg triplet repeat binding protein 1  LMO4 - lim domain only 4  PSMC6 - proteasome (prosome, macropain) 26s subunit, atpase, 6  TAF6L - taf6-like rna polymerase ii, p300/cbp-associated factor (pcaf)-associated factor, 65kda  PSMD1 - proteasome (prosome, macropain) 26s subunit, non-atpase, 1  CAMK1 - calcium/calmodulin-dependent protein kinase i  PSMC4 - proteasome (prosome, macropain) 26s subunit, atpase, 4  SOX18 - sry (sex determining region y)-box 18  CBX4 - chromobox homolog 4  POLR3G - polymerase (rna) iii (dna directed) polypeptide g (32kd)  PSMC2 - proteasome (prosome, macropain) 26s subunit, atpase, 2  PSMD9 - proteasome (prosome, macropain) 26s subunit, non-atpase, 9  PSMD10 - proteasome (prosome, macropain) 26s subunit, non-atpase, 10  PSMD7 - proteasome (prosome, macropain) 26s subunit, non-atpase, 7  PSMD8 - proteasome (prosome, macropain) 26s subunit, non-atpase, 8  CSRNP1 - cysteine-serine-rich nuclear protein 1  PSMD5 - proteasome (prosome, macropain) 26s subunit, non-atpase, 5  TERF2IP - telomeric repeat binding factor 2, interacting protein  PSMD2 - proteasome (prosome, macropain) 26s subunit, non-atpase, 2  PSMD12 - proteasome (prosome, macropain) 26s subunit, non-atpase, 12  PSMD13 - proteasome (prosome, macropain) 26s subunit, non-atpase, 13  ZNF25 - zinc finger protein 25  PTEN - phosphatase and tensin homolog  LAMTOR5 - late endosomal/lysosomal adaptor, mapk and mtor activator 5  STK16 - serine/threonine kinase 16  JMY - junction mediating and regulatory protein, p53 cofactor  ITGA3 - integrin, alpha 3 (antigen cd49c, alpha 3 subunit of vla-3 receptor)  MYBBP1A - myb binding protein (p160) 1a  FBLN5 - fibulin 5  PTGIS - prostaglandin i2 (prostacyclin) synthase  CIB1 - calcium and integrin binding 1 (calmyrin)  CASK - calcium/calmodulin-dependent serine protein kinase (maguk family)  DDX17 - dead (asp-glu-ala-asp) box helicase 17  SUDS3 - suppressor of defective silencing 3 homolog (s. cerevisiae)  KAT5 - k(lysine) acetyltransferase 5  ZFP69 - zfp69 zinc finger protein  CEBPG - ccaat/enhancer binding protein (c/ebp), gamma  SKAP1 - src kinase associated phosphoprotein 1  CDK5 - cyclin-dependent kinase 5  GZF1 - gdnf-inducible zinc finger protein 1  CDK9 - cyclin-dependent kinase 9  CDK8 - cyclin-dependent kinase 8  INSR - insulin receptor  LIN54 - lin-54 homolog (c. elegans)  INSM1 - insulinoma-associated 1  CDK7 - cyclin-dependent kinase 7  CDK13 - cyclin-dependent kinase 13  CDKN1C - cyclin-dependent kinase inhibitor 1c (p57, kip2)  CDKN1B - cyclin-dependent kinase inhibitor 1b (p27, kip1)  PDCD4 - programmed cell death 4 (neoplastic transformation inhibitor)  PCBP3 - poly(rc) binding protein 3  RFXANK - regulatory factor x-associated ankyrin-containing protein  PTPN2 - protein tyrosine phosphatase, non-receptor type 2  IRF2 - interferon regulatory factor 2  KLF7 - kruppel-like factor 7 (ubiquitous)  IRF3 - interferon regulatory factor 3  HTATIP2 - hiv-1 tat interactive protein 2, 30kda  RUVBL1 - ruvb-like 1 (e. coli)  IKZF5 - ikaros family zinc finger 5 (pegasus)  MED19 - mediator complex subunit 19  ITGA6 - integrin, alpha 6  IRAK2 - interleukin-1 receptor-associated kinase 2  CDK2 - cyclin-dependent kinase 2  IRAK1 - interleukin-1 receptor-associated kinase 1  PURA - purine-rich element binding protein a  RRN3 - rrn3 rna polymerase i transcription factor homolog (s. cerevisiae)  PURB - purine-rich element binding protein b  ZSCAN31 - zinc finger and scan domain containing 31  KIAA1324L - kiaa1324-like  NSD1 - nuclear receptor binding set domain protein 1  ZMYND11 - zinc finger, mynd-type containing 11  ZBTB11 - zinc finger and btb domain containing 11  SRSF10 - serine/arginine-rich splicing factor 10  PTPRK - protein tyrosine phosphatase, receptor type, k  RNF25 - ring finger protein 25  PTPRN - protein tyrosine phosphatase, receptor type, n  DKK3 - dickkopf wnt signaling pathway inhibitor 3  MMS19 - mms19 nucleotide excision repair homolog (s. cerevisiae)  PEX2 - peroxisomal biogenesis factor 2  MPHOSPH8 - m-phase phosphoprotein 8  KDM5B - lysine (k)-specific demethylase 5b  PXN - paxillin  SNX5 - sorting nexin 5  ZNF777 - zinc finger protein 777  ID2 - inhibitor of dna binding 2, dominant negative helix-loop-helix protein  ID3 - inhibitor of dna binding 3, dominant negative helix-loop-helix protein  CHD4 - chromodomain helicase dna binding protein 4  CHD2 - chromodomain helicase dna binding protein 2  STK36 - serine/threonine kinase 36  CHD3 - chromodomain helicase dna binding protein 3  MTERFD2 - mterf domain containing 2  CHD1 - chromodomain helicase dna binding protein 1  IRF2BPL - interferon regulatory factor 2 binding protein-like  JAZF1 - jazf zinc finger 1  QARS - glutaminyl-trna synthetase  MYPOP - myb-related transcription factor, partner of profilin  RAD21 - rad21 homolog (s. pombe)  FOXK1 - forkhead box k1  PARP14 - poly (adp-ribose) polymerase family, member 14  PAF1 - paf1, rna polymerase ii associated factor, homolog (s. cerevisiae)  ZNF181 - zinc finger protein 181  ZNF268 - zinc finger protein 268  INO80 - ino80 complex subunit  ZNF513 - zinc finger protein 513  IGHMBP2 - immunoglobulin mu binding protein 2  ZNF282 - zinc finger protein 282  GMEB1 - glucocorticoid modulatory element binding protein 1  RBPJ - recombination signal binding protein for immunoglobulin kappa j region  MIER2 - mesoderm induction early response 1, family member 2  NCK2 - nck adaptor protein 2  RARG - retinoic acid receptor, gamma  RARB - retinoic acid receptor, beta  DLL4 - delta-like 4 (drosophila)  ZBTB9 - zinc finger and btb domain containing 9  USP21 - ubiquitin specific peptidase 21  ZBTB12 - zinc finger and btb domain containing 12  CLU - clusterin  KHDRBS1 - kh domain containing, rna binding, signal transduction associated 1  TAF8 - taf8 rna polymerase ii, tata box binding protein (tbp)-associated factor, 43kda  MAGEL2 - mage-like 2  CTCF - ccctc-binding factor (zinc finger protein)  JMJD1C - jumonji domain containing 1c  IGBP1 - immunoglobulin (cd79a) binding protein 1  RBL2 - retinoblastoma-like 2 (p130)  RAI1 - retinoic acid induced 1  RBBP7 - retinoblastoma binding protein 7  SMARCA5 - swi/snf related, matrix associated, actin dependent regulator of chromatin, subfamily a, member 5  RBBP8 - retinoblastoma binding protein 8  MAP3K2 - mitogen-activated protein kinase kinase kinase 2  OGT - o-linked n-acetylglucosamine (glcnac) transferase  RB1 - retinoblastoma 1  PELP1 - proline, glutamate and leucine rich protein 1  ARID4A - at rich interactive domain 4a (rbp1-like)  SND1 - staphylococcal nuclease and tudor domain containing 1  KDM5A - lysine (k)-specific demethylase 5a  NUP35 - nucleoporin 35kda  CUL2 - cullin 2  ZNF667 - zinc finger protein 667  CUL3 - cullin 3  CKS1B - cdc28 protein kinase regulatory subunit 1b  NFAT5 - nuclear factor of activated t-cells 5, tonicity-responsive  FOXP1 - forkhead box p1  CHUK - conserved helix-loop-helix ubiquitous kinase  SUPT3H - suppressor of ty 3 homolog (s. cerevisiae)  BEND6 - ben domain containing 6  ZNF572 - zinc finger protein 572  ZMYND8 - zinc finger, mynd-type containing 8  PCGF5 - polycomb group ring finger 5  ANKRA2 - ankyrin repeat, family a (rfxank-like), 2  WAC - ww domain containing adaptor with coiled-coil  RBAK - rb-associated krab zinc finger  NFATC2IP - nuclear factor of activated t-cells, cytoplasmic, calcineurin-dependent 2 interacting protein  ZNF414 - zinc finger protein 414  PHF21A - phd finger protein 21a  ZNF771 - zinc finger protein 771  PATZ1 - poz (btb) and at hook containing zinc finger 1  IER2 - immediate early response 2  SPP1 - secreted phosphoprotein 1  NCOR1 - nuclear receptor corepressor 1  SSBP2 - single-stranded dna binding protein 2  KPNA6 - karyopherin alpha 6 (importin alpha 7)  NCOR2 - nuclear receptor corepressor 2  MTPN - myotrophin  CIRH1A - cirrhosis, autosomal recessive 1a (cirhin)  DOT1L - dot1-like histone h3k79 methyltransferase  MIER1 - mesoderm induction early response 1, transcriptional regulator  ZNF382 - zinc finger protein 382  ZNF705A - zinc finger protein 705a  EFCAB7 - ef-hand calcium binding domain 7  SP3 - sp3 transcription factor  L3MBTL3 - l(3)mbt-like 3 (drosophila)  SP4 - sp4 transcription factor  SP1 - sp1 transcription factor  ZNF410 - zinc finger protein 410  SP2 - sp2 transcription factor  ZNF333 - zinc finger protein 333  CIR1 - corepressor interacting with rbpj, 1  SCAND1 - scan domain containing 1  ARL2BP - adp-ribosylation factor-like 2 binding protein  SOX4 - sry (sex determining region y)-box 4  H2AFY - h2a histone family, member y  WBP2 - ww domain binding protein 2  GTF2IRD1 - gtf2i repeat domain containing 1  FADS1 - fatty acid desaturase 1  ZNF527 - zinc finger protein 527  HES4 - hairy and enhancer of split 4 (drosophila)  LEMD3 - lem domain containing 3  CREBL2 - camp responsive element binding protein-like 2  GATAD1 - gata zinc finger domain containing 1  ATF6B - activating transcription factor 6 beta  HOPX - hop homeobox  CREBBP - creb binding protein  ZNF316 - zinc finger protein 316  ZC3H8 - zinc finger ccch-type containing 8  SOX13 - sry (sex determining region y)-box 13  ATF2 - activating transcription factor 2  LMO1 - lim domain only 1 (rhombotin 1)  CREB1 - camp responsive element binding protein 1  IER5 - immediate early response 5  NACC2 - nacc family member 2, ben and btb (poz) domain containing  DDX58 - dead (asp-glu-ala-asp) box polypeptide 58  CRABP2 - cellular retinoic acid binding protein 2  CLOCK - clock circadian regulator  KLF3 - kruppel-like factor 3 (basic)  RPRD1B - regulation of nuclear pre-mrna domain containing 1b  DDX41 - dead (asp-glu-ala-asp) box polypeptide 41  PLK3 - polo-like kinase 3  SNAPC4 - small nuclear rna activating complex, polypeptide 4, 190kda  LITAF - lipopolysaccharide-induced tnf factor  SNCA - synuclein, alpha (non a4 component of amyloid precursor)  TBPL1 - tbp-like 1  ZBED5 - zinc finger, bed-type containing 5  CREBZF - creb/atf bzip transcription factor  ZFP62 - zfp62 zinc finger protein  ZBTB41 - zinc finger and btb domain containing 41  EID1 - ep300 interacting inhibitor of differentiation 1  TP53INP2 - tumor protein p53 inducible nuclear protein 2  SMARCE1 - swi/snf related, matrix associated, actin dependent regulator of chromatin, subfamily e, member 1  SMO - smoothened, frizzled family receptor  ZGPAT - zinc finger, ccch-type with g patch domain  ACTL6B - actin-like 6b  SMARCB1 - swi/snf related, matrix associated, actin dependent regulator of chromatin, subfamily b, member 1  SMARCC1 - swi/snf related, matrix associated, actin dependent regulator of chromatin, subfamily c, member 1  HLTF - helicase-like transcription factor  SSBP3 - single stranded dna binding protein 3  SNX6 - sorting nexin 6  SMARCA4 - swi/snf related, matrix associated, actin dependent regulator of chromatin, subfamily a, member 4  SMARCD2 - swi/snf related, matrix associated, actin dependent regulator of chromatin, subfamily d, member 2  SMARCD3 - swi/snf related, matrix associated, actin dependent regulator of chromatin, subfamily d, member 3  SMARCC2 - swi/snf related, matrix associated, actin dependent regulator of chromatin, subfamily c, member 2  SMARCD1 - swi/snf related, matrix associated, actin dependent regulator of chromatin, subfamily d, member 1  WIZ - widely interspaced zinc finger motifs  MED20 - mediator complex subunit 20  AFAP1L2 - actin filament associated protein 1-like 2  SMARCA1 - swi/snf related, matrix associated, actin dependent regulator of chromatin, subfamily a, member 1  SMARCA2 - swi/snf related, matrix associated, actin dependent regulator of chromatin, subfamily a, member 2  CRLF3 - cytokine receptor-like factor 3  MAPK8IP1 - mitogen-activated protein kinase 8 interacting protein 1  SGK3 - serum/glucocorticoid regulated kinase family, member 3  CACTIN - cactin, spliceosome c complex subunit  KMT2C - lysine (k)-specific methyltransferase 2c  GLYR1 - glyoxylate reductase 1 homolog (arabidopsis)  ZBTB7A - zinc finger and btb domain containing 7a  DPY30 - dpy-30 homolog (c. elegans)  PPIL4 - peptidylprolyl isomerase (cyclophilin)-like 4  ZNF462 - zinc finger protein 462  MBTPS2 - membrane-bound transcription factor peptidase, site 2  MAGED1 - melanoma antigen family d, 1  GLIS2 - glis family zinc finger 2  IRF2BP2 - interferon regulatory factor 2 binding protein 2  MED27 - mediator complex subunit 27  MED26 - mediator complex subunit 26  CHD6 - chromodomain helicase dna binding protein 6  SENP2 - sumo1/sentrin/smt3 specific peptidase 2  MED17 - mediator complex subunit 17  MED23 - mediator complex subunit 23  ZNF580 - zinc finger protein 580  ZNF318 - zinc finger protein 318  CDYL - chromodomain protein, y-like  PRPF6 - pre-mrna processing factor 6  GTF2IRD2 - gtf2i repeat domain containing 2  KDM2B - lysine (k)-specific demethylase 2b  SUPT6H - suppressor of ty 6 homolog (s. cerevisiae)  ZNF710 - zinc finger protein 710  CBY1 - chibby homolog 1 (drosophila)  CTBP1 - c-terminal binding protein 1  CTBP2 - c-terminal binding protein 2  SUPT4H1 - suppressor of ty 4 homolog 1 (s. cerevisiae)  ING4 - inhibitor of growth family, member 4  SUPT5H - suppressor of ty 5 homolog (s. cerevisiae)  EIF2AK3 - eukaryotic translation initiation factor 2-alpha kinase 3  INSIG2 - insulin induced gene 2  CTNNB1 - catenin (cadherin-associated protein), beta 1, 88kda  RLIM - ring finger protein, lim domain interacting  SAV1 - salvador homolog 1 (drosophila)  KRAS - kirsten rat sarcoma viral oncogene homolog  RECQL5 - recq protein-like 5  CUX1 - cut-like homeobox 1  TGFBRAP1 - transforming growth factor, beta receptor associated protein 1  ZNF706 - zinc finger protein 706  CIAO1 - cytosolic iron-sulfur protein assembly 1  NIF3L1 - nif3 ngg1 interacting factor 3-like 1 (s. cerevisiae)  NEK4 - nima-related kinase 4  STK3 - serine/threonine kinase 3  PCGF1 - polycomb group ring finger 1  FKBP8 - fk506 binding protein 8, 38kda  NPNT - nephronectin  GPBP1L1 - gc-rich promoter binding protein 1-like 1  PHF20L1 - phd finger protein 20-like 1  MED21 - mediator complex subunit 21  MED10 - mediator complex subunit 10  CYLD - cylindromatosis (turban tumor syndrome)  KIT - v-kit hardy-zuckerman 4 feline sarcoma viral oncogene homolog  ZFP28 - zfp28 zinc finger protein  TGIF2 - tgfb-induced factor homeobox 2  BCL6B - b-cell cll/lymphoma 6, member b  CRK - v-crk avian sarcoma virus ct10 oncogene homolog  NIPBL - nipped-b homolog (drosophila)  STAT2 - signal transducer and activator of transcription 2, 113kda  CRY1 - cryptochrome 1 (photolyase-like)  STAT1 - signal transducer and activator of transcription 1, 91kda  CRY2 - cryptochrome 2 (photolyase-like)  STAT3 - signal transducer and activator of transcription 3 (acute-phase response factor)  JUP - junction plakoglobin  STAT5A - signal transducer and activator of transcription 5a  ASF1A - anti-silencing function 1a histone chaperone  STAT6 - signal transducer and activator of transcription 6, interleukin-4 induced  JAK2 - janus kinase 2  ING5 - inhibitor of growth family, member 5  BRMS1 - breast cancer metastasis suppressor 1  ZNF219 - zinc finger protein 219  ZNF496 - zinc finger protein 496  CRYM - crystallin, mu  SS18 - synovial sarcoma translocation, chromosome 18  PHF20 - phd finger protein 20  PHF5A - phd finger protein 5a  GPATCH3 - g patch domain containing 3  ZNHIT3 - zinc finger, hit-type containing 3  ZNF397 - zinc finger protein 397  TRIP4 - thyroid hormone receptor interactor 4  HMGN3 - high mobility group nucleosomal binding domain 3  TAB3 - tgf-beta activated kinase 1/map3k7 binding protein 3  PHF6 - phd finger protein 6  ZNF235 - zinc finger protein 235  COPS2 - cop9 signalosome subunit 2  ZNF335 - zinc finger protein 335  BAMBI - bmp and activin membrane-bound inhibitor  HAVCR2 - hepatitis a virus cellular receptor 2  ZNF639 - zinc finger protein 639  SRI - sorcin  SRC - v-src avian sarcoma (schmidt-ruppin a-2) viral oncogene homolog  SARNP - sap domain containing ribonucleoprotein  BRMS1L - breast cancer metastasis-suppressor 1-like  LEF1 - lymphoid enhancer-binding factor 1  DNAJB5 - dnaj (hsp40) homolog, subfamily b, member 5  SREBF2 - sterol regulatory element binding transcription factor 2  UBE2O - ubiquitin-conjugating enzyme e2o  CNOT8 - ccr4-not transcription complex, subunit 8  PRDX5 - peroxiredoxin 5  CDK12 - cyclin-dependent kinase 12  DHX9 - deah (asp-glu-ala-his) box helicase 9  KANK2 - kn motif and ankyrin repeat domains 2  TRAPPC2 - trafficking protein particle complex 2  ZNF280B - zinc finger protein 280b  DDX5 - dead (asp-glu-ala-asp) box helicase 5  DDX3X - dead (asp-glu-ala-asp) box helicase 3, x-linked  DDX1 - dead (asp-glu-ala-asp) box helicase 1  ARID4B - at rich interactive domain 4b (rbp1-like)  SIN3A - sin3 transcription regulator family member a  MAP3K10 - mitogen-activated protein kinase kinase kinase 10  WWTR1 - ww domain containing transcription regulator 1  SET - set nuclear oncogene  ZNF304 - zinc finger protein 304  KDM3B - lysine (k)-specific demethylase 3b  ITGB1BP1 - integrin beta 1 binding protein 1  RSF1 - remodeling and spacing factor 1  ZNF286A - zinc finger protein 286a  MLLT6 - myeloid/lymphoid or mixed-lineage leukemia (trithorax homolog, drosophila); translocated to, 6  PBXIP1 - pre-b-cell leukemia homeobox interacting protein 1  NOL11 - nucleolar protein 11  MLLT3 - myeloid/lymphoid or mixed-lineage leukemia (trithorax homolog, drosophila); translocated to, 3  MED14 - mediator complex subunit 14  MLLT1 - myeloid/lymphoid or mixed-lineage leukemia (trithorax homolog, drosophila); translocated to, 1  DBP - d site of albumin promoter (albumin d-box) binding protein  ZBTB22 - zinc finger and btb domain containing 22  KMT2A - lysine (k)-specific methyltransferase 2a  NELFB - negative elongation factor complex member b  RPS6KA5 - ribosomal protein s6 kinase, 90kda, polypeptide 5  SUFU - suppressor of fused homolog (drosophila)  AHCTF1 - at hook containing transcription factor 1  ZBTB38 - zinc finger and btb domain containing 38  NLK - nemo-like kinase  UIMC1 - ubiquitin interaction motif containing 1  MNAT1 - mnat cdk-activating kinase assembly factor 1  ZNF551 - zinc finger protein 551  GATAD2B - gata zinc finger domain containing 2b  MTA2 - metastasis associated 1 family, member 2  NOLC1 - nucleolar and coiled-body phosphoprotein 1  ZNF473 - zinc finger protein 473  EIF2A - eukaryotic translation initiation factor 2a, 65kda  CD200 - cd200 molecule  WWOX - ww domain containing oxidoreductase  MNT - mnt, max dimerization protein  CCPG1 - cell cycle progression 1  CNOT6 - ccr4-not transcription complex, subunit 6  ZNF451 - zinc finger protein 451  SPTY2D1 - spt2, suppressor of ty, domain containing 1 (s. cerevisiae)  SS18L1 - synovial sarcoma translocation gene on chromosome 18-like 1  SCAF4 - sr-related ctd-associated factor 4  CHD5 - chromodomain helicase dna binding protein 5  MECP2 - methyl cpg binding protein 2 (rett syndrome)  PELO - pelota homolog (drosophila)  MEF2A - myocyte enhancer factor 2a  ARID1B - at rich interactive domain 1b (swi1-like)  PRICKLE1 - prickle homolog 1 (drosophila)  MED30 - mediator complex subunit 30  ZBED1 - zinc finger, bed-type containing 1  MKL2 - mkl/myocardin-like 2  MEIS2 - meis homeobox 2  BRWD1 - bromodomain and wd repeat domain containing 1  ZNF664 - zinc finger protein 664  MEIS1 - meis homeobox 1  MAP3K5 - mitogen-activated protein kinase kinase kinase 5  SLC33A1 - solute carrier family 33 (acetyl-coa transporter), member 1  MEN1 - multiple endocrine neoplasia i  WDR75 - wd repeat domain 75  DAPK3 - death-associated protein kinase 3  DAXX - death-domain associated protein  UTP15 - utp15, u3 small nucleolar ribonucleoprotein, homolog (s. cerevisiae)  ZZZ3 - zinc finger, zz-type containing 3  HINFP - histone h4 transcription factor  KAT8 - k(lysine) acetyltransferase 8  ZNF644 - zinc finger protein 644  DGKQ - diacylglycerol kinase, theta 110kda  MTA3 - metastasis associated 1 family, member 3  ARHGEF2 - rho/rac guanine nucleotide exchange factor (gef) 2  ZNF608 - zinc finger protein 608  MAVS - mitochondrial antiviral signaling protein  DAP - death-associated protein  SARS - seryl-trna synthetase  SATB1 - satb homeobox 1  MBD3 - methyl-cpg binding domain protein 3  RPTOR - regulatory associated protein of mtor, complex 1  SAFB - scaffold attachment factor b  PTOV1 - prostate tumor overexpressed 1  ZNF394 - zinc finger protein 394  SALL2 - sal-like 2 (drosophila)  CBFA2T2 - core-binding factor, runt domain, alpha subunit 2; translocated to, 2  DRD2 - dopamine receptor d2  EPC2 - enhancer of polycomb homolog 2 (drosophila)  CXXC5 - cxxc finger protein 5  PHF19 - phd finger protein 19  SLC9A1 - solute carrier family 9, subfamily a (nhe1, cation proton antiporter 1), member 1  IRF2BP1 - interferon regulatory factor 2 binding protein 1  RQCD1 - rcd1 required for cell differentiation1 homolog (s. pombe)  EAF1 - ell associated factor 1  ATN1 - atrophin 1  MARS - methionyl-trna synthetase  IL23A - interleukin 23, alpha subunit p19  C10orf137 - chromosome 10 open reading frame 137  SIRT6 - sirtuin 6  MTA1 - metastasis associated 1  DR1 - down-regulator of transcription 1, tbp-binding (negative cofactor 2)  PPHLN1 - periphilin 1  SKI - v-ski avian sarcoma viral oncogene homolog  SKIL - ski-like oncogene  MBD1 - methyl-cpg binding domain protein 1  DYRK1A - dual-specificity tyrosine-(y)-phosphorylation regulated kinase 1a  SFMBT1 - scm-like with four mbt domains 1  MKL1 - megakaryoblastic leukemia (translocation) 1  MAX - myc associated factor x  ZMIZ2 - zinc finger, miz-type containing 2  DVL2 - dishevelled segment polarity protein 2  DVL3 - dishevelled segment polarity protein 3  LDB2 - lim domain binding 2  ZFP90 - zfp90 zinc finger protein  FNIP2 - folliculin interacting protein 2  HOMEZ - homeobox and leucine zipper encoding  NELFCD - negative elongation factor complex member c/d  EBF4 - early b-cell factor 4  ASH2L - ash2 (absent, small, or homeotic)-like (drosophila)  ZFHX2 - zinc finger homeobox 2  ZSCAN29 - zinc finger and scan domain containing 29  CTDSPL2 - ctd (carboxy-terminal domain, rna polymerase ii, polypeptide a) small phosphatase like 2  THAP2 - thap domain containing, apoptosis associated protein 2  DTX1 - deltex homolog 1 (drosophila)  ITCH - itchy e3 ubiquitin protein ligase  ZNF484 - zinc finger protein 484  L3MBTL2 - l(3)mbt-like 2 (drosophila)  DLG1 - discs, large homolog 1 (drosophila)  LRP6 - low density lipoprotein receptor-related protein 6  ZNF445 - zinc finger protein 445  SHOX2 - short stature homeobox 2  AIP - aryl hydrocarbon receptor interacting protein  SIAH2 - siah e3 ubiquitin protein ligase 2  TRAPPC9 - trafficking protein particle complex 9  LUM - lumican  BTAF1 - btaf1 rna polymerase ii, b-tfiid transcription factor-associated, 170kda  POGK - pogo transposable element with krab domain  UBA3 - ubiquitin-like modifier activating enzyme 3  DHFR - dihydrofolate reductase  SESN2 - sestrin 2  PARP9 - poly (adp-ribose) polymerase family, member 9  PHF12 - phd finger protein 12  RNF8 - ring finger protein 8, e3 ubiquitin protein ligase  BAZ1B - bromodomain adjacent to zinc finger domain, 1b  CALCOCO1 - calcium binding and coiled-coil domain 1  SFPQ - splicing factor proline/glutamine-rich  ZBTB4 - zinc finger and btb domain containing 4  SFRP1 - secreted frizzled-related protein 1  SFRP2 - secreted frizzled-related protein 2  MXD1 - max dimerization protein 1  MED15 - mediator complex subunit 15  SFRP4 - secreted frizzled-related protein 4  PIAS4 - protein inhibitor of activated stat, 4  TRIM33 - tripartite motif containing 33  SRRT - serrate rna effector molecule homolog (arabidopsis)  SMAD2 - smad family member 2  SRSF2 - serine/arginine-rich splicing factor 2  GMEB2 - glucocorticoid modulatory element binding protein 2  DNM2 - dynamin 2  SPAG8 - sperm associated antigen 8  DNMT1 - dna (cytosine-5-)-methyltransferase 1  HDAC7 - histone deacetylase 7  KLF16 - kruppel-like factor 16  ARMCX3 - armadillo repeat containing, x-linked 3  DNMT3A - dna (cytosine-5-)-methyltransferase 3 alpha  TDP2 - tyrosyl-dna phosphodiesterase 2  EIF2AK4 - eukaryotic translation initiation factor 2 alpha kinase 4  LARP7 - la ribonucleoprotein domain family, member 7  CHD8 - chromodomain helicase dna binding protein 8  RPS6KA4 - ribosomal protein s6 kinase, 90kda, polypeptide 4  MAFG - v-maf avian musculoaponeurotic fibrosarcoma oncogene homolog g  TAF9B - taf9b rna polymerase ii, tata box binding protein (tbp)-associated factor, 31kda  NOC2L - nucleolar complex associated 2 homolog (s. cerevisiae)  SGK1 - serum/glucocorticoid regulated kinase 1  SMAD4 - smad family member 4  ZNF317 - zinc finger protein 317  H1FX - h1 histone family, member x  SMAD5 - smad family member 5  SMAD7 - smad family member 7  WASL - wiskott-aldrich syndrome-like  SAP30BP - sap30 binding protein  ZNF565 - zinc finger protein 565  MEIS3 - meis homeobox 3  YAF2 - yy1 associated factor 2  TAGLN3 - transgelin 3  SIX5 - six homeobox 5  NDN - necdin, melanoma antigen (mage) family member  MAPRE3 - microtubule-associated protein, rp/eb family, member 3  ZNF582 - zinc finger protein 582  NCK1 - nck adaptor protein 1  BRD7 - bromodomain containing 7  NCL - nucleolin  ELK1 - elk1, member of ets oncogene family  CEBPZ - ccaat/enhancer binding protein (c/ebp), zeta  TICAM1 - toll-like receptor adaptor molecule 1  ELK3 - elk3, ets-domain protein (srf accessory protein 2)  ELK4 - elk4, ets-domain protein (srf accessory protein 1)  SNW1 - snw domain containing 1  CCNL2 - cyclin l2  SCAP - srebf chaperone  TRIM28 - tripartite motif containing 28  EPAS1 - endothelial pas domain protein 1  EP300 - e1a binding protein p300  ATF6 - activating transcription factor 6  TBK1 - tank-binding kinase 1  ZNF197 - zinc finger protein 197  ENO1 - enolase 1, (alpha)  TDRD3 - tudor domain containing 3  EIF4A2 - eukaryotic translation initiation factor 4a2  EGR2 - early growth response 2  TRIM11 - tripartite motif containing 11  PPP1R12A - protein phosphatase 1, regulatory subunit 12a  EGR3 - early growth response 3  MYT1 - myelin transcription factor 1  ALYREF - aly/ref export factor  NAB1 - ngfi-a binding protein 1 (egr1 binding protein 1)  CSRNP2 - cysteine-serine-rich nuclear protein 2  SENP1 - sumo1/sentrin specific peptidase 1  RNF41 - ring finger protein 41  NAB2 - ngfi-a binding protein 2 (egr1 binding protein 2)  TRIM32 - tripartite motif containing 32  TSHZ1 - teashirt zinc finger homeobox 1  KDM2A - lysine (k)-specific demethylase 2a  PSME3 - proteasome (prosome, macropain) activator subunit 3 (pa28 gamma; ki)  ZNF326 - zinc finger protein 326  ELF1 - e74-like factor 1 (ets domain transcription factor)  ELF2 - e74-like factor 2 (ets domain transcription factor)  TCF25 - transcription factor 25 (basic helix-loop-helix)  TSG101 - tumor susceptibility gene 101  TRIM13 - tripartite motif containing 13  TRIM8 - tripartite motif containing 8  MYO6 - myosin vi  LBH - limb bud and heart development  ABT1 - activator of basal transcription 1  TOPORS - topoisomerase i binding, arginine/serine-rich, e3 ubiquitin protein ligase  CNOT1 - ccr4-not transcription complex, subunit 1  EFNA1 - ephrin-a1  SPEN - spen homolog, transcriptional regulator (drosophila)  CTNNBIP1 - catenin, beta interacting protein 1  MYD88 - myeloid differentiation primary response 88  ZNF362 - zinc finger protein 362  BBX - bobby sox homolog (drosophila)  PHC1 - polyhomeotic homolog 1 (drosophila)  TP53BP1 - tumor protein p53 binding protein 1  HCFC2 - host cell factor c2  PHC2 - polyhomeotic homolog 2 (drosophila)  EGR1 - early growth response 1  ZNF292 - zinc finger protein 292  CNOT7 - ccr4-not transcription complex, subunit 7  EGFR - epidermal growth factor receptor  MXI1 - max interactor 1, dimerization protein  MEGF8 - multiple egf-like-domains 8  PQBP1 - polyglutamine binding protein 1  CELSR2 - cadherin, egf lag seven-pass g-type receptor 2  KDM1A - lysine (k)-specific demethylase 1a  ATXN7L3 - ataxin 7-like 3  ACTR2 - arp2 actin-related protein 2 homolog (yeast)  ACTR3 - arp3 actin-related protein 3 homolog (yeast)  NCOA6 - nuclear receptor coactivator 6  E2F6 - e2f transcription factor 6  TSFM - ts translation elongation factor, mitochondrial  E4F1 - e4f transcription factor 1  ZHX3 - zinc fingers and homeoboxes 3  E2F4 - e2f transcription factor 4, p107/p130-binding  E2F5 - e2f transcription factor 5, p130-binding  ENY2 - enhancer of yellow 2 homolog (drosophila)  TRIM37 - tripartite motif containing 37  C11orf30 - chromosome 11 open reading frame 30  RRP1B - ribosomal rna processing 1b  ZNF558 - zinc finger protein 558  EDNRB - endothelin receptor type b  ZNF263 - zinc finger protein 263  TRAF1 - tnf receptor-associated factor 1  S1PR1 - sphingosine-1-phosphate receptor 1  TRAF3 - tnf receptor-associated factor 3  TRAF2 - tnf receptor-associated factor 2  PURG - purine-rich element binding protein g  NR2C1 - nuclear receptor subfamily 2, group c, member 1  SETX - senataxin  ZBTB47 - zinc finger and btb domain containing 47  NR2C2 - nuclear receptor subfamily 2, group c, member 2  MED25 - mediator complex subunit 25  TPR - translocated promoter region, nuclear basket protein  ZNF609 - zinc finger protein 609  ERC1 - elks/rab6-interacting/cast family member 1  MED6 - mediator complex subunit 6  PPRC1 - peroxisome proliferator-activated receptor gamma, coactivator-related 1  NAT14 - n-acetyltransferase 14 (gcn5-related, putative)  KDM4C - lysine (k)-specific demethylase 4c  ZNF423 - zinc finger protein 423  REXO4 - rex4, rna exonuclease 4 homolog (s. cerevisiae)  CAMK1D - calcium/calmodulin-dependent protein kinase id  DMTF1 - cyclin d binding myb-like transcription factor 1  USF1 - upstream transcription factor 1  NUP107 - nucleoporin 107kda  ZBTB43 - zinc finger and btb domain containing 43  ZBTB33 - zinc finger and btb domain containing 33  PITHD1 - pith (c-terminal proteasome-interacting domain of thioredoxin-like) domain containing 1  BPTF - bromodomain phd finger transcription factor  HDAC5 - histone deacetylase 5  MTF1 - metal-regulatory transcription factor 1  SRA1 - steroid receptor rna activator 1  HDAC6 - histone deacetylase 6  TAB2 - tgf-beta activated kinase 1/map3k7 binding protein 2  NR1H2 - nuclear receptor subfamily 1, group h, member 2  ETV3 - ets variant 3  CAMTA2 - calmodulin binding transcription activator 2  RNF187 - ring finger protein 187  ETV5 - ets variant 5  POGZ - pogo transposable element with znf domain  TWSG1 - twisted gastrulation homolog 1 (drosophila)  MT3 - metallothionein 3  ETV6 - ets variant 6  PLXND1 - plexin d1  VHL - von hippel-lindau tumor suppressor, e3 ubiquitin protein ligase  MED16 - mediator complex subunit 16  KDM6B - lysine (k)-specific demethylase 6b  PHF8 - phd finger protein 8  EZR - ezrin  EWSR1 - ews rna-binding protein 1  TRIM41 - tripartite motif containing 41  TBX20 - t-box 20  KCTD1 - potassium channel tetramerization domain containing 1  CCDC22 - coiled-coil domain containing 22  CREB3L1 - camp responsive element binding protein 3-like 1  EZH1 - enhancer of zeste homolog 1 (drosophila)  CIC - capicua transcriptional repressor  CCNL1 - cyclin l1  F2R - coagulation factor ii (thrombin) receptor  DNAJB6 - dnaj (hsp40) homolog, subfamily b, member 6  WDR43 - wd repeat domain 43  ERF - ets2 repressor factor  ZNF575 - zinc finger protein 575  CITED1 - cbp/p300-interacting transactivator, with glu/asp-rich carboxy-terminal domain, 1  ZBTB5 - zinc finger and btb domain containing 5  ZBTB40 - zinc finger and btb domain containing 40  ELMSAN1 - elm2 and myb/sant-like domain containing 1  THAP11 - thap domain containing 11  ZNF248 - zinc finger protein 248  SUPT7L - suppressor of ty 7 (s. cerevisiae)-like  UBB - ubiquitin b  RGCC - regulator of cell cycle  RCOR1 - rest corepressor 1  TULP3 - tubby like protein 3  ZNFX1 - zinc finger, nfx1-type containing 1  HIRA - histone cell cycle regulator  COMMD1 - copper metabolism (murr1) domain containing 1  TTC5 - tetratricopeptide repeat domain 5  ETV1 - ets variant 1  ETS1 - v-ets avian erythroblastosis virus e26 oncogene homolog 1  ZMIZ1 - zinc finger, miz-type containing 1  USP3 - ubiquitin specific peptidase 3  UBP1 - upstream binding protein 1 (lbp-1a)  SUMO1 - small ubiquitin-like modifier 1  UBTF - upstream binding transcription factor, rna polymerase i  USP15 - ubiquitin specific peptidase 15  PELI1 - pellino e3 ubiquitin protein ligase 1  CHURC1 - churchill domain containing 1  SMURF1 - smad specific e3 ubiquitin protein ligase 1  ZC3H4 - zinc finger ccch-type containing 4  MED4 - mediator complex subunit 4  JMJD6 - jumonji domain containing 6  THOC1 - tho complex 1  SETD2 - set domain containing 2  UBE2I - ubiquitin-conjugating enzyme e2i  RBX1 - ring-box 1, e3 ubiquitin protein ligase  THRAP3 - thyroid hormone receptor associated protein 3  UBE2L3 - ubiquitin-conjugating enzyme e2l 3  MED12 - mediator complex subunit 12  MED13 - mediator complex subunit 13  UBE2N - ubiquitin-conjugating enzyme e2n  UBE2V1 - ubiquitin-conjugating enzyme e2 variant 1  PSMD6 - proteasome (prosome, macropain) 26s subunit, non-atpase, 6  VPS72 - vacuolar protein sorting 72 homolog (s. cerevisiae)  MED24 - mediator complex subunit 24  MGA - mga, max dimerization protein  ZC3H6 - zinc finger ccch-type containing 6  DTX3L - deltex 3-like (drosophila)  SETDB1 - set domain, bifurcated 1  ZNF34 - zinc finger protein 34  ZEB1 - zinc finger e-box binding homeobox 1  WWC3 - wwc family member 3  ORC2 - origin recognition complex, subunit 2  TCF12 - transcription factor 12  ZNF354A - zinc finger protein 354a  TOX4 - tox high mobility group box family member 4  ZNF436 - zinc finger protein 436  CAMTA1 - calmodulin binding transcription activator 1  TCF20 - transcription factor 20 (ar1)  FLI1 - fli-1 proto-oncogene, ets transcription factor  RNF141 - ring finger protein 141  FLNA - filamin a, alpha  AGRN - agrin  PLCB1 - phospholipase c, beta 1 (phosphoinositide-specific)  FOXS1 - forkhead box s1  FOXO3 - forkhead box o3  HDAC8 - histone deacetylase 8  ASH1L - ash1 (absent, small, or homeotic)-like (drosophila)  SIN3B - sin3 transcription regulator family member b  G3BP2 - gtpase activating protein (sh3 domain) binding protein 2  NACC1 - nucleus accumbens associated 1, ben and btb (poz) domain containing  HR - hair growth associated  MED8 - mediator complex subunit 8  CAND1 - cullin-associated and neddylation-dissociated 1  TDG - thymine-dna glycosylase  OGG1 - 8-oxoguanine dna glycosylase  PRDX2 - peroxiredoxin 2  ZSCAN16 - zinc finger and scan domain containing 16  EAPP - e2f-associated phosphoprotein  FBXW11 - f-box and wd repeat domain containing 11  FAM208A - family with sequence similarity 208, member a  KDM3A - lysine (k)-specific demethylase 3a  FHL2 - four and a half lim domains 2  FGFR2 - fibroblast growth factor receptor 2  TAF9 - taf9 rna polymerase ii, tata box binding protein (tbp)-associated factor, 32kda  TAF7 - taf7 rna polymerase ii, tata box binding protein (tbp)-associated factor, 55kda  TAF6 - taf6 rna polymerase ii, tata box binding protein (tbp)-associated factor, 80kda  FGFR1 - fibroblast growth factor receptor 1  FGF9 - fibroblast growth factor 9  TADA2A - transcriptional adaptor 2a  DDR2 - discoidin domain receptor tyrosine kinase 2  TACC1 - transforming, acidic coiled-coil containing protein 1  DAB2IP - dab2 interacting protein  BCLAF1 - bcl2-associated transcription factor 1  NUMA1 - nuclear mitotic apparatus protein 1  TIRAP - toll-interleukin 1 receptor (tir) domain containing adaptor protein  FGF1 - fibroblast growth factor 1 (acidic)  FGF2 - fibroblast growth factor 2 (basic)  GMNN - geminin, dna replication inhibitor  NUP98 - nucleoporin 98kda  NR4A2 - nuclear receptor subfamily 4, group a, member 2  USP22 - ubiquitin specific peptidase 22  ZNF593 - zinc finger protein 593  ZBTB7B - zinc finger and btb domain containing 7b  TARBP1 - tar (hiv-1) rna binding protein 1  FER - fer (fps/fes related) tyrosine kinase  ZNF691 - zinc finger protein 691  MAP3K7 - mitogen-activated protein kinase kinase kinase 7  CREBRF - creb3 regulatory factor  ZNF623 - zinc finger protein 623  ADNP - activity-dependent neuroprotector homeobox  NPM1 - nucleophosmin (nucleolar phosphoprotein b23, numatrin)  ARHGEF11 - rho guanine nucleotide exchange factor (gef) 11  KMT2E - lysine (k)-specific methyltransferase 2e  MED13L - mediator complex subunit 13-like  ZBTB24 - zinc finger and btb domain containing 24  ZEB2 - zinc finger e-box binding homeobox 2  THAP4 - thap domain containing 4  ZNF395 - zinc finger protein 395  MYNN - myoneurin  CRTC1 - creb regulated transcription coactivator 1  UFL1 - ufm1-specific ligase 1  TCF3 - transcription factor 3  DMAP1 - dna methyltransferase 1 associated protein 1  TCEB3 - transcription elongation factor b (siii), polypeptide 3 (110kda, elongin a)  TCF4 - transcription factor 4  EFEMP1 - egf containing fibulin-like extracellular matrix protein 1  NKRF - nfkb repressing factor  TSC22D2 - tsc22 domain family, member 2  ZNF629 - zinc finger protein 629  FBN1 - fibrillin 1  LANCL2 - lanc lantibiotic synthetase component c-like 2 (bacterial)  NFXL1 - nuclear transcription factor, x-box binding-like 1  NRF1 - nuclear respiratory factor 1  TCEA2 - transcription elongation factor a (sii), 2  ERBB2IP - erbb2 interacting protein  TCEA1 - transcription elongation factor a (sii), 1  NOP2 - nop2 nucleolar protein  VPRBP - vpr (hiv-1) binding protein  NONO - non-pou domain containing, octamer-binding  ITGB3BP - integrin beta 3 binding protein (beta3-endonexin)  RYBP - ring1 and yy1 binding protein  MTOR - mechanistic target of rapamycin (serine/threonine kinase)  MDFIC - myod family inhibitor domain containing  PAXBP1 - pax3 and pax7 binding protein 1  TLE1 - transducin-like enhancer of split 1 (e(sp1) homolog, drosophila)  TLE3 - transducin-like enhancer of split 3 (e(sp1) homolog, drosophila)  NOTCH4 - notch 4  IFT74 - intraflagellar transport 74 homolog (chlamydomonas)  TLE4 - transducin-like enhancer of split 4 (e(sp1) homolog, drosophila)  CEP290 - centrosomal protein 290kda  NPAT - nuclear protein, ataxia-telangiectasia locus  TLR3 - toll-like receptor 3  CNOT2 - ccr4-not transcription complex, subunit 2  NAA15 - n(alpha)-acetyltransferase 15, nata auxiliary subunit  CNOT3 - ccr4-not transcription complex, subunit 3  NOTCH1 - notch 1  BAZ2B - bromodomain adjacent to zinc finger domain, 2b  SIRT1 - sirtuin 1  NOTCH3 - notch 3  ST18 - suppression of tumorigenicity 18 (breast carcinoma) (zinc finger protein)  MKX - mohawk homeobox  NOTCH2 - notch 2  NFYA - nuclear transcription factor y, alpha  TMF1 - tata element modulatory factor 1  HDAC4 - histone deacetylase 4  TOX - thymocyte selection-associated high mobility group box  ACTR8 - arp8 actin-related protein 8 homolog (yeast)  TMSB4X - thymosin beta 4, x-linked  CBX6 - chromobox homolog 6  NFYC - nuclear transcription factor y, gamma  HEY1 - hairy/enhancer-of-split related with yrpw motif 1  CXXC1 - cxxc finger protein 1  ZFYVE16 - zinc finger, fyve domain containing 16  TMPO - thymopoietin  NFKBIA - nuclear factor of kappa light polypeptide gene enhancer in b-cells inhibitor, alpha  NFKB1 - nuclear factor of kappa light polypeptide gene enhancer in b-cells 1  CBX5 - chromobox homolog 5  KMT2B - lysine (k)-specific methyltransferase 2b  BRD4 - bromodomain containing 4  NFKBIB - nuclear factor of kappa light polypeptide gene enhancer in b-cells inhibitor, beta  NFKBIE - nuclear factor of kappa light polypeptide gene enhancer in b-cells inhibitor, epsilon  TARDBP - tar dna binding protein  BCL9L - b-cell cll/lymphoma 9-like  TNFRSF1A - tumor necrosis factor receptor superfamily, member 1a  NKX2-2 - nk2 homeobox 2  ZNF606 - zinc finger protein 606  HDAC9 - histone deacetylase 9  ZNF614 - zinc finger protein 614  ZFP2 - zfp2 zinc finger protein  TMBIM6 - transmembrane bax inhibitor motif containing 6  SUZ12 - suz12 polycomb repressive complex 2 subunit  NEO1 - neogenin 1  POFUT1 - protein o-fucosyltransferase 1  TEF - thyrotrophic embryonic factor  RCOR2 - rest corepressor 2  TFAM - transcription factor a, mitochondrial  SMARCAL1 - swi/snf related, matrix associated, actin dependent regulator of chromatin, subfamily a-like 1  NEDD8 - neural precursor cell expressed, developmentally down-regulated 8  CTR9 - ctr9, paf1/rna polymerase ii complex component  PPM1F - protein phosphatase, mg2+/mn2+ dependent, 1f  TFDP1 - transcription factor dp-1  NFE2L2 - nuclear factor (erythroid-derived 2)-like 2  EPC1 - enhancer of polycomb homolog 1 (drosophila)  NR2F2 - nuclear receptor subfamily 2, group f, member 2  RASL11A - ras-like, family 11, member a  MORF4L2 - mortality factor 4 like 2  CBX7 - chromobox homolog 7  TFE3 - transcription factor binding to ighm enhancer 3  NFATC4 - nuclear factor of activated t-cells, cytoplasmic, calcineurin-dependent 4  NR2F1 - nuclear receptor subfamily 2, group f, member 1  TFCP2 - transcription factor cp2  NFIA - nuclear factor i/a  TGFA - transforming growth factor, alpha  ZNF740 - zinc finger protein 740  HEY2 - hairy/enhancer-of-split related with yrpw motif 2  FNIP1 - folliculin interacting protein 1  CDK5RAP3 - cdk5 regulatory subunit associated protein 3  TGFBR1 - transforming growth factor, beta receptor 1  RBFOX2 - rna binding protein, fox-1 homolog (c. elegans) 2  TGFB2 - transforming growth factor, beta 2  TGFB3 - transforming growth factor, beta 3  TGFB1 - transforming growth factor, beta 1  TGFB1I1 - transforming growth factor beta 1 induced transcript 1  ZNF746 - zinc finger protein 746  TP53INP1 - tumor protein p53 inducible nuclear protein 1  VGLL4 - vestigial like 4 (drosophila)  H2AFV - h2a histone family, member v  LRRC14 - leucine rich repeat containing 14  MEPCE - methylphosphate capping enzyme  RNF20 - ring finger protein 20, e3 ubiquitin protein ligase  KDM4A - lysine (k)-specific demethylase 4a  TGFBR3 - transforming growth factor, beta receptor iii  FOSL2 - fos-like antigen 2  PHF14 - phd finger protein 14  FOSB - fbj murine osteosarcoma viral oncogene homolog b  FOS - fbj murine osteosarcoma viral oncogene homolog  KAT6B - k(lysine) acetyltransferase 6b  HP1BP3 - heterochromatin protein 1, binding protein 3  ZNF432 - zinc finger protein 432  SAFB2 - scaffold attachment factor b2  MYEF2 - myelin expression factor 2  LPIN2 - lipin 2  THRA - thyroid hormone receptor, alpha |
| GO:0006355 | regulation of transcription, DNA-templated | 4.31E-9 | 1.54E-6 | 1.08 (10334,1938,6996,1418) | [+] Show genes  ELL - elongation factor rna polymerase ii  EXOSC9 - exosome component 9  ZNF239 - zinc finger protein 239  MSL3 - male-specific lethal 3 homolog (drosophila)  TADA1 - transcriptional adaptor 1  CBX1 - chromobox homolog 1  ABCA2 - atp-binding cassette, sub-family a (abc1), member 2  ZNF821 - zinc finger protein 821  ABL1 - c-abl oncogene 1, non-receptor tyrosine kinase  PHC3 - polyhomeotic homolog 3 (drosophila)  PML - promyelocytic leukemia  MLLT11 - myeloid/lymphoid or mixed-lineage leukemia (trithorax homolog, drosophila); translocated to, 11  SUPT20H - suppressor of ty 20 homolog (s. cerevisiae)  YWHAQ - tyrosine 3-monooxygenase/tryptophan 5-monooxygenase activation protein, theta polypeptide  KAT2A - k(lysine) acetyltransferase 2a  NRIP1 - nuclear receptor interacting protein 1  NCOA3 - nuclear receptor coactivator 3  BRD8 - bromodomain containing 8  WWC2 - ww and c2 domain containing 2  CHD7 - chromodomain helicase dna binding protein 7  LYAR - ly1 antibody reactive  ZNF354B - zinc finger protein 354b  TCERG1 - transcription elongation regulator 1  TAF15 - taf15 rna polymerase ii, tata box binding protein (tbp)-associated factor, 68kda  EHMT2 - euchromatic histone-lysine n-methyltransferase 2  SPIN1 - spindlin 1  TASP1 - taspase, threonine aspartase, 1  SUB1 - sub1 homolog (s. cerevisiae)  MORF4L1 - mortality factor 4 like 1  ZNF511 - zinc finger protein 511  PPID - peptidylprolyl isomerase d  ACTL6A - actin-like 6a  AXIN1 - axin 1  TRRAP - transformation/transcription domain-associated protein  PICALM - phosphatidylinositol binding clathrin assembly protein  ACVR2A - activin a receptor, type iia  ACVRL1 - activin a receptor type ii-like 1  ACVR1 - activin a receptor, type i  DLL1 - delta-like 1 (drosophila)  ARID1A - at rich interactive domain 1a (swi-like)  ACVR1B - activin a receptor, type ib  SETD6 - set domain containing 6  FZD6 - frizzled family receptor 6  ACTR5 - arp5 actin-related protein 5 homolog (yeast)  ZNF331 - zinc finger protein 331  CDC45 - cell division cycle 45  MED1 - mediator complex subunit 1  GCLC - glutamate-cysteine ligase, catalytic subunit  FZD4 - frizzled family receptor 4  FZD1 - frizzled family receptor 1  BAP1 - brca1 associated protein-1 (ubiquitin carboxy-terminal hydrolase)  ADCY1 - adenylate cyclase 1 (brain)  ZNF613 - zinc finger protein 613  NUP85 - nucleoporin 85kda  PPARD - peroxisome proliferator-activated receptor delta  KDM5C - lysine (k)-specific demethylase 5c  USP9X - ubiquitin specific peptidase 9, x-linked  RBM10 - rna binding motif protein 10  MAML3 - mastermind-like 3 (drosophila)  POLR2K - polymerase (rna) ii (dna directed) polypeptide k, 7.0kda  PARP1 - poly (adp-ribose) polymerase 1  H2AFY2 - h2a histone family, member y2  PPP1R13L - protein phosphatase 1, regulatory subunit 13 like  HES6 - hairy and enhancer of split 6 (drosophila)  SMPD3 - sphingomyelin phosphodiesterase 3, neutral membrane (neutral sphingomyelinase ii)  POLR2A - polymerase (rna) ii (dna directed) polypeptide a, 220kda  RUVBL2 - ruvb-like 2 (e. coli)  TTC21B - tetratricopeptide repeat domain 21b  CDK5RAP2 - cdk5 regulatory subunit associated protein 2  RCOR3 - rest corepressor 3  SLTM - safb-like, transcription modulator  NELFE - negative elongation factor complex member e  EHMT1 - euchromatic histone-lysine n-methyltransferase 1  AES - amino-terminal enhancer of split  NR5A1 - nuclear receptor subfamily 5, group a, member 1  NUP133 - nucleoporin 133kda  FUS - fused in sarcoma  NAA40 - n(alpha)-acetyltransferase 40, natd catalytic subunit, homolog (s. cerevisiae)  JAG1 - jagged 1  PRDM4 - pr domain containing 4  PPP3R1 - protein phosphatase 3, regulatory subunit b, alpha  ATF7IP - activating transcription factor 7 interacting protein  ZNF689 - zinc finger protein 689  LRIF1 - ligand dependent nuclear receptor interacting factor 1  PPP2R5B - protein phosphatase 2, regulatory subunit b', beta  AKT1 - v-akt murine thymoma viral oncogene homolog 1  ZNF653 - zinc finger protein 653  USP7 - ubiquitin specific peptidase 7 (herpes virus-associated)  PCID2 - pci domain containing 2  XRCC6 - x-ray repair complementing defective repair in chinese hamster cells 6  RNF168 - ring finger protein 168, e3 ubiquitin protein ligase  GABPB1 - ga binding protein transcription factor, beta subunit 1  PPP2R1A - protein phosphatase 2, regulatory subunit a, alpha  GABPA - ga binding protein transcription factor, alpha subunit 60kda  WWP2 - ww domain containing e3 ubiquitin protein ligase 2  AHR - aryl hydrocarbon receptor  ZNF526 - zinc finger protein 526  FOXP4 - forkhead box p4  PPP2CA - protein phosphatase 2, catalytic subunit, alpha isozyme  RPAP2 - rna polymerase ii associated protein 2  ZNF83 - zinc finger protein 83  DDX39B - dead (asp-glu-ala-asp) box polypeptide 39b  KANSL3 - kat8 regulatory nsl complex subunit 3  PRNP - prion protein  ATF7 - activating transcription factor 7  KMT2D - lysine (k)-specific methyltransferase 2d  AMFR - autocrine motility factor receptor, e3 ubiquitin protein ligase  PRKRIR - protein-kinase, interferon-inducible double stranded rna dependent inhibitor, repressor of (p58 repressor)  ZNF419 - zinc finger protein 419  MAP2K5 - mitogen-activated protein kinase kinase 5  MAP2K7 - mitogen-activated protein kinase kinase 7  ZNF692 - zinc finger protein 692  PIM2 - pim-2 oncogene  EIF2AK2 - eukaryotic translation initiation factor 2-alpha kinase 2  YEATS4 - yeats domain containing 4  MAP2K1 - mitogen-activated protein kinase kinase 1  MAP2K2 - mitogen-activated protein kinase kinase 2  SNIP1 - smad nuclear interacting protein 1  ZFPM1 - zinc finger protein, fog family member 1  ZNF212 - zinc finger protein 212  MAPK10 - mitogen-activated protein kinase 10  EID2 - ep300 interacting inhibitor of differentiation 2  MAPK9 - mitogen-activated protein kinase 9  ZNF383 - zinc finger protein 383  MAPK11 - mitogen-activated protein kinase 11  MAPK8 - mitogen-activated protein kinase 8  MAPK7 - mitogen-activated protein kinase 7  GCN1L1 - gcn1 general control of amino-acid synthesis 1-like 1 (yeast)  GATA2 - gata binding protein 2  MAPK3 - mitogen-activated protein kinase 3  MAPK1 - mitogen-activated protein kinase 1  KAT6A - k(lysine) acetyltransferase 6a  ADIRF - adipogenesis regulatory factor  PRKD1 - protein kinase d1  ANXA3 - annexin a3  PRKCH - protein kinase c, eta  PRKCI - protein kinase c, iota  CCDC85B - coiled-coil domain containing 85b  NR4A3 - nuclear receptor subfamily 4, group a, member 3  PRKCB - protein kinase c, beta  BRD3 - bromodomain containing 3  GAS6 - growth arrest-specific 6  PRKAR1A - protein kinase, camp-dependent, regulatory, type i, alpha  CARF - calcium responsive transcription factor  MLLT10 - myeloid/lymphoid or mixed-lineage leukemia (trithorax homolog, drosophila); translocated to, 10  ZNF408 - zinc finger protein 408  DEDD2 - death effector domain containing 2  YEATS2 - yeats domain containing 2  APEX1 - apex nuclease (multifunctional dna repair enzyme) 1  DHX36 - deah (asp-glu-ala-his) box polypeptide 36  ZNF189 - zinc finger protein 189  ZSCAN26 - zinc finger and scan domain containing 26  ZNF605 - zinc finger protein 605  RBM23 - rna binding motif protein 23  XIAP - x-linked inhibitor of apoptosis  ZNF177 - zinc finger protein 177  KLF12 - kruppel-like factor 12  ZNF175 - zinc finger protein 175  ZNF174 - zinc finger protein 174  ZNF184 - zinc finger protein 184  SSBP4 - single stranded dna binding protein 4  GTF2F1 - general transcription factor iif, polypeptide 1, 74kda  PBX2 - pre-b-cell leukemia homeobox 2  PRMT6 - protein arginine methyltransferase 6  PBX3 - pre-b-cell leukemia homeobox 3  ZNF148 - zinc finger protein 148  PBX1 - pre-b-cell leukemia homeobox 1  ZBTB17 - zinc finger and btb domain containing 17  BEND5 - ben domain containing 5  GTF2H2 - general transcription factor iih, polypeptide 2, 44kda  GTF2H3 - general transcription factor iih, polypeptide 3, 34kda  TRIM26 - tripartite motif containing 26  GTF2H1 - general transcription factor iih, polypeptide 1, 62kda  VEZF1 - vascular endothelial zinc finger 1  PCBD1 - pterin-4 alpha-carbinolamine dehydratase/dimerization cofactor of hepatocyte nuclear factor 1 alpha  ZNF142 - zinc finger protein 142  MCPH1 - microcephalin 1  ZNF140 - zinc finger protein 140  APBB2 - amyloid beta (a4) precursor protein-binding, family b, member 2  GTF2B - general transcription factor iib  PCGF2 - polycomb group ring finger 2  CHP1 - calcineurin-like ef-hand protein 1  APBB1 - amyloid beta (a4) precursor protein-binding, family b, member 1 (fe65)  ZNF132 - zinc finger protein 132  ZNF131 - zinc finger protein 131  RIF1 - rap1 interacting factor homolog (yeast)  ZNF134 - zinc finger protein 134  ZNF133 - zinc finger protein 133  TBL1XR1 - transducin (beta)-like 1 x-linked receptor 1  GTF2I - general transcription factor iii  GTF2H4 - general transcription factor iih, polypeptide 4, 52kda  KIAA1551 - kiaa1551  PA2G4 - proliferation-associated 2g4, 38kda  BRF1 - brf1, rna polymerase iii transcription initiation factor 90 kda subunit  RPRD1A - regulation of nuclear pre-mrna domain containing 1a  ZHX1 - zinc fingers and homeoboxes 1  ARF4 - adp-ribosylation factor 4  ZNF79 - zinc finger protein 79  SBNO1 - strawberry notch homolog 1 (drosophila)  IRAK3 - interleukin-1 receptor-associated kinase 3  SETD5 - set domain containing 5  APP - amyloid beta (a4) precursor protein  ZNF70 - zinc finger protein 70  BBS7 - bardet-biedl syndrome 7  DDX20 - dead (asp-glu-ala-asp) box polypeptide 20  ZNF75D - zinc finger protein 75d  PAM - peptidylglycine alpha-amidating monooxygenase  ZNF76 - zinc finger protein 76  H3F3A - h3 histone, family 3a  ARNTL - aryl hydrocarbon receptor nuclear translocator-like  ARNT - aryl hydrocarbon receptor nuclear translocator  BRPF1 - bromodomain and phd finger containing, 1  H2AFZ - h2a histone family, member z  FZD5 - frizzled family receptor 5  SAP130 - sin3a-associated protein, 130kda  H2AFX - h2a histone family, member x  PAX8 - paired box 8  SUPT16H - suppressor of ty 16 homolog (s. cerevisiae)  MIER3 - mesoderm induction early response 1, family member 3  ARRB2 - arrestin, beta 2  ARRB1 - arrestin, beta 1  CDC73 - cell division cycle 73  PDGFB - platelet-derived growth factor beta polypeptide  NKAP - nfkb activating protein  THAP5 - thap domain containing 5  STRAP - serine/threonine kinase receptor associated protein  RHOA - ras homolog family member a  PSIP1 - pc4 and sfrs1 interacting protein 1  MED9 - mediator complex subunit 9  FSTL1 - follistatin-like 1  LRP8 - low density lipoprotein receptor-related protein 8, apolipoprotein e receptor  BAZ2A - bromodomain adjacent to zinc finger domain, 2a  HCFC1 - host cell factor c1 (vp16-accessory protein)  PCNA - proliferating cell nuclear antigen  PRDM2 - pr domain containing 2, with znf domain  CHMP1A - charged multivesicular body protein 1a  MAP3K12 - mitogen-activated protein kinase kinase kinase 12  JDP2 - jun dimerization protein 2  CDK16 - cyclin-dependent kinase 16  AKIRIN2 - akirin 2  HEATR1 - heat repeat containing 1  CDK17 - cyclin-dependent kinase 17  LEO1 - leo1, paf1/rna polymerase ii complex component, homolog (s. cerevisiae)  ERLIN2 - er lipid raft associated 2  ZNF226 - zinc finger protein 226  PWP1 - pwp1 homolog (s. cerevisiae)  PDE2A - phosphodiesterase 2a, cgmp-stimulated  HDAC2 - histone deacetylase 2  HMBOX1 - homeobox containing 1  HDGF - hepatoma-derived growth factor  ZNF213 - zinc finger protein 213  THAP1 - thap domain containing, apoptosis associated protein 1  HDAC1 - histone deacetylase 1  ZNF205 - zinc finger protein 205  HCLS1 - hematopoietic cell-specific lyn substrate 1  PKIG - protein kinase (camp-dependent, catalytic) inhibitor gamma  ZNF207 - zinc finger protein 207  KAT7 - k(lysine) acetyltransferase 7  ASCL1 - achaete-scute complex homolog 1 (drosophila)  ELP3 - elongator acetyltransferase complex subunit 3  RGMB - rgm domain family, member b  SBNO2 - strawberry notch homolog 2 (drosophila)  ZNF444 - zinc finger protein 444  XPA - xeroderma pigmentosum, complementation group a  ZBTB1 - zinc finger and btb domain containing 1  XPC - xeroderma pigmentosum, complementation group c  FBXW7 - f-box and wd repeat domain containing 7, e3 ubiquitin protein ligase  XPO1 - exportin 1 (crm1 homolog, yeast)  GOLGB1 - golgin b1  BAHD1 - bromo adjacent homology domain containing 1  PAGR1 - paxip1 associated glutamate-rich protein 1  ZHX2 - zinc fingers and homeoboxes 2  MORC2 - morc family cw-type zinc finger 2  PHF2 - phd finger protein 2  PHF1 - phd finger protein 1  FOXJ3 - forkhead box j3  RERE - arginine-glutamic acid dipeptide (re) repeats  ZXDC - zxd family zinc finger c  XBP1 - x-box binding protein 1  ZNF367 - zinc finger protein 367  BHLHE41 - basic helix-loop-helix family, member e41  ZFHX3 - zinc finger homeobox 3  PEX14 - peroxisomal biogenesis factor 14  WFS1 - wolfram syndrome 1 (wolframin)  WHSC1 - wolf-hirschhorn syndrome candidate 1  ADNP2 - adnp homeobox 2  NELFA - negative elongation factor complex member a  AEBP2 - ae binding protein 2  IMPACT - impact rwd domain protein  ZNF507 - zinc finger protein 507  LGR4 - leucine-rich repeat containing g protein-coupled receptor 4  ARID2 - at rich interactive domain 2 (arid, rfx-like)  ATP2B4 - atpase, ca++ transporting, plasma membrane 4  ZNF652 - zinc finger protein 652  SCAF8 - sr-related ctd-associated factor 8  PKD1 - polycystic kidney disease 1 (autosomal dominant)  PKD2 - polycystic kidney disease 2 (autosomal dominant)  ZNF35 - zinc finger protein 35  ZKSCAN1 - zinc finger with krab and scan domains 1  FBXL15 - f-box and leucine-rich repeat protein 15  MTF2 - metal response element binding transcription factor 2  ZNF576 - zinc finger protein 576  ZNF33B - zinc finger protein 33b  ZNF32 - zinc finger protein 32  BACH1 - btb and cnc homology 1, basic leucine zipper transcription factor 1  PIN1 - peptidylprolyl cis/trans isomerase, nima-interacting 1  PIK3R2 - phosphoinositide-3-kinase, regulatory subunit 2 (beta)  ZNF45 - zinc finger protein 45  MZF1 - myeloid zinc finger 1  PIK3R1 - phosphoinositide-3-kinase, regulatory subunit 1 (alpha)  ZNF18 - zinc finger protein 18  ASXL2 - additional sex combs like 2 (drosophila)  ZNF16 - zinc finger protein 16  PLAGL1 - pleiomorphic adenoma gene-like 1  ELP2 - elongator acetyltransferase complex subunit 2  ASXL1 - additional sex combs like 1 (drosophila)  ZNF384 - zinc finger protein 384  TNIP2 - tnfaip3 interacting protein 2  ATRX - alpha thalassemia/mental retardation syndrome x-linked  ZNF24 - zinc finger protein 24  PKNOX1 - pbx/knotted 1 homeobox 1  ZFPL1 - zinc finger protein-like 1  ZFX - zinc finger protein, x-linked  TMEM100 - transmembrane protein 100  PHF10 - phd finger protein 10  PSPC1 - paraspeckle component 1  ZBTB14 - zinc finger and btb domain containing 14  ZNF711 - zinc finger protein 711  ZNF7 - zinc finger protein 7  ZNF8 - zinc finger protein 8  BCL7A - b-cell cll/lymphoma 7a  ZNF2 - zinc finger protein 2  MRGBP - mrg/morf4l binding protein  ZNF3 - zinc finger protein 3  BCL9 - b-cell cll/lymphoma 9  YES1 - v-yes-1 yamaguchi sarcoma viral oncogene homolog 1  BRF2 - brf2, rna polymerase iii transcription initiation factor 50 kda subunit  PIM1 - pim-1 oncogene  XRCC5 - x-ray repair complementing defective repair in chinese hamster cells 5 (double-strand-break rejoining)  ECD - ecdysoneless homolog (drosophila)  CCND1 - cyclin d1  GSK3A - glycogen synthase kinase 3 alpha  PARK7 - parkinson protein 7  C3orf33 - chromosome 3 open reading frame 33  YWHAB - tyrosine 3-monooxygenase/tryptophan 5-monooxygenase activation protein, beta polypeptide  CCNK - cyclin k  TRIM27 - tripartite motif containing 27  HSF2 - heat shock transcription factor 2  SAP18 - sin3a-associated protein, 18kda  TRIM24 - tripartite motif containing 24  TAF1D - tata box binding protein (tbp)-associated factor, rna polymerase i, d, 41kda  TSHZ2 - teashirt zinc finger homeobox 2  RFXAP - regulatory factor x-associated protein  CREG1 - cellular repressor of e1a-stimulated genes 1  RFX5 - regulatory factor x, 5 (influences hla class ii expression)  WHSC1L1 - wolf-hirschhorn syndrome candidate 1-like 1  FLCN - folliculin  HSBP1 - heat shock factor binding protein 1  RFX1 - regulatory factor x, 1 (influences hla class ii expression)  RELA - v-rel avian reticuloendotheliosis viral oncogene homolog a  APBB3 - amyloid beta (a4) precursor protein-binding, family b, member 3  PRMT2 - protein arginine methyltransferase 2  BCOR - bcl6 corepressor  ZFP36L2 - zfp36 ring finger protein-like 2  ZFP36L1 - zfp36 ring finger protein-like 1  RET - ret proto-oncogene  HESX1 - hesx homeobox 1  SAP30 - sin3a-associated protein, 30kda  DPF2 - d4, zinc and double phd fingers family 2  CC2D1A - coiled-coil and c2 domain containing 1a  RLF - rearranged l-myc fusion  RING1 - ring finger protein 1  CFLAR - casp8 and fadd-like apoptosis regulator  MED11 - mediator complex subunit 11  BMI1 - bmi1 polycomb ring finger oncogene  PCGF3 - polycomb group ring finger 3  WDR77 - wd repeat domain 77  LDB1 - lim domain binding 1  BMPR1A - bone morphogenetic protein receptor, type ia  BMPR2 - bone morphogenetic protein receptor, type ii (serine/threonine kinase)  BMPR1B - bone morphogenetic protein receptor, type ib  GON4L - gon-4-like (c. elegans)  KAT2B - k(lysine) acetyltransferase 2b  TSC22D1 - tsc22 domain family, member 1  CRTC2 - creb regulated transcription coactivator 2  BMP7 - bone morphogenetic protein 7  PSMD14 - proteasome (prosome, macropain) 26s subunit, non-atpase, 14  ZNF259 - zinc finger protein 259  FLOT1 - flotillin 1  TAX1BP1 - tax1 (human t-cell leukemia virus type i) binding protein 1  BCAS3 - breast carcinoma amplified sequence 3  CC2D1B - coiled-coil and c2 domain containing 1b  AHI1 - abelson helper integration site 1  THAP8 - thap domain containing 8  RNF4 - ring finger protein 4  BRD2 - bromodomain containing 2  RNF2 - ring finger protein 2  BUD31 - bud31 homolog (s. cerevisiae)  ZNF280D - zinc finger protein 280d  GATAD2A - gata zinc finger domain containing 2a  RNF6 - ring finger protein (c3h2c3 type) 6  ZNF655 - zinc finger protein 655  RNF111 - ring finger protein 111  TRAK2 - trafficking protein, kinesin binding 2  TET2 - tet methylcytosine dioxygenase 2  HSPD1 - heat shock 60kda protein 1 (chaperonin)  DNAJB1 - dnaj (hsp40) homolog, subfamily b, member 1  MED18 - mediator complex subunit 18  ZNF48 - zinc finger protein 48  DENND4A - denn/madd domain containing 4a  BCL10 - b-cell cll/lymphoma 10  MBTD1 - mbt domain containing 1  FUBP3 - far upstream element (fuse) binding protein 3  BTRC - beta-transducin repeat containing e3 ubiquitin protein ligase  ZMPSTE24 - zinc metallopeptidase ste24  HSPA8 - heat shock 70kda protein 8  TRIM44 - tripartite motif containing 44  TAB1 - tgf-beta activated kinase 1/map3k7 binding protein 1  PPIE - peptidylprolyl isomerase e (cyclophilin e)  MCRS1 - microspherule protein 1  HMGN1 - high mobility group nucleosome binding domain 1  HMGB3 - high mobility group box 3  NRDE2 - nrde-2, necessary for rna interference, domain containing  HMGB2 - high mobility group box 2  HMGB1 - high mobility group box 1  C1D - c1d nuclear receptor corepressor  BRD9 - bromodomain containing 9  TNKS - tankyrase, trf1-interacting ankyrin-related adp-ribose polymerase  SLC30A9 - solute carrier family 30 (zinc transporter), member 9  ZBTB10 - zinc finger and btb domain containing 10  AGO1 - argonaute risc catalytic component 1  MAD2L2 - mad2 mitotic arrest deficient-like 2 (yeast)  CAPRIN2 - caprin family member 2  PID1 - phosphotyrosine interaction domain containing 1  PHIP - pleckstrin homology domain interacting protein  ZBTB48 - zinc finger and btb domain containing 48  ZNF787 - zinc finger protein 787  NRARP - notch-regulated ankyrin repeat protein  HYAL2 - hyaluronoglucosaminidase 2  GPBP1 - gc-rich promoter binding protein 1  TRIM38 - tripartite motif containing 38  TADA3 - transcriptional adaptor 3  CALR - calreticulin  ZNF792 - zinc finger protein 792  HFE - hemochromatosis  WTIP - wilms tumor 1 interacting protein  NCOA2 - nuclear receptor coactivator 2  HINT1 - histidine triad nucleotide binding protein 1  ELP4 - elongator acetyltransferase complex subunit 4  HIVEP1 - human immunodeficiency virus type i enhancer binding protein 1  CREB3 - camp responsive element binding protein 3  HIF1A - hypoxia inducible factor 1, alpha subunit (basic helix-loop-helix transcription factor)  LPIN3 - lipin 3  CITED2 - cbp/p300-interacting transactivator, with glu/asp-rich carboxy-terminal domain, 2  TRADD - tnfrsf1a-associated via death domain  CCNT2 - cyclin t2  MYCBP - myc binding protein  EED - embryonic ectoderm development  URI1 - uri1, prefoldin-like chaperone  CCND3 - cyclin d3  CCNT1 - cyclin t1  CCNH - cyclin h  HMG20A - high mobility group 20a  BANP - btg3 associated nuclear protein  RIPK1 - receptor (tnfrsf)-interacting serine-threonine kinase 1  HNRNPU - heterogeneous nuclear ribonucleoprotein u (scaffold attachment factor a)  HNRNPK - heterogeneous nuclear ribonucleoprotein k  RPS6KA1 - ribosomal protein s6 kinase, 90kda, polypeptide 1  CBFA2T3 - core-binding factor, runt domain, alpha subunit 2; translocated to, 3  CBFB - core-binding factor, beta subunit  HNRNPA2B1 - heterogeneous nuclear ribonucleoprotein a2/b1  RIPK2 - receptor-interacting serine-threonine kinase 2  HNRNPAB - heterogeneous nuclear ribonucleoprotein a/b  RXRB - retinoid x receptor, beta  PIAS3 - protein inhibitor of activated stat, 3  HNRNPD - heterogeneous nuclear ribonucleoprotein d (au-rich element rna binding protein 1, 37kda)  CAV1 - caveolin 1, caveolae protein, 22kda  CCNC - cyclin c  RBM14 - rna binding motif protein 14  ZNF584 - zinc finger protein 584  HMGA1 - high mobility group at-hook 1  FAM129B - family with sequence similarity 129, member b  PRMT5 - protein arginine methyltransferase 5  NR4A1 - nuclear receptor subfamily 4, group a, member 1  RFX7 - regulatory factor x, 7  HTATSF1 - hiv-1 tat specific factor 1  FOXK2 - forkhead box k2  HAT1 - histone acetyltransferase 1  CDK11B - cyclin-dependent kinase 11b  CDC5L - cell division cycle 5-like  MXD4 - max dimerization protein 4  CRTC3 - creb regulated transcription coactivator 3  RBM15 - rna binding motif protein 15  HTRA1 - htra serine peptidase 1  POLR3F - polymerase (rna) iii (dna directed) polypeptide f, 39 kda  HEXIM1 - hexamethylene bis-acetamide inducible 1  ERLIN1 - er lipid raft associated 1  RBMX - rna binding motif protein, x-linked  SPIN2A - spindlin family, member 2a  NCOA7 - nuclear receptor coactivator 7  PSEN1 - presenilin 1  ING2 - inhibitor of growth family, member 2  ZNF574 - zinc finger protein 574  PPM1D - protein phosphatase, mg2+/mn2+ dependent, 1d  CREB3L2 - camp responsive element binding protein 3-like 2  USP16 - ubiquitin specific peptidase 16  PSMA1 - proteasome (prosome, macropain) subunit, alpha type, 1  IPPK - inositol 1,3,4,5,6-pentakisphosphate 2-kinase  PSMA2 - proteasome (prosome, macropain) subunit, alpha type, 2  ILF2 - interleukin enhancer binding factor 2  ILF3 - interleukin enhancer binding factor 3, 90kda  ILK - integrin-linked kinase  CD81 - cd81 molecule  PIAS1 - protein inhibitor of activated stat, 1  CAMKK2 - calcium/calmodulin-dependent protein kinase kinase 2, beta  PSMB1 - proteasome (prosome, macropain) subunit, beta type, 1  SMYD3 - set and mynd domain containing 3  SLCO3A1 - solute carrier organic anion transporter family, member 3a1  SMURF2 - smad specific e3 ubiquitin protein ligase 2  PSMA5 - proteasome (prosome, macropain) subunit, alpha type, 5  PSMA3 - proteasome (prosome, macropain) subunit, alpha type, 3  PSMB10 - proteasome (prosome, macropain) subunit, beta type, 10  RIPPLY2 - ripply transcriptional repressor 2  CGGBP1 - cgg triplet repeat binding protein 1  LMO4 - lim domain only 4  TAF6L - taf6-like rna polymerase ii, p300/cbp-associated factor (pcaf)-associated factor, 65kda  PSMC6 - proteasome (prosome, macropain) 26s subunit, atpase, 6  PSMD1 - proteasome (prosome, macropain) 26s subunit, non-atpase, 1  CAMK1 - calcium/calmodulin-dependent protein kinase i  PSMC4 - proteasome (prosome, macropain) 26s subunit, atpase, 4  SOX18 - sry (sex determining region y)-box 18  CBX4 - chromobox homolog 4  POLR3G - polymerase (rna) iii (dna directed) polypeptide g (32kd)  PSMC2 - proteasome (prosome, macropain) 26s subunit, atpase, 2  PSMD9 - proteasome (prosome, macropain) 26s subunit, non-atpase, 9  PSMD10 - proteasome (prosome, macropain) 26s subunit, non-atpase, 10  PSMD7 - proteasome (prosome, macropain) 26s subunit, non-atpase, 7  PSMD8 - proteasome (prosome, macropain) 26s subunit, non-atpase, 8  CSRNP1 - cysteine-serine-rich nuclear protein 1  PSMD5 - proteasome (prosome, macropain) 26s subunit, non-atpase, 5  PSMD2 - proteasome (prosome, macropain) 26s subunit, non-atpase, 2  TERF2IP - telomeric repeat binding factor 2, interacting protein  PSMD12 - proteasome (prosome, macropain) 26s subunit, non-atpase, 12  PSMD13 - proteasome (prosome, macropain) 26s subunit, non-atpase, 13  ZNF25 - zinc finger protein 25  LAMTOR5 - late endosomal/lysosomal adaptor, mapk and mtor activator 5  PTEN - phosphatase and tensin homolog  STK16 - serine/threonine kinase 16  JMY - junction mediating and regulatory protein, p53 cofactor  ITGA3 - integrin, alpha 3 (antigen cd49c, alpha 3 subunit of vla-3 receptor)  MYBBP1A - myb binding protein (p160) 1a  FBLN5 - fibulin 5  CIB1 - calcium and integrin binding 1 (calmyrin)  PTGIS - prostaglandin i2 (prostacyclin) synthase  CASK - calcium/calmodulin-dependent serine protein kinase (maguk family)  DDX17 - dead (asp-glu-ala-asp) box helicase 17  SUDS3 - suppressor of defective silencing 3 homolog (s. cerevisiae)  KAT5 - k(lysine) acetyltransferase 5  ZFP69 - zfp69 zinc finger protein  CEBPG - ccaat/enhancer binding protein (c/ebp), gamma  SKAP1 - src kinase associated phosphoprotein 1  CDK5 - cyclin-dependent kinase 5  GZF1 - gdnf-inducible zinc finger protein 1  CDK9 - cyclin-dependent kinase 9  CDK8 - cyclin-dependent kinase 8  INSR - insulin receptor  LIN54 - lin-54 homolog (c. elegans)  INSM1 - insulinoma-associated 1  CDK7 - cyclin-dependent kinase 7  CDK13 - cyclin-dependent kinase 13  CDKN1C - cyclin-dependent kinase inhibitor 1c (p57, kip2)  CDKN1B - cyclin-dependent kinase inhibitor 1b (p27, kip1)  PDCD4 - programmed cell death 4 (neoplastic transformation inhibitor)  PCBP3 - poly(rc) binding protein 3  RFXANK - regulatory factor x-associated ankyrin-containing protein  PTPN2 - protein tyrosine phosphatase, non-receptor type 2  IRF2 - interferon regulatory factor 2  KLF7 - kruppel-like factor 7 (ubiquitous)  IRF3 - interferon regulatory factor 3  HTATIP2 - hiv-1 tat interactive protein 2, 30kda  RUVBL1 - ruvb-like 1 (e. coli)  IKZF5 - ikaros family zinc finger 5 (pegasus)  MED19 - mediator complex subunit 19  ITGA6 - integrin, alpha 6  IRAK2 - interleukin-1 receptor-associated kinase 2  CDK2 - cyclin-dependent kinase 2  IRAK1 - interleukin-1 receptor-associated kinase 1  RRN3 - rrn3 rna polymerase i transcription factor homolog (s. cerevisiae)  PURA - purine-rich element binding protein a  PURB - purine-rich element binding protein b  ZSCAN31 - zinc finger and scan domain containing 31  KIAA1324L - kiaa1324-like  NSD1 - nuclear receptor binding set domain protein 1  ZMYND11 - zinc finger, mynd-type containing 11  ZBTB11 - zinc finger and btb domain containing 11  PTPRK - protein tyrosine phosphatase, receptor type, k  SRSF10 - serine/arginine-rich splicing factor 10  RNF25 - ring finger protein 25  PTPRN - protein tyrosine phosphatase, receptor type, n  DKK3 - dickkopf wnt signaling pathway inhibitor 3  MMS19 - mms19 nucleotide excision repair homolog (s. cerevisiae)  PEX2 - peroxisomal biogenesis factor 2  KDM5B - lysine (k)-specific demethylase 5b  MPHOSPH8 - m-phase phosphoprotein 8  SNX5 - sorting nexin 5  ZNF777 - zinc finger protein 777  ID2 - inhibitor of dna binding 2, dominant negative helix-loop-helix protein  ID3 - inhibitor of dna binding 3, dominant negative helix-loop-helix protein  CHD4 - chromodomain helicase dna binding protein 4  CHD2 - chromodomain helicase dna binding protein 2  STK36 - serine/threonine kinase 36  CHD3 - chromodomain helicase dna binding protein 3  CHD1 - chromodomain helicase dna binding protein 1  MTERFD2 - mterf domain containing 2  IRF2BPL - interferon regulatory factor 2 binding protein-like  JAZF1 - jazf zinc finger 1  QARS - glutaminyl-trna synthetase  MYPOP - myb-related transcription factor, partner of profilin  RAD21 - rad21 homolog (s. pombe)  FOXK1 - forkhead box k1  ZNF181 - zinc finger protein 181  PAF1 - paf1, rna polymerase ii associated factor, homolog (s. cerevisiae)  ZNF268 - zinc finger protein 268  INO80 - ino80 complex subunit  ZNF513 - zinc finger protein 513  IGHMBP2 - immunoglobulin mu binding protein 2  ZNF282 - zinc finger protein 282  GMEB1 - glucocorticoid modulatory element binding protein 1  RBPJ - recombination signal binding protein for immunoglobulin kappa j region  MIER2 - mesoderm induction early response 1, family member 2  NCK2 - nck adaptor protein 2  RARG - retinoic acid receptor, gamma  RARB - retinoic acid receptor, beta  DLL4 - delta-like 4 (drosophila)  ZBTB9 - zinc finger and btb domain containing 9  USP21 - ubiquitin specific peptidase 21  ZBTB12 - zinc finger and btb domain containing 12  KHDRBS1 - kh domain containing, rna binding, signal transduction associated 1  CLU - clusterin  TAF8 - taf8 rna polymerase ii, tata box binding protein (tbp)-associated factor, 43kda  MAGEL2 - mage-like 2  CTCF - ccctc-binding factor (zinc finger protein)  JMJD1C - jumonji domain containing 1c  IGBP1 - immunoglobulin (cd79a) binding protein 1  RAI1 - retinoic acid induced 1  RBL2 - retinoblastoma-like 2 (p130)  RBBP7 - retinoblastoma binding protein 7  SMARCA5 - swi/snf related, matrix associated, actin dependent regulator of chromatin, subfamily a, member 5  RBBP8 - retinoblastoma binding protein 8  MAP3K2 - mitogen-activated protein kinase kinase kinase 2  OGT - o-linked n-acetylglucosamine (glcnac) transferase  RB1 - retinoblastoma 1  PELP1 - proline, glutamate and leucine rich protein 1  ARID4A - at rich interactive domain 4a (rbp1-like)  KDM5A - lysine (k)-specific demethylase 5a  NUP35 - nucleoporin 35kda  CUL2 - cullin 2  ZNF667 - zinc finger protein 667  CUL3 - cullin 3  NFAT5 - nuclear factor of activated t-cells 5, tonicity-responsive  CKS1B - cdc28 protein kinase regulatory subunit 1b  FOXP1 - forkhead box p1  CHUK - conserved helix-loop-helix ubiquitous kinase  SUPT3H - suppressor of ty 3 homolog (s. cerevisiae)  ZNF572 - zinc finger protein 572  ZMYND8 - zinc finger, mynd-type containing 8  PCGF5 - polycomb group ring finger 5  ANKRA2 - ankyrin repeat, family a (rfxank-like), 2  WAC - ww domain containing adaptor with coiled-coil  RBAK - rb-associated krab zinc finger  NFATC2IP - nuclear factor of activated t-cells, cytoplasmic, calcineurin-dependent 2 interacting protein  ZNF414 - zinc finger protein 414  PHF21A - phd finger protein 21a  ZNF771 - zinc finger protein 771  PATZ1 - poz (btb) and at hook containing zinc finger 1  IER2 - immediate early response 2  SPP1 - secreted phosphoprotein 1  NCOR1 - nuclear receptor corepressor 1  SSBP2 - single-stranded dna binding protein 2  KPNA6 - karyopherin alpha 6 (importin alpha 7)  NCOR2 - nuclear receptor corepressor 2  MTPN - myotrophin  CIRH1A - cirrhosis, autosomal recessive 1a (cirhin)  DOT1L - dot1-like histone h3k79 methyltransferase  MIER1 - mesoderm induction early response 1, transcriptional regulator  ZNF382 - zinc finger protein 382  ZNF705A - zinc finger protein 705a  EFCAB7 - ef-hand calcium binding domain 7  SP3 - sp3 transcription factor  L3MBTL3 - l(3)mbt-like 3 (drosophila)  SP4 - sp4 transcription factor  SP1 - sp1 transcription factor  SP2 - sp2 transcription factor  ZNF410 - zinc finger protein 410  ZNF333 - zinc finger protein 333  CIR1 - corepressor interacting with rbpj, 1  SOX4 - sry (sex determining region y)-box 4  H2AFY - h2a histone family, member y  WBP2 - ww domain binding protein 2  GTF2IRD1 - gtf2i repeat domain containing 1  FADS1 - fatty acid desaturase 1  ZNF527 - zinc finger protein 527  HES4 - hairy and enhancer of split 4 (drosophila)  LEMD3 - lem domain containing 3  GATAD1 - gata zinc finger domain containing 1  CREBL2 - camp responsive element binding protein-like 2  ATF6B - activating transcription factor 6 beta  HOPX - hop homeobox  CREBBP - creb binding protein  ZNF316 - zinc finger protein 316  ZC3H8 - zinc finger ccch-type containing 8  SOX13 - sry (sex determining region y)-box 13  ATF2 - activating transcription factor 2  LMO1 - lim domain only 1 (rhombotin 1)  CREB1 - camp responsive element binding protein 1  IER5 - immediate early response 5  NACC2 - nacc family member 2, ben and btb (poz) domain containing  DDX58 - dead (asp-glu-ala-asp) box polypeptide 58  CRABP2 - cellular retinoic acid binding protein 2  CLOCK - clock circadian regulator  KLF3 - kruppel-like factor 3 (basic)  RPRD1B - regulation of nuclear pre-mrna domain containing 1b  DDX41 - dead (asp-glu-ala-asp) box polypeptide 41  PLK3 - polo-like kinase 3  SNAPC4 - small nuclear rna activating complex, polypeptide 4, 190kda  LITAF - lipopolysaccharide-induced tnf factor  SNCA - synuclein, alpha (non a4 component of amyloid precursor)  TBPL1 - tbp-like 1  ZBED5 - zinc finger, bed-type containing 5  CREBZF - creb/atf bzip transcription factor  ZFP62 - zfp62 zinc finger protein  ZBTB41 - zinc finger and btb domain containing 41  EID1 - ep300 interacting inhibitor of differentiation 1  TP53INP2 - tumor protein p53 inducible nuclear protein 2  SMARCE1 - swi/snf related, matrix associated, actin dependent regulator of chromatin, subfamily e, member 1  SMO - smoothened, frizzled family receptor  ZGPAT - zinc finger, ccch-type with g patch domain  ACTL6B - actin-like 6b  SMARCB1 - swi/snf related, matrix associated, actin dependent regulator of chromatin, subfamily b, member 1  SMARCC1 - swi/snf related, matrix associated, actin dependent regulator of chromatin, subfamily c, member 1  HLTF - helicase-like transcription factor  SMARCA4 - swi/snf related, matrix associated, actin dependent regulator of chromatin, subfamily a, member 4  SNX6 - sorting nexin 6  SSBP3 - single stranded dna binding protein 3  SMARCD2 - swi/snf related, matrix associated, actin dependent regulator of chromatin, subfamily d, member 2  SMARCD3 - swi/snf related, matrix associated, actin dependent regulator of chromatin, subfamily d, member 3  SMARCC2 - swi/snf related, matrix associated, actin dependent regulator of chromatin, subfamily c, member 2  SMARCD1 - swi/snf related, matrix associated, actin dependent regulator of chromatin, subfamily d, member 1  WIZ - widely interspaced zinc finger motifs  MED20 - mediator complex subunit 20  AFAP1L2 - actin filament associated protein 1-like 2  SMARCA1 - swi/snf related, matrix associated, actin dependent regulator of chromatin, subfamily a, member 1  SMARCA2 - swi/snf related, matrix associated, actin dependent regulator of chromatin, subfamily a, member 2  CRLF3 - cytokine receptor-like factor 3  MAPK8IP1 - mitogen-activated protein kinase 8 interacting protein 1  SGK3 - serum/glucocorticoid regulated kinase family, member 3  CACTIN - cactin, spliceosome c complex subunit  KMT2C - lysine (k)-specific methyltransferase 2c  GLYR1 - glyoxylate reductase 1 homolog (arabidopsis)  ZBTB7A - zinc finger and btb domain containing 7a  DPY30 - dpy-30 homolog (c. elegans)  ZNF462 - zinc finger protein 462  PPIL4 - peptidylprolyl isomerase (cyclophilin)-like 4  MBTPS2 - membrane-bound transcription factor peptidase, site 2  MAGED1 - melanoma antigen family d, 1  GLIS2 - glis family zinc finger 2  IRF2BP2 - interferon regulatory factor 2 binding protein 2  MED27 - mediator complex subunit 27  MED26 - mediator complex subunit 26  CHD6 - chromodomain helicase dna binding protein 6  SENP2 - sumo1/sentrin/smt3 specific peptidase 2  MED17 - mediator complex subunit 17  MED23 - mediator complex subunit 23  ZNF580 - zinc finger protein 580  ZNF318 - zinc finger protein 318  PRPF6 - pre-mrna processing factor 6  GTF2IRD2 - gtf2i repeat domain containing 2  KDM2B - lysine (k)-specific demethylase 2b  SUPT6H - suppressor of ty 6 homolog (s. cerevisiae)  ZNF710 - zinc finger protein 710  CBY1 - chibby homolog 1 (drosophila)  CTBP1 - c-terminal binding protein 1  SUPT4H1 - suppressor of ty 4 homolog 1 (s. cerevisiae)  CTBP2 - c-terminal binding protein 2  ING4 - inhibitor of growth family, member 4  SUPT5H - suppressor of ty 5 homolog (s. cerevisiae)  INSIG2 - insulin induced gene 2  EIF2AK3 - eukaryotic translation initiation factor 2-alpha kinase 3  CTNNB1 - catenin (cadherin-associated protein), beta 1, 88kda  RLIM - ring finger protein, lim domain interacting  SAV1 - salvador homolog 1 (drosophila)  RECQL5 - recq protein-like 5  KRAS - kirsten rat sarcoma viral oncogene homolog  CUX1 - cut-like homeobox 1  ZNF706 - zinc finger protein 706  TGFBRAP1 - transforming growth factor, beta receptor associated protein 1  CIAO1 - cytosolic iron-sulfur protein assembly 1  NIF3L1 - nif3 ngg1 interacting factor 3-like 1 (s. cerevisiae)  NEK4 - nima-related kinase 4  STK3 - serine/threonine kinase 3  PCGF1 - polycomb group ring finger 1  NPNT - nephronectin  FKBP8 - fk506 binding protein 8, 38kda  GPBP1L1 - gc-rich promoter binding protein 1-like 1  PHF20L1 - phd finger protein 20-like 1  MED10 - mediator complex subunit 10  MED21 - mediator complex subunit 21  CYLD - cylindromatosis (turban tumor syndrome)  ZFP28 - zfp28 zinc finger protein  KIT - v-kit hardy-zuckerman 4 feline sarcoma viral oncogene homolog  TGIF2 - tgfb-induced factor homeobox 2  BCL6B - b-cell cll/lymphoma 6, member b  CRK - v-crk avian sarcoma virus ct10 oncogene homolog  NIPBL - nipped-b homolog (drosophila)  CRY1 - cryptochrome 1 (photolyase-like)  STAT2 - signal transducer and activator of transcription 2, 113kda  STAT1 - signal transducer and activator of transcription 1, 91kda  STAT3 - signal transducer and activator of transcription 3 (acute-phase response factor)  CRY2 - cryptochrome 2 (photolyase-like)  JUP - junction plakoglobin  ASF1A - anti-silencing function 1a histone chaperone  STAT5A - signal transducer and activator of transcription 5a  STAT6 - signal transducer and activator of transcription 6, interleukin-4 induced  JAK2 - janus kinase 2  ING5 - inhibitor of growth family, member 5  BRMS1 - breast cancer metastasis suppressor 1  ZNF219 - zinc finger protein 219  CRYM - crystallin, mu  ZNF496 - zinc finger protein 496  SS18 - synovial sarcoma translocation, chromosome 18  PHF20 - phd finger protein 20  PHF5A - phd finger protein 5a  GPATCH3 - g patch domain containing 3  ZNF397 - zinc finger protein 397  ZNHIT3 - zinc finger, hit-type containing 3  TRIP4 - thyroid hormone receptor interactor 4  HMGN3 - high mobility group nucleosomal binding domain 3  TAB3 - tgf-beta activated kinase 1/map3k7 binding protein 3  PHF6 - phd finger protein 6  ZNF235 - zinc finger protein 235  COPS2 - cop9 signalosome subunit 2  BAMBI - bmp and activin membrane-bound inhibitor  ZNF335 - zinc finger protein 335  HAVCR2 - hepatitis a virus cellular receptor 2  ZNF639 - zinc finger protein 639  SRI - sorcin  SRC - v-src avian sarcoma (schmidt-ruppin a-2) viral oncogene homolog  SARNP - sap domain containing ribonucleoprotein  LEF1 - lymphoid enhancer-binding factor 1  BRMS1L - breast cancer metastasis-suppressor 1-like  DNAJB5 - dnaj (hsp40) homolog, subfamily b, member 5  SREBF2 - sterol regulatory element binding transcription factor 2  UBE2O - ubiquitin-conjugating enzyme e2o  CNOT8 - ccr4-not transcription complex, subunit 8  PRDX5 - peroxiredoxin 5  CDK12 - cyclin-dependent kinase 12  DHX9 - deah (asp-glu-ala-his) box helicase 9  TRAPPC2 - trafficking protein particle complex 2  KANK2 - kn motif and ankyrin repeat domains 2  ZNF280B - zinc finger protein 280b  DDX5 - dead (asp-glu-ala-asp) box helicase 5  DDX3X - dead (asp-glu-ala-asp) box helicase 3, x-linked  ARID4B - at rich interactive domain 4b (rbp1-like)  SIN3A - sin3 transcription regulator family member a  MAP3K10 - mitogen-activated protein kinase kinase kinase 10  SET - set nuclear oncogene  WWTR1 - ww domain containing transcription regulator 1  ZNF304 - zinc finger protein 304  ITGB1BP1 - integrin beta 1 binding protein 1  KDM3B - lysine (k)-specific demethylase 3b  RSF1 - remodeling and spacing factor 1  ZNF286A - zinc finger protein 286a  MLLT6 - myeloid/lymphoid or mixed-lineage leukemia (trithorax homolog, drosophila); translocated to, 6  NOL11 - nucleolar protein 11  PBXIP1 - pre-b-cell leukemia homeobox interacting protein 1  MLLT3 - myeloid/lymphoid or mixed-lineage leukemia (trithorax homolog, drosophila); translocated to, 3  MLLT1 - myeloid/lymphoid or mixed-lineage leukemia (trithorax homolog, drosophila); translocated to, 1  MED14 - mediator complex subunit 14  DBP - d site of albumin promoter (albumin d-box) binding protein  KMT2A - lysine (k)-specific methyltransferase 2a  ZBTB22 - zinc finger and btb domain containing 22  NELFB - negative elongation factor complex member b  RPS6KA5 - ribosomal protein s6 kinase, 90kda, polypeptide 5  SUFU - suppressor of fused homolog (drosophila)  AHCTF1 - at hook containing transcription factor 1  ZBTB38 - zinc finger and btb domain containing 38  NLK - nemo-like kinase  MNAT1 - mnat cdk-activating kinase assembly factor 1  UIMC1 - ubiquitin interaction motif containing 1  GATAD2B - gata zinc finger domain containing 2b  ZNF551 - zinc finger protein 551  MTA2 - metastasis associated 1 family, member 2  NOLC1 - nucleolar and coiled-body phosphoprotein 1  ZNF473 - zinc finger protein 473  EIF2A - eukaryotic translation initiation factor 2a, 65kda  WWOX - ww domain containing oxidoreductase  CD200 - cd200 molecule  MNT - mnt, max dimerization protein  CCPG1 - cell cycle progression 1  ZNF451 - zinc finger protein 451  SPTY2D1 - spt2, suppressor of ty, domain containing 1 (s. cerevisiae)  SCAF4 - sr-related ctd-associated factor 4  SS18L1 - synovial sarcoma translocation gene on chromosome 18-like 1  CHD5 - chromodomain helicase dna binding protein 5  PELO - pelota homolog (drosophila)  MECP2 - methyl cpg binding protein 2 (rett syndrome)  MEF2A - myocyte enhancer factor 2a  ARID1B - at rich interactive domain 1b (swi1-like)  PRICKLE1 - prickle homolog 1 (drosophila)  MED30 - mediator complex subunit 30  ZBED1 - zinc finger, bed-type containing 1  MKL2 - mkl/myocardin-like 2  MEIS2 - meis homeobox 2  BRWD1 - bromodomain and wd repeat domain containing 1  ZNF664 - zinc finger protein 664  MEIS1 - meis homeobox 1  MAP3K5 - mitogen-activated protein kinase kinase kinase 5  SLC33A1 - solute carrier family 33 (acetyl-coa transporter), member 1  MEN1 - multiple endocrine neoplasia i  WDR75 - wd repeat domain 75  DAPK3 - death-associated protein kinase 3  DAXX - death-domain associated protein  UTP15 - utp15, u3 small nucleolar ribonucleoprotein, homolog (s. cerevisiae)  ZZZ3 - zinc finger, zz-type containing 3  HINFP - histone h4 transcription factor  KAT8 - k(lysine) acetyltransferase 8  ZNF644 - zinc finger protein 644  MTA3 - metastasis associated 1 family, member 3  DGKQ - diacylglycerol kinase, theta 110kda  ARHGEF2 - rho/rac guanine nucleotide exchange factor (gef) 2  ZNF608 - zinc finger protein 608  DAP - death-associated protein  MAVS - mitochondrial antiviral signaling protein  SARS - seryl-trna synthetase  SATB1 - satb homeobox 1  MBD3 - methyl-cpg binding domain protein 3  RPTOR - regulatory associated protein of mtor, complex 1  SAFB - scaffold attachment factor b  PTOV1 - prostate tumor overexpressed 1  ZNF394 - zinc finger protein 394  SALL2 - sal-like 2 (drosophila)  CBFA2T2 - core-binding factor, runt domain, alpha subunit 2; translocated to, 2  EPC2 - enhancer of polycomb homolog 2 (drosophila)  DRD2 - dopamine receptor d2  CXXC5 - cxxc finger protein 5  PHF19 - phd finger protein 19  SLC9A1 - solute carrier family 9, subfamily a (nhe1, cation proton antiporter 1), member 1  IRF2BP1 - interferon regulatory factor 2 binding protein 1  EAF1 - ell associated factor 1  ATN1 - atrophin 1  IL23A - interleukin 23, alpha subunit p19  MARS - methionyl-trna synthetase  C10orf137 - chromosome 10 open reading frame 137  MTA1 - metastasis associated 1  SIRT6 - sirtuin 6  DR1 - down-regulator of transcription 1, tbp-binding (negative cofactor 2)  PPHLN1 - periphilin 1  SKI - v-ski avian sarcoma viral oncogene homolog  SKIL - ski-like oncogene  MBD1 - methyl-cpg binding domain protein 1  DYRK1A - dual-specificity tyrosine-(y)-phosphorylation regulated kinase 1a  SFMBT1 - scm-like with four mbt domains 1  MKL1 - megakaryoblastic leukemia (translocation) 1  MAX - myc associated factor x  ZMIZ2 - zinc finger, miz-type containing 2  DVL2 - dishevelled segment polarity protein 2  DVL3 - dishevelled segment polarity protein 3  LDB2 - lim domain binding 2  ZFP90 - zfp90 zinc finger protein  FNIP2 - folliculin interacting protein 2  HOMEZ - homeobox and leucine zipper encoding  NELFCD - negative elongation factor complex member c/d  EBF4 - early b-cell factor 4  ASH2L - ash2 (absent, small, or homeotic)-like (drosophila)  ZFHX2 - zinc finger homeobox 2  CTDSPL2 - ctd (carboxy-terminal domain, rna polymerase ii, polypeptide a) small phosphatase like 2  ZSCAN29 - zinc finger and scan domain containing 29  THAP2 - thap domain containing, apoptosis associated protein 2  ITCH - itchy e3 ubiquitin protein ligase  ZNF484 - zinc finger protein 484  L3MBTL2 - l(3)mbt-like 2 (drosophila)  DLG1 - discs, large homolog 1 (drosophila)  LRP6 - low density lipoprotein receptor-related protein 6  ZNF445 - zinc finger protein 445  SHOX2 - short stature homeobox 2  TRAPPC9 - trafficking protein particle complex 9  LUM - lumican  BTAF1 - btaf1 rna polymerase ii, b-tfiid transcription factor-associated, 170kda  POGK - pogo transposable element with krab domain  UBA3 - ubiquitin-like modifier activating enzyme 3  DHFR - dihydrofolate reductase  SESN2 - sestrin 2  PARP9 - poly (adp-ribose) polymerase family, member 9  PHF12 - phd finger protein 12  RNF8 - ring finger protein 8, e3 ubiquitin protein ligase  BAZ1B - bromodomain adjacent to zinc finger domain, 1b  CALCOCO1 - calcium binding and coiled-coil domain 1  ZBTB4 - zinc finger and btb domain containing 4  SFPQ - splicing factor proline/glutamine-rich  SFRP1 - secreted frizzled-related protein 1  SFRP2 - secreted frizzled-related protein 2  MED15 - mediator complex subunit 15  MXD1 - max dimerization protein 1  SFRP4 - secreted frizzled-related protein 4  PIAS4 - protein inhibitor of activated stat, 4  TRIM33 - tripartite motif containing 33  SMAD2 - smad family member 2  SRRT - serrate rna effector molecule homolog (arabidopsis)  DNM2 - dynamin 2  GMEB2 - glucocorticoid modulatory element binding protein 2  DNMT1 - dna (cytosine-5-)-methyltransferase 1  SPAG8 - sperm associated antigen 8  KLF16 - kruppel-like factor 16  HDAC7 - histone deacetylase 7  ARMCX3 - armadillo repeat containing, x-linked 3  DNMT3A - dna (cytosine-5-)-methyltransferase 3 alpha  EIF2AK4 - eukaryotic translation initiation factor 2 alpha kinase 4  LARP7 - la ribonucleoprotein domain family, member 7  CHD8 - chromodomain helicase dna binding protein 8  MAFG - v-maf avian musculoaponeurotic fibrosarcoma oncogene homolog g  RPS6KA4 - ribosomal protein s6 kinase, 90kda, polypeptide 4  TAF9B - taf9b rna polymerase ii, tata box binding protein (tbp)-associated factor, 31kda  SGK1 - serum/glucocorticoid regulated kinase 1  NOC2L - nucleolar complex associated 2 homolog (s. cerevisiae)  SMAD4 - smad family member 4  ZNF317 - zinc finger protein 317  SMAD5 - smad family member 5  H1FX - h1 histone family, member x  SMAD7 - smad family member 7  WASL - wiskott-aldrich syndrome-like  ZNF565 - zinc finger protein 565  SAP30BP - sap30 binding protein  MEIS3 - meis homeobox 3  YAF2 - yy1 associated factor 2  SIX5 - six homeobox 5  TAGLN3 - transgelin 3  NDN - necdin, melanoma antigen (mage) family member  MAPRE3 - microtubule-associated protein, rp/eb family, member 3  ZNF582 - zinc finger protein 582  BRD7 - bromodomain containing 7  NCK1 - nck adaptor protein 1  NCL - nucleolin  ELK1 - elk1, member of ets oncogene family  CEBPZ - ccaat/enhancer binding protein (c/ebp), zeta  TICAM1 - toll-like receptor adaptor molecule 1  ELK3 - elk3, ets-domain protein (srf accessory protein 2)  ELK4 - elk4, ets-domain protein (srf accessory protein 1)  SNW1 - snw domain containing 1  CCNL2 - cyclin l2  SCAP - srebf chaperone  TRIM28 - tripartite motif containing 28  EPAS1 - endothelial pas domain protein 1  EP300 - e1a binding protein p300  ATF6 - activating transcription factor 6  TBK1 - tank-binding kinase 1  ZNF197 - zinc finger protein 197  ENO1 - enolase 1, (alpha)  EGR2 - early growth response 2  PPP1R12A - protein phosphatase 1, regulatory subunit 12a  TRIM11 - tripartite motif containing 11  EGR3 - early growth response 3  MYT1 - myelin transcription factor 1  ALYREF - aly/ref export factor  SENP1 - sumo1/sentrin specific peptidase 1  CSRNP2 - cysteine-serine-rich nuclear protein 2  NAB1 - ngfi-a binding protein 1 (egr1 binding protein 1)  NAB2 - ngfi-a binding protein 2 (egr1 binding protein 2)  RNF41 - ring finger protein 41  TRIM32 - tripartite motif containing 32  TSHZ1 - teashirt zinc finger homeobox 1  KDM2A - lysine (k)-specific demethylase 2a  PSME3 - proteasome (prosome, macropain) activator subunit 3 (pa28 gamma; ki)  ZNF326 - zinc finger protein 326  ELF1 - e74-like factor 1 (ets domain transcription factor)  ELF2 - e74-like factor 2 (ets domain transcription factor)  TCF25 - transcription factor 25 (basic helix-loop-helix)  TSG101 - tumor susceptibility gene 101  TRIM13 - tripartite motif containing 13  TRIM8 - tripartite motif containing 8  MYO6 - myosin vi  ABT1 - activator of basal transcription 1  LBH - limb bud and heart development  TOPORS - topoisomerase i binding, arginine/serine-rich, e3 ubiquitin protein ligase  CNOT1 - ccr4-not transcription complex, subunit 1  EFNA1 - ephrin-a1  SPEN - spen homolog, transcriptional regulator (drosophila)  CTNNBIP1 - catenin, beta interacting protein 1  MYD88 - myeloid differentiation primary response 88  ZNF362 - zinc finger protein 362  BBX - bobby sox homolog (drosophila)  PHC1 - polyhomeotic homolog 1 (drosophila)  TP53BP1 - tumor protein p53 binding protein 1  HCFC2 - host cell factor c2  PHC2 - polyhomeotic homolog 2 (drosophila)  EGR1 - early growth response 1  ZNF292 - zinc finger protein 292  CNOT7 - ccr4-not transcription complex, subunit 7  EGFR - epidermal growth factor receptor  MXI1 - max interactor 1, dimerization protein  MEGF8 - multiple egf-like-domains 8  PQBP1 - polyglutamine binding protein 1  CELSR2 - cadherin, egf lag seven-pass g-type receptor 2  KDM1A - lysine (k)-specific demethylase 1a  ATXN7L3 - ataxin 7-like 3  ACTR2 - arp2 actin-related protein 2 homolog (yeast)  ACTR3 - arp3 actin-related protein 3 homolog (yeast)  NCOA6 - nuclear receptor coactivator 6  E2F6 - e2f transcription factor 6  TSFM - ts translation elongation factor, mitochondrial  E4F1 - e4f transcription factor 1  ZHX3 - zinc fingers and homeoboxes 3  E2F4 - e2f transcription factor 4, p107/p130-binding  E2F5 - e2f transcription factor 5, p130-binding  ENY2 - enhancer of yellow 2 homolog (drosophila)  TRIM37 - tripartite motif containing 37  C11orf30 - chromosome 11 open reading frame 30  RRP1B - ribosomal rna processing 1b  ZNF558 - zinc finger protein 558  EDNRB - endothelin receptor type b  ZNF263 - zinc finger protein 263  TRAF1 - tnf receptor-associated factor 1  S1PR1 - sphingosine-1-phosphate receptor 1  TRAF3 - tnf receptor-associated factor 3  TRAF2 - tnf receptor-associated factor 2  PURG - purine-rich element binding protein g  NR2C1 - nuclear receptor subfamily 2, group c, member 1  SETX - senataxin  ZBTB47 - zinc finger and btb domain containing 47  NR2C2 - nuclear receptor subfamily 2, group c, member 2  MED25 - mediator complex subunit 25  TPR - translocated promoter region, nuclear basket protein  ZNF609 - zinc finger protein 609  ERC1 - elks/rab6-interacting/cast family member 1  MED6 - mediator complex subunit 6  PPRC1 - peroxisome proliferator-activated receptor gamma, coactivator-related 1  NAT14 - n-acetyltransferase 14 (gcn5-related, putative)  KDM4C - lysine (k)-specific demethylase 4c  ZNF423 - zinc finger protein 423  REXO4 - rex4, rna exonuclease 4 homolog (s. cerevisiae)  CAMK1D - calcium/calmodulin-dependent protein kinase id  DMTF1 - cyclin d binding myb-like transcription factor 1  USF1 - upstream transcription factor 1  NUP107 - nucleoporin 107kda  ZBTB43 - zinc finger and btb domain containing 43  ZBTB33 - zinc finger and btb domain containing 33  PITHD1 - pith (c-terminal proteasome-interacting domain of thioredoxin-like) domain containing 1  BPTF - bromodomain phd finger transcription factor  MTF1 - metal-regulatory transcription factor 1  HDAC5 - histone deacetylase 5  HDAC6 - histone deacetylase 6  TAB2 - tgf-beta activated kinase 1/map3k7 binding protein 2  NR1H2 - nuclear receptor subfamily 1, group h, member 2  ETV3 - ets variant 3  CAMTA2 - calmodulin binding transcription activator 2  RNF187 - ring finger protein 187  ETV5 - ets variant 5  POGZ - pogo transposable element with znf domain  TWSG1 - twisted gastrulation homolog 1 (drosophila)  MT3 - metallothionein 3  ETV6 - ets variant 6  PLXND1 - plexin d1  VHL - von hippel-lindau tumor suppressor, e3 ubiquitin protein ligase  MED16 - mediator complex subunit 16  KDM6B - lysine (k)-specific demethylase 6b  PHF8 - phd finger protein 8  EZR - ezrin  EWSR1 - ews rna-binding protein 1  TRIM41 - tripartite motif containing 41  TBX20 - t-box 20  KCTD1 - potassium channel tetramerization domain containing 1  CCDC22 - coiled-coil domain containing 22  CREB3L1 - camp responsive element binding protein 3-like 1  EZH1 - enhancer of zeste homolog 1 (drosophila)  CIC - capicua transcriptional repressor  CCNL1 - cyclin l1  F2R - coagulation factor ii (thrombin) receptor  DNAJB6 - dnaj (hsp40) homolog, subfamily b, member 6  WDR43 - wd repeat domain 43  ERF - ets2 repressor factor  ZNF575 - zinc finger protein 575  CITED1 - cbp/p300-interacting transactivator, with glu/asp-rich carboxy-terminal domain, 1  ZBTB5 - zinc finger and btb domain containing 5  ZBTB40 - zinc finger and btb domain containing 40  ELMSAN1 - elm2 and myb/sant-like domain containing 1  THAP11 - thap domain containing 11  ZNF248 - zinc finger protein 248  UBB - ubiquitin b  RGCC - regulator of cell cycle  RCOR1 - rest corepressor 1  TULP3 - tubby like protein 3  ZNFX1 - zinc finger, nfx1-type containing 1  HIRA - histone cell cycle regulator  COMMD1 - copper metabolism (murr1) domain containing 1  TTC5 - tetratricopeptide repeat domain 5  ETV1 - ets variant 1  ETS1 - v-ets avian erythroblastosis virus e26 oncogene homolog 1  ZMIZ1 - zinc finger, miz-type containing 1  USP3 - ubiquitin specific peptidase 3  UBP1 - upstream binding protein 1 (lbp-1a)  SUMO1 - small ubiquitin-like modifier 1  UBTF - upstream binding transcription factor, rna polymerase i  USP15 - ubiquitin specific peptidase 15  PELI1 - pellino e3 ubiquitin protein ligase 1  CHURC1 - churchill domain containing 1  SMURF1 - smad specific e3 ubiquitin protein ligase 1  ZC3H4 - zinc finger ccch-type containing 4  MED4 - mediator complex subunit 4  JMJD6 - jumonji domain containing 6  THOC1 - tho complex 1  SETD2 - set domain containing 2  UBE2I - ubiquitin-conjugating enzyme e2i  RBX1 - ring-box 1, e3 ubiquitin protein ligase  THRAP3 - thyroid hormone receptor associated protein 3  UBE2L3 - ubiquitin-conjugating enzyme e2l 3  MED12 - mediator complex subunit 12  MED13 - mediator complex subunit 13  UBE2N - ubiquitin-conjugating enzyme e2n  UBE2V1 - ubiquitin-conjugating enzyme e2 variant 1  PSMD6 - proteasome (prosome, macropain) 26s subunit, non-atpase, 6  VPS72 - vacuolar protein sorting 72 homolog (s. cerevisiae)  MED24 - mediator complex subunit 24  MGA - mga, max dimerization protein  ZC3H6 - zinc finger ccch-type containing 6  DTX3L - deltex 3-like (drosophila)  ZNF34 - zinc finger protein 34  SETDB1 - set domain, bifurcated 1  ZEB1 - zinc finger e-box binding homeobox 1  WWC3 - wwc family member 3  ORC2 - origin recognition complex, subunit 2  TCF12 - transcription factor 12  ZNF354A - zinc finger protein 354a  TOX4 - tox high mobility group box family member 4  ZNF436 - zinc finger protein 436  CAMTA1 - calmodulin binding transcription activator 1  TCF20 - transcription factor 20 (ar1)  FLI1 - fli-1 proto-oncogene, ets transcription factor  RNF141 - ring finger protein 141  FLNA - filamin a, alpha  AGRN - agrin  PLCB1 - phospholipase c, beta 1 (phosphoinositide-specific)  FOXS1 - forkhead box s1  FOXO3 - forkhead box o3  HDAC8 - histone deacetylase 8  ASH1L - ash1 (absent, small, or homeotic)-like (drosophila)  SIN3B - sin3 transcription regulator family member b  G3BP2 - gtpase activating protein (sh3 domain) binding protein 2  NACC1 - nucleus accumbens associated 1, ben and btb (poz) domain containing  HR - hair growth associated  MED8 - mediator complex subunit 8  CAND1 - cullin-associated and neddylation-dissociated 1  TDG - thymine-dna glycosylase  OGG1 - 8-oxoguanine dna glycosylase  PRDX2 - peroxiredoxin 2  ZSCAN16 - zinc finger and scan domain containing 16  EAPP - e2f-associated phosphoprotein  FBXW11 - f-box and wd repeat domain containing 11  FAM208A - family with sequence similarity 208, member a  KDM3A - lysine (k)-specific demethylase 3a  FHL2 - four and a half lim domains 2  FGFR2 - fibroblast growth factor receptor 2  TAF9 - taf9 rna polymerase ii, tata box binding protein (tbp)-associated factor, 32kda  TAF7 - taf7 rna polymerase ii, tata box binding protein (tbp)-associated factor, 55kda  TAF6 - taf6 rna polymerase ii, tata box binding protein (tbp)-associated factor, 80kda  FGFR1 - fibroblast growth factor receptor 1  FGF9 - fibroblast growth factor 9  TADA2A - transcriptional adaptor 2a  DDR2 - discoidin domain receptor tyrosine kinase 2  NUMA1 - nuclear mitotic apparatus protein 1  BCLAF1 - bcl2-associated transcription factor 1  DAB2IP - dab2 interacting protein  TIRAP - toll-interleukin 1 receptor (tir) domain containing adaptor protein  FGF1 - fibroblast growth factor 1 (acidic)  FGF2 - fibroblast growth factor 2 (basic)  GMNN - geminin, dna replication inhibitor  NR4A2 - nuclear receptor subfamily 4, group a, member 2  USP22 - ubiquitin specific peptidase 22  ZNF593 - zinc finger protein 593  ZBTB7B - zinc finger and btb domain containing 7b  TARBP1 - tar (hiv-1) rna binding protein 1  FER - fer (fps/fes related) tyrosine kinase  ZNF691 - zinc finger protein 691  MAP3K7 - mitogen-activated protein kinase kinase kinase 7  CREBRF - creb3 regulatory factor  ZNF623 - zinc finger protein 623  ADNP - activity-dependent neuroprotector homeobox  NPM1 - nucleophosmin (nucleolar phosphoprotein b23, numatrin)  ARHGEF11 - rho guanine nucleotide exchange factor (gef) 11  KMT2E - lysine (k)-specific methyltransferase 2e  MED13L - mediator complex subunit 13-like  ZBTB24 - zinc finger and btb domain containing 24  THAP4 - thap domain containing 4  ZEB2 - zinc finger e-box binding homeobox 2  ZNF395 - zinc finger protein 395  MYNN - myoneurin  CRTC1 - creb regulated transcription coactivator 1  UFL1 - ufm1-specific ligase 1  TCF3 - transcription factor 3  DMAP1 - dna methyltransferase 1 associated protein 1  TCEB3 - transcription elongation factor b (siii), polypeptide 3 (110kda, elongin a)  TCF4 - transcription factor 4  EFEMP1 - egf containing fibulin-like extracellular matrix protein 1  NKRF - nfkb repressing factor  TSC22D2 - tsc22 domain family, member 2  ZNF629 - zinc finger protein 629  FBN1 - fibrillin 1  LANCL2 - lanc lantibiotic synthetase component c-like 2 (bacterial)  NRF1 - nuclear respiratory factor 1  NFXL1 - nuclear transcription factor, x-box binding-like 1  TCEA2 - transcription elongation factor a (sii), 2  ERBB2IP - erbb2 interacting protein  TCEA1 - transcription elongation factor a (sii), 1  NOP2 - nop2 nucleolar protein  NONO - non-pou domain containing, octamer-binding  VPRBP - vpr (hiv-1) binding protein  ITGB3BP - integrin beta 3 binding protein (beta3-endonexin)  RYBP - ring1 and yy1 binding protein  MTOR - mechanistic target of rapamycin (serine/threonine kinase)  MDFIC - myod family inhibitor domain containing  PAXBP1 - pax3 and pax7 binding protein 1  TLE1 - transducin-like enhancer of split 1 (e(sp1) homolog, drosophila)  TLE3 - transducin-like enhancer of split 3 (e(sp1) homolog, drosophila)  IFT74 - intraflagellar transport 74 homolog (chlamydomonas)  NOTCH4 - notch 4  TLE4 - transducin-like enhancer of split 4 (e(sp1) homolog, drosophila)  NPAT - nuclear protein, ataxia-telangiectasia locus  CEP290 - centrosomal protein 290kda  TLR3 - toll-like receptor 3  CNOT2 - ccr4-not transcription complex, subunit 2  NAA15 - n(alpha)-acetyltransferase 15, nata auxiliary subunit  CNOT3 - ccr4-not transcription complex, subunit 3  BAZ2B - bromodomain adjacent to zinc finger domain, 2b  NOTCH1 - notch 1  SIRT1 - sirtuin 1  ST18 - suppression of tumorigenicity 18 (breast carcinoma) (zinc finger protein)  NOTCH3 - notch 3  MKX - mohawk homeobox  NOTCH2 - notch 2  NFYA - nuclear transcription factor y, alpha  TMF1 - tata element modulatory factor 1  HDAC4 - histone deacetylase 4  TOX - thymocyte selection-associated high mobility group box  ACTR8 - arp8 actin-related protein 8 homolog (yeast)  TMSB4X - thymosin beta 4, x-linked  CBX6 - chromobox homolog 6  NFYC - nuclear transcription factor y, gamma  HEY1 - hairy/enhancer-of-split related with yrpw motif 1  ZFYVE16 - zinc finger, fyve domain containing 16  CXXC1 - cxxc finger protein 1  TMPO - thymopoietin  NFKBIA - nuclear factor of kappa light polypeptide gene enhancer in b-cells inhibitor, alpha  NFKB1 - nuclear factor of kappa light polypeptide gene enhancer in b-cells 1  CBX5 - chromobox homolog 5  KMT2B - lysine (k)-specific methyltransferase 2b  BRD4 - bromodomain containing 4  NFKBIB - nuclear factor of kappa light polypeptide gene enhancer in b-cells inhibitor, beta  NFKBIE - nuclear factor of kappa light polypeptide gene enhancer in b-cells inhibitor, epsilon  TARDBP - tar dna binding protein  BCL9L - b-cell cll/lymphoma 9-like  TNFRSF1A - tumor necrosis factor receptor superfamily, member 1a  NKX2-2 - nk2 homeobox 2  HDAC9 - histone deacetylase 9  ZNF606 - zinc finger protein 606  ZNF614 - zinc finger protein 614  ZFP2 - zfp2 zinc finger protein  TMBIM6 - transmembrane bax inhibitor motif containing 6  SUZ12 - suz12 polycomb repressive complex 2 subunit  NEO1 - neogenin 1  POFUT1 - protein o-fucosyltransferase 1  TEF - thyrotrophic embryonic factor  RCOR2 - rest corepressor 2  TFAM - transcription factor a, mitochondrial  SMARCAL1 - swi/snf related, matrix associated, actin dependent regulator of chromatin, subfamily a-like 1  NEDD8 - neural precursor cell expressed, developmentally down-regulated 8  CTR9 - ctr9, paf1/rna polymerase ii complex component  PPM1F - protein phosphatase, mg2+/mn2+ dependent, 1f  TFDP1 - transcription factor dp-1  EPC1 - enhancer of polycomb homolog 1 (drosophila)  NFE2L2 - nuclear factor (erythroid-derived 2)-like 2  NR2F2 - nuclear receptor subfamily 2, group f, member 2  MORF4L2 - mortality factor 4 like 2  RASL11A - ras-like, family 11, member a  TFE3 - transcription factor binding to ighm enhancer 3  CBX7 - chromobox homolog 7  NFATC4 - nuclear factor of activated t-cells, cytoplasmic, calcineurin-dependent 4  NR2F1 - nuclear receptor subfamily 2, group f, member 1  TFCP2 - transcription factor cp2  NFIA - nuclear factor i/a  ZNF740 - zinc finger protein 740  TGFA - transforming growth factor, alpha  FNIP1 - folliculin interacting protein 1  HEY2 - hairy/enhancer-of-split related with yrpw motif 2  CDK5RAP3 - cdk5 regulatory subunit associated protein 3  TGFBR1 - transforming growth factor, beta receptor 1  RBFOX2 - rna binding protein, fox-1 homolog (c. elegans) 2  TGFB2 - transforming growth factor, beta 2  TGFB3 - transforming growth factor, beta 3  TGFB1 - transforming growth factor, beta 1  TGFB1I1 - transforming growth factor beta 1 induced transcript 1  ZNF746 - zinc finger protein 746  TP53INP1 - tumor protein p53 inducible nuclear protein 1  VGLL4 - vestigial like 4 (drosophila)  H2AFV - h2a histone family, member v  LRRC14 - leucine rich repeat containing 14  MEPCE - methylphosphate capping enzyme  RNF20 - ring finger protein 20, e3 ubiquitin protein ligase  KDM4A - lysine (k)-specific demethylase 4a  TGFBR3 - transforming growth factor, beta receptor iii  FOSL2 - fos-like antigen 2  PHF14 - phd finger protein 14  FOSB - fbj murine osteosarcoma viral oncogene homolog b  FOS - fbj murine osteosarcoma viral oncogene homolog  KAT6B - k(lysine) acetyltransferase 6b  HP1BP3 - heterochromatin protein 1, binding protein 3  ZNF432 - zinc finger protein 432  SAFB2 - scaffold attachment factor b2  MYEF2 - myelin expression factor 2  LPIN2 - lipin 2  THRA - thyroid hormone receptor, alpha |
| GO:0033044 | regulation of chromosome organization | 4.68E-9 | 1.63E-6 | 1.23 (10334,264,6996,220) | [+] Show genes  EXOSC10 - exosome component 10  BRD7 - bromodomain containing 7  LIG4 - ligase iv, dna, atp-dependent  NAF1 - nuclear assembly factor 1 ribonucleoprotein  WHSC1L1 - wolf-hirschhorn syndrome candidate 1-like 1  FLCN - folliculin  CENPV - centromere protein v  SNW1 - snw domain containing 1  BCOR - bcl6 corepressor  TRIM28 - tripartite motif containing 28  PML - promyelocytic leukemia  OBFC1 - oligonucleotide/oligosaccharide-binding fold containing 1  MIER1 - mesoderm induction early response 1, transcriptional regulator  UPF1 - upf1 regulator of nonsense transcripts homolog (yeast)  NBN - nibrin  KAT2A - k(lysine) acetyltransferase 2a  CCT5 - chaperonin containing tcp1, subunit 5 (epsilon)  SLX4 - slx4 structure-specific endonuclease subunit  H2AFY - h2a histone family, member y  WBP2 - ww domain binding protein 2  SPHK2 - sphingosine kinase 2  OTUB1 - otu domain, ubiquitin aldehyde binding 1  LMNA - lamin a/c  CDC16 - cell division cycle 16  ANAPC5 - anaphase promoting complex subunit 5  ANAPC7 - anaphase promoting complex subunit 7  SNCA - synuclein, alpha (non a4 component of amyloid precursor)  EID1 - ep300 interacting inhibitor of differentiation 1  NABP2 - nucleic acid binding protein 2  RNF4 - ring finger protein 4  CDC45 - cell division cycle 45  KDM1A - lysine (k)-specific demethylase 1a  AXIN2 - axin 2  SMARCB1 - swi/snf related, matrix associated, actin dependent regulator of chromatin, subfamily b, member 1  UBR5 - ubiquitin protein ligase e3 component n-recognin 5  ANAPC4 - anaphase promoting complex subunit 4  CCT3 - chaperonin containing tcp1, subunit 3 (gamma)  SMG1 - smg1 phosphatidylinositol 3-kinase-related kinase  ATG5 - autophagy related 5  RAD50 - rad50 homolog (s. cerevisiae)  PARP1 - poly (adp-ribose) polymerase 1  GLYR1 - glyoxylate reductase 1 homolog (arabidopsis)  AKAP8 - a kinase (prka) anchor protein 8  ZMPSTE24 - zinc metallopeptidase ste24  TPR - translocated promoter region, nuclear basket protein  WAPAL - wings apart-like homolog (drosophila)  RUVBL2 - ruvb-like 2 (e. coli)  KDM4C - lysine (k)-specific demethylase 4c  CDK5RAP2 - cdk5 regulatory subunit associated protein 2  NELFE - negative elongation factor complex member e  SUPT6H - suppressor of ty 6 homolog (s. cerevisiae)  NAA40 - n(alpha)-acetyltransferase 40, natd catalytic subunit, homolog (s. cerevisiae)  CTBP1 - c-terminal binding protein 1  TNKS - tankyrase, trf1-interacting ankyrin-related adp-ribose polymerase  ATF7IP - activating transcription factor 7 interacting protein  DYNC1LI1 - dynein, cytoplasmic 1, light intermediate chain 1  MAD2L2 - mad2 mitotic arrest deficient-like 2 (yeast)  CTNNB1 - catenin (cadherin-associated protein), beta 1, 88kda  BECN1 - beclin 1, autophagy related  USP7 - ubiquitin specific peptidase 7 (herpes virus-associated)  PCID2 - pci domain containing 2  PHF8 - phd finger protein 8  SMC5 - structural maintenance of chromosomes 5  RIOK2 - rio kinase 2  ACD - adrenocortical dysplasia homolog (mouse)  TADA3 - transcriptional adaptor 3  TRAPPC12 - trafficking protein particle complex 12  CDC23 - cell division cycle 23  PPP1R10 - protein phosphatase 1, regulatory subunit 10  RMI2 - recq mediated genome instability 2  CCT6A - chaperonin containing tcp1, subunit 6a (zeta 1)  ERCC4 - excision repair cross-complementing rodent repair deficiency, complementation group 4  PINX1 - pin2/terf1 interacting, telomerase inhibitor 1  IWS1 - iws1 homolog (s. cerevisiae)  NIPBL - nipped-b homolog (drosophila)  EED - embryonic ectoderm development  ASF1A - anti-silencing function 1a histone chaperone  HNRNPU - heterogeneous nuclear ribonucleoprotein u (scaffold attachment factor a)  SSRP1 - structure specific recognition protein 1  FBXO4 - f-box protein 4  HIRA - histone cell cycle regulator  TINF2 - terf1 (trf1)-interacting nuclear factor 2  MAP2K7 - mitogen-activated protein kinase kinase 7  TRIP12 - thyroid hormone receptor interactor 12  MAPK8 - mitogen-activated protein kinase 8  HNRNPA2B1 - heterogeneous nuclear ribonucleoprotein a2/b1  MAPK3 - mitogen-activated protein kinase 3  HNRNPA1 - heterogeneous nuclear ribonucleoprotein a1  GATA2 - gata binding protein 2  MAPK1 - mitogen-activated protein kinase 1  HNRNPC - heterogeneous nuclear ribonucleoprotein c (c1/c2)  KLHL22 - kelch-like family member 22  HNRNPD - heterogeneous nuclear ribonucleoprotein d (au-rich element rna binding protein 1, 37kda)  SSBP1 - single-stranded dna binding protein 1, mitochondrial  ZNF335 - zinc finger protein 335  PRKD1 - protein kinase d1  TLK2 - tousled-like kinase 2  SRC - v-src avian sarcoma (schmidt-ruppin a-2) viral oncogene homolog  HMGA1 - high mobility group at-hook 1  MRE11A - mre11 meiotic recombination 11 homolog a (s. cerevisiae)  UBE2N - ubiquitin-conjugating enzyme e2n  PKIB - protein kinase (camp-dependent, catalytic) inhibitor beta  DHX36 - deah (asp-glu-ala-his) box polypeptide 36  FMR1 - fragile x mental retardation 1  PARN - poly(a)-specific ribonuclease  TCP1 - t-complex 1  SETDB1 - set domain, bifurcated 1  SIN3A - sin3 transcription regulator family member a  TLK1 - tousled-like kinase 1  CDC27 - cell division cycle 27  SET - set nuclear oncogene  SETMAR - set domain and mariner transposase fusion gene  PRMT6 - protein arginine methyltransferase 6  ZNF304 - zinc finger protein 304  PARG - poly (adp-ribose) glycohydrolase  GTF2H2 - general transcription factor iih, polypeptide 2, 44kda  ING2 - inhibitor of growth family, member 2  MLLT6 - myeloid/lymphoid or mixed-lineage leukemia (trithorax homolog, drosophila); translocated to, 6  MCPH1 - microcephalin 1  MLLT3 - myeloid/lymphoid or mixed-lineage leukemia (trithorax homolog, drosophila); translocated to, 3  APC - adenomatous polyposis coli  HDAC8 - histone deacetylase 8  KMT2A - lysine (k)-specific methyltransferase 2a  POT1 - protection of telomeres 1  RIF1 - rap1 interacting factor homolog (yeast)  RPS6KA5 - ribosomal protein s6 kinase, 90kda, polypeptide 5  MAPKAPK5 - mitogen-activated protein kinase-activated protein kinase 5  KIAA1551 - kiaa1551  MAP1S - microtubule-associated protein 1s  MNAT1 - mnat cdk-activating kinase assembly factor 1  SMG6 - smg6 nonsense mediated mrna decay factor  SETD5 - set domain containing 5  TNKS2 - tankyrase, trf1-interacting ankyrin-related adp-ribose polymerase 2  KDM3A - lysine (k)-specific demethylase 3a  FAM208A - family with sequence similarity 208, member a  WDR61 - wd repeat domain 61  ANAPC1 - anaphase promoting complex subunit 1  TERF2IP - telomeric repeat binding factor 2, interacting protein  H3F3A - h3 histone, family 3a  TAF7 - taf7 rna polymerase ii, tata box binding protein (tbp)-associated factor, 55kda  DCP2 - decapping mrna 2  NSMCE2 - non-smc element 2, mms21 homolog (s. cerevisiae)  ZNF451 - zinc finger protein 451  TADA2A - transcriptional adaptor 2a  ARRB1 - arrestin, beta 1  SPTY2D1 - spt2, suppressor of ty, domain containing 1 (s. cerevisiae)  MECP2 - methyl cpg binding protein 2 (rett syndrome)  BUB3 - bub3 mitotic checkpoint protein  NUMA1 - nuclear mitotic apparatus protein 1  ZW10 - zw10 kinetochore protein  ZBTB7B - zinc finger and btb domain containing 7b  FEN1 - flap structure-specific endonuclease 1  MAP3K4 - mitogen-activated protein kinase kinase kinase 4  WDR70 - wd repeat domain 70  PYGO2 - pygopus homolog 2 (drosophila)  CDK9 - cyclin-dependent kinase 9  JDP2 - jun dimerization protein 2  CCT7 - chaperonin containing tcp1, subunit 7 (eta)  KMT2E - lysine (k)-specific methyltransferase 2e  CCT2 - chaperonin containing tcp1, subunit 2 (beta)  CCT4 - chaperonin containing tcp1, subunit 4 (delta)  RNF40 - ring finger protein 40, e3 ubiquitin protein ligase  HMBOX1 - homeobox containing 1  KAT7 - k(lysine) acetyltransferase 7  ZNF207 - zinc finger protein 207  PAPD5 - pap associated domain containing 5  PHF19 - phd finger protein 19  NSD1 - nuclear receptor binding set domain protein 1  CDC26 - cell division cycle 26  PAXBP1 - pax3 and pax7 binding protein 1  XRCC1 - x-ray repair complementing defective repair in chinese hamster cells 1  MORC2 - morc family cw-type zinc finger 2  PHF2 - phd finger protein 2  MAPT - microtubule-associated protein tau  PHF1 - phd finger protein 1  LRRK2 - leucine-rich repeat kinase 2  MPHOSPH8 - m-phase phosphoprotein 8  SIRT6 - sirtuin 6  SIRT1 - sirtuin 1  CTC1 - cts telomere maintenance complex component 1  PPHLN1 - periphilin 1  MAPK15 - mitogen-activated protein kinase 15  SKI - v-ski avian sarcoma viral oncogene homolog  CHTOP - chromatin target of prmt1  KMT2B - lysine (k)-specific methyltransferase 2b  BRD4 - bromodomain containing 4  RAD21 - rad21 homolog (s. pombe)  SENP6 - sumo1/sentrin specific peptidase 6  MCM2 - minichromosome maintenance complex component 2  PAF1 - paf1, rna polymerase ii associated factor, homolog (s. cerevisiae)  SART3 - squamous cell carcinoma antigen recognized by t cells 3  MAD1L1 - mad1 mitotic arrest deficient-like 1 (yeast)  SETD1A - set domain containing 1a  DKC1 - dyskeratosis congenita 1, dyskerin  MTF2 - metal response element binding transcription factor 2  CCT8 - chaperonin containing tcp1, subunit 8 (theta)  NAT10 - n-acetyltransferase 10 (gcn5-related)  TERF1 - telomeric repeat binding factor (nima-interacting) 1  TERF2 - telomeric repeat binding factor 2  CTR9 - ctr9, paf1/rna polymerase ii complex component  C6orf89 - chromosome 6 open reading frame 89  ATRX - alpha thalassemia/mental retardation syndrome x-linked  GNL3L - guanine nucleotide binding protein-like 3 (nucleolar)-like  IK - ik cytokine, down-regulator of hla ii  CTCF - ccctc-binding factor (zinc finger protein)  YLPM1 - ylp motif containing 1  SFPQ - splicing factor proline/glutamine-rich  OGT - o-linked n-acetylglucosamine (glcnac) transferase  RB1 - retinoblastoma 1  DNMT1 - dna (cytosine-5-)-methyltransferase 1  KDM5A - lysine (k)-specific demethylase 5a  RNF20 - ring finger protein 20, e3 ubiquitin protein ligase  RPS6KA4 - ribosomal protein s6 kinase, 90kda, polypeptide 4  CUL3 - cullin 3  XRCC5 - x-ray repair complementing defective repair in chinese hamster cells 5 (double-strand-break rejoining)  NOC2L - nucleolar complex associated 2 homolog (s. cerevisiae)  SMAD4 - smad family member 4  H1FX - h1 histone family, member x  PARK7 - parkinson protein 7  PNKP - polynucleotide kinase 3'-phosphatase |
| GO:0051641 | cellular localization | 5.94E-9 | 2.02E-6 | 1.09 (10334,1725,6996,1268) | [+] Show genes  AGAP2 - arfgap with gtpase domain, ankyrin repeat and ph domain 2  EXOSC10 - exosome component 10  SYMPK - symplekin  AP3M2 - adaptor-related protein complex 3, mu 2 subunit  WLS - wntless homolog (drosophila)  SEC61B - sec61 beta subunit  ABCA1 - atp-binding cassette, sub-family a (abc1), member 1  TOMM34 - translocase of outer mitochondrial membrane 34  GEM - gtp binding protein overexpressed in skeletal muscle  OS9 - osteosarcoma amplified 9, endoplasmic reticulum lectin  HAUS7 - haus augmin-like complex, subunit 7  RAB3IP - rab3a interacting protein  TMED2 - transmembrane emp24 domain trafficking protein 2  SSX2IP - synovial sarcoma, x breakpoint 2 interacting protein  ERP29 - endoplasmic reticulum protein 29  NUDT19 - nudix (nucleoside diphosphate linked moiety x)-type motif 19  PML - promyelocytic leukemia  ACACA - acetyl-coa carboxylase alpha  ACOT2 - acyl-coa thioesterase 2  PMM1 - phosphomannomutase 1  RAB40B - rab40b, member ras oncogene family  MKKS - mckusick-kaufman syndrome  YWHAQ - tyrosine 3-monooxygenase/tryptophan 5-monooxygenase activation protein, theta polypeptide  KAT2A - k(lysine) acetyltransferase 2a  TMED10 - transmembrane emp24-like trafficking protein 10 (yeast)  TBC1D22B - tbc1 domain family, member 22b  ANP32A - acidic (leucine-rich) nuclear phosphoprotein 32 family, member a  AP3B2 - adaptor-related protein complex 3, beta 2 subunit  NOP9 - nop9 nucleolar protein  SYBU - syntabulin (syntaxin-interacting)  ACOX1 - acyl-coa oxidase 1, palmitoyl  PLRG1 - pleiotropic regulator 1  PLXNA2 - plexin a2  PABPN1 - poly(a) binding protein, nuclear 1  RNPS1 - rna binding protein s1, serine-rich domain  GJA1 - gap junction protein, alpha 1, 43kda  KIF16B - kinesin family member 16b  EHD1 - eh-domain containing 1  ZDHHC7 - zinc finger, dhhc-type containing 7  AP1AR - adaptor-related protein complex 1 associated regulatory protein  PICALM - phosphatidylinositol binding clathrin assembly protein  ACTN4 - actinin, alpha 4  RAB10 - rab10, member ras oncogene family  GLE1 - gle1 rna export mediator  TMCO6 - transmembrane and coiled-coil domains 6  CPSF4 - cleavage and polyadenylation specific factor 4, 30kda  MED1 - mediator complex subunit 1  AXIN2 - axin 2  NUP85 - nucleoporin 85kda  USP9X - ubiquitin specific peptidase 9, x-linked  ADPRH - adp-ribosylarginine hydrolase  H2AFY2 - h2a histone family, member y2  CEP76 - centrosomal protein 76kda  SNAP47 - synaptosomal-associated protein, 47kda  POLR2D - polymerase (rna) ii (dna directed) polypeptide d  RUVBL2 - ruvb-like 2 (e. coli)  AP2A2 - adaptor-related protein complex 2, alpha 2 subunit  TMEM30A - transmembrane protein 30a  AP2A1 - adaptor-related protein complex 2, alpha 1 subunit  AP2B1 - adaptor-related protein complex 2, beta 1 subunit  AP1B1 - adaptor-related protein complex 1, beta 1 subunit  CDK5RAP2 - cdk5 regulatory subunit associated protein 2  TTC21B - tetratricopeptide repeat domain 21b  AP1G1 - adaptor-related protein complex 1, gamma 1 subunit  PRKACA - protein kinase, camp-dependent, catalytic, alpha  NUP133 - nucleoporin 133kda  AP5M1 - adaptor-related protein complex 5, mu 1 subunit  AGK - acylglycerol kinase  NUPL2 - nucleoporin like 2  VPS35 - vacuolar protein sorting 35 homolog (s. cerevisiae)  KIF3A - kinesin family member 3a  TOR1AIP2 - torsin a interacting protein 2  GEMIN6 - gem (nuclear organelle) associated protein 6  FGFR1OP - fgfr1 oncogene partner  RAB3C - rab3c, member ras oncogene family  CNTRL - centriolin  AKT1 - v-akt murine thymoma viral oncogene homolog 1  RAB7A - rab7a, member ras oncogene family  PCID2 - pci domain containing 2  BBS12 - bardet-biedl syndrome 12  PPP2R1A - protein phosphatase 2, regulatory subunit a, alpha  EVI5L - ecotropic viral integration site 5-like  SLBP - stem-loop binding protein  TBC1D23 - tbc1 domain family, member 23  RER1 - rer1 retention in endoplasmic reticulum 1 homolog (s. cerevisiae)  PPP1R10 - protein phosphatase 1, regulatory subunit 10  TCTN2 - tectonic family member 2  EXOC1 - exocyst complex component 1  IFT122 - intraflagellar transport 122 homolog (chlamydomonas)  TMEM115 - transmembrane protein 115  DDX39B - dead (asp-glu-ala-asp) box polypeptide 39b  SCYL2 - scy1-like 2 (s. cerevisiae)  GAK - cyclin g associated kinase  PEX26 - peroxisomal biogenesis factor 26  TBC1D17 - tbc1 domain family, member 17  RAB35 - rab35, member ras oncogene family  IFT27 - intraflagellar transport 27 homolog (chlamydomonas)  DENND4C - denn/madd domain containing 4c  NPLOC4 - nuclear protein localization 4 homolog (s. cerevisiae)  KDELR2 - kdel (lys-asp-glu-leu) endoplasmic reticulum protein retention receptor 2  AAAS - achalasia, adrenocortical insufficiency, alacrimia  RNF126 - ring finger protein 126  IFT88 - intraflagellar transport 88 homolog (chlamydomonas)  RAB31 - rab31, member ras oncogene family  RNF157 - ring finger protein 157  MAPK10 - mitogen-activated protein kinase 10  WDR11 - wd repeat domain 11  GEMIN7 - gem (nuclear organelle) associated protein 7  MAPRE2 - microtubule-associated protein, rp/eb family, member 2  OSBPL8 - oxysterol binding protein-like 8  MICALL2 - mical-like 2  NDC1 - ndc1 transmembrane nucleoporin  HAUS1 - haus augmin-like complex, subunit 1  CCDC88A - coiled-coil domain containing 88a  IPO9 - importin 9  STAM - signal transducing adaptor molecule (sh3 domain and itam motif) 1  PRKCI - protein kinase c, iota  ANXA2 - annexin a2  NUP214 - nucleoporin 214kda  NR4A3 - nuclear receptor subfamily 4, group a, member 3  PRKAR2B - protein kinase, camp-dependent, regulatory, type ii, beta  GAS6 - growth arrest-specific 6  NCOA4 - nuclear receptor coactivator 4  PACS1 - phosphofurin acidic cluster sorting protein 1  PRKAG1 - protein kinase, amp-activated, gamma 1 non-catalytic subunit  DDX19B - dead (asp-glu-ala-asp) box polypeptide 19b  MSTO1 - misato 1, mitochondrial distribution and morphology regulator  SDAD1 - sda1 domain containing 1  SYNRG - synergin, gamma  TBCCD1 - tbcc domain containing 1  PACSIN2 - protein kinase c and casein kinase substrate in neurons 2  DCTN3 - dynactin 3 (p22)  MCPH1 - microcephalin 1  APBA1 - amyloid beta (a4) precursor protein-binding, family a, member 1  SEC61A2 - sec61 alpha 2 subunit (s. cerevisiae)  XPOT - exportin, trna  CHP1 - calcineurin-like ef-hand protein 1  VPS13D - vacuolar protein sorting 13 homolog d (s. cerevisiae)  SEC63 - sec63 homolog (s. cerevisiae)  ARCN1 - archain 1  PDCD10 - programmed cell death 10  TRIM23 - tripartite motif containing 23  APPL2 - adaptor protein, phosphotyrosine interaction, ph domain and leucine zipper containing 2  ARF1 - adp-ribosylation factor 1  IPO4 - importin 4  ARF4 - adp-ribosylation factor 4  MAP1S - microtubule-associated protein 1s  PAFAH1B1 - platelet-activating factor acetylhydrolase 1b, regulatory subunit 1 (45kda)  GOLPH3L - golgi phosphoprotein 3-like  ARL8B - adp-ribosylation factor-like 8b  AKAP13 - a kinase (prka) anchor protein 13  APP - amyloid beta (a4) precursor protein  AKAP11 - a kinase (prka) anchor protein 11  ZDHHC14 - zinc finger, dhhc-type containing 14  BBS7 - bardet-biedl syndrome 7  DDX20 - dead (asp-glu-ala-asp) box polypeptide 20  PALM - paralemmin  ARNTL - aryl hydrocarbon receptor nuclear translocator-like  CEP250 - centrosomal protein 250kda  TCTN1 - tectonic family member 1  TRAPPC6B - trafficking protein particle complex 6b  ARL3 - adp-ribosylation factor-like 3  MUL1 - mitochondrial e3 ubiquitin protein ligase 1  ARL1 - adp-ribosylation factor-like 1  TUBA1A - tubulin, alpha 1a  CORO7 - coronin 7  IFT57 - intraflagellar transport 57 homolog (chlamydomonas)  SEC23IP - sec23 interacting protein  ALMS1 - alstrom syndrome 1  RHOB - ras homolog family member b  FAM83H - family with sequence similarity 83, member h  ARF6 - adp-ribosylation factor 6  PSIP1 - pc4 and sfrs1 interacting protein 1  ARF5 - adp-ribosylation factor 5  EVI5 - ecotropic viral integration site 5  ARHGAP1 - rho gtpase activating protein 1  WDR60 - wd repeat domain 60  ASNA1 - arsa arsenite transporter, atp-binding, homolog 1 (bacterial)  AP4S1 - adaptor-related protein complex 4, sigma 1 subunit  CHMP1A - charged multivesicular body protein 1a  PLEKHJ1 - pleckstrin homology domain containing, family j member 1  MAGOHB - mago-nashi homolog b (drosophila)  PCM1 - pericentriolar material 1  RABL2B - rab, member of ras oncogene family-like 2b  FRMD6 - ferm domain containing 6  TBC1D8 - tbc1 domain family, member 8 (with gram domain)  WRAP53 - wd repeat containing, antisense to tp53  CDC37 - cell division cycle 37  KPTN - kaptin (actin binding protein)  HTT - huntingtin  RIC3 - ric3 acetylcholine receptor chaperone  HAUS2 - haus augmin-like complex, subunit 2  DDX19A - dead (asp-glu-ala-asp) box polypeptide 19a  DENND3 - denn/madd domain containing 3  XPA - xeroderma pigmentosum, complementation group a  GOLGA2 - golgin a2  HAUS3 - haus augmin-like complex, subunit 3  XPO1 - exportin 1 (crm1 homolog, yeast)  GOLGB1 - golgin b1  RPH3A - rabphilin 3a homolog (mouse)  ATP1B3 - atpase, na+/k+ transporting, beta 3 polypeptide  CCDC91 - coiled-coil domain containing 91  ATP1B1 - atpase, na+/k+ transporting, beta 1 polypeptide  LIN7C - lin-7 homolog c (c. elegans)  MON1B - mon1 secretory trafficking family member b  LRRK2 - leucine-rich repeat kinase 2  BLOC1S4 - biogenesis of lysosomal organelles complex-1, subunit 4, cappuccino  CLSTN1 - calsyntenin 1  RSG1 - rem2 and rab-like small gtpase 1  AP5S1 - adaptor-related protein complex 5, sigma 1 subunit  DZIP1 - daz interacting zinc finger protein 1  SEC31A - sec31 homolog a (s. cerevisiae)  WRB - tryptophan rich basic protein  PEX6 - peroxisomal biogenesis factor 6  PEX7 - peroxisomal biogenesis factor 7  ATG14 - autophagy related 14  LSG1 - large 60s subunit nuclear export gtpase 1  SH3PXD2B - sh3 and px domains 2b  PEX12 - peroxisomal biogenesis factor 12  PEX13 - peroxisomal biogenesis factor 13  ATP6AP1 - atpase, h+ transporting, lysosomal accessory protein 1  PEX14 - peroxisomal biogenesis factor 14  RILPL2 - rab interacting lysosomal protein-like 2  LMTK2 - lemur tyrosine kinase 2  ICK - intestinal cell (mak-like) kinase  WDR33 - wd repeat domain 33  PEX1 - peroxisomal biogenesis factor 1  PAOX - polyamine oxidase (exo-n4-amino)  PKD1 - polycystic kidney disease 1 (autosomal dominant)  BAX - bcl2-associated x protein  THOC6 - tho complex 6 homolog (drosophila)  COPZ1 - coatomer protein complex, subunit zeta 1  COPG1 - coatomer protein complex, subunit gamma 1  BAD - bcl2-associated agonist of cell death  PIK3R1 - phosphoinositide-3-kinase, regulatory subunit 1 (alpha)  PDZD8 - pdz domain containing 8  COX18 - cox18 cytochrome c oxidase assembly factor  KIF1A - kinesin family member 1a  ZFYVE27 - zinc finger, fyve domain containing 27  PKP2 - plakophilin 2  COG2 - component of oligomeric golgi complex 2  PEF1 - penta-ef-hand domain containing 1  ATRX - alpha thalassemia/mental retardation syndrome x-linked  CASC3 - cancer susceptibility candidate 3  GABARAPL2 - gaba(a) receptor-associated protein-like 2  GRIK5 - glutamate receptor, ionotropic, kainate 5  VPS53 - vacuolar protein sorting 53 homolog (s. cerevisiae)  RABL3 - rab, member of ras oncogene family-like 3  EXOC3 - exocyst complex component 3  GRIK2 - glutamate receptor, ionotropic, kainate 2  GABARAP - gaba(a) receptor-associated protein  U2AF2 - u2 small nuclear rna auxiliary factor 2  BCR - breakpoint cluster region  RHOT1 - ras homolog family member t1  XRCC5 - x-ray repair complementing defective repair in chinese hamster cells 5 (double-strand-break rejoining)  PIK3C3 - phosphatidylinositol 3-kinase, catalytic subunit type 3  YWHAG - tyrosine 3-monooxygenase/tryptophan 5-monooxygenase activation protein, gamma polypeptide  COPE - coatomer protein complex, subunit epsilon  YWHAZ - tyrosine 3-monooxygenase/tryptophan 5-monooxygenase activation protein, zeta polypeptide  BBS4 - bardet-biedl syndrome 4  BBS2 - bardet-biedl syndrome 2  YWHAE - tyrosine 3-monooxygenase/tryptophan 5-monooxygenase activation protein, epsilon polypeptide  VPS45 - vacuolar protein sorting 45 homolog (s. cerevisiae)  YWHAB - tyrosine 3-monooxygenase/tryptophan 5-monooxygenase activation protein, beta polypeptide  TBC1D20 - tbc1 domain family, member 20  DST - dystonin  TRIM27 - tripartite motif containing 27  DNAJA1 - dnaj (hsp40) homolog, subfamily a, member 1  BET1 - bet1 golgi vesicular membrane trafficking protein  BRAF - v-raf murine sarcoma viral oncogene homolog b  FLCN - folliculin  MKS1 - meckel syndrome, type 1  NRP1 - neuropilin 1  LIN7A - lin-7 homolog a (c. elegans)  KATNB1 - katanin p80 (wd repeat containing) subunit b 1  RDX - radixin  ARL13B - adp-ribosylation factor-like 13b  AGFG1 - arfgap with fg repeats 1  ALKBH5 - alkb, alkylation repair homolog 5 (e. coli)  UPF1 - upf1 regulator of nonsense transcripts homolog (yeast)  DERL1 - derlin 1  DSCR3 - down syndrome critical region gene 3  RANBP6 - ran binding protein 6  RRAGB - ras-related gtp binding b  FNBP1L - formin binding protein 1-like  SYNGAP1 - synaptic ras gtpase activating protein 1  TFG - trk-fused gene  DEF8 - differentially expressed in fdcp 8 homolog (mouse)  SYNJ1 - synaptojanin 1  RNF215 - ring finger protein 215  BMP7 - bone morphogenetic protein 7  ABCA7 - atp-binding cassette, sub-family a (abc1), member 7  FLOT1 - flotillin 1  NDE1 - nude neurodevelopment protein 1  VPS51 - vacuolar protein sorting 51 homolog (s. cerevisiae)  ABCE1 - atp-binding cassette, sub-family e (oabp), member 1  PTTG1IP - pituitary tumor-transforming 1 interacting protein  VPS13C - vacuolar protein sorting 13 homolog c (s. cerevisiae)  MIS12 - mis12 kinetochore complex component  TMEM50B - transmembrane protein 50b  KXD1 - kxdl motif containing 1  ARL8A - adp-ribosylation factor-like 8a  NABP2 - nucleic acid binding protein 2  HAUS6 - haus augmin-like complex, subunit 6  STX6 - syntaxin 6  AP1M1 - adaptor-related protein complex 1, mu 1 subunit  TMEM201 - transmembrane protein 201  AP1G2 - adaptor-related protein complex 1, gamma 2 subunit  AP1S2 - adaptor-related protein complex 1, sigma 2 subunit  AHI1 - abelson helper integration site 1  AP3S2 - adaptor-related protein complex 3, sigma 2 subunit  U2AF1L4 - u2 small nuclear rna auxiliary factor 1-like 4  AFTPH - aftiphilin  MCM3AP - minichromosome maintenance complex component 3 associated protein  GPHN - gephyrin  TMEM108 - transmembrane protein 108  RABEPK - rab9 effector protein with kelch motifs  ROCK1 - rho-associated, coiled-coil containing protein kinase 1  HERC2 - hect and rld domain containing e3 ubiquitin protein ligase 2  TRAK2 - trafficking protein, kinesin binding 2  TSPO - translocator protein (18kda)  SRRM1 - serine/arginine repetitive matrix 1  SPRY1 - sprouty homolog 1, antagonist of fgf signaling (drosophila)  SPRY2 - sprouty homolog 2 (drosophila)  HSPD1 - heat shock 60kda protein 1 (chaperonin)  STAM2 - signal transducing adaptor molecule (sh3 domain and itam motif) 2  HSP90AA1 - heat shock protein 90kda alpha (cytosolic), class a member 1  RAMP2 - receptor (g protein-coupled) activity modifying protein 2  RAMP3 - receptor (g protein-coupled) activity modifying protein 3  AP3D1 - adaptor-related protein complex 3, delta 1 subunit  RAMP1 - receptor (g protein-coupled) activity modifying protein 1  ZMPSTE24 - zinc metallopeptidase ste24  HSPA4 - heat shock 70kda protein 4  HSPA9 - heat shock 70kda protein 9 (mortalin)  BAIAP3 - bai1-associated protein 3  WASF1 - was protein family, member 1  HSPA8 - heat shock 70kda protein 8  CAPZA1 - capping protein (actin filament) muscle z-line, alpha 1  TOMM40 - translocase of outer mitochondrial membrane 40 homolog (yeast)  RPL17 - ribosomal protein l17  TIMM17A - translocase of inner mitochondrial membrane 17 homolog a (yeast)  LAMTOR3 - late endosomal/lysosomal adaptor, mapk and mtor activator 3  RPL15 - ribosomal protein l15  VPS37C - vacuolar protein sorting 37 homolog c (s. cerevisiae)  MPPE1 - metallophosphoesterase 1  RPL11 - ribosomal protein l11  RPL9 - ribosomal protein l9  TIMM10B - translocase of inner mitochondrial membrane 10 homolog b (yeast)  NOL8 - nucleolar protein 8  COG5 - component of oligomeric golgi complex 5  RPL7 - ribosomal protein l7  DYNLL1 - dynein, light chain, lc8-type 1  PIBF1 - progesterone immunomodulatory binding factor 1  TNKS - tankyrase, trf1-interacting ankyrin-related adp-ribose polymerase  RPL3 - ribosomal protein l3  HEATR3 - heat repeat containing 3  TIMM9 - translocase of inner mitochondrial membrane 9 homolog (yeast)  RPA1 - replication protein a1, 70kda  RPA2 - replication protein a2, 32kda  CAPZB - capping protein (actin filament) muscle z-line, beta  RPS4X - ribosomal protein s4, x-linked  BECN1 - beclin 1, autophagy related  SEC23A - sec23 homolog a (s. cerevisiae)  SRSF9 - serine/arginine-rich splicing factor 9  RPS4Y1 - ribosomal protein s4, y-linked 1  HK1 - hexokinase 1  SEC23B - sec23 homolog b (s. cerevisiae)  NDRG4 - ndrg family member 4  STX17 - syntaxin 17  CACNB3 - calcium channel, voltage-dependent, beta 3 subunit  CACNB2 - calcium channel, voltage-dependent, beta 2 subunit  ACD - adrenocortical dysplasia homolog (mouse)  LAMTOR1 - late endosomal/lysosomal adaptor, mapk and mtor activator 1  NOL6 - nucleolar protein 6 (rna-associated)  CALR - calreticulin  UNC13B - unc-13 homolog b (c. elegans)  CDC23 - cell division cycle 23  VPS33A - vacuolar protein sorting 33 homolog a (s. cerevisiae)  HIP1 - huntingtin interacting protein 1  RPL32 - ribosomal protein l32  STK25 - serine/threonine kinase 25  UPF3B - upf3 regulator of nonsense transcripts homolog b (yeast)  UPF3A - upf3 regulator of nonsense transcripts homolog a (yeast)  VTI1B - vesicle transport through interaction with t-snares 1b  HIF1A - hypoxia inducible factor 1, alpha subunit (basic helix-loop-helix transcription factor)  MBTPS1 - membrane-bound transcription factor peptidase, site 1  ARFGAP3 - adp-ribosylation factor gtpase activating protein 3  RPS23 - ribosomal protein s23  SNX3 - sorting nexin 3  PINX1 - pin2/terf1 interacting, telomerase inhibitor 1  SNX4 - sorting nexin 4  TSNARE1 - t-snare domain containing 1  TUBA1B - tubulin, alpha 1b  TUBB - tubulin, beta class i  GBF1 - golgi brefeldin a resistant guanine nucleotide exchange factor 1  BANP - btg3 associated nuclear protein  HNRNPU - heterogeneous nuclear ribonucleoprotein u (scaffold attachment factor a)  GEMIN8 - gem (nuclear organelle) associated protein 8  FBXO22 - f-box protein 22  RPS15 - ribosomal protein s15  RPS12 - ribosomal protein s12  VPS33B - vacuolar protein sorting 33 homolog b (yeast)  TUBB4B - tubulin, beta 4b class ivb  TINF2 - terf1 (trf1)-interacting nuclear factor 2  NRBP2 - nuclear receptor binding protein 2  ARV1 - arv1 homolog (s. cerevisiae)  CBL - cbl proto-oncogene, e3 ubiquitin protein ligase  HNRNPA2B1 - heterogeneous nuclear ribonucleoprotein a2/b1  RAB11A - rab11a, member ras oncogene family  HNRNPA1 - heterogeneous nuclear ribonucleoprotein a1  CAV2 - caveolin 2  CAV1 - caveolin 1, caveolae protein, 22kda  NAPA - n-ethylmaleimide-sensitive factor attachment protein, alpha  NAPG - n-ethylmaleimide-sensitive factor attachment protein, gamma  SEC24B - sec24 family, member b (s. cerevisiae)  HMGA1 - high mobility group at-hook 1  CMTM6 - cklf-like marvel transmembrane domain containing 6  TBC1D15 - tbc1 domain family, member 15  PSAP - prosaposin  CDH2 - cadherin 2, type 1, n-cadherin (neuronal)  DESI1 - desumoylating isopeptidase 1  PEX3 - peroxisomal biogenesis factor 3  SGSM3 - small g protein signaling modulator 3  RANBP3 - ran binding protein 3  PPFIA2 - protein tyrosine phosphatase, receptor type, f polypeptide (ptprf), interacting protein (liprin), alpha 2  TOR1B - torsin family 1, member b (torsin b)  PSEN2 - presenilin 2 (alzheimer disease 4)  RABGEF1 - rab guanine nucleotide exchange factor (gef) 1  TOLLIP - toll interacting protein  CREB3L2 - camp responsive element binding protein 3-like 2  PRPF19 - pre-mrna processing factor 19  CD81 - cd81 molecule  STARD4 - star-related lipid transfer (start) domain containing 4  SCARB2 - scavenger receptor class b, member 2  EXOC5 - exocyst complex component 5  BLZF1 - basic leucine zipper nuclear factor 1  AP3B1 - adaptor-related protein complex 3, beta 1 subunit  YKT6 - ykt6 v-snare homolog (s. cerevisiae)  PPFIA3 - protein tyrosine phosphatase, receptor type, f polypeptide (ptprf), interacting protein (liprin), alpha 3  AGPS - alkylglycerone phosphate synthase  CAMK1 - calcium/calmodulin-dependent protein kinase i  IL10RA - interleukin 10 receptor, alpha  GDAP1 - ganglioside induced differentiation associated protein 1  GORASP1 - golgi reassembly stacking protein 1, 65kda  TSPAN33 - tetraspanin 33  GRPEL2 - grpe-like 2, mitochondrial (e. coli)  GAS2L1 - growth arrest-specific 2 like 1  TERF2IP - telomeric repeat binding factor 2, interacting protein  PITRM1 - pitrilysin metallopeptidase 1  VPS16 - vacuolar protein sorting 16 homolog (s. cerevisiae)  DCTN2 - dynactin 2 (p50)  CETN2 - centrin, ef-hand protein, 2  LAMTOR5 - late endosomal/lysosomal adaptor, mapk and mtor activator 5  TM9SF1 - transmembrane 9 superfamily member 1  APPBP2 - amyloid beta precursor protein (cytoplasmic tail) binding protein 2  TRAM1L1 - translocation associated membrane protein 1-like 1  FBLN5 - fibulin 5  ARFIP1 - adp-ribosylation factor interacting protein 1  ITGB1 - integrin, beta 1 (fibronectin receptor, beta polypeptide, antigen cd29 includes mdf2, msk12)  PRPF18 - pre-mrna processing factor 18  HYOU1 - hypoxia up-regulated 1  ZDHHC6 - zinc finger, dhhc-type containing 6  IPO8 - importin 8  PTK2 - protein tyrosine kinase 2  IPO7 - importin 7  EIF6 - eukaryotic translation initiation factor 6  BBS9 - bardet-biedl syndrome 9  SLU7 - slu7 splicing factor homolog (s. cerevisiae)  SKAP1 - src kinase associated phosphoprotein 1  CDK5 - cyclin-dependent kinase 5  ATG3 - autophagy related 3  PTN - pleiotrophin  KLHL20 - kelch-like family member 20  AKTIP - akt interacting protein  NPC2 - niemann-pick disease, type c2  CADPS - ca++-dependent secretion activator  MPP5 - membrane protein, palmitoylated 5 (maguk p55 subfamily member 5)  HTATIP2 - hiv-1 tat interactive protein 2, 30kda  USO1 - uso1 vesicle transport factor  SCLT1 - sodium channel and clathrin linker 1  PURA - purine-rich element binding protein a  TRAPPC3 - trafficking protein particle complex 3  LRWD1 - leucine-rich repeats and wd repeat domain containing 1  TMEM106B - transmembrane protein 106b  SRSF10 - serine/arginine-rich splicing factor 10  PTPRK - protein tyrosine phosphatase, receptor type, k  TBC1D13 - tbc1 domain family, member 13  LYST - lysosomal trafficking regulator  LEPROT - leptin receptor overlapping transcript  AHCYL1 - adenosylhomocysteinase-like 1  PEX2 - peroxisomal biogenesis factor 2  CHML - choroideremia-like (rab escort protein 2)  NUP50 - nucleoporin 50kda  CHM - choroideremia (rab escort protein 1)  CHCHD4 - coiled-coil-helix-coiled-coil-helix domain containing 4  GIPC1 - gipc pdz domain containing family, member 1  SNX5 - sorting nexin 5  CHGA - chromogranin a (parathyroid secretory protein 1)  INVS - inversin  PVRL2 - poliovirus receptor-related 2 (herpesvirus entry mediator b)  RAB6A - rab6a, member ras oncogene family  PIP5K1A - phosphatidylinositol-4-phosphate 5-kinase, type i, alpha  C17orf75 - chromosome 17 open reading frame 75  RAB5A - rab5a, member ras oncogene family  RAB5B - rab5b, member ras oncogene family  RAB3A - rab3a, member ras oncogene family  MTERFD2 - mterf domain containing 2  RAB3B - rab3b, member ras oncogene family  CPLX2 - complexin 2  RAB1A - rab1a, member ras oncogene family  RRAGC - ras-related gtp binding c  SDCCAG3 - serologically defined colon cancer antigen 3  RAD21 - rad21 homolog (s. pombe)  SEC24A - sec24 family, member a (s. cerevisiae)  BRAT1 - brca1-associated atm activator 1  PAF1 - paf1, rna polymerase ii associated factor, homolog (s. cerevisiae)  RAC1 - ras-related c3 botulinum toxin substrate 1 (rho family, small gtp binding protein rac1)  RAB5C - rab5c, member ras oncogene family  TMED4 - transmembrane emp24 protein transport domain containing 4  LIN7B - lin-7 homolog b (c. elegans)  VPS4A - vacuolar protein sorting 4 homolog a (s. cerevisiae)  RAB27A - rab27a, member ras oncogene family  SNX16 - sorting nexin 16  COPG2 - coatomer protein complex, subunit gamma 2  RALA - v-ral simian leukemia viral oncogene homolog a (ras related)  TOMM7 - translocase of outer mitochondrial membrane 7 homolog (yeast)  RANBP2 - ran binding protein 2  RAP1A - rap1a, member of ras oncogene family  CLTC - clathrin, heavy chain (hc)  SEC22A - sec22 vesicle trafficking protein homolog a (s. cerevisiae)  AP3M1 - adaptor-related protein complex 3, mu 1 subunit  LRRC4 - leucine rich repeat containing 4  GNPAT - glyceronephosphate o-acyltransferase  EXOC6 - exocyst complex component 6  CLN5 - ceroid-lipofuscinosis, neuronal 5  SGTB - small glutamine-rich tetratricopeptide repeat (tpr)-containing, beta  DCTN6 - dynactin 6  BBS5 - bardet-biedl syndrome 5  RRAGA - ras-related gtp binding a  EEA1 - early endosome antigen 1  CLTB - clathrin, light chain b  CLTA - clathrin, light chain a  CLU - clusterin  STX7 - syntaxin 7  MAGEL2 - mage-like 2  GOLPH3 - golgi phosphoprotein 3 (coat-protein)  RAP2A - rap2a, member of ras oncogene family  IK - ik cytokine, down-regulator of hla ii  TPP1 - tripeptidyl peptidase i  CTCF - ccctc-binding factor (zinc finger protein)  HEATR5B - heat repeat containing 5b  AP1S1 - adaptor-related protein complex 1, sigma 1 subunit  TMEM150A - transmembrane protein 150a  RB1 - retinoblastoma 1  ARL5B - adp-ribosylation factor-like 5b  RAE1 - rae1 rna export 1 homolog (s. pombe)  LAT - linker for activation of t cells  AP2M1 - adaptor-related protein complex 2, mu 1 subunit  ANKRD54 - ankyrin repeat domain 54  GEMIN2 - gem (nuclear organelle) associated protein 2  NSG1 - neuron specific gene family member 1  NUP35 - nucleoporin 35kda  VPS41 - vacuolar protein sorting 41 homolog (s. cerevisiae)  DOC2B - double c2-like domains, beta  B9D1 - b9 protein domain 1  CUL3 - cullin 3  CCDC93 - coiled-coil domain containing 93  SGSM1 - small g protein signaling modulator 1  CHMP4B - charged multivesicular body protein 4b  PIGU - phosphatidylinositol glycan anchor biosynthesis, class u  CACNG4 - calcium channel, voltage-dependent, gamma subunit 4  AP4B1 - adaptor-related protein complex 4, beta 1 subunit  RSRC1 - arginine/serine-rich coiled-coil 1  TMEM87B - transmembrane protein 87b  SPTAN1 - spectrin, alpha, non-erythrocytic 1  CEP89 - centrosomal protein 89kda  TBRG1 - transforming growth factor beta regulator 1  WDR19 - wd repeat domain 19  SPTBN4 - spectrin, beta, non-erythrocytic 4  AKAP12 - a kinase (prka) anchor protein 12  COG8 - component of oligomeric golgi complex 8  VPS37A - vacuolar protein sorting 37 homolog a (s. cerevisiae)  EPG5 - ectopic p-granules autophagy protein 5 homolog (c. elegans)  CORO1C - coronin, actin binding protein, 1c  ZDHHC3 - zinc finger, dhhc-type containing 3  KPNA6 - karyopherin alpha 6 (importin alpha 7)  LDLR - low density lipoprotein receptor  RABGAP1 - rab gtpase activating protein 1  SPG7 - spastic paraplegia 7 (pure and complicated autosomal recessive)  HOOK3 - hook microtubule-tethering protein 3  DENND1A - denn/madd domain containing 1a  LZTS2 - leucine zipper, putative tumor suppressor 2  SPAST - spastin  REEP2 - receptor accessory protein 2  FAM53C - family with sequence similarity 53, member c  ABCG1 - atp-binding cassette, sub-family g (white), member 1  LTV1 - ltv1 homolog (s. cerevisiae)  ERGIC2 - ergic and golgi 2  CPT1A - carnitine palmitoyltransferase 1a (liver)  TICAM2 - toll-like receptor adaptor molecule 2  RAB3D - rab3d, member ras oncogene family  SYVN1 - synovial apoptosis inhibitor 1, synoviolin  VPS26A - vacuolar protein sorting 26 homolog a (s. pombe)  SEC22B - sec22 vesicle trafficking protein homolog b (s. cerevisiae) (gene/pseudogene)  H2AFY - h2a histone family, member y  CPE - carboxypeptidase e  GOSR2 - golgi snap receptor complex member 2  BET1L - bet1 golgi vesicular membrane trafficking protein-like  UBAP1 - ubiquitin associated protein 1  UBXN2B - ubx domain protein 2b  RAB40C - rab40c, member ras oncogene family  SPIRE2 - spire actin nucleation factor 2  PREPL - prolyl endopeptidase-like  NACC2 - nacc family member 2, ben and btb (poz) domain containing  SNX2 - sorting nexin 2  LMNA - lamin a/c  DNAJC27 - dnaj (hsp40) homolog, subfamily c, member 27  SNX1 - sorting nexin 1  TMEM50A - transmembrane protein 50a  LMAN1 - lectin, mannose-binding, 1  LLGL1 - lethal giant larvae homolog 1 (drosophila)  SERAC1 - serine active site containing 1  DCTN5 - dynactin 5 (p25)  MARK4 - map/microtubule affinity-regulating kinase 4  PRKAG2 - protein kinase, amp-activated, gamma 2 non-catalytic subunit  SNRPD1 - small nuclear ribonucleoprotein d1 polypeptide 16kda  SNRPF - small nuclear ribonucleoprotein polypeptide f  SNCA - synuclein, alpha (non a4 component of amyloid precursor)  TRAPPC1 - trafficking protein particle complex 1  SNRPB - small nuclear ribonucleoprotein polypeptides b and b1  VPS4B - vacuolar protein sorting 4 homolog b (s. cerevisiae)  GOSR1 - golgi snap receptor complex member 1  SNAP25 - synaptosomal-associated protein, 25kda  SRPRB - signal recognition particle receptor, b subunit  BAG4 - bcl2-associated athanogene 4  TRAPPC4 - trafficking protein particle complex 4  SMN1 - survival of motor neuron 1, telomeric  SMO - smoothened, frizzled family receptor  LAMP2 - lysosomal-associated membrane protein 2  ARFIP2 - adp-ribosylation factor interacting protein 2  SNX6 - sorting nexin 6  ATCAY - ataxia, cerebellar, cayman type  MID1IP1 - mid1 interacting protein 1  REPS1 - ralbp1 associated eps domain containing 1  ROCK2 - rho-associated, coiled-coil containing protein kinase 2  ATP6V1D - atpase, h+ transporting, lysosomal 34kda, v1 subunit d  SH3BP4 - sh3-domain binding protein 4  STX8 - syntaxin 8  ZBTB7A - zinc finger and btb domain containing 7a  EPS15L1 - epidermal growth factor receptor pathway substrate 15-like 1  COPB1 - coatomer protein complex, subunit beta 1  RAB38 - rab38, member ras oncogene family  COPA - coatomer protein complex, subunit alpha  DPY30 - dpy-30 homolog (c. elegans)  LAMA5 - laminin, alpha 5  FBF1 - fas (tnfrsf6) binding factor 1  CDC40 - cell division cycle 40  STX12 - syntaxin 12  RPH3AL - rabphilin 3a-like (without c2 domains)  SYN1 - synapsin i  NAGPA - n-acetylglucosamine-1-phosphodiester alpha-n-acetylglucosaminidase  SYN2 - synapsin ii  DCTN4 - dynactin 4 (p62)  FAM126A - family with sequence similarity 126, member a  VPS28 - vacuolar protein sorting 28 homolog (s. cerevisiae)  KIF5C - kinesin family member 5c  KIF3C - kinesin family member 3c  SYT1 - synaptotagmin i  RPF2 - ribosome production factor 2 homolog (s. cerevisiae)  KIF5B - kinesin family member 5b  SYT5 - synaptotagmin v  KLHL12 - kelch-like family member 12  KIF5A - kinesin family member 5a  SYT4 - synaptotagmin iv  PICK1 - protein interacting with prkca 1  SUN2 - sad1 and unc84 domain containing 2  CTNNA1 - catenin (cadherin-associated protein), alpha 1, 102kda  CACNG7 - calcium channel, voltage-dependent, gamma subunit 7  CTBP2 - c-terminal binding protein 2  DYNC1LI1 - dynein, cytoplasmic 1, light intermediate chain 1  VAMP2 - vesicle-associated membrane protein 2 (synaptobrevin 2)  CCDC41 - coiled-coil domain containing 41  RAB3GAP2 - rab3 gtpase activating protein subunit 2 (non-catalytic)  CTNNB1 - catenin (cadherin-associated protein), beta 1, 88kda  PPIL2 - peptidylprolyl isomerase (cyclophilin)-like 2  STXBP1 - syntaxin binding protein 1  ASB3 - ankyrin repeat and socs box containing 3  STX5 - syntaxin 5  SAV1 - salvador homolog 1 (drosophila)  STX4 - syntaxin 4  STX3 - syntaxin 3  STX1A - syntaxin 1a (brain)  GOLGA7 - golgin a7  TNPO1 - transportin 1  MGARP - mitochondria-localized glutamic acid-rich protein  IER3IP1 - immediate early response 3 interacting protein 1  TGFBRAP1 - transforming growth factor, beta receptor associated protein 1  FUT10 - fucosyltransferase 10 (alpha (1,3) fucosyltransferase)  KPNA2 - karyopherin alpha 2 (rag cohort 1, importin alpha 1)  KPNB1 - karyopherin (importin) beta 1  KPNA1 - karyopherin alpha 1 (importin alpha 5)  STXBP3 - syntaxin binding protein 3  ZDHHC9 - zinc finger, dhhc-type containing 9  SYT3 - synaptotagmin iii  TRAPPC12 - trafficking protein particle complex 12  CRIPT - cysteine-rich pdz-binding protein  FYTTD1 - forty-two-three domain containing 1  TUBA1C - tubulin, alpha 1c  PAN3 - pan3 poly(a) specific ribonuclease subunit homolog (s. cerevisiae)  PEX16 - peroxisomal biogenesis factor 16  KIT - v-kit hardy-zuckerman 4 feline sarcoma viral oncogene homolog  EXOC4 - exocyst complex component 4  CNEP1R1 - ctd nuclear envelope phosphatase 1 regulatory subunit 1  SH3GLB1 - sh3-domain grb2-like endophilin b1  RPAIN - rpa interacting protein  RAB28 - rab28, member ras oncogene family  TMEM216 - transmembrane protein 216  POLDIP3 - polymerase (dna-directed), delta interacting protein 3  LHFPL4 - lipoma hmgic fusion partner-like 4  NIPBL - nipped-b homolog (drosophila)  RAB9A - rab9a, member ras oncogene family  TRAPPC11 - trafficking protein particle complex 11  COG4 - component of oligomeric golgi complex 4  RAB26 - rab26, member ras oncogene family  PDZD11 - pdz domain containing 11  STAT3 - signal transducer and activator of transcription 3 (acute-phase response factor)  JUP - junction plakoglobin  SSR3 - signal sequence receptor, gamma (translocon-associated protein gamma)  COG1 - component of oligomeric golgi complex 1  SPCS3 - signal peptidase complex subunit 3 homolog (s. cerevisiae)  KIF3B - kinesin family member 3b  ZFYVE9 - zinc finger, fyve domain containing 9  TM9SF2 - transmembrane 9 superfamily member 2  NAPB - n-ethylmaleimide-sensitive factor attachment protein, beta  SRP68 - signal recognition particle 68kda  SNX33 - sorting nexin 33  SRP54 - signal recognition particle 54kda  FBXO7 - f-box protein 7  CSE1L - cse1 chromosome segregation 1-like (yeast)  SRP19 - signal recognition particle 19kda  SRP14 - signal recognition particle 14kda (homologous alu rna binding protein)  BLOC1S5 - biogenesis of lysosomal organelles complex-1, subunit 5, muted  SRP72 - signal recognition particle 72kda  SSR1 - signal sequence receptor, alpha  ZDHHC2 - zinc finger, dhhc-type containing 2  SSB - sjogren syndrome antigen b (autoantigen la)  SNAP29 - synaptosomal-associated protein, 29kda  THOC3 - tho complex 3  VAMP3 - vesicle-associated membrane protein 3  CSNK1D - casein kinase 1, delta  SPTBN1 - spectrin, beta, non-erythrocytic 1  MIA3 - melanoma inhibitory activity family, member 3  IPO11 - importin 11  TAOK2 - tao kinase 2  SARNP - sap domain containing ribonucleoprotein  VIPAS39 - vps33b interacting protein, apical-basolateral polarity regulator, spe-39 homolog  VPS25 - vacuolar protein sorting 25 homolog (s. cerevisiae)  ZDHHC12 - zinc finger, dhhc-type containing 12  UBE2O - ubiquitin-conjugating enzyme e2o  SRP9 - signal recognition particle 9kda  TMEM107 - transmembrane protein 107  MON1A - mon1 secretory trafficking family member a  DHX9 - deah (asp-glu-ala-his) box helicase 9  SEL1L - sel-1 suppressor of lin-12-like (c. elegans)  TRAPPC2 - trafficking protein particle complex 2  SRSF11 - serine/arginine-rich splicing factor 11  DDX3X - dead (asp-glu-ala-asp) box helicase 3, x-linked  SEC13 - sec13 homolog (s. cerevisiae)  DDX1 - dead (asp-glu-ala-asp) box helicase 1  RAB8B - rab8b, member ras oncogene family  SIN3A - sin3 transcription regulator family member a  ERGIC1 - endoplasmic reticulum-golgi intermediate compartment (ergic) 1  SYT17 - synaptotagmin xvii  FAM98A - family with sequence similarity 98, member a  SDCBP - syndecan binding protein (syntenin)  COPB2 - coatomer protein complex, subunit beta 2 (beta prime)  HEATR5A - heat repeat containing 5a  MLH1 - mutl homolog 1, colon cancer, nonpolyposis type 2 (e. coli)  RHOT2 - ras homolog family member t2  DCTN1 - dynactin 1  PTPN23 - protein tyrosine phosphatase, non-receptor type 23  ITGB1BP1 - integrin beta 1 binding protein 1  MCM9 - minichromosome maintenance complex component 9  GEMIN5 - gem (nuclear organelle) associated protein 5  ZDHHC24 - zinc finger, dhhc-type containing 24  WDR34 - wd repeat domain 34  MYRIP - myosin viia and rab interacting protein  PHAX - phosphorylated adaptor for rna export  CPSF3 - cleavage and polyadenylation specific factor 3, 73kda  POT1 - protection of telomeres 1  TRAPPC2L - trafficking protein particle complex 2-like  SCYL1 - scy1-like 1 (s. cerevisiae)  RAB22A - rab22a, member ras oncogene family  VPS29 - vacuolar protein sorting 29 homolog (s. cerevisiae)  RAB11B - rab11b, member ras oncogene family  SYT9 - synaptotagmin ix  RAB23 - rab23, member ras oncogene family  ZDHHC20 - zinc finger, dhhc-type containing 20  DNM3 - dynamin 3  ANKRD27 - ankyrin repeat domain 27 (vps9 domain)  VAPB - vamp (vesicle-associated membrane protein)-associated protein b and c  BUB3 - bub3 mitotic checkpoint protein  MCFD2 - multiple coagulation factor deficiency 2  REPS2 - ralbp1 associated eps domain containing 2  MEF2A - myocyte enhancer factor 2a  PRICKLE1 - prickle homolog 1 (drosophila)  ESYT2 - extended synaptotagmin-like protein 2  RAB24 - rab24, member ras oncogene family  ZW10 - zw10 kinetochore protein  FAM160A2 - family with sequence similarity 160, member a2  CPSF2 - cleavage and polyadenylation specific factor 2, 100kda  DCLK1 - doublecortin-like kinase 1  RAB8A - rab8a, member ras oncogene family  TBC1D10B - tbc1 domain family, member 10b  GORASP2 - golgi reassembly stacking protein 2, 55kda  COG6 - component of oligomeric golgi complex 6  CEP78 - centrosomal protein 78kda  XPO5 - exportin 5  C2CD3 - c2 calcium-dependent domain containing 3  LRSAM1 - leucine rich repeat and sterile alpha motif containing 1  AP4M1 - adaptor-related protein complex 4, mu 1 subunit  ARHGEF2 - rho/rac guanine nucleotide exchange factor (gef) 2  S100A10 - s100 calcium binding protein a10  CHMP2B - charged multivesicular body protein 2b  STX18 - syntaxin 18  ARL6 - adp-ribosylation factor-like 6  TBC1D14 - tbc1 domain family, member 14  SYNGR1 - synaptogyrin 1  HGS - hepatocyte growth factor-regulated tyrosine kinase substrate  NEMF - nuclear export mediator factor  TMEM87A - transmembrane protein 87a  VPS52 - vacuolar protein sorting 52 homolog (s. cerevisiae)  VTA1 - vesicle (multivesicular body) trafficking 1  RABEP1 - rabaptin, rab gtpase binding effector protein 1  COG3 - component of oligomeric golgi complex 3  MAL - mal, t-cell differentiation protein  MAGOH - mago-nashi homolog, proliferation-associated (drosophila)  MICALL1 - mical-like 1  TCTN3 - tectonic family member 3  NKD2 - naked cuticle homolog 2 (drosophila)  NLGN2 - neuroligin 2  TRAF3IP1 - tnf receptor-associated factor 3 interacting protein 1  RAB6B - rab6b, member ras oncogene family  MARK1 - map/microtubule affinity-regulating kinase 1  MAPT - microtubule-associated protein tau  SHANK3 - sh3 and multiple ankyrin repeat domains 3  MAP6 - microtubule-associated protein 6  WIPI2 - wd repeat domain, phosphoinositide interacting 2  RAB14 - rab14, member ras oncogene family  MAP1B - microtubule-associated protein 1b  LATS1 - large tumor suppressor kinase 1  TTC7B - tetratricopeptide repeat domain 7b  MAP1A - microtubule-associated protein 1a  PRPF31 - pre-mrna processing factor 31  RAB33B - rab33b, member ras oncogene family  VPS54 - vacuolar protein sorting 54 homolog (s. cerevisiae)  TANC2 - tetratricopeptide repeat, ankyrin repeat and coiled-coil containing 2  PPHLN1 - periphilin 1  DYNLRB2 - dynein, light chain, roadblock-type 2  DYNLRB1 - dynein, light chain, roadblock-type 1  MAPK15 - mitogen-activated protein kinase 15  NUP43 - nucleoporin 43kda  TOR1A - torsin family 1, member a (torsin a)  CHTOP - chromatin target of prmt1  ARHGAP21 - rho gtpase activating protein 21  GGA1 - golgi-associated, gamma adaptin ear containing, arf binding protein 1  DVL2 - dishevelled segment polarity protein 2  TOR1AIP1 - torsin a interacting protein 1  CHMP5 - charged multivesicular body protein 5  DOCK7 - dedicator of cytokinesis 7  APPL1 - adaptor protein, phosphotyrosine interaction, ph domain and leucine zipper containing 1  TTBK2 - tau tubulin kinase 2  ANKFY1 - ankyrin repeat and fyve domain containing 1  SYT7 - synaptotagmin vii  MAP7 - microtubule-associated protein 7  KIAA1468 - kiaa1468  DLG3 - discs, large homolog 3 (drosophila)  DLG4 - discs, large homolog 4 (drosophila)  VPS18 - vacuolar protein sorting 18 homolog (s. cerevisiae)  DLG1 - discs, large homolog 1 (drosophila)  BLOC1S6 - biogenesis of lysosomal organelles complex-1, subunit 6, pallidin  RPL14 - ribosomal protein l14  DYNC2LI1 - dynein, cytoplasmic 2, light intermediate chain 1  LRP6 - low density lipoprotein receptor-related protein 6  STXBP4 - syntaxin binding protein 4  AIP - aryl hydrocarbon receptor interacting protein  TOM1L2 - target of myb1-like 2 (chicken)  ARL5A - adp-ribosylation factor-like 5a  LYN - v-yes-1 yamaguchi sarcoma viral related oncogene homolog  SPAG9 - sperm associated antigen 9  JAM3 - junctional adhesion molecule 3  TMED6 - transmembrane emp24 protein transport domain containing 6  CHMP3 - charged multivesicular body protein 3  SFPQ - splicing factor proline/glutamine-rich  DYNC1H1 - dynein, cytoplasmic 1, heavy chain 1  DYNC1I1 - dynein, cytoplasmic 1, intermediate chain 1  DYNC1I2 - dynein, cytoplasmic 1, intermediate chain 2  DYNC1LI2 - dynein, cytoplasmic 1, light intermediate chain 2  RAB34 - rab34, member ras oncogene family  SRSF1 - serine/arginine-rich splicing factor 1  DNM2 - dynamin 2  SRSF2 - serine/arginine-rich splicing factor 2  SRSF3 - serine/arginine-rich splicing factor 3  SRSF4 - serine/arginine-rich splicing factor 4  ARMCX3 - armadillo repeat containing, x-linked 3  SRSF5 - serine/arginine-rich splicing factor 5  SRSF6 - serine/arginine-rich splicing factor 6  SRSF7 - serine/arginine-rich splicing factor 7  M6PR - mannose-6-phosphate receptor (cation dependent)  ALS2 - amyotrophic lateral sclerosis 2 (juvenile)  ERGIC3 - ergic and golgi 3  HPS4 - hermansky-pudlak syndrome 4  SCG5 - secretogranin v (7b2 protein)  MZT1 - mitotic spindle organizing protein 1  SGTA - small glutamine-rich tetratricopeptide repeat (tpr)-containing, alpha  IFT172 - intraflagellar transport 172 homolog (chlamydomonas)  RUNDC1 - run domain containing 1  ITSN1 - intersectin 1 (sh3 domain protein)  SMAD7 - smad family member 7  WASL - wiskott-aldrich syndrome-like  MAPRE1 - microtubule-associated protein, rp/eb family, member 1  NCBP2 - nuclear cap binding protein subunit 2, 20kda  NCBP1 - nuclear cap binding protein subunit 1, 80kda  MARK2 - map/microtubule affinity-regulating kinase 2  CHMP4C - charged multivesicular body protein 4c  MAPRE3 - microtubule-associated protein, rp/eb family, member 3  TSPAN14 - tetraspanin 14  TSC1 - tuberous sclerosis 1  CTTN - cortactin  AKAP9 - a kinase (prka) anchor protein (yotiao) 9  KIFAP3 - kinesin-associated protein 3  DDX25 - dead (asp-glu-ala-asp) box helicase 25  SPIRE1 - spire actin nucleation factor 1  IFT46 - intraflagellar transport 46 homolog (chlamydomonas)  SCAP - srebf chaperone  SCG3 - secretogranin iii  TM9SF3 - transmembrane 9 superfamily member 3  DNAJC15 - dnaj (hsp40) homolog, subfamily c, member 15  NUBP1 - nucleotide binding protein 1  RAB18 - rab18, member ras oncogene family  RAB3GAP1 - rab3 gtpase activating protein subunit 1 (catalytic)  TIMM50 - translocase of inner mitochondrial membrane 50 homolog (s. cerevisiae)  TUB - tubby bipartite transcription factor  GRIPAP1 - grip1 associated protein 1  MYO10 - myosin x  CNIH - cornichon homolog (drosophila)  EIF4E - eukaryotic translation initiation factor 4e  TUBG1 - tubulin, gamma 1  ZDHHC1 - zinc finger, dhhc-type containing 1  EGR2 - early growth response 2  LMAN2L - lectin, mannose-binding 2-like  TTC30A - tetratricopeptide repeat domain 30a  ALYREF - aly/ref export factor  NDEL1 - nude neurodevelopment protein 1-like 1  NACA - nascent polypeptide-associated complex alpha subunit  SAR1A - sar1 homolog a (s. cerevisiae)  ELAVL1 - elav (embryonic lethal, abnormal vision, drosophila)-like 1 (hu antigen r)  TMEM129 - transmembrane protein 129  AZI1 - 5-azacytidine induced 1  BTBD8 - btb (poz) domain containing 8  STRADA - ste20-related kinase adaptor alpha  NUTF2 - nuclear transport factor 2  MYO1D - myosin id  TSG101 - tumor susceptibility gene 101  EFR3B - efr3 homolog b (s. cerevisiae)  TSC2 - tuberous sclerosis 2  MYO5A - myosin va (heavy chain 12, myoxin)  MYO1E - myosin ie  MYO6 - myosin vi  SNX27 - sorting nexin family member 27  TOPORS - topoisomerase i binding, arginine/serine-rich, e3 ubiquitin protein ligase  FIP1L1 - factor interacting with papola and cpsf1  MVB12A - multivesicular body subunit 12a  RAB1B - rab1b, member ras oncogene family  SNX15 - sorting nexin 15  EIF2D - eukaryotic translation initiation factor 2d  RAB21 - rab21, member ras oncogene family  SEH1L - seh1-like (s. cerevisiae)  MYH10 - myosin, heavy chain 10, non-muscle  SNX11 - sorting nexin 11  PTPRU - protein tyrosine phosphatase, receptor type, u  MYH9 - myosin, heavy chain 9, non-muscle  SNUPN - snurportin 1  SNX10 - sorting nexin 10  EGFR - epidermal growth factor receptor  COL4A3BP - collagen, type iv, alpha 3 (goodpasture antigen) binding protein  DOPEY1 - dopey family member 1  MX1 - myxovirus (influenza virus) resistance 1, interferon-inducible protein p78 (mouse)  GPSM2 - g-protein signaling modulator 2  GOLGA7B - golgin a7 family, member b  TSPAN5 - tetraspanin 5  CPSF1 - cleavage and polyadenylation specific factor 1, 160kda  ACTR2 - arp2 actin-related protein 2 homolog (yeast)  UNC13A - unc-13 homolog a (c. elegans)  ACTR3 - arp3 actin-related protein 3 homolog (yeast)  RBM15B - rna binding motif protein 15b  CLUAP1 - clusterin associated protein 1  SMG1 - smg1 phosphatidylinositol 3-kinase-related kinase  MFF - mitochondrial fission factor  NRBP1 - nuclear receptor binding protein 1  TNIK - traf2 and nck interacting kinase  ENY2 - enhancer of yellow 2 homolog (drosophila)  FAM126B - family with sequence similarity 126, member b  XPO7 - exportin 7  MON2 - mon2 homolog (s. cerevisiae)  LRPPRC - leucine-rich pentatricopeptide repeat containing  ACTR1A - arp1 actin-related protein 1 homolog a, centractin alpha (yeast)  CPLX3 - complexin 3  HSP90B1 - heat shock protein 90kda beta (grp94), member 1  ARL4A - adp-ribosylation factor-like 4a  TIMM22 - translocase of inner mitochondrial membrane 22 homolog (yeast)  ARL4C - adp-ribosylation factor-like 4c  SEC61A1 - sec61 alpha 1 subunit (s. cerevisiae)  BCAP31 - b-cell receptor-associated protein 31  TBC1D9B - tbc1 domain family, member 9b (with gram domain)  SNX12 - sorting nexin 12  TPR - translocated promoter region, nuclear basket protein  WAPAL - wings apart-like homolog (drosophila)  BLOC1S3 - biogenesis of lysosomal organelles complex-1, subunit 3  GGA2 - golgi-associated, gamma adaptin ear containing, arf binding protein 2  ERC1 - elks/rab6-interacting/cast family member 1  ZNF423 - zinc finger protein 423  BOD1 - biorientation of chromosomes in cell division 1  PIGW - phosphatidylinositol glycan anchor biosynthesis, class w  NUP107 - nucleoporin 107kda  GOPC - golgi-associated pdz and coiled-coil motif containing  KIF1B - kinesin family member 1b  TBC1D2B - tbc1 domain family, member 2b  PDCD6 - programmed cell death 6  MTM1 - myotubularin 1  NFASC - neurofascin  USP4 - ubiquitin specific peptidase 4 (proto-oncogene)  HDAC6 - histone deacetylase 6  OCLN - occludin  CLASP2 - cytoplasmic linker associated protein 2  HMGXB4 - hmg box domain containing 4  ETV5 - ets variant 5  TOM1 - target of myb1 (chicken)  EZR - ezrin  PIGK - phosphatidylinositol glycan anchor biosynthesis, class k  EPB41L3 - erythrocyte membrane protein band 4.1-like 3  ESAM - endothelial cell adhesion molecule  CCDC22 - coiled-coil domain containing 22  EXOC8 - exocyst complex component 8  UVRAG - uv radiation resistance associated  DNM1L - dynamin 1-like  AP5B1 - adaptor-related protein complex 5, beta 1 subunit  DNAJB6 - dnaj (hsp40) homolog, subfamily b, member 6  VCL - vinculin  VCP - valosin containing protein  TBC1D9 - tbc1 domain family, member 9 (with gram domain)  MAPK8IP3 - mitogen-activated protein kinase 8 interacting protein 3  MSH2 - muts homolog 2, colon cancer, nonpolyposis type 1 (e. coli)  CITED1 - cbp/p300-interacting transactivator, with glu/asp-rich carboxy-terminal domain, 1  NUP205 - nucleoporin 205kda  YTHDC1 - yth domain containing 1  THOC2 - tho complex 2  GGA3 - golgi-associated, gamma adaptin ear containing, arf binding protein 3  CEP68 - centrosomal protein 68kda  SEC16A - sec16 homolog a (s. cerevisiae)  CHMP7 - charged multivesicular body protein 7  UBB - ubiquitin b  CORO1B - coronin, actin binding protein, 1b  IFT81 - intraflagellar transport 81 homolog (chlamydomonas)  SGPP1 - sphingosine-1-phosphate phosphatase 1  TULP3 - tubby like protein 3  MESDC2 - mesoderm development candidate 2  SPCS1 - signal peptidase complex subunit 1 homolog (s. cerevisiae)  RBM8A - rna binding motif protein 8a  FAF2 - fas associated factor family member 2  TPGS1 - tubulin polyglutamylase complex subunit 1  POLR2M - polymerase (rna) ii (dna directed) polypeptide m  COG7 - component of oligomeric golgi complex 7  STXBP6 - syntaxin binding protein 6 (amisyn)  ARL6IP1 - adp-ribosylation factor-like 6 interacting protein 1  TIMM21 - translocase of inner mitochondrial membrane 21 homolog (yeast)  SCYL3 - scy1-like 3 (s. cerevisiae)  SUMO1 - small ubiquitin-like modifier 1  UCHL1 - ubiquitin carboxyl-terminal esterase l1 (ubiquitin thiolesterase)  CHMP4A - charged multivesicular body protein 4a  RRS1 - rrs1 ribosome biogenesis regulator homolog (s. cerevisiae)  XPO6 - exportin 6  SYT11 - synaptotagmin xi  SMURF1 - smad specific e3 ubiquitin protein ligase 1  THOC1 - tho complex 1  MYADM - myeloid-associated differentiation marker  TBC1D1 - tbc1 (tre-2/usp6, bub2, cdc16) domain family, member 1  UBE2D2 - ubiquitin-conjugating enzyme e2d 2  STX2 - syntaxin 2  NUP153 - nucleoporin 153kda  TBC1D12 - tbc1 domain family, member 12  TMEM230 - transmembrane protein 230  EXOC6B - exocyst complex component 6b  WDR91 - wd repeat domain 91  NUP210 - nucleoporin 210kda  EPS15 - epidermal growth factor receptor pathway substrate 15  CHAMP1 - chromosome alignment maintaining phosphoprotein 1  DTX3L - deltex 3-like (drosophila)  B9D2 - b9 protein domain 2  FNTA - farnesyltransferase, caax box, alpha  SCFD1 - sec1 family domain containing 1  SEC24D - sec24 family, member d (s. cerevisiae)  TLK1 - tousled-like kinase 1  DENND5A - denn/madd domain containing 5a  PLEKHM1 - pleckstrin homology domain containing, family m (with run domain) member 1  OSBP - oxysterol binding protein  C2CD5 - c2 calcium-dependent domain containing 5  FLNA - filamin a, alpha  AGRN - agrin  ACTR10 - actin-related protein 10 homolog (s. cerevisiae)  PACS2 - phosphofurin acidic cluster sorting protein 2  RAB15 - rab15, member ras oncogene family  TBC1D25 - tbc1 domain family, member 25  SPATA7 - spermatogenesis associated 7  KLHL21 - kelch-like family member 21  DCP1A - decapping mrna 1a  AP5Z1 - adaptor-related protein complex 5, zeta 1 subunit  SCN3B - sodium channel, voltage-gated, type iii, beta subunit  BICD2 - bicaudal d homolog 2 (drosophila)  ZDHHC22 - zinc finger, dhhc-type containing 22  VPS26B - vacuolar protein sorting 26 homolog b (s. pombe)  SGSM2 - small g protein signaling modulator 2  MGRN1 - mahogunin ring finger 1, e3 ubiquitin protein ligase  ARHGAP44 - rho gtpase activating protein 44  ODF2 - outer dense fiber of sperm tails 2  KIF13B - kinesin family member 13b  TMED5 - transmembrane emp24 protein transport domain containing 5  OSBPL2 - oxysterol binding protein-like 2  SMG6 - smg6 nonsense mediated mrna decay factor  FBXW11 - f-box and wd repeat domain containing 11  EXOSC3 - exosome component 3  TNKS2 - tankyrase, trf1-interacting ankyrin-related adp-ribose polymerase 2  VPS11 - vacuolar protein sorting 11 homolog (s. cerevisiae)  SMG7 - smg7 nonsense mediated mrna decay factor  FAM208A - family with sequence similarity 208, member a  OPA1 - optic atrophy 1 (autosomal dominant)  CLEC16A - c-type lectin domain family 16, member a  TMED7 - transmembrane emp24 protein transport domain containing 7  UBXN6 - ubx domain protein 6  TECPR2 - tectonin beta-propeller repeat containing 2  DYNLT1 - dynein, light chain, tctex-type 1  STX1B - syntaxin 1b  NUP160 - nucleoporin 160kda  IFT43 - intraflagellar transport 43 homolog (chlamydomonas)  FIS1 - fission 1 (mitochondrial outer membrane) homolog (s. cerevisiae)  CKAP5 - cytoskeleton associated protein 5  HAUS5 - haus augmin-like complex, subunit 5  NSF - n-ethylmaleimide-sensitive factor  FGF13 - fibroblast growth factor 13  SPCS2 - signal peptidase complex subunit 2 homolog (s. cerevisiae)  ESYT1 - extended synaptotagmin-like protein 1  FGF9 - fibroblast growth factor 9  BCAP29 - b-cell receptor-associated protein 29  TOMM20 - translocase of outer mitochondrial membrane 20 homolog (yeast)  VPS39 - vacuolar protein sorting 39 homolog (s. cerevisiae)  NSFL1C - nsfl1 (p97) cofactor (p47)  NUMA1 - nuclear mitotic apparatus protein 1  EIF4A3 - eukaryotic translation initiation factor 4a3  NUP88 - nucleoporin 88kda  CLASP1 - cytoplasmic linker associated protein 1  NUP98 - nucleoporin 98kda  ATG13 - autophagy related 13  TM9SF4 - transmembrane 9 superfamily protein member 4  SZT2 - seizure threshold 2 homolog (mouse)  TAP2 - transporter 2, atp-binding cassette, sub-family b (mdr/tap)  TBC1D30 - tbc1 domain family, member 30  TMEM167A - transmembrane protein 167a  RIMS3 - regulating synaptic membrane exocytosis 3  SNX17 - sorting nexin 17  DHX38 - deah (asp-glu-ala-his) box polypeptide 38  FAM45A - family with sequence similarity 45, member a  TBC1D5 - tbc1 domain family, member 5  CEP120 - centrosomal protein 120kda  PIP5K1C - phosphatidylinositol-4-phosphate 5-kinase, type i, gamma  RGP1 - rgp1 retrograde golgi transport homolog (s. cerevisiae)  NMD3 - nmd3 ribosome export adaptor  NPM1 - nucleophosmin (nucleolar phosphoprotein b23, numatrin)  NPHP1 - nephronophthisis 1 (juvenile)  NUDCD3 - nudc domain containing 3  SCC4 - sister chromatid cohesion 4  SPICE1 - spindle and centriole associated protein 1  SFI1 - sfi1 homolog, spindle assembly associated (yeast)  TRIM9 - tripartite motif containing 9  DUSP16 - dual specificity phosphatase 16  OBSL1 - obscurin-like 1  IFT52 - intraflagellar transport 52 homolog (chlamydomonas)  PAQR3 - progestin and adipoq receptor family member iii  RCC2 - regulator of chromosome condensation 2  GRIP2 - glutamate receptor interacting protein 2  NUDT7 - nudix (nucleoside diphosphate linked moiety x)-type motif 7  ERBB2IP - erbb2 interacting protein  RAB11FIP3 - rab11 family interacting protein 3 (class ii)  SPG11 - spastic paraplegia 11 (autosomal recessive)  TMED3 - transmembrane emp24 protein transport domain containing 3  MTOR - mechanistic target of rapamycin (serine/threonine kinase)  TNPO2 - transportin 2  BLOC1S2 - biogenesis of lysosomal organelles complex-1, subunit 2  IFT74 - intraflagellar transport 74 homolog (chlamydomonas)  ATP13A2 - atpase type 13a2  HERPUD1 - homocysteine-inducible, endoplasmic reticulum stress-inducible, ubiquitin-like domain member 1  USP6NL - usp6 n-terminal like  MYO19 - myosin xix  SEC62 - sec62 homolog (s. cerevisiae)  CEP290 - centrosomal protein 290kda  EXOSC2 - exosome component 2  RIMS2 - regulating synaptic membrane exocytosis 2  PACSIN1 - protein kinase c and casein kinase substrate in neurons 1  CEP57 - centrosomal protein 57kda  PQLC1 - pq loop repeat containing 1  TRAPPC10 - trafficking protein particle complex 10  THOC7 - tho complex 7 homolog (drosophila)  ZFYVE16 - zinc finger, fyve domain containing 16  CENPT - centromere protein t  ICMT - isoprenylcysteine carboxyl methyltransferase  NFKBIA - nuclear factor of kappa light polypeptide gene enhancer in b-cells inhibitor, alpha  TRAM1 - translocation associated membrane protein 1  SNPH - syntaphilin  PIK3R4 - phosphoinositide-3-kinase, regulatory subunit 4  IFT140 - intraflagellar transport 140 homolog (chlamydomonas)  TNFRSF1A - tumor necrosis factor receptor superfamily, member 1a  TBCK - tbc1 domain containing kinase  EHD2 - eh-domain containing 2  EHD3 - eh-domain containing 3  EHD4 - eh-domain containing 4  CCP110 - centriolar coiled coil protein 110kda  IQCB1 - iq motif containing b1  KCNIP4 - kv channel interacting protein 4  EIF4ENIF1 - eukaryotic translation initiation factor 4e nuclear import factor 1  NUP188 - nucleoporin 188kda  DNAJC5 - dnaj (hsp40) homolog, subfamily c, member 5  GCC2 - grip and coiled-coil domain containing 2  MRAP2 - melanocortin 2 receptor accessory protein 2  NEFL - neurofilament, light polypeptide  TERF1 - telomeric repeat binding factor (nima-interacting) 1  TERF2 - telomeric repeat binding factor 2  WRAP73 - wd repeat containing, antisense to tp73  SNX18 - sorting nexin 18  CEP70 - centrosomal protein 70kda  LEPROTL1 - leptin receptor overlapping transcript-like 1  ITSN2 - intersectin 2  BCCIP - brca2 and cdkn1a interacting protein  FEZ1 - fasciculation and elongation protein zeta 1 (zygin i)  PES1 - pescadillo ribosomal biogenesis factor 1  SEC61G - sec61 gamma subunit  NUP155 - nucleoporin 155kda  TGFA - transforming growth factor, alpha  SEC24C - sec24 family, member c (s. cerevisiae)  CEP41 - centrosomal protein 41kda  HEY2 - hairy/enhancer-of-split related with yrpw motif 2  FAM91A1 - family with sequence similarity 91, member a1  MACF1 - microtubule-actin crosslinking factor 1  CROCC - ciliary rootlet coiled-coil, rootletin  VPS37D - vacuolar protein sorting 37 homolog d (s. cerevisiae)  RAPGEF2 - rap guanine nucleotide exchange factor (gef) 2  TGFB1 - transforming growth factor, beta 1  C16orf70 - chromosome 16 open reading frame 70  TSPAN15 - tetraspanin 15  FAM21A - family with sequence similarity 21, member a  NUP93 - nucleoporin 93kda  CEP63 - centrosomal protein 63kda  CALY - calcyon neuron-specific vesicular protein  GEMIN4 - gem (nuclear organelle) associated protein 4  ACAP2 - arfgap with coiled-coil, ankyrin repeat and ph domains 2  WDR45B - wd repeat domain 45b  SYTL4 - synaptotagmin-like 4  TIA1 - tia1 cytotoxic granule-associated rna binding protein  THY1 - thy-1 cell surface antigen  TNPO3 - transportin 3 |
| GO:0022406 | membrane docking | 7.45E-9 | 2.47E-6 | 1.30 (10334,149,6996,131) | [+] Show genes  MAPRE1 - microtubule-associated protein, rp/eb family, member 1  AKAP9 - a kinase (prka) anchor protein (yotiao) 9  B9D2 - b9 protein domain 2  CEP89 - centrosomal protein 89kda  SCFD1 - sec1 family domain containing 1  RAB8B - rab8b, member ras oncogene family  MKS1 - meckel syndrome, type 1  HAUS7 - haus augmin-like complex, subunit 7  RAB3IP - rab3a interacting protein  DCTN1 - dynactin 1  PSEN2 - presenilin 2 (alzheimer disease 4)  DCTN3 - dynactin 3 (p22)  PACS2 - phosphofurin acidic cluster sorting protein 2  CHP1 - calcineurin-like ef-hand protein 1  RAB15 - rab15, member ras oncogene family  TUBG1 - tubulin, gamma 1  RAB3D - rab3d, member ras oncogene family  YKT6 - ykt6 v-snare homolog (s. cerevisiae)  ODF2 - outer dense fiber of sperm tails 2  PPFIA3 - protein tyrosine phosphatase, receptor type, f polypeptide (ptprf), interacting protein (liprin), alpha 3  PAFAH1B1 - platelet-activating factor acetylhydrolase 1b, regulatory subunit 1 (45kda)  AZI1 - 5-azacytidine induced 1  VPS11 - vacuolar protein sorting 11 homolog (s. cerevisiae)  STX1B - syntaxin 1b  MARK4 - map/microtubule affinity-regulating kinase 4  CEP250 - centrosomal protein 250kda  TCTN1 - tectonic family member 1  NDE1 - nude neurodevelopment protein 1  CKAP5 - cytoskeleton associated protein 5  HAUS5 - haus augmin-like complex, subunit 5  NSF - n-ethylmaleimide-sensitive factor  ESYT1 - extended synaptotagmin-like protein 1  DCTN2 - dynactin 2 (p50)  TUBA1A - tubulin, alpha 1a  CETN2 - centrin, ef-hand protein, 2  VAPB - vamp (vesicle-associated membrane protein)-associated protein b and c  ALMS1 - alstrom syndrome 1  RAB10 - rab10, member ras oncogene family  CLASP1 - cytoplasmic linker associated protein 1  ESYT2 - extended synaptotagmin-like protein 2  SNAP25 - synaptosomal-associated protein, 25kda  HAUS6 - haus augmin-like complex, subunit 6  STX6 - syntaxin 6  AHI1 - abelson helper integration site 1  UNC13A - unc-13 homolog a (c. elegans)  RABEPK - rab9 effector protein with kelch motifs  RAB8A - rab8a, member ras oncogene family  ROCK1 - rho-associated, coiled-coil containing protein kinase 1  CEP78 - centrosomal protein 78kda  C2CD3 - c2 calcium-dependent domain containing 3  NPHP1 - nephronophthisis 1 (juvenile)  PCM1 - pericentriolar material 1  SFI1 - sfi1 homolog, spindle assembly associated (yeast)  HSP90AA1 - heat shock protein 90kda alpha (cytosolic), class a member 1  STX8 - syntaxin 8  CEP76 - centrosomal protein 76kda  ACTR1A - arp1 actin-related protein 1 homolog a, centractin alpha (yeast)  USO1 - uso1 vesicle transport factor  SCLT1 - sodium channel and clathrin linker 1  FBF1 - fas (tnfrsf6) binding factor 1  STX12 - syntaxin 12  HAUS2 - haus augmin-like complex, subunit 2  CDK5RAP2 - cdk5 regulatory subunit associated protein 2  PRKACA - protein kinase, camp-dependent, catalytic, alpha  TCTN3 - tectonic family member 3  HAUS3 - haus augmin-like complex, subunit 3  SYT1 - synaptotagmin i  CEP290 - centrosomal protein 290kda  DYNLL1 - dynein, light chain, lc8-type 1  CTBP2 - c-terminal binding protein 2  FGFR1OP - fgfr1 oncogene partner  CEP57 - centrosomal protein 57kda  CCDC41 - coiled-coil domain containing 41  CNTRL - centriolin  RAB3C - rab3c, member ras oncogene family  ATG14 - autophagy related 14  STXBP1 - syntaxin binding protein 1  STX5 - syntaxin 5  STX4 - syntaxin 4  STX3 - syntaxin 3  RAB7A - rab7a, member ras oncogene family  STX1A - syntaxin 1a (brain)  RAB3A - rab3a, member ras oncogene family  RAB3B - rab3b, member ras oncogene family  NDRG4 - ndrg family member 4  STX17 - syntaxin 17  EZR - ezrin  PPP2R1A - protein phosphatase 2, regulatory subunit a, alpha  CPLX2 - complexin 2  SNPH - syntaphilin  STXBP3 - syntaxin binding protein 3  TTBK2 - tau tubulin kinase 2  UNC13B - unc-13 homolog b (c. elegans)  TCTN2 - tectonic family member 2  VPS33A - vacuolar protein sorting 33 homolog a (s. cerevisiae)  VCAM1 - vascular cell adhesion molecule 1  PEX16 - peroxisomal biogenesis factor 16  EXOC4 - exocyst complex component 4  VTI1B - vesicle transport through interaction with t-snares 1b  CCP110 - centriolar coiled coil protein 110kda  MSN - moesin  IQCB1 - iq motif containing b1  SNX3 - sorting nexin 3  TMEM216 - transmembrane protein 216  TSNARE1 - t-snare domain containing 1  VPS18 - vacuolar protein sorting 18 homolog (s. cerevisiae)  TUBB - tubulin, beta class i  BLOC1S6 - biogenesis of lysosomal organelles complex-1, subunit 6, pallidin  EXOC6 - exocyst complex component 6  CEP70 - centrosomal protein 70kda  PDZD8 - pdz domain containing 8  VPS33B - vacuolar protein sorting 33 homolog b (yeast)  TUBB4B - tubulin, beta 4b class ivb  STX7 - syntaxin 7  CEP41 - centrosomal protein 41kda  DYNC1H1 - dynein, cytoplasmic 1, heavy chain 1  DYNC1I2 - dynein, cytoplasmic 1, intermediate chain 2  RAB11A - rab11a, member ras oncogene family  CAV2 - caveolin 2  CEP63 - centrosomal protein 63kda  SNAP29 - synaptosomal-associated protein, 29kda  HAUS1 - haus augmin-like complex, subunit 1  VAMP3 - vesicle-associated membrane protein 3  CSNK1D - casein kinase 1, delta  B9D1 - b9 protein domain 1  STX2 - syntaxin 2  PRKAR2B - protein kinase, camp-dependent, regulatory, type ii, beta  YWHAG - tyrosine 3-monooxygenase/tryptophan 5-monooxygenase activation protein, gamma polypeptide  EXOC6B - exocyst complex component 6b  VPS45 - vacuolar protein sorting 45 homolog (s. cerevisiae)  YWHAE - tyrosine 3-monooxygenase/tryptophan 5-monooxygenase activation protein, epsilon polypeptide |
| GO:0006405 | RNA export from nucleus | 7.45E-9 | 2.41E-6 | 1.34 (10334,115,6996,104) | [+] Show genes  DDX19B - dead (asp-glu-ala-asp) box polypeptide 19b  NCBP2 - nuclear cap binding protein subunit 2, 20kda  NCBP1 - nuclear cap binding protein subunit 1, 80kda  SRSF11 - serine/arginine-rich splicing factor 11  SEC13 - sec13 homolog (s. cerevisiae)  TSC1 - tuberous sclerosis 1  SDAD1 - sda1 domain containing 1  SYMPK - symplekin  DDX25 - dead (asp-glu-ala-asp) box helicase 25  ALKBH5 - alkb, alkylation repair homolog 5 (e. coli)  AGFG1 - arfgap with fg repeats 1  UPF1 - upf1 regulator of nonsense transcripts homolog (yeast)  XPOT - exportin, trna  PHAX - phosphorylated adaptor for rna export  LTV1 - ltv1 homolog (s. cerevisiae)  CPSF3 - cleavage and polyadenylation specific factor 3, 73kda  EIF4E - eukaryotic translation initiation factor 4e  NOP9 - nop9 nucleolar protein  ALYREF - aly/ref export factor  PABPN1 - poly(a) binding protein, nuclear 1  RNPS1 - rna binding protein s1, serine-rich domain  SMG6 - smg6 nonsense mediated mrna decay factor  SMG7 - smg7 nonsense mediated mrna decay factor  NUP160 - nucleoporin 160kda  FIP1L1 - factor interacting with papola and cpsf1  ABCE1 - atp-binding cassette, sub-family e (oabp), member 1  SEH1L - seh1-like (s. cerevisiae)  EIF4A3 - eukaryotic translation initiation factor 4a3  NUP88 - nucleoporin 88kda  NUP98 - nucleoporin 98kda  GLE1 - gle1 rna export mediator  CPSF4 - cleavage and polyadenylation specific factor 4, 30kda  U2AF1L4 - u2 small nuclear rna auxiliary factor 1-like 4  DHX38 - deah (asp-glu-ala-his) box polypeptide 38  CPSF1 - cleavage and polyadenylation specific factor 1, 160kda  CPSF2 - cleavage and polyadenylation specific factor 2, 100kda  MCM3AP - minichromosome maintenance complex component 3 associated protein  RBM15B - rna binding motif protein 15b  NUP85 - nucleoporin 85kda  EIF6 - eukaryotic translation initiation factor 6  SLU7 - slu7 splicing factor homolog (s. cerevisiae)  XPO5 - exportin 5  NPM1 - nucleophosmin (nucleolar phosphoprotein b23, numatrin)  NMD3 - nmd3 ribosome export adaptor  SMG1 - smg1 phosphatidylinositol 3-kinase-related kinase  SRRM1 - serine/arginine repetitive matrix 1  MAGOHB - mago-nashi homolog b (drosophila)  ENY2 - enhancer of yellow 2 homolog (drosophila)  POLR2D - polymerase (rna) ii (dna directed) polypeptide d  CDC40 - cell division cycle 40  TPR - translocated promoter region, nuclear basket protein  DDX19A - dead (asp-glu-ala-asp) box polypeptide 19a  MAGOH - mago-nashi homolog, proliferation-associated (drosophila)  NUP133 - nucleoporin 133kda  XPO1 - exportin 1 (crm1 homolog, yeast)  NUP107 - nucleoporin 107kda  NUPL2 - nucleoporin like 2  NUP50 - nucleoporin 50kda  LSG1 - large 60s subunit nuclear export gtpase 1  SRSF9 - serine/arginine-rich splicing factor 9  THOC7 - tho complex 7 homolog (drosophila)  NUP43 - nucleoporin 43kda  PCID2 - pci domain containing 2  SLBP - stem-loop binding protein  CHTOP - chromatin target of prmt1  WDR33 - wd repeat domain 33  NOL6 - nucleolar protein 6 (rna-associated)  FYTTD1 - forty-two-three domain containing 1  UPF3B - upf3 regulator of nonsense transcripts homolog b (yeast)  DDX39B - dead (asp-glu-ala-asp) box polypeptide 39b  NUP188 - nucleoporin 188kda  NUP205 - nucleoporin 205kda  RANBP2 - ran binding protein 2  POLDIP3 - polymerase (dna-directed), delta interacting protein 3  YTHDC1 - yth domain containing 1  THOC2 - tho complex 2  THOC6 - tho complex 6 homolog (drosophila)  AAAS - achalasia, adrenocortical insufficiency, alacrimia  RPS15 - ribosomal protein s15  NUP155 - nucleoporin 155kda  RBM8A - rna binding motif protein 8a  CASC3 - cancer susceptibility candidate 3  HNRNPA2B1 - heterogeneous nuclear ribonucleoprotein a2/b1  SRSF1 - serine/arginine-rich splicing factor 1  U2AF2 - u2 small nuclear rna auxiliary factor 2  HNRNPA1 - heterogeneous nuclear ribonucleoprotein a1  SRSF2 - serine/arginine-rich splicing factor 2  SRSF3 - serine/arginine-rich splicing factor 3  SRSF4 - serine/arginine-rich splicing factor 4  RRS1 - rrs1 ribosome biogenesis regulator homolog (s. cerevisiae)  NUP93 - nucleoporin 93kda  SRSF5 - serine/arginine-rich splicing factor 5  SRSF6 - serine/arginine-rich splicing factor 6  RAE1 - rae1 rna export 1 homolog (s. pombe)  SRSF7 - serine/arginine-rich splicing factor 7  NDC1 - ndc1 transmembrane nucleoporin  NUP35 - nucleoporin 35kda  SSB - sjogren syndrome antigen b (autoantigen la)  THOC1 - tho complex 1  THOC3 - tho complex 3  NUP214 - nucleoporin 214kda  SARNP - sap domain containing ribonucleoprotein  NUP153 - nucleoporin 153kda  NUP210 - nucleoporin 210kda |
| GO:0050658 | RNA transport | 8.02E-9 | 2.54E-6 | 1.29 (10334,158,6996,138) | [+] Show genes  DHX9 - deah (asp-glu-ala-his) box helicase 9  DDX19B - dead (asp-glu-ala-asp) box polypeptide 19b  NCBP2 - nuclear cap binding protein subunit 2, 20kda  SRSF11 - serine/arginine-rich splicing factor 11  NCBP1 - nuclear cap binding protein subunit 1, 80kda  FMR1 - fragile x mental retardation 1  SEC13 - sec13 homolog (s. cerevisiae)  SDAD1 - sda1 domain containing 1  TSC1 - tuberous sclerosis 1  SYMPK - symplekin  DDX25 - dead (asp-glu-ala-asp) box helicase 25  ZFP36L1 - zfp36 ring finger protein-like 1  ARC - activity-regulated cytoskeleton-associated protein  ALKBH5 - alkb, alkylation repair homolog 5 (e. coli)  AGFG1 - arfgap with fg repeats 1  UPF1 - upf1 regulator of nonsense transcripts homolog (yeast)  XPOT - exportin, trna  PHAX - phosphorylated adaptor for rna export  LTV1 - ltv1 homolog (s. cerevisiae)  CPSF3 - cleavage and polyadenylation specific factor 3, 73kda  EIF4E - eukaryotic translation initiation factor 4e  AHCTF1 - at hook containing transcription factor 1  NOP9 - nop9 nucleolar protein  G3BP2 - gtpase activating protein (sh3 domain) binding protein 2  BICD2 - bicaudal d homolog 2 (drosophila)  ALYREF - aly/ref export factor  PABPN1 - poly(a) binding protein, nuclear 1  RNPS1 - rna binding protein s1, serine-rich domain  SMG6 - smg6 nonsense mediated mrna decay factor  NUTF2 - nuclear transport factor 2  SMG7 - smg7 nonsense mediated mrna decay factor  FIP1L1 - factor interacting with papola and cpsf1  NUP160 - nucleoporin 160kda  FLOT1 - flotillin 1  CKAP5 - cytoskeleton associated protein 5  ABCE1 - atp-binding cassette, sub-family e (oabp), member 1  SEH1L - seh1-like (s. cerevisiae)  ATXN2 - ataxin 2  CETN2 - centrin, ef-hand protein, 2  TOMM20 - translocase of outer mitochondrial membrane 20 homolog (yeast)  CETN3 - centrin, ef-hand protein, 3  SNUPN - snurportin 1  EIF4A3 - eukaryotic translation initiation factor 4a3  NUP88 - nucleoporin 88kda  NUP98 - nucleoporin 98kda  KHSRP - kh-type splicing regulatory protein  GLE1 - gle1 rna export mediator  CPSF4 - cleavage and polyadenylation specific factor 4, 30kda  U2AF1L4 - u2 small nuclear rna auxiliary factor 1-like 4  MCM3AP - minichromosome maintenance complex component 3 associated protein  CPSF2 - cleavage and polyadenylation specific factor 2, 100kda  CPSF1 - cleavage and polyadenylation specific factor 1, 160kda  DHX38 - deah (asp-glu-ala-his) box polypeptide 38  RBM15B - rna binding motif protein 15b  NUP85 - nucleoporin 85kda  EIF6 - eukaryotic translation initiation factor 6  SLU7 - slu7 splicing factor homolog (s. cerevisiae)  XPO5 - exportin 5  NMD3 - nmd3 ribosome export adaptor  NPM1 - nucleophosmin (nucleolar phosphoprotein b23, numatrin)  SMG1 - smg1 phosphatidylinositol 3-kinase-related kinase  SRRM1 - serine/arginine repetitive matrix 1  MAGOHB - mago-nashi homolog b (drosophila)  ENY2 - enhancer of yellow 2 homolog (drosophila)  LRPPRC - leucine-rich pentatricopeptide repeat containing  POLR2D - polymerase (rna) ii (dna directed) polypeptide d  CDC40 - cell division cycle 40  TPR - translocated promoter region, nuclear basket protein  SIDT2 - sid1 transmembrane family, member 2  PURA - purine-rich element binding protein a  DDX19A - dead (asp-glu-ala-asp) box polypeptide 19a  SENP2 - sumo1/sentrin/smt3 specific peptidase 2  MAGOH - mago-nashi homolog, proliferation-associated (drosophila)  NUP133 - nucleoporin 133kda  KIF5C - kinesin family member 5c  NUP107 - nucleoporin 107kda  XPO1 - exportin 1 (crm1 homolog, yeast)  NUPL2 - nucleoporin like 2  SUPT6H - suppressor of ty 6 homolog (s. cerevisiae)  NUP50 - nucleoporin 50kda  TNKS - tankyrase, trf1-interacting ankyrin-related adp-ribose polymerase  PARP11 - poly (adp-ribose) polymerase family, member 11  LSG1 - large 60s subunit nuclear export gtpase 1  SRSF9 - serine/arginine-rich splicing factor 9  THOC7 - tho complex 7 homolog (drosophila)  NUP43 - nucleoporin 43kda  PCID2 - pci domain containing 2  SLBP - stem-loop binding protein  CHTOP - chromatin target of prmt1  WDR33 - wd repeat domain 33  NOL6 - nucleolar protein 6 (rna-associated)  ZC3H3 - zinc finger ccch-type containing 3  FYTTD1 - forty-two-three domain containing 1  UPF3B - upf3 regulator of nonsense transcripts homolog b (yeast)  UPF3A - upf3 regulator of nonsense transcripts homolog a (yeast)  DDX39B - dead (asp-glu-ala-asp) box polypeptide 39b  NUP188 - nucleoporin 188kda  RANBP2 - ran binding protein 2  NUP205 - nucleoporin 205kda  POLDIP3 - polymerase (dna-directed), delta interacting protein 3  PNPT1 - polyribonucleotide nucleotidyltransferase 1  IWS1 - iws1 homolog (s. cerevisiae)  YTHDC1 - yth domain containing 1  THOC2 - tho complex 2  THOC6 - tho complex 6 homolog (drosophila)  TERF2 - telomeric repeat binding factor 2  RFTN2 - raftlin family member 2  HNRNPU - heterogeneous nuclear ribonucleoprotein u (scaffold attachment factor a)  AAAS - achalasia, adrenocortical insufficiency, alacrimia  RPS15 - ribosomal protein s15  NUP155 - nucleoporin 155kda  RBM8A - rna binding motif protein 8a  CASC3 - cancer susceptibility candidate 3  SFPQ - splicing factor proline/glutamine-rich  HNRNPA2B1 - heterogeneous nuclear ribonucleoprotein a2/b1  SRSF1 - serine/arginine-rich splicing factor 1  U2AF2 - u2 small nuclear rna auxiliary factor 2  HNRNPA1 - heterogeneous nuclear ribonucleoprotein a1  SRSF2 - serine/arginine-rich splicing factor 2  SRSF3 - serine/arginine-rich splicing factor 3  NUP93 - nucleoporin 93kda  SRSF4 - serine/arginine-rich splicing factor 4  RRS1 - rrs1 ribosome biogenesis regulator homolog (s. cerevisiae)  SRSF5 - serine/arginine-rich splicing factor 5  RAE1 - rae1 rna export 1 homolog (s. pombe)  SRSF6 - serine/arginine-rich splicing factor 6  SRSF7 - serine/arginine-rich splicing factor 7  TGFBR2 - transforming growth factor, beta receptor ii (70/80kda)  NUP35 - nucleoporin 35kda  SSB - sjogren syndrome antigen b (autoantigen la)  NDC1 - ndc1 transmembrane nucleoporin  THOC1 - tho complex 1  THOC3 - tho complex 3  MRPL18 - mitochondrial ribosomal protein l18  NUP214 - nucleoporin 214kda  SARNP - sap domain containing ribonucleoprotein  NUP153 - nucleoporin 153kda  NUP210 - nucleoporin 210kda |
| GO:0050657 | nucleic acid transport | 8.02E-9 | 2.48E-6 | 1.29 (10334,158,6996,138) | [+] Show genes  DHX9 - deah (asp-glu-ala-his) box helicase 9  DDX19B - dead (asp-glu-ala-asp) box polypeptide 19b  NCBP2 - nuclear cap binding protein subunit 2, 20kda  FMR1 - fragile x mental retardation 1  NCBP1 - nuclear cap binding protein subunit 1, 80kda  SRSF11 - serine/arginine-rich splicing factor 11  TSC1 - tuberous sclerosis 1  SDAD1 - sda1 domain containing 1  SEC13 - sec13 homolog (s. cerevisiae)  SYMPK - symplekin  DDX25 - dead (asp-glu-ala-asp) box helicase 25  ZFP36L1 - zfp36 ring finger protein-like 1  ARC - activity-regulated cytoskeleton-associated protein  ALKBH5 - alkb, alkylation repair homolog 5 (e. coli)  AGFG1 - arfgap with fg repeats 1  UPF1 - upf1 regulator of nonsense transcripts homolog (yeast)  XPOT - exportin, trna  PHAX - phosphorylated adaptor for rna export  LTV1 - ltv1 homolog (s. cerevisiae)  CPSF3 - cleavage and polyadenylation specific factor 3, 73kda  EIF4E - eukaryotic translation initiation factor 4e  AHCTF1 - at hook containing transcription factor 1  NOP9 - nop9 nucleolar protein  G3BP2 - gtpase activating protein (sh3 domain) binding protein 2  BICD2 - bicaudal d homolog 2 (drosophila)  ALYREF - aly/ref export factor  PABPN1 - poly(a) binding protein, nuclear 1  RNPS1 - rna binding protein s1, serine-rich domain  SMG6 - smg6 nonsense mediated mrna decay factor  NUTF2 - nuclear transport factor 2  SMG7 - smg7 nonsense mediated mrna decay factor  NUP160 - nucleoporin 160kda  FIP1L1 - factor interacting with papola and cpsf1  CKAP5 - cytoskeleton associated protein 5  FLOT1 - flotillin 1  ABCE1 - atp-binding cassette, sub-family e (oabp), member 1  ATXN2 - ataxin 2  SEH1L - seh1-like (s. cerevisiae)  CETN2 - centrin, ef-hand protein, 2  TOMM20 - translocase of outer mitochondrial membrane 20 homolog (yeast)  CETN3 - centrin, ef-hand protein, 3  SNUPN - snurportin 1  EIF4A3 - eukaryotic translation initiation factor 4a3  NUP88 - nucleoporin 88kda  NUP98 - nucleoporin 98kda  KHSRP - kh-type splicing regulatory protein  GLE1 - gle1 rna export mediator  CPSF4 - cleavage and polyadenylation specific factor 4, 30kda  U2AF1L4 - u2 small nuclear rna auxiliary factor 1-like 4  MCM3AP - minichromosome maintenance complex component 3 associated protein  CPSF2 - cleavage and polyadenylation specific factor 2, 100kda  CPSF1 - cleavage and polyadenylation specific factor 1, 160kda  DHX38 - deah (asp-glu-ala-his) box polypeptide 38  RBM15B - rna binding motif protein 15b  NUP85 - nucleoporin 85kda  EIF6 - eukaryotic translation initiation factor 6  SLU7 - slu7 splicing factor homolog (s. cerevisiae)  XPO5 - exportin 5  NMD3 - nmd3 ribosome export adaptor  NPM1 - nucleophosmin (nucleolar phosphoprotein b23, numatrin)  SMG1 - smg1 phosphatidylinositol 3-kinase-related kinase  SRRM1 - serine/arginine repetitive matrix 1  MAGOHB - mago-nashi homolog b (drosophila)  ENY2 - enhancer of yellow 2 homolog (drosophila)  LRPPRC - leucine-rich pentatricopeptide repeat containing  POLR2D - polymerase (rna) ii (dna directed) polypeptide d  CDC40 - cell division cycle 40  TPR - translocated promoter region, nuclear basket protein  SIDT2 - sid1 transmembrane family, member 2  PURA - purine-rich element binding protein a  DDX19A - dead (asp-glu-ala-asp) box polypeptide 19a  SENP2 - sumo1/sentrin/smt3 specific peptidase 2  MAGOH - mago-nashi homolog, proliferation-associated (drosophila)  NUP133 - nucleoporin 133kda  KIF5C - kinesin family member 5c  NUP107 - nucleoporin 107kda  XPO1 - exportin 1 (crm1 homolog, yeast)  NUPL2 - nucleoporin like 2  SUPT6H - suppressor of ty 6 homolog (s. cerevisiae)  NUP50 - nucleoporin 50kda  TNKS - tankyrase, trf1-interacting ankyrin-related adp-ribose polymerase  PARP11 - poly (adp-ribose) polymerase family, member 11  LSG1 - large 60s subunit nuclear export gtpase 1  THOC7 - tho complex 7 homolog (drosophila)  SRSF9 - serine/arginine-rich splicing factor 9  NUP43 - nucleoporin 43kda  PCID2 - pci domain containing 2  SLBP - stem-loop binding protein  CHTOP - chromatin target of prmt1  WDR33 - wd repeat domain 33  NOL6 - nucleolar protein 6 (rna-associated)  ZC3H3 - zinc finger ccch-type containing 3  FYTTD1 - forty-two-three domain containing 1  UPF3B - upf3 regulator of nonsense transcripts homolog b (yeast)  DDX39B - dead (asp-glu-ala-asp) box polypeptide 39b  UPF3A - upf3 regulator of nonsense transcripts homolog a (yeast)  NUP188 - nucleoporin 188kda  IWS1 - iws1 homolog (s. cerevisiae)  PNPT1 - polyribonucleotide nucleotidyltransferase 1  POLDIP3 - polymerase (dna-directed), delta interacting protein 3  NUP205 - nucleoporin 205kda  RANBP2 - ran binding protein 2  YTHDC1 - yth domain containing 1  THOC2 - tho complex 2  THOC6 - tho complex 6 homolog (drosophila)  RFTN2 - raftlin family member 2  TERF2 - telomeric repeat binding factor 2  HNRNPU - heterogeneous nuclear ribonucleoprotein u (scaffold attachment factor a)  AAAS - achalasia, adrenocortical insufficiency, alacrimia  RPS15 - ribosomal protein s15  NUP155 - nucleoporin 155kda  RBM8A - rna binding motif protein 8a  CASC3 - cancer susceptibility candidate 3  SFPQ - splicing factor proline/glutamine-rich  U2AF2 - u2 small nuclear rna auxiliary factor 2  SRSF1 - serine/arginine-rich splicing factor 1  HNRNPA2B1 - heterogeneous nuclear ribonucleoprotein a2/b1  SRSF2 - serine/arginine-rich splicing factor 2  HNRNPA1 - heterogeneous nuclear ribonucleoprotein a1  SRSF3 - serine/arginine-rich splicing factor 3  RRS1 - rrs1 ribosome biogenesis regulator homolog (s. cerevisiae)  SRSF4 - serine/arginine-rich splicing factor 4  NUP93 - nucleoporin 93kda  SRSF5 - serine/arginine-rich splicing factor 5  SRSF6 - serine/arginine-rich splicing factor 6  RAE1 - rae1 rna export 1 homolog (s. pombe)  SRSF7 - serine/arginine-rich splicing factor 7  TGFBR2 - transforming growth factor, beta receptor ii (70/80kda)  NDC1 - ndc1 transmembrane nucleoporin  SSB - sjogren syndrome antigen b (autoantigen la)  NUP35 - nucleoporin 35kda  THOC1 - tho complex 1  THOC3 - tho complex 3  MRPL18 - mitochondrial ribosomal protein l18  NUP214 - nucleoporin 214kda  SARNP - sap domain containing ribonucleoprotein  NUP153 - nucleoporin 153kda  NUP210 - nucleoporin 210kda |
| GO:0051236 | establishment of RNA localization | 8.02E-9 | 2.42E-6 | 1.29 (10334,158,6996,138) | [+] Show genes  DHX9 - deah (asp-glu-ala-his) box helicase 9  DDX19B - dead (asp-glu-ala-asp) box polypeptide 19b  NCBP2 - nuclear cap binding protein subunit 2, 20kda  FMR1 - fragile x mental retardation 1  NCBP1 - nuclear cap binding protein subunit 1, 80kda  SRSF11 - serine/arginine-rich splicing factor 11  TSC1 - tuberous sclerosis 1  SDAD1 - sda1 domain containing 1  SEC13 - sec13 homolog (s. cerevisiae)  SYMPK - symplekin  DDX25 - dead (asp-glu-ala-asp) box helicase 25  ZFP36L1 - zfp36 ring finger protein-like 1  ARC - activity-regulated cytoskeleton-associated protein  ALKBH5 - alkb, alkylation repair homolog 5 (e. coli)  AGFG1 - arfgap with fg repeats 1  UPF1 - upf1 regulator of nonsense transcripts homolog (yeast)  XPOT - exportin, trna  PHAX - phosphorylated adaptor for rna export  LTV1 - ltv1 homolog (s. cerevisiae)  CPSF3 - cleavage and polyadenylation specific factor 3, 73kda  EIF4E - eukaryotic translation initiation factor 4e  AHCTF1 - at hook containing transcription factor 1  NOP9 - nop9 nucleolar protein  G3BP2 - gtpase activating protein (sh3 domain) binding protein 2  BICD2 - bicaudal d homolog 2 (drosophila)  ALYREF - aly/ref export factor  PABPN1 - poly(a) binding protein, nuclear 1  RNPS1 - rna binding protein s1, serine-rich domain  SMG6 - smg6 nonsense mediated mrna decay factor  NUTF2 - nuclear transport factor 2  SMG7 - smg7 nonsense mediated mrna decay factor  NUP160 - nucleoporin 160kda  FIP1L1 - factor interacting with papola and cpsf1  CKAP5 - cytoskeleton associated protein 5  FLOT1 - flotillin 1  ABCE1 - atp-binding cassette, sub-family e (oabp), member 1  ATXN2 - ataxin 2  SEH1L - seh1-like (s. cerevisiae)  CETN2 - centrin, ef-hand protein, 2  TOMM20 - translocase of outer mitochondrial membrane 20 homolog (yeast)  CETN3 - centrin, ef-hand protein, 3  SNUPN - snurportin 1  EIF4A3 - eukaryotic translation initiation factor 4a3  NUP88 - nucleoporin 88kda  NUP98 - nucleoporin 98kda  KHSRP - kh-type splicing regulatory protein  GLE1 - gle1 rna export mediator  CPSF4 - cleavage and polyadenylation specific factor 4, 30kda  U2AF1L4 - u2 small nuclear rna auxiliary factor 1-like 4  MCM3AP - minichromosome maintenance complex component 3 associated protein  CPSF2 - cleavage and polyadenylation specific factor 2, 100kda  CPSF1 - cleavage and polyadenylation specific factor 1, 160kda  DHX38 - deah (asp-glu-ala-his) box polypeptide 38  RBM15B - rna binding motif protein 15b  NUP85 - nucleoporin 85kda  EIF6 - eukaryotic translation initiation factor 6  SLU7 - slu7 splicing factor homolog (s. cerevisiae)  XPO5 - exportin 5  NMD3 - nmd3 ribosome export adaptor  NPM1 - nucleophosmin (nucleolar phosphoprotein b23, numatrin)  SMG1 - smg1 phosphatidylinositol 3-kinase-related kinase  SRRM1 - serine/arginine repetitive matrix 1  MAGOHB - mago-nashi homolog b (drosophila)  ENY2 - enhancer of yellow 2 homolog (drosophila)  LRPPRC - leucine-rich pentatricopeptide repeat containing  POLR2D - polymerase (rna) ii (dna directed) polypeptide d  CDC40 - cell division cycle 40  TPR - translocated promoter region, nuclear basket protein  SIDT2 - sid1 transmembrane family, member 2  PURA - purine-rich element binding protein a  DDX19A - dead (asp-glu-ala-asp) box polypeptide 19a  SENP2 - sumo1/sentrin/smt3 specific peptidase 2  MAGOH - mago-nashi homolog, proliferation-associated (drosophila)  NUP133 - nucleoporin 133kda  KIF5C - kinesin family member 5c  NUP107 - nucleoporin 107kda  XPO1 - exportin 1 (crm1 homolog, yeast)  NUPL2 - nucleoporin like 2  SUPT6H - suppressor of ty 6 homolog (s. cerevisiae)  NUP50 - nucleoporin 50kda  TNKS - tankyrase, trf1-interacting ankyrin-related adp-ribose polymerase  PARP11 - poly (adp-ribose) polymerase family, member 11  LSG1 - large 60s subunit nuclear export gtpase 1  THOC7 - tho complex 7 homolog (drosophila)  SRSF9 - serine/arginine-rich splicing factor 9  NUP43 - nucleoporin 43kda  PCID2 - pci domain containing 2  SLBP - stem-loop binding protein  CHTOP - chromatin target of prmt1  WDR33 - wd repeat domain 33  NOL6 - nucleolar protein 6 (rna-associated)  ZC3H3 - zinc finger ccch-type containing 3  FYTTD1 - forty-two-three domain containing 1  UPF3B - upf3 regulator of nonsense transcripts homolog b (yeast)  DDX39B - dead (asp-glu-ala-asp) box polypeptide 39b  UPF3A - upf3 regulator of nonsense transcripts homolog a (yeast)  NUP188 - nucleoporin 188kda  IWS1 - iws1 homolog (s. cerevisiae)  PNPT1 - polyribonucleotide nucleotidyltransferase 1  POLDIP3 - polymerase (dna-directed), delta interacting protein 3  NUP205 - nucleoporin 205kda  RANBP2 - ran binding protein 2  YTHDC1 - yth domain containing 1  THOC2 - tho complex 2  THOC6 - tho complex 6 homolog (drosophila)  RFTN2 - raftlin family member 2  TERF2 - telomeric repeat binding factor 2  HNRNPU - heterogeneous nuclear ribonucleoprotein u (scaffold attachment factor a)  AAAS - achalasia, adrenocortical insufficiency, alacrimia  RPS15 - ribosomal protein s15  NUP155 - nucleoporin 155kda  RBM8A - rna binding motif protein 8a  CASC3 - cancer susceptibility candidate 3  SFPQ - splicing factor proline/glutamine-rich  U2AF2 - u2 small nuclear rna auxiliary factor 2  SRSF1 - serine/arginine-rich splicing factor 1  HNRNPA2B1 - heterogeneous nuclear ribonucleoprotein a2/b1  SRSF2 - serine/arginine-rich splicing factor 2  HNRNPA1 - heterogeneous nuclear ribonucleoprotein a1  SRSF3 - serine/arginine-rich splicing factor 3  RRS1 - rrs1 ribosome biogenesis regulator homolog (s. cerevisiae)  SRSF4 - serine/arginine-rich splicing factor 4  NUP93 - nucleoporin 93kda  SRSF5 - serine/arginine-rich splicing factor 5  SRSF6 - serine/arginine-rich splicing factor 6  RAE1 - rae1 rna export 1 homolog (s. pombe)  SRSF7 - serine/arginine-rich splicing factor 7  TGFBR2 - transforming growth factor, beta receptor ii (70/80kda)  NDC1 - ndc1 transmembrane nucleoporin  SSB - sjogren syndrome antigen b (autoantigen la)  NUP35 - nucleoporin 35kda  THOC1 - tho complex 1  THOC3 - tho complex 3  MRPL18 - mitochondrial ribosomal protein l18  NUP214 - nucleoporin 214kda  SARNP - sap domain containing ribonucleoprotein  NUP153 - nucleoporin 153kda  NUP210 - nucleoporin 210kda |
| GO:2001141 | regulation of RNA biosynthetic process | 8.52E-9 | 2.52E-6 | 1.08 (10334,1984,6996,1448) | [+] Show genes  ELL - elongation factor rna polymerase ii  EXOSC9 - exosome component 9  ZNF239 - zinc finger protein 239  MSL3 - male-specific lethal 3 homolog (drosophila)  TADA1 - transcriptional adaptor 1  CBX1 - chromobox homolog 1  ABCA2 - atp-binding cassette, sub-family a (abc1), member 2  ABL1 - c-abl oncogene 1, non-receptor tyrosine kinase  ZNF821 - zinc finger protein 821  PHC3 - polyhomeotic homolog 3 (drosophila)  PML - promyelocytic leukemia  MLLT11 - myeloid/lymphoid or mixed-lineage leukemia (trithorax homolog, drosophila); translocated to, 11  SUPT20H - suppressor of ty 20 homolog (s. cerevisiae)  YWHAQ - tyrosine 3-monooxygenase/tryptophan 5-monooxygenase activation protein, theta polypeptide  KAT2A - k(lysine) acetyltransferase 2a  NRIP1 - nuclear receptor interacting protein 1  NCOA3 - nuclear receptor coactivator 3  BRD8 - bromodomain containing 8  WWC2 - ww and c2 domain containing 2  CHD7 - chromodomain helicase dna binding protein 7  LYAR - ly1 antibody reactive  TCERG1 - transcription elongation regulator 1  ZNF354B - zinc finger protein 354b  TAF15 - taf15 rna polymerase ii, tata box binding protein (tbp)-associated factor, 68kda  EHMT2 - euchromatic histone-lysine n-methyltransferase 2  SPIN1 - spindlin 1  TASP1 - taspase, threonine aspartase, 1  SUB1 - sub1 homolog (s. cerevisiae)  MORF4L1 - mortality factor 4 like 1  ZNF511 - zinc finger protein 511  PPID - peptidylprolyl isomerase d  ACTL6A - actin-like 6a  AXIN1 - axin 1  ACTN1 - actinin, alpha 1  TRRAP - transformation/transcription domain-associated protein  PICALM - phosphatidylinositol binding clathrin assembly protein  ACTN4 - actinin, alpha 4  ACVR2A - activin a receptor, type iia  ACVRL1 - activin a receptor type ii-like 1  ACVR1 - activin a receptor, type i  DLL1 - delta-like 1 (drosophila)  ARID1A - at rich interactive domain 1a (swi-like)  ACVR1B - activin a receptor, type ib  SETD6 - set domain containing 6  FZD6 - frizzled family receptor 6  ACTR5 - arp5 actin-related protein 5 homolog (yeast)  ZNF331 - zinc finger protein 331  CDC45 - cell division cycle 45  MED1 - mediator complex subunit 1  FZD4 - frizzled family receptor 4  GCLC - glutamate-cysteine ligase, catalytic subunit  FZD1 - frizzled family receptor 1  BAP1 - brca1 associated protein-1 (ubiquitin carboxy-terminal hydrolase)  ADCY1 - adenylate cyclase 1 (brain)  ZNF613 - zinc finger protein 613  NUP85 - nucleoporin 85kda  PPARD - peroxisome proliferator-activated receptor delta  KDM5C - lysine (k)-specific demethylase 5c  USP9X - ubiquitin specific peptidase 9, x-linked  RBM10 - rna binding motif protein 10  MAML3 - mastermind-like 3 (drosophila)  POLR2K - polymerase (rna) ii (dna directed) polypeptide k, 7.0kda  PARP1 - poly (adp-ribose) polymerase 1  H2AFY2 - h2a histone family, member y2  PHF17 - phd finger protein 17  PPP1R13L - protein phosphatase 1, regulatory subunit 13 like  HES6 - hairy and enhancer of split 6 (drosophila)  SMPD3 - sphingomyelin phosphodiesterase 3, neutral membrane (neutral sphingomyelinase ii)  RUVBL2 - ruvb-like 2 (e. coli)  POLR2A - polymerase (rna) ii (dna directed) polypeptide a, 220kda  TTC21B - tetratricopeptide repeat domain 21b  CDK5RAP2 - cdk5 regulatory subunit associated protein 2  RCOR3 - rest corepressor 3  SLTM - safb-like, transcription modulator  NELFE - negative elongation factor complex member e  EHMT1 - euchromatic histone-lysine n-methyltransferase 1  AES - amino-terminal enhancer of split  NR5A1 - nuclear receptor subfamily 5, group a, member 1  NUP133 - nucleoporin 133kda  FUS - fused in sarcoma  NAA40 - n(alpha)-acetyltransferase 40, natd catalytic subunit, homolog (s. cerevisiae)  JAG1 - jagged 1  PRDM4 - pr domain containing 4  PPP3R1 - protein phosphatase 3, regulatory subunit b, alpha  ATF7IP - activating transcription factor 7 interacting protein  ZNF689 - zinc finger protein 689  LRIF1 - ligand dependent nuclear receptor interacting factor 1  PPP2R5B - protein phosphatase 2, regulatory subunit b', beta  AKT1 - v-akt murine thymoma viral oncogene homolog 1  ZNF653 - zinc finger protein 653  USP7 - ubiquitin specific peptidase 7 (herpes virus-associated)  PCID2 - pci domain containing 2  XRCC6 - x-ray repair complementing defective repair in chinese hamster cells 6  RNF168 - ring finger protein 168, e3 ubiquitin protein ligase  GABPB1 - ga binding protein transcription factor, beta subunit 1  PPP2R1A - protein phosphatase 2, regulatory subunit a, alpha  GABPA - ga binding protein transcription factor, alpha subunit 60kda  WWP2 - ww domain containing e3 ubiquitin protein ligase 2  AHR - aryl hydrocarbon receptor  ZNF526 - zinc finger protein 526  FOXP4 - forkhead box p4  PPP2CA - protein phosphatase 2, catalytic subunit, alpha isozyme  RPAP2 - rna polymerase ii associated protein 2  ZNF83 - zinc finger protein 83  DDX39B - dead (asp-glu-ala-asp) box polypeptide 39b  KANSL3 - kat8 regulatory nsl complex subunit 3  PRNP - prion protein  ATF7 - activating transcription factor 7  KMT2D - lysine (k)-specific methyltransferase 2d  AMFR - autocrine motility factor receptor, e3 ubiquitin protein ligase  PRKRIR - protein-kinase, interferon-inducible double stranded rna dependent inhibitor, repressor of (p58 repressor)  ZNF419 - zinc finger protein 419  MAP2K5 - mitogen-activated protein kinase kinase 5  MAP2K7 - mitogen-activated protein kinase kinase 7  EIF2AK2 - eukaryotic translation initiation factor 2-alpha kinase 2  PIM2 - pim-2 oncogene  ZNF692 - zinc finger protein 692  YEATS4 - yeats domain containing 4  MAP2K1 - mitogen-activated protein kinase kinase 1  MAP2K2 - mitogen-activated protein kinase kinase 2  SNIP1 - smad nuclear interacting protein 1  ZFPM1 - zinc finger protein, fog family member 1  ZNF212 - zinc finger protein 212  MAPK10 - mitogen-activated protein kinase 10  EID2 - ep300 interacting inhibitor of differentiation 2  MAPK9 - mitogen-activated protein kinase 9  ZNF383 - zinc finger protein 383  MAPK11 - mitogen-activated protein kinase 11  MAPK8 - mitogen-activated protein kinase 8  MAPK7 - mitogen-activated protein kinase 7  GCN1L1 - gcn1 general control of amino-acid synthesis 1-like 1 (yeast)  GATA2 - gata binding protein 2  MAPK3 - mitogen-activated protein kinase 3  MAPK1 - mitogen-activated protein kinase 1  KAT6A - k(lysine) acetyltransferase 6a  PRKD1 - protein kinase d1  ADIRF - adipogenesis regulatory factor  ANXA3 - annexin a3  PRKCH - protein kinase c, eta  PRKCI - protein kinase c, iota  CCDC85B - coiled-coil domain containing 85b  NR4A3 - nuclear receptor subfamily 4, group a, member 3  PRKCB - protein kinase c, beta  BRD3 - bromodomain containing 3  GAS6 - growth arrest-specific 6  PRKAR1A - protein kinase, camp-dependent, regulatory, type i, alpha  CARF - calcium responsive transcription factor  NCOA4 - nuclear receptor coactivator 4  MLLT10 - myeloid/lymphoid or mixed-lineage leukemia (trithorax homolog, drosophila); translocated to, 10  ZNF408 - zinc finger protein 408  DEDD2 - death effector domain containing 2  YEATS2 - yeats domain containing 2  APEX1 - apex nuclease (multifunctional dna repair enzyme) 1  DHX36 - deah (asp-glu-ala-his) box polypeptide 36  ZNF189 - zinc finger protein 189  ZSCAN26 - zinc finger and scan domain containing 26  RBM23 - rna binding motif protein 23  ZNF605 - zinc finger protein 605  XIAP - x-linked inhibitor of apoptosis  BIRC2 - baculoviral iap repeat containing 2  ZNF177 - zinc finger protein 177  KLF12 - kruppel-like factor 12  ZNF175 - zinc finger protein 175  ZNF174 - zinc finger protein 174  ZNF184 - zinc finger protein 184  SSBP4 - single stranded dna binding protein 4  GTF2F1 - general transcription factor iif, polypeptide 1, 74kda  PBX2 - pre-b-cell leukemia homeobox 2  PRMT6 - protein arginine methyltransferase 6  PBX3 - pre-b-cell leukemia homeobox 3  ZNF148 - zinc finger protein 148  PBX1 - pre-b-cell leukemia homeobox 1  ZBTB17 - zinc finger and btb domain containing 17  BEND5 - ben domain containing 5  GTF2H2 - general transcription factor iih, polypeptide 2, 44kda  GTF2H3 - general transcription factor iih, polypeptide 3, 34kda  TRIM26 - tripartite motif containing 26  GTF2H1 - general transcription factor iih, polypeptide 1, 62kda  VEZF1 - vascular endothelial zinc finger 1  PCBD1 - pterin-4 alpha-carbinolamine dehydratase/dimerization cofactor of hepatocyte nuclear factor 1 alpha  ZNF142 - zinc finger protein 142  MCPH1 - microcephalin 1  ZNF140 - zinc finger protein 140  APBB2 - amyloid beta (a4) precursor protein-binding, family b, member 2  GTF2B - general transcription factor iib  PCGF2 - polycomb group ring finger 2  APBB1 - amyloid beta (a4) precursor protein-binding, family b, member 1 (fe65)  CHP1 - calcineurin-like ef-hand protein 1  ZNF132 - zinc finger protein 132  ZNF131 - zinc finger protein 131  RIF1 - rap1 interacting factor homolog (yeast)  ZNF134 - zinc finger protein 134  ZNF133 - zinc finger protein 133  TBL1XR1 - transducin (beta)-like 1 x-linked receptor 1  GTF2I - general transcription factor iii  GTF2H4 - general transcription factor iih, polypeptide 4, 52kda  KIAA1551 - kiaa1551  PA2G4 - proliferation-associated 2g4, 38kda  BRF1 - brf1, rna polymerase iii transcription initiation factor 90 kda subunit  RPRD1A - regulation of nuclear pre-mrna domain containing 1a  ZHX1 - zinc fingers and homeoboxes 1  ARF4 - adp-ribosylation factor 4  ZNF79 - zinc finger protein 79  SBNO1 - strawberry notch homolog 1 (drosophila)  IRAK3 - interleukin-1 receptor-associated kinase 3  SETD5 - set domain containing 5  APP - amyloid beta (a4) precursor protein  ZNF70 - zinc finger protein 70  BBS7 - bardet-biedl syndrome 7  DDX20 - dead (asp-glu-ala-asp) box polypeptide 20  ZNF75D - zinc finger protein 75d  PAM - peptidylglycine alpha-amidating monooxygenase  ZNF76 - zinc finger protein 76  H3F3A - h3 histone, family 3a  ARNTL - aryl hydrocarbon receptor nuclear translocator-like  ARNT - aryl hydrocarbon receptor nuclear translocator  BRPF1 - bromodomain and phd finger containing, 1  H2AFZ - h2a histone family, member z  FZD5 - frizzled family receptor 5  SAP130 - sin3a-associated protein, 130kda  H2AFX - h2a histone family, member x  PAX8 - paired box 8  SUPT16H - suppressor of ty 16 homolog (s. cerevisiae)  MIER3 - mesoderm induction early response 1, family member 3  ARRB2 - arrestin, beta 2  ARRB1 - arrestin, beta 1  CDC73 - cell division cycle 73  PDGFB - platelet-derived growth factor beta polypeptide  NKAP - nfkb activating protein  THAP5 - thap domain containing 5  STRAP - serine/threonine kinase receptor associated protein  RHOA - ras homolog family member a  PSIP1 - pc4 and sfrs1 interacting protein 1  MED9 - mediator complex subunit 9  FSTL1 - follistatin-like 1  LRP8 - low density lipoprotein receptor-related protein 8, apolipoprotein e receptor  BAZ2A - bromodomain adjacent to zinc finger domain, 2a  HCFC1 - host cell factor c1 (vp16-accessory protein)  PCNA - proliferating cell nuclear antigen  PRDM2 - pr domain containing 2, with znf domain  CHMP1A - charged multivesicular body protein 1a  MAP3K12 - mitogen-activated protein kinase kinase kinase 12  JDP2 - jun dimerization protein 2  CDK16 - cyclin-dependent kinase 16  AKIRIN2 - akirin 2  HEATR1 - heat repeat containing 1  CDK17 - cyclin-dependent kinase 17  LEO1 - leo1, paf1/rna polymerase ii complex component, homolog (s. cerevisiae)  ERLIN2 - er lipid raft associated 2  ZNF226 - zinc finger protein 226  PWP1 - pwp1 homolog (s. cerevisiae)  PDE2A - phosphodiesterase 2a, cgmp-stimulated  HDAC2 - histone deacetylase 2  HMBOX1 - homeobox containing 1  ZCCHC12 - zinc finger, cchc domain containing 12  HDGF - hepatoma-derived growth factor  ZNF213 - zinc finger protein 213  THAP1 - thap domain containing, apoptosis associated protein 1  HDAC1 - histone deacetylase 1  ZNF205 - zinc finger protein 205  HCLS1 - hematopoietic cell-specific lyn substrate 1  PKIG - protein kinase (camp-dependent, catalytic) inhibitor gamma  ZNF207 - zinc finger protein 207  KAT7 - k(lysine) acetyltransferase 7  ASCL1 - achaete-scute complex homolog 1 (drosophila)  ELP3 - elongator acetyltransferase complex subunit 3  RGMB - rgm domain family, member b  SBNO2 - strawberry notch homolog 2 (drosophila)  ZNF444 - zinc finger protein 444  XPA - xeroderma pigmentosum, complementation group a  ZBTB1 - zinc finger and btb domain containing 1  XPC - xeroderma pigmentosum, complementation group c  FBXW7 - f-box and wd repeat domain containing 7, e3 ubiquitin protein ligase  XPO1 - exportin 1 (crm1 homolog, yeast)  GOLGB1 - golgin b1  BAHD1 - bromo adjacent homology domain containing 1  PAGR1 - paxip1 associated glutamate-rich protein 1  ZHX2 - zinc fingers and homeoboxes 2  MORC2 - morc family cw-type zinc finger 2  PHF2 - phd finger protein 2  PHF1 - phd finger protein 1  FOXJ3 - forkhead box j3  RERE - arginine-glutamic acid dipeptide (re) repeats  ZXDC - zxd family zinc finger c  XBP1 - x-box binding protein 1  ZNF367 - zinc finger protein 367  BHLHE41 - basic helix-loop-helix family, member e41  ZFHX3 - zinc finger homeobox 3  PEX14 - peroxisomal biogenesis factor 14  WFS1 - wolfram syndrome 1 (wolframin)  WHSC1 - wolf-hirschhorn syndrome candidate 1  ADNP2 - adnp homeobox 2  NELFA - negative elongation factor complex member a  AEBP2 - ae binding protein 2  IMPACT - impact rwd domain protein  ZNF507 - zinc finger protein 507  LGR4 - leucine-rich repeat containing g protein-coupled receptor 4  ARID2 - at rich interactive domain 2 (arid, rfx-like)  ATP2B4 - atpase, ca++ transporting, plasma membrane 4  ZNF652 - zinc finger protein 652  SCAF8 - sr-related ctd-associated factor 8  PKD1 - polycystic kidney disease 1 (autosomal dominant)  PKD2 - polycystic kidney disease 2 (autosomal dominant)  ZNF35 - zinc finger protein 35  ZKSCAN1 - zinc finger with krab and scan domains 1  FBXL15 - f-box and leucine-rich repeat protein 15  MTF2 - metal response element binding transcription factor 2  ZNF33B - zinc finger protein 33b  ZNF576 - zinc finger protein 576  ZNF32 - zinc finger protein 32  BACH1 - btb and cnc homology 1, basic leucine zipper transcription factor 1  PIN1 - peptidylprolyl cis/trans isomerase, nima-interacting 1  PIK3R2 - phosphoinositide-3-kinase, regulatory subunit 2 (beta)  ZNF45 - zinc finger protein 45  MZF1 - myeloid zinc finger 1  PIK3R1 - phosphoinositide-3-kinase, regulatory subunit 1 (alpha)  ZNF18 - zinc finger protein 18  ASXL2 - additional sex combs like 2 (drosophila)  ZNF16 - zinc finger protein 16  PLAGL1 - pleiomorphic adenoma gene-like 1  ELP2 - elongator acetyltransferase complex subunit 2  ASXL1 - additional sex combs like 1 (drosophila)  ZNF384 - zinc finger protein 384  TNIP2 - tnfaip3 interacting protein 2  ATRX - alpha thalassemia/mental retardation syndrome x-linked  ZNF24 - zinc finger protein 24  PKNOX1 - pbx/knotted 1 homeobox 1  ZFPL1 - zinc finger protein-like 1  ZFX - zinc finger protein, x-linked  TMEM100 - transmembrane protein 100  PHF10 - phd finger protein 10  SF1 - splicing factor 1  PSPC1 - paraspeckle component 1  ZBTB14 - zinc finger and btb domain containing 14  ZNF711 - zinc finger protein 711  ZNF7 - zinc finger protein 7  ZNF8 - zinc finger protein 8  BCL7A - b-cell cll/lymphoma 7a  ZNF2 - zinc finger protein 2  MRGBP - mrg/morf4l binding protein  ZNF3 - zinc finger protein 3  BCL9 - b-cell cll/lymphoma 9  YES1 - v-yes-1 yamaguchi sarcoma viral oncogene homolog 1  BRF2 - brf2, rna polymerase iii transcription initiation factor 50 kda subunit  PIM1 - pim-1 oncogene  XRCC5 - x-ray repair complementing defective repair in chinese hamster cells 5 (double-strand-break rejoining)  ECD - ecdysoneless homolog (drosophila)  CCND1 - cyclin d1  GSK3A - glycogen synthase kinase 3 alpha  PARK7 - parkinson protein 7  C3orf33 - chromosome 3 open reading frame 33  YWHAB - tyrosine 3-monooxygenase/tryptophan 5-monooxygenase activation protein, beta polypeptide  CCNK - cyclin k  TRIM27 - tripartite motif containing 27  HSF2 - heat shock transcription factor 2  SAP18 - sin3a-associated protein, 18kda  TRIM24 - tripartite motif containing 24  TAF1D - tata box binding protein (tbp)-associated factor, rna polymerase i, d, 41kda  TSHZ2 - teashirt zinc finger homeobox 2  RFXAP - regulatory factor x-associated protein  CREG1 - cellular repressor of e1a-stimulated genes 1  RFX5 - regulatory factor x, 5 (influences hla class ii expression)  WHSC1L1 - wolf-hirschhorn syndrome candidate 1-like 1  FLCN - folliculin  HSBP1 - heat shock factor binding protein 1  RFX1 - regulatory factor x, 1 (influences hla class ii expression)  APBB3 - amyloid beta (a4) precursor protein-binding, family b, member 3  RELA - v-rel avian reticuloendotheliosis viral oncogene homolog a  PRMT2 - protein arginine methyltransferase 2  BCOR - bcl6 corepressor  ZFP36L2 - zfp36 ring finger protein-like 2  ZFP36L1 - zfp36 ring finger protein-like 1  RET - ret proto-oncogene  HESX1 - hesx homeobox 1  SAP30 - sin3a-associated protein, 30kda  DPF2 - d4, zinc and double phd fingers family 2  CC2D1A - coiled-coil and c2 domain containing 1a  RLF - rearranged l-myc fusion  RING1 - ring finger protein 1  CFLAR - casp8 and fadd-like apoptosis regulator  MED11 - mediator complex subunit 11  BMI1 - bmi1 polycomb ring finger oncogene  PCGF3 - polycomb group ring finger 3  LDB1 - lim domain binding 1  WDR77 - wd repeat domain 77  BMPR1A - bone morphogenetic protein receptor, type ia  BMPR2 - bone morphogenetic protein receptor, type ii (serine/threonine kinase)  BMPR1B - bone morphogenetic protein receptor, type ib  GON4L - gon-4-like (c. elegans)  KAT2B - k(lysine) acetyltransferase 2b  TSC22D1 - tsc22 domain family, member 1  CRTC2 - creb regulated transcription coactivator 2  BMP7 - bone morphogenetic protein 7  PSMD14 - proteasome (prosome, macropain) 26s subunit, non-atpase, 14  ZNF259 - zinc finger protein 259  FLOT1 - flotillin 1  TAX1BP1 - tax1 (human t-cell leukemia virus type i) binding protein 1  BCAS3 - breast carcinoma amplified sequence 3  CC2D1B - coiled-coil and c2 domain containing 1b  AHI1 - abelson helper integration site 1  THAP8 - thap domain containing 8  RNF4 - ring finger protein 4  BRD2 - bromodomain containing 2  RNF2 - ring finger protein 2  BUD31 - bud31 homolog (s. cerevisiae)  ZNF280D - zinc finger protein 280d  RNF6 - ring finger protein (c3h2c3 type) 6  GATAD2A - gata zinc finger domain containing 2a  ZNF655 - zinc finger protein 655  RNF111 - ring finger protein 111  TRAK2 - trafficking protein, kinesin binding 2  TET2 - tet methylcytosine dioxygenase 2  HSPD1 - heat shock 60kda protein 1 (chaperonin)  DNAJB1 - dnaj (hsp40) homolog, subfamily b, member 1  MED18 - mediator complex subunit 18  ZNF48 - zinc finger protein 48  DENND4A - denn/madd domain containing 4a  BCL10 - b-cell cll/lymphoma 10  MBTD1 - mbt domain containing 1  FUBP3 - far upstream element (fuse) binding protein 3  BTRC - beta-transducin repeat containing e3 ubiquitin protein ligase  ZMPSTE24 - zinc metallopeptidase ste24  HSPA8 - heat shock 70kda protein 8  TRIM44 - tripartite motif containing 44  TAB1 - tgf-beta activated kinase 1/map3k7 binding protein 1  PPIE - peptidylprolyl isomerase e (cyclophilin e)  MCRS1 - microspherule protein 1  HMGN1 - high mobility group nucleosome binding domain 1  HMGB3 - high mobility group box 3  NRDE2 - nrde-2, necessary for rna interference, domain containing  HMGB2 - high mobility group box 2  HMGB1 - high mobility group box 1  C1D - c1d nuclear receptor corepressor  BRD9 - bromodomain containing 9  TNKS - tankyrase, trf1-interacting ankyrin-related adp-ribose polymerase  SLC30A9 - solute carrier family 30 (zinc transporter), member 9  ZBTB10 - zinc finger and btb domain containing 10  AGO1 - argonaute risc catalytic component 1  MAD2L2 - mad2 mitotic arrest deficient-like 2 (yeast)  CAPRIN2 - caprin family member 2  PID1 - phosphotyrosine interaction domain containing 1  PHIP - pleckstrin homology domain interacting protein  ZBTB48 - zinc finger and btb domain containing 48  ZNF787 - zinc finger protein 787  NRARP - notch-regulated ankyrin repeat protein  HYAL2 - hyaluronoglucosaminidase 2  GPBP1 - gc-rich promoter binding protein 1  TRIM38 - tripartite motif containing 38  TADA3 - transcriptional adaptor 3  CALR - calreticulin  ZNF792 - zinc finger protein 792  HFE - hemochromatosis  WTIP - wilms tumor 1 interacting protein  NCOA2 - nuclear receptor coactivator 2  HINT1 - histidine triad nucleotide binding protein 1  HIVEP1 - human immunodeficiency virus type i enhancer binding protein 1  ELP4 - elongator acetyltransferase complex subunit 4  CREB3 - camp responsive element binding protein 3  HIF1A - hypoxia inducible factor 1, alpha subunit (basic helix-loop-helix transcription factor)  LPIN3 - lipin 3  CITED2 - cbp/p300-interacting transactivator, with glu/asp-rich carboxy-terminal domain, 2  TRADD - tnfrsf1a-associated via death domain  CCNT2 - cyclin t2  MYCBP - myc binding protein  EED - embryonic ectoderm development  URI1 - uri1, prefoldin-like chaperone  CCND3 - cyclin d3  CCNT1 - cyclin t1  CCNH - cyclin h  HMG20A - high mobility group 20a  BANP - btg3 associated nuclear protein  HNRNPU - heterogeneous nuclear ribonucleoprotein u (scaffold attachment factor a)  RIPK1 - receptor (tnfrsf)-interacting serine-threonine kinase 1  HNRNPK - heterogeneous nuclear ribonucleoprotein k  RPS6KA1 - ribosomal protein s6 kinase, 90kda, polypeptide 1  CBFA2T3 - core-binding factor, runt domain, alpha subunit 2; translocated to, 3  CBFB - core-binding factor, beta subunit  HNRNPA2B1 - heterogeneous nuclear ribonucleoprotein a2/b1  HNRNPAB - heterogeneous nuclear ribonucleoprotein a/b  RIPK2 - receptor-interacting serine-threonine kinase 2  RXRB - retinoid x receptor, beta  PIAS3 - protein inhibitor of activated stat, 3  HNRNPD - heterogeneous nuclear ribonucleoprotein d (au-rich element rna binding protein 1, 37kda)  CAV1 - caveolin 1, caveolae protein, 22kda  CCNC - cyclin c  RBM14 - rna binding motif protein 14  HMGA1 - high mobility group at-hook 1  ZNF584 - zinc finger protein 584  FAM129B - family with sequence similarity 129, member b  PRMT5 - protein arginine methyltransferase 5  NR4A1 - nuclear receptor subfamily 4, group a, member 1  RFX7 - regulatory factor x, 7  FOXK2 - forkhead box k2  HTATSF1 - hiv-1 tat specific factor 1  HAT1 - histone acetyltransferase 1  CDK11B - cyclin-dependent kinase 11b  CDC5L - cell division cycle 5-like  MXD4 - max dimerization protein 4  CRTC3 - creb regulated transcription coactivator 3  RBM15 - rna binding motif protein 15  HTRA1 - htra serine peptidase 1  POLR3F - polymerase (rna) iii (dna directed) polypeptide f, 39 kda  HEXIM1 - hexamethylene bis-acetamide inducible 1  ERLIN1 - er lipid raft associated 1  RBMX - rna binding motif protein, x-linked  SPIN2A - spindlin family, member 2a  NCOA7 - nuclear receptor coactivator 7  PSEN1 - presenilin 1  ING2 - inhibitor of growth family, member 2  PPM1D - protein phosphatase, mg2+/mn2+ dependent, 1d  ZNF574 - zinc finger protein 574  CREB3L2 - camp responsive element binding protein 3-like 2  USP16 - ubiquitin specific peptidase 16  PSMA1 - proteasome (prosome, macropain) subunit, alpha type, 1  IPPK - inositol 1,3,4,5,6-pentakisphosphate 2-kinase  PSMA2 - proteasome (prosome, macropain) subunit, alpha type, 2  ILF2 - interleukin enhancer binding factor 2  ILF3 - interleukin enhancer binding factor 3, 90kda  ILK - integrin-linked kinase  CD81 - cd81 molecule  PIAS1 - protein inhibitor of activated stat, 1  CAMKK2 - calcium/calmodulin-dependent protein kinase kinase 2, beta  PSMB1 - proteasome (prosome, macropain) subunit, beta type, 1  SLCO3A1 - solute carrier organic anion transporter family, member 3a1  SMYD3 - set and mynd domain containing 3  SMURF2 - smad specific e3 ubiquitin protein ligase 2  PSMA5 - proteasome (prosome, macropain) subunit, alpha type, 5  PSMA3 - proteasome (prosome, macropain) subunit, alpha type, 3  PSMB10 - proteasome (prosome, macropain) subunit, beta type, 10  RIPPLY2 - ripply transcriptional repressor 2  CGGBP1 - cgg triplet repeat binding protein 1  LMO4 - lim domain only 4  PSMC6 - proteasome (prosome, macropain) 26s subunit, atpase, 6  TAF6L - taf6-like rna polymerase ii, p300/cbp-associated factor (pcaf)-associated factor, 65kda  PSMD1 - proteasome (prosome, macropain) 26s subunit, non-atpase, 1  CAMK1 - calcium/calmodulin-dependent protein kinase i  PSMC4 - proteasome (prosome, macropain) 26s subunit, atpase, 4  SOX18 - sry (sex determining region y)-box 18  CBX4 - chromobox homolog 4  POLR3G - polymerase (rna) iii (dna directed) polypeptide g (32kd)  PSMC2 - proteasome (prosome, macropain) 26s subunit, atpase, 2  PSMD9 - proteasome (prosome, macropain) 26s subunit, non-atpase, 9  PSMD10 - proteasome (prosome, macropain) 26s subunit, non-atpase, 10  PSMD7 - proteasome (prosome, macropain) 26s subunit, non-atpase, 7  PSMD8 - proteasome (prosome, macropain) 26s subunit, non-atpase, 8  CSRNP1 - cysteine-serine-rich nuclear protein 1  PSMD5 - proteasome (prosome, macropain) 26s subunit, non-atpase, 5  TERF2IP - telomeric repeat binding factor 2, interacting protein  PSMD2 - proteasome (prosome, macropain) 26s subunit, non-atpase, 2  PSMD12 - proteasome (prosome, macropain) 26s subunit, non-atpase, 12  PSMD13 - proteasome (prosome, macropain) 26s subunit, non-atpase, 13  ZNF25 - zinc finger protein 25  PTEN - phosphatase and tensin homolog  LAMTOR5 - late endosomal/lysosomal adaptor, mapk and mtor activator 5  STK16 - serine/threonine kinase 16  JMY - junction mediating and regulatory protein, p53 cofactor  ITGA3 - integrin, alpha 3 (antigen cd49c, alpha 3 subunit of vla-3 receptor)  MYBBP1A - myb binding protein (p160) 1a  FBLN5 - fibulin 5  PTGIS - prostaglandin i2 (prostacyclin) synthase  CIB1 - calcium and integrin binding 1 (calmyrin)  CASK - calcium/calmodulin-dependent serine protein kinase (maguk family)  DDX17 - dead (asp-glu-ala-asp) box helicase 17  SUDS3 - suppressor of defective silencing 3 homolog (s. cerevisiae)  KAT5 - k(lysine) acetyltransferase 5  ZFP69 - zfp69 zinc finger protein  CEBPG - ccaat/enhancer binding protein (c/ebp), gamma  SKAP1 - src kinase associated phosphoprotein 1  CDK5 - cyclin-dependent kinase 5  GZF1 - gdnf-inducible zinc finger protein 1  CDK9 - cyclin-dependent kinase 9  CDK8 - cyclin-dependent kinase 8  INSR - insulin receptor  LIN54 - lin-54 homolog (c. elegans)  INSM1 - insulinoma-associated 1  CDK7 - cyclin-dependent kinase 7  CDK13 - cyclin-dependent kinase 13  CDKN1C - cyclin-dependent kinase inhibitor 1c (p57, kip2)  CDKN1B - cyclin-dependent kinase inhibitor 1b (p27, kip1)  PDCD4 - programmed cell death 4 (neoplastic transformation inhibitor)  PCBP3 - poly(rc) binding protein 3  RFXANK - regulatory factor x-associated ankyrin-containing protein  PTPN2 - protein tyrosine phosphatase, non-receptor type 2  IRF2 - interferon regulatory factor 2  KLF7 - kruppel-like factor 7 (ubiquitous)  IRF3 - interferon regulatory factor 3  HTATIP2 - hiv-1 tat interactive protein 2, 30kda  IKZF5 - ikaros family zinc finger 5 (pegasus)  RUVBL1 - ruvb-like 1 (e. coli)  MED19 - mediator complex subunit 19  ITGA6 - integrin, alpha 6  IRAK2 - interleukin-1 receptor-associated kinase 2  CDK2 - cyclin-dependent kinase 2  IRAK1 - interleukin-1 receptor-associated kinase 1  RRN3 - rrn3 rna polymerase i transcription factor homolog (s. cerevisiae)  PURA - purine-rich element binding protein a  PURB - purine-rich element binding protein b  ZSCAN31 - zinc finger and scan domain containing 31  KIAA1324L - kiaa1324-like  NSD1 - nuclear receptor binding set domain protein 1  ZMYND11 - zinc finger, mynd-type containing 11  ZBTB11 - zinc finger and btb domain containing 11  SRSF10 - serine/arginine-rich splicing factor 10  PTPRK - protein tyrosine phosphatase, receptor type, k  RNF25 - ring finger protein 25  PTPRN - protein tyrosine phosphatase, receptor type, n  DKK3 - dickkopf wnt signaling pathway inhibitor 3  MMS19 - mms19 nucleotide excision repair homolog (s. cerevisiae)  PEX2 - peroxisomal biogenesis factor 2  KDM5B - lysine (k)-specific demethylase 5b  MPHOSPH8 - m-phase phosphoprotein 8  PXN - paxillin  SNX5 - sorting nexin 5  ID2 - inhibitor of dna binding 2, dominant negative helix-loop-helix protein  ZNF777 - zinc finger protein 777  ID3 - inhibitor of dna binding 3, dominant negative helix-loop-helix protein  CHD4 - chromodomain helicase dna binding protein 4  CHD2 - chromodomain helicase dna binding protein 2  STK36 - serine/threonine kinase 36  CHD3 - chromodomain helicase dna binding protein 3  MTERFD2 - mterf domain containing 2  CHD1 - chromodomain helicase dna binding protein 1  IRF2BPL - interferon regulatory factor 2 binding protein-like  JAZF1 - jazf zinc finger 1  QARS - glutaminyl-trna synthetase  MYPOP - myb-related transcription factor, partner of profilin  RAD21 - rad21 homolog (s. pombe)  FOXK1 - forkhead box k1  PARP14 - poly (adp-ribose) polymerase family, member 14  ZNF181 - zinc finger protein 181  PAF1 - paf1, rna polymerase ii associated factor, homolog (s. cerevisiae)  ZNF268 - zinc finger protein 268  INO80 - ino80 complex subunit  ZNF513 - zinc finger protein 513  IGHMBP2 - immunoglobulin mu binding protein 2  ZNF282 - zinc finger protein 282  GMEB1 - glucocorticoid modulatory element binding protein 1  RBPJ - recombination signal binding protein for immunoglobulin kappa j region  MIER2 - mesoderm induction early response 1, family member 2  NCK2 - nck adaptor protein 2  RARG - retinoic acid receptor, gamma  RARB - retinoic acid receptor, beta  DLL4 - delta-like 4 (drosophila)  USP21 - ubiquitin specific peptidase 21  ZBTB9 - zinc finger and btb domain containing 9  ZBTB12 - zinc finger and btb domain containing 12  CLU - clusterin  KHDRBS1 - kh domain containing, rna binding, signal transduction associated 1  TAF8 - taf8 rna polymerase ii, tata box binding protein (tbp)-associated factor, 43kda  MAGEL2 - mage-like 2  CTCF - ccctc-binding factor (zinc finger protein)  IGBP1 - immunoglobulin (cd79a) binding protein 1  JMJD1C - jumonji domain containing 1c  RBL2 - retinoblastoma-like 2 (p130)  RAI1 - retinoic acid induced 1  RBBP7 - retinoblastoma binding protein 7  SMARCA5 - swi/snf related, matrix associated, actin dependent regulator of chromatin, subfamily a, member 5  RBBP8 - retinoblastoma binding protein 8  MAP3K2 - mitogen-activated protein kinase kinase kinase 2  OGT - o-linked n-acetylglucosamine (glcnac) transferase  RB1 - retinoblastoma 1  PELP1 - proline, glutamate and leucine rich protein 1  ARID4A - at rich interactive domain 4a (rbp1-like)  SND1 - staphylococcal nuclease and tudor domain containing 1  KDM5A - lysine (k)-specific demethylase 5a  NUP35 - nucleoporin 35kda  CUL2 - cullin 2  CUL3 - cullin 3  ZNF667 - zinc finger protein 667  CKS1B - cdc28 protein kinase regulatory subunit 1b  NFAT5 - nuclear factor of activated t-cells 5, tonicity-responsive  FOXP1 - forkhead box p1  CHUK - conserved helix-loop-helix ubiquitous kinase  SUPT3H - suppressor of ty 3 homolog (s. cerevisiae)  BEND6 - ben domain containing 6  ZNF572 - zinc finger protein 572  ZMYND8 - zinc finger, mynd-type containing 8  PCGF5 - polycomb group ring finger 5  ANKRA2 - ankyrin repeat, family a (rfxank-like), 2  WAC - ww domain containing adaptor with coiled-coil  RBAK - rb-associated krab zinc finger  NFATC2IP - nuclear factor of activated t-cells, cytoplasmic, calcineurin-dependent 2 interacting protein  PHF21A - phd finger protein 21a  ZNF414 - zinc finger protein 414  ZNF771 - zinc finger protein 771  PATZ1 - poz (btb) and at hook containing zinc finger 1  IER2 - immediate early response 2  SPP1 - secreted phosphoprotein 1  NCOR1 - nuclear receptor corepressor 1  SSBP2 - single-stranded dna binding protein 2  KPNA6 - karyopherin alpha 6 (importin alpha 7)  NCOR2 - nuclear receptor corepressor 2  MTPN - myotrophin  CIRH1A - cirrhosis, autosomal recessive 1a (cirhin)  DOT1L - dot1-like histone h3k79 methyltransferase  MIER1 - mesoderm induction early response 1, transcriptional regulator  ZNF382 - zinc finger protein 382  ZNF705A - zinc finger protein 705a  EFCAB7 - ef-hand calcium binding domain 7  SP3 - sp3 transcription factor  L3MBTL3 - l(3)mbt-like 3 (drosophila)  SP4 - sp4 transcription factor  SP1 - sp1 transcription factor  SP2 - sp2 transcription factor  ZNF410 - zinc finger protein 410  ZNF333 - zinc finger protein 333  CIR1 - corepressor interacting with rbpj, 1  SCAND1 - scan domain containing 1  ARL2BP - adp-ribosylation factor-like 2 binding protein  SOX4 - sry (sex determining region y)-box 4  H2AFY - h2a histone family, member y  WBP2 - ww domain binding protein 2  GTF2IRD1 - gtf2i repeat domain containing 1  FADS1 - fatty acid desaturase 1  ZNF527 - zinc finger protein 527  HES4 - hairy and enhancer of split 4 (drosophila)  LEMD3 - lem domain containing 3  CREBL2 - camp responsive element binding protein-like 2  GATAD1 - gata zinc finger domain containing 1  ATF6B - activating transcription factor 6 beta  HOPX - hop homeobox  CREBBP - creb binding protein  SOX13 - sry (sex determining region y)-box 13  ZC3H8 - zinc finger ccch-type containing 8  ZNF316 - zinc finger protein 316  ATF2 - activating transcription factor 2  LMO1 - lim domain only 1 (rhombotin 1)  CREB1 - camp responsive element binding protein 1  NACC2 - nacc family member 2, ben and btb (poz) domain containing  IER5 - immediate early response 5  DDX58 - dead (asp-glu-ala-asp) box polypeptide 58  CRABP2 - cellular retinoic acid binding protein 2  CLOCK - clock circadian regulator  KLF3 - kruppel-like factor 3 (basic)  RPRD1B - regulation of nuclear pre-mrna domain containing 1b  DDX41 - dead (asp-glu-ala-asp) box polypeptide 41  PLK3 - polo-like kinase 3  SNAPC4 - small nuclear rna activating complex, polypeptide 4, 190kda  LITAF - lipopolysaccharide-induced tnf factor  SNCA - synuclein, alpha (non a4 component of amyloid precursor)  TBPL1 - tbp-like 1  ZBED5 - zinc finger, bed-type containing 5  CREBZF - creb/atf bzip transcription factor  ZBTB41 - zinc finger and btb domain containing 41  ZFP62 - zfp62 zinc finger protein  EID1 - ep300 interacting inhibitor of differentiation 1  TP53INP2 - tumor protein p53 inducible nuclear protein 2  SMARCE1 - swi/snf related, matrix associated, actin dependent regulator of chromatin, subfamily e, member 1  SMO - smoothened, frizzled family receptor  ZGPAT - zinc finger, ccch-type with g patch domain  ACTL6B - actin-like 6b  SMARCB1 - swi/snf related, matrix associated, actin dependent regulator of chromatin, subfamily b, member 1  SMARCC1 - swi/snf related, matrix associated, actin dependent regulator of chromatin, subfamily c, member 1  HLTF - helicase-like transcription factor  SNX6 - sorting nexin 6  SMARCA4 - swi/snf related, matrix associated, actin dependent regulator of chromatin, subfamily a, member 4  SSBP3 - single stranded dna binding protein 3  SMARCD2 - swi/snf related, matrix associated, actin dependent regulator of chromatin, subfamily d, member 2  SMARCD3 - swi/snf related, matrix associated, actin dependent regulator of chromatin, subfamily d, member 3  SMARCC2 - swi/snf related, matrix associated, actin dependent regulator of chromatin, subfamily c, member 2  SMARCD1 - swi/snf related, matrix associated, actin dependent regulator of chromatin, subfamily d, member 1  WIZ - widely interspaced zinc finger motifs  MED20 - mediator complex subunit 20  AFAP1L2 - actin filament associated protein 1-like 2  SMARCA1 - swi/snf related, matrix associated, actin dependent regulator of chromatin, subfamily a, member 1  SMARCA2 - swi/snf related, matrix associated, actin dependent regulator of chromatin, subfamily a, member 2  MAPK8IP1 - mitogen-activated protein kinase 8 interacting protein 1  CRLF3 - cytokine receptor-like factor 3  SGK3 - serum/glucocorticoid regulated kinase family, member 3  CACTIN - cactin, spliceosome c complex subunit  KMT2C - lysine (k)-specific methyltransferase 2c  GLYR1 - glyoxylate reductase 1 homolog (arabidopsis)  ZBTB7A - zinc finger and btb domain containing 7a  DPY30 - dpy-30 homolog (c. elegans)  PPIL4 - peptidylprolyl isomerase (cyclophilin)-like 4  ZNF462 - zinc finger protein 462  MBTPS2 - membrane-bound transcription factor peptidase, site 2  IRF2BP2 - interferon regulatory factor 2 binding protein 2  GLIS2 - glis family zinc finger 2  MAGED1 - melanoma antigen family d, 1  MED27 - mediator complex subunit 27  MED26 - mediator complex subunit 26  CHD6 - chromodomain helicase dna binding protein 6  SENP2 - sumo1/sentrin/smt3 specific peptidase 2  MED17 - mediator complex subunit 17  MED23 - mediator complex subunit 23  ZNF580 - zinc finger protein 580  ZNF318 - zinc finger protein 318  CDYL - chromodomain protein, y-like  PRPF6 - pre-mrna processing factor 6  GTF2IRD2 - gtf2i repeat domain containing 2  KDM2B - lysine (k)-specific demethylase 2b  SUPT6H - suppressor of ty 6 homolog (s. cerevisiae)  ZNF710 - zinc finger protein 710  CBY1 - chibby homolog 1 (drosophila)  CTBP1 - c-terminal binding protein 1  SUPT4H1 - suppressor of ty 4 homolog 1 (s. cerevisiae)  CTBP2 - c-terminal binding protein 2  ING4 - inhibitor of growth family, member 4  SUPT5H - suppressor of ty 5 homolog (s. cerevisiae)  INSIG2 - insulin induced gene 2  EIF2AK3 - eukaryotic translation initiation factor 2-alpha kinase 3  CTNNB1 - catenin (cadherin-associated protein), beta 1, 88kda  RLIM - ring finger protein, lim domain interacting  SAV1 - salvador homolog 1 (drosophila)  RECQL5 - recq protein-like 5  KRAS - kirsten rat sarcoma viral oncogene homolog  CUX1 - cut-like homeobox 1  ZNF706 - zinc finger protein 706  TGFBRAP1 - transforming growth factor, beta receptor associated protein 1  CIAO1 - cytosolic iron-sulfur protein assembly 1  NIF3L1 - nif3 ngg1 interacting factor 3-like 1 (s. cerevisiae)  NEK4 - nima-related kinase 4  STK3 - serine/threonine kinase 3  PCGF1 - polycomb group ring finger 1  FKBP8 - fk506 binding protein 8, 38kda  NPNT - nephronectin  GPBP1L1 - gc-rich promoter binding protein 1-like 1  PHF20L1 - phd finger protein 20-like 1  MED10 - mediator complex subunit 10  MED21 - mediator complex subunit 21  CYLD - cylindromatosis (turban tumor syndrome)  ZFP28 - zfp28 zinc finger protein  KIT - v-kit hardy-zuckerman 4 feline sarcoma viral oncogene homolog  TGIF2 - tgfb-induced factor homeobox 2  BCL6B - b-cell cll/lymphoma 6, member b  CRK - v-crk avian sarcoma virus ct10 oncogene homolog  NIPBL - nipped-b homolog (drosophila)  CRY1 - cryptochrome 1 (photolyase-like)  STAT2 - signal transducer and activator of transcription 2, 113kda  STAT1 - signal transducer and activator of transcription 1, 91kda  STAT3 - signal transducer and activator of transcription 3 (acute-phase response factor)  CRY2 - cryptochrome 2 (photolyase-like)  JUP - junction plakoglobin  STAT5A - signal transducer and activator of transcription 5a  ASF1A - anti-silencing function 1a histone chaperone  STAT6 - signal transducer and activator of transcription 6, interleukin-4 induced  JAK2 - janus kinase 2  ING5 - inhibitor of growth family, member 5  BRMS1 - breast cancer metastasis suppressor 1  ZNF219 - zinc finger protein 219  CRYM - crystallin, mu  ZNF496 - zinc finger protein 496  SS18 - synovial sarcoma translocation, chromosome 18  PHF20 - phd finger protein 20  PHF5A - phd finger protein 5a  GPATCH3 - g patch domain containing 3  ZNF397 - zinc finger protein 397  ZNHIT3 - zinc finger, hit-type containing 3  TRIP4 - thyroid hormone receptor interactor 4  HMGN3 - high mobility group nucleosomal binding domain 3  TAB3 - tgf-beta activated kinase 1/map3k7 binding protein 3  PHF6 - phd finger protein 6  ZNF235 - zinc finger protein 235  COPS2 - cop9 signalosome subunit 2  ZNF335 - zinc finger protein 335  BAMBI - bmp and activin membrane-bound inhibitor  HAVCR2 - hepatitis a virus cellular receptor 2  ZNF639 - zinc finger protein 639  SRI - sorcin  SRC - v-src avian sarcoma (schmidt-ruppin a-2) viral oncogene homolog  SARNP - sap domain containing ribonucleoprotein  BRMS1L - breast cancer metastasis-suppressor 1-like  LEF1 - lymphoid enhancer-binding factor 1  DNAJB5 - dnaj (hsp40) homolog, subfamily b, member 5  SREBF2 - sterol regulatory element binding transcription factor 2  UBE2O - ubiquitin-conjugating enzyme e2o  CNOT8 - ccr4-not transcription complex, subunit 8  PRDX5 - peroxiredoxin 5  CDK12 - cyclin-dependent kinase 12  DHX9 - deah (asp-glu-ala-his) box helicase 9  KANK2 - kn motif and ankyrin repeat domains 2  TRAPPC2 - trafficking protein particle complex 2  ZNF280B - zinc finger protein 280b  DDX5 - dead (asp-glu-ala-asp) box helicase 5  DDX3X - dead (asp-glu-ala-asp) box helicase 3, x-linked  DDX1 - dead (asp-glu-ala-asp) box helicase 1  ARID4B - at rich interactive domain 4b (rbp1-like)  SIN3A - sin3 transcription regulator family member a  MAP3K10 - mitogen-activated protein kinase kinase kinase 10  WWTR1 - ww domain containing transcription regulator 1  SET - set nuclear oncogene  ZNF304 - zinc finger protein 304  ITGB1BP1 - integrin beta 1 binding protein 1  KDM3B - lysine (k)-specific demethylase 3b  RSF1 - remodeling and spacing factor 1  ZNF286A - zinc finger protein 286a  MLLT6 - myeloid/lymphoid or mixed-lineage leukemia (trithorax homolog, drosophila); translocated to, 6  PBXIP1 - pre-b-cell leukemia homeobox interacting protein 1  NOL11 - nucleolar protein 11  MLLT3 - myeloid/lymphoid or mixed-lineage leukemia (trithorax homolog, drosophila); translocated to, 3  MLLT1 - myeloid/lymphoid or mixed-lineage leukemia (trithorax homolog, drosophila); translocated to, 1  MED14 - mediator complex subunit 14  DBP - d site of albumin promoter (albumin d-box) binding protein  KMT2A - lysine (k)-specific methyltransferase 2a  ZBTB22 - zinc finger and btb domain containing 22  NELFB - negative elongation factor complex member b  RPS6KA5 - ribosomal protein s6 kinase, 90kda, polypeptide 5  SUFU - suppressor of fused homolog (drosophila)  AHCTF1 - at hook containing transcription factor 1  ZBTB38 - zinc finger and btb domain containing 38  NLK - nemo-like kinase  UIMC1 - ubiquitin interaction motif containing 1  MNAT1 - mnat cdk-activating kinase assembly factor 1  GATAD2B - gata zinc finger domain containing 2b  ZNF551 - zinc finger protein 551  MTA2 - metastasis associated 1 family, member 2  NOLC1 - nucleolar and coiled-body phosphoprotein 1  ZNF473 - zinc finger protein 473  EIF2A - eukaryotic translation initiation factor 2a, 65kda  WWOX - ww domain containing oxidoreductase  CD200 - cd200 molecule  MNT - mnt, max dimerization protein  CCPG1 - cell cycle progression 1  CNOT6 - ccr4-not transcription complex, subunit 6  ZNF451 - zinc finger protein 451  SPTY2D1 - spt2, suppressor of ty, domain containing 1 (s. cerevisiae)  SCAF4 - sr-related ctd-associated factor 4  SS18L1 - synovial sarcoma translocation gene on chromosome 18-like 1  CHD5 - chromodomain helicase dna binding protein 5  MECP2 - methyl cpg binding protein 2 (rett syndrome)  PELO - pelota homolog (drosophila)  MEF2A - myocyte enhancer factor 2a  ARID1B - at rich interactive domain 1b (swi1-like)  PRICKLE1 - prickle homolog 1 (drosophila)  MED30 - mediator complex subunit 30  ZBED1 - zinc finger, bed-type containing 1  MKL2 - mkl/myocardin-like 2  MEIS2 - meis homeobox 2  BRWD1 - bromodomain and wd repeat domain containing 1  ZNF664 - zinc finger protein 664  MEIS1 - meis homeobox 1  MAP3K5 - mitogen-activated protein kinase kinase kinase 5  SLC33A1 - solute carrier family 33 (acetyl-coa transporter), member 1  MEN1 - multiple endocrine neoplasia i  WDR75 - wd repeat domain 75  DAPK3 - death-associated protein kinase 3  DAXX - death-domain associated protein  UTP15 - utp15, u3 small nucleolar ribonucleoprotein, homolog (s. cerevisiae)  ZZZ3 - zinc finger, zz-type containing 3  HINFP - histone h4 transcription factor  KAT8 - k(lysine) acetyltransferase 8  ZNF644 - zinc finger protein 644  MTA3 - metastasis associated 1 family, member 3  DGKQ - diacylglycerol kinase, theta 110kda  ARHGEF2 - rho/rac guanine nucleotide exchange factor (gef) 2  ZNF608 - zinc finger protein 608  DAP - death-associated protein  MAVS - mitochondrial antiviral signaling protein  SARS - seryl-trna synthetase  SATB1 - satb homeobox 1  MBD3 - methyl-cpg binding domain protein 3  RPTOR - regulatory associated protein of mtor, complex 1  SAFB - scaffold attachment factor b  PTOV1 - prostate tumor overexpressed 1  ZNF394 - zinc finger protein 394  SALL2 - sal-like 2 (drosophila)  CBFA2T2 - core-binding factor, runt domain, alpha subunit 2; translocated to, 2  DRD2 - dopamine receptor d2  EPC2 - enhancer of polycomb homolog 2 (drosophila)  CXXC5 - cxxc finger protein 5  PHF19 - phd finger protein 19  SLC9A1 - solute carrier family 9, subfamily a (nhe1, cation proton antiporter 1), member 1  IRF2BP1 - interferon regulatory factor 2 binding protein 1  RQCD1 - rcd1 required for cell differentiation1 homolog (s. pombe)  EAF1 - ell associated factor 1  ATN1 - atrophin 1  IL23A - interleukin 23, alpha subunit p19  MARS - methionyl-trna synthetase  C10orf137 - chromosome 10 open reading frame 137  MTA1 - metastasis associated 1  SIRT6 - sirtuin 6  DR1 - down-regulator of transcription 1, tbp-binding (negative cofactor 2)  PPHLN1 - periphilin 1  SKI - v-ski avian sarcoma viral oncogene homolog  SKIL - ski-like oncogene  MBD1 - methyl-cpg binding domain protein 1  DYRK1A - dual-specificity tyrosine-(y)-phosphorylation regulated kinase 1a  SFMBT1 - scm-like with four mbt domains 1  MKL1 - megakaryoblastic leukemia (translocation) 1  MAX - myc associated factor x  ZMIZ2 - zinc finger, miz-type containing 2  DVL2 - dishevelled segment polarity protein 2  DVL3 - dishevelled segment polarity protein 3  LDB2 - lim domain binding 2  ZFP90 - zfp90 zinc finger protein  FNIP2 - folliculin interacting protein 2  HOMEZ - homeobox and leucine zipper encoding  NELFCD - negative elongation factor complex member c/d  EBF4 - early b-cell factor 4  ASH2L - ash2 (absent, small, or homeotic)-like (drosophila)  ZFHX2 - zinc finger homeobox 2  CTDSPL2 - ctd (carboxy-terminal domain, rna polymerase ii, polypeptide a) small phosphatase like 2  ZSCAN29 - zinc finger and scan domain containing 29  THAP2 - thap domain containing, apoptosis associated protein 2  DTX1 - deltex homolog 1 (drosophila)  ITCH - itchy e3 ubiquitin protein ligase  ZNF484 - zinc finger protein 484  L3MBTL2 - l(3)mbt-like 2 (drosophila)  DLG1 - discs, large homolog 1 (drosophila)  LRP6 - low density lipoprotein receptor-related protein 6  ZNF445 - zinc finger protein 445  SHOX2 - short stature homeobox 2  AIP - aryl hydrocarbon receptor interacting protein  SIAH2 - siah e3 ubiquitin protein ligase 2  TRAPPC9 - trafficking protein particle complex 9  LUM - lumican  BTAF1 - btaf1 rna polymerase ii, b-tfiid transcription factor-associated, 170kda  POGK - pogo transposable element with krab domain  UBA3 - ubiquitin-like modifier activating enzyme 3  DHFR - dihydrofolate reductase  SESN2 - sestrin 2  PARP9 - poly (adp-ribose) polymerase family, member 9  PHF12 - phd finger protein 12  RNF8 - ring finger protein 8, e3 ubiquitin protein ligase  BAZ1B - bromodomain adjacent to zinc finger domain, 1b  CALCOCO1 - calcium binding and coiled-coil domain 1  ZBTB4 - zinc finger and btb domain containing 4  SFPQ - splicing factor proline/glutamine-rich  SFRP1 - secreted frizzled-related protein 1  SFRP2 - secreted frizzled-related protein 2  MXD1 - max dimerization protein 1  MED15 - mediator complex subunit 15  SFRP4 - secreted frizzled-related protein 4  PIAS4 - protein inhibitor of activated stat, 4  TRIM33 - tripartite motif containing 33  SMAD2 - smad family member 2  SRRT - serrate rna effector molecule homolog (arabidopsis)  SRSF2 - serine/arginine-rich splicing factor 2  GMEB2 - glucocorticoid modulatory element binding protein 2  DNM2 - dynamin 2  DNMT1 - dna (cytosine-5-)-methyltransferase 1  SPAG8 - sperm associated antigen 8  HDAC7 - histone deacetylase 7  KLF16 - kruppel-like factor 16  ARMCX3 - armadillo repeat containing, x-linked 3  DNMT3A - dna (cytosine-5-)-methyltransferase 3 alpha  TDP2 - tyrosyl-dna phosphodiesterase 2  EIF2AK4 - eukaryotic translation initiation factor 2 alpha kinase 4  LARP7 - la ribonucleoprotein domain family, member 7  CHD8 - chromodomain helicase dna binding protein 8  MAFG - v-maf avian musculoaponeurotic fibrosarcoma oncogene homolog g  RPS6KA4 - ribosomal protein s6 kinase, 90kda, polypeptide 4  TAF9B - taf9b rna polymerase ii, tata box binding protein (tbp)-associated factor, 31kda  NOC2L - nucleolar complex associated 2 homolog (s. cerevisiae)  SGK1 - serum/glucocorticoid regulated kinase 1  SMAD4 - smad family member 4  ZNF317 - zinc finger protein 317  SMAD5 - smad family member 5  H1FX - h1 histone family, member x  SMAD7 - smad family member 7  WASL - wiskott-aldrich syndrome-like  ZNF565 - zinc finger protein 565  SAP30BP - sap30 binding protein  MEIS3 - meis homeobox 3  YAF2 - yy1 associated factor 2  TAGLN3 - transgelin 3  SIX5 - six homeobox 5  NDN - necdin, melanoma antigen (mage) family member  MAPRE3 - microtubule-associated protein, rp/eb family, member 3  ZNF582 - zinc finger protein 582  NCK1 - nck adaptor protein 1  BRD7 - bromodomain containing 7  NCL - nucleolin  ELK1 - elk1, member of ets oncogene family  CEBPZ - ccaat/enhancer binding protein (c/ebp), zeta  TICAM1 - toll-like receptor adaptor molecule 1  ELK3 - elk3, ets-domain protein (srf accessory protein 2)  ELK4 - elk4, ets-domain protein (srf accessory protein 1)  SNW1 - snw domain containing 1  CCNL2 - cyclin l2  SCAP - srebf chaperone  TRIM28 - tripartite motif containing 28  EPAS1 - endothelial pas domain protein 1  EP300 - e1a binding protein p300  ATF6 - activating transcription factor 6  TBK1 - tank-binding kinase 1  ZNF197 - zinc finger protein 197  ENO1 - enolase 1, (alpha)  TDRD3 - tudor domain containing 3  EIF4A2 - eukaryotic translation initiation factor 4a2  EGR2 - early growth response 2  TRIM11 - tripartite motif containing 11  PPP1R12A - protein phosphatase 1, regulatory subunit 12a  EGR3 - early growth response 3  MYT1 - myelin transcription factor 1  ALYREF - aly/ref export factor  NAB1 - ngfi-a binding protein 1 (egr1 binding protein 1)  SENP1 - sumo1/sentrin specific peptidase 1  CSRNP2 - cysteine-serine-rich nuclear protein 2  NAB2 - ngfi-a binding protein 2 (egr1 binding protein 2)  RNF41 - ring finger protein 41  TRIM32 - tripartite motif containing 32  TSHZ1 - teashirt zinc finger homeobox 1  KDM2A - lysine (k)-specific demethylase 2a  PSME3 - proteasome (prosome, macropain) activator subunit 3 (pa28 gamma; ki)  ZNF326 - zinc finger protein 326  ELF1 - e74-like factor 1 (ets domain transcription factor)  ELF2 - e74-like factor 2 (ets domain transcription factor)  TCF25 - transcription factor 25 (basic helix-loop-helix)  TSG101 - tumor susceptibility gene 101  TRIM13 - tripartite motif containing 13  TRIM8 - tripartite motif containing 8  MYO6 - myosin vi  LBH - limb bud and heart development  ABT1 - activator of basal transcription 1  TOPORS - topoisomerase i binding, arginine/serine-rich, e3 ubiquitin protein ligase  CNOT1 - ccr4-not transcription complex, subunit 1  EFNA1 - ephrin-a1  SPEN - spen homolog, transcriptional regulator (drosophila)  CTNNBIP1 - catenin, beta interacting protein 1  MYD88 - myeloid differentiation primary response 88  ZNF362 - zinc finger protein 362  BBX - bobby sox homolog (drosophila)  PHC1 - polyhomeotic homolog 1 (drosophila)  TP53BP1 - tumor protein p53 binding protein 1  HCFC2 - host cell factor c2  PHC2 - polyhomeotic homolog 2 (drosophila)  EGR1 - early growth response 1  ZNF292 - zinc finger protein 292  CNOT7 - ccr4-not transcription complex, subunit 7  EGFR - epidermal growth factor receptor  MXI1 - max interactor 1, dimerization protein  MEGF8 - multiple egf-like-domains 8  PQBP1 - polyglutamine binding protein 1  CELSR2 - cadherin, egf lag seven-pass g-type receptor 2  KDM1A - lysine (k)-specific demethylase 1a  ATXN7L3 - ataxin 7-like 3  ACTR2 - arp2 actin-related protein 2 homolog (yeast)  ACTR3 - arp3 actin-related protein 3 homolog (yeast)  NCOA6 - nuclear receptor coactivator 6  E2F6 - e2f transcription factor 6  TSFM - ts translation elongation factor, mitochondrial  E4F1 - e4f transcription factor 1  ZHX3 - zinc fingers and homeoboxes 3  E2F4 - e2f transcription factor 4, p107/p130-binding  E2F5 - e2f transcription factor 5, p130-binding  ENY2 - enhancer of yellow 2 homolog (drosophila)  TRIM37 - tripartite motif containing 37  C11orf30 - chromosome 11 open reading frame 30  RRP1B - ribosomal rna processing 1b  ZNF558 - zinc finger protein 558  EDNRB - endothelin receptor type b  ZNF263 - zinc finger protein 263  TRAF1 - tnf receptor-associated factor 1  S1PR1 - sphingosine-1-phosphate receptor 1  TRAF3 - tnf receptor-associated factor 3  TRAF2 - tnf receptor-associated factor 2  PURG - purine-rich element binding protein g  NR2C1 - nuclear receptor subfamily 2, group c, member 1  SETX - senataxin  ZBTB47 - zinc finger and btb domain containing 47  NR2C2 - nuclear receptor subfamily 2, group c, member 2  MED25 - mediator complex subunit 25  TPR - translocated promoter region, nuclear basket protein  ZNF609 - zinc finger protein 609  ERC1 - elks/rab6-interacting/cast family member 1  MED6 - mediator complex subunit 6  PPRC1 - peroxisome proliferator-activated receptor gamma, coactivator-related 1  NAT14 - n-acetyltransferase 14 (gcn5-related, putative)  KDM4C - lysine (k)-specific demethylase 4c  ZNF423 - zinc finger protein 423  REXO4 - rex4, rna exonuclease 4 homolog (s. cerevisiae)  CAMK1D - calcium/calmodulin-dependent protein kinase id  DMTF1 - cyclin d binding myb-like transcription factor 1  USF1 - upstream transcription factor 1  NUP107 - nucleoporin 107kda  ZBTB43 - zinc finger and btb domain containing 43  ZBTB33 - zinc finger and btb domain containing 33  BPTF - bromodomain phd finger transcription factor  PITHD1 - pith (c-terminal proteasome-interacting domain of thioredoxin-like) domain containing 1  HDAC5 - histone deacetylase 5  MTF1 - metal-regulatory transcription factor 1  SRA1 - steroid receptor rna activator 1  HDAC6 - histone deacetylase 6  NR1H2 - nuclear receptor subfamily 1, group h, member 2  TAB2 - tgf-beta activated kinase 1/map3k7 binding protein 2  ETV3 - ets variant 3  CAMTA2 - calmodulin binding transcription activator 2  RNF187 - ring finger protein 187  ETV5 - ets variant 5  POGZ - pogo transposable element with znf domain  TWSG1 - twisted gastrulation homolog 1 (drosophila)  MT3 - metallothionein 3  ETV6 - ets variant 6  PLXND1 - plexin d1  VHL - von hippel-lindau tumor suppressor, e3 ubiquitin protein ligase  MED16 - mediator complex subunit 16  KDM6B - lysine (k)-specific demethylase 6b  EZR - ezrin  PHF8 - phd finger protein 8  TBX20 - t-box 20  EWSR1 - ews rna-binding protein 1  TRIM41 - tripartite motif containing 41  KCTD1 - potassium channel tetramerization domain containing 1  CCDC22 - coiled-coil domain containing 22  CREB3L1 - camp responsive element binding protein 3-like 1  EZH1 - enhancer of zeste homolog 1 (drosophila)  CIC - capicua transcriptional repressor  CCNL1 - cyclin l1  F2R - coagulation factor ii (thrombin) receptor  DNAJB6 - dnaj (hsp40) homolog, subfamily b, member 6  WDR43 - wd repeat domain 43  ERF - ets2 repressor factor  ZNF575 - zinc finger protein 575  CITED1 - cbp/p300-interacting transactivator, with glu/asp-rich carboxy-terminal domain, 1  ZBTB5 - zinc finger and btb domain containing 5  ZBTB40 - zinc finger and btb domain containing 40  ELMSAN1 - elm2 and myb/sant-like domain containing 1  THAP11 - thap domain containing 11  ZNF248 - zinc finger protein 248  SUPT7L - suppressor of ty 7 (s. cerevisiae)-like  UBB - ubiquitin b  RGCC - regulator of cell cycle  RCOR1 - rest corepressor 1  TULP3 - tubby like protein 3  ZNFX1 - zinc finger, nfx1-type containing 1  HIRA - histone cell cycle regulator  COMMD1 - copper metabolism (murr1) domain containing 1  TTC5 - tetratricopeptide repeat domain 5  ETV1 - ets variant 1  ETS1 - v-ets avian erythroblastosis virus e26 oncogene homolog 1  ZMIZ1 - zinc finger, miz-type containing 1  USP3 - ubiquitin specific peptidase 3  UBP1 - upstream binding protein 1 (lbp-1a)  SUMO1 - small ubiquitin-like modifier 1  UBTF - upstream binding transcription factor, rna polymerase i  USP15 - ubiquitin specific peptidase 15  PELI1 - pellino e3 ubiquitin protein ligase 1  CHURC1 - churchill domain containing 1  SMURF1 - smad specific e3 ubiquitin protein ligase 1  ZC3H4 - zinc finger ccch-type containing 4  MED4 - mediator complex subunit 4  JMJD6 - jumonji domain containing 6  THOC1 - tho complex 1  SETD2 - set domain containing 2  UBE2I - ubiquitin-conjugating enzyme e2i  RBX1 - ring-box 1, e3 ubiquitin protein ligase  THRAP3 - thyroid hormone receptor associated protein 3  UBE2L3 - ubiquitin-conjugating enzyme e2l 3  MED12 - mediator complex subunit 12  MED13 - mediator complex subunit 13  UBE2N - ubiquitin-conjugating enzyme e2n  UBE2V1 - ubiquitin-conjugating enzyme e2 variant 1  PSMD6 - proteasome (prosome, macropain) 26s subunit, non-atpase, 6  VPS72 - vacuolar protein sorting 72 homolog (s. cerevisiae)  MED24 - mediator complex subunit 24  MGA - mga, max dimerization protein  ZC3H6 - zinc finger ccch-type containing 6  DTX3L - deltex 3-like (drosophila)  ZNF34 - zinc finger protein 34  SETDB1 - set domain, bifurcated 1  ZEB1 - zinc finger e-box binding homeobox 1  WWC3 - wwc family member 3  ORC2 - origin recognition complex, subunit 2  TCF12 - transcription factor 12  ZNF354A - zinc finger protein 354a  TOX4 - tox high mobility group box family member 4  ZNF436 - zinc finger protein 436  CAMTA1 - calmodulin binding transcription activator 1  TCF20 - transcription factor 20 (ar1)  FLI1 - fli-1 proto-oncogene, ets transcription factor  RNF141 - ring finger protein 141  FLNA - filamin a, alpha  AGRN - agrin  PLCB1 - phospholipase c, beta 1 (phosphoinositide-specific)  FOXS1 - forkhead box s1  FOXO3 - forkhead box o3  HDAC8 - histone deacetylase 8  ASH1L - ash1 (absent, small, or homeotic)-like (drosophila)  SIN3B - sin3 transcription regulator family member b  G3BP2 - gtpase activating protein (sh3 domain) binding protein 2  NACC1 - nucleus accumbens associated 1, ben and btb (poz) domain containing  HR - hair growth associated  MED8 - mediator complex subunit 8  CAND1 - cullin-associated and neddylation-dissociated 1  TDG - thymine-dna glycosylase  OGG1 - 8-oxoguanine dna glycosylase  PRDX2 - peroxiredoxin 2  ZSCAN16 - zinc finger and scan domain containing 16  EAPP - e2f-associated phosphoprotein  FBXW11 - f-box and wd repeat domain containing 11  FAM208A - family with sequence similarity 208, member a  KDM3A - lysine (k)-specific demethylase 3a  FHL2 - four and a half lim domains 2  FGFR2 - fibroblast growth factor receptor 2  TAF9 - taf9 rna polymerase ii, tata box binding protein (tbp)-associated factor, 32kda  TAF7 - taf7 rna polymerase ii, tata box binding protein (tbp)-associated factor, 55kda  TAF6 - taf6 rna polymerase ii, tata box binding protein (tbp)-associated factor, 80kda  FGFR1 - fibroblast growth factor receptor 1  FGF9 - fibroblast growth factor 9  TADA2A - transcriptional adaptor 2a  DDR2 - discoidin domain receptor tyrosine kinase 2  TACC1 - transforming, acidic coiled-coil containing protein 1  BCLAF1 - bcl2-associated transcription factor 1  DAB2IP - dab2 interacting protein  NUMA1 - nuclear mitotic apparatus protein 1  TIRAP - toll-interleukin 1 receptor (tir) domain containing adaptor protein  FGF1 - fibroblast growth factor 1 (acidic)  FGF2 - fibroblast growth factor 2 (basic)  GMNN - geminin, dna replication inhibitor  NUP98 - nucleoporin 98kda  NR4A2 - nuclear receptor subfamily 4, group a, member 2  USP22 - ubiquitin specific peptidase 22  ZNF593 - zinc finger protein 593  ZBTB7B - zinc finger and btb domain containing 7b  TARBP1 - tar (hiv-1) rna binding protein 1  FER - fer (fps/fes related) tyrosine kinase  ZNF691 - zinc finger protein 691  MAP3K7 - mitogen-activated protein kinase kinase kinase 7  CREBRF - creb3 regulatory factor  ZNF623 - zinc finger protein 623  ADNP - activity-dependent neuroprotector homeobox  NPM1 - nucleophosmin (nucleolar phosphoprotein b23, numatrin)  NMD3 - nmd3 ribosome export adaptor  ARHGEF11 - rho guanine nucleotide exchange factor (gef) 11  KMT2E - lysine (k)-specific methyltransferase 2e  MED13L - mediator complex subunit 13-like  ZBTB24 - zinc finger and btb domain containing 24  ZEB2 - zinc finger e-box binding homeobox 2  THAP4 - thap domain containing 4  ZNF395 - zinc finger protein 395  MYNN - myoneurin  CRTC1 - creb regulated transcription coactivator 1  UFL1 - ufm1-specific ligase 1  TCF3 - transcription factor 3  DMAP1 - dna methyltransferase 1 associated protein 1  TCEB3 - transcription elongation factor b (siii), polypeptide 3 (110kda, elongin a)  TCF4 - transcription factor 4  NKRF - nfkb repressing factor  EFEMP1 - egf containing fibulin-like extracellular matrix protein 1  TSC22D2 - tsc22 domain family, member 2  ZNF629 - zinc finger protein 629  FBN1 - fibrillin 1  LANCL2 - lanc lantibiotic synthetase component c-like 2 (bacterial)  NRF1 - nuclear respiratory factor 1  NFXL1 - nuclear transcription factor, x-box binding-like 1  TCEA2 - transcription elongation factor a (sii), 2  ERBB2IP - erbb2 interacting protein  TCEA1 - transcription elongation factor a (sii), 1  NOP2 - nop2 nucleolar protein  NONO - non-pou domain containing, octamer-binding  VPRBP - vpr (hiv-1) binding protein  ITGB3BP - integrin beta 3 binding protein (beta3-endonexin)  RYBP - ring1 and yy1 binding protein  MTOR - mechanistic target of rapamycin (serine/threonine kinase)  MDFIC - myod family inhibitor domain containing  PAXBP1 - pax3 and pax7 binding protein 1  TLE1 - transducin-like enhancer of split 1 (e(sp1) homolog, drosophila)  TLE3 - transducin-like enhancer of split 3 (e(sp1) homolog, drosophila)  NOTCH4 - notch 4  IFT74 - intraflagellar transport 74 homolog (chlamydomonas)  TLE4 - transducin-like enhancer of split 4 (e(sp1) homolog, drosophila)  NPAT - nuclear protein, ataxia-telangiectasia locus  CEP290 - centrosomal protein 290kda  TLR3 - toll-like receptor 3  CNOT2 - ccr4-not transcription complex, subunit 2  NAA15 - n(alpha)-acetyltransferase 15, nata auxiliary subunit  CNOT3 - ccr4-not transcription complex, subunit 3  NOTCH1 - notch 1  BAZ2B - bromodomain adjacent to zinc finger domain, 2b  SIRT1 - sirtuin 1  NOTCH3 - notch 3  ST18 - suppression of tumorigenicity 18 (breast carcinoma) (zinc finger protein)  MKX - mohawk homeobox  NOTCH2 - notch 2  NFYA - nuclear transcription factor y, alpha  TMF1 - tata element modulatory factor 1  HDAC4 - histone deacetylase 4  TOX - thymocyte selection-associated high mobility group box  CBX6 - chromobox homolog 6  ACTR8 - arp8 actin-related protein 8 homolog (yeast)  TMSB4X - thymosin beta 4, x-linked  NFYC - nuclear transcription factor y, gamma  HEY1 - hairy/enhancer-of-split related with yrpw motif 1  ZFYVE16 - zinc finger, fyve domain containing 16  CXXC1 - cxxc finger protein 1  TMPO - thymopoietin  NFKBIA - nuclear factor of kappa light polypeptide gene enhancer in b-cells inhibitor, alpha  NFKB1 - nuclear factor of kappa light polypeptide gene enhancer in b-cells 1  CBX5 - chromobox homolog 5  KMT2B - lysine (k)-specific methyltransferase 2b  BRD4 - bromodomain containing 4  NFKBIB - nuclear factor of kappa light polypeptide gene enhancer in b-cells inhibitor, beta  NFKBIE - nuclear factor of kappa light polypeptide gene enhancer in b-cells inhibitor, epsilon  TARDBP - tar dna binding protein  BCL9L - b-cell cll/lymphoma 9-like  TNFRSF1A - tumor necrosis factor receptor superfamily, member 1a  NKX2-2 - nk2 homeobox 2  HDAC9 - histone deacetylase 9  ZNF606 - zinc finger protein 606  TNFRSF1B - tumor necrosis factor receptor superfamily, member 1b  ZNF614 - zinc finger protein 614  ZFP2 - zfp2 zinc finger protein  TMBIM6 - transmembrane bax inhibitor motif containing 6  SUZ12 - suz12 polycomb repressive complex 2 subunit  NEO1 - neogenin 1  TEF - thyrotrophic embryonic factor  POFUT1 - protein o-fucosyltransferase 1  RCOR2 - rest corepressor 2  TFAM - transcription factor a, mitochondrial  SMARCAL1 - swi/snf related, matrix associated, actin dependent regulator of chromatin, subfamily a-like 1  NEDD8 - neural precursor cell expressed, developmentally down-regulated 8  CTR9 - ctr9, paf1/rna polymerase ii complex component  PPM1F - protein phosphatase, mg2+/mn2+ dependent, 1f  TFDP1 - transcription factor dp-1  EPC1 - enhancer of polycomb homolog 1 (drosophila)  NFE2L2 - nuclear factor (erythroid-derived 2)-like 2  NR2F2 - nuclear receptor subfamily 2, group f, member 2  MORF4L2 - mortality factor 4 like 2  RASL11A - ras-like, family 11, member a  TFE3 - transcription factor binding to ighm enhancer 3  CBX7 - chromobox homolog 7  NFATC4 - nuclear factor of activated t-cells, cytoplasmic, calcineurin-dependent 4  NR2F1 - nuclear receptor subfamily 2, group f, member 1  TFCP2 - transcription factor cp2  NFIA - nuclear factor i/a  TGFA - transforming growth factor, alpha  ZNF740 - zinc finger protein 740  FNIP1 - folliculin interacting protein 1  HEY2 - hairy/enhancer-of-split related with yrpw motif 2  CDK5RAP3 - cdk5 regulatory subunit associated protein 3  TGFBR1 - transforming growth factor, beta receptor 1  TGFB2 - transforming growth factor, beta 2  RBFOX2 - rna binding protein, fox-1 homolog (c. elegans) 2  TGFB3 - transforming growth factor, beta 3  TGFB1 - transforming growth factor, beta 1  TGFB1I1 - transforming growth factor beta 1 induced transcript 1  ZNF746 - zinc finger protein 746  TP53INP1 - tumor protein p53 inducible nuclear protein 1  VGLL4 - vestigial like 4 (drosophila)  H2AFV - h2a histone family, member v  LRRC14 - leucine rich repeat containing 14  MEPCE - methylphosphate capping enzyme  RNF20 - ring finger protein 20, e3 ubiquitin protein ligase  KDM4A - lysine (k)-specific demethylase 4a  TGFBR3 - transforming growth factor, beta receptor iii  FOSL2 - fos-like antigen 2  PHF14 - phd finger protein 14  FOSB - fbj murine osteosarcoma viral oncogene homolog b  FOS - fbj murine osteosarcoma viral oncogene homolog  KAT6B - k(lysine) acetyltransferase 6b  HP1BP3 - heterochromatin protein 1, binding protein 3  ZNF432 - zinc finger protein 432  SAFB2 - scaffold attachment factor b2  MYEF2 - myelin expression factor 2  LPIN2 - lipin 2  THRA - thyroid hormone receptor, alpha |
| GO:0006281 | DNA repair | 9.33E-9 | 2.71E-6 | 1.20 (10334,345,6996,280) | [+] Show genes  RFC5 - replication factor c (activator 1) 5, 36.5kda  RFC2 - replication factor c (activator 1) 2, 40kda  PMS2 - pms2 postmeiotic segregation increased 2 (s. cerevisiae)  RFC3 - replication factor c (activator 1) 3, 38kda  LIG4 - ligase iv, dna, atp-dependent  FAN1 - fancd2/fanci-associated nuclease 1  FBXO18 - f-box protein, helicase, 18  LIG1 - ligase i, dna, atp-dependent  LIG3 - ligase iii, dna, atp-dependent  ABL1 - c-abl oncogene 1, non-receptor tyrosine kinase  MGME1 - mitochondrial genome maintenance exonuclease 1  TRIM28 - tripartite motif containing 28  EP300 - e1a binding protein p300  RECQL - recq protein-like (dna helicase q1-like)  KIN - kin, antigenic determinant of reca protein homolog (mouse)  DOT1L - dot1-like histone h3k79 methyltransferase  UPF1 - upf1 regulator of nonsense transcripts homolog (yeast)  NBN - nibrin  PMS1 - pms1 postmeiotic segregation increased 1 (s. cerevisiae)  UBQLN4 - ubiquilin 4  ALKBH1 - alkb, alkylation repair homolog 1 (e. coli)  MUM1 - melanoma associated antigen (mutated) 1  SLX4 - slx4 structure-specific endonuclease subunit  CHD1L - chromodomain helicase dna binding protein 1-like  TFPT - tcf3 (e2a) fusion partner (in childhood leukemia)  OTUB1 - otu domain, ubiquitin aldehyde binding 1  KDM2A - lysine (k)-specific demethylase 2a  MORF4L1 - mortality factor 4 like 1  SMUG1 - single-strand-selective monofunctional uracil-dna glycosylase 1  PSMD14 - proteasome (prosome, macropain) 26s subunit, non-atpase, 14  POLK - polymerase (dna directed) kappa  ACTL6A - actin-like 6a  INIP - ints3 and nabp interacting protein  TRRAP - transformation/transcription domain-associated protein  C1orf86 - chromosome 1 open reading frame 86  TP53BP1 - tumor protein p53 binding protein 1  RNF138 - ring finger protein 138, e3 ubiquitin protein ligase  HUWE1 - hect, uba and wwe domain containing 1, e3 ubiquitin protein ligase  NABP2 - nucleic acid binding protein 2  HUS1 - hus1 checkpoint homolog (s. pombe)  ACTR5 - arp5 actin-related protein 5 homolog (yeast)  RBM17 - rna binding motif protein 17  CDC45 - cell division cycle 45  INO80B - ino80 complex subunit b  CDC7 - cell division cycle 7  SMC1A - structural maintenance of chromosomes 1a  UBR5 - ubiquitin protein ligase e3 component n-recognin 5  HERC2 - hect and rld domain containing e3 ubiquitin protein ligase 2  POLR2K - polymerase (rna) ii (dna directed) polypeptide k, 7.0kda  RNF111 - ring finger protein 111  SMG1 - smg1 phosphatidylinositol 3-kinase-related kinase  UCHL5 - ubiquitin carboxyl-terminal hydrolase l5  XAB2 - xpa binding protein 2  MUTYH - muty homolog (e. coli)  PDS5B - pds5, regulator of cohesion maintenance, homolog b (s. cerevisiae)  NSMCE1 - non-smc element 1 homolog (s. cerevisiae)  RAD50 - rad50 homolog (s. cerevisiae)  C11orf30 - chromosome 11 open reading frame 30  POLH - polymerase (dna directed), eta  PARP1 - poly (adp-ribose) polymerase 1  C7orf49 - chromosome 7 open reading frame 49  POLD2 - polymerase (dna directed), delta 2, accessory subunit  TAOK3 - tao kinase 3  POLD1 - polymerase (dna directed), delta 1, catalytic subunit  FZR1 - fizzy/cell division cycle 20 related 1 (drosophila)  ZBTB7A - zinc finger and btb domain containing 7a  ZMPSTE24 - zinc metallopeptidase ste24  MBD4 - methyl-cpg binding domain protein 4  STUB1 - stip1 homology and u-box containing protein 1, e3 ubiquitin protein ligase  SETX - senataxin  POLR2G - polymerase (rna) ii (dna directed) polypeptide g  POLR2F - polymerase (rna) ii (dna directed) polypeptide f  POLR2E - polymerase (rna) ii (dna directed) polypeptide e, 25kda  POLR2D - polymerase (rna) ii (dna directed) polypeptide d  POLR2C - polymerase (rna) ii (dna directed) polypeptide c, 33kda  POLR2B - polymerase (rna) ii (dna directed) polypeptide b, 140kda  POLR2A - polymerase (rna) ii (dna directed) polypeptide a, 220kda  PARP4 - poly (adp-ribose) polymerase family, member 4  RUVBL2 - ruvb-like 2 (e. coli)  PPIE - peptidylprolyl isomerase e (cyclophilin e)  REXO4 - rex4, rna exonuclease 4 homolog (s. cerevisiae)  MCRS1 - microspherule protein 1  HMGN1 - high mobility group nucleosome binding domain 1  USP1 - ubiquitin specific peptidase 1  FANCE - fanconi anemia, complementation group e  SLC30A9 - solute carrier family 30 (zinc transporter), member 9  UNG - uracil-dna glycosylase  PPP5C - protein phosphatase 5, catalytic subunit  RPA3 - replication protein a3, 14kda  MAD2L2 - mad2 mitotic arrest deficient-like 2 (yeast)  RPA1 - replication protein a1, 70kda  C19orf40 - chromosome 19 open reading frame 40  RPA2 - replication protein a2, 32kda  PARP2 - poly (adp-ribose) polymerase 2  RECQL5 - recq protein-like 5  USP7 - ubiquitin specific peptidase 7 (herpes virus-associated)  XRCC6 - x-ray repair complementing defective repair in chinese hamster cells 6  RNF168 - ring finger protein 168, e3 ubiquitin protein ligase  SMC5 - structural maintenance of chromosomes 5  CHAF1A - chromatin assembly factor 1, subunit a (p150)  SUV420H1 - suppressor of variegation 4-20 homolog 1 (drosophila)  UVRAG - uv radiation resistance associated  EYA3 - eyes absent homolog 3 (drosophila)  TOPBP1 - topoisomerase (dna) ii binding protein 1  ENDOV - endonuclease v  INTS3 - integrator complex subunit 3  RMI2 - recq mediated genome instability 2  SUV420H2 - suppressor of variegation 4-20 homolog 2 (drosophila)  VCP - valosin containing protein  RPAIN - rpa interacting protein  MSH3 - muts homolog 3 (e. coli)  MSH2 - muts homolog 2, colon cancer, nonpolyposis type 1 (e. coli)  ERCC5 - excision repair cross-complementing rodent repair deficiency, complementation group 5  ERCC4 - excision repair cross-complementing rodent repair deficiency, complementation group 4  ERCC3 - excision repair cross-complementing rodent repair deficiency, complementation group 3  NIPBL - nipped-b homolog (drosophila)  UBE2A - ubiquitin-conjugating enzyme e2a  INO80C - ino80 complex subunit c  ASF1A - anti-silencing function 1a histone chaperone  NPLOC4 - nuclear protein localization 4 homolog (s. cerevisiae)  CCNH - cyclin h  UBB - ubiquitin b  SWSAP1 - swim-type zinc finger 7 associated protein 1  SSRP1 - structure specific recognition protein 1  TTC5 - tetratricopeptide repeat domain 5  COMMD1 - copper metabolism (murr1) domain containing 1  FBXO6 - f-box protein 6  PAPD7 - pap associated domain containing 7  BOD1L1 - biorientation of chromosomes in cell division 1-like 1  TRIP12 - thyroid hormone receptor interactor 12  USP3 - ubiquitin specific peptidase 3  SUMO1 - small ubiquitin-like modifier 1  BABAM1 - brisc and brca1 a complex member 1  UBE2T - ubiquitin-conjugating enzyme e2t (putative)  ZNF830 - zinc finger protein 830  COPS6 - cop9 signalosome subunit 6  COPS2 - cop9 signalosome subunit 2  ADPRHL2 - adp-ribosylhydrolase like 2  ZSWIM7 - zinc finger, swim-type containing 7  SETD2 - set domain containing 2  RBX1 - ring-box 1, e3 ubiquitin protein ligase  HMGA1 - high mobility group at-hook 1  MRE11A - mre11 meiotic recombination 11 homolog a (s. cerevisiae)  UBE2V2 - ubiquitin-conjugating enzyme e2 variant 2  ERCC2 - excision repair cross-complementing rodent repair deficiency, complementation group 2  NABP1 - nucleic acid binding protein 1  SHPRH - snf2 histone linker phd ring helicase, e3 ubiquitin protein ligase  UBE2N - ubiquitin-conjugating enzyme e2n  UBE2V1 - ubiquitin-conjugating enzyme e2 variant 1  APEX1 - apex nuclease (multifunctional dna repair enzyme) 1  CDC5L - cell division cycle 5-like  SMC6 - structural maintenance of chromosomes 6  DTX3L - deltex 3-like (drosophila)  DDX1 - dead (asp-glu-ala-asp) box helicase 1  SPRTN - sprt-like n-terminal domain  RFWD3 - ring finger and wd repeat domain 3  TREX1 - three prime repair exonuclease 1  RNF113A - ring finger protein 113a  SAMHD1 - sam domain and hd domain 1  PDS5A - pds5, regulator of cohesion maintenance, homolog a (s. cerevisiae)  PRMT6 - protein arginine methyltransferase 6  SETMAR - set domain and mariner transposase fusion gene  DDB1 - damage-specific dna binding protein 1, 127kda  MLH1 - mutl homolog 1, colon cancer, nonpolyposis type 2 (e. coli)  ERCC6L2 - excision repair cross-complementing rodent repair deficiency, complementation group 6-like 2  NEIL1 - nei endonuclease viii-like 1 (e. coli)  GTF2H2 - general transcription factor iih, polypeptide 2, 44kda  GTF2H3 - general transcription factor iih, polypeptide 3, 34kda  MCM9 - minichromosome maintenance complex component 9  GTF2H1 - general transcription factor iih, polypeptide 1, 62kda  POLL - polymerase (dna directed), lambda  PRPF19 - pre-mrna processing factor 19  MSH6 - muts homolog 6 (e. coli)  APBB1 - amyloid beta (a4) precursor protein-binding, family b, member 1 (fe65)  CDC14B - cell division cycle 14b  RIF1 - rap1 interacting factor homolog (yeast)  POLG2 - polymerase (dna directed), gamma 2, accessory subunit  AP5Z1 - adaptor-related protein complex 5, zeta 1 subunit  GTF2H4 - general transcription factor iih, polypeptide 4, 52kda  RCHY1 - ring finger and chy zinc finger domain containing 1, e3 ubiquitin protein ligase  POLI - polymerase (dna directed) iota  UIMC1 - ubiquitin interaction motif containing 1  MNAT1 - mnat cdk-activating kinase assembly factor 1  TDG - thymine-dna glycosylase  COPS7B - cop9 signalosome subunit 7b  OGG1 - 8-oxoguanine dna glycosylase  COPS3 - cop9 signalosome subunit 3  APITD1 - apoptosis-inducing, taf9-like domain 1  MCM7 - minichromosome maintenance complex component 7  H2AFX - h2a histone family, member x  RNASEH2A - ribonuclease h2, subunit a  NSMCE2 - non-smc element 2, mms21 homolog (s. cerevisiae)  SUPT16H - suppressor of ty 16 homolog (s. cerevisiae)  CETN2 - centrin, ef-hand protein, 2  SMCHD1 - structural maintenance of chromosomes flexible hinge domain containing 1  JMY - junction mediating and regulatory protein, p53 cofactor  CIB1 - calcium and integrin binding 1 (calmyrin)  FEN1 - flap structure-specific endonuclease 1  KAT5 - k(lysine) acetyltransferase 5  MEN1 - multiple endocrine neoplasia i  PCNA - proliferating cell nuclear antigen  INO80E - ino80 complex subunit e  NPM1 - nucleophosmin (nucleolar phosphoprotein b23, numatrin)  CDK9 - cyclin-dependent kinase 9  CDK7 - cyclin-dependent kinase 7  HINFP - histone h4 transcription factor  FANCL - fanconi anemia, complementation group l  APEX2 - apex nuclease (apurinic/apyrimidinic endonuclease) 2  WRAP53 - wd repeat containing, antisense to tp53  RUVBL1 - ruvb-like 1 (e. coli)  DMAP1 - dna methyltransferase 1 associated protein 1  CDK2 - cyclin-dependent kinase 2  KAT7 - k(lysine) acetyltransferase 7  ATRIP - atr interacting protein  TCEA1 - transcription elongation factor a (sii), 1  RAD1 - rad1 homolog (s. pombe)  NONO - non-pou domain containing, octamer-binding  EPC2 - enhancer of polycomb homolog 2 (drosophila)  XPA - xeroderma pigmentosum, complementation group a  ZBTB1 - zinc finger and btb domain containing 1  XPC - xeroderma pigmentosum, complementation group c  MUS81 - mus81 structure-specific endonuclease subunit  XRCC1 - x-ray repair complementing defective repair in chinese hamster cells 1  TAOK1 - tao kinase 1  SMC3 - structural maintenance of chromosomes 3  PAGR1 - paxip1 associated glutamate-rich protein 1  NDNL2 - necdin-like 2  MMS19 - mms19 nucleotide excision repair homolog (s. cerevisiae)  AQR - aquarius homolog (mouse)  MTA1 - metastasis associated 1  SIRT6 - sirtuin 6  AP5S1 - adaptor-related protein complex 5, sigma 1 subunit  SIRT1 - sirtuin 1  TNKS1BP1 - tankyrase 1 binding protein 1, 182kda  NFRKB - nuclear factor related to kappab binding protein  USP10 - ubiquitin specific peptidase 10  ACTR8 - arp8 actin-related protein 8 homolog (yeast)  WHSC1 - wolf-hirschhorn syndrome candidate 1  REV1 - rev1, polymerase (dna directed)  WDR33 - wd repeat domain 33  WDR48 - wd repeat domain 48  MCM3 - minichromosome maintenance complex component 3  MCM6 - minichromosome maintenance complex component 6  RAD21 - rad21 homolog (s. pombe)  MCM5 - minichromosome maintenance complex component 5  RAD17 - rad17 homolog (s. pombe)  MCM2 - minichromosome maintenance complex component 2  INO80 - ino80 complex subunit  MDC1 - mediator of dna-damage checkpoint 1  IGHMBP2 - immunoglobulin mu binding protein 2  RRM2B - ribonucleotide reductase m2 b (tp53 inducible)  SMARCAL1 - swi/snf related, matrix associated, actin dependent regulator of chromatin, subfamily a-like 1  BACH1 - btb and cnc homology 1, basic leucine zipper transcription factor 1  NEIL2 - nei endonuclease viii-like 2 (e. coli)  BRCC3 - brca1/brca2-containing complex, subunit 3  EXD2 - exonuclease 3'-5' domain containing 2  FAM35A - family with sequence similarity 35, member a  MORF4L2 - mortality factor 4 like 2  BCCIP - brca2 and cdkn1a interacting protein  ZFYVE26 - zinc finger, fyve domain containing 26  PARP9 - poly (adp-ribose) polymerase family, member 9  RNF8 - ring finger protein 8, e3 ubiquitin protein ligase  ATRX - alpha thalassemia/mental retardation syndrome x-linked  UVSSA - uv-stimulated scaffold protein a  MLH3 - mutl homolog 3 (e. coli)  SFPQ - splicing factor proline/glutamine-rich  RBBP8 - retinoblastoma binding protein 8  PIAS4 - protein inhibitor of activated stat, 4  TDP2 - tyrosyl-dna phosphodiesterase 2  CUL4B - cullin 4b  ERCC8 - excision repair cross-complementing rodent repair deficiency, complementation group 8  UBE2W - ubiquitin-conjugating enzyme e2w (putative)  XRCC5 - x-ray repair complementing defective repair in chinese hamster cells 5 (double-strand-break rejoining)  COPS7A - cop9 signalosome subunit 7a  XRCC4 - x-ray repair complementing defective repair in chinese hamster cells 4  POLD3 - polymerase (dna-directed), delta 3, accessory subunit  EME1 - essential meiotic structure-specific endonuclease 1  PARK7 - parkinson protein 7  KIAA0430 - kiaa0430  PNKP - polynucleotide kinase 3'-phosphatase |
| GO:0070925 | organelle assembly | 1.27E-8 | 3.61E-6 | 1.19 (10334,359,6996,290) | [+] Show genes  MAPRE1 - microtubule-associated protein, rp/eb family, member 1  TBC1D20 - tbc1 domain family, member 20  CHMP4C - charged multivesicular body protein 4c  MAPRE3 - microtubule-associated protein, rp/eb family, member 3  G3BP1 - gtpase activating protein (sh3 domain) binding protein 1  CEP89 - centrosomal protein 89kda  WDR19 - wd repeat domain 19  ATAT1 - alpha tubulin acetyltransferase 1  ABLIM1 - actin binding lim protein 1  GORAB - golgin, rab6-interacting  IFT46 - intraflagellar transport 46 homolog (chlamydomonas)  VPS37A - vacuolar protein sorting 37 homolog a (s. cerevisiae)  MKS1 - meckel syndrome, type 1  NCOR1 - nuclear receptor corepressor 1  HAUS7 - haus augmin-like complex, subunit 7  VMP1 - vacuole membrane protein 1  SSX2IP - synovial sarcoma, x breakpoint 2 interacting protein  ARL13B - adp-ribosylation factor-like 13b  AGFG1 - arfgap with fg repeats 1  MKKS - mckusick-kaufman syndrome  CCDC28B - coiled-coil domain containing 28b  KIF27 - kinesin family member 27  TMEM17 - transmembrane protein 17  FNBP1L - formin binding protein 1-like  CFLAR - casp8 and fadd-like apoptosis regulator  PTPDC1 - protein tyrosine phosphatase domain containing 1  EIF2S1 - eukaryotic translation initiation factor 2, subunit 1 alpha, 35kda  TMEM41B - transmembrane protein 41b  UBXN2B - ubx domain protein 2b  TMOD2 - tropomodulin 2 (neuronal)  AZI1 - 5-azacytidine induced 1  TSG101 - tumor susceptibility gene 101  AMBRA1 - autophagy/beclin-1 regulator 1  EHD1 - eh-domain containing 1  AKAP4 - a kinase (prka) anchor protein 4  FITM2 - fat storage-inducing transmembrane protein 2  MVB12A - multivesicular body subunit 12a  RAB1B - rab1b, member ras oncogene family  CEP19 - centrosomal protein 19kda  GABARAPL1 - gaba(a) receptor-associated protein like 1  MYH10 - myosin, heavy chain 10, non-muscle  MIS12 - mis12 kinetochore complex component  TBPL1 - tbp-like 1  MAP9 - microtubule-associated protein 9  SNX10 - sorting nexin 10  VPS4B - vacuolar protein sorting 4 homolog b (s. cerevisiae)  MAP1LC3A - microtubule-associated protein 1 light chain 3 alpha  HAUS6 - haus augmin-like complex, subunit 6  NIP7 - nip7, nucleolar pre-rrna processing protein  CNOT7 - ccr4-not transcription complex, subunit 7  CELSR2 - cadherin, egf lag seven-pass g-type receptor 2  AHI1 - abelson helper integration site 1  TP53INP2 - tumor protein p53 inducible nuclear protein 2  ACTR2 - arp2 actin-related protein 2 homolog (yeast)  ACTR3 - arp3 actin-related protein 3 homolog (yeast)  SMC1A - structural maintenance of chromosomes 1a  CLUAP1 - clusterin associated protein 1  E2F4 - e2f transcription factor 4, p107/p130-binding  STAM2 - signal transducing adaptor molecule (sh3 domain and itam motif) 2  ATG5 - autophagy related 5  ATP6V1D - atpase, h+ transporting, lysosomal 34kda, v1 subunit d  CNTROB - centrobin, centrosomal brca2 interacting protein  RAB38 - rab38, member ras oncogene family  TUBGCP2 - tubulin, gamma complex associated protein 2  LAMA5 - laminin, alpha 5  STAG1 - stromal antigen 1  FBF1 - fas (tnfrsf6) binding factor 1  STX12 - syntaxin 12  CDK5RAP2 - cdk5 regulatory subunit associated protein 2  VPS28 - vacuolar protein sorting 28 homolog (s. cerevisiae)  ATG16L1 - autophagy related 16-like 1 (s. cerevisiae)  VPS37C - vacuolar protein sorting 37 homolog c (s. cerevisiae)  KIF2A - kinesin heavy chain member 2a  KIF3A - kinesin family member 3a  PARVA - parvin, alpha  PIBF1 - progesterone immunomodulatory binding factor 1  DYNLL1 - dynein, light chain, lc8-type 1  TNKS - tankyrase, trf1-interacting ankyrin-related adp-ribose polymerase  PDCD6IP - programmed cell death 6 interacting protein  CCDC41 - coiled-coil domain containing 41  HDAC6 - histone deacetylase 6  CLASP2 - cytoplasmic linker associated protein 2  BECN1 - beclin 1, autophagy related  POGZ - pogo transposable element with znf domain  RAB7A - rab7a, member ras oncogene family  EZR - ezrin  TMEM237 - transmembrane protein 237  UNC119B - unc-119 homolog b (c. elegans)  SBDS - shwachman-bodian-diamond syndrome  KPNB1 - karyopherin (importin) beta 1  KIFC2 - kinesin family member c2  MPV17L2 - mpv17 mitochondrial membrane protein-like 2  BSCL2 - berardinelli-seip congenital lipodystrophy 2 (seipin)  TCTEX1D2 - tctex1 domain containing 2  TCTN2 - tectonic family member 2  IFT122 - intraflagellar transport 122 homolog (chlamydomonas)  C12orf44 - chromosome 12 open reading frame 44  RPS23 - ribosomal protein s23  TMEM216 - transmembrane protein 216  TUBB - tubulin, beta class i  TUBGCP5 - tubulin, gamma complex associated protein 5  DBNL - drebrin-like  CHMP7 - charged multivesicular body protein 7  IFT81 - intraflagellar transport 81 homolog (chlamydomonas)  AAAS - achalasia, adrenocortical insufficiency, alacrimia  KIF3B - kinesin family member 3b  IFT88 - intraflagellar transport 88 homolog (chlamydomonas)  TXNDC15 - thioredoxin domain containing 15  CDS2 - cdp-diacylglycerol synthase (phosphatidate cytidylyltransferase) 2  RAB11A - rab11a, member ras oncogene family  WDR11 - wd repeat domain 11  CHMP4A - charged multivesicular body protein 4a  TROVE2 - trove domain family, member 2  MAPRE2 - microtubule-associated protein, rp/eb family, member 2  PRRC2C - proline-rich coiled-coil 2c  GALNT11 - udp-n-acetyl-alpha-d-galactosamine:polypeptide n-acetylgalactosaminyltransferase 11 (galnac-t11)  SNAP29 - synaptosomal-associated protein, 29kda  HAUS1 - haus augmin-like complex, subunit 1  ATXN10 - ataxin 10  STAM - signal transducing adaptor molecule (sh3 domain and itam motif) 1  TUBGCP3 - tubulin, gamma complex associated protein 3  SQLE - squalene epoxidase  CSNK1D - casein kinase 1, delta  RBM14 - rna binding motif protein 14  VPS25 - vacuolar protein sorting 25 homolog (s. cerevisiae)  TMEM107 - transmembrane protein 107  DDX6 - dead (asp-glu-ala-asp) box helicase 6  MSTO1 - misato 1, mitochondrial distribution and morphology regulator  CCDC78 - coiled-coil domain containing 78  XIAP - x-linked inhibitor of apoptosis  DDX3X - dead (asp-glu-ala-asp) box helicase 3, x-linked  ATXN2L - ataxin 2-like  B9D2 - b9 protein domain 2  GSN - gelsolin  WWTR1 - ww domain containing transcription regulator 1  FLII - flightless i homolog (drosophila)  DCTN1 - dynactin 1  FLNA - filamin a, alpha  PTPN23 - protein tyrosine phosphatase, non-receptor type 23  PSEN1 - presenilin 1  WDR34 - wd repeat domain 34  FOPNL - fgfr1op n-terminal like  SHANK1 - sh3 and multiple ankyrin repeat domains 1  PACS2 - phosphofurin acidic cluster sorting protein 2  CDC14B - cell division cycle 14b  UBAP2L - ubiquitin associated protein 2-like  EXOC5 - exocyst complex component 5  G3BP2 - gtpase activating protein (sh3 domain) binding protein 2  PATL1 - protein associated with topoisomerase ii homolog 1 (yeast)  OCRL - oculocerebrorenal syndrome of lowe  PAFAH1B1 - platelet-activating factor acetylhydrolase 1b, regulatory subunit 1 (45kda)  RAB23 - rab23, member ras oncogene family  EIF2A - eukaryotic translation initiation factor 2a, 65kda  CD34 - cd34 molecule  BBS7 - bardet-biedl syndrome 7  IFT43 - intraflagellar transport 43 homolog (chlamydomonas)  APITD1 - apoptosis-inducing, taf9-like domain 1  HIAT1 - hippocampus abundant transcript 1  CEP250 - centrosomal protein 250kda  TCTN1 - tectonic family member 1  ARL3 - adp-ribosylation factor-like 3  ATG7 - autophagy related 7  HAUS5 - haus augmin-like complex, subunit 5  CENPC - centromere protein c  KIAA0586 - kiaa0586  TUBGCP4 - tubulin, gamma complex associated protein 4  ATXN2 - ataxin 2  PTEN - phosphatase and tensin homolog  CETN2 - centrin, ef-hand protein, 2  PDGFRB - platelet-derived growth factor receptor, beta polypeptide  IFT57 - intraflagellar transport 57 homolog (chlamydomonas)  TMEM231 - transmembrane protein 231  PDGFRA - platelet-derived growth factor receptor, alpha polypeptide  IST1 - increased sodium tolerance 1 homolog (yeast)  NSFL1C - nsfl1 (p97) cofactor (p47)  NUMA1 - nuclear mitotic apparatus protein 1  MEF2A - myocyte enhancer factor 2a  CLASP1 - cytoplasmic linker associated protein 1  ATG13 - autophagy related 13  RHOA - ras homolog family member a  ATG2B - autophagy related 2b  BBS9 - bardet-biedl syndrome 9  EIF6 - eukaryotic translation initiation factor 6  TEKT2 - tektin 2 (testicular)  WDR60 - wd repeat domain 60  RAB8A - rab8a, member ras oncogene family  GORASP2 - golgi reassembly stacking protein 2, 55kda  CHMP1A - charged multivesicular body protein 1a  ATG3 - autophagy related 3  NPM1 - nucleophosmin (nucleolar phosphoprotein b23, numatrin)  C2CD3 - c2 calcium-dependent domain containing 3  TTC8 - tetratricopeptide repeat domain 8  NUDCD3 - nudc domain containing 3  PCM1 - pericentriolar material 1  ZFYVE1 - zinc finger, fyve domain containing 1  FAM161A - family with sequence similarity 161, member a  LSM3 - lsm3 homolog, u6 small nuclear rna associated (s. cerevisiae)  SPICE1 - spindle and centriole associated protein 1  RABL2B - rab, member of ras oncogene family-like 2b  ATG12 - autophagy related 12  CHMP2B - charged multivesicular body protein 2b  ARL6 - adp-ribosylation factor-like 6  PUM2 - pumilio homolog 2 (drosophila)  HGS - hepatocyte growth factor-regulated tyrosine kinase substrate  SCLT1 - sodium channel and clathrin linker 1  RB1CC1 - rb1-inducible coiled-coil 1  OBSL1 - obscurin-like 1  IFT52 - intraflagellar transport 52 homolog (chlamydomonas)  CDK2 - cyclin-dependent kinase 2  ZNF207 - zinc finger protein 207  HAUS2 - haus augmin-like complex, subunit 2  UBQLN1 - ubiquilin 1  VTA1 - vesicle (multivesicular body) trafficking 1  TUBGCP6 - tubulin, gamma complex associated protein 6  ARMC9 - armadillo repeat containing 9  POC1B - poc1 centriolar protein b  TCTN3 - tectonic family member 3  GOLGA2 - golgin a2  TMEM138 - transmembrane protein 138  HAUS3 - haus augmin-like complex, subunit 3  TRAF3IP1 - tnf receptor-associated factor 3 interacting protein 1  SMC3 - structural maintenance of chromosomes 3  UBQLN2 - ubiquilin 2  IFT74 - intraflagellar transport 74 homolog (chlamydomonas)  MAPT - microtubule-associated protein tau  SHANK3 - sh3 and multiple ankyrin repeat domains 3  WIPI2 - wd repeat domain, phosphoinositide interacting 2  RAB14 - rab14, member ras oncogene family  ATP6V0D1 - atpase, h+ transporting, lysosomal 38kda, v0 subunit d1  CEP290 - centrosomal protein 290kda  RSG1 - rem2 and rab-like small gtpase 1  DZIP1 - daz interacting zinc finger protein 1  RAB33B - rab33b, member ras oncogene family  NOTCH1 - notch 1  PVRL2 - poliovirus receptor-related 2 (herpesvirus entry mediator b)  TMF1 - tata element modulatory factor 1  ATG14 - autophagy related 14  SH3PXD2B - sh3 and px domains 2b  EDC3 - enhancer of mrna decapping 3  STK36 - serine/threonine kinase 36  RILPL2 - rab interacting lysosomal protein-like 2  CENPT - centromere protein t  MTERFD2 - mterf domain containing 2  RAB1A - rab1a, member ras oncogene family  ICK - intestinal cell (mak-like) kinase  CHMP5 - charged multivesicular body protein 5  IFT140 - intraflagellar transport 140 homolog (chlamydomonas)  TTBK2 - tau tubulin kinase 2  NME5 - nme/nm23 family member 5  VPS4A - vacuolar protein sorting 4 homolog a (s. cerevisiae)  INO80 - ino80 complex subunit  EHD2 - eh-domain containing 2  EHD3 - eh-domain containing 3  CCP110 - centriolar coiled coil protein 110kda  LSM14A - lsm14a, scd6 homolog a (s. cerevisiae)  EHD4 - eh-domain containing 4  IQCB1 - iq motif containing b1  RSPH9 - radial spoke head 9 homolog (chlamydomonas)  NEK1 - nima-related kinase 1  TMEM80 - transmembrane protein 80  WRAP73 - wd repeat containing, antisense to tp73  ARHGEF10 - rho guanine nucleotide exchange factor (gef) 10  BCCIP - brca2 and cdkn1a interacting protein  BBS5 - bardet-biedl syndrome 5  CHMP3 - charged multivesicular body protein 3  DHX37 - deah (asp-glu-ala-his) box polypeptide 37  STX7 - syntaxin 7  CEP41 - centrosomal protein 41kda  OGFOD1 - 2-oxoglutarate and iron-dependent oxygenase domain containing 1  GABARAPL2 - gaba(a) receptor-associated protein-like 2  DYNC1H1 - dynein, cytoplasmic 1, heavy chain 1  VPS37D - vacuolar protein sorting 37 homolog d (s. cerevisiae)  GABARAP - gaba(a) receptor-associated protein  ATG16L2 - autophagy related 16-like 2 (s. cerevisiae)  STAG2 - stromal antigen 2  TP53INP1 - tumor protein p53 inducible nuclear protein 1  CEP63 - centrosomal protein 63kda  B9D1 - b9 protein domain 1  WDR45B - wd repeat domain 45b  HPS4 - hermansky-pudlak syndrome 4  PIK3C3 - phosphatidylinositol 3-kinase, catalytic subunit type 3  MZT1 - mitotic spindle organizing protein 1  CIRBP - cold inducible rna binding protein  CHMP4B - charged multivesicular body protein 4b  IFT172 - intraflagellar transport 172 homolog (chlamydomonas)  DHX29 - deah (asp-glu-ala-his) box polypeptide 29  CCDC66 - coiled-coil domain containing 66  ASAP1 - arfgap with sh3 domain, ankyrin repeat and ph domain 1  BBS4 - bardet-biedl syndrome 4  BBS2 - bardet-biedl syndrome 2 |
| GO:0031323 | regulation of cellular metabolic process | 1.38E-8 | 3.83E-6 | 1.05 (10334,3825,6996,2717) | [+] Show genes  AGAP2 - arfgap with gtpase domain, ankyrin repeat and ph domain 2  EXOSC9 - exosome component 9  MSL3 - male-specific lethal 3 homolog (drosophila)  EXOSC10 - exosome component 10  TADA1 - transcriptional adaptor 1  HNRNPA0 - heterogeneous nuclear ribonucleoprotein a0  AARS - alanyl-trna synthetase  CBX1 - chromobox homolog 1  PPP1R9A - protein phosphatase 1, regulatory subunit 9a  ABCA2 - atp-binding cassette, sub-family a (abc1), member 2  OS9 - osteosarcoma amplified 9, endoplasmic reticulum lectin  ABL1 - c-abl oncogene 1, non-receptor tyrosine kinase  ZNF821 - zinc finger protein 821  ERP29 - endoplasmic reticulum protein 29  PML - promyelocytic leukemia  MLLT11 - myeloid/lymphoid or mixed-lineage leukemia (trithorax homolog, drosophila); translocated to, 11  CNOT11 - ccr4-not transcription complex, subunit 11  ACACA - acetyl-coa carboxylase alpha  SUPT20H - suppressor of ty 20 homolog (s. cerevisiae)  YWHAQ - tyrosine 3-monooxygenase/tryptophan 5-monooxygenase activation protein, theta polypeptide  TMED10 - transmembrane emp24-like trafficking protein 10 (yeast)  BRD8 - bromodomain containing 8  ACLY - atp citrate lyase  ACHE - acetylcholinesterase  RFFL - ring finger and fyve-like domain containing e3 ubiquitin protein ligase  CHD7 - chromodomain helicase dna binding protein 7  LYAR - ly1 antibody reactive  PAPOLA - poly(a) polymerase alpha  TCERG1 - transcription elongation regulator 1  ZNF354B - zinc finger protein 354b  RNPS1 - rna binding protein s1, serine-rich domain  ACTB - actin, beta  OTUB1 - otu domain, ubiquitin aldehyde binding 1  EHMT2 - euchromatic histone-lysine n-methyltransferase 2  PLAUR - plasminogen activator, urokinase receptor  SPIN1 - spindlin 1  TASP1 - taspase, threonine aspartase, 1  PLCL1 - phospholipase c-like 1  PRR5 - proline rich 5 (renal)  SUB1 - sub1 homolog (s. cerevisiae)  KIF16B - kinesin family member 16b  MORF4L1 - mortality factor 4 like 1  AMBRA1 - autophagy/beclin-1 regulator 1  ZNF511 - zinc finger protein 511  PPID - peptidylprolyl isomerase d  CAMK2N1 - calcium/calmodulin-dependent protein kinase ii inhibitor 1  ACTL6A - actin-like 6a  ACTN1 - actinin, alpha 1  AP1AR - adaptor-related protein complex 1 associated regulatory protein  USP19 - ubiquitin specific peptidase 19  ACTN4 - actinin, alpha 4  ACVR2A - activin a receptor, type iia  ACVRL1 - activin a receptor type ii-like 1  PARL - presenilin associated, rhomboid-like  FAM122A - family with sequence similarity 122a  ACVR1 - activin a receptor, type i  ACVR1B - activin a receptor, type ib  ADAR - adenosine deaminase, rna-specific  ADARB1 - adenosine deaminase, rna-specific, b1  ADCY6 - adenylate cyclase 6  CPSF4 - cleavage and polyadenylation specific factor 4, 30kda  ZNF331 - zinc finger protein 331  ADCY5 - adenylate cyclase 5  MED1 - mediator complex subunit 1  ADCY1 - adenylate cyclase 1 (brain)  ADCY3 - adenylate cyclase 3  RAB39B - rab39b, member ras oncogene family  PPARD - peroxisome proliferator-activated receptor delta  MAML3 - mastermind-like 3 (drosophila)  ADCYAP1R1 - adenylate cyclase activating polypeptide 1 (pituitary) receptor type i  POLR2K - polymerase (rna) ii (dna directed) polypeptide k, 7.0kda  FAM129A - family with sequence similarity 129, member a  PARP1 - poly (adp-ribose) polymerase 1  POLH - polymerase (dna directed), eta  ADPRH - adp-ribosylarginine hydrolase  H2AFY2 - h2a histone family, member y2  PPP1R13L - protein phosphatase 1, regulatory subunit 13 like  TNFRSF19 - tumor necrosis factor receptor superfamily, member 19  HES6 - hairy and enhancer of split 6 (drosophila)  POLR2G - polymerase (rna) ii (dna directed) polypeptide g  POLR2D - polymerase (rna) ii (dna directed) polypeptide d  SMPD3 - sphingomyelin phosphodiesterase 3, neutral membrane (neutral sphingomyelinase ii)  PARP4 - poly (adp-ribose) polymerase family, member 4  RUVBL2 - ruvb-like 2 (e. coli)  POLR2A - polymerase (rna) ii (dna directed) polypeptide a, 220kda  AP2A1 - adaptor-related protein complex 2, alpha 1 subunit  CDK5RAP2 - cdk5 regulatory subunit associated protein 2  PRKAB1 - protein kinase, amp-activated, beta 1 non-catalytic subunit  RCOR3 - rest corepressor 3  PRKACB - protein kinase, camp-dependent, catalytic, beta  PRKACA - protein kinase, camp-dependent, catalytic, alpha  AES - amino-terminal enhancer of split  NUP133 - nucleoporin 133kda  NUPL2 - nucleoporin like 2  FAM46B - family with sequence similarity 46, member b  VPS35 - vacuolar protein sorting 35 homolog (s. cerevisiae)  JAG1 - jagged 1  EDEM2 - er degradation enhancer, mannosidase alpha-like 2  PRCP - prolylcarboxypeptidase (angiotensinase c)  CHFR - checkpoint with forkhead and ring finger domains, e3 ubiquitin protein ligase  PRDM4 - pr domain containing 4  PPP3R1 - protein phosphatase 3, regulatory subunit b, alpha  ATF7IP - activating transcription factor 7 interacting protein  FGFR1OP - fgfr1 oncogene partner  PPP5C - protein phosphatase 5, catalytic subunit  ZNF689 - zinc finger protein 689  PPP2R5D - protein phosphatase 2, regulatory subunit b', delta  LRIF1 - ligand dependent nuclear receptor interacting factor 1  PPP2R5C - protein phosphatase 2, regulatory subunit b', gamma  PPP2R5B - protein phosphatase 2, regulatory subunit b', beta  WDFY2 - wd repeat and fyve domain containing 2  ZNF653 - zinc finger protein 653  AKT1 - v-akt murine thymoma viral oncogene homolog 1  PPP4C - protein phosphatase 4, catalytic subunit  PCID2 - pci domain containing 2  PPP2R5E - protein phosphatase 2, regulatory subunit b', epsilon isoform  PPP2R2A - protein phosphatase 2, regulatory subunit b, alpha  PPP2R1B - protein phosphatase 2, regulatory subunit a, beta  CPSF6 - cleavage and polyadenylation specific factor 6, 68kda  RIOK2 - rio kinase 2  PPP2R1A - protein phosphatase 2, regulatory subunit a, alpha  NUDT21 - nudix (nucleoside diphosphate linked moiety x)-type motif 21  WWP2 - ww domain containing e3 ubiquitin protein ligase 2  AHR - aryl hydrocarbon receptor  PPP2R2C - protein phosphatase 2, regulatory subunit b, gamma  PPP2R2B - protein phosphatase 2, regulatory subunit b, beta  PPP1R7 - protein phosphatase 1, regulatory subunit 7  ZNF526 - zinc finger protein 526  PPP1R8 - protein phosphatase 1, regulatory subunit 8  FOXP4 - forkhead box p4  PPP2CA - protein phosphatase 2, catalytic subunit, alpha isozyme  PPP1R10 - protein phosphatase 1, regulatory subunit 10  DUSP14 - dual specificity phosphatase 14  EXOC1 - exocyst complex component 1  RMI2 - recq mediated genome instability 2  PPP1R2 - protein phosphatase 1, regulatory (inhibitor) subunit 2  ZNF83 - zinc finger protein 83  ESCO1 - establishment of sister chromatid cohesion n-acetyltransferase 1  KANSL3 - kat8 regulatory nsl complex subunit 3  IWS1 - iws1 homolog (s. cerevisiae)  SCYL2 - scy1-like 2 (s. cerevisiae)  PRNP - prion protein  ATF7 - activating transcription factor 7  AMFR - autocrine motility factor receptor, e3 ubiquitin protein ligase  DNAJC3 - dnaj (hsp40) homolog, subfamily c, member 3  PRKRIR - protein-kinase, interferon-inducible double stranded rna dependent inhibitor, repressor of (p58 repressor)  PAPD7 - pap associated domain containing 7  MAP2K5 - mitogen-activated protein kinase kinase 5  MAP2K7 - mitogen-activated protein kinase kinase 7  PIM2 - pim-2 oncogene  ZNF692 - zinc finger protein 692  EIF2AK2 - eukaryotic translation initiation factor 2-alpha kinase 2  MAP2K1 - mitogen-activated protein kinase kinase 1  MAP2K2 - mitogen-activated protein kinase kinase 2  EIF4E3 - eukaryotic translation initiation factor 4e family member 3  MAPK10 - mitogen-activated protein kinase 10  MAPK9 - mitogen-activated protein kinase 9  MAPK11 - mitogen-activated protein kinase 11  MAPK8 - mitogen-activated protein kinase 8  MAPK7 - mitogen-activated protein kinase 7  GCN1L1 - gcn1 general control of amino-acid synthesis 1-like 1 (yeast)  MAPK3 - mitogen-activated protein kinase 3  CISD2 - cdgsh iron sulfur domain 2  CCNI - cyclin i  MAPK1 - mitogen-activated protein kinase 1  OSBPL7 - oxysterol binding protein-like 7  OSBPL8 - oxysterol binding protein-like 8  ADIRF - adipogenesis regulatory factor  NDC1 - ndc1 transmembrane nucleoporin  PRKD1 - protein kinase d1  ANXA3 - annexin a3  CCDC88A - coiled-coil domain containing 88a  PRKCH - protein kinase c, eta  TLK2 - tousled-like kinase 2  PRKCI - protein kinase c, iota  CCDC85B - coiled-coil domain containing 85b  ANXA2 - annexin a2  PRKCE - protein kinase c, epsilon  PRKCB - protein kinase c, beta  PRKCD - protein kinase c, delta  RBM22 - rna binding motif protein 22  PRKAR2B - protein kinase, camp-dependent, regulatory, type ii, beta  PRKCA - protein kinase c, alpha  VAC14 - vac14 homolog (s. cerevisiae)  PRKAR1B - protein kinase, camp-dependent, regulatory, type i, beta  NSUN5 - nop2/sun domain family, member 5  PRKAR1A - protein kinase, camp-dependent, regulatory, type i, alpha  YEATS2 - yeats domain containing 2  PKIB - protein kinase (camp-dependent, catalytic) inhibitor beta  PRKAG1 - protein kinase, amp-activated, gamma 1 non-catalytic subunit  APEX1 - apex nuclease (multifunctional dna repair enzyme) 1  ZNF605 - zinc finger protein 605  DUSP12 - dual specificity phosphatase 12  RBM23 - rna binding motif protein 23  XIAP - x-linked inhibitor of apoptosis  BIRC3 - baculoviral iap repeat containing 3  NOXO1 - nadph oxidase organizer 1  BIRC2 - baculoviral iap repeat containing 2  PARN - poly(a)-specific ribonuclease  KLF12 - kruppel-like factor 12  APLP2 - amyloid beta (a4) precursor-like protein 2  TREX1 - three prime repair exonuclease 1  SHQ1 - shq1, h/aca ribonucleoprotein assembly factor  PBX2 - pre-b-cell leukemia homeobox 2  PRMT6 - protein arginine methyltransferase 6  PBX3 - pre-b-cell leukemia homeobox 3  PBX1 - pre-b-cell leukemia homeobox 1  PCBP2 - poly(rc) binding protein 2  PCBD1 - pterin-4 alpha-carbinolamine dehydratase/dimerization cofactor of hepatocyte nuclear factor 1 alpha  APBB2 - amyloid beta (a4) precursor protein-binding, family b, member 2  DCUN1D3 - dcn1, defective in cullin neddylation 1, domain containing 3  SMG8 - smg8 nonsense mediated mrna decay factor  APC - adenomatous polyposis coli  APBB1 - amyloid beta (a4) precursor protein-binding, family b, member 1 (fe65)  CHP1 - calcineurin-like ef-hand protein 1  RIF1 - rap1 interacting factor homolog (yeast)  VPS13D - vacuolar protein sorting 13 homolog d (s. cerevisiae)  POLG2 - polymerase (dna directed), gamma 2, accessory subunit  RNF139 - ring finger protein 139  PDCD10 - programmed cell death 10  KIAA1551 - kiaa1551  PEBP1 - phosphatidylethanolamine binding protein 1  PA2G4 - proliferation-associated 2g4, 38kda  APPL2 - adaptor protein, phosphotyrosine interaction, ph domain and leucine zipper containing 2  ARF1 - adp-ribosylation factor 1  RPRD1A - regulation of nuclear pre-mrna domain containing 1a  ARF4 - adp-ribosylation factor 4  ZHX1 - zinc fingers and homeoboxes 1  PAFAH1B1 - platelet-activating factor acetylhydrolase 1b, regulatory subunit 1 (45kda)  PAFAH1B2 - platelet-activating factor acetylhydrolase 1b, catalytic subunit 2 (30kda)  SBNO1 - strawberry notch homolog 1 (drosophila)  PPP1R42 - protein phosphatase 1, regulatory subunit 42  APOD - apolipoprotein d  IRAK3 - interleukin-1 receptor-associated kinase 3  PRDX1 - peroxiredoxin 1  AKAP13 - a kinase (prka) anchor protein 13  SETD5 - set domain containing 5  AKAP11 - a kinase (prka) anchor protein 11  APP - amyloid beta (a4) precursor protein  BBS7 - bardet-biedl syndrome 7  DDX20 - dead (asp-glu-ala-asp) box polypeptide 20  PAK2 - p21 protein (cdc42/rac)-activated kinase 2  PAK3 - p21 protein (cdc42/rac)-activated kinase 3  PAM - peptidylglycine alpha-amidating monooxygenase  ARNTL - aryl hydrocarbon receptor nuclear translocator-like  OXR1 - oxidation resistance 1  PDGFA - platelet-derived growth factor alpha polypeptide  ARNT - aryl hydrocarbon receptor nuclear translocator  CELF3 - cugbp, elav-like family member 3  PDE1B - phosphodiesterase 1b, calmodulin-dependent  MAP4K5 - mitogen-activated protein kinase kinase kinase kinase 5  PDPR - pyruvate dehydrogenase phosphatase regulatory subunit  SUPT16H - suppressor of ty 16 homolog (s. cerevisiae)  IFT57 - intraflagellar transport 57 homolog (chlamydomonas)  PDGFRB - platelet-derived growth factor receptor, beta polypeptide  ARRB2 - arrestin, beta 2  ARRB1 - arrestin, beta 1  PDGFRA - platelet-derived growth factor receptor, alpha polypeptide  PDGFB - platelet-derived growth factor beta polypeptide  SAMD4B - sterile alpha motif domain containing 4b  PDPK1 - 3-phosphoinositide dependent protein kinase-1  ARG2 - arginase 2  FAM107A - family with sequence similarity 107, member a  RHOA - ras homolog family member a  STRAP - serine/threonine kinase receptor associated protein  PSIP1 - pc4 and sfrs1 interacting protein 1  PDK3 - pyruvate dehydrogenase kinase, isozyme 3  MED9 - mediator complex subunit 9  FSTL1 - follistatin-like 1  SLC2A6 - solute carrier family 2 (facilitated glucose transporter), member 6  PECAM1 - platelet/endothelial cell adhesion molecule 1  WDR6 - wd repeat domain 6  BAZ2A - bromodomain adjacent to zinc finger domain, 2a  WDR70 - wd repeat domain 70  PCNA - proliferating cell nuclear antigen  CHMP1A - charged multivesicular body protein 1a  JDP2 - jun dimerization protein 2  CDK16 - cyclin-dependent kinase 16  AKIRIN2 - akirin 2  HEATR1 - heat repeat containing 1  CDK17 - cyclin-dependent kinase 17  LEO1 - leo1, paf1/rna polymerase ii complex component, homolog (s. cerevisiae)  ASPH - aspartate beta-hydroxylase  ERLIN2 - er lipid raft associated 2  PDCD2 - programmed cell death 2  PWP1 - pwp1 homolog (s. cerevisiae)  WRAP53 - wd repeat containing, antisense to tp53  CDC37 - cell division cycle 37  SOCS4 - suppressor of cytokine signaling 4  PDE2A - phosphodiesterase 2a, cgmp-stimulated  GLMN - glomulin, fkbp associated protein  THAP1 - thap domain containing, apoptosis associated protein 1  PKIG - protein kinase (camp-dependent, catalytic) inhibitor gamma  KAT7 - k(lysine) acetyltransferase 7  ASCL1 - achaete-scute complex homolog 1 (drosophila)  ELP3 - elongator acetyltransferase complex subunit 3  RGMB - rgm domain family, member b  SBNO2 - strawberry notch homolog 2 (drosophila)  ZNF444 - zinc finger protein 444  ATP2A3 - atpase, ca++ transporting, ubiquitous  ZBTB1 - zinc finger and btb domain containing 1  ZNF365 - zinc finger protein 365  PGAM1 - phosphoglycerate mutase 1 (brain)  ATP1A1 - atpase, na+/k+ transporting, alpha 1 polypeptide  RNF180 - ring finger protein 180  PFN2 - profilin 2  FBXW7 - f-box and wd repeat domain containing 7, e3 ubiquitin protein ligase  PGF - placental growth factor  BAHD1 - bromo adjacent homology domain containing 1  DIS3 - dis3 mitotic control homolog (s. cerevisiae)  ZHX2 - zinc fingers and homeoboxes 2  ANKRD6 - ankyrin repeat domain 6  MORC2 - morc family cw-type zinc finger 2  PHF2 - phd finger protein 2  PHF1 - phd finger protein 1  LRRK2 - leucine-rich repeat kinase 2  FOXJ3 - forkhead box j3  RERE - arginine-glutamic acid dipeptide (re) repeats  DNAJC24 - dnaj (hsp40) homolog, subfamily c, member 24  LARP6 - la ribonucleoprotein domain family, member 6  ZFHX3 - zinc finger homeobox 3  SPPL3 - signal peptide peptidase like 3  ATP6V1E1 - atpase, h+ transporting, lysosomal 31kda, v1 subunit e1  ATG14 - autophagy related 14  ATP6V0B - atpase, h+ transporting, lysosomal 21kda, v0 subunit b  ATP6V0A1 - atpase, h+ transporting, lysosomal v0 subunit a1  ATP6AP1 - atpase, h+ transporting, lysosomal accessory protein 1  ATP7A - atpase, cu++ transporting, alpha polypeptide  PEX14 - peroxisomal biogenesis factor 14  PPP6R1 - protein phosphatase 6, regulatory subunit 1  ADNP2 - adnp homeobox 2  LMTK2 - lemur tyrosine kinase 2  ATP6V1A - atpase, h+ transporting, lysosomal 70kda, v1 subunit a  AEBP2 - ae binding protein 2  ATP6V1B2 - atpase, h+ transporting, lysosomal 56/58kda, v1 subunit b2  ATP6V0C - atpase, h+ transporting, lysosomal 16kda, v0 subunit c  ATP6V1C1 - atpase, h+ transporting, lysosomal 42kda, v1 subunit c1  PPM1E - protein phosphatase, mg2+/mn2+ dependent, 1e  ZNF507 - zinc finger protein 507  IMPACT - impact rwd domain protein  LGR4 - leucine-rich repeat containing g protein-coupled receptor 4  ATP2B4 - atpase, ca++ transporting, plasma membrane 4  ZNF652 - zinc finger protein 652  RELL2 - relt-like 2  BAX - bcl2-associated x protein  SCAF8 - sr-related ctd-associated factor 8  SMU1 - smu-1 suppressor of mec-8 and unc-52 homolog (c. elegans)  PKD1 - polycystic kidney disease 1 (autosomal dominant)  PKD2 - polycystic kidney disease 2 (autosomal dominant)  PUF60 - poly-u binding splicing factor 60kda  MTF2 - metal response element binding transcription factor 2  BAK1 - bcl2-antagonist/killer 1  PIP4K2A - phosphatidylinositol-5-phosphate 4-kinase, type ii, alpha  NAT10 - n-acetyltransferase 10 (gcn5-related)  BACH1 - btb and cnc homology 1, basic leucine zipper transcription factor 1  BAD - bcl2-associated agonist of cell death  PIN1 - peptidylprolyl cis/trans isomerase, nima-interacting 1  PIK3R2 - phosphoinositide-3-kinase, regulatory subunit 2 (beta)  DPH3 - diphthamide biosynthesis 3  PIK3R1 - phosphoinositide-3-kinase, regulatory subunit 1 (alpha)  TMEM39A - transmembrane protein 39a  PLAT - plasminogen activator, tissue  ASXL2 - additional sex combs like 2 (drosophila)  AXL - axl receptor tyrosine kinase  PLAGL1 - pleiomorphic adenoma gene-like 1  ELP2 - elongator acetyltransferase complex subunit 2  ATRX - alpha thalassemia/mental retardation syndrome x-linked  PEF1 - penta-ef-hand domain containing 1  PKNOX1 - pbx/knotted 1 homeobox 1  PKM - pyruvate kinase, muscle  CASC3 - cancer susceptibility candidate 3  OGFOD1 - 2-oxoglutarate and iron-dependent oxygenase domain containing 1  GABARAPL2 - gaba(a) receptor-associated protein-like 2  SERPINI1 - serpin peptidase inhibitor, clade i (neuroserpin), member 1  EXOSC8 - exosome component 8  TMEM100 - transmembrane protein 100  PHF10 - phd finger protein 10  RABL3 - rab, member of ras oncogene family-like 3  SERPINE2 - serpin peptidase inhibitor, clade e (nexin, plasminogen activator inhibitor type 1), member 2  SERPINB8 - serpin peptidase inhibitor, clade b (ovalbumin), member 8  PSPC1 - paraspeckle component 1  U2AF2 - u2 small nuclear rna auxiliary factor 2  NUDT15 - nudix (nucleoside diphosphate linked moiety x)-type motif 15  TUSC2 - tumor suppressor candidate 2  BCR - breakpoint cluster region  PHKG2 - phosphorylase kinase, gamma 2 (testis)  WDR41 - wd repeat domain 41  BCL7A - b-cell cll/lymphoma 7a  STK38 - serine/threonine kinase 38  MRGBP - mrg/morf4l binding protein  BCL9 - b-cell cll/lymphoma 9  PIK3CB - phosphatidylinositol-4,5-bisphosphate 3-kinase, catalytic subunit beta  PIK3CA - phosphatidylinositol-4,5-bisphosphate 3-kinase, catalytic subunit alpha  RPUSD3 - rna pseudouridylate synthase domain containing 3  PPP6R3 - protein phosphatase 6, regulatory subunit 3  PIM1 - pim-1 oncogene  BRF2 - brf2, rna polymerase iii transcription initiation factor 50 kda subunit  BCL2 - b-cell cll/lymphoma 2  ECD - ecdysoneless homolog (drosophila)  CCND1 - cyclin d1  PARK7 - parkinson protein 7  PNKP - polynucleotide kinase 3'-phosphatase  C3orf33 - chromosome 3 open reading frame 33  TRIM27 - tripartite motif containing 27  RFC5 - replication factor c (activator 1) 5, 36.5kda  BNIP3L - bcl2/adenovirus e1b 19kda interacting protein 3-like  RFC2 - replication factor c (activator 1) 2, 40kda  RFC3 - replication factor c (activator 1) 3, 38kda  SAP18 - sin3a-associated protein, 18kda  TSHZ2 - teashirt zinc finger homeobox 2  RFXAP - regulatory factor x-associated protein  SEMA4C - sema domain, immunoglobulin domain (ig), transmembrane domain (tm) and short cytoplasmic domain, (semaphorin) 4c  MAEA - macrophage erythroblast attacher  RFX5 - regulatory factor x, 5 (influences hla class ii expression)  BRAF - v-raf murine sarcoma viral oncogene homolog b  WHSC1L1 - wolf-hirschhorn syndrome candidate 1-like 1  EIF1B - eukaryotic translation initiation factor 1b  RFX1 - regulatory factor x, 1 (influences hla class ii expression)  RELA - v-rel avian reticuloendotheliosis viral oncogene homolog a  APBB3 - amyloid beta (a4) precursor protein-binding, family b, member 3  BCOR - bcl6 corepressor  ZFP36L2 - zfp36 ring finger protein-like 2  RDX - radixin  PAK4 - p21 protein (cdc42/rac)-activated kinase 4  ZFP36L1 - zfp36 ring finger protein-like 1  RET - ret proto-oncogene  ALKBH5 - alkb, alkylation repair homolog 5 (e. coli)  DPF2 - d4, zinc and double phd fingers family 2  SH3RF3 - sh3 domain containing ring finger 3  DUSP15 - dual specificity phosphatase 15  UPF1 - upf1 regulator of nonsense transcripts homolog (yeast)  NSUN2 - nop2/sun rna methyltransferase family, member 2  CC2D1A - coiled-coil and c2 domain containing 1a  RLF - rearranged l-myc fusion  RRAGB - ras-related gtp binding b  RING1 - ring finger protein 1  CNPY2 - canopy fgf signaling regulator 2  DRAM2 - dna-damage regulated autophagy modulator 2  BMI1 - bmi1 polycomb ring finger oncogene  PCGF3 - polycomb group ring finger 3  RGS4 - regulator of g-protein signaling 4  RGS3 - regulator of g-protein signaling 3  RHEB - ras homolog enriched in brain  BMPR1A - bone morphogenetic protein receptor, type ia  BMPR2 - bone morphogenetic protein receptor, type ii (serine/threonine kinase)  GON4L - gon-4-like (c. elegans)  BMPR1B - bone morphogenetic protein receptor, type ib  BMP7 - bone morphogenetic protein 7  FITM2 - fat storage-inducing transmembrane protein 2  ABCA7 - atp-binding cassette, sub-family a (abc1), member 7  PSMD14 - proteasome (prosome, macropain) 26s subunit, non-atpase, 14  RNF19B - ring finger protein 19b  FLOT1 - flotillin 1  CTDSPL - ctd (carboxy-terminal domain, rna polymerase ii, polypeptide a) small phosphatase-like  BCAS3 - breast carcinoma amplified sequence 3  ABCE1 - atp-binding cassette, sub-family e (oabp), member 1  LDLRAD4 - low density lipoprotein receptor class a domain containing 4  VPS13C - vacuolar protein sorting 13 homolog c (s. cerevisiae)  PTTG1IP - pituitary tumor-transforming 1 interacting protein  COQ7 - coenzyme q7 homolog, ubiquinone (yeast)  UHMK1 - u2af homology motif (uhm) kinase 1  AHI1 - abelson helper integration site 1  RNF4 - ring finger protein 4  BRD2 - bromodomain containing 2  RNF2 - ring finger protein 2  HNRNPR - heterogeneous nuclear ribonucleoprotein r  ZNF280D - zinc finger protein 280d  GATAD2A - gata zinc finger domain containing 2a  RNF6 - ring finger protein (c3h2c3 type) 6  CALCOCO2 - calcium binding and coiled-coil domain 2  ROBO1 - roundabout, axon guidance receptor, homolog 1 (drosophila)  ROCK1 - rho-associated, coiled-coil containing protein kinase 1  RNF111 - ring finger protein 111  TSPO - translocator protein (18kda)  SPRY1 - sprouty homolog 1, antagonist of fgf signaling (drosophila)  TET2 - tet methylcytosine dioxygenase 2  SPRY2 - sprouty homolog 2 (drosophila)  MED18 - mediator complex subunit 18  DENND4A - denn/madd domain containing 4a  MBTD1 - mbt domain containing 1  SF3B4 - splicing factor 3b, subunit 4, 49kda  RAMP3 - receptor (g protein-coupled) activity modifying protein 3  RAMP1 - receptor (g protein-coupled) activity modifying protein 1  AKAP8 - a kinase (prka) anchor protein 8  ZMPSTE24 - zinc metallopeptidase ste24  C5AR1 - complement component 5a receptor 1  STUB1 - stip1 homology and u-box containing protein 1, e3 ubiquitin protein ligase  TRIM44 - tripartite motif containing 44  TAB1 - tgf-beta activated kinase 1/map3k7 binding protein 1  VAV3 - vav 3 guanine nucleotide exchange factor  PPIE - peptidylprolyl isomerase e (cyclophilin e)  CAPNS1 - calpain, small subunit 1  CAPN2 - calpain 2, (m/ii) large subunit  FAM3C - family with sequence similarity 3, member c  CAPN1 - calpain 1, (mu/i) large subunit  MCRS1 - microspherule protein 1  ZER1 - zyg-11 related, cell cycle regulator  NRDE2 - nrde-2, necessary for rna interference, domain containing  TIMM17A - translocase of inner mitochondrial membrane 17 homolog a (yeast)  CAMLG - calcium modulating ligand  C1D - c1d nuclear receptor corepressor  RPL11 - ribosomal protein l11  PTCD3 - pentatricopeptide repeat domain 3  CASP8 - caspase 8, apoptosis-related cysteine peptidase  CASP9 - caspase 9, apoptosis-related cysteine peptidase  PIBF1 - progesterone immunomodulatory binding factor 1  SLC30A9 - solute carrier family 30 (zinc transporter), member 9  CASP2 - caspase 2, apoptosis-related cysteine peptidase  CASP3 - caspase 3, apoptosis-related cysteine peptidase  MAD2L2 - mad2 mitotic arrest deficient-like 2 (yeast)  PID1 - phosphotyrosine interaction domain containing 1  PHIP - pleckstrin homology domain interacting protein  RPA2 - replication protein a2, 32kda  RPS4X - ribosomal protein s4, x-linked  PPP2R3C - protein phosphatase 2, regulatory subunit b'', gamma  NRARP - notch-regulated ankyrin repeat protein  ZNF787 - zinc finger protein 787  RMND1 - required for meiotic nuclear division 1 homolog (s. cerevisiae)  LAMTOR1 - late endosomal/lysosomal adaptor, mapk and mtor activator 1  DDR1 - discoidin domain receptor tyrosine kinase 1  TRIM38 - tripartite motif containing 38  TADA3 - transcriptional adaptor 3  CALR - calreticulin  CALM2 - calmodulin 2 (phosphorylase kinase, delta)  ZNF792 - zinc finger protein 792  WTIP - wilms tumor 1 interacting protein  NCOA2 - nuclear receptor coactivator 2  SYNCRIP - synaptotagmin binding, cytoplasmic rna interacting protein  STK25 - serine/threonine kinase 25  CREB3 - camp responsive element binding protein 3  CRTAP - cartilage associated protein  MFSD12 - major facilitator superfamily domain containing 12  CITED2 - cbp/p300-interacting transactivator, with glu/asp-rich carboxy-terminal domain, 2  CCT6A - chaperonin containing tcp1, subunit 6a (zeta 1)  CCNT2 - cyclin t2  PINX1 - pin2/terf1 interacting, telomerase inhibitor 1  CLN6 - ceroid-lipofuscinosis, neuronal 6, late infantile, variant  CCND3 - cyclin d3  CCNT1 - cyclin t1  HMG20A - high mobility group 20a  CCNH - cyclin h  CCNG2 - cyclin g2  BANP - btg3 associated nuclear protein  TIPIN - timeless interacting protein  ACER2 - alkaline ceramidase 2  DLC1 - deleted in liver cancer 1  TRNAU1AP - trna selenocysteine 1 associated protein 1  NRBP2 - nuclear receptor binding protein 2  RPS6KA1 - ribosomal protein s6 kinase, 90kda, polypeptide 1  PARP16 - poly (adp-ribose) polymerase family, member 16  RPS6KB1 - ribosomal protein s6 kinase, 70kda, polypeptide 1  RPS6KB2 - ribosomal protein s6 kinase, 70kda, polypeptide 2  CBFA2T3 - core-binding factor, runt domain, alpha subunit 2; translocated to, 3  CBFB - core-binding factor, beta subunit  RAPGEF3 - rap guanine nucleotide exchange factor (gef) 3  CBL - cbl proto-oncogene, e3 ubiquitin protein ligase  SERPINH1 - serpin peptidase inhibitor, clade h (heat shock protein 47), member 1, (collagen binding protein 1)  RXRB - retinoid x receptor, beta  CAV2 - caveolin 2  PIAS3 - protein inhibitor of activated stat, 3  CAV1 - caveolin 1, caveolae protein, 22kda  ANKRD13B - ankyrin repeat domain 13b  RBM14 - rna binding motif protein 14  CCNC - cyclin c  CCND2 - cyclin d2  TRMT10C - trna methyltransferase 10 homolog c (s. cerevisiae)  SPON1 - spondin 1, extracellular matrix protein  PRMT5 - protein arginine methyltransferase 5  RRAS - related ras viral (r-ras) oncogene homolog  CD2BP2 - cd2 (cytoplasmic tail) binding protein 2  CHTF8 - ctf8, chromosome transmission fidelity factor 8 homolog (s. cerevisiae)  CDK11B - cyclin-dependent kinase 11b  CDC5L - cell division cycle 5-like  MXD4 - max dimerization protein 4  HTRA1 - htra serine peptidase 1  PSAP - prosaposin  POLR3F - polymerase (rna) iii (dna directed) polypeptide f, 39 kda  CDH3 - cadherin 3, type 1, p-cadherin (placental)  CDH2 - cadherin 2, type 1, n-cadherin (neuronal)  HEXIM1 - hexamethylene bis-acetamide inducible 1  ERLIN1 - er lipid raft associated 1  SPIN2A - spindlin family, member 2a  ANKIB1 - ankyrin repeat and ibr domain containing 1  NCOA7 - nuclear receptor coactivator 7  POMT1 - protein-o-mannosyltransferase 1  PSEN1 - presenilin 1  TOLLIP - toll interacting protein  USP16 - ubiquitin specific peptidase 16  PSMA1 - proteasome (prosome, macropain) subunit, alpha type, 1  NLE1 - notchless homolog 1 (drosophila)  SRSF12 - serine/arginine-rich splicing factor 12  PSMA2 - proteasome (prosome, macropain) subunit, alpha type, 2  CD81 - cd81 molecule  CAMKK2 - calcium/calmodulin-dependent protein kinase kinase 2, beta  PSMB1 - proteasome (prosome, macropain) subunit, beta type, 1  STARD4 - star-related lipid transfer (start) domain containing 4  PSMA5 - proteasome (prosome, macropain) subunit, alpha type, 5  SCARB2 - scavenger receptor class b, member 2  NPRL2 - nitrogen permease regulator-like 2 (s. cerevisiae)  PSMA3 - proteasome (prosome, macropain) subunit, alpha type, 3  PSMB10 - proteasome (prosome, macropain) subunit, beta type, 10  KHDRBS3 - kh domain containing, rna binding, signal transduction associated 3  RIPPLY2 - ripply transcriptional repressor 2  CD47 - cd47 molecule  SPINT2 - serine peptidase inhibitor, kunitz type, 2  CD44 - cd44 molecule (indian blood group)  PSMC6 - proteasome (prosome, macropain) 26s subunit, atpase, 6  FAM173B - family with sequence similarity 173, member b  TAF6L - taf6-like rna polymerase ii, p300/cbp-associated factor (pcaf)-associated factor, 65kda  PSMD1 - proteasome (prosome, macropain) 26s subunit, non-atpase, 1  PSMC4 - proteasome (prosome, macropain) 26s subunit, atpase, 4  SOX18 - sry (sex determining region y)-box 18  POLR3G - polymerase (rna) iii (dna directed) polypeptide g (32kd)  PSMC2 - proteasome (prosome, macropain) 26s subunit, atpase, 2  PSMD9 - proteasome (prosome, macropain) 26s subunit, non-atpase, 9  PSMD10 - proteasome (prosome, macropain) 26s subunit, non-atpase, 10  CD34 - cd34 molecule  PSMD7 - proteasome (prosome, macropain) 26s subunit, non-atpase, 7  PSMD8 - proteasome (prosome, macropain) 26s subunit, non-atpase, 8  DNAJC10 - dnaj (hsp40) homolog, subfamily c, member 10  PSMD5 - proteasome (prosome, macropain) 26s subunit, non-atpase, 5  TERF2IP - telomeric repeat binding factor 2, interacting protein  PSMD2 - proteasome (prosome, macropain) 26s subunit, non-atpase, 2  SLC38A2 - solute carrier family 38, member 2  PSMD12 - proteasome (prosome, macropain) 26s subunit, non-atpase, 12  PSMD13 - proteasome (prosome, macropain) 26s subunit, non-atpase, 13  ATG7 - autophagy related 7  PRRC1 - proline-rich coiled-coil 1  PTBP1 - polypyrimidine tract binding protein 1  PTEN - phosphatase and tensin homolog  LAMTOR5 - late endosomal/lysosomal adaptor, mapk and mtor activator 5  ANP32B - acidic (leucine-rich) nuclear phosphoprotein 32 family, member b  JMY - junction mediating and regulatory protein, p53 cofactor  MYBBP1A - myb binding protein (p160) 1a  FBLN5 - fibulin 5  PTGIS - prostaglandin i2 (prostacyclin) synthase  CIB1 - calcium and integrin binding 1 (calmyrin)  DDX17 - dead (asp-glu-ala-asp) box helicase 17  KAT5 - k(lysine) acetyltransferase 5  ZFP69 - zfp69 zinc finger protein  PTK2 - protein tyrosine kinase 2  IPO7 - importin 7  CEBPG - ccaat/enhancer binding protein (c/ebp), gamma  CDK5 - cyclin-dependent kinase 5  CDK4 - cyclin-dependent kinase 4  TWF1 - twinfilin actin-binding protein 1  PTN - pleiotrophin  CDK9 - cyclin-dependent kinase 9  CCT7 - chaperonin containing tcp1, subunit 7 (eta)  CDK8 - cyclin-dependent kinase 8  LIN54 - lin-54 homolog (c. elegans)  CDK7 - cyclin-dependent kinase 7  CDKN1C - cyclin-dependent kinase inhibitor 1c (p57, kip2)  NPC2 - niemann-pick disease, type c2  SPOPL - speckle-type poz protein-like  CDKN1B - cyclin-dependent kinase inhibitor 1b (p27, kip1)  CCT2 - chaperonin containing tcp1, subunit 2 (beta)  CCT4 - chaperonin containing tcp1, subunit 4 (delta)  PCBP3 - poly(rc) binding protein 3  PTPN3 - protein tyrosine phosphatase, non-receptor type 3  CDKN2D - cyclin-dependent kinase inhibitor 2d (p19, inhibits cdk4)  PTPN2 - protein tyrosine phosphatase, non-receptor type 2  PTPN1 - protein tyrosine phosphatase, non-receptor type 1  PTPN11 - protein tyrosine phosphatase, non-receptor type 11  HTATIP2 - hiv-1 tat interactive protein 2, 30kda  ARL6IP5 - adp-ribosylation-like factor 6 interacting protein 5  AGPAT1 - 1-acylglycerol-3-phosphate o-acyltransferase 1  PTPN13 - protein tyrosine phosphatase, non-receptor type 13 (apo-1/cd95 (fas)-associated phosphatase)  SPTLC1 - serine palmitoyltransferase, long chain base subunit 1  CDK2 - cyclin-dependent kinase 2  ARFGEF1 - adp-ribosylation factor guanine nucleotide-exchange factor 1 (brefeldin a-inhibited)  RRN3 - rrn3 rna polymerase i transcription factor homolog (s. cerevisiae)  PURA - purine-rich element binding protein a  PURB - purine-rich element binding protein b  PDP1 - pyruvate dehyrogenase phosphatase catalytic subunit 1  GTPBP2 - gtp binding protein 2  CHRNB2 - cholinergic receptor, nicotinic, beta 2 (neuronal)  NUDT16 - nudix (nucleoside diphosphate linked moiety x)-type motif 16  PTPRR - protein tyrosine phosphatase, receptor type, r  ZMYND11 - zinc finger, mynd-type containing 11  PTPRK - protein tyrosine phosphatase, receptor type, k  SRSF10 - serine/arginine-rich splicing factor 10  PTPRN - protein tyrosine phosphatase, receptor type, n  RELL1 - relt-like 1  JMJD8 - jumonji domain containing 8  PLK2 - polo-like kinase 2  AHCYL1 - adenosylhomocysteinase-like 1  PEX2 - peroxisomal biogenesis factor 2  NUP50 - nucleoporin 50kda  MPHOSPH8 - m-phase phosphoprotein 8  KDM5B - lysine (k)-specific demethylase 5b  PXN - paxillin  GIPC1 - gipc pdz domain containing family, member 1  MAP4K2 - mitogen-activated protein kinase kinase kinase kinase 2  RAB5A - rab5a, member ras oncogene family  CHD4 - chromodomain helicase dna binding protein 4  CHD2 - chromodomain helicase dna binding protein 2  CHD3 - chromodomain helicase dna binding protein 3  FRS2 - fibroblast growth factor receptor substrate 2  MTERFD2 - mterf domain containing 2  CHD1 - chromodomain helicase dna binding protein 1  RAB1A - rab1a, member ras oncogene family  QARS - glutaminyl-trna synthetase  NOXA1 - nadph oxidase activator 1  MYPOP - myb-related transcription factor, partner of profilin  RAD21 - rad21 homolog (s. pombe)  RAD17 - rad17 homolog (s. pombe)  SEC24A - sec24 family, member a (s. cerevisiae)  PARP14 - poly (adp-ribose) polymerase family, member 14  ZNF181 - zinc finger protein 181  PAF1 - paf1, rna polymerase ii associated factor, homolog (s. cerevisiae)  RAC1 - ras-related c3 botulinum toxin substrate 1 (rho family, small gtp binding protein rac1)  ZNF268 - zinc finger protein 268  INO80 - ino80 complex subunit  ZNF513 - zinc finger protein 513  RAB27A - rab27a, member ras oncogene family  NDFIP2 - nedd4 family interacting protein 2  RALB - v-ral simian leukemia viral oncogene homolog b  RC3H2 - ring finger and ccch-type domains 2  GMEB1 - glucocorticoid modulatory element binding protein 1  TOMM7 - translocase of outer mitochondrial membrane 7 homolog (yeast)  RANBP2 - ran binding protein 2  CCT8 - chaperonin containing tcp1, subunit 8 (theta)  RAP1A - rap1a, member of ras oncogene family  CLTC - clathrin, heavy chain (hc)  MIER2 - mesoderm induction early response 1, family member 2  FAM35A - family with sequence similarity 35, member a  RARG - retinoic acid receptor, gamma  RARB - retinoic acid receptor, beta  DLL4 - delta-like 4 (drosophila)  RRAGA - ras-related gtp binding a  RASA1 - ras p21 protein activator (gtpase activating protein) 1  NECAB2 - n-terminal ef-hand calcium binding protein 2  RAP1B - rap1b, member of ras oncogene family  CELF1 - cugbp, elav-like family member 1  CLU - clusterin  KHDRBS1 - kh domain containing, rna binding, signal transduction associated 1  GNL3L - guanine nucleotide binding protein-like 3 (nucleolar)-like  CLK1 - cdc-like kinase 1  TAF8 - taf8 rna polymerase ii, tata box binding protein (tbp)-associated factor, 43kda  MAGEL2 - mage-like 2  CELF2 - cugbp, elav-like family member 2  RAP2B - rap2b, member of ras oncogene family  RAP2A - rap2a, member of ras oncogene family  CLK2 - cdc-like kinase 2  DDX49 - dead (asp-glu-ala-asp) box polypeptide 49  CTCF - ccctc-binding factor (zinc finger protein)  CLK3 - cdc-like kinase 3  RBL2 - retinoblastoma-like 2 (p130)  RAI1 - retinoic acid induced 1  RBBP7 - retinoblastoma binding protein 7  RBBP8 - retinoblastoma binding protein 8  MAP3K2 - mitogen-activated protein kinase kinase kinase 2  RBM3 - rna binding motif (rnp1, rrm) protein 3  RBM4 - rna binding motif protein 4  RB1 - retinoblastoma 1  TMEM150A - transmembrane protein 150a  ARID4A - at rich interactive domain 4a (rbp1-like)  ANKRD54 - ankyrin repeat domain 54  RBBP6 - retinoblastoma binding protein 6  KDM5A - lysine (k)-specific demethylase 5a  NUP35 - nucleoporin 35kda  PUS7 - pseudouridylate synthase 7 homolog (s. cerevisiae)  ERCC8 - excision repair cross-complementing rodent repair deficiency, complementation group 8  EXOSC4 - exosome component 4  ANKRD13D - ankyrin repeat domain 13 family, member d  PTGES3 - prostaglandin e synthase 3 (cytosolic)  NFAT5 - nuclear factor of activated t-cells 5, tonicity-responsive  CKS1B - cdc28 protein kinase regulatory subunit 1b  CIRBP - cold inducible rna binding protein  CHMP4B - charged multivesicular body protein 4b  DHX29 - deah (asp-glu-ala-his) box polypeptide 29  CHUK - conserved helix-loop-helix ubiquitous kinase  CERS1 - ceramide synthase 1  ZMYND8 - zinc finger, mynd-type containing 8  ANKRA2 - ankyrin repeat, family a (rfxank-like), 2  RBAK - rb-associated krab zinc finger  NFATC2IP - nuclear factor of activated t-cells, cytoplasmic, calcineurin-dependent 2 interacting protein  ATAD1 - atpase family, aaa domain containing 1  SPTBN4 - spectrin, beta, non-erythrocytic 4  FBXO18 - f-box protein, helicase, 18  PATZ1 - poz (btb) and at hook containing zinc finger 1  METTL14 - methyltransferase like 14  SPOCK1 - sparc/osteonectin, cwcv and kazal-like domains proteoglycan (testican) 1  SPP1 - secreted phosphoprotein 1  CORO1C - coronin, actin binding protein, 1c  SSBP2 - single-stranded dna binding protein 2  KPNA6 - karyopherin alpha 6 (importin alpha 7)  FIZ1 - flt3-interacting zinc finger 1  COX11 - cytochrome c oxidase assembly homolog 11 (yeast)  CIRH1A - cirrhosis, autosomal recessive 1a (cirhin)  COX7A2 - cytochrome c oxidase subunit viia polypeptide 2 (liver)  MIER1 - mesoderm induction early response 1, transcriptional regulator  PPP1R15B - protein phosphatase 1, regulatory subunit 15b  CWC22 - cwc22 spliceosome-associated protein homolog (s. cerevisiae)  ZNF382 - zinc finger protein 382  ZNF705A - zinc finger protein 705a  SP3 - sp3 transcription factor  SP4 - sp4 transcription factor  SP1 - sp1 transcription factor  CPT1A - carnitine palmitoyltransferase 1a (liver)  ZNF410 - zinc finger protein 410  SP2 - sp2 transcription factor  ARL2BP - adp-ribosylation factor-like 2 binding protein  DDAH2 - dimethylarginine dimethylaminohydrolase 2  SOX4 - sry (sex determining region y)-box 4  TIPRL - tip41, tor signaling pathway regulator-like (s. cerevisiae)  GTPBP4 - gtp binding protein 4  WBP2 - ww domain binding protein 2  ORC3 - origin recognition complex, subunit 3  MAGI3 - membrane associated guanylate kinase, ww and pdz domain containing 3  SON - son dna binding protein  HES4 - hairy and enhancer of split 4 (drosophila)  LEMD3 - lem domain containing 3  GATAD1 - gata zinc finger domain containing 1  CREBL2 - camp responsive element binding protein-like 2  ATF6B - activating transcription factor 6 beta  SOAT1 - sterol o-acyltransferase 1  CREBBP - creb binding protein  ATF2 - activating transcription factor 2  CREB1 - camp responsive element binding protein 1  DDX58 - dead (asp-glu-ala-asp) box polypeptide 58  CRABP2 - cellular retinoic acid binding protein 2  RPRD1B - regulation of nuclear pre-mrna domain containing 1b  PLK3 - polo-like kinase 3  PPP1R16A - protein phosphatase 1, regulatory subunit 16a  SNAPC4 - small nuclear rna activating complex, polypeptide 4, 190kda  SNCA - synuclein, alpha (non a4 component of amyloid precursor)  SNRNP70 - small nuclear ribonucleoprotein 70kda (u1)  ZBED5 - zinc finger, bed-type containing 5  SNRPA - small nuclear ribonucleoprotein polypeptide a  CREBZF - creb/atf bzip transcription factor  SUMO2 - small ubiquitin-like modifier 2  EID1 - ep300 interacting inhibitor of differentiation 1  TP53INP2 - tumor protein p53 inducible nuclear protein 2  PTBP2 - polypyrimidine tract binding protein 2  SMARCE1 - swi/snf related, matrix associated, actin dependent regulator of chromatin, subfamily e, member 1  RBM17 - rna binding motif protein 17  SMO - smoothened, frizzled family receptor  CNR1 - cannabinoid receptor 1 (brain)  SMPD1 - sphingomyelin phosphodiesterase 1, acid lysosomal  CNTN1 - contactin 1  SMARCB1 - swi/snf related, matrix associated, actin dependent regulator of chromatin, subfamily b, member 1  SMARCC1 - swi/snf related, matrix associated, actin dependent regulator of chromatin, subfamily c, member 1  HLTF - helicase-like transcription factor  SMARCA4 - swi/snf related, matrix associated, actin dependent regulator of chromatin, subfamily a, member 4  SNX6 - sorting nexin 6  SSBP3 - single stranded dna binding protein 3  SMARCD2 - swi/snf related, matrix associated, actin dependent regulator of chromatin, subfamily d, member 2  ATCAY - ataxia, cerebellar, cayman type  SMARCD3 - swi/snf related, matrix associated, actin dependent regulator of chromatin, subfamily d, member 3  SMARCC2 - swi/snf related, matrix associated, actin dependent regulator of chromatin, subfamily c, member 2  SMARCD1 - swi/snf related, matrix associated, actin dependent regulator of chromatin, subfamily d, member 1  WIZ - widely interspaced zinc finger motifs  MID1IP1 - mid1 interacting protein 1  RBM25 - rna binding motif protein 25  HSPBP1 - hspa (heat shock 70kda) binding protein, cytoplasmic cochaperone 1  SMARCA1 - swi/snf related, matrix associated, actin dependent regulator of chromatin, subfamily a, member 1  SMARCA2 - swi/snf related, matrix associated, actin dependent regulator of chromatin, subfamily a, member 2  SGK3 - serum/glucocorticoid regulated kinase family, member 3  CACTIN - cactin, spliceosome c complex subunit  SH3BP4 - sh3-domain binding protein 4  KMT2C - lysine (k)-specific methyltransferase 2c  RAB38 - rab38, member ras oncogene family  ZNF462 - zinc finger protein 462  PPIL4 - peptidylprolyl isomerase (cyclophilin)-like 4  SLC18A2 - solute carrier family 18 (vesicular monoamine transporter), member 2  TFIP11 - tuftelin interacting protein 11  SENP2 - sumo1/sentrin/smt3 specific peptidase 2  CSTB - cystatin b (stefin b)  CST3 - cystatin c  SHC2 - shc (src homology 2 domain containing) transforming protein 2  ZNF318 - zinc finger protein 318  PRPF6 - pre-mrna processing factor 6  KDM2B - lysine (k)-specific demethylase 2b  SUPT6H - suppressor of ty 6 homolog (s. cerevisiae)  CTGF - connective tissue growth factor  SUPV3L1 - suppressor of var1, 3-like 1 (s. cerevisiae)  CBY1 - chibby homolog 1 (drosophila)  CACNG7 - calcium channel, voltage-dependent, gamma subunit 7  DSTYK - dual serine/threonine and tyrosine protein kinase  CTBP1 - c-terminal binding protein 1  SUPT4H1 - suppressor of ty 4 homolog 1 (s. cerevisiae)  CTBP2 - c-terminal binding protein 2  SUPT5H - suppressor of ty 5 homolog (s. cerevisiae)  MFSD8 - major facilitator superfamily domain containing 8  RAB3GAP2 - rab3 gtpase activating protein subunit 2 (non-catalytic)  CTNNB1 - catenin (cadherin-associated protein), beta 1, 88kda  SAV1 - salvador homolog 1 (drosophila)  CTSD - cathepsin d  ELOVL5 - elovl fatty acid elongase 5  CUX1 - cut-like homeobox 1  USP30 - ubiquitin specific peptidase 30  NIF3L1 - nif3 ngg1 interacting factor 3-like 1 (s. cerevisiae)  NEK4 - nima-related kinase 4  STK3 - serine/threonine kinase 3  MPV17L2 - mpv17 mitochondrial membrane protein-like 2  FAM3A - family with sequence similarity 3, member a  PCGF1 - polycomb group ring finger 1  NPNT - nephronectin  FKBP8 - fk506 binding protein 8, 38kda  GPBP1L1 - gc-rich promoter binding protein 1-like 1  MTCH1 - mitochondrial carrier 1  CYLD - cylindromatosis (turban tumor syndrome)  SUV420H2 - suppressor of variegation 4-20 homolog 2 (drosophila)  EXOC4 - exocyst complex component 4  TGIF2 - tgfb-induced factor homeobox 2  CNEP1R1 - ctd nuclear envelope phosphatase 1 regulatory subunit 1  STK4 - serine/threonine kinase 4  BCL6B - b-cell cll/lymphoma 6, member b  ST5 - suppression of tumorigenicity 5  C12orf44 - chromosome 12 open reading frame 44  CRKL - v-crk avian sarcoma virus ct10 oncogene homolog-like  CRK - v-crk avian sarcoma virus ct10 oncogene homolog  EEFSEC - eukaryotic elongation factor, selenocysteine-trna-specific  CELF6 - cugbp, elav-like family member 6  NIPBL - nipped-b homolog (drosophila)  STAT2 - signal transducer and activator of transcription 2, 113kda  CRY1 - cryptochrome 1 (photolyase-like)  STAT1 - signal transducer and activator of transcription 1, 91kda  STAT3 - signal transducer and activator of transcription 3 (acute-phase response factor)  CRY2 - cryptochrome 2 (photolyase-like)  STAT5A - signal transducer and activator of transcription 5a  ASF1A - anti-silencing function 1a histone chaperone  STAT6 - signal transducer and activator of transcription 6, interleukin-4 induced  PARM1 - prostate androgen-regulated mucin-like protein 1  BRMS1 - breast cancer metastasis suppressor 1  BOD1L1 - biorientation of chromosomes in cell division 1-like 1  SCOC - short coiled-coil protein  CRYM - crystallin, mu  ZNF496 - zinc finger protein 496  SS18 - synovial sarcoma translocation, chromosome 18  PHF5A - phd finger protein 5a  CSF1R - colony stimulating factor 1 receptor  GPATCH3 - g patch domain containing 3  SNX33 - sorting nexin 33  FBXO7 - f-box protein 7  CSF1 - colony stimulating factor 1 (macrophage)  SRPK2 - srsf protein kinase 2  SRPK1 - srsf protein kinase 1  TAB3 - tgf-beta activated kinase 1/map3k7 binding protein 3  KLHL22 - kelch-like family member 22  ZNF335 - zinc finger protein 335  BAMBI - bmp and activin membrane-bound inhibitor  HAVCR2 - hepatitis a virus cellular receptor 2  CSK - c-src tyrosine kinase  CSNK1A1 - casein kinase 1, alpha 1  ZC3H10 - zinc finger ccch-type containing 10  CSNK1D - casein kinase 1, delta  CSNK2A2 - casein kinase 2, alpha prime polypeptide  SRI - sorcin  SRC - v-src avian sarcoma (schmidt-ruppin a-2) viral oncogene homolog  TNFAIP8 - tumor necrosis factor, alpha-induced protein 8  CSNK2A1 - casein kinase 2, alpha 1 polypeptide  DNAJB5 - dnaj (hsp40) homolog, subfamily b, member 5  SREBF2 - sterol regulatory element binding transcription factor 2  ARIH1 - ariadne homolog, ubiquitin-conjugating enzyme e2 binding protein, 1 (drosophila)  UBE2O - ubiquitin-conjugating enzyme e2o  NSUN3 - nop2/sun domain family, member 3  SRP9 - signal recognition particle 9kda  FBXL2 - f-box and leucine-rich repeat protein 2  PRDX5 - peroxiredoxin 5  BACE2 - beta-site app-cleaving enzyme 2  RNF169 - ring finger protein 169  DHX9 - deah (asp-glu-ala-his) box helicase 9  TRAPPC2 - trafficking protein particle complex 2  KANK2 - kn motif and ankyrin repeat domains 2  DDX5 - dead (asp-glu-ala-asp) box helicase 5  SEC13 - sec13 homolog (s. cerevisiae)  DDX3X - dead (asp-glu-ala-asp) box helicase 3, x-linked  PNKD - paroxysmal nonkinesigenic dyskinesia  DDX1 - dead (asp-glu-ala-asp) box helicase 1  SIN3A - sin3 transcription regulator family member a  DPH6 - diphthamine biosynthesis 6  KIAA1244 - kiaa1244  MIDN - midnolin  SDCBP - syndecan binding protein (syntenin)  SET - set nuclear oncogene  WWTR1 - ww domain containing transcription regulator 1  SETMAR - set domain and mariner transposase fusion gene  DDB1 - damage-specific dna binding protein 1, 127kda  ZNF304 - zinc finger protein 304  MAP2K4 - mitogen-activated protein kinase kinase 4  RCN3 - reticulocalbin 3, ef-hand calcium binding domain  ZNF286A - zinc finger protein 286a  GEMIN5 - gem (nuclear organelle) associated protein 5  PBXIP1 - pre-b-cell leukemia homeobox interacting protein 1  NOL11 - nucleolar protein 11  DBP - d site of albumin promoter (albumin d-box) binding protein  NELFB - negative elongation factor complex member b  POT1 - protection of telomeres 1  AHCTF1 - at hook containing transcription factor 1  CLK4 - cdc-like kinase 4  CNOT10 - ccr4-not transcription complex, subunit 10  ZBTB38 - zinc finger and btb domain containing 38  RNF19A - ring finger protein 19a, e3 ubiquitin protein ligase  RCHY1 - ring finger and chy zinc finger domain containing 1, e3 ubiquitin protein ligase  GATAD2B - gata zinc finger domain containing 2b  ZNF551 - zinc finger protein 551  DFFA - dna fragmentation factor, 45kda, alpha polypeptide  KCTD13 - potassium channel tetramerization domain containing 13  ZNF473 - zinc finger protein 473  YTHDF3 - yth domain family, member 3  BIRC6 - baculoviral iap repeat containing 6  WDR18 - wd repeat domain 18  CNOT6 - ccr4-not transcription complex, subunit 6  LTN1 - listerin e3 ubiquitin protein ligase 1  ATXN2 - ataxin 2  ZNF451 - zinc finger protein 451  TMEM150C - transmembrane protein 150c  ZNF622 - zinc finger protein 622  SCAF4 - sr-related ctd-associated factor 4  SS18L1 - synovial sarcoma translocation gene on chromosome 18-like 1  CHD5 - chromodomain helicase dna binding protein 5  ARID1B - at rich interactive domain 1b (swi1-like)  SCO1 - sco1 cytochrome c oxidase assembly protein  TMEM98 - transmembrane protein 98  MED30 - mediator complex subunit 30  MKL2 - mkl/myocardin-like 2  HEG1 - heart development protein with egf-like domains 1  NSMF - nmda receptor synaptonuclear signaling and neuronal migration factor  NLN - neurolysin (metallopeptidase m3 family)  PYGO2 - pygopus homolog 2 (drosophila)  DAPK3 - death-associated protein kinase 3  DAXX - death-domain associated protein  ANGEL2 - angel homolog 2 (drosophila)  ZZZ3 - zinc finger, zz-type containing 3  KIDINS220 - kinase d-interacting substrate, 220kda  HINFP - histone h4 transcription factor  DAG1 - dystroglycan 1 (dystrophin-associated glycoprotein 1)  LRSAM1 - leucine rich repeat and sterile alpha motif containing 1  MTA3 - metastasis associated 1 family, member 3  DGKQ - diacylglycerol kinase, theta 110kda  ZNF608 - zinc finger protein 608  DAPK1 - death-associated protein kinase 1  CLIP3 - cap-gly domain containing linker protein 3  MAVS - mitochondrial antiviral signaling protein  DAP - death-associated protein  IBTK - inhibitor of bruton agammaglobulinemia tyrosine kinase  SARS - seryl-trna synthetase  TBC1D14 - tbc1 domain family, member 14  SATB1 - satb homeobox 1  NGDN - neuroguidin, eif4e binding protein  RPTOR - regulatory associated protein of mtor, complex 1  SAFB - scaffold attachment factor b  SH2B1 - sh2b adaptor protein 1  SALL2 - sal-like 2 (drosophila)  SERBP1 - serpine1 mrna binding protein 1  EPC2 - enhancer of polycomb homolog 2 (drosophila)  DRD2 - dopamine receptor d2  PHF19 - phd finger protein 19  SLC9A1 - solute carrier family 9, subfamily a (nhe1, cation proton antiporter 1), member 1  NKD2 - naked cuticle homolog 2 (drosophila)  IRF2BP1 - interferon regulatory factor 2 binding protein 1  SLC8A1 - solute carrier family 8 (sodium/calcium exchanger), member 1  TRAF3IP1 - tnf receptor-associated factor 3 interacting protein 1  CNOT6L - ccr4-not transcription complex, subunit 6-like  EAF1 - ell associated factor 1  TAOK1 - tao kinase 1  SLC8A2 - solute carrier family 8 (sodium/calcium exchanger), member 2  PDP2 - pyruvate dehyrogenase phosphatase catalytic subunit 2  ATN1 - atrophin 1  DPH2 - dph2 homolog (s. cerevisiae)  ARRDC3 - arrestin domain containing 3  DPH1 - diphthamide biosynthesis 1  DOM3Z - dom-3 homolog z (c. elegans)  SEMA6A - sema domain, transmembrane domain (tm), and cytoplasmic domain, (semaphorin) 6a  C10orf137 - chromosome 10 open reading frame 137  DR1 - down-regulator of transcription 1, tbp-binding (negative cofactor 2)  DUSP8 - dual specificity phosphatase 8  TNKS1BP1 - tankyrase 1 binding protein 1, 182kda  MAPK15 - mitogen-activated protein kinase 15  GPSM1 - g-protein signaling modulator 1  SKI - v-ski avian sarcoma viral oncogene homolog  SKIL - ski-like oncogene  UNK - unkempt homolog (drosophila)  DUSP5 - dual specificity phosphatase 5  DUSP6 - dual specificity phosphatase 6  SKP1 - s-phase kinase-associated protein 1  DUSP7 - dual specificity phosphatase 7  DYRK1A - dual-specificity tyrosine-(y)-phosphorylation regulated kinase 1a  MKL1 - megakaryoblastic leukemia (translocation) 1  CHTOP - chromatin target of prmt1  DVL2 - dishevelled segment polarity protein 2  DVL3 - dishevelled segment polarity protein 3  WDR48 - wd repeat domain 48  GIGYF2 - grb10 interacting gyf protein 2  USP36 - ubiquitin specific peptidase 36  FNIP2 - folliculin interacting protein 2  APPL1 - adaptor protein, phosphotyrosine interaction, ph domain and leucine zipper containing 1  DOCK7 - dedicator of cytokinesis 7  HOMEZ - homeobox and leucine zipper encoding  EBF4 - early b-cell factor 4  ANKRD17 - ankyrin repeat domain 17  ZFHX2 - zinc finger homeobox 2  HBEGF - heparin-binding egf-like growth factor  KIAA1462 - kiaa1462  LSM14A - lsm14a, scd6 homolog a (s. cerevisiae)  DTX1 - deltex homolog 1 (drosophila)  DKC1 - dyskeratosis congenita 1, dyskerin  DLG3 - discs, large homolog 3 (drosophila)  PNPT1 - polyribonucleotide nucleotidyltransferase 1  DLG4 - discs, large homolog 4 (drosophila)  DLG1 - discs, large homolog 1 (drosophila)  ANKRD13A - ankyrin repeat domain 13a  SHOX2 - short stature homeobox 2  FBXW8 - f-box and wd repeat domain containing 8  SH3RF1 - sh3 domain containing ring finger 1  DHCR24 - 24-dehydrocholesterol reductase  SIAH2 - siah e3 ubiquitin protein ligase 2  POGK - pogo transposable element with krab domain  B3GAT3 - beta-1,3-glucuronyltransferase 3 (glucuronosyltransferase i)  DHFR - dihydrofolate reductase  PHF12 - phd finger protein 12  RICTOR - rptor independent companion of mtor, complex 2  FBXL5 - f-box and leucine-rich repeat protein 5  NQO1 - nad(p)h dehydrogenase, quinone 1  FBXO2 - f-box protein 2  CALCOCO1 - calcium binding and coiled-coil domain 1  ZBTB4 - zinc finger and btb domain containing 4  SFPQ - splicing factor proline/glutamine-rich  SFRP1 - secreted frizzled-related protein 1  SFRP2 - secreted frizzled-related protein 2  SFRP4 - secreted frizzled-related protein 4  SRSF1 - serine/arginine-rich splicing factor 1  SRSF2 - serine/arginine-rich splicing factor 2  GMEB2 - glucocorticoid modulatory element binding protein 2  DNM2 - dynamin 2  SRSF3 - serine/arginine-rich splicing factor 3  DNMT1 - dna (cytosine-5-)-methyltransferase 1  SPAG8 - sperm associated antigen 8  SRSF4 - serine/arginine-rich splicing factor 4  TRDMT1 - trna aspartic acid methyltransferase 1  SRSF5 - serine/arginine-rich splicing factor 5  DNMT3A - dna (cytosine-5-)-methyltransferase 3 alpha  SRSF6 - serine/arginine-rich splicing factor 6  SRSF7 - serine/arginine-rich splicing factor 7  EIF2AK4 - eukaryotic translation initiation factor 2 alpha kinase 4  SFSWAP - splicing factor, suppressor of white-apricot homolog (drosophila)  ALS2 - amyotrophic lateral sclerosis 2 (juvenile)  TRA2B - transformer 2 beta homolog (drosophila)  CHD8 - chromodomain helicase dna binding protein 8  DOCK3 - dedicator of cytokinesis 3  SGK1 - serum/glucocorticoid regulated kinase 1  TNRC6C - trinucleotide repeat containing 6c  NOC2L - nucleolar complex associated 2 homolog (s. cerevisiae)  ZNF317 - zinc finger protein 317  SGTA - small glutamine-rich tetratricopeptide repeat (tpr)-containing, alpha  USP37 - ubiquitin specific peptidase 37  ITSN1 - intersectin 1 (sh3 domain protein)  PPP1R3F - protein phosphatase 1, regulatory subunit 3f  SMARCAD1 - swi/snf-related, matrix-associated actin-dependent regulator of chromatin, subfamily a, containing dead/h box 1  MEIS3 - meis homeobox 3  NCBP2 - nuclear cap binding protein subunit 2, 20kda  MARK2 - map/microtubule affinity-regulating kinase 2  MAPRE3 - microtubule-associated protein, rp/eb family, member 3  TSC1 - tuberous sclerosis 1  CTTN - cortactin  ELK1 - elk1, member of ets oncogene family  SPIRE1 - spire actin nucleation factor 1  NAF1 - nuclear assembly factor 1 ribonucleoprotein  ELK3 - elk3, ets-domain protein (srf accessory protein 2)  ELK4 - elk4, ets-domain protein (srf accessory protein 1)  SNW1 - snw domain containing 1  SCAP - srebf chaperone  ENSA - endosulfine alpha  WRNIP1 - werner helicase interacting protein 1  EPAS1 - endothelial pas domain protein 1  DPH7 - diphthamide biosynthesis 7  EP300 - e1a binding protein p300  HABP4 - hyaluronan binding protein 4  ATF6 - activating transcription factor 6  SRRD - srr1 domain containing  UBQLN4 - ubiquilin 4  ENO1 - enolase 1, (alpha)  MOB1B - mob kinase activator 1b  RAB3GAP1 - rab3 gtpase activating protein subunit 1 (catalytic)  EIF4A2 - eukaryotic translation initiation factor 4a2  CELF4 - cugbp, elav-like family member 4  EIF4E - eukaryotic translation initiation factor 4e  DAPL1 - death associated protein-like 1  EGR2 - early growth response 2  EGR3 - early growth response 3  CCT5 - chaperonin containing tcp1, subunit 5 (epsilon)  TTF1 - transcription termination factor, rna polymerase i  ZC3HAV1 - zinc finger ccch-type, antiviral 1  EIF2S1 - eukaryotic translation initiation factor 2, subunit 1 alpha, 35kda  GPR137 - g protein-coupled receptor 137  TRIM32 - tripartite motif containing 32  EIF2B1 - eukaryotic translation initiation factor 2b, subunit 1 alpha, 26kda  SPHK2 - sphingosine kinase 2  EIF2S3 - eukaryotic translation initiation factor 2, subunit 3 gamma, 52kda  SERPINB1 - serpin peptidase inhibitor, clade b (ovalbumin), member 1  KDM2A - lysine (k)-specific demethylase 2a  ELAVL1 - elav (embryonic lethal, abnormal vision, drosophila)-like 1 (hu antigen r)  ZNF326 - zinc finger protein 326  ELF1 - e74-like factor 1 (ets domain transcription factor)  ELAVL4 - elav (embryonic lethal, abnormal vision, drosophila)-like 4  STRADA - ste20-related kinase adaptor alpha  ELF2 - e74-like factor 2 (ets domain transcription factor)  TSG101 - tumor susceptibility gene 101  TCF25 - transcription factor 25 (basic helix-loop-helix)  EIF4G2 - eukaryotic translation initiation factor 4 gamma, 2  TSC2 - tuberous sclerosis 2  EFNA1 - ephrin-a1  CNOT1 - ccr4-not transcription complex, subunit 1  EEF2 - eukaryotic translation elongation factor 2  EXOSC7 - exosome component 7  CTNNBIP1 - catenin, beta interacting protein 1  SPEN - spen homolog, transcriptional regulator (drosophila)  RNF149 - ring finger protein 149  KLHDC10 - kelch domain containing 10  EEF1A1 - eukaryotic translation elongation factor 1 alpha 1  EEF1A2 - eukaryotic translation elongation factor 1 alpha 2  BBX - bobby sox homolog (drosophila)  TP53BP1 - tumor protein p53 binding protein 1  PHC1 - polyhomeotic homolog 1 (drosophila)  PHC2 - polyhomeotic homolog 2 (drosophila)  EGR1 - early growth response 1  ZNF292 - zinc finger protein 292  EGFR - epidermal growth factor receptor  MEGF8 - multiple egf-like-domains 8  USP33 - ubiquitin specific peptidase 33  CELSR2 - cadherin, egf lag seven-pass g-type receptor 2  FAM20C - family with sequence similarity 20, member c  KDM1A - lysine (k)-specific demethylase 1a  ATXN7L3 - ataxin 7-like 3  EFNB2 - ephrin-b2  EFNA5 - ephrin-a5  HNRNPLL - heterogeneous nuclear ribonucleoprotein l-like  NCOA6 - nuclear receptor coactivator 6  CCT3 - chaperonin containing tcp1, subunit 3 (gamma)  E2F6 - e2f transcription factor 6  E4F1 - e4f transcription factor 1  SMG1 - smg1 phosphatidylinositol 3-kinase-related kinase  ZHX3 - zinc fingers and homeoboxes 3  TNIK - traf2 and nck interacting kinase  E2F4 - e2f transcription factor 4, p107/p130-binding  E2F5 - e2f transcription factor 5, p130-binding  ENY2 - enhancer of yellow 2 homolog (drosophila)  C11orf30 - chromosome 11 open reading frame 30  RRP1B - ribosomal rna processing 1b  EDNRB - endothelin receptor type b  MYCBP2 - myc binding protein 2, e3 ubiquitin protein ligase  TRAF1 - tnf receptor-associated factor 1  LPAR1 - lysophosphatidic acid receptor 1  HSP90B1 - heat shock protein 90kda beta (grp94), member 1  S1PR1 - sphingosine-1-phosphate receptor 1  TRAF3 - tnf receptor-associated factor 3  PMEPA1 - prostate transmembrane protein, androgen induced 1  TRAF2 - tnf receptor-associated factor 2  NR2C1 - nuclear receptor subfamily 2, group c, member 1  ZBTB47 - zinc finger and btb domain containing 47  SETX - senataxin  NR2C2 - nuclear receptor subfamily 2, group c, member 2  ECE1 - endothelin converting enzyme 1  TPR - translocated promoter region, nuclear basket protein  ZNF609 - zinc finger protein 609  WAPAL - wings apart-like homolog (drosophila)  LXN - latexin  ERC1 - elks/rab6-interacting/cast family member 1  PPRC1 - peroxisome proliferator-activated receptor gamma, coactivator-related 1  NAT14 - n-acetyltransferase 14 (gcn5-related, putative)  KDM4C - lysine (k)-specific demethylase 4c  ZNF423 - zinc finger protein 423  REXO4 - rex4, rna exonuclease 4 homolog (s. cerevisiae)  BOD1 - biorientation of chromosomes in cell division 1  CAMK1D - calcium/calmodulin-dependent protein kinase id  TMEM259 - transmembrane protein 259  USF1 - upstream transcription factor 1  NUP107 - nucleoporin 107kda  DERL3 - derlin 3  USP1 - ubiquitin specific peptidase 1  ZBTB43 - zinc finger and btb domain containing 43  PTK2B - protein tyrosine kinase 2 beta  ACSL3 - acyl-coa synthetase long-chain family member 3  MRPS27 - mitochondrial ribosomal protein s27  PITHD1 - pith (c-terminal proteasome-interacting domain of thioredoxin-like) domain containing 1  BPTF - bromodomain phd finger transcription factor  TNRC6B - trinucleotide repeat containing 6b  FASN - fatty acid synthase  USP4 - ubiquitin specific peptidase 4 (proto-oncogene)  RNF185 - ring finger protein 185  NR1H2 - nuclear receptor subfamily 1, group h, member 2  TAB2 - tgf-beta activated kinase 1/map3k7 binding protein 2  FBLN1 - fibulin 1  CAMTA2 - calmodulin binding transcription activator 2  ETV3 - ets variant 3  ETV5 - ets variant 5  ZNF598 - zinc finger protein 598  TWSG1 - twisted gastrulation homolog 1 (drosophila)  POGZ - pogo transposable element with znf domain  PLXND1 - plexin d1  ETV6 - ets variant 6  VHL - von hippel-lindau tumor suppressor, e3 ubiquitin protein ligase  VIM - vimentin  KDM6B - lysine (k)-specific demethylase 6b  EZR - ezrin  PHF8 - phd finger protein 8  TBX20 - t-box 20  TRIM41 - tripartite motif containing 41  EWSR1 - ews rna-binding protein 1  KCTD1 - potassium channel tetramerization domain containing 1  PCBP4 - poly(rc) binding protein 4  ANKLE2 - ankyrin repeat and lem domain containing 2  DCUN1D4 - dcn1, defective in cullin neddylation 1, domain containing 4  CREB3L1 - camp responsive element binding protein 3-like 1  UVRAG - uv radiation resistance associated  ACKR3 - atypical chemokine receptor 3  ZC3H3 - zinc finger ccch-type containing 3  EYA3 - eyes absent homolog 3 (drosophila)  VAV2 - vav 2 guanine nucleotide exchange factor  EZH1 - enhancer of zeste homolog 1 (drosophila)  GRAMD4 - gram domain containing 4  CCNL1 - cyclin l1  CIC - capicua transcriptional repressor  F2R - coagulation factor ii (thrombin) receptor  VCP - valosin containing protein  F3 - coagulation factor iii (thromboplastin, tissue factor)  WDR43 - wd repeat domain 43  MAPK8IP3 - mitogen-activated protein kinase 8 interacting protein 3  ZNF575 - zinc finger protein 575  ERF - ets2 repressor factor  ERCC4 - excision repair cross-complementing rodent repair deficiency, complementation group 4  NUP205 - nucleoporin 205kda  YTHDC1 - yth domain containing 1  ELMSAN1 - elm2 and myb/sant-like domain containing 1  GGA3 - golgi-associated, gamma adaptin ear containing, arf binding protein 3  THAP11 - thap domain containing 11  EMC10 - er membrane protein complex subunit 10  ERN1 - endoplasmic reticulum to nucleus signaling 1  ZNF248 - zinc finger protein 248  UBB - ubiquitin b  PPP1R37 - protein phosphatase 1, regulatory subunit 37  GPD1L - glycerol-3-phosphate dehydrogenase 1-like  ARRDC4 - arrestin domain containing 4  LARP4B - la ribonucleoprotein domain family, member 4b  RCOR1 - rest corepressor 1  TULP3 - tubby like protein 3  HIRA - histone cell cycle regulator  ZNFX1 - zinc finger, nfx1-type containing 1  TTC5 - tetratricopeptide repeat domain 5  TYRO3 - tyro3 protein tyrosine kinase  ETV1 - ets variant 1  ETS1 - v-ets avian erythroblastosis virus e26 oncogene homolog 1  SLC39A10 - solute carrier family 39 (zinc transporter), member 10  ZMIZ1 - zinc finger, miz-type containing 1  ETF1 - eukaryotic translation termination factor 1  CYFIP1 - cytoplasmic fmr1 interacting protein 1  ARL6IP1 - adp-ribosylation factor-like 6 interacting protein 1  DMTN - dematin actin binding protein  UBP1 - upstream binding protein 1 (lbp-1a)  SUMO1 - small ubiquitin-like modifier 1  UCHL1 - ubiquitin carboxyl-terminal esterase l1 (ubiquitin thiolesterase)  FAM168A - family with sequence similarity 168, member a  UBTF - upstream binding transcription factor, rna polymerase i  ZNF830 - zinc finger protein 830  PELI1 - pellino e3 ubiquitin protein ligase 1  CHURC1 - churchill domain containing 1  SMURF1 - smad specific e3 ubiquitin protein ligase 1  ZC3H4 - zinc finger ccch-type containing 4  JMJD6 - jumonji domain containing 6  MYADM - myeloid-associated differentiation marker  UBE2I - ubiquitin-conjugating enzyme e2i  EPRS - glutamyl-prolyl-trna synthetase  EPHB6 - eph receptor b6  UBE2V2 - ubiquitin-conjugating enzyme e2 variant 2  TBC1D12 - tbc1 domain family, member 12  ERCC2 - excision repair cross-complementing rodent repair deficiency, complementation group 2  UBE2L3 - ubiquitin-conjugating enzyme e2l 3  NUP210 - nucleoporin 210kda  EPS15 - epidermal growth factor receptor pathway substrate 15  UBE2N - ubiquitin-conjugating enzyme e2n  UBE2V1 - ubiquitin-conjugating enzyme e2 variant 1  VPS72 - vacuolar protein sorting 72 homolog (s. cerevisiae)  PSENEN - presenilin enhancer 2 homolog (c. elegans)  FMR1 - fragile x mental retardation 1  EXOC7 - exocyst complex component 7  PIK3IP1 - phosphoinositide-3-kinase interacting protein 1  TCOF1 - treacher collins-franceschetti syndrome 1  MGA - mga, max dimerization protein  TCP1 - t-complex 1  FNTA - farnesyltransferase, caax box, alpha  WWC3 - wwc family member 3  ZEB1 - zinc finger e-box binding homeobox 1  FLT1 - fms-related tyrosine kinase 1  SCFD1 - sec1 family domain containing 1  TCF12 - transcription factor 12  ZNF354A - zinc finger protein 354a  TCF20 - transcription factor 20 (ar1)  CAMTA1 - calmodulin binding transcription activator 1  PDS5A - pds5, regulator of cohesion maintenance, homolog a (s. cerevisiae)  TMEM106A - transmembrane protein 106a  FLI1 - fli-1 proto-oncogene, ets transcription factor  FLNA - filamin a, alpha  PLCB1 - phospholipase c, beta 1 (phosphoinositide-specific)  ARC - activity-regulated cytoskeleton-associated protein  FOXS1 - forkhead box s1  PHLPP1 - ph domain and leucine rich repeat protein phosphatase 1  FOXO3 - forkhead box o3  HDAC8 - histone deacetylase 8  LARP4 - la ribonucleoprotein domain family, member 4  ASH1L - ash1 (absent, small, or homeotic)-like (drosophila)  SIN3B - sin3 transcription regulator family member b  FKBP15 - fk506 binding protein 15, 133kda  DCP1A - decapping mrna 1a  NACC1 - nucleus accumbens associated 1, ben and btb (poz) domain containing  VPS26B - vacuolar protein sorting 26 homolog b (s. pombe)  HR - hair growth associated  MED8 - mediator complex subunit 8  CAND1 - cullin-associated and neddylation-dissociated 1  FKBP1A - fk506 binding protein 1a, 12kda  TDG - thymine-dna glycosylase  SMG6 - smg6 nonsense mediated mrna decay factor  PRDX2 - peroxiredoxin 2  FKBP1B - fk506 binding protein 1b, 12.6 kda  EAPP - e2f-associated phosphoprotein  FBXW11 - f-box and wd repeat domain containing 11  VPS11 - vacuolar protein sorting 11 homolog (s. cerevisiae)  KDM3A - lysine (k)-specific demethylase 3a  FAM208A - family with sequence similarity 208, member a  CLEC16A - c-type lectin domain family 16, member a  FHL2 - four and a half lim domains 2  FHIT - fragile histidine triad  SLC24A5 - solute carrier family 24 (sodium/potassium/calcium exchanger), member 5  PPP1R11 - protein phosphatase 1, regulatory (inhibitor) subunit 11  NUP160 - nucleoporin 160kda  TAF9 - taf9 rna polymerase ii, tata box binding protein (tbp)-associated factor, 32kda  FGFR2 - fibroblast growth factor receptor 2  TAF7 - taf7 rna polymerase ii, tata box binding protein (tbp)-associated factor, 55kda  FGF13 - fibroblast growth factor 13  TAF6 - taf6 rna polymerase ii, tata box binding protein (tbp)-associated factor, 80kda  FGFR1 - fibroblast growth factor receptor 1  TADA2A - transcriptional adaptor 2a  FGF9 - fibroblast growth factor 9  SMCHD1 - structural maintenance of chromosomes flexible hinge domain containing 1  ADAM17 - adam metallopeptidase domain 17  TACC1 - transforming, acidic coiled-coil containing protein 1  TIRAP - toll-interleukin 1 receptor (tir) domain containing adaptor protein  FGF1 - fibroblast growth factor 1 (acidic)  FGF2 - fibroblast growth factor 2 (basic)  SZT2 - seizure threshold 2 homolog (mouse)  USP22 - ubiquitin specific peptidase 22  SASH1 - sam and sh3 domain containing 1  TARBP1 - tar (hiv-1) rna binding protein 1  FER - fer (fps/fes related) tyrosine kinase  MAP3K7 - mitogen-activated protein kinase kinase kinase 7  PDGFC - platelet derived growth factor c  ADNP - activity-dependent neuroprotector homeobox  KMT2E - lysine (k)-specific methyltransferase 2e  MED13L - mediator complex subunit 13-like  PGP - phosphoglycolate phosphatase  NCSTN - nicastrin  FKTN - fukutin  ZNF395 - zinc finger protein 395  MYNN - myoneurin  TAZ - tafazzin  CRTC1 - creb regulated transcription coactivator 1  UFL1 - ufm1-specific ligase 1  TCF3 - transcription factor 3  PUM2 - pumilio homolog 2 (drosophila)  TCEB3 - transcription elongation factor b (siii), polypeptide 3 (110kda, elongin a)  DMAP1 - dna methyltransferase 1 associated protein 1  TCF4 - transcription factor 4  LARP1 - la ribonucleoprotein domain family, member 1  NKRF - nfkb repressing factor  EFEMP1 - egf containing fibulin-like extracellular matrix protein 1  ZNF629 - zinc finger protein 629  FBN1 - fibrillin 1  LANCL2 - lanc lantibiotic synthetase component c-like 2 (bacterial)  TCEA2 - transcription elongation factor a (sii), 2  TCEA1 - transcription elongation factor a (sii), 1  ERBB2IP - erbb2 interacting protein  TIE1 - tyrosine kinase with immunoglobulin-like and egf-like domains 1  TIMP1 - timp metallopeptidase inhibitor 1  CAMK2N2 - calcium/calmodulin-dependent protein kinase ii inhibitor 2  TIMP2 - timp metallopeptidase inhibitor 2  TIMP3 - timp metallopeptidase inhibitor 3  TIMP4 - timp metallopeptidase inhibitor 4  ITGB3BP - integrin beta 3 binding protein (beta3-endonexin)  SYAP1 - synapse associated protein 1  RYBP - ring1 and yy1 binding protein  ORMDL1 - orm1-like 1 (s. cerevisiae)  MTOR - mechanistic target of rapamycin (serine/threonine kinase)  PAXBP1 - pax3 and pax7 binding protein 1  TLE1 - transducin-like enhancer of split 1 (e(sp1) homolog, drosophila)  TLE3 - transducin-like enhancer of split 3 (e(sp1) homolog, drosophila)  TLE4 - transducin-like enhancer of split 4 (e(sp1) homolog, drosophila)  NDNL2 - necdin-like 2  ATP13A2 - atpase type 13a2  TLR3 - toll-like receptor 3  EXOSC2 - exosome component 2  SIRT1 - sirtuin 1  MKX - mohawk homeobox  TMF1 - tata element modulatory factor 1  GPR137B - g protein-coupled receptor 137b  TMSB4X - thymosin beta 4, x-linked  ACTR8 - arp8 actin-related protein 8 homolog (yeast)  CBX6 - chromobox homolog 6  HEY1 - hairy/enhancer-of-split related with yrpw motif 1  TMPO - thymopoietin  CBX5 - chromobox homolog 5  BRD4 - bromodomain containing 4  TNFAIP1 - tumor necrosis factor, alpha-induced protein 1 (endothelial)  BCL9L - b-cell cll/lymphoma 9-like  TARDBP - tar dna binding protein  TNFRSF1A - tumor necrosis factor receptor superfamily, member 1a  TNFRSF1B - tumor necrosis factor receptor superfamily, member 1b  TMBIM6 - transmembrane bax inhibitor motif containing 6  PRKCDBP - protein kinase c, delta binding protein  EIF4ENIF1 - eukaryotic translation initiation factor 4e nuclear import factor 1  TEK - tek tyrosine kinase, endothelial  NUP188 - nucleoporin 188kda  SUZ12 - suz12 polycomb repressive complex 2 subunit  TEF - thyrotrophic embryonic factor  POFUT1 - protein o-fucosyltransferase 1  RCOR2 - rest corepressor 2  TFAM - transcription factor a, mitochondrial  DIABLO - diablo, iap-binding mitochondrial protein  INPP5E - inositol polyphosphate-5-phosphatase, 72 kda  TERF1 - telomeric repeat binding factor (nima-interacting) 1  TERF2 - telomeric repeat binding factor 2  TESK1 - testis-specific kinase 1  TFDP1 - transcription factor dp-1  NR2F2 - nuclear receptor subfamily 2, group f, member 2  TFE3 - transcription factor binding to ighm enhancer 3  CBX7 - chromobox homolog 7  BCCIP - brca2 and cdkn1a interacting protein  ISCU - iron-sulfur cluster assembly enzyme  NR2F1 - nuclear receptor subfamily 2, group f, member 1  TFCP2 - transcription factor cp2  TGFA - transforming growth factor, alpha  ZNF740 - zinc finger protein 740  RIMBP2 - rims binding protein 2  FNIP1 - folliculin interacting protein 1  HEY2 - hairy/enhancer-of-split related with yrpw motif 2  TGFBR1 - transforming growth factor, beta receptor 1  YLPM1 - ylp motif containing 1  ATP6V0A2 - atpase, h+ transporting, lysosomal v0 subunit a2  TGFB2 - transforming growth factor, beta 2  RBFOX2 - rna binding protein, fox-1 homolog (c. elegans) 2  TGFB3 - transforming growth factor, beta 3  TGFB1 - transforming growth factor, beta 1  TGFB1I1 - transforming growth factor beta 1 induced transcript 1  MAPK8IP2 - mitogen-activated protein kinase 8 interacting protein 2  TP53INP1 - tumor protein p53 inducible nuclear protein 1  H2AFV - h2a histone family, member v  MEPCE - methylphosphate capping enzyme  RNF20 - ring finger protein 20, e3 ubiquitin protein ligase  TGFBR2 - transforming growth factor, beta receptor ii (70/80kda)  TGFBR3 - transforming growth factor, beta receptor iii  FOSL2 - fos-like antigen 2  FOSB - fbj murine osteosarcoma viral oncogene homolog b  FOS - fbj murine osteosarcoma viral oncogene homolog  THBS1 - thrombospondin 1  CABIN1 - calcineurin binding protein 1  KAT6B - k(lysine) acetyltransferase 6b  TIA1 - tia1 cytotoxic granule-associated rna binding protein  METTL3 - methyltransferase like 3  THY1 - thy-1 cell surface antigen  THRA - thyroid hormone receptor, alpha  MMD - monocyte to macrophage differentiation-associated  ELL - elongation factor rna polymerase ii  ZNF239 - zinc finger protein 239  GFRA2 - gdnf family receptor alpha 2  SYMPK - symplekin  PHC3 - polyhomeotic homolog 3 (drosophila)  FAM192A - family with sequence similarity 192, member a  OBFC1 - oligonucleotide/oligosaccharide-binding fold containing 1  NRIP1 - nuclear receptor interacting protein 1  KAT2A - k(lysine) acetyltransferase 2a  NCOA3 - nuclear receptor coactivator 3  PANK2 - pantothenate kinase 2  ANP32A - acidic (leucine-rich) nuclear phosphoprotein 32 family, member a  GLA - galactosidase, alpha  WWC2 - ww and c2 domain containing 2  PABPN1 - poly(a) binding protein, nuclear 1  TAF15 - taf15 rna polymerase ii, tata box binding protein (tbp)-associated factor, 68kda  GJA1 - gap junction protein, alpha 1, 43kda  NPRL3 - nitrogen permease regulator-like 3 (s. cerevisiae)  TMTC3 - transmembrane and tetratricopeptide repeat containing 3  AXIN1 - axin 1  TRRAP - transformation/transcription domain-associated protein  PICALM - phosphatidylinositol binding clathrin assembly protein  GGNBP2 - gametogenetin binding protein 2  CAAP1 - caspase activity and apoptosis inhibitor 1  LPCAT1 - lysophosphatidylcholine acyltransferase 1  ZC3H14 - zinc finger ccch-type containing 14  ARID1A - at rich interactive domain 1a (swi-like)  DLL1 - delta-like 1 (drosophila)  SETD6 - set domain containing 6  GLG1 - golgi glycoprotein 1  FZD6 - frizzled family receptor 6  GLE1 - gle1 rna export mediator  FZD8 - frizzled family receptor 8  ACTR5 - arp5 actin-related protein 5 homolog (yeast)  CDC45 - cell division cycle 45  FZD4 - frizzled family receptor 4  GCLC - glutamate-cysteine ligase, catalytic subunit  FZD1 - frizzled family receptor 1  BAP1 - brca1 associated protein-1 (ubiquitin carboxy-terminal hydrolase)  ZNF613 - zinc finger protein 613  AXIN2 - axin 2  CDC7 - cell division cycle 7  NUP85 - nucleoporin 85kda  KDM5C - lysine (k)-specific demethylase 5c  USP9X - ubiquitin specific peptidase 9, x-linked  GNG3 - guanine nucleotide binding protein (g protein), gamma 3  RBM10 - rna binding motif protein 10  GNAQ - guanine nucleotide binding protein (g protein), q polypeptide  GNAI3 - guanine nucleotide binding protein (g protein), alpha inhibiting activity polypeptide 3  GNAI2 - guanine nucleotide binding protein (g protein), alpha inhibiting activity polypeptide 2  PHF17 - phd finger protein 17  GNA12 - guanine nucleotide binding protein (g protein) alpha 12  GMFB - glia maturation factor, beta  CNST - consortin, connexin sorting protein  TTC21B - tetratricopeptide repeat domain 21b  SLTM - safb-like, transcription modulator  NELFE - negative elongation factor complex member e  EHMT1 - euchromatic histone-lysine n-methyltransferase 1  NR5A1 - nuclear receptor subfamily 5, group a, member 1  FUS - fused in sarcoma  NAA40 - n(alpha)-acetyltransferase 40, natd catalytic subunit, homolog (s. cerevisiae)  PIP4K2C - phosphatidylinositol-5-phosphate 4-kinase, type ii, gamma  USP7 - ubiquitin specific peptidase 7 (herpes virus-associated)  RNF168 - ring finger protein 168, e3 ubiquitin protein ligase  XRCC6 - x-ray repair complementing defective repair in chinese hamster cells 6  GABPB1 - ga binding protein transcription factor, beta subunit 1  GABPA - ga binding protein transcription factor, alpha subunit 60kda  CPSF7 - cleavage and polyadenylation specific factor 7, 59kda  FYN - fyn oncogene related to src, fgr, yes  RPAP2 - rna polymerase ii associated protein 2  G6PD - glucose-6-phosphate dehydrogenase  DDX39B - dead (asp-glu-ala-asp) box polypeptide 39b  USP5 - ubiquitin specific peptidase 5 (isopeptidase t)  KMT2D - lysine (k)-specific methyltransferase 2d  SPRED1 - sprouty-related, evh1 domain containing 1  AAAS - achalasia, adrenocortical insufficiency, alacrimia  ZNF419 - zinc finger protein 419  CDK2AP1 - cyclin-dependent kinase 2 associated protein 1  IFT88 - intraflagellar transport 88 homolog (chlamydomonas)  YEATS4 - yeats domain containing 4  SNIP1 - smad nuclear interacting protein 1  ZFPM1 - zinc finger protein, fog family member 1  ZNF212 - zinc finger protein 212  EID2 - ep300 interacting inhibitor of differentiation 2  GBA - glucosidase, beta, acid  ZNF383 - zinc finger protein 383  GATA2 - gata binding protein 2  KAT6A - k(lysine) acetyltransferase 6a  NUP214 - nucleoporin 214kda  NR4A3 - nuclear receptor subfamily 4, group a, member 3  BRD3 - bromodomain containing 3  SHOC2 - soc-2 suppressor of clear homolog (c. elegans)  GAS6 - growth arrest-specific 6  CARF - calcium responsive transcription factor  NCOA4 - nuclear receptor coactivator 4  MLLT10 - myeloid/lymphoid or mixed-lineage leukemia (trithorax homolog, drosophila); translocated to, 10  ZNF408 - zinc finger protein 408  DEDD2 - death effector domain containing 2  FASTKD1 - fast kinase domains 1  DHX36 - deah (asp-glu-ala-his) box polypeptide 36  ZNF189 - zinc finger protein 189  ZSCAN26 - zinc finger and scan domain containing 26  GSTP1 - glutathione s-transferase pi 1  ZNF177 - zinc finger protein 177  GSPT1 - g1 to s phase transition 1  ZNF175 - zinc finger protein 175  GSN - gelsolin  ZNF174 - zinc finger protein 174  ZNF184 - zinc finger protein 184  SSBP4 - single stranded dna binding protein 4  GTF2F1 - general transcription factor iif, polypeptide 1, 74kda  ZNF148 - zinc finger protein 148  ZBTB17 - zinc finger and btb domain containing 17  BEND5 - ben domain containing 5  NEIL1 - nei endonuclease viii-like 1 (e. coli)  GTF2H2 - general transcription factor iih, polypeptide 2, 44kda  TRIM26 - tripartite motif containing 26  GTF2H3 - general transcription factor iih, polypeptide 3, 34kda  VEZF1 - vascular endothelial zinc finger 1  GTF2H1 - general transcription factor iih, polypeptide 1, 62kda  ZNF142 - zinc finger protein 142  MCPH1 - microcephalin 1  ZNF140 - zinc finger protein 140  GTF2B - general transcription factor iib  PCGF2 - polycomb group ring finger 2  MSH6 - muts homolog 6 (e. coli)  ZNF132 - zinc finger protein 132  ZNF131 - zinc finger protein 131  ZNF134 - zinc finger protein 134  ZNF133 - zinc finger protein 133  TBL1XR1 - transducin (beta)-like 1 x-linked receptor 1  GTF2I - general transcription factor iii  GTF2H4 - general transcription factor iih, polypeptide 4, 52kda  PRKRIP1 - prkr interacting protein 1 (il11 inducible)  BRF1 - brf1, rna polymerase iii transcription initiation factor 90 kda subunit  ZNF79 - zinc finger protein 79  ZYG11B - zyg-11 family member b, cell cycle regulator  ZNF70 - zinc finger protein 70  RBM12B - rna binding motif protein 12b  ZNF75D - zinc finger protein 75d  ZNF76 - zinc finger protein 76  H3F3A - h3 histone, family 3a  BRPF1 - bromodomain and phd finger containing, 1  H2AFZ - h2a histone family, member z  SAP130 - sin3a-associated protein, 130kda  FZD5 - frizzled family receptor 5  DCP2 - decapping mrna 2  H2AFX - h2a histone family, member x  CXCR4 - chemokine (c-x-c motif) receptor 4  MUL1 - mitochondrial e3 ubiquitin protein ligase 1  PAPD4 - pap associated domain containing 4  PAX8 - paired box 8  MIER3 - mesoderm induction early response 1, family member 3  CDC73 - cell division cycle 73  NKAP - nfkb activating protein  THAP5 - thap domain containing 5  LRP8 - low density lipoprotein receptor-related protein 8, apolipoprotein e receptor  HCFC1 - host cell factor c1 (vp16-accessory protein)  PRDM2 - pr domain containing 2, with znf domain  MAP3K12 - mitogen-activated protein kinase kinase kinase 12  ZNF226 - zinc finger protein 226  HDAC2 - histone deacetylase 2  HMBOX1 - homeobox containing 1  ZCCHC12 - zinc finger, cchc domain containing 12  HDGF - hepatoma-derived growth factor  ZNF213 - zinc finger protein 213  HTT - huntingtin  HDAC1 - histone deacetylase 1  ZNF205 - zinc finger protein 205  HCLS1 - hematopoietic cell-specific lyn substrate 1  ZNF207 - zinc finger protein 207  XPA - xeroderma pigmentosum, complementation group a  XPC - xeroderma pigmentosum, complementation group c  GOLGA2 - golgin a2  XPO1 - exportin 1 (crm1 homolog, yeast)  GOLGB1 - golgin b1  XRCC1 - x-ray repair complementing defective repair in chinese hamster cells 1  PAGR1 - paxip1 associated glutamate-rich protein 1  ZXDC - zxd family zinc finger c  AAED1 - ahpc/tsa antioxidant enzyme domain containing 1  XBP1 - x-box binding protein 1  ZNF367 - zinc finger protein 367  BHLHE41 - basic helix-loop-helix family, member e41  EIF4H - eukaryotic translation initiation factor 4h  WFS1 - wolfram syndrome 1 (wolframin)  WHSC1 - wolf-hirschhorn syndrome candidate 1  NELFA - negative elongation factor complex member a  VRK2 - vaccinia related kinase 2  ARID2 - at rich interactive domain 2 (arid, rfx-like)  WARS - tryptophanyl-trna synthetase  PAOX - polyamine oxidase (exo-n4-amino)  ZNF35 - zinc finger protein 35  ZKSCAN1 - zinc finger with krab and scan domains 1  FBXL15 - f-box and leucine-rich repeat protein 15  ZNF33B - zinc finger protein 33b  ZNF576 - zinc finger protein 576  ZNF32 - zinc finger protein 32  ZNF45 - zinc finger protein 45  BRCC3 - brca1/brca2-containing complex, subunit 3  MZF1 - myeloid zinc finger 1  ZNF18 - zinc finger protein 18  ZNF16 - zinc finger protein 16  GRB2 - growth factor receptor-bound protein 2  ASXL1 - additional sex combs like 1 (drosophila)  ZNF384 - zinc finger protein 384  TNIP2 - tnfaip3 interacting protein 2  RBM42 - rna binding motif protein 42  ZNF24 - zinc finger protein 24  ZFPL1 - zinc finger protein-like 1  ZFX - zinc finger protein, x-linked  SF1 - splicing factor 1  GRIK2 - glutamate receptor, ionotropic, kainate 2  ZBTB14 - zinc finger and btb domain containing 14  ZNF711 - zinc finger protein 711  ZNF7 - zinc finger protein 7  ZNF8 - zinc finger protein 8  ZNF2 - zinc finger protein 2  ZNF3 - zinc finger protein 3  YES1 - v-yes-1 yamaguchi sarcoma viral oncogene homolog 1  XRCC5 - x-ray repair complementing defective repair in chinese hamster cells 5 (double-strand-break rejoining)  YWHAG - tyrosine 3-monooxygenase/tryptophan 5-monooxygenase activation protein, gamma polypeptide  GRSF1 - g-rich rna sequence binding factor 1  GSK3B - glycogen synthase kinase 3 beta  YWHAZ - tyrosine 3-monooxygenase/tryptophan 5-monooxygenase activation protein, zeta polypeptide  GSK3A - glycogen synthase kinase 3 alpha  YWHAE - tyrosine 3-monooxygenase/tryptophan 5-monooxygenase activation protein, epsilon polypeptide  YWHAB - tyrosine 3-monooxygenase/tryptophan 5-monooxygenase activation protein, beta polypeptide  CCNK - cyclin k  TRIM65 - tripartite motif containing 65  MAPKAP1 - mitogen-activated protein kinase associated protein 1  DNAJB2 - dnaj (hsp40) homolog, subfamily b, member 2  DNAJA1 - dnaj (hsp40) homolog, subfamily a, member 1  HSF2 - heat shock transcription factor 2  TRIM24 - tripartite motif containing 24  TAF1D - tata box binding protein (tbp)-associated factor, rna polymerase i, d, 41kda  CREG1 - cellular repressor of e1a-stimulated genes 1  CHAC1 - chac, cation transport regulator homolog 1 (e. coli)  FLCN - folliculin  HSBP1 - heat shock factor binding protein 1  NRP1 - neuropilin 1  PRMT1 - protein arginine methyltransferase 1  IQGAP1 - iq motif containing gtpase activating protein 1  PRMT2 - protein arginine methyltransferase 2  HESX1 - hesx homeobox 1  SAP30 - sin3a-associated protein, 30kda  PHF23 - phd finger protein 23  DERL1 - derlin 1  METTL16 - methyltransferase like 16  FTO - fat mass and obesity associated  ALKBH1 - alkb, alkylation repair homolog 1 (e. coli)  HPRT1 - hypoxanthine phosphoribosyltransferase 1  SPRED2 - sprouty-related, evh1 domain containing 2  CFLAR - casp8 and fadd-like apoptosis regulator  SYNGAP1 - synaptic ras gtpase activating protein 1  SECISBP2 - secis binding protein 2  MED11 - mediator complex subunit 11  LDB1 - lim domain binding 1  WDR77 - wd repeat domain 77  DSCC1 - dna replication and sister chromatid cohesion 1  KAT2B - k(lysine) acetyltransferase 2b  TSC22D1 - tsc22 domain family, member 1  CRTC2 - creb regulated transcription coactivator 2  DDA1 - det1 and ddb1 associated 1  ZNF259 - zinc finger protein 259  TAX1BP1 - tax1 (human t-cell leukemia virus type i) binding protein 1  CDC123 - cell division cycle 123  CC2D1B - coiled-coil and c2 domain containing 1b  DBNDD1 - dysbindin (dystrobrevin binding protein 1) domain containing 1  MTMR2 - myotubularin related protein 2  NABP2 - nucleic acid binding protein 2  MTMR3 - myotubularin related protein 3  HUS1 - hus1 checkpoint homolog (s. pombe)  EIF2B2 - eukaryotic translation initiation factor 2b, subunit 2 beta, 39kda  THAP8 - thap domain containing 8  CADM4 - cell adhesion molecule 4  BUD31 - bud31 homolog (s. cerevisiae)  ZNF655 - zinc finger protein 655  EIF2B5 - eukaryotic translation initiation factor 2b, subunit 5 epsilon, 82kda  HERC1 - hect and rld domain containing e3 ubiquitin protein ligase family member 1  TRAK2 - trafficking protein, kinesin binding 2  MTMR9 - myotubularin related protein 9  HSPD1 - heat shock 60kda protein 1 (chaperonin)  DNAJB1 - dnaj (hsp40) homolog, subfamily b, member 1  ZNF48 - zinc finger protein 48  BCL10 - b-cell cll/lymphoma 10  FUBP3 - far upstream element (fuse) binding protein 3  HSP90AA1 - heat shock protein 90kda alpha (cytosolic), class a member 1  CDK5R2 - cyclin-dependent kinase 5, regulatory subunit 2 (p39)  C7orf49 - chromosome 7 open reading frame 49  BTRC - beta-transducin repeat containing e3 ubiquitin protein ligase  HSPA8 - heat shock 70kda protein 8  EIF4EBP3 - eukaryotic translation initiation factor 4e binding protein 3  WNK1 - wnk lysine deficient protein kinase 1  HMGN1 - high mobility group nucleosome binding domain 1  UBE2Z - ubiquitin-conjugating enzyme e2z  HMGB3 - high mobility group box 3  NUMB - numb homolog (drosophila)  HMGB2 - high mobility group box 2  LAMTOR3 - late endosomal/lysosomal adaptor, mapk and mtor activator 3  HMGB1 - high mobility group box 1  EIF3C - eukaryotic translation initiation factor 3, subunit c  PHACTR4 - phosphatase and actin regulator 4  BRD9 - bromodomain containing 9  EIF3D - eukaryotic translation initiation factor 3, subunit d  EIF3A - eukaryotic translation initiation factor 3, subunit a  EIF3B - eukaryotic translation initiation factor 3, subunit b  IRS2 - insulin receptor substrate 2  DYNLL1 - dynein, light chain, lc8-type 1  TNKS - tankyrase, trf1-interacting ankyrin-related adp-ribose polymerase  EIF4G3 - eukaryotic translation initiation factor 4 gamma, 3  DAZAP1 - daz associated protein 1  ZBTB10 - zinc finger and btb domain containing 10  AGO1 - argonaute risc catalytic component 1  LATS2 - large tumor suppressor kinase 2  CAPRIN2 - caprin family member 2  ZBTB48 - zinc finger and btb domain containing 48  SRSF9 - serine/arginine-rich splicing factor 9  PEA15 - phosphoprotein enriched in astrocytes 15  NDRG4 - ndrg family member 4  HYAL2 - hyaluronoglucosaminidase 2  ACD - adrenocortical dysplasia homolog (mouse)  GPBP1 - gc-rich promoter binding protein 1  HFE - hemochromatosis  HIP1 - huntingtin interacting protein 1  UBE2K - ubiquitin-conjugating enzyme e2k  HINT1 - histidine triad nucleotide binding protein 1  ELP4 - elongator acetyltransferase complex subunit 4  HIVEP1 - human immunodeficiency virus type i enhancer binding protein 1  UPF3B - upf3 regulator of nonsense transcripts homolog b (yeast)  UPF3A - upf3 regulator of nonsense transcripts homolog a (yeast)  HIF1A - hypoxia inducible factor 1, alpha subunit (basic helix-loop-helix transcription factor)  LPIN3 - lipin 3  TRADD - tnfrsf1a-associated via death domain  MYCBP - myc binding protein  EED - embryonic ectoderm development  URI1 - uri1, prefoldin-like chaperone  RIPK1 - receptor (tnfrsf)-interacting serine-threonine kinase 1  HNRNPU - heterogeneous nuclear ribonucleoprotein u (scaffold attachment factor a)  HNRNPK - heterogeneous nuclear ribonucleoprotein k  FBXO22 - f-box protein 22  HNRNPL - heterogeneous nuclear ribonucleoprotein l  TNFSF13 - tumor necrosis factor (ligand) superfamily, member 13  FBXO4 - f-box protein 4  TINF2 - terf1 (trf1)-interacting nuclear factor 2  ADAM9 - adam metallopeptidase domain 9  P2RY12 - purinergic receptor p2y, g-protein coupled, 12  HNRNPA2B1 - heterogeneous nuclear ribonucleoprotein a2/b1  HNRNPA1 - heterogeneous nuclear ribonucleoprotein a1  HNRNPC - heterogeneous nuclear ribonucleoprotein c (c1/c2)  RIPK2 - receptor-interacting serine-threonine kinase 2  HNRNPAB - heterogeneous nuclear ribonucleoprotein a/b  HNRNPF - heterogeneous nuclear ribonucleoprotein f  HNRNPD - heterogeneous nuclear ribonucleoprotein d (au-rich element rna binding protein 1, 37kda)  HNRNPH1 - heterogeneous nuclear ribonucleoprotein h1 (h)  HNRNPH3 - heterogeneous nuclear ribonucleoprotein h3 (2h9)  MTMR1 - myotubularin related protein 1  HNRNPH2 - heterogeneous nuclear ribonucleoprotein h2 (h')  HMGCR - 3-hydroxy-3-methylglutaryl-coa reductase  AIDA - axin interactor, dorsalization associated  USP46 - ubiquitin specific peptidase 46  ZNF584 - zinc finger protein 584  HMGA1 - high mobility group at-hook 1  FAM129B - family with sequence similarity 129, member b  NR4A1 - nuclear receptor subfamily 4, group a, member 1  PDE12 - phosphodiesterase 12  RFX7 - regulatory factor x, 7  DEPTOR - dep domain containing mtor-interacting protein  FOXK2 - forkhead box k2  HTATSF1 - hiv-1 tat specific factor 1  HAT1 - histone acetyltransferase 1  EIF3K - eukaryotic translation initiation factor 3, subunit k  RPS6KA6 - ribosomal protein s6 kinase, 90kda, polypeptide 6  CRTC3 - creb regulated transcription coactivator 3  RBM15 - rna binding motif protein 15  TNRC6A - trinucleotide repeat containing 6a  ENC1 - ectodermal-neural cortex 1 (with btb domain)  RBMX - rna binding motif protein, x-linked  INHA - inhibin, alpha  PIK3R3 - phosphoinositide-3-kinase, regulatory subunit 3 (gamma)  DESI1 - desumoylating isopeptidase 1  PARG - poly (adp-ribose) glycohydrolase  HTRA2 - htra serine peptidase 2  STK39 - serine threonine kinase 39  ING2 - inhibitor of growth family, member 2  PPM1D - protein phosphatase, mg2+/mn2+ dependent, 1d  ZNF574 - zinc finger protein 574  CREB3L2 - camp responsive element binding protein 3-like 2  PCSK1N - proprotein convertase subtilisin/kexin type 1 inhibitor  IPPK - inositol 1,3,4,5,6-pentakisphosphate 2-kinase  UBE2S - ubiquitin-conjugating enzyme e2s  ILF2 - interleukin enhancer binding factor 2  PRPF19 - pre-mrna processing factor 19  ILF3 - interleukin enhancer binding factor 3, 90kda  MAP4K3 - mitogen-activated protein kinase kinase kinase kinase 3  ILK - integrin-linked kinase  CDC14B - cell division cycle 14b  PIAS1 - protein inhibitor of activated stat, 1  SMYD3 - set and mynd domain containing 3  SLCO3A1 - solute carrier organic anion transporter family, member 3a1  SMURF2 - smad specific e3 ubiquitin protein ligase 2  MAPKAPK5 - mitogen-activated protein kinase-activated protein kinase 5  CGGBP1 - cgg triplet repeat binding protein 1  LMO4 - lim domain only 4  CAMK1 - calcium/calmodulin-dependent protein kinase i  IL10RA - interleukin 10 receptor, alpha  CBX4 - chromobox homolog 4  GORASP1 - golgi reassembly stacking protein 1, 65kda  IL6ST - interleukin 6 signal transducer (gp130, oncostatin m receptor)  CSRNP1 - cysteine-serine-rich nuclear protein 1  DGKZ - diacylglycerol kinase, zeta  C5AR2 - complement component 5a receptor 2  SERP1 - stress-associated endoplasmic reticulum protein 1  ZNF25 - zinc finger protein 25  STK16 - serine/threonine kinase 16  CCNY - cyclin y  ITGA1 - integrin, alpha 1  ITGA3 - integrin, alpha 3 (antigen cd49c, alpha 3 subunit of vla-3 receptor)  MADD - map-kinase activating death domain  KHSRP - kh-type splicing regulatory protein  ERLEC1 - endoplasmic reticulum lectin 1  CASK - calcium/calmodulin-dependent serine protein kinase (maguk family)  ITGAV - integrin, alpha v  SUDS3 - suppressor of defective silencing 3 homolog (s. cerevisiae)  CDK10 - cyclin-dependent kinase 10  TPCN2 - two pore segment channel 2  EIF6 - eukaryotic translation initiation factor 6  SKAP1 - src kinase associated phosphoprotein 1  GZF1 - gdnf-inducible zinc finger protein 1  INSR - insulin receptor  INSM1 - insulinoma-associated 1  CDK13 - cyclin-dependent kinase 13  AKTIP - akt interacting protein  PDCD4 - programmed cell death 4 (neoplastic transformation inhibitor)  RFXANK - regulatory factor x-associated ankyrin-containing protein  CSDC2 - cold shock domain containing c2, rna binding  KLF7 - kruppel-like factor 7 (ubiquitous)  IRF2 - interferon regulatory factor 2  IRF3 - interferon regulatory factor 3  IKZF5 - ikaros family zinc finger 5 (pegasus)  RUVBL1 - ruvb-like 1 (e. coli)  IREB2 - iron-responsive element binding protein 2  DIMT1 - dim1 dimethyladenosine transferase 1 homolog (s. cerevisiae)  MED19 - mediator complex subunit 19  ITGA6 - integrin, alpha 6  IRAK2 - interleukin-1 receptor-associated kinase 2  IRAK1 - interleukin-1 receptor-associated kinase 1  CACYBP - calcyclin binding protein  ZSCAN31 - zinc finger and scan domain containing 31  EIF2AK1 - eukaryotic translation initiation factor 2-alpha kinase 1  PAPD5 - pap associated domain containing 5  KIAA1324L - kiaa1324-like  NSD1 - nuclear receptor binding set domain protein 1  RFWD2 - ring finger and wd repeat domain 2, e3 ubiquitin protein ligase  ZBTB11 - zinc finger and btb domain containing 11  RNF25 - ring finger protein 25  KCTD20 - potassium channel tetramerization domain containing 20  MMS19 - mms19 nucleotide excision repair homolog (s. cerevisiae)  DKK3 - dickkopf wnt signaling pathway inhibitor 3  SNX5 - sorting nexin 5  IFNGR1 - interferon gamma receptor 1  ZNF777 - zinc finger protein 777  ID2 - inhibitor of dna binding 2, dominant negative helix-loop-helix protein  ID3 - inhibitor of dna binding 3, dominant negative helix-loop-helix protein  BRPF3 - bromodomain and phd finger containing, 3  STK36 - serine/threonine kinase 36  IRF2BPL - interferon regulatory factor 2 binding protein-like  JAZF1 - jazf zinc finger 1  PLA2G6 - phospholipase a2, group vi (cytosolic, calcium-independent)  RRAGC - ras-related gtp binding c  FOXK1 - forkhead box k1  BRAT1 - brca1-associated atm activator 1  MAGEF1 - melanoma antigen family f, 1  ZNF282 - zinc finger protein 282  IGHMBP2 - immunoglobulin mu binding protein 2  CHORDC1 - cysteine and histidine-rich domain (chord) containing 1  RBPJ - recombination signal binding protein for immunoglobulin kappa j region  NSMAF - neutral sphingomyelinase (n-smase) activation associated factor  NCK2 - nck adaptor protein 2  PABPC1 - poly(a) binding protein, cytoplasmic 1  TRUB2 - trub pseudouridine (psi) synthase homolog 2 (e. coli)  CYFIP2 - cytoplasmic fmr1 interacting protein 2  C6orf89 - chromosome 6 open reading frame 89  RBM26 - rna binding motif protein 26  ZBTB9 - zinc finger and btb domain containing 9  USP21 - ubiquitin specific peptidase 21  ULK1 - unc-51 like autophagy activating kinase 1  ZBTB12 - zinc finger and btb domain containing 12  BCAR3 - breast cancer anti-estrogen resistance 3  NGFRAP1 - nerve growth factor receptor (tnfrsf16) associated protein 1  NPTN - neuroplastin  PHACTR1 - phosphatase and actin regulator 1  IK - ik cytokine, down-regulator of hla ii  JMJD1C - jumonji domain containing 1c  IGBP1 - immunoglobulin (cd79a) binding protein 1  SMARCA5 - swi/snf related, matrix associated, actin dependent regulator of chromatin, subfamily a, member 5  IGF1R - insulin-like growth factor 1 receptor  OGT - o-linked n-acetylglucosamine (glcnac) transferase  PELP1 - proline, glutamate and leucine rich protein 1  RAE1 - rae1 rna export 1 homolog (s. pombe)  LAT - linker for activation of t cells  NSG1 - neuron specific gene family member 1  TSPYL2 - tspy-like 2  IGFBP6 - insulin-like growth factor binding protein 6  SND1 - staphylococcal nuclease and tudor domain containing 1  CUL2 - cullin 2  PCIF1 - pdx1 c-terminal inhibiting factor 1  CUL3 - cullin 3  ZNF667 - zinc finger protein 667  FOXP1 - forkhead box p1  SUPT3H - suppressor of ty 3 homolog (s. cerevisiae)  BEND6 - ben domain containing 6  ZNF572 - zinc finger protein 572  PCGF5 - polycomb group ring finger 5  WTAP - wilms tumor 1 associated protein  WAC - ww domain containing adaptor with coiled-coil  ZNF414 - zinc finger protein 414  RBM39 - rna binding motif protein 39  PHF21A - phd finger protein 21a  ZNF771 - zinc finger protein 771  LIMK1 - lim domain kinase 1  AKAP12 - a kinase (prka) anchor protein 12  IER2 - immediate early response 2  LIG3 - ligase iii, dna, atp-dependent  NCOR1 - nuclear receptor corepressor 1  LDLR - low density lipoprotein receptor  NCOR2 - nuclear receptor corepressor 2  MTPN - myotrophin  CARTPT - cart prepropeptide  DOT1L - dot1-like histone h3k79 methyltransferase  ABCG1 - atp-binding cassette, sub-family g (white), member 1  EFCAB7 - ef-hand calcium binding domain 7  ATP6V1G1 - atpase, h+ transporting, lysosomal 13kda, v1 subunit g1  L3MBTL3 - l(3)mbt-like 3 (drosophila)  ZNF333 - zinc finger protein 333  CIR1 - corepressor interacting with rbpj, 1  SCAND1 - scan domain containing 1  VPS26A - vacuolar protein sorting 26 homolog a (s. pombe)  SLX4 - slx4 structure-specific endonuclease subunit  SEC22B - sec22 vesicle trafficking protein homolog b (s. cerevisiae) (gene/pseudogene)  H2AFY - h2a histone family, member y  GTF2IRD1 - gtf2i repeat domain containing 1  FADS1 - fatty acid desaturase 1  GTPBP1 - gtp binding protein 1  ZNF527 - zinc finger protein 527  H6PD - hexose-6-phosphate dehydrogenase (glucose 1-dehydrogenase)  SPIRE2 - spire actin nucleation factor 2  HOPX - hop homeobox  SOX13 - sry (sex determining region y)-box 13  ZC3H8 - zinc finger ccch-type containing 8  ZNF316 - zinc finger protein 316  LMO1 - lim domain only 1 (rhombotin 1)  IER5 - immediate early response 5  NACC2 - nacc family member 2, ben and btb (poz) domain containing  LMNA - lamin a/c  DNAJC27 - dnaj (hsp40) homolog, subfamily c, member 27  CLOCK - clock circadian regulator  KLF3 - kruppel-like factor 3 (basic)  PRKAG2 - protein kinase, amp-activated, gamma 2 non-catalytic subunit  DDX41 - dead (asp-glu-ala-asp) box polypeptide 41  FXR2 - fragile x mental retardation, autosomal homolog 2  LITAF - lipopolysaccharide-induced tnf factor  SPTLC2 - serine palmitoyltransferase, long chain base subunit 2  TBPL1 - tbp-like 1  YTHDF2 - yth domain family, member 2  ZBTB41 - zinc finger and btb domain containing 41  ZFP62 - zfp62 zinc finger protein  BAG5 - bcl2-associated athanogene 5  TMEM59 - transmembrane protein 59  BAG4 - bcl2-associated athanogene 4  PPME1 - protein phosphatase methylesterase 1  ZGPAT - zinc finger, ccch-type with g patch domain  ACTL6B - actin-like 6b  UBR5 - ubiquitin protein ligase e3 component n-recognin 5  IL27RA - interleukin 27 receptor, alpha  EIF4E2 - eukaryotic translation initiation factor 4e family member 2  MED20 - mediator complex subunit 20  UCHL5 - ubiquitin carboxyl-terminal hydrolase l5  AFAP1L2 - actin filament associated protein 1-like 2  ATG5 - autophagy related 5  ROCK2 - rho-associated, coiled-coil containing protein kinase 2  ATRAID - all-trans retinoic acid-induced differentiation factor  ATP6V1D - atpase, h+ transporting, lysosomal 34kda, v1 subunit d  ANGPT4 - angiopoietin 4  MAPK8IP1 - mitogen-activated protein kinase 8 interacting protein 1  CRLF3 - cytokine receptor-like factor 3  NGRN - neugrin, neurite outgrowth associated  TAOK3 - tao kinase 3  GLYR1 - glyoxylate reductase 1 homolog (arabidopsis)  FZR1 - fizzy/cell division cycle 20 related 1 (drosophila)  ZBTB7A - zinc finger and btb domain containing 7a  DPY30 - dpy-30 homolog (c. elegans)  MBTPS2 - membrane-bound transcription factor peptidase, site 2  IRF2BP2 - interferon regulatory factor 2 binding protein 2  GLIS2 - glis family zinc finger 2  MAGED1 - melanoma antigen family d, 1  MED27 - mediator complex subunit 27  MED26 - mediator complex subunit 26  CHD6 - chromodomain helicase dna binding protein 6  MED17 - mediator complex subunit 17  MED23 - mediator complex subunit 23  VPS28 - vacuolar protein sorting 28 homolog (s. cerevisiae)  ZNF580 - zinc finger protein 580  ANTXR1 - anthrax toxin receptor 1  CDYL - chromodomain protein, y-like  GTF2IRD2 - gtf2i repeat domain containing 2  ZNF710 - zinc finger protein 710  KDR - kinase insert domain receptor (a type iii receptor tyrosine kinase)  PICK1 - protein interacting with prkca 1  ING4 - inhibitor of growth family, member 4  EIF2AK3 - eukaryotic translation initiation factor 2-alpha kinase 3  INSIG2 - insulin induced gene 2  ITM2B - integral membrane protein 2b  MAP4K4 - mitogen-activated protein kinase kinase kinase kinase 4  RHBDD1 - rhomboid domain containing 1  RLIM - ring finger protein, lim domain interacting  SOGA1 - suppressor of glucose, autophagy associated 1  TRAF7 - tnf receptor-associated factor 7, e3 ubiquitin protein ligase  RECQL5 - recq protein-like 5  KRAS - kirsten rat sarcoma viral oncogene homolog  TNPO1 - transportin 1  TGFBRAP1 - transforming growth factor, beta receptor associated protein 1  ZNF706 - zinc finger protein 706  CIAO1 - cytosolic iron-sulfur protein assembly 1  KPNA2 - karyopherin alpha 2 (rag cohort 1, importin alpha 1)  WDR24 - wd repeat domain 24  KPNA1 - karyopherin alpha 1 (importin alpha 5)  CAMKK1 - calcium/calmodulin-dependent protein kinase kinase 1, alpha  WFDC3 - wap four-disulfide core domain 3  SUV420H1 - suppressor of variegation 4-20 homolog 1 (drosophila)  PHF20L1 - phd finger protein 20-like 1  MED10 - mediator complex subunit 10  MED21 - mediator complex subunit 21  KIT - v-kit hardy-zuckerman 4 feline sarcoma viral oncogene homolog  ZFP28 - zfp28 zinc finger protein  ABHD5 - abhydrolase domain containing 5  SH3GLB1 - sh3-domain grb2-like endophilin b1  POLDIP3 - polymerase (dna-directed), delta interacting protein 3  NOA1 - nitric oxide associated 1  DCUN1D5 - dcn1, defective in cullin neddylation 1, domain containing 5  SLIT2 - slit homolog 2 (drosophila)  PAIP2 - poly(a) binding protein interacting protein 2  JUP - junction plakoglobin  CRIM1 - cysteine rich transmembrane bmp regulator 1 (chordin-like)  JAK2 - janus kinase 2  ING5 - inhibitor of growth family, member 5  JAK3 - janus kinase 3  SLC25A33 - solute carrier family 25 (pyrimidine nucleotide carrier), member 33  ZNF219 - zinc finger protein 219  DNAJC30 - dnaj (hsp40) homolog, subfamily c, member 30  BTBD10 - btb (poz) domain containing 10  ITPR1 - inositol 1,4,5-trisphosphate receptor, type 1  PHF20 - phd finger protein 20  TRIP12 - thyroid hormone receptor interactor 12  NUDT16L1 - nudix (nucleoside diphosphate linked moiety x)-type motif 16-like 1  KCNK2 - potassium channel, subfamily k, member 2  ZNF397 - zinc finger protein 397  ZNHIT3 - zinc finger, hit-type containing 3  TRIP4 - thyroid hormone receptor interactor 4  HMGN3 - high mobility group nucleosomal binding domain 3  PHF6 - phd finger protein 6  ZNF235 - zinc finger protein 235  COPS2 - cop9 signalosome subunit 2  VAMP3 - vesicle-associated membrane protein 3  ZNF639 - zinc finger protein 639  TAOK2 - tao kinase 2  SARNP - sap domain containing ribonucleoprotein  BRMS1L - breast cancer metastasis-suppressor 1-like  LEF1 - lymphoid enhancer-binding factor 1  VPS25 - vacuolar protein sorting 25 homolog (s. cerevisiae)  SMCR8 - smith-magenis syndrome chromosome region, candidate 8  CNOT8 - ccr4-not transcription complex, subunit 8  SIRPA - signal-regulatory protein alpha  CDK12 - cyclin-dependent kinase 12  ZNF280B - zinc finger protein 280b  S1PR2 - sphingosine-1-phosphate receptor 2  SNX25 - sorting nexin 25  ARID4B - at rich interactive domain 4b (rbp1-like)  SOCS6 - suppressor of cytokine signaling 6  SPRTN - sprt-like n-terminal domain  MIF - macrophage migration inhibitory factor (glycosylation-inhibiting factor)  CCNI2 - cyclin i family, member 2  MAP3K10 - mitogen-activated protein kinase kinase kinase 10  DUSP19 - dual specificity phosphatase 19  MLH1 - mutl homolog 1, colon cancer, nonpolyposis type 2 (e. coli)  MKLN1 - muskelin 1, intracellular mediator containing kelch motifs  ITGB1BP1 - integrin beta 1 binding protein 1  KDM3B - lysine (k)-specific demethylase 3b  RSF1 - remodeling and spacing factor 1  MLLT6 - myeloid/lymphoid or mixed-lineage leukemia (trithorax homolog, drosophila); translocated to, 6  SAMD8 - sterile alpha motif domain containing 8  MLLT3 - myeloid/lymphoid or mixed-lineage leukemia (trithorax homolog, drosophila); translocated to, 3  MED14 - mediator complex subunit 14  MLLT1 - myeloid/lymphoid or mixed-lineage leukemia (trithorax homolog, drosophila); translocated to, 1  MAP3K11 - mitogen-activated protein kinase kinase kinase 11  ZBTB22 - zinc finger and btb domain containing 22  KMT2A - lysine (k)-specific methyltransferase 2a  RPS6KA5 - ribosomal protein s6 kinase, 90kda, polypeptide 5  CCDC8 - coiled-coil domain containing 8  SUFU - suppressor of fused homolog (drosophila)  GPRC5B - g protein-coupled receptor, family c, group 5, member b  STK17A - serine/threonine kinase 17a  MMP14 - matrix metallopeptidase 14 (membrane-inserted)  MFHAS1 - malignant fibrous histiocytoma amplified sequence 1  NLK - nemo-like kinase  UIMC1 - ubiquitin interaction motif containing 1  MNAT1 - mnat cdk-activating kinase assembly factor 1  MTA2 - metastasis associated 1 family, member 2  NOLC1 - nucleolar and coiled-body phosphoprotein 1  EIF2A - eukaryotic translation initiation factor 2a, 65kda  CD200 - cd200 molecule  WWOX - ww domain containing oxidoreductase  MNT - mnt, max dimerization protein  CCPG1 - cell cycle progression 1  SESN3 - sestrin 3  MCM7 - minichromosome maintenance complex component 7  NSRP1 - nuclear speckle splicing regulatory protein 1  ME1 - malic enzyme 1, nadp(+)-dependent, cytosolic  SPTY2D1 - spt2, suppressor of ty, domain containing 1 (s. cerevisiae)  MECP2 - methyl cpg binding protein 2 (rett syndrome)  PELO - pelota homolog (drosophila)  MEF2A - myocyte enhancer factor 2a  WDR54 - wd repeat domain 54  ME2 - malic enzyme 2, nad(+)-dependent, mitochondrial  PRICKLE1 - prickle homolog 1 (drosophila)  ZBED1 - zinc finger, bed-type containing 1  BCDIN3D - bcdin3 domain containing  MEIS2 - meis homeobox 2  BRWD1 - bromodomain and wd repeat domain containing 1  ZNF664 - zinc finger protein 664  MEIS1 - meis homeobox 1  MAP3K4 - mitogen-activated protein kinase kinase kinase 4  MAP3K5 - mitogen-activated protein kinase kinase kinase 5  SLC33A1 - solute carrier family 33 (acetyl-coa transporter), member 1  MAP3K1 - mitogen-activated protein kinase kinase kinase 1, e3 ubiquitin protein ligase  MAP3K3 - mitogen-activated protein kinase kinase kinase 3  MEN1 - multiple endocrine neoplasia i  WDR75 - wd repeat domain 75  RAB8A - rab8a, member ras oncogene family  COX7A2L - cytochrome c oxidase subunit viia polypeptide 2 like  UTP15 - utp15, u3 small nucleolar ribonucleoprotein, homolog (s. cerevisiae)  FAM161A - family with sequence similarity 161, member a  ZFYVE1 - zinc finger, fyve domain containing 1  KAT8 - k(lysine) acetyltransferase 8  ZNF644 - zinc finger protein 644  ARHGEF2 - rho/rac guanine nucleotide exchange factor (gef) 2  PPP1R1B - protein phosphatase 1, regulatory (inhibitor) subunit 1b  HGS - hepatocyte growth factor-regulated tyrosine kinase substrate  MGAT5 - mannosyl (alpha-1,6-)-glycoprotein beta-1,6-n-acetyl-glucosaminyltransferase  NEMF - nuclear export mediator factor  LACC1 - laccase (multicopper oxidoreductase) domain containing 1  MBD3 - methyl-cpg binding domain protein 3  KITLG - kit ligand  PTOV1 - prostate tumor overexpressed 1  ZNF394 - zinc finger protein 394  CBFA2T2 - core-binding factor, runt domain, alpha subunit 2; translocated to, 2  MAGOH - mago-nashi homolog, proliferation-associated (drosophila)  GSKIP - gsk3b interacting protein  CXXC5 - cxxc finger protein 5  RQCD1 - rcd1 required for cell differentiation1 homolog (s. pombe)  SMC3 - structural maintenance of chromosomes 3  MARS - methionyl-trna synthetase  IL23A - interleukin 23, alpha subunit p19  MAPT - microtubule-associated protein tau  ATP6V0D1 - atpase, h+ transporting, lysosomal 38kda, v0 subunit d1  LATS1 - large tumor suppressor kinase 1  MTA1 - metastasis associated 1  MAP1A - microtubule-associated protein 1a  SIRT6 - sirtuin 6  MTMR4 - myotubularin related protein 4  PPHLN1 - periphilin 1  USP14 - ubiquitin specific peptidase 14 (trna-guanine transglycosylase)  USP10 - ubiquitin specific peptidase 10  UBE2J1 - ubiquitin-conjugating enzyme e2, j1  NUP43 - nucleoporin 43kda  UNC119 - unc-119 homolog (c. elegans)  MBD1 - methyl-cpg binding domain protein 1  PKMYT1 - protein kinase, membrane associated tyrosine/threonine 1  SFMBT1 - scm-like with four mbt domains 1  MAX - myc associated factor x  ZMIZ2 - zinc finger, miz-type containing 2  LDB2 - lim domain binding 2  TRMT112 - trna methyltransferase 11-2 homolog (s. cerevisiae)  CHMP5 - charged multivesicular body protein 5  ZFP90 - zfp90 zinc finger protein  NELFCD - negative elongation factor complex member c/d  CDAN1 - codanin 1  DIRAS3 - diras family, gtp-binding ras-like 3  ASH2L - ash2 (absent, small, or homeotic)-like (drosophila)  ZSCAN29 - zinc finger and scan domain containing 29  CTDSPL2 - ctd (carboxy-terminal domain, rna polymerase ii, polypeptide a) small phosphatase like 2  PTPLAD1 - protein tyrosine phosphatase-like a domain containing 1  THAP2 - thap domain containing, apoptosis associated protein 2  LRPAP1 - low density lipoprotein receptor-related protein associated protein 1  ITCH - itchy e3 ubiquitin protein ligase  ZNF484 - zinc finger protein 484  L3MBTL2 - l(3)mbt-like 2 (drosophila)  IL34 - interleukin 34  LRP1 - low density lipoprotein receptor-related protein 1  RIOK1 - rio kinase 1  LRP6 - low density lipoprotein receptor-related protein 6  ZNF445 - zinc finger protein 445  AIP - aryl hydrocarbon receptor interacting protein  NUB1 - negative regulator of ubiquitin-like proteins 1  TRAPPC9 - trafficking protein particle complex 9  ASB1 - ankyrin repeat and socs box containing 1  LUM - lumican  BTAF1 - btaf1 rna polymerase ii, b-tfiid transcription factor-associated, 170kda  LYN - v-yes-1 yamaguchi sarcoma viral related oncogene homolog  SPAG9 - sperm associated antigen 9  UBA3 - ubiquitin-like modifier activating enzyme 3  RBFOX3 - rna binding protein, fox-1 homolog (c. elegans) 3  SESN2 - sestrin 2  PARP9 - poly (adp-ribose) polymerase family, member 9  HIP1R - huntingtin interacting protein 1 related  CDK5RAP1 - cdk5 regulatory subunit associated protein 1  RNF8 - ring finger protein 8, e3 ubiquitin protein ligase  BAZ1B - bromodomain adjacent to zinc finger domain, 1b  AZIN1 - antizyme inhibitor 1  MXD1 - max dimerization protein 1  MED15 - mediator complex subunit 15  PIAS4 - protein inhibitor of activated stat, 4  TRIM33 - tripartite motif containing 33  SRRT - serrate rna effector molecule homolog (arabidopsis)  SMAD2 - smad family member 2  MBIP - map3k12 binding inhibitory protein 1  KLF16 - kruppel-like factor 16  HDAC7 - histone deacetylase 7  ARMCX3 - armadillo repeat containing, x-linked 3  TDP2 - tyrosyl-dna phosphodiesterase 2  CAPRIN1 - cell cycle associated protein 1  NBR1 - neighbor of brca1 gene 1  LARP7 - la ribonucleoprotein domain family, member 7  RPS6KA4 - ribosomal protein s6 kinase, 90kda, polypeptide 4  MAFG - v-maf avian musculoaponeurotic fibrosarcoma oncogene homolog g  ATP6V1H - atpase, h+ transporting, lysosomal 50/57kda, v1 subunit h  DPH5 - diphthamide biosynthesis 5  TAF9B - taf9b rna polymerase ii, tata box binding protein (tbp)-associated factor, 31kda  SMAD4 - smad family member 4  NBAS - neuroblastoma amplified sequence  H1FX - h1 histone family, member x  SMAD5 - smad family member 5  SMAD7 - smad family member 7  WASL - wiskott-aldrich syndrome-like  RBM4B - rna binding motif protein 4b  SAP30BP - sap30 binding protein  ZNF565 - zinc finger protein 565  MYLIP - myosin regulatory light chain interacting protein  YAF2 - yy1 associated factor 2  NCBP1 - nuclear cap binding protein subunit 1, 80kda  TAGLN3 - transgelin 3  SIX5 - six homeobox 5  ITM2C - integral membrane protein 2c  NDN - necdin, melanoma antigen (mage) family member  ZNF582 - zinc finger protein 582  AKAP9 - a kinase (prka) anchor protein (yotiao) 9  NCK1 - nck adaptor protein 1  BRD7 - bromodomain containing 7  CAB39L - calcium binding protein 39-like  DDX25 - dead (asp-glu-ala-asp) box helicase 25  NCL - nucleolin  MBNL2 - muscleblind-like splicing regulator 2  CEBPZ - ccaat/enhancer binding protein (c/ebp), zeta  TICAM1 - toll-like receptor adaptor molecule 1  CCNL2 - cyclin l2  HNRNPM - heterogeneous nuclear ribonucleoprotein m  ORMDL2 - orm1-like 2 (s. cerevisiae)  TRIM28 - tripartite motif containing 28  DROSHA - drosha, ribonuclease type iii  ATP6AP2 - atpase, h+ transporting, lysosomal accessory protein 2  DNAJC15 - dnaj (hsp40) homolog, subfamily c, member 15  TBK1 - tank-binding kinase 1  NBN - nibrin  ZNF197 - zinc finger protein 197  TDRD3 - tudor domain containing 3  RBM7 - rna binding motif protein 7  RBM5 - rna binding motif protein 5  TRIM11 - tripartite motif containing 11  PPP1R12A - protein phosphatase 1, regulatory subunit 12a  TNK2 - tyrosine kinase, non-receptor, 2  MYT1 - myelin transcription factor 1  ALYREF - aly/ref export factor  CSRNP2 - cysteine-serine-rich nuclear protein 2  SENP1 - sumo1/sentrin specific peptidase 1  NAB1 - ngfi-a binding protein 1 (egr1 binding protein 1)  RNF41 - ring finger protein 41  NAB2 - ngfi-a binding protein 2 (egr1 binding protein 2)  TSHZ1 - teashirt zinc finger homeobox 1  PRMT3 - protein arginine methyltransferase 3  ANKRD13C - ankyrin repeat domain 13c  PSME3 - proteasome (prosome, macropain) activator subunit 3 (pa28 gamma; ki)  MPHOSPH10 - m-phase phosphoprotein 10 (u3 small nucleolar ribonucleoprotein)  TNFAIP8L3 - tumor necrosis factor, alpha-induced protein 8-like 3  MYO1D - myosin id  TRIM13 - tripartite motif containing 13  TRIM8 - tripartite motif containing 8  MYO6 - myosin vi  LBH - limb bud and heart development  ABT1 - activator of basal transcription 1  TOPORS - topoisomerase i binding, arginine/serine-rich, e3 ubiquitin protein ligase  RAB1B - rab1b, member ras oncogene family  MYD88 - myeloid differentiation primary response 88  SEH1L - seh1-like (s. cerevisiae)  ZNF362 - zinc finger protein 362  MYH9 - myosin, heavy chain 9, non-muscle  HUWE1 - hect, uba and wwe domain containing 1, e3 ubiquitin protein ligase  HCFC2 - host cell factor c2  CNOT7 - ccr4-not transcription complex, subunit 7  MXI1 - max interactor 1, dimerization protein  PQBP1 - polyglutamine binding protein 1  TRA2A - transformer 2 alpha homolog (drosophila)  ACTR2 - arp2 actin-related protein 2 homolog (yeast)  ACTR3 - arp3 actin-related protein 3 homolog (yeast)  RBM15B - rna binding motif protein 15b  CTDSP2 - ctd (carboxy-terminal domain, rna polymerase ii, polypeptide a) small phosphatase 2  TSFM - ts translation elongation factor, mitochondrial  FEM1B - fem-1 homolog b (c. elegans)  RAD50 - rad50 homolog (s. cerevisiae)  TRIM37 - tripartite motif containing 37  ZNF558 - zinc finger protein 558  LRPPRC - leucine-rich pentatricopeptide repeat containing  ZNF263 - zinc finger protein 263  SPRY4 - sprouty homolog 4 (drosophila)  PURG - purine-rich element binding protein g  BCAP31 - b-cell receptor-associated protein 31  RBM12 - rna binding motif protein 12  MED25 - mediator complex subunit 25  SNX12 - sorting nexin 12  TRAP1 - tnf receptor-associated protein 1  MED6 - mediator complex subunit 6  ABI1 - abl-interactor 1  PTBP3 - polypyrimidine tract binding protein 3  PPP4R1 - protein phosphatase 4, regulatory subunit 1  DMTF1 - cyclin d binding myb-like transcription factor 1  SH2B3 - sh2b adaptor protein 3  MTIF2 - mitochondrial translational initiation factor 2  PDCD6 - programmed cell death 6  MTM1 - myotubularin 1  ZBTB33 - zinc finger and btb domain containing 33  TANK - traf family member-associated nfkb activator  HDAC5 - histone deacetylase 5  MTF1 - metal-regulatory transcription factor 1  SRA1 - steroid receptor rna activator 1  HDAC6 - histone deacetylase 6  OCLN - occludin  RNF187 - ring finger protein 187  SH2D3C - sh2 domain containing 3c  MT3 - metallothionein 3  MED16 - mediator complex subunit 16  CCDC22 - coiled-coil domain containing 22  SAE1 - sumo1 activating enzyme subunit 1  EXOC8 - exocyst complex component 8  DNM1L - dynamin 1-like  RANBP9 - ran binding protein 9  DNAJB6 - dnaj (hsp40) homolog, subfamily b, member 6  UBA2 - ubiquitin-like modifier activating enzyme 2  MSN - moesin  MSH3 - muts homolog 3 (e. coli)  MSH2 - muts homolog 2, colon cancer, nonpolyposis type 1 (e. coli)  LPGAT1 - lysophosphatidylglycerol acyltransferase 1  CITED1 - cbp/p300-interacting transactivator, with glu/asp-rich carboxy-terminal domain, 1  ZBTB5 - zinc finger and btb domain containing 5  ZBTB40 - zinc finger and btb domain containing 40  DBNL - drebrin-like  SUPT7L - suppressor of ty 7 (s. cerevisiae)-like  RGCC - regulator of cell cycle  COMMD1 - copper metabolism (murr1) domain containing 1  RBM8A - rna binding motif protein 8a  USP3 - ubiquitin specific peptidase 3  BABAM1 - brisc and brca1 a complex member 1  CHMP4A - charged multivesicular body protein 4a  METTL5 - methyltransferase like 5  USP15 - ubiquitin specific peptidase 15  MED4 - mediator complex subunit 4  THOC1 - tho complex 1  SETD2 - set domain containing 2  RBX1 - ring-box 1, e3 ubiquitin protein ligase  DUSP18 - dual specificity phosphatase 18  MRE11A - mre11 meiotic recombination 11 homolog a (s. cerevisiae)  NUP153 - nucleoporin 153kda  WDR91 - wd repeat domain 91  THRAP3 - thyroid hormone receptor associated protein 3  MED12 - mediator complex subunit 12  MED13 - mediator complex subunit 13  PSMD6 - proteasome (prosome, macropain) 26s subunit, non-atpase, 6  LRIG2 - leucine-rich repeats and immunoglobulin-like domains 2  GLTPD1 - glycolipid transfer protein domain containing 1  MED24 - mediator complex subunit 24  ZC3H6 - zinc finger ccch-type containing 6  DTX3L - deltex 3-like (drosophila)  PJA2 - praja ring finger 2, e3 ubiquitin protein ligase  ZNF34 - zinc finger protein 34  SETDB1 - set domain, bifurcated 1  ORC2 - origin recognition complex, subunit 2  PARD6A - par-6 partitioning defective 6 homolog alpha (c. elegans)  ORC5 - origin recognition complex, subunit 5  TOX4 - tox high mobility group box family member 4  ZNF436 - zinc finger protein 436  RNF141 - ring finger protein 141  PRR7 - proline rich 7 (synaptic)  AGRN - agrin  EPM2AIP1 - epm2a (laforin) interacting protein 1  PPP4R2 - protein phosphatase 4, regulatory subunit 2  NDFIP1 - nedd4 family interacting protein 1  PPP1R26 - protein phosphatase 1, regulatory subunit 26  GLIPR2 - gli pathogenesis-related 2  TBC1D25 - tbc1 domain family, member 25  RBM19 - rna binding motif protein 19  OAZ2 - ornithine decarboxylase antizyme 2  OAZ1 - ornithine decarboxylase antizyme 1  G3BP2 - gtpase activating protein (sh3 domain) binding protein 2  ODC1 - ornithine decarboxylase 1  CCNYL1 - cyclin y-like 1  OGG1 - 8-oxoguanine dna glycosylase  ZSCAN16 - zinc finger and scan domain containing 16  TNKS2 - tankyrase, trf1-interacting ankyrin-related adp-ribose polymerase 2  EXOSC3 - exosome component 3  SMG7 - smg7 nonsense mediated mrna decay factor  WDR61 - wd repeat domain 61  APITD1 - apoptosis-inducing, taf9-like domain 1  EXOSC1 - exosome component 1  FIS1 - fission 1 (mitochondrial outer membrane) homolog (s. cerevisiae)  NSF - n-ethylmaleimide-sensitive factor  DAZAP2 - daz associated protein 2  DDR2 - discoidin domain receptor tyrosine kinase 2  IST1 - increased sodium tolerance 1 homolog (yeast)  BCLAF1 - bcl2-associated transcription factor 1  DAB2IP - dab2 interacting protein  NUMA1 - nuclear mitotic apparatus protein 1  EIF4A3 - eukaryotic translation initiation factor 4a3  NUP88 - nucleoporin 88kda  ATG13 - autophagy related 13  NUP98 - nucleoporin 98kda  GMNN - geminin, dna replication inhibitor  SLC38A9 - solute carrier family 38, member 9  NR4A2 - nuclear receptor subfamily 4, group a, member 2  ZNF593 - zinc finger protein 593  ZBTB7B - zinc finger and btb domain containing 7b  MATR3 - matrin 3  NVL - nuclear vcp-like  TBC1D5 - tbc1 domain family, member 5  ZNF691 - zinc finger protein 691  RNF144A - ring finger protein 144a  CREBRF - creb3 regulatory factor  ZNF623 - zinc finger protein 623  RGP1 - rgp1 retrograde golgi transport homolog (s. cerevisiae)  NPM1 - nucleophosmin (nucleolar phosphoprotein b23, numatrin)  NMD3 - nmd3 ribosome export adaptor  ARHGEF11 - rho guanine nucleotide exchange factor (gef) 11  ZBTB24 - zinc finger and btb domain containing 24  ZEB2 - zinc finger e-box binding homeobox 2  THAP4 - thap domain containing 4  SFI1 - sfi1 homolog, spindle assembly associated (yeast)  CTIF - cbp80/20-dependent translation initiation factor  DUSP16 - dual specificity phosphatase 16  RNF40 - ring finger protein 40, e3 ubiquitin protein ligase  SPOCK2 - sparc/osteonectin, cwcv and kazal-like domains proteoglycan (testican) 2  RB1CC1 - rb1-inducible coiled-coil 1  TSC22D2 - tsc22 domain family, member 2  PAQR3 - progestin and adipoq receptor family member iii  SH3BP5L - sh3-binding domain protein 5-like  ADIPOR1 - adiponectin receptor 1  NFXL1 - nuclear transcription factor, x-box binding-like 1  NRF1 - nuclear respiratory factor 1  SH3D19 - sh3 domain containing 19  UBQLN1 - ubiquilin 1  DONSON - downstream neighbor of son  NRBF2 - nuclear receptor binding factor 2  NOP2 - nop2 nucleolar protein  VPRBP - vpr (hiv-1) binding protein  NONO - non-pou domain containing, octamer-binding  RNF34 - ring finger protein 34, e3 ubiquitin protein ligase  MDFIC - myod family inhibitor domain containing  UBQLN2 - ubiquilin 2  IFT74 - intraflagellar transport 74 homolog (chlamydomonas)  NOTCH4 - notch 4  NOVA2 - neuro-oncological ventral antigen 2  HERPUD1 - homocysteine-inducible, endoplasmic reticulum stress-inducible, ubiquitin-like domain member 1  NOVA1 - neuro-oncological ventral antigen 1  CEP290 - centrosomal protein 290kda  NPAT - nuclear protein, ataxia-telangiectasia locus  CNOT2 - ccr4-not transcription complex, subunit 2  NAA15 - n(alpha)-acetyltransferase 15, nata auxiliary subunit  PUM1 - pumilio homolog 1 (drosophila)  NOS3 - nitric oxide synthase 3 (endothelial cell)  PPP6R2 - protein phosphatase 6, regulatory subunit 2  CNOT3 - ccr4-not transcription complex, subunit 3  NOTCH1 - notch 1  BAZ2B - bromodomain adjacent to zinc finger domain, 2b  CTC1 - cts telomere maintenance complex component 1  ST18 - suppression of tumorigenicity 18 (breast carcinoma) (zinc finger protein)  NOTCH3 - notch 3  DHX34 - deah (asp-glu-ala-his) box polypeptide 34  NOTCH2 - notch 2  NFYA - nuclear transcription factor y, alpha  HDAC4 - histone deacetylase 4  TOX - thymocyte selection-associated high mobility group box  NFYC - nuclear transcription factor y, gamma  ZFYVE16 - zinc finger, fyve domain containing 16  CXXC1 - cxxc finger protein 1  NFKBIA - nuclear factor of kappa light polypeptide gene enhancer in b-cells inhibitor, alpha  NFKB1 - nuclear factor of kappa light polypeptide gene enhancer in b-cells 1  KMT2B - lysine (k)-specific methyltransferase 2b  NFKBIB - nuclear factor of kappa light polypeptide gene enhancer in b-cells inhibitor, beta  NFKBIE - nuclear factor of kappa light polypeptide gene enhancer in b-cells inhibitor, epsilon  PIK3R4 - phosphoinositide-3-kinase, regulatory subunit 4  PHACTR2 - phosphatase and actin regulator 2  NKX2-2 - nk2 homeobox 2  CNKSR3 - cnksr family member 3  HDAC9 - histone deacetylase 9  ZNF606 - zinc finger protein 606  SART3 - squamous cell carcinoma antigen recognized by t cells 3  ZNF614 - zinc finger protein 614  EHD4 - eh-domain containing 4  ZFP2 - zfp2 zinc finger protein  SOCS7 - suppressor of cytokine signaling 7  NEK3 - nima-related kinase 3  MINK1 - misshapen-like kinase 1  SOCS5 - suppressor of cytokine signaling 5  NEO1 - neogenin 1  SMARCAL1 - swi/snf related, matrix associated, actin dependent regulator of chromatin, subfamily a-like 1  NEDD8 - neural precursor cell expressed, developmentally down-regulated 8  NEDD9 - neural precursor cell expressed, developmentally down-regulated 9  CTR9 - ctr9, paf1/rna polymerase ii complex component  PPM1F - protein phosphatase, mg2+/mn2+ dependent, 1f  CPEB4 - cytoplasmic polyadenylation element binding protein 4  EPC1 - enhancer of polycomb homolog 1 (drosophila)  NFE2L2 - nuclear factor (erythroid-derived 2)-like 2  MORF4L2 - mortality factor 4 like 2  RASL11A - ras-like, family 11, member a  KLHL15 - kelch-like family member 15  NFATC4 - nuclear factor of activated t-cells, cytoplasmic, calcineurin-dependent 4  PDGFD - platelet derived growth factor d  FEZ1 - fasciculation and elongation protein zeta 1 (zygin i)  FEZ2 - fasciculation and elongation protein zeta 2 (zygin ii)  NUP155 - nucleoporin 155kda  NFIA - nuclear factor i/a  NF1 - neurofibromin 1  CDK5RAP3 - cdk5 regulatory subunit associated protein 3  EDEM1 - er degradation enhancer, mannosidase alpha-like 1  RAPGEF2 - rap guanine nucleotide exchange factor (gef) 2  NUP93 - nucleoporin 93kda  ATP6V0E2 - atpase, h+ transporting v0 subunit e2  ZNF746 - zinc finger protein 746  VGLL4 - vestigial like 4 (drosophila)  LRRC14 - leucine rich repeat containing 14  DEPDC5 - dep domain containing 5  KDM4A - lysine (k)-specific demethylase 4a  PHF14 - phd finger protein 14  RDH10 - retinol dehydrogenase 10 (all-trans)  HP1BP3 - heterochromatin protein 1, binding protein 3  ZNF432 - zinc finger protein 432  SAFB2 - scaffold attachment factor b2  KIAA0430 - kiaa0430  MYEF2 - myelin expression factor 2  LPIN2 - lipin 2 |
| GO:0051052 | regulation of DNA metabolic process | 1.83E-8 | 4.99E-6 | 1.21 (10334,306,6996,250) | [+] Show genes  SMARCAD1 - swi/snf-related, matrix-associated actin-dependent regulator of chromatin, subfamily a, containing dead/h box 1  RFC5 - replication factor c (activator 1) 5, 36.5kda  RFC2 - replication factor c (activator 1) 2, 40kda  EXOSC10 - exosome component 10  RFC3 - replication factor c (activator 1) 3, 38kda  SPIRE1 - spire actin nucleation factor 1  NAF1 - nuclear assembly factor 1 ribonucleoprotein  FBXO18 - f-box protein, helicase, 18  FLCN - folliculin  LIG3 - ligase iii, dna, atp-dependent  WRNIP1 - werner helicase interacting protein 1  TRIM28 - tripartite motif containing 28  PML - promyelocytic leukemia  OBFC1 - oligonucleotide/oligosaccharide-binding fold containing 1  UPF1 - upf1 regulator of nonsense transcripts homolog (yeast)  NBN - nibrin  UBQLN4 - ubiquilin 4  RLF - rearranged l-myc fusion  CCT5 - chaperonin containing tcp1, subunit 5 (epsilon)  SLX4 - slx4 structure-specific endonuclease subunit  TTF1 - transcription termination factor, rna polymerase i  ALYREF - aly/ref export factor  GTPBP4 - gtp binding protein 4  OTUB1 - otu domain, ubiquitin aldehyde binding 1  ORC3 - origin recognition complex, subunit 3  EHMT2 - euchromatic histone-lysine n-methyltransferase 2  GJA1 - gap junction protein, alpha 1, 43kda  SPIRE2 - spire actin nucleation factor 2  SUB1 - sub1 homolog (s. cerevisiae)  DSCC1 - dna replication and sister chromatid cohesion 1  BMPR2 - bone morphogenetic protein receptor, type ii (serine/threonine kinase)  ACVRL1 - activin a receptor type ii-like 1  TP53BP1 - tumor protein p53 binding protein 1  EGFR - epidermal growth factor receptor  NABP2 - nucleic acid binding protein 2  HUS1 - hus1 checkpoint homolog (s. pombe)  KDM1A - lysine (k)-specific demethylase 1a  ACTR2 - arp2 actin-related protein 2 homolog (yeast)  AXIN2 - axin 2  CDC7 - cell division cycle 7  UBR5 - ubiquitin protein ligase e3 component n-recognin 5  USP9X - ubiquitin specific peptidase 9, x-linked  IL27RA - interleukin 27 receptor, alpha  CCT3 - chaperonin containing tcp1, subunit 3 (gamma)  SMG1 - smg1 phosphatidylinositol 3-kinase-related kinase  WIZ - widely interspaced zinc finger motifs  RAD50 - rad50 homolog (s. cerevisiae)  PARP1 - poly (adp-ribose) polymerase 1  POLH - polymerase (dna directed), eta  HSP90AA1 - heat shock protein 90kda alpha (cytosolic), class a member 1  C7orf49 - chromosome 7 open reading frame 49  ZMPSTE24 - zinc metallopeptidase ste24  WAPAL - wings apart-like homolog (drosophila)  PARP4 - poly (adp-ribose) polymerase family, member 4  TFIP11 - tuftelin interacting protein 11  SENP2 - sumo1/sentrin/smt3 specific peptidase 2  CST3 - cystatin c  HMGB1 - high mobility group box 1  USP1 - ubiquitin specific peptidase 1  FUS - fused in sarcoma  SUPT6H - suppressor of ty 6 homolog (s. cerevisiae)  PTK2B - protein tyrosine kinase 2 beta  TNKS - tankyrase, trf1-interacting ankyrin-related adp-ribose polymerase  ATF7IP - activating transcription factor 7 interacting protein  MAD2L2 - mad2 mitotic arrest deficient-like 2 (yeast)  CTNNB1 - catenin (cadherin-associated protein), beta 1, 88kda  RPA2 - replication protein a2, 32kda  RECQL5 - recq protein-like 5  AKT1 - v-akt murine thymoma viral oncogene homolog 1  PPP4C - protein phosphatase 4, catalytic subunit  USP7 - ubiquitin specific peptidase 7 (herpes virus-associated)  RNF168 - ring finger protein 168, e3 ubiquitin protein ligase  PPP2R1A - protein phosphatase 2, regulatory subunit a, alpha  KPNA2 - karyopherin alpha 2 (rag cohort 1, importin alpha 1)  ACD - adrenocortical dysplasia homolog (mouse)  KPNA1 - karyopherin alpha 1 (importin alpha 5)  SUV420H1 - suppressor of variegation 4-20 homolog 1 (drosophila)  EYA3 - eyes absent homolog 3 (drosophila)  PPP2CA - protein phosphatase 2, catalytic subunit, alpha isozyme  PPP1R10 - protein phosphatase 1, regulatory subunit 10  RMI2 - recq mediated genome instability 2  SUV420H2 - suppressor of variegation 4-20 homolog 2 (drosophila)  DDX39B - dead (asp-glu-ala-asp) box polypeptide 39b  ESCO1 - establishment of sister chromatid cohesion n-acetyltransferase 1  CCT6A - chaperonin containing tcp1, subunit 6a (zeta 1)  MSH3 - muts homolog 3 (e. coli)  MSH2 - muts homolog 2, colon cancer, nonpolyposis type 1 (e. coli)  ERCC4 - excision repair cross-complementing rodent repair deficiency, complementation group 4  PINX1 - pin2/terf1 interacting, telomerase inhibitor 1  STAT6 - signal transducer and activator of transcription 6, interleukin-4 induced  HNRNPU - heterogeneous nuclear ribonucleoprotein u (scaffold attachment factor a)  RGCC - regulator of cell cycle  PARM1 - prostate androgen-regulated mucin-like protein 1  TNFSF13 - tumor necrosis factor (ligand) superfamily, member 13  FBXO4 - f-box protein 4  TIPIN - timeless interacting protein  TINF2 - terf1 (trf1)-interacting nuclear factor 2  MAP2K7 - mitogen-activated protein kinase kinase 7  TRIP12 - thyroid hormone receptor interactor 12  KCNK2 - potassium channel, subfamily k, member 2  NUDT16L1 - nudix (nucleoside diphosphate linked moiety x)-type motif 16-like 1  BABAM1 - brisc and brca1 a complex member 1  FAM168A - family with sequence similarity 168, member a  HNRNPA2B1 - heterogeneous nuclear ribonucleoprotein a2/b1  HNRNPA1 - heterogeneous nuclear ribonucleoprotein a1  MAPK3 - mitogen-activated protein kinase 3  ZNF830 - zinc finger protein 830  HNRNPC - heterogeneous nuclear ribonucleoprotein c (c1/c2)  MAPK1 - mitogen-activated protein kinase 1  HNRNPD - heterogeneous nuclear ribonucleoprotein d (au-rich element rna binding protein 1, 37kda)  THOC1 - tho complex 1  CCDC88A - coiled-coil domain containing 88a  ANXA3 - annexin a3  SETD2 - set domain containing 2  SRC - v-src avian sarcoma (schmidt-ruppin a-2) viral oncogene homolog  FAM129B - family with sequence similarity 129, member b  PRKCD - protein kinase c, delta  MRE11A - mre11 meiotic recombination 11 homolog a (s. cerevisiae)  UBE2V2 - ubiquitin-conjugating enzyme e2 variant 2  PRMT5 - protein arginine methyltransferase 5  ERCC2 - excision repair cross-complementing rodent repair deficiency, complementation group 2  CHTF8 - ctf8, chromosome transmission fidelity factor 8 homolog (s. cerevisiae)  PKIB - protein kinase (camp-dependent, catalytic) inhibitor beta  UBE2N - ubiquitin-conjugating enzyme e2n  UBE2V1 - ubiquitin-conjugating enzyme e2 variant 1  DHX9 - deah (asp-glu-ala-his) box helicase 9  RNF169 - ring finger protein 169  DHX36 - deah (asp-glu-ala-his) box polypeptide 36  DTX3L - deltex 3-like (drosophila)  PARN - poly(a)-specific ribonuclease  TCP1 - t-complex 1  SETDB1 - set domain, bifurcated 1  ORC5 - origin recognition complex, subunit 5  PDS5A - pds5, regulator of cohesion maintenance, homolog a (s. cerevisiae)  SETMAR - set domain and mariner transposase fusion gene  MLH1 - mutl homolog 1, colon cancer, nonpolyposis type 2 (e. coli)  MAP2K4 - mitogen-activated protein kinase kinase 4  PARG - poly (adp-ribose) glycohydrolase  ZNF304 - zinc finger protein 304  PPP4R2 - protein phosphatase 4, regulatory subunit 2  NDFIP1 - nedd4 family interacting protein 1  MSH6 - muts homolog 6 (e. coli)  HDAC8 - histone deacetylase 8  APBB1 - amyloid beta (a4) precursor protein-binding, family b, member 1 (fe65)  KMT2A - lysine (k)-specific methyltransferase 2a  POT1 - protection of telomeres 1  RIF1 - rap1 interacting factor homolog (yeast)  MAPKAPK5 - mitogen-activated protein kinase-activated protein kinase 5  POLG2 - polymerase (dna directed), gamma 2, accessory subunit  GTF2I - general transcription factor iii  KIAA1551 - kiaa1551  ZBTB38 - zinc finger and btb domain containing 38  UIMC1 - ubiquitin interaction motif containing 1  DFFA - dna fragmentation factor, 45kda, alpha polypeptide  SMG6 - smg6 nonsense mediated mrna decay factor  KCTD13 - potassium channel tetramerization domain containing 13  OGG1 - 8-oxoguanine dna glycosylase  EXOSC3 - exosome component 3  TNKS2 - tankyrase, trf1-interacting ankyrin-related adp-ribose polymerase 2  FAM208A - family with sequence similarity 208, member a  PAK3 - p21 protein (cdc42/rac)-activated kinase 3  WDR18 - wd repeat domain 18  TERF2IP - telomeric repeat binding factor 2, interacting protein  ATG7 - autophagy related 7  H2AFX - h2a histone family, member x  DCP2 - decapping mrna 2  FGFR1 - fibroblast growth factor receptor 1  SMCHD1 - structural maintenance of chromosomes flexible hinge domain containing 1  PDGFRB - platelet-derived growth factor receptor, beta polypeptide  ARRB2 - arrestin, beta 2  PDGFB - platelet-derived growth factor beta polypeptide  MECP2 - methyl cpg binding protein 2 (rett syndrome)  FGF2 - fibroblast growth factor 2 (basic)  GMNN - geminin, dna replication inhibitor  NVL - nuclear vcp-like  MAP3K4 - mitogen-activated protein kinase kinase kinase 4  CEBPG - ccaat/enhancer binding protein (c/ebp), gamma  WDR70 - wd repeat domain 70  MEN1 - multiple endocrine neoplasia i  PCNA - proliferating cell nuclear antigen  CDK9 - cyclin-dependent kinase 9  NPM1 - nucleophosmin (nucleolar phosphoprotein b23, numatrin)  CCT7 - chaperonin containing tcp1, subunit 7 (eta)  CCT2 - chaperonin containing tcp1, subunit 2 (beta)  CCT4 - chaperonin containing tcp1, subunit 4 (delta)  WRAP53 - wd repeat containing, antisense to tp53  HMBOX1 - homeobox containing 1  MBD3 - methyl-cpg binding domain protein 3  SH2B1 - sh2b adaptor protein 1  CDK2 - cyclin-dependent kinase 2  KAT7 - k(lysine) acetyltransferase 7  DONSON - downstream neighbor of son  CACYBP - calcyclin binding protein  PAPD5 - pap associated domain containing 5  NUDT16 - nudix (nucleoside diphosphate linked moiety x)-type motif 16  ZNF365 - zinc finger protein 365  XRCC1 - x-ray repair complementing defective repair in chinese hamster cells 1  SMC3 - structural maintenance of chromosomes 3  MORC2 - morc family cw-type zinc finger 2  MMS19 - mms19 nucleotide excision repair homolog (s. cerevisiae)  MPHOSPH8 - m-phase phosphoprotein 8  SIRT6 - sirtuin 6  SIRT1 - sirtuin 1  CTC1 - cts telomere maintenance complex component 1  PPHLN1 - periphilin 1  BRPF3 - bromodomain and phd finger containing, 3  ID3 - inhibitor of dna binding 3, dominant negative helix-loop-helix protein  MAPK15 - mitogen-activated protein kinase 15  TOX - thymocyte selection-associated high mobility group box  MBD1 - methyl-cpg binding domain protein 1  WHSC1 - wolf-hirschhorn syndrome candidate 1  TNFAIP1 - tumor necrosis factor, alpha-induced protein 1 (endothelial)  RAD17 - rad17 homolog (s. pombe)  CDAN1 - codanin 1  ANKRD17 - ankyrin repeat domain 17  MAGEF1 - melanoma antigen family f, 1  ARID2 - at rich interactive domain 2 (arid, rfx-like)  RAC1 - ras-related c3 botulinum toxin substrate 1 (rho family, small gtp binding protein rac1)  INO80 - ino80 complex subunit  BAX - bcl2-associated x protein  DKC1 - dyskeratosis congenita 1, dyskerin  CCT8 - chaperonin containing tcp1, subunit 8 (theta)  NAT10 - n-acetyltransferase 10 (gcn5-related)  SMARCAL1 - swi/snf related, matrix associated, actin dependent regulator of chromatin, subfamily a-like 1  TERF1 - telomeric repeat binding factor (nima-interacting) 1  TERF2 - telomeric repeat binding factor 2  FAM35A - family with sequence similarity 35, member a  BRCC3 - brca1/brca2-containing complex, subunit 3  TFDP1 - transcription factor dp-1  KLHL15 - kelch-like family member 15  PARP9 - poly (adp-ribose) polymerase family, member 9  BCAR3 - breast cancer anti-estrogen resistance 3  GNL3L - guanine nucleotide binding protein-like 3 (nucleolar)-like  ATRX - alpha thalassemia/mental retardation syndrome x-linked  RNF8 - ring finger protein 8, e3 ubiquitin protein ligase  YLPM1 - ylp motif containing 1  TGFB1 - transforming growth factor, beta 1  DNMT1 - dna (cytosine-5-)-methyltransferase 1  ZNF711 - zinc finger protein 711  RBBP6 - retinoblastoma binding protein 6  TSPYL2 - tspy-like 2  ERCC8 - excision repair cross-complementing rodent repair deficiency, complementation group 8  PIK3CA - phosphatidylinositol-4,5-bisphosphate 3-kinase, catalytic subunit alpha  PTGES3 - prostaglandin e synthase 3 (cytosolic)  XRCC5 - x-ray repair complementing defective repair in chinese hamster cells 5 (double-strand-break rejoining)  GSK3A - glycogen synthase kinase 3 alpha  USP37 - ubiquitin specific peptidase 37  H1FX - h1 histone family, member x  PNKP - polynucleotide kinase 3'-phosphatase  CERS1 - ceramide synthase 1 |
| GO:0140056 | organelle localization by membrane tethering | 2.38E-8 | 6.38E-6 | 1.30 (10334,140,6996,123) | [+] Show genes  MAPRE1 - microtubule-associated protein, rp/eb family, member 1  AKAP9 - a kinase (prka) anchor protein (yotiao) 9  CEP89 - centrosomal protein 89kda  B9D2 - b9 protein domain 2  SCFD1 - sec1 family domain containing 1  RAB8B - rab8b, member ras oncogene family  MKS1 - meckel syndrome, type 1  HAUS7 - haus augmin-like complex, subunit 7  RAB3IP - rab3a interacting protein  DCTN1 - dynactin 1  PSEN2 - presenilin 2 (alzheimer disease 4)  DCTN3 - dynactin 3 (p22)  PACS2 - phosphofurin acidic cluster sorting protein 2  RAB15 - rab15, member ras oncogene family  TUBG1 - tubulin, gamma 1  RAB3D - rab3d, member ras oncogene family  YKT6 - ykt6 v-snare homolog (s. cerevisiae)  ODF2 - outer dense fiber of sperm tails 2  PPFIA3 - protein tyrosine phosphatase, receptor type, f polypeptide (ptprf), interacting protein (liprin), alpha 3  PAFAH1B1 - platelet-activating factor acetylhydrolase 1b, regulatory subunit 1 (45kda)  AZI1 - 5-azacytidine induced 1  VPS11 - vacuolar protein sorting 11 homolog (s. cerevisiae)  STX1B - syntaxin 1b  MARK4 - map/microtubule affinity-regulating kinase 4  CEP250 - centrosomal protein 250kda  TCTN1 - tectonic family member 1  CKAP5 - cytoskeleton associated protein 5  NDE1 - nude neurodevelopment protein 1  HAUS5 - haus augmin-like complex, subunit 5  NSF - n-ethylmaleimide-sensitive factor  DCTN2 - dynactin 2 (p50)  ESYT1 - extended synaptotagmin-like protein 1  TUBA1A - tubulin, alpha 1a  CETN2 - centrin, ef-hand protein, 2  VAPB - vamp (vesicle-associated membrane protein)-associated protein b and c  ALMS1 - alstrom syndrome 1  RAB10 - rab10, member ras oncogene family  CLASP1 - cytoplasmic linker associated protein 1  HAUS6 - haus augmin-like complex, subunit 6  SNAP25 - synaptosomal-associated protein, 25kda  ESYT2 - extended synaptotagmin-like protein 2  STX6 - syntaxin 6  AHI1 - abelson helper integration site 1  UNC13A - unc-13 homolog a (c. elegans)  RABEPK - rab9 effector protein with kelch motifs  RAB8A - rab8a, member ras oncogene family  CEP78 - centrosomal protein 78kda  C2CD3 - c2 calcium-dependent domain containing 3  NPHP1 - nephronophthisis 1 (juvenile)  PCM1 - pericentriolar material 1  SFI1 - sfi1 homolog, spindle assembly associated (yeast)  HSP90AA1 - heat shock protein 90kda alpha (cytosolic), class a member 1  STX8 - syntaxin 8  CEP76 - centrosomal protein 76kda  ACTR1A - arp1 actin-related protein 1 homolog a, centractin alpha (yeast)  USO1 - uso1 vesicle transport factor  SCLT1 - sodium channel and clathrin linker 1  FBF1 - fas (tnfrsf6) binding factor 1  STX12 - syntaxin 12  HAUS2 - haus augmin-like complex, subunit 2  CDK5RAP2 - cdk5 regulatory subunit associated protein 2  PRKACA - protein kinase, camp-dependent, catalytic, alpha  TCTN3 - tectonic family member 3  HAUS3 - haus augmin-like complex, subunit 3  SYT1 - synaptotagmin i  CEP290 - centrosomal protein 290kda  DYNLL1 - dynein, light chain, lc8-type 1  CTBP2 - c-terminal binding protein 2  FGFR1OP - fgfr1 oncogene partner  CEP57 - centrosomal protein 57kda  CCDC41 - coiled-coil domain containing 41  CNTRL - centriolin  RAB3C - rab3c, member ras oncogene family  ATG14 - autophagy related 14  STXBP1 - syntaxin binding protein 1  STX5 - syntaxin 5  STX4 - syntaxin 4  STX3 - syntaxin 3  STX1A - syntaxin 1a (brain)  RAB3A - rab3a, member ras oncogene family  RAB3B - rab3b, member ras oncogene family  NDRG4 - ndrg family member 4  STX17 - syntaxin 17  PPP2R1A - protein phosphatase 2, regulatory subunit a, alpha  SNPH - syntaphilin  CPLX2 - complexin 2  STXBP3 - syntaxin binding protein 3  UNC13B - unc-13 homolog b (c. elegans)  TTBK2 - tau tubulin kinase 2  VPS33A - vacuolar protein sorting 33 homolog a (s. cerevisiae)  TCTN2 - tectonic family member 2  EXOC4 - exocyst complex component 4  VTI1B - vesicle transport through interaction with t-snares 1b  CCP110 - centriolar coiled coil protein 110kda  IQCB1 - iq motif containing b1  TMEM216 - transmembrane protein 216  VPS18 - vacuolar protein sorting 18 homolog (s. cerevisiae)  TSNARE1 - t-snare domain containing 1  TUBB - tubulin, beta class i  BLOC1S6 - biogenesis of lysosomal organelles complex-1, subunit 6, pallidin  EXOC6 - exocyst complex component 6  CEP70 - centrosomal protein 70kda  PDZD8 - pdz domain containing 8  TUBB4B - tubulin, beta 4b class ivb  VPS33B - vacuolar protein sorting 33 homolog b (yeast)  STX7 - syntaxin 7  CEP41 - centrosomal protein 41kda  DYNC1H1 - dynein, cytoplasmic 1, heavy chain 1  DYNC1I2 - dynein, cytoplasmic 1, intermediate chain 2  RAB11A - rab11a, member ras oncogene family  CAV2 - caveolin 2  CEP63 - centrosomal protein 63kda  HAUS1 - haus augmin-like complex, subunit 1  SNAP29 - synaptosomal-associated protein, 29kda  CSNK1D - casein kinase 1, delta  VAMP3 - vesicle-associated membrane protein 3  B9D1 - b9 protein domain 1  STX2 - syntaxin 2  PRKAR2B - protein kinase, camp-dependent, regulatory, type ii, beta  YWHAG - tyrosine 3-monooxygenase/tryptophan 5-monooxygenase activation protein, gamma polypeptide  EXOC6B - exocyst complex component 6b  YWHAE - tyrosine 3-monooxygenase/tryptophan 5-monooxygenase activation protein, epsilon polypeptide  VPS45 - vacuolar protein sorting 45 homolog (s. cerevisiae) |
| GO:0018205 | peptidyl-lysine modification | 2.43E-8 | 6.39E-6 | 1.23 (10334,244,6996,203) | [+] Show genes  MSL3 - male-specific lethal 3 homolog (drosophila)  TADA1 - transcriptional adaptor 1  ATAT1 - alpha tubulin acetyltransferase 1  WHSC1L1 - wolf-hirschhorn syndrome candidate 1-like 1  METTL22 - methyltransferase like 22  TRIM28 - tripartite motif containing 28  EP300 - e1a binding protein p300  DPF2 - d4, zinc and double phd fingers family 2  DOT1L - dot1-like histone h3k79 methyltransferase  KAT2A - k(lysine) acetyltransferase 2a  NCOA3 - nuclear receptor coactivator 3  LOXL1 - lysyl oxidase-like 1  RLF - rearranged l-myc fusion  BRD8 - bromodomain containing 8  METTL12 - methyltransferase like 12  PLOD2 - procollagen-lysine, 2-oxoglutarate 5-dioxygenase 2  PLOD1 - procollagen-lysine, 2-oxoglutarate 5-dioxygenase 1  EGR2 - early growth response 2  SENP1 - sumo1/sentrin specific peptidase 1  SPHK2 - sphingosine kinase 2  EHMT2 - euchromatic histone-lysine n-methyltransferase 2  LDB1 - lim domain binding 1  CREBBP - creb binding protein  MORF4L1 - mortality factor 4 like 1  ATF2 - activating transcription factor 2  USPL1 - ubiquitin specific peptidase like 1  KAT2B - k(lysine) acetyltransferase 2b  CLOCK - clock circadian regulator  TOPORS - topoisomerase i binding, arginine/serine-rich, e3 ubiquitin protein ligase  CRTC2 - creb regulated transcription coactivator 2  ACTL6A - actin-like 6a  TRRAP - transformation/transcription domain-associated protein  SEH1L - seh1-like (s. cerevisiae)  SETD6 - set domain containing 6  SUMO2 - small ubiquitin-like modifier 2  NAA60 - n(alpha)-acetyltransferase 60, natf catalytic subunit  MCM3AP - minichromosome maintenance complex component 3 associated protein  SUMO3 - small ubiquitin-like modifier 3  NUP85 - nucleoporin 85kda  ACTL6B - actin-like 6b  TET2 - tet methylcytosine dioxygenase 2  TRPM4 - transient receptor potential cation channel, subfamily m, member 4  PHF17 - phd finger protein 17  KMT2C - lysine (k)-specific methyltransferase 2c  ZMPSTE24 - zinc metallopeptidase ste24  DPY30 - dpy-30 homolog (c. elegans)  SETD1B - set domain containing 1b  TPR - translocated promoter region, nuclear basket protein  RUVBL2 - ruvb-like 2 (e. coli)  SENP2 - sumo1/sentrin/smt3 specific peptidase 2  EHMT1 - euchromatic histone-lysine n-methyltransferase 1  NUP133 - nucleoporin 133kda  MCRS1 - microspherule protein 1  NUP107 - nucleoporin 107kda  CAMKMT - calmodulin-lysine n-methyltransferase  NUPL2 - nucleoporin like 2  SENP5 - sumo1/sentrin specific peptidase 5  NAA40 - n(alpha)-acetyltransferase 40, natd catalytic subunit, homolog (s. cerevisiae)  ING4 - inhibitor of growth family, member 4  KANSL1 - kat8 regulatory nsl complex subunit 1  HDAC6 - histone deacetylase 6  STX1A - syntaxin 1a (brain)  TADA3 - transcriptional adaptor 3  SAE1 - sumo1 activating enzyme subunit 1  KDM6A - lysine (k)-specific demethylase 6a  SUV420H1 - suppressor of variegation 4-20 homolog 1 (drosophila)  BRD1 - bromodomain containing 1  EZH1 - enhancer of zeste homolog 1 (drosophila)  PHF20L1 - phd finger protein 20-like 1  SUV420H2 - suppressor of variegation 4-20 homolog 2 (drosophila)  UBA2 - ubiquitin-like modifier activating enzyme 2  TTLL12 - tubulin tyrosine ligase-like family, member 12  KANSL1L - kat8 regulatory nsl complex subunit 1-like  ESCO1 - establishment of sister chromatid cohesion n-acetyltransferase 1  KANSL3 - kat8 regulatory nsl complex subunit 3  NUP205 - nucleoporin 205kda  LIAS - lipoic acid synthetase  EED - embryonic ectoderm development  SUPT7L - suppressor of ty 7 (s. cerevisiae)-like  KMT2D - lysine (k)-specific methyltransferase 2d  AAAS - achalasia, adrenocortical insufficiency, alacrimia  ING5 - inhibitor of growth family, member 5  VPS33B - vacuolar protein sorting 33 homolog b (yeast)  YEATS4 - yeats domain containing 4  PHF20 - phd finger protein 20  SUMO1 - small ubiquitin-like modifier 1  PIAS3 - protein inhibitor of activated stat, 3  KANSL2 - kat8 regulatory nsl complex subunit 2  KAT6A - k(lysine) acetyltransferase 6a  ZNF335 - zinc finger protein 335  NDC1 - ndc1 transmembrane nucleoporin  JMJD6 - jumonji domain containing 6  SETD2 - set domain containing 2  UBE2I - ubiquitin-conjugating enzyme e2i  NUP214 - nucleoporin 214kda  VIPAS39 - vps33b interacting protein, apical-basolateral polarity regulator, spe-39 homolog  LEF1 - lymphoid enhancer-binding factor 1  NUP153 - nucleoporin 153kda  GTF3C4 - general transcription factor iiic, polypeptide 4, 90kda  NUP210 - nucleoporin 210kda  YEATS2 - yeats domain containing 2  HAT1 - histone acetyltransferase 1  ARID4B - at rich interactive domain 4b (rbp1-like)  SETDB1 - set domain, bifurcated 1  RNF113A - ring finger protein 113a  SETMAR - set domain and mariner transposase fusion gene  MSL2 - male-specific lethal 2 homolog (drosophila)  MEAF6 - myst/esa1-associated factor 6  SENP7 - sumo1/sentrin specific peptidase 7  RSF1 - remodeling and spacing factor 1  CSRP2BP - csrp2 binding protein  GTF2B - general transcription factor iib  PCGF2 - polycomb group ring finger 2  KMT2A - lysine (k)-specific methyltransferase 2a  ASH1L - ash1 (absent, small, or homeotic)-like (drosophila)  APBB1 - amyloid beta (a4) precursor protein-binding, family b, member 1 (fe65)  PIAS1 - protein inhibitor of activated stat, 1  SMYD3 - set and mynd domain containing 3  METTL21A - methyltransferase like 21a  FAM173B - family with sequence similarity 173, member b  TAF6L - taf6-like rna polymerase ii, p300/cbp-associated factor (pcaf)-associated factor, 65kda  CBX4 - chromobox homolog 4  SETD5 - set domain containing 5  KDM3A - lysine (k)-specific demethylase 3a  WDR61 - wd repeat domain 61  NUP160 - nucleoporin 160kda  TAF9 - taf9 rna polymerase ii, tata box binding protein (tbp)-associated factor, 32kda  BRPF1 - bromodomain and phd finger containing, 1  LEPREL2 - leprecan-like 2  MUL1 - mitochondrial e3 ubiquitin protein ligase 1  TAF6 - taf6 rna polymerase ii, tata box binding protein (tbp)-associated factor, 80kda  NSMCE2 - non-smc element 2, mms21 homolog (s. cerevisiae)  ZNF451 - zinc finger protein 451  TADA2A - transcriptional adaptor 2a  SETD4 - set domain containing 4  ARRB1 - arrestin, beta 1  CHD5 - chromodomain helicase dna binding protein 5  PHF15 - phd finger protein 15  MECP2 - methyl cpg binding protein 2 (rett syndrome)  NUP88 - nucleoporin 88kda  NUP98 - nucleoporin 98kda  USP22 - ubiquitin specific peptidase 22  ZBED1 - zinc finger, bed-type containing 1  KAT5 - k(lysine) acetyltransferase 5  MAP3K7 - mitogen-activated protein kinase kinase kinase 7  HCFC1 - host cell factor c1 (vp16-accessory protein)  MEN1 - multiple endocrine neoplasia i  KMT2E - lysine (k)-specific methyltransferase 2e  ZZZ3 - zinc finger, zz-type containing 3  KAT8 - k(lysine) acetyltransferase 8  PWP1 - pwp1 homolog (s. cerevisiae)  RUVBL1 - ruvb-like 1 (e. coli)  DMAP1 - dna methyltransferase 1 associated protein 1  MBD3 - methyl-cpg binding domain protein 3  KAT7 - k(lysine) acetyltransferase 7  EPC2 - enhancer of polycomb homolog 2 (drosophila)  NSD1 - nuclear receptor binding set domain protein 1  PAGR1 - paxip1 associated glutamate-rich protein 1  DOHH - deoxyhypusine hydroxylase/monooxygenase  NUP50 - nucleoporin 50kda  DR1 - down-regulator of transcription 1, tbp-binding (negative cofactor 2)  SIRT1 - sirtuin 1  BRPF3 - bromodomain and phd finger containing, 3  HDAC4 - histone deacetylase 4  ATP7A - atpase, cu++ transporting, alpha polypeptide  NUP43 - nucleoporin 43kda  CXXC1 - cxxc finger protein 1  WHSC1 - wolf-hirschhorn syndrome candidate 1  KMT2B - lysine (k)-specific methyltransferase 2b  TRMT112 - trna methyltransferase 11-2 homolog (s. cerevisiae)  SENP6 - sumo1/sentrin specific peptidase 6  ASH2L - ash2 (absent, small, or homeotic)-like (drosophila)  MSL1 - male-specific lethal 1 homolog (drosophila)  HDAC9 - histone deacetylase 9  SETD1A - set domain containing 1a  NUP188 - nucleoporin 188kda  SUZ12 - suz12 polycomb repressive complex 2 subunit  RANBP2 - ran binding protein 2  RANGAP1 - ran gtpase activating protein 1  CTR9 - ctr9, paf1/rna polymerase ii complex component  EPC1 - enhancer of polycomb homolog 1 (drosophila)  MORF4L2 - mortality factor 4 like 2  EP400 - e1a binding protein p400  DHPS - deoxyhypusine synthase  NUP155 - nucleoporin 155kda  ING3 - inhibitor of growth family, member 3  PHF10 - phd finger protein 10  PIAS4 - protein inhibitor of activated stat, 4  OGT - o-linked n-acetylglucosamine (glcnac) transferase  MBIP - map3k12 binding inhibitory protein 1  ZNF711 - zinc finger protein 711  ARID4A - at rich interactive domain 4a (rbp1-like)  NUP93 - nucleoporin 93kda  RAE1 - rae1 rna export 1 homolog (s. pombe)  RBBP5 - retinoblastoma binding protein 5  MRGBP - mrg/morf4l binding protein  NUP35 - nucleoporin 35kda  PLOD3 - procollagen-lysine, 2-oxoglutarate 5-dioxygenase 3  PHF14 - phd finger protein 14  KAT6B - k(lysine) acetyltransferase 6b  PARK7 - parkinson protein 7  SUPT3H - suppressor of ty 3 homolog (s. cerevisiae)  SENP3 - sumo1/sentrin/smt3 specific peptidase 3 |
| GO:0016197 | endosomal transport | 2.99E-8 | 7.71E-6 | 1.26 (10334,184,6996,157) | [+] Show genes  TRIM27 - tripartite motif containing 27  TMEM87B - transmembrane protein 87b  CHMP4C - charged multivesicular body protein 4c  AGAP2 - arfgap with gtpase domain, ankyrin repeat and ph domain 2  DENND5A - denn/madd domain containing 5a  ABCA1 - atp-binding cassette, sub-family a (abc1), member 1  EPG5 - ectopic p-granules autophagy protein 5 homolog (c. elegans)  VPS37A - vacuolar protein sorting 37 homolog a (s. cerevisiae)  CORO1C - coronin, actin binding protein, 1c  HEATR5A - heat repeat containing 5a  DCTN1 - dynactin 1  PTPN23 - protein tyrosine phosphatase, non-receptor type 23  DENND1A - denn/madd domain containing 1a  DSCR3 - down syndrome critical region gene 3  GRIPAP1 - grip1 associated protein 1  VPS26A - vacuolar protein sorting 26 homolog a (s. pombe)  AP5Z1 - adaptor-related protein complex 5, zeta 1 subunit  VPS26B - vacuolar protein sorting 26 homolog b (s. pombe)  SGSM2 - small g protein signaling modulator 2  ARHGAP44 - rho gtpase activating protein 44  YKT6 - ykt6 v-snare homolog (s. cerevisiae)  VPS29 - vacuolar protein sorting 29 homolog (s. cerevisiae)  GOSR2 - golgi snap receptor complex member 2  BET1L - bet1 golgi vesicular membrane trafficking protein-like  UBAP1 - ubiquitin associated protein 1  RAB11B - rab11b, member ras oncogene family  ARL8B - adp-ribosylation factor-like 8b  PREPL - prolyl endopeptidase-like  TSG101 - tumor susceptibility gene 101  SNX2 - sorting nexin 2  CLEC16A - c-type lectin domain family 16, member a  SNX1 - sorting nexin 1  EHD1 - eh-domain containing 1  TMEM50A - transmembrane protein 50a  UBXN6 - ubx domain protein 6  SNX27 - sorting nexin family member 27  MVB12A - multivesicular body subunit 12a  VPS51 - vacuolar protein sorting 51 homolog (s. cerevisiae)  ARL1 - adp-ribosylation factor-like 1  ANKRD27 - ankyrin repeat domain 27 (vps9 domain)  PICALM - phosphatidylinositol binding clathrin assembly protein  VPS16 - vacuolar protein sorting 16 homolog (s. cerevisiae)  VPS39 - vacuolar protein sorting 39 homolog (s. cerevisiae)  ALMS1 - alstrom syndrome 1  TMEM50B - transmembrane protein 50b  NUMA1 - nuclear mitotic apparatus protein 1  VPS4B - vacuolar protein sorting 4 homolog b (s. cerevisiae)  RAB10 - rab10, member ras oncogene family  REPS2 - ralbp1 associated eps domain containing 2  GOSR1 - golgi snap receptor complex member 1  STX6 - syntaxin 6  ARF6 - adp-ribosylation factor 6  EVI5 - ecotropic viral integration site 5  SNX17 - sorting nexin 17  FAM45A - family with sequence similarity 45, member a  ARHGAP1 - rho gtpase activating protein 1  DCLK1 - doublecortin-like kinase 1  TBC1D5 - tbc1 domain family, member 5  RAB8A - rab8a, member ras oncogene family  TBC1D10B - tbc1 domain family, member 10b  CHMP1A - charged multivesicular body protein 1a  SNX6 - sorting nexin 6  RGP1 - rgp1 retrograde golgi transport homolog (s. cerevisiae)  PLEKHJ1 - pleckstrin homology domain containing, family j member 1  STAM2 - signal transducing adaptor molecule (sh3 domain and itam motif) 2  REPS1 - ralbp1 associated eps domain containing 1  CHMP2B - charged multivesicular body protein 2b  TBC1D14 - tbc1 domain family, member 14  HGS - hepatocyte growth factor-regulated tyrosine kinase substrate  EPS15L1 - epidermal growth factor receptor pathway substrate 15-like 1  ARL4C - adp-ribosylation factor-like 4c  DPY30 - dpy-30 homolog (c. elegans)  TMEM87A - transmembrane protein 87a  VPS52 - vacuolar protein sorting 52 homolog (s. cerevisiae)  SNX12 - sorting nexin 12  GRIP2 - glutamate receptor interacting protein 2  BAIAP3 - bai1-associated protein 3  ERC1 - elks/rab6-interacting/cast family member 1  VTA1 - vesicle (multivesicular body) trafficking 1  RAB11FIP3 - rab11 family interacting protein 3 (class ii)  MICALL1 - mical-like 1  VPS28 - vacuolar protein sorting 28 homolog (s. cerevisiae)  AP5M1 - adaptor-related protein complex 5, mu 1 subunit  VPS37C - vacuolar protein sorting 37 homolog c (s. cerevisiae)  BLOC1S2 - biogenesis of lysosomal organelles complex-1, subunit 2  VPS35 - vacuolar protein sorting 35 homolog (s. cerevisiae)  RAB6B - rab6b, member ras oncogene family  LYST - lysosomal trafficking regulator  LEPROT - leptin receptor overlapping transcript  RAB14 - rab14, member ras oncogene family  AP5S1 - adaptor-related protein complex 5, sigma 1 subunit  VPS54 - vacuolar protein sorting 54 homolog (s. cerevisiae)  SNX5 - sorting nexin 5  PQLC1 - pq loop repeat containing 1  TRAPPC10 - trafficking protein particle complex 10  RAB6A - rab6a, member ras oncogene family  STX5 - syntaxin 5  RAB7A - rab7a, member ras oncogene family  TOM1 - target of myb1 (chicken)  ZFYVE16 - zinc finger, fyve domain containing 16  LMTK2 - lemur tyrosine kinase 2  GGA1 - golgi-associated, gamma adaptin ear containing, arf binding protein 1  LAMTOR1 - late endosomal/lysosomal adaptor, mapk and mtor activator 1  CCDC22 - coiled-coil domain containing 22  TBC1D23 - tbc1 domain family, member 23  SDCCAG3 - serologically defined colon cancer antigen 3  CHMP5 - charged multivesicular body protein 5  AP5B1 - adaptor-related protein complex 5, beta 1 subunit  VCP - valosin containing protein  EHD2 - eh-domain containing 2  VPS4A - vacuolar protein sorting 4 homolog a (s. cerevisiae)  ANKFY1 - ankyrin repeat and fyve domain containing 1  EHD3 - eh-domain containing 3  VTI1B - vesicle transport through interaction with t-snares 1b  EHD4 - eh-domain containing 4  SNX3 - sorting nexin 3  SNX4 - sorting nexin 4  RAB9A - rab9a, member ras oncogene family  GGA3 - golgi-associated, gamma adaptin ear containing, arf binding protein 3  CLTC - clathrin, heavy chain (hc)  GCC2 - grip and coiled-coil domain containing 2  TBC1D17 - tbc1 domain family, member 17  RAB35 - rab35, member ras oncogene family  CHMP7 - charged multivesicular body protein 7  SNX18 - sorting nexin 18  GBF1 - golgi brefeldin a resistant guanine nucleotide exchange factor 1  UBB - ubiquitin b  CLN5 - ceroid-lipofuscinosis, neuronal 5  LEPROTL1 - leptin receptor overlapping transcript-like 1  ITSN2 - intersectin 2  RNF126 - ring finger protein 126  SPAG9 - sperm associated antigen 9  CHMP3 - charged multivesicular body protein 3  MAGEL2 - mage-like 2  ZFYVE9 - zinc finger, fyve domain containing 9  HEATR5B - heat repeat containing 5b  SNX33 - sorting nexin 33  VPS37D - vacuolar protein sorting 37 homolog d (s. cerevisiae)  VPS53 - vacuolar protein sorting 53 homolog (s. cerevisiae)  RAB11A - rab11a, member ras oncogene family  AP1S1 - adaptor-related protein complex 1, sigma 1 subunit  CHMP4A - charged multivesicular body protein 4a  FAM21A - family with sequence similarity 21, member a  NSG1 - neuron specific gene family member 1  CALY - calcyon neuron-specific vesicular protein  MICALL2 - mical-like 2  ALS2 - amyotrophic lateral sclerosis 2 (juvenile)  STAM - signal transducing adaptor molecule (sh3 domain and itam motif) 1  ACAP2 - arfgap with coiled-coil, ankyrin repeat and ph domains 2  VAMP3 - vesicle-associated membrane protein 3  CCDC93 - coiled-coil domain containing 93  CHMP4B - charged multivesicular body protein 4b  VPS25 - vacuolar protein sorting 25 homolog (s. cerevisiae)  CMTM6 - cklf-like marvel transmembrane domain containing 6  UBE2O - ubiquitin-conjugating enzyme e2o  ITSN1 - intersectin 1 (sh3 domain protein)  EPS15 - epidermal growth factor receptor pathway substrate 15 |
| GO:0043484 | regulation of RNA splicing | 3.67E-8 | 9.28E-6 | 1.31 (10334,129,6996,114) | [+] Show genes  CDK12 - cyclin-dependent kinase 12  RBM23 - rna binding motif protein 23  NCBP1 - nuclear cap binding protein subunit 1, 80kda  FMR1 - fragile x mental retardation 1  WTAP - wilms tumor 1 associated protein  DDX5 - dead (asp-glu-ala-asp) box helicase 5  SAP18 - sin3a-associated protein, 18kda  RBM39 - rna binding motif protein 39  NCL - nucleolin  RBM15 - rna binding motif protein 15  MBNL2 - muscleblind-like splicing regulator 2  RBMX - rna binding motif protein, x-linked  SNW1 - snw domain containing 1  HABP4 - hyaluronan binding protein 4  SRSF12 - serine/arginine-rich splicing factor 12  CWC22 - cwc22 spliceosome-associated protein homolog (s. cerevisiae)  PRPF19 - pre-mrna processing factor 19  METTL16 - methyltransferase like 16  RBM19 - rna binding motif protein 19  CELF4 - cugbp, elav-like family member 4  RBM7 - rna binding motif protein 7  RBM5 - rna binding motif protein 5  KHDRBS3 - kh domain containing, rna binding, signal transduction associated 3  CLK4 - cdc-like kinase 4  RNPS1 - rna binding protein s1, serine-rich domain  SON - son dna binding protein  ZNF326 - zinc finger protein 326  RBM12B - rna binding motif protein 12b  SLC38A2 - solute carrier family 38, member 2  CELF3 - cugbp, elav-like family member 3  ZNF259 - zinc finger protein 259  NSRP1 - nuclear speckle splicing regulatory protein 1  FXR2 - fragile x mental retardation, autosomal homolog 2  PTBP1 - polypyrimidine tract binding protein 1  SNRNP70 - small nuclear ribonucleoprotein 70kda (u1)  NUP98 - nucleoporin 98kda  PQBP1 - polyglutamine binding protein 1  DDX17 - dead (asp-glu-ala-asp) box helicase 17  PTBP2 - polypyrimidine tract binding protein 2  TRA2A - transformer 2 alpha homolog (drosophila)  RBM17 - rna binding motif protein 17  RBM15B - rna binding motif protein 15b  HNRNPLL - heterogeneous nuclear ribonucleoprotein l-like  RBM10 - rna binding motif protein 10  RBM25 - rna binding motif protein 25  RRP1B - ribosomal rna processing 1b  SF3B4 - splicing factor 3b, subunit 4, 49kda  ZBTB7A - zinc finger and btb domain containing 7a  SETX - senataxin  RBM12 - rna binding motif protein 12  POLR2A - polymerase (rna) ii (dna directed) polypeptide a, 220kda  HSPA8 - heat shock 70kda protein 8  MAGOH - mago-nashi homolog, proliferation-associated (drosophila)  PTBP3 - polypyrimidine tract binding protein 3  SRSF10 - serine/arginine-rich splicing factor 10  FUS - fused in sarcoma  NOVA2 - neuro-oncological ventral antigen 2  NOVA1 - neuro-oncological ventral antigen 1  DAZAP1 - daz associated protein 1  SRSF9 - serine/arginine-rich splicing factor 9  DYRK1A - dual-specificity tyrosine-(y)-phosphorylation regulated kinase 1a  PCBP4 - poly(rc) binding protein 4  SART3 - squamous cell carcinoma antigen recognized by t cells 3  TMBIM6 - transmembrane bax inhibitor motif containing 6  SMU1 - smu-1 suppressor of mec-8 and unc-52 homolog (c. elegans)  PUF60 - poly-u binding splicing factor 60kda  CELF6 - cugbp, elav-like family member 6  YTHDC1 - yth domain containing 1  ERN1 - endoplasmic reticulum to nucleus signaling 1  PIK3R1 - phosphoinositide-3-kinase, regulatory subunit 1 (alpha)  HNRNPU - heterogeneous nuclear ribonucleoprotein u (scaffold attachment factor a)  HNRNPK - heterogeneous nuclear ribonucleoprotein k  HNRNPL - heterogeneous nuclear ribonucleoprotein l  RBFOX3 - rna binding protein, fox-1 homolog (c. elegans) 3  CELF1 - cugbp, elav-like family member 1  KHDRBS1 - kh domain containing, rna binding, signal transduction associated 1  RBM8A - rna binding motif protein 8a  CLK1 - cdc-like kinase 1  RBM42 - rna binding motif protein 42  CELF2 - cugbp, elav-like family member 2  CLK2 - cdc-like kinase 2  IK - ik cytokine, down-regulator of hla ii  CLK3 - cdc-like kinase 3  RBFOX2 - rna binding protein, fox-1 homolog (c. elegans) 2  SF1 - splicing factor 1  SRPK2 - srsf protein kinase 2  HNRNPA2B1 - heterogeneous nuclear ribonucleoprotein a2/b1  U2AF2 - u2 small nuclear rna auxiliary factor 2  SRPK1 - srsf protein kinase 1  SRSF1 - serine/arginine-rich splicing factor 1  RBM3 - rna binding motif (rnp1, rrm) protein 3  RBM4 - rna binding motif protein 4  HNRNPA1 - heterogeneous nuclear ribonucleoprotein a1  SRSF2 - serine/arginine-rich splicing factor 2  SRSF3 - serine/arginine-rich splicing factor 3  SRSF4 - serine/arginine-rich splicing factor 4  SRSF5 - serine/arginine-rich splicing factor 5  HNRNPF - heterogeneous nuclear ribonucleoprotein f  SRSF6 - serine/arginine-rich splicing factor 6  SRSF7 - serine/arginine-rich splicing factor 7  HNRNPH1 - heterogeneous nuclear ribonucleoprotein h1 (h)  SFSWAP - splicing factor, suppressor of white-apricot homolog (drosophila)  TRA2B - transformer 2 beta homolog (drosophila)  HNRNPH3 - heterogeneous nuclear ribonucleoprotein h3 (2h9)  JMJD6 - jumonji domain containing 6  LARP7 - la ribonucleoprotein domain family, member 7  HNRNPH2 - heterogeneous nuclear ribonucleoprotein h2 (h')  ZC3H10 - zinc finger ccch-type containing 10  CIRBP - cold inducible rna binding protein  RBM22 - rna binding motif protein 22  TIA1 - tia1 cytotoxic granule-associated rna binding protein  GRSF1 - g-rich rna sequence binding factor 1  THRAP3 - thyroid hormone receptor associated protein 3  RBM4B - rna binding motif protein 4b |
| GO:0006913 | nucleocytoplasmic transport | 3.85E-8 | 9.57E-6 | 1.24 (10334,217,6996,182) | [+] Show genes  RSRC1 - arginine/serine-rich coiled-coil 1  DDX19B - dead (asp-glu-ala-asp) box polypeptide 19b  NCBP2 - nuclear cap binding protein subunit 2, 20kda  SRSF11 - serine/arginine-rich splicing factor 11  NCBP1 - nuclear cap binding protein subunit 1, 80kda  SEC13 - sec13 homolog (s. cerevisiae)  SDAD1 - sda1 domain containing 1  TSC1 - tuberous sclerosis 1  SYMPK - symplekin  DDX25 - dead (asp-glu-ala-asp) box helicase 25  DESI1 - desumoylating isopeptidase 1  KPNA6 - karyopherin alpha 6 (importin alpha 7)  RANBP3 - ran binding protein 3  PML - promyelocytic leukemia  GEMIN5 - gem (nuclear organelle) associated protein 5  AGFG1 - arfgap with fg repeats 1  ALKBH5 - alkb, alkylation repair homolog 5 (e. coli)  LZTS2 - leucine zipper, putative tumor suppressor 2  UPF1 - upf1 regulator of nonsense transcripts homolog (yeast)  FAM53C - family with sequence similarity 53, member c  XPOT - exportin, trna  RAB18 - rab18, member ras oncogene family  PHAX - phosphorylated adaptor for rna export  CHP1 - calcineurin-like ef-hand protein 1  CPSF3 - cleavage and polyadenylation specific factor 3, 73kda  LTV1 - ltv1 homolog (s. cerevisiae)  RANBP6 - ran binding protein 6  ANP32A - acidic (leucine-rich) nuclear phosphoprotein 32 family, member a  EIF4E - eukaryotic translation initiation factor 4e  NOP9 - nop9 nucleolar protein  EGR2 - early growth response 2  APPL2 - adaptor protein, phosphotyrosine interaction, ph domain and leucine zipper containing 2  ALYREF - aly/ref export factor  IPO4 - importin 4  PABPN1 - poly(a) binding protein, nuclear 1  RNPS1 - rna binding protein s1, serine-rich domain  CAMK1 - calcium/calmodulin-dependent protein kinase i  ELAVL1 - elav (embryonic lethal, abnormal vision, drosophila)-like 1 (hu antigen r)  SMG6 - smg6 nonsense mediated mrna decay factor  STRADA - ste20-related kinase adaptor alpha  AKAP13 - a kinase (prka) anchor protein 13  NUTF2 - nuclear transport factor 2  TSC2 - tuberous sclerosis 2  SMG7 - smg7 nonsense mediated mrna decay factor  DDX20 - dead (asp-glu-ala-asp) box polypeptide 20  LMNA - lamin a/c  NUP160 - nucleoporin 160kda  FIP1L1 - factor interacting with papola and cpsf1  ARNTL - aryl hydrocarbon receptor nuclear translocator-like  SNRPD1 - small nuclear ribonucleoprotein d1 polypeptide 16kda  SNRPF - small nuclear ribonucleoprotein polypeptide f  ABCE1 - atp-binding cassette, sub-family e (oabp), member 1  SEH1L - seh1-like (s. cerevisiae)  FGF9 - fibroblast growth factor 9  PTTG1IP - pituitary tumor-transforming 1 interacting protein  SNUPN - snurportin 1  SNRPB - small nuclear ribonucleoprotein polypeptides b and b1  EIF4A3 - eukaryotic translation initiation factor 4a3  NUP88 - nucleoporin 88kda  NUP98 - nucleoporin 98kda  PRICKLE1 - prickle homolog 1 (drosophila)  GLE1 - gle1 rna export mediator  TMCO6 - transmembrane and coiled-coil domains 6  CPSF4 - cleavage and polyadenylation specific factor 4, 30kda  SMN1 - survival of motor neuron 1, telomeric  U2AF1L4 - u2 small nuclear rna auxiliary factor 1-like 4  MED1 - mediator complex subunit 1  CPSF2 - cleavage and polyadenylation specific factor 2, 100kda  DHX38 - deah (asp-glu-ala-his) box polypeptide 38  MCM3AP - minichromosome maintenance complex component 3 associated protein  CPSF1 - cleavage and polyadenylation specific factor 1, 160kda  IPO8 - importin 8  RBM15B - rna binding motif protein 15b  IPO7 - importin 7  NUP85 - nucleoporin 85kda  EIF6 - eukaryotic translation initiation factor 6  SLU7 - slu7 splicing factor homolog (s. cerevisiae)  CDK5 - cyclin-dependent kinase 5  XPO5 - exportin 5  NPM1 - nucleophosmin (nucleolar phosphoprotein b23, numatrin)  NMD3 - nmd3 ribosome export adaptor  SMG1 - smg1 phosphatidylinositol 3-kinase-related kinase  SRRM1 - serine/arginine repetitive matrix 1  MAGOHB - mago-nashi homolog b (drosophila)  ENY2 - enhancer of yellow 2 homolog (drosophila)  XPO7 - exportin 7  HTATIP2 - hiv-1 tat interactive protein 2, 30kda  DUSP16 - dual specificity phosphatase 16  NEMF - nuclear export mediator factor  POLR2D - polymerase (rna) ii (dna directed) polypeptide d  CDC40 - cell division cycle 40  TPR - translocated promoter region, nuclear basket protein  HSPA9 - heat shock 70kda protein 9 (mortalin)  DDX19A - dead (asp-glu-ala-asp) box polypeptide 19a  MAGOH - mago-nashi homolog, proliferation-associated (drosophila)  NUP133 - nucleoporin 133kda  XPO1 - exportin 1 (crm1 homolog, yeast)  NUP107 - nucleoporin 107kda  NUPL2 - nucleoporin like 2  TNPO2 - transportin 2  AHCYL1 - adenosylhomocysteinase-like 1  LRRK2 - leucine-rich repeat kinase 2  NUP50 - nucleoporin 50kda  GEMIN6 - gem (nuclear organelle) associated protein 6  HEATR3 - heat repeat containing 3  LSG1 - large 60s subunit nuclear export gtpase 1  THOC7 - tho complex 7 homolog (drosophila)  SRSF9 - serine/arginine-rich splicing factor 9  NUP43 - nucleoporin 43kda  AKT1 - v-akt murine thymoma viral oncogene homolog 1  PCID2 - pci domain containing 2  TNPO1 - transportin 1  NFKBIA - nuclear factor of kappa light polypeptide gene enhancer in b-cells inhibitor, alpha  SLBP - stem-loop binding protein  CHTOP - chromatin target of prmt1  KPNA2 - karyopherin alpha 2 (rag cohort 1, importin alpha 1)  KPNB1 - karyopherin (importin) beta 1  KPNA1 - karyopherin alpha 1 (importin alpha 5)  WDR33 - wd repeat domain 33  NOL6 - nucleolar protein 6 (rna-associated)  CALR - calreticulin  APPL1 - adaptor protein, phosphotyrosine interaction, ph domain and leucine zipper containing 1  PPP1R10 - protein phosphatase 1, regulatory subunit 10  FYTTD1 - forty-two-three domain containing 1  UPF3B - upf3 regulator of nonsense transcripts homolog b (yeast)  UPF3A - upf3 regulator of nonsense transcripts homolog a (yeast)  DDX39B - dead (asp-glu-ala-asp) box polypeptide 39b  PKD1 - polycystic kidney disease 1 (autosomal dominant)  RPAIN - rpa interacting protein  MBTPS1 - membrane-bound transcription factor peptidase, site 1  EIF4ENIF1 - eukaryotic translation initiation factor 4e nuclear import factor 1  NUP188 - nucleoporin 188kda  CITED1 - cbp/p300-interacting transactivator, with glu/asp-rich carboxy-terminal domain, 1  RANBP2 - ran binding protein 2  NUP205 - nucleoporin 205kda  POLDIP3 - polymerase (dna-directed), delta interacting protein 3  YTHDC1 - yth domain containing 1  THOC2 - tho complex 2  THOC6 - tho complex 6 homolog (drosophila)  STAT3 - signal transducer and activator of transcription 3 (acute-phase response factor)  PIK3R1 - phosphoinositide-3-kinase, regulatory subunit 1 (alpha)  GEMIN8 - gem (nuclear organelle) associated protein 8  FBXO22 - f-box protein 22  AAAS - achalasia, adrenocortical insufficiency, alacrimia  RPS15 - ribosomal protein s15  NUP155 - nucleoporin 155kda  RBM8A - rna binding motif protein 8a  CASC3 - cancer susceptibility candidate 3  CSE1L - cse1 chromosome segregation 1-like (yeast)  U2AF2 - u2 small nuclear rna auxiliary factor 2  SRSF1 - serine/arginine-rich splicing factor 1  TGFB1 - transforming growth factor, beta 1  HNRNPA2B1 - heterogeneous nuclear ribonucleoprotein a2/b1  SRSF2 - serine/arginine-rich splicing factor 2  GEMIN7 - gem (nuclear organelle) associated protein 7  HNRNPA1 - heterogeneous nuclear ribonucleoprotein a1  SRSF3 - serine/arginine-rich splicing factor 3  NUP93 - nucleoporin 93kda  SRSF4 - serine/arginine-rich splicing factor 4  RRS1 - rrs1 ribosome biogenesis regulator homolog (s. cerevisiae)  SRSF5 - serine/arginine-rich splicing factor 5  RAE1 - rae1 rna export 1 homolog (s. pombe)  SRSF6 - serine/arginine-rich splicing factor 6  ANKRD54 - ankyrin repeat domain 54  XPO6 - exportin 6  GEMIN2 - gem (nuclear organelle) associated protein 2  SRSF7 - serine/arginine-rich splicing factor 7  SMURF1 - smad specific e3 ubiquitin protein ligase 1  GEMIN4 - gem (nuclear organelle) associated protein 4  NUP35 - nucleoporin 35kda  SSB - sjogren syndrome antigen b (autoantigen la)  NDC1 - ndc1 transmembrane nucleoporin  THOC1 - tho complex 1  IPO9 - importin 9  THOC3 - tho complex 3  NUP214 - nucleoporin 214kda  IPO11 - importin 11  SARNP - sap domain containing ribonucleoprotein  NUP153 - nucleoporin 153kda  TNPO3 - transportin 3  NUP210 - nucleoporin 210kda  PRKAG1 - protein kinase, amp-activated, gamma 1 non-catalytic subunit |
| GO:0051169 | nuclear transport | 5E-8 | 1.22E-5 | 1.24 (10334,220,6996,184) | [+] Show genes  RSRC1 - arginine/serine-rich coiled-coil 1  DDX19B - dead (asp-glu-ala-asp) box polypeptide 19b  NCBP2 - nuclear cap binding protein subunit 2, 20kda  SRSF11 - serine/arginine-rich splicing factor 11  NCBP1 - nuclear cap binding protein subunit 1, 80kda  SEC13 - sec13 homolog (s. cerevisiae)  SDAD1 - sda1 domain containing 1  TSC1 - tuberous sclerosis 1  SYMPK - symplekin  DDX25 - dead (asp-glu-ala-asp) box helicase 25  DESI1 - desumoylating isopeptidase 1  KPNA6 - karyopherin alpha 6 (importin alpha 7)  RANBP3 - ran binding protein 3  PML - promyelocytic leukemia  GEMIN5 - gem (nuclear organelle) associated protein 5  AGFG1 - arfgap with fg repeats 1  ALKBH5 - alkb, alkylation repair homolog 5 (e. coli)  LZTS2 - leucine zipper, putative tumor suppressor 2  UPF1 - upf1 regulator of nonsense transcripts homolog (yeast)  FAM53C - family with sequence similarity 53, member c  XPOT - exportin, trna  RAB18 - rab18, member ras oncogene family  PHAX - phosphorylated adaptor for rna export  CHP1 - calcineurin-like ef-hand protein 1  CPSF3 - cleavage and polyadenylation specific factor 3, 73kda  LTV1 - ltv1 homolog (s. cerevisiae)  RANBP6 - ran binding protein 6  EIF4E - eukaryotic translation initiation factor 4e  ANP32A - acidic (leucine-rich) nuclear phosphoprotein 32 family, member a  NOP9 - nop9 nucleolar protein  EGR2 - early growth response 2  APPL2 - adaptor protein, phosphotyrosine interaction, ph domain and leucine zipper containing 2  ALYREF - aly/ref export factor  IPO4 - importin 4  PABPN1 - poly(a) binding protein, nuclear 1  RNPS1 - rna binding protein s1, serine-rich domain  CAMK1 - calcium/calmodulin-dependent protein kinase i  ELAVL1 - elav (embryonic lethal, abnormal vision, drosophila)-like 1 (hu antigen r)  SMG6 - smg6 nonsense mediated mrna decay factor  STRADA - ste20-related kinase adaptor alpha  AKAP13 - a kinase (prka) anchor protein 13  NUTF2 - nuclear transport factor 2  TSC2 - tuberous sclerosis 2  SMG7 - smg7 nonsense mediated mrna decay factor  DDX20 - dead (asp-glu-ala-asp) box polypeptide 20  LMNA - lamin a/c  NUP160 - nucleoporin 160kda  FIP1L1 - factor interacting with papola and cpsf1  ARNTL - aryl hydrocarbon receptor nuclear translocator-like  SNRPD1 - small nuclear ribonucleoprotein d1 polypeptide 16kda  SNRPF - small nuclear ribonucleoprotein polypeptide f  ABCE1 - atp-binding cassette, sub-family e (oabp), member 1  SEH1L - seh1-like (s. cerevisiae)  FGF9 - fibroblast growth factor 9  PTTG1IP - pituitary tumor-transforming 1 interacting protein  SNUPN - snurportin 1  SNRPB - small nuclear ribonucleoprotein polypeptides b and b1  EIF4A3 - eukaryotic translation initiation factor 4a3  NUP88 - nucleoporin 88kda  NUP98 - nucleoporin 98kda  PRICKLE1 - prickle homolog 1 (drosophila)  PSIP1 - pc4 and sfrs1 interacting protein 1  GLE1 - gle1 rna export mediator  TMCO6 - transmembrane and coiled-coil domains 6  CPSF4 - cleavage and polyadenylation specific factor 4, 30kda  SMN1 - survival of motor neuron 1, telomeric  MED1 - mediator complex subunit 1  U2AF1L4 - u2 small nuclear rna auxiliary factor 1-like 4  CPSF2 - cleavage and polyadenylation specific factor 2, 100kda  DHX38 - deah (asp-glu-ala-his) box polypeptide 38  CPSF1 - cleavage and polyadenylation specific factor 1, 160kda  MCM3AP - minichromosome maintenance complex component 3 associated protein  IPO8 - importin 8  RBM15B - rna binding motif protein 15b  IPO7 - importin 7  NUP85 - nucleoporin 85kda  EIF6 - eukaryotic translation initiation factor 6  SLU7 - slu7 splicing factor homolog (s. cerevisiae)  CDK5 - cyclin-dependent kinase 5  XPO5 - exportin 5  NPM1 - nucleophosmin (nucleolar phosphoprotein b23, numatrin)  NMD3 - nmd3 ribosome export adaptor  SMG1 - smg1 phosphatidylinositol 3-kinase-related kinase  SRRM1 - serine/arginine repetitive matrix 1  MAGOHB - mago-nashi homolog b (drosophila)  ENY2 - enhancer of yellow 2 homolog (drosophila)  XPO7 - exportin 7  HTATIP2 - hiv-1 tat interactive protein 2, 30kda  DUSP16 - dual specificity phosphatase 16  NEMF - nuclear export mediator factor  CDC40 - cell division cycle 40  POLR2D - polymerase (rna) ii (dna directed) polypeptide d  TPR - translocated promoter region, nuclear basket protein  HSPA9 - heat shock 70kda protein 9 (mortalin)  DDX19A - dead (asp-glu-ala-asp) box polypeptide 19a  MAGOH - mago-nashi homolog, proliferation-associated (drosophila)  NUP133 - nucleoporin 133kda  XPO1 - exportin 1 (crm1 homolog, yeast)  NUP107 - nucleoporin 107kda  NUPL2 - nucleoporin like 2  TNPO2 - transportin 2  AHCYL1 - adenosylhomocysteinase-like 1  LRRK2 - leucine-rich repeat kinase 2  NUP50 - nucleoporin 50kda  GEMIN6 - gem (nuclear organelle) associated protein 6  HEATR3 - heat repeat containing 3  LSG1 - large 60s subunit nuclear export gtpase 1  THOC7 - tho complex 7 homolog (drosophila)  SRSF9 - serine/arginine-rich splicing factor 9  NUP43 - nucleoporin 43kda  AKT1 - v-akt murine thymoma viral oncogene homolog 1  PCID2 - pci domain containing 2  TNPO1 - transportin 1  NFKBIA - nuclear factor of kappa light polypeptide gene enhancer in b-cells inhibitor, alpha  SLBP - stem-loop binding protein  CHTOP - chromatin target of prmt1  KPNA2 - karyopherin alpha 2 (rag cohort 1, importin alpha 1)  KPNB1 - karyopherin (importin) beta 1  KPNA1 - karyopherin alpha 1 (importin alpha 5)  WDR33 - wd repeat domain 33  NOL6 - nucleolar protein 6 (rna-associated)  CALR - calreticulin  APPL1 - adaptor protein, phosphotyrosine interaction, ph domain and leucine zipper containing 1  PPP1R10 - protein phosphatase 1, regulatory subunit 10  FYTTD1 - forty-two-three domain containing 1  UPF3B - upf3 regulator of nonsense transcripts homolog b (yeast)  DDX39B - dead (asp-glu-ala-asp) box polypeptide 39b  UPF3A - upf3 regulator of nonsense transcripts homolog a (yeast)  PKD1 - polycystic kidney disease 1 (autosomal dominant)  MBTPS1 - membrane-bound transcription factor peptidase, site 1  RPAIN - rpa interacting protein  EIF4ENIF1 - eukaryotic translation initiation factor 4e nuclear import factor 1  NUP188 - nucleoporin 188kda  CITED1 - cbp/p300-interacting transactivator, with glu/asp-rich carboxy-terminal domain, 1  RANBP2 - ran binding protein 2  NUP205 - nucleoporin 205kda  POLDIP3 - polymerase (dna-directed), delta interacting protein 3  YTHDC1 - yth domain containing 1  THOC2 - tho complex 2  THOC6 - tho complex 6 homolog (drosophila)  STAT3 - signal transducer and activator of transcription 3 (acute-phase response factor)  PIK3R1 - phosphoinositide-3-kinase, regulatory subunit 1 (alpha)  GEMIN8 - gem (nuclear organelle) associated protein 8  FBXO22 - f-box protein 22  AAAS - achalasia, adrenocortical insufficiency, alacrimia  RPS15 - ribosomal protein s15  NUP155 - nucleoporin 155kda  RBM8A - rna binding motif protein 8a  CASC3 - cancer susceptibility candidate 3  CSE1L - cse1 chromosome segregation 1-like (yeast)  TGFB1 - transforming growth factor, beta 1  SRSF1 - serine/arginine-rich splicing factor 1  U2AF2 - u2 small nuclear rna auxiliary factor 2  HNRNPA2B1 - heterogeneous nuclear ribonucleoprotein a2/b1  SRSF2 - serine/arginine-rich splicing factor 2  GEMIN7 - gem (nuclear organelle) associated protein 7  HNRNPA1 - heterogeneous nuclear ribonucleoprotein a1  SRSF3 - serine/arginine-rich splicing factor 3  SRSF4 - serine/arginine-rich splicing factor 4  NUP93 - nucleoporin 93kda  RRS1 - rrs1 ribosome biogenesis regulator homolog (s. cerevisiae)  SRSF5 - serine/arginine-rich splicing factor 5  ANKRD54 - ankyrin repeat domain 54  SRSF6 - serine/arginine-rich splicing factor 6  RAE1 - rae1 rna export 1 homolog (s. pombe)  XPO6 - exportin 6  SRSF7 - serine/arginine-rich splicing factor 7  GEMIN2 - gem (nuclear organelle) associated protein 2  SMURF1 - smad specific e3 ubiquitin protein ligase 1  GEMIN4 - gem (nuclear organelle) associated protein 4  NUP35 - nucleoporin 35kda  SSB - sjogren syndrome antigen b (autoantigen la)  NDC1 - ndc1 transmembrane nucleoporin  THOC1 - tho complex 1  IPO9 - importin 9  THOC3 - tho complex 3  NUP214 - nucleoporin 214kda  IPO11 - importin 11  HMGA1 - high mobility group at-hook 1  SARNP - sap domain containing ribonucleoprotein  NUP153 - nucleoporin 153kda  TNPO3 - transportin 3  NUP210 - nucleoporin 210kda  PRKAG1 - protein kinase, amp-activated, gamma 1 non-catalytic subunit |
| GO:0051171 | regulation of nitrogen compound metabolic process | 5.07E-8 | 1.22E-5 | 1.05 (10334,3580,6996,2544) | [+] Show genes  AGAP2 - arfgap with gtpase domain, ankyrin repeat and ph domain 2  EXOSC9 - exosome component 9  MSL3 - male-specific lethal 3 homolog (drosophila)  EXOSC10 - exosome component 10  TADA1 - transcriptional adaptor 1  AARS - alanyl-trna synthetase  HNRNPA0 - heterogeneous nuclear ribonucleoprotein a0  CBX1 - chromobox homolog 1  PPP1R9A - protein phosphatase 1, regulatory subunit 9a  ABCA2 - atp-binding cassette, sub-family a (abc1), member 2  OS9 - osteosarcoma amplified 9, endoplasmic reticulum lectin  ABL1 - c-abl oncogene 1, non-receptor tyrosine kinase  ZNF821 - zinc finger protein 821  ERP29 - endoplasmic reticulum protein 29  PML - promyelocytic leukemia  MLLT11 - myeloid/lymphoid or mixed-lineage leukemia (trithorax homolog, drosophila); translocated to, 11  CNOT11 - ccr4-not transcription complex, subunit 11  SUPT20H - suppressor of ty 20 homolog (s. cerevisiae)  YWHAQ - tyrosine 3-monooxygenase/tryptophan 5-monooxygenase activation protein, theta polypeptide  TMED10 - transmembrane emp24-like trafficking protein 10 (yeast)  BRD8 - bromodomain containing 8  RFFL - ring finger and fyve-like domain containing e3 ubiquitin protein ligase  CHD7 - chromodomain helicase dna binding protein 7  LYAR - ly1 antibody reactive  PAPOLA - poly(a) polymerase alpha  TCERG1 - transcription elongation regulator 1  ZNF354B - zinc finger protein 354b  RNPS1 - rna binding protein s1, serine-rich domain  ACTB - actin, beta  OTUB1 - otu domain, ubiquitin aldehyde binding 1  EHMT2 - euchromatic histone-lysine n-methyltransferase 2  PLAUR - plasminogen activator, urokinase receptor  SPIN1 - spindlin 1  TASP1 - taspase, threonine aspartase, 1  PRR5 - proline rich 5 (renal)  PLCL1 - phospholipase c-like 1  SUB1 - sub1 homolog (s. cerevisiae)  MORF4L1 - mortality factor 4 like 1  ZNF511 - zinc finger protein 511  PPID - peptidylprolyl isomerase d  CAMK2N1 - calcium/calmodulin-dependent protein kinase ii inhibitor 1  ACTL6A - actin-like 6a  ACTN1 - actinin, alpha 1  USP19 - ubiquitin specific peptidase 19  ACTN4 - actinin, alpha 4  ACVR2A - activin a receptor, type iia  ACVRL1 - activin a receptor type ii-like 1  PARL - presenilin associated, rhomboid-like  FAM122A - family with sequence similarity 122a  ACVR1 - activin a receptor, type i  ACVR1B - activin a receptor, type ib  ADAR - adenosine deaminase, rna-specific  ADARB1 - adenosine deaminase, rna-specific, b1  ADCY6 - adenylate cyclase 6  CPSF4 - cleavage and polyadenylation specific factor 4, 30kda  ZNF331 - zinc finger protein 331  ADCY5 - adenylate cyclase 5  MED1 - mediator complex subunit 1  ADCY1 - adenylate cyclase 1 (brain)  ADCY3 - adenylate cyclase 3  PPARD - peroxisome proliferator-activated receptor delta  MAML3 - mastermind-like 3 (drosophila)  POLR2K - polymerase (rna) ii (dna directed) polypeptide k, 7.0kda  FAM129A - family with sequence similarity 129, member a  PARP1 - poly (adp-ribose) polymerase 1  POLH - polymerase (dna directed), eta  H2AFY2 - h2a histone family, member y2  PPP1R13L - protein phosphatase 1, regulatory subunit 13 like  TNFRSF19 - tumor necrosis factor receptor superfamily, member 19  HES6 - hairy and enhancer of split 6 (drosophila)  POLR2G - polymerase (rna) ii (dna directed) polypeptide g  SMPD3 - sphingomyelin phosphodiesterase 3, neutral membrane (neutral sphingomyelinase ii)  POLR2D - polymerase (rna) ii (dna directed) polypeptide d  RUVBL2 - ruvb-like 2 (e. coli)  PARP4 - poly (adp-ribose) polymerase family, member 4  POLR2A - polymerase (rna) ii (dna directed) polypeptide a, 220kda  AP2A1 - adaptor-related protein complex 2, alpha 1 subunit  CDK5RAP2 - cdk5 regulatory subunit associated protein 2  RCOR3 - rest corepressor 3  PRKACB - protein kinase, camp-dependent, catalytic, beta  PRKACA - protein kinase, camp-dependent, catalytic, alpha  AES - amino-terminal enhancer of split  NUP133 - nucleoporin 133kda  NUPL2 - nucleoporin like 2  FAM46B - family with sequence similarity 46, member b  VPS35 - vacuolar protein sorting 35 homolog (s. cerevisiae)  JAG1 - jagged 1  EDEM2 - er degradation enhancer, mannosidase alpha-like 2  CHFR - checkpoint with forkhead and ring finger domains, e3 ubiquitin protein ligase  PRDM4 - pr domain containing 4  PPP3R1 - protein phosphatase 3, regulatory subunit b, alpha  ATF7IP - activating transcription factor 7 interacting protein  FGFR1OP - fgfr1 oncogene partner  PPP5C - protein phosphatase 5, catalytic subunit  ZNF689 - zinc finger protein 689  PPP2R5D - protein phosphatase 2, regulatory subunit b', delta  LRIF1 - ligand dependent nuclear receptor interacting factor 1  PPP2R5C - protein phosphatase 2, regulatory subunit b', gamma  PPP2R5B - protein phosphatase 2, regulatory subunit b', beta  WDFY2 - wd repeat and fyve domain containing 2  AKT1 - v-akt murine thymoma viral oncogene homolog 1  ZNF653 - zinc finger protein 653  PPP4C - protein phosphatase 4, catalytic subunit  PCID2 - pci domain containing 2  PPP2R5E - protein phosphatase 2, regulatory subunit b', epsilon isoform  PPP2R2A - protein phosphatase 2, regulatory subunit b, alpha  PPP2R1B - protein phosphatase 2, regulatory subunit a, beta  CPSF6 - cleavage and polyadenylation specific factor 6, 68kda  PPP2R1A - protein phosphatase 2, regulatory subunit a, alpha  RIOK2 - rio kinase 2  NUDT21 - nudix (nucleoside diphosphate linked moiety x)-type motif 21  WWP2 - ww domain containing e3 ubiquitin protein ligase 2  AHR - aryl hydrocarbon receptor  PPP2R2C - protein phosphatase 2, regulatory subunit b, gamma  PPP2R2B - protein phosphatase 2, regulatory subunit b, beta  PPP1R7 - protein phosphatase 1, regulatory subunit 7  ZNF526 - zinc finger protein 526  PPP1R8 - protein phosphatase 1, regulatory subunit 8  FOXP4 - forkhead box p4  PPP2CA - protein phosphatase 2, catalytic subunit, alpha isozyme  PPP1R10 - protein phosphatase 1, regulatory subunit 10  DUSP14 - dual specificity phosphatase 14  RMI2 - recq mediated genome instability 2  PPP1R2 - protein phosphatase 1, regulatory (inhibitor) subunit 2  ZNF83 - zinc finger protein 83  ESCO1 - establishment of sister chromatid cohesion n-acetyltransferase 1  KANSL3 - kat8 regulatory nsl complex subunit 3  IWS1 - iws1 homolog (s. cerevisiae)  PRNP - prion protein  ATF7 - activating transcription factor 7  AMFR - autocrine motility factor receptor, e3 ubiquitin protein ligase  DNAJC3 - dnaj (hsp40) homolog, subfamily c, member 3  PRKRIR - protein-kinase, interferon-inducible double stranded rna dependent inhibitor, repressor of (p58 repressor)  PAPD7 - pap associated domain containing 7  MAP2K5 - mitogen-activated protein kinase kinase 5  MAP2K7 - mitogen-activated protein kinase kinase 7  PIM2 - pim-2 oncogene  EIF2AK2 - eukaryotic translation initiation factor 2-alpha kinase 2  ZNF692 - zinc finger protein 692  MAP2K1 - mitogen-activated protein kinase kinase 1  EIF4E3 - eukaryotic translation initiation factor 4e family member 3  MAP2K2 - mitogen-activated protein kinase kinase 2  MAPK10 - mitogen-activated protein kinase 10  MAPK9 - mitogen-activated protein kinase 9  MAPK11 - mitogen-activated protein kinase 11  MAPK8 - mitogen-activated protein kinase 8  MAPK7 - mitogen-activated protein kinase 7  GCN1L1 - gcn1 general control of amino-acid synthesis 1-like 1 (yeast)  MAPK3 - mitogen-activated protein kinase 3  CCNI - cyclin i  MAPK1 - mitogen-activated protein kinase 1  OSBPL7 - oxysterol binding protein-like 7  OSBPL8 - oxysterol binding protein-like 8  ADIRF - adipogenesis regulatory factor  NDC1 - ndc1 transmembrane nucleoporin  PRKD1 - protein kinase d1  ANXA3 - annexin a3  CCDC88A - coiled-coil domain containing 88a  PRKCH - protein kinase c, eta  TLK2 - tousled-like kinase 2  PRKCI - protein kinase c, iota  CCDC85B - coiled-coil domain containing 85b  ANXA2 - annexin a2  PRKCE - protein kinase c, epsilon  PRKCB - protein kinase c, beta  PRKCD - protein kinase c, delta  RBM22 - rna binding motif protein 22  PRKAR2B - protein kinase, camp-dependent, regulatory, type ii, beta  PRKCA - protein kinase c, alpha  PRKAR1B - protein kinase, camp-dependent, regulatory, type i, beta  NSUN5 - nop2/sun domain family, member 5  PRKAR1A - protein kinase, camp-dependent, regulatory, type i, alpha  PKIB - protein kinase (camp-dependent, catalytic) inhibitor beta  YEATS2 - yeats domain containing 2  PRKAG1 - protein kinase, amp-activated, gamma 1 non-catalytic subunit  APEX1 - apex nuclease (multifunctional dna repair enzyme) 1  ZNF605 - zinc finger protein 605  RBM23 - rna binding motif protein 23  XIAP - x-linked inhibitor of apoptosis  BIRC3 - baculoviral iap repeat containing 3  BIRC2 - baculoviral iap repeat containing 2  PARN - poly(a)-specific ribonuclease  KLF12 - kruppel-like factor 12  TREX1 - three prime repair exonuclease 1  APLP2 - amyloid beta (a4) precursor-like protein 2  SHQ1 - shq1, h/aca ribonucleoprotein assembly factor  PBX2 - pre-b-cell leukemia homeobox 2  PRMT6 - protein arginine methyltransferase 6  PBX3 - pre-b-cell leukemia homeobox 3  PBX1 - pre-b-cell leukemia homeobox 1  PCBP2 - poly(rc) binding protein 2  PCBD1 - pterin-4 alpha-carbinolamine dehydratase/dimerization cofactor of hepatocyte nuclear factor 1 alpha  APBB2 - amyloid beta (a4) precursor protein-binding, family b, member 2  DCUN1D3 - dcn1, defective in cullin neddylation 1, domain containing 3  SMG8 - smg8 nonsense mediated mrna decay factor  APC - adenomatous polyposis coli  APBB1 - amyloid beta (a4) precursor protein-binding, family b, member 1 (fe65)  CHP1 - calcineurin-like ef-hand protein 1  RIF1 - rap1 interacting factor homolog (yeast)  POLG2 - polymerase (dna directed), gamma 2, accessory subunit  RNF139 - ring finger protein 139  PDCD10 - programmed cell death 10  KIAA1551 - kiaa1551  PEBP1 - phosphatidylethanolamine binding protein 1  PA2G4 - proliferation-associated 2g4, 38kda  RPRD1A - regulation of nuclear pre-mrna domain containing 1a  ZHX1 - zinc fingers and homeoboxes 1  ARF4 - adp-ribosylation factor 4  PAFAH1B1 - platelet-activating factor acetylhydrolase 1b, regulatory subunit 1 (45kda)  SBNO1 - strawberry notch homolog 1 (drosophila)  APOD - apolipoprotein d  IRAK3 - interleukin-1 receptor-associated kinase 3  PRDX1 - peroxiredoxin 1  AKAP13 - a kinase (prka) anchor protein 13  SETD5 - set domain containing 5  APP - amyloid beta (a4) precursor protein  BBS7 - bardet-biedl syndrome 7  DDX20 - dead (asp-glu-ala-asp) box polypeptide 20  PAK2 - p21 protein (cdc42/rac)-activated kinase 2  PAK3 - p21 protein (cdc42/rac)-activated kinase 3  PAM - peptidylglycine alpha-amidating monooxygenase  ARNTL - aryl hydrocarbon receptor nuclear translocator-like  PDGFA - platelet-derived growth factor alpha polypeptide  OXR1 - oxidation resistance 1  ARNT - aryl hydrocarbon receptor nuclear translocator  CELF3 - cugbp, elav-like family member 3  PDE1B - phosphodiesterase 1b, calmodulin-dependent  MAP4K5 - mitogen-activated protein kinase kinase kinase kinase 5  PDPR - pyruvate dehydrogenase phosphatase regulatory subunit  SUPT16H - suppressor of ty 16 homolog (s. cerevisiae)  PDGFRB - platelet-derived growth factor receptor, beta polypeptide  IFT57 - intraflagellar transport 57 homolog (chlamydomonas)  ARRB2 - arrestin, beta 2  ARRB1 - arrestin, beta 1  PDGFRA - platelet-derived growth factor receptor, alpha polypeptide  PDGFB - platelet-derived growth factor beta polypeptide  SAMD4B - sterile alpha motif domain containing 4b  PDPK1 - 3-phosphoinositide dependent protein kinase-1  FAM107A - family with sequence similarity 107, member a  STRAP - serine/threonine kinase receptor associated protein  RHOA - ras homolog family member a  PSIP1 - pc4 and sfrs1 interacting protein 1  PDK3 - pyruvate dehydrogenase kinase, isozyme 3  MED9 - mediator complex subunit 9  FSTL1 - follistatin-like 1  SLC2A6 - solute carrier family 2 (facilitated glucose transporter), member 6  PECAM1 - platelet/endothelial cell adhesion molecule 1  BAZ2A - bromodomain adjacent to zinc finger domain, 2a  WDR70 - wd repeat domain 70  PCNA - proliferating cell nuclear antigen  CHMP1A - charged multivesicular body protein 1a  JDP2 - jun dimerization protein 2  CDK16 - cyclin-dependent kinase 16  AKIRIN2 - akirin 2  HEATR1 - heat repeat containing 1  CDK17 - cyclin-dependent kinase 17  ASPH - aspartate beta-hydroxylase  LEO1 - leo1, paf1/rna polymerase ii complex component, homolog (s. cerevisiae)  ERLIN2 - er lipid raft associated 2  PDCD2 - programmed cell death 2  PWP1 - pwp1 homolog (s. cerevisiae)  WRAP53 - wd repeat containing, antisense to tp53  SOCS4 - suppressor of cytokine signaling 4  CDC37 - cell division cycle 37  PDE2A - phosphodiesterase 2a, cgmp-stimulated  GLMN - glomulin, fkbp associated protein  THAP1 - thap domain containing, apoptosis associated protein 1  PKIG - protein kinase (camp-dependent, catalytic) inhibitor gamma  ASCL1 - achaete-scute complex homolog 1 (drosophila)  KAT7 - k(lysine) acetyltransferase 7  ELP3 - elongator acetyltransferase complex subunit 3  RGMB - rgm domain family, member b  SBNO2 - strawberry notch homolog 2 (drosophila)  ZNF444 - zinc finger protein 444  ATP2A3 - atpase, ca++ transporting, ubiquitous  ZBTB1 - zinc finger and btb domain containing 1  ZNF365 - zinc finger protein 365  PGAM1 - phosphoglycerate mutase 1 (brain)  PFN2 - profilin 2  RNF180 - ring finger protein 180  FBXW7 - f-box and wd repeat domain containing 7, e3 ubiquitin protein ligase  PGF - placental growth factor  BAHD1 - bromo adjacent homology domain containing 1  DIS3 - dis3 mitotic control homolog (s. cerevisiae)  ZHX2 - zinc fingers and homeoboxes 2  ANKRD6 - ankyrin repeat domain 6  MORC2 - morc family cw-type zinc finger 2  PHF2 - phd finger protein 2  PHF1 - phd finger protein 1  LRRK2 - leucine-rich repeat kinase 2  FOXJ3 - forkhead box j3  RERE - arginine-glutamic acid dipeptide (re) repeats  DNAJC24 - dnaj (hsp40) homolog, subfamily c, member 24  LARP6 - la ribonucleoprotein domain family, member 6  ZFHX3 - zinc finger homeobox 3  SPPL3 - signal peptide peptidase like 3  ATG14 - autophagy related 14  ATP6AP1 - atpase, h+ transporting, lysosomal accessory protein 1  ATP7A - atpase, cu++ transporting, alpha polypeptide  PEX14 - peroxisomal biogenesis factor 14  PPP6R1 - protein phosphatase 6, regulatory subunit 1  ADNP2 - adnp homeobox 2  LMTK2 - lemur tyrosine kinase 2  AEBP2 - ae binding protein 2  PPM1E - protein phosphatase, mg2+/mn2+ dependent, 1e  ZNF507 - zinc finger protein 507  IMPACT - impact rwd domain protein  LGR4 - leucine-rich repeat containing g protein-coupled receptor 4  ATP2B4 - atpase, ca++ transporting, plasma membrane 4  ZNF652 - zinc finger protein 652  RELL2 - relt-like 2  SCAF8 - sr-related ctd-associated factor 8  BAX - bcl2-associated x protein  SMU1 - smu-1 suppressor of mec-8 and unc-52 homolog (c. elegans)  PKD1 - polycystic kidney disease 1 (autosomal dominant)  PKD2 - polycystic kidney disease 2 (autosomal dominant)  PUF60 - poly-u binding splicing factor 60kda  MTF2 - metal response element binding transcription factor 2  BAK1 - bcl2-antagonist/killer 1  NAT10 - n-acetyltransferase 10 (gcn5-related)  BACH1 - btb and cnc homology 1, basic leucine zipper transcription factor 1  BAD - bcl2-associated agonist of cell death  PIN1 - peptidylprolyl cis/trans isomerase, nima-interacting 1  PIK3R2 - phosphoinositide-3-kinase, regulatory subunit 2 (beta)  DPH3 - diphthamide biosynthesis 3  PIK3R1 - phosphoinositide-3-kinase, regulatory subunit 1 (alpha)  PLAT - plasminogen activator, tissue  ASXL2 - additional sex combs like 2 (drosophila)  PLAGL1 - pleiomorphic adenoma gene-like 1  ELP2 - elongator acetyltransferase complex subunit 2  PEF1 - penta-ef-hand domain containing 1  ATRX - alpha thalassemia/mental retardation syndrome x-linked  PKNOX1 - pbx/knotted 1 homeobox 1  CASC3 - cancer susceptibility candidate 3  PKM - pyruvate kinase, muscle  OGFOD1 - 2-oxoglutarate and iron-dependent oxygenase domain containing 1  GABARAPL2 - gaba(a) receptor-associated protein-like 2  EXOSC8 - exosome component 8  SERPINI1 - serpin peptidase inhibitor, clade i (neuroserpin), member 1  TMEM100 - transmembrane protein 100  PHF10 - phd finger protein 10  RABL3 - rab, member of ras oncogene family-like 3  SERPINE2 - serpin peptidase inhibitor, clade e (nexin, plasminogen activator inhibitor type 1), member 2  SERPINB8 - serpin peptidase inhibitor, clade b (ovalbumin), member 8  U2AF2 - u2 small nuclear rna auxiliary factor 2  PSPC1 - paraspeckle component 1  NUDT15 - nudix (nucleoside diphosphate linked moiety x)-type motif 15  BCR - breakpoint cluster region  STK38 - serine/threonine kinase 38  BCL7A - b-cell cll/lymphoma 7a  MRGBP - mrg/morf4l binding protein  BCL9 - b-cell cll/lymphoma 9  PIK3CB - phosphatidylinositol-4,5-bisphosphate 3-kinase, catalytic subunit beta  RPUSD3 - rna pseudouridylate synthase domain containing 3  PIK3CA - phosphatidylinositol-4,5-bisphosphate 3-kinase, catalytic subunit alpha  PPP6R3 - protein phosphatase 6, regulatory subunit 3  BRF2 - brf2, rna polymerase iii transcription initiation factor 50 kda subunit  PIM1 - pim-1 oncogene  BCL2 - b-cell cll/lymphoma 2  ECD - ecdysoneless homolog (drosophila)  CCND1 - cyclin d1  PARK7 - parkinson protein 7  PNKP - polynucleotide kinase 3'-phosphatase  C3orf33 - chromosome 3 open reading frame 33  TRIM27 - tripartite motif containing 27  RFC5 - replication factor c (activator 1) 5, 36.5kda  RFC2 - replication factor c (activator 1) 2, 40kda  SAP18 - sin3a-associated protein, 18kda  RFC3 - replication factor c (activator 1) 3, 38kda  TSHZ2 - teashirt zinc finger homeobox 2  RFXAP - regulatory factor x-associated protein  SEMA4C - sema domain, immunoglobulin domain (ig), transmembrane domain (tm) and short cytoplasmic domain, (semaphorin) 4c  BRAF - v-raf murine sarcoma viral oncogene homolog b  RFX5 - regulatory factor x, 5 (influences hla class ii expression)  WHSC1L1 - wolf-hirschhorn syndrome candidate 1-like 1  EIF1B - eukaryotic translation initiation factor 1b  RFX1 - regulatory factor x, 1 (influences hla class ii expression)  APBB3 - amyloid beta (a4) precursor protein-binding, family b, member 3  RELA - v-rel avian reticuloendotheliosis viral oncogene homolog a  BCOR - bcl6 corepressor  ZFP36L2 - zfp36 ring finger protein-like 2  PAK4 - p21 protein (cdc42/rac)-activated kinase 4  ZFP36L1 - zfp36 ring finger protein-like 1  RDX - radixin  RET - ret proto-oncogene  DUSP15 - dual specificity phosphatase 15  DPF2 - d4, zinc and double phd fingers family 2  ALKBH5 - alkb, alkylation repair homolog 5 (e. coli)  SH3RF3 - sh3 domain containing ring finger 3  UPF1 - upf1 regulator of nonsense transcripts homolog (yeast)  NSUN2 - nop2/sun rna methyltransferase family, member 2  CC2D1A - coiled-coil and c2 domain containing 1a  RLF - rearranged l-myc fusion  RING1 - ring finger protein 1  CNPY2 - canopy fgf signaling regulator 2  BMI1 - bmi1 polycomb ring finger oncogene  PCGF3 - polycomb group ring finger 3  RGS4 - regulator of g-protein signaling 4  RGS3 - regulator of g-protein signaling 3  BMPR1A - bone morphogenetic protein receptor, type ia  BMPR2 - bone morphogenetic protein receptor, type ii (serine/threonine kinase)  BMPR1B - bone morphogenetic protein receptor, type ib  GON4L - gon-4-like (c. elegans)  BMP7 - bone morphogenetic protein 7  ABCA7 - atp-binding cassette, sub-family a (abc1), member 7  PSMD14 - proteasome (prosome, macropain) 26s subunit, non-atpase, 14  FLOT1 - flotillin 1  RNF19B - ring finger protein 19b  CTDSPL - ctd (carboxy-terminal domain, rna polymerase ii, polypeptide a) small phosphatase-like  BCAS3 - breast carcinoma amplified sequence 3  ABCE1 - atp-binding cassette, sub-family e (oabp), member 1  LDLRAD4 - low density lipoprotein receptor class a domain containing 4  PTTG1IP - pituitary tumor-transforming 1 interacting protein  UHMK1 - u2af homology motif (uhm) kinase 1  AHI1 - abelson helper integration site 1  RNF4 - ring finger protein 4  BRD2 - bromodomain containing 2  RNF2 - ring finger protein 2  HNRNPR - heterogeneous nuclear ribonucleoprotein r  ZNF280D - zinc finger protein 280d  GATAD2A - gata zinc finger domain containing 2a  RNF6 - ring finger protein (c3h2c3 type) 6  ROBO1 - roundabout, axon guidance receptor, homolog 1 (drosophila)  ROCK1 - rho-associated, coiled-coil containing protein kinase 1  RNF111 - ring finger protein 111  TSPO - translocator protein (18kda)  SPRY1 - sprouty homolog 1, antagonist of fgf signaling (drosophila)  SPRY2 - sprouty homolog 2 (drosophila)  TET2 - tet methylcytosine dioxygenase 2  MED18 - mediator complex subunit 18  DENND4A - denn/madd domain containing 4a  MBTD1 - mbt domain containing 1  SF3B4 - splicing factor 3b, subunit 4, 49kda  RAMP3 - receptor (g protein-coupled) activity modifying protein 3  RAMP1 - receptor (g protein-coupled) activity modifying protein 1  AKAP8 - a kinase (prka) anchor protein 8  ZMPSTE24 - zinc metallopeptidase ste24  C5AR1 - complement component 5a receptor 1  STUB1 - stip1 homology and u-box containing protein 1, e3 ubiquitin protein ligase  TRIM44 - tripartite motif containing 44  TAB1 - tgf-beta activated kinase 1/map3k7 binding protein 1  PPIE - peptidylprolyl isomerase e (cyclophilin e)  CAPN2 - calpain 2, (m/ii) large subunit  MCRS1 - microspherule protein 1  ZER1 - zyg-11 related, cell cycle regulator  NRDE2 - nrde-2, necessary for rna interference, domain containing  CAMLG - calcium modulating ligand  TIMM17A - translocase of inner mitochondrial membrane 17 homolog a (yeast)  C1D - c1d nuclear receptor corepressor  RPL11 - ribosomal protein l11  PTCD3 - pentatricopeptide repeat domain 3  CASP8 - caspase 8, apoptosis-related cysteine peptidase  CASP9 - caspase 9, apoptosis-related cysteine peptidase  PIBF1 - progesterone immunomodulatory binding factor 1  SLC30A9 - solute carrier family 30 (zinc transporter), member 9  CASP2 - caspase 2, apoptosis-related cysteine peptidase  CASP3 - caspase 3, apoptosis-related cysteine peptidase  MAD2L2 - mad2 mitotic arrest deficient-like 2 (yeast)  PID1 - phosphotyrosine interaction domain containing 1  RPA2 - replication protein a2, 32kda  PHIP - pleckstrin homology domain interacting protein  RPS4X - ribosomal protein s4, x-linked  PPP2R3C - protein phosphatase 2, regulatory subunit b'', gamma  NRARP - notch-regulated ankyrin repeat protein  ZNF787 - zinc finger protein 787  RMND1 - required for meiotic nuclear division 1 homolog (s. cerevisiae)  LAMTOR1 - late endosomal/lysosomal adaptor, mapk and mtor activator 1  TRIM38 - tripartite motif containing 38  TADA3 - transcriptional adaptor 3  CALR - calreticulin  ZNF792 - zinc finger protein 792  CALM2 - calmodulin 2 (phosphorylase kinase, delta)  WTIP - wilms tumor 1 interacting protein  NCOA2 - nuclear receptor coactivator 2  SYNCRIP - synaptotagmin binding, cytoplasmic rna interacting protein  STK25 - serine/threonine kinase 25  CREB3 - camp responsive element binding protein 3  CRTAP - cartilage associated protein  CITED2 - cbp/p300-interacting transactivator, with glu/asp-rich carboxy-terminal domain, 2  CCT6A - chaperonin containing tcp1, subunit 6a (zeta 1)  CCNT2 - cyclin t2  PINX1 - pin2/terf1 interacting, telomerase inhibitor 1  CLN6 - ceroid-lipofuscinosis, neuronal 6, late infantile, variant  CCND3 - cyclin d3  CCNT1 - cyclin t1  CCNH - cyclin h  HMG20A - high mobility group 20a  CCNG2 - cyclin g2  BANP - btg3 associated nuclear protein  TIPIN - timeless interacting protein  DLC1 - deleted in liver cancer 1  ACER2 - alkaline ceramidase 2  TRNAU1AP - trna selenocysteine 1 associated protein 1  RPS6KA1 - ribosomal protein s6 kinase, 90kda, polypeptide 1  PARP16 - poly (adp-ribose) polymerase family, member 16  RPS6KB1 - ribosomal protein s6 kinase, 70kda, polypeptide 1  RPS6KB2 - ribosomal protein s6 kinase, 70kda, polypeptide 2  CBFA2T3 - core-binding factor, runt domain, alpha subunit 2; translocated to, 3  CBFB - core-binding factor, beta subunit  CBL - cbl proto-oncogene, e3 ubiquitin protein ligase  RAPGEF3 - rap guanine nucleotide exchange factor (gef) 3  SERPINH1 - serpin peptidase inhibitor, clade h (heat shock protein 47), member 1, (collagen binding protein 1)  PIAS3 - protein inhibitor of activated stat, 3  CAV2 - caveolin 2  RXRB - retinoid x receptor, beta  CAV1 - caveolin 1, caveolae protein, 22kda  RBM14 - rna binding motif protein 14  CCNC - cyclin c  CCND2 - cyclin d2  TRMT10C - trna methyltransferase 10 homolog c (s. cerevisiae)  SPON1 - spondin 1, extracellular matrix protein  PRMT5 - protein arginine methyltransferase 5  RRAS - related ras viral (r-ras) oncogene homolog  CHTF8 - ctf8, chromosome transmission fidelity factor 8 homolog (s. cerevisiae)  CDK11B - cyclin-dependent kinase 11b  CDC5L - cell division cycle 5-like  MXD4 - max dimerization protein 4  HTRA1 - htra serine peptidase 1  POLR3F - polymerase (rna) iii (dna directed) polypeptide f, 39 kda  PSAP - prosaposin  CDH2 - cadherin 2, type 1, n-cadherin (neuronal)  HEXIM1 - hexamethylene bis-acetamide inducible 1  ERLIN1 - er lipid raft associated 1  SPIN2A - spindlin family, member 2a  NCOA7 - nuclear receptor coactivator 7  ANKIB1 - ankyrin repeat and ibr domain containing 1  POMT1 - protein-o-mannosyltransferase 1  PSEN1 - presenilin 1  TOLLIP - toll interacting protein  USP16 - ubiquitin specific peptidase 16  SRSF12 - serine/arginine-rich splicing factor 12  PSMA1 - proteasome (prosome, macropain) subunit, alpha type, 1  NLE1 - notchless homolog 1 (drosophila)  PSMA2 - proteasome (prosome, macropain) subunit, alpha type, 2  CD81 - cd81 molecule  CAMKK2 - calcium/calmodulin-dependent protein kinase kinase 2, beta  PSMB1 - proteasome (prosome, macropain) subunit, beta type, 1  PSMA5 - proteasome (prosome, macropain) subunit, alpha type, 5  PSMA3 - proteasome (prosome, macropain) subunit, alpha type, 3  KHDRBS3 - kh domain containing, rna binding, signal transduction associated 3  RIPPLY2 - ripply transcriptional repressor 2  PSMB10 - proteasome (prosome, macropain) subunit, beta type, 10  CD47 - cd47 molecule  CD44 - cd44 molecule (indian blood group)  SPINT2 - serine peptidase inhibitor, kunitz type, 2  TAF6L - taf6-like rna polymerase ii, p300/cbp-associated factor (pcaf)-associated factor, 65kda  FAM173B - family with sequence similarity 173, member b  PSMC6 - proteasome (prosome, macropain) 26s subunit, atpase, 6  PSMD1 - proteasome (prosome, macropain) 26s subunit, non-atpase, 1  PSMC4 - proteasome (prosome, macropain) 26s subunit, atpase, 4  SOX18 - sry (sex determining region y)-box 18  POLR3G - polymerase (rna) iii (dna directed) polypeptide g (32kd)  PSMC2 - proteasome (prosome, macropain) 26s subunit, atpase, 2  PSMD9 - proteasome (prosome, macropain) 26s subunit, non-atpase, 9  CD34 - cd34 molecule  PSMD10 - proteasome (prosome, macropain) 26s subunit, non-atpase, 10  PSMD7 - proteasome (prosome, macropain) 26s subunit, non-atpase, 7  DNAJC10 - dnaj (hsp40) homolog, subfamily c, member 10  PSMD8 - proteasome (prosome, macropain) 26s subunit, non-atpase, 8  PSMD5 - proteasome (prosome, macropain) 26s subunit, non-atpase, 5  TERF2IP - telomeric repeat binding factor 2, interacting protein  PSMD2 - proteasome (prosome, macropain) 26s subunit, non-atpase, 2  SLC38A2 - solute carrier family 38, member 2  PSMD12 - proteasome (prosome, macropain) 26s subunit, non-atpase, 12  PSMD13 - proteasome (prosome, macropain) 26s subunit, non-atpase, 13  ATG7 - autophagy related 7  PRRC1 - proline-rich coiled-coil 1  PTBP1 - polypyrimidine tract binding protein 1  PTEN - phosphatase and tensin homolog  LAMTOR5 - late endosomal/lysosomal adaptor, mapk and mtor activator 5  JMY - junction mediating and regulatory protein, p53 cofactor  ANP32B - acidic (leucine-rich) nuclear phosphoprotein 32 family, member b  MYBBP1A - myb binding protein (p160) 1a  FBLN5 - fibulin 5  PTGIS - prostaglandin i2 (prostacyclin) synthase  CIB1 - calcium and integrin binding 1 (calmyrin)  DDX17 - dead (asp-glu-ala-asp) box helicase 17  KAT5 - k(lysine) acetyltransferase 5  PTK2 - protein tyrosine kinase 2  ZFP69 - zfp69 zinc finger protein  IPO7 - importin 7  CEBPG - ccaat/enhancer binding protein (c/ebp), gamma  CDK5 - cyclin-dependent kinase 5  CDK4 - cyclin-dependent kinase 4  TWF1 - twinfilin actin-binding protein 1  PTN - pleiotrophin  CDK9 - cyclin-dependent kinase 9  CCT7 - chaperonin containing tcp1, subunit 7 (eta)  CDK8 - cyclin-dependent kinase 8  LIN54 - lin-54 homolog (c. elegans)  CDK7 - cyclin-dependent kinase 7  CDKN1C - cyclin-dependent kinase inhibitor 1c (p57, kip2)  SPOPL - speckle-type poz protein-like  CDKN1B - cyclin-dependent kinase inhibitor 1b (p27, kip1)  CCT2 - chaperonin containing tcp1, subunit 2 (beta)  CCT4 - chaperonin containing tcp1, subunit 4 (delta)  PCBP3 - poly(rc) binding protein 3  PTPN3 - protein tyrosine phosphatase, non-receptor type 3  CDKN2D - cyclin-dependent kinase inhibitor 2d (p19, inhibits cdk4)  PTPN2 - protein tyrosine phosphatase, non-receptor type 2  PTPN1 - protein tyrosine phosphatase, non-receptor type 1  PTPN11 - protein tyrosine phosphatase, non-receptor type 11  HTATIP2 - hiv-1 tat interactive protein 2, 30kda  ARL6IP5 - adp-ribosylation-like factor 6 interacting protein 5  PTPN13 - protein tyrosine phosphatase, non-receptor type 13 (apo-1/cd95 (fas)-associated phosphatase)  CDK2 - cyclin-dependent kinase 2  ARFGEF1 - adp-ribosylation factor guanine nucleotide-exchange factor 1 (brefeldin a-inhibited)  PURA - purine-rich element binding protein a  RRN3 - rrn3 rna polymerase i transcription factor homolog (s. cerevisiae)  PDP1 - pyruvate dehyrogenase phosphatase catalytic subunit 1  PURB - purine-rich element binding protein b  GTPBP2 - gtp binding protein 2  CHRNB2 - cholinergic receptor, nicotinic, beta 2 (neuronal)  NUDT16 - nudix (nucleoside diphosphate linked moiety x)-type motif 16  PTPRR - protein tyrosine phosphatase, receptor type, r  ZMYND11 - zinc finger, mynd-type containing 11  PTPRK - protein tyrosine phosphatase, receptor type, k  SRSF10 - serine/arginine-rich splicing factor 10  PTPRN - protein tyrosine phosphatase, receptor type, n  RELL1 - relt-like 1  JMJD8 - jumonji domain containing 8  PLK2 - polo-like kinase 2  AHCYL1 - adenosylhomocysteinase-like 1  PEX2 - peroxisomal biogenesis factor 2  NUP50 - nucleoporin 50kda  MPHOSPH8 - m-phase phosphoprotein 8  KDM5B - lysine (k)-specific demethylase 5b  PXN - paxillin  GIPC1 - gipc pdz domain containing family, member 1  MAP4K2 - mitogen-activated protein kinase kinase kinase kinase 2  CHD4 - chromodomain helicase dna binding protein 4  CHD2 - chromodomain helicase dna binding protein 2  CHD3 - chromodomain helicase dna binding protein 3  CHD1 - chromodomain helicase dna binding protein 1  MTERFD2 - mterf domain containing 2  FRS2 - fibroblast growth factor receptor substrate 2  RAB1A - rab1a, member ras oncogene family  QARS - glutaminyl-trna synthetase  MYPOP - myb-related transcription factor, partner of profilin  RAD21 - rad21 homolog (s. pombe)  RAD17 - rad17 homolog (s. pombe)  SEC24A - sec24 family, member a (s. cerevisiae)  PARP14 - poly (adp-ribose) polymerase family, member 14  PAF1 - paf1, rna polymerase ii associated factor, homolog (s. cerevisiae)  ZNF181 - zinc finger protein 181  RAC1 - ras-related c3 botulinum toxin substrate 1 (rho family, small gtp binding protein rac1)  ZNF268 - zinc finger protein 268  INO80 - ino80 complex subunit  ZNF513 - zinc finger protein 513  NDFIP2 - nedd4 family interacting protein 2  RALB - v-ral simian leukemia viral oncogene homolog b  RC3H2 - ring finger and ccch-type domains 2  GMEB1 - glucocorticoid modulatory element binding protein 1  RANBP2 - ran binding protein 2  CCT8 - chaperonin containing tcp1, subunit 8 (theta)  RAP1A - rap1a, member of ras oncogene family  CLTC - clathrin, heavy chain (hc)  MIER2 - mesoderm induction early response 1, family member 2  FAM35A - family with sequence similarity 35, member a  RARG - retinoic acid receptor, gamma  RARB - retinoic acid receptor, beta  DLL4 - delta-like 4 (drosophila)  RASA1 - ras p21 protein activator (gtpase activating protein) 1  NECAB2 - n-terminal ef-hand calcium binding protein 2  RAP1B - rap1b, member of ras oncogene family  CELF1 - cugbp, elav-like family member 1  KHDRBS1 - kh domain containing, rna binding, signal transduction associated 1  CLU - clusterin  GNL3L - guanine nucleotide binding protein-like 3 (nucleolar)-like  TAF8 - taf8 rna polymerase ii, tata box binding protein (tbp)-associated factor, 43kda  CLK1 - cdc-like kinase 1  MAGEL2 - mage-like 2  CELF2 - cugbp, elav-like family member 2  RAP2B - rap2b, member of ras oncogene family  RAP2A - rap2a, member of ras oncogene family  CLK2 - cdc-like kinase 2  DDX49 - dead (asp-glu-ala-asp) box polypeptide 49  CLK3 - cdc-like kinase 3  CTCF - ccctc-binding factor (zinc finger protein)  RBL2 - retinoblastoma-like 2 (p130)  RAI1 - retinoic acid induced 1  RBBP7 - retinoblastoma binding protein 7  RBBP8 - retinoblastoma binding protein 8  MAP3K2 - mitogen-activated protein kinase kinase kinase 2  RBM3 - rna binding motif (rnp1, rrm) protein 3  RBM4 - rna binding motif protein 4  RB1 - retinoblastoma 1  ARID4A - at rich interactive domain 4a (rbp1-like)  ANKRD54 - ankyrin repeat domain 54  RBBP6 - retinoblastoma binding protein 6  KDM5A - lysine (k)-specific demethylase 5a  NUP35 - nucleoporin 35kda  PUS7 - pseudouridylate synthase 7 homolog (s. cerevisiae)  ERCC8 - excision repair cross-complementing rodent repair deficiency, complementation group 8  EXOSC4 - exosome component 4  PTGES3 - prostaglandin e synthase 3 (cytosolic)  NFAT5 - nuclear factor of activated t-cells 5, tonicity-responsive  CKS1B - cdc28 protein kinase regulatory subunit 1b  CIRBP - cold inducible rna binding protein  DHX29 - deah (asp-glu-ala-his) box polypeptide 29  CHUK - conserved helix-loop-helix ubiquitous kinase  CERS1 - ceramide synthase 1  ZMYND8 - zinc finger, mynd-type containing 8  ANKRA2 - ankyrin repeat, family a (rfxank-like), 2  RBAK - rb-associated krab zinc finger  NFATC2IP - nuclear factor of activated t-cells, cytoplasmic, calcineurin-dependent 2 interacting protein  SPTBN4 - spectrin, beta, non-erythrocytic 4  FBXO18 - f-box protein, helicase, 18  PATZ1 - poz (btb) and at hook containing zinc finger 1  SPOCK1 - sparc/osteonectin, cwcv and kazal-like domains proteoglycan (testican) 1  METTL14 - methyltransferase like 14  SPP1 - secreted phosphoprotein 1  CORO1C - coronin, actin binding protein, 1c  SSBP2 - single-stranded dna binding protein 2  KPNA6 - karyopherin alpha 6 (importin alpha 7)  FIZ1 - flt3-interacting zinc finger 1  CIRH1A - cirrhosis, autosomal recessive 1a (cirhin)  COX7A2 - cytochrome c oxidase subunit viia polypeptide 2 (liver)  MIER1 - mesoderm induction early response 1, transcriptional regulator  PPP1R15B - protein phosphatase 1, regulatory subunit 15b  CWC22 - cwc22 spliceosome-associated protein homolog (s. cerevisiae)  ZNF382 - zinc finger protein 382  ZNF705A - zinc finger protein 705a  SP3 - sp3 transcription factor  SP4 - sp4 transcription factor  SP1 - sp1 transcription factor  SP2 - sp2 transcription factor  ZNF410 - zinc finger protein 410  ARL2BP - adp-ribosylation factor-like 2 binding protein  DDAH2 - dimethylarginine dimethylaminohydrolase 2  SOX4 - sry (sex determining region y)-box 4  TIPRL - tip41, tor signaling pathway regulator-like (s. cerevisiae)  GTPBP4 - gtp binding protein 4  WBP2 - ww domain binding protein 2  ORC3 - origin recognition complex, subunit 3  FBXL20 - f-box and leucine-rich repeat protein 20  MAGI3 - membrane associated guanylate kinase, ww and pdz domain containing 3  SON - son dna binding protein  HES4 - hairy and enhancer of split 4 (drosophila)  LEMD3 - lem domain containing 3  GATAD1 - gata zinc finger domain containing 1  CREBL2 - camp responsive element binding protein-like 2  ATF6B - activating transcription factor 6 beta  SOAT1 - sterol o-acyltransferase 1  CREBBP - creb binding protein  ATF2 - activating transcription factor 2  CREB1 - camp responsive element binding protein 1  DDX58 - dead (asp-glu-ala-asp) box polypeptide 58  SNX1 - sorting nexin 1  CRABP2 - cellular retinoic acid binding protein 2  RPRD1B - regulation of nuclear pre-mrna domain containing 1b  PLK3 - polo-like kinase 3  PPP1R16A - protein phosphatase 1, regulatory subunit 16a  SNAPC4 - small nuclear rna activating complex, polypeptide 4, 190kda  SNCA - synuclein, alpha (non a4 component of amyloid precursor)  SNRNP70 - small nuclear ribonucleoprotein 70kda (u1)  SNRPA - small nuclear ribonucleoprotein polypeptide a  ZBED5 - zinc finger, bed-type containing 5  CREBZF - creb/atf bzip transcription factor  SUMO2 - small ubiquitin-like modifier 2  EID1 - ep300 interacting inhibitor of differentiation 1  TP53INP2 - tumor protein p53 inducible nuclear protein 2  PTBP2 - polypyrimidine tract binding protein 2  SMARCE1 - swi/snf related, matrix associated, actin dependent regulator of chromatin, subfamily e, member 1  RBM17 - rna binding motif protein 17  SMO - smoothened, frizzled family receptor  SMPD1 - sphingomyelin phosphodiesterase 1, acid lysosomal  CNTN1 - contactin 1  SMARCB1 - swi/snf related, matrix associated, actin dependent regulator of chromatin, subfamily b, member 1  SMARCC1 - swi/snf related, matrix associated, actin dependent regulator of chromatin, subfamily c, member 1  HLTF - helicase-like transcription factor  SNX6 - sorting nexin 6  SMARCA4 - swi/snf related, matrix associated, actin dependent regulator of chromatin, subfamily a, member 4  SSBP3 - single stranded dna binding protein 3  SMARCD2 - swi/snf related, matrix associated, actin dependent regulator of chromatin, subfamily d, member 2  ATCAY - ataxia, cerebellar, cayman type  SMARCD3 - swi/snf related, matrix associated, actin dependent regulator of chromatin, subfamily d, member 3  SMARCC2 - swi/snf related, matrix associated, actin dependent regulator of chromatin, subfamily c, member 2  SMARCD1 - swi/snf related, matrix associated, actin dependent regulator of chromatin, subfamily d, member 1  WIZ - widely interspaced zinc finger motifs  RBM25 - rna binding motif protein 25  HSPBP1 - hspa (heat shock 70kda) binding protein, cytoplasmic cochaperone 1  SMARCA1 - swi/snf related, matrix associated, actin dependent regulator of chromatin, subfamily a, member 1  SMARCA2 - swi/snf related, matrix associated, actin dependent regulator of chromatin, subfamily a, member 2  SGK3 - serum/glucocorticoid regulated kinase family, member 3  CACTIN - cactin, spliceosome c complex subunit  KMT2C - lysine (k)-specific methyltransferase 2c  RAB38 - rab38, member ras oncogene family  ZNF462 - zinc finger protein 462  PPIL4 - peptidylprolyl isomerase (cyclophilin)-like 4  TFIP11 - tuftelin interacting protein 11  SENP2 - sumo1/sentrin/smt3 specific peptidase 2  CSTB - cystatin b (stefin b)  CST3 - cystatin c  SHC2 - shc (src homology 2 domain containing) transforming protein 2  ZNF318 - zinc finger protein 318  PRPF6 - pre-mrna processing factor 6  KDM2B - lysine (k)-specific demethylase 2b  SUPT6H - suppressor of ty 6 homolog (s. cerevisiae)  CTGF - connective tissue growth factor  SUPV3L1 - suppressor of var1, 3-like 1 (s. cerevisiae)  CBY1 - chibby homolog 1 (drosophila)  CACNG7 - calcium channel, voltage-dependent, gamma subunit 7  DSTYK - dual serine/threonine and tyrosine protein kinase  CTBP1 - c-terminal binding protein 1  SUPT4H1 - suppressor of ty 4 homolog 1 (s. cerevisiae)  CTBP2 - c-terminal binding protein 2  SUPT5H - suppressor of ty 5 homolog (s. cerevisiae)  MFSD8 - major facilitator superfamily domain containing 8  RAB3GAP2 - rab3 gtpase activating protein subunit 2 (non-catalytic)  CTNNB1 - catenin (cadherin-associated protein), beta 1, 88kda  STX5 - syntaxin 5  SAV1 - salvador homolog 1 (drosophila)  CTSD - cathepsin d  CUX1 - cut-like homeobox 1  NIF3L1 - nif3 ngg1 interacting factor 3-like 1 (s. cerevisiae)  NEK4 - nima-related kinase 4  STK3 - serine/threonine kinase 3  MPV17L2 - mpv17 mitochondrial membrane protein-like 2  PCGF1 - polycomb group ring finger 1  NPNT - nephronectin  FKBP8 - fk506 binding protein 8, 38kda  GPBP1L1 - gc-rich promoter binding protein 1-like 1  MTCH1 - mitochondrial carrier 1  SUV420H2 - suppressor of variegation 4-20 homolog 2 (drosophila)  CYLD - cylindromatosis (turban tumor syndrome)  TGIF2 - tgfb-induced factor homeobox 2  STK4 - serine/threonine kinase 4  CNEP1R1 - ctd nuclear envelope phosphatase 1 regulatory subunit 1  BCL6B - b-cell cll/lymphoma 6, member b  ST5 - suppression of tumorigenicity 5  CRKL - v-crk avian sarcoma virus ct10 oncogene homolog-like  CRK - v-crk avian sarcoma virus ct10 oncogene homolog  EEFSEC - eukaryotic elongation factor, selenocysteine-trna-specific  CELF6 - cugbp, elav-like family member 6  NIPBL - nipped-b homolog (drosophila)  STAT2 - signal transducer and activator of transcription 2, 113kda  CRY1 - cryptochrome 1 (photolyase-like)  STAT1 - signal transducer and activator of transcription 1, 91kda  RAB26 - rab26, member ras oncogene family  STAT3 - signal transducer and activator of transcription 3 (acute-phase response factor)  CRY2 - cryptochrome 2 (photolyase-like)  STAT5A - signal transducer and activator of transcription 5a  ASF1A - anti-silencing function 1a histone chaperone  STAT6 - signal transducer and activator of transcription 6, interleukin-4 induced  PARM1 - prostate androgen-regulated mucin-like protein 1  BRMS1 - breast cancer metastasis suppressor 1  BOD1L1 - biorientation of chromosomes in cell division 1-like 1  CRYM - crystallin, mu  ZNF496 - zinc finger protein 496  SS18 - synovial sarcoma translocation, chromosome 18  PHF5A - phd finger protein 5a  CSF1R - colony stimulating factor 1 receptor  SNX33 - sorting nexin 33  GPATCH3 - g patch domain containing 3  CSF1 - colony stimulating factor 1 (macrophage)  FBXO7 - f-box protein 7  SRPK2 - srsf protein kinase 2  SRPK1 - srsf protein kinase 1  TAB3 - tgf-beta activated kinase 1/map3k7 binding protein 3  ZNF335 - zinc finger protein 335  BAMBI - bmp and activin membrane-bound inhibitor  CSK - c-src tyrosine kinase  HAVCR2 - hepatitis a virus cellular receptor 2  CSNK1A1 - casein kinase 1, alpha 1  ZC3H10 - zinc finger ccch-type containing 10  CSNK1D - casein kinase 1, delta  CSNK2A2 - casein kinase 2, alpha prime polypeptide  SRI - sorcin  SRC - v-src avian sarcoma (schmidt-ruppin a-2) viral oncogene homolog  TNFAIP8 - tumor necrosis factor, alpha-induced protein 8  CSNK2A1 - casein kinase 2, alpha 1 polypeptide  DNAJB5 - dnaj (hsp40) homolog, subfamily b, member 5  SREBF2 - sterol regulatory element binding transcription factor 2  ARIH1 - ariadne homolog, ubiquitin-conjugating enzyme e2 binding protein, 1 (drosophila)  UBE2O - ubiquitin-conjugating enzyme e2o  NSUN3 - nop2/sun domain family, member 3  SRP9 - signal recognition particle 9kda  PRDX5 - peroxiredoxin 5  BACE2 - beta-site app-cleaving enzyme 2  RNF169 - ring finger protein 169  DHX9 - deah (asp-glu-ala-his) box helicase 9  TRAPPC2 - trafficking protein particle complex 2  KANK2 - kn motif and ankyrin repeat domains 2  DDX5 - dead (asp-glu-ala-asp) box helicase 5  SEC13 - sec13 homolog (s. cerevisiae)  DDX3X - dead (asp-glu-ala-asp) box helicase 3, x-linked  PNKD - paroxysmal nonkinesigenic dyskinesia  DDX1 - dead (asp-glu-ala-asp) box helicase 1  DPH6 - diphthamine biosynthesis 6  SIN3A - sin3 transcription regulator family member a  SDCBP - syndecan binding protein (syntenin)  SET - set nuclear oncogene  WWTR1 - ww domain containing transcription regulator 1  SETMAR - set domain and mariner transposase fusion gene  DDB1 - damage-specific dna binding protein 1, 127kda  MAP2K4 - mitogen-activated protein kinase kinase 4  ZNF304 - zinc finger protein 304  RCN3 - reticulocalbin 3, ef-hand calcium binding domain  ZNF286A - zinc finger protein 286a  GEMIN5 - gem (nuclear organelle) associated protein 5  PBXIP1 - pre-b-cell leukemia homeobox interacting protein 1  NOL11 - nucleolar protein 11  DBP - d site of albumin promoter (albumin d-box) binding protein  NELFB - negative elongation factor complex member b  POT1 - protection of telomeres 1  AHCTF1 - at hook containing transcription factor 1  CLK4 - cdc-like kinase 4  CNOT10 - ccr4-not transcription complex, subunit 10  ZBTB38 - zinc finger and btb domain containing 38  RNF19A - ring finger protein 19a, e3 ubiquitin protein ligase  RCHY1 - ring finger and chy zinc finger domain containing 1, e3 ubiquitin protein ligase  GATAD2B - gata zinc finger domain containing 2b  DFFA - dna fragmentation factor, 45kda, alpha polypeptide  ZNF551 - zinc finger protein 551  KCTD13 - potassium channel tetramerization domain containing 13  ZNF473 - zinc finger protein 473  BIRC6 - baculoviral iap repeat containing 6  YTHDF3 - yth domain family, member 3  PLEKHG5 - pleckstrin homology domain containing, family g (with rhogef domain) member 5  WDR18 - wd repeat domain 18  CNOT6 - ccr4-not transcription complex, subunit 6  LTN1 - listerin e3 ubiquitin protein ligase 1  ATXN2 - ataxin 2  ZNF451 - zinc finger protein 451  ZNF622 - zinc finger protein 622  SCAF4 - sr-related ctd-associated factor 4  SS18L1 - synovial sarcoma translocation gene on chromosome 18-like 1  CHD5 - chromodomain helicase dna binding protein 5  SCO1 - sco1 cytochrome c oxidase assembly protein  ARID1B - at rich interactive domain 1b (swi1-like)  TMEM98 - transmembrane protein 98  MED30 - mediator complex subunit 30  MKL2 - mkl/myocardin-like 2  HEG1 - heart development protein with egf-like domains 1  NSMF - nmda receptor synaptonuclear signaling and neuronal migration factor  PYGO2 - pygopus homolog 2 (drosophila)  DAPK3 - death-associated protein kinase 3  DAXX - death-domain associated protein  ANGEL2 - angel homolog 2 (drosophila)  C2CD3 - c2 calcium-dependent domain containing 3  ZZZ3 - zinc finger, zz-type containing 3  KIDINS220 - kinase d-interacting substrate, 220kda  HINFP - histone h4 transcription factor  DAG1 - dystroglycan 1 (dystrophin-associated glycoprotein 1)  MTA3 - metastasis associated 1 family, member 3  DGKQ - diacylglycerol kinase, theta 110kda  ZNF608 - zinc finger protein 608  CLIP3 - cap-gly domain containing linker protein 3  DAPK1 - death-associated protein kinase 1  MAVS - mitochondrial antiviral signaling protein  IBTK - inhibitor of bruton agammaglobulinemia tyrosine kinase  DAP - death-associated protein  TIPARP - tcdd-inducible poly(adp-ribose) polymerase  CYP51A1 - cytochrome p450, family 51, subfamily a, polypeptide 1  HACE1 - hect domain and ankyrin repeat containing e3 ubiquitin protein ligase 1  SARS - seryl-trna synthetase  SATB1 - satb homeobox 1  NGDN - neuroguidin, eif4e binding protein  RPTOR - regulatory associated protein of mtor, complex 1  SAFB - scaffold attachment factor b  SH2B1 - sh2b adaptor protein 1  SALL2 - sal-like 2 (drosophila)  SERBP1 - serpine1 mrna binding protein 1  EPC2 - enhancer of polycomb homolog 2 (drosophila)  DRD2 - dopamine receptor d2  PHF19 - phd finger protein 19  SLC9A1 - solute carrier family 9, subfamily a (nhe1, cation proton antiporter 1), member 1  NKD2 - naked cuticle homolog 2 (drosophila)  IRF2BP1 - interferon regulatory factor 2 binding protein 1  SLC8A1 - solute carrier family 8 (sodium/calcium exchanger), member 1  TRAF3IP1 - tnf receptor-associated factor 3 interacting protein 1  CNOT6L - ccr4-not transcription complex, subunit 6-like  EAF1 - ell associated factor 1  TAOK1 - tao kinase 1  SLC8A2 - solute carrier family 8 (sodium/calcium exchanger), member 2  PDP2 - pyruvate dehyrogenase phosphatase catalytic subunit 2  ATN1 - atrophin 1  DPH2 - dph2 homolog (s. cerevisiae)  ARRDC3 - arrestin domain containing 3  DPH1 - diphthamide biosynthesis 1  DOM3Z - dom-3 homolog z (c. elegans)  SEMA6A - sema domain, transmembrane domain (tm), and cytoplasmic domain, (semaphorin) 6a  C10orf137 - chromosome 10 open reading frame 137  DR1 - down-regulator of transcription 1, tbp-binding (negative cofactor 2)  DUSP8 - dual specificity phosphatase 8  TNKS1BP1 - tankyrase 1 binding protein 1, 182kda  MAPK15 - mitogen-activated protein kinase 15  SKI - v-ski avian sarcoma viral oncogene homolog  SKIL - ski-like oncogene  DUSP5 - dual specificity phosphatase 5  UNK - unkempt homolog (drosophila)  DUSP6 - dual specificity phosphatase 6  SKP1 - s-phase kinase-associated protein 1  DUSP7 - dual specificity phosphatase 7  DYRK1A - dual-specificity tyrosine-(y)-phosphorylation regulated kinase 1a  MKL1 - megakaryoblastic leukemia (translocation) 1  CHTOP - chromatin target of prmt1  GGA1 - golgi-associated, gamma adaptin ear containing, arf binding protein 1  DVL2 - dishevelled segment polarity protein 2  DVL3 - dishevelled segment polarity protein 3  WDR48 - wd repeat domain 48  GIGYF2 - grb10 interacting gyf protein 2  USP36 - ubiquitin specific peptidase 36  FNIP2 - folliculin interacting protein 2  DOCK7 - dedicator of cytokinesis 7  HOMEZ - homeobox and leucine zipper encoding  EBF4 - early b-cell factor 4  ANKRD17 - ankyrin repeat domain 17  ZFHX2 - zinc finger homeobox 2  HBEGF - heparin-binding egf-like growth factor  KIAA1462 - kiaa1462  LSM14A - lsm14a, scd6 homolog a (s. cerevisiae)  DTX1 - deltex homolog 1 (drosophila)  DKC1 - dyskeratosis congenita 1, dyskerin  DLG3 - discs, large homolog 3 (drosophila)  DLG4 - discs, large homolog 4 (drosophila)  PNPT1 - polyribonucleotide nucleotidyltransferase 1  DLG1 - discs, large homolog 1 (drosophila)  SHOX2 - short stature homeobox 2  FBXW8 - f-box and wd repeat domain containing 8  SH3RF1 - sh3 domain containing ring finger 1  DHCR24 - 24-dehydrocholesterol reductase  SIAH2 - siah e3 ubiquitin protein ligase 2  POGK - pogo transposable element with krab domain  B3GAT3 - beta-1,3-glucuronyltransferase 3 (glucuronosyltransferase i)  DHFR - dihydrofolate reductase  RICTOR - rptor independent companion of mtor, complex 2  PHF12 - phd finger protein 12  FBXL5 - f-box and leucine-rich repeat protein 5  NQO1 - nad(p)h dehydrogenase, quinone 1  FBXO2 - f-box protein 2  CALCOCO1 - calcium binding and coiled-coil domain 1  ZBTB4 - zinc finger and btb domain containing 4  SFPQ - splicing factor proline/glutamine-rich  SFRP1 - secreted frizzled-related protein 1  SFRP2 - secreted frizzled-related protein 2  SFRP4 - secreted frizzled-related protein 4  SRSF1 - serine/arginine-rich splicing factor 1  SRSF2 - serine/arginine-rich splicing factor 2  DNM2 - dynamin 2  GMEB2 - glucocorticoid modulatory element binding protein 2  SRSF3 - serine/arginine-rich splicing factor 3  DNMT1 - dna (cytosine-5-)-methyltransferase 1  SPAG8 - sperm associated antigen 8  SRSF4 - serine/arginine-rich splicing factor 4  TRDMT1 - trna aspartic acid methyltransferase 1  SRSF5 - serine/arginine-rich splicing factor 5  DNMT3A - dna (cytosine-5-)-methyltransferase 3 alpha  SRSF6 - serine/arginine-rich splicing factor 6  SRSF7 - serine/arginine-rich splicing factor 7  EIF2AK4 - eukaryotic translation initiation factor 2 alpha kinase 4  SFSWAP - splicing factor, suppressor of white-apricot homolog (drosophila)  ALS2 - amyotrophic lateral sclerosis 2 (juvenile)  TRA2B - transformer 2 beta homolog (drosophila)  CHD8 - chromodomain helicase dna binding protein 8  DOCK3 - dedicator of cytokinesis 3  TNRC6C - trinucleotide repeat containing 6c  SGK1 - serum/glucocorticoid regulated kinase 1  NOC2L - nucleolar complex associated 2 homolog (s. cerevisiae)  SGTA - small glutamine-rich tetratricopeptide repeat (tpr)-containing, alpha  ZNF317 - zinc finger protein 317  USP37 - ubiquitin specific peptidase 37  SMARCAD1 - swi/snf-related, matrix-associated actin-dependent regulator of chromatin, subfamily a, containing dead/h box 1  MEIS3 - meis homeobox 3  NCBP2 - nuclear cap binding protein subunit 2, 20kda  MARK2 - map/microtubule affinity-regulating kinase 2  MAPRE3 - microtubule-associated protein, rp/eb family, member 3  TSC1 - tuberous sclerosis 1  ELK1 - elk1, member of ets oncogene family  SPIRE1 - spire actin nucleation factor 1  NAF1 - nuclear assembly factor 1 ribonucleoprotein  ELK3 - elk3, ets-domain protein (srf accessory protein 2)  ELK4 - elk4, ets-domain protein (srf accessory protein 1)  SNW1 - snw domain containing 1  ENSA - endosulfine alpha  SCAP - srebf chaperone  WRNIP1 - werner helicase interacting protein 1  EPAS1 - endothelial pas domain protein 1  DPH7 - diphthamide biosynthesis 7  EP300 - e1a binding protein p300  HABP4 - hyaluronan binding protein 4  ATF6 - activating transcription factor 6  SRRD - srr1 domain containing  UBQLN4 - ubiquilin 4  MOB1B - mob kinase activator 1b  ENO1 - enolase 1, (alpha)  RAB3GAP1 - rab3 gtpase activating protein subunit 1 (catalytic)  EIF4A2 - eukaryotic translation initiation factor 4a2  CELF4 - cugbp, elav-like family member 4  EIF4E - eukaryotic translation initiation factor 4e  EGR2 - early growth response 2  CCT5 - chaperonin containing tcp1, subunit 5 (epsilon)  EGR3 - early growth response 3  TTF1 - transcription termination factor, rna polymerase i  ZC3HAV1 - zinc finger ccch-type, antiviral 1  EIF2S1 - eukaryotic translation initiation factor 2, subunit 1 alpha, 35kda  EIF2B1 - eukaryotic translation initiation factor 2b, subunit 1 alpha, 26kda  TRIM32 - tripartite motif containing 32  SPHK2 - sphingosine kinase 2  EIF2S3 - eukaryotic translation initiation factor 2, subunit 3 gamma, 52kda  SERPINB1 - serpin peptidase inhibitor, clade b (ovalbumin), member 1  KDM2A - lysine (k)-specific demethylase 2a  ELAVL1 - elav (embryonic lethal, abnormal vision, drosophila)-like 1 (hu antigen r)  ZNF326 - zinc finger protein 326  ELF1 - e74-like factor 1 (ets domain transcription factor)  STRADA - ste20-related kinase adaptor alpha  ELAVL4 - elav (embryonic lethal, abnormal vision, drosophila)-like 4  ELF2 - e74-like factor 2 (ets domain transcription factor)  TSG101 - tumor susceptibility gene 101  EIF4G2 - eukaryotic translation initiation factor 4 gamma, 2  TCF25 - transcription factor 25 (basic helix-loop-helix)  TSC2 - tuberous sclerosis 2  EFNA1 - ephrin-a1  CNOT1 - ccr4-not transcription complex, subunit 1  EEF2 - eukaryotic translation elongation factor 2  EXOSC7 - exosome component 7  CTNNBIP1 - catenin, beta interacting protein 1  SPEN - spen homolog, transcriptional regulator (drosophila)  RNF149 - ring finger protein 149  KLHDC10 - kelch domain containing 10  EEF1A1 - eukaryotic translation elongation factor 1 alpha 1  EEF1A2 - eukaryotic translation elongation factor 1 alpha 2  BBX - bobby sox homolog (drosophila)  TP53BP1 - tumor protein p53 binding protein 1  PHC1 - polyhomeotic homolog 1 (drosophila)  PHC2 - polyhomeotic homolog 2 (drosophila)  EGR1 - early growth response 1  ZNF292 - zinc finger protein 292  EGFR - epidermal growth factor receptor  MEGF8 - multiple egf-like-domains 8  CELSR2 - cadherin, egf lag seven-pass g-type receptor 2  KDM1A - lysine (k)-specific demethylase 1a  ATXN7L3 - ataxin 7-like 3  UNC13A - unc-13 homolog a (c. elegans)  EFNA5 - ephrin-a5  HNRNPLL - heterogeneous nuclear ribonucleoprotein l-like  NCOA6 - nuclear receptor coactivator 6  CCT3 - chaperonin containing tcp1, subunit 3 (gamma)  E2F6 - e2f transcription factor 6  SMG1 - smg1 phosphatidylinositol 3-kinase-related kinase  E4F1 - e4f transcription factor 1  ZHX3 - zinc fingers and homeoboxes 3  TNIK - traf2 and nck interacting kinase  E2F4 - e2f transcription factor 4, p107/p130-binding  E2F5 - e2f transcription factor 5, p130-binding  ENY2 - enhancer of yellow 2 homolog (drosophila)  C11orf30 - chromosome 11 open reading frame 30  RRP1B - ribosomal rna processing 1b  EDNRB - endothelin receptor type b  MYCBP2 - myc binding protein 2, e3 ubiquitin protein ligase  TRAF1 - tnf receptor-associated factor 1  LPAR1 - lysophosphatidic acid receptor 1  HSP90B1 - heat shock protein 90kda beta (grp94), member 1  S1PR1 - sphingosine-1-phosphate receptor 1  TRAF3 - tnf receptor-associated factor 3  PMEPA1 - prostate transmembrane protein, androgen induced 1  TRAF2 - tnf receptor-associated factor 2  NR2C1 - nuclear receptor subfamily 2, group c, member 1  ZBTB47 - zinc finger and btb domain containing 47  SETX - senataxin  NR2C2 - nuclear receptor subfamily 2, group c, member 2  TPR - translocated promoter region, nuclear basket protein  ZNF609 - zinc finger protein 609  WAPAL - wings apart-like homolog (drosophila)  LXN - latexin  ERC1 - elks/rab6-interacting/cast family member 1  PPRC1 - peroxisome proliferator-activated receptor gamma, coactivator-related 1  NAT14 - n-acetyltransferase 14 (gcn5-related, putative)  KDM4C - lysine (k)-specific demethylase 4c  ZNF423 - zinc finger protein 423  REXO4 - rex4, rna exonuclease 4 homolog (s. cerevisiae)  BOD1 - biorientation of chromosomes in cell division 1  CAMK1D - calcium/calmodulin-dependent protein kinase id  TMEM259 - transmembrane protein 259  USF1 - upstream transcription factor 1  NUP107 - nucleoporin 107kda  DERL3 - derlin 3  USP1 - ubiquitin specific peptidase 1  ZBTB43 - zinc finger and btb domain containing 43  PTK2B - protein tyrosine kinase 2 beta  MRPS27 - mitochondrial ribosomal protein s27  ACSL3 - acyl-coa synthetase long-chain family member 3  PITHD1 - pith (c-terminal proteasome-interacting domain of thioredoxin-like) domain containing 1  TNRC6B - trinucleotide repeat containing 6b  BPTF - bromodomain phd finger transcription factor  USP4 - ubiquitin specific peptidase 4 (proto-oncogene)  RNF185 - ring finger protein 185  NR1H2 - nuclear receptor subfamily 1, group h, member 2  TAB2 - tgf-beta activated kinase 1/map3k7 binding protein 2  FBLN1 - fibulin 1  CAMTA2 - calmodulin binding transcription activator 2  ETV3 - ets variant 3  ZNF598 - zinc finger protein 598  ETV5 - ets variant 5  TWSG1 - twisted gastrulation homolog 1 (drosophila)  POGZ - pogo transposable element with znf domain  PLXND1 - plexin d1  ETV6 - ets variant 6  VHL - von hippel-lindau tumor suppressor, e3 ubiquitin protein ligase  VIM - vimentin  KDM6B - lysine (k)-specific demethylase 6b  EZR - ezrin  PHF8 - phd finger protein 8  TBX20 - t-box 20  EWSR1 - ews rna-binding protein 1  TRIM41 - tripartite motif containing 41  PCBP4 - poly(rc) binding protein 4  KCTD1 - potassium channel tetramerization domain containing 1  ANKLE2 - ankyrin repeat and lem domain containing 2  DCUN1D4 - dcn1, defective in cullin neddylation 1, domain containing 4  CREB3L1 - camp responsive element binding protein 3-like 1  ACKR3 - atypical chemokine receptor 3  UVRAG - uv radiation resistance associated  ZC3H3 - zinc finger ccch-type containing 3  EYA3 - eyes absent homolog 3 (drosophila)  EZH1 - enhancer of zeste homolog 1 (drosophila)  GRAMD4 - gram domain containing 4  CCNL1 - cyclin l1  CIC - capicua transcriptional repressor  F2R - coagulation factor ii (thrombin) receptor  VCP - valosin containing protein  F3 - coagulation factor iii (thromboplastin, tissue factor)  WDR43 - wd repeat domain 43  MAPK8IP3 - mitogen-activated protein kinase 8 interacting protein 3  ZNF575 - zinc finger protein 575  ERF - ets2 repressor factor  ERCC4 - excision repair cross-complementing rodent repair deficiency, complementation group 4  NUP205 - nucleoporin 205kda  YTHDC1 - yth domain containing 1  GGA3 - golgi-associated, gamma adaptin ear containing, arf binding protein 3  ELMSAN1 - elm2 and myb/sant-like domain containing 1  THAP11 - thap domain containing 11  EMC10 - er membrane protein complex subunit 10  ERN1 - endoplasmic reticulum to nucleus signaling 1  ZNF248 - zinc finger protein 248  UBB - ubiquitin b  PPP1R37 - protein phosphatase 1, regulatory subunit 37  GPD1L - glycerol-3-phosphate dehydrogenase 1-like  ARRDC4 - arrestin domain containing 4  LARP4B - la ribonucleoprotein domain family, member 4b  RCOR1 - rest corepressor 1  TULP3 - tubby like protein 3  HIRA - histone cell cycle regulator  ZNFX1 - zinc finger, nfx1-type containing 1  TTC5 - tetratricopeptide repeat domain 5  ETV1 - ets variant 1  ETS1 - v-ets avian erythroblastosis virus e26 oncogene homolog 1  ATG4B - autophagy related 4b, cysteine peptidase  SLC39A10 - solute carrier family 39 (zinc transporter), member 10  ZMIZ1 - zinc finger, miz-type containing 1  ETF1 - eukaryotic translation termination factor 1  CYFIP1 - cytoplasmic fmr1 interacting protein 1  ARL6IP1 - adp-ribosylation factor-like 6 interacting protein 1  DMTN - dematin actin binding protein  UBP1 - upstream binding protein 1 (lbp-1a)  SUMO1 - small ubiquitin-like modifier 1  UCHL1 - ubiquitin carboxyl-terminal esterase l1 (ubiquitin thiolesterase)  FAM168A - family with sequence similarity 168, member a  UBTF - upstream binding transcription factor, rna polymerase i  ZNF830 - zinc finger protein 830  PELI1 - pellino e3 ubiquitin protein ligase 1  CHURC1 - churchill domain containing 1  SMURF1 - smad specific e3 ubiquitin protein ligase 1  ZC3H4 - zinc finger ccch-type containing 4  JMJD6 - jumonji domain containing 6  MYADM - myeloid-associated differentiation marker  UBE2I - ubiquitin-conjugating enzyme e2i  EPRS - glutamyl-prolyl-trna synthetase  UBE2V2 - ubiquitin-conjugating enzyme e2 variant 2  ERCC2 - excision repair cross-complementing rodent repair deficiency, complementation group 2  UBE2L3 - ubiquitin-conjugating enzyme e2l 3  NUP210 - nucleoporin 210kda  UBE2N - ubiquitin-conjugating enzyme e2n  UBE2V1 - ubiquitin-conjugating enzyme e2 variant 1  VPS72 - vacuolar protein sorting 72 homolog (s. cerevisiae)  PSENEN - presenilin enhancer 2 homolog (c. elegans)  FMR1 - fragile x mental retardation 1  TCOF1 - treacher collins-franceschetti syndrome 1  MGA - mga, max dimerization protein  TCP1 - t-complex 1  FNTA - farnesyltransferase, caax box, alpha  WWC3 - wwc family member 3  ZEB1 - zinc finger e-box binding homeobox 1  FLT1 - fms-related tyrosine kinase 1  TCF12 - transcription factor 12  ZNF354A - zinc finger protein 354a  TCF20 - transcription factor 20 (ar1)  CAMTA1 - calmodulin binding transcription activator 1  PDS5A - pds5, regulator of cohesion maintenance, homolog a (s. cerevisiae)  TMEM106A - transmembrane protein 106a  FLI1 - fli-1 proto-oncogene, ets transcription factor  FLNA - filamin a, alpha  PLCB1 - phospholipase c, beta 1 (phosphoinositide-specific)  FOXS1 - forkhead box s1  PHLPP1 - ph domain and leucine rich repeat protein phosphatase 1  FOXO3 - forkhead box o3  HDAC8 - histone deacetylase 8  LARP4 - la ribonucleoprotein domain family, member 4  ASH1L - ash1 (absent, small, or homeotic)-like (drosophila)  SIN3B - sin3 transcription regulator family member b  DCP1A - decapping mrna 1a  NACC1 - nucleus accumbens associated 1, ben and btb (poz) domain containing  HR - hair growth associated  MED8 - mediator complex subunit 8  CAND1 - cullin-associated and neddylation-dissociated 1  FKBP1A - fk506 binding protein 1a, 12kda  TDG - thymine-dna glycosylase  SMG6 - smg6 nonsense mediated mrna decay factor  PRDX2 - peroxiredoxin 2  FKBP1B - fk506 binding protein 1b, 12.6 kda  EAPP - e2f-associated phosphoprotein  FBXW11 - f-box and wd repeat domain containing 11  VPS11 - vacuolar protein sorting 11 homolog (s. cerevisiae)  KDM3A - lysine (k)-specific demethylase 3a  FAM208A - family with sequence similarity 208, member a  CLEC16A - c-type lectin domain family 16, member a  FHL2 - four and a half lim domains 2  FHIT - fragile histidine triad  PPP1R11 - protein phosphatase 1, regulatory (inhibitor) subunit 11  NUP160 - nucleoporin 160kda  TAF9 - taf9 rna polymerase ii, tata box binding protein (tbp)-associated factor, 32kda  FGFR2 - fibroblast growth factor receptor 2  TAF7 - taf7 rna polymerase ii, tata box binding protein (tbp)-associated factor, 55kda  FGF13 - fibroblast growth factor 13  TAF6 - taf6 rna polymerase ii, tata box binding protein (tbp)-associated factor, 80kda  FGFR1 - fibroblast growth factor receptor 1  TADA2A - transcriptional adaptor 2a  FGF9 - fibroblast growth factor 9  SMCHD1 - structural maintenance of chromosomes flexible hinge domain containing 1  ADAM17 - adam metallopeptidase domain 17  TACC1 - transforming, acidic coiled-coil containing protein 1  FGF1 - fibroblast growth factor 1 (acidic)  TIRAP - toll-interleukin 1 receptor (tir) domain containing adaptor protein  FGF2 - fibroblast growth factor 2 (basic)  USP22 - ubiquitin specific peptidase 22  SASH1 - sam and sh3 domain containing 1  TARBP1 - tar (hiv-1) rna binding protein 1  FER - fer (fps/fes related) tyrosine kinase  MAP3K7 - mitogen-activated protein kinase kinase kinase 7  PDGFC - platelet derived growth factor c  ADNP - activity-dependent neuroprotector homeobox  KMT2E - lysine (k)-specific methyltransferase 2e  MED13L - mediator complex subunit 13-like  NCSTN - nicastrin  FKTN - fukutin  ZNF395 - zinc finger protein 395  TAZ - tafazzin  MYNN - myoneurin  CRTC1 - creb regulated transcription coactivator 1  UFL1 - ufm1-specific ligase 1  TCF3 - transcription factor 3  PUM2 - pumilio homolog 2 (drosophila)  TCEB3 - transcription elongation factor b (siii), polypeptide 3 (110kda, elongin a)  DMAP1 - dna methyltransferase 1 associated protein 1  TCF4 - transcription factor 4  LARP1 - la ribonucleoprotein domain family, member 1  NKRF - nfkb repressing factor  EFEMP1 - egf containing fibulin-like extracellular matrix protein 1  ZNF629 - zinc finger protein 629  FBN1 - fibrillin 1  LANCL2 - lanc lantibiotic synthetase component c-like 2 (bacterial)  TCEA2 - transcription elongation factor a (sii), 2  TCEA1 - transcription elongation factor a (sii), 1  ERBB2IP - erbb2 interacting protein  TIMP1 - timp metallopeptidase inhibitor 1  CAMK2N2 - calcium/calmodulin-dependent protein kinase ii inhibitor 2  TIMP2 - timp metallopeptidase inhibitor 2  TIMP3 - timp metallopeptidase inhibitor 3  TIMP4 - timp metallopeptidase inhibitor 4  ITGB3BP - integrin beta 3 binding protein (beta3-endonexin)  SYAP1 - synapse associated protein 1  ORMDL1 - orm1-like 1 (s. cerevisiae)  RYBP - ring1 and yy1 binding protein  MTOR - mechanistic target of rapamycin (serine/threonine kinase)  PAXBP1 - pax3 and pax7 binding protein 1  TLE1 - transducin-like enhancer of split 1 (e(sp1) homolog, drosophila)  TLE3 - transducin-like enhancer of split 3 (e(sp1) homolog, drosophila)  TLE4 - transducin-like enhancer of split 4 (e(sp1) homolog, drosophila)  NDNL2 - necdin-like 2  ATP13A2 - atpase type 13a2  TLR3 - toll-like receptor 3  EXOSC2 - exosome component 2  SIRT1 - sirtuin 1  MKX - mohawk homeobox  TMF1 - tata element modulatory factor 1  TMSB4X - thymosin beta 4, x-linked  CBX6 - chromobox homolog 6  ACTR8 - arp8 actin-related protein 8 homolog (yeast)  HEY1 - hairy/enhancer-of-split related with yrpw motif 1  TMPO - thymopoietin  CBX5 - chromobox homolog 5  BRD4 - bromodomain containing 4  TNFAIP1 - tumor necrosis factor, alpha-induced protein 1 (endothelial)  BCL9L - b-cell cll/lymphoma 9-like  TARDBP - tar dna binding protein  TNFRSF1A - tumor necrosis factor receptor superfamily, member 1a  TNFRSF1B - tumor necrosis factor receptor superfamily, member 1b  SF3B3 - splicing factor 3b, subunit 3, 130kda  TMBIM6 - transmembrane bax inhibitor motif containing 6  PRKCDBP - protein kinase c, delta binding protein  EIF4ENIF1 - eukaryotic translation initiation factor 4e nuclear import factor 1  TEK - tek tyrosine kinase, endothelial  NUP188 - nucleoporin 188kda  SUZ12 - suz12 polycomb repressive complex 2 subunit  TEF - thyrotrophic embryonic factor  POFUT1 - protein o-fucosyltransferase 1  RCOR2 - rest corepressor 2  TFAM - transcription factor a, mitochondrial  DIABLO - diablo, iap-binding mitochondrial protein  INPP5E - inositol polyphosphate-5-phosphatase, 72 kda  TERF1 - telomeric repeat binding factor (nima-interacting) 1  TERF2 - telomeric repeat binding factor 2  TESK1 - testis-specific kinase 1  TFDP1 - transcription factor dp-1  NR2F2 - nuclear receptor subfamily 2, group f, member 2  TFE3 - transcription factor binding to ighm enhancer 3  CBX7 - chromobox homolog 7  BCCIP - brca2 and cdkn1a interacting protein  ISCU - iron-sulfur cluster assembly enzyme  NR2F1 - nuclear receptor subfamily 2, group f, member 1  TFCP2 - transcription factor cp2  TGFA - transforming growth factor, alpha  ZNF740 - zinc finger protein 740  FNIP1 - folliculin interacting protein 1  HEY2 - hairy/enhancer-of-split related with yrpw motif 2  TGFBR1 - transforming growth factor, beta receptor 1  YLPM1 - ylp motif containing 1  TGFB2 - transforming growth factor, beta 2  RBFOX2 - rna binding protein, fox-1 homolog (c. elegans) 2  TGFB3 - transforming growth factor, beta 3  TGFB1 - transforming growth factor, beta 1  TGFB1I1 - transforming growth factor beta 1 induced transcript 1  MAPK8IP2 - mitogen-activated protein kinase 8 interacting protein 2  TP53INP1 - tumor protein p53 inducible nuclear protein 1  H2AFV - h2a histone family, member v  MEPCE - methylphosphate capping enzyme  RNF20 - ring finger protein 20, e3 ubiquitin protein ligase  TGFBR2 - transforming growth factor, beta receptor ii (70/80kda)  TGFBR3 - transforming growth factor, beta receptor iii  FOSL2 - fos-like antigen 2  FOSB - fbj murine osteosarcoma viral oncogene homolog b  FOS - fbj murine osteosarcoma viral oncogene homolog  THBS1 - thrombospondin 1  CABIN1 - calcineurin binding protein 1  KAT6B - k(lysine) acetyltransferase 6b  TIA1 - tia1 cytotoxic granule-associated rna binding protein  METTL3 - methyltransferase like 3  THY1 - thy-1 cell surface antigen  THRA - thyroid hormone receptor, alpha  MMD - monocyte to macrophage differentiation-associated  ELL - elongation factor rna polymerase ii  ZNF239 - zinc finger protein 239  GFRA2 - gdnf family receptor alpha 2  SYMPK - symplekin  PHC3 - polyhomeotic homolog 3 (drosophila)  FAM192A - family with sequence similarity 192, member a  OBFC1 - oligonucleotide/oligosaccharide-binding fold containing 1  NRIP1 - nuclear receptor interacting protein 1  KAT2A - k(lysine) acetyltransferase 2a  NCOA3 - nuclear receptor coactivator 3  ANP32A - acidic (leucine-rich) nuclear phosphoprotein 32 family, member a  GLA - galactosidase, alpha  WWC2 - ww and c2 domain containing 2  PABPN1 - poly(a) binding protein, nuclear 1  TAF15 - taf15 rna polymerase ii, tata box binding protein (tbp)-associated factor, 68kda  GJA1 - gap junction protein, alpha 1, 43kda  TMTC3 - transmembrane and tetratricopeptide repeat containing 3  AXIN1 - axin 1  TRRAP - transformation/transcription domain-associated protein  PICALM - phosphatidylinositol binding clathrin assembly protein  GGNBP2 - gametogenetin binding protein 2  CAAP1 - caspase activity and apoptosis inhibitor 1  LPCAT1 - lysophosphatidylcholine acyltransferase 1  ZC3H14 - zinc finger ccch-type containing 14  ARID1A - at rich interactive domain 1a (swi-like)  DLL1 - delta-like 1 (drosophila)  SETD6 - set domain containing 6  GLG1 - golgi glycoprotein 1  FZD6 - frizzled family receptor 6  GLE1 - gle1 rna export mediator  ACTR5 - arp5 actin-related protein 5 homolog (yeast)  FZD8 - frizzled family receptor 8  CDC45 - cell division cycle 45  FZD4 - frizzled family receptor 4  GCLC - glutamate-cysteine ligase, catalytic subunit  FZD1 - frizzled family receptor 1  BAP1 - brca1 associated protein-1 (ubiquitin carboxy-terminal hydrolase)  AXIN2 - axin 2  ZNF613 - zinc finger protein 613  NUP85 - nucleoporin 85kda  CDC7 - cell division cycle 7  KDM5C - lysine (k)-specific demethylase 5c  USP9X - ubiquitin specific peptidase 9, x-linked  GNG3 - guanine nucleotide binding protein (g protein), gamma 3  RBM10 - rna binding motif protein 10  GNAQ - guanine nucleotide binding protein (g protein), q polypeptide  GNAI2 - guanine nucleotide binding protein (g protein), alpha inhibiting activity polypeptide 2  PHF17 - phd finger protein 17  GNA12 - guanine nucleotide binding protein (g protein) alpha 12  GMFB - glia maturation factor, beta  TTC21B - tetratricopeptide repeat domain 21b  SLTM - safb-like, transcription modulator  NELFE - negative elongation factor complex member e  EHMT1 - euchromatic histone-lysine n-methyltransferase 1  NR5A1 - nuclear receptor subfamily 5, group a, member 1  FUS - fused in sarcoma  NAA40 - n(alpha)-acetyltransferase 40, natd catalytic subunit, homolog (s. cerevisiae)  RAB7A - rab7a, member ras oncogene family  USP7 - ubiquitin specific peptidase 7 (herpes virus-associated)  XRCC6 - x-ray repair complementing defective repair in chinese hamster cells 6  RNF168 - ring finger protein 168, e3 ubiquitin protein ligase  GABPB1 - ga binding protein transcription factor, beta subunit 1  GABPA - ga binding protein transcription factor, alpha subunit 60kda  CPSF7 - cleavage and polyadenylation specific factor 7, 59kda  FYN - fyn oncogene related to src, fgr, yes  RPAP2 - rna polymerase ii associated protein 2  G6PD - glucose-6-phosphate dehydrogenase  DDX39B - dead (asp-glu-ala-asp) box polypeptide 39b  USP5 - ubiquitin specific peptidase 5 (isopeptidase t)  KMT2D - lysine (k)-specific methyltransferase 2d  SPRED1 - sprouty-related, evh1 domain containing 1  AAAS - achalasia, adrenocortical insufficiency, alacrimia  ZNF419 - zinc finger protein 419  CDK2AP1 - cyclin-dependent kinase 2 associated protein 1  YEATS4 - yeats domain containing 4  SNIP1 - smad nuclear interacting protein 1  ZFPM1 - zinc finger protein, fog family member 1  ZNF212 - zinc finger protein 212  EID2 - ep300 interacting inhibitor of differentiation 2  GBA - glucosidase, beta, acid  ZNF383 - zinc finger protein 383  GATA2 - gata binding protein 2  KAT6A - k(lysine) acetyltransferase 6a  NUP214 - nucleoporin 214kda  NR4A3 - nuclear receptor subfamily 4, group a, member 3  BRD3 - bromodomain containing 3  SHOC2 - soc-2 suppressor of clear homolog (c. elegans)  GAS6 - growth arrest-specific 6  CARF - calcium responsive transcription factor  NCOA4 - nuclear receptor coactivator 4  ZNF408 - zinc finger protein 408  MLLT10 - myeloid/lymphoid or mixed-lineage leukemia (trithorax homolog, drosophila); translocated to, 10  DEDD2 - death effector domain containing 2  FASTKD1 - fast kinase domains 1  DHX36 - deah (asp-glu-ala-his) box polypeptide 36  ZNF189 - zinc finger protein 189  ZSCAN26 - zinc finger and scan domain containing 26  GSTP1 - glutathione s-transferase pi 1  ZNF177 - zinc finger protein 177  GSPT1 - g1 to s phase transition 1  ZNF175 - zinc finger protein 175  GSN - gelsolin  ZNF174 - zinc finger protein 174  ZNF184 - zinc finger protein 184  SSBP4 - single stranded dna binding protein 4  GTF2F1 - general transcription factor iif, polypeptide 1, 74kda  ZNF148 - zinc finger protein 148  BEND5 - ben domain containing 5  ZBTB17 - zinc finger and btb domain containing 17  NEIL1 - nei endonuclease viii-like 1 (e. coli)  GTF2H2 - general transcription factor iih, polypeptide 2, 44kda  TRIM26 - tripartite motif containing 26  GTF2H3 - general transcription factor iih, polypeptide 3, 34kda  VEZF1 - vascular endothelial zinc finger 1  GTF2H1 - general transcription factor iih, polypeptide 1, 62kda  ZNF142 - zinc finger protein 142  MCPH1 - microcephalin 1  ZNF140 - zinc finger protein 140  GTF2B - general transcription factor iib  PCGF2 - polycomb group ring finger 2  MSH6 - muts homolog 6 (e. coli)  ZNF132 - zinc finger protein 132  ZNF131 - zinc finger protein 131  ZNF134 - zinc finger protein 134  ZNF133 - zinc finger protein 133  TBL1XR1 - transducin (beta)-like 1 x-linked receptor 1  GTF2I - general transcription factor iii  GTF2H4 - general transcription factor iih, polypeptide 4, 52kda  PRKRIP1 - prkr interacting protein 1 (il11 inducible)  BRF1 - brf1, rna polymerase iii transcription initiation factor 90 kda subunit  ZNF79 - zinc finger protein 79  ZYG11B - zyg-11 family member b, cell cycle regulator  ZNF70 - zinc finger protein 70  RBM12B - rna binding motif protein 12b  ZNF75D - zinc finger protein 75d  ZNF76 - zinc finger protein 76  H3F3A - h3 histone, family 3a  BRPF1 - bromodomain and phd finger containing, 1  H2AFZ - h2a histone family, member z  SAP130 - sin3a-associated protein, 130kda  FZD5 - frizzled family receptor 5  H2AFX - h2a histone family, member x  DCP2 - decapping mrna 2  MUL1 - mitochondrial e3 ubiquitin protein ligase 1  CXCR4 - chemokine (c-x-c motif) receptor 4  PAPD4 - pap associated domain containing 4  PAX8 - paired box 8  MIER3 - mesoderm induction early response 1, family member 3  CDC73 - cell division cycle 73  NKAP - nfkb activating protein  THAP5 - thap domain containing 5  LRP8 - low density lipoprotein receptor-related protein 8, apolipoprotein e receptor  HCFC1 - host cell factor c1 (vp16-accessory protein)  PRDM2 - pr domain containing 2, with znf domain  MAP3K12 - mitogen-activated protein kinase kinase kinase 12  ZNF226 - zinc finger protein 226  HDAC2 - histone deacetylase 2  HMBOX1 - homeobox containing 1  ZCCHC12 - zinc finger, cchc domain containing 12  HDGF - hepatoma-derived growth factor  ZNF213 - zinc finger protein 213  HTT - huntingtin  HDAC1 - histone deacetylase 1  ZNF205 - zinc finger protein 205  HCLS1 - hematopoietic cell-specific lyn substrate 1  ZNF207 - zinc finger protein 207  XPA - xeroderma pigmentosum, complementation group a  XPC - xeroderma pigmentosum, complementation group c  GOLGA2 - golgin a2  XPO1 - exportin 1 (crm1 homolog, yeast)  GOLGB1 - golgin b1  XRCC1 - x-ray repair complementing defective repair in chinese hamster cells 1  PAGR1 - paxip1 associated glutamate-rich protein 1  ZXDC - zxd family zinc finger c  AAED1 - ahpc/tsa antioxidant enzyme domain containing 1  XBP1 - x-box binding protein 1  ZNF367 - zinc finger protein 367  BHLHE41 - basic helix-loop-helix family, member e41  EIF4H - eukaryotic translation initiation factor 4h  WFS1 - wolfram syndrome 1 (wolframin)  WHSC1 - wolf-hirschhorn syndrome candidate 1  NELFA - negative elongation factor complex member a  VRK2 - vaccinia related kinase 2  ARID2 - at rich interactive domain 2 (arid, rfx-like)  WARS - tryptophanyl-trna synthetase  PAOX - polyamine oxidase (exo-n4-amino)  ZNF35 - zinc finger protein 35  ZKSCAN1 - zinc finger with krab and scan domains 1  FBXL15 - f-box and leucine-rich repeat protein 15  ZNF33B - zinc finger protein 33b  ZNF576 - zinc finger protein 576  ZNF32 - zinc finger protein 32  ZNF45 - zinc finger protein 45  BRCC3 - brca1/brca2-containing complex, subunit 3  MZF1 - myeloid zinc finger 1  ZNF18 - zinc finger protein 18  ZNF16 - zinc finger protein 16  GRB2 - growth factor receptor-bound protein 2  ASXL1 - additional sex combs like 1 (drosophila)  ZNF384 - zinc finger protein 384  TNIP2 - tnfaip3 interacting protein 2  RBM42 - rna binding motif protein 42  ZNF24 - zinc finger protein 24  ZFPL1 - zinc finger protein-like 1  ZFX - zinc finger protein, x-linked  SF1 - splicing factor 1  GRIK2 - glutamate receptor, ionotropic, kainate 2  ZBTB14 - zinc finger and btb domain containing 14  ZNF711 - zinc finger protein 711  ZNF7 - zinc finger protein 7  ZNF8 - zinc finger protein 8  ZNF2 - zinc finger protein 2  ZNF3 - zinc finger protein 3  YES1 - v-yes-1 yamaguchi sarcoma viral oncogene homolog 1  XRCC5 - x-ray repair complementing defective repair in chinese hamster cells 5 (double-strand-break rejoining)  YWHAG - tyrosine 3-monooxygenase/tryptophan 5-monooxygenase activation protein, gamma polypeptide  GRSF1 - g-rich rna sequence binding factor 1  GSK3B - glycogen synthase kinase 3 beta  YWHAZ - tyrosine 3-monooxygenase/tryptophan 5-monooxygenase activation protein, zeta polypeptide  GSK3A - glycogen synthase kinase 3 alpha  YWHAE - tyrosine 3-monooxygenase/tryptophan 5-monooxygenase activation protein, epsilon polypeptide  YWHAB - tyrosine 3-monooxygenase/tryptophan 5-monooxygenase activation protein, beta polypeptide  CCNK - cyclin k  MAPKAP1 - mitogen-activated protein kinase associated protein 1  DNAJB2 - dnaj (hsp40) homolog, subfamily b, member 2  DNAJA1 - dnaj (hsp40) homolog, subfamily a, member 1  HSF2 - heat shock transcription factor 2  TRIM24 - tripartite motif containing 24  TAF1D - tata box binding protein (tbp)-associated factor, rna polymerase i, d, 41kda  CREG1 - cellular repressor of e1a-stimulated genes 1  CHAC1 - chac, cation transport regulator homolog 1 (e. coli)  HSBP1 - heat shock factor binding protein 1  FLCN - folliculin  NRP1 - neuropilin 1  PRMT1 - protein arginine methyltransferase 1  IQGAP1 - iq motif containing gtpase activating protein 1  PRMT2 - protein arginine methyltransferase 2  HESX1 - hesx homeobox 1  SAP30 - sin3a-associated protein, 30kda  PHF23 - phd finger protein 23  DERL1 - derlin 1  METTL16 - methyltransferase like 16  ALKBH1 - alkb, alkylation repair homolog 1 (e. coli)  FTO - fat mass and obesity associated  HPRT1 - hypoxanthine phosphoribosyltransferase 1  SPRED2 - sprouty-related, evh1 domain containing 2  CFLAR - casp8 and fadd-like apoptosis regulator  SYNGAP1 - synaptic ras gtpase activating protein 1  SECISBP2 - secis binding protein 2  MED11 - mediator complex subunit 11  LDB1 - lim domain binding 1  WDR77 - wd repeat domain 77  DSCC1 - dna replication and sister chromatid cohesion 1  KAT2B - k(lysine) acetyltransferase 2b  TSC22D1 - tsc22 domain family, member 1  CRTC2 - creb regulated transcription coactivator 2  DDA1 - det1 and ddb1 associated 1  ZNF259 - zinc finger protein 259  TAX1BP1 - tax1 (human t-cell leukemia virus type i) binding protein 1  CDC123 - cell division cycle 123  CC2D1B - coiled-coil and c2 domain containing 1b  DBNDD1 - dysbindin (dystrobrevin binding protein 1) domain containing 1  NABP2 - nucleic acid binding protein 2  HUS1 - hus1 checkpoint homolog (s. pombe)  EIF2B2 - eukaryotic translation initiation factor 2b, subunit 2 beta, 39kda  THAP8 - thap domain containing 8  CADM4 - cell adhesion molecule 4  BUD31 - bud31 homolog (s. cerevisiae)  ZNF655 - zinc finger protein 655  EIF2B5 - eukaryotic translation initiation factor 2b, subunit 5 epsilon, 82kda  TRAK2 - trafficking protein, kinesin binding 2  HSPD1 - heat shock 60kda protein 1 (chaperonin)  DNAJB1 - dnaj (hsp40) homolog, subfamily b, member 1  ZNF48 - zinc finger protein 48  BCL10 - b-cell cll/lymphoma 10  FUBP3 - far upstream element (fuse) binding protein 3  HSP90AA1 - heat shock protein 90kda alpha (cytosolic), class a member 1  C7orf49 - chromosome 7 open reading frame 49  CDK5R2 - cyclin-dependent kinase 5, regulatory subunit 2 (p39)  BTRC - beta-transducin repeat containing e3 ubiquitin protein ligase  HSPA8 - heat shock 70kda protein 8  EIF4EBP3 - eukaryotic translation initiation factor 4e binding protein 3  WNK1 - wnk lysine deficient protein kinase 1  HMGN1 - high mobility group nucleosome binding domain 1  UBE2Z - ubiquitin-conjugating enzyme e2z  HMGB3 - high mobility group box 3  HMGB2 - high mobility group box 2  LAMTOR3 - late endosomal/lysosomal adaptor, mapk and mtor activator 3  HMGB1 - high mobility group box 1  EIF3C - eukaryotic translation initiation factor 3, subunit c  PHACTR4 - phosphatase and actin regulator 4  BRD9 - bromodomain containing 9  EIF3D - eukaryotic translation initiation factor 3, subunit d  EIF3A - eukaryotic translation initiation factor 3, subunit a  EIF3B - eukaryotic translation initiation factor 3, subunit b  TNKS - tankyrase, trf1-interacting ankyrin-related adp-ribose polymerase  EIF4G3 - eukaryotic translation initiation factor 4 gamma, 3  DAZAP1 - daz associated protein 1  ZBTB10 - zinc finger and btb domain containing 10  AGO1 - argonaute risc catalytic component 1  LATS2 - large tumor suppressor kinase 2  CAPRIN2 - caprin family member 2  ZBTB48 - zinc finger and btb domain containing 48  SRSF9 - serine/arginine-rich splicing factor 9  PEA15 - phosphoprotein enriched in astrocytes 15  NDRG4 - ndrg family member 4  HYAL2 - hyaluronoglucosaminidase 2  ACD - adrenocortical dysplasia homolog (mouse)  GPBP1 - gc-rich promoter binding protein 1  HFE - hemochromatosis  HIP1 - huntingtin interacting protein 1  UBE2K - ubiquitin-conjugating enzyme e2k  HINT1 - histidine triad nucleotide binding protein 1  ELP4 - elongator acetyltransferase complex subunit 4  HIVEP1 - human immunodeficiency virus type i enhancer binding protein 1  UPF3B - upf3 regulator of nonsense transcripts homolog b (yeast)  UPF3A - upf3 regulator of nonsense transcripts homolog a (yeast)  HIF1A - hypoxia inducible factor 1, alpha subunit (basic helix-loop-helix transcription factor)  LPIN3 - lipin 3  TRADD - tnfrsf1a-associated via death domain  SNX3 - sorting nexin 3  MYCBP - myc binding protein  EED - embryonic ectoderm development  URI1 - uri1, prefoldin-like chaperone  RIPK1 - receptor (tnfrsf)-interacting serine-threonine kinase 1  HNRNPU - heterogeneous nuclear ribonucleoprotein u (scaffold attachment factor a)  FBXO22 - f-box protein 22  HNRNPK - heterogeneous nuclear ribonucleoprotein k  HNRNPL - heterogeneous nuclear ribonucleoprotein l  TNFSF13 - tumor necrosis factor (ligand) superfamily, member 13  TNFSF12 - tumor necrosis factor (ligand) superfamily, member 12  FBXO4 - f-box protein 4  TINF2 - terf1 (trf1)-interacting nuclear factor 2  ADAM9 - adam metallopeptidase domain 9  HNRNPA2B1 - heterogeneous nuclear ribonucleoprotein a2/b1  HNRNPA1 - heterogeneous nuclear ribonucleoprotein a1  HNRNPC - heterogeneous nuclear ribonucleoprotein c (c1/c2)  RIPK2 - receptor-interacting serine-threonine kinase 2  HNRNPAB - heterogeneous nuclear ribonucleoprotein a/b  HNRNPF - heterogeneous nuclear ribonucleoprotein f  HNRNPD - heterogeneous nuclear ribonucleoprotein d (au-rich element rna binding protein 1, 37kda)  HNRNPH1 - heterogeneous nuclear ribonucleoprotein h1 (h)  HNRNPH3 - heterogeneous nuclear ribonucleoprotein h3 (2h9)  HNRNPH2 - heterogeneous nuclear ribonucleoprotein h2 (h')  HMGCR - 3-hydroxy-3-methylglutaryl-coa reductase  AIDA - axin interactor, dorsalization associated  ZNF584 - zinc finger protein 584  HMGA1 - high mobility group at-hook 1  FAM129B - family with sequence similarity 129, member b  NR4A1 - nuclear receptor subfamily 4, group a, member 1  PDE12 - phosphodiesterase 12  RFX7 - regulatory factor x, 7  DEPTOR - dep domain containing mtor-interacting protein  HTATSF1 - hiv-1 tat specific factor 1  FOXK2 - forkhead box k2  HAT1 - histone acetyltransferase 1  EIF3K - eukaryotic translation initiation factor 3, subunit k  RPS6KA6 - ribosomal protein s6 kinase, 90kda, polypeptide 6  CRTC3 - creb regulated transcription coactivator 3  RBM15 - rna binding motif protein 15  TNRC6A - trinucleotide repeat containing 6a  ENC1 - ectodermal-neural cortex 1 (with btb domain)  RBMX - rna binding motif protein, x-linked  INHA - inhibin, alpha  PIK3R3 - phosphoinositide-3-kinase, regulatory subunit 3 (gamma)  DESI1 - desumoylating isopeptidase 1  SGSM3 - small g protein signaling modulator 3  PARG - poly (adp-ribose) glycohydrolase  HTRA2 - htra serine peptidase 2  STK39 - serine threonine kinase 39  ING2 - inhibitor of growth family, member 2  PPM1D - protein phosphatase, mg2+/mn2+ dependent, 1d  ZNF574 - zinc finger protein 574  CREB3L2 - camp responsive element binding protein 3-like 2  PCSK1N - proprotein convertase subtilisin/kexin type 1 inhibitor  IPPK - inositol 1,3,4,5,6-pentakisphosphate 2-kinase  UBE2S - ubiquitin-conjugating enzyme e2s  ILF2 - interleukin enhancer binding factor 2  ILF3 - interleukin enhancer binding factor 3, 90kda  PRPF19 - pre-mrna processing factor 19  MAP4K3 - mitogen-activated protein kinase kinase kinase kinase 3  ILK - integrin-linked kinase  CDC14B - cell division cycle 14b  PIAS1 - protein inhibitor of activated stat, 1  SMYD3 - set and mynd domain containing 3  SLCO3A1 - solute carrier organic anion transporter family, member 3a1  SMURF2 - smad specific e3 ubiquitin protein ligase 2  MAPKAPK5 - mitogen-activated protein kinase-activated protein kinase 5  CGGBP1 - cgg triplet repeat binding protein 1  LMO4 - lim domain only 4  CAMK1 - calcium/calmodulin-dependent protein kinase i  CBX4 - chromobox homolog 4  GORASP1 - golgi reassembly stacking protein 1, 65kda  IL6ST - interleukin 6 signal transducer (gp130, oncostatin m receptor)  CSRNP1 - cysteine-serine-rich nuclear protein 1  C5AR2 - complement component 5a receptor 2  SERP1 - stress-associated endoplasmic reticulum protein 1  ZNF25 - zinc finger protein 25  STK16 - serine/threonine kinase 16  CCNY - cyclin y  ITGA1 - integrin, alpha 1  ITGA3 - integrin, alpha 3 (antigen cd49c, alpha 3 subunit of vla-3 receptor)  MADD - map-kinase activating death domain  KHSRP - kh-type splicing regulatory protein  ERLEC1 - endoplasmic reticulum lectin 1  CASK - calcium/calmodulin-dependent serine protein kinase (maguk family)  ITGAV - integrin, alpha v  SUDS3 - suppressor of defective silencing 3 homolog (s. cerevisiae)  CDK10 - cyclin-dependent kinase 10  EIF6 - eukaryotic translation initiation factor 6  SKAP1 - src kinase associated phosphoprotein 1  GZF1 - gdnf-inducible zinc finger protein 1  INSR - insulin receptor  INSM1 - insulinoma-associated 1  CDK13 - cyclin-dependent kinase 13  AKTIP - akt interacting protein  PDCD4 - programmed cell death 4 (neoplastic transformation inhibitor)  RFXANK - regulatory factor x-associated ankyrin-containing protein  INPP5B - inositol polyphosphate-5-phosphatase, 75kda  CSDC2 - cold shock domain containing c2, rna binding  KLF7 - kruppel-like factor 7 (ubiquitous)  IRF2 - interferon regulatory factor 2  IRF3 - interferon regulatory factor 3  IKZF5 - ikaros family zinc finger 5 (pegasus)  RUVBL1 - ruvb-like 1 (e. coli)  IREB2 - iron-responsive element binding protein 2  DIMT1 - dim1 dimethyladenosine transferase 1 homolog (s. cerevisiae)  MED19 - mediator complex subunit 19  ITGA6 - integrin, alpha 6  IRAK2 - interleukin-1 receptor-associated kinase 2  IRAK1 - interleukin-1 receptor-associated kinase 1  CACYBP - calcyclin binding protein  ZSCAN31 - zinc finger and scan domain containing 31  EIF2AK1 - eukaryotic translation initiation factor 2-alpha kinase 1  PAPD5 - pap associated domain containing 5  KIAA1324L - kiaa1324-like  NSD1 - nuclear receptor binding set domain protein 1  RFWD2 - ring finger and wd repeat domain 2, e3 ubiquitin protein ligase  ZBTB11 - zinc finger and btb domain containing 11  RNF25 - ring finger protein 25  MMS19 - mms19 nucleotide excision repair homolog (s. cerevisiae)  DKK3 - dickkopf wnt signaling pathway inhibitor 3  SNX5 - sorting nexin 5  IFNGR1 - interferon gamma receptor 1  ZNF777 - zinc finger protein 777  ID2 - inhibitor of dna binding 2, dominant negative helix-loop-helix protein  BRPF3 - bromodomain and phd finger containing, 3  ID3 - inhibitor of dna binding 3, dominant negative helix-loop-helix protein  STK36 - serine/threonine kinase 36  IRF2BPL - interferon regulatory factor 2 binding protein-like  JAZF1 - jazf zinc finger 1  PLA2G6 - phospholipase a2, group vi (cytosolic, calcium-independent)  FOXK1 - forkhead box k1  BRAT1 - brca1-associated atm activator 1  MAGEF1 - melanoma antigen family f, 1  LTBP4 - latent transforming growth factor beta binding protein 4  ZNF282 - zinc finger protein 282  IGHMBP2 - immunoglobulin mu binding protein 2  CHORDC1 - cysteine and histidine-rich domain (chord) containing 1  RBPJ - recombination signal binding protein for immunoglobulin kappa j region  NSMAF - neutral sphingomyelinase (n-smase) activation associated factor  NCK2 - nck adaptor protein 2  PABPC1 - poly(a) binding protein, cytoplasmic 1  TRUB2 - trub pseudouridine (psi) synthase homolog 2 (e. coli)  SPOP - speckle-type poz protein  C6orf89 - chromosome 6 open reading frame 89  CYFIP2 - cytoplasmic fmr1 interacting protein 2  USP21 - ubiquitin specific peptidase 21  ZBTB9 - zinc finger and btb domain containing 9  ZBTB12 - zinc finger and btb domain containing 12  BCAR3 - breast cancer anti-estrogen resistance 3  NGFRAP1 - nerve growth factor receptor (tnfrsf16) associated protein 1  NPTN - neuroplastin  PHACTR1 - phosphatase and actin regulator 1  IK - ik cytokine, down-regulator of hla ii  JMJD1C - jumonji domain containing 1c  IGBP1 - immunoglobulin (cd79a) binding protein 1  SMARCA5 - swi/snf related, matrix associated, actin dependent regulator of chromatin, subfamily a, member 5  IGF1R - insulin-like growth factor 1 receptor  OGT - o-linked n-acetylglucosamine (glcnac) transferase  PELP1 - proline, glutamate and leucine rich protein 1  RAE1 - rae1 rna export 1 homolog (s. pombe)  LAT - linker for activation of t cells  TSPYL2 - tspy-like 2  IGFBP6 - insulin-like growth factor binding protein 6  SND1 - staphylococcal nuclease and tudor domain containing 1  CUL4B - cullin 4b  CUL2 - cullin 2  PCIF1 - pdx1 c-terminal inhibiting factor 1  CUL3 - cullin 3  ZNF667 - zinc finger protein 667  FOXP1 - forkhead box p1  SUPT3H - suppressor of ty 3 homolog (s. cerevisiae)  BEND6 - ben domain containing 6  ZNF572 - zinc finger protein 572  PCGF5 - polycomb group ring finger 5  WTAP - wilms tumor 1 associated protein  WAC - ww domain containing adaptor with coiled-coil  ZNF414 - zinc finger protein 414  RBM39 - rna binding motif protein 39  PHF21A - phd finger protein 21a  ZNF771 - zinc finger protein 771  LIMK1 - lim domain kinase 1  AKAP12 - a kinase (prka) anchor protein 12  IER2 - immediate early response 2  LIG3 - ligase iii, dna, atp-dependent  NCOR1 - nuclear receptor corepressor 1  LDLR - low density lipoprotein receptor  NCOR2 - nuclear receptor corepressor 2  MTPN - myotrophin  CARTPT - cart prepropeptide  DOT1L - dot1-like histone h3k79 methyltransferase  ABCG1 - atp-binding cassette, sub-family g (white), member 1  EFCAB7 - ef-hand calcium binding domain 7  L3MBTL3 - l(3)mbt-like 3 (drosophila)  ZNF333 - zinc finger protein 333  CIR1 - corepressor interacting with rbpj, 1  SCAND1 - scan domain containing 1  SLX4 - slx4 structure-specific endonuclease subunit  SEC22B - sec22 vesicle trafficking protein homolog b (s. cerevisiae) (gene/pseudogene)  H2AFY - h2a histone family, member y  GTF2IRD1 - gtf2i repeat domain containing 1  FADS1 - fatty acid desaturase 1  GTPBP1 - gtp binding protein 1  ZNF527 - zinc finger protein 527  SPIRE2 - spire actin nucleation factor 2  HOPX - hop homeobox  SOX13 - sry (sex determining region y)-box 13  ZC3H8 - zinc finger ccch-type containing 8  ZNF316 - zinc finger protein 316  LMO1 - lim domain only 1 (rhombotin 1)  IER5 - immediate early response 5  NACC2 - nacc family member 2, ben and btb (poz) domain containing  DNAJC27 - dnaj (hsp40) homolog, subfamily c, member 27  LMNA - lamin a/c  CLOCK - clock circadian regulator  KLF3 - kruppel-like factor 3 (basic)  PRKAG2 - protein kinase, amp-activated, gamma 2 non-catalytic subunit  DDX41 - dead (asp-glu-ala-asp) box polypeptide 41  FXR2 - fragile x mental retardation, autosomal homolog 2  LITAF - lipopolysaccharide-induced tnf factor  TBPL1 - tbp-like 1  YTHDF2 - yth domain family, member 2  ZBTB41 - zinc finger and btb domain containing 41  ZFP62 - zfp62 zinc finger protein  BAG5 - bcl2-associated athanogene 5  TMEM59 - transmembrane protein 59  BAG4 - bcl2-associated athanogene 4  PPME1 - protein phosphatase methylesterase 1  ZGPAT - zinc finger, ccch-type with g patch domain  ACTL6B - actin-like 6b  UBR5 - ubiquitin protein ligase e3 component n-recognin 5  IL27RA - interleukin 27 receptor, alpha  EIF4E2 - eukaryotic translation initiation factor 4e family member 2  MED20 - mediator complex subunit 20  UCHL5 - ubiquitin carboxyl-terminal hydrolase l5  AFAP1L2 - actin filament associated protein 1-like 2  ATG5 - autophagy related 5  ROCK2 - rho-associated, coiled-coil containing protein kinase 2  ATRAID - all-trans retinoic acid-induced differentiation factor  ANGPT4 - angiopoietin 4  MAPK8IP1 - mitogen-activated protein kinase 8 interacting protein 1  CRLF3 - cytokine receptor-like factor 3  NGRN - neugrin, neurite outgrowth associated  TAOK3 - tao kinase 3  GLYR1 - glyoxylate reductase 1 homolog (arabidopsis)  FZR1 - fizzy/cell division cycle 20 related 1 (drosophila)  ZBTB7A - zinc finger and btb domain containing 7a  DPY30 - dpy-30 homolog (c. elegans)  MBTPS2 - membrane-bound transcription factor peptidase, site 2  IRF2BP2 - interferon regulatory factor 2 binding protein 2  GLIS2 - glis family zinc finger 2  MAGED1 - melanoma antigen family d, 1  MED27 - mediator complex subunit 27  MED26 - mediator complex subunit 26  CHD6 - chromodomain helicase dna binding protein 6  MED17 - mediator complex subunit 17  MED23 - mediator complex subunit 23  VPS28 - vacuolar protein sorting 28 homolog (s. cerevisiae)  ZNF580 - zinc finger protein 580  ANTXR1 - anthrax toxin receptor 1  CDYL - chromodomain protein, y-like  GTF2IRD2 - gtf2i repeat domain containing 2  ZNF710 - zinc finger protein 710  KDR - kinase insert domain receptor (a type iii receptor tyrosine kinase)  ING4 - inhibitor of growth family, member 4  EIF2AK3 - eukaryotic translation initiation factor 2-alpha kinase 3  INSIG2 - insulin induced gene 2  ITM2B - integral membrane protein 2b  MAP4K4 - mitogen-activated protein kinase kinase kinase kinase 4  RHBDD1 - rhomboid domain containing 1  RLIM - ring finger protein, lim domain interacting  TRAF7 - tnf receptor-associated factor 7, e3 ubiquitin protein ligase  RECQL5 - recq protein-like 5  KRAS - kirsten rat sarcoma viral oncogene homolog  TNPO1 - transportin 1  TGFBRAP1 - transforming growth factor, beta receptor associated protein 1  ZNF706 - zinc finger protein 706  CIAO1 - cytosolic iron-sulfur protein assembly 1  KPNA2 - karyopherin alpha 2 (rag cohort 1, importin alpha 1)  KPNA1 - karyopherin alpha 1 (importin alpha 5)  CAMKK1 - calcium/calmodulin-dependent protein kinase kinase 1, alpha  WFDC3 - wap four-disulfide core domain 3  SUV420H1 - suppressor of variegation 4-20 homolog 1 (drosophila)  PHF20L1 - phd finger protein 20-like 1  MED10 - mediator complex subunit 10  MED21 - mediator complex subunit 21  KIT - v-kit hardy-zuckerman 4 feline sarcoma viral oncogene homolog  ZFP28 - zfp28 zinc finger protein  POLDIP3 - polymerase (dna-directed), delta interacting protein 3  DCUN1D5 - dcn1, defective in cullin neddylation 1, domain containing 5  SLIT2 - slit homolog 2 (drosophila)  PAIP2 - poly(a) binding protein interacting protein 2  JUP - junction plakoglobin  CRIM1 - cysteine rich transmembrane bmp regulator 1 (chordin-like)  JAK2 - janus kinase 2  ING5 - inhibitor of growth family, member 5  JAK3 - janus kinase 3  SLC25A33 - solute carrier family 25 (pyrimidine nucleotide carrier), member 33  ZNF219 - zinc finger protein 219  DNAJC30 - dnaj (hsp40) homolog, subfamily c, member 30  PHF20 - phd finger protein 20  TRIP12 - thyroid hormone receptor interactor 12  NUDT16L1 - nudix (nucleoside diphosphate linked moiety x)-type motif 16-like 1  KCNK2 - potassium channel, subfamily k, member 2  ZNF397 - zinc finger protein 397  ZNHIT3 - zinc finger, hit-type containing 3  TRIP4 - thyroid hormone receptor interactor 4  HMGN3 - high mobility group nucleosomal binding domain 3  PHF6 - phd finger protein 6  ZNF235 - zinc finger protein 235  COPS2 - cop9 signalosome subunit 2  ZDHHC2 - zinc finger, dhhc-type containing 2  ZNF639 - zinc finger protein 639  TAOK2 - tao kinase 2  SARNP - sap domain containing ribonucleoprotein  BRMS1L - breast cancer metastasis-suppressor 1-like  LEF1 - lymphoid enhancer-binding factor 1  VPS25 - vacuolar protein sorting 25 homolog (s. cerevisiae)  SMCR8 - smith-magenis syndrome chromosome region, candidate 8  CNOT8 - ccr4-not transcription complex, subunit 8  CDK12 - cyclin-dependent kinase 12  SIRPA - signal-regulatory protein alpha  ZNF280B - zinc finger protein 280b  S1PR2 - sphingosine-1-phosphate receptor 2  SNX25 - sorting nexin 25  ARID4B - at rich interactive domain 4b (rbp1-like)  SPRTN - sprt-like n-terminal domain  MIF - macrophage migration inhibitory factor (glycosylation-inhibiting factor)  CCNI2 - cyclin i family, member 2  MAP3K10 - mitogen-activated protein kinase kinase kinase 10  DUSP19 - dual specificity phosphatase 19  MLH1 - mutl homolog 1, colon cancer, nonpolyposis type 2 (e. coli)  ITGB1BP1 - integrin beta 1 binding protein 1  KDM3B - lysine (k)-specific demethylase 3b  RSF1 - remodeling and spacing factor 1  MLLT6 - myeloid/lymphoid or mixed-lineage leukemia (trithorax homolog, drosophila); translocated to, 6  SAMD8 - sterile alpha motif domain containing 8  MLLT3 - myeloid/lymphoid or mixed-lineage leukemia (trithorax homolog, drosophila); translocated to, 3  MED14 - mediator complex subunit 14  MLLT1 - myeloid/lymphoid or mixed-lineage leukemia (trithorax homolog, drosophila); translocated to, 1  MAP3K11 - mitogen-activated protein kinase kinase kinase 11  ZBTB22 - zinc finger and btb domain containing 22  KMT2A - lysine (k)-specific methyltransferase 2a  RPS6KA5 - ribosomal protein s6 kinase, 90kda, polypeptide 5  SUFU - suppressor of fused homolog (drosophila)  GPRC5B - g protein-coupled receptor, family c, group 5, member b  MMP14 - matrix metallopeptidase 14 (membrane-inserted)  MFHAS1 - malignant fibrous histiocytoma amplified sequence 1  NLK - nemo-like kinase  MNAT1 - mnat cdk-activating kinase assembly factor 1  UIMC1 - ubiquitin interaction motif containing 1  MTA2 - metastasis associated 1 family, member 2  NOLC1 - nucleolar and coiled-body phosphoprotein 1  EIF2A - eukaryotic translation initiation factor 2a, 65kda  LDLRAD3 - low density lipoprotein receptor class a domain containing 3  CD200 - cd200 molecule  WWOX - ww domain containing oxidoreductase  MNT - mnt, max dimerization protein  CCPG1 - cell cycle progression 1  CD46 - cd46 molecule, complement regulatory protein  NSRP1 - nuclear speckle splicing regulatory protein 1  ME1 - malic enzyme 1, nadp(+)-dependent, cytosolic  SPTY2D1 - spt2, suppressor of ty, domain containing 1 (s. cerevisiae)  PELO - pelota homolog (drosophila)  MECP2 - methyl cpg binding protein 2 (rett syndrome)  MEF2A - myocyte enhancer factor 2a  WDR54 - wd repeat domain 54  ME2 - malic enzyme 2, nad(+)-dependent, mitochondrial  PRICKLE1 - prickle homolog 1 (drosophila)  ZBED1 - zinc finger, bed-type containing 1  BCDIN3D - bcdin3 domain containing  MEIS2 - meis homeobox 2  BRWD1 - bromodomain and wd repeat domain containing 1  ZNF664 - zinc finger protein 664  MEIS1 - meis homeobox 1  MAP3K4 - mitogen-activated protein kinase kinase kinase 4  MAP3K5 - mitogen-activated protein kinase kinase kinase 5  SLC33A1 - solute carrier family 33 (acetyl-coa transporter), member 1  MAP3K1 - mitogen-activated protein kinase kinase kinase 1, e3 ubiquitin protein ligase  MAP3K3 - mitogen-activated protein kinase kinase kinase 3  MEN1 - multiple endocrine neoplasia i  WDR75 - wd repeat domain 75  COX7A2L - cytochrome c oxidase subunit viia polypeptide 2 like  UTP15 - utp15, u3 small nucleolar ribonucleoprotein, homolog (s. cerevisiae)  FAM161A - family with sequence similarity 161, member a  KAT8 - k(lysine) acetyltransferase 8  ZNF644 - zinc finger protein 644  ARHGEF2 - rho/rac guanine nucleotide exchange factor (gef) 2  PPP1R1B - protein phosphatase 1, regulatory (inhibitor) subunit 1b  HGS - hepatocyte growth factor-regulated tyrosine kinase substrate  MGAT5 - mannosyl (alpha-1,6-)-glycoprotein beta-1,6-n-acetyl-glucosaminyltransferase  NEMF - nuclear export mediator factor  LACC1 - laccase (multicopper oxidoreductase) domain containing 1  MBD3 - methyl-cpg binding domain protein 3  KITLG - kit ligand  PTOV1 - prostate tumor overexpressed 1  ZNF394 - zinc finger protein 394  CBFA2T2 - core-binding factor, runt domain, alpha subunit 2; translocated to, 2  MAGOH - mago-nashi homolog, proliferation-associated (drosophila)  GSKIP - gsk3b interacting protein  CXXC5 - cxxc finger protein 5  RQCD1 - rcd1 required for cell differentiation1 homolog (s. pombe)  SMC3 - structural maintenance of chromosomes 3  MARS - methionyl-trna synthetase  IL23A - interleukin 23, alpha subunit p19  MAPT - microtubule-associated protein tau  LATS1 - large tumor suppressor kinase 1  MTA1 - metastasis associated 1  MAP1A - microtubule-associated protein 1a  SIRT6 - sirtuin 6  USP8 - ubiquitin specific peptidase 8  PPHLN1 - periphilin 1  USP14 - ubiquitin specific peptidase 14 (trna-guanine transglycosylase)  UBE2J1 - ubiquitin-conjugating enzyme e2, j1  NUP43 - nucleoporin 43kda  UNC119 - unc-119 homolog (c. elegans)  MBD1 - methyl-cpg binding domain protein 1  PKMYT1 - protein kinase, membrane associated tyrosine/threonine 1  SFMBT1 - scm-like with four mbt domains 1  MAX - myc associated factor x  ZMIZ2 - zinc finger, miz-type containing 2  LDB2 - lim domain binding 2  TRMT112 - trna methyltransferase 11-2 homolog (s. cerevisiae)  ZFP90 - zfp90 zinc finger protein  NELFCD - negative elongation factor complex member c/d  CDAN1 - codanin 1  DIRAS3 - diras family, gtp-binding ras-like 3  ASH2L - ash2 (absent, small, or homeotic)-like (drosophila)  ZSCAN29 - zinc finger and scan domain containing 29  CTDSPL2 - ctd (carboxy-terminal domain, rna polymerase ii, polypeptide a) small phosphatase like 2  PTPLAD1 - protein tyrosine phosphatase-like a domain containing 1  THAP2 - thap domain containing, apoptosis associated protein 2  ITCH - itchy e3 ubiquitin protein ligase  ZNF484 - zinc finger protein 484  L3MBTL2 - l(3)mbt-like 2 (drosophila)  IL34 - interleukin 34  LRP1 - low density lipoprotein receptor-related protein 1  RIOK1 - rio kinase 1  LRP6 - low density lipoprotein receptor-related protein 6  ZNF445 - zinc finger protein 445  AIP - aryl hydrocarbon receptor interacting protein  NUB1 - negative regulator of ubiquitin-like proteins 1  TRAPPC9 - trafficking protein particle complex 9  ASB1 - ankyrin repeat and socs box containing 1  LUM - lumican  BTAF1 - btaf1 rna polymerase ii, b-tfiid transcription factor-associated, 170kda  LYN - v-yes-1 yamaguchi sarcoma viral related oncogene homolog  SPAG9 - sperm associated antigen 9  UBA3 - ubiquitin-like modifier activating enzyme 3  RBFOX3 - rna binding protein, fox-1 homolog (c. elegans) 3  SESN2 - sestrin 2  PARP9 - poly (adp-ribose) polymerase family, member 9  HIP1R - huntingtin interacting protein 1 related  CDK5RAP1 - cdk5 regulatory subunit associated protein 1  RNF8 - ring finger protein 8, e3 ubiquitin protein ligase  BAZ1B - bromodomain adjacent to zinc finger domain, 1b  AZIN1 - antizyme inhibitor 1  MED15 - mediator complex subunit 15  MXD1 - max dimerization protein 1  PIAS4 - protein inhibitor of activated stat, 4  TRIM33 - tripartite motif containing 33  SRRT - serrate rna effector molecule homolog (arabidopsis)  SMAD2 - smad family member 2  MBIP - map3k12 binding inhibitory protein 1  KLF16 - kruppel-like factor 16  HDAC7 - histone deacetylase 7  ARMCX3 - armadillo repeat containing, x-linked 3  TDP2 - tyrosyl-dna phosphodiesterase 2  CAPRIN1 - cell cycle associated protein 1  NBR1 - neighbor of brca1 gene 1  LARP7 - la ribonucleoprotein domain family, member 7  RPS6KA4 - ribosomal protein s6 kinase, 90kda, polypeptide 4  MAFG - v-maf avian musculoaponeurotic fibrosarcoma oncogene homolog g  DPH5 - diphthamide biosynthesis 5  TAF9B - taf9b rna polymerase ii, tata box binding protein (tbp)-associated factor, 31kda  SMAD4 - smad family member 4  NBAS - neuroblastoma amplified sequence  H1FX - h1 histone family, member x  SMAD5 - smad family member 5  SMAD7 - smad family member 7  WASL - wiskott-aldrich syndrome-like  RBM4B - rna binding motif protein 4b  SAP30BP - sap30 binding protein  ZNF565 - zinc finger protein 565  MYLIP - myosin regulatory light chain interacting protein  YAF2 - yy1 associated factor 2  NCBP1 - nuclear cap binding protein subunit 1, 80kda  SIX5 - six homeobox 5  TAGLN3 - transgelin 3  ITM2C - integral membrane protein 2c  NDN - necdin, melanoma antigen (mage) family member  ZNF582 - zinc finger protein 582  AKAP9 - a kinase (prka) anchor protein (yotiao) 9  NCK1 - nck adaptor protein 1  BRD7 - bromodomain containing 7  CAB39L - calcium binding protein 39-like  NCL - nucleolin  DDX25 - dead (asp-glu-ala-asp) box helicase 25  MBNL2 - muscleblind-like splicing regulator 2  CEBPZ - ccaat/enhancer binding protein (c/ebp), zeta  TICAM1 - toll-like receptor adaptor molecule 1  CCNL2 - cyclin l2  HNRNPM - heterogeneous nuclear ribonucleoprotein m  ORMDL2 - orm1-like 2 (s. cerevisiae)  TRIM28 - tripartite motif containing 28  DROSHA - drosha, ribonuclease type iii  ATP6AP2 - atpase, h+ transporting, lysosomal accessory protein 2  DNAJC15 - dnaj (hsp40) homolog, subfamily c, member 15  TBK1 - tank-binding kinase 1  NBN - nibrin  ZNF197 - zinc finger protein 197  TDRD3 - tudor domain containing 3  RBM7 - rna binding motif protein 7  RBM5 - rna binding motif protein 5  TRIM11 - tripartite motif containing 11  PPP1R12A - protein phosphatase 1, regulatory subunit 12a  TNK2 - tyrosine kinase, non-receptor, 2  MYT1 - myelin transcription factor 1  ALYREF - aly/ref export factor  CSRNP2 - cysteine-serine-rich nuclear protein 2  SENP1 - sumo1/sentrin specific peptidase 1  NAB1 - ngfi-a binding protein 1 (egr1 binding protein 1)  RNF41 - ring finger protein 41  NAB2 - ngfi-a binding protein 2 (egr1 binding protein 2)  TSHZ1 - teashirt zinc finger homeobox 1  PRMT3 - protein arginine methyltransferase 3  PSME3 - proteasome (prosome, macropain) activator subunit 3 (pa28 gamma; ki)  TNFAIP8L3 - tumor necrosis factor, alpha-induced protein 8-like 3  TRIM13 - tripartite motif containing 13  TRIM8 - tripartite motif containing 8  MYO6 - myosin vi  LBH - limb bud and heart development  ABT1 - activator of basal transcription 1  TOPORS - topoisomerase i binding, arginine/serine-rich, e3 ubiquitin protein ligase  RAB1B - rab1b, member ras oncogene family  MYD88 - myeloid differentiation primary response 88  SEH1L - seh1-like (s. cerevisiae)  ZNF362 - zinc finger protein 362  MYH9 - myosin, heavy chain 9, non-muscle  HUWE1 - hect, uba and wwe domain containing 1, e3 ubiquitin protein ligase  HCFC2 - host cell factor c2  CNOT7 - ccr4-not transcription complex, subunit 7  MXI1 - max interactor 1, dimerization protein  PQBP1 - polyglutamine binding protein 1  TRA2A - transformer 2 alpha homolog (drosophila)  ACTR2 - arp2 actin-related protein 2 homolog (yeast)  ACTR3 - arp3 actin-related protein 3 homolog (yeast)  RBM15B - rna binding motif protein 15b  CTDSP2 - ctd (carboxy-terminal domain, rna polymerase ii, polypeptide a) small phosphatase 2  TSFM - ts translation elongation factor, mitochondrial  FEM1B - fem-1 homolog b (c. elegans)  RAD50 - rad50 homolog (s. cerevisiae)  TRIM37 - tripartite motif containing 37  ZNF558 - zinc finger protein 558  LRPPRC - leucine-rich pentatricopeptide repeat containing  ZNF263 - zinc finger protein 263  SPRY4 - sprouty homolog 4 (drosophila)  PURG - purine-rich element binding protein g  BCAP31 - b-cell receptor-associated protein 31  RBM12 - rna binding motif protein 12  MED25 - mediator complex subunit 25  SNX12 - sorting nexin 12  TRAP1 - tnf receptor-associated protein 1  MED6 - mediator complex subunit 6  ABI1 - abl-interactor 1  PTBP3 - polypyrimidine tract binding protein 3  PPP4R1 - protein phosphatase 4, regulatory subunit 1  DMTF1 - cyclin d binding myb-like transcription factor 1  SH2B3 - sh2b adaptor protein 3  MTIF2 - mitochondrial translational initiation factor 2  PDCD6 - programmed cell death 6  MTM1 - myotubularin 1  ZBTB33 - zinc finger and btb domain containing 33  TANK - traf family member-associated nfkb activator  HDAC5 - histone deacetylase 5  MTF1 - metal-regulatory transcription factor 1  SRA1 - steroid receptor rna activator 1  HDAC6 - histone deacetylase 6  OCLN - occludin  RNF187 - ring finger protein 187  SH2D3C - sh2 domain containing 3c  MT3 - metallothionein 3  MED16 - mediator complex subunit 16  CCDC22 - coiled-coil domain containing 22  SAE1 - sumo1 activating enzyme subunit 1  DNM1L - dynamin 1-like  RANBP9 - ran binding protein 9  DNAJB6 - dnaj (hsp40) homolog, subfamily b, member 6  UBA2 - ubiquitin-like modifier activating enzyme 2  MSN - moesin  MSH3 - muts homolog 3 (e. coli)  MSH2 - muts homolog 2, colon cancer, nonpolyposis type 1 (e. coli)  CITED1 - cbp/p300-interacting transactivator, with glu/asp-rich carboxy-terminal domain, 1  ZBTB5 - zinc finger and btb domain containing 5  ZBTB40 - zinc finger and btb domain containing 40  DBNL - drebrin-like  SUPT7L - suppressor of ty 7 (s. cerevisiae)-like  RGCC - regulator of cell cycle  COMMD1 - copper metabolism (murr1) domain containing 1  RBM8A - rna binding motif protein 8a  USP3 - ubiquitin specific peptidase 3  BABAM1 - brisc and brca1 a complex member 1  METTL5 - methyltransferase like 5  USP15 - ubiquitin specific peptidase 15  MED4 - mediator complex subunit 4  THOC1 - tho complex 1  SETD2 - set domain containing 2  RBX1 - ring-box 1, e3 ubiquitin protein ligase  DUSP18 - dual specificity phosphatase 18  MRE11A - mre11 meiotic recombination 11 homolog a (s. cerevisiae)  NUP153 - nucleoporin 153kda  WDR91 - wd repeat domain 91  THRAP3 - thyroid hormone receptor associated protein 3  MED12 - mediator complex subunit 12  MED13 - mediator complex subunit 13  PSMD6 - proteasome (prosome, macropain) 26s subunit, non-atpase, 6  LRIG2 - leucine-rich repeats and immunoglobulin-like domains 2  MED24 - mediator complex subunit 24  ZC3H6 - zinc finger ccch-type containing 6  DTX3L - deltex 3-like (drosophila)  PJA2 - praja ring finger 2, e3 ubiquitin protein ligase  AREL1 - apoptosis resistant e3 ubiquitin protein ligase 1  ZNF34 - zinc finger protein 34  SETDB1 - set domain, bifurcated 1  ORC2 - origin recognition complex, subunit 2  PARD6A - par-6 partitioning defective 6 homolog alpha (c. elegans)  ORC5 - origin recognition complex, subunit 5  TOX4 - tox high mobility group box family member 4  ZNF436 - zinc finger protein 436  RNF141 - ring finger protein 141  PRR7 - proline rich 7 (synaptic)  AGRN - agrin  PPP4R2 - protein phosphatase 4, regulatory subunit 2  NDFIP1 - nedd4 family interacting protein 1  PPP1R26 - protein phosphatase 1, regulatory subunit 26  GLIPR2 - gli pathogenesis-related 2  RBM19 - rna binding motif protein 19  OAZ2 - ornithine decarboxylase antizyme 2  OAZ1 - ornithine decarboxylase antizyme 1  G3BP2 - gtpase activating protein (sh3 domain) binding protein 2  ODC1 - ornithine decarboxylase 1  CCNYL1 - cyclin y-like 1  OGG1 - 8-oxoguanine dna glycosylase  ZSCAN16 - zinc finger and scan domain containing 16  TNKS2 - tankyrase, trf1-interacting ankyrin-related adp-ribose polymerase 2  EXOSC3 - exosome component 3  WDR61 - wd repeat domain 61  APITD1 - apoptosis-inducing, taf9-like domain 1  EXOSC1 - exosome component 1  FIS1 - fission 1 (mitochondrial outer membrane) homolog (s. cerevisiae)  NSF - n-ethylmaleimide-sensitive factor  DAZAP2 - daz associated protein 2  DDR2 - discoidin domain receptor tyrosine kinase 2  IST1 - increased sodium tolerance 1 homolog (yeast)  BCLAF1 - bcl2-associated transcription factor 1  NUMA1 - nuclear mitotic apparatus protein 1  DAB2IP - dab2 interacting protein  EIF4A3 - eukaryotic translation initiation factor 4a3  NUP88 - nucleoporin 88kda  ATG13 - autophagy related 13  NUP98 - nucleoporin 98kda  GMNN - geminin, dna replication inhibitor  NR4A2 - nuclear receptor subfamily 4, group a, member 2  ZNF593 - zinc finger protein 593  ZBTB7B - zinc finger and btb domain containing 7b  MATR3 - matrin 3  NVL - nuclear vcp-like  ZNF691 - zinc finger protein 691  RNF144A - ring finger protein 144a  CREBRF - creb3 regulatory factor  ZNF623 - zinc finger protein 623  RGP1 - rgp1 retrograde golgi transport homolog (s. cerevisiae)  NPM1 - nucleophosmin (nucleolar phosphoprotein b23, numatrin)  NMD3 - nmd3 ribosome export adaptor  ARHGEF11 - rho guanine nucleotide exchange factor (gef) 11  ZBTB24 - zinc finger and btb domain containing 24  ZEB2 - zinc finger e-box binding homeobox 2  THAP4 - thap domain containing 4  CTIF - cbp80/20-dependent translation initiation factor  DUSP16 - dual specificity phosphatase 16  RNF40 - ring finger protein 40, e3 ubiquitin protein ligase  SPOCK2 - sparc/osteonectin, cwcv and kazal-like domains proteoglycan (testican) 2  RB1CC1 - rb1-inducible coiled-coil 1  IFT52 - intraflagellar transport 52 homolog (chlamydomonas)  TSC22D2 - tsc22 domain family, member 2  PAQR3 - progestin and adipoq receptor family member iii  SH3BP5L - sh3-binding domain protein 5-like  NRF1 - nuclear respiratory factor 1  NFXL1 - nuclear transcription factor, x-box binding-like 1  SH3D19 - sh3 domain containing 19  UBQLN1 - ubiquilin 1  DONSON - downstream neighbor of son  NOP2 - nop2 nucleolar protein  VPRBP - vpr (hiv-1) binding protein  NONO - non-pou domain containing, octamer-binding  RNF34 - ring finger protein 34, e3 ubiquitin protein ligase  MDFIC - myod family inhibitor domain containing  UBQLN2 - ubiquilin 2  IFT74 - intraflagellar transport 74 homolog (chlamydomonas)  NOTCH4 - notch 4  NOVA2 - neuro-oncological ventral antigen 2  HERPUD1 - homocysteine-inducible, endoplasmic reticulum stress-inducible, ubiquitin-like domain member 1  NOVA1 - neuro-oncological ventral antigen 1  CEP290 - centrosomal protein 290kda  NPAT - nuclear protein, ataxia-telangiectasia locus  CNOT2 - ccr4-not transcription complex, subunit 2  NAA15 - n(alpha)-acetyltransferase 15, nata auxiliary subunit  PUM1 - pumilio homolog 1 (drosophila)  NOS3 - nitric oxide synthase 3 (endothelial cell)  PPP6R2 - protein phosphatase 6, regulatory subunit 2  CNOT3 - ccr4-not transcription complex, subunit 3  BAZ2B - bromodomain adjacent to zinc finger domain, 2b  NOTCH1 - notch 1  CTC1 - cts telomere maintenance complex component 1  ST18 - suppression of tumorigenicity 18 (breast carcinoma) (zinc finger protein)  NOTCH3 - notch 3  DHX34 - deah (asp-glu-ala-his) box polypeptide 34  NOTCH2 - notch 2  NFYA - nuclear transcription factor y, alpha  HDAC4 - histone deacetylase 4  TOX - thymocyte selection-associated high mobility group box  NFYC - nuclear transcription factor y, gamma  ZFYVE16 - zinc finger, fyve domain containing 16  CXXC1 - cxxc finger protein 1  NFKBIA - nuclear factor of kappa light polypeptide gene enhancer in b-cells inhibitor, alpha  NFKB1 - nuclear factor of kappa light polypeptide gene enhancer in b-cells 1  KMT2B - lysine (k)-specific methyltransferase 2b  NFKBIB - nuclear factor of kappa light polypeptide gene enhancer in b-cells inhibitor, beta  NFKBIE - nuclear factor of kappa light polypeptide gene enhancer in b-cells inhibitor, epsilon  PHACTR2 - phosphatase and actin regulator 2  NKX2-2 - nk2 homeobox 2  CNKSR3 - cnksr family member 3  HDAC9 - histone deacetylase 9  ZNF606 - zinc finger protein 606  SART3 - squamous cell carcinoma antigen recognized by t cells 3  ZNF614 - zinc finger protein 614  EHD4 - eh-domain containing 4  ZFP2 - zfp2 zinc finger protein  MINK1 - misshapen-like kinase 1  NEK3 - nima-related kinase 3  SOCS5 - suppressor of cytokine signaling 5  NEO1 - neogenin 1  SMARCAL1 - swi/snf related, matrix associated, actin dependent regulator of chromatin, subfamily a-like 1  NEDD8 - neural precursor cell expressed, developmentally down-regulated 8  NEDD9 - neural precursor cell expressed, developmentally down-regulated 9  CTR9 - ctr9, paf1/rna polymerase ii complex component  PPM1F - protein phosphatase, mg2+/mn2+ dependent, 1f  CPEB4 - cytoplasmic polyadenylation element binding protein 4  EPC1 - enhancer of polycomb homolog 1 (drosophila)  NFE2L2 - nuclear factor (erythroid-derived 2)-like 2  MORF4L2 - mortality factor 4 like 2  RASL11A - ras-like, family 11, member a  KLHL15 - kelch-like family member 15  NFATC4 - nuclear factor of activated t-cells, cytoplasmic, calcineurin-dependent 4  PDGFD - platelet derived growth factor d  NUP155 - nucleoporin 155kda  NFIA - nuclear factor i/a  NF1 - neurofibromin 1  CDK5RAP3 - cdk5 regulatory subunit associated protein 3  EDEM1 - er degradation enhancer, mannosidase alpha-like 1  RAPGEF2 - rap guanine nucleotide exchange factor (gef) 2  NUP93 - nucleoporin 93kda  ZNF746 - zinc finger protein 746  VGLL4 - vestigial like 4 (drosophila)  LRRC14 - leucine rich repeat containing 14  KDM4A - lysine (k)-specific demethylase 4a  PHF14 - phd finger protein 14  HP1BP3 - heterochromatin protein 1, binding protein 3  ZNF432 - zinc finger protein 432  SAFB2 - scaffold attachment factor b2  MYEF2 - myelin expression factor 2  LPIN2 - lipin 2 |
| GO:0033043 | regulation of organelle organization | 6.31E-8 | 1.49E-5 | 1.11 (10334,963,6996,724) | [+] Show genes  ZMYND8 - zinc finger, mynd-type containing 8  SPTAN1 - spectrin, alpha, non-erythrocytic 1  EXOSC10 - exosome component 10  LIG4 - ligase iv, dna, atp-dependent  ATAT1 - alpha tubulin acetyltransferase 1  LRRC16A - leucine rich repeat containing 16a  CD2AP - cd2-associated protein  SPTBN4 - spectrin, beta, non-erythrocytic 4  LIMK1 - lim domain kinase 1  PPP1R9A - protein phosphatase 1, regulatory subunit 9a  LIG3 - ligase iii, dna, atp-dependent  PLXNB1 - plexin b1  PLXNA3 - plexin a3  ABL1 - c-abl oncogene 1, non-receptor tyrosine kinase  MTPN - myotrophin  PML - promyelocytic leukemia  MLLT11 - myeloid/lymphoid or mixed-lineage leukemia (trithorax homolog, drosophila); translocated to, 11  OBFC1 - oligonucleotide/oligosaccharide-binding fold containing 1  SPAST - spastin  MKKS - mckusick-kaufman syndrome  MIER1 - mesoderm induction early response 1, transcriptional regulator  YWHAQ - tyrosine 3-monooxygenase/tryptophan 5-monooxygenase activation protein, theta polypeptide  KAT2A - k(lysine) acetyltransferase 2a  GBA2 - glucosidase, beta (bile acid) 2  SLX4 - slx4 structure-specific endonuclease subunit  SEC22B - sec22 vesicle trafficking protein homolog b (s. cerevisiae) (gene/pseudogene)  H2AFY - h2a histone family, member y  WBP2 - ww domain binding protein 2  OTUB1 - otu domain, ubiquitin aldehyde binding 1  UBXN2B - ubx domain protein 2b  PLAUR - plasminogen activator, urokinase receptor  ASAP3 - arfgap with sh3 domain, ankyrin repeat and ph domain 3  RND2 - rho family gtpase 2  SPIRE2 - spire actin nucleation factor 2  ACTG1 - actin, gamma 1  NPRL3 - nitrogen permease regulator-like 3 (s. cerevisiae)  CGNL1 - cingulin-like 1  LMNA - lamin a/c  LMAN1 - lectin, mannose-binding, 1  MARK4 - map/microtubule affinity-regulating kinase 4  AP1AR - adaptor-related protein complex 1 associated regulatory protein  ANAPC5 - anaphase promoting complex subunit 5  ANAPC7 - anaphase promoting complex subunit 7  SNCA - synuclein, alpha (non a4 component of amyloid precursor)  RHOU - ras homolog family member u  PARL - presenilin associated, rhomboid-like  FSCN1 - fascin homolog 1, actin-bundling protein (strongylocentrotus purpuratus)  NPEPPS - aminopeptidase puromycin sensitive  MAP9 - microtubule-associated protein 9  VPS4B - vacuolar protein sorting 4 homolog b (s. cerevisiae)  EID1 - ep300 interacting inhibitor of differentiation 1  BAG4 - bcl2-associated athanogene 4  CDC45 - cell division cycle 45  GCLC - glutamate-cysteine ligase, catalytic subunit  BAP1 - brca1 associated protein-1 (ubiquitin carboxy-terminal hydrolase)  AXIN2 - axin 2  SMARCB1 - swi/snf related, matrix associated, actin dependent regulator of chromatin, subfamily b, member 1  UBR5 - ubiquitin protein ligase e3 component n-recognin 5  ADD2 - adducin 2 (beta)  ARFIP2 - adp-ribosylation factor interacting protein 2  ADD3 - adducin 3 (gamma)  STMN1 - stathmin 1  ADD1 - adducin 1 (alpha)  MID1IP1 - mid1 interacting protein 1  ATG5 - autophagy related 5  ROCK2 - rho-associated, coiled-coil containing protein kinase 2  LCP1 - lymphocyte cytosolic protein 1 (l-plastin)  PARP1 - poly (adp-ribose) polymerase 1  ADPRH - adp-ribosylarginine hydrolase  GNAI1 - guanine nucleotide binding protein (g protein), alpha inhibiting activity polypeptide 1  CNTROB - centrobin, centrosomal brca2 interacting protein  CEP76 - centrosomal protein 76kda  FZR1 - fizzy/cell division cycle 20 related 1 (drosophila)  GLYR1 - glyoxylate reductase 1 homolog (arabidopsis)  GMFB - glia maturation factor, beta  SMPD3 - sphingomyelin phosphodiesterase 3, neutral membrane (neutral sphingomyelinase ii)  TMEFF2 - transmembrane protein with egf-like and two follistatin-like domains 2  RUVBL2 - ruvb-like 2 (e. coli)  TTC21B - tetratricopeptide repeat domain 21b  CDK5RAP2 - cdk5 regulatory subunit associated protein 2  EML2 - echinoderm microtubule associated protein like 2  NELFE - negative elongation factor complex member e  NAT6 - n-acetyltransferase 6 (gcn5-related)  TAC1 - tachykinin, precursor 1  SYT1 - synaptotagmin i  KIF5B - kinesin family member 5b  SYT4 - synaptotagmin iv  VPS35 - vacuolar protein sorting 35 homolog (s. cerevisiae)  SUPT6H - suppressor of ty 6 homolog (s. cerevisiae)  CTGF - connective tissue growth factor  KDR - kinase insert domain receptor (a type iii receptor tyrosine kinase)  PICK1 - protein interacting with prkca 1  NAA40 - n(alpha)-acetyltransferase 40, natd catalytic subunit, homolog (s. cerevisiae)  CTBP1 - c-terminal binding protein 1  PPP3R1 - protein phosphatase 3, regulatory subunit b, alpha  ATF7IP - activating transcription factor 7 interacting protein  PIP4K2C - phosphatidylinositol-5-phosphate 4-kinase, type ii, gamma  DYNC1LI1 - dynein, cytoplasmic 1, light intermediate chain 1  CTNNA2 - catenin (cadherin-associated protein), alpha 2  UBL5 - ubiquitin-like 5  RAB3GAP2 - rab3 gtpase activating protein subunit 2 (non-catalytic)  CTNNB1 - catenin (cadherin-associated protein), beta 1, 88kda  CNTRL - centriolin  STXBP1 - syntaxin binding protein 1  STX5 - syntaxin 5  RAB7A - rab7a, member ras oncogene family  AKT1 - v-akt murine thymoma viral oncogene homolog 1  USP7 - ubiquitin specific peptidase 7 (herpes virus-associated)  PCID2 - pci domain containing 2  MGARP - mitochondria-localized glutamic acid-rich protein  USP30 - ubiquitin specific peptidase 30  RIOK2 - rio kinase 2  EVI5L - ecotropic viral integration site 5-like  TRIOBP - trio and f-actin binding protein  TRAPPC12 - trafficking protein particle complex 12  CRIPT - cysteine-rich pdz-binding protein  TCTEX1D2 - tctex1 domain containing 2  PPP1R10 - protein phosphatase 1, regulatory subunit 10  RMI2 - recq mediated genome instability 2  PAN3 - pan3 poly(a) specific ribonuclease subunit homolog (s. cerevisiae)  CYLD - cylindromatosis (turban tumor syndrome)  CDKL5 - cyclin-dependent kinase-like 5  SH3GLB1 - sh3-domain grb2-like endophilin b1  STMN2 - stathmin-like 2  CRK - v-crk avian sarcoma virus ct10 oncogene homolog  IWS1 - iws1 homolog (s. cerevisiae)  NIPBL - nipped-b homolog (drosophila)  TCHP - trichoplein, keratin filament binding  STAT2 - signal transducer and activator of transcription 2, 113kda  SLIT2 - slit homolog 2 (drosophila)  ASF1A - anti-silencing function 1a histone chaperone  KCTD17 - potassium channel tetramerization domain containing 17  SSRP1 - structure specific recognition protein 1  MAP2K7 - mitogen-activated protein kinase kinase 7  IFT88 - intraflagellar transport 88 homolog (chlamydomonas)  MAP2K1 - mitogen-activated protein kinase kinase 1  MAP2K2 - mitogen-activated protein kinase kinase 2  DSTN - destrin (actin depolymerizing factor)  CSF1R - colony stimulating factor 1 receptor  FBXO7 - f-box protein 7  MAPK9 - mitogen-activated protein kinase 9  TRIP12 - thyroid hormone receptor interactor 12  GBA - glucosidase, beta, acid  KATNBL1 - katanin p80 subunit b-like 1  MAPK8 - mitogen-activated protein kinase 8  GATA2 - gata binding protein 2  MAPK3 - mitogen-activated protein kinase 3  MAPK1 - mitogen-activated protein kinase 1  MAPRE2 - microtubule-associated protein, rp/eb family, member 2  KLHL22 - kelch-like family member 22  MICALL2 - mical-like 2  SSBP1 - single-stranded dna binding protein 1, mitochondrial  ZNF335 - zinc finger protein 335  PRKD1 - protein kinase d1  CCDC88A - coiled-coil domain containing 88a  STAM - signal transducing adaptor molecule (sh3 domain and itam motif) 1  SPTBN1 - spectrin, beta, non-erythrocytic 1  TLK2 - tousled-like kinase 2  CSNK2A2 - casein kinase 2, alpha prime polypeptide  PRKCE - protein kinase c, epsilon  ANXA2 - annexin a2  SRC - v-src avian sarcoma (schmidt-ruppin a-2) viral oncogene homolog  TAOK2 - tao kinase 2  PRKCD - protein kinase c, delta  SREBF2 - sterol regulatory element binding transcription factor 2  SLC25A5 - solute carrier family 25 (mitochondrial carrier; adenine nucleotide translocator), member 5  SMCR8 - smith-magenis syndrome chromosome region, candidate 8  PRKAR1A - protein kinase, camp-dependent, regulatory, type i, alpha  PKIB - protein kinase (camp-dependent, catalytic) inhibitor beta  DHX36 - deah (asp-glu-ala-his) box polypeptide 36  ATXN2L - ataxin 2-like  PARN - poly(a)-specific ribonuclease  TREX1 - three prime repair exonuclease 1  GSN - gelsolin  SIN3A - sin3 transcription regulator family member a  SDCBP - syndecan binding protein (syntenin)  TMEM33 - transmembrane protein 33  SET - set nuclear oncogene  SETMAR - set domain and mariner transposase fusion gene  PRMT6 - protein arginine methyltransferase 6  RHOT2 - ras homolog family member t2  ZNF304 - zinc finger protein 304  DCTN1 - dynactin 1  GTF2H2 - general transcription factor iih, polypeptide 2, 44kda  CLIC4 - chloride intracellular channel 4  ITGB1BP1 - integrin beta 1 binding protein 1  MLLT6 - myeloid/lymphoid or mixed-lineage leukemia (trithorax homolog, drosophila); translocated to, 6  MCPH1 - microcephalin 1  MLLT3 - myeloid/lymphoid or mixed-lineage leukemia (trithorax homolog, drosophila); translocated to, 3  DBN1 - drebrin 1  ATL3 - atlastin gtpase 3  APC - adenomatous polyposis coli  KMT2A - lysine (k)-specific methyltransferase 2a  POT1 - protection of telomeres 1  RIF1 - rap1 interacting factor homolog (yeast)  VPS13D - vacuolar protein sorting 13 homolog d (s. cerevisiae)  RPS6KA5 - ribosomal protein s6 kinase, 90kda, polypeptide 5  CCDC8 - coiled-coil domain containing 8  KIAA1551 - kiaa1551  ARF1 - adp-ribosylation factor 1  MAP1S - microtubule-associated protein 1s  MNAT1 - mnat cdk-activating kinase assembly factor 1  PAFAH1B1 - platelet-activating factor acetylhydrolase 1b, regulatory subunit 1 (45kda)  SYT9 - synaptotagmin ix  AKAP13 - a kinase (prka) anchor protein 13  SETD5 - set domain containing 5  PAK2 - p21 protein (cdc42/rac)-activated kinase 2  PAK3 - p21 protein (cdc42/rac)-activated kinase 3  PAM - peptidylglycine alpha-amidating monooxygenase  CNOT6 - ccr4-not transcription complex, subunit 6  PDGFA - platelet-derived growth factor alpha polypeptide  CEP250 - centrosomal protein 250kda  H3F3A - h3 histone, family 3a  ARL2 - adp-ribosylation factor-like 2  FZD5 - frizzled family receptor 5  DCP2 - decapping mrna 2  MUL1 - mitochondrial e3 ubiquitin protein ligase 1  ANKRD27 - ankyrin repeat domain 27 (vps9 domain)  C10orf76 - chromosome 10 open reading frame 76  ATXN2 - ataxin 2  NSMCE2 - non-smc element 2, mms21 homolog (s. cerevisiae)  ZNF451 - zinc finger protein 451  PDGFRB - platelet-derived growth factor receptor, beta polypeptide  ARRB2 - arrestin, beta 2  SPTY2D1 - spt2, suppressor of ty, domain containing 1 (s. cerevisiae)  ARRB1 - arrestin, beta 1  ALMS1 - alstrom syndrome 1  PDGFRA - platelet-derived growth factor receptor, alpha polypeptide  SKA2 - spindle and kinetochore associated complex subunit 2  PDGFB - platelet-derived growth factor beta polypeptide  BUB3 - bub3 mitotic checkpoint protein  MECP2 - methyl cpg binding protein 2 (rett syndrome)  RHOB - ras homolog family member b  RHOC - ras homolog family member c  FAM107A - family with sequence similarity 107, member a  ODF2L - outer dense fiber of sperm tails 2-like  ZW10 - zw10 kinetochore protein  RHOA - ras homolog family member a  ARF6 - adp-ribosylation factor 6  ARHGDIA - rho gdp dissociation inhibitor (gdi) alpha  MAP3K4 - mitogen-activated protein kinase kinase kinase 4  RND3 - rho family gtpase 3  WDR70 - wd repeat domain 70  PYGO2 - pygopus homolog 2 (drosophila)  DAPK3 - death-associated protein kinase 3  CHMP1A - charged multivesicular body protein 1a  ARHGAP17 - rho gtpase activating protein 17  JDP2 - jun dimerization protein 2  LRSAM1 - leucine rich repeat and sterile alpha motif containing 1  ARHGEF2 - rho/rac guanine nucleotide exchange factor (gef) 2  S100A10 - s100 calcium binding protein a10  CLIP3 - cap-gly domain containing linker protein 3  MAVS - mitochondrial antiviral signaling protein  CHMP2B - charged multivesicular body protein 2b  CDC37 - cell division cycle 37  PDE2A - phosphodiesterase 2a, cgmp-stimulated  STX18 - syntaxin 18  TBC1D14 - tbc1 domain family, member 14  HMBOX1 - homeobox containing 1  HTT - huntingtin  SH2B1 - sh2b adaptor protein 1  ZNF205 - zinc finger protein 205  HCLS1 - hematopoietic cell-specific lyn substrate 1  KAT7 - k(lysine) acetyltransferase 7  ZNF207 - zinc finger protein 207  PHF19 - phd finger protein 19  CDC26 - cell division cycle 26  SLC9A1 - solute carrier family 9, subfamily a (nhe1, cation proton antiporter 1), member 1  PFN2 - profilin 2  TRAF3IP1 - tnf receptor-associated factor 3 interacting protein 1  FBXW7 - f-box and wd repeat domain containing 7, e3 ubiquitin protein ligase  CNOT6L - ccr4-not transcription complex, subunit 6-like  XPO1 - exportin 1 (crm1 homolog, yeast)  TAOK1 - tao kinase 1  XRCC1 - x-ray repair complementing defective repair in chinese hamster cells 1  SYDE1 - synapse defective 1, rho gtpase, homolog 1 (c. elegans)  MORC2 - morc family cw-type zinc finger 2  PHF2 - phd finger protein 2  SHANK3 - sh3 and multiple ankyrin repeat domains 3  MAPT - microtubule-associated protein tau  PHF1 - phd finger protein 1  MAP6 - microtubule-associated protein 6  LRRK2 - leucine-rich repeat kinase 2  MAP2 - microtubule-associated protein 2  MNS1 - meiosis-specific nuclear structural 1  LATS1 - large tumor suppressor kinase 1  MAP1B - microtubule-associated protein 1b  SIRT6 - sirtuin 6  MAP1A - microtubule-associated protein 1a  DZIP1 - daz interacting zinc finger protein 1  RSG1 - rem2 and rab-like small gtpase 1  RAB33B - rab33b, member ras oncogene family  GPM6B - glycoprotein m6b  PPHLN1 - periphilin 1  LIMA1 - lim domain and actin binding 1  PREX1 - phosphatidylinositol-3,4,5-trisphosphate-dependent rac exchange factor 1  MAPK15 - mitogen-activated protein kinase 15  SH3PXD2B - sh3 and px domains 2b  DIXDC1 - dix domain containing 1  SKI - v-ski avian sarcoma viral oncogene homolog  EVL - enah/vasp-like  DYRK1A - dual-specificity tyrosine-(y)-phosphorylation regulated kinase 1a  PKMYT1 - protein kinase, membrane associated tyrosine/threonine 1  CHTOP - chromatin target of prmt1  PPM1E - protein phosphatase, mg2+/mn2+ dependent, 1e  USP36 - ubiquitin specific peptidase 36  CHMP5 - charged multivesicular body protein 5  FNIP2 - folliculin interacting protein 2  SENP6 - sumo1/sentrin specific peptidase 6  TTBK2 - tau tubulin kinase 2  MCM2 - minichromosome maintenance complex component 2  PFDN2 - prefoldin subunit 2  SYT7 - synaptotagmin vii  BAX - bcl2-associated x protein  PKD1 - polycystic kidney disease 1 (autosomal dominant)  DKC1 - dyskeratosis congenita 1, dyskerin  MTF2 - metal response element binding transcription factor 2  BAK1 - bcl2-antagonist/killer 1  DLG1 - discs, large homolog 1 (drosophila)  VPS18 - vacuolar protein sorting 18 homolog (s. cerevisiae)  PIP4K2A - phosphatidylinositol-5-phosphate 4-kinase, type ii, alpha  LRP1 - low density lipoprotein receptor-related protein 1  NAT10 - n-acetyltransferase 10 (gcn5-related)  DYNC2LI1 - dynein, cytoplasmic 2, light intermediate chain 1  BAD - bcl2-associated agonist of cell death  PIN1 - peptidylprolyl cis/trans isomerase, nima-interacting 1  FSD1 - fibronectin type iii and spry domain containing 1  PIK3R1 - phosphoinositide-3-kinase, regulatory subunit 1 (alpha)  TOM1L2 - target of myb1-like 2 (chicken)  TMEM39A - transmembrane protein 39a  SEMA5A - sema domain, seven thrombospondin repeats (type 1 and type 1-like), transmembrane domain (tm) and short cytoplasmic domain, (semaphorin) 5a  SCIN - scinderin  GRB2 - growth factor receptor-bound protein 2  JAM3 - junctional adhesion molecule 3  RICTOR - rptor independent companion of mtor, complex 2  HIP1R - huntingtin interacting protein 1 related  CHMP3 - charged multivesicular body protein 3  ATRX - alpha thalassemia/mental retardation syndrome x-linked  TWF2 - twinfilin actin-binding protein 2  GRIK5 - glutamate receptor, ionotropic, kainate 5  SFPQ - splicing factor proline/glutamine-rich  SFRP1 - secreted frizzled-related protein 1  DYNC1H1 - dynein, cytoplasmic 1, heavy chain 1  DNM2 - dynamin 2  DNMT1 - dna (cytosine-5-)-methyltransferase 1  ZNF711 - zinc finger protein 711  ARHGAP35 - rho gtpase activating protein 35  FAM49B - family with sequence similarity 49, member b  RPS6KA4 - ribosomal protein s6 kinase, 90kda, polypeptide 4  RHOT1 - ras homolog family member t1  HPS4 - hermansky-pudlak syndrome 4  BCL2 - b-cell cll/lymphoma 2  XRCC5 - x-ray repair complementing defective repair in chinese hamster cells 5 (double-strand-break rejoining)  BCL2L2 - bcl2-like 2  NOC2L - nucleolar complex associated 2 homolog (s. cerevisiae)  BCL2L1 - bcl2-like 1  SMAD4 - smad family member 4  YWHAG - tyrosine 3-monooxygenase/tryptophan 5-monooxygenase activation protein, gamma polypeptide  GSK3B - glycogen synthase kinase 3 beta  H1FX - h1 histone family, member x  PARK7 - parkinson protein 7  GSK3A - glycogen synthase kinase 3 alpha  YWHAZ - tyrosine 3-monooxygenase/tryptophan 5-monooxygenase activation protein, zeta polypeptide  BBS4 - bardet-biedl syndrome 4  PNKP - polynucleotide kinase 3'-phosphatase  WASL - wiskott-aldrich syndrome-like  YWHAE - tyrosine 3-monooxygenase/tryptophan 5-monooxygenase activation protein, epsilon polypeptide  YWHAB - tyrosine 3-monooxygenase/tryptophan 5-monooxygenase activation protein, beta polypeptide  MAPRE1 - microtubule-associated protein, rp/eb family, member 1  TRIM27 - tripartite motif containing 27  MARK2 - map/microtubule affinity-regulating kinase 2  CHMP4C - charged multivesicular body protein 4c  BNIP3L - bcl2/adenovirus e1b 19kda interacting protein 3-like  MAPRE3 - microtubule-associated protein, rp/eb family, member 3  CTTN - cortactin  TSC1 - tuberous sclerosis 1  G3BP1 - gtpase activating protein (sh3 domain) binding protein 1  BRD7 - bromodomain containing 7  AKAP9 - a kinase (prka) anchor protein (yotiao) 9  NCK1 - nck adaptor protein 1  SPIRE1 - spire actin nucleation factor 1  NAF1 - nuclear assembly factor 1 ribonucleoprotein  ELN - elastin  WHSC1L1 - wolf-hirschhorn syndrome candidate 1-like 1  FLCN - folliculin  ABI2 - abl-interactor 2  CENPV - centromere protein v  SNW1 - snw domain containing 1  NRP1 - neuropilin 1  TRIM28 - tripartite motif containing 28  IQGAP1 - iq motif containing gtpase activating protein 1  BCOR - bcl6 corepressor  EP300 - e1a binding protein p300  KATNB1 - katanin p80 (wd repeat containing) subunit b 1  RDX - radixin  PHF23 - phd finger protein 23  WASF2 - was protein family, member 2  UPF1 - upf1 regulator of nonsense transcripts homolog (yeast)  ENO2 - enolase 2 (gamma, neuronal)  NBN - nibrin  NUBP1 - nucleotide binding protein 1  UBQLN4 - ubiquilin 4  ENO1 - enolase 1, (alpha)  RAB3GAP1 - rab3 gtpase activating protein subunit 1 (catalytic)  FAM49A - family with sequence similarity 49, member a  CCT5 - chaperonin containing tcp1, subunit 5 (epsilon)  RNF41 - ring finger protein 41  SPHK2 - sphingosine kinase 2  SAR1A - sar1 homolog a (s. cerevisiae)  SYNJ1 - synaptojanin 1  AZI1 - 5-azacytidine induced 1  TMOD2 - tropomodulin 2 (neuronal)  RGS4 - regulator of g-protein signaling 4  TSG101 - tumor susceptibility gene 101  TSC2 - tuberous sclerosis 2  KAT2B - k(lysine) acetyltransferase 2b  BMP7 - bone morphogenetic protein 7  CDC16 - cell division cycle 16  CNOT1 - ccr4-not transcription complex, subunit 1  RAB1B - rab1b, member ras oncogene family  ARPC5L - actin related protein 2/3 complex, subunit 5-like  BCAS3 - breast carcinoma amplified sequence 3  VPS13C - vacuolar protein sorting 13 homolog c (s. cerevisiae)  HUWE1 - hect, uba and wwe domain containing 1, e3 ubiquitin protein ligase  TP53BP2 - tumor protein p53 binding protein, 2  ARPC5 - actin related protein 2/3 complex, subunit 5, 16kda  NABP2 - nucleic acid binding protein 2  MTMR3 - myotubularin related protein 3  GPSM2 - g-protein signaling modulator 2  RNF4 - ring finger protein 4  KDM1A - lysine (k)-specific demethylase 1a  ACTR2 - arp2 actin-related protein 2 homolog (yeast)  ACTR3 - arp3 actin-related protein 3 homolog (yeast)  EFNA5 - ephrin-a5  ARPC3 - actin related protein 2/3 complex, subunit 3, 21kda  CALCOCO2 - calcium binding and coiled-coil domain 2  ARPC4 - actin related protein 2/3 complex, subunit 4, 20kda  ROCK1 - rho-associated, coiled-coil containing protein kinase 1  ARPC2 - actin related protein 2/3 complex, subunit 2, 34kda  CCT3 - chaperonin containing tcp1, subunit 3 (gamma)  ANAPC4 - anaphase promoting complex subunit 4  SMG1 - smg1 phosphatidylinositol 3-kinase-related kinase  TSPO - translocator protein (18kda)  MFF - mitochondrial fission factor  RAD50 - rad50 homolog (s. cerevisiae)  TRIM37 - tripartite motif containing 37  CDK2AP2 - cyclin-dependent kinase 2 associated protein 2  CDC42EP5 - cdc42 effector protein (rho gtpase binding) 5  MYCBP2 - myc binding protein 2, e3 ubiquitin protein ligase  LPAR1 - lysophosphatidic acid receptor 1  S1PR1 - sphingosine-1-phosphate receptor 1  CPLX3 - complexin 3  AKAP8 - a kinase (prka) anchor protein 8  ZMPSTE24 - zinc metallopeptidase ste24  TPR - translocated promoter region, nuclear basket protein  WAPAL - wings apart-like homolog (drosophila)  WASF1 - was protein family, member 1  CCSAP - centriole, cilia and spindle-associated protein  CAPZA1 - capping protein (actin filament) muscle z-line, alpha 1  KDM4C - lysine (k)-specific demethylase 4c  CAPN2 - calpain 2, (m/ii) large subunit  CASP8 - caspase 8, apoptosis-related cysteine peptidase  PTK2B - protein tyrosine kinase 2 beta  MTM1 - myotubularin 1  TNKS - tankyrase, trf1-interacting ankyrin-related adp-ribose polymerase  CUL9 - cullin 9  BAIAP2 - bai1-associated protein 2  PDCD6IP - programmed cell death 6 interacting protein  MAD2L2 - mad2 mitotic arrest deficient-like 2 (yeast)  PID1 - phosphotyrosine interaction domain containing 1  OCLN - occludin  HDAC6 - histone deacetylase 6  PHIP - pleckstrin homology domain interacting protein  CAPZB - capping protein (actin filament) muscle z-line, beta  CLASP2 - cytoplasmic linker associated protein 2  BECN1 - beclin 1, autophagy related  PLSCR3 - phospholipid scramblase 3  EZR - ezrin  PHF8 - phd finger protein 8  SMC5 - structural maintenance of chromosomes 5  ESAM - endothelial cell adhesion molecule  ACD - adrenocortical dysplasia homolog (mouse)  TADA3 - transcriptional adaptor 3  SAE1 - sumo1 activating enzyme subunit 1  TMEM135 - transmembrane protein 135  CALR - calreticulin  UVRAG - uv radiation resistance associated  DNM1L - dynamin 1-like  VASP - vasodilator-stimulated phosphoprotein  CDC23 - cell division cycle 23  VAT1 - vesicle amine transport protein 1 homolog (t. californica)  PDXP - pyridoxal (pyridoxine, vitamin b6) phosphatase  MSN - moesin  HIF1A - hypoxia inducible factor 1, alpha subunit (basic helix-loop-helix transcription factor)  SLC35F6 - solute carrier family 35, member f6  CCT6A - chaperonin containing tcp1, subunit 6a (zeta 1)  ERCC4 - excision repair cross-complementing rodent repair deficiency, complementation group 4  PINX1 - pin2/terf1 interacting, telomerase inhibitor 1  PAN2 - pan2 poly(a) specific ribonuclease subunit homolog (s. cerevisiae)  EED - embryonic ectoderm development  DBNL - drebrin-like  PHLDB1 - pleckstrin homology-like domain, family b, member 1  CORO1B - coronin, actin binding protein, 1b  HNRNPU - heterogeneous nuclear ribonucleoprotein u (scaffold attachment factor a)  WDR1 - wd repeat domain 1  KANK1 - kn motif and ankyrin repeat domains 1  RGCC - regulator of cell cycle  SSH3 - slingshot protein phosphatase 3  CORO2B - coronin, actin binding protein, 2b  HIRA - histone cell cycle regulator  TMEM14A - transmembrane protein 14a  FBXO4 - f-box protein 4  DLC1 - deleted in liver cancer 1  TINF2 - terf1 (trf1)-interacting nuclear factor 2  CYFIP1 - cytoplasmic fmr1 interacting protein 1  ARL6IP1 - adp-ribosylation factor-like 6 interacting protein 1  STXBP6 - syntaxin binding protein 6 (amisyn)  DMTN - dematin actin binding protein  FAM206A - family with sequence similarity 206, member a  RAPGEF3 - rap guanine nucleotide exchange factor (gef) 3  HNRNPA2B1 - heterogeneous nuclear ribonucleoprotein a2/b1  HNRNPA1 - heterogeneous nuclear ribonucleoprotein a1  CHMP4A - charged multivesicular body protein 4a  HNRNPC - heterogeneous nuclear ribonucleoprotein c (c1/c2)  CAV2 - caveolin 2  HNRNPD - heterogeneous nuclear ribonucleoprotein d (au-rich element rna binding protein 1, 37kda)  SMURF1 - smad specific e3 ubiquitin protein ligase 1  SYT11 - synaptotagmin xi  MYADM - myeloid-associated differentiation marker  SEC24B - sec24 family, member b (s. cerevisiae)  RBM14 - rna binding motif protein 14  HMGA1 - high mobility group at-hook 1  MRE11A - mre11 meiotic recombination 11 homolog a (s. cerevisiae)  TBC1D12 - tbc1 domain family, member 12  ADCK1 - aarf domain containing kinase 1  PRMT5 - protein arginine methyltransferase 5  UBE2L3 - ubiquitin-conjugating enzyme e2l 3  EPS8 - epidermal growth factor receptor pathway substrate 8  UBE2N - ubiquitin-conjugating enzyme e2n  FMR1 - fragile x mental retardation 1  CAMSAP2 - calmodulin regulated spectrin-associated protein family, member 2  TCP1 - t-complex 1  SETDB1 - set domain, bifurcated 1  FCHSD2 - fch and double sh3 domains 2  BRK1 - brick1, scar/wave actin-nucleating complex subunit  SCFD1 - sec1 family domain containing 1  CDH2 - cadherin 2, type 1, n-cadherin (neuronal)  DDHD2 - ddhd domain containing 2  TLK1 - tousled-like kinase 1  CDC27 - cell division cycle 27  STMN3 - stathmin-like 3  FLII - flightless i homolog (drosophila)  PARG - poly (adp-ribose) glycohydrolase  HTRA2 - htra serine peptidase 2  C2CD5 - c2 calcium-dependent domain containing 5  FLNA - filamin a, alpha  PPFIA1 - protein tyrosine phosphatase, receptor type, f polypeptide (ptprf), interacting protein (liprin), alpha 1  ING2 - inhibitor of growth family, member 2  PLCB1 - phospholipase c, beta 1 (phosphoinositide-specific)  CDC42EP3 - cdc42 effector protein (rho gtpase binding) 3  SHANK1 - sh3 and multiple ankyrin repeat domains 1  HDAC8 - histone deacetylase 8  ILK - integrin-linked kinase  SMCR7L - smith-magenis syndrome chromosome region, candidate 7-like  TBC1D25 - tbc1 domain family, member 25  CDC14B - cell division cycle 14b  CAMKK2 - calcium/calmodulin-dependent protein kinase kinase 2, beta  NPRL2 - nitrogen permease regulator-like 2 (s. cerevisiae)  SCARB2 - scavenger receptor class b, member 2  MAPKAPK5 - mitogen-activated protein kinase-activated protein kinase 5  FKBP4 - fk506 binding protein 4, 59kda  G3BP2 - gtpase activating protein (sh3 domain) binding protein 2  BICD2 - bicaudal d homolog 2 (drosophila)  CD47 - cd47 molecule  ARHGAP44 - rho gtpase activating protein 44  SMG6 - smg6 nonsense mediated mrna decay factor  TNKS2 - tankyrase, trf1-interacting ankyrin-related adp-ribose polymerase 2  VPS11 - vacuolar protein sorting 11 homolog (s. cerevisiae)  PSMD10 - proteasome (prosome, macropain) 26s subunit, non-atpase, 10  FAM208A - family with sequence similarity 208, member a  WDR61 - wd repeat domain 61  KDM3A - lysine (k)-specific demethylase 3a  OPA1 - optic atrophy 1 (autosomal dominant)  CLEC16A - c-type lectin domain family 16, member a  ANAPC1 - anaphase promoting complex subunit 1  DYNLT1 - dynein, light chain, tctex-type 1  GAS2L1 - growth arrest-specific 2 like 1  TERF2IP - telomeric repeat binding factor 2, interacting protein  TAF7 - taf7 rna polymerase ii, tata box binding protein (tbp)-associated factor, 55kda  FIS1 - fission 1 (mitochondrial outer membrane) homolog (s. cerevisiae)  CKAP5 - cytoskeleton associated protein 5  ATG7 - autophagy related 7  FGF13 - fibroblast growth factor 13  VPS16 - vacuolar protein sorting 16 homolog (s. cerevisiae)  TADA2A - transcriptional adaptor 2a  JMY - junction mediating and regulatory protein, p53 cofactor  NSFL1C - nsfl1 (p97) cofactor (p47)  CFL1 - cofilin 1 (non-muscle)  NUMA1 - nuclear mitotic apparatus protein 1  CLASP1 - cytoplasmic linker associated protein 1  ATG13 - autophagy related 13  ZBTB7B - zinc finger and btb domain containing 7b  CIB1 - calcium and integrin binding 1 (calmyrin)  TBC1D30 - tbc1 domain family, member 30  FEN1 - flap structure-specific endonuclease 1  CDK10 - cyclin-dependent kinase 10  ARFIP1 - adp-ribosylation factor interacting protein 1  FER - fer (fps/fes related) tyrosine kinase  PTK2 - protein tyrosine kinase 2  ZDHHC6 - zinc finger, dhhc-type containing 6  CEP120 - centrosomal protein 120kda  CDK5 - cyclin-dependent kinase 5  TWF1 - twinfilin actin-binding protein 1  NPM1 - nucleophosmin (nucleolar phosphoprotein b23, numatrin)  ATG3 - autophagy related 3  RND1 - rho family gtpase 1  CDK9 - cyclin-dependent kinase 9  CCT7 - chaperonin containing tcp1, subunit 7 (eta)  KMT2E - lysine (k)-specific methyltransferase 2e  INSR - insulin receptor  CDK13 - cyclin-dependent kinase 13  TBCD - tubulin folding cofactor d  ZEB2 - zinc finger e-box binding homeobox 2  CDKN1B - cyclin-dependent kinase inhibitor 1b (p27, kip1)  CCT2 - chaperonin containing tcp1, subunit 2 (beta)  CCT4 - chaperonin containing tcp1, subunit 4 (delta)  SPICE1 - spindle and centriole associated protein 1  ARPC1A - actin related protein 2/3 complex, subunit 1a, 41kda  TRIM9 - tripartite motif containing 9  PPP1R13B - protein phosphatase 1, regulatory subunit 13b  RNF40 - ring finger protein 40, e3 ubiquitin protein ligase  DDHD1 - ddhd domain containing 1  OBSL1 - obscurin-like 1  PTPRD - protein tyrosine phosphatase, receptor type, d  CUL7 - cullin 7  ARFGEF1 - adp-ribosylation factor guanine nucleotide-exchange factor 1 (brefeldin a-inhibited)  LRRC4B - leucine rich repeat containing 4b  RAD1 - rad1 homolog (s. pombe)  NMT1 - n-myristoyltransferase 1  NCKAP1 - nck-associated protein 1  TJP1 - tight junction protein 1  PAPD5 - pap associated domain containing 5  NSD1 - nuclear receptor binding set domain protein 1  MTOR - mechanistic target of rapamycin (serine/threonine kinase)  PTPRS - protein tyrosine phosphatase, receptor type, s  PAXBP1 - pax3 and pax7 binding protein 1  UBQLN2 - ubiquilin 2  ATP13A2 - atpase type 13a2  PLK2 - polo-like kinase 2  LEPROT - leptin receptor overlapping transcript  MYO19 - myosin xix  USP6NL - usp6 n-terminal like  MPHOSPH8 - m-phase phosphoprotein 8  PXN - paxillin  CNOT2 - ccr4-not transcription complex, subunit 2  SIRT1 - sirtuin 1  CTC1 - cts telomere maintenance complex component 1  NOTCH2 - notch 2  RAB5A - rab5a, member ras oncogene family  TMSB4X - thymosin beta 4, x-linked  RAB3A - rab3a, member ras oncogene family  CPLX2 - complexin 2  KMT2B - lysine (k)-specific methyltransferase 2b  PLA2G6 - phospholipase a2, group vi (cytosolic, calcium-independent)  WASF3 - was protein family, member 3  BRD4 - bromodomain containing 4  SDCCAG3 - serologically defined colon cancer antigen 3  IFT140 - intraflagellar transport 140 homolog (chlamydomonas)  RAD21 - rad21 homolog (s. pombe)  RAC3 - ras-related c3 botulinum toxin substrate 3 (rho family, small gtp binding protein rac3)  PAF1 - paf1, rna polymerase ii associated factor, homolog (s. cerevisiae)  RAC1 - ras-related c3 botulinum toxin substrate 1 (rho family, small gtp binding protein rac1)  SART3 - squamous cell carcinoma antigen recognized by t cells 3  MAD1L1 - mad1 mitotic arrest deficient-like 1 (yeast)  ARHGAP18 - rho gtpase activating protein 18  SETD1A - set domain containing 1a  EHD3 - eh-domain containing 3  CCP110 - centriolar coiled coil protein 110kda  TEK - tek tyrosine kinase, endothelial  RALB - v-ral simian leukemia viral oncogene homolog b  CHORDC1 - cysteine and histidine-rich domain (chord) containing 1  TOMM7 - translocase of outer mitochondrial membrane 7 homolog (yeast)  CCT8 - chaperonin containing tcp1, subunit 8 (theta)  CLTC - clathrin, heavy chain (hc)  NCK2 - nck adaptor protein 2  TERF1 - telomeric repeat binding factor (nima-interacting) 1  TERF2 - telomeric repeat binding factor 2  WRAP73 - wd repeat containing, antisense to tp73  CTR9 - ctr9, paf1/rna polymerase ii complex component  CEP70 - centrosomal protein 70kda  PPM1F - protein phosphatase, mg2+/mn2+ dependent, 1f  TESK1 - testis-specific kinase 1  TFDP1 - transcription factor dp-1  C6orf89 - chromosome 6 open reading frame 89  CYFIP2 - cytoplasmic fmr1 interacting protein 2  ARHGEF10 - rho guanine nucleotide exchange factor (gef) 10  ULK1 - unc-51 like autophagy activating kinase 1  FEZ1 - fasciculation and elongation protein zeta 1 (zygin i)  CAMSAP1 - calmodulin regulated spectrin-associated protein 1  FEZ2 - fasciculation and elongation protein zeta 2 (zygin ii)  RASA1 - ras p21 protein activator (gtpase activating protein) 1  FXN - frataxin  CLU - clusterin  TGFA - transforming growth factor, alpha  GNL3L - guanine nucleotide binding protein-like 3 (nucleolar)-like  MAGEL2 - mage-like 2  GOLPH3 - golgi phosphoprotein 3 (coat-protein)  FNIP1 - folliculin interacting protein 1  IK - ik cytokine, down-regulator of hla ii  CTCF - ccctc-binding factor (zinc finger protein)  TGFBR1 - transforming growth factor, beta receptor 1  CROCC - ciliary rootlet coiled-coil, rootletin  YLPM1 - ylp motif containing 1  TGFB3 - transforming growth factor, beta 3  OGT - o-linked n-acetylglucosamine (glcnac) transferase  RB1 - retinoblastoma 1  RAE1 - rae1 rna export 1 homolog (s. pombe)  RNF20 - ring finger protein 20, e3 ubiquitin protein ligase  KDM5A - lysine (k)-specific demethylase 5a  DRG1 - developmentally regulated gtp binding protein 1  DOC2B - double c2-like domains, beta  CUL3 - cullin 3  CHMP4B - charged multivesicular body protein 4b  ASAP1 - arfgap with sh3 domain, ankyrin repeat and ph domain 1 |
| GO:0010564 | regulation of cell cycle process | 6.73E-8 | 1.56E-5 | 1.15 (10334,504,6996,394) | [+] Show genes  HAUS7 - haus augmin-like complex, subunit 7  PHC3 - polyhomeotic homolog 3 (drosophila)  PML - promyelocytic leukemia  CNOT11 - ccr4-not transcription complex, subunit 11  SPAST - spastin  KAT2A - k(lysine) acetyltransferase 2a  BRSK1 - br serine/threonine kinase 1  ZFYVE19 - zinc finger, fyve domain containing 19  SOX4 - sry (sex determining region y)-box 4  PLRG1 - pleiotropic regulator 1  H2AFY - h2a histone family, member y  UBXN2B - ubx domain protein 2b  EHMT2 - euchromatic histone-lysine n-methyltransferase 2  NACC2 - nacc family member 2, ben and btb (poz) domain containing  MARK4 - map/microtubule affinity-regulating kinase 4  RPRD1B - regulation of nuclear pre-mrna domain containing 1b  ADAMTS1 - adam metallopeptidase with thrombospondin type 1 motif, 1  PLK3 - polo-like kinase 3  ANAPC5 - anaphase promoting complex subunit 5  ANAPC7 - anaphase promoting complex subunit 7  USP19 - ubiquitin specific peptidase 19  YTHDF2 - yth domain family, member 2  MAP9 - microtubule-associated protein 9  VPS4B - vacuolar protein sorting 4 homolog b (s. cerevisiae)  MED1 - mediator complex subunit 1  CDC45 - cell division cycle 45  AXIN2 - axin 2  CDC7 - cell division cycle 7  SMARCD3 - swi/snf related, matrix associated, actin dependent regulator of chromatin, subfamily d, member 3  WIZ - widely interspaced zinc finger motifs  ROCK2 - rho-associated, coiled-coil containing protein kinase 2  CRLF3 - cytokine receptor-like factor 3  GNAI1 - guanine nucleotide binding protein (g protein), alpha inhibiting activity polypeptide 1  PHF17 - phd finger protein 17  TAOK3 - tao kinase 3  CEP76 - centrosomal protein 76kda  FZR1 - fizzy/cell division cycle 20 related 1 (drosophila)  SMPD3 - sphingomyelin phosphodiesterase 3, neutral membrane (neutral sphingomyelinase ii)  SENP2 - sumo1/sentrin/smt3 specific peptidase 2  CDK5RAP2 - cdk5 regulatory subunit associated protein 2  PRKACA - protein kinase, camp-dependent, catalytic, alpha  EHMT1 - euchromatic histone-lysine n-methyltransferase 1  CTGF - connective tissue growth factor  CIT - citron (rho-interacting, serine/threonine kinase 21)  DYNC1LI1 - dynein, cytoplasmic 1, light intermediate chain 1  FGFR1OP - fgfr1 oncogene partner  UHRF2 - ubiquitin-like with phd and ring finger domains 2, e3 ubiquitin protein ligase  SDE2 - sde2 telomere maintenance homolog (s. pombe)  CTNNB1 - catenin (cadherin-associated protein), beta 1, 88kda  CNTRL - centriolin  PPP2R5B - protein phosphatase 2, regulatory subunit b', beta  AKT1 - v-akt murine thymoma viral oncogene homolog 1  CSPP1 - centrosome and spindle pole associated protein 1  PCID2 - pci domain containing 2  RIOK2 - rio kinase 2  PPP2R1A - protein phosphatase 2, regulatory subunit a, alpha  TXLNG - taxilin gamma  PPP1R10 - protein phosphatase 1, regulatory subunit 10  AURKC - aurora kinase c  TOPBP1 - topoisomerase (dna) ii binding protein 1  RMI2 - recq mediated genome instability 2  SH3GLB1 - sh3-domain grb2-like endophilin b1  NIPBL - nipped-b homolog (drosophila)  KCNA5 - potassium voltage-gated channel, shaker-related subfamily, member 5  PRPF40A - prp40 pre-mrna processing factor 40 homolog a (s. cerevisiae)  SLC25A33 - solute carrier family 25 (pyrimidine nucleotide carrier), member 33  KIF3B - kinesin family member 3b  RINT1 - rad50 interactor 1  MIIP - migration and invasion inhibitory protein  FBXO7 - f-box protein 7  KLHL22 - kelch-like family member 22  HAUS1 - haus augmin-like complex, subunit 1  PKN2 - protein kinase n2  CSNK1D - casein kinase 1, delta  CSNK2A2 - casein kinase 2, alpha prime polypeptide  PRKCE - protein kinase c, epsilon  TAOK2 - tao kinase 2  CSNK2A1 - casein kinase 2, alpha 1 polypeptide  PRKAR2B - protein kinase, camp-dependent, regulatory, type ii, beta  CNOT8 - ccr4-not transcription complex, subunit 8  PRKAR1A - protein kinase, camp-dependent, regulatory, type i, alpha  APEX1 - apex nuclease (multifunctional dna repair enzyme) 1  KANK2 - kn motif and ankyrin repeat domains 2  DDX3X - dead (asp-glu-ala-asp) box helicase 3, x-linked  SYF2 - syf2 pre-mrna-splicing factor  RNF112 - ring finger protein 112  RFWD3 - ring finger and wd repeat domain 3  SIN3A - sin3 transcription regulator family member a  MIF - macrophage migration inhibitory factor (glycosylation-inhibiting factor)  DDB1 - damage-specific dna binding protein 1, 127kda  SETMAR - set domain and mariner transposase fusion gene  PBX1 - pre-b-cell leukemia homeobox 1  ZBTB17 - zinc finger and btb domain containing 17  DCTN1 - dynactin 1  DCTN3 - dynactin 3 (p22)  MCPH1 - microcephalin 1  DCUN1D3 - dcn1, defective in cullin neddylation 1, domain containing 3  APC - adenomatous polyposis coli  PCGF2 - polycomb group ring finger 2  CCDC8 - coiled-coil domain containing 8  AHCTF1 - at hook containing transcription factor 1  KLHL13 - kelch-like family member 13  CNOT10 - ccr4-not transcription complex, subunit 10  APPL2 - adaptor protein, phosphotyrosine interaction, ph domain and leucine zipper containing 2  UIMC1 - ubiquitin interaction motif containing 1  PAFAH1B1 - platelet-activating factor acetylhydrolase 1b, regulatory subunit 1 (45kda)  APP - amyloid beta (a4) precursor protein  BIRC6 - baculoviral iap repeat containing 6  CEP250 - centrosomal protein 250kda  CNOT6 - ccr4-not transcription complex, subunit 6  NSMCE2 - non-smc element 2, mms21 homolog (s. cerevisiae)  TUBA1A - tubulin, alpha 1a  PDGFRB - platelet-derived growth factor receptor, beta polypeptide  ALMS1 - alstrom syndrome 1  PDGFB - platelet-derived growth factor beta polypeptide  CDC73 - cell division cycle 73  BUB3 - bub3 mitotic checkpoint protein  MECP2 - methyl cpg binding protein 2 (rett syndrome)  FAM107A - family with sequence similarity 107, member a  ZW10 - zw10 kinetochore protein  RHOA - ras homolog family member a  KNSTRN - kinetochore-localized astrin/spag5 binding protein  MEN1 - multiple endocrine neoplasia i  DAPK3 - death-associated protein kinase 3  PCNA - proliferating cell nuclear antigen  CHMP1A - charged multivesicular body protein 1a  CEP78 - centrosomal protein 78kda  PCM1 - pericentriolar material 1  MTA3 - metastasis associated 1 family, member 3  CHMP2B - charged multivesicular body protein 2b  RPTOR - regulatory associated protein of mtor, complex 1  SH2B1 - sh2b adaptor protein 1  ZNF207 - zinc finger protein 207  HAUS2 - haus augmin-like complex, subunit 2  DRD2 - dopamine receptor d2  CDC26 - cell division cycle 26  XPC - xeroderma pigmentosum, complementation group c  HAUS3 - haus augmin-like complex, subunit 3  FBXW7 - f-box and wd repeat domain containing 7, e3 ubiquitin protein ligase  XPO1 - exportin 1 (crm1 homolog, yeast)  RQCD1 - rcd1 required for cell differentiation1 homolog (s. pombe)  CNOT6L - ccr4-not transcription complex, subunit 6-like  TAOK1 - tao kinase 1  PAGR1 - paxip1 associated glutamate-rich protein 1  TNKS1BP1 - tankyrase 1 binding protein 1, 182kda  MAPK15 - mitogen-activated protein kinase 15  WEE1 - wee1 homolog (s. pombe)  SKP1 - s-phase kinase-associated protein 1  PKMYT1 - protein kinase, membrane associated tyrosine/threonine 1  MAX - myc associated factor x  GIGYF2 - grb10 interacting gyf protein 2  CHMP5 - charged multivesicular body protein 5  APPL1 - adaptor protein, phosphotyrosine interaction, ph domain and leucine zipper containing 1  SENP6 - sumo1/sentrin specific peptidase 6  ANKRD17 - ankyrin repeat domain 17  ATP2B4 - atpase, ca++ transporting, plasma membrane 4  PKD1 - polycystic kidney disease 1 (autosomal dominant)  BAX - bcl2-associated x protein  PKD2 - polycystic kidney disease 2 (autosomal dominant)  L3MBTL2 - l(3)mbt-like 2 (drosophila)  PIN1 - peptidylprolyl cis/trans isomerase, nima-interacting 1  STXBP4 - syntaxin binding protein 4  BRCC3 - brca1/brca2-containing complex, subunit 3  FSD1 - fibronectin type iii and spry domain containing 1  TOM1L2 - target of myb1-like 2 (chicken)  ZNF16 - zinc finger protein 16  PLAGL1 - pleiomorphic adenoma gene-like 1  CHMP3 - charged multivesicular body protein 3  ATRX - alpha thalassemia/mental retardation syndrome x-linked  SFPQ - splicing factor proline/glutamine-rich  DYNC1H1 - dynein, cytoplasmic 1, heavy chain 1  SFRP1 - secreted frizzled-related protein 1  DYNC1I2 - dynein, cytoplasmic 1, intermediate chain 2  ZNF711 - zinc finger protein 711  BCL2 - b-cell cll/lymphoma 2  PIK3C3 - phosphatidylinositol 3-kinase, catalytic subunit type 3  BCL2L1 - bcl2-like 1  ECD - ecdysoneless homolog (drosophila)  YWHAG - tyrosine 3-monooxygenase/tryptophan 5-monooxygenase activation protein, gamma polypeptide  CCND1 - cyclin d1  BBS4 - bardet-biedl syndrome 4  YWHAE - tyrosine 3-monooxygenase/tryptophan 5-monooxygenase activation protein, epsilon polypeptide  MAPRE1 - microtubule-associated protein, rp/eb family, member 1  YAF2 - yy1 associated factor 2  CHMP4C - charged multivesicular body protein 4c  BRD7 - bromodomain containing 7  AKAP9 - a kinase (prka) anchor protein (yotiao) 9  FLCN - folliculin  CENPV - centromere protein v  RACGAP1 - rac gtpase activating protein 1  PRMT1 - protein arginine methyltransferase 1  PRMT2 - protein arginine methyltransferase 2  EP300 - e1a binding protein p300  ZFP36L2 - zfp36 ring finger protein-like 2  RDX - radixin  ZFP36L1 - zfp36 ring finger protein-like 1  NBN - nibrin  NUBP1 - nucleotide binding protein 1  TUBG1 - tubulin, gamma 1  RING1 - ring finger protein 1  BMI1 - bmi1 polycomb ring finger oncogene  PSME3 - proteasome (prosome, macropain) activator subunit 3 (pa28 gamma; ki)  AZI1 - 5-azacytidine induced 1  KAT2B - k(lysine) acetyltransferase 2b  BMP7 - bone morphogenetic protein 7  CDC16 - cell division cycle 16  PSMD14 - proteasome (prosome, macropain) 26s subunit, non-atpase, 14  CNOT1 - ccr4-not transcription complex, subunit 1  NDE1 - nude neurodevelopment protein 1  CTDSPL - ctd (carboxy-terminal domain, rna polymerase ii, polypeptide a) small phosphatase-like  NAE1 - nedd8 activating enzyme e1 subunit 1  PHC1 - polyhomeotic homolog 1 (drosophila)  HAUS6 - haus augmin-like complex, subunit 6  EGFR - epidermal growth factor receptor  CNOT7 - ccr4-not transcription complex, subunit 7  HUS1 - hus1 checkpoint homolog (s. pombe)  GPSM2 - g-protein signaling modulator 2  RNF4 - ring finger protein 4  RNF2 - ring finger protein 2  ZNF655 - zinc finger protein 655  CTDSP2 - ctd (carboxy-terminal domain, rna polymerase ii, polypeptide a) small phosphatase 2  ANAPC4 - anaphase promoting complex subunit 4  E2F6 - e2f transcription factor 6  E4F1 - e4f transcription factor 1  E2F4 - e2f transcription factor 4, p107/p130-binding  TRIM37 - tripartite motif containing 37  CDK2AP2 - cyclin-dependent kinase 2 associated protein 2  HSP90AA1 - heat shock protein 90kda alpha (cytosolic), class a member 1  ACTR1A - arp1 actin-related protein 1 homolog a, centractin alpha (yeast)  BTRC - beta-transducin repeat containing e3 ubiquitin protein ligase  ZMPSTE24 - zinc metallopeptidase ste24  TPR - translocated promoter region, nuclear basket protein  MED25 - mediator complex subunit 25  WAPAL - wings apart-like homolog (drosophila)  CCSAP - centriole, cilia and spindle-associated protein  DYNLL1 - dynein, light chain, lc8-type 1  TNKS - tankyrase, trf1-interacting ankyrin-related adp-ribose polymerase  AVEN - apoptosis, caspase activation inhibitor  CASP2 - caspase 2, apoptosis-related cysteine peptidase  CUL9 - cullin 9  MAD2L2 - mad2 mitotic arrest deficient-like 2 (yeast)  PDCD6IP - programmed cell death 6 interacting protein  PHIP - pleckstrin homology domain interacting protein  RPA2 - replication protein a2, 32kda  BECN1 - beclin 1, autophagy related  GIT1 - g protein-coupled receptor kinase interacting arfgap 1  SMC5 - structural maintenance of chromosomes 5  PCBP4 - poly(rc) binding protein 4  CALR - calreticulin  UVRAG - uv radiation resistance associated  CALM2 - calmodulin 2 (phosphorylase kinase, delta)  CDC23 - cell division cycle 23  PDXP - pyridoxal (pyridoxine, vitamin b6) phosphatase  PINX1 - pin2/terf1 interacting, telomerase inhibitor 1  ERCC3 - excision repair cross-complementing rodent repair deficiency, complementation group 3  TUBB - tubulin, beta class i  EED - embryonic ectoderm development  CCND3 - cyclin d3  HNRNPU - heterogeneous nuclear ribonucleoprotein u (scaffold attachment factor a)  RIPK1 - receptor (tnfrsf)-interacting serine-threonine kinase 1  ANAPC10 - anaphase promoting complex subunit 10  RGCC - regulator of cell cycle  TIPIN - timeless interacting protein  TUBB4B - tubulin, beta 4b class ivb  BABAM1 - brisc and brca1 a complex member 1  RAB11A - rab11a, member ras oncogene family  ZNF830 - zinc finger protein 830  CAV2 - caveolin 2  TTL - tubulin tyrosine ligase  SETD2 - set domain containing 2  RBM14 - rna binding motif protein 14  RBX1 - ring-box 1, e3 ubiquitin protein ligase  CCND2 - cyclin d2  MRE11A - mre11 meiotic recombination 11 homolog a (s. cerevisiae)  HMMR - hyaluronan-mediated motility receptor (rhamm)  PRMT5 - protein arginine methyltransferase 5  ERCC2 - excision repair cross-complementing rodent repair deficiency, complementation group 2  PSMD6 - proteasome (prosome, macropain) 26s subunit, non-atpase, 6  MGA - mga, max dimerization protein  CDC27 - cell division cycle 27  PKP4 - plakophilin 4  PLCB1 - phospholipase c, beta 1 (phosphoinositide-specific)  PSMA1 - proteasome (prosome, macropain) subunit, alpha type, 1  PSMA2 - proteasome (prosome, macropain) subunit, alpha type, 2  HDAC8 - histone deacetylase 8  CDC14B - cell division cycle 14b  PSMB1 - proteasome (prosome, macropain) subunit, beta type, 1  KLHL21 - kelch-like family member 21  PSMA5 - proteasome (prosome, macropain) subunit, alpha type, 5  PSMA3 - proteasome (prosome, macropain) subunit, alpha type, 3  PSMB10 - proteasome (prosome, macropain) subunit, beta type, 10  ODF2 - outer dense fiber of sperm tails 2  PSMC6 - proteasome (prosome, macropain) 26s subunit, atpase, 6  PSMD1 - proteasome (prosome, macropain) 26s subunit, non-atpase, 1  PSMC4 - proteasome (prosome, macropain) 26s subunit, atpase, 4  PSMC2 - proteasome (prosome, macropain) 26s subunit, atpase, 2  PSMD9 - proteasome (prosome, macropain) 26s subunit, non-atpase, 9  PSMD10 - proteasome (prosome, macropain) 26s subunit, non-atpase, 10  KLHL18 - kelch-like family member 18  PSMD7 - proteasome (prosome, macropain) 26s subunit, non-atpase, 7  PSMD8 - proteasome (prosome, macropain) 26s subunit, non-atpase, 8  ANAPC1 - anaphase promoting complex subunit 1  PSMD5 - proteasome (prosome, macropain) 26s subunit, non-atpase, 5  PSMD2 - proteasome (prosome, macropain) 26s subunit, non-atpase, 2  PSMD12 - proteasome (prosome, macropain) 26s subunit, non-atpase, 12  CKAP5 - cytoskeleton associated protein 5  HAUS5 - haus augmin-like complex, subunit 5  PSMD13 - proteasome (prosome, macropain) 26s subunit, non-atpase, 13  FGFR1 - fibroblast growth factor receptor 1  DCTN2 - dynactin 2 (p50)  PTEN - phosphatase and tensin homolog  CETN2 - centrin, ef-hand protein, 2  ADAM17 - adam metallopeptidase domain 17  NSFL1C - nsfl1 (p97) cofactor (p47)  NUMA1 - nuclear mitotic apparatus protein 1  DAB2IP - dab2 interacting protein  CLASP1 - cytoplasmic linker associated protein 1  MYBBP1A - myb binding protein (p160) 1a  FEN1 - flap structure-specific endonuclease 1  CDK10 - cyclin-dependent kinase 10  CEP120 - centrosomal protein 120kda  CDK5 - cyclin-dependent kinase 5  CDK4 - cyclin-dependent kinase 4  NPM1 - nucleophosmin (nucleolar phosphoprotein b23, numatrin)  CDK9 - cyclin-dependent kinase 9  KMT2E - lysine (k)-specific methyltransferase 2e  INSR - insulin receptor  INSM1 - insulinoma-associated 1  CDK13 - cyclin-dependent kinase 13  CDKN1B - cyclin-dependent kinase inhibitor 1b (p27, kip1)  CDKN2D - cyclin-dependent kinase inhibitor 2d (p19, inhibits cdk4)  SPICE1 - spindle and centriole associated protein 1  SFI1 - sfi1 homolog, spindle assembly associated (yeast)  KLHL9 - kelch-like family member 9  OBSL1 - obscurin-like 1  RCC2 - regulator of chromosome condensation 2  CUL7 - cullin 7  CDK2 - cyclin-dependent kinase 2  RAD1 - rad1 homolog (s. pombe)  DONSON - downstream neighbor of son  RAB11FIP3 - rab11 family interacting protein 3 (class ii)  NUDT16 - nudix (nucleoside diphosphate linked moiety x)-type motif 16  RYBP - ring1 and yy1 binding protein  PLK2 - polo-like kinase 2  MYO19 - myosin xix  CEP290 - centrosomal protein 290kda  CNOT2 - ccr4-not transcription complex, subunit 2  CNOT4 - ccr4-not transcription complex, subunit 4  CNOT3 - ccr4-not transcription complex, subunit 3  GIPC1 - gipc pdz domain containing family, member 1  CEP57 - centrosomal protein 57kda  CTC1 - cts telomere maintenance complex component 1  ID2 - inhibitor of dna binding 2, dominant negative helix-loop-helix protein  CBX5 - chromobox homolog 5  BRD4 - bromodomain containing 4  SDCCAG3 - serologically defined colon cancer antigen 3  PIK3R4 - phosphoinositide-3-kinase, regulatory subunit 4  RAD21 - rad21 homolog (s. pombe)  RAD17 - rad17 homolog (s. pombe)  PAF1 - paf1, rna polymerase ii associated factor, homolog (s. cerevisiae)  ZNF268 - zinc finger protein 268  MAD1L1 - mad1 mitotic arrest deficient-like 1 (yeast)  INO80 - ino80 complex subunit  VPS4A - vacuolar protein sorting 4 homolog a (s. cerevisiae)  CCP110 - centriolar coiled coil protein 110kda  SUZ12 - suz12 polycomb repressive complex 2 subunit  CHORDC1 - cysteine and histidine-rich domain (chord) containing 1  CLTC - clathrin, heavy chain (hc)  TERF1 - telomeric repeat binding factor (nima-interacting) 1  DYRK3 - dual-specificity tyrosine-(y)-phosphorylation regulated kinase 3  TERF2 - telomeric repeat binding factor 2  CEP70 - centrosomal protein 70kda  TFDP1 - transcription factor dp-1  EPC1 - enhancer of polycomb homolog 1 (drosophila)  ZFYVE26 - zinc finger, fyve domain containing 26  TGFA - transforming growth factor, alpha  CEP41 - centrosomal protein 41kda  IK - ik cytokine, down-regulator of hla ii  CTCF - ccctc-binding factor (zinc finger protein)  CDK5RAP3 - cdk5 regulatory subunit associated protein 3  RBL2 - retinoblastoma-like 2 (p130)  RBBP7 - retinoblastoma binding protein 7  RBBP8 - retinoblastoma binding protein 8  RB1 - retinoblastoma 1  RAE1 - rae1 rna export 1 homolog (s. pombe)  CEP63 - centrosomal protein 63kda  MEPCE - methylphosphate capping enzyme  SND1 - staphylococcal nuclease and tudor domain containing 1  RBBP4 - retinoblastoma binding protein 4  CUL4B - cullin 4b  DRG1 - developmentally regulated gtp binding protein 1  CUL1 - cullin 1  CUL3 - cullin 3  CHMP4B - charged multivesicular body protein 4b |
| GO:0048024 | regulation of mRNA splicing, via spliceosome | 1.03E-7 | 2.34E-5 | 1.34 (10334,96,6996,87) | [+] Show genes  RBM23 - rna binding motif protein 23  NCBP1 - nuclear cap binding protein subunit 1, 80kda  FMR1 - fragile x mental retardation 1  WTAP - wilms tumor 1 associated protein  DDX5 - dead (asp-glu-ala-asp) box helicase 5  SAP18 - sin3a-associated protein, 18kda  MAGOH - mago-nashi homolog, proliferation-associated (drosophila)  RBM39 - rna binding motif protein 39  NCL - nucleolin  PTBP3 - polypyrimidine tract binding protein 3  RBM15 - rna binding motif protein 15  MBNL2 - muscleblind-like splicing regulator 2  SRSF10 - serine/arginine-rich splicing factor 10  RBMX - rna binding motif protein, x-linked  SNW1 - snw domain containing 1  NOVA2 - neuro-oncological ventral antigen 2  NOVA1 - neuro-oncological ventral antigen 1  DAZAP1 - daz associated protein 1  SRSF12 - serine/arginine-rich splicing factor 12  CWC22 - cwc22 spliceosome-associated protein homolog (s. cerevisiae)  PRPF19 - pre-mrna processing factor 19  METTL16 - methyltransferase like 16  SRSF9 - serine/arginine-rich splicing factor 9  CELF4 - cugbp, elav-like family member 4  RBM19 - rna binding motif protein 19  RBM7 - rna binding motif protein 7  RBM5 - rna binding motif protein 5  KHDRBS3 - kh domain containing, rna binding, signal transduction associated 3  DYRK1A - dual-specificity tyrosine-(y)-phosphorylation regulated kinase 1a  PCBP4 - poly(rc) binding protein 4  RNPS1 - rna binding protein s1, serine-rich domain  SON - son dna binding protein  SART3 - squamous cell carcinoma antigen recognized by t cells 3  SMU1 - smu-1 suppressor of mec-8 and unc-52 homolog (c. elegans)  CELF3 - cugbp, elav-like family member 3  PUF60 - poly-u binding splicing factor 60kda  NSRP1 - nuclear speckle splicing regulatory protein 1  CELF6 - cugbp, elav-like family member 6  FXR2 - fragile x mental retardation, autosomal homolog 2  YTHDC1 - yth domain containing 1  PTBP1 - polypyrimidine tract binding protein 1  SNRNP70 - small nuclear ribonucleoprotein 70kda (u1)  HNRNPU - heterogeneous nuclear ribonucleoprotein u (scaffold attachment factor a)  HNRNPK - heterogeneous nuclear ribonucleoprotein k  NUP98 - nucleoporin 98kda  HNRNPL - heterogeneous nuclear ribonucleoprotein l  RBFOX3 - rna binding protein, fox-1 homolog (c. elegans) 3  CELF1 - cugbp, elav-like family member 1  DDX17 - dead (asp-glu-ala-asp) box helicase 17  TRA2A - transformer 2 alpha homolog (drosophila)  RBM17 - rna binding motif protein 17  RBM8A - rna binding motif protein 8a  KHDRBS1 - kh domain containing, rna binding, signal transduction associated 1  RBM42 - rna binding motif protein 42  CELF2 - cugbp, elav-like family member 2  RBM15B - rna binding motif protein 15b  IK - ik cytokine, down-regulator of hla ii  RBM10 - rna binding motif protein 10  SF1 - splicing factor 1  RBFOX2 - rna binding protein, fox-1 homolog (c. elegans) 2  SRPK2 - srsf protein kinase 2  RBM3 - rna binding motif (rnp1, rrm) protein 3  HNRNPA2B1 - heterogeneous nuclear ribonucleoprotein a2/b1  U2AF2 - u2 small nuclear rna auxiliary factor 2  SRSF1 - serine/arginine-rich splicing factor 1  SRPK1 - srsf protein kinase 1  SRSF2 - serine/arginine-rich splicing factor 2  HNRNPA1 - heterogeneous nuclear ribonucleoprotein a1  RBM4 - rna binding motif protein 4  SRSF3 - serine/arginine-rich splicing factor 3  SRSF4 - serine/arginine-rich splicing factor 4  RBM25 - rna binding motif protein 25  SRSF5 - serine/arginine-rich splicing factor 5  SRSF6 - serine/arginine-rich splicing factor 6  SRSF7 - serine/arginine-rich splicing factor 7  SFSWAP - splicing factor, suppressor of white-apricot homolog (drosophila)  TRA2B - transformer 2 beta homolog (drosophila)  JMJD6 - jumonji domain containing 6  LARP7 - la ribonucleoprotein domain family, member 7  SF3B4 - splicing factor 3b, subunit 4, 49kda  ZC3H10 - zinc finger ccch-type containing 10  ZBTB7A - zinc finger and btb domain containing 7a  CIRBP - cold inducible rna binding protein  TIA1 - tia1 cytotoxic granule-associated rna binding protein  THRAP3 - thyroid hormone receptor associated protein 3  HSPA8 - heat shock 70kda protein 8  RBM4B - rna binding motif protein 4b |
| GO:0051726 | regulation of cell cycle | 1.26E-7 | 2.83E-5 | 1.12 (10334,811,6996,614) | [+] Show genes  WAC - ww domain containing adaptor with coiled-coil  TBRG1 - transforming growth factor beta regulator 1  BTG3 - btg family, member 3  HAUS7 - haus augmin-like complex, subunit 7  ABL1 - c-abl oncogene 1, non-receptor tyrosine kinase  PHC3 - polyhomeotic homolog 3 (drosophila)  PML - promyelocytic leukemia  CNOT11 - ccr4-not transcription complex, subunit 11  LFNG - lfng o-fucosylpeptide 3-beta-n-acetylglucosaminyltransferase  SPAST - spastin  DOT1L - dot1-like histone h3k79 methyltransferase  KAT2A - k(lysine) acetyltransferase 2a  BRSK1 - br serine/threonine kinase 1  ZFYVE19 - zinc finger, fyve domain containing 19  SOX4 - sry (sex determining region y)-box 4  PLRG1 - pleiotropic regulator 1  TIPRL - tip41, tor signaling pathway regulator-like (s. cerevisiae)  H2AFY - h2a histone family, member y  GTPBP4 - gtp binding protein 4  ACTB - actin, beta  UBXN2B - ubx domain protein 2b  EHMT2 - euchromatic histone-lysine n-methyltransferase 2  SON - son dna binding protein  ATF2 - activating transcription factor 2  NACC2 - nacc family member 2, ben and btb (poz) domain containing  CLOCK - clock circadian regulator  CCNDBP1 - cyclin d-type binding-protein 1  MARK4 - map/microtubule affinity-regulating kinase 4  PRKAG2 - protein kinase, amp-activated, gamma 2 non-catalytic subunit  CAMK2N1 - calcium/calmodulin-dependent protein kinase ii inhibitor 1  RPRD1B - regulation of nuclear pre-mrna domain containing 1b  ADAMTS1 - adam metallopeptidase with thrombospondin type 1 motif, 1  PLK3 - polo-like kinase 3  ANAPC5 - anaphase promoting complex subunit 5  USP19 - ubiquitin specific peptidase 19  ANAPC7 - anaphase promoting complex subunit 7  YTHDF2 - yth domain family, member 2  MAP9 - microtubule-associated protein 9  VPS4B - vacuolar protein sorting 4 homolog b (s. cerevisiae)  ADARB1 - adenosine deaminase, rna-specific, b1  CDC45 - cell division cycle 45  MED1 - mediator complex subunit 1  BAP1 - brca1 associated protein-1 (ubiquitin carboxy-terminal hydrolase)  AXIN2 - axin 2  CDC7 - cell division cycle 7  SMARCD3 - swi/snf related, matrix associated, actin dependent regulator of chromatin, subfamily d, member 3  WIZ - widely interspaced zinc finger motifs  AFAP1L2 - actin filament associated protein 1-like 2  ROCK2 - rho-associated, coiled-coil containing protein kinase 2  CRLF3 - cytokine receptor-like factor 3  GNAI1 - guanine nucleotide binding protein (g protein), alpha inhibiting activity polypeptide 1  PHF17 - phd finger protein 17  CEP76 - centrosomal protein 76kda  TAOK3 - tao kinase 3  FZR1 - fizzy/cell division cycle 20 related 1 (drosophila)  SMPD3 - sphingomyelin phosphodiesterase 3, neutral membrane (neutral sphingomyelinase ii)  PRKAB1 - protein kinase, amp-activated, beta 1 non-catalytic subunit  CDK5RAP2 - cdk5 regulatory subunit associated protein 2  SENP2 - sumo1/sentrin/smt3 specific peptidase 2  PRKACB - protein kinase, camp-dependent, catalytic, beta  PRKACA - protein kinase, camp-dependent, catalytic, alpha  WDR12 - wd repeat domain 12  EHMT1 - euchromatic histone-lysine n-methyltransferase 1  CTGF - connective tissue growth factor  PRCC - papillary renal cell carcinoma (translocation-associated)  CTBP1 - c-terminal binding protein 1  CHFR - checkpoint with forkhead and ring finger domains, e3 ubiquitin protein ligase  ZWINT - zw10 interacting kinetochore protein  ING4 - inhibitor of growth family, member 4  CIT - citron (rho-interacting, serine/threonine kinase 21)  BEX2 - brain expressed x-linked 2  FGFR1OP - fgfr1 oncogene partner  DYNC1LI1 - dynein, cytoplasmic 1, light intermediate chain 1  UHRF2 - ubiquitin-like with phd and ring finger domains 2, e3 ubiquitin protein ligase  SDE2 - sde2 telomere maintenance homolog (s. pombe)  FZD3 - frizzled family receptor 3  CTNNB1 - catenin (cadherin-associated protein), beta 1, 88kda  CNTRL - centriolin  PPP2R5B - protein phosphatase 2, regulatory subunit b', beta  AKT1 - v-akt murine thymoma viral oncogene homolog 1  CSPP1 - centrosome and spindle pole associated protein 1  PCID2 - pci domain containing 2  PPP2R1A - protein phosphatase 2, regulatory subunit a, alpha  RIOK2 - rio kinase 2  TXLNG - taxilin gamma  PPP1R10 - protein phosphatase 1, regulatory subunit 10  AURKC - aurora kinase c  TOPBP1 - topoisomerase (dna) ii binding protein 1  RMI2 - recq mediated genome instability 2  PPM1G - protein phosphatase, mg2+/mn2+ dependent, 1g  CYLD - cylindromatosis (turban tumor syndrome)  CDKL5 - cyclin-dependent kinase-like 5  DDX39B - dead (asp-glu-ala-asp) box polypeptide 39b  SH3GLB1 - sh3-domain grb2-like endophilin b1  NIPBL - nipped-b homolog (drosophila)  KCNA5 - potassium voltage-gated channel, shaker-related subfamily, member 5  CRY1 - cryptochrome 1 (photolyase-like)  STAT3 - signal transducer and activator of transcription 3 (acute-phase response factor)  PRNP - prion protein  PRPF40A - prp40 pre-mrna processing factor 40 homolog a (s. cerevisiae)  SLC25A33 - solute carrier family 25 (pyrimidine nucleotide carrier), member 33  RINT1 - rad50 interactor 1  KIF3B - kinesin family member 3b  PIM2 - pim-2 oncogene  MAP2K1 - mitogen-activated protein kinase kinase 1  MIIP - migration and invasion inhibitory protein  FBXO7 - f-box protein 7  SRPK2 - srsf protein kinase 2  CCNI - cyclin i  KLHL22 - kelch-like family member 22  HAUS1 - haus augmin-like complex, subunit 1  PKN2 - protein kinase n2  CSNK1D - casein kinase 1, delta  NUP214 - nucleoporin 214kda  PRKCE - protein kinase c, epsilon  CSNK2A2 - casein kinase 2, alpha prime polypeptide  NR4A3 - nuclear receptor subfamily 4, group a, member 3  TAOK2 - tao kinase 2  SRC - v-src avian sarcoma (schmidt-ruppin a-2) viral oncogene homolog  CSNK2A1 - casein kinase 2, alpha 1 polypeptide  PRKAR2B - protein kinase, camp-dependent, regulatory, type ii, beta  PRKCA - protein kinase c, alpha  CNOT8 - ccr4-not transcription complex, subunit 8  PRKAR1A - protein kinase, camp-dependent, regulatory, type i, alpha  PRKAG1 - protein kinase, amp-activated, gamma 1 non-catalytic subunit  CDK12 - cyclin-dependent kinase 12  APEX1 - apex nuclease (multifunctional dna repair enzyme) 1  TMEM8B - transmembrane protein 8b  KANK2 - kn motif and ankyrin repeat domains 2  DDX3X - dead (asp-glu-ala-asp) box helicase 3, x-linked  SYF2 - syf2 pre-mrna-splicing factor  BIRC2 - baculoviral iap repeat containing 2  RNF112 - ring finger protein 112  RFWD3 - ring finger and wd repeat domain 3  SIN3A - sin3 transcription regulator family member a  MIF - macrophage migration inhibitory factor (glycosylation-inhibiting factor)  SDCBP - syndecan binding protein (syntenin)  CCNI2 - cyclin i family, member 2  DDB1 - damage-specific dna binding protein 1, 127kda  SETMAR - set domain and mariner transposase fusion gene  PBX1 - pre-b-cell leukemia homeobox 1  DCTN1 - dynactin 1  ZBTB17 - zinc finger and btb domain containing 17  GTF2H1 - general transcription factor iih, polypeptide 1, 62kda  DCTN3 - dynactin 3 (p22)  MCPH1 - microcephalin 1  DCUN1D3 - dcn1, defective in cullin neddylation 1, domain containing 3  APBB2 - amyloid beta (a4) precursor protein-binding, family b, member 2  APC - adenomatous polyposis coli  PCGF2 - polycomb group ring finger 2  APBB1 - amyloid beta (a4) precursor protein-binding, family b, member 1 (fe65)  HECA - headcase homolog (drosophila)  CCDC8 - coiled-coil domain containing 8  AHCTF1 - at hook containing transcription factor 1  KLHL13 - kelch-like family member 13  CNOT10 - ccr4-not transcription complex, subunit 10  APPL2 - adaptor protein, phosphotyrosine interaction, ph domain and leucine zipper containing 2  MNAT1 - mnat cdk-activating kinase assembly factor 1  UIMC1 - ubiquitin interaction motif containing 1  PAFAH1B1 - platelet-activating factor acetylhydrolase 1b, regulatory subunit 1 (45kda)  APP - amyloid beta (a4) precursor protein  BIRC6 - baculoviral iap repeat containing 6  MNT - mnt, max dimerization protein  CCPG1 - cell cycle progression 1  CNOT6 - ccr4-not transcription complex, subunit 6  ARNTL - aryl hydrocarbon receptor nuclear translocator-like  CEP250 - centrosomal protein 250kda  H2AFX - h2a histone family, member x  NSMCE2 - non-smc element 2, mms21 homolog (s. cerevisiae)  TUBA1A - tubulin, alpha 1a  PDGFRB - platelet-derived growth factor receptor, beta polypeptide  ALMS1 - alstrom syndrome 1  CDC73 - cell division cycle 73  PDGFB - platelet-derived growth factor beta polypeptide  MECP2 - methyl cpg binding protein 2 (rett syndrome)  BUB3 - bub3 mitotic checkpoint protein  RHOB - ras homolog family member b  FAM107A - family with sequence similarity 107, member a  ZW10 - zw10 kinetochore protein  THAP5 - thap domain containing 5  RHOA - ras homolog family member a  KNSTRN - kinetochore-localized astrin/spag5 binding protein  MEIS2 - meis homeobox 2  WDR6 - wd repeat domain 6  MEN1 - multiple endocrine neoplasia i  HCFC1 - host cell factor c1 (vp16-accessory protein)  ASNS - asparagine synthetase (glutamine-hydrolyzing)  DAPK3 - death-associated protein kinase 3  PCNA - proliferating cell nuclear antigen  CHMP1A - charged multivesicular body protein 1a  CEP78 - centrosomal protein 78kda  ANGEL2 - angel homolog 2 (drosophila)  BRINP1 - bone morphogenetic protein/retinoic acid inducible neural-specific 1  PCM1 - pericentriolar material 1  HINFP - histone h4 transcription factor  CDK16 - cyclin-dependent kinase 16  CDK17 - cyclin-dependent kinase 17  MTA3 - metastasis associated 1 family, member 3  CHMP2B - charged multivesicular body protein 2b  CDC37 - cell division cycle 37  PLA2G16 - phospholipase a2, group xvi  RPTOR - regulatory associated protein of mtor, complex 1  THAP1 - thap domain containing, apoptosis associated protein 1  SH2B1 - sh2b adaptor protein 1  ASCL1 - achaete-scute complex homolog 1 (drosophila)  ZNF207 - zinc finger protein 207  ATRIP - atr interacting protein  HAUS2 - haus augmin-like complex, subunit 2  PGGT1B - protein geranylgeranyltransferase type i, beta subunit  DRD2 - dopamine receptor d2  CDC26 - cell division cycle 26  XPC - xeroderma pigmentosum, complementation group c  HAUS3 - haus augmin-like complex, subunit 3  FBXW7 - f-box and wd repeat domain containing 7, e3 ubiquitin protein ligase  RQCD1 - rcd1 required for cell differentiation1 homolog (s. pombe)  CNOT6L - ccr4-not transcription complex, subunit 6-like  XPO1 - exportin 1 (crm1 homolog, yeast)  TAOK1 - tao kinase 1  PAGR1 - paxip1 associated glutamate-rich protein 1  LATS1 - large tumor suppressor kinase 1  ZFHX3 - zinc finger homeobox 3  TNKS1BP1 - tankyrase 1 binding protein 1, 182kda  MAPK15 - mitogen-activated protein kinase 15  SKIL - ski-like oncogene  WEE1 - wee1 homolog (s. pombe)  SKP1 - s-phase kinase-associated protein 1  PKMYT1 - protein kinase, membrane associated tyrosine/threonine 1  MAX - myc associated factor x  GIGYF2 - grb10 interacting gyf protein 2  CHMP5 - charged multivesicular body protein 5  APPL1 - adaptor protein, phosphotyrosine interaction, ph domain and leucine zipper containing 1  SENP6 - sumo1/sentrin specific peptidase 6  VASH1 - vasohibin 1  DIRAS3 - diras family, gtp-binding ras-like 3  ANKRD17 - ankyrin repeat domain 17  ATP2B4 - atpase, ca++ transporting, plasma membrane 4  PKD1 - polycystic kidney disease 1 (autosomal dominant)  BAX - bcl2-associated x protein  GRK5 - g protein-coupled receptor kinase 5  PKD2 - polycystic kidney disease 2 (autosomal dominant)  PNPT1 - polyribonucleotide nucleotidyltransferase 1  L3MBTL2 - l(3)mbt-like 2 (drosophila)  BAK1 - bcl2-antagonist/killer 1  DLG1 - discs, large homolog 1 (drosophila)  PIN1 - peptidylprolyl cis/trans isomerase, nima-interacting 1  LRP6 - low density lipoprotein receptor-related protein 6  STXBP4 - syntaxin binding protein 4  BRCC3 - brca1/brca2-containing complex, subunit 3  FSD1 - fibronectin type iii and spry domain containing 1  DHCR24 - 24-dehydrocholesterol reductase  TOM1L2 - target of myb1-like 2 (chicken)  FBXL3 - f-box and leucine-rich repeat protein 3  ZNF16 - zinc finger protein 16  PLAGL1 - pleiomorphic adenoma gene-like 1  UBA3 - ubiquitin-like modifier activating enzyme 3  CDK5RAP1 - cdk5 regulatory subunit associated protein 1  CHMP3 - charged multivesicular body protein 3  ATRX - alpha thalassemia/mental retardation syndrome x-linked  SIPA1 - signal-induced proliferation-associated 1  SFPQ - splicing factor proline/glutamine-rich  DYNC1H1 - dynein, cytoplasmic 1, heavy chain 1  SFRP1 - secreted frizzled-related protein 1  DYNC1I2 - dynein, cytoplasmic 1, intermediate chain 2  ZNF711 - zinc finger protein 711  SRSF5 - serine/arginine-rich splicing factor 5  BCR - breakpoint cluster region  EIF2AK4 - eukaryotic translation initiation factor 2 alpha kinase 4  CDKL2 - cyclin-dependent kinase-like 2 (cdc2-related kinase)  BCL2 - b-cell cll/lymphoma 2  PIK3C3 - phosphatidylinositol 3-kinase, catalytic subunit type 3  ECD - ecdysoneless homolog (drosophila)  BCL2L1 - bcl2-like 1  YWHAG - tyrosine 3-monooxygenase/tryptophan 5-monooxygenase activation protein, gamma polypeptide  CCND1 - cyclin d1  EME1 - essential meiotic structure-specific endonuclease 1  BBS4 - bardet-biedl syndrome 4  YWHAE - tyrosine 3-monooxygenase/tryptophan 5-monooxygenase activation protein, epsilon polypeptide  CCNK - cyclin k  MAPRE1 - microtubule-associated protein, rp/eb family, member 1  YAF2 - yy1 associated factor 2  CHMP4C - charged multivesicular body protein 4c  MAPRE3 - microtubule-associated protein, rp/eb family, member 3  TSC1 - tuberous sclerosis 1  AKAP9 - a kinase (prka) anchor protein (yotiao) 9  BRD7 - bromodomain containing 7  CAB39L - calcium binding protein 39-like  MAEA - macrophage erythroblast attacher  FLCN - folliculin  RACGAP1 - rac gtpase activating protein 1  CENPV - centromere protein v  CDKL4 - cyclin-dependent kinase-like 4  CCNL2 - cyclin l2  PRMT1 - protein arginine methyltransferase 1  IQGAP1 - iq motif containing gtpase activating protein 1  PRMT2 - protein arginine methyltransferase 2  EP300 - e1a binding protein p300  ZFP36L2 - zfp36 ring finger protein-like 2  RDX - radixin  ZFP36L1 - zfp36 ring finger protein-like 1  NSUN2 - nop2/sun rna methyltransferase family, member 2  NBN - nibrin  NUBP1 - nucleotide binding protein 1  RRAGB - ras-related gtp binding b  EIF4E - eukaryotic translation initiation factor 4e  TUBG1 - tubulin, gamma 1  RING1 - ring finger protein 1  TRIM32 - tripartite motif containing 32  BMI1 - bmi1 polycomb ring finger oncogene  PSME3 - proteasome (prosome, macropain) activator subunit 3 (pa28 gamma; ki)  AZI1 - 5-azacytidine induced 1  STRADA - ste20-related kinase adaptor alpha  TSG101 - tumor susceptibility gene 101  EIF4G2 - eukaryotic translation initiation factor 4 gamma, 2  RHEB - ras homolog enriched in brain  TSC2 - tuberous sclerosis 2  KAT2B - k(lysine) acetyltransferase 2b  FBXL12 - f-box and leucine-rich repeat protein 12  BMP7 - bone morphogenetic protein 7  CDC16 - cell division cycle 16  PSMD14 - proteasome (prosome, macropain) 26s subunit, non-atpase, 14  CNOT1 - ccr4-not transcription complex, subunit 1  NDE1 - nude neurodevelopment protein 1  CTDSPL - ctd (carboxy-terminal domain, rna polymerase ii, polypeptide a) small phosphatase-like  NAE1 - nedd8 activating enzyme e1 subunit 1  CDC123 - cell division cycle 123  CABLES2 - cdk5 and abl enzyme substrate 2  PHC1 - polyhomeotic homolog 1 (drosophila)  TP53BP1 - tumor protein p53 binding protein 1  TP53BP2 - tumor protein p53 binding protein, 2  CNOT7 - ccr4-not transcription complex, subunit 7  EGFR - epidermal growth factor receptor  TOP2B - topoisomerase (dna) ii beta 180kda  HAUS6 - haus augmin-like complex, subunit 6  NABP2 - nucleic acid binding protein 2  HUS1 - hus1 checkpoint homolog (s. pombe)  UHMK1 - u2af homology motif (uhm) kinase 1  GPSM2 - g-protein signaling modulator 2  RNF4 - ring finger protein 4  RNF2 - ring finger protein 2  ZNF655 - zinc finger protein 655  CTDSP2 - ctd (carboxy-terminal domain, rna polymerase ii, polypeptide a) small phosphatase 2  ANAPC4 - anaphase promoting complex subunit 4  E2F6 - e2f transcription factor 6  E4F1 - e4f transcription factor 1  FEM1B - fem-1 homolog b (c. elegans)  E2F4 - e2f transcription factor 4, p107/p130-binding  E2F5 - e2f transcription factor 5, p130-binding  TRIM37 - tripartite motif containing 37  CDK2AP2 - cyclin-dependent kinase 2 associated protein 2  HSP90AA1 - heat shock protein 90kda alpha (cytosolic), class a member 1  CDK5R2 - cyclin-dependent kinase 5, regulatory subunit 2 (p39)  ACTR1A - arp1 actin-related protein 1 homolog a, centractin alpha (yeast)  ZMPSTE24 - zinc metallopeptidase ste24  BTRC - beta-transducin repeat containing e3 ubiquitin protein ligase  MED25 - mediator complex subunit 25  TPR - translocated promoter region, nuclear basket protein  WAPAL - wings apart-like homolog (drosophila)  HSPA8 - heat shock 70kda protein 8  CCSAP - centriole, cilia and spindle-associated protein  TRIM35 - tripartite motif containing 35  ZWILCH - zwilch kinetochore protein  LAMTOR3 - late endosomal/lysosomal adaptor, mapk and mtor activator 3  CDK19 - cyclin-dependent kinase 19  PHACTR4 - phosphatase and actin regulator 4  STK33 - serine/threonine kinase 33  DYNLL1 - dynein, light chain, lc8-type 1  TNKS - tankyrase, trf1-interacting ankyrin-related adp-ribose polymerase  AVEN - apoptosis, caspase activation inhibitor  CASP2 - caspase 2, apoptosis-related cysteine peptidase  CUL9 - cullin 9  CASP3 - caspase 3, apoptosis-related cysteine peptidase  RPA3 - replication protein a3, 14kda  PDCD6IP - programmed cell death 6 interacting protein  MAD2L2 - mad2 mitotic arrest deficient-like 2 (yeast)  LATS2 - large tumor suppressor kinase 2  SRA1 - steroid receptor rna activator 1  PHIP - pleckstrin homology domain interacting protein  RPA2 - replication protein a2, 32kda  BECN1 - beclin 1, autophagy related  PEA15 - phosphoprotein enriched in astrocytes 15  GIT1 - g protein-coupled receptor kinase interacting arfgap 1  SMC5 - structural maintenance of chromosomes 5  PCBP4 - poly(rc) binding protein 4  LAMTOR1 - late endosomal/lysosomal adaptor, mapk and mtor activator 1  CALR - calreticulin  UVRAG - uv radiation resistance associated  CALM2 - calmodulin 2 (phosphorylase kinase, delta)  CDC23 - cell division cycle 23  CCNL1 - cyclin l1  INTS3 - integrator complex subunit 3  PDXP - pyridoxal (pyridoxine, vitamin b6) phosphatase  TTLL12 - tubulin tyrosine ligase-like family, member 12  CITED2 - cbp/p300-interacting transactivator, with glu/asp-rich carboxy-terminal domain, 2  MSH2 - muts homolog 2, colon cancer, nonpolyposis type 1 (e. coli)  CCNT2 - cyclin t2  PINX1 - pin2/terf1 interacting, telomerase inhibitor 1  ERCC3 - excision repair cross-complementing rodent repair deficiency, complementation group 3  LIN52 - lin-52 homolog (c. elegans)  TUBB - tubulin, beta class i  EED - embryonic ectoderm development  CCND3 - cyclin d3  CCNT1 - cyclin t1  ERN1 - endoplasmic reticulum to nucleus signaling 1  CCNH - cyclin h  CCNG2 - cyclin g2  GBF1 - golgi brefeldin a resistant guanine nucleotide exchange factor 1  RIPK1 - receptor (tnfrsf)-interacting serine-threonine kinase 1  HNRNPU - heterogeneous nuclear ribonucleoprotein u (scaffold attachment factor a)  RGCC - regulator of cell cycle  ANAPC10 - anaphase promoting complex subunit 10  FBXO4 - f-box protein 4  TIPIN - timeless interacting protein  FBXO6 - f-box protein 6  RPS6KA2 - ribosomal protein s6 kinase, 90kda, polypeptide 2  TUBB4B - tubulin, beta 4b class ivb  RPS6KB1 - ribosomal protein s6 kinase, 70kda, polypeptide 1  BABAM1 - brisc and brca1 a complex member 1  RAB11A - rab11a, member ras oncogene family  ZNF830 - zinc finger protein 830  CAV2 - caveolin 2  TTL - tubulin tyrosine ligase  THOC1 - tho complex 1  YTHDC2 - yth domain containing 2  SETD2 - set domain containing 2  RBX1 - ring-box 1, e3 ubiquitin protein ligase  RBM14 - rna binding motif protein 14  CCNC - cyclin c  CCND2 - cyclin d2  MRE11A - mre11 meiotic recombination 11 homolog a (s. cerevisiae)  HMMR - hyaluronan-mediated motility receptor (rhamm)  PRMT5 - protein arginine methyltransferase 5  ERCC2 - excision repair cross-complementing rodent repair deficiency, complementation group 2  NABP1 - nucleic acid binding protein 1  NR4A1 - nuclear receptor subfamily 4, group a, member 1  PSMD6 - proteasome (prosome, macropain) 26s subunit, non-atpase, 6  MGA - mga, max dimerization protein  CDK11B - cyclin-dependent kinase 11b  HEXIM1 - hexamethylene bis-acetamide inducible 1  CDC27 - cell division cycle 27  INHA - inhibin, alpha  PKP4 - plakophilin 4  BOP1 - block of proliferation 1  SGSM3 - small g protein signaling modulator 3  SPIN2A - spindlin family, member 2a  HTRA2 - htra serine peptidase 2  PLCB1 - phospholipase c, beta 1 (phosphoinositide-specific)  USP16 - ubiquitin specific peptidase 16  NLE1 - notchless homolog 1 (drosophila)  PSMA1 - proteasome (prosome, macropain) subunit, alpha type, 1  PSMA2 - proteasome (prosome, macropain) subunit, alpha type, 2  HDAC8 - histone deacetylase 8  ILK - integrin-linked kinase  CDC14B - cell division cycle 14b  PSMB1 - proteasome (prosome, macropain) subunit, beta type, 1  KLHL21 - kelch-like family member 21  PSMA5 - proteasome (prosome, macropain) subunit, alpha type, 5  PSMA3 - proteasome (prosome, macropain) subunit, alpha type, 3  PSMB10 - proteasome (prosome, macropain) subunit, beta type, 10  CCNYL1 - cyclin y-like 1  ODF2 - outer dense fiber of sperm tails 2  PSMC6 - proteasome (prosome, macropain) 26s subunit, atpase, 6  PSMD1 - proteasome (prosome, macropain) 26s subunit, non-atpase, 1  PSMC4 - proteasome (prosome, macropain) 26s subunit, atpase, 4  PSMC2 - proteasome (prosome, macropain) 26s subunit, atpase, 2  PSMD9 - proteasome (prosome, macropain) 26s subunit, non-atpase, 9  DYNLT3 - dynein, light chain, tctex-type 3  KLHL18 - kelch-like family member 18  PSMD10 - proteasome (prosome, macropain) 26s subunit, non-atpase, 10  PSMD7 - proteasome (prosome, macropain) 26s subunit, non-atpase, 7  PSMD8 - proteasome (prosome, macropain) 26s subunit, non-atpase, 8  ANAPC1 - anaphase promoting complex subunit 1  GAS2L1 - growth arrest-specific 2 like 1  PSMD5 - proteasome (prosome, macropain) 26s subunit, non-atpase, 5  PSMD2 - proteasome (prosome, macropain) 26s subunit, non-atpase, 2  FGFR2 - fibroblast growth factor receptor 2  PSMD12 - proteasome (prosome, macropain) 26s subunit, non-atpase, 12  CKAP5 - cytoskeleton associated protein 5  HAUS5 - haus augmin-like complex, subunit 5  PSMD13 - proteasome (prosome, macropain) 26s subunit, non-atpase, 13  TAF6 - taf6 rna polymerase ii, tata box binding protein (tbp)-associated factor, 80kda  FGFR1 - fibroblast growth factor receptor 1  DCTN2 - dynactin 2 (p50)  LAMTOR5 - late endosomal/lysosomal adaptor, mapk and mtor activator 5  PTEN - phosphatase and tensin homolog  CETN2 - centrin, ef-hand protein, 2  JMY - junction mediating and regulatory protein, p53 cofactor  ADAM17 - adam metallopeptidase domain 17  CCNY - cyclin y  NSFL1C - nsfl1 (p97) cofactor (p47)  NUMA1 - nuclear mitotic apparatus protein 1  DAB2IP - dab2 interacting protein  CLASP1 - cytoplasmic linker associated protein 1  GMNN - geminin, dna replication inhibitor  MADD - map-kinase activating death domain  MYBBP1A - myb binding protein (p160) 1a  SLC38A9 - solute carrier family 38, member 9  USP22 - ubiquitin specific peptidase 22  FEN1 - flap structure-specific endonuclease 1  CDK10 - cyclin-dependent kinase 10  ITGB1 - integrin, beta 1 (fibronectin receptor, beta polypeptide, antigen cd29 includes mdf2, msk12)  CEP120 - centrosomal protein 120kda  IPO7 - importin 7  CDK5 - cyclin-dependent kinase 5  CDK4 - cyclin-dependent kinase 4  NPM1 - nucleophosmin (nucleolar phosphoprotein b23, numatrin)  CDK9 - cyclin-dependent kinase 9  KMT2E - lysine (k)-specific methyltransferase 2e  CDK8 - cyclin-dependent kinase 8  LIN54 - lin-54 homolog (c. elegans)  INSR - insulin receptor  CDK7 - cyclin-dependent kinase 7  INSM1 - insulinoma-associated 1  CDKN1C - cyclin-dependent kinase inhibitor 1c (p57, kip2)  CDK13 - cyclin-dependent kinase 13  CDKN1B - cyclin-dependent kinase inhibitor 1b (p27, kip1)  PDCD4 - programmed cell death 4 (neoplastic transformation inhibitor)  SPICE1 - spindle and centriole associated protein 1  PTPN3 - protein tyrosine phosphatase, non-receptor type 3  CDKN2D - cyclin-dependent kinase inhibitor 2d (p19, inhibits cdk4)  SFI1 - sfi1 homolog, spindle assembly associated (yeast)  PTPN11 - protein tyrosine phosphatase, non-receptor type 11  KLHL9 - kelch-like family member 9  PPP1R13B - protein phosphatase 1, regulatory subunit 13b  OBSL1 - obscurin-like 1  CUL7 - cullin 7  RCC2 - regulator of chromosome condensation 2  CDK2 - cyclin-dependent kinase 2  RAD1 - rad1 homolog (s. pombe)  DONSON - downstream neighbor of son  RAB11FIP3 - rab11 family interacting protein 3 (class ii)  TIMP2 - timp metallopeptidase inhibitor 2  NUDT16 - nudix (nucleoside diphosphate linked moiety x)-type motif 16  RYBP - ring1 and yy1 binding protein  MTOR - mechanistic target of rapamycin (serine/threonine kinase)  MUS81 - mus81 structure-specific endonuclease subunit  PTPRK - protein tyrosine phosphatase, receptor type, k  PLK2 - polo-like kinase 2  MYO19 - myosin xix  CEP290 - centrosomal protein 290kda  CNOT2 - ccr4-not transcription complex, subunit 2  PUM1 - pumilio homolog 1 (drosophila)  CNOT4 - ccr4-not transcription complex, subunit 4  CNOT3 - ccr4-not transcription complex, subunit 3  GIPC1 - gipc pdz domain containing family, member 1  SIRT1 - sirtuin 1  CEP57 - centrosomal protein 57kda  NOTCH1 - notch 1  CTC1 - cts telomere maintenance complex component 1  NOTCH2 - notch 2  ID2 - inhibitor of dna binding 2, dominant negative helix-loop-helix protein  ID3 - inhibitor of dna binding 3, dominant negative helix-loop-helix protein  CBX5 - chromobox homolog 5  BRD4 - bromodomain containing 4  RRAGC - ras-related gtp binding c  SDCCAG3 - serologically defined colon cancer antigen 3  PIK3R4 - phosphoinositide-3-kinase, regulatory subunit 4  TARDBP - tar dna binding protein  RAD21 - rad21 homolog (s. pombe)  RAD17 - rad17 homolog (s. pombe)  PAF1 - paf1, rna polymerase ii associated factor, homolog (s. cerevisiae)  ZNF268 - zinc finger protein 268  MAD1L1 - mad1 mitotic arrest deficient-like 1 (yeast)  VPS4A - vacuolar protein sorting 4 homolog a (s. cerevisiae)  INO80 - ino80 complex subunit  CCP110 - centriolar coiled coil protein 110kda  MDC1 - mediator of dna-damage checkpoint 1  SUZ12 - suz12 polycomb repressive complex 2 subunit  CHORDC1 - cysteine and histidine-rich domain (chord) containing 1  RPRM - reprimo, tp53 dependent g2 arrest mediator candidate  CLTC - clathrin, heavy chain (hc)  MOK - mok protein kinase  TERF1 - telomeric repeat binding factor (nima-interacting) 1  TERF2 - telomeric repeat binding factor 2  DYRK3 - dual-specificity tyrosine-(y)-phosphorylation regulated kinase 3  CEP70 - centrosomal protein 70kda  TFDP1 - transcription factor dp-1  C6orf89 - chromosome 6 open reading frame 89  NR2F2 - nuclear receptor subfamily 2, group f, member 2  EPC1 - enhancer of polycomb homolog 1 (drosophila)  CGREF1 - cell growth regulator with ef-hand domain 1  CGRRF1 - cell growth regulator with ring finger domain 1  BCCIP - brca2 and cdkn1a interacting protein  RRAGA - ras-related gtp binding a  PES1 - pescadillo ribosomal biogenesis factor 1  ZFYVE26 - zinc finger, fyve domain containing 26  CLIC1 - chloride intracellular channel 1  TGFA - transforming growth factor, alpha  CEP41 - centrosomal protein 41kda  IK - ik cytokine, down-regulator of hla ii  CTCF - ccctc-binding factor (zinc finger protein)  TGFBR1 - transforming growth factor, beta receptor 1  CDK5RAP3 - cdk5 regulatory subunit associated protein 3  RBL2 - retinoblastoma-like 2 (p130)  RBBP7 - retinoblastoma binding protein 7  RBBP8 - retinoblastoma binding protein 8  TGFB2 - transforming growth factor, beta 2  TGFB1 - transforming growth factor, beta 1  RB1 - retinoblastoma 1  TP53INP1 - tumor protein p53 inducible nuclear protein 1  RAE1 - rae1 rna export 1 homolog (s. pombe)  CEP63 - centrosomal protein 63kda  MEPCE - methylphosphate capping enzyme  CDK20 - cyclin-dependent kinase 20  TSPYL2 - tspy-like 2  SND1 - staphylococcal nuclease and tudor domain containing 1  RBBP4 - retinoblastoma binding protein 4  CUL4B - cullin 4b  DRG1 - developmentally regulated gtp binding protein 1  CUL1 - cullin 1  THBS1 - thrombospondin 1  CUL3 - cullin 3  CKS1B - cdc28 protein kinase regulatory subunit 1b  CHMP4B - charged multivesicular body protein 4b  METTL3 - methyltransferase like 3 |
| GO:0120036 | plasma membrane bounded cell projection organization | 1.47E-7 | 3.24E-5 | 1.17 (10334,410,6996,324) | [+] Show genes  EMP1 - epithelial membrane protein 1  MARK2 - map/microtubule affinity-regulating kinase 2  NDN - necdin, melanoma antigen (mage) family member  CTTN - cortactin  NCK1 - nck adaptor protein 1  KIFAP3 - kinesin-associated protein 3  CEP89 - centrosomal protein 89kda  WDR19 - wd repeat domain 19  ABLIM1 - actin binding lim protein 1  ATAT1 - alpha tubulin acetyltransferase 1  SAMD14 - sterile alpha motif domain containing 14  CD2AP - cd2-associated protein  LRRC16A - leucine rich repeat containing 16a  GORAB - golgin, rab6-interacting  IFT46 - intraflagellar transport 46 homolog (chlamydomonas)  PPP1R9A - protein phosphatase 1, regulatory subunit 9a  MKS1 - meckel syndrome, type 1  NRP1 - neuropilin 1  ABL1 - c-abl oncogene 1, non-receptor tyrosine kinase  SSX2IP - synovial sarcoma, x breakpoint 2 interacting protein  PRMT1 - protein arginine methyltransferase 1  RDX - radixin  PMP22 - peripheral myelin protein 22  ARL13B - adp-ribosylation factor-like 13b  MKKS - mckusick-kaufman syndrome  WASF2 - was protein family, member 2  CCDC28B - coiled-coil domain containing 28b  KIF27 - kinesin family member 27  MTSS1L - metastasis suppressor 1-like  ALKBH1 - alkb, alkylation repair homolog 1 (e. coli)  TMEM17 - transmembrane protein 17  FNBP1L - formin binding protein 1-like  PTPDC1 - protein tyrosine phosphatase domain containing 1  SYNGAP1 - synaptic ras gtpase activating protein 1  TRIM32 - tripartite motif containing 32  MYLK - myosin light chain kinase  AZI1 - 5-azacytidine induced 1  BTBD8 - btb (poz) domain containing 8  SNX2 - sorting nexin 2  DOCK10 - dedicator of cytokinesis 10  SNX1 - sorting nexin 1  EHD1 - eh-domain containing 1  BMP7 - bone morphogenetic protein 7  AKAP4 - a kinase (prka) anchor protein 4  MARK4 - map/microtubule affinity-regulating kinase 4  ZNF259 - zinc finger protein 259  CEP19 - centrosomal protein 19kda  ARHGEF7 - rho guanine nucleotide exchange factor (gef) 7  MYH10 - myosin, heavy chain 10, non-muscle  FSCN1 - fascin homolog 1, actin-bundling protein (strongylocentrotus purpuratus)  MYH9 - myosin, heavy chain 9, non-muscle  SNX10 - sorting nexin 10  ARPC5 - actin related protein 2/3 complex, subunit 5, 16kda  MTMR2 - myotubularin related protein 2  MEGF8 - multiple egf-like-domains 8  UHMK1 - u2af homology motif (uhm) kinase 1  CELSR2 - cadherin, egf lag seven-pass g-type receptor 2  PQBP1 - polyglutamine binding protein 1  AHI1 - abelson helper integration site 1  BAG4 - bcl2-associated athanogene 4  TSPAN2 - tetraspanin 2  TRAPPC4 - trafficking protein particle complex 4  CNR1 - cannabinoid receptor 1 (brain)  ACTR2 - arp2 actin-related protein 2 homolog (yeast)  ACTR3 - arp3 actin-related protein 3 homolog (yeast)  NUP85 - nucleoporin 85kda  CNTN1 - contactin 1  ACTL6B - actin-like 6b  TMEM108 - transmembrane protein 108  ROCK1 - rho-associated, coiled-coil containing protein kinase 1  ARFIP2 - adp-ribosylation factor interacting protein 2  CLUAP1 - clusterin associated protein 1  HERC1 - hect and rld domain containing e3 ubiquitin protein ligase family member 1  ATCAY - ataxia, cerebellar, cayman type  STMN1 - stathmin 1  TSPO - translocator protein (18kda)  TNIK - traf2 and nck interacting kinase  E2F4 - e2f transcription factor 4, p107/p130-binding  ATP6V1D - atpase, h+ transporting, lysosomal 34kda, v1 subunit d  L1CAM - l1 cell adhesion molecule  LPAR1 - lysophosphatidic acid receptor 1  S1PR1 - sphingosine-1-phosphate receptor 1  LAMA5 - laminin, alpha 5  FBF1 - fas (tnfrsf6) binding factor 1  UBE4B - ubiquitination factor e4b  WASF1 - was protein family, member 1  BLOC1S3 - biogenesis of lysosomal organelles complex-1, subunit 3  VAV3 - vav 3 guanine nucleotide exchange factor  ABI1 - abl-interactor 1  PLEKHA1 - pleckstrin homology domain containing, family a (phosphoinositide binding specific) member 1  HMGB1 - high mobility group box 1  ARHGEF6 - rac/cdc42 guanine nucleotide exchange factor (gef) 6  PICK1 - protein interacting with prkca 1  CTNNA1 - catenin (cadherin-associated protein), alpha 1, 102kda  KIF3A - kinesin family member 3a  PTK2B - protein tyrosine kinase 2 beta  PARVA - parvin, alpha  DYNLL1 - dynein, light chain, lc8-type 1  PIBF1 - progesterone immunomodulatory binding factor 1  CTNND2 - catenin (cadherin-associated protein), delta 2  CASP3 - caspase 3, apoptosis-related cysteine peptidase  BAIAP2 - bai1-associated protein 2  CCDC41 - coiled-coil domain containing 41  HDAC6 - histone deacetylase 6  STX3 - syntaxin 3  MANF - mesencephalic astrocyte-derived neurotrophic factor  STRN - striatin, calmodulin binding protein  TMEM237 - transmembrane protein 237  EZR - ezrin  UNC119B - unc-119 homolog b (c. elegans)  DDR1 - discoidin domain receptor tyrosine kinase 1  TBC1D23 - tbc1 domain family, member 23  TRIOBP - trio and f-actin binding protein  TCTEX1D2 - tctex1 domain containing 2  VAV2 - vav 2 guanine nucleotide exchange factor  TCTN2 - tectonic family member 2  NCDN - neurochondrin  IFT122 - intraflagellar transport 122 homolog (chlamydomonas)  FYN - fyn oncogene related to src, fgr, yes  SLC25A46 - solute carrier family 25, member 46  VCL - vinculin  TTC17 - tetratricopeptide repeat domain 17  KIT - v-kit hardy-zuckerman 4 feline sarcoma viral oncogene homolog  GLDN - gliomedin  MAPK8IP3 - mitogen-activated protein kinase 8 interacting protein 3  STMN2 - stathmin-like 2  CRKL - v-crk avian sarcoma virus ct10 oncogene homolog-like  CRK - v-crk avian sarcoma virus ct10 oncogene homolog  TMEM216 - transmembrane protein 216  RAB35 - rab35, member ras oncogene family  IFT27 - intraflagellar transport 27 homolog (chlamydomonas)  DBNL - drebrin-like  PRNP - prion protein  GPRIN1 - g protein regulated inducer of neurite outgrowth 1  CORO1B - coronin, actin binding protein, 1b  IFT81 - intraflagellar transport 81 homolog (chlamydomonas)  JAK2 - janus kinase 2  DFNB31 - deafness, autosomal recessive 31  IFT88 - intraflagellar transport 88 homolog (chlamydomonas)  CYFIP1 - cytoplasmic fmr1 interacting protein 1  CSF1R - colony stimulating factor 1 receptor  DMTN - dematin actin binding protein  TXNDC15 - thioredoxin domain containing 15  P2RY12 - purinergic receptor p2y, g-protein coupled, 12  BLOC1S5 - biogenesis of lysosomal organelles complex-1, subunit 5, muted  RAB11A - rab11a, member ras oncogene family  WDR11 - wd repeat domain 11  TROVE2 - trove domain family, member 2  GALNT11 - udp-n-acetyl-alpha-d-galactosamine:polypeptide n-acetylgalactosaminyltransferase 11 (galnac-t11)  MICALL2 - mical-like 2  SNAP29 - synaptosomal-associated protein, 29kda  CCDC88A - coiled-coil domain containing 88a  ATXN10 - ataxin 10  SEC24B - sec24 family, member b (s. cerevisiae)  CSNK1D - casein kinase 1, delta  PLEKHO1 - pleckstrin homology domain containing, family o member 1  TMEM107 - transmembrane protein 107  S1PR2 - sphingosine-1-phosphate receptor 2  CAMSAP2 - calmodulin regulated spectrin-associated protein family, member 2  B9D2 - b9 protein domain 2  GSN - gelsolin  SDCBP - syndecan binding protein (syntenin)  WWTR1 - ww domain containing transcription regulator 1  STMN3 - stathmin-like 3  DCTN1 - dynactin 1  FLNA - filamin a, alpha  PTPN23 - protein tyrosine phosphatase, non-receptor type 23  PSEN1 - presenilin 1  WDR34 - wd repeat domain 34  FOPNL - fgfr1op n-terminal like  ARC - activity-regulated cytoskeleton-associated protein  SHANK1 - sh3 and multiple ankyrin repeat domains 1  CDC14B - cell division cycle 14b  EXOC5 - exocyst complex component 5  OCRL - oculocerebrorenal syndrome of lowe  ARF1 - adp-ribosylation factor 1  UNC5A - unc-5 homolog a (c. elegans)  ODF2 - outer dense fiber of sperm tails 2  MAP1S - microtubule-associated protein 1s  APOD - apolipoprotein d  RAB23 - rab23, member ras oncogene family  APP - amyloid beta (a4) precursor protein  BBS7 - bardet-biedl syndrome 7  DYNLT1 - dynein, light chain, tctex-type 1  PAK3 - p21 protein (cdc42/rac)-activated kinase 3  IFT43 - intraflagellar transport 43 homolog (chlamydomonas)  CEP250 - centrosomal protein 250kda  TCTN1 - tectonic family member 1  ARL3 - adp-ribosylation factor-like 3  DNM3 - dynamin 3  KIAA0586 - kiaa0586  AMIGO1 - adhesion molecule with ig-like domain 1  PTEN - phosphatase and tensin homolog  PDGFRB - platelet-derived growth factor receptor, beta polypeptide  IFT57 - intraflagellar transport 57 homolog (chlamydomonas)  TMEM231 - transmembrane protein 231  ITGA3 - integrin, alpha 3 (antigen cd49c, alpha 3 subunit of vla-3 receptor)  SS18L1 - synovial sarcoma translocation gene on chromosome 18-like 1  TBC1D24 - tbc1 domain family, member 24  MECP2 - methyl cpg binding protein 2 (rett syndrome)  ARID1B - at rich interactive domain 1b (swi1-like)  ARF6 - adp-ribosylation factor 6  FGD1 - fyve, rhogef and ph domain containing 1  ITGB1 - integrin, beta 1 (fibronectin receptor, beta polypeptide, antigen cd29 includes mdf2, msk12)  BBS9 - bardet-biedl syndrome 9  TEKT2 - tektin 2 (testicular)  CDK5 - cyclin-dependent kinase 5  RAB8A - rab8a, member ras oncogene family  WDR60 - wd repeat domain 60  INPPL1 - inositol polyphosphate phosphatase-like 1  CEP78 - centrosomal protein 78kda  PTN - pleiotrophin  C2CD3 - c2 calcium-dependent domain containing 3  INSR - insulin receptor  NUDCD3 - nudc domain containing 3  TTC8 - tetratricopeptide repeat domain 8  PCM1 - pericentriolar material 1  CDK16 - cyclin-dependent kinase 16  DAG1 - dystroglycan 1 (dystrophin-associated glycoprotein 1)  FAM161A - family with sequence similarity 161, member a  RABL2B - rab, member of ras oncogene family-like 2b  PTPN11 - protein tyrosine phosphatase, non-receptor type 11  CNTN4 - contactin 4  HDAC2 - histone deacetylase 2  ARL6 - adp-ribosylation factor-like 6  FGD5 - fyve, rhogef and ph domain containing 5  SCLT1 - sodium channel and clathrin linker 1  IFT52 - intraflagellar transport 52 homolog (chlamydomonas)  SH2B1 - sh2b adaptor protein 1  ITGA6 - integrin, alpha 6  ARFGEF1 - adp-ribosylation factor guanine nucleotide-exchange factor 1 (brefeldin a-inhibited)  NRCAM - neuronal cell adhesion molecule  PTPRF - protein tyrosine phosphatase, receptor type, f  ARMC9 - armadillo repeat containing 9  MICALL1 - mical-like 1  POC1B - poc1 centriolar protein b  TCTN3 - tectonic family member 3  ATP1A3 - atpase, na+/k+ transporting, alpha 3 polypeptide  ZNF365 - zinc finger protein 365  TMEM138 - transmembrane protein 138  LRP12 - low density lipoprotein receptor-related protein 12  MTOR - mechanistic target of rapamycin (serine/threonine kinase)  TRAF3IP1 - tnf receptor-associated factor 3 interacting protein 1  ATP1B2 - atpase, na+/k+ transporting, beta 2 polypeptide  PTPRK - protein tyrosine phosphatase, receptor type, k  PTPRM - protein tyrosine phosphatase, receptor type, m  BLOC1S2 - biogenesis of lysosomal organelles complex-1, subunit 2  IFT74 - intraflagellar transport 74 homolog (chlamydomonas)  MAPT - microtubule-associated protein tau  SHANK3 - sh3 and multiple ankyrin repeat domains 3  BLOC1S4 - biogenesis of lysosomal organelles complex-1, subunit 4, cappuccino  CEP290 - centrosomal protein 290kda  MAP2 - microtubule-associated protein 2  MNS1 - meiosis-specific nuclear structural 1  ATP6V0D1 - atpase, h+ transporting, lysosomal 38kda, v0 subunit d1  MAP1B - microtubule-associated protein 1b  MAP1A - microtubule-associated protein 1a  RSG1 - rem2 and rab-like small gtpase 1  DZIP1 - daz interacting zinc finger protein 1  NPY - neuropeptide y  NOTCH1 - notch 1  FAM161B - family with sequence similarity 161, member b  GPM6B - glycoprotein m6b  PVRL2 - poliovirus receptor-related 2 (herpesvirus entry mediator b)  LIMA1 - lim domain and actin binding 1  PIP5K1A - phosphatidylinositol-4-phosphate 5-kinase, type i, alpha  STK36 - serine/threonine kinase 36  RILPL2 - rab interacting lysosomal protein-like 2  FRYL - fry-like  TOR1A - torsin family 1, member a (torsin a)  MATN2 - matrilin 2  WASF3 - was protein family, member 3  TANC1 - tetratricopeptide repeat, ankyrin repeat and coiled-coil containing 1  ICK - intestinal cell (mak-like) kinase  IFT140 - intraflagellar transport 140 homolog (chlamydomonas)  DOCK7 - dedicator of cytokinesis 7  TTBK2 - tau tubulin kinase 2  MCF2 - mcf.2 cell line derived transforming sequence  RAC3 - ras-related c3 botulinum toxin substrate 3 (rho family, small gtp binding protein rac3)  RAC1 - ras-related c3 botulinum toxin substrate 1 (rho family, small gtp binding protein rac1)  NME5 - nme/nm23 family member 5  EHD2 - eh-domain containing 2  EHD3 - eh-domain containing 3  CCP110 - centriolar coiled coil protein 110kda  EHD4 - eh-domain containing 4  IQCB1 - iq motif containing b1  PKD2 - polycystic kidney disease 2 (autosomal dominant)  DLG4 - discs, large homolog 4 (drosophila)  RSPH9 - radial spoke head 9 homolog (chlamydomonas)  NEFL - neurofilament, light polypeptide  BLOC1S6 - biogenesis of lysosomal organelles complex-1, subunit 6, pallidin  NEK1 - nima-related kinase 1  NCK2 - nck adaptor protein 2  TMEM80 - transmembrane protein 80  PHGDH - phosphoglycerate dehydrogenase  SEMA5A - sema domain, seven thrombospondin repeats (type 1 and type 1-like), transmembrane domain (tm) and short cytoplasmic domain, (semaphorin) 5a  BBS5 - bardet-biedl syndrome 5  USP21 - ubiquitin specific peptidase 21  LYN - v-yes-1 yamaguchi sarcoma viral related oncogene homolog  ULK1 - unc-51 like autophagy activating kinase 1  JAM3 - junctional adhesion molecule 3  CAMSAP1 - calmodulin regulated spectrin-associated protein 1  DHFR - dihydrofolate reductase  ZFYVE27 - zinc finger, fyve domain containing 27  CEP41 - centrosomal protein 41kda  GOLPH3 - golgi phosphoprotein 3 (coat-protein)  RAP2A - rap2a, member of ras oncogene family  CROCC - ciliary rootlet coiled-coil, rootletin  IGF1R - insulin-like growth factor 1 receptor  RAPGEF2 - rap guanine nucleotide exchange factor (gef) 2  ARHGEF4 - rho guanine nucleotide exchange factor (gef) 4  RB1 - retinoblastoma 1  RTN4RL1 - reticulon 4 receptor-like 1  ARHGAP35 - rho gtpase activating protein 35  CUL4B - cullin 4b  B9D1 - b9 protein domain 1  BCL2 - b-cell cll/lymphoma 2  IFT172 - intraflagellar transport 172 homolog (chlamydomonas)  CCDC66 - coiled-coil domain containing 66  GSK3B - glycogen synthase kinase 3 beta  ASAP1 - arfgap with sh3 domain, ankyrin repeat and ph domain 1  BBS4 - bardet-biedl syndrome 4  BBS2 - bardet-biedl syndrome 2  WASL - wiskott-aldrich syndrome-like |
| GO:2001252 | positive regulation of chromosome organization | 1.55E-7 | 3.37E-5 | 1.28 (10334,142,6996,123) | [+] Show genes  DHX36 - deah (asp-glu-ala-his) box polypeptide 36  FMR1 - fragile x mental retardation 1  BRD7 - bromodomain containing 7  TCP1 - t-complex 1  PARN - poly(a)-specific ribonuclease  LIG4 - ligase iv, dna, atp-dependent  SETDB1 - set domain, bifurcated 1  NAF1 - nuclear assembly factor 1 ribonucleoprotein  WHSC1L1 - wolf-hirschhorn syndrome candidate 1-like 1  SIN3A - sin3 transcription regulator family member a  CDC27 - cell division cycle 27  SNW1 - snw domain containing 1  TRIM28 - tripartite motif containing 28  ZNF304 - zinc finger protein 304  GTF2H2 - general transcription factor iih, polypeptide 2, 44kda  PML - promyelocytic leukemia  ING2 - inhibitor of growth family, member 2  MIER1 - mesoderm induction early response 1, transcriptional regulator  NBN - nibrin  KAT2A - k(lysine) acetyltransferase 2a  KMT2A - lysine (k)-specific methyltransferase 2a  POT1 - protection of telomeres 1  RIF1 - rap1 interacting factor homolog (yeast)  RPS6KA5 - ribosomal protein s6 kinase, 90kda, polypeptide 5  MAPKAPK5 - mitogen-activated protein kinase-activated protein kinase 5  CCT5 - chaperonin containing tcp1, subunit 5 (epsilon)  KIAA1551 - kiaa1551  SLX4 - slx4 structure-specific endonuclease subunit  H2AFY - h2a histone family, member y  SPHK2 - sphingosine kinase 2  WBP2 - ww domain binding protein 2  TNKS2 - tankyrase, trf1-interacting ankyrin-related adp-ribose polymerase 2  FAM208A - family with sequence similarity 208, member a  WDR61 - wd repeat domain 61  LMNA - lamin a/c  CDC16 - cell division cycle 16  ANAPC5 - anaphase promoting complex subunit 5  ANAPC7 - anaphase promoting complex subunit 7  NSMCE2 - non-smc element 2, mms21 homolog (s. cerevisiae)  TADA2A - transcriptional adaptor 2a  ARRB1 - arrestin, beta 1  NUMA1 - nuclear mitotic apparatus protein 1  MECP2 - methyl cpg binding protein 2 (rett syndrome)  NABP2 - nucleic acid binding protein 2  ZBTB7B - zinc finger and btb domain containing 7b  FEN1 - flap structure-specific endonuclease 1  KDM1A - lysine (k)-specific demethylase 1a  MAP3K4 - mitogen-activated protein kinase kinase kinase 4  SMARCB1 - swi/snf related, matrix associated, actin dependent regulator of chromatin, subfamily b, member 1  JDP2 - jun dimerization protein 2  CDK9 - cyclin-dependent kinase 9  CCT7 - chaperonin containing tcp1, subunit 7 (eta)  CCT3 - chaperonin containing tcp1, subunit 3 (gamma)  ANAPC4 - anaphase promoting complex subunit 4  KMT2E - lysine (k)-specific methyltransferase 2e  CCT2 - chaperonin containing tcp1, subunit 2 (beta)  CCT4 - chaperonin containing tcp1, subunit 4 (delta)  RAD50 - rad50 homolog (s. cerevisiae)  RNF40 - ring finger protein 40, e3 ubiquitin protein ligase  GLYR1 - glyoxylate reductase 1 homolog (arabidopsis)  HMBOX1 - homeobox containing 1  AKAP8 - a kinase (prka) anchor protein 8  TPR - translocated promoter region, nuclear basket protein  KAT7 - k(lysine) acetyltransferase 7  RUVBL2 - ruvb-like 2 (e. coli)  NELFE - negative elongation factor complex member e  PHF19 - phd finger protein 19  PAXBP1 - pax3 and pax7 binding protein 1  MORC2 - morc family cw-type zinc finger 2  PHF1 - phd finger protein 1  LRRK2 - leucine-rich repeat kinase 2  CTBP1 - c-terminal binding protein 1  MPHOSPH8 - m-phase phosphoprotein 8  TNKS - tankyrase, trf1-interacting ankyrin-related adp-ribose polymerase  SIRT6 - sirtuin 6  ATF7IP - activating transcription factor 7 interacting protein  SIRT1 - sirtuin 1  CTNNB1 - catenin (cadherin-associated protein), beta 1, 88kda  PPHLN1 - periphilin 1  BECN1 - beclin 1, autophagy related  MAPK15 - mitogen-activated protein kinase 15  SMC5 - structural maintenance of chromosomes 5  CHTOP - chromatin target of prmt1  ACD - adrenocortical dysplasia homolog (mouse)  BRD4 - bromodomain containing 4  RAD21 - rad21 homolog (s. pombe)  CDC23 - cell division cycle 23  PAF1 - paf1, rna polymerase ii associated factor, homolog (s. cerevisiae)  PPP1R10 - protein phosphatase 1, regulatory subunit 10  SART3 - squamous cell carcinoma antigen recognized by t cells 3  CCT6A - chaperonin containing tcp1, subunit 6a (zeta 1)  DKC1 - dyskeratosis congenita 1, dyskerin  MTF2 - metal response element binding transcription factor 2  NIPBL - nipped-b homolog (drosophila)  CCT8 - chaperonin containing tcp1, subunit 8 (theta)  EED - embryonic ectoderm development  TERF1 - telomeric repeat binding factor (nima-interacting) 1  CTR9 - ctr9, paf1/rna polymerase ii complex component  HNRNPU - heterogeneous nuclear ribonucleoprotein u (scaffold attachment factor a)  C6orf89 - chromosome 6 open reading frame 89  FBXO4 - f-box protein 4  ATRX - alpha thalassemia/mental retardation syndrome x-linked  MAP2K7 - mitogen-activated protein kinase kinase 7  SFPQ - splicing factor proline/glutamine-rich  HNRNPA2B1 - heterogeneous nuclear ribonucleoprotein a2/b1  MAPK3 - mitogen-activated protein kinase 3  HNRNPA1 - heterogeneous nuclear ribonucleoprotein a1  OGT - o-linked n-acetylglucosamine (glcnac) transferase  RB1 - retinoblastoma 1  MAPK1 - mitogen-activated protein kinase 1  DNMT1 - dna (cytosine-5-)-methyltransferase 1  HNRNPD - heterogeneous nuclear ribonucleoprotein d (au-rich element rna binding protein 1, 37kda)  SSBP1 - single-stranded dna binding protein 1, mitochondrial  RNF20 - ring finger protein 20, e3 ubiquitin protein ligase  PRKD1 - protein kinase d1  RPS6KA4 - ribosomal protein s6 kinase, 90kda, polypeptide 4  XRCC5 - x-ray repair complementing defective repair in chinese hamster cells 5 (double-strand-break rejoining)  CUL3 - cullin 3  MRE11A - mre11 meiotic recombination 11 homolog a (s. cerevisiae)  SMAD4 - smad family member 4  PNKP - polynucleotide kinase 3'-phosphatase  PKIB - protein kinase (camp-dependent, catalytic) inhibitor beta  UBE2N - ubiquitin-conjugating enzyme e2n |
| GO:0120031 | plasma membrane bounded cell projection assembly | 1.68E-7 | 3.6E-5 | 1.24 (10334,202,6996,169) | [+] Show genes  EMP1 - epithelial membrane protein 1  S1PR2 - sphingosine-1-phosphate receptor 2  CTTN - cortactin  NCK1 - nck adaptor protein 1  CEP89 - centrosomal protein 89kda  B9D2 - b9 protein domain 2  WDR19 - wd repeat domain 19  ABLIM1 - actin binding lim protein 1  ATAT1 - alpha tubulin acetyltransferase 1  GSN - gelsolin  LRRC16A - leucine rich repeat containing 16a  CD2AP - cd2-associated protein  GORAB - golgin, rab6-interacting  IFT46 - intraflagellar transport 46 homolog (chlamydomonas)  SDCBP - syndecan binding protein (syntenin)  MKS1 - meckel syndrome, type 1  WWTR1 - ww domain containing transcription regulator 1  NRP1 - neuropilin 1  SSX2IP - synovial sarcoma, x breakpoint 2 interacting protein  ABL1 - c-abl oncogene 1, non-receptor tyrosine kinase  DCTN1 - dynactin 1  PTPN23 - protein tyrosine phosphatase, non-receptor type 23  FLNA - filamin a, alpha  RDX - radixin  WDR34 - wd repeat domain 34  FOPNL - fgfr1op n-terminal like  ARL13B - adp-ribosylation factor-like 13b  PMP22 - peripheral myelin protein 22  MKKS - mckusick-kaufman syndrome  CCDC28B - coiled-coil domain containing 28b  WASF2 - was protein family, member 2  KIF27 - kinesin family member 27  MTSS1L - metastasis suppressor 1-like  CDC14B - cell division cycle 14b  EXOC5 - exocyst complex component 5  TMEM17 - transmembrane protein 17  FNBP1L - formin binding protein 1-like  OCRL - oculocerebrorenal syndrome of lowe  PTPDC1 - protein tyrosine phosphatase domain containing 1  AZI1 - 5-azacytidine induced 1  MYLK - myosin light chain kinase  RAB23 - rab23, member ras oncogene family  BBS7 - bardet-biedl syndrome 7  EHD1 - eh-domain containing 1  AKAP4 - a kinase (prka) anchor protein 4  IFT43 - intraflagellar transport 43 homolog (chlamydomonas)  CEP250 - centrosomal protein 250kda  TCTN1 - tectonic family member 1  ARL3 - adp-ribosylation factor-like 3  CEP19 - centrosomal protein 19kda  DNM3 - dynamin 3  KIAA0586 - kiaa0586  ARHGEF7 - rho guanine nucleotide exchange factor (gef) 7  MYH10 - myosin, heavy chain 10, non-muscle  IFT57 - intraflagellar transport 57 homolog (chlamydomonas)  PDGFRB - platelet-derived growth factor receptor, beta polypeptide  FSCN1 - fascin homolog 1, actin-bundling protein (strongylocentrotus purpuratus)  TMEM231 - transmembrane protein 231  SNX10 - sorting nexin 10  ARID1B - at rich interactive domain 1b (swi1-like)  ARF6 - adp-ribosylation factor 6  CELSR2 - cadherin, egf lag seven-pass g-type receptor 2  FGD1 - fyve, rhogef and ph domain containing 1  AHI1 - abelson helper integration site 1  BAG4 - bcl2-associated athanogene 4  ITGB1 - integrin, beta 1 (fibronectin receptor, beta polypeptide, antigen cd29 includes mdf2, msk12)  ACTR2 - arp2 actin-related protein 2 homolog (yeast)  ACTR3 - arp3 actin-related protein 3 homolog (yeast)  NUP85 - nucleoporin 85kda  BBS9 - bardet-biedl syndrome 9  TEKT2 - tektin 2 (testicular)  WDR60 - wd repeat domain 60  RAB8A - rab8a, member ras oncogene family  ROCK1 - rho-associated, coiled-coil containing protein kinase 1  ARFIP2 - adp-ribosylation factor interacting protein 2  INPPL1 - inositol polyphosphate phosphatase-like 1  CLUAP1 - clusterin associated protein 1  C2CD3 - c2 calcium-dependent domain containing 3  NUDCD3 - nudc domain containing 3  PCM1 - pericentriolar material 1  TTC8 - tetratricopeptide repeat domain 8  FAM161A - family with sequence similarity 161, member a  TNIK - traf2 and nck interacting kinase  E2F4 - e2f transcription factor 4, p107/p130-binding  RABL2B - rab, member of ras oncogene family-like 2b  ATP6V1D - atpase, h+ transporting, lysosomal 34kda, v1 subunit d  ARL6 - adp-ribosylation factor-like 6  LPAR1 - lysophosphatidic acid receptor 1  S1PR1 - sphingosine-1-phosphate receptor 1  FGD5 - fyve, rhogef and ph domain containing 5  SCLT1 - sodium channel and clathrin linker 1  LAMA5 - laminin, alpha 5  IFT52 - intraflagellar transport 52 homolog (chlamydomonas)  SH2B1 - sh2b adaptor protein 1  FBF1 - fas (tnfrsf6) binding factor 1  ITGA6 - integrin, alpha 6  VAV3 - vav 3 guanine nucleotide exchange factor  ARMC9 - armadillo repeat containing 9  POC1B - poc1 centriolar protein b  TCTN3 - tectonic family member 3  TMEM138 - transmembrane protein 138  TRAF3IP1 - tnf receptor-associated factor 3 interacting protein 1  ARHGEF6 - rac/cdc42 guanine nucleotide exchange factor (gef) 6  IFT74 - intraflagellar transport 74 homolog (chlamydomonas)  KIF3A - kinesin family member 3a  PARVA - parvin, alpha  CEP290 - centrosomal protein 290kda  ATP6V0D1 - atpase, h+ transporting, lysosomal 38kda, v0 subunit d1  PIBF1 - progesterone immunomodulatory binding factor 1  DYNLL1 - dynein, light chain, lc8-type 1  DZIP1 - daz interacting zinc finger protein 1  RSG1 - rem2 and rab-like small gtpase 1  NOTCH1 - notch 1  CCDC41 - coiled-coil domain containing 41  HDAC6 - histone deacetylase 6  PIP5K1A - phosphatidylinositol-4-phosphate 5-kinase, type i, alpha  STK36 - serine/threonine kinase 36  RILPL2 - rab interacting lysosomal protein-like 2  EZR - ezrin  TMEM237 - transmembrane protein 237  UNC119B - unc-119 homolog b (c. elegans)  WASF3 - was protein family, member 3  ICK - intestinal cell (mak-like) kinase  IFT140 - intraflagellar transport 140 homolog (chlamydomonas)  TTBK2 - tau tubulin kinase 2  TCTEX1D2 - tctex1 domain containing 2  VAV2 - vav 2 guanine nucleotide exchange factor  TCTN2 - tectonic family member 2  RAC1 - ras-related c3 botulinum toxin substrate 1 (rho family, small gtp binding protein rac1)  IFT122 - intraflagellar transport 122 homolog (chlamydomonas)  NME5 - nme/nm23 family member 5  VCL - vinculin  EHD2 - eh-domain containing 2  KIT - v-kit hardy-zuckerman 4 feline sarcoma viral oncogene homolog  EHD3 - eh-domain containing 3  EHD4 - eh-domain containing 4  IQCB1 - iq motif containing b1  TMEM216 - transmembrane protein 216  RSPH9 - radial spoke head 9 homolog (chlamydomonas)  NEK1 - nima-related kinase 1  NCK2 - nck adaptor protein 2  DBNL - drebrin-like  TMEM80 - transmembrane protein 80  IFT81 - intraflagellar transport 81 homolog (chlamydomonas)  BBS5 - bardet-biedl syndrome 5  IFT88 - intraflagellar transport 88 homolog (chlamydomonas)  CEP41 - centrosomal protein 41kda  GOLPH3 - golgi phosphoprotein 3 (coat-protein)  RAP2A - rap2a, member of ras oncogene family  CYFIP1 - cytoplasmic fmr1 interacting protein 1  DMTN - dematin actin binding protein  TXNDC15 - thioredoxin domain containing 15  RAPGEF2 - rap guanine nucleotide exchange factor (gef) 2  P2RY12 - purinergic receptor p2y, g-protein coupled, 12  ARHGEF4 - rho guanine nucleotide exchange factor (gef) 4  WDR11 - wd repeat domain 11  TROVE2 - trove domain family, member 2  GALNT11 - udp-n-acetyl-alpha-d-galactosamine:polypeptide n-acetylgalactosaminyltransferase 11 (galnac-t11)  CCDC88A - coiled-coil domain containing 88a  SNAP29 - synaptosomal-associated protein, 29kda  ATXN10 - ataxin 10  CSNK1D - casein kinase 1, delta  B9D1 - b9 protein domain 1  IFT172 - intraflagellar transport 172 homolog (chlamydomonas)  CCDC66 - coiled-coil domain containing 66  ASAP1 - arfgap with sh3 domain, ankyrin repeat and ph domain 1  BBS4 - bardet-biedl syndrome 4  BBS2 - bardet-biedl syndrome 2  TMEM107 - transmembrane protein 107 |
| GO:0006996 | organelle organization | 1.8E-7 | 3.79E-5 | 1.08 (10334,1690,6996,1233) | [+] Show genes  AFG3L2 - afg3 atpase family member 3-like 2 (s. cerevisiae)  AGAP2 - arfgap with gtpase domain, ankyrin repeat and ph domain 2  ATAT1 - alpha tubulin acetyltransferase 1  ABCA1 - atp-binding cassette, sub-family a (abc1), member 1  PPP1R9A - protein phosphatase 1, regulatory subunit 9a  GEM - gtp binding protein overexpressed in skeletal muscle  HAUS7 - haus augmin-like complex, subunit 7  SSX2IP - synovial sarcoma, x breakpoint 2 interacting protein  ABL1 - c-abl oncogene 1, non-receptor tyrosine kinase  TMED2 - transmembrane emp24 domain trafficking protein 2  NUDT19 - nudix (nucleoside diphosphate linked moiety x)-type motif 19  ABR - active bcr-related  PML - promyelocytic leukemia  ACOT2 - acyl-coa thioesterase 2  OBFC1 - oligonucleotide/oligosaccharide-binding fold containing 1  MKKS - mckusick-kaufman syndrome  YWHAQ - tyrosine 3-monooxygenase/tryptophan 5-monooxygenase activation protein, theta polypeptide  LURAP1 - leucine rich adaptor protein 1  KIF27 - kinesin family member 27  ASCC3 - activating signal cointegrator 1 complex subunit 3  KAT2A - k(lysine) acetyltransferase 2a  TMED10 - transmembrane emp24-like trafficking protein 10 (yeast)  PANK2 - pantothenate kinase 2  SMPD4 - sphingomyelin phosphodiesterase 4, neutral membrane (neutral sphingomyelinase-3)  TRAFD1 - traf-type zinc finger domain containing 1  ACOX1 - acyl-coa oxidase 1, palmitoyl  CHD7 - chromodomain helicase dna binding protein 7  ACTB - actin, beta  GJA1 - gap junction protein, alpha 1, 43kda  RND2 - rho family gtpase 2  ACTG1 - actin, gamma 1  PLD2 - phospholipase d2  EHD1 - eh-domain containing 1  AMBRA1 - autophagy/beclin-1 regulator 1  PPID - peptidylprolyl isomerase d  PPIA - peptidylprolyl isomerase a (cyclophilin a)  MCMBP - minichromosome maintenance complex binding protein  PICALM - phosphatidylinositol binding clathrin assembly protein  MAP9 - microtubule-associated protein 9  RAB10 - rab10, member ras oncogene family  GCLM - glutamate-cysteine ligase, modifier subunit  GCLC - glutamate-cysteine ligase, catalytic subunit  AXIN2 - axin 2  SMC1A - structural maintenance of chromosomes 1a  ADD2 - adducin 2 (beta)  POLRMT - polymerase (rna) mitochondrial (dna directed)  USP9X - ubiquitin specific peptidase 9, x-linked  ADD1 - adducin 1 (alpha)  GNAI3 - guanine nucleotide binding protein (g protein), alpha inhibiting activity polypeptide 3  PARP1 - poly (adp-ribose) polymerase 1  NOP10 - nop10 ribonucleoprotein  CNTROB - centrobin, centrosomal brca2 interacting protein  POLD2 - polymerase (dna directed), delta 2, accessory subunit  SNAP47 - synaptosomal-associated protein, 47kda  POLD1 - polymerase (dna directed), delta 1, catalytic subunit  TUBGCP2 - tubulin, gamma complex associated protein 2  SMPD3 - sphingomyelin phosphodiesterase 3, neutral membrane (neutral sphingomyelinase ii)  RUVBL2 - ruvb-like 2 (e. coli)  CDK5RAP2 - cdk5 regulatory subunit associated protein 2  KATNA1 - katanin p60 (atpase containing) subunit a 1  AP1G1 - adaptor-related protein complex 1, gamma 1 subunit  NUP133 - nucleoporin 133kda  SRGN - serglycin  AGK - acylglycerol kinase  PRIM2 - primase, dna, polypeptide 2 (58kda)  CAMKMT - calmodulin-lysine n-methyltransferase  PRIM1 - primase, dna, polypeptide 1 (49kda)  VPS35 - vacuolar protein sorting 35 homolog (s. cerevisiae)  KIF3A - kinesin family member 3a  TOR1AIP2 - torsin a interacting protein 2  PARVA - parvin, alpha  ZWINT - zw10 interacting kinetochore protein  PPP3R1 - protein phosphatase 3, regulatory subunit b, alpha  CIT - citron (rho-interacting, serine/threonine kinase 21)  FGFR1OP - fgfr1 oncogene partner  PPP6C - protein phosphatase 6, catalytic subunit  DNAJC11 - dnaj (hsp40) homolog, subfamily c, member 11  AKT1 - v-akt murine thymoma viral oncogene homolog 1  RAB7A - rab7a, member ras oncogene family  GAB1 - grb2-associated binding protein 1  PCID2 - pci domain containing 2  GAA - glucosidase, alpha; acid  XRCC6 - x-ray repair complementing defective repair in chinese hamster cells 6  PPP2R1A - protein phosphatase 2, regulatory subunit a, alpha  GABPB1 - ga binding protein transcription factor, beta subunit 1  GABPA - ga binding protein transcription factor, alpha subunit 60kda  TRIOBP - trio and f-actin binding protein  PPP2CB - protein phosphatase 2, catalytic subunit, beta isozyme  TCTN2 - tectonic family member 2  IFT122 - intraflagellar transport 122 homolog (chlamydomonas)  RMI2 - recq mediated genome instability 2  TTC17 - tetratricopeptide repeat domain 17  UBA5 - ubiquitin-like modifier activating enzyme 5  LYSMD3 - lysm, putative peptidoglycan-binding, domain containing 3  STMN2 - stathmin-like 2  ESCO1 - establishment of sister chromatid cohesion n-acetyltransferase 1  TDRKH - tudor and kh domain containing  GAK - cyclin g associated kinase  TUBGCP5 - tubulin, gamma complex associated protein 5  PEX26 - peroxisomal biogenesis factor 26  TTLL7 - tubulin tyrosine ligase-like family, member 7  FMNL2 - formin-like 2  NPLOC4 - nuclear protein localization 4 homolog (s. cerevisiae)  AAAS - achalasia, adrenocortical insufficiency, alacrimia  PRPF40A - prp40 pre-mrna processing factor 40 homolog a (s. cerevisiae)  PAPD7 - pap associated domain containing 7  TMEM127 - transmembrane protein 127  PIM2 - pim-2 oncogene  IFT88 - intraflagellar transport 88 homolog (chlamydomonas)  RAB31 - rab31, member ras oncogene family  YEATS4 - yeats domain containing 4  ZFAND1 - zinc finger, an1-type domain 1  ZFPM1 - zinc finger protein, fog family member 1  IMMT - inner membrane protein, mitochondrial  TXNDC15 - thioredoxin domain containing 15  GBA - glucosidase, beta, acid  MAPK8 - mitogen-activated protein kinase 8  WDR11 - wd repeat domain 11  CISD2 - cdgsh iron sulfur domain 2  MAPRE2 - microtubule-associated protein, rp/eb family, member 2  COPS6 - cop9 signalosome subunit 6  KAT6A - k(lysine) acetyltransferase 6a  MICALL2 - mical-like 2  PRKD1 - protein kinase d1  NDC1 - ndc1 transmembrane nucleoporin  CCDC88A - coiled-coil domain containing 88a  HAUS1 - haus augmin-like complex, subunit 1  STAM - signal transducing adaptor molecule (sh3 domain and itam motif) 1  PRKCI - protein kinase c, iota  ANXA2 - annexin a2  SLC25A5 - solute carrier family 25 (mitochondrial carrier; adenine nucleotide translocator), member 5  PRKAR1A - protein kinase, camp-dependent, regulatory, type i, alpha  CCDC6 - coiled-coil domain containing 6  APEX1 - apex nuclease (multifunctional dna repair enzyme) 1  DHX36 - deah (asp-glu-ala-his) box polypeptide 36  MSTO1 - misato 1, mitochondrial distribution and morphology regulator  CCDC78 - coiled-coil domain containing 78  XIAP - x-linked inhibitor of apoptosis  SDAD1 - sda1 domain containing 1  ATXN2L - ataxin 2-like  SMC6 - structural maintenance of chromosomes 6  RNF112 - ring finger protein 112  RFWD3 - ring finger and wd repeat domain 3  TREX1 - three prime repair exonuclease 1  GSN - gelsolin  TMEM33 - transmembrane protein 33  GTF2F2 - general transcription factor iif, polypeptide 2, 30kda  GTF2H2 - general transcription factor iih, polypeptide 2, 44kda  GTF2H3 - general transcription factor iih, polypeptide 3, 34kda  PACSIN2 - protein kinase c and casein kinase substrate in neurons 2  GTF2H1 - general transcription factor iih, polypeptide 1, 62kda  FOPNL - fgfr1op n-terminal like  MCPH1 - microcephalin 1  MAP7D3 - map7 domain containing 3  CHP1 - calcineurin-like ef-hand protein 1  RIF1 - rap1 interacting factor homolog (yeast)  VPS13D - vacuolar protein sorting 13 homolog d (s. cerevisiae)  SLC25A36 - solute carrier family 25 (pyrimidine nucleotide carrier ), member 36  HPS5 - hermansky-pudlak syndrome 5  POLG2 - polymerase (dna directed), gamma 2, accessory subunit  PDCD10 - programmed cell death 10  GTF2H4 - general transcription factor iih, polypeptide 4, 52kda  ARF1 - adp-ribosylation factor 1  MAP1S - microtubule-associated protein 1s  PAFAH1B1 - platelet-activating factor acetylhydrolase 1b, regulatory subunit 1 (45kda)  GOLPH3L - golgi phosphoprotein 3-like  TMEM170A - transmembrane protein 170a  AKAP11 - a kinase (prka) anchor protein 11  BBS7 - bardet-biedl syndrome 7  PALM - paralemmin  PDGFA - platelet-derived growth factor alpha polypeptide  CEP250 - centrosomal protein 250kda  H3F3A - h3 histone, family 3a  CELF3 - cugbp, elav-like family member 3  TCTN1 - tectonic family member 1  NISCH - nischarin  ARL3 - adp-ribosylation factor-like 3  TRAPPC6B - trafficking protein particle complex 6b  ARL1 - adp-ribosylation factor-like 1  MUL1 - mitochondrial e3 ubiquitin protein ligase 1  NSMCE2 - non-smc element 2, mms21 homolog (s. cerevisiae)  TUBA1A - tubulin, alpha 1a  PDGFRB - platelet-derived growth factor receptor, beta polypeptide  IFT57 - intraflagellar transport 57 homolog (chlamydomonas)  ARSB - arylsulfatase b  SEC23IP - sec23 interacting protein  TMEM231 - transmembrane protein 231  ARRB1 - arrestin, beta 1  PDGFRA - platelet-derived growth factor receptor, alpha polypeptide  RHOB - ras homolog family member b  FAM83H - family with sequence similarity 83, member h  RHOC - ras homolog family member c  PDPK1 - 3-phosphoinositide dependent protein kinase-1  RHOA - ras homolog family member a  ARF6 - adp-ribosylation factor 6  ATG2B - autophagy related 2b  RND3 - rho family gtpase 3  HCFC1 - host cell factor c1 (vp16-accessory protein)  WDR60 - wd repeat domain 60  PCNA - proliferating cell nuclear antigen  CHMP1A - charged multivesicular body protein 1a  PLEKHJ1 - pleckstrin homology domain containing, family j member 1  ZYX - zyxin  TTC8 - tetratricopeptide repeat domain 8  PCM1 - pericentriolar material 1  RABL2B - rab, member of ras oncogene family-like 2b  FRMD6 - ferm domain containing 6  WRAP53 - wd repeat containing, antisense to tp53  CAPN10 - calpain 10  PLA2G16 - phospholipase a2, group xvi  HTT - huntingtin  ZNF205 - zinc finger protein 205  ZNF207 - zinc finger protein 207  HAUS2 - haus augmin-like complex, subunit 2  XPA - xeroderma pigmentosum, complementation group a  XPC - xeroderma pigmentosum, complementation group c  GOLGA2 - golgin a2  ZNF365 - zinc finger protein 365  HAUS3 - haus augmin-like complex, subunit 3  PFN2 - profilin 2  FBXW7 - f-box and wd repeat domain containing 7, e3 ubiquitin protein ligase  GOLGB1 - golgin b1  XRCC1 - x-ray repair complementing defective repair in chinese hamster cells 1  HIGD2A - hig1 hypoxia inducible domain family, member 2a  LRRK2 - leucine-rich repeat kinase 2  BLOC1S4 - biogenesis of lysosomal organelles complex-1, subunit 4, cappuccino  MNS1 - meiosis-specific nuclear structural 1  RSG1 - rem2 and rab-like small gtpase 1  DZIP1 - daz interacting zinc finger protein 1  SEC31A - sec31 homolog a (s. cerevisiae)  PEX6 - peroxisomal biogenesis factor 6  PEX7 - peroxisomal biogenesis factor 7  ATG14 - autophagy related 14  CLIP2 - cap-gly domain containing linker protein 2  SH3PXD2B - sh3 and px domains 2b  PEX12 - peroxisomal biogenesis factor 12  PEX13 - peroxisomal biogenesis factor 13  WEE1 - wee1 homolog (s. pombe)  PEX14 - peroxisomal biogenesis factor 14  ATP7A - atpase, cu++ transporting, alpha polypeptide  RILPL2 - rab interacting lysosomal protein-like 2  PPP6R1 - protein phosphatase 6, regulatory subunit 1  ICK - intestinal cell (mak-like) kinase  PEX1 - peroxisomal biogenesis factor 1  VRK1 - vaccinia related kinase 1  PI4K2A - phosphatidylinositol 4-kinase type 2 alpha  PAOX - polyamine oxidase (exo-n4-amino)  BAX - bcl2-associated x protein  PKD2 - polycystic kidney disease 2 (autosomal dominant)  BAK1 - bcl2-antagonist/killer 1  BAD - bcl2-associated agonist of cell death  FSD1 - fibronectin type iii and spry domain containing 1  PDZD8 - pdz domain containing 8  MRAS - muscle ras oncogene homolog  COX18 - cox18 cytochrome c oxidase assembly factor  GRB2 - growth factor receptor-bound protein 2  GNPTAB - n-acetylglucosamine-1-phosphate transferase, alpha and beta subunits  PKP2 - plakophilin 2  ZFYVE27 - zinc finger, fyve domain containing 27  PEF1 - penta-ef-hand domain containing 1  ATRX - alpha thalassemia/mental retardation syndrome x-linked  COG2 - component of oligomeric golgi complex 2  OGFOD1 - 2-oxoglutarate and iron-dependent oxygenase domain containing 1  GABARAPL2 - gaba(a) receptor-associated protein-like 2  SF1 - splicing factor 1  SERPINE2 - serpin peptidase inhibitor, clade e (nexin, plasminogen activator inhibitor type 1), member 2  GABARAP - gaba(a) receptor-associated protein  ARHGAP35 - rho gtpase activating protein 35  BCR - breakpoint cluster region  RHOT1 - ras homolog family member t1  PPP6R3 - protein phosphatase 6, regulatory subunit 3  PIK3C2B - phosphatidylinositol-4-phosphate 3-kinase, catalytic subunit type 2 beta  BCL2 - b-cell cll/lymphoma 2  XRCC5 - x-ray repair complementing defective repair in chinese hamster cells 5 (double-strand-break rejoining)  BCL2L2 - bcl2-like 2  PIK3C3 - phosphatidylinositol 3-kinase, catalytic subunit type 3  BCL2L1 - bcl2-like 1  YWHAG - tyrosine 3-monooxygenase/tryptophan 5-monooxygenase activation protein, gamma polypeptide  CCDC66 - coiled-coil domain containing 66  GSK3B - glycogen synthase kinase 3 beta  YWHAZ - tyrosine 3-monooxygenase/tryptophan 5-monooxygenase activation protein, zeta polypeptide  GSK3A - glycogen synthase kinase 3 alpha  PARK7 - parkinson protein 7  BBS4 - bardet-biedl syndrome 4  BBS2 - bardet-biedl syndrome 2  YWHAE - tyrosine 3-monooxygenase/tryptophan 5-monooxygenase activation protein, epsilon polypeptide  YWHAB - tyrosine 3-monooxygenase/tryptophan 5-monooxygenase activation protein, beta polypeptide  TBC1D20 - tbc1 domain family, member 20  DST - dystonin  MAPKAP1 - mitogen-activated protein kinase associated protein 1  RFC5 - replication factor c (activator 1) 5, 36.5kda  BNIP3L - bcl2/adenovirus e1b 19kda interacting protein 3-like  RNF26 - ring finger protein 26  RFC2 - replication factor c (activator 1) 2, 40kda  BNIP1 - bcl2/adenovirus e1b 19kda interacting protein 1  RFC3 - replication factor c (activator 1) 3, 38kda  BET1 - bet1 golgi vesicular membrane trafficking protein  MAEA - macrophage erythroblast attacher  PEX11B - peroxisomal biogenesis factor 11 beta  PEX11A - peroxisomal biogenesis factor 11 alpha  CENPV - centromere protein v  MKS1 - meckel syndrome, type 1  NRP1 - neuropilin 1  KLHDC8B - kelch domain containing 8b  RECQL - recq protein-like (dna helicase q1-like)  KATNB1 - katanin p80 (wd repeat containing) subunit b 1  PAK4 - p21 protein (cdc42/rac)-activated kinase 4  ARL13B - adp-ribosylation factor-like 13b  AGFG1 - arfgap with fg repeats 1  CCDC28B - coiled-coil domain containing 28b  UPF1 - upf1 regulator of nonsense transcripts homolog (yeast)  RTN3 - reticulon 3  APOO - apolipoprotein o  RLF - rearranged l-myc fusion  TMEM17 - transmembrane protein 17  FAM174B - family with sequence similarity 174, member b  CFLAR - casp8 and fadd-like apoptosis regulator  MTX3 - metaxin 3  FNBP1L - formin binding protein 1-like  SYNGAP1 - synaptic ras gtpase activating protein 1  TFG - trk-fused gene  LDB1 - lim domain binding 1  DSCC1 - dna replication and sister chromatid cohesion 1  AKAP4 - a kinase (prka) anchor protein 4  FITM2 - fat storage-inducing transmembrane protein 2  TRPM7 - transient receptor potential cation channel, subfamily m, member 7  ZNF259 - zinc finger protein 259  NDE1 - nude neurodevelopment protein 1  VPS51 - vacuolar protein sorting 51 homolog (s. cerevisiae)  BCAS3 - breast carcinoma amplified sequence 3  ARHGEF7 - rho guanine nucleotide exchange factor (gef) 7  VPS13C - vacuolar protein sorting 13 homolog c (s. cerevisiae)  MIS12 - mis12 kinetochore complex component  FMNL1 - formin-like 1  HAUS6 - haus augmin-like complex, subunit 6  STX6 - syntaxin 6  AP1M1 - adaptor-related protein complex 1, mu 1 subunit  HUS1 - hus1 checkpoint homolog (s. pombe)  DYM - dymeclin  AHI1 - abelson helper integration site 1  TMEM108 - transmembrane protein 108  ROCK1 - rho-associated, coiled-coil containing protein kinase 1  TRAK2 - trafficking protein, kinesin binding 2  SNX19 - sorting nexin 19  SPRY1 - sprouty homolog 1, antagonist of fgf signaling (drosophila)  SPRY2 - sprouty homolog 2 (drosophila)  HSPD1 - heat shock 60kda protein 1 (chaperonin)  STAM2 - signal transducing adaptor molecule (sh3 domain and itam motif) 2  HSP90AA1 - heat shock protein 90kda alpha (cytosolic), class a member 1  AP3D1 - adaptor-related protein complex 3, delta 1 subunit  AKAP8 - a kinase (prka) anchor protein 8  ZMPSTE24 - zinc metallopeptidase ste24  HSPA4 - heat shock 70kda protein 4  STAG1 - stromal antigen 1  BAIAP3 - bai1-associated protein 3  HSPA9 - heat shock 70kda protein 9 (mortalin)  WASF1 - was protein family, member 1  CAPZA1 - capping protein (actin filament) muscle z-line, alpha 1  CCSAP - centriole, cilia and spindle-associated protein  VAV3 - vav 3 guanine nucleotide exchange factor  ATG16L1 - autophagy related 16-like 1 (s. cerevisiae)  HMGB3 - high mobility group box 3  HMGB2 - high mobility group box 2  HMGB1 - high mobility group box 1  VPS37C - vacuolar protein sorting 37 homolog c (s. cerevisiae)  MTIF3 - mitochondrial translational initiation factor 3  PHACTR4 - phosphatase and actin regulator 4  CASP8 - caspase 8, apoptosis-related cysteine peptidase  DYNLL1 - dynein, light chain, lc8-type 1  PIBF1 - progesterone immunomodulatory binding factor 1  TNKS - tankyrase, trf1-interacting ankyrin-related adp-ribose polymerase  RPA3 - replication protein a3, 14kda  TIMM9 - translocase of inner mitochondrial membrane 9 homolog (yeast)  RPA1 - replication protein a1, 70kda  PID1 - phosphotyrosine interaction domain containing 1  RPA2 - replication protein a2, 32kda  PHIP - pleckstrin homology domain interacting protein  CAPZB - capping protein (actin filament) muscle z-line, beta  ZBTB48 - zinc finger and btb domain containing 48  BECN1 - beclin 1, autophagy related  SEC23A - sec23 homolog a (s. cerevisiae)  VAMP4 - vesicle-associated membrane protein 4  PPP2R3C - protein phosphatase 2, regulatory subunit b'', gamma  STX17 - syntaxin 17  TMEM237 - transmembrane protein 237  ACD - adrenocortical dysplasia homolog (mouse)  LAMTOR1 - late endosomal/lysosomal adaptor, mapk and mtor activator 1  TMEM135 - transmembrane protein 135  CALR - calreticulin  NAIF1 - nuclear apoptosis inducing factor 1  UNC13B - unc-13 homolog b (c. elegans)  CALM2 - calmodulin 2 (phosphorylase kinase, delta)  BSCL2 - berardinelli-seip congenital lipodystrophy 2 (seipin)  WTIP - wilms tumor 1 interacting protein  STK25 - serine/threonine kinase 25  VTI1B - vesicle transport through interaction with t-snares 1b  MBTPS1 - membrane-bound transcription factor peptidase, site 1  ARFGAP3 - adp-ribosylation factor gtpase activating protein 3  SLC35F6 - solute carrier family 35, member f6  RPS23 - ribosomal protein s23  SNX3 - sorting nexin 3  PINX1 - pin2/terf1 interacting, telomerase inhibitor 1  CLN6 - ceroid-lipofuscinosis, neuronal 6, late infantile, variant  TSNARE1 - t-snare domain containing 1  TUBA1B - tubulin, alpha 1b  TUBB - tubulin, beta class i  GBF1 - golgi brefeldin a resistant guanine nucleotide exchange factor 1  SSH3 - slingshot protein phosphatase 3  CORO2B - coronin, actin binding protein, 2b  DLC1 - deleted in liver cancer 1  VPS33B - vacuolar protein sorting 33 homolog b (yeast)  TUBB4B - tubulin, beta 4b class ivb  TINF2 - terf1 (trf1)-interacting nuclear factor 2  TUBB3 - tubulin, beta 3 class iii  CDS2 - cdp-diacylglycerol synthase (phosphatidate cytidylyltransferase) 2  HNRNPA2B1 - heterogeneous nuclear ribonucleoprotein a2/b1  RAB11A - rab11a, member ras oncogene family  CAV2 - caveolin 2  CAV1 - caveolin 1, caveolae protein, 22kda  NAPA - n-ethylmaleimide-sensitive factor attachment protein, alpha  TUBGCP3 - tubulin, gamma complex associated protein 3  SEC24B - sec24 family, member b (s. cerevisiae)  RBM14 - rna binding motif protein 14  HMGA1 - high mobility group at-hook 1  PRMT5 - protein arginine methyltransferase 5  CHTF8 - ctf8, chromosome transmission fidelity factor 8 homolog (s. cerevisiae)  HAT1 - histone acetyltransferase 1  RAB30 - rab30, member ras oncogene family  PEX3 - peroxisomal biogenesis factor 3  HTRA2 - htra serine peptidase 2  TOR1B - torsin family 1, member b (torsin b)  PSEN1 - presenilin 1  CCSER2 - coiled-coil serine-rich protein 2  ING2 - inhibitor of growth family, member 2  USP16 - ubiquitin specific peptidase 16  SMCR7L - smith-magenis syndrome chromosome region, candidate 7-like  CDC14B - cell division cycle 14b  EXOC5 - exocyst complex component 5  BLZF1 - basic leucine zipper nuclear factor 1  AP3B1 - adaptor-related protein complex 3, beta 1 subunit  PATL1 - protein associated with topoisomerase ii homolog 1 (yeast)  EML4 - echinoderm microtubule associated protein like 4  MTX2 - metaxin 2  AGPS - alkylglycerone phosphate synthase  COPS7B - cop9 signalosome subunit 7b  GDAP1 - ganglioside induced differentiation associated protein 1  COPS3 - cop9 signalosome subunit 3  GORASP1 - golgi reassembly stacking protein 1, 65kda  CD34 - cd34 molecule  GAR1 - gar1 ribonucleoprotein  GAS2L1 - growth arrest-specific 2 like 1  TERF2IP - telomeric repeat binding factor 2, interacting protein  ORAOV1 - oral cancer overexpressed 1  HIAT1 - hippocampus abundant transcript 1  PSMD13 - proteasome (prosome, macropain) 26s subunit, non-atpase, 13  ATG7 - autophagy related 7  CENPC - centromere protein c  TUBGCP4 - tubulin, gamma complex associated protein 4  VPS16 - vacuolar protein sorting 16 homolog (s. cerevisiae)  DCTN2 - dynactin 2 (p50)  KLHL17 - kelch-like family member 17  CETN2 - centrin, ef-hand protein, 2  PTEN - phosphatase and tensin homolog  CFL1 - cofilin 1 (non-muscle)  ITGB5 - integrin, beta 5  PTGFRN - prostaglandin f2 receptor inhibitor  PDLIM4 - pdz and lim domain 4  CIB1 - calcium and integrin binding 1 (calmyrin)  TBCEL - tubulin folding cofactor e-like  ITGB1 - integrin, beta 1 (fibronectin receptor, beta polypeptide, antigen cd29 includes mdf2, msk12)  DENR - density-regulated protein  PTK2 - protein tyrosine kinase 2  TPCN2 - two pore segment channel 2  BBS9 - bardet-biedl syndrome 9  EIF6 - eukaryotic translation initiation factor 6  PTK7 - protein tyrosine kinase 7  TEKT2 - tektin 2 (testicular)  CDK5 - cyclin-dependent kinase 5  ARAP3 - arfgap with rhogap domain, ankyrin repeat and ph domain 3  ATG3 - autophagy related 3  RND1 - rho family gtpase 1  AKTIP - akt interacting protein  KLHL20 - kelch-like family member 20  LSM3 - lsm3 homolog, u6 small nuclear rna associated (s. cerevisiae)  PTPN1 - protein tyrosine phosphatase, non-receptor type 1  ARPC1A - actin related protein 2/3 complex, subunit 1a, 41kda  RUVBL1 - ruvb-like 1 (e. coli)  USO1 - uso1 vesicle transport factor  SCLT1 - sodium channel and clathrin linker 1  ARFGEF2 - adp-ribosylation factor guanine nucleotide-exchange factor 2 (brefeldin a-inhibited)  ARFGEF1 - adp-ribosylation factor guanine nucleotide-exchange factor 1 (brefeldin a-inhibited)  CDK2 - cyclin-dependent kinase 2  PURA - purine-rich element binding protein a  RRN3 - rrn3 rna polymerase i transcription factor homolog (s. cerevisiae)  NCKAP1 - nck-associated protein 1  TRAPPC3 - trafficking protein particle complex 3  TMEM106B - transmembrane protein 106b  PTPRN - protein tyrosine phosphatase, receptor type, n  PLK2 - polo-like kinase 2  LYST - lysosomal trafficking regulator  PEX2 - peroxisomal biogenesis factor 2  PDLIM2 - pdz and lim domain 2 (mystique)  ABCD3 - atp-binding cassette, sub-family d (ald), member 3  CHGA - chromogranin a (parathyroid secretory protein 1)  PVRL2 - poliovirus receptor-related 2 (herpesvirus entry mediator b)  PIP5K1A - phosphatidylinositol-4-phosphate 5-kinase, type i, alpha  CHD4 - chromodomain helicase dna binding protein 4  RAB5B - rab5b, member ras oncogene family  CHD2 - chromodomain helicase dna binding protein 2  STK36 - serine/threonine kinase 36  CHD3 - chromodomain helicase dna binding protein 3  RAB3A - rab3a, member ras oncogene family  CHD1 - chromodomain helicase dna binding protein 1  MTERFD2 - mterf domain containing 2  RAB2A - rab2a, member ras oncogene family  RAB1A - rab1a, member ras oncogene family  WASF3 - was protein family, member 3  THSD7A - thrombospondin, type i, domain containing 7a  TUBG2 - tubulin, gamma 2  RAD21 - rad21 homolog (s. pombe)  SEC24A - sec24 family, member a (s. cerevisiae)  RAC3 - ras-related c3 botulinum toxin substrate 3 (rho family, small gtp binding protein rac3)  RAC1 - ras-related c3 botulinum toxin substrate 1 (rho family, small gtp binding protein rac1)  TMED4 - transmembrane emp24 protein transport domain containing 4  NME5 - nme/nm23 family member 5  VPS4A - vacuolar protein sorting 4 homolog a (s. cerevisiae)  INO80 - ino80 complex subunit  RAB27A - rab27a, member ras oncogene family  RALA - v-ral simian leukemia viral oncogene homolog a (ras related)  IGHMBP2 - immunoglobulin mu binding protein 2  RSPH9 - radial spoke head 9 homolog (chlamydomonas)  EPB41L4A - erythrocyte membrane protein band 4.1 like 4a  GNPAT - glyceronephosphate o-acyltransferase  DYRK3 - dual-specificity tyrosine-(y)-phosphorylation regulated kinase 3  CLN5 - ceroid-lipofuscinosis, neuronal 5  DCTN6 - dynactin 6  BBS5 - bardet-biedl syndrome 5  EEA1 - early endosome antigen 1  CLU - clusterin  RAP1GDS1 - rap1, gtp-gdp dissociation stimulator 1  STX7 - syntaxin 7  GOLPH3 - golgi phosphoprotein 3 (coat-protein)  PHACTR1 - phosphatase and actin regulator 1  RAP2A - rap2a, member of ras oncogene family  MLH3 - mutl homolog 3 (e. coli)  TPP1 - tripeptidyl peptidase i  REEP3 - receptor accessory protein 3  ATP2C1 - atpase, ca++ transporting, type 2c, member 1  CDC42BPA - cdc42 binding protein kinase alpha (dmpk-like)  FAM171A1 - family with sequence similarity 171, member a1  STAG2 - stromal antigen 2  RAE1 - rae1 rna export 1 homolog (s. pombe)  AP2M1 - adaptor-related protein complex 2, mu 1 subunit  NUP35 - nucleoporin 35kda  CUL4B - cullin 4b  VPS41 - vacuolar protein sorting 41 homolog (s. cerevisiae)  ERCC8 - excision repair cross-complementing rodent repair deficiency, complementation group 8  B9D1 - b9 protein domain 1  YME1L1 - yme1-like 1 atpase  PTGES3 - prostaglandin e synthase 3 (cytosolic)  CUL3 - cullin 3  POLD3 - polymerase (dna-directed), delta 3, accessory subunit  CHMP4B - charged multivesicular body protein 4b  CIRBP - cold inducible rna binding protein  DHX29 - deah (asp-glu-ala-his) box polypeptide 29  SPTAN1 - spectrin, alpha, non-erythrocytic 1  CEP89 - centrosomal protein 89kda  LIG4 - ligase iv, dna, atp-dependent  HPS3 - hermansky-pudlak syndrome 3  WDR19 - wd repeat domain 19  ABLIM1 - actin binding lim protein 1  LIMK1 - lim domain kinase 1  CD2AP - cd2-associated protein  SPTBN4 - spectrin, beta, non-erythrocytic 4  FBXO18 - f-box protein, helicase, 18  VPS37A - vacuolar protein sorting 37 homolog a (s. cerevisiae)  CORO1C - coronin, actin binding protein, 1c  NCOR1 - nuclear receptor corepressor 1  SPRYD3 - spry domain containing 3  SPG7 - spastic paraplegia 7 (pure and complicated autosomal recessive)  HOOK3 - hook microtubule-tethering protein 3  COX7A2 - cytochrome c oxidase subunit viia polypeptide 2 (liver)  LZTS2 - leucine zipper, putative tumor suppressor 2  SPAST - spastin  DOT1L - dot1-like histone h3k79 methyltransferase  REEP2 - receptor accessory protein 2  NCKAP5L - nck-associated protein 5-like  LETM1 - leucine zipper-ef-hand containing transmembrane protein 1  IQSEC3 - iq motif and sec7 domain 3  SYCE1L - synaptonemal complex central element protein 1-like  WDR73 - wd repeat domain 73  TICAM2 - toll-like receptor adaptor molecule 2  BRSK1 - br serine/threonine kinase 1  SLX4 - slx4 structure-specific endonuclease subunit  CHD1L - chromodomain helicase dna binding protein 1-like  SEC22B - sec22 vesicle trafficking protein homolog b (s. cerevisiae) (gene/pseudogene)  PTPDC1 - protein tyrosine phosphatase domain containing 1  GOSR2 - golgi snap receptor complex member 2  TMEM41B - transmembrane protein 41b  UBXN2B - ubx domain protein 2b  SON - son dna binding protein  HEBP2 - heme binding protein 2  SPIRE2 - spire actin nucleation factor 2  LIMK2 - lim domain kinase 2  GOLM1 - golgi membrane protein 1  ATF2 - activating transcription factor 2  CDC42BPB - cdc42 binding protein kinase beta (dmpk-like)  JAGN1 - jagunal homolog 1 (drosophila)  CREB1 - camp responsive element binding protein 1  LMNA - lamin a/c  LMAN1 - lectin, mannose-binding, 1  LLGL1 - lethal giant larvae homolog 1 (drosophila)  MARK4 - map/microtubule affinity-regulating kinase 4  CEP19 - centrosomal protein 19kda  GABARAPL1 - gaba(a) receptor-associated protein like 1  PLK3 - polo-like kinase 3  CNN1 - calponin 1, basic, smooth muscle  RHOU - ras homolog family member u  FSCN1 - fascin homolog 1, actin-bundling protein (strongylocentrotus purpuratus)  TBPL1 - tbp-like 1  TRAPPC1 - trafficking protein particle complex 1  VPS4B - vacuolar protein sorting 4 homolog b (s. cerevisiae)  MAP1LC3A - microtubule-associated protein 1 light chain 3 alpha  GOSR1 - golgi snap receptor complex member 1  NIP7 - nip7, nucleolar pre-rrna processing protein  SNAP25 - synaptosomal-associated protein, 25kda  BAG5 - bcl2-associated athanogene 5  TP53INP2 - tumor protein p53 inducible nuclear protein 2  TRAPPC4 - trafficking protein particle complex 4  CNN3 - calponin 3, acidic  CNN2 - calponin 2  CNP - 2',3'-cyclic nucleotide 3' phosphodiesterase  ACTL6B - actin-like 6b  ARFIP2 - adp-ribosylation factor interacting protein 2  STMN1 - stathmin 1  ATCAY - ataxia, cerebellar, cayman type  TEX264 - testis expressed 264  ATG5 - autophagy related 5  ROCK2 - rho-associated, coiled-coil containing protein kinase 2  SMARCA1 - swi/snf related, matrix associated, actin dependent regulator of chromatin, subfamily a, member 1  ATP6V1D - atpase, h+ transporting, lysosomal 34kda, v1 subunit d  FUNDC1 - fun14 domain containing 1  STX8 - syntaxin 8  RAB38 - rab38, member ras oncogene family  LAMA5 - laminin, alpha 5  FBF1 - fas (tnfrsf6) binding factor 1  STX12 - syntaxin 12  TFIP11 - tuftelin interacting protein 11  NAGPA - n-acetylglucosamine-1-phosphodiester alpha-n-acetylglucosaminidase  SYP - synaptophysin  CHD6 - chromodomain helicase dna binding protein 6  POLR1B - polymerase (rna) i polypeptide b, 128kda  EML2 - echinoderm microtubule associated protein like 2  NAT6 - n-acetyltransferase 6 (gcn5-related)  VPS28 - vacuolar protein sorting 28 homolog (s. cerevisiae)  KIFC3 - kinesin family member c3  KIF5C - kinesin family member 5c  ANTXR1 - anthrax toxin receptor 1  KIF2A - kinesin heavy chain member 2a  KIF5B - kinesin family member 5b  KLHL12 - kelch-like family member 12  SYT4 - synaptotagmin iv  SUPV3L1 - suppressor of var1, 3-like 1 (s. cerevisiae)  SUN2 - sad1 and unc84 domain containing 2  EML3 - echinoderm microtubule associated protein like 3  EIF2AK3 - eukaryotic translation initiation factor 2-alpha kinase 3  DYNC1LI1 - dynein, cytoplasmic 1, light intermediate chain 1  VAMP2 - vesicle-associated membrane protein 2 (synaptobrevin 2)  CTNNA2 - catenin (cadherin-associated protein), alpha 2  CCDC41 - coiled-coil domain containing 41  MFSD8 - major facilitator superfamily domain containing 8  PITPNB - phosphatidylinositol transfer protein, beta  STXBP1 - syntaxin binding protein 1  STX5 - syntaxin 5  STX4 - syntaxin 4  RECQL5 - recq protein-like 5  KRAS - kirsten rat sarcoma viral oncogene homolog  STX3 - syntaxin 3  STX1A - syntaxin 1a (brain)  USP30 - ubiquitin specific peptidase 30  TGFBRAP1 - transforming growth factor, beta receptor associated protein 1  UNC119B - unc-119 homolog b (c. elegans)  SBDS - shwachman-bodian-diamond syndrome  KPNB1 - karyopherin (importin) beta 1  RMDN1 - regulator of microtubule dynamics 1  MPV17L2 - mpv17 mitochondrial membrane protein-like 2  TRAPPC12 - trafficking protein particle complex 12  KLC1 - kinesin light chain 1  CRIPT - cysteine-rich pdz-binding protein  TCTEX1D2 - tctex1 domain containing 2  CXADR - coxsackie virus and adenovirus receptor  AURKC - aurora kinase c  TFB1M - transcription factor b1, mitochondrial  TUBA1C - tubulin, alpha 1c  KIT - v-kit hardy-zuckerman 4 feline sarcoma viral oncogene homolog  PEX16 - peroxisomal biogenesis factor 16  LONP1 - lon peptidase 1, mitochondrial  C12orf44 - chromosome 12 open reading frame 44  CRK - v-crk avian sarcoma virus ct10 oncogene homolog  TMEM216 - transmembrane protein 216  CRMP1 - collapsin response mediator protein 1  NIPBL - nipped-b homolog (drosophila)  COG4 - component of oligomeric golgi complex 4  TRAPPC11 - trafficking protein particle complex 11  STAT3 - signal transducer and activator of transcription 3 (acute-phase response factor)  TMEM175 - transmembrane protein 175  COG1 - component of oligomeric golgi complex 1  SLC25A33 - solute carrier family 25 (pyrimidine nucleotide carrier), member 33  ITPA - inosine triphosphatase (nucleoside triphosphate pyrophosphatase)  KIF3B - kinesin family member 3b  MAST4 - microtubule associated serine/threonine kinase family member 4  SS18 - synovial sarcoma translocation, chromosome 18  CHCHD6 - coiled-coil-helix-coiled-coil-helix domain containing 6  FBXO7 - f-box protein 7  SNX33 - sorting nexin 33  LRCH3 - leucine-rich repeats and calponin homology (ch) domain containing 3  SRPK2 - srsf protein kinase 2  BLOC1S5 - biogenesis of lysosomal organelles complex-1, subunit 5, muted  SRPK1 - srsf protein kinase 1  TROVE2 - trove domain family, member 2  KLHL22 - kelch-like family member 22  GALNT11 - udp-n-acetyl-alpha-d-galactosamine:polypeptide n-acetylgalactosaminyltransferase 11 (galnac-t11)  COPS2 - cop9 signalosome subunit 2  ZDHHC2 - zinc finger, dhhc-type containing 2  SSBP1 - single-stranded dna binding protein 1, mitochondrial  SNAP29 - synaptosomal-associated protein, 29kda  ATXN10 - ataxin 10  SQLE - squalene epoxidase  TTLL1 - tubulin tyrosine ligase-like family, member 1  CSNK1A1 - casein kinase 1, alpha 1  VAMP3 - vesicle-associated membrane protein 3  SPTBN1 - spectrin, beta, non-erythrocytic 1  CSNK1D - casein kinase 1, delta  MIA3 - melanoma inhibitory activity family, member 3  TAOK2 - tao kinase 2  SRC - v-src avian sarcoma (schmidt-ruppin a-2) viral oncogene homolog  VPS25 - vacuolar protein sorting 25 homolog (s. cerevisiae)  CSRP1 - cysteine and glycine-rich protein 1  TUBE1 - tubulin, epsilon 1  TMEM107 - transmembrane protein 107  CSRP2 - cysteine and glycine-rich protein 2  DHX9 - deah (asp-glu-ala-his) box helicase 9  TRAPPC2 - trafficking protein particle complex 2  DDX6 - dead (asp-glu-ala-asp) box helicase 6  S1PR2 - sphingosine-1-phosphate receptor 2  DDX3X - dead (asp-glu-ala-asp) box helicase 3, x-linked  SEC13 - sec13 homolog (s. cerevisiae)  DDX1 - dead (asp-glu-ala-asp) box helicase 1  CCDC120 - coiled-coil domain containing 120  RAB8B - rab8b, member ras oncogene family  ATP8A2 - atpase, aminophospholipid transporter, class i, type 8a, member 2  KIAA1244 - kiaa1244  SDCBP - syndecan binding protein (syntenin)  WWTR1 - ww domain containing transcription regulator 1  DDB1 - damage-specific dna binding protein 1, 127kda  MLH1 - mutl homolog 1, colon cancer, nonpolyposis type 2 (e. coli)  RHOT2 - ras homolog family member t2  DCTN1 - dynactin 1  MKLN1 - muskelin 1, intracellular mediator containing kelch motifs  PTPN23 - protein tyrosine phosphatase, non-receptor type 23  MCM9 - minichromosome maintenance complex component 9  CYTH2 - cytohesin 2  WDR34 - wd repeat domain 34  DBN1 - drebrin 1  ATL3 - atlastin gtpase 3  POT1 - protection of telomeres 1  TRAPPC2L - trafficking protein particle complex 2-like  CCDC8 - coiled-coil domain containing 8  AHCTF1 - at hook containing transcription factor 1  RAB22A - rab22a, member ras oncogene family  KATNAL1 - katanin p60 subunit a-like 1  RNF19A - ring finger protein 19a, e3 ubiquitin protein ligase  NOLC1 - nucleolar and coiled-body phosphoprotein 1  RAB23 - rab23, member ras oncogene family  KCTD13 - potassium channel tetramerization domain containing 13  EIF2A - eukaryotic translation initiation factor 2a, 65kda  ZMYM3 - zinc finger, mym-type 3  MCM7 - minichromosome maintenance complex component 7  ZMYM4 - zinc finger, mym-type 4  DNM3 - dynamin 3  ATXN2 - ataxin 2  SIPA1L1 - signal-induced proliferation-associated 1 like 1  VAPB - vamp (vesicle-associated membrane protein)-associated protein b and c  CHD5 - chromodomain helicase dna binding protein 5  BUB3 - bub3 mitotic checkpoint protein  PELO - pelota homolog (drosophila)  MECP2 - methyl cpg binding protein 2 (rett syndrome)  MCFD2 - multiple coagulation factor deficiency 2  MEF2A - myocyte enhancer factor 2a  ZW10 - zw10 kinetochore protein  KNSTRN - kinetochore-localized astrin/spag5 binding protein  BRWD1 - bromodomain and wd repeat domain containing 1  FAM160A2 - family with sequence similarity 160, member a2  PYGO2 - pygopus homolog 2 (drosophila)  RAB8A - rab8a, member ras oncogene family  GORASP2 - golgi reassembly stacking protein 2, 55kda  CEP78 - centrosomal protein 78kda  COX7A2L - cytochrome c oxidase subunit viia polypeptide 2 like  C2CD3 - c2 calcium-dependent domain containing 3  ZFYVE1 - zinc finger, fyve domain containing 1  FAM161A - family with sequence similarity 161, member a  DAG1 - dystroglycan 1 (dystrophin-associated glycoprotein 1)  ARHGEF2 - rho/rac guanine nucleotide exchange factor (gef) 2  S100A10 - s100 calcium binding protein a10  CLIP3 - cap-gly domain containing linker protein 3  TUBB2B - tubulin, beta 2b class iib  ATG12 - autophagy related 12  STARD13 - star-related lipid transfer (start) domain containing 13  CHMP2B - charged multivesicular body protein 2b  HACE1 - hect domain and ankyrin repeat containing e3 ubiquitin protein ligase 1  ARL6 - adp-ribosylation factor-like 6  HGS - hepatocyte growth factor-regulated tyrosine kinase substrate  VTA1 - vesicle (multivesicular body) trafficking 1  SERBP1 - serpine1 mrna binding protein 1  COG3 - component of oligomeric golgi complex 3  TUBGCP6 - tubulin, gamma complex associated protein 6  TCTN3 - tectonic family member 3  TMEM138 - transmembrane protein 138  NKD2 - naked cuticle homolog 2 (drosophila)  SLC9A1 - solute carrier family 9, subfamily a (nhe1, cation proton antiporter 1), member 1  MICAL3 - microtubule associated monooxygenase, calponin and lim domain containing 3  TRAF3IP1 - tnf receptor-associated factor 3 interacting protein 1  NCKIPSD - nck interacting protein with sh3 domain  SMC3 - structural maintenance of chromosomes 3  TAOK1 - tao kinase 1  MARK3 - map/microtubule affinity-regulating kinase 3  SYDE1 - synapse defective 1, rho gtpase, homolog 1 (c. elegans)  MARK1 - map/microtubule affinity-regulating kinase 1  MAPT - microtubule-associated protein tau  SHANK3 - sh3 and multiple ankyrin repeat domains 3  MAP6 - microtubule-associated protein 6  INA - internexin neuronal intermediate filament protein, alpha  WIPI2 - wd repeat domain, phosphoinositide interacting 2  ATP6V0D1 - atpase, h+ transporting, lysosomal 38kda, v0 subunit d1  RAB14 - rab14, member ras oncogene family  MAP2 - microtubule-associated protein 2  LATS1 - large tumor suppressor kinase 1  MAP1B - microtubule-associated protein 1b  SEMA6A - sema domain, transmembrane domain (tm), and cytoplasmic domain, (semaphorin) 6a  MAP1A - microtubule-associated protein 1a  STRIP1 - striatin interacting protein 1  RAB33B - rab33b, member ras oncogene family  VPS54 - vacuolar protein sorting 54 homolog (s. cerevisiae)  DPYSL2 - dihydropyrimidinase-like 2  FAM161B - family with sequence similarity 161, member b  MAN2A1 - mannosidase, alpha, class 2a, member 1  USP8 - ubiquitin specific peptidase 8  TNKS1BP1 - tankyrase 1 binding protein 1, 182kda  MAPK15 - mitogen-activated protein kinase 15  TOR1A - torsin family 1, member a (torsin a)  MKL1 - megakaryoblastic leukemia (translocation) 1  ARHGAP21 - rho gtpase activating protein 21  SYT13 - synaptotagmin xiii  UFC1 - ubiquitin-fold modifier conjugating enzyme 1  MCM3 - minichromosome maintenance complex component 3  MCM6 - minichromosome maintenance complex component 6  CHMP5 - charged multivesicular body protein 5  USP36 - ubiquitin specific peptidase 36  MCM5 - minichromosome maintenance complex component 5  DOCK7 - dedicator of cytokinesis 7  TTBK2 - tau tubulin kinase 2  MCM2 - minichromosome maintenance complex component 2  RANBP10 - ran binding protein 10  ANKFY1 - ankyrin repeat and fyve domain containing 1  LSM14A - lsm14a, scd6 homolog a (s. cerevisiae)  SYT7 - synaptotagmin vii  MAP7 - microtubule-associated protein 7  DKC1 - dyskeratosis congenita 1, dyskerin  DLG3 - discs, large homolog 3 (drosophila)  DLG4 - discs, large homolog 4 (drosophila)  PNPT1 - polyribonucleotide nucleotidyltransferase 1  DLG1 - discs, large homolog 1 (drosophila)  VPS18 - vacuolar protein sorting 18 homolog (s. cerevisiae)  BLOC1S6 - biogenesis of lysosomal organelles complex-1, subunit 6, pallidin  RSPH1 - radial spoke head 1 homolog (chlamydomonas)  FBXW8 - f-box and wd repeat domain containing 8  TRAPPC9 - trafficking protein particle complex 9  TPPP3 - tubulin polymerization-promoting protein family member 3  HIP1R - huntingtin interacting protein 1 related  RICTOR - rptor independent companion of mtor, complex 2  TMED6 - transmembrane emp24 protein transport domain containing 6  CHMP3 - charged multivesicular body protein 3  DHX37 - deah (asp-glu-ala-his) box polypeptide 37  SIPA1 - signal-induced proliferation-associated 1  DYNC1H1 - dynein, cytoplasmic 1, heavy chain 1  DYNC1LI2 - dynein, cytoplasmic 1, light intermediate chain 2  RAB34 - rab34, member ras oncogene family  ATG16L2 - autophagy related 16-like 2 (s. cerevisiae)  DNM2 - dynamin 2  EPB41L5 - erythrocyte membrane protein band 4.1 like 5  ALS2 - amyotrophic lateral sclerosis 2 (juvenile)  CHD8 - chromodomain helicase dna binding protein 8  HPS4 - hermansky-pudlak syndrome 4  MZT1 - mitotic spindle organizing protein 1  IFT172 - intraflagellar transport 172 homolog (chlamydomonas)  NAV1 - neuron navigator 1  H1FX - h1 histone family, member x  WASL - wiskott-aldrich syndrome-like  SMARCAD1 - swi/snf-related, matrix-associated actin-dependent regulator of chromatin, subfamily a, containing dead/h box 1  MAPRE1 - microtubule-associated protein, rp/eb family, member 1  MARK2 - map/microtubule affinity-regulating kinase 2  CHMP4C - charged multivesicular body protein 4c  MAPRE3 - microtubule-associated protein, rp/eb family, member 3  G3BP1 - gtpase activating protein (sh3 domain) binding protein 1  CTTN - cortactin  AKAP9 - a kinase (prka) anchor protein (yotiao) 9  KIFAP3 - kinesin-associated protein 3  SPIRE1 - spire actin nucleation factor 1  GORAB - golgin, rab6-interacting  ELN - elastin  IFT46 - intraflagellar transport 46 homolog (chlamydomonas)  EML1 - echinoderm microtubule associated protein like 1  WDR47 - wd repeat domain 47  ABI2 - abl-interactor 2  RACGAP1 - rac gtpase activating protein 1  VMP1 - vacuole membrane protein 1  NAGLU - n-acetylglucosaminidase, alpha  MGME1 - mitochondrial genome maintenance exonuclease 1  WRNIP1 - werner helicase interacting protein 1  EPAS1 - endothelial pas domain protein 1  HABP4 - hyaluronan binding protein 4  WASF2 - was protein family, member 2  NBN - nibrin  RAB18 - rab18, member ras oncogene family  TIMM50 - translocase of inner mitochondrial membrane 50 homolog (s. cerevisiae)  RAB3GAP1 - rab3 gtpase activating protein subunit 1 (catalytic)  NCAPH2 - non-smc condensin ii complex, subunit h2  CNIH - cornichon homolog (drosophila)  TUBB2A - tubulin, beta 2a class iia  TUBG1 - tubulin, gamma 1  LMAN2L - lectin, mannose-binding 2-like  NDEL1 - nude neurodevelopment protein 1-like 1  EIF2S1 - eukaryotic translation initiation factor 2, subunit 1 alpha, 35kda  TRIM32 - tripartite motif containing 32  SAR1A - sar1 homolog a (s. cerevisiae)  TMOD2 - tropomodulin 2 (neuronal)  AZI1 - 5-azacytidine induced 1  TSG101 - tumor susceptibility gene 101  USPL1 - ubiquitin specific peptidase like 1  MAST1 - microtubule associated serine/threonine kinase 1  ACIN1 - apoptotic chromatin condensation inducer 1  CCDC47 - coiled-coil domain containing 47  MVB12A - multivesicular body subunit 12a  RAB1B - rab1b, member ras oncogene family  EIF2D - eukaryotic translation initiation factor 2d  SEH1L - seh1-like (s. cerevisiae)  MYH10 - myosin, heavy chain 10, non-muscle  DAAM1 - dishevelled associated activator of morphogenesis 1  MYH11 - myosin, heavy chain 11, smooth muscle  SNX11 - sorting nexin 11  MYH9 - myosin, heavy chain 9, non-muscle  HUWE1 - hect, uba and wwe domain containing 1, e3 ubiquitin protein ligase  TP53BP2 - tumor protein p53 binding protein, 2  SNX10 - sorting nexin 10  ARPC5 - actin related protein 2/3 complex, subunit 5, 16kda  TOP3A - topoisomerase (dna) iii alpha  CNOT7 - ccr4-not transcription complex, subunit 7  TOP2B - topoisomerase (dna) ii beta 180kda  COL4A3BP - collagen, type iv, alpha 3 (goodpasture antigen) binding protein  MX1 - myxovirus (influenza virus) resistance 1, interferon-inducible protein p78 (mouse)  MAST3 - microtubule associated serine/threonine kinase 3  CELSR2 - cadherin, egf lag seven-pass g-type receptor 2  TOP1 - topoisomerase (dna) i  GPSM2 - g-protein signaling modulator 2  ACTR2 - arp2 actin-related protein 2 homolog (yeast)  UNC13A - unc-13 homolog a (c. elegans)  ACTR3 - arp3 actin-related protein 3 homolog (yeast)  CLUAP1 - clusterin associated protein 1  MTX1 - metaxin 1  MFF - mitochondrial fission factor  TNIK - traf2 and nck interacting kinase  PHF13 - phd finger protein 13  E2F4 - e2f transcription factor 4, p107/p130-binding  PDS5B - pds5, regulator of cohesion maintenance, homolog b (s. cerevisiae)  RAD50 - rad50 homolog (s. cerevisiae)  S1PR1 - sphingosine-1-phosphate receptor 1  TIMM22 - translocase of inner mitochondrial membrane 22 homolog (yeast)  SEC61A1 - sec61 alpha 1 subunit (s. cerevisiae)  SETX - senataxin  SHARPIN - shank-associated rh domain interactor  TPR - translocated promoter region, nuclear basket protein  BLOC1S3 - biogenesis of lysosomal organelles complex-1, subunit 3  PPRC1 - peroxisome proliferator-activated receptor gamma, coactivator-related 1  AKT3 - v-akt murine thymoma viral oncogene homolog 3  BOD1 - biorientation of chromosomes in cell division 1  SIPA1L3 - signal-induced proliferation-associated 1 like 3  TTLL5 - tubulin tyrosine ligase-like family, member 5  ARHGAP26 - rho gtpase activating protein 26  NUP107 - nucleoporin 107kda  IQSEC2 - iq motif and sec7 domain 2  GOPC - golgi-associated pdz and coiled-coil motif containing  MTIF2 - mitochondrial translational initiation factor 2  PDCD6 - programmed cell death 6  PTK2B - protein tyrosine kinase 2 beta  MTM1 - myotubularin 1  CUL9 - cullin 9  PDCD6IP - programmed cell death 6 interacting protein  HDAC6 - histone deacetylase 6  CLASP2 - cytoplasmic linker associated protein 2  POGZ - pogo transposable element with znf domain  RAD54L2 - rad54-like 2 (s. cerevisiae)  VIM - vimentin  EZR - ezrin  SMC5 - structural maintenance of chromosomes 5  EPB41L3 - erythrocyte membrane protein band 4.1-like 3  KIFC2 - kinesin family member c2  FMNL3 - formin-like 3  EXOC8 - exocyst complex component 8  UVRAG - uv radiation resistance associated  DNM1L - dynamin 1-like  VASP - vasodilator-stimulated phosphoprotein  RANBP9 - ran binding protein 9  F2R - coagulation factor ii (thrombin) receptor  DNAJB6 - dnaj (hsp40) homolog, subfamily b, member 6  SLC25A46 - solute carrier family 25, member 46  VCP - valosin containing protein  SMC4 - structural maintenance of chromosomes 4  MSN - moesin  ATP8B2 - atpase, aminophospholipid transporter, class i, type 8b, member 2  MSH3 - muts homolog 3 (e. coli)  MSH2 - muts homolog 2, colon cancer, nonpolyposis type 1 (e. coli)  MCOLN1 - mucolipin 1  ERCC4 - excision repair cross-complementing rodent repair deficiency, complementation group 4  NUP205 - nucleoporin 205kda  ERCC3 - excision repair cross-complementing rodent repair deficiency, complementation group 3  SEC16A - sec16 homolog a (s. cerevisiae)  NCAPD2 - non-smc condensin i complex, subunit d2  DBNL - drebrin-like  CHMP7 - charged multivesicular body protein 7  UBB - ubiquitin b  CORO1B - coronin, actin binding protein, 1b  WDR1 - wd repeat domain 1  KANK1 - kn motif and ankyrin repeat domains 1  IFT81 - intraflagellar transport 81 homolog (chlamydomonas)  TMEM14A - transmembrane protein 14a  HIRA - histone cell cycle regulator  FAF2 - fas associated factor family member 2  TPGS1 - tubulin polyglutamylase complex subunit 1  ETS1 - v-ets avian erythroblastosis virus e26 oncogene homolog 1  COG7 - component of oligomeric golgi complex 7  ARL6IP1 - adp-ribosylation factor-like 6 interacting protein 1  DMTN - dematin actin binding protein  EPB41L1 - erythrocyte membrane protein band 4.1-like 1  EPB41 - erythrocyte membrane protein band 4.1 (elliptocytosis 1, rh-linked)  SUMO1 - small ubiquitin-like modifier 1  EPB41L2 - erythrocyte membrane protein band 4.1-like 2  CHMP4A - charged multivesicular body protein 4a  ZNF830 - zinc finger protein 830  PRRC2C - proline-rich coiled-coil 2c  TTL - tubulin tyrosine ligase  GOLGA5 - golgin a5  SYT11 - synaptotagmin xi  SETD2 - set domain containing 2  UBE2D2 - ubiquitin-conjugating enzyme e2d 2  RBX1 - ring-box 1, e3 ubiquitin protein ligase  MRE11A - mre11 meiotic recombination 11 homolog a (s. cerevisiae)  STX2 - syntaxin 2  NUP153 - nucleoporin 153kda  ADCK1 - aarf domain containing kinase 1  ERCC2 - excision repair cross-complementing rodent repair deficiency, complementation group 2  EPS8 - epidermal growth factor receptor pathway substrate 8  EPS15 - epidermal growth factor receptor pathway substrate 15  CAMSAP2 - calmodulin regulated spectrin-associated protein family, member 2  B9D2 - b9 protein domain 2  PLK1S1 - polo-like kinase 1 substrate 1  SCFD1 - sec1 family domain containing 1  SEC24D - sec24 family, member d (s. cerevisiae)  DDHD2 - ddhd domain containing 2  PDS5A - pds5, regulator of cohesion maintenance, homolog a (s. cerevisiae)  STMN3 - stathmin-like 3  ELMO1 - engulfment and cell motility 1  FLII - flightless i homolog (drosophila)  C2CD5 - c2 calcium-dependent domain containing 5  FLNA - filamin a, alpha  FLNB - filamin b, beta  AGRN - agrin  ARC - activity-regulated cytoskeleton-associated protein  SHANK1 - sh3 and multiple ankyrin repeat domains 1  CEP350 - centrosomal protein 350kda  PACS2 - phosphofurin acidic cluster sorting protein 2  LARP4 - la ribonucleoprotein domain family, member 4  HDAC8 - histone deacetylase 8  ANKRD28 - ankyrin repeat domain 28  SPATA7 - spermatogenesis associated 7  UBAP2L - ubiquitin associated protein 2-like  NCAPD3 - non-smc condensin ii complex, subunit d3  G3BP2 - gtpase activating protein (sh3 domain) binding protein 2  EHBP1 - eh domain binding protein 1  OCRL - oculocerebrorenal syndrome of lowe  BICD2 - bicaudal d homolog 2 (drosophila)  ODF2 - outer dense fiber of sperm tails 2  TMED5 - transmembrane emp24 protein transport domain containing 5  REEP4 - receptor accessory protein 4  FBXW11 - f-box and wd repeat domain containing 11  VPS11 - vacuolar protein sorting 11 homolog (s. cerevisiae)  KDM3A - lysine (k)-specific demethylase 3a  OPA1 - optic atrophy 1 (autosomal dominant)  TMED7 - transmembrane emp24 protein transport domain containing 7  DYNLT1 - dynein, light chain, tctex-type 1  FIG4 - fig4 homolog, sac1 lipid phosphatase domain containing (s. cerevisiae)  SNAP91 - synaptosomal-associated protein, 91kda  STX1B - syntaxin 1b  APITD1 - apoptosis-inducing, taf9-like domain 1  IFT43 - intraflagellar transport 43 homolog (chlamydomonas)  FIS1 - fission 1 (mitochondrial outer membrane) homolog (s. cerevisiae)  CKAP5 - cytoskeleton associated protein 5  VPS8 - vacuolar protein sorting 8 homolog (s. cerevisiae)  HAUS5 - haus augmin-like complex, subunit 5  NSF - n-ethylmaleimide-sensitive factor  KIAA0586 - kiaa0586  FGF13 - fibroblast growth factor 13  SYNE1 - spectrin repeat containing, nuclear envelope 1  TACC1 - transforming, acidic coiled-coil containing protein 1  IST1 - increased sodium tolerance 1 homolog (yeast)  VPS39 - vacuolar protein sorting 39 homolog (s. cerevisiae)  NSFL1C - nsfl1 (p97) cofactor (p47)  NUMA1 - nuclear mitotic apparatus protein 1  CLASP1 - cytoplasmic linker associated protein 1  NUP98 - nucleoporin 98kda  ATG13 - autophagy related 13  TAP2 - transporter 2, atp-binding cassette, sub-family b (mdr/tap)  TAPBP - tap binding protein (tapasin)  FGD1 - fyve, rhogef and ph domain containing 1  FEN1 - flap structure-specific endonuclease 1  ATL1 - atlastin gtpase 1  FER - fer (fps/fes related) tyrosine kinase  CEP120 - centrosomal protein 120kda  DNAJC13 - dnaj (hsp40) homolog, subfamily c, member 13  EFHC1 - ef-hand domain (c-terminal) containing 1  PIP5K1C - phosphatidylinositol-4-phosphate 5-kinase, type i, gamma  ARHGEF17 - rho guanine nucleotide exchange factor (gef) 17  NPM1 - nucleophosmin (nucleolar phosphoprotein b23, numatrin)  ARHGEF11 - rho guanine nucleotide exchange factor (gef) 11  NPHP1 - nephronophthisis 1 (juvenile)  SIK3 - sik family kinase 3  NUDCD3 - nudc domain containing 3  SPECC1L - sperm antigen with calponin homology and coiled-coil domains 1-like  TBCE - tubulin folding cofactor e  TBCD - tubulin folding cofactor d  SPICE1 - spindle and centriole associated protein 1  TAZ - tafazzin  UFL1 - ufm1-specific ligase 1  PPP1R13B - protein phosphatase 1, regulatory subunit 13b  PUM2 - pumilio homolog 2 (drosophila)  FGD5 - fyve, rhogef and ph domain containing 5  RB1CC1 - rb1-inducible coiled-coil 1  OBSL1 - obscurin-like 1  IFT52 - intraflagellar transport 52 homolog (chlamydomonas)  CUL7 - cullin 7  NRF1 - nuclear respiratory factor 1  SH3D19 - sh3 domain containing 19  NUDT7 - nudix (nucleoside diphosphate linked moiety x)-type motif 7  ERBB2IP - erbb2 interacting protein  CHD9 - chromodomain helicase dna binding protein 9  UBQLN1 - ubiquilin 1  NMT1 - n-myristoyltransferase 1  SPG11 - spastic paraplegia 11 (autosomal recessive)  ARMC9 - armadillo repeat containing 9  SLC2A8 - solute carrier family 2 (facilitated glucose transporter), member 8  POC1B - poc1 centriolar protein b  TMED3 - transmembrane emp24 protein transport domain containing 3  TJP1 - tight junction protein 1  MTOR - mechanistic target of rapamycin (serine/threonine kinase)  UBQLN2 - ubiquilin 2  BLOC1S2 - biogenesis of lysosomal organelles complex-1, subunit 2  IFT74 - intraflagellar transport 74 homolog (chlamydomonas)  ATP13A2 - atpase type 13a2  TLN1 - talin 1  USP6NL - usp6 n-terminal like  CEP290 - centrosomal protein 290kda  NOS3 - nitric oxide synthase 3 (endothelial cell)  PACSIN1 - protein kinase c and casein kinase substrate in neurons 1  CEP57 - centrosomal protein 57kda  NOTCH1 - notch 1  CTC1 - cts telomere maintenance complex component 1  AMOTL1 - angiomotin like 1  TRAPPC10 - trafficking protein particle complex 10  TMF1 - tata element modulatory factor 1  EDC3 - enhancer of mrna decapping 3  CENPT - centromere protein t  ZFYVE16 - zinc finger, fyve domain containing 16  TNFAIP1 - tumor necrosis factor, alpha-induced protein 1 (endothelial)  IFT140 - intraflagellar transport 140 homolog (chlamydomonas)  PHACTR2 - phosphatase and actin regulator 2  TBCK - tbc1 domain containing kinase  SLK - ste20-like kinase  TJAP1 - tight junction associated protein 1 (peripheral)  VCPIP1 - valosin containing protein (p97)/p47 complex interacting protein 1  EHD2 - eh-domain containing 2  EHD3 - eh-domain containing 3  EHD4 - eh-domain containing 4  CCP110 - centriolar coiled coil protein 110kda  PRKCDBP - protein kinase c, delta binding protein  IQCB1 - iq motif containing b1  MINK1 - misshapen-like kinase 1  TTLL4 - tubulin tyrosine ligase-like family, member 4  GCC2 - grip and coiled-coil domain containing 2  NEFL - neurofilament, light polypeptide  TFAM - transcription factor a, mitochondrial  SMARCAL1 - swi/snf related, matrix associated, actin dependent regulator of chromatin, subfamily a-like 1  MTFR1 - mitochondrial fission regulator 1  NEK1 - nima-related kinase 1  TERF1 - telomeric repeat binding factor (nima-interacting) 1  NEDD9 - neural precursor cell expressed, developmentally down-regulated 9  TMEM80 - transmembrane protein 80  TERF2 - telomeric repeat binding factor 2  WRAP73 - wd repeat containing, antisense to tp73  NEFM - neurofilament, medium polypeptide  TESK1 - testis-specific kinase 1  TFDP1 - transcription factor dp-1  ARHGEF10 - rho guanine nucleotide exchange factor (gef) 10  BCCIP - brca2 and cdkn1a interacting protein  FEZ1 - fasciculation and elongation protein zeta 1 (zygin i)  PES1 - pescadillo ribosomal biogenesis factor 1  CAMSAP1 - calmodulin regulated spectrin-associated protein 1  NUP155 - nucleoporin 155kda  FXN - frataxin  TGFA - transforming growth factor, alpha  SEC24C - sec24 family, member c (s. cerevisiae)  NF1 - neurofibromin 1  CEP41 - centrosomal protein 41kda  POLE4 - polymerase (dna-directed), epsilon 4, accessory subunit  MACF1 - microtubule-actin crosslinking factor 1  CROCC - ciliary rootlet coiled-coil, rootletin  VPS37D - vacuolar protein sorting 37 homolog d (s. cerevisiae)  NUP93 - nucleoporin 93kda  TP53INP1 - tumor protein p53 inducible nuclear protein 1  CEP63 - centrosomal protein 63kda  WDR45B - wd repeat domain 45b  COPS7A - cop9 signalosome subunit 7a  ASAP1 - arfgap with sh3 domain, ankyrin repeat and ph domain 1  THY1 - thy-1 cell surface antigen  KIAA0430 - kiaa0430 |
| GO:0016071 | mRNA metabolic process | 1.99E-7 | 4.14E-5 | 1.14 (10334,566,6996,437) | [+] Show genes  RSRC1 - arginine/serine-rich coiled-coil 1  EXOSC9 - exosome component 9  WTAP - wilms tumor 1 associated protein  EXOSC10 - exosome component 10  ZCCHC8 - zinc finger, cchc domain containing 8  SYMPK - symplekin  RNPC3 - rna-binding region (rnp1, rrm) containing 3  RBM39 - rna binding motif protein 39  SF3A3 - splicing factor 3a, subunit 3, 60kda  HNRNPA0 - heterogeneous nuclear ribonucleoprotein a0  PNN - pinin, desmosome associated protein  METTL14 - methyltransferase like 14  SF3A2 - splicing factor 3a, subunit 2, 66kda  CNOT11 - ccr4-not transcription complex, subunit 11  CWC22 - cwc22 spliceosome-associated protein homolog (s. cerevisiae)  PNRC2 - proline-rich nuclear receptor coactivator 2  TXNL4A - thioredoxin-like 4a  CIR1 - corepressor interacting with rbpj, 1  PLRG1 - pleiotropic regulator 1  LSM2 - lsm2 homolog, u6 small nuclear rna associated (s. cerevisiae)  PAPOLA - poly(a) polymerase alpha  TCERG1 - transcription elongation regulator 1  PABPN1 - poly(a) binding protein, nuclear 1  RNPS1 - rna binding protein s1, serine-rich domain  SON - son dna binding protein  SUGP1 - surp and g patch domain containing 1  PRPF38A - prp38 pre-mrna processing factor 38 (yeast) domain containing a  SNRPN - small nuclear ribonucleoprotein polypeptide n  SNRPB2 - small nuclear ribonucleoprotein polypeptide b  RPRD1B - regulation of nuclear pre-mrna domain containing 1b  SNRPD1 - small nuclear ribonucleoprotein d1 polypeptide 16kda  DDX41 - dead (asp-glu-ala-asp) box polypeptide 41  SNRPF - small nuclear ribonucleoprotein polypeptide f  SNRNP70 - small nuclear ribonucleoprotein 70kda (u1)  YTHDF2 - yth domain family, member 2  SNRPA - small nuclear ribonucleoprotein polypeptide a  SNRPA1 - small nuclear ribonucleoprotein polypeptide a'  SNRPB - small nuclear ribonucleoprotein polypeptides b and b1  ADAR - adenosine deaminase, rna-specific  ADARB1 - adenosine deaminase, rna-specific, b1  CPSF4 - cleavage and polyadenylation specific factor 4, 30kda  RBM17 - rna binding motif protein 17  MED1 - mediator complex subunit 1  PPARD - peroxisome proliferator-activated receptor delta  RBM10 - rna binding motif protein 10  POLR2K - polymerase (rna) ii (dna directed) polypeptide k, 7.0kda  LSM5 - lsm5 homolog, u6 small nuclear rna associated (s. cerevisiae)  TSEN15 - tsen15 trna splicing endonuclease subunit  RBM25 - rna binding motif protein 25  EDC4 - enhancer of mrna decapping 4  CACTIN - cactin, spliceosome c complex subunit  CRNKL1 - crooked neck pre-mrna splicing factor-like 1 (drosophila)  POLR2G - polymerase (rna) ii (dna directed) polypeptide g  POLR2F - polymerase (rna) ii (dna directed) polypeptide f  POLR2E - polymerase (rna) ii (dna directed) polypeptide e, 25kda  POLR2D - polymerase (rna) ii (dna directed) polypeptide d  SCAF1 - sr-related ctd-associated factor 1  CDC40 - cell division cycle 40  POLR2C - polymerase (rna) ii (dna directed) polypeptide c, 33kda  POLR2B - polymerase (rna) ii (dna directed) polypeptide b, 140kda  POLR2A - polymerase (rna) ii (dna directed) polypeptide a, 220kda  HNRNPUL1 - heterogeneous nuclear ribonucleoprotein u-like 1  TFIP11 - tuftelin interacting protein 11  CSTF1 - cleavage stimulation factor, 3' pre-rna, subunit 1, 50kda  PRKACA - protein kinase, camp-dependent, catalytic, alpha  DBR1 - debranching rna lariats 1  PRPF40B - prp40 pre-mrna processing factor 40 homolog b (s. cerevisiae)  MRTO4 - mrna turnover 4 homolog (s. cerevisiae)  CSTF3 - cleavage stimulation factor, 3' pre-rna, subunit 3, 77kda  CSTF2 - cleavage stimulation factor, 3' pre-rna, subunit 2, 64kda  PRPF6 - pre-mrna processing factor 6  FUS - fused in sarcoma  SUPT6H - suppressor of ty 6 homolog (s. cerevisiae)  SUPV3L1 - suppressor of var1, 3-like 1 (s. cerevisiae)  FRG1 - fshd region gene 1  METTL8 - methyltransferase like 8  PRCC - papillary renal cell carcinoma (translocation-associated)  CLASRP - clk4-associating serine/arginine rich protein  SUPT4H1 - suppressor of ty 4 homolog 1 (s. cerevisiae)  SUPT5H - suppressor of ty 5 homolog (s. cerevisiae)  GEMIN6 - gem (nuclear organelle) associated protein 6  UBL5 - ubiquitin-like 5  SNRNP35 - small nuclear ribonucleoprotein 35kda (u11/u12)  PPP2R2A - protein phosphatase 2, regulatory subunit b, alpha  CMTR2 - cap methyltransferase 2  PPP2R1A - protein phosphatase 2, regulatory subunit a, alpha  CPSF6 - cleavage and polyadenylation specific factor 6, 68kda  SLBP - stem-loop binding protein  NUDT21 - nudix (nucleoside diphosphate linked moiety x)-type motif 21  PPP1R8 - protein phosphatase 1, regulatory subunit 8  PPP2CA - protein phosphatase 2, catalytic subunit, alpha isozyme  CPSF7 - cleavage and polyadenylation specific factor 7, 59kda  DDX23 - dead (asp-glu-ala-asp) box polypeptide 23  CBLL1 - cbl proto-oncogene-like 1, e3 ubiquitin protein ligase  PAN3 - pan3 poly(a) specific ribonuclease subunit homolog (s. cerevisiae)  SNRNP40 - small nuclear ribonucleoprotein 40kda (u5)  DDX39B - dead (asp-glu-ala-asp) box polypeptide 39b  ZRANB2 - zinc finger, ran-binding domain containing 2  POLDIP3 - polymerase (dna-directed), delta interacting protein 3  IWS1 - iws1 homolog (s. cerevisiae)  BUD13 - bud13 homolog (s. cerevisiae)  STAT3 - signal transducer and activator of transcription 3 (acute-phase response factor)  PRPF40A - prp40 pre-mrna processing factor 40 homolog a (s. cerevisiae)  PAPD7 - pap associated domain containing 7  DHX35 - deah (asp-glu-ala-his) box polypeptide 35  PHF5A - phd finger protein 5a  SNIP1 - smad nuclear interacting protein 1  GEMIN7 - gem (nuclear organelle) associated protein 7  DDX47 - dead (asp-glu-ala-asp) box polypeptide 47  CLP1 - cleavage and polyadenylation factor i subunit 1  SSB - sjogren syndrome antigen b (autoantigen la)  THOC3 - tho complex 3  EFTUD2 - elongation factor tu gtp binding domain containing 2  NT5C3B - 5'-nucleotidase, cytosolic iiib  CCDC94 - coiled-coil domain containing 94  SARNP - sap domain containing ribonucleoprotein  RBM22 - rna binding motif protein 22  CNOT8 - ccr4-not transcription complex, subunit 8  SF3B2 - splicing factor 3b, subunit 2, 145kda  CDK12 - cyclin-dependent kinase 12  DHX9 - deah (asp-glu-ala-his) box helicase 9  MTPAP - mitochondrial poly(a) polymerase  DHX8 - deah (asp-glu-ala-his) box polypeptide 8  DDX6 - dead (asp-glu-ala-asp) box helicase 6  RBM23 - rna binding motif protein 23  SRSF11 - serine/arginine-rich splicing factor 11  DDX5 - dead (asp-glu-ala-asp) box helicase 5  PARN - poly(a)-specific ribonuclease  SYF2 - syf2 pre-mrna-splicing factor  GSPT1 - g1 to s phase transition 1  ZCCHC6 - zinc finger, cchc domain containing 6  RNF113A - ring finger protein 113a  GTF2F1 - general transcription factor iif, polypeptide 1, 74kda  GTF2F2 - general transcription factor iif, polypeptide 2, 30kda  MLH1 - mutl homolog 1, colon cancer, nonpolyposis type 2 (e. coli)  GTF2H2 - general transcription factor iih, polypeptide 2, 44kda  GTF2H3 - general transcription factor iih, polypeptide 3, 34kda  PCBP2 - poly(rc) binding protein 2  GTF2H1 - general transcription factor iih, polypeptide 1, 62kda  GEMIN5 - gem (nuclear organelle) associated protein 5  SMG8 - smg8 nonsense mediated mrna decay factor  CPSF3 - cleavage and polyadenylation specific factor 3, 73kda  NAA38 - n(alpha)-acetyltransferase 38, natc auxiliary subunit  LSM7 - lsm7 homolog, u6 small nuclear rna associated (s. cerevisiae)  GTF2H4 - general transcription factor iih, polypeptide 4, 52kda  CNOT10 - ccr4-not transcription complex, subunit 10  PRKRIP1 - prkr interacting protein 1 (il11 inducible)  RPRD1A - regulation of nuclear pre-mrna domain containing 1a  MNAT1 - mnat cdk-activating kinase assembly factor 1  ZNF473 - zinc finger protein 473  APP - amyloid beta (a4) precursor protein  WBP11 - ww domain binding protein 11  DHX15 - deah (asp-glu-ala-his) box helicase 15  CNOT6 - ccr4-not transcription complex, subunit 6  DCP2 - decapping mrna 2  NSRP1 - nuclear speckle splicing regulatory protein 1  CWF19L2 - cwf19-like 2, cell cycle control (s. pombe)  PAPD4 - pap associated domain containing 4  SCAF4 - sr-related ctd-associated factor 4  CDC73 - cell division cycle 73  PELO - pelota homolog (drosophila)  GPATCH1 - g patch domain containing 1  SAMD4B - sterile alpha motif domain containing 4b  PPIL3 - peptidylprolyl isomerase (cyclophilin)-like 3  CPSF2 - cleavage and polyadenylation specific factor 2, 100kda  LSM6 - lsm6 homolog, u6 small nuclear rna associated (s. cerevisiae)  SCAF11 - sr-related ctd-associated factor 11  MAGOHB - mago-nashi homolog b (drosophila)  MFAP1 - microfibrillar-associated protein 1  LEO1 - leo1, paf1/rna polymerase ii complex component, homolog (s. cerevisiae)  PRPF38B - prp38 pre-mrna processing factor 38 (yeast) domain containing b  RBM28 - rna binding motif protein 28  SNRNP25 - small nuclear ribonucleoprotein 25kda (u11/u12)  MAGOH - mago-nashi homolog, proliferation-associated (drosophila)  ZBTB1 - zinc finger and btb domain containing 1  PRPF3 - pre-mrna processing factor 3  PRPF4 - prp4 pre-mrna processing factor 4 homolog (yeast)  CNOT6L - ccr4-not transcription complex, subunit 6-like  RQCD1 - rcd1 required for cell differentiation1 homolog (s. pombe)  DIS3 - dis3 mitotic control homolog (s. cerevisiae)  ZHX2 - zinc fingers and homeoboxes 2  PUS3 - pseudouridylate synthase 3  DOM3Z - dom-3 homolog z (c. elegans)  PRPF31 - pre-mrna processing factor 31  TNKS1BP1 - tankyrase 1 binding protein 1, 182kda  SKIV2L - superkiller viralicidic activity 2-like (s. cerevisiae)  CHTOP - chromatin target of prmt1  SART1 - squamous cell carcinoma antigen recognized by t cells  WDR33 - wd repeat domain 33  ZCRB1 - zinc finger cchc-type and rna binding motif 1  DCP1B - decapping mrna 1b  CWC15 - cwc15 spliceosome-associated protein homolog (s. cerevisiae)  NUDT12 - nudix (nucleoside diphosphate linked moiety x)-type motif 12  SMU1 - smu-1 suppressor of mec-8 and unc-52 homolog (c. elegans)  SCAF8 - sr-related ctd-associated factor 8  PPIL1 - peptidylprolyl isomerase (cyclophilin)-like 1  PUF60 - poly-u binding splicing factor 60kda  DKC1 - dyskeratosis congenita 1, dyskerin  PNPT1 - polyribonucleotide nucleotidyltransferase 1  RAVER2 - ribonucleoprotein, ptb-binding 2  THOC6 - tho complex 6 homolog (drosophila)  RPL14 - ribosomal protein l14  XRN2 - 5'-3' exoribonuclease 2  RBFOX3 - rna binding protein, fox-1 homolog (c. elegans) 3  CASC3 - cancer susceptibility candidate 3  EXOSC8 - exosome component 8  SFPQ - splicing factor proline/glutamine-rich  PHRF1 - phd and ring finger domains 1  PCF11 - pcf11 cleavage and polyadenylation factor subunit  SF1 - splicing factor 1  PSPC1 - paraspeckle component 1  U2AF2 - u2 small nuclear rna auxiliary factor 2  SRSF1 - serine/arginine-rich splicing factor 1  SRRT - serrate rna effector molecule homolog (arabidopsis)  SRSF2 - serine/arginine-rich splicing factor 2  SRSF3 - serine/arginine-rich splicing factor 3  SRSF4 - serine/arginine-rich splicing factor 4  SRSF5 - serine/arginine-rich splicing factor 5  SRSF6 - serine/arginine-rich splicing factor 6  SRSF7 - serine/arginine-rich splicing factor 7  SFSWAP - splicing factor, suppressor of white-apricot homolog (drosophila)  TRA2B - transformer 2 beta homolog (drosophila)  LARP7 - la ribonucleoprotein domain family, member 7  RPUSD3 - rna pseudouridylate synthase domain containing 3  DDX42 - dead (asp-glu-ala-asp) box helicase 42  ECD - ecdysoneless homolog (drosophila)  CWF19L1 - cwf19-like 1, cell cycle control (s. pombe)  GRSF1 - g-rich rna sequence binding factor 1  NBAS - neuroblastoma amplified sequence  TRMT6 - trna methyltransferase 6 homolog (s. cerevisiae)  RBM4B - rna binding motif protein 4b  NCBP2 - nuclear cap binding protein subunit 2, 20kda  SMNDC1 - survival motor neuron domain containing 1  NCBP1 - nuclear cap binding protein subunit 1, 80kda  BCAS2 - breast carcinoma amplified sequence 2  CWC27 - cwc27 spliceosome-associated protein homolog (s. cerevisiae)  SAP18 - sin3a-associated protein, 18kda  MBNL2 - muscleblind-like splicing regulator 2  SUGP2 - surp and g patch domain containing 2  SF3A1 - splicing factor 3a, subunit 1, 120kda  SNW1 - snw domain containing 1  CWC25 - cwc25 spliceosome-associated protein homolog (s. cerevisiae)  HNRNPM - heterogeneous nuclear ribonucleoprotein m  KIN - kin, antigenic determinant of reca protein homolog (mouse)  ZFP36L2 - zfp36 ring finger protein-like 2  ZFP36L1 - zfp36 ring finger protein-like 1  ALKBH5 - alkb, alkylation repair homolog 5 (e. coli)  HABP4 - hyaluronan binding protein 4  NSUN2 - nop2/sun rna methyltransferase family, member 2  UPF1 - upf1 regulator of nonsense transcripts homolog (yeast)  METTL16 - methyltransferase like 16  CELF4 - cugbp, elav-like family member 4  RBM6 - rna binding motif protein 6  RBM5 - rna binding motif protein 5  ALYREF - aly/ref export factor  ELAVL1 - elav (embryonic lethal, abnormal vision, drosophila)-like 1 (hu antigen r)  ZNF326 - zinc finger protein 326  ELAVL4 - elav (embryonic lethal, abnormal vision, drosophila)-like 4  PDCD11 - programmed cell death 11  FIP1L1 - factor interacting with papola and cpsf1  ACIN1 - apoptotic chromatin condensation inducer 1  CNOT1 - ccr4-not transcription complex, subunit 1  ZNF259 - zinc finger protein 259  EXOSC7 - exosome component 7  SPEN - spen homolog, transcriptional regulator (drosophila)  PDCD7 - programmed cell death 7  SCNM1 - sodium channel modifier 1  PRPF4B - prp4 pre-mrna processing factor 4 homolog b (yeast)  CNOT7 - ccr4-not transcription complex, subunit 7  TSEN34 - tsen34 trna splicing endonuclease subunit  PQBP1 - polyglutamine binding protein 1  KDM1A - lysine (k)-specific demethylase 1a  U2AF1L4 - u2 small nuclear rna auxiliary factor 1-like 4  TRA2A - transformer 2 alpha homolog (drosophila)  CPSF1 - cleavage and polyadenylation specific factor 1, 160kda  SF3B5 - splicing factor 3b, subunit 5, 10kda  HNRNPR - heterogeneous nuclear ribonucleoprotein r  BUD31 - bud31 homolog (s. cerevisiae)  RBM15B - rna binding motif protein 15b  SNRNP200 - small nuclear ribonucleoprotein 200kda (u5)  HNRNPLL - heterogeneous nuclear ribonucleoprotein l-like  SMG1 - smg1 phosphatidylinositol 3-kinase-related kinase  SRRM1 - serine/arginine repetitive matrix 1  XAB2 - xpa binding protein 2  RRP1B - ribosomal rna processing 1b  SF3B4 - splicing factor 3b, subunit 4, 49kda  CMTR1 - cap methyltransferase 1  C5AR1 - complement component 5a receptor 1  HSPA8 - heat shock 70kda protein 8  PPIE - peptidylprolyl isomerase e (cyclophilin e)  PTBP3 - polypyrimidine tract binding protein 3  RPL17 - ribosomal protein l17  ZC3H13 - zinc finger ccch-type containing 13  RPL15 - ribosomal protein l15  RPL11 - ribosomal protein l11  RPL9 - ribosomal protein l9  RPL7 - ribosomal protein l7  PPIH - peptidylprolyl isomerase h (cyclophilin h)  RPL3 - ribosomal protein l3  AGO1 - argonaute risc catalytic component 1  RPS4X - ribosomal protein s4, x-linked  SRSF9 - serine/arginine-rich splicing factor 9  RPS4Y1 - ribosomal protein s4, y-linked 1  DCPS - decapping enzyme, scavenger  ZC3H3 - zinc finger ccch-type containing 3  SYNCRIP - synaptotagmin binding, cytoplasmic rna interacting protein  RPL32 - ribosomal protein l32  UPF3B - upf3 regulator of nonsense transcripts homolog b (yeast)  UPF3A - upf3 regulator of nonsense transcripts homolog a (yeast)  RPS23 - ribosomal protein s23  PAPOLG - poly(a) polymerase gamma  RAVER1 - ribonucleoprotein, ptb-binding 1  PAN2 - pan2 poly(a) specific ribonuclease subunit homolog (s. cerevisiae)  ERCC3 - excision repair cross-complementing rodent repair deficiency, complementation group 3  YTHDC1 - yth domain containing 1  THOC2 - tho complex 2  RNGTT - rna guanylyltransferase and 5'-phosphatase  CCNH - cyclin h  ERN1 - endoplasmic reticulum to nucleus signaling 1  RNMT - rna (guanine-7-) methyltransferase  HNRNPU - heterogeneous nuclear ribonucleoprotein u (scaffold attachment factor a)  HNRNPK - heterogeneous nuclear ribonucleoprotein k  HNRNPL - heterogeneous nuclear ribonucleoprotein l  RPS15 - ribosomal protein s15  RPS12 - ribosomal protein s12  RBM8A - rna binding motif protein 8a  ETF1 - eukaryotic translation termination factor 1  HNRNPA2B1 - heterogeneous nuclear ribonucleoprotein a2/b1  HNRNPA1 - heterogeneous nuclear ribonucleoprotein a1  HNRNPC - heterogeneous nuclear ribonucleoprotein c (c1/c2)  ZNF830 - zinc finger protein 830  HNRNPF - heterogeneous nuclear ribonucleoprotein f  HNRNPD - heterogeneous nuclear ribonucleoprotein d (au-rich element rna binding protein 1, 37kda)  HNRNPH1 - heterogeneous nuclear ribonucleoprotein h1 (h)  HNRNPH3 - heterogeneous nuclear ribonucleoprotein h3 (2h9)  HNRNPH2 - heterogeneous nuclear ribonucleoprotein h2 (h')  JMJD6 - jumonji domain containing 6  THOC1 - tho complex 1  TUT1 - terminal uridylyl transferase 1, u6 snrna-specific  TRMT10C - trna methyltransferase 10 homolog c (s. cerevisiae)  ERCC2 - excision repair cross-complementing rodent repair deficiency, complementation group 2  CD2BP2 - cd2 (cytoplasmic tail) binding protein 2  THRAP3 - thyroid hormone receptor associated protein 3  PDE12 - phosphodiesterase 12  RBM27 - rna binding motif protein 27  HTATSF1 - hiv-1 tat specific factor 1  FMR1 - fragile x mental retardation 1  CDC5L - cell division cycle 5-like  RBM15 - rna binding motif protein 15  RBMX - rna binding motif protein, x-linked  HNRNPA3 - heterogeneous nuclear ribonucleoprotein a3  RPRD2 - regulation of nuclear pre-mrna domain containing 2  FLNA - filamin a, alpha  SRSF12 - serine/arginine-rich splicing factor 12  PPP4R2 - protein phosphatase 4, regulatory subunit 2  PRPF8 - pre-mrna processing factor 8  PRPF19 - pre-mrna processing factor 19  RBM19 - rna binding motif protein 19  KHDRBS3 - kh domain containing, rna binding, signal transduction associated 3  DCP1A - decapping mrna 1a  PATL1 - protein associated with topoisomerase ii homolog 1 (yeast)  DDX46 - dead (asp-glu-ala-asp) box polypeptide 46  SMG6 - smg6 nonsense mediated mrna decay factor  EXOSC3 - exosome component 3  WDR61 - wd repeat domain 61  SMG7 - smg7 nonsense mediated mrna decay factor  CSTF2T - cleavage stimulation factor, 3' pre-rna, subunit 2, 64kda, tau variant  EXOSC1 - exosome component 1  U2SURP - u2 snrnp-associated surp domain containing  PTBP1 - polypyrimidine tract binding protein 1  EIF4A3 - eukaryotic translation initiation factor 4a3  KHSRP - kh-type splicing regulatory protein  DDX17 - dead (asp-glu-ala-asp) box helicase 17  CHERP - calcium homeostasis endoplasmic reticulum protein  DHX38 - deah (asp-glu-ala-his) box polypeptide 38  PRPF18 - pre-mrna processing factor 18  GPKOW - g patch domain and kow motifs  SMG9 - smg9 nonsense mediated mrna decay factor  CEBPG - ccaat/enhancer binding protein (c/ebp), gamma  ZCCHC11 - zinc finger, cchc domain containing 11  SLU7 - slu7 splicing factor homolog (s. cerevisiae)  CDK7 - cyclin-dependent kinase 7  CDK13 - cyclin-dependent kinase 13  PCBP3 - poly(rc) binding protein 3  LSM3 - lsm3 homolog, u6 small nuclear rna associated (s. cerevisiae)  CSDC2 - cold shock domain containing c2, rna binding  TSEN54 - tsen54 trna splicing endonuclease subunit  CTIF - cbp80/20-dependent translation initiation factor  GTPBP2 - gtp binding protein 2  NONO - non-pou domain containing, octamer-binding  PAPD5 - pap associated domain containing 5  NUDT16 - nudix (nucleoside diphosphate linked moiety x)-type motif 16  SRSF10 - serine/arginine-rich splicing factor 10  PPWD1 - peptidylprolyl isomerase domain and wd repeat containing 1  NOVA2 - neuro-oncological ventral antigen 2  AHCYL1 - adenosylhomocysteinase-like 1  NOVA1 - neuro-oncological ventral antigen 1  AQR - aquarius homolog (mouse)  EXOSC2 - exosome component 2  CNOT2 - ccr4-not transcription complex, subunit 2  CNOT4 - ccr4-not transcription complex, subunit 4  CNOT3 - ccr4-not transcription complex, subunit 3  DHX34 - deah (asp-glu-ala-his) box polypeptide 34  THOC7 - tho complex 7 homolog (drosophila)  EDC3 - enhancer of mrna decapping 3  ZMAT2 - zinc finger, matrin-type 2  TARDBP - tar dna binding protein  SNRNP48 - small nuclear ribonucleoprotein 48kda (u11/u12)  PAF1 - paf1, rna polymerase ii associated factor, homolog (s. cerevisiae)  NHP2L1 - nhp2 non-histone chromosome protein 2-like 1 (s. cerevisiae)  ZNRD1 - zinc ribbon domain containing 1  SART3 - squamous cell carcinoma antigen recognized by t cells 3  SF3B1 - splicing factor 3b, subunit 1, 155kda  SF3B3 - splicing factor 3b, subunit 3, 130kda  RC3H2 - ring finger and ccch-type domains 2  TTC37 - tetratricopeptide repeat domain 37  PABPC1 - poly(a) binding protein, cytoplasmic 1  TRUB2 - trub pseudouridine (psi) synthase homolog 2 (e. coli)  SKIV2L2 - superkiller viralicidic activity 2-like 2 (s. cerevisiae)  RBM26 - rna binding motif protein 26  TFCP2 - transcription factor cp2  CELF1 - cugbp, elav-like family member 1  KHDRBS1 - kh domain containing, rna binding, signal transduction associated 1  IK - ik cytokine, down-regulator of hla ii  RBFOX2 - rna binding protein, fox-1 homolog (c. elegans) 2  DIS3L2 - dis3 mitotic control homolog (s. cerevisiae)-like 2  RBM4 - rna binding motif protein 4  CTNNBL1 - catenin, beta like 1  GEMIN2 - gem (nuclear organelle) associated protein 2  RBBP6 - retinoblastoma binding protein 6  PUS7 - pseudouridylate synthase 7 homolog (s. cerevisiae)  DHX16 - deah (asp-glu-ala-his) box polypeptide 16  EXOSC4 - exosome component 4  PCIF1 - pdx1 c-terminal inhibiting factor 1  SRRM2 - serine/arginine repetitive matrix 2  USP39 - ubiquitin specific peptidase 39  METTL3 - methyltransferase like 3 |
| GO:0099003 | vesicle-mediated transport in synapse | 2.28E-7 | 4.66E-5 | 1.35 (10334,83,6996,76) | [+] Show genes  AP2B1 - adaptor-related protein complex 2, beta 1 subunit  SYP - synaptophysin  SPG11 - spastic paraplegia 11 (autosomal recessive)  AP3M2 - adaptor-related protein complex 3, mu 2 subunit  NLGN2 - neuroligin 2  CANX - calnexin  KIF5C - kinesin family member 5c  SYT1 - synaptotagmin i  SYT5 - synaptotagmin v  KIF5B - kinesin family member 5b  SYT17 - synaptotagmin xvii  KIF5A - kinesin family member 5a  BLOC1S2 - biogenesis of lysosomal organelles complex-1, subunit 2  VPS35 - vacuolar protein sorting 35 homolog (s. cerevisiae)  LIN7A - lin-7 homolog a (c. elegans)  BLOC1S4 - biogenesis of lysosomal organelles complex-1, subunit 4, cappuccino  CLSTN1 - calsyntenin 1  RIMS2 - regulating synaptic membrane exocytosis 2  DENND1A - denn/madd domain containing 1a  PACSIN1 - protein kinase c and casein kinase substrate in neurons 1  VAMP2 - vesicle-associated membrane protein 2 (synaptobrevin 2)  CTNNB1 - catenin (cadherin-associated protein), beta 1, 88kda  STXBP1 - syntaxin binding protein 1  STX1A - syntaxin 1a (brain)  AP3B2 - adaptor-related protein complex 3, beta 2 subunit  RAB3A - rab3a, member ras oncogene family  TOR1A - torsin family 1, member a (torsin a)  AP3B1 - adaptor-related protein complex 3, beta 1 subunit  CPLX2 - complexin 2  SYT3 - synaptotagmin iii  ACTB - actin, beta  SYNJ1 - synaptojanin 1  SYT9 - synaptotagmin ix  UNC13B - unc-13 homolog b (c. elegans)  DNM1L - dynamin 1-like  BTBD8 - btb (poz) domain containing 8  ACTG1 - actin, gamma 1  STX1B - syntaxin 1b  RAB27A - rab27a, member ras oncogene family  SNAP91 - synaptosomal-associated protein, 91kda  SYT7 - synaptotagmin vii  DNM3 - dynamin 3  PICALM - phosphatidylinositol binding clathrin assembly protein  DNAJC5 - dnaj (hsp40) homolog, subfamily c, member 5  SNCA - synuclein, alpha (non a4 component of amyloid precursor)  BLOC1S6 - biogenesis of lysosomal organelles complex-1, subunit 6, pallidin  SNCG - synuclein, gamma (breast cancer-specific protein 1)  AP3M1 - adaptor-related protein complex 3, mu 1 subunit  ITSN2 - intersectin 2  SNAP25 - synaptosomal-associated protein, 25kda  ARF6 - adp-ribosylation factor 6  MX1 - myxovirus (influenza virus) resistance 1, interferon-inducible protein p78 (mouse)  SNCB - synuclein, beta  AP3S2 - adaptor-related protein complex 3, sigma 2 subunit  RIMS3 - regulating synaptic membrane exocytosis 3  UNC13A - unc-13 homolog a (c. elegans)  PIP5K1C - phosphatidylinositol-4-phosphate 5-kinase, type i, gamma  MAPK10 - mitogen-activated protein kinase 10  CDK5 - cyclin-dependent kinase 5  RAB8A - rab8a, member ras oncogene family  BLOC1S5 - biogenesis of lysosomal organelles complex-1, subunit 5, muted  RAB11A - rab11a, member ras oncogene family  DNM2 - dynamin 2  AMPH - amphiphysin  CADPS - ca++-dependent secretion activator  CALY - calcyon neuron-specific vesicular protein  NSG1 - neuron specific gene family member 1  TRIM9 - tripartite motif containing 9  DOC2B - double c2-like domains, beta  AP3D1 - adaptor-related protein complex 3, delta 1 subunit  CPLX3 - complexin 3  TMEM230 - transmembrane protein 230  ITSN1 - intersectin 1 (sh3 domain protein)  EPS15 - epidermal growth factor receptor pathway substrate 15  GRIP2 - glutamate receptor interacting protein 2  BLOC1S3 - biogenesis of lysosomal organelles complex-1, subunit 3 |
| GO:0031326 | regulation of cellular biosynthetic process | 2.47E-7 | 4.97E-5 | 1.06 (10334,2505,6996,1798) | [+] Show genes  AGAP2 - arfgap with gtpase domain, ankyrin repeat and ph domain 2  EXOSC9 - exosome component 9  EXOSC10 - exosome component 10  MSL3 - male-specific lethal 3 homolog (drosophila)  TADA1 - transcriptional adaptor 1  AARS - alanyl-trna synthetase  CBX1 - chromobox homolog 1  ABCA2 - atp-binding cassette, sub-family a (abc1), member 2  ABL1 - c-abl oncogene 1, non-receptor tyrosine kinase  ZNF821 - zinc finger protein 821  PML - promyelocytic leukemia  CNOT11 - ccr4-not transcription complex, subunit 11  MLLT11 - myeloid/lymphoid or mixed-lineage leukemia (trithorax homolog, drosophila); translocated to, 11  SUPT20H - suppressor of ty 20 homolog (s. cerevisiae)  YWHAQ - tyrosine 3-monooxygenase/tryptophan 5-monooxygenase activation protein, theta polypeptide  BRD8 - bromodomain containing 8  CHD7 - chromodomain helicase dna binding protein 7  LYAR - ly1 antibody reactive  ZNF354B - zinc finger protein 354b  TCERG1 - transcription elongation regulator 1  EHMT2 - euchromatic histone-lysine n-methyltransferase 2  SPIN1 - spindlin 1  TASP1 - taspase, threonine aspartase, 1  SUB1 - sub1 homolog (s. cerevisiae)  MORF4L1 - mortality factor 4 like 1  ZNF511 - zinc finger protein 511  PPID - peptidylprolyl isomerase d  ACTL6A - actin-like 6a  ACTN1 - actinin, alpha 1  ACTN4 - actinin, alpha 4  ACVR2A - activin a receptor, type iia  ACVRL1 - activin a receptor type ii-like 1  ACVR1 - activin a receptor, type i  ACVR1B - activin a receptor, type ib  ZNF331 - zinc finger protein 331  MED1 - mediator complex subunit 1  ADCY1 - adenylate cyclase 1 (brain)  PPARD - peroxisome proliferator-activated receptor delta  MAML3 - mastermind-like 3 (drosophila)  POLR2K - polymerase (rna) ii (dna directed) polypeptide k, 7.0kda  ADCYAP1R1 - adenylate cyclase activating polypeptide 1 (pituitary) receptor type i  FAM129A - family with sequence similarity 129, member a  PARP1 - poly (adp-ribose) polymerase 1  H2AFY2 - h2a histone family, member y2  PPP1R13L - protein phosphatase 1, regulatory subunit 13 like  HES6 - hairy and enhancer of split 6 (drosophila)  POLR2G - polymerase (rna) ii (dna directed) polypeptide g  POLR2D - polymerase (rna) ii (dna directed) polypeptide d  SMPD3 - sphingomyelin phosphodiesterase 3, neutral membrane (neutral sphingomyelinase ii)  RUVBL2 - ruvb-like 2 (e. coli)  POLR2A - polymerase (rna) ii (dna directed) polypeptide a, 220kda  PARP4 - poly (adp-ribose) polymerase family, member 4  AP2A1 - adaptor-related protein complex 2, alpha 1 subunit  CDK5RAP2 - cdk5 regulatory subunit associated protein 2  RCOR3 - rest corepressor 3  AES - amino-terminal enhancer of split  NUP133 - nucleoporin 133kda  NUPL2 - nucleoporin like 2  VPS35 - vacuolar protein sorting 35 homolog (s. cerevisiae)  JAG1 - jagged 1  PRDM4 - pr domain containing 4  PPP3R1 - protein phosphatase 3, regulatory subunit b, alpha  ATF7IP - activating transcription factor 7 interacting protein  ZNF689 - zinc finger protein 689  LRIF1 - ligand dependent nuclear receptor interacting factor 1  PPP2R5B - protein phosphatase 2, regulatory subunit b', beta  ZNF653 - zinc finger protein 653  AKT1 - v-akt murine thymoma viral oncogene homolog 1  PCID2 - pci domain containing 2  PPP2R1A - protein phosphatase 2, regulatory subunit a, alpha  WWP2 - ww domain containing e3 ubiquitin protein ligase 2  AHR - aryl hydrocarbon receptor  ZNF526 - zinc finger protein 526  FOXP4 - forkhead box p4  PPP2CA - protein phosphatase 2, catalytic subunit, alpha isozyme  ZNF83 - zinc finger protein 83  ESCO1 - establishment of sister chromatid cohesion n-acetyltransferase 1  KANSL3 - kat8 regulatory nsl complex subunit 3  PRNP - prion protein  ATF7 - activating transcription factor 7  AMFR - autocrine motility factor receptor, e3 ubiquitin protein ligase  DNAJC3 - dnaj (hsp40) homolog, subfamily c, member 3  PRKRIR - protein-kinase, interferon-inducible double stranded rna dependent inhibitor, repressor of (p58 repressor)  MAP2K5 - mitogen-activated protein kinase kinase 5  MAP2K7 - mitogen-activated protein kinase kinase 7  ZNF692 - zinc finger protein 692  PIM2 - pim-2 oncogene  EIF2AK2 - eukaryotic translation initiation factor 2-alpha kinase 2  MAP2K1 - mitogen-activated protein kinase kinase 1  EIF4E3 - eukaryotic translation initiation factor 4e family member 3  MAP2K2 - mitogen-activated protein kinase kinase 2  MAPK10 - mitogen-activated protein kinase 10  MAPK9 - mitogen-activated protein kinase 9  MAPK11 - mitogen-activated protein kinase 11  MAPK8 - mitogen-activated protein kinase 8  MAPK7 - mitogen-activated protein kinase 7  GCN1L1 - gcn1 general control of amino-acid synthesis 1-like 1 (yeast)  MAPK3 - mitogen-activated protein kinase 3  MAPK1 - mitogen-activated protein kinase 1  PRKD1 - protein kinase d1  ADIRF - adipogenesis regulatory factor  NDC1 - ndc1 transmembrane nucleoporin  ANXA3 - annexin a3  CCDC88A - coiled-coil domain containing 88a  PRKCH - protein kinase c, eta  PRKCI - protein kinase c, iota  CCDC85B - coiled-coil domain containing 85b  PRKCB - protein kinase c, beta  PRKCD - protein kinase c, delta  NSUN5 - nop2/sun domain family, member 5  PRKAR1A - protein kinase, camp-dependent, regulatory, type i, alpha  YEATS2 - yeats domain containing 2  PKIB - protein kinase (camp-dependent, catalytic) inhibitor beta  PRKAG1 - protein kinase, amp-activated, gamma 1 non-catalytic subunit  APEX1 - apex nuclease (multifunctional dna repair enzyme) 1  RBM23 - rna binding motif protein 23  ZNF605 - zinc finger protein 605  XIAP - x-linked inhibitor of apoptosis  PARN - poly(a)-specific ribonuclease  BIRC2 - baculoviral iap repeat containing 2  KLF12 - kruppel-like factor 12  TREX1 - three prime repair exonuclease 1  PBX2 - pre-b-cell leukemia homeobox 2  PRMT6 - protein arginine methyltransferase 6  PBX3 - pre-b-cell leukemia homeobox 3  PBX1 - pre-b-cell leukemia homeobox 1  PCBD1 - pterin-4 alpha-carbinolamine dehydratase/dimerization cofactor of hepatocyte nuclear factor 1 alpha  APBB2 - amyloid beta (a4) precursor protein-binding, family b, member 2  CHP1 - calcineurin-like ef-hand protein 1  APBB1 - amyloid beta (a4) precursor protein-binding, family b, member 1 (fe65)  RIF1 - rap1 interacting factor homolog (yeast)  POLG2 - polymerase (dna directed), gamma 2, accessory subunit  RNF139 - ring finger protein 139  KIAA1551 - kiaa1551  PA2G4 - proliferation-associated 2g4, 38kda  RPRD1A - regulation of nuclear pre-mrna domain containing 1a  ZHX1 - zinc fingers and homeoboxes 1  ARF4 - adp-ribosylation factor 4  SBNO1 - strawberry notch homolog 1 (drosophila)  IRAK3 - interleukin-1 receptor-associated kinase 3  SETD5 - set domain containing 5  APP - amyloid beta (a4) precursor protein  BBS7 - bardet-biedl syndrome 7  DDX20 - dead (asp-glu-ala-asp) box polypeptide 20  PAK3 - p21 protein (cdc42/rac)-activated kinase 3  PAM - peptidylglycine alpha-amidating monooxygenase  ARNTL - aryl hydrocarbon receptor nuclear translocator-like  PDGFA - platelet-derived growth factor alpha polypeptide  ARNT - aryl hydrocarbon receptor nuclear translocator  PDPR - pyruvate dehydrogenase phosphatase regulatory subunit  SUPT16H - suppressor of ty 16 homolog (s. cerevisiae)  PDGFRB - platelet-derived growth factor receptor, beta polypeptide  ARRB2 - arrestin, beta 2  ARRB1 - arrestin, beta 1  PDGFB - platelet-derived growth factor beta polypeptide  SAMD4B - sterile alpha motif domain containing 4b  STRAP - serine/threonine kinase receptor associated protein  RHOA - ras homolog family member a  PSIP1 - pc4 and sfrs1 interacting protein 1  PDK3 - pyruvate dehydrogenase kinase, isozyme 3  MED9 - mediator complex subunit 9  FSTL1 - follistatin-like 1  SLC2A6 - solute carrier family 2 (facilitated glucose transporter), member 6  BAZ2A - bromodomain adjacent to zinc finger domain, 2a  PCNA - proliferating cell nuclear antigen  CHMP1A - charged multivesicular body protein 1a  JDP2 - jun dimerization protein 2  CDK16 - cyclin-dependent kinase 16  AKIRIN2 - akirin 2  HEATR1 - heat repeat containing 1  CDK17 - cyclin-dependent kinase 17  LEO1 - leo1, paf1/rna polymerase ii complex component, homolog (s. cerevisiae)  ERLIN2 - er lipid raft associated 2  PWP1 - pwp1 homolog (s. cerevisiae)  WRAP53 - wd repeat containing, antisense to tp53  PDE2A - phosphodiesterase 2a, cgmp-stimulated  THAP1 - thap domain containing, apoptosis associated protein 1  PKIG - protein kinase (camp-dependent, catalytic) inhibitor gamma  ELP3 - elongator acetyltransferase complex subunit 3  ASCL1 - achaete-scute complex homolog 1 (drosophila)  KAT7 - k(lysine) acetyltransferase 7  RGMB - rgm domain family, member b  SBNO2 - strawberry notch homolog 2 (drosophila)  ZNF444 - zinc finger protein 444  ZBTB1 - zinc finger and btb domain containing 1  ZNF365 - zinc finger protein 365  PGAM1 - phosphoglycerate mutase 1 (brain)  ATP1A1 - atpase, na+/k+ transporting, alpha 1 polypeptide  FBXW7 - f-box and wd repeat domain containing 7, e3 ubiquitin protein ligase  BAHD1 - bromo adjacent homology domain containing 1  ZHX2 - zinc fingers and homeoboxes 2  PHF2 - phd finger protein 2  MORC2 - morc family cw-type zinc finger 2  PHF1 - phd finger protein 1  FOXJ3 - forkhead box j3  RERE - arginine-glutamic acid dipeptide (re) repeats  DNAJC24 - dnaj (hsp40) homolog, subfamily c, member 24  LARP6 - la ribonucleoprotein domain family, member 6  ZFHX3 - zinc finger homeobox 3  PEX14 - peroxisomal biogenesis factor 14  ADNP2 - adnp homeobox 2  AEBP2 - ae binding protein 2  IMPACT - impact rwd domain protein  ZNF507 - zinc finger protein 507  LGR4 - leucine-rich repeat containing g protein-coupled receptor 4  ATP2B4 - atpase, ca++ transporting, plasma membrane 4  ZNF652 - zinc finger protein 652  SCAF8 - sr-related ctd-associated factor 8  PKD1 - polycystic kidney disease 1 (autosomal dominant)  PKD2 - polycystic kidney disease 2 (autosomal dominant)  MTF2 - metal response element binding transcription factor 2  NAT10 - n-acetyltransferase 10 (gcn5-related)  BACH1 - btb and cnc homology 1, basic leucine zipper transcription factor 1  PIN1 - peptidylprolyl cis/trans isomerase, nima-interacting 1  PIK3R2 - phosphoinositide-3-kinase, regulatory subunit 2 (beta)  DPH3 - diphthamide biosynthesis 3  PIK3R1 - phosphoinositide-3-kinase, regulatory subunit 1 (alpha)  ASXL2 - additional sex combs like 2 (drosophila)  PLAGL1 - pleiomorphic adenoma gene-like 1  ELP2 - elongator acetyltransferase complex subunit 2  ATRX - alpha thalassemia/mental retardation syndrome x-linked  PKNOX1 - pbx/knotted 1 homeobox 1  CASC3 - cancer susceptibility candidate 3  PKM - pyruvate kinase, muscle  OGFOD1 - 2-oxoglutarate and iron-dependent oxygenase domain containing 1  TMEM100 - transmembrane protein 100  PHF10 - phd finger protein 10  RABL3 - rab, member of ras oncogene family-like 3  PSPC1 - paraspeckle component 1  BCL7A - b-cell cll/lymphoma 7a  MRGBP - mrg/morf4l binding protein  BCL9 - b-cell cll/lymphoma 9  RPUSD3 - rna pseudouridylate synthase domain containing 3  PIM1 - pim-1 oncogene  BRF2 - brf2, rna polymerase iii transcription initiation factor 50 kda subunit  BCL2 - b-cell cll/lymphoma 2  ECD - ecdysoneless homolog (drosophila)  CCND1 - cyclin d1  PARK7 - parkinson protein 7  PNKP - polynucleotide kinase 3'-phosphatase  C3orf33 - chromosome 3 open reading frame 33  TRIM27 - tripartite motif containing 27  RFC5 - replication factor c (activator 1) 5, 36.5kda  RFC2 - replication factor c (activator 1) 2, 40kda  SAP18 - sin3a-associated protein, 18kda  RFC3 - replication factor c (activator 1) 3, 38kda  TSHZ2 - teashirt zinc finger homeobox 2  MAEA - macrophage erythroblast attacher  RFXAP - regulatory factor x-associated protein  RFX5 - regulatory factor x, 5 (influences hla class ii expression)  WHSC1L1 - wolf-hirschhorn syndrome candidate 1-like 1  EIF1B - eukaryotic translation initiation factor 1b  RFX1 - regulatory factor x, 1 (influences hla class ii expression)  RELA - v-rel avian reticuloendotheliosis viral oncogene homolog a  APBB3 - amyloid beta (a4) precursor protein-binding, family b, member 3  BCOR - bcl6 corepressor  ZFP36L2 - zfp36 ring finger protein-like 2  ZFP36L1 - zfp36 ring finger protein-like 1  RET - ret proto-oncogene  DPF2 - d4, zinc and double phd fingers family 2  UPF1 - upf1 regulator of nonsense transcripts homolog (yeast)  CC2D1A - coiled-coil and c2 domain containing 1a  RLF - rearranged l-myc fusion  RING1 - ring finger protein 1  BMI1 - bmi1 polycomb ring finger oncogene  PCGF3 - polycomb group ring finger 3  BMPR1A - bone morphogenetic protein receptor, type ia  BMPR2 - bone morphogenetic protein receptor, type ii (serine/threonine kinase)  BMPR1B - bone morphogenetic protein receptor, type ib  GON4L - gon-4-like (c. elegans)  BMP7 - bone morphogenetic protein 7  FITM2 - fat storage-inducing transmembrane protein 2  ABCA7 - atp-binding cassette, sub-family a (abc1), member 7  PSMD14 - proteasome (prosome, macropain) 26s subunit, non-atpase, 14  FLOT1 - flotillin 1  BCAS3 - breast carcinoma amplified sequence 3  UHMK1 - u2af homology motif (uhm) kinase 1  AHI1 - abelson helper integration site 1  RNF4 - ring finger protein 4  BRD2 - bromodomain containing 2  RNF2 - ring finger protein 2  HNRNPR - heterogeneous nuclear ribonucleoprotein r  ZNF280D - zinc finger protein 280d  GATAD2A - gata zinc finger domain containing 2a  RNF6 - ring finger protein (c3h2c3 type) 6  ROCK1 - rho-associated, coiled-coil containing protein kinase 1  RNF111 - ring finger protein 111  TSPO - translocator protein (18kda)  TET2 - tet methylcytosine dioxygenase 2  MED18 - mediator complex subunit 18  DENND4A - denn/madd domain containing 4a  MBTD1 - mbt domain containing 1  RAMP1 - receptor (g protein-coupled) activity modifying protein 1  ZMPSTE24 - zinc metallopeptidase ste24  TRIM44 - tripartite motif containing 44  TAB1 - tgf-beta activated kinase 1/map3k7 binding protein 1  PPIE - peptidylprolyl isomerase e (cyclophilin e)  FAM3C - family with sequence similarity 3, member c  CAPN2 - calpain 2, (m/ii) large subunit  MCRS1 - microspherule protein 1  NRDE2 - nrde-2, necessary for rna interference, domain containing  C1D - c1d nuclear receptor corepressor  PTCD3 - pentatricopeptide repeat domain 3  PIBF1 - progesterone immunomodulatory binding factor 1  SLC30A9 - solute carrier family 30 (zinc transporter), member 9  MAD2L2 - mad2 mitotic arrest deficient-like 2 (yeast)  PID1 - phosphotyrosine interaction domain containing 1  PHIP - pleckstrin homology domain interacting protein  RPS4X - ribosomal protein s4, x-linked  ZNF787 - zinc finger protein 787  NRARP - notch-regulated ankyrin repeat protein  RMND1 - required for meiotic nuclear division 1 homolog (s. cerevisiae)  TRIM38 - tripartite motif containing 38  TADA3 - transcriptional adaptor 3  CALR - calreticulin  ZNF792 - zinc finger protein 792  WTIP - wilms tumor 1 interacting protein  NCOA2 - nuclear receptor coactivator 2  SYNCRIP - synaptotagmin binding, cytoplasmic rna interacting protein  CREB3 - camp responsive element binding protein 3  MFSD12 - major facilitator superfamily domain containing 12  CITED2 - cbp/p300-interacting transactivator, with glu/asp-rich carboxy-terminal domain, 2  CCT6A - chaperonin containing tcp1, subunit 6a (zeta 1)  CCNT2 - cyclin t2  PINX1 - pin2/terf1 interacting, telomerase inhibitor 1  CCND3 - cyclin d3  CCNT1 - cyclin t1  CCNH - cyclin h  HMG20A - high mobility group 20a  BANP - btg3 associated nuclear protein  TIPIN - timeless interacting protein  ACER2 - alkaline ceramidase 2  TRNAU1AP - trna selenocysteine 1 associated protein 1  RPS6KA1 - ribosomal protein s6 kinase, 90kda, polypeptide 1  RPS6KB1 - ribosomal protein s6 kinase, 70kda, polypeptide 1  RPS6KB2 - ribosomal protein s6 kinase, 70kda, polypeptide 2  CBFA2T3 - core-binding factor, runt domain, alpha subunit 2; translocated to, 3  CBFB - core-binding factor, beta subunit  RXRB - retinoid x receptor, beta  PIAS3 - protein inhibitor of activated stat, 3  CAV1 - caveolin 1, caveolae protein, 22kda  CCNC - cyclin c  RBM14 - rna binding motif protein 14  TRMT10C - trna methyltransferase 10 homolog c (s. cerevisiae)  PRMT5 - protein arginine methyltransferase 5  CHTF8 - ctf8, chromosome transmission fidelity factor 8 homolog (s. cerevisiae)  CDK11B - cyclin-dependent kinase 11b  MXD4 - max dimerization protein 4  CDC5L - cell division cycle 5-like  HTRA1 - htra serine peptidase 1  CDH3 - cadherin 3, type 1, p-cadherin (placental)  POLR3F - polymerase (rna) iii (dna directed) polypeptide f, 39 kda  HEXIM1 - hexamethylene bis-acetamide inducible 1  ERLIN1 - er lipid raft associated 1  SPIN2A - spindlin family, member 2a  NCOA7 - nuclear receptor coactivator 7  POMT1 - protein-o-mannosyltransferase 1  PSEN1 - presenilin 1  USP16 - ubiquitin specific peptidase 16  PSMA1 - proteasome (prosome, macropain) subunit, alpha type, 1  PSMA2 - proteasome (prosome, macropain) subunit, alpha type, 2  CD81 - cd81 molecule  CAMKK2 - calcium/calmodulin-dependent protein kinase kinase 2, beta  PSMB1 - proteasome (prosome, macropain) subunit, beta type, 1  STARD4 - star-related lipid transfer (start) domain containing 4  PSMA5 - proteasome (prosome, macropain) subunit, alpha type, 5  PSMA3 - proteasome (prosome, macropain) subunit, alpha type, 3  RIPPLY2 - ripply transcriptional repressor 2  PSMB10 - proteasome (prosome, macropain) subunit, beta type, 10  CD47 - cd47 molecule  PSMC6 - proteasome (prosome, macropain) 26s subunit, atpase, 6  TAF6L - taf6-like rna polymerase ii, p300/cbp-associated factor (pcaf)-associated factor, 65kda  FAM173B - family with sequence similarity 173, member b  PSMD1 - proteasome (prosome, macropain) 26s subunit, non-atpase, 1  PSMC4 - proteasome (prosome, macropain) 26s subunit, atpase, 4  SOX18 - sry (sex determining region y)-box 18  POLR3G - polymerase (rna) iii (dna directed) polypeptide g (32kd)  PSMC2 - proteasome (prosome, macropain) 26s subunit, atpase, 2  PSMD9 - proteasome (prosome, macropain) 26s subunit, non-atpase, 9  CD34 - cd34 molecule  PSMD10 - proteasome (prosome, macropain) 26s subunit, non-atpase, 10  PSMD7 - proteasome (prosome, macropain) 26s subunit, non-atpase, 7  PSMD8 - proteasome (prosome, macropain) 26s subunit, non-atpase, 8  PSMD5 - proteasome (prosome, macropain) 26s subunit, non-atpase, 5  PSMD2 - proteasome (prosome, macropain) 26s subunit, non-atpase, 2  TERF2IP - telomeric repeat binding factor 2, interacting protein  PSMD12 - proteasome (prosome, macropain) 26s subunit, non-atpase, 12  ATG7 - autophagy related 7  PSMD13 - proteasome (prosome, macropain) 26s subunit, non-atpase, 13  PTBP1 - polypyrimidine tract binding protein 1  LAMTOR5 - late endosomal/lysosomal adaptor, mapk and mtor activator 5  PTEN - phosphatase and tensin homolog  JMY - junction mediating and regulatory protein, p53 cofactor  MYBBP1A - myb binding protein (p160) 1a  FBLN5 - fibulin 5  CIB1 - calcium and integrin binding 1 (calmyrin)  PTGIS - prostaglandin i2 (prostacyclin) synthase  DDX17 - dead (asp-glu-ala-asp) box helicase 17  KAT5 - k(lysine) acetyltransferase 5  ZFP69 - zfp69 zinc finger protein  CEBPG - ccaat/enhancer binding protein (c/ebp), gamma  CDK5 - cyclin-dependent kinase 5  CDK4 - cyclin-dependent kinase 4  CCT7 - chaperonin containing tcp1, subunit 7 (eta)  CDK9 - cyclin-dependent kinase 9  CDK8 - cyclin-dependent kinase 8  LIN54 - lin-54 homolog (c. elegans)  CDK7 - cyclin-dependent kinase 7  CDKN1C - cyclin-dependent kinase inhibitor 1c (p57, kip2)  CCT2 - chaperonin containing tcp1, subunit 2 (beta)  CDKN1B - cyclin-dependent kinase inhibitor 1b (p27, kip1)  CCT4 - chaperonin containing tcp1, subunit 4 (delta)  PCBP3 - poly(rc) binding protein 3  PTPN2 - protein tyrosine phosphatase, non-receptor type 2  HTATIP2 - hiv-1 tat interactive protein 2, 30kda  ARFGEF1 - adp-ribosylation factor guanine nucleotide-exchange factor 1 (brefeldin a-inhibited)  CDK2 - cyclin-dependent kinase 2  RRN3 - rrn3 rna polymerase i transcription factor homolog (s. cerevisiae)  PURA - purine-rich element binding protein a  PDP1 - pyruvate dehyrogenase phosphatase catalytic subunit 1  PURB - purine-rich element binding protein b  GTPBP2 - gtp binding protein 2  ZMYND11 - zinc finger, mynd-type containing 11  PTPRK - protein tyrosine phosphatase, receptor type, k  SRSF10 - serine/arginine-rich splicing factor 10  PTPRN - protein tyrosine phosphatase, receptor type, n  JMJD8 - jumonji domain containing 8  NUP50 - nucleoporin 50kda  PEX2 - peroxisomal biogenesis factor 2  KDM5B - lysine (k)-specific demethylase 5b  MPHOSPH8 - m-phase phosphoprotein 8  PXN - paxillin  GIPC1 - gipc pdz domain containing family, member 1  CHD4 - chromodomain helicase dna binding protein 4  CHD2 - chromodomain helicase dna binding protein 2  CHD3 - chromodomain helicase dna binding protein 3  CHD1 - chromodomain helicase dna binding protein 1  MTERFD2 - mterf domain containing 2  QARS - glutaminyl-trna synthetase  MYPOP - myb-related transcription factor, partner of profilin  RAD21 - rad21 homolog (s. pombe)  RAD17 - rad17 homolog (s. pombe)  PARP14 - poly (adp-ribose) polymerase family, member 14  ZNF181 - zinc finger protein 181  PAF1 - paf1, rna polymerase ii associated factor, homolog (s. cerevisiae)  RAC1 - ras-related c3 botulinum toxin substrate 1 (rho family, small gtp binding protein rac1)  ZNF268 - zinc finger protein 268  INO80 - ino80 complex subunit  ZNF513 - zinc finger protein 513  GMEB1 - glucocorticoid modulatory element binding protein 1  RANBP2 - ran binding protein 2  CCT8 - chaperonin containing tcp1, subunit 8 (theta)  CLTC - clathrin, heavy chain (hc)  MIER2 - mesoderm induction early response 1, family member 2  RARG - retinoic acid receptor, gamma  RARB - retinoic acid receptor, beta  DLL4 - delta-like 4 (drosophila)  NECAB2 - n-terminal ef-hand calcium binding protein 2  CELF1 - cugbp, elav-like family member 1  KHDRBS1 - kh domain containing, rna binding, signal transduction associated 1  CLU - clusterin  GNL3L - guanine nucleotide binding protein-like 3 (nucleolar)-like  TAF8 - taf8 rna polymerase ii, tata box binding protein (tbp)-associated factor, 43kda  MAGEL2 - mage-like 2  CLK2 - cdc-like kinase 2  CTCF - ccctc-binding factor (zinc finger protein)  RAI1 - retinoic acid induced 1  RBL2 - retinoblastoma-like 2 (p130)  RBBP7 - retinoblastoma binding protein 7  RBBP8 - retinoblastoma binding protein 8  MAP3K2 - mitogen-activated protein kinase kinase kinase 2  RBM3 - rna binding motif (rnp1, rrm) protein 3  RBM4 - rna binding motif protein 4  RB1 - retinoblastoma 1  ARID4A - at rich interactive domain 4a (rbp1-like)  RBBP6 - retinoblastoma binding protein 6  KDM5A - lysine (k)-specific demethylase 5a  NUP35 - nucleoporin 35kda  PUS7 - pseudouridylate synthase 7 homolog (s. cerevisiae)  PTGES3 - prostaglandin e synthase 3 (cytosolic)  NFAT5 - nuclear factor of activated t-cells 5, tonicity-responsive  CKS1B - cdc28 protein kinase regulatory subunit 1b  CIRBP - cold inducible rna binding protein  DHX29 - deah (asp-glu-ala-his) box polypeptide 29  CHUK - conserved helix-loop-helix ubiquitous kinase  CERS1 - ceramide synthase 1  ZMYND8 - zinc finger, mynd-type containing 8  ANKRA2 - ankyrin repeat, family a (rfxank-like), 2  RBAK - rb-associated krab zinc finger  NFATC2IP - nuclear factor of activated t-cells, cytoplasmic, calcineurin-dependent 2 interacting protein  FBXO18 - f-box protein, helicase, 18  PATZ1 - poz (btb) and at hook containing zinc finger 1  METTL14 - methyltransferase like 14  SPP1 - secreted phosphoprotein 1  SSBP2 - single-stranded dna binding protein 2  KPNA6 - karyopherin alpha 6 (importin alpha 7)  CIRH1A - cirrhosis, autosomal recessive 1a (cirhin)  PPP1R15B - protein phosphatase 1, regulatory subunit 15b  MIER1 - mesoderm induction early response 1, transcriptional regulator  ZNF382 - zinc finger protein 382  ZNF705A - zinc finger protein 705a  SP3 - sp3 transcription factor  SP4 - sp4 transcription factor  SP1 - sp1 transcription factor  ZNF410 - zinc finger protein 410  SP2 - sp2 transcription factor  ARL2BP - adp-ribosylation factor-like 2 binding protein  DDAH2 - dimethylarginine dimethylaminohydrolase 2  SOX4 - sry (sex determining region y)-box 4  GTPBP4 - gtp binding protein 4  WBP2 - ww domain binding protein 2  ORC3 - origin recognition complex, subunit 3  HES4 - hairy and enhancer of split 4 (drosophila)  LEMD3 - lem domain containing 3  CREBL2 - camp responsive element binding protein-like 2  GATAD1 - gata zinc finger domain containing 1  ATF6B - activating transcription factor 6 beta  CREBBP - creb binding protein  SOAT1 - sterol o-acyltransferase 1  ATF2 - activating transcription factor 2  CREB1 - camp responsive element binding protein 1  DDX58 - dead (asp-glu-ala-asp) box polypeptide 58  CRABP2 - cellular retinoic acid binding protein 2  RPRD1B - regulation of nuclear pre-mrna domain containing 1b  PLK3 - polo-like kinase 3  SNAPC4 - small nuclear rna activating complex, polypeptide 4, 190kda  SNCA - synuclein, alpha (non a4 component of amyloid precursor)  ZBED5 - zinc finger, bed-type containing 5  CREBZF - creb/atf bzip transcription factor  EID1 - ep300 interacting inhibitor of differentiation 1  TP53INP2 - tumor protein p53 inducible nuclear protein 2  PTBP2 - polypyrimidine tract binding protein 2  SMARCE1 - swi/snf related, matrix associated, actin dependent regulator of chromatin, subfamily e, member 1  SMO - smoothened, frizzled family receptor  SMARCB1 - swi/snf related, matrix associated, actin dependent regulator of chromatin, subfamily b, member 1  SMARCC1 - swi/snf related, matrix associated, actin dependent regulator of chromatin, subfamily c, member 1  HLTF - helicase-like transcription factor  SMARCA4 - swi/snf related, matrix associated, actin dependent regulator of chromatin, subfamily a, member 4  SNX6 - sorting nexin 6  SSBP3 - single stranded dna binding protein 3  SMARCD2 - swi/snf related, matrix associated, actin dependent regulator of chromatin, subfamily d, member 2  SMARCD3 - swi/snf related, matrix associated, actin dependent regulator of chromatin, subfamily d, member 3  SMARCC2 - swi/snf related, matrix associated, actin dependent regulator of chromatin, subfamily c, member 2  SMARCD1 - swi/snf related, matrix associated, actin dependent regulator of chromatin, subfamily d, member 1  WIZ - widely interspaced zinc finger motifs  MID1IP1 - mid1 interacting protein 1  SMARCA1 - swi/snf related, matrix associated, actin dependent regulator of chromatin, subfamily a, member 1  SMARCA2 - swi/snf related, matrix associated, actin dependent regulator of chromatin, subfamily a, member 2  SGK3 - serum/glucocorticoid regulated kinase family, member 3  CACTIN - cactin, spliceosome c complex subunit  KMT2C - lysine (k)-specific methyltransferase 2c  RAB38 - rab38, member ras oncogene family  ZNF462 - zinc finger protein 462  PPIL4 - peptidylprolyl isomerase (cyclophilin)-like 4  SENP2 - sumo1/sentrin/smt3 specific peptidase 2  CST3 - cystatin c  ZNF318 - zinc finger protein 318  PRPF6 - pre-mrna processing factor 6  KDM2B - lysine (k)-specific demethylase 2b  SUPT6H - suppressor of ty 6 homolog (s. cerevisiae)  CBY1 - chibby homolog 1 (drosophila)  CTBP1 - c-terminal binding protein 1  SUPT4H1 - suppressor of ty 4 homolog 1 (s. cerevisiae)  CTBP2 - c-terminal binding protein 2  SUPT5H - suppressor of ty 5 homolog (s. cerevisiae)  RAB3GAP2 - rab3 gtpase activating protein subunit 2 (non-catalytic)  CTNNB1 - catenin (cadherin-associated protein), beta 1, 88kda  SAV1 - salvador homolog 1 (drosophila)  ELOVL5 - elovl fatty acid elongase 5  CUX1 - cut-like homeobox 1  NIF3L1 - nif3 ngg1 interacting factor 3-like 1 (s. cerevisiae)  NEK4 - nima-related kinase 4  STK3 - serine/threonine kinase 3  MPV17L2 - mpv17 mitochondrial membrane protein-like 2  PCGF1 - polycomb group ring finger 1  FAM3A - family with sequence similarity 3, member a  NPNT - nephronectin  FKBP8 - fk506 binding protein 8, 38kda  GPBP1L1 - gc-rich promoter binding protein 1-like 1  CYLD - cylindromatosis (turban tumor syndrome)  TGIF2 - tgfb-induced factor homeobox 2  CNEP1R1 - ctd nuclear envelope phosphatase 1 regulatory subunit 1  BCL6B - b-cell cll/lymphoma 6, member b  CRK - v-crk avian sarcoma virus ct10 oncogene homolog  EEFSEC - eukaryotic elongation factor, selenocysteine-trna-specific  NIPBL - nipped-b homolog (drosophila)  CRY1 - cryptochrome 1 (photolyase-like)  STAT2 - signal transducer and activator of transcription 2, 113kda  STAT1 - signal transducer and activator of transcription 1, 91kda  CRY2 - cryptochrome 2 (photolyase-like)  STAT3 - signal transducer and activator of transcription 3 (acute-phase response factor)  ASF1A - anti-silencing function 1a histone chaperone  STAT5A - signal transducer and activator of transcription 5a  STAT6 - signal transducer and activator of transcription 6, interleukin-4 induced  PARM1 - prostate androgen-regulated mucin-like protein 1  BRMS1 - breast cancer metastasis suppressor 1  CRYM - crystallin, mu  ZNF496 - zinc finger protein 496  SS18 - synovial sarcoma translocation, chromosome 18  PHF5A - phd finger protein 5a  GPATCH3 - g patch domain containing 3  TAB3 - tgf-beta activated kinase 1/map3k7 binding protein 3  BAMBI - bmp and activin membrane-bound inhibitor  ZNF335 - zinc finger protein 335  HAVCR2 - hepatitis a virus cellular receptor 2  SRI - sorcin  SRC - v-src avian sarcoma (schmidt-ruppin a-2) viral oncogene homolog  DNAJB5 - dnaj (hsp40) homolog, subfamily b, member 5  SREBF2 - sterol regulatory element binding transcription factor 2  UBE2O - ubiquitin-conjugating enzyme e2o  NSUN3 - nop2/sun domain family, member 3  SRP9 - signal recognition particle 9kda  PRDX5 - peroxiredoxin 5  BACE2 - beta-site app-cleaving enzyme 2  DHX9 - deah (asp-glu-ala-his) box helicase 9  TRAPPC2 - trafficking protein particle complex 2  KANK2 - kn motif and ankyrin repeat domains 2  DDX5 - dead (asp-glu-ala-asp) box helicase 5  DDX3X - dead (asp-glu-ala-asp) box helicase 3, x-linked  SEC13 - sec13 homolog (s. cerevisiae)  DDX1 - dead (asp-glu-ala-asp) box helicase 1  SIN3A - sin3 transcription regulator family member a  DPH6 - diphthamine biosynthesis 6  SET - set nuclear oncogene  WWTR1 - ww domain containing transcription regulator 1  DDB1 - damage-specific dna binding protein 1, 127kda  ZNF304 - zinc finger protein 304  MAP2K4 - mitogen-activated protein kinase kinase 4  ZNF286A - zinc finger protein 286a  GEMIN5 - gem (nuclear organelle) associated protein 5  NOL11 - nucleolar protein 11  PBXIP1 - pre-b-cell leukemia homeobox interacting protein 1  DBP - d site of albumin promoter (albumin d-box) binding protein  NELFB - negative elongation factor complex member b  POT1 - protection of telomeres 1  AHCTF1 - at hook containing transcription factor 1  CNOT10 - ccr4-not transcription complex, subunit 10  ZBTB38 - zinc finger and btb domain containing 38  GATAD2B - gata zinc finger domain containing 2b  ZNF551 - zinc finger protein 551  ZNF473 - zinc finger protein 473  KCTD13 - potassium channel tetramerization domain containing 13  YTHDF3 - yth domain family, member 3  WDR18 - wd repeat domain 18  CNOT6 - ccr4-not transcription complex, subunit 6  LTN1 - listerin e3 ubiquitin protein ligase 1  ATXN2 - ataxin 2  ZNF451 - zinc finger protein 451  SCAF4 - sr-related ctd-associated factor 4  SS18L1 - synovial sarcoma translocation gene on chromosome 18-like 1  CHD5 - chromodomain helicase dna binding protein 5  ARID1B - at rich interactive domain 1b (swi1-like)  MED30 - mediator complex subunit 30  MKL2 - mkl/myocardin-like 2  DAPK3 - death-associated protein kinase 3  DAXX - death-domain associated protein  ZZZ3 - zinc finger, zz-type containing 3  HINFP - histone h4 transcription factor  MTA3 - metastasis associated 1 family, member 3  DGKQ - diacylglycerol kinase, theta 110kda  ZNF608 - zinc finger protein 608  DAPK1 - death-associated protein kinase 1  DAP - death-associated protein  MAVS - mitochondrial antiviral signaling protein  SARS - seryl-trna synthetase  SATB1 - satb homeobox 1  NGDN - neuroguidin, eif4e binding protein  RPTOR - regulatory associated protein of mtor, complex 1  SAFB - scaffold attachment factor b  SH2B1 - sh2b adaptor protein 1  SALL2 - sal-like 2 (drosophila)  EPC2 - enhancer of polycomb homolog 2 (drosophila)  DRD2 - dopamine receptor d2  PHF19 - phd finger protein 19  SLC9A1 - solute carrier family 9, subfamily a (nhe1, cation proton antiporter 1), member 1  IRF2BP1 - interferon regulatory factor 2 binding protein 1  EAF1 - ell associated factor 1  CNOT6L - ccr4-not transcription complex, subunit 6-like  PDP2 - pyruvate dehyrogenase phosphatase catalytic subunit 2  ATN1 - atrophin 1  DPH2 - dph2 homolog (s. cerevisiae)  DPH1 - diphthamide biosynthesis 1  C10orf137 - chromosome 10 open reading frame 137  DR1 - down-regulator of transcription 1, tbp-binding (negative cofactor 2)  MAPK15 - mitogen-activated protein kinase 15  SKI - v-ski avian sarcoma viral oncogene homolog  SKIL - ski-like oncogene  UNK - unkempt homolog (drosophila)  DYRK1A - dual-specificity tyrosine-(y)-phosphorylation regulated kinase 1a  MKL1 - megakaryoblastic leukemia (translocation) 1  DVL2 - dishevelled segment polarity protein 2  DVL3 - dishevelled segment polarity protein 3  GIGYF2 - grb10 interacting gyf protein 2  FNIP2 - folliculin interacting protein 2  APPL1 - adaptor protein, phosphotyrosine interaction, ph domain and leucine zipper containing 1  HOMEZ - homeobox and leucine zipper encoding  EBF4 - early b-cell factor 4  ANKRD17 - ankyrin repeat domain 17  ZFHX2 - zinc finger homeobox 2  HBEGF - heparin-binding egf-like growth factor  LSM14A - lsm14a, scd6 homolog a (s. cerevisiae)  DTX1 - deltex homolog 1 (drosophila)  DKC1 - dyskeratosis congenita 1, dyskerin  DLG1 - discs, large homolog 1 (drosophila)  SHOX2 - short stature homeobox 2  SIAH2 - siah e3 ubiquitin protein ligase 2  POGK - pogo transposable element with krab domain  DHFR - dihydrofolate reductase  PHF12 - phd finger protein 12  CALCOCO1 - calcium binding and coiled-coil domain 1  ZBTB4 - zinc finger and btb domain containing 4  SFPQ - splicing factor proline/glutamine-rich  SFRP1 - secreted frizzled-related protein 1  SFRP2 - secreted frizzled-related protein 2  SFRP4 - secreted frizzled-related protein 4  DNM2 - dynamin 2  GMEB2 - glucocorticoid modulatory element binding protein 2  SRSF2 - serine/arginine-rich splicing factor 2  DNMT1 - dna (cytosine-5-)-methyltransferase 1  SPAG8 - sperm associated antigen 8  DNMT3A - dna (cytosine-5-)-methyltransferase 3 alpha  EIF2AK4 - eukaryotic translation initiation factor 2 alpha kinase 4  CHD8 - chromodomain helicase dna binding protein 8  SGK1 - serum/glucocorticoid regulated kinase 1  NOC2L - nucleolar complex associated 2 homolog (s. cerevisiae)  TNRC6C - trinucleotide repeat containing 6c  ZNF317 - zinc finger protein 317  USP37 - ubiquitin specific peptidase 37  PPP1R3F - protein phosphatase 1, regulatory subunit 3f  MEIS3 - meis homeobox 3  NCBP2 - nuclear cap binding protein subunit 2, 20kda  MAPRE3 - microtubule-associated protein, rp/eb family, member 3  TSC1 - tuberous sclerosis 1  ELK1 - elk1, member of ets oncogene family  NAF1 - nuclear assembly factor 1 ribonucleoprotein  ELK3 - elk3, ets-domain protein (srf accessory protein 2)  ELK4 - elk4, ets-domain protein (srf accessory protein 1)  SNW1 - snw domain containing 1  SCAP - srebf chaperone  WRNIP1 - werner helicase interacting protein 1  EPAS1 - endothelial pas domain protein 1  DPH7 - diphthamide biosynthesis 7  EP300 - e1a binding protein p300  HABP4 - hyaluronan binding protein 4  ATF6 - activating transcription factor 6  SRRD - srr1 domain containing  ENO1 - enolase 1, (alpha)  RAB3GAP1 - rab3 gtpase activating protein subunit 1 (catalytic)  EIF4A2 - eukaryotic translation initiation factor 4a2  EIF4E - eukaryotic translation initiation factor 4e  CELF4 - cugbp, elav-like family member 4  EGR2 - early growth response 2  EGR3 - early growth response 3  CCT5 - chaperonin containing tcp1, subunit 5 (epsilon)  TTF1 - transcription termination factor, rna polymerase i  EIF2S1 - eukaryotic translation initiation factor 2, subunit 1 alpha, 35kda  TRIM32 - tripartite motif containing 32  EIF2B1 - eukaryotic translation initiation factor 2b, subunit 1 alpha, 26kda  EIF2S3 - eukaryotic translation initiation factor 2, subunit 3 gamma, 52kda  SPHK2 - sphingosine kinase 2  KDM2A - lysine (k)-specific demethylase 2a  ELAVL1 - elav (embryonic lethal, abnormal vision, drosophila)-like 1 (hu antigen r)  ELF1 - e74-like factor 1 (ets domain transcription factor)  ZNF326 - zinc finger protein 326  ELF2 - e74-like factor 2 (ets domain transcription factor)  TSG101 - tumor susceptibility gene 101  TCF25 - transcription factor 25 (basic helix-loop-helix)  EIF4G2 - eukaryotic translation initiation factor 4 gamma, 2  CNOT1 - ccr4-not transcription complex, subunit 1  EFNA1 - ephrin-a1  EEF2 - eukaryotic translation elongation factor 2  SPEN - spen homolog, transcriptional regulator (drosophila)  CTNNBIP1 - catenin, beta interacting protein 1  BBX - bobby sox homolog (drosophila)  PHC1 - polyhomeotic homolog 1 (drosophila)  TP53BP1 - tumor protein p53 binding protein 1  PHC2 - polyhomeotic homolog 2 (drosophila)  EGR1 - early growth response 1  ZNF292 - zinc finger protein 292  EGFR - epidermal growth factor receptor  MEGF8 - multiple egf-like-domains 8  CELSR2 - cadherin, egf lag seven-pass g-type receptor 2  KDM1A - lysine (k)-specific demethylase 1a  ATXN7L3 - ataxin 7-like 3  HNRNPLL - heterogeneous nuclear ribonucleoprotein l-like  NCOA6 - nuclear receptor coactivator 6  CCT3 - chaperonin containing tcp1, subunit 3 (gamma)  E2F6 - e2f transcription factor 6  E4F1 - e4f transcription factor 1  ZHX3 - zinc fingers and homeoboxes 3  E2F4 - e2f transcription factor 4, p107/p130-binding  E2F5 - e2f transcription factor 5, p130-binding  ENY2 - enhancer of yellow 2 homolog (drosophila)  C11orf30 - chromosome 11 open reading frame 30  RRP1B - ribosomal rna processing 1b  EDNRB - endothelin receptor type b  TRAF1 - tnf receptor-associated factor 1  S1PR1 - sphingosine-1-phosphate receptor 1  TRAF3 - tnf receptor-associated factor 3  TRAF2 - tnf receptor-associated factor 2  NR2C1 - nuclear receptor subfamily 2, group c, member 1  SETX - senataxin  ZBTB47 - zinc finger and btb domain containing 47  NR2C2 - nuclear receptor subfamily 2, group c, member 2  TPR - translocated promoter region, nuclear basket protein  ZNF609 - zinc finger protein 609  WAPAL - wings apart-like homolog (drosophila)  ERC1 - elks/rab6-interacting/cast family member 1  PPRC1 - peroxisome proliferator-activated receptor gamma, coactivator-related 1  NAT14 - n-acetyltransferase 14 (gcn5-related, putative)  KDM4C - lysine (k)-specific demethylase 4c  ZNF423 - zinc finger protein 423  REXO4 - rex4, rna exonuclease 4 homolog (s. cerevisiae)  CAMK1D - calcium/calmodulin-dependent protein kinase id  USF1 - upstream transcription factor 1  NUP107 - nucleoporin 107kda  ZBTB43 - zinc finger and btb domain containing 43  PTK2B - protein tyrosine kinase 2 beta  ACSL3 - acyl-coa synthetase long-chain family member 3  MRPS27 - mitochondrial ribosomal protein s27  TNRC6B - trinucleotide repeat containing 6b  PITHD1 - pith (c-terminal proteasome-interacting domain of thioredoxin-like) domain containing 1  BPTF - bromodomain phd finger transcription factor  TAB2 - tgf-beta activated kinase 1/map3k7 binding protein 2  NR1H2 - nuclear receptor subfamily 1, group h, member 2  ETV3 - ets variant 3  CAMTA2 - calmodulin binding transcription activator 2  ZNF598 - zinc finger protein 598  ETV5 - ets variant 5  POGZ - pogo transposable element with znf domain  TWSG1 - twisted gastrulation homolog 1 (drosophila)  ETV6 - ets variant 6  PLXND1 - plexin d1  VHL - von hippel-lindau tumor suppressor, e3 ubiquitin protein ligase  VIM - vimentin  KDM6B - lysine (k)-specific demethylase 6b  PHF8 - phd finger protein 8  EZR - ezrin  EWSR1 - ews rna-binding protein 1  TRIM41 - tripartite motif containing 41  TBX20 - t-box 20  KCTD1 - potassium channel tetramerization domain containing 1  CREB3L1 - camp responsive element binding protein 3-like 1  EZH1 - enhancer of zeste homolog 1 (drosophila)  CIC - capicua transcriptional repressor  CCNL1 - cyclin l1  F2R - coagulation factor ii (thrombin) receptor  VCP - valosin containing protein  WDR43 - wd repeat domain 43  ERF - ets2 repressor factor  ZNF575 - zinc finger protein 575  NUP205 - nucleoporin 205kda  ELMSAN1 - elm2 and myb/sant-like domain containing 1  THAP11 - thap domain containing 11  ZNF248 - zinc finger protein 248  UBB - ubiquitin b  LARP4B - la ribonucleoprotein domain family, member 4b  RCOR1 - rest corepressor 1  TULP3 - tubby like protein 3  ZNFX1 - zinc finger, nfx1-type containing 1  HIRA - histone cell cycle regulator  TTC5 - tetratricopeptide repeat domain 5  ETV1 - ets variant 1  ETS1 - v-ets avian erythroblastosis virus e26 oncogene homolog 1  ETF1 - eukaryotic translation termination factor 1  ZMIZ1 - zinc finger, miz-type containing 1  CYFIP1 - cytoplasmic fmr1 interacting protein 1  UBP1 - upstream binding protein 1 (lbp-1a)  SUMO1 - small ubiquitin-like modifier 1  UBTF - upstream binding transcription factor, rna polymerase i  ZNF830 - zinc finger protein 830  PELI1 - pellino e3 ubiquitin protein ligase 1  CHURC1 - churchill domain containing 1  SMURF1 - smad specific e3 ubiquitin protein ligase 1  ZC3H4 - zinc finger ccch-type containing 4  JMJD6 - jumonji domain containing 6  EPRS - glutamyl-prolyl-trna synthetase  UBE2I - ubiquitin-conjugating enzyme e2i  NUP210 - nucleoporin 210kda  UBE2L3 - ubiquitin-conjugating enzyme e2l 3  UBE2N - ubiquitin-conjugating enzyme e2n  UBE2V1 - ubiquitin-conjugating enzyme e2 variant 1  VPS72 - vacuolar protein sorting 72 homolog (s. cerevisiae)  FMR1 - fragile x mental retardation 1  TCOF1 - treacher collins-franceschetti syndrome 1  MGA - mga, max dimerization protein  TCP1 - t-complex 1  ZEB1 - zinc finger e-box binding homeobox 1  WWC3 - wwc family member 3  TCF12 - transcription factor 12  ZNF354A - zinc finger protein 354a  CAMTA1 - calmodulin binding transcription activator 1  TCF20 - transcription factor 20 (ar1)  PDS5A - pds5, regulator of cohesion maintenance, homolog a (s. cerevisiae)  FLI1 - fli-1 proto-oncogene, ets transcription factor  FLNA - filamin a, alpha  PLCB1 - phospholipase c, beta 1 (phosphoinositide-specific)  FOXS1 - forkhead box s1  FOXO3 - forkhead box o3  LARP4 - la ribonucleoprotein domain family, member 4  HDAC8 - histone deacetylase 8  ASH1L - ash1 (absent, small, or homeotic)-like (drosophila)  SIN3B - sin3 transcription regulator family member b  NACC1 - nucleus accumbens associated 1, ben and btb (poz) domain containing  HR - hair growth associated  MED8 - mediator complex subunit 8  CAND1 - cullin-associated and neddylation-dissociated 1  TDG - thymine-dna glycosylase  SMG6 - smg6 nonsense mediated mrna decay factor  PRDX2 - peroxiredoxin 2  EAPP - e2f-associated phosphoprotein  FBXW11 - f-box and wd repeat domain containing 11  FAM208A - family with sequence similarity 208, member a  KDM3A - lysine (k)-specific demethylase 3a  FHL2 - four and a half lim domains 2  SLC24A5 - solute carrier family 24 (sodium/potassium/calcium exchanger), member 5  NUP160 - nucleoporin 160kda  FGFR2 - fibroblast growth factor receptor 2  TAF9 - taf9 rna polymerase ii, tata box binding protein (tbp)-associated factor, 32kda  TAF7 - taf7 rna polymerase ii, tata box binding protein (tbp)-associated factor, 55kda  TAF6 - taf6 rna polymerase ii, tata box binding protein (tbp)-associated factor, 80kda  FGFR1 - fibroblast growth factor receptor 1  FGF9 - fibroblast growth factor 9  TADA2A - transcriptional adaptor 2a  TACC1 - transforming, acidic coiled-coil containing protein 1  TIRAP - toll-interleukin 1 receptor (tir) domain containing adaptor protein  FGF1 - fibroblast growth factor 1 (acidic)  FGF2 - fibroblast growth factor 2 (basic)  USP22 - ubiquitin specific peptidase 22  TARBP1 - tar (hiv-1) rna binding protein 1  FER - fer (fps/fes related) tyrosine kinase  MAP3K7 - mitogen-activated protein kinase kinase kinase 7  ADNP - activity-dependent neuroprotector homeobox  KMT2E - lysine (k)-specific methyltransferase 2e  MED13L - mediator complex subunit 13-like  PGP - phosphoglycolate phosphatase  NCSTN - nicastrin  FKTN - fukutin  ZNF395 - zinc finger protein 395  TAZ - tafazzin  MYNN - myoneurin  CRTC1 - creb regulated transcription coactivator 1  UFL1 - ufm1-specific ligase 1  TCF3 - transcription factor 3  PUM2 - pumilio homolog 2 (drosophila)  DMAP1 - dna methyltransferase 1 associated protein 1  TCEB3 - transcription elongation factor b (siii), polypeptide 3 (110kda, elongin a)  LARP1 - la ribonucleoprotein domain family, member 1  TCF4 - transcription factor 4  EFEMP1 - egf containing fibulin-like extracellular matrix protein 1  NKRF - nfkb repressing factor  ZNF629 - zinc finger protein 629  FBN1 - fibrillin 1  LANCL2 - lanc lantibiotic synthetase component c-like 2 (bacterial)  TCEA2 - transcription elongation factor a (sii), 2  ERBB2IP - erbb2 interacting protein  TCEA1 - transcription elongation factor a (sii), 1  ITGB3BP - integrin beta 3 binding protein (beta3-endonexin)  ORMDL1 - orm1-like 1 (s. cerevisiae)  RYBP - ring1 and yy1 binding protein  MTOR - mechanistic target of rapamycin (serine/threonine kinase)  PAXBP1 - pax3 and pax7 binding protein 1  TLE1 - transducin-like enhancer of split 1 (e(sp1) homolog, drosophila)  TLE3 - transducin-like enhancer of split 3 (e(sp1) homolog, drosophila)  TLE4 - transducin-like enhancer of split 4 (e(sp1) homolog, drosophila)  TLR3 - toll-like receptor 3  SIRT1 - sirtuin 1  MKX - mohawk homeobox  TMF1 - tata element modulatory factor 1  TMSB4X - thymosin beta 4, x-linked  ACTR8 - arp8 actin-related protein 8 homolog (yeast)  CBX6 - chromobox homolog 6  HEY1 - hairy/enhancer-of-split related with yrpw motif 1  TMPO - thymopoietin  CBX5 - chromobox homolog 5  BRD4 - bromodomain containing 4  TNFAIP1 - tumor necrosis factor, alpha-induced protein 1 (endothelial)  TARDBP - tar dna binding protein  BCL9L - b-cell cll/lymphoma 9-like  TNFRSF1A - tumor necrosis factor receptor superfamily, member 1a  TNFRSF1B - tumor necrosis factor receptor superfamily, member 1b  TMBIM6 - transmembrane bax inhibitor motif containing 6  NUP188 - nucleoporin 188kda  EIF4ENIF1 - eukaryotic translation initiation factor 4e nuclear import factor 1  SUZ12 - suz12 polycomb repressive complex 2 subunit  POFUT1 - protein o-fucosyltransferase 1  TEF - thyrotrophic embryonic factor  RCOR2 - rest corepressor 2  TFAM - transcription factor a, mitochondrial  INPP5E - inositol polyphosphate-5-phosphatase, 72 kda  TERF1 - telomeric repeat binding factor (nima-interacting) 1  TERF2 - telomeric repeat binding factor 2  TFDP1 - transcription factor dp-1  NR2F2 - nuclear receptor subfamily 2, group f, member 2  TFE3 - transcription factor binding to ighm enhancer 3  CBX7 - chromobox homolog 7  NR2F1 - nuclear receptor subfamily 2, group f, member 1  TFCP2 - transcription factor cp2  ZNF740 - zinc finger protein 740  TGFA - transforming growth factor, alpha  FNIP1 - folliculin interacting protein 1  HEY2 - hairy/enhancer-of-split related with yrpw motif 2  TGFBR1 - transforming growth factor, beta receptor 1  TGFB2 - transforming growth factor, beta 2  RBFOX2 - rna binding protein, fox-1 homolog (c. elegans) 2  TGFB3 - transforming growth factor, beta 3  TGFB1 - transforming growth factor, beta 1  TGFB1I1 - transforming growth factor beta 1 induced transcript 1  TP53INP1 - tumor protein p53 inducible nuclear protein 1  H2AFV - h2a histone family, member v  MEPCE - methylphosphate capping enzyme  RNF20 - ring finger protein 20, e3 ubiquitin protein ligase  TGFBR3 - transforming growth factor, beta receptor iii  FOSL2 - fos-like antigen 2  FOSB - fbj murine osteosarcoma viral oncogene homolog b  FOS - fbj murine osteosarcoma viral oncogene homolog  THBS1 - thrombospondin 1  KAT6B - k(lysine) acetyltransferase 6b  TIA1 - tia1 cytotoxic granule-associated rna binding protein  METTL3 - methyltransferase like 3  THRA - thyroid hormone receptor, alpha  ELL - elongation factor rna polymerase ii  ZNF239 - zinc finger protein 239  PHC3 - polyhomeotic homolog 3 (drosophila)  OBFC1 - oligonucleotide/oligosaccharide-binding fold containing 1  NRIP1 - nuclear receptor interacting protein 1  KAT2A - k(lysine) acetyltransferase 2a  NCOA3 - nuclear receptor coactivator 3  GLA - galactosidase, alpha  WWC2 - ww and c2 domain containing 2  TAF15 - taf15 rna polymerase ii, tata box binding protein (tbp)-associated factor, 68kda  GJA1 - gap junction protein, alpha 1, 43kda  AXIN1 - axin 1  TRRAP - transformation/transcription domain-associated protein  PICALM - phosphatidylinositol binding clathrin assembly protein  LPCAT1 - lysophosphatidylcholine acyltransferase 1  ARID1A - at rich interactive domain 1a (swi-like)  DLL1 - delta-like 1 (drosophila)  SETD6 - set domain containing 6  FZD6 - frizzled family receptor 6  GLE1 - gle1 rna export mediator  ACTR5 - arp5 actin-related protein 5 homolog (yeast)  CDC45 - cell division cycle 45  FZD4 - frizzled family receptor 4  GCLC - glutamate-cysteine ligase, catalytic subunit  FZD1 - frizzled family receptor 1  BAP1 - brca1 associated protein-1 (ubiquitin carboxy-terminal hydrolase)  ZNF613 - zinc finger protein 613  NUP85 - nucleoporin 85kda  CDC7 - cell division cycle 7  KDM5C - lysine (k)-specific demethylase 5c  USP9X - ubiquitin specific peptidase 9, x-linked  RBM10 - rna binding motif protein 10  PHF17 - phd finger protein 17  TTC21B - tetratricopeptide repeat domain 21b  SLTM - safb-like, transcription modulator  NELFE - negative elongation factor complex member e  EHMT1 - euchromatic histone-lysine n-methyltransferase 1  NR5A1 - nuclear receptor subfamily 5, group a, member 1  FUS - fused in sarcoma  NAA40 - n(alpha)-acetyltransferase 40, natd catalytic subunit, homolog (s. cerevisiae)  USP7 - ubiquitin specific peptidase 7 (herpes virus-associated)  RNF168 - ring finger protein 168, e3 ubiquitin protein ligase  XRCC6 - x-ray repair complementing defective repair in chinese hamster cells 6  GABPB1 - ga binding protein transcription factor, beta subunit 1  GABPA - ga binding protein transcription factor, alpha subunit 60kda  FYN - fyn oncogene related to src, fgr, yes  RPAP2 - rna polymerase ii associated protein 2  DDX39B - dead (asp-glu-ala-asp) box polypeptide 39b  KMT2D - lysine (k)-specific methyltransferase 2d  AAAS - achalasia, adrenocortical insufficiency, alacrimia  ZNF419 - zinc finger protein 419  YEATS4 - yeats domain containing 4  ZFPM1 - zinc finger protein, fog family member 1  SNIP1 - smad nuclear interacting protein 1  ZNF212 - zinc finger protein 212  EID2 - ep300 interacting inhibitor of differentiation 2  GBA - glucosidase, beta, acid  ZNF383 - zinc finger protein 383  GATA2 - gata binding protein 2  KAT6A - k(lysine) acetyltransferase 6a  NUP214 - nucleoporin 214kda  NR4A3 - nuclear receptor subfamily 4, group a, member 3  BRD3 - bromodomain containing 3  GAS6 - growth arrest-specific 6  NCOA4 - nuclear receptor coactivator 4  CARF - calcium responsive transcription factor  DEDD2 - death effector domain containing 2  ZNF408 - zinc finger protein 408  MLLT10 - myeloid/lymphoid or mixed-lineage leukemia (trithorax homolog, drosophila); translocated to, 10  DHX36 - deah (asp-glu-ala-his) box polypeptide 36  ZNF189 - zinc finger protein 189  ZSCAN26 - zinc finger and scan domain containing 26  GSPT1 - g1 to s phase transition 1  ZNF177 - zinc finger protein 177  ZNF175 - zinc finger protein 175  ZNF174 - zinc finger protein 174  ZNF184 - zinc finger protein 184  SSBP4 - single stranded dna binding protein 4  GTF2F1 - general transcription factor iif, polypeptide 1, 74kda  ZNF148 - zinc finger protein 148  BEND5 - ben domain containing 5  ZBTB17 - zinc finger and btb domain containing 17  GTF2H2 - general transcription factor iih, polypeptide 2, 44kda  TRIM26 - tripartite motif containing 26  GTF2H3 - general transcription factor iih, polypeptide 3, 34kda  VEZF1 - vascular endothelial zinc finger 1  GTF2H1 - general transcription factor iih, polypeptide 1, 62kda  ZNF142 - zinc finger protein 142  MCPH1 - microcephalin 1  ZNF140 - zinc finger protein 140  GTF2B - general transcription factor iib  PCGF2 - polycomb group ring finger 2  ZNF132 - zinc finger protein 132  ZNF131 - zinc finger protein 131  ZNF134 - zinc finger protein 134  ZNF133 - zinc finger protein 133  TBL1XR1 - transducin (beta)-like 1 x-linked receptor 1  GTF2I - general transcription factor iii  GTF2H4 - general transcription factor iih, polypeptide 4, 52kda  BRF1 - brf1, rna polymerase iii transcription initiation factor 90 kda subunit  ZNF79 - zinc finger protein 79  ZNF70 - zinc finger protein 70  ZNF75D - zinc finger protein 75d  ZNF76 - zinc finger protein 76  H3F3A - h3 histone, family 3a  BRPF1 - bromodomain and phd finger containing, 1  H2AFZ - h2a histone family, member z  DCP2 - decapping mrna 2  H2AFX - h2a histone family, member x  SAP130 - sin3a-associated protein, 130kda  FZD5 - frizzled family receptor 5  PAX8 - paired box 8  MIER3 - mesoderm induction early response 1, family member 3  CDC73 - cell division cycle 73  NKAP - nfkb activating protein  THAP5 - thap domain containing 5  LRP8 - low density lipoprotein receptor-related protein 8, apolipoprotein e receptor  HCFC1 - host cell factor c1 (vp16-accessory protein)  PRDM2 - pr domain containing 2, with znf domain  MAP3K12 - mitogen-activated protein kinase kinase kinase 12  ZNF226 - zinc finger protein 226  HDAC2 - histone deacetylase 2  HMBOX1 - homeobox containing 1  ZCCHC12 - zinc finger, cchc domain containing 12  HDGF - hepatoma-derived growth factor  ZNF213 - zinc finger protein 213  HDAC1 - histone deacetylase 1  ZNF205 - zinc finger protein 205  HCLS1 - hematopoietic cell-specific lyn substrate 1  ZNF207 - zinc finger protein 207  XPA - xeroderma pigmentosum, complementation group a  XPC - xeroderma pigmentosum, complementation group c  GOLGA2 - golgin a2  XPO1 - exportin 1 (crm1 homolog, yeast)  GOLGB1 - golgin b1  PAGR1 - paxip1 associated glutamate-rich protein 1  ZXDC - zxd family zinc finger c  AAED1 - ahpc/tsa antioxidant enzyme domain containing 1  XBP1 - x-box binding protein 1  ZNF367 - zinc finger protein 367  BHLHE41 - basic helix-loop-helix family, member e41  EIF4H - eukaryotic translation initiation factor 4h  WFS1 - wolfram syndrome 1 (wolframin)  WHSC1 - wolf-hirschhorn syndrome candidate 1  NELFA - negative elongation factor complex member a  ARID2 - at rich interactive domain 2 (arid, rfx-like)  PAOX - polyamine oxidase (exo-n4-amino)  ZNF35 - zinc finger protein 35  ZKSCAN1 - zinc finger with krab and scan domains 1  FBXL15 - f-box and leucine-rich repeat protein 15  ZNF576 - zinc finger protein 576  ZNF33B - zinc finger protein 33b  ZNF32 - zinc finger protein 32  ZNF45 - zinc finger protein 45  MZF1 - myeloid zinc finger 1  ZNF18 - zinc finger protein 18  ZNF16 - zinc finger protein 16  ASXL1 - additional sex combs like 1 (drosophila)  ZNF384 - zinc finger protein 384  TNIP2 - tnfaip3 interacting protein 2  ZNF24 - zinc finger protein 24  ZFPL1 - zinc finger protein-like 1  ZFX - zinc finger protein, x-linked  SF1 - splicing factor 1  ZBTB14 - zinc finger and btb domain containing 14  ZNF711 - zinc finger protein 711  ZNF7 - zinc finger protein 7  ZNF8 - zinc finger protein 8  ZNF2 - zinc finger protein 2  ZNF3 - zinc finger protein 3  YES1 - v-yes-1 yamaguchi sarcoma viral oncogene homolog 1  XRCC5 - x-ray repair complementing defective repair in chinese hamster cells 5 (double-strand-break rejoining)  GSK3B - glycogen synthase kinase 3 beta  GSK3A - glycogen synthase kinase 3 alpha  YWHAB - tyrosine 3-monooxygenase/tryptophan 5-monooxygenase activation protein, beta polypeptide  CCNK - cyclin k  HSF2 - heat shock transcription factor 2  TRIM24 - tripartite motif containing 24  TAF1D - tata box binding protein (tbp)-associated factor, rna polymerase i, d, 41kda  CREG1 - cellular repressor of e1a-stimulated genes 1  HSBP1 - heat shock factor binding protein 1  FLCN - folliculin  PRMT1 - protein arginine methyltransferase 1  PRMT2 - protein arginine methyltransferase 2  HESX1 - hesx homeobox 1  SAP30 - sin3a-associated protein, 30kda  METTL16 - methyltransferase like 16  FTO - fat mass and obesity associated  ALKBH1 - alkb, alkylation repair homolog 1 (e. coli)  CFLAR - casp8 and fadd-like apoptosis regulator  SECISBP2 - secis binding protein 2  MED11 - mediator complex subunit 11  WDR77 - wd repeat domain 77  LDB1 - lim domain binding 1  DSCC1 - dna replication and sister chromatid cohesion 1  KAT2B - k(lysine) acetyltransferase 2b  TSC22D1 - tsc22 domain family, member 1  CRTC2 - creb regulated transcription coactivator 2  ZNF259 - zinc finger protein 259  TAX1BP1 - tax1 (human t-cell leukemia virus type i) binding protein 1  CDC123 - cell division cycle 123  CC2D1B - coiled-coil and c2 domain containing 1b  HUS1 - hus1 checkpoint homolog (s. pombe)  THAP8 - thap domain containing 8  EIF2B2 - eukaryotic translation initiation factor 2b, subunit 2 beta, 39kda  BUD31 - bud31 homolog (s. cerevisiae)  ZNF655 - zinc finger protein 655  EIF2B5 - eukaryotic translation initiation factor 2b, subunit 5 epsilon, 82kda  TRAK2 - trafficking protein, kinesin binding 2  HSPD1 - heat shock 60kda protein 1 (chaperonin)  DNAJB1 - dnaj (hsp40) homolog, subfamily b, member 1  ZNF48 - zinc finger protein 48  BCL10 - b-cell cll/lymphoma 10  FUBP3 - far upstream element (fuse) binding protein 3  HSP90AA1 - heat shock protein 90kda alpha (cytosolic), class a member 1  BTRC - beta-transducin repeat containing e3 ubiquitin protein ligase  HSPA8 - heat shock 70kda protein 8  EIF4EBP3 - eukaryotic translation initiation factor 4e binding protein 3  HMGN1 - high mobility group nucleosome binding domain 1  HMGB3 - high mobility group box 3  HMGB2 - high mobility group box 2  HMGB1 - high mobility group box 1  EIF3C - eukaryotic translation initiation factor 3, subunit c  BRD9 - bromodomain containing 9  EIF3D - eukaryotic translation initiation factor 3, subunit d  EIF3B - eukaryotic translation initiation factor 3, subunit b  IRS2 - insulin receptor substrate 2  TNKS - tankyrase, trf1-interacting ankyrin-related adp-ribose polymerase  EIF4G3 - eukaryotic translation initiation factor 4 gamma, 3  ZBTB10 - zinc finger and btb domain containing 10  AGO1 - argonaute risc catalytic component 1  CAPRIN2 - caprin family member 2  ZBTB48 - zinc finger and btb domain containing 48  HYAL2 - hyaluronoglucosaminidase 2  ACD - adrenocortical dysplasia homolog (mouse)  GPBP1 - gc-rich promoter binding protein 1  HFE - hemochromatosis  HINT1 - histidine triad nucleotide binding protein 1  ELP4 - elongator acetyltransferase complex subunit 4  HIVEP1 - human immunodeficiency virus type i enhancer binding protein 1  UPF3B - upf3 regulator of nonsense transcripts homolog b (yeast)  UPF3A - upf3 regulator of nonsense transcripts homolog a (yeast)  HIF1A - hypoxia inducible factor 1, alpha subunit (basic helix-loop-helix transcription factor)  LPIN3 - lipin 3  TRADD - tnfrsf1a-associated via death domain  MYCBP - myc binding protein  EED - embryonic ectoderm development  URI1 - uri1, prefoldin-like chaperone  RIPK1 - receptor (tnfrsf)-interacting serine-threonine kinase 1  HNRNPU - heterogeneous nuclear ribonucleoprotein u (scaffold attachment factor a)  HNRNPK - heterogeneous nuclear ribonucleoprotein k  HNRNPL - heterogeneous nuclear ribonucleoprotein l  FBXO4 - f-box protein 4  TINF2 - terf1 (trf1)-interacting nuclear factor 2  HNRNPA2B1 - heterogeneous nuclear ribonucleoprotein a2/b1  HNRNPA1 - heterogeneous nuclear ribonucleoprotein a1  HNRNPC - heterogeneous nuclear ribonucleoprotein c (c1/c2)  RIPK2 - receptor-interacting serine-threonine kinase 2  HNRNPAB - heterogeneous nuclear ribonucleoprotein a/b  HNRNPD - heterogeneous nuclear ribonucleoprotein d (au-rich element rna binding protein 1, 37kda)  ZNF584 - zinc finger protein 584  HMGA1 - high mobility group at-hook 1  FAM129B - family with sequence similarity 129, member b  NR4A1 - nuclear receptor subfamily 4, group a, member 1  RFX7 - regulatory factor x, 7  HTATSF1 - hiv-1 tat specific factor 1  FOXK2 - forkhead box k2  HAT1 - histone acetyltransferase 1  EIF3K - eukaryotic translation initiation factor 3, subunit k  CRTC3 - creb regulated transcription coactivator 3  RBM15 - rna binding motif protein 15  TNRC6A - trinucleotide repeat containing 6a  ENC1 - ectodermal-neural cortex 1 (with btb domain)  RBMX - rna binding motif protein, x-linked  ING2 - inhibitor of growth family, member 2  ZNF574 - zinc finger protein 574  PPM1D - protein phosphatase, mg2+/mn2+ dependent, 1d  CREB3L2 - camp responsive element binding protein 3-like 2  IPPK - inositol 1,3,4,5,6-pentakisphosphate 2-kinase  ILF2 - interleukin enhancer binding factor 2  ILF3 - interleukin enhancer binding factor 3, 90kda  ILK - integrin-linked kinase  PIAS1 - protein inhibitor of activated stat, 1  SMYD3 - set and mynd domain containing 3  SLCO3A1 - solute carrier organic anion transporter family, member 3a1  SMURF2 - smad specific e3 ubiquitin protein ligase 2  MAPKAPK5 - mitogen-activated protein kinase-activated protein kinase 5  CGGBP1 - cgg triplet repeat binding protein 1  LMO4 - lim domain only 4  CAMK1 - calcium/calmodulin-dependent protein kinase i  CBX4 - chromobox homolog 4  CSRNP1 - cysteine-serine-rich nuclear protein 1  SERP1 - stress-associated endoplasmic reticulum protein 1  ZNF25 - zinc finger protein 25  STK16 - serine/threonine kinase 16  ITGA3 - integrin, alpha 3 (antigen cd49c, alpha 3 subunit of vla-3 receptor)  KHSRP - kh-type splicing regulatory protein  CASK - calcium/calmodulin-dependent serine protein kinase (maguk family)  SUDS3 - suppressor of defective silencing 3 homolog (s. cerevisiae)  EIF6 - eukaryotic translation initiation factor 6  SKAP1 - src kinase associated phosphoprotein 1  GZF1 - gdnf-inducible zinc finger protein 1  INSR - insulin receptor  INSM1 - insulinoma-associated 1  CDK13 - cyclin-dependent kinase 13  PDCD4 - programmed cell death 4 (neoplastic transformation inhibitor)  RFXANK - regulatory factor x-associated ankyrin-containing protein  IRF2 - interferon regulatory factor 2  KLF7 - kruppel-like factor 7 (ubiquitous)  IRF3 - interferon regulatory factor 3  RUVBL1 - ruvb-like 1 (e. coli)  IKZF5 - ikaros family zinc finger 5 (pegasus)  IREB2 - iron-responsive element binding protein 2  MED19 - mediator complex subunit 19  ITGA6 - integrin, alpha 6  IRAK2 - interleukin-1 receptor-associated kinase 2  IRAK1 - interleukin-1 receptor-associated kinase 1  CACYBP - calcyclin binding protein  ZSCAN31 - zinc finger and scan domain containing 31  EIF2AK1 - eukaryotic translation initiation factor 2-alpha kinase 1  KIAA1324L - kiaa1324-like  PAPD5 - pap associated domain containing 5  NSD1 - nuclear receptor binding set domain protein 1  ZBTB11 - zinc finger and btb domain containing 11  RNF25 - ring finger protein 25  MMS19 - mms19 nucleotide excision repair homolog (s. cerevisiae)  DKK3 - dickkopf wnt signaling pathway inhibitor 3  SNX5 - sorting nexin 5  ZNF777 - zinc finger protein 777  ID2 - inhibitor of dna binding 2, dominant negative helix-loop-helix protein  ID3 - inhibitor of dna binding 3, dominant negative helix-loop-helix protein  BRPF3 - bromodomain and phd finger containing, 3  STK36 - serine/threonine kinase 36  IRF2BPL - interferon regulatory factor 2 binding protein-like  JAZF1 - jazf zinc finger 1  PLA2G6 - phospholipase a2, group vi (cytosolic, calcium-independent)  FOXK1 - forkhead box k1  IGHMBP2 - immunoglobulin mu binding protein 2  ZNF282 - zinc finger protein 282  RBPJ - recombination signal binding protein for immunoglobulin kappa j region  NSMAF - neutral sphingomyelinase (n-smase) activation associated factor  NCK2 - nck adaptor protein 2  PABPC1 - poly(a) binding protein, cytoplasmic 1  TRUB2 - trub pseudouridine (psi) synthase homolog 2 (e. coli)  ZBTB9 - zinc finger and btb domain containing 9  USP21 - ubiquitin specific peptidase 21  ZBTB12 - zinc finger and btb domain containing 12  BCAR3 - breast cancer anti-estrogen resistance 3  JMJD1C - jumonji domain containing 1c  IGBP1 - immunoglobulin (cd79a) binding protein 1  SMARCA5 - swi/snf related, matrix associated, actin dependent regulator of chromatin, subfamily a, member 5  OGT - o-linked n-acetylglucosamine (glcnac) transferase  PELP1 - proline, glutamate and leucine rich protein 1  RAE1 - rae1 rna export 1 homolog (s. pombe)  TSPYL2 - tspy-like 2  SND1 - staphylococcal nuclease and tudor domain containing 1  CUL2 - cullin 2  PCIF1 - pdx1 c-terminal inhibiting factor 1  ZNF667 - zinc finger protein 667  CUL3 - cullin 3  FOXP1 - forkhead box p1  SUPT3H - suppressor of ty 3 homolog (s. cerevisiae)  BEND6 - ben domain containing 6  ZNF572 - zinc finger protein 572  PCGF5 - polycomb group ring finger 5  WAC - ww domain containing adaptor with coiled-coil  ZNF414 - zinc finger protein 414  PHF21A - phd finger protein 21a  ZNF771 - zinc finger protein 771  AKAP12 - a kinase (prka) anchor protein 12  IER2 - immediate early response 2  LIG3 - ligase iii, dna, atp-dependent  NCOR1 - nuclear receptor corepressor 1  LDLR - low density lipoprotein receptor  NCOR2 - nuclear receptor corepressor 2  MTPN - myotrophin  DOT1L - dot1-like histone h3k79 methyltransferase  EFCAB7 - ef-hand calcium binding domain 7  L3MBTL3 - l(3)mbt-like 3 (drosophila)  ZNF333 - zinc finger protein 333  SCAND1 - scan domain containing 1  CIR1 - corepressor interacting with rbpj, 1  H2AFY - h2a histone family, member y  GTF2IRD1 - gtf2i repeat domain containing 1  FADS1 - fatty acid desaturase 1  ZNF527 - zinc finger protein 527  H6PD - hexose-6-phosphate dehydrogenase (glucose 1-dehydrogenase)  HOPX - hop homeobox  ZNF316 - zinc finger protein 316  ZC3H8 - zinc finger ccch-type containing 8  SOX13 - sry (sex determining region y)-box 13  LMO1 - lim domain only 1 (rhombotin 1)  IER5 - immediate early response 5  NACC2 - nacc family member 2, ben and btb (poz) domain containing  CLOCK - clock circadian regulator  KLF3 - kruppel-like factor 3 (basic)  PRKAG2 - protein kinase, amp-activated, gamma 2 non-catalytic subunit  DDX41 - dead (asp-glu-ala-asp) box polypeptide 41  FXR2 - fragile x mental retardation, autosomal homolog 2  LITAF - lipopolysaccharide-induced tnf factor  TBPL1 - tbp-like 1  YTHDF2 - yth domain family, member 2  ZFP62 - zfp62 zinc finger protein  ZBTB41 - zinc finger and btb domain containing 41  TMEM59 - transmembrane protein 59  ZGPAT - zinc finger, ccch-type with g patch domain  ACTL6B - actin-like 6b  EIF4E2 - eukaryotic translation initiation factor 4e family member 2  MED20 - mediator complex subunit 20  AFAP1L2 - actin filament associated protein 1-like 2  ROCK2 - rho-associated, coiled-coil containing protein kinase 2  CRLF3 - cytokine receptor-like factor 3  MAPK8IP1 - mitogen-activated protein kinase 8 interacting protein 1  NGRN - neugrin, neurite outgrowth associated  GLYR1 - glyoxylate reductase 1 homolog (arabidopsis)  ZBTB7A - zinc finger and btb domain containing 7a  DPY30 - dpy-30 homolog (c. elegans)  MBTPS2 - membrane-bound transcription factor peptidase, site 2  MAGED1 - melanoma antigen family d, 1  GLIS2 - glis family zinc finger 2  IRF2BP2 - interferon regulatory factor 2 binding protein 2  MED27 - mediator complex subunit 27  MED26 - mediator complex subunit 26  CHD6 - chromodomain helicase dna binding protein 6  MED17 - mediator complex subunit 17  MED23 - mediator complex subunit 23  ZNF580 - zinc finger protein 580  CDYL - chromodomain protein, y-like  GTF2IRD2 - gtf2i repeat domain containing 2  ZNF710 - zinc finger protein 710  ING4 - inhibitor of growth family, member 4  EIF2AK3 - eukaryotic translation initiation factor 2-alpha kinase 3  INSIG2 - insulin induced gene 2  ITM2B - integral membrane protein 2b  RLIM - ring finger protein, lim domain interacting  SOGA1 - suppressor of glucose, autophagy associated 1  KRAS - kirsten rat sarcoma viral oncogene homolog  RECQL5 - recq protein-like 5  TGFBRAP1 - transforming growth factor, beta receptor associated protein 1  ZNF706 - zinc finger protein 706  CIAO1 - cytosolic iron-sulfur protein assembly 1  PHF20L1 - phd finger protein 20-like 1  MED21 - mediator complex subunit 21  MED10 - mediator complex subunit 10  KIT - v-kit hardy-zuckerman 4 feline sarcoma viral oncogene homolog  ZFP28 - zfp28 zinc finger protein  POLDIP3 - polymerase (dna-directed), delta interacting protein 3  PAIP2 - poly(a) binding protein interacting protein 2  JUP - junction plakoglobin  JAK2 - janus kinase 2  ING5 - inhibitor of growth family, member 5  JAK3 - janus kinase 3  ZNF219 - zinc finger protein 219  DNAJC30 - dnaj (hsp40) homolog, subfamily c, member 30  PHF20 - phd finger protein 20  KCNK2 - potassium channel, subfamily k, member 2  ZNHIT3 - zinc finger, hit-type containing 3  ZNF397 - zinc finger protein 397  TRIP4 - thyroid hormone receptor interactor 4  HMGN3 - high mobility group nucleosomal binding domain 3  PHF6 - phd finger protein 6  ZNF235 - zinc finger protein 235  COPS2 - cop9 signalosome subunit 2  ZNF639 - zinc finger protein 639  SARNP - sap domain containing ribonucleoprotein  BRMS1L - breast cancer metastasis-suppressor 1-like  LEF1 - lymphoid enhancer-binding factor 1  CNOT8 - ccr4-not transcription complex, subunit 8  SIRPA - signal-regulatory protein alpha  CDK12 - cyclin-dependent kinase 12  ZNF280B - zinc finger protein 280b  ARID4B - at rich interactive domain 4b (rbp1-like)  MAP3K10 - mitogen-activated protein kinase kinase kinase 10  KDM3B - lysine (k)-specific demethylase 3b  ITGB1BP1 - integrin beta 1 binding protein 1  RSF1 - remodeling and spacing factor 1  MLLT6 - myeloid/lymphoid or mixed-lineage leukemia (trithorax homolog, drosophila); translocated to, 6  SAMD8 - sterile alpha motif domain containing 8  MLLT3 - myeloid/lymphoid or mixed-lineage leukemia (trithorax homolog, drosophila); translocated to, 3  MED14 - mediator complex subunit 14  MLLT1 - myeloid/lymphoid or mixed-lineage leukemia (trithorax homolog, drosophila); translocated to, 1  ZBTB22 - zinc finger and btb domain containing 22  KMT2A - lysine (k)-specific methyltransferase 2a  RPS6KA5 - ribosomal protein s6 kinase, 90kda, polypeptide 5  SUFU - suppressor of fused homolog (drosophila)  NLK - nemo-like kinase  UIMC1 - ubiquitin interaction motif containing 1  MNAT1 - mnat cdk-activating kinase assembly factor 1  MTA2 - metastasis associated 1 family, member 2  NOLC1 - nucleolar and coiled-body phosphoprotein 1  EIF2A - eukaryotic translation initiation factor 2a, 65kda  CD200 - cd200 molecule  WWOX - ww domain containing oxidoreductase  MNT - mnt, max dimerization protein  CCPG1 - cell cycle progression 1  SPTY2D1 - spt2, suppressor of ty, domain containing 1 (s. cerevisiae)  MECP2 - methyl cpg binding protein 2 (rett syndrome)  PELO - pelota homolog (drosophila)  MEF2A - myocyte enhancer factor 2a  PRICKLE1 - prickle homolog 1 (drosophila)  ZBED1 - zinc finger, bed-type containing 1  MEIS2 - meis homeobox 2  BRWD1 - bromodomain and wd repeat domain containing 1  ZNF664 - zinc finger protein 664  MEIS1 - meis homeobox 1  MAP3K4 - mitogen-activated protein kinase kinase kinase 4  MAP3K5 - mitogen-activated protein kinase kinase kinase 5  SLC33A1 - solute carrier family 33 (acetyl-coa transporter), member 1  MEN1 - multiple endocrine neoplasia i  WDR75 - wd repeat domain 75  UTP15 - utp15, u3 small nucleolar ribonucleoprotein, homolog (s. cerevisiae)  KAT8 - k(lysine) acetyltransferase 8  ZNF644 - zinc finger protein 644  ARHGEF2 - rho/rac guanine nucleotide exchange factor (gef) 2  NEMF - nuclear export mediator factor  MBD3 - methyl-cpg binding domain protein 3  PTOV1 - prostate tumor overexpressed 1  ZNF394 - zinc finger protein 394  CBFA2T2 - core-binding factor, runt domain, alpha subunit 2; translocated to, 2  MAGOH - mago-nashi homolog, proliferation-associated (drosophila)  CXXC5 - cxxc finger protein 5  RQCD1 - rcd1 required for cell differentiation1 homolog (s. pombe)  SMC3 - structural maintenance of chromosomes 3  IL23A - interleukin 23, alpha subunit p19  MARS - methionyl-trna synthetase  SIRT6 - sirtuin 6  MTA1 - metastasis associated 1  PPHLN1 - periphilin 1  UBE2J1 - ubiquitin-conjugating enzyme e2, j1  NUP43 - nucleoporin 43kda  MBD1 - methyl-cpg binding domain protein 1  SFMBT1 - scm-like with four mbt domains 1  MAX - myc associated factor x  ZMIZ2 - zinc finger, miz-type containing 2  LDB2 - lim domain binding 2  ZFP90 - zfp90 zinc finger protein  CDAN1 - codanin 1  NELFCD - negative elongation factor complex member c/d  ASH2L - ash2 (absent, small, or homeotic)-like (drosophila)  CTDSPL2 - ctd (carboxy-terminal domain, rna polymerase ii, polypeptide a) small phosphatase like 2  ZSCAN29 - zinc finger and scan domain containing 29  THAP2 - thap domain containing, apoptosis associated protein 2  ITCH - itchy e3 ubiquitin protein ligase  ZNF484 - zinc finger protein 484  L3MBTL2 - l(3)mbt-like 2 (drosophila)  LRP6 - low density lipoprotein receptor-related protein 6  ZNF445 - zinc finger protein 445  AIP - aryl hydrocarbon receptor interacting protein  TRAPPC9 - trafficking protein particle complex 9  ASB1 - ankyrin repeat and socs box containing 1  LUM - lumican  BTAF1 - btaf1 rna polymerase ii, b-tfiid transcription factor-associated, 170kda  UBA3 - ubiquitin-like modifier activating enzyme 3  SESN2 - sestrin 2  CDK5RAP1 - cdk5 regulatory subunit associated protein 1  PARP9 - poly (adp-ribose) polymerase family, member 9  RNF8 - ring finger protein 8, e3 ubiquitin protein ligase  BAZ1B - bromodomain adjacent to zinc finger domain, 1b  MED15 - mediator complex subunit 15  MXD1 - max dimerization protein 1  PIAS4 - protein inhibitor of activated stat, 4  TRIM33 - tripartite motif containing 33  SMAD2 - smad family member 2  SRRT - serrate rna effector molecule homolog (arabidopsis)  KLF16 - kruppel-like factor 16  HDAC7 - histone deacetylase 7  ARMCX3 - armadillo repeat containing, x-linked 3  TDP2 - tyrosyl-dna phosphodiesterase 2  CAPRIN1 - cell cycle associated protein 1  LARP7 - la ribonucleoprotein domain family, member 7  MAFG - v-maf avian musculoaponeurotic fibrosarcoma oncogene homolog g  RPS6KA4 - ribosomal protein s6 kinase, 90kda, polypeptide 4  DPH5 - diphthamide biosynthesis 5  TAF9B - taf9b rna polymerase ii, tata box binding protein (tbp)-associated factor, 31kda  SMAD4 - smad family member 4  SMAD5 - smad family member 5  H1FX - h1 histone family, member x  SMAD7 - smad family member 7  WASL - wiskott-aldrich syndrome-like  RBM4B - rna binding motif protein 4b  ZNF565 - zinc finger protein 565  SAP30BP - sap30 binding protein  YAF2 - yy1 associated factor 2  NCBP1 - nuclear cap binding protein subunit 1, 80kda  SIX5 - six homeobox 5  TAGLN3 - transgelin 3  ITM2C - integral membrane protein 2c  NDN - necdin, melanoma antigen (mage) family member  ZNF582 - zinc finger protein 582  BRD7 - bromodomain containing 7  NCK1 - nck adaptor protein 1  DDX25 - dead (asp-glu-ala-asp) box helicase 25  NCL - nucleolin  CEBPZ - ccaat/enhancer binding protein (c/ebp), zeta  TICAM1 - toll-like receptor adaptor molecule 1  CCNL2 - cyclin l2  ORMDL2 - orm1-like 2 (s. cerevisiae)  TRIM28 - tripartite motif containing 28  TBK1 - tank-binding kinase 1  NBN - nibrin  ZNF197 - zinc finger protein 197  TDRD3 - tudor domain containing 3  PPP1R12A - protein phosphatase 1, regulatory subunit 12a  TRIM11 - tripartite motif containing 11  MYT1 - myelin transcription factor 1  ALYREF - aly/ref export factor  SENP1 - sumo1/sentrin specific peptidase 1  CSRNP2 - cysteine-serine-rich nuclear protein 2  NAB1 - ngfi-a binding protein 1 (egr1 binding protein 1)  NAB2 - ngfi-a binding protein 2 (egr1 binding protein 2)  RNF41 - ring finger protein 41  TSHZ1 - teashirt zinc finger homeobox 1  PSME3 - proteasome (prosome, macropain) activator subunit 3 (pa28 gamma; ki)  TRIM13 - tripartite motif containing 13  TRIM8 - tripartite motif containing 8  MYO6 - myosin vi  ABT1 - activator of basal transcription 1  LBH - limb bud and heart development  TOPORS - topoisomerase i binding, arginine/serine-rich, e3 ubiquitin protein ligase  MYD88 - myeloid differentiation primary response 88  SEH1L - seh1-like (s. cerevisiae)  ZNF362 - zinc finger protein 362  HCFC2 - host cell factor c2  CNOT7 - ccr4-not transcription complex, subunit 7  MXI1 - max interactor 1, dimerization protein  PQBP1 - polyglutamine binding protein 1  ACTR2 - arp2 actin-related protein 2 homolog (yeast)  ACTR3 - arp3 actin-related protein 3 homolog (yeast)  TSFM - ts translation elongation factor, mitochondrial  TRIM37 - tripartite motif containing 37  ZNF558 - zinc finger protein 558  LRPPRC - leucine-rich pentatricopeptide repeat containing  ZNF263 - zinc finger protein 263  PURG - purine-rich element binding protein g  MED25 - mediator complex subunit 25  TRAP1 - tnf receptor-associated protein 1  MED6 - mediator complex subunit 6  PTBP3 - polypyrimidine tract binding protein 3  DMTF1 - cyclin d binding myb-like transcription factor 1  MTIF2 - mitochondrial translational initiation factor 2  ZBTB33 - zinc finger and btb domain containing 33  MTF1 - metal-regulatory transcription factor 1  HDAC5 - histone deacetylase 5  SRA1 - steroid receptor rna activator 1  HDAC6 - histone deacetylase 6  RNF187 - ring finger protein 187  MT3 - metallothionein 3  MED16 - mediator complex subunit 16  CCDC22 - coiled-coil domain containing 22  DNAJB6 - dnaj (hsp40) homolog, subfamily b, member 6  MSH3 - muts homolog 3 (e. coli)  CITED1 - cbp/p300-interacting transactivator, with glu/asp-rich carboxy-terminal domain, 1  LPGAT1 - lysophosphatidylglycerol acyltransferase 1  ZBTB5 - zinc finger and btb domain containing 5  ZBTB40 - zinc finger and btb domain containing 40  SUPT7L - suppressor of ty 7 (s. cerevisiae)-like  RGCC - regulator of cell cycle  COMMD1 - copper metabolism (murr1) domain containing 1  RBM8A - rna binding motif protein 8a  USP3 - ubiquitin specific peptidase 3  METTL5 - methyltransferase like 5  USP15 - ubiquitin specific peptidase 15  MED4 - mediator complex subunit 4  THOC1 - tho complex 1  SETD2 - set domain containing 2  RBX1 - ring-box 1, e3 ubiquitin protein ligase  NUP153 - nucleoporin 153kda  THRAP3 - thyroid hormone receptor associated protein 3  MED12 - mediator complex subunit 12  MED13 - mediator complex subunit 13  PSMD6 - proteasome (prosome, macropain) 26s subunit, non-atpase, 6  MED24 - mediator complex subunit 24  ZC3H6 - zinc finger ccch-type containing 6  DTX3L - deltex 3-like (drosophila)  ZNF34 - zinc finger protein 34  SETDB1 - set domain, bifurcated 1  ORC2 - origin recognition complex, subunit 2  ORC5 - origin recognition complex, subunit 5  TOX4 - tox high mobility group box family member 4  ZNF436 - zinc finger protein 436  RNF141 - ring finger protein 141  EPM2AIP1 - epm2a (laforin) interacting protein 1  AGRN - agrin  G3BP2 - gtpase activating protein (sh3 domain) binding protein 2  OGG1 - 8-oxoguanine dna glycosylase  ZSCAN16 - zinc finger and scan domain containing 16  TNKS2 - tankyrase, trf1-interacting ankyrin-related adp-ribose polymerase 2  DDR2 - discoidin domain receptor tyrosine kinase 2  NUMA1 - nuclear mitotic apparatus protein 1  BCLAF1 - bcl2-associated transcription factor 1  DAB2IP - dab2 interacting protein  NUP88 - nucleoporin 88kda  EIF4A3 - eukaryotic translation initiation factor 4a3  NUP98 - nucleoporin 98kda  GMNN - geminin, dna replication inhibitor  NR4A2 - nuclear receptor subfamily 4, group a, member 2  ZNF593 - zinc finger protein 593  ZBTB7B - zinc finger and btb domain containing 7b  MATR3 - matrin 3  NVL - nuclear vcp-like  ZNF691 - zinc finger protein 691  CREBRF - creb3 regulatory factor  ZNF623 - zinc finger protein 623  NPM1 - nucleophosmin (nucleolar phosphoprotein b23, numatrin)  NMD3 - nmd3 ribosome export adaptor  ARHGEF11 - rho guanine nucleotide exchange factor (gef) 11  ZBTB24 - zinc finger and btb domain containing 24  THAP4 - thap domain containing 4  ZEB2 - zinc finger e-box binding homeobox 2  CTIF - cbp80/20-dependent translation initiation factor  TSC22D2 - tsc22 domain family, member 2  NRF1 - nuclear respiratory factor 1  NFXL1 - nuclear transcription factor, x-box binding-like 1  ADIPOR1 - adiponectin receptor 1  DONSON - downstream neighbor of son  NOP2 - nop2 nucleolar protein  NONO - non-pou domain containing, octamer-binding  VPRBP - vpr (hiv-1) binding protein  MDFIC - myod family inhibitor domain containing  IFT74 - intraflagellar transport 74 homolog (chlamydomonas)  NOTCH4 - notch 4  NPAT - nuclear protein, ataxia-telangiectasia locus  CEP290 - centrosomal protein 290kda  CNOT2 - ccr4-not transcription complex, subunit 2  NOS3 - nitric oxide synthase 3 (endothelial cell)  PUM1 - pumilio homolog 1 (drosophila)  NAA15 - n(alpha)-acetyltransferase 15, nata auxiliary subunit  CNOT3 - ccr4-not transcription complex, subunit 3  BAZ2B - bromodomain adjacent to zinc finger domain, 2b  NOTCH1 - notch 1  CTC1 - cts telomere maintenance complex component 1  ST18 - suppression of tumorigenicity 18 (breast carcinoma) (zinc finger protein)  NOTCH3 - notch 3  NOTCH2 - notch 2  NFYA - nuclear transcription factor y, alpha  HDAC4 - histone deacetylase 4  TOX - thymocyte selection-associated high mobility group box  NFYC - nuclear transcription factor y, gamma  ZFYVE16 - zinc finger, fyve domain containing 16  CXXC1 - cxxc finger protein 1  NFKBIA - nuclear factor of kappa light polypeptide gene enhancer in b-cells inhibitor, alpha  NFKB1 - nuclear factor of kappa light polypeptide gene enhancer in b-cells 1  KMT2B - lysine (k)-specific methyltransferase 2b  NFKBIB - nuclear factor of kappa light polypeptide gene enhancer in b-cells inhibitor, beta  NFKBIE - nuclear factor of kappa light polypeptide gene enhancer in b-cells inhibitor, epsilon  NKX2-2 - nk2 homeobox 2  HDAC9 - histone deacetylase 9  ZNF606 - zinc finger protein 606  ZNF614 - zinc finger protein 614  ZFP2 - zfp2 zinc finger protein  NEO1 - neogenin 1  SMARCAL1 - swi/snf related, matrix associated, actin dependent regulator of chromatin, subfamily a-like 1  NEDD8 - neural precursor cell expressed, developmentally down-regulated 8  CTR9 - ctr9, paf1/rna polymerase ii complex component  PPM1F - protein phosphatase, mg2+/mn2+ dependent, 1f  CPEB4 - cytoplasmic polyadenylation element binding protein 4  EPC1 - enhancer of polycomb homolog 1 (drosophila)  NFE2L2 - nuclear factor (erythroid-derived 2)-like 2  MORF4L2 - mortality factor 4 like 2  RASL11A - ras-like, family 11, member a  NFATC4 - nuclear factor of activated t-cells, cytoplasmic, calcineurin-dependent 4  NUP155 - nucleoporin 155kda  NFIA - nuclear factor i/a  CDK5RAP3 - cdk5 regulatory subunit associated protein 3  RAPGEF2 - rap guanine nucleotide exchange factor (gef) 2  NUP93 - nucleoporin 93kda  ZNF746 - zinc finger protein 746  VGLL4 - vestigial like 4 (drosophila)  LRRC14 - leucine rich repeat containing 14  KDM4A - lysine (k)-specific demethylase 4a  PHF14 - phd finger protein 14  RDH10 - retinol dehydrogenase 10 (all-trans)  HP1BP3 - heterochromatin protein 1, binding protein 3  ZNF432 - zinc finger protein 432  SAFB2 - scaffold attachment factor b2  MYEF2 - myelin expression factor 2  LPIN2 - lipin 2 |
| GO:0016573 | histone acetylation | 2.5E-7 | 4.96E-5 | 1.33 (10334,93,6996,84) | [+] Show genes  HAT1 - histone acetyltransferase 1  MSL3 - male-specific lethal 3 homolog (drosophila)  EPC2 - enhancer of polycomb homolog 2 (drosophila)  TADA1 - transcriptional adaptor 1  MCRS1 - microspherule protein 1  MSL2 - male-specific lethal 2 homolog (drosophila)  NAA40 - n(alpha)-acetyltransferase 40, natd catalytic subunit, homolog (s. cerevisiae)  MEAF6 - myst/esa1-associated factor 6  EP300 - e1a binding protein p300  RSF1 - remodeling and spacing factor 1  ING4 - inhibitor of growth family, member 4  DPF2 - d4, zinc and double phd fingers family 2  DR1 - down-regulator of transcription 1, tbp-binding (negative cofactor 2)  CSRP2BP - csrp2 binding protein  KANSL1 - kat8 regulatory nsl complex subunit 1  GTF2B - general transcription factor iib  KAT2A - k(lysine) acetyltransferase 2a  PCGF2 - polycomb group ring finger 2  KMT2A - lysine (k)-specific methyltransferase 2a  APBB1 - amyloid beta (a4) precursor protein-binding, family b, member 1 (fe65)  NCOA3 - nuclear receptor coactivator 3  BRPF3 - bromodomain and phd finger containing, 3  BRD8 - bromodomain containing 8  SPHK2 - sphingosine kinase 2  TADA3 - transcriptional adaptor 3  TAF6L - taf6-like rna polymerase ii, p300/cbp-associated factor (pcaf)-associated factor, 65kda  BRD1 - bromodomain containing 1  LDB1 - lim domain binding 1  CREBBP - creb binding protein  PHF20L1 - phd finger protein 20-like 1  MORF4L1 - mortality factor 4 like 1  ATF2 - activating transcription factor 2  MSL1 - male-specific lethal 1 homolog (drosophila)  KAT2B - k(lysine) acetyltransferase 2b  CLOCK - clock circadian regulator  CRTC2 - creb regulated transcription coactivator 2  KANSL1L - kat8 regulatory nsl complex subunit 1-like  TAF9 - taf9 rna polymerase ii, tata box binding protein (tbp)-associated factor, 32kda  KANSL3 - kat8 regulatory nsl complex subunit 3  BRPF1 - bromodomain and phd finger containing, 1  ACTL6A - actin-like 6a  TRRAP - transformation/transcription domain-associated protein  TAF6 - taf6 rna polymerase ii, tata box binding protein (tbp)-associated factor, 80kda  TADA2A - transcriptional adaptor 2a  ARRB1 - arrestin, beta 1  CHD5 - chromodomain helicase dna binding protein 5  SUPT7L - suppressor of ty 7 (s. cerevisiae)-like  PHF15 - phd finger protein 15  MECP2 - methyl cpg binding protein 2 (rett syndrome)  EPC1 - enhancer of polycomb homolog 1 (drosophila)  MORF4L2 - mortality factor 4 like 2  EP400 - e1a binding protein p400  USP22 - ubiquitin specific peptidase 22  ING5 - inhibitor of growth family, member 5  NAA60 - n(alpha)-acetyltransferase 60, natf catalytic subunit  MCM3AP - minichromosome maintenance complex component 3 associated protein  KAT5 - k(lysine) acetyltransferase 5  YEATS4 - yeats domain containing 4  MAP3K7 - mitogen-activated protein kinase kinase kinase 7  ING3 - inhibitor of growth family, member 3  PHF20 - phd finger protein 20  ACTL6B - actin-like 6b  HCFC1 - host cell factor c1 (vp16-accessory protein)  PHF10 - phd finger protein 10  OGT - o-linked n-acetylglucosamine (glcnac) transferase  ZZZ3 - zinc finger, zz-type containing 3  MBIP - map3k12 binding inhibitory protein 1  KANSL2 - kat8 regulatory nsl complex subunit 2  KAT6A - k(lysine) acetyltransferase 6a  KAT8 - k(lysine) acetyltransferase 8  MRGBP - mrg/morf4l binding protein  PHF14 - phd finger protein 14  RUVBL1 - ruvb-like 1 (e. coli)  PHF17 - phd finger protein 17  DMAP1 - dna methyltransferase 1 associated protein 1  ZMPSTE24 - zinc metallopeptidase ste24  KAT6B - k(lysine) acetyltransferase 6b  LEF1 - lymphoid enhancer-binding factor 1  MBD3 - methyl-cpg binding domain protein 3  GTF3C4 - general transcription factor iiic, polypeptide 4, 90kda  SUPT3H - suppressor of ty 3 homolog (s. cerevisiae)  KAT7 - k(lysine) acetyltransferase 7  YEATS2 - yeats domain containing 2  RUVBL2 - ruvb-like 2 (e. coli) |
| GO:0034660 | ncRNA metabolic process | 2.72E-7 | 5.33E-5 | 1.16 (10334,436,6996,342) | [+] Show genes  CCNK - cyclin k  ELL - elongation factor rna polymerase ii  CDKAL1 - cdk5 regulatory subunit associated protein 1-like 1  NCBP2 - nuclear cap binding protein subunit 2, 20kda  EXOSC9 - exosome component 9  NCBP1 - nuclear cap binding protein subunit 1, 80kda  EXOSC10 - exosome component 10  ZCCHC8 - zinc finger, cchc domain containing 8  MKI67IP - mki67 (fha domain) interacting nucleolar phosphoprotein  AARS - alanyl-trna synthetase  NAF1 - nuclear assembly factor 1 ribonucleoprotein  TRMT1L - trna methyltransferase 1 homolog (s. cerevisiae)-like  ELL2 - elongation factor, rna polymerase ii, 2  TEX10 - testis expressed 10  SNAPC5 - small nuclear rna activating complex, polypeptide 5, 19kda  DTD1 - d-tyrosyl-trna deacylase 1  DROSHA - drosha, ribonuclease type iii  CIRH1A - cirrhosis, autosomal recessive 1a (cirhin)  EBNA1BP2 - ebna1 binding protein 2  NARS - asparaginyl-trna synthetase  NSUN2 - nop2/sun rna methyltransferase family, member 2  FTSJ3 - ftsj homolog 3 (e. coli)  METTL16 - methyltransferase like 16  SP1 - sp1 transcription factor  ALKBH1 - alkb, alkylation repair homolog 1 (e. coli)  RBM7 - rna binding motif protein 7  ELP6 - elongator acetyltransferase complex subunit 6  NOP9 - nop9 nucleolar protein  RPP25L - ribonuclease p/mrp 25kda subunit-like  CHD7 - chromodomain helicase dna binding protein 7  LYAR - ly1 antibody reactive  GTPBP4 - gtp binding protein 4  INTS4 - integrator complex subunit 4  NOC4L - nucleolar complex associated 4 homolog (s. cerevisiae)  FBLL1 - fibrillarin-like 1  SPIN1 - spindlin 1  MPHOSPH6 - m-phase phosphoprotein 6  MPHOSPH10 - m-phase phosphoprotein 10 (u3 small nucleolar ribonucleoprotein)  ZC3H8 - zinc finger ccch-type containing 8  URM1 - ubiquitin related modifier 1  WARS2 - tryptophanyl trna synthetase 2, mitochondrial  PDCD11 - programmed cell death 11  USPL1 - ubiquitin specific peptidase like 1  ABT1 - activator of basal transcription 1  WDR55 - wd repeat domain 55  THUMPD1 - thump domain containing 1  RPRD1B - regulation of nuclear pre-mrna domain containing 1b  LAS1L - las1-like (s. cerevisiae)  EXOSC7 - exosome component 7  ISG20L2 - interferon stimulated exonuclease gene 20kda-like 2  SNAPC4 - small nuclear rna activating complex, polypeptide 4, 190kda  IARS - isoleucyl-trna synthetase  MAK16 - mak16 homolog (s. cerevisiae)  EEF1E1 - eukaryotic translation elongation factor 1 epsilon 1  TRMT10B - trna methyltransferase 10 homolog b (s. cerevisiae)  ADAR - adenosine deaminase, rna-specific  TRIT1 - trna isopentenyltransferase 1  TRMT10A - trna methyltransferase 10 homolog a (s. cerevisiae)  NABP2 - nucleic acid binding protein 2  TSEN34 - tsen34 trna splicing endonuclease subunit  SNAPC2 - small nuclear rna activating complex, polypeptide 2, 45kda  WDR3 - wd repeat domain 3  SNAPC1 - small nuclear rna activating complex, polypeptide 1, 43kda  SNAPC3 - small nuclear rna activating complex, polypeptide 3, 50kda  CPSF4 - cleavage and polyadenylation specific factor 4, 30kda  CPSF1 - cleavage and polyadenylation specific factor 1, 160kda  ERI3 - eri1 exoribonuclease family member 3  PPA1 - pyrophosphatase (inorganic) 1  IMP4 - imp4, u3 small nucleolar ribonucleoprotein, homolog (yeast)  BYSL - bystin-like  POLR2K - polymerase (rna) ii (dna directed) polypeptide k, 7.0kda  TSEN15 - tsen15 trna splicing endonuclease subunit  TYW3 - trna-yw synthesizing protein 3 homolog (s. cerevisiae)  RRP1B - ribosomal rna processing 1b  NOP10 - nop10 ribonucleoprotein  POLR2G - polymerase (rna) ii (dna directed) polypeptide g  NOL10 - nucleolar protein 10  DUS3L - dihydrouridine synthase 3-like (s. cerevisiae)  POLR2F - polymerase (rna) ii (dna directed) polypeptide f  POLR2E - polymerase (rna) ii (dna directed) polypeptide e, 25kda  MARS2 - methionyl-trna synthetase 2, mitochondrial  POLR2D - polymerase (rna) ii (dna directed) polypeptide d  POLR2C - polymerase (rna) ii (dna directed) polypeptide c, 33kda  POLR2B - polymerase (rna) ii (dna directed) polypeptide b, 140kda  POLR2A - polymerase (rna) ii (dna directed) polypeptide a, 220kda  RPP14 - ribonuclease p/mrp 14kda subunit  INTS9 - integrator complex subunit 9  FTSJ1 - ftsj rna methyltransferase homolog 1 (e. coli)  POLR1B - polymerase (rna) i polypeptide b, 128kda  KRR1 - krr1, small subunit (ssu) processome component, homolog (yeast)  REXO4 - rex4, rna exonuclease 4 homolog (s. cerevisiae)  WDR12 - wd repeat domain 12  INTS12 - integrator complex subunit 12  MRTO4 - mrna turnover 4 homolog (s. cerevisiae)  C1D - c1d nuclear receptor corepressor  RPF2 - ribosome production factor 2 homolog (s. cerevisiae)  CSTF2 - cleavage stimulation factor, 3' pre-rna, subunit 2, 64kda  RPL11 - ribosomal protein l11  TRMT12 - trna methyltransferase 12 homolog (s. cerevisiae)  FRG1 - fshd region gene 1  NOL8 - nucleolar protein 8  METTL8 - methyltransferase like 8  RPL7 - ribosomal protein l7  INTS6 - integrator complex subunit 6  GTPBP3 - gtp binding protein 3 (mitochondrial)  AGO1 - argonaute risc catalytic component 1  FARSA - phenylalanyl-trna synthetase, alpha subunit  CARS - cysteinyl-trna synthetase  TRMT11 - trna methyltransferase 11 homolog (s. cerevisiae)  MRPL1 - mitochondrial ribosomal protein l1  UTP3 - utp3, small subunit (ssu) processome component, homolog (s. cerevisiae)  RIOK2 - rio kinase 2  SBDS - shwachman-bodian-diamond syndrome  DIS3L - dis3 mitotic control homolog (s. cerevisiae)-like  DDX52 - dead (asp-glu-ala-asp) box polypeptide 52  NOL6 - nucleolar protein 6 (rna-associated)  DDX51 - dead (asp-glu-ala-asp) box polypeptide 51  FARSB - phenylalanyl-trna synthetase, beta subunit  VARS - valyl-trna synthetase  RBFA - ribosome binding factor a (putative)  MRPL44 - mitochondrial ribosomal protein l44  TFB1M - transcription factor b1, mitochondrial  INTS3 - integrator complex subunit 3  ELP4 - elongator acetyltransferase complex subunit 4  RPAP2 - rna polymerase ii associated protein 2  WDR43 - wd repeat domain 43  RARS2 - arginyl-trna synthetase 2, mitochondrial  CCNT2 - cyclin t2  TDRKH - tudor and kh domain containing  NPM3 - nucleophosmin/nucleoplasmin 3  CCNT1 - cyclin t1  THG1L - trna-histidine guanylyltransferase 1-like (s. cerevisiae)  NARS2 - asparaginyl-trna synthetase 2, mitochondrial (putative)  NOB1 - nin1/rpn12 binding protein 1 homolog (s. cerevisiae)  VARS2 - valyl-trna synthetase 2, mitochondrial  RPS15 - ribosomal protein s15  MRPS11 - mitochondrial ribosomal protein s11  DDX27 - dead (asp-glu-ala-asp) box polypeptide 27  PAPD7 - pap associated domain containing 7  INTS8 - integrator complex subunit 8  MDN1 - mdn1, midasin homolog (yeast)  NSA2 - nsa2 ribosome biogenesis homolog (s. cerevisiae)  TSR1 - tsr1, 20s rrna accumulation, homolog (s. cerevisiae)  GTF3C5 - general transcription factor iiic, polypeptide 5, 63kda  ZNHIT3 - zinc finger, hit-type containing 3  HNRNPA2B1 - heterogeneous nuclear ribonucleoprotein a2/b1  METTL5 - methyltransferase like 5  DDX47 - dead (asp-glu-ala-asp) box polypeptide 47  CLP1 - cleavage and polyadenylation factor i subunit 1  SARS2 - seryl-trna synthetase 2, mitochondrial  SSB - sjogren syndrome antigen b (autoantigen la)  EPRS - glutamyl-prolyl-trna synthetase  TUT1 - terminal uridylyl transferase 1, u6 snrna-specific  TRMT10C - trna methyltransferase 10 homolog c (s. cerevisiae)  THADA - thyroid adenoma associated  GTF3C4 - general transcription factor iiic, polypeptide 4, 90kda  ERCC2 - excision repair cross-complementing rodent repair deficiency, complementation group 2  NABP1 - nucleic acid binding protein 1  NSUN5 - nop2/sun domain family, member 5  MTO1 - mitochondrial trna translation optimization 1  GTF3C3 - general transcription factor iiic, polypeptide 3, 102kda  NSUN3 - nop2/sun domain family, member 3  DEDD2 - death effector domain containing 2  GARS - glycyl-trna synthetase  IKBKAP - inhibitor of kappa light polypeptide gene enhancer in b-cells, kinase complex-associated protein  TSR2 - tsr2, 20s rrna accumulation, homolog (s. cerevisiae)  TCOF1 - treacher collins-franceschetti syndrome 1  HENMT1 - hen1 methyltransferase homolog 1 (arabidopsis)  DDX1 - dead (asp-glu-ala-asp) box helicase 1  DALRD3 - dalr anticodon binding domain containing 3  TBL3 - transducin (beta)-like 3  PARN - poly(a)-specific ribonuclease  DARS2 - aspartyl-trna synthetase 2, mitochondrial  INTS5 - integrator complex subunit 5  ZCCHC6 - zinc finger, cchc domain containing 6  RNF113A - ring finger protein 113a  GTF2F1 - general transcription factor iif, polypeptide 1, 74kda  GTF2F2 - general transcription factor iif, polypeptide 2, 30kda  BOP1 - block of proliferation 1  GTF2E1 - general transcription factor iie, polypeptide 1, alpha 56kda  THUMPD2 - thump domain containing 2  GTF2E2 - general transcription factor iie, polypeptide 2, beta 34kda  RPRD2 - regulation of nuclear pre-mrna domain containing 2  INTS10 - integrator complex subunit 10  NOL11 - nucleolar protein 11  GTF2A2 - general transcription factor iia, 2, 12kda  GTF2B - general transcription factor iib  UTP20 - utp20, small subunit (ssu) processome component, homolog (yeast)  THUMPD3 - thump domain containing 3  WDR46 - wd repeat domain 46  GTF2A1 - general transcription factor iia, 1, 19/37kda  PHAX - phosphorylated adaptor for rna export  USB1 - u6 snrna biogenesis 1  GTF3C2 - general transcription factor iiic, polypeptide 2, beta 110kda  WDR36 - wd repeat domain 36  UTP6 - utp6, small subunit (ssu) processome component, homolog (yeast)  GTF3A - general transcription factor iiia  ADAT2 - adenosine deaminase, trna-specific 2  PA2G4 - proliferation-associated 2g4, 38kda  RPUSD1 - rna pseudouridylate synthase domain containing 1  RPRD1A - regulation of nuclear pre-mrna domain containing 1a  BRF1 - brf1, rna polymerase iii transcription initiation factor 90 kda subunit  NOL9 - nucleolar protein 9  GTF3C1 - general transcription factor iiic, polypeptide 1, alpha 220kda  NOLC1 - nucleolar and coiled-body phosphoprotein 1  EXOSC3 - exosome component 3  GAR1 - gar1 ribonucleoprotein  DCAF13 - ddb1 and cul4 associated factor 13  DDX10 - dead (asp-glu-ala-asp) box polypeptide 10  WDR18 - wd repeat domain 18  WBP11 - ww domain binding protein 11  EXOSC1 - exosome component 1  TAF9 - taf9 rna polymerase ii, tata box binding protein (tbp)-associated factor, 32kda  BMS1 - bms1 ribosome biogenesis factor  CELF3 - cugbp, elav-like family member 3  FAM98B - family with sequence similarity 98, member b  RRP15 - ribosomal rna processing 15 homolog (s. cerevisiae)  TAF6 - taf6 rna polymerase ii, tata box binding protein (tbp)-associated factor, 80kda  PRKRA - protein kinase, interferon-inducible double stranded rna dependent activator  ERI1 - exoribonuclease 1  YARS - tyrosyl-trna synthetase  PELO - pelota homolog (drosophila)  EIF4A3 - eukaryotic translation initiation factor 4a3  ZBTB8OS - zinc finger and btb domain containing 8 opposite strand  TARS - threonyl-trna synthetase  RRP1 - ribosomal rna processing 1  HARS - histidyl-trna synthetase  DDX21 - dead (asp-glu-ala-asp) box helicase 21  KHSRP - kh-type splicing regulatory protein  BCDIN3D - bcdin3 domain containing  TARBP1 - tar (hiv-1) rna binding protein 1  DDX17 - dead (asp-glu-ala-asp) box helicase 17  POLR1E - polymerase (rna) i polypeptide e, 53kda  NVL - nuclear vcp-like  TAF13 - taf13 rna polymerase ii, tata box binding protein (tbp)-associated factor, 18kda  ZCCHC11 - zinc finger, cchc domain containing 11  NOP56 - nop56 ribonucleoprotein  EIF6 - eukaryotic translation initiation factor 6  DARS - aspartyl-trna synthetase  LARS2 - leucyl-trna synthetase 2, mitochondrial  INTS2 - integrator complex subunit 2  WDR75 - wd repeat domain 75  LSM6 - lsm6 homolog, u6 small nuclear rna associated (s. cerevisiae)  XPO5 - exportin 5  CDK9 - cyclin-dependent kinase 9  CDK7 - cyclin-dependent kinase 7  UTP15 - utp15, u3 small nucleolar ribonucleoprotein, homolog (s. cerevisiae)  MTFMT - mitochondrial methionyl-trna formyltransferase  METTL1 - methyltransferase like 1  TARSL2 - threonyl-trna synthetase-like 2  HEATR1 - heat repeat containing 1  FCF1 - fcf1 rrna-processing protein  AARS2 - alanyl-trna synthetase 2, mitochondrial  RPL7L1 - ribosomal protein l7-like 1  TSEN54 - tsen54 trna splicing endonuclease subunit  SARS - seryl-trna synthetase  RPP38 - ribonuclease p/mrp 38kda subunit  NGDN - neuroguidin, eif4e binding protein  MOCS3 - molybdenum cofactor synthesis 3  DIMT1 - dim1 dimethyladenosine transferase 1 homolog (s. cerevisiae)  TRNT1 - trna nucleotidyl transferase, cca-adding, 1  UTP18 - utp18 small subunit (ssu) processome component homolog (yeast)  RRNAD1 - ribosomal rna adenine dimethylase domain containing 1  TOE1 - target of egr1, member 1 (nuclear)  NOP14 - nop14 nucleolar protein  ELP3 - elongator acetyltransferase complex subunit 3  WDR4 - wd repeat domain 4  RRP9 - ribosomal rna processing 9, small subunit (ssu) processome component, homolog (yeast)  NOP2 - nop2 nucleolar protein  ZNHIT6 - zinc finger, hit-type containing 6  METTL6 - methyltransferase like 6  PAPD5 - pap associated domain containing 5  POP4 - processing of precursor 4, ribonuclease p/mrp subunit (s. cerevisiae)  NUDT16 - nudix (nucleoside diphosphate linked moiety x)-type motif 16  LARS - leucyl-trna synthetase  DIS3 - dis3 mitotic control homolog (s. cerevisiae)  PUS3 - pseudouridylate synthase 3  MARS - methionyl-trna synthetase  MAPT - microtubule-associated protein tau  EXOSC2 - exosome component 2  TRMT5 - trna methyltransferase 5  MTERFD2 - mterf domain containing 2  SRFBP1 - serum response factor binding protein 1  SART1 - squamous cell carcinoma antigen recognized by t cells  OSGEPL1 - o-sialoglycoprotein endopeptidase-like 1  QARS - glutaminyl-trna synthetase  METTL2A - methyltransferase like 2a  HARS2 - histidyl-trna synthetase 2, mitochondrial  TRMT112 - trna methyltransferase 11-2 homolog (s. cerevisiae)  RPP40 - ribonuclease p/mrp 40kda subunit  RTCB - rna 2',3'-cyclic phosphate and 5'-oh ligase  NHP2L1 - nhp2 non-histone chromosome protein 2-like 1 (s. cerevisiae)  WARS - tryptophanyl-trna synthetase  DDX56 - dead (asp-glu-ala-asp) box helicase 56  DKC1 - dyskeratosis congenita 1, dyskerin  NAT10 - n-acetyltransferase 10 (gcn5-related)  RPL14 - ribosomal protein l14  RIOK1 - rio kinase 1  DPH3 - diphthamide biosynthesis 3  GTF3C6 - general transcription factor iiic, polypeptide 6, alpha 35kda  SKIV2L2 - superkiller viralicidic activity 2-like 2 (s. cerevisiae)  TYW1 - trna-yw synthesizing protein 1 homolog (s. cerevisiae)  RARS - arginyl-trna synthetase  XRN2 - 5'-3' exoribonuclease 2  ELP2 - elongator acetyltransferase complex subunit 2  PES1 - pescadillo ribosomal biogenesis factor 1  CDK5RAP1 - cdk5 regulatory subunit associated protein 1  TAF8 - taf8 rna polymerase ii, tata box binding protein (tbp)-associated factor, 43kda  DHX37 - deah (asp-glu-ala-his) box polypeptide 37  PSTK - phosphoseryl-trna kinase  DDX49 - dead (asp-glu-ala-asp) box polypeptide 49  PIH1D2 - pih1 domain containing 2  IMP3 - imp3, u3 small nucleolar ribonucleoprotein, homolog (yeast)  ESF1 - esf1, nucleolar pre-rrna processing protein, homolog (s. cerevisiae)  TAF1B - tata box binding protein (tbp)-associated factor, rna polymerase i, b, 63kda  EXOSC8 - exosome component 8  KIAA0391 - kiaa0391  DIS3L2 - dis3 mitotic control homolog (s. cerevisiae)-like 2  SMAD2 - smad family member 2  SRRT - serrate rna effector molecule homolog (arabidopsis)  DIEXF - digestive organ expansion factor homolog (zebrafish)  PELP1 - proline, glutamate and leucine rich protein 1  TRDMT1 - trna aspartic acid methyltransferase 1  TYW5 - trna-yw synthesizing protein 5  MEPCE - methylphosphate capping enzyme  DGCR8 - dgcr8 microprocessor complex subunit  GEMIN4 - gem (nuclear organelle) associated protein 4  SND1 - staphylococcal nuclease and tudor domain containing 1  LARP7 - la ribonucleoprotein domain family, member 7  PUS7 - pseudouridylate synthase 7 homolog (s. cerevisiae)  RPUSD3 - rna pseudouridylate synthase domain containing 3  EXOSC4 - exosome component 4  ANKRD16 - ankyrin repeat domain 16  RSL1D1 - ribosomal l1 domain containing 1  GATC - glutamyl-trna(gln) amidotransferase, subunit c  TARS2 - threonyl-trna synthetase 2, mitochondrial (putative)  GRSF1 - g-rich rna sequence binding factor 1  METTL3 - methyltransferase like 3  INTS1 - integrator complex subunit 1  TRMT6 - trna methyltransferase 6 homolog (s. cerevisiae)  SENP3 - sumo1/sentrin/smt3 specific peptidase 3  QRSL1 - glutaminyl-trna synthase (glutamine-hydrolyzing)-like 1 |
| GO:0051276 | chromosome organization | 3.04E-7 | 5.87E-5 | 1.22 (10334,232,6996,191) | [+] Show genes  APEX1 - apex nuclease (multifunctional dna repair enzyme) 1  DHX9 - deah (asp-glu-ala-his) box helicase 9  SMARCAD1 - swi/snf-related, matrix-associated actin-dependent regulator of chromatin, subfamily a, containing dead/h box 1  DHX36 - deah (asp-glu-ala-his) box polypeptide 36  HAT1 - histone acetyltransferase 1  RFC5 - replication factor c (activator 1) 5, 36.5kda  MSTO1 - misato 1, mitochondrial distribution and morphology regulator  RFC2 - replication factor c (activator 1) 2, 40kda  DDX3X - dead (asp-glu-ala-asp) box helicase 3, x-linked  G3BP1 - gtpase activating protein (sh3 domain) binding protein 1  RFC3 - replication factor c (activator 1) 3, 38kda  SMC6 - structural maintenance of chromosomes 6  DDX1 - dead (asp-glu-ala-asp) box helicase 1  LIG4 - ligase iv, dna, atp-dependent  RFWD3 - ring finger and wd repeat domain 3  TREX1 - three prime repair exonuclease 1  FBXO18 - f-box protein, helicase, 18  CENPV - centromere protein v  GEM - gtp binding protein overexpressed in skeletal muscle  PDS5A - pds5, regulator of cohesion maintenance, homolog a (s. cerevisiae)  DDB1 - damage-specific dna binding protein 1, 127kda  GTF2F2 - general transcription factor iif, polypeptide 2, 30kda  MLH1 - mutl homolog 1, colon cancer, nonpolyposis type 2 (e. coli)  ABL1 - c-abl oncogene 1, non-receptor tyrosine kinase  WRNIP1 - werner helicase interacting protein 1  GTF2H2 - general transcription factor iih, polypeptide 2, 44kda  GTF2H3 - general transcription factor iih, polypeptide 3, 34kda  RECQL - recq protein-like (dna helicase q1-like)  MCM9 - minichromosome maintenance complex component 9  GTF2H1 - general transcription factor iih, polypeptide 1, 62kda  OBFC1 - oligonucleotide/oligosaccharide-binding fold containing 1  DOT1L - dot1-like histone h3k79 methyltransferase  UPF1 - upf1 regulator of nonsense transcripts homolog (yeast)  NBN - nibrin  HDAC8 - histone deacetylase 8  ASCC3 - activating signal cointegrator 1 complex subunit 3  POT1 - protection of telomeres 1  NCAPH2 - non-smc condensin ii complex, subunit h2  SYCE1L - synaptonemal complex central element protein 1-like  RIF1 - rap1 interacting factor homolog (yeast)  RLF - rearranged l-myc fusion  TUBG1 - tubulin, gamma 1  NCAPD3 - non-smc condensin ii complex, subunit d3  GTF2H4 - general transcription factor iih, polypeptide 4, 52kda  SLX4 - slx4 structure-specific endonuclease subunit  CHD1L - chromodomain helicase dna binding protein 1-like  CHD7 - chromodomain helicase dna binding protein 7  COPS7B - cop9 signalosome subunit 7b  COPS3 - cop9 signalosome subunit 3  LDB1 - lim domain binding 1  GAR1 - gar1 ribonucleoprotein  DSCC1 - dna replication and sister chromatid cohesion 1  TERF2IP - telomeric repeat binding factor 2, interacting protein  ORAOV1 - oral cancer overexpressed 1  APITD1 - apoptosis-inducing, taf9-like domain 1  ACIN1 - apoptotic chromatin condensation inducer 1  H3F3A - h3 histone, family 3a  MCM7 - minichromosome maintenance complex component 7  CENPC - centromere protein c  MCMBP - minichromosome maintenance complex binding protein  NSMCE2 - non-smc element 2, mms21 homolog (s. cerevisiae)  CETN2 - centrin, ef-hand protein, 2  MIS12 - mis12 kinetochore complex component  CHD5 - chromodomain helicase dna binding protein 5  BUB3 - bub3 mitotic checkpoint protein  PELO - pelota homolog (drosophila)  TOP3A - topoisomerase (dna) iii alpha  TOP2B - topoisomerase (dna) ii beta 180kda  ZW10 - zw10 kinetochore protein  HUS1 - hus1 checkpoint homolog (s. pombe)  KNSTRN - kinetochore-localized astrin/spag5 binding protein  TOP1 - topoisomerase (dna) i  FEN1 - flap structure-specific endonuclease 1  AXIN2 - axin 2  PCNA - proliferating cell nuclear antigen  SMC1A - structural maintenance of chromosomes 1a  CHMP1A - charged multivesicular body protein 1a  PHF13 - phd finger protein 13  PDS5B - pds5, regulator of cohesion maintenance, homolog b (s. cerevisiae)  SMARCA1 - swi/snf related, matrix associated, actin dependent regulator of chromatin, subfamily a, member 1  RAD50 - rad50 homolog (s. cerevisiae)  PARP1 - poly (adp-ribose) polymerase 1  RUVBL1 - ruvb-like 1 (e. coli)  WRAP53 - wd repeat containing, antisense to tp53  HSP90AA1 - heat shock protein 90kda alpha (cytosolic), class a member 1  NOP10 - nop10 ribonucleoprotein  POLD2 - polymerase (dna directed), delta 2, accessory subunit  POLD1 - polymerase (dna directed), delta 1, catalytic subunit  AKAP8 - a kinase (prka) anchor protein 8  ZMPSTE24 - zinc metallopeptidase ste24  SETX - senataxin  STAG1 - stromal antigen 1  ZNF207 - zinc finger protein 207  RUVBL2 - ruvb-like 2 (e. coli)  CHD9 - chromodomain helicase dna binding protein 9  PURA - purine-rich element binding protein a  TFIP11 - tuftelin interacting protein 11  CHD6 - chromodomain helicase dna binding protein 6  BOD1 - biorientation of chromosomes in cell division 1  XPA - xeroderma pigmentosum, complementation group a  NUP133 - nucleoporin 133kda  ZNF365 - zinc finger protein 365  XPC - xeroderma pigmentosum, complementation group c  HMGB3 - high mobility group box 3  FBXW7 - f-box and wd repeat domain containing 7, e3 ubiquitin protein ligase  HMGB2 - high mobility group box 2  HMGB1 - high mobility group box 1  NUP107 - nucleoporin 107kda  XRCC1 - x-ray repair complementing defective repair in chinese hamster cells 1  SMC3 - structural maintenance of chromosomes 3  PRIM2 - primase, dna, polypeptide 2 (58kda)  PRIM1 - primase, dna, polypeptide 1 (49kda)  SUPV3L1 - suppressor of var1, 3-like 1 (s. cerevisiae)  LATS1 - large tumor suppressor kinase 1  ZWINT - zw10 interacting kinetochore protein  RPA3 - replication protein a3, 14kda  CTC1 - cts telomere maintenance complex component 1  RPA1 - replication protein a1, 70kda  RPA2 - replication protein a2, 32kda  TNKS1BP1 - tankyrase 1 binding protein 1, 182kda  ZBTB48 - zinc finger and btb domain containing 48  CHD4 - chromodomain helicase dna binding protein 4  POGZ - pogo transposable element with znf domain  CHD2 - chromodomain helicase dna binding protein 2  RECQL5 - recq protein-like 5  CHD3 - chromodomain helicase dna binding protein 3  RAD54L2 - rad54-like 2 (s. cerevisiae)  PCID2 - pci domain containing 2  CHD1 - chromodomain helicase dna binding protein 1  CENPT - centromere protein t  XRCC6 - x-ray repair complementing defective repair in chinese hamster cells 6  PPP2R1A - protein phosphatase 2, regulatory subunit a, alpha  SMC5 - structural maintenance of chromosomes 5  ACD - adrenocortical dysplasia homolog (mouse)  MCM3 - minichromosome maintenance complex component 3  MCM6 - minichromosome maintenance complex component 6  TUBG2 - tubulin, gamma 2  RAD21 - rad21 homolog (s. pombe)  MCM5 - minichromosome maintenance complex component 5  MCM2 - minichromosome maintenance complex component 2  RMI2 - recq mediated genome instability 2  INO80 - ino80 complex subunit  SMC4 - structural maintenance of chromosomes 4  ESCO1 - establishment of sister chromatid cohesion n-acetyltransferase 1  IGHMBP2 - immunoglobulin mu binding protein 2  MSH3 - muts homolog 3 (e. coli)  MSH2 - muts homolog 2, colon cancer, nonpolyposis type 1 (e. coli)  DKC1 - dyskeratosis congenita 1, dyskerin  PINX1 - pin2/terf1 interacting, telomerase inhibitor 1  ERCC4 - excision repair cross-complementing rodent repair deficiency, complementation group 4  ERCC3 - excision repair cross-complementing rodent repair deficiency, complementation group 3  NIPBL - nipped-b homolog (drosophila)  SMARCAL1 - swi/snf related, matrix associated, actin dependent regulator of chromatin, subfamily a-like 1  NCAPD2 - non-smc condensin i complex, subunit d2  TERF1 - telomeric repeat binding factor (nima-interacting) 1  TERF2 - telomeric repeat binding factor 2  WRAP73 - wd repeat containing, antisense to tp73  UBB - ubiquitin b  HIRA - histone cell cycle regulator  PAPD7 - pap associated domain containing 7  NUP155 - nucleoporin 155kda  TINF2 - terf1 (trf1)-interacting nuclear factor 2  ATRX - alpha thalassemia/mental retardation syndrome x-linked  ITPA - inosine triphosphatase (nucleoside triphosphate pyrophosphatase)  POLE4 - polymerase (dna-directed), epsilon 4, accessory subunit  MLH3 - mutl homolog 3 (e. coli)  ZFPM1 - zinc finger protein, fog family member 1  SRPK1 - srsf protein kinase 1  HNRNPA2B1 - heterogeneous nuclear ribonucleoprotein a2/b1  ZNF830 - zinc finger protein 830  STAG2 - stromal antigen 2  KLHL22 - kelch-like family member 22  RAE1 - rae1 rna export 1 homolog (s. pombe)  COPS6 - cop9 signalosome subunit 6  KAT6A - k(lysine) acetyltransferase 6a  COPS2 - cop9 signalosome subunit 2  SSBP1 - single-stranded dna binding protein 1, mitochondrial  CHD8 - chromodomain helicase dna binding protein 8  NDC1 - ndc1 transmembrane nucleoporin  ERCC8 - excision repair cross-complementing rodent repair deficiency, complementation group 8  CUL4B - cullin 4b  PTGES3 - prostaglandin e synthase 3 (cytosolic)  RBX1 - ring-box 1, e3 ubiquitin protein ligase  XRCC5 - x-ray repair complementing defective repair in chinese hamster cells 5 (double-strand-break rejoining)  HMGA1 - high mobility group at-hook 1  COPS7A - cop9 signalosome subunit 7a  MRE11A - mre11 meiotic recombination 11 homolog a (s. cerevisiae)  POLD3 - polymerase (dna-directed), delta 3, accessory subunit  H1FX - h1 histone family, member x  ERCC2 - excision repair cross-complementing rodent repair deficiency, complementation group 2  CHTF8 - ctf8, chromosome transmission fidelity factor 8 homolog (s. cerevisiae) |
| GO:0060255 | regulation of macromolecule metabolic process | 5.32E-7 | 1.01E-4 | 1.04 (10334,3770,6996,2664) | [+] Show genes  AGAP2 - arfgap with gtpase domain, ankyrin repeat and ph domain 2  EXOSC9 - exosome component 9  MSL3 - male-specific lethal 3 homolog (drosophila)  EXOSC10 - exosome component 10  TADA1 - transcriptional adaptor 1  HNRNPA0 - heterogeneous nuclear ribonucleoprotein a0  AARS - alanyl-trna synthetase  CBX1 - chromobox homolog 1  PPP1R9A - protein phosphatase 1, regulatory subunit 9a  ABCA2 - atp-binding cassette, sub-family a (abc1), member 2  OS9 - osteosarcoma amplified 9, endoplasmic reticulum lectin  TMED2 - transmembrane emp24 domain trafficking protein 2  ABL1 - c-abl oncogene 1, non-receptor tyrosine kinase  ZNF821 - zinc finger protein 821  ERP29 - endoplasmic reticulum protein 29  PML - promyelocytic leukemia  MLLT11 - myeloid/lymphoid or mixed-lineage leukemia (trithorax homolog, drosophila); translocated to, 11  CNOT11 - ccr4-not transcription complex, subunit 11  SUPT20H - suppressor of ty 20 homolog (s. cerevisiae)  YWHAQ - tyrosine 3-monooxygenase/tryptophan 5-monooxygenase activation protein, theta polypeptide  TMED10 - transmembrane emp24-like trafficking protein 10 (yeast)  PNRC2 - proline-rich nuclear receptor coactivator 2  BRD8 - bromodomain containing 8  ACHE - acetylcholinesterase  RFFL - ring finger and fyve-like domain containing e3 ubiquitin protein ligase  CHD7 - chromodomain helicase dna binding protein 7  LYAR - ly1 antibody reactive  PAPOLA - poly(a) polymerase alpha  ZNF354B - zinc finger protein 354b  TCERG1 - transcription elongation regulator 1  RNPS1 - rna binding protein s1, serine-rich domain  ACTB - actin, beta  OTUB1 - otu domain, ubiquitin aldehyde binding 1  EHMT2 - euchromatic histone-lysine n-methyltransferase 2  PLAUR - plasminogen activator, urokinase receptor  SPIN1 - spindlin 1  TASP1 - taspase, threonine aspartase, 1  PLCL1 - phospholipase c-like 1  PRR5 - proline rich 5 (renal)  SUB1 - sub1 homolog (s. cerevisiae)  KIF16B - kinesin family member 16b  MORF4L1 - mortality factor 4 like 1  ACTG1 - actin, gamma 1  ZNF511 - zinc finger protein 511  ACTG2 - actin, gamma 2, smooth muscle, enteric  CAMK2N1 - calcium/calmodulin-dependent protein kinase ii inhibitor 1  PPID - peptidylprolyl isomerase d  ACTL6A - actin-like 6a  ACTN1 - actinin, alpha 1  AP1AR - adaptor-related protein complex 1 associated regulatory protein  USP19 - ubiquitin specific peptidase 19  ACTN4 - actinin, alpha 4  ACVR2A - activin a receptor, type iia  ACVRL1 - activin a receptor type ii-like 1  PARL - presenilin associated, rhomboid-like  FAM122A - family with sequence similarity 122a  ACVR1 - activin a receptor, type i  DNAJA4 - dnaj (hsp40) homolog, subfamily a, member 4  ACVR1B - activin a receptor, type ib  ADAR - adenosine deaminase, rna-specific  ADARB1 - adenosine deaminase, rna-specific, b1  CPSF4 - cleavage and polyadenylation specific factor 4, 30kda  ADCY6 - adenylate cyclase 6  ZNF331 - zinc finger protein 331  ADCY5 - adenylate cyclase 5  MED1 - mediator complex subunit 1  ADCY1 - adenylate cyclase 1 (brain)  ADCY3 - adenylate cyclase 3  PPARD - peroxisome proliferator-activated receptor delta  MAML3 - mastermind-like 3 (drosophila)  POLR2K - polymerase (rna) ii (dna directed) polypeptide k, 7.0kda  FAM129A - family with sequence similarity 129, member a  PARP1 - poly (adp-ribose) polymerase 1  POLH - polymerase (dna directed), eta  H2AFY2 - h2a histone family, member y2  PPP1R13L - protein phosphatase 1, regulatory subunit 13 like  TNFRSF19 - tumor necrosis factor receptor superfamily, member 19  HES6 - hairy and enhancer of split 6 (drosophila)  POLR2G - polymerase (rna) ii (dna directed) polypeptide g  POLR2F - polymerase (rna) ii (dna directed) polypeptide f  POLR2E - polymerase (rna) ii (dna directed) polypeptide e, 25kda  SMPD3 - sphingomyelin phosphodiesterase 3, neutral membrane (neutral sphingomyelinase ii)  POLR2D - polymerase (rna) ii (dna directed) polypeptide d  POLR2C - polymerase (rna) ii (dna directed) polypeptide c, 33kda  POLR2B - polymerase (rna) ii (dna directed) polypeptide b, 140kda  PARP4 - poly (adp-ribose) polymerase family, member 4  RUVBL2 - ruvb-like 2 (e. coli)  POLR2A - polymerase (rna) ii (dna directed) polypeptide a, 220kda  AP2A1 - adaptor-related protein complex 2, alpha 1 subunit  CDK5RAP2 - cdk5 regulatory subunit associated protein 2  PRKAB1 - protein kinase, amp-activated, beta 1 non-catalytic subunit  PRKACB - protein kinase, camp-dependent, catalytic, beta  RCOR3 - rest corepressor 3  PRKACA - protein kinase, camp-dependent, catalytic, alpha  AES - amino-terminal enhancer of split  NUP133 - nucleoporin 133kda  NUPL2 - nucleoporin like 2  FAM46B - family with sequence similarity 46, member b  VPS35 - vacuolar protein sorting 35 homolog (s. cerevisiae)  JAG1 - jagged 1  EDEM2 - er degradation enhancer, mannosidase alpha-like 2  CHFR - checkpoint with forkhead and ring finger domains, e3 ubiquitin protein ligase  PRDM4 - pr domain containing 4  PPP3R1 - protein phosphatase 3, regulatory subunit b, alpha  ATF7IP - activating transcription factor 7 interacting protein  FGFR1OP - fgfr1 oncogene partner  PPP5C - protein phosphatase 5, catalytic subunit  ZNF689 - zinc finger protein 689  PPP2R5D - protein phosphatase 2, regulatory subunit b', delta  PPP2R5C - protein phosphatase 2, regulatory subunit b', gamma  LRIF1 - ligand dependent nuclear receptor interacting factor 1  PPP2R5B - protein phosphatase 2, regulatory subunit b', beta  WDFY2 - wd repeat and fyve domain containing 2  ZNF653 - zinc finger protein 653  AKT1 - v-akt murine thymoma viral oncogene homolog 1  PPP4C - protein phosphatase 4, catalytic subunit  PCID2 - pci domain containing 2  PPP2R5E - protein phosphatase 2, regulatory subunit b', epsilon isoform  PPP2R2A - protein phosphatase 2, regulatory subunit b, alpha  PPP2R1B - protein phosphatase 2, regulatory subunit a, beta  CPSF6 - cleavage and polyadenylation specific factor 6, 68kda  RIOK2 - rio kinase 2  PPP2R1A - protein phosphatase 2, regulatory subunit a, alpha  NUDT21 - nudix (nucleoside diphosphate linked moiety x)-type motif 21  WWP2 - ww domain containing e3 ubiquitin protein ligase 2  AHR - aryl hydrocarbon receptor  PPP2R2C - protein phosphatase 2, regulatory subunit b, gamma  PPP2R2B - protein phosphatase 2, regulatory subunit b, beta  PPP1R7 - protein phosphatase 1, regulatory subunit 7  ZNF526 - zinc finger protein 526  PPP1R8 - protein phosphatase 1, regulatory subunit 8  FOXP4 - forkhead box p4  PPP2CA - protein phosphatase 2, catalytic subunit, alpha isozyme  PPP2CB - protein phosphatase 2, catalytic subunit, beta isozyme  PPP1R10 - protein phosphatase 1, regulatory subunit 10  DUSP14 - dual specificity phosphatase 14  RMI2 - recq mediated genome instability 2  PPP1R2 - protein phosphatase 1, regulatory (inhibitor) subunit 2  ZNF83 - zinc finger protein 83  PPP1CC - protein phosphatase 1, catalytic subunit, gamma isozyme  ESCO1 - establishment of sister chromatid cohesion n-acetyltransferase 1  KANSL3 - kat8 regulatory nsl complex subunit 3  TDRKH - tudor and kh domain containing  IWS1 - iws1 homolog (s. cerevisiae)  SCYL2 - scy1-like 2 (s. cerevisiae)  PRNP - prion protein  ATF7 - activating transcription factor 7  AMFR - autocrine motility factor receptor, e3 ubiquitin protein ligase  DNAJC3 - dnaj (hsp40) homolog, subfamily c, member 3  PRKRIR - protein-kinase, interferon-inducible double stranded rna dependent inhibitor, repressor of (p58 repressor)  PAPD7 - pap associated domain containing 7  MAP2K5 - mitogen-activated protein kinase kinase 5  MAP2K7 - mitogen-activated protein kinase kinase 7  PIM2 - pim-2 oncogene  ZNF692 - zinc finger protein 692  EIF2AK2 - eukaryotic translation initiation factor 2-alpha kinase 2  MAPK13 - mitogen-activated protein kinase 13  MAP2K1 - mitogen-activated protein kinase kinase 1  EIF4E3 - eukaryotic translation initiation factor 4e family member 3  MAP2K2 - mitogen-activated protein kinase kinase 2  MAPK10 - mitogen-activated protein kinase 10  MAPK9 - mitogen-activated protein kinase 9  MAPK11 - mitogen-activated protein kinase 11  MAPK8 - mitogen-activated protein kinase 8  MAPK7 - mitogen-activated protein kinase 7  GCN1L1 - gcn1 general control of amino-acid synthesis 1-like 1 (yeast)  MAPK3 - mitogen-activated protein kinase 3  CCNI - cyclin i  MAPK1 - mitogen-activated protein kinase 1  OSBPL7 - oxysterol binding protein-like 7  OSBPL8 - oxysterol binding protein-like 8  ADIRF - adipogenesis regulatory factor  NDC1 - ndc1 transmembrane nucleoporin  PRKD1 - protein kinase d1  ANXA3 - annexin a3  CCDC88A - coiled-coil domain containing 88a  NT5C3B - 5'-nucleotidase, cytosolic iiib  PRKCH - protein kinase c, eta  TLK2 - tousled-like kinase 2  PRKCI - protein kinase c, iota  CCDC85B - coiled-coil domain containing 85b  ANXA2 - annexin a2  PRKCE - protein kinase c, epsilon  PRKCB - protein kinase c, beta  PRKCD - protein kinase c, delta  RBM22 - rna binding motif protein 22  PRKAR2B - protein kinase, camp-dependent, regulatory, type ii, beta  PRKCA - protein kinase c, alpha  PRKAR1B - protein kinase, camp-dependent, regulatory, type i, beta  NSUN5 - nop2/sun domain family, member 5  PRKAR1A - protein kinase, camp-dependent, regulatory, type i, alpha  YEATS2 - yeats domain containing 2  PKIB - protein kinase (camp-dependent, catalytic) inhibitor beta  PRKAG1 - protein kinase, amp-activated, gamma 1 non-catalytic subunit  APEX1 - apex nuclease (multifunctional dna repair enzyme) 1  MTPAP - mitochondrial poly(a) polymerase  ZNF605 - zinc finger protein 605  RBM23 - rna binding motif protein 23  XIAP - x-linked inhibitor of apoptosis  BIRC3 - baculoviral iap repeat containing 3  BIRC2 - baculoviral iap repeat containing 2  PARN - poly(a)-specific ribonuclease  KLF12 - kruppel-like factor 12  APLP2 - amyloid beta (a4) precursor-like protein 2  SHQ1 - shq1, h/aca ribonucleoprotein assembly factor  PBX2 - pre-b-cell leukemia homeobox 2  PRMT6 - protein arginine methyltransferase 6  PBX3 - pre-b-cell leukemia homeobox 3  PBX1 - pre-b-cell leukemia homeobox 1  PCBP2 - poly(rc) binding protein 2  PCBD1 - pterin-4 alpha-carbinolamine dehydratase/dimerization cofactor of hepatocyte nuclear factor 1 alpha  APBA1 - amyloid beta (a4) precursor protein-binding, family a, member 1  DCUN1D3 - dcn1, defective in cullin neddylation 1, domain containing 3  APBB2 - amyloid beta (a4) precursor protein-binding, family b, member 2  SMG8 - smg8 nonsense mediated mrna decay factor  APC - adenomatous polyposis coli  APBB1 - amyloid beta (a4) precursor protein-binding, family b, member 1 (fe65)  CHP1 - calcineurin-like ef-hand protein 1  RIF1 - rap1 interacting factor homolog (yeast)  POLG2 - polymerase (dna directed), gamma 2, accessory subunit  RNF139 - ring finger protein 139  PDCD10 - programmed cell death 10  KIAA1551 - kiaa1551  PEBP1 - phosphatidylethanolamine binding protein 1  PA2G4 - proliferation-associated 2g4, 38kda  ARF1 - adp-ribosylation factor 1  RPRD1A - regulation of nuclear pre-mrna domain containing 1a  ARF4 - adp-ribosylation factor 4  ZHX1 - zinc fingers and homeoboxes 1  PAFAH1B1 - platelet-activating factor acetylhydrolase 1b, regulatory subunit 1 (45kda)  SBNO1 - strawberry notch homolog 1 (drosophila)  APOD - apolipoprotein d  IRAK3 - interleukin-1 receptor-associated kinase 3  PRDX1 - peroxiredoxin 1  AKAP13 - a kinase (prka) anchor protein 13  SETD5 - set domain containing 5  APP - amyloid beta (a4) precursor protein  BBS7 - bardet-biedl syndrome 7  DDX20 - dead (asp-glu-ala-asp) box polypeptide 20  PAK2 - p21 protein (cdc42/rac)-activated kinase 2  PAK3 - p21 protein (cdc42/rac)-activated kinase 3  PAM - peptidylglycine alpha-amidating monooxygenase  ARNTL - aryl hydrocarbon receptor nuclear translocator-like  PDGFA - platelet-derived growth factor alpha polypeptide  OXR1 - oxidation resistance 1  CELF3 - cugbp, elav-like family member 3  ARNT - aryl hydrocarbon receptor nuclear translocator  MAP4K5 - mitogen-activated protein kinase kinase kinase kinase 5  SUPT16H - suppressor of ty 16 homolog (s. cerevisiae)  IFT57 - intraflagellar transport 57 homolog (chlamydomonas)  PDGFRB - platelet-derived growth factor receptor, beta polypeptide  ARRB2 - arrestin, beta 2  ARRB1 - arrestin, beta 1  PDGFRA - platelet-derived growth factor receptor, alpha polypeptide  PDGFB - platelet-derived growth factor beta polypeptide  SAMD4B - sterile alpha motif domain containing 4b  PDPK1 - 3-phosphoinositide dependent protein kinase-1  FAM107A - family with sequence similarity 107, member a  RHOA - ras homolog family member a  STRAP - serine/threonine kinase receptor associated protein  PSIP1 - pc4 and sfrs1 interacting protein 1  MED9 - mediator complex subunit 9  FSTL1 - follistatin-like 1  PECAM1 - platelet/endothelial cell adhesion molecule 1  BAZ2A - bromodomain adjacent to zinc finger domain, 2a  WDR70 - wd repeat domain 70  PCNA - proliferating cell nuclear antigen  LSM6 - lsm6 homolog, u6 small nuclear rna associated (s. cerevisiae)  CHMP1A - charged multivesicular body protein 1a  JDP2 - jun dimerization protein 2  MAGOHB - mago-nashi homolog b (drosophila)  CDK16 - cyclin-dependent kinase 16  AKIRIN2 - akirin 2  HEATR1 - heat repeat containing 1  CDK17 - cyclin-dependent kinase 17  LEO1 - leo1, paf1/rna polymerase ii complex component, homolog (s. cerevisiae)  ASPH - aspartate beta-hydroxylase  ERLIN2 - er lipid raft associated 2  PDCD2 - programmed cell death 2  PWP1 - pwp1 homolog (s. cerevisiae)  WRAP53 - wd repeat containing, antisense to tp53  SOCS4 - suppressor of cytokine signaling 4  CDC37 - cell division cycle 37  PDE2A - phosphodiesterase 2a, cgmp-stimulated  GLMN - glomulin, fkbp associated protein  THAP1 - thap domain containing, apoptosis associated protein 1  PKIG - protein kinase (camp-dependent, catalytic) inhibitor gamma  KAT7 - k(lysine) acetyltransferase 7  ASCL1 - achaete-scute complex homolog 1 (drosophila)  ELP3 - elongator acetyltransferase complex subunit 3  RGMB - rgm domain family, member b  PGGT1B - protein geranylgeranyltransferase type i, beta subunit  SBNO2 - strawberry notch homolog 2 (drosophila)  ZNF444 - zinc finger protein 444  ATP2A3 - atpase, ca++ transporting, ubiquitous  ZBTB1 - zinc finger and btb domain containing 1  ZNF365 - zinc finger protein 365  PGAM1 - phosphoglycerate mutase 1 (brain)  RNF180 - ring finger protein 180  PFN2 - profilin 2  FBXW7 - f-box and wd repeat domain containing 7, e3 ubiquitin protein ligase  PGF - placental growth factor  BAHD1 - bromo adjacent homology domain containing 1  ATP1B1 - atpase, na+/k+ transporting, beta 1 polypeptide  DIS3 - dis3 mitotic control homolog (s. cerevisiae)  ZHX2 - zinc fingers and homeoboxes 2  ANKRD6 - ankyrin repeat domain 6  MORC2 - morc family cw-type zinc finger 2  PHF2 - phd finger protein 2  PHF1 - phd finger protein 1  LRRK2 - leucine-rich repeat kinase 2  FOXJ3 - forkhead box j3  RERE - arginine-glutamic acid dipeptide (re) repeats  DNAJC24 - dnaj (hsp40) homolog, subfamily c, member 24  LARP6 - la ribonucleoprotein domain family, member 6  ZFHX3 - zinc finger homeobox 3  SPPL3 - signal peptide peptidase like 3  ATG14 - autophagy related 14  SH3PXD2B - sh3 and px domains 2b  ATP6AP1 - atpase, h+ transporting, lysosomal accessory protein 1  ATP7A - atpase, cu++ transporting, alpha polypeptide  PEX14 - peroxisomal biogenesis factor 14  PPP6R1 - protein phosphatase 6, regulatory subunit 1  ADNP2 - adnp homeobox 2  LMTK2 - lemur tyrosine kinase 2  AEBP2 - ae binding protein 2  ICK - intestinal cell (mak-like) kinase  PPM1E - protein phosphatase, mg2+/mn2+ dependent, 1e  ZNF507 - zinc finger protein 507  IMPACT - impact rwd domain protein  LGR4 - leucine-rich repeat containing g protein-coupled receptor 4  ATP2B4 - atpase, ca++ transporting, plasma membrane 4  ZNF652 - zinc finger protein 652  RELL2 - relt-like 2  BAX - bcl2-associated x protein  SCAF8 - sr-related ctd-associated factor 8  PKD1 - polycystic kidney disease 1 (autosomal dominant)  SMU1 - smu-1 suppressor of mec-8 and unc-52 homolog (c. elegans)  PKD2 - polycystic kidney disease 2 (autosomal dominant)  PUF60 - poly-u binding splicing factor 60kda  MTF2 - metal response element binding transcription factor 2  BAK1 - bcl2-antagonist/killer 1  NAT10 - n-acetyltransferase 10 (gcn5-related)  BACH1 - btb and cnc homology 1, basic leucine zipper transcription factor 1  BAD - bcl2-associated agonist of cell death  PIN1 - peptidylprolyl cis/trans isomerase, nima-interacting 1  PIK3R2 - phosphoinositide-3-kinase, regulatory subunit 2 (beta)  DPH3 - diphthamide biosynthesis 3  PIK3R1 - phosphoinositide-3-kinase, regulatory subunit 1 (alpha)  PLAT - plasminogen activator, tissue  ASXL2 - additional sex combs like 2 (drosophila)  PLAGL1 - pleiomorphic adenoma gene-like 1  XRN2 - 5'-3' exoribonuclease 2  ELP2 - elongator acetyltransferase complex subunit 2  ATRX - alpha thalassemia/mental retardation syndrome x-linked  PEF1 - penta-ef-hand domain containing 1  PKNOX1 - pbx/knotted 1 homeobox 1  CASC3 - cancer susceptibility candidate 3  PKM - pyruvate kinase, muscle  OGFOD1 - 2-oxoglutarate and iron-dependent oxygenase domain containing 1  GABARAPL2 - gaba(a) receptor-associated protein-like 2  EXOSC8 - exosome component 8  SERPINI1 - serpin peptidase inhibitor, clade i (neuroserpin), member 1  TMEM100 - transmembrane protein 100  ATXN7L3B - ataxin 7-like 3b  PHF10 - phd finger protein 10  RABL3 - rab, member of ras oncogene family-like 3  SERPINE2 - serpin peptidase inhibitor, clade e (nexin, plasminogen activator inhibitor type 1), member 2  SERPINB8 - serpin peptidase inhibitor, clade b (ovalbumin), member 8  U2AF2 - u2 small nuclear rna auxiliary factor 2  PSPC1 - paraspeckle component 1  NUDT15 - nudix (nucleoside diphosphate linked moiety x)-type motif 15  PHKG2 - phosphorylase kinase, gamma 2 (testis)  STK38 - serine/threonine kinase 38  BCL7A - b-cell cll/lymphoma 7a  MRGBP - mrg/morf4l binding protein  BCL9 - b-cell cll/lymphoma 9  PIK3CB - phosphatidylinositol-4,5-bisphosphate 3-kinase, catalytic subunit beta  RPUSD3 - rna pseudouridylate synthase domain containing 3  PIK3CA - phosphatidylinositol-4,5-bisphosphate 3-kinase, catalytic subunit alpha  PPP6R3 - protein phosphatase 6, regulatory subunit 3  BRF2 - brf2, rna polymerase iii transcription initiation factor 50 kda subunit  PIM1 - pim-1 oncogene  BCL2 - b-cell cll/lymphoma 2  ECD - ecdysoneless homolog (drosophila)  CCND1 - cyclin d1  PARK7 - parkinson protein 7  BBS4 - bardet-biedl syndrome 4  PNKP - polynucleotide kinase 3'-phosphatase  BBS2 - bardet-biedl syndrome 2  C3orf33 - chromosome 3 open reading frame 33  TRIM27 - tripartite motif containing 27  RFC5 - replication factor c (activator 1) 5, 36.5kda  RFC2 - replication factor c (activator 1) 2, 40kda  SAP18 - sin3a-associated protein, 18kda  RFC3 - replication factor c (activator 1) 3, 38kda  TSHZ2 - teashirt zinc finger homeobox 2  SEMA4C - sema domain, immunoglobulin domain (ig), transmembrane domain (tm) and short cytoplasmic domain, (semaphorin) 4c  RFXAP - regulatory factor x-associated protein  BRAF - v-raf murine sarcoma viral oncogene homolog b  RFX5 - regulatory factor x, 5 (influences hla class ii expression)  WHSC1L1 - wolf-hirschhorn syndrome candidate 1-like 1  EIF1B - eukaryotic translation initiation factor 1b  RFX1 - regulatory factor x, 1 (influences hla class ii expression)  APBB3 - amyloid beta (a4) precursor protein-binding, family b, member 3  RELA - v-rel avian reticuloendotheliosis viral oncogene homolog a  BCOR - bcl6 corepressor  ZFP36L2 - zfp36 ring finger protein-like 2  PAK4 - p21 protein (cdc42/rac)-activated kinase 4  ZFP36L1 - zfp36 ring finger protein-like 1  RDX - radixin  RET - ret proto-oncogene  DUSP15 - dual specificity phosphatase 15  SH3RF3 - sh3 domain containing ring finger 3  ALKBH5 - alkb, alkylation repair homolog 5 (e. coli)  DPF2 - d4, zinc and double phd fingers family 2  NSUN2 - nop2/sun rna methyltransferase family, member 2  UPF1 - upf1 regulator of nonsense transcripts homolog (yeast)  CC2D1A - coiled-coil and c2 domain containing 1a  RLF - rearranged l-myc fusion  RING1 - ring finger protein 1  CNPY2 - canopy fgf signaling regulator 2  BMI1 - bmi1 polycomb ring finger oncogene  PCGF3 - polycomb group ring finger 3  RGS4 - regulator of g-protein signaling 4  RGS3 - regulator of g-protein signaling 3  BMPR1A - bone morphogenetic protein receptor, type ia  BMPR2 - bone morphogenetic protein receptor, type ii (serine/threonine kinase)  BMPR1B - bone morphogenetic protein receptor, type ib  GON4L - gon-4-like (c. elegans)  BMP7 - bone morphogenetic protein 7  ABCA7 - atp-binding cassette, sub-family a (abc1), member 7  PSMD14 - proteasome (prosome, macropain) 26s subunit, non-atpase, 14  RNF19B - ring finger protein 19b  FLOT1 - flotillin 1  CTDSPL - ctd (carboxy-terminal domain, rna polymerase ii, polypeptide a) small phosphatase-like  BCAS3 - breast carcinoma amplified sequence 3  ABCE1 - atp-binding cassette, sub-family e (oabp), member 1  LDLRAD4 - low density lipoprotein receptor class a domain containing 4  PTTG1IP - pituitary tumor-transforming 1 interacting protein  COQ7 - coenzyme q7 homolog, ubiquinone (yeast)  UHMK1 - u2af homology motif (uhm) kinase 1  AHI1 - abelson helper integration site 1  RNF4 - ring finger protein 4  BRD2 - bromodomain containing 2  RNF2 - ring finger protein 2  HNRNPR - heterogeneous nuclear ribonucleoprotein r  ZNF280D - zinc finger protein 280d  GATAD2A - gata zinc finger domain containing 2a  RNF6 - ring finger protein (c3h2c3 type) 6  ROBO1 - roundabout, axon guidance receptor, homolog 1 (drosophila)  ROCK1 - rho-associated, coiled-coil containing protein kinase 1  ROM1 - retinal outer segment membrane protein 1  RNF111 - ring finger protein 111  TSPO - translocator protein (18kda)  SPRY1 - sprouty homolog 1, antagonist of fgf signaling (drosophila)  TET2 - tet methylcytosine dioxygenase 2  SPRY2 - sprouty homolog 2 (drosophila)  MED18 - mediator complex subunit 18  DENND4A - denn/madd domain containing 4a  MBTD1 - mbt domain containing 1  SF3B4 - splicing factor 3b, subunit 4, 49kda  RAMP2 - receptor (g protein-coupled) activity modifying protein 2  RAMP3 - receptor (g protein-coupled) activity modifying protein 3  RAMP1 - receptor (g protein-coupled) activity modifying protein 1  AKAP8 - a kinase (prka) anchor protein 8  ZMPSTE24 - zinc metallopeptidase ste24  STUB1 - stip1 homology and u-box containing protein 1, e3 ubiquitin protein ligase  C5AR1 - complement component 5a receptor 1  TRIM44 - tripartite motif containing 44  TAB1 - tgf-beta activated kinase 1/map3k7 binding protein 1  PPIE - peptidylprolyl isomerase e (cyclophilin e)  MCRS1 - microspherule protein 1  ZER1 - zyg-11 related, cell cycle regulator  NRDE2 - nrde-2, necessary for rna interference, domain containing  RPL17 - ribosomal protein l17  TIMM17A - translocase of inner mitochondrial membrane 17 homolog a (yeast)  CAMLG - calcium modulating ligand  RPL15 - ribosomal protein l15  OLFM1 - olfactomedin 1  C1D - c1d nuclear receptor corepressor  RPL11 - ribosomal protein l11  PTCD3 - pentatricopeptide repeat domain 3  RPL9 - ribosomal protein l9  CASP8 - caspase 8, apoptosis-related cysteine peptidase  RPL7 - ribosomal protein l7  CASP9 - caspase 9, apoptosis-related cysteine peptidase  PIBF1 - progesterone immunomodulatory binding factor 1  SLC30A9 - solute carrier family 30 (zinc transporter), member 9  CASP2 - caspase 2, apoptosis-related cysteine peptidase  RPL3 - ribosomal protein l3  CASP3 - caspase 3, apoptosis-related cysteine peptidase  MAD2L2 - mad2 mitotic arrest deficient-like 2 (yeast)  PID1 - phosphotyrosine interaction domain containing 1  PHIP - pleckstrin homology domain interacting protein  RPA2 - replication protein a2, 32kda  RPS4X - ribosomal protein s4, x-linked  RPS4Y1 - ribosomal protein s4, y-linked 1  PPP2R3C - protein phosphatase 2, regulatory subunit b'', gamma  NRARP - notch-regulated ankyrin repeat protein  ZNF787 - zinc finger protein 787  RMND1 - required for meiotic nuclear division 1 homolog (s. cerevisiae)  LAMTOR1 - late endosomal/lysosomal adaptor, mapk and mtor activator 1  TRIM38 - tripartite motif containing 38  TADA3 - transcriptional adaptor 3  CALR - calreticulin  ZNF792 - zinc finger protein 792  CALM2 - calmodulin 2 (phosphorylase kinase, delta)  WTIP - wilms tumor 1 interacting protein  NCOA2 - nuclear receptor coactivator 2  SYNCRIP - synaptotagmin binding, cytoplasmic rna interacting protein  RPL32 - ribosomal protein l32  STK25 - serine/threonine kinase 25  CREB3 - camp responsive element binding protein 3  CRTAP - cartilage associated protein  CITED2 - cbp/p300-interacting transactivator, with glu/asp-rich carboxy-terminal domain, 2  RPS23 - ribosomal protein s23  CCT6A - chaperonin containing tcp1, subunit 6a (zeta 1)  CCNT2 - cyclin t2  PINX1 - pin2/terf1 interacting, telomerase inhibitor 1  CLN6 - ceroid-lipofuscinosis, neuronal 6, late infantile, variant  CCND3 - cyclin d3  CCNT1 - cyclin t1  HMG20A - high mobility group 20a  CCNH - cyclin h  CCNG2 - cyclin g2  BANP - btg3 associated nuclear protein  RPS15 - ribosomal protein s15  TIPIN - timeless interacting protein  RPS12 - ribosomal protein s12  ACER2 - alkaline ceramidase 2  DLC1 - deleted in liver cancer 1  TRNAU1AP - trna selenocysteine 1 associated protein 1  RPS6KA1 - ribosomal protein s6 kinase, 90kda, polypeptide 1  PARP16 - poly (adp-ribose) polymerase family, member 16  RPS6KB1 - ribosomal protein s6 kinase, 70kda, polypeptide 1  RPS6KB2 - ribosomal protein s6 kinase, 70kda, polypeptide 2  CBFA2T3 - core-binding factor, runt domain, alpha subunit 2; translocated to, 3  CBFB - core-binding factor, beta subunit  RAPGEF3 - rap guanine nucleotide exchange factor (gef) 3  CBL - cbl proto-oncogene, e3 ubiquitin protein ligase  SERPINH1 - serpin peptidase inhibitor, clade h (heat shock protein 47), member 1, (collagen binding protein 1)  RXRB - retinoid x receptor, beta  CAV2 - caveolin 2  PIAS3 - protein inhibitor of activated stat, 3  CAV1 - caveolin 1, caveolae protein, 22kda  ANKRD13B - ankyrin repeat domain 13b  CCNC - cyclin c  RBM14 - rna binding motif protein 14  CCND2 - cyclin d2  TRMT10C - trna methyltransferase 10 homolog c (s. cerevisiae)  SPON1 - spondin 1, extracellular matrix protein  PRMT5 - protein arginine methyltransferase 5  RRAS - related ras viral (r-ras) oncogene homolog  CHTF8 - ctf8, chromosome transmission fidelity factor 8 homolog (s. cerevisiae)  CDK11B - cyclin-dependent kinase 11b  CDC5L - cell division cycle 5-like  MXD4 - max dimerization protein 4  HTRA1 - htra serine peptidase 1  PSAP - prosaposin  CDH3 - cadherin 3, type 1, p-cadherin (placental)  POLR3F - polymerase (rna) iii (dna directed) polypeptide f, 39 kda  CDH2 - cadherin 2, type 1, n-cadherin (neuronal)  HEXIM1 - hexamethylene bis-acetamide inducible 1  ERLIN1 - er lipid raft associated 1  SPIN2A - spindlin family, member 2a  ANKIB1 - ankyrin repeat and ibr domain containing 1  NCOA7 - nuclear receptor coactivator 7  POMT1 - protein-o-mannosyltransferase 1  PSEN1 - presenilin 1  TOLLIP - toll interacting protein  USP16 - ubiquitin specific peptidase 16  NLE1 - notchless homolog 1 (drosophila)  PSMA1 - proteasome (prosome, macropain) subunit, alpha type, 1  SRSF12 - serine/arginine-rich splicing factor 12  PSMA2 - proteasome (prosome, macropain) subunit, alpha type, 2  CD81 - cd81 molecule  CAMKK2 - calcium/calmodulin-dependent protein kinase kinase 2, beta  PSMB1 - proteasome (prosome, macropain) subunit, beta type, 1  PSMA5 - proteasome (prosome, macropain) subunit, alpha type, 5  SCARB2 - scavenger receptor class b, member 2  PSMA3 - proteasome (prosome, macropain) subunit, alpha type, 3  PSMB10 - proteasome (prosome, macropain) subunit, beta type, 10  KHDRBS3 - kh domain containing, rna binding, signal transduction associated 3  RIPPLY2 - ripply transcriptional repressor 2  CD44 - cd44 molecule (indian blood group)  SPINT2 - serine peptidase inhibitor, kunitz type, 2  PSMC6 - proteasome (prosome, macropain) 26s subunit, atpase, 6  TAF6L - taf6-like rna polymerase ii, p300/cbp-associated factor (pcaf)-associated factor, 65kda  PSMD1 - proteasome (prosome, macropain) 26s subunit, non-atpase, 1  PSMC4 - proteasome (prosome, macropain) 26s subunit, atpase, 4  SOX18 - sry (sex determining region y)-box 18  POLR3G - polymerase (rna) iii (dna directed) polypeptide g (32kd)  PSMC2 - proteasome (prosome, macropain) 26s subunit, atpase, 2  PSMD9 - proteasome (prosome, macropain) 26s subunit, non-atpase, 9  PSMD10 - proteasome (prosome, macropain) 26s subunit, non-atpase, 10  CD34 - cd34 molecule  PSMD7 - proteasome (prosome, macropain) 26s subunit, non-atpase, 7  DNAJC10 - dnaj (hsp40) homolog, subfamily c, member 10  PSMD8 - proteasome (prosome, macropain) 26s subunit, non-atpase, 8  PSMD5 - proteasome (prosome, macropain) 26s subunit, non-atpase, 5  TERF2IP - telomeric repeat binding factor 2, interacting protein  PSMD2 - proteasome (prosome, macropain) 26s subunit, non-atpase, 2  SLC38A2 - solute carrier family 38, member 2  PSMD12 - proteasome (prosome, macropain) 26s subunit, non-atpase, 12  PSMD13 - proteasome (prosome, macropain) 26s subunit, non-atpase, 13  ATG7 - autophagy related 7  PRRC1 - proline-rich coiled-coil 1  PTBP1 - polypyrimidine tract binding protein 1  PTEN - phosphatase and tensin homolog  LAMTOR5 - late endosomal/lysosomal adaptor, mapk and mtor activator 5  ANP32B - acidic (leucine-rich) nuclear phosphoprotein 32 family, member b  JMY - junction mediating and regulatory protein, p53 cofactor  MYBBP1A - myb binding protein (p160) 1a  FBLN5 - fibulin 5  PTGIS - prostaglandin i2 (prostacyclin) synthase  CIB1 - calcium and integrin binding 1 (calmyrin)  DDX17 - dead (asp-glu-ala-asp) box helicase 17  KAT5 - k(lysine) acetyltransferase 5  ZFP69 - zfp69 zinc finger protein  PTK2 - protein tyrosine kinase 2  IPO8 - importin 8  IPO7 - importin 7  CEBPG - ccaat/enhancer binding protein (c/ebp), gamma  CDK5 - cyclin-dependent kinase 5  CDK4 - cyclin-dependent kinase 4  TWF1 - twinfilin actin-binding protein 1  PTN - pleiotrophin  CCT7 - chaperonin containing tcp1, subunit 7 (eta)  CDK9 - cyclin-dependent kinase 9  CDK8 - cyclin-dependent kinase 8  LIN54 - lin-54 homolog (c. elegans)  CDK7 - cyclin-dependent kinase 7  CDKN1C - cyclin-dependent kinase inhibitor 1c (p57, kip2)  SPOPL - speckle-type poz protein-like  CCT2 - chaperonin containing tcp1, subunit 2 (beta)  CDKN1B - cyclin-dependent kinase inhibitor 1b (p27, kip1)  CCT4 - chaperonin containing tcp1, subunit 4 (delta)  PCBP3 - poly(rc) binding protein 3  PTPN3 - protein tyrosine phosphatase, non-receptor type 3  CDKN2D - cyclin-dependent kinase inhibitor 2d (p19, inhibits cdk4)  PTPN2 - protein tyrosine phosphatase, non-receptor type 2  PTPN1 - protein tyrosine phosphatase, non-receptor type 1  PTPN11 - protein tyrosine phosphatase, non-receptor type 11  HTATIP2 - hiv-1 tat interactive protein 2, 30kda  ARL6IP5 - adp-ribosylation-like factor 6 interacting protein 5  PTPN13 - protein tyrosine phosphatase, non-receptor type 13 (apo-1/cd95 (fas)-associated phosphatase)  ARFGEF1 - adp-ribosylation factor guanine nucleotide-exchange factor 1 (brefeldin a-inhibited)  CDK2 - cyclin-dependent kinase 2  PURA - purine-rich element binding protein a  RRN3 - rrn3 rna polymerase i transcription factor homolog (s. cerevisiae)  PURB - purine-rich element binding protein b  GTPBP2 - gtp binding protein 2  NUDT16 - nudix (nucleoside diphosphate linked moiety x)-type motif 16  PTPRR - protein tyrosine phosphatase, receptor type, r  ZMYND11 - zinc finger, mynd-type containing 11  PTPRK - protein tyrosine phosphatase, receptor type, k  SRSF10 - serine/arginine-rich splicing factor 10  PTPRN - protein tyrosine phosphatase, receptor type, n  RELL1 - relt-like 1  PLK2 - polo-like kinase 2  AHCYL1 - adenosylhomocysteinase-like 1  PEX2 - peroxisomal biogenesis factor 2  NUP50 - nucleoporin 50kda  MPHOSPH8 - m-phase phosphoprotein 8  KDM5B - lysine (k)-specific demethylase 5b  PXN - paxillin  GIPC1 - gipc pdz domain containing family, member 1  MAP4K2 - mitogen-activated protein kinase kinase kinase kinase 2  CHD4 - chromodomain helicase dna binding protein 4  CHD2 - chromodomain helicase dna binding protein 2  CHD3 - chromodomain helicase dna binding protein 3  FRS2 - fibroblast growth factor receptor substrate 2  CHD1 - chromodomain helicase dna binding protein 1  MTERFD2 - mterf domain containing 2  RAB1A - rab1a, member ras oncogene family  QARS - glutaminyl-trna synthetase  HSPH1 - heat shock 105kda/110kda protein 1  MYPOP - myb-related transcription factor, partner of profilin  RAD21 - rad21 homolog (s. pombe)  RAD17 - rad17 homolog (s. pombe)  SEC24A - sec24 family, member a (s. cerevisiae)  PARP14 - poly (adp-ribose) polymerase family, member 14  PAF1 - paf1, rna polymerase ii associated factor, homolog (s. cerevisiae)  ZNF181 - zinc finger protein 181  RAC1 - ras-related c3 botulinum toxin substrate 1 (rho family, small gtp binding protein rac1)  ZNF268 - zinc finger protein 268  INO80 - ino80 complex subunit  ZNF513 - zinc finger protein 513  NDFIP2 - nedd4 family interacting protein 2  RAB27A - rab27a, member ras oncogene family  RALB - v-ral simian leukemia viral oncogene homolog b  RC3H2 - ring finger and ccch-type domains 2  GMEB1 - glucocorticoid modulatory element binding protein 1  RANBP2 - ran binding protein 2  CCT8 - chaperonin containing tcp1, subunit 8 (theta)  RAP1A - rap1a, member of ras oncogene family  CLTC - clathrin, heavy chain (hc)  MIER2 - mesoderm induction early response 1, family member 2  MOK - mok protein kinase  FAM35A - family with sequence similarity 35, member a  RARG - retinoic acid receptor, gamma  RARB - retinoic acid receptor, beta  DLL4 - delta-like 4 (drosophila)  RASA1 - ras p21 protein activator (gtpase activating protein) 1  NECAB2 - n-terminal ef-hand calcium binding protein 2  RAP1B - rap1b, member of ras oncogene family  CELF1 - cugbp, elav-like family member 1  CLU - clusterin  KHDRBS1 - kh domain containing, rna binding, signal transduction associated 1  GNL3L - guanine nucleotide binding protein-like 3 (nucleolar)-like  TAF8 - taf8 rna polymerase ii, tata box binding protein (tbp)-associated factor, 43kda  CLK1 - cdc-like kinase 1  MAGEL2 - mage-like 2  CELF2 - cugbp, elav-like family member 2  RAP2B - rap2b, member of ras oncogene family  RAP2A - rap2a, member of ras oncogene family  CLK2 - cdc-like kinase 2  DDX49 - dead (asp-glu-ala-asp) box polypeptide 49  CTCF - ccctc-binding factor (zinc finger protein)  CLK3 - cdc-like kinase 3  PRMT7 - protein arginine methyltransferase 7  RBL2 - retinoblastoma-like 2 (p130)  RAI1 - retinoic acid induced 1  RBBP7 - retinoblastoma binding protein 7  RBBP8 - retinoblastoma binding protein 8  MAP3K2 - mitogen-activated protein kinase kinase kinase 2  RBM3 - rna binding motif (rnp1, rrm) protein 3  DIS3L2 - dis3 mitotic control homolog (s. cerevisiae)-like 2  RBM4 - rna binding motif protein 4  RB1 - retinoblastoma 1  ARID4A - at rich interactive domain 4a (rbp1-like)  ANKRD54 - ankyrin repeat domain 54  RBBP6 - retinoblastoma binding protein 6  KDM5A - lysine (k)-specific demethylase 5a  RBBP4 - retinoblastoma binding protein 4  NUP35 - nucleoporin 35kda  PUS7 - pseudouridylate synthase 7 homolog (s. cerevisiae)  ERCC8 - excision repair cross-complementing rodent repair deficiency, complementation group 8  EXOSC4 - exosome component 4  ANKRD13D - ankyrin repeat domain 13 family, member d  PTGES3 - prostaglandin e synthase 3 (cytosolic)  CKS1B - cdc28 protein kinase regulatory subunit 1b  NFAT5 - nuclear factor of activated t-cells 5, tonicity-responsive  CIRBP - cold inducible rna binding protein  DHX29 - deah (asp-glu-ala-his) box polypeptide 29  CHUK - conserved helix-loop-helix ubiquitous kinase  CERS1 - ceramide synthase 1  ZMYND8 - zinc finger, mynd-type containing 8  ANKRA2 - ankyrin repeat, family a (rfxank-like), 2  RBAK - rb-associated krab zinc finger  NFATC2IP - nuclear factor of activated t-cells, cytoplasmic, calcineurin-dependent 2 interacting protein  ATAD1 - atpase family, aaa domain containing 1  SPTBN4 - spectrin, beta, non-erythrocytic 4  PATZ1 - poz (btb) and at hook containing zinc finger 1  FBXO18 - f-box protein, helicase, 18  SPOCK1 - sparc/osteonectin, cwcv and kazal-like domains proteoglycan (testican) 1  METTL14 - methyltransferase like 14  SPP1 - secreted phosphoprotein 1  CORO1C - coronin, actin binding protein, 1c  SSBP2 - single-stranded dna binding protein 2  KPNA6 - karyopherin alpha 6 (importin alpha 7)  FIZ1 - flt3-interacting zinc finger 1  CIRH1A - cirrhosis, autosomal recessive 1a (cirhin)  MIER1 - mesoderm induction early response 1, transcriptional regulator  PPP1R15B - protein phosphatase 1, regulatory subunit 15b  CWC22 - cwc22 spliceosome-associated protein homolog (s. cerevisiae)  ZNF382 - zinc finger protein 382  ZNF705A - zinc finger protein 705a  SP3 - sp3 transcription factor  SP4 - sp4 transcription factor  SP1 - sp1 transcription factor  ZNF410 - zinc finger protein 410  SP2 - sp2 transcription factor  ARL2BP - adp-ribosylation factor-like 2 binding protein  SOX4 - sry (sex determining region y)-box 4  TIPRL - tip41, tor signaling pathway regulator-like (s. cerevisiae)  GTPBP4 - gtp binding protein 4  LSM2 - lsm2 homolog, u6 small nuclear rna associated (s. cerevisiae)  WBP2 - ww domain binding protein 2  FBXL20 - f-box and leucine-rich repeat protein 20  ORC3 - origin recognition complex, subunit 3  SON - son dna binding protein  MAGI3 - membrane associated guanylate kinase, ww and pdz domain containing 3  HES4 - hairy and enhancer of split 4 (drosophila)  LEMD3 - lem domain containing 3  GATAD1 - gata zinc finger domain containing 1  CREBL2 - camp responsive element binding protein-like 2  ATF6B - activating transcription factor 6 beta  SOAT1 - sterol o-acyltransferase 1  CREBBP - creb binding protein  ATF2 - activating transcription factor 2  CREB1 - camp responsive element binding protein 1  DDX58 - dead (asp-glu-ala-asp) box polypeptide 58  SNX1 - sorting nexin 1  CRABP2 - cellular retinoic acid binding protein 2  RPRD1B - regulation of nuclear pre-mrna domain containing 1b  PLK3 - polo-like kinase 3  PPP1R16A - protein phosphatase 1, regulatory subunit 16a  SNAPC4 - small nuclear rna activating complex, polypeptide 4, 190kda  SNCA - synuclein, alpha (non a4 component of amyloid precursor)  SNRNP70 - small nuclear ribonucleoprotein 70kda (u1)  SNRPA - small nuclear ribonucleoprotein polypeptide a  ZBED5 - zinc finger, bed-type containing 5  CREBZF - creb/atf bzip transcription factor  SUMO2 - small ubiquitin-like modifier 2  EID1 - ep300 interacting inhibitor of differentiation 1  TP53INP2 - tumor protein p53 inducible nuclear protein 2  PTBP2 - polypyrimidine tract binding protein 2  SMARCE1 - swi/snf related, matrix associated, actin dependent regulator of chromatin, subfamily e, member 1  RBM17 - rna binding motif protein 17  SMO - smoothened, frizzled family receptor  SMPD1 - sphingomyelin phosphodiesterase 1, acid lysosomal  CNTN1 - contactin 1  SMARCB1 - swi/snf related, matrix associated, actin dependent regulator of chromatin, subfamily b, member 1  SMARCC1 - swi/snf related, matrix associated, actin dependent regulator of chromatin, subfamily c, member 1  HLTF - helicase-like transcription factor  SNX6 - sorting nexin 6  SMARCA4 - swi/snf related, matrix associated, actin dependent regulator of chromatin, subfamily a, member 4  SSBP3 - single stranded dna binding protein 3  SMARCD2 - swi/snf related, matrix associated, actin dependent regulator of chromatin, subfamily d, member 2  LSM5 - lsm5 homolog, u6 small nuclear rna associated (s. cerevisiae)  SMARCD3 - swi/snf related, matrix associated, actin dependent regulator of chromatin, subfamily d, member 3  SMARCC2 - swi/snf related, matrix associated, actin dependent regulator of chromatin, subfamily c, member 2  SMARCD1 - swi/snf related, matrix associated, actin dependent regulator of chromatin, subfamily d, member 1  SLIT3 - slit homolog 3 (drosophila)  WIZ - widely interspaced zinc finger motifs  RBM25 - rna binding motif protein 25  HSPBP1 - hspa (heat shock 70kda) binding protein, cytoplasmic cochaperone 1  SMARCA1 - swi/snf related, matrix associated, actin dependent regulator of chromatin, subfamily a, member 1  SMARCA2 - swi/snf related, matrix associated, actin dependent regulator of chromatin, subfamily a, member 2  EDC4 - enhancer of mrna decapping 4  SGK3 - serum/glucocorticoid regulated kinase family, member 3  CACTIN - cactin, spliceosome c complex subunit  KMT2C - lysine (k)-specific methyltransferase 2c  ZNF462 - zinc finger protein 462  PPIL4 - peptidylprolyl isomerase (cyclophilin)-like 4  TMEFF2 - transmembrane protein with egf-like and two follistatin-like domains 2  TFIP11 - tuftelin interacting protein 11  SENP2 - sumo1/sentrin/smt3 specific peptidase 2  CSTB - cystatin b (stefin b)  CST3 - cystatin c  SHC2 - shc (src homology 2 domain containing) transforming protein 2  ZNF318 - zinc finger protein 318  PRPF6 - pre-mrna processing factor 6  KDM2B - lysine (k)-specific demethylase 2b  SUPT6H - suppressor of ty 6 homolog (s. cerevisiae)  CTGF - connective tissue growth factor  SUPV3L1 - suppressor of var1, 3-like 1 (s. cerevisiae)  CBY1 - chibby homolog 1 (drosophila)  CACNG7 - calcium channel, voltage-dependent, gamma subunit 7  DSTYK - dual serine/threonine and tyrosine protein kinase  CTBP1 - c-terminal binding protein 1  SUPT4H1 - suppressor of ty 4 homolog 1 (s. cerevisiae)  CTBP2 - c-terminal binding protein 2  SUPT5H - suppressor of ty 5 homolog (s. cerevisiae)  MFSD8 - major facilitator superfamily domain containing 8  RAB3GAP2 - rab3 gtpase activating protein subunit 2 (non-catalytic)  CTNNB1 - catenin (cadherin-associated protein), beta 1, 88kda  STX5 - syntaxin 5  SAV1 - salvador homolog 1 (drosophila)  CTSD - cathepsin d  CUX1 - cut-like homeobox 1  NIF3L1 - nif3 ngg1 interacting factor 3-like 1 (s. cerevisiae)  NEK4 - nima-related kinase 4  STK3 - serine/threonine kinase 3  MPV17L2 - mpv17 mitochondrial membrane protein-like 2  PCGF1 - polycomb group ring finger 1  NPNT - nephronectin  FKBP8 - fk506 binding protein 8, 38kda  GPBP1L1 - gc-rich promoter binding protein 1-like 1  MTCH1 - mitochondrial carrier 1  PAN3 - pan3 poly(a) specific ribonuclease subunit homolog (s. cerevisiae)  CYLD - cylindromatosis (turban tumor syndrome)  SUV420H2 - suppressor of variegation 4-20 homolog 2 (drosophila)  TGIF2 - tgfb-induced factor homeobox 2  CNEP1R1 - ctd nuclear envelope phosphatase 1 regulatory subunit 1  STK4 - serine/threonine kinase 4  BCL6B - b-cell cll/lymphoma 6, member b  ST5 - suppression of tumorigenicity 5  CRKL - v-crk avian sarcoma virus ct10 oncogene homolog-like  CRK - v-crk avian sarcoma virus ct10 oncogene homolog  EEFSEC - eukaryotic elongation factor, selenocysteine-trna-specific  CELF6 - cugbp, elav-like family member 6  NIPBL - nipped-b homolog (drosophila)  STAT2 - signal transducer and activator of transcription 2, 113kda  CRY1 - cryptochrome 1 (photolyase-like)  STAT1 - signal transducer and activator of transcription 1, 91kda  RAB26 - rab26, member ras oncogene family  STAT3 - signal transducer and activator of transcription 3 (acute-phase response factor)  CRY2 - cryptochrome 2 (photolyase-like)  STAT5A - signal transducer and activator of transcription 5a  ASF1A - anti-silencing function 1a histone chaperone  STAT6 - signal transducer and activator of transcription 6, interleukin-4 induced  SGMS1 - sphingomyelin synthase 1  PARM1 - prostate androgen-regulated mucin-like protein 1  BRMS1 - breast cancer metastasis suppressor 1  BOD1L1 - biorientation of chromosomes in cell division 1-like 1  DFNB31 - deafness, autosomal recessive 31  ZNF496 - zinc finger protein 496  CRYM - crystallin, mu  SS18 - synovial sarcoma translocation, chromosome 18  PHF5A - phd finger protein 5a  CSF1R - colony stimulating factor 1 receptor  GPATCH3 - g patch domain containing 3  SNX33 - sorting nexin 33  FBXO7 - f-box protein 7  CSF1 - colony stimulating factor 1 (macrophage)  SRPK2 - srsf protein kinase 2  SRPK1 - srsf protein kinase 1  TAB3 - tgf-beta activated kinase 1/map3k7 binding protein 3  TROVE2 - trove domain family, member 2  ZNF335 - zinc finger protein 335  BAMBI - bmp and activin membrane-bound inhibitor  SSB - sjogren syndrome antigen b (autoantigen la)  CSK - c-src tyrosine kinase  HAVCR2 - hepatitis a virus cellular receptor 2  ZC3H10 - zinc finger ccch-type containing 10  CSNK1A1 - casein kinase 1, alpha 1  CSNK1D - casein kinase 1, delta  CSNK2A2 - casein kinase 2, alpha prime polypeptide  SRI - sorcin  SRC - v-src avian sarcoma (schmidt-ruppin a-2) viral oncogene homolog  TNFAIP8 - tumor necrosis factor, alpha-induced protein 8  CSNK2A1 - casein kinase 2, alpha 1 polypeptide  DNAJB5 - dnaj (hsp40) homolog, subfamily b, member 5  SREBF2 - sterol regulatory element binding transcription factor 2  ARIH1 - ariadne homolog, ubiquitin-conjugating enzyme e2 binding protein, 1 (drosophila)  UBE2O - ubiquitin-conjugating enzyme e2o  NSUN3 - nop2/sun domain family, member 3  SRP9 - signal recognition particle 9kda  PRDX5 - peroxiredoxin 5  BACE2 - beta-site app-cleaving enzyme 2  RNF169 - ring finger protein 169  DHX9 - deah (asp-glu-ala-his) box helicase 9  TRAPPC2 - trafficking protein particle complex 2  KANK2 - kn motif and ankyrin repeat domains 2  DDX6 - dead (asp-glu-ala-asp) box helicase 6  DDX5 - dead (asp-glu-ala-asp) box helicase 5  SEC13 - sec13 homolog (s. cerevisiae)  DDX3X - dead (asp-glu-ala-asp) box helicase 3, x-linked  DDX1 - dead (asp-glu-ala-asp) box helicase 1  DPH6 - diphthamine biosynthesis 6  SIN3A - sin3 transcription regulator family member a  SDCBP - syndecan binding protein (syntenin)  FAM98A - family with sequence similarity 98, member a  SET - set nuclear oncogene  WWTR1 - ww domain containing transcription regulator 1  SETMAR - set domain and mariner transposase fusion gene  DDB1 - damage-specific dna binding protein 1, 127kda  ZNF304 - zinc finger protein 304  MAP2K4 - mitogen-activated protein kinase kinase 4  RCN3 - reticulocalbin 3, ef-hand calcium binding domain  ZNF286A - zinc finger protein 286a  GEMIN5 - gem (nuclear organelle) associated protein 5  PBXIP1 - pre-b-cell leukemia homeobox interacting protein 1  NOL11 - nucleolar protein 11  DBP - d site of albumin promoter (albumin d-box) binding protein  NELFB - negative elongation factor complex member b  POT1 - protection of telomeres 1  AHCTF1 - at hook containing transcription factor 1  CLK4 - cdc-like kinase 4  CNOT10 - ccr4-not transcription complex, subunit 10  ZBTB38 - zinc finger and btb domain containing 38  RNF19A - ring finger protein 19a, e3 ubiquitin protein ligase  RCHY1 - ring finger and chy zinc finger domain containing 1, e3 ubiquitin protein ligase  GATAD2B - gata zinc finger domain containing 2b  DFFA - dna fragmentation factor, 45kda, alpha polypeptide  ZNF551 - zinc finger protein 551  POLR1A - polymerase (rna) i polypeptide a, 194kda  KCTD13 - potassium channel tetramerization domain containing 13  ZNF473 - zinc finger protein 473  BIRC6 - baculoviral iap repeat containing 6  YTHDF3 - yth domain family, member 3  PLEKHG5 - pleckstrin homology domain containing, family g (with rhogef domain) member 5  WDR18 - wd repeat domain 18  CNOT6 - ccr4-not transcription complex, subunit 6  LTN1 - listerin e3 ubiquitin protein ligase 1  ATXN2 - ataxin 2  ZNF451 - zinc finger protein 451  ZNF622 - zinc finger protein 622  ERI1 - exoribonuclease 1  SCAF4 - sr-related ctd-associated factor 4  SS18L1 - synovial sarcoma translocation gene on chromosome 18-like 1  CHD5 - chromodomain helicase dna binding protein 5  SCO1 - sco1 cytochrome c oxidase assembly protein  ARID1B - at rich interactive domain 1b (swi1-like)  TMEM98 - transmembrane protein 98  MED30 - mediator complex subunit 30  MKL2 - mkl/myocardin-like 2  HEG1 - heart development protein with egf-like domains 1  NSMF - nmda receptor synaptonuclear signaling and neuronal migration factor  PYGO2 - pygopus homolog 2 (drosophila)  DAPK3 - death-associated protein kinase 3  XPO5 - exportin 5  ANGEL2 - angel homolog 2 (drosophila)  DAXX - death-domain associated protein  C2CD3 - c2 calcium-dependent domain containing 3  ZZZ3 - zinc finger, zz-type containing 3  KIDINS220 - kinase d-interacting substrate, 220kda  HINFP - histone h4 transcription factor  DAG1 - dystroglycan 1 (dystrophin-associated glycoprotein 1)  MTA3 - metastasis associated 1 family, member 3  DGKQ - diacylglycerol kinase, theta 110kda  ZNF608 - zinc finger protein 608  DAPK1 - death-associated protein kinase 1  CLIP3 - cap-gly domain containing linker protein 3  MAVS - mitochondrial antiviral signaling protein  IBTK - inhibitor of bruton agammaglobulinemia tyrosine kinase  DAP - death-associated protein  TIPARP - tcdd-inducible poly(adp-ribose) polymerase  CYP51A1 - cytochrome p450, family 51, subfamily a, polypeptide 1  SARS - seryl-trna synthetase  HACE1 - hect domain and ankyrin repeat containing e3 ubiquitin protein ligase 1  SATB1 - satb homeobox 1  NGDN - neuroguidin, eif4e binding protein  RPTOR - regulatory associated protein of mtor, complex 1  SAFB - scaffold attachment factor b  SH2B1 - sh2b adaptor protein 1  SALL2 - sal-like 2 (drosophila)  SERBP1 - serpine1 mrna binding protein 1  DRD2 - dopamine receptor d2  EPC2 - enhancer of polycomb homolog 2 (drosophila)  PHF19 - phd finger protein 19  SLC9A1 - solute carrier family 9, subfamily a (nhe1, cation proton antiporter 1), member 1  NKD2 - naked cuticle homolog 2 (drosophila)  IRF2BP1 - interferon regulatory factor 2 binding protein 1  SLC8A1 - solute carrier family 8 (sodium/calcium exchanger), member 1  TRAF3IP1 - tnf receptor-associated factor 3 interacting protein 1  CNOT6L - ccr4-not transcription complex, subunit 6-like  EAF1 - ell associated factor 1  SLC8A2 - solute carrier family 8 (sodium/calcium exchanger), member 2  TAOK1 - tao kinase 1  ATN1 - atrophin 1  DPH2 - dph2 homolog (s. cerevisiae)  ARRDC3 - arrestin domain containing 3  DPH1 - diphthamide biosynthesis 1  DOM3Z - dom-3 homolog z (c. elegans)  SEMA6A - sema domain, transmembrane domain (tm), and cytoplasmic domain, (semaphorin) 6a  C10orf137 - chromosome 10 open reading frame 137  DR1 - down-regulator of transcription 1, tbp-binding (negative cofactor 2)  DUSP8 - dual specificity phosphatase 8  TNKS1BP1 - tankyrase 1 binding protein 1, 182kda  MAPK15 - mitogen-activated protein kinase 15  SKI - v-ski avian sarcoma viral oncogene homolog  SKIL - ski-like oncogene  DUSP5 - dual specificity phosphatase 5  UNK - unkempt homolog (drosophila)  SKIV2L - superkiller viralicidic activity 2-like (s. cerevisiae)  DUSP6 - dual specificity phosphatase 6  SKP1 - s-phase kinase-associated protein 1  DUSP7 - dual specificity phosphatase 7  DYRK1A - dual-specificity tyrosine-(y)-phosphorylation regulated kinase 1a  MKL1 - megakaryoblastic leukemia (translocation) 1  CHTOP - chromatin target of prmt1  GGA1 - golgi-associated, gamma adaptin ear containing, arf binding protein 1  DVL2 - dishevelled segment polarity protein 2  DVL3 - dishevelled segment polarity protein 3  WDR48 - wd repeat domain 48  GIGYF2 - grb10 interacting gyf protein 2  USP36 - ubiquitin specific peptidase 36  FNIP2 - folliculin interacting protein 2  DOCK7 - dedicator of cytokinesis 7  HOMEZ - homeobox and leucine zipper encoding  EBF4 - early b-cell factor 4  ANKRD17 - ankyrin repeat domain 17  ZFHX2 - zinc finger homeobox 2  HBEGF - heparin-binding egf-like growth factor  KIAA1462 - kiaa1462  LSM14A - lsm14a, scd6 homolog a (s. cerevisiae)  DTX1 - deltex homolog 1 (drosophila)  DKC1 - dyskeratosis congenita 1, dyskerin  DLG3 - discs, large homolog 3 (drosophila)  DLG4 - discs, large homolog 4 (drosophila)  PNPT1 - polyribonucleotide nucleotidyltransferase 1  DLG1 - discs, large homolog 1 (drosophila)  ANKRD13A - ankyrin repeat domain 13a  SHOX2 - short stature homeobox 2  FBXW8 - f-box and wd repeat domain containing 8  SH3RF1 - sh3 domain containing ring finger 1  DHCR24 - 24-dehydrocholesterol reductase  PHGDH - phosphoglycerate dehydrogenase  SIAH2 - siah e3 ubiquitin protein ligase 2  POGK - pogo transposable element with krab domain  B3GAT3 - beta-1,3-glucuronyltransferase 3 (glucuronosyltransferase i)  DHFR - dihydrofolate reductase  RICTOR - rptor independent companion of mtor, complex 2  PHF12 - phd finger protein 12  FBXL5 - f-box and leucine-rich repeat protein 5  FBXO2 - f-box protein 2  CALCOCO1 - calcium binding and coiled-coil domain 1  SFPQ - splicing factor proline/glutamine-rich  ZBTB4 - zinc finger and btb domain containing 4  SFRP1 - secreted frizzled-related protein 1  SFRP2 - secreted frizzled-related protein 2  SFRP4 - secreted frizzled-related protein 4  SRSF1 - serine/arginine-rich splicing factor 1  SRSF2 - serine/arginine-rich splicing factor 2  DNM2 - dynamin 2  GMEB2 - glucocorticoid modulatory element binding protein 2  SRSF3 - serine/arginine-rich splicing factor 3  EPB41L5 - erythrocyte membrane protein band 4.1 like 5  DNMT1 - dna (cytosine-5-)-methyltransferase 1  SPAG8 - sperm associated antigen 8  SRSF4 - serine/arginine-rich splicing factor 4  TRDMT1 - trna aspartic acid methyltransferase 1  SRSF5 - serine/arginine-rich splicing factor 5  DNMT3A - dna (cytosine-5-)-methyltransferase 3 alpha  SRSF6 - serine/arginine-rich splicing factor 6  SRSF7 - serine/arginine-rich splicing factor 7  EIF2AK4 - eukaryotic translation initiation factor 2 alpha kinase 4  SFSWAP - splicing factor, suppressor of white-apricot homolog (drosophila)  TRA2B - transformer 2 beta homolog (drosophila)  ALS2 - amyotrophic lateral sclerosis 2 (juvenile)  CHD8 - chromodomain helicase dna binding protein 8  DOCK3 - dedicator of cytokinesis 3  TNRC6C - trinucleotide repeat containing 6c  SGK1 - serum/glucocorticoid regulated kinase 1  NOC2L - nucleolar complex associated 2 homolog (s. cerevisiae)  SGTA - small glutamine-rich tetratricopeptide repeat (tpr)-containing, alpha  ZNF317 - zinc finger protein 317  USP37 - ubiquitin specific peptidase 37  PPP1R3F - protein phosphatase 1, regulatory subunit 3f  SMARCAD1 - swi/snf-related, matrix-associated actin-dependent regulator of chromatin, subfamily a, containing dead/h box 1  MEIS3 - meis homeobox 3  NCBP2 - nuclear cap binding protein subunit 2, 20kda  MARK2 - map/microtubule affinity-regulating kinase 2  MAPRE3 - microtubule-associated protein, rp/eb family, member 3  TSC1 - tuberous sclerosis 1  ELK1 - elk1, member of ets oncogene family  SPIRE1 - spire actin nucleation factor 1  NAF1 - nuclear assembly factor 1 ribonucleoprotein  ELK3 - elk3, ets-domain protein (srf accessory protein 2)  ELK4 - elk4, ets-domain protein (srf accessory protein 1)  SNW1 - snw domain containing 1  ENSA - endosulfine alpha  SCAP - srebf chaperone  WRNIP1 - werner helicase interacting protein 1  EPAS1 - endothelial pas domain protein 1  DPH7 - diphthamide biosynthesis 7  EP300 - e1a binding protein p300  HABP4 - hyaluronan binding protein 4  ATF6 - activating transcription factor 6  UBQLN4 - ubiquilin 4  ENO1 - enolase 1, (alpha)  MOB1B - mob kinase activator 1b  RAB3GAP1 - rab3 gtpase activating protein subunit 1 (catalytic)  EIF4A2 - eukaryotic translation initiation factor 4a2  CELF4 - cugbp, elav-like family member 4  EIF4E - eukaryotic translation initiation factor 4e  EGR2 - early growth response 2  EGR3 - early growth response 3  CCT5 - chaperonin containing tcp1, subunit 5 (epsilon)  TTF1 - transcription termination factor, rna polymerase i  ZC3HAV1 - zinc finger ccch-type, antiviral 1  EIF2S1 - eukaryotic translation initiation factor 2, subunit 1 alpha, 35kda  EIF2B1 - eukaryotic translation initiation factor 2b, subunit 1 alpha, 26kda  TRIM32 - tripartite motif containing 32  SPHK2 - sphingosine kinase 2  EIF2S3 - eukaryotic translation initiation factor 2, subunit 3 gamma, 52kda  SERPINB1 - serpin peptidase inhibitor, clade b (ovalbumin), member 1  KDM2A - lysine (k)-specific demethylase 2a  ELAVL1 - elav (embryonic lethal, abnormal vision, drosophila)-like 1 (hu antigen r)  ZNF326 - zinc finger protein 326  ELF1 - e74-like factor 1 (ets domain transcription factor)  ELAVL4 - elav (embryonic lethal, abnormal vision, drosophila)-like 4  STRADA - ste20-related kinase adaptor alpha  ELF2 - e74-like factor 2 (ets domain transcription factor)  TSG101 - tumor susceptibility gene 101  EIF4G2 - eukaryotic translation initiation factor 4 gamma, 2  TCF25 - transcription factor 25 (basic helix-loop-helix)  TSC2 - tuberous sclerosis 2  CNOT1 - ccr4-not transcription complex, subunit 1  EFNA1 - ephrin-a1  EEF2 - eukaryotic translation elongation factor 2  EXOSC7 - exosome component 7  CTNNBIP1 - catenin, beta interacting protein 1  SPEN - spen homolog, transcriptional regulator (drosophila)  RNF149 - ring finger protein 149  KLHDC10 - kelch domain containing 10  EEF1A1 - eukaryotic translation elongation factor 1 alpha 1  EEF1A2 - eukaryotic translation elongation factor 1 alpha 2  BBX - bobby sox homolog (drosophila)  TP53BP1 - tumor protein p53 binding protein 1  PHC1 - polyhomeotic homolog 1 (drosophila)  PHC2 - polyhomeotic homolog 2 (drosophila)  EGR1 - early growth response 1  ZNF292 - zinc finger protein 292  EGFR - epidermal growth factor receptor  MEGF8 - multiple egf-like-domains 8  CELSR2 - cadherin, egf lag seven-pass g-type receptor 2  TOP1 - topoisomerase (dna) i  KDM1A - lysine (k)-specific demethylase 1a  ATXN7L3 - ataxin 7-like 3  EFNB2 - ephrin-b2  UNC13A - unc-13 homolog a (c. elegans)  EFNA5 - ephrin-a5  HNRNPLL - heterogeneous nuclear ribonucleoprotein l-like  NCOA6 - nuclear receptor coactivator 6  CCT3 - chaperonin containing tcp1, subunit 3 (gamma)  E2F6 - e2f transcription factor 6  SMG1 - smg1 phosphatidylinositol 3-kinase-related kinase  E4F1 - e4f transcription factor 1  ZHX3 - zinc fingers and homeoboxes 3  TNIK - traf2 and nck interacting kinase  E2F4 - e2f transcription factor 4, p107/p130-binding  E2F5 - e2f transcription factor 5, p130-binding  ENY2 - enhancer of yellow 2 homolog (drosophila)  C11orf30 - chromosome 11 open reading frame 30  RRP1B - ribosomal rna processing 1b  EDNRB - endothelin receptor type b  MYCBP2 - myc binding protein 2, e3 ubiquitin protein ligase  TRAF1 - tnf receptor-associated factor 1  LPAR1 - lysophosphatidic acid receptor 1  HSP90B1 - heat shock protein 90kda beta (grp94), member 1  S1PR1 - sphingosine-1-phosphate receptor 1  PMEPA1 - prostate transmembrane protein, androgen induced 1  TRAF3 - tnf receptor-associated factor 3  TRAF2 - tnf receptor-associated factor 2  NR2C1 - nuclear receptor subfamily 2, group c, member 1  SETX - senataxin  ZBTB47 - zinc finger and btb domain containing 47  NR2C2 - nuclear receptor subfamily 2, group c, member 2  ECE1 - endothelin converting enzyme 1  TPR - translocated promoter region, nuclear basket protein  ZNF609 - zinc finger protein 609  WAPAL - wings apart-like homolog (drosophila)  LXN - latexin  ERC1 - elks/rab6-interacting/cast family member 1  PPRC1 - peroxisome proliferator-activated receptor gamma, coactivator-related 1  NAT14 - n-acetyltransferase 14 (gcn5-related, putative)  KDM4C - lysine (k)-specific demethylase 4c  ZNF423 - zinc finger protein 423  REXO4 - rex4, rna exonuclease 4 homolog (s. cerevisiae)  BOD1 - biorientation of chromosomes in cell division 1  CAMK1D - calcium/calmodulin-dependent protein kinase id  TMEM259 - transmembrane protein 259  USF1 - upstream transcription factor 1  NUP107 - nucleoporin 107kda  DERL3 - derlin 3  USP1 - ubiquitin specific peptidase 1  KIF1B - kinesin family member 1b  ZBTB43 - zinc finger and btb domain containing 43  PTK2B - protein tyrosine kinase 2 beta  MRPS27 - mitochondrial ribosomal protein s27  PITHD1 - pith (c-terminal proteasome-interacting domain of thioredoxin-like) domain containing 1  TNRC6B - trinucleotide repeat containing 6b  BPTF - bromodomain phd finger transcription factor  USP4 - ubiquitin specific peptidase 4 (proto-oncogene)  RNF185 - ring finger protein 185  NR1H2 - nuclear receptor subfamily 1, group h, member 2  FBLN1 - fibulin 1  TAB2 - tgf-beta activated kinase 1/map3k7 binding protein 2  ETV3 - ets variant 3  CAMTA2 - calmodulin binding transcription activator 2  ZNF598 - zinc finger protein 598  ETV5 - ets variant 5  TWSG1 - twisted gastrulation homolog 1 (drosophila)  POGZ - pogo transposable element with znf domain  ETV6 - ets variant 6  PLXND1 - plexin d1  VHL - von hippel-lindau tumor suppressor, e3 ubiquitin protein ligase  VIM - vimentin  KDM6B - lysine (k)-specific demethylase 6b  EZR - ezrin  PHF8 - phd finger protein 8  TBX20 - t-box 20  EWSR1 - ews rna-binding protein 1  TRIM41 - tripartite motif containing 41  PCBP4 - poly(rc) binding protein 4  KCTD1 - potassium channel tetramerization domain containing 1  ANKLE2 - ankyrin repeat and lem domain containing 2  KDM6A - lysine (k)-specific demethylase 6a  DCUN1D4 - dcn1, defective in cullin neddylation 1, domain containing 4  CREB3L1 - camp responsive element binding protein 3-like 1  ACKR3 - atypical chemokine receptor 3  UVRAG - uv radiation resistance associated  ZC3H3 - zinc finger ccch-type containing 3  EYA3 - eyes absent homolog 3 (drosophila)  EZH1 - enhancer of zeste homolog 1 (drosophila)  GRAMD4 - gram domain containing 4  CCNL1 - cyclin l1  CIC - capicua transcriptional repressor  F2R - coagulation factor ii (thrombin) receptor  VCP - valosin containing protein  F3 - coagulation factor iii (thromboplastin, tissue factor)  WDR43 - wd repeat domain 43  MAPK8IP3 - mitogen-activated protein kinase 8 interacting protein 3  ZNF575 - zinc finger protein 575  ERF - ets2 repressor factor  ERCC4 - excision repair cross-complementing rodent repair deficiency, complementation group 4  NUP205 - nucleoporin 205kda  YTHDC1 - yth domain containing 1  THOC2 - tho complex 2  GGA3 - golgi-associated, gamma adaptin ear containing, arf binding protein 3  ELMSAN1 - elm2 and myb/sant-like domain containing 1  THAP11 - thap domain containing 11  EMC10 - er membrane protein complex subunit 10  ERN1 - endoplasmic reticulum to nucleus signaling 1  ZNF248 - zinc finger protein 248  PPP1R37 - protein phosphatase 1, regulatory subunit 37  UBB - ubiquitin b  GPD1L - glycerol-3-phosphate dehydrogenase 1-like  ARRDC4 - arrestin domain containing 4  LARP4B - la ribonucleoprotein domain family, member 4b  RCOR1 - rest corepressor 1  TULP3 - tubby like protein 3  DIP2A - dip2 disco-interacting protein 2 homolog a (drosophila)  ZNFX1 - zinc finger, nfx1-type containing 1  HIRA - histone cell cycle regulator  TTC5 - tetratricopeptide repeat domain 5  ETV1 - ets variant 1  ETS1 - v-ets avian erythroblastosis virus e26 oncogene homolog 1  ATG4B - autophagy related 4b, cysteine peptidase  SLC39A10 - solute carrier family 39 (zinc transporter), member 10  ZMIZ1 - zinc finger, miz-type containing 1  ETF1 - eukaryotic translation termination factor 1  CYFIP1 - cytoplasmic fmr1 interacting protein 1  ARL6IP1 - adp-ribosylation factor-like 6 interacting protein 1  DMTN - dematin actin binding protein  UBP1 - upstream binding protein 1 (lbp-1a)  SUMO1 - small ubiquitin-like modifier 1  UCHL1 - ubiquitin carboxyl-terminal esterase l1 (ubiquitin thiolesterase)  FAM168A - family with sequence similarity 168, member a  UBTF - upstream binding transcription factor, rna polymerase i  ZNF830 - zinc finger protein 830  PELI1 - pellino e3 ubiquitin protein ligase 1  CHURC1 - churchill domain containing 1  SMURF1 - smad specific e3 ubiquitin protein ligase 1  ZC3H4 - zinc finger ccch-type containing 4  JMJD6 - jumonji domain containing 6  MYADM - myeloid-associated differentiation marker  UBE2I - ubiquitin-conjugating enzyme e2i  EPRS - glutamyl-prolyl-trna synthetase  UBE2V2 - ubiquitin-conjugating enzyme e2 variant 2  ERCC2 - excision repair cross-complementing rodent repair deficiency, complementation group 2  UBE2L3 - ubiquitin-conjugating enzyme e2l 3  NUP210 - nucleoporin 210kda  EPS15 - epidermal growth factor receptor pathway substrate 15  UBE2N - ubiquitin-conjugating enzyme e2n  UBE2V1 - ubiquitin-conjugating enzyme e2 variant 1  ZC3H7B - zinc finger ccch-type containing 7b  VPS72 - vacuolar protein sorting 72 homolog (s. cerevisiae)  PSENEN - presenilin enhancer 2 homolog (c. elegans)  FMR1 - fragile x mental retardation 1  TCOF1 - treacher collins-franceschetti syndrome 1  MGA - mga, max dimerization protein  TCP1 - t-complex 1  FNTA - farnesyltransferase, caax box, alpha  ZEB1 - zinc finger e-box binding homeobox 1  WWC3 - wwc family member 3  FLT1 - fms-related tyrosine kinase 1  TCF12 - transcription factor 12  ZNF354A - zinc finger protein 354a  TCF20 - transcription factor 20 (ar1)  CAMTA1 - calmodulin binding transcription activator 1  PDS5A - pds5, regulator of cohesion maintenance, homolog a (s. cerevisiae)  TMEM106A - transmembrane protein 106a  FLI1 - fli-1 proto-oncogene, ets transcription factor  FLNA - filamin a, alpha  PLCB1 - phospholipase c, beta 1 (phosphoinositide-specific)  ARC - activity-regulated cytoskeleton-associated protein  FOXS1 - forkhead box s1  PHLPP1 - ph domain and leucine rich repeat protein phosphatase 1  FOXO3 - forkhead box o3  HDAC8 - histone deacetylase 8  LARP4 - la ribonucleoprotein domain family, member 4  ASH1L - ash1 (absent, small, or homeotic)-like (drosophila)  SIN3B - sin3 transcription regulator family member b  DCP1A - decapping mrna 1a  NACC1 - nucleus accumbens associated 1, ben and btb (poz) domain containing  HR - hair growth associated  MED8 - mediator complex subunit 8  CAND1 - cullin-associated and neddylation-dissociated 1  FKBP1A - fk506 binding protein 1a, 12kda  TDG - thymine-dna glycosylase  SMG6 - smg6 nonsense mediated mrna decay factor  PRDX2 - peroxiredoxin 2  FKBP1B - fk506 binding protein 1b, 12.6 kda  EAPP - e2f-associated phosphoprotein  FBXW11 - f-box and wd repeat domain containing 11  POFUT2 - protein o-fucosyltransferase 2  VPS11 - vacuolar protein sorting 11 homolog (s. cerevisiae)  KDM3A - lysine (k)-specific demethylase 3a  FAM208A - family with sequence similarity 208, member a  CLEC16A - c-type lectin domain family 16, member a  FHL2 - four and a half lim domains 2  FHIT - fragile histidine triad  STX1B - syntaxin 1b  PPP1R11 - protein phosphatase 1, regulatory (inhibitor) subunit 11  NUP160 - nucleoporin 160kda  TAF9 - taf9 rna polymerase ii, tata box binding protein (tbp)-associated factor, 32kda  FGFR2 - fibroblast growth factor receptor 2  TAF7 - taf7 rna polymerase ii, tata box binding protein (tbp)-associated factor, 55kda  FAM98B - family with sequence similarity 98, member b  FGF13 - fibroblast growth factor 13  TAF6 - taf6 rna polymerase ii, tata box binding protein (tbp)-associated factor, 80kda  FGFR1 - fibroblast growth factor receptor 1  TADA2A - transcriptional adaptor 2a  FGF9 - fibroblast growth factor 9  SMCHD1 - structural maintenance of chromosomes flexible hinge domain containing 1  ADAM17 - adam metallopeptidase domain 17  TACC1 - transforming, acidic coiled-coil containing protein 1  TIRAP - toll-interleukin 1 receptor (tir) domain containing adaptor protein  FGF1 - fibroblast growth factor 1 (acidic)  FGF2 - fibroblast growth factor 2 (basic)  USP22 - ubiquitin specific peptidase 22  TAPBP - tap binding protein (tapasin)  SASH1 - sam and sh3 domain containing 1  TARBP1 - tar (hiv-1) rna binding protein 1  FER - fer (fps/fes related) tyrosine kinase  SMG9 - smg9 nonsense mediated mrna decay factor  MAP3K7 - mitogen-activated protein kinase kinase kinase 7  PDGFC - platelet derived growth factor c  ZCCHC11 - zinc finger, cchc domain containing 11  ADNP - activity-dependent neuroprotector homeobox  KMT2E - lysine (k)-specific methyltransferase 2e  MED13L - mediator complex subunit 13-like  NCSTN - nicastrin  FKTN - fukutin  ZNF395 - zinc finger protein 395  MYNN - myoneurin  CRTC1 - creb regulated transcription coactivator 1  UFL1 - ufm1-specific ligase 1  TCF3 - transcription factor 3  PUM2 - pumilio homolog 2 (drosophila)  DMAP1 - dna methyltransferase 1 associated protein 1  TCEB3 - transcription elongation factor b (siii), polypeptide 3 (110kda, elongin a)  TCF4 - transcription factor 4  LARP1 - la ribonucleoprotein domain family, member 1  NKRF - nfkb repressing factor  EFEMP1 - egf containing fibulin-like extracellular matrix protein 1  ZNF629 - zinc finger protein 629  FBN1 - fibrillin 1  LANCL2 - lanc lantibiotic synthetase component c-like 2 (bacterial)  TCEA2 - transcription elongation factor a (sii), 2  ERBB2IP - erbb2 interacting protein  TCEA1 - transcription elongation factor a (sii), 1  TIMP1 - timp metallopeptidase inhibitor 1  CAMK2N2 - calcium/calmodulin-dependent protein kinase ii inhibitor 2  TIMP2 - timp metallopeptidase inhibitor 2  TIMP3 - timp metallopeptidase inhibitor 3  TIMP4 - timp metallopeptidase inhibitor 4  ITGB3BP - integrin beta 3 binding protein (beta3-endonexin)  TDRD7 - tudor domain containing 7  SYAP1 - synapse associated protein 1  RYBP - ring1 and yy1 binding protein  ORMDL1 - orm1-like 1 (s. cerevisiae)  MTOR - mechanistic target of rapamycin (serine/threonine kinase)  PAXBP1 - pax3 and pax7 binding protein 1  TLE1 - transducin-like enhancer of split 1 (e(sp1) homolog, drosophila)  TLE3 - transducin-like enhancer of split 3 (e(sp1) homolog, drosophila)  TLE4 - transducin-like enhancer of split 4 (e(sp1) homolog, drosophila)  NDNL2 - necdin-like 2  ATP13A2 - atpase type 13a2  TLR3 - toll-like receptor 3  EXOSC2 - exosome component 2  SIRT1 - sirtuin 1  MKX - mohawk homeobox  TMF1 - tata element modulatory factor 1  TMSB4X - thymosin beta 4, x-linked  CBX6 - chromobox homolog 6  ACTR8 - arp8 actin-related protein 8 homolog (yeast)  HEY1 - hairy/enhancer-of-split related with yrpw motif 1  TMPO - thymopoietin  CBX5 - chromobox homolog 5  BRD4 - bromodomain containing 4  TNFAIP1 - tumor necrosis factor, alpha-induced protein 1 (endothelial)  BCL9L - b-cell cll/lymphoma 9-like  TARDBP - tar dna binding protein  TNFRSF1A - tumor necrosis factor receptor superfamily, member 1a  TNFRSF1B - tumor necrosis factor receptor superfamily, member 1b  SF3B1 - splicing factor 3b, subunit 1, 155kda  SF3B3 - splicing factor 3b, subunit 3, 130kda  TMBIM6 - transmembrane bax inhibitor motif containing 6  PRKCDBP - protein kinase c, delta binding protein  EIF4ENIF1 - eukaryotic translation initiation factor 4e nuclear import factor 1  TEK - tek tyrosine kinase, endothelial  NUP188 - nucleoporin 188kda  SUZ12 - suz12 polycomb repressive complex 2 subunit  TEF - thyrotrophic embryonic factor  POFUT1 - protein o-fucosyltransferase 1  RCOR2 - rest corepressor 2  TFAM - transcription factor a, mitochondrial  DIABLO - diablo, iap-binding mitochondrial protein  INPP5E - inositol polyphosphate-5-phosphatase, 72 kda  TERF1 - telomeric repeat binding factor (nima-interacting) 1  TERF2 - telomeric repeat binding factor 2  TESK1 - testis-specific kinase 1  TFDP1 - transcription factor dp-1  NR2F2 - nuclear receptor subfamily 2, group f, member 2  TFE3 - transcription factor binding to ighm enhancer 3  CBX7 - chromobox homolog 7  BCCIP - brca2 and cdkn1a interacting protein  NR2F1 - nuclear receptor subfamily 2, group f, member 1  TFCP2 - transcription factor cp2  TGFA - transforming growth factor, alpha  ZNF740 - zinc finger protein 740  FNIP1 - folliculin interacting protein 1  HEY2 - hairy/enhancer-of-split related with yrpw motif 2  TGFBR1 - transforming growth factor, beta receptor 1  YLPM1 - ylp motif containing 1  TGFB2 - transforming growth factor, beta 2  RBFOX2 - rna binding protein, fox-1 homolog (c. elegans) 2  TGFB3 - transforming growth factor, beta 3  TGFB1 - transforming growth factor, beta 1  TGFB1I1 - transforming growth factor beta 1 induced transcript 1  MAPK8IP2 - mitogen-activated protein kinase 8 interacting protein 2  TP53INP1 - tumor protein p53 inducible nuclear protein 1  H2AFV - h2a histone family, member v  MEPCE - methylphosphate capping enzyme  RNF20 - ring finger protein 20, e3 ubiquitin protein ligase  TGFBR2 - transforming growth factor, beta receptor ii (70/80kda)  TGFBR3 - transforming growth factor, beta receptor iii  FOSL2 - fos-like antigen 2  FOSB - fbj murine osteosarcoma viral oncogene homolog b  FOS - fbj murine osteosarcoma viral oncogene homolog  THBS1 - thrombospondin 1  CABIN1 - calcineurin binding protein 1  KAT6B - k(lysine) acetyltransferase 6b  TIA1 - tia1 cytotoxic granule-associated rna binding protein  THY1 - thy-1 cell surface antigen  METTL3 - methyltransferase like 3  THRA - thyroid hormone receptor, alpha  MMD - monocyte to macrophage differentiation-associated  ELL - elongation factor rna polymerase ii  GFPT1 - glutamine--fructose-6-phosphate transaminase 1  ZNF239 - zinc finger protein 239  GFRA2 - gdnf family receptor alpha 2  SYMPK - symplekin  PHC3 - polyhomeotic homolog 3 (drosophila)  FAM192A - family with sequence similarity 192, member a  OBFC1 - oligonucleotide/oligosaccharide-binding fold containing 1  MKKS - mckusick-kaufman syndrome  NRIP1 - nuclear receptor interacting protein 1  KAT2A - k(lysine) acetyltransferase 2a  NCOA3 - nuclear receptor coactivator 3  ANP32A - acidic (leucine-rich) nuclear phosphoprotein 32 family, member a  WWC2 - ww and c2 domain containing 2  PABPN1 - poly(a) binding protein, nuclear 1  TAF15 - taf15 rna polymerase ii, tata box binding protein (tbp)-associated factor, 68kda  GJA1 - gap junction protein, alpha 1, 43kda  TMTC3 - transmembrane and tetratricopeptide repeat containing 3  AXIN1 - axin 1  TRRAP - transformation/transcription domain-associated protein  PICALM - phosphatidylinositol binding clathrin assembly protein  GGNBP2 - gametogenetin binding protein 2  CAAP1 - caspase activity and apoptosis inhibitor 1  LPCAT1 - lysophosphatidylcholine acyltransferase 1  ZC3H14 - zinc finger ccch-type containing 14  ARID1A - at rich interactive domain 1a (swi-like)  DLL1 - delta-like 1 (drosophila)  SETD6 - set domain containing 6  GLG1 - golgi glycoprotein 1  FZD6 - frizzled family receptor 6  GLE1 - gle1 rna export mediator  ACTR5 - arp5 actin-related protein 5 homolog (yeast)  FZD8 - frizzled family receptor 8  CDC45 - cell division cycle 45  FZD4 - frizzled family receptor 4  GCLC - glutamate-cysteine ligase, catalytic subunit  FZD1 - frizzled family receptor 1  BAP1 - brca1 associated protein-1 (ubiquitin carboxy-terminal hydrolase)  ZNF613 - zinc finger protein 613  AXIN2 - axin 2  NUP85 - nucleoporin 85kda  CDC7 - cell division cycle 7  KDM5C - lysine (k)-specific demethylase 5c  USP9X - ubiquitin specific peptidase 9, x-linked  GNG3 - guanine nucleotide binding protein (g protein), gamma 3  RBM10 - rna binding motif protein 10  GNAS - gnas complex locus  GNAQ - guanine nucleotide binding protein (g protein), q polypeptide  GNAI2 - guanine nucleotide binding protein (g protein), alpha inhibiting activity polypeptide 2  PHF17 - phd finger protein 17  GNA12 - guanine nucleotide binding protein (g protein) alpha 12  GMFB - glia maturation factor, beta  TTC21B - tetratricopeptide repeat domain 21b  SLTM - safb-like, transcription modulator  NELFE - negative elongation factor complex member e  EHMT1 - euchromatic histone-lysine n-methyltransferase 1  NR5A1 - nuclear receptor subfamily 5, group a, member 1  FUS - fused in sarcoma  NAA40 - n(alpha)-acetyltransferase 40, natd catalytic subunit, homolog (s. cerevisiae)  RAB7A - rab7a, member ras oncogene family  USP7 - ubiquitin specific peptidase 7 (herpes virus-associated)  RNF168 - ring finger protein 168, e3 ubiquitin protein ligase  XRCC6 - x-ray repair complementing defective repair in chinese hamster cells 6  GABPB1 - ga binding protein transcription factor, beta subunit 1  GABPA - ga binding protein transcription factor, alpha subunit 60kda  CPSF7 - cleavage and polyadenylation specific factor 7, 59kda  FUT8 - fucosyltransferase 8 (alpha (1,6) fucosyltransferase)  FYN - fyn oncogene related to src, fgr, yes  RPAP2 - rna polymerase ii associated protein 2  G6PD - glucose-6-phosphate dehydrogenase  DDX39B - dead (asp-glu-ala-asp) box polypeptide 39b  USP5 - ubiquitin specific peptidase 5 (isopeptidase t)  KMT2D - lysine (k)-specific methyltransferase 2d  SPRED1 - sprouty-related, evh1 domain containing 1  AAAS - achalasia, adrenocortical insufficiency, alacrimia  ZNF419 - zinc finger protein 419  CDK2AP1 - cyclin-dependent kinase 2 associated protein 1  YEATS4 - yeats domain containing 4  SNIP1 - smad nuclear interacting protein 1  ZFPM1 - zinc finger protein, fog family member 1  ZNF212 - zinc finger protein 212  EID2 - ep300 interacting inhibitor of differentiation 2  ZNF383 - zinc finger protein 383  GBA - glucosidase, beta, acid  GATA2 - gata binding protein 2  KAT6A - k(lysine) acetyltransferase 6a  NUP214 - nucleoporin 214kda  NR4A3 - nuclear receptor subfamily 4, group a, member 3  BRD3 - bromodomain containing 3  SHOC2 - soc-2 suppressor of clear homolog (c. elegans)  GAS6 - growth arrest-specific 6  NCOA4 - nuclear receptor coactivator 4  CARF - calcium responsive transcription factor  MLLT10 - myeloid/lymphoid or mixed-lineage leukemia (trithorax homolog, drosophila); translocated to, 10  ZNF408 - zinc finger protein 408  DEDD2 - death effector domain containing 2  FASTKD1 - fast kinase domains 1  DHX36 - deah (asp-glu-ala-his) box polypeptide 36  ZNF189 - zinc finger protein 189  ZSCAN26 - zinc finger and scan domain containing 26  GSTP1 - glutathione s-transferase pi 1  ZNF177 - zinc finger protein 177  GSPT1 - g1 to s phase transition 1  ZNF175 - zinc finger protein 175  GSN - gelsolin  ZNF174 - zinc finger protein 174  ZNF184 - zinc finger protein 184  ZCCHC6 - zinc finger, cchc domain containing 6  SSBP4 - single stranded dna binding protein 4  GTF2F1 - general transcription factor iif, polypeptide 1, 74kda  ZNF148 - zinc finger protein 148  ZBTB17 - zinc finger and btb domain containing 17  BEND5 - ben domain containing 5  NEIL1 - nei endonuclease viii-like 1 (e. coli)  GTF2H2 - general transcription factor iih, polypeptide 2, 44kda  TRIM26 - tripartite motif containing 26  GTF2H3 - general transcription factor iih, polypeptide 3, 34kda  VEZF1 - vascular endothelial zinc finger 1  GTF2H1 - general transcription factor iih, polypeptide 1, 62kda  ZNF142 - zinc finger protein 142  MCPH1 - microcephalin 1  ZNF140 - zinc finger protein 140  GTF2B - general transcription factor iib  PCGF2 - polycomb group ring finger 2  MSH6 - muts homolog 6 (e. coli)  ZNF132 - zinc finger protein 132  ZNF131 - zinc finger protein 131  ZNF134 - zinc finger protein 134  ZNF133 - zinc finger protein 133  TBL1XR1 - transducin (beta)-like 1 x-linked receptor 1  GTF2I - general transcription factor iii  GTF2H4 - general transcription factor iih, polypeptide 4, 52kda  PRKRIP1 - prkr interacting protein 1 (il11 inducible)  BRF1 - brf1, rna polymerase iii transcription initiation factor 90 kda subunit  ZNF79 - zinc finger protein 79  ZYG11B - zyg-11 family member b, cell cycle regulator  ZNF70 - zinc finger protein 70  RBM12B - rna binding motif protein 12b  ZNF75D - zinc finger protein 75d  ZNF76 - zinc finger protein 76  H3F3A - h3 histone, family 3a  BRPF1 - bromodomain and phd finger containing, 1  H2AFZ - h2a histone family, member z  FZD5 - frizzled family receptor 5  SAP130 - sin3a-associated protein, 130kda  DCP2 - decapping mrna 2  H2AFX - h2a histone family, member x  MUL1 - mitochondrial e3 ubiquitin protein ligase 1  CXCR4 - chemokine (c-x-c motif) receptor 4  PAPD4 - pap associated domain containing 4  PAX8 - paired box 8  MIER3 - mesoderm induction early response 1, family member 3  CDC73 - cell division cycle 73  NKAP - nfkb activating protein  THAP5 - thap domain containing 5  LRP8 - low density lipoprotein receptor-related protein 8, apolipoprotein e receptor  HCFC1 - host cell factor c1 (vp16-accessory protein)  PRDM2 - pr domain containing 2, with znf domain  MAP3K12 - mitogen-activated protein kinase kinase kinase 12  ZNF226 - zinc finger protein 226  HDAC2 - histone deacetylase 2  HMBOX1 - homeobox containing 1  HDGF - hepatoma-derived growth factor  ZCCHC12 - zinc finger, cchc domain containing 12  ZNF213 - zinc finger protein 213  HTT - huntingtin  HDAC1 - histone deacetylase 1  ZNF205 - zinc finger protein 205  HCLS1 - hematopoietic cell-specific lyn substrate 1  ZNF207 - zinc finger protein 207  XPA - xeroderma pigmentosum, complementation group a  XPC - xeroderma pigmentosum, complementation group c  GOLGA2 - golgin a2  XPO1 - exportin 1 (crm1 homolog, yeast)  GOLGB1 - golgin b1  XRCC1 - x-ray repair complementing defective repair in chinese hamster cells 1  PAGR1 - paxip1 associated glutamate-rich protein 1  ZXDC - zxd family zinc finger c  AAED1 - ahpc/tsa antioxidant enzyme domain containing 1  XBP1 - x-box binding protein 1  ZNF367 - zinc finger protein 367  BHLHE41 - basic helix-loop-helix family, member e41  EIF4H - eukaryotic translation initiation factor 4h  WFS1 - wolfram syndrome 1 (wolframin)  WHSC1 - wolf-hirschhorn syndrome candidate 1  NELFA - negative elongation factor complex member a  VRK2 - vaccinia related kinase 2  DCP1B - decapping mrna 1b  ARID2 - at rich interactive domain 2 (arid, rfx-like)  WARS - tryptophanyl-trna synthetase  ZNF35 - zinc finger protein 35  ZKSCAN1 - zinc finger with krab and scan domains 1  FBXL15 - f-box and leucine-rich repeat protein 15  ZNF576 - zinc finger protein 576  ZNF33B - zinc finger protein 33b  ZNF32 - zinc finger protein 32  ZNF45 - zinc finger protein 45  BRCC3 - brca1/brca2-containing complex, subunit 3  MZF1 - myeloid zinc finger 1  ZNF18 - zinc finger protein 18  ZNF16 - zinc finger protein 16  GRB2 - growth factor receptor-bound protein 2  ASXL1 - additional sex combs like 1 (drosophila)  ZNF384 - zinc finger protein 384  TNIP2 - tnfaip3 interacting protein 2  RBM42 - rna binding motif protein 42  ZNF24 - zinc finger protein 24  ZFPL1 - zinc finger protein-like 1  ZFX - zinc finger protein, x-linked  SF1 - splicing factor 1  GRIK2 - glutamate receptor, ionotropic, kainate 2  ZBTB14 - zinc finger and btb domain containing 14  ZNF711 - zinc finger protein 711  ZNF7 - zinc finger protein 7  ZNF8 - zinc finger protein 8  ZNF2 - zinc finger protein 2  ZNF3 - zinc finger protein 3  YES1 - v-yes-1 yamaguchi sarcoma viral oncogene homolog 1  XRCC5 - x-ray repair complementing defective repair in chinese hamster cells 5 (double-strand-break rejoining)  YWHAG - tyrosine 3-monooxygenase/tryptophan 5-monooxygenase activation protein, gamma polypeptide  GRSF1 - g-rich rna sequence binding factor 1  GSK3B - glycogen synthase kinase 3 beta  YWHAZ - tyrosine 3-monooxygenase/tryptophan 5-monooxygenase activation protein, zeta polypeptide  GSK3A - glycogen synthase kinase 3 alpha  YWHAE - tyrosine 3-monooxygenase/tryptophan 5-monooxygenase activation protein, epsilon polypeptide  YWHAB - tyrosine 3-monooxygenase/tryptophan 5-monooxygenase activation protein, beta polypeptide  CCNK - cyclin k  MAPKAP1 - mitogen-activated protein kinase associated protein 1  DNAJB2 - dnaj (hsp40) homolog, subfamily b, member 2  DNAJA1 - dnaj (hsp40) homolog, subfamily a, member 1  HSF2 - heat shock transcription factor 2  TRIM24 - tripartite motif containing 24  TAF1D - tata box binding protein (tbp)-associated factor, rna polymerase i, d, 41kda  CREG1 - cellular repressor of e1a-stimulated genes 1  CHAC1 - chac, cation transport regulator homolog 1 (e. coli)  HSBP1 - heat shock factor binding protein 1  FLCN - folliculin  NRP1 - neuropilin 1  PRMT1 - protein arginine methyltransferase 1  IQGAP1 - iq motif containing gtpase activating protein 1  PRMT2 - protein arginine methyltransferase 2  HESX1 - hesx homeobox 1  SAP30 - sin3a-associated protein, 30kda  PHF23 - phd finger protein 23  DERL1 - derlin 1  METTL16 - methyltransferase like 16  FTO - fat mass and obesity associated  ALKBH1 - alkb, alkylation repair homolog 1 (e. coli)  SPRED2 - sprouty-related, evh1 domain containing 2  CFLAR - casp8 and fadd-like apoptosis regulator  SYNGAP1 - synaptic ras gtpase activating protein 1  SECISBP2 - secis binding protein 2  MED11 - mediator complex subunit 11  WDR77 - wd repeat domain 77  LDB1 - lim domain binding 1  ALDH1A2 - aldehyde dehydrogenase 1 family, member a2  DSCC1 - dna replication and sister chromatid cohesion 1  KAT2B - k(lysine) acetyltransferase 2b  TSC22D1 - tsc22 domain family, member 1  CRTC2 - creb regulated transcription coactivator 2  DDA1 - det1 and ddb1 associated 1  ZNF259 - zinc finger protein 259  FUBP1 - far upstream element (fuse) binding protein 1  TAX1BP1 - tax1 (human t-cell leukemia virus type i) binding protein 1  CDC123 - cell division cycle 123  CC2D1B - coiled-coil and c2 domain containing 1b  DBNDD1 - dysbindin (dystrobrevin binding protein 1) domain containing 1  MTMR2 - myotubularin related protein 2  NABP2 - nucleic acid binding protein 2  MTMR3 - myotubularin related protein 3  HUS1 - hus1 checkpoint homolog (s. pombe)  CPNE1 - copine i  EIF2B2 - eukaryotic translation initiation factor 2b, subunit 2 beta, 39kda  THAP8 - thap domain containing 8  CADM4 - cell adhesion molecule 4  BUD31 - bud31 homolog (s. cerevisiae)  ZNF655 - zinc finger protein 655  EIF2B5 - eukaryotic translation initiation factor 2b, subunit 5 epsilon, 82kda  TRAK2 - trafficking protein, kinesin binding 2  MTMR9 - myotubularin related protein 9  HSPD1 - heat shock 60kda protein 1 (chaperonin)  DNAJB1 - dnaj (hsp40) homolog, subfamily b, member 1  ZNF48 - zinc finger protein 48  BCL10 - b-cell cll/lymphoma 10  FUBP3 - far upstream element (fuse) binding protein 3  HSP90AA1 - heat shock protein 90kda alpha (cytosolic), class a member 1  C7orf49 - chromosome 7 open reading frame 49  CDK5R2 - cyclin-dependent kinase 5, regulatory subunit 2 (p39)  BTRC - beta-transducin repeat containing e3 ubiquitin protein ligase  HSPA8 - heat shock 70kda protein 8  WNK1 - wnk lysine deficient protein kinase 1  EIF4EBP3 - eukaryotic translation initiation factor 4e binding protein 3  HMGN1 - high mobility group nucleosome binding domain 1  UBE2Z - ubiquitin-conjugating enzyme e2z  HMGB3 - high mobility group box 3  NUMB - numb homolog (drosophila)  HMGB2 - high mobility group box 2  LAMTOR3 - late endosomal/lysosomal adaptor, mapk and mtor activator 3  HMGB1 - high mobility group box 1  EIF3C - eukaryotic translation initiation factor 3, subunit c  PHACTR4 - phosphatase and actin regulator 4  BRD9 - bromodomain containing 9  EIF3D - eukaryotic translation initiation factor 3, subunit d  EIF3A - eukaryotic translation initiation factor 3, subunit a  EIF3B - eukaryotic translation initiation factor 3, subunit b  IRS2 - insulin receptor substrate 2  TNKS - tankyrase, trf1-interacting ankyrin-related adp-ribose polymerase  EIF4G3 - eukaryotic translation initiation factor 4 gamma, 3  DAZAP1 - daz associated protein 1  ZBTB10 - zinc finger and btb domain containing 10  AGO1 - argonaute risc catalytic component 1  LATS2 - large tumor suppressor kinase 2  CAPRIN2 - caprin family member 2  ZBTB48 - zinc finger and btb domain containing 48  SRSF9 - serine/arginine-rich splicing factor 9  PEA15 - phosphoprotein enriched in astrocytes 15  NDRG4 - ndrg family member 4  HYAL2 - hyaluronoglucosaminidase 2  ACD - adrenocortical dysplasia homolog (mouse)  GPBP1 - gc-rich promoter binding protein 1  HFE - hemochromatosis  HIP1 - huntingtin interacting protein 1  UBE2K - ubiquitin-conjugating enzyme e2k  HINT1 - histidine triad nucleotide binding protein 1  HIVEP1 - human immunodeficiency virus type i enhancer binding protein 1  ELP4 - elongator acetyltransferase complex subunit 4  UPF3B - upf3 regulator of nonsense transcripts homolog b (yeast)  UPF3A - upf3 regulator of nonsense transcripts homolog a (yeast)  HIF1A - hypoxia inducible factor 1, alpha subunit (basic helix-loop-helix transcription factor)  LPIN3 - lipin 3  TRADD - tnfrsf1a-associated via death domain  SNX3 - sorting nexin 3  MYCBP - myc binding protein  EED - embryonic ectoderm development  URI1 - uri1, prefoldin-like chaperone  RIPK1 - receptor (tnfrsf)-interacting serine-threonine kinase 1  HNRNPU - heterogeneous nuclear ribonucleoprotein u (scaffold attachment factor a)  FBXO22 - f-box protein 22  HNRNPK - heterogeneous nuclear ribonucleoprotein k  HNRNPL - heterogeneous nuclear ribonucleoprotein l  TNFSF13 - tumor necrosis factor (ligand) superfamily, member 13  TNFSF12 - tumor necrosis factor (ligand) superfamily, member 12  FBXO4 - f-box protein 4  TINF2 - terf1 (trf1)-interacting nuclear factor 2  ADAM9 - adam metallopeptidase domain 9  HNRNPA2B1 - heterogeneous nuclear ribonucleoprotein a2/b1  HNRNPA1 - heterogeneous nuclear ribonucleoprotein a1  HNRNPC - heterogeneous nuclear ribonucleoprotein c (c1/c2)  RIPK2 - receptor-interacting serine-threonine kinase 2  HNRNPAB - heterogeneous nuclear ribonucleoprotein a/b  HNRNPF - heterogeneous nuclear ribonucleoprotein f  HNRNPD - heterogeneous nuclear ribonucleoprotein d (au-rich element rna binding protein 1, 37kda)  HNRNPH1 - heterogeneous nuclear ribonucleoprotein h1 (h)  HNRNPH3 - heterogeneous nuclear ribonucleoprotein h3 (2h9)  MTMR1 - myotubularin related protein 1  HNRNPH2 - heterogeneous nuclear ribonucleoprotein h2 (h')  HMGCR - 3-hydroxy-3-methylglutaryl-coa reductase  AIDA - axin interactor, dorsalization associated  USP46 - ubiquitin specific peptidase 46  HMGA1 - high mobility group at-hook 1  ZNF584 - zinc finger protein 584  FAM129B - family with sequence similarity 129, member b  NR4A1 - nuclear receptor subfamily 4, group a, member 1  PDE12 - phosphodiesterase 12  RFX7 - regulatory factor x, 7  DEPTOR - dep domain containing mtor-interacting protein  FOXK2 - forkhead box k2  HTATSF1 - hiv-1 tat specific factor 1  HAT1 - histone acetyltransferase 1  EIF3K - eukaryotic translation initiation factor 3, subunit k  RPS6KA6 - ribosomal protein s6 kinase, 90kda, polypeptide 6  CRTC3 - creb regulated transcription coactivator 3  RBM15 - rna binding motif protein 15  TNRC6A - trinucleotide repeat containing 6a  ENC1 - ectodermal-neural cortex 1 (with btb domain)  RBMX - rna binding motif protein, x-linked  INHA - inhibin, alpha  PIK3R3 - phosphoinositide-3-kinase, regulatory subunit 3 (gamma)  DESI1 - desumoylating isopeptidase 1  SGSM3 - small g protein signaling modulator 3  PARG - poly (adp-ribose) glycohydrolase  HTRA2 - htra serine peptidase 2  STK39 - serine threonine kinase 39  ING2 - inhibitor of growth family, member 2  ZNF574 - zinc finger protein 574  PPM1D - protein phosphatase, mg2+/mn2+ dependent, 1d  CREB3L2 - camp responsive element binding protein 3-like 2  PCSK1N - proprotein convertase subtilisin/kexin type 1 inhibitor  IPPK - inositol 1,3,4,5,6-pentakisphosphate 2-kinase  UBE2S - ubiquitin-conjugating enzyme e2s  ILF2 - interleukin enhancer binding factor 2  PRPF19 - pre-mrna processing factor 19  ILF3 - interleukin enhancer binding factor 3, 90kda  MAP4K3 - mitogen-activated protein kinase kinase kinase kinase 3  ILK - integrin-linked kinase  CDC14B - cell division cycle 14b  PIAS1 - protein inhibitor of activated stat, 1  SMYD3 - set and mynd domain containing 3  SLCO3A1 - solute carrier organic anion transporter family, member 3a1  SMURF2 - smad specific e3 ubiquitin protein ligase 2  MAPKAPK5 - mitogen-activated protein kinase-activated protein kinase 5  PATL1 - protein associated with topoisomerase ii homolog 1 (yeast)  CGGBP1 - cgg triplet repeat binding protein 1  LMO4 - lim domain only 4  CAMK1 - calcium/calmodulin-dependent protein kinase i  CBX4 - chromobox homolog 4  GORASP1 - golgi reassembly stacking protein 1, 65kda  IL6ST - interleukin 6 signal transducer (gp130, oncostatin m receptor)  CSRNP1 - cysteine-serine-rich nuclear protein 1  C5AR2 - complement component 5a receptor 2  SERP1 - stress-associated endoplasmic reticulum protein 1  ZNF25 - zinc finger protein 25  STK16 - serine/threonine kinase 16  CCNY - cyclin y  ITGA1 - integrin, alpha 1  ITGA3 - integrin, alpha 3 (antigen cd49c, alpha 3 subunit of vla-3 receptor)  MADD - map-kinase activating death domain  KHSRP - kh-type splicing regulatory protein  ERLEC1 - endoplasmic reticulum lectin 1  CASK - calcium/calmodulin-dependent serine protein kinase (maguk family)  ITGAV - integrin, alpha v  POLR1E - polymerase (rna) i polypeptide e, 53kda  SUDS3 - suppressor of defective silencing 3 homolog (s. cerevisiae)  CDK10 - cyclin-dependent kinase 10  EIF6 - eukaryotic translation initiation factor 6  SKAP1 - src kinase associated phosphoprotein 1  GZF1 - gdnf-inducible zinc finger protein 1  INPPL1 - inositol polyphosphate phosphatase-like 1  INSR - insulin receptor  INSM1 - insulinoma-associated 1  CDK13 - cyclin-dependent kinase 13  AKTIP - akt interacting protein  PDCD4 - programmed cell death 4 (neoplastic transformation inhibitor)  LSM3 - lsm3 homolog, u6 small nuclear rna associated (s. cerevisiae)  RFXANK - regulatory factor x-associated ankyrin-containing protein  INPP5B - inositol polyphosphate-5-phosphatase, 75kda  CSDC2 - cold shock domain containing c2, rna binding  KLF7 - kruppel-like factor 7 (ubiquitous)  IRF2 - interferon regulatory factor 2  IRF3 - interferon regulatory factor 3  RUVBL1 - ruvb-like 1 (e. coli)  IKZF5 - ikaros family zinc finger 5 (pegasus)  IREB2 - iron-responsive element binding protein 2  DIMT1 - dim1 dimethyladenosine transferase 1 homolog (s. cerevisiae)  MED19 - mediator complex subunit 19  ITGA6 - integrin, alpha 6  IRAK2 - interleukin-1 receptor-associated kinase 2  IRAK1 - interleukin-1 receptor-associated kinase 1  CACYBP - calcyclin binding protein  ZSCAN31 - zinc finger and scan domain containing 31  EIF2AK1 - eukaryotic translation initiation factor 2-alpha kinase 1  PAPD5 - pap associated domain containing 5  KIAA1324L - kiaa1324-like  NSD1 - nuclear receptor binding set domain protein 1  RFWD2 - ring finger and wd repeat domain 2, e3 ubiquitin protein ligase  ZBTB11 - zinc finger and btb domain containing 11  RNF25 - ring finger protein 25  AFF4 - af4/fmr2 family, member 4  MMS19 - mms19 nucleotide excision repair homolog (s. cerevisiae)  DKK3 - dickkopf wnt signaling pathway inhibitor 3  SNX5 - sorting nexin 5  IFNGR1 - interferon gamma receptor 1  ID2 - inhibitor of dna binding 2, dominant negative helix-loop-helix protein  ZNF777 - zinc finger protein 777  BRPF3 - bromodomain and phd finger containing, 3  ID3 - inhibitor of dna binding 3, dominant negative helix-loop-helix protein  STK36 - serine/threonine kinase 36  IRF2BPL - interferon regulatory factor 2 binding protein-like  JAZF1 - jazf zinc finger 1  PLA2G6 - phospholipase a2, group vi (cytosolic, calcium-independent)  FOXK1 - forkhead box k1  BRAT1 - brca1-associated atm activator 1  MAGEF1 - melanoma antigen family f, 1  LTBP4 - latent transforming growth factor beta binding protein 4  ZNF282 - zinc finger protein 282  IGHMBP2 - immunoglobulin mu binding protein 2  CHORDC1 - cysteine and histidine-rich domain (chord) containing 1  RBPJ - recombination signal binding protein for immunoglobulin kappa j region  NSMAF - neutral sphingomyelinase (n-smase) activation associated factor  NCK2 - nck adaptor protein 2  PABPC1 - poly(a) binding protein, cytoplasmic 1  TRUB2 - trub pseudouridine (psi) synthase homolog 2 (e. coli)  SPOP - speckle-type poz protein  C6orf89 - chromosome 6 open reading frame 89  CYFIP2 - cytoplasmic fmr1 interacting protein 2  USP21 - ubiquitin specific peptidase 21  ZBTB9 - zinc finger and btb domain containing 9  ZBTB12 - zinc finger and btb domain containing 12  BCAR3 - breast cancer anti-estrogen resistance 3  NGFRAP1 - nerve growth factor receptor (tnfrsf16) associated protein 1  NPTN - neuroplastin  PHACTR1 - phosphatase and actin regulator 1  IK - ik cytokine, down-regulator of hla ii  IGBP1 - immunoglobulin (cd79a) binding protein 1  JMJD1C - jumonji domain containing 1c  SMARCA5 - swi/snf related, matrix associated, actin dependent regulator of chromatin, subfamily a, member 5  IGF1R - insulin-like growth factor 1 receptor  OGT - o-linked n-acetylglucosamine (glcnac) transferase  PELP1 - proline, glutamate and leucine rich protein 1  RAE1 - rae1 rna export 1 homolog (s. pombe)  LAT - linker for activation of t cells  NSG1 - neuron specific gene family member 1  TSPYL2 - tspy-like 2  IGFBP6 - insulin-like growth factor binding protein 6  SND1 - staphylococcal nuclease and tudor domain containing 1  CUL4B - cullin 4b  CUL2 - cullin 2  PCIF1 - pdx1 c-terminal inhibiting factor 1  ZNF667 - zinc finger protein 667  CUL3 - cullin 3  FOXP1 - forkhead box p1  SUPT3H - suppressor of ty 3 homolog (s. cerevisiae)  BEND6 - ben domain containing 6  ZUFSP - zinc finger with ufm1-specific peptidase domain  ZNF572 - zinc finger protein 572  PCGF5 - polycomb group ring finger 5  WTAP - wilms tumor 1 associated protein  WAC - ww domain containing adaptor with coiled-coil  ZNF414 - zinc finger protein 414  RBM39 - rna binding motif protein 39  PHF21A - phd finger protein 21a  ZNF771 - zinc finger protein 771  LIMK1 - lim domain kinase 1  AKAP12 - a kinase (prka) anchor protein 12  IER2 - immediate early response 2  LIG3 - ligase iii, dna, atp-dependent  NCOR1 - nuclear receptor corepressor 1  LDLR - low density lipoprotein receptor  NCOR2 - nuclear receptor corepressor 2  MTPN - myotrophin  CARTPT - cart prepropeptide  DOT1L - dot1-like histone h3k79 methyltransferase  ABCG1 - atp-binding cassette, sub-family g (white), member 1  EFCAB7 - ef-hand calcium binding domain 7  L3MBTL3 - l(3)mbt-like 3 (drosophila)  APBA3 - amyloid beta (a4) precursor protein-binding, family a, member 3  ZNF333 - zinc finger protein 333  CIR1 - corepressor interacting with rbpj, 1  SCAND1 - scan domain containing 1  SLX4 - slx4 structure-specific endonuclease subunit  SEC22B - sec22 vesicle trafficking protein homolog b (s. cerevisiae) (gene/pseudogene)  H2AFY - h2a histone family, member y  GTF2IRD1 - gtf2i repeat domain containing 1  FADS1 - fatty acid desaturase 1  GTPBP1 - gtp binding protein 1  ZNF527 - zinc finger protein 527  SPIRE2 - spire actin nucleation factor 2  HOPX - hop homeobox  ZNF316 - zinc finger protein 316  ZC3H8 - zinc finger ccch-type containing 8  SOX13 - sry (sex determining region y)-box 13  LMO1 - lim domain only 1 (rhombotin 1)  NACC2 - nacc family member 2, ben and btb (poz) domain containing  IER5 - immediate early response 5  DNAJC27 - dnaj (hsp40) homolog, subfamily c, member 27  LMNA - lamin a/c  CLOCK - clock circadian regulator  KLF3 - kruppel-like factor 3 (basic)  PRKAG2 - protein kinase, amp-activated, gamma 2 non-catalytic subunit  DDX41 - dead (asp-glu-ala-asp) box polypeptide 41  FXR2 - fragile x mental retardation, autosomal homolog 2  LITAF - lipopolysaccharide-induced tnf factor  TBPL1 - tbp-like 1  YTHDF2 - yth domain family, member 2  ZBTB41 - zinc finger and btb domain containing 41  ZFP62 - zfp62 zinc finger protein  BAG5 - bcl2-associated athanogene 5  TMEM59 - transmembrane protein 59  BAG4 - bcl2-associated athanogene 4  POLR1C - polymerase (rna) i polypeptide c, 30kda  PPME1 - protein phosphatase methylesterase 1  ZGPAT - zinc finger, ccch-type with g patch domain  ACTL6B - actin-like 6b  UBR5 - ubiquitin protein ligase e3 component n-recognin 5  IL27RA - interleukin 27 receptor, alpha  EIF4E2 - eukaryotic translation initiation factor 4e family member 2  MED20 - mediator complex subunit 20  UCHL5 - ubiquitin carboxyl-terminal hydrolase l5  ATG5 - autophagy related 5  AFAP1L2 - actin filament associated protein 1-like 2  ROCK2 - rho-associated, coiled-coil containing protein kinase 2  ATRAID - all-trans retinoic acid-induced differentiation factor  ANGPT4 - angiopoietin 4  MAPK8IP1 - mitogen-activated protein kinase 8 interacting protein 1  CRLF3 - cytokine receptor-like factor 3  NGRN - neugrin, neurite outgrowth associated  TAOK3 - tao kinase 3  GLYR1 - glyoxylate reductase 1 homolog (arabidopsis)  FZR1 - fizzy/cell division cycle 20 related 1 (drosophila)  ZBTB7A - zinc finger and btb domain containing 7a  DPY30 - dpy-30 homolog (c. elegans)  MBTPS2 - membrane-bound transcription factor peptidase, site 2  IRF2BP2 - interferon regulatory factor 2 binding protein 2  GLIS2 - glis family zinc finger 2  MAGED1 - melanoma antigen family d, 1  MED27 - mediator complex subunit 27  MED26 - mediator complex subunit 26  CHD6 - chromodomain helicase dna binding protein 6  POLR1B - polymerase (rna) i polypeptide b, 128kda  MED17 - mediator complex subunit 17  MED23 - mediator complex subunit 23  VPS28 - vacuolar protein sorting 28 homolog (s. cerevisiae)  ZNF580 - zinc finger protein 580  ANTXR1 - anthrax toxin receptor 1  MRTO4 - mrna turnover 4 homolog (s. cerevisiae)  CDYL - chromodomain protein, y-like  GTF2IRD2 - gtf2i repeat domain containing 2  ZNF710 - zinc finger protein 710  KDR - kinase insert domain receptor (a type iii receptor tyrosine kinase)  PICK1 - protein interacting with prkca 1  ING4 - inhibitor of growth family, member 4  EIF2AK3 - eukaryotic translation initiation factor 2-alpha kinase 3  INSIG2 - insulin induced gene 2  ITM2B - integral membrane protein 2b  MAP4K4 - mitogen-activated protein kinase kinase kinase kinase 4  RHBDD1 - rhomboid domain containing 1  RLIM - ring finger protein, lim domain interacting  TRAF7 - tnf receptor-associated factor 7, e3 ubiquitin protein ligase  RECQL5 - recq protein-like 5  KRAS - kirsten rat sarcoma viral oncogene homolog  TNPO1 - transportin 1  TGFBRAP1 - transforming growth factor, beta receptor associated protein 1  ZNF706 - zinc finger protein 706  CIAO1 - cytosolic iron-sulfur protein assembly 1  KPNA2 - karyopherin alpha 2 (rag cohort 1, importin alpha 1)  KPNA1 - karyopherin alpha 1 (importin alpha 5)  CAMKK1 - calcium/calmodulin-dependent protein kinase kinase 1, alpha  SUV420H1 - suppressor of variegation 4-20 homolog 1 (drosophila)  WFDC3 - wap four-disulfide core domain 3  PHF20L1 - phd finger protein 20-like 1  MED21 - mediator complex subunit 21  MED10 - mediator complex subunit 10  ZFP28 - zfp28 zinc finger protein  KIT - v-kit hardy-zuckerman 4 feline sarcoma viral oncogene homolog  POLDIP3 - polymerase (dna-directed), delta interacting protein 3  DCUN1D5 - dcn1, defective in cullin neddylation 1, domain containing 5  SLIT2 - slit homolog 2 (drosophila)  PAIP2 - poly(a) binding protein interacting protein 2  JUP - junction plakoglobin  CRIM1 - cysteine rich transmembrane bmp regulator 1 (chordin-like)  JAK2 - janus kinase 2  ING5 - inhibitor of growth family, member 5  JAK3 - janus kinase 3  ZNF219 - zinc finger protein 219  PHF20 - phd finger protein 20  TRIP12 - thyroid hormone receptor interactor 12  NUDT16L1 - nudix (nucleoside diphosphate linked moiety x)-type motif 16-like 1  KCNK2 - potassium channel, subfamily k, member 2  ZNHIT3 - zinc finger, hit-type containing 3  ZNF397 - zinc finger protein 397  TRIP4 - thyroid hormone receptor interactor 4  HMGN3 - high mobility group nucleosomal binding domain 3  PHF6 - phd finger protein 6  ZNF235 - zinc finger protein 235  COPS2 - cop9 signalosome subunit 2  ZDHHC2 - zinc finger, dhhc-type containing 2  VAMP3 - vesicle-associated membrane protein 3  ZNF639 - zinc finger protein 639  TAOK2 - tao kinase 2  SARNP - sap domain containing ribonucleoprotein  BRMS1L - breast cancer metastasis-suppressor 1-like  LEF1 - lymphoid enhancer-binding factor 1  VPS25 - vacuolar protein sorting 25 homolog (s. cerevisiae)  SMCR8 - smith-magenis syndrome chromosome region, candidate 8  CNOT8 - ccr4-not transcription complex, subunit 8  SIRPA - signal-regulatory protein alpha  CDK12 - cyclin-dependent kinase 12  ZNF280B - zinc finger protein 280b  S1PR2 - sphingosine-1-phosphate receptor 2  SNX25 - sorting nexin 25  ARID4B - at rich interactive domain 4b (rbp1-like)  SPRTN - sprt-like n-terminal domain  MIF - macrophage migration inhibitory factor (glycosylation-inhibiting factor)  CCNI2 - cyclin i family, member 2  MAP3K10 - mitogen-activated protein kinase kinase kinase 10  DUSP19 - dual specificity phosphatase 19  MLH1 - mutl homolog 1, colon cancer, nonpolyposis type 2 (e. coli)  MKLN1 - muskelin 1, intracellular mediator containing kelch motifs  ITGB1BP1 - integrin beta 1 binding protein 1  KDM3B - lysine (k)-specific demethylase 3b  RSF1 - remodeling and spacing factor 1  MLLT6 - myeloid/lymphoid or mixed-lineage leukemia (trithorax homolog, drosophila); translocated to, 6  SAMD8 - sterile alpha motif domain containing 8  MLLT3 - myeloid/lymphoid or mixed-lineage leukemia (trithorax homolog, drosophila); translocated to, 3  MED14 - mediator complex subunit 14  MLLT1 - myeloid/lymphoid or mixed-lineage leukemia (trithorax homolog, drosophila); translocated to, 1  MAP3K11 - mitogen-activated protein kinase kinase kinase 11  ZBTB22 - zinc finger and btb domain containing 22  KMT2A - lysine (k)-specific methyltransferase 2a  RPS6KA5 - ribosomal protein s6 kinase, 90kda, polypeptide 5  SUFU - suppressor of fused homolog (drosophila)  LSM7 - lsm7 homolog, u6 small nuclear rna associated (s. cerevisiae)  GPRC5B - g protein-coupled receptor, family c, group 5, member b  MMP14 - matrix metallopeptidase 14 (membrane-inserted)  MFHAS1 - malignant fibrous histiocytoma amplified sequence 1  NLK - nemo-like kinase  MNAT1 - mnat cdk-activating kinase assembly factor 1  UIMC1 - ubiquitin interaction motif containing 1  MTA2 - metastasis associated 1 family, member 2  NOLC1 - nucleolar and coiled-body phosphoprotein 1  EIF2A - eukaryotic translation initiation factor 2a, 65kda  LDLRAD3 - low density lipoprotein receptor class a domain containing 3  WWOX - ww domain containing oxidoreductase  CD200 - cd200 molecule  MNT - mnt, max dimerization protein  CCPG1 - cell cycle progression 1  CD46 - cd46 molecule, complement regulatory protein  NSRP1 - nuclear speckle splicing regulatory protein 1  SPTY2D1 - spt2, suppressor of ty, domain containing 1 (s. cerevisiae)  PELO - pelota homolog (drosophila)  MECP2 - methyl cpg binding protein 2 (rett syndrome)  MEF2A - myocyte enhancer factor 2a  WDR54 - wd repeat domain 54  PRICKLE1 - prickle homolog 1 (drosophila)  DDX21 - dead (asp-glu-ala-asp) box helicase 21  ZBED1 - zinc finger, bed-type containing 1  BCDIN3D - bcdin3 domain containing  MEIS2 - meis homeobox 2  BRWD1 - bromodomain and wd repeat domain containing 1  ZNF664 - zinc finger protein 664  MEIS1 - meis homeobox 1  MAP3K4 - mitogen-activated protein kinase kinase kinase 4  MAP3K5 - mitogen-activated protein kinase kinase kinase 5  SLC33A1 - solute carrier family 33 (acetyl-coa transporter), member 1  MAP3K1 - mitogen-activated protein kinase kinase kinase 1, e3 ubiquitin protein ligase  MAP3K3 - mitogen-activated protein kinase kinase kinase 3  MEN1 - multiple endocrine neoplasia i  WDR75 - wd repeat domain 75  UTP15 - utp15, u3 small nucleolar ribonucleoprotein, homolog (s. cerevisiae)  FAM161A - family with sequence similarity 161, member a  KAT8 - k(lysine) acetyltransferase 8  ZNF644 - zinc finger protein 644  ARHGEF2 - rho/rac guanine nucleotide exchange factor (gef) 2  PPP1R1B - protein phosphatase 1, regulatory (inhibitor) subunit 1b  HGS - hepatocyte growth factor-regulated tyrosine kinase substrate  MGAT5 - mannosyl (alpha-1,6-)-glycoprotein beta-1,6-n-acetyl-glucosaminyltransferase  NEMF - nuclear export mediator factor  MBD3 - methyl-cpg binding domain protein 3  KITLG - kit ligand  PTOV1 - prostate tumor overexpressed 1  ZNF394 - zinc finger protein 394  CBFA2T2 - core-binding factor, runt domain, alpha subunit 2; translocated to, 2  MAGOH - mago-nashi homolog, proliferation-associated (drosophila)  GSKIP - gsk3b interacting protein  CXXC5 - cxxc finger protein 5  RQCD1 - rcd1 required for cell differentiation1 homolog (s. pombe)  SMC3 - structural maintenance of chromosomes 3  IL23A - interleukin 23, alpha subunit p19  MARS - methionyl-trna synthetase  MAPT - microtubule-associated protein tau  LATS1 - large tumor suppressor kinase 1  MTA1 - metastasis associated 1  MAP1A - microtubule-associated protein 1a  SIRT6 - sirtuin 6  MTMR4 - myotubularin related protein 4  USP8 - ubiquitin specific peptidase 8  PPHLN1 - periphilin 1  USP14 - ubiquitin specific peptidase 14 (trna-guanine transglycosylase)  UBE2J1 - ubiquitin-conjugating enzyme e2, j1  NUP43 - nucleoporin 43kda  UNC119 - unc-119 homolog (c. elegans)  MBD1 - methyl-cpg binding domain protein 1  PKMYT1 - protein kinase, membrane associated tyrosine/threonine 1  SFMBT1 - scm-like with four mbt domains 1  MAX - myc associated factor x  ZMIZ2 - zinc finger, miz-type containing 2  LDB2 - lim domain binding 2  TRMT112 - trna methyltransferase 11-2 homolog (s. cerevisiae)  CHMP5 - charged multivesicular body protein 5  ZFP90 - zfp90 zinc finger protein  CDAN1 - codanin 1  NELFCD - negative elongation factor complex member c/d  DIRAS3 - diras family, gtp-binding ras-like 3  ASH2L - ash2 (absent, small, or homeotic)-like (drosophila)  CTDSPL2 - ctd (carboxy-terminal domain, rna polymerase ii, polypeptide a) small phosphatase like 2  ZSCAN29 - zinc finger and scan domain containing 29  NUDT12 - nudix (nucleoside diphosphate linked moiety x)-type motif 12  PTPLAD1 - protein tyrosine phosphatase-like a domain containing 1  THAP2 - thap domain containing, apoptosis associated protein 2  LRPAP1 - low density lipoprotein receptor-related protein associated protein 1  ITCH - itchy e3 ubiquitin protein ligase  ZNF484 - zinc finger protein 484  L3MBTL2 - l(3)mbt-like 2 (drosophila)  IL34 - interleukin 34  LRP1 - low density lipoprotein receptor-related protein 1  RPL14 - ribosomal protein l14  RIOK1 - rio kinase 1  LRP6 - low density lipoprotein receptor-related protein 6  ZNF445 - zinc finger protein 445  AIP - aryl hydrocarbon receptor interacting protein  LRP3 - low density lipoprotein receptor-related protein 3  NUB1 - negative regulator of ubiquitin-like proteins 1  TRAPPC9 - trafficking protein particle complex 9  ASB1 - ankyrin repeat and socs box containing 1  LUM - lumican  BTAF1 - btaf1 rna polymerase ii, b-tfiid transcription factor-associated, 170kda  LYN - v-yes-1 yamaguchi sarcoma viral related oncogene homolog  SPAG9 - sperm associated antigen 9  UBA3 - ubiquitin-like modifier activating enzyme 3  RBFOX3 - rna binding protein, fox-1 homolog (c. elegans) 3  SESN2 - sestrin 2  PARP9 - poly (adp-ribose) polymerase family, member 9  HIP1R - huntingtin interacting protein 1 related  CDK5RAP1 - cdk5 regulatory subunit associated protein 1  RNF8 - ring finger protein 8, e3 ubiquitin protein ligase  BAZ1B - bromodomain adjacent to zinc finger domain, 1b  TAF1B - tata box binding protein (tbp)-associated factor, rna polymerase i, b, 63kda  AZIN1 - antizyme inhibitor 1  MED15 - mediator complex subunit 15  MXD1 - max dimerization protein 1  PIAS4 - protein inhibitor of activated stat, 4  TRIM33 - tripartite motif containing 33  SMAD2 - smad family member 2  SRRT - serrate rna effector molecule homolog (arabidopsis)  MBIP - map3k12 binding inhibitory protein 1  KLF16 - kruppel-like factor 16  HDAC7 - histone deacetylase 7  ARMCX3 - armadillo repeat containing, x-linked 3  TDP2 - tyrosyl-dna phosphodiesterase 2  CAPRIN1 - cell cycle associated protein 1  TAF1C - tata box binding protein (tbp)-associated factor, rna polymerase i, c, 110kda  NBR1 - neighbor of brca1 gene 1  LARP7 - la ribonucleoprotein domain family, member 7  RPS6KA4 - ribosomal protein s6 kinase, 90kda, polypeptide 4  MAFG - v-maf avian musculoaponeurotic fibrosarcoma oncogene homolog g  DPH5 - diphthamide biosynthesis 5  TAF9B - taf9b rna polymerase ii, tata box binding protein (tbp)-associated factor, 31kda  SMAD4 - smad family member 4  NBAS - neuroblastoma amplified sequence  H1FX - h1 histone family, member x  SMAD5 - smad family member 5  SMAD7 - smad family member 7  WASL - wiskott-aldrich syndrome-like  RBM4B - rna binding motif protein 4b  ZNF565 - zinc finger protein 565  SAP30BP - sap30 binding protein  MYLIP - myosin regulatory light chain interacting protein  YAF2 - yy1 associated factor 2  NCBP1 - nuclear cap binding protein subunit 1, 80kda  SIX5 - six homeobox 5  TAGLN3 - transgelin 3  ITM2C - integral membrane protein 2c  NDN - necdin, melanoma antigen (mage) family member  ZNF582 - zinc finger protein 582  AKAP9 - a kinase (prka) anchor protein (yotiao) 9  BRD7 - bromodomain containing 7  NCK1 - nck adaptor protein 1  CAB39L - calcium binding protein 39-like  DDX25 - dead (asp-glu-ala-asp) box helicase 25  NCL - nucleolin  MBNL2 - muscleblind-like splicing regulator 2  CEBPZ - ccaat/enhancer binding protein (c/ebp), zeta  TICAM1 - toll-like receptor adaptor molecule 1  CCNL2 - cyclin l2  HNRNPM - heterogeneous nuclear ribonucleoprotein m  ORMDL2 - orm1-like 2 (s. cerevisiae)  TRIM28 - tripartite motif containing 28  DROSHA - drosha, ribonuclease type iii  ATP6AP2 - atpase, h+ transporting, lysosomal accessory protein 2  TBK1 - tank-binding kinase 1  NBN - nibrin  ZNF197 - zinc finger protein 197  TDRD3 - tudor domain containing 3  RBM7 - rna binding motif protein 7  RBM5 - rna binding motif protein 5  TRIM11 - tripartite motif containing 11  PPP1R12A - protein phosphatase 1, regulatory subunit 12a  TNK2 - tyrosine kinase, non-receptor, 2  MYT1 - myelin transcription factor 1  ALYREF - aly/ref export factor  CSRNP2 - cysteine-serine-rich nuclear protein 2  SENP1 - sumo1/sentrin specific peptidase 1  NAB1 - ngfi-a binding protein 1 (egr1 binding protein 1)  RNF41 - ring finger protein 41  NAB2 - ngfi-a binding protein 2 (egr1 binding protein 2)  TSHZ1 - teashirt zinc finger homeobox 1  PRMT3 - protein arginine methyltransferase 3  ANKRD13C - ankyrin repeat domain 13c  PSME3 - proteasome (prosome, macropain) activator subunit 3 (pa28 gamma; ki)  TNFAIP8L3 - tumor necrosis factor, alpha-induced protein 8-like 3  TRIM13 - tripartite motif containing 13  TRIM8 - tripartite motif containing 8  MYO6 - myosin vi  LBH - limb bud and heart development  ABT1 - activator of basal transcription 1  TOPORS - topoisomerase i binding, arginine/serine-rich, e3 ubiquitin protein ligase  RAB1B - rab1b, member ras oncogene family  MYD88 - myeloid differentiation primary response 88  SEH1L - seh1-like (s. cerevisiae)  ZNF362 - zinc finger protein 362  MYH9 - myosin, heavy chain 9, non-muscle  HUWE1 - hect, uba and wwe domain containing 1, e3 ubiquitin protein ligase  HCFC2 - host cell factor c2  CNOT7 - ccr4-not transcription complex, subunit 7  MXI1 - max interactor 1, dimerization protein  PQBP1 - polyglutamine binding protein 1  TRA2A - transformer 2 alpha homolog (drosophila)  ACTR2 - arp2 actin-related protein 2 homolog (yeast)  ACTR3 - arp3 actin-related protein 3 homolog (yeast)  RBM15B - rna binding motif protein 15b  CTDSP2 - ctd (carboxy-terminal domain, rna polymerase ii, polypeptide a) small phosphatase 2  TSFM - ts translation elongation factor, mitochondrial  FEM1B - fem-1 homolog b (c. elegans)  RAD50 - rad50 homolog (s. cerevisiae)  TRIM37 - tripartite motif containing 37  ZNF558 - zinc finger protein 558  LRPPRC - leucine-rich pentatricopeptide repeat containing  ZNF263 - zinc finger protein 263  SPRY4 - sprouty homolog 4 (drosophila)  PURG - purine-rich element binding protein g  BCAP31 - b-cell receptor-associated protein 31  RBM12 - rna binding motif protein 12  MED25 - mediator complex subunit 25  SNX12 - sorting nexin 12  TRAP1 - tnf receptor-associated protein 1  MED6 - mediator complex subunit 6  ABI1 - abl-interactor 1  RNF207 - ring finger protein 207  PTBP3 - polypyrimidine tract binding protein 3  PPP4R1 - protein phosphatase 4, regulatory subunit 1  DMTF1 - cyclin d binding myb-like transcription factor 1  SH2B3 - sh2b adaptor protein 3  MTIF2 - mitochondrial translational initiation factor 2  PDCD6 - programmed cell death 6  MTM1 - myotubularin 1  ZBTB33 - zinc finger and btb domain containing 33  TANK - traf family member-associated nfkb activator  HDAC5 - histone deacetylase 5  MTF1 - metal-regulatory transcription factor 1  SRA1 - steroid receptor rna activator 1  HDAC6 - histone deacetylase 6  OCLN - occludin  RNF187 - ring finger protein 187  SH2D3C - sh2 domain containing 3c  DCPS - decapping enzyme, scavenger  MT3 - metallothionein 3  MED16 - mediator complex subunit 16  CCDC22 - coiled-coil domain containing 22  SAE1 - sumo1 activating enzyme subunit 1  DNM1L - dynamin 1-like  RANBP9 - ran binding protein 9  DNAJB6 - dnaj (hsp40) homolog, subfamily b, member 6  UBA2 - ubiquitin-like modifier activating enzyme 2  MSN - moesin  MSH3 - muts homolog 3 (e. coli)  MSH2 - muts homolog 2, colon cancer, nonpolyposis type 1 (e. coli)  CITED1 - cbp/p300-interacting transactivator, with glu/asp-rich carboxy-terminal domain, 1  ZBTB5 - zinc finger and btb domain containing 5  PAN2 - pan2 poly(a) specific ribonuclease subunit homolog (s. cerevisiae)  ZBTB40 - zinc finger and btb domain containing 40  DBNL - drebrin-like  SUPT7L - suppressor of ty 7 (s. cerevisiae)-like  RGCC - regulator of cell cycle  COMMD1 - copper metabolism (murr1) domain containing 1  RBM8A - rna binding motif protein 8a  HELZ - helicase with zinc finger  USP3 - ubiquitin specific peptidase 3  BABAM1 - brisc and brca1 a complex member 1  METTL5 - methyltransferase like 5  USP15 - ubiquitin specific peptidase 15  MED4 - mediator complex subunit 4  THOC1 - tho complex 1  SETD2 - set domain containing 2  RBX1 - ring-box 1, e3 ubiquitin protein ligase  DUSP18 - dual specificity phosphatase 18  ZC3H7A - zinc finger ccch-type containing 7a  MRE11A - mre11 meiotic recombination 11 homolog a (s. cerevisiae)  NUP153 - nucleoporin 153kda  WDR91 - wd repeat domain 91  THRAP3 - thyroid hormone receptor associated protein 3  MED12 - mediator complex subunit 12  MED13 - mediator complex subunit 13  PSMD6 - proteasome (prosome, macropain) 26s subunit, non-atpase, 6  LRIG2 - leucine-rich repeats and immunoglobulin-like domains 2  MED24 - mediator complex subunit 24  ZC3H6 - zinc finger ccch-type containing 6  DTX3L - deltex 3-like (drosophila)  PJA2 - praja ring finger 2, e3 ubiquitin protein ligase  AREL1 - apoptosis resistant e3 ubiquitin protein ligase 1  SETDB1 - set domain, bifurcated 1  ZNF34 - zinc finger protein 34  ORC2 - origin recognition complex, subunit 2  PARD6A - par-6 partitioning defective 6 homolog alpha (c. elegans)  ORC5 - origin recognition complex, subunit 5  TOX4 - tox high mobility group box family member 4  ZNF436 - zinc finger protein 436  RNF141 - ring finger protein 141  PRR7 - proline rich 7 (synaptic)  AGRN - agrin  EPM2AIP1 - epm2a (laforin) interacting protein 1  PPP4R2 - protein phosphatase 4, regulatory subunit 2  NDFIP1 - nedd4 family interacting protein 1  PPP1R26 - protein phosphatase 1, regulatory subunit 26  GLIPR2 - gli pathogenesis-related 2  RBM19 - rna binding motif protein 19  OAZ2 - ornithine decarboxylase antizyme 2  OAZ1 - ornithine decarboxylase antizyme 1  G3BP2 - gtpase activating protein (sh3 domain) binding protein 2  ODC1 - ornithine decarboxylase 1  CCNYL1 - cyclin y-like 1  OGG1 - 8-oxoguanine dna glycosylase  ZSCAN16 - zinc finger and scan domain containing 16  TNKS2 - tankyrase, trf1-interacting ankyrin-related adp-ribose polymerase 2  EXOSC3 - exosome component 3  SMG7 - smg7 nonsense mediated mrna decay factor  WDR61 - wd repeat domain 61  APITD1 - apoptosis-inducing, taf9-like domain 1  EXOSC1 - exosome component 1  FIS1 - fission 1 (mitochondrial outer membrane) homolog (s. cerevisiae)  NSF - n-ethylmaleimide-sensitive factor  DAZAP2 - daz associated protein 2  DDR2 - discoidin domain receptor tyrosine kinase 2  IST1 - increased sodium tolerance 1 homolog (yeast)  BCLAF1 - bcl2-associated transcription factor 1  NUMA1 - nuclear mitotic apparatus protein 1  DAB2IP - dab2 interacting protein  EIF4A3 - eukaryotic translation initiation factor 4a3  NUP88 - nucleoporin 88kda  ATG13 - autophagy related 13  NUP98 - nucleoporin 98kda  GMNN - geminin, dna replication inhibitor  NR4A2 - nuclear receptor subfamily 4, group a, member 2  ZNF593 - zinc finger protein 593  ZBTB7B - zinc finger and btb domain containing 7b  MATR3 - matrin 3  NVL - nuclear vcp-like  TBC1D5 - tbc1 domain family, member 5  ZNF691 - zinc finger protein 691  RNF144A - ring finger protein 144a  CREBRF - creb3 regulatory factor  ZNF623 - zinc finger protein 623  RGP1 - rgp1 retrograde golgi transport homolog (s. cerevisiae)  NPM1 - nucleophosmin (nucleolar phosphoprotein b23, numatrin)  NMD3 - nmd3 ribosome export adaptor  ARHGEF11 - rho guanine nucleotide exchange factor (gef) 11  ZBTB24 - zinc finger and btb domain containing 24  ZEB2 - zinc finger e-box binding homeobox 2  THAP4 - thap domain containing 4  CTIF - cbp80/20-dependent translation initiation factor  DUSP16 - dual specificity phosphatase 16  RNF40 - ring finger protein 40, e3 ubiquitin protein ligase  SPOCK2 - sparc/osteonectin, cwcv and kazal-like domains proteoglycan (testican) 2  RB1CC1 - rb1-inducible coiled-coil 1  IFT52 - intraflagellar transport 52 homolog (chlamydomonas)  TSC22D2 - tsc22 domain family, member 2  PAQR3 - progestin and adipoq receptor family member iii  SH3BP5L - sh3-binding domain protein 5-like  NRF1 - nuclear respiratory factor 1  NFXL1 - nuclear transcription factor, x-box binding-like 1  SH3D19 - sh3 domain containing 19  UBQLN1 - ubiquilin 1  DONSON - downstream neighbor of son  NOP2 - nop2 nucleolar protein  VPRBP - vpr (hiv-1) binding protein  NONO - non-pou domain containing, octamer-binding  RNF34 - ring finger protein 34, e3 ubiquitin protein ligase  NME1 - nme/nm23 nucleoside diphosphate kinase 1  MDFIC - myod family inhibitor domain containing  UBQLN2 - ubiquilin 2  IFT74 - intraflagellar transport 74 homolog (chlamydomonas)  NOTCH4 - notch 4  NOVA2 - neuro-oncological ventral antigen 2  HERPUD1 - homocysteine-inducible, endoplasmic reticulum stress-inducible, ubiquitin-like domain member 1  NOVA1 - neuro-oncological ventral antigen 1  CEP290 - centrosomal protein 290kda  NPAT - nuclear protein, ataxia-telangiectasia locus  RIMS2 - regulating synaptic membrane exocytosis 2  CNOT2 - ccr4-not transcription complex, subunit 2  NAA15 - n(alpha)-acetyltransferase 15, nata auxiliary subunit  PUM1 - pumilio homolog 1 (drosophila)  NOS3 - nitric oxide synthase 3 (endothelial cell)  PPP6R2 - protein phosphatase 6, regulatory subunit 2  CNOT4 - ccr4-not transcription complex, subunit 4  CNOT3 - ccr4-not transcription complex, subunit 3  BAZ2B - bromodomain adjacent to zinc finger domain, 2b  NOTCH1 - notch 1  ST18 - suppression of tumorigenicity 18 (breast carcinoma) (zinc finger protein)  CTC1 - cts telomere maintenance complex component 1  NOTCH3 - notch 3  DHX34 - deah (asp-glu-ala-his) box polypeptide 34  NOTCH2 - notch 2  NFYA - nuclear transcription factor y, alpha  HDAC4 - histone deacetylase 4  TOX - thymocyte selection-associated high mobility group box  EDC3 - enhancer of mrna decapping 3  NFYC - nuclear transcription factor y, gamma  ZFYVE16 - zinc finger, fyve domain containing 16  CXXC1 - cxxc finger protein 1  NFKBIA - nuclear factor of kappa light polypeptide gene enhancer in b-cells inhibitor, alpha  NFKB1 - nuclear factor of kappa light polypeptide gene enhancer in b-cells 1  KMT2B - lysine (k)-specific methyltransferase 2b  NFKBIB - nuclear factor of kappa light polypeptide gene enhancer in b-cells inhibitor, beta  NFKBIE - nuclear factor of kappa light polypeptide gene enhancer in b-cells inhibitor, epsilon  PHACTR2 - phosphatase and actin regulator 2  NKX2-2 - nk2 homeobox 2  CNKSR3 - cnksr family member 3  ZNF606 - zinc finger protein 606  HDAC9 - histone deacetylase 9  ZNRD1 - zinc ribbon domain containing 1  SART3 - squamous cell carcinoma antigen recognized by t cells 3  ZNF614 - zinc finger protein 614  EHD4 - eh-domain containing 4  ZFP2 - zfp2 zinc finger protein  TTC37 - tetratricopeptide repeat domain 37  MINK1 - misshapen-like kinase 1  NEK3 - nima-related kinase 3  SOCS5 - suppressor of cytokine signaling 5  NEO1 - neogenin 1  SMARCAL1 - swi/snf related, matrix associated, actin dependent regulator of chromatin, subfamily a-like 1  NEDD8 - neural precursor cell expressed, developmentally down-regulated 8  NEDD9 - neural precursor cell expressed, developmentally down-regulated 9  CTR9 - ctr9, paf1/rna polymerase ii complex component  PPM1F - protein phosphatase, mg2+/mn2+ dependent, 1f  CPEB4 - cytoplasmic polyadenylation element binding protein 4  EPC1 - enhancer of polycomb homolog 1 (drosophila)  NFE2L2 - nuclear factor (erythroid-derived 2)-like 2  RASL11A - ras-like, family 11, member a  MORF4L2 - mortality factor 4 like 2  KLHL15 - kelch-like family member 15  NFATC4 - nuclear factor of activated t-cells, cytoplasmic, calcineurin-dependent 4  PDGFD - platelet derived growth factor d  NUP155 - nucleoporin 155kda  NFIA - nuclear factor i/a  NF1 - neurofibromin 1  CDK5RAP3 - cdk5 regulatory subunit associated protein 3  EDEM1 - er degradation enhancer, mannosidase alpha-like 1  RAPGEF2 - rap guanine nucleotide exchange factor (gef) 2  NUP93 - nucleoporin 93kda  ZNF746 - zinc finger protein 746  VGLL4 - vestigial like 4 (drosophila)  LRRC14 - leucine rich repeat containing 14  KDM4A - lysine (k)-specific demethylase 4a  PHF14 - phd finger protein 14  HP1BP3 - heterochromatin protein 1, binding protein 3  ZNF432 - zinc finger protein 432  SAFB2 - scaffold attachment factor b2  KIAA0430 - kiaa0430  MYEF2 - myelin expression factor 2  LPIN2 - lipin 2 |
| GO:0010468 | regulation of gene expression | 6.01E-7 | 1.13E-4 | 1.06 (10334,2726,6996,1947) | [+] Show genes  EXOSC9 - exosome component 9  MSL3 - male-specific lethal 3 homolog (drosophila)  EXOSC10 - exosome component 10  TADA1 - transcriptional adaptor 1  HNRNPA0 - heterogeneous nuclear ribonucleoprotein a0  AARS - alanyl-trna synthetase  CBX1 - chromobox homolog 1  ABCA2 - atp-binding cassette, sub-family a (abc1), member 2  ABL1 - c-abl oncogene 1, non-receptor tyrosine kinase  ZNF821 - zinc finger protein 821  TMED2 - transmembrane emp24 domain trafficking protein 2  ERP29 - endoplasmic reticulum protein 29  PML - promyelocytic leukemia  CNOT11 - ccr4-not transcription complex, subunit 11  MLLT11 - myeloid/lymphoid or mixed-lineage leukemia (trithorax homolog, drosophila); translocated to, 11  SUPT20H - suppressor of ty 20 homolog (s. cerevisiae)  YWHAQ - tyrosine 3-monooxygenase/tryptophan 5-monooxygenase activation protein, theta polypeptide  PNRC2 - proline-rich nuclear receptor coactivator 2  BRD8 - bromodomain containing 8  CHD7 - chromodomain helicase dna binding protein 7  LYAR - ly1 antibody reactive  PAPOLA - poly(a) polymerase alpha  ZNF354B - zinc finger protein 354b  TCERG1 - transcription elongation regulator 1  ACTB - actin, beta  RNPS1 - rna binding protein s1, serine-rich domain  EHMT2 - euchromatic histone-lysine n-methyltransferase 2  TASP1 - taspase, threonine aspartase, 1  SPIN1 - spindlin 1  SUB1 - sub1 homolog (s. cerevisiae)  MORF4L1 - mortality factor 4 like 1  ACTG1 - actin, gamma 1  ZNF511 - zinc finger protein 511  ACTG2 - actin, gamma 2, smooth muscle, enteric  PPID - peptidylprolyl isomerase d  CAMK2N1 - calcium/calmodulin-dependent protein kinase ii inhibitor 1  ACTL6A - actin-like 6a  ACVR2A - activin a receptor, type iia  ACVRL1 - activin a receptor type ii-like 1  ACVR1 - activin a receptor, type i  DNAJA4 - dnaj (hsp40) homolog, subfamily a, member 4  ACVR1B - activin a receptor, type ib  ADAR - adenosine deaminase, rna-specific  ZNF331 - zinc finger protein 331  CPSF4 - cleavage and polyadenylation specific factor 4, 30kda  MED1 - mediator complex subunit 1  ADCY1 - adenylate cyclase 1 (brain)  PPARD - peroxisome proliferator-activated receptor delta  MAML3 - mastermind-like 3 (drosophila)  POLR2K - polymerase (rna) ii (dna directed) polypeptide k, 7.0kda  FAM129A - family with sequence similarity 129, member a  PARP1 - poly (adp-ribose) polymerase 1  H2AFY2 - h2a histone family, member y2  PPP1R13L - protein phosphatase 1, regulatory subunit 13 like  HES6 - hairy and enhancer of split 6 (drosophila)  POLR2G - polymerase (rna) ii (dna directed) polypeptide g  POLR2F - polymerase (rna) ii (dna directed) polypeptide f  POLR2E - polymerase (rna) ii (dna directed) polypeptide e, 25kda  POLR2D - polymerase (rna) ii (dna directed) polypeptide d  SMPD3 - sphingomyelin phosphodiesterase 3, neutral membrane (neutral sphingomyelinase ii)  POLR2C - polymerase (rna) ii (dna directed) polypeptide c, 33kda  POLR2B - polymerase (rna) ii (dna directed) polypeptide b, 140kda  POLR2A - polymerase (rna) ii (dna directed) polypeptide a, 220kda  RUVBL2 - ruvb-like 2 (e. coli)  PRKAB1 - protein kinase, amp-activated, beta 1 non-catalytic subunit  CDK5RAP2 - cdk5 regulatory subunit associated protein 2  PRKACB - protein kinase, camp-dependent, catalytic, beta  RCOR3 - rest corepressor 3  PRKACA - protein kinase, camp-dependent, catalytic, alpha  AES - amino-terminal enhancer of split  NUP133 - nucleoporin 133kda  NUPL2 - nucleoporin like 2  FAM46B - family with sequence similarity 46, member b  VPS35 - vacuolar protein sorting 35 homolog (s. cerevisiae)  JAG1 - jagged 1  PRDM4 - pr domain containing 4  PPP3R1 - protein phosphatase 3, regulatory subunit b, alpha  ATF7IP - activating transcription factor 7 interacting protein  ZNF689 - zinc finger protein 689  LRIF1 - ligand dependent nuclear receptor interacting factor 1  PPP2R5B - protein phosphatase 2, regulatory subunit b', beta  ZNF653 - zinc finger protein 653  AKT1 - v-akt murine thymoma viral oncogene homolog 1  PCID2 - pci domain containing 2  PPP2R2A - protein phosphatase 2, regulatory subunit b, alpha  CPSF6 - cleavage and polyadenylation specific factor 6, 68kda  RIOK2 - rio kinase 2  PPP2R1A - protein phosphatase 2, regulatory subunit a, alpha  NUDT21 - nudix (nucleoside diphosphate linked moiety x)-type motif 21  WWP2 - ww domain containing e3 ubiquitin protein ligase 2  AHR - aryl hydrocarbon receptor  ZNF526 - zinc finger protein 526  FOXP4 - forkhead box p4  PPP2CA - protein phosphatase 2, catalytic subunit, alpha isozyme  PPP2CB - protein phosphatase 2, catalytic subunit, beta isozyme  ZNF83 - zinc finger protein 83  PPP1CC - protein phosphatase 1, catalytic subunit, gamma isozyme  KANSL3 - kat8 regulatory nsl complex subunit 3  IWS1 - iws1 homolog (s. cerevisiae)  TDRKH - tudor and kh domain containing  PRNP - prion protein  ATF7 - activating transcription factor 7  AMFR - autocrine motility factor receptor, e3 ubiquitin protein ligase  DNAJC3 - dnaj (hsp40) homolog, subfamily c, member 3  PRKRIR - protein-kinase, interferon-inducible double stranded rna dependent inhibitor, repressor of (p58 repressor)  PAPD7 - pap associated domain containing 7  MAP2K5 - mitogen-activated protein kinase kinase 5  MAP2K7 - mitogen-activated protein kinase kinase 7  EIF2AK2 - eukaryotic translation initiation factor 2-alpha kinase 2  PIM2 - pim-2 oncogene  ZNF692 - zinc finger protein 692  MAPK13 - mitogen-activated protein kinase 13  MAP2K1 - mitogen-activated protein kinase kinase 1  MAP2K2 - mitogen-activated protein kinase kinase 2  EIF4E3 - eukaryotic translation initiation factor 4e family member 3  MAPK10 - mitogen-activated protein kinase 10  MAPK9 - mitogen-activated protein kinase 9  MAPK11 - mitogen-activated protein kinase 11  MAPK8 - mitogen-activated protein kinase 8  MAPK7 - mitogen-activated protein kinase 7  GCN1L1 - gcn1 general control of amino-acid synthesis 1-like 1 (yeast)  MAPK3 - mitogen-activated protein kinase 3  MAPK1 - mitogen-activated protein kinase 1  PRKD1 - protein kinase d1  ADIRF - adipogenesis regulatory factor  NDC1 - ndc1 transmembrane nucleoporin  ANXA3 - annexin a3  PRKCH - protein kinase c, eta  NT5C3B - 5'-nucleotidase, cytosolic iiib  PRKCI - protein kinase c, iota  CCDC85B - coiled-coil domain containing 85b  PRKCB - protein kinase c, beta  PRKCD - protein kinase c, delta  RBM22 - rna binding motif protein 22  PRKCA - protein kinase c, alpha  NSUN5 - nop2/sun domain family, member 5  PRKAR1A - protein kinase, camp-dependent, regulatory, type i, alpha  YEATS2 - yeats domain containing 2  PRKAG1 - protein kinase, amp-activated, gamma 1 non-catalytic subunit  APEX1 - apex nuclease (multifunctional dna repair enzyme) 1  MTPAP - mitochondrial poly(a) polymerase  RBM23 - rna binding motif protein 23  ZNF605 - zinc finger protein 605  XIAP - x-linked inhibitor of apoptosis  BIRC3 - baculoviral iap repeat containing 3  PARN - poly(a)-specific ribonuclease  BIRC2 - baculoviral iap repeat containing 2  KLF12 - kruppel-like factor 12  SHQ1 - shq1, h/aca ribonucleoprotein assembly factor  PBX2 - pre-b-cell leukemia homeobox 2  PRMT6 - protein arginine methyltransferase 6  PBX3 - pre-b-cell leukemia homeobox 3  PBX1 - pre-b-cell leukemia homeobox 1  PCBP2 - poly(rc) binding protein 2  PCBD1 - pterin-4 alpha-carbinolamine dehydratase/dimerization cofactor of hepatocyte nuclear factor 1 alpha  APBA1 - amyloid beta (a4) precursor protein-binding, family a, member 1  APBB2 - amyloid beta (a4) precursor protein-binding, family b, member 2  SMG8 - smg8 nonsense mediated mrna decay factor  CHP1 - calcineurin-like ef-hand protein 1  APBB1 - amyloid beta (a4) precursor protein-binding, family b, member 1 (fe65)  RIF1 - rap1 interacting factor homolog (yeast)  RNF139 - ring finger protein 139  PDCD10 - programmed cell death 10  KIAA1551 - kiaa1551  PA2G4 - proliferation-associated 2g4, 38kda  RPRD1A - regulation of nuclear pre-mrna domain containing 1a  ZHX1 - zinc fingers and homeoboxes 1  ARF4 - adp-ribosylation factor 4  SBNO1 - strawberry notch homolog 1 (drosophila)  IRAK3 - interleukin-1 receptor-associated kinase 3  SETD5 - set domain containing 5  APP - amyloid beta (a4) precursor protein  BBS7 - bardet-biedl syndrome 7  DDX20 - dead (asp-glu-ala-asp) box polypeptide 20  PAM - peptidylglycine alpha-amidating monooxygenase  ARNTL - aryl hydrocarbon receptor nuclear translocator-like  ARNT - aryl hydrocarbon receptor nuclear translocator  CELF3 - cugbp, elav-like family member 3  SUPT16H - suppressor of ty 16 homolog (s. cerevisiae)  ARRB2 - arrestin, beta 2  ARRB1 - arrestin, beta 1  PDGFB - platelet-derived growth factor beta polypeptide  SAMD4B - sterile alpha motif domain containing 4b  STRAP - serine/threonine kinase receptor associated protein  RHOA - ras homolog family member a  PSIP1 - pc4 and sfrs1 interacting protein 1  MED9 - mediator complex subunit 9  FSTL1 - follistatin-like 1  BAZ2A - bromodomain adjacent to zinc finger domain, 2a  PCNA - proliferating cell nuclear antigen  CHMP1A - charged multivesicular body protein 1a  LSM6 - lsm6 homolog, u6 small nuclear rna associated (s. cerevisiae)  JDP2 - jun dimerization protein 2  MAGOHB - mago-nashi homolog b (drosophila)  CDK16 - cyclin-dependent kinase 16  AKIRIN2 - akirin 2  HEATR1 - heat repeat containing 1  CDK17 - cyclin-dependent kinase 17  LEO1 - leo1, paf1/rna polymerase ii complex component, homolog (s. cerevisiae)  ERLIN2 - er lipid raft associated 2  PWP1 - pwp1 homolog (s. cerevisiae)  CDC37 - cell division cycle 37  PDE2A - phosphodiesterase 2a, cgmp-stimulated  GLMN - glomulin, fkbp associated protein  THAP1 - thap domain containing, apoptosis associated protein 1  PKIG - protein kinase (camp-dependent, catalytic) inhibitor gamma  KAT7 - k(lysine) acetyltransferase 7  ASCL1 - achaete-scute complex homolog 1 (drosophila)  ELP3 - elongator acetyltransferase complex subunit 3  RGMB - rgm domain family, member b  SBNO2 - strawberry notch homolog 2 (drosophila)  ZNF444 - zinc finger protein 444  ZBTB1 - zinc finger and btb domain containing 1  FBXW7 - f-box and wd repeat domain containing 7, e3 ubiquitin protein ligase  BAHD1 - bromo adjacent homology domain containing 1  ATP1B1 - atpase, na+/k+ transporting, beta 1 polypeptide  DIS3 - dis3 mitotic control homolog (s. cerevisiae)  ZHX2 - zinc fingers and homeoboxes 2  MORC2 - morc family cw-type zinc finger 2  PHF2 - phd finger protein 2  PHF1 - phd finger protein 1  LRRK2 - leucine-rich repeat kinase 2  FOXJ3 - forkhead box j3  RERE - arginine-glutamic acid dipeptide (re) repeats  DNAJC24 - dnaj (hsp40) homolog, subfamily c, member 24  LARP6 - la ribonucleoprotein domain family, member 6  ZFHX3 - zinc finger homeobox 3  ATG14 - autophagy related 14  SH3PXD2B - sh3 and px domains 2b  ATP7A - atpase, cu++ transporting, alpha polypeptide  PEX14 - peroxisomal biogenesis factor 14  ADNP2 - adnp homeobox 2  AEBP2 - ae binding protein 2  ICK - intestinal cell (mak-like) kinase  IMPACT - impact rwd domain protein  ZNF507 - zinc finger protein 507  LGR4 - leucine-rich repeat containing g protein-coupled receptor 4  ATP2B4 - atpase, ca++ transporting, plasma membrane 4  ZNF652 - zinc finger protein 652  SMU1 - smu-1 suppressor of mec-8 and unc-52 homolog (c. elegans)  SCAF8 - sr-related ctd-associated factor 8  PKD1 - polycystic kidney disease 1 (autosomal dominant)  PKD2 - polycystic kidney disease 2 (autosomal dominant)  PUF60 - poly-u binding splicing factor 60kda  MTF2 - metal response element binding transcription factor 2  BAK1 - bcl2-antagonist/killer 1  NAT10 - n-acetyltransferase 10 (gcn5-related)  BACH1 - btb and cnc homology 1, basic leucine zipper transcription factor 1  PIN1 - peptidylprolyl cis/trans isomerase, nima-interacting 1  PIK3R2 - phosphoinositide-3-kinase, regulatory subunit 2 (beta)  DPH3 - diphthamide biosynthesis 3  PIK3R1 - phosphoinositide-3-kinase, regulatory subunit 1 (alpha)  ASXL2 - additional sex combs like 2 (drosophila)  PLAGL1 - pleiomorphic adenoma gene-like 1  XRN2 - 5'-3' exoribonuclease 2  ELP2 - elongator acetyltransferase complex subunit 2  ATRX - alpha thalassemia/mental retardation syndrome x-linked  PKNOX1 - pbx/knotted 1 homeobox 1  CASC3 - cancer susceptibility candidate 3  PKM - pyruvate kinase, muscle  OGFOD1 - 2-oxoglutarate and iron-dependent oxygenase domain containing 1  EXOSC8 - exosome component 8  TMEM100 - transmembrane protein 100  ATXN7L3B - ataxin 7-like 3b  PHF10 - phd finger protein 10  SERPINE2 - serpin peptidase inhibitor, clade e (nexin, plasminogen activator inhibitor type 1), member 2  U2AF2 - u2 small nuclear rna auxiliary factor 2  PSPC1 - paraspeckle component 1  BCL7A - b-cell cll/lymphoma 7a  MRGBP - mrg/morf4l binding protein  BCL9 - b-cell cll/lymphoma 9  PIK3CB - phosphatidylinositol-4,5-bisphosphate 3-kinase, catalytic subunit beta  PIK3CA - phosphatidylinositol-4,5-bisphosphate 3-kinase, catalytic subunit alpha  RPUSD3 - rna pseudouridylate synthase domain containing 3  BRF2 - brf2, rna polymerase iii transcription initiation factor 50 kda subunit  PIM1 - pim-1 oncogene  BCL2 - b-cell cll/lymphoma 2  ECD - ecdysoneless homolog (drosophila)  CCND1 - cyclin d1  PARK7 - parkinson protein 7  BBS4 - bardet-biedl syndrome 4  BBS2 - bardet-biedl syndrome 2  C3orf33 - chromosome 3 open reading frame 33  TRIM27 - tripartite motif containing 27  SAP18 - sin3a-associated protein, 18kda  TSHZ2 - teashirt zinc finger homeobox 2  RFXAP - regulatory factor x-associated protein  BRAF - v-raf murine sarcoma viral oncogene homolog b  RFX5 - regulatory factor x, 5 (influences hla class ii expression)  WHSC1L1 - wolf-hirschhorn syndrome candidate 1-like 1  EIF1B - eukaryotic translation initiation factor 1b  RFX1 - regulatory factor x, 1 (influences hla class ii expression)  APBB3 - amyloid beta (a4) precursor protein-binding, family b, member 3  RELA - v-rel avian reticuloendotheliosis viral oncogene homolog a  BCOR - bcl6 corepressor  ZFP36L2 - zfp36 ring finger protein-like 2  RDX - radixin  ZFP36L1 - zfp36 ring finger protein-like 1  RET - ret proto-oncogene  ALKBH5 - alkb, alkylation repair homolog 5 (e. coli)  DPF2 - d4, zinc and double phd fingers family 2  NSUN2 - nop2/sun rna methyltransferase family, member 2  UPF1 - upf1 regulator of nonsense transcripts homolog (yeast)  CC2D1A - coiled-coil and c2 domain containing 1a  RLF - rearranged l-myc fusion  RING1 - ring finger protein 1  CNPY2 - canopy fgf signaling regulator 2  BMI1 - bmi1 polycomb ring finger oncogene  PCGF3 - polycomb group ring finger 3  BMPR1A - bone morphogenetic protein receptor, type ia  BMPR2 - bone morphogenetic protein receptor, type ii (serine/threonine kinase)  GON4L - gon-4-like (c. elegans)  BMPR1B - bone morphogenetic protein receptor, type ib  BMP7 - bone morphogenetic protein 7  PSMD14 - proteasome (prosome, macropain) 26s subunit, non-atpase, 14  FLOT1 - flotillin 1  BCAS3 - breast carcinoma amplified sequence 3  COQ7 - coenzyme q7 homolog, ubiquinone (yeast)  UHMK1 - u2af homology motif (uhm) kinase 1  AHI1 - abelson helper integration site 1  RNF4 - ring finger protein 4  BRD2 - bromodomain containing 2  RNF2 - ring finger protein 2  HNRNPR - heterogeneous nuclear ribonucleoprotein r  ZNF280D - zinc finger protein 280d  RNF6 - ring finger protein (c3h2c3 type) 6  GATAD2A - gata zinc finger domain containing 2a  ROBO1 - roundabout, axon guidance receptor, homolog 1 (drosophila)  ROCK1 - rho-associated, coiled-coil containing protein kinase 1  ROM1 - retinal outer segment membrane protein 1  RNF111 - ring finger protein 111  SPRY2 - sprouty homolog 2 (drosophila)  TET2 - tet methylcytosine dioxygenase 2  MED18 - mediator complex subunit 18  DENND4A - denn/madd domain containing 4a  MBTD1 - mbt domain containing 1  SF3B4 - splicing factor 3b, subunit 4, 49kda  RAMP2 - receptor (g protein-coupled) activity modifying protein 2  RAMP3 - receptor (g protein-coupled) activity modifying protein 3  ZMPSTE24 - zinc metallopeptidase ste24  C5AR1 - complement component 5a receptor 1  TRIM44 - tripartite motif containing 44  TAB1 - tgf-beta activated kinase 1/map3k7 binding protein 1  PPIE - peptidylprolyl isomerase e (cyclophilin e)  MCRS1 - microspherule protein 1  NRDE2 - nrde-2, necessary for rna interference, domain containing  RPL17 - ribosomal protein l17  TIMM17A - translocase of inner mitochondrial membrane 17 homolog a (yeast)  RPL15 - ribosomal protein l15  OLFM1 - olfactomedin 1  C1D - c1d nuclear receptor corepressor  RPL11 - ribosomal protein l11  RPL9 - ribosomal protein l9  PTCD3 - pentatricopeptide repeat domain 3  RPL7 - ribosomal protein l7  SLC30A9 - solute carrier family 30 (zinc transporter), member 9  RPL3 - ribosomal protein l3  MAD2L2 - mad2 mitotic arrest deficient-like 2 (yeast)  PID1 - phosphotyrosine interaction domain containing 1  PHIP - pleckstrin homology domain interacting protein  RPS4X - ribosomal protein s4, x-linked  RPS4Y1 - ribosomal protein s4, y-linked 1  NRARP - notch-regulated ankyrin repeat protein  ZNF787 - zinc finger protein 787  RMND1 - required for meiotic nuclear division 1 homolog (s. cerevisiae)  TRIM38 - tripartite motif containing 38  TADA3 - transcriptional adaptor 3  CALR - calreticulin  ZNF792 - zinc finger protein 792  WTIP - wilms tumor 1 interacting protein  NCOA2 - nuclear receptor coactivator 2  SYNCRIP - synaptotagmin binding, cytoplasmic rna interacting protein  RPL32 - ribosomal protein l32  CREB3 - camp responsive element binding protein 3  CITED2 - cbp/p300-interacting transactivator, with glu/asp-rich carboxy-terminal domain, 2  RPS23 - ribosomal protein s23  CCNT2 - cyclin t2  CCND3 - cyclin d3  CCNT1 - cyclin t1  HMG20A - high mobility group 20a  CCNH - cyclin h  BANP - btg3 associated nuclear protein  RPS15 - ribosomal protein s15  RPS12 - ribosomal protein s12  TRNAU1AP - trna selenocysteine 1 associated protein 1  RPS6KA1 - ribosomal protein s6 kinase, 90kda, polypeptide 1  RPS6KB1 - ribosomal protein s6 kinase, 70kda, polypeptide 1  RPS6KB2 - ribosomal protein s6 kinase, 70kda, polypeptide 2  CBFA2T3 - core-binding factor, runt domain, alpha subunit 2; translocated to, 3  CBFB - core-binding factor, beta subunit  PIAS3 - protein inhibitor of activated stat, 3  RXRB - retinoid x receptor, beta  CAV1 - caveolin 1, caveolae protein, 22kda  RBM14 - rna binding motif protein 14  CCNC - cyclin c  TRMT10C - trna methyltransferase 10 homolog c (s. cerevisiae)  SPON1 - spondin 1, extracellular matrix protein  PRMT5 - protein arginine methyltransferase 5  CDK11B - cyclin-dependent kinase 11b  MXD4 - max dimerization protein 4  CDC5L - cell division cycle 5-like  HTRA1 - htra serine peptidase 1  POLR3F - polymerase (rna) iii (dna directed) polypeptide f, 39 kda  CDH3 - cadherin 3, type 1, p-cadherin (placental)  HEXIM1 - hexamethylene bis-acetamide inducible 1  ERLIN1 - er lipid raft associated 1  SPIN2A - spindlin family, member 2a  NCOA7 - nuclear receptor coactivator 7  PSEN1 - presenilin 1  USP16 - ubiquitin specific peptidase 16  PSMA1 - proteasome (prosome, macropain) subunit, alpha type, 1  SRSF12 - serine/arginine-rich splicing factor 12  PSMA2 - proteasome (prosome, macropain) subunit, alpha type, 2  CD81 - cd81 molecule  CAMKK2 - calcium/calmodulin-dependent protein kinase kinase 2, beta  PSMB1 - proteasome (prosome, macropain) subunit, beta type, 1  PSMA5 - proteasome (prosome, macropain) subunit, alpha type, 5  PSMA3 - proteasome (prosome, macropain) subunit, alpha type, 3  RIPPLY2 - ripply transcriptional repressor 2  PSMB10 - proteasome (prosome, macropain) subunit, beta type, 10  KHDRBS3 - kh domain containing, rna binding, signal transduction associated 3  PSMC6 - proteasome (prosome, macropain) 26s subunit, atpase, 6  TAF6L - taf6-like rna polymerase ii, p300/cbp-associated factor (pcaf)-associated factor, 65kda  PSMD1 - proteasome (prosome, macropain) 26s subunit, non-atpase, 1  PSMC4 - proteasome (prosome, macropain) 26s subunit, atpase, 4  SOX18 - sry (sex determining region y)-box 18  POLR3G - polymerase (rna) iii (dna directed) polypeptide g (32kd)  PSMC2 - proteasome (prosome, macropain) 26s subunit, atpase, 2  PSMD9 - proteasome (prosome, macropain) 26s subunit, non-atpase, 9  PSMD10 - proteasome (prosome, macropain) 26s subunit, non-atpase, 10  CD34 - cd34 molecule  PSMD7 - proteasome (prosome, macropain) 26s subunit, non-atpase, 7  PSMD8 - proteasome (prosome, macropain) 26s subunit, non-atpase, 8  PSMD5 - proteasome (prosome, macropain) 26s subunit, non-atpase, 5  TERF2IP - telomeric repeat binding factor 2, interacting protein  PSMD2 - proteasome (prosome, macropain) 26s subunit, non-atpase, 2  SLC38A2 - solute carrier family 38, member 2  PSMD12 - proteasome (prosome, macropain) 26s subunit, non-atpase, 12  PSMD13 - proteasome (prosome, macropain) 26s subunit, non-atpase, 13  PTBP1 - polypyrimidine tract binding protein 1  PTEN - phosphatase and tensin homolog  LAMTOR5 - late endosomal/lysosomal adaptor, mapk and mtor activator 5  JMY - junction mediating and regulatory protein, p53 cofactor  MYBBP1A - myb binding protein (p160) 1a  FBLN5 - fibulin 5  PTGIS - prostaglandin i2 (prostacyclin) synthase  CIB1 - calcium and integrin binding 1 (calmyrin)  DDX17 - dead (asp-glu-ala-asp) box helicase 17  KAT5 - k(lysine) acetyltransferase 5  ZFP69 - zfp69 zinc finger protein  IPO8 - importin 8  CEBPG - ccaat/enhancer binding protein (c/ebp), gamma  CDK5 - cyclin-dependent kinase 5  CDK4 - cyclin-dependent kinase 4  CDK9 - cyclin-dependent kinase 9  CDK8 - cyclin-dependent kinase 8  LIN54 - lin-54 homolog (c. elegans)  CDK7 - cyclin-dependent kinase 7  CDKN1C - cyclin-dependent kinase inhibitor 1c (p57, kip2)  CDKN1B - cyclin-dependent kinase inhibitor 1b (p27, kip1)  PCBP3 - poly(rc) binding protein 3  PTPN2 - protein tyrosine phosphatase, non-receptor type 2  HTATIP2 - hiv-1 tat interactive protein 2, 30kda  CDK2 - cyclin-dependent kinase 2  PURA - purine-rich element binding protein a  RRN3 - rrn3 rna polymerase i transcription factor homolog (s. cerevisiae)  PURB - purine-rich element binding protein b  GTPBP2 - gtp binding protein 2  NUDT16 - nudix (nucleoside diphosphate linked moiety x)-type motif 16  ZMYND11 - zinc finger, mynd-type containing 11  SRSF10 - serine/arginine-rich splicing factor 10  PTPRK - protein tyrosine phosphatase, receptor type, k  PTPRN - protein tyrosine phosphatase, receptor type, n  AHCYL1 - adenosylhomocysteinase-like 1  PEX2 - peroxisomal biogenesis factor 2  NUP50 - nucleoporin 50kda  MPHOSPH8 - m-phase phosphoprotein 8  KDM5B - lysine (k)-specific demethylase 5b  CHD4 - chromodomain helicase dna binding protein 4  CHD2 - chromodomain helicase dna binding protein 2  CHD3 - chromodomain helicase dna binding protein 3  MTERFD2 - mterf domain containing 2  CHD1 - chromodomain helicase dna binding protein 1  QARS - glutaminyl-trna synthetase  MYPOP - myb-related transcription factor, partner of profilin  RAD21 - rad21 homolog (s. pombe)  PARP14 - poly (adp-ribose) polymerase family, member 14  PAF1 - paf1, rna polymerase ii associated factor, homolog (s. cerevisiae)  ZNF181 - zinc finger protein 181  ZNF268 - zinc finger protein 268  INO80 - ino80 complex subunit  ZNF513 - zinc finger protein 513  RAB27A - rab27a, member ras oncogene family  NDFIP2 - nedd4 family interacting protein 2  GMEB1 - glucocorticoid modulatory element binding protein 1  RC3H2 - ring finger and ccch-type domains 2  RANBP2 - ran binding protein 2  MIER2 - mesoderm induction early response 1, family member 2  MOK - mok protein kinase  RARG - retinoic acid receptor, gamma  RARB - retinoic acid receptor, beta  DLL4 - delta-like 4 (drosophila)  CELF1 - cugbp, elav-like family member 1  CLU - clusterin  KHDRBS1 - kh domain containing, rna binding, signal transduction associated 1  TAF8 - taf8 rna polymerase ii, tata box binding protein (tbp)-associated factor, 43kda  CLK1 - cdc-like kinase 1  MAGEL2 - mage-like 2  CELF2 - cugbp, elav-like family member 2  CLK2 - cdc-like kinase 2  CTCF - ccctc-binding factor (zinc finger protein)  DDX49 - dead (asp-glu-ala-asp) box polypeptide 49  CLK3 - cdc-like kinase 3  PRMT7 - protein arginine methyltransferase 7  RBL2 - retinoblastoma-like 2 (p130)  RAI1 - retinoic acid induced 1  RBBP7 - retinoblastoma binding protein 7  RBBP8 - retinoblastoma binding protein 8  MAP3K2 - mitogen-activated protein kinase kinase kinase 2  RBM3 - rna binding motif (rnp1, rrm) protein 3  DIS3L2 - dis3 mitotic control homolog (s. cerevisiae)-like 2  RBM4 - rna binding motif protein 4  RB1 - retinoblastoma 1  ARID4A - at rich interactive domain 4a (rbp1-like)  KDM5A - lysine (k)-specific demethylase 5a  NUP35 - nucleoporin 35kda  RBBP4 - retinoblastoma binding protein 4  PUS7 - pseudouridylate synthase 7 homolog (s. cerevisiae)  EXOSC4 - exosome component 4  CKS1B - cdc28 protein kinase regulatory subunit 1b  NFAT5 - nuclear factor of activated t-cells 5, tonicity-responsive  CIRBP - cold inducible rna binding protein  DHX29 - deah (asp-glu-ala-his) box polypeptide 29  CHUK - conserved helix-loop-helix ubiquitous kinase  ZMYND8 - zinc finger, mynd-type containing 8  ANKRA2 - ankyrin repeat, family a (rfxank-like), 2  RBAK - rb-associated krab zinc finger  NFATC2IP - nuclear factor of activated t-cells, cytoplasmic, calcineurin-dependent 2 interacting protein  PATZ1 - poz (btb) and at hook containing zinc finger 1  METTL14 - methyltransferase like 14  SPP1 - secreted phosphoprotein 1  SSBP2 - single-stranded dna binding protein 2  KPNA6 - karyopherin alpha 6 (importin alpha 7)  CIRH1A - cirrhosis, autosomal recessive 1a (cirhin)  MIER1 - mesoderm induction early response 1, transcriptional regulator  PPP1R15B - protein phosphatase 1, regulatory subunit 15b  ZNF382 - zinc finger protein 382  CWC22 - cwc22 spliceosome-associated protein homolog (s. cerevisiae)  ZNF705A - zinc finger protein 705a  SP3 - sp3 transcription factor  SP4 - sp4 transcription factor  SP1 - sp1 transcription factor  SP2 - sp2 transcription factor  ZNF410 - zinc finger protein 410  SOX4 - sry (sex determining region y)-box 4  LSM2 - lsm2 homolog, u6 small nuclear rna associated (s. cerevisiae)  WBP2 - ww domain binding protein 2  SON - son dna binding protein  HES4 - hairy and enhancer of split 4 (drosophila)  LEMD3 - lem domain containing 3  GATAD1 - gata zinc finger domain containing 1  CREBL2 - camp responsive element binding protein-like 2  ATF6B - activating transcription factor 6 beta  CREBBP - creb binding protein  ATF2 - activating transcription factor 2  CREB1 - camp responsive element binding protein 1  DDX58 - dead (asp-glu-ala-asp) box polypeptide 58  CRABP2 - cellular retinoic acid binding protein 2  RPRD1B - regulation of nuclear pre-mrna domain containing 1b  PLK3 - polo-like kinase 3  SNAPC4 - small nuclear rna activating complex, polypeptide 4, 190kda  SNCA - synuclein, alpha (non a4 component of amyloid precursor)  SNRNP70 - small nuclear ribonucleoprotein 70kda (u1)  ZBED5 - zinc finger, bed-type containing 5  SNRPA - small nuclear ribonucleoprotein polypeptide a  CREBZF - creb/atf bzip transcription factor  EID1 - ep300 interacting inhibitor of differentiation 1  TP53INP2 - tumor protein p53 inducible nuclear protein 2  PTBP2 - polypyrimidine tract binding protein 2  SMARCE1 - swi/snf related, matrix associated, actin dependent regulator of chromatin, subfamily e, member 1  RBM17 - rna binding motif protein 17  SMO - smoothened, frizzled family receptor  CNTN1 - contactin 1  SMARCB1 - swi/snf related, matrix associated, actin dependent regulator of chromatin, subfamily b, member 1  SMARCC1 - swi/snf related, matrix associated, actin dependent regulator of chromatin, subfamily c, member 1  HLTF - helicase-like transcription factor  SMARCA4 - swi/snf related, matrix associated, actin dependent regulator of chromatin, subfamily a, member 4  SNX6 - sorting nexin 6  SSBP3 - single stranded dna binding protein 3  SMARCD2 - swi/snf related, matrix associated, actin dependent regulator of chromatin, subfamily d, member 2  LSM5 - lsm5 homolog, u6 small nuclear rna associated (s. cerevisiae)  SMARCD3 - swi/snf related, matrix associated, actin dependent regulator of chromatin, subfamily d, member 3  SMARCC2 - swi/snf related, matrix associated, actin dependent regulator of chromatin, subfamily c, member 2  SMARCD1 - swi/snf related, matrix associated, actin dependent regulator of chromatin, subfamily d, member 1  WIZ - widely interspaced zinc finger motifs  SLIT3 - slit homolog 3 (drosophila)  RBM25 - rna binding motif protein 25  SMARCA1 - swi/snf related, matrix associated, actin dependent regulator of chromatin, subfamily a, member 1  SMARCA2 - swi/snf related, matrix associated, actin dependent regulator of chromatin, subfamily a, member 2  EDC4 - enhancer of mrna decapping 4  SGK3 - serum/glucocorticoid regulated kinase family, member 3  CACTIN - cactin, spliceosome c complex subunit  KMT2C - lysine (k)-specific methyltransferase 2c  ZNF462 - zinc finger protein 462  PPIL4 - peptidylprolyl isomerase (cyclophilin)-like 4  SENP2 - sumo1/sentrin/smt3 specific peptidase 2  ZNF318 - zinc finger protein 318  PRPF6 - pre-mrna processing factor 6  KDM2B - lysine (k)-specific demethylase 2b  SUPT6H - suppressor of ty 6 homolog (s. cerevisiae)  CTGF - connective tissue growth factor  CBY1 - chibby homolog 1 (drosophila)  SUPV3L1 - suppressor of var1, 3-like 1 (s. cerevisiae)  CACNG7 - calcium channel, voltage-dependent, gamma subunit 7  CTBP1 - c-terminal binding protein 1  SUPT4H1 - suppressor of ty 4 homolog 1 (s. cerevisiae)  CTBP2 - c-terminal binding protein 2  SUPT5H - suppressor of ty 5 homolog (s. cerevisiae)  CTNNB1 - catenin (cadherin-associated protein), beta 1, 88kda  SAV1 - salvador homolog 1 (drosophila)  CUX1 - cut-like homeobox 1  NIF3L1 - nif3 ngg1 interacting factor 3-like 1 (s. cerevisiae)  NEK4 - nima-related kinase 4  STK3 - serine/threonine kinase 3  MPV17L2 - mpv17 mitochondrial membrane protein-like 2  PCGF1 - polycomb group ring finger 1  NPNT - nephronectin  FKBP8 - fk506 binding protein 8, 38kda  GPBP1L1 - gc-rich promoter binding protein 1-like 1  CYLD - cylindromatosis (turban tumor syndrome)  PAN3 - pan3 poly(a) specific ribonuclease subunit homolog (s. cerevisiae)  TGIF2 - tgfb-induced factor homeobox 2  BCL6B - b-cell cll/lymphoma 6, member b  CRKL - v-crk avian sarcoma virus ct10 oncogene homolog-like  CRK - v-crk avian sarcoma virus ct10 oncogene homolog  EEFSEC - eukaryotic elongation factor, selenocysteine-trna-specific  CELF6 - cugbp, elav-like family member 6  NIPBL - nipped-b homolog (drosophila)  CRY1 - cryptochrome 1 (photolyase-like)  STAT2 - signal transducer and activator of transcription 2, 113kda  STAT1 - signal transducer and activator of transcription 1, 91kda  STAT3 - signal transducer and activator of transcription 3 (acute-phase response factor)  CRY2 - cryptochrome 2 (photolyase-like)  ASF1A - anti-silencing function 1a histone chaperone  STAT5A - signal transducer and activator of transcription 5a  STAT6 - signal transducer and activator of transcription 6, interleukin-4 induced  SGMS1 - sphingomyelin synthase 1  BRMS1 - breast cancer metastasis suppressor 1  DFNB31 - deafness, autosomal recessive 31  CRYM - crystallin, mu  ZNF496 - zinc finger protein 496  SS18 - synovial sarcoma translocation, chromosome 18  PHF5A - phd finger protein 5a  GPATCH3 - g patch domain containing 3  CSF1 - colony stimulating factor 1 (macrophage)  SRPK2 - srsf protein kinase 2  SRPK1 - srsf protein kinase 1  TAB3 - tgf-beta activated kinase 1/map3k7 binding protein 3  TROVE2 - trove domain family, member 2  BAMBI - bmp and activin membrane-bound inhibitor  ZNF335 - zinc finger protein 335  HAVCR2 - hepatitis a virus cellular receptor 2  SSB - sjogren syndrome antigen b (autoantigen la)  ZC3H10 - zinc finger ccch-type containing 10  CSNK1D - casein kinase 1, delta  SRI - sorcin  SRC - v-src avian sarcoma (schmidt-ruppin a-2) viral oncogene homolog  DNAJB5 - dnaj (hsp40) homolog, subfamily b, member 5  SREBF2 - sterol regulatory element binding transcription factor 2  UBE2O - ubiquitin-conjugating enzyme e2o  NSUN3 - nop2/sun domain family, member 3  SRP9 - signal recognition particle 9kda  PRDX5 - peroxiredoxin 5  DHX9 - deah (asp-glu-ala-his) box helicase 9  TRAPPC2 - trafficking protein particle complex 2  KANK2 - kn motif and ankyrin repeat domains 2  DDX6 - dead (asp-glu-ala-asp) box helicase 6  DDX5 - dead (asp-glu-ala-asp) box helicase 5  DDX3X - dead (asp-glu-ala-asp) box helicase 3, x-linked  SEC13 - sec13 homolog (s. cerevisiae)  DDX1 - dead (asp-glu-ala-asp) box helicase 1  SIN3A - sin3 transcription regulator family member a  DPH6 - diphthamine biosynthesis 6  FAM98A - family with sequence similarity 98, member a  SET - set nuclear oncogene  WWTR1 - ww domain containing transcription regulator 1  ZNF304 - zinc finger protein 304  ZNF286A - zinc finger protein 286a  GEMIN5 - gem (nuclear organelle) associated protein 5  NOL11 - nucleolar protein 11  PBXIP1 - pre-b-cell leukemia homeobox interacting protein 1  DBP - d site of albumin promoter (albumin d-box) binding protein  NELFB - negative elongation factor complex member b  AHCTF1 - at hook containing transcription factor 1  CLK4 - cdc-like kinase 4  CNOT10 - ccr4-not transcription complex, subunit 10  ZBTB38 - zinc finger and btb domain containing 38  GATAD2B - gata zinc finger domain containing 2b  ZNF551 - zinc finger protein 551  POLR1A - polymerase (rna) i polypeptide a, 194kda  ZNF473 - zinc finger protein 473  YTHDF3 - yth domain family, member 3  CNOT6 - ccr4-not transcription complex, subunit 6  LTN1 - listerin e3 ubiquitin protein ligase 1  ATXN2 - ataxin 2  ZNF451 - zinc finger protein 451  ERI1 - exoribonuclease 1  SCAF4 - sr-related ctd-associated factor 4  SS18L1 - synovial sarcoma translocation gene on chromosome 18-like 1  CHD5 - chromodomain helicase dna binding protein 5  ARID1B - at rich interactive domain 1b (swi1-like)  TMEM98 - transmembrane protein 98  MED30 - mediator complex subunit 30  MKL2 - mkl/myocardin-like 2  DAPK3 - death-associated protein kinase 3  DAXX - death-domain associated protein  ANGEL2 - angel homolog 2 (drosophila)  XPO5 - exportin 5  ZZZ3 - zinc finger, zz-type containing 3  HINFP - histone h4 transcription factor  MTA3 - metastasis associated 1 family, member 3  DGKQ - diacylglycerol kinase, theta 110kda  ZNF608 - zinc finger protein 608  DAPK1 - death-associated protein kinase 1  DAP - death-associated protein  MAVS - mitochondrial antiviral signaling protein  TIPARP - tcdd-inducible poly(adp-ribose) polymerase  SARS - seryl-trna synthetase  SATB1 - satb homeobox 1  NGDN - neuroguidin, eif4e binding protein  RPTOR - regulatory associated protein of mtor, complex 1  SAFB - scaffold attachment factor b  SALL2 - sal-like 2 (drosophila)  SERBP1 - serpine1 mrna binding protein 1  EPC2 - enhancer of polycomb homolog 2 (drosophila)  DRD2 - dopamine receptor d2  PHF19 - phd finger protein 19  SLC9A1 - solute carrier family 9, subfamily a (nhe1, cation proton antiporter 1), member 1  NKD2 - naked cuticle homolog 2 (drosophila)  IRF2BP1 - interferon regulatory factor 2 binding protein 1  SLC8A1 - solute carrier family 8 (sodium/calcium exchanger), member 1  EAF1 - ell associated factor 1  CNOT6L - ccr4-not transcription complex, subunit 6-like  SLC8A2 - solute carrier family 8 (sodium/calcium exchanger), member 2  ATN1 - atrophin 1  DPH2 - dph2 homolog (s. cerevisiae)  DPH1 - diphthamide biosynthesis 1  DOM3Z - dom-3 homolog z (c. elegans)  C10orf137 - chromosome 10 open reading frame 137  DR1 - down-regulator of transcription 1, tbp-binding (negative cofactor 2)  TNKS1BP1 - tankyrase 1 binding protein 1, 182kda  MAPK15 - mitogen-activated protein kinase 15  SKI - v-ski avian sarcoma viral oncogene homolog  SKIL - ski-like oncogene  UNK - unkempt homolog (drosophila)  SKIV2L - superkiller viralicidic activity 2-like (s. cerevisiae)  DYRK1A - dual-specificity tyrosine-(y)-phosphorylation regulated kinase 1a  MKL1 - megakaryoblastic leukemia (translocation) 1  DVL2 - dishevelled segment polarity protein 2  DVL3 - dishevelled segment polarity protein 3  GIGYF2 - grb10 interacting gyf protein 2  USP36 - ubiquitin specific peptidase 36  FNIP2 - folliculin interacting protein 2  HOMEZ - homeobox and leucine zipper encoding  EBF4 - early b-cell factor 4  ZFHX2 - zinc finger homeobox 2  LSM14A - lsm14a, scd6 homolog a (s. cerevisiae)  DKC1 - dyskeratosis congenita 1, dyskerin  PNPT1 - polyribonucleotide nucleotidyltransferase 1  DLG1 - discs, large homolog 1 (drosophila)  SHOX2 - short stature homeobox 2  PHGDH - phosphoglycerate dehydrogenase  POGK - pogo transposable element with krab domain  DHFR - dihydrofolate reductase  RICTOR - rptor independent companion of mtor, complex 2  PHF12 - phd finger protein 12  CALCOCO1 - calcium binding and coiled-coil domain 1  SFPQ - splicing factor proline/glutamine-rich  ZBTB4 - zinc finger and btb domain containing 4  SFRP1 - secreted frizzled-related protein 1  SFRP2 - secreted frizzled-related protein 2  SFRP4 - secreted frizzled-related protein 4  SRSF1 - serine/arginine-rich splicing factor 1  SRSF2 - serine/arginine-rich splicing factor 2  DNM2 - dynamin 2  GMEB2 - glucocorticoid modulatory element binding protein 2  SRSF3 - serine/arginine-rich splicing factor 3  EPB41L5 - erythrocyte membrane protein band 4.1 like 5  DNMT1 - dna (cytosine-5-)-methyltransferase 1  SPAG8 - sperm associated antigen 8  SRSF4 - serine/arginine-rich splicing factor 4  TRDMT1 - trna aspartic acid methyltransferase 1  SRSF5 - serine/arginine-rich splicing factor 5  DNMT3A - dna (cytosine-5-)-methyltransferase 3 alpha  SRSF6 - serine/arginine-rich splicing factor 6  SRSF7 - serine/arginine-rich splicing factor 7  EIF2AK4 - eukaryotic translation initiation factor 2 alpha kinase 4  SFSWAP - splicing factor, suppressor of white-apricot homolog (drosophila)  TRA2B - transformer 2 beta homolog (drosophila)  CHD8 - chromodomain helicase dna binding protein 8  TNRC6C - trinucleotide repeat containing 6c  SGK1 - serum/glucocorticoid regulated kinase 1  NOC2L - nucleolar complex associated 2 homolog (s. cerevisiae)  ZNF317 - zinc finger protein 317  MEIS3 - meis homeobox 3  NCBP2 - nuclear cap binding protein subunit 2, 20kda  MAPRE3 - microtubule-associated protein, rp/eb family, member 3  TSC1 - tuberous sclerosis 1  ELK1 - elk1, member of ets oncogene family  NAF1 - nuclear assembly factor 1 ribonucleoprotein  ELK3 - elk3, ets-domain protein (srf accessory protein 2)  ELK4 - elk4, ets-domain protein (srf accessory protein 1)  SNW1 - snw domain containing 1  SCAP - srebf chaperone  EPAS1 - endothelial pas domain protein 1  DPH7 - diphthamide biosynthesis 7  EP300 - e1a binding protein p300  HABP4 - hyaluronan binding protein 4  ATF6 - activating transcription factor 6  ENO1 - enolase 1, (alpha)  RAB3GAP1 - rab3 gtpase activating protein subunit 1 (catalytic)  EIF4A2 - eukaryotic translation initiation factor 4a2  EIF4E - eukaryotic translation initiation factor 4e  CELF4 - cugbp, elav-like family member 4  EGR2 - early growth response 2  EGR3 - early growth response 3  ZC3HAV1 - zinc finger ccch-type, antiviral 1  EIF2S1 - eukaryotic translation initiation factor 2, subunit 1 alpha, 35kda  EIF2B1 - eukaryotic translation initiation factor 2b, subunit 1 alpha, 26kda  TRIM32 - tripartite motif containing 32  SPHK2 - sphingosine kinase 2  EIF2S3 - eukaryotic translation initiation factor 2, subunit 3 gamma, 52kda  KDM2A - lysine (k)-specific demethylase 2a  ELAVL1 - elav (embryonic lethal, abnormal vision, drosophila)-like 1 (hu antigen r)  ZNF326 - zinc finger protein 326  ELF1 - e74-like factor 1 (ets domain transcription factor)  ELAVL4 - elav (embryonic lethal, abnormal vision, drosophila)-like 4  ELF2 - e74-like factor 2 (ets domain transcription factor)  EIF4G2 - eukaryotic translation initiation factor 4 gamma, 2  TCF25 - transcription factor 25 (basic helix-loop-helix)  TSG101 - tumor susceptibility gene 101  CNOT1 - ccr4-not transcription complex, subunit 1  EFNA1 - ephrin-a1  EEF2 - eukaryotic translation elongation factor 2  EXOSC7 - exosome component 7  SPEN - spen homolog, transcriptional regulator (drosophila)  CTNNBIP1 - catenin, beta interacting protein 1  BBX - bobby sox homolog (drosophila)  PHC1 - polyhomeotic homolog 1 (drosophila)  TP53BP1 - tumor protein p53 binding protein 1  PHC2 - polyhomeotic homolog 2 (drosophila)  EGR1 - early growth response 1  ZNF292 - zinc finger protein 292  EGFR - epidermal growth factor receptor  MEGF8 - multiple egf-like-domains 8  CELSR2 - cadherin, egf lag seven-pass g-type receptor 2  TOP1 - topoisomerase (dna) i  KDM1A - lysine (k)-specific demethylase 1a  ATXN7L3 - ataxin 7-like 3  HNRNPLL - heterogeneous nuclear ribonucleoprotein l-like  NCOA6 - nuclear receptor coactivator 6  E2F6 - e2f transcription factor 6  SMG1 - smg1 phosphatidylinositol 3-kinase-related kinase  E4F1 - e4f transcription factor 1  ZHX3 - zinc fingers and homeoboxes 3  E2F4 - e2f transcription factor 4, p107/p130-binding  E2F5 - e2f transcription factor 5, p130-binding  ENY2 - enhancer of yellow 2 homolog (drosophila)  C11orf30 - chromosome 11 open reading frame 30  RRP1B - ribosomal rna processing 1b  EDNRB - endothelin receptor type b  MYCBP2 - myc binding protein 2, e3 ubiquitin protein ligase  TRAF1 - tnf receptor-associated factor 1  S1PR1 - sphingosine-1-phosphate receptor 1  TRAF3 - tnf receptor-associated factor 3  TRAF2 - tnf receptor-associated factor 2  NR2C1 - nuclear receptor subfamily 2, group c, member 1  SETX - senataxin  ZBTB47 - zinc finger and btb domain containing 47  NR2C2 - nuclear receptor subfamily 2, group c, member 2  TPR - translocated promoter region, nuclear basket protein  ZNF609 - zinc finger protein 609  ERC1 - elks/rab6-interacting/cast family member 1  PPRC1 - peroxisome proliferator-activated receptor gamma, coactivator-related 1  NAT14 - n-acetyltransferase 14 (gcn5-related, putative)  KDM4C - lysine (k)-specific demethylase 4c  ZNF423 - zinc finger protein 423  REXO4 - rex4, rna exonuclease 4 homolog (s. cerevisiae)  CAMK1D - calcium/calmodulin-dependent protein kinase id  USF1 - upstream transcription factor 1  NUP107 - nucleoporin 107kda  KIF1B - kinesin family member 1b  ZBTB43 - zinc finger and btb domain containing 43  PTK2B - protein tyrosine kinase 2 beta  MRPS27 - mitochondrial ribosomal protein s27  TNRC6B - trinucleotide repeat containing 6b  PITHD1 - pith (c-terminal proteasome-interacting domain of thioredoxin-like) domain containing 1  BPTF - bromodomain phd finger transcription factor  TAB2 - tgf-beta activated kinase 1/map3k7 binding protein 2  NR1H2 - nuclear receptor subfamily 1, group h, member 2  ETV3 - ets variant 3  CAMTA2 - calmodulin binding transcription activator 2  ZNF598 - zinc finger protein 598  ETV5 - ets variant 5  POGZ - pogo transposable element with znf domain  TWSG1 - twisted gastrulation homolog 1 (drosophila)  ETV6 - ets variant 6  PLXND1 - plexin d1  VHL - von hippel-lindau tumor suppressor, e3 ubiquitin protein ligase  VIM - vimentin  KDM6B - lysine (k)-specific demethylase 6b  PHF8 - phd finger protein 8  EZR - ezrin  EWSR1 - ews rna-binding protein 1  TRIM41 - tripartite motif containing 41  TBX20 - t-box 20  PCBP4 - poly(rc) binding protein 4  KCTD1 - potassium channel tetramerization domain containing 1  KDM6A - lysine (k)-specific demethylase 6a  CREB3L1 - camp responsive element binding protein 3-like 1  ZC3H3 - zinc finger ccch-type containing 3  EZH1 - enhancer of zeste homolog 1 (drosophila)  CIC - capicua transcriptional repressor  CCNL1 - cyclin l1  F2R - coagulation factor ii (thrombin) receptor  F3 - coagulation factor iii (thromboplastin, tissue factor)  WDR43 - wd repeat domain 43  ERF - ets2 repressor factor  ZNF575 - zinc finger protein 575  NUP205 - nucleoporin 205kda  YTHDC1 - yth domain containing 1  THOC2 - tho complex 2  ELMSAN1 - elm2 and myb/sant-like domain containing 1  THAP11 - thap domain containing 11  ERN1 - endoplasmic reticulum to nucleus signaling 1  ZNF248 - zinc finger protein 248  UBB - ubiquitin b  LARP4B - la ribonucleoprotein domain family, member 4b  RCOR1 - rest corepressor 1  DIP2A - dip2 disco-interacting protein 2 homolog a (drosophila)  TULP3 - tubby like protein 3  ZNFX1 - zinc finger, nfx1-type containing 1  HIRA - histone cell cycle regulator  TTC5 - tetratricopeptide repeat domain 5  ETV1 - ets variant 1  ETS1 - v-ets avian erythroblastosis virus e26 oncogene homolog 1  ETF1 - eukaryotic translation termination factor 1  ZMIZ1 - zinc finger, miz-type containing 1  CYFIP1 - cytoplasmic fmr1 interacting protein 1  UBP1 - upstream binding protein 1 (lbp-1a)  SUMO1 - small ubiquitin-like modifier 1  UBTF - upstream binding transcription factor, rna polymerase i  PELI1 - pellino e3 ubiquitin protein ligase 1  CHURC1 - churchill domain containing 1  SMURF1 - smad specific e3 ubiquitin protein ligase 1  ZC3H4 - zinc finger ccch-type containing 4  JMJD6 - jumonji domain containing 6  MYADM - myeloid-associated differentiation marker  EPRS - glutamyl-prolyl-trna synthetase  UBE2I - ubiquitin-conjugating enzyme e2i  NUP210 - nucleoporin 210kda  UBE2L3 - ubiquitin-conjugating enzyme e2l 3  UBE2N - ubiquitin-conjugating enzyme e2n  UBE2V1 - ubiquitin-conjugating enzyme e2 variant 1  ZC3H7B - zinc finger ccch-type containing 7b  VPS72 - vacuolar protein sorting 72 homolog (s. cerevisiae)  FMR1 - fragile x mental retardation 1  TCOF1 - treacher collins-franceschetti syndrome 1  MGA - mga, max dimerization protein  ZEB1 - zinc finger e-box binding homeobox 1  WWC3 - wwc family member 3  TCF12 - transcription factor 12  ZNF354A - zinc finger protein 354a  CAMTA1 - calmodulin binding transcription activator 1  TCF20 - transcription factor 20 (ar1)  FLI1 - fli-1 proto-oncogene, ets transcription factor  FLNA - filamin a, alpha  PLCB1 - phospholipase c, beta 1 (phosphoinositide-specific)  FOXS1 - forkhead box s1  FOXO3 - forkhead box o3  LARP4 - la ribonucleoprotein domain family, member 4  HDAC8 - histone deacetylase 8  ASH1L - ash1 (absent, small, or homeotic)-like (drosophila)  SIN3B - sin3 transcription regulator family member b  DCP1A - decapping mrna 1a  NACC1 - nucleus accumbens associated 1, ben and btb (poz) domain containing  HR - hair growth associated  MED8 - mediator complex subunit 8  CAND1 - cullin-associated and neddylation-dissociated 1  TDG - thymine-dna glycosylase  SMG6 - smg6 nonsense mediated mrna decay factor  PRDX2 - peroxiredoxin 2  EAPP - e2f-associated phosphoprotein  FBXW11 - f-box and wd repeat domain containing 11  POFUT2 - protein o-fucosyltransferase 2  FAM208A - family with sequence similarity 208, member a  KDM3A - lysine (k)-specific demethylase 3a  FHL2 - four and a half lim domains 2  STX1B - syntaxin 1b  NUP160 - nucleoporin 160kda  FGFR2 - fibroblast growth factor receptor 2  TAF9 - taf9 rna polymerase ii, tata box binding protein (tbp)-associated factor, 32kda  TAF7 - taf7 rna polymerase ii, tata box binding protein (tbp)-associated factor, 55kda  FAM98B - family with sequence similarity 98, member b  TAF6 - taf6 rna polymerase ii, tata box binding protein (tbp)-associated factor, 80kda  FGFR1 - fibroblast growth factor receptor 1  FGF9 - fibroblast growth factor 9  TADA2A - transcriptional adaptor 2a  SMCHD1 - structural maintenance of chromosomes flexible hinge domain containing 1  TIRAP - toll-interleukin 1 receptor (tir) domain containing adaptor protein  FGF1 - fibroblast growth factor 1 (acidic)  FGF2 - fibroblast growth factor 2 (basic)  USP22 - ubiquitin specific peptidase 22  TAPBP - tap binding protein (tapasin)  TARBP1 - tar (hiv-1) rna binding protein 1  FER - fer (fps/fes related) tyrosine kinase  SMG9 - smg9 nonsense mediated mrna decay factor  MAP3K7 - mitogen-activated protein kinase kinase kinase 7  ZCCHC11 - zinc finger, cchc domain containing 11  ADNP - activity-dependent neuroprotector homeobox  KMT2E - lysine (k)-specific methyltransferase 2e  MED13L - mediator complex subunit 13-like  ZNF395 - zinc finger protein 395  MYNN - myoneurin  CRTC1 - creb regulated transcription coactivator 1  UFL1 - ufm1-specific ligase 1  TCF3 - transcription factor 3  PUM2 - pumilio homolog 2 (drosophila)  DMAP1 - dna methyltransferase 1 associated protein 1  TCEB3 - transcription elongation factor b (siii), polypeptide 3 (110kda, elongin a)  LARP1 - la ribonucleoprotein domain family, member 1  TCF4 - transcription factor 4  EFEMP1 - egf containing fibulin-like extracellular matrix protein 1  NKRF - nfkb repressing factor  ZNF629 - zinc finger protein 629  FBN1 - fibrillin 1  LANCL2 - lanc lantibiotic synthetase component c-like 2 (bacterial)  TCEA2 - transcription elongation factor a (sii), 2  ERBB2IP - erbb2 interacting protein  TCEA1 - transcription elongation factor a (sii), 1  ITGB3BP - integrin beta 3 binding protein (beta3-endonexin)  TDRD7 - tudor domain containing 7  RYBP - ring1 and yy1 binding protein  MTOR - mechanistic target of rapamycin (serine/threonine kinase)  PAXBP1 - pax3 and pax7 binding protein 1  TLE1 - transducin-like enhancer of split 1 (e(sp1) homolog, drosophila)  TLE3 - transducin-like enhancer of split 3 (e(sp1) homolog, drosophila)  TLE4 - transducin-like enhancer of split 4 (e(sp1) homolog, drosophila)  ATP13A2 - atpase type 13a2  TLR3 - toll-like receptor 3  EXOSC2 - exosome component 2  SIRT1 - sirtuin 1  MKX - mohawk homeobox  TMF1 - tata element modulatory factor 1  ACTR8 - arp8 actin-related protein 8 homolog (yeast)  TMSB4X - thymosin beta 4, x-linked  CBX6 - chromobox homolog 6  HEY1 - hairy/enhancer-of-split related with yrpw motif 1  TMPO - thymopoietin  CBX5 - chromobox homolog 5  BRD4 - bromodomain containing 4  TARDBP - tar dna binding protein  BCL9L - b-cell cll/lymphoma 9-like  TNFRSF1A - tumor necrosis factor receptor superfamily, member 1a  TNFRSF1B - tumor necrosis factor receptor superfamily, member 1b  SF3B1 - splicing factor 3b, subunit 1, 155kda  PRKCDBP - protein kinase c, delta binding protein  TMBIM6 - transmembrane bax inhibitor motif containing 6  NUP188 - nucleoporin 188kda  EIF4ENIF1 - eukaryotic translation initiation factor 4e nuclear import factor 1  SUZ12 - suz12 polycomb repressive complex 2 subunit  POFUT1 - protein o-fucosyltransferase 1  TEF - thyrotrophic embryonic factor  RCOR2 - rest corepressor 2  TFAM - transcription factor a, mitochondrial  INPP5E - inositol polyphosphate-5-phosphatase, 72 kda  TERF2 - telomeric repeat binding factor 2  TFDP1 - transcription factor dp-1  NR2F2 - nuclear receptor subfamily 2, group f, member 2  TFE3 - transcription factor binding to ighm enhancer 3  CBX7 - chromobox homolog 7  NR2F1 - nuclear receptor subfamily 2, group f, member 1  TFCP2 - transcription factor cp2  ZNF740 - zinc finger protein 740  TGFA - transforming growth factor, alpha  FNIP1 - folliculin interacting protein 1  HEY2 - hairy/enhancer-of-split related with yrpw motif 2  TGFBR1 - transforming growth factor, beta receptor 1  TGFB2 - transforming growth factor, beta 2  RBFOX2 - rna binding protein, fox-1 homolog (c. elegans) 2  TGFB3 - transforming growth factor, beta 3  TGFB1 - transforming growth factor, beta 1  TGFB1I1 - transforming growth factor beta 1 induced transcript 1  TP53INP1 - tumor protein p53 inducible nuclear protein 1  H2AFV - h2a histone family, member v  MEPCE - methylphosphate capping enzyme  TGFBR2 - transforming growth factor, beta receptor ii (70/80kda)  RNF20 - ring finger protein 20, e3 ubiquitin protein ligase  TGFBR3 - transforming growth factor, beta receptor iii  FOSL2 - fos-like antigen 2  FOSB - fbj murine osteosarcoma viral oncogene homolog b  FOS - fbj murine osteosarcoma viral oncogene homolog  THBS1 - thrombospondin 1  KAT6B - k(lysine) acetyltransferase 6b  TIA1 - tia1 cytotoxic granule-associated rna binding protein  METTL3 - methyltransferase like 3  THRA - thyroid hormone receptor, alpha  ELL - elongation factor rna polymerase ii  GFPT1 - glutamine--fructose-6-phosphate transaminase 1  ZNF239 - zinc finger protein 239  PHC3 - polyhomeotic homolog 3 (drosophila)  MKKS - mckusick-kaufman syndrome  NRIP1 - nuclear receptor interacting protein 1  KAT2A - k(lysine) acetyltransferase 2a  NCOA3 - nuclear receptor coactivator 3  ANP32A - acidic (leucine-rich) nuclear phosphoprotein 32 family, member a  WWC2 - ww and c2 domain containing 2  PABPN1 - poly(a) binding protein, nuclear 1  TAF15 - taf15 rna polymerase ii, tata box binding protein (tbp)-associated factor, 68kda  GJA1 - gap junction protein, alpha 1, 43kda  AXIN1 - axin 1  TRRAP - transformation/transcription domain-associated protein  PICALM - phosphatidylinositol binding clathrin assembly protein  GGNBP2 - gametogenetin binding protein 2  ZC3H14 - zinc finger ccch-type containing 14  ARID1A - at rich interactive domain 1a (swi-like)  DLL1 - delta-like 1 (drosophila)  SETD6 - set domain containing 6  GLG1 - golgi glycoprotein 1  FZD6 - frizzled family receptor 6  GLE1 - gle1 rna export mediator  ACTR5 - arp5 actin-related protein 5 homolog (yeast)  CDC45 - cell division cycle 45  FZD4 - frizzled family receptor 4  GCLC - glutamate-cysteine ligase, catalytic subunit  FZD1 - frizzled family receptor 1  BAP1 - brca1 associated protein-1 (ubiquitin carboxy-terminal hydrolase)  AXIN2 - axin 2  ZNF613 - zinc finger protein 613  NUP85 - nucleoporin 85kda  KDM5C - lysine (k)-specific demethylase 5c  USP9X - ubiquitin specific peptidase 9, x-linked  RBM10 - rna binding motif protein 10  TTC21B - tetratricopeptide repeat domain 21b  SLTM - safb-like, transcription modulator  NELFE - negative elongation factor complex member e  EHMT1 - euchromatic histone-lysine n-methyltransferase 1  NR5A1 - nuclear receptor subfamily 5, group a, member 1  FUS - fused in sarcoma  NAA40 - n(alpha)-acetyltransferase 40, natd catalytic subunit, homolog (s. cerevisiae)  USP7 - ubiquitin specific peptidase 7 (herpes virus-associated)  RNF168 - ring finger protein 168, e3 ubiquitin protein ligase  XRCC6 - x-ray repair complementing defective repair in chinese hamster cells 6  GABPB1 - ga binding protein transcription factor, beta subunit 1  GABPA - ga binding protein transcription factor, alpha subunit 60kda  FUT8 - fucosyltransferase 8 (alpha (1,6) fucosyltransferase)  CPSF7 - cleavage and polyadenylation specific factor 7, 59kda  FYN - fyn oncogene related to src, fgr, yes  RPAP2 - rna polymerase ii associated protein 2  DDX39B - dead (asp-glu-ala-asp) box polypeptide 39b  KMT2D - lysine (k)-specific methyltransferase 2d  AAAS - achalasia, adrenocortical insufficiency, alacrimia  ZNF419 - zinc finger protein 419  YEATS4 - yeats domain containing 4  ZFPM1 - zinc finger protein, fog family member 1  SNIP1 - smad nuclear interacting protein 1  ZNF212 - zinc finger protein 212  EID2 - ep300 interacting inhibitor of differentiation 2  ZNF383 - zinc finger protein 383  GATA2 - gata binding protein 2  KAT6A - k(lysine) acetyltransferase 6a  NUP214 - nucleoporin 214kda  NR4A3 - nuclear receptor subfamily 4, group a, member 3  BRD3 - bromodomain containing 3  GAS6 - growth arrest-specific 6  CARF - calcium responsive transcription factor  DEDD2 - death effector domain containing 2  ZNF408 - zinc finger protein 408  MLLT10 - myeloid/lymphoid or mixed-lineage leukemia (trithorax homolog, drosophila); translocated to, 10  FASTKD1 - fast kinase domains 1  DHX36 - deah (asp-glu-ala-his) box polypeptide 36  ZNF189 - zinc finger protein 189  ZSCAN26 - zinc finger and scan domain containing 26  GSPT1 - g1 to s phase transition 1  ZNF177 - zinc finger protein 177  GSN - gelsolin  ZNF175 - zinc finger protein 175  ZNF174 - zinc finger protein 174  ZCCHC6 - zinc finger, cchc domain containing 6  ZNF184 - zinc finger protein 184  SSBP4 - single stranded dna binding protein 4  GTF2F1 - general transcription factor iif, polypeptide 1, 74kda  ZNF148 - zinc finger protein 148  BEND5 - ben domain containing 5  ZBTB17 - zinc finger and btb domain containing 17  GTF2H2 - general transcription factor iih, polypeptide 2, 44kda  TRIM26 - tripartite motif containing 26  GTF2H3 - general transcription factor iih, polypeptide 3, 34kda  VEZF1 - vascular endothelial zinc finger 1  GTF2H1 - general transcription factor iih, polypeptide 1, 62kda  ZNF142 - zinc finger protein 142  MCPH1 - microcephalin 1  ZNF140 - zinc finger protein 140  GTF2B - general transcription factor iib  PCGF2 - polycomb group ring finger 2  ZNF132 - zinc finger protein 132  ZNF131 - zinc finger protein 131  ZNF134 - zinc finger protein 134  ZNF133 - zinc finger protein 133  TBL1XR1 - transducin (beta)-like 1 x-linked receptor 1  GTF2I - general transcription factor iii  GTF2H4 - general transcription factor iih, polypeptide 4, 52kda  BRF1 - brf1, rna polymerase iii transcription initiation factor 90 kda subunit  ZNF79 - zinc finger protein 79  ZNF70 - zinc finger protein 70  RBM12B - rna binding motif protein 12b  ZNF75D - zinc finger protein 75d  ZNF76 - zinc finger protein 76  H3F3A - h3 histone, family 3a  BRPF1 - bromodomain and phd finger containing, 1  H2AFZ - h2a histone family, member z  DCP2 - decapping mrna 2  H2AFX - h2a histone family, member x  SAP130 - sin3a-associated protein, 130kda  FZD5 - frizzled family receptor 5  PAPD4 - pap associated domain containing 4  PAX8 - paired box 8  MIER3 - mesoderm induction early response 1, family member 3  CDC73 - cell division cycle 73  NKAP - nfkb activating protein  THAP5 - thap domain containing 5  LRP8 - low density lipoprotein receptor-related protein 8, apolipoprotein e receptor  HCFC1 - host cell factor c1 (vp16-accessory protein)  PRDM2 - pr domain containing 2, with znf domain  MAP3K12 - mitogen-activated protein kinase kinase kinase 12  ZNF226 - zinc finger protein 226  HDAC2 - histone deacetylase 2  HMBOX1 - homeobox containing 1  HDGF - hepatoma-derived growth factor  ZNF213 - zinc finger protein 213  HDAC1 - histone deacetylase 1  HCLS1 - hematopoietic cell-specific lyn substrate 1  ZNF205 - zinc finger protein 205  ZNF207 - zinc finger protein 207  XPA - xeroderma pigmentosum, complementation group a  XPC - xeroderma pigmentosum, complementation group c  GOLGB1 - golgin b1  XPO1 - exportin 1 (crm1 homolog, yeast)  PAGR1 - paxip1 associated glutamate-rich protein 1  ZXDC - zxd family zinc finger c  XBP1 - x-box binding protein 1  ZNF367 - zinc finger protein 367  BHLHE41 - basic helix-loop-helix family, member e41  EIF4H - eukaryotic translation initiation factor 4h  WFS1 - wolfram syndrome 1 (wolframin)  WHSC1 - wolf-hirschhorn syndrome candidate 1  NELFA - negative elongation factor complex member a  DCP1B - decapping mrna 1b  ARID2 - at rich interactive domain 2 (arid, rfx-like)  WARS - tryptophanyl-trna synthetase  ZNF35 - zinc finger protein 35  ZKSCAN1 - zinc finger with krab and scan domains 1  FBXL15 - f-box and leucine-rich repeat protein 15  ZNF33B - zinc finger protein 33b  ZNF576 - zinc finger protein 576  ZNF32 - zinc finger protein 32  ZNF45 - zinc finger protein 45  MZF1 - myeloid zinc finger 1  ZNF18 - zinc finger protein 18  ZNF16 - zinc finger protein 16  ASXL1 - additional sex combs like 1 (drosophila)  ZNF384 - zinc finger protein 384  TNIP2 - tnfaip3 interacting protein 2  RBM42 - rna binding motif protein 42  ZNF24 - zinc finger protein 24  ZFPL1 - zinc finger protein-like 1  ZFX - zinc finger protein, x-linked  SF1 - splicing factor 1  ZBTB14 - zinc finger and btb domain containing 14  ZNF711 - zinc finger protein 711  ZNF7 - zinc finger protein 7  ZNF8 - zinc finger protein 8  ZNF2 - zinc finger protein 2  ZNF3 - zinc finger protein 3  YES1 - v-yes-1 yamaguchi sarcoma viral oncogene homolog 1  XRCC5 - x-ray repair complementing defective repair in chinese hamster cells 5 (double-strand-break rejoining)  GRSF1 - g-rich rna sequence binding factor 1  GSK3B - glycogen synthase kinase 3 beta  YWHAZ - tyrosine 3-monooxygenase/tryptophan 5-monooxygenase activation protein, zeta polypeptide  GSK3A - glycogen synthase kinase 3 alpha  YWHAB - tyrosine 3-monooxygenase/tryptophan 5-monooxygenase activation protein, beta polypeptide  CCNK - cyclin k  HSF2 - heat shock transcription factor 2  TRIM24 - tripartite motif containing 24  TAF1D - tata box binding protein (tbp)-associated factor, rna polymerase i, d, 41kda  CREG1 - cellular repressor of e1a-stimulated genes 1  CHAC1 - chac, cation transport regulator homolog 1 (e. coli)  HSBP1 - heat shock factor binding protein 1  FLCN - folliculin  PRMT2 - protein arginine methyltransferase 2  HESX1 - hesx homeobox 1  SAP30 - sin3a-associated protein, 30kda  METTL16 - methyltransferase like 16  ALKBH1 - alkb, alkylation repair homolog 1 (e. coli)  FTO - fat mass and obesity associated  CFLAR - casp8 and fadd-like apoptosis regulator  SECISBP2 - secis binding protein 2  MED11 - mediator complex subunit 11  LDB1 - lim domain binding 1  WDR77 - wd repeat domain 77  ALDH1A2 - aldehyde dehydrogenase 1 family, member a2  KAT2B - k(lysine) acetyltransferase 2b  TSC22D1 - tsc22 domain family, member 1  CRTC2 - creb regulated transcription coactivator 2  ZNF259 - zinc finger protein 259  FUBP1 - far upstream element (fuse) binding protein 1  TAX1BP1 - tax1 (human t-cell leukemia virus type i) binding protein 1  CDC123 - cell division cycle 123  CC2D1B - coiled-coil and c2 domain containing 1b  CPNE1 - copine i  EIF2B2 - eukaryotic translation initiation factor 2b, subunit 2 beta, 39kda  THAP8 - thap domain containing 8  BUD31 - bud31 homolog (s. cerevisiae)  EIF2B5 - eukaryotic translation initiation factor 2b, subunit 5 epsilon, 82kda  ZNF655 - zinc finger protein 655  TRAK2 - trafficking protein, kinesin binding 2  HSPD1 - heat shock 60kda protein 1 (chaperonin)  DNAJB1 - dnaj (hsp40) homolog, subfamily b, member 1  ZNF48 - zinc finger protein 48  BCL10 - b-cell cll/lymphoma 10  FUBP3 - far upstream element (fuse) binding protein 3  BTRC - beta-transducin repeat containing e3 ubiquitin protein ligase  HSPA8 - heat shock 70kda protein 8  EIF4EBP3 - eukaryotic translation initiation factor 4e binding protein 3  HMGN1 - high mobility group nucleosome binding domain 1  HMGB3 - high mobility group box 3  HMGB2 - high mobility group box 2  HMGB1 - high mobility group box 1  EIF3C - eukaryotic translation initiation factor 3, subunit c  EIF3D - eukaryotic translation initiation factor 3, subunit d  BRD9 - bromodomain containing 9  EIF3B - eukaryotic translation initiation factor 3, subunit b  TNKS - tankyrase, trf1-interacting ankyrin-related adp-ribose polymerase  EIF4G3 - eukaryotic translation initiation factor 4 gamma, 3  DAZAP1 - daz associated protein 1  ZBTB10 - zinc finger and btb domain containing 10  AGO1 - argonaute risc catalytic component 1  CAPRIN2 - caprin family member 2  ZBTB48 - zinc finger and btb domain containing 48  SRSF9 - serine/arginine-rich splicing factor 9  HYAL2 - hyaluronoglucosaminidase 2  GPBP1 - gc-rich promoter binding protein 1  HFE - hemochromatosis  HINT1 - histidine triad nucleotide binding protein 1  HIVEP1 - human immunodeficiency virus type i enhancer binding protein 1  ELP4 - elongator acetyltransferase complex subunit 4  UPF3B - upf3 regulator of nonsense transcripts homolog b (yeast)  UPF3A - upf3 regulator of nonsense transcripts homolog a (yeast)  HIF1A - hypoxia inducible factor 1, alpha subunit (basic helix-loop-helix transcription factor)  LPIN3 - lipin 3  TRADD - tnfrsf1a-associated via death domain  MYCBP - myc binding protein  EED - embryonic ectoderm development  URI1 - uri1, prefoldin-like chaperone  HNRNPU - heterogeneous nuclear ribonucleoprotein u (scaffold attachment factor a)  RIPK1 - receptor (tnfrsf)-interacting serine-threonine kinase 1  HNRNPK - heterogeneous nuclear ribonucleoprotein k  HNRNPL - heterogeneous nuclear ribonucleoprotein l  TNFSF13 - tumor necrosis factor (ligand) superfamily, member 13  FBXO4 - f-box protein 4  HNRNPA2B1 - heterogeneous nuclear ribonucleoprotein a2/b1  HNRNPA1 - heterogeneous nuclear ribonucleoprotein a1  HNRNPC - heterogeneous nuclear ribonucleoprotein c (c1/c2)  HNRNPAB - heterogeneous nuclear ribonucleoprotein a/b  RIPK2 - receptor-interacting serine-threonine kinase 2  HNRNPF - heterogeneous nuclear ribonucleoprotein f  HNRNPD - heterogeneous nuclear ribonucleoprotein d (au-rich element rna binding protein 1, 37kda)  HNRNPH1 - heterogeneous nuclear ribonucleoprotein h1 (h)  HNRNPH3 - heterogeneous nuclear ribonucleoprotein h3 (2h9)  HNRNPH2 - heterogeneous nuclear ribonucleoprotein h2 (h')  HMGA1 - high mobility group at-hook 1  ZNF584 - zinc finger protein 584  FAM129B - family with sequence similarity 129, member b  NR4A1 - nuclear receptor subfamily 4, group a, member 1  PDE12 - phosphodiesterase 12  RFX7 - regulatory factor x, 7  FOXK2 - forkhead box k2  HTATSF1 - hiv-1 tat specific factor 1  EIF3K - eukaryotic translation initiation factor 3, subunit k  HAT1 - histone acetyltransferase 1  CRTC3 - creb regulated transcription coactivator 3  RBM15 - rna binding motif protein 15  TNRC6A - trinucleotide repeat containing 6a  ENC1 - ectodermal-neural cortex 1 (with btb domain)  RBMX - rna binding motif protein, x-linked  PIK3R3 - phosphoinositide-3-kinase, regulatory subunit 3 (gamma)  ING2 - inhibitor of growth family, member 2  PPM1D - protein phosphatase, mg2+/mn2+ dependent, 1d  ZNF574 - zinc finger protein 574  CREB3L2 - camp responsive element binding protein 3-like 2  IPPK - inositol 1,3,4,5,6-pentakisphosphate 2-kinase  ILF2 - interleukin enhancer binding factor 2  PRPF19 - pre-mrna processing factor 19  ILF3 - interleukin enhancer binding factor 3, 90kda  ILK - integrin-linked kinase  PIAS1 - protein inhibitor of activated stat, 1  SLCO3A1 - solute carrier organic anion transporter family, member 3a1  SMYD3 - set and mynd domain containing 3  MAPKAPK5 - mitogen-activated protein kinase-activated protein kinase 5  SMURF2 - smad specific e3 ubiquitin protein ligase 2  PATL1 - protein associated with topoisomerase ii homolog 1 (yeast)  CGGBP1 - cgg triplet repeat binding protein 1  LMO4 - lim domain only 4  CAMK1 - calcium/calmodulin-dependent protein kinase i  CBX4 - chromobox homolog 4  CSRNP1 - cysteine-serine-rich nuclear protein 1  C5AR2 - complement component 5a receptor 2  SERP1 - stress-associated endoplasmic reticulum protein 1  ZNF25 - zinc finger protein 25  STK16 - serine/threonine kinase 16  ITGA3 - integrin, alpha 3 (antigen cd49c, alpha 3 subunit of vla-3 receptor)  KHSRP - kh-type splicing regulatory protein  CASK - calcium/calmodulin-dependent serine protein kinase (maguk family)  POLR1E - polymerase (rna) i polypeptide e, 53kda  CDK10 - cyclin-dependent kinase 10  SUDS3 - suppressor of defective silencing 3 homolog (s. cerevisiae)  EIF6 - eukaryotic translation initiation factor 6  SKAP1 - src kinase associated phosphoprotein 1  GZF1 - gdnf-inducible zinc finger protein 1  INPPL1 - inositol polyphosphate phosphatase-like 1  INSR - insulin receptor  INSM1 - insulinoma-associated 1  CDK13 - cyclin-dependent kinase 13  PDCD4 - programmed cell death 4 (neoplastic transformation inhibitor)  LSM3 - lsm3 homolog, u6 small nuclear rna associated (s. cerevisiae)  INPP5B - inositol polyphosphate-5-phosphatase, 75kda  RFXANK - regulatory factor x-associated ankyrin-containing protein  CSDC2 - cold shock domain containing c2, rna binding  KLF7 - kruppel-like factor 7 (ubiquitous)  IRF2 - interferon regulatory factor 2  IRF3 - interferon regulatory factor 3  IREB2 - iron-responsive element binding protein 2  IKZF5 - ikaros family zinc finger 5 (pegasus)  RUVBL1 - ruvb-like 1 (e. coli)  DIMT1 - dim1 dimethyladenosine transferase 1 homolog (s. cerevisiae)  MED19 - mediator complex subunit 19  ITGA6 - integrin, alpha 6  IRAK2 - interleukin-1 receptor-associated kinase 2  IRAK1 - interleukin-1 receptor-associated kinase 1  EIF2AK1 - eukaryotic translation initiation factor 2-alpha kinase 1  ZSCAN31 - zinc finger and scan domain containing 31  PAPD5 - pap associated domain containing 5  KIAA1324L - kiaa1324-like  NSD1 - nuclear receptor binding set domain protein 1  ZBTB11 - zinc finger and btb domain containing 11  RNF25 - ring finger protein 25  AFF4 - af4/fmr2 family, member 4  MMS19 - mms19 nucleotide excision repair homolog (s. cerevisiae)  DKK3 - dickkopf wnt signaling pathway inhibitor 3  SNX5 - sorting nexin 5  IFNGR1 - interferon gamma receptor 1  ID2 - inhibitor of dna binding 2, dominant negative helix-loop-helix protein  ZNF777 - zinc finger protein 777  ID3 - inhibitor of dna binding 3, dominant negative helix-loop-helix protein  STK36 - serine/threonine kinase 36  IRF2BPL - interferon regulatory factor 2 binding protein-like  JAZF1 - jazf zinc finger 1  FOXK1 - forkhead box k1  ZNF282 - zinc finger protein 282  IGHMBP2 - immunoglobulin mu binding protein 2  RBPJ - recombination signal binding protein for immunoglobulin kappa j region  PABPC1 - poly(a) binding protein, cytoplasmic 1  NCK2 - nck adaptor protein 2  TRUB2 - trub pseudouridine (psi) synthase homolog 2 (e. coli)  USP21 - ubiquitin specific peptidase 21  ZBTB9 - zinc finger and btb domain containing 9  ZBTB12 - zinc finger and btb domain containing 12  IK - ik cytokine, down-regulator of hla ii  IGBP1 - immunoglobulin (cd79a) binding protein 1  JMJD1C - jumonji domain containing 1c  SMARCA5 - swi/snf related, matrix associated, actin dependent regulator of chromatin, subfamily a, member 5  OGT - o-linked n-acetylglucosamine (glcnac) transferase  PELP1 - proline, glutamate and leucine rich protein 1  RAE1 - rae1 rna export 1 homolog (s. pombe)  SND1 - staphylococcal nuclease and tudor domain containing 1  PCIF1 - pdx1 c-terminal inhibiting factor 1  CUL2 - cullin 2  CUL3 - cullin 3  ZNF667 - zinc finger protein 667  FOXP1 - forkhead box p1  SUPT3H - suppressor of ty 3 homolog (s. cerevisiae)  ZUFSP - zinc finger with ufm1-specific peptidase domain  ZNF572 - zinc finger protein 572  PCGF5 - polycomb group ring finger 5  WTAP - wilms tumor 1 associated protein  WAC - ww domain containing adaptor with coiled-coil  RBM39 - rna binding motif protein 39  PHF21A - phd finger protein 21a  ZNF414 - zinc finger protein 414  ZNF771 - zinc finger protein 771  IER2 - immediate early response 2  NCOR1 - nuclear receptor corepressor 1  LDLR - low density lipoprotein receptor  MTPN - myotrophin  NCOR2 - nuclear receptor corepressor 2  CARTPT - cart prepropeptide  DOT1L - dot1-like histone h3k79 methyltransferase  EFCAB7 - ef-hand calcium binding domain 7  L3MBTL3 - l(3)mbt-like 3 (drosophila)  APBA3 - amyloid beta (a4) precursor protein-binding, family a, member 3  ZNF333 - zinc finger protein 333  CIR1 - corepressor interacting with rbpj, 1  H2AFY - h2a histone family, member y  FADS1 - fatty acid desaturase 1  GTF2IRD1 - gtf2i repeat domain containing 1  GTPBP1 - gtp binding protein 1  ZNF527 - zinc finger protein 527  HOPX - hop homeobox  SOX13 - sry (sex determining region y)-box 13  ZC3H8 - zinc finger ccch-type containing 8  ZNF316 - zinc finger protein 316  LMO1 - lim domain only 1 (rhombotin 1)  NACC2 - nacc family member 2, ben and btb (poz) domain containing  IER5 - immediate early response 5  LMNA - lamin a/c  CLOCK - clock circadian regulator  KLF3 - kruppel-like factor 3 (basic)  DDX41 - dead (asp-glu-ala-asp) box polypeptide 41  FXR2 - fragile x mental retardation, autosomal homolog 2  LITAF - lipopolysaccharide-induced tnf factor  YTHDF2 - yth domain family, member 2  TBPL1 - tbp-like 1  ZBTB41 - zinc finger and btb domain containing 41  ZFP62 - zfp62 zinc finger protein  TMEM59 - transmembrane protein 59  POLR1C - polymerase (rna) i polypeptide c, 30kda  ZGPAT - zinc finger, ccch-type with g patch domain  ACTL6B - actin-like 6b  UBR5 - ubiquitin protein ligase e3 component n-recognin 5  EIF4E2 - eukaryotic translation initiation factor 4e family member 2  MED20 - mediator complex subunit 20  AFAP1L2 - actin filament associated protein 1-like 2  ROCK2 - rho-associated, coiled-coil containing protein kinase 2  ATRAID - all-trans retinoic acid-induced differentiation factor  MAPK8IP1 - mitogen-activated protein kinase 8 interacting protein 1  CRLF3 - cytokine receptor-like factor 3  NGRN - neugrin, neurite outgrowth associated  GLYR1 - glyoxylate reductase 1 homolog (arabidopsis)  ZBTB7A - zinc finger and btb domain containing 7a  DPY30 - dpy-30 homolog (c. elegans)  MBTPS2 - membrane-bound transcription factor peptidase, site 2  IRF2BP2 - interferon regulatory factor 2 binding protein 2  GLIS2 - glis family zinc finger 2  MAGED1 - melanoma antigen family d, 1  MED27 - mediator complex subunit 27  MED26 - mediator complex subunit 26  CHD6 - chromodomain helicase dna binding protein 6  POLR1B - polymerase (rna) i polypeptide b, 128kda  MED17 - mediator complex subunit 17  MED23 - mediator complex subunit 23  ZNF580 - zinc finger protein 580  MRTO4 - mrna turnover 4 homolog (s. cerevisiae)  CDYL - chromodomain protein, y-like  GTF2IRD2 - gtf2i repeat domain containing 2  ZNF710 - zinc finger protein 710  KDR - kinase insert domain receptor (a type iii receptor tyrosine kinase)  ING4 - inhibitor of growth family, member 4  EIF2AK3 - eukaryotic translation initiation factor 2-alpha kinase 3  INSIG2 - insulin induced gene 2  RLIM - ring finger protein, lim domain interacting  RHBDD1 - rhomboid domain containing 1  KRAS - kirsten rat sarcoma viral oncogene homolog  RECQL5 - recq protein-like 5  TNPO1 - transportin 1  TGFBRAP1 - transforming growth factor, beta receptor associated protein 1  ZNF706 - zinc finger protein 706  CIAO1 - cytosolic iron-sulfur protein assembly 1  PHF20L1 - phd finger protein 20-like 1  MED21 - mediator complex subunit 21  MED10 - mediator complex subunit 10  KIT - v-kit hardy-zuckerman 4 feline sarcoma viral oncogene homolog  ZFP28 - zfp28 zinc finger protein  POLDIP3 - polymerase (dna-directed), delta interacting protein 3  PAIP2 - poly(a) binding protein interacting protein 2  JUP - junction plakoglobin  JAK2 - janus kinase 2  ING5 - inhibitor of growth family, member 5  ZNF219 - zinc finger protein 219  PHF20 - phd finger protein 20  ZNHIT3 - zinc finger, hit-type containing 3  ZNF397 - zinc finger protein 397  TRIP4 - thyroid hormone receptor interactor 4  HMGN3 - high mobility group nucleosomal binding domain 3  PHF6 - phd finger protein 6  ZNF235 - zinc finger protein 235  COPS2 - cop9 signalosome subunit 2  ZNF639 - zinc finger protein 639  SARNP - sap domain containing ribonucleoprotein  BRMS1L - breast cancer metastasis-suppressor 1-like  LEF1 - lymphoid enhancer-binding factor 1  SMCR8 - smith-magenis syndrome chromosome region, candidate 8  CNOT8 - ccr4-not transcription complex, subunit 8  CDK12 - cyclin-dependent kinase 12  SIRPA - signal-regulatory protein alpha  ZNF280B - zinc finger protein 280b  ARID4B - at rich interactive domain 4b (rbp1-like)  MIF - macrophage migration inhibitory factor (glycosylation-inhibiting factor)  MAP3K10 - mitogen-activated protein kinase kinase kinase 10  MLH1 - mutl homolog 1, colon cancer, nonpolyposis type 2 (e. coli)  KDM3B - lysine (k)-specific demethylase 3b  ITGB1BP1 - integrin beta 1 binding protein 1  RSF1 - remodeling and spacing factor 1  MLLT6 - myeloid/lymphoid or mixed-lineage leukemia (trithorax homolog, drosophila); translocated to, 6  MLLT3 - myeloid/lymphoid or mixed-lineage leukemia (trithorax homolog, drosophila); translocated to, 3  MED14 - mediator complex subunit 14  MLLT1 - myeloid/lymphoid or mixed-lineage leukemia (trithorax homolog, drosophila); translocated to, 1  ZBTB22 - zinc finger and btb domain containing 22  KMT2A - lysine (k)-specific methyltransferase 2a  RPS6KA5 - ribosomal protein s6 kinase, 90kda, polypeptide 5  SUFU - suppressor of fused homolog (drosophila)  LSM7 - lsm7 homolog, u6 small nuclear rna associated (s. cerevisiae)  MMP14 - matrix metallopeptidase 14 (membrane-inserted)  NLK - nemo-like kinase  UIMC1 - ubiquitin interaction motif containing 1  MNAT1 - mnat cdk-activating kinase assembly factor 1  MTA2 - metastasis associated 1 family, member 2  NOLC1 - nucleolar and coiled-body phosphoprotein 1  EIF2A - eukaryotic translation initiation factor 2a, 65kda  LDLRAD3 - low density lipoprotein receptor class a domain containing 3  CD200 - cd200 molecule  WWOX - ww domain containing oxidoreductase  MNT - mnt, max dimerization protein  CCPG1 - cell cycle progression 1  CD46 - cd46 molecule, complement regulatory protein  NSRP1 - nuclear speckle splicing regulatory protein 1  SPTY2D1 - spt2, suppressor of ty, domain containing 1 (s. cerevisiae)  MECP2 - methyl cpg binding protein 2 (rett syndrome)  PELO - pelota homolog (drosophila)  MEF2A - myocyte enhancer factor 2a  PRICKLE1 - prickle homolog 1 (drosophila)  DDX21 - dead (asp-glu-ala-asp) box helicase 21  ZBED1 - zinc finger, bed-type containing 1  BCDIN3D - bcdin3 domain containing  MEIS2 - meis homeobox 2  BRWD1 - bromodomain and wd repeat domain containing 1  ZNF664 - zinc finger protein 664  MEIS1 - meis homeobox 1  MAP3K4 - mitogen-activated protein kinase kinase kinase 4  MAP3K5 - mitogen-activated protein kinase kinase kinase 5  SLC33A1 - solute carrier family 33 (acetyl-coa transporter), member 1  MEN1 - multiple endocrine neoplasia i  WDR75 - wd repeat domain 75  UTP15 - utp15, u3 small nucleolar ribonucleoprotein, homolog (s. cerevisiae)  KAT8 - k(lysine) acetyltransferase 8  ZNF644 - zinc finger protein 644  ARHGEF2 - rho/rac guanine nucleotide exchange factor (gef) 2  HGS - hepatocyte growth factor-regulated tyrosine kinase substrate  NEMF - nuclear export mediator factor  MBD3 - methyl-cpg binding domain protein 3  PTOV1 - prostate tumor overexpressed 1  ZNF394 - zinc finger protein 394  CBFA2T2 - core-binding factor, runt domain, alpha subunit 2; translocated to, 2  MAGOH - mago-nashi homolog, proliferation-associated (drosophila)  CXXC5 - cxxc finger protein 5  RQCD1 - rcd1 required for cell differentiation1 homolog (s. pombe)  MARS - methionyl-trna synthetase  IL23A - interleukin 23, alpha subunit p19  MAPT - microtubule-associated protein tau  SIRT6 - sirtuin 6  MTA1 - metastasis associated 1  PPHLN1 - periphilin 1  NUP43 - nucleoporin 43kda  MBD1 - methyl-cpg binding domain protein 1  SFMBT1 - scm-like with four mbt domains 1  MAX - myc associated factor x  ZMIZ2 - zinc finger, miz-type containing 2  TRMT112 - trna methyltransferase 11-2 homolog (s. cerevisiae)  LDB2 - lim domain binding 2  ZFP90 - zfp90 zinc finger protein  NELFCD - negative elongation factor complex member c/d  DIRAS3 - diras family, gtp-binding ras-like 3  ASH2L - ash2 (absent, small, or homeotic)-like (drosophila)  ZSCAN29 - zinc finger and scan domain containing 29  CTDSPL2 - ctd (carboxy-terminal domain, rna polymerase ii, polypeptide a) small phosphatase like 2  NUDT12 - nudix (nucleoside diphosphate linked moiety x)-type motif 12  THAP2 - thap domain containing, apoptosis associated protein 2  ITCH - itchy e3 ubiquitin protein ligase  ZNF484 - zinc finger protein 484  L3MBTL2 - l(3)mbt-like 2 (drosophila)  IL34 - interleukin 34  RPL14 - ribosomal protein l14  RIOK1 - rio kinase 1  LRP6 - low density lipoprotein receptor-related protein 6  ZNF445 - zinc finger protein 445  LRP3 - low density lipoprotein receptor-related protein 3  TRAPPC9 - trafficking protein particle complex 9  LUM - lumican  BTAF1 - btaf1 rna polymerase ii, b-tfiid transcription factor-associated, 170kda  UBA3 - ubiquitin-like modifier activating enzyme 3  RBFOX3 - rna binding protein, fox-1 homolog (c. elegans) 3  SESN2 - sestrin 2  PARP9 - poly (adp-ribose) polymerase family, member 9  CDK5RAP1 - cdk5 regulatory subunit associated protein 1  RNF8 - ring finger protein 8, e3 ubiquitin protein ligase  BAZ1B - bromodomain adjacent to zinc finger domain, 1b  TAF1B - tata box binding protein (tbp)-associated factor, rna polymerase i, b, 63kda  MXD1 - max dimerization protein 1  MED15 - mediator complex subunit 15  PIAS4 - protein inhibitor of activated stat, 4  TRIM33 - tripartite motif containing 33  SRRT - serrate rna effector molecule homolog (arabidopsis)  SMAD2 - smad family member 2  HDAC7 - histone deacetylase 7  KLF16 - kruppel-like factor 16  ARMCX3 - armadillo repeat containing, x-linked 3  CAPRIN1 - cell cycle associated protein 1  LARP7 - la ribonucleoprotein domain family, member 7  TAF1C - tata box binding protein (tbp)-associated factor, rna polymerase i, c, 110kda  RPS6KA4 - ribosomal protein s6 kinase, 90kda, polypeptide 4  MAFG - v-maf avian musculoaponeurotic fibrosarcoma oncogene homolog g  DPH5 - diphthamide biosynthesis 5  TAF9B - taf9b rna polymerase ii, tata box binding protein (tbp)-associated factor, 31kda  SMAD4 - smad family member 4  NBAS - neuroblastoma amplified sequence  H1FX - h1 histone family, member x  SMAD5 - smad family member 5  SMAD7 - smad family member 7  WASL - wiskott-aldrich syndrome-like  RBM4B - rna binding motif protein 4b  SAP30BP - sap30 binding protein  ZNF565 - zinc finger protein 565  YAF2 - yy1 associated factor 2  TAGLN3 - transgelin 3  SIX5 - six homeobox 5  NCBP1 - nuclear cap binding protein subunit 1, 80kda  NDN - necdin, melanoma antigen (mage) family member  ZNF582 - zinc finger protein 582  NCK1 - nck adaptor protein 1  BRD7 - bromodomain containing 7  NCL - nucleolin  DDX25 - dead (asp-glu-ala-asp) box helicase 25  MBNL2 - muscleblind-like splicing regulator 2  CEBPZ - ccaat/enhancer binding protein (c/ebp), zeta  TICAM1 - toll-like receptor adaptor molecule 1  CCNL2 - cyclin l2  HNRNPM - heterogeneous nuclear ribonucleoprotein m  TRIM28 - tripartite motif containing 28  DROSHA - drosha, ribonuclease type iii  TBK1 - tank-binding kinase 1  ZNF197 - zinc finger protein 197  RBM7 - rna binding motif protein 7  RBM5 - rna binding motif protein 5  TRIM11 - tripartite motif containing 11  PPP1R12A - protein phosphatase 1, regulatory subunit 12a  MYT1 - myelin transcription factor 1  ALYREF - aly/ref export factor  NAB1 - ngfi-a binding protein 1 (egr1 binding protein 1)  CSRNP2 - cysteine-serine-rich nuclear protein 2  SENP1 - sumo1/sentrin specific peptidase 1  RNF41 - ring finger protein 41  NAB2 - ngfi-a binding protein 2 (egr1 binding protein 2)  TSHZ1 - teashirt zinc finger homeobox 1  PSME3 - proteasome (prosome, macropain) activator subunit 3 (pa28 gamma; ki)  TRIM13 - tripartite motif containing 13  TRIM8 - tripartite motif containing 8  MYO6 - myosin vi  LBH - limb bud and heart development  ABT1 - activator of basal transcription 1  TOPORS - topoisomerase i binding, arginine/serine-rich, e3 ubiquitin protein ligase  MYD88 - myeloid differentiation primary response 88  SEH1L - seh1-like (s. cerevisiae)  ZNF362 - zinc finger protein 362  MYH9 - myosin, heavy chain 9, non-muscle  HCFC2 - host cell factor c2  HUWE1 - hect, uba and wwe domain containing 1, e3 ubiquitin protein ligase  CNOT7 - ccr4-not transcription complex, subunit 7  MXI1 - max interactor 1, dimerization protein  PQBP1 - polyglutamine binding protein 1  TRA2A - transformer 2 alpha homolog (drosophila)  ACTR2 - arp2 actin-related protein 2 homolog (yeast)  ACTR3 - arp3 actin-related protein 3 homolog (yeast)  RBM15B - rna binding motif protein 15b  TSFM - ts translation elongation factor, mitochondrial  TRIM37 - tripartite motif containing 37  ZNF558 - zinc finger protein 558  LRPPRC - leucine-rich pentatricopeptide repeat containing  ZNF263 - zinc finger protein 263  PURG - purine-rich element binding protein g  RBM12 - rna binding motif protein 12  MED25 - mediator complex subunit 25  SNX12 - sorting nexin 12  TRAP1 - tnf receptor-associated protein 1  MED6 - mediator complex subunit 6  RNF207 - ring finger protein 207  PTBP3 - polypyrimidine tract binding protein 3  DMTF1 - cyclin d binding myb-like transcription factor 1  MTIF2 - mitochondrial translational initiation factor 2  ZBTB33 - zinc finger and btb domain containing 33  HDAC5 - histone deacetylase 5  MTF1 - metal-regulatory transcription factor 1  HDAC6 - histone deacetylase 6  OCLN - occludin  RNF187 - ring finger protein 187  DCPS - decapping enzyme, scavenger  MT3 - metallothionein 3  MED16 - mediator complex subunit 16  CCDC22 - coiled-coil domain containing 22  DNM1L - dynamin 1-like  DNAJB6 - dnaj (hsp40) homolog, subfamily b, member 6  MSN - moesin  CITED1 - cbp/p300-interacting transactivator, with glu/asp-rich carboxy-terminal domain, 1  ZBTB5 - zinc finger and btb domain containing 5  PAN2 - pan2 poly(a) specific ribonuclease subunit homolog (s. cerevisiae)  ZBTB40 - zinc finger and btb domain containing 40  RGCC - regulator of cell cycle  COMMD1 - copper metabolism (murr1) domain containing 1  RBM8A - rna binding motif protein 8a  HELZ - helicase with zinc finger  USP3 - ubiquitin specific peptidase 3  METTL5 - methyltransferase like 5  USP15 - ubiquitin specific peptidase 15  MED4 - mediator complex subunit 4  THOC1 - tho complex 1  SETD2 - set domain containing 2  RBX1 - ring-box 1, e3 ubiquitin protein ligase  ZC3H7A - zinc finger ccch-type containing 7a  NUP153 - nucleoporin 153kda  THRAP3 - thyroid hormone receptor associated protein 3  MED12 - mediator complex subunit 12  MED13 - mediator complex subunit 13  PSMD6 - proteasome (prosome, macropain) 26s subunit, non-atpase, 6  MED24 - mediator complex subunit 24  ZC3H6 - zinc finger ccch-type containing 6  DTX3L - deltex 3-like (drosophila)  SETDB1 - set domain, bifurcated 1  ZNF34 - zinc finger protein 34  ORC2 - origin recognition complex, subunit 2  TOX4 - tox high mobility group box family member 4  ZNF436 - zinc finger protein 436  RNF141 - ring finger protein 141  AGRN - agrin  NDFIP1 - nedd4 family interacting protein 1  RBM19 - rna binding motif protein 19  G3BP2 - gtpase activating protein (sh3 domain) binding protein 2  OGG1 - 8-oxoguanine dna glycosylase  ZSCAN16 - zinc finger and scan domain containing 16  EXOSC3 - exosome component 3  SMG7 - smg7 nonsense mediated mrna decay factor  WDR61 - wd repeat domain 61  EXOSC1 - exosome component 1  DDR2 - discoidin domain receptor tyrosine kinase 2  DAB2IP - dab2 interacting protein  BCLAF1 - bcl2-associated transcription factor 1  NUMA1 - nuclear mitotic apparatus protein 1  EIF4A3 - eukaryotic translation initiation factor 4a3  NUP88 - nucleoporin 88kda  GMNN - geminin, dna replication inhibitor  NUP98 - nucleoporin 98kda  NR4A2 - nuclear receptor subfamily 4, group a, member 2  ZNF593 - zinc finger protein 593  ZBTB7B - zinc finger and btb domain containing 7b  MATR3 - matrin 3  ZNF691 - zinc finger protein 691  CREBRF - creb3 regulatory factor  ZNF623 - zinc finger protein 623  NPM1 - nucleophosmin (nucleolar phosphoprotein b23, numatrin)  ARHGEF11 - rho guanine nucleotide exchange factor (gef) 11  ZBTB24 - zinc finger and btb domain containing 24  ZEB2 - zinc finger e-box binding homeobox 2  THAP4 - thap domain containing 4  CTIF - cbp80/20-dependent translation initiation factor  RNF40 - ring finger protein 40, e3 ubiquitin protein ligase  IFT52 - intraflagellar transport 52 homolog (chlamydomonas)  TSC22D2 - tsc22 domain family, member 2  NFXL1 - nuclear transcription factor, x-box binding-like 1  NRF1 - nuclear respiratory factor 1  NOP2 - nop2 nucleolar protein  VPRBP - vpr (hiv-1) binding protein  NONO - non-pou domain containing, octamer-binding  MDFIC - myod family inhibitor domain containing  NME1 - nme/nm23 nucleoside diphosphate kinase 1  NOTCH4 - notch 4  IFT74 - intraflagellar transport 74 homolog (chlamydomonas)  NOVA2 - neuro-oncological ventral antigen 2  NOVA1 - neuro-oncological ventral antigen 1  CEP290 - centrosomal protein 290kda  NPAT - nuclear protein, ataxia-telangiectasia locus  CNOT2 - ccr4-not transcription complex, subunit 2  RIMS2 - regulating synaptic membrane exocytosis 2  NAA15 - n(alpha)-acetyltransferase 15, nata auxiliary subunit  NOS3 - nitric oxide synthase 3 (endothelial cell)  PUM1 - pumilio homolog 1 (drosophila)  CNOT4 - ccr4-not transcription complex, subunit 4  CNOT3 - ccr4-not transcription complex, subunit 3  NOTCH1 - notch 1  BAZ2B - bromodomain adjacent to zinc finger domain, 2b  NOTCH3 - notch 3  ST18 - suppression of tumorigenicity 18 (breast carcinoma) (zinc finger protein)  NOTCH2 - notch 2  DHX34 - deah (asp-glu-ala-his) box polypeptide 34  NFYA - nuclear transcription factor y, alpha  HDAC4 - histone deacetylase 4  TOX - thymocyte selection-associated high mobility group box  EDC3 - enhancer of mrna decapping 3  NFYC - nuclear transcription factor y, gamma  CXXC1 - cxxc finger protein 1  ZFYVE16 - zinc finger, fyve domain containing 16  NFKBIA - nuclear factor of kappa light polypeptide gene enhancer in b-cells inhibitor, alpha  NFKB1 - nuclear factor of kappa light polypeptide gene enhancer in b-cells 1  KMT2B - lysine (k)-specific methyltransferase 2b  NFKBIB - nuclear factor of kappa light polypeptide gene enhancer in b-cells inhibitor, beta  NFKBIE - nuclear factor of kappa light polypeptide gene enhancer in b-cells inhibitor, epsilon  NKX2-2 - nk2 homeobox 2  ZNF606 - zinc finger protein 606  HDAC9 - histone deacetylase 9  ZNRD1 - zinc ribbon domain containing 1  SART3 - squamous cell carcinoma antigen recognized by t cells 3  ZNF614 - zinc finger protein 614  ZFP2 - zfp2 zinc finger protein  TTC37 - tetratricopeptide repeat domain 37  NEO1 - neogenin 1  SMARCAL1 - swi/snf related, matrix associated, actin dependent regulator of chromatin, subfamily a-like 1  NEDD8 - neural precursor cell expressed, developmentally down-regulated 8  CTR9 - ctr9, paf1/rna polymerase ii complex component  PPM1F - protein phosphatase, mg2+/mn2+ dependent, 1f  CPEB4 - cytoplasmic polyadenylation element binding protein 4  NFE2L2 - nuclear factor (erythroid-derived 2)-like 2  EPC1 - enhancer of polycomb homolog 1 (drosophila)  RASL11A - ras-like, family 11, member a  MORF4L2 - mortality factor 4 like 2  NFATC4 - nuclear factor of activated t-cells, cytoplasmic, calcineurin-dependent 4  NUP155 - nucleoporin 155kda  NFIA - nuclear factor i/a  NF1 - neurofibromin 1  CDK5RAP3 - cdk5 regulatory subunit associated protein 3  NUP93 - nucleoporin 93kda  ZNF746 - zinc finger protein 746  VGLL4 - vestigial like 4 (drosophila)  LRRC14 - leucine rich repeat containing 14  KDM4A - lysine (k)-specific demethylase 4a  PHF14 - phd finger protein 14  HP1BP3 - heterochromatin protein 1, binding protein 3  ZNF432 - zinc finger protein 432  SAFB2 - scaffold attachment factor b2  MYEF2 - myelin expression factor 2  KIAA0430 - kiaa0430  LPIN2 - lipin 2 |
| GO:0009889 | regulation of biosynthetic process | 6.41E-7 | 1.19E-4 | 1.06 (10334,2564,6996,1835) | [+] Show genes  AGAP2 - arfgap with gtpase domain, ankyrin repeat and ph domain 2  EXOSC9 - exosome component 9  EXOSC10 - exosome component 10  MSL3 - male-specific lethal 3 homolog (drosophila)  TADA1 - transcriptional adaptor 1  AARS - alanyl-trna synthetase  CBX1 - chromobox homolog 1  ABCA2 - atp-binding cassette, sub-family a (abc1), member 2  ZNF821 - zinc finger protein 821  ABL1 - c-abl oncogene 1, non-receptor tyrosine kinase  PML - promyelocytic leukemia  MLLT11 - myeloid/lymphoid or mixed-lineage leukemia (trithorax homolog, drosophila); translocated to, 11  CNOT11 - ccr4-not transcription complex, subunit 11  ACACA - acetyl-coa carboxylase alpha  SUPT20H - suppressor of ty 20 homolog (s. cerevisiae)  YWHAQ - tyrosine 3-monooxygenase/tryptophan 5-monooxygenase activation protein, theta polypeptide  BRD8 - bromodomain containing 8  CHD7 - chromodomain helicase dna binding protein 7  LYAR - ly1 antibody reactive  TCERG1 - transcription elongation regulator 1  ZNF354B - zinc finger protein 354b  EHMT2 - euchromatic histone-lysine n-methyltransferase 2  SPIN1 - spindlin 1  TASP1 - taspase, threonine aspartase, 1  SUB1 - sub1 homolog (s. cerevisiae)  MORF4L1 - mortality factor 4 like 1  ZNF511 - zinc finger protein 511  PPID - peptidylprolyl isomerase d  ACTL6A - actin-like 6a  ACTN1 - actinin, alpha 1  ACTN4 - actinin, alpha 4  ACVR2A - activin a receptor, type iia  ACVRL1 - activin a receptor type ii-like 1  ACVR1 - activin a receptor, type i  ACVR1B - activin a receptor, type ib  ZNF331 - zinc finger protein 331  MED1 - mediator complex subunit 1  ADCY1 - adenylate cyclase 1 (brain)  PPARD - peroxisome proliferator-activated receptor delta  MAML3 - mastermind-like 3 (drosophila)  POLR2K - polymerase (rna) ii (dna directed) polypeptide k, 7.0kda  ADCYAP1R1 - adenylate cyclase activating polypeptide 1 (pituitary) receptor type i  FAM129A - family with sequence similarity 129, member a  PARP1 - poly (adp-ribose) polymerase 1  H2AFY2 - h2a histone family, member y2  PPP1R13L - protein phosphatase 1, regulatory subunit 13 like  HES6 - hairy and enhancer of split 6 (drosophila)  POLR2G - polymerase (rna) ii (dna directed) polypeptide g  SMPD3 - sphingomyelin phosphodiesterase 3, neutral membrane (neutral sphingomyelinase ii)  POLR2D - polymerase (rna) ii (dna directed) polypeptide d  RUVBL2 - ruvb-like 2 (e. coli)  POLR2A - polymerase (rna) ii (dna directed) polypeptide a, 220kda  PARP4 - poly (adp-ribose) polymerase family, member 4  AP2A1 - adaptor-related protein complex 2, alpha 1 subunit  CDK5RAP2 - cdk5 regulatory subunit associated protein 2  RCOR3 - rest corepressor 3  AES - amino-terminal enhancer of split  NUP133 - nucleoporin 133kda  NUPL2 - nucleoporin like 2  VPS35 - vacuolar protein sorting 35 homolog (s. cerevisiae)  JAG1 - jagged 1  PRDM4 - pr domain containing 4  PPP3R1 - protein phosphatase 3, regulatory subunit b, alpha  ATF7IP - activating transcription factor 7 interacting protein  ZNF689 - zinc finger protein 689  LRIF1 - ligand dependent nuclear receptor interacting factor 1  PPP2R5B - protein phosphatase 2, regulatory subunit b', beta  AKT1 - v-akt murine thymoma viral oncogene homolog 1  ZNF653 - zinc finger protein 653  PCID2 - pci domain containing 2  PPP2R1A - protein phosphatase 2, regulatory subunit a, alpha  WWP2 - ww domain containing e3 ubiquitin protein ligase 2  AHR - aryl hydrocarbon receptor  ZNF526 - zinc finger protein 526  FOXP4 - forkhead box p4  PPP2CA - protein phosphatase 2, catalytic subunit, alpha isozyme  ZNF83 - zinc finger protein 83  ESCO1 - establishment of sister chromatid cohesion n-acetyltransferase 1  KANSL3 - kat8 regulatory nsl complex subunit 3  PRNP - prion protein  ATF7 - activating transcription factor 7  AMFR - autocrine motility factor receptor, e3 ubiquitin protein ligase  DNAJC3 - dnaj (hsp40) homolog, subfamily c, member 3  PRKRIR - protein-kinase, interferon-inducible double stranded rna dependent inhibitor, repressor of (p58 repressor)  MAP2K5 - mitogen-activated protein kinase kinase 5  MAP2K7 - mitogen-activated protein kinase kinase 7  EIF2AK2 - eukaryotic translation initiation factor 2-alpha kinase 2  PIM2 - pim-2 oncogene  ZNF692 - zinc finger protein 692  MAP2K1 - mitogen-activated protein kinase kinase 1  MAP2K2 - mitogen-activated protein kinase kinase 2  EIF4E3 - eukaryotic translation initiation factor 4e family member 3  MAPK10 - mitogen-activated protein kinase 10  MAPK9 - mitogen-activated protein kinase 9  MAPK11 - mitogen-activated protein kinase 11  MAPK8 - mitogen-activated protein kinase 8  MAPK7 - mitogen-activated protein kinase 7  GCN1L1 - gcn1 general control of amino-acid synthesis 1-like 1 (yeast)  MAPK3 - mitogen-activated protein kinase 3  MAPK1 - mitogen-activated protein kinase 1  NDC1 - ndc1 transmembrane nucleoporin  ADIRF - adipogenesis regulatory factor  PRKD1 - protein kinase d1  CCDC88A - coiled-coil domain containing 88a  ANXA3 - annexin a3  PRKCH - protein kinase c, eta  PRKCI - protein kinase c, iota  CCDC85B - coiled-coil domain containing 85b  PRKCB - protein kinase c, beta  PRKCD - protein kinase c, delta  NSUN5 - nop2/sun domain family, member 5  PRKAR1A - protein kinase, camp-dependent, regulatory, type i, alpha  YEATS2 - yeats domain containing 2  PKIB - protein kinase (camp-dependent, catalytic) inhibitor beta  PRKAG1 - protein kinase, amp-activated, gamma 1 non-catalytic subunit  APEX1 - apex nuclease (multifunctional dna repair enzyme) 1  RBM23 - rna binding motif protein 23  ZNF605 - zinc finger protein 605  XIAP - x-linked inhibitor of apoptosis  PARN - poly(a)-specific ribonuclease  BIRC2 - baculoviral iap repeat containing 2  KLF12 - kruppel-like factor 12  TREX1 - three prime repair exonuclease 1  PBX2 - pre-b-cell leukemia homeobox 2  PRMT6 - protein arginine methyltransferase 6  PBX3 - pre-b-cell leukemia homeobox 3  PBX1 - pre-b-cell leukemia homeobox 1  PCBD1 - pterin-4 alpha-carbinolamine dehydratase/dimerization cofactor of hepatocyte nuclear factor 1 alpha  APBB2 - amyloid beta (a4) precursor protein-binding, family b, member 2  CHP1 - calcineurin-like ef-hand protein 1  APBB1 - amyloid beta (a4) precursor protein-binding, family b, member 1 (fe65)  RIF1 - rap1 interacting factor homolog (yeast)  POLG2 - polymerase (dna directed), gamma 2, accessory subunit  RNF139 - ring finger protein 139  KIAA1551 - kiaa1551  PA2G4 - proliferation-associated 2g4, 38kda  RPRD1A - regulation of nuclear pre-mrna domain containing 1a  ZHX1 - zinc fingers and homeoboxes 1  ARF4 - adp-ribosylation factor 4  SBNO1 - strawberry notch homolog 1 (drosophila)  IRAK3 - interleukin-1 receptor-associated kinase 3  SETD5 - set domain containing 5  APP - amyloid beta (a4) precursor protein  BBS7 - bardet-biedl syndrome 7  DDX20 - dead (asp-glu-ala-asp) box polypeptide 20  PAK3 - p21 protein (cdc42/rac)-activated kinase 3  PAM - peptidylglycine alpha-amidating monooxygenase  ARNTL - aryl hydrocarbon receptor nuclear translocator-like  PDGFA - platelet-derived growth factor alpha polypeptide  ARNT - aryl hydrocarbon receptor nuclear translocator  PDPR - pyruvate dehydrogenase phosphatase regulatory subunit  SUPT16H - suppressor of ty 16 homolog (s. cerevisiae)  PDGFRB - platelet-derived growth factor receptor, beta polypeptide  ARRB2 - arrestin, beta 2  ARRB1 - arrestin, beta 1  PDGFB - platelet-derived growth factor beta polypeptide  SAMD4B - sterile alpha motif domain containing 4b  ARG2 - arginase 2  STRAP - serine/threonine kinase receptor associated protein  RHOA - ras homolog family member a  PSIP1 - pc4 and sfrs1 interacting protein 1  PDK3 - pyruvate dehydrogenase kinase, isozyme 3  MED9 - mediator complex subunit 9  FSTL1 - follistatin-like 1  SLC2A6 - solute carrier family 2 (facilitated glucose transporter), member 6  BAZ2A - bromodomain adjacent to zinc finger domain, 2a  PCNA - proliferating cell nuclear antigen  CHMP1A - charged multivesicular body protein 1a  JDP2 - jun dimerization protein 2  CDK16 - cyclin-dependent kinase 16  AKIRIN2 - akirin 2  HEATR1 - heat repeat containing 1  CDK17 - cyclin-dependent kinase 17  LEO1 - leo1, paf1/rna polymerase ii complex component, homolog (s. cerevisiae)  ERLIN2 - er lipid raft associated 2  PWP1 - pwp1 homolog (s. cerevisiae)  WRAP53 - wd repeat containing, antisense to tp53  PDE2A - phosphodiesterase 2a, cgmp-stimulated  THAP1 - thap domain containing, apoptosis associated protein 1  PKIG - protein kinase (camp-dependent, catalytic) inhibitor gamma  ASAH1 - n-acylsphingosine amidohydrolase (acid ceramidase) 1  ELP3 - elongator acetyltransferase complex subunit 3  ASCL1 - achaete-scute complex homolog 1 (drosophila)  KAT7 - k(lysine) acetyltransferase 7  RGMB - rgm domain family, member b  PGGT1B - protein geranylgeranyltransferase type i, beta subunit  SBNO2 - strawberry notch homolog 2 (drosophila)  ZNF444 - zinc finger protein 444  ZBTB1 - zinc finger and btb domain containing 1  ZNF365 - zinc finger protein 365  PGAM1 - phosphoglycerate mutase 1 (brain)  ATP1A1 - atpase, na+/k+ transporting, alpha 1 polypeptide  FBXW7 - f-box and wd repeat domain containing 7, e3 ubiquitin protein ligase  BAHD1 - bromo adjacent homology domain containing 1  ZHX2 - zinc fingers and homeoboxes 2  MORC2 - morc family cw-type zinc finger 2  PHF2 - phd finger protein 2  PHF1 - phd finger protein 1  LRRK2 - leucine-rich repeat kinase 2  FOXJ3 - forkhead box j3  RERE - arginine-glutamic acid dipeptide (re) repeats  DNAJC24 - dnaj (hsp40) homolog, subfamily c, member 24  LARP6 - la ribonucleoprotein domain family, member 6  ZFHX3 - zinc finger homeobox 3  PEX14 - peroxisomal biogenesis factor 14  ADNP2 - adnp homeobox 2  AEBP2 - ae binding protein 2  IMPACT - impact rwd domain protein  ZNF507 - zinc finger protein 507  LGR4 - leucine-rich repeat containing g protein-coupled receptor 4  ATP2B4 - atpase, ca++ transporting, plasma membrane 4  ZNF652 - zinc finger protein 652  SCAF8 - sr-related ctd-associated factor 8  PKD1 - polycystic kidney disease 1 (autosomal dominant)  PKD2 - polycystic kidney disease 2 (autosomal dominant)  MTF2 - metal response element binding transcription factor 2  NAT10 - n-acetyltransferase 10 (gcn5-related)  BACH1 - btb and cnc homology 1, basic leucine zipper transcription factor 1  PIN1 - peptidylprolyl cis/trans isomerase, nima-interacting 1  PIK3R2 - phosphoinositide-3-kinase, regulatory subunit 2 (beta)  DPH3 - diphthamide biosynthesis 3  PIK3R1 - phosphoinositide-3-kinase, regulatory subunit 1 (alpha)  ASXL2 - additional sex combs like 2 (drosophila)  PLAGL1 - pleiomorphic adenoma gene-like 1  ELP2 - elongator acetyltransferase complex subunit 2  ATRX - alpha thalassemia/mental retardation syndrome x-linked  PKNOX1 - pbx/knotted 1 homeobox 1  CASC3 - cancer susceptibility candidate 3  PKM - pyruvate kinase, muscle  OGFOD1 - 2-oxoglutarate and iron-dependent oxygenase domain containing 1  TMEM100 - transmembrane protein 100  PHF10 - phd finger protein 10  RABL3 - rab, member of ras oncogene family-like 3  PSPC1 - paraspeckle component 1  BCL7A - b-cell cll/lymphoma 7a  MRGBP - mrg/morf4l binding protein  BCL9 - b-cell cll/lymphoma 9  RPUSD3 - rna pseudouridylate synthase domain containing 3  PIM1 - pim-1 oncogene  BRF2 - brf2, rna polymerase iii transcription initiation factor 50 kda subunit  BCL2 - b-cell cll/lymphoma 2  ECD - ecdysoneless homolog (drosophila)  CCND1 - cyclin d1  PARK7 - parkinson protein 7  PNKP - polynucleotide kinase 3'-phosphatase  C3orf33 - chromosome 3 open reading frame 33  TRIM27 - tripartite motif containing 27  RFC5 - replication factor c (activator 1) 5, 36.5kda  RFC2 - replication factor c (activator 1) 2, 40kda  SAP18 - sin3a-associated protein, 18kda  RFC3 - replication factor c (activator 1) 3, 38kda  TSHZ2 - teashirt zinc finger homeobox 2  MAEA - macrophage erythroblast attacher  RFXAP - regulatory factor x-associated protein  RFX5 - regulatory factor x, 5 (influences hla class ii expression)  WHSC1L1 - wolf-hirschhorn syndrome candidate 1-like 1  EIF1B - eukaryotic translation initiation factor 1b  RFX1 - regulatory factor x, 1 (influences hla class ii expression)  APBB3 - amyloid beta (a4) precursor protein-binding, family b, member 3  RELA - v-rel avian reticuloendotheliosis viral oncogene homolog a  BCOR - bcl6 corepressor  ZFP36L2 - zfp36 ring finger protein-like 2  ZFP36L1 - zfp36 ring finger protein-like 1  RET - ret proto-oncogene  DPF2 - d4, zinc and double phd fingers family 2  UPF1 - upf1 regulator of nonsense transcripts homolog (yeast)  CC2D1A - coiled-coil and c2 domain containing 1a  RLF - rearranged l-myc fusion  RING1 - ring finger protein 1  CNPY2 - canopy fgf signaling regulator 2  BMI1 - bmi1 polycomb ring finger oncogene  PCGF3 - polycomb group ring finger 3  BMPR1A - bone morphogenetic protein receptor, type ia  BMPR2 - bone morphogenetic protein receptor, type ii (serine/threonine kinase)  GON4L - gon-4-like (c. elegans)  BMPR1B - bone morphogenetic protein receptor, type ib  BMP7 - bone morphogenetic protein 7  ABCA7 - atp-binding cassette, sub-family a (abc1), member 7  FITM2 - fat storage-inducing transmembrane protein 2  PSMD14 - proteasome (prosome, macropain) 26s subunit, non-atpase, 14  FLOT1 - flotillin 1  BCAS3 - breast carcinoma amplified sequence 3  UHMK1 - u2af homology motif (uhm) kinase 1  AHI1 - abelson helper integration site 1  RNF4 - ring finger protein 4  BRD2 - bromodomain containing 2  RNF2 - ring finger protein 2  HNRNPR - heterogeneous nuclear ribonucleoprotein r  ZNF280D - zinc finger protein 280d  RNF6 - ring finger protein (c3h2c3 type) 6  GATAD2A - gata zinc finger domain containing 2a  ROCK1 - rho-associated, coiled-coil containing protein kinase 1  RNF111 - ring finger protein 111  TSPO - translocator protein (18kda)  TET2 - tet methylcytosine dioxygenase 2  MED18 - mediator complex subunit 18  DENND4A - denn/madd domain containing 4a  MBTD1 - mbt domain containing 1  RAMP1 - receptor (g protein-coupled) activity modifying protein 1  ZMPSTE24 - zinc metallopeptidase ste24  TRIM44 - tripartite motif containing 44  TAB1 - tgf-beta activated kinase 1/map3k7 binding protein 1  PPIE - peptidylprolyl isomerase e (cyclophilin e)  CAPN2 - calpain 2, (m/ii) large subunit  FAM3C - family with sequence similarity 3, member c  MCRS1 - microspherule protein 1  NRDE2 - nrde-2, necessary for rna interference, domain containing  C1D - c1d nuclear receptor corepressor  PTCD3 - pentatricopeptide repeat domain 3  PIBF1 - progesterone immunomodulatory binding factor 1  SLC30A9 - solute carrier family 30 (zinc transporter), member 9  MAD2L2 - mad2 mitotic arrest deficient-like 2 (yeast)  PID1 - phosphotyrosine interaction domain containing 1  PHIP - pleckstrin homology domain interacting protein  RPS4X - ribosomal protein s4, x-linked  NRARP - notch-regulated ankyrin repeat protein  ZNF787 - zinc finger protein 787  RMND1 - required for meiotic nuclear division 1 homolog (s. cerevisiae)  TRIM38 - tripartite motif containing 38  TADA3 - transcriptional adaptor 3  CALR - calreticulin  ZNF792 - zinc finger protein 792  WTIP - wilms tumor 1 interacting protein  NCOA2 - nuclear receptor coactivator 2  SYNCRIP - synaptotagmin binding, cytoplasmic rna interacting protein  CREB3 - camp responsive element binding protein 3  MFSD12 - major facilitator superfamily domain containing 12  CITED2 - cbp/p300-interacting transactivator, with glu/asp-rich carboxy-terminal domain, 2  CCT6A - chaperonin containing tcp1, subunit 6a (zeta 1)  CCNT2 - cyclin t2  PINX1 - pin2/terf1 interacting, telomerase inhibitor 1  CCND3 - cyclin d3  CCNT1 - cyclin t1  HMG20A - high mobility group 20a  CCNH - cyclin h  BANP - btg3 associated nuclear protein  TIPIN - timeless interacting protein  ACER2 - alkaline ceramidase 2  TRNAU1AP - trna selenocysteine 1 associated protein 1  RPS6KA1 - ribosomal protein s6 kinase, 90kda, polypeptide 1  RPS6KB1 - ribosomal protein s6 kinase, 70kda, polypeptide 1  RPS6KB2 - ribosomal protein s6 kinase, 70kda, polypeptide 2  CBFA2T3 - core-binding factor, runt domain, alpha subunit 2; translocated to, 3  CBFB - core-binding factor, beta subunit  PIAS3 - protein inhibitor of activated stat, 3  RXRB - retinoid x receptor, beta  CAV1 - caveolin 1, caveolae protein, 22kda  RBM14 - rna binding motif protein 14  CCNC - cyclin c  TRMT10C - trna methyltransferase 10 homolog c (s. cerevisiae)  PRMT5 - protein arginine methyltransferase 5  CHTF8 - ctf8, chromosome transmission fidelity factor 8 homolog (s. cerevisiae)  CDK11B - cyclin-dependent kinase 11b  CDC5L - cell division cycle 5-like  MXD4 - max dimerization protein 4  HTRA1 - htra serine peptidase 1  POLR3F - polymerase (rna) iii (dna directed) polypeptide f, 39 kda  CDH3 - cadherin 3, type 1, p-cadherin (placental)  HEXIM1 - hexamethylene bis-acetamide inducible 1  ERLIN1 - er lipid raft associated 1  SPIN2A - spindlin family, member 2a  NCOA7 - nuclear receptor coactivator 7  POMT1 - protein-o-mannosyltransferase 1  PSEN1 - presenilin 1  USP16 - ubiquitin specific peptidase 16  PSMA1 - proteasome (prosome, macropain) subunit, alpha type, 1  PSMA2 - proteasome (prosome, macropain) subunit, alpha type, 2  CD81 - cd81 molecule  CAMKK2 - calcium/calmodulin-dependent protein kinase kinase 2, beta  PSMB1 - proteasome (prosome, macropain) subunit, beta type, 1  STARD4 - star-related lipid transfer (start) domain containing 4  PSMA5 - proteasome (prosome, macropain) subunit, alpha type, 5  PSMA3 - proteasome (prosome, macropain) subunit, alpha type, 3  PSMB10 - proteasome (prosome, macropain) subunit, beta type, 10  RIPPLY2 - ripply transcriptional repressor 2  CD47 - cd47 molecule  FAM173B - family with sequence similarity 173, member b  TAF6L - taf6-like rna polymerase ii, p300/cbp-associated factor (pcaf)-associated factor, 65kda  PSMC6 - proteasome (prosome, macropain) 26s subunit, atpase, 6  PSMD1 - proteasome (prosome, macropain) 26s subunit, non-atpase, 1  PSMC4 - proteasome (prosome, macropain) 26s subunit, atpase, 4  SOX18 - sry (sex determining region y)-box 18  POLR3G - polymerase (rna) iii (dna directed) polypeptide g (32kd)  PSMC2 - proteasome (prosome, macropain) 26s subunit, atpase, 2  PSMD9 - proteasome (prosome, macropain) 26s subunit, non-atpase, 9  CD34 - cd34 molecule  PSMD10 - proteasome (prosome, macropain) 26s subunit, non-atpase, 10  PSMD7 - proteasome (prosome, macropain) 26s subunit, non-atpase, 7  PSMD8 - proteasome (prosome, macropain) 26s subunit, non-atpase, 8  PSMD5 - proteasome (prosome, macropain) 26s subunit, non-atpase, 5  TERF2IP - telomeric repeat binding factor 2, interacting protein  PSMD2 - proteasome (prosome, macropain) 26s subunit, non-atpase, 2  PSMD12 - proteasome (prosome, macropain) 26s subunit, non-atpase, 12  PSMD13 - proteasome (prosome, macropain) 26s subunit, non-atpase, 13  ATG7 - autophagy related 7  PTBP1 - polypyrimidine tract binding protein 1  PTEN - phosphatase and tensin homolog  LAMTOR5 - late endosomal/lysosomal adaptor, mapk and mtor activator 5  JMY - junction mediating and regulatory protein, p53 cofactor  MYBBP1A - myb binding protein (p160) 1a  FBLN5 - fibulin 5  PTGIS - prostaglandin i2 (prostacyclin) synthase  CIB1 - calcium and integrin binding 1 (calmyrin)  DDX17 - dead (asp-glu-ala-asp) box helicase 17  KAT5 - k(lysine) acetyltransferase 5  ZFP69 - zfp69 zinc finger protein  CEBPG - ccaat/enhancer binding protein (c/ebp), gamma  CDK5 - cyclin-dependent kinase 5  CDK4 - cyclin-dependent kinase 4  CDK9 - cyclin-dependent kinase 9  CCT7 - chaperonin containing tcp1, subunit 7 (eta)  CDK8 - cyclin-dependent kinase 8  LIN54 - lin-54 homolog (c. elegans)  CDK7 - cyclin-dependent kinase 7  CDKN1C - cyclin-dependent kinase inhibitor 1c (p57, kip2)  CDKN1B - cyclin-dependent kinase inhibitor 1b (p27, kip1)  CCT2 - chaperonin containing tcp1, subunit 2 (beta)  CCT4 - chaperonin containing tcp1, subunit 4 (delta)  PCBP3 - poly(rc) binding protein 3  PTPN2 - protein tyrosine phosphatase, non-receptor type 2  HTATIP2 - hiv-1 tat interactive protein 2, 30kda  CDK2 - cyclin-dependent kinase 2  ARFGEF1 - adp-ribosylation factor guanine nucleotide-exchange factor 1 (brefeldin a-inhibited)  PURA - purine-rich element binding protein a  RRN3 - rrn3 rna polymerase i transcription factor homolog (s. cerevisiae)  PURB - purine-rich element binding protein b  PDP1 - pyruvate dehyrogenase phosphatase catalytic subunit 1  GTPBP2 - gtp binding protein 2  ZMYND11 - zinc finger, mynd-type containing 11  SRSF10 - serine/arginine-rich splicing factor 10  PTPRK - protein tyrosine phosphatase, receptor type, k  PTPRN - protein tyrosine phosphatase, receptor type, n  JMJD8 - jumonji domain containing 8  PEX2 - peroxisomal biogenesis factor 2  NUP50 - nucleoporin 50kda  MPHOSPH8 - m-phase phosphoprotein 8  KDM5B - lysine (k)-specific demethylase 5b  PXN - paxillin  GIPC1 - gipc pdz domain containing family, member 1  CHD4 - chromodomain helicase dna binding protein 4  CHD2 - chromodomain helicase dna binding protein 2  CHD3 - chromodomain helicase dna binding protein 3  MTERFD2 - mterf domain containing 2  CHD1 - chromodomain helicase dna binding protein 1  QARS - glutaminyl-trna synthetase  HSPH1 - heat shock 105kda/110kda protein 1  MYPOP - myb-related transcription factor, partner of profilin  RAD21 - rad21 homolog (s. pombe)  RAD17 - rad17 homolog (s. pombe)  SEC24A - sec24 family, member a (s. cerevisiae)  PARP14 - poly (adp-ribose) polymerase family, member 14  PAF1 - paf1, rna polymerase ii associated factor, homolog (s. cerevisiae)  ZNF181 - zinc finger protein 181  RAC1 - ras-related c3 botulinum toxin substrate 1 (rho family, small gtp binding protein rac1)  ZNF268 - zinc finger protein 268  INO80 - ino80 complex subunit  ZNF513 - zinc finger protein 513  RAB27A - rab27a, member ras oncogene family  GMEB1 - glucocorticoid modulatory element binding protein 1  RANBP2 - ran binding protein 2  CCT8 - chaperonin containing tcp1, subunit 8 (theta)  RAP1A - rap1a, member of ras oncogene family  CLTC - clathrin, heavy chain (hc)  MIER2 - mesoderm induction early response 1, family member 2  RARG - retinoic acid receptor, gamma  RARB - retinoic acid receptor, beta  DLL4 - delta-like 4 (drosophila)  CELF1 - cugbp, elav-like family member 1  NECAB2 - n-terminal ef-hand calcium binding protein 2  CLU - clusterin  KHDRBS1 - kh domain containing, rna binding, signal transduction associated 1  TAF8 - taf8 rna polymerase ii, tata box binding protein (tbp)-associated factor, 43kda  GNL3L - guanine nucleotide binding protein-like 3 (nucleolar)-like  MAGEL2 - mage-like 2  CLK2 - cdc-like kinase 2  CTCF - ccctc-binding factor (zinc finger protein)  RBL2 - retinoblastoma-like 2 (p130)  RAI1 - retinoic acid induced 1  RBBP7 - retinoblastoma binding protein 7  RBBP8 - retinoblastoma binding protein 8  MAP3K2 - mitogen-activated protein kinase kinase kinase 2  RBM3 - rna binding motif (rnp1, rrm) protein 3  RBM4 - rna binding motif protein 4  RB1 - retinoblastoma 1  ARID4A - at rich interactive domain 4a (rbp1-like)  RBBP6 - retinoblastoma binding protein 6  KDM5A - lysine (k)-specific demethylase 5a  NUP35 - nucleoporin 35kda  PUS7 - pseudouridylate synthase 7 homolog (s. cerevisiae)  PTGES3 - prostaglandin e synthase 3 (cytosolic)  CKS1B - cdc28 protein kinase regulatory subunit 1b  NFAT5 - nuclear factor of activated t-cells 5, tonicity-responsive  CIRBP - cold inducible rna binding protein  DHX29 - deah (asp-glu-ala-his) box polypeptide 29  CHUK - conserved helix-loop-helix ubiquitous kinase  CERS1 - ceramide synthase 1  ZMYND8 - zinc finger, mynd-type containing 8  ANKRA2 - ankyrin repeat, family a (rfxank-like), 2  RBAK - rb-associated krab zinc finger  NFATC2IP - nuclear factor of activated t-cells, cytoplasmic, calcineurin-dependent 2 interacting protein  PATZ1 - poz (btb) and at hook containing zinc finger 1  FBXO18 - f-box protein, helicase, 18  METTL14 - methyltransferase like 14  SPP1 - secreted phosphoprotein 1  SSBP2 - single-stranded dna binding protein 2  KPNA6 - karyopherin alpha 6 (importin alpha 7)  CIRH1A - cirrhosis, autosomal recessive 1a (cirhin)  MIER1 - mesoderm induction early response 1, transcriptional regulator  PPP1R15B - protein phosphatase 1, regulatory subunit 15b  ZNF382 - zinc finger protein 382  ZNF705A - zinc finger protein 705a  SP3 - sp3 transcription factor  SP4 - sp4 transcription factor  SP1 - sp1 transcription factor  SP2 - sp2 transcription factor  ZNF410 - zinc finger protein 410  ARL2BP - adp-ribosylation factor-like 2 binding protein  DDAH2 - dimethylarginine dimethylaminohydrolase 2  SOX4 - sry (sex determining region y)-box 4  GTPBP4 - gtp binding protein 4  WBP2 - ww domain binding protein 2  ORC3 - origin recognition complex, subunit 3  HES4 - hairy and enhancer of split 4 (drosophila)  LEMD3 - lem domain containing 3  CREBL2 - camp responsive element binding protein-like 2  GATAD1 - gata zinc finger domain containing 1  ATF6B - activating transcription factor 6 beta  CREBBP - creb binding protein  SOAT1 - sterol o-acyltransferase 1  ATF2 - activating transcription factor 2  CREB1 - camp responsive element binding protein 1  DDX58 - dead (asp-glu-ala-asp) box polypeptide 58  CRABP2 - cellular retinoic acid binding protein 2  RPRD1B - regulation of nuclear pre-mrna domain containing 1b  PLK3 - polo-like kinase 3  SNAPC4 - small nuclear rna activating complex, polypeptide 4, 190kda  SNCA - synuclein, alpha (non a4 component of amyloid precursor)  ZBED5 - zinc finger, bed-type containing 5  CREBZF - creb/atf bzip transcription factor  EID1 - ep300 interacting inhibitor of differentiation 1  TP53INP2 - tumor protein p53 inducible nuclear protein 2  PTBP2 - polypyrimidine tract binding protein 2  SMARCE1 - swi/snf related, matrix associated, actin dependent regulator of chromatin, subfamily e, member 1  SMO - smoothened, frizzled family receptor  SMARCB1 - swi/snf related, matrix associated, actin dependent regulator of chromatin, subfamily b, member 1  SMARCC1 - swi/snf related, matrix associated, actin dependent regulator of chromatin, subfamily c, member 1  HLTF - helicase-like transcription factor  SMARCA4 - swi/snf related, matrix associated, actin dependent regulator of chromatin, subfamily a, member 4  SNX6 - sorting nexin 6  SSBP3 - single stranded dna binding protein 3  SMARCD2 - swi/snf related, matrix associated, actin dependent regulator of chromatin, subfamily d, member 2  SMARCD3 - swi/snf related, matrix associated, actin dependent regulator of chromatin, subfamily d, member 3  SMARCC2 - swi/snf related, matrix associated, actin dependent regulator of chromatin, subfamily c, member 2  SMARCD1 - swi/snf related, matrix associated, actin dependent regulator of chromatin, subfamily d, member 1  WIZ - widely interspaced zinc finger motifs  MID1IP1 - mid1 interacting protein 1  SMARCA1 - swi/snf related, matrix associated, actin dependent regulator of chromatin, subfamily a, member 1  SMARCA2 - swi/snf related, matrix associated, actin dependent regulator of chromatin, subfamily a, member 2  SGK3 - serum/glucocorticoid regulated kinase family, member 3  CACTIN - cactin, spliceosome c complex subunit  KMT2C - lysine (k)-specific methyltransferase 2c  RAB38 - rab38, member ras oncogene family  ZNF462 - zinc finger protein 462  PPIL4 - peptidylprolyl isomerase (cyclophilin)-like 4  TMEFF2 - transmembrane protein with egf-like and two follistatin-like domains 2  SLC18A2 - solute carrier family 18 (vesicular monoamine transporter), member 2  SENP2 - sumo1/sentrin/smt3 specific peptidase 2  CST3 - cystatin c  ZNF318 - zinc finger protein 318  PRPF6 - pre-mrna processing factor 6  KDM2B - lysine (k)-specific demethylase 2b  SUPT6H - suppressor of ty 6 homolog (s. cerevisiae)  CTGF - connective tissue growth factor  CBY1 - chibby homolog 1 (drosophila)  CTBP1 - c-terminal binding protein 1  SUPT4H1 - suppressor of ty 4 homolog 1 (s. cerevisiae)  CTBP2 - c-terminal binding protein 2  SUPT5H - suppressor of ty 5 homolog (s. cerevisiae)  RAB3GAP2 - rab3 gtpase activating protein subunit 2 (non-catalytic)  CTNNB1 - catenin (cadherin-associated protein), beta 1, 88kda  SAV1 - salvador homolog 1 (drosophila)  ELOVL5 - elovl fatty acid elongase 5  CUX1 - cut-like homeobox 1  NIF3L1 - nif3 ngg1 interacting factor 3-like 1 (s. cerevisiae)  NEK4 - nima-related kinase 4  STK3 - serine/threonine kinase 3  MPV17L2 - mpv17 mitochondrial membrane protein-like 2  PCGF1 - polycomb group ring finger 1  FAM3A - family with sequence similarity 3, member a  NPNT - nephronectin  FKBP8 - fk506 binding protein 8, 38kda  GPBP1L1 - gc-rich promoter binding protein 1-like 1  CYLD - cylindromatosis (turban tumor syndrome)  TGIF2 - tgfb-induced factor homeobox 2  CNEP1R1 - ctd nuclear envelope phosphatase 1 regulatory subunit 1  BCL6B - b-cell cll/lymphoma 6, member b  CRK - v-crk avian sarcoma virus ct10 oncogene homolog  EEFSEC - eukaryotic elongation factor, selenocysteine-trna-specific  NIPBL - nipped-b homolog (drosophila)  CRY1 - cryptochrome 1 (photolyase-like)  STAT2 - signal transducer and activator of transcription 2, 113kda  STAT1 - signal transducer and activator of transcription 1, 91kda  CRY2 - cryptochrome 2 (photolyase-like)  STAT3 - signal transducer and activator of transcription 3 (acute-phase response factor)  ASF1A - anti-silencing function 1a histone chaperone  STAT5A - signal transducer and activator of transcription 5a  STAT6 - signal transducer and activator of transcription 6, interleukin-4 induced  PARM1 - prostate androgen-regulated mucin-like protein 1  BRMS1 - breast cancer metastasis suppressor 1  CRYM - crystallin, mu  ZNF496 - zinc finger protein 496  SS18 - synovial sarcoma translocation, chromosome 18  PHF5A - phd finger protein 5a  GPATCH3 - g patch domain containing 3  TAB3 - tgf-beta activated kinase 1/map3k7 binding protein 3  BAMBI - bmp and activin membrane-bound inhibitor  ZNF335 - zinc finger protein 335  HAVCR2 - hepatitis a virus cellular receptor 2  SQLE - squalene epoxidase  SRI - sorcin  SRC - v-src avian sarcoma (schmidt-ruppin a-2) viral oncogene homolog  DNAJB5 - dnaj (hsp40) homolog, subfamily b, member 5  SREBF2 - sterol regulatory element binding transcription factor 2  UBE2O - ubiquitin-conjugating enzyme e2o  NSUN3 - nop2/sun domain family, member 3  SRP9 - signal recognition particle 9kda  PRDX5 - peroxiredoxin 5  BACE2 - beta-site app-cleaving enzyme 2  DHX9 - deah (asp-glu-ala-his) box helicase 9  TRAPPC2 - trafficking protein particle complex 2  KANK2 - kn motif and ankyrin repeat domains 2  DDX5 - dead (asp-glu-ala-asp) box helicase 5  DDX3X - dead (asp-glu-ala-asp) box helicase 3, x-linked  SEC13 - sec13 homolog (s. cerevisiae)  DDX1 - dead (asp-glu-ala-asp) box helicase 1  SIN3A - sin3 transcription regulator family member a  DPH6 - diphthamine biosynthesis 6  SET - set nuclear oncogene  WWTR1 - ww domain containing transcription regulator 1  DDB1 - damage-specific dna binding protein 1, 127kda  ZNF304 - zinc finger protein 304  MAP2K4 - mitogen-activated protein kinase kinase 4  ZNF286A - zinc finger protein 286a  GEMIN5 - gem (nuclear organelle) associated protein 5  NOL11 - nucleolar protein 11  PBXIP1 - pre-b-cell leukemia homeobox interacting protein 1  DBP - d site of albumin promoter (albumin d-box) binding protein  NELFB - negative elongation factor complex member b  POT1 - protection of telomeres 1  AHCTF1 - at hook containing transcription factor 1  ABHD6 - abhydrolase domain containing 6  CNOT10 - ccr4-not transcription complex, subunit 10  ZBTB38 - zinc finger and btb domain containing 38  GATAD2B - gata zinc finger domain containing 2b  ZNF551 - zinc finger protein 551  ZNF473 - zinc finger protein 473  KCTD13 - potassium channel tetramerization domain containing 13  YTHDF3 - yth domain family, member 3  WDR18 - wd repeat domain 18  CNOT6 - ccr4-not transcription complex, subunit 6  LTN1 - listerin e3 ubiquitin protein ligase 1  ATXN2 - ataxin 2  ZNF451 - zinc finger protein 451  SCAF4 - sr-related ctd-associated factor 4  SS18L1 - synovial sarcoma translocation gene on chromosome 18-like 1  CHD5 - chromodomain helicase dna binding protein 5  ARID1B - at rich interactive domain 1b (swi1-like)  MED30 - mediator complex subunit 30  MKL2 - mkl/myocardin-like 2  NLN - neurolysin (metallopeptidase m3 family)  DAPK3 - death-associated protein kinase 3  DAXX - death-domain associated protein  ZZZ3 - zinc finger, zz-type containing 3  HINFP - histone h4 transcription factor  MTA3 - metastasis associated 1 family, member 3  DGKQ - diacylglycerol kinase, theta 110kda  ZNF608 - zinc finger protein 608  DAPK1 - death-associated protein kinase 1  DAP - death-associated protein  MAVS - mitochondrial antiviral signaling protein  CYP51A1 - cytochrome p450, family 51, subfamily a, polypeptide 1  SARS - seryl-trna synthetase  SATB1 - satb homeobox 1  NGDN - neuroguidin, eif4e binding protein  RPTOR - regulatory associated protein of mtor, complex 1  SAFB - scaffold attachment factor b  SH2B1 - sh2b adaptor protein 1  SALL2 - sal-like 2 (drosophila)  EPC2 - enhancer of polycomb homolog 2 (drosophila)  DRD2 - dopamine receptor d2  PHF19 - phd finger protein 19  SLC9A1 - solute carrier family 9, subfamily a (nhe1, cation proton antiporter 1), member 1  IRF2BP1 - interferon regulatory factor 2 binding protein 1  EAF1 - ell associated factor 1  CNOT6L - ccr4-not transcription complex, subunit 6-like  PDP2 - pyruvate dehyrogenase phosphatase catalytic subunit 2  ATN1 - atrophin 1  DPH2 - dph2 homolog (s. cerevisiae)  DPH1 - diphthamide biosynthesis 1  C10orf137 - chromosome 10 open reading frame 137  DR1 - down-regulator of transcription 1, tbp-binding (negative cofactor 2)  MAPK15 - mitogen-activated protein kinase 15  SKI - v-ski avian sarcoma viral oncogene homolog  SKIL - ski-like oncogene  UNK - unkempt homolog (drosophila)  DYRK1A - dual-specificity tyrosine-(y)-phosphorylation regulated kinase 1a  MKL1 - megakaryoblastic leukemia (translocation) 1  DVL2 - dishevelled segment polarity protein 2  DVL3 - dishevelled segment polarity protein 3  GIGYF2 - grb10 interacting gyf protein 2  FNIP2 - folliculin interacting protein 2  APPL1 - adaptor protein, phosphotyrosine interaction, ph domain and leucine zipper containing 1  HOMEZ - homeobox and leucine zipper encoding  EBF4 - early b-cell factor 4  ANKRD17 - ankyrin repeat domain 17  ZFHX2 - zinc finger homeobox 2  HBEGF - heparin-binding egf-like growth factor  LSM14A - lsm14a, scd6 homolog a (s. cerevisiae)  DTX1 - deltex homolog 1 (drosophila)  DKC1 - dyskeratosis congenita 1, dyskerin  DLG1 - discs, large homolog 1 (drosophila)  SHOX2 - short stature homeobox 2  SIAH2 - siah e3 ubiquitin protein ligase 2  DHCR7 - 7-dehydrocholesterol reductase  POGK - pogo transposable element with krab domain  DHFR - dihydrofolate reductase  PHF12 - phd finger protein 12  CALCOCO1 - calcium binding and coiled-coil domain 1  ZBTB4 - zinc finger and btb domain containing 4  SFPQ - splicing factor proline/glutamine-rich  SFRP1 - secreted frizzled-related protein 1  SFRP2 - secreted frizzled-related protein 2  SFRP4 - secreted frizzled-related protein 4  DNM2 - dynamin 2  GMEB2 - glucocorticoid modulatory element binding protein 2  SRSF2 - serine/arginine-rich splicing factor 2  DNMT1 - dna (cytosine-5-)-methyltransferase 1  SPAG8 - sperm associated antigen 8  DNMT3A - dna (cytosine-5-)-methyltransferase 3 alpha  EIF2AK4 - eukaryotic translation initiation factor 2 alpha kinase 4  CHD8 - chromodomain helicase dna binding protein 8  SGK1 - serum/glucocorticoid regulated kinase 1  NOC2L - nucleolar complex associated 2 homolog (s. cerevisiae)  TNRC6C - trinucleotide repeat containing 6c  ZNF317 - zinc finger protein 317  USP37 - ubiquitin specific peptidase 37  PPP1R3F - protein phosphatase 1, regulatory subunit 3f  MEIS3 - meis homeobox 3  NCBP2 - nuclear cap binding protein subunit 2, 20kda  MAPRE3 - microtubule-associated protein, rp/eb family, member 3  TSC1 - tuberous sclerosis 1  ELK1 - elk1, member of ets oncogene family  NAF1 - nuclear assembly factor 1 ribonucleoprotein  ELK3 - elk3, ets-domain protein (srf accessory protein 2)  ELK4 - elk4, ets-domain protein (srf accessory protein 1)  SNW1 - snw domain containing 1  SCAP - srebf chaperone  WRNIP1 - werner helicase interacting protein 1  EPAS1 - endothelial pas domain protein 1  DPH7 - diphthamide biosynthesis 7  EP300 - e1a binding protein p300  HABP4 - hyaluronan binding protein 4  ATF6 - activating transcription factor 6  SRRD - srr1 domain containing  ENO1 - enolase 1, (alpha)  RAB3GAP1 - rab3 gtpase activating protein subunit 1 (catalytic)  EIF4A2 - eukaryotic translation initiation factor 4a2  EIF4E - eukaryotic translation initiation factor 4e  CELF4 - cugbp, elav-like family member 4  EGR2 - early growth response 2  CCT5 - chaperonin containing tcp1, subunit 5 (epsilon)  EGR3 - early growth response 3  TTF1 - transcription termination factor, rna polymerase i  EIF2S1 - eukaryotic translation initiation factor 2, subunit 1 alpha, 35kda  EIF2B1 - eukaryotic translation initiation factor 2b, subunit 1 alpha, 26kda  TRIM32 - tripartite motif containing 32  SPHK2 - sphingosine kinase 2  EIF2S3 - eukaryotic translation initiation factor 2, subunit 3 gamma, 52kda  KDM2A - lysine (k)-specific demethylase 2a  ELAVL1 - elav (embryonic lethal, abnormal vision, drosophila)-like 1 (hu antigen r)  ZNF326 - zinc finger protein 326  ELF1 - e74-like factor 1 (ets domain transcription factor)  ELF2 - e74-like factor 2 (ets domain transcription factor)  EIF4G2 - eukaryotic translation initiation factor 4 gamma, 2  TCF25 - transcription factor 25 (basic helix-loop-helix)  TSG101 - tumor susceptibility gene 101  CNOT1 - ccr4-not transcription complex, subunit 1  EFNA1 - ephrin-a1  EEF2 - eukaryotic translation elongation factor 2  SPEN - spen homolog, transcriptional regulator (drosophila)  CTNNBIP1 - catenin, beta interacting protein 1  BBX - bobby sox homolog (drosophila)  PHC1 - polyhomeotic homolog 1 (drosophila)  TP53BP1 - tumor protein p53 binding protein 1  PHC2 - polyhomeotic homolog 2 (drosophila)  EGR1 - early growth response 1  ZNF292 - zinc finger protein 292  EGFR - epidermal growth factor receptor  MEGF8 - multiple egf-like-domains 8  CELSR2 - cadherin, egf lag seven-pass g-type receptor 2  KDM1A - lysine (k)-specific demethylase 1a  ATXN7L3 - ataxin 7-like 3  HNRNPLL - heterogeneous nuclear ribonucleoprotein l-like  NCOA6 - nuclear receptor coactivator 6  CCT3 - chaperonin containing tcp1, subunit 3 (gamma)  E2F6 - e2f transcription factor 6  E4F1 - e4f transcription factor 1  ZHX3 - zinc fingers and homeoboxes 3  E2F4 - e2f transcription factor 4, p107/p130-binding  E2F5 - e2f transcription factor 5, p130-binding  ENY2 - enhancer of yellow 2 homolog (drosophila)  C11orf30 - chromosome 11 open reading frame 30  RRP1B - ribosomal rna processing 1b  EDNRB - endothelin receptor type b  TRAF1 - tnf receptor-associated factor 1  S1PR1 - sphingosine-1-phosphate receptor 1  TRAF3 - tnf receptor-associated factor 3  TRAF2 - tnf receptor-associated factor 2  NR2C1 - nuclear receptor subfamily 2, group c, member 1  SETX - senataxin  ZBTB47 - zinc finger and btb domain containing 47  NR2C2 - nuclear receptor subfamily 2, group c, member 2  TPR - translocated promoter region, nuclear basket protein  ZNF609 - zinc finger protein 609  WAPAL - wings apart-like homolog (drosophila)  ERC1 - elks/rab6-interacting/cast family member 1  PPRC1 - peroxisome proliferator-activated receptor gamma, coactivator-related 1  NAT14 - n-acetyltransferase 14 (gcn5-related, putative)  KDM4C - lysine (k)-specific demethylase 4c  ZNF423 - zinc finger protein 423  REXO4 - rex4, rna exonuclease 4 homolog (s. cerevisiae)  CAMK1D - calcium/calmodulin-dependent protein kinase id  USF1 - upstream transcription factor 1  NUP107 - nucleoporin 107kda  ZBTB43 - zinc finger and btb domain containing 43  PTK2B - protein tyrosine kinase 2 beta  ACSL3 - acyl-coa synthetase long-chain family member 3  MRPS27 - mitochondrial ribosomal protein s27  TNRC6B - trinucleotide repeat containing 6b  PITHD1 - pith (c-terminal proteasome-interacting domain of thioredoxin-like) domain containing 1  BPTF - bromodomain phd finger transcription factor  FASN - fatty acid synthase  TAB2 - tgf-beta activated kinase 1/map3k7 binding protein 2  NR1H2 - nuclear receptor subfamily 1, group h, member 2  ETV3 - ets variant 3  CAMTA2 - calmodulin binding transcription activator 2  ZNF598 - zinc finger protein 598  ETV5 - ets variant 5  POGZ - pogo transposable element with znf domain  TWSG1 - twisted gastrulation homolog 1 (drosophila)  ETV6 - ets variant 6  PLXND1 - plexin d1  VHL - von hippel-lindau tumor suppressor, e3 ubiquitin protein ligase  VIM - vimentin  KDM6B - lysine (k)-specific demethylase 6b  PHF8 - phd finger protein 8  EZR - ezrin  EWSR1 - ews rna-binding protein 1  TRIM41 - tripartite motif containing 41  TBX20 - t-box 20  KCTD1 - potassium channel tetramerization domain containing 1  CREB3L1 - camp responsive element binding protein 3-like 1  EZH1 - enhancer of zeste homolog 1 (drosophila)  CIC - capicua transcriptional repressor  CCNL1 - cyclin l1  F2R - coagulation factor ii (thrombin) receptor  VCP - valosin containing protein  WDR43 - wd repeat domain 43  ERF - ets2 repressor factor  ZNF575 - zinc finger protein 575  NUP205 - nucleoporin 205kda  ELMSAN1 - elm2 and myb/sant-like domain containing 1  THAP11 - thap domain containing 11  ZNF248 - zinc finger protein 248  UBB - ubiquitin b  LARP4B - la ribonucleoprotein domain family, member 4b  RCOR1 - rest corepressor 1  TULP3 - tubby like protein 3  ZNFX1 - zinc finger, nfx1-type containing 1  HIRA - histone cell cycle regulator  TTC5 - tetratricopeptide repeat domain 5  ETV1 - ets variant 1  ETS1 - v-ets avian erythroblastosis virus e26 oncogene homolog 1  ETF1 - eukaryotic translation termination factor 1  ZMIZ1 - zinc finger, miz-type containing 1  CYFIP1 - cytoplasmic fmr1 interacting protein 1  UBP1 - upstream binding protein 1 (lbp-1a)  SUMO1 - small ubiquitin-like modifier 1  UBTF - upstream binding transcription factor, rna polymerase i  ZNF830 - zinc finger protein 830  PELI1 - pellino e3 ubiquitin protein ligase 1  CHURC1 - churchill domain containing 1  SMURF1 - smad specific e3 ubiquitin protein ligase 1  ZC3H4 - zinc finger ccch-type containing 4  JMJD6 - jumonji domain containing 6  EPRS - glutamyl-prolyl-trna synthetase  UBE2I - ubiquitin-conjugating enzyme e2i  NUP210 - nucleoporin 210kda  UBE2L3 - ubiquitin-conjugating enzyme e2l 3  UBE2N - ubiquitin-conjugating enzyme e2n  UBE2V1 - ubiquitin-conjugating enzyme e2 variant 1  VPS72 - vacuolar protein sorting 72 homolog (s. cerevisiae)  FMR1 - fragile x mental retardation 1  TCOF1 - treacher collins-franceschetti syndrome 1  MGA - mga, max dimerization protein  TCP1 - t-complex 1  ZEB1 - zinc finger e-box binding homeobox 1  WWC3 - wwc family member 3  TCF12 - transcription factor 12  ZNF354A - zinc finger protein 354a  CAMTA1 - calmodulin binding transcription activator 1  TCF20 - transcription factor 20 (ar1)  TMEM106A - transmembrane protein 106a  PDS5A - pds5, regulator of cohesion maintenance, homolog a (s. cerevisiae)  FLI1 - fli-1 proto-oncogene, ets transcription factor  FLNA - filamin a, alpha  PLCB1 - phospholipase c, beta 1 (phosphoinositide-specific)  FOXS1 - forkhead box s1  FOXO3 - forkhead box o3  LARP4 - la ribonucleoprotein domain family, member 4  HDAC8 - histone deacetylase 8  ASH1L - ash1 (absent, small, or homeotic)-like (drosophila)  SIN3B - sin3 transcription regulator family member b  NACC1 - nucleus accumbens associated 1, ben and btb (poz) domain containing  HR - hair growth associated  MED8 - mediator complex subunit 8  CAND1 - cullin-associated and neddylation-dissociated 1  TDG - thymine-dna glycosylase  SMG6 - smg6 nonsense mediated mrna decay factor  PRDX2 - peroxiredoxin 2  EAPP - e2f-associated phosphoprotein  FBXW11 - f-box and wd repeat domain containing 11  FAM208A - family with sequence similarity 208, member a  KDM3A - lysine (k)-specific demethylase 3a  FHL2 - four and a half lim domains 2  SLC24A5 - solute carrier family 24 (sodium/potassium/calcium exchanger), member 5  NUP160 - nucleoporin 160kda  FGFR2 - fibroblast growth factor receptor 2  TAF9 - taf9 rna polymerase ii, tata box binding protein (tbp)-associated factor, 32kda  TAF7 - taf7 rna polymerase ii, tata box binding protein (tbp)-associated factor, 55kda  TAF6 - taf6 rna polymerase ii, tata box binding protein (tbp)-associated factor, 80kda  FGFR1 - fibroblast growth factor receptor 1  CYGB - cytoglobin  FGF9 - fibroblast growth factor 9  TADA2A - transcriptional adaptor 2a  TACC1 - transforming, acidic coiled-coil containing protein 1  TIRAP - toll-interleukin 1 receptor (tir) domain containing adaptor protein  FGF1 - fibroblast growth factor 1 (acidic)  FGF2 - fibroblast growth factor 2 (basic)  USP22 - ubiquitin specific peptidase 22  TARBP1 - tar (hiv-1) rna binding protein 1  FER - fer (fps/fes related) tyrosine kinase  MAP3K7 - mitogen-activated protein kinase kinase kinase 7  ADNP - activity-dependent neuroprotector homeobox  KMT2E - lysine (k)-specific methyltransferase 2e  MED13L - mediator complex subunit 13-like  FDFT1 - farnesyl-diphosphate farnesyltransferase 1  PGP - phosphoglycolate phosphatase  NCSTN - nicastrin  FKTN - fukutin  ZNF395 - zinc finger protein 395  TAZ - tafazzin  MYNN - myoneurin  CRTC1 - creb regulated transcription coactivator 1  UFL1 - ufm1-specific ligase 1  TCF3 - transcription factor 3  PUM2 - pumilio homolog 2 (drosophila)  DMAP1 - dna methyltransferase 1 associated protein 1  TCEB3 - transcription elongation factor b (siii), polypeptide 3 (110kda, elongin a)  LARP1 - la ribonucleoprotein domain family, member 1  TCF4 - transcription factor 4  EFEMP1 - egf containing fibulin-like extracellular matrix protein 1  NKRF - nfkb repressing factor  ZNF629 - zinc finger protein 629  FBN1 - fibrillin 1  LANCL2 - lanc lantibiotic synthetase component c-like 2 (bacterial)  TCEA2 - transcription elongation factor a (sii), 2  ERBB2IP - erbb2 interacting protein  TCEA1 - transcription elongation factor a (sii), 1  ITGB3BP - integrin beta 3 binding protein (beta3-endonexin)  ORMDL1 - orm1-like 1 (s. cerevisiae)  RYBP - ring1 and yy1 binding protein  MTOR - mechanistic target of rapamycin (serine/threonine kinase)  PAXBP1 - pax3 and pax7 binding protein 1  TLE1 - transducin-like enhancer of split 1 (e(sp1) homolog, drosophila)  TLE3 - transducin-like enhancer of split 3 (e(sp1) homolog, drosophila)  TLE4 - transducin-like enhancer of split 4 (e(sp1) homolog, drosophila)  TLR3 - toll-like receptor 3  SIRT1 - sirtuin 1  MKX - mohawk homeobox  TMF1 - tata element modulatory factor 1  TMSB4X - thymosin beta 4, x-linked  ACTR8 - arp8 actin-related protein 8 homolog (yeast)  CBX6 - chromobox homolog 6  HEY1 - hairy/enhancer-of-split related with yrpw motif 1  TMPO - thymopoietin  CBX5 - chromobox homolog 5  BRD4 - bromodomain containing 4  TNFAIP1 - tumor necrosis factor, alpha-induced protein 1 (endothelial)  TARDBP - tar dna binding protein  BCL9L - b-cell cll/lymphoma 9-like  TNFRSF1A - tumor necrosis factor receptor superfamily, member 1a  TNFRSF1B - tumor necrosis factor receptor superfamily, member 1b  TMBIM6 - transmembrane bax inhibitor motif containing 6  NUP188 - nucleoporin 188kda  EIF4ENIF1 - eukaryotic translation initiation factor 4e nuclear import factor 1  SUZ12 - suz12 polycomb repressive complex 2 subunit  POFUT1 - protein o-fucosyltransferase 1  TEF - thyrotrophic embryonic factor  RCOR2 - rest corepressor 2  TFAM - transcription factor a, mitochondrial  INPP5E - inositol polyphosphate-5-phosphatase, 72 kda  TERF1 - telomeric repeat binding factor (nima-interacting) 1  TERF2 - telomeric repeat binding factor 2  TFDP1 - transcription factor dp-1  NR2F2 - nuclear receptor subfamily 2, group f, member 2  TFE3 - transcription factor binding to ighm enhancer 3  CBX7 - chromobox homolog 7  NR2F1 - nuclear receptor subfamily 2, group f, member 1  TFCP2 - transcription factor cp2  ZNF740 - zinc finger protein 740  TGFA - transforming growth factor, alpha  FNIP1 - folliculin interacting protein 1  HEY2 - hairy/enhancer-of-split related with yrpw motif 2  TGFBR1 - transforming growth factor, beta receptor 1  TGFB2 - transforming growth factor, beta 2  RBFOX2 - rna binding protein, fox-1 homolog (c. elegans) 2  TGFB3 - transforming growth factor, beta 3  TGFB1 - transforming growth factor, beta 1  TGFB1I1 - transforming growth factor beta 1 induced transcript 1  TP53INP1 - tumor protein p53 inducible nuclear protein 1  H2AFV - h2a histone family, member v  MEPCE - methylphosphate capping enzyme  RNF20 - ring finger protein 20, e3 ubiquitin protein ligase  TGFBR3 - transforming growth factor, beta receptor iii  FOSL2 - fos-like antigen 2  FOSB - fbj murine osteosarcoma viral oncogene homolog b  FOS - fbj murine osteosarcoma viral oncogene homolog  THBS1 - thrombospondin 1  KAT6B - k(lysine) acetyltransferase 6b  TIA1 - tia1 cytotoxic granule-associated rna binding protein  METTL3 - methyltransferase like 3  THRA - thyroid hormone receptor, alpha  ELL - elongation factor rna polymerase ii  ZNF239 - zinc finger protein 239  PHC3 - polyhomeotic homolog 3 (drosophila)  OBFC1 - oligonucleotide/oligosaccharide-binding fold containing 1  NRIP1 - nuclear receptor interacting protein 1  KAT2A - k(lysine) acetyltransferase 2a  NCOA3 - nuclear receptor coactivator 3  GLA - galactosidase, alpha  WWC2 - ww and c2 domain containing 2  TAF15 - taf15 rna polymerase ii, tata box binding protein (tbp)-associated factor, 68kda  GJA1 - gap junction protein, alpha 1, 43kda  AXIN1 - axin 1  TRRAP - transformation/transcription domain-associated protein  PICALM - phosphatidylinositol binding clathrin assembly protein  LPCAT1 - lysophosphatidylcholine acyltransferase 1  ARID1A - at rich interactive domain 1a (swi-like)  DLL1 - delta-like 1 (drosophila)  SETD6 - set domain containing 6  FZD6 - frizzled family receptor 6  GLE1 - gle1 rna export mediator  ACTR5 - arp5 actin-related protein 5 homolog (yeast)  CDC45 - cell division cycle 45  FZD4 - frizzled family receptor 4  GCLC - glutamate-cysteine ligase, catalytic subunit  FZD1 - frizzled family receptor 1  BAP1 - brca1 associated protein-1 (ubiquitin carboxy-terminal hydrolase)  ZNF613 - zinc finger protein 613  NUP85 - nucleoporin 85kda  CDC7 - cell division cycle 7  KDM5C - lysine (k)-specific demethylase 5c  USP9X - ubiquitin specific peptidase 9, x-linked  RBM10 - rna binding motif protein 10  PHF17 - phd finger protein 17  TTC21B - tetratricopeptide repeat domain 21b  SLTM - safb-like, transcription modulator  NELFE - negative elongation factor complex member e  EHMT1 - euchromatic histone-lysine n-methyltransferase 1  NR5A1 - nuclear receptor subfamily 5, group a, member 1  FUS - fused in sarcoma  NAA40 - n(alpha)-acetyltransferase 40, natd catalytic subunit, homolog (s. cerevisiae)  USP7 - ubiquitin specific peptidase 7 (herpes virus-associated)  RNF168 - ring finger protein 168, e3 ubiquitin protein ligase  XRCC6 - x-ray repair complementing defective repair in chinese hamster cells 6  GABPB1 - ga binding protein transcription factor, beta subunit 1  GABPA - ga binding protein transcription factor, alpha subunit 60kda  FYN - fyn oncogene related to src, fgr, yes  RPAP2 - rna polymerase ii associated protein 2  DDX39B - dead (asp-glu-ala-asp) box polypeptide 39b  KMT2D - lysine (k)-specific methyltransferase 2d  AAAS - achalasia, adrenocortical insufficiency, alacrimia  ZNF419 - zinc finger protein 419  YEATS4 - yeats domain containing 4  ZFPM1 - zinc finger protein, fog family member 1  SNIP1 - smad nuclear interacting protein 1  ZNF212 - zinc finger protein 212  EID2 - ep300 interacting inhibitor of differentiation 2  GBA - glucosidase, beta, acid  ZNF383 - zinc finger protein 383  GATA2 - gata binding protein 2  KAT6A - k(lysine) acetyltransferase 6a  NUP214 - nucleoporin 214kda  NR4A3 - nuclear receptor subfamily 4, group a, member 3  BRD3 - bromodomain containing 3  GAS6 - growth arrest-specific 6  NCOA4 - nuclear receptor coactivator 4  CARF - calcium responsive transcription factor  DEDD2 - death effector domain containing 2  ZNF408 - zinc finger protein 408  MLLT10 - myeloid/lymphoid or mixed-lineage leukemia (trithorax homolog, drosophila); translocated to, 10  DHX36 - deah (asp-glu-ala-his) box polypeptide 36  ZNF189 - zinc finger protein 189  ZSCAN26 - zinc finger and scan domain containing 26  GSTP1 - glutathione s-transferase pi 1  GSPT1 - g1 to s phase transition 1  ZNF177 - zinc finger protein 177  ZNF175 - zinc finger protein 175  ZNF174 - zinc finger protein 174  ZNF184 - zinc finger protein 184  SSBP4 - single stranded dna binding protein 4  GTF2F1 - general transcription factor iif, polypeptide 1, 74kda  ZNF148 - zinc finger protein 148  ZBTB17 - zinc finger and btb domain containing 17  BEND5 - ben domain containing 5  GTF2H2 - general transcription factor iih, polypeptide 2, 44kda  GTF2H3 - general transcription factor iih, polypeptide 3, 34kda  TRIM26 - tripartite motif containing 26  GTF2H1 - general transcription factor iih, polypeptide 1, 62kda  VEZF1 - vascular endothelial zinc finger 1  ZNF142 - zinc finger protein 142  MCPH1 - microcephalin 1  ZNF140 - zinc finger protein 140  GTF2B - general transcription factor iib  PCGF2 - polycomb group ring finger 2  ZNF132 - zinc finger protein 132  ZNF131 - zinc finger protein 131  ZNF134 - zinc finger protein 134  ZNF133 - zinc finger protein 133  TBL1XR1 - transducin (beta)-like 1 x-linked receptor 1  GTF2I - general transcription factor iii  GTF2H4 - general transcription factor iih, polypeptide 4, 52kda  BRF1 - brf1, rna polymerase iii transcription initiation factor 90 kda subunit  ZNF79 - zinc finger protein 79  ZNF70 - zinc finger protein 70  ZNF75D - zinc finger protein 75d  ZNF76 - zinc finger protein 76  H3F3A - h3 histone, family 3a  BRPF1 - bromodomain and phd finger containing, 1  H2AFZ - h2a histone family, member z  FZD5 - frizzled family receptor 5  SAP130 - sin3a-associated protein, 130kda  H2AFX - h2a histone family, member x  DCP2 - decapping mrna 2  PAX8 - paired box 8  MIER3 - mesoderm induction early response 1, family member 3  CDC73 - cell division cycle 73  NKAP - nfkb activating protein  THAP5 - thap domain containing 5  LRP8 - low density lipoprotein receptor-related protein 8, apolipoprotein e receptor  HCFC1 - host cell factor c1 (vp16-accessory protein)  PRDM2 - pr domain containing 2, with znf domain  MAP3K12 - mitogen-activated protein kinase kinase kinase 12  ZNF226 - zinc finger protein 226  HDAC2 - histone deacetylase 2  HMBOX1 - homeobox containing 1  HDGF - hepatoma-derived growth factor  ZCCHC12 - zinc finger, cchc domain containing 12  ZNF213 - zinc finger protein 213  HDAC1 - histone deacetylase 1  HCLS1 - hematopoietic cell-specific lyn substrate 1  ZNF205 - zinc finger protein 205  ZNF207 - zinc finger protein 207  XPA - xeroderma pigmentosum, complementation group a  GOLGA2 - golgin a2  XPC - xeroderma pigmentosum, complementation group c  GOLGB1 - golgin b1  XPO1 - exportin 1 (crm1 homolog, yeast)  PAGR1 - paxip1 associated glutamate-rich protein 1  ZXDC - zxd family zinc finger c  AAED1 - ahpc/tsa antioxidant enzyme domain containing 1  XBP1 - x-box binding protein 1  ZNF367 - zinc finger protein 367  BHLHE41 - basic helix-loop-helix family, member e41  EIF4H - eukaryotic translation initiation factor 4h  WFS1 - wolfram syndrome 1 (wolframin)  WHSC1 - wolf-hirschhorn syndrome candidate 1  NELFA - negative elongation factor complex member a  ARID2 - at rich interactive domain 2 (arid, rfx-like)  PAOX - polyamine oxidase (exo-n4-amino)  ZNF35 - zinc finger protein 35  ZKSCAN1 - zinc finger with krab and scan domains 1  FBXL15 - f-box and leucine-rich repeat protein 15  ZNF33B - zinc finger protein 33b  ZNF576 - zinc finger protein 576  ZNF32 - zinc finger protein 32  ZNF45 - zinc finger protein 45  MZF1 - myeloid zinc finger 1  ZNF18 - zinc finger protein 18  ZNF16 - zinc finger protein 16  ASXL1 - additional sex combs like 1 (drosophila)  ZNF384 - zinc finger protein 384  TNIP2 - tnfaip3 interacting protein 2  ZNF24 - zinc finger protein 24  ZFPL1 - zinc finger protein-like 1  ZFX - zinc finger protein, x-linked  SF1 - splicing factor 1  ZBTB14 - zinc finger and btb domain containing 14  ZNF711 - zinc finger protein 711  ZNF7 - zinc finger protein 7  ZNF8 - zinc finger protein 8  ZNF2 - zinc finger protein 2  ZNF3 - zinc finger protein 3  YES1 - v-yes-1 yamaguchi sarcoma viral oncogene homolog 1  XRCC5 - x-ray repair complementing defective repair in chinese hamster cells 5 (double-strand-break rejoining)  GSK3B - glycogen synthase kinase 3 beta  GSK3A - glycogen synthase kinase 3 alpha  YWHAB - tyrosine 3-monooxygenase/tryptophan 5-monooxygenase activation protein, beta polypeptide  CCNK - cyclin k  HSF2 - heat shock transcription factor 2  TRIM24 - tripartite motif containing 24  TAF1D - tata box binding protein (tbp)-associated factor, rna polymerase i, d, 41kda  CREG1 - cellular repressor of e1a-stimulated genes 1  FLCN - folliculin  HSBP1 - heat shock factor binding protein 1  PRMT1 - protein arginine methyltransferase 1  PRMT2 - protein arginine methyltransferase 2  HESX1 - hesx homeobox 1  SAP30 - sin3a-associated protein, 30kda  METTL16 - methyltransferase like 16  ALKBH1 - alkb, alkylation repair homolog 1 (e. coli)  FTO - fat mass and obesity associated  CFLAR - casp8 and fadd-like apoptosis regulator  SECISBP2 - secis binding protein 2  MED11 - mediator complex subunit 11  LDB1 - lim domain binding 1  WDR77 - wd repeat domain 77  DSCC1 - dna replication and sister chromatid cohesion 1  KAT2B - k(lysine) acetyltransferase 2b  ELOVL6 - elovl fatty acid elongase 6  TSC22D1 - tsc22 domain family, member 1  CRTC2 - creb regulated transcription coactivator 2  ZNF259 - zinc finger protein 259  TAX1BP1 - tax1 (human t-cell leukemia virus type i) binding protein 1  CDC123 - cell division cycle 123  CC2D1B - coiled-coil and c2 domain containing 1b  HUS1 - hus1 checkpoint homolog (s. pombe)  EIF2B2 - eukaryotic translation initiation factor 2b, subunit 2 beta, 39kda  THAP8 - thap domain containing 8  BUD31 - bud31 homolog (s. cerevisiae)  EIF2B5 - eukaryotic translation initiation factor 2b, subunit 5 epsilon, 82kda  ZNF655 - zinc finger protein 655  TRAK2 - trafficking protein, kinesin binding 2  HSPD1 - heat shock 60kda protein 1 (chaperonin)  DNAJB1 - dnaj (hsp40) homolog, subfamily b, member 1  ZNF48 - zinc finger protein 48  BCL10 - b-cell cll/lymphoma 10  FUBP3 - far upstream element (fuse) binding protein 3  HSP90AA1 - heat shock protein 90kda alpha (cytosolic), class a member 1  BTRC - beta-transducin repeat containing e3 ubiquitin protein ligase  HSPA8 - heat shock 70kda protein 8  EIF4EBP3 - eukaryotic translation initiation factor 4e binding protein 3  HMGN1 - high mobility group nucleosome binding domain 1  HMGB3 - high mobility group box 3  HMGB2 - high mobility group box 2  HMGB1 - high mobility group box 1  EIF3C - eukaryotic translation initiation factor 3, subunit c  EIF3D - eukaryotic translation initiation factor 3, subunit d  BRD9 - bromodomain containing 9  EIF3B - eukaryotic translation initiation factor 3, subunit b  IRS2 - insulin receptor substrate 2  TNKS - tankyrase, trf1-interacting ankyrin-related adp-ribose polymerase  EIF4G3 - eukaryotic translation initiation factor 4 gamma, 3  ZBTB10 - zinc finger and btb domain containing 10  AGO1 - argonaute risc catalytic component 1  CAPRIN2 - caprin family member 2  ZBTB48 - zinc finger and btb domain containing 48  HYAL2 - hyaluronoglucosaminidase 2  ACD - adrenocortical dysplasia homolog (mouse)  GPBP1 - gc-rich promoter binding protein 1  HFE - hemochromatosis  HINT1 - histidine triad nucleotide binding protein 1  HIVEP1 - human immunodeficiency virus type i enhancer binding protein 1  ELP4 - elongator acetyltransferase complex subunit 4  UPF3B - upf3 regulator of nonsense transcripts homolog b (yeast)  UPF3A - upf3 regulator of nonsense transcripts homolog a (yeast)  HIF1A - hypoxia inducible factor 1, alpha subunit (basic helix-loop-helix transcription factor)  MBTPS1 - membrane-bound transcription factor peptidase, site 1  LPIN3 - lipin 3  TRADD - tnfrsf1a-associated via death domain  MYCBP - myc binding protein  EED - embryonic ectoderm development  URI1 - uri1, prefoldin-like chaperone  HNRNPU - heterogeneous nuclear ribonucleoprotein u (scaffold attachment factor a)  RIPK1 - receptor (tnfrsf)-interacting serine-threonine kinase 1  HNRNPK - heterogeneous nuclear ribonucleoprotein k  HNRNPL - heterogeneous nuclear ribonucleoprotein l  FBXO4 - f-box protein 4  TINF2 - terf1 (trf1)-interacting nuclear factor 2  HNRNPA2B1 - heterogeneous nuclear ribonucleoprotein a2/b1  HNRNPA1 - heterogeneous nuclear ribonucleoprotein a1  HNRNPC - heterogeneous nuclear ribonucleoprotein c (c1/c2)  HNRNPAB - heterogeneous nuclear ribonucleoprotein a/b  RIPK2 - receptor-interacting serine-threonine kinase 2  HNRNPD - heterogeneous nuclear ribonucleoprotein d (au-rich element rna binding protein 1, 37kda)  HMGCR - 3-hydroxy-3-methylglutaryl-coa reductase  HMGCS1 - 3-hydroxy-3-methylglutaryl-coa synthase 1 (soluble)  HMGA1 - high mobility group at-hook 1  ZNF584 - zinc finger protein 584  FAM129B - family with sequence similarity 129, member b  NR4A1 - nuclear receptor subfamily 4, group a, member 1  RFX7 - regulatory factor x, 7  FOXK2 - forkhead box k2  HTATSF1 - hiv-1 tat specific factor 1  EIF3K - eukaryotic translation initiation factor 3, subunit k  HAT1 - histone acetyltransferase 1  CRTC3 - creb regulated transcription coactivator 3  RBM15 - rna binding motif protein 15  TNRC6A - trinucleotide repeat containing 6a  ENC1 - ectodermal-neural cortex 1 (with btb domain)  RBMX - rna binding motif protein, x-linked  ING2 - inhibitor of growth family, member 2  PPM1D - protein phosphatase, mg2+/mn2+ dependent, 1d  ZNF574 - zinc finger protein 574  CREB3L2 - camp responsive element binding protein 3-like 2  IPPK - inositol 1,3,4,5,6-pentakisphosphate 2-kinase  ILF2 - interleukin enhancer binding factor 2  ILF3 - interleukin enhancer binding factor 3, 90kda  ILK - integrin-linked kinase  PIAS1 - protein inhibitor of activated stat, 1  SLCO3A1 - solute carrier organic anion transporter family, member 3a1  SMYD3 - set and mynd domain containing 3  MAPKAPK5 - mitogen-activated protein kinase-activated protein kinase 5  SMURF2 - smad specific e3 ubiquitin protein ligase 2  CGGBP1 - cgg triplet repeat binding protein 1  LMO4 - lim domain only 4  CAMK1 - calcium/calmodulin-dependent protein kinase i  CBX4 - chromobox homolog 4  CSRNP1 - cysteine-serine-rich nuclear protein 1  SERP1 - stress-associated endoplasmic reticulum protein 1  ZNF25 - zinc finger protein 25  STK16 - serine/threonine kinase 16  ITGA3 - integrin, alpha 3 (antigen cd49c, alpha 3 subunit of vla-3 receptor)  KHSRP - kh-type splicing regulatory protein  CASK - calcium/calmodulin-dependent serine protein kinase (maguk family)  ITGAV - integrin, alpha v  SUDS3 - suppressor of defective silencing 3 homolog (s. cerevisiae)  EIF6 - eukaryotic translation initiation factor 6  SKAP1 - src kinase associated phosphoprotein 1  GZF1 - gdnf-inducible zinc finger protein 1  INSR - insulin receptor  INSM1 - insulinoma-associated 1  CDK13 - cyclin-dependent kinase 13  PDCD4 - programmed cell death 4 (neoplastic transformation inhibitor)  RFXANK - regulatory factor x-associated ankyrin-containing protein  KLF7 - kruppel-like factor 7 (ubiquitous)  IRF2 - interferon regulatory factor 2  IRF3 - interferon regulatory factor 3  IREB2 - iron-responsive element binding protein 2  IKZF5 - ikaros family zinc finger 5 (pegasus)  RUVBL1 - ruvb-like 1 (e. coli)  MED19 - mediator complex subunit 19  ITGA6 - integrin, alpha 6  IRAK2 - interleukin-1 receptor-associated kinase 2  IRAK1 - interleukin-1 receptor-associated kinase 1  CACYBP - calcyclin binding protein  EIF2AK1 - eukaryotic translation initiation factor 2-alpha kinase 1  ZSCAN31 - zinc finger and scan domain containing 31  PAPD5 - pap associated domain containing 5  KIAA1324L - kiaa1324-like  NSD1 - nuclear receptor binding set domain protein 1  ZBTB11 - zinc finger and btb domain containing 11  RNF25 - ring finger protein 25  DKK3 - dickkopf wnt signaling pathway inhibitor 3  MMS19 - mms19 nucleotide excision repair homolog (s. cerevisiae)  SNX5 - sorting nexin 5  ID2 - inhibitor of dna binding 2, dominant negative helix-loop-helix protein  ZNF777 - zinc finger protein 777  BRPF3 - bromodomain and phd finger containing, 3  ID3 - inhibitor of dna binding 3, dominant negative helix-loop-helix protein  STK36 - serine/threonine kinase 36  IRF2BPL - interferon regulatory factor 2 binding protein-like  JAZF1 - jazf zinc finger 1  PLA2G6 - phospholipase a2, group vi (cytosolic, calcium-independent)  FOXK1 - forkhead box k1  IDI1 - isopentenyl-diphosphate delta isomerase 1  ZNF282 - zinc finger protein 282  IGHMBP2 - immunoglobulin mu binding protein 2  RBPJ - recombination signal binding protein for immunoglobulin kappa j region  NSMAF - neutral sphingomyelinase (n-smase) activation associated factor  PABPC1 - poly(a) binding protein, cytoplasmic 1  NCK2 - nck adaptor protein 2  TRUB2 - trub pseudouridine (psi) synthase homolog 2 (e. coli)  USP21 - ubiquitin specific peptidase 21  ZBTB9 - zinc finger and btb domain containing 9  ZBTB12 - zinc finger and btb domain containing 12  BCAR3 - breast cancer anti-estrogen resistance 3  IGBP1 - immunoglobulin (cd79a) binding protein 1  JMJD1C - jumonji domain containing 1c  SMARCA5 - swi/snf related, matrix associated, actin dependent regulator of chromatin, subfamily a, member 5  OGT - o-linked n-acetylglucosamine (glcnac) transferase  PELP1 - proline, glutamate and leucine rich protein 1  RAE1 - rae1 rna export 1 homolog (s. pombe)  TSPYL2 - tspy-like 2  SND1 - staphylococcal nuclease and tudor domain containing 1  PCIF1 - pdx1 c-terminal inhibiting factor 1  CUL2 - cullin 2  CUL3 - cullin 3  ZNF667 - zinc finger protein 667  FOXP1 - forkhead box p1  SUPT3H - suppressor of ty 3 homolog (s. cerevisiae)  BEND6 - ben domain containing 6  ZNF572 - zinc finger protein 572  PCGF5 - polycomb group ring finger 5  WAC - ww domain containing adaptor with coiled-coil  PHF21A - phd finger protein 21a  ZNF414 - zinc finger protein 414  ZNF771 - zinc finger protein 771  AKAP12 - a kinase (prka) anchor protein 12  IER2 - immediate early response 2  LIG3 - ligase iii, dna, atp-dependent  NCOR1 - nuclear receptor corepressor 1  LDLR - low density lipoprotein receptor  MTPN - myotrophin  NCOR2 - nuclear receptor corepressor 2  DOT1L - dot1-like histone h3k79 methyltransferase  ABCG1 - atp-binding cassette, sub-family g (white), member 1  EFCAB7 - ef-hand calcium binding domain 7  L3MBTL3 - l(3)mbt-like 3 (drosophila)  ZNF333 - zinc finger protein 333  CIR1 - corepressor interacting with rbpj, 1  SCAND1 - scan domain containing 1  H2AFY - h2a histone family, member y  FADS1 - fatty acid desaturase 1  GTF2IRD1 - gtf2i repeat domain containing 1  ZNF527 - zinc finger protein 527  H6PD - hexose-6-phosphate dehydrogenase (glucose 1-dehydrogenase)  HOPX - hop homeobox  SOX13 - sry (sex determining region y)-box 13  ZC3H8 - zinc finger ccch-type containing 8  ZNF316 - zinc finger protein 316  LMO1 - lim domain only 1 (rhombotin 1)  NACC2 - nacc family member 2, ben and btb (poz) domain containing  IER5 - immediate early response 5  CLOCK - clock circadian regulator  KLF3 - kruppel-like factor 3 (basic)  PRKAG2 - protein kinase, amp-activated, gamma 2 non-catalytic subunit  DDX41 - dead (asp-glu-ala-asp) box polypeptide 41  SUCO - sun domain containing ossification factor  FXR2 - fragile x mental retardation, autosomal homolog 2  LITAF - lipopolysaccharide-induced tnf factor  YTHDF2 - yth domain family, member 2  TBPL1 - tbp-like 1  ZBTB41 - zinc finger and btb domain containing 41  ZFP62 - zfp62 zinc finger protein  TMEM59 - transmembrane protein 59  ZGPAT - zinc finger, ccch-type with g patch domain  ACTL6B - actin-like 6b  EIF4E2 - eukaryotic translation initiation factor 4e family member 2  MED20 - mediator complex subunit 20  AFAP1L2 - actin filament associated protein 1-like 2  ROCK2 - rho-associated, coiled-coil containing protein kinase 2  MAPK8IP1 - mitogen-activated protein kinase 8 interacting protein 1  CRLF3 - cytokine receptor-like factor 3  NGRN - neugrin, neurite outgrowth associated  GLYR1 - glyoxylate reductase 1 homolog (arabidopsis)  ZBTB7A - zinc finger and btb domain containing 7a  DPY30 - dpy-30 homolog (c. elegans)  MBTPS2 - membrane-bound transcription factor peptidase, site 2  IRF2BP2 - interferon regulatory factor 2 binding protein 2  GLIS2 - glis family zinc finger 2  MAGED1 - melanoma antigen family d, 1  MED27 - mediator complex subunit 27  MED26 - mediator complex subunit 26  CHD6 - chromodomain helicase dna binding protein 6  MED17 - mediator complex subunit 17  MED23 - mediator complex subunit 23  ZNF580 - zinc finger protein 580  CDYL - chromodomain protein, y-like  GTF2IRD2 - gtf2i repeat domain containing 2  KDR - kinase insert domain receptor (a type iii receptor tyrosine kinase)  ZNF710 - zinc finger protein 710  ING4 - inhibitor of growth family, member 4  INSIG2 - insulin induced gene 2  EIF2AK3 - eukaryotic translation initiation factor 2-alpha kinase 3  GGPS1 - geranylgeranyl diphosphate synthase 1  ITM2B - integral membrane protein 2b  RLIM - ring finger protein, lim domain interacting  SOGA1 - suppressor of glucose, autophagy associated 1  RECQL5 - recq protein-like 5  KRAS - kirsten rat sarcoma viral oncogene homolog  ZNF706 - zinc finger protein 706  TGFBRAP1 - transforming growth factor, beta receptor associated protein 1  CIAO1 - cytosolic iron-sulfur protein assembly 1  KPNB1 - karyopherin (importin) beta 1  PHF20L1 - phd finger protein 20-like 1  MED10 - mediator complex subunit 10  MED21 - mediator complex subunit 21  ZFP28 - zfp28 zinc finger protein  KIT - v-kit hardy-zuckerman 4 feline sarcoma viral oncogene homolog  POLDIP3 - polymerase (dna-directed), delta interacting protein 3  PAIP2 - poly(a) binding protein interacting protein 2  JUP - junction plakoglobin  JAK2 - janus kinase 2  JAK3 - janus kinase 3  ING5 - inhibitor of growth family, member 5  SLC25A33 - solute carrier family 25 (pyrimidine nucleotide carrier), member 33  ZNF219 - zinc finger protein 219  DNAJC30 - dnaj (hsp40) homolog, subfamily c, member 30  PHF20 - phd finger protein 20  KCNK2 - potassium channel, subfamily k, member 2  ZNF397 - zinc finger protein 397  ZNHIT3 - zinc finger, hit-type containing 3  TRIP4 - thyroid hormone receptor interactor 4  HMGN3 - high mobility group nucleosomal binding domain 3  PHF6 - phd finger protein 6  ZNF235 - zinc finger protein 235  COPS2 - cop9 signalosome subunit 2  ZNF639 - zinc finger protein 639  SARNP - sap domain containing ribonucleoprotein  LEF1 - lymphoid enhancer-binding factor 1  BRMS1L - breast cancer metastasis-suppressor 1-like  CNOT8 - ccr4-not transcription complex, subunit 8  CDK12 - cyclin-dependent kinase 12  SIRPA - signal-regulatory protein alpha  ZNF280B - zinc finger protein 280b  ARID4B - at rich interactive domain 4b (rbp1-like)  MAP3K10 - mitogen-activated protein kinase kinase kinase 10  ITGB1BP1 - integrin beta 1 binding protein 1  KDM3B - lysine (k)-specific demethylase 3b  RSF1 - remodeling and spacing factor 1  MLLT6 - myeloid/lymphoid or mixed-lineage leukemia (trithorax homolog, drosophila); translocated to, 6  SAMD8 - sterile alpha motif domain containing 8  MLLT3 - myeloid/lymphoid or mixed-lineage leukemia (trithorax homolog, drosophila); translocated to, 3  MLLT1 - myeloid/lymphoid or mixed-lineage leukemia (trithorax homolog, drosophila); translocated to, 1  MED14 - mediator complex subunit 14  ZBTB22 - zinc finger and btb domain containing 22  KMT2A - lysine (k)-specific methyltransferase 2a  RPS6KA5 - ribosomal protein s6 kinase, 90kda, polypeptide 5  SUFU - suppressor of fused homolog (drosophila)  NLK - nemo-like kinase  UIMC1 - ubiquitin interaction motif containing 1  MNAT1 - mnat cdk-activating kinase assembly factor 1  MTA2 - metastasis associated 1 family, member 2  NOLC1 - nucleolar and coiled-body phosphoprotein 1  EIF2A - eukaryotic translation initiation factor 2a, 65kda  CD200 - cd200 molecule  WWOX - ww domain containing oxidoreductase  MNT - mnt, max dimerization protein  CCPG1 - cell cycle progression 1  SPTY2D1 - spt2, suppressor of ty, domain containing 1 (s. cerevisiae)  MECP2 - methyl cpg binding protein 2 (rett syndrome)  PELO - pelota homolog (drosophila)  MEF2A - myocyte enhancer factor 2a  PRICKLE1 - prickle homolog 1 (drosophila)  ZBED1 - zinc finger, bed-type containing 1  MEIS2 - meis homeobox 2  BRWD1 - bromodomain and wd repeat domain containing 1  ZNF664 - zinc finger protein 664  MEIS1 - meis homeobox 1  MAP3K4 - mitogen-activated protein kinase kinase kinase 4  MAP3K5 - mitogen-activated protein kinase kinase kinase 5  SLC33A1 - solute carrier family 33 (acetyl-coa transporter), member 1  MEN1 - multiple endocrine neoplasia i  WDR75 - wd repeat domain 75  UTP15 - utp15, u3 small nucleolar ribonucleoprotein, homolog (s. cerevisiae)  KAT8 - k(lysine) acetyltransferase 8  ZNF644 - zinc finger protein 644  ARHGEF2 - rho/rac guanine nucleotide exchange factor (gef) 2  NEMF - nuclear export mediator factor  MBD3 - methyl-cpg binding domain protein 3  PTOV1 - prostate tumor overexpressed 1  ZNF394 - zinc finger protein 394  CBFA2T2 - core-binding factor, runt domain, alpha subunit 2; translocated to, 2  MAGOH - mago-nashi homolog, proliferation-associated (drosophila)  CXXC5 - cxxc finger protein 5  RQCD1 - rcd1 required for cell differentiation1 homolog (s. pombe)  SMC3 - structural maintenance of chromosomes 3  MARS - methionyl-trna synthetase  IL23A - interleukin 23, alpha subunit p19  MTA1 - metastasis associated 1  SIRT6 - sirtuin 6  PPHLN1 - periphilin 1  UBE2J1 - ubiquitin-conjugating enzyme e2, j1  NUP43 - nucleoporin 43kda  MBD1 - methyl-cpg binding domain protein 1  SFMBT1 - scm-like with four mbt domains 1  MAX - myc associated factor x  ZMIZ2 - zinc finger, miz-type containing 2  LDB2 - lim domain binding 2  ZFP90 - zfp90 zinc finger protein  NELFCD - negative elongation factor complex member c/d  CDAN1 - codanin 1  ASH2L - ash2 (absent, small, or homeotic)-like (drosophila)  ZSCAN29 - zinc finger and scan domain containing 29  CTDSPL2 - ctd (carboxy-terminal domain, rna polymerase ii, polypeptide a) small phosphatase like 2  THAP2 - thap domain containing, apoptosis associated protein 2  LSS - lanosterol synthase (2,3-oxidosqualene-lanosterol cyclase)  ITCH - itchy e3 ubiquitin protein ligase  ZNF484 - zinc finger protein 484  L3MBTL2 - l(3)mbt-like 2 (drosophila)  LRP6 - low density lipoprotein receptor-related protein 6  ZNF445 - zinc finger protein 445  AIP - aryl hydrocarbon receptor interacting protein  ASB1 - ankyrin repeat and socs box containing 1  TRAPPC9 - trafficking protein particle complex 9  LUM - lumican  BTAF1 - btaf1 rna polymerase ii, b-tfiid transcription factor-associated, 170kda  UBA3 - ubiquitin-like modifier activating enzyme 3  SESN2 - sestrin 2  PARP9 - poly (adp-ribose) polymerase family, member 9  CDK5RAP1 - cdk5 regulatory subunit associated protein 1  RNF8 - ring finger protein 8, e3 ubiquitin protein ligase  BAZ1B - bromodomain adjacent to zinc finger domain, 1b  MXD1 - max dimerization protein 1  MED15 - mediator complex subunit 15  PIAS4 - protein inhibitor of activated stat, 4  TRIM33 - tripartite motif containing 33  SRRT - serrate rna effector molecule homolog (arabidopsis)  SMAD2 - smad family member 2  HDAC7 - histone deacetylase 7  KLF16 - kruppel-like factor 16  ARMCX3 - armadillo repeat containing, x-linked 3  TDP2 - tyrosyl-dna phosphodiesterase 2  CAPRIN1 - cell cycle associated protein 1  LARP7 - la ribonucleoprotein domain family, member 7  RPS6KA4 - ribosomal protein s6 kinase, 90kda, polypeptide 4  MAFG - v-maf avian musculoaponeurotic fibrosarcoma oncogene homolog g  DPH5 - diphthamide biosynthesis 5  TAF9B - taf9b rna polymerase ii, tata box binding protein (tbp)-associated factor, 31kda  SMAD4 - smad family member 4  H1FX - h1 histone family, member x  SMAD5 - smad family member 5  SMAD7 - smad family member 7  WASL - wiskott-aldrich syndrome-like  RBM4B - rna binding motif protein 4b  SAP30BP - sap30 binding protein  ZNF565 - zinc finger protein 565  YAF2 - yy1 associated factor 2  TAGLN3 - transgelin 3  SIX5 - six homeobox 5  NCBP1 - nuclear cap binding protein subunit 1, 80kda  NDN - necdin, melanoma antigen (mage) family member  ITM2C - integral membrane protein 2c  ZNF582 - zinc finger protein 582  NCK1 - nck adaptor protein 1  BRD7 - bromodomain containing 7  NCL - nucleolin  DDX25 - dead (asp-glu-ala-asp) box helicase 25  CEBPZ - ccaat/enhancer binding protein (c/ebp), zeta  TICAM1 - toll-like receptor adaptor molecule 1  CCNL2 - cyclin l2  ORMDL2 - orm1-like 2 (s. cerevisiae)  TRIM28 - tripartite motif containing 28  TBK1 - tank-binding kinase 1  NBN - nibrin  ZNF197 - zinc finger protein 197  TDRD3 - tudor domain containing 3  TRIM11 - tripartite motif containing 11  PPP1R12A - protein phosphatase 1, regulatory subunit 12a  MYT1 - myelin transcription factor 1  ALYREF - aly/ref export factor  NAB1 - ngfi-a binding protein 1 (egr1 binding protein 1)  CSRNP2 - cysteine-serine-rich nuclear protein 2  SENP1 - sumo1/sentrin specific peptidase 1  RNF41 - ring finger protein 41  NAB2 - ngfi-a binding protein 2 (egr1 binding protein 2)  TSHZ1 - teashirt zinc finger homeobox 1  ANKRD13C - ankyrin repeat domain 13c  PSME3 - proteasome (prosome, macropain) activator subunit 3 (pa28 gamma; ki)  TRIM13 - tripartite motif containing 13  TRIM8 - tripartite motif containing 8  MYO6 - myosin vi  LBH - limb bud and heart development  ABT1 - activator of basal transcription 1  TOPORS - topoisomerase i binding, arginine/serine-rich, e3 ubiquitin protein ligase  MYD88 - myeloid differentiation primary response 88  SEH1L - seh1-like (s. cerevisiae)  ZNF362 - zinc finger protein 362  HCFC2 - host cell factor c2  CNOT7 - ccr4-not transcription complex, subunit 7  MXI1 - max interactor 1, dimerization protein  PQBP1 - polyglutamine binding protein 1  ACTR2 - arp2 actin-related protein 2 homolog (yeast)  ACTR3 - arp3 actin-related protein 3 homolog (yeast)  TSFM - ts translation elongation factor, mitochondrial  TRIM37 - tripartite motif containing 37  ZNF558 - zinc finger protein 558  LRPPRC - leucine-rich pentatricopeptide repeat containing  ZNF263 - zinc finger protein 263  PURG - purine-rich element binding protein g  MED25 - mediator complex subunit 25  TRAP1 - tnf receptor-associated protein 1  MED6 - mediator complex subunit 6  PTBP3 - polypyrimidine tract binding protein 3  DMTF1 - cyclin d binding myb-like transcription factor 1  MTIF2 - mitochondrial translational initiation factor 2  ZBTB33 - zinc finger and btb domain containing 33  HDAC5 - histone deacetylase 5  MTF1 - metal-regulatory transcription factor 1  SRA1 - steroid receptor rna activator 1  HDAC6 - histone deacetylase 6  RNF187 - ring finger protein 187  MT3 - metallothionein 3  MED16 - mediator complex subunit 16  CCDC22 - coiled-coil domain containing 22  DNAJB6 - dnaj (hsp40) homolog, subfamily b, member 6  MSH3 - muts homolog 3 (e. coli)  LPGAT1 - lysophosphatidylglycerol acyltransferase 1  CITED1 - cbp/p300-interacting transactivator, with glu/asp-rich carboxy-terminal domain, 1  ZBTB5 - zinc finger and btb domain containing 5  ZBTB40 - zinc finger and btb domain containing 40  SUPT7L - suppressor of ty 7 (s. cerevisiae)-like  RGCC - regulator of cell cycle  COMMD1 - copper metabolism (murr1) domain containing 1  RBM8A - rna binding motif protein 8a  USP3 - ubiquitin specific peptidase 3  METTL5 - methyltransferase like 5  USP15 - ubiquitin specific peptidase 15  MED4 - mediator complex subunit 4  THOC1 - tho complex 1  SETD2 - set domain containing 2  RBX1 - ring-box 1, e3 ubiquitin protein ligase  NUP153 - nucleoporin 153kda  THRAP3 - thyroid hormone receptor associated protein 3  MED12 - mediator complex subunit 12  MED13 - mediator complex subunit 13  PSMD6 - proteasome (prosome, macropain) 26s subunit, non-atpase, 6  MED24 - mediator complex subunit 24  ZC3H6 - zinc finger ccch-type containing 6  DTX3L - deltex 3-like (drosophila)  SETDB1 - set domain, bifurcated 1  ZNF34 - zinc finger protein 34  ORC2 - origin recognition complex, subunit 2  ORC5 - origin recognition complex, subunit 5  TOX4 - tox high mobility group box family member 4  ZNF436 - zinc finger protein 436  RNF141 - ring finger protein 141  AGRN - agrin  EPM2AIP1 - epm2a (laforin) interacting protein 1  G3BP2 - gtpase activating protein (sh3 domain) binding protein 2  OGG1 - 8-oxoguanine dna glycosylase  ZSCAN16 - zinc finger and scan domain containing 16  TNKS2 - tankyrase, trf1-interacting ankyrin-related adp-ribose polymerase 2  DDR2 - discoidin domain receptor tyrosine kinase 2  DAB2IP - dab2 interacting protein  BCLAF1 - bcl2-associated transcription factor 1  NUMA1 - nuclear mitotic apparatus protein 1  EIF4A3 - eukaryotic translation initiation factor 4a3  NUP88 - nucleoporin 88kda  GMNN - geminin, dna replication inhibitor  NUP98 - nucleoporin 98kda  NR4A2 - nuclear receptor subfamily 4, group a, member 2  ZNF593 - zinc finger protein 593  ZBTB7B - zinc finger and btb domain containing 7b  MATR3 - matrin 3  NVL - nuclear vcp-like  ZNF691 - zinc finger protein 691  CREBRF - creb3 regulatory factor  ZNF623 - zinc finger protein 623  NMD3 - nmd3 ribosome export adaptor  NPM1 - nucleophosmin (nucleolar phosphoprotein b23, numatrin)  ARHGEF11 - rho guanine nucleotide exchange factor (gef) 11  ZBTB24 - zinc finger and btb domain containing 24  ZEB2 - zinc finger e-box binding homeobox 2  THAP4 - thap domain containing 4  CTIF - cbp80/20-dependent translation initiation factor  TSC22D2 - tsc22 domain family, member 2  ADIPOR1 - adiponectin receptor 1  NFXL1 - nuclear transcription factor, x-box binding-like 1  NRF1 - nuclear respiratory factor 1  DONSON - downstream neighbor of son  NOP2 - nop2 nucleolar protein  VPRBP - vpr (hiv-1) binding protein  NONO - non-pou domain containing, octamer-binding  MDFIC - myod family inhibitor domain containing  NOTCH4 - notch 4  IFT74 - intraflagellar transport 74 homolog (chlamydomonas)  CEP290 - centrosomal protein 290kda  NPAT - nuclear protein, ataxia-telangiectasia locus  CNOT2 - ccr4-not transcription complex, subunit 2  NAA15 - n(alpha)-acetyltransferase 15, nata auxiliary subunit  PUM1 - pumilio homolog 1 (drosophila)  NOS3 - nitric oxide synthase 3 (endothelial cell)  CNOT3 - ccr4-not transcription complex, subunit 3  NOTCH1 - notch 1  BAZ2B - bromodomain adjacent to zinc finger domain, 2b  NOTCH3 - notch 3  ST18 - suppression of tumorigenicity 18 (breast carcinoma) (zinc finger protein)  CTC1 - cts telomere maintenance complex component 1  NOTCH2 - notch 2  NFYA - nuclear transcription factor y, alpha  HDAC4 - histone deacetylase 4  TOX - thymocyte selection-associated high mobility group box  NFYC - nuclear transcription factor y, gamma  CXXC1 - cxxc finger protein 1  ZFYVE16 - zinc finger, fyve domain containing 16  NFKBIA - nuclear factor of kappa light polypeptide gene enhancer in b-cells inhibitor, alpha  NFKB1 - nuclear factor of kappa light polypeptide gene enhancer in b-cells 1  KMT2B - lysine (k)-specific methyltransferase 2b  NFKBIB - nuclear factor of kappa light polypeptide gene enhancer in b-cells inhibitor, beta  NFKBIE - nuclear factor of kappa light polypeptide gene enhancer in b-cells inhibitor, epsilon  NKX2-2 - nk2 homeobox 2  ZNF606 - zinc finger protein 606  HDAC9 - histone deacetylase 9  ZNF614 - zinc finger protein 614  ZFP2 - zfp2 zinc finger protein  NEO1 - neogenin 1  SMARCAL1 - swi/snf related, matrix associated, actin dependent regulator of chromatin, subfamily a-like 1  NEDD8 - neural precursor cell expressed, developmentally down-regulated 8  CTR9 - ctr9, paf1/rna polymerase ii complex component  PPM1F - protein phosphatase, mg2+/mn2+ dependent, 1f  CPEB4 - cytoplasmic polyadenylation element binding protein 4  NFE2L2 - nuclear factor (erythroid-derived 2)-like 2  EPC1 - enhancer of polycomb homolog 1 (drosophila)  RASL11A - ras-like, family 11, member a  MORF4L2 - mortality factor 4 like 2  NFATC4 - nuclear factor of activated t-cells, cytoplasmic, calcineurin-dependent 4  NUP155 - nucleoporin 155kda  NFIA - nuclear factor i/a  CDK5RAP3 - cdk5 regulatory subunit associated protein 3  RAPGEF2 - rap guanine nucleotide exchange factor (gef) 2  NUP93 - nucleoporin 93kda  ZNF746 - zinc finger protein 746  VGLL4 - vestigial like 4 (drosophila)  LRRC14 - leucine rich repeat containing 14  KDM4A - lysine (k)-specific demethylase 4a  PHF14 - phd finger protein 14  RDH10 - retinol dehydrogenase 10 (all-trans)  HP1BP3 - heterochromatin protein 1, binding protein 3  ZNF432 - zinc finger protein 432  SAFB2 - scaffold attachment factor b2  MYEF2 - myelin expression factor 2  LPIN2 - lipin 2 |
| GO:0031503 | protein-containing complex localization | 7.05E-7 | 1.29E-4 | 1.31 (10334,104,6996,92) | [+] Show genes  PURA - purine-rich element binding protein a  AP2B1 - adaptor-related protein complex 2, beta 1 subunit  TTC21B - tetratricopeptide repeat domain 21b  SDAD1 - sda1 domain containing 1  KIFAP3 - kinesin-associated protein 3  NUP133 - nucleoporin 133kda  WDR19 - wd repeat domain 19  KIF5C - kinesin family member 5c  TRAF3IP1 - tnf receptor-associated factor 3 interacting protein 1  IFT46 - intraflagellar transport 46 homolog (chlamydomonas)  KIF3C - kinesin family member 3c  XPO1 - exportin 1 (crm1 homolog, yeast)  KIF5B - kinesin family member 5b  KIF5A - kinesin family member 5a  VPS35 - vacuolar protein sorting 35 homolog (s. cerevisiae)  IFT74 - intraflagellar transport 74 homolog (chlamydomonas)  SSX2IP - synovial sarcoma, x breakpoint 2 interacting protein  CACNG7 - calcium channel, voltage-dependent, gamma subunit 7  KIF3A - kinesin family member 3a  DYNLL1 - dynein, light chain, lc8-type 1  CLSTN1 - calsyntenin 1  WDR34 - wd repeat domain 34  LTV1 - ltv1 homolog (s. cerevisiae)  TUB - tubby bipartite transcription factor  DYNLRB2 - dynein, light chain, roadblock-type 2  LSG1 - large 60s subunit nuclear export gtpase 1  GRIPAP1 - grip1 associated protein 1  DYNLRB1 - dynein, light chain, roadblock-type 1  KLHL21 - kelch-like family member 21  TNPO1 - transportin 1  NOP9 - nop9 nucleolar protein  BBS12 - bardet-biedl syndrome 12  FKBP4 - fk506 binding protein 4, 59kda  EZR - ezrin  TTC30A - tetratricopeptide repeat domain 30a  ARHGAP44 - rho gtpase activating protein 44  ICK - intestinal cell (mak-like) kinase  IFT140 - intraflagellar transport 140 homolog (chlamydomonas)  AZI1 - 5-azacytidine induced 1  EXOC1 - exocyst complex component 1  HIP1 - huntingtin interacting protein 1  IFT122 - intraflagellar transport 122 homolog (chlamydomonas)  NACC2 - nacc family member 2, ben and btb (poz) domain containing  IFT43 - intraflagellar transport 43 homolog (chlamydomonas)  ARL3 - adp-ribosylation factor-like 3  DLG3 - discs, large homolog 3 (drosophila)  DNM3 - dynamin 3  DLG4 - discs, large homolog 4 (drosophila)  DLG1 - discs, large homolog 1 (drosophila)  ABCE1 - atp-binding cassette, sub-family e (oabp), member 1  SEH1L - seh1-like (s. cerevisiae)  DYNC2LI1 - dynein, cytoplasmic 2, light intermediate chain 1  IFT27 - intraflagellar transport 27 homolog (chlamydomonas)  IFT57 - intraflagellar transport 57 homolog (chlamydomonas)  TERF2 - telomeric repeat binding factor 2  HNRNPU - heterogeneous nuclear ribonucleoprotein u (scaffold attachment factor a)  NUP88 - nucleoporin 88kda  IFT81 - intraflagellar transport 81 homolog (chlamydomonas)  SNAP25 - synaptosomal-associated protein, 25kda  RPS15 - ribosomal protein s15  MX1 - myxovirus (influenza virus) resistance 1, interferon-inducible protein p78 (mouse)  KIF3B - kinesin family member 3b  IFT88 - intraflagellar transport 88 homolog (chlamydomonas)  GPHN - gephyrin  EIF6 - eukaryotic translation initiation factor 6  MAPK10 - mitogen-activated protein kinase 10  STXBP6 - syntaxin binding protein 6 (amisyn)  SFPQ - splicing factor proline/glutamine-rich  WDR60 - wd repeat domain 60  RAB8A - rab8a, member ras oncogene family  CLUAP1 - clusterin associated protein 1  EXOC3 - exocyst complex component 3  NPM1 - nucleophosmin (nucleolar phosphoprotein b23, numatrin)  NMD3 - nmd3 ribosome export adaptor  RAB11A - rab11a, member ras oncogene family  DNM2 - dynamin 2  PCM1 - pericentriolar material 1  RRS1 - rrs1 ribosome biogenesis regulator homolog (s. cerevisiae)  NSG1 - neuron specific gene family member 1  CALY - calcyon neuron-specific vesicular protein  RABL2B - rab, member of ras oncogene family-like 2b  NDC1 - ndc1 transmembrane nucleoporin  AP3D1 - adaptor-related protein complex 3, delta 1 subunit  IFT172 - intraflagellar transport 172 homolog (chlamydomonas)  MZT1 - mitotic spindle organizing protein 1  IFT52 - intraflagellar transport 52 homolog (chlamydomonas)  RCC2 - regulator of chromosome condensation 2  CACNG4 - calcium channel, voltage-dependent, gamma subunit 4  SMAD7 - smad family member 7  EPS15 - epidermal growth factor receptor pathway substrate 15  GRIP2 - glutamate receptor interacting protein 2  WASL - wiskott-aldrich syndrome-like |
| GO:0032774 | RNA biosynthetic process | 7.56E-7 | 1.37E-4 | 1.16 (10334,411,6996,322) | [+] Show genes  CCNK - cyclin k  ELL - elongation factor rna polymerase ii  NCBP2 - nuclear cap binding protein subunit 2, 20kda  NCBP1 - nuclear cap binding protein subunit 1, 80kda  SYMPK - symplekin  TRIM24 - tripartite motif containing 24  TAF1D - tata box binding protein (tbp)-associated factor, rna polymerase i, d, 41kda  MKI67IP - mki67 (fha domain) interacting nucleolar phosphoprotein  RELA - v-rel avian reticuloendotheliosis viral oncogene homolog a  SNW1 - snw domain containing 1  ELL2 - elongation factor, rna polymerase ii, 2  TRIM28 - tripartite motif containing 28  SNAPC5 - small nuclear rna activating complex, polypeptide 5, 19kda  SP1 - sp1 transcription factor  EGR2 - early growth response 2  TTF1 - transcription termination factor, rna polymerase i  INTS4 - integrator complex subunit 4  PAPOLA - poly(a) polymerase alpha  PABPN1 - poly(a) binding protein, nuclear 1  TAF15 - taf15 rna polymerase ii, tata box binding protein (tbp)-associated factor, 68kda  RNPS1 - rna binding protein s1, serine-rich domain  GTF2IRD1 - gtf2i repeat domain containing 1  SPIN1 - spindlin 1  CREBL2 - camp responsive element binding protein-like 2  LDB1 - lim domain binding 1  CREBBP - creb binding protein  ZC3H8 - zinc finger ccch-type containing 8  CREB1 - camp responsive element binding protein 1  KAT2B - k(lysine) acetyltransferase 2b  USPL1 - ubiquitin specific peptidase like 1  ABT1 - activator of basal transcription 1  TSC22D1 - tsc22 domain family, member 1  TOPORS - topoisomerase i binding, arginine/serine-rich, e3 ubiquitin protein ligase  FIP1L1 - factor interacting with papola and cpsf1  RPRD1B - regulation of nuclear pre-mrna domain containing 1b  SNRPF - small nuclear ribonucleoprotein polypeptide f  SNAPC4 - small nuclear rna activating complex, polypeptide 4, 190kda  TBPL1 - tbp-like 1  SNRPB - small nuclear ribonucleoprotein polypeptides b and b1  NABP2 - nucleic acid binding protein 2  SNAPC2 - small nuclear rna activating complex, polypeptide 2, 45kda  SNAPC1 - small nuclear rna activating complex, polypeptide 1, 43kda  SNAPC3 - small nuclear rna activating complex, polypeptide 3, 50kda  POLR1C - polymerase (rna) i polypeptide c, 30kda  SMN1 - survival of motor neuron 1, telomeric  CPSF4 - cleavage and polyadenylation specific factor 4, 30kda  MED1 - mediator complex subunit 1  CPSF1 - cleavage and polyadenylation specific factor 1, 160kda  PPARD - peroxisome proliferator-activated receptor delta  NCOA6 - nuclear receptor coactivator 6  POLRMT - polymerase (rna) mitochondrial (dna directed)  MAML3 - mastermind-like 3 (drosophila)  POLR2K - polymerase (rna) ii (dna directed) polypeptide k, 7.0kda  SMARCD3 - swi/snf related, matrix associated, actin dependent regulator of chromatin, subfamily d, member 3  NRBP1 - nuclear receptor binding protein 1  MED20 - mediator complex subunit 20  XAB2 - xpa binding protein 2  COL4A2 - collagen, type iv, alpha 2  ENY2 - enhancer of yellow 2 homolog (drosophila)  MED18 - mediator complex subunit 18  PARP1 - poly (adp-ribose) polymerase 1  FUBP3 - far upstream element (fuse) binding protein 3  PPP1R13L - protein phosphatase 1, regulatory subunit 13 like  ZBTB7A - zinc finger and btb domain containing 7a  C5AR1 - complement component 5a receptor 1  NR2C1 - nuclear receptor subfamily 2, group c, member 1  SETX - senataxin  POLR2G - polymerase (rna) ii (dna directed) polypeptide g  POLR2F - polymerase (rna) ii (dna directed) polypeptide f  TCEANC2 - transcription elongation factor a (sii) n-terminal and central domain containing 2  NR2C2 - nuclear receptor subfamily 2, group c, member 2  POLR2E - polymerase (rna) ii (dna directed) polypeptide e, 25kda  POLR2D - polymerase (rna) ii (dna directed) polypeptide d  SCAF1 - sr-related ctd-associated factor 1  MED25 - mediator complex subunit 25  POLR2C - polymerase (rna) ii (dna directed) polypeptide c, 33kda  POLR2B - polymerase (rna) ii (dna directed) polypeptide b, 140kda  POLR2A - polymerase (rna) ii (dna directed) polypeptide a, 220kda  MED27 - mediator complex subunit 27  MED6 - mediator complex subunit 6  MED26 - mediator complex subunit 26  NAT14 - n-acetyltransferase 14 (gcn5-related, putative)  INTS9 - integrator complex subunit 9  CSTF1 - cleavage stimulation factor, 3' pre-rna, subunit 1, 50kda  POLR1B - polymerase (rna) i polypeptide b, 128kda  NELFE - negative elongation factor complex member e  MED17 - mediator complex subunit 17  MED23 - mediator complex subunit 23  NR5A1 - nuclear receptor subfamily 5, group a, member 1  INTS12 - integrator complex subunit 12  CSTF3 - cleavage stimulation factor, 3' pre-rna, subunit 3, 77kda  CSTF2 - cleavage stimulation factor, 3' pre-rna, subunit 2, 64kda  PRIM2 - primase, dna, polypeptide 2 (58kda)  PRIM1 - primase, dna, polypeptide 1 (49kda)  SUPT6H - suppressor of ty 6 homolog (s. cerevisiae)  CTGF - connective tissue growth factor  POLR3A - polymerase (rna) iii (dna directed) polypeptide a, 155kda  SUPT4H1 - suppressor of ty 4 homolog 1 (s. cerevisiae)  INTS6 - integrator complex subunit 6  SUPT5H - suppressor of ty 5 homolog (s. cerevisiae)  PRDM4 - pr domain containing 4  PPP5C - protein phosphatase 5, catalytic subunit  MTF1 - metal-regulatory transcription factor 1  NR1H2 - nuclear receptor subfamily 1, group h, member 2  PCID2 - pci domain containing 2  MED16 - mediator complex subunit 16  SLBP - stem-loop binding protein  NUDT21 - nudix (nucleoside diphosphate linked moiety x)-type motif 21  GPBP1 - gc-rich promoter binding protein 1  CPSF7 - cleavage and polyadenylation specific factor 7, 59kda  NR2C2AP - nuclear receptor 2c2-associated protein  DIDO1 - death inducer-obliterator 1  GPBP1L1 - gc-rich promoter binding protein 1-like 1  INTS3 - integrator complex subunit 3  ELP4 - elongator acetyltransferase complex subunit 4  RPAP2 - rna polymerase ii associated protein 2  MED10 - mediator complex subunit 10  MED21 - mediator complex subunit 21  HSF2BP - heat shock transcription factor 2 binding protein  CCNT2 - cyclin t2  LIN52 - lin-52 homolog (c. elegans)  ERCC3 - excision repair cross-complementing rodent repair deficiency, complementation group 3  IWS1 - iws1 homolog (s. cerevisiae)  NPM3 - nucleophosmin/nucleoplasmin 3  STAT3 - signal transducer and activator of transcription 3 (acute-phase response factor)  CCNT1 - cyclin t1  RNGTT - rna guanylyltransferase and 5'-phosphatase  CCNH - cyclin h  RNMT - rna (guanine-7-) methyltransferase  POLR3GL - polymerase (rna) iii (dna directed) polypeptide g (32kd)-like  SSRP1 - structure specific recognition protein 1  HIRA - histone cell cycle regulator  SLC25A33 - solute carrier family 25 (pyrimidine nucleotide carrier), member 33  ETV1 - ets variant 1  INTS8 - integrator complex subunit 8  POLR2M - polymerase (rna) ii (dna directed) polypeptide m  CBFA2T3 - core-binding factor, runt domain, alpha subunit 2; translocated to, 3  CBFB - core-binding factor, beta subunit  GTF3C5 - general transcription factor iiic, polypeptide 5, 63kda  POLR3E - polymerase (rna) iii (dna directed) polypeptide e (80kd)  UBTF - upstream binding transcription factor, rna polymerase i  MAPK3 - mitogen-activated protein kinase 3  TROVE2 - trove domain family, member 2  RXRB - retinoid x receptor, beta  COPS2 - cop9 signalosome subunit 2  CLP1 - cleavage and polyadenylation factor i subunit 1  SARS2 - seryl-trna synthetase 2, mitochondrial  MED4 - mediator complex subunit 4  SETD2 - set domain containing 2  POLR3B - polymerase (rna) iii (dna directed) polypeptide b  CCNC - cyclin c  NR4A3 - nuclear receptor subfamily 4, group a, member 3  PRMT5 - protein arginine methyltransferase 5  GTF3C4 - general transcription factor iiic, polypeptide 4, 90kda  NABP1 - nucleic acid binding protein 1  NR4A1 - nuclear receptor subfamily 4, group a, member 1  ERCC2 - excision repair cross-complementing rodent repair deficiency, complementation group 2  GTF3C3 - general transcription factor iiic, polypeptide 3, 102kda  CNOT8 - ccr4-not transcription complex, subunit 8  MED12 - mediator complex subunit 12  MED13 - mediator complex subunit 13  DHX9 - deah (asp-glu-ala-his) box helicase 9  CDK12 - cyclin-dependent kinase 12  MED24 - mediator complex subunit 24  TCOF1 - treacher collins-franceschetti syndrome 1  DDX5 - dead (asp-glu-ala-asp) box helicase 5  POLR3F - polymerase (rna) iii (dna directed) polypeptide f, 39 kda  INTS5 - integrator complex subunit 5  RBMX - rna binding motif protein, x-linked  GTF2F1 - general transcription factor iif, polypeptide 1, 74kda  WWTR1 - ww domain containing transcription regulator 1  GTF2F2 - general transcription factor iif, polypeptide 2, 30kda  GTF2E1 - general transcription factor iie, polypeptide 1, alpha 56kda  GTF2E2 - general transcription factor iie, polypeptide 2, beta 34kda  FLNA - filamin a, alpha  RPRD2 - regulation of nuclear pre-mrna domain containing 2  GTF2H2 - general transcription factor iih, polypeptide 2, 44kda  GTF2H3 - general transcription factor iih, polypeptide 3, 34kda  INTS10 - integrator complex subunit 10  RSF1 - remodeling and spacing factor 1  GTF2H1 - general transcription factor iih, polypeptide 1, 62kda  PPM1D - protein phosphatase, mg2+/mn2+ dependent, 1d  MLLT3 - myeloid/lymphoid or mixed-lineage leukemia (trithorax homolog, drosophila); translocated to, 3  MED14 - mediator complex subunit 14  GTF2A2 - general transcription factor iia, 2, 12kda  MLLT1 - myeloid/lymphoid or mixed-lineage leukemia (trithorax homolog, drosophila); translocated to, 1  FOXO3 - forkhead box o3  GTF2B - general transcription factor iib  GTF2A1 - general transcription factor iia, 1, 19/37kda  PHAX - phosphorylated adaptor for rna export  NELFB - negative elongation factor complex member b  CPSF3 - cleavage and polyadenylation specific factor 3, 73kda  GTF3C2 - general transcription factor iiic, polypeptide 2, beta 110kda  GTF2I - general transcription factor iii  GTF2H4 - general transcription factor iih, polypeptide 4, 52kda  GTF3A - general transcription factor iiia  LMO4 - lim domain only 4  BRF1 - brf1, rna polymerase iii transcription initiation factor 90 kda subunit  RPRD1A - regulation of nuclear pre-mrna domain containing 1a  MED8 - mediator complex subunit 8  GTF3C1 - general transcription factor iiic, polypeptide 1, alpha 220kda  TAF6L - taf6-like rna polymerase ii, p300/cbp-associated factor (pcaf)-associated factor, 65kda  MNAT1 - mnat cdk-activating kinase assembly factor 1  POLR1A - polymerase (rna) i polypeptide a, 194kda  POLR3G - polymerase (rna) iii (dna directed) polypeptide g (32kd)  ZNF473 - zinc finger protein 473  WDR61 - wd repeat domain 61  MNT - mnt, max dimerization protein  CSTF2T - cleavage stimulation factor, 3' pre-rna, subunit 2, 64kda, tau variant  POLR3K - polymerase (rna) iii (dna directed) polypeptide k, 12.3 kda  TAF9 - taf9 rna polymerase ii, tata box binding protein (tbp)-associated factor, 32kda  TAF7 - taf7 rna polymerase ii, tata box binding protein (tbp)-associated factor, 55kda  CELF3 - cugbp, elav-like family member 3  TAF6 - taf6 rna polymerase ii, tata box binding protein (tbp)-associated factor, 80kda  PAX8 - paired box 8  SUPT16H - suppressor of ty 16 homolog (s. cerevisiae)  PTEN - phosphatase and tensin homolog  TAF4 - taf4 rna polymerase ii, tata box binding protein (tbp)-associated factor, 135kda  TAF2 - taf2 rna polymerase ii, tata box binding protein (tbp)-associated factor, 150kda  ARRB2 - arrestin, beta 2  ARRB1 - arrestin, beta 1  CDC73 - cell division cycle 73  MECP2 - methyl cpg binding protein 2 (rett syndrome)  MEF2A - myocyte enhancer factor 2a  NR4A2 - nuclear receptor subfamily 4, group a, member 2  MED30 - mediator complex subunit 30  DDX21 - dead (asp-glu-ala-asp) box helicase 21  ZBTB7B - zinc finger and btb domain containing 7b  POLR1E - polymerase (rna) i polypeptide e, 53kda  RPAP1 - rna polymerase ii associated protein 1  CPSF2 - cleavage and polyadenylation specific factor 2, 100kda  TAF13 - taf13 rna polymerase ii, tata box binding protein (tbp)-associated factor, 18kda  BAZ2A - bromodomain adjacent to zinc finger domain, 2a  INTS2 - integrator complex subunit 2  PTMA - prothymosin, alpha  CDK4 - cyclin-dependent kinase 4  CDK9 - cyclin-dependent kinase 9  CDK8 - cyclin-dependent kinase 8  CDK7 - cyclin-dependent kinase 7  HINFP - histone h4 transcription factor  CDK13 - cyclin-dependent kinase 13  LEO1 - leo1, paf1/rna polymerase ii complex component, homolog (s. cerevisiae)  PPP1R1B - protein phosphatase 1, regulatory (inhibitor) subunit 1b  PWP1 - pwp1 homolog (s. cerevisiae)  SARS - seryl-trna synthetase  POLR1D - polymerase (rna) i polypeptide d, 16kda  TCEB3 - transcription elongation factor b (siii), polypeptide 3 (110kda, elongin a)  THAP1 - thap domain containing, apoptosis associated protein 1  TCEA2 - transcription elongation factor a (sii), 2  CRCP - cgrp receptor component  TCEA1 - transcription elongation factor a (sii), 1  RRN3 - rrn3 rna polymerase i transcription factor homolog (s. cerevisiae)  CBFA2T2 - core-binding factor, runt domain, alpha subunit 2; translocated to, 2  NRBF2 - nuclear receptor binding factor 2  ZBTB1 - zinc finger and btb domain containing 1  EAF1 - ell associated factor 1  MARS - methionyl-trna synthetase  AFF4 - af4/fmr2 family, member 4  NOTCH4 - notch 4  MMS19 - mms19 nucleotide excision repair homolog (s. cerevisiae)  DR1 - down-regulator of transcription 1, tbp-binding (negative cofactor 2)  XBP1 - x-box binding protein 1  NOTCH1 - notch 1  NOTCH3 - notch 3  NOTCH2 - notch 2  NFYA - nuclear transcription factor y, alpha  NFRKB - nuclear factor related to kappab binding protein  SKI - v-ski avian sarcoma viral oncogene homolog  MBD1 - methyl-cpg binding domain protein 1  MTERFD2 - mterf domain containing 2  PHF3 - phd finger protein 3  NELFA - negative elongation factor complex member a  NFKB1 - nuclear factor of kappa light polypeptide gene enhancer in b-cells 1  NFKBIB - nuclear factor of kappa light polypeptide gene enhancer in b-cells inhibitor, beta  WDR33 - wd repeat domain 33  RRAGC - ras-related gtp binding c  TARDBP - tar dna binding protein  NELFCD - negative elongation factor complex member c/d  PAF1 - paf1, rna polymerase ii associated factor, homolog (s. cerevisiae)  ZNRD1 - zinc ribbon domain containing 1  INO80 - ino80 complex subunit  DTX1 - deltex homolog 1 (drosophila)  SCAF8 - sr-related ctd-associated factor 8  BAX - bcl2-associated x protein  RBPJ - recombination signal binding protein for immunoglobulin kappa j region  TFAM - transcription factor a, mitochondrial  ZNF45 - zinc finger protein 45  CTR9 - ctr9, paf1/rna polymerase ii complex component  GTF3C6 - general transcription factor iiic, polypeptide 6, alpha 35kda  TFDP1 - transcription factor dp-1  RARG - retinoic acid receptor, gamma  EPC1 - enhancer of polycomb homolog 1 (drosophila)  RARB - retinoic acid receptor, beta  ASXL2 - additional sex combs like 2 (drosophila)  XRN2 - 5'-3' exoribonuclease 2  ELP2 - elongator acetyltransferase complex subunit 2  ASXL1 - additional sex combs like 1 (drosophila)  NR2F1 - nuclear receptor subfamily 2, group f, member 1  TFCP2 - transcription factor cp2  TAF8 - taf8 rna polymerase ii, tata box binding protein (tbp)-associated factor, 43kda  PKNOX1 - pbx/knotted 1 homeobox 1  PSTK - phosphoseryl-trna kinase  TAF1B - tata box binding protein (tbp)-associated factor, rna polymerase i, b, 63kda  PHRF1 - phd and ring finger domains 1  SMARCA5 - swi/snf related, matrix associated, actin dependent regulator of chromatin, subfamily a, member 5  PCF11 - pcf11 cleavage and polyadenylation factor subunit  MED15 - mediator complex subunit 15  SRRT - serrate rna effector molecule homolog (arabidopsis)  GMEB2 - glucocorticoid modulatory element binding protein 2  TAF1C - tata box binding protein (tbp)-associated factor, rna polymerase i, c, 110kda  FOSB - fbj murine osteosarcoma viral oncogene homolog b  FOS - fbj murine osteosarcoma viral oncogene homolog  DRG1 - developmentally regulated gtp binding protein 1  BRF2 - brf2, rna polymerase iii transcription initiation factor 50 kda subunit  TAF9B - taf9b rna polymerase ii, tata box binding protein (tbp)-associated factor, 31kda  NFAT5 - nuclear factor of activated t-cells 5, tonicity-responsive  POLR3H - polymerase (rna) iii (dna directed) polypeptide h (22.9kd)  SMAD4 - smad family member 4  CCND1 - cyclin d1  SUPT3H - suppressor of ty 3 homolog (s. cerevisiae)  THRA - thyroid hormone receptor, alpha  INTS1 - integrator complex subunit 1 |
| GO:0032465 | regulation of cytokinesis | 9.83E-7 | 1.75E-4 | 1.42 (10334,50,6996,48) | [+] Show genes  RAB11FIP3 - rab11 family interacting protein 3 (class ii)  CHMP4C - charged multivesicular body protein 4c  PLK3 - polo-like kinase 3  DRD2 - dopamine receptor d2  CETN2 - centrin, ef-hand protein, 2  PIN1 - peptidylprolyl cis/trans isomerase, nima-interacting 1  FLCN - folliculin  MAP9 - microtubule-associated protein 9  FSD1 - fibronectin type iii and spry domain containing 1  RACGAP1 - rac gtpase activating protein 1  CENPV - centromere protein v  PKP4 - plakophilin 4  PLK2 - polo-like kinase 2  RHOA - ras homolog family member a  MYO19 - myosin xix  PRPF40A - prp40 pre-mrna processing factor 40 homolog a (s. cerevisiae)  ZFYVE26 - zinc finger, fyve domain containing 26  KIF3B - kinesin family member 3b  SPAST - spastin  CIT - citron (rho-interacting, serine/threonine kinase 21)  GIPC1 - gipc pdz domain containing family, member 1  CDC14B - cell division cycle 14b  BECN1 - beclin 1, autophagy related  KLHL21 - kelch-like family member 21  RAB11A - rab11a, member ras oncogene family  CSPP1 - centrosome and spindle pole associated protein 1  GIT1 - g protein-coupled receptor kinase interacting arfgap 1  AHCTF1 - at hook containing transcription factor 1  ZFYVE19 - zinc finger, fyve domain containing 19  KLHL13 - kelch-like family member 13  SDCCAG3 - serologically defined colon cancer antigen 3  PKN2 - protein kinase n2  PIK3R4 - phosphoinositide-3-kinase, regulatory subunit 4  SETD2 - set domain containing 2  UVRAG - uv radiation resistance associated  KLHL9 - kelch-like family member 9  PRKCE - protein kinase c, epsilon  CALM2 - calmodulin 2 (phosphorylase kinase, delta)  PIK3C3 - phosphatidylinositol 3-kinase, catalytic subunit type 3  CUL3 - cullin 3  BCL2L1 - bcl2-like 1  AURKC - aurora kinase c  BIRC6 - baculoviral iap repeat containing 6  PDXP - pyridoxal (pyridoxine, vitamin b6) phosphatase  BBS4 - bardet-biedl syndrome 4  VPS4A - vacuolar protein sorting 4 homolog a (s. cerevisiae)  CCP110 - centriolar coiled coil protein 110kda  SH3GLB1 - sh3-domain grb2-like endophilin b1 |
| GO:0070647 | protein modification by small protein conjugation or removal | 1.09E-6 | 1.92E-4 | 1.11 (10334,741,6996,559) | [+] Show genes  PCGF5 - polycomb group ring finger 5  MEX3C - mex-3 rna binding family member c  WAC - ww domain containing adaptor with coiled-coil  MKRN1 - makorin ring finger protein 1  OTUD5 - otu domain containing 5  MKRN2 - makorin ring finger protein 2  USP54 - ubiquitin specific peptidase 54  FBXO18 - f-box protein, helicase, 18  OS9 - osteosarcoma amplified 9, endoplasmic reticulum lectin  LZTR1 - leucine-zipper-like transcription regulator 1  PML - promyelocytic leukemia  RAB40B - rab40b, member ras oncogene family  KAT2A - k(lysine) acetyltransferase 2a  UBE2Q1 - ubiquitin-conjugating enzyme e2q family member 1  FBXL18 - f-box and leucine-rich repeat protein 18  LNPEP - leucyl/cystinyl aminopeptidase  SYVN1 - synovial apoptosis inhibitor 1, synoviolin  BFAR - bifunctional apoptosis regulator  RFFL - ring finger and fyve-like domain containing e3 ubiquitin protein ligase  NEURL4 - neuralized homolog 4 (drosophila)  ACTB - actin, beta  OTUB1 - otu domain, ubiquitin aldehyde binding 1  FBXL20 - f-box and leucine-rich repeat protein 20  RAB40C - rab40c, member ras oncogene family  DDX58 - dead (asp-glu-ala-asp) box polypeptide 58  ACTL6A - actin-like 6a  TRRAP - transformation/transcription domain-associated protein  ANAPC5 - anaphase promoting complex subunit 5  USP20 - ubiquitin specific peptidase 20  ANAPC7 - anaphase promoting complex subunit 7  USP19 - ubiquitin specific peptidase 19  NPEPPS - aminopeptidase puromycin sensitive  RNF138 - ring finger protein 138, e3 ubiquitin protein ligase  SUMO2 - small ubiquitin-like modifier 2  ACTR5 - arp5 actin-related protein 5 homolog (yeast)  MED1 - mediator complex subunit 1  BAP1 - brca1 associated protein-1 (ubiquitin carboxy-terminal hydrolase)  NUP85 - nucleoporin 85kda  SUMO3 - small ubiquitin-like modifier 3  BRAP - brca1 associated protein  UBR5 - ubiquitin protein ligase e3 component n-recognin 5  HLTF - helicase-like transcription factor  USP9X - ubiquitin specific peptidase 9, x-linked  USP11 - ubiquitin specific peptidase 11  MED20 - mediator complex subunit 20  UCHL5 - ubiquitin carboxyl-terminal hydrolase l5  FZR1 - fizzy/cell division cycle 20 related 1 (drosophila)  IRF2BP2 - interferon regulatory factor 2 binding protein 2  MED27 - mediator complex subunit 27  SENP2 - sumo1/sentrin/smt3 specific peptidase 2  USP32 - ubiquitin specific peptidase 32  NUP133 - nucleoporin 133kda  ASB7 - ankyrin repeat and socs box containing 7  ASB8 - ankyrin repeat and socs box containing 8  KLHL12 - kelch-like family member 12  KDM2B - lysine (k)-specific demethylase 2b  NUPL2 - nucleoporin like 2  ASB6 - ankyrin repeat and socs box containing 6  CHFR - checkpoint with forkhead and ring finger domains, e3 ubiquitin protein ligase  USP48 - ubiquitin specific peptidase 48  UHRF2 - ubiquitin-like with phd and ring finger domains 2, e3 ubiquitin protein ligase  SDE2 - sde2 telomere maintenance homolog (s. pombe)  CTNNB1 - catenin (cadherin-associated protein), beta 1, 88kda  RLIM - ring finger protein, lim domain interacting  PPIL2 - peptidylprolyl isomerase (cyclophilin)-like 2  ASB3 - ankyrin repeat and socs box containing 3  TRAF7 - tnf receptor-associated factor 7, e3 ubiquitin protein ligase  AKT1 - v-akt murine thymoma viral oncogene homolog 1  STX1A - syntaxin 1a (brain)  USP7 - ubiquitin specific peptidase 7 (herpes virus-associated)  LNX1 - ligand of numb-protein x 1, e3 ubiquitin protein ligase  RNF168 - ring finger protein 168, e3 ubiquitin protein ligase  USP30 - ubiquitin specific peptidase 30  WWP2 - ww domain containing e3 ubiquitin protein ligase 2  PCGF1 - polycomb group ring finger 1  MBD5 - methyl-cpg binding domain protein 5  CBLL1 - cbl proto-oncogene-like 1, e3 ubiquitin protein ligase  CYLD - cylindromatosis (turban tumor syndrome)  MED10 - mediator complex subunit 10  MED21 - mediator complex subunit 21  UBA5 - ubiquitin-like modifier activating enzyme 5  HECTD1 - hect domain containing e3 ubiquitin protein ligase 1  DCUN1D5 - dcn1, defective in cullin neddylation 1, domain containing 5  UBE4A - ubiquitination factor e4a  FBXW9 - f-box and wd repeat domain containing 9  USP5 - ubiquitin specific peptidase 5 (isopeptidase t)  ANAPC13 - anaphase promoting complex subunit 13  AMFR - autocrine motility factor receptor, e3 ubiquitin protein ligase  AAAS - achalasia, adrenocortical insufficiency, alacrimia  RNF126 - ring finger protein 126  DCAF12 - ddb1 and cul4 associated factor 12  ANKRD39 - ankyrin repeat domain 39  RNF157 - ring finger protein 157  FBXO7 - f-box protein 7  TRIM59 - tripartite motif containing 59  TRIP12 - thyroid hormone receptor interactor 12  KLHL22 - kelch-like family member 22  COPS6 - cop9 signalosome subunit 6  COPS2 - cop9 signalosome subunit 2  NDC1 - ndc1 transmembrane nucleoporin  STAM - signal transducing adaptor molecule (sh3 domain and itam motif) 1  CRBN - cereblon  RNF214 - ring finger protein 214  NUP214 - nucleoporin 214kda  KLHL36 - kelch-like family member 36  ARIH1 - ariadne homolog, ubiquitin-conjugating enzyme e2 binding protein, 1 (drosophila)  UBE2O - ubiquitin-conjugating enzyme e2o  FBXL2 - f-box and leucine-rich repeat protein 2  SHPRH - snf2 histone linker phd ring helicase, e3 ubiquitin protein ligase  RNF169 - ring finger protein 169  UBR7 - ubiquitin protein ligase e3 component n-recognin 7 (putative)  KLHL2 - kelch-like family member 2  FBXO33 - f-box protein 33  XIAP - x-linked inhibitor of apoptosis  KCTD10 - potassium channel tetramerization domain containing 10  BIRC3 - baculoviral iap repeat containing 3  BIRC2 - baculoviral iap repeat containing 2  RNF112 - ring finger protein 112  RFWD3 - ring finger and wd repeat domain 3  SOCS6 - suppressor of cytokine signaling 6  RNF113A - ring finger protein 113a  RSPRY1 - ring finger and spry domain containing 1  WWTR1 - ww domain containing transcription regulator 1  DDB1 - damage-specific dna binding protein 1, 127kda  MIB2 - mindbomb e3 ubiquitin protein ligase 2  HECTD3 - hect domain containing e3 ubiquitin protein ligase 3  MSL2 - male-specific lethal 2 homolog (drosophila)  TRIM26 - tripartite motif containing 26  SENP7 - sumo1/sentrin specific peptidase 7  USP27X - ubiquitin specific peptidase 27, x-linked  DCUN1D3 - dcn1, defective in cullin neddylation 1, domain containing 3  RNF220 - ring finger protein 220  APC - adenomatous polyposis coli  PCGF2 - polycomb group ring finger 2  RNF139 - ring finger protein 139  KLHL13 - kelch-like family member 13  TRIM23 - tripartite motif containing 23  RNF19A - ring finger protein 19a, e3 ubiquitin protein ligase  RCHY1 - ring finger and chy zinc finger domain containing 1, e3 ubiquitin protein ligase  UIMC1 - ubiquitin interaction motif containing 1  KCTD13 - potassium channel tetramerization domain containing 13  DCAF13 - ddb1 and cul4 associated factor 13  BIRC6 - baculoviral iap repeat containing 6  HIST1H2BB - histone cluster 1, h2bb  LTN1 - listerin e3 ubiquitin protein ligase 1  MUL1 - mitochondrial e3 ubiquitin protein ligase 1  NSMCE2 - non-smc element 2, mms21 homolog (s. cerevisiae)  ZNF451 - zinc finger protein 451  RNF103 - ring finger protein 103  ARRB2 - arrestin, beta 2  ARRB1 - arrestin, beta 1  CDC73 - cell division cycle 73  MED30 - mediator complex subunit 30  RHOA - ras homolog family member a  ZBED1 - zinc finger, bed-type containing 1  USP31 - ubiquitin specific peptidase 31  DCAF15 - ddb1 and cul4 associated factor 15  HCFC1 - host cell factor c1 (vp16-accessory protein)  PCNA - proliferating cell nuclear antigen  USP42 - ubiquitin specific peptidase 42  LRSAM1 - leucine rich repeat and sterile alpha motif containing 1  LEO1 - leo1, paf1/rna polymerase ii complex component, homolog (s. cerevisiae)  FANCL - fanconi anemia, complementation group l  FBXL14 - f-box and leucine-rich repeat protein 14  HACE1 - hect domain and ankyrin repeat containing e3 ubiquitin protein ligase 1  SOCS4 - suppressor of cytokine signaling 4  MIB1 - mindbomb e3 ubiquitin protein ligase 1  HGS - hepatocyte growth factor-regulated tyrosine kinase substrate  ZC3HC1 - zinc finger, c3hc-type containing 1  TRPC4AP - transient receptor potential cation channel, subfamily c, member 4 associated protein  KLHL42 - kelch-like family member 42  CDC26 - cell division cycle 26  IRF2BP1 - interferon regulatory factor 2 binding protein 1  UBOX5 - u-box domain containing 5  RNF180 - ring finger protein 180  FBXW7 - f-box and wd repeat domain containing 7, e3 ubiquitin protein ligase  RNF121 - ring finger protein 121  KLHL8 - kelch-like family member 8  USP35 - ubiquitin specific peptidase 35  WSB1 - wd repeat and socs box containing 1  USP8 - ubiquitin specific peptidase 8  ZNRF2 - zinc and ring finger 2  USP14 - ubiquitin specific peptidase 14 (trna-guanine transglycosylase)  PEX12 - peroxisomal biogenesis factor 12  USP10 - ubiquitin specific peptidase 10  UBE2J1 - ubiquitin-conjugating enzyme e2, j1  PEX13 - peroxisomal biogenesis factor 13  PEX14 - peroxisomal biogenesis factor 14  NUP43 - nucleoporin 43kda  SKP1 - s-phase kinase-associated protein 1  TOR1A - torsin family 1, member a (torsin a)  DTX3 - deltex homolog 3 (drosophila)  HERC4 - hect and rld domain containing e3 ubiquitin protein ligase 4  WDR48 - wd repeat domain 48  UFC1 - ubiquitin-fold modifier conjugating enzyme 1  USP36 - ubiquitin specific peptidase 36  SENP6 - sumo1/sentrin specific peptidase 6  DTX1 - deltex homolog 1 (drosophila)  UBA6 - ubiquitin-like modifier activating enzyme 6  ITCH - itchy e3 ubiquitin protein ligase  FBXL16 - f-box and leucine-rich repeat protein 16  FBXL15 - f-box and leucine-rich repeat protein 15  USP40 - ubiquitin specific peptidase 40  OTUD6B - otu domain containing 6b  BRCC3 - brca1/brca2-containing complex, subunit 3  FBXW8 - f-box and wd repeat domain containing 8  FBXO25 - f-box protein 25  SH3RF1 - sh3 domain containing ring finger 1  NUB1 - negative regulator of ubiquitin-like proteins 1  SIAH2 - siah e3 ubiquitin protein ligase 2  ASXL2 - additional sex combs like 2 (drosophila)  ASB1 - ankyrin repeat and socs box containing 1  SIAH1 - siah e3 ubiquitin protein ligase 1  FBXL3 - f-box and leucine-rich repeat protein 3  ASXL1 - additional sex combs like 1 (drosophila)  UBE2M - ubiquitin-conjugating enzyme e2m  TNIP2 - tnfaip3 interacting protein 2  UBA3 - ubiquitin-like modifier activating enzyme 3  FBXL5 - f-box and leucine-rich repeat protein 5  PEF1 - penta-ef-hand domain containing 1  RNF8 - ring finger protein 8, e3 ubiquitin protein ligase  FBXO2 - f-box protein 2  UVSSA - uv-stimulated scaffold protein a  RNF13 - ring finger protein 13  PELI3 - pellino e3 ubiquitin protein ligase family member 3  PIAS4 - protein inhibitor of activated stat, 4  FBXW2 - f-box and wd repeat domain containing 2  TRIM33 - tripartite motif containing 33  SMAD2 - smad family member 2  FBXW12 - f-box and wd repeat domain containing 12  KCTD11 - potassium channel tetramerization domain containing 11  RHOT1 - ras homolog family member t1  TAF9B - taf9b rna polymerase ii, tata box binding protein (tbp)-associated factor, 31kda  UBE2W - ubiquitin-conjugating enzyme e2w (putative)  BCL2 - b-cell cll/lymphoma 2  DCAF10 - ddb1 and cul4 associated factor 10  SMAD4 - smad family member 4  USP37 - ubiquitin specific peptidase 37  SMAD7 - smad family member 7  SENP3 - sumo1/sentrin/smt3 specific peptidase 3  TRIM27 - tripartite motif containing 27  MYLIP - myosin regulatory light chain interacting protein  RNF26 - ring finger protein 26  TRIM24 - tripartite motif containing 24  TRIM28 - tripartite motif containing 28  BCOR - bcl6 corepressor  EPAS1 - endothelial pas domain protein 1  EP300 - e1a binding protein p300  FBXO45 - f-box protein 45  DCAF16 - ddb1 and cul4 associated factor 16  SH3RF3 - sh3 domain containing ring finger 3  DERL1 - derlin 1  DCAF5 - ddb1 and cul4 associated factor 5  KCTD6 - potassium channel tetramerization domain containing 6  NHLRC3 - nhl repeat containing 3  RING1 - ring finger protein 1  EGR2 - early growth response 2  TTC3 - tetratricopeptide repeat domain 3  TRIM11 - tripartite motif containing 11  SENP1 - sumo1/sentrin specific peptidase 1  TFPT - tcf3 (e2a) fusion partner (in childhood leukemia)  RNF41 - ring finger protein 41  TRIM32 - tripartite motif containing 32  MED11 - mediator complex subunit 11  BMI1 - bmi1 polycomb ring finger oncogene  TMEM129 - transmembrane protein 129  PSME3 - proteasome (prosome, macropain) activator subunit 3 (pa28 gamma; ki)  RNF215 - ring finger protein 215  PCGF3 - polycomb group ring finger 3  TSG101 - tumor susceptibility gene 101  URM1 - ubiquitin related modifier 1  TRIM13 - tripartite motif containing 13  TRIM8 - tripartite motif containing 8  KAT2B - k(lysine) acetyltransferase 2b  USPL1 - ubiquitin specific peptidase like 1  FBXL12 - f-box and leucine-rich repeat protein 12  TOPORS - topoisomerase i binding, arginine/serine-rich, e3 ubiquitin protein ligase  DDA1 - det1 and ddb1 associated 1  CDC16 - cell division cycle 16  PSMD14 - proteasome (prosome, macropain) 26s subunit, non-atpase, 14  RNF19B - ring finger protein 19b  FBXO21 - f-box protein 21  NAE1 - nedd8 activating enzyme e1 subunit 1  GID4 - gid complex subunit 4  RNF149 - ring finger protein 149  SEH1L - seh1-like (s. cerevisiae)  TULP4 - tubby like protein 4  PHC1 - polyhomeotic homolog 1 (drosophila)  HUWE1 - hect, uba and wwe domain containing 1, e3 ubiquitin protein ligase  USP33 - ubiquitin specific peptidase 33  RNF4 - ring finger protein 4  DCAF7 - ddb1 and cul4 associated factor 7  INO80B - ino80 complex subunit b  RNF2 - ring finger protein 2  ATXN7L3 - ataxin 7-like 3  RNF6 - ring finger protein (c3h2c3 type) 6  HERC3 - hect and rld domain containing e3 ubiquitin protein ligase 3  UBE2Q2 - ubiquitin-conjugating enzyme e2q family member 2  HERC2 - hect and rld domain containing e3 ubiquitin protein ligase 2  RNF111 - ring finger protein 111  ANAPC4 - anaphase promoting complex subunit 4  HERC1 - hect and rld domain containing e3 ubiquitin protein ligase family member 1  E4F1 - e4f transcription factor 1  RNF170 - ring finger protein 170  FEM1B - fem-1 homolog b (c. elegans)  STAM2 - signal transducing adaptor molecule (sh3 domain and itam motif) 2  TRPM4 - transient receptor potential cation channel, subfamily m, member 4  ENY2 - enhancer of yellow 2 homolog (drosophila)  NSMCE1 - non-smc element 1 homolog (s. cerevisiae)  TRIM37 - tripartite motif containing 37  BCL10 - b-cell cll/lymphoma 10  MYCBP2 - myc binding protein 2, e3 ubiquitin protein ligase  TRAF1 - tnf receptor-associated factor 1  TRAF3 - tnf receptor-associated factor 3  BTRC - beta-transducin repeat containing e3 ubiquitin protein ligase  TRAF2 - tnf receptor-associated factor 2  STUB1 - stip1 homology and u-box containing protein 1, e3 ubiquitin protein ligase  FEM1C - fem-1 homolog c (c. elegans)  SHARPIN - shank-associated rh domain interactor  TPR - translocated promoter region, nuclear basket protein  UBE4B - ubiquitination factor e4b  UBR1 - ubiquitin protein ligase e3 component n-recognin 1  TRIM44 - tripartite motif containing 44  TAB1 - tgf-beta activated kinase 1/map3k7 binding protein 1  USP12 - ubiquitin specific peptidase 12  TRIM35 - tripartite motif containing 35  MCRS1 - microspherule protein 1  UBE2Z - ubiquitin-conjugating enzyme e2z  NUP107 - nucleoporin 107kda  USP1 - ubiquitin specific peptidase 1  PDCD6 - programmed cell death 6  SENP5 - sumo1/sentrin specific peptidase 5  TNKS - tankyrase, trf1-interacting ankyrin-related adp-ribose polymerase  PCNP - pest proteolytic signal containing nuclear protein  CUL9 - cullin 9  USP4 - ubiquitin specific peptidase 4 (proto-oncogene)  EIF3F - eukaryotic translation initiation factor 3, subunit f  RNF185 - ring finger protein 185  HDAC6 - histone deacetylase 6  BECN1 - beclin 1, autophagy related  RNF187 - ring finger protein 187  ZNF598 - zinc finger protein 598  SPSB3 - spla/ryanodine receptor domain and socs box containing 3  VHL - von hippel-lindau tumor suppressor, e3 ubiquitin protein ligase  UBE2E3 - ubiquitin-conjugating enzyme e2e 3  HERC6 - hect and rld domain containing e3 ubiquitin protein ligase family member 6  TRIM41 - tripartite motif containing 41  TRIM38 - tripartite motif containing 38  TADA3 - transcriptional adaptor 3  CCDC22 - coiled-coil domain containing 22  SAE1 - sumo1 activating enzyme subunit 1  DCUN1D4 - dcn1, defective in cullin neddylation 1, domain containing 4  CDC23 - cell division cycle 23  UBE2K - ubiquitin-conjugating enzyme e2k  TRIM47 - tripartite motif containing 47  VCP - valosin containing protein  LRRC41 - leucine rich repeat containing 41  HIF1A - hypoxia inducible factor 1, alpha subunit (basic helix-loop-helix transcription factor)  UBA2 - ubiquitin-like modifier activating enzyme 2  JOSD1 - josephin domain containing 1  SNX3 - sorting nexin 3  NUP205 - nucleoporin 205kda  WDR20 - wd repeat domain 20  UBE2A - ubiquitin-conjugating enzyme e2a  INO80C - ino80 complex subunit c  UBA1 - ubiquitin-like modifier activating enzyme 1  UBB - ubiquitin b  RIPK1 - receptor (tnfrsf)-interacting serine-threonine kinase 1  FBXO10 - f-box protein 10  ARRDC4 - arrestin domain containing 4  FBXO38 - f-box protein 38  ANAPC10 - anaphase promoting complex subunit 10  FBXO22 - f-box protein 22  FBXO4 - f-box protein 4  COMMD1 - copper metabolism (murr1) domain containing 1  FBXO6 - f-box protein 6  FBXO3 - f-box protein 3  RNF125 - ring finger protein 125, e3 ubiquitin protein ligase  UCHL3 - ubiquitin carboxyl-terminal esterase l3 (ubiquitin thiolesterase)  CBFB - core-binding factor, beta subunit  USP3 - ubiquitin specific peptidase 3  BABAM1 - brisc and brca1 a complex member 1  SUMO1 - small ubiquitin-like modifier 1  CBL - cbl proto-oncogene, e3 ubiquitin protein ligase  UBE2T - ubiquitin-conjugating enzyme e2t (putative)  UCHL1 - ubiquitin carboxyl-terminal esterase l1 (ubiquitin thiolesterase)  PELI1 - pellino e3 ubiquitin protein ligase 1  PIAS3 - protein inhibitor of activated stat, 3  USP15 - ubiquitin specific peptidase 15  SMURF1 - smad specific e3 ubiquitin protein ligase 1  UBE2R2 - ubiquitin-conjugating enzyme e2r 2  UBE2H - ubiquitin-conjugating enzyme e2h  FBXO15 - f-box protein 15  RCE1 - rce1 homolog, prenyl protein protease (s. cerevisiae)  UBE2I - ubiquitin-conjugating enzyme e2i  UBE2D2 - ubiquitin-conjugating enzyme e2d 2  RBX1 - ring-box 1, e3 ubiquitin protein ligase  USP46 - ubiquitin specific peptidase 46  FBXO28 - f-box protein 28  UBE2V2 - ubiquitin-conjugating enzyme e2 variant 2  LONRF1 - lon peptidase n-terminal domain and ring finger 1  NUP153 - nucleoporin 153kda  UBE2L3 - ubiquitin-conjugating enzyme e2l 3  NUP210 - nucleoporin 210kda  MED12 - mediator complex subunit 12  UBE2N - ubiquitin-conjugating enzyme e2n  UBE2V1 - ubiquitin-conjugating enzyme e2 variant 1  PSMD6 - proteasome (prosome, macropain) 26s subunit, non-atpase, 6  WDSUB1 - wd repeat, sterile alpha motif and u-box domain containing 1  TRIM3 - tripartite motif containing 3  FOXK2 - forkhead box k2  DTX3L - deltex 3-like (drosophila)  PJA2 - praja ring finger 2, e3 ubiquitin protein ligase  AREL1 - apoptosis resistant e3 ubiquitin protein ligase 1  STAMBP - stam binding protein  ENC1 - ectodermal-neural cortex 1 (with btb domain)  CDC27 - cell division cycle 27  DESI1 - desumoylating isopeptidase 1  RNF141 - ring finger protein 141  ANKIB1 - ankyrin repeat and ibr domain containing 1  WSB2 - wd repeat and socs box containing 2  USP16 - ubiquitin specific peptidase 16  PSMA1 - proteasome (prosome, macropain) subunit, alpha type, 1  PSMA2 - proteasome (prosome, macropain) subunit, alpha type, 2  RNF216 - ring finger protein 216  UBE2S - ubiquitin-conjugating enzyme e2s  RNF38 - ring finger protein 38  PRPF19 - pre-mrna processing factor 19  PIAS1 - protein inhibitor of activated stat, 1  PSMB1 - proteasome (prosome, macropain) subunit, beta type, 1  SMURF2 - smad specific e3 ubiquitin protein ligase 2  PSMA5 - proteasome (prosome, macropain) subunit, alpha type, 5  KLHL21 - kelch-like family member 21  PSMA3 - proteasome (prosome, macropain) subunit, alpha type, 3  PSMB10 - proteasome (prosome, macropain) subunit, beta type, 10  MGRN1 - mahogunin ring finger 1, e3 ubiquitin protein ligase  MED8 - mediator complex subunit 8  PSMC6 - proteasome (prosome, macropain) 26s subunit, atpase, 6  CAND1 - cullin-associated and neddylation-dissociated 1  PSMD1 - proteasome (prosome, macropain) 26s subunit, non-atpase, 1  PSMC4 - proteasome (prosome, macropain) 26s subunit, atpase, 4  COPS7B - cop9 signalosome subunit 7b  CBX4 - chromobox homolog 4  FBXW11 - f-box and wd repeat domain containing 11  PSMC2 - proteasome (prosome, macropain) 26s subunit, atpase, 2  COPS3 - cop9 signalosome subunit 3  TNKS2 - tankyrase, trf1-interacting ankyrin-related adp-ribose polymerase 2  PSMD9 - proteasome (prosome, macropain) 26s subunit, non-atpase, 9  PSMD10 - proteasome (prosome, macropain) 26s subunit, non-atpase, 10  KLHL18 - kelch-like family member 18  PSMD7 - proteasome (prosome, macropain) 26s subunit, non-atpase, 7  WDR61 - wd repeat domain 61  PSMD8 - proteasome (prosome, macropain) 26s subunit, non-atpase, 8  ANAPC1 - anaphase promoting complex subunit 1  RNF130 - ring finger protein 130  PSMD5 - proteasome (prosome, macropain) 26s subunit, non-atpase, 5  PSMD2 - proteasome (prosome, macropain) 26s subunit, non-atpase, 2  PPP1R11 - protein phosphatase 1, regulatory (inhibitor) subunit 11  NUP160 - nucleoporin 160kda  PSMD12 - proteasome (prosome, macropain) 26s subunit, non-atpase, 12  UBR4 - ubiquitin protein ligase e3 component n-recognin 4  ATG7 - autophagy related 7  PSMD13 - proteasome (prosome, macropain) 26s subunit, non-atpase, 13  MBD6 - methyl-cpg binding domain protein 6  KLHL7 - kelch-like family member 7  KLHL17 - kelch-like family member 17  PTEN - phosphatase and tensin homolog  TOMM20 - translocase of outer mitochondrial membrane 20 homolog (yeast)  PHF15 - phd finger protein 15  NUP88 - nucleoporin 88kda  NUP98 - nucleoporin 98kda  USP22 - ubiquitin specific peptidase 22  RNF115 - ring finger protein 115  SASH1 - sam and sh3 domain containing 1  TRIM2 - tripartite motif containing 2  SUDS3 - suppressor of defective silencing 3 homolog (s. cerevisiae)  MAP3K7 - mitogen-activated protein kinase kinase kinase 7  RNF144A - ring finger protein 144a  INO80E - ino80 complex subunit e  RNF114 - ring finger protein 114  ATG3 - autophagy related 3  SPATA2 - spermatogenesis associated 2  KLHL20 - kelch-like family member 20  AKTIP - akt interacting protein  SPOPL - speckle-type poz protein-like  UFL1 - ufm1-specific ligase 1  RUVBL1 - ruvb-like 1 (e. coli)  TRIM9 - tripartite motif containing 9  KLHL9 - kelch-like family member 9  RNF40 - ring finger protein 40, e3 ubiquitin protein ligase  MOCS3 - molybdenum cofactor synthesis 3  CUL7 - cullin 7  RNF208 - ring finger protein 208  USP24 - ubiquitin specific peptidase 24  VPRBP - vpr (hiv-1) binding protein  RFWD2 - ring finger and wd repeat domain 2, e3 ubiquitin protein ligase  RYBP - ring1 and yy1 binding protein  RNF34 - ring finger protein 34, e3 ubiquitin protein ligase  RNF25 - ring finger protein 25  FBXO11 - f-box protein 11  SPSB1 - spla/ryanodine receptor domain and socs box containing 1  HERPUD1 - homocysteine-inducible, endoplasmic reticulum stress-inducible, ubiquitin-like domain member 1  PEX2 - peroxisomal biogenesis factor 2  NUP50 - nucleoporin 50kda  CNOT4 - ccr4-not transcription complex, subunit 4  SIRT1 - sirtuin 1  HDAC4 - histone deacetylase 4  NFRKB - nuclear factor related to kappab binding protein  ACTR8 - arp8 actin-related protein 8 homolog (yeast)  IRF2BPL - interferon regulatory factor 2 binding protein-like  NFKBIA - nuclear factor of kappa light polypeptide gene enhancer in b-cells inhibitor, alpha  FBXO44 - f-box protein 44  TNFAIP1 - tumor necrosis factor, alpha-induced protein 1 (endothelial)  FOXK1 - forkhead box k1  PAF1 - paf1, rna polymerase ii associated factor, homolog (s. cerevisiae)  VCPIP1 - valosin containing protein (p97)/p47 complex interacting protein 1  MAGEF1 - melanoma antigen family f, 1  USP34 - ubiquitin specific peptidase 34  FBXL19 - f-box and leucine-rich repeat protein 19  RNF145 - ring finger protein 145  INO80 - ino80 complex subunit  CCP110 - centriolar coiled coil protein 110kda  SOCS7 - suppressor of cytokine signaling 7  KCTD21 - potassium channel tetramerization domain containing 21  NUP188 - nucleoporin 188kda  SUZ12 - suz12 polycomb repressive complex 2 subunit  RC3H2 - ring finger and ccch-type domains 2  RANBP2 - ran binding protein 2  RANGAP1 - ran gtpase activating protein 1  SOCS5 - suppressor of cytokine signaling 5  USP53 - ubiquitin specific peptidase 53  NEDD8 - neural precursor cell expressed, developmentally down-regulated 8  RNF11 - ring finger protein 11  CTR9 - ctr9, paf1/rna polymerase ii complex component  SPOP - speckle-type poz protein  NFE2L2 - nuclear factor (erythroid-derived 2)-like 2  KLHL15 - kelch-like family member 15  USP21 - ubiquitin specific peptidase 21  RRAGA - ras-related gtp binding a  NUP155 - nucleoporin 155kda  MAGEL2 - mage-like 2  CDK5RAP3 - cdk5 regulatory subunit associated protein 3  TGFBR1 - transforming growth factor, beta receptor 1  DCAF8 - ddb1 and cul4 associated factor 8  OGT - o-linked n-acetylglucosamine (glcnac) transferase  NUP93 - nucleoporin 93kda  RAE1 - rae1 rna export 1 homolog (s. pombe)  RBBP6 - retinoblastoma binding protein 6  RNF20 - ring finger protein 20, e3 ubiquitin protein ligase  NUP35 - nucleoporin 35kda  CUL4B - cullin 4b  ERCC8 - excision repair cross-complementing rodent repair deficiency, complementation group 8  CUL1 - cullin 1  CUL2 - cullin 2  CUL3 - cullin 3  COPS7A - cop9 signalosome subunit 7a  USP39 - ubiquitin specific peptidase 39  DZIP3 - daz interacting zinc finger protein 3  SUPT3H - suppressor of ty 3 homolog (s. cerevisiae) |
| GO:0051028 | mRNA transport | 1.18E-6 | 2.06E-4 | 1.28 (10334,125,6996,108) | [+] Show genes  DHX9 - deah (asp-glu-ala-his) box helicase 9  DDX19B - dead (asp-glu-ala-asp) box polypeptide 19b  NCBP2 - nuclear cap binding protein subunit 2, 20kda  NCBP1 - nuclear cap binding protein subunit 1, 80kda  SRSF11 - serine/arginine-rich splicing factor 11  FMR1 - fragile x mental retardation 1  SEC13 - sec13 homolog (s. cerevisiae)  SYMPK - symplekin  DDX25 - dead (asp-glu-ala-asp) box helicase 25  ZFP36L1 - zfp36 ring finger protein-like 1  AGFG1 - arfgap with fg repeats 1  ALKBH5 - alkb, alkylation repair homolog 5 (e. coli)  ARC - activity-regulated cytoskeleton-associated protein  UPF1 - upf1 regulator of nonsense transcripts homolog (yeast)  CPSF3 - cleavage and polyadenylation specific factor 3, 73kda  EIF4E - eukaryotic translation initiation factor 4e  AHCTF1 - at hook containing transcription factor 1  G3BP2 - gtpase activating protein (sh3 domain) binding protein 2  BICD2 - bicaudal d homolog 2 (drosophila)  ALYREF - aly/ref export factor  PABPN1 - poly(a) binding protein, nuclear 1  RNPS1 - rna binding protein s1, serine-rich domain  SMG6 - smg6 nonsense mediated mrna decay factor  NUTF2 - nuclear transport factor 2  SMG7 - smg7 nonsense mediated mrna decay factor  FIP1L1 - factor interacting with papola and cpsf1  NUP160 - nucleoporin 160kda  SEH1L - seh1-like (s. cerevisiae)  CETN2 - centrin, ef-hand protein, 2  CETN3 - centrin, ef-hand protein, 3  EIF4A3 - eukaryotic translation initiation factor 4a3  NUP88 - nucleoporin 88kda  NUP98 - nucleoporin 98kda  KHSRP - kh-type splicing regulatory protein  GLE1 - gle1 rna export mediator  CPSF4 - cleavage and polyadenylation specific factor 4, 30kda  U2AF1L4 - u2 small nuclear rna auxiliary factor 1-like 4  MCM3AP - minichromosome maintenance complex component 3 associated protein  CPSF2 - cleavage and polyadenylation specific factor 2, 100kda  CPSF1 - cleavage and polyadenylation specific factor 1, 160kda  DHX38 - deah (asp-glu-ala-his) box polypeptide 38  RBM15B - rna binding motif protein 15b  NUP85 - nucleoporin 85kda  SLU7 - slu7 splicing factor homolog (s. cerevisiae)  SMG1 - smg1 phosphatidylinositol 3-kinase-related kinase  SRRM1 - serine/arginine repetitive matrix 1  MAGOHB - mago-nashi homolog b (drosophila)  ENY2 - enhancer of yellow 2 homolog (drosophila)  LRPPRC - leucine-rich pentatricopeptide repeat containing  CDC40 - cell division cycle 40  POLR2D - polymerase (rna) ii (dna directed) polypeptide d  TPR - translocated promoter region, nuclear basket protein  DDX19A - dead (asp-glu-ala-asp) box polypeptide 19a  SENP2 - sumo1/sentrin/smt3 specific peptidase 2  MAGOH - mago-nashi homolog, proliferation-associated (drosophila)  NUP133 - nucleoporin 133kda  KIF5C - kinesin family member 5c  XPO1 - exportin 1 (crm1 homolog, yeast)  NUP107 - nucleoporin 107kda  NUPL2 - nucleoporin like 2  SUPT6H - suppressor of ty 6 homolog (s. cerevisiae)  NUP50 - nucleoporin 50kda  TNKS - tankyrase, trf1-interacting ankyrin-related adp-ribose polymerase  PARP11 - poly (adp-ribose) polymerase family, member 11  SRSF9 - serine/arginine-rich splicing factor 9  THOC7 - tho complex 7 homolog (drosophila)  NUP43 - nucleoporin 43kda  PCID2 - pci domain containing 2  SLBP - stem-loop binding protein  CHTOP - chromatin target of prmt1  WDR33 - wd repeat domain 33  ZC3H3 - zinc finger ccch-type containing 3  FYTTD1 - forty-two-three domain containing 1  UPF3B - upf3 regulator of nonsense transcripts homolog b (yeast)  UPF3A - upf3 regulator of nonsense transcripts homolog a (yeast)  DDX39B - dead (asp-glu-ala-asp) box polypeptide 39b  NUP188 - nucleoporin 188kda  IWS1 - iws1 homolog (s. cerevisiae)  POLDIP3 - polymerase (dna-directed), delta interacting protein 3  NUP205 - nucleoporin 205kda  RANBP2 - ran binding protein 2  YTHDC1 - yth domain containing 1  THOC2 - tho complex 2  THOC6 - tho complex 6 homolog (drosophila)  AAAS - achalasia, adrenocortical insufficiency, alacrimia  NUP155 - nucleoporin 155kda  RBM8A - rna binding motif protein 8a  CASC3 - cancer susceptibility candidate 3  U2AF2 - u2 small nuclear rna auxiliary factor 2  SRSF1 - serine/arginine-rich splicing factor 1  HNRNPA2B1 - heterogeneous nuclear ribonucleoprotein a2/b1  HNRNPA1 - heterogeneous nuclear ribonucleoprotein a1  SRSF2 - serine/arginine-rich splicing factor 2  SRSF3 - serine/arginine-rich splicing factor 3  NUP93 - nucleoporin 93kda  SRSF4 - serine/arginine-rich splicing factor 4  SRSF5 - serine/arginine-rich splicing factor 5  RAE1 - rae1 rna export 1 homolog (s. pombe)  SRSF6 - serine/arginine-rich splicing factor 6  SRSF7 - serine/arginine-rich splicing factor 7  NDC1 - ndc1 transmembrane nucleoporin  NUP35 - nucleoporin 35kda  THOC1 - tho complex 1  THOC3 - tho complex 3  NUP214 - nucleoporin 214kda  SARNP - sap domain containing ribonucleoprotein  NUP153 - nucleoporin 153kda  NUP210 - nucleoporin 210kda |
| GO:0006406 | mRNA export from nucleus | 1.31E-6 | 2.25E-4 | 1.31 (10334,97,6996,86) | [+] Show genes  DDX19B - dead (asp-glu-ala-asp) box polypeptide 19b  DDX19A - dead (asp-glu-ala-asp) box polypeptide 19a  NCBP2 - nuclear cap binding protein subunit 2, 20kda  NCBP1 - nuclear cap binding protein subunit 1, 80kda  SRSF11 - serine/arginine-rich splicing factor 11  SEC13 - sec13 homolog (s. cerevisiae)  MAGOH - mago-nashi homolog, proliferation-associated (drosophila)  SYMPK - symplekin  DDX25 - dead (asp-glu-ala-asp) box helicase 25  NUP133 - nucleoporin 133kda  NUP107 - nucleoporin 107kda  NUPL2 - nucleoporin like 2  NUP50 - nucleoporin 50kda  AGFG1 - arfgap with fg repeats 1  ALKBH5 - alkb, alkylation repair homolog 5 (e. coli)  UPF1 - upf1 regulator of nonsense transcripts homolog (yeast)  CPSF3 - cleavage and polyadenylation specific factor 3, 73kda  SRSF9 - serine/arginine-rich splicing factor 9  THOC7 - tho complex 7 homolog (drosophila)  EIF4E - eukaryotic translation initiation factor 4e  NUP43 - nucleoporin 43kda  PCID2 - pci domain containing 2  SLBP - stem-loop binding protein  CHTOP - chromatin target of prmt1  ALYREF - aly/ref export factor  WDR33 - wd repeat domain 33  PABPN1 - poly(a) binding protein, nuclear 1  RNPS1 - rna binding protein s1, serine-rich domain  SMG6 - smg6 nonsense mediated mrna decay factor  FYTTD1 - forty-two-three domain containing 1  SMG7 - smg7 nonsense mediated mrna decay factor  UPF3B - upf3 regulator of nonsense transcripts homolog b (yeast)  DDX39B - dead (asp-glu-ala-asp) box polypeptide 39b  FIP1L1 - factor interacting with papola and cpsf1  NUP160 - nucleoporin 160kda  NUP188 - nucleoporin 188kda  RANBP2 - ran binding protein 2  NUP205 - nucleoporin 205kda  POLDIP3 - polymerase (dna-directed), delta interacting protein 3  YTHDC1 - yth domain containing 1  THOC2 - tho complex 2  THOC6 - tho complex 6 homolog (drosophila)  SEH1L - seh1-like (s. cerevisiae)  EIF4A3 - eukaryotic translation initiation factor 4a3  NUP88 - nucleoporin 88kda  AAAS - achalasia, adrenocortical insufficiency, alacrimia  NUP98 - nucleoporin 98kda  GLE1 - gle1 rna export mediator  NUP155 - nucleoporin 155kda  CPSF4 - cleavage and polyadenylation specific factor 4, 30kda  RBM8A - rna binding motif protein 8a  U2AF1L4 - u2 small nuclear rna auxiliary factor 1-like 4  MCM3AP - minichromosome maintenance complex component 3 associated protein  CPSF2 - cleavage and polyadenylation specific factor 2, 100kda  CPSF1 - cleavage and polyadenylation specific factor 1, 160kda  DHX38 - deah (asp-glu-ala-his) box polypeptide 38  CASC3 - cancer susceptibility candidate 3  RBM15B - rna binding motif protein 15b  NUP85 - nucleoporin 85kda  SLU7 - slu7 splicing factor homolog (s. cerevisiae)  SMG1 - smg1 phosphatidylinositol 3-kinase-related kinase  HNRNPA2B1 - heterogeneous nuclear ribonucleoprotein a2/b1  SRSF1 - serine/arginine-rich splicing factor 1  U2AF2 - u2 small nuclear rna auxiliary factor 2  SRRM1 - serine/arginine repetitive matrix 1  SRSF2 - serine/arginine-rich splicing factor 2  MAGOHB - mago-nashi homolog b (drosophila)  SRSF3 - serine/arginine-rich splicing factor 3  SRSF4 - serine/arginine-rich splicing factor 4  NUP93 - nucleoporin 93kda  SRSF5 - serine/arginine-rich splicing factor 5  RAE1 - rae1 rna export 1 homolog (s. pombe)  SRSF6 - serine/arginine-rich splicing factor 6  SRSF7 - serine/arginine-rich splicing factor 7  ENY2 - enhancer of yellow 2 homolog (drosophila)  NDC1 - ndc1 transmembrane nucleoporin  NUP35 - nucleoporin 35kda  THOC1 - tho complex 1  THOC3 - tho complex 3  NUP214 - nucleoporin 214kda  SARNP - sap domain containing ribonucleoprotein  NUP153 - nucleoporin 153kda  NUP210 - nucleoporin 210kda  POLR2D - polymerase (rna) ii (dna directed) polypeptide d  CDC40 - cell division cycle 40  TPR - translocated promoter region, nuclear basket protein |
| GO:0018393 | internal peptidyl-lysine acetylation | 1.73E-6 | 2.93E-4 | 1.31 (10334,96,6996,85) | [+] Show genes  HAT1 - histone acetyltransferase 1  MSL3 - male-specific lethal 3 homolog (drosophila)  EPC2 - enhancer of polycomb homolog 2 (drosophila)  TADA1 - transcriptional adaptor 1  MCRS1 - microspherule protein 1  ATAT1 - alpha tubulin acetyltransferase 1  MSL2 - male-specific lethal 2 homolog (drosophila)  NAA40 - n(alpha)-acetyltransferase 40, natd catalytic subunit, homolog (s. cerevisiae)  MEAF6 - myst/esa1-associated factor 6  EP300 - e1a binding protein p300  RSF1 - remodeling and spacing factor 1  ING4 - inhibitor of growth family, member 4  DPF2 - d4, zinc and double phd fingers family 2  DR1 - down-regulator of transcription 1, tbp-binding (negative cofactor 2)  CSRP2BP - csrp2 binding protein  KANSL1 - kat8 regulatory nsl complex subunit 1  GTF2B - general transcription factor iib  PCGF2 - polycomb group ring finger 2  KAT2A - k(lysine) acetyltransferase 2a  NCOA3 - nuclear receptor coactivator 3  APBB1 - amyloid beta (a4) precursor protein-binding, family b, member 1 (fe65)  KMT2A - lysine (k)-specific methyltransferase 2a  BRPF3 - bromodomain and phd finger containing, 3  BRD8 - bromodomain containing 8  TADA3 - transcriptional adaptor 3  SPHK2 - sphingosine kinase 2  TAF6L - taf6-like rna polymerase ii, p300/cbp-associated factor (pcaf)-associated factor, 65kda  BRD1 - bromodomain containing 1  LDB1 - lim domain binding 1  CREBBP - creb binding protein  PHF20L1 - phd finger protein 20-like 1  MORF4L1 - mortality factor 4 like 1  ATF2 - activating transcription factor 2  MSL1 - male-specific lethal 1 homolog (drosophila)  KAT2B - k(lysine) acetyltransferase 2b  CLOCK - clock circadian regulator  CRTC2 - creb regulated transcription coactivator 2  KANSL1L - kat8 regulatory nsl complex subunit 1-like  TAF9 - taf9 rna polymerase ii, tata box binding protein (tbp)-associated factor, 32kda  KANSL3 - kat8 regulatory nsl complex subunit 3  BRPF1 - bromodomain and phd finger containing, 1  ACTL6A - actin-like 6a  TRRAP - transformation/transcription domain-associated protein  TAF6 - taf6 rna polymerase ii, tata box binding protein (tbp)-associated factor, 80kda  TADA2A - transcriptional adaptor 2a  ARRB1 - arrestin, beta 1  PHF15 - phd finger protein 15  SUPT7L - suppressor of ty 7 (s. cerevisiae)-like  CHD5 - chromodomain helicase dna binding protein 5  MECP2 - methyl cpg binding protein 2 (rett syndrome)  EPC1 - enhancer of polycomb homolog 1 (drosophila)  MORF4L2 - mortality factor 4 like 2  EP400 - e1a binding protein p400  USP22 - ubiquitin specific peptidase 22  ING5 - inhibitor of growth family, member 5  NAA60 - n(alpha)-acetyltransferase 60, natf catalytic subunit  KAT5 - k(lysine) acetyltransferase 5  MCM3AP - minichromosome maintenance complex component 3 associated protein  YEATS4 - yeats domain containing 4  ING3 - inhibitor of growth family, member 3  MAP3K7 - mitogen-activated protein kinase kinase kinase 7  ACTL6B - actin-like 6b  PHF20 - phd finger protein 20  HCFC1 - host cell factor c1 (vp16-accessory protein)  PHF10 - phd finger protein 10  ZZZ3 - zinc finger, zz-type containing 3  OGT - o-linked n-acetylglucosamine (glcnac) transferase  MBIP - map3k12 binding inhibitory protein 1  KANSL2 - kat8 regulatory nsl complex subunit 2  KAT8 - k(lysine) acetyltransferase 8  KAT6A - k(lysine) acetyltransferase 6a  MRGBP - mrg/morf4l binding protein  PHF14 - phd finger protein 14  RUVBL1 - ruvb-like 1 (e. coli)  PHF17 - phd finger protein 17  DMAP1 - dna methyltransferase 1 associated protein 1  KAT6B - k(lysine) acetyltransferase 6b  ZMPSTE24 - zinc metallopeptidase ste24  MBD3 - methyl-cpg binding domain protein 3  LEF1 - lymphoid enhancer-binding factor 1  GTF3C4 - general transcription factor iiic, polypeptide 4, 90kda  SUPT3H - suppressor of ty 3 homolog (s. cerevisiae)  YEATS2 - yeats domain containing 2  KAT7 - k(lysine) acetyltransferase 7  RUVBL2 - ruvb-like 2 (e. coli) |
| GO:0006357 | regulation of transcription by RNA polymerase II | 1.74E-6 | 2.91E-4 | 1.08 (10334,1446,6996,1055) | [+] Show genes  ELL - elongation factor rna polymerase ii  EXOSC9 - exosome component 9  ZNF239 - zinc finger protein 239  TADA1 - transcriptional adaptor 1  ABCA2 - atp-binding cassette, sub-family a (abc1), member 2  ZNF821 - zinc finger protein 821  ABL1 - c-abl oncogene 1, non-receptor tyrosine kinase  SUPT20H - suppressor of ty 20 homolog (s. cerevisiae)  NRIP1 - nuclear receptor interacting protein 1  KAT2A - k(lysine) acetyltransferase 2a  NCOA3 - nuclear receptor coactivator 3  BRD8 - bromodomain containing 8  CHD7 - chromodomain helicase dna binding protein 7  WWC2 - ww and c2 domain containing 2  LYAR - ly1 antibody reactive  TCERG1 - transcription elongation regulator 1  ZNF354B - zinc finger protein 354b  EHMT2 - euchromatic histone-lysine n-methyltransferase 2  SUB1 - sub1 homolog (s. cerevisiae)  ZNF511 - zinc finger protein 511  PPID - peptidylprolyl isomerase d  ACTL6A - actin-like 6a  ACVR2A - activin a receptor, type iia  ACVRL1 - activin a receptor type ii-like 1  ACVR1 - activin a receptor, type i  ARID1A - at rich interactive domain 1a (swi-like)  ACVR1B - activin a receptor, type ib  DLL1 - delta-like 1 (drosophila)  MED1 - mediator complex subunit 1  FZD1 - frizzled family receptor 1  ZNF613 - zinc finger protein 613  PPARD - peroxisome proliferator-activated receptor delta  USP9X - ubiquitin specific peptidase 9, x-linked  MAML3 - mastermind-like 3 (drosophila)  RBM10 - rna binding motif protein 10  PARP1 - poly (adp-ribose) polymerase 1  H2AFY2 - h2a histone family, member y2  PPP1R13L - protein phosphatase 1, regulatory subunit 13 like  HES6 - hairy and enhancer of split 6 (drosophila)  SMPD3 - sphingomyelin phosphodiesterase 3, neutral membrane (neutral sphingomyelinase ii)  RUVBL2 - ruvb-like 2 (e. coli)  TTC21B - tetratricopeptide repeat domain 21b  RCOR3 - rest corepressor 3  SLTM - safb-like, transcription modulator  NELFE - negative elongation factor complex member e  AES - amino-terminal enhancer of split  EHMT1 - euchromatic histone-lysine n-methyltransferase 1  NR5A1 - nuclear receptor subfamily 5, group a, member 1  FUS - fused in sarcoma  JAG1 - jagged 1  PRDM4 - pr domain containing 4  PPP3R1 - protein phosphatase 3, regulatory subunit b, alpha  ATF7IP - activating transcription factor 7 interacting protein  ZNF689 - zinc finger protein 689  PPP2R5B - protein phosphatase 2, regulatory subunit b', beta  AKT1 - v-akt murine thymoma viral oncogene homolog 1  ZNF653 - zinc finger protein 653  RNF168 - ring finger protein 168, e3 ubiquitin protein ligase  XRCC6 - x-ray repair complementing defective repair in chinese hamster cells 6  GABPB1 - ga binding protein transcription factor, beta subunit 1  GABPA - ga binding protein transcription factor, alpha subunit 60kda  WWP2 - ww domain containing e3 ubiquitin protein ligase 2  AHR - aryl hydrocarbon receptor  ZNF526 - zinc finger protein 526  FOXP4 - forkhead box p4  RPAP2 - rna polymerase ii associated protein 2  ZNF83 - zinc finger protein 83  KANSL3 - kat8 regulatory nsl complex subunit 3  ATF7 - activating transcription factor 7  KMT2D - lysine (k)-specific methyltransferase 2d  AMFR - autocrine motility factor receptor, e3 ubiquitin protein ligase  PRKRIR - protein-kinase, interferon-inducible double stranded rna dependent inhibitor, repressor of (p58 repressor)  ZNF419 - zinc finger protein 419  MAP2K5 - mitogen-activated protein kinase kinase 5  ZNF692 - zinc finger protein 692  YEATS4 - yeats domain containing 4  ZFPM1 - zinc finger protein, fog family member 1  SNIP1 - smad nuclear interacting protein 1  MAPK10 - mitogen-activated protein kinase 10  ZNF212 - zinc finger protein 212  EID2 - ep300 interacting inhibitor of differentiation 2  ZNF383 - zinc finger protein 383  MAPK7 - mitogen-activated protein kinase 7  GCN1L1 - gcn1 general control of amino-acid synthesis 1-like 1 (yeast)  MAPK3 - mitogen-activated protein kinase 3  GATA2 - gata binding protein 2  KAT6A - k(lysine) acetyltransferase 6a  ADIRF - adipogenesis regulatory factor  PRKD1 - protein kinase d1  NR4A3 - nuclear receptor subfamily 4, group a, member 3  PRKCB - protein kinase c, beta  BRD3 - bromodomain containing 3  PRKAR1A - protein kinase, camp-dependent, regulatory, type i, alpha  CARF - calcium responsive transcription factor  YEATS2 - yeats domain containing 2  MLLT10 - myeloid/lymphoid or mixed-lineage leukemia (trithorax homolog, drosophila); translocated to, 10  ZNF408 - zinc finger protein 408  APEX1 - apex nuclease (multifunctional dna repair enzyme) 1  DHX36 - deah (asp-glu-ala-his) box polypeptide 36  ZNF189 - zinc finger protein 189  ZSCAN26 - zinc finger and scan domain containing 26  ZNF605 - zinc finger protein 605  XIAP - x-linked inhibitor of apoptosis  KLF12 - kruppel-like factor 12  ZNF177 - zinc finger protein 177  ZNF175 - zinc finger protein 175  ZNF174 - zinc finger protein 174  ZNF184 - zinc finger protein 184  SSBP4 - single stranded dna binding protein 4  PBX2 - pre-b-cell leukemia homeobox 2  GTF2F1 - general transcription factor iif, polypeptide 1, 74kda  PBX3 - pre-b-cell leukemia homeobox 3  PRMT6 - protein arginine methyltransferase 6  PBX1 - pre-b-cell leukemia homeobox 1  ZNF148 - zinc finger protein 148  ZBTB17 - zinc finger and btb domain containing 17  GTF2H2 - general transcription factor iih, polypeptide 2, 44kda  GTF2H3 - general transcription factor iih, polypeptide 3, 34kda  VEZF1 - vascular endothelial zinc finger 1  GTF2H1 - general transcription factor iih, polypeptide 1, 62kda  ZNF142 - zinc finger protein 142  MCPH1 - microcephalin 1  ZNF140 - zinc finger protein 140  GTF2B - general transcription factor iib  PCGF2 - polycomb group ring finger 2  APBB1 - amyloid beta (a4) precursor protein-binding, family b, member 1 (fe65)  ZNF132 - zinc finger protein 132  ZNF131 - zinc finger protein 131  RIF1 - rap1 interacting factor homolog (yeast)  ZNF134 - zinc finger protein 134  ZNF133 - zinc finger protein 133  TBL1XR1 - transducin (beta)-like 1 x-linked receptor 1  GTF2I - general transcription factor iii  GTF2H4 - general transcription factor iih, polypeptide 4, 52kda  RPRD1A - regulation of nuclear pre-mrna domain containing 1a  ARF4 - adp-ribosylation factor 4  ZHX1 - zinc fingers and homeoboxes 1  ZNF79 - zinc finger protein 79  APP - amyloid beta (a4) precursor protein  ZNF70 - zinc finger protein 70  BBS7 - bardet-biedl syndrome 7  DDX20 - dead (asp-glu-ala-asp) box polypeptide 20  ZNF75D - zinc finger protein 75d  PAM - peptidylglycine alpha-amidating monooxygenase  ZNF76 - zinc finger protein 76  ARNTL - aryl hydrocarbon receptor nuclear translocator-like  ARNT - aryl hydrocarbon receptor nuclear translocator  H2AFZ - h2a histone family, member z  FZD5 - frizzled family receptor 5  SAP130 - sin3a-associated protein, 130kda  PAX8 - paired box 8  SUPT16H - suppressor of ty 16 homolog (s. cerevisiae)  MIER3 - mesoderm induction early response 1, family member 3  ARRB1 - arrestin, beta 1  CDC73 - cell division cycle 73  PDGFB - platelet-derived growth factor beta polypeptide  NKAP - nfkb activating protein  RHOA - ras homolog family member a  STRAP - serine/threonine kinase receptor associated protein  THAP5 - thap domain containing 5  PSIP1 - pc4 and sfrs1 interacting protein 1  MED9 - mediator complex subunit 9  FSTL1 - follistatin-like 1  HCFC1 - host cell factor c1 (vp16-accessory protein)  PCNA - proliferating cell nuclear antigen  PRDM2 - pr domain containing 2, with znf domain  JDP2 - jun dimerization protein 2  AKIRIN2 - akirin 2  LEO1 - leo1, paf1/rna polymerase ii complex component, homolog (s. cerevisiae)  ERLIN2 - er lipid raft associated 2  ZNF226 - zinc finger protein 226  PDE2A - phosphodiesterase 2a, cgmp-stimulated  HDAC2 - histone deacetylase 2  HMBOX1 - homeobox containing 1  HDGF - hepatoma-derived growth factor  ZNF213 - zinc finger protein 213  THAP1 - thap domain containing, apoptosis associated protein 1  HDAC1 - histone deacetylase 1  PKIG - protein kinase (camp-dependent, catalytic) inhibitor gamma  ZNF205 - zinc finger protein 205  HCLS1 - hematopoietic cell-specific lyn substrate 1  ELP3 - elongator acetyltransferase complex subunit 3  KAT7 - k(lysine) acetyltransferase 7  ASCL1 - achaete-scute complex homolog 1 (drosophila)  RGMB - rgm domain family, member b  SBNO2 - strawberry notch homolog 2 (drosophila)  ZNF444 - zinc finger protein 444  ZBTB1 - zinc finger and btb domain containing 1  XPA - xeroderma pigmentosum, complementation group a  FBXW7 - f-box and wd repeat domain containing 7, e3 ubiquitin protein ligase  XPO1 - exportin 1 (crm1 homolog, yeast)  PAGR1 - paxip1 associated glutamate-rich protein 1  ZHX2 - zinc fingers and homeoboxes 2  FOXJ3 - forkhead box j3  RERE - arginine-glutamic acid dipeptide (re) repeats  ZXDC - zxd family zinc finger c  XBP1 - x-box binding protein 1  ZNF367 - zinc finger protein 367  BHLHE41 - basic helix-loop-helix family, member e41  ZFHX3 - zinc finger homeobox 3  WFS1 - wolfram syndrome 1 (wolframin)  WHSC1 - wolf-hirschhorn syndrome candidate 1  ADNP2 - adnp homeobox 2  NELFA - negative elongation factor complex member a  AEBP2 - ae binding protein 2  IMPACT - impact rwd domain protein  ZNF507 - zinc finger protein 507  ATP2B4 - atpase, ca++ transporting, plasma membrane 4  ZNF652 - zinc finger protein 652  SCAF8 - sr-related ctd-associated factor 8  PKD1 - polycystic kidney disease 1 (autosomal dominant)  PKD2 - polycystic kidney disease 2 (autosomal dominant)  ZNF35 - zinc finger protein 35  ZKSCAN1 - zinc finger with krab and scan domains 1  FBXL15 - f-box and leucine-rich repeat protein 15  MTF2 - metal response element binding transcription factor 2  ZNF576 - zinc finger protein 576  ZNF33B - zinc finger protein 33b  ZNF32 - zinc finger protein 32  BACH1 - btb and cnc homology 1, basic leucine zipper transcription factor 1  PIN1 - peptidylprolyl cis/trans isomerase, nima-interacting 1  PIK3R2 - phosphoinositide-3-kinase, regulatory subunit 2 (beta)  ZNF45 - zinc finger protein 45  MZF1 - myeloid zinc finger 1  PIK3R1 - phosphoinositide-3-kinase, regulatory subunit 1 (alpha)  ZNF18 - zinc finger protein 18  ASXL2 - additional sex combs like 2 (drosophila)  PLAGL1 - pleiomorphic adenoma gene-like 1  ZNF16 - zinc finger protein 16  ELP2 - elongator acetyltransferase complex subunit 2  ASXL1 - additional sex combs like 1 (drosophila)  ZNF384 - zinc finger protein 384  TNIP2 - tnfaip3 interacting protein 2  ATRX - alpha thalassemia/mental retardation syndrome x-linked  PKNOX1 - pbx/knotted 1 homeobox 1  ZNF24 - zinc finger protein 24  TMEM100 - transmembrane protein 100  ZFX - zinc finger protein, x-linked  PHF10 - phd finger protein 10  ZBTB14 - zinc finger and btb domain containing 14  ZNF711 - zinc finger protein 711  ZNF7 - zinc finger protein 7  ZNF8 - zinc finger protein 8  MRGBP - mrg/morf4l binding protein  ZNF2 - zinc finger protein 2  ZNF3 - zinc finger protein 3  BCL9 - b-cell cll/lymphoma 9  YES1 - v-yes-1 yamaguchi sarcoma viral oncogene homolog 1  ECD - ecdysoneless homolog (drosophila)  CCND1 - cyclin d1  PARK7 - parkinson protein 7  GSK3A - glycogen synthase kinase 3 alpha  CCNK - cyclin k  TRIM27 - tripartite motif containing 27  HSF2 - heat shock transcription factor 2  SAP18 - sin3a-associated protein, 18kda  TSHZ2 - teashirt zinc finger homeobox 2  RFXAP - regulatory factor x-associated protein  CREG1 - cellular repressor of e1a-stimulated genes 1  RFX5 - regulatory factor x, 5 (influences hla class ii expression)  FLCN - folliculin  HSBP1 - heat shock factor binding protein 1  RFX1 - regulatory factor x, 1 (influences hla class ii expression)  RELA - v-rel avian reticuloendotheliosis viral oncogene homolog a  BCOR - bcl6 corepressor  ZFP36L2 - zfp36 ring finger protein-like 2  ZFP36L1 - zfp36 ring finger protein-like 1  HESX1 - hesx homeobox 1  DPF2 - d4, zinc and double phd fingers family 2  SAP30 - sin3a-associated protein, 30kda  CC2D1A - coiled-coil and c2 domain containing 1a  RLF - rearranged l-myc fusion  MED11 - mediator complex subunit 11  BMI1 - bmi1 polycomb ring finger oncogene  PCGF3 - polycomb group ring finger 3  LDB1 - lim domain binding 1  WDR77 - wd repeat domain 77  BMPR1A - bone morphogenetic protein receptor, type ia  BMPR2 - bone morphogenetic protein receptor, type ii (serine/threonine kinase)  BMPR1B - bone morphogenetic protein receptor, type ib  KAT2B - k(lysine) acetyltransferase 2b  TSC22D1 - tsc22 domain family, member 1  BMP7 - bone morphogenetic protein 7  CRTC2 - creb regulated transcription coactivator 2  PSMD14 - proteasome (prosome, macropain) 26s subunit, non-atpase, 14  BCAS3 - breast carcinoma amplified sequence 3  CC2D1B - coiled-coil and c2 domain containing 1b  AHI1 - abelson helper integration site 1  RNF4 - ring finger protein 4  THAP8 - thap domain containing 8  BRD2 - bromodomain containing 2  RNF2 - ring finger protein 2  BUD31 - bud31 homolog (s. cerevisiae)  GATAD2A - gata zinc finger domain containing 2a  ZNF655 - zinc finger protein 655  RNF111 - ring finger protein 111  TRAK2 - trafficking protein, kinesin binding 2  TET2 - tet methylcytosine dioxygenase 2  HSPD1 - heat shock 60kda protein 1 (chaperonin)  DNAJB1 - dnaj (hsp40) homolog, subfamily b, member 1  MED18 - mediator complex subunit 18  ZNF48 - zinc finger protein 48  FUBP3 - far upstream element (fuse) binding protein 3  MCRS1 - microspherule protein 1  HMGN1 - high mobility group nucleosome binding domain 1  HMGB3 - high mobility group box 3  HMGB2 - high mobility group box 2  HMGB1 - high mobility group box 1  BRD9 - bromodomain containing 9  TNKS - tankyrase, trf1-interacting ankyrin-related adp-ribose polymerase  SLC30A9 - solute carrier family 30 (zinc transporter), member 9  ZBTB10 - zinc finger and btb domain containing 10  AGO1 - argonaute risc catalytic component 1  MAD2L2 - mad2 mitotic arrest deficient-like 2 (yeast)  PID1 - phosphotyrosine interaction domain containing 1  CAPRIN2 - caprin family member 2  PHIP - pleckstrin homology domain interacting protein  ZBTB48 - zinc finger and btb domain containing 48  NRARP - notch-regulated ankyrin repeat protein  ZNF787 - zinc finger protein 787  HYAL2 - hyaluronoglucosaminidase 2  TADA3 - transcriptional adaptor 3  CALR - calreticulin  ZNF792 - zinc finger protein 792  HFE - hemochromatosis  NCOA2 - nuclear receptor coactivator 2  HIVEP1 - human immunodeficiency virus type i enhancer binding protein 1  ELP4 - elongator acetyltransferase complex subunit 4  CREB3 - camp responsive element binding protein 3  HIF1A - hypoxia inducible factor 1, alpha subunit (basic helix-loop-helix transcription factor)  CITED2 - cbp/p300-interacting transactivator, with glu/asp-rich carboxy-terminal domain, 2  LPIN3 - lipin 3  CCNT2 - cyclin t2  EED - embryonic ectoderm development  CCND3 - cyclin d3  URI1 - uri1, prefoldin-like chaperone  CCNT1 - cyclin t1  CCNH - cyclin h  HMG20A - high mobility group 20a  HNRNPU - heterogeneous nuclear ribonucleoprotein u (scaffold attachment factor a)  RIPK1 - receptor (tnfrsf)-interacting serine-threonine kinase 1  HNRNPK - heterogeneous nuclear ribonucleoprotein k  RPS6KA1 - ribosomal protein s6 kinase, 90kda, polypeptide 1  CBFB - core-binding factor, beta subunit  HNRNPA2B1 - heterogeneous nuclear ribonucleoprotein a2/b1  RIPK2 - receptor-interacting serine-threonine kinase 2  PIAS3 - protein inhibitor of activated stat, 3  RXRB - retinoid x receptor, beta  CAV1 - caveolin 1, caveolae protein, 22kda  CCNC - cyclin c  RBM14 - rna binding motif protein 14  HMGA1 - high mobility group at-hook 1  ZNF584 - zinc finger protein 584  NR4A1 - nuclear receptor subfamily 4, group a, member 1  RFX7 - regulatory factor x, 7  FOXK2 - forkhead box k2  HTATSF1 - hiv-1 tat specific factor 1  MXD4 - max dimerization protein 4  CDC5L - cell division cycle 5-like  CRTC3 - creb regulated transcription coactivator 3  HTRA1 - htra serine peptidase 1  RBM15 - rna binding motif protein 15  HEXIM1 - hexamethylene bis-acetamide inducible 1  ERLIN1 - er lipid raft associated 1  RBMX - rna binding motif protein, x-linked  NCOA7 - nuclear receptor coactivator 7  PSEN1 - presenilin 1  ZNF574 - zinc finger protein 574  USP16 - ubiquitin specific peptidase 16  CREB3L2 - camp responsive element binding protein 3-like 2  PSMA1 - proteasome (prosome, macropain) subunit, alpha type, 1  PSMA2 - proteasome (prosome, macropain) subunit, alpha type, 2  ILK - integrin-linked kinase  CD81 - cd81 molecule  PIAS1 - protein inhibitor of activated stat, 1  PSMB1 - proteasome (prosome, macropain) subunit, beta type, 1  SMYD3 - set and mynd domain containing 3  PSMA5 - proteasome (prosome, macropain) subunit, alpha type, 5  SMURF2 - smad specific e3 ubiquitin protein ligase 2  PSMA3 - proteasome (prosome, macropain) subunit, alpha type, 3  PSMB10 - proteasome (prosome, macropain) subunit, beta type, 10  RIPPLY2 - ripply transcriptional repressor 2  CGGBP1 - cgg triplet repeat binding protein 1  LMO4 - lim domain only 4  TAF6L - taf6-like rna polymerase ii, p300/cbp-associated factor (pcaf)-associated factor, 65kda  PSMC6 - proteasome (prosome, macropain) 26s subunit, atpase, 6  PSMD1 - proteasome (prosome, macropain) 26s subunit, non-atpase, 1  CAMK1 - calcium/calmodulin-dependent protein kinase i  PSMC4 - proteasome (prosome, macropain) 26s subunit, atpase, 4  SOX18 - sry (sex determining region y)-box 18  CBX4 - chromobox homolog 4  PSMC2 - proteasome (prosome, macropain) 26s subunit, atpase, 2  PSMD9 - proteasome (prosome, macropain) 26s subunit, non-atpase, 9  PSMD10 - proteasome (prosome, macropain) 26s subunit, non-atpase, 10  PSMD7 - proteasome (prosome, macropain) 26s subunit, non-atpase, 7  PSMD8 - proteasome (prosome, macropain) 26s subunit, non-atpase, 8  CSRNP1 - cysteine-serine-rich nuclear protein 1  PSMD5 - proteasome (prosome, macropain) 26s subunit, non-atpase, 5  PSMD2 - proteasome (prosome, macropain) 26s subunit, non-atpase, 2  PSMD12 - proteasome (prosome, macropain) 26s subunit, non-atpase, 12  PSMD13 - proteasome (prosome, macropain) 26s subunit, non-atpase, 13  ZNF25 - zinc finger protein 25  JMY - junction mediating and regulatory protein, p53 cofactor  STK16 - serine/threonine kinase 16  ITGA3 - integrin, alpha 3 (antigen cd49c, alpha 3 subunit of vla-3 receptor)  FBLN5 - fibulin 5  CASK - calcium/calmodulin-dependent serine protein kinase (maguk family)  DDX17 - dead (asp-glu-ala-asp) box helicase 17  SUDS3 - suppressor of defective silencing 3 homolog (s. cerevisiae)  KAT5 - k(lysine) acetyltransferase 5  ZFP69 - zfp69 zinc finger protein  CEBPG - ccaat/enhancer binding protein (c/ebp), gamma  SKAP1 - src kinase associated phosphoprotein 1  GZF1 - gdnf-inducible zinc finger protein 1  CDK9 - cyclin-dependent kinase 9  CDK8 - cyclin-dependent kinase 8  INSM1 - insulinoma-associated 1  CDK7 - cyclin-dependent kinase 7  CDK13 - cyclin-dependent kinase 13  PDCD4 - programmed cell death 4 (neoplastic transformation inhibitor)  PCBP3 - poly(rc) binding protein 3  RFXANK - regulatory factor x-associated ankyrin-containing protein  PTPN2 - protein tyrosine phosphatase, non-receptor type 2  KLF7 - kruppel-like factor 7 (ubiquitous)  IRF2 - interferon regulatory factor 2  HTATIP2 - hiv-1 tat interactive protein 2, 30kda  IRF3 - interferon regulatory factor 3  RUVBL1 - ruvb-like 1 (e. coli)  IKZF5 - ikaros family zinc finger 5 (pegasus)  MED19 - mediator complex subunit 19  ITGA6 - integrin, alpha 6  CDK2 - cyclin-dependent kinase 2  PURA - purine-rich element binding protein a  PURB - purine-rich element binding protein b  ZSCAN31 - zinc finger and scan domain containing 31  KIAA1324L - kiaa1324-like  NSD1 - nuclear receptor binding set domain protein 1  ZMYND11 - zinc finger, mynd-type containing 11  PTPRN - protein tyrosine phosphatase, receptor type, n  PEX2 - peroxisomal biogenesis factor 2  KDM5B - lysine (k)-specific demethylase 5b  ZNF777 - zinc finger protein 777  ID2 - inhibitor of dna binding 2, dominant negative helix-loop-helix protein  ID3 - inhibitor of dna binding 3, dominant negative helix-loop-helix protein  CHD4 - chromodomain helicase dna binding protein 4  CHD2 - chromodomain helicase dna binding protein 2  CHD3 - chromodomain helicase dna binding protein 3  CHD1 - chromodomain helicase dna binding protein 1  IRF2BPL - interferon regulatory factor 2 binding protein-like  JAZF1 - jazf zinc finger 1  MYPOP - myb-related transcription factor, partner of profilin  RAD21 - rad21 homolog (s. pombe)  FOXK1 - forkhead box k1  ZNF181 - zinc finger protein 181  PAF1 - paf1, rna polymerase ii associated factor, homolog (s. cerevisiae)  ZNF268 - zinc finger protein 268  INO80 - ino80 complex subunit  ZNF513 - zinc finger protein 513  ZNF282 - zinc finger protein 282  IGHMBP2 - immunoglobulin mu binding protein 2  GMEB1 - glucocorticoid modulatory element binding protein 1  RBPJ - recombination signal binding protein for immunoglobulin kappa j region  MIER2 - mesoderm induction early response 1, family member 2  NCK2 - nck adaptor protein 2  RARG - retinoic acid receptor, gamma  RARB - retinoic acid receptor, beta  DLL4 - delta-like 4 (drosophila)  ZBTB9 - zinc finger and btb domain containing 9  ZBTB12 - zinc finger and btb domain containing 12  KHDRBS1 - kh domain containing, rna binding, signal transduction associated 1  CTCF - ccctc-binding factor (zinc finger protein)  RBL2 - retinoblastoma-like 2 (p130)  JMJD1C - jumonji domain containing 1c  RAI1 - retinoic acid induced 1  IGBP1 - immunoglobulin (cd79a) binding protein 1  RBBP7 - retinoblastoma binding protein 7  RBBP8 - retinoblastoma binding protein 8  SMARCA5 - swi/snf related, matrix associated, actin dependent regulator of chromatin, subfamily a, member 5  OGT - o-linked n-acetylglucosamine (glcnac) transferase  RB1 - retinoblastoma 1  PELP1 - proline, glutamate and leucine rich protein 1  ARID4A - at rich interactive domain 4a (rbp1-like)  KDM5A - lysine (k)-specific demethylase 5a  CUL2 - cullin 2  ZNF667 - zinc finger protein 667  CUL3 - cullin 3  NFAT5 - nuclear factor of activated t-cells 5, tonicity-responsive  FOXP1 - forkhead box p1  CHUK - conserved helix-loop-helix ubiquitous kinase  ZNF572 - zinc finger protein 572  PCGF5 - polycomb group ring finger 5  ANKRA2 - ankyrin repeat, family a (rfxank-like), 2  RBAK - rb-associated krab zinc finger  NFATC2IP - nuclear factor of activated t-cells, cytoplasmic, calcineurin-dependent 2 interacting protein  ZNF414 - zinc finger protein 414  PHF21A - phd finger protein 21a  ZNF771 - zinc finger protein 771  PATZ1 - poz (btb) and at hook containing zinc finger 1  IER2 - immediate early response 2  NCOR1 - nuclear receptor corepressor 1  SSBP2 - single-stranded dna binding protein 2  KPNA6 - karyopherin alpha 6 (importin alpha 7)  NCOR2 - nuclear receptor corepressor 2  MIER1 - mesoderm induction early response 1, transcriptional regulator  DOT1L - dot1-like histone h3k79 methyltransferase  ZNF382 - zinc finger protein 382  ZNF705A - zinc finger protein 705a  SP3 - sp3 transcription factor  EFCAB7 - ef-hand calcium binding domain 7  SP4 - sp4 transcription factor  SP1 - sp1 transcription factor  ZNF410 - zinc finger protein 410  SP2 - sp2 transcription factor  ZNF333 - zinc finger protein 333  CIR1 - corepressor interacting with rbpj, 1  SOX4 - sry (sex determining region y)-box 4  H2AFY - h2a histone family, member y  WBP2 - ww domain binding protein 2  GTF2IRD1 - gtf2i repeat domain containing 1  HES4 - hairy and enhancer of split 4 (drosophila)  ZNF527 - zinc finger protein 527  LEMD3 - lem domain containing 3  CREBL2 - camp responsive element binding protein-like 2  ATF6B - activating transcription factor 6 beta  HOPX - hop homeobox  CREBBP - creb binding protein  ZNF316 - zinc finger protein 316  ATF2 - activating transcription factor 2  ZC3H8 - zinc finger ccch-type containing 8  SOX13 - sry (sex determining region y)-box 13  LMO1 - lim domain only 1 (rhombotin 1)  CREB1 - camp responsive element binding protein 1  NACC2 - nacc family member 2, ben and btb (poz) domain containing  DDX58 - dead (asp-glu-ala-asp) box polypeptide 58  IER5 - immediate early response 5  CLOCK - clock circadian regulator  KLF3 - kruppel-like factor 3 (basic)  RPRD1B - regulation of nuclear pre-mrna domain containing 1b  DDX41 - dead (asp-glu-ala-asp) box polypeptide 41  PLK3 - polo-like kinase 3  SNAPC4 - small nuclear rna activating complex, polypeptide 4, 190kda  SNCA - synuclein, alpha (non a4 component of amyloid precursor)  LITAF - lipopolysaccharide-induced tnf factor  TBPL1 - tbp-like 1  ZBED5 - zinc finger, bed-type containing 5  CREBZF - creb/atf bzip transcription factor  ZBTB41 - zinc finger and btb domain containing 41  ZFP62 - zfp62 zinc finger protein  EID1 - ep300 interacting inhibitor of differentiation 1  SMARCE1 - swi/snf related, matrix associated, actin dependent regulator of chromatin, subfamily e, member 1  SMO - smoothened, frizzled family receptor  ZGPAT - zinc finger, ccch-type with g patch domain  ACTL6B - actin-like 6b  SMARCB1 - swi/snf related, matrix associated, actin dependent regulator of chromatin, subfamily b, member 1  SMARCC1 - swi/snf related, matrix associated, actin dependent regulator of chromatin, subfamily c, member 1  HLTF - helicase-like transcription factor  SSBP3 - single stranded dna binding protein 3  SMARCA4 - swi/snf related, matrix associated, actin dependent regulator of chromatin, subfamily a, member 4  SMARCD2 - swi/snf related, matrix associated, actin dependent regulator of chromatin, subfamily d, member 2  SMARCD3 - swi/snf related, matrix associated, actin dependent regulator of chromatin, subfamily d, member 3  SMARCC2 - swi/snf related, matrix associated, actin dependent regulator of chromatin, subfamily c, member 2  SMARCD1 - swi/snf related, matrix associated, actin dependent regulator of chromatin, subfamily d, member 1  MED20 - mediator complex subunit 20  SMARCA1 - swi/snf related, matrix associated, actin dependent regulator of chromatin, subfamily a, member 1  SMARCA2 - swi/snf related, matrix associated, actin dependent regulator of chromatin, subfamily a, member 2  CRLF3 - cytokine receptor-like factor 3  KMT2C - lysine (k)-specific methyltransferase 2c  GLYR1 - glyoxylate reductase 1 homolog (arabidopsis)  ZBTB7A - zinc finger and btb domain containing 7a  ZNF462 - zinc finger protein 462  PPIL4 - peptidylprolyl isomerase (cyclophilin)-like 4  MBTPS2 - membrane-bound transcription factor peptidase, site 2  MAGED1 - melanoma antigen family d, 1  GLIS2 - glis family zinc finger 2  IRF2BP2 - interferon regulatory factor 2 binding protein 2  MED27 - mediator complex subunit 27  MED26 - mediator complex subunit 26  SENP2 - sumo1/sentrin/smt3 specific peptidase 2  CHD6 - chromodomain helicase dna binding protein 6  MED17 - mediator complex subunit 17  MED23 - mediator complex subunit 23  ZNF580 - zinc finger protein 580  PRPF6 - pre-mrna processing factor 6  GTF2IRD2 - gtf2i repeat domain containing 2  KDM2B - lysine (k)-specific demethylase 2b  SUPT6H - suppressor of ty 6 homolog (s. cerevisiae)  ZNF710 - zinc finger protein 710  CTBP1 - c-terminal binding protein 1  CTBP2 - c-terminal binding protein 2  SUPT4H1 - suppressor of ty 4 homolog 1 (s. cerevisiae)  SUPT5H - suppressor of ty 5 homolog (s. cerevisiae)  INSIG2 - insulin induced gene 2  CTNNB1 - catenin (cadherin-associated protein), beta 1, 88kda  RLIM - ring finger protein, lim domain interacting  RECQL5 - recq protein-like 5  CUX1 - cut-like homeobox 1  CIAO1 - cytosolic iron-sulfur protein assembly 1  PCGF1 - polycomb group ring finger 1  NPNT - nephronectin  FKBP8 - fk506 binding protein 8, 38kda  PHF20L1 - phd finger protein 20-like 1  MED21 - mediator complex subunit 21  MED10 - mediator complex subunit 10  KIT - v-kit hardy-zuckerman 4 feline sarcoma viral oncogene homolog  ZFP28 - zfp28 zinc finger protein  TGIF2 - tgfb-induced factor homeobox 2  BCL6B - b-cell cll/lymphoma 6, member b  CRK - v-crk avian sarcoma virus ct10 oncogene homolog  NIPBL - nipped-b homolog (drosophila)  STAT2 - signal transducer and activator of transcription 2, 113kda  CRY1 - cryptochrome 1 (photolyase-like)  STAT1 - signal transducer and activator of transcription 1, 91kda  STAT3 - signal transducer and activator of transcription 3 (acute-phase response factor)  CRY2 - cryptochrome 2 (photolyase-like)  STAT5A - signal transducer and activator of transcription 5a  JUP - junction plakoglobin  STAT6 - signal transducer and activator of transcription 6, interleukin-4 induced  BRMS1 - breast cancer metastasis suppressor 1  ZNF219 - zinc finger protein 219  ZNF496 - zinc finger protein 496  CRYM - crystallin, mu  SS18 - synovial sarcoma translocation, chromosome 18  PHF20 - phd finger protein 20  ZNF397 - zinc finger protein 397  HMGN3 - high mobility group nucleosomal binding domain 3  PHF6 - phd finger protein 6  ZNF235 - zinc finger protein 235  COPS2 - cop9 signalosome subunit 2  ZNF335 - zinc finger protein 335  ZNF639 - zinc finger protein 639  SARNP - sap domain containing ribonucleoprotein  DNAJB5 - dnaj (hsp40) homolog, subfamily b, member 5  LEF1 - lymphoid enhancer-binding factor 1  BRMS1L - breast cancer metastasis-suppressor 1-like  SREBF2 - sterol regulatory element binding transcription factor 2  UBE2O - ubiquitin-conjugating enzyme e2o  DHX9 - deah (asp-glu-ala-his) box helicase 9  CDK12 - cyclin-dependent kinase 12  KANK2 - kn motif and ankyrin repeat domains 2  DDX5 - dead (asp-glu-ala-asp) box helicase 5  DDX3X - dead (asp-glu-ala-asp) box helicase 3, x-linked  ARID4B - at rich interactive domain 4b (rbp1-like)  SIN3A - sin3 transcription regulator family member a  WWTR1 - ww domain containing transcription regulator 1  ZNF304 - zinc finger protein 304  KDM3B - lysine (k)-specific demethylase 3b  ITGB1BP1 - integrin beta 1 binding protein 1  RSF1 - remodeling and spacing factor 1  ZNF286A - zinc finger protein 286a  MLLT6 - myeloid/lymphoid or mixed-lineage leukemia (trithorax homolog, drosophila); translocated to, 6  MLLT3 - myeloid/lymphoid or mixed-lineage leukemia (trithorax homolog, drosophila); translocated to, 3  MLLT1 - myeloid/lymphoid or mixed-lineage leukemia (trithorax homolog, drosophila); translocated to, 1  MED14 - mediator complex subunit 14  DBP - d site of albumin promoter (albumin d-box) binding protein  ZBTB22 - zinc finger and btb domain containing 22  KMT2A - lysine (k)-specific methyltransferase 2a  NELFB - negative elongation factor complex member b  RPS6KA5 - ribosomal protein s6 kinase, 90kda, polypeptide 5  SUFU - suppressor of fused homolog (drosophila)  AHCTF1 - at hook containing transcription factor 1  ZBTB38 - zinc finger and btb domain containing 38  MNAT1 - mnat cdk-activating kinase assembly factor 1  ZNF551 - zinc finger protein 551  GATAD2B - gata zinc finger domain containing 2b  MTA2 - metastasis associated 1 family, member 2  ZNF473 - zinc finger protein 473  EIF2A - eukaryotic translation initiation factor 2a, 65kda  WWOX - ww domain containing oxidoreductase  MNT - mnt, max dimerization protein  CCPG1 - cell cycle progression 1  ZNF451 - zinc finger protein 451  SS18L1 - synovial sarcoma translocation gene on chromosome 18-like 1  SCAF4 - sr-related ctd-associated factor 4  CHD5 - chromodomain helicase dna binding protein 5  MECP2 - methyl cpg binding protein 2 (rett syndrome)  PELO - pelota homolog (drosophila)  ARID1B - at rich interactive domain 1b (swi1-like)  MEF2A - myocyte enhancer factor 2a  MED30 - mediator complex subunit 30  ZBED1 - zinc finger, bed-type containing 1  MKL2 - mkl/myocardin-like 2  MEIS2 - meis homeobox 2  BRWD1 - bromodomain and wd repeat domain containing 1  MEIS1 - meis homeobox 1  SLC33A1 - solute carrier family 33 (acetyl-coa transporter), member 1  MEN1 - multiple endocrine neoplasia i  ZZZ3 - zinc finger, zz-type containing 3  HINFP - histone h4 transcription factor  KAT8 - k(lysine) acetyltransferase 8  DGKQ - diacylglycerol kinase, theta 110kda  MTA3 - metastasis associated 1 family, member 3  ARHGEF2 - rho/rac guanine nucleotide exchange factor (gef) 2  ZNF608 - zinc finger protein 608  MAVS - mitochondrial antiviral signaling protein  SARS - seryl-trna synthetase  SATB1 - satb homeobox 1  MBD3 - methyl-cpg binding domain protein 3  SAFB - scaffold attachment factor b  PTOV1 - prostate tumor overexpressed 1  ZNF394 - zinc finger protein 394  SALL2 - sal-like 2 (drosophila)  CBFA2T2 - core-binding factor, runt domain, alpha subunit 2; translocated to, 2  EPC2 - enhancer of polycomb homolog 2 (drosophila)  DRD2 - dopamine receptor d2  CXXC5 - cxxc finger protein 5  SLC9A1 - solute carrier family 9, subfamily a (nhe1, cation proton antiporter 1), member 1  IRF2BP1 - interferon regulatory factor 2 binding protein 1  ATN1 - atrophin 1  IL23A - interleukin 23, alpha subunit p19  MTA1 - metastasis associated 1  SIRT6 - sirtuin 6  DR1 - down-regulator of transcription 1, tbp-binding (negative cofactor 2)  SKI - v-ski avian sarcoma viral oncogene homolog  SKIL - ski-like oncogene  MKL1 - megakaryoblastic leukemia (translocation) 1  MAX - myc associated factor x  DVL2 - dishevelled segment polarity protein 2  ZMIZ2 - zinc finger, miz-type containing 2  DVL3 - dishevelled segment polarity protein 3  LDB2 - lim domain binding 2  ZFP90 - zfp90 zinc finger protein  FNIP2 - folliculin interacting protein 2  HOMEZ - homeobox and leucine zipper encoding  EBF4 - early b-cell factor 4  NELFCD - negative elongation factor complex member c/d  ASH2L - ash2 (absent, small, or homeotic)-like (drosophila)  ZFHX2 - zinc finger homeobox 2  ZSCAN29 - zinc finger and scan domain containing 29  CTDSPL2 - ctd (carboxy-terminal domain, rna polymerase ii, polypeptide a) small phosphatase like 2  THAP2 - thap domain containing, apoptosis associated protein 2  ZNF484 - zinc finger protein 484  DLG1 - discs, large homolog 1 (drosophila)  LRP6 - low density lipoprotein receptor-related protein 6  SHOX2 - short stature homeobox 2  ZNF445 - zinc finger protein 445  LUM - lumican  SESN2 - sestrin 2  PHF12 - phd finger protein 12  PARP9 - poly (adp-ribose) polymerase family, member 9  RNF8 - ring finger protein 8, e3 ubiquitin protein ligase  BAZ1B - bromodomain adjacent to zinc finger domain, 1b  CALCOCO1 - calcium binding and coiled-coil domain 1  SFPQ - splicing factor proline/glutamine-rich  ZBTB4 - zinc finger and btb domain containing 4  SFRP1 - secreted frizzled-related protein 1  SFRP2 - secreted frizzled-related protein 2  MED15 - mediator complex subunit 15  MXD1 - max dimerization protein 1  SFRP4 - secreted frizzled-related protein 4  PIAS4 - protein inhibitor of activated stat, 4  TRIM33 - tripartite motif containing 33  GMEB2 - glucocorticoid modulatory element binding protein 2  SMAD2 - smad family member 2  SPAG8 - sperm associated antigen 8  DNMT1 - dna (cytosine-5-)-methyltransferase 1  HDAC7 - histone deacetylase 7  KLF16 - kruppel-like factor 16  ARMCX3 - armadillo repeat containing, x-linked 3  DNMT3A - dna (cytosine-5-)-methyltransferase 3 alpha  CHD8 - chromodomain helicase dna binding protein 8  LARP7 - la ribonucleoprotein domain family, member 7  RPS6KA4 - ribosomal protein s6 kinase, 90kda, polypeptide 4  MAFG - v-maf avian musculoaponeurotic fibrosarcoma oncogene homolog g  TAF9B - taf9b rna polymerase ii, tata box binding protein (tbp)-associated factor, 31kda  NOC2L - nucleolar complex associated 2 homolog (s. cerevisiae)  ZNF317 - zinc finger protein 317  SMAD4 - smad family member 4  SMAD5 - smad family member 5  SMAD7 - smad family member 7  WASL - wiskott-aldrich syndrome-like  ZNF565 - zinc finger protein 565  MEIS3 - meis homeobox 3  TAGLN3 - transgelin 3  SIX5 - six homeobox 5  NDN - necdin, melanoma antigen (mage) family member  ZNF582 - zinc finger protein 582  NCK1 - nck adaptor protein 1  BRD7 - bromodomain containing 7  NCL - nucleolin  ELK1 - elk1, member of ets oncogene family  CEBPZ - ccaat/enhancer binding protein (c/ebp), zeta  ELK3 - elk3, ets-domain protein (srf accessory protein 2)  ELK4 - elk4, ets-domain protein (srf accessory protein 1)  SNW1 - snw domain containing 1  SCAP - srebf chaperone  CCNL2 - cyclin l2  TRIM28 - tripartite motif containing 28  EPAS1 - endothelial pas domain protein 1  EP300 - e1a binding protein p300  ATF6 - activating transcription factor 6  TBK1 - tank-binding kinase 1  ENO1 - enolase 1, (alpha)  ZNF197 - zinc finger protein 197  EGR2 - early growth response 2  PPP1R12A - protein phosphatase 1, regulatory subunit 12a  EGR3 - early growth response 3  MYT1 - myelin transcription factor 1  SENP1 - sumo1/sentrin specific peptidase 1  CSRNP2 - cysteine-serine-rich nuclear protein 2  TSHZ1 - teashirt zinc finger homeobox 1  PSME3 - proteasome (prosome, macropain) activator subunit 3 (pa28 gamma; ki)  ELF1 - e74-like factor 1 (ets domain transcription factor)  ELF2 - e74-like factor 2 (ets domain transcription factor)  TCF25 - transcription factor 25 (basic helix-loop-helix)  MYO6 - myosin vi  ABT1 - activator of basal transcription 1  EFNA1 - ephrin-a1  CNOT1 - ccr4-not transcription complex, subunit 1  CTNNBIP1 - catenin, beta interacting protein 1  SPEN - spen homolog, transcriptional regulator (drosophila)  ZNF362 - zinc finger protein 362  BBX - bobby sox homolog (drosophila)  TP53BP1 - tumor protein p53 binding protein 1  HCFC2 - host cell factor c2  EGR1 - early growth response 1  ZNF292 - zinc finger protein 292  EGFR - epidermal growth factor receptor  CNOT7 - ccr4-not transcription complex, subunit 7  MEGF8 - multiple egf-like-domains 8  MXI1 - max interactor 1, dimerization protein  KDM1A - lysine (k)-specific demethylase 1a  ATXN7L3 - ataxin 7-like 3  ACTR2 - arp2 actin-related protein 2 homolog (yeast)  ACTR3 - arp3 actin-related protein 3 homolog (yeast)  NCOA6 - nuclear receptor coactivator 6  E2F6 - e2f transcription factor 6  E4F1 - e4f transcription factor 1  ZHX3 - zinc fingers and homeoboxes 3  E2F4 - e2f transcription factor 4, p107/p130-binding  E2F5 - e2f transcription factor 5, p130-binding  ENY2 - enhancer of yellow 2 homolog (drosophila)  TRIM37 - tripartite motif containing 37  RRP1B - ribosomal rna processing 1b  ZNF558 - zinc finger protein 558  EDNRB - endothelin receptor type b  ZNF263 - zinc finger protein 263  S1PR1 - sphingosine-1-phosphate receptor 1  PURG - purine-rich element binding protein g  NR2C1 - nuclear receptor subfamily 2, group c, member 1  SETX - senataxin  ZBTB47 - zinc finger and btb domain containing 47  NR2C2 - nuclear receptor subfamily 2, group c, member 2  MED25 - mediator complex subunit 25  TPR - translocated promoter region, nuclear basket protein  ZNF609 - zinc finger protein 609  MED6 - mediator complex subunit 6  PPRC1 - peroxisome proliferator-activated receptor gamma, coactivator-related 1  KDM4C - lysine (k)-specific demethylase 4c  ZNF423 - zinc finger protein 423  USF1 - upstream transcription factor 1  DMTF1 - cyclin d binding myb-like transcription factor 1  ZBTB43 - zinc finger and btb domain containing 43  ZBTB33 - zinc finger and btb domain containing 33  BPTF - bromodomain phd finger transcription factor  HDAC5 - histone deacetylase 5  MTF1 - metal-regulatory transcription factor 1  NR1H2 - nuclear receptor subfamily 1, group h, member 2  ETV3 - ets variant 3  CAMTA2 - calmodulin binding transcription activator 2  ETV5 - ets variant 5  TWSG1 - twisted gastrulation homolog 1 (drosophila)  POGZ - pogo transposable element with znf domain  MT3 - metallothionein 3  ETV6 - ets variant 6  PLXND1 - plexin d1  VHL - von hippel-lindau tumor suppressor, e3 ubiquitin protein ligase  MED16 - mediator complex subunit 16  KDM6B - lysine (k)-specific demethylase 6b  EZR - ezrin  TBX20 - t-box 20  KCTD1 - potassium channel tetramerization domain containing 1  CREB3L1 - camp responsive element binding protein 3-like 1  EZH1 - enhancer of zeste homolog 1 (drosophila)  CIC - capicua transcriptional repressor  CCNL1 - cyclin l1  WDR43 - wd repeat domain 43  ERF - ets2 repressor factor  ZNF575 - zinc finger protein 575  CITED1 - cbp/p300-interacting transactivator, with glu/asp-rich carboxy-terminal domain, 1  ZBTB5 - zinc finger and btb domain containing 5  ZBTB40 - zinc finger and btb domain containing 40  ELMSAN1 - elm2 and myb/sant-like domain containing 1  THAP11 - thap domain containing 11  ZNF248 - zinc finger protein 248  UBB - ubiquitin b  RGCC - regulator of cell cycle  RCOR1 - rest corepressor 1  ZNFX1 - zinc finger, nfx1-type containing 1  HIRA - histone cell cycle regulator  TTC5 - tetratricopeptide repeat domain 5  ETV1 - ets variant 1  ETS1 - v-ets avian erythroblastosis virus e26 oncogene homolog 1  ZMIZ1 - zinc finger, miz-type containing 1  USP3 - ubiquitin specific peptidase 3  UBP1 - upstream binding protein 1 (lbp-1a)  SUMO1 - small ubiquitin-like modifier 1  UBTF - upstream binding transcription factor, rna polymerase i  USP15 - ubiquitin specific peptidase 15  SMURF1 - smad specific e3 ubiquitin protein ligase 1  ZC3H4 - zinc finger ccch-type containing 4  MED4 - mediator complex subunit 4  JMJD6 - jumonji domain containing 6  UBE2I - ubiquitin-conjugating enzyme e2i  RBX1 - ring-box 1, e3 ubiquitin protein ligase  THRAP3 - thyroid hormone receptor associated protein 3  MED12 - mediator complex subunit 12  MED13 - mediator complex subunit 13  PSMD6 - proteasome (prosome, macropain) 26s subunit, non-atpase, 6  VPS72 - vacuolar protein sorting 72 homolog (s. cerevisiae)  MED24 - mediator complex subunit 24  MGA - mga, max dimerization protein  ZC3H6 - zinc finger ccch-type containing 6  WWC3 - wwc family member 3  ZEB1 - zinc finger e-box binding homeobox 1  ZNF34 - zinc finger protein 34  ORC2 - origin recognition complex, subunit 2  TCF12 - transcription factor 12  ZNF354A - zinc finger protein 354a  TOX4 - tox high mobility group box family member 4  ZNF436 - zinc finger protein 436  TCF20 - transcription factor 20 (ar1)  CAMTA1 - calmodulin binding transcription activator 1  FLI1 - fli-1 proto-oncogene, ets transcription factor  AGRN - agrin  FOXS1 - forkhead box s1  FOXO3 - forkhead box o3  HDAC8 - histone deacetylase 8  ASH1L - ash1 (absent, small, or homeotic)-like (drosophila)  SIN3B - sin3 transcription regulator family member b  NACC1 - nucleus accumbens associated 1, ben and btb (poz) domain containing  HR - hair growth associated  MED8 - mediator complex subunit 8  CAND1 - cullin-associated and neddylation-dissociated 1  TDG - thymine-dna glycosylase  EAPP - e2f-associated phosphoprotein  ZSCAN16 - zinc finger and scan domain containing 16  KDM3A - lysine (k)-specific demethylase 3a  FHL2 - four and a half lim domains 2  FGFR2 - fibroblast growth factor receptor 2  TAF9 - taf9 rna polymerase ii, tata box binding protein (tbp)-associated factor, 32kda  TAF7 - taf7 rna polymerase ii, tata box binding protein (tbp)-associated factor, 55kda  TAF6 - taf6 rna polymerase ii, tata box binding protein (tbp)-associated factor, 80kda  FGFR1 - fibroblast growth factor receptor 1  FGF9 - fibroblast growth factor 9  TADA2A - transcriptional adaptor 2a  NUMA1 - nuclear mitotic apparatus protein 1  DAB2IP - dab2 interacting protein  FGF1 - fibroblast growth factor 1 (acidic)  BCLAF1 - bcl2-associated transcription factor 1  FGF2 - fibroblast growth factor 2 (basic)  NR4A2 - nuclear receptor subfamily 4, group a, member 2  ZNF593 - zinc finger protein 593  ZBTB7B - zinc finger and btb domain containing 7b  TARBP1 - tar (hiv-1) rna binding protein 1  ZNF691 - zinc finger protein 691  CREBRF - creb3 regulatory factor  ZNF623 - zinc finger protein 623  ADNP - activity-dependent neuroprotector homeobox  NPM1 - nucleophosmin (nucleolar phosphoprotein b23, numatrin)  MED13L - mediator complex subunit 13-like  ZBTB24 - zinc finger and btb domain containing 24  ZEB2 - zinc finger e-box binding homeobox 2  THAP4 - thap domain containing 4  ZNF395 - zinc finger protein 395  MYNN - myoneurin  CRTC1 - creb regulated transcription coactivator 1  TCF3 - transcription factor 3  DMAP1 - dna methyltransferase 1 associated protein 1  TCEB3 - transcription elongation factor b (siii), polypeptide 3 (110kda, elongin a)  TCF4 - transcription factor 4  NKRF - nfkb repressing factor  FBN1 - fibrillin 1  TSC22D2 - tsc22 domain family, member 2  ZNF629 - zinc finger protein 629  TCEA2 - transcription elongation factor a (sii), 2  NRF1 - nuclear respiratory factor 1  NFXL1 - nuclear transcription factor, x-box binding-like 1  TCEA1 - transcription elongation factor a (sii), 1  NOP2 - nop2 nucleolar protein  VPRBP - vpr (hiv-1) binding protein  RYBP - ring1 and yy1 binding protein  PAXBP1 - pax3 and pax7 binding protein 1  NOTCH4 - notch 4  TLE4 - transducin-like enhancer of split 4 (e(sp1) homolog, drosophila)  IFT74 - intraflagellar transport 74 homolog (chlamydomonas)  TLR3 - toll-like receptor 3  NPAT - nuclear protein, ataxia-telangiectasia locus  CNOT2 - ccr4-not transcription complex, subunit 2  NOTCH1 - notch 1  SIRT1 - sirtuin 1  BAZ2B - bromodomain adjacent to zinc finger domain, 2b  MKX - mohawk homeobox  NOTCH3 - notch 3  ST18 - suppression of tumorigenicity 18 (breast carcinoma) (zinc finger protein)  NOTCH2 - notch 2  NFYA - nuclear transcription factor y, alpha  TMF1 - tata element modulatory factor 1  HDAC4 - histone deacetylase 4  TOX - thymocyte selection-associated high mobility group box  CBX6 - chromobox homolog 6  HEY1 - hairy/enhancer-of-split related with yrpw motif 1  NFYC - nuclear transcription factor y, gamma  ZFYVE16 - zinc finger, fyve domain containing 16  NFKBIA - nuclear factor of kappa light polypeptide gene enhancer in b-cells inhibitor, alpha  NFKB1 - nuclear factor of kappa light polypeptide gene enhancer in b-cells 1  CBX5 - chromobox homolog 5  BRD4 - bromodomain containing 4  BCL9L - b-cell cll/lymphoma 9-like  TNFRSF1A - tumor necrosis factor receptor superfamily, member 1a  NKX2-2 - nk2 homeobox 2  HDAC9 - histone deacetylase 9  ZNF606 - zinc finger protein 606  ZNF614 - zinc finger protein 614  ZFP2 - zfp2 zinc finger protein  TMBIM6 - transmembrane bax inhibitor motif containing 6  SUZ12 - suz12 polycomb repressive complex 2 subunit  NEO1 - neogenin 1  TEF - thyrotrophic embryonic factor  RCOR2 - rest corepressor 2  TFAM - transcription factor a, mitochondrial  SMARCAL1 - swi/snf related, matrix associated, actin dependent regulator of chromatin, subfamily a-like 1  NEDD8 - neural precursor cell expressed, developmentally down-regulated 8  CTR9 - ctr9, paf1/rna polymerase ii complex component  TFDP1 - transcription factor dp-1  NFE2L2 - nuclear factor (erythroid-derived 2)-like 2  EPC1 - enhancer of polycomb homolog 1 (drosophila)  NR2F2 - nuclear receptor subfamily 2, group f, member 2  CBX7 - chromobox homolog 7  TFE3 - transcription factor binding to ighm enhancer 3  MORF4L2 - mortality factor 4 like 2  NFATC4 - nuclear factor of activated t-cells, cytoplasmic, calcineurin-dependent 4  NR2F1 - nuclear receptor subfamily 2, group f, member 1  TFCP2 - transcription factor cp2  NFIA - nuclear factor i/a  ZNF740 - zinc finger protein 740  TGFA - transforming growth factor, alpha  FNIP1 - folliculin interacting protein 1  HEY2 - hairy/enhancer-of-split related with yrpw motif 2  CDK5RAP3 - cdk5 regulatory subunit associated protein 3  TGFB2 - transforming growth factor, beta 2  TGFB3 - transforming growth factor, beta 3  TGFB1 - transforming growth factor, beta 1  ZNF746 - zinc finger protein 746  MEPCE - methylphosphate capping enzyme  TGFBR3 - transforming growth factor, beta receptor iii  FOSL2 - fos-like antigen 2  FOSB - fbj murine osteosarcoma viral oncogene homolog b  PHF14 - phd finger protein 14  FOS - fbj murine osteosarcoma viral oncogene homolog  KAT6B - k(lysine) acetyltransferase 6b  ZNF432 - zinc finger protein 432  SAFB2 - scaffold attachment factor b2  MYEF2 - myelin expression factor 2  THRA - thyroid hormone receptor, alpha  LPIN2 - lipin 2 |
| GO:1902850 | microtubule cytoskeleton organization involved in mitosis | 1.82E-6 | 3.01E-4 | 1.35 (10334,71,6996,65) | [+] Show genes  CDK5RAP2 - cdk5 regulatory subunit associated protein 2  MSTO1 - misato 1, mitochondrial distribution and morphology regulator  XIAP - x-linked inhibitor of apoptosis  GOLGA2 - golgin a2  SMC3 - structural maintenance of chromosomes 3  KIF2A - kinesin heavy chain member 2a  EML1 - echinoderm microtubule associated protein like 1  PLK2 - polo-like kinase 2  SUN2 - sad1 and unc84 domain containing 2  DCTN1 - dynactin 1  FLNA - filamin a, alpha  PIBF1 - progesterone immunomodulatory binding factor 1  TNKS - tankyrase, trf1-interacting ankyrin-related adp-ribose polymerase  MCPH1 - microcephalin 1  SPAST - spastin  CLASP2 - cytoplasmic linker associated protein 2  TUBG1 - tubulin, gamma 1  SBDS - shwachman-bodian-diamond syndrome  NDEL1 - nude neurodevelopment protein 1-like 1  KPNB1 - karyopherin (importin) beta 1  KIFC2 - kinesin family member c2  RMDN1 - regulator of microtubule dynamics 1  TUBG2 - tubulin, gamma 2  PAFAH1B1 - platelet-activating factor acetylhydrolase 1b, regulatory subunit 1 (45kda)  UBXN2B - ubx domain protein 2b  GJA1 - gap junction protein, alpha 1, 43kda  FBXW11 - f-box and wd repeat domain containing 11  AURKC - aurora kinase c  VCP - valosin containing protein  DYNLT1 - dynein, light chain, tctex-type 1  NDE1 - nude neurodevelopment protein 1  CKAP5 - cytoskeleton associated protein 5  DCTN2 - dynactin 2 (p50)  TACC1 - transforming, acidic coiled-coil containing protein 1  WRAP73 - wd repeat containing, antisense to tp73  MAP9 - microtubule-associated protein 9  NSFL1C - nsfl1 (p97) cofactor (p47)  MECP2 - methyl cpg binding protein 2 (rett syndrome)  NUMA1 - nuclear mitotic apparatus protein 1  ARHGEF10 - rho guanine nucleotide exchange factor (gef) 10  CLASP1 - cytoplasmic linker associated protein 1  AAAS - achalasia, adrenocortical insufficiency, alacrimia  RHOA - ras homolog family member a  ZW10 - zw10 kinetochore protein  DCTN6 - dynactin 6  BCCIP - brca2 and cdkn1a interacting protein  GPSM2 - g-protein signaling modulator 2  KIF3B - kinesin family member 3b  ITGB1 - integrin, beta 1 (fibronectin receptor, beta polypeptide, antigen cd29 includes mdf2, msk12)  EFHC1 - ef-hand domain (c-terminal) containing 1  SMC1A - structural maintenance of chromosomes 1a  DYNC1H1 - dynein, cytoplasmic 1, heavy chain 1  STMN1 - stathmin 1  RAB11A - rab11a, member ras oncogene family  SPRY1 - sprouty homolog 1, antagonist of fgf signaling (drosophila)  SPRY2 - sprouty homolog 2 (drosophila)  STAG2 - stromal antigen 2  TBCE - tubulin folding cofactor e  SPICE1 - spindle and centriole associated protein 1  ARHGEF2 - rho/rac guanine nucleotide exchange factor (gef) 2  SETD2 - set domain containing 2  MZT1 - mitotic spindle organizing protein 1  HTT - huntingtin  STAG1 - stromal antigen 1  ZNF207 - zinc finger protein 207 |
| GO:0007018 | microtubule-based movement | 1.98E-6 | 3.24E-4 | 1.25 (10334,149,6996,126) | [+] Show genes  DST - dystonin  KIFAP3 - kinesin-associated protein 3  AP3M2 - adaptor-related protein complex 3, mu 2 subunit  WDR19 - wd repeat domain 19  IFT46 - intraflagellar transport 46 homolog (chlamydomonas)  KIF21A - kinesin family member 21a  RACGAP1 - rac gtpase activating protein 1  RHOT2 - ras homolog family member t2  SSX2IP - synovial sarcoma, x breakpoint 2 interacting protein  SPG7 - spastic paraplegia 7 (pure and complicated autosomal recessive)  KATNB1 - katanin p80 (wd repeat containing) subunit b 1  WDR34 - wd repeat domain 34  APBA1 - amyloid beta (a4) precursor protein-binding, family a, member 1  ACTR10 - actin-related protein 10 homolog (s. cerevisiae)  SPAST - spastin  KIF27 - kinesin family member 27  TUB - tubby bipartite transcription factor  AP3B2 - adaptor-related protein complex 3, beta 2 subunit  DPCD - deleted in primary ciliary dyskinesia homolog (mouse)  SYBU - syntabulin (syntaxin-interacting)  AP3B1 - adaptor-related protein complex 3, beta 1 subunit  TTC30A - tetratricopeptide repeat domain 30a  BICD2 - bicaudal d homolog 2 (drosophila)  NDEL1 - nude neurodevelopment protein 1-like 1  KIF13B - kinesin family member 13b  MAP1S - microtubule-associated protein 1s  PAFAH1B1 - platelet-activating factor acetylhydrolase 1b, regulatory subunit 1 (45kda)  AZI1 - 5-azacytidine induced 1  ARL8B - adp-ribosylation factor-like 8b  FBXW11 - f-box and wd repeat domain containing 11  KIF16B - kinesin family member 16b  APP - amyloid beta (a4) precursor protein  OPA1 - optic atrophy 1 (autosomal dominant)  IFT43 - intraflagellar transport 43 homolog (chlamydomonas)  NDE1 - nude neurodevelopment protein 1  ARL3 - adp-ribosylation factor-like 3  RAB21 - rab21, member ras oncogene family  IFT57 - intraflagellar transport 57 homolog (chlamydomonas)  KTN1 - kinectin 1 (kinesin receptor)  ARL8A - adp-ribosylation factor-like 8a  TMEM201 - transmembrane protein 201  CELSR2 - cadherin, egf lag seven-pass g-type receptor 2  AP3S2 - adaptor-related protein complex 3, sigma 2 subunit  ACTR2 - arp2 actin-related protein 2 homolog (yeast)  ACTR3 - arp3 actin-related protein 3 homolog (yeast)  TMEM108 - transmembrane protein 108  TEKT2 - tektin 2 (testicular)  WDR60 - wd repeat domain 60  SPA17 - sperm autoantigenic protein 17  CLUAP1 - clusterin associated protein 1  TRAK2 - trafficking protein, kinesin binding 2  PCM1 - pericentriolar material 1  RABL2B - rab, member of ras oncogene family-like 2b  LRPPRC - leucine-rich pentatricopeptide repeat containing  AP3D1 - adaptor-related protein complex 3, delta 1 subunit  IFT52 - intraflagellar transport 52 homolog (chlamydomonas)  HTT - huntingtin  WASF1 - was protein family, member 1  HSPA8 - heat shock 70kda protein 8  BLOC1S3 - biogenesis of lysosomal organelles complex-1, subunit 3  PURA - purine-rich element binding protein a  TTC21B - tetratricopeptide repeat domain 21b  SPG11 - spastic paraplegia 11 (autosomal recessive)  KIFC3 - kinesin family member c3  KIF5C - kinesin family member 5c  TRAF3IP1 - tnf receptor-associated factor 3 interacting protein 1  KIF3C - kinesin family member 3c  KIF2A - kinesin heavy chain member 2a  KIF5B - kinesin family member 5b  KIF1B - kinesin family member 1b  KIF5A - kinesin family member 5a  BLOC1S2 - biogenesis of lysosomal organelles complex-1, subunit 2  IFT74 - intraflagellar transport 74 homolog (chlamydomonas)  SUN2 - sad1 and unc84 domain containing 2  MAPT - microtubule-associated protein tau  KIF3A - kinesin family member 3a  BLOC1S4 - biogenesis of lysosomal organelles complex-1, subunit 4, cappuccino  DYNLL1 - dynein, light chain, lc8-type 1  MAP1B - microtubule-associated protein 1b  MAP1A - microtubule-associated protein 1a  DYNC1LI1 - dynein, cytoplasmic 1, light intermediate chain 1  RAB6A - rab6a, member ras oncogene family  DYNLRB2 - dynein, light chain, roadblock-type 2  DYNLRB1 - dynein, light chain, roadblock-type 1  PEX14 - peroxisomal biogenesis factor 14  STK36 - serine/threonine kinase 36  TNPO1 - transportin 1  MGARP - mitochondria-localized glutamic acid-rich protein  BBS12 - bardet-biedl syndrome 12  RAB1A - rab1a, member ras oncogene family  ARHGAP21 - rho gtpase activating protein 21  KPNB1 - karyopherin (importin) beta 1  ICK - intestinal cell (mak-like) kinase  KIFC2 - kinesin family member c2  KLC1 - kinesin light chain 1  IFT140 - intraflagellar transport 140 homolog (chlamydomonas)  IFT122 - intraflagellar transport 122 homolog (chlamydomonas)  NME5 - nme/nm23 family member 5  HIF1A - hypoxia inducible factor 1, alpha subunit (basic helix-loop-helix transcription factor)  MAPK8IP3 - mitogen-activated protein kinase 8 interacting protein 3  COPG2 - coatomer protein complex, subunit gamma 2  RSPH9 - radial spoke head 9 homolog (chlamydomonas)  NEFL - neurofilament, light polypeptide  BLOC1S6 - biogenesis of lysosomal organelles complex-1, subunit 6, pallidin  COPG1 - coatomer protein complex, subunit gamma 1  AP3M1 - adaptor-related protein complex 3, mu 1 subunit  IFT27 - intraflagellar transport 27 homolog (chlamydomonas)  DYNC2LI1 - dynein, cytoplasmic 2, light intermediate chain 1  TERF2 - telomeric repeat binding factor 2  UBB - ubiquitin b  HNRNPU - heterogeneous nuclear ribonucleoprotein u (scaffold attachment factor a)  IFT81 - intraflagellar transport 81 homolog (chlamydomonas)  KIF1A - kinesin family member 1a  KIF3B - kinesin family member 3b  IFT88 - intraflagellar transport 88 homolog (chlamydomonas)  SFPQ - splicing factor proline/glutamine-rich  DYNC1H1 - dynein, cytoplasmic 1, heavy chain 1  DYNC1I1 - dynein, cytoplasmic 1, intermediate chain 1  DYNC1I2 - dynein, cytoplasmic 1, intermediate chain 2  BLOC1S5 - biogenesis of lysosomal organelles complex-1, subunit 5, muted  DYNC1LI2 - dynein, cytoplasmic 1, light intermediate chain 2  UCHL1 - ubiquitin carboxyl-terminal esterase l1 (ubiquitin thiolesterase)  KLC2 - kinesin light chain 2  RHOT1 - ras homolog family member t1  TTLL1 - tubulin tyrosine ligase-like family, member 1  IFT172 - intraflagellar transport 172 homolog (chlamydomonas) |
| GO:0120039 | plasma membrane bounded cell projection morphogenesis | 2.2E-6 | 3.56E-4 | 1.24 (10334,157,6996,132) | [+] Show genes  AFG3L2 - afg3 atpase family member 3-like 2 (s. cerevisiae)  CTTN - cortactin  SPTBN4 - spectrin, beta, non-erythrocytic 4  ATP8A2 - atpase, aminophospholipid transporter, class i, type 8a, member 2  NRP1 - neuropilin 1  PLXNB1 - plexin b1  FBXO45 - f-box protein 45  SPAST - spastin  ARC - activity-regulated cytoskeleton-associated protein  SHANK1 - sh3 and multiple ankyrin repeat domains 1  WASF2 - was protein family, member 2  DBN1 - drebrin 1  ILK - integrin-linked kinase  APBB1 - amyloid beta (a4) precursor protein-binding, family b, member 1 (fe65)  NUMBL - numb homolog (drosophila)-like  HPRT1 - hypoxanthine phosphoribosyltransferase 1  BRSK1 - br serine/threonine kinase 1  NDEL1 - nude neurodevelopment protein 1-like 1  MAP1S - microtubule-associated protein 1s  ACTB - actin, beta  PAFAH1B1 - platelet-activating factor acetylhydrolase 1b, regulatory subunit 1 (45kda)  GJA1 - gap junction protein, alpha 1, 43kda  APP - amyloid beta (a4) precursor protein  CREB1 - camp responsive element binding protein 1  SNX2 - sorting nexin 2  DOCK10 - dedicator of cytokinesis 10  SNX1 - sorting nexin 1  DYNLT1 - dynein, light chain, tctex-type 1  PAK3 - p21 protein (cdc42/rac)-activated kinase 3  GAS7 - growth arrest-specific 7  BMP7 - bone morphogenetic protein 7  LLGL1 - lethal giant larvae homolog 1 (drosophila)  FGFR2 - fibroblast growth factor receptor 2  TCTN1 - tectonic family member 1  FLOT1 - flotillin 1  ANKRD27 - ankyrin repeat domain 27 (vps9 domain)  PICALM - phosphatidylinositol binding clathrin assembly protein  AMIGO1 - adhesion molecule with ig-like domain 1  PTEN - phosphatase and tensin homolog  ITGA1 - integrin, alpha 1  RAB10 - rab10, member ras oncogene family  DAB2IP - dab2 interacting protein  MEF2A - myocyte enhancer factor 2a  TOP2B - topoisomerase (dna) ii beta 180kda  EGFR - epidermal growth factor receptor  ADARB1 - adenosine deaminase, rna-specific, b1  SZT2 - seizure threshold 2 homolog (mouse)  NR4A2 - nuclear receptor subfamily 4, group a, member 2  RHOA - ras homolog family member a  CELSR2 - cadherin, egf lag seven-pass g-type receptor 2  ATL1 - atlastin gtpase 1  ITGB1 - integrin, beta 1 (fibronectin receptor, beta polypeptide, antigen cd29 includes mdf2, msk12)  CNP - 2',3'-cyclic nucleotide 3' phosphodiesterase  ADCY1 - adenylate cyclase 1 (brain)  DCLK1 - doublecortin-like kinase 1  PTK2 - protein tyrosine kinase 2  CDK5 - cyclin-dependent kinase 5  RAB8A - rab8a, member ras oncogene family  ROCK1 - rho-associated, coiled-coil containing protein kinase 1  S100B - s100 calcium binding protein b  PTN - pleiotrophin  STMN1 - stathmin 1  TRAK2 - trafficking protein, kinesin binding 2  KIDINS220 - kinase d-interacting substrate, 220kda  TNIK - traf2 and nck interacting kinase  ZEB2 - zinc finger e-box binding homeobox 2  SLC11A2 - solute carrier family 11 (proton-coupled divalent metal ion transporter), member 2  KLF7 - kruppel-like factor 7 (ubiquitous)  HSP90AA1 - heat shock protein 90kda alpha (cytosolic), class a member 1  CNTN4 - contactin 4  MYCBP2 - myc binding protein 2, e3 ubiquitin protein ligase  TAOK3 - tao kinase 3  CDH11 - cadherin 11, type 2, ob-cadherin (osteoblast)  NPTX1 - neuronal pentraxin i  WASF1 - was protein family, member 1  NRCAM - neuronal cell adhesion molecule  NCKAP1 - nck-associated protein 1  ABI1 - abl-interactor 1  CHRNB2 - cholinergic receptor, nicotinic, beta 2 (neuronal)  DRD2 - dopamine receptor d2  BTBD3 - btb (poz) domain containing 3  TMEM106B - transmembrane protein 106b  ZNF365 - zinc finger protein 365  NUMB - numb homolog (drosophila)  TAOK1 - tao kinase 1  SHANK3 - sh3 and multiple ankyrin repeat domains 3  LRRK2 - leucine-rich repeat kinase 2  MAP6 - microtubule-associated protein 6  MAP2 - microtubule-associated protein 2  RERE - arginine-glutamic acid dipeptide (re) repeats  MAP1B - microtubule-associated protein 1b  MAP1A - microtubule-associated protein 1a  CTNND2 - catenin (cadherin-associated protein), delta 2  PACSIN1 - protein kinase c and casein kinase substrate in neurons 1  BAIAP2 - bai1-associated protein 2  CTNNA2 - catenin (cadherin-associated protein), alpha 2  MAP4K4 - mitogen-activated protein kinase kinase kinase kinase 4  HDAC6 - histone deacetylase 6  NYAP1 - neuronal tyrosine-phosphorylated phosphoinositide-3-kinase adaptor 1  WEE1 - wee1 homolog (s. pombe)  ATP7A - atpase, cu++ transporting, alpha polypeptide  RAB3A - rab3a, member ras oncogene family  SLC9A6 - solute carrier family 9, subfamily a (nhe6, cation proton antiporter 6), member 6  LMTK2 - lemur tyrosine kinase 2  EPB41L3 - erythrocyte membrane protein band 4.1-like 3  DOCK7 - dedicator of cytokinesis 7  FYN - fyn oncogene related to src, fgr, yes  SLC25A46 - solute carrier family 25, member 46  PITPNA - phosphatidylinositol transfer protein, alpha  NEK3 - nima-related kinase 3  DLG4 - discs, large homolog 4 (drosophila)  MINK1 - misshapen-like kinase 1  NEFL - neurofilament, light polypeptide  DBNL - drebrin-like  UBB - ubiquitin b  PHACTR1 - phosphatase and actin regulator 1  FAM206A - family with sequence similarity 206, member a  RBFOX2 - rna binding protein, fox-1 homolog (c. elegans) 2  DNM2 - dynamin 2  MAPK8IP2 - mitogen-activated protein kinase 8 interacting protein 2  ARHGAP35 - rho gtpase activating protein 35  ZNF335 - zinc finger protein 335  ALS2 - amyotrophic lateral sclerosis 2 (juvenile)  EPHB6 - eph receptor b6  ZSWIM6 - zinc finger, swim-type containing 6  BCL2 - b-cell cll/lymphoma 2  TAOK2 - tao kinase 2  SGK1 - serum/glucocorticoid regulated kinase 1  B4GALT6 - udp-gal:betaglcnac beta 1,4- galactosyltransferase, polypeptide 6  PLEKHO1 - pleckstrin homology domain containing, family o member 1  WASL - wiskott-aldrich syndrome-like  B4GALT5 - udp-gal:betaglcnac beta 1,4- galactosyltransferase, polypeptide 5 |
| GO:0006139 | nucleobase-containing compound metabolic process | 2.2E-6 | 3.52E-4 | 1.06 (10334,2078,6996,1494) | [+] Show genes  ELL - elongation factor rna polymerase ii  GFPT1 - glutamine--fructose-6-phosphate transaminase 1  EXOSC9 - exosome component 9  EXOSC10 - exosome component 10  PMS2 - pms2 postmeiotic segregation increased 2 (s. cerevisiae)  ZCCHC8 - zinc finger, cchc domain containing 8  SYMPK - symplekin  RNPC3 - rna-binding region (rnp1, rrm) containing 3  SF3A3 - splicing factor 3a, subunit 3, 60kda  SCD5 - stearoyl-coa desaturase 5  HNRNPA0 - heterogeneous nuclear ribonucleoprotein a0  AARS - alanyl-trna synthetase  PNN - pinin, desmosome associated protein  SF3A2 - splicing factor 3a, subunit 2, 66kda  ABL1 - c-abl oncogene 1, non-receptor tyrosine kinase  CNOT11 - ccr4-not transcription complex, subunit 11  OBFC1 - oligonucleotide/oligosaccharide-binding fold containing 1  ACOT2 - acyl-coa thioesterase 2  ACACA - acetyl-coa carboxylase alpha  PMM1 - phosphomannomutase 1  ELOVL7 - elovl fatty acid elongase 7  EBNA1BP2 - ebna1 binding protein 2  NAGK - n-acetylglucosamine kinase  PMS1 - pms1 postmeiotic segregation increased 1 (s. cerevisiae)  ASCC3 - activating signal cointegrator 1 complex subunit 3  FTSJ3 - ftsj homolog 3 (e. coli)  PANK2 - pantothenate kinase 2  PNRC2 - proline-rich nuclear receptor coactivator 2  TXNL4A - thioredoxin-like 4a  ACLY - atp citrate lyase  NOP9 - nop9 nucleolar protein  CHD7 - chromodomain helicase dna binding protein 7  PLRG1 - pleiotropic regulator 1  LYAR - ly1 antibody reactive  PAPOLA - poly(a) polymerase alpha  PABPN1 - poly(a) binding protein, nuclear 1  TCERG1 - transcription elongation regulator 1  TAF15 - taf15 rna polymerase ii, tata box binding protein (tbp)-associated factor, 68kda  RNPS1 - rna binding protein s1, serine-rich domain  OTUB1 - otu domain, ubiquitin aldehyde binding 1  EHMT2 - euchromatic histone-lysine n-methyltransferase 2  SPIN1 - spindlin 1  MORF4L1 - mortality factor 4 like 1  THUMPD1 - thump domain containing 1  ACTL6A - actin-like 6a  TRRAP - transformation/transcription domain-associated protein  MCMBP - minichromosome maintenance complex binding protein  PPIA - peptidylprolyl isomerase a (cyclophilin a)  TRMT10B - trna methyltransferase 10 homolog b (s. cerevisiae)  ADAR - adenosine deaminase, rna-specific  ADARB1 - adenosine deaminase, rna-specific, b1  WDR3 - wd repeat domain 3  ACTR5 - arp5 actin-related protein 5 homolog (yeast)  CPSF4 - cleavage and polyadenylation specific factor 4, 30kda  ADCY6 - adenylate cyclase 6  CDC45 - cell division cycle 45  ADCY5 - adenylate cyclase 5  MED1 - mediator complex subunit 1  AK8 - adenylate kinase 8  PPAT - phosphoribosyl pyrophosphate amidotransferase  ADCY1 - adenylate cyclase 1 (brain)  AXIN2 - axin 2  PPA1 - pyrophosphatase (inorganic) 1  CDC7 - cell division cycle 7  ADCY3 - adenylate cyclase 3  PPARD - peroxisome proliferator-activated receptor delta  SMC1A - structural maintenance of chromosomes 1a  POLRMT - polymerase (rna) mitochondrial (dna directed)  RBM10 - rna binding motif protein 10  MAML3 - mastermind-like 3 (drosophila)  POLR2K - polymerase (rna) ii (dna directed) polypeptide k, 7.0kda  TSEN15 - tsen15 trna splicing endonuclease subunit  GNAS - gnas complex locus  GNAI3 - guanine nucleotide binding protein (g protein), alpha inhibiting activity polypeptide 3  PARP1 - poly (adp-ribose) polymerase 1  POLH - polymerase (dna directed), eta  PPP1R13L - protein phosphatase 1, regulatory subunit 13 like  NOP10 - nop10 ribonucleoprotein  POLD2 - polymerase (dna directed), delta 2, accessory subunit  POLD1 - polymerase (dna directed), delta 1, catalytic subunit  NOL10 - nucleolar protein 10  POLR2G - polymerase (rna) ii (dna directed) polypeptide g  ELAC1 - elac ribonuclease z 1  POLR2F - polymerase (rna) ii (dna directed) polypeptide f  POLR2E - polymerase (rna) ii (dna directed) polypeptide e, 25kda  SMPD3 - sphingomyelin phosphodiesterase 3, neutral membrane (neutral sphingomyelinase ii)  POLR2D - polymerase (rna) ii (dna directed) polypeptide d  POLR2C - polymerase (rna) ii (dna directed) polypeptide c, 33kda  POLR2B - polymerase (rna) ii (dna directed) polypeptide b, 140kda  RUVBL2 - ruvb-like 2 (e. coli)  PARP4 - poly (adp-ribose) polymerase family, member 4  POLR2A - polymerase (rna) ii (dna directed) polypeptide a, 220kda  HNRNPUL1 - heterogeneous nuclear ribonucleoprotein u-like 1  OGDHL - oxoglutarate dehydrogenase-like  RPP14 - ribonuclease p/mrp 14kda subunit  INTS9 - integrator complex subunit 9  KRR1 - krr1, small subunit (ssu) processome component, homolog (yeast)  PRKACA - protein kinase, camp-dependent, catalytic, alpha  NELFE - negative elongation factor complex member e  EHMT1 - euchromatic histone-lysine n-methyltransferase 1  WDR12 - wd repeat domain 12  NR5A1 - nuclear receptor subfamily 5, group a, member 1  ADSS - adenylosuccinate synthase  PRIM2 - primase, dna, polypeptide 2 (58kda)  FUS - fused in sarcoma  PRIM1 - primase, dna, polypeptide 1 (49kda)  FRG1 - fshd region gene 1  METTL8 - methyltransferase like 8  PRCC - papillary renal cell carcinoma (translocation-associated)  POLR3A - polymerase (rna) iii (dna directed) polypeptide a, 155kda  CLASRP - clk4-associating serine/arginine rich protein  GEMIN6 - gem (nuclear organelle) associated protein 6  PRDM4 - pr domain containing 4  N4BP2 - nedd4 binding protein 2  ATF7IP - activating transcription factor 7 interacting protein  PPP5C - protein phosphatase 5, catalytic subunit  UHRF2 - ubiquitin-like with phd and ring finger domains 2, e3 ubiquitin protein ligase  PPT1 - palmitoyl-protein thioesterase 1  SDE2 - sde2 telomere maintenance homolog (s. pombe)  CSGALNACT1 - chondroitin sulfate n-acetylgalactosaminyltransferase 1  USP7 - ubiquitin specific peptidase 7 (herpes virus-associated)  SNRNP35 - small nuclear ribonucleoprotein 35kda (u11/u12)  PCID2 - pci domain containing 2  RNF168 - ring finger protein 168, e3 ubiquitin protein ligase  XRCC6 - x-ray repair complementing defective repair in chinese hamster cells 6  PPP2R2A - protein phosphatase 2, regulatory subunit b, alpha  CMTR2 - cap methyltransferase 2  SLBP - stem-loop binding protein  RIOK2 - rio kinase 2  PPP2R1A - protein phosphatase 2, regulatory subunit a, alpha  CPSF6 - cleavage and polyadenylation specific factor 6, 68kda  NUDT21 - nudix (nucleoside diphosphate linked moiety x)-type motif 21  DIS3L - dis3 mitotic control homolog (s. cerevisiae)-like  DDX52 - dead (asp-glu-ala-asp) box polypeptide 52  DDX51 - dead (asp-glu-ala-asp) box polypeptide 51  PPP1R8 - protein phosphatase 1, regulatory subunit 8  RBFA - ribosome binding factor a (putative)  PPP2CA - protein phosphatase 2, catalytic subunit, alpha isozyme  CPSF7 - cleavage and polyadenylation specific factor 7, 59kda  FUT8 - fucosyltransferase 8 (alpha (1,6) fucosyltransferase)  DIDO1 - death inducer-obliterator 1  CBLL1 - cbl proto-oncogene-like 1, e3 ubiquitin protein ligase  NUDT18 - nudix (nucleoside diphosphate linked moiety x)-type motif 18  TOPBP1 - topoisomerase (dna) ii binding protein 1  RMI2 - recq mediated genome instability 2  RPAP2 - rna polymerase ii associated protein 2  DCAKD - dephospho-coa kinase domain containing  G6PD - glucose-6-phosphate dehydrogenase  HSF2BP - heat shock transcription factor 2 binding protein  DDX39B - dead (asp-glu-ala-asp) box polypeptide 39b  PRPSAP2 - phosphoribosyl pyrophosphate synthetase-associated protein 2  PRPSAP1 - phosphoribosyl pyrophosphate synthetase-associated protein 1  IWS1 - iws1 homolog (s. cerevisiae)  TDRKH - tudor and kh domain containing  PRPS2 - phosphoribosyl pyrophosphate synthetase 2  PRPS1 - phosphoribosyl pyrophosphate synthetase 1  NARS2 - asparaginyl-trna synthetase 2, mitochondrial (putative)  NPLOC4 - nuclear protein localization 4 homolog (s. cerevisiae)  ALDOC - aldolase c, fructose-bisphosphate  ADAL - adenosine deaminase-like  AMD1 - adenosylmethionine decarboxylase 1  PRPF40A - prp40 pre-mrna processing factor 40 homolog a (s. cerevisiae)  PAPD7 - pap associated domain containing 7  DDX27 - dead (asp-glu-ala-asp) box polypeptide 27  CDK2AP1 - cyclin-dependent kinase 2 associated protein 1  INTS8 - integrator complex subunit 8  SNORA8 - small nucleolar rna, h/aca box 8  SNIP1 - smad nuclear interacting protein 1  TSR1 - tsr1, 20s rrna accumulation, homolog (s. cerevisiae)  POLR3E - polymerase (rna) iii (dna directed) polypeptide e (80kd)  GEMIN7 - gem (nuclear organelle) associated protein 7  MAPK3 - mitogen-activated protein kinase 3  MAPK1 - mitogen-activated protein kinase 1  COPS6 - cop9 signalosome subunit 6  FAR2 - fatty acyl coa reductase 2  AMPD2 - adenosine monophosphate deaminase 2  CLP1 - cleavage and polyadenylation factor i subunit 1  CCDC88A - coiled-coil domain containing 88a  NT5C3B - 5'-nucleotidase, cytosolic iiib  CCDC94 - coiled-coil domain containing 94  POLR3B - polymerase (rna) iii (dna directed) polypeptide b  NR4A3 - nuclear receptor subfamily 4, group a, member 3  RBM22 - rna binding motif protein 22  NSUN5 - nop2/sun domain family, member 5  GART - phosphoribosylglycinamide formyltransferase, phosphoribosylglycinamide synthetase, phosphoribosylaminoimidazole synthetase  DEDD2 - death effector domain containing 2  SF3B2 - splicing factor 3b, subunit 2, 145kda  GARS - glycyl-trna synthetase  FASTKD1 - fast kinase domains 1  APEX1 - apex nuclease (multifunctional dna repair enzyme) 1  DHX36 - deah (asp-glu-ala-his) box polypeptide 36  MTPAP - mitochondrial poly(a) polymerase  RBM23 - rna binding motif protein 23  SMC6 - structural maintenance of chromosomes 6  DALRD3 - dalr anticodon binding domain containing 3  PARN - poly(a)-specific ribonuclease  GSR - glutathione reductase  GSPT1 - g1 to s phase transition 1  RFWD3 - ring finger and wd repeat domain 3  TREX1 - three prime repair exonuclease 1  DARS2 - aspartyl-trna synthetase 2, mitochondrial  ZCCHC6 - zinc finger, cchc domain containing 6  RNF113A - ring finger protein 113a  GTF2F1 - general transcription factor iif, polypeptide 1, 74kda  GTF2F2 - general transcription factor iif, polypeptide 2, 30kda  PRMT6 - protein arginine methyltransferase 6  GTF2E1 - general transcription factor iie, polypeptide 1, alpha 56kda  GTF2E2 - general transcription factor iie, polypeptide 2, beta 34kda  GTF2H2 - general transcription factor iih, polypeptide 2, 44kda  NEIL1 - nei endonuclease viii-like 1 (e. coli)  GTF2H3 - general transcription factor iih, polypeptide 3, 34kda  INTS10 - integrator complex subunit 10  PCBP2 - poly(rc) binding protein 2  GTF2H1 - general transcription factor iih, polypeptide 1, 62kda  PANK3 - pantothenate kinase 3  NUDT2 - nudix (nucleoside diphosphate linked moiety x)-type motif 2  GTF2A2 - general transcription factor iia, 2, 12kda  SMG8 - smg8 nonsense mediated mrna decay factor  GTF2B - general transcription factor iib  MSH6 - muts homolog 6 (e. coli)  USB1 - u6 snrna biogenesis 1  GTF2A1 - general transcription factor iia, 1, 19/37kda  APBB1 - amyloid beta (a4) precursor protein-binding, family b, member 1 (fe65)  GTF3C2 - general transcription factor iiic, polypeptide 2, beta 110kda  RIF1 - rap1 interacting factor homolog (yeast)  PPCS - phosphopantothenoylcysteine synthetase  GUCY1B3 - guanylate cyclase 1, soluble, beta 3  GUCY1A3 - guanylate cyclase 1, soluble, alpha 3  POLG2 - polymerase (dna directed), gamma 2, accessory subunit  GTF2I - general transcription factor iii  GTF2H4 - general transcription factor iih, polypeptide 4, 52kda  PRKRIP1 - prkr interacting protein 1 (il11 inducible)  GTF3A - general transcription factor iiia  PA2G4 - proliferation-associated 2g4, 38kda  NOL9 - nucleolar protein 9  BRF1 - brf1, rna polymerase iii transcription initiation factor 90 kda subunit  RPRD1A - regulation of nuclear pre-mrna domain containing 1a  CNTD1 - cyclin n-terminal domain containing 1  GTF3C1 - general transcription factor iiic, polypeptide 1, alpha 220kda  MAP1S - microtubule-associated protein 1s  POLI - polymerase (dna directed) iota  APP - amyloid beta (a4) precursor protein  DDX20 - dead (asp-glu-ala-asp) box polypeptide 20  CELF3 - cugbp, elav-like family member 3  PDE9A - phosphodiesterase 9a  H2AFX - h2a histone family, member x  DCP2 - decapping mrna 2  SNORA43 - small nucleolar rna, h/aca box 43  PAPD4 - pap associated domain containing 4  PAX8 - paired box 8  NSMCE2 - non-smc element 2, mms21 homolog (s. cerevisiae)  SUPT16H - suppressor of ty 16 homolog (s. cerevisiae)  ARRB2 - arrestin, beta 2  ARRB1 - arrestin, beta 1  CDC73 - cell division cycle 73  GPATCH1 - g patch domain containing 1  SAMD4B - sterile alpha motif domain containing 4b  RHOA - ras homolog family member a  HARS - histidyl-trna synthetase  BAZ2A - bromodomain adjacent to zinc finger domain, 2a  PCNA - proliferating cell nuclear antigen  LSM6 - lsm6 homolog, u6 small nuclear rna associated (s. cerevisiae)  MAGOHB - mago-nashi homolog b (drosophila)  NUDT4 - nudix (nucleoside diphosphate linked moiety x)-type motif 4  MTFMT - mitochondrial methionyl-trna formyltransferase  HEATR1 - heat repeat containing 1  TARSL2 - threonyl-trna synthetase-like 2  LEO1 - leo1, paf1/rna polymerase ii complex component, homolog (s. cerevisiae)  RPL7L1 - ribosomal protein l7-like 1  FANCL - fanconi anemia, complementation group l  PRPF38B - prp38 pre-mrna processing factor 38 (yeast) domain containing b  SRBD1 - s1 rna binding domain 1  PWP1 - pwp1 homolog (s. cerevisiae)  WRAP53 - wd repeat containing, antisense to tp53  PDE2A - phosphodiesterase 2a, cgmp-stimulated  SNRNP25 - small nuclear ribonucleoprotein 25kda (u11/u12)  RBM28 - rna binding motif protein 28  THAP1 - thap domain containing, apoptosis associated protein 1  HDAC1 - histone deacetylase 1  PGBD5 - piggybac transposable element derived 5  SNORA11 - small nucleolar rna, h/aca box 11  ELP3 - elongator acetyltransferase complex subunit 3  KAT7 - k(lysine) acetyltransferase 7  PGM3 - phosphoglucomutase 3  RFK - riboflavin kinase  XPA - xeroderma pigmentosum, complementation group a  ZBTB1 - zinc finger and btb domain containing 1  XPC - xeroderma pigmentosum, complementation group c  PGAM1 - phosphoglycerate mutase 1 (brain)  ZNF365 - zinc finger protein 365  PGD - phosphogluconate dehydrogenase  XRCC1 - x-ray repair complementing defective repair in chinese hamster cells 1  PAGR1 - paxip1 associated glutamate-rich protein 1  ATP1B1 - atpase, na+/k+ transporting, beta 1 polypeptide  DIS3 - dis3 mitotic control homolog (s. cerevisiae)  SNORD86 - small nucleolar rna, c/d box 86  ZHX2 - zinc fingers and homeoboxes 2  ATIC - 5-aminoimidazole-4-carboxamide ribonucleotide formyltransferase/imp cyclohydrolase  LRRK2 - leucine-rich repeat kinase 2  AP5S1 - adaptor-related protein complex 5, sigma 1 subunit  GPD2 - glycerol-3-phosphate dehydrogenase 2 (mitochondrial)  XBP1 - x-box binding protein 1  LARP6 - la ribonucleoprotein domain family, member 6  ENPP4 - ectonucleotide pyrophosphatase/phosphodiesterase 4 (putative)  WHSC1 - wolf-hirschhorn syndrome candidate 1  NELFA - negative elongation factor complex member a  ATP6V1A - atpase, h+ transporting, lysosomal 70kda, v1 subunit a  ZFC3H1 - zinc finger, c3h1-type containing  SNORD12 - small nucleolar rna, c/d box 12  ATP6V1B2 - atpase, h+ transporting, lysosomal 56/58kda, v1 subunit b2  ALKBH2 - alkb, alkylation repair homolog 2 (e. coli)  WDR33 - wd repeat domain 33  PFKL - phosphofructokinase, liver  DCP1B - decapping mrna 1b  PFKP - phosphofructokinase, platelet  PFAS - phosphoribosylformylglycinamidine synthase  WARS - tryptophanyl-trna synthetase  SMU1 - smu-1 suppressor of mec-8 and unc-52 homolog (c. elegans)  BAX - bcl2-associated x protein  SCAF8 - sr-related ctd-associated factor 8  PUF60 - poly-u binding splicing factor 60kda  PANK4 - pantothenate kinase 4  THOC6 - tho complex 6 homolog (drosophila)  RAVER2 - ribonucleoprotein, ptb-binding 2  NAT10 - n-acetyltransferase 10 (gcn5-related)  BACH1 - btb and cnc homology 1, basic leucine zipper transcription factor 1  BAD - bcl2-associated agonist of cell death  ZNF45 - zinc finger protein 45  DPH3 - diphthamide biosynthesis 3  BRCC3 - brca1/brca2-containing complex, subunit 3  EXD2 - exonuclease 3'-5' domain containing 2  TYW1 - trna-yw synthesizing protein 1 homolog (s. cerevisiae)  ASXL2 - additional sex combs like 2 (drosophila)  XRN2 - 5'-3' exoribonuclease 2  ELP2 - elongator acetyltransferase complex subunit 2  ASXL1 - additional sex combs like 1 (drosophila)  ATRX - alpha thalassemia/mental retardation syndrome x-linked  PKNOX1 - pbx/knotted 1 homeobox 1  CASC3 - cancer susceptibility candidate 3  PKM - pyruvate kinase, muscle  PSTK - phosphoseryl-trna kinase  EXOSC8 - exosome component 8  PIH1D2 - pih1 domain containing 2  IMP3 - imp3, u3 small nucleolar ribonucleoprotein, homolog (yeast)  SF1 - splicing factor 1  PSPC1 - paraspeckle component 1  U2AF2 - u2 small nuclear rna auxiliary factor 2  NUDT15 - nudix (nucleoside diphosphate linked moiety x)-type motif 15  ACOT7 - acyl-coa thioesterase 7  RPUSD3 - rna pseudouridylate synthase domain containing 3  DDX42 - dead (asp-glu-ala-asp) box helicase 42  BRF2 - brf2, rna polymerase iii transcription initiation factor 50 kda subunit  UBE2W - ubiquitin-conjugating enzyme e2w (putative)  XRCC5 - x-ray repair complementing defective repair in chinese hamster cells 5 (double-strand-break rejoining)  XRCC4 - x-ray repair complementing defective repair in chinese hamster cells 4  ECD - ecdysoneless homolog (drosophila)  POLR3H - polymerase (rna) iii (dna directed) polypeptide h (22.9kd)  GRSF1 - g-rich rna sequence binding factor 1  CWF19L1 - cwf19-like 1, cell cycle control (s. pombe)  CCND1 - cyclin d1  GSK3A - glycogen synthase kinase 3 alpha  PARK7 - parkinson protein 7  PNKP - polynucleotide kinase 3'-phosphatase  PGM2 - phosphoglucomutase 2  QRSL1 - glutaminyl-trna synthase (glutamine-hydrolyzing)-like 1  CCNK - cyclin k  CDKAL1 - cdk5 regulatory subunit associated protein 1-like 1  SMNDC1 - survival motor neuron domain containing 1  BCAS2 - breast carcinoma amplified sequence 2  RFC5 - replication factor c (activator 1) 5, 36.5kda  CWC27 - cwc27 spliceosome-associated protein homolog (s. cerevisiae)  RFC2 - replication factor c (activator 1) 2, 40kda  SAP18 - sin3a-associated protein, 18kda  RFC3 - replication factor c (activator 1) 3, 38kda  TRIM24 - tripartite motif containing 24  TAF1D - tata box binding protein (tbp)-associated factor, rna polymerase i, d, 41kda  SNORD24 - small nucleolar rna, c/d box 24  SF3A1 - splicing factor 3a, subunit 1, 120kda  CWC25 - cwc25 spliceosome-associated protein homolog (s. cerevisiae)  RELA - v-rel avian reticuloendotheliosis viral oncogene homolog a  TEX10 - testis expressed 10  SNAPC5 - small nuclear rna activating complex, polypeptide 5, 19kda  SNORD55 - small nucleolar rna, c/d box 55  RECQL - recq protein-like (dna helicase q1-like)  ZFP36L2 - zfp36 ring finger protein-like 2  ZFP36L1 - zfp36 ring finger protein-like 1  ALKBH5 - alkb, alkylation repair homolog 5 (e. coli)  UPF1 - upf1 regulator of nonsense transcripts homolog (yeast)  NSUN2 - nop2/sun rna methyltransferase family, member 2  METTL16 - methyltransferase like 16  ALKBH1 - alkb, alkylation repair homolog 1 (e. coli)  FTO - fat mass and obesity associated  RLF - rearranged l-myc fusion  HPRT1 - hypoxanthine phosphoribosyltransferase 1  ELP6 - elongator acetyltransferase complex subunit 6  NOC4L - nucleolar complex associated 4 homolog (s. cerevisiae)  FBLL1 - fibrillarin-like 1  TATDN3 - tatd dnase domain containing 3  PCGF3 - polycomb group ring finger 3  LDB1 - lim domain binding 1  DSCC1 - dna replication and sister chromatid cohesion 1  WARS2 - tryptophanyl trna synthetase 2, mitochondrial  KAT2B - k(lysine) acetyltransferase 2b  ELOVL6 - elovl fatty acid elongase 6  TSC22D1 - tsc22 domain family, member 1  WDR55 - wd repeat domain 55  PSMD14 - proteasome (prosome, macropain) 26s subunit, non-atpase, 14  ZNF259 - zinc finger protein 259  GIN1 - gypsy retrotransposon integrase 1  SCNM1 - sodium channel modifier 1  C1orf86 - chromosome 1 open reading frame 86  IARS - isoleucyl-trna synthetase  PRPF4B - prp4 pre-mrna processing factor 4 homolog b (yeast)  TRIT1 - trna isopentenyltransferase 1  NABP2 - nucleic acid binding protein 2  HUS1 - hus1 checkpoint homolog (s. pombe)  TSEN34 - tsen34 trna splicing endonuclease subunit  RNASE6 - ribonuclease, rnase a family, k6  U2AF1L4 - u2 small nuclear rna auxiliary factor 1-like 4  HNRNPR - heterogeneous nuclear ribonucleoprotein r  ERI3 - eri1 exoribonuclease family member 3  BUD31 - bud31 homolog (s. cerevisiae)  GPHN - gephyrin  GATAD2A - gata zinc finger domain containing 2a  HERC2 - hect and rld domain containing e3 ubiquitin protein ligase 2  RNF111 - ring finger protein 111  BYSL - bystin-like  SRRM1 - serine/arginine repetitive matrix 1  TET2 - tet methylcytosine dioxygenase 2  HSPD1 - heat shock 60kda protein 1 (chaperonin)  NSMCE1 - non-smc element 1 homolog (s. cerevisiae)  MED18 - mediator complex subunit 18  TYW3 - trna-yw synthesizing protein 3 homolog (s. cerevisiae)  FUBP3 - far upstream element (fuse) binding protein 3  SF3B4 - splicing factor 3b, subunit 4, 49kda  HSP90AA1 - heat shock protein 90kda alpha (cytosolic), class a member 1  C7orf49 - chromosome 7 open reading frame 49  FUK - fucokinase  ZMPSTE24 - zinc metallopeptidase ste24  MBD4 - methyl-cpg binding domain protein 4  STUB1 - stip1 homology and u-box containing protein 1, e3 ubiquitin protein ligase  C5AR1 - complement component 5a receptor 1  TCEANC2 - transcription elongation factor a (sii) n-terminal and central domain containing 2  HSPA8 - heat shock 70kda protein 8  NADK - nad kinase  PPIE - peptidylprolyl isomerase e (cyclophilin e)  MCRS1 - microspherule protein 1  HMGN1 - high mobility group nucleosome binding domain 1  HMGB3 - high mobility group box 3  HMGB2 - high mobility group box 2  RPL17 - ribosomal protein l17  HMGB1 - high mobility group box 1  RPL15 - ribosomal protein l15  C1D - c1d nuclear receptor corepressor  TRMT12 - trna methyltransferase 12 homolog (s. cerevisiae)  RPL11 - ribosomal protein l11  RPL9 - ribosomal protein l9  NOL8 - nucleolar protein 8  RPL7 - ribosomal protein l7  INTS6 - integrator complex subunit 6  PPIH - peptidylprolyl isomerase h (cyclophilin h)  SLC30A9 - solute carrier family 30 (zinc transporter), member 9  CASP3 - caspase 3, apoptosis-related cysteine peptidase  RPL3 - ribosomal protein l3  AGO1 - argonaute risc catalytic component 1  CARS - cysteinyl-trna synthetase  RPA3 - replication protein a3, 14kda  MAD2L2 - mad2 mitotic arrest deficient-like 2 (yeast)  RPA1 - replication protein a1, 70kda  RPA2 - replication protein a2, 32kda  RPS4X - ribosomal protein s4, x-linked  ZBTB48 - zinc finger and btb domain containing 48  CAD - carbamoyl-phosphate synthetase 2, aspartate transcarbamylase, and dihydroorotase  SRSF9 - serine/arginine-rich splicing factor 9  RPS4Y1 - ribosomal protein s4, y-linked 1  HK1 - hexokinase 1  MRPL1 - mitochondrial ribosomal protein l1  ACD - adrenocortical dysplasia homolog (mouse)  GPBP1 - gc-rich promoter binding protein 1  NOL6 - nucleolar protein 6 (rna-associated)  KRI1 - kri1 homolog (s. cerevisiae)  DGAT1 - diacylglycerol o-acyltransferase 1  MRPL44 - mitochondrial ribosomal protein l44  NR2C2AP - nuclear receptor 2c2-associated protein  SYNCRIP - synaptotagmin binding, cytoplasmic rna interacting protein  HINT1 - histidine triad nucleotide binding protein 1  INTS3 - integrator complex subunit 3  RPL32 - ribosomal protein l32  ELP4 - elongator acetyltransferase complex subunit 4  UPF3B - upf3 regulator of nonsense transcripts homolog b (yeast)  UPF3A - upf3 regulator of nonsense transcripts homolog a (yeast)  RPS23 - ribosomal protein s23  PAPOLG - poly(a) polymerase gamma  RAVER1 - ribonucleoprotein, ptb-binding 1  CCNT2 - cyclin t2  PINX1 - pin2/terf1 interacting, telomerase inhibitor 1  BPNT1 - 3'(2'), 5'-bisphosphate nucleotidase 1  NMRK1 - nicotinamide riboside kinase 1  AK5 - adenylate kinase 5  NPM3 - nucleophosmin/nucleoplasmin 3  EED - embryonic ectoderm development  INO80C - ino80 complex subunit c  THG1L - trna-histidine guanylyltransferase 1-like (s. cerevisiae)  CCNT1 - cyclin t1  RNGTT - rna guanylyltransferase and 5'-phosphatase  CCNH - cyclin h  RNMT - rna (guanine-7-) methyltransferase  HNRNPU - heterogeneous nuclear ribonucleoprotein u (scaffold attachment factor a)  SWSAP1 - swim-type zinc finger 7 associated protein 1  HNRNPK - heterogeneous nuclear ribonucleoprotein k  HNRNPL - heterogeneous nuclear ribonucleoprotein l  RPS15 - ribosomal protein s15  RPS12 - ribosomal protein s12  TIPIN - timeless interacting protein  FBXO6 - f-box protein 6  MRPS11 - mitochondrial ribosomal protein s11  TINF2 - terf1 (trf1)-interacting nuclear factor 2  PARP16 - poly (adp-ribose) polymerase family, member 16  CBFA2T3 - core-binding factor, runt domain, alpha subunit 2; translocated to, 3  CDS2 - cdp-diacylglycerol synthase (phosphatidate cytidylyltransferase) 2  CBFB - core-binding factor, beta subunit  NSA2 - nsa2 ribosome biogenesis homolog (s. cerevisiae)  ELOVL1 - elovl fatty acid elongase 1  HNRNPA2B1 - heterogeneous nuclear ribonucleoprotein a2/b1  HNRNPA1 - heterogeneous nuclear ribonucleoprotein a1  HNRNPC - heterogeneous nuclear ribonucleoprotein c (c1/c2)  HNRNPF - heterogeneous nuclear ribonucleoprotein f  RXRB - retinoid x receptor, beta  HNRNPD - heterogeneous nuclear ribonucleoprotein d (au-rich element rna binding protein 1, 37kda)  HNRNPH1 - heterogeneous nuclear ribonucleoprotein h1 (h)  GNPNAT1 - glucosamine-phosphate n-acetyltransferase 1  ADPRHL2 - adp-ribosylhydrolase like 2  HNRNPH3 - heterogeneous nuclear ribonucleoprotein h3 (2h9)  HNRNPH2 - heterogeneous nuclear ribonucleoprotein h2 (h')  SARS2 - seryl-trna synthetase 2, mitochondrial  ZSWIM7 - zinc finger, swim-type containing 7  HMGCR - 3-hydroxy-3-methylglutaryl-coa reductase  TUT1 - terminal uridylyl transferase 1, u6 snrna-specific  HMGCS1 - 3-hydroxy-3-methylglutaryl-coa synthase 1 (soluble)  IMPAD1 - inositol monophosphatase domain containing 1  CCNC - cyclin c  HMGA1 - high mobility group at-hook 1  TRMT10C - trna methyltransferase 10 homolog c (s. cerevisiae)  PTPLB - protein tyrosine phosphatase-like (proline instead of catalytic arginine), member b  PRMT5 - protein arginine methyltransferase 5  NR4A1 - nuclear receptor subfamily 4, group a, member 1  NABP1 - nucleic acid binding protein 1  PDE12 - phosphodiesterase 12  CD2BP2 - cd2 (cytoplasmic tail) binding protein 2  CHTF8 - ctf8, chromosome transmission fidelity factor 8 homolog (s. cerevisiae)  RRM1 - ribonucleotide reductase m1  CDIPT - cdp-diacylglycerol--inositol 3-phosphatidyltransferase  RBM27 - rna binding motif protein 27  IKBKAP - inhibitor of kappa light polypeptide gene enhancer in b-cells, kinase complex-associated protein  FOXK2 - forkhead box k2  HTATSF1 - hiv-1 tat specific factor 1  PAICS - phosphoribosylaminoimidazole carboxylase, phosphoribosylaminoimidazole succinocarboxamide synthetase  ZNF638 - zinc finger protein 638  CDC5L - cell division cycle 5-like  TBL3 - transducin (beta)-like 3  RBM15 - rna binding motif protein 15  POLR3F - polymerase (rna) iii (dna directed) polypeptide f, 39 kda  RBMX - rna binding motif protein, x-linked  HNRNPA3 - heterogeneous nuclear ribonucleoprotein a3  PARG - poly (adp-ribose) glycohydrolase  PPM1D - protein phosphatase, mg2+/mn2+ dependent, 1d  IMPDH1 - imp (inosine 5'-monophosphate) dehydrogenase 1  POLL - polymerase (dna directed), lambda  IMPDH2 - imp (inosine 5'-monophosphate) dehydrogenase 2  SRSF12 - serine/arginine-rich splicing factor 12  PRPF8 - pre-mrna processing factor 8  PRPF19 - pre-mrna processing factor 19  UTP20 - utp20, small subunit (ssu) processome component, homolog (yeast)  CDC14B - cell division cycle 14b  ENTPD6 - ectonucleoside triphosphate diphosphohydrolase 6 (putative)  NANS - n-acetylneuraminic acid synthase  WDR36 - wd repeat domain 36  KHDRBS3 - kh domain containing, rna binding, signal transduction associated 3  PATL1 - protein associated with topoisomerase ii homolog 1 (yeast)  ADAT2 - adenosine deaminase, trna-specific 2  LMO4 - lim domain only 4  ENTPD3 - ectonucleoside triphosphate diphosphohydrolase 3  TAF6L - taf6-like rna polymerase ii, p300/cbp-associated factor (pcaf)-associated factor, 65kda  COPS7B - cop9 signalosome subunit 7b  POLR3G - polymerase (rna) iii (dna directed) polypeptide g (32kd)  COPS3 - cop9 signalosome subunit 3  GAR1 - gar1 ribonucleoprotein  TERF2IP - telomeric repeat binding factor 2, interacting protein  ORAOV1 - oral cancer overexpressed 1  RNASEH2A - ribonuclease h2, subunit a  PRKRA - protein kinase, interferon-inducible double stranded rna dependent activator  NADK2 - nad kinase 2, mitochondrial  PTBP1 - polypyrimidine tract binding protein 1  CETN2 - centrin, ef-hand protein, 2  PTEN - phosphatase and tensin homolog  JMY - junction mediating and regulatory protein, p53 cofactor  YARS - tyrosyl-trna synthetase  ZBTB8OS - zinc finger and btb domain containing 8 opposite strand  RRP1 - ribosomal rna processing 1  KHSRP - kh-type splicing regulatory protein  CIB1 - calcium and integrin binding 1 (calmyrin)  PTGIS - prostaglandin i2 (prostacyclin) synthase  CHRAC1 - chromatin accessibility complex 1  CASK - calcium/calmodulin-dependent serine protein kinase (maguk family)  DDX17 - dead (asp-glu-ala-asp) box helicase 17  POLR1E - polymerase (rna) i polypeptide e, 53kda  CHERP - calcium homeostasis endoplasmic reticulum protein  PRPF18 - pre-mrna processing factor 18  KAT5 - k(lysine) acetyltransferase 5  GPKOW - g patch domain and kow motifs  EIF6 - eukaryotic translation initiation factor 6  CEBPG - ccaat/enhancer binding protein (c/ebp), gamma  NOP56 - nop56 ribonucleoprotein  SLU7 - slu7 splicing factor homolog (s. cerevisiae)  CDK4 - cyclin-dependent kinase 4  PTMA - prothymosin, alpha  CDK9 - cyclin-dependent kinase 9  RNASET2 - ribonuclease t2  CDK8 - cyclin-dependent kinase 8  RTCA - rna 3'-terminal phosphate cyclase  CDK7 - cyclin-dependent kinase 7  PDE8B - phosphodiesterase 8b  CDK13 - cyclin-dependent kinase 13  GNPDA2 - glucosamine-6-phosphate deaminase 2  LSM3 - lsm3 homolog, u6 small nuclear rna associated (s. cerevisiae)  PCBP3 - poly(rc) binding protein 3  CDKN2D - cyclin-dependent kinase inhibitor 2d (p19, inhibits cdk4)  CSDC2 - cold shock domain containing c2, rna binding  APEX2 - apex nuclease (apurinic/apyrimidinic endonuclease) 2  RUVBL1 - ruvb-like 1 (e. coli)  RPP38 - ribonuclease p/mrp 38kda subunit  AGPAT1 - 1-acylglycerol-3-phosphate o-acyltransferase 1  MOCS3 - molybdenum cofactor synthesis 3  DIMT1 - dim1 dimethyladenosine transferase 1 homolog (s. cerevisiae)  CRCP - cgrp receptor component  CDK2 - cyclin-dependent kinase 2  NOP14 - nop14 nucleolar protein  TAMM41 - tam41, mitochondrial translocator assembly and maintenance protein, homolog (s. cerevisiae)  WDR4 - wd repeat domain 4  RAD1 - rad1 homolog (s. pombe)  RRN3 - rrn3 rna polymerase i transcription factor homolog (s. cerevisiae)  PURA - purine-rich element binding protein a  GTPBP2 - gtp binding protein 2  LRWD1 - leucine-rich repeats and wd repeat domain containing 1  ZNHIT6 - zinc finger, hit-type containing 6  METTL6 - methyltransferase like 6  PAPD5 - pap associated domain containing 5  NUDT16 - nudix (nucleoside diphosphate linked moiety x)-type motif 16  POP4 - processing of precursor 4, ribonuclease p/mrp subunit (s. cerevisiae)  PDE7B - phosphodiesterase 7b  SRSF10 - serine/arginine-rich splicing factor 10  AFF4 - af4/fmr2 family, member 4  AHCYL1 - adenosylhomocysteinase-like 1  MMS19 - mms19 nucleotide excision repair homolog (s. cerevisiae)  MTERFD2 - mterf domain containing 2  OSGEPL1 - o-sialoglycoprotein endopeptidase-like 1  QARS - glutaminyl-trna synthetase  METTL2A - methyltransferase like 2a  RRAGC - ras-related gtp binding c  RAD21 - rad21 homolog (s. pombe)  FOXK1 - forkhead box k1  RAD17 - rad17 homolog (s. pombe)  PAF1 - paf1, rna polymerase ii associated factor, homolog (s. cerevisiae)  RPP40 - ribonuclease p/mrp 40kda subunit  NME5 - nme/nm23 family member 5  INO80 - ino80 complex subunit  DDX56 - dead (asp-glu-ala-asp) box helicase 56  IGHMBP2 - immunoglobulin mu binding protein 2  RC3H2 - ring finger and ccch-type domains 2  RBPJ - recombination signal binding protein for immunoglobulin kappa j region  OARD1 - o-acyl-adp-ribose deacylase 1  PABPC1 - poly(a) binding protein, cytoplasmic 1  TRUB2 - trub pseudouridine (psi) synthase homolog 2 (e. coli)  FAM35A - family with sequence similarity 35, member a  DUSP11 - dual specificity phosphatase 11 (rna/rnp complex 1-interacting)  RARG - retinoic acid receptor, gamma  RBM26 - rna binding motif protein 26  RARB - retinoic acid receptor, beta  RARS - arginyl-trna synthetase  CELF1 - cugbp, elav-like family member 1  KHDRBS1 - kh domain containing, rna binding, signal transduction associated 1  RBKS - ribokinase  TAF8 - taf8 rna polymerase ii, tata box binding protein (tbp)-associated factor, 43kda  CELF2 - cugbp, elav-like family member 2  MLH3 - mutl homolog 3 (e. coli)  IK - ik cytokine, down-regulator of hla ii  MCCC2 - methylcrotonoyl-coa carboxylase 2 (beta)  CTCF - ccctc-binding factor (zinc finger protein)  DDX49 - dead (asp-glu-ala-asp) box polypeptide 49  PRMT7 - protein arginine methyltransferase 7  RBBP7 - retinoblastoma binding protein 7  SMARCA5 - swi/snf related, matrix associated, actin dependent regulator of chromatin, subfamily a, member 5  RBBP8 - retinoblastoma binding protein 8  RBMS1 - rna binding motif, single stranded interacting protein 1  CMPK2 - cytidine monophosphate (ump-cmp) kinase 2, mitochondrial  RBM3 - rna binding motif (rnp1, rrm) protein 3  DIS3L2 - dis3 mitotic control homolog (s. cerevisiae)-like 2  RBM4 - rna binding motif protein 4  DIEXF - digestive organ expansion factor homolog (zebrafish)  PELP1 - proline, glutamate and leucine rich protein 1  ARID4A - at rich interactive domain 4a (rbp1-like)  GEMIN2 - gem (nuclear organelle) associated protein 2  TYW5 - trna-yw synthesizing protein 5  RBBP6 - retinoblastoma binding protein 6  SND1 - staphylococcal nuclease and tudor domain containing 1  DGCR8 - dgcr8 microprocessor complex subunit  RBBP4 - retinoblastoma binding protein 4  CUL4B - cullin 4b  ERCC8 - excision repair cross-complementing rodent repair deficiency, complementation group 8  PUS7 - pseudouridylate synthase 7 homolog (s. cerevisiae)  DHX16 - deah (asp-glu-ala-his) box polypeptide 16  EXOSC4 - exosome component 4  ANKRD16 - ankyrin repeat domain 16  PCIF1 - pdx1 c-terminal inhibiting factor 1  PTGES3 - prostaglandin e synthase 3 (cytosolic)  NFAT5 - nuclear factor of activated t-cells 5, tonicity-responsive  TPST2 - tyrosylprotein sulfotransferase 2  POLD3 - polymerase (dna-directed), delta 3, accessory subunit  USP39 - ubiquitin specific peptidase 39  SUPT3H - suppressor of ty 3 homolog (s. cerevisiae)  TPST1 - tyrosylprotein sulfotransferase 1  RSRC1 - arginine/serine-rich coiled-coil 1  PCGF5 - polycomb group ring finger 5  WTAP - wilms tumor 1 associated protein  ENTPD4 - ectonucleoside triphosphate diphosphohydrolase 4  RBM39 - rna binding motif protein 39  LIG4 - ligase iv, dna, atp-dependent  TBRG1 - transforming growth factor beta regulator 1  MKI67IP - mki67 (fha domain) interacting nucleolar phosphoprotein  FBXO18 - f-box protein, helicase, 18  METTL14 - methyltransferase like 14  LIG1 - ligase i, dna, atp-dependent  LIG3 - ligase iii, dna, atp-dependent  VPS9D1 - vps9 domain containing 1  COX7A2 - cytochrome c oxidase subunit viia polypeptide 2 (liver)  CIRH1A - cirrhosis, autosomal recessive 1a (cirhin)  DOT1L - dot1-like histone h3k79 methyltransferase  CWC22 - cwc22 spliceosome-associated protein homolog (s. cerevisiae)  UAP1 - udp-n-acteylglucosamine pyrophosphorylase 1  SP1 - sp1 transcription factor  CIR1 - corepressor interacting with rbpj, 1  MUM1 - melanoma associated antigen (mutated) 1  RPP25L - ribonuclease p/mrp 25kda subunit-like  SLX4 - slx4 structure-specific endonuclease subunit  CHD1L - chromodomain helicase dna binding protein 1-like  GTPBP4 - gtp binding protein 4  LSM2 - lsm2 homolog, u6 small nuclear rna associated (s. cerevisiae)  GMPR2 - guanosine monophosphate reductase 2  ACOT9 - acyl-coa thioesterase 9  GTF2IRD1 - gtf2i repeat domain containing 1  ORC3 - origin recognition complex, subunit 3  GTPBP1 - gtp binding protein 1  MAGI3 - membrane associated guanylate kinase, ww and pdz domain containing 3  SON - son dna binding protein  ORC6 - origin recognition complex, subunit 6  H6PD - hexose-6-phosphate dehydrogenase (glucose 1-dehydrogenase)  CREBL2 - camp responsive element binding protein-like 2  CREBBP - creb binding protein  ZC3H8 - zinc finger ccch-type containing 8  SUGP1 - surp and g patch domain containing 1  CREB1 - camp responsive element binding protein 1  PRPF38A - prp38 pre-mrna processing factor 38 (yeast) domain containing a  SMUG1 - single-strand-selective monofunctional uracil-dna glycosylase 1  SNRPN - small nuclear ribonucleoprotein polypeptide n  PRKAG2 - protein kinase, amp-activated, gamma 2 non-catalytic subunit  SNRPB2 - small nuclear ribonucleoprotein polypeptide b  POLK - polymerase (dna directed) kappa  RPRD1B - regulation of nuclear pre-mrna domain containing 1b  SNRPD1 - small nuclear ribonucleoprotein d1 polypeptide 16kda  DDX41 - dead (asp-glu-ala-asp) box polypeptide 41  INIP - ints3 and nabp interacting protein  SNRPF - small nuclear ribonucleoprotein polypeptide f  SNAPC4 - small nuclear rna activating complex, polypeptide 4, 190kda  TBPL1 - tbp-like 1  YTHDF2 - yth domain family, member 2  SNRNP70 - small nuclear ribonucleoprotein 70kda (u1)  MAK16 - mak16 homolog (s. cerevisiae)  SNRPA - small nuclear ribonucleoprotein polypeptide a  EEF1E1 - eukaryotic translation elongation factor 1 epsilon 1  RNF138 - ring finger protein 138, e3 ubiquitin protein ligase  SNRPA1 - small nuclear ribonucleoprotein polypeptide a'  SNRPB - small nuclear ribonucleoprotein polypeptides b and b1  SNAPC2 - small nuclear rna activating complex, polypeptide 2, 45kda  SNAPC1 - small nuclear rna activating complex, polypeptide 1, 43kda  SNAPC3 - small nuclear rna activating complex, polypeptide 3, 50kda  POLR1C - polymerase (rna) i polypeptide c, 30kda  SMN1 - survival of motor neuron 1, telomeric  RBM17 - rna binding motif protein 17  CNP - 2',3'-cyclic nucleotide 3' phosphodiesterase  HEMK1 - hemk methyltransferase family member 1  TP53I3 - tumor protein p53 inducible protein 3  SMARCB1 - swi/snf related, matrix associated, actin dependent regulator of chromatin, subfamily b, member 1  UBR5 - ubiquitin protein ligase e3 component n-recognin 5  LSM5 - lsm5 homolog, u6 small nuclear rna associated (s. cerevisiae)  SMARCD3 - swi/snf related, matrix associated, actin dependent regulator of chromatin, subfamily d, member 3  MED20 - mediator complex subunit 20  UCHL5 - ubiquitin carboxyl-terminal hydrolase l5  COL4A2 - collagen, type iv, alpha 2  RBM25 - rna binding motif protein 25  SMARCA1 - swi/snf related, matrix associated, actin dependent regulator of chromatin, subfamily a, member 1  PLD3 - phospholipase d family, member 3  EDC4 - enhancer of mrna decapping 4  CACTIN - cactin, spliceosome c complex subunit  TAOK3 - tao kinase 3  FZR1 - fizzy/cell division cycle 20 related 1 (drosophila)  ZBTB7A - zinc finger and btb domain containing 7a  CRNKL1 - crooked neck pre-mrna splicing factor-like 1 (drosophila)  UPRT - uracil phosphoribosyltransferase (fur1) homolog (s. cerevisiae)  CDC40 - cell division cycle 40  SCAF1 - sr-related ctd-associated factor 1  MED27 - mediator complex subunit 27  MED26 - mediator complex subunit 26  TFIP11 - tuftelin interacting protein 11  CSTF1 - cleavage stimulation factor, 3' pre-rna, subunit 1, 50kda  POLR1B - polymerase (rna) i polypeptide b, 128kda  FTSJ1 - ftsj rna methyltransferase homolog 1 (e. coli)  DBR1 - debranching rna lariats 1  MED17 - mediator complex subunit 17  MED23 - mediator complex subunit 23  PRPF40B - prp40 pre-mrna processing factor 40 homolog b (s. cerevisiae)  MRTO4 - mrna turnover 4 homolog (s. cerevisiae)  CSTF3 - cleavage stimulation factor, 3' pre-rna, subunit 3, 77kda  RPF2 - ribosome production factor 2 homolog (s. cerevisiae)  CSTF2 - cleavage stimulation factor, 3' pre-rna, subunit 2, 64kda  PRPF6 - pre-mrna processing factor 6  CTGF - connective tissue growth factor  SUPT6H - suppressor of ty 6 homolog (s. cerevisiae)  SUPV3L1 - suppressor of var1, 3-like 1 (s. cerevisiae)  PICK1 - protein interacting with prkca 1  HSD17B12 - hydroxysteroid (17-beta) dehydrogenase 12  SUPT4H1 - suppressor of ty 4 homolog 1 (s. cerevisiae)  ING4 - inhibitor of growth family, member 4  SUPT5H - suppressor of ty 5 homolog (s. cerevisiae)  GTPBP3 - gtp binding protein 3 (mitochondrial)  CTPS1 - ctp synthase 1  UBL5 - ubiquitin-like 5  FAR1 - fatty acyl coa reductase 1  TRMT11 - trna methyltransferase 11 homolog (s. cerevisiae)  RECQL5 - recq protein-like 5  ELOVL5 - elovl fatty acid elongase 5  KPNA2 - karyopherin alpha 2 (rag cohort 1, importin alpha 1)  KPNB1 - karyopherin (importin) beta 1  SBDS - shwachman-bodian-diamond syndrome  KPNA1 - karyopherin alpha 1 (importin alpha 5)  SUV420H1 - suppressor of variegation 4-20 homolog 1 (drosophila)  ELOVL4 - elovl fatty acid elongase 4  LACTB2 - lactamase, beta 2  DDX23 - dead (asp-glu-ala-asp) box polypeptide 23  GPBP1L1 - gc-rich promoter binding protein 1-like 1  TFB1M - transcription factor b1, mitochondrial  TJP2 - tight junction protein 2  MED10 - mediator complex subunit 10  MED21 - mediator complex subunit 21  PAN3 - pan3 poly(a) specific ribonuclease subunit homolog (s. cerevisiae)  SUV420H2 - suppressor of variegation 4-20 homolog 2 (drosophila)  SNRNP40 - small nuclear ribonucleoprotein 40kda (u5)  ZRANB2 - zinc finger, ran-binding domain containing 2  RPAIN - rpa interacting protein  LONP1 - lon peptidase 1, mitochondrial  SULT4A1 - sulfotransferase family 4a, member 1  POLDIP3 - polymerase (dna-directed), delta interacting protein 3  CRMP1 - collapsin response mediator protein 1  AGPAT9 - 1-acylglycerol-3-phosphate o-acyltransferase 9  NIPBL - nipped-b homolog (drosophila)  BUD13 - bud13 homolog (s. cerevisiae)  STAT3 - signal transducer and activator of transcription 3 (acute-phase response factor)  ASF1A - anti-silencing function 1a histone chaperone  PPIG - peptidylprolyl isomerase g (cyclophilin g)  POLR3GL - polymerase (rna) iii (dna directed) polypeptide g (32kd)-like  SSRP1 - structure specific recognition protein 1  ING5 - inhibitor of growth family, member 5  SLC25A33 - solute carrier family 25 (pyrimidine nucleotide carrier), member 33  BOD1L1 - biorientation of chromosomes in cell division 1-like 1  DNAJC30 - dnaj (hsp40) homolog, subfamily c, member 30  ITPA - inosine triphosphatase (nucleoside triphosphate pyrophosphatase)  PPT2 - palmitoyl-protein thioesterase 2  DHX35 - deah (asp-glu-ala-his) box polypeptide 35  PHF5A - phd finger protein 5a  TRIP12 - thyroid hormone receptor interactor 12  GTF3C5 - general transcription factor iiic, polypeptide 5, 63kda  NUDT16L1 - nudix (nucleoside diphosphate linked moiety x)-type motif 16-like 1  ZNHIT3 - zinc finger, hit-type containing 3  SRPK2 - srsf protein kinase 2  SRPK1 - srsf protein kinase 1  PGLS - 6-phosphogluconolactonase  UTP23 - utp23, small subunit (ssu) processome component, homolog (yeast)  TROVE2 - trove domain family, member 2  COPS2 - cop9 signalosome subunit 2  DDX47 - dead (asp-glu-ala-asp) box polypeptide 47  SSBP1 - single-stranded dna binding protein 1, mitochondrial  SSB - sjogren syndrome antigen b (autoantigen la)  ACBD6 - acyl-coa binding domain containing 6  THOC3 - tho complex 3  EFTUD2 - elongation factor tu gtp binding domain containing 2  SARNP - sap domain containing ribonucleoprotein  LEF1 - lymphoid enhancer-binding factor 1  GTF3C4 - general transcription factor iiic, polypeptide 4, 90kda  THADA - thyroid adenoma associated  GTF3C3 - general transcription factor iiic, polypeptide 3, 102kda  MTO1 - mitochondrial trna translation optimization 1  CNOT8 - ccr4-not transcription complex, subunit 8  NSUN3 - nop2/sun domain family, member 3  SHPRH - snf2 histone linker phd ring helicase, e3 ubiquitin protein ligase  CDK12 - cyclin-dependent kinase 12  SNORD31 - small nucleolar rna, c/d box 31  DHX9 - deah (asp-glu-ala-his) box helicase 9  SNORD29 - small nucleolar rna, c/d box 29  DHX8 - deah (asp-glu-ala-his) box polypeptide 8  TSR2 - tsr2, 20s rrna accumulation, homolog (s. cerevisiae)  SRSF11 - serine/arginine-rich splicing factor 11  DDX6 - dead (asp-glu-ala-asp) box helicase 6  DDX5 - dead (asp-glu-ala-asp) box helicase 5  LUC7L3 - luc7-like 3 (s. cerevisiae)  DDX3X - dead (asp-glu-ala-asp) box helicase 3, x-linked  DDX1 - dead (asp-glu-ala-asp) box helicase 1  ARID4B - at rich interactive domain 4b (rbp1-like)  SYF2 - syf2 pre-mrna-splicing factor  SPRTN - sprt-like n-terminal domain  SNORD22 - small nucleolar rna, c/d box 22  SIN3A - sin3 transcription regulator family member a  SNORD30 - small nucleolar rna, c/d box 30  SAMHD1 - sam domain and hd domain 1  WWTR1 - ww domain containing transcription regulator 1  SET - set nuclear oncogene  DDB1 - damage-specific dna binding protein 1, 127kda  SETMAR - set domain and mariner transposase fusion gene  MLH1 - mutl homolog 1, colon cancer, nonpolyposis type 2 (e. coli)  RSF1 - remodeling and spacing factor 1  MCM9 - minichromosome maintenance complex component 9  GEMIN5 - gem (nuclear organelle) associated protein 5  NOL11 - nucleolar protein 11  DCK - deoxycytidine kinase  MLLT3 - myeloid/lymphoid or mixed-lineage leukemia (trithorax homolog, drosophila); translocated to, 3  MED14 - mediator complex subunit 14  MLLT1 - myeloid/lymphoid or mixed-lineage leukemia (trithorax homolog, drosophila); translocated to, 1  WDR46 - wd repeat domain 46  THUMPD3 - thump domain containing 3  PHAX - phosphorylated adaptor for rna export  NELFB - negative elongation factor complex member b  CPSF3 - cleavage and polyadenylation specific factor 3, 73kda  POT1 - protection of telomeres 1  NAA38 - n(alpha)-acetyltransferase 38, natc auxiliary subunit  LSM7 - lsm7 homolog, u6 small nuclear rna associated (s. cerevisiae)  CNOT10 - ccr4-not transcription complex, subunit 10  LCLAT1 - lysocardiolipin acyltransferase 1  RCHY1 - ring finger and chy zinc finger domain containing 1, e3 ubiquitin protein ligase  TATDN1 - tatd dnase domain containing 1  MNAT1 - mnat cdk-activating kinase assembly factor 1  UIMC1 - ubiquitin interaction motif containing 1  DFFA - dna fragmentation factor, 45kda, alpha polypeptide  MTA2 - metastasis associated 1 family, member 2  POLR1A - polymerase (rna) i polypeptide a, 194kda  NOLC1 - nucleolar and coiled-body phosphoprotein 1  RAB23 - rab23, member ras oncogene family  ZNF473 - zinc finger protein 473  KCTD13 - potassium channel tetramerization domain containing 13  REXO1 - rex1, rna exonuclease 1 homolog (s. cerevisiae)  DCAF13 - ddb1 and cul4 associated factor 13  MNT - mnt, max dimerization protein  CMPK1 - cytidine monophosphate (ump-cmp) kinase 1, cytosolic  POLR3K - polymerase (rna) iii (dna directed) polypeptide k, 12.3 kda  DDX10 - dead (asp-glu-ala-asp) box polypeptide 10  WBP11 - ww domain binding protein 11  WDR18 - wd repeat domain 18  DHX15 - deah (asp-glu-ala-his) box helicase 15  CNOT6 - ccr4-not transcription complex, subunit 6  MCM7 - minichromosome maintenance complex component 7  NSRP1 - nuclear speckle splicing regulatory protein 1  CWF19L2 - cwf19-like 2, cell cycle control (s. pombe)  ATXN2 - ataxin 2  MDH1 - malate dehydrogenase 1, nad (soluble)  ERI1 - exoribonuclease 1  ME1 - malic enzyme 1, nadp(+)-dependent, cytosolic  SCAF4 - sr-related ctd-associated factor 4  MECP2 - methyl cpg binding protein 2 (rett syndrome)  PELO - pelota homolog (drosophila)  MEF2A - myocyte enhancer factor 2a  SLC35B2 - solute carrier family 35 (adenosine 3'-phospho 5'-phosphosulfate transporter), member b2  MED30 - mediator complex subunit 30  DDX21 - dead (asp-glu-ala-asp) box helicase 21  PPIL3 - peptidylprolyl isomerase (cyclophilin)-like 3  BCDIN3D - bcdin3 domain containing  MTHFD2L - methylenetetrahydrofolate dehydrogenase (nadp+ dependent) 2-like  RPAP1 - rna polymerase ii associated protein 1  CPSF2 - cleavage and polyadenylation specific factor 2, 100kda  MEN1 - multiple endocrine neoplasia i  DARS - aspartyl-trna synthetase  WDR75 - wd repeat domain 75  INTS2 - integrator complex subunit 2  NUDT9 - nudix (nucleoside diphosphate linked moiety x)-type motif 9  ANGEL2 - angel homolog 2 (drosophila)  XPO5 - exportin 5  SCAF11 - sr-related ctd-associated factor 11  UTP15 - utp15, u3 small nucleolar ribonucleoprotein, homolog (s. cerevisiae)  METTL1 - methyltransferase like 1  HINFP - histone h4 transcription factor  MFAP1 - microfibrillar-associated protein 1  REXO2 - rna exonuclease 2  AARS2 - alanyl-trna synthetase 2, mitochondrial  RNASEH2C - ribonuclease h2, subunit c  PPP1R1B - protein phosphatase 1, regulatory (inhibitor) subunit 1b  MGAT1 - mannosyl (alpha-1,3-)-glycoprotein beta-1,2-n-acetylglucosaminyltransferase  SARS - seryl-trna synthetase  NGDN - neuroguidin, eif4e binding protein  MBD3 - methyl-cpg binding domain protein 3  NYNRIN - nyn domain and retroviral integrase containing  ATRIP - atr interacting protein  UCK1 - uridine-cytidine kinase 1  CBFA2T2 - core-binding factor, runt domain, alpha subunit 2; translocated to, 2  RRP9 - ribosomal rna processing 9, small subunit (ssu) processome component, homolog (yeast)  MAGOH - mago-nashi homolog, proliferation-associated (drosophila)  EPC2 - enhancer of polycomb homolog 2 (drosophila)  PRPF3 - pre-mrna processing factor 3  LARS - leucyl-trna synthetase  PRPF4 - prp4 pre-mrna processing factor 4 homolog (yeast)  RQCD1 - rcd1 required for cell differentiation1 homolog (s. pombe)  EAF1 - ell associated factor 1  CNOT6L - ccr4-not transcription complex, subunit 6-like  SMC3 - structural maintenance of chromosomes 3  TAOK1 - tao kinase 1  MARS - methionyl-trna synthetase  PUS3 - pseudouridylate synthase 3  MAPT - microtubule-associated protein tau  DPAGT1 - dolichyl-phosphate (udp-n-acetylglucosamine) n-acetylglucosaminephosphotransferase 1 (glcnac-1-p transferase)  RNASEH1 - ribonuclease h1  DOM3Z - dom-3 homolog z (c. elegans)  SIRT6 - sirtuin 6  MTA1 - metastasis associated 1  PRPF31 - pre-mrna processing factor 31  DR1 - down-regulator of transcription 1, tbp-binding (negative cofactor 2)  DPYSL2 - dihydropyrimidinase-like 2  TRMT5 - trna methyltransferase 5  TNKS1BP1 - tankyrase 1 binding protein 1, 182kda  USP10 - ubiquitin specific peptidase 10  SKI - v-ski avian sarcoma viral oncogene homolog  MBD1 - methyl-cpg binding domain protein 1  SKIV2L - superkiller viralicidic activity 2-like (s. cerevisiae)  REV1 - rev1, polymerase (dna directed)  SART1 - squamous cell carcinoma antigen recognized by t cells  CHTOP - chromatin target of prmt1  ZCRB1 - zinc finger cchc-type and rna binding motif 1  WDR48 - wd repeat domain 48  TRMT112 - trna methyltransferase 11-2 homolog (s. cerevisiae)  MCM3 - minichromosome maintenance complex component 3  MCM6 - minichromosome maintenance complex component 6  RTFDC1 - replication termination factor 2 domain containing 1  MCM5 - minichromosome maintenance complex component 5  NELFCD - negative elongation factor complex member c/d  CWC15 - cwc15 spliceosome-associated protein homolog (s. cerevisiae)  MCM2 - minichromosome maintenance complex component 2  DIRAS3 - diras family, gtp-binding ras-like 3  RTCB - rna 2',3'-cyclic phosphate and 5'-oh ligase  NUDT12 - nudix (nucleoside diphosphate linked moiety x)-type motif 12  DTX1 - deltex homolog 1 (drosophila)  PPIL1 - peptidylprolyl isomerase (cyclophilin)-like 1  NFS1 - nfs1 cysteine desulfurase  DKC1 - dyskeratosis congenita 1, dyskerin  PAPSS1 - 3'-phosphoadenosine 5'-phosphosulfate synthase 1  PNPT1 - polyribonucleotide nucleotidyltransferase 1  DLG1 - discs, large homolog 1 (drosophila)  RPL14 - ribosomal protein l14  RIOK1 - rio kinase 1  ZNF445 - zinc finger protein 445  RNASEK - ribonuclease, rnase k  NEIL2 - nei endonuclease viii-like 2 (e. coli)  DGUOK - deoxyguanosine kinase  RBFOX3 - rna binding protein, fox-1 homolog (c. elegans) 3  SESN2 - sestrin 2  PARP9 - poly (adp-ribose) polymerase family, member 9  CDK5RAP1 - cdk5 regulatory subunit associated protein 1  RNF8 - ring finger protein 8, e3 ubiquitin protein ligase  DHX37 - deah (asp-glu-ala-his) box polypeptide 37  UVSSA - uv-stimulated scaffold protein a  TAF1B - tata box binding protein (tbp)-associated factor, rna polymerase i, b, 63kda  ESF1 - esf1, nucleolar pre-rrna processing protein, homolog (s. cerevisiae)  DNASE2 - deoxyribonuclease ii, lysosomal  SFPQ - splicing factor proline/glutamine-rich  PHRF1 - phd and ring finger domains 1  PCF11 - pcf11 cleavage and polyadenylation factor subunit  MED15 - mediator complex subunit 15  PIAS4 - protein inhibitor of activated stat, 4  SRSF1 - serine/arginine-rich splicing factor 1  SRRT - serrate rna effector molecule homolog (arabidopsis)  SMAD2 - smad family member 2  GMEB2 - glucocorticoid modulatory element binding protein 2  SRSF2 - serine/arginine-rich splicing factor 2  SRSF3 - serine/arginine-rich splicing factor 3  DNMT1 - dna (cytosine-5-)-methyltransferase 1  SRSF4 - serine/arginine-rich splicing factor 4  TRDMT1 - trna aspartic acid methyltransferase 1  SRSF5 - serine/arginine-rich splicing factor 5  DNMT3A - dna (cytosine-5-)-methyltransferase 3 alpha  TDP2 - tyrosyl-dna phosphodiesterase 2  TMTC1 - transmembrane and tetratricopeptide repeat containing 1  SRSF6 - serine/arginine-rich splicing factor 6  SRSF7 - serine/arginine-rich splicing factor 7  SFSWAP - splicing factor, suppressor of white-apricot homolog (drosophila)  TRA2B - transformer 2 beta homolog (drosophila)  TAF1C - tata box binding protein (tbp)-associated factor, rna polymerase i, c, 110kda  LARP7 - la ribonucleoprotein domain family, member 7  TAF9B - taf9b rna polymerase ii, tata box binding protein (tbp)-associated factor, 31kda  RSL1D1 - ribosomal l1 domain containing 1  SMAD4 - smad family member 4  NBAS - neuroblastoma amplified sequence  EME1 - essential meiotic structure-specific endonuclease 1  TRMT6 - trna methyltransferase 6 homolog (s. cerevisiae)  INTS1 - integrator complex subunit 1  RBM4B - rna binding motif protein 4b  SENP3 - sumo1/sentrin/smt3 specific peptidase 3  SMARCAD1 - swi/snf-related, matrix-associated actin-dependent regulator of chromatin, subfamily a, containing dead/h box 1  NCBP2 - nuclear cap binding protein subunit 2, 20kda  NCBP1 - nuclear cap binding protein subunit 1, 80kda  NDN - necdin, melanoma antigen (mage) family member  TSN - translin  G3BP1 - gtpase activating protein (sh3 domain) binding protein 1  MBNL2 - muscleblind-like splicing regulator 2  FAN1 - fancd2/fanci-associated nuclease 1  SUGP2 - surp and g patch domain containing 2  NAF1 - nuclear assembly factor 1 ribonucleoprotein  TRMT1L - trna methyltransferase 1 homolog (s. cerevisiae)-like  SNW1 - snw domain containing 1  HNRNPM - heterogeneous nuclear ribonucleoprotein m  WRNIP1 - werner helicase interacting protein 1  ELL2 - elongation factor, rna polymerase ii, 2  MGME1 - mitochondrial genome maintenance exonuclease 1  TRIM28 - tripartite motif containing 28  DTD1 - d-tyrosyl-trna deacylase 1  DROSHA - drosha, ribonuclease type iii  KIN - kin, antigenic determinant of reca protein homolog (mouse)  EP300 - e1a binding protein p300  NAP1L1 - nucleosome assembly protein 1-like 1  NASP - nuclear autoantigenic sperm protein (histone-binding)  NARS - asparaginyl-trna synthetase  HABP4 - hyaluronan binding protein 4  AGPAT4 - 1-acylglycerol-3-phosphate o-acyltransferase 4  ENO2 - enolase 2 (gamma, neuronal)  NBN - nibrin  AGPAT3 - 1-acylglycerol-3-phosphate o-acyltransferase 3  ENO1 - enolase 1, (alpha)  UBQLN4 - ubiquilin 4  OLA1 - obg-like atpase 1  CELF4 - cugbp, elav-like family member 4  RBM7 - rna binding motif protein 7  RBM6 - rna binding motif protein 6  RBM5 - rna binding motif protein 5  UGGT1 - udp-glucose glycoprotein glucosyltransferase 1  NT5C2 - 5'-nucleotidase, cytosolic ii  EGR2 - early growth response 2  TTF1 - transcription termination factor, rna polymerase i  ALYREF - aly/ref export factor  INTS4 - integrator complex subunit 4  TFPT - tcf3 (e2a) fusion partner (in childhood leukemia)  KDM2A - lysine (k)-specific demethylase 2a  ELAVL1 - elav (embryonic lethal, abnormal vision, drosophila)-like 1 (hu antigen r)  CCDC130 - coiled-coil domain containing 130  MPHOSPH6 - m-phase phosphoprotein 6  ZNF326 - zinc finger protein 326  MPHOSPH10 - m-phase phosphoprotein 10 (u3 small nucleolar ribonucleoprotein)  ELAVL4 - elav (embryonic lethal, abnormal vision, drosophila)-like 4  CDADC1 - cytidine and dcmp deaminase domain containing 1  URM1 - ubiquitin related modifier 1  USPL1 - ubiquitin specific peptidase like 1  PDCD11 - programmed cell death 11  ABT1 - activator of basal transcription 1  TOPORS - topoisomerase i binding, arginine/serine-rich, e3 ubiquitin protein ligase  TSNAX - translin-associated factor x  FIP1L1 - factor interacting with papola and cpsf1  ACIN1 - apoptotic chromatin condensation inducer 1  CNOT1 - ccr4-not transcription complex, subunit 1  LAS1L - las1-like (s. cerevisiae)  TPMT - thiopurine s-methyltransferase  EXOSC7 - exosome component 7  SPEN - spen homolog, transcriptional regulator (drosophila)  ISG20L2 - interferon stimulated exonuclease gene 20kda-like 2  PDCD7 - programmed cell death 7  NME7 - nme/nm23 family member 7  TP53BP1 - tumor protein p53 binding protein 1  HUWE1 - hect, uba and wwe domain containing 1, e3 ubiquitin protein ligase  TRMT10A - trna methyltransferase 10 homolog a (s. cerevisiae)  TOP3A - topoisomerase (dna) iii alpha  CNOT7 - ccr4-not transcription complex, subunit 7  TOP2B - topoisomerase (dna) ii beta 180kda  PQBP1 - polyglutamine binding protein 1  FAM20C - family with sequence similarity 20, member c  TOP1 - topoisomerase (dna) i  TRA2A - transformer 2 alpha homolog (drosophila)  KDM1A - lysine (k)-specific demethylase 1a  INO80B - ino80 complex subunit b  CPSF1 - cleavage and polyadenylation specific factor 1, 160kda  SF3B5 - splicing factor 3b, subunit 5, 10kda  ADPGK - adp-dependent glucokinase  RBM15B - rna binding motif protein 15b  SNRNP200 - small nuclear ribonucleoprotein 200kda (u5)  HNRNPLL - heterogeneous nuclear ribonucleoprotein l-like  NCOA6 - nuclear receptor coactivator 6  IMP4 - imp4, u3 small nucleolar ribonucleoprotein, homolog (yeast)  NMNAT2 - nicotinamide nucleotide adenylyltransferase 2  SMG1 - smg1 phosphatidylinositol 3-kinase-related kinase  E4F1 - e4f transcription factor 1  NT5M - 5',3'-nucleotidase, mitochondrial  ENDOD1 - endonuclease domain containing 1  NRBP1 - nuclear receptor binding protein 1  MUTYH - muty homolog (e. coli)  XAB2 - xpa binding protein 2  PDS5B - pds5, regulator of cohesion maintenance, homolog b (s. cerevisiae)  ENY2 - enhancer of yellow 2 homolog (drosophila)  RAD50 - rad50 homolog (s. cerevisiae)  C11orf30 - chromosome 11 open reading frame 30  RRP1B - ribosomal rna processing 1b  GMPPB - gdp-mannose pyrophosphorylase b  CMTR1 - cap methyltransferase 1  NR2C1 - nuclear receptor subfamily 2, group c, member 1  SETX - senataxin  DUS3L - dihydrouridine synthase 3-like (s. cerevisiae)  NR2C2 - nuclear receptor subfamily 2, group c, member 2  MARS2 - methionyl-trna synthetase 2, mitochondrial  MED25 - mediator complex subunit 25  MED6 - mediator complex subunit 6  NAT14 - n-acetyltransferase 14 (gcn5-related, putative)  UQCRH - ubiquinol-cytochrome c reductase hinge protein  MTRR - 5-methyltetrahydrofolate-homocysteine methyltransferase reductase  REXO4 - rex4, rna exonuclease 4 homolog (s. cerevisiae)  PTBP3 - polypyrimidine tract binding protein 3  INTS12 - integrator complex subunit 12  ZC3H13 - zinc finger ccch-type containing 13  SARM1 - sterile alpha and tir motif containing 1  USP1 - ubiquitin specific peptidase 1  UAP1L1 - udp-n-acteylglucosamine pyrophosphorylase 1-like 1  FANCE - fanconi anemia, complementation group e  ENTPD7 - ectonucleoside triphosphate diphosphohydrolase 7  GNE - glucosamine (udp-n-acetyl)-2-epimerase/n-acetylmannosamine kinase  ACSL3 - acyl-coa synthetase long-chain family member 3  ACSL4 - acyl-coa synthetase long-chain family member 4  SNORD107 - small nucleolar rna, c/d box 107  UNG - uracil-dna glycosylase  GNPDA1 - glucosamine-6-phosphate deaminase 1  UCK2 - uridine-cytidine kinase 2  UMPS - uridine monophosphate synthetase  MTF1 - metal-regulatory transcription factor 1  FARSA - phenylalanyl-trna synthetase, alpha subunit  FASN - fatty acid synthase  NR1H2 - nuclear receptor subfamily 1, group h, member 2  C19orf40 - chromosome 19 open reading frame 40  PARP2 - poly (adp-ribose) polymerase 2  DCPS - decapping enzyme, scavenger  UTP3 - utp3, small subunit (ssu) processome component, homolog (s. cerevisiae)  MED16 - mediator complex subunit 16  SMC5 - structural maintenance of chromosomes 5  CHAF1A - chromatin assembly factor 1, subunit a (p150)  DDX24 - dead (asp-glu-ala-asp) box helicase 24  EXTL2 - exostosin-like glycosyltransferase 2  FARSB - phenylalanyl-trna synthetase, beta subunit  ZC3H3 - zinc finger ccch-type containing 3  UVRAG - uv radiation resistance associated  VARS - valyl-trna synthetase  EYA3 - eyes absent homolog 3 (drosophila)  CCNL1 - cyclin l1  ENDOV - endonuclease v  VCP - valosin containing protein  WDR43 - wd repeat domain 43  RARS2 - arginyl-trna synthetase 2, mitochondrial  MSH3 - muts homolog 3 (e. coli)  MSH2 - muts homolog 2, colon cancer, nonpolyposis type 1 (e. coli)  ERCC5 - excision repair cross-complementing rodent repair deficiency, complementation group 5  ERCC4 - excision repair cross-complementing rodent repair deficiency, complementation group 4  PAN2 - pan2 poly(a) specific ribonuclease subunit homolog (s. cerevisiae)  ERCC3 - excision repair cross-complementing rodent repair deficiency, complementation group 3  LIN52 - lin-52 homolog (c. elegans)  YTHDC1 - yth domain containing 1  THOC2 - tho complex 2  ASTE1 - asteroid homolog 1 (drosophila)  UBE2A - ubiquitin-conjugating enzyme e2a  NOB1 - nin1/rpn12 binding protein 1 homolog (s. cerevisiae)  ERN1 - endoplasmic reticulum to nucleus signaling 1  ERH - enhancer of rudimentary homolog (drosophila)  GPD1L - glycerol-3-phosphate dehydrogenase 1-like  UBB - ubiquitin b  VARS2 - valyl-trna synthetase 2, mitochondrial  GFPT2 - glutamine-fructose-6-phosphate transaminase 2  HIRA - histone cell cycle regulator  COMMD1 - copper metabolism (murr1) domain containing 1  TTC5 - tetratricopeptide repeat domain 5  SNORA3 - small nucleolar rna, h/aca box 3  RBM8A - rna binding motif protein 8a  ETV1 - ets variant 1  MDN1 - mdn1, midasin homolog (yeast)  POLR2M - polymerase (rna) ii (dna directed) polypeptide m  ETF1 - eukaryotic translation termination factor 1  TXNRD1 - thioredoxin reductase 1  ACSBG1 - acyl-coa synthetase bubblegum family member 1  USP3 - ubiquitin specific peptidase 3  BABAM1 - brisc and brca1 a complex member 1  SUMO1 - small ubiquitin-like modifier 1  UBE2T - ubiquitin-conjugating enzyme e2t (putative)  SLC23A2 - solute carrier family 23 (ascorbic acid transporter), member 2  UBTF - upstream binding transcription factor, rna polymerase i  ZNF830 - zinc finger protein 830  METTL5 - methyltransferase like 5  RRS1 - rrs1 ribosome biogenesis regulator homolog (s. cerevisiae)  MED4 - mediator complex subunit 4  JMJD6 - jumonji domain containing 6  THOC1 - tho complex 1  SETD2 - set domain containing 2  EPRS - glutamyl-prolyl-trna synthetase  RBX1 - ring-box 1, e3 ubiquitin protein ligase  ZC3H7A - zinc finger ccch-type containing 7a  MRE11A - mre11 meiotic recombination 11 homolog a (s. cerevisiae)  UBE2V2 - ubiquitin-conjugating enzyme e2 variant 2  ERCC2 - excision repair cross-complementing rodent repair deficiency, complementation group 2  THRAP3 - thyroid hormone receptor associated protein 3  MED12 - mediator complex subunit 12  MPI - mannose phosphate isomerase  MED13 - mediator complex subunit 13  UBE2N - ubiquitin-conjugating enzyme e2n  UBE2V1 - ubiquitin-conjugating enzyme e2 variant 1  ZC3H7B - zinc finger ccch-type containing 7b  MED24 - mediator complex subunit 24  FMR1 - fragile x mental retardation 1  TCOF1 - treacher collins-franceschetti syndrome 1  HENMT1 - hen1 methyltransferase homolog 1 (arabidopsis)  DTX3L - deltex 3-like (drosophila)  ORC4 - origin recognition complex, subunit 4  INTS5 - integrator complex subunit 5  ORC2 - origin recognition complex, subunit 2  ORC5 - origin recognition complex, subunit 5  PDS5A - pds5, regulator of cohesion maintenance, homolog a (s. cerevisiae)  BOP1 - block of proliferation 1  THUMPD2 - thump domain containing 2  ERCC6L2 - excision repair cross-complementing rodent repair deficiency, complementation group 6-like 2  RPRD2 - regulation of nuclear pre-mrna domain containing 2  FLNA - filamin a, alpha  PPP4R2 - protein phosphatase 4, regulatory subunit 2  FOXO3 - forkhead box o3  RBM19 - rna binding motif protein 19  UTP6 - utp6, small subunit (ssu) processome component, homolog (yeast)  DCP1A - decapping mrna 1a  AP5Z1 - adaptor-related protein complex 5, zeta 1 subunit  ACSL6 - acyl-coa synthetase long-chain family member 6  RPUSD1 - rna pseudouridylate synthase domain containing 1  MED8 - mediator complex subunit 8  DDX46 - dead (asp-glu-ala-asp) box polypeptide 46  TDG - thymine-dna glycosylase  SLC35B3 - solute carrier family 35 (adenosine 3'-phospho 5'-phosphosulfate transporter), member b3  SMG6 - smg6 nonsense mediated mrna decay factor  OGG1 - 8-oxoguanine dna glycosylase  EXOSC3 - exosome component 3  WDR61 - wd repeat domain 61  SMG7 - smg7 nonsense mediated mrna decay factor  OPA1 - optic atrophy 1 (autosomal dominant)  CSTF2T - cleavage stimulation factor, 3' pre-rna, subunit 2, 64kda, tau variant  FHIT - fragile histidine triad  APITD1 - apoptosis-inducing, taf9-like domain 1  EXOSC1 - exosome component 1  TAF9 - taf9 rna polymerase ii, tata box binding protein (tbp)-associated factor, 32kda  BMS1 - bms1 ribosome biogenesis factor  TAF7 - taf7 rna polymerase ii, tata box binding protein (tbp)-associated factor, 55kda  FAM98B - family with sequence similarity 98, member b  RRP15 - ribosomal rna processing 15 homolog (s. cerevisiae)  KHNYN - kh and nyn domain containing  TAF6 - taf6 rna polymerase ii, tata box binding protein (tbp)-associated factor, 80kda  U2SURP - u2 snrnp-associated surp domain containing  SMCHD1 - structural maintenance of chromosomes flexible hinge domain containing 1  TAF4 - taf4 rna polymerase ii, tata box binding protein (tbp)-associated factor, 135kda  TAF2 - taf2 rna polymerase ii, tata box binding protein (tbp)-associated factor, 150kda  NTHL1 - nth endonuclease iii-like 1 (e. coli)  EIF4A3 - eukaryotic translation initiation factor 4a3  TARS - threonyl-trna synthetase  NR4A2 - nuclear receptor subfamily 4, group a, member 2  ZBTB7B - zinc finger and btb domain containing 7b  TARBP1 - tar (hiv-1) rna binding protein 1  FEN1 - flap structure-specific endonuclease 1  TALDO1 - transaldolase 1  DHX38 - deah (asp-glu-ala-his) box polypeptide 38  NVL - nuclear vcp-like  SMG9 - smg9 nonsense mediated mrna decay factor  TAF13 - taf13 rna polymerase ii, tata box binding protein (tbp)-associated factor, 18kda  ZCCHC11 - zinc finger, cchc domain containing 11  DERA - deoxyribose-phosphate aldolase (putative)  LARS2 - leucyl-trna synthetase 2, mitochondrial  INO80E - ino80 complex subunit e  NPM1 - nucleophosmin (nucleolar phosphoprotein b23, numatrin)  KMT2E - lysine (k)-specific methyltransferase 2e  ACSS2 - acyl-coa synthetase short-chain family member 2  GINS1 - gins complex subunit 1 (psf1 homolog)  FCF1 - fcf1 rrna-processing protein  AHCYL2 - adenosylhomocysteinase-like 2  TSEN54 - tsen54 trna splicing endonuclease subunit  CTIF - cbp80/20-dependent translation initiation factor  TCF3 - transcription factor 3  PUM2 - pumilio homolog 2 (drosophila)  POLR1D - polymerase (rna) i polypeptide d, 16kda  DMAP1 - dna methyltransferase 1 associated protein 1  TCEB3 - transcription elongation factor b (siii), polypeptide 3 (110kda, elongin a)  TRNT1 - trna nucleotidyl transferase, cca-adding, 1  UTP18 - utp18 small subunit (ssu) processome component homolog (yeast)  RRNAD1 - ribosomal rna adenine dimethylase domain containing 1  TOE1 - target of egr1, member 1 (nuclear)  TCEA2 - transcription elongation factor a (sii), 2  ANGEL1 - angel homolog 1 (drosophila)  NUDT7 - nudix (nucleoside diphosphate linked moiety x)-type motif 7  SIDT2 - sid1 transmembrane family, member 2  TCEA1 - transcription elongation factor a (sii), 1  DONSON - downstream neighbor of son  NRBF2 - nuclear receptor binding factor 2  NOP2 - nop2 nucleolar protein  VPRBP - vpr (hiv-1) binding protein  NONO - non-pou domain containing, octamer-binding  TK1 - thymidine kinase 1, soluble  TK2 - thymidine kinase 2, mitochondrial  MTOR - mechanistic target of rapamycin (serine/threonine kinase)  TKT - transketolase  NME1 - nme/nm23 nucleoside diphosphate kinase 1  MUS81 - mus81 structure-specific endonuclease subunit  SNORD118 - small nucleolar rna, c/d box 118  PPWD1 - peptidylprolyl isomerase domain and wd repeat containing 1  NOTCH4 - notch 4  NDNL2 - necdin-like 2  NOVA2 - neuro-oncological ventral antigen 2  NOVA1 - neuro-oncological ventral antigen 1  AQR - aquarius homolog (mouse)  EXOSC2 - exosome component 2  CNOT2 - ccr4-not transcription complex, subunit 2  PUM1 - pumilio homolog 1 (drosophila)  CNOT4 - ccr4-not transcription complex, subunit 4  CNOT3 - ccr4-not transcription complex, subunit 3  NOTCH1 - notch 1  SIRT1 - sirtuin 1  NOTCH3 - notch 3  CTC1 - cts telomere maintenance complex component 1  NOTCH2 - notch 2  DHX34 - deah (asp-glu-ala-his) box polypeptide 34  NFYA - nuclear transcription factor y, alpha  NFRKB - nuclear factor related to kappab binding protein  THOC7 - tho complex 7 homolog (drosophila)  UXS1 - udp-glucuronate decarboxylase 1  EDC3 - enhancer of mrna decapping 3  ACTR8 - arp8 actin-related protein 8 homolog (yeast)  ZMAT2 - zinc finger, matrin-type 2  PHF3 - phd finger protein 3  NFKB1 - nuclear factor of kappa light polypeptide gene enhancer in b-cells 1  SRFBP1 - serum response factor binding protein 1  NFKBIB - nuclear factor of kappa light polypeptide gene enhancer in b-cells inhibitor, beta  HARS2 - histidyl-trna synthetase 2, mitochondrial  TNFAIP1 - tumor necrosis factor, alpha-induced protein 1 (endothelial)  TARDBP - tar dna binding protein  SNRNP48 - small nuclear ribonucleoprotein 48kda (u11/u12)  NHP2L1 - nhp2 non-histone chromosome protein 2-like 1 (s. cerevisiae)  ZNRD1 - zinc ribbon domain containing 1  SART3 - squamous cell carcinoma antigen recognized by t cells 3  SF3B1 - splicing factor 3b, subunit 1, 155kda  SF3B3 - splicing factor 3b, subunit 3, 130kda  MDC1 - mediator of dna-damage checkpoint 1  TTC37 - tetratricopeptide repeat domain 37  PGM2L1 - phosphoglucomutase 2-like 1  CTPS2 - ctp synthase 2  RRM2B - ribonucleotide reductase m2 b (tp53 inducible)  TFAM - transcription factor a, mitochondrial  SMARCAL1 - swi/snf related, matrix associated, actin dependent regulator of chromatin, subfamily a-like 1  HARBI1 - harbinger transposase derived 1  TERF1 - telomeric repeat binding factor (nima-interacting) 1  TERF2 - telomeric repeat binding factor 2  CTR9 - ctr9, paf1/rna polymerase ii complex component  SKIV2L2 - superkiller viralicidic activity 2-like 2 (s. cerevisiae)  GTF3C6 - general transcription factor iiic, polypeptide 6, alpha 35kda  TFDP1 - transcription factor dp-1  EPC1 - enhancer of polycomb homolog 1 (drosophila)  MORF4L2 - mortality factor 4 like 2  BCCIP - brca2 and cdkn1a interacting protein  PES1 - pescadillo ribosomal biogenesis factor 1  NR2F1 - nuclear receptor subfamily 2, group f, member 1  TFCP2 - transcription factor cp2  ZFYVE26 - zinc finger, fyve domain containing 26  FXN - frataxin  TGS1 - trimethylguanosine synthase 1  NFIA - nuclear factor i/a  POLE4 - polymerase (dna-directed), epsilon 4, accessory subunit  KIAA0391 - kiaa0391  RBFOX2 - rna binding protein, fox-1 homolog (c. elegans) 2  TGFB1 - transforming growth factor, beta 1  CTNNBL1 - catenin, beta like 1  MEPCE - methylphosphate capping enzyme  GEMIN4 - gem (nuclear organelle) associated protein 4  FOSB - fbj murine osteosarcoma viral oncogene homolog b  FOS - fbj murine osteosarcoma viral oncogene homolog  DRG1 - developmentally regulated gtp binding protein 1  SRRM2 - serine/arginine repetitive matrix 2  COPS7A - cop9 signalosome subunit 7a  GATC - glutamyl-trna(gln) amidotransferase, subunit c  TARS2 - threonyl-trna synthetase 2, mitochondrial (putative)  AK3 - adenylate kinase 3  ACSF2 - acyl-coa synthetase family member 2  METTL3 - methyltransferase like 3  KIAA0430 - kiaa0430  THRA - thyroid hormone receptor, alpha |
| GO:0032990 | cell part morphogenesis | 2.65E-6 | 4.19E-4 | 1.22 (10334,181,6996,150) | [+] Show genes  AFG3L2 - afg3 atpase family member 3-like 2 (s. cerevisiae)  CTTN - cortactin  SPTBN4 - spectrin, beta, non-erythrocytic 4  ATP8A2 - atpase, aminophospholipid transporter, class i, type 8a, member 2  NRP1 - neuropilin 1  PLXNB1 - plexin b1  PACSIN2 - protein kinase c and casein kinase substrate in neurons 2  FBXO45 - f-box protein 45  SPAST - spastin  ARC - activity-regulated cytoskeleton-associated protein  WASF2 - was protein family, member 2  SHANK1 - sh3 and multiple ankyrin repeat domains 1  DBN1 - drebrin 1  ILK - integrin-linked kinase  APBB1 - amyloid beta (a4) precursor protein-binding, family b, member 1 (fe65)  PANK2 - pantothenate kinase 2  NUMBL - numb homolog (drosophila)-like  HPRT1 - hypoxanthine phosphoribosyltransferase 1  BRSK1 - br serine/threonine kinase 1  POLG2 - polymerase (dna directed), gamma 2, accessory subunit  NDEL1 - nude neurodevelopment protein 1-like 1  MAP1S - microtubule-associated protein 1s  ACTB - actin, beta  PAFAH1B1 - platelet-activating factor acetylhydrolase 1b, regulatory subunit 1 (45kda)  GJA1 - gap junction protein, alpha 1, 43kda  APP - amyloid beta (a4) precursor protein  CREB1 - camp responsive element binding protein 1  DOCK10 - dedicator of cytokinesis 10  SNX2 - sorting nexin 2  OPA1 - optic atrophy 1 (autosomal dominant)  PAK3 - p21 protein (cdc42/rac)-activated kinase 3  DYNLT1 - dynein, light chain, tctex-type 1  SNX1 - sorting nexin 1  LLGL1 - lethal giant larvae homolog 1 (drosophila)  BMP7 - bone morphogenetic protein 7  GAS7 - growth arrest-specific 7  FGFR2 - fibroblast growth factor receptor 2  TCTN1 - tectonic family member 1  FLOT1 - flotillin 1  FIS1 - fission 1 (mitochondrial outer membrane) homolog (s. cerevisiae)  PICALM - phosphatidylinositol binding clathrin assembly protein  ANKRD27 - ankyrin repeat domain 27 (vps9 domain)  AMIGO1 - adhesion molecule with ig-like domain 1  PTEN - phosphatase and tensin homolog  ITGA1 - integrin, alpha 1  DAB2IP - dab2 interacting protein  RAB10 - rab10, member ras oncogene family  MEF2A - myocyte enhancer factor 2a  EGFR - epidermal growth factor receptor  TOP2B - topoisomerase (dna) ii beta 180kda  COL4A3BP - collagen, type iv, alpha 3 (goodpasture antigen) binding protein  RHOA - ras homolog family member a  NR4A2 - nuclear receptor subfamily 4, group a, member 2  SZT2 - seizure threshold 2 homolog (mouse)  ADARB1 - adenosine deaminase, rna-specific, b1  CELSR2 - cadherin, egf lag seven-pass g-type receptor 2  ATL1 - atlastin gtpase 1  ITGB1 - integrin, beta 1 (fibronectin receptor, beta polypeptide, antigen cd29 includes mdf2, msk12)  CNP - 2',3'-cyclic nucleotide 3' phosphodiesterase  ADCY1 - adenylate cyclase 1 (brain)  DCLK1 - doublecortin-like kinase 1  PTK2 - protein tyrosine kinase 2  CDK5 - cyclin-dependent kinase 5  RAB8A - rab8a, member ras oncogene family  ROCK1 - rho-associated, coiled-coil containing protein kinase 1  S100B - s100 calcium binding protein b  PTN - pleiotrophin  STMN1 - stathmin 1  TRAK2 - trafficking protein, kinesin binding 2  MFF - mitochondrial fission factor  KIDINS220 - kinase d-interacting substrate, 220kda  TNIK - traf2 and nck interacting kinase  ZEB2 - zinc finger e-box binding homeobox 2  KLF7 - kruppel-like factor 7 (ubiquitous)  SLC11A2 - solute carrier family 11 (proton-coupled divalent metal ion transporter), member 2  CNTN4 - contactin 4  HSP90AA1 - heat shock protein 90kda alpha (cytosolic), class a member 1  MYCBP2 - myc binding protein 2, e3 ubiquitin protein ligase  TAOK3 - tao kinase 3  CDH11 - cadherin 11, type 2, ob-cadherin (osteoblast)  NPTX1 - neuronal pentraxin i  NRCAM - neuronal cell adhesion molecule  WASF1 - was protein family, member 1  NCKAP1 - nck-associated protein 1  ABI1 - abl-interactor 1  DRD2 - dopamine receptor d2  CHRNB2 - cholinergic receptor, nicotinic, beta 2 (neuronal)  BTBD3 - btb (poz) domain containing 3  TMEM106B - transmembrane protein 106b  ZNF365 - zinc finger protein 365  NUMB - numb homolog (drosophila)  TAOK1 - tao kinase 1  SUPV3L1 - suppressor of var1, 3-like 1 (s. cerevisiae)  SHANK3 - sh3 and multiple ankyrin repeat domains 3  LRRK2 - leucine-rich repeat kinase 2  MAP6 - microtubule-associated protein 6  RERE - arginine-glutamic acid dipeptide (re) repeats  MAP2 - microtubule-associated protein 2  MTM1 - myotubularin 1  MAP1B - microtubule-associated protein 1b  MAP1A - microtubule-associated protein 1a  CTNND2 - catenin (cadherin-associated protein), delta 2  PACSIN1 - protein kinase c and casein kinase substrate in neurons 1  CTNNA2 - catenin (cadherin-associated protein), alpha 2  BAIAP2 - bai1-associated protein 2  PID1 - phosphotyrosine interaction domain containing 1  MAP4K4 - mitogen-activated protein kinase kinase kinase kinase 4  HDAC6 - histone deacetylase 6  PVRL2 - poliovirus receptor-related 2 (herpesvirus entry mediator b)  NYAP1 - neuronal tyrosine-phosphorylated phosphoinositide-3-kinase adaptor 1  WEE1 - wee1 homolog (s. pombe)  ATP7A - atpase, cu++ transporting, alpha polypeptide  RAB3A - rab3a, member ras oncogene family  SLC9A6 - solute carrier family 9, subfamily a (nhe6, cation proton antiporter 6), member 6  LMTK2 - lemur tyrosine kinase 2  EPB41L3 - erythrocyte membrane protein band 4.1-like 3  DOCK7 - dedicator of cytokinesis 7  DNM1L - dynamin 1-like  FYN - fyn oncogene related to src, fgr, yes  SLC25A46 - solute carrier family 25, member 46  BAX - bcl2-associated x protein  PITPNA - phosphatidylinositol transfer protein, alpha  PNPT1 - polyribonucleotide nucleotidyltransferase 1  NEK3 - nima-related kinase 3  DLG4 - discs, large homolog 4 (drosophila)  MINK1 - misshapen-like kinase 1  NEFL - neurofilament, light polypeptide  DBNL - drebrin-like  UBB - ubiquitin b  FEZ1 - fasciculation and elongation protein zeta 1 (zygin i)  PHACTR1 - phosphatase and actin regulator 1  IMMT - inner membrane protein, mitochondrial  FAM206A - family with sequence similarity 206, member a  RBFOX2 - rna binding protein, fox-1 homolog (c. elegans) 2  DNM2 - dynamin 2  MAPK8IP2 - mitogen-activated protein kinase 8 interacting protein 2  ARHGAP35 - rho gtpase activating protein 35  SSBP1 - single-stranded dna binding protein 1, mitochondrial  ZNF335 - zinc finger protein 335  ALS2 - amyotrophic lateral sclerosis 2 (juvenile)  EPHB6 - eph receptor b6  ZSWIM6 - zinc finger, swim-type containing 6  BCL2 - b-cell cll/lymphoma 2  TAOK2 - tao kinase 2  BCL2L1 - bcl2-like 1  SGK1 - serum/glucocorticoid regulated kinase 1  B4GALT6 - udp-gal:betaglcnac beta 1,4- galactosyltransferase, polypeptide 6  PLEKHO1 - pleckstrin homology domain containing, family o member 1  WASL - wiskott-aldrich syndrome-like  B4GALT5 - udp-gal:betaglcnac beta 1,4- galactosyltransferase, polypeptide 5 |
| GO:0018394 | peptidyl-lysine acetylation | 2.75E-6 | 4.29E-4 | 1.30 (10334,99,6996,87) | [+] Show genes  HAT1 - histone acetyltransferase 1  MSL3 - male-specific lethal 3 homolog (drosophila)  EPC2 - enhancer of polycomb homolog 2 (drosophila)  TADA1 - transcriptional adaptor 1  MCRS1 - microspherule protein 1  ATAT1 - alpha tubulin acetyltransferase 1  NAA40 - n(alpha)-acetyltransferase 40, natd catalytic subunit, homolog (s. cerevisiae)  MSL2 - male-specific lethal 2 homolog (drosophila)  MEAF6 - myst/esa1-associated factor 6  EP300 - e1a binding protein p300  RSF1 - remodeling and spacing factor 1  ING4 - inhibitor of growth family, member 4  DR1 - down-regulator of transcription 1, tbp-binding (negative cofactor 2)  DPF2 - d4, zinc and double phd fingers family 2  CSRP2BP - csrp2 binding protein  KANSL1 - kat8 regulatory nsl complex subunit 1  SIRT1 - sirtuin 1  GTF2B - general transcription factor iib  KAT2A - k(lysine) acetyltransferase 2a  PCGF2 - polycomb group ring finger 2  NCOA3 - nuclear receptor coactivator 3  APBB1 - amyloid beta (a4) precursor protein-binding, family b, member 1 (fe65)  KMT2A - lysine (k)-specific methyltransferase 2a  BRPF3 - bromodomain and phd finger containing, 3  BRD8 - bromodomain containing 8  TADA3 - transcriptional adaptor 3  SPHK2 - sphingosine kinase 2  TAF6L - taf6-like rna polymerase ii, p300/cbp-associated factor (pcaf)-associated factor, 65kda  BRD1 - bromodomain containing 1  LDB1 - lim domain binding 1  PHF20L1 - phd finger protein 20-like 1  CREBBP - creb binding protein  MORF4L1 - mortality factor 4 like 1  ATF2 - activating transcription factor 2  MSL1 - male-specific lethal 1 homolog (drosophila)  KAT2B - k(lysine) acetyltransferase 2b  CLOCK - clock circadian regulator  CRTC2 - creb regulated transcription coactivator 2  ESCO1 - establishment of sister chromatid cohesion n-acetyltransferase 1  KANSL1L - kat8 regulatory nsl complex subunit 1-like  TAF9 - taf9 rna polymerase ii, tata box binding protein (tbp)-associated factor, 32kda  KANSL3 - kat8 regulatory nsl complex subunit 3  ACTL6A - actin-like 6a  BRPF1 - bromodomain and phd finger containing, 1  TRRAP - transformation/transcription domain-associated protein  TAF6 - taf6 rna polymerase ii, tata box binding protein (tbp)-associated factor, 80kda  TADA2A - transcriptional adaptor 2a  ARRB1 - arrestin, beta 1  SUPT7L - suppressor of ty 7 (s. cerevisiae)-like  PHF15 - phd finger protein 15  CHD5 - chromodomain helicase dna binding protein 5  MECP2 - methyl cpg binding protein 2 (rett syndrome)  EPC1 - enhancer of polycomb homolog 1 (drosophila)  MORF4L2 - mortality factor 4 like 2  EP400 - e1a binding protein p400  USP22 - ubiquitin specific peptidase 22  ING5 - inhibitor of growth family, member 5  NAA60 - n(alpha)-acetyltransferase 60, natf catalytic subunit  MCM3AP - minichromosome maintenance complex component 3 associated protein  KAT5 - k(lysine) acetyltransferase 5  YEATS4 - yeats domain containing 4  ING3 - inhibitor of growth family, member 3  MAP3K7 - mitogen-activated protein kinase kinase kinase 7  PHF20 - phd finger protein 20  ACTL6B - actin-like 6b  HCFC1 - host cell factor c1 (vp16-accessory protein)  PHF10 - phd finger protein 10  OGT - o-linked n-acetylglucosamine (glcnac) transferase  ZZZ3 - zinc finger, zz-type containing 3  MBIP - map3k12 binding inhibitory protein 1  KANSL2 - kat8 regulatory nsl complex subunit 2  KAT6A - k(lysine) acetyltransferase 6a  KAT8 - k(lysine) acetyltransferase 8  MRGBP - mrg/morf4l binding protein  PHF14 - phd finger protein 14  RUVBL1 - ruvb-like 1 (e. coli)  PHF17 - phd finger protein 17  DMAP1 - dna methyltransferase 1 associated protein 1  KAT6B - k(lysine) acetyltransferase 6b  ZMPSTE24 - zinc metallopeptidase ste24  LEF1 - lymphoid enhancer-binding factor 1  MBD3 - methyl-cpg binding domain protein 3  GTF3C4 - general transcription factor iiic, polypeptide 4, 90kda  SUPT3H - suppressor of ty 3 homolog (s. cerevisiae)  YEATS2 - yeats domain containing 2  KAT7 - k(lysine) acetyltransferase 7  RUVBL2 - ruvb-like 2 (e. coli) |
| GO:0000226 | microtubule cytoskeleton organization | 2.83E-6 | 4.38E-4 | 1.19 (10334,256,6996,206) | [+] Show genes  MAPRE1 - microtubule-associated protein, rp/eb family, member 1  DST - dystonin  MARK2 - map/microtubule affinity-regulating kinase 2  MAPRE3 - microtubule-associated protein, rp/eb family, member 3  AKAP9 - a kinase (prka) anchor protein (yotiao) 9  SPIRE1 - spire actin nucleation factor 1  EML1 - echinoderm microtubule associated protein like 1  WDR47 - wd repeat domain 47  HAUS7 - haus augmin-like complex, subunit 7  NCOR1 - nuclear receptor corepressor 1  HOOK3 - hook microtubule-tethering protein 3  KATNB1 - katanin p80 (wd repeat containing) subunit b 1  LZTS2 - leucine zipper, putative tumor suppressor 2  SPAST - spastin  NCKAP5L - nck-associated protein 5-like  WDR73 - wd repeat domain 73  TUBB2A - tubulin, beta 2a class iia  TUBG1 - tubulin, gamma 1  BRSK1 - br serine/threonine kinase 1  NDEL1 - nude neurodevelopment protein 1-like 1  UBXN2B - ubx domain protein 2b  SON - son dna binding protein  GJA1 - gap junction protein, alpha 1, 43kda  SPIRE2 - spire actin nucleation factor 2  LIMK2 - lim domain kinase 2  LMNA - lamin a/c  LLGL1 - lethal giant larvae homolog 1 (drosophila)  MARK4 - map/microtubule affinity-regulating kinase 4  ZNF259 - zinc finger protein 259  CEP19 - centrosomal protein 19kda  NDE1 - nude neurodevelopment protein 1  PLK3 - polo-like kinase 3  MYH9 - myosin, heavy chain 9, non-muscle  MAP9 - microtubule-associated protein 9  VPS4B - vacuolar protein sorting 4 homolog b (s. cerevisiae)  HAUS6 - haus augmin-like complex, subunit 6  GPSM2 - g-protein signaling modulator 2  CNP - 2',3'-cyclic nucleotide 3' phosphodiesterase  SMC1A - structural maintenance of chromosomes 1a  STMN1 - stathmin 1  SPRY1 - sprouty homolog 1, antagonist of fgf signaling (drosophila)  SPRY2 - sprouty homolog 2 (drosophila)  TUBGCP2 - tubulin, gamma complex associated protein 2  STAG1 - stromal antigen 1  CCSAP - centriole, cilia and spindle-associated protein  CDK5RAP2 - cdk5 regulatory subunit associated protein 2  KATNA1 - katanin p60 (atpase containing) subunit a 1  EML2 - echinoderm microtubule associated protein like 2  TTLL5 - tubulin tyrosine ligase-like family, member 5  KIF2A - kinesin heavy chain member 2a  SUN2 - sad1 and unc84 domain containing 2  KIF3A - kinesin family member 3a  PIBF1 - progesterone immunomodulatory binding factor 1  TNKS - tankyrase, trf1-interacting ankyrin-related adp-ribose polymerase  EML3 - echinoderm microtubule associated protein like 3  FGFR1OP - fgfr1 oncogene partner  CUL9 - cullin 9  DYNC1LI1 - dynein, cytoplasmic 1, light intermediate chain 1  CLASP2 - cytoplasmic linker associated protein 2  VAMP4 - vesicle-associated membrane protein 4  PPP2R3C - protein phosphatase 2, regulatory subunit b'', gamma  EZR - ezrin  SBDS - shwachman-bodian-diamond syndrome  KPNB1 - karyopherin (importin) beta 1  KIFC2 - kinesin family member c2  RMDN1 - regulator of microtubule dynamics 1  UVRAG - uv radiation resistance associated  CRIPT - cysteine-rich pdz-binding protein  CALM2 - calmodulin 2 (phosphorylase kinase, delta)  AURKC - aurora kinase c  RANBP9 - ran binding protein 9  TUBA1C - tubulin, alpha 1c  VCP - valosin containing protein  STMN2 - stathmin-like 2  CRMP1 - collapsin response mediator protein 1  TUBA1B - tubulin, alpha 1b  TUBB - tubulin, beta class i  TUBGCP5 - tubulin, gamma complex associated protein 5  TTLL7 - tubulin tyrosine ligase-like family, member 7  AAAS - achalasia, adrenocortical insufficiency, alacrimia  TUBB4B - tubulin, beta 4b class ivb  KIF3B - kinesin family member 3b  TUBB3 - tubulin, beta 3 class iii  TPGS1 - tubulin polyglutamylase complex subunit 1  SS18 - synovial sarcoma translocation, chromosome 18  RAB11A - rab11a, member ras oncogene family  MAPRE2 - microtubule-associated protein, rp/eb family, member 2  TTL - tubulin tyrosine ligase  CCDC88A - coiled-coil domain containing 88a  HAUS1 - haus augmin-like complex, subunit 1  TUBGCP3 - tubulin, gamma complex associated protein 3  SETD2 - set domain containing 2  TTLL1 - tubulin tyrosine ligase-like family, member 1  CSNK1D - casein kinase 1, delta  TUBE1 - tubulin, epsilon 1  MSTO1 - misato 1, mitochondrial distribution and morphology regulator  XIAP - x-linked inhibitor of apoptosis  CAMSAP2 - calmodulin regulated spectrin-associated protein family, member 2  CCDC120 - coiled-coil domain containing 120  PLK1S1 - polo-like kinase 1 substrate 1  STMN3 - stathmin-like 3  DCTN1 - dynactin 1  FLNA - filamin a, alpha  CCSER2 - coiled-coil serine-rich protein 2  FOPNL - fgfr1op n-terminal like  MCPH1 - microcephalin 1  CEP350 - centrosomal protein 350kda  MAP7D3 - map7 domain containing 3  CHP1 - calcineurin-like ef-hand protein 1  SPATA7 - spermatogenesis associated 7  CDC14B - cell division cycle 14b  CCDC8 - coiled-coil domain containing 8  BICD2 - bicaudal d homolog 2 (drosophila)  KATNAL1 - katanin p60 subunit a-like 1  EML4 - echinoderm microtubule associated protein like 4  RNF19A - ring finger protein 19a, e3 ubiquitin protein ligase  MAP1S - microtubule-associated protein 1s  PAFAH1B1 - platelet-activating factor acetylhydrolase 1b, regulatory subunit 1 (45kda)  FBXW11 - f-box and wd repeat domain containing 11  DYNLT1 - dynein, light chain, tctex-type 1  GAS2L1 - growth arrest-specific 2 like 1  CKAP5 - cytoskeleton associated protein 5  HAUS5 - haus augmin-like complex, subunit 5  TUBGCP4 - tubulin, gamma complex associated protein 4  FGF13 - fibroblast growth factor 13  DCTN2 - dynactin 2 (p50)  TUBA1A - tubulin, alpha 1a  TACC1 - transforming, acidic coiled-coil containing protein 1  NSFL1C - nsfl1 (p97) cofactor (p47)  NUMA1 - nuclear mitotic apparatus protein 1  MECP2 - methyl cpg binding protein 2 (rett syndrome)  CLASP1 - cytoplasmic linker associated protein 1  ZW10 - zw10 kinetochore protein  RHOA - ras homolog family member a  KNSTRN - kinetochore-localized astrin/spag5 binding protein  CIB1 - calcium and integrin binding 1 (calmyrin)  TBCEL - tubulin folding cofactor e-like  FER - fer (fps/fes related) tyrosine kinase  ITGB1 - integrin, beta 1 (fibronectin receptor, beta polypeptide, antigen cd29 includes mdf2, msk12)  PTK2 - protein tyrosine kinase 2  EFHC1 - ef-hand domain (c-terminal) containing 1  CEP120 - centrosomal protein 120kda  CDK5 - cyclin-dependent kinase 5  SIK3 - sik family kinase 3  PCM1 - pericentriolar material 1  DAG1 - dystroglycan 1 (dystrophin-associated glycoprotein 1)  TBCE - tubulin folding cofactor e  TBCD - tubulin folding cofactor d  SPICE1 - spindle and centriole associated protein 1  ARHGEF2 - rho/rac guanine nucleotide exchange factor (gef) 2  CLIP3 - cap-gly domain containing linker protein 3  TUBB2B - tubulin, beta 2b class iib  OBSL1 - obscurin-like 1  HTT - huntingtin  CUL7 - cullin 7  ZNF207 - zinc finger protein 207  HAUS2 - haus augmin-like complex, subunit 2  TUBGCP6 - tubulin, gamma complex associated protein 6  GOLGA2 - golgin a2  HAUS3 - haus augmin-like complex, subunit 3  TAOK1 - tao kinase 1  SMC3 - structural maintenance of chromosomes 3  MARK3 - map/microtubule affinity-regulating kinase 3  MARK1 - map/microtubule affinity-regulating kinase 1  PLK2 - polo-like kinase 2  MAPT - microtubule-associated protein tau  MAP6 - microtubule-associated protein 6  MAP2 - microtubule-associated protein 2  MAP1B - microtubule-associated protein 1b  MAP1A - microtubule-associated protein 1a  CEP57 - centrosomal protein 57kda  CLIP2 - cap-gly domain containing linker protein 2  WEE1 - wee1 homolog (s. pombe)  PEX14 - peroxisomal biogenesis factor 14  CHD3 - chromodomain helicase dna binding protein 3  TUBG2 - tubulin, gamma 2  DOCK7 - dedicator of cytokinesis 7  TTBK2 - tau tubulin kinase 2  SLK - ste20-like kinase  INO80 - ino80 complex subunit  MAP7 - microtubule-associated protein 7  TTLL4 - tubulin tyrosine ligase-like family, member 4  RSPH9 - radial spoke head 9 homolog (chlamydomonas)  DLG1 - discs, large homolog 1 (drosophila)  GCC2 - grip and coiled-coil domain containing 2  NEFL - neurofilament, light polypeptide  RSPH1 - radial spoke head 1 homolog (chlamydomonas)  WRAP73 - wd repeat containing, antisense to tp73  FSD1 - fibronectin type iii and spry domain containing 1  ARHGEF10 - rho guanine nucleotide exchange factor (gef) 10  BCCIP - brca2 and cdkn1a interacting protein  DCTN6 - dynactin 6  TPPP3 - tubulin polymerization-promoting protein family member 3  CAMSAP1 - calmodulin regulated spectrin-associated protein 1  ATRX - alpha thalassemia/mental retardation syndrome x-linked  DYNC1H1 - dynein, cytoplasmic 1, heavy chain 1  DYNC1LI2 - dynein, cytoplasmic 1, light intermediate chain 2  GABARAP - gaba(a) receptor-associated protein  STAG2 - stromal antigen 2  CEP63 - centrosomal protein 63kda  IFT172 - intraflagellar transport 172 homolog (chlamydomonas)  MZT1 - mitotic spindle organizing protein 1  CCDC66 - coiled-coil domain containing 66  NAV1 - neuron navigator 1  BBS4 - bardet-biedl syndrome 4  BBS2 - bardet-biedl syndrome 2 |
| GO:0097711 | ciliary basal body-plasma membrane docking | 3.48E-6 | 5.33E-4 | 1.34 (10334,74,6996,67) | [+] Show genes  MAPRE1 - microtubule-associated protein, rp/eb family, member 1  CDK5RAP2 - cdk5 regulatory subunit associated protein 2  PRKACA - protein kinase, camp-dependent, catalytic, alpha  AKAP9 - a kinase (prka) anchor protein (yotiao) 9  CEP89 - centrosomal protein 89kda  B9D2 - b9 protein domain 2  TCTN3 - tectonic family member 3  HAUS3 - haus augmin-like complex, subunit 3  MKS1 - meckel syndrome, type 1  HAUS7 - haus augmin-like complex, subunit 7  RAB3IP - rab3a interacting protein  DCTN1 - dynactin 1  DYNLL1 - dynein, light chain, lc8-type 1  CEP290 - centrosomal protein 290kda  DCTN3 - dynactin 3 (p22)  FGFR1OP - fgfr1 oncogene partner  CCDC41 - coiled-coil domain containing 41  CEP57 - centrosomal protein 57kda  CNTRL - centriolin  TUBG1 - tubulin, gamma 1  PPP2R1A - protein phosphatase 2, regulatory subunit a, alpha  ODF2 - outer dense fiber of sperm tails 2  PAFAH1B1 - platelet-activating factor acetylhydrolase 1b, regulatory subunit 1 (45kda)  AZI1 - 5-azacytidine induced 1  TTBK2 - tau tubulin kinase 2  TCTN2 - tectonic family member 2  CCP110 - centriolar coiled coil protein 110kda  MARK4 - map/microtubule affinity-regulating kinase 4  CEP250 - centrosomal protein 250kda  TCTN1 - tectonic family member 1  IQCB1 - iq motif containing b1  CKAP5 - cytoskeleton associated protein 5  NDE1 - nude neurodevelopment protein 1  HAUS5 - haus augmin-like complex, subunit 5  TMEM216 - transmembrane protein 216  TUBB - tubulin, beta class i  DCTN2 - dynactin 2 (p50)  TUBA1A - tubulin, alpha 1a  CETN2 - centrin, ef-hand protein, 2  ALMS1 - alstrom syndrome 1  CEP70 - centrosomal protein 70kda  CLASP1 - cytoplasmic linker associated protein 1  HAUS6 - haus augmin-like complex, subunit 6  AHI1 - abelson helper integration site 1  TUBB4B - tubulin, beta 4b class ivb  CEP41 - centrosomal protein 41kda  DYNC1H1 - dynein, cytoplasmic 1, heavy chain 1  CEP78 - centrosomal protein 78kda  DYNC1I2 - dynein, cytoplasmic 1, intermediate chain 2  C2CD3 - c2 calcium-dependent domain containing 3  NPHP1 - nephronophthisis 1 (juvenile)  RAB11A - rab11a, member ras oncogene family  PCM1 - pericentriolar material 1  CEP63 - centrosomal protein 63kda  HAUS1 - haus augmin-like complex, subunit 1  SFI1 - sfi1 homolog, spindle assembly associated (yeast)  HSP90AA1 - heat shock protein 90kda alpha (cytosolic), class a member 1  CSNK1D - casein kinase 1, delta  B9D1 - b9 protein domain 1  ACTR1A - arp1 actin-related protein 1 homolog a, centractin alpha (yeast)  CEP76 - centrosomal protein 76kda  PRKAR2B - protein kinase, camp-dependent, regulatory, type ii, beta  SCLT1 - sodium channel and clathrin linker 1  YWHAG - tyrosine 3-monooxygenase/tryptophan 5-monooxygenase activation protein, gamma polypeptide  FBF1 - fas (tnfrsf6) binding factor 1  YWHAE - tyrosine 3-monooxygenase/tryptophan 5-monooxygenase activation protein, epsilon polypeptide  HAUS2 - haus augmin-like complex, subunit 2 |
| GO:0006351 | transcription, DNA-templated | 3.53E-6 | 5.35E-4 | 1.18 (10334,274,6996,219) | [+] Show genes  CCNK - cyclin k  ELL - elongation factor rna polymerase ii  NCBP2 - nuclear cap binding protein subunit 2, 20kda  NCBP1 - nuclear cap binding protein subunit 1, 80kda  TRIM24 - tripartite motif containing 24  MKI67IP - mki67 (fha domain) interacting nucleolar phosphoprotein  RELA - v-rel avian reticuloendotheliosis viral oncogene homolog a  ELL2 - elongation factor, rna polymerase ii, 2  SNAPC5 - small nuclear rna activating complex, polypeptide 5, 19kda  SP1 - sp1 transcription factor  EGR2 - early growth response 2  INTS4 - integrator complex subunit 4  TAF15 - taf15 rna polymerase ii, tata box binding protein (tbp)-associated factor, 68kda  GTF2IRD1 - gtf2i repeat domain containing 1  RNPS1 - rna binding protein s1, serine-rich domain  SPIN1 - spindlin 1  CREBL2 - camp responsive element binding protein-like 2  LDB1 - lim domain binding 1  ZC3H8 - zinc finger ccch-type containing 8  CREB1 - camp responsive element binding protein 1  USPL1 - ubiquitin specific peptidase like 1  TSC22D1 - tsc22 domain family, member 1  ABT1 - activator of basal transcription 1  TOPORS - topoisomerase i binding, arginine/serine-rich, e3 ubiquitin protein ligase  RPRD1B - regulation of nuclear pre-mrna domain containing 1b  SNAPC4 - small nuclear rna activating complex, polypeptide 4, 190kda  TBPL1 - tbp-like 1  NABP2 - nucleic acid binding protein 2  SNAPC2 - small nuclear rna activating complex, polypeptide 2, 45kda  SNAPC1 - small nuclear rna activating complex, polypeptide 1, 43kda  SNAPC3 - small nuclear rna activating complex, polypeptide 3, 50kda  POLR1C - polymerase (rna) i polypeptide c, 30kda  MED1 - mediator complex subunit 1  PPARD - peroxisome proliferator-activated receptor delta  POLRMT - polymerase (rna) mitochondrial (dna directed)  POLR2K - polymerase (rna) ii (dna directed) polypeptide k, 7.0kda  SMARCD3 - swi/snf related, matrix associated, actin dependent regulator of chromatin, subfamily d, member 3  MED20 - mediator complex subunit 20  COL4A2 - collagen, type iv, alpha 2  XAB2 - xpa binding protein 2  PARP1 - poly (adp-ribose) polymerase 1  FUBP3 - far upstream element (fuse) binding protein 3  PPP1R13L - protein phosphatase 1, regulatory subunit 13 like  ZBTB7A - zinc finger and btb domain containing 7a  C5AR1 - complement component 5a receptor 1  POLR2G - polymerase (rna) ii (dna directed) polypeptide g  POLR2F - polymerase (rna) ii (dna directed) polypeptide f  TCEANC2 - transcription elongation factor a (sii) n-terminal and central domain containing 2  POLR2E - polymerase (rna) ii (dna directed) polypeptide e, 25kda  SCAF1 - sr-related ctd-associated factor 1  POLR2D - polymerase (rna) ii (dna directed) polypeptide d  POLR2C - polymerase (rna) ii (dna directed) polypeptide c, 33kda  POLR2B - polymerase (rna) ii (dna directed) polypeptide b, 140kda  POLR2A - polymerase (rna) ii (dna directed) polypeptide a, 220kda  INTS9 - integrator complex subunit 9  POLR1B - polymerase (rna) i polypeptide b, 128kda  NELFE - negative elongation factor complex member e  INTS12 - integrator complex subunit 12  SUPT6H - suppressor of ty 6 homolog (s. cerevisiae)  POLR3A - polymerase (rna) iii (dna directed) polypeptide a, 155kda  SUPT4H1 - suppressor of ty 4 homolog 1 (s. cerevisiae)  SUPT5H - suppressor of ty 5 homolog (s. cerevisiae)  INTS6 - integrator complex subunit 6  PRDM4 - pr domain containing 4  PPP5C - protein phosphatase 5, catalytic subunit  MTF1 - metal-regulatory transcription factor 1  MED16 - mediator complex subunit 16  GPBP1 - gc-rich promoter binding protein 1  GPBP1L1 - gc-rich promoter binding protein 1-like 1  DIDO1 - death inducer-obliterator 1  INTS3 - integrator complex subunit 3  RPAP2 - rna polymerase ii associated protein 2  HSF2BP - heat shock transcription factor 2 binding protein  CCNT2 - cyclin t2  IWS1 - iws1 homolog (s. cerevisiae)  ERCC3 - excision repair cross-complementing rodent repair deficiency, complementation group 3  LIN52 - lin-52 homolog (c. elegans)  NPM3 - nucleophosmin/nucleoplasmin 3  STAT3 - signal transducer and activator of transcription 3 (acute-phase response factor)  CCNT1 - cyclin t1  CCNH - cyclin h  RNGTT - rna guanylyltransferase and 5'-phosphatase  RNMT - rna (guanine-7-) methyltransferase  POLR3GL - polymerase (rna) iii (dna directed) polypeptide g (32kd)-like  SSRP1 - structure specific recognition protein 1  HIRA - histone cell cycle regulator  SLC25A33 - solute carrier family 25 (pyrimidine nucleotide carrier), member 33  ETV1 - ets variant 1  INTS8 - integrator complex subunit 8  CBFA2T3 - core-binding factor, runt domain, alpha subunit 2; translocated to, 3  CBFB - core-binding factor, beta subunit  GTF3C5 - general transcription factor iiic, polypeptide 5, 63kda  POLR3E - polymerase (rna) iii (dna directed) polypeptide e (80kd)  UBTF - upstream binding transcription factor, rna polymerase i  TROVE2 - trove domain family, member 2  COPS2 - cop9 signalosome subunit 2  MED4 - mediator complex subunit 4  POLR3B - polymerase (rna) iii (dna directed) polypeptide b  GTF3C4 - general transcription factor iiic, polypeptide 4, 90kda  ERCC2 - excision repair cross-complementing rodent repair deficiency, complementation group 2  GTF3C3 - general transcription factor iiic, polypeptide 3, 102kda  NABP1 - nucleic acid binding protein 1  CNOT8 - ccr4-not transcription complex, subunit 8  DDX5 - dead (asp-glu-ala-asp) box helicase 5  TCOF1 - treacher collins-franceschetti syndrome 1  POLR3F - polymerase (rna) iii (dna directed) polypeptide f, 39 kda  INTS5 - integrator complex subunit 5  RBMX - rna binding motif protein, x-linked  GTF2F1 - general transcription factor iif, polypeptide 1, 74kda  GTF2F2 - general transcription factor iif, polypeptide 2, 30kda  GTF2E1 - general transcription factor iie, polypeptide 1, alpha 56kda  GTF2E2 - general transcription factor iie, polypeptide 2, beta 34kda  RPRD2 - regulation of nuclear pre-mrna domain containing 2  FLNA - filamin a, alpha  GTF2H2 - general transcription factor iih, polypeptide 2, 44kda  INTS10 - integrator complex subunit 10  GTF2H3 - general transcription factor iih, polypeptide 3, 34kda  GTF2H1 - general transcription factor iih, polypeptide 1, 62kda  MLLT3 - myeloid/lymphoid or mixed-lineage leukemia (trithorax homolog, drosophila); translocated to, 3  GTF2A2 - general transcription factor iia, 2, 12kda  MLLT1 - myeloid/lymphoid or mixed-lineage leukemia (trithorax homolog, drosophila); translocated to, 1  FOXO3 - forkhead box o3  GTF2B - general transcription factor iib  PHAX - phosphorylated adaptor for rna export  GTF2A1 - general transcription factor iia, 1, 19/37kda  NELFB - negative elongation factor complex member b  GTF3C2 - general transcription factor iiic, polypeptide 2, beta 110kda  GTF2I - general transcription factor iii  GTF2H4 - general transcription factor iih, polypeptide 4, 52kda  GTF3A - general transcription factor iiia  LMO4 - lim domain only 4  RPRD1A - regulation of nuclear pre-mrna domain containing 1a  BRF1 - brf1, rna polymerase iii transcription initiation factor 90 kda subunit  GTF3C1 - general transcription factor iiic, polypeptide 1, alpha 220kda  MNAT1 - mnat cdk-activating kinase assembly factor 1  POLR3G - polymerase (rna) iii (dna directed) polypeptide g (32kd)  WDR61 - wd repeat domain 61  MNT - mnt, max dimerization protein  POLR3K - polymerase (rna) iii (dna directed) polypeptide k, 12.3 kda  TAF9 - taf9 rna polymerase ii, tata box binding protein (tbp)-associated factor, 32kda  CELF3 - cugbp, elav-like family member 3  TAF7 - taf7 rna polymerase ii, tata box binding protein (tbp)-associated factor, 55kda  TAF6 - taf6 rna polymerase ii, tata box binding protein (tbp)-associated factor, 80kda  PAX8 - paired box 8  SUPT16H - suppressor of ty 16 homolog (s. cerevisiae)  TAF4 - taf4 rna polymerase ii, tata box binding protein (tbp)-associated factor, 135kda  TAF2 - taf2 rna polymerase ii, tata box binding protein (tbp)-associated factor, 150kda  ARRB2 - arrestin, beta 2  ARRB1 - arrestin, beta 1  CDC73 - cell division cycle 73  MEF2A - myocyte enhancer factor 2a  NR4A2 - nuclear receptor subfamily 4, group a, member 2  DDX21 - dead (asp-glu-ala-asp) box helicase 21  ZBTB7B - zinc finger and btb domain containing 7b  POLR1E - polymerase (rna) i polypeptide e, 53kda  RPAP1 - rna polymerase ii associated protein 1  TAF13 - taf13 rna polymerase ii, tata box binding protein (tbp)-associated factor, 18kda  BAZ2A - bromodomain adjacent to zinc finger domain, 2a  INTS2 - integrator complex subunit 2  PTMA - prothymosin, alpha  CDK9 - cyclin-dependent kinase 9  CDK7 - cyclin-dependent kinase 7  HINFP - histone h4 transcription factor  LEO1 - leo1, paf1/rna polymerase ii complex component, homolog (s. cerevisiae)  PPP1R1B - protein phosphatase 1, regulatory (inhibitor) subunit 1b  PWP1 - pwp1 homolog (s. cerevisiae)  POLR1D - polymerase (rna) i polypeptide d, 16kda  TCEB3 - transcription elongation factor b (siii), polypeptide 3 (110kda, elongin a)  THAP1 - thap domain containing, apoptosis associated protein 1  CRCP - cgrp receptor component  TCEA1 - transcription elongation factor a (sii), 1  CBFA2T2 - core-binding factor, runt domain, alpha subunit 2; translocated to, 2  ZBTB1 - zinc finger and btb domain containing 1  EAF1 - ell associated factor 1  MARS - methionyl-trna synthetase  AFF4 - af4/fmr2 family, member 4  MMS19 - mms19 nucleotide excision repair homolog (s. cerevisiae)  DR1 - down-regulator of transcription 1, tbp-binding (negative cofactor 2)  XBP1 - x-box binding protein 1  NFYA - nuclear transcription factor y, alpha  NFRKB - nuclear factor related to kappab binding protein  SKI - v-ski avian sarcoma viral oncogene homolog  MBD1 - methyl-cpg binding domain protein 1  MTERFD2 - mterf domain containing 2  PHF3 - phd finger protein 3  NELFA - negative elongation factor complex member a  NFKB1 - nuclear factor of kappa light polypeptide gene enhancer in b-cells 1  NFKBIB - nuclear factor of kappa light polypeptide gene enhancer in b-cells inhibitor, beta  RRAGC - ras-related gtp binding c  TARDBP - tar dna binding protein  NELFCD - negative elongation factor complex member c/d  PAF1 - paf1, rna polymerase ii associated factor, homolog (s. cerevisiae)  INO80 - ino80 complex subunit  DTX1 - deltex homolog 1 (drosophila)  TFAM - transcription factor a, mitochondrial  CTR9 - ctr9, paf1/rna polymerase ii complex component  GTF3C6 - general transcription factor iiic, polypeptide 6, alpha 35kda  TFDP1 - transcription factor dp-1  EPC1 - enhancer of polycomb homolog 1 (drosophila)  ASXL2 - additional sex combs like 2 (drosophila)  ASXL1 - additional sex combs like 1 (drosophila)  TFCP2 - transcription factor cp2  TAF8 - taf8 rna polymerase ii, tata box binding protein (tbp)-associated factor, 43kda  PKNOX1 - pbx/knotted 1 homeobox 1  TAF1B - tata box binding protein (tbp)-associated factor, rna polymerase i, b, 63kda  PHRF1 - phd and ring finger domains 1  SRRT - serrate rna effector molecule homolog (arabidopsis)  GMEB2 - glucocorticoid modulatory element binding protein 2  TAF1C - tata box binding protein (tbp)-associated factor, rna polymerase i, c, 110kda  FOSB - fbj murine osteosarcoma viral oncogene homolog b  FOS - fbj murine osteosarcoma viral oncogene homolog  DRG1 - developmentally regulated gtp binding protein 1  TAF9B - taf9b rna polymerase ii, tata box binding protein (tbp)-associated factor, 31kda  NFAT5 - nuclear factor of activated t-cells 5, tonicity-responsive  POLR3H - polymerase (rna) iii (dna directed) polypeptide h (22.9kd)  SMAD4 - smad family member 4  SUPT3H - suppressor of ty 3 homolog (s. cerevisiae)  INTS1 - integrator complex subunit 1  THRA - thyroid hormone receptor, alpha |
| GO:0097659 | nucleic acid-templated transcription | 3.53E-6 | 5.29E-4 | 1.18 (10334,274,6996,219) | [+] Show genes  CCNK - cyclin k  ELL - elongation factor rna polymerase ii  NCBP2 - nuclear cap binding protein subunit 2, 20kda  NCBP1 - nuclear cap binding protein subunit 1, 80kda  TRIM24 - tripartite motif containing 24  MKI67IP - mki67 (fha domain) interacting nucleolar phosphoprotein  RELA - v-rel avian reticuloendotheliosis viral oncogene homolog a  ELL2 - elongation factor, rna polymerase ii, 2  SNAPC5 - small nuclear rna activating complex, polypeptide 5, 19kda  SP1 - sp1 transcription factor  EGR2 - early growth response 2  INTS4 - integrator complex subunit 4  TAF15 - taf15 rna polymerase ii, tata box binding protein (tbp)-associated factor, 68kda  RNPS1 - rna binding protein s1, serine-rich domain  GTF2IRD1 - gtf2i repeat domain containing 1  SPIN1 - spindlin 1  CREBL2 - camp responsive element binding protein-like 2  LDB1 - lim domain binding 1  ZC3H8 - zinc finger ccch-type containing 8  CREB1 - camp responsive element binding protein 1  USPL1 - ubiquitin specific peptidase like 1  TSC22D1 - tsc22 domain family, member 1  ABT1 - activator of basal transcription 1  TOPORS - topoisomerase i binding, arginine/serine-rich, e3 ubiquitin protein ligase  RPRD1B - regulation of nuclear pre-mrna domain containing 1b  SNAPC4 - small nuclear rna activating complex, polypeptide 4, 190kda  TBPL1 - tbp-like 1  NABP2 - nucleic acid binding protein 2  SNAPC2 - small nuclear rna activating complex, polypeptide 2, 45kda  SNAPC1 - small nuclear rna activating complex, polypeptide 1, 43kda  SNAPC3 - small nuclear rna activating complex, polypeptide 3, 50kda  POLR1C - polymerase (rna) i polypeptide c, 30kda  MED1 - mediator complex subunit 1  PPARD - peroxisome proliferator-activated receptor delta  POLRMT - polymerase (rna) mitochondrial (dna directed)  POLR2K - polymerase (rna) ii (dna directed) polypeptide k, 7.0kda  SMARCD3 - swi/snf related, matrix associated, actin dependent regulator of chromatin, subfamily d, member 3  MED20 - mediator complex subunit 20  XAB2 - xpa binding protein 2  COL4A2 - collagen, type iv, alpha 2  PARP1 - poly (adp-ribose) polymerase 1  FUBP3 - far upstream element (fuse) binding protein 3  PPP1R13L - protein phosphatase 1, regulatory subunit 13 like  ZBTB7A - zinc finger and btb domain containing 7a  C5AR1 - complement component 5a receptor 1  POLR2G - polymerase (rna) ii (dna directed) polypeptide g  POLR2F - polymerase (rna) ii (dna directed) polypeptide f  POLR2E - polymerase (rna) ii (dna directed) polypeptide e, 25kda  TCEANC2 - transcription elongation factor a (sii) n-terminal and central domain containing 2  POLR2D - polymerase (rna) ii (dna directed) polypeptide d  SCAF1 - sr-related ctd-associated factor 1  POLR2C - polymerase (rna) ii (dna directed) polypeptide c, 33kda  POLR2B - polymerase (rna) ii (dna directed) polypeptide b, 140kda  POLR2A - polymerase (rna) ii (dna directed) polypeptide a, 220kda  INTS9 - integrator complex subunit 9  POLR1B - polymerase (rna) i polypeptide b, 128kda  NELFE - negative elongation factor complex member e  INTS12 - integrator complex subunit 12  SUPT6H - suppressor of ty 6 homolog (s. cerevisiae)  POLR3A - polymerase (rna) iii (dna directed) polypeptide a, 155kda  SUPT4H1 - suppressor of ty 4 homolog 1 (s. cerevisiae)  INTS6 - integrator complex subunit 6  SUPT5H - suppressor of ty 5 homolog (s. cerevisiae)  PRDM4 - pr domain containing 4  PPP5C - protein phosphatase 5, catalytic subunit  MTF1 - metal-regulatory transcription factor 1  MED16 - mediator complex subunit 16  GPBP1 - gc-rich promoter binding protein 1  DIDO1 - death inducer-obliterator 1  GPBP1L1 - gc-rich promoter binding protein 1-like 1  INTS3 - integrator complex subunit 3  RPAP2 - rna polymerase ii associated protein 2  HSF2BP - heat shock transcription factor 2 binding protein  CCNT2 - cyclin t2  LIN52 - lin-52 homolog (c. elegans)  ERCC3 - excision repair cross-complementing rodent repair deficiency, complementation group 3  IWS1 - iws1 homolog (s. cerevisiae)  NPM3 - nucleophosmin/nucleoplasmin 3  STAT3 - signal transducer and activator of transcription 3 (acute-phase response factor)  CCNT1 - cyclin t1  CCNH - cyclin h  RNGTT - rna guanylyltransferase and 5'-phosphatase  RNMT - rna (guanine-7-) methyltransferase  POLR3GL - polymerase (rna) iii (dna directed) polypeptide g (32kd)-like  SSRP1 - structure specific recognition protein 1  HIRA - histone cell cycle regulator  SLC25A33 - solute carrier family 25 (pyrimidine nucleotide carrier), member 33  ETV1 - ets variant 1  INTS8 - integrator complex subunit 8  CBFA2T3 - core-binding factor, runt domain, alpha subunit 2; translocated to, 3  CBFB - core-binding factor, beta subunit  GTF3C5 - general transcription factor iiic, polypeptide 5, 63kda  POLR3E - polymerase (rna) iii (dna directed) polypeptide e (80kd)  UBTF - upstream binding transcription factor, rna polymerase i  TROVE2 - trove domain family, member 2  COPS2 - cop9 signalosome subunit 2  MED4 - mediator complex subunit 4  POLR3B - polymerase (rna) iii (dna directed) polypeptide b  GTF3C4 - general transcription factor iiic, polypeptide 4, 90kda  ERCC2 - excision repair cross-complementing rodent repair deficiency, complementation group 2  GTF3C3 - general transcription factor iiic, polypeptide 3, 102kda  NABP1 - nucleic acid binding protein 1  CNOT8 - ccr4-not transcription complex, subunit 8  DDX5 - dead (asp-glu-ala-asp) box helicase 5  TCOF1 - treacher collins-franceschetti syndrome 1  POLR3F - polymerase (rna) iii (dna directed) polypeptide f, 39 kda  INTS5 - integrator complex subunit 5  RBMX - rna binding motif protein, x-linked  GTF2F1 - general transcription factor iif, polypeptide 1, 74kda  GTF2F2 - general transcription factor iif, polypeptide 2, 30kda  GTF2E1 - general transcription factor iie, polypeptide 1, alpha 56kda  GTF2E2 - general transcription factor iie, polypeptide 2, beta 34kda  RPRD2 - regulation of nuclear pre-mrna domain containing 2  FLNA - filamin a, alpha  GTF2H2 - general transcription factor iih, polypeptide 2, 44kda  INTS10 - integrator complex subunit 10  GTF2H3 - general transcription factor iih, polypeptide 3, 34kda  GTF2H1 - general transcription factor iih, polypeptide 1, 62kda  MLLT3 - myeloid/lymphoid or mixed-lineage leukemia (trithorax homolog, drosophila); translocated to, 3  GTF2A2 - general transcription factor iia, 2, 12kda  MLLT1 - myeloid/lymphoid or mixed-lineage leukemia (trithorax homolog, drosophila); translocated to, 1  FOXO3 - forkhead box o3  GTF2B - general transcription factor iib  PHAX - phosphorylated adaptor for rna export  GTF2A1 - general transcription factor iia, 1, 19/37kda  NELFB - negative elongation factor complex member b  GTF3C2 - general transcription factor iiic, polypeptide 2, beta 110kda  GTF2I - general transcription factor iii  GTF2H4 - general transcription factor iih, polypeptide 4, 52kda  GTF3A - general transcription factor iiia  LMO4 - lim domain only 4  RPRD1A - regulation of nuclear pre-mrna domain containing 1a  BRF1 - brf1, rna polymerase iii transcription initiation factor 90 kda subunit  GTF3C1 - general transcription factor iiic, polypeptide 1, alpha 220kda  MNAT1 - mnat cdk-activating kinase assembly factor 1  POLR3G - polymerase (rna) iii (dna directed) polypeptide g (32kd)  WDR61 - wd repeat domain 61  MNT - mnt, max dimerization protein  POLR3K - polymerase (rna) iii (dna directed) polypeptide k, 12.3 kda  TAF9 - taf9 rna polymerase ii, tata box binding protein (tbp)-associated factor, 32kda  CELF3 - cugbp, elav-like family member 3  TAF7 - taf7 rna polymerase ii, tata box binding protein (tbp)-associated factor, 55kda  TAF6 - taf6 rna polymerase ii, tata box binding protein (tbp)-associated factor, 80kda  PAX8 - paired box 8  SUPT16H - suppressor of ty 16 homolog (s. cerevisiae)  TAF4 - taf4 rna polymerase ii, tata box binding protein (tbp)-associated factor, 135kda  TAF2 - taf2 rna polymerase ii, tata box binding protein (tbp)-associated factor, 150kda  ARRB2 - arrestin, beta 2  ARRB1 - arrestin, beta 1  CDC73 - cell division cycle 73  MEF2A - myocyte enhancer factor 2a  NR4A2 - nuclear receptor subfamily 4, group a, member 2  DDX21 - dead (asp-glu-ala-asp) box helicase 21  ZBTB7B - zinc finger and btb domain containing 7b  POLR1E - polymerase (rna) i polypeptide e, 53kda  RPAP1 - rna polymerase ii associated protein 1  TAF13 - taf13 rna polymerase ii, tata box binding protein (tbp)-associated factor, 18kda  BAZ2A - bromodomain adjacent to zinc finger domain, 2a  INTS2 - integrator complex subunit 2  PTMA - prothymosin, alpha  CDK9 - cyclin-dependent kinase 9  CDK7 - cyclin-dependent kinase 7  HINFP - histone h4 transcription factor  LEO1 - leo1, paf1/rna polymerase ii complex component, homolog (s. cerevisiae)  PPP1R1B - protein phosphatase 1, regulatory (inhibitor) subunit 1b  PWP1 - pwp1 homolog (s. cerevisiae)  TCEB3 - transcription elongation factor b (siii), polypeptide 3 (110kda, elongin a)  POLR1D - polymerase (rna) i polypeptide d, 16kda  THAP1 - thap domain containing, apoptosis associated protein 1  CRCP - cgrp receptor component  TCEA1 - transcription elongation factor a (sii), 1  CBFA2T2 - core-binding factor, runt domain, alpha subunit 2; translocated to, 2  ZBTB1 - zinc finger and btb domain containing 1  EAF1 - ell associated factor 1  MARS - methionyl-trna synthetase  AFF4 - af4/fmr2 family, member 4  MMS19 - mms19 nucleotide excision repair homolog (s. cerevisiae)  DR1 - down-regulator of transcription 1, tbp-binding (negative cofactor 2)  XBP1 - x-box binding protein 1  NFYA - nuclear transcription factor y, alpha  NFRKB - nuclear factor related to kappab binding protein  SKI - v-ski avian sarcoma viral oncogene homolog  MBD1 - methyl-cpg binding domain protein 1  MTERFD2 - mterf domain containing 2  PHF3 - phd finger protein 3  NELFA - negative elongation factor complex member a  NFKB1 - nuclear factor of kappa light polypeptide gene enhancer in b-cells 1  NFKBIB - nuclear factor of kappa light polypeptide gene enhancer in b-cells inhibitor, beta  RRAGC - ras-related gtp binding c  TARDBP - tar dna binding protein  NELFCD - negative elongation factor complex member c/d  PAF1 - paf1, rna polymerase ii associated factor, homolog (s. cerevisiae)  INO80 - ino80 complex subunit  DTX1 - deltex homolog 1 (drosophila)  TFAM - transcription factor a, mitochondrial  CTR9 - ctr9, paf1/rna polymerase ii complex component  GTF3C6 - general transcription factor iiic, polypeptide 6, alpha 35kda  TFDP1 - transcription factor dp-1  EPC1 - enhancer of polycomb homolog 1 (drosophila)  ASXL2 - additional sex combs like 2 (drosophila)  ASXL1 - additional sex combs like 1 (drosophila)  TFCP2 - transcription factor cp2  TAF8 - taf8 rna polymerase ii, tata box binding protein (tbp)-associated factor, 43kda  PKNOX1 - pbx/knotted 1 homeobox 1  TAF1B - tata box binding protein (tbp)-associated factor, rna polymerase i, b, 63kda  PHRF1 - phd and ring finger domains 1  GMEB2 - glucocorticoid modulatory element binding protein 2  SRRT - serrate rna effector molecule homolog (arabidopsis)  TAF1C - tata box binding protein (tbp)-associated factor, rna polymerase i, c, 110kda  FOSB - fbj murine osteosarcoma viral oncogene homolog b  FOS - fbj murine osteosarcoma viral oncogene homolog  DRG1 - developmentally regulated gtp binding protein 1  TAF9B - taf9b rna polymerase ii, tata box binding protein (tbp)-associated factor, 31kda  NFAT5 - nuclear factor of activated t-cells 5, tonicity-responsive  SMAD4 - smad family member 4  POLR3H - polymerase (rna) iii (dna directed) polypeptide h (22.9kd)  SUPT3H - suppressor of ty 3 homolog (s. cerevisiae)  THRA - thyroid hormone receptor, alpha  INTS1 - integrator complex subunit 1 |
| GO:0006475 | internal protein amino acid acetylation | 3.59E-6 | 5.31E-4 | 1.30 (10334,98,6996,86) | [+] Show genes  HAT1 - histone acetyltransferase 1  MSL3 - male-specific lethal 3 homolog (drosophila)  EPC2 - enhancer of polycomb homolog 2 (drosophila)  TADA1 - transcriptional adaptor 1  MCRS1 - microspherule protein 1  ATAT1 - alpha tubulin acetyltransferase 1  MAPT - microtubule-associated protein tau  NAA40 - n(alpha)-acetyltransferase 40, natd catalytic subunit, homolog (s. cerevisiae)  MSL2 - male-specific lethal 2 homolog (drosophila)  MEAF6 - myst/esa1-associated factor 6  EP300 - e1a binding protein p300  RSF1 - remodeling and spacing factor 1  ING4 - inhibitor of growth family, member 4  DR1 - down-regulator of transcription 1, tbp-binding (negative cofactor 2)  DPF2 - d4, zinc and double phd fingers family 2  CSRP2BP - csrp2 binding protein  KANSL1 - kat8 regulatory nsl complex subunit 1  GTF2B - general transcription factor iib  KAT2A - k(lysine) acetyltransferase 2a  PCGF2 - polycomb group ring finger 2  NCOA3 - nuclear receptor coactivator 3  APBB1 - amyloid beta (a4) precursor protein-binding, family b, member 1 (fe65)  KMT2A - lysine (k)-specific methyltransferase 2a  BRPF3 - bromodomain and phd finger containing, 3  BRD8 - bromodomain containing 8  TADA3 - transcriptional adaptor 3  SPHK2 - sphingosine kinase 2  TAF6L - taf6-like rna polymerase ii, p300/cbp-associated factor (pcaf)-associated factor, 65kda  BRD1 - bromodomain containing 1  LDB1 - lim domain binding 1  PHF20L1 - phd finger protein 20-like 1  CREBBP - creb binding protein  MORF4L1 - mortality factor 4 like 1  ATF2 - activating transcription factor 2  MSL1 - male-specific lethal 1 homolog (drosophila)  KAT2B - k(lysine) acetyltransferase 2b  CLOCK - clock circadian regulator  CRTC2 - creb regulated transcription coactivator 2  KANSL1L - kat8 regulatory nsl complex subunit 1-like  TAF9 - taf9 rna polymerase ii, tata box binding protein (tbp)-associated factor, 32kda  KANSL3 - kat8 regulatory nsl complex subunit 3  ACTL6A - actin-like 6a  BRPF1 - bromodomain and phd finger containing, 1  TRRAP - transformation/transcription domain-associated protein  TAF6 - taf6 rna polymerase ii, tata box binding protein (tbp)-associated factor, 80kda  TADA2A - transcriptional adaptor 2a  ARRB1 - arrestin, beta 1  SUPT7L - suppressor of ty 7 (s. cerevisiae)-like  PHF15 - phd finger protein 15  CHD5 - chromodomain helicase dna binding protein 5  MECP2 - methyl cpg binding protein 2 (rett syndrome)  EPC1 - enhancer of polycomb homolog 1 (drosophila)  MORF4L2 - mortality factor 4 like 2  EP400 - e1a binding protein p400  USP22 - ubiquitin specific peptidase 22  ING5 - inhibitor of growth family, member 5  NAA60 - n(alpha)-acetyltransferase 60, natf catalytic subunit  MCM3AP - minichromosome maintenance complex component 3 associated protein  KAT5 - k(lysine) acetyltransferase 5  YEATS4 - yeats domain containing 4  ING3 - inhibitor of growth family, member 3  MAP3K7 - mitogen-activated protein kinase kinase kinase 7  PHF20 - phd finger protein 20  ACTL6B - actin-like 6b  HCFC1 - host cell factor c1 (vp16-accessory protein)  PHF10 - phd finger protein 10  OGT - o-linked n-acetylglucosamine (glcnac) transferase  ZZZ3 - zinc finger, zz-type containing 3  MBIP - map3k12 binding inhibitory protein 1  KANSL2 - kat8 regulatory nsl complex subunit 2  KAT6A - k(lysine) acetyltransferase 6a  KAT8 - k(lysine) acetyltransferase 8  MRGBP - mrg/morf4l binding protein  PHF14 - phd finger protein 14  RUVBL1 - ruvb-like 1 (e. coli)  PHF17 - phd finger protein 17  DMAP1 - dna methyltransferase 1 associated protein 1  KAT6B - k(lysine) acetyltransferase 6b  ZMPSTE24 - zinc metallopeptidase ste24  LEF1 - lymphoid enhancer-binding factor 1  MBD3 - methyl-cpg binding domain protein 3  GTF3C4 - general transcription factor iiic, polypeptide 4, 90kda  SUPT3H - suppressor of ty 3 homolog (s. cerevisiae)  YEATS2 - yeats domain containing 2  KAT7 - k(lysine) acetyltransferase 7  RUVBL2 - ruvb-like 2 (e. coli) |
| GO:0016050 | vesicle organization | 3.64E-6 | 5.33E-4 | 1.19 (10334,243,6996,196) | [+] Show genes  TBC1D20 - tbc1 domain family, member 20  CHMP4C - charged multivesicular body protein 4c  RNF26 - ring finger protein 26  BET1 - bet1 golgi vesicular membrane trafficking protein  CD2AP - cd2-associated protein  ABCA1 - atp-binding cassette, sub-family a (abc1), member 1  VPS37A - vacuolar protein sorting 37 homolog a (s. cerevisiae)  CORO1C - coronin, actin binding protein, 1c  TMED2 - transmembrane emp24 domain trafficking protein 2  HOOK3 - hook microtubule-tethering protein 3  AGFG1 - arfgap with fg repeats 1  TMED10 - transmembrane emp24-like trafficking protein 10 (yeast)  CNIH - cornichon homolog (drosophila)  FNBP1L - formin binding protein 1-like  SEC22B - sec22 vesicle trafficking protein homolog b (s. cerevisiae) (gene/pseudogene)  TFG - trk-fused gene  GOSR2 - golgi snap receptor complex member 2  SAR1A - sar1 homolog a (s. cerevisiae)  TSG101 - tumor susceptibility gene 101  CREB1 - camp responsive element binding protein 1  LMAN1 - lectin, mannose-binding, 1  MVB12A - multivesicular body subunit 12a  RAB1B - rab1b, member ras oncogene family  PICALM - phosphatidylinositol binding clathrin assembly protein  SNX11 - sorting nexin 11  TRAPPC1 - trafficking protein particle complex 1  TBPL1 - tbp-like 1  VPS4B - vacuolar protein sorting 4 homolog b (s. cerevisiae)  SNX10 - sorting nexin 10  SNAP25 - synaptosomal-associated protein, 25kda  GOSR1 - golgi snap receptor complex member 1  STX6 - syntaxin 6  AP1M1 - adaptor-related protein complex 1, mu 1 subunit  MX1 - myxovirus (influenza virus) resistance 1, interferon-inducible protein p78 (mouse)  TRAPPC4 - trafficking protein particle complex 4  UNC13A - unc-13 homolog a (c. elegans)  SNX19 - sorting nexin 19  STAM2 - signal transducing adaptor molecule (sh3 domain and itam motif) 2  GNAI3 - guanine nucleotide binding protein (g protein), alpha inhibiting activity polypeptide 3  STX8 - syntaxin 8  AP3D1 - adaptor-related protein complex 3, delta 1 subunit  SNAP47 - synaptosomal-associated protein, 47kda  RAB38 - rab38, member ras oncogene family  STX12 - syntaxin 12  BAIAP3 - bai1-associated protein 3  BLOC1S3 - biogenesis of lysosomal organelles complex-1, subunit 3  SYP - synaptophysin  VAV3 - vav 3 guanine nucleotide exchange factor  AP1G1 - adaptor-related protein complex 1, gamma 1 subunit  VPS28 - vacuolar protein sorting 28 homolog (s. cerevisiae)  SRGN - serglycin  VPS37C - vacuolar protein sorting 37 homolog c (s. cerevisiae)  KLHL12 - kelch-like family member 12  SYT4 - synaptotagmin iv  PDCD6 - programmed cell death 6  VAMP2 - vesicle-associated membrane protein 2 (synaptobrevin 2)  PPP6C - protein phosphatase 6, catalytic subunit  PDCD6IP - programmed cell death 6 interacting protein  SEC23A - sec23 homolog a (s. cerevisiae)  STXBP1 - syntaxin binding protein 1  STX5 - syntaxin 5  STX4 - syntaxin 4  STX3 - syntaxin 3  STX1A - syntaxin 1a (brain)  RAB7A - rab7a, member ras oncogene family  STX17 - syntaxin 17  TGFBRAP1 - transforming growth factor, beta receptor associated protein 1  LAMTOR1 - late endosomal/lysosomal adaptor, mapk and mtor activator 1  CALR - calreticulin  EXOC8 - exocyst complex component 8  UNC13B - unc-13 homolog b (c. elegans)  F2R - coagulation factor ii (thrombin) receptor  VTI1B - vesicle transport through interaction with t-snares 1b  ARFGAP3 - adp-ribosylation factor gtpase activating protein 3  SNX3 - sorting nexin 3  TSNARE1 - t-snare domain containing 1  SEC16A - sec16 homolog a (s. cerevisiae)  CHMP7 - charged multivesicular body protein 7  GBF1 - golgi brefeldin a resistant guanine nucleotide exchange factor 1  TMEM175 - transmembrane protein 175  TMEM127 - transmembrane protein 127  VPS33B - vacuolar protein sorting 33 homolog b (yeast)  SNX33 - sorting nexin 33  BLOC1S5 - biogenesis of lysosomal organelles complex-1, subunit 5, muted  RAB11A - rab11a, member ras oncogene family  CHMP4A - charged multivesicular body protein 4a  CAV2 - caveolin 2  CAV1 - caveolin 1, caveolae protein, 22kda  NAPA - n-ethylmaleimide-sensitive factor attachment protein, alpha  SYT11 - synaptotagmin xi  ZDHHC2 - zinc finger, dhhc-type containing 2  SNAP29 - synaptosomal-associated protein, 29kda  STAM - signal transducing adaptor molecule (sh3 domain and itam motif) 1  SEC24B - sec24 family, member b (s. cerevisiae)  VAMP3 - vesicle-associated membrane protein 3  CSNK1D - casein kinase 1, delta  PRKCI - protein kinase c, iota  ANXA2 - annexin a2  STX2 - syntaxin 2  VPS25 - vacuolar protein sorting 25 homolog (s. cerevisiae)  EPS15 - epidermal growth factor receptor pathway substrate 15  TRAPPC2 - trafficking protein particle complex 2  SEC13 - sec13 homolog (s. cerevisiae)  SCFD1 - sec1 family domain containing 1  SEC24D - sec24 family, member d (s. cerevisiae)  RAB8B - rab8b, member ras oncogene family  C2CD5 - c2 calcium-dependent domain containing 5  ANKRD28 - ankyrin repeat domain 28  TRAPPC2L - trafficking protein particle complex 2-like  AP3B1 - adaptor-related protein complex 3, beta 1 subunit  RAB22A - rab22a, member ras oncogene family  ARF1 - adp-ribosylation factor 1  GOLPH3L - golgi phosphoprotein 3-like  PAFAH1B1 - platelet-activating factor acetylhydrolase 1b, regulatory subunit 1 (45kda)  GORASP1 - golgi reassembly stacking protein 1, 65kda  CD34 - cd34 molecule  VPS11 - vacuolar protein sorting 11 homolog (s. cerevisiae)  SNAP91 - synaptosomal-associated protein, 91kda  STX1B - syntaxin 1b  HIAT1 - hippocampus abundant transcript 1  VPS8 - vacuolar protein sorting 8 homolog (s. cerevisiae)  TRAPPC6B - trafficking protein particle complex 6b  DNM3 - dynamin 3  NSF - n-ethylmaleimide-sensitive factor  SEC23IP - sec23 interacting protein  VAPB - vamp (vesicle-associated membrane protein)-associated protein b and c  VPS39 - vacuolar protein sorting 39 homolog (s. cerevisiae)  IST1 - increased sodium tolerance 1 homolog (yeast)  MCFD2 - multiple coagulation factor deficiency 2  TAP2 - transporter 2, atp-binding cassette, sub-family b (mdr/tap)  TAPBP - tap binding protein (tapasin)  FAM160A2 - family with sequence similarity 160, member a2  DNAJC13 - dnaj (hsp40) homolog, subfamily c, member 13  RAB8A - rab8a, member ras oncogene family  CHMP1A - charged multivesicular body protein 1a  PLEKHJ1 - pleckstrin homology domain containing, family j member 1  AKTIP - akt interacting protein  S100A10 - s100 calcium binding protein a10  CHMP2B - charged multivesicular body protein 2b  USO1 - uso1 vesicle transport factor  HGS - hepatocyte growth factor-regulated tyrosine kinase substrate  ARFGEF2 - adp-ribosylation factor guanine nucleotide-exchange factor 2 (brefeldin a-inhibited)  VTA1 - vesicle (multivesicular body) trafficking 1  SPG11 - spastic paraplegia 11 (autosomal recessive)  TRAPPC3 - trafficking protein particle complex 3  NKD2 - naked cuticle homolog 2 (drosophila)  GOLGA2 - golgin a2  PTPRN - protein tyrosine phosphatase, receptor type, n  BLOC1S2 - biogenesis of lysosomal organelles complex-1, subunit 2  LYST - lysosomal trafficking regulator  ATP13A2 - atpase type 13a2  BLOC1S4 - biogenesis of lysosomal organelles complex-1, subunit 4, cappuccino  RAB14 - rab14, member ras oncogene family  SEC31A - sec31 homolog a (s. cerevisiae)  PVRL2 - poliovirus receptor-related 2 (herpesvirus entry mediator b)  USP8 - ubiquitin specific peptidase 8  TRAPPC10 - trafficking protein particle complex 10  TMF1 - tata element modulatory factor 1  RAB5B - rab5b, member ras oncogene family  RAB3A - rab3a, member ras oncogene family  ZFYVE16 - zinc finger, fyve domain containing 16  PPP6R1 - protein phosphatase 6, regulatory subunit 1  RAB1A - rab1a, member ras oncogene family  SYT13 - synaptotagmin xiii  CHMP5 - charged multivesicular body protein 5  PI4K2A - phosphatidylinositol 4-kinase type 2 alpha  SEC24A - sec24 family, member a (s. cerevisiae)  VPS4A - vacuolar protein sorting 4 homolog a (s. cerevisiae)  ANKFY1 - ankyrin repeat and fyve domain containing 1  RAB27A - rab27a, member ras oncogene family  SYT7 - synaptotagmin vii  DLG4 - discs, large homolog 4 (drosophila)  VPS18 - vacuolar protein sorting 18 homolog (s. cerevisiae)  BLOC1S6 - biogenesis of lysosomal organelles complex-1, subunit 6, pallidin  TRAPPC9 - trafficking protein particle complex 9  EEA1 - early endosome antigen 1  TGFA - transforming growth factor, alpha  PEF1 - penta-ef-hand domain containing 1  CHMP3 - charged multivesicular body protein 3  SEC24C - sec24 family, member c (s. cerevisiae)  STX7 - syntaxin 7  GOLPH3 - golgi phosphoprotein 3 (coat-protein)  VPS37D - vacuolar protein sorting 37 homolog d (s. cerevisiae)  SERPINE2 - serpin peptidase inhibitor, clade e (nexin, plasminogen activator inhibitor type 1), member 2  RAB34 - rab34, member ras oncogene family  DNM2 - dynamin 2  AP2M1 - adaptor-related protein complex 2, mu 1 subunit  ALS2 - amyotrophic lateral sclerosis 2 (juvenile)  VPS41 - vacuolar protein sorting 41 homolog (s. cerevisiae)  PPP6R3 - protein phosphatase 6, regulatory subunit 3  HPS4 - hermansky-pudlak syndrome 4  BCL2 - b-cell cll/lymphoma 2  PIK3C3 - phosphatidylinositol 3-kinase, catalytic subunit type 3  CUL3 - cullin 3  CHMP4B - charged multivesicular body protein 4b  WASL - wiskott-aldrich syndrome-like |
| GO:0045934 | negative regulation of nucleobase-containing compound metabolic process | 4.28E-6 | 6.2E-4 | 1.09 (10334,956,6996,708) | [+] Show genes  ZMYND8 - zinc finger, mynd-type containing 8  RBAK - rb-associated krab zinc finger  ZNF239 - zinc finger protein 239  MSL3 - male-specific lethal 3 homolog (drosophila)  EXOSC10 - exosome component 10  PHF21A - phd finger protein 21a  HNRNPA0 - heterogeneous nuclear ribonucleoprotein a0  CBX1 - chromobox homolog 1  FBXO18 - f-box protein, helicase, 18  PATZ1 - poz (btb) and at hook containing zinc finger 1  LIG3 - ligase iii, dna, atp-dependent  NCOR1 - nuclear receptor corepressor 1  NCOR2 - nuclear receptor corepressor 2  PML - promyelocytic leukemia  PHC3 - polyhomeotic homolog 3 (drosophila)  OBFC1 - oligonucleotide/oligosaccharide-binding fold containing 1  MIER1 - mesoderm induction early response 1, transcriptional regulator  DOT1L - dot1-like histone h3k79 methyltransferase  YWHAQ - tyrosine 3-monooxygenase/tryptophan 5-monooxygenase activation protein, theta polypeptide  NRIP1 - nuclear receptor interacting protein 1  ZNF705A - zinc finger protein 705a  SP3 - sp3 transcription factor  L3MBTL3 - l(3)mbt-like 3 (drosophila)  SP2 - sp2 transcription factor  CIR1 - corepressor interacting with rbpj, 1  SLX4 - slx4 structure-specific endonuclease subunit  WWC2 - ww and c2 domain containing 2  LYAR - ly1 antibody reactive  GTPBP4 - gtp binding protein 4  H2AFY - h2a histone family, member y  ZNF354B - zinc finger protein 354b  TCERG1 - transcription elongation regulator 1  TAF15 - taf15 rna polymerase ii, tata box binding protein (tbp)-associated factor, 68kda  RNPS1 - rna binding protein s1, serine-rich domain  GTF2IRD1 - gtf2i repeat domain containing 1  OTUB1 - otu domain, ubiquitin aldehyde binding 1  EHMT2 - euchromatic histone-lysine n-methyltransferase 2  GJA1 - gap junction protein, alpha 1, 43kda  SUB1 - sub1 homolog (s. cerevisiae)  MORF4L1 - mortality factor 4 like 1  CREBBP - creb binding protein  SOX13 - sry (sex determining region y)-box 13  ZC3H8 - zinc finger ccch-type containing 8  ATF2 - activating transcription factor 2  CREB1 - camp responsive element binding protein 1  LMO1 - lim domain only 1 (rhombotin 1)  NACC2 - nacc family member 2, ben and btb (poz) domain containing  CLOCK - clock circadian regulator  KLF3 - kruppel-like factor 3 (basic)  PPID - peptidylprolyl isomerase d  PLK3 - polo-like kinase 3  SNCA - synuclein, alpha (non a4 component of amyloid precursor)  ACVRL1 - activin a receptor type ii-like 1  ZC3H14 - zinc finger ccch-type containing 14  CREBZF - creb/atf bzip transcription factor  ARID1A - at rich interactive domain 1a (swi-like)  EID1 - ep300 interacting inhibitor of differentiation 1  PTBP2 - polypyrimidine tract binding protein 2  MED1 - mediator complex subunit 1  SMARCE1 - swi/snf related, matrix associated, actin dependent regulator of chromatin, subfamily e, member 1  GCLC - glutamate-cysteine ligase, catalytic subunit  SMO - smoothened, frizzled family receptor  FZD1 - frizzled family receptor 1  BAP1 - brca1 associated protein-1 (ubiquitin carboxy-terminal hydrolase)  ZGPAT - zinc finger, ccch-type with g patch domain  AXIN2 - axin 2  PPARD - peroxisome proliferator-activated receptor delta  KDM5C - lysine (k)-specific demethylase 5c  UBR5 - ubiquitin protein ligase e3 component n-recognin 5  USP9X - ubiquitin specific peptidase 9, x-linked  RBM10 - rna binding motif protein 10  SMARCA4 - swi/snf related, matrix associated, actin dependent regulator of chromatin, subfamily a, member 4  SNX6 - sorting nexin 6  SMARCC2 - swi/snf related, matrix associated, actin dependent regulator of chromatin, subfamily c, member 2  SMARCA2 - swi/snf related, matrix associated, actin dependent regulator of chromatin, subfamily a, member 2  PARP1 - poly (adp-ribose) polymerase 1  H2AFY2 - h2a histone family, member y2  PPP1R13L - protein phosphatase 1, regulatory subunit 13 like  ZBTB7A - zinc finger and btb domain containing 7a  DPY30 - dpy-30 homolog (c. elegans)  RUVBL2 - ruvb-like 2 (e. coli)  IRF2BP2 - interferon regulatory factor 2 binding protein 2  GLIS2 - glis family zinc finger 2  MAGED1 - melanoma antigen family d, 1  TFIP11 - tuftelin interacting protein 11  RCOR3 - rest corepressor 3  NELFE - negative elongation factor complex member e  AES - amino-terminal enhancer of split  EHMT1 - euchromatic histone-lysine n-methyltransferase 1  ZNF318 - zinc finger protein 318  CDYL - chromodomain protein, y-like  KDM2B - lysine (k)-specific demethylase 2b  FUS - fused in sarcoma  FAM46B - family with sequence similarity 46, member b  CBY1 - chibby homolog 1 (drosophila)  CTBP1 - c-terminal binding protein 1  CTBP2 - c-terminal binding protein 2  SUPT4H1 - suppressor of ty 4 homolog 1 (s. cerevisiae)  ING4 - inhibitor of growth family, member 4  SUPT5H - suppressor of ty 5 homolog (s. cerevisiae)  ATF7IP - activating transcription factor 7 interacting protein  CTNNB1 - catenin (cadherin-associated protein), beta 1, 88kda  RLIM - ring finger protein, lim domain interacting  RECQL5 - recq protein-like 5  ZNF653 - zinc finger protein 653  RNF168 - ring finger protein 168, e3 ubiquitin protein ligase  XRCC6 - x-ray repair complementing defective repair in chinese hamster cells 6  CUX1 - cut-like homeobox 1  ZNF706 - zinc finger protein 706  GABPA - ga binding protein transcription factor, alpha subunit 60kda  WWP2 - ww domain containing e3 ubiquitin protein ligase 2  AHR - aryl hydrocarbon receptor  NIF3L1 - nif3 ngg1 interacting factor 3-like 1 (s. cerevisiae)  FOXP4 - forkhead box p4  PCGF1 - polycomb group ring finger 1  RMI2 - recq mediated genome instability 2  TGIF2 - tgfb-induced factor homeobox 2  BCL6B - b-cell cll/lymphoma 6, member b  NIPBL - nipped-b homolog (drosophila)  CRY1 - cryptochrome 1 (photolyase-like)  STAT1 - signal transducer and activator of transcription 1, 91kda  CRY2 - cryptochrome 2 (photolyase-like)  STAT3 - signal transducer and activator of transcription 3 (acute-phase response factor)  STAT6 - signal transducer and activator of transcription 6, interleukin-4 induced  KMT2D - lysine (k)-specific methyltransferase 2d  BRMS1 - breast cancer metastasis suppressor 1  PAPD7 - pap associated domain containing 7  MAP2K5 - mitogen-activated protein kinase kinase 5  ZNF219 - zinc finger protein 219  CRYM - crystallin, mu  ZNF692 - zinc finger protein 692  ZFPM1 - zinc finger protein, fog family member 1  MAPK10 - mitogen-activated protein kinase 10  EID2 - ep300 interacting inhibitor of differentiation 2  TRIP12 - thyroid hormone receptor interactor 12  NUDT16L1 - nudix (nucleoside diphosphate linked moiety x)-type motif 16-like 1  KCNK2 - potassium channel, subfamily k, member 2  GATA2 - gata binding protein 2  PHF6 - phd finger protein 6  COPS2 - cop9 signalosome subunit 2  KAT6A - k(lysine) acetyltransferase 6a  ZC3H10 - zinc finger ccch-type containing 10  ZNF639 - zinc finger protein 639  CCDC85B - coiled-coil domain containing 85b  SRC - v-src avian sarcoma (schmidt-ruppin a-2) viral oncogene homolog  NR4A3 - nuclear receptor subfamily 4, group a, member 3  SARNP - sap domain containing ribonucleoprotein  DNAJB5 - dnaj (hsp40) homolog, subfamily b, member 5  LEF1 - lymphoid enhancer-binding factor 1  BRMS1L - breast cancer metastasis-suppressor 1-like  SREBF2 - sterol regulatory element binding transcription factor 2  GAS6 - growth arrest-specific 6  PRDX5 - peroxiredoxin 5  DEDD2 - death effector domain containing 2  YEATS2 - yeats domain containing 2  DHX9 - deah (asp-glu-ala-his) box helicase 9  RNF169 - ring finger protein 169  APEX1 - apex nuclease (multifunctional dna repair enzyme) 1  DHX36 - deah (asp-glu-ala-his) box polypeptide 36  KANK2 - kn motif and ankyrin repeat domains 2  DDX5 - dead (asp-glu-ala-asp) box helicase 5  PARN - poly(a)-specific ribonuclease  ZNF177 - zinc finger protein 177  KLF12 - kruppel-like factor 12  ZNF175 - zinc finger protein 175  ZNF174 - zinc finger protein 174  SIN3A - sin3 transcription regulator family member a  SHQ1 - shq1, h/aca ribonucleoprotein assembly factor  WWTR1 - ww domain containing transcription regulator 1  SET - set nuclear oncogene  MAP3K10 - mitogen-activated protein kinase kinase kinase 10  PRMT6 - protein arginine methyltransferase 6  MLH1 - mutl homolog 1, colon cancer, nonpolyposis type 2 (e. coli)  ZNF148 - zinc finger protein 148  BEND5 - ben domain containing 5  NEIL1 - nei endonuclease viii-like 1 (e. coli)  RSF1 - remodeling and spacing factor 1  PBXIP1 - pre-b-cell leukemia homeobox interacting protein 1  MCPH1 - microcephalin 1  ZNF140 - zinc finger protein 140  MSH6 - muts homolog 6 (e. coli)  PCGF2 - polycomb group ring finger 2  NELFB - negative elongation factor complex member b  KMT2A - lysine (k)-specific methyltransferase 2a  APBB1 - amyloid beta (a4) precursor protein-binding, family b, member 1 (fe65)  POT1 - protection of telomeres 1  RIF1 - rap1 interacting factor homolog (yeast)  RPS6KA5 - ribosomal protein s6 kinase, 90kda, polypeptide 5  ZNF133 - zinc finger protein 133  SUFU - suppressor of fused homolog (drosophila)  TBL1XR1 - transducin (beta)-like 1 x-linked receptor 1  PA2G4 - proliferation-associated 2g4, 38kda  ZBTB38 - zinc finger and btb domain containing 38  ZHX1 - zinc fingers and homeoboxes 1  ZNF79 - zinc finger protein 79  UIMC1 - ubiquitin interaction motif containing 1  GATAD2B - gata zinc finger domain containing 2b  DFFA - dna fragmentation factor, 45kda, alpha polypeptide  MTA2 - metastasis associated 1 family, member 2  WWOX - ww domain containing oxidoreductase  DDX20 - dead (asp-glu-ala-asp) box polypeptide 20  MNT - mnt, max dimerization protein  H3F3A - h3 histone, family 3a  ARNTL - aryl hydrocarbon receptor nuclear translocator-like  H2AFZ - h2a histone family, member z  DCP2 - decapping mrna 2  H2AFX - h2a histone family, member x  SAP130 - sin3a-associated protein, 130kda  PAPD4 - pap associated domain containing 4  ZNF451 - zinc finger protein 451  MIER3 - mesoderm induction early response 1, family member 3  SCAF4 - sr-related ctd-associated factor 4  PDGFB - platelet-derived growth factor beta polypeptide  CDC73 - cell division cycle 73  MECP2 - methyl cpg binding protein 2 (rett syndrome)  MEF2A - myocyte enhancer factor 2a  NKAP - nfkb activating protein  PRICKLE1 - prickle homolog 1 (drosophila)  THAP5 - thap domain containing 5  STRAP - serine/threonine kinase receptor associated protein  RHOA - ras homolog family member a  BCDIN3D - bcdin3 domain containing  MEIS2 - meis homeobox 2  BAZ2A - bromodomain adjacent to zinc finger domain, 2a  MEN1 - multiple endocrine neoplasia i  HCFC1 - host cell factor c1 (vp16-accessory protein)  PCNA - proliferating cell nuclear antigen  CHMP1A - charged multivesicular body protein 1a  PRDM2 - pr domain containing 2, with znf domain  DAXX - death-domain associated protein  ANGEL2 - angel homolog 2 (drosophila)  JDP2 - jun dimerization protein 2  HINFP - histone h4 transcription factor  AKIRIN2 - akirin 2  KAT8 - k(lysine) acetyltransferase 8  MTA3 - metastasis associated 1 family, member 3  ZNF608 - zinc finger protein 608  DAP - death-associated protein  SARS - seryl-trna synthetase  PDE2A - phosphodiesterase 2a, cgmp-stimulated  HDAC2 - histone deacetylase 2  SATB1 - satb homeobox 1  HMBOX1 - homeobox containing 1  HDGF - hepatoma-derived growth factor  MBD3 - methyl-cpg binding domain protein 3  THAP1 - thap domain containing, apoptosis associated protein 1  HDAC1 - histone deacetylase 1  HCLS1 - hematopoietic cell-specific lyn substrate 1  ZNF205 - zinc finger protein 205  PKIG - protein kinase (camp-dependent, catalytic) inhibitor gamma  SALL2 - sal-like 2 (drosophila)  KAT7 - k(lysine) acetyltransferase 7  ASCL1 - achaete-scute complex homolog 1 (drosophila)  CBFA2T2 - core-binding factor, runt domain, alpha subunit 2; translocated to, 2  SBNO2 - strawberry notch homolog 2 (drosophila)  CXXC5 - cxxc finger protein 5  ZBTB1 - zinc finger and btb domain containing 1  IRF2BP1 - interferon regulatory factor 2 binding protein 1  XPO1 - exportin 1 (crm1 homolog, yeast)  XRCC1 - x-ray repair complementing defective repair in chinese hamster cells 1  BAHD1 - bromo adjacent homology domain containing 1  ATN1 - atrophin 1  ZHX2 - zinc fingers and homeoboxes 2  RERE - arginine-glutamic acid dipeptide (re) repeats  MTA1 - metastasis associated 1  SIRT6 - sirtuin 6  DR1 - down-regulator of transcription 1, tbp-binding (negative cofactor 2)  PPHLN1 - periphilin 1  BHLHE41 - basic helix-loop-helix family, member e41  ZFHX3 - zinc finger homeobox 3  SKI - v-ski avian sarcoma viral oncogene homolog  SKIL - ski-like oncogene  PEX14 - peroxisomal biogenesis factor 14  MBD1 - methyl-cpg binding domain protein 1  WFS1 - wolfram syndrome 1 (wolframin)  DYRK1A - dual-specificity tyrosine-(y)-phosphorylation regulated kinase 1a  WHSC1 - wolf-hirschhorn syndrome candidate 1  NELFA - negative elongation factor complex member a  SFMBT1 - scm-like with four mbt domains 1  MAX - myc associated factor x  AEBP2 - ae binding protein 2  LDB2 - lim domain binding 2  ZFP90 - zfp90 zinc finger protein  FNIP2 - folliculin interacting protein 2  IMPACT - impact rwd domain protein  NELFCD - negative elongation factor complex member c/d  CDAN1 - codanin 1  LGR4 - leucine-rich repeat containing g protein-coupled receptor 4  ZFHX2 - zinc finger homeobox 2  SCAF8 - sr-related ctd-associated factor 8  DKC1 - dyskeratosis congenita 1, dyskerin  MTF2 - metal response element binding transcription factor 2  L3MBTL2 - l(3)mbt-like 2 (drosophila)  DLG1 - discs, large homolog 1 (drosophila)  NAT10 - n-acetyltransferase 10 (gcn5-related)  BACH1 - btb and cnc homology 1, basic leucine zipper transcription factor 1  MZF1 - myeloid zinc finger 1  SIAH2 - siah e3 ubiquitin protein ligase 2  BTAF1 - btaf1 rna polymerase ii, b-tfiid transcription factor-associated, 170kda  UBA3 - ubiquitin-like modifier activating enzyme 3  PARP9 - poly (adp-ribose) polymerase family, member 9  PHF12 - phd finger protein 12  RNF8 - ring finger protein 8, e3 ubiquitin protein ligase  RBM42 - rna binding motif protein 42  ATRX - alpha thalassemia/mental retardation syndrome x-linked  ZNF24 - zinc finger protein 24  ZBTB4 - zinc finger and btb domain containing 4  SFPQ - splicing factor proline/glutamine-rich  SFRP1 - secreted frizzled-related protein 1  PHF10 - phd finger protein 10  SFRP2 - secreted frizzled-related protein 2  MXD1 - max dimerization protein 1  SF1 - splicing factor 1  PIAS4 - protein inhibitor of activated stat, 4  TRIM33 - tripartite motif containing 33  U2AF2 - u2 small nuclear rna auxiliary factor 2  PSPC1 - paraspeckle component 1  SMAD2 - smad family member 2  SRSF2 - serine/arginine-rich splicing factor 2  ZBTB14 - zinc finger and btb domain containing 14  DNMT1 - dna (cytosine-5-)-methyltransferase 1  KLF16 - kruppel-like factor 16  HDAC7 - histone deacetylase 7  SRSF4 - serine/arginine-rich splicing factor 4  TRDMT1 - trna aspartic acid methyltransferase 1  DNMT3A - dna (cytosine-5-)-methyltransferase 3 alpha  ZNF8 - zinc finger protein 8  TDP2 - tyrosyl-dna phosphodiesterase 2  SRSF6 - serine/arginine-rich splicing factor 6  SRSF7 - serine/arginine-rich splicing factor 7  SFSWAP - splicing factor, suppressor of white-apricot homolog (drosophila)  BCL7A - b-cell cll/lymphoma 7a  CHD8 - chromodomain helicase dna binding protein 8  ZNF3 - zinc finger protein 3  TAF9B - taf9b rna polymerase ii, tata box binding protein (tbp)-associated factor, 31kda  XRCC5 - x-ray repair complementing defective repair in chinese hamster cells 5 (double-strand-break rejoining)  NOC2L - nucleolar complex associated 2 homolog (s. cerevisiae)  SMAD4 - smad family member 4  NBAS - neuroblastoma amplified sequence  CCND1 - cyclin d1  SMAD5 - smad family member 5  H1FX - h1 histone family, member x  GSK3A - glycogen synthase kinase 3 alpha  SMAD7 - smad family member 7  YWHAB - tyrosine 3-monooxygenase/tryptophan 5-monooxygenase activation protein, beta polypeptide  TRIM27 - tripartite motif containing 27  YAF2 - yy1 associated factor 2  SIX5 - six homeobox 5  TAGLN3 - transgelin 3  ZNF582 - zinc finger protein 582  SAP18 - sin3a-associated protein, 18kda  BRD7 - bromodomain containing 7  NCK1 - nck adaptor protein 1  TRIM24 - tripartite motif containing 24  CREG1 - cellular repressor of e1a-stimulated genes 1  NAF1 - nuclear assembly factor 1 ribonucleoprotein  RFX5 - regulatory factor x, 5 (influences hla class ii expression)  ELK3 - elk3, ets-domain protein (srf accessory protein 2)  FLCN - folliculin  HSBP1 - heat shock factor binding protein 1  ELK4 - elk4, ets-domain protein (srf accessory protein 1)  SNW1 - snw domain containing 1  RELA - v-rel avian reticuloendotheliosis viral oncogene homolog a  TRIM28 - tripartite motif containing 28  PRMT2 - protein arginine methyltransferase 2  BCOR - bcl6 corepressor  EP300 - e1a binding protein p300  HESX1 - hesx homeobox 1  DPF2 - d4, zinc and double phd fingers family 2  SAP30 - sin3a-associated protein, 30kda  DNAJC15 - dnaj (hsp40) homolog, subfamily c, member 15  NSUN2 - nop2/sun rna methyltransferase family, member 2  NBN - nibrin  ENO1 - enolase 1, (alpha)  UBQLN4 - ubiquilin 4  CC2D1A - coiled-coil and c2 domain containing 1a  EIF4A2 - eukaryotic translation initiation factor 4a2  CELF4 - cugbp, elav-like family member 4  RING1 - ring finger protein 1  TRIM11 - tripartite motif containing 11  TTF1 - transcription termination factor, rna polymerase i  NAB1 - ngfi-a binding protein 1 (egr1 binding protein 1)  SECISBP2 - secis binding protein 2  NAB2 - ngfi-a binding protein 2 (egr1 binding protein 2)  BMI1 - bmi1 polycomb ring finger oncogene  KDM2A - lysine (k)-specific demethylase 2a  ELAVL1 - elav (embryonic lethal, abnormal vision, drosophila)-like 1 (hu antigen r)  ELAVL4 - elav (embryonic lethal, abnormal vision, drosophila)-like 4  LDB1 - lim domain binding 1  TCF25 - transcription factor 25 (basic helix-loop-helix)  TSG101 - tumor susceptibility gene 101  BMPR2 - bone morphogenetic protein receptor, type ii (serine/threonine kinase)  GON4L - gon-4-like (c. elegans)  KAT2B - k(lysine) acetyltransferase 2b  LBH - limb bud and heart development  BMP7 - bone morphogenetic protein 7  CNOT1 - ccr4-not transcription complex, subunit 1  EFNA1 - ephrin-a1  SPEN - spen homolog, transcriptional regulator (drosophila)  CTNNBIP1 - catenin, beta interacting protein 1  MYD88 - myeloid differentiation primary response 88  ABCE1 - atp-binding cassette, sub-family e (oabp), member 1  BBX - bobby sox homolog (drosophila)  PHC1 - polyhomeotic homolog 1 (drosophila)  TP53BP1 - tumor protein p53 binding protein 1  CC2D1B - coiled-coil and c2 domain containing 1b  HCFC2 - host cell factor c2  PHC2 - polyhomeotic homolog 2 (drosophila)  EGR1 - early growth response 1  CNOT7 - ccr4-not transcription complex, subunit 7  MXI1 - max interactor 1, dimerization protein  HUS1 - hus1 checkpoint homolog (s. pombe)  KDM1A - lysine (k)-specific demethylase 1a  RNF2 - ring finger protein 2  GATAD2A - gata zinc finger domain containing 2a  E2F6 - e2f transcription factor 6  E4F1 - e4f transcription factor 1  TSPO - translocator protein (18kda)  ZHX3 - zinc fingers and homeoboxes 3  E2F4 - e2f transcription factor 4, p107/p130-binding  E2F5 - e2f transcription factor 5, p130-binding  RAD50 - rad50 homolog (s. cerevisiae)  DNAJB1 - dnaj (hsp40) homolog, subfamily b, member 1  TRIM37 - tripartite motif containing 37  MBTD1 - mbt domain containing 1  ZNF558 - zinc finger protein 558  EDNRB - endothelin receptor type b  LRPPRC - leucine-rich pentatricopeptide repeat containing  ZNF263 - zinc finger protein 263  C7orf49 - chromosome 7 open reading frame 49  ZMPSTE24 - zinc metallopeptidase ste24  BTRC - beta-transducin repeat containing e3 ubiquitin protein ligase  NR2C1 - nuclear receptor subfamily 2, group c, member 1  PURG - purine-rich element binding protein g  NR2C2 - nuclear receptor subfamily 2, group c, member 2  MED25 - mediator complex subunit 25  TPR - translocated promoter region, nuclear basket protein  WAPAL - wings apart-like homolog (drosophila)  HSPA8 - heat shock 70kda protein 8  ZNF423 - zinc finger protein 423  PTBP3 - polypyrimidine tract binding protein 3  NRDE2 - nrde-2, necessary for rna interference, domain containing  HMGB2 - high mobility group box 2  HMGB1 - high mobility group box 1  C1D - c1d nuclear receptor corepressor  TNKS - tankyrase, trf1-interacting ankyrin-related adp-ribose polymerase  ZBTB33 - zinc finger and btb domain containing 33  BPTF - bromodomain phd finger transcription factor  HDAC5 - histone deacetylase 5  MAD2L2 - mad2 mitotic arrest deficient-like 2 (yeast)  PID1 - phosphotyrosine interaction domain containing 1  NR1H2 - nuclear receptor subfamily 1, group h, member 2  HDAC6 - histone deacetylase 6  ETV3 - ets variant 3  SRSF9 - serine/arginine-rich splicing factor 9  ETV6 - ets variant 6  MT3 - metallothionein 3  VHL - von hippel-lindau tumor suppressor, e3 ubiquitin protein ligase  EZR - ezrin  NRARP - notch-regulated ankyrin repeat protein  TBX20 - t-box 20  ACD - adrenocortical dysplasia homolog (mouse)  KCTD1 - potassium channel tetramerization domain containing 1  PCBP4 - poly(rc) binding protein 4  CREB3L1 - camp responsive element binding protein 3-like 1  CALR - calreticulin  WTIP - wilms tumor 1 interacting protein  EZH1 - enhancer of zeste homolog 1 (drosophila)  NCOA2 - nuclear receptor coactivator 2  CIC - capicua transcriptional repressor  SYNCRIP - synaptotagmin binding, cytoplasmic rna interacting protein  DNAJB6 - dnaj (hsp40) homolog, subfamily b, member 6  HIVEP1 - human immunodeficiency virus type i enhancer binding protein 1  ERF - ets2 repressor factor  CITED2 - cbp/p300-interacting transactivator, with glu/asp-rich carboxy-terminal domain, 2  MSH3 - muts homolog 3 (e. coli)  MSH2 - muts homolog 2, colon cancer, nonpolyposis type 1 (e. coli)  CITED1 - cbp/p300-interacting transactivator, with glu/asp-rich carboxy-terminal domain, 1  ZBTB5 - zinc finger and btb domain containing 5  ERCC4 - excision repair cross-complementing rodent repair deficiency, complementation group 4  PINX1 - pin2/terf1 interacting, telomerase inhibitor 1  ELMSAN1 - elm2 and myb/sant-like domain containing 1  THAP11 - thap domain containing 11  EED - embryonic ectoderm development  URI1 - uri1, prefoldin-like chaperone  CCND3 - cyclin d3  CCNT1 - cyclin t1  HMG20A - high mobility group 20a  ZNF248 - zinc finger protein 248  UBB - ubiquitin b  HNRNPU - heterogeneous nuclear ribonucleoprotein u (scaffold attachment factor a)  HNRNPK - heterogeneous nuclear ribonucleoprotein k  RCOR1 - rest corepressor 1  HNRNPL - heterogeneous nuclear ribonucleoprotein l  ZNFX1 - zinc finger, nfx1-type containing 1  HIRA - histone cell cycle regulator  TIPIN - timeless interacting protein  TINF2 - terf1 (trf1)-interacting nuclear factor 2  CBFA2T3 - core-binding factor, runt domain, alpha subunit 2; translocated to, 3  CBFB - core-binding factor, beta subunit  USP3 - ubiquitin specific peptidase 3  SUMO1 - small ubiquitin-like modifier 1  HNRNPA2B1 - heterogeneous nuclear ribonucleoprotein a2/b1  HNRNPA1 - heterogeneous nuclear ribonucleoprotein a1  ZNF830 - zinc finger protein 830  HNRNPC - heterogeneous nuclear ribonucleoprotein c (c1/c2)  PIAS3 - protein inhibitor of activated stat, 3  CAV1 - caveolin 1, caveolae protein, 22kda  HNRNPD - heterogeneous nuclear ribonucleoprotein d (au-rich element rna binding protein 1, 37kda)  ZC3H4 - zinc finger ccch-type containing 4  THOC1 - tho complex 1  UBE2I - ubiquitin-conjugating enzyme e2i  HMGA1 - high mobility group at-hook 1  FAM129B - family with sequence similarity 129, member b  PRMT5 - protein arginine methyltransferase 5  THRAP3 - thyroid hormone receptor associated protein 3  PDE12 - phosphodiesterase 12  VPS72 - vacuolar protein sorting 72 homolog (s. cerevisiae)  FOXK2 - forkhead box k2  FMR1 - fragile x mental retardation 1  HAT1 - histone acetyltransferase 1  MGA - mga, max dimerization protein  ZC3H6 - zinc finger ccch-type containing 6  MXD4 - max dimerization protein 4  ZEB1 - zinc finger e-box binding homeobox 1  WWC3 - wwc family member 3  ORC2 - origin recognition complex, subunit 2  HEXIM1 - hexamethylene bis-acetamide inducible 1  ZNF436 - zinc finger protein 436  RBMX - rna binding motif protein, x-linked  PDS5A - pds5, regulator of cohesion maintenance, homolog a (s. cerevisiae)  FLNA - filamin a, alpha  PSEN1 - presenilin 1  PPM1D - protein phosphatase, mg2+/mn2+ dependent, 1d  PLCB1 - phospholipase c, beta 1 (phosphoinositide-specific)  SRSF12 - serine/arginine-rich splicing factor 12  FOXS1 - forkhead box s1  NDFIP1 - nedd4 family interacting protein 1  FOXO3 - forkhead box o3  ILF3 - interleukin enhancer binding factor 3, 90kda  HDAC8 - histone deacetylase 8  SIN3B - sin3 transcription regulator family member b  PIAS1 - protein inhibitor of activated stat, 1  SMURF2 - smad specific e3 ubiquitin protein ligase 2  NACC1 - nucleus accumbens associated 1, ben and btb (poz) domain containing  RIPPLY2 - ripply transcriptional repressor 2  CGGBP1 - cgg triplet repeat binding protein 1  HR - hair growth associated  TDG - thymine-dna glycosylase  SMG6 - smg6 nonsense mediated mrna decay factor  SOX18 - sry (sex determining region y)-box 18  CBX4 - chromobox homolog 4  OGG1 - 8-oxoguanine dna glycosylase  FBXW11 - f-box and wd repeat domain containing 11  EAPP - e2f-associated phosphoprotein  TNKS2 - tankyrase, trf1-interacting ankyrin-related adp-ribose polymerase 2  PSMD10 - proteasome (prosome, macropain) 26s subunit, non-atpase, 10  FHL2 - four and a half lim domains 2  TERF2IP - telomeric repeat binding factor 2, interacting protein  FGFR2 - fibroblast growth factor receptor 2  TAF7 - taf7 rna polymerase ii, tata box binding protein (tbp)-associated factor, 55kda  ATG7 - autophagy related 7  FGFR1 - fibroblast growth factor receptor 1  FGF9 - fibroblast growth factor 9  PTBP1 - polypyrimidine tract binding protein 1  SMCHD1 - structural maintenance of chromosomes flexible hinge domain containing 1  ZNF25 - zinc finger protein 25  TIRAP - toll-interleukin 1 receptor (tir) domain containing adaptor protein  BCLAF1 - bcl2-associated transcription factor 1  DAB2IP - dab2 interacting protein  GMNN - geminin, dna replication inhibitor  MYBBP1A - myb binding protein (p160) 1a  NR4A2 - nuclear receptor subfamily 4, group a, member 2  FBLN5 - fibulin 5  ZBTB7B - zinc finger and btb domain containing 7b  SUDS3 - suppressor of defective silencing 3 homolog (s. cerevisiae)  KAT5 - k(lysine) acetyltransferase 5  ZFP69 - zfp69 zinc finger protein  CDK5 - cyclin-dependent kinase 5  GZF1 - gdnf-inducible zinc finger protein 1  CDK9 - cyclin-dependent kinase 9  INSM1 - insulinoma-associated 1  CDKN1C - cyclin-dependent kinase inhibitor 1c (p57, kip2)  ZEB2 - zinc finger e-box binding homeobox 2  CDKN1B - cyclin-dependent kinase inhibitor 1b (p27, kip1)  PDCD4 - programmed cell death 4 (neoplastic transformation inhibitor)  PCBP3 - poly(rc) binding protein 3  PTPN2 - protein tyrosine phosphatase, non-receptor type 2  KLF7 - kruppel-like factor 7 (ubiquitous)  IRF2 - interferon regulatory factor 2  IRF3 - interferon regulatory factor 3  IKZF5 - ikaros family zinc finger 5 (pegasus)  TCF3 - transcription factor 3  RNF40 - ring finger protein 40, e3 ubiquitin protein ligase  DMAP1 - dna methyltransferase 1 associated protein 1  LARP1 - la ribonucleoprotein domain family, member 1  NKRF - nfkb repressing factor  LANCL2 - lanc lantibiotic synthetase component c-like 2 (bacterial)  CDK2 - cyclin-dependent kinase 2  DONSON - downstream neighbor of son  PURA - purine-rich element binding protein a  PURB - purine-rich element binding protein b  NONO - non-pou domain containing, octamer-binding  VPRBP - vpr (hiv-1) binding protein  PAPD5 - pap associated domain containing 5  NUDT16 - nudix (nucleoside diphosphate linked moiety x)-type motif 16  NSD1 - nuclear receptor binding set domain protein 1  RYBP - ring1 and yy1 binding protein  MTOR - mechanistic target of rapamycin (serine/threonine kinase)  MDFIC - myod family inhibitor domain containing  PAXBP1 - pax3 and pax7 binding protein 1  ZMYND11 - zinc finger, mynd-type containing 11  TLE1 - transducin-like enhancer of split 1 (e(sp1) homolog, drosophila)  PTPRK - protein tyrosine phosphatase, receptor type, k  SRSF10 - serine/arginine-rich splicing factor 10  TLE3 - transducin-like enhancer of split 3 (e(sp1) homolog, drosophila)  NOTCH4 - notch 4  TLE4 - transducin-like enhancer of split 4 (e(sp1) homolog, drosophila)  PEX2 - peroxisomal biogenesis factor 2  DKK3 - dickkopf wnt signaling pathway inhibitor 3  NPAT - nuclear protein, ataxia-telangiectasia locus  MPHOSPH8 - m-phase phosphoprotein 8  KDM5B - lysine (k)-specific demethylase 5b  CNOT2 - ccr4-not transcription complex, subunit 2  NOTCH1 - notch 1  SIRT1 - sirtuin 1  MKX - mohawk homeobox  NOTCH3 - notch 3  CTC1 - cts telomere maintenance complex component 1  DHX34 - deah (asp-glu-ala-his) box polypeptide 34  NOTCH2 - notch 2  ID2 - inhibitor of dna binding 2, dominant negative helix-loop-helix protein  ID3 - inhibitor of dna binding 3, dominant negative helix-loop-helix protein  HDAC4 - histone deacetylase 4  CBX6 - chromobox homolog 6  HEY1 - hairy/enhancer-of-split related with yrpw motif 1  IRF2BPL - interferon regulatory factor 2 binding protein-like  JAZF1 - jazf zinc finger 1  NFKB1 - nuclear factor of kappa light polypeptide gene enhancer in b-cells 1  CBX5 - chromobox homolog 5  QARS - glutaminyl-trna synthetase  MYPOP - myb-related transcription factor, partner of profilin  TARDBP - tar dna binding protein  RAD17 - rad17 homolog (s. pombe)  FOXK1 - forkhead box k1  PARP14 - poly (adp-ribose) polymerase family, member 14  MAGEF1 - melanoma antigen family f, 1  PAF1 - paf1, rna polymerase ii associated factor, homolog (s. cerevisiae)  HDAC9 - histone deacetylase 9  ZNF268 - zinc finger protein 268  ZNF614 - zinc finger protein 614  TMBIM6 - transmembrane bax inhibitor motif containing 6  IGHMBP2 - immunoglobulin mu binding protein 2  ZNF282 - zinc finger protein 282  SUZ12 - suz12 polycomb repressive complex 2 subunit  RBPJ - recombination signal binding protein for immunoglobulin kappa j region  RCOR2 - rest corepressor 2  MIER2 - mesoderm induction early response 1, family member 2  SMARCAL1 - swi/snf related, matrix associated, actin dependent regulator of chromatin, subfamily a-like 1  PABPC1 - poly(a) binding protein, cytoplasmic 1  NCK2 - nck adaptor protein 2  TERF1 - telomeric repeat binding factor (nima-interacting) 1  TERF2 - telomeric repeat binding factor 2  CTR9 - ctr9, paf1/rna polymerase ii complex component  FAM35A - family with sequence similarity 35, member a  PPM1F - protein phosphatase, mg2+/mn2+ dependent, 1f  RARG - retinoic acid receptor, gamma  EPC1 - enhancer of polycomb homolog 1 (drosophila)  NR2F2 - nuclear receptor subfamily 2, group f, member 2  RARB - retinoic acid receptor, beta  CBX7 - chromobox homolog 7  MORF4L2 - mortality factor 4 like 2  DLL4 - delta-like 4 (drosophila)  NFATC4 - nuclear factor of activated t-cells, cytoplasmic, calcineurin-dependent 4  KLHL15 - kelch-like family member 15  NR2F1 - nuclear receptor subfamily 2, group f, member 1  KHDRBS1 - kh domain containing, rna binding, signal transduction associated 1  NFIA - nuclear factor i/a  GNL3L - guanine nucleotide binding protein-like 3 (nucleolar)-like  MAGEL2 - mage-like 2  FNIP1 - folliculin interacting protein 1  HEY2 - hairy/enhancer-of-split related with yrpw motif 2  CTCF - ccctc-binding factor (zinc finger protein)  IGBP1 - immunoglobulin (cd79a) binding protein 1  RBBP7 - retinoblastoma binding protein 7  RBBP8 - retinoblastoma binding protein 8  SMARCA5 - swi/snf related, matrix associated, actin dependent regulator of chromatin, subfamily a, member 5  RBFOX2 - rna binding protein, fox-1 homolog (c. elegans) 2  TGFB1 - transforming growth factor, beta 1  RB1 - retinoblastoma 1  ARID4A - at rich interactive domain 4a (rbp1-like)  ZNF746 - zinc finger protein 746  VGLL4 - vestigial like 4 (drosophila)  H2AFV - h2a histone family, member v  MEPCE - methylphosphate capping enzyme  TSPYL2 - tspy-like 2  RNF20 - ring finger protein 20, e3 ubiquitin protein ligase  KDM5A - lysine (k)-specific demethylase 5a  KDM4A - lysine (k)-specific demethylase 4a  FOSB - fbj murine osteosarcoma viral oncogene homolog b  PHF14 - phd finger protein 14  CUL3 - cullin 3  KAT6B - k(lysine) acetyltransferase 6b  CIRBP - cold inducible rna binding protein  FOXP1 - forkhead box p1  BEND6 - ben domain containing 6  THRA - thyroid hormone receptor, alpha  CERS1 - ceramide synthase 1 |
| GO:0042147 | retrograde transport, endosome to Golgi | 4.55E-6 | 6.53E-4 | 1.35 (10334,68,6996,62) | [+] Show genes  ERC1 - elks/rab6-interacting/cast family member 1  TRIM27 - tripartite motif containing 27  TMEM87B - transmembrane protein 87b  DENND5A - denn/madd domain containing 5a  VPS35 - vacuolar protein sorting 35 homolog (s. cerevisiae)  RAB6B - rab6b, member ras oncogene family  HEATR5A - heat repeat containing 5a  DCTN1 - dynactin 1  VPS54 - vacuolar protein sorting 54 homolog (s. cerevisiae)  SNX5 - sorting nexin 5  PQLC1 - pq loop repeat containing 1  TRAPPC10 - trafficking protein particle complex 10  RAB6A - rab6a, member ras oncogene family  STX5 - syntaxin 5  RAB7A - rab7a, member ras oncogene family  VPS26A - vacuolar protein sorting 26 homolog a (s. pombe)  VPS26B - vacuolar protein sorting 26 homolog b (s. pombe)  SGSM2 - small g protein signaling modulator 2  GGA1 - golgi-associated, gamma adaptin ear containing, arf binding protein 1  YKT6 - ykt6 v-snare homolog (s. cerevisiae)  VPS29 - vacuolar protein sorting 29 homolog (s. cerevisiae)  BET1L - bet1 golgi vesicular membrane trafficking protein-like  GOSR2 - golgi snap receptor complex member 2  TBC1D23 - tbc1 domain family, member 23  PREPL - prolyl endopeptidase-like  SNX2 - sorting nexin 2  SNX1 - sorting nexin 1  EHD3 - eh-domain containing 3  ANKFY1 - ankyrin repeat and fyve domain containing 1  VTI1B - vesicle transport through interaction with t-snares 1b  VPS51 - vacuolar protein sorting 51 homolog (s. cerevisiae)  SNX3 - sorting nexin 3  ARL1 - adp-ribosylation factor-like 1  RAB9A - rab9a, member ras oncogene family  GCC2 - grip and coiled-coil domain containing 2  CLTC - clathrin, heavy chain (hc)  TBC1D17 - tbc1 domain family, member 17  GBF1 - golgi brefeldin a resistant guanine nucleotide exchange factor 1  NUMA1 - nuclear mitotic apparatus protein 1  CLN5 - ceroid-lipofuscinosis, neuronal 5  GOSR1 - golgi snap receptor complex member 1  STX6 - syntaxin 6  RNF126 - ring finger protein 126  SPAG9 - sperm associated antigen 9  EVI5 - ecotropic viral integration site 5  MAGEL2 - mage-like 2  TBC1D5 - tbc1 domain family, member 5  HEATR5B - heat repeat containing 5b  TBC1D10B - tbc1 domain family, member 10b  SNX6 - sorting nexin 6  VPS53 - vacuolar protein sorting 53 homolog (s. cerevisiae)  RGP1 - rgp1 retrograde golgi transport homolog (s. cerevisiae)  PLEKHJ1 - pleckstrin homology domain containing, family j member 1  AP1S1 - adaptor-related protein complex 1, sigma 1 subunit  FAM21A - family with sequence similarity 21, member a  VAMP3 - vesicle-associated membrane protein 3  TBC1D14 - tbc1 domain family, member 14  TMEM87A - transmembrane protein 87a  VPS52 - vacuolar protein sorting 52 homolog (s. cerevisiae)  UBE2O - ubiquitin-conjugating enzyme e2o  SNX12 - sorting nexin 12  BAIAP3 - bai1-associated protein 3 |
| GO:0032446 | protein modification by small protein conjugation | 4.65E-6 | 6.61E-4 | 1.12 (10334,596,6996,452) | [+] Show genes  MEX3C - mex-3 rna binding family member c  PCGF5 - polycomb group ring finger 5  WAC - ww domain containing adaptor with coiled-coil  MKRN1 - makorin ring finger protein 1  MKRN2 - makorin ring finger protein 2  FBXO18 - f-box protein, helicase, 18  OS9 - osteosarcoma amplified 9, endoplasmic reticulum lectin  PML - promyelocytic leukemia  LZTR1 - leucine-zipper-like transcription regulator 1  RAB40B - rab40b, member ras oncogene family  UBE2Q1 - ubiquitin-conjugating enzyme e2q family member 1  FBXL18 - f-box and leucine-rich repeat protein 18  LNPEP - leucyl/cystinyl aminopeptidase  SYVN1 - synovial apoptosis inhibitor 1, synoviolin  BFAR - bifunctional apoptosis regulator  RFFL - ring finger and fyve-like domain containing e3 ubiquitin protein ligase  NEURL4 - neuralized homolog 4 (drosophila)  FBXL20 - f-box and leucine-rich repeat protein 20  RAB40C - rab40c, member ras oncogene family  ANAPC5 - anaphase promoting complex subunit 5  ANAPC7 - anaphase promoting complex subunit 7  NPEPPS - aminopeptidase puromycin sensitive  RNF138 - ring finger protein 138, e3 ubiquitin protein ligase  SUMO2 - small ubiquitin-like modifier 2  MED1 - mediator complex subunit 1  SUMO3 - small ubiquitin-like modifier 3  NUP85 - nucleoporin 85kda  BRAP - brca1 associated protein  UBR5 - ubiquitin protein ligase e3 component n-recognin 5  USP9X - ubiquitin specific peptidase 9, x-linked  HLTF - helicase-like transcription factor  MED20 - mediator complex subunit 20  FZR1 - fizzy/cell division cycle 20 related 1 (drosophila)  IRF2BP2 - interferon regulatory factor 2 binding protein 2  MED27 - mediator complex subunit 27  SENP2 - sumo1/sentrin/smt3 specific peptidase 2  NUP133 - nucleoporin 133kda  ASB7 - ankyrin repeat and socs box containing 7  ASB8 - ankyrin repeat and socs box containing 8  KDM2B - lysine (k)-specific demethylase 2b  KLHL12 - kelch-like family member 12  NUPL2 - nucleoporin like 2  ASB6 - ankyrin repeat and socs box containing 6  CHFR - checkpoint with forkhead and ring finger domains, e3 ubiquitin protein ligase  UHRF2 - ubiquitin-like with phd and ring finger domains 2, e3 ubiquitin protein ligase  SDE2 - sde2 telomere maintenance homolog (s. pombe)  CTNNB1 - catenin (cadherin-associated protein), beta 1, 88kda  RLIM - ring finger protein, lim domain interacting  PPIL2 - peptidylprolyl isomerase (cyclophilin)-like 2  ASB3 - ankyrin repeat and socs box containing 3  TRAF7 - tnf receptor-associated factor 7, e3 ubiquitin protein ligase  AKT1 - v-akt murine thymoma viral oncogene homolog 1  STX1A - syntaxin 1a (brain)  USP7 - ubiquitin specific peptidase 7 (herpes virus-associated)  LNX1 - ligand of numb-protein x 1, e3 ubiquitin protein ligase  RNF168 - ring finger protein 168, e3 ubiquitin protein ligase  WWP2 - ww domain containing e3 ubiquitin protein ligase 2  PCGF1 - polycomb group ring finger 1  CBLL1 - cbl proto-oncogene-like 1, e3 ubiquitin protein ligase  MED10 - mediator complex subunit 10  MED21 - mediator complex subunit 21  UBA5 - ubiquitin-like modifier activating enzyme 5  HECTD1 - hect domain containing e3 ubiquitin protein ligase 1  DCUN1D5 - dcn1, defective in cullin neddylation 1, domain containing 5  UBE4A - ubiquitination factor e4a  FBXW9 - f-box and wd repeat domain containing 9  USP5 - ubiquitin specific peptidase 5 (isopeptidase t)  ANAPC13 - anaphase promoting complex subunit 13  AMFR - autocrine motility factor receptor, e3 ubiquitin protein ligase  AAAS - achalasia, adrenocortical insufficiency, alacrimia  RNF126 - ring finger protein 126  DCAF12 - ddb1 and cul4 associated factor 12  ANKRD39 - ankyrin repeat domain 39  RNF157 - ring finger protein 157  FBXO7 - f-box protein 7  TRIP12 - thyroid hormone receptor interactor 12  TRIM59 - tripartite motif containing 59  KLHL22 - kelch-like family member 22  NDC1 - ndc1 transmembrane nucleoporin  CRBN - cereblon  RNF214 - ring finger protein 214  NUP214 - nucleoporin 214kda  KLHL36 - kelch-like family member 36  ARIH1 - ariadne homolog, ubiquitin-conjugating enzyme e2 binding protein, 1 (drosophila)  UBE2O - ubiquitin-conjugating enzyme e2o  FBXL2 - f-box and leucine-rich repeat protein 2  SHPRH - snf2 histone linker phd ring helicase, e3 ubiquitin protein ligase  RNF169 - ring finger protein 169  UBR7 - ubiquitin protein ligase e3 component n-recognin 7 (putative)  KLHL2 - kelch-like family member 2  FBXO33 - f-box protein 33  XIAP - x-linked inhibitor of apoptosis  KCTD10 - potassium channel tetramerization domain containing 10  BIRC3 - baculoviral iap repeat containing 3  BIRC2 - baculoviral iap repeat containing 2  RNF112 - ring finger protein 112  SOCS6 - suppressor of cytokine signaling 6  RFWD3 - ring finger and wd repeat domain 3  RNF113A - ring finger protein 113a  RSPRY1 - ring finger and spry domain containing 1  WWTR1 - ww domain containing transcription regulator 1  DDB1 - damage-specific dna binding protein 1, 127kda  HECTD3 - hect domain containing e3 ubiquitin protein ligase 3  MIB2 - mindbomb e3 ubiquitin protein ligase 2  MSL2 - male-specific lethal 2 homolog (drosophila)  TRIM26 - tripartite motif containing 26  DCUN1D3 - dcn1, defective in cullin neddylation 1, domain containing 3  RNF220 - ring finger protein 220  PCGF2 - polycomb group ring finger 2  RNF139 - ring finger protein 139  KLHL13 - kelch-like family member 13  TRIM23 - tripartite motif containing 23  RNF19A - ring finger protein 19a, e3 ubiquitin protein ligase  RCHY1 - ring finger and chy zinc finger domain containing 1, e3 ubiquitin protein ligase  KCTD13 - potassium channel tetramerization domain containing 13  DCAF13 - ddb1 and cul4 associated factor 13  BIRC6 - baculoviral iap repeat containing 6  HIST1H2BB - histone cluster 1, h2bb  LTN1 - listerin e3 ubiquitin protein ligase 1  MUL1 - mitochondrial e3 ubiquitin protein ligase 1  NSMCE2 - non-smc element 2, mms21 homolog (s. cerevisiae)  ZNF451 - zinc finger protein 451  RNF103 - ring finger protein 103  ARRB2 - arrestin, beta 2  ARRB1 - arrestin, beta 1  CDC73 - cell division cycle 73  MED30 - mediator complex subunit 30  ZBED1 - zinc finger, bed-type containing 1  DCAF15 - ddb1 and cul4 associated factor 15  PCNA - proliferating cell nuclear antigen  LRSAM1 - leucine rich repeat and sterile alpha motif containing 1  LEO1 - leo1, paf1/rna polymerase ii complex component, homolog (s. cerevisiae)  FBXL14 - f-box and leucine-rich repeat protein 14  FANCL - fanconi anemia, complementation group l  HACE1 - hect domain and ankyrin repeat containing e3 ubiquitin protein ligase 1  SOCS4 - suppressor of cytokine signaling 4  MIB1 - mindbomb e3 ubiquitin protein ligase 1  ZC3HC1 - zinc finger, c3hc-type containing 1  KLHL42 - kelch-like family member 42  TRPC4AP - transient receptor potential cation channel, subfamily c, member 4 associated protein  CDC26 - cell division cycle 26  RNF180 - ring finger protein 180  UBOX5 - u-box domain containing 5  IRF2BP1 - interferon regulatory factor 2 binding protein 1  FBXW7 - f-box and wd repeat domain containing 7, e3 ubiquitin protein ligase  RNF121 - ring finger protein 121  KLHL8 - kelch-like family member 8  WSB1 - wd repeat and socs box containing 1  ZNRF2 - zinc and ring finger 2  PEX12 - peroxisomal biogenesis factor 12  UBE2J1 - ubiquitin-conjugating enzyme e2, j1  PEX13 - peroxisomal biogenesis factor 13  PEX14 - peroxisomal biogenesis factor 14  NUP43 - nucleoporin 43kda  SKP1 - s-phase kinase-associated protein 1  DTX3 - deltex homolog 3 (drosophila)  HERC4 - hect and rld domain containing e3 ubiquitin protein ligase 4  UFC1 - ubiquitin-fold modifier conjugating enzyme 1  SENP6 - sumo1/sentrin specific peptidase 6  DTX1 - deltex homolog 1 (drosophila)  UBA6 - ubiquitin-like modifier activating enzyme 6  FBXL16 - f-box and leucine-rich repeat protein 16  ITCH - itchy e3 ubiquitin protein ligase  FBXL15 - f-box and leucine-rich repeat protein 15  FBXW8 - f-box and wd repeat domain containing 8  SH3RF1 - sh3 domain containing ring finger 1  FBXO25 - f-box protein 25  NUB1 - negative regulator of ubiquitin-like proteins 1  SIAH2 - siah e3 ubiquitin protein ligase 2  SIAH1 - siah e3 ubiquitin protein ligase 1  ASB1 - ankyrin repeat and socs box containing 1  FBXL3 - f-box and leucine-rich repeat protein 3  UBE2M - ubiquitin-conjugating enzyme e2m  UBA3 - ubiquitin-like modifier activating enzyme 3  FBXL5 - f-box and leucine-rich repeat protein 5  RNF8 - ring finger protein 8, e3 ubiquitin protein ligase  PEF1 - penta-ef-hand domain containing 1  FBXO2 - f-box protein 2  UVSSA - uv-stimulated scaffold protein a  PELI3 - pellino e3 ubiquitin protein ligase family member 3  RNF13 - ring finger protein 13  FBXW2 - f-box and wd repeat domain containing 2  PIAS4 - protein inhibitor of activated stat, 4  TRIM33 - tripartite motif containing 33  FBXW12 - f-box and wd repeat domain containing 12  KCTD11 - potassium channel tetramerization domain containing 11  BCL2 - b-cell cll/lymphoma 2  UBE2W - ubiquitin-conjugating enzyme e2w (putative)  DCAF10 - ddb1 and cul4 associated factor 10  MYLIP - myosin regulatory light chain interacting protein  TRIM27 - tripartite motif containing 27  RNF26 - ring finger protein 26  TRIM24 - tripartite motif containing 24  TRIM28 - tripartite motif containing 28  BCOR - bcl6 corepressor  EPAS1 - endothelial pas domain protein 1  FBXO45 - f-box protein 45  DCAF16 - ddb1 and cul4 associated factor 16  SH3RF3 - sh3 domain containing ring finger 3  DERL1 - derlin 1  DCAF5 - ddb1 and cul4 associated factor 5  KCTD6 - potassium channel tetramerization domain containing 6  NHLRC3 - nhl repeat containing 3  RING1 - ring finger protein 1  EGR2 - early growth response 2  TTC3 - tetratricopeptide repeat domain 3  TRIM11 - tripartite motif containing 11  SENP1 - sumo1/sentrin specific peptidase 1  RNF41 - ring finger protein 41  TRIM32 - tripartite motif containing 32  MED11 - mediator complex subunit 11  BMI1 - bmi1 polycomb ring finger oncogene  TMEM129 - transmembrane protein 129  PSME3 - proteasome (prosome, macropain) activator subunit 3 (pa28 gamma; ki)  RNF215 - ring finger protein 215  PCGF3 - polycomb group ring finger 3  TSG101 - tumor susceptibility gene 101  URM1 - ubiquitin related modifier 1  TRIM13 - tripartite motif containing 13  TRIM8 - tripartite motif containing 8  FBXL12 - f-box and leucine-rich repeat protein 12  TOPORS - topoisomerase i binding, arginine/serine-rich, e3 ubiquitin protein ligase  DDA1 - det1 and ddb1 associated 1  CDC16 - cell division cycle 16  PSMD14 - proteasome (prosome, macropain) 26s subunit, non-atpase, 14  RNF19B - ring finger protein 19b  FBXO21 - f-box protein 21  NAE1 - nedd8 activating enzyme e1 subunit 1  GID4 - gid complex subunit 4  RNF149 - ring finger protein 149  SEH1L - seh1-like (s. cerevisiae)  TULP4 - tubby like protein 4  PHC1 - polyhomeotic homolog 1 (drosophila)  HUWE1 - hect, uba and wwe domain containing 1, e3 ubiquitin protein ligase  RNF4 - ring finger protein 4  DCAF7 - ddb1 and cul4 associated factor 7  RNF2 - ring finger protein 2  ATXN7L3 - ataxin 7-like 3  RNF6 - ring finger protein (c3h2c3 type) 6  HERC3 - hect and rld domain containing e3 ubiquitin protein ligase 3  UBE2Q2 - ubiquitin-conjugating enzyme e2q family member 2  HERC2 - hect and rld domain containing e3 ubiquitin protein ligase 2  RNF111 - ring finger protein 111  ANAPC4 - anaphase promoting complex subunit 4  HERC1 - hect and rld domain containing e3 ubiquitin protein ligase family member 1  E4F1 - e4f transcription factor 1  RNF170 - ring finger protein 170  FEM1B - fem-1 homolog b (c. elegans)  TRPM4 - transient receptor potential cation channel, subfamily m, member 4  NSMCE1 - non-smc element 1 homolog (s. cerevisiae)  TRIM37 - tripartite motif containing 37  BCL10 - b-cell cll/lymphoma 10  MYCBP2 - myc binding protein 2, e3 ubiquitin protein ligase  TRAF1 - tnf receptor-associated factor 1  TRAF3 - tnf receptor-associated factor 3  BTRC - beta-transducin repeat containing e3 ubiquitin protein ligase  TRAF2 - tnf receptor-associated factor 2  STUB1 - stip1 homology and u-box containing protein 1, e3 ubiquitin protein ligase  FEM1C - fem-1 homolog c (c. elegans)  SHARPIN - shank-associated rh domain interactor  TPR - translocated promoter region, nuclear basket protein  UBE4B - ubiquitination factor e4b  UBR1 - ubiquitin protein ligase e3 component n-recognin 1  TRIM44 - tripartite motif containing 44  TRIM35 - tripartite motif containing 35  UBE2Z - ubiquitin-conjugating enzyme e2z  NUP107 - nucleoporin 107kda  PDCD6 - programmed cell death 6  SENP5 - sumo1/sentrin specific peptidase 5  TNKS - tankyrase, trf1-interacting ankyrin-related adp-ribose polymerase  PCNP - pest proteolytic signal containing nuclear protein  CUL9 - cullin 9  RNF185 - ring finger protein 185  HDAC6 - histone deacetylase 6  RNF187 - ring finger protein 187  ZNF598 - zinc finger protein 598  SPSB3 - spla/ryanodine receptor domain and socs box containing 3  VHL - von hippel-lindau tumor suppressor, e3 ubiquitin protein ligase  UBE2E3 - ubiquitin-conjugating enzyme e2e 3  HERC6 - hect and rld domain containing e3 ubiquitin protein ligase family member 6  TRIM41 - tripartite motif containing 41  TRIM38 - tripartite motif containing 38  CCDC22 - coiled-coil domain containing 22  SAE1 - sumo1 activating enzyme subunit 1  DCUN1D4 - dcn1, defective in cullin neddylation 1, domain containing 4  CDC23 - cell division cycle 23  UBE2K - ubiquitin-conjugating enzyme e2k  TRIM47 - tripartite motif containing 47  VCP - valosin containing protein  LRRC41 - leucine rich repeat containing 41  HIF1A - hypoxia inducible factor 1, alpha subunit (basic helix-loop-helix transcription factor)  UBA2 - ubiquitin-like modifier activating enzyme 2  NUP205 - nucleoporin 205kda  UBE2A - ubiquitin-conjugating enzyme e2a  UBA1 - ubiquitin-like modifier activating enzyme 1  UBB - ubiquitin b  FBXO10 - f-box protein 10  FBXO38 - f-box protein 38  ANAPC10 - anaphase promoting complex subunit 10  ARRDC4 - arrestin domain containing 4  FBXO22 - f-box protein 22  FBXO4 - f-box protein 4  COMMD1 - copper metabolism (murr1) domain containing 1  FBXO6 - f-box protein 6  FBXO3 - f-box protein 3  RNF125 - ring finger protein 125, e3 ubiquitin protein ligase  UCHL3 - ubiquitin carboxyl-terminal esterase l3 (ubiquitin thiolesterase)  CBFB - core-binding factor, beta subunit  CBL - cbl proto-oncogene, e3 ubiquitin protein ligase  SUMO1 - small ubiquitin-like modifier 1  UBE2T - ubiquitin-conjugating enzyme e2t (putative)  PELI1 - pellino e3 ubiquitin protein ligase 1  PIAS3 - protein inhibitor of activated stat, 3  SMURF1 - smad specific e3 ubiquitin protein ligase 1  UBE2R2 - ubiquitin-conjugating enzyme e2r 2  UBE2H - ubiquitin-conjugating enzyme e2h  FBXO15 - f-box protein 15  UBE2I - ubiquitin-conjugating enzyme e2i  UBE2D2 - ubiquitin-conjugating enzyme e2d 2  RBX1 - ring-box 1, e3 ubiquitin protein ligase  FBXO28 - f-box protein 28  UBE2V2 - ubiquitin-conjugating enzyme e2 variant 2  LONRF1 - lon peptidase n-terminal domain and ring finger 1  NUP153 - nucleoporin 153kda  UBE2L3 - ubiquitin-conjugating enzyme e2l 3  NUP210 - nucleoporin 210kda  MED12 - mediator complex subunit 12  UBE2N - ubiquitin-conjugating enzyme e2n  UBE2V1 - ubiquitin-conjugating enzyme e2 variant 1  PSMD6 - proteasome (prosome, macropain) 26s subunit, non-atpase, 6  WDSUB1 - wd repeat, sterile alpha motif and u-box domain containing 1  TRIM3 - tripartite motif containing 3  DTX3L - deltex 3-like (drosophila)  PJA2 - praja ring finger 2, e3 ubiquitin protein ligase  AREL1 - apoptosis resistant e3 ubiquitin protein ligase 1  ENC1 - ectodermal-neural cortex 1 (with btb domain)  CDC27 - cell division cycle 27  RNF141 - ring finger protein 141  ANKIB1 - ankyrin repeat and ibr domain containing 1  WSB2 - wd repeat and socs box containing 2  PSMA1 - proteasome (prosome, macropain) subunit, alpha type, 1  PSMA2 - proteasome (prosome, macropain) subunit, alpha type, 2  RNF216 - ring finger protein 216  UBE2S - ubiquitin-conjugating enzyme e2s  RNF38 - ring finger protein 38  PRPF19 - pre-mrna processing factor 19  PIAS1 - protein inhibitor of activated stat, 1  PSMB1 - proteasome (prosome, macropain) subunit, beta type, 1  SMURF2 - smad specific e3 ubiquitin protein ligase 2  PSMA5 - proteasome (prosome, macropain) subunit, alpha type, 5  KLHL21 - kelch-like family member 21  PSMA3 - proteasome (prosome, macropain) subunit, alpha type, 3  PSMB10 - proteasome (prosome, macropain) subunit, beta type, 10  MGRN1 - mahogunin ring finger 1, e3 ubiquitin protein ligase  MED8 - mediator complex subunit 8  PSMC6 - proteasome (prosome, macropain) 26s subunit, atpase, 6  CAND1 - cullin-associated and neddylation-dissociated 1  PSMD1 - proteasome (prosome, macropain) 26s subunit, non-atpase, 1  PSMC4 - proteasome (prosome, macropain) 26s subunit, atpase, 4  CBX4 - chromobox homolog 4  FBXW11 - f-box and wd repeat domain containing 11  PSMC2 - proteasome (prosome, macropain) 26s subunit, atpase, 2  TNKS2 - tankyrase, trf1-interacting ankyrin-related adp-ribose polymerase 2  PSMD9 - proteasome (prosome, macropain) 26s subunit, non-atpase, 9  PSMD10 - proteasome (prosome, macropain) 26s subunit, non-atpase, 10  KLHL18 - kelch-like family member 18  PSMD7 - proteasome (prosome, macropain) 26s subunit, non-atpase, 7  WDR61 - wd repeat domain 61  PSMD8 - proteasome (prosome, macropain) 26s subunit, non-atpase, 8  ANAPC1 - anaphase promoting complex subunit 1  RNF130 - ring finger protein 130  PSMD5 - proteasome (prosome, macropain) 26s subunit, non-atpase, 5  PSMD2 - proteasome (prosome, macropain) 26s subunit, non-atpase, 2  PPP1R11 - protein phosphatase 1, regulatory (inhibitor) subunit 11  NUP160 - nucleoporin 160kda  PSMD12 - proteasome (prosome, macropain) 26s subunit, non-atpase, 12  UBR4 - ubiquitin protein ligase e3 component n-recognin 4  ATG7 - autophagy related 7  PSMD13 - proteasome (prosome, macropain) 26s subunit, non-atpase, 13  KLHL7 - kelch-like family member 7  KLHL17 - kelch-like family member 17  PHF15 - phd finger protein 15  NUP88 - nucleoporin 88kda  NUP98 - nucleoporin 98kda  RNF115 - ring finger protein 115  USP22 - ubiquitin specific peptidase 22  SASH1 - sam and sh3 domain containing 1  TRIM2 - tripartite motif containing 2  RNF144A - ring finger protein 144a  ATG3 - autophagy related 3  RNF114 - ring finger protein 114  AKTIP - akt interacting protein  KLHL20 - kelch-like family member 20  SPOPL - speckle-type poz protein-like  UFL1 - ufm1-specific ligase 1  KLHL9 - kelch-like family member 9  TRIM9 - tripartite motif containing 9  RNF40 - ring finger protein 40, e3 ubiquitin protein ligase  MOCS3 - molybdenum cofactor synthesis 3  CUL7 - cullin 7  RNF208 - ring finger protein 208  VPRBP - vpr (hiv-1) binding protein  RFWD2 - ring finger and wd repeat domain 2, e3 ubiquitin protein ligase  RYBP - ring1 and yy1 binding protein  RNF34 - ring finger protein 34, e3 ubiquitin protein ligase  RNF25 - ring finger protein 25  FBXO11 - f-box protein 11  HERPUD1 - homocysteine-inducible, endoplasmic reticulum stress-inducible, ubiquitin-like domain member 1  SPSB1 - spla/ryanodine receptor domain and socs box containing 1  NUP50 - nucleoporin 50kda  PEX2 - peroxisomal biogenesis factor 2  CNOT4 - ccr4-not transcription complex, subunit 4  SIRT1 - sirtuin 1  HDAC4 - histone deacetylase 4  IRF2BPL - interferon regulatory factor 2 binding protein-like  TNFAIP1 - tumor necrosis factor, alpha-induced protein 1 (endothelial)  FBXO44 - f-box protein 44  MAGEF1 - melanoma antigen family f, 1  VCPIP1 - valosin containing protein (p97)/p47 complex interacting protein 1  PAF1 - paf1, rna polymerase ii associated factor, homolog (s. cerevisiae)  FBXL19 - f-box and leucine-rich repeat protein 19  RNF145 - ring finger protein 145  SOCS7 - suppressor of cytokine signaling 7  KCTD21 - potassium channel tetramerization domain containing 21  NUP188 - nucleoporin 188kda  SUZ12 - suz12 polycomb repressive complex 2 subunit  RC3H2 - ring finger and ccch-type domains 2  RANBP2 - ran binding protein 2  RANGAP1 - ran gtpase activating protein 1  SOCS5 - suppressor of cytokine signaling 5  NEDD8 - neural precursor cell expressed, developmentally down-regulated 8  RNF11 - ring finger protein 11  CTR9 - ctr9, paf1/rna polymerase ii complex component  SPOP - speckle-type poz protein  NFE2L2 - nuclear factor (erythroid-derived 2)-like 2  KLHL15 - kelch-like family member 15  RRAGA - ras-related gtp binding a  NUP155 - nucleoporin 155kda  MAGEL2 - mage-like 2  CDK5RAP3 - cdk5 regulatory subunit associated protein 3  DCAF8 - ddb1 and cul4 associated factor 8  NUP93 - nucleoporin 93kda  RAE1 - rae1 rna export 1 homolog (s. pombe)  RBBP6 - retinoblastoma binding protein 6  RNF20 - ring finger protein 20, e3 ubiquitin protein ligase  NUP35 - nucleoporin 35kda  ERCC8 - excision repair cross-complementing rodent repair deficiency, complementation group 8  CUL4B - cullin 4b  CUL1 - cullin 1  CUL2 - cullin 2  CUL3 - cullin 3  DZIP3 - daz interacting zinc finger protein 3 |
| GO:0030031 | cell projection assembly | 4.93E-6 | 6.93E-4 | 1.20 (10334,214,6996,174) | [+] Show genes  EMP1 - epithelial membrane protein 1  S1PR2 - sphingosine-1-phosphate receptor 2  CTTN - cortactin  NCK1 - nck adaptor protein 1  CEP89 - centrosomal protein 89kda  B9D2 - b9 protein domain 2  WDR19 - wd repeat domain 19  ABLIM1 - actin binding lim protein 1  ATAT1 - alpha tubulin acetyltransferase 1  GSN - gelsolin  LRRC16A - leucine rich repeat containing 16a  CD2AP - cd2-associated protein  GORAB - golgin, rab6-interacting  IFT46 - intraflagellar transport 46 homolog (chlamydomonas)  SDCBP - syndecan binding protein (syntenin)  MKS1 - meckel syndrome, type 1  WWTR1 - ww domain containing transcription regulator 1  NRP1 - neuropilin 1  ABL1 - c-abl oncogene 1, non-receptor tyrosine kinase  SSX2IP - synovial sarcoma, x breakpoint 2 interacting protein  DCTN1 - dynactin 1  PTPN23 - protein tyrosine phosphatase, non-receptor type 23  FLNA - filamin a, alpha  RDX - radixin  WDR34 - wd repeat domain 34  FOPNL - fgfr1op n-terminal like  PMP22 - peripheral myelin protein 22  ARL13B - adp-ribosylation factor-like 13b  WASF2 - was protein family, member 2  CCDC28B - coiled-coil domain containing 28b  MKKS - mckusick-kaufman syndrome  KIF27 - kinesin family member 27  MTSS1L - metastasis suppressor 1-like  CDC14B - cell division cycle 14b  EXOC5 - exocyst complex component 5  TMEM17 - transmembrane protein 17  OCRL - oculocerebrorenal syndrome of lowe  FNBP1L - formin binding protein 1-like  PTPDC1 - protein tyrosine phosphatase domain containing 1  AZI1 - 5-azacytidine induced 1  MYLK - myosin light chain kinase  RAB23 - rab23, member ras oncogene family  BBS7 - bardet-biedl syndrome 7  EHD1 - eh-domain containing 1  AKAP4 - a kinase (prka) anchor protein 4  IFT43 - intraflagellar transport 43 homolog (chlamydomonas)  CEP250 - centrosomal protein 250kda  PDGFA - platelet-derived growth factor alpha polypeptide  TCTN1 - tectonic family member 1  ARL3 - adp-ribosylation factor-like 3  CEP19 - centrosomal protein 19kda  DNM3 - dynamin 3  KIAA0586 - kiaa0586  ARHGEF7 - rho guanine nucleotide exchange factor (gef) 7  RHOU - ras homolog family member u  MYH10 - myosin, heavy chain 10, non-muscle  IFT57 - intraflagellar transport 57 homolog (chlamydomonas)  PDGFRB - platelet-derived growth factor receptor, beta polypeptide  FSCN1 - fascin homolog 1, actin-bundling protein (strongylocentrotus purpuratus)  TMEM231 - transmembrane protein 231  SNX10 - sorting nexin 10  ARID1B - at rich interactive domain 1b (swi1-like)  ARF6 - adp-ribosylation factor 6  CELSR2 - cadherin, egf lag seven-pass g-type receptor 2  FGD1 - fyve, rhogef and ph domain containing 1  AHI1 - abelson helper integration site 1  BAG4 - bcl2-associated athanogene 4  ITGB1 - integrin, beta 1 (fibronectin receptor, beta polypeptide, antigen cd29 includes mdf2, msk12)  ACTR2 - arp2 actin-related protein 2 homolog (yeast)  ACTR3 - arp3 actin-related protein 3 homolog (yeast)  NUP85 - nucleoporin 85kda  BBS9 - bardet-biedl syndrome 9  TEKT2 - tektin 2 (testicular)  WDR60 - wd repeat domain 60  RAB8A - rab8a, member ras oncogene family  ARFIP2 - adp-ribosylation factor interacting protein 2  ROCK1 - rho-associated, coiled-coil containing protein kinase 1  INPPL1 - inositol polyphosphate phosphatase-like 1  CLUAP1 - clusterin associated protein 1  C2CD3 - c2 calcium-dependent domain containing 3  NUDCD3 - nudc domain containing 3  PCM1 - pericentriolar material 1  TTC8 - tetratricopeptide repeat domain 8  FAM161A - family with sequence similarity 161, member a  TNIK - traf2 and nck interacting kinase  E2F4 - e2f transcription factor 4, p107/p130-binding  RABL2B - rab, member of ras oncogene family-like 2b  ATP6V1D - atpase, h+ transporting, lysosomal 34kda, v1 subunit d  ARL6 - adp-ribosylation factor-like 6  LPAR1 - lysophosphatidic acid receptor 1  S1PR1 - sphingosine-1-phosphate receptor 1  FGD5 - fyve, rhogef and ph domain containing 5  SCLT1 - sodium channel and clathrin linker 1  LAMA5 - laminin, alpha 5  IFT52 - intraflagellar transport 52 homolog (chlamydomonas)  SH2B1 - sh2b adaptor protein 1  FBF1 - fas (tnfrsf6) binding factor 1  ITGA6 - integrin, alpha 6  NCKAP1 - nck-associated protein 1  VAV3 - vav 3 guanine nucleotide exchange factor  ARMC9 - armadillo repeat containing 9  POC1B - poc1 centriolar protein b  TCTN3 - tectonic family member 3  TMEM138 - transmembrane protein 138  TRAF3IP1 - tnf receptor-associated factor 3 interacting protein 1  ARHGEF6 - rac/cdc42 guanine nucleotide exchange factor (gef) 6  IFT74 - intraflagellar transport 74 homolog (chlamydomonas)  KIF3A - kinesin family member 3a  PARVA - parvin, alpha  CEP290 - centrosomal protein 290kda  ATP6V0D1 - atpase, h+ transporting, lysosomal 38kda, v0 subunit d1  DYNLL1 - dynein, light chain, lc8-type 1  PIBF1 - progesterone immunomodulatory binding factor 1  DZIP1 - daz interacting zinc finger protein 1  RSG1 - rem2 and rab-like small gtpase 1  NOTCH1 - notch 1  CCDC41 - coiled-coil domain containing 41  HDAC6 - histone deacetylase 6  PIP5K1A - phosphatidylinositol-4-phosphate 5-kinase, type i, alpha  STK36 - serine/threonine kinase 36  RILPL2 - rab interacting lysosomal protein-like 2  TMEM237 - transmembrane protein 237  EZR - ezrin  UNC119B - unc-119 homolog b (c. elegans)  WASF3 - was protein family, member 3  ICK - intestinal cell (mak-like) kinase  IFT140 - intraflagellar transport 140 homolog (chlamydomonas)  TTBK2 - tau tubulin kinase 2  TCTEX1D2 - tctex1 domain containing 2  RAC3 - ras-related c3 botulinum toxin substrate 3 (rho family, small gtp binding protein rac3)  VAV2 - vav 2 guanine nucleotide exchange factor  TCTN2 - tectonic family member 2  RAC1 - ras-related c3 botulinum toxin substrate 1 (rho family, small gtp binding protein rac1)  IFT122 - intraflagellar transport 122 homolog (chlamydomonas)  NME5 - nme/nm23 family member 5  VCL - vinculin  EHD2 - eh-domain containing 2  KIT - v-kit hardy-zuckerman 4 feline sarcoma viral oncogene homolog  EHD3 - eh-domain containing 3  EHD4 - eh-domain containing 4  IQCB1 - iq motif containing b1  TMEM216 - transmembrane protein 216  RSPH9 - radial spoke head 9 homolog (chlamydomonas)  NEK1 - nima-related kinase 1  NCK2 - nck adaptor protein 2  DBNL - drebrin-like  TMEM80 - transmembrane protein 80  SCIN - scinderin  IFT81 - intraflagellar transport 81 homolog (chlamydomonas)  BBS5 - bardet-biedl syndrome 5  IFT88 - intraflagellar transport 88 homolog (chlamydomonas)  CEP41 - centrosomal protein 41kda  GOLPH3 - golgi phosphoprotein 3 (coat-protein)  RAP2A - rap2a, member of ras oncogene family  CYFIP1 - cytoplasmic fmr1 interacting protein 1  DMTN - dematin actin binding protein  TXNDC15 - thioredoxin domain containing 15  RAPGEF2 - rap guanine nucleotide exchange factor (gef) 2  P2RY12 - purinergic receptor p2y, g-protein coupled, 12  ARHGEF4 - rho guanine nucleotide exchange factor (gef) 4  WDR11 - wd repeat domain 11  TROVE2 - trove domain family, member 2  GALNT11 - udp-n-acetyl-alpha-d-galactosamine:polypeptide n-acetylgalactosaminyltransferase 11 (galnac-t11)  SNAP29 - synaptosomal-associated protein, 29kda  CCDC88A - coiled-coil domain containing 88a  ATXN10 - ataxin 10  CSNK1D - casein kinase 1, delta  B9D1 - b9 protein domain 1  IFT172 - intraflagellar transport 172 homolog (chlamydomonas)  CCDC66 - coiled-coil domain containing 66  ASAP1 - arfgap with sh3 domain, ankyrin repeat and ph domain 1  BBS4 - bardet-biedl syndrome 4  BBS2 - bardet-biedl syndrome 2  TMEM107 - transmembrane protein 107 |
| GO:0071824 | protein-DNA complex subunit organization | 4.94E-6 | 6.87E-4 | 1.24 (10334,145,6996,122) | [+] Show genes  VPS72 - vacuolar protein sorting 72 homolog (s. cerevisiae)  HAT1 - histone acetyltransferase 1  ANP32E - acidic (leucine-rich) nuclear phosphoprotein 32 family, member e  TSPYL4 - tspy-like 4  CENPV - centromere protein v  SET - set nuclear oncogene  DDB1 - damage-specific dna binding protein 1, 127kda  GTF2E1 - general transcription factor iie, polypeptide 1, alpha 56kda  GTF2H2 - general transcription factor iih, polypeptide 2, 44kda  GTF2H3 - general transcription factor iih, polypeptide 3, 34kda  RSF1 - remodeling and spacing factor 1  NAP1L1 - nucleosome assembly protein 1-like 1  GTF2H1 - general transcription factor iih, polypeptide 1, 62kda  NASP - nuclear autoantigenic sperm protein (histone-binding)  NAP1L4 - nucleosome assembly protein 1-like 4  GTF2A2 - general transcription factor iia, 2, 12kda  GTF2B - general transcription factor iib  PIAS1 - protein inhibitor of activated stat, 1  SMYD3 - set and mynd domain containing 3  BDP1 - b double prime 1, subunit of rna polymerase iii transcription initiation factor iiib  GTF2H4 - general transcription factor iih, polypeptide 4, 52kda  CHD1L - chromodomain helicase dna binding protein 1-like  H2AFY - h2a histone family, member y  HIST3H2BB - histone cluster 3, h2bb  BRF1 - brf1, rna polymerase iii transcription initiation factor 90 kda subunit  IPO4 - importin 4  TSPYL1 - tspy-like 1  MNAT1 - mnat cdk-activating kinase assembly factor 1  ORC3 - origin recognition complex, subunit 3  MLF1IP - mlf1 interacting protein  APITD1 - apoptosis-inducing, taf9-like domain 1  H3F3A - h3 histone, family 3a  TAF7 - taf7 rna polymerase ii, tata box binding protein (tbp)-associated factor, 55kda  HIST1H2BB - histone cluster 1, h2bb  MCM7 - minichromosome maintenance complex component 7  H2AFX - h2a histone family, member x  CENPC - centromere protein c  CENPM - centromere protein m  SUPT16H - suppressor of ty 16 homolog (s. cerevisiae)  CETN2 - centrin, ef-hand protein, 2  ANP32B - acidic (leucine-rich) nuclear phosphoprotein 32 family, member b  SPTY2D1 - spt2, suppressor of ty, domain containing 1 (s. cerevisiae)  ARID1A - at rich interactive domain 1a (swi-like)  CHD5 - chromodomain helicase dna binding protein 5  GMNN - geminin, dna replication inhibitor  UBN1 - ubinuclein 1  POLR1E - polymerase (rna) i polypeptide e, 53kda  CDC45 - cell division cycle 45  SMARCE1 - swi/snf related, matrix associated, actin dependent regulator of chromatin, subfamily e, member 1  NAA60 - n(alpha)-acetyltransferase 60, natf catalytic subunit  BRD2 - bromodomain containing 2  MCM3AP - minichromosome maintenance complex component 3 associated protein  ACTR6 - arp6 actin-related protein 6 homolog (yeast)  BAZ2A - bromodomain adjacent to zinc finger domain, 2a  SMARCB1 - swi/snf related, matrix associated, actin dependent regulator of chromatin, subfamily b, member 1  SMARCC1 - swi/snf related, matrix associated, actin dependent regulator of chromatin, subfamily c, member 1  PTMA - prothymosin, alpha  SMARCA4 - swi/snf related, matrix associated, actin dependent regulator of chromatin, subfamily a, member 4  DAXX - death-domain associated protein  SMARCD2 - swi/snf related, matrix associated, actin dependent regulator of chromatin, subfamily d, member 2  NPM1 - nucleophosmin (nucleolar phosphoprotein b23, numatrin)  SMARCD3 - swi/snf related, matrix associated, actin dependent regulator of chromatin, subfamily d, member 3  SMARCC2 - swi/snf related, matrix associated, actin dependent regulator of chromatin, subfamily c, member 2  SMARCD1 - swi/snf related, matrix associated, actin dependent regulator of chromatin, subfamily d, member 1  CDK7 - cyclin-dependent kinase 7  PARP1 - poly (adp-ribose) polymerase 1  H2AFY2 - h2a histone family, member y2  RUVBL1 - ruvb-like 1 (e. coli)  DMAP1 - dna methyltransferase 1 associated protein 1  TCF4 - transcription factor 4  NAP1L5 - nucleosome assembly protein 1-like 5  RRN3 - rrn3 rna polymerase i transcription factor homolog (s. cerevisiae)  ITGB3BP - integrin beta 3 binding protein (beta3-endonexin)  XPA - xeroderma pigmentosum, complementation group a  XPC - xeroderma pigmentosum, complementation group c  HMGB2 - high mobility group box 2  SUPT6H - suppressor of ty 6 homolog (s. cerevisiae)  BPTF - bromodomain phd finger transcription factor  DR1 - down-regulator of transcription 1, tbp-binding (negative cofactor 2)  RPA3 - replication protein a3, 14kda  RPA1 - replication protein a1, 70kda  RPA2 - replication protein a2, 32kda  CENPT - centromere protein t  CHAF1A - chromatin assembly factor 1, subunit a (p150)  MCM3 - minichromosome maintenance complex component 3  MCM6 - minichromosome maintenance complex component 6  MCM5 - minichromosome maintenance complex component 5  MCM2 - minichromosome maintenance complex component 2  PAF1 - paf1, rna polymerase ii associated factor, homolog (s. cerevisiae)  ARID2 - at rich interactive domain 2 (arid, rfx-like)  SART3 - squamous cell carcinoma antigen recognized by t cells 3  INO80 - ino80 complex subunit  ERCC5 - excision repair cross-complementing rodent repair deficiency, complementation group 5  ERCC3 - excision repair cross-complementing rodent repair deficiency, complementation group 3  ASF1A - anti-silencing function 1a histone chaperone  CCNH - cyclin h  UBB - ubiquitin b  HIRA - histone cell cycle regulator  CENPO - centromere protein o  ATRX - alpha thalassemia/mental retardation syndrome x-linked  RNF8 - ring finger protein 8, e3 ubiquitin protein ligase  HEY2 - hairy/enhancer-of-split related with yrpw motif 2  CTCF - ccctc-binding factor (zinc finger protein)  TAF1B - tata box binding protein (tbp)-associated factor, rna polymerase i, b, 63kda  RBBP7 - retinoblastoma binding protein 7  SMARCA5 - swi/snf related, matrix associated, actin dependent regulator of chromatin, subfamily a, member 5  UBTF - upstream binding transcription factor, rna polymerase i  KAT6A - k(lysine) acetyltransferase 6a  TSPYL2 - tspy-like 2  RBBP4 - retinoblastoma binding protein 4  TAF1C - tata box binding protein (tbp)-associated factor, rna polymerase i, c, 110kda  CUL4B - cullin 4b  SETD2 - set domain containing 2  BRF2 - brf2, rna polymerase iii transcription initiation factor 50 kda subunit  RBX1 - ring-box 1, e3 ubiquitin protein ligase  HMGA1 - high mobility group at-hook 1  CABIN1 - calcineurin binding protein 1  KAT6B - k(lysine) acetyltransferase 6b  HP1BP3 - heterochromatin protein 1, binding protein 3  H1FX - h1 histone family, member x  ERCC2 - excision repair cross-complementing rodent repair deficiency, complementation group 2  SHPRH - snf2 histone linker phd ring helicase, e3 ubiquitin protein ligase |
| GO:0015931 | nucleobase-containing compound transport | 5.07E-6 | 6.99E-4 | 1.21 (10334,198,6996,162) | [+] Show genes  DHX9 - deah (asp-glu-ala-his) box helicase 9  DDX19B - dead (asp-glu-ala-asp) box polypeptide 19b  NCBP2 - nuclear cap binding protein subunit 2, 20kda  NCBP1 - nuclear cap binding protein subunit 1, 80kda  SRSF11 - serine/arginine-rich splicing factor 11  FMR1 - fragile x mental retardation 1  TSC1 - tuberous sclerosis 1  SEC13 - sec13 homolog (s. cerevisiae)  SDAD1 - sda1 domain containing 1  SYMPK - symplekin  SLC35A4 - solute carrier family 35, member a4  DDX25 - dead (asp-glu-ala-asp) box helicase 25  EPG5 - ectopic p-granules autophagy protein 5 homolog (c. elegans)  ZFP36L1 - zfp36 ring finger protein-like 1  ALKBH5 - alkb, alkylation repair homolog 5 (e. coli)  AGFG1 - arfgap with fg repeats 1  ARC - activity-regulated cytoskeleton-associated protein  UPF1 - upf1 regulator of nonsense transcripts homolog (yeast)  XPOT - exportin, trna  PHAX - phosphorylated adaptor for rna export  LTV1 - ltv1 homolog (s. cerevisiae)  CPSF3 - cleavage and polyadenylation specific factor 3, 73kda  SLC25A36 - solute carrier family 25 (pyrimidine nucleotide carrier ), member 36  EIF4E - eukaryotic translation initiation factor 4e  AHCTF1 - at hook containing transcription factor 1  NOP9 - nop9 nucleolar protein  G3BP2 - gtpase activating protein (sh3 domain) binding protein 2  BICD2 - bicaudal d homolog 2 (drosophila)  ALYREF - aly/ref export factor  PABPN1 - poly(a) binding protein, nuclear 1  RNPS1 - rna binding protein s1, serine-rich domain  SLC35B3 - solute carrier family 35 (adenosine 3'-phospho 5'-phosphosulfate transporter), member b3  SMG6 - smg6 nonsense mediated mrna decay factor  GJA1 - gap junction protein, alpha 1, 43kda  NUTF2 - nuclear transport factor 2  SMG7 - smg7 nonsense mediated mrna decay factor  NUP160 - nucleoporin 160kda  FIP1L1 - factor interacting with papola and cpsf1  CKAP5 - cytoskeleton associated protein 5  FLOT1 - flotillin 1  ABCE1 - atp-binding cassette, sub-family e (oabp), member 1  ATXN2 - ataxin 2  SEH1L - seh1-like (s. cerevisiae)  CETN2 - centrin, ef-hand protein, 2  TOMM20 - translocase of outer mitochondrial membrane 20 homolog (yeast)  CETN3 - centrin, ef-hand protein, 3  SNUPN - snurportin 1  NUP88 - nucleoporin 88kda  EIF4A3 - eukaryotic translation initiation factor 4a3  NUP98 - nucleoporin 98kda  SLC35B2 - solute carrier family 35 (adenosine 3'-phospho 5'-phosphosulfate transporter), member b2  KHSRP - kh-type splicing regulatory protein  GLE1 - gle1 rna export mediator  CPSF4 - cleavage and polyadenylation specific factor 4, 30kda  U2AF1L4 - u2 small nuclear rna auxiliary factor 1-like 4  SLC35B1 - solute carrier family 35, member b1  DHX38 - deah (asp-glu-ala-his) box polypeptide 38  CPSF2 - cleavage and polyadenylation specific factor 2, 100kda  CPSF1 - cleavage and polyadenylation specific factor 1, 160kda  MCM3AP - minichromosome maintenance complex component 3 associated protein  RBM15B - rna binding motif protein 15b  SLC33A1 - solute carrier family 33 (acetyl-coa transporter), member 1  NUP85 - nucleoporin 85kda  EIF6 - eukaryotic translation initiation factor 6  SLU7 - slu7 splicing factor homolog (s. cerevisiae)  SLC25A29 - solute carrier family 25 (mitochondrial carnitine/acylcarnitine carrier), member 29  XPO5 - exportin 5  NPM1 - nucleophosmin (nucleolar phosphoprotein b23, numatrin)  NMD3 - nmd3 ribosome export adaptor  SMG1 - smg1 phosphatidylinositol 3-kinase-related kinase  SRRM1 - serine/arginine repetitive matrix 1  MAGOHB - mago-nashi homolog b (drosophila)  SLC25A32 - solute carrier family 25 (mitochondrial folate carrier), member 32  ENY2 - enhancer of yellow 2 homolog (drosophila)  SLC25A24 - solute carrier family 25 (mitochondrial carrier; phosphate carrier), member 24  SLC35E3 - solute carrier family 35, member e3  LRPPRC - leucine-rich pentatricopeptide repeat containing  SLC35A1 - solute carrier family 35 (cmp-sialic acid transporter), member a1  CDC40 - cell division cycle 40  POLR2D - polymerase (rna) ii (dna directed) polypeptide d  TPR - translocated promoter region, nuclear basket protein  SIDT2 - sid1 transmembrane family, member 2  DDX19A - dead (asp-glu-ala-asp) box polypeptide 19a  PURA - purine-rich element binding protein a  SENP2 - sumo1/sentrin/smt3 specific peptidase 2  MAGOH - mago-nashi homolog, proliferation-associated (drosophila)  NUP133 - nucleoporin 133kda  KIF5C - kinesin family member 5c  XPO1 - exportin 1 (crm1 homolog, yeast)  NUP107 - nucleoporin 107kda  NUPL2 - nucleoporin like 2  SUPT6H - suppressor of ty 6 homolog (s. cerevisiae)  NUP50 - nucleoporin 50kda  TNKS - tankyrase, trf1-interacting ankyrin-related adp-ribose polymerase  SLC35A5 - solute carrier family 35, member a5  PARP11 - poly (adp-ribose) polymerase family, member 11  LSG1 - large 60s subunit nuclear export gtpase 1  THOC7 - tho complex 7 homolog (drosophila)  SRSF9 - serine/arginine-rich splicing factor 9  SLC35C1 - solute carrier family 35 (gdp-fucose transporter), member c1  NUP43 - nucleoporin 43kda  PCID2 - pci domain containing 2  SLC29A4 - solute carrier family 29 (equilibrative nucleoside transporter), member 4  SLBP - stem-loop binding protein  CHTOP - chromatin target of prmt1  SLC25A17 - solute carrier family 25 (mitochondrial carrier; peroxisomal membrane protein, 34kda), member 17  WDR33 - wd repeat domain 33  NOL6 - nucleolar protein 6 (rna-associated)  ZC3H3 - zinc finger ccch-type containing 3  FYTTD1 - forty-two-three domain containing 1  SLC25A43 - solute carrier family 25, member 43  UPF3B - upf3 regulator of nonsense transcripts homolog b (yeast)  SLC25A19 - solute carrier family 25 (mitochondrial thiamine pyrophosphate carrier), member 19  DDX39B - dead (asp-glu-ala-asp) box polypeptide 39b  UPF3A - upf3 regulator of nonsense transcripts homolog a (yeast)  NUP188 - nucleoporin 188kda  PNPT1 - polyribonucleotide nucleotidyltransferase 1  RANBP2 - ran binding protein 2  NUP205 - nucleoporin 205kda  POLDIP3 - polymerase (dna-directed), delta interacting protein 3  IWS1 - iws1 homolog (s. cerevisiae)  YTHDC1 - yth domain containing 1  THOC2 - tho complex 2  THOC6 - tho complex 6 homolog (drosophila)  RFTN2 - raftlin family member 2  TERF2 - telomeric repeat binding factor 2  HNRNPU - heterogeneous nuclear ribonucleoprotein u (scaffold attachment factor a)  AAAS - achalasia, adrenocortical insufficiency, alacrimia  RPS15 - ribosomal protein s15  NUP155 - nucleoporin 155kda  SLC25A33 - solute carrier family 25 (pyrimidine nucleotide carrier), member 33  RBM8A - rna binding motif protein 8a  CASC3 - cancer susceptibility candidate 3  SFPQ - splicing factor proline/glutamine-rich  U2AF2 - u2 small nuclear rna auxiliary factor 2  SRSF1 - serine/arginine-rich splicing factor 1  HNRNPA2B1 - heterogeneous nuclear ribonucleoprotein a2/b1  SRSF2 - serine/arginine-rich splicing factor 2  HNRNPA1 - heterogeneous nuclear ribonucleoprotein a1  SRSF3 - serine/arginine-rich splicing factor 3  SRSF4 - serine/arginine-rich splicing factor 4  NUP93 - nucleoporin 93kda  RRS1 - rrs1 ribosome biogenesis regulator homolog (s. cerevisiae)  SRSF5 - serine/arginine-rich splicing factor 5  SRSF6 - serine/arginine-rich splicing factor 6  RAE1 - rae1 rna export 1 homolog (s. pombe)  SRSF7 - serine/arginine-rich splicing factor 7  TGFBR2 - transforming growth factor, beta receptor ii (70/80kda)  SLC35A2 - solute carrier family 35 (udp-galactose transporter), member a2  NUP35 - nucleoporin 35kda  SSB - sjogren syndrome antigen b (autoantigen la)  NDC1 - ndc1 transmembrane nucleoporin  THOC1 - tho complex 1  THOC3 - tho complex 3  MRPL18 - mitochondrial ribosomal protein l18  NUP214 - nucleoporin 214kda  SLC25A44 - solute carrier family 25, member 44  SARNP - sap domain containing ribonucleoprotein  NUP153 - nucleoporin 153kda  SLC25A5 - solute carrier family 25 (mitochondrial carrier; adenine nucleotide translocator), member 5  SLC25A16 - solute carrier family 25 (mitochondrial carrier; graves disease autoantigen), member 16  NUP210 - nucleoporin 210kda |
| GO:0006338 | chromatin remodeling | 5.73E-6 | 7.82E-4 | 1.26 (10334,123,6996,105) | [+] Show genes  SMARCAD1 - swi/snf-related, matrix-associated actin-dependent regulator of chromatin, subfamily a, containing dead/h box 1  VPS72 - vacuolar protein sorting 72 homolog (s. cerevisiae)  ANP32E - acidic (leucine-rich) nuclear phosphoprotein 32 family, member e  CENPV - centromere protein v  RSF1 - remodeling and spacing factor 1  NASP - nuclear autoantigenic sperm protein (histone-binding)  KAT2A - k(lysine) acetyltransferase 2a  PBRM1 - polybromo 1  CHD1L - chromodomain helicase dna binding protein 1-like  CHD7 - chromodomain helicase dna binding protein 7  TAF6L - taf6-like rna polymerase ii, p300/cbp-associated factor (pcaf)-associated factor, 65kda  ACTB - actin, beta  GATAD2B - gata zinc finger domain containing 2b  MTA2 - metastasis associated 1 family, member 2  GATAD1 - gata zinc finger domain containing 1  MLF1IP - mlf1 interacting protein  KAT2B - k(lysine) acetyltransferase 2b  APITD1 - apoptosis-inducing, taf9-like domain 1  H3F3A - h3 histone, family 3a  ACTL6A - actin-like 6a  CENPC - centromere protein c  CENPM - centromere protein m  TADA2A - transcriptional adaptor 2a  SMCHD1 - structural maintenance of chromosomes flexible hinge domain containing 1  SPTY2D1 - spt2, suppressor of ty, domain containing 1 (s. cerevisiae)  ARID1A - at rich interactive domain 1a (swi-like)  CHD5 - chromodomain helicase dna binding protein 5  HCFC2 - host cell factor c2  ARID1B - at rich interactive domain 1b (swi1-like)  CHRAC1 - chromatin accessibility complex 1  ACTR5 - arp5 actin-related protein 5 homolog (yeast)  KDM4B - lysine (k)-specific demethylase 4b  TOP1 - topoisomerase (dna) i  SMARCE1 - swi/snf related, matrix associated, actin dependent regulator of chromatin, subfamily e, member 1  INO80B - ino80 complex subunit b  ACTL6B - actin-like 6b  ACTR6 - arp6 actin-related protein 6 homolog (yeast)  BAZ2A - bromodomain adjacent to zinc finger domain, 2a  SMARCB1 - swi/snf related, matrix associated, actin dependent regulator of chromatin, subfamily b, member 1  HCFC1 - host cell factor c1 (vp16-accessory protein)  KDM5C - lysine (k)-specific demethylase 5c  SMARCC1 - swi/snf related, matrix associated, actin dependent regulator of chromatin, subfamily c, member 1  PTMA - prothymosin, alpha  INO80E - ino80 complex subunit e  SMARCA4 - swi/snf related, matrix associated, actin dependent regulator of chromatin, subfamily a, member 4  DAXX - death-domain associated protein  SMARCD2 - swi/snf related, matrix associated, actin dependent regulator of chromatin, subfamily d, member 2  NPM1 - nucleophosmin (nucleolar phosphoprotein b23, numatrin)  SMARCD3 - swi/snf related, matrix associated, actin dependent regulator of chromatin, subfamily d, member 3  SMARCC2 - swi/snf related, matrix associated, actin dependent regulator of chromatin, subfamily c, member 2  SMARCD1 - swi/snf related, matrix associated, actin dependent regulator of chromatin, subfamily d, member 1  SMARCA1 - swi/snf related, matrix associated, actin dependent regulator of chromatin, subfamily a, member 1  SMARCA2 - swi/snf related, matrix associated, actin dependent regulator of chromatin, subfamily a, member 2  RUVBL1 - ruvb-like 1 (e. coli)  HDAC2 - histone deacetylase 2  SATB1 - satb homeobox 1  ZBTB7A - zinc finger and btb domain containing 7a  DMAP1 - dna methyltransferase 1 associated protein 1  MBD3 - methyl-cpg binding domain protein 3  HDAC1 - histone deacetylase 1  RUVBL2 - ruvb-like 2 (e. coli)  KDM4C - lysine (k)-specific demethylase 4c  ITGB3BP - integrin beta 3 binding protein (beta3-endonexin)  ZBTB1 - zinc finger and btb domain containing 1  HMGB3 - high mobility group box 3  HMGB2 - high mobility group box 2  HMGB1 - high mobility group box 1  BAHD1 - bromo adjacent homology domain containing 1  MORC2 - morc family cw-type zinc finger 2  KDM5B - lysine (k)-specific demethylase 5b  RERE - arginine-glutamic acid dipeptide (re) repeats  BPTF - bromodomain phd finger transcription factor  DR1 - down-regulator of transcription 1, tbp-binding (negative cofactor 2)  HDAC5 - histone deacetylase 5  SIRT1 - sirtuin 1  BAZ2B - bromodomain adjacent to zinc finger domain, 2b  CTNNB1 - catenin (cadherin-associated protein), beta 1, 88kda  CHD4 - chromodomain helicase dna binding protein 4  HDAC4 - histone deacetylase 4  CHD3 - chromodomain helicase dna binding protein 3  ACTR8 - arp8 actin-related protein 8 homolog (yeast)  CENPT - centromere protein t  CHD1 - chromodomain helicase dna binding protein 1  KDM6B - lysine (k)-specific demethylase 6b  ZMIZ2 - zinc finger, miz-type containing 2  KDM6A - lysine (k)-specific demethylase 6a  EZH1 - enhancer of zeste homolog 1 (drosophila)  INO80 - ino80 complex subunit  NPM3 - nucleophosmin/nucleoplasmin 3  TFAM - transcription factor a, mitochondrial  INO80C - ino80 complex subunit c  CENPO - centromere protein o  ATRX - alpha thalassemia/mental retardation syndrome x-linked  RNF8 - ring finger protein 8, e3 ubiquitin protein ligase  BAZ1B - bromodomain adjacent to zinc finger domain, 1b  ZMIZ1 - zinc finger, miz-type containing 1  RBBP7 - retinoblastoma binding protein 7  SMARCA5 - swi/snf related, matrix associated, actin dependent regulator of chromatin, subfamily a, member 5  HNRNPC - heterogeneous nuclear ribonucleoprotein c (c1/c2)  RB1 - retinoblastoma 1  KDM5A - lysine (k)-specific demethylase 5a  CHD8 - chromodomain helicase dna binding protein 8  RBBP4 - retinoblastoma binding protein 4  KDM4A - lysine (k)-specific demethylase 4a  HMGA1 - high mobility group at-hook 1 |
| GO:0006302 | double-strand break repair | 6.09E-6 | 8.23E-4 | 1.27 (10334,114,6996,98) | [+] Show genes  DDX1 - dead (asp-glu-ala-asp) box helicase 1  DTX3L - deltex 3-like (drosophila)  LIG4 - ligase iv, dna, atp-dependent  RFWD3 - ring finger and wd repeat domain 3  FAN1 - fancd2/fanci-associated nuclease 1  FBXO18 - f-box protein, helicase, 18  SAMHD1 - sam domain and hd domain 1  LIG3 - ligase iii, dna, atp-dependent  SETMAR - set domain and mariner transposase fusion gene  MLH1 - mutl homolog 1, colon cancer, nonpolyposis type 2 (e. coli)  RECQL - recq protein-like (dna helicase q1-like)  MCM9 - minichromosome maintenance complex component 9  POLL - polymerase (dna directed), lambda  NBN - nibrin  PRPF19 - pre-mrna processing factor 19  APBB1 - amyloid beta (a4) precursor protein-binding, family b, member 1 (fe65)  RIF1 - rap1 interacting factor homolog (yeast)  AP5Z1 - adaptor-related protein complex 5, zeta 1 subunit  SLX4 - slx4 structure-specific endonuclease subunit  UIMC1 - ubiquitin interaction motif containing 1  KDM2A - lysine (k)-specific demethylase 2a  MORF4L1 - mortality factor 4 like 1  PSMD14 - proteasome (prosome, macropain) 26s subunit, non-atpase, 14  MCM7 - minichromosome maintenance complex component 7  H2AFX - h2a histone family, member x  NSMCE2 - non-smc element 2, mms21 homolog (s. cerevisiae)  SMCHD1 - structural maintenance of chromosomes flexible hinge domain containing 1  TP53BP1 - tumor protein p53 binding protein 1  RNF138 - ring finger protein 138, e3 ubiquitin protein ligase  NABP2 - nucleic acid binding protein 2  HUS1 - hus1 checkpoint homolog (s. pombe)  CIB1 - calcium and integrin binding 1 (calmyrin)  ACTR5 - arp5 actin-related protein 5 homolog (yeast)  CDC45 - cell division cycle 45  FEN1 - flap structure-specific endonuclease 1  KAT5 - k(lysine) acetyltransferase 5  CDC7 - cell division cycle 7  HERC2 - hect and rld domain containing e3 ubiquitin protein ligase 2  NSMCE1 - non-smc element 1 homolog (s. cerevisiae)  RAD50 - rad50 homolog (s. cerevisiae)  PARP1 - poly (adp-ribose) polymerase 1  C7orf49 - chromosome 7 open reading frame 49  ZBTB7A - zinc finger and btb domain containing 7a  SETX - senataxin  MUS81 - mus81 structure-specific endonuclease subunit  XRCC1 - x-ray repair complementing defective repair in chinese hamster cells 1  MTA1 - metastasis associated 1  AP5S1 - adaptor-related protein complex 5, sigma 1 subunit  RPA3 - replication protein a3, 14kda  MAD2L2 - mad2 mitotic arrest deficient-like 2 (yeast)  RPA1 - replication protein a1, 70kda  RPA2 - replication protein a2, 32kda  PARP2 - poly (adp-ribose) polymerase 2  TNKS1BP1 - tankyrase 1 binding protein 1, 182kda  RECQL5 - recq protein-like 5  ACTR8 - arp8 actin-related protein 8 homolog (yeast)  RNF168 - ring finger protein 168, e3 ubiquitin protein ligase  XRCC6 - x-ray repair complementing defective repair in chinese hamster cells 6  WHSC1 - wolf-hirschhorn syndrome candidate 1  SMC5 - structural maintenance of chromosomes 5  WDR48 - wd repeat domain 48  MCM3 - minichromosome maintenance complex component 3  MCM6 - minichromosome maintenance complex component 6  UVRAG - uv radiation resistance associated  MCM5 - minichromosome maintenance complex component 5  RAD21 - rad21 homolog (s. pombe)  EYA3 - eyes absent homolog 3 (drosophila)  MCM2 - minichromosome maintenance complex component 2  VCP - valosin containing protein  INO80 - ino80 complex subunit  MDC1 - mediator of dna-damage checkpoint 1  MSH2 - muts homolog 2, colon cancer, nonpolyposis type 1 (e. coli)  ERCC4 - excision repair cross-complementing rodent repair deficiency, complementation group 4  NIPBL - nipped-b homolog (drosophila)  SMARCAL1 - swi/snf related, matrix associated, actin dependent regulator of chromatin, subfamily a-like 1  EXD2 - exonuclease 3'-5' domain containing 2  BRCC3 - brca1/brca2-containing complex, subunit 3  SWSAP1 - swim-type zinc finger 7 associated protein 1  PAPD7 - pap associated domain containing 7  ZFYVE26 - zinc finger, fyve domain containing 26  PARP9 - poly (adp-ribose) polymerase family, member 9  RNF8 - ring finger protein 8, e3 ubiquitin protein ligase  SFPQ - splicing factor proline/glutamine-rich  RBBP8 - retinoblastoma binding protein 8  SUMO1 - small ubiquitin-like modifier 1  BABAM1 - brisc and brca1 a complex member 1  PIAS4 - protein inhibitor of activated stat, 4  TDP2 - tyrosyl-dna phosphodiesterase 2  ERCC8 - excision repair cross-complementing rodent repair deficiency, complementation group 8  ZSWIM7 - zinc finger, swim-type containing 7  XRCC5 - x-ray repair complementing defective repair in chinese hamster cells 5 (double-strand-break rejoining)  MRE11A - mre11 meiotic recombination 11 homolog a (s. cerevisiae)  XRCC4 - x-ray repair complementing defective repair in chinese hamster cells 4  UBE2V2 - ubiquitin-conjugating enzyme e2 variant 2  EME1 - essential meiotic structure-specific endonuclease 1  NABP1 - nucleic acid binding protein 1  KIAA0430 - kiaa0430  UBE2N - ubiquitin-conjugating enzyme e2n |
| GO:0006974 | cellular response to DNA damage stimulus | 6.37E-6 | 8.53E-4 | 1.13 (10334,537,6996,409) | [+] Show genes  WAC - ww domain containing adaptor with coiled-coil  PMS2 - pms2 postmeiotic segregation increased 2 (s. cerevisiae)  LIG4 - ligase iv, dna, atp-dependent  FBXO18 - f-box protein, helicase, 18  CBX1 - chromobox homolog 1  LIG1 - ligase i, dna, atp-dependent  LIG3 - ligase iii, dna, atp-dependent  ABL1 - c-abl oncogene 1, non-receptor tyrosine kinase  PML - promyelocytic leukemia  CNOT11 - ccr4-not transcription complex, subunit 11  DOT1L - dot1-like histone h3k79 methyltransferase  PMS1 - pms1 postmeiotic segregation increased 1 (s. cerevisiae)  BRSK1 - br serine/threonine kinase 1  MUM1 - melanoma associated antigen (mutated) 1  SOX4 - sry (sex determining region y)-box 4  SLX4 - slx4 structure-specific endonuclease subunit  CHD1L - chromodomain helicase dna binding protein 1-like  TIPRL - tip41, tor signaling pathway regulator-like (s. cerevisiae)  OTUB1 - otu domain, ubiquitin aldehyde binding 1  MORF4L1 - mortality factor 4 like 1  ATF2 - activating transcription factor 2  CLOCK - clock circadian regulator  SMUG1 - single-strand-selective monofunctional uracil-dna glycosylase 1  POLK - polymerase (dna directed) kappa  ACTL6A - actin-like 6a  INIP - ints3 and nabp interacting protein  TRRAP - transformation/transcription domain-associated protein  PLK3 - polo-like kinase 3  RNF138 - ring finger protein 138, e3 ubiquitin protein ligase  ACTR5 - arp5 actin-related protein 5 homolog (yeast)  RBM17 - rna binding motif protein 17  CDC45 - cell division cycle 45  CDC7 - cell division cycle 7  SMC1A - structural maintenance of chromosomes 1a  UBR5 - ubiquitin protein ligase e3 component n-recognin 5  POLR2K - polymerase (rna) ii (dna directed) polypeptide k, 7.0kda  UCHL5 - ubiquitin carboxyl-terminal hydrolase l5  PARP1 - poly (adp-ribose) polymerase 1  POLH - polymerase (dna directed), eta  TAOK3 - tao kinase 3  POLD2 - polymerase (dna directed), delta 2, accessory subunit  FZR1 - fizzy/cell division cycle 20 related 1 (drosophila)  POLD1 - polymerase (dna directed), delta 1, catalytic subunit  ZBTB7A - zinc finger and btb domain containing 7a  POLR2G - polymerase (rna) ii (dna directed) polypeptide g  POLR2F - polymerase (rna) ii (dna directed) polypeptide f  POLR2E - polymerase (rna) ii (dna directed) polypeptide e, 25kda  POLR2D - polymerase (rna) ii (dna directed) polypeptide d  POLR2C - polymerase (rna) ii (dna directed) polypeptide c, 33kda  POLR2B - polymerase (rna) ii (dna directed) polypeptide b, 140kda  POLR2A - polymerase (rna) ii (dna directed) polypeptide a, 220kda  PARP4 - poly (adp-ribose) polymerase family, member 4  RUVBL2 - ruvb-like 2 (e. coli)  TFIP11 - tuftelin interacting protein 11  ING4 - inhibitor of growth family, member 4  PPP5C - protein phosphatase 5, catalytic subunit  RECQL5 - recq protein-like 5  AKT1 - v-akt murine thymoma viral oncogene homolog 1  USP7 - ubiquitin specific peptidase 7 (herpes virus-associated)  RNF168 - ring finger protein 168, e3 ubiquitin protein ligase  XRCC6 - x-ray repair complementing defective repair in chinese hamster cells 6  NEK4 - nima-related kinase 4  SUV420H1 - suppressor of variegation 4-20 homolog 1 (drosophila)  TOPBP1 - topoisomerase (dna) ii binding protein 1  RMI2 - recq mediated genome instability 2  SUV420H2 - suppressor of variegation 4-20 homolog 2 (drosophila)  RPAIN - rpa interacting protein  NIPBL - nipped-b homolog (drosophila)  CRY1 - cryptochrome 1 (photolyase-like)  SHISA5 - shisa family member 5  NPLOC4 - nuclear protein localization 4 homolog (s. cerevisiae)  ASF1A - anti-silencing function 1a histone chaperone  SSRP1 - structure specific recognition protein 1  ZDHHC16 - zinc finger, dhhc-type containing 16  PAPD7 - pap associated domain containing 7  BOD1L1 - biorientation of chromosomes in cell division 1-like 1  CHCHD6 - coiled-coil-helix-coiled-coil-helix domain containing 6  TRIP12 - thyroid hormone receptor interactor 12  MAPK3 - mitogen-activated protein kinase 3  MAPK1 - mitogen-activated protein kinase 1  COPS6 - cop9 signalosome subunit 6  COPS2 - cop9 signalosome subunit 2  TLK2 - tousled-like kinase 2  TAOK2 - tao kinase 2  CNOT8 - ccr4-not transcription complex, subunit 8  SHPRH - snf2 histone linker phd ring helicase, e3 ubiquitin protein ligase  RNF169 - ring finger protein 169  APEX1 - apex nuclease (multifunctional dna repair enzyme) 1  XIAP - x-linked inhibitor of apoptosis  SMC6 - structural maintenance of chromosomes 6  DDX1 - dead (asp-glu-ala-asp) box helicase 1  SYF2 - syf2 pre-mrna-splicing factor  RFWD3 - ring finger and wd repeat domain 3  SPRTN - sprt-like n-terminal domain  TREX1 - three prime repair exonuclease 1  MIF - macrophage migration inhibitory factor (glycosylation-inhibiting factor)  RNF113A - ring finger protein 113a  SAMHD1 - sam domain and hd domain 1  DDB1 - damage-specific dna binding protein 1, 127kda  SETMAR - set domain and mariner transposase fusion gene  PRMT6 - protein arginine methyltransferase 6  MLH1 - mutl homolog 1, colon cancer, nonpolyposis type 2 (e. coli)  GTF2H2 - general transcription factor iih, polypeptide 2, 44kda  NEIL1 - nei endonuclease viii-like 1 (e. coli)  GTF2H3 - general transcription factor iih, polypeptide 3, 34kda  MCM9 - minichromosome maintenance complex component 9  GTF2H1 - general transcription factor iih, polypeptide 1, 62kda  APC - adenomatous polyposis coli  MSH6 - muts homolog 6 (e. coli)  APBB1 - amyloid beta (a4) precursor protein-binding, family b, member 1 (fe65)  RIF1 - rap1 interacting factor homolog (yeast)  POLG2 - polymerase (dna directed), gamma 2, accessory subunit  GTF2H4 - general transcription factor iih, polypeptide 4, 52kda  CNOT10 - ccr4-not transcription complex, subunit 10  ZBTB38 - zinc finger and btb domain containing 38  RCHY1 - ring finger and chy zinc finger domain containing 1, e3 ubiquitin protein ligase  MNAT1 - mnat cdk-activating kinase assembly factor 1  UIMC1 - ubiquitin interaction motif containing 1  POLI - polymerase (dna directed) iota  CNOT6 - ccr4-not transcription complex, subunit 6  MCM7 - minichromosome maintenance complex component 7  H2AFX - h2a histone family, member x  NSMCE2 - non-smc element 2, mms21 homolog (s. cerevisiae)  SUPT16H - suppressor of ty 16 homolog (s. cerevisiae)  MEN1 - multiple endocrine neoplasia i  PCNA - proliferating cell nuclear antigen  HINFP - histone h4 transcription factor  FANCL - fanconi anemia, complementation group l  WRAP53 - wd repeat containing, antisense to tp53  KAT7 - k(lysine) acetyltransferase 7  ATRIP - atr interacting protein  EPC2 - enhancer of polycomb homolog 2 (drosophila)  GNL1 - guanine nucleotide binding protein-like 1  XPA - xeroderma pigmentosum, complementation group a  ZBTB1 - zinc finger and btb domain containing 1  XPC - xeroderma pigmentosum, complementation group c  RQCD1 - rcd1 required for cell differentiation1 homolog (s. pombe)  CNOT6L - ccr4-not transcription complex, subunit 6-like  XRCC1 - x-ray repair complementing defective repair in chinese hamster cells 1  SMC3 - structural maintenance of chromosomes 3  TAOK1 - tao kinase 1  PAGR1 - paxip1 associated glutamate-rich protein 1  MORC2 - morc family cw-type zinc finger 2  PHF1 - phd finger protein 1  MTA1 - metastasis associated 1  SIRT6 - sirtuin 6  AP5S1 - adaptor-related protein complex 5, sigma 1 subunit  TNKS1BP1 - tankyrase 1 binding protein 1, 182kda  MAPK15 - mitogen-activated protein kinase 15  USP10 - ubiquitin specific peptidase 10  WHSC1 - wolf-hirschhorn syndrome candidate 1  REV1 - rev1, polymerase (dna directed)  WDR33 - wd repeat domain 33  WDR48 - wd repeat domain 48  MCM3 - minichromosome maintenance complex component 3  GIGYF2 - grb10 interacting gyf protein 2  MCM6 - minichromosome maintenance complex component 6  MCM5 - minichromosome maintenance complex component 5  FNIP2 - folliculin interacting protein 2  MCM2 - minichromosome maintenance complex component 2  ASH2L - ash2 (absent, small, or homeotic)-like (drosophila)  BAX - bcl2-associated x protein  UBA6 - ubiquitin-like modifier activating enzyme 6  BAK1 - bcl2-antagonist/killer 1  BACH1 - btb and cnc homology 1, basic leucine zipper transcription factor 1  BAD - bcl2-associated agonist of cell death  STXBP4 - syntaxin binding protein 4  NEIL2 - nei endonuclease viii-like 2 (e. coli)  BRCC3 - brca1/brca2-containing complex, subunit 3  EXD2 - exonuclease 3'-5' domain containing 2  PIK3R1 - phosphoinositide-3-kinase, regulatory subunit 1 (alpha)  GRB2 - growth factor receptor-bound protein 2  PLAGL1 - pleiomorphic adenoma gene-like 1  LYN - v-yes-1 yamaguchi sarcoma viral related oncogene homolog  SESN2 - sestrin 2  PARP9 - poly (adp-ribose) polymerase family, member 9  RNF8 - ring finger protein 8, e3 ubiquitin protein ligase  ATRX - alpha thalassemia/mental retardation syndrome x-linked  BAZ1B - bromodomain adjacent to zinc finger domain, 1b  UVSSA - uv-stimulated scaffold protein a  SFPQ - splicing factor proline/glutamine-rich  ZBTB4 - zinc finger and btb domain containing 4  PIAS4 - protein inhibitor of activated stat, 4  TDP2 - tyrosyl-dna phosphodiesterase 2  EIF2AK4 - eukaryotic translation initiation factor 2 alpha kinase 4  UBE2W - ubiquitin-conjugating enzyme e2w (putative)  BCL2 - b-cell cll/lymphoma 2  XRCC5 - x-ray repair complementing defective repair in chinese hamster cells 5 (double-strand-break rejoining)  BCL2L2 - bcl2-like 2  XRCC4 - x-ray repair complementing defective repair in chinese hamster cells 4  BCL2L1 - bcl2-like 1  SGK1 - serum/glucocorticoid regulated kinase 1  CCND1 - cyclin d1  EME1 - essential meiotic structure-specific endonuclease 1  PARK7 - parkinson protein 7  PNKP - polynucleotide kinase 3'-phosphatase  CCNK - cyclin k  RFC5 - replication factor c (activator 1) 5, 36.5kda  RFC2 - replication factor c (activator 1) 2, 40kda  RFC3 - replication factor c (activator 1) 3, 38kda  FAN1 - fancd2/fanci-associated nuclease 1  SNW1 - snw domain containing 1  PRMT1 - protein arginine methyltransferase 1  MGME1 - mitochondrial genome maintenance exonuclease 1  TRIM28 - tripartite motif containing 28  KIN - kin, antigenic determinant of reca protein homolog (mouse)  RECQL - recq protein-like (dna helicase q1-like)  EP300 - e1a binding protein p300  FBXO45 - f-box protein 45  UPF1 - upf1 regulator of nonsense transcripts homolog (yeast)  NBN - nibrin  UBQLN4 - ubiquilin 4  ALKBH1 - alkb, alkylation repair homolog 1 (e. coli)  TFPT - tcf3 (e2a) fusion partner (in childhood leukemia)  KDM2A - lysine (k)-specific demethylase 2a  MYO6 - myosin vi  TOPORS - topoisomerase i binding, arginine/serine-rich, e3 ubiquitin protein ligase  TMEM109 - transmembrane protein 109  PSMD14 - proteasome (prosome, macropain) 26s subunit, non-atpase, 14  CNOT1 - ccr4-not transcription complex, subunit 1  C1orf86 - chromosome 1 open reading frame 86  TP53BP1 - tumor protein p53 binding protein 1  HUWE1 - hect, uba and wwe domain containing 1, e3 ubiquitin protein ligase  CNOT7 - ccr4-not transcription complex, subunit 7  NABP2 - nucleic acid binding protein 2  HUS1 - hus1 checkpoint homolog (s. pombe)  INO80B - ino80 complex subunit b  NCOA6 - nuclear receptor coactivator 6  HERC2 - hect and rld domain containing e3 ubiquitin protein ligase 2  RNF111 - ring finger protein 111  SMG1 - smg1 phosphatidylinositol 3-kinase-related kinase  E2F4 - e2f transcription factor 4, p107/p130-binding  XAB2 - xpa binding protein 2  MUTYH - muty homolog (e. coli)  PDS5B - pds5, regulator of cohesion maintenance, homolog b (s. cerevisiae)  NSMCE1 - non-smc element 1 homolog (s. cerevisiae)  RAD50 - rad50 homolog (s. cerevisiae)  C11orf30 - chromosome 11 open reading frame 30  C7orf49 - chromosome 7 open reading frame 49  ZMPSTE24 - zinc metallopeptidase ste24  MBD4 - methyl-cpg binding domain protein 4  STUB1 - stip1 homology and u-box containing protein 1, e3 ubiquitin protein ligase  SETX - senataxin  VAV3 - vav 3 guanine nucleotide exchange factor  PPIE - peptidylprolyl isomerase e (cyclophilin e)  REXO4 - rex4, rna exonuclease 4 homolog (s. cerevisiae)  MCRS1 - microspherule protein 1  HMGN1 - high mobility group nucleosome binding domain 1  USP1 - ubiquitin specific peptidase 1  FANCE - fanconi anemia, complementation group e  STK33 - serine/threonine kinase 33  CASP9 - caspase 9, apoptosis-related cysteine peptidase  TANK - traf family member-associated nfkb activator  SLC30A9 - solute carrier family 30 (zinc transporter), member 9  UNG - uracil-dna glycosylase  CASP2 - caspase 2, apoptosis-related cysteine peptidase  CASP3 - caspase 3, apoptosis-related cysteine peptidase  RPA3 - replication protein a3, 14kda  MAD2L2 - mad2 mitotic arrest deficient-like 2 (yeast)  RPA1 - replication protein a1, 70kda  C19orf40 - chromosome 19 open reading frame 40  RPA2 - replication protein a2, 32kda  PARP2 - poly (adp-ribose) polymerase 2  PEA15 - phosphoprotein enriched in astrocytes 15  HIPK1 - homeodomain interacting protein kinase 1  SMC5 - structural maintenance of chromosomes 5  ACD - adrenocortical dysplasia homolog (mouse)  PCBP4 - poly(rc) binding protein 4  CHAF1A - chromatin assembly factor 1, subunit a (p150)  UVRAG - uv radiation resistance associated  EYA3 - eyes absent homolog 3 (drosophila)  ENDOV - endonuclease v  INTS3 - integrator complex subunit 3  VCP - valosin containing protein  MSH3 - muts homolog 3 (e. coli)  MSH2 - muts homolog 2, colon cancer, nonpolyposis type 1 (e. coli)  ERCC5 - excision repair cross-complementing rodent repair deficiency, complementation group 5  ERCC4 - excision repair cross-complementing rodent repair deficiency, complementation group 4  ERCC3 - excision repair cross-complementing rodent repair deficiency, complementation group 3  ZBTB40 - zinc finger and btb domain containing 40  UBE2A - ubiquitin-conjugating enzyme e2a  INO80C - ino80 complex subunit c  UBA1 - ubiquitin-like modifier activating enzyme 1  CCNH - cyclin h  UBB - ubiquitin b  RGCC - regulator of cell cycle  SWSAP1 - swim-type zinc finger 7 associated protein 1  NDRG1 - n-myc downstream regulated 1  TIPIN - timeless interacting protein  TTC5 - tetratricopeptide repeat domain 5  COMMD1 - copper metabolism (murr1) domain containing 1  ACER2 - alkaline ceramidase 2  MRPS11 - mitochondrial ribosomal protein s11  FBXO6 - f-box protein 6  USP3 - ubiquitin specific peptidase 3  CBL - cbl proto-oncogene, e3 ubiquitin protein ligase  SUMO1 - small ubiquitin-like modifier 1  BABAM1 - brisc and brca1 a complex member 1  UBE2T - ubiquitin-conjugating enzyme e2t (putative)  ZNF830 - zinc finger protein 830  ADPRHL2 - adp-ribosylhydrolase like 2  ZSWIM7 - zinc finger, swim-type containing 7  SETD2 - set domain containing 2  RBX1 - ring-box 1, e3 ubiquitin protein ligase  HMGA1 - high mobility group at-hook 1  MRE11A - mre11 meiotic recombination 11 homolog a (s. cerevisiae)  UBE2V2 - ubiquitin-conjugating enzyme e2 variant 2  ERCC2 - excision repair cross-complementing rodent repair deficiency, complementation group 2  NABP1 - nucleic acid binding protein 1  UBE2N - ubiquitin-conjugating enzyme e2n  UBE2V1 - ubiquitin-conjugating enzyme e2 variant 1  FMR1 - fragile x mental retardation 1  CDC5L - cell division cycle 5-like  DTX3L - deltex 3-like (drosophila)  RPS6KA6 - ribosomal protein s6 kinase, 90kda, polypeptide 6  TLK1 - tousled-like kinase 1  PDS5A - pds5, regulator of cohesion maintenance, homolog a (s. cerevisiae)  PARG - poly (adp-ribose) glycohydrolase  HTRA2 - htra serine peptidase 2  ERCC6L2 - excision repair cross-complementing rodent repair deficiency, complementation group 6-like 2  PSEN1 - presenilin 1  PPM1D - protein phosphatase, mg2+/mn2+ dependent, 1d  USP16 - ubiquitin specific peptidase 16  POLL - polymerase (dna directed), lambda  FOXO3 - forkhead box o3  PRPF19 - pre-mrna processing factor 19  CDC14B - cell division cycle 14b  AP5Z1 - adaptor-related protein complex 5, zeta 1 subunit  TDG - thymine-dna glycosylase  COPS7B - cop9 signalosome subunit 7b  OGG1 - 8-oxoguanine dna glycosylase  COPS3 - cop9 signalosome subunit 3  TERF2IP - telomeric repeat binding factor 2, interacting protein  APITD1 - apoptosis-inducing, taf9-like domain 1  TAF9 - taf9 rna polymerase ii, tata box binding protein (tbp)-associated factor, 32kda  RNASEH2A - ribonuclease h2, subunit a  SMCHD1 - structural maintenance of chromosomes flexible hinge domain containing 1  CETN2 - centrin, ef-hand protein, 2  JMY - junction mediating and regulatory protein, p53 cofactor  CIB1 - calcium and integrin binding 1 (calmyrin)  FEN1 - flap structure-specific endonuclease 1  KAT5 - k(lysine) acetyltransferase 5  INO80E - ino80 complex subunit e  CDK9 - cyclin-dependent kinase 9  NPM1 - nucleophosmin (nucleolar phosphoprotein b23, numatrin)  CDK7 - cyclin-dependent kinase 7  CDKN1B - cyclin-dependent kinase inhibitor 1b (p27, kip1)  APEX2 - apex nuclease (apurinic/apyrimidinic endonuclease) 2  IRF3 - interferon regulatory factor 3  UFL1 - ufm1-specific ligase 1  PTPN11 - protein tyrosine phosphatase, non-receptor type 11  RUVBL1 - ruvb-like 1 (e. coli)  ZMAT3 - zinc finger, matrin-type 3  DMAP1 - dna methyltransferase 1 associated protein 1  CDK2 - cyclin-dependent kinase 2  TCEA1 - transcription elongation factor a (sii), 1  WDR4 - wd repeat domain 4  RAD1 - rad1 homolog (s. pombe)  DONSON - downstream neighbor of son  NONO - non-pou domain containing, octamer-binding  CDIP1 - cell death-inducing p53 target 1  MUS81 - mus81 structure-specific endonuclease subunit  NDNL2 - necdin-like 2  PLK2 - polo-like kinase 2  MMS19 - mms19 nucleotide excision repair homolog (s. cerevisiae)  AQR - aquarius homolog (mouse)  CNOT2 - ccr4-not transcription complex, subunit 2  CNOT4 - ccr4-not transcription complex, subunit 4  CNOT3 - ccr4-not transcription complex, subunit 3  SIRT1 - sirtuin 1  CTC1 - cts telomere maintenance complex component 1  NFRKB - nuclear factor related to kappab binding protein  CHD2 - chromodomain helicase dna binding protein 2  ACTR8 - arp8 actin-related protein 8 homolog (yeast)  CBX5 - chromobox homolog 5  BRD4 - bromodomain containing 4  RAD21 - rad21 homolog (s. pombe)  TNFRSF1A - tumor necrosis factor receptor superfamily, member 1a  RAD17 - rad17 homolog (s. pombe)  BRAT1 - brca1-associated atm activator 1  TNFRSF1B - tumor necrosis factor receptor superfamily, member 1b  INO80 - ino80 complex subunit  MDC1 - mediator of dna-damage checkpoint 1  IGHMBP2 - immunoglobulin mu binding protein 2  RRM2B - ribonucleotide reductase m2 b (tp53 inducible)  SMARCAL1 - swi/snf related, matrix associated, actin dependent regulator of chromatin, subfamily a-like 1  OARD1 - o-acyl-adp-ribose deacylase 1  NEK1 - nima-related kinase 1  TERF2 - telomeric repeat binding factor 2  FAM35A - family with sequence similarity 35, member a  TFDP1 - transcription factor dp-1  MORF4L2 - mortality factor 4 like 2  NFATC4 - nuclear factor of activated t-cells, cytoplasmic, calcineurin-dependent 4  BCCIP - brca2 and cdkn1a interacting protein  ZFYVE26 - zinc finger, fyve domain containing 26  MLH3 - mutl homolog 3 (e. coli)  CDK5RAP3 - cdk5 regulatory subunit associated protein 3  RBL2 - retinoblastoma-like 2 (p130)  RBBP8 - retinoblastoma binding protein 8  CEP63 - centrosomal protein 63kda  RBBP5 - retinoblastoma binding protein 5  RBBP6 - retinoblastoma binding protein 6  CUL4B - cullin 4b  ERCC8 - excision repair cross-complementing rodent repair deficiency, complementation group 8  COPS7A - cop9 signalosome subunit 7a  POLD3 - polymerase (dna-directed), delta 3, accessory subunit  FOXP1 - forkhead box p1  METTL3 - methyltransferase like 3  KIAA0430 - kiaa0430 |
| GO:0048812 | neuron projection morphogenesis | 6.68E-6 | 8.86E-4 | 1.23 (10334,152,6996,127) | [+] Show genes  AFG3L2 - afg3 atpase family member 3-like 2 (s. cerevisiae)  CTTN - cortactin  SPTBN4 - spectrin, beta, non-erythrocytic 4  ATP8A2 - atpase, aminophospholipid transporter, class i, type 8a, member 2  NRP1 - neuropilin 1  PLXNB1 - plexin b1  FBXO45 - f-box protein 45  SPAST - spastin  ARC - activity-regulated cytoskeleton-associated protein  SHANK1 - sh3 and multiple ankyrin repeat domains 1  DBN1 - drebrin 1  ILK - integrin-linked kinase  APBB1 - amyloid beta (a4) precursor protein-binding, family b, member 1 (fe65)  NUMBL - numb homolog (drosophila)-like  HPRT1 - hypoxanthine phosphoribosyltransferase 1  BRSK1 - br serine/threonine kinase 1  NDEL1 - nude neurodevelopment protein 1-like 1  MAP1S - microtubule-associated protein 1s  ACTB - actin, beta  PAFAH1B1 - platelet-activating factor acetylhydrolase 1b, regulatory subunit 1 (45kda)  GJA1 - gap junction protein, alpha 1, 43kda  APP - amyloid beta (a4) precursor protein  CREB1 - camp responsive element binding protein 1  DOCK10 - dedicator of cytokinesis 10  PAK3 - p21 protein (cdc42/rac)-activated kinase 3  DYNLT1 - dynein, light chain, tctex-type 1  LLGL1 - lethal giant larvae homolog 1 (drosophila)  BMP7 - bone morphogenetic protein 7  GAS7 - growth arrest-specific 7  FGFR2 - fibroblast growth factor receptor 2  TCTN1 - tectonic family member 1  FLOT1 - flotillin 1  PICALM - phosphatidylinositol binding clathrin assembly protein  ANKRD27 - ankyrin repeat domain 27 (vps9 domain)  AMIGO1 - adhesion molecule with ig-like domain 1  PTEN - phosphatase and tensin homolog  ITGA1 - integrin, alpha 1  RAB10 - rab10, member ras oncogene family  DAB2IP - dab2 interacting protein  MEF2A - myocyte enhancer factor 2a  TOP2B - topoisomerase (dna) ii beta 180kda  EGFR - epidermal growth factor receptor  ADARB1 - adenosine deaminase, rna-specific, b1  SZT2 - seizure threshold 2 homolog (mouse)  NR4A2 - nuclear receptor subfamily 4, group a, member 2  RHOA - ras homolog family member a  CELSR2 - cadherin, egf lag seven-pass g-type receptor 2  ATL1 - atlastin gtpase 1  ITGB1 - integrin, beta 1 (fibronectin receptor, beta polypeptide, antigen cd29 includes mdf2, msk12)  CNP - 2',3'-cyclic nucleotide 3' phosphodiesterase  ADCY1 - adenylate cyclase 1 (brain)  DCLK1 - doublecortin-like kinase 1  PTK2 - protein tyrosine kinase 2  CDK5 - cyclin-dependent kinase 5  RAB8A - rab8a, member ras oncogene family  ROCK1 - rho-associated, coiled-coil containing protein kinase 1  S100B - s100 calcium binding protein b  PTN - pleiotrophin  STMN1 - stathmin 1  TRAK2 - trafficking protein, kinesin binding 2  KIDINS220 - kinase d-interacting substrate, 220kda  TNIK - traf2 and nck interacting kinase  ZEB2 - zinc finger e-box binding homeobox 2  SLC11A2 - solute carrier family 11 (proton-coupled divalent metal ion transporter), member 2  KLF7 - kruppel-like factor 7 (ubiquitous)  HSP90AA1 - heat shock protein 90kda alpha (cytosolic), class a member 1  CNTN4 - contactin 4  MYCBP2 - myc binding protein 2, e3 ubiquitin protein ligase  TAOK3 - tao kinase 3  CDH11 - cadherin 11, type 2, ob-cadherin (osteoblast)  NPTX1 - neuronal pentraxin i  NRCAM - neuronal cell adhesion molecule  NCKAP1 - nck-associated protein 1  ABI1 - abl-interactor 1  CHRNB2 - cholinergic receptor, nicotinic, beta 2 (neuronal)  DRD2 - dopamine receptor d2  BTBD3 - btb (poz) domain containing 3  TMEM106B - transmembrane protein 106b  ZNF365 - zinc finger protein 365  NUMB - numb homolog (drosophila)  TAOK1 - tao kinase 1  SHANK3 - sh3 and multiple ankyrin repeat domains 3  MAP6 - microtubule-associated protein 6  LRRK2 - leucine-rich repeat kinase 2  RERE - arginine-glutamic acid dipeptide (re) repeats  MAP2 - microtubule-associated protein 2  MAP1B - microtubule-associated protein 1b  MAP1A - microtubule-associated protein 1a  CTNND2 - catenin (cadherin-associated protein), delta 2  PACSIN1 - protein kinase c and casein kinase substrate in neurons 1  CTNNA2 - catenin (cadherin-associated protein), alpha 2  BAIAP2 - bai1-associated protein 2  MAP4K4 - mitogen-activated protein kinase kinase kinase kinase 4  HDAC6 - histone deacetylase 6  NYAP1 - neuronal tyrosine-phosphorylated phosphoinositide-3-kinase adaptor 1  WEE1 - wee1 homolog (s. pombe)  ATP7A - atpase, cu++ transporting, alpha polypeptide  RAB3A - rab3a, member ras oncogene family  LMTK2 - lemur tyrosine kinase 2  SLC9A6 - solute carrier family 9, subfamily a (nhe6, cation proton antiporter 6), member 6  EPB41L3 - erythrocyte membrane protein band 4.1-like 3  DOCK7 - dedicator of cytokinesis 7  FYN - fyn oncogene related to src, fgr, yes  SLC25A46 - solute carrier family 25, member 46  PITPNA - phosphatidylinositol transfer protein, alpha  MINK1 - misshapen-like kinase 1  DLG4 - discs, large homolog 4 (drosophila)  NEK3 - nima-related kinase 3  NEFL - neurofilament, light polypeptide  DBNL - drebrin-like  UBB - ubiquitin b  PHACTR1 - phosphatase and actin regulator 1  FAM206A - family with sequence similarity 206, member a  RBFOX2 - rna binding protein, fox-1 homolog (c. elegans) 2  MAPK8IP2 - mitogen-activated protein kinase 8 interacting protein 2  DNM2 - dynamin 2  ARHGAP35 - rho gtpase activating protein 35  ALS2 - amyotrophic lateral sclerosis 2 (juvenile)  ZNF335 - zinc finger protein 335  EPHB6 - eph receptor b6  BCL2 - b-cell cll/lymphoma 2  ZSWIM6 - zinc finger, swim-type containing 6  TAOK2 - tao kinase 2  SGK1 - serum/glucocorticoid regulated kinase 1  B4GALT6 - udp-gal:betaglcnac beta 1,4- galactosyltransferase, polypeptide 6  WASL - wiskott-aldrich syndrome-like  B4GALT5 - udp-gal:betaglcnac beta 1,4- galactosyltransferase, polypeptide 5 |
| GO:0033036 | macromolecule localization | 6.96E-6 | 9.14E-4 | 1.07 (10334,1470,6996,1067) | [+] Show genes  AGAP1 - arfgap with gtpase domain, ankyrin repeat and ph domain 1  AGAP2 - arfgap with gtpase domain, ankyrin repeat and ph domain 2  EXOSC10 - exosome component 10  AP3M2 - adaptor-related protein complex 3, mu 2 subunit  GDI1 - gdp dissociation inhibitor 1  GDI2 - gdp dissociation inhibitor 2  WLS - wntless homolog (drosophila)  SEC61B - sec61 beta subunit  ABCA1 - atp-binding cassette, sub-family a (abc1), member 1  TOMM34 - translocase of outer mitochondrial membrane 34  OS9 - osteosarcoma amplified 9, endoplasmic reticulum lectin  RAB3IP - rab3a interacting protein  TMED2 - transmembrane emp24 domain trafficking protein 2  ABL1 - c-abl oncogene 1, non-receptor tyrosine kinase  NUDT19 - nudix (nucleoside diphosphate linked moiety x)-type motif 19  ERP29 - endoplasmic reticulum protein 29  PML - promyelocytic leukemia  ACOT2 - acyl-coa thioesterase 2  PMM1 - phosphomannomutase 1  RAB40B - rab40b, member ras oncogene family  YWHAQ - tyrosine 3-monooxygenase/tryptophan 5-monooxygenase activation protein, theta polypeptide  UBE2Q1 - ubiquitin-conjugating enzyme e2q family member 1  TMED10 - transmembrane emp24-like trafficking protein 10 (yeast)  TBC1D22B - tbc1 domain family, member 22b  AP3B2 - adaptor-related protein complex 3, beta 2 subunit  NOP9 - nop9 nucleolar protein  ACOX1 - acyl-coa oxidase 1, palmitoyl  PLRG1 - pleiotropic regulator 1  ACTB - actin, beta  RAB37 - rab37, member ras oncogene family  GJA1 - gap junction protein, alpha 1, 43kda  ACTG1 - actin, gamma 1  EHD1 - eh-domain containing 1  ZDHHC7 - zinc finger, dhhc-type containing 7  GRASP - grp1 (general receptor for phosphoinositides 1)-associated scaffold protein  PPID - peptidylprolyl isomerase d  AP1AR - adaptor-related protein complex 1 associated regulatory protein  ACTN4 - actinin, alpha 4  RAB10 - rab10, member ras oncogene family  TMCO6 - transmembrane and coiled-coil domains 6  MED1 - mediator complex subunit 1  AXIN2 - axin 2  RAB39B - rab39b, member ras oncogene family  NUP85 - nucleoporin 85kda  USP9X - ubiquitin specific peptidase 9, x-linked  ADPRH - adp-ribosylarginine hydrolase  H2AFY2 - h2a histone family, member y2  RUVBL2 - ruvb-like 2 (e. coli)  TMEM30A - transmembrane protein 30a  AP2A2 - adaptor-related protein complex 2, alpha 2 subunit  AP2A1 - adaptor-related protein complex 2, alpha 1 subunit  AP2B1 - adaptor-related protein complex 2, beta 1 subunit  TTC21B - tetratricopeptide repeat domain 21b  AP1B1 - adaptor-related protein complex 1, beta 1 subunit  AP1G1 - adaptor-related protein complex 1, gamma 1 subunit  NUP133 - nucleoporin 133kda  AP5M1 - adaptor-related protein complex 5, mu 1 subunit  AGK - acylglycerol kinase  NUPL2 - nucleoporin like 2  VPS35 - vacuolar protein sorting 35 homolog (s. cerevisiae)  TOR1AIP2 - torsin a interacting protein 2  KIF3A - kinesin family member 3a  GPR89A - g protein-coupled receptor 89a  PPT1 - palmitoyl-protein thioesterase 1  RAB3C - rab3c, member ras oncogene family  AKT1 - v-akt murine thymoma viral oncogene homolog 1  RAB7A - rab7a, member ras oncogene family  PCID2 - pci domain containing 2  EVI5L - ecotropic viral integration site 5-like  EXOC2 - exocyst complex component 2  RER1 - rer1 retention in endoplasmic reticulum 1 homolog (s. cerevisiae)  PPP1R10 - protein phosphatase 1, regulatory subunit 10  TCTN2 - tectonic family member 2  EXOC1 - exocyst complex component 1  IFT122 - intraflagellar transport 122 homolog (chlamydomonas)  RAPGEF4 - rap guanine nucleotide exchange factor (gef) 4  TMEM115 - transmembrane protein 115  GAK - cyclin g associated kinase  STK11IP - serine/threonine kinase 11 interacting protein  PEX26 - peroxisomal biogenesis factor 26  TBC1D17 - tbc1 domain family, member 17  RAB35 - rab35, member ras oncogene family  IFT27 - intraflagellar transport 27 homolog (chlamydomonas)  DENND4C - denn/madd domain containing 4c  NPLOC4 - nuclear protein localization 4 homolog (s. cerevisiae)  KDELR2 - kdel (lys-asp-glu-leu) endoplasmic reticulum protein retention receptor 2  RAB31 - rab31, member ras oncogene family  MAPK10 - mitogen-activated protein kinase 10  MAPK9 - mitogen-activated protein kinase 9  C1QTNF5 - c1q and tumor necrosis factor related protein 5  WDR11 - wd repeat domain 11  MAPRE2 - microtubule-associated protein, rp/eb family, member 2  OSBPL8 - oxysterol binding protein-like 8  NECAP2 - necap endocytosis associated 2  NDC1 - ndc1 transmembrane nucleoporin  STAM - signal transducing adaptor molecule (sh3 domain and itam motif) 1  IPO9 - importin 9  PRKCI - protein kinase c, iota  ANXA2 - annexin a2  NUP214 - nucleoporin 214kda  PRKCB - protein kinase c, beta  GAS6 - growth arrest-specific 6  NCOA4 - nuclear receptor coactivator 4  PACS1 - phosphofurin acidic cluster sorting protein 1  SDAD1 - sda1 domain containing 1  TREX1 - three prime repair exonuclease 1  SYNRG - synergin, gamma  PACSIN2 - protein kinase c and casein kinase substrate in neurons 2  APBA1 - amyloid beta (a4) precursor protein-binding, family a, member 1  MCPH1 - microcephalin 1  SEC61A2 - sec61 alpha 2 subunit (s. cerevisiae)  CHP1 - calcineurin-like ef-hand protein 1  VPS13D - vacuolar protein sorting 13 homolog d (s. cerevisiae)  SEC63 - sec63 homolog (s. cerevisiae)  PRAF2 - pra1 domain family, member 2  AAGAB - alpha- and gamma-adaptin binding protein  ARCN1 - archain 1  TRIM23 - tripartite motif containing 23  APPL2 - adaptor protein, phosphotyrosine interaction, ph domain and leucine zipper containing 2  ARF1 - adp-ribosylation factor 1  IPO4 - importin 4  ARF4 - adp-ribosylation factor 4  PAFAH1B1 - platelet-activating factor acetylhydrolase 1b, regulatory subunit 1 (45kda)  GOLPH3L - golgi phosphoprotein 3-like  ARL8B - adp-ribosylation factor-like 8b  AKAP11 - a kinase (prka) anchor protein 11  AKAP10 - a kinase (prka) anchor protein 10  ZDHHC14 - zinc finger, dhhc-type containing 14  BBS7 - bardet-biedl syndrome 7  PALM - paralemmin  CEP250 - centrosomal protein 250kda  ARNTL - aryl hydrocarbon receptor nuclear translocator-like  TCTN1 - tectonic family member 1  ARL3 - adp-ribosylation factor-like 3  SCG2 - secretogranin ii  ARL1 - adp-ribosylation factor-like 1  CORO7 - coronin 7  SEC23IP - sec23 interacting protein  ARRB2 - arrestin, beta 2  ARRB1 - arrestin, beta 1  RHOB - ras homolog family member b  FAM83H - family with sequence similarity 83, member h  ARF6 - adp-ribosylation factor 6  ARF5 - adp-ribosylation factor 5  EVI5 - ecotropic viral integration site 5  ARHGAP1 - rho gtpase activating protein 1  AP4S1 - adaptor-related protein complex 4, sigma 1 subunit  ASNA1 - arsa arsenite transporter, atp-binding, homolog 1 (bacterial)  CHMP1A - charged multivesicular body protein 1a  TTC8 - tetratricopeptide repeat domain 8  PCM1 - pericentriolar material 1  RABL2B - rab, member of ras oncogene family-like 2b  FRMD6 - ferm domain containing 6  TBC1D8 - tbc1 domain family, member 8 (with gram domain)  WRAP53 - wd repeat containing, antisense to tp53  CDC37 - cell division cycle 37  KPTN - kaptin (actin binding protein)  TANGO6 - transport and golgi organization 6 homolog (drosophila)  RIC3 - ric3 acetylcholine receptor chaperone  SLC15A4 - solute carrier family 15 (oligopeptide transporter), member 4  XPA - xeroderma pigmentosum, complementation group a  GOLGA2 - golgin a2  UEVLD - uev and lactate/malate dehyrogenase domains  RPH3A - rabphilin 3a homolog (mouse)  XPO1 - exportin 1 (crm1 homolog, yeast)  GOLGB1 - golgin b1  ATP1B3 - atpase, na+/k+ transporting, beta 3 polypeptide  CCDC91 - coiled-coil domain containing 91  ATP1B1 - atpase, na+/k+ transporting, beta 1 polypeptide  LIN7C - lin-7 homolog c (c. elegans)  MON1B - mon1 secretory trafficking family member b  LRRK2 - leucine-rich repeat kinase 2  CLSTN1 - calsyntenin 1  AP5S1 - adaptor-related protein complex 5, sigma 1 subunit  RSG1 - rem2 and rab-like small gtpase 1  DZIP1 - daz interacting zinc finger protein 1  SCAMP5 - secretory carrier membrane protein 5  SEC31A - sec31 homolog a (s. cerevisiae)  WRB - tryptophan rich basic protein  XBP1 - x-box binding protein 1  GPM6B - glycoprotein m6b  ATP6V1E1 - atpase, h+ transporting, lysosomal 31kda, v1 subunit e1  PEX6 - peroxisomal biogenesis factor 6  LSG1 - large 60s subunit nuclear export gtpase 1  ATP6V0B - atpase, h+ transporting, lysosomal 21kda, v0 subunit b  PEX7 - peroxisomal biogenesis factor 7  SH3PXD2B - sh3 and px domains 2b  ATP6V0A1 - atpase, h+ transporting, lysosomal v0 subunit a1  PEX12 - peroxisomal biogenesis factor 12  ATP6AP1 - atpase, h+ transporting, lysosomal accessory protein 1  PEX13 - peroxisomal biogenesis factor 13  PEX14 - peroxisomal biogenesis factor 14  RILPL2 - rab interacting lysosomal protein-like 2  LMTK2 - lemur tyrosine kinase 2  ATP6V1A - atpase, h+ transporting, lysosomal 70kda, v1 subunit a  ATP6V1B2 - atpase, h+ transporting, lysosomal 56/58kda, v1 subunit b2  ATP6V0C - atpase, h+ transporting, lysosomal 16kda, v0 subunit c  PEX1 - peroxisomal biogenesis factor 1  ATP6V1C1 - atpase, h+ transporting, lysosomal 42kda, v1 subunit c1  RAB11FIP2 - rab11 family interacting protein 2 (class i)  PAOX - polyamine oxidase (exo-n4-amino)  BAX - bcl2-associated x protein  PKD1 - polycystic kidney disease 1 (autosomal dominant)  COPZ1 - coatomer protein complex, subunit zeta 1  COPG1 - coatomer protein complex, subunit gamma 1  BAD - bcl2-associated agonist of cell death  PIK3R2 - phosphoinositide-3-kinase, regulatory subunit 2 (beta)  PIK3R1 - phosphoinositide-3-kinase, regulatory subunit 1 (alpha)  COX18 - cox18 cytochrome c oxidase assembly factor  GNPTAB - n-acetylglucosamine-1-phosphate transferase, alpha and beta subunits  PKP2 - plakophilin 2  ZFYVE27 - zinc finger, fyve domain containing 27  KIF1A - kinesin family member 1a  ATRX - alpha thalassemia/mental retardation syndrome x-linked  COG2 - component of oligomeric golgi complex 2  CASC3 - cancer susceptibility candidate 3  GABARAPL2 - gaba(a) receptor-associated protein-like 2  GRIK5 - glutamate receptor, ionotropic, kainate 5  VPS53 - vacuolar protein sorting 53 homolog (s. cerevisiae)  EXOC3 - exocyst complex component 3  RABL3 - rab, member of ras oncogene family-like 3  GRIK2 - glutamate receptor, ionotropic, kainate 2  GABARAP - gaba(a) receptor-associated protein  BCR - breakpoint cluster region  DDX42 - dead (asp-glu-ala-asp) box helicase 42  XRCC5 - x-ray repair complementing defective repair in chinese hamster cells 5 (double-strand-break rejoining)  PIK3C3 - phosphatidylinositol 3-kinase, catalytic subunit type 3  YWHAG - tyrosine 3-monooxygenase/tryptophan 5-monooxygenase activation protein, gamma polypeptide  COPE - coatomer protein complex, subunit epsilon  YWHAZ - tyrosine 3-monooxygenase/tryptophan 5-monooxygenase activation protein, zeta polypeptide  PARK7 - parkinson protein 7  BBS4 - bardet-biedl syndrome 4  BBS2 - bardet-biedl syndrome 2  YWHAE - tyrosine 3-monooxygenase/tryptophan 5-monooxygenase activation protein, epsilon polypeptide  VPS45 - vacuolar protein sorting 45 homolog (s. cerevisiae)  YWHAB - tyrosine 3-monooxygenase/tryptophan 5-monooxygenase activation protein, beta polypeptide  DNAJA1 - dnaj (hsp40) homolog, subfamily a, member 1  RNF26 - ring finger protein 26  BET1 - bet1 golgi vesicular membrane trafficking protein  BRAF - v-raf murine sarcoma viral oncogene homolog b  NRP1 - neuropilin 1  LIN7A - lin-7 homolog a (c. elegans)  KATNB1 - katanin p80 (wd repeat containing) subunit b 1  RDX - radixin  ARL13B - adp-ribosylation factor-like 13b  DERL1 - derlin 1  DSCR3 - down syndrome critical region gene 3  RRAGB - ras-related gtp binding b  RANBP6 - ran binding protein 6  MTX3 - metaxin 3  SYNGAP1 - synaptic ras gtpase activating protein 1  RNF215 - ring finger protein 215  BMP7 - bone morphogenetic protein 7  AKAP4 - a kinase (prka) anchor protein 4  ABCA7 - atp-binding cassette, sub-family a (abc1), member 7  FLOT1 - flotillin 1  VPS51 - vacuolar protein sorting 51 homolog (s. cerevisiae)  ABCE1 - atp-binding cassette, sub-family e (oabp), member 1  PTTG1IP - pituitary tumor-transforming 1 interacting protein  VPS13C - vacuolar protein sorting 13 homolog c (s. cerevisiae)  MIS12 - mis12 kinetochore complex component  ARL8A - adp-ribosylation factor-like 8a  NABP2 - nucleic acid binding protein 2  STX6 - syntaxin 6  AP1M1 - adaptor-related protein complex 1, mu 1 subunit  AP1G2 - adaptor-related protein complex 1, gamma 2 subunit  AP1S2 - adaptor-related protein complex 1, sigma 2 subunit  AHI1 - abelson helper integration site 1  AP3S2 - adaptor-related protein complex 3, sigma 2 subunit  AFTPH - aftiphilin  GPHN - gephyrin  ROCK1 - rho-associated, coiled-coil containing protein kinase 1  HERC2 - hect and rld domain containing e3 ubiquitin protein ligase 2  TSPO - translocator protein (18kda)  TRAK2 - trafficking protein, kinesin binding 2  SNX19 - sorting nexin 19  HSPD1 - heat shock 60kda protein 1 (chaperonin)  STAM2 - signal transducing adaptor molecule (sh3 domain and itam motif) 2  HSP90AA1 - heat shock protein 90kda alpha (cytosolic), class a member 1  RAMP2 - receptor (g protein-coupled) activity modifying protein 2  RAMP3 - receptor (g protein-coupled) activity modifying protein 3  RAMP1 - receptor (g protein-coupled) activity modifying protein 1  AP3D1 - adaptor-related protein complex 3, delta 1 subunit  AKAP8 - a kinase (prka) anchor protein 8  HSPA4 - heat shock 70kda protein 4  HSPA9 - heat shock 70kda protein 9 (mortalin)  HSPA8 - heat shock 70kda protein 8  TOMM40 - translocase of outer mitochondrial membrane 40 homolog (yeast)  ATG16L1 - autophagy related 16-like 1 (s. cerevisiae)  CANX - calnexin  TIMM17A - translocase of inner mitochondrial membrane 17 homolog a (yeast)  RPL17 - ribosomal protein l17  RPL15 - ribosomal protein l15  VPS37C - vacuolar protein sorting 37 homolog c (s. cerevisiae)  LAMTOR3 - late endosomal/lysosomal adaptor, mapk and mtor activator 3  HMGB1 - high mobility group box 1  RPL11 - ribosomal protein l11  RPL9 - ribosomal protein l9  NOL8 - nucleolar protein 8  TIMM10B - translocase of inner mitochondrial membrane 10 homolog b (yeast)  COG5 - component of oligomeric golgi complex 5  RPL7 - ribosomal protein l7  PIBF1 - progesterone immunomodulatory binding factor 1  TNKS - tankyrase, trf1-interacting ankyrin-related adp-ribose polymerase  HEATR3 - heat repeat containing 3  RPL3 - ribosomal protein l3  BAIAP2 - bai1-associated protein 2  RPA1 - replication protein a1, 70kda  TIMM9 - translocase of inner mitochondrial membrane 9 homolog (yeast)  RPA2 - replication protein a2, 32kda  RPS4X - ribosomal protein s4, x-linked  SEC23A - sec23 homolog a (s. cerevisiae)  RPS4Y1 - ribosomal protein s4, y-linked 1  HK1 - hexokinase 1  SEC23B - sec23 homolog b (s. cerevisiae)  STX17 - syntaxin 17  CACNB3 - calcium channel, voltage-dependent, beta 3 subunit  CACNB2 - calcium channel, voltage-dependent, beta 2 subunit  ACD - adrenocortical dysplasia homolog (mouse)  LAMTOR1 - late endosomal/lysosomal adaptor, mapk and mtor activator 1  CALR - calreticulin  HFE - hemochromatosis  VPS33A - vacuolar protein sorting 33 homolog a (s. cerevisiae)  HIP1 - huntingtin interacting protein 1  RPL32 - ribosomal protein l32  VTI1B - vesicle transport through interaction with t-snares 1b  MBTPS1 - membrane-bound transcription factor peptidase, site 1  RPS23 - ribosomal protein s23  ARFGAP3 - adp-ribosylation factor gtpase activating protein 3  PINX1 - pin2/terf1 interacting, telomerase inhibitor 1  SNX3 - sorting nexin 3  SNX4 - sorting nexin 4  TSNARE1 - t-snare domain containing 1  BANP - btg3 associated nuclear protein  GBF1 - golgi brefeldin a resistant guanine nucleotide exchange factor 1  HNRNPU - heterogeneous nuclear ribonucleoprotein u (scaffold attachment factor a)  RPS15 - ribosomal protein s15  RPS12 - ribosomal protein s12  VPS33B - vacuolar protein sorting 33 homolog b (yeast)  TINF2 - terf1 (trf1)-interacting nuclear factor 2  RAB11A - rab11a, member ras oncogene family  PORCN - porcupine homolog (drosophila)  CAV1 - caveolin 1, caveolae protein, 22kda  NAPA - n-ethylmaleimide-sensitive factor attachment protein, alpha  NAPG - n-ethylmaleimide-sensitive factor attachment protein, gamma  SEC24B - sec24 family, member b (s. cerevisiae)  CMTM6 - cklf-like marvel transmembrane domain containing 6  TRIM3 - tripartite motif containing 3  LRBA - lps-responsive vesicle trafficking, beach and anchor containing  TBC1D15 - tbc1 domain family, member 15  PSAP - prosaposin  CDH2 - cadherin 2, type 1, n-cadherin (neuronal)  DESI1 - desumoylating isopeptidase 1  STXBP5 - syntaxin binding protein 5 (tomosyn)  PEX3 - peroxisomal biogenesis factor 3  SGSM3 - small g protein signaling modulator 3  RANBP3 - ran binding protein 3  TOR1B - torsin family 1, member b (torsin b)  PSEN1 - presenilin 1  TOLLIP - toll interacting protein  RABGEF1 - rab guanine nucleotide exchange factor (gef) 1  PRPF19 - pre-mrna processing factor 19  CD81 - cd81 molecule  SMYD3 - set and mynd domain containing 3  SCARB2 - scavenger receptor class b, member 2  EXOC5 - exocyst complex component 5  BLZF1 - basic leucine zipper nuclear factor 1  AP3B1 - adaptor-related protein complex 3, beta 1 subunit  YKT6 - ykt6 v-snare homolog (s. cerevisiae)  MTX2 - metaxin 2  AGPS - alkylglycerone phosphate synthase  IL10RA - interleukin 10 receptor, alpha  GDAP1 - ganglioside induced differentiation associated protein 1  GORASP1 - golgi reassembly stacking protein 1, 65kda  TSPAN33 - tetraspanin 33  GRPEL2 - grpe-like 2, mitochondrial (e. coli)  GAS2L1 - growth arrest-specific 2 like 1  TERF2IP - telomeric repeat binding factor 2, interacting protein  PITRM1 - pitrilysin metallopeptidase 1  ATG7 - autophagy related 7  SERP1 - stress-associated endoplasmic reticulum protein 1  VPS16 - vacuolar protein sorting 16 homolog (s. cerevisiae)  DCTN2 - dynactin 2 (p50)  LAMTOR5 - late endosomal/lysosomal adaptor, mapk and mtor activator 5  CETN2 - centrin, ef-hand protein, 2  CETN3 - centrin, ef-hand protein, 3  TM9SF1 - transmembrane 9 superfamily member 1  APPBP2 - amyloid beta precursor protein (cytoplasmic tail) binding protein 2  TRAM1L1 - translocation associated membrane protein 1-like 1  FBLN5 - fibulin 5  ITGAV - integrin, alpha v  ARFIP1 - adp-ribosylation factor interacting protein 1  PRPF18 - pre-mrna processing factor 18  ZDHHC6 - zinc finger, dhhc-type containing 6  IPO8 - importin 8  IPO7 - importin 7  EIF6 - eukaryotic translation initiation factor 6  BBS9 - bardet-biedl syndrome 9  SLU7 - slu7 splicing factor homolog (s. cerevisiae)  SKAP1 - src kinase associated phosphoprotein 1  CDK5 - cyclin-dependent kinase 5  ATG3 - autophagy related 3  PTN - pleiotrophin  AKTIP - akt interacting protein  KLHL20 - kelch-like family member 20  CCT2 - chaperonin containing tcp1, subunit 2 (beta)  CADPS - ca++-dependent secretion activator  MPP5 - membrane protein, palmitoylated 5 (maguk p55 subfamily member 5)  CCT4 - chaperonin containing tcp1, subunit 4 (delta)  USO1 - uso1 vesicle transport factor  ZMAT3 - zinc finger, matrin-type 3  ARFGEF2 - adp-ribosylation factor guanine nucleotide-exchange factor 2 (brefeldin a-inhibited)  ARFGEF1 - adp-ribosylation factor guanine nucleotide-exchange factor 1 (brefeldin a-inhibited)  PURA - purine-rich element binding protein a  LRWD1 - leucine-rich repeats and wd repeat domain containing 1  ZNHIT6 - zinc finger, hit-type containing 6  PTPRK - protein tyrosine phosphatase, receptor type, k  TBC1D13 - tbc1 domain family, member 13  PTPRN - protein tyrosine phosphatase, receptor type, n  LYST - lysosomal trafficking regulator  AHCYL1 - adenosylhomocysteinase-like 1  CHML - choroideremia-like (rab escort protein 2)  NUP50 - nucleoporin 50kda  PEX2 - peroxisomal biogenesis factor 2  CHM - choroideremia (rab escort protein 1)  CHCHD4 - coiled-coil-helix-coiled-coil-helix domain containing 4  GIPC1 - gipc pdz domain containing family, member 1  PVRL1 - poliovirus receptor-related 1 (herpesvirus entry mediator c)  SNX5 - sorting nexin 5  CHGA - chromogranin a (parathyroid secretory protein 1)  INVS - inversin  RAB6A - rab6a, member ras oncogene family  PIP5K1A - phosphatidylinositol-4-phosphate 5-kinase, type i, alpha  C17orf75 - chromosome 17 open reading frame 75  RAB5A - rab5a, member ras oncogene family  RAB5B - rab5b, member ras oncogene family  RAB4A - rab4a, member ras oncogene family  RAB3A - rab3a, member ras oncogene family  MTERFD2 - mterf domain containing 2  RAB3B - rab3b, member ras oncogene family  RAB2A - rab2a, member ras oncogene family  RAB1A - rab1a, member ras oncogene family  SDCCAG3 - serologically defined colon cancer antigen 3  RRAGC - ras-related gtp binding c  RAD21 - rad21 homolog (s. pombe)  SEC24A - sec24 family, member a (s. cerevisiae)  PAF1 - paf1, rna polymerase ii associated factor, homolog (s. cerevisiae)  RAB5C - rab5c, member ras oncogene family  RABIF - rab interacting factor  TMED4 - transmembrane emp24 protein transport domain containing 4  LIN7B - lin-7 homolog b (c. elegans)  VPS4A - vacuolar protein sorting 4 homolog a (s. cerevisiae)  RAB27A - rab27a, member ras oncogene family  COPG2 - coatomer protein complex, subunit gamma 2  SNX16 - sorting nexin 16  NBEA - neurobeachin  TOMM7 - translocase of outer mitochondrial membrane 7 homolog (yeast)  RANBP2 - ran binding protein 2  RAP1A - rap1a, member of ras oncogene family  CLTC - clathrin, heavy chain (hc)  SEC22A - sec22 vesicle trafficking protein homolog a (s. cerevisiae)  LRRC4 - leucine rich repeat containing 4  AP3M1 - adaptor-related protein complex 3, mu 1 subunit  GNPAT - glyceronephosphate o-acyltransferase  EXOC6 - exocyst complex component 6  SGTB - small glutamine-rich tetratricopeptide repeat (tpr)-containing, beta  BBS5 - bardet-biedl syndrome 5  ULK1 - unc-51 like autophagy activating kinase 1  RRAGA - ras-related gtp binding a  CLTB - clathrin, light chain b  CLTA - clathrin, light chain a  CLU - clusterin  STX7 - syntaxin 7  GOLPH3 - golgi phosphoprotein 3 (coat-protein)  RAP2A - rap2a, member of ras oncogene family  MLH3 - mutl homolog 3 (e. coli)  TPP1 - tripeptidyl peptidase i  IK - ik cytokine, down-regulator of hla ii  CTCF - ccctc-binding factor (zinc finger protein)  HEATR5B - heat repeat containing 5b  AP1S1 - adaptor-related protein complex 1, sigma 1 subunit  TMEM150A - transmembrane protein 150a  RB1 - retinoblastoma 1  AP2M1 - adaptor-related protein complex 2, mu 1 subunit  ARL5B - adp-ribosylation factor-like 5b  NSG1 - neuron specific gene family member 1  NUP35 - nucleoporin 35kda  VPS41 - vacuolar protein sorting 41 homolog (s. cerevisiae)  DOC2B - double c2-like domains, beta  CCDC93 - coiled-coil domain containing 93  SGSM1 - small g protein signaling modulator 1  CHMP4B - charged multivesicular body protein 4b  PIGU - phosphatidylinositol glycan anchor biosynthesis, class u  CACNG4 - calcium channel, voltage-dependent, gamma subunit 4  AP4B1 - adaptor-related protein complex 4, beta 1 subunit  TBRG1 - transforming growth factor beta regulator 1  PITPNM1 - phosphatidylinositol transfer protein, membrane-associated 1  WDR19 - wd repeat domain 19  PDIA4 - protein disulfide isomerase family a, member 4  SPTBN4 - spectrin, beta, non-erythrocytic 4  AKAP12 - a kinase (prka) anchor protein 12  CORO1C - coronin, actin binding protein, 1c  COG8 - component of oligomeric golgi complex 8  VPS37A - vacuolar protein sorting 37 homolog a (s. cerevisiae)  ZDHHC3 - zinc finger, dhhc-type containing 3  KPNA6 - karyopherin alpha 6 (importin alpha 7)  RABGAP1 - rab gtpase activating protein 1  HOOK3 - hook microtubule-tethering protein 3  RAB36 - rab36, member ras oncogene family  DENND1A - denn/madd domain containing 1a  REEP2 - receptor accessory protein 2  FAM53C - family with sequence similarity 53, member c  ABCG1 - atp-binding cassette, sub-family g (white), member 1  LTV1 - ltv1 homolog (s. cerevisiae)  ATP6V1G1 - atpase, h+ transporting, lysosomal 13kda, v1 subunit g1  CADM3 - cell adhesion molecule 3  APBA3 - amyloid beta (a4) precursor protein-binding, family a, member 3  TICAM2 - toll-like receptor adaptor molecule 2  RAB3D - rab3d, member ras oncogene family  ATG4C - autophagy related 4c, cysteine peptidase  SYVN1 - synovial apoptosis inhibitor 1, synoviolin  VPS26A - vacuolar protein sorting 26 homolog a (s. pombe)  SEC22B - sec22 vesicle trafficking protein homolog b (s. cerevisiae) (gene/pseudogene)  RAB2B - rab2b, member ras oncogene family  CPE - carboxypeptidase e  H2AFY - h2a histone family, member y  WBP2 - ww domain binding protein 2  BET1L - bet1 golgi vesicular membrane trafficking protein-like  GOSR2 - golgi snap receptor complex member 2  UBAP1 - ubiquitin associated protein 1  RAB40C - rab40c, member ras oncogene family  SPIRE2 - spire actin nucleation factor 2  PREPL - prolyl endopeptidase-like  JAGN1 - jagunal homolog 1 (drosophila)  SNX2 - sorting nexin 2  NACC2 - nacc family member 2, ben and btb (poz) domain containing  DNAJC27 - dnaj (hsp40) homolog, subfamily c, member 27  SNX1 - sorting nexin 1  LMNA - lamin a/c  SNCG - synuclein, gamma (breast cancer-specific protein 1)  KTN1 - kinectin 1 (kinesin receptor)  SCAMP1 - secretory carrier membrane protein 1  VPS4B - vacuolar protein sorting 4 homolog b (s. cerevisiae)  GOSR1 - golgi snap receptor complex member 1  SRPRB - signal recognition particle receptor, b subunit  BAG4 - bcl2-associated athanogene 4  SMO - smoothened, frizzled family receptor  LAMP2 - lysosomal-associated membrane protein 2  ARFIP2 - adp-ribosylation factor interacting protein 2  SNX6 - sorting nexin 6  SNX7 - sorting nexin 7  ROCK2 - rho-associated, coiled-coil containing protein kinase 2  ATP6V1D - atpase, h+ transporting, lysosomal 34kda, v1 subunit d  SH3BP4 - sh3-domain binding protein 4  STX8 - syntaxin 8  COPB1 - coatomer protein complex, subunit beta 1  ZBTB7A - zinc finger and btb domain containing 7a  COPA - coatomer protein complex, subunit alpha  RAB38 - rab38, member ras oncogene family  LAMA5 - laminin, alpha 5  STX12 - syntaxin 12  RPH3AL - rabphilin 3a-like (without c2 domains)  NAGPA - n-acetylglucosamine-1-phosphodiester alpha-n-acetylglucosaminidase  SENP2 - sumo1/sentrin/smt3 specific peptidase 2  PLEKHA1 - pleckstrin homology domain containing, family a (phosphoinositide binding specific) member 1  FAM126A - family with sequence similarity 126, member a  CYB5R4 - cytochrome b5 reductase 4  VPS28 - vacuolar protein sorting 28 homolog (s. cerevisiae)  KIF5C - kinesin family member 5c  PRPF6 - pre-mrna processing factor 6  RPF2 - ribosome production factor 2 homolog (s. cerevisiae)  KIF5B - kinesin family member 5b  KIF5A - kinesin family member 5a  CBY1 - chibby homolog 1 (drosophila)  PICK1 - protein interacting with prkca 1  CTNNA1 - catenin (cadherin-associated protein), alpha 1, 102kda  CACNG7 - calcium channel, voltage-dependent, gamma subunit 7  VAMP2 - vesicle-associated membrane protein 2 (synaptobrevin 2)  RAB3GAP2 - rab3 gtpase activating protein subunit 2 (non-catalytic)  CCDC41 - coiled-coil domain containing 41  CTNNB1 - catenin (cadherin-associated protein), beta 1, 88kda  STXBP1 - syntaxin binding protein 1  PPIL2 - peptidylprolyl isomerase (cyclophilin)-like 2  STX5 - syntaxin 5  ASB3 - ankyrin repeat and socs box containing 3  STX4 - syntaxin 4  SAV1 - salvador homolog 1 (drosophila)  STX3 - syntaxin 3  STX1A - syntaxin 1a (brain)  GOLGA7 - golgin a7  MGARP - mitochondria-localized glutamic acid-rich protein  TNPO1 - transportin 1  TGFBRAP1 - transforming growth factor, beta receptor associated protein 1  FUT10 - fucosyltransferase 10 (alpha (1,3) fucosyltransferase)  UNC119B - unc-119 homolog b (c. elegans)  KPNA2 - karyopherin alpha 2 (rag cohort 1, importin alpha 1)  KPNB1 - karyopherin (importin) beta 1  KPNA1 - karyopherin alpha 1 (importin alpha 5)  STXBP3 - syntaxin binding protein 3  ZDHHC9 - zinc finger, dhhc-type containing 9  CRIPT - cysteine-rich pdz-binding protein  NPNT - nephronectin  PAN3 - pan3 poly(a) specific ribonuclease subunit homolog (s. cerevisiae)  PEX16 - peroxisomal biogenesis factor 16  EXOC4 - exocyst complex component 4  CNEP1R1 - ctd nuclear envelope phosphatase 1 regulatory subunit 1  SH3GLB1 - sh3-domain grb2-like endophilin b1  RPAIN - rpa interacting protein  RAB28 - rab28, member ras oncogene family  LHFPL4 - lipoma hmgic fusion partner-like 4  NIPBL - nipped-b homolog (drosophila)  RAB9A - rab9a, member ras oncogene family  COG4 - component of oligomeric golgi complex 4  RAB26 - rab26, member ras oncogene family  PDZD11 - pdz domain containing 11  STAT3 - signal transducer and activator of transcription 3 (acute-phase response factor)  JUP - junction plakoglobin  SSR3 - signal sequence receptor, gamma (translocon-associated protein gamma)  SFT2D3 - sft2 domain containing 3  COG1 - component of oligomeric golgi complex 1  SPCS3 - signal peptidase complex subunit 3 homolog (s. cerevisiae)  DFNB31 - deafness, autosomal recessive 31  RINT1 - rad50 interactor 1  TM9SF2 - transmembrane 9 superfamily member 2  NAPB - n-ethylmaleimide-sensitive factor attachment protein, beta  SRP68 - signal recognition particle 68kda  SNX33 - sorting nexin 33  SRP54 - signal recognition particle 54kda  FBXO7 - f-box protein 7  SRP19 - signal recognition particle 19kda  CSE1L - cse1 chromosome segregation 1-like (yeast)  SRP14 - signal recognition particle 14kda (homologous alu rna binding protein)  ZNHIT3 - zinc finger, hit-type containing 3  SRP72 - signal recognition particle 72kda  SSR1 - signal sequence receptor, alpha  ZDHHC2 - zinc finger, dhhc-type containing 2  SSB - sjogren syndrome antigen b (autoantigen la)  SNAP29 - synaptosomal-associated protein, 29kda  SPTBN1 - spectrin, beta, non-erythrocytic 1  VAMP3 - vesicle-associated membrane protein 3  CSNK1D - casein kinase 1, delta  MIA3 - melanoma inhibitory activity family, member 3  SFT2D2 - sft2 domain containing 2  IPO11 - importin 11  TAOK2 - tao kinase 2  VIPAS39 - vps33b interacting protein, apical-basolateral polarity regulator, spe-39 homolog  ZDHHC12 - zinc finger, dhhc-type containing 12  VPS25 - vacuolar protein sorting 25 homolog (s. cerevisiae)  SRP9 - signal recognition particle 9kda  TMEM107 - transmembrane protein 107  MON1A - mon1 secretory trafficking family member a  DHX9 - deah (asp-glu-ala-his) box helicase 9  SEL1L - sel-1 suppressor of lin-12-like (c. elegans)  ATP6V1F - atpase, h+ transporting, lysosomal 14kda, v1 subunit f  SEC13 - sec13 homolog (s. cerevisiae)  DDX3X - dead (asp-glu-ala-asp) box helicase 3, x-linked  DDX1 - dead (asp-glu-ala-asp) box helicase 1  SNX25 - sorting nexin 25  SNX14 - sorting nexin 14  CCDC120 - coiled-coil domain containing 120  SIN3A - sin3 transcription regulator family member a  RAB8B - rab8b, member ras oncogene family  SDCBP - syndecan binding protein (syntenin)  COPB2 - coatomer protein complex, subunit beta 2 (beta prime)  HEATR5A - heat repeat containing 5a  PTPN23 - protein tyrosine phosphatase, non-receptor type 23  ITGB1BP1 - integrin beta 1 binding protein 1  RCN3 - reticulocalbin 3, ef-hand calcium binding domain  MCM9 - minichromosome maintenance complex component 9  ZDHHC24 - zinc finger, dhhc-type containing 24  MYRIP - myosin viia and rab interacting protein  PHAX - phosphorylated adaptor for rna export  POT1 - protection of telomeres 1  SCYL1 - scy1-like 1 (s. cerevisiae)  AHCTF1 - at hook containing transcription factor 1  RAB22A - rab22a, member ras oncogene family  VPS29 - vacuolar protein sorting 29 homolog (s. cerevisiae)  RAB11B - rab11b, member ras oncogene family  RAB23 - rab23, member ras oncogene family  DLG5 - discs, large homolog 5 (drosophila)  ZDHHC20 - zinc finger, dhhc-type containing 20  ANKRD27 - ankyrin repeat domain 27 (vps9 domain)  MCFD2 - multiple coagulation factor deficiency 2  MECP2 - methyl cpg binding protein 2 (rett syndrome)  BUB3 - bub3 mitotic checkpoint protein  PRICKLE1 - prickle homolog 1 (drosophila)  RAB24 - rab24, member ras oncogene family  ZW10 - zw10 kinetochore protein  HEG1 - heart development protein with egf-like domains 1  FAM160A2 - family with sequence similarity 160, member a2  TBC1D10B - tbc1 domain family, member 10b  RAB8A - rab8a, member ras oncogene family  COG6 - component of oligomeric golgi complex 6  GORASP2 - golgi reassembly stacking protein 2, 55kda  XPO5 - exportin 5  C2CD3 - c2 calcium-dependent domain containing 3  SLC7A6OS - solute carrier family 7, member 6 opposite strand  HINFP - histone h4 transcription factor  LRSAM1 - leucine rich repeat and sterile alpha motif containing 1  AP4M1 - adaptor-related protein complex 4, mu 1 subunit  ARHGEF2 - rho/rac guanine nucleotide exchange factor (gef) 2  CLIP3 - cap-gly domain containing linker protein 3  S100A10 - s100 calcium binding protein a10  NECAP1 - necap endocytosis associated 1  CHMP2B - charged multivesicular body protein 2b  STX18 - syntaxin 18  TBC1D14 - tbc1 domain family, member 14  ARL6 - adp-ribosylation factor-like 6  SYNGR1 - synaptogyrin 1  HGS - hepatocyte growth factor-regulated tyrosine kinase substrate  VPS52 - vacuolar protein sorting 52 homolog (s. cerevisiae)  UNC50 - unc-50 homolog (c. elegans)  VTA1 - vesicle (multivesicular body) trafficking 1  RABEP1 - rabaptin, rab gtpase binding effector protein 1  MAL - mal, t-cell differentiation protein  COG3 - component of oligomeric golgi complex 3  MICALL1 - mical-like 1  DRD2 - dopamine receptor d2  NLGN2 - neuroligin 2  NKD2 - naked cuticle homolog 2 (drosophila)  DNAJC14 - dnaj (hsp40) homolog, subfamily c, member 14  RAB6B - rab6b, member ras oncogene family  ARRDC3 - arrestin domain containing 3  SHANK3 - sh3 and multiple ankyrin repeat domains 3  WIPI2 - wd repeat domain, phosphoinositide interacting 2  RAB14 - rab14, member ras oncogene family  ATP6V0D1 - atpase, h+ transporting, lysosomal 38kda, v0 subunit d1  LATS1 - large tumor suppressor kinase 1  TTC7B - tetratricopeptide repeat domain 7b  MAP1A - microtubule-associated protein 1a  RAB33B - rab33b, member ras oncogene family  VPS54 - vacuolar protein sorting 54 homolog (s. cerevisiae)  PPHLN1 - periphilin 1  MAPK15 - mitogen-activated protein kinase 15  UNC119 - unc-119 homolog (c. elegans)  TOR1A - torsin family 1, member a (torsin a)  GGA1 - golgi-associated, gamma adaptin ear containing, arf binding protein 1  DVL2 - dishevelled segment polarity protein 2  TOR1AIP1 - torsin a interacting protein 1  CHMP5 - charged multivesicular body protein 5  APPL1 - adaptor protein, phosphotyrosine interaction, ph domain and leucine zipper containing 1  CDAN1 - codanin 1  MCC - mutated in colorectal cancers  MAP7 - microtubule-associated protein 7  DKC1 - dyskeratosis congenita 1, dyskerin  DLG3 - discs, large homolog 3 (drosophila)  DLG4 - discs, large homolog 4 (drosophila)  VPS18 - vacuolar protein sorting 18 homolog (s. cerevisiae)  DLG1 - discs, large homolog 1 (drosophila)  LRP1 - low density lipoprotein receptor-related protein 1  RPL14 - ribosomal protein l14  LRP6 - low density lipoprotein receptor-related protein 6  STXBP4 - syntaxin binding protein 4  AIP - aryl hydrocarbon receptor interacting protein  TOM1L2 - target of myb1-like 2 (chicken)  DHCR24 - 24-dehydrocholesterol reductase  ARL5A - adp-ribosylation factor-like 5a  LYN - v-yes-1 yamaguchi sarcoma viral related oncogene homolog  JAM3 - junctional adhesion molecule 3  TMED6 - transmembrane emp24 protein transport domain containing 6  CHMP3 - charged multivesicular body protein 3  SFPQ - splicing factor proline/glutamine-rich  RAB34 - rab34, member ras oncogene family  DNM2 - dynamin 2  SMAD2 - smad family member 2  ATG16L2 - autophagy related 16-like 2 (s. cerevisiae)  ARMCX3 - armadillo repeat containing, x-linked 3  M6PR - mannose-6-phosphate receptor (cation dependent)  ALS2 - amyotrophic lateral sclerosis 2 (juvenile)  PLOD3 - procollagen-lysine, 2-oxoglutarate 5-dioxygenase 3  ATP6V1H - atpase, h+ transporting, lysosomal 50/57kda, v1 subunit h  HPS4 - hermansky-pudlak syndrome 4  SCG5 - secretogranin v (7b2 protein)  SGTA - small glutamine-rich tetratricopeptide repeat (tpr)-containing, alpha  MZT1 - mitotic spindle organizing protein 1  CUTA - cuta divalent cation tolerance homolog (e. coli)  NBAS - neuroblastoma amplified sequence  RUNDC1 - run domain containing 1  LSR - lipolysis stimulated lipoprotein receptor  ITSN1 - intersectin 1 (sh3 domain protein)  SMAD7 - smad family member 7  WASL - wiskott-aldrich syndrome-like  MAPRE1 - microtubule-associated protein, rp/eb family, member 1  CHMP4C - charged multivesicular body protein 4c  MAPRE3 - microtubule-associated protein, rp/eb family, member 3  TSPAN14 - tetraspanin 14  CTTN - cortactin  SPIRE1 - spire actin nucleation factor 1  SCG3 - secretogranin iii  NASP - nuclear autoantigenic sperm protein (histone-binding)  TM9SF3 - transmembrane 9 superfamily member 3  DNAJC15 - dnaj (hsp40) homolog, subfamily c, member 15  NUBP1 - nucleotide binding protein 1  RAB18 - rab18, member ras oncogene family  TIMM50 - translocase of inner mitochondrial membrane 50 homolog (s. cerevisiae)  RAB3GAP1 - rab3 gtpase activating protein subunit 1 (catalytic)  TUB - tubby bipartite transcription factor  GRIPAP1 - grip1 associated protein 1  YIPF5 - yip1 domain family, member 5  ZDHHC1 - zinc finger, dhhc-type containing 1  EGR2 - early growth response 2  LMAN2L - lectin, mannose-binding 2-like  NACA - nascent polypeptide-associated complex alpha subunit  SPHK2 - sphingosine kinase 2  SAR1A - sar1 homolog a (s. cerevisiae)  ELAVL1 - elav (embryonic lethal, abnormal vision, drosophila)-like 1 (hu antigen r)  TMEM129 - transmembrane protein 129  AZI1 - 5-azacytidine induced 1  STRADA - ste20-related kinase adaptor alpha  NUTF2 - nuclear transport factor 2  TSG101 - tumor susceptibility gene 101  MYO1D - myosin id  TSC2 - tuberous sclerosis 2  EFR3B - efr3 homolog b (s. cerevisiae)  CALCRL - calcitonin receptor-like  MYO5A - myosin va (heavy chain 12, myoxin)  MYO6 - myosin vi  TOPORS - topoisomerase i binding, arginine/serine-rich, e3 ubiquitin protein ligase  SNX27 - sorting nexin family member 27  MVB12A - multivesicular body subunit 12a  RAB1B - rab1b, member ras oncogene family  SNX15 - sorting nexin 15  EIF2D - eukaryotic translation initiation factor 2d  SCAMP2 - secretory carrier membrane protein 2  SCAMP3 - secretory carrier membrane protein 3  RAB21 - rab21, member ras oncogene family  SEH1L - seh1-like (s. cerevisiae)  PTPRU - protein tyrosine phosphatase, receptor type, u  SNX11 - sorting nexin 11  MYH9 - myosin, heavy chain 9, non-muscle  SNUPN - snurportin 1  SNX30 - sorting nexin family member 30  SNX10 - sorting nexin 10  EGFR - epidermal growth factor receptor  DOPEY1 - dopey family member 1  GOLGA7B - golgin a7 family, member b  TSPAN5 - tetraspanin 5  MTX1 - metaxin 1  MFF - mitochondrial fission factor  TNIK - traf2 and nck interacting kinase  FAM126B - family with sequence similarity 126, member b  XPO7 - exportin 7  MON2 - mon2 homolog (s. cerevisiae)  CPLX3 - complexin 3  HSP90B1 - heat shock protein 90kda beta (grp94), member 1  TIMM22 - translocase of inner mitochondrial membrane 22 homolog (yeast)  ARL4A - adp-ribosylation factor-like 4a  SEC61A1 - sec61 alpha 1 subunit (s. cerevisiae)  ARL4C - adp-ribosylation factor-like 4c  BCAP31 - b-cell receptor-associated protein 31  TBC1D9B - tbc1 domain family, member 9b (with gram domain)  TPR - translocated promoter region, nuclear basket protein  SNX12 - sorting nexin 12  WAPAL - wings apart-like homolog (drosophila)  GGA2 - golgi-associated, gamma adaptin ear containing, arf binding protein 2  BLOC1S3 - biogenesis of lysosomal organelles complex-1, subunit 3  ERC1 - elks/rab6-interacting/cast family member 1  ZNF423 - zinc finger protein 423  BOD1 - biorientation of chromosomes in cell division 1  PIGW - phosphatidylinositol glycan anchor biosynthesis, class w  NUP107 - nucleoporin 107kda  GOPC - golgi-associated pdz and coiled-coil motif containing  KIF1B - kinesin family member 1b  TBC1D2B - tbc1 domain family, member 2b  PDCD6 - programmed cell death 6  MTM1 - myotubularin 1  NFASC - neurofascin  PARP11 - poly (adp-ribose) polymerase family, member 11  PDCD6IP - programmed cell death 6 interacting protein  USP4 - ubiquitin specific peptidase 4 (proto-oncogene)  OCLN - occludin  HDAC6 - histone deacetylase 6  CLASP2 - cytoplasmic linker associated protein 2  ETV5 - ets variant 5  VGF - vgf nerve growth factor inducible  TOM1 - target of myb1 (chicken)  EZR - ezrin  EPB41L3 - erythrocyte membrane protein band 4.1-like 3  PIGK - phosphatidylinositol glycan anchor biosynthesis, class k  ESAM - endothelial cell adhesion molecule  CCDC22 - coiled-coil domain containing 22  EXOC8 - exocyst complex component 8  DNM1L - dynamin 1-like  AP5B1 - adaptor-related protein complex 5, beta 1 subunit  DNAJB6 - dnaj (hsp40) homolog, subfamily b, member 6  VCL - vinculin  VCP - valosin containing protein  TBC1D9 - tbc1 domain family, member 9 (with gram domain)  MAPK8IP3 - mitogen-activated protein kinase 8 interacting protein 3  MSH2 - muts homolog 2, colon cancer, nonpolyposis type 1 (e. coli)  MCOLN1 - mucolipin 1  ERCC3 - excision repair cross-complementing rodent repair deficiency, complementation group 3  GGA3 - golgi-associated, gamma adaptin ear containing, arf binding protein 3  CEP68 - centrosomal protein 68kda  SEC16A - sec16 homolog a (s. cerevisiae)  CHMP7 - charged multivesicular body protein 7  UBB - ubiquitin b  CORO1B - coronin, actin binding protein, 1b  ARRDC4 - arrestin domain containing 4  TULP3 - tubby like protein 3  MESDC2 - mesoderm development candidate 2  COMMD1 - copper metabolism (murr1) domain containing 1  SPCS1 - signal peptidase complex subunit 1 homolog (s. cerevisiae)  FAF2 - fas associated factor family member 2  ATG4B - autophagy related 4b, cysteine peptidase  COG7 - component of oligomeric golgi complex 7  SNX24 - sorting nexin 24  STXBP6 - syntaxin binding protein 6 (amisyn)  ARL6IP1 - adp-ribosylation factor-like 6 interacting protein 1  TIMM21 - translocase of inner mitochondrial membrane 21 homolog (yeast)  DMTN - dematin actin binding protein  SCYL3 - scy1-like 3 (s. cerevisiae)  PKDCC - protein kinase domain containing, cytoplasmic  SUMO1 - small ubiquitin-like modifier 1  CHMP4A - charged multivesicular body protein 4a  RRS1 - rrs1 ribosome biogenesis regulator homolog (s. cerevisiae)  XPO6 - exportin 6  SMURF1 - smad specific e3 ubiquitin protein ligase 1  MYADM - myeloid-associated differentiation marker  UBE2D2 - ubiquitin-conjugating enzyme e2d 2  TBC1D1 - tbc1 (tre-2/usp6, bub2, cdc16) domain family, member 1  STX2 - syntaxin 2  NUP153 - nucleoporin 153kda  TBC1D12 - tbc1 domain family, member 12  EXOC6B - exocyst complex component 6b  EXOC7 - exocyst complex component 7  CHAMP1 - chromosome alignment maintaining phosphoprotein 1  DTX3L - deltex 3-like (drosophila)  FNTA - farnesyltransferase, caax box, alpha  TCP1 - t-complex 1  FCHSD2 - fch and double sh3 domains 2  SCFD1 - sec1 family domain containing 1  SEC24D - sec24 family, member d (s. cerevisiae)  TLK1 - tousled-like kinase 1  USE1 - unconventional snare in the er 1 homolog (s. cerevisiae)  PLEKHM1 - pleckstrin homology domain containing, family m (with run domain) member 1  TMEM106A - transmembrane protein 106a  SFT2D1 - sft2 domain containing 1  FLNA - filamin a, alpha  C2CD5 - c2 calcium-dependent domain containing 5  AGRN - agrin  SHANK1 - sh3 and multiple ankyrin repeat domains 1  PACS2 - phosphofurin acidic cluster sorting protein 2  RAB15 - rab15, member ras oncogene family  SPATA7 - spermatogenesis associated 7  TBC1D25 - tbc1 domain family, member 25  KLHL21 - kelch-like family member 21  SCAMP4 - secretory carrier membrane protein 4  DCP1A - decapping mrna 1a  EHBP1 - eh domain binding protein 1  AP5Z1 - adaptor-related protein complex 5, zeta 1 subunit  BICD2 - bicaudal d homolog 2 (drosophila)  SCN3B - sodium channel, voltage-gated, type iii, beta subunit  ZDHHC22 - zinc finger, dhhc-type containing 22  VPS26B - vacuolar protein sorting 26 homolog b (s. pombe)  SGSM2 - small g protein signaling modulator 2  ARHGAP44 - rho gtpase activating protein 44  ODF2 - outer dense fiber of sperm tails 2  KIF13B - kinesin family member 13b  TMED5 - transmembrane emp24 protein transport domain containing 5  FKBP1B - fk506 binding protein 1b, 12.6 kda  TNKS2 - tankyrase, trf1-interacting ankyrin-related adp-ribose polymerase 2  EXOSC3 - exosome component 3  VPS11 - vacuolar protein sorting 11 homolog (s. cerevisiae)  FAM208A - family with sequence similarity 208, member a  TMED7 - transmembrane emp24 protein transport domain containing 7  DYNLT1 - dynein, light chain, tctex-type 1  TECPR2 - tectonin beta-propeller repeat containing 2  SNAP91 - synaptosomal-associated protein, 91kda  STX1B - syntaxin 1b  FIS1 - fission 1 (mitochondrial outer membrane) homolog (s. cerevisiae)  VPS8 - vacuolar protein sorting 8 homolog (s. cerevisiae)  FGF13 - fibroblast growth factor 13  NSF - n-ethylmaleimide-sensitive factor  SPCS2 - signal peptidase complex subunit 2 homolog (s. cerevisiae)  TVP23B - trans-golgi network vesicle protein 23 homolog b (s. cerevisiae)  FGF9 - fibroblast growth factor 9  BCAP29 - b-cell receptor-associated protein 29  TOMM20 - translocase of outer mitochondrial membrane 20 homolog (yeast)  GOLT1B - golgi transport 1b  IST1 - increased sodium tolerance 1 homolog (yeast)  VPS39 - vacuolar protein sorting 39 homolog (s. cerevisiae)  NUMA1 - nuclear mitotic apparatus protein 1  NUP88 - nucleoporin 88kda  ATG13 - autophagy related 13  NUP98 - nucleoporin 98kda  TM9SF4 - transmembrane 9 superfamily protein member 4  SZT2 - seizure threshold 2 homolog (mouse)  TAP2 - transporter 2, atp-binding cassette, sub-family b (mdr/tap)  TBC1D30 - tbc1 domain family, member 30  TMEM167A - transmembrane protein 167a  SNX17 - sorting nexin 17  FAM45A - family with sequence similarity 45, member a  TBC1D5 - tbc1 domain family, member 5  DNAJC13 - dnaj (hsp40) homolog, subfamily c, member 13  ZDHHC17 - zinc finger, dhhc-type containing 17  NPM1 - nucleophosmin (nucleolar phosphoprotein b23, numatrin)  NMD3 - nmd3 ribosome export adaptor  NUDCD3 - nudc domain containing 3  SCC4 - sister chromatid cohesion 4  DUSP16 - dual specificity phosphatase 16  OBSL1 - obscurin-like 1  PAQR3 - progestin and adipoq receptor family member iii  RCC2 - regulator of chromosome condensation 2  GRIP2 - glutamate receptor interacting protein 2  NUDT7 - nudix (nucleoside diphosphate linked moiety x)-type motif 7  NRCAM - neuronal cell adhesion molecule  ERBB2IP - erbb2 interacting protein  RAB11FIP3 - rab11 family interacting protein 3 (class ii)  TMED3 - transmembrane emp24 protein transport domain containing 3  TJP1 - tight junction protein 1  TNPO2 - transportin 2  ATP13A2 - atpase type 13a2  HERPUD1 - homocysteine-inducible, endoplasmic reticulum stress-inducible, ubiquitin-like domain member 1  USP6NL - usp6 n-terminal like  SEC62 - sec62 homolog (s. cerevisiae)  CEP290 - centrosomal protein 290kda  EXOSC2 - exosome component 2  RIMS2 - regulating synaptic membrane exocytosis 2  PACSIN1 - protein kinase c and casein kinase substrate in neurons 1  ABCB9 - atp-binding cassette, sub-family b (mdr/tap), member 9  CADPS2 - ca++-dependent secretion activator 2  ZFYVE16 - zinc finger, fyve domain containing 16  CENPT - centromere protein t  ICMT - isoprenylcysteine carboxyl methyltransferase  NFKBIA - nuclear factor of kappa light polypeptide gene enhancer in b-cells inhibitor, alpha  TRAM1 - translocation associated membrane protein 1  PIK3R4 - phosphoinositide-3-kinase, regulatory subunit 4  IFT140 - intraflagellar transport 140 homolog (chlamydomonas)  TNFRSF1A - tumor necrosis factor receptor superfamily, member 1a  TBCK - tbc1 domain containing kinase  EHD2 - eh-domain containing 2  EHD3 - eh-domain containing 3  EHD4 - eh-domain containing 4  NUP188 - nucleoporin 188kda  KCNIP4 - kv channel interacting protein 4  EIF4ENIF1 - eukaryotic translation initiation factor 4e nuclear import factor 1  GCC2 - grip and coiled-coil domain containing 2  MRAP2 - melanocortin 2 receptor accessory protein 2  NEDD8 - neural precursor cell expressed, developmentally down-regulated 8  TERF2 - telomeric repeat binding factor 2  WRAP73 - wd repeat containing, antisense to tp73  SNX18 - sorting nexin 18  PES1 - pescadillo ribosomal biogenesis factor 1  SEC61G - sec61 gamma subunit  NUP155 - nucleoporin 155kda  SEC24C - sec24 family, member c (s. cerevisiae)  CEP41 - centrosomal protein 41kda  HEY2 - hairy/enhancer-of-split related with yrpw motif 2  MACF1 - microtubule-actin crosslinking factor 1  FAM91A1 - family with sequence similarity 91, member a1  CHST11 - carbohydrate (chondroitin 4) sulfotransferase 11  CROCC - ciliary rootlet coiled-coil, rootletin  VPS37D - vacuolar protein sorting 37 homolog d (s. cerevisiae)  ATP6V0A2 - atpase, h+ transporting, lysosomal v0 subunit a2  RAPGEF2 - rap guanine nucleotide exchange factor (gef) 2  TGFB1 - transforming growth factor, beta 1  TSPAN15 - tetraspanin 15  C16orf70 - chromosome 16 open reading frame 70  NUP93 - nucleoporin 93kda  FAM21A - family with sequence similarity 21, member a  ATP6V0E2 - atpase, h+ transporting v0 subunit e2  WDR45B - wd repeat domain 45b  SYTL4 - synaptotagmin-like 4  TIA1 - tia1 cytotoxic granule-associated rna binding protein  THY1 - thy-1 cell surface antigen  TNPO3 - transportin 3 |
| GO:0048858 | cell projection morphogenesis | 7.07E-6 | 9.19E-4 | 1.23 (10334,160,6996,133) | [+] Show genes  AFG3L2 - afg3 atpase family member 3-like 2 (s. cerevisiae)  CTTN - cortactin  SPTBN4 - spectrin, beta, non-erythrocytic 4  ATP8A2 - atpase, aminophospholipid transporter, class i, type 8a, member 2  PLXNB1 - plexin b1  NRP1 - neuropilin 1  FBXO45 - f-box protein 45  PACSIN2 - protein kinase c and casein kinase substrate in neurons 2  ARC - activity-regulated cytoskeleton-associated protein  SPAST - spastin  SHANK1 - sh3 and multiple ankyrin repeat domains 1  WASF2 - was protein family, member 2  DBN1 - drebrin 1  ILK - integrin-linked kinase  APBB1 - amyloid beta (a4) precursor protein-binding, family b, member 1 (fe65)  NUMBL - numb homolog (drosophila)-like  HPRT1 - hypoxanthine phosphoribosyltransferase 1  BRSK1 - br serine/threonine kinase 1  NDEL1 - nude neurodevelopment protein 1-like 1  MAP1S - microtubule-associated protein 1s  ACTB - actin, beta  PAFAH1B1 - platelet-activating factor acetylhydrolase 1b, regulatory subunit 1 (45kda)  GJA1 - gap junction protein, alpha 1, 43kda  APP - amyloid beta (a4) precursor protein  CREB1 - camp responsive element binding protein 1  DOCK10 - dedicator of cytokinesis 10  SNX2 - sorting nexin 2  PAK3 - p21 protein (cdc42/rac)-activated kinase 3  DYNLT1 - dynein, light chain, tctex-type 1  SNX1 - sorting nexin 1  LLGL1 - lethal giant larvae homolog 1 (drosophila)  BMP7 - bone morphogenetic protein 7  GAS7 - growth arrest-specific 7  FGFR2 - fibroblast growth factor receptor 2  TCTN1 - tectonic family member 1  FLOT1 - flotillin 1  PICALM - phosphatidylinositol binding clathrin assembly protein  ANKRD27 - ankyrin repeat domain 27 (vps9 domain)  AMIGO1 - adhesion molecule with ig-like domain 1  PTEN - phosphatase and tensin homolog  ITGA1 - integrin, alpha 1  DAB2IP - dab2 interacting protein  RAB10 - rab10, member ras oncogene family  MEF2A - myocyte enhancer factor 2a  TOP2B - topoisomerase (dna) ii beta 180kda  EGFR - epidermal growth factor receptor  SZT2 - seizure threshold 2 homolog (mouse)  NR4A2 - nuclear receptor subfamily 4, group a, member 2  RHOA - ras homolog family member a  ADARB1 - adenosine deaminase, rna-specific, b1  CELSR2 - cadherin, egf lag seven-pass g-type receptor 2  ATL1 - atlastin gtpase 1  ITGB1 - integrin, beta 1 (fibronectin receptor, beta polypeptide, antigen cd29 includes mdf2, msk12)  CNP - 2',3'-cyclic nucleotide 3' phosphodiesterase  ADCY1 - adenylate cyclase 1 (brain)  DCLK1 - doublecortin-like kinase 1  PTK2 - protein tyrosine kinase 2  CDK5 - cyclin-dependent kinase 5  RAB8A - rab8a, member ras oncogene family  ROCK1 - rho-associated, coiled-coil containing protein kinase 1  S100B - s100 calcium binding protein b  PTN - pleiotrophin  STMN1 - stathmin 1  TRAK2 - trafficking protein, kinesin binding 2  KIDINS220 - kinase d-interacting substrate, 220kda  TNIK - traf2 and nck interacting kinase  ZEB2 - zinc finger e-box binding homeobox 2  SLC11A2 - solute carrier family 11 (proton-coupled divalent metal ion transporter), member 2  KLF7 - kruppel-like factor 7 (ubiquitous)  HSP90AA1 - heat shock protein 90kda alpha (cytosolic), class a member 1  CNTN4 - contactin 4  MYCBP2 - myc binding protein 2, e3 ubiquitin protein ligase  TAOK3 - tao kinase 3  CDH11 - cadherin 11, type 2, ob-cadherin (osteoblast)  NPTX1 - neuronal pentraxin i  WASF1 - was protein family, member 1  NRCAM - neuronal cell adhesion molecule  NCKAP1 - nck-associated protein 1  ABI1 - abl-interactor 1  CHRNB2 - cholinergic receptor, nicotinic, beta 2 (neuronal)  DRD2 - dopamine receptor d2  BTBD3 - btb (poz) domain containing 3  TMEM106B - transmembrane protein 106b  ZNF365 - zinc finger protein 365  NUMB - numb homolog (drosophila)  TAOK1 - tao kinase 1  SHANK3 - sh3 and multiple ankyrin repeat domains 3  MAP6 - microtubule-associated protein 6  LRRK2 - leucine-rich repeat kinase 2  RERE - arginine-glutamic acid dipeptide (re) repeats  MAP2 - microtubule-associated protein 2  MAP1B - microtubule-associated protein 1b  MAP1A - microtubule-associated protein 1a  CTNND2 - catenin (cadherin-associated protein), delta 2  PACSIN1 - protein kinase c and casein kinase substrate in neurons 1  CTNNA2 - catenin (cadherin-associated protein), alpha 2  BAIAP2 - bai1-associated protein 2  HDAC6 - histone deacetylase 6  MAP4K4 - mitogen-activated protein kinase kinase kinase kinase 4  NYAP1 - neuronal tyrosine-phosphorylated phosphoinositide-3-kinase adaptor 1  ATP7A - atpase, cu++ transporting, alpha polypeptide  WEE1 - wee1 homolog (s. pombe)  RAB3A - rab3a, member ras oncogene family  LMTK2 - lemur tyrosine kinase 2  SLC9A6 - solute carrier family 9, subfamily a (nhe6, cation proton antiporter 6), member 6  EPB41L3 - erythrocyte membrane protein band 4.1-like 3  DOCK7 - dedicator of cytokinesis 7  FYN - fyn oncogene related to src, fgr, yes  SLC25A46 - solute carrier family 25, member 46  PITPNA - phosphatidylinositol transfer protein, alpha  MINK1 - misshapen-like kinase 1  DLG4 - discs, large homolog 4 (drosophila)  NEK3 - nima-related kinase 3  NEFL - neurofilament, light polypeptide  DBNL - drebrin-like  UBB - ubiquitin b  PHACTR1 - phosphatase and actin regulator 1  FAM206A - family with sequence similarity 206, member a  RBFOX2 - rna binding protein, fox-1 homolog (c. elegans) 2  MAPK8IP2 - mitogen-activated protein kinase 8 interacting protein 2  DNM2 - dynamin 2  ARHGAP35 - rho gtpase activating protein 35  ALS2 - amyotrophic lateral sclerosis 2 (juvenile)  ZNF335 - zinc finger protein 335  EPHB6 - eph receptor b6  BCL2 - b-cell cll/lymphoma 2  ZSWIM6 - zinc finger, swim-type containing 6  TAOK2 - tao kinase 2  SGK1 - serum/glucocorticoid regulated kinase 1  B4GALT6 - udp-gal:betaglcnac beta 1,4- galactosyltransferase, polypeptide 6  PLEKHO1 - pleckstrin homology domain containing, family o member 1  WASL - wiskott-aldrich syndrome-like  B4GALT5 - udp-gal:betaglcnac beta 1,4- galactosyltransferase, polypeptide 5 |
| GO:0008104 | protein localization | 7.88E-6 | 1.02E-3 | 1.07 (10334,1452,6996,1054) | [+] Show genes  AGAP1 - arfgap with gtpase domain, ankyrin repeat and ph domain 1  AGAP2 - arfgap with gtpase domain, ankyrin repeat and ph domain 2  AP3M2 - adaptor-related protein complex 3, mu 2 subunit  GDI1 - gdp dissociation inhibitor 1  WLS - wntless homolog (drosophila)  GDI2 - gdp dissociation inhibitor 2  ABCA1 - atp-binding cassette, sub-family a (abc1), member 1  SEC61B - sec61 beta subunit  TOMM34 - translocase of outer mitochondrial membrane 34  OS9 - osteosarcoma amplified 9, endoplasmic reticulum lectin  RAB3IP - rab3a interacting protein  ABL1 - c-abl oncogene 1, non-receptor tyrosine kinase  TMED2 - transmembrane emp24 domain trafficking protein 2  NUDT19 - nudix (nucleoside diphosphate linked moiety x)-type motif 19  ERP29 - endoplasmic reticulum protein 29  PML - promyelocytic leukemia  ACOT2 - acyl-coa thioesterase 2  PMM1 - phosphomannomutase 1  RAB40B - rab40b, member ras oncogene family  YWHAQ - tyrosine 3-monooxygenase/tryptophan 5-monooxygenase activation protein, theta polypeptide  TMED10 - transmembrane emp24-like trafficking protein 10 (yeast)  UBE2Q1 - ubiquitin-conjugating enzyme e2q family member 1  TBC1D22B - tbc1 domain family, member 22b  AP3B2 - adaptor-related protein complex 3, beta 2 subunit  NOP9 - nop9 nucleolar protein  ACOX1 - acyl-coa oxidase 1, palmitoyl  PLRG1 - pleiotropic regulator 1  ACTB - actin, beta  RAB37 - rab37, member ras oncogene family  GJA1 - gap junction protein, alpha 1, 43kda  ACTG1 - actin, gamma 1  EHD1 - eh-domain containing 1  ZDHHC7 - zinc finger, dhhc-type containing 7  GRASP - grp1 (general receptor for phosphoinositides 1)-associated scaffold protein  PPID - peptidylprolyl isomerase d  AP1AR - adaptor-related protein complex 1 associated regulatory protein  ACTN4 - actinin, alpha 4  RAB10 - rab10, member ras oncogene family  TMCO6 - transmembrane and coiled-coil domains 6  MED1 - mediator complex subunit 1  AXIN2 - axin 2  RAB39B - rab39b, member ras oncogene family  NUP85 - nucleoporin 85kda  USP9X - ubiquitin specific peptidase 9, x-linked  ADPRH - adp-ribosylarginine hydrolase  H2AFY2 - h2a histone family, member y2  RUVBL2 - ruvb-like 2 (e. coli)  AP2A2 - adaptor-related protein complex 2, alpha 2 subunit  TMEM30A - transmembrane protein 30a  AP2A1 - adaptor-related protein complex 2, alpha 1 subunit  AP2B1 - adaptor-related protein complex 2, beta 1 subunit  TTC21B - tetratricopeptide repeat domain 21b  AP1B1 - adaptor-related protein complex 1, beta 1 subunit  AP1G1 - adaptor-related protein complex 1, gamma 1 subunit  NUP133 - nucleoporin 133kda  AP5M1 - adaptor-related protein complex 5, mu 1 subunit  AGK - acylglycerol kinase  NUPL2 - nucleoporin like 2  VPS35 - vacuolar protein sorting 35 homolog (s. cerevisiae)  TOR1AIP2 - torsin a interacting protein 2  KIF3A - kinesin family member 3a  GPR89A - g protein-coupled receptor 89a  PPT1 - palmitoyl-protein thioesterase 1  RAB3C - rab3c, member ras oncogene family  AKT1 - v-akt murine thymoma viral oncogene homolog 1  RAB7A - rab7a, member ras oncogene family  EVI5L - ecotropic viral integration site 5-like  EXOC2 - exocyst complex component 2  RER1 - rer1 retention in endoplasmic reticulum 1 homolog (s. cerevisiae)  TCTN2 - tectonic family member 2  PPP1R10 - protein phosphatase 1, regulatory subunit 10  EXOC1 - exocyst complex component 1  IFT122 - intraflagellar transport 122 homolog (chlamydomonas)  RAPGEF4 - rap guanine nucleotide exchange factor (gef) 4  TMEM115 - transmembrane protein 115  GAK - cyclin g associated kinase  STK11IP - serine/threonine kinase 11 interacting protein  PEX26 - peroxisomal biogenesis factor 26  TBC1D17 - tbc1 domain family, member 17  RAB35 - rab35, member ras oncogene family  IFT27 - intraflagellar transport 27 homolog (chlamydomonas)  DENND4C - denn/madd domain containing 4c  KDELR2 - kdel (lys-asp-glu-leu) endoplasmic reticulum protein retention receptor 2  NPLOC4 - nuclear protein localization 4 homolog (s. cerevisiae)  RAB31 - rab31, member ras oncogene family  MAPK10 - mitogen-activated protein kinase 10  MAPK9 - mitogen-activated protein kinase 9  C1QTNF5 - c1q and tumor necrosis factor related protein 5  WDR11 - wd repeat domain 11  MAPRE2 - microtubule-associated protein, rp/eb family, member 2  OSBPL8 - oxysterol binding protein-like 8  NECAP2 - necap endocytosis associated 2  NDC1 - ndc1 transmembrane nucleoporin  IPO9 - importin 9  STAM - signal transducing adaptor molecule (sh3 domain and itam motif) 1  PRKCI - protein kinase c, iota  NUP214 - nucleoporin 214kda  ANXA2 - annexin a2  PRKCB - protein kinase c, beta  GAS6 - growth arrest-specific 6  NCOA4 - nuclear receptor coactivator 4  PACS1 - phosphofurin acidic cluster sorting protein 1  SDAD1 - sda1 domain containing 1  TREX1 - three prime repair exonuclease 1  SYNRG - synergin, gamma  PACSIN2 - protein kinase c and casein kinase substrate in neurons 2  APBA1 - amyloid beta (a4) precursor protein-binding, family a, member 1  MCPH1 - microcephalin 1  SEC61A2 - sec61 alpha 2 subunit (s. cerevisiae)  CHP1 - calcineurin-like ef-hand protein 1  VPS13D - vacuolar protein sorting 13 homolog d (s. cerevisiae)  SEC63 - sec63 homolog (s. cerevisiae)  PRAF2 - pra1 domain family, member 2  AAGAB - alpha- and gamma-adaptin binding protein  ARCN1 - archain 1  TRIM23 - tripartite motif containing 23  APPL2 - adaptor protein, phosphotyrosine interaction, ph domain and leucine zipper containing 2  ARF1 - adp-ribosylation factor 1  IPO4 - importin 4  ARF4 - adp-ribosylation factor 4  PAFAH1B1 - platelet-activating factor acetylhydrolase 1b, regulatory subunit 1 (45kda)  GOLPH3L - golgi phosphoprotein 3-like  ARL8B - adp-ribosylation factor-like 8b  AKAP11 - a kinase (prka) anchor protein 11  AKAP10 - a kinase (prka) anchor protein 10  ZDHHC14 - zinc finger, dhhc-type containing 14  BBS7 - bardet-biedl syndrome 7  PALM - paralemmin  CEP250 - centrosomal protein 250kda  ARNTL - aryl hydrocarbon receptor nuclear translocator-like  TCTN1 - tectonic family member 1  ARL3 - adp-ribosylation factor-like 3  SCG2 - secretogranin ii  ARL1 - adp-ribosylation factor-like 1  SEC23IP - sec23 interacting protein  CORO7 - coronin 7  ARRB2 - arrestin, beta 2  ARRB1 - arrestin, beta 1  RHOB - ras homolog family member b  FAM83H - family with sequence similarity 83, member h  ARF6 - adp-ribosylation factor 6  ARF5 - adp-ribosylation factor 5  EVI5 - ecotropic viral integration site 5  ARHGAP1 - rho gtpase activating protein 1  AP4S1 - adaptor-related protein complex 4, sigma 1 subunit  ASNA1 - arsa arsenite transporter, atp-binding, homolog 1 (bacterial)  CHMP1A - charged multivesicular body protein 1a  TTC8 - tetratricopeptide repeat domain 8  PCM1 - pericentriolar material 1  RABL2B - rab, member of ras oncogene family-like 2b  FRMD6 - ferm domain containing 6  TBC1D8 - tbc1 domain family, member 8 (with gram domain)  WRAP53 - wd repeat containing, antisense to tp53  CDC37 - cell division cycle 37  KPTN - kaptin (actin binding protein)  TANGO6 - transport and golgi organization 6 homolog (drosophila)  RIC3 - ric3 acetylcholine receptor chaperone  SLC15A4 - solute carrier family 15 (oligopeptide transporter), member 4  XPA - xeroderma pigmentosum, complementation group a  GOLGA2 - golgin a2  UEVLD - uev and lactate/malate dehyrogenase domains  RPH3A - rabphilin 3a homolog (mouse)  XPO1 - exportin 1 (crm1 homolog, yeast)  GOLGB1 - golgin b1  ATP1B3 - atpase, na+/k+ transporting, beta 3 polypeptide  CCDC91 - coiled-coil domain containing 91  ATP1B1 - atpase, na+/k+ transporting, beta 1 polypeptide  LIN7C - lin-7 homolog c (c. elegans)  MON1B - mon1 secretory trafficking family member b  LRRK2 - leucine-rich repeat kinase 2  CLSTN1 - calsyntenin 1  AP5S1 - adaptor-related protein complex 5, sigma 1 subunit  RSG1 - rem2 and rab-like small gtpase 1  DZIP1 - daz interacting zinc finger protein 1  SEC31A - sec31 homolog a (s. cerevisiae)  SCAMP5 - secretory carrier membrane protein 5  WRB - tryptophan rich basic protein  XBP1 - x-box binding protein 1  GPM6B - glycoprotein m6b  ATP6V1E1 - atpase, h+ transporting, lysosomal 31kda, v1 subunit e1  PEX6 - peroxisomal biogenesis factor 6  ATP6V0B - atpase, h+ transporting, lysosomal 21kda, v0 subunit b  LSG1 - large 60s subunit nuclear export gtpase 1  PEX7 - peroxisomal biogenesis factor 7  SH3PXD2B - sh3 and px domains 2b  ATP6V0A1 - atpase, h+ transporting, lysosomal v0 subunit a1  PEX12 - peroxisomal biogenesis factor 12  ATP6AP1 - atpase, h+ transporting, lysosomal accessory protein 1  PEX13 - peroxisomal biogenesis factor 13  PEX14 - peroxisomal biogenesis factor 14  RILPL2 - rab interacting lysosomal protein-like 2  LMTK2 - lemur tyrosine kinase 2  ATP6V1A - atpase, h+ transporting, lysosomal 70kda, v1 subunit a  ATP6V1B2 - atpase, h+ transporting, lysosomal 56/58kda, v1 subunit b2  ATP6V0C - atpase, h+ transporting, lysosomal 16kda, v0 subunit c  PEX1 - peroxisomal biogenesis factor 1  ATP6V1C1 - atpase, h+ transporting, lysosomal 42kda, v1 subunit c1  RAB11FIP2 - rab11 family interacting protein 2 (class i)  PAOX - polyamine oxidase (exo-n4-amino)  BAX - bcl2-associated x protein  PKD1 - polycystic kidney disease 1 (autosomal dominant)  COPZ1 - coatomer protein complex, subunit zeta 1  COPG1 - coatomer protein complex, subunit gamma 1  BAD - bcl2-associated agonist of cell death  PIK3R2 - phosphoinositide-3-kinase, regulatory subunit 2 (beta)  PIK3R1 - phosphoinositide-3-kinase, regulatory subunit 1 (alpha)  COX18 - cox18 cytochrome c oxidase assembly factor  GNPTAB - n-acetylglucosamine-1-phosphate transferase, alpha and beta subunits  PKP2 - plakophilin 2  ZFYVE27 - zinc finger, fyve domain containing 27  KIF1A - kinesin family member 1a  ATRX - alpha thalassemia/mental retardation syndrome x-linked  COG2 - component of oligomeric golgi complex 2  GABARAPL2 - gaba(a) receptor-associated protein-like 2  GRIK5 - glutamate receptor, ionotropic, kainate 5  VPS53 - vacuolar protein sorting 53 homolog (s. cerevisiae)  EXOC3 - exocyst complex component 3  RABL3 - rab, member of ras oncogene family-like 3  GRIK2 - glutamate receptor, ionotropic, kainate 2  GABARAP - gaba(a) receptor-associated protein  BCR - breakpoint cluster region  DDX42 - dead (asp-glu-ala-asp) box helicase 42  XRCC5 - x-ray repair complementing defective repair in chinese hamster cells 5 (double-strand-break rejoining)  PIK3C3 - phosphatidylinositol 3-kinase, catalytic subunit type 3  YWHAG - tyrosine 3-monooxygenase/tryptophan 5-monooxygenase activation protein, gamma polypeptide  COPE - coatomer protein complex, subunit epsilon  PARK7 - parkinson protein 7  YWHAZ - tyrosine 3-monooxygenase/tryptophan 5-monooxygenase activation protein, zeta polypeptide  BBS4 - bardet-biedl syndrome 4  BBS2 - bardet-biedl syndrome 2  YWHAE - tyrosine 3-monooxygenase/tryptophan 5-monooxygenase activation protein, epsilon polypeptide  VPS45 - vacuolar protein sorting 45 homolog (s. cerevisiae)  YWHAB - tyrosine 3-monooxygenase/tryptophan 5-monooxygenase activation protein, beta polypeptide  DNAJA1 - dnaj (hsp40) homolog, subfamily a, member 1  RNF26 - ring finger protein 26  BET1 - bet1 golgi vesicular membrane trafficking protein  BRAF - v-raf murine sarcoma viral oncogene homolog b  NRP1 - neuropilin 1  LIN7A - lin-7 homolog a (c. elegans)  KATNB1 - katanin p80 (wd repeat containing) subunit b 1  RDX - radixin  ARL13B - adp-ribosylation factor-like 13b  DERL1 - derlin 1  DSCR3 - down syndrome critical region gene 3  RANBP6 - ran binding protein 6  RRAGB - ras-related gtp binding b  MTX3 - metaxin 3  SYNGAP1 - synaptic ras gtpase activating protein 1  RNF215 - ring finger protein 215  BMP7 - bone morphogenetic protein 7  AKAP4 - a kinase (prka) anchor protein 4  ABCA7 - atp-binding cassette, sub-family a (abc1), member 7  FLOT1 - flotillin 1  VPS51 - vacuolar protein sorting 51 homolog (s. cerevisiae)  ABCE1 - atp-binding cassette, sub-family e (oabp), member 1  VPS13C - vacuolar protein sorting 13 homolog c (s. cerevisiae)  PTTG1IP - pituitary tumor-transforming 1 interacting protein  MIS12 - mis12 kinetochore complex component  ARL8A - adp-ribosylation factor-like 8a  NABP2 - nucleic acid binding protein 2  STX6 - syntaxin 6  AP1M1 - adaptor-related protein complex 1, mu 1 subunit  AP1G2 - adaptor-related protein complex 1, gamma 2 subunit  AP1S2 - adaptor-related protein complex 1, sigma 2 subunit  AHI1 - abelson helper integration site 1  AP3S2 - adaptor-related protein complex 3, sigma 2 subunit  AFTPH - aftiphilin  GPHN - gephyrin  ROCK1 - rho-associated, coiled-coil containing protein kinase 1  HERC2 - hect and rld domain containing e3 ubiquitin protein ligase 2  TSPO - translocator protein (18kda)  TRAK2 - trafficking protein, kinesin binding 2  SNX19 - sorting nexin 19  HSPD1 - heat shock 60kda protein 1 (chaperonin)  STAM2 - signal transducing adaptor molecule (sh3 domain and itam motif) 2  HSP90AA1 - heat shock protein 90kda alpha (cytosolic), class a member 1  RAMP2 - receptor (g protein-coupled) activity modifying protein 2  RAMP3 - receptor (g protein-coupled) activity modifying protein 3  AP3D1 - adaptor-related protein complex 3, delta 1 subunit  RAMP1 - receptor (g protein-coupled) activity modifying protein 1  AKAP8 - a kinase (prka) anchor protein 8  HSPA4 - heat shock 70kda protein 4  HSPA9 - heat shock 70kda protein 9 (mortalin)  HSPA8 - heat shock 70kda protein 8  TOMM40 - translocase of outer mitochondrial membrane 40 homolog (yeast)  ATG16L1 - autophagy related 16-like 1 (s. cerevisiae)  CANX - calnexin  RPL17 - ribosomal protein l17  TIMM17A - translocase of inner mitochondrial membrane 17 homolog a (yeast)  LAMTOR3 - late endosomal/lysosomal adaptor, mapk and mtor activator 3  VPS37C - vacuolar protein sorting 37 homolog c (s. cerevisiae)  RPL15 - ribosomal protein l15  HMGB1 - high mobility group box 1  RPL11 - ribosomal protein l11  RPL9 - ribosomal protein l9  TIMM10B - translocase of inner mitochondrial membrane 10 homolog b (yeast)  NOL8 - nucleolar protein 8  COG5 - component of oligomeric golgi complex 5  RPL7 - ribosomal protein l7  PIBF1 - progesterone immunomodulatory binding factor 1  TNKS - tankyrase, trf1-interacting ankyrin-related adp-ribose polymerase  RPL3 - ribosomal protein l3  HEATR3 - heat repeat containing 3  BAIAP2 - bai1-associated protein 2  TIMM9 - translocase of inner mitochondrial membrane 9 homolog (yeast)  RPA1 - replication protein a1, 70kda  RPA2 - replication protein a2, 32kda  RPS4X - ribosomal protein s4, x-linked  SEC23A - sec23 homolog a (s. cerevisiae)  RPS4Y1 - ribosomal protein s4, y-linked 1  HK1 - hexokinase 1  SEC23B - sec23 homolog b (s. cerevisiae)  STX17 - syntaxin 17  CACNB3 - calcium channel, voltage-dependent, beta 3 subunit  CACNB2 - calcium channel, voltage-dependent, beta 2 subunit  ACD - adrenocortical dysplasia homolog (mouse)  LAMTOR1 - late endosomal/lysosomal adaptor, mapk and mtor activator 1  CALR - calreticulin  HFE - hemochromatosis  VPS33A - vacuolar protein sorting 33 homolog a (s. cerevisiae)  HIP1 - huntingtin interacting protein 1  RPL32 - ribosomal protein l32  VTI1B - vesicle transport through interaction with t-snares 1b  MBTPS1 - membrane-bound transcription factor peptidase, site 1  RPS23 - ribosomal protein s23  ARFGAP3 - adp-ribosylation factor gtpase activating protein 3  PINX1 - pin2/terf1 interacting, telomerase inhibitor 1  SNX3 - sorting nexin 3  SNX4 - sorting nexin 4  TSNARE1 - t-snare domain containing 1  GBF1 - golgi brefeldin a resistant guanine nucleotide exchange factor 1  BANP - btg3 associated nuclear protein  HNRNPU - heterogeneous nuclear ribonucleoprotein u (scaffold attachment factor a)  RPS15 - ribosomal protein s15  RPS12 - ribosomal protein s12  VPS33B - vacuolar protein sorting 33 homolog b (yeast)  TINF2 - terf1 (trf1)-interacting nuclear factor 2  RAB11A - rab11a, member ras oncogene family  PORCN - porcupine homolog (drosophila)  CAV1 - caveolin 1, caveolae protein, 22kda  NAPA - n-ethylmaleimide-sensitive factor attachment protein, alpha  NAPG - n-ethylmaleimide-sensitive factor attachment protein, gamma  SEC24B - sec24 family, member b (s. cerevisiae)  CMTM6 - cklf-like marvel transmembrane domain containing 6  TRIM3 - tripartite motif containing 3  LRBA - lps-responsive vesicle trafficking, beach and anchor containing  PSAP - prosaposin  TBC1D15 - tbc1 domain family, member 15  CDH2 - cadherin 2, type 1, n-cadherin (neuronal)  STXBP5 - syntaxin binding protein 5 (tomosyn)  DESI1 - desumoylating isopeptidase 1  PEX3 - peroxisomal biogenesis factor 3  SGSM3 - small g protein signaling modulator 3  RANBP3 - ran binding protein 3  TOR1B - torsin family 1, member b (torsin b)  PSEN1 - presenilin 1  RABGEF1 - rab guanine nucleotide exchange factor (gef) 1  TOLLIP - toll interacting protein  PRPF19 - pre-mrna processing factor 19  CD81 - cd81 molecule  SMYD3 - set and mynd domain containing 3  SCARB2 - scavenger receptor class b, member 2  EXOC5 - exocyst complex component 5  BLZF1 - basic leucine zipper nuclear factor 1  AP3B1 - adaptor-related protein complex 3, beta 1 subunit  YKT6 - ykt6 v-snare homolog (s. cerevisiae)  MTX2 - metaxin 2  AGPS - alkylglycerone phosphate synthase  IL10RA - interleukin 10 receptor, alpha  GDAP1 - ganglioside induced differentiation associated protein 1  GORASP1 - golgi reassembly stacking protein 1, 65kda  TSPAN33 - tetraspanin 33  GRPEL2 - grpe-like 2, mitochondrial (e. coli)  GAS2L1 - growth arrest-specific 2 like 1  TERF2IP - telomeric repeat binding factor 2, interacting protein  PITRM1 - pitrilysin metallopeptidase 1  ATG7 - autophagy related 7  SERP1 - stress-associated endoplasmic reticulum protein 1  VPS16 - vacuolar protein sorting 16 homolog (s. cerevisiae)  DCTN2 - dynactin 2 (p50)  LAMTOR5 - late endosomal/lysosomal adaptor, mapk and mtor activator 5  CETN2 - centrin, ef-hand protein, 2  CETN3 - centrin, ef-hand protein, 3  TM9SF1 - transmembrane 9 superfamily member 1  APPBP2 - amyloid beta precursor protein (cytoplasmic tail) binding protein 2  TRAM1L1 - translocation associated membrane protein 1-like 1  FBLN5 - fibulin 5  ITGAV - integrin, alpha v  ARFIP1 - adp-ribosylation factor interacting protein 1  IPO8 - importin 8  ZDHHC6 - zinc finger, dhhc-type containing 6  IPO7 - importin 7  BBS9 - bardet-biedl syndrome 9  EIF6 - eukaryotic translation initiation factor 6  SLU7 - slu7 splicing factor homolog (s. cerevisiae)  CDK5 - cyclin-dependent kinase 5  SKAP1 - src kinase associated phosphoprotein 1  PTN - pleiotrophin  ATG3 - autophagy related 3  KLHL20 - kelch-like family member 20  AKTIP - akt interacting protein  CADPS - ca++-dependent secretion activator  MPP5 - membrane protein, palmitoylated 5 (maguk p55 subfamily member 5)  USO1 - uso1 vesicle transport factor  ZMAT3 - zinc finger, matrin-type 3  ARFGEF2 - adp-ribosylation factor guanine nucleotide-exchange factor 2 (brefeldin a-inhibited)  ARFGEF1 - adp-ribosylation factor guanine nucleotide-exchange factor 1 (brefeldin a-inhibited)  PURA - purine-rich element binding protein a  LRWD1 - leucine-rich repeats and wd repeat domain containing 1  PTPRK - protein tyrosine phosphatase, receptor type, k  TBC1D13 - tbc1 domain family, member 13  PTPRN - protein tyrosine phosphatase, receptor type, n  LYST - lysosomal trafficking regulator  AHCYL1 - adenosylhomocysteinase-like 1  CHML - choroideremia-like (rab escort protein 2)  NUP50 - nucleoporin 50kda  PEX2 - peroxisomal biogenesis factor 2  CHM - choroideremia (rab escort protein 1)  CHCHD4 - coiled-coil-helix-coiled-coil-helix domain containing 4  GIPC1 - gipc pdz domain containing family, member 1  SNX5 - sorting nexin 5  PVRL1 - poliovirus receptor-related 1 (herpesvirus entry mediator c)  CHGA - chromogranin a (parathyroid secretory protein 1)  INVS - inversin  RAB6A - rab6a, member ras oncogene family  PIP5K1A - phosphatidylinositol-4-phosphate 5-kinase, type i, alpha  RAB5A - rab5a, member ras oncogene family  C17orf75 - chromosome 17 open reading frame 75  RAB5B - rab5b, member ras oncogene family  RAB4A - rab4a, member ras oncogene family  RAB3A - rab3a, member ras oncogene family  RAB3B - rab3b, member ras oncogene family  MTERFD2 - mterf domain containing 2  RAB2A - rab2a, member ras oncogene family  RAB1A - rab1a, member ras oncogene family  SDCCAG3 - serologically defined colon cancer antigen 3  RRAGC - ras-related gtp binding c  RAD21 - rad21 homolog (s. pombe)  SEC24A - sec24 family, member a (s. cerevisiae)  PAF1 - paf1, rna polymerase ii associated factor, homolog (s. cerevisiae)  RAB5C - rab5c, member ras oncogene family  TMED4 - transmembrane emp24 protein transport domain containing 4  RABIF - rab interacting factor  LIN7B - lin-7 homolog b (c. elegans)  VPS4A - vacuolar protein sorting 4 homolog a (s. cerevisiae)  RAB27A - rab27a, member ras oncogene family  COPG2 - coatomer protein complex, subunit gamma 2  SNX16 - sorting nexin 16  NBEA - neurobeachin  TOMM7 - translocase of outer mitochondrial membrane 7 homolog (yeast)  RANBP2 - ran binding protein 2  RAP1A - rap1a, member of ras oncogene family  CLTC - clathrin, heavy chain (hc)  SEC22A - sec22 vesicle trafficking protein homolog a (s. cerevisiae)  LRRC4 - leucine rich repeat containing 4  AP3M1 - adaptor-related protein complex 3, mu 1 subunit  GNPAT - glyceronephosphate o-acyltransferase  EXOC6 - exocyst complex component 6  SGTB - small glutamine-rich tetratricopeptide repeat (tpr)-containing, beta  BBS5 - bardet-biedl syndrome 5  ULK1 - unc-51 like autophagy activating kinase 1  RRAGA - ras-related gtp binding a  CLTB - clathrin, light chain b  CLTA - clathrin, light chain a  CLU - clusterin  STX7 - syntaxin 7  GOLPH3 - golgi phosphoprotein 3 (coat-protein)  RAP2A - rap2a, member of ras oncogene family  MLH3 - mutl homolog 3 (e. coli)  TPP1 - tripeptidyl peptidase i  IK - ik cytokine, down-regulator of hla ii  CTCF - ccctc-binding factor (zinc finger protein)  HEATR5B - heat repeat containing 5b  AP1S1 - adaptor-related protein complex 1, sigma 1 subunit  TMEM150A - transmembrane protein 150a  RB1 - retinoblastoma 1  AP2M1 - adaptor-related protein complex 2, mu 1 subunit  ARL5B - adp-ribosylation factor-like 5b  NSG1 - neuron specific gene family member 1  NUP35 - nucleoporin 35kda  VPS41 - vacuolar protein sorting 41 homolog (s. cerevisiae)  DOC2B - double c2-like domains, beta  CCDC93 - coiled-coil domain containing 93  SGSM1 - small g protein signaling modulator 1  CHMP4B - charged multivesicular body protein 4b  PIGU - phosphatidylinositol glycan anchor biosynthesis, class u  CACNG4 - calcium channel, voltage-dependent, gamma subunit 4  AP4B1 - adaptor-related protein complex 4, beta 1 subunit  TBRG1 - transforming growth factor beta regulator 1  PITPNM1 - phosphatidylinositol transfer protein, membrane-associated 1  WDR19 - wd repeat domain 19  PDIA4 - protein disulfide isomerase family a, member 4  SPTBN4 - spectrin, beta, non-erythrocytic 4  AKAP12 - a kinase (prka) anchor protein 12  COG8 - component of oligomeric golgi complex 8  CORO1C - coronin, actin binding protein, 1c  VPS37A - vacuolar protein sorting 37 homolog a (s. cerevisiae)  ZDHHC3 - zinc finger, dhhc-type containing 3  KPNA6 - karyopherin alpha 6 (importin alpha 7)  RABGAP1 - rab gtpase activating protein 1  HOOK3 - hook microtubule-tethering protein 3  RAB36 - rab36, member ras oncogene family  DENND1A - denn/madd domain containing 1a  REEP2 - receptor accessory protein 2  FAM53C - family with sequence similarity 53, member c  ABCG1 - atp-binding cassette, sub-family g (white), member 1  CADM3 - cell adhesion molecule 3  ATP6V1G1 - atpase, h+ transporting, lysosomal 13kda, v1 subunit g1  LTV1 - ltv1 homolog (s. cerevisiae)  APBA3 - amyloid beta (a4) precursor protein-binding, family a, member 3  TICAM2 - toll-like receptor adaptor molecule 2  RAB3D - rab3d, member ras oncogene family  SYVN1 - synovial apoptosis inhibitor 1, synoviolin  ATG4C - autophagy related 4c, cysteine peptidase  VPS26A - vacuolar protein sorting 26 homolog a (s. pombe)  SEC22B - sec22 vesicle trafficking protein homolog b (s. cerevisiae) (gene/pseudogene)  RAB2B - rab2b, member ras oncogene family  CPE - carboxypeptidase e  H2AFY - h2a histone family, member y  WBP2 - ww domain binding protein 2  BET1L - bet1 golgi vesicular membrane trafficking protein-like  GOSR2 - golgi snap receptor complex member 2  UBAP1 - ubiquitin associated protein 1  RAB40C - rab40c, member ras oncogene family  SPIRE2 - spire actin nucleation factor 2  PREPL - prolyl endopeptidase-like  JAGN1 - jagunal homolog 1 (drosophila)  SNX2 - sorting nexin 2  NACC2 - nacc family member 2, ben and btb (poz) domain containing  SNX1 - sorting nexin 1  DNAJC27 - dnaj (hsp40) homolog, subfamily c, member 27  LMNA - lamin a/c  SNCG - synuclein, gamma (breast cancer-specific protein 1)  KTN1 - kinectin 1 (kinesin receptor)  SCAMP1 - secretory carrier membrane protein 1  VPS4B - vacuolar protein sorting 4 homolog b (s. cerevisiae)  GOSR1 - golgi snap receptor complex member 1  SRPRB - signal recognition particle receptor, b subunit  BAG4 - bcl2-associated athanogene 4  SMO - smoothened, frizzled family receptor  LAMP2 - lysosomal-associated membrane protein 2  ARFIP2 - adp-ribosylation factor interacting protein 2  SNX6 - sorting nexin 6  SNX7 - sorting nexin 7  ROCK2 - rho-associated, coiled-coil containing protein kinase 2  ATP6V1D - atpase, h+ transporting, lysosomal 34kda, v1 subunit d  SH3BP4 - sh3-domain binding protein 4  STX8 - syntaxin 8  COPB1 - coatomer protein complex, subunit beta 1  ZBTB7A - zinc finger and btb domain containing 7a  COPA - coatomer protein complex, subunit alpha  RAB38 - rab38, member ras oncogene family  LAMA5 - laminin, alpha 5  STX12 - syntaxin 12  RPH3AL - rabphilin 3a-like (without c2 domains)  NAGPA - n-acetylglucosamine-1-phosphodiester alpha-n-acetylglucosaminidase  SENP2 - sumo1/sentrin/smt3 specific peptidase 2  PLEKHA1 - pleckstrin homology domain containing, family a (phosphoinositide binding specific) member 1  FAM126A - family with sequence similarity 126, member a  CYB5R4 - cytochrome b5 reductase 4  VPS28 - vacuolar protein sorting 28 homolog (s. cerevisiae)  KIF5C - kinesin family member 5c  RPF2 - ribosome production factor 2 homolog (s. cerevisiae)  KIF5B - kinesin family member 5b  KIF5A - kinesin family member 5a  CBY1 - chibby homolog 1 (drosophila)  PICK1 - protein interacting with prkca 1  CTNNA1 - catenin (cadherin-associated protein), alpha 1, 102kda  CACNG7 - calcium channel, voltage-dependent, gamma subunit 7  VAMP2 - vesicle-associated membrane protein 2 (synaptobrevin 2)  RAB3GAP2 - rab3 gtpase activating protein subunit 2 (non-catalytic)  CCDC41 - coiled-coil domain containing 41  CTNNB1 - catenin (cadherin-associated protein), beta 1, 88kda  STXBP1 - syntaxin binding protein 1  PPIL2 - peptidylprolyl isomerase (cyclophilin)-like 2  STX5 - syntaxin 5  ASB3 - ankyrin repeat and socs box containing 3  STX4 - syntaxin 4  SAV1 - salvador homolog 1 (drosophila)  STX3 - syntaxin 3  STX1A - syntaxin 1a (brain)  GOLGA7 - golgin a7  MGARP - mitochondria-localized glutamic acid-rich protein  TNPO1 - transportin 1  FUT10 - fucosyltransferase 10 (alpha (1,3) fucosyltransferase)  TGFBRAP1 - transforming growth factor, beta receptor associated protein 1  UNC119B - unc-119 homolog b (c. elegans)  KPNA2 - karyopherin alpha 2 (rag cohort 1, importin alpha 1)  KPNB1 - karyopherin (importin) beta 1  KPNA1 - karyopherin alpha 1 (importin alpha 5)  STXBP3 - syntaxin binding protein 3  ZDHHC9 - zinc finger, dhhc-type containing 9  CRIPT - cysteine-rich pdz-binding protein  NPNT - nephronectin  PAN3 - pan3 poly(a) specific ribonuclease subunit homolog (s. cerevisiae)  PEX16 - peroxisomal biogenesis factor 16  EXOC4 - exocyst complex component 4  CNEP1R1 - ctd nuclear envelope phosphatase 1 regulatory subunit 1  SH3GLB1 - sh3-domain grb2-like endophilin b1  RPAIN - rpa interacting protein  RAB28 - rab28, member ras oncogene family  LHFPL4 - lipoma hmgic fusion partner-like 4  NIPBL - nipped-b homolog (drosophila)  RAB9A - rab9a, member ras oncogene family  COG4 - component of oligomeric golgi complex 4  RAB26 - rab26, member ras oncogene family  PDZD11 - pdz domain containing 11  STAT3 - signal transducer and activator of transcription 3 (acute-phase response factor)  JUP - junction plakoglobin  SSR3 - signal sequence receptor, gamma (translocon-associated protein gamma)  COG1 - component of oligomeric golgi complex 1  SFT2D3 - sft2 domain containing 3  SPCS3 - signal peptidase complex subunit 3 homolog (s. cerevisiae)  RINT1 - rad50 interactor 1  DFNB31 - deafness, autosomal recessive 31  TM9SF2 - transmembrane 9 superfamily member 2  NAPB - n-ethylmaleimide-sensitive factor attachment protein, beta  SRP68 - signal recognition particle 68kda  SNX33 - sorting nexin 33  SRP54 - signal recognition particle 54kda  FBXO7 - f-box protein 7  SRP19 - signal recognition particle 19kda  CSE1L - cse1 chromosome segregation 1-like (yeast)  SRP14 - signal recognition particle 14kda (homologous alu rna binding protein)  SRP72 - signal recognition particle 72kda  SSR1 - signal sequence receptor, alpha  ZDHHC2 - zinc finger, dhhc-type containing 2  SSB - sjogren syndrome antigen b (autoantigen la)  SNAP29 - synaptosomal-associated protein, 29kda  SPTBN1 - spectrin, beta, non-erythrocytic 1  VAMP3 - vesicle-associated membrane protein 3  CSNK1D - casein kinase 1, delta  MIA3 - melanoma inhibitory activity family, member 3  SFT2D2 - sft2 domain containing 2  IPO11 - importin 11  TAOK2 - tao kinase 2  VIPAS39 - vps33b interacting protein, apical-basolateral polarity regulator, spe-39 homolog  ZDHHC12 - zinc finger, dhhc-type containing 12  VPS25 - vacuolar protein sorting 25 homolog (s. cerevisiae)  SRP9 - signal recognition particle 9kda  TMEM107 - transmembrane protein 107  MON1A - mon1 secretory trafficking family member a  DHX9 - deah (asp-glu-ala-his) box helicase 9  SEL1L - sel-1 suppressor of lin-12-like (c. elegans)  ATP6V1F - atpase, h+ transporting, lysosomal 14kda, v1 subunit f  SEC13 - sec13 homolog (s. cerevisiae)  DDX3X - dead (asp-glu-ala-asp) box helicase 3, x-linked  DDX1 - dead (asp-glu-ala-asp) box helicase 1  CCDC120 - coiled-coil domain containing 120  SNX14 - sorting nexin 14  SNX25 - sorting nexin 25  SIN3A - sin3 transcription regulator family member a  RAB8B - rab8b, member ras oncogene family  SDCBP - syndecan binding protein (syntenin)  HEATR5A - heat repeat containing 5a  COPB2 - coatomer protein complex, subunit beta 2 (beta prime)  PTPN23 - protein tyrosine phosphatase, non-receptor type 23  ITGB1BP1 - integrin beta 1 binding protein 1  RCN3 - reticulocalbin 3, ef-hand calcium binding domain  MCM9 - minichromosome maintenance complex component 9  ZDHHC24 - zinc finger, dhhc-type containing 24  MYRIP - myosin viia and rab interacting protein  PHAX - phosphorylated adaptor for rna export  POT1 - protection of telomeres 1  SCYL1 - scy1-like 1 (s. cerevisiae)  AHCTF1 - at hook containing transcription factor 1  RAB22A - rab22a, member ras oncogene family  VPS29 - vacuolar protein sorting 29 homolog (s. cerevisiae)  RAB11B - rab11b, member ras oncogene family  RAB23 - rab23, member ras oncogene family  DLG5 - discs, large homolog 5 (drosophila)  ZDHHC20 - zinc finger, dhhc-type containing 20  ANKRD27 - ankyrin repeat domain 27 (vps9 domain)  MECP2 - methyl cpg binding protein 2 (rett syndrome)  MCFD2 - multiple coagulation factor deficiency 2  BUB3 - bub3 mitotic checkpoint protein  PRICKLE1 - prickle homolog 1 (drosophila)  RAB24 - rab24, member ras oncogene family  ZW10 - zw10 kinetochore protein  HEG1 - heart development protein with egf-like domains 1  FAM160A2 - family with sequence similarity 160, member a2  TBC1D10B - tbc1 domain family, member 10b  RAB8A - rab8a, member ras oncogene family  COG6 - component of oligomeric golgi complex 6  GORASP2 - golgi reassembly stacking protein 2, 55kda  XPO5 - exportin 5  C2CD3 - c2 calcium-dependent domain containing 3  SLC7A6OS - solute carrier family 7, member 6 opposite strand  HINFP - histone h4 transcription factor  LRSAM1 - leucine rich repeat and sterile alpha motif containing 1  AP4M1 - adaptor-related protein complex 4, mu 1 subunit  CLIP3 - cap-gly domain containing linker protein 3  ARHGEF2 - rho/rac guanine nucleotide exchange factor (gef) 2  S100A10 - s100 calcium binding protein a10  NECAP1 - necap endocytosis associated 1  CHMP2B - charged multivesicular body protein 2b  TBC1D14 - tbc1 domain family, member 14  STX18 - syntaxin 18  ARL6 - adp-ribosylation factor-like 6  SYNGR1 - synaptogyrin 1  HGS - hepatocyte growth factor-regulated tyrosine kinase substrate  VPS52 - vacuolar protein sorting 52 homolog (s. cerevisiae)  UNC50 - unc-50 homolog (c. elegans)  VTA1 - vesicle (multivesicular body) trafficking 1  RABEP1 - rabaptin, rab gtpase binding effector protein 1  MAL - mal, t-cell differentiation protein  COG3 - component of oligomeric golgi complex 3  MICALL1 - mical-like 1  DRD2 - dopamine receptor d2  NLGN2 - neuroligin 2  NKD2 - naked cuticle homolog 2 (drosophila)  DNAJC14 - dnaj (hsp40) homolog, subfamily c, member 14  RAB6B - rab6b, member ras oncogene family  ARRDC3 - arrestin domain containing 3  SHANK3 - sh3 and multiple ankyrin repeat domains 3  WIPI2 - wd repeat domain, phosphoinositide interacting 2  ATP6V0D1 - atpase, h+ transporting, lysosomal 38kda, v0 subunit d1  RAB14 - rab14, member ras oncogene family  LATS1 - large tumor suppressor kinase 1  TTC7B - tetratricopeptide repeat domain 7b  MAP1A - microtubule-associated protein 1a  RAB33B - rab33b, member ras oncogene family  VPS54 - vacuolar protein sorting 54 homolog (s. cerevisiae)  PPHLN1 - periphilin 1  MAPK15 - mitogen-activated protein kinase 15  UNC119 - unc-119 homolog (c. elegans)  TOR1A - torsin family 1, member a (torsin a)  GGA1 - golgi-associated, gamma adaptin ear containing, arf binding protein 1  DVL2 - dishevelled segment polarity protein 2  TOR1AIP1 - torsin a interacting protein 1  CHMP5 - charged multivesicular body protein 5  APPL1 - adaptor protein, phosphotyrosine interaction, ph domain and leucine zipper containing 1  CDAN1 - codanin 1  MCC - mutated in colorectal cancers  MAP7 - microtubule-associated protein 7  DLG3 - discs, large homolog 3 (drosophila)  DLG4 - discs, large homolog 4 (drosophila)  VPS18 - vacuolar protein sorting 18 homolog (s. cerevisiae)  DLG1 - discs, large homolog 1 (drosophila)  LRP1 - low density lipoprotein receptor-related protein 1  RPL14 - ribosomal protein l14  LRP6 - low density lipoprotein receptor-related protein 6  STXBP4 - syntaxin binding protein 4  AIP - aryl hydrocarbon receptor interacting protein  DHCR24 - 24-dehydrocholesterol reductase  TOM1L2 - target of myb1-like 2 (chicken)  ARL5A - adp-ribosylation factor-like 5a  LYN - v-yes-1 yamaguchi sarcoma viral related oncogene homolog  JAM3 - junctional adhesion molecule 3  CHMP3 - charged multivesicular body protein 3  TMED6 - transmembrane emp24 protein transport domain containing 6  SFPQ - splicing factor proline/glutamine-rich  RAB34 - rab34, member ras oncogene family  ATG16L2 - autophagy related 16-like 2 (s. cerevisiae)  SMAD2 - smad family member 2  DNM2 - dynamin 2  ARMCX3 - armadillo repeat containing, x-linked 3  M6PR - mannose-6-phosphate receptor (cation dependent)  ALS2 - amyotrophic lateral sclerosis 2 (juvenile)  PLOD3 - procollagen-lysine, 2-oxoglutarate 5-dioxygenase 3  ATP6V1H - atpase, h+ transporting, lysosomal 50/57kda, v1 subunit h  HPS4 - hermansky-pudlak syndrome 4  SCG5 - secretogranin v (7b2 protein)  CUTA - cuta divalent cation tolerance homolog (e. coli)  MZT1 - mitotic spindle organizing protein 1  SGTA - small glutamine-rich tetratricopeptide repeat (tpr)-containing, alpha  NBAS - neuroblastoma amplified sequence  RUNDC1 - run domain containing 1  LSR - lipolysis stimulated lipoprotein receptor  ITSN1 - intersectin 1 (sh3 domain protein)  SMAD7 - smad family member 7  WASL - wiskott-aldrich syndrome-like  MAPRE1 - microtubule-associated protein, rp/eb family, member 1  CHMP4C - charged multivesicular body protein 4c  MAPRE3 - microtubule-associated protein, rp/eb family, member 3  TSPAN14 - tetraspanin 14  CTTN - cortactin  SPIRE1 - spire actin nucleation factor 1  SCG3 - secretogranin iii  NASP - nuclear autoantigenic sperm protein (histone-binding)  TM9SF3 - transmembrane 9 superfamily member 3  DNAJC15 - dnaj (hsp40) homolog, subfamily c, member 15  NUBP1 - nucleotide binding protein 1  RAB18 - rab18, member ras oncogene family  TIMM50 - translocase of inner mitochondrial membrane 50 homolog (s. cerevisiae)  RAB3GAP1 - rab3 gtpase activating protein subunit 1 (catalytic)  TUB - tubby bipartite transcription factor  GRIPAP1 - grip1 associated protein 1  YIPF5 - yip1 domain family, member 5  ZDHHC1 - zinc finger, dhhc-type containing 1  EGR2 - early growth response 2  LMAN2L - lectin, mannose-binding 2-like  NACA - nascent polypeptide-associated complex alpha subunit  SPHK2 - sphingosine kinase 2  SAR1A - sar1 homolog a (s. cerevisiae)  ELAVL1 - elav (embryonic lethal, abnormal vision, drosophila)-like 1 (hu antigen r)  TMEM129 - transmembrane protein 129  AZI1 - 5-azacytidine induced 1  STRADA - ste20-related kinase adaptor alpha  NUTF2 - nuclear transport factor 2  MYO1D - myosin id  TSG101 - tumor susceptibility gene 101  TSC2 - tuberous sclerosis 2  EFR3B - efr3 homolog b (s. cerevisiae)  CALCRL - calcitonin receptor-like  MYO5A - myosin va (heavy chain 12, myoxin)  MYO6 - myosin vi  TOPORS - topoisomerase i binding, arginine/serine-rich, e3 ubiquitin protein ligase  SNX27 - sorting nexin family member 27  MVB12A - multivesicular body subunit 12a  RAB1B - rab1b, member ras oncogene family  EIF2D - eukaryotic translation initiation factor 2d  SNX15 - sorting nexin 15  SCAMP2 - secretory carrier membrane protein 2  SCAMP3 - secretory carrier membrane protein 3  RAB21 - rab21, member ras oncogene family  SEH1L - seh1-like (s. cerevisiae)  PTPRU - protein tyrosine phosphatase, receptor type, u  SNX11 - sorting nexin 11  MYH9 - myosin, heavy chain 9, non-muscle  SNUPN - snurportin 1  SNX30 - sorting nexin family member 30  SNX10 - sorting nexin 10  EGFR - epidermal growth factor receptor  DOPEY1 - dopey family member 1  GOLGA7B - golgin a7 family, member b  TSPAN5 - tetraspanin 5  MTX1 - metaxin 1  MFF - mitochondrial fission factor  TNIK - traf2 and nck interacting kinase  FAM126B - family with sequence similarity 126, member b  XPO7 - exportin 7  MON2 - mon2 homolog (s. cerevisiae)  HSP90B1 - heat shock protein 90kda beta (grp94), member 1  CPLX3 - complexin 3  TIMM22 - translocase of inner mitochondrial membrane 22 homolog (yeast)  ARL4A - adp-ribosylation factor-like 4a  SEC61A1 - sec61 alpha 1 subunit (s. cerevisiae)  ARL4C - adp-ribosylation factor-like 4c  BCAP31 - b-cell receptor-associated protein 31  TBC1D9B - tbc1 domain family, member 9b (with gram domain)  TPR - translocated promoter region, nuclear basket protein  SNX12 - sorting nexin 12  WAPAL - wings apart-like homolog (drosophila)  GGA2 - golgi-associated, gamma adaptin ear containing, arf binding protein 2  BLOC1S3 - biogenesis of lysosomal organelles complex-1, subunit 3  ERC1 - elks/rab6-interacting/cast family member 1  ZNF423 - zinc finger protein 423  BOD1 - biorientation of chromosomes in cell division 1  PIGW - phosphatidylinositol glycan anchor biosynthesis, class w  NUP107 - nucleoporin 107kda  GOPC - golgi-associated pdz and coiled-coil motif containing  KIF1B - kinesin family member 1b  TBC1D2B - tbc1 domain family, member 2b  PDCD6 - programmed cell death 6  MTM1 - myotubularin 1  NFASC - neurofascin  PARP11 - poly (adp-ribose) polymerase family, member 11  PDCD6IP - programmed cell death 6 interacting protein  USP4 - ubiquitin specific peptidase 4 (proto-oncogene)  OCLN - occludin  HDAC6 - histone deacetylase 6  CLASP2 - cytoplasmic linker associated protein 2  ETV5 - ets variant 5  VGF - vgf nerve growth factor inducible  TOM1 - target of myb1 (chicken)  EZR - ezrin  EPB41L3 - erythrocyte membrane protein band 4.1-like 3  PIGK - phosphatidylinositol glycan anchor biosynthesis, class k  ESAM - endothelial cell adhesion molecule  CCDC22 - coiled-coil domain containing 22  EXOC8 - exocyst complex component 8  DNM1L - dynamin 1-like  AP5B1 - adaptor-related protein complex 5, beta 1 subunit  DNAJB6 - dnaj (hsp40) homolog, subfamily b, member 6  VCL - vinculin  VCP - valosin containing protein  TBC1D9 - tbc1 domain family, member 9 (with gram domain)  MAPK8IP3 - mitogen-activated protein kinase 8 interacting protein 3  MSH2 - muts homolog 2, colon cancer, nonpolyposis type 1 (e. coli)  MCOLN1 - mucolipin 1  ERCC3 - excision repair cross-complementing rodent repair deficiency, complementation group 3  GGA3 - golgi-associated, gamma adaptin ear containing, arf binding protein 3  CEP68 - centrosomal protein 68kda  SEC16A - sec16 homolog a (s. cerevisiae)  CHMP7 - charged multivesicular body protein 7  UBB - ubiquitin b  CORO1B - coronin, actin binding protein, 1b  ARRDC4 - arrestin domain containing 4  TULP3 - tubby like protein 3  MESDC2 - mesoderm development candidate 2  COMMD1 - copper metabolism (murr1) domain containing 1  SPCS1 - signal peptidase complex subunit 1 homolog (s. cerevisiae)  FAF2 - fas associated factor family member 2  ATG4B - autophagy related 4b, cysteine peptidase  COG7 - component of oligomeric golgi complex 7  SNX24 - sorting nexin 24  STXBP6 - syntaxin binding protein 6 (amisyn)  ARL6IP1 - adp-ribosylation factor-like 6 interacting protein 1  TIMM21 - translocase of inner mitochondrial membrane 21 homolog (yeast)  DMTN - dematin actin binding protein  SCYL3 - scy1-like 3 (s. cerevisiae)  PKDCC - protein kinase domain containing, cytoplasmic  SUMO1 - small ubiquitin-like modifier 1  CHMP4A - charged multivesicular body protein 4a  RRS1 - rrs1 ribosome biogenesis regulator homolog (s. cerevisiae)  XPO6 - exportin 6  SMURF1 - smad specific e3 ubiquitin protein ligase 1  MYADM - myeloid-associated differentiation marker  UBE2D2 - ubiquitin-conjugating enzyme e2d 2  TBC1D1 - tbc1 (tre-2/usp6, bub2, cdc16) domain family, member 1  STX2 - syntaxin 2  NUP153 - nucleoporin 153kda  TBC1D12 - tbc1 domain family, member 12  EXOC6B - exocyst complex component 6b  EXOC7 - exocyst complex component 7  CHAMP1 - chromosome alignment maintaining phosphoprotein 1  DTX3L - deltex 3-like (drosophila)  FNTA - farnesyltransferase, caax box, alpha  TCP1 - t-complex 1  SCFD1 - sec1 family domain containing 1  FCHSD2 - fch and double sh3 domains 2  SEC24D - sec24 family, member d (s. cerevisiae)  TLK1 - tousled-like kinase 1  USE1 - unconventional snare in the er 1 homolog (s. cerevisiae)  TMEM106A - transmembrane protein 106a  PLEKHM1 - pleckstrin homology domain containing, family m (with run domain) member 1  SFT2D1 - sft2 domain containing 1  FLNA - filamin a, alpha  C2CD5 - c2 calcium-dependent domain containing 5  AGRN - agrin  SHANK1 - sh3 and multiple ankyrin repeat domains 1  PACS2 - phosphofurin acidic cluster sorting protein 2  RAB15 - rab15, member ras oncogene family  SPATA7 - spermatogenesis associated 7  TBC1D25 - tbc1 domain family, member 25  KLHL21 - kelch-like family member 21  SCAMP4 - secretory carrier membrane protein 4  DCP1A - decapping mrna 1a  EHBP1 - eh domain binding protein 1  AP5Z1 - adaptor-related protein complex 5, zeta 1 subunit  BICD2 - bicaudal d homolog 2 (drosophila)  SCN3B - sodium channel, voltage-gated, type iii, beta subunit  ZDHHC22 - zinc finger, dhhc-type containing 22  VPS26B - vacuolar protein sorting 26 homolog b (s. pombe)  SGSM2 - small g protein signaling modulator 2  ARHGAP44 - rho gtpase activating protein 44  KIF13B - kinesin family member 13b  ODF2 - outer dense fiber of sperm tails 2  TMED5 - transmembrane emp24 protein transport domain containing 5  FKBP1B - fk506 binding protein 1b, 12.6 kda  TNKS2 - tankyrase, trf1-interacting ankyrin-related adp-ribose polymerase 2  VPS11 - vacuolar protein sorting 11 homolog (s. cerevisiae)  FAM208A - family with sequence similarity 208, member a  TECPR2 - tectonin beta-propeller repeat containing 2  DYNLT1 - dynein, light chain, tctex-type 1  TMED7 - transmembrane emp24 protein transport domain containing 7  STX1B - syntaxin 1b  SNAP91 - synaptosomal-associated protein, 91kda  FIS1 - fission 1 (mitochondrial outer membrane) homolog (s. cerevisiae)  VPS8 - vacuolar protein sorting 8 homolog (s. cerevisiae)  FGF13 - fibroblast growth factor 13  NSF - n-ethylmaleimide-sensitive factor  SPCS2 - signal peptidase complex subunit 2 homolog (s. cerevisiae)  TVP23B - trans-golgi network vesicle protein 23 homolog b (s. cerevisiae)  FGF9 - fibroblast growth factor 9  BCAP29 - b-cell receptor-associated protein 29  TOMM20 - translocase of outer mitochondrial membrane 20 homolog (yeast)  GOLT1B - golgi transport 1b  VPS39 - vacuolar protein sorting 39 homolog (s. cerevisiae)  IST1 - increased sodium tolerance 1 homolog (yeast)  NUMA1 - nuclear mitotic apparatus protein 1  NUP88 - nucleoporin 88kda  NUP98 - nucleoporin 98kda  ATG13 - autophagy related 13  TM9SF4 - transmembrane 9 superfamily protein member 4  SZT2 - seizure threshold 2 homolog (mouse)  TAP2 - transporter 2, atp-binding cassette, sub-family b (mdr/tap)  TBC1D30 - tbc1 domain family, member 30  TMEM167A - transmembrane protein 167a  SNX17 - sorting nexin 17  FAM45A - family with sequence similarity 45, member a  TBC1D5 - tbc1 domain family, member 5  DNAJC13 - dnaj (hsp40) homolog, subfamily c, member 13  NMD3 - nmd3 ribosome export adaptor  NPM1 - nucleophosmin (nucleolar phosphoprotein b23, numatrin)  ZDHHC17 - zinc finger, dhhc-type containing 17  NUDCD3 - nudc domain containing 3  SCC4 - sister chromatid cohesion 4  DUSP16 - dual specificity phosphatase 16  OBSL1 - obscurin-like 1  RCC2 - regulator of chromosome condensation 2  PAQR3 - progestin and adipoq receptor family member iii  GRIP2 - glutamate receptor interacting protein 2  NUDT7 - nudix (nucleoside diphosphate linked moiety x)-type motif 7  ERBB2IP - erbb2 interacting protein  NRCAM - neuronal cell adhesion molecule  RAB11FIP3 - rab11 family interacting protein 3 (class ii)  TMED3 - transmembrane emp24 protein transport domain containing 3  TJP1 - tight junction protein 1  TNPO2 - transportin 2  ATP13A2 - atpase type 13a2  HERPUD1 - homocysteine-inducible, endoplasmic reticulum stress-inducible, ubiquitin-like domain member 1  USP6NL - usp6 n-terminal like  SEC62 - sec62 homolog (s. cerevisiae)  CEP290 - centrosomal protein 290kda  RIMS2 - regulating synaptic membrane exocytosis 2  PACSIN1 - protein kinase c and casein kinase substrate in neurons 1  ABCB9 - atp-binding cassette, sub-family b (mdr/tap), member 9  CADPS2 - ca++-dependent secretion activator 2  ICMT - isoprenylcysteine carboxyl methyltransferase  CENPT - centromere protein t  ZFYVE16 - zinc finger, fyve domain containing 16  TRAM1 - translocation associated membrane protein 1  NFKBIA - nuclear factor of kappa light polypeptide gene enhancer in b-cells inhibitor, alpha  PIK3R4 - phosphoinositide-3-kinase, regulatory subunit 4  IFT140 - intraflagellar transport 140 homolog (chlamydomonas)  TNFRSF1A - tumor necrosis factor receptor superfamily, member 1a  TBCK - tbc1 domain containing kinase  EHD2 - eh-domain containing 2  EHD3 - eh-domain containing 3  EHD4 - eh-domain containing 4  KCNIP4 - kv channel interacting protein 4  NUP188 - nucleoporin 188kda  EIF4ENIF1 - eukaryotic translation initiation factor 4e nuclear import factor 1  MRAP2 - melanocortin 2 receptor accessory protein 2  GCC2 - grip and coiled-coil domain containing 2  NEDD8 - neural precursor cell expressed, developmentally down-regulated 8  TERF2 - telomeric repeat binding factor 2  WRAP73 - wd repeat containing, antisense to tp73  SNX18 - sorting nexin 18  PES1 - pescadillo ribosomal biogenesis factor 1  SEC61G - sec61 gamma subunit  NUP155 - nucleoporin 155kda  SEC24C - sec24 family, member c (s. cerevisiae)  CEP41 - centrosomal protein 41kda  HEY2 - hairy/enhancer-of-split related with yrpw motif 2  MACF1 - microtubule-actin crosslinking factor 1  FAM91A1 - family with sequence similarity 91, member a1  CROCC - ciliary rootlet coiled-coil, rootletin  VPS37D - vacuolar protein sorting 37 homolog d (s. cerevisiae)  ATP6V0A2 - atpase, h+ transporting, lysosomal v0 subunit a2  RAPGEF2 - rap guanine nucleotide exchange factor (gef) 2  TGFB1 - transforming growth factor, beta 1  C16orf70 - chromosome 16 open reading frame 70  TSPAN15 - tetraspanin 15  FAM21A - family with sequence similarity 21, member a  NUP93 - nucleoporin 93kda  ATP6V0E2 - atpase, h+ transporting v0 subunit e2  WDR45B - wd repeat domain 45b  SYTL4 - synaptotagmin-like 4  TIA1 - tia1 cytotoxic granule-associated rna binding protein  THY1 - thy-1 cell surface antigen  TNPO3 - transportin 3 |
| GO:0032392 | DNA geometric change | 7.89E-6 | 1.01E-3 | 1.31 (10334,81,6996,72) | [+] Show genes  DHX9 - deah (asp-glu-ala-his) box helicase 9  CHD9 - chromodomain helicase dna binding protein 9  SMARCAD1 - swi/snf-related, matrix-associated actin-dependent regulator of chromatin, subfamily a, containing dead/h box 1  DHX36 - deah (asp-glu-ala-his) box polypeptide 36  PURA - purine-rich element binding protein a  CHD6 - chromodomain helicase dna binding protein 6  RFC5 - replication factor c (activator 1) 5, 36.5kda  RFC2 - replication factor c (activator 1) 2, 40kda  RFC3 - replication factor c (activator 1) 3, 38kda  G3BP1 - gtpase activating protein (sh3 domain) binding protein 1  DDX3X - dead (asp-glu-ala-asp) box helicase 3, x-linked  DDX1 - dead (asp-glu-ala-asp) box helicase 1  XPA - xeroderma pigmentosum, complementation group a  XPC - xeroderma pigmentosum, complementation group c  TREX1 - three prime repair exonuclease 1  HMGB3 - high mobility group box 3  HMGB2 - high mobility group box 2  FBXO18 - f-box protein, helicase, 18  HMGB1 - high mobility group box 1  DDB1 - damage-specific dna binding protein 1, 127kda  GTF2F2 - general transcription factor iif, polypeptide 2, 30kda  SUPV3L1 - suppressor of var1, 3-like 1 (s. cerevisiae)  WRNIP1 - werner helicase interacting protein 1  GTF2H2 - general transcription factor iih, polypeptide 2, 44kda  RECQL - recq protein-like (dna helicase q1-like)  GTF2H3 - general transcription factor iih, polypeptide 3, 34kda  MCM9 - minichromosome maintenance complex component 9  GTF2H1 - general transcription factor iih, polypeptide 1, 62kda  NBN - nibrin  ASCC3 - activating signal cointegrator 1 complex subunit 3  RPA1 - replication protein a1, 70kda  POT1 - protection of telomeres 1  CHD4 - chromodomain helicase dna binding protein 4  CHD2 - chromodomain helicase dna binding protein 2  RECQL5 - recq protein-like 5  CHD3 - chromodomain helicase dna binding protein 3  RAD54L2 - rad54-like 2 (s. cerevisiae)  CHD1 - chromodomain helicase dna binding protein 1  XRCC6 - x-ray repair complementing defective repair in chinese hamster cells 6  GTF2H4 - general transcription factor iih, polypeptide 4, 52kda  CHD7 - chromodomain helicase dna binding protein 7  CHD1L - chromodomain helicase dna binding protein 1-like  MCM3 - minichromosome maintenance complex component 3  MCM6 - minichromosome maintenance complex component 6  MCM5 - minichromosome maintenance complex component 5  MCM2 - minichromosome maintenance complex component 2  DSCC1 - dna replication and sister chromatid cohesion 1  IGHMBP2 - immunoglobulin mu binding protein 2  MCM7 - minichromosome maintenance complex component 7  ERCC3 - excision repair cross-complementing rodent repair deficiency, complementation group 3  SMARCAL1 - swi/snf related, matrix associated, actin dependent regulator of chromatin, subfamily a-like 1  CETN2 - centrin, ef-hand protein, 2  UBB - ubiquitin b  CHD5 - chromodomain helicase dna binding protein 5  ATRX - alpha thalassemia/mental retardation syndrome x-linked  HNRNPA2B1 - heterogeneous nuclear ribonucleoprotein a2/b1  SMARCA1 - swi/snf related, matrix associated, actin dependent regulator of chromatin, subfamily a, member 1  RAD50 - rad50 homolog (s. cerevisiae)  SSBP1 - single-stranded dna binding protein 1, mitochondrial  CHD8 - chromodomain helicase dna binding protein 8  ERCC8 - excision repair cross-complementing rodent repair deficiency, complementation group 8  PARP1 - poly (adp-ribose) polymerase 1  CUL4B - cullin 4b  RUVBL1 - ruvb-like 1 (e. coli)  RBX1 - ring-box 1, e3 ubiquitin protein ligase  XRCC5 - x-ray repair complementing defective repair in chinese hamster cells 5 (double-strand-break rejoining)  HMGA1 - high mobility group at-hook 1  MRE11A - mre11 meiotic recombination 11 homolog a (s. cerevisiae)  SETX - senataxin  ERCC2 - excision repair cross-complementing rodent repair deficiency, complementation group 2  CHTF8 - ctf8, chromosome transmission fidelity factor 8 homolog (s. cerevisiae)  RUVBL2 - ruvb-like 2 (e. coli) |
| GO:0016073 | snRNA metabolic process | 7.89E-6 | 9.98E-4 | 1.31 (10334,81,6996,72) | [+] Show genes  CCNK - cyclin k  ELL - elongation factor rna polymerase ii  NCBP2 - nuclear cap binding protein subunit 2, 20kda  INTS9 - integrator complex subunit 9  EXOSC9 - exosome component 9  NCBP1 - nuclear cap binding protein subunit 1, 80kda  EXOSC10 - exosome component 10  ZCCHC8 - zinc finger, cchc domain containing 8  INTS12 - integrator complex subunit 12  INTS5 - integrator complex subunit 5  GTF2F1 - general transcription factor iif, polypeptide 1, 74kda  GTF2F2 - general transcription factor iif, polypeptide 2, 30kda  ELL2 - elongation factor, rna polymerase ii, 2  GTF2E1 - general transcription factor iie, polypeptide 1, alpha 56kda  SNAPC5 - small nuclear rna activating complex, polypeptide 5, 19kda  GTF2E2 - general transcription factor iie, polypeptide 2, beta 34kda  RPRD2 - regulation of nuclear pre-mrna domain containing 2  INTS10 - integrator complex subunit 10  EXOSC2 - exosome component 2  INTS6 - integrator complex subunit 6  GTF2A2 - general transcription factor iia, 2, 12kda  GTF2B - general transcription factor iib  GTF2A1 - general transcription factor iia, 1, 19/37kda  PHAX - phosphorylated adaptor for rna export  USB1 - u6 snrna biogenesis 1  METTL16 - methyltransferase like 16  SP1 - sp1 transcription factor  RBM7 - rna binding motif protein 7  INTS4 - integrator complex subunit 4  RPRD1A - regulation of nuclear pre-mrna domain containing 1a  EXOSC3 - exosome component 3  ZC3H8 - zinc finger ccch-type containing 8  INTS3 - integrator complex subunit 3  RPAP2 - rna polymerase ii associated protein 2  USPL1 - ubiquitin specific peptidase like 1  TAF9 - taf9 rna polymerase ii, tata box binding protein (tbp)-associated factor, 32kda  RPRD1B - regulation of nuclear pre-mrna domain containing 1b  DKC1 - dyskeratosis congenita 1, dyskerin  EXOSC7 - exosome component 7  CCNT2 - cyclin t2  TAF6 - taf6 rna polymerase ii, tata box binding protein (tbp)-associated factor, 80kda  SNAPC4 - small nuclear rna activating complex, polypeptide 4, 190kda  CCNT1 - cyclin t1  SKIV2L2 - superkiller viralicidic activity 2-like 2 (s. cerevisiae)  NABP2 - nucleic acid binding protein 2  SNAPC2 - small nuclear rna activating complex, polypeptide 2, 45kda  SNAPC1 - small nuclear rna activating complex, polypeptide 1, 43kda  SNAPC3 - small nuclear rna activating complex, polypeptide 3, 50kda  INTS8 - integrator complex subunit 8  TAF8 - taf8 rna polymerase ii, tata box binding protein (tbp)-associated factor, 43kda  TAF13 - taf13 rna polymerase ii, tata box binding protein (tbp)-associated factor, 18kda  EXOSC8 - exosome component 8  INTS2 - integrator complex subunit 2  POLR2K - polymerase (rna) ii (dna directed) polypeptide k, 7.0kda  CDK9 - cyclin-dependent kinase 9  SRRT - serrate rna effector molecule homolog (arabidopsis)  CDK7 - cyclin-dependent kinase 7  MEPCE - methylphosphate capping enzyme  LARP7 - la ribonucleoprotein domain family, member 7  EXOSC4 - exosome component 4  NOP10 - nop10 ribonucleoprotein  TUT1 - terminal uridylyl transferase 1, u6 snrna-specific  POLR2G - polymerase (rna) ii (dna directed) polypeptide g  POLR2F - polymerase (rna) ii (dna directed) polypeptide f  POLR2E - polymerase (rna) ii (dna directed) polypeptide e, 25kda  NABP1 - nucleic acid binding protein 1  TOE1 - target of egr1, member 1 (nuclear)  POLR2D - polymerase (rna) ii (dna directed) polypeptide d  POLR2C - polymerase (rna) ii (dna directed) polypeptide c, 33kda  INTS1 - integrator complex subunit 1  POLR2B - polymerase (rna) ii (dna directed) polypeptide b, 140kda  POLR2A - polymerase (rna) ii (dna directed) polypeptide a, 220kda |
| GO:0032508 | DNA duplex unwinding | 1.1E-5 | 1.38E-3 | 1.32 (10334,75,6996,67) | [+] Show genes  CHD9 - chromodomain helicase dna binding protein 9  SMARCAD1 - swi/snf-related, matrix-associated actin-dependent regulator of chromatin, subfamily a, containing dead/h box 1  DHX9 - deah (asp-glu-ala-his) box helicase 9  PURA - purine-rich element binding protein a  DHX36 - deah (asp-glu-ala-his) box polypeptide 36  CHD6 - chromodomain helicase dna binding protein 6  RFC5 - replication factor c (activator 1) 5, 36.5kda  RFC2 - replication factor c (activator 1) 2, 40kda  G3BP1 - gtpase activating protein (sh3 domain) binding protein 1  DDX3X - dead (asp-glu-ala-asp) box helicase 3, x-linked  RFC3 - replication factor c (activator 1) 3, 38kda  DDX1 - dead (asp-glu-ala-asp) box helicase 1  XPA - xeroderma pigmentosum, complementation group a  XPC - xeroderma pigmentosum, complementation group c  TREX1 - three prime repair exonuclease 1  FBXO18 - f-box protein, helicase, 18  SUPV3L1 - suppressor of var1, 3-like 1 (s. cerevisiae)  GTF2F2 - general transcription factor iif, polypeptide 2, 30kda  DDB1 - damage-specific dna binding protein 1, 127kda  WRNIP1 - werner helicase interacting protein 1  GTF2H2 - general transcription factor iih, polypeptide 2, 44kda  GTF2H3 - general transcription factor iih, polypeptide 3, 34kda  RECQL - recq protein-like (dna helicase q1-like)  MCM9 - minichromosome maintenance complex component 9  GTF2H1 - general transcription factor iih, polypeptide 1, 62kda  NBN - nibrin  ASCC3 - activating signal cointegrator 1 complex subunit 3  RPA1 - replication protein a1, 70kda  POT1 - protection of telomeres 1  CHD4 - chromodomain helicase dna binding protein 4  CHD2 - chromodomain helicase dna binding protein 2  RECQL5 - recq protein-like 5  CHD3 - chromodomain helicase dna binding protein 3  RAD54L2 - rad54-like 2 (s. cerevisiae)  CHD1 - chromodomain helicase dna binding protein 1  XRCC6 - x-ray repair complementing defective repair in chinese hamster cells 6  GTF2H4 - general transcription factor iih, polypeptide 4, 52kda  CHD7 - chromodomain helicase dna binding protein 7  CHD1L - chromodomain helicase dna binding protein 1-like  MCM3 - minichromosome maintenance complex component 3  MCM6 - minichromosome maintenance complex component 6  MCM5 - minichromosome maintenance complex component 5  MCM2 - minichromosome maintenance complex component 2  DSCC1 - dna replication and sister chromatid cohesion 1  IGHMBP2 - immunoglobulin mu binding protein 2  MCM7 - minichromosome maintenance complex component 7  ERCC3 - excision repair cross-complementing rodent repair deficiency, complementation group 3  CETN2 - centrin, ef-hand protein, 2  SMARCAL1 - swi/snf related, matrix associated, actin dependent regulator of chromatin, subfamily a-like 1  CHD5 - chromodomain helicase dna binding protein 5  UBB - ubiquitin b  ATRX - alpha thalassemia/mental retardation syndrome x-linked  RAD50 - rad50 homolog (s. cerevisiae)  SSBP1 - single-stranded dna binding protein 1, mitochondrial  CHD8 - chromodomain helicase dna binding protein 8  CUL4B - cullin 4b  PARP1 - poly (adp-ribose) polymerase 1  ERCC8 - excision repair cross-complementing rodent repair deficiency, complementation group 8  RUVBL1 - ruvb-like 1 (e. coli)  RBX1 - ring-box 1, e3 ubiquitin protein ligase  HMGA1 - high mobility group at-hook 1  XRCC5 - x-ray repair complementing defective repair in chinese hamster cells 5 (double-strand-break rejoining)  MRE11A - mre11 meiotic recombination 11 homolog a (s. cerevisiae)  SETX - senataxin  ERCC2 - excision repair cross-complementing rodent repair deficiency, complementation group 2  CHTF8 - ctf8, chromosome transmission fidelity factor 8 homolog (s. cerevisiae)  RUVBL2 - ruvb-like 2 (e. coli) |
| GO:0043967 | histone H4 acetylation | 1.1E-5 | 1.37E-3 | 1.39 (10334,49,6996,46) | [+] Show genes  KANSL1L - kat8 regulatory nsl complex subunit 1-like  KANSL3 - kat8 regulatory nsl complex subunit 3  ACTL6A - actin-like 6a  HAT1 - histone acetyltransferase 1  MSL3 - male-specific lethal 3 homolog (drosophila)  TRRAP - transformation/transcription domain-associated protein  MCRS1 - microspherule protein 1  CHD5 - chromodomain helicase dna binding protein 5  PHF15 - phd finger protein 15  EPC1 - enhancer of polycomb homolog 1 (drosophila)  MORF4L2 - mortality factor 4 like 2  NAA40 - n(alpha)-acetyltransferase 40, natd catalytic subunit, homolog (s. cerevisiae)  MSL2 - male-specific lethal 2 homolog (drosophila)  EP400 - e1a binding protein p400  USP22 - ubiquitin specific peptidase 22  MEAF6 - myst/esa1-associated factor 6  EP300 - e1a binding protein p300  ING5 - inhibitor of growth family, member 5  ING4 - inhibitor of growth family, member 4  NAA60 - n(alpha)-acetyltransferase 60, natf catalytic subunit  KAT5 - k(lysine) acetyltransferase 5  YEATS4 - yeats domain containing 4  KANSL1 - kat8 regulatory nsl complex subunit 1  ING3 - inhibitor of growth family, member 3  KAT2A - k(lysine) acetyltransferase 2a  ACTL6B - actin-like 6b  PHF20 - phd finger protein 20  APBB1 - amyloid beta (a4) precursor protein-binding, family b, member 1 (fe65)  KMT2A - lysine (k)-specific methyltransferase 2a  HCFC1 - host cell factor c1 (vp16-accessory protein)  BRD8 - bromodomain containing 8  ZZZ3 - zinc finger, zz-type containing 3  OGT - o-linked n-acetylglucosamine (glcnac) transferase  KANSL2 - kat8 regulatory nsl complex subunit 2  KAT6A - k(lysine) acetyltransferase 6a  KAT8 - k(lysine) acetyltransferase 8  TADA3 - transcriptional adaptor 3  RUVBL1 - ruvb-like 1 (e. coli)  PHF17 - phd finger protein 17  DMAP1 - dna methyltransferase 1 associated protein 1  KAT6B - k(lysine) acetyltransferase 6b  LEF1 - lymphoid enhancer-binding factor 1  MORF4L1 - mortality factor 4 like 1  MSL1 - male-specific lethal 1 homolog (drosophila)  KAT7 - k(lysine) acetyltransferase 7  RUVBL2 - ruvb-like 2 (e. coli) |
| GO:0097479 | synaptic vesicle localization | 1.1E-5 | 1.36E-3 | 1.39 (10334,49,6996,46) | [+] Show genes  SYN1 - synapsin i  SPG11 - spastic paraplegia 11 (autosomal recessive)  SYN2 - synapsin ii  DNAJC5 - dnaj (hsp40) homolog, subfamily c, member 5  AP3M2 - adaptor-related protein complex 3, mu 2 subunit  SNCA - synuclein, alpha (non a4 component of amyloid precursor)  BLOC1S6 - biogenesis of lysosomal organelles complex-1, subunit 6, pallidin  KIF5C - kinesin family member 5c  AP3M1 - adaptor-related protein complex 3, mu 1 subunit  SYT1 - synaptotagmin i  KIF5B - kinesin family member 5b  SYT17 - synaptotagmin xvii  SYT5 - synaptotagmin v  KIF5A - kinesin family member 5a  BLOC1S2 - biogenesis of lysosomal organelles complex-1, subunit 2  SNAP25 - synaptosomal-associated protein, 25kda  LIN7A - lin-7 homolog a (c. elegans)  BLOC1S4 - biogenesis of lysosomal organelles complex-1, subunit 4, cappuccino  AP3S2 - adaptor-related protein complex 3, sigma 2 subunit  RIMS2 - regulating synaptic membrane exocytosis 2  RIMS3 - regulating synaptic membrane exocytosis 3  VAMP2 - vesicle-associated membrane protein 2 (synaptobrevin 2)  CTNNB1 - catenin (cadherin-associated protein), beta 1, 88kda  PIP5K1C - phosphatidylinositol-4-phosphate 5-kinase, type i, gamma  CDK5 - cyclin-dependent kinase 5  BLOC1S5 - biogenesis of lysosomal organelles complex-1, subunit 5, muted  STX1A - syntaxin 1a (brain)  RAB3A - rab3a, member ras oncogene family  AP3B2 - adaptor-related protein complex 3, beta 2 subunit  DNM2 - dynamin 2  TOR1A - torsin family 1, member a (torsin a)  AP3B1 - adaptor-related protein complex 3, beta 1 subunit  CADPS - ca++-dependent secretion activator  CPLX2 - complexin 2  SYT3 - synaptotagmin iii  DOC2B - double c2-like domains, beta  TRIM9 - tripartite motif containing 9  SYNJ1 - synaptojanin 1  SYT9 - synaptotagmin ix  CPLX3 - complexin 3  AP3D1 - adaptor-related protein complex 3, delta 1 subunit  TMEM230 - transmembrane protein 230  STX1B - syntaxin 1b  RAB27A - rab27a, member ras oncogene family  BLOC1S3 - biogenesis of lysosomal organelles complex-1, subunit 3  SYT7 - synaptotagmin vii |
| GO:0051054 | positive regulation of DNA metabolic process | 1.12E-5 | 1.37E-3 | 1.22 (10334,170,6996,140) | [+] Show genes  DHX9 - deah (asp-glu-ala-his) box helicase 9  DHX36 - deah (asp-glu-ala-his) box polypeptide 36  RFC5 - replication factor c (activator 1) 5, 36.5kda  RFC2 - replication factor c (activator 1) 2, 40kda  RFC3 - replication factor c (activator 1) 3, 38kda  DTX3L - deltex 3-like (drosophila)  PARN - poly(a)-specific ribonuclease  TCP1 - t-complex 1  SPIRE1 - spire actin nucleation factor 1  SETDB1 - set domain, bifurcated 1  NAF1 - nuclear assembly factor 1 ribonucleoprotein  SETMAR - set domain and mariner transposase fusion gene  MLH1 - mutl homolog 1, colon cancer, nonpolyposis type 2 (e. coli)  ZNF304 - zinc finger protein 304  MAP2K4 - mitogen-activated protein kinase kinase 4  TRIM28 - tripartite motif containing 28  PML - promyelocytic leukemia  OBFC1 - oligonucleotide/oligosaccharide-binding fold containing 1  NBN - nibrin  APBB1 - amyloid beta (a4) precursor protein-binding, family b, member 1 (fe65)  POT1 - protection of telomeres 1  RIF1 - rap1 interacting factor homolog (yeast)  MAPKAPK5 - mitogen-activated protein kinase-activated protein kinase 5  POLG2 - polymerase (dna directed), gamma 2, accessory subunit  GTF2I - general transcription factor iii  CCT5 - chaperonin containing tcp1, subunit 5 (epsilon)  SLX4 - slx4 structure-specific endonuclease subunit  KIAA1551 - kiaa1551  UIMC1 - ubiquitin interaction motif containing 1  SPIRE2 - spire actin nucleation factor 2  KCTD13 - potassium channel tetramerization domain containing 13  TNKS2 - tankyrase, trf1-interacting ankyrin-related adp-ribose polymerase 2  EXOSC3 - exosome component 3  DSCC1 - dna replication and sister chromatid cohesion 1  FAM208A - family with sequence similarity 208, member a  PAK3 - p21 protein (cdc42/rac)-activated kinase 3  H2AFX - h2a histone family, member x  FGFR1 - fibroblast growth factor receptor 1  SMCHD1 - structural maintenance of chromosomes flexible hinge domain containing 1  PDGFRB - platelet-derived growth factor receptor, beta polypeptide  ARRB2 - arrestin, beta 2  TP53BP1 - tumor protein p53 binding protein 1  PDGFB - platelet-derived growth factor beta polypeptide  MECP2 - methyl cpg binding protein 2 (rett syndrome)  FGF2 - fibroblast growth factor 2 (basic)  EGFR - epidermal growth factor receptor  NABP2 - nucleic acid binding protein 2  ACTR2 - arp2 actin-related protein 2 homolog (yeast)  MAP3K4 - mitogen-activated protein kinase kinase kinase 4  NVL - nuclear vcp-like  CDC7 - cell division cycle 7  CEBPG - ccaat/enhancer binding protein (c/ebp), gamma  PCNA - proliferating cell nuclear antigen  USP9X - ubiquitin specific peptidase 9, x-linked  CCT7 - chaperonin containing tcp1, subunit 7 (eta)  CCT3 - chaperonin containing tcp1, subunit 3 (gamma)  WIZ - widely interspaced zinc finger motifs  CCT2 - chaperonin containing tcp1, subunit 2 (beta)  CCT4 - chaperonin containing tcp1, subunit 4 (delta)  RAD50 - rad50 homolog (s. cerevisiae)  PARP1 - poly (adp-ribose) polymerase 1  HSP90AA1 - heat shock protein 90kda alpha (cytosolic), class a member 1  WRAP53 - wd repeat containing, antisense to tp53  HMBOX1 - homeobox containing 1  CDK2 - cyclin-dependent kinase 2  KAT7 - k(lysine) acetyltransferase 7  CACYBP - calcyclin binding protein  CST3 - cystatin c  NUDT16 - nudix (nucleoside diphosphate linked moiety x)-type motif 16  HMGB1 - high mobility group box 1  XRCC1 - x-ray repair complementing defective repair in chinese hamster cells 1  FUS - fused in sarcoma  MORC2 - morc family cw-type zinc finger 2  MMS19 - mms19 nucleotide excision repair homolog (s. cerevisiae)  PTK2B - protein tyrosine kinase 2 beta  MPHOSPH8 - m-phase phosphoprotein 8  TNKS - tankyrase, trf1-interacting ankyrin-related adp-ribose polymerase  SIRT6 - sirtuin 6  ATF7IP - activating transcription factor 7 interacting protein  SIRT1 - sirtuin 1  MAD2L2 - mad2 mitotic arrest deficient-like 2 (yeast)  CTC1 - cts telomere maintenance complex component 1  PPHLN1 - periphilin 1  CTNNB1 - catenin (cadherin-associated protein), beta 1, 88kda  BRPF3 - bromodomain and phd finger containing, 3  MAPK15 - mitogen-activated protein kinase 15  TOX - thymocyte selection-associated high mobility group box  AKT1 - v-akt murine thymoma viral oncogene homolog 1  USP7 - ubiquitin specific peptidase 7 (herpes virus-associated)  RNF168 - ring finger protein 168, e3 ubiquitin protein ligase  WHSC1 - wolf-hirschhorn syndrome candidate 1  ACD - adrenocortical dysplasia homolog (mouse)  TNFAIP1 - tumor necrosis factor, alpha-induced protein 1 (endothelial)  SUV420H1 - suppressor of variegation 4-20 homolog 1 (drosophila)  EYA3 - eyes absent homolog 3 (drosophila)  PPP1R10 - protein phosphatase 1, regulatory subunit 10  ARID2 - at rich interactive domain 2 (arid, rfx-like)  RAC1 - ras-related c3 botulinum toxin substrate 1 (rho family, small gtp binding protein rac1)  SUV420H2 - suppressor of variegation 4-20 homolog 2 (drosophila)  INO80 - ino80 complex subunit  DDX39B - dead (asp-glu-ala-asp) box polypeptide 39b  BAX - bcl2-associated x protein  CCT6A - chaperonin containing tcp1, subunit 6a (zeta 1)  MSH2 - muts homolog 2, colon cancer, nonpolyposis type 1 (e. coli)  DKC1 - dyskeratosis congenita 1, dyskerin  CCT8 - chaperonin containing tcp1, subunit 8 (theta)  FAM35A - family with sequence similarity 35, member a  BRCC3 - brca1/brca2-containing complex, subunit 3  STAT6 - signal transducer and activator of transcription 6, interleukin-4 induced  PARM1 - prostate androgen-regulated mucin-like protein 1  RGCC - regulator of cell cycle  TNFSF13 - tumor necrosis factor (ligand) superfamily, member 13  FBXO4 - f-box protein 4  PARP9 - poly (adp-ribose) polymerase family, member 9  BCAR3 - breast cancer anti-estrogen resistance 3  RNF8 - ring finger protein 8, e3 ubiquitin protein ligase  ATRX - alpha thalassemia/mental retardation syndrome x-linked  MAP2K7 - mitogen-activated protein kinase kinase 7  BABAM1 - brisc and brca1 a complex member 1  HNRNPA2B1 - heterogeneous nuclear ribonucleoprotein a2/b1  FAM168A - family with sequence similarity 168, member a  TGFB1 - transforming growth factor, beta 1  MAPK3 - mitogen-activated protein kinase 3  HNRNPA1 - heterogeneous nuclear ribonucleoprotein a1  MAPK1 - mitogen-activated protein kinase 1  ZNF711 - zinc finger protein 711  DNMT1 - dna (cytosine-5-)-methyltransferase 1  HNRNPD - heterogeneous nuclear ribonucleoprotein d (au-rich element rna binding protein 1, 37kda)  ERCC8 - excision repair cross-complementing rodent repair deficiency, complementation group 8  ANXA3 - annexin a3  PTGES3 - prostaglandin e synthase 3 (cytosolic)  XRCC5 - x-ray repair complementing defective repair in chinese hamster cells 5 (double-strand-break rejoining)  SRC - v-src avian sarcoma (schmidt-ruppin a-2) viral oncogene homolog  MRE11A - mre11 meiotic recombination 11 homolog a (s. cerevisiae)  PRKCD - protein kinase c, delta  ERCC2 - excision repair cross-complementing rodent repair deficiency, complementation group 2  PNKP - polynucleotide kinase 3'-phosphatase  PKIB - protein kinase (camp-dependent, catalytic) inhibitor beta  CHTF8 - ctf8, chromosome transmission fidelity factor 8 homolog (s. cerevisiae)  UBE2N - ubiquitin-conjugating enzyme e2n |
| GO:0043631 | RNA polyadenylation | 1.2E-5 | 1.45E-3 | 1.48 (10334,29,6996,29) | [+] Show genes  SCAF8 - sr-related ctd-associated factor 8  MTPAP - mitochondrial poly(a) polymerase  PAPOLG - poly(a) polymerase gamma  PNPT1 - polyribonucleotide nucleotidyltransferase 1  SYMPK - symplekin  PAPD5 - pap associated domain containing 5  PAPD4 - pap associated domain containing 4  PABPC1 - poly(a) binding protein, cytoplasmic 1  CSTF3 - cleavage stimulation factor, 3' pre-rna, subunit 3, 77kda  ZCCHC6 - zinc finger, cchc domain containing 6  CSTF2 - cleavage stimulation factor, 3' pre-rna, subunit 2, 64kda  CDC73 - cell division cycle 73  AHCYL1 - adenosylhomocysteinase-like 1  PAPD7 - pap associated domain containing 7  CPSF1 - cleavage and polyadenylation specific factor 1, 160kda  CPSF2 - cleavage and polyadenylation specific factor 2, 100kda  ZCCHC11 - zinc finger, cchc domain containing 11  CPSF3 - cleavage and polyadenylation specific factor 3, 73kda  PCF11 - pcf11 cleavage and polyadenylation factor subunit  NUDT21 - nudix (nucleoside diphosphate linked moiety x)-type motif 21  LEO1 - leo1, paf1/rna polymerase ii complex component, homolog (s. cerevisiae)  CLP1 - cleavage and polyadenylation factor i subunit 1  PAPOLA - poly(a) polymerase alpha  WDR33 - wd repeat domain 33  TUT1 - terminal uridylyl transferase 1, u6 snrna-specific  PAF1 - paf1, rna polymerase ii associated factor, homolog (s. cerevisiae)  APP - amyloid beta (a4) precursor protein  GRSF1 - g-rich rna sequence binding factor 1  FIP1L1 - factor interacting with papola and cpsf1 |
| GO:0080090 | regulation of primary metabolic process | 1.26E-5 | 1.51E-3 | 1.04 (10334,3716,6996,2612) | [+] Show genes  AGAP2 - arfgap with gtpase domain, ankyrin repeat and ph domain 2  EXOSC9 - exosome component 9  MSL3 - male-specific lethal 3 homolog (drosophila)  EXOSC10 - exosome component 10  TADA1 - transcriptional adaptor 1  HNRNPA0 - heterogeneous nuclear ribonucleoprotein a0  AARS - alanyl-trna synthetase  CBX1 - chromobox homolog 1  ABCA1 - atp-binding cassette, sub-family a (abc1), member 1  PPP1R9A - protein phosphatase 1, regulatory subunit 9a  ABCA2 - atp-binding cassette, sub-family a (abc1), member 2  OS9 - osteosarcoma amplified 9, endoplasmic reticulum lectin  ABL1 - c-abl oncogene 1, non-receptor tyrosine kinase  ZNF821 - zinc finger protein 821  ERP29 - endoplasmic reticulum protein 29  PML - promyelocytic leukemia  MLLT11 - myeloid/lymphoid or mixed-lineage leukemia (trithorax homolog, drosophila); translocated to, 11  CNOT11 - ccr4-not transcription complex, subunit 11  ACACA - acetyl-coa carboxylase alpha  SUPT20H - suppressor of ty 20 homolog (s. cerevisiae)  YWHAQ - tyrosine 3-monooxygenase/tryptophan 5-monooxygenase activation protein, theta polypeptide  TMED10 - transmembrane emp24-like trafficking protein 10 (yeast)  BRD8 - bromodomain containing 8  ACOX1 - acyl-coa oxidase 1, palmitoyl  RFFL - ring finger and fyve-like domain containing e3 ubiquitin protein ligase  CHD7 - chromodomain helicase dna binding protein 7  LYAR - ly1 antibody reactive  PAPOLA - poly(a) polymerase alpha  ZNF354B - zinc finger protein 354b  TCERG1 - transcription elongation regulator 1  ACTB - actin, beta  RNPS1 - rna binding protein s1, serine-rich domain  OTUB1 - otu domain, ubiquitin aldehyde binding 1  EHMT2 - euchromatic histone-lysine n-methyltransferase 2  PLAUR - plasminogen activator, urokinase receptor  SPIN1 - spindlin 1  TASP1 - taspase, threonine aspartase, 1  PRR5 - proline rich 5 (renal)  PLCL1 - phospholipase c-like 1  SUB1 - sub1 homolog (s. cerevisiae)  MORF4L1 - mortality factor 4 like 1  AMBRA1 - autophagy/beclin-1 regulator 1  ZNF511 - zinc finger protein 511  CAMK2N1 - calcium/calmodulin-dependent protein kinase ii inhibitor 1  PPID - peptidylprolyl isomerase d  ACTL6A - actin-like 6a  ACTN1 - actinin, alpha 1  ACTN4 - actinin, alpha 4  USP19 - ubiquitin specific peptidase 19  ACVR2A - activin a receptor, type iia  ACVRL1 - activin a receptor type ii-like 1  PARL - presenilin associated, rhomboid-like  FAM122A - family with sequence similarity 122a  ACVR1 - activin a receptor, type i  ACVR1B - activin a receptor, type ib  ADAR - adenosine deaminase, rna-specific  ADARB1 - adenosine deaminase, rna-specific, b1  CPSF4 - cleavage and polyadenylation specific factor 4, 30kda  ADCY6 - adenylate cyclase 6  ZNF331 - zinc finger protein 331  ADCY5 - adenylate cyclase 5  MED1 - mediator complex subunit 1  ADCY1 - adenylate cyclase 1 (brain)  ADCY3 - adenylate cyclase 3  PPARD - peroxisome proliferator-activated receptor delta  MAML3 - mastermind-like 3 (drosophila)  ADCYAP1R1 - adenylate cyclase activating polypeptide 1 (pituitary) receptor type i  POLR2K - polymerase (rna) ii (dna directed) polypeptide k, 7.0kda  FAM129A - family with sequence similarity 129, member a  PARP1 - poly (adp-ribose) polymerase 1  POLH - polymerase (dna directed), eta  H2AFY2 - h2a histone family, member y2  PPP1R13L - protein phosphatase 1, regulatory subunit 13 like  TNFRSF19 - tumor necrosis factor receptor superfamily, member 19  HES6 - hairy and enhancer of split 6 (drosophila)  POLR2G - polymerase (rna) ii (dna directed) polypeptide g  SMPD3 - sphingomyelin phosphodiesterase 3, neutral membrane (neutral sphingomyelinase ii)  POLR2D - polymerase (rna) ii (dna directed) polypeptide d  RUVBL2 - ruvb-like 2 (e. coli)  PARP4 - poly (adp-ribose) polymerase family, member 4  POLR2A - polymerase (rna) ii (dna directed) polypeptide a, 220kda  AP2A1 - adaptor-related protein complex 2, alpha 1 subunit  CDK5RAP2 - cdk5 regulatory subunit associated protein 2  PRKACB - protein kinase, camp-dependent, catalytic, beta  RCOR3 - rest corepressor 3  PRKACA - protein kinase, camp-dependent, catalytic, alpha  AES - amino-terminal enhancer of split  NUP133 - nucleoporin 133kda  NUPL2 - nucleoporin like 2  FAM46B - family with sequence similarity 46, member b  VPS35 - vacuolar protein sorting 35 homolog (s. cerevisiae)  JAG1 - jagged 1  EDEM2 - er degradation enhancer, mannosidase alpha-like 2  CHFR - checkpoint with forkhead and ring finger domains, e3 ubiquitin protein ligase  PRDM4 - pr domain containing 4  PPP3R1 - protein phosphatase 3, regulatory subunit b, alpha  ATF7IP - activating transcription factor 7 interacting protein  FGFR1OP - fgfr1 oncogene partner  PPP5C - protein phosphatase 5, catalytic subunit  ZNF689 - zinc finger protein 689  PPP2R5D - protein phosphatase 2, regulatory subunit b', delta  PPP2R5C - protein phosphatase 2, regulatory subunit b', gamma  LRIF1 - ligand dependent nuclear receptor interacting factor 1  PPP2R5B - protein phosphatase 2, regulatory subunit b', beta  WDFY2 - wd repeat and fyve domain containing 2  AKT1 - v-akt murine thymoma viral oncogene homolog 1  ZNF653 - zinc finger protein 653  PPP4C - protein phosphatase 4, catalytic subunit  PCID2 - pci domain containing 2  PPP2R5E - protein phosphatase 2, regulatory subunit b', epsilon isoform  PPP2R2A - protein phosphatase 2, regulatory subunit b, alpha  PPP2R1B - protein phosphatase 2, regulatory subunit a, beta  CPSF6 - cleavage and polyadenylation specific factor 6, 68kda  PPP2R1A - protein phosphatase 2, regulatory subunit a, alpha  RIOK2 - rio kinase 2  NUDT21 - nudix (nucleoside diphosphate linked moiety x)-type motif 21  WWP2 - ww domain containing e3 ubiquitin protein ligase 2  AHR - aryl hydrocarbon receptor  PPP2R2C - protein phosphatase 2, regulatory subunit b, gamma  PPP2R2B - protein phosphatase 2, regulatory subunit b, beta  PPP1R7 - protein phosphatase 1, regulatory subunit 7  ZNF526 - zinc finger protein 526  PPP1R8 - protein phosphatase 1, regulatory subunit 8  FOXP4 - forkhead box p4  PPP2CA - protein phosphatase 2, catalytic subunit, alpha isozyme  PPP1R10 - protein phosphatase 1, regulatory subunit 10  DUSP14 - dual specificity phosphatase 14  RMI2 - recq mediated genome instability 2  PPP1R2 - protein phosphatase 1, regulatory (inhibitor) subunit 2  ZNF83 - zinc finger protein 83  ESCO1 - establishment of sister chromatid cohesion n-acetyltransferase 1  KANSL3 - kat8 regulatory nsl complex subunit 3  IWS1 - iws1 homolog (s. cerevisiae)  PRNP - prion protein  ATF7 - activating transcription factor 7  AMFR - autocrine motility factor receptor, e3 ubiquitin protein ligase  DNAJC3 - dnaj (hsp40) homolog, subfamily c, member 3  PRKRIR - protein-kinase, interferon-inducible double stranded rna dependent inhibitor, repressor of (p58 repressor)  PAPD7 - pap associated domain containing 7  MAP2K5 - mitogen-activated protein kinase kinase 5  MAP2K7 - mitogen-activated protein kinase kinase 7  PIM2 - pim-2 oncogene  EIF2AK2 - eukaryotic translation initiation factor 2-alpha kinase 2  ZNF692 - zinc finger protein 692  MAP2K1 - mitogen-activated protein kinase kinase 1  MAP2K2 - mitogen-activated protein kinase kinase 2  EIF4E3 - eukaryotic translation initiation factor 4e family member 3  MAPK10 - mitogen-activated protein kinase 10  MAPK9 - mitogen-activated protein kinase 9  MAPK11 - mitogen-activated protein kinase 11  MAPK8 - mitogen-activated protein kinase 8  MAPK7 - mitogen-activated protein kinase 7  GCN1L1 - gcn1 general control of amino-acid synthesis 1-like 1 (yeast)  MAPK3 - mitogen-activated protein kinase 3  CCNI - cyclin i  MAPK1 - mitogen-activated protein kinase 1  OSBPL7 - oxysterol binding protein-like 7  OSBPL8 - oxysterol binding protein-like 8  ADIRF - adipogenesis regulatory factor  PRKD1 - protein kinase d1  NDC1 - ndc1 transmembrane nucleoporin  ANXA3 - annexin a3  CCDC88A - coiled-coil domain containing 88a  PRKCH - protein kinase c, eta  TLK2 - tousled-like kinase 2  PRKCI - protein kinase c, iota  ANXA2 - annexin a2  CCDC85B - coiled-coil domain containing 85b  PRKCE - protein kinase c, epsilon  PRKCB - protein kinase c, beta  PRKCD - protein kinase c, delta  PRKAR2B - protein kinase, camp-dependent, regulatory, type ii, beta  RBM22 - rna binding motif protein 22  PRKCA - protein kinase c, alpha  PRKAR1B - protein kinase, camp-dependent, regulatory, type i, beta  NSUN5 - nop2/sun domain family, member 5  PRKAR1A - protein kinase, camp-dependent, regulatory, type i, alpha  PKIB - protein kinase (camp-dependent, catalytic) inhibitor beta  YEATS2 - yeats domain containing 2  PRKAG1 - protein kinase, amp-activated, gamma 1 non-catalytic subunit  APEX1 - apex nuclease (multifunctional dna repair enzyme) 1  DUSP12 - dual specificity phosphatase 12  ZNF605 - zinc finger protein 605  RBM23 - rna binding motif protein 23  XIAP - x-linked inhibitor of apoptosis  BIRC3 - baculoviral iap repeat containing 3  BIRC2 - baculoviral iap repeat containing 2  PARN - poly(a)-specific ribonuclease  KLF12 - kruppel-like factor 12  TREX1 - three prime repair exonuclease 1  APLP2 - amyloid beta (a4) precursor-like protein 2  SHQ1 - shq1, h/aca ribonucleoprotein assembly factor  PBX2 - pre-b-cell leukemia homeobox 2  PRMT6 - protein arginine methyltransferase 6  PBX3 - pre-b-cell leukemia homeobox 3  PBX1 - pre-b-cell leukemia homeobox 1  PCBP2 - poly(rc) binding protein 2  PCBD1 - pterin-4 alpha-carbinolamine dehydratase/dimerization cofactor of hepatocyte nuclear factor 1 alpha  DCUN1D3 - dcn1, defective in cullin neddylation 1, domain containing 3  APBB2 - amyloid beta (a4) precursor protein-binding, family b, member 2  SMG8 - smg8 nonsense mediated mrna decay factor  APC - adenomatous polyposis coli  CHP1 - calcineurin-like ef-hand protein 1  APBB1 - amyloid beta (a4) precursor protein-binding, family b, member 1 (fe65)  RIF1 - rap1 interacting factor homolog (yeast)  POLG2 - polymerase (dna directed), gamma 2, accessory subunit  RNF139 - ring finger protein 139  PDCD10 - programmed cell death 10  PEBP1 - phosphatidylethanolamine binding protein 1  KIAA1551 - kiaa1551  PA2G4 - proliferation-associated 2g4, 38kda  APPL2 - adaptor protein, phosphotyrosine interaction, ph domain and leucine zipper containing 2  ARF1 - adp-ribosylation factor 1  RPRD1A - regulation of nuclear pre-mrna domain containing 1a  ARF4 - adp-ribosylation factor 4  ZHX1 - zinc fingers and homeoboxes 1  PAFAH1B1 - platelet-activating factor acetylhydrolase 1b, regulatory subunit 1 (45kda)  SBNO1 - strawberry notch homolog 1 (drosophila)  APOD - apolipoprotein d  IRAK3 - interleukin-1 receptor-associated kinase 3  PRDX1 - peroxiredoxin 1  AKAP13 - a kinase (prka) anchor protein 13  SETD5 - set domain containing 5  APP - amyloid beta (a4) precursor protein  BBS7 - bardet-biedl syndrome 7  DDX20 - dead (asp-glu-ala-asp) box polypeptide 20  PAK2 - p21 protein (cdc42/rac)-activated kinase 2  PAK3 - p21 protein (cdc42/rac)-activated kinase 3  PAM - peptidylglycine alpha-amidating monooxygenase  ARNTL - aryl hydrocarbon receptor nuclear translocator-like  PDGFA - platelet-derived growth factor alpha polypeptide  OXR1 - oxidation resistance 1  CELF3 - cugbp, elav-like family member 3  ARNT - aryl hydrocarbon receptor nuclear translocator  PDE1B - phosphodiesterase 1b, calmodulin-dependent  MAP4K5 - mitogen-activated protein kinase kinase kinase kinase 5  PDPR - pyruvate dehydrogenase phosphatase regulatory subunit  SUPT16H - suppressor of ty 16 homolog (s. cerevisiae)  PDGFRB - platelet-derived growth factor receptor, beta polypeptide  IFT57 - intraflagellar transport 57 homolog (chlamydomonas)  ARRB2 - arrestin, beta 2  ARRB1 - arrestin, beta 1  PDGFRA - platelet-derived growth factor receptor, alpha polypeptide  PDGFB - platelet-derived growth factor beta polypeptide  SAMD4B - sterile alpha motif domain containing 4b  PDPK1 - 3-phosphoinositide dependent protein kinase-1  FAM107A - family with sequence similarity 107, member a  RHOA - ras homolog family member a  STRAP - serine/threonine kinase receptor associated protein  PSIP1 - pc4 and sfrs1 interacting protein 1  PDK3 - pyruvate dehydrogenase kinase, isozyme 3  MED9 - mediator complex subunit 9  FSTL1 - follistatin-like 1  SLC2A6 - solute carrier family 2 (facilitated glucose transporter), member 6  PECAM1 - platelet/endothelial cell adhesion molecule 1  BAZ2A - bromodomain adjacent to zinc finger domain, 2a  WDR70 - wd repeat domain 70  PCNA - proliferating cell nuclear antigen  CHMP1A - charged multivesicular body protein 1a  JDP2 - jun dimerization protein 2  CDK16 - cyclin-dependent kinase 16  AKIRIN2 - akirin 2  HEATR1 - heat repeat containing 1  CDK17 - cyclin-dependent kinase 17  LEO1 - leo1, paf1/rna polymerase ii complex component, homolog (s. cerevisiae)  ASPH - aspartate beta-hydroxylase  ERLIN2 - er lipid raft associated 2  PDCD2 - programmed cell death 2  PWP1 - pwp1 homolog (s. cerevisiae)  WRAP53 - wd repeat containing, antisense to tp53  CDC37 - cell division cycle 37  SOCS4 - suppressor of cytokine signaling 4  PDE2A - phosphodiesterase 2a, cgmp-stimulated  GLMN - glomulin, fkbp associated protein  THAP1 - thap domain containing, apoptosis associated protein 1  PKIG - protein kinase (camp-dependent, catalytic) inhibitor gamma  ASAH1 - n-acylsphingosine amidohydrolase (acid ceramidase) 1  PDE3B - phosphodiesterase 3b, cgmp-inhibited  ASCL1 - achaete-scute complex homolog 1 (drosophila)  KAT7 - k(lysine) acetyltransferase 7  ELP3 - elongator acetyltransferase complex subunit 3  RGMB - rgm domain family, member b  SBNO2 - strawberry notch homolog 2 (drosophila)  ZNF444 - zinc finger protein 444  ATP2A3 - atpase, ca++ transporting, ubiquitous  ZBTB1 - zinc finger and btb domain containing 1  ZNF365 - zinc finger protein 365  PGAM1 - phosphoglycerate mutase 1 (brain)  ATP1A1 - atpase, na+/k+ transporting, alpha 1 polypeptide  PFN2 - profilin 2  RNF180 - ring finger protein 180  FBXW7 - f-box and wd repeat domain containing 7, e3 ubiquitin protein ligase  PGF - placental growth factor  BAHD1 - bromo adjacent homology domain containing 1  DIS3 - dis3 mitotic control homolog (s. cerevisiae)  ZHX2 - zinc fingers and homeoboxes 2  ANKRD6 - ankyrin repeat domain 6  MORC2 - morc family cw-type zinc finger 2  PHF2 - phd finger protein 2  PHF1 - phd finger protein 1  FOXJ3 - forkhead box j3  LRRK2 - leucine-rich repeat kinase 2  RERE - arginine-glutamic acid dipeptide (re) repeats  DNAJC24 - dnaj (hsp40) homolog, subfamily c, member 24  LARP6 - la ribonucleoprotein domain family, member 6  ZFHX3 - zinc finger homeobox 3  SPPL3 - signal peptide peptidase like 3  ATG14 - autophagy related 14  ATP6AP1 - atpase, h+ transporting, lysosomal accessory protein 1  ATP7A - atpase, cu++ transporting, alpha polypeptide  PEX14 - peroxisomal biogenesis factor 14  PPP6R1 - protein phosphatase 6, regulatory subunit 1  ADNP2 - adnp homeobox 2  LMTK2 - lemur tyrosine kinase 2  AEBP2 - ae binding protein 2  PPM1E - protein phosphatase, mg2+/mn2+ dependent, 1e  ZNF507 - zinc finger protein 507  IMPACT - impact rwd domain protein  LGR4 - leucine-rich repeat containing g protein-coupled receptor 4  ATP2B4 - atpase, ca++ transporting, plasma membrane 4  ZNF652 - zinc finger protein 652  RELL2 - relt-like 2  SCAF8 - sr-related ctd-associated factor 8  BAX - bcl2-associated x protein  PKD1 - polycystic kidney disease 1 (autosomal dominant)  SMU1 - smu-1 suppressor of mec-8 and unc-52 homolog (c. elegans)  PKD2 - polycystic kidney disease 2 (autosomal dominant)  PUF60 - poly-u binding splicing factor 60kda  MTF2 - metal response element binding transcription factor 2  BAK1 - bcl2-antagonist/killer 1  NAT10 - n-acetyltransferase 10 (gcn5-related)  BACH1 - btb and cnc homology 1, basic leucine zipper transcription factor 1  BAD - bcl2-associated agonist of cell death  PIN1 - peptidylprolyl cis/trans isomerase, nima-interacting 1  PIK3R2 - phosphoinositide-3-kinase, regulatory subunit 2 (beta)  DPH3 - diphthamide biosynthesis 3  PIK3R1 - phosphoinositide-3-kinase, regulatory subunit 1 (alpha)  PLAT - plasminogen activator, tissue  ASXL2 - additional sex combs like 2 (drosophila)  PLAGL1 - pleiomorphic adenoma gene-like 1  ELP2 - elongator acetyltransferase complex subunit 2  ATRX - alpha thalassemia/mental retardation syndrome x-linked  PEF1 - penta-ef-hand domain containing 1  PKNOX1 - pbx/knotted 1 homeobox 1  CASC3 - cancer susceptibility candidate 3  PKM - pyruvate kinase, muscle  OGFOD1 - 2-oxoglutarate and iron-dependent oxygenase domain containing 1  GABARAPL2 - gaba(a) receptor-associated protein-like 2  EXOSC8 - exosome component 8  SERPINI1 - serpin peptidase inhibitor, clade i (neuroserpin), member 1  TMEM100 - transmembrane protein 100  PHF10 - phd finger protein 10  RABL3 - rab, member of ras oncogene family-like 3  SERPINE2 - serpin peptidase inhibitor, clade e (nexin, plasminogen activator inhibitor type 1), member 2  SERPINB8 - serpin peptidase inhibitor, clade b (ovalbumin), member 8  U2AF2 - u2 small nuclear rna auxiliary factor 2  PSPC1 - paraspeckle component 1  NUDT15 - nudix (nucleoside diphosphate linked moiety x)-type motif 15  PHKG2 - phosphorylase kinase, gamma 2 (testis)  BCL7A - b-cell cll/lymphoma 7a  STK38 - serine/threonine kinase 38  MRGBP - mrg/morf4l binding protein  BCL9 - b-cell cll/lymphoma 9  PIK3CB - phosphatidylinositol-4,5-bisphosphate 3-kinase, catalytic subunit beta  PIK3CA - phosphatidylinositol-4,5-bisphosphate 3-kinase, catalytic subunit alpha  RPUSD3 - rna pseudouridylate synthase domain containing 3  PPP6R3 - protein phosphatase 6, regulatory subunit 3  BRF2 - brf2, rna polymerase iii transcription initiation factor 50 kda subunit  PIM1 - pim-1 oncogene  BCL2 - b-cell cll/lymphoma 2  ECD - ecdysoneless homolog (drosophila)  CCND1 - cyclin d1  PARK7 - parkinson protein 7  BBS4 - bardet-biedl syndrome 4  PNKP - polynucleotide kinase 3'-phosphatase  C3orf33 - chromosome 3 open reading frame 33  TRIM27 - tripartite motif containing 27  RFC5 - replication factor c (activator 1) 5, 36.5kda  RFC2 - replication factor c (activator 1) 2, 40kda  SAP18 - sin3a-associated protein, 18kda  RFC3 - replication factor c (activator 1) 3, 38kda  TSHZ2 - teashirt zinc finger homeobox 2  MAEA - macrophage erythroblast attacher  SEMA4C - sema domain, immunoglobulin domain (ig), transmembrane domain (tm) and short cytoplasmic domain, (semaphorin) 4c  RFXAP - regulatory factor x-associated protein  BRAF - v-raf murine sarcoma viral oncogene homolog b  RFX5 - regulatory factor x, 5 (influences hla class ii expression)  WHSC1L1 - wolf-hirschhorn syndrome candidate 1-like 1  EIF1B - eukaryotic translation initiation factor 1b  RFX1 - regulatory factor x, 1 (influences hla class ii expression)  APBB3 - amyloid beta (a4) precursor protein-binding, family b, member 3  RELA - v-rel avian reticuloendotheliosis viral oncogene homolog a  BCOR - bcl6 corepressor  ZFP36L2 - zfp36 ring finger protein-like 2  ZFP36L1 - zfp36 ring finger protein-like 1  PAK4 - p21 protein (cdc42/rac)-activated kinase 4  RDX - radixin  RET - ret proto-oncogene  DUSP15 - dual specificity phosphatase 15  SH3RF3 - sh3 domain containing ring finger 3  DPF2 - d4, zinc and double phd fingers family 2  ALKBH5 - alkb, alkylation repair homolog 5 (e. coli)  UPF1 - upf1 regulator of nonsense transcripts homolog (yeast)  NSUN2 - nop2/sun rna methyltransferase family, member 2  CC2D1A - coiled-coil and c2 domain containing 1a  RLF - rearranged l-myc fusion  RING1 - ring finger protein 1  CNPY2 - canopy fgf signaling regulator 2  BMI1 - bmi1 polycomb ring finger oncogene  PCGF3 - polycomb group ring finger 3  RGS4 - regulator of g-protein signaling 4  RGS3 - regulator of g-protein signaling 3  BMPR1A - bone morphogenetic protein receptor, type ia  BMPR2 - bone morphogenetic protein receptor, type ii (serine/threonine kinase)  BMPR1B - bone morphogenetic protein receptor, type ib  GON4L - gon-4-like (c. elegans)  BMP7 - bone morphogenetic protein 7  FITM2 - fat storage-inducing transmembrane protein 2  ABCA7 - atp-binding cassette, sub-family a (abc1), member 7  PSMD14 - proteasome (prosome, macropain) 26s subunit, non-atpase, 14  RNF19B - ring finger protein 19b  FLOT1 - flotillin 1  CTDSPL - ctd (carboxy-terminal domain, rna polymerase ii, polypeptide a) small phosphatase-like  BCAS3 - breast carcinoma amplified sequence 3  ABCE1 - atp-binding cassette, sub-family e (oabp), member 1  LDLRAD4 - low density lipoprotein receptor class a domain containing 4  PTTG1IP - pituitary tumor-transforming 1 interacting protein  UHMK1 - u2af homology motif (uhm) kinase 1  AHI1 - abelson helper integration site 1  RNF4 - ring finger protein 4  BRD2 - bromodomain containing 2  RNF2 - ring finger protein 2  HNRNPR - heterogeneous nuclear ribonucleoprotein r  ZNF280D - zinc finger protein 280d  GATAD2A - gata zinc finger domain containing 2a  RNF6 - ring finger protein (c3h2c3 type) 6  ROBO1 - roundabout, axon guidance receptor, homolog 1 (drosophila)  ROCK1 - rho-associated, coiled-coil containing protein kinase 1  RNF111 - ring finger protein 111  TSPO - translocator protein (18kda)  SPRY1 - sprouty homolog 1, antagonist of fgf signaling (drosophila)  SPRY2 - sprouty homolog 2 (drosophila)  TET2 - tet methylcytosine dioxygenase 2  MED18 - mediator complex subunit 18  DENND4A - denn/madd domain containing 4a  MBTD1 - mbt domain containing 1  SF3B4 - splicing factor 3b, subunit 4, 49kda  RAMP3 - receptor (g protein-coupled) activity modifying protein 3  RAMP1 - receptor (g protein-coupled) activity modifying protein 1  AKAP8 - a kinase (prka) anchor protein 8  ZMPSTE24 - zinc metallopeptidase ste24  STUB1 - stip1 homology and u-box containing protein 1, e3 ubiquitin protein ligase  C5AR1 - complement component 5a receptor 1  TRIM44 - tripartite motif containing 44  TAB1 - tgf-beta activated kinase 1/map3k7 binding protein 1  VAV3 - vav 3 guanine nucleotide exchange factor  PPIE - peptidylprolyl isomerase e (cyclophilin e)  CAPN2 - calpain 2, (m/ii) large subunit  FAM3C - family with sequence similarity 3, member c  MCRS1 - microspherule protein 1  ZER1 - zyg-11 related, cell cycle regulator  NRDE2 - nrde-2, necessary for rna interference, domain containing  CAMLG - calcium modulating ligand  TIMM17A - translocase of inner mitochondrial membrane 17 homolog a (yeast)  C1D - c1d nuclear receptor corepressor  RPL11 - ribosomal protein l11  PTCD3 - pentatricopeptide repeat domain 3  CASP8 - caspase 8, apoptosis-related cysteine peptidase  CASP9 - caspase 9, apoptosis-related cysteine peptidase  PIBF1 - progesterone immunomodulatory binding factor 1  SLC30A9 - solute carrier family 30 (zinc transporter), member 9  CASP2 - caspase 2, apoptosis-related cysteine peptidase  CASP3 - caspase 3, apoptosis-related cysteine peptidase  MAD2L2 - mad2 mitotic arrest deficient-like 2 (yeast)  PID1 - phosphotyrosine interaction domain containing 1  RPA2 - replication protein a2, 32kda  PHIP - pleckstrin homology domain interacting protein  RPS4X - ribosomal protein s4, x-linked  PPP2R3C - protein phosphatase 2, regulatory subunit b'', gamma  NRARP - notch-regulated ankyrin repeat protein  ZNF787 - zinc finger protein 787  RMND1 - required for meiotic nuclear division 1 homolog (s. cerevisiae)  LAMTOR1 - late endosomal/lysosomal adaptor, mapk and mtor activator 1  TRIM38 - tripartite motif containing 38  TADA3 - transcriptional adaptor 3  CALR - calreticulin  CALM2 - calmodulin 2 (phosphorylase kinase, delta)  ZNF792 - zinc finger protein 792  WTIP - wilms tumor 1 interacting protein  NCOA2 - nuclear receptor coactivator 2  SYNCRIP - synaptotagmin binding, cytoplasmic rna interacting protein  STK25 - serine/threonine kinase 25  CREB3 - camp responsive element binding protein 3  CRTAP - cartilage associated protein  CITED2 - cbp/p300-interacting transactivator, with glu/asp-rich carboxy-terminal domain, 2  CCT6A - chaperonin containing tcp1, subunit 6a (zeta 1)  CCNT2 - cyclin t2  PINX1 - pin2/terf1 interacting, telomerase inhibitor 1  CLN6 - ceroid-lipofuscinosis, neuronal 6, late infantile, variant  CCND3 - cyclin d3  CCNT1 - cyclin t1  CCNH - cyclin h  HMG20A - high mobility group 20a  CCNG2 - cyclin g2  BANP - btg3 associated nuclear protein  TIPIN - timeless interacting protein  ACER2 - alkaline ceramidase 2  DLC1 - deleted in liver cancer 1  TRNAU1AP - trna selenocysteine 1 associated protein 1  RPS6KA1 - ribosomal protein s6 kinase, 90kda, polypeptide 1  PARP16 - poly (adp-ribose) polymerase family, member 16  RPS6KB1 - ribosomal protein s6 kinase, 70kda, polypeptide 1  RPS6KB2 - ribosomal protein s6 kinase, 70kda, polypeptide 2  CBFA2T3 - core-binding factor, runt domain, alpha subunit 2; translocated to, 3  CBFB - core-binding factor, beta subunit  RAPGEF3 - rap guanine nucleotide exchange factor (gef) 3  CBL - cbl proto-oncogene, e3 ubiquitin protein ligase  SERPINH1 - serpin peptidase inhibitor, clade h (heat shock protein 47), member 1, (collagen binding protein 1)  RXRB - retinoid x receptor, beta  CAV2 - caveolin 2  PIAS3 - protein inhibitor of activated stat, 3  CAV1 - caveolin 1, caveolae protein, 22kda  CCNC - cyclin c  RBM14 - rna binding motif protein 14  CCND2 - cyclin d2  TRMT10C - trna methyltransferase 10 homolog c (s. cerevisiae)  SPON1 - spondin 1, extracellular matrix protein  PRMT5 - protein arginine methyltransferase 5  RRAS - related ras viral (r-ras) oncogene homolog  CHTF8 - ctf8, chromosome transmission fidelity factor 8 homolog (s. cerevisiae)  CDK11B - cyclin-dependent kinase 11b  MXD4 - max dimerization protein 4  CDC5L - cell division cycle 5-like  HTRA1 - htra serine peptidase 1  PSAP - prosaposin  POLR3F - polymerase (rna) iii (dna directed) polypeptide f, 39 kda  CDH2 - cadherin 2, type 1, n-cadherin (neuronal)  HEXIM1 - hexamethylene bis-acetamide inducible 1  ERLIN1 - er lipid raft associated 1  SPIN2A - spindlin family, member 2a  ANKIB1 - ankyrin repeat and ibr domain containing 1  NCOA7 - nuclear receptor coactivator 7  POMT1 - protein-o-mannosyltransferase 1  PSEN1 - presenilin 1  TOLLIP - toll interacting protein  USP16 - ubiquitin specific peptidase 16  PSMA1 - proteasome (prosome, macropain) subunit, alpha type, 1  NLE1 - notchless homolog 1 (drosophila)  SRSF12 - serine/arginine-rich splicing factor 12  PSMA2 - proteasome (prosome, macropain) subunit, alpha type, 2  CD81 - cd81 molecule  CAMKK2 - calcium/calmodulin-dependent protein kinase kinase 2, beta  PSMB1 - proteasome (prosome, macropain) subunit, beta type, 1  STARD4 - star-related lipid transfer (start) domain containing 4  PSMA5 - proteasome (prosome, macropain) subunit, alpha type, 5  SCARB2 - scavenger receptor class b, member 2  PSMA3 - proteasome (prosome, macropain) subunit, alpha type, 3  PSMB10 - proteasome (prosome, macropain) subunit, beta type, 10  RIPPLY2 - ripply transcriptional repressor 2  KHDRBS3 - kh domain containing, rna binding, signal transduction associated 3  SPINT2 - serine peptidase inhibitor, kunitz type, 2  CD44 - cd44 molecule (indian blood group)  PSMC6 - proteasome (prosome, macropain) 26s subunit, atpase, 6  FAM173B - family with sequence similarity 173, member b  TAF6L - taf6-like rna polymerase ii, p300/cbp-associated factor (pcaf)-associated factor, 65kda  PSMD1 - proteasome (prosome, macropain) 26s subunit, non-atpase, 1  PSMC4 - proteasome (prosome, macropain) 26s subunit, atpase, 4  SOX18 - sry (sex determining region y)-box 18  POLR3G - polymerase (rna) iii (dna directed) polypeptide g (32kd)  PSMC2 - proteasome (prosome, macropain) 26s subunit, atpase, 2  PSMD9 - proteasome (prosome, macropain) 26s subunit, non-atpase, 9  PSMD10 - proteasome (prosome, macropain) 26s subunit, non-atpase, 10  PSMD7 - proteasome (prosome, macropain) 26s subunit, non-atpase, 7  PSMD8 - proteasome (prosome, macropain) 26s subunit, non-atpase, 8  DNAJC10 - dnaj (hsp40) homolog, subfamily c, member 10  PSMD5 - proteasome (prosome, macropain) 26s subunit, non-atpase, 5  PSMD2 - proteasome (prosome, macropain) 26s subunit, non-atpase, 2  TERF2IP - telomeric repeat binding factor 2, interacting protein  SLC38A2 - solute carrier family 38, member 2  PSMD12 - proteasome (prosome, macropain) 26s subunit, non-atpase, 12  PSMD13 - proteasome (prosome, macropain) 26s subunit, non-atpase, 13  ATG7 - autophagy related 7  PRRC1 - proline-rich coiled-coil 1  PTBP1 - polypyrimidine tract binding protein 1  PTEN - phosphatase and tensin homolog  LAMTOR5 - late endosomal/lysosomal adaptor, mapk and mtor activator 5  ANP32B - acidic (leucine-rich) nuclear phosphoprotein 32 family, member b  JMY - junction mediating and regulatory protein, p53 cofactor  MYBBP1A - myb binding protein (p160) 1a  FBLN5 - fibulin 5  PTGIS - prostaglandin i2 (prostacyclin) synthase  CIB1 - calcium and integrin binding 1 (calmyrin)  DDX17 - dead (asp-glu-ala-asp) box helicase 17  KAT5 - k(lysine) acetyltransferase 5  ZFP69 - zfp69 zinc finger protein  PTK2 - protein tyrosine kinase 2  IPO7 - importin 7  CEBPG - ccaat/enhancer binding protein (c/ebp), gamma  CDK5 - cyclin-dependent kinase 5  CDK4 - cyclin-dependent kinase 4  TWF1 - twinfilin actin-binding protein 1  PTN - pleiotrophin  CDK9 - cyclin-dependent kinase 9  CCT7 - chaperonin containing tcp1, subunit 7 (eta)  CDK8 - cyclin-dependent kinase 8  LIN54 - lin-54 homolog (c. elegans)  CDK7 - cyclin-dependent kinase 7  CDKN1C - cyclin-dependent kinase inhibitor 1c (p57, kip2)  NPC2 - niemann-pick disease, type c2  SPOPL - speckle-type poz protein-like  CDKN1B - cyclin-dependent kinase inhibitor 1b (p27, kip1)  CCT2 - chaperonin containing tcp1, subunit 2 (beta)  CCT4 - chaperonin containing tcp1, subunit 4 (delta)  PCBP3 - poly(rc) binding protein 3  PTPN3 - protein tyrosine phosphatase, non-receptor type 3  CDKN2D - cyclin-dependent kinase inhibitor 2d (p19, inhibits cdk4)  PTPN2 - protein tyrosine phosphatase, non-receptor type 2  PTPN1 - protein tyrosine phosphatase, non-receptor type 1  PTPN11 - protein tyrosine phosphatase, non-receptor type 11  HTATIP2 - hiv-1 tat interactive protein 2, 30kda  ARL6IP5 - adp-ribosylation-like factor 6 interacting protein 5  PTPN13 - protein tyrosine phosphatase, non-receptor type 13 (apo-1/cd95 (fas)-associated phosphatase)  ARFGEF1 - adp-ribosylation factor guanine nucleotide-exchange factor 1 (brefeldin a-inhibited)  CDK2 - cyclin-dependent kinase 2  RRN3 - rrn3 rna polymerase i transcription factor homolog (s. cerevisiae)  PURA - purine-rich element binding protein a  PURB - purine-rich element binding protein b  PDP1 - pyruvate dehyrogenase phosphatase catalytic subunit 1  GTPBP2 - gtp binding protein 2  CHRNB2 - cholinergic receptor, nicotinic, beta 2 (neuronal)  NUDT16 - nudix (nucleoside diphosphate linked moiety x)-type motif 16  PTPRR - protein tyrosine phosphatase, receptor type, r  ZMYND11 - zinc finger, mynd-type containing 11  PTPRK - protein tyrosine phosphatase, receptor type, k  SRSF10 - serine/arginine-rich splicing factor 10  PTPRN - protein tyrosine phosphatase, receptor type, n  RELL1 - relt-like 1  JMJD8 - jumonji domain containing 8  PLK2 - polo-like kinase 2  AHCYL1 - adenosylhomocysteinase-like 1  PEX2 - peroxisomal biogenesis factor 2  NUP50 - nucleoporin 50kda  MPHOSPH8 - m-phase phosphoprotein 8  KDM5B - lysine (k)-specific demethylase 5b  PXN - paxillin  GIPC1 - gipc pdz domain containing family, member 1  MAP4K2 - mitogen-activated protein kinase kinase kinase kinase 2  CHD4 - chromodomain helicase dna binding protein 4  CHD2 - chromodomain helicase dna binding protein 2  CHD3 - chromodomain helicase dna binding protein 3  FRS2 - fibroblast growth factor receptor substrate 2  MTERFD2 - mterf domain containing 2  CHD1 - chromodomain helicase dna binding protein 1  RAB1A - rab1a, member ras oncogene family  QARS - glutaminyl-trna synthetase  MYPOP - myb-related transcription factor, partner of profilin  RAD21 - rad21 homolog (s. pombe)  RAD17 - rad17 homolog (s. pombe)  SEC24A - sec24 family, member a (s. cerevisiae)  PARP14 - poly (adp-ribose) polymerase family, member 14  ZNF181 - zinc finger protein 181  PAF1 - paf1, rna polymerase ii associated factor, homolog (s. cerevisiae)  RAC1 - ras-related c3 botulinum toxin substrate 1 (rho family, small gtp binding protein rac1)  ZNF268 - zinc finger protein 268  INO80 - ino80 complex subunit  ZNF513 - zinc finger protein 513  NDFIP2 - nedd4 family interacting protein 2  RALB - v-ral simian leukemia viral oncogene homolog b  RC3H2 - ring finger and ccch-type domains 2  GMEB1 - glucocorticoid modulatory element binding protein 1  RANBP2 - ran binding protein 2  CCT8 - chaperonin containing tcp1, subunit 8 (theta)  RAP1A - rap1a, member of ras oncogene family  CLTC - clathrin, heavy chain (hc)  MIER2 - mesoderm induction early response 1, family member 2  FAM35A - family with sequence similarity 35, member a  RARG - retinoic acid receptor, gamma  RARB - retinoic acid receptor, beta  DLL4 - delta-like 4 (drosophila)  RASA1 - ras p21 protein activator (gtpase activating protein) 1  RAP1B - rap1b, member of ras oncogene family  NECAB2 - n-terminal ef-hand calcium binding protein 2  CELF1 - cugbp, elav-like family member 1  KHDRBS1 - kh domain containing, rna binding, signal transduction associated 1  CLU - clusterin  GNL3L - guanine nucleotide binding protein-like 3 (nucleolar)-like  CLK1 - cdc-like kinase 1  TAF8 - taf8 rna polymerase ii, tata box binding protein (tbp)-associated factor, 43kda  MAGEL2 - mage-like 2  CELF2 - cugbp, elav-like family member 2  RAP2B - rap2b, member of ras oncogene family  RAP2A - rap2a, member of ras oncogene family  CLK2 - cdc-like kinase 2  DDX49 - dead (asp-glu-ala-asp) box polypeptide 49  CTCF - ccctc-binding factor (zinc finger protein)  CLK3 - cdc-like kinase 3  RBL2 - retinoblastoma-like 2 (p130)  RAI1 - retinoic acid induced 1  RBBP7 - retinoblastoma binding protein 7  RBBP8 - retinoblastoma binding protein 8  MAP3K2 - mitogen-activated protein kinase kinase kinase 2  RBM3 - rna binding motif (rnp1, rrm) protein 3  RBM4 - rna binding motif protein 4  RB1 - retinoblastoma 1  ARID4A - at rich interactive domain 4a (rbp1-like)  ANKRD54 - ankyrin repeat domain 54  RBBP6 - retinoblastoma binding protein 6  KDM5A - lysine (k)-specific demethylase 5a  NUP35 - nucleoporin 35kda  PUS7 - pseudouridylate synthase 7 homolog (s. cerevisiae)  ERCC8 - excision repair cross-complementing rodent repair deficiency, complementation group 8  EXOSC4 - exosome component 4  PTGES3 - prostaglandin e synthase 3 (cytosolic)  CKS1B - cdc28 protein kinase regulatory subunit 1b  NFAT5 - nuclear factor of activated t-cells 5, tonicity-responsive  CIRBP - cold inducible rna binding protein  DHX29 - deah (asp-glu-ala-his) box polypeptide 29  CHUK - conserved helix-loop-helix ubiquitous kinase  CERS1 - ceramide synthase 1  ZMYND8 - zinc finger, mynd-type containing 8  ANKRA2 - ankyrin repeat, family a (rfxank-like), 2  RBAK - rb-associated krab zinc finger  NFATC2IP - nuclear factor of activated t-cells, cytoplasmic, calcineurin-dependent 2 interacting protein  SPTBN4 - spectrin, beta, non-erythrocytic 4  FBXO18 - f-box protein, helicase, 18  PATZ1 - poz (btb) and at hook containing zinc finger 1  SPOCK1 - sparc/osteonectin, cwcv and kazal-like domains proteoglycan (testican) 1  METTL14 - methyltransferase like 14  SPP1 - secreted phosphoprotein 1  CORO1C - coronin, actin binding protein, 1c  SSBP2 - single-stranded dna binding protein 2  KPNA6 - karyopherin alpha 6 (importin alpha 7)  FIZ1 - flt3-interacting zinc finger 1  COX11 - cytochrome c oxidase assembly homolog 11 (yeast)  COX7A2 - cytochrome c oxidase subunit viia polypeptide 2 (liver)  CIRH1A - cirrhosis, autosomal recessive 1a (cirhin)  MIER1 - mesoderm induction early response 1, transcriptional regulator  PPP1R15B - protein phosphatase 1, regulatory subunit 15b  CWC22 - cwc22 spliceosome-associated protein homolog (s. cerevisiae)  ZNF382 - zinc finger protein 382  ZNF705A - zinc finger protein 705a  SP3 - sp3 transcription factor  SP4 - sp4 transcription factor  SP1 - sp1 transcription factor  CPT1A - carnitine palmitoyltransferase 1a (liver)  ZNF410 - zinc finger protein 410  SP2 - sp2 transcription factor  ARL2BP - adp-ribosylation factor-like 2 binding protein  SOX4 - sry (sex determining region y)-box 4  TIPRL - tip41, tor signaling pathway regulator-like (s. cerevisiae)  GTPBP4 - gtp binding protein 4  WBP2 - ww domain binding protein 2  FBXL20 - f-box and leucine-rich repeat protein 20  ORC3 - origin recognition complex, subunit 3  SON - son dna binding protein  MAGI3 - membrane associated guanylate kinase, ww and pdz domain containing 3  HES4 - hairy and enhancer of split 4 (drosophila)  LEMD3 - lem domain containing 3  GATAD1 - gata zinc finger domain containing 1  CREBL2 - camp responsive element binding protein-like 2  ATF6B - activating transcription factor 6 beta  SOAT1 - sterol o-acyltransferase 1  CREBBP - creb binding protein  ATF2 - activating transcription factor 2  CREB1 - camp responsive element binding protein 1  DDX58 - dead (asp-glu-ala-asp) box polypeptide 58  SNX1 - sorting nexin 1  CRABP2 - cellular retinoic acid binding protein 2  RPRD1B - regulation of nuclear pre-mrna domain containing 1b  PLK3 - polo-like kinase 3  PPP1R16A - protein phosphatase 1, regulatory subunit 16a  SNAPC4 - small nuclear rna activating complex, polypeptide 4, 190kda  SNCA - synuclein, alpha (non a4 component of amyloid precursor)  SNRNP70 - small nuclear ribonucleoprotein 70kda (u1)  ZBED5 - zinc finger, bed-type containing 5  SNRPA - small nuclear ribonucleoprotein polypeptide a  CREBZF - creb/atf bzip transcription factor  SUMO2 - small ubiquitin-like modifier 2  EID1 - ep300 interacting inhibitor of differentiation 1  TP53INP2 - tumor protein p53 inducible nuclear protein 2  PTBP2 - polypyrimidine tract binding protein 2  SMARCE1 - swi/snf related, matrix associated, actin dependent regulator of chromatin, subfamily e, member 1  RBM17 - rna binding motif protein 17  SMO - smoothened, frizzled family receptor  CNR1 - cannabinoid receptor 1 (brain)  SMPD1 - sphingomyelin phosphodiesterase 1, acid lysosomal  CNTN1 - contactin 1  SMARCB1 - swi/snf related, matrix associated, actin dependent regulator of chromatin, subfamily b, member 1  SMARCC1 - swi/snf related, matrix associated, actin dependent regulator of chromatin, subfamily c, member 1  HLTF - helicase-like transcription factor  SMARCA4 - swi/snf related, matrix associated, actin dependent regulator of chromatin, subfamily a, member 4  SNX6 - sorting nexin 6  SSBP3 - single stranded dna binding protein 3  SMARCD2 - swi/snf related, matrix associated, actin dependent regulator of chromatin, subfamily d, member 2  ATCAY - ataxia, cerebellar, cayman type  SMARCD3 - swi/snf related, matrix associated, actin dependent regulator of chromatin, subfamily d, member 3  SMARCC2 - swi/snf related, matrix associated, actin dependent regulator of chromatin, subfamily c, member 2  SMARCD1 - swi/snf related, matrix associated, actin dependent regulator of chromatin, subfamily d, member 1  WIZ - widely interspaced zinc finger motifs  MID1IP1 - mid1 interacting protein 1  RBM25 - rna binding motif protein 25  HSPBP1 - hspa (heat shock 70kda) binding protein, cytoplasmic cochaperone 1  SMARCA1 - swi/snf related, matrix associated, actin dependent regulator of chromatin, subfamily a, member 1  SMARCA2 - swi/snf related, matrix associated, actin dependent regulator of chromatin, subfamily a, member 2  SGK3 - serum/glucocorticoid regulated kinase family, member 3  CACTIN - cactin, spliceosome c complex subunit  KMT2C - lysine (k)-specific methyltransferase 2c  RAB38 - rab38, member ras oncogene family  ZNF462 - zinc finger protein 462  PPIL4 - peptidylprolyl isomerase (cyclophilin)-like 4  TFIP11 - tuftelin interacting protein 11  SENP2 - sumo1/sentrin/smt3 specific peptidase 2  CSTB - cystatin b (stefin b)  CST3 - cystatin c  SHC2 - shc (src homology 2 domain containing) transforming protein 2  ZNF318 - zinc finger protein 318  PRPF6 - pre-mrna processing factor 6  KDM2B - lysine (k)-specific demethylase 2b  SUPT6H - suppressor of ty 6 homolog (s. cerevisiae)  CTGF - connective tissue growth factor  SUPV3L1 - suppressor of var1, 3-like 1 (s. cerevisiae)  CBY1 - chibby homolog 1 (drosophila)  CACNG7 - calcium channel, voltage-dependent, gamma subunit 7  DSTYK - dual serine/threonine and tyrosine protein kinase  CTBP1 - c-terminal binding protein 1  SUPT4H1 - suppressor of ty 4 homolog 1 (s. cerevisiae)  CTBP2 - c-terminal binding protein 2  SUPT5H - suppressor of ty 5 homolog (s. cerevisiae)  MFSD8 - major facilitator superfamily domain containing 8  RAB3GAP2 - rab3 gtpase activating protein subunit 2 (non-catalytic)  CTNNB1 - catenin (cadherin-associated protein), beta 1, 88kda  STX5 - syntaxin 5  SAV1 - salvador homolog 1 (drosophila)  CTSD - cathepsin d  ELOVL5 - elovl fatty acid elongase 5  CUX1 - cut-like homeobox 1  NIF3L1 - nif3 ngg1 interacting factor 3-like 1 (s. cerevisiae)  NEK4 - nima-related kinase 4  STK3 - serine/threonine kinase 3  MPV17L2 - mpv17 mitochondrial membrane protein-like 2  FAM3A - family with sequence similarity 3, member a  PCGF1 - polycomb group ring finger 1  NPNT - nephronectin  FKBP8 - fk506 binding protein 8, 38kda  GPBP1L1 - gc-rich promoter binding protein 1-like 1  MTCH1 - mitochondrial carrier 1  SUV420H2 - suppressor of variegation 4-20 homolog 2 (drosophila)  CYLD - cylindromatosis (turban tumor syndrome)  TGIF2 - tgfb-induced factor homeobox 2  CNEP1R1 - ctd nuclear envelope phosphatase 1 regulatory subunit 1  STK4 - serine/threonine kinase 4  BCL6B - b-cell cll/lymphoma 6, member b  ST5 - suppression of tumorigenicity 5  CRKL - v-crk avian sarcoma virus ct10 oncogene homolog-like  CRK - v-crk avian sarcoma virus ct10 oncogene homolog  EEFSEC - eukaryotic elongation factor, selenocysteine-trna-specific  CELF6 - cugbp, elav-like family member 6  NIPBL - nipped-b homolog (drosophila)  STAT2 - signal transducer and activator of transcription 2, 113kda  CRY1 - cryptochrome 1 (photolyase-like)  STAT1 - signal transducer and activator of transcription 1, 91kda  RAB26 - rab26, member ras oncogene family  STAT3 - signal transducer and activator of transcription 3 (acute-phase response factor)  CRY2 - cryptochrome 2 (photolyase-like)  STAT5A - signal transducer and activator of transcription 5a  ASF1A - anti-silencing function 1a histone chaperone  STAT6 - signal transducer and activator of transcription 6, interleukin-4 induced  PARM1 - prostate androgen-regulated mucin-like protein 1  BRMS1 - breast cancer metastasis suppressor 1  BOD1L1 - biorientation of chromosomes in cell division 1-like 1  ZNF496 - zinc finger protein 496  CRYM - crystallin, mu  SS18 - synovial sarcoma translocation, chromosome 18  PHF5A - phd finger protein 5a  CSF1R - colony stimulating factor 1 receptor  GPATCH3 - g patch domain containing 3  SNX33 - sorting nexin 33  FBXO7 - f-box protein 7  CSF1 - colony stimulating factor 1 (macrophage)  SRPK2 - srsf protein kinase 2  SRPK1 - srsf protein kinase 1  TAB3 - tgf-beta activated kinase 1/map3k7 binding protein 3  ZNF335 - zinc finger protein 335  BAMBI - bmp and activin membrane-bound inhibitor  CSK - c-src tyrosine kinase  HAVCR2 - hepatitis a virus cellular receptor 2  SQLE - squalene epoxidase  ZC3H10 - zinc finger ccch-type containing 10  CSNK1A1 - casein kinase 1, alpha 1  CSNK1D - casein kinase 1, delta  CSNK2A2 - casein kinase 2, alpha prime polypeptide  SRI - sorcin  SRC - v-src avian sarcoma (schmidt-ruppin a-2) viral oncogene homolog  TNFAIP8 - tumor necrosis factor, alpha-induced protein 8  CSNK2A1 - casein kinase 2, alpha 1 polypeptide  DNAJB5 - dnaj (hsp40) homolog, subfamily b, member 5  SREBF2 - sterol regulatory element binding transcription factor 2  ARIH1 - ariadne homolog, ubiquitin-conjugating enzyme e2 binding protein, 1 (drosophila)  UBE2O - ubiquitin-conjugating enzyme e2o  NSUN3 - nop2/sun domain family, member 3  SRP9 - signal recognition particle 9kda  PRDX5 - peroxiredoxin 5  BACE2 - beta-site app-cleaving enzyme 2  RNF169 - ring finger protein 169  DHX9 - deah (asp-glu-ala-his) box helicase 9  TRAPPC2 - trafficking protein particle complex 2  KANK2 - kn motif and ankyrin repeat domains 2  DDX5 - dead (asp-glu-ala-asp) box helicase 5  SEC13 - sec13 homolog (s. cerevisiae)  DDX3X - dead (asp-glu-ala-asp) box helicase 3, x-linked  PNKD - paroxysmal nonkinesigenic dyskinesia  DDX1 - dead (asp-glu-ala-asp) box helicase 1  SIN3A - sin3 transcription regulator family member a  DPH6 - diphthamine biosynthesis 6  MIDN - midnolin  SDCBP - syndecan binding protein (syntenin)  SET - set nuclear oncogene  WWTR1 - ww domain containing transcription regulator 1  SETMAR - set domain and mariner transposase fusion gene  DDB1 - damage-specific dna binding protein 1, 127kda  MAP2K4 - mitogen-activated protein kinase kinase 4  ZNF304 - zinc finger protein 304  RCN3 - reticulocalbin 3, ef-hand calcium binding domain  ZNF286A - zinc finger protein 286a  GEMIN5 - gem (nuclear organelle) associated protein 5  PBXIP1 - pre-b-cell leukemia homeobox interacting protein 1  NOL11 - nucleolar protein 11  DBP - d site of albumin promoter (albumin d-box) binding protein  NELFB - negative elongation factor complex member b  POT1 - protection of telomeres 1  ABHD6 - abhydrolase domain containing 6  AHCTF1 - at hook containing transcription factor 1  CLK4 - cdc-like kinase 4  CNOT10 - ccr4-not transcription complex, subunit 10  ZBTB38 - zinc finger and btb domain containing 38  RNF19A - ring finger protein 19a, e3 ubiquitin protein ligase  RCHY1 - ring finger and chy zinc finger domain containing 1, e3 ubiquitin protein ligase  GATAD2B - gata zinc finger domain containing 2b  ZNF551 - zinc finger protein 551  DFFA - dna fragmentation factor, 45kda, alpha polypeptide  KCTD13 - potassium channel tetramerization domain containing 13  ZNF473 - zinc finger protein 473  BIRC6 - baculoviral iap repeat containing 6  YTHDF3 - yth domain family, member 3  PLEKHG5 - pleckstrin homology domain containing, family g (with rhogef domain) member 5  WDR18 - wd repeat domain 18  CNOT6 - ccr4-not transcription complex, subunit 6  LTN1 - listerin e3 ubiquitin protein ligase 1  ATXN2 - ataxin 2  ZNF451 - zinc finger protein 451  ZNF622 - zinc finger protein 622  SCAF4 - sr-related ctd-associated factor 4  SS18L1 - synovial sarcoma translocation gene on chromosome 18-like 1  CHD5 - chromodomain helicase dna binding protein 5  ARID1B - at rich interactive domain 1b (swi1-like)  SCO1 - sco1 cytochrome c oxidase assembly protein  TMEM98 - transmembrane protein 98  MED30 - mediator complex subunit 30  MKL2 - mkl/myocardin-like 2  HEG1 - heart development protein with egf-like domains 1  NSMF - nmda receptor synaptonuclear signaling and neuronal migration factor  NLN - neurolysin (metallopeptidase m3 family)  PYGO2 - pygopus homolog 2 (drosophila)  DAPK3 - death-associated protein kinase 3  ANGEL2 - angel homolog 2 (drosophila)  DAXX - death-domain associated protein  C2CD3 - c2 calcium-dependent domain containing 3  ZZZ3 - zinc finger, zz-type containing 3  KIDINS220 - kinase d-interacting substrate, 220kda  HINFP - histone h4 transcription factor  DAG1 - dystroglycan 1 (dystrophin-associated glycoprotein 1)  MTA3 - metastasis associated 1 family, member 3  DGKQ - diacylglycerol kinase, theta 110kda  ZNF608 - zinc finger protein 608  DAPK1 - death-associated protein kinase 1  CLIP3 - cap-gly domain containing linker protein 3  MAVS - mitochondrial antiviral signaling protein  DAP - death-associated protein  IBTK - inhibitor of bruton agammaglobulinemia tyrosine kinase  TIPARP - tcdd-inducible poly(adp-ribose) polymerase  CYP51A1 - cytochrome p450, family 51, subfamily a, polypeptide 1  SARS - seryl-trna synthetase  HACE1 - hect domain and ankyrin repeat containing e3 ubiquitin protein ligase 1  SATB1 - satb homeobox 1  NGDN - neuroguidin, eif4e binding protein  RPTOR - regulatory associated protein of mtor, complex 1  SAFB - scaffold attachment factor b  SH2B1 - sh2b adaptor protein 1  SALL2 - sal-like 2 (drosophila)  SERBP1 - serpine1 mrna binding protein 1  EPC2 - enhancer of polycomb homolog 2 (drosophila)  DRD2 - dopamine receptor d2  PHF19 - phd finger protein 19  SLC9A1 - solute carrier family 9, subfamily a (nhe1, cation proton antiporter 1), member 1  NKD2 - naked cuticle homolog 2 (drosophila)  IRF2BP1 - interferon regulatory factor 2 binding protein 1  SLC8A1 - solute carrier family 8 (sodium/calcium exchanger), member 1  TRAF3IP1 - tnf receptor-associated factor 3 interacting protein 1  CNOT6L - ccr4-not transcription complex, subunit 6-like  EAF1 - ell associated factor 1  SLC8A2 - solute carrier family 8 (sodium/calcium exchanger), member 2  TAOK1 - tao kinase 1  PDP2 - pyruvate dehyrogenase phosphatase catalytic subunit 2  ATN1 - atrophin 1  DPH2 - dph2 homolog (s. cerevisiae)  ARRDC3 - arrestin domain containing 3  DPH1 - diphthamide biosynthesis 1  DOM3Z - dom-3 homolog z (c. elegans)  SEMA6A - sema domain, transmembrane domain (tm), and cytoplasmic domain, (semaphorin) 6a  C10orf137 - chromosome 10 open reading frame 137  DR1 - down-regulator of transcription 1, tbp-binding (negative cofactor 2)  DUSP8 - dual specificity phosphatase 8  TNKS1BP1 - tankyrase 1 binding protein 1, 182kda  MAPK15 - mitogen-activated protein kinase 15  SKI - v-ski avian sarcoma viral oncogene homolog  SKIL - ski-like oncogene  DUSP5 - dual specificity phosphatase 5  UNK - unkempt homolog (drosophila)  DUSP6 - dual specificity phosphatase 6  SKP1 - s-phase kinase-associated protein 1  DUSP7 - dual specificity phosphatase 7  DYRK1A - dual-specificity tyrosine-(y)-phosphorylation regulated kinase 1a  MKL1 - megakaryoblastic leukemia (translocation) 1  CHTOP - chromatin target of prmt1  GGA1 - golgi-associated, gamma adaptin ear containing, arf binding protein 1  DVL2 - dishevelled segment polarity protein 2  DVL3 - dishevelled segment polarity protein 3  WDR48 - wd repeat domain 48  GIGYF2 - grb10 interacting gyf protein 2  USP36 - ubiquitin specific peptidase 36  FNIP2 - folliculin interacting protein 2  DOCK7 - dedicator of cytokinesis 7  HOMEZ - homeobox and leucine zipper encoding  EBF4 - early b-cell factor 4  ANKRD17 - ankyrin repeat domain 17  ZFHX2 - zinc finger homeobox 2  HBEGF - heparin-binding egf-like growth factor  KIAA1462 - kiaa1462  LSM14A - lsm14a, scd6 homolog a (s. cerevisiae)  DTX1 - deltex homolog 1 (drosophila)  DKC1 - dyskeratosis congenita 1, dyskerin  DLG3 - discs, large homolog 3 (drosophila)  PNPT1 - polyribonucleotide nucleotidyltransferase 1  DLG4 - discs, large homolog 4 (drosophila)  DLG1 - discs, large homolog 1 (drosophila)  SHOX2 - short stature homeobox 2  FBXW8 - f-box and wd repeat domain containing 8  SH3RF1 - sh3 domain containing ring finger 1  DHCR24 - 24-dehydrocholesterol reductase  SIAH2 - siah e3 ubiquitin protein ligase 2  DHCR7 - 7-dehydrocholesterol reductase  POGK - pogo transposable element with krab domain  B3GAT3 - beta-1,3-glucuronyltransferase 3 (glucuronosyltransferase i)  DHFR - dihydrofolate reductase  PHF12 - phd finger protein 12  RICTOR - rptor independent companion of mtor, complex 2  FBXL5 - f-box and leucine-rich repeat protein 5  NQO1 - nad(p)h dehydrogenase, quinone 1  FBXO2 - f-box protein 2  CALCOCO1 - calcium binding and coiled-coil domain 1  SFPQ - splicing factor proline/glutamine-rich  ZBTB4 - zinc finger and btb domain containing 4  SFRP1 - secreted frizzled-related protein 1  SFRP2 - secreted frizzled-related protein 2  SFRP4 - secreted frizzled-related protein 4  SRSF1 - serine/arginine-rich splicing factor 1  SRSF2 - serine/arginine-rich splicing factor 2  GMEB2 - glucocorticoid modulatory element binding protein 2  DNM2 - dynamin 2  SRSF3 - serine/arginine-rich splicing factor 3  SPAG8 - sperm associated antigen 8  DNMT1 - dna (cytosine-5-)-methyltransferase 1  SRSF4 - serine/arginine-rich splicing factor 4  TRDMT1 - trna aspartic acid methyltransferase 1  SRSF5 - serine/arginine-rich splicing factor 5  DNMT3A - dna (cytosine-5-)-methyltransferase 3 alpha  SRSF6 - serine/arginine-rich splicing factor 6  SRSF7 - serine/arginine-rich splicing factor 7  EIF2AK4 - eukaryotic translation initiation factor 2 alpha kinase 4  SFSWAP - splicing factor, suppressor of white-apricot homolog (drosophila)  TRA2B - transformer 2 beta homolog (drosophila)  ALS2 - amyotrophic lateral sclerosis 2 (juvenile)  CHD8 - chromodomain helicase dna binding protein 8  DOCK3 - dedicator of cytokinesis 3  SGK1 - serum/glucocorticoid regulated kinase 1  TNRC6C - trinucleotide repeat containing 6c  NOC2L - nucleolar complex associated 2 homolog (s. cerevisiae)  ZNF317 - zinc finger protein 317  SGTA - small glutamine-rich tetratricopeptide repeat (tpr)-containing, alpha  USP37 - ubiquitin specific peptidase 37  PPP1R3F - protein phosphatase 1, regulatory subunit 3f  SMARCAD1 - swi/snf-related, matrix-associated actin-dependent regulator of chromatin, subfamily a, containing dead/h box 1  MEIS3 - meis homeobox 3  NCBP2 - nuclear cap binding protein subunit 2, 20kda  MARK2 - map/microtubule affinity-regulating kinase 2  MAPRE3 - microtubule-associated protein, rp/eb family, member 3  TSC1 - tuberous sclerosis 1  ELK1 - elk1, member of ets oncogene family  SPIRE1 - spire actin nucleation factor 1  NAF1 - nuclear assembly factor 1 ribonucleoprotein  ELK3 - elk3, ets-domain protein (srf accessory protein 2)  ELK4 - elk4, ets-domain protein (srf accessory protein 1)  SNW1 - snw domain containing 1  SCAP - srebf chaperone  ENSA - endosulfine alpha  WRNIP1 - werner helicase interacting protein 1  EPAS1 - endothelial pas domain protein 1  EP300 - e1a binding protein p300  DPH7 - diphthamide biosynthesis 7  HABP4 - hyaluronan binding protein 4  ATF6 - activating transcription factor 6  UBQLN4 - ubiquilin 4  ENO1 - enolase 1, (alpha)  MOB1B - mob kinase activator 1b  RAB3GAP1 - rab3 gtpase activating protein subunit 1 (catalytic)  EIF4A2 - eukaryotic translation initiation factor 4a2  CELF4 - cugbp, elav-like family member 4  EIF4E - eukaryotic translation initiation factor 4e  EGR2 - early growth response 2  EGR3 - early growth response 3  CCT5 - chaperonin containing tcp1, subunit 5 (epsilon)  ZC3HAV1 - zinc finger ccch-type, antiviral 1  TTF1 - transcription termination factor, rna polymerase i  EIF2S1 - eukaryotic translation initiation factor 2, subunit 1 alpha, 35kda  TRIM32 - tripartite motif containing 32  EIF2B1 - eukaryotic translation initiation factor 2b, subunit 1 alpha, 26kda  SPHK2 - sphingosine kinase 2  EIF2S3 - eukaryotic translation initiation factor 2, subunit 3 gamma, 52kda  SERPINB1 - serpin peptidase inhibitor, clade b (ovalbumin), member 1  KDM2A - lysine (k)-specific demethylase 2a  ELAVL1 - elav (embryonic lethal, abnormal vision, drosophila)-like 1 (hu antigen r)  ZNF326 - zinc finger protein 326  ELF1 - e74-like factor 1 (ets domain transcription factor)  ELAVL4 - elav (embryonic lethal, abnormal vision, drosophila)-like 4  STRADA - ste20-related kinase adaptor alpha  ELF2 - e74-like factor 2 (ets domain transcription factor)  TSG101 - tumor susceptibility gene 101  TCF25 - transcription factor 25 (basic helix-loop-helix)  EIF4G2 - eukaryotic translation initiation factor 4 gamma, 2  TSC2 - tuberous sclerosis 2  EFNA1 - ephrin-a1  CNOT1 - ccr4-not transcription complex, subunit 1  EEF2 - eukaryotic translation elongation factor 2  EXOSC7 - exosome component 7  CTNNBIP1 - catenin, beta interacting protein 1  SPEN - spen homolog, transcriptional regulator (drosophila)  RNF149 - ring finger protein 149  KLHDC10 - kelch domain containing 10  EEF1A1 - eukaryotic translation elongation factor 1 alpha 1  EEF1A2 - eukaryotic translation elongation factor 1 alpha 2  BBX - bobby sox homolog (drosophila)  TP53BP1 - tumor protein p53 binding protein 1  PHC1 - polyhomeotic homolog 1 (drosophila)  PHC2 - polyhomeotic homolog 2 (drosophila)  EGR1 - early growth response 1  ZNF292 - zinc finger protein 292  EGFR - epidermal growth factor receptor  MEGF8 - multiple egf-like-domains 8  CELSR2 - cadherin, egf lag seven-pass g-type receptor 2  KDM1A - lysine (k)-specific demethylase 1a  ATXN7L3 - ataxin 7-like 3  UNC13A - unc-13 homolog a (c. elegans)  EFNA5 - ephrin-a5  HNRNPLL - heterogeneous nuclear ribonucleoprotein l-like  NCOA6 - nuclear receptor coactivator 6  CCT3 - chaperonin containing tcp1, subunit 3 (gamma)  E2F6 - e2f transcription factor 6  E4F1 - e4f transcription factor 1  SMG1 - smg1 phosphatidylinositol 3-kinase-related kinase  ZHX3 - zinc fingers and homeoboxes 3  TNIK - traf2 and nck interacting kinase  E2F4 - e2f transcription factor 4, p107/p130-binding  E2F5 - e2f transcription factor 5, p130-binding  ENY2 - enhancer of yellow 2 homolog (drosophila)  C11orf30 - chromosome 11 open reading frame 30  RRP1B - ribosomal rna processing 1b  EDNRB - endothelin receptor type b  MYCBP2 - myc binding protein 2, e3 ubiquitin protein ligase  TRAF1 - tnf receptor-associated factor 1  LPAR1 - lysophosphatidic acid receptor 1  HSP90B1 - heat shock protein 90kda beta (grp94), member 1  S1PR1 - sphingosine-1-phosphate receptor 1  TRAF3 - tnf receptor-associated factor 3  PMEPA1 - prostate transmembrane protein, androgen induced 1  TRAF2 - tnf receptor-associated factor 2  NR2C1 - nuclear receptor subfamily 2, group c, member 1  SETX - senataxin  ZBTB47 - zinc finger and btb domain containing 47  NR2C2 - nuclear receptor subfamily 2, group c, member 2  TPR - translocated promoter region, nuclear basket protein  ZNF609 - zinc finger protein 609  WAPAL - wings apart-like homolog (drosophila)  LXN - latexin  ERC1 - elks/rab6-interacting/cast family member 1  PPRC1 - peroxisome proliferator-activated receptor gamma, coactivator-related 1  NAT14 - n-acetyltransferase 14 (gcn5-related, putative)  KDM4C - lysine (k)-specific demethylase 4c  ZNF423 - zinc finger protein 423  REXO4 - rex4, rna exonuclease 4 homolog (s. cerevisiae)  BOD1 - biorientation of chromosomes in cell division 1  CAMK1D - calcium/calmodulin-dependent protein kinase id  TMEM259 - transmembrane protein 259  USF1 - upstream transcription factor 1  NUP107 - nucleoporin 107kda  DERL3 - derlin 3  USP1 - ubiquitin specific peptidase 1  ZBTB43 - zinc finger and btb domain containing 43  PTK2B - protein tyrosine kinase 2 beta  ACSL3 - acyl-coa synthetase long-chain family member 3  MRPS27 - mitochondrial ribosomal protein s27  PITHD1 - pith (c-terminal proteasome-interacting domain of thioredoxin-like) domain containing 1  BPTF - bromodomain phd finger transcription factor  TNRC6B - trinucleotide repeat containing 6b  FASN - fatty acid synthase  USP4 - ubiquitin specific peptidase 4 (proto-oncogene)  RNF185 - ring finger protein 185  NR1H2 - nuclear receptor subfamily 1, group h, member 2  FBLN1 - fibulin 1  TAB2 - tgf-beta activated kinase 1/map3k7 binding protein 2  ETV3 - ets variant 3  CAMTA2 - calmodulin binding transcription activator 2  ETV5 - ets variant 5  ZNF598 - zinc finger protein 598  TWSG1 - twisted gastrulation homolog 1 (drosophila)  POGZ - pogo transposable element with znf domain  ETV6 - ets variant 6  PLXND1 - plexin d1  VHL - von hippel-lindau tumor suppressor, e3 ubiquitin protein ligase  VIM - vimentin  KDM6B - lysine (k)-specific demethylase 6b  EZR - ezrin  PHF8 - phd finger protein 8  TBX20 - t-box 20  TRIM41 - tripartite motif containing 41  EWSR1 - ews rna-binding protein 1  KCTD1 - potassium channel tetramerization domain containing 1  PCBP4 - poly(rc) binding protein 4  ANKLE2 - ankyrin repeat and lem domain containing 2  DCUN1D4 - dcn1, defective in cullin neddylation 1, domain containing 4  CREB3L1 - camp responsive element binding protein 3-like 1  ACKR3 - atypical chemokine receptor 3  UVRAG - uv radiation resistance associated  ZC3H3 - zinc finger ccch-type containing 3  EYA3 - eyes absent homolog 3 (drosophila)  VAV2 - vav 2 guanine nucleotide exchange factor  EZH1 - enhancer of zeste homolog 1 (drosophila)  GRAMD4 - gram domain containing 4  CCNL1 - cyclin l1  CIC - capicua transcriptional repressor  F2R - coagulation factor ii (thrombin) receptor  VCP - valosin containing protein  F3 - coagulation factor iii (thromboplastin, tissue factor)  WDR43 - wd repeat domain 43  MAPK8IP3 - mitogen-activated protein kinase 8 interacting protein 3  ZNF575 - zinc finger protein 575  ERF - ets2 repressor factor  ERCC4 - excision repair cross-complementing rodent repair deficiency, complementation group 4  NUP205 - nucleoporin 205kda  YTHDC1 - yth domain containing 1  ELMSAN1 - elm2 and myb/sant-like domain containing 1  GGA3 - golgi-associated, gamma adaptin ear containing, arf binding protein 3  THAP11 - thap domain containing 11  EMC10 - er membrane protein complex subunit 10  ERN1 - endoplasmic reticulum to nucleus signaling 1  ZNF248 - zinc finger protein 248  UBB - ubiquitin b  PPP1R37 - protein phosphatase 1, regulatory subunit 37  GPD1L - glycerol-3-phosphate dehydrogenase 1-like  ARRDC4 - arrestin domain containing 4  LARP4B - la ribonucleoprotein domain family, member 4b  RCOR1 - rest corepressor 1  TULP3 - tubby like protein 3  ZNFX1 - zinc finger, nfx1-type containing 1  HIRA - histone cell cycle regulator  TTC5 - tetratricopeptide repeat domain 5  RGL1 - ral guanine nucleotide dissociation stimulator-like 1  ETV1 - ets variant 1  ETS1 - v-ets avian erythroblastosis virus e26 oncogene homolog 1  ATG4B - autophagy related 4b, cysteine peptidase  SLC39A10 - solute carrier family 39 (zinc transporter), member 10  ZMIZ1 - zinc finger, miz-type containing 1  ETF1 - eukaryotic translation termination factor 1  TXNRD1 - thioredoxin reductase 1  CYFIP1 - cytoplasmic fmr1 interacting protein 1  ARL6IP1 - adp-ribosylation factor-like 6 interacting protein 1  DMTN - dematin actin binding protein  UBP1 - upstream binding protein 1 (lbp-1a)  SUMO1 - small ubiquitin-like modifier 1  UCHL1 - ubiquitin carboxyl-terminal esterase l1 (ubiquitin thiolesterase)  FAM168A - family with sequence similarity 168, member a  UBTF - upstream binding transcription factor, rna polymerase i  ZNF830 - zinc finger protein 830  PELI1 - pellino e3 ubiquitin protein ligase 1  CHURC1 - churchill domain containing 1  SMURF1 - smad specific e3 ubiquitin protein ligase 1  ZC3H4 - zinc finger ccch-type containing 4  JMJD6 - jumonji domain containing 6  MYADM - myeloid-associated differentiation marker  UBE2I - ubiquitin-conjugating enzyme e2i  EPRS - glutamyl-prolyl-trna synthetase  UBE2V2 - ubiquitin-conjugating enzyme e2 variant 2  ERCC2 - excision repair cross-complementing rodent repair deficiency, complementation group 2  UBE2L3 - ubiquitin-conjugating enzyme e2l 3  NUP210 - nucleoporin 210kda  UBE2N - ubiquitin-conjugating enzyme e2n  UBE2V1 - ubiquitin-conjugating enzyme e2 variant 1  VPS72 - vacuolar protein sorting 72 homolog (s. cerevisiae)  PSENEN - presenilin enhancer 2 homolog (c. elegans)  PIK3IP1 - phosphoinositide-3-kinase interacting protein 1  FMR1 - fragile x mental retardation 1  TCOF1 - treacher collins-franceschetti syndrome 1  MGA - mga, max dimerization protein  TCP1 - t-complex 1  FNTA - farnesyltransferase, caax box, alpha  ZEB1 - zinc finger e-box binding homeobox 1  WWC3 - wwc family member 3  FLT1 - fms-related tyrosine kinase 1  TCF12 - transcription factor 12  ZNF354A - zinc finger protein 354a  TCF20 - transcription factor 20 (ar1)  CAMTA1 - calmodulin binding transcription activator 1  PDS5A - pds5, regulator of cohesion maintenance, homolog a (s. cerevisiae)  TMEM106A - transmembrane protein 106a  FLI1 - fli-1 proto-oncogene, ets transcription factor  FLNA - filamin a, alpha  PLCB1 - phospholipase c, beta 1 (phosphoinositide-specific)  FOXS1 - forkhead box s1  PHLPP1 - ph domain and leucine rich repeat protein phosphatase 1  FOXO3 - forkhead box o3  HDAC8 - histone deacetylase 8  LARP4 - la ribonucleoprotein domain family, member 4  ASH1L - ash1 (absent, small, or homeotic)-like (drosophila)  SIN3B - sin3 transcription regulator family member b  DCP1A - decapping mrna 1a  NACC1 - nucleus accumbens associated 1, ben and btb (poz) domain containing  HR - hair growth associated  MED8 - mediator complex subunit 8  CAND1 - cullin-associated and neddylation-dissociated 1  FKBP1A - fk506 binding protein 1a, 12kda  TDG - thymine-dna glycosylase  SMG6 - smg6 nonsense mediated mrna decay factor  PRDX2 - peroxiredoxin 2  FKBP1B - fk506 binding protein 1b, 12.6 kda  EAPP - e2f-associated phosphoprotein  FBXW11 - f-box and wd repeat domain containing 11  VPS11 - vacuolar protein sorting 11 homolog (s. cerevisiae)  KDM3A - lysine (k)-specific demethylase 3a  FAM208A - family with sequence similarity 208, member a  CLEC16A - c-type lectin domain family 16, member a  FHL2 - four and a half lim domains 2  FHIT - fragile histidine triad  PPP1R11 - protein phosphatase 1, regulatory (inhibitor) subunit 11  NUP160 - nucleoporin 160kda  TAF9 - taf9 rna polymerase ii, tata box binding protein (tbp)-associated factor, 32kda  FGFR2 - fibroblast growth factor receptor 2  TAF7 - taf7 rna polymerase ii, tata box binding protein (tbp)-associated factor, 55kda  FGF13 - fibroblast growth factor 13  TAF6 - taf6 rna polymerase ii, tata box binding protein (tbp)-associated factor, 80kda  FGFR1 - fibroblast growth factor receptor 1  TADA2A - transcriptional adaptor 2a  FGF9 - fibroblast growth factor 9  SMCHD1 - structural maintenance of chromosomes flexible hinge domain containing 1  ADAM17 - adam metallopeptidase domain 17  TACC1 - transforming, acidic coiled-coil containing protein 1  FGF1 - fibroblast growth factor 1 (acidic)  TIRAP - toll-interleukin 1 receptor (tir) domain containing adaptor protein  FGF2 - fibroblast growth factor 2 (basic)  USP22 - ubiquitin specific peptidase 22  SASH1 - sam and sh3 domain containing 1  TARBP1 - tar (hiv-1) rna binding protein 1  FER - fer (fps/fes related) tyrosine kinase  PDGFC - platelet derived growth factor c  MAP3K7 - mitogen-activated protein kinase kinase kinase 7  ADNP - activity-dependent neuroprotector homeobox  KMT2E - lysine (k)-specific methyltransferase 2e  MED13L - mediator complex subunit 13-like  FDFT1 - farnesyl-diphosphate farnesyltransferase 1  PGP - phosphoglycolate phosphatase  NCSTN - nicastrin  FKTN - fukutin  ZNF395 - zinc finger protein 395  MYNN - myoneurin  TAZ - tafazzin  CRTC1 - creb regulated transcription coactivator 1  UFL1 - ufm1-specific ligase 1  TCF3 - transcription factor 3  PUM2 - pumilio homolog 2 (drosophila)  DMAP1 - dna methyltransferase 1 associated protein 1  TCEB3 - transcription elongation factor b (siii), polypeptide 3 (110kda, elongin a)  TCF4 - transcription factor 4  LARP1 - la ribonucleoprotein domain family, member 1  NKRF - nfkb repressing factor  EFEMP1 - egf containing fibulin-like extracellular matrix protein 1  FBN1 - fibrillin 1  ZNF629 - zinc finger protein 629  LANCL2 - lanc lantibiotic synthetase component c-like 2 (bacterial)  TCEA2 - transcription elongation factor a (sii), 2  ERBB2IP - erbb2 interacting protein  TCEA1 - transcription elongation factor a (sii), 1  TIMP1 - timp metallopeptidase inhibitor 1  CAMK2N2 - calcium/calmodulin-dependent protein kinase ii inhibitor 2  TIMP2 - timp metallopeptidase inhibitor 2  TIMP3 - timp metallopeptidase inhibitor 3  TIMP4 - timp metallopeptidase inhibitor 4  ITGB3BP - integrin beta 3 binding protein (beta3-endonexin)  SYAP1 - synapse associated protein 1  ORMDL1 - orm1-like 1 (s. cerevisiae)  RYBP - ring1 and yy1 binding protein  MTOR - mechanistic target of rapamycin (serine/threonine kinase)  PAXBP1 - pax3 and pax7 binding protein 1  TLE1 - transducin-like enhancer of split 1 (e(sp1) homolog, drosophila)  TLE3 - transducin-like enhancer of split 3 (e(sp1) homolog, drosophila)  TLE4 - transducin-like enhancer of split 4 (e(sp1) homolog, drosophila)  NDNL2 - necdin-like 2  ATP13A2 - atpase type 13a2  TLR3 - toll-like receptor 3  EXOSC2 - exosome component 2  SIRT1 - sirtuin 1  MKX - mohawk homeobox  TMF1 - tata element modulatory factor 1  TMSB4X - thymosin beta 4, x-linked  CBX6 - chromobox homolog 6  ACTR8 - arp8 actin-related protein 8 homolog (yeast)  HEY1 - hairy/enhancer-of-split related with yrpw motif 1  TMPO - thymopoietin  CBX5 - chromobox homolog 5  BRD4 - bromodomain containing 4  TNFAIP1 - tumor necrosis factor, alpha-induced protein 1 (endothelial)  BCL9L - b-cell cll/lymphoma 9-like  TARDBP - tar dna binding protein  TNFRSF1A - tumor necrosis factor receptor superfamily, member 1a  TNFRSF1B - tumor necrosis factor receptor superfamily, member 1b  SF3B3 - splicing factor 3b, subunit 3, 130kda  TMBIM6 - transmembrane bax inhibitor motif containing 6  PRKCDBP - protein kinase c, delta binding protein  TEK - tek tyrosine kinase, endothelial  EIF4ENIF1 - eukaryotic translation initiation factor 4e nuclear import factor 1  NUP188 - nucleoporin 188kda  SUZ12 - suz12 polycomb repressive complex 2 subunit  TEF - thyrotrophic embryonic factor  POFUT1 - protein o-fucosyltransferase 1  RCOR2 - rest corepressor 2  TFAM - transcription factor a, mitochondrial  DIABLO - diablo, iap-binding mitochondrial protein  INPP5E - inositol polyphosphate-5-phosphatase, 72 kda  TERF1 - telomeric repeat binding factor (nima-interacting) 1  TERF2 - telomeric repeat binding factor 2  TESK1 - testis-specific kinase 1  TFDP1 - transcription factor dp-1  NR2F2 - nuclear receptor subfamily 2, group f, member 2  TFE3 - transcription factor binding to ighm enhancer 3  CBX7 - chromobox homolog 7  BCCIP - brca2 and cdkn1a interacting protein  ISCU - iron-sulfur cluster assembly enzyme  NR2F1 - nuclear receptor subfamily 2, group f, member 1  TFCP2 - transcription factor cp2  TGS1 - trimethylguanosine synthase 1  ZNF740 - zinc finger protein 740  TGFA - transforming growth factor, alpha  FNIP1 - folliculin interacting protein 1  HEY2 - hairy/enhancer-of-split related with yrpw motif 2  YLPM1 - ylp motif containing 1  TGFBR1 - transforming growth factor, beta receptor 1  TGFB2 - transforming growth factor, beta 2  RBFOX2 - rna binding protein, fox-1 homolog (c. elegans) 2  TGFB3 - transforming growth factor, beta 3  TGFB1 - transforming growth factor, beta 1  TGFB1I1 - transforming growth factor beta 1 induced transcript 1  MAPK8IP2 - mitogen-activated protein kinase 8 interacting protein 2  TP53INP1 - tumor protein p53 inducible nuclear protein 1  H2AFV - h2a histone family, member v  MEPCE - methylphosphate capping enzyme  TGFBR2 - transforming growth factor, beta receptor ii (70/80kda)  RNF20 - ring finger protein 20, e3 ubiquitin protein ligase  TGFBR3 - transforming growth factor, beta receptor iii  FOSL2 - fos-like antigen 2  FOSB - fbj murine osteosarcoma viral oncogene homolog b  FOS - fbj murine osteosarcoma viral oncogene homolog  THBS1 - thrombospondin 1  CABIN1 - calcineurin binding protein 1  KAT6B - k(lysine) acetyltransferase 6b  TIA1 - tia1 cytotoxic granule-associated rna binding protein  METTL3 - methyltransferase like 3  THY1 - thy-1 cell surface antigen  THRA - thyroid hormone receptor, alpha  MMD - monocyte to macrophage differentiation-associated  ELL - elongation factor rna polymerase ii  ZNF239 - zinc finger protein 239  GFRA2 - gdnf family receptor alpha 2  SYMPK - symplekin  PHC3 - polyhomeotic homolog 3 (drosophila)  FAM192A - family with sequence similarity 192, member a  OBFC1 - oligonucleotide/oligosaccharide-binding fold containing 1  NRIP1 - nuclear receptor interacting protein 1  KAT2A - k(lysine) acetyltransferase 2a  NCOA3 - nuclear receptor coactivator 3  PANK2 - pantothenate kinase 2  ANP32A - acidic (leucine-rich) nuclear phosphoprotein 32 family, member a  WWC2 - ww and c2 domain containing 2  PABPN1 - poly(a) binding protein, nuclear 1  TAF15 - taf15 rna polymerase ii, tata box binding protein (tbp)-associated factor, 68kda  GJA1 - gap junction protein, alpha 1, 43kda  TMTC3 - transmembrane and tetratricopeptide repeat containing 3  AXIN1 - axin 1  TRRAP - transformation/transcription domain-associated protein  PICALM - phosphatidylinositol binding clathrin assembly protein  GGNBP2 - gametogenetin binding protein 2  CAAP1 - caspase activity and apoptosis inhibitor 1  LPCAT1 - lysophosphatidylcholine acyltransferase 1  ZC3H14 - zinc finger ccch-type containing 14  ARID1A - at rich interactive domain 1a (swi-like)  DLL1 - delta-like 1 (drosophila)  SETD6 - set domain containing 6  GLG1 - golgi glycoprotein 1  TTC39B - tetratricopeptide repeat domain 39b  FZD6 - frizzled family receptor 6  GLE1 - gle1 rna export mediator  ACTR5 - arp5 actin-related protein 5 homolog (yeast)  FZD8 - frizzled family receptor 8  CDC45 - cell division cycle 45  FZD4 - frizzled family receptor 4  GCLC - glutamate-cysteine ligase, catalytic subunit  FZD1 - frizzled family receptor 1  BAP1 - brca1 associated protein-1 (ubiquitin carboxy-terminal hydrolase)  ZNF613 - zinc finger protein 613  AXIN2 - axin 2  CDC7 - cell division cycle 7  NUP85 - nucleoporin 85kda  KDM5C - lysine (k)-specific demethylase 5c  USP9X - ubiquitin specific peptidase 9, x-linked  GNG3 - guanine nucleotide binding protein (g protein), gamma 3  RBM10 - rna binding motif protein 10  GNAQ - guanine nucleotide binding protein (g protein), q polypeptide  GNAI2 - guanine nucleotide binding protein (g protein), alpha inhibiting activity polypeptide 2  PHF17 - phd finger protein 17  GNA12 - guanine nucleotide binding protein (g protein) alpha 12  GMFB - glia maturation factor, beta  TTC21B - tetratricopeptide repeat domain 21b  SLTM - safb-like, transcription modulator  NELFE - negative elongation factor complex member e  EHMT1 - euchromatic histone-lysine n-methyltransferase 1  NR5A1 - nuclear receptor subfamily 5, group a, member 1  FUS - fused in sarcoma  NAA40 - n(alpha)-acetyltransferase 40, natd catalytic subunit, homolog (s. cerevisiae)  RAB7A - rab7a, member ras oncogene family  USP7 - ubiquitin specific peptidase 7 (herpes virus-associated)  XRCC6 - x-ray repair complementing defective repair in chinese hamster cells 6  RNF168 - ring finger protein 168, e3 ubiquitin protein ligase  GABPB1 - ga binding protein transcription factor, beta subunit 1  GABPA - ga binding protein transcription factor, alpha subunit 60kda  CPSF7 - cleavage and polyadenylation specific factor 7, 59kda  FYN - fyn oncogene related to src, fgr, yes  RPAP2 - rna polymerase ii associated protein 2  G6PD - glucose-6-phosphate dehydrogenase  DDX39B - dead (asp-glu-ala-asp) box polypeptide 39b  USP5 - ubiquitin specific peptidase 5 (isopeptidase t)  KMT2D - lysine (k)-specific methyltransferase 2d  SPRED1 - sprouty-related, evh1 domain containing 1  AAAS - achalasia, adrenocortical insufficiency, alacrimia  ZNF419 - zinc finger protein 419  CDK2AP1 - cyclin-dependent kinase 2 associated protein 1  YEATS4 - yeats domain containing 4  SNIP1 - smad nuclear interacting protein 1  ZFPM1 - zinc finger protein, fog family member 1  ZNF212 - zinc finger protein 212  EID2 - ep300 interacting inhibitor of differentiation 2  GBA - glucosidase, beta, acid  ZNF383 - zinc finger protein 383  GATA2 - gata binding protein 2  KAT6A - k(lysine) acetyltransferase 6a  NUP214 - nucleoporin 214kda  NR4A3 - nuclear receptor subfamily 4, group a, member 3  BRD3 - bromodomain containing 3  SHOC2 - soc-2 suppressor of clear homolog (c. elegans)  GAS6 - growth arrest-specific 6  CARF - calcium responsive transcription factor  NCOA4 - nuclear receptor coactivator 4  ZNF408 - zinc finger protein 408  MLLT10 - myeloid/lymphoid or mixed-lineage leukemia (trithorax homolog, drosophila); translocated to, 10  DEDD2 - death effector domain containing 2  FASTKD1 - fast kinase domains 1  DHX36 - deah (asp-glu-ala-his) box polypeptide 36  ZNF189 - zinc finger protein 189  ZSCAN26 - zinc finger and scan domain containing 26  GSTP1 - glutathione s-transferase pi 1  ZNF177 - zinc finger protein 177  GSPT1 - g1 to s phase transition 1  ZNF175 - zinc finger protein 175  GSN - gelsolin  ZNF174 - zinc finger protein 174  ZNF184 - zinc finger protein 184  SSBP4 - single stranded dna binding protein 4  GTF2F1 - general transcription factor iif, polypeptide 1, 74kda  ZNF148 - zinc finger protein 148  BEND5 - ben domain containing 5  ZBTB17 - zinc finger and btb domain containing 17  NEIL1 - nei endonuclease viii-like 1 (e. coli)  GTF2H2 - general transcription factor iih, polypeptide 2, 44kda  TRIM26 - tripartite motif containing 26  GTF2H3 - general transcription factor iih, polypeptide 3, 34kda  VEZF1 - vascular endothelial zinc finger 1  GTF2H1 - general transcription factor iih, polypeptide 1, 62kda  ZNF142 - zinc finger protein 142  MCPH1 - microcephalin 1  ZNF140 - zinc finger protein 140  GTF2B - general transcription factor iib  PCGF2 - polycomb group ring finger 2  MSH6 - muts homolog 6 (e. coli)  ZNF132 - zinc finger protein 132  ZNF131 - zinc finger protein 131  ZNF134 - zinc finger protein 134  ZNF133 - zinc finger protein 133  TBL1XR1 - transducin (beta)-like 1 x-linked receptor 1  GTF2I - general transcription factor iii  GTF2H4 - general transcription factor iih, polypeptide 4, 52kda  PRKRIP1 - prkr interacting protein 1 (il11 inducible)  BRF1 - brf1, rna polymerase iii transcription initiation factor 90 kda subunit  ZNF79 - zinc finger protein 79  ZYG11B - zyg-11 family member b, cell cycle regulator  ZNF70 - zinc finger protein 70  RBM12B - rna binding motif protein 12b  ZNF75D - zinc finger protein 75d  ZNF76 - zinc finger protein 76  H3F3A - h3 histone, family 3a  BRPF1 - bromodomain and phd finger containing, 1  H2AFZ - h2a histone family, member z  FZD5 - frizzled family receptor 5  SAP130 - sin3a-associated protein, 130kda  DCP2 - decapping mrna 2  H2AFX - h2a histone family, member x  MUL1 - mitochondrial e3 ubiquitin protein ligase 1  CXCR4 - chemokine (c-x-c motif) receptor 4  PAPD4 - pap associated domain containing 4  PAX8 - paired box 8  MIER3 - mesoderm induction early response 1, family member 3  CDC73 - cell division cycle 73  NKAP - nfkb activating protein  THAP5 - thap domain containing 5  LRP8 - low density lipoprotein receptor-related protein 8, apolipoprotein e receptor  HCFC1 - host cell factor c1 (vp16-accessory protein)  PRDM2 - pr domain containing 2, with znf domain  MAP3K12 - mitogen-activated protein kinase kinase kinase 12  ZNF226 - zinc finger protein 226  HDAC2 - histone deacetylase 2  HMBOX1 - homeobox containing 1  HDGF - hepatoma-derived growth factor  ZCCHC12 - zinc finger, cchc domain containing 12  ZNF213 - zinc finger protein 213  HTT - huntingtin  HDAC1 - histone deacetylase 1  ZNF205 - zinc finger protein 205  HCLS1 - hematopoietic cell-specific lyn substrate 1  ZNF207 - zinc finger protein 207  XPA - xeroderma pigmentosum, complementation group a  XPC - xeroderma pigmentosum, complementation group c  GOLGA2 - golgin a2  XPO1 - exportin 1 (crm1 homolog, yeast)  GOLGB1 - golgin b1  XRCC1 - x-ray repair complementing defective repair in chinese hamster cells 1  PAGR1 - paxip1 associated glutamate-rich protein 1  ZXDC - zxd family zinc finger c  AAED1 - ahpc/tsa antioxidant enzyme domain containing 1  XBP1 - x-box binding protein 1  ZNF367 - zinc finger protein 367  BHLHE41 - basic helix-loop-helix family, member e41  EIF4H - eukaryotic translation initiation factor 4h  WFS1 - wolfram syndrome 1 (wolframin)  WHSC1 - wolf-hirschhorn syndrome candidate 1  NELFA - negative elongation factor complex member a  VRK2 - vaccinia related kinase 2  ARID2 - at rich interactive domain 2 (arid, rfx-like)  WARS - tryptophanyl-trna synthetase  PAOX - polyamine oxidase (exo-n4-amino)  ZNF35 - zinc finger protein 35  ZKSCAN1 - zinc finger with krab and scan domains 1  FBXL15 - f-box and leucine-rich repeat protein 15  ZNF576 - zinc finger protein 576  ZNF33B - zinc finger protein 33b  ZNF32 - zinc finger protein 32  ZNF45 - zinc finger protein 45  BRCC3 - brca1/brca2-containing complex, subunit 3  MZF1 - myeloid zinc finger 1  ZNF18 - zinc finger protein 18  ZNF16 - zinc finger protein 16  GRB2 - growth factor receptor-bound protein 2  ASXL1 - additional sex combs like 1 (drosophila)  ZNF384 - zinc finger protein 384  TNIP2 - tnfaip3 interacting protein 2  RBM42 - rna binding motif protein 42  ZNF24 - zinc finger protein 24  ZFPL1 - zinc finger protein-like 1  ZFX - zinc finger protein, x-linked  SF1 - splicing factor 1  GRIK2 - glutamate receptor, ionotropic, kainate 2  ZBTB14 - zinc finger and btb domain containing 14  ZNF711 - zinc finger protein 711  ZNF7 - zinc finger protein 7  ZNF8 - zinc finger protein 8  ZNF2 - zinc finger protein 2  ZNF3 - zinc finger protein 3  YES1 - v-yes-1 yamaguchi sarcoma viral oncogene homolog 1  XRCC5 - x-ray repair complementing defective repair in chinese hamster cells 5 (double-strand-break rejoining)  YWHAG - tyrosine 3-monooxygenase/tryptophan 5-monooxygenase activation protein, gamma polypeptide  GRSF1 - g-rich rna sequence binding factor 1  GSK3B - glycogen synthase kinase 3 beta  YWHAZ - tyrosine 3-monooxygenase/tryptophan 5-monooxygenase activation protein, zeta polypeptide  GSK3A - glycogen synthase kinase 3 alpha  YWHAE - tyrosine 3-monooxygenase/tryptophan 5-monooxygenase activation protein, epsilon polypeptide  YWHAB - tyrosine 3-monooxygenase/tryptophan 5-monooxygenase activation protein, beta polypeptide  CCNK - cyclin k  MAPKAP1 - mitogen-activated protein kinase associated protein 1  DNAJB2 - dnaj (hsp40) homolog, subfamily b, member 2  DNAJA1 - dnaj (hsp40) homolog, subfamily a, member 1  HSF2 - heat shock transcription factor 2  TRIM24 - tripartite motif containing 24  TAF1D - tata box binding protein (tbp)-associated factor, rna polymerase i, d, 41kda  CREG1 - cellular repressor of e1a-stimulated genes 1  CHAC1 - chac, cation transport regulator homolog 1 (e. coli)  PEX11A - peroxisomal biogenesis factor 11 alpha  HSBP1 - heat shock factor binding protein 1  FLCN - folliculin  NRP1 - neuropilin 1  PRMT1 - protein arginine methyltransferase 1  IQGAP1 - iq motif containing gtpase activating protein 1  PRMT2 - protein arginine methyltransferase 2  HESX1 - hesx homeobox 1  PHF23 - phd finger protein 23  SAP30 - sin3a-associated protein, 30kda  DERL1 - derlin 1  METTL16 - methyltransferase like 16  ALKBH1 - alkb, alkylation repair homolog 1 (e. coli)  FTO - fat mass and obesity associated  HPRT1 - hypoxanthine phosphoribosyltransferase 1  SPRED2 - sprouty-related, evh1 domain containing 2  CFLAR - casp8 and fadd-like apoptosis regulator  SYNGAP1 - synaptic ras gtpase activating protein 1  SECISBP2 - secis binding protein 2  MED11 - mediator complex subunit 11  LDB1 - lim domain binding 1  WDR77 - wd repeat domain 77  DSCC1 - dna replication and sister chromatid cohesion 1  KAT2B - k(lysine) acetyltransferase 2b  ELOVL6 - elovl fatty acid elongase 6  TSC22D1 - tsc22 domain family, member 1  CRTC2 - creb regulated transcription coactivator 2  DDA1 - det1 and ddb1 associated 1  ZNF259 - zinc finger protein 259  TAX1BP1 - tax1 (human t-cell leukemia virus type i) binding protein 1  CDC123 - cell division cycle 123  CC2D1B - coiled-coil and c2 domain containing 1b  DBNDD1 - dysbindin (dystrobrevin binding protein 1) domain containing 1  MTMR2 - myotubularin related protein 2  NABP2 - nucleic acid binding protein 2  MTMR3 - myotubularin related protein 3  HUS1 - hus1 checkpoint homolog (s. pombe)  EIF2B2 - eukaryotic translation initiation factor 2b, subunit 2 beta, 39kda  THAP8 - thap domain containing 8  CADM4 - cell adhesion molecule 4  BUD31 - bud31 homolog (s. cerevisiae)  EIF2B5 - eukaryotic translation initiation factor 2b, subunit 5 epsilon, 82kda  ZNF655 - zinc finger protein 655  TRAK2 - trafficking protein, kinesin binding 2  MTMR9 - myotubularin related protein 9  HSPD1 - heat shock 60kda protein 1 (chaperonin)  DNAJB1 - dnaj (hsp40) homolog, subfamily b, member 1  ZNF48 - zinc finger protein 48  BCL10 - b-cell cll/lymphoma 10  FUBP3 - far upstream element (fuse) binding protein 3  HSP90AA1 - heat shock protein 90kda alpha (cytosolic), class a member 1  CDK5R2 - cyclin-dependent kinase 5, regulatory subunit 2 (p39)  C7orf49 - chromosome 7 open reading frame 49  BTRC - beta-transducin repeat containing e3 ubiquitin protein ligase  HSPA8 - heat shock 70kda protein 8  WNK1 - wnk lysine deficient protein kinase 1  EIF4EBP3 - eukaryotic translation initiation factor 4e binding protein 3  HMGN1 - high mobility group nucleosome binding domain 1  UBE2Z - ubiquitin-conjugating enzyme e2z  HMGB3 - high mobility group box 3  HMGB2 - high mobility group box 2  LAMTOR3 - late endosomal/lysosomal adaptor, mapk and mtor activator 3  HMGB1 - high mobility group box 1  PHACTR4 - phosphatase and actin regulator 4  EIF3C - eukaryotic translation initiation factor 3, subunit c  EIF3D - eukaryotic translation initiation factor 3, subunit d  BRD9 - bromodomain containing 9  EIF3A - eukaryotic translation initiation factor 3, subunit a  EIF3B - eukaryotic translation initiation factor 3, subunit b  IRS2 - insulin receptor substrate 2  TNKS - tankyrase, trf1-interacting ankyrin-related adp-ribose polymerase  EIF4G3 - eukaryotic translation initiation factor 4 gamma, 3  DAZAP1 - daz associated protein 1  ZBTB10 - zinc finger and btb domain containing 10  AGO1 - argonaute risc catalytic component 1  LATS2 - large tumor suppressor kinase 2  CAPRIN2 - caprin family member 2  ZBTB48 - zinc finger and btb domain containing 48  SRSF9 - serine/arginine-rich splicing factor 9  PEA15 - phosphoprotein enriched in astrocytes 15  NDRG4 - ndrg family member 4  HYAL2 - hyaluronoglucosaminidase 2  ACD - adrenocortical dysplasia homolog (mouse)  GPBP1 - gc-rich promoter binding protein 1  BSCL2 - berardinelli-seip congenital lipodystrophy 2 (seipin)  HFE - hemochromatosis  HIP1 - huntingtin interacting protein 1  UBE2K - ubiquitin-conjugating enzyme e2k  HINT1 - histidine triad nucleotide binding protein 1  HIVEP1 - human immunodeficiency virus type i enhancer binding protein 1  ELP4 - elongator acetyltransferase complex subunit 4  UPF3B - upf3 regulator of nonsense transcripts homolog b (yeast)  UPF3A - upf3 regulator of nonsense transcripts homolog a (yeast)  HIF1A - hypoxia inducible factor 1, alpha subunit (basic helix-loop-helix transcription factor)  MBTPS1 - membrane-bound transcription factor peptidase, site 1  LPIN3 - lipin 3  TRADD - tnfrsf1a-associated via death domain  SNX3 - sorting nexin 3  MYCBP - myc binding protein  EED - embryonic ectoderm development  URI1 - uri1, prefoldin-like chaperone  RIPK1 - receptor (tnfrsf)-interacting serine-threonine kinase 1  HNRNPU - heterogeneous nuclear ribonucleoprotein u (scaffold attachment factor a)  HNRNPK - heterogeneous nuclear ribonucleoprotein k  FBXO22 - f-box protein 22  HNRNPL - heterogeneous nuclear ribonucleoprotein l  TNFSF13 - tumor necrosis factor (ligand) superfamily, member 13  TNFSF12 - tumor necrosis factor (ligand) superfamily, member 12  FBXO4 - f-box protein 4  TINF2 - terf1 (trf1)-interacting nuclear factor 2  ADAM9 - adam metallopeptidase domain 9  ARV1 - arv1 homolog (s. cerevisiae)  P2RY12 - purinergic receptor p2y, g-protein coupled, 12  HNRNPA2B1 - heterogeneous nuclear ribonucleoprotein a2/b1  HNRNPA1 - heterogeneous nuclear ribonucleoprotein a1  HNRNPC - heterogeneous nuclear ribonucleoprotein c (c1/c2)  RIPK2 - receptor-interacting serine-threonine kinase 2  HNRNPAB - heterogeneous nuclear ribonucleoprotein a/b  HNRNPF - heterogeneous nuclear ribonucleoprotein f  HNRNPD - heterogeneous nuclear ribonucleoprotein d (au-rich element rna binding protein 1, 37kda)  HNRNPH1 - heterogeneous nuclear ribonucleoprotein h1 (h)  HNRNPH3 - heterogeneous nuclear ribonucleoprotein h3 (2h9)  MTMR1 - myotubularin related protein 1  HNRNPH2 - heterogeneous nuclear ribonucleoprotein h2 (h')  HMGCR - 3-hydroxy-3-methylglutaryl-coa reductase  HMGCS1 - 3-hydroxy-3-methylglutaryl-coa synthase 1 (soluble)  AIDA - axin interactor, dorsalization associated  HMGA1 - high mobility group at-hook 1  ZNF584 - zinc finger protein 584  FAM129B - family with sequence similarity 129, member b  NR4A1 - nuclear receptor subfamily 4, group a, member 1  PDE12 - phosphodiesterase 12  RFX7 - regulatory factor x, 7  DEPTOR - dep domain containing mtor-interacting protein  HTATSF1 - hiv-1 tat specific factor 1  FOXK2 - forkhead box k2  HAT1 - histone acetyltransferase 1  EIF3K - eukaryotic translation initiation factor 3, subunit k  RPS6KA6 - ribosomal protein s6 kinase, 90kda, polypeptide 6  CRTC3 - creb regulated transcription coactivator 3  RBM15 - rna binding motif protein 15  TNRC6A - trinucleotide repeat containing 6a  ENC1 - ectodermal-neural cortex 1 (with btb domain)  RBMX - rna binding motif protein, x-linked  INHA - inhibin, alpha  PIK3R3 - phosphoinositide-3-kinase, regulatory subunit 3 (gamma)  DESI1 - desumoylating isopeptidase 1  SGSM3 - small g protein signaling modulator 3  PARG - poly (adp-ribose) glycohydrolase  HTRA2 - htra serine peptidase 2  STK39 - serine threonine kinase 39  ING2 - inhibitor of growth family, member 2  ZNF574 - zinc finger protein 574  PPM1D - protein phosphatase, mg2+/mn2+ dependent, 1d  CREB3L2 - camp responsive element binding protein 3-like 2  PCSK1N - proprotein convertase subtilisin/kexin type 1 inhibitor  IPPK - inositol 1,3,4,5,6-pentakisphosphate 2-kinase  ILF2 - interleukin enhancer binding factor 2  UBE2S - ubiquitin-conjugating enzyme e2s  PRPF19 - pre-mrna processing factor 19  ILF3 - interleukin enhancer binding factor 3, 90kda  MAP4K3 - mitogen-activated protein kinase kinase kinase kinase 3  ILK - integrin-linked kinase  CDC14B - cell division cycle 14b  PIAS1 - protein inhibitor of activated stat, 1  SMYD3 - set and mynd domain containing 3  SLCO3A1 - solute carrier organic anion transporter family, member 3a1  MAPKAPK5 - mitogen-activated protein kinase-activated protein kinase 5  SMURF2 - smad specific e3 ubiquitin protein ligase 2  CGGBP1 - cgg triplet repeat binding protein 1  LMO4 - lim domain only 4  CAMK1 - calcium/calmodulin-dependent protein kinase i  CBX4 - chromobox homolog 4  GORASP1 - golgi reassembly stacking protein 1, 65kda  IL6ST - interleukin 6 signal transducer (gp130, oncostatin m receptor)  CSRNP1 - cysteine-serine-rich nuclear protein 1  DGKZ - diacylglycerol kinase, zeta  C5AR2 - complement component 5a receptor 2  SERP1 - stress-associated endoplasmic reticulum protein 1  ZNF25 - zinc finger protein 25  STK16 - serine/threonine kinase 16  ITGA1 - integrin, alpha 1  CCNY - cyclin y  ITGA3 - integrin, alpha 3 (antigen cd49c, alpha 3 subunit of vla-3 receptor)  MADD - map-kinase activating death domain  KHSRP - kh-type splicing regulatory protein  ERLEC1 - endoplasmic reticulum lectin 1  CASK - calcium/calmodulin-dependent serine protein kinase (maguk family)  ITGAV - integrin, alpha v  CDK10 - cyclin-dependent kinase 10  SUDS3 - suppressor of defective silencing 3 homolog (s. cerevisiae)  EIF6 - eukaryotic translation initiation factor 6  SKAP1 - src kinase associated phosphoprotein 1  GZF1 - gdnf-inducible zinc finger protein 1  INSR - insulin receptor  INSM1 - insulinoma-associated 1  AKTIP - akt interacting protein  CDK13 - cyclin-dependent kinase 13  PDCD4 - programmed cell death 4 (neoplastic transformation inhibitor)  RFXANK - regulatory factor x-associated ankyrin-containing protein  INPP5B - inositol polyphosphate-5-phosphatase, 75kda  CSDC2 - cold shock domain containing c2, rna binding  KLF7 - kruppel-like factor 7 (ubiquitous)  IRF2 - interferon regulatory factor 2  IRF3 - interferon regulatory factor 3  RUVBL1 - ruvb-like 1 (e. coli)  IKZF5 - ikaros family zinc finger 5 (pegasus)  IREB2 - iron-responsive element binding protein 2  DIMT1 - dim1 dimethyladenosine transferase 1 homolog (s. cerevisiae)  MED19 - mediator complex subunit 19  ITGA6 - integrin, alpha 6  IRAK2 - interleukin-1 receptor-associated kinase 2  IRAK1 - interleukin-1 receptor-associated kinase 1  CACYBP - calcyclin binding protein  ZSCAN31 - zinc finger and scan domain containing 31  EIF2AK1 - eukaryotic translation initiation factor 2-alpha kinase 1  PAPD5 - pap associated domain containing 5  KIAA1324L - kiaa1324-like  NSD1 - nuclear receptor binding set domain protein 1  RFWD2 - ring finger and wd repeat domain 2, e3 ubiquitin protein ligase  ZBTB11 - zinc finger and btb domain containing 11  RNF25 - ring finger protein 25  MMS19 - mms19 nucleotide excision repair homolog (s. cerevisiae)  DKK3 - dickkopf wnt signaling pathway inhibitor 3  SNX5 - sorting nexin 5  IFNGR1 - interferon gamma receptor 1  ZNF777 - zinc finger protein 777  ID2 - inhibitor of dna binding 2, dominant negative helix-loop-helix protein  ID3 - inhibitor of dna binding 3, dominant negative helix-loop-helix protein  BRPF3 - bromodomain and phd finger containing, 3  STK36 - serine/threonine kinase 36  IRF2BPL - interferon regulatory factor 2 binding protein-like  JAZF1 - jazf zinc finger 1  PLA2G6 - phospholipase a2, group vi (cytosolic, calcium-independent)  FOXK1 - forkhead box k1  BRAT1 - brca1-associated atm activator 1  MAGEF1 - melanoma antigen family f, 1  IDI1 - isopentenyl-diphosphate delta isomerase 1  LTBP4 - latent transforming growth factor beta binding protein 4  ZNF282 - zinc finger protein 282  IGHMBP2 - immunoglobulin mu binding protein 2  CHORDC1 - cysteine and histidine-rich domain (chord) containing 1  RBPJ - recombination signal binding protein for immunoglobulin kappa j region  NSMAF - neutral sphingomyelinase (n-smase) activation associated factor  NCK2 - nck adaptor protein 2  PABPC1 - poly(a) binding protein, cytoplasmic 1  TRUB2 - trub pseudouridine (psi) synthase homolog 2 (e. coli)  SPOP - speckle-type poz protein  CYFIP2 - cytoplasmic fmr1 interacting protein 2  C6orf89 - chromosome 6 open reading frame 89  ZBTB9 - zinc finger and btb domain containing 9  USP21 - ubiquitin specific peptidase 21  ZBTB12 - zinc finger and btb domain containing 12  BCAR3 - breast cancer anti-estrogen resistance 3  NGFRAP1 - nerve growth factor receptor (tnfrsf16) associated protein 1  PHACTR1 - phosphatase and actin regulator 1  NPTN - neuroplastin  IK - ik cytokine, down-regulator of hla ii  JMJD1C - jumonji domain containing 1c  IGBP1 - immunoglobulin (cd79a) binding protein 1  SMARCA5 - swi/snf related, matrix associated, actin dependent regulator of chromatin, subfamily a, member 5  IGF1R - insulin-like growth factor 1 receptor  OGT - o-linked n-acetylglucosamine (glcnac) transferase  PELP1 - proline, glutamate and leucine rich protein 1  RAE1 - rae1 rna export 1 homolog (s. pombe)  LAT - linker for activation of t cells  TSPYL2 - tspy-like 2  IGFBP6 - insulin-like growth factor binding protein 6  SND1 - staphylococcal nuclease and tudor domain containing 1  CUL4B - cullin 4b  PCIF1 - pdx1 c-terminal inhibiting factor 1  CUL2 - cullin 2  CUL3 - cullin 3  ZNF667 - zinc finger protein 667  FOXP1 - forkhead box p1  SUPT3H - suppressor of ty 3 homolog (s. cerevisiae)  BEND6 - ben domain containing 6  ZNF572 - zinc finger protein 572  PCGF5 - polycomb group ring finger 5  WTAP - wilms tumor 1 associated protein  WAC - ww domain containing adaptor with coiled-coil  RBM39 - rna binding motif protein 39  ZNF414 - zinc finger protein 414  PHF21A - phd finger protein 21a  ZNF771 - zinc finger protein 771  LIMK1 - lim domain kinase 1  AKAP12 - a kinase (prka) anchor protein 12  IER2 - immediate early response 2  LIG3 - ligase iii, dna, atp-dependent  NCOR1 - nuclear receptor corepressor 1  LDLR - low density lipoprotein receptor  NCOR2 - nuclear receptor corepressor 2  MTPN - myotrophin  CARTPT - cart prepropeptide  DOT1L - dot1-like histone h3k79 methyltransferase  ABCG1 - atp-binding cassette, sub-family g (white), member 1  EFCAB7 - ef-hand calcium binding domain 7  L3MBTL3 - l(3)mbt-like 3 (drosophila)  ZNF333 - zinc finger protein 333  CIR1 - corepressor interacting with rbpj, 1  SCAND1 - scan domain containing 1  SLX4 - slx4 structure-specific endonuclease subunit  SEC22B - sec22 vesicle trafficking protein homolog b (s. cerevisiae) (gene/pseudogene)  H2AFY - h2a histone family, member y  GTF2IRD1 - gtf2i repeat domain containing 1  FADS1 - fatty acid desaturase 1  GTPBP1 - gtp binding protein 1  ZNF527 - zinc finger protein 527  H6PD - hexose-6-phosphate dehydrogenase (glucose 1-dehydrogenase)  SPIRE2 - spire actin nucleation factor 2  HOPX - hop homeobox  ZNF316 - zinc finger protein 316  ZC3H8 - zinc finger ccch-type containing 8  SOX13 - sry (sex determining region y)-box 13  GOLM1 - golgi membrane protein 1  LMO1 - lim domain only 1 (rhombotin 1)  NACC2 - nacc family member 2, ben and btb (poz) domain containing  IER5 - immediate early response 5  LMNA - lamin a/c  DNAJC27 - dnaj (hsp40) homolog, subfamily c, member 27  CLOCK - clock circadian regulator  KLF3 - kruppel-like factor 3 (basic)  PRKAG2 - protein kinase, amp-activated, gamma 2 non-catalytic subunit  DDX41 - dead (asp-glu-ala-asp) box polypeptide 41  FXR2 - fragile x mental retardation, autosomal homolog 2  LITAF - lipopolysaccharide-induced tnf factor  TBPL1 - tbp-like 1  YTHDF2 - yth domain family, member 2  ZBTB41 - zinc finger and btb domain containing 41  ZFP62 - zfp62 zinc finger protein  BAG5 - bcl2-associated athanogene 5  TMEM59 - transmembrane protein 59  BAG4 - bcl2-associated athanogene 4  PPME1 - protein phosphatase methylesterase 1  ZGPAT - zinc finger, ccch-type with g patch domain  ACTL6B - actin-like 6b  UBR5 - ubiquitin protein ligase e3 component n-recognin 5  IL27RA - interleukin 27 receptor, alpha  EIF4E2 - eukaryotic translation initiation factor 4e family member 2  MED20 - mediator complex subunit 20  UCHL5 - ubiquitin carboxyl-terminal hydrolase l5  ATG5 - autophagy related 5  AFAP1L2 - actin filament associated protein 1-like 2  ROCK2 - rho-associated, coiled-coil containing protein kinase 2  ATRAID - all-trans retinoic acid-induced differentiation factor  ANGPT4 - angiopoietin 4  MAPK8IP1 - mitogen-activated protein kinase 8 interacting protein 1  CRLF3 - cytokine receptor-like factor 3  NGRN - neugrin, neurite outgrowth associated  TAOK3 - tao kinase 3  GLYR1 - glyoxylate reductase 1 homolog (arabidopsis)  FZR1 - fizzy/cell division cycle 20 related 1 (drosophila)  ZBTB7A - zinc finger and btb domain containing 7a  DPY30 - dpy-30 homolog (c. elegans)  MBTPS2 - membrane-bound transcription factor peptidase, site 2  MAGED1 - melanoma antigen family d, 1  GLIS2 - glis family zinc finger 2  IRF2BP2 - interferon regulatory factor 2 binding protein 2  MED27 - mediator complex subunit 27  MED26 - mediator complex subunit 26  CHD6 - chromodomain helicase dna binding protein 6  MED17 - mediator complex subunit 17  MED23 - mediator complex subunit 23  VPS28 - vacuolar protein sorting 28 homolog (s. cerevisiae)  ZNF580 - zinc finger protein 580  ANTXR1 - anthrax toxin receptor 1  CDYL - chromodomain protein, y-like  GTF2IRD2 - gtf2i repeat domain containing 2  ZNF710 - zinc finger protein 710  KDR - kinase insert domain receptor (a type iii receptor tyrosine kinase)  ING4 - inhibitor of growth family, member 4  EIF2AK3 - eukaryotic translation initiation factor 2-alpha kinase 3  INSIG2 - insulin induced gene 2  GGPS1 - geranylgeranyl diphosphate synthase 1  ITM2B - integral membrane protein 2b  MAP4K4 - mitogen-activated protein kinase kinase kinase kinase 4  RHBDD1 - rhomboid domain containing 1  RLIM - ring finger protein, lim domain interacting  SOGA1 - suppressor of glucose, autophagy associated 1  TRAF7 - tnf receptor-associated factor 7, e3 ubiquitin protein ligase  RECQL5 - recq protein-like 5  KRAS - kirsten rat sarcoma viral oncogene homolog  TNPO1 - transportin 1  TGFBRAP1 - transforming growth factor, beta receptor associated protein 1  ZNF706 - zinc finger protein 706  CIAO1 - cytosolic iron-sulfur protein assembly 1  KPNA2 - karyopherin alpha 2 (rag cohort 1, importin alpha 1)  KPNB1 - karyopherin (importin) beta 1  KPNA1 - karyopherin alpha 1 (importin alpha 5)  CAMKK1 - calcium/calmodulin-dependent protein kinase kinase 1, alpha  SUV420H1 - suppressor of variegation 4-20 homolog 1 (drosophila)  WFDC3 - wap four-disulfide core domain 3  PHF20L1 - phd finger protein 20-like 1  MED21 - mediator complex subunit 21  MED10 - mediator complex subunit 10  ZFP28 - zfp28 zinc finger protein  KIT - v-kit hardy-zuckerman 4 feline sarcoma viral oncogene homolog  ABHD5 - abhydrolase domain containing 5  POLDIP3 - polymerase (dna-directed), delta interacting protein 3  DCUN1D5 - dcn1, defective in cullin neddylation 1, domain containing 5  SLIT2 - slit homolog 2 (drosophila)  PAIP2 - poly(a) binding protein interacting protein 2  JUP - junction plakoglobin  CRIM1 - cysteine rich transmembrane bmp regulator 1 (chordin-like)  JAK2 - janus kinase 2  ING5 - inhibitor of growth family, member 5  JAK3 - janus kinase 3  SLC25A33 - solute carrier family 25 (pyrimidine nucleotide carrier), member 33  ZNF219 - zinc finger protein 219  DNAJC30 - dnaj (hsp40) homolog, subfamily c, member 30  PHF20 - phd finger protein 20  TRIP12 - thyroid hormone receptor interactor 12  NUDT16L1 - nudix (nucleoside diphosphate linked moiety x)-type motif 16-like 1  KCNK2 - potassium channel, subfamily k, member 2  ZNHIT3 - zinc finger, hit-type containing 3  ZNF397 - zinc finger protein 397  TRIP4 - thyroid hormone receptor interactor 4  HMGN3 - high mobility group nucleosomal binding domain 3  PHF6 - phd finger protein 6  ZNF235 - zinc finger protein 235  COPS2 - cop9 signalosome subunit 2  ZDHHC2 - zinc finger, dhhc-type containing 2  ZNF639 - zinc finger protein 639  TAOK2 - tao kinase 2  SARNP - sap domain containing ribonucleoprotein  BRMS1L - breast cancer metastasis-suppressor 1-like  LEF1 - lymphoid enhancer-binding factor 1  VPS25 - vacuolar protein sorting 25 homolog (s. cerevisiae)  SMCR8 - smith-magenis syndrome chromosome region, candidate 8  CNOT8 - ccr4-not transcription complex, subunit 8  CDK12 - cyclin-dependent kinase 12  SIRPA - signal-regulatory protein alpha  ZNF280B - zinc finger protein 280b  S1PR2 - sphingosine-1-phosphate receptor 2  SNX25 - sorting nexin 25  ARID4B - at rich interactive domain 4b (rbp1-like)  SPRTN - sprt-like n-terminal domain  SOCS6 - suppressor of cytokine signaling 6  MIF - macrophage migration inhibitory factor (glycosylation-inhibiting factor)  CCNI2 - cyclin i family, member 2  MAP3K10 - mitogen-activated protein kinase kinase kinase 10  DUSP19 - dual specificity phosphatase 19  MLH1 - mutl homolog 1, colon cancer, nonpolyposis type 2 (e. coli)  ITGB1BP1 - integrin beta 1 binding protein 1  KDM3B - lysine (k)-specific demethylase 3b  RSF1 - remodeling and spacing factor 1  MLLT6 - myeloid/lymphoid or mixed-lineage leukemia (trithorax homolog, drosophila); translocated to, 6  SAMD8 - sterile alpha motif domain containing 8  MLLT3 - myeloid/lymphoid or mixed-lineage leukemia (trithorax homolog, drosophila); translocated to, 3  MED14 - mediator complex subunit 14  MLLT1 - myeloid/lymphoid or mixed-lineage leukemia (trithorax homolog, drosophila); translocated to, 1  MAP3K11 - mitogen-activated protein kinase kinase kinase 11  ZBTB22 - zinc finger and btb domain containing 22  KMT2A - lysine (k)-specific methyltransferase 2a  RPS6KA5 - ribosomal protein s6 kinase, 90kda, polypeptide 5  SUFU - suppressor of fused homolog (drosophila)  GPRC5B - g protein-coupled receptor, family c, group 5, member b  MMP14 - matrix metallopeptidase 14 (membrane-inserted)  MFHAS1 - malignant fibrous histiocytoma amplified sequence 1  NLK - nemo-like kinase  UIMC1 - ubiquitin interaction motif containing 1  MNAT1 - mnat cdk-activating kinase assembly factor 1  MTA2 - metastasis associated 1 family, member 2  NOLC1 - nucleolar and coiled-body phosphoprotein 1  EIF2A - eukaryotic translation initiation factor 2a, 65kda  LDLRAD3 - low density lipoprotein receptor class a domain containing 3  WWOX - ww domain containing oxidoreductase  CD200 - cd200 molecule  MNT - mnt, max dimerization protein  CCPG1 - cell cycle progression 1  CD46 - cd46 molecule, complement regulatory protein  NSRP1 - nuclear speckle splicing regulatory protein 1  ME1 - malic enzyme 1, nadp(+)-dependent, cytosolic  SPTY2D1 - spt2, suppressor of ty, domain containing 1 (s. cerevisiae)  PELO - pelota homolog (drosophila)  MECP2 - methyl cpg binding protein 2 (rett syndrome)  MEF2A - myocyte enhancer factor 2a  WDR54 - wd repeat domain 54  ME2 - malic enzyme 2, nad(+)-dependent, mitochondrial  PRICKLE1 - prickle homolog 1 (drosophila)  ZBED1 - zinc finger, bed-type containing 1  BCDIN3D - bcdin3 domain containing  MEIS2 - meis homeobox 2  BRWD1 - bromodomain and wd repeat domain containing 1  ZNF664 - zinc finger protein 664  MEIS1 - meis homeobox 1  MAP3K4 - mitogen-activated protein kinase kinase kinase 4  MAP3K5 - mitogen-activated protein kinase kinase kinase 5  SLC33A1 - solute carrier family 33 (acetyl-coa transporter), member 1  MAP3K1 - mitogen-activated protein kinase kinase kinase 1, e3 ubiquitin protein ligase  MAP3K3 - mitogen-activated protein kinase kinase kinase 3  MEN1 - multiple endocrine neoplasia i  WDR75 - wd repeat domain 75  COX7A2L - cytochrome c oxidase subunit viia polypeptide 2 like  UTP15 - utp15, u3 small nucleolar ribonucleoprotein, homolog (s. cerevisiae)  FAM161A - family with sequence similarity 161, member a  KAT8 - k(lysine) acetyltransferase 8  ZNF644 - zinc finger protein 644  ARHGEF2 - rho/rac guanine nucleotide exchange factor (gef) 2  PPP1R1B - protein phosphatase 1, regulatory (inhibitor) subunit 1b  HGS - hepatocyte growth factor-regulated tyrosine kinase substrate  MGAT5 - mannosyl (alpha-1,6-)-glycoprotein beta-1,6-n-acetyl-glucosaminyltransferase  NEMF - nuclear export mediator factor  LACC1 - laccase (multicopper oxidoreductase) domain containing 1  MBD3 - methyl-cpg binding domain protein 3  KITLG - kit ligand  PTOV1 - prostate tumor overexpressed 1  ZNF394 - zinc finger protein 394  CBFA2T2 - core-binding factor, runt domain, alpha subunit 2; translocated to, 2  MAGOH - mago-nashi homolog, proliferation-associated (drosophila)  GSKIP - gsk3b interacting protein  CXXC5 - cxxc finger protein 5  RQCD1 - rcd1 required for cell differentiation1 homolog (s. pombe)  SMC3 - structural maintenance of chromosomes 3  MARS - methionyl-trna synthetase  IL23A - interleukin 23, alpha subunit p19  MAPT - microtubule-associated protein tau  LATS1 - large tumor suppressor kinase 1  MTA1 - metastasis associated 1  SIRT6 - sirtuin 6  MAP1A - microtubule-associated protein 1a  MTMR4 - myotubularin related protein 4  USP8 - ubiquitin specific peptidase 8  PPHLN1 - periphilin 1  USP14 - ubiquitin specific peptidase 14 (trna-guanine transglycosylase)  UBE2J1 - ubiquitin-conjugating enzyme e2, j1  UNC119 - unc-119 homolog (c. elegans)  NUP43 - nucleoporin 43kda  MBD1 - methyl-cpg binding domain protein 1  PKMYT1 - protein kinase, membrane associated tyrosine/threonine 1  SFMBT1 - scm-like with four mbt domains 1  MAX - myc associated factor x  ZMIZ2 - zinc finger, miz-type containing 2  LDB2 - lim domain binding 2  TRMT112 - trna methyltransferase 11-2 homolog (s. cerevisiae)  ZFP90 - zfp90 zinc finger protein  CDAN1 - codanin 1  NELFCD - negative elongation factor complex member c/d  DIRAS3 - diras family, gtp-binding ras-like 3  ASH2L - ash2 (absent, small, or homeotic)-like (drosophila)  CTDSPL2 - ctd (carboxy-terminal domain, rna polymerase ii, polypeptide a) small phosphatase like 2  ZSCAN29 - zinc finger and scan domain containing 29  PTPLAD1 - protein tyrosine phosphatase-like a domain containing 1  THAP2 - thap domain containing, apoptosis associated protein 2  LSS - lanosterol synthase (2,3-oxidosqualene-lanosterol cyclase)  ITCH - itchy e3 ubiquitin protein ligase  ZNF484 - zinc finger protein 484  L3MBTL2 - l(3)mbt-like 2 (drosophila)  IL34 - interleukin 34  LRP1 - low density lipoprotein receptor-related protein 1  RIOK1 - rio kinase 1  LRP6 - low density lipoprotein receptor-related protein 6  ZNF445 - zinc finger protein 445  AIP - aryl hydrocarbon receptor interacting protein  NUB1 - negative regulator of ubiquitin-like proteins 1  TRAPPC9 - trafficking protein particle complex 9  ASB1 - ankyrin repeat and socs box containing 1  LUM - lumican  BTAF1 - btaf1 rna polymerase ii, b-tfiid transcription factor-associated, 170kda  LYN - v-yes-1 yamaguchi sarcoma viral related oncogene homolog  SPAG9 - sperm associated antigen 9  UBA3 - ubiquitin-like modifier activating enzyme 3  RBFOX3 - rna binding protein, fox-1 homolog (c. elegans) 3  SESN2 - sestrin 2  HIP1R - huntingtin interacting protein 1 related  PARP9 - poly (adp-ribose) polymerase family, member 9  CDK5RAP1 - cdk5 regulatory subunit associated protein 1  RNF8 - ring finger protein 8, e3 ubiquitin protein ligase  BAZ1B - bromodomain adjacent to zinc finger domain, 1b  AZIN1 - antizyme inhibitor 1  MXD1 - max dimerization protein 1  MED15 - mediator complex subunit 15  PIAS4 - protein inhibitor of activated stat, 4  TRIM33 - tripartite motif containing 33  SMAD2 - smad family member 2  SRRT - serrate rna effector molecule homolog (arabidopsis)  MBIP - map3k12 binding inhibitory protein 1  KLF16 - kruppel-like factor 16  HDAC7 - histone deacetylase 7  ARMCX3 - armadillo repeat containing, x-linked 3  TDP2 - tyrosyl-dna phosphodiesterase 2  CAPRIN1 - cell cycle associated protein 1  LARP7 - la ribonucleoprotein domain family, member 7  NBR1 - neighbor of brca1 gene 1  RPS6KA4 - ribosomal protein s6 kinase, 90kda, polypeptide 4  MAFG - v-maf avian musculoaponeurotic fibrosarcoma oncogene homolog g  DPH5 - diphthamide biosynthesis 5  TAF9B - taf9b rna polymerase ii, tata box binding protein (tbp)-associated factor, 31kda  SMAD4 - smad family member 4  NBAS - neuroblastoma amplified sequence  H1FX - h1 histone family, member x  SMAD5 - smad family member 5  LSR - lipolysis stimulated lipoprotein receptor  SMAD7 - smad family member 7  WASL - wiskott-aldrich syndrome-like  RBM4B - rna binding motif protein 4b  ZNF565 - zinc finger protein 565  SAP30BP - sap30 binding protein  MYLIP - myosin regulatory light chain interacting protein  YAF2 - yy1 associated factor 2  TAGLN3 - transgelin 3  SIX5 - six homeobox 5  NCBP1 - nuclear cap binding protein subunit 1, 80kda  ITM2C - integral membrane protein 2c  NDN - necdin, melanoma antigen (mage) family member  ZNF582 - zinc finger protein 582  AKAP9 - a kinase (prka) anchor protein (yotiao) 9  BRD7 - bromodomain containing 7  NCK1 - nck adaptor protein 1  CAB39L - calcium binding protein 39-like  DDX25 - dead (asp-glu-ala-asp) box helicase 25  NCL - nucleolin  MBNL2 - muscleblind-like splicing regulator 2  CEBPZ - ccaat/enhancer binding protein (c/ebp), zeta  TICAM1 - toll-like receptor adaptor molecule 1  CCNL2 - cyclin l2  HNRNPM - heterogeneous nuclear ribonucleoprotein m  ORMDL2 - orm1-like 2 (s. cerevisiae)  TRIM28 - tripartite motif containing 28  DROSHA - drosha, ribonuclease type iii  ATP6AP2 - atpase, h+ transporting, lysosomal accessory protein 2  DNAJC15 - dnaj (hsp40) homolog, subfamily c, member 15  TBK1 - tank-binding kinase 1  NBN - nibrin  ZNF197 - zinc finger protein 197  TDRD3 - tudor domain containing 3  RBM7 - rna binding motif protein 7  RBM5 - rna binding motif protein 5  TRIM11 - tripartite motif containing 11  PPP1R12A - protein phosphatase 1, regulatory subunit 12a  TNK2 - tyrosine kinase, non-receptor, 2  MYT1 - myelin transcription factor 1  ALYREF - aly/ref export factor  CSRNP2 - cysteine-serine-rich nuclear protein 2  NAB1 - ngfi-a binding protein 1 (egr1 binding protein 1)  SENP1 - sumo1/sentrin specific peptidase 1  RNF41 - ring finger protein 41  NAB2 - ngfi-a binding protein 2 (egr1 binding protein 2)  TSHZ1 - teashirt zinc finger homeobox 1  PRMT3 - protein arginine methyltransferase 3  PSME3 - proteasome (prosome, macropain) activator subunit 3 (pa28 gamma; ki)  TNFAIP8L3 - tumor necrosis factor, alpha-induced protein 8-like 3  TRIM13 - tripartite motif containing 13  TRIM8 - tripartite motif containing 8  MYO6 - myosin vi  LBH - limb bud and heart development  ABT1 - activator of basal transcription 1  TOPORS - topoisomerase i binding, arginine/serine-rich, e3 ubiquitin protein ligase  RAB1B - rab1b, member ras oncogene family  MYD88 - myeloid differentiation primary response 88  SEH1L - seh1-like (s. cerevisiae)  ZNF362 - zinc finger protein 362  MYH9 - myosin, heavy chain 9, non-muscle  HUWE1 - hect, uba and wwe domain containing 1, e3 ubiquitin protein ligase  HCFC2 - host cell factor c2  CNOT7 - ccr4-not transcription complex, subunit 7  MXI1 - max interactor 1, dimerization protein  PQBP1 - polyglutamine binding protein 1  TRA2A - transformer 2 alpha homolog (drosophila)  ACTR2 - arp2 actin-related protein 2 homolog (yeast)  ACTR3 - arp3 actin-related protein 3 homolog (yeast)  RBM15B - rna binding motif protein 15b  CTDSP2 - ctd (carboxy-terminal domain, rna polymerase ii, polypeptide a) small phosphatase 2  TSFM - ts translation elongation factor, mitochondrial  FEM1B - fem-1 homolog b (c. elegans)  RAD50 - rad50 homolog (s. cerevisiae)  TRIM37 - tripartite motif containing 37  ZNF558 - zinc finger protein 558  LRPPRC - leucine-rich pentatricopeptide repeat containing  ZNF263 - zinc finger protein 263  SPRY4 - sprouty homolog 4 (drosophila)  PURG - purine-rich element binding protein g  BCAP31 - b-cell receptor-associated protein 31  RBM12 - rna binding motif protein 12  MED25 - mediator complex subunit 25  SNX12 - sorting nexin 12  TRAP1 - tnf receptor-associated protein 1  MED6 - mediator complex subunit 6  ABI1 - abl-interactor 1  PTBP3 - polypyrimidine tract binding protein 3  PPP4R1 - protein phosphatase 4, regulatory subunit 1  DMTF1 - cyclin d binding myb-like transcription factor 1  SH2B3 - sh2b adaptor protein 3  MTIF2 - mitochondrial translational initiation factor 2  PDCD6 - programmed cell death 6  MTM1 - myotubularin 1  ZBTB33 - zinc finger and btb domain containing 33  TANK - traf family member-associated nfkb activator  HDAC5 - histone deacetylase 5  MTF1 - metal-regulatory transcription factor 1  SRA1 - steroid receptor rna activator 1  HDAC6 - histone deacetylase 6  OCLN - occludin  RNF187 - ring finger protein 187  SH2D3C - sh2 domain containing 3c  MT3 - metallothionein 3  MED16 - mediator complex subunit 16  CCDC22 - coiled-coil domain containing 22  SAE1 - sumo1 activating enzyme subunit 1  DNM1L - dynamin 1-like  RANBP9 - ran binding protein 9  DNAJB6 - dnaj (hsp40) homolog, subfamily b, member 6  UBA2 - ubiquitin-like modifier activating enzyme 2  MSN - moesin  MSH3 - muts homolog 3 (e. coli)  MSH2 - muts homolog 2, colon cancer, nonpolyposis type 1 (e. coli)  LPGAT1 - lysophosphatidylglycerol acyltransferase 1  CITED1 - cbp/p300-interacting transactivator, with glu/asp-rich carboxy-terminal domain, 1  ZBTB5 - zinc finger and btb domain containing 5  ZBTB40 - zinc finger and btb domain containing 40  DBNL - drebrin-like  SUPT7L - suppressor of ty 7 (s. cerevisiae)-like  RGCC - regulator of cell cycle  COMMD1 - copper metabolism (murr1) domain containing 1  RBM8A - rna binding motif protein 8a  USP3 - ubiquitin specific peptidase 3  BABAM1 - brisc and brca1 a complex member 1  METTL5 - methyltransferase like 5  USP15 - ubiquitin specific peptidase 15  MED4 - mediator complex subunit 4  THOC1 - tho complex 1  SETD2 - set domain containing 2  RBX1 - ring-box 1, e3 ubiquitin protein ligase  DUSP18 - dual specificity phosphatase 18  MRE11A - mre11 meiotic recombination 11 homolog a (s. cerevisiae)  NUP153 - nucleoporin 153kda  WDR91 - wd repeat domain 91  THRAP3 - thyroid hormone receptor associated protein 3  MED12 - mediator complex subunit 12  MED13 - mediator complex subunit 13  PSMD6 - proteasome (prosome, macropain) 26s subunit, non-atpase, 6  LRIG2 - leucine-rich repeats and immunoglobulin-like domains 2  MED24 - mediator complex subunit 24  ZC3H6 - zinc finger ccch-type containing 6  DTX3L - deltex 3-like (drosophila)  PJA2 - praja ring finger 2, e3 ubiquitin protein ligase  AREL1 - apoptosis resistant e3 ubiquitin protein ligase 1  SETDB1 - set domain, bifurcated 1  ZNF34 - zinc finger protein 34  ORC2 - origin recognition complex, subunit 2  PARD6A - par-6 partitioning defective 6 homolog alpha (c. elegans)  ORC5 - origin recognition complex, subunit 5  TOX4 - tox high mobility group box family member 4  ZNF436 - zinc finger protein 436  RNF141 - ring finger protein 141  PRR7 - proline rich 7 (synaptic)  EPM2AIP1 - epm2a (laforin) interacting protein 1  AGRN - agrin  PPP4R2 - protein phosphatase 4, regulatory subunit 2  NDFIP1 - nedd4 family interacting protein 1  PPP1R26 - protein phosphatase 1, regulatory subunit 26  GLIPR2 - gli pathogenesis-related 2  RBM19 - rna binding motif protein 19  OAZ2 - ornithine decarboxylase antizyme 2  OAZ1 - ornithine decarboxylase antizyme 1  G3BP2 - gtpase activating protein (sh3 domain) binding protein 2  ODC1 - ornithine decarboxylase 1  CCNYL1 - cyclin y-like 1  OGG1 - 8-oxoguanine dna glycosylase  ZSCAN16 - zinc finger and scan domain containing 16  TNKS2 - tankyrase, trf1-interacting ankyrin-related adp-ribose polymerase 2  EXOSC3 - exosome component 3  WDR61 - wd repeat domain 61  APITD1 - apoptosis-inducing, taf9-like domain 1  EXOSC1 - exosome component 1  FIS1 - fission 1 (mitochondrial outer membrane) homolog (s. cerevisiae)  NSF - n-ethylmaleimide-sensitive factor  DAZAP2 - daz associated protein 2  DDR2 - discoidin domain receptor tyrosine kinase 2  IST1 - increased sodium tolerance 1 homolog (yeast)  BCLAF1 - bcl2-associated transcription factor 1  NUMA1 - nuclear mitotic apparatus protein 1  DAB2IP - dab2 interacting protein  EIF4A3 - eukaryotic translation initiation factor 4a3  NUP88 - nucleoporin 88kda  ATG13 - autophagy related 13  GMNN - geminin, dna replication inhibitor  NUP98 - nucleoporin 98kda  NR4A2 - nuclear receptor subfamily 4, group a, member 2  ZNF593 - zinc finger protein 593  ZBTB7B - zinc finger and btb domain containing 7b  MATR3 - matrin 3  NVL - nuclear vcp-like  ZNF691 - zinc finger protein 691  RNF144A - ring finger protein 144a  CREBRF - creb3 regulatory factor  ZNF623 - zinc finger protein 623  RGP1 - rgp1 retrograde golgi transport homolog (s. cerevisiae)  NMD3 - nmd3 ribosome export adaptor  NPM1 - nucleophosmin (nucleolar phosphoprotein b23, numatrin)  ARHGEF11 - rho guanine nucleotide exchange factor (gef) 11  ZBTB24 - zinc finger and btb domain containing 24  ZEB2 - zinc finger e-box binding homeobox 2  THAP4 - thap domain containing 4  CTIF - cbp80/20-dependent translation initiation factor  DUSP16 - dual specificity phosphatase 16  RNF40 - ring finger protein 40, e3 ubiquitin protein ligase  SPOCK2 - sparc/osteonectin, cwcv and kazal-like domains proteoglycan (testican) 2  RB1CC1 - rb1-inducible coiled-coil 1  IFT52 - intraflagellar transport 52 homolog (chlamydomonas)  TSC22D2 - tsc22 domain family, member 2  PAQR3 - progestin and adipoq receptor family member iii  SH3BP5L - sh3-binding domain protein 5-like  NFXL1 - nuclear transcription factor, x-box binding-like 1  NRF1 - nuclear respiratory factor 1  ADIPOR1 - adiponectin receptor 1  SH3D19 - sh3 domain containing 19  CHD9 - chromodomain helicase dna binding protein 9  UBQLN1 - ubiquilin 1  DONSON - downstream neighbor of son  NRBF2 - nuclear receptor binding factor 2  NOP2 - nop2 nucleolar protein  VPRBP - vpr (hiv-1) binding protein  NONO - non-pou domain containing, octamer-binding  RNF34 - ring finger protein 34, e3 ubiquitin protein ligase  MDFIC - myod family inhibitor domain containing  UBQLN2 - ubiquilin 2  IFT74 - intraflagellar transport 74 homolog (chlamydomonas)  NOTCH4 - notch 4  NOVA2 - neuro-oncological ventral antigen 2  HERPUD1 - homocysteine-inducible, endoplasmic reticulum stress-inducible, ubiquitin-like domain member 1  NOVA1 - neuro-oncological ventral antigen 1  CEP290 - centrosomal protein 290kda  NPAT - nuclear protein, ataxia-telangiectasia locus  CNOT2 - ccr4-not transcription complex, subunit 2  PUM1 - pumilio homolog 1 (drosophila)  NAA15 - n(alpha)-acetyltransferase 15, nata auxiliary subunit  NOS3 - nitric oxide synthase 3 (endothelial cell)  PPP6R2 - protein phosphatase 6, regulatory subunit 2  CNOT3 - ccr4-not transcription complex, subunit 3  BAZ2B - bromodomain adjacent to zinc finger domain, 2b  NOTCH1 - notch 1  ST18 - suppression of tumorigenicity 18 (breast carcinoma) (zinc finger protein)  CTC1 - cts telomere maintenance complex component 1  NOTCH3 - notch 3  DHX34 - deah (asp-glu-ala-his) box polypeptide 34  NOTCH2 - notch 2  NFYA - nuclear transcription factor y, alpha  HDAC4 - histone deacetylase 4  TOX - thymocyte selection-associated high mobility group box  NFYC - nuclear transcription factor y, gamma  ZFYVE16 - zinc finger, fyve domain containing 16  CXXC1 - cxxc finger protein 1  NFKBIA - nuclear factor of kappa light polypeptide gene enhancer in b-cells inhibitor, alpha  NFKB1 - nuclear factor of kappa light polypeptide gene enhancer in b-cells 1  KMT2B - lysine (k)-specific methyltransferase 2b  NFKBIB - nuclear factor of kappa light polypeptide gene enhancer in b-cells inhibitor, beta  NFKBIE - nuclear factor of kappa light polypeptide gene enhancer in b-cells inhibitor, epsilon  PIK3R4 - phosphoinositide-3-kinase, regulatory subunit 4  PHACTR2 - phosphatase and actin regulator 2  NKX2-2 - nk2 homeobox 2  CNKSR3 - cnksr family member 3  ZNF606 - zinc finger protein 606  HDAC9 - histone deacetylase 9  SART3 - squamous cell carcinoma antigen recognized by t cells 3  ZNF614 - zinc finger protein 614  EHD4 - eh-domain containing 4  ZFP2 - zfp2 zinc finger protein  SOCS7 - suppressor of cytokine signaling 7  NEK3 - nima-related kinase 3  MINK1 - misshapen-like kinase 1  SOCS5 - suppressor of cytokine signaling 5  NEO1 - neogenin 1  SMARCAL1 - swi/snf related, matrix associated, actin dependent regulator of chromatin, subfamily a-like 1  NEDD8 - neural precursor cell expressed, developmentally down-regulated 8  NEDD9 - neural precursor cell expressed, developmentally down-regulated 9  CTR9 - ctr9, paf1/rna polymerase ii complex component  PPM1F - protein phosphatase, mg2+/mn2+ dependent, 1f  CPEB4 - cytoplasmic polyadenylation element binding protein 4  EPC1 - enhancer of polycomb homolog 1 (drosophila)  NFE2L2 - nuclear factor (erythroid-derived 2)-like 2  RASL11A - ras-like, family 11, member a  MORF4L2 - mortality factor 4 like 2  KLHL15 - kelch-like family member 15  NFATC4 - nuclear factor of activated t-cells, cytoplasmic, calcineurin-dependent 4  PDGFD - platelet derived growth factor d  NUP155 - nucleoporin 155kda  NFIA - nuclear factor i/a  NF1 - neurofibromin 1  CDK5RAP3 - cdk5 regulatory subunit associated protein 3  EDEM1 - er degradation enhancer, mannosidase alpha-like 1  RAPGEF2 - rap guanine nucleotide exchange factor (gef) 2  NUP93 - nucleoporin 93kda  ZNF746 - zinc finger protein 746  VGLL4 - vestigial like 4 (drosophila)  LRRC14 - leucine rich repeat containing 14  KDM4A - lysine (k)-specific demethylase 4a  PHF14 - phd finger protein 14  RDH10 - retinol dehydrogenase 10 (all-trans)  HP1BP3 - heterochromatin protein 1, binding protein 3  ZNF432 - zinc finger protein 432  SAFB2 - scaffold attachment factor b2  MYEF2 - myelin expression factor 2  LPIN2 - lipin 2 |
| GO:0043044 | ATP-dependent chromatin remodeling | 1.33E-5 | 1.58E-3 | 1.37 (10334,54,6996,50) | [+] Show genes  SMARCAD1 - swi/snf-related, matrix-associated actin-dependent regulator of chromatin, subfamily a, containing dead/h box 1  VPS72 - vacuolar protein sorting 72 homolog (s. cerevisiae)  ANP32E - acidic (leucine-rich) nuclear phosphoprotein 32 family, member e  ITGB3BP - integrin beta 3 binding protein (beta3-endonexin)  RSF1 - remodeling and spacing factor 1  NASP - nuclear autoantigenic sperm protein (histone-binding)  CHD4 - chromodomain helicase dna binding protein 4  ACTR8 - arp8 actin-related protein 8 homolog (yeast)  CHD3 - chromodomain helicase dna binding protein 3  CENPT - centromere protein t  PBRM1 - polybromo 1  ACTB - actin, beta  GATAD2B - gata zinc finger domain containing 2b  MTA2 - metastasis associated 1 family, member 2  MLF1IP - mlf1 interacting protein  INO80 - ino80 complex subunit  APITD1 - apoptosis-inducing, taf9-like domain 1  ACTL6A - actin-like 6a  CENPC - centromere protein c  CENPM - centromere protein m  SPTY2D1 - spt2, suppressor of ty, domain containing 1 (s. cerevisiae)  ARID1A - at rich interactive domain 1a (swi-like)  CHD5 - chromodomain helicase dna binding protein 5  ARID1B - at rich interactive domain 1b (swi1-like)  ACTR5 - arp5 actin-related protein 5 homolog (yeast)  CENPO - centromere protein o  SMARCE1 - swi/snf related, matrix associated, actin dependent regulator of chromatin, subfamily e, member 1  RNF8 - ring finger protein 8, e3 ubiquitin protein ligase  INO80B - ino80 complex subunit b  ACTR6 - arp6 actin-related protein 6 homolog (yeast)  ACTL6B - actin-like 6b  SMARCB1 - swi/snf related, matrix associated, actin dependent regulator of chromatin, subfamily b, member 1  SMARCC1 - swi/snf related, matrix associated, actin dependent regulator of chromatin, subfamily c, member 1  PTMA - prothymosin, alpha  RBBP7 - retinoblastoma binding protein 7  SMARCA5 - swi/snf related, matrix associated, actin dependent regulator of chromatin, subfamily a, member 5  SMARCA4 - swi/snf related, matrix associated, actin dependent regulator of chromatin, subfamily a, member 4  NPM1 - nucleophosmin (nucleolar phosphoprotein b23, numatrin)  SMARCD2 - swi/snf related, matrix associated, actin dependent regulator of chromatin, subfamily d, member 2  SMARCC2 - swi/snf related, matrix associated, actin dependent regulator of chromatin, subfamily c, member 2  HNRNPC - heterogeneous nuclear ribonucleoprotein c (c1/c2)  SMARCA1 - swi/snf related, matrix associated, actin dependent regulator of chromatin, subfamily a, member 1  SMARCA2 - swi/snf related, matrix associated, actin dependent regulator of chromatin, subfamily a, member 2  RBBP4 - retinoblastoma binding protein 4  CHD8 - chromodomain helicase dna binding protein 8  RUVBL1 - ruvb-like 1 (e. coli)  HDAC2 - histone deacetylase 2  DMAP1 - dna methyltransferase 1 associated protein 1  MBD3 - methyl-cpg binding domain protein 3  HDAC1 - histone deacetylase 1 |
| GO:0000381 | regulation of alternative mRNA splicing, via spliceosome | 1.52E-5 | 1.79E-3 | 1.34 (10334,64,6996,58) | [+] Show genes  FMR1 - fragile x mental retardation 1  WTAP - wilms tumor 1 associated protein  DDX5 - dead (asp-glu-ala-asp) box helicase 5  MAGOH - mago-nashi homolog, proliferation-associated (drosophila)  SAP18 - sin3a-associated protein, 18kda  RBM15 - rna binding motif protein 15  MBNL2 - muscleblind-like splicing regulator 2  SRSF10 - serine/arginine-rich splicing factor 10  RBMX - rna binding motif protein, x-linked  NOVA2 - neuro-oncological ventral antigen 2  NOVA1 - neuro-oncological ventral antigen 1  SRSF12 - serine/arginine-rich splicing factor 12  SRSF9 - serine/arginine-rich splicing factor 9  RBM19 - rna binding motif protein 19  CELF4 - cugbp, elav-like family member 4  RBM7 - rna binding motif protein 7  RBM5 - rna binding motif protein 5  KHDRBS3 - kh domain containing, rna binding, signal transduction associated 3  DYRK1A - dual-specificity tyrosine-(y)-phosphorylation regulated kinase 1a  RNPS1 - rna binding protein s1, serine-rich domain  SART3 - squamous cell carcinoma antigen recognized by t cells 3  SMU1 - smu-1 suppressor of mec-8 and unc-52 homolog (c. elegans)  CELF3 - cugbp, elav-like family member 3  PUF60 - poly-u binding splicing factor 60kda  NSRP1 - nuclear speckle splicing regulatory protein 1  CELF6 - cugbp, elav-like family member 6  YTHDC1 - yth domain containing 1  FXR2 - fragile x mental retardation, autosomal homolog 2  PTBP1 - polypyrimidine tract binding protein 1  HNRNPU - heterogeneous nuclear ribonucleoprotein u (scaffold attachment factor a)  HNRNPL - heterogeneous nuclear ribonucleoprotein l  RBFOX3 - rna binding protein, fox-1 homolog (c. elegans) 3  DDX17 - dead (asp-glu-ala-asp) box helicase 17  CELF1 - cugbp, elav-like family member 1  RBM17 - rna binding motif protein 17  RBM8A - rna binding motif protein 8a  KHDRBS1 - kh domain containing, rna binding, signal transduction associated 1  CELF2 - cugbp, elav-like family member 2  RBM15B - rna binding motif protein 15b  IK - ik cytokine, down-regulator of hla ii  RBM10 - rna binding motif protein 10  SF1 - splicing factor 1  RBFOX2 - rna binding protein, fox-1 homolog (c. elegans) 2  SRSF1 - serine/arginine-rich splicing factor 1  SRSF2 - serine/arginine-rich splicing factor 2  HNRNPA1 - heterogeneous nuclear ribonucleoprotein a1  RBM4 - rna binding motif protein 4  SRSF3 - serine/arginine-rich splicing factor 3  SRSF4 - serine/arginine-rich splicing factor 4  SRSF5 - serine/arginine-rich splicing factor 5  RBM25 - rna binding motif protein 25  SRSF6 - serine/arginine-rich splicing factor 6  SRSF7 - serine/arginine-rich splicing factor 7  TRA2B - transformer 2 beta homolog (drosophila)  ZC3H10 - zinc finger ccch-type containing 10  ZBTB7A - zinc finger and btb domain containing 7a  THRAP3 - thyroid hormone receptor associated protein 3  RBM4B - rna binding motif protein 4b |
| GO:0065004 | protein-DNA complex assembly | 1.75E-5 | 2.05E-3 | 1.26 (10334,114,6996,97) | [+] Show genes  HAT1 - histone acetyltransferase 1  TSPYL4 - tspy-like 4  CENPV - centromere protein v  SET - set nuclear oncogene  DDB1 - damage-specific dna binding protein 1, 127kda  GTF2H2 - general transcription factor iih, polypeptide 2, 44kda  GTF2H3 - general transcription factor iih, polypeptide 3, 34kda  NAP1L1 - nucleosome assembly protein 1-like 1  RSF1 - remodeling and spacing factor 1  GTF2H1 - general transcription factor iih, polypeptide 1, 62kda  NASP - nuclear autoantigenic sperm protein (histone-binding)  NAP1L4 - nucleosome assembly protein 1-like 4  GTF2A2 - general transcription factor iia, 2, 12kda  GTF2B - general transcription factor iib  PIAS1 - protein inhibitor of activated stat, 1  SMYD3 - set and mynd domain containing 3  BDP1 - b double prime 1, subunit of rna polymerase iii transcription initiation factor iiib  GTF2H4 - general transcription factor iih, polypeptide 4, 52kda  CHD1L - chromodomain helicase dna binding protein 1-like  BRF1 - brf1, rna polymerase iii transcription initiation factor 90 kda subunit  HIST3H2BB - histone cluster 3, h2bb  H2AFY - h2a histone family, member y  IPO4 - importin 4  TSPYL1 - tspy-like 1  MNAT1 - mnat cdk-activating kinase assembly factor 1  ORC3 - origin recognition complex, subunit 3  MLF1IP - mlf1 interacting protein  APITD1 - apoptosis-inducing, taf9-like domain 1  H3F3A - h3 histone, family 3a  HIST1H2BB - histone cluster 1, h2bb  TAF7 - taf7 rna polymerase ii, tata box binding protein (tbp)-associated factor, 55kda  MCM7 - minichromosome maintenance complex component 7  H2AFX - h2a histone family, member x  CENPC - centromere protein c  CENPM - centromere protein m  CETN2 - centrin, ef-hand protein, 2  ANP32B - acidic (leucine-rich) nuclear phosphoprotein 32 family, member b  SPTY2D1 - spt2, suppressor of ty, domain containing 1 (s. cerevisiae)  GMNN - geminin, dna replication inhibitor  UBN1 - ubinuclein 1  BRD2 - bromodomain containing 2  NAA60 - n(alpha)-acetyltransferase 60, natf catalytic subunit  POLR1E - polymerase (rna) i polypeptide e, 53kda  CDC45 - cell division cycle 45  BAZ2A - bromodomain adjacent to zinc finger domain, 2a  SMARCB1 - swi/snf related, matrix associated, actin dependent regulator of chromatin, subfamily b, member 1  DAXX - death-domain associated protein  SMARCA4 - swi/snf related, matrix associated, actin dependent regulator of chromatin, subfamily a, member 4  NPM1 - nucleophosmin (nucleolar phosphoprotein b23, numatrin)  CDK7 - cyclin-dependent kinase 7  PARP1 - poly (adp-ribose) polymerase 1  H2AFY2 - h2a histone family, member y2  RUVBL1 - ruvb-like 1 (e. coli)  TCF4 - transcription factor 4  NAP1L5 - nucleosome assembly protein 1-like 5  RRN3 - rrn3 rna polymerase i transcription factor homolog (s. cerevisiae)  ITGB3BP - integrin beta 3 binding protein (beta3-endonexin)  XPA - xeroderma pigmentosum, complementation group a  XPC - xeroderma pigmentosum, complementation group c  HMGB2 - high mobility group box 2  DR1 - down-regulator of transcription 1, tbp-binding (negative cofactor 2)  RPA3 - replication protein a3, 14kda  RPA1 - replication protein a1, 70kda  RPA2 - replication protein a2, 32kda  CENPT - centromere protein t  CHAF1A - chromatin assembly factor 1, subunit a (p150)  MCM3 - minichromosome maintenance complex component 3  MCM6 - minichromosome maintenance complex component 6  MCM5 - minichromosome maintenance complex component 5  MCM2 - minichromosome maintenance complex component 2  SART3 - squamous cell carcinoma antigen recognized by t cells 3  ERCC5 - excision repair cross-complementing rodent repair deficiency, complementation group 5  ERCC3 - excision repair cross-complementing rodent repair deficiency, complementation group 3  CCNH - cyclin h  ASF1A - anti-silencing function 1a histone chaperone  UBB - ubiquitin b  HIRA - histone cell cycle regulator  CENPO - centromere protein o  ATRX - alpha thalassemia/mental retardation syndrome x-linked  HEY2 - hairy/enhancer-of-split related with yrpw motif 2  TAF1B - tata box binding protein (tbp)-associated factor, rna polymerase i, b, 63kda  RBBP7 - retinoblastoma binding protein 7  SMARCA5 - swi/snf related, matrix associated, actin dependent regulator of chromatin, subfamily a, member 5  UBTF - upstream binding transcription factor, rna polymerase i  KAT6A - k(lysine) acetyltransferase 6a  TSPYL2 - tspy-like 2  RBBP4 - retinoblastoma binding protein 4  TAF1C - tata box binding protein (tbp)-associated factor, rna polymerase i, c, 110kda  CUL4B - cullin 4b  BRF2 - brf2, rna polymerase iii transcription initiation factor 50 kda subunit  RBX1 - ring-box 1, e3 ubiquitin protein ligase  CABIN1 - calcineurin binding protein 1  KAT6B - k(lysine) acetyltransferase 6b  HP1BP3 - heterochromatin protein 1, binding protein 3  H1FX - h1 histone family, member x  ERCC2 - excision repair cross-complementing rodent repair deficiency, complementation group 2  SHPRH - snf2 histone linker phd ring helicase, e3 ubiquitin protein ligase |
| GO:0006378 | mRNA polyadenylation | 1.77E-5 | 2.06E-3 | 1.48 (10334,28,6996,28) | [+] Show genes  SCAF8 - sr-related ctd-associated factor 8  MTPAP - mitochondrial poly(a) polymerase  PAPOLG - poly(a) polymerase gamma  PNPT1 - polyribonucleotide nucleotidyltransferase 1  SYMPK - symplekin  PAPD4 - pap associated domain containing 4  PAPD5 - pap associated domain containing 5  PABPC1 - poly(a) binding protein, cytoplasmic 1  CSTF3 - cleavage stimulation factor, 3' pre-rna, subunit 3, 77kda  CSTF2 - cleavage stimulation factor, 3' pre-rna, subunit 2, 64kda  ZCCHC6 - zinc finger, cchc domain containing 6  CDC73 - cell division cycle 73  AHCYL1 - adenosylhomocysteinase-like 1  CPSF1 - cleavage and polyadenylation specific factor 1, 160kda  CPSF2 - cleavage and polyadenylation specific factor 2, 100kda  ZCCHC11 - zinc finger, cchc domain containing 11  CPSF3 - cleavage and polyadenylation specific factor 3, 73kda  PCF11 - pcf11 cleavage and polyadenylation factor subunit  NUDT21 - nudix (nucleoside diphosphate linked moiety x)-type motif 21  LEO1 - leo1, paf1/rna polymerase ii complex component, homolog (s. cerevisiae)  CLP1 - cleavage and polyadenylation factor i subunit 1  PAPOLA - poly(a) polymerase alpha  WDR33 - wd repeat domain 33  TUT1 - terminal uridylyl transferase 1, u6 snrna-specific  PAF1 - paf1, rna polymerase ii associated factor, homolog (s. cerevisiae)  APP - amyloid beta (a4) precursor protein  GRSF1 - g-rich rna sequence binding factor 1  FIP1L1 - factor interacting with papola and cpsf1 |
| GO:0016482 | cytosolic transport | 1.87E-5 | 2.16E-3 | 1.25 (10334,118,6996,100) | [+] Show genes  TRIM27 - tripartite motif containing 27  TMEM87B - transmembrane protein 87b  DENND5A - denn/madd domain containing 5a  HEATR5A - heat repeat containing 5a  DCTN1 - dynactin 1  PTPN23 - protein tyrosine phosphatase, non-receptor type 23  PPFIA2 - protein tyrosine phosphatase, receptor type, f polypeptide (ptprf), interacting protein (liprin), alpha 2  HOOK3 - hook microtubule-tethering protein 3  SYBU - syntabulin (syntaxin-interacting)  VPS26A - vacuolar protein sorting 26 homolog a (s. pombe)  VPS26B - vacuolar protein sorting 26 homolog b (s. pombe)  SGSM2 - small g protein signaling modulator 2  YKT6 - ykt6 v-snare homolog (s. cerevisiae)  VPS29 - vacuolar protein sorting 29 homolog (s. cerevisiae)  GOSR2 - golgi snap receptor complex member 2  BET1L - bet1 golgi vesicular membrane trafficking protein-like  KIF16B - kinesin family member 16b  MYO1D - myosin id  PREPL - prolyl endopeptidase-like  SNX2 - sorting nexin 2  SNX1 - sorting nexin 1  VPS51 - vacuolar protein sorting 51 homolog (s. cerevisiae)  ARL1 - adp-ribosylation factor-like 1  ANKRD27 - ankyrin repeat domain 27 (vps9 domain)  VPS13C - vacuolar protein sorting 13 homolog c (s. cerevisiae)  CORO7 - coronin 7  NUMA1 - nuclear mitotic apparatus protein 1  GOSR1 - golgi snap receptor complex member 1  STX6 - syntaxin 6  DOPEY1 - dopey family member 1  EVI5 - ecotropic viral integration site 5  FAM160A2 - family with sequence similarity 160, member a2  ACTR2 - arp2 actin-related protein 2 homolog (yeast)  TBC1D5 - tbc1 domain family, member 5  TBC1D10B - tbc1 domain family, member 10b  SNX6 - sorting nexin 6  RGP1 - rgp1 retrograde golgi transport homolog (s. cerevisiae)  PLEKHJ1 - pleckstrin homology domain containing, family j member 1  KLHL20 - kelch-like family member 20  AKTIP - akt interacting protein  AP4M1 - adaptor-related protein complex 4, mu 1 subunit  MON2 - mon2 homolog (s. cerevisiae)  STX8 - syntaxin 8  TBC1D14 - tbc1 domain family, member 14  TMEM87A - transmembrane protein 87a  VPS52 - vacuolar protein sorting 52 homolog (s. cerevisiae)  SNX12 - sorting nexin 12  BAIAP3 - bai1-associated protein 3  ERC1 - elks/rab6-interacting/cast family member 1  AP2A1 - adaptor-related protein complex 2, alpha 1 subunit  AP1G1 - adaptor-related protein complex 1, gamma 1 subunit  SRSF10 - serine/arginine-rich splicing factor 10  KIF5B - kinesin family member 5b  CCDC91 - coiled-coil domain containing 91  KIF1B - kinesin family member 1b  KIF5A - kinesin family member 5a  SYT4 - synaptotagmin iv  VPS35 - vacuolar protein sorting 35 homolog (s. cerevisiae)  RAB6B - rab6b, member ras oncogene family  RAB14 - rab14, member ras oncogene family  VPS54 - vacuolar protein sorting 54 homolog (s. cerevisiae)  SNX5 - sorting nexin 5  TANC2 - tetratricopeptide repeat, ankyrin repeat and coiled-coil containing 2  RAB6A - rab6a, member ras oncogene family  TRAPPC10 - trafficking protein particle complex 10  PQLC1 - pq loop repeat containing 1  BECN1 - beclin 1, autophagy related  RAB5A - rab5a, member ras oncogene family  STX5 - syntaxin 5  RAB7A - rab7a, member ras oncogene family  LMTK2 - lemur tyrosine kinase 2  GGA1 - golgi-associated, gamma adaptin ear containing, arf binding protein 1  TBC1D23 - tbc1 domain family, member 23  EHD3 - eh-domain containing 3  ANKFY1 - ankyrin repeat and fyve domain containing 1  VTI1B - vesicle transport through interaction with t-snares 1b  SYT7 - synaptotagmin vii  SNX16 - sorting nexin 16  SNX3 - sorting nexin 3  GAK - cyclin g associated kinase  RAB9A - rab9a, member ras oncogene family  GCC2 - grip and coiled-coil domain containing 2  CLTC - clathrin, heavy chain (hc)  TBC1D17 - tbc1 domain family, member 17  GBF1 - golgi brefeldin a resistant guanine nucleotide exchange factor 1  CLN5 - ceroid-lipofuscinosis, neuronal 5  RNF126 - ring finger protein 126  SPAG9 - sperm associated antigen 9  EEA1 - early endosome antigen 1  KIF1A - kinesin family member 1a  MAGEL2 - mage-like 2  HEATR5B - heat repeat containing 5b  VPS53 - vacuolar protein sorting 53 homolog (s. cerevisiae)  AP1S1 - adaptor-related protein complex 1, sigma 1 subunit  FAM21A - family with sequence similarity 21, member a  VAMP3 - vesicle-associated membrane protein 3  PIK3C3 - phosphatidylinositol 3-kinase, catalytic subunit type 3  UBE2O - ubiquitin-conjugating enzyme e2o  WDR91 - wd repeat domain 91  EPS15 - epidermal growth factor receptor pathway substrate 15 |
| GO:0048489 | synaptic vesicle transport | 2.14E-5 | 2.44E-3 | 1.38 (10334,47,6996,44) | [+] Show genes  SPG11 - spastic paraplegia 11 (autosomal recessive)  DNAJC5 - dnaj (hsp40) homolog, subfamily c, member 5  AP3M2 - adaptor-related protein complex 3, mu 2 subunit  SNCA - synuclein, alpha (non a4 component of amyloid precursor)  BLOC1S6 - biogenesis of lysosomal organelles complex-1, subunit 6, pallidin  KIF5C - kinesin family member 5c  AP3M1 - adaptor-related protein complex 3, mu 1 subunit  SYT1 - synaptotagmin i  SYT5 - synaptotagmin v  SYT17 - synaptotagmin xvii  KIF5B - kinesin family member 5b  KIF5A - kinesin family member 5a  BLOC1S2 - biogenesis of lysosomal organelles complex-1, subunit 2  SNAP25 - synaptosomal-associated protein, 25kda  LIN7A - lin-7 homolog a (c. elegans)  BLOC1S4 - biogenesis of lysosomal organelles complex-1, subunit 4, cappuccino  AP3S2 - adaptor-related protein complex 3, sigma 2 subunit  RIMS2 - regulating synaptic membrane exocytosis 2  RIMS3 - regulating synaptic membrane exocytosis 3  VAMP2 - vesicle-associated membrane protein 2 (synaptobrevin 2)  CTNNB1 - catenin (cadherin-associated protein), beta 1, 88kda  PIP5K1C - phosphatidylinositol-4-phosphate 5-kinase, type i, gamma  CDK5 - cyclin-dependent kinase 5  STX1A - syntaxin 1a (brain)  BLOC1S5 - biogenesis of lysosomal organelles complex-1, subunit 5, muted  AP3B2 - adaptor-related protein complex 3, beta 2 subunit  RAB3A - rab3a, member ras oncogene family  DNM2 - dynamin 2  AP3B1 - adaptor-related protein complex 3, beta 1 subunit  TOR1A - torsin family 1, member a (torsin a)  CPLX2 - complexin 2  CADPS - ca++-dependent secretion activator  SYT3 - synaptotagmin iii  DOC2B - double c2-like domains, beta  TRIM9 - tripartite motif containing 9  SYNJ1 - synaptojanin 1  SYT9 - synaptotagmin ix  CPLX3 - complexin 3  AP3D1 - adaptor-related protein complex 3, delta 1 subunit  TMEM230 - transmembrane protein 230  STX1B - syntaxin 1b  RAB27A - rab27a, member ras oncogene family  SYT7 - synaptotagmin vii  BLOC1S3 - biogenesis of lysosomal organelles complex-1, subunit 3 |
| GO:0097480 | establishment of synaptic vesicle localization | 2.14E-5 | 2.42E-3 | 1.38 (10334,47,6996,44) | [+] Show genes  SPG11 - spastic paraplegia 11 (autosomal recessive)  DNAJC5 - dnaj (hsp40) homolog, subfamily c, member 5  AP3M2 - adaptor-related protein complex 3, mu 2 subunit  KIF5C - kinesin family member 5c  BLOC1S6 - biogenesis of lysosomal organelles complex-1, subunit 6, pallidin  SNCA - synuclein, alpha (non a4 component of amyloid precursor)  AP3M1 - adaptor-related protein complex 3, mu 1 subunit  SYT1 - synaptotagmin i  KIF5B - kinesin family member 5b  SYT17 - synaptotagmin xvii  SYT5 - synaptotagmin v  KIF5A - kinesin family member 5a  BLOC1S2 - biogenesis of lysosomal organelles complex-1, subunit 2  SNAP25 - synaptosomal-associated protein, 25kda  LIN7A - lin-7 homolog a (c. elegans)  BLOC1S4 - biogenesis of lysosomal organelles complex-1, subunit 4, cappuccino  AP3S2 - adaptor-related protein complex 3, sigma 2 subunit  RIMS2 - regulating synaptic membrane exocytosis 2  RIMS3 - regulating synaptic membrane exocytosis 3  VAMP2 - vesicle-associated membrane protein 2 (synaptobrevin 2)  CTNNB1 - catenin (cadherin-associated protein), beta 1, 88kda  PIP5K1C - phosphatidylinositol-4-phosphate 5-kinase, type i, gamma  CDK5 - cyclin-dependent kinase 5  BLOC1S5 - biogenesis of lysosomal organelles complex-1, subunit 5, muted  STX1A - syntaxin 1a (brain)  RAB3A - rab3a, member ras oncogene family  AP3B2 - adaptor-related protein complex 3, beta 2 subunit  DNM2 - dynamin 2  TOR1A - torsin family 1, member a (torsin a)  AP3B1 - adaptor-related protein complex 3, beta 1 subunit  CADPS - ca++-dependent secretion activator  CPLX2 - complexin 2  SYT3 - synaptotagmin iii  SYNJ1 - synaptojanin 1  TRIM9 - tripartite motif containing 9  DOC2B - double c2-like domains, beta  SYT9 - synaptotagmin ix  AP3D1 - adaptor-related protein complex 3, delta 1 subunit  CPLX3 - complexin 3  TMEM230 - transmembrane protein 230  RAB27A - rab27a, member ras oncogene family  STX1B - syntaxin 1b  BLOC1S3 - biogenesis of lysosomal organelles complex-1, subunit 3  SYT7 - synaptotagmin vii |
| GO:0048193 | Golgi vesicle transport | 2.21E-5 | 2.49E-3 | 1.16 (10334,282,6996,222) | [+] Show genes  TBC1D20 - tbc1 domain family, member 20  SPTAN1 - spectrin, alpha, non-erythrocytic 1  BNIP1 - bcl2/adenovirus e1b 19kda interacting protein 1  KIFAP3 - kinesin-associated protein 3  BET1 - bet1 golgi vesicular membrane trafficking protein  SPIRE1 - spire actin nucleation factor 1  SPTBN4 - spectrin, beta, non-erythrocytic 4  COG8 - component of oligomeric golgi complex 8  RACGAP1 - rac gtpase activating protein 1  TMED2 - transmembrane emp24 domain trafficking protein 2  SPAST - spastin  TMED10 - transmembrane emp24-like trafficking protein 10 (yeast)  ERGIC2 - ergic and golgi 2  CNIH - cornichon homolog (drosophila)  TICAM2 - toll-like receptor adaptor molecule 2  LMAN2L - lectin, mannose-binding 2-like  STEAP2 - steap family member 2, metalloreductase  SEC22B - sec22 vesicle trafficking protein homolog b (s. cerevisiae) (gene/pseudogene)  TFG - trk-fused gene  BET1L - bet1 golgi vesicular membrane trafficking protein-like  SAR1A - sar1 homolog a (s. cerevisiae)  GOSR2 - golgi snap receptor complex member 2  RNF215 - ring finger protein 215  SPIRE2 - spire actin nucleation factor 2  KIF16B - kinesin family member 16b  PREPL - prolyl endopeptidase-like  MYO5A - myosin va (heavy chain 12, myoxin)  SNX2 - sorting nexin 2  SNX1 - sorting nexin 1  LMAN1 - lectin, mannose-binding, 1  LLGL1 - lethal giant larvae homolog 1 (drosophila)  DCTN5 - dynactin 5 (p25)  RAB1B - rab1b, member ras oncogene family  VPS51 - vacuolar protein sorting 51 homolog (s. cerevisiae)  SCAMP2 - secretory carrier membrane protein 2  SCAMP3 - secretory carrier membrane protein 3  VPS13C - vacuolar protein sorting 13 homolog c (s. cerevisiae)  ATP9A - atpase, class ii, type 9a  TRAPPC1 - trafficking protein particle complex 1  SCAMP1 - secretory carrier membrane protein 1  RAB10 - rab10, member ras oncogene family  GOSR1 - golgi snap receptor complex member 1  STX6 - syntaxin 6  DOPEY1 - dopey family member 1  AP1G2 - adaptor-related protein complex 1, gamma 2 subunit  TRAPPC4 - trafficking protein particle complex 4  NRBP1 - nuclear receptor binding protein 1  MON2 - mon2 homolog (s. cerevisiae)  ACTR1A - arp1 actin-related protein 1 homolog a, centractin alpha (yeast)  AP3D1 - adaptor-related protein complex 3, delta 1 subunit  COPB1 - coatomer protein complex, subunit beta 1  COPA - coatomer protein complex, subunit alpha  BCAP31 - b-cell receptor-associated protein 31  SNX12 - sorting nexin 12  GGA2 - golgi-associated, gamma adaptin ear containing, arf binding protein 2  AP2A1 - adaptor-related protein complex 2, alpha 1 subunit  CAPZA1 - capping protein (actin filament) muscle z-line, alpha 1  DCTN4 - dynactin 4 (p62)  AP1G1 - adaptor-related protein complex 1, gamma 1 subunit  KIF3C - kinesin family member 3c  KIF2A - kinesin heavy chain member 2a  MPPE1 - metallophosphoesterase 1  GOPC - golgi-associated pdz and coiled-coil motif containing  KLHL12 - kelch-like family member 12  KIF5A - kinesin family member 5a  PICK1 - protein interacting with prkca 1  PDCD6 - programmed cell death 6  KIF3A - kinesin family member 3a  COG5 - component of oligomeric golgi complex 5  DYNLL1 - dynein, light chain, lc8-type 1  VAMP2 - vesicle-associated membrane protein 2 (synaptobrevin 2)  DYNC1LI1 - dynein, cytoplasmic 1, light intermediate chain 1  CAPZB - capping protein (actin filament) muscle z-line, beta  PITPNB - phosphatidylinositol transfer protein, beta  SEC23A - sec23 homolog a (s. cerevisiae)  STX5 - syntaxin 5  STX4 - syntaxin 4  GOLGA7 - golgin a7  STX17 - syntaxin 17  CUX1 - cut-like homeobox 1  IER3IP1 - immediate early response 3 interacting protein 1  CCDC22 - coiled-coil domain containing 22  EXOC2 - exocyst complex component 2  EXOC8 - exocyst complex component 8  UVRAG - uv radiation resistance associated  KLC1 - kinesin light chain 1  RER1 - rer1 retention in endoplasmic reticulum 1 homolog (s. cerevisiae)  TRAPPC12 - trafficking protein particle complex 12  EXOC1 - exocyst complex component 1  TMEM115 - transmembrane protein 115  VCP - valosin containing protein  EXOC4 - exocyst complex component 4  VTI1B - vesicle transport through interaction with t-snares 1b  ARFGAP3 - adp-ribosylation factor gtpase activating protein 3  SNX3 - sorting nexin 3  GAK - cyclin g associated kinase  GGA3 - golgi-associated, gamma adaptin ear containing, arf binding protein 3  TRAPPC11 - trafficking protein particle complex 11  COG4 - component of oligomeric golgi complex 4  RAB26 - rab26, member ras oncogene family  SEC16A - sec16 homolog a (s. cerevisiae)  KDELR2 - kdel (lys-asp-glu-leu) endoplasmic reticulum protein retention receptor 2  GBF1 - golgi brefeldin a resistant guanine nucleotide exchange factor 1  COG1 - component of oligomeric golgi complex 1  COMMD1 - copper metabolism (murr1) domain containing 1  NRBP2 - nuclear receptor binding protein 2  KIF3B - kinesin family member 3b  RINT1 - rad50 interactor 1  RAB31 - rab31, member ras oncogene family  COG7 - component of oligomeric golgi complex 7  STXBP6 - syntaxin binding protein 6 (amisyn)  KLC2 - kinesin light chain 2  ATP9B - atpase, class ii, type 9b  NAPA - n-ethylmaleimide-sensitive factor attachment protein, alpha  GOLGA5 - golgin a5  NAPG - n-ethylmaleimide-sensitive factor attachment protein, gamma  OSBPL5 - oxysterol binding protein-like 5  PRKD1 - protein kinase d1  SEC24B - sec24 family, member b (s. cerevisiae)  SPTBN1 - spectrin, beta, non-erythrocytic 1  VAMP3 - vesicle-associated membrane protein 3  MIA3 - melanoma inhibitory activity family, member 3  EXOC6B - exocyst complex component 6b  GAS6 - growth arrest-specific 6  EPS15 - epidermal growth factor receptor pathway substrate 15  TRAPPC2 - trafficking protein particle complex 2  SCFD1 - sec1 family domain containing 1  SEC24D - sec24 family, member d (s. cerevisiae)  ERGIC1 - endoplasmic reticulum-golgi intermediate compartment (ergic) 1  USE1 - unconventional snare in the er 1 homolog (s. cerevisiae)  COPB2 - coatomer protein complex, subunit beta 2 (beta prime)  DCTN1 - dynactin 1  CYTH3 - cytohesin 3  DCTN3 - dynactin 3 (p22)  CREB3L2 - camp responsive element binding protein 3-like 2  ACTR10 - actin-related protein 10 homolog (s. cerevisiae)  SCYL1 - scy1-like 1 (s. cerevisiae)  TRAPPC2L - trafficking protein particle complex 2-like  EXOC5 - exocyst complex component 5  BLZF1 - basic leucine zipper nuclear factor 1  ARCN1 - archain 1  BICD2 - bicaudal d homolog 2 (drosophila)  SGSM2 - small g protein signaling modulator 2  YKT6 - ykt6 v-snare homolog (s. cerevisiae)  ARF4 - adp-ribosylation factor 4  TMED5 - transmembrane emp24 protein transport domain containing 5  GOLPH3L - golgi phosphoprotein 3-like  GORASP1 - golgi reassembly stacking protein 1, 65kda  TMED7 - transmembrane emp24 protein transport domain containing 7  TRAPPC6B - trafficking protein particle complex 6b  ARL3 - adp-ribosylation factor-like 3  ARL1 - adp-ribosylation factor-like 1  NSF - n-ethylmaleimide-sensitive factor  DCTN2 - dynactin 2 (p50)  BCAP29 - b-cell receptor-associated protein 29  CORO7 - coronin 7  VAPB - vamp (vesicle-associated membrane protein)-associated protein b and c  MCFD2 - multiple coagulation factor deficiency 2  NUMA1 - nuclear mitotic apparatus protein 1  ZW10 - zw10 kinetochore protein  TAPBP - tap binding protein (tapasin)  ARF5 - adp-ribosylation factor 5  HYOU1 - hypoxia up-regulated 1  COG6 - component of oligomeric golgi complex 6  KLHL20 - kelch-like family member 20  AP4M1 - adaptor-related protein complex 4, mu 1 subunit  TBC1D14 - tbc1 domain family, member 14  STX18 - syntaxin 18  USO1 - uso1 vesicle transport factor  HTT - huntingtin  VPS52 - vacuolar protein sorting 52 homolog (s. cerevisiae)  ARFGEF2 - adp-ribosylation factor guanine nucleotide-exchange factor 2 (brefeldin a-inhibited)  RABEP1 - rabaptin, rab gtpase binding effector protein 1  COG3 - component of oligomeric golgi complex 3  TRAPPC3 - trafficking protein particle complex 3  TMED3 - transmembrane emp24 protein transport domain containing 3  GOLGA2 - golgin a2  GOLGB1 - golgin b1  CCDC91 - coiled-coil domain containing 91  GOLGA3 - golgin a3  RAB6B - rab6b, member ras oncogene family  RAB14 - rab14, member ras oncogene family  SEC31A - sec31 homolog a (s. cerevisiae)  RAB33B - rab33b, member ras oncogene family  VPS54 - vacuolar protein sorting 54 homolog (s. cerevisiae)  RAB6A - rab6a, member ras oncogene family  TRAPPC10 - trafficking protein particle complex 10  RAB1A - rab1a, member ras oncogene family  GGA1 - golgi-associated, gamma adaptin ear containing, arf binding protein 1  SEC24A - sec24 family, member a (s. cerevisiae)  TMED4 - transmembrane emp24 protein transport domain containing 4  RABIF - rab interacting factor  ANKFY1 - ankyrin repeat and fyve domain containing 1  EHD3 - eh-domain containing 3  COPG2 - coatomer protein complex, subunit gamma 2  GCC2 - grip and coiled-coil domain containing 2  COPZ1 - coatomer protein complex, subunit zeta 1  COPG1 - coatomer protein complex, subunit gamma 1  SEC22A - sec22 vesicle trafficking protein homolog a (s. cerevisiae)  EXOC6 - exocyst complex component 6  DCTN6 - dynactin 6  COG2 - component of oligomeric golgi complex 2  PEF1 - penta-ef-hand domain containing 1  TMED6 - transmembrane emp24 protein transport domain containing 6  TGFA - transforming growth factor, alpha  SEC24C - sec24 family, member c (s. cerevisiae)  GOLPH3 - golgi phosphoprotein 3 (coat-protein)  MACF1 - microtubule-actin crosslinking factor 1  GABARAPL2 - gaba(a) receptor-associated protein-like 2  DYNC1H1 - dynein, cytoplasmic 1, heavy chain 1  DYNC1I1 - dynein, cytoplasmic 1, intermediate chain 1  DYNC1I2 - dynein, cytoplasmic 1, intermediate chain 2  DYNC1LI2 - dynein, cytoplasmic 1, light intermediate chain 2  RAB34 - rab34, member ras oncogene family  DNM2 - dynamin 2  C16orf70 - chromosome 16 open reading frame 70  ERGIC3 - ergic and golgi 3  CCDC93 - coiled-coil domain containing 93  CUL3 - cullin 3  NBAS - neuroblastoma amplified sequence  COPE - coatomer protein complex, subunit epsilon  BBS2 - bardet-biedl syndrome 2 |
| GO:0034728 | nucleosome organization | 2.55E-5 | 2.83E-3 | 1.29 (10334,86,6996,75) | [+] Show genes  VPS72 - vacuolar protein sorting 72 homolog (s. cerevisiae)  ANP32E - acidic (leucine-rich) nuclear phosphoprotein 32 family, member e  HAT1 - histone acetyltransferase 1  ITGB3BP - integrin beta 3 binding protein (beta3-endonexin)  TSPYL4 - tspy-like 4  HMGB2 - high mobility group box 2  SUPT6H - suppressor of ty 6 homolog (s. cerevisiae)  SET - set nuclear oncogene  NAP1L1 - nucleosome assembly protein 1-like 1  RSF1 - remodeling and spacing factor 1  NASP - nuclear autoantigenic sperm protein (histone-binding)  BPTF - bromodomain phd finger transcription factor  NAP1L4 - nucleosome assembly protein 1-like 4  SMYD3 - set and mynd domain containing 3  CENPT - centromere protein t  HIST3H2BB - histone cluster 3, h2bb  H2AFY - h2a histone family, member y  CHAF1A - chromatin assembly factor 1, subunit a (p150)  IPO4 - importin 4  TSPYL1 - tspy-like 1  MCM2 - minichromosome maintenance complex component 2  PAF1 - paf1, rna polymerase ii associated factor, homolog (s. cerevisiae)  MLF1IP - mlf1 interacting protein  ARID2 - at rich interactive domain 2 (arid, rfx-like)  SART3 - squamous cell carcinoma antigen recognized by t cells 3  INO80 - ino80 complex subunit  APITD1 - apoptosis-inducing, taf9-like domain 1  H3F3A - h3 histone, family 3a  HIST1H2BB - histone cluster 1, h2bb  H2AFX - h2a histone family, member x  CENPC - centromere protein c  CENPM - centromere protein m  SUPT16H - suppressor of ty 16 homolog (s. cerevisiae)  ANP32B - acidic (leucine-rich) nuclear phosphoprotein 32 family, member b  ASF1A - anti-silencing function 1a histone chaperone  SPTY2D1 - spt2, suppressor of ty, domain containing 1 (s. cerevisiae)  ARID1A - at rich interactive domain 1a (swi-like)  CHD5 - chromodomain helicase dna binding protein 5  HIRA - histone cell cycle regulator  UBN1 - ubinuclein 1  BRD2 - bromodomain containing 2  NAA60 - n(alpha)-acetyltransferase 60, natf catalytic subunit  SMARCE1 - swi/snf related, matrix associated, actin dependent regulator of chromatin, subfamily e, member 1  CENPO - centromere protein o  ATRX - alpha thalassemia/mental retardation syndrome x-linked  RNF8 - ring finger protein 8, e3 ubiquitin protein ligase  MCM3AP - minichromosome maintenance complex component 3 associated protein  CTCF - ccctc-binding factor (zinc finger protein)  ACTR6 - arp6 actin-related protein 6 homolog (yeast)  SMARCB1 - swi/snf related, matrix associated, actin dependent regulator of chromatin, subfamily b, member 1  SMARCC1 - swi/snf related, matrix associated, actin dependent regulator of chromatin, subfamily c, member 1  PTMA - prothymosin, alpha  RBBP7 - retinoblastoma binding protein 7  SMARCA5 - swi/snf related, matrix associated, actin dependent regulator of chromatin, subfamily a, member 5  SMARCA4 - swi/snf related, matrix associated, actin dependent regulator of chromatin, subfamily a, member 4  DAXX - death-domain associated protein  NPM1 - nucleophosmin (nucleolar phosphoprotein b23, numatrin)  SMARCD2 - swi/snf related, matrix associated, actin dependent regulator of chromatin, subfamily d, member 2  SMARCD3 - swi/snf related, matrix associated, actin dependent regulator of chromatin, subfamily d, member 3  SMARCC2 - swi/snf related, matrix associated, actin dependent regulator of chromatin, subfamily c, member 2  SMARCD1 - swi/snf related, matrix associated, actin dependent regulator of chromatin, subfamily d, member 1  KAT6A - k(lysine) acetyltransferase 6a  TSPYL2 - tspy-like 2  RBBP4 - retinoblastoma binding protein 4  RUVBL1 - ruvb-like 1 (e. coli)  H2AFY2 - h2a histone family, member y2  SETD2 - set domain containing 2  DMAP1 - dna methyltransferase 1 associated protein 1  HMGA1 - high mobility group at-hook 1  CABIN1 - calcineurin binding protein 1  KAT6B - k(lysine) acetyltransferase 6b  HP1BP3 - heterochromatin protein 1, binding protein 3  H1FX - h1 histone family, member x  NAP1L5 - nucleosome assembly protein 1-like 5  SHPRH - snf2 histone linker phd ring helicase, e3 ubiquitin protein ligase |
| GO:0032204 | regulation of telomere maintenance | 2.57E-5 | 2.84E-3 | 1.31 (10334,72,6996,64) | [+] Show genes  DHX36 - deah (asp-glu-ala-his) box polypeptide 36  EXOSC10 - exosome component 10  PARN - poly(a)-specific ribonuclease  PAPD5 - pap associated domain containing 5  TCP1 - t-complex 1  NAF1 - nuclear assembly factor 1 ribonucleoprotein  XRCC1 - x-ray repair complementing defective repair in chinese hamster cells 1  PML - promyelocytic leukemia  TNKS - tankyrase, trf1-interacting ankyrin-related adp-ribose polymerase  SIRT6 - sirtuin 6  OBFC1 - oligonucleotide/oligosaccharide-binding fold containing 1  UPF1 - upf1 regulator of nonsense transcripts homolog (yeast)  NBN - nibrin  CTC1 - cts telomere maintenance complex component 1  HDAC8 - histone deacetylase 8  CTNNB1 - catenin (cadherin-associated protein), beta 1, 88kda  POT1 - protection of telomeres 1  MAPK15 - mitogen-activated protein kinase 15  MAPKAPK5 - mitogen-activated protein kinase-activated protein kinase 5  USP7 - ubiquitin specific peptidase 7 (herpes virus-associated)  CCT5 - chaperonin containing tcp1, subunit 5 (epsilon)  SLX4 - slx4 structure-specific endonuclease subunit  ACD - adrenocortical dysplasia homolog (mouse)  SMG6 - smg6 nonsense mediated mrna decay factor  PPP1R10 - protein phosphatase 1, regulatory subunit 10  TNKS2 - tankyrase, trf1-interacting ankyrin-related adp-ribose polymerase 2  TERF2IP - telomeric repeat binding factor 2, interacting protein  CCT6A - chaperonin containing tcp1, subunit 6a (zeta 1)  DKC1 - dyskeratosis congenita 1, dyskerin  PINX1 - pin2/terf1 interacting, telomerase inhibitor 1  DCP2 - decapping mrna 2  ERCC4 - excision repair cross-complementing rodent repair deficiency, complementation group 4  CCT8 - chaperonin containing tcp1, subunit 8 (theta)  NAT10 - n-acetyltransferase 10 (gcn5-related)  TERF1 - telomeric repeat binding factor (nima-interacting) 1  TERF2 - telomeric repeat binding factor 2  HNRNPU - heterogeneous nuclear ribonucleoprotein u (scaffold attachment factor a)  NABP2 - nucleic acid binding protein 2  FBXO4 - f-box protein 4  TINF2 - terf1 (trf1)-interacting nuclear factor 2  ATRX - alpha thalassemia/mental retardation syndrome x-linked  GNL3L - guanine nucleotide binding protein-like 3 (nucleolar)-like  MAP2K7 - mitogen-activated protein kinase kinase 7  MAP3K4 - mitogen-activated protein kinase kinase kinase 4  YLPM1 - ylp motif containing 1  CCT7 - chaperonin containing tcp1, subunit 7 (eta)  CCT3 - chaperonin containing tcp1, subunit 3 (gamma)  SMG1 - smg1 phosphatidylinositol 3-kinase-related kinase  HNRNPA2B1 - heterogeneous nuclear ribonucleoprotein a2/b1  MAPK3 - mitogen-activated protein kinase 3  HNRNPA1 - heterogeneous nuclear ribonucleoprotein a1  MAPK1 - mitogen-activated protein kinase 1  HNRNPC - heterogeneous nuclear ribonucleoprotein c (c1/c2)  CCT2 - chaperonin containing tcp1, subunit 2 (beta)  HNRNPD - heterogeneous nuclear ribonucleoprotein d (au-rich element rna binding protein 1, 37kda)  CCT4 - chaperonin containing tcp1, subunit 4 (delta)  RAD50 - rad50 homolog (s. cerevisiae)  PARP1 - poly (adp-ribose) polymerase 1  HMBOX1 - homeobox containing 1  SRC - v-src avian sarcoma (schmidt-ruppin a-2) viral oncogene homolog  XRCC5 - x-ray repair complementing defective repair in chinese hamster cells 5 (double-strand-break rejoining)  MRE11A - mre11 meiotic recombination 11 homolog a (s. cerevisiae)  PNKP - polynucleotide kinase 3'-phosphatase  PKIB - protein kinase (camp-dependent, catalytic) inhibitor beta |
| GO:0051649 | establishment of localization in cell | 2.71E-5 | 2.97E-3 | 1.07 (10334,1381,6996,1000) | [+] Show genes  AGAP2 - arfgap with gtpase domain, ankyrin repeat and ph domain 2  SYMPK - symplekin  AP3M2 - adaptor-related protein complex 3, mu 2 subunit  WLS - wntless homolog (drosophila)  SEC61B - sec61 beta subunit  ABCA1 - atp-binding cassette, sub-family a (abc1), member 1  TOMM34 - translocase of outer mitochondrial membrane 34  GEM - gtp binding protein overexpressed in skeletal muscle  OS9 - osteosarcoma amplified 9, endoplasmic reticulum lectin  RAB3IP - rab3a interacting protein  TMED2 - transmembrane emp24 domain trafficking protein 2  SSX2IP - synovial sarcoma, x breakpoint 2 interacting protein  NUDT19 - nudix (nucleoside diphosphate linked moiety x)-type motif 19  ERP29 - endoplasmic reticulum protein 29  PML - promyelocytic leukemia  ACACA - acetyl-coa carboxylase alpha  ACOT2 - acyl-coa thioesterase 2  PMM1 - phosphomannomutase 1  MKKS - mckusick-kaufman syndrome  YWHAQ - tyrosine 3-monooxygenase/tryptophan 5-monooxygenase activation protein, theta polypeptide  TMED10 - transmembrane emp24-like trafficking protein 10 (yeast)  ANP32A - acidic (leucine-rich) nuclear phosphoprotein 32 family, member a  TBC1D22B - tbc1 domain family, member 22b  AP3B2 - adaptor-related protein complex 3, beta 2 subunit  NOP9 - nop9 nucleolar protein  ACOX1 - acyl-coa oxidase 1, palmitoyl  SYBU - syntabulin (syntaxin-interacting)  PABPN1 - poly(a) binding protein, nuclear 1  RNPS1 - rna binding protein s1, serine-rich domain  ACTB - actin, beta  GJA1 - gap junction protein, alpha 1, 43kda  KIF16B - kinesin family member 16b  ACTG1 - actin, gamma 1  PLD2 - phospholipase d2  EHD1 - eh-domain containing 1  ZDHHC7 - zinc finger, dhhc-type containing 7  PICALM - phosphatidylinositol binding clathrin assembly protein  ACTN4 - actinin, alpha 4  RAB10 - rab10, member ras oncogene family  GLE1 - gle1 rna export mediator  TMCO6 - transmembrane and coiled-coil domains 6  CPSF4 - cleavage and polyadenylation specific factor 4, 30kda  MED1 - mediator complex subunit 1  NUP85 - nucleoporin 85kda  USP9X - ubiquitin specific peptidase 9, x-linked  ADPRH - adp-ribosylarginine hydrolase  SNAP47 - synaptosomal-associated protein, 47kda  POLR2D - polymerase (rna) ii (dna directed) polypeptide d  AP2A2 - adaptor-related protein complex 2, alpha 2 subunit  AP2A1 - adaptor-related protein complex 2, alpha 1 subunit  AP2B1 - adaptor-related protein complex 2, beta 1 subunit  CDK5RAP2 - cdk5 regulatory subunit associated protein 2  TTC21B - tetratricopeptide repeat domain 21b  AP1B1 - adaptor-related protein complex 1, beta 1 subunit  AP1G1 - adaptor-related protein complex 1, gamma 1 subunit  NUP133 - nucleoporin 133kda  AP5M1 - adaptor-related protein complex 5, mu 1 subunit  AGK - acylglycerol kinase  NUPL2 - nucleoporin like 2  VPS35 - vacuolar protein sorting 35 homolog (s. cerevisiae)  KIF3A - kinesin family member 3a  GEMIN6 - gem (nuclear organelle) associated protein 6  ZWINT - zw10 interacting kinetochore protein  PPT1 - palmitoyl-protein thioesterase 1  RAB7A - rab7a, member ras oncogene family  AKT1 - v-akt murine thymoma viral oncogene homolog 1  PCID2 - pci domain containing 2  BBS12 - bardet-biedl syndrome 12  SLBP - stem-loop binding protein  EVI5L - ecotropic viral integration site 5-like  TBC1D23 - tbc1 domain family, member 23  PPP1R10 - protein phosphatase 1, regulatory subunit 10  IFT122 - intraflagellar transport 122 homolog (chlamydomonas)  TMEM115 - transmembrane protein 115  DDX39B - dead (asp-glu-ala-asp) box polypeptide 39b  SCYL2 - scy1-like 2 (s. cerevisiae)  GAK - cyclin g associated kinase  TBC1D17 - tbc1 domain family, member 17  PEX26 - peroxisomal biogenesis factor 26  RAB35 - rab35, member ras oncogene family  IFT27 - intraflagellar transport 27 homolog (chlamydomonas)  NPLOC4 - nuclear protein localization 4 homolog (s. cerevisiae)  KDELR2 - kdel (lys-asp-glu-leu) endoplasmic reticulum protein retention receptor 2  AAAS - achalasia, adrenocortical insufficiency, alacrimia  RNF126 - ring finger protein 126  IFT88 - intraflagellar transport 88 homolog (chlamydomonas)  RAB31 - rab31, member ras oncogene family  RNF157 - ring finger protein 157  MAPK10 - mitogen-activated protein kinase 10  GEMIN7 - gem (nuclear organelle) associated protein 7  WDR11 - wd repeat domain 11  AMPH - amphiphysin  MICALL2 - mical-like 2  NDC1 - ndc1 transmembrane nucleoporin  CCDC88A - coiled-coil domain containing 88a  IPO9 - importin 9  STAM - signal transducing adaptor molecule (sh3 domain and itam motif) 1  PRKCI - protein kinase c, iota  NUP214 - nucleoporin 214kda  NR4A3 - nuclear receptor subfamily 4, group a, member 3  GAS6 - growth arrest-specific 6  NCOA4 - nuclear receptor coactivator 4  PRKAG1 - protein kinase, amp-activated, gamma 1 non-catalytic subunit  DDX19B - dead (asp-glu-ala-asp) box polypeptide 19b  SDAD1 - sda1 domain containing 1  SYNRG - synergin, gamma  DCTN3 - dynactin 3 (p22)  MCPH1 - microcephalin 1  APBA1 - amyloid beta (a4) precursor protein-binding, family a, member 1  SEC61A2 - sec61 alpha 2 subunit (s. cerevisiae)  XPOT - exportin, trna  CHP1 - calcineurin-like ef-hand protein 1  VPS13D - vacuolar protein sorting 13 homolog d (s. cerevisiae)  SEC63 - sec63 homolog (s. cerevisiae)  ARCN1 - archain 1  PDCD10 - programmed cell death 10  TRIM23 - tripartite motif containing 23  APPL2 - adaptor protein, phosphotyrosine interaction, ph domain and leucine zipper containing 2  ARF1 - adp-ribosylation factor 1  IPO4 - importin 4  ARF4 - adp-ribosylation factor 4  MAP1S - microtubule-associated protein 1s  PAFAH1B1 - platelet-activating factor acetylhydrolase 1b, regulatory subunit 1 (45kda)  ARL8B - adp-ribosylation factor-like 8b  AKAP13 - a kinase (prka) anchor protein 13  APP - amyloid beta (a4) precursor protein  ZDHHC14 - zinc finger, dhhc-type containing 14  BBS7 - bardet-biedl syndrome 7  DDX20 - dead (asp-glu-ala-asp) box polypeptide 20  ARNTL - aryl hydrocarbon receptor nuclear translocator-like  TRAPPC6B - trafficking protein particle complex 6b  ARL3 - adp-ribosylation factor-like 3  ARL1 - adp-ribosylation factor-like 1  TUBA1A - tubulin, alpha 1a  CORO7 - coronin 7  IFT57 - intraflagellar transport 57 homolog (chlamydomonas)  SEC23IP - sec23 interacting protein  ALMS1 - alstrom syndrome 1  RHOB - ras homolog family member b  PSIP1 - pc4 and sfrs1 interacting protein 1  ARF6 - adp-ribosylation factor 6  ARF5 - adp-ribosylation factor 5  EVI5 - ecotropic viral integration site 5  ARHGAP1 - rho gtpase activating protein 1  AP4S1 - adaptor-related protein complex 4, sigma 1 subunit  ASNA1 - arsa arsenite transporter, atp-binding, homolog 1 (bacterial)  WDR60 - wd repeat domain 60  CHMP1A - charged multivesicular body protein 1a  PLEKHJ1 - pleckstrin homology domain containing, family j member 1  MAGOHB - mago-nashi homolog b (drosophila)  PCM1 - pericentriolar material 1  RABL2B - rab, member of ras oncogene family-like 2b  TBC1D8 - tbc1 domain family, member 8 (with gram domain)  CDC37 - cell division cycle 37  HTT - huntingtin  DDX19A - dead (asp-glu-ala-asp) box polypeptide 19a  DENND3 - denn/madd domain containing 3  GOLGA2 - golgin a2  RPH3A - rabphilin 3a homolog (mouse)  XPO1 - exportin 1 (crm1 homolog, yeast)  GOLGB1 - golgin b1  CCDC91 - coiled-coil domain containing 91  ATP1B1 - atpase, na+/k+ transporting, beta 1 polypeptide  LIN7C - lin-7 homolog c (c. elegans)  MON1B - mon1 secretory trafficking family member b  LRRK2 - leucine-rich repeat kinase 2  BLOC1S4 - biogenesis of lysosomal organelles complex-1, subunit 4, cappuccino  CLSTN1 - calsyntenin 1  AP5S1 - adaptor-related protein complex 5, sigma 1 subunit  SEC31A - sec31 homolog a (s. cerevisiae)  WRB - tryptophan rich basic protein  PEX6 - peroxisomal biogenesis factor 6  LSG1 - large 60s subunit nuclear export gtpase 1  ATG14 - autophagy related 14  PEX7 - peroxisomal biogenesis factor 7  PEX12 - peroxisomal biogenesis factor 12  PEX13 - peroxisomal biogenesis factor 13  PEX14 - peroxisomal biogenesis factor 14  RILPL2 - rab interacting lysosomal protein-like 2  LMTK2 - lemur tyrosine kinase 2  ICK - intestinal cell (mak-like) kinase  WDR33 - wd repeat domain 33  PEX1 - peroxisomal biogenesis factor 1  RAB11FIP2 - rab11 family interacting protein 2 (class i)  ATP2B4 - atpase, ca++ transporting, plasma membrane 4  PAOX - polyamine oxidase (exo-n4-amino)  BAX - bcl2-associated x protein  PKD1 - polycystic kidney disease 1 (autosomal dominant)  PKD2 - polycystic kidney disease 2 (autosomal dominant)  RASA3 - ras p21 protein activator 3  THOC6 - tho complex 6 homolog (drosophila)  COPZ1 - coatomer protein complex, subunit zeta 1  COPG1 - coatomer protein complex, subunit gamma 1  BAD - bcl2-associated agonist of cell death  PIK3R1 - phosphoinositide-3-kinase, regulatory subunit 1 (alpha)  COX18 - cox18 cytochrome c oxidase assembly factor  KIF1A - kinesin family member 1a  COG2 - component of oligomeric golgi complex 2  PEF1 - penta-ef-hand domain containing 1  CASC3 - cancer susceptibility candidate 3  GRIK5 - glutamate receptor, ionotropic, kainate 5  VPS53 - vacuolar protein sorting 53 homolog (s. cerevisiae)  RABL3 - rab, member of ras oncogene family-like 3  GABARAP - gaba(a) receptor-associated protein  U2AF2 - u2 small nuclear rna auxiliary factor 2  BCR - breakpoint cluster region  RHOT1 - ras homolog family member t1  PIK3C3 - phosphatidylinositol 3-kinase, catalytic subunit type 3  YWHAG - tyrosine 3-monooxygenase/tryptophan 5-monooxygenase activation protein, gamma polypeptide  COPE - coatomer protein complex, subunit epsilon  YWHAZ - tyrosine 3-monooxygenase/tryptophan 5-monooxygenase activation protein, zeta polypeptide  PARK7 - parkinson protein 7  BBS4 - bardet-biedl syndrome 4  BBS2 - bardet-biedl syndrome 2  VPS45 - vacuolar protein sorting 45 homolog (s. cerevisiae)  YWHAE - tyrosine 3-monooxygenase/tryptophan 5-monooxygenase activation protein, epsilon polypeptide  YWHAB - tyrosine 3-monooxygenase/tryptophan 5-monooxygenase activation protein, beta polypeptide  TRIM27 - tripartite motif containing 27  TBC1D20 - tbc1 domain family, member 20  DST - dystonin  BET1 - bet1 golgi vesicular membrane trafficking protein  LIN7A - lin-7 homolog a (c. elegans)  KATNB1 - katanin p80 (wd repeat containing) subunit b 1  ALKBH5 - alkb, alkylation repair homolog 5 (e. coli)  AGFG1 - arfgap with fg repeats 1  UPF1 - upf1 regulator of nonsense transcripts homolog (yeast)  DERL1 - derlin 1  DSCR3 - down syndrome critical region gene 3  RANBP6 - ran binding protein 6  FNBP1L - formin binding protein 1-like  TFG - trk-fused gene  SYNJ1 - synaptojanin 1  RNF215 - ring finger protein 215  NDE1 - nude neurodevelopment protein 1  VPS51 - vacuolar protein sorting 51 homolog (s. cerevisiae)  ABCE1 - atp-binding cassette, sub-family e (oabp), member 1  VPS13C - vacuolar protein sorting 13 homolog c (s. cerevisiae)  PTTG1IP - pituitary tumor-transforming 1 interacting protein  TMEM50B - transmembrane protein 50b  ARL8A - adp-ribosylation factor-like 8a  STX6 - syntaxin 6  AP1M1 - adaptor-related protein complex 1, mu 1 subunit  AP1G2 - adaptor-related protein complex 1, gamma 2 subunit  TMEM201 - transmembrane protein 201  AP1S2 - adaptor-related protein complex 1, sigma 2 subunit  AP3S2 - adaptor-related protein complex 3, sigma 2 subunit  U2AF1L4 - u2 small nuclear rna auxiliary factor 1-like 4  AFTPH - aftiphilin  MCM3AP - minichromosome maintenance complex component 3 associated protein  CACNA1C - calcium channel, voltage-dependent, l type, alpha 1c subunit  TMEM108 - transmembrane protein 108  HERC2 - hect and rld domain containing e3 ubiquitin protein ligase 2  TRAK2 - trafficking protein, kinesin binding 2  TSPO - translocator protein (18kda)  SRRM1 - serine/arginine repetitive matrix 1  SPRY1 - sprouty homolog 1, antagonist of fgf signaling (drosophila)  SPRY2 - sprouty homolog 2 (drosophila)  STAM2 - signal transducing adaptor molecule (sh3 domain and itam motif) 2  HSPD1 - heat shock 60kda protein 1 (chaperonin)  RAMP2 - receptor (g protein-coupled) activity modifying protein 2  HSP90AA1 - heat shock protein 90kda alpha (cytosolic), class a member 1  RAMP3 - receptor (g protein-coupled) activity modifying protein 3  AP3D1 - adaptor-related protein complex 3, delta 1 subunit  RAMP1 - receptor (g protein-coupled) activity modifying protein 1  ZMPSTE24 - zinc metallopeptidase ste24  HSPA4 - heat shock 70kda protein 4  BAIAP3 - bai1-associated protein 3  HSPA9 - heat shock 70kda protein 9 (mortalin)  WASF1 - was protein family, member 1  HSPA8 - heat shock 70kda protein 8  CAPZA1 - capping protein (actin filament) muscle z-line, alpha 1  TOMM40 - translocase of outer mitochondrial membrane 40 homolog (yeast)  CANX - calnexin  TIMM17A - translocase of inner mitochondrial membrane 17 homolog a (yeast)  RPL17 - ribosomal protein l17  VPS37C - vacuolar protein sorting 37 homolog c (s. cerevisiae)  RPL15 - ribosomal protein l15  MPPE1 - metallophosphoesterase 1  RPL11 - ribosomal protein l11  RPL9 - ribosomal protein l9  TIMM10B - translocase of inner mitochondrial membrane 10 homolog b (yeast)  COG5 - component of oligomeric golgi complex 5  RPL7 - ribosomal protein l7  DYNLL1 - dynein, light chain, lc8-type 1  PIBF1 - progesterone immunomodulatory binding factor 1  HEATR3 - heat repeat containing 3  RPL3 - ribosomal protein l3  TIMM9 - translocase of inner mitochondrial membrane 9 homolog (yeast)  CAPZB - capping protein (actin filament) muscle z-line, beta  RPS4X - ribosomal protein s4, x-linked  SEC23A - sec23 homolog a (s. cerevisiae)  BECN1 - beclin 1, autophagy related  SRSF9 - serine/arginine-rich splicing factor 9  RPS4Y1 - ribosomal protein s4, y-linked 1  SEC23B - sec23 homolog b (s. cerevisiae)  STX17 - syntaxin 17  CACNB3 - calcium channel, voltage-dependent, beta 3 subunit  ACD - adrenocortical dysplasia homolog (mouse)  LAMTOR1 - late endosomal/lysosomal adaptor, mapk and mtor activator 1  NOL6 - nucleolar protein 6 (rna-associated)  CALR - calreticulin  UNC13B - unc-13 homolog b (c. elegans)  CDC23 - cell division cycle 23  VPS33A - vacuolar protein sorting 33 homolog a (s. cerevisiae)  HIP1 - huntingtin interacting protein 1  RPL32 - ribosomal protein l32  STK25 - serine/threonine kinase 25  UPF3B - upf3 regulator of nonsense transcripts homolog b (yeast)  VTI1B - vesicle transport through interaction with t-snares 1b  UPF3A - upf3 regulator of nonsense transcripts homolog a (yeast)  HIF1A - hypoxia inducible factor 1, alpha subunit (basic helix-loop-helix transcription factor)  MBTPS1 - membrane-bound transcription factor peptidase, site 1  ARFGAP3 - adp-ribosylation factor gtpase activating protein 3  RPS23 - ribosomal protein s23  SNX3 - sorting nexin 3  PINX1 - pin2/terf1 interacting, telomerase inhibitor 1  SNX4 - sorting nexin 4  TUBA1B - tubulin, alpha 1b  TSNARE1 - t-snare domain containing 1  TUBB - tubulin, beta class i  GBF1 - golgi brefeldin a resistant guanine nucleotide exchange factor 1  HNRNPU - heterogeneous nuclear ribonucleoprotein u (scaffold attachment factor a)  GEMIN8 - gem (nuclear organelle) associated protein 8  FBXO22 - f-box protein 22  RPS15 - ribosomal protein s15  RPS12 - ribosomal protein s12  VPS33B - vacuolar protein sorting 33 homolog b (yeast)  NRBP2 - nuclear receptor binding protein 2  ARV1 - arv1 homolog (s. cerevisiae)  CBL - cbl proto-oncogene, e3 ubiquitin protein ligase  HNRNPA2B1 - heterogeneous nuclear ribonucleoprotein a2/b1  RAB11A - rab11a, member ras oncogene family  HNRNPA1 - heterogeneous nuclear ribonucleoprotein a1  NAPA - n-ethylmaleimide-sensitive factor attachment protein, alpha  NAPG - n-ethylmaleimide-sensitive factor attachment protein, gamma  SEC24B - sec24 family, member b (s. cerevisiae)  HMGA1 - high mobility group at-hook 1  CMTM6 - cklf-like marvel transmembrane domain containing 6  TBC1D15 - tbc1 domain family, member 15  PSAP - prosaposin  DESI1 - desumoylating isopeptidase 1  STXBP5 - syntaxin binding protein 5 (tomosyn)  PEX3 - peroxisomal biogenesis factor 3  SGSM3 - small g protein signaling modulator 3  RANBP3 - ran binding protein 3  PPFIA2 - protein tyrosine phosphatase, receptor type, f polypeptide (ptprf), interacting protein (liprin), alpha 2  PPFIA1 - protein tyrosine phosphatase, receptor type, f polypeptide (ptprf), interacting protein (liprin), alpha 1  RABGEF1 - rab guanine nucleotide exchange factor (gef) 1  CREB3L2 - camp responsive element binding protein 3-like 2  STARD4 - star-related lipid transfer (start) domain containing 4  SCARB2 - scavenger receptor class b, member 2  AP3B1 - adaptor-related protein complex 3, beta 1 subunit  YKT6 - ykt6 v-snare homolog (s. cerevisiae)  PPFIA3 - protein tyrosine phosphatase, receptor type, f polypeptide (ptprf), interacting protein (liprin), alpha 3  AGPS - alkylglycerone phosphate synthase  CAMK1 - calcium/calmodulin-dependent protein kinase i  GDAP1 - ganglioside induced differentiation associated protein 1  GORASP1 - golgi reassembly stacking protein 1, 65kda  GRPEL2 - grpe-like 2, mitochondrial (e. coli)  PITRM1 - pitrilysin metallopeptidase 1  VPS16 - vacuolar protein sorting 16 homolog (s. cerevisiae)  DCTN2 - dynactin 2 (p50)  APPBP2 - amyloid beta precursor protein (cytoplasmic tail) binding protein 2  TRAM1L1 - translocation associated membrane protein 1-like 1  CASK - calcium/calmodulin-dependent serine protein kinase (maguk family)  ARFIP1 - adp-ribosylation factor interacting protein 1  CHERP - calcium homeostasis endoplasmic reticulum protein  ITGB1 - integrin, beta 1 (fibronectin receptor, beta polypeptide, antigen cd29 includes mdf2, msk12)  HYOU1 - hypoxia up-regulated 1  PTK2 - protein tyrosine kinase 2  ZDHHC6 - zinc finger, dhhc-type containing 6  IPO8 - importin 8  IPO7 - importin 7  EIF6 - eukaryotic translation initiation factor 6  TPCN2 - two pore segment channel 2  SLU7 - slu7 splicing factor homolog (s. cerevisiae)  CDK5 - cyclin-dependent kinase 5  ATG3 - autophagy related 3  KLHL20 - kelch-like family member 20  NPC2 - niemann-pick disease, type c2  AKTIP - akt interacting protein  CADPS - ca++-dependent secretion activator  HTATIP2 - hiv-1 tat interactive protein 2, 30kda  USO1 - uso1 vesicle transport factor  PURA - purine-rich element binding protein a  TRAPPC3 - trafficking protein particle complex 3  FAM155B - family with sequence similarity 155, member b  SRSF10 - serine/arginine-rich splicing factor 10  TBC1D13 - tbc1 domain family, member 13  PTPRN - protein tyrosine phosphatase, receptor type, n  LYST - lysosomal trafficking regulator  AHCYL1 - adenosylhomocysteinase-like 1  LEPROT - leptin receptor overlapping transcript  NUP50 - nucleoporin 50kda  CHML - choroideremia-like (rab escort protein 2)  PEX2 - peroxisomal biogenesis factor 2  CHM - choroideremia (rab escort protein 1)  CHCHD4 - coiled-coil-helix-coiled-coil-helix domain containing 4  GIPC1 - gipc pdz domain containing family, member 1  CHGA - chromogranin a (parathyroid secretory protein 1)  SNX5 - sorting nexin 5  PVRL2 - poliovirus receptor-related 2 (herpesvirus entry mediator b)  RAB6A - rab6a, member ras oncogene family  RAB5A - rab5a, member ras oncogene family  C17orf75 - chromosome 17 open reading frame 75  RAB5B - rab5b, member ras oncogene family  RAB3A - rab3a, member ras oncogene family  MTERFD2 - mterf domain containing 2  RAB1A - rab1a, member ras oncogene family  CPLX2 - complexin 2  SDCCAG3 - serologically defined colon cancer antigen 3  SEC24A - sec24 family, member a (s. cerevisiae)  RAB5C - rab5c, member ras oncogene family  TMED4 - transmembrane emp24 protein transport domain containing 4  LIN7B - lin-7 homolog b (c. elegans)  VPS4A - vacuolar protein sorting 4 homolog a (s. cerevisiae)  RAB27A - rab27a, member ras oncogene family  SNX16 - sorting nexin 16  COPG2 - coatomer protein complex, subunit gamma 2  TOMM7 - translocase of outer mitochondrial membrane 7 homolog (yeast)  RANBP2 - ran binding protein 2  CLTC - clathrin, heavy chain (hc)  SEC22A - sec22 vesicle trafficking protein homolog a (s. cerevisiae)  AP3M1 - adaptor-related protein complex 3, mu 1 subunit  GNPAT - glyceronephosphate o-acyltransferase  CLN5 - ceroid-lipofuscinosis, neuronal 5  SGTB - small glutamine-rich tetratricopeptide repeat (tpr)-containing, beta  BBS5 - bardet-biedl syndrome 5  DCTN6 - dynactin 6  EEA1 - early endosome antigen 1  CLTB - clathrin, light chain b  CLTA - clathrin, light chain a  CLU - clusterin  MAGEL2 - mage-like 2  STX7 - syntaxin 7  HEATR5B - heat repeat containing 5b  AP1S1 - adaptor-related protein complex 1, sigma 1 subunit  LAT - linker for activation of t cells  AP2M1 - adaptor-related protein complex 2, mu 1 subunit  ARL5B - adp-ribosylation factor-like 5b  RAE1 - rae1 rna export 1 homolog (s. pombe)  ANKRD54 - ankyrin repeat domain 54  NSG1 - neuron specific gene family member 1  GEMIN2 - gem (nuclear organelle) associated protein 2  NUP35 - nucleoporin 35kda  VPS41 - vacuolar protein sorting 41 homolog (s. cerevisiae)  DOC2B - double c2-like domains, beta  CCDC93 - coiled-coil domain containing 93  CUL3 - cullin 3  SGSM1 - small g protein signaling modulator 1  CHMP4B - charged multivesicular body protein 4b  CACNG4 - calcium channel, voltage-dependent, gamma subunit 4  AP4B1 - adaptor-related protein complex 4, beta 1 subunit  RSRC1 - arginine/serine-rich coiled-coil 1  TMEM87B - transmembrane protein 87b  SPTAN1 - spectrin, alpha, non-erythrocytic 1  WDR19 - wd repeat domain 19  SPTBN4 - spectrin, beta, non-erythrocytic 4  AKAP12 - a kinase (prka) anchor protein 12  COG8 - component of oligomeric golgi complex 8  CORO1C - coronin, actin binding protein, 1c  VPS37A - vacuolar protein sorting 37 homolog a (s. cerevisiae)  EPG5 - ectopic p-granules autophagy protein 5 homolog (c. elegans)  ZDHHC3 - zinc finger, dhhc-type containing 3  KPNA6 - karyopherin alpha 6 (importin alpha 7)  RABGAP1 - rab gtpase activating protein 1  LDLR - low density lipoprotein receptor  SPG7 - spastic paraplegia 7 (pure and complicated autosomal recessive)  HOOK3 - hook microtubule-tethering protein 3  DENND1A - denn/madd domain containing 1a  SPAST - spastin  LZTS2 - leucine zipper, putative tumor suppressor 2  REEP2 - receptor accessory protein 2  FAM53C - family with sequence similarity 53, member c  ABCG1 - atp-binding cassette, sub-family g (white), member 1  LETM1 - leucine zipper-ef-hand containing transmembrane protein 1  LTV1 - ltv1 homolog (s. cerevisiae)  ERGIC2 - ergic and golgi 2  CPT1A - carnitine palmitoyltransferase 1a (liver)  TICAM2 - toll-like receptor adaptor molecule 2  BRSK1 - br serine/threonine kinase 1  SYVN1 - synovial apoptosis inhibitor 1, synoviolin  VPS26A - vacuolar protein sorting 26 homolog a (s. pombe)  SEC22B - sec22 vesicle trafficking protein homolog b (s. cerevisiae) (gene/pseudogene)  GOSR2 - golgi snap receptor complex member 2  BET1L - bet1 golgi vesicular membrane trafficking protein-like  UBAP1 - ubiquitin associated protein 1  UBXN2B - ubx domain protein 2b  JPH4 - junctophilin 4  SPIRE2 - spire actin nucleation factor 2  PREPL - prolyl endopeptidase-like  SNX2 - sorting nexin 2  LMNA - lamin a/c  DNAJC27 - dnaj (hsp40) homolog, subfamily c, member 27  TMEM50A - transmembrane protein 50a  SNX1 - sorting nexin 1  LMAN1 - lectin, mannose-binding, 1  SERAC1 - serine active site containing 1  LLGL1 - lethal giant larvae homolog 1 (drosophila)  DCTN5 - dynactin 5 (p25)  PRKAG2 - protein kinase, amp-activated, gamma 2 non-catalytic subunit  SNRPD1 - small nuclear ribonucleoprotein d1 polypeptide 16kda  SNRPF - small nuclear ribonucleoprotein polypeptide f  SNCA - synuclein, alpha (non a4 component of amyloid precursor)  SNCG - synuclein, gamma (breast cancer-specific protein 1)  TRAPPC1 - trafficking protein particle complex 1  SNRPB - small nuclear ribonucleoprotein polypeptides b and b1  VPS4B - vacuolar protein sorting 4 homolog b (s. cerevisiae)  GOSR1 - golgi snap receptor complex member 1  SNAP25 - synaptosomal-associated protein, 25kda  SRPRB - signal recognition particle receptor, b subunit  SNCB - synuclein, beta  TRAPPC4 - trafficking protein particle complex 4  SMN1 - survival of motor neuron 1, telomeric  LAMP2 - lysosomal-associated membrane protein 2  ARFIP2 - adp-ribosylation factor interacting protein 2  SNX6 - sorting nexin 6  MID1IP1 - mid1 interacting protein 1  REPS1 - ralbp1 associated eps domain containing 1  STX8 - syntaxin 8  COPB1 - coatomer protein complex, subunit beta 1  EPS15L1 - epidermal growth factor receptor pathway substrate 15-like 1  COPA - coatomer protein complex, subunit alpha  RAB38 - rab38, member ras oncogene family  DPY30 - dpy-30 homolog (c. elegans)  STX12 - syntaxin 12  CDC40 - cell division cycle 40  RPH3AL - rabphilin 3a-like (without c2 domains)  SLC18A2 - solute carrier family 18 (vesicular monoamine transporter), member 2  SYN1 - synapsin i  NAGPA - n-acetylglucosamine-1-phosphodiester alpha-n-acetylglucosaminidase  SYP - synaptophysin  SYN2 - synapsin ii  DCTN4 - dynactin 4 (p62)  VPS28 - vacuolar protein sorting 28 homolog (s. cerevisiae)  KIF5C - kinesin family member 5c  KIF3C - kinesin family member 3c  SYT1 - synaptotagmin i  KLHL12 - kelch-like family member 12  KIF5B - kinesin family member 5b  SYT5 - synaptotagmin v  KIF5A - kinesin family member 5a  SYT4 - synaptotagmin iv  PICK1 - protein interacting with prkca 1  SUN2 - sad1 and unc84 domain containing 2  CACNG7 - calcium channel, voltage-dependent, gamma subunit 7  SLC24A2 - solute carrier family 24 (sodium/potassium/calcium exchanger), member 2  DYNC1LI1 - dynein, cytoplasmic 1, light intermediate chain 1  VAMP2 - vesicle-associated membrane protein 2 (synaptobrevin 2)  RAB3GAP2 - rab3 gtpase activating protein subunit 2 (non-catalytic)  CCDC41 - coiled-coil domain containing 41  CTNNB1 - catenin (cadherin-associated protein), beta 1, 88kda  STXBP1 - syntaxin binding protein 1  STX5 - syntaxin 5  STX4 - syntaxin 4  STX3 - syntaxin 3  STX1A - syntaxin 1a (brain)  GOLGA7 - golgin a7  TNPO1 - transportin 1  MGARP - mitochondria-localized glutamic acid-rich protein  IER3IP1 - immediate early response 3 interacting protein 1  FUT10 - fucosyltransferase 10 (alpha (1,3) fucosyltransferase)  TGFBRAP1 - transforming growth factor, beta receptor associated protein 1  KPNA2 - karyopherin alpha 2 (rag cohort 1, importin alpha 1)  KPNB1 - karyopherin (importin) beta 1  KPNA1 - karyopherin alpha 1 (importin alpha 5)  STXBP3 - syntaxin binding protein 3  ZDHHC9 - zinc finger, dhhc-type containing 9  SYT3 - synaptotagmin iii  TRAPPC12 - trafficking protein particle complex 12  TUBA1C - tubulin, alpha 1c  FYTTD1 - forty-two-three domain containing 1  PAN3 - pan3 poly(a) specific ribonuclease subunit homolog (s. cerevisiae)  EXOC4 - exocyst complex component 4  PEX16 - peroxisomal biogenesis factor 16  KIT - v-kit hardy-zuckerman 4 feline sarcoma viral oncogene homolog  RPAIN - rpa interacting protein  RAB28 - rab28, member ras oncogene family  POLDIP3 - polymerase (dna-directed), delta interacting protein 3  RAB9A - rab9a, member ras oncogene family  COG4 - component of oligomeric golgi complex 4  TRAPPC11 - trafficking protein particle complex 11  PDZD11 - pdz domain containing 11  STAT3 - signal transducer and activator of transcription 3 (acute-phase response factor)  SSR3 - signal sequence receptor, gamma (translocon-associated protein gamma)  COG1 - component of oligomeric golgi complex 1  SPCS3 - signal peptidase complex subunit 3 homolog (s. cerevisiae)  KIF3B - kinesin family member 3b  ZFYVE9 - zinc finger, fyve domain containing 9  ITPR1 - inositol 1,4,5-trisphosphate receptor, type 1  ITPR2 - inositol 1,4,5-trisphosphate receptor, type 2  SRP68 - signal recognition particle 68kda  NAPB - n-ethylmaleimide-sensitive factor attachment protein, beta  FBXO7 - f-box protein 7  SRP54 - signal recognition particle 54kda  SNX33 - sorting nexin 33  SRP19 - signal recognition particle 19kda  CSE1L - cse1 chromosome segregation 1-like (yeast)  SRP14 - signal recognition particle 14kda (homologous alu rna binding protein)  BLOC1S5 - biogenesis of lysosomal organelles complex-1, subunit 5, muted  SRP72 - signal recognition particle 72kda  SSR1 - signal sequence receptor, alpha  ZDHHC2 - zinc finger, dhhc-type containing 2  SSB - sjogren syndrome antigen b (autoantigen la)  THOC3 - tho complex 3  SPTBN1 - spectrin, beta, non-erythrocytic 1  VAMP3 - vesicle-associated membrane protein 3  MIA3 - melanoma inhibitory activity family, member 3  IPO11 - importin 11  TAOK2 - tao kinase 2  SARNP - sap domain containing ribonucleoprotein  VIPAS39 - vps33b interacting protein, apical-basolateral polarity regulator, spe-39 homolog  VPS25 - vacuolar protein sorting 25 homolog (s. cerevisiae)  ZDHHC12 - zinc finger, dhhc-type containing 12  UBE2O - ubiquitin-conjugating enzyme e2o  SRP9 - signal recognition particle 9kda  MON1A - mon1 secretory trafficking family member a  SEL1L - sel-1 suppressor of lin-12-like (c. elegans)  TRAPPC2 - trafficking protein particle complex 2  SRSF11 - serine/arginine-rich splicing factor 11  SEC13 - sec13 homolog (s. cerevisiae)  RAB8B - rab8b, member ras oncogene family  ERGIC1 - endoplasmic reticulum-golgi intermediate compartment (ergic) 1  SDCBP - syndecan binding protein (syntenin)  SYT17 - synaptotagmin xvii  COPB2 - coatomer protein complex, subunit beta 2 (beta prime)  HEATR5A - heat repeat containing 5a  RHOT2 - ras homolog family member t2  JPH3 - junctophilin 3  MLH1 - mutl homolog 1, colon cancer, nonpolyposis type 2 (e. coli)  DCTN1 - dynactin 1  PTPN23 - protein tyrosine phosphatase, non-receptor type 23  WDR34 - wd repeat domain 34  ZDHHC24 - zinc finger, dhhc-type containing 24  GEMIN5 - gem (nuclear organelle) associated protein 5  MYRIP - myosin viia and rab interacting protein  PHAX - phosphorylated adaptor for rna export  CPSF3 - cleavage and polyadenylation specific factor 3, 73kda  TRAPPC2L - trafficking protein particle complex 2-like  RAB22A - rab22a, member ras oncogene family  VPS29 - vacuolar protein sorting 29 homolog (s. cerevisiae)  RAB11B - rab11b, member ras oncogene family  SYT9 - synaptotagmin ix  RAB23 - rab23, member ras oncogene family  ZDHHC20 - zinc finger, dhhc-type containing 20  DNM3 - dynamin 3  ANKRD27 - ankyrin repeat domain 27 (vps9 domain)  VAPB - vamp (vesicle-associated membrane protein)-associated protein b and c  MCFD2 - multiple coagulation factor deficiency 2  REPS2 - ralbp1 associated eps domain containing 2  PRICKLE1 - prickle homolog 1 (drosophila)  RAB24 - rab24, member ras oncogene family  ZW10 - zw10 kinetochore protein  FAM160A2 - family with sequence similarity 160, member a2  CPSF2 - cleavage and polyadenylation specific factor 2, 100kda  DCLK1 - doublecortin-like kinase 1  TBC1D10B - tbc1 domain family, member 10b  RAB8A - rab8a, member ras oncogene family  COG6 - component of oligomeric golgi complex 6  XPO5 - exportin 5  AP4M1 - adaptor-related protein complex 4, mu 1 subunit  ARHGEF2 - rho/rac guanine nucleotide exchange factor (gef) 2  IBTK - inhibitor of bruton agammaglobulinemia tyrosine kinase  CHMP2B - charged multivesicular body protein 2b  TBC1D14 - tbc1 domain family, member 14  ARL6 - adp-ribosylation factor-like 6  STX18 - syntaxin 18  SYNGR1 - synaptogyrin 1  HGS - hepatocyte growth factor-regulated tyrosine kinase substrate  NEMF - nuclear export mediator factor  TMEM87A - transmembrane protein 87a  VPS52 - vacuolar protein sorting 52 homolog (s. cerevisiae)  VTA1 - vesicle (multivesicular body) trafficking 1  COG3 - component of oligomeric golgi complex 3  MICALL1 - mical-like 1  MAGOH - mago-nashi homolog, proliferation-associated (drosophila)  DRD2 - dopamine receptor d2  NLGN2 - neuroligin 2  TRAF3IP1 - tnf receptor-associated factor 3 interacting protein 1  SLC8A1 - solute carrier family 8 (sodium/calcium exchanger), member 1  SLC8A2 - solute carrier family 8 (sodium/calcium exchanger), member 2  RAB6B - rab6b, member ras oncogene family  MARK1 - map/microtubule affinity-regulating kinase 1  MAPT - microtubule-associated protein tau  MAP6 - microtubule-associated protein 6  RAB14 - rab14, member ras oncogene family  MAP1B - microtubule-associated protein 1b  MAP1A - microtubule-associated protein 1a  VPS54 - vacuolar protein sorting 54 homolog (s. cerevisiae)  TANC2 - tetratricopeptide repeat, ankyrin repeat and coiled-coil containing 2  DYNLRB2 - dynein, light chain, roadblock-type 2  DYNLRB1 - dynein, light chain, roadblock-type 1  NUP43 - nucleoporin 43kda  SLC29A4 - solute carrier family 29 (equilibrative nucleoside transporter), member 4  TOR1A - torsin family 1, member a (torsin a)  CHTOP - chromatin target of prmt1  GGA1 - golgi-associated, gamma adaptin ear containing, arf binding protein 1  ARHGAP21 - rho gtpase activating protein 21  CHMP5 - charged multivesicular body protein 5  DOCK7 - dedicator of cytokinesis 7  APPL1 - adaptor protein, phosphotyrosine interaction, ph domain and leucine zipper containing 1  ANKFY1 - ankyrin repeat and fyve domain containing 1  SYT7 - synaptotagmin vii  KIAA1468 - kiaa1468  VPS18 - vacuolar protein sorting 18 homolog (s. cerevisiae)  DLG1 - discs, large homolog 1 (drosophila)  BLOC1S6 - biogenesis of lysosomal organelles complex-1, subunit 6, pallidin  RPL14 - ribosomal protein l14  DYNC2LI1 - dynein, cytoplasmic 2, light intermediate chain 1  LRP6 - low density lipoprotein receptor-related protein 6  STXBP4 - syntaxin binding protein 4  AIP - aryl hydrocarbon receptor interacting protein  TOM1L2 - target of myb1-like 2 (chicken)  ARL5A - adp-ribosylation factor-like 5a  LYN - v-yes-1 yamaguchi sarcoma viral related oncogene homolog  SPAG9 - sperm associated antigen 9  TMED6 - transmembrane emp24 protein transport domain containing 6  CHMP3 - charged multivesicular body protein 3  SFPQ - splicing factor proline/glutamine-rich  DYNC1H1 - dynein, cytoplasmic 1, heavy chain 1  DYNC1I1 - dynein, cytoplasmic 1, intermediate chain 1  DYNC1I2 - dynein, cytoplasmic 1, intermediate chain 2  DYNC1LI2 - dynein, cytoplasmic 1, light intermediate chain 2  SRSF1 - serine/arginine-rich splicing factor 1  SRSF2 - serine/arginine-rich splicing factor 2  DNM2 - dynamin 2  SRSF3 - serine/arginine-rich splicing factor 3  SRSF4 - serine/arginine-rich splicing factor 4  SRSF5 - serine/arginine-rich splicing factor 5  SRSF6 - serine/arginine-rich splicing factor 6  SRSF7 - serine/arginine-rich splicing factor 7  M6PR - mannose-6-phosphate receptor (cation dependent)  ALS2 - amyotrophic lateral sclerosis 2 (juvenile)  ERGIC3 - ergic and golgi 3  HPS4 - hermansky-pudlak syndrome 4  SCG5 - secretogranin v (7b2 protein)  IFT172 - intraflagellar transport 172 homolog (chlamydomonas)  SGTA - small glutamine-rich tetratricopeptide repeat (tpr)-containing, alpha  RUNDC1 - run domain containing 1  ITSN1 - intersectin 1 (sh3 domain protein)  WASL - wiskott-aldrich syndrome-like  NCBP2 - nuclear cap binding protein subunit 2, 20kda  NCBP1 - nuclear cap binding protein subunit 1, 80kda  CHMP4C - charged multivesicular body protein 4c  CTTN - cortactin  TSC1 - tuberous sclerosis 1  KIFAP3 - kinesin-associated protein 3  DDX25 - dead (asp-glu-ala-asp) box helicase 25  SPIRE1 - spire actin nucleation factor 1  IFT46 - intraflagellar transport 46 homolog (chlamydomonas)  SCAP - srebf chaperone  DNAJC15 - dnaj (hsp40) homolog, subfamily c, member 15  RAB18 - rab18, member ras oncogene family  TIMM50 - translocase of inner mitochondrial membrane 50 homolog (s. cerevisiae)  TUB - tubby bipartite transcription factor  GRIPAP1 - grip1 associated protein 1  MYO10 - myosin x  CNIH - cornichon homolog (drosophila)  EIF4E - eukaryotic translation initiation factor 4e  ZDHHC1 - zinc finger, dhhc-type containing 1  EGR2 - early growth response 2  LMAN2L - lectin, mannose-binding 2-like  TTC30A - tetratricopeptide repeat domain 30a  ALYREF - aly/ref export factor  NDEL1 - nude neurodevelopment protein 1-like 1  NACA - nascent polypeptide-associated complex alpha subunit  SAR1A - sar1 homolog a (s. cerevisiae)  TMEM129 - transmembrane protein 129  ELAVL1 - elav (embryonic lethal, abnormal vision, drosophila)-like 1 (hu antigen r)  AZI1 - 5-azacytidine induced 1  BTBD8 - btb (poz) domain containing 8  STRADA - ste20-related kinase adaptor alpha  NUTF2 - nuclear transport factor 2  MYO1D - myosin id  TSG101 - tumor susceptibility gene 101  TSC2 - tuberous sclerosis 2  MYO5A - myosin va (heavy chain 12, myoxin)  MYO1E - myosin ie  MYO6 - myosin vi  SNX27 - sorting nexin family member 27  FIP1L1 - factor interacting with papola and cpsf1  MVB12A - multivesicular body subunit 12a  RAB1B - rab1b, member ras oncogene family  EIF2D - eukaryotic translation initiation factor 2d  SNX15 - sorting nexin 15  RAB21 - rab21, member ras oncogene family  SEH1L - seh1-like (s. cerevisiae)  MYH10 - myosin, heavy chain 10, non-muscle  SNX11 - sorting nexin 11  MYH9 - myosin, heavy chain 9, non-muscle  SNUPN - snurportin 1  SNX10 - sorting nexin 10  COL4A3BP - collagen, type iv, alpha 3 (goodpasture antigen) binding protein  DOPEY1 - dopey family member 1  MX1 - myxovirus (influenza virus) resistance 1, interferon-inducible protein p78 (mouse)  GPSM2 - g-protein signaling modulator 2  GOLGA7B - golgin a7 family, member b  UNC13A - unc-13 homolog a (c. elegans)  ACTR2 - arp2 actin-related protein 2 homolog (yeast)  CPSF1 - cleavage and polyadenylation specific factor 1, 160kda  ACTR3 - arp3 actin-related protein 3 homolog (yeast)  RBM15B - rna binding motif protein 15b  CLUAP1 - clusterin associated protein 1  SMG1 - smg1 phosphatidylinositol 3-kinase-related kinase  MFF - mitochondrial fission factor  NRBP1 - nuclear receptor binding protein 1  ENY2 - enhancer of yellow 2 homolog (drosophila)  XPO7 - exportin 7  MON2 - mon2 homolog (s. cerevisiae)  LRPPRC - leucine-rich pentatricopeptide repeat containing  ACTR1A - arp1 actin-related protein 1 homolog a, centractin alpha (yeast)  CPLX3 - complexin 3  HSP90B1 - heat shock protein 90kda beta (grp94), member 1  TIMM22 - translocase of inner mitochondrial membrane 22 homolog (yeast)  ARL4A - adp-ribosylation factor-like 4a  SEC61A1 - sec61 alpha 1 subunit (s. cerevisiae)  ARL4C - adp-ribosylation factor-like 4c  BCAP31 - b-cell receptor-associated protein 31  TBC1D9B - tbc1 domain family, member 9b (with gram domain)  TPR - translocated promoter region, nuclear basket protein  SNX12 - sorting nexin 12  GGA2 - golgi-associated, gamma adaptin ear containing, arf binding protein 2  BLOC1S3 - biogenesis of lysosomal organelles complex-1, subunit 3  ERC1 - elks/rab6-interacting/cast family member 1  BOD1 - biorientation of chromosomes in cell division 1  NUP107 - nucleoporin 107kda  GOPC - golgi-associated pdz and coiled-coil motif containing  KIF1B - kinesin family member 1b  TBC1D2B - tbc1 domain family, member 2b  PDCD6 - programmed cell death 6  MTM1 - myotubularin 1  HDAC6 - histone deacetylase 6  CLASP2 - cytoplasmic linker associated protein 2  HMGXB4 - hmg box domain containing 4  TOM1 - target of myb1 (chicken)  EZR - ezrin  CCDC22 - coiled-coil domain containing 22  DNM1L - dynamin 1-like  AP5B1 - adaptor-related protein complex 5, beta 1 subunit  F2R - coagulation factor ii (thrombin) receptor  VCP - valosin containing protein  TBC1D9 - tbc1 domain family, member 9 (with gram domain)  MAPK8IP3 - mitogen-activated protein kinase 8 interacting protein 3  MCOLN1 - mucolipin 1  CITED1 - cbp/p300-interacting transactivator, with glu/asp-rich carboxy-terminal domain, 1  NUP205 - nucleoporin 205kda  YTHDC1 - yth domain containing 1  THOC2 - tho complex 2  GGA3 - golgi-associated, gamma adaptin ear containing, arf binding protein 3  SEC16A - sec16 homolog a (s. cerevisiae)  CHMP7 - charged multivesicular body protein 7  UBB - ubiquitin b  IFT81 - intraflagellar transport 81 homolog (chlamydomonas)  SGPP1 - sphingosine-1-phosphate phosphatase 1  SPCS1 - signal peptidase complex subunit 1 homolog (s. cerevisiae)  RBM8A - rna binding motif protein 8a  FAF2 - fas associated factor family member 2  COG7 - component of oligomeric golgi complex 7  ARL6IP1 - adp-ribosylation factor-like 6 interacting protein 1  TIMM21 - translocase of inner mitochondrial membrane 21 homolog (yeast)  UCHL1 - ubiquitin carboxyl-terminal esterase l1 (ubiquitin thiolesterase)  CHMP4A - charged multivesicular body protein 4a  RRS1 - rrs1 ribosome biogenesis regulator homolog (s. cerevisiae)  FAM155A - family with sequence similarity 155, member a  XPO6 - exportin 6  SMURF1 - smad specific e3 ubiquitin protein ligase 1  SYT11 - synaptotagmin xi  THOC1 - tho complex 1  TBC1D1 - tbc1 (tre-2/usp6, bub2, cdc16) domain family, member 1  UBE2D2 - ubiquitin-conjugating enzyme e2d 2  STX2 - syntaxin 2  TBC1D12 - tbc1 domain family, member 12  NUP153 - nucleoporin 153kda  TMEM230 - transmembrane protein 230  WDR91 - wd repeat domain 91  NUP210 - nucleoporin 210kda  EPS15 - epidermal growth factor receptor pathway substrate 15  DTX3L - deltex 3-like (drosophila)  SCFD1 - sec1 family domain containing 1  SEC24D - sec24 family, member d (s. cerevisiae)  TLK1 - tousled-like kinase 1  DENND5A - denn/madd domain containing 5a  OSBP - oxysterol binding protein  C2CD5 - c2 calcium-dependent domain containing 5  ACTR10 - actin-related protein 10 homolog (s. cerevisiae)  TBC1D25 - tbc1 domain family, member 25  AP5Z1 - adaptor-related protein complex 5, zeta 1 subunit  ZDHHC22 - zinc finger, dhhc-type containing 22  BICD2 - bicaudal d homolog 2 (drosophila)  VPS26B - vacuolar protein sorting 26 homolog b (s. pombe)  MGRN1 - mahogunin ring finger 1, e3 ubiquitin protein ligase  SGSM2 - small g protein signaling modulator 2  ARHGAP44 - rho gtpase activating protein 44  KIF13B - kinesin family member 13b  TMED5 - transmembrane emp24 protein transport domain containing 5  SMG6 - smg6 nonsense mediated mrna decay factor  OSBPL2 - oxysterol binding protein-like 2  FKBP1B - fk506 binding protein 1b, 12.6 kda  FBXW11 - f-box and wd repeat domain containing 11  VPS11 - vacuolar protein sorting 11 homolog (s. cerevisiae)  SMG7 - smg7 nonsense mediated mrna decay factor  OPA1 - optic atrophy 1 (autosomal dominant)  CLEC16A - c-type lectin domain family 16, member a  DYNLT1 - dynein, light chain, tctex-type 1  TECPR2 - tectonin beta-propeller repeat containing 2  UBXN6 - ubx domain protein 6  TMED7 - transmembrane emp24 protein transport domain containing 7  STX1B - syntaxin 1b  SNAP91 - synaptosomal-associated protein, 91kda  NUP160 - nucleoporin 160kda  IFT43 - intraflagellar transport 43 homolog (chlamydomonas)  FIS1 - fission 1 (mitochondrial outer membrane) homolog (s. cerevisiae)  NSF - n-ethylmaleimide-sensitive factor  SPCS2 - signal peptidase complex subunit 2 homolog (s. cerevisiae)  FGF9 - fibroblast growth factor 9  BCAP29 - b-cell receptor-associated protein 29  TOMM20 - translocase of outer mitochondrial membrane 20 homolog (yeast)  VPS39 - vacuolar protein sorting 39 homolog (s. cerevisiae)  NSFL1C - nsfl1 (p97) cofactor (p47)  NUMA1 - nuclear mitotic apparatus protein 1  CLASP1 - cytoplasmic linker associated protein 1  FGF2 - fibroblast growth factor 2 (basic)  EIF4A3 - eukaryotic translation initiation factor 4a3  NUP88 - nucleoporin 88kda  NUP98 - nucleoporin 98kda  TAP2 - transporter 2, atp-binding cassette, sub-family b (mdr/tap)  TBC1D30 - tbc1 domain family, member 30  TMEM167A - transmembrane protein 167a  RIMS3 - regulating synaptic membrane exocytosis 3  SNX17 - sorting nexin 17  DHX38 - deah (asp-glu-ala-his) box polypeptide 38  FAM45A - family with sequence similarity 45, member a  TBC1D5 - tbc1 domain family, member 5  CEP120 - centrosomal protein 120kda  PIP5K1C - phosphatidylinositol-4-phosphate 5-kinase, type i, gamma  NPM1 - nucleophosmin (nucleolar phosphoprotein b23, numatrin)  NMD3 - nmd3 ribosome export adaptor  RGP1 - rgp1 retrograde golgi transport homolog (s. cerevisiae)  SPICE1 - spindle and centriole associated protein 1  TRIM9 - tripartite motif containing 9  DUSP16 - dual specificity phosphatase 16  IFT52 - intraflagellar transport 52 homolog (chlamydomonas)  GRIP2 - glutamate receptor interacting protein 2  NUDT7 - nudix (nucleoside diphosphate linked moiety x)-type motif 7  ERBB2IP - erbb2 interacting protein  RAB11FIP3 - rab11 family interacting protein 3 (class ii)  SPG11 - spastic paraplegia 11 (autosomal recessive)  TMED3 - transmembrane emp24 protein transport domain containing 3  TNPO2 - transportin 2  BLOC1S2 - biogenesis of lysosomal organelles complex-1, subunit 2  IFT74 - intraflagellar transport 74 homolog (chlamydomonas)  ERO1L - ero1-like (s. cerevisiae)  HERPUD1 - homocysteine-inducible, endoplasmic reticulum stress-inducible, ubiquitin-like domain member 1  USP6NL - usp6 n-terminal like  MYO19 - myosin xix  SEC62 - sec62 homolog (s. cerevisiae)  RIMS2 - regulating synaptic membrane exocytosis 2  PACSIN1 - protein kinase c and casein kinase substrate in neurons 1  PQLC1 - pq loop repeat containing 1  TRAPPC10 - trafficking protein particle complex 10  THOC7 - tho complex 7 homolog (drosophila)  ICMT - isoprenylcysteine carboxyl methyltransferase  ZFYVE16 - zinc finger, fyve domain containing 16  TRAM1 - translocation associated membrane protein 1  NFKBIA - nuclear factor of kappa light polypeptide gene enhancer in b-cells inhibitor, alpha  SNPH - syntaphilin  PIK3R4 - phosphoinositide-3-kinase, regulatory subunit 4  IFT140 - intraflagellar transport 140 homolog (chlamydomonas)  TBCK - tbc1 domain containing kinase  EHD2 - eh-domain containing 2  EHD3 - eh-domain containing 3  EHD4 - eh-domain containing 4  EIF4ENIF1 - eukaryotic translation initiation factor 4e nuclear import factor 1  NUP188 - nucleoporin 188kda  DNAJC5 - dnaj (hsp40) homolog, subfamily c, member 5  GCC2 - grip and coiled-coil domain containing 2  NEFL - neurofilament, light polypeptide  TERF1 - telomeric repeat binding factor (nima-interacting) 1  TERF2 - telomeric repeat binding factor 2  SNX18 - sorting nexin 18  LEPROTL1 - leptin receptor overlapping transcript-like 1  ITSN2 - intersectin 2  BCCIP - brca2 and cdkn1a interacting protein  FEZ1 - fasciculation and elongation protein zeta 1 (zygin i)  SEC61G - sec61 gamma subunit  NUP155 - nucleoporin 155kda  TGFA - transforming growth factor, alpha  SEC24C - sec24 family, member c (s. cerevisiae)  NF1 - neurofibromin 1  FAM91A1 - family with sequence similarity 91, member a1  VPS37D - vacuolar protein sorting 37 homolog d (s. cerevisiae)  TGFB1 - transforming growth factor, beta 1  TH - tyrosine hydroxylase  NUP93 - nucleoporin 93kda  FAM21A - family with sequence similarity 21, member a  CALY - calcyon neuron-specific vesicular protein  GEMIN4 - gem (nuclear organelle) associated protein 4  ACAP2 - arfgap with coiled-coil, ankyrin repeat and ph domains 2  SYTL4 - synaptotagmin-like 4  TNPO3 - transportin 3 |
| GO:0006473 | protein acetylation | 2.81E-5 | 3.05E-3 | 1.25 (10334,112,6996,95) | [+] Show genes  HAT1 - histone acetyltransferase 1  MSL3 - male-specific lethal 3 homolog (drosophila)  EPC2 - enhancer of polycomb homolog 2 (drosophila)  NAT6 - n-acetyltransferase 6 (gcn5-related)  TADA1 - transcriptional adaptor 1  MCRS1 - microspherule protein 1  ATAT1 - alpha tubulin acetyltransferase 1  NAA40 - n(alpha)-acetyltransferase 40, natd catalytic subunit, homolog (s. cerevisiae)  MAPT - microtubule-associated protein tau  MSL2 - male-specific lethal 2 homolog (drosophila)  MEAF6 - myst/esa1-associated factor 6  EP300 - e1a binding protein p300  RSF1 - remodeling and spacing factor 1  ING4 - inhibitor of growth family, member 4  NAA15 - n(alpha)-acetyltransferase 15, nata auxiliary subunit  DR1 - down-regulator of transcription 1, tbp-binding (negative cofactor 2)  DPF2 - d4, zinc and double phd fingers family 2  CSRP2BP - csrp2 binding protein  KANSL1 - kat8 regulatory nsl complex subunit 1  SIRT1 - sirtuin 1  GTF2B - general transcription factor iib  PCGF2 - polycomb group ring finger 2  KAT2A - k(lysine) acetyltransferase 2a  KMT2A - lysine (k)-specific methyltransferase 2a  APBB1 - amyloid beta (a4) precursor protein-binding, family b, member 1 (fe65)  NCOA3 - nuclear receptor coactivator 3  BRPF3 - bromodomain and phd finger containing, 3  BRD8 - bromodomain containing 8  NAA20 - n(alpha)-acetyltransferase 20, natb catalytic subunit  NAA25 - n(alpha)-acetyltransferase 25, natb auxiliary subunit  TADA3 - transcriptional adaptor 3  SPHK2 - sphingosine kinase 2  TAF6L - taf6-like rna polymerase ii, p300/cbp-associated factor (pcaf)-associated factor, 65kda  BRD1 - bromodomain containing 1  LDB1 - lim domain binding 1  PHF20L1 - phd finger protein 20-like 1  CREBBP - creb binding protein  MORF4L1 - mortality factor 4 like 1  DSCC1 - dna replication and sister chromatid cohesion 1  ATF2 - activating transcription factor 2  MSL1 - male-specific lethal 1 homolog (drosophila)  KAT2B - k(lysine) acetyltransferase 2b  CLOCK - clock circadian regulator  CRTC2 - creb regulated transcription coactivator 2  KANSL1L - kat8 regulatory nsl complex subunit 1-like  ESCO1 - establishment of sister chromatid cohesion n-acetyltransferase 1  TAF9 - taf9 rna polymerase ii, tata box binding protein (tbp)-associated factor, 32kda  KANSL3 - kat8 regulatory nsl complex subunit 3  BRPF1 - bromodomain and phd finger containing, 1  ACTL6A - actin-like 6a  TRRAP - transformation/transcription domain-associated protein  TAF6 - taf6 rna polymerase ii, tata box binding protein (tbp)-associated factor, 80kda  TADA2A - transcriptional adaptor 2a  ARRB1 - arrestin, beta 1  SUPT7L - suppressor of ty 7 (s. cerevisiae)-like  PHF15 - phd finger protein 15  CHD5 - chromodomain helicase dna binding protein 5  MECP2 - methyl cpg binding protein 2 (rett syndrome)  EPC1 - enhancer of polycomb homolog 1 (drosophila)  MORF4L2 - mortality factor 4 like 2  EP400 - e1a binding protein p400  USP22 - ubiquitin specific peptidase 22  ING5 - inhibitor of growth family, member 5  NAA35 - n(alpha)-acetyltransferase 35, natc auxiliary subunit  NAA60 - n(alpha)-acetyltransferase 60, natf catalytic subunit  MCM3AP - minichromosome maintenance complex component 3 associated protein  KAT5 - k(lysine) acetyltransferase 5  YEATS4 - yeats domain containing 4  ING3 - inhibitor of growth family, member 3  MAP3K7 - mitogen-activated protein kinase kinase kinase 7  PHF20 - phd finger protein 20  ACTL6B - actin-like 6b  HCFC1 - host cell factor c1 (vp16-accessory protein)  PHF10 - phd finger protein 10  OGT - o-linked n-acetylglucosamine (glcnac) transferase  ZZZ3 - zinc finger, zz-type containing 3  MBIP - map3k12 binding inhibitory protein 1  KANSL2 - kat8 regulatory nsl complex subunit 2  KAT6A - k(lysine) acetyltransferase 6a  KAT8 - k(lysine) acetyltransferase 8  MRGBP - mrg/morf4l binding protein  PHF14 - phd finger protein 14  RUVBL1 - ruvb-like 1 (e. coli)  NAT9 - n-acetyltransferase 9 (gcn5-related, putative)  PHF17 - phd finger protein 17  DMAP1 - dna methyltransferase 1 associated protein 1  KAT6B - k(lysine) acetyltransferase 6b  ZMPSTE24 - zinc metallopeptidase ste24  LEF1 - lymphoid enhancer-binding factor 1  MBD3 - methyl-cpg binding domain protein 3  GTF3C4 - general transcription factor iiic, polypeptide 4, 90kda  SUPT3H - suppressor of ty 3 homolog (s. cerevisiae)  YEATS2 - yeats domain containing 2  KAT7 - k(lysine) acetyltransferase 7  RUVBL2 - ruvb-like 2 (e. coli) |
| GO:0099111 | microtubule-based transport | 3.14E-5 | 3.39E-3 | 1.24 (10334,120,6996,101) | [+] Show genes  DST - dystonin  KIFAP3 - kinesin-associated protein 3  AP3M2 - adaptor-related protein complex 3, mu 2 subunit  WDR19 - wd repeat domain 19  IFT46 - intraflagellar transport 46 homolog (chlamydomonas)  RHOT2 - ras homolog family member t2  SSX2IP - synovial sarcoma, x breakpoint 2 interacting protein  SPG7 - spastic paraplegia 7 (pure and complicated autosomal recessive)  WDR34 - wd repeat domain 34  APBA1 - amyloid beta (a4) precursor protein-binding, family a, member 1  ACTR10 - actin-related protein 10 homolog (s. cerevisiae)  SPAST - spastin  TUB - tubby bipartite transcription factor  AP3B2 - adaptor-related protein complex 3, beta 2 subunit  SYBU - syntabulin (syntaxin-interacting)  AP3B1 - adaptor-related protein complex 3, beta 1 subunit  TTC30A - tetratricopeptide repeat domain 30a  BICD2 - bicaudal d homolog 2 (drosophila)  NDEL1 - nude neurodevelopment protein 1-like 1  MAP1S - microtubule-associated protein 1s  PAFAH1B1 - platelet-activating factor acetylhydrolase 1b, regulatory subunit 1 (45kda)  AZI1 - 5-azacytidine induced 1  ARL8B - adp-ribosylation factor-like 8b  FBXW11 - f-box and wd repeat domain containing 11  APP - amyloid beta (a4) precursor protein  OPA1 - optic atrophy 1 (autosomal dominant)  IFT43 - intraflagellar transport 43 homolog (chlamydomonas)  NDE1 - nude neurodevelopment protein 1  ARL3 - adp-ribosylation factor-like 3  RAB21 - rab21, member ras oncogene family  IFT57 - intraflagellar transport 57 homolog (chlamydomonas)  ARL8A - adp-ribosylation factor-like 8a  TMEM201 - transmembrane protein 201  AP3S2 - adaptor-related protein complex 3, sigma 2 subunit  TMEM108 - transmembrane protein 108  WDR60 - wd repeat domain 60  CLUAP1 - clusterin associated protein 1  TRAK2 - trafficking protein, kinesin binding 2  PCM1 - pericentriolar material 1  RABL2B - rab, member of ras oncogene family-like 2b  LRPPRC - leucine-rich pentatricopeptide repeat containing  AP3D1 - adaptor-related protein complex 3, delta 1 subunit  IFT52 - intraflagellar transport 52 homolog (chlamydomonas)  HTT - huntingtin  WASF1 - was protein family, member 1  BLOC1S3 - biogenesis of lysosomal organelles complex-1, subunit 3  HSPA8 - heat shock 70kda protein 8  PURA - purine-rich element binding protein a  TTC21B - tetratricopeptide repeat domain 21b  SPG11 - spastic paraplegia 11 (autosomal recessive)  KIF5C - kinesin family member 5c  TRAF3IP1 - tnf receptor-associated factor 3 interacting protein 1  KIF3C - kinesin family member 3c  KIF5B - kinesin family member 5b  KIF5A - kinesin family member 5a  KIF1B - kinesin family member 1b  BLOC1S2 - biogenesis of lysosomal organelles complex-1, subunit 2  IFT74 - intraflagellar transport 74 homolog (chlamydomonas)  SUN2 - sad1 and unc84 domain containing 2  MAPT - microtubule-associated protein tau  KIF3A - kinesin family member 3a  BLOC1S4 - biogenesis of lysosomal organelles complex-1, subunit 4, cappuccino  DYNLL1 - dynein, light chain, lc8-type 1  MAP1B - microtubule-associated protein 1b  MAP1A - microtubule-associated protein 1a  RAB6A - rab6a, member ras oncogene family  DYNLRB2 - dynein, light chain, roadblock-type 2  DYNLRB1 - dynein, light chain, roadblock-type 1  PEX14 - peroxisomal biogenesis factor 14  TNPO1 - transportin 1  MGARP - mitochondria-localized glutamic acid-rich protein  BBS12 - bardet-biedl syndrome 12  RAB1A - rab1a, member ras oncogene family  ARHGAP21 - rho gtpase activating protein 21  ICK - intestinal cell (mak-like) kinase  IFT140 - intraflagellar transport 140 homolog (chlamydomonas)  IFT122 - intraflagellar transport 122 homolog (chlamydomonas)  MAPK8IP3 - mitogen-activated protein kinase 8 interacting protein 3  HIF1A - hypoxia inducible factor 1, alpha subunit (basic helix-loop-helix transcription factor)  COPG2 - coatomer protein complex, subunit gamma 2  BLOC1S6 - biogenesis of lysosomal organelles complex-1, subunit 6, pallidin  NEFL - neurofilament, light polypeptide  COPG1 - coatomer protein complex, subunit gamma 1  AP3M1 - adaptor-related protein complex 3, mu 1 subunit  IFT27 - intraflagellar transport 27 homolog (chlamydomonas)  DYNC2LI1 - dynein, cytoplasmic 2, light intermediate chain 1  TERF2 - telomeric repeat binding factor 2  UBB - ubiquitin b  HNRNPU - heterogeneous nuclear ribonucleoprotein u (scaffold attachment factor a)  IFT81 - intraflagellar transport 81 homolog (chlamydomonas)  KIF1A - kinesin family member 1a  KIF3B - kinesin family member 3b  IFT88 - intraflagellar transport 88 homolog (chlamydomonas)  SFPQ - splicing factor proline/glutamine-rich  DYNC1H1 - dynein, cytoplasmic 1, heavy chain 1  DYNC1I1 - dynein, cytoplasmic 1, intermediate chain 1  DYNC1I2 - dynein, cytoplasmic 1, intermediate chain 2  BLOC1S5 - biogenesis of lysosomal organelles complex-1, subunit 5, muted  UCHL1 - ubiquitin carboxyl-terminal esterase l1 (ubiquitin thiolesterase)  RHOT1 - ras homolog family member t1  IFT172 - intraflagellar transport 172 homolog (chlamydomonas) |
| GO:0010970 | transport along microtubule | 3.14E-5 | 3.36E-3 | 1.24 (10334,120,6996,101) | [+] Show genes  DST - dystonin  KIFAP3 - kinesin-associated protein 3  AP3M2 - adaptor-related protein complex 3, mu 2 subunit  WDR19 - wd repeat domain 19  IFT46 - intraflagellar transport 46 homolog (chlamydomonas)  RHOT2 - ras homolog family member t2  SSX2IP - synovial sarcoma, x breakpoint 2 interacting protein  SPG7 - spastic paraplegia 7 (pure and complicated autosomal recessive)  WDR34 - wd repeat domain 34  ACTR10 - actin-related protein 10 homolog (s. cerevisiae)  APBA1 - amyloid beta (a4) precursor protein-binding, family a, member 1  SPAST - spastin  TUB - tubby bipartite transcription factor  AP3B2 - adaptor-related protein complex 3, beta 2 subunit  SYBU - syntabulin (syntaxin-interacting)  AP3B1 - adaptor-related protein complex 3, beta 1 subunit  BICD2 - bicaudal d homolog 2 (drosophila)  TTC30A - tetratricopeptide repeat domain 30a  NDEL1 - nude neurodevelopment protein 1-like 1  MAP1S - microtubule-associated protein 1s  PAFAH1B1 - platelet-activating factor acetylhydrolase 1b, regulatory subunit 1 (45kda)  AZI1 - 5-azacytidine induced 1  ARL8B - adp-ribosylation factor-like 8b  FBXW11 - f-box and wd repeat domain containing 11  APP - amyloid beta (a4) precursor protein  OPA1 - optic atrophy 1 (autosomal dominant)  IFT43 - intraflagellar transport 43 homolog (chlamydomonas)  ARL3 - adp-ribosylation factor-like 3  NDE1 - nude neurodevelopment protein 1  RAB21 - rab21, member ras oncogene family  IFT57 - intraflagellar transport 57 homolog (chlamydomonas)  ARL8A - adp-ribosylation factor-like 8a  TMEM201 - transmembrane protein 201  AP3S2 - adaptor-related protein complex 3, sigma 2 subunit  TMEM108 - transmembrane protein 108  WDR60 - wd repeat domain 60  CLUAP1 - clusterin associated protein 1  TRAK2 - trafficking protein, kinesin binding 2  PCM1 - pericentriolar material 1  RABL2B - rab, member of ras oncogene family-like 2b  LRPPRC - leucine-rich pentatricopeptide repeat containing  AP3D1 - adaptor-related protein complex 3, delta 1 subunit  IFT52 - intraflagellar transport 52 homolog (chlamydomonas)  HTT - huntingtin  WASF1 - was protein family, member 1  BLOC1S3 - biogenesis of lysosomal organelles complex-1, subunit 3  HSPA8 - heat shock 70kda protein 8  PURA - purine-rich element binding protein a  TTC21B - tetratricopeptide repeat domain 21b  SPG11 - spastic paraplegia 11 (autosomal recessive)  KIF5C - kinesin family member 5c  TRAF3IP1 - tnf receptor-associated factor 3 interacting protein 1  KIF3C - kinesin family member 3c  KIF5B - kinesin family member 5b  KIF5A - kinesin family member 5a  KIF1B - kinesin family member 1b  BLOC1S2 - biogenesis of lysosomal organelles complex-1, subunit 2  IFT74 - intraflagellar transport 74 homolog (chlamydomonas)  SUN2 - sad1 and unc84 domain containing 2  MAPT - microtubule-associated protein tau  KIF3A - kinesin family member 3a  BLOC1S4 - biogenesis of lysosomal organelles complex-1, subunit 4, cappuccino  DYNLL1 - dynein, light chain, lc8-type 1  MAP1B - microtubule-associated protein 1b  MAP1A - microtubule-associated protein 1a  RAB6A - rab6a, member ras oncogene family  DYNLRB2 - dynein, light chain, roadblock-type 2  DYNLRB1 - dynein, light chain, roadblock-type 1  PEX14 - peroxisomal biogenesis factor 14  TNPO1 - transportin 1  MGARP - mitochondria-localized glutamic acid-rich protein  BBS12 - bardet-biedl syndrome 12  RAB1A - rab1a, member ras oncogene family  ARHGAP21 - rho gtpase activating protein 21  ICK - intestinal cell (mak-like) kinase  IFT140 - intraflagellar transport 140 homolog (chlamydomonas)  IFT122 - intraflagellar transport 122 homolog (chlamydomonas)  MAPK8IP3 - mitogen-activated protein kinase 8 interacting protein 3  HIF1A - hypoxia inducible factor 1, alpha subunit (basic helix-loop-helix transcription factor)  COPG2 - coatomer protein complex, subunit gamma 2  COPG1 - coatomer protein complex, subunit gamma 1  NEFL - neurofilament, light polypeptide  BLOC1S6 - biogenesis of lysosomal organelles complex-1, subunit 6, pallidin  AP3M1 - adaptor-related protein complex 3, mu 1 subunit  DYNC2LI1 - dynein, cytoplasmic 2, light intermediate chain 1  IFT27 - intraflagellar transport 27 homolog (chlamydomonas)  TERF2 - telomeric repeat binding factor 2  UBB - ubiquitin b  HNRNPU - heterogeneous nuclear ribonucleoprotein u (scaffold attachment factor a)  IFT81 - intraflagellar transport 81 homolog (chlamydomonas)  KIF1A - kinesin family member 1a  KIF3B - kinesin family member 3b  IFT88 - intraflagellar transport 88 homolog (chlamydomonas)  SFPQ - splicing factor proline/glutamine-rich  DYNC1H1 - dynein, cytoplasmic 1, heavy chain 1  DYNC1I1 - dynein, cytoplasmic 1, intermediate chain 1  DYNC1I2 - dynein, cytoplasmic 1, intermediate chain 2  BLOC1S5 - biogenesis of lysosomal organelles complex-1, subunit 5, muted  UCHL1 - ubiquitin carboxyl-terminal esterase l1 (ubiquitin thiolesterase)  RHOT1 - ras homolog family member t1  IFT172 - intraflagellar transport 172 homolog (chlamydomonas) |
| GO:0032784 | regulation of DNA-templated transcription, elongation | 3.22E-5 | 3.42E-3 | 1.40 (10334,40,6996,38) | [+] Show genes  CCNK - cyclin k  CDK12 - cyclin-dependent kinase 12  SCAF8 - sr-related ctd-associated factor 8  ELL - elongation factor rna polymerase ii  HTATSF1 - hiv-1 tat specific factor 1  CCNT2 - cyclin t2  NELFE - negative elongation factor complex member e  HMGN1 - high mobility group nucleosome binding domain 1  SUPT16H - suppressor of ty 16 homolog (s. cerevisiae)  CCNT1 - cyclin t1  ZMYND11 - zinc finger, mynd-type containing 11  CDC73 - cell division cycle 73  HNRNPU - heterogeneous nuclear ribonucleoprotein u (scaffold attachment factor a)  SUPT6H - suppressor of ty 6 homolog (s. cerevisiae)  GTF2F1 - general transcription factor iif, polypeptide 1, 74kda  SUPT4H1 - suppressor of ty 4 homolog 1 (s. cerevisiae)  SUPT5H - suppressor of ty 5 homolog (s. cerevisiae)  RNF8 - ring finger protein 8, e3 ubiquitin protein ligase  NELFB - negative elongation factor complex member b  RECQL5 - recq protein-like 5  TSFM - ts translation elongation factor, mitochondrial  RNF168 - ring finger protein 168, e3 ubiquitin protein ligase  NELFA - negative elongation factor complex member a  CDK13 - cyclin-dependent kinase 13  ALYREF - aly/ref export factor  LEO1 - leo1, paf1/rna polymerase ii complex component, homolog (s. cerevisiae)  BRD4 - bromodomain containing 4  TCERG1 - transcription elongation regulator 1  THOC1 - tho complex 1  ZNF326 - zinc finger protein 326  NELFCD - negative elongation factor complex member c/d  EAPP - e2f-associated phosphoprotein  LDB1 - lim domain binding 1  SETD5 - set domain containing 5  WDR43 - wd repeat domain 43  TCEA2 - transcription elongation factor a (sii), 2  DDX39B - dead (asp-glu-ala-asp) box polypeptide 39b  KAT7 - k(lysine) acetyltransferase 7 |
| GO:0007030 | Golgi organization | 3.26E-5 | 3.44E-3 | 1.27 (10334,94,6996,81) | [+] Show genes  TBC1D20 - tbc1 domain family, member 20  COG3 - component of oligomeric golgi complex 3  TMED3 - transmembrane emp24 protein transport domain containing 3  KIFC3 - kinesin family member c3  GOLGA2 - golgin a2  GOLGB1 - golgin b1  RAB30 - rab30, member ras oncogene family  VMP1 - vacuole membrane protein 1  TMED2 - transmembrane emp24 domain trafficking protein 2  LRRK2 - leucine-rich repeat kinase 2  USP6NL - usp6 n-terminal like  ATL3 - atlastin gtpase 3  TMED10 - transmembrane emp24-like trafficking protein 10 (yeast)  CLASP2 - cytoplasmic linker associated protein 2  STX5 - syntaxin 5  TICAM2 - toll-like receptor adaptor molecule 2  VAMP4 - vesicle-associated membrane protein 4  BLZF1 - basic leucine zipper nuclear factor 1  STX17 - syntaxin 17  RAB2A - rab2a, member ras oncogene family  PDCD10 - programmed cell death 10  LMAN2L - lectin, mannose-binding 2-like  FAM174B - family with sequence similarity 174, member b  RAB1A - rab1a, member ras oncogene family  ARHGAP21 - rho gtpase activating protein 21  VRK1 - vaccinia related kinase 1  TMED5 - transmembrane emp24 protein transport domain containing 5  UBXN2B - ubx domain protein 2b  GOLPH3L - golgi phosphoprotein 3-like  PI4K2A - phosphatidylinositol 4-kinase type 2 alpha  TRAPPC12 - trafficking protein particle complex 12  TJAP1 - tight junction associated protein 1 (peripheral)  VCPIP1 - valosin containing protein (p97)/p47 complex interacting protein 1  GORASP1 - golgi reassembly stacking protein 1, 65kda  TMED4 - transmembrane emp24 protein transport domain containing 4  STK25 - serine/threonine kinase 25  TMED7 - transmembrane emp24 protein transport domain containing 7  LMAN1 - lectin, mannose-binding, 1  LYSMD3 - lysm, putative peptidoglycan-binding, domain containing 3  ATP8B2 - atpase, aminophospholipid transporter, class i, type 8b, member 2  VPS51 - vacuolar protein sorting 51 homolog (s. cerevisiae)  ARL1 - adp-ribosylation factor-like 1  BCAS3 - breast carcinoma amplified sequence 3  GAK - cyclin g associated kinase  PLK3 - polo-like kinase 3  COG4 - component of oligomeric golgi complex 4  TRAPPC11 - trafficking protein particle complex 11  GCC2 - grip and coiled-coil domain containing 2  ARHGEF7 - rho guanine nucleotide exchange factor (gef) 7  SEC16A - sec16 homolog a (s. cerevisiae)  SEC23IP - sec23 interacting protein  SYNE1 - spectrin repeat containing, nuclear envelope 1  NPLOC4 - nuclear protein localization 4 homolog (s. cerevisiae)  FBXW8 - f-box and wd repeat domain containing 8  GBF1 - golgi brefeldin a resistant guanine nucleotide exchange factor 1  NSFL1C - nsfl1 (p97) cofactor (p47)  HUWE1 - hect, uba and wwe domain containing 1, e3 ubiquitin protein ligase  NUMA1 - nuclear mitotic apparatus protein 1  CLASP1 - cytoplasmic linker associated protein 1  STX6 - syntaxin 6  ZW10 - zw10 kinetochore protein  BAG5 - bcl2-associated athanogene 5  DYM - dymeclin  COG1 - component of oligomeric golgi complex 1  TMED6 - transmembrane emp24 protein transport domain containing 6  COG2 - component of oligomeric golgi complex 2  GOLPH3 - golgi phosphoprotein 3 (coat-protein)  COG7 - component of oligomeric golgi complex 7  GORASP2 - golgi reassembly stacking protein 2, 55kda  GOLGA5 - golgin a5  PRKD1 - protein kinase d1  HACE1 - hect domain and ankyrin repeat containing e3 ubiquitin protein ligase 1  CSNK1A1 - casein kinase 1, alpha 1  CSNK1D - casein kinase 1, delta  USO1 - uso1 vesicle transport factor  OBSL1 - obscurin-like 1  PRMT5 - protein arginine methyltransferase 5  HTT - huntingtin  YWHAZ - tyrosine 3-monooxygenase/tryptophan 5-monooxygenase activation protein, zeta polypeptide  CUL7 - cullin 7  ARFGEF1 - adp-ribosylation factor guanine nucleotide-exchange factor 1 (brefeldin a-inhibited) |
| GO:0045666 | positive regulation of neuron differentiation | 3.93E-5 | 4.11E-3 | 1.17 (10334,241,6996,191) | [+] Show genes  ZMYND8 - zinc finger, mynd-type containing 8  DHX36 - deah (asp-glu-ala-his) box polypeptide 36  MARK2 - map/microtubule affinity-regulating kinase 2  FMR1 - fragile x mental retardation 1  NCK1 - nck adaptor protein 1  RNF112 - ring finger protein 112  ZEB1 - zinc finger e-box binding homeobox 1  GDI1 - gdp dissociation inhibitor 1  LIMK1 - lim domain kinase 1  TCF12 - transcription factor 12  SIN3A - sin3 transcription regulator family member a  ENC1 - ectodermal-neural cortex 1 (with btb domain)  PPP1R9A - protein phosphatase 1, regulatory subunit 9a  ATP8A2 - atpase, aminophospholipid transporter, class i, type 8a, member 2  SYT17 - synaptotagmin xvii  SF3A2 - splicing factor 3a, subunit 2, 66kda  PLXNB1 - plexin b1  PLXNA3 - plexin a3  NRP1 - neuropilin 1  IQGAP1 - iq motif containing gtpase activating protein 1  KATNB1 - katanin p80 (wd repeat containing) subunit b 1  EP300 - e1a binding protein p300  PSEN1 - presenilin 1  RET - ret proto-oncogene  CREB3L2 - camp responsive element binding protein 3-like 2  SHANK1 - sh3 and multiple ankyrin repeat domains 1  DBN1 - drebrin 1  PRPF19 - pre-mrna processing factor 19  ILK - integrin-linked kinase  APBB1 - amyloid beta (a4) precursor protein-binding, family b, member 1 (fe65)  SCARB2 - scavenger receptor class b, member 2  GPRC5B - g protein-coupled receptor, family c, group 5, member b  CFLAR - casp8 and fadd-like apoptosis regulator  NDEL1 - nude neurodevelopment protein 1-like 1  PLXNA1 - plexin a1  ARF1 - adp-ribosylation factor 1  PLXNA2 - plexin a2  TRIM32 - tripartite motif containing 32  PAFAH1B1 - platelet-activating factor acetylhydrolase 1b, regulatory subunit 1 (45kda)  CAMK1 - calcium/calmodulin-dependent protein kinase i  FKBP1B - fk506 binding protein 1b, 12.6 kda  RND2 - rho family gtpase 2  BMPR2 - bone morphogenetic protein receptor, type ii (serine/threonine kinase)  OPA1 - optic atrophy 1 (autosomal dominant)  DYNLT1 - dynein, light chain, tctex-type 1  DLG5 - discs, large homolog 5 (drosophila)  PAK3 - p21 protein (cdc42/rac)-activated kinase 3  CRABP2 - cellular retinoic acid binding protein 2  FIG4 - fig4 homolog, sac1 lipid phosphatase domain containing (s. cerevisiae)  BMP7 - bone morphogenetic protein 7  CXCR4 - chemokine (c-x-c motif) receptor 4  MUL1 - mitochondrial e3 ubiquitin protein ligase 1  ANKRD27 - ankyrin repeat domain 27 (vps9 domain)  RAB21 - rab21, member ras oncogene family  FGFR1 - fibroblast growth factor receptor 1  AMIGO1 - adhesion molecule with ig-like domain 1  PTEN - phosphatase and tensin homolog  ARSB - arylsulfatase b  ITGA3 - integrin, alpha 3 (antigen cd49c, alpha 3 subunit of vla-3 receptor)  IST1 - increased sodium tolerance 1 homolog (yeast)  SS18L1 - synovial sarcoma translocation gene on chromosome 18-like 1  METRN - meteorin, glial cell differentiation regulator  DAB2IP - dab2 interacting protein  RHOA - ras homolog family member a  MEGF8 - multiple egf-like-domains 8  CPNE1 - copine i  ARHGDIA - rho gdp dissociation inhibitor (gdi) alpha  KDM1A - lysine (k)-specific demethylase 1a  CNR1 - cannabinoid receptor 1 (brain)  FZD4 - frizzled family receptor 4  FZD1 - frizzled family receptor 1  UNC13A - unc-13 homolog a (c. elegans)  ACTR2 - arp2 actin-related protein 2 homolog (yeast)  LRP8 - low density lipoprotein receptor-related protein 8, apolipoprotein e receptor  EFNA5 - ephrin-a5  PARP6 - poly (adp-ribose) polymerase family, member 6  CNTN1 - contactin 1  PTK7 - protein tyrosine kinase 7  ROBO1 - roundabout, axon guidance receptor, homolog 1 (drosophila)  ROBO2 - roundabout, axon guidance receptor, homolog 2 (drosophila)  ADNP - activity-dependent neuroprotector homeobox  TWF1 - twinfilin actin-binding protein 1  ZC4H2 - zinc finger, c4h2 domain containing  PTN - pleiotrophin  BRINP1 - bone morphogenetic protein/retinoic acid inducible neural-specific 1  KIDINS220 - kinase d-interacting substrate, 220kda  ZEB2 - zinc finger e-box binding homeobox 2  ARHGEF2 - rho/rac guanine nucleotide exchange factor (gef) 2  TUBB2B - tubulin, beta 2b class iib  CRTC1 - creb regulated transcription coactivator 1  NDNF - neuron-derived neurotrophic factor  TCF3 - transcription factor 3  L1CAM - l1 cell adhesion molecule  LPAR1 - lysophosphatidic acid receptor 1  TCF4 - transcription factor 4  PTPRD - protein tyrosine phosphatase, receptor type, d  OBSL1 - obscurin-like 1  SETX - senataxin  CUL7 - cullin 7  ITGA6 - integrin, alpha 6  ASCL1 - achaete-scute complex homolog 1 (drosophila)  NRCAM - neuronal cell adhesion molecule  TMEM30A - transmembrane protein 30a  CBFA2T2 - core-binding factor, runt domain, alpha subunit 2; translocated to, 2  RRN3 - rrn3 rna polymerase i transcription factor homolog (s. cerevisiae)  AP2A1 - adaptor-related protein complex 2, alpha 1 subunit  KDM4C - lysine (k)-specific demethylase 4c  TIMP2 - timp metallopeptidase inhibitor 2  RUFY3 - run and fyve domain containing 3  TMEM106B - transmembrane protein 106b  CAMK1D - calcium/calmodulin-dependent protein kinase id  MTOR - mechanistic target of rapamycin (serine/threonine kinase)  NME1 - nme/nm23 nucleoside diphosphate kinase 1  ATP1B2 - atpase, na+/k+ transporting, beta 2 polypeptide  SYT1 - synaptotagmin i  NCKIPSD - nck interacting protein with sh3 domain  CAMK2B - calcium/calmodulin-dependent protein kinase ii beta  SYT4 - synaptotagmin iv  SHANK3 - sh3 and multiple ankyrin repeat domains 3  MAPT - microtubule-associated protein tau  CACNG7 - calcium channel, voltage-dependent, gamma subunit 7  MAP6 - microtubule-associated protein 6  PTK2B - protein tyrosine kinase 2 beta  MAP1B - microtubule-associated protein 1b  RIMS2 - regulating synaptic membrane exocytosis 2  PACSIN1 - protein kinase c and casein kinase substrate in neurons 1  DPYSL3 - dihydropyrimidinase-like 3  BAIAP2 - bai1-associated protein 2  CAPRIN2 - caprin family member 2  ETV5 - ets variant 5  PPP2R5B - protein phosphatase 2, regulatory subunit b', beta  TOX - thymocyte selection-associated high mobility group box  SKIL - ski-like oncogene  PLXND1 - plexin d1  NDRG4 - ndrg family member 4  CUX1 - cut-like homeobox 1  DVL2 - dishevelled segment polarity protein 2  DVL3 - dishevelled segment polarity protein 3  SYT3 - synaptotagmin iii  IMPACT - impact rwd domain protein  DNM1L - dynamin 1-like  NKX2-2 - nk2 homeobox 2  FYN - fyn oncogene related to src, fgr, yes  STK25 - serine/threonine kinase 25  DDX56 - dead (asp-glu-ala-asp) box helicase 56  TGIF2 - tgfb-induced factor homeobox 2  CDKL5 - cyclin-dependent kinase-like 5  STMN2 - stathmin-like 2  SH3GL3 - sh3-domain grb2-like 3  SNX3 - sorting nexin 3  DLG4 - discs, large homolog 4 (drosophila)  RAP1A - rap1a, member of ras oncogene family  RASAL1 - ras protein activator like 1 (gap1 like)  LRP1 - low density lipoprotein receptor-related protein 1  NEFL - neurofilament, light polypeptide  SLIT2 - slit homolog 2 (drosophila)  DBNL - drebrin-like  SHOX2 - short stature homeobox 2  FBXW8 - f-box and wd repeat domain containing 8  NFE2L2 - nuclear factor (erythroid-derived 2)-like 2  SEMA5A - sema domain, seven thrombospondin repeats (type 1 and type 1-like), transmembrane domain (tm) and short cytoplasmic domain, (semaphorin) 5a  ITSN2 - intersectin 2  RARB - retinoic acid receptor, beta  FBXO38 - f-box protein 38  LYN - v-yes-1 yamaguchi sarcoma viral related oncogene homolog  SPAG9 - sperm associated antigen 9  FEZ1 - fasciculation and elongation protein zeta 1 (zygin i)  ZFYVE27 - zinc finger, fyve domain containing 27  NPTN - neuroplastin  MAP2K1 - mitogen-activated protein kinase kinase 1  RNF157 - ring finger protein 157  MACF1 - microtubule-actin crosslinking factor 1  TWF2 - twinfilin actin-binding protein 2  CYFIP1 - cytoplasmic fmr1 interacting protein 1  SERPINI1 - serpin peptidase inhibitor, clade i (neuroserpin), member 1  CYB5D2 - cytochrome b5 domain containing 2  RAPGEF2 - rap guanine nucleotide exchange factor (gef) 2  RAB11A - rab11a, member ras oncogene family  GATA2 - gata binding protein 2  ARHGAP35 - rho gtpase activating protein 35  TENM3 - teneurin transmembrane protein 3  SMURF1 - smad specific e3 ubiquitin protein ligase 1  CAPRIN1 - cell cycle associated protein 1  PRKD1 - protein kinase d1  KCTD11 - potassium channel tetramerization domain containing 11  CSNK1D - casein kinase 1, delta  PRKCI - protein kinase c, iota  BCL2 - b-cell cll/lymphoma 2  ITSN1 - intersectin 1 (sh3 domain protein)  BEND6 - ben domain containing 6  MMD - monocyte to macrophage differentiation-associated |
| GO:0046907 | intracellular transport | 4.08E-5 | 4.24E-3 | 1.07 (10334,1247,6996,905) | [+] Show genes  AGAP2 - arfgap with gtpase domain, ankyrin repeat and ph domain 2  SYMPK - symplekin  AP3M2 - adaptor-related protein complex 3, mu 2 subunit  WLS - wntless homolog (drosophila)  ABCA1 - atp-binding cassette, sub-family a (abc1), member 1  SEC61B - sec61 beta subunit  TOMM34 - translocase of outer mitochondrial membrane 34  OS9 - osteosarcoma amplified 9, endoplasmic reticulum lectin  RAB3IP - rab3a interacting protein  SSX2IP - synovial sarcoma, x breakpoint 2 interacting protein  TMED2 - transmembrane emp24 domain trafficking protein 2  NUDT19 - nudix (nucleoside diphosphate linked moiety x)-type motif 19  ERP29 - endoplasmic reticulum protein 29  PML - promyelocytic leukemia  ACOT2 - acyl-coa thioesterase 2  ACACA - acetyl-coa carboxylase alpha  PMM1 - phosphomannomutase 1  MKKS - mckusick-kaufman syndrome  YWHAQ - tyrosine 3-monooxygenase/tryptophan 5-monooxygenase activation protein, theta polypeptide  TMED10 - transmembrane emp24-like trafficking protein 10 (yeast)  TBC1D22B - tbc1 domain family, member 22b  ANP32A - acidic (leucine-rich) nuclear phosphoprotein 32 family, member a  AP3B2 - adaptor-related protein complex 3, beta 2 subunit  NOP9 - nop9 nucleolar protein  SYBU - syntabulin (syntaxin-interacting)  ACOX1 - acyl-coa oxidase 1, palmitoyl  PABPN1 - poly(a) binding protein, nuclear 1  RNPS1 - rna binding protein s1, serine-rich domain  KIF16B - kinesin family member 16b  EHD1 - eh-domain containing 1  ZDHHC7 - zinc finger, dhhc-type containing 7  PICALM - phosphatidylinositol binding clathrin assembly protein  ACTN4 - actinin, alpha 4  RAB10 - rab10, member ras oncogene family  GLE1 - gle1 rna export mediator  TMCO6 - transmembrane and coiled-coil domains 6  CPSF4 - cleavage and polyadenylation specific factor 4, 30kda  MED1 - mediator complex subunit 1  NUP85 - nucleoporin 85kda  USP9X - ubiquitin specific peptidase 9, x-linked  ADPRH - adp-ribosylarginine hydrolase  SNAP47 - synaptosomal-associated protein, 47kda  POLR2D - polymerase (rna) ii (dna directed) polypeptide d  AP2A2 - adaptor-related protein complex 2, alpha 2 subunit  AP2A1 - adaptor-related protein complex 2, alpha 1 subunit  AP2B1 - adaptor-related protein complex 2, beta 1 subunit  TTC21B - tetratricopeptide repeat domain 21b  AP1B1 - adaptor-related protein complex 1, beta 1 subunit  AP1G1 - adaptor-related protein complex 1, gamma 1 subunit  NUP133 - nucleoporin 133kda  AP5M1 - adaptor-related protein complex 5, mu 1 subunit  AGK - acylglycerol kinase  NUPL2 - nucleoporin like 2  VPS35 - vacuolar protein sorting 35 homolog (s. cerevisiae)  KIF3A - kinesin family member 3a  GEMIN6 - gem (nuclear organelle) associated protein 6  AKT1 - v-akt murine thymoma viral oncogene homolog 1  RAB7A - rab7a, member ras oncogene family  PCID2 - pci domain containing 2  BBS12 - bardet-biedl syndrome 12  EVI5L - ecotropic viral integration site 5-like  SLBP - stem-loop binding protein  TBC1D23 - tbc1 domain family, member 23  PPP1R10 - protein phosphatase 1, regulatory subunit 10  IFT122 - intraflagellar transport 122 homolog (chlamydomonas)  TMEM115 - transmembrane protein 115  DDX39B - dead (asp-glu-ala-asp) box polypeptide 39b  GAK - cyclin g associated kinase  SCYL2 - scy1-like 2 (s. cerevisiae)  PEX26 - peroxisomal biogenesis factor 26  TBC1D17 - tbc1 domain family, member 17  RAB35 - rab35, member ras oncogene family  IFT27 - intraflagellar transport 27 homolog (chlamydomonas)  KDELR2 - kdel (lys-asp-glu-leu) endoplasmic reticulum protein retention receptor 2  NPLOC4 - nuclear protein localization 4 homolog (s. cerevisiae)  AAAS - achalasia, adrenocortical insufficiency, alacrimia  RNF126 - ring finger protein 126  IFT88 - intraflagellar transport 88 homolog (chlamydomonas)  RAB31 - rab31, member ras oncogene family  RNF157 - ring finger protein 157  MAPK10 - mitogen-activated protein kinase 10  WDR11 - wd repeat domain 11  GEMIN7 - gem (nuclear organelle) associated protein 7  MICALL2 - mical-like 2  NDC1 - ndc1 transmembrane nucleoporin  CCDC88A - coiled-coil domain containing 88a  STAM - signal transducing adaptor molecule (sh3 domain and itam motif) 1  IPO9 - importin 9  PRKCI - protein kinase c, iota  NUP214 - nucleoporin 214kda  NR4A3 - nuclear receptor subfamily 4, group a, member 3  GAS6 - growth arrest-specific 6  NCOA4 - nuclear receptor coactivator 4  PRKAG1 - protein kinase, amp-activated, gamma 1 non-catalytic subunit  DDX19B - dead (asp-glu-ala-asp) box polypeptide 19b  SDAD1 - sda1 domain containing 1  SYNRG - synergin, gamma  DCTN3 - dynactin 3 (p22)  APBA1 - amyloid beta (a4) precursor protein-binding, family a, member 1  SEC61A2 - sec61 alpha 2 subunit (s. cerevisiae)  XPOT - exportin, trna  CHP1 - calcineurin-like ef-hand protein 1  VPS13D - vacuolar protein sorting 13 homolog d (s. cerevisiae)  SEC63 - sec63 homolog (s. cerevisiae)  ARCN1 - archain 1  TRIM23 - tripartite motif containing 23  APPL2 - adaptor protein, phosphotyrosine interaction, ph domain and leucine zipper containing 2  ARF1 - adp-ribosylation factor 1  IPO4 - importin 4  ARF4 - adp-ribosylation factor 4  MAP1S - microtubule-associated protein 1s  PAFAH1B1 - platelet-activating factor acetylhydrolase 1b, regulatory subunit 1 (45kda)  ARL8B - adp-ribosylation factor-like 8b  AKAP13 - a kinase (prka) anchor protein 13  APP - amyloid beta (a4) precursor protein  ZDHHC14 - zinc finger, dhhc-type containing 14  BBS7 - bardet-biedl syndrome 7  DDX20 - dead (asp-glu-ala-asp) box polypeptide 20  ARNTL - aryl hydrocarbon receptor nuclear translocator-like  ARL3 - adp-ribosylation factor-like 3  TRAPPC6B - trafficking protein particle complex 6b  ARL1 - adp-ribosylation factor-like 1  TUBA1A - tubulin, alpha 1a  SEC23IP - sec23 interacting protein  IFT57 - intraflagellar transport 57 homolog (chlamydomonas)  CORO7 - coronin 7  ALMS1 - alstrom syndrome 1  RHOB - ras homolog family member b  PSIP1 - pc4 and sfrs1 interacting protein 1  ARF6 - adp-ribosylation factor 6  ARF5 - adp-ribosylation factor 5  EVI5 - ecotropic viral integration site 5  ARHGAP1 - rho gtpase activating protein 1  AP4S1 - adaptor-related protein complex 4, sigma 1 subunit  ASNA1 - arsa arsenite transporter, atp-binding, homolog 1 (bacterial)  WDR60 - wd repeat domain 60  CHMP1A - charged multivesicular body protein 1a  PLEKHJ1 - pleckstrin homology domain containing, family j member 1  MAGOHB - mago-nashi homolog b (drosophila)  PCM1 - pericentriolar material 1  RABL2B - rab, member of ras oncogene family-like 2b  TBC1D8 - tbc1 domain family, member 8 (with gram domain)  CDC37 - cell division cycle 37  HTT - huntingtin  DDX19A - dead (asp-glu-ala-asp) box polypeptide 19a  DENND3 - denn/madd domain containing 3  GOLGA2 - golgin a2  XPO1 - exportin 1 (crm1 homolog, yeast)  RPH3A - rabphilin 3a homolog (mouse)  GOLGB1 - golgin b1  CCDC91 - coiled-coil domain containing 91  ATP1B1 - atpase, na+/k+ transporting, beta 1 polypeptide  MON1B - mon1 secretory trafficking family member b  LRRK2 - leucine-rich repeat kinase 2  BLOC1S4 - biogenesis of lysosomal organelles complex-1, subunit 4, cappuccino  CLSTN1 - calsyntenin 1  AP5S1 - adaptor-related protein complex 5, sigma 1 subunit  SEC31A - sec31 homolog a (s. cerevisiae)  WRB - tryptophan rich basic protein  PEX6 - peroxisomal biogenesis factor 6  LSG1 - large 60s subunit nuclear export gtpase 1  ATG14 - autophagy related 14  PEX7 - peroxisomal biogenesis factor 7  PEX12 - peroxisomal biogenesis factor 12  PEX13 - peroxisomal biogenesis factor 13  PEX14 - peroxisomal biogenesis factor 14  RILPL2 - rab interacting lysosomal protein-like 2  LMTK2 - lemur tyrosine kinase 2  ICK - intestinal cell (mak-like) kinase  WDR33 - wd repeat domain 33  PEX1 - peroxisomal biogenesis factor 1  PAOX - polyamine oxidase (exo-n4-amino)  PKD1 - polycystic kidney disease 1 (autosomal dominant)  THOC6 - tho complex 6 homolog (drosophila)  COPZ1 - coatomer protein complex, subunit zeta 1  COPG1 - coatomer protein complex, subunit gamma 1  PIK3R1 - phosphoinositide-3-kinase, regulatory subunit 1 (alpha)  KIF1A - kinesin family member 1a  PEF1 - penta-ef-hand domain containing 1  COG2 - component of oligomeric golgi complex 2  CASC3 - cancer susceptibility candidate 3  VPS53 - vacuolar protein sorting 53 homolog (s. cerevisiae)  RABL3 - rab, member of ras oncogene family-like 3  GABARAP - gaba(a) receptor-associated protein  U2AF2 - u2 small nuclear rna auxiliary factor 2  BCR - breakpoint cluster region  RHOT1 - ras homolog family member t1  PIK3C3 - phosphatidylinositol 3-kinase, catalytic subunit type 3  YWHAG - tyrosine 3-monooxygenase/tryptophan 5-monooxygenase activation protein, gamma polypeptide  COPE - coatomer protein complex, subunit epsilon  YWHAZ - tyrosine 3-monooxygenase/tryptophan 5-monooxygenase activation protein, zeta polypeptide  BBS4 - bardet-biedl syndrome 4  BBS2 - bardet-biedl syndrome 2  VPS45 - vacuolar protein sorting 45 homolog (s. cerevisiae)  YWHAE - tyrosine 3-monooxygenase/tryptophan 5-monooxygenase activation protein, epsilon polypeptide  YWHAB - tyrosine 3-monooxygenase/tryptophan 5-monooxygenase activation protein, beta polypeptide  TRIM27 - tripartite motif containing 27  TBC1D20 - tbc1 domain family, member 20  DST - dystonin  BET1 - bet1 golgi vesicular membrane trafficking protein  LIN7A - lin-7 homolog a (c. elegans)  KATNB1 - katanin p80 (wd repeat containing) subunit b 1  ALKBH5 - alkb, alkylation repair homolog 5 (e. coli)  AGFG1 - arfgap with fg repeats 1  UPF1 - upf1 regulator of nonsense transcripts homolog (yeast)  DERL1 - derlin 1  DSCR3 - down syndrome critical region gene 3  RANBP6 - ran binding protein 6  FNBP1L - formin binding protein 1-like  TFG - trk-fused gene  SYNJ1 - synaptojanin 1  RNF215 - ring finger protein 215  NDE1 - nude neurodevelopment protein 1  VPS51 - vacuolar protein sorting 51 homolog (s. cerevisiae)  ABCE1 - atp-binding cassette, sub-family e (oabp), member 1  VPS13C - vacuolar protein sorting 13 homolog c (s. cerevisiae)  PTTG1IP - pituitary tumor-transforming 1 interacting protein  TMEM50B - transmembrane protein 50b  ARL8A - adp-ribosylation factor-like 8a  STX6 - syntaxin 6  AP1M1 - adaptor-related protein complex 1, mu 1 subunit  AP1G2 - adaptor-related protein complex 1, gamma 2 subunit  TMEM201 - transmembrane protein 201  AP1S2 - adaptor-related protein complex 1, sigma 2 subunit  AP3S2 - adaptor-related protein complex 3, sigma 2 subunit  U2AF1L4 - u2 small nuclear rna auxiliary factor 1-like 4  AFTPH - aftiphilin  MCM3AP - minichromosome maintenance complex component 3 associated protein  TMEM108 - transmembrane protein 108  HERC2 - hect and rld domain containing e3 ubiquitin protein ligase 2  TRAK2 - trafficking protein, kinesin binding 2  TSPO - translocator protein (18kda)  SRRM1 - serine/arginine repetitive matrix 1  STAM2 - signal transducing adaptor molecule (sh3 domain and itam motif) 2  HSPD1 - heat shock 60kda protein 1 (chaperonin)  RAMP2 - receptor (g protein-coupled) activity modifying protein 2  HSP90AA1 - heat shock protein 90kda alpha (cytosolic), class a member 1  RAMP3 - receptor (g protein-coupled) activity modifying protein 3  AP3D1 - adaptor-related protein complex 3, delta 1 subunit  RAMP1 - receptor (g protein-coupled) activity modifying protein 1  ZMPSTE24 - zinc metallopeptidase ste24  HSPA4 - heat shock 70kda protein 4  BAIAP3 - bai1-associated protein 3  HSPA9 - heat shock 70kda protein 9 (mortalin)  HSPA8 - heat shock 70kda protein 8  WASF1 - was protein family, member 1  CAPZA1 - capping protein (actin filament) muscle z-line, alpha 1  TOMM40 - translocase of outer mitochondrial membrane 40 homolog (yeast)  TIMM17A - translocase of inner mitochondrial membrane 17 homolog a (yeast)  RPL17 - ribosomal protein l17  VPS37C - vacuolar protein sorting 37 homolog c (s. cerevisiae)  RPL15 - ribosomal protein l15  MPPE1 - metallophosphoesterase 1  RPL11 - ribosomal protein l11  RPL9 - ribosomal protein l9  TIMM10B - translocase of inner mitochondrial membrane 10 homolog b (yeast)  COG5 - component of oligomeric golgi complex 5  RPL7 - ribosomal protein l7  DYNLL1 - dynein, light chain, lc8-type 1  HEATR3 - heat repeat containing 3  RPL3 - ribosomal protein l3  TIMM9 - translocase of inner mitochondrial membrane 9 homolog (yeast)  CAPZB - capping protein (actin filament) muscle z-line, beta  RPS4X - ribosomal protein s4, x-linked  BECN1 - beclin 1, autophagy related  SEC23A - sec23 homolog a (s. cerevisiae)  SRSF9 - serine/arginine-rich splicing factor 9  RPS4Y1 - ribosomal protein s4, y-linked 1  SEC23B - sec23 homolog b (s. cerevisiae)  STX17 - syntaxin 17  ACD - adrenocortical dysplasia homolog (mouse)  LAMTOR1 - late endosomal/lysosomal adaptor, mapk and mtor activator 1  NOL6 - nucleolar protein 6 (rna-associated)  CALR - calreticulin  VPS33A - vacuolar protein sorting 33 homolog a (s. cerevisiae)  HIP1 - huntingtin interacting protein 1  RPL32 - ribosomal protein l32  UPF3B - upf3 regulator of nonsense transcripts homolog b (yeast)  VTI1B - vesicle transport through interaction with t-snares 1b  UPF3A - upf3 regulator of nonsense transcripts homolog a (yeast)  HIF1A - hypoxia inducible factor 1, alpha subunit (basic helix-loop-helix transcription factor)  MBTPS1 - membrane-bound transcription factor peptidase, site 1  ARFGAP3 - adp-ribosylation factor gtpase activating protein 3  RPS23 - ribosomal protein s23  SNX3 - sorting nexin 3  SNX4 - sorting nexin 4  TUBA1B - tubulin, alpha 1b  TSNARE1 - t-snare domain containing 1  TUBB - tubulin, beta class i  GBF1 - golgi brefeldin a resistant guanine nucleotide exchange factor 1  HNRNPU - heterogeneous nuclear ribonucleoprotein u (scaffold attachment factor a)  GEMIN8 - gem (nuclear organelle) associated protein 8  FBXO22 - f-box protein 22  RPS15 - ribosomal protein s15  RPS12 - ribosomal protein s12  VPS33B - vacuolar protein sorting 33 homolog b (yeast)  NRBP2 - nuclear receptor binding protein 2  ARV1 - arv1 homolog (s. cerevisiae)  CBL - cbl proto-oncogene, e3 ubiquitin protein ligase  RAB11A - rab11a, member ras oncogene family  HNRNPA2B1 - heterogeneous nuclear ribonucleoprotein a2/b1  HNRNPA1 - heterogeneous nuclear ribonucleoprotein a1  NAPA - n-ethylmaleimide-sensitive factor attachment protein, alpha  NAPG - n-ethylmaleimide-sensitive factor attachment protein, gamma  SEC24B - sec24 family, member b (s. cerevisiae)  HMGA1 - high mobility group at-hook 1  CMTM6 - cklf-like marvel transmembrane domain containing 6  TBC1D15 - tbc1 domain family, member 15  PSAP - prosaposin  DESI1 - desumoylating isopeptidase 1  PEX3 - peroxisomal biogenesis factor 3  SGSM3 - small g protein signaling modulator 3  RANBP3 - ran binding protein 3  PPFIA2 - protein tyrosine phosphatase, receptor type, f polypeptide (ptprf), interacting protein (liprin), alpha 2  RABGEF1 - rab guanine nucleotide exchange factor (gef) 1  CREB3L2 - camp responsive element binding protein 3-like 2  STARD4 - star-related lipid transfer (start) domain containing 4  SCARB2 - scavenger receptor class b, member 2  AP3B1 - adaptor-related protein complex 3, beta 1 subunit  YKT6 - ykt6 v-snare homolog (s. cerevisiae)  AGPS - alkylglycerone phosphate synthase  CAMK1 - calcium/calmodulin-dependent protein kinase i  GDAP1 - ganglioside induced differentiation associated protein 1  GORASP1 - golgi reassembly stacking protein 1, 65kda  GRPEL2 - grpe-like 2, mitochondrial (e. coli)  PITRM1 - pitrilysin metallopeptidase 1  VPS16 - vacuolar protein sorting 16 homolog (s. cerevisiae)  DCTN2 - dynactin 2 (p50)  APPBP2 - amyloid beta precursor protein (cytoplasmic tail) binding protein 2  TRAM1L1 - translocation associated membrane protein 1-like 1  ARFIP1 - adp-ribosylation factor interacting protein 1  HYOU1 - hypoxia up-regulated 1  PTK2 - protein tyrosine kinase 2  ZDHHC6 - zinc finger, dhhc-type containing 6  IPO8 - importin 8  IPO7 - importin 7  EIF6 - eukaryotic translation initiation factor 6  SLU7 - slu7 splicing factor homolog (s. cerevisiae)  CDK5 - cyclin-dependent kinase 5  ATG3 - autophagy related 3  KLHL20 - kelch-like family member 20  NPC2 - niemann-pick disease, type c2  AKTIP - akt interacting protein  CADPS - ca++-dependent secretion activator  HTATIP2 - hiv-1 tat interactive protein 2, 30kda  USO1 - uso1 vesicle transport factor  PURA - purine-rich element binding protein a  TRAPPC3 - trafficking protein particle complex 3  SRSF10 - serine/arginine-rich splicing factor 10  TBC1D13 - tbc1 domain family, member 13  LYST - lysosomal trafficking regulator  LEPROT - leptin receptor overlapping transcript  AHCYL1 - adenosylhomocysteinase-like 1  PEX2 - peroxisomal biogenesis factor 2  CHML - choroideremia-like (rab escort protein 2)  NUP50 - nucleoporin 50kda  CHM - choroideremia (rab escort protein 1)  CHCHD4 - coiled-coil-helix-coiled-coil-helix domain containing 4  GIPC1 - gipc pdz domain containing family, member 1  SNX5 - sorting nexin 5  CHGA - chromogranin a (parathyroid secretory protein 1)  RAB6A - rab6a, member ras oncogene family  RAB5A - rab5a, member ras oncogene family  C17orf75 - chromosome 17 open reading frame 75  RAB5B - rab5b, member ras oncogene family  RAB3A - rab3a, member ras oncogene family  MTERFD2 - mterf domain containing 2  CPLX2 - complexin 2  RAB1A - rab1a, member ras oncogene family  SDCCAG3 - serologically defined colon cancer antigen 3  SEC24A - sec24 family, member a (s. cerevisiae)  RAB5C - rab5c, member ras oncogene family  TMED4 - transmembrane emp24 protein transport domain containing 4  VPS4A - vacuolar protein sorting 4 homolog a (s. cerevisiae)  RAB27A - rab27a, member ras oncogene family  COPG2 - coatomer protein complex, subunit gamma 2  SNX16 - sorting nexin 16  TOMM7 - translocase of outer mitochondrial membrane 7 homolog (yeast)  RANBP2 - ran binding protein 2  CLTC - clathrin, heavy chain (hc)  SEC22A - sec22 vesicle trafficking protein homolog a (s. cerevisiae)  AP3M1 - adaptor-related protein complex 3, mu 1 subunit  GNPAT - glyceronephosphate o-acyltransferase  CLN5 - ceroid-lipofuscinosis, neuronal 5  SGTB - small glutamine-rich tetratricopeptide repeat (tpr)-containing, beta  BBS5 - bardet-biedl syndrome 5  DCTN6 - dynactin 6  CLTB - clathrin, light chain b  EEA1 - early endosome antigen 1  CLTA - clathrin, light chain a  CLU - clusterin  STX7 - syntaxin 7  MAGEL2 - mage-like 2  HEATR5B - heat repeat containing 5b  AP1S1 - adaptor-related protein complex 1, sigma 1 subunit  ANKRD54 - ankyrin repeat domain 54  RAE1 - rae1 rna export 1 homolog (s. pombe)  ARL5B - adp-ribosylation factor-like 5b  AP2M1 - adaptor-related protein complex 2, mu 1 subunit  LAT - linker for activation of t cells  GEMIN2 - gem (nuclear organelle) associated protein 2  NSG1 - neuron specific gene family member 1  NUP35 - nucleoporin 35kda  VPS41 - vacuolar protein sorting 41 homolog (s. cerevisiae)  DOC2B - double c2-like domains, beta  CCDC93 - coiled-coil domain containing 93  CUL3 - cullin 3  SGSM1 - small g protein signaling modulator 1  CHMP4B - charged multivesicular body protein 4b  CACNG4 - calcium channel, voltage-dependent, gamma subunit 4  AP4B1 - adaptor-related protein complex 4, beta 1 subunit  RSRC1 - arginine/serine-rich coiled-coil 1  TMEM87B - transmembrane protein 87b  SPTAN1 - spectrin, alpha, non-erythrocytic 1  WDR19 - wd repeat domain 19  SPTBN4 - spectrin, beta, non-erythrocytic 4  AKAP12 - a kinase (prka) anchor protein 12  EPG5 - ectopic p-granules autophagy protein 5 homolog (c. elegans)  VPS37A - vacuolar protein sorting 37 homolog a (s. cerevisiae)  CORO1C - coronin, actin binding protein, 1c  COG8 - component of oligomeric golgi complex 8  ZDHHC3 - zinc finger, dhhc-type containing 3  KPNA6 - karyopherin alpha 6 (importin alpha 7)  LDLR - low density lipoprotein receptor  RABGAP1 - rab gtpase activating protein 1  SPG7 - spastic paraplegia 7 (pure and complicated autosomal recessive)  HOOK3 - hook microtubule-tethering protein 3  DENND1A - denn/madd domain containing 1a  LZTS2 - leucine zipper, putative tumor suppressor 2  SPAST - spastin  REEP2 - receptor accessory protein 2  FAM53C - family with sequence similarity 53, member c  ABCG1 - atp-binding cassette, sub-family g (white), member 1  LTV1 - ltv1 homolog (s. cerevisiae)  ERGIC2 - ergic and golgi 2  CPT1A - carnitine palmitoyltransferase 1a (liver)  TICAM2 - toll-like receptor adaptor molecule 2  SYVN1 - synovial apoptosis inhibitor 1, synoviolin  VPS26A - vacuolar protein sorting 26 homolog a (s. pombe)  SEC22B - sec22 vesicle trafficking protein homolog b (s. cerevisiae) (gene/pseudogene)  BET1L - bet1 golgi vesicular membrane trafficking protein-like  GOSR2 - golgi snap receptor complex member 2  UBAP1 - ubiquitin associated protein 1  SPIRE2 - spire actin nucleation factor 2  PREPL - prolyl endopeptidase-like  SNX2 - sorting nexin 2  SNX1 - sorting nexin 1  TMEM50A - transmembrane protein 50a  DNAJC27 - dnaj (hsp40) homolog, subfamily c, member 27  LMNA - lamin a/c  LMAN1 - lectin, mannose-binding, 1  SERAC1 - serine active site containing 1  DCTN5 - dynactin 5 (p25)  PRKAG2 - protein kinase, amp-activated, gamma 2 non-catalytic subunit  SNRPD1 - small nuclear ribonucleoprotein d1 polypeptide 16kda  SNRPF - small nuclear ribonucleoprotein polypeptide f  SNCA - synuclein, alpha (non a4 component of amyloid precursor)  TRAPPC1 - trafficking protein particle complex 1  SNRPB - small nuclear ribonucleoprotein polypeptides b and b1  VPS4B - vacuolar protein sorting 4 homolog b (s. cerevisiae)  SNAP25 - synaptosomal-associated protein, 25kda  GOSR1 - golgi snap receptor complex member 1  SRPRB - signal recognition particle receptor, b subunit  SMN1 - survival of motor neuron 1, telomeric  TRAPPC4 - trafficking protein particle complex 4  LAMP2 - lysosomal-associated membrane protein 2  ARFIP2 - adp-ribosylation factor interacting protein 2  SNX6 - sorting nexin 6  MID1IP1 - mid1 interacting protein 1  REPS1 - ralbp1 associated eps domain containing 1  STX8 - syntaxin 8  EPS15L1 - epidermal growth factor receptor pathway substrate 15-like 1  COPB1 - coatomer protein complex, subunit beta 1  RAB38 - rab38, member ras oncogene family  COPA - coatomer protein complex, subunit alpha  DPY30 - dpy-30 homolog (c. elegans)  CDC40 - cell division cycle 40  STX12 - syntaxin 12  RPH3AL - rabphilin 3a-like (without c2 domains)  NAGPA - n-acetylglucosamine-1-phosphodiester alpha-n-acetylglucosaminidase  DCTN4 - dynactin 4 (p62)  VPS28 - vacuolar protein sorting 28 homolog (s. cerevisiae)  KIF5C - kinesin family member 5c  SYT1 - synaptotagmin i  KIF3C - kinesin family member 3c  SYT5 - synaptotagmin v  KIF5B - kinesin family member 5b  KLHL12 - kelch-like family member 12  KIF5A - kinesin family member 5a  SYT4 - synaptotagmin iv  SUN2 - sad1 and unc84 domain containing 2  PICK1 - protein interacting with prkca 1  CACNG7 - calcium channel, voltage-dependent, gamma subunit 7  VAMP2 - vesicle-associated membrane protein 2 (synaptobrevin 2)  DYNC1LI1 - dynein, cytoplasmic 1, light intermediate chain 1  RAB3GAP2 - rab3 gtpase activating protein subunit 2 (non-catalytic)  CTNNB1 - catenin (cadherin-associated protein), beta 1, 88kda  STXBP1 - syntaxin binding protein 1  STX5 - syntaxin 5  STX4 - syntaxin 4  STX3 - syntaxin 3  STX1A - syntaxin 1a (brain)  MGARP - mitochondria-localized glutamic acid-rich protein  TNPO1 - transportin 1  GOLGA7 - golgin a7  IER3IP1 - immediate early response 3 interacting protein 1  TGFBRAP1 - transforming growth factor, beta receptor associated protein 1  FUT10 - fucosyltransferase 10 (alpha (1,3) fucosyltransferase)  KPNA2 - karyopherin alpha 2 (rag cohort 1, importin alpha 1)  KPNB1 - karyopherin (importin) beta 1  KPNA1 - karyopherin alpha 1 (importin alpha 5)  STXBP3 - syntaxin binding protein 3  ZDHHC9 - zinc finger, dhhc-type containing 9  SYT3 - synaptotagmin iii  TRAPPC12 - trafficking protein particle complex 12  FYTTD1 - forty-two-three domain containing 1  TUBA1C - tubulin, alpha 1c  PAN3 - pan3 poly(a) specific ribonuclease subunit homolog (s. cerevisiae)  KIT - v-kit hardy-zuckerman 4 feline sarcoma viral oncogene homolog  PEX16 - peroxisomal biogenesis factor 16  EXOC4 - exocyst complex component 4  RPAIN - rpa interacting protein  RAB28 - rab28, member ras oncogene family  POLDIP3 - polymerase (dna-directed), delta interacting protein 3  RAB9A - rab9a, member ras oncogene family  TRAPPC11 - trafficking protein particle complex 11  COG4 - component of oligomeric golgi complex 4  STAT3 - signal transducer and activator of transcription 3 (acute-phase response factor)  SSR3 - signal sequence receptor, gamma (translocon-associated protein gamma)  COG1 - component of oligomeric golgi complex 1  SPCS3 - signal peptidase complex subunit 3 homolog (s. cerevisiae)  KIF3B - kinesin family member 3b  ZFYVE9 - zinc finger, fyve domain containing 9  NAPB - n-ethylmaleimide-sensitive factor attachment protein, beta  SRP68 - signal recognition particle 68kda  SNX33 - sorting nexin 33  SRP54 - signal recognition particle 54kda  FBXO7 - f-box protein 7  CSE1L - cse1 chromosome segregation 1-like (yeast)  SRP19 - signal recognition particle 19kda  SRP14 - signal recognition particle 14kda (homologous alu rna binding protein)  BLOC1S5 - biogenesis of lysosomal organelles complex-1, subunit 5, muted  SRP72 - signal recognition particle 72kda  SSR1 - signal sequence receptor, alpha  ZDHHC2 - zinc finger, dhhc-type containing 2  SSB - sjogren syndrome antigen b (autoantigen la)  THOC3 - tho complex 3  VAMP3 - vesicle-associated membrane protein 3  SPTBN1 - spectrin, beta, non-erythrocytic 1  MIA3 - melanoma inhibitory activity family, member 3  IPO11 - importin 11  TAOK2 - tao kinase 2  SARNP - sap domain containing ribonucleoprotein  VIPAS39 - vps33b interacting protein, apical-basolateral polarity regulator, spe-39 homolog  ZDHHC12 - zinc finger, dhhc-type containing 12  VPS25 - vacuolar protein sorting 25 homolog (s. cerevisiae)  UBE2O - ubiquitin-conjugating enzyme e2o  SRP9 - signal recognition particle 9kda  MON1A - mon1 secretory trafficking family member a  SEL1L - sel-1 suppressor of lin-12-like (c. elegans)  TRAPPC2 - trafficking protein particle complex 2  SRSF11 - serine/arginine-rich splicing factor 11  SEC13 - sec13 homolog (s. cerevisiae)  ERGIC1 - endoplasmic reticulum-golgi intermediate compartment (ergic) 1  RAB8B - rab8b, member ras oncogene family  SYT17 - synaptotagmin xvii  SDCBP - syndecan binding protein (syntenin)  HEATR5A - heat repeat containing 5a  COPB2 - coatomer protein complex, subunit beta 2 (beta prime)  RHOT2 - ras homolog family member t2  DCTN1 - dynactin 1  PTPN23 - protein tyrosine phosphatase, non-receptor type 23  GEMIN5 - gem (nuclear organelle) associated protein 5  ZDHHC24 - zinc finger, dhhc-type containing 24  WDR34 - wd repeat domain 34  MYRIP - myosin viia and rab interacting protein  PHAX - phosphorylated adaptor for rna export  CPSF3 - cleavage and polyadenylation specific factor 3, 73kda  TRAPPC2L - trafficking protein particle complex 2-like  RAB22A - rab22a, member ras oncogene family  VPS29 - vacuolar protein sorting 29 homolog (s. cerevisiae)  RAB11B - rab11b, member ras oncogene family  SYT9 - synaptotagmin ix  RAB23 - rab23, member ras oncogene family  ZDHHC20 - zinc finger, dhhc-type containing 20  ANKRD27 - ankyrin repeat domain 27 (vps9 domain)  VAPB - vamp (vesicle-associated membrane protein)-associated protein b and c  MCFD2 - multiple coagulation factor deficiency 2  REPS2 - ralbp1 associated eps domain containing 2  PRICKLE1 - prickle homolog 1 (drosophila)  RAB24 - rab24, member ras oncogene family  ZW10 - zw10 kinetochore protein  CPSF2 - cleavage and polyadenylation specific factor 2, 100kda  FAM160A2 - family with sequence similarity 160, member a2  DCLK1 - doublecortin-like kinase 1  RAB8A - rab8a, member ras oncogene family  TBC1D10B - tbc1 domain family, member 10b  COG6 - component of oligomeric golgi complex 6  XPO5 - exportin 5  AP4M1 - adaptor-related protein complex 4, mu 1 subunit  ARHGEF2 - rho/rac guanine nucleotide exchange factor (gef) 2  CHMP2B - charged multivesicular body protein 2b  STX18 - syntaxin 18  ARL6 - adp-ribosylation factor-like 6  TBC1D14 - tbc1 domain family, member 14  SYNGR1 - synaptogyrin 1  HGS - hepatocyte growth factor-regulated tyrosine kinase substrate  NEMF - nuclear export mediator factor  TMEM87A - transmembrane protein 87a  VPS52 - vacuolar protein sorting 52 homolog (s. cerevisiae)  VTA1 - vesicle (multivesicular body) trafficking 1  COG3 - component of oligomeric golgi complex 3  MAGOH - mago-nashi homolog, proliferation-associated (drosophila)  MICALL1 - mical-like 1  TRAF3IP1 - tnf receptor-associated factor 3 interacting protein 1  RAB6B - rab6b, member ras oncogene family  MAPT - microtubule-associated protein tau  MAP6 - microtubule-associated protein 6  RAB14 - rab14, member ras oncogene family  MAP1B - microtubule-associated protein 1b  MAP1A - microtubule-associated protein 1a  VPS54 - vacuolar protein sorting 54 homolog (s. cerevisiae)  TANC2 - tetratricopeptide repeat, ankyrin repeat and coiled-coil containing 2  DYNLRB2 - dynein, light chain, roadblock-type 2  DYNLRB1 - dynein, light chain, roadblock-type 1  NUP43 - nucleoporin 43kda  TOR1A - torsin family 1, member a (torsin a)  CHTOP - chromatin target of prmt1  ARHGAP21 - rho gtpase activating protein 21  GGA1 - golgi-associated, gamma adaptin ear containing, arf binding protein 1  CHMP5 - charged multivesicular body protein 5  APPL1 - adaptor protein, phosphotyrosine interaction, ph domain and leucine zipper containing 1  DOCK7 - dedicator of cytokinesis 7  ANKFY1 - ankyrin repeat and fyve domain containing 1  SYT7 - synaptotagmin vii  KIAA1468 - kiaa1468  VPS18 - vacuolar protein sorting 18 homolog (s. cerevisiae)  BLOC1S6 - biogenesis of lysosomal organelles complex-1, subunit 6, pallidin  RPL14 - ribosomal protein l14  DYNC2LI1 - dynein, cytoplasmic 2, light intermediate chain 1  LRP6 - low density lipoprotein receptor-related protein 6  STXBP4 - syntaxin binding protein 4  AIP - aryl hydrocarbon receptor interacting protein  TOM1L2 - target of myb1-like 2 (chicken)  ARL5A - adp-ribosylation factor-like 5a  LYN - v-yes-1 yamaguchi sarcoma viral related oncogene homolog  SPAG9 - sperm associated antigen 9  CHMP3 - charged multivesicular body protein 3  TMED6 - transmembrane emp24 protein transport domain containing 6  SFPQ - splicing factor proline/glutamine-rich  DYNC1H1 - dynein, cytoplasmic 1, heavy chain 1  DYNC1I1 - dynein, cytoplasmic 1, intermediate chain 1  DYNC1I2 - dynein, cytoplasmic 1, intermediate chain 2  DYNC1LI2 - dynein, cytoplasmic 1, light intermediate chain 2  SRSF1 - serine/arginine-rich splicing factor 1  DNM2 - dynamin 2  SRSF2 - serine/arginine-rich splicing factor 2  SRSF3 - serine/arginine-rich splicing factor 3  SRSF4 - serine/arginine-rich splicing factor 4  SRSF5 - serine/arginine-rich splicing factor 5  SRSF6 - serine/arginine-rich splicing factor 6  SRSF7 - serine/arginine-rich splicing factor 7  M6PR - mannose-6-phosphate receptor (cation dependent)  ALS2 - amyotrophic lateral sclerosis 2 (juvenile)  ERGIC3 - ergic and golgi 3  HPS4 - hermansky-pudlak syndrome 4  SCG5 - secretogranin v (7b2 protein)  SGTA - small glutamine-rich tetratricopeptide repeat (tpr)-containing, alpha  IFT172 - intraflagellar transport 172 homolog (chlamydomonas)  RUNDC1 - run domain containing 1  ITSN1 - intersectin 1 (sh3 domain protein)  WASL - wiskott-aldrich syndrome-like  NCBP2 - nuclear cap binding protein subunit 2, 20kda  NCBP1 - nuclear cap binding protein subunit 1, 80kda  CHMP4C - charged multivesicular body protein 4c  TSC1 - tuberous sclerosis 1  CTTN - cortactin  KIFAP3 - kinesin-associated protein 3  DDX25 - dead (asp-glu-ala-asp) box helicase 25  SPIRE1 - spire actin nucleation factor 1  IFT46 - intraflagellar transport 46 homolog (chlamydomonas)  SCAP - srebf chaperone  DNAJC15 - dnaj (hsp40) homolog, subfamily c, member 15  RAB18 - rab18, member ras oncogene family  TIMM50 - translocase of inner mitochondrial membrane 50 homolog (s. cerevisiae)  TUB - tubby bipartite transcription factor  GRIPAP1 - grip1 associated protein 1  MYO10 - myosin x  CNIH - cornichon homolog (drosophila)  EIF4E - eukaryotic translation initiation factor 4e  ZDHHC1 - zinc finger, dhhc-type containing 1  EGR2 - early growth response 2  TTC30A - tetratricopeptide repeat domain 30a  LMAN2L - lectin, mannose-binding 2-like  ALYREF - aly/ref export factor  NDEL1 - nude neurodevelopment protein 1-like 1  NACA - nascent polypeptide-associated complex alpha subunit  SAR1A - sar1 homolog a (s. cerevisiae)  ELAVL1 - elav (embryonic lethal, abnormal vision, drosophila)-like 1 (hu antigen r)  TMEM129 - transmembrane protein 129  AZI1 - 5-azacytidine induced 1  STRADA - ste20-related kinase adaptor alpha  BTBD8 - btb (poz) domain containing 8  NUTF2 - nuclear transport factor 2  MYO1D - myosin id  TSG101 - tumor susceptibility gene 101  TSC2 - tuberous sclerosis 2  MYO5A - myosin va (heavy chain 12, myoxin)  MYO1E - myosin ie  MYO6 - myosin vi  SNX27 - sorting nexin family member 27  FIP1L1 - factor interacting with papola and cpsf1  MVB12A - multivesicular body subunit 12a  RAB1B - rab1b, member ras oncogene family  EIF2D - eukaryotic translation initiation factor 2d  SNX15 - sorting nexin 15  RAB21 - rab21, member ras oncogene family  SEH1L - seh1-like (s. cerevisiae)  MYH10 - myosin, heavy chain 10, non-muscle  SNX11 - sorting nexin 11  SNUPN - snurportin 1  SNX10 - sorting nexin 10  COL4A3BP - collagen, type iv, alpha 3 (goodpasture antigen) binding protein  DOPEY1 - dopey family member 1  GOLGA7B - golgin a7 family, member b  CPSF1 - cleavage and polyadenylation specific factor 1, 160kda  ACTR2 - arp2 actin-related protein 2 homolog (yeast)  RBM15B - rna binding motif protein 15b  CLUAP1 - clusterin associated protein 1  SMG1 - smg1 phosphatidylinositol 3-kinase-related kinase  MFF - mitochondrial fission factor  NRBP1 - nuclear receptor binding protein 1  ENY2 - enhancer of yellow 2 homolog (drosophila)  XPO7 - exportin 7  MON2 - mon2 homolog (s. cerevisiae)  LRPPRC - leucine-rich pentatricopeptide repeat containing  ACTR1A - arp1 actin-related protein 1 homolog a, centractin alpha (yeast)  CPLX3 - complexin 3  HSP90B1 - heat shock protein 90kda beta (grp94), member 1  TIMM22 - translocase of inner mitochondrial membrane 22 homolog (yeast)  ARL4A - adp-ribosylation factor-like 4a  SEC61A1 - sec61 alpha 1 subunit (s. cerevisiae)  ARL4C - adp-ribosylation factor-like 4c  BCAP31 - b-cell receptor-associated protein 31  TBC1D9B - tbc1 domain family, member 9b (with gram domain)  TPR - translocated promoter region, nuclear basket protein  SNX12 - sorting nexin 12  GGA2 - golgi-associated, gamma adaptin ear containing, arf binding protein 2  BLOC1S3 - biogenesis of lysosomal organelles complex-1, subunit 3  ERC1 - elks/rab6-interacting/cast family member 1  NUP107 - nucleoporin 107kda  GOPC - golgi-associated pdz and coiled-coil motif containing  KIF1B - kinesin family member 1b  TBC1D2B - tbc1 domain family, member 2b  PDCD6 - programmed cell death 6  MTM1 - myotubularin 1  HDAC6 - histone deacetylase 6  HMGXB4 - hmg box domain containing 4  TOM1 - target of myb1 (chicken)  CCDC22 - coiled-coil domain containing 22  DNM1L - dynamin 1-like  AP5B1 - adaptor-related protein complex 5, beta 1 subunit  VCP - valosin containing protein  TBC1D9 - tbc1 domain family, member 9 (with gram domain)  MAPK8IP3 - mitogen-activated protein kinase 8 interacting protein 3  CITED1 - cbp/p300-interacting transactivator, with glu/asp-rich carboxy-terminal domain, 1  NUP205 - nucleoporin 205kda  YTHDC1 - yth domain containing 1  GGA3 - golgi-associated, gamma adaptin ear containing, arf binding protein 3  THOC2 - tho complex 2  SEC16A - sec16 homolog a (s. cerevisiae)  CHMP7 - charged multivesicular body protein 7  UBB - ubiquitin b  IFT81 - intraflagellar transport 81 homolog (chlamydomonas)  SGPP1 - sphingosine-1-phosphate phosphatase 1  SPCS1 - signal peptidase complex subunit 1 homolog (s. cerevisiae)  FAF2 - fas associated factor family member 2  RBM8A - rna binding motif protein 8a  COG7 - component of oligomeric golgi complex 7  ARL6IP1 - adp-ribosylation factor-like 6 interacting protein 1  TIMM21 - translocase of inner mitochondrial membrane 21 homolog (yeast)  UCHL1 - ubiquitin carboxyl-terminal esterase l1 (ubiquitin thiolesterase)  CHMP4A - charged multivesicular body protein 4a  RRS1 - rrs1 ribosome biogenesis regulator homolog (s. cerevisiae)  XPO6 - exportin 6  SMURF1 - smad specific e3 ubiquitin protein ligase 1  THOC1 - tho complex 1  UBE2D2 - ubiquitin-conjugating enzyme e2d 2  TBC1D1 - tbc1 (tre-2/usp6, bub2, cdc16) domain family, member 1  STX2 - syntaxin 2  NUP153 - nucleoporin 153kda  TBC1D12 - tbc1 domain family, member 12  TMEM230 - transmembrane protein 230  WDR91 - wd repeat domain 91  NUP210 - nucleoporin 210kda  EPS15 - epidermal growth factor receptor pathway substrate 15  DTX3L - deltex 3-like (drosophila)  SCFD1 - sec1 family domain containing 1  SEC24D - sec24 family, member d (s. cerevisiae)  DENND5A - denn/madd domain containing 5a  TLK1 - tousled-like kinase 1  OSBP - oxysterol binding protein  C2CD5 - c2 calcium-dependent domain containing 5  ACTR10 - actin-related protein 10 homolog (s. cerevisiae)  TBC1D25 - tbc1 domain family, member 25  AP5Z1 - adaptor-related protein complex 5, zeta 1 subunit  VPS26B - vacuolar protein sorting 26 homolog b (s. pombe)  BICD2 - bicaudal d homolog 2 (drosophila)  ZDHHC22 - zinc finger, dhhc-type containing 22  SGSM2 - small g protein signaling modulator 2  MGRN1 - mahogunin ring finger 1, e3 ubiquitin protein ligase  ARHGAP44 - rho gtpase activating protein 44  KIF13B - kinesin family member 13b  TMED5 - transmembrane emp24 protein transport domain containing 5  OSBPL2 - oxysterol binding protein-like 2  SMG6 - smg6 nonsense mediated mrna decay factor  FBXW11 - f-box and wd repeat domain containing 11  VPS11 - vacuolar protein sorting 11 homolog (s. cerevisiae)  SMG7 - smg7 nonsense mediated mrna decay factor  CLEC16A - c-type lectin domain family 16, member a  OPA1 - optic atrophy 1 (autosomal dominant)  TMED7 - transmembrane emp24 protein transport domain containing 7  DYNLT1 - dynein, light chain, tctex-type 1  UBXN6 - ubx domain protein 6  TECPR2 - tectonin beta-propeller repeat containing 2  STX1B - syntaxin 1b  IFT43 - intraflagellar transport 43 homolog (chlamydomonas)  NUP160 - nucleoporin 160kda  FIS1 - fission 1 (mitochondrial outer membrane) homolog (s. cerevisiae)  NSF - n-ethylmaleimide-sensitive factor  SPCS2 - signal peptidase complex subunit 2 homolog (s. cerevisiae)  FGF9 - fibroblast growth factor 9  BCAP29 - b-cell receptor-associated protein 29  TOMM20 - translocase of outer mitochondrial membrane 20 homolog (yeast)  VPS39 - vacuolar protein sorting 39 homolog (s. cerevisiae)  NUMA1 - nuclear mitotic apparatus protein 1  NUP88 - nucleoporin 88kda  EIF4A3 - eukaryotic translation initiation factor 4a3  NUP98 - nucleoporin 98kda  TAP2 - transporter 2, atp-binding cassette, sub-family b (mdr/tap)  TBC1D30 - tbc1 domain family, member 30  TMEM167A - transmembrane protein 167a  RIMS3 - regulating synaptic membrane exocytosis 3  SNX17 - sorting nexin 17  FAM45A - family with sequence similarity 45, member a  DHX38 - deah (asp-glu-ala-his) box polypeptide 38  TBC1D5 - tbc1 domain family, member 5  CEP120 - centrosomal protein 120kda  PIP5K1C - phosphatidylinositol-4-phosphate 5-kinase, type i, gamma  RGP1 - rgp1 retrograde golgi transport homolog (s. cerevisiae)  NMD3 - nmd3 ribosome export adaptor  NPM1 - nucleophosmin (nucleolar phosphoprotein b23, numatrin)  TRIM9 - tripartite motif containing 9  DUSP16 - dual specificity phosphatase 16  IFT52 - intraflagellar transport 52 homolog (chlamydomonas)  GRIP2 - glutamate receptor interacting protein 2  NUDT7 - nudix (nucleoside diphosphate linked moiety x)-type motif 7  ERBB2IP - erbb2 interacting protein  RAB11FIP3 - rab11 family interacting protein 3 (class ii)  SPG11 - spastic paraplegia 11 (autosomal recessive)  TMED3 - transmembrane emp24 protein transport domain containing 3  BLOC1S2 - biogenesis of lysosomal organelles complex-1, subunit 2  TNPO2 - transportin 2  IFT74 - intraflagellar transport 74 homolog (chlamydomonas)  HERPUD1 - homocysteine-inducible, endoplasmic reticulum stress-inducible, ubiquitin-like domain member 1  MYO19 - myosin xix  USP6NL - usp6 n-terminal like  SEC62 - sec62 homolog (s. cerevisiae)  RIMS2 - regulating synaptic membrane exocytosis 2  TRAPPC10 - trafficking protein particle complex 10  PQLC1 - pq loop repeat containing 1  THOC7 - tho complex 7 homolog (drosophila)  ZFYVE16 - zinc finger, fyve domain containing 16  ICMT - isoprenylcysteine carboxyl methyltransferase  NFKBIA - nuclear factor of kappa light polypeptide gene enhancer in b-cells inhibitor, alpha  TRAM1 - translocation associated membrane protein 1  PIK3R4 - phosphoinositide-3-kinase, regulatory subunit 4  IFT140 - intraflagellar transport 140 homolog (chlamydomonas)  TBCK - tbc1 domain containing kinase  EHD2 - eh-domain containing 2  EHD3 - eh-domain containing 3  EHD4 - eh-domain containing 4  NUP188 - nucleoporin 188kda  EIF4ENIF1 - eukaryotic translation initiation factor 4e nuclear import factor 1  DNAJC5 - dnaj (hsp40) homolog, subfamily c, member 5  GCC2 - grip and coiled-coil domain containing 2  NEFL - neurofilament, light polypeptide  TERF2 - telomeric repeat binding factor 2  SNX18 - sorting nexin 18  LEPROTL1 - leptin receptor overlapping transcript-like 1  ITSN2 - intersectin 2  SEC61G - sec61 gamma subunit  NUP155 - nucleoporin 155kda  TGFA - transforming growth factor, alpha  SEC24C - sec24 family, member c (s. cerevisiae)  FAM91A1 - family with sequence similarity 91, member a1  VPS37D - vacuolar protein sorting 37 homolog d (s. cerevisiae)  TGFB1 - transforming growth factor, beta 1  FAM21A - family with sequence similarity 21, member a  NUP93 - nucleoporin 93kda  CALY - calcyon neuron-specific vesicular protein  GEMIN4 - gem (nuclear organelle) associated protein 4  ACAP2 - arfgap with coiled-coil, ankyrin repeat and ph domains 2  SYTL4 - synaptotagmin-like 4  TNPO3 - transportin 3 |
| GO:2000779 | regulation of double-strand break repair | 4.7E-5 | 4.85E-3 | 1.36 (10334,50,6996,46) | [+] Show genes  RNF169 - ring finger protein 169  TFIP11 - tuftelin interacting protein 11  DTX3L - deltex 3-like (drosophila)  NUDT16 - nudix (nucleoside diphosphate linked moiety x)-type motif 16  SPIRE1 - spire actin nucleation factor 1  ZNF365 - zinc finger protein 365  SMCHD1 - structural maintenance of chromosomes flexible hinge domain containing 1  FBXO18 - f-box protein, helicase, 18  FAM35A - family with sequence similarity 35, member a  TP53BP1 - tumor protein p53 binding protein 1  FUS - fused in sarcoma  SETMAR - set domain and mariner transposase fusion gene  KLHL15 - kelch-like family member 15  MMS19 - mms19 nucleotide excision repair homolog (s. cerevisiae)  PML - promyelocytic leukemia  SIRT6 - sirtuin 6  KDM1A - lysine (k)-specific demethylase 1a  PARP9 - poly (adp-ribose) polymerase family, member 9  PPP4R2 - protein phosphatase 4, regulatory subunit 2  ACTR2 - arp2 actin-related protein 2 homolog (yeast)  MAD2L2 - mad2 mitotic arrest deficient-like 2 (yeast)  UBQLN4 - ubiquilin 4  WDR70 - wd repeat domain 70  RPA2 - replication protein a2, 32kda  POT1 - protection of telomeres 1  UBR5 - ubiquitin protein ligase e3 component n-recognin 5  RIF1 - rap1 interacting factor homolog (yeast)  TRIP12 - thyroid hormone receptor interactor 12  NUDT16L1 - nudix (nucleoside diphosphate linked moiety x)-type motif 16-like 1  RECQL5 - recq protein-like 5  PPP4C - protein phosphatase 4, catalytic subunit  WHSC1 - wolf-hirschhorn syndrome candidate 1  PARP1 - poly (adp-ribose) polymerase 1  SETD2 - set domain containing 2  WRAP53 - wd repeat containing, antisense to tp53  SUV420H1 - suppressor of variegation 4-20 homolog 1 (drosophila)  OTUB1 - otu domain, ubiquitin aldehyde binding 1  C7orf49 - chromosome 7 open reading frame 49  SPIRE2 - spire actin nucleation factor 2  OGG1 - 8-oxoguanine dna glycosylase  MAGEF1 - melanoma antigen family f, 1  ARID2 - at rich interactive domain 2 (arid, rfx-like)  RMI2 - recq mediated genome instability 2  SUV420H2 - suppressor of variegation 4-20 homolog 2 (drosophila)  PNKP - polynucleotide kinase 3'-phosphatase  TERF2IP - telomeric repeat binding factor 2, interacting protein |
| GO:0050769 | positive regulation of neurogenesis | 4.75E-5 | 4.86E-3 | 1.15 (10334,303,6996,236) | [+] Show genes  ZMYND8 - zinc finger, mynd-type containing 8  MARK2 - map/microtubule affinity-regulating kinase 2  NCK1 - nck adaptor protein 1  LIG4 - ligase iv, dna, atp-dependent  GDI1 - gdp dissociation inhibitor 1  LIMK1 - lim domain kinase 1  PPP1R9A - protein phosphatase 1, regulatory subunit 9a  SF3A2 - splicing factor 3a, subunit 2, 66kda  SNW1 - snw domain containing 1  RELA - v-rel avian reticuloendotheliosis viral oncogene homolog a  PLXNB1 - plexin b1  PLXNA3 - plexin a3  NRP1 - neuropilin 1  IQGAP1 - iq motif containing gtpase activating protein 1  EP300 - e1a binding protein p300  KATNB1 - katanin p80 (wd repeat containing) subunit b 1  NAP1L1 - nucleosome assembly protein 1-like 1  RET - ret proto-oncogene  EGR2 - early growth response 2  CFLAR - casp8 and fadd-like apoptosis regulator  NDEL1 - nude neurodevelopment protein 1-like 1  PLXNA1 - plexin a1  PLXNA2 - plexin a2  TRIM32 - tripartite motif containing 32  RND2 - rho family gtpase 2  RHEB - ras homolog enriched in brain  BMPR2 - bone morphogenetic protein receptor, type ii (serine/threonine kinase)  CRABP2 - cellular retinoic acid binding protein 2  BMP7 - bone morphogenetic protein 7  SPEN - spen homolog, transcriptional regulator (drosophila)  RAB21 - rab21, member ras oncogene family  METRN - meteorin, glial cell differentiation regulator  MEGF8 - multiple egf-like-domains 8  CPNE1 - copine i  KDM1A - lysine (k)-specific demethylase 1a  SMO - smoothened, frizzled family receptor  FZD4 - frizzled family receptor 4  CNR1 - cannabinoid receptor 1 (brain)  ACTR2 - arp2 actin-related protein 2 homolog (yeast)  UNC13A - unc-13 homolog a (c. elegans)  FZD1 - frizzled family receptor 1  EFNA5 - ephrin-a5  PARP6 - poly (adp-ribose) polymerase family, member 6  CNTN1 - contactin 1  ROBO1 - roundabout, axon guidance receptor, homolog 1 (drosophila)  ROBO2 - roundabout, axon guidance receptor, homolog 2 (drosophila)  TSPO - translocator protein (18kda)  SMARCD3 - swi/snf related, matrix associated, actin dependent regulator of chromatin, subfamily d, member 3  L1CAM - l1 cell adhesion molecule  LPAR1 - lysophosphatidic acid receptor 1  SETX - senataxin  ZNF609 - zinc finger protein 609  TMEM30A - transmembrane protein 30a  AP2A1 - adaptor-related protein complex 2, alpha 1 subunit  KDM4C - lysine (k)-specific demethylase 4c  CAMK1D - calcium/calmodulin-dependent protein kinase id  NUMB - numb homolog (drosophila)  SYT1 - synaptotagmin i  CAMK2B - calcium/calmodulin-dependent protein kinase ii beta  SYT4 - synaptotagmin iv  CACNG7 - calcium channel, voltage-dependent, gamma subunit 7  PTK2B - protein tyrosine kinase 2 beta  BAIAP2 - bai1-associated protein 2  CAPRIN2 - caprin family member 2  CTNNB1 - catenin (cadherin-associated protein), beta 1, 88kda  FZD3 - frizzled family receptor 3  PPP2R5B - protein phosphatase 2, regulatory subunit b', beta  ETV5 - ets variant 5  PLXND1 - plexin d1  NDRG4 - ndrg family member 4  CUX1 - cut-like homeobox 1  SYT3 - synaptotagmin iii  DNM1L - dynamin 1-like  FYN - fyn oncogene related to src, fgr, yes  STK25 - serine/threonine kinase 25  KIT - v-kit hardy-zuckerman 4 feline sarcoma viral oncogene homolog  CDKL5 - cyclin-dependent kinase-like 5  TGIF2 - tgfb-induced factor homeobox 2  PPP1CC - protein phosphatase 1, catalytic subunit, gamma isozyme  STMN2 - stathmin-like 2  HIF1A - hypoxia inducible factor 1, alpha subunit (basic helix-loop-helix transcription factor)  CRKL - v-crk avian sarcoma virus ct10 oncogene homolog-like  SNX3 - sorting nexin 3  NIPBL - nipped-b homolog (drosophila)  SLIT2 - slit homolog 2 (drosophila)  DBNL - drebrin-like  FBXO38 - f-box protein 38  RNF157 - ring finger protein 157  MAP2K1 - mitogen-activated protein kinase kinase 1  CYFIP1 - cytoplasmic fmr1 interacting protein 1  CYB5D2 - cytochrome b5 domain containing 2  CSF1 - colony stimulating factor 1 (macrophage)  P2RY12 - purinergic receptor p2y, g-protein coupled, 12  RAB11A - rab11a, member ras oncogene family  GATA2 - gata binding protein 2  TENM3 - teneurin transmembrane protein 3  SMURF1 - smad specific e3 ubiquitin protein ligase 1  ZNF335 - zinc finger protein 335  PRKD1 - protein kinase d1  PRKCH - protein kinase c, eta  PRKCI - protein kinase c, iota  CSNK1D - casein kinase 1, delta  PRMT5 - protein arginine methyltransferase 5  DHX36 - deah (asp-glu-ala-his) box polypeptide 36  FMR1 - fragile x mental retardation 1  RNF112 - ring finger protein 112  ZEB1 - zinc finger e-box binding homeobox 1  TCF12 - transcription factor 12  SIN3A - sin3 transcription regulator family member a  ENC1 - ectodermal-neural cortex 1 (with btb domain)  ATP8A2 - atpase, aminophospholipid transporter, class i, type 8a, member 2  SYT17 - synaptotagmin xvii  FLNA - filamin a, alpha  PSEN1 - presenilin 1  CREB3L2 - camp responsive element binding protein 3-like 2  SHANK1 - sh3 and multiple ankyrin repeat domains 1  DBN1 - drebrin 1  PRPF19 - pre-mrna processing factor 19  FAIM - fas apoptotic inhibitory molecule  ILK - integrin-linked kinase  APBB1 - amyloid beta (a4) precursor protein-binding, family b, member 1 (fe65)  NUMBL - numb homolog (drosophila)-like  SCARB2 - scavenger receptor class b, member 2  GPRC5B - g protein-coupled receptor, family c, group 5, member b  ARF1 - adp-ribosylation factor 1  CAMK1 - calcium/calmodulin-dependent protein kinase i  PAFAH1B1 - platelet-activating factor acetylhydrolase 1b, regulatory subunit 1 (45kda)  FKBP1B - fk506 binding protein 1b, 12.6 kda  OPA1 - optic atrophy 1 (autosomal dominant)  IL6ST - interleukin 6 signal transducer (gp130, oncostatin m receptor)  PAK3 - p21 protein (cdc42/rac)-activated kinase 3  DLG5 - discs, large homolog 5 (drosophila)  DYNLT1 - dynein, light chain, tctex-type 1  FIG4 - fig4 homolog, sac1 lipid phosphatase domain containing (s. cerevisiae)  MUL1 - mitochondrial e3 ubiquitin protein ligase 1  CXCR4 - chemokine (c-x-c motif) receptor 4  ANKRD27 - ankyrin repeat domain 27 (vps9 domain)  FGFR1 - fibroblast growth factor receptor 1  AMIGO1 - adhesion molecule with ig-like domain 1  PTEN - phosphatase and tensin homolog  ARSB - arylsulfatase b  ITGA3 - integrin, alpha 3 (antigen cd49c, alpha 3 subunit of vla-3 receptor)  IST1 - increased sodium tolerance 1 homolog (yeast)  SS18L1 - synovial sarcoma translocation gene on chromosome 18-like 1  DAB2IP - dab2 interacting protein  RHOA - ras homolog family member a  ARHGDIA - rho gdp dissociation inhibitor (gdi) alpha  NSMF - nmda receptor synaptonuclear signaling and neuronal migration factor  LRP8 - low density lipoprotein receptor-related protein 8, apolipoprotein e receptor  PTK7 - protein tyrosine kinase 7  ADNP - activity-dependent neuroprotector homeobox  TWF1 - twinfilin actin-binding protein 1  ZC4H2 - zinc finger, c4h2 domain containing  BRINP1 - bone morphogenetic protein/retinoic acid inducible neural-specific 1  PTN - pleiotrophin  KIDINS220 - kinase d-interacting substrate, 220kda  DAG1 - dystroglycan 1 (dystrophin-associated glycoprotein 1)  ZEB2 - zinc finger e-box binding homeobox 2  ARHGEF2 - rho/rac guanine nucleotide exchange factor (gef) 2  TUBB2B - tubulin, beta 2b class iib  CRTC1 - creb regulated transcription coactivator 1  UFL1 - ufm1-specific ligase 1  NDNF - neuron-derived neurotrophic factor  TCF3 - transcription factor 3  HDAC2 - histone deacetylase 2  TCF4 - transcription factor 4  PTPRD - protein tyrosine phosphatase, receptor type, d  OBSL1 - obscurin-like 1  CUL7 - cullin 7  HDAC1 - histone deacetylase 1  ITGA6 - integrin, alpha 6  ASCL1 - achaete-scute complex homolog 1 (drosophila)  NRCAM - neuronal cell adhesion molecule  CBFA2T2 - core-binding factor, runt domain, alpha subunit 2; translocated to, 2  RRN3 - rrn3 rna polymerase i transcription factor homolog (s. cerevisiae)  TIMP2 - timp metallopeptidase inhibitor 2  RUFY3 - run and fyve domain containing 3  DRD2 - dopamine receptor d2  TMEM106B - transmembrane protein 106b  ZNF365 - zinc finger protein 365  MTOR - mechanistic target of rapamycin (serine/threonine kinase)  NME1 - nme/nm23 nucleoside diphosphate kinase 1  ATP1B2 - atpase, na+/k+ transporting, beta 2 polypeptide  NCKIPSD - nck interacting protein with sh3 domain  MAPT - microtubule-associated protein tau  SHANK3 - sh3 and multiple ankyrin repeat domains 3  MAP6 - microtubule-associated protein 6  MAP1B - microtubule-associated protein 1b  SEMA6A - sema domain, transmembrane domain (tm), and cytoplasmic domain, (semaphorin) 6a  RIMS2 - regulating synaptic membrane exocytosis 2  PACSIN1 - protein kinase c and casein kinase substrate in neurons 1  DPYSL3 - dihydropyrimidinase-like 3  NOTCH1 - notch 1  MAN2A1 - mannosidase, alpha, class 2a, member 1  ID2 - inhibitor of dna binding 2, dominant negative helix-loop-helix protein  TOX - thymocyte selection-associated high mobility group box  SKIL - ski-like oncogene  DVL2 - dishevelled segment polarity protein 2  DVL3 - dishevelled segment polarity protein 3  IMPACT - impact rwd domain protein  NKX2-2 - nk2 homeobox 2  TNFRSF1B - tumor necrosis factor receptor superfamily, member 1b  DDX56 - dead (asp-glu-ala-asp) box helicase 56  SH3GL3 - sh3-domain grb2-like 3  DLG4 - discs, large homolog 4 (drosophila)  IL34 - interleukin 34  RAP1A - rap1a, member of ras oncogene family  LRP1 - low density lipoprotein receptor-related protein 1  RASAL1 - ras protein activator like 1 (gap1 like)  NEFL - neurofilament, light polypeptide  SHOX2 - short stature homeobox 2  FBXW8 - f-box and wd repeat domain containing 8  RARB - retinoic acid receptor, beta  ITSN2 - intersectin 2  SEMA5A - sema domain, seven thrombospondin repeats (type 1 and type 1-like), transmembrane domain (tm) and short cytoplasmic domain, (semaphorin) 5a  NFE2L2 - nuclear factor (erythroid-derived 2)-like 2  LYN - v-yes-1 yamaguchi sarcoma viral related oncogene homolog  SPAG9 - sperm associated antigen 9  FEZ1 - fasciculation and elongation protein zeta 1 (zygin i)  ZFYVE27 - zinc finger, fyve domain containing 27  NPTN - neuroplastin  MACF1 - microtubule-actin crosslinking factor 1  TWF2 - twinfilin actin-binding protein 2  SERPINI1 - serpin peptidase inhibitor, clade i (neuroserpin), member 1  SERPINE2 - serpin peptidase inhibitor, clade e (nexin, plasminogen activator inhibitor type 1), member 2  RAPGEF2 - rap guanine nucleotide exchange factor (gef) 2  TGFB1 - transforming growth factor, beta 1  SRRT - serrate rna effector molecule homolog (arabidopsis)  ARHGAP35 - rho gtpase activating protein 35  CAPRIN1 - cell cycle associated protein 1  KCTD11 - potassium channel tetramerization domain containing 11  BCL2 - b-cell cll/lymphoma 2  XRCC5 - x-ray repair complementing defective repair in chinese hamster cells 5 (double-strand-break rejoining)  ITSN1 - intersectin 1 (sh3 domain protein)  BEND6 - ben domain containing 6  MMD - monocyte to macrophage differentiation-associated |
| GO:0090305 | nucleic acid phosphodiester bond hydrolysis | 5.03E-5 | 5.11E-3 | 1.18 (10334,213,6996,170) | [+] Show genes  APEX1 - apex nuclease (multifunctional dna repair enzyme) 1  NCBP2 - nuclear cap binding protein subunit 2, 20kda  RFC5 - replication factor c (activator 1) 5, 36.5kda  EXOSC9 - exosome component 9  NCBP1 - nuclear cap binding protein subunit 1, 80kda  RFC2 - replication factor c (activator 1) 2, 40kda  EXOSC10 - exosome component 10  TSN - translin  G3BP1 - gtpase activating protein (sh3 domain) binding protein 1  PMS2 - pms2 postmeiotic segregation increased 2 (s. cerevisiae)  RFC3 - replication factor c (activator 1) 3, 38kda  DDX1 - dead (asp-glu-ala-asp) box helicase 1  TBL3 - transducin (beta)-like 3  PARN - poly(a)-specific ribonuclease  FAN1 - fancd2/fanci-associated nuclease 1  TREX1 - three prime repair exonuclease 1  FBXO18 - f-box protein, helicase, 18  SAMHD1 - sam domain and hd domain 1  SETMAR - set domain and mariner transposase fusion gene  DDB1 - damage-specific dna binding protein 1, 127kda  MGME1 - mitochondrial genome maintenance exonuclease 1  BOP1 - block of proliferation 1  DROSHA - drosha, ribonuclease type iii  GTF2H2 - general transcription factor iih, polypeptide 2, 44kda  GTF2H3 - general transcription factor iih, polypeptide 3, 34kda  GTF2H1 - general transcription factor iih, polypeptide 1, 62kda  UTP20 - utp20, small subunit (ssu) processome component, homolog (yeast)  USB1 - u6 snrna biogenesis 1  CPSF3 - cleavage and polyadenylation specific factor 3, 73kda  PNRC2 - proline-rich nuclear receptor coactivator 2  NOP9 - nop9 nucleolar protein  DCP1A - decapping mrna 1a  GTF2H4 - general transcription factor iih, polypeptide 4, 52kda  CHD1L - chromodomain helicase dna binding protein 1-like  NOL9 - nucleolar protein 9  TATDN1 - tatd dnase domain containing 1  TATDN3 - tatd dnase domain containing 3  DFFA - dna fragmentation factor, 45kda, alpha polypeptide  SMG6 - smg6 nonsense mediated mrna decay factor  OGG1 - 8-oxoguanine dna glycosylase  REXO1 - rex1, rna exonuclease 1 homolog (s. cerevisiae)  EXOSC3 - exosome component 3  CSTF2T - cleavage stimulation factor, 3' pre-rna, subunit 2, 64kda, tau variant  POLR3K - polymerase (rna) iii (dna directed) polypeptide k, 12.3 kda  ABT1 - activator of basal transcription 1  TSNAX - translin-associated factor x  EXOSC1 - exosome component 1  FIP1L1 - factor interacting with papola and cpsf1  CNOT6 - ccr4-not transcription complex, subunit 6  BMS1 - bms1 ribosome biogenesis factor  CNOT1 - ccr4-not transcription complex, subunit 1  POLK - polymerase (dna directed) kappa  EXOSC7 - exosome component 7  LAS1L - las1-like (s. cerevisiae)  DCP2 - decapping mrna 2  RNASEH2A - ribonuclease h2, subunit a  ISG20L2 - interferon stimulated exonuclease gene 20kda-like 2  KHNYN - kh and nyn domain containing  ERI1 - exoribonuclease 1  NTHL1 - nth endonuclease iii-like 1 (e. coli)  PELO - pelota homolog (drosophila)  CNOT7 - ccr4-not transcription complex, subunit 7  TSEN34 - tsen34 trna splicing endonuclease subunit  RNASE6 - ribonuclease, rnase a family, k6  CPSF4 - cleavage and polyadenylation specific factor 4, 30kda  FEN1 - flap structure-specific endonuclease 1  CPSF2 - cleavage and polyadenylation specific factor 2, 100kda  CPSF1 - cleavage and polyadenylation specific factor 1, 160kda  ERI3 - eri1 exoribonuclease family member 3  PCNA - proliferating cell nuclear antigen  ANGEL2 - angel homolog 2 (drosophila)  RNASET2 - ribonuclease t2  TSEN15 - tsen15 trna splicing endonuclease subunit  ENDOD1 - endonuclease domain containing 1  FCF1 - fcf1 rrna-processing protein  REXO2 - rna exonuclease 2  RAD50 - rad50 homolog (s. cerevisiae)  PLD3 - phospholipase d family, member 3  EDC4 - enhancer of mrna decapping 4  APEX2 - apex nuclease (apurinic/apyrimidinic endonuclease) 2  PARP1 - poly (adp-ribose) polymerase 1  POLD2 - polymerase (dna directed), delta 2, accessory subunit  RPP38 - ribonuclease p/mrp 38kda subunit  POLD1 - polymerase (dna directed), delta 1, catalytic subunit  ELAC1 - elac ribonuclease z 1  TOE1 - target of egr1, member 1 (nuclear)  NYNRIN - nyn domain and retroviral integrase containing  PGBD5 - piggybac transposable element derived 5  ANGEL1 - angel homolog 1 (drosophila)  NOP14 - nop14 nucleolar protein  RAD1 - rad1 homolog (s. pombe)  RPP14 - ribonuclease p/mrp 14kda subunit  DBR1 - debranching rna lariats 1  REXO4 - rex4, rna exonuclease 4 homolog (s. cerevisiae)  XPA - xeroderma pigmentosum, complementation group a  POP4 - processing of precursor 4, ribonuclease p/mrp subunit (s. cerevisiae)  NUDT16 - nudix (nucleoside diphosphate linked moiety x)-type motif 16  XPC - xeroderma pigmentosum, complementation group c  HMGB2 - high mobility group box 2  MUS81 - mus81 structure-specific endonuclease subunit  CNOT6L - ccr4-not transcription complex, subunit 6-like  HMGB1 - high mobility group box 1  CSTF3 - cleavage stimulation factor, 3' pre-rna, subunit 3, 77kda  CSTF2 - cleavage stimulation factor, 3' pre-rna, subunit 2, 64kda  DIS3 - dis3 mitotic control homolog (s. cerevisiae)  RNASEH1 - ribonuclease h1  DOM3Z - dom-3 homolog z (c. elegans)  EXOSC2 - exosome component 2  CNOT2 - ccr4-not transcription complex, subunit 2  N4BP2 - nedd4 binding protein 2  CNOT3 - ccr4-not transcription complex, subunit 3  CASP3 - caspase 3, apoptosis-related cysteine peptidase  AGO1 - argonaute risc catalytic component 1  RPA3 - replication protein a3, 14kda  RPA1 - replication protein a1, 70kda  RPA2 - replication protein a2, 32kda  EDC3 - enhancer of mrna decapping 3  DCPS - decapping enzyme, scavenger  CPSF6 - cleavage and polyadenylation specific factor 6, 68kda  NUDT21 - nudix (nucleoside diphosphate linked moiety x)-type motif 21  KPNB1 - karyopherin (importin) beta 1  KPNA1 - karyopherin alpha 1 (importin alpha 5)  DIS3L - dis3 mitotic control homolog (s. cerevisiae)-like  PPP1R8 - protein phosphatase 1, regulatory subunit 8  KRI1 - kri1 homolog (s. cerevisiae)  LACTB2 - lactamase, beta 2  CPSF7 - cleavage and polyadenylation specific factor 7, 59kda  DCP1B - decapping mrna 1b  MRPL44 - mitochondrial ribosomal protein l44  RPP40 - ribonuclease p/mrp 40kda subunit  ENDOV - endonuclease v  ZNRD1 - zinc ribbon domain containing 1  PAN3 - pan3 poly(a) specific ribonuclease subunit homolog (s. cerevisiae)  ERCC5 - excision repair cross-complementing rodent repair deficiency, complementation group 5  ERCC4 - excision repair cross-complementing rodent repair deficiency, complementation group 4  PNPT1 - polyribonucleotide nucleotidyltransferase 1  PAN2 - pan2 poly(a) specific ribonuclease subunit homolog (s. cerevisiae)  ERCC3 - excision repair cross-complementing rodent repair deficiency, complementation group 3  ASTE1 - asteroid homolog 1 (drosophila)  HARBI1 - harbinger transposase derived 1  RNASEK - ribonuclease, rnase k  ERN1 - endoplasmic reticulum to nucleus signaling 1  NOB1 - nin1/rpn12 binding protein 1 homolog (s. cerevisiae)  EXD2 - exonuclease 3'-5' domain containing 2  UBB - ubiquitin b  XRN2 - 5'-3' exoribonuclease 2  EXOSC8 - exosome component 8  DNASE2 - deoxyribonuclease ii, lysosomal  PCF11 - pcf11 cleavage and polyadenylation factor subunit  RBBP8 - retinoblastoma binding protein 8  KIAA0391 - kiaa0391  TSR1 - tsr1, 20s rrna accumulation, homolog (s. cerevisiae)  NUDT16L1 - nudix (nucleoside diphosphate linked moiety x)-type motif 16-like 1  DIS3L2 - dis3 mitotic control homolog (s. cerevisiae)-like 2  UTP23 - utp23, small subunit (ssu) processome component, homolog (yeast)  RRS1 - rrs1 ribosome biogenesis regulator homolog (s. cerevisiae)  TDP2 - tyrosyl-dna phosphodiesterase 2  SND1 - staphylococcal nuclease and tudor domain containing 1  DGCR8 - dgcr8 microprocessor complex subunit  CUL4B - cullin 4b  EXOSC4 - exosome component 4  TUT1 - terminal uridylyl transferase 1, u6 snrna-specific  RBX1 - ring-box 1, e3 ubiquitin protein ligase  MRE11A - mre11 meiotic recombination 11 homolog a (s. cerevisiae)  POLD3 - polymerase (dna-directed), delta 3, accessory subunit  EME1 - essential meiotic structure-specific endonuclease 1  ERCC2 - excision repair cross-complementing rodent repair deficiency, complementation group 2  PDE12 - phosphodiesterase 12  CNOT8 - ccr4-not transcription complex, subunit 8  PNKP - polynucleotide kinase 3'-phosphatase |
| GO:0006366 | transcription by RNA polymerase II | 5.38E-5 | 5.42E-3 | 1.20 (10334,174,6996,141) | [+] Show genes  CCNK - cyclin k  ELL - elongation factor rna polymerase ii  NCBP2 - nuclear cap binding protein subunit 2, 20kda  NCBP1 - nuclear cap binding protein subunit 1, 80kda  DDX5 - dead (asp-glu-ala-asp) box helicase 5  TRIM24 - tripartite motif containing 24  INTS5 - integrator complex subunit 5  RBMX - rna binding motif protein, x-linked  GTF2F1 - general transcription factor iif, polypeptide 1, 74kda  GTF2F2 - general transcription factor iif, polypeptide 2, 30kda  GTF2E1 - general transcription factor iie, polypeptide 1, alpha 56kda  ELL2 - elongation factor, rna polymerase ii, 2  SNAPC5 - small nuclear rna activating complex, polypeptide 5, 19kda  GTF2E2 - general transcription factor iie, polypeptide 2, beta 34kda  RPRD2 - regulation of nuclear pre-mrna domain containing 2  FLNA - filamin a, alpha  GTF2H2 - general transcription factor iih, polypeptide 2, 44kda  INTS10 - integrator complex subunit 10  GTF2H3 - general transcription factor iih, polypeptide 3, 34kda  GTF2H1 - general transcription factor iih, polypeptide 1, 62kda  MLLT3 - myeloid/lymphoid or mixed-lineage leukemia (trithorax homolog, drosophila); translocated to, 3  GTF2A2 - general transcription factor iia, 2, 12kda  MLLT1 - myeloid/lymphoid or mixed-lineage leukemia (trithorax homolog, drosophila); translocated to, 1  GTF2B - general transcription factor iib  NELFB - negative elongation factor complex member b  PHAX - phosphorylated adaptor for rna export  GTF2A1 - general transcription factor iia, 1, 19/37kda  SP1 - sp1 transcription factor  EGR2 - early growth response 2  GTF2I - general transcription factor iii  GTF2H4 - general transcription factor iih, polypeptide 4, 52kda  LMO4 - lim domain only 4  RPRD1A - regulation of nuclear pre-mrna domain containing 1a  INTS4 - integrator complex subunit 4  TAF15 - taf15 rna polymerase ii, tata box binding protein (tbp)-associated factor, 68kda  MNAT1 - mnat cdk-activating kinase assembly factor 1  GTF2IRD1 - gtf2i repeat domain containing 1  ZC3H8 - zinc finger ccch-type containing 8  CREB1 - camp responsive element binding protein 1  WDR61 - wd repeat domain 61  MNT - mnt, max dimerization protein  TSC22D1 - tsc22 domain family, member 1  ABT1 - activator of basal transcription 1  TAF9 - taf9 rna polymerase ii, tata box binding protein (tbp)-associated factor, 32kda  RPRD1B - regulation of nuclear pre-mrna domain containing 1b  TAF7 - taf7 rna polymerase ii, tata box binding protein (tbp)-associated factor, 55kda  TAF6 - taf6 rna polymerase ii, tata box binding protein (tbp)-associated factor, 80kda  SNAPC4 - small nuclear rna activating complex, polypeptide 4, 190kda  SUPT16H - suppressor of ty 16 homolog (s. cerevisiae)  TAF4 - taf4 rna polymerase ii, tata box binding protein (tbp)-associated factor, 135kda  TAF2 - taf2 rna polymerase ii, tata box binding protein (tbp)-associated factor, 150kda  TBPL1 - tbp-like 1  ARRB2 - arrestin, beta 2  ARRB1 - arrestin, beta 1  CDC73 - cell division cycle 73  NABP2 - nucleic acid binding protein 2  DDX21 - dead (asp-glu-ala-asp) box helicase 21  SNAPC2 - small nuclear rna activating complex, polypeptide 2, 45kda  SNAPC1 - small nuclear rna activating complex, polypeptide 1, 43kda  ZBTB7B - zinc finger and btb domain containing 7b  SNAPC3 - small nuclear rna activating complex, polypeptide 3, 50kda  RPAP1 - rna polymerase ii associated protein 1  MED1 - mediator complex subunit 1  TAF13 - taf13 rna polymerase ii, tata box binding protein (tbp)-associated factor, 18kda  INTS2 - integrator complex subunit 2  CDK9 - cyclin-dependent kinase 9  POLR2K - polymerase (rna) ii (dna directed) polypeptide k, 7.0kda  CDK7 - cyclin-dependent kinase 7  MED20 - mediator complex subunit 20  LEO1 - leo1, paf1/rna polymerase ii complex component, homolog (s. cerevisiae)  PARP1 - poly (adp-ribose) polymerase 1  TCEB3 - transcription elongation factor b (siii), polypeptide 3 (110kda, elongin a)  C5AR1 - complement component 5a receptor 1  POLR2G - polymerase (rna) ii (dna directed) polypeptide g  POLR2F - polymerase (rna) ii (dna directed) polypeptide f  POLR2E - polymerase (rna) ii (dna directed) polypeptide e, 25kda  SCAF1 - sr-related ctd-associated factor 1  POLR2D - polymerase (rna) ii (dna directed) polypeptide d  POLR2C - polymerase (rna) ii (dna directed) polypeptide c, 33kda  POLR2B - polymerase (rna) ii (dna directed) polypeptide b, 140kda  TCEA1 - transcription elongation factor a (sii), 1  POLR2A - polymerase (rna) ii (dna directed) polypeptide a, 220kda  INTS9 - integrator complex subunit 9  NELFE - negative elongation factor complex member e  ZBTB1 - zinc finger and btb domain containing 1  INTS12 - integrator complex subunit 12  EAF1 - ell associated factor 1  SUPT6H - suppressor of ty 6 homolog (s. cerevisiae)  AFF4 - af4/fmr2 family, member 4  SUPT4H1 - suppressor of ty 4 homolog 1 (s. cerevisiae)  SUPT5H - suppressor of ty 5 homolog (s. cerevisiae)  INTS6 - integrator complex subunit 6  PRDM4 - pr domain containing 4  DR1 - down-regulator of transcription 1, tbp-binding (negative cofactor 2)  XBP1 - x-box binding protein 1  NFYA - nuclear transcription factor y, alpha  NFRKB - nuclear factor related to kappab binding protein  MBD1 - methyl-cpg binding domain protein 1  MED16 - mediator complex subunit 16  NELFA - negative elongation factor complex member a  NFKB1 - nuclear factor of kappa light polypeptide gene enhancer in b-cells 1  TARDBP - tar dna binding protein  NELFCD - negative elongation factor complex member c/d  PAF1 - paf1, rna polymerase ii associated factor, homolog (s. cerevisiae)  INTS3 - integrator complex subunit 3  RPAP2 - rna polymerase ii associated protein 2  HSF2BP - heat shock transcription factor 2 binding protein  DTX1 - deltex homolog 1 (drosophila)  CCNT2 - cyclin t2  IWS1 - iws1 homolog (s. cerevisiae)  ERCC3 - excision repair cross-complementing rodent repair deficiency, complementation group 3  STAT3 - signal transducer and activator of transcription 3 (acute-phase response factor)  CCNT1 - cyclin t1  CCNH - cyclin h  RNGTT - rna guanylyltransferase and 5'-phosphatase  CTR9 - ctr9, paf1/rna polymerase ii complex component  RNMT - rna (guanine-7-) methyltransferase  TFDP1 - transcription factor dp-1  SSRP1 - structure specific recognition protein 1  TFCP2 - transcription factor cp2  ETV1 - ets variant 1  TAF8 - taf8 rna polymerase ii, tata box binding protein (tbp)-associated factor, 43kda  INTS8 - integrator complex subunit 8  PKNOX1 - pbx/knotted 1 homeobox 1  PHRF1 - phd and ring finger domains 1  CBFB - core-binding factor, beta subunit  SRRT - serrate rna effector molecule homolog (arabidopsis)  GMEB2 - glucocorticoid modulatory element binding protein 2  COPS2 - cop9 signalosome subunit 2  MED4 - mediator complex subunit 4  TAF1C - tata box binding protein (tbp)-associated factor, rna polymerase i, c, 110kda  FOSB - fbj murine osteosarcoma viral oncogene homolog b  FOS - fbj murine osteosarcoma viral oncogene homolog  TAF9B - taf9b rna polymerase ii, tata box binding protein (tbp)-associated factor, 31kda  NFAT5 - nuclear factor of activated t-cells 5, tonicity-responsive  SMAD4 - smad family member 4  NABP1 - nucleic acid binding protein 1  ERCC2 - excision repair cross-complementing rodent repair deficiency, complementation group 2  SUPT3H - suppressor of ty 3 homolog (s. cerevisiae)  INTS1 - integrator complex subunit 1  THRA - thyroid hormone receptor, alpha |
| GO:0006275 | regulation of DNA replication | 5.39E-5 | 5.39E-3 | 1.30 (10334,74,6996,65) | [+] Show genes  DHX9 - deah (asp-glu-ala-his) box helicase 9  DONSON - downstream neighbor of son  CACYBP - calcyclin binding protein  SENP2 - sumo1/sentrin/smt3 specific peptidase 2  RFC5 - replication factor c (activator 1) 5, 36.5kda  RFC2 - replication factor c (activator 1) 2, 40kda  CST3 - cystatin c  RFC3 - replication factor c (activator 1) 3, 38kda  ZNF365 - zinc finger protein 365  FBXO18 - f-box protein, helicase, 18  SMC3 - structural maintenance of chromosomes 3  ORC5 - origin recognition complex, subunit 5  LIG3 - ligase iii, dna, atp-dependent  PDS5A - pds5, regulator of cohesion maintenance, homolog a (s. cerevisiae)  WRNIP1 - werner helicase interacting protein 1  MAP2K4 - mitogen-activated protein kinase kinase 4  OBFC1 - oligonucleotide/oligosaccharide-binding fold containing 1  NBN - nibrin  CTC1 - cts telomere maintenance complex component 1  BRPF3 - bromodomain and phd finger containing, 3  ID3 - inhibitor of dna binding 3, dominant negative helix-loop-helix protein  POLG2 - polymerase (dna directed), gamma 2, accessory subunit  PPP2R1A - protein phosphatase 2, regulatory subunit a, alpha  TTF1 - transcription termination factor, rna polymerase i  ZBTB38 - zinc finger and btb domain containing 38  GTPBP4 - gtp binding protein 4  TNFAIP1 - tumor necrosis factor, alpha-induced protein 1 (endothelial)  ORC3 - origin recognition complex, subunit 3  EHMT2 - euchromatic histone-lysine n-methyltransferase 2  PPP2CA - protein phosphatase 2, catalytic subunit, alpha isozyme  RAD17 - rad17 homolog (s. pombe)  CDAN1 - codanin 1  ANKRD17 - ankyrin repeat domain 17  KCTD13 - potassium channel tetramerization domain containing 13  RAC1 - ras-related c3 botulinum toxin substrate 1 (rho family, small gtp binding protein rac1)  DSCC1 - dna replication and sister chromatid cohesion 1  INO80 - ino80 complex subunit  WDR18 - wd repeat domain 18  ESCO1 - establishment of sister chromatid cohesion n-acetyltransferase 1  MSH3 - muts homolog 3 (e. coli)  ATG7 - autophagy related 7  FGFR1 - fibroblast growth factor receptor 1  ACVRL1 - activin a receptor type ii-like 1  SMARCAL1 - swi/snf related, matrix associated, actin dependent regulator of chromatin, subfamily a-like 1  TERF1 - telomeric repeat binding factor (nima-interacting) 1  TERF2 - telomeric repeat binding factor 2  GMNN - geminin, dna replication inhibitor  EGFR - epidermal growth factor receptor  HUS1 - hus1 checkpoint homolog (s. pombe)  TIPIN - timeless interacting protein  BCAR3 - breast cancer anti-estrogen resistance 3  ATRX - alpha thalassemia/mental retardation syndrome x-linked  CDC7 - cell division cycle 7  PCNA - proliferating cell nuclear antigen  WIZ - widely interspaced zinc finger motifs  ZNF830 - zinc finger protein 830  TSPYL2 - tspy-like 2  RBBP6 - retinoblastoma binding protein 6  CCDC88A - coiled-coil domain containing 88a  ZMPSTE24 - zinc metallopeptidase ste24  USP37 - ubiquitin specific peptidase 37  CDK2 - cyclin-dependent kinase 2  KAT7 - k(lysine) acetyltransferase 7  WAPAL - wings apart-like homolog (drosophila)  CHTF8 - ctf8, chromosome transmission fidelity factor 8 homolog (s. cerevisiae) |
| GO:0033554 | cellular response to stress | 5.65E-5 | 5.62E-3 | 1.07 (10334,1200,6996,871) | [+] Show genes  GFPT1 - glutamine--fructose-6-phosphate transaminase 1  DNAJB14 - dnaj (hsp40) homolog, subfamily b, member 14  AGAP3 - arfgap with gtpase domain, ankyrin repeat and ph domain 3  PMS2 - pms2 postmeiotic segregation increased 2 (s. cerevisiae)  CBX1 - chromobox homolog 1  SEC61B - sec61 beta subunit  OS9 - osteosarcoma amplified 9, endoplasmic reticulum lectin  ABL1 - c-abl oncogene 1, non-receptor tyrosine kinase  PML - promyelocytic leukemia  CNOT11 - ccr4-not transcription complex, subunit 11  PMS1 - pms1 postmeiotic segregation increased 1 (s. cerevisiae)  OTUB1 - otu domain, ubiquitin aldehyde binding 1  EHMT2 - euchromatic histone-lysine n-methyltransferase 2  NPRL3 - nitrogen permease regulator-like 3 (s. cerevisiae)  MORF4L1 - mortality factor 4 like 1  TMTC3 - transmembrane and tetratricopeptide repeat containing 3  AMBRA1 - autophagy/beclin-1 regulator 1  ACTL6A - actin-like 6a  TRRAP - transformation/transcription domain-associated protein  USP19 - ubiquitin specific peptidase 19  PYROXD1 - pyridine nucleotide-disulphide oxidoreductase domain 1  ACTR5 - arp5 actin-related protein 5 homolog (yeast)  CDC45 - cell division cycle 45  CDC7 - cell division cycle 7  PPARD - peroxisome proliferator-activated receptor delta  SMC1A - structural maintenance of chromosomes 1a  POLR2K - polymerase (rna) ii (dna directed) polypeptide k, 7.0kda  ADD1 - adducin 1 (alpha)  FAM129A - family with sequence similarity 129, member a  GNB1 - guanine nucleotide binding protein (g protein), beta polypeptide 1  POLH - polymerase (dna directed), eta  PARP1 - poly (adp-ribose) polymerase 1  TNFRSF19 - tumor necrosis factor receptor superfamily, member 19  POLD2 - polymerase (dna directed), delta 2, accessory subunit  POLD1 - polymerase (dna directed), delta 1, catalytic subunit  POLR2G - polymerase (rna) ii (dna directed) polypeptide g  POLR2F - polymerase (rna) ii (dna directed) polypeptide f  POLR2E - polymerase (rna) ii (dna directed) polypeptide e, 25kda  SMPD3 - sphingomyelin phosphodiesterase 3, neutral membrane (neutral sphingomyelinase ii)  POLR2D - polymerase (rna) ii (dna directed) polypeptide d  POLR2C - polymerase (rna) ii (dna directed) polypeptide c, 33kda  POLR2B - polymerase (rna) ii (dna directed) polypeptide b, 140kda  POLR2A - polymerase (rna) ii (dna directed) polypeptide a, 220kda  PARP4 - poly (adp-ribose) polymerase family, member 4  RUVBL2 - ruvb-like 2 (e. coli)  PRKACA - protein kinase, camp-dependent, catalytic, alpha  EDEM2 - er degradation enhancer, mannosidase alpha-like 2  PPP5C - protein phosphatase 5, catalytic subunit  PPP2R5B - protein phosphatase 2, regulatory subunit b', beta  AKT1 - v-akt murine thymoma viral oncogene homolog 1  USP7 - ubiquitin specific peptidase 7 (herpes virus-associated)  XRCC6 - x-ray repair complementing defective repair in chinese hamster cells 6  RNF168 - ring finger protein 168, e3 ubiquitin protein ligase  ALDH3B1 - aldehyde dehydrogenase 3 family, member b1  PPP2CB - protein phosphatase 2, catalytic subunit, beta isozyme  TOPBP1 - topoisomerase (dna) ii binding protein 1  RMI2 - recq mediated genome instability 2  UBA5 - ubiquitin-like modifier activating enzyme 5  G6PD - glucose-6-phosphate dehydrogenase  WDR59 - wd repeat domain 59  AKR1B1 - aldo-keto reductase family 1, member b1 (aldose reductase)  NPLOC4 - nuclear protein localization 4 homolog (s. cerevisiae)  AMFR - autocrine motility factor receptor, e3 ubiquitin protein ligase  DNAJC3 - dnaj (hsp40) homolog, subfamily c, member 3  PAPD7 - pap associated domain containing 7  MAP2K5 - mitogen-activated protein kinase kinase 5  MAP2K7 - mitogen-activated protein kinase kinase 7  EIF2AK2 - eukaryotic translation initiation factor 2-alpha kinase 2  MAPK13 - mitogen-activated protein kinase 13  MAP2K1 - mitogen-activated protein kinase kinase 1  MAPK10 - mitogen-activated protein kinase 10  MAPK9 - mitogen-activated protein kinase 9  GBA - glucosidase, beta, acid  MAPK11 - mitogen-activated protein kinase 11  MAPK8 - mitogen-activated protein kinase 8  MAPK7 - mitogen-activated protein kinase 7  GPX8 - glutathione peroxidase 8 (putative)  GCN1L1 - gcn1 general control of amino-acid synthesis 1-like 1 (yeast)  MAPK3 - mitogen-activated protein kinase 3  MAPK1 - mitogen-activated protein kinase 1  COPS6 - cop9 signalosome subunit 6  KAT6A - k(lysine) acetyltransferase 6a  UBXN8 - ubx domain protein 8  PRKD1 - protein kinase d1  TLK2 - tousled-like kinase 2  PRKCE - protein kinase c, epsilon  VASN - vasorin  PRKCD - protein kinase c, delta  GAS6 - growth arrest-specific 6  PRKAG1 - protein kinase, amp-activated, gamma 1 non-catalytic subunit  APEX1 - apex nuclease (multifunctional dna repair enzyme) 1  DHX36 - deah (asp-glu-ala-his) box polypeptide 36  GSTP1 - glutathione s-transferase pi 1  XIAP - x-linked inhibitor of apoptosis  SMC6 - structural maintenance of chromosomes 6  GSR - glutathione reductase  RFWD3 - ring finger and wd repeat domain 3  TREX1 - three prime repair exonuclease 1  RNF113A - ring finger protein 113a  TMEM33 - transmembrane protein 33  PRMT6 - protein arginine methyltransferase 6  ZBTB17 - zinc finger and btb domain containing 17  MAN1B1 - mannosidase, alpha, class 1b, member 1  GTF2H2 - general transcription factor iih, polypeptide 2, 44kda  NEIL1 - nei endonuclease viii-like 1 (e. coli)  GTF2H3 - general transcription factor iih, polypeptide 3, 34kda  GTF2H1 - general transcription factor iih, polypeptide 1, 62kda  NUDT2 - nudix (nucleoside diphosphate linked moiety x)-type motif 2  APC - adenomatous polyposis coli  PCGF2 - polycomb group ring finger 2  MSH6 - muts homolog 6 (e. coli)  APBB1 - amyloid beta (a4) precursor protein-binding, family b, member 1 (fe65)  RIF1 - rap1 interacting factor homolog (yeast)  GUCY1B3 - guanylate cyclase 1, soluble, beta 3  POLG2 - polymerase (dna directed), gamma 2, accessory subunit  RNF139 - ring finger protein 139  GTF2H4 - general transcription factor iih, polypeptide 4, 52kda  POLI - polymerase (dna directed) iota  APOD - apolipoprotein d  PRDX1 - peroxiredoxin 1  PAK2 - p21 protein (cdc42/rac)-activated kinase 2  PAK3 - p21 protein (cdc42/rac)-activated kinase 3  OXR1 - oxidation resistance 1  ARNTL - aryl hydrocarbon receptor nuclear translocator-like  ARNT - aryl hydrocarbon receptor nuclear translocator  H2AFX - h2a histone family, member x  MUL1 - mitochondrial e3 ubiquitin protein ligase 1  NSMCE2 - non-smc element 2, mms21 homolog (s. cerevisiae)  SUPT16H - suppressor of ty 16 homolog (s. cerevisiae)  RNF103 - ring finger protein 103  PDGFRB - platelet-derived growth factor receptor, beta polypeptide  PDGFRA - platelet-derived growth factor receptor, alpha polypeptide  RHOB - ras homolog family member b  RHOA - ras homolog family member a  ZNF277 - zinc finger protein 277  ASNS - asparagine synthetase (glutamine-hydrolyzing)  ASNA1 - arsa arsenite transporter, atp-binding, homolog 1 (bacterial)  PCNA - proliferating cell nuclear antigen  MAP3K12 - mitogen-activated protein kinase kinase kinase 12  FICD - fic domain containing  FANCL - fanconi anemia, complementation group l  ERLIN2 - er lipid raft associated 2  WRAP53 - wd repeat containing, antisense to tp53  NDNF - neuron-derived neurotrophic factor  CDC37 - cell division cycle 37  HDAC2 - histone deacetylase 2  HDGF - hepatoma-derived growth factor  KPTN - kaptin (actin binding protein)  LRRC8D - leucine rich repeat containing 8 family, member d  ANKZF1 - ankyrin repeat and zinc finger domain containing 1  KAT7 - k(lysine) acetyltransferase 7  ATP2A3 - atpase, ca++ transporting, ubiquitous  GNL1 - guanine nucleotide binding protein-like 1  XPA - xeroderma pigmentosum, complementation group a  ZBTB1 - zinc finger and btb domain containing 1  XPC - xeroderma pigmentosum, complementation group c  XRCC1 - x-ray repair complementing defective repair in chinese hamster cells 1  RNF121 - ring finger protein 121  PAGR1 - paxip1 associated glutamate-rich protein 1  MORC2 - morc family cw-type zinc finger 2  PHF1 - phd finger protein 1  LRRK2 - leucine-rich repeat kinase 2  AP5S1 - adaptor-related protein complex 5, sigma 1 subunit  SCAMP5 - secretory carrier membrane protein 5  SEC31A - sec31 homolog a (s. cerevisiae)  XBP1 - x-box binding protein 1  ATG14 - autophagy related 14  ATP7A - atpase, cu++ transporting, alpha polypeptide  WFS1 - wolfram syndrome 1 (wolframin)  ADNP2 - adnp homeobox 2  PENK - proenkephalin  WHSC1 - wolf-hirschhorn syndrome candidate 1  WDR33 - wd repeat domain 33  IMPACT - impact rwd domain protein  VRK2 - vaccinia related kinase 2  BAX - bcl2-associated x protein  PKD2 - polycystic kidney disease 2 (autosomal dominant)  UBA6 - ubiquitin-like modifier activating enzyme 6  BAK1 - bcl2-antagonist/killer 1  GPX3 - glutathione peroxidase 3 (plasma)  BACH1 - btb and cnc homology 1, basic leucine zipper transcription factor 1  BAD - bcl2-associated agonist of cell death  PIK3R2 - phosphoinositide-3-kinase, regulatory subunit 2 (beta)  BRCC3 - brca1/brca2-containing complex, subunit 3  EXD2 - exonuclease 3'-5' domain containing 2  PIK3R1 - phosphoinositide-3-kinase, regulatory subunit 1 (alpha)  AXL - axl receptor tyrosine kinase  GRB2 - growth factor receptor-bound protein 2  PLAGL1 - pleiomorphic adenoma gene-like 1  TNIP2 - tnfaip3 interacting protein 2  ATRX - alpha thalassemia/mental retardation syndrome x-linked  GABARAPL2 - gaba(a) receptor-associated protein-like 2  GABARAP - gaba(a) receptor-associated protein  NUDT15 - nudix (nucleoside diphosphate linked moiety x)-type motif 15  BRF2 - brf2, rna polymerase iii transcription initiation factor 50 kda subunit  PIK3C2B - phosphatidylinositol-4-phosphate 3-kinase, catalytic subunit type 2 beta  BCL2 - b-cell cll/lymphoma 2  UBE2W - ubiquitin-conjugating enzyme e2w (putative)  PIK3C3 - phosphatidylinositol 3-kinase, catalytic subunit type 3  BCL2L2 - bcl2-like 2  XRCC5 - x-ray repair complementing defective repair in chinese hamster cells 5 (double-strand-break rejoining)  BCL2L1 - bcl2-like 1  XRCC4 - x-ray repair complementing defective repair in chinese hamster cells 4  CCND1 - cyclin d1  GSK3B - glycogen synthase kinase 3 beta  PARK7 - parkinson protein 7  GSK3A - glycogen synthase kinase 3 alpha  PNKP - polynucleotide kinase 3'-phosphatase  YWHAE - tyrosine 3-monooxygenase/tryptophan 5-monooxygenase activation protein, epsilon polypeptide  PDIA3 - protein disulfide isomerase family a, member 3  CCNK - cyclin k  DNAJB2 - dnaj (hsp40) homolog, subfamily b, member 2  BNIP3L - bcl2/adenovirus e1b 19kda interacting protein 3-like  RFC5 - replication factor c (activator 1) 5, 36.5kda  RFC2 - replication factor c (activator 1) 2, 40kda  RFC3 - replication factor c (activator 1) 3, 38kda  CHAC1 - chac, cation transport regulator homolog 1 (e. coli)  FLCN - folliculin  HSBP1 - heat shock factor binding protein 1  RELA - v-rel avian reticuloendotheliosis viral oncogene homolog a  PRMT1 - protein arginine methyltransferase 1  RECQL - recq protein-like (dna helicase q1-like)  ZFP36L1 - zfp36 ring finger protein-like 1  FBXO45 - f-box protein 45  PAK4 - p21 protein (cdc42/rac)-activated kinase 4  UPF1 - upf1 regulator of nonsense transcripts homolog (yeast)  DERL1 - derlin 1  RRAGB - ras-related gtp binding b  ALKBH1 - alkb, alkylation repair homolog 1 (e. coli)  CFLAR - casp8 and fadd-like apoptosis regulator  TMUB2 - transmembrane and ubiquitin-like domain containing 2  BMPR2 - bone morphogenetic protein receptor, type ii (serine/threonine kinase)  BMP7 - bone morphogenetic protein 7  TMEM109 - transmembrane protein 109  PSMD14 - proteasome (prosome, macropain) 26s subunit, non-atpase, 14  FLOT1 - flotillin 1  C1orf86 - chromosome 1 open reading frame 86  NABP2 - nucleic acid binding protein 2  MTMR3 - myotubularin related protein 3  CA2 - carbonic anhydrase ii  HUS1 - hus1 checkpoint homolog (s. pombe)  EIF2B5 - eukaryotic translation initiation factor 2b, subunit 5 epsilon, 82kda  HERC2 - hect and rld domain containing e3 ubiquitin protein ligase 2  RNF111 - ring finger protein 111  TSPO - translocator protein (18kda)  DNAJB12 - dnaj (hsp40) homolog, subfamily b, member 12  HSPD1 - heat shock 60kda protein 1 (chaperonin)  NSMCE1 - non-smc element 1 homolog (s. cerevisiae)  DNAJB1 - dnaj (hsp40) homolog, subfamily b, member 1  HSP90AA1 - heat shock protein 90kda alpha (cytosolic), class a member 1  C7orf49 - chromosome 7 open reading frame 49  ZMPSTE24 - zinc metallopeptidase ste24  BTRC - beta-transducin repeat containing e3 ubiquitin protein ligase  MBD4 - methyl-cpg binding domain protein 4  STUB1 - stip1 homology and u-box containing protein 1, e3 ubiquitin protein ligase  UBE4B - ubiquitination factor e4b  HSPA9 - heat shock 70kda protein 9 (mortalin)  HSPA8 - heat shock 70kda protein 8  TAB1 - tgf-beta activated kinase 1/map3k7 binding protein 1  VAV3 - vav 3 guanine nucleotide exchange factor  PPIE - peptidylprolyl isomerase e (cyclophilin e)  MCRS1 - microspherule protein 1  HMGN1 - high mobility group nucleosome binding domain 1  CANX - calnexin  FKBP14 - fk506 binding protein 14, 22 kda  CASP9 - caspase 9, apoptosis-related cysteine peptidase  STK33 - serine/threonine kinase 33  SLC30A9 - solute carrier family 30 (zinc transporter), member 9  CASP2 - caspase 2, apoptosis-related cysteine peptidase  CASP3 - caspase 3, apoptosis-related cysteine peptidase  RPA3 - replication protein a3, 14kda  MAD2L2 - mad2 mitotic arrest deficient-like 2 (yeast)  RPA1 - replication protein a1, 70kda  RPA2 - replication protein a2, 32kda  BECN1 - beclin 1, autophagy related  PEA15 - phosphoprotein enriched in astrocytes 15  HIPK1 - homeodomain interacting protein kinase 1  ACD - adrenocortical dysplasia homolog (mouse)  CALR - calreticulin  HFE - hemochromatosis  TBL2 - transducin (beta)-like 2  UBE2K - ubiquitin-conjugating enzyme e2k  INTS3 - integrator complex subunit 3  CREB3 - camp responsive element binding protein 3  HIF1A - hypoxia inducible factor 1, alpha subunit (basic helix-loop-helix transcription factor)  CITED2 - cbp/p300-interacting transactivator, with glu/asp-rich carboxy-terminal domain, 2  MBTPS1 - membrane-bound transcription factor peptidase, site 1  INO80C - ino80 complex subunit c  CCNH - cyclin h  RIPK1 - receptor (tnfrsf)-interacting serine-threonine kinase 1  SWSAP1 - swim-type zinc finger 7 associated protein 1  FBXO22 - f-box protein 22  HNRNPL - heterogeneous nuclear ribonucleoprotein l  NDRG1 - n-myc downstream regulated 1  TIPIN - timeless interacting protein  FBXO6 - f-box protein 6  MRPS11 - mitochondrial ribosomal protein s11  ACER2 - alkaline ceramidase 2  RPS6KA1 - ribosomal protein s6 kinase, 90kda, polypeptide 1  PARP16 - poly (adp-ribose) polymerase family, member 16  CBL - cbl proto-oncogene, e3 ubiquitin protein ligase  HNRNPA1 - heterogeneous nuclear ribonucleoprotein a1  RIPK2 - receptor-interacting serine-threonine kinase 2  CAV1 - caveolin 1, caveolae protein, 22kda  HNRNPD - heterogeneous nuclear ribonucleoprotein d (au-rich element rna binding protein 1, 37kda)  ADPRHL2 - adp-ribosylhydrolase like 2  DNAJC18 - dnaj (hsp40) homolog, subfamily c, member 18  ZSWIM7 - zinc finger, swim-type containing 7  HMGA1 - high mobility group at-hook 1  NABP1 - nucleic acid binding protein 1  STT3B - stt3b, subunit of the oligosaccharyltransferase complex (catalytic)  CDC5L - cell division cycle 5-like  RPS6KA6 - ribosomal protein s6 kinase, 90kda, polypeptide 6  TNRC6A - trinucleotide repeat containing 6a  ERLIN1 - er lipid raft associated 1  HTRA2 - htra serine peptidase 2  PARG - poly (adp-ribose) glycohydrolase  STK39 - serine threonine kinase 39  PSEN1 - presenilin 1  PPM1D - protein phosphatase, mg2+/mn2+ dependent, 1d  POLL - polymerase (dna directed), lambda  USP16 - ubiquitin specific peptidase 16  CREB3L2 - camp responsive element binding protein 3-like 2  PSMA1 - proteasome (prosome, macropain) subunit, alpha type, 1  PSMA2 - proteasome (prosome, macropain) subunit, alpha type, 2  PRPF19 - pre-mrna processing factor 19  MAP4K3 - mitogen-activated protein kinase kinase kinase kinase 3  CDC14B - cell division cycle 14b  CAMKK2 - calcium/calmodulin-dependent protein kinase kinase 2, beta  PSMB1 - proteasome (prosome, macropain) subunit, beta type, 1  NPRL2 - nitrogen permease regulator-like 2 (s. cerevisiae)  MAPKAPK5 - mitogen-activated protein kinase-activated protein kinase 5  PSMA5 - proteasome (prosome, macropain) subunit, alpha type, 5  PSMA3 - proteasome (prosome, macropain) subunit, alpha type, 3  PSMB10 - proteasome (prosome, macropain) subunit, beta type, 10  PSMC6 - proteasome (prosome, macropain) 26s subunit, atpase, 6  PSMD1 - proteasome (prosome, macropain) 26s subunit, non-atpase, 1  PSMC4 - proteasome (prosome, macropain) 26s subunit, atpase, 4  COPS7B - cop9 signalosome subunit 7b  COPS3 - cop9 signalosome subunit 3  PSMC2 - proteasome (prosome, macropain) 26s subunit, atpase, 2  PSMD9 - proteasome (prosome, macropain) 26s subunit, non-atpase, 9  PSMD10 - proteasome (prosome, macropain) 26s subunit, non-atpase, 10  PSMD7 - proteasome (prosome, macropain) 26s subunit, non-atpase, 7  PSMD8 - proteasome (prosome, macropain) 26s subunit, non-atpase, 8  DNAJC10 - dnaj (hsp40) homolog, subfamily c, member 10  GAS2L1 - growth arrest-specific 2 like 1  PSMD5 - proteasome (prosome, macropain) 26s subunit, non-atpase, 5  TERF2IP - telomeric repeat binding factor 2, interacting protein  PSMD2 - proteasome (prosome, macropain) 26s subunit, non-atpase, 2  SLC38A2 - solute carrier family 38, member 2  PSMD12 - proteasome (prosome, macropain) 26s subunit, non-atpase, 12  ATG7 - autophagy related 7  PSMD13 - proteasome (prosome, macropain) 26s subunit, non-atpase, 13  SERP1 - stress-associated endoplasmic reticulum protein 1  RNASEH2A - ribonuclease h2, subunit a  PRKRA - protein kinase, interferon-inducible double stranded rna dependent activator  PTEN - phosphatase and tensin homolog  CETN2 - centrin, ef-hand protein, 2  JMY - junction mediating and regulatory protein, p53 cofactor  PRDX4 - peroxiredoxin 4  MYBBP1A - myb binding protein (p160) 1a  PTGIS - prostaglandin i2 (prostacyclin) synthase  CIB1 - calcium and integrin binding 1 (calmyrin)  ERLEC1 - endoplasmic reticulum lectin 1  CLGN - calmegin  KAT5 - k(lysine) acetyltransferase 5  HYOU1 - hypoxia up-regulated 1  SLU7 - slu7 splicing factor homolog (s. cerevisiae)  CDK9 - cyclin-dependent kinase 9  PTN - pleiotrophin  CDK7 - cyclin-dependent kinase 7  CDKN1B - cyclin-dependent kinase inhibitor 1b (p27, kip1)  PTPN1 - protein tyrosine phosphatase, non-receptor type 1  APEX2 - apex nuclease (apurinic/apyrimidinic endonuclease) 2  PTPN11 - protein tyrosine phosphatase, non-receptor type 11  IRF3 - interferon regulatory factor 3  RUVBL1 - ruvb-like 1 (e. coli)  ZMAT3 - zinc finger, matrin-type 3  CDK2 - cyclin-dependent kinase 2  IRAK2 - interleukin-1 receptor-associated kinase 2  IRAK1 - interleukin-1 receptor-associated kinase 1  PTPRF - protein tyrosine phosphatase, receptor type, f  RAD1 - rad1 homolog (s. pombe)  WDR4 - wd repeat domain 4  EIF2AK1 - eukaryotic translation initiation factor 2-alpha kinase 1  PTPRK - protein tyrosine phosphatase, receptor type, k  PYCR1 - pyrroline-5-carboxylate reductase 1  PLK2 - polo-like kinase 2  MMS19 - mms19 nucleotide excision repair homolog (s. cerevisiae)  PXN - paxillin  HERPUD2 - herpud family member 2  ID2 - inhibitor of dna binding 2, dominant negative helix-loop-helix protein  MAP4K2 - mitogen-activated protein kinase kinase kinase kinase 2  CHD2 - chromodomain helicase dna binding protein 2  PLA2G6 - phospholipase a2, group vi (cytosolic, calcium-independent)  RRAGC - ras-related gtp binding c  RAD21 - rad21 homolog (s. pombe)  RAD17 - rad17 homolog (s. pombe)  BRAT1 - brca1-associated atm activator 1  INO80 - ino80 complex subunit  IGHMBP2 - immunoglobulin mu binding protein 2  RALB - v-ral simian leukemia viral oncogene homolog b  STK24 - serine/threonine kinase 24  TOMM7 - translocase of outer mitochondrial membrane 7 homolog (yeast)  RBPJ - recombination signal binding protein for immunoglobulin kappa j region  OARD1 - o-acyl-adp-ribose deacylase 1  NCK2 - nck adaptor protein 2  FAM35A - family with sequence similarity 35, member a  SGTB - small glutamine-rich tetratricopeptide repeat (tpr)-containing, beta  ULK1 - unc-51 like autophagy activating kinase 1  RRAGA - ras-related gtp binding a  TPP1 - tripeptidyl peptidase i  MLH3 - mutl homolog 3 (e. coli)  RBL2 - retinoblastoma-like 2 (p130)  RBBP7 - retinoblastoma binding protein 7  RBBP8 - retinoblastoma binding protein 8  TMCO1 - transmembrane and coiled-coil domains 1  RBM4 - rna binding motif protein 4  RBBP5 - retinoblastoma binding protein 5  TSPYL2 - tspy-like 2  RBBP6 - retinoblastoma binding protein 6  ERCC8 - excision repair cross-complementing rodent repair deficiency, complementation group 8  CUL4B - cullin 4b  VPS41 - vacuolar protein sorting 41 homolog (s. cerevisiae)  CUL1 - cullin 1  CUL2 - cullin 2  POLD3 - polymerase (dna-directed), delta 3, accessory subunit  B3GAT1 - beta-1,3-glucuronyltransferase 1 (glucuronosyltransferase p)  FOXP1 - forkhead box p1  CHUK - conserved helix-loop-helix ubiquitous kinase  WAC - ww domain containing adaptor with coiled-coil  LIG4 - ligase iv, dna, atp-dependent  PDIA4 - protein disulfide isomerase family a, member 4  FBXO18 - f-box protein, helicase, 18  LIG1 - ligase i, dna, atp-dependent  LIG3 - ligase iii, dna, atp-dependent  CARTPT - cart prepropeptide  PPP1R15B - protein phosphatase 1, regulatory subunit 15b  DOT1L - dot1-like histone h3k79 methyltransferase  MUM1 - melanoma associated antigen (mutated) 1  BRSK1 - br serine/threonine kinase 1  SYVN1 - synovial apoptosis inhibitor 1, synoviolin  SLX4 - slx4 structure-specific endonuclease subunit  SOX4 - sry (sex determining region y)-box 4  CHD1L - chromodomain helicase dna binding protein 1-like  TIPRL - tip41, tor signaling pathway regulator-like (s. cerevisiae)  GOSR2 - golgi snap receptor complex member 2  FADS1 - fatty acid desaturase 1  ATF6B - activating transcription factor 6 beta  CREBBP - creb binding protein  ATF2 - activating transcription factor 2  IER5 - immediate early response 5  LMNA - lamin a/c  CLOCK - clock circadian regulator  SMUG1 - single-strand-selective monofunctional uracil-dna glycosylase 1  PRKAG2 - protein kinase, amp-activated, gamma 2 non-catalytic subunit  POLK - polymerase (dna directed) kappa  GABARAPL1 - gaba(a) receptor-associated protein like 1  INIP - ints3 and nabp interacting protein  PLK3 - polo-like kinase 3  SNCA - synuclein, alpha (non a4 component of amyloid precursor)  NPEPPS - aminopeptidase puromycin sensitive  RNF138 - ring finger protein 138, e3 ubiquitin protein ligase  MAP1LC3A - microtubule-associated protein 1 light chain 3 alpha  SRPRB - signal recognition particle receptor, b subunit  RBM17 - rna binding motif protein 17  LAMP2 - lysosomal-associated membrane protein 2  UBR5 - ubiquitin protein ligase e3 component n-recognin 5  UCHL5 - ubiquitin carboxyl-terminal hydrolase l5  ATG5 - autophagy related 5  ANGPT4 - angiopoietin 4  TAOK3 - tao kinase 3  FZR1 - fizzy/cell division cycle 20 related 1 (drosophila)  ZBTB7A - zinc finger and btb domain containing 7a  MBTPS2 - membrane-bound transcription factor peptidase, site 2  TFIP11 - tuftelin interacting protein 11  CHD6 - chromodomain helicase dna binding protein 6  CST3 - cystatin c  PLEKHA1 - pleckstrin homology domain containing, family a (phosphoinositide binding specific) member 1  ZNF580 - zinc finger protein 580  ARHGEF6 - rac/cdc42 guanine nucleotide exchange factor (gef) 6  PICK1 - protein interacting with prkca 1  TMEM117 - transmembrane protein 117  CTNNA1 - catenin (cadherin-associated protein), alpha 1, 102kda  ING4 - inhibitor of growth family, member 4  INSIG2 - insulin induced gene 2  EIF2AK3 - eukaryotic translation initiation factor 2-alpha kinase 3  KCNK3 - potassium channel, subfamily k, member 3  IRAK4 - interleukin-1 receptor-associated kinase 4  MAP4K4 - mitogen-activated protein kinase kinase kinase kinase 4  RHBDD1 - rhomboid domain containing 1  STX4 - syntaxin 4  LRRC8C - leucine rich repeat containing 8 family, member c  RECQL5 - recq protein-like 5  MGARP - mitochondria-localized glutamic acid-rich protein  WDR24 - wd repeat domain 24  CTSV - cathepsin v  NEK4 - nima-related kinase 4  STK3 - serine/threonine kinase 3  SUV420H1 - suppressor of variegation 4-20 homolog 1 (drosophila)  HSPA13 - heat shock protein 70kda family, member 13  STAU1 - staufen double-stranded rna binding protein 1  STC1 - stanniocalcin 1  SUV420H2 - suppressor of variegation 4-20 homolog 2 (drosophila)  STK4 - serine/threonine kinase 4  SH3GLB1 - sh3-domain grb2-like endophilin b1  RPAIN - rpa interacting protein  LONP1 - lon peptidase 1, mitochondrial  CRKL - v-crk avian sarcoma virus ct10 oncogene homolog-like  CRK - v-crk avian sarcoma virus ct10 oncogene homolog  NIPBL - nipped-b homolog (drosophila)  CRY1 - cryptochrome 1 (photolyase-like)  SHISA5 - shisa family member 5  UBE4A - ubiquitination factor e4a  ASF1A - anti-silencing function 1a histone chaperone  STAT6 - signal transducer and activator of transcription 6, interleukin-4 induced  SSRP1 - structure specific recognition protein 1  ZDHHC16 - zinc finger, dhhc-type containing 16  JAK2 - janus kinase 2  BOD1L1 - biorientation of chromosomes in cell division 1-like 1  ITPR1 - inositol 1,4,5-trisphosphate receptor, type 1  CHCHD6 - coiled-coil-helix-coiled-coil-helix domain containing 6  TRIP12 - thyroid hormone receptor interactor 12  KCNK2 - potassium channel, subfamily k, member 2  TAB3 - tgf-beta activated kinase 1/map3k7 binding protein 3  SSR1 - signal sequence receptor, alpha  COPS2 - cop9 signalosome subunit 2  SRXN1 - sulfiredoxin 1  TAOK2 - tao kinase 2  SRC - v-src avian sarcoma (schmidt-ruppin a-2) viral oncogene homolog  SREBF2 - sterol regulatory element binding transcription factor 2  HSPA14 - heat shock 70kda protein 14  CNOT8 - ccr4-not transcription complex, subunit 8  PRDX5 - peroxiredoxin 5  SHPRH - snf2 histone linker phd ring helicase, e3 ubiquitin protein ligase  SIRPA - signal-regulatory protein alpha  RNF169 - ring finger protein 169  SEL1L - sel-1 suppressor of lin-12-like (c. elegans)  DDX3X - dead (asp-glu-ala-asp) box helicase 3, x-linked  DDX1 - dead (asp-glu-ala-asp) box helicase 1  SYF2 - syf2 pre-mrna-splicing factor  TM7SF3 - transmembrane 7 superfamily member 3  SPRTN - sprt-like n-terminal domain  SIN3A - sin3 transcription regulator family member a  MIF - macrophage migration inhibitory factor (glycosylation-inhibiting factor)  SAMHD1 - sam domain and hd domain 1  MAP3K10 - mitogen-activated protein kinase kinase kinase 10  DDB1 - damage-specific dna binding protein 1, 127kda  SETMAR - set domain and mariner transposase fusion gene  MLH1 - mutl homolog 1, colon cancer, nonpolyposis type 2 (e. coli)  DCTN1 - dynactin 1  MAP2K4 - mitogen-activated protein kinase kinase 4  RCN3 - reticulocalbin 3, ef-hand calcium binding domain  MCM9 - minichromosome maintenance complex component 9  MAP3K11 - mitogen-activated protein kinase kinase kinase 11  MMP2 - matrix metallopeptidase 2 (gelatinase a, 72kda gelatinase, 72kda type iv collagenase)  CNOT10 - ccr4-not transcription complex, subunit 10  ZBTB38 - zinc finger and btb domain containing 38  RCHY1 - ring finger and chy zinc finger domain containing 1, e3 ubiquitin protein ligase  MNAT1 - mnat cdk-activating kinase assembly factor 1  UIMC1 - ubiquitin interaction motif containing 1  DNAJB11 - dnaj (hsp40) homolog, subfamily b, member 11  EIF2A - eukaryotic translation initiation factor 2a, 65kda  RHBDD2 - rhomboid domain containing 2  CNOT6 - ccr4-not transcription complex, subunit 6  SESN3 - sestrin 3  MCM7 - minichromosome maintenance complex component 7  XPR1 - xenotropic and polytropic retrovirus receptor 1  DNAJB9 - dnaj (hsp40) homolog, subfamily b, member 9  VAPB - vamp (vesicle-associated membrane protein)-associated protein b and c  MAP3K5 - mitogen-activated protein kinase kinase kinase 5  MEN1 - multiple endocrine neoplasia i  GORASP2 - golgi reassembly stacking protein 2, 55kda  S100B - s100 calcium binding protein b  DAXX - death-domain associated protein  HINFP - histone h4 transcription factor  DAG1 - dystroglycan 1 (dystrophin-associated glycoprotein 1)  ZFYVE1 - zinc finger, fyve domain containing 1  ULK3 - unc-51 like kinase 3  DAPK1 - death-associated protein kinase 1  ARHGEF2 - rho/rac guanine nucleotide exchange factor (gef) 2  DAP - death-associated protein  RPTOR - regulatory associated protein of mtor, complex 1  ATRIP - atr interacting protein  JKAMP - jnk1/mapk8-associated membrane protein  EPC2 - enhancer of polycomb homolog 2 (drosophila)  SLC9A1 - solute carrier family 9, subfamily a (nhe1, cation proton antiporter 1), member 1  LARS - leucyl-trna synthetase  SLC8A1 - solute carrier family 8 (sodium/calcium exchanger), member 1  RQCD1 - rcd1 required for cell differentiation1 homolog (s. pombe)  CNOT6L - ccr4-not transcription complex, subunit 6-like  SMC3 - structural maintenance of chromosomes 3  TAOK1 - tao kinase 1  MARS - methionyl-trna synthetase  MAPT - microtubule-associated protein tau  WIPI2 - wd repeat domain, phosphoinositide interacting 2  ATP6V0D1 - atpase, h+ transporting, lysosomal 38kda, v0 subunit d1  MAP1B - microtubule-associated protein 1b  MTA1 - metastasis associated 1  SIRT6 - sirtuin 6  TNKS1BP1 - tankyrase 1 binding protein 1, 182kda  MAPK15 - mitogen-activated protein kinase 15  USP10 - ubiquitin specific peptidase 10  UBE2J1 - ubiquitin-conjugating enzyme e2, j1  SKP1 - s-phase kinase-associated protein 1  REV1 - rev1, polymerase (dna directed)  TOR1A - torsin family 1, member a (torsin a)  MATN2 - matrilin 2  SLC2A1 - solute carrier family 2 (facilitated glucose transporter), member 1  MAX - myc associated factor x  UFC1 - ubiquitin-fold modifier conjugating enzyme 1  WDR48 - wd repeat domain 48  MCM3 - minichromosome maintenance complex component 3  GIGYF2 - grb10 interacting gyf protein 2  MCM6 - minichromosome maintenance complex component 6  MCM5 - minichromosome maintenance complex component 5  FNIP2 - folliculin interacting protein 2  MCM2 - minichromosome maintenance complex component 2  ASH2L - ash2 (absent, small, or homeotic)-like (drosophila)  PNPT1 - polyribonucleotide nucleotidyltransferase 1  STXBP4 - syntaxin binding protein 4  NEIL2 - nei endonuclease viii-like 2 (e. coli)  LYN - v-yes-1 yamaguchi sarcoma viral related oncogene homolog  JAM3 - junctional adhesion molecule 3  DHFR - dihydrofolate reductase  SESN2 - sestrin 2  PARP9 - poly (adp-ribose) polymerase family, member 9  NQO1 - nad(p)h dehydrogenase, quinone 1  RNF8 - ring finger protein 8, e3 ubiquitin protein ligase  FBXO2 - f-box protein 2  BAZ1B - bromodomain adjacent to zinc finger domain, 1b  SIPA1 - signal-induced proliferation-associated 1  UVSSA - uv-stimulated scaffold protein a  SFPQ - splicing factor proline/glutamine-rich  ZBTB4 - zinc finger and btb domain containing 4  SFRP1 - secreted frizzled-related protein 1  PIAS4 - protein inhibitor of activated stat, 4  DNM2 - dynamin 2  RTN4RL1 - reticulon 4 receptor-like 1  DNMT3A - dna (cytosine-5-)-methyltransferase 3 alpha  TDP2 - tyrosyl-dna phosphodiesterase 2  EIF2AK4 - eukaryotic translation initiation factor 2 alpha kinase 4  SGK1 - serum/glucocorticoid regulated kinase 1  SGTA - small glutamine-rich tetratricopeptide repeat (tpr)-containing, alpha  EME1 - essential meiotic structure-specific endonuclease 1  PLA2R1 - phospholipase a2 receptor 1, 180kda  NCK1 - nck adaptor protein 1  FAN1 - fancd2/fanci-associated nuclease 1  SNW1 - snw domain containing 1  SCAP - srebf chaperone  MGME1 - mitochondrial genome maintenance exonuclease 1  TRIM28 - tripartite motif containing 28  EPAS1 - endothelial pas domain protein 1  EP300 - e1a binding protein p300  KIN - kin, antigenic determinant of reca protein homolog (mouse)  ATF6 - activating transcription factor 6  DNAJC15 - dnaj (hsp40) homolog, subfamily c, member 15  NBN - nibrin  UBQLN4 - ubiquilin 4  DAPL1 - death associated protein-like 1  UGGT1 - udp-glucose glycoprotein glucosyltransferase 1  EIF2S1 - eukaryotic translation initiation factor 2, subunit 1 alpha, 35kda  TFPT - tcf3 (e2a) fusion partner (in childhood leukemia)  CLPB - clpb caseinolytic peptidase b homolog (e. coli)  KDM2A - lysine (k)-specific demethylase 2a  TMEM129 - transmembrane protein 129  PSME3 - proteasome (prosome, macropain) activator subunit 3 (pa28 gamma; ki)  MYLK - myosin light chain kinase  TRIM13 - tripartite motif containing 13  MYO6 - myosin vi  TOPORS - topoisomerase i binding, arginine/serine-rich, e3 ubiquitin protein ligase  CCDC47 - coiled-coil domain containing 47  CNOT1 - ccr4-not transcription complex, subunit 1  EEF2 - eukaryotic translation elongation factor 2  SEH1L - seh1-like (s. cerevisiae)  PYCR2 - pyrroline-5-carboxylate reductase family, member 2  TP53BP1 - tumor protein p53 binding protein 1  HUWE1 - hect, uba and wwe domain containing 1, e3 ubiquitin protein ligase  EGR1 - early growth response 1  EGFR - epidermal growth factor receptor  CNOT7 - ccr4-not transcription complex, subunit 7  COL4A3BP - collagen, type iv, alpha 3 (goodpasture antigen) binding protein  USP33 - ubiquitin specific peptidase 33  INO80B - ino80 complex subunit b  CTDSP2 - ctd (carboxy-terminal domain, rna polymerase ii, polypeptide a) small phosphatase 2  NCOA6 - nuclear receptor coactivator 6  SMG1 - smg1 phosphatidylinositol 3-kinase-related kinase  TNIK - traf2 and nck interacting kinase  MUTYH - muty homolog (e. coli)  XAB2 - xpa binding protein 2  E2F4 - e2f transcription factor 4, p107/p130-binding  PDS5B - pds5, regulator of cohesion maintenance, homolog b (s. cerevisiae)  RAD50 - rad50 homolog (s. cerevisiae)  SLC25A24 - solute carrier family 25 (mitochondrial carrier; phosphate carrier), member 24  C11orf30 - chromosome 11 open reading frame 30  CDC42EP5 - cdc42 effector protein (rho gtpase binding) 5  ERP44 - endoplasmic reticulum protein 44  HSP90B1 - heat shock protein 90kda beta (grp94), member 1  TRAF2 - tnf receptor-associated factor 2  SETX - senataxin  PDIA6 - protein disulfide isomerase family a, member 6  TPR - translocated promoter region, nuclear basket protein  REXO4 - rex4, rna exonuclease 4 homolog (s. cerevisiae)  TMEM259 - transmembrane protein 259  SLC12A6 - solute carrier family 12 (potassium/chloride transporter), member 6  DERL3 - derlin 3  USP1 - ubiquitin specific peptidase 1  FANCE - fanconi anemia, complementation group e  PDCD6 - programmed cell death 6  PTK2B - protein tyrosine kinase 2 beta  TANK - traf family member-associated nfkb activator  UNG - uracil-dna glycosylase  RNF185 - ring finger protein 185  HDAC6 - histone deacetylase 6  TAB2 - tgf-beta activated kinase 1/map3k7 binding protein 2  C19orf40 - chromosome 19 open reading frame 40  PARP2 - poly (adp-ribose) polymerase 2  ETV5 - ets variant 5  SH2D3C - sh2 domain containing 3c  MT3 - metallothionein 3  VHL - von hippel-lindau tumor suppressor, e3 ubiquitin protein ligase  KDM6B - lysine (k)-specific demethylase 6b  SMC5 - structural maintenance of chromosomes 5  PCBP4 - poly(rc) binding protein 4  CHAF1A - chromatin assembly factor 1, subunit a (p150)  EXTL2 - exostosin-like glycosyltransferase 2  EXTL3 - exostosin-like glycosyltransferase 3  CREB3L1 - camp responsive element binding protein 3-like 1  UVRAG - uv radiation resistance associated  EYA3 - eyes absent homolog 3 (drosophila)  ENDOV - endonuclease v  VCP - valosin containing protein  MAPK8IP3 - mitogen-activated protein kinase 8 interacting protein 3  MSH3 - muts homolog 3 (e. coli)  MSH2 - muts homolog 2, colon cancer, nonpolyposis type 1 (e. coli)  ERCC5 - excision repair cross-complementing rodent repair deficiency, complementation group 5  ERCC4 - excision repair cross-complementing rodent repair deficiency, complementation group 4  ERCC3 - excision repair cross-complementing rodent repair deficiency, complementation group 3  ZBTB40 - zinc finger and btb domain containing 40  SEC16A - sec16 homolog a (s. cerevisiae)  UBE2A - ubiquitin-conjugating enzyme e2a  UBA1 - ubiquitin-like modifier activating enzyme 1  ERN1 - endoplasmic reticulum to nucleus signaling 1  UBB - ubiquitin b  RGCC - regulator of cell cycle  TMX1 - thioredoxin-related transmembrane protein 1  OXSR1 - oxidative stress responsive 1  TTC5 - tetratricopeptide repeat domain 5  COMMD1 - copper metabolism (murr1) domain containing 1  FAF2 - fas associated factor family member 2  ETS1 - v-ets avian erythroblastosis virus e26 oncogene homolog 1  UBXN4 - ubx domain protein 4  TXNRD1 - thioredoxin reductase 1  USP3 - ubiquitin specific peptidase 3  SUMO1 - small ubiquitin-like modifier 1  BABAM1 - brisc and brca1 a complex member 1  UBE2T - ubiquitin-conjugating enzyme e2t (putative)  ZNF830 - zinc finger protein 830  SMURF1 - smad specific e3 ubiquitin protein ligase 1  SETD2 - set domain containing 2  RBX1 - ring-box 1, e3 ubiquitin protein ligase  STX2 - syntaxin 2  MRE11A - mre11 meiotic recombination 11 homolog a (s. cerevisiae)  UBE2V2 - ubiquitin-conjugating enzyme e2 variant 2  ERCC2 - excision repair cross-complementing rodent repair deficiency, complementation group 2  UBE2N - ubiquitin-conjugating enzyme e2n  UBE2V1 - ubiquitin-conjugating enzyme e2 variant 1  PSMD6 - proteasome (prosome, macropain) 26s subunit, non-atpase, 6  FMR1 - fragile x mental retardation 1  DTX3L - deltex 3-like (drosophila)  ITFG2 - integrin alpha fg-gap repeat containing 2  TLK1 - tousled-like kinase 1  PDS5A - pds5, regulator of cohesion maintenance, homolog a (s. cerevisiae)  ERCC6L2 - excision repair cross-complementing rodent repair deficiency, complementation group 6-like 2  SIK2 - salt-inducible kinase 2  FOXO3 - forkhead box o3  AP5Z1 - adaptor-related protein complex 5, zeta 1 subunit  TDG - thymine-dna glycosylase  OGG1 - 8-oxoguanine dna glycosylase  PRDX2 - peroxiredoxin 2  FBXW11 - f-box and wd repeat domain containing 11  CLEC16A - c-type lectin domain family 16, member a  OPA1 - optic atrophy 1 (autosomal dominant)  UBXN6 - ubx domain protein 6  APITD1 - apoptosis-inducing, taf9-like domain 1  TAF9 - taf9 rna polymerase ii, tata box binding protein (tbp)-associated factor, 32kda  SMCHD1 - structural maintenance of chromosomes flexible hinge domain containing 1  BCLAF1 - bcl2-associated transcription factor 1  DAB2IP - dab2 interacting protein  FGF1 - fibroblast growth factor 1 (acidic)  ATG13 - autophagy related 13  SZT2 - seizure threshold 2 homolog (mouse)  NR4A2 - nuclear receptor subfamily 4, group a, member 2  FEN1 - flap structure-specific endonuclease 1  FER - fer (fps/fes related) tyrosine kinase  MAP3K7 - mitogen-activated protein kinase kinase kinase 7  CREBRF - creb3 regulatory factor  INO80E - ino80 complex subunit e  NPM1 - nucleophosmin (nucleolar phosphoprotein b23, numatrin)  UFL1 - ufm1-specific ligase 1  DMAP1 - dna methyltransferase 1 associated protein 1  CUL7 - cullin 7  TCEA1 - transcription elongation factor a (sii), 1  UBQLN1 - ubiquilin 1  DONSON - downstream neighbor of son  NRBF2 - nuclear receptor binding factor 2  NONO - non-pou domain containing, octamer-binding  CDIP1 - cell death-inducing p53 target 1  MTOR - mechanistic target of rapamycin (serine/threonine kinase)  RNF34 - ring finger protein 34, e3 ubiquitin protein ligase  MUS81 - mus81 structure-specific endonuclease subunit  UBQLN2 - ubiquilin 2  NDNL2 - necdin-like 2  ATP13A2 - atpase type 13a2  TLN1 - talin 1  ERO1L - ero1-like (s. cerevisiae)  HERPUD1 - homocysteine-inducible, endoplasmic reticulum stress-inducible, ubiquitin-like domain member 1  AQR - aquarius homolog (mouse)  CNOT2 - ccr4-not transcription complex, subunit 2  CNOT4 - ccr4-not transcription complex, subunit 4  CNOT3 - ccr4-not transcription complex, subunit 3  SIRT1 - sirtuin 1  NOTCH1 - notch 1  CTC1 - cts telomere maintenance complex component 1  NFRKB - nuclear factor related to kappab binding protein  CADPS2 - ca++-dependent secretion activator 2  ACTR8 - arp8 actin-related protein 8 homolog (yeast)  CXXC1 - cxxc finger protein 1  NFKBIA - nuclear factor of kappa light polypeptide gene enhancer in b-cells inhibitor, alpha  CBX5 - chromobox homolog 5  NFKB1 - nuclear factor of kappa light polypeptide gene enhancer in b-cells 1  BRD4 - bromodomain containing 4  FBXO44 - f-box protein 44  PIK3R4 - phosphoinositide-3-kinase, regulatory subunit 4  TARDBP - tar dna binding protein  TNFRSF1A - tumor necrosis factor receptor superfamily, member 1a  TNFRSF1B - tumor necrosis factor receptor superfamily, member 1b  MDC1 - mediator of dna-damage checkpoint 1  TMBIM6 - transmembrane bax inhibitor motif containing 6  MINK1 - misshapen-like kinase 1  SOCS5 - suppressor of cytokine signaling 5  RRM2B - ribonucleotide reductase m2 b (tp53 inducible)  NEFL - neurofilament, light polypeptide  SMARCAL1 - swi/snf related, matrix associated, actin dependent regulator of chromatin, subfamily a-like 1  NEK1 - nima-related kinase 1  TERF2 - telomeric repeat binding factor 2  CPEB4 - cytoplasmic polyadenylation element binding protein 4  TFDP1 - transcription factor dp-1  NFE2L2 - nuclear factor (erythroid-derived 2)-like 2  MORF4L2 - mortality factor 4 like 2  NFATC4 - nuclear factor of activated t-cells, cytoplasmic, calcineurin-dependent 4  BCCIP - brca2 and cdkn1a interacting protein  PDGFD - platelet derived growth factor d  ZFYVE26 - zinc finger, fyve domain containing 26  FXN - frataxin  NF1 - neurofibromin 1  FNIP1 - folliculin interacting protein 1  CDK5RAP3 - cdk5 regulatory subunit associated protein 3  EDEM1 - er degradation enhancer, mannosidase alpha-like 1  MAPK8IP2 - mitogen-activated protein kinase 8 interacting protein 2  TP53INP1 - tumor protein p53 inducible nuclear protein 1  CEP63 - centrosomal protein 63kda  DEPDC5 - dep domain containing 5  FOS - fbj murine osteosarcoma viral oncogene homolog  WDR45B - wd repeat domain 45b  THBS1 - thrombospondin 1  COPS7A - cop9 signalosome subunit 7a  HP1BP3 - heterochromatin protein 1, binding protein 3  METTL3 - methyltransferase like 3  KIAA0430 - kiaa0430 |
| GO:0045935 | positive regulation of nucleobase-containing compound metabolic process | 5.8E-5 | 5.73E-3 | 1.07 (10334,1203,6996,873) | [+] Show genes  ELL - elongation factor rna polymerase ii  EXOSC9 - exosome component 9  TADA1 - transcriptional adaptor 1  ABL1 - c-abl oncogene 1, non-receptor tyrosine kinase  PML - promyelocytic leukemia  MLLT11 - myeloid/lymphoid or mixed-lineage leukemia (trithorax homolog, drosophila); translocated to, 11  OBFC1 - oligonucleotide/oligosaccharide-binding fold containing 1  NRIP1 - nuclear receptor interacting protein 1  KAT2A - k(lysine) acetyltransferase 2a  NCOA3 - nuclear receptor coactivator 3  BRD8 - bromodomain containing 8  CHD7 - chromodomain helicase dna binding protein 7  TCERG1 - transcription elongation regulator 1  TAF15 - taf15 rna polymerase ii, tata box binding protein (tbp)-associated factor, 68kda  SPIN1 - spindlin 1  TASP1 - taspase, threonine aspartase, 1  SUB1 - sub1 homolog (s. cerevisiae)  ACTL6A - actin-like 6a  AXIN1 - axin 1  ACTN1 - actinin, alpha 1  PICALM - phosphatidylinositol binding clathrin assembly protein  ACTN4 - actinin, alpha 4  ACVR2A - activin a receptor, type iia  ACVRL1 - activin a receptor type ii-like 1  ACVR1 - activin a receptor, type i  ARID1A - at rich interactive domain 1a (swi-like)  ACVR1B - activin a receptor, type ib  DLL1 - delta-like 1 (drosophila)  MED1 - mediator complex subunit 1  FZD4 - frizzled family receptor 4  FZD1 - frizzled family receptor 1  NUP85 - nucleoporin 85kda  CDC7 - cell division cycle 7  PPARD - peroxisome proliferator-activated receptor delta  USP9X - ubiquitin specific peptidase 9, x-linked  MAML3 - mastermind-like 3 (drosophila)  PARP1 - poly (adp-ribose) polymerase 1  PHF17 - phd finger protein 17  POLR2G - polymerase (rna) ii (dna directed) polypeptide g  RUVBL2 - ruvb-like 2 (e. coli)  POLR2A - polymerase (rna) ii (dna directed) polypeptide a, 220kda  CDK5RAP2 - cdk5 regulatory subunit associated protein 2  NELFE - negative elongation factor complex member e  NR5A1 - nuclear receptor subfamily 5, group a, member 1  FUS - fused in sarcoma  JAG1 - jagged 1  PRDM4 - pr domain containing 4  PPP3R1 - protein phosphatase 3, regulatory subunit b, alpha  ATF7IP - activating transcription factor 7 interacting protein  PPP2R5B - protein phosphatase 2, regulatory subunit b', beta  AKT1 - v-akt murine thymoma viral oncogene homolog 1  USP7 - ubiquitin specific peptidase 7 (herpes virus-associated)  PCID2 - pci domain containing 2  RNF168 - ring finger protein 168, e3 ubiquitin protein ligase  XRCC6 - x-ray repair complementing defective repair in chinese hamster cells 6  RIOK2 - rio kinase 2  GABPB1 - ga binding protein transcription factor, beta subunit 1  NUDT21 - nudix (nucleoside diphosphate linked moiety x)-type motif 21  GABPA - ga binding protein transcription factor, alpha subunit 60kda  WWP2 - ww domain containing e3 ubiquitin protein ligase 2  AHR - aryl hydrocarbon receptor  PPP1R10 - protein phosphatase 1, regulatory subunit 10  DDX39B - dead (asp-glu-ala-asp) box polypeptide 39b  KANSL3 - kat8 regulatory nsl complex subunit 3  KMT2D - lysine (k)-specific methyltransferase 2d  MAP2K5 - mitogen-activated protein kinase kinase 5  MAP2K7 - mitogen-activated protein kinase kinase 7  PIM2 - pim-2 oncogene  YEATS4 - yeats domain containing 4  MAP2K1 - mitogen-activated protein kinase kinase 1  MAP2K2 - mitogen-activated protein kinase kinase 2  ZFPM1 - zinc finger protein, fog family member 1  MAPK7 - mitogen-activated protein kinase 7  GCN1L1 - gcn1 general control of amino-acid synthesis 1-like 1 (yeast)  MAPK3 - mitogen-activated protein kinase 3  GATA2 - gata binding protein 2  MAPK1 - mitogen-activated protein kinase 1  KAT6A - k(lysine) acetyltransferase 6a  PRKD1 - protein kinase d1  ADIRF - adipogenesis regulatory factor  ANXA3 - annexin a3  PRKCB - protein kinase c, beta  NR4A3 - nuclear receptor subfamily 4, group a, member 3  PRKCD - protein kinase c, delta  RBM22 - rna binding motif protein 22  CARF - calcium responsive transcription factor  NCOA4 - nuclear receptor coactivator 4  PKIB - protein kinase (camp-dependent, catalytic) inhibitor beta  MLLT10 - myeloid/lymphoid or mixed-lineage leukemia (trithorax homolog, drosophila); translocated to, 10  APEX1 - apex nuclease (multifunctional dna repair enzyme) 1  DHX36 - deah (asp-glu-ala-his) box polypeptide 36  RBM23 - rna binding motif protein 23  PARN - poly(a)-specific ribonuclease  BIRC2 - baculoviral iap repeat containing 2  ZNF175 - zinc finger protein 175  SSBP4 - single stranded dna binding protein 4  GTF2F1 - general transcription factor iif, polypeptide 1, 74kda  PBX2 - pre-b-cell leukemia homeobox 2  PBX3 - pre-b-cell leukemia homeobox 3  PBX1 - pre-b-cell leukemia homeobox 1  ZBTB17 - zinc finger and btb domain containing 17  PCBD1 - pterin-4 alpha-carbinolamine dehydratase/dimerization cofactor of hepatocyte nuclear factor 1 alpha  GTF2H1 - general transcription factor iih, polypeptide 1, 62kda  VEZF1 - vascular endothelial zinc finger 1  GTF2B - general transcription factor iib  APBB1 - amyloid beta (a4) precursor protein-binding, family b, member 1 (fe65)  ZNF131 - zinc finger protein 131  RIF1 - rap1 interacting factor homolog (yeast)  TBL1XR1 - transducin (beta)-like 1 x-linked receptor 1  POLG2 - polymerase (dna directed), gamma 2, accessory subunit  GTF2I - general transcription factor iii  KIAA1551 - kiaa1551  BRF1 - brf1, rna polymerase iii transcription initiation factor 90 kda subunit  ARF4 - adp-ribosylation factor 4  APP - amyloid beta (a4) precursor protein  PAK3 - p21 protein (cdc42/rac)-activated kinase 3  ZNF76 - zinc finger protein 76  ARNTL - aryl hydrocarbon receptor nuclear translocator-like  CELF3 - cugbp, elav-like family member 3  ARNT - aryl hydrocarbon receptor nuclear translocator  BRPF1 - bromodomain and phd finger containing, 1  H2AFZ - h2a histone family, member z  FZD5 - frizzled family receptor 5  H2AFX - h2a histone family, member x  PAX8 - paired box 8  SUPT16H - suppressor of ty 16 homolog (s. cerevisiae)  PDGFRB - platelet-derived growth factor receptor, beta polypeptide  ARRB2 - arrestin, beta 2  ARRB1 - arrestin, beta 1  CDC73 - cell division cycle 73  PDGFB - platelet-derived growth factor beta polypeptide  HCFC1 - host cell factor c1 (vp16-accessory protein)  PCNA - proliferating cell nuclear antigen  PRDM2 - pr domain containing 2, with znf domain  MAP3K12 - mitogen-activated protein kinase kinase kinase 12  AKIRIN2 - akirin 2  HEATR1 - heat repeat containing 1  LEO1 - leo1, paf1/rna polymerase ii complex component, homolog (s. cerevisiae)  ERLIN2 - er lipid raft associated 2  PWP1 - pwp1 homolog (s. cerevisiae)  WRAP53 - wd repeat containing, antisense to tp53  HDAC2 - histone deacetylase 2  HMBOX1 - homeobox containing 1  ZCCHC12 - zinc finger, cchc domain containing 12  HDAC1 - histone deacetylase 1  HCLS1 - hematopoietic cell-specific lyn substrate 1  ASCL1 - achaete-scute complex homolog 1 (drosophila)  KAT7 - k(lysine) acetyltransferase 7  RGMB - rgm domain family, member b  SBNO2 - strawberry notch homolog 2 (drosophila)  XPA - xeroderma pigmentosum, complementation group a  XPC - xeroderma pigmentosum, complementation group c  XRCC1 - x-ray repair complementing defective repair in chinese hamster cells 1  PAGR1 - paxip1 associated glutamate-rich protein 1  PHF2 - phd finger protein 2  MORC2 - morc family cw-type zinc finger 2  FOXJ3 - forkhead box j3  RERE - arginine-glutamic acid dipeptide (re) repeats  ZXDC - zxd family zinc finger c  AAED1 - ahpc/tsa antioxidant enzyme domain containing 1  XBP1 - x-box binding protein 1  ZFHX3 - zinc finger homeobox 3  WHSC1 - wolf-hirschhorn syndrome candidate 1  ZNF507 - zinc finger protein 507  LGR4 - leucine-rich repeat containing g protein-coupled receptor 4  ARID2 - at rich interactive domain 2 (arid, rfx-like)  BAX - bcl2-associated x protein  PKD1 - polycystic kidney disease 1 (autosomal dominant)  SCAF8 - sr-related ctd-associated factor 8  PKD2 - polycystic kidney disease 2 (autosomal dominant)  MTF2 - metal response element binding transcription factor 2  BACH1 - btb and cnc homology 1, basic leucine zipper transcription factor 1  PIN1 - peptidylprolyl cis/trans isomerase, nima-interacting 1  PIK3R2 - phosphoinositide-3-kinase, regulatory subunit 2 (beta)  BRCC3 - brca1/brca2-containing complex, subunit 3  PIK3R1 - phosphoinositide-3-kinase, regulatory subunit 1 (alpha)  MZF1 - myeloid zinc finger 1  ASXL2 - additional sex combs like 2 (drosophila)  PLAGL1 - pleiomorphic adenoma gene-like 1  ASXL1 - additional sex combs like 1 (drosophila)  TNIP2 - tnfaip3 interacting protein 2  ATRX - alpha thalassemia/mental retardation syndrome x-linked  PKNOX1 - pbx/knotted 1 homeobox 1  ZNF24 - zinc finger protein 24  PHF10 - phd finger protein 10  U2AF2 - u2 small nuclear rna auxiliary factor 2  ZNF711 - zinc finger protein 711  BCL9 - b-cell cll/lymphoma 9  YES1 - v-yes-1 yamaguchi sarcoma viral oncogene homolog 1  PIM1 - pim-1 oncogene  XRCC5 - x-ray repair complementing defective repair in chinese hamster cells 5 (double-strand-break rejoining)  ECD - ecdysoneless homolog (drosophila)  GRSF1 - g-rich rna sequence binding factor 1  GSK3A - glycogen synthase kinase 3 alpha  PARK7 - parkinson protein 7  PNKP - polynucleotide kinase 3'-phosphatase  CCNK - cyclin k  RFC5 - replication factor c (activator 1) 5, 36.5kda  HSF2 - heat shock transcription factor 2  RFC2 - replication factor c (activator 1) 2, 40kda  RFC3 - replication factor c (activator 1) 3, 38kda  TRIM24 - tripartite motif containing 24  RFXAP - regulatory factor x-associated protein  RFX5 - regulatory factor x, 5 (influences hla class ii expression)  WHSC1L1 - wolf-hirschhorn syndrome candidate 1-like 1  FLCN - folliculin  RELA - v-rel avian reticuloendotheliosis viral oncogene homolog a  PRMT2 - protein arginine methyltransferase 2  ZFP36L2 - zfp36 ring finger protein-like 2  ZFP36L1 - zfp36 ring finger protein-like 1  RET - ret proto-oncogene  DPF2 - d4, zinc and double phd fingers family 2  UPF1 - upf1 regulator of nonsense transcripts homolog (yeast)  METTL16 - methyltransferase like 16  FTO - fat mass and obesity associated  RLF - rearranged l-myc fusion  LDB1 - lim domain binding 1  WDR77 - wd repeat domain 77  BMPR1A - bone morphogenetic protein receptor, type ia  DSCC1 - dna replication and sister chromatid cohesion 1  BMPR2 - bone morphogenetic protein receptor, type ii (serine/threonine kinase)  BMPR1B - bone morphogenetic protein receptor, type ib  KAT2B - k(lysine) acetyltransferase 2b  BMP7 - bone morphogenetic protein 7  CRTC2 - creb regulated transcription coactivator 2  ZNF259 - zinc finger protein 259  BCAS3 - breast carcinoma amplified sequence 3  NABP2 - nucleic acid binding protein 2  AHI1 - abelson helper integration site 1  RNF4 - ring finger protein 4  HNRNPR - heterogeneous nuclear ribonucleoprotein r  BUD31 - bud31 homolog (s. cerevisiae)  RNF6 - ring finger protein (c3h2c3 type) 6  ROCK1 - rho-associated, coiled-coil containing protein kinase 1  RNF111 - ring finger protein 111  TET2 - tet methylcytosine dioxygenase 2  BCL10 - b-cell cll/lymphoma 10  SF3B4 - splicing factor 3b, subunit 4, 49kda  FUBP3 - far upstream element (fuse) binding protein 3  HSP90AA1 - heat shock protein 90kda alpha (cytosolic), class a member 1  BTRC - beta-transducin repeat containing e3 ubiquitin protein ligase  HSPA8 - heat shock 70kda protein 8  TRIM44 - tripartite motif containing 44  MCRS1 - microspherule protein 1  HMGN1 - high mobility group nucleosome binding domain 1  HMGB3 - high mobility group box 3  HMGB2 - high mobility group box 2  HMGB1 - high mobility group box 1  TNKS - tankyrase, trf1-interacting ankyrin-related adp-ribose polymerase  DAZAP1 - daz associated protein 1  SLC30A9 - solute carrier family 30 (zinc transporter), member 9  AGO1 - argonaute risc catalytic component 1  MAD2L2 - mad2 mitotic arrest deficient-like 2 (yeast)  CAPRIN2 - caprin family member 2  PID1 - phosphotyrosine interaction domain containing 1  PHIP - pleckstrin homology domain interacting protein  ZBTB48 - zinc finger and btb domain containing 48  HYAL2 - hyaluronoglucosaminidase 2  ACD - adrenocortical dysplasia homolog (mouse)  GPBP1 - gc-rich promoter binding protein 1  TADA3 - transcriptional adaptor 3  NCOA2 - nuclear receptor coactivator 2  HIVEP1 - human immunodeficiency virus type i enhancer binding protein 1  CREB3 - camp responsive element binding protein 3  HIF1A - hypoxia inducible factor 1, alpha subunit (basic helix-loop-helix transcription factor)  LPIN3 - lipin 3  CITED2 - cbp/p300-interacting transactivator, with glu/asp-rich carboxy-terminal domain, 2  CCT6A - chaperonin containing tcp1, subunit 6a (zeta 1)  CCNT2 - cyclin t2  MYCBP - myc binding protein  CCNT1 - cyclin t1  BANP - btg3 associated nuclear protein  HNRNPU - heterogeneous nuclear ribonucleoprotein u (scaffold attachment factor a)  RIPK1 - receptor (tnfrsf)-interacting serine-threonine kinase 1  HNRNPK - heterogeneous nuclear ribonucleoprotein k  TNFSF13 - tumor necrosis factor (ligand) superfamily, member 13  FBXO4 - f-box protein 4  RPS6KA1 - ribosomal protein s6 kinase, 90kda, polypeptide 1  CBFB - core-binding factor, beta subunit  HNRNPA2B1 - heterogeneous nuclear ribonucleoprotein a2/b1  HNRNPA1 - heterogeneous nuclear ribonucleoprotein a1  RIPK2 - receptor-interacting serine-threonine kinase 2  HNRNPAB - heterogeneous nuclear ribonucleoprotein a/b  RXRB - retinoid x receptor, beta  HNRNPD - heterogeneous nuclear ribonucleoprotein d (au-rich element rna binding protein 1, 37kda)  RBM14 - rna binding motif protein 14  CCNC - cyclin c  HMGA1 - high mobility group at-hook 1  FAM129B - family with sequence similarity 129, member b  NR4A1 - nuclear receptor subfamily 4, group a, member 1  CHTF8 - ctf8, chromosome transmission fidelity factor 8 homolog (s. cerevisiae)  FOXK2 - forkhead box k2  CDC5L - cell division cycle 5-like  CRTC3 - creb regulated transcription coactivator 3  RBM15 - rna binding motif protein 15  TNRC6A - trinucleotide repeat containing 6a  ERLIN1 - er lipid raft associated 1  RBMX - rna binding motif protein, x-linked  NCOA7 - nuclear receptor coactivator 7  PSEN1 - presenilin 1  ING2 - inhibitor of growth family, member 2  CREB3L2 - camp responsive element binding protein 3-like 2  USP16 - ubiquitin specific peptidase 16  IPPK - inositol 1,3,4,5,6-pentakisphosphate 2-kinase  ILF2 - interleukin enhancer binding factor 2  ILF3 - interleukin enhancer binding factor 3, 90kda  PRPF19 - pre-mrna processing factor 19  CD81 - cd81 molecule  ILK - integrin-linked kinase  CAMKK2 - calcium/calmodulin-dependent protein kinase kinase 2, beta  PIAS1 - protein inhibitor of activated stat, 1  SMYD3 - set and mynd domain containing 3  MAPKAPK5 - mitogen-activated protein kinase-activated protein kinase 5  LMO4 - lim domain only 4  PSMC6 - proteasome (prosome, macropain) 26s subunit, atpase, 6  TAF6L - taf6-like rna polymerase ii, p300/cbp-associated factor (pcaf)-associated factor, 65kda  PSMC4 - proteasome (prosome, macropain) 26s subunit, atpase, 4  CAMK1 - calcium/calmodulin-dependent protein kinase i  SOX18 - sry (sex determining region y)-box 18  PSMC2 - proteasome (prosome, macropain) 26s subunit, atpase, 2  PSMD9 - proteasome (prosome, macropain) 26s subunit, non-atpase, 9  CSRNP1 - cysteine-serine-rich nuclear protein 1  SLC38A2 - solute carrier family 38, member 2  JMY - junction mediating and regulatory protein, p53 cofactor  STK16 - serine/threonine kinase 16  FBLN5 - fibulin 5  KHSRP - kh-type splicing regulatory protein  CASK - calcium/calmodulin-dependent serine protein kinase (maguk family)  DDX17 - dead (asp-glu-ala-asp) box helicase 17  KAT5 - k(lysine) acetyltransferase 5  CEBPG - ccaat/enhancer binding protein (c/ebp), gamma  SKAP1 - src kinase associated phosphoprotein 1  CDK9 - cyclin-dependent kinase 9  CCT7 - chaperonin containing tcp1, subunit 7 (eta)  CDK8 - cyclin-dependent kinase 8  INSR - insulin receptor  CDK7 - cyclin-dependent kinase 7  CDKN1C - cyclin-dependent kinase inhibitor 1c (p57, kip2)  CDK13 - cyclin-dependent kinase 13  CCT2 - chaperonin containing tcp1, subunit 2 (beta)  CCT4 - chaperonin containing tcp1, subunit 4 (delta)  RFXANK - regulatory factor x-associated ankyrin-containing protein  IRF2 - interferon regulatory factor 2  KLF7 - kruppel-like factor 7 (ubiquitous)  IRF3 - interferon regulatory factor 3  HTATIP2 - hiv-1 tat interactive protein 2, 30kda  RUVBL1 - ruvb-like 1 (e. coli)  DIMT1 - dim1 dimethyladenosine transferase 1 homolog (s. cerevisiae)  MED19 - mediator complex subunit 19  ITGA6 - integrin, alpha 6  CDK2 - cyclin-dependent kinase 2  RRN3 - rrn3 rna polymerase i transcription factor homolog (s. cerevisiae)  CACYBP - calcyclin binding protein  NSD1 - nuclear receptor binding set domain protein 1  NUDT16 - nudix (nucleoside diphosphate linked moiety x)-type motif 16  PTPRN - protein tyrosine phosphatase, receptor type, n  MMS19 - mms19 nucleotide excision repair homolog (s. cerevisiae)  MPHOSPH8 - m-phase phosphoprotein 8  SNX5 - sorting nexin 5  ID2 - inhibitor of dna binding 2, dominant negative helix-loop-helix protein  ID3 - inhibitor of dna binding 3, dominant negative helix-loop-helix protein  BRPF3 - bromodomain and phd finger containing, 3  IRF2BPL - interferon regulatory factor 2 binding protein-like  FOXK1 - forkhead box k1  PAF1 - paf1, rna polymerase ii associated factor, homolog (s. cerevisiae)  RAC1 - ras-related c3 botulinum toxin substrate 1 (rho family, small gtp binding protein rac1)  ZNF268 - zinc finger protein 268  INO80 - ino80 complex subunit  ZNF513 - zinc finger protein 513  GMEB1 - glucocorticoid modulatory element binding protein 1  CCT8 - chaperonin containing tcp1, subunit 8 (theta)  RBPJ - recombination signal binding protein for immunoglobulin kappa j region  NCK2 - nck adaptor protein 2  PABPC1 - poly(a) binding protein, cytoplasmic 1  FAM35A - family with sequence similarity 35, member a  RARG - retinoic acid receptor, gamma  RARB - retinoic acid receptor, beta  USP21 - ubiquitin specific peptidase 21  BCAR3 - breast cancer anti-estrogen resistance 3  TAF8 - taf8 rna polymerase ii, tata box binding protein (tbp)-associated factor, 43kda  CTCF - ccctc-binding factor (zinc finger protein)  RAI1 - retinoic acid induced 1  SMARCA5 - swi/snf related, matrix associated, actin dependent regulator of chromatin, subfamily a, member 5  MAP3K2 - mitogen-activated protein kinase kinase kinase 2  RBM3 - rna binding motif (rnp1, rrm) protein 3  OGT - o-linked n-acetylglucosamine (glcnac) transferase  RB1 - retinoblastoma 1  ARID4A - at rich interactive domain 4a (rbp1-like)  PELP1 - proline, glutamate and leucine rich protein 1  KDM5A - lysine (k)-specific demethylase 5a  ERCC8 - excision repair cross-complementing rodent repair deficiency, complementation group 8  PTGES3 - prostaglandin e synthase 3 (cytosolic)  NFAT5 - nuclear factor of activated t-cells 5, tonicity-responsive  CIRBP - cold inducible rna binding protein  FOXP1 - forkhead box p1  CHUK - conserved helix-loop-helix ubiquitous kinase  SUPT3H - suppressor of ty 3 homolog (s. cerevisiae)  PCGF5 - polycomb group ring finger 5  WAC - ww domain containing adaptor with coiled-coil  NFATC2IP - nuclear factor of activated t-cells, cytoplasmic, calcineurin-dependent 2 interacting protein  PATZ1 - poz (btb) and at hook containing zinc finger 1  METTL14 - methyltransferase like 14  IER2 - immediate early response 2  SPP1 - secreted phosphoprotein 1  SSBP2 - single-stranded dna binding protein 2  KPNA6 - karyopherin alpha 6 (importin alpha 7)  DOT1L - dot1-like histone h3k79 methyltransferase  SP3 - sp3 transcription factor  EFCAB7 - ef-hand calcium binding domain 7  SP1 - sp1 transcription factor  SCAND1 - scan domain containing 1  ARL2BP - adp-ribosylation factor-like 2 binding protein  SLX4 - slx4 structure-specific endonuclease subunit  SOX4 - sry (sex determining region y)-box 4  WBP2 - ww domain binding protein 2  GTPBP1 - gtp binding protein 1  SPIRE2 - spire actin nucleation factor 2  CREBL2 - camp responsive element binding protein-like 2  ATF6B - activating transcription factor 6 beta  CREBBP - creb binding protein  ATF2 - activating transcription factor 2  SOX13 - sry (sex determining region y)-box 13  ZC3H8 - zinc finger ccch-type containing 8  LMO1 - lim domain only 1 (rhombotin 1)  CREB1 - camp responsive element binding protein 1  DDX58 - dead (asp-glu-ala-asp) box polypeptide 58  IER5 - immediate early response 5  CLOCK - clock circadian regulator  RPRD1B - regulation of nuclear pre-mrna domain containing 1b  DDX41 - dead (asp-glu-ala-asp) box polypeptide 41  LITAF - lipopolysaccharide-induced tnf factor  SNRNP70 - small nuclear ribonucleoprotein 70kda (u1)  YTHDF2 - yth domain family, member 2  TBPL1 - tbp-like 1  TP53INP2 - tumor protein p53 inducible nuclear protein 2  SMARCE1 - swi/snf related, matrix associated, actin dependent regulator of chromatin, subfamily e, member 1  SMO - smoothened, frizzled family receptor  ACTL6B - actin-like 6b  SMARCB1 - swi/snf related, matrix associated, actin dependent regulator of chromatin, subfamily b, member 1  SMARCC1 - swi/snf related, matrix associated, actin dependent regulator of chromatin, subfamily c, member 1  HLTF - helicase-like transcription factor  SMARCA4 - swi/snf related, matrix associated, actin dependent regulator of chromatin, subfamily a, member 4  SSBP3 - single stranded dna binding protein 3  SMARCD2 - swi/snf related, matrix associated, actin dependent regulator of chromatin, subfamily d, member 2  SMARCD3 - swi/snf related, matrix associated, actin dependent regulator of chromatin, subfamily d, member 3  SMARCC2 - swi/snf related, matrix associated, actin dependent regulator of chromatin, subfamily c, member 2  SMARCD1 - swi/snf related, matrix associated, actin dependent regulator of chromatin, subfamily d, member 1  WIZ - widely interspaced zinc finger motifs  MED20 - mediator complex subunit 20  AFAP1L2 - actin filament associated protein 1-like 2  ROCK2 - rho-associated, coiled-coil containing protein kinase 2  SMARCA1 - swi/snf related, matrix associated, actin dependent regulator of chromatin, subfamily a, member 1  SMARCA2 - swi/snf related, matrix associated, actin dependent regulator of chromatin, subfamily a, member 2  CRLF3 - cytokine receptor-like factor 3  KMT2C - lysine (k)-specific methyltransferase 2c  GLYR1 - glyoxylate reductase 1 homolog (arabidopsis)  ZNF462 - zinc finger protein 462  MBTPS2 - membrane-bound transcription factor peptidase, site 2  MAGED1 - melanoma antigen family d, 1  GLIS2 - glis family zinc finger 2  MED27 - mediator complex subunit 27  MED26 - mediator complex subunit 26  CHD6 - chromodomain helicase dna binding protein 6  SENP2 - sumo1/sentrin/smt3 specific peptidase 2  CST3 - cystatin c  MED17 - mediator complex subunit 17  MED23 - mediator complex subunit 23  ZNF318 - zinc finger protein 318  PRPF6 - pre-mrna processing factor 6  SUPT6H - suppressor of ty 6 homolog (s. cerevisiae)  SUPV3L1 - suppressor of var1, 3-like 1 (s. cerevisiae)  CTBP2 - c-terminal binding protein 2  SUPT4H1 - suppressor of ty 4 homolog 1 (s. cerevisiae)  ING4 - inhibitor of growth family, member 4  SUPT5H - suppressor of ty 5 homolog (s. cerevisiae)  INSIG2 - insulin induced gene 2  EIF2AK3 - eukaryotic translation initiation factor 2-alpha kinase 3  CTNNB1 - catenin (cadherin-associated protein), beta 1, 88kda  NIF3L1 - nif3 ngg1 interacting factor 3-like 1 (s. cerevisiae)  NEK4 - nima-related kinase 4  SUV420H1 - suppressor of variegation 4-20 homolog 1 (drosophila)  NPNT - nephronectin  GPBP1L1 - gc-rich promoter binding protein 1-like 1  SUV420H2 - suppressor of variegation 4-20 homolog 2 (drosophila)  MED10 - mediator complex subunit 10  MED21 - mediator complex subunit 21  NIPBL - nipped-b homolog (drosophila)  STAT2 - signal transducer and activator of transcription 2, 113kda  STAT1 - signal transducer and activator of transcription 1, 91kda  STAT3 - signal transducer and activator of transcription 3 (acute-phase response factor)  ASF1A - anti-silencing function 1a histone chaperone  JUP - junction plakoglobin  STAT5A - signal transducer and activator of transcription 5a  STAT6 - signal transducer and activator of transcription 6, interleukin-4 induced  PARM1 - prostate androgen-regulated mucin-like protein 1  ING5 - inhibitor of growth family, member 5  ZNF496 - zinc finger protein 496  SS18 - synovial sarcoma translocation, chromosome 18  PHF5A - phd finger protein 5a  GPATCH3 - g patch domain containing 3  TRIP4 - thyroid hormone receptor interactor 4  HMGN3 - high mobility group nucleosomal binding domain 3  ZNF335 - zinc finger protein 335  BAMBI - bmp and activin membrane-bound inhibitor  ZNF639 - zinc finger protein 639  SRC - v-src avian sarcoma (schmidt-ruppin a-2) viral oncogene homolog  LEF1 - lymphoid enhancer-binding factor 1  SREBF2 - sterol regulatory element binding transcription factor 2  CNOT8 - ccr4-not transcription complex, subunit 8  CDK12 - cyclin-dependent kinase 12  DHX9 - deah (asp-glu-ala-his) box helicase 9  DDX5 - dead (asp-glu-ala-asp) box helicase 5  DDX3X - dead (asp-glu-ala-asp) box helicase 3, x-linked  ARID4B - at rich interactive domain 4b (rbp1-like)  SIN3A - sin3 transcription regulator family member a  WWTR1 - ww domain containing transcription regulator 1  SETMAR - set domain and mariner transposase fusion gene  MLH1 - mutl homolog 1, colon cancer, nonpolyposis type 2 (e. coli)  ZNF304 - zinc finger protein 304  MAP2K4 - mitogen-activated protein kinase kinase 4  ITGB1BP1 - integrin beta 1 binding protein 1  RSF1 - remodeling and spacing factor 1  NOL11 - nucleolar protein 11  MLLT6 - myeloid/lymphoid or mixed-lineage leukemia (trithorax homolog, drosophila); translocated to, 6  MLLT3 - myeloid/lymphoid or mixed-lineage leukemia (trithorax homolog, drosophila); translocated to, 3  MED14 - mediator complex subunit 14  DBP - d site of albumin promoter (albumin d-box) binding protein  KMT2A - lysine (k)-specific methyltransferase 2a  POT1 - protection of telomeres 1  RPS6KA5 - ribosomal protein s6 kinase, 90kda, polypeptide 5  ZBTB38 - zinc finger and btb domain containing 38  UIMC1 - ubiquitin interaction motif containing 1  MTA2 - metastasis associated 1 family, member 2  NOLC1 - nucleolar and coiled-body phosphoprotein 1  KCTD13 - potassium channel tetramerization domain containing 13  EIF2A - eukaryotic translation initiation factor 2a, 65kda  YTHDF3 - yth domain family, member 3  WWOX - ww domain containing oxidoreductase  CCPG1 - cell cycle progression 1  CNOT6 - ccr4-not transcription complex, subunit 6  ZNF451 - zinc finger protein 451  SS18L1 - synovial sarcoma translocation gene on chromosome 18-like 1  MECP2 - methyl cpg binding protein 2 (rett syndrome)  ARID1B - at rich interactive domain 1b (swi1-like)  MEF2A - myocyte enhancer factor 2a  MED30 - mediator complex subunit 30  MKL2 - mkl/myocardin-like 2  ZBED1 - zinc finger, bed-type containing 1  MEIS2 - meis homeobox 2  MEIS1 - meis homeobox 1  MAP3K4 - mitogen-activated protein kinase kinase kinase 4  MAP3K5 - mitogen-activated protein kinase kinase kinase 5  MEN1 - multiple endocrine neoplasia i  WDR75 - wd repeat domain 75  DAXX - death-domain associated protein  UTP15 - utp15, u3 small nucleolar ribonucleoprotein, homolog (s. cerevisiae)  HINFP - histone h4 transcription factor  KAT8 - k(lysine) acetyltransferase 8  MTA3 - metastasis associated 1 family, member 3  ARHGEF2 - rho/rac guanine nucleotide exchange factor (gef) 2  MAVS - mitochondrial antiviral signaling protein  RPTOR - regulatory associated protein of mtor, complex 1  SAFB - scaffold attachment factor b  PTOV1 - prostate tumor overexpressed 1  SALL2 - sal-like 2 (drosophila)  DRD2 - dopamine receptor d2  SLC9A1 - solute carrier family 9, subfamily a (nhe1, cation proton antiporter 1), member 1  RQCD1 - rcd1 required for cell differentiation1 homolog (s. pombe)  CNOT6L - ccr4-not transcription complex, subunit 6-like  MARS - methionyl-trna synthetase  IL23A - interleukin 23, alpha subunit p19  DOM3Z - dom-3 homolog z (c. elegans)  C10orf137 - chromosome 10 open reading frame 137  MTA1 - metastasis associated 1  SIRT6 - sirtuin 6  DR1 - down-regulator of transcription 1, tbp-binding (negative cofactor 2)  PPHLN1 - periphilin 1  MAPK15 - mitogen-activated protein kinase 15  SKI - v-ski avian sarcoma viral oncogene homolog  DYRK1A - dual-specificity tyrosine-(y)-phosphorylation regulated kinase 1a  MKL1 - megakaryoblastic leukemia (translocation) 1  MAX - myc associated factor x  ZMIZ2 - zinc finger, miz-type containing 2  DVL2 - dishevelled segment polarity protein 2  DVL3 - dishevelled segment polarity protein 3  LDB2 - lim domain binding 2  GIGYF2 - grb10 interacting gyf protein 2  TRMT112 - trna methyltransferase 11-2 homolog (s. cerevisiae)  ZFP90 - zfp90 zinc finger protein  EBF4 - early b-cell factor 4  ASH2L - ash2 (absent, small, or homeotic)-like (drosophila)  ZFHX2 - zinc finger homeobox 2  DTX1 - deltex homolog 1 (drosophila)  DKC1 - dyskeratosis congenita 1, dyskerin  PNPT1 - polyribonucleotide nucleotidyltransferase 1  LRP6 - low density lipoprotein receptor-related protein 6  RIOK1 - rio kinase 1  AIP - aryl hydrocarbon receptor interacting protein  LUM - lumican  SESN2 - sestrin 2  PARP9 - poly (adp-ribose) polymerase family, member 9  RNF8 - ring finger protein 8, e3 ubiquitin protein ligase  CALCOCO1 - calcium binding and coiled-coil domain 1  SFPQ - splicing factor proline/glutamine-rich  SFRP1 - secreted frizzled-related protein 1  SFRP2 - secreted frizzled-related protein 2  SMAD2 - smad family member 2  GMEB2 - glucocorticoid modulatory element binding protein 2  DNM2 - dynamin 2  SPAG8 - sperm associated antigen 8  DNMT1 - dna (cytosine-5-)-methyltransferase 1  ARMCX3 - armadillo repeat containing, x-linked 3  SRSF5 - serine/arginine-rich splicing factor 5  TRA2B - transformer 2 beta homolog (drosophila)  LARP7 - la ribonucleoprotein domain family, member 7  CHD8 - chromodomain helicase dna binding protein 8  RPS6KA4 - ribosomal protein s6 kinase, 90kda, polypeptide 4  MAFG - v-maf avian musculoaponeurotic fibrosarcoma oncogene homolog g  TNRC6C - trinucleotide repeat containing 6c  SMAD4 - smad family member 4  SMAD5 - smad family member 5  H1FX - h1 histone family, member x  SMAD7 - smad family member 7  WASL - wiskott-aldrich syndrome-like  MEIS3 - meis homeobox 3  NCBP2 - nuclear cap binding protein subunit 2, 20kda  YAF2 - yy1 associated factor 2  NCBP1 - nuclear cap binding protein subunit 1, 80kda  SIX5 - six homeobox 5  NDN - necdin, melanoma antigen (mage) family member  MAPRE3 - microtubule-associated protein, rp/eb family, member 3  NCK1 - nck adaptor protein 1  BRD7 - bromodomain containing 7  NCL - nucleolin  ELK1 - elk1, member of ets oncogene family  SPIRE1 - spire actin nucleation factor 1  NAF1 - nuclear assembly factor 1 ribonucleoprotein  CEBPZ - ccaat/enhancer binding protein (c/ebp), zeta  ELK3 - elk3, ets-domain protein (srf accessory protein 2)  ELK4 - elk4, ets-domain protein (srf accessory protein 1)  SNW1 - snw domain containing 1  SCAP - srebf chaperone  TRIM28 - tripartite motif containing 28  EPAS1 - endothelial pas domain protein 1  EP300 - e1a binding protein p300  HABP4 - hyaluronan binding protein 4  ATF6 - activating transcription factor 6  TBK1 - tank-binding kinase 1  NBN - nibrin  ENO1 - enolase 1, (alpha)  TDRD3 - tudor domain containing 3  CELF4 - cugbp, elav-like family member 4  EGR2 - early growth response 2  PPP1R12A - protein phosphatase 1, regulatory subunit 12a  CCT5 - chaperonin containing tcp1, subunit 5 (epsilon)  ZC3HAV1 - zinc finger ccch-type, antiviral 1  ALYREF - aly/ref export factor  SENP1 - sumo1/sentrin specific peptidase 1  CSRNP2 - cysteine-serine-rich nuclear protein 2  TRIM32 - tripartite motif containing 32  ELF1 - e74-like factor 1 (ets domain transcription factor)  ELF2 - e74-like factor 2 (ets domain transcription factor)  TSG101 - tumor susceptibility gene 101  MYO6 - myosin vi  ABT1 - activator of basal transcription 1  LBH - limb bud and heart development  TOPORS - topoisomerase i binding, arginine/serine-rich, e3 ubiquitin protein ligase  CNOT1 - ccr4-not transcription complex, subunit 1  TP53BP1 - tumor protein p53 binding protein 1  HCFC2 - host cell factor c2  EGR1 - early growth response 1  ZNF292 - zinc finger protein 292  CNOT7 - ccr4-not transcription complex, subunit 7  EGFR - epidermal growth factor receptor  PQBP1 - polyglutamine binding protein 1  TRA2A - transformer 2 alpha homolog (drosophila)  KDM1A - lysine (k)-specific demethylase 1a  ATXN7L3 - ataxin 7-like 3  ACTR2 - arp2 actin-related protein 2 homolog (yeast)  ACTR3 - arp3 actin-related protein 3 homolog (yeast)  HNRNPLL - heterogeneous nuclear ribonucleoprotein l-like  NCOA6 - nuclear receptor coactivator 6  E2F6 - e2f transcription factor 6  CCT3 - chaperonin containing tcp1, subunit 3 (gamma)  E4F1 - e4f transcription factor 1  E2F4 - e2f transcription factor 4, p107/p130-binding  E2F5 - e2f transcription factor 5, p130-binding  ENY2 - enhancer of yellow 2 homolog (drosophila)  RAD50 - rad50 homolog (s. cerevisiae)  RRP1B - ribosomal rna processing 1b  S1PR1 - sphingosine-1-phosphate receptor 1  SETX - senataxin  NR2C2 - nuclear receptor subfamily 2, group c, member 2  MED25 - mediator complex subunit 25  ZNF609 - zinc finger protein 609  PPRC1 - peroxisome proliferator-activated receptor gamma, coactivator-related 1  MED6 - mediator complex subunit 6  NAT14 - n-acetyltransferase 14 (gcn5-related, putative)  ZNF423 - zinc finger protein 423  USF1 - upstream transcription factor 1  PTK2B - protein tyrosine kinase 2 beta  TNRC6B - trinucleotide repeat containing 6b  BPTF - bromodomain phd finger transcription factor  PITHD1 - pith (c-terminal proteasome-interacting domain of thioredoxin-like) domain containing 1  HDAC5 - histone deacetylase 5  MTF1 - metal-regulatory transcription factor 1  SRA1 - steroid receptor rna activator 1  NR1H2 - nuclear receptor subfamily 1, group h, member 2  CAMTA2 - calmodulin binding transcription activator 2  RNF187 - ring finger protein 187  ETV5 - ets variant 5  POGZ - pogo transposable element with znf domain  MT3 - metallothionein 3  PLXND1 - plexin d1  ETV6 - ets variant 6  VHL - von hippel-lindau tumor suppressor, e3 ubiquitin protein ligase  MED16 - mediator complex subunit 16  KDM6B - lysine (k)-specific demethylase 6b  PHF8 - phd finger protein 8  TBX20 - t-box 20  CREB3L1 - camp responsive element binding protein 3-like 1  EYA3 - eyes absent homolog 3 (drosophila)  EZH1 - enhancer of zeste homolog 1 (drosophila)  F2R - coagulation factor ii (thrombin) receptor  VCP - valosin containing protein  WDR43 - wd repeat domain 43  MSH2 - muts homolog 2, colon cancer, nonpolyposis type 1 (e. coli)  CITED1 - cbp/p300-interacting transactivator, with glu/asp-rich carboxy-terminal domain, 1  ERN1 - endoplasmic reticulum to nucleus signaling 1  UBB - ubiquitin b  SUPT7L - suppressor of ty 7 (s. cerevisiae)-like  RGCC - regulator of cell cycle  TTC5 - tetratricopeptide repeat domain 5  ETV1 - ets variant 1  ETS1 - v-ets avian erythroblastosis virus e26 oncogene homolog 1  ZMIZ1 - zinc finger, miz-type containing 1  UBP1 - upstream binding protein 1 (lbp-1a)  BABAM1 - brisc and brca1 a complex member 1  FAM168A - family with sequence similarity 168, member a  UBTF - upstream binding transcription factor, rna polymerase i  CHURC1 - churchill domain containing 1  JMJD6 - jumonji domain containing 6  MED4 - mediator complex subunit 4  THOC1 - tho complex 1  MRE11A - mre11 meiotic recombination 11 homolog a (s. cerevisiae)  ERCC2 - excision repair cross-complementing rodent repair deficiency, complementation group 2  THRAP3 - thyroid hormone receptor associated protein 3  UBE2L3 - ubiquitin-conjugating enzyme e2l 3  MED12 - mediator complex subunit 12  MED13 - mediator complex subunit 13  UBE2N - ubiquitin-conjugating enzyme e2n  UBE2V1 - ubiquitin-conjugating enzyme e2 variant 1  MED24 - mediator complex subunit 24  MGA - mga, max dimerization protein  DTX3L - deltex 3-like (drosophila)  TCP1 - t-complex 1  ZEB1 - zinc finger e-box binding homeobox 1  SETDB1 - set domain, bifurcated 1  TCF12 - transcription factor 12  TCF20 - transcription factor 20 (ar1)  FLI1 - fli-1 proto-oncogene, ets transcription factor  AGRN - agrin  PLCB1 - phospholipase c, beta 1 (phosphoinositide-specific)  FOXO3 - forkhead box o3  HDAC8 - histone deacetylase 8  ASH1L - ash1 (absent, small, or homeotic)-like (drosophila)  CAND1 - cullin-associated and neddylation-dissociated 1  FBXW11 - f-box and wd repeat domain containing 11  EAPP - e2f-associated phosphoprotein  TNKS2 - tankyrase, trf1-interacting ankyrin-related adp-ribose polymerase 2  EXOSC3 - exosome component 3  FAM208A - family with sequence similarity 208, member a  KDM3A - lysine (k)-specific demethylase 3a  FGFR2 - fibroblast growth factor receptor 2  TAF9 - taf9 rna polymerase ii, tata box binding protein (tbp)-associated factor, 32kda  TAF7 - taf7 rna polymerase ii, tata box binding protein (tbp)-associated factor, 55kda  TAF6 - taf6 rna polymerase ii, tata box binding protein (tbp)-associated factor, 80kda  FGFR1 - fibroblast growth factor receptor 1  TADA2A - transcriptional adaptor 2a  SMCHD1 - structural maintenance of chromosomes flexible hinge domain containing 1  TACC1 - transforming, acidic coiled-coil containing protein 1  DAB2IP - dab2 interacting protein  BCLAF1 - bcl2-associated transcription factor 1  FGF1 - fibroblast growth factor 1 (acidic)  FGF2 - fibroblast growth factor 2 (basic)  NUP98 - nucleoporin 98kda  NR4A2 - nuclear receptor subfamily 4, group a, member 2  USP22 - ubiquitin specific peptidase 22  ZNF593 - zinc finger protein 593  ZBTB7B - zinc finger and btb domain containing 7b  NVL - nuclear vcp-like  CREBRF - creb3 regulatory factor  NPM1 - nucleophosmin (nucleolar phosphoprotein b23, numatrin)  NMD3 - nmd3 ribosome export adaptor  KMT2E - lysine (k)-specific methyltransferase 2e  ARHGEF11 - rho guanine nucleotide exchange factor (gef) 11  ZEB2 - zinc finger e-box binding homeobox 2  TAZ - tafazzin  CRTC1 - creb regulated transcription coactivator 1  TCF3 - transcription factor 3  TCF4 - transcription factor 4  NKRF - nfkb repressing factor  NRF1 - nuclear respiratory factor 1  TCEA2 - transcription elongation factor a (sii), 2  TCEA1 - transcription elongation factor a (sii), 1  RYBP - ring1 and yy1 binding protein  MTOR - mechanistic target of rapamycin (serine/threonine kinase)  PAXBP1 - pax3 and pax7 binding protein 1  MDFIC - myod family inhibitor domain containing  NOTCH4 - notch 4  IFT74 - intraflagellar transport 74 homolog (chlamydomonas)  NPAT - nuclear protein, ataxia-telangiectasia locus  TLR3 - toll-like receptor 3  CEP290 - centrosomal protein 290kda  NOS3 - nitric oxide synthase 3 (endothelial cell)  NAA15 - n(alpha)-acetyltransferase 15, nata auxiliary subunit  PUM1 - pumilio homolog 1 (drosophila)  SIRT1 - sirtuin 1  NOTCH1 - notch 1  CTC1 - cts telomere maintenance complex component 1  NOTCH3 - notch 3  MKX - mohawk homeobox  ST18 - suppression of tumorigenicity 18 (breast carcinoma) (zinc finger protein)  NFYA - nuclear transcription factor y, alpha  TMF1 - tata element modulatory factor 1  HDAC4 - histone deacetylase 4  TOX - thymocyte selection-associated high mobility group box  TMSB4X - thymosin beta 4, x-linked  HEY1 - hairy/enhancer-of-split related with yrpw motif 1  NFYC - nuclear transcription factor y, gamma  CXXC1 - cxxc finger protein 1  NFKBIA - nuclear factor of kappa light polypeptide gene enhancer in b-cells inhibitor, alpha  NFKB1 - nuclear factor of kappa light polypeptide gene enhancer in b-cells 1  KMT2B - lysine (k)-specific methyltransferase 2b  BRD4 - bromodomain containing 4  NFKBIB - nuclear factor of kappa light polypeptide gene enhancer in b-cells inhibitor, beta  TNFAIP1 - tumor necrosis factor, alpha-induced protein 1 (endothelial)  BCL9L - b-cell cll/lymphoma 9-like  TARDBP - tar dna binding protein  TNFRSF1A - tumor necrosis factor receptor superfamily, member 1a  TNFRSF1B - tumor necrosis factor receptor superfamily, member 1b  TEF - thyrotrophic embryonic factor  TFAM - transcription factor a, mitochondrial  TFDP1 - transcription factor dp-1  NFE2L2 - nuclear factor (erythroid-derived 2)-like 2  EPC1 - enhancer of polycomb homolog 1 (drosophila)  NR2F2 - nuclear receptor subfamily 2, group f, member 2  RASL11A - ras-like, family 11, member a  MORF4L2 - mortality factor 4 like 2  TFE3 - transcription factor binding to ighm enhancer 3  ISCU - iron-sulfur cluster assembly enzyme  NFATC4 - nuclear factor of activated t-cells, cytoplasmic, calcineurin-dependent 4  NR2F1 - nuclear receptor subfamily 2, group f, member 1  TFCP2 - transcription factor cp2  NFIA - nuclear factor i/a  HEY2 - hairy/enhancer-of-split related with yrpw motif 2  CDK5RAP3 - cdk5 regulatory subunit associated protein 3  TGFBR1 - transforming growth factor, beta receptor 1  TGFB2 - transforming growth factor, beta 2  TGFB3 - transforming growth factor, beta 3  TGFB1 - transforming growth factor, beta 1  TGFB1I1 - transforming growth factor beta 1 induced transcript 1  ZNF746 - zinc finger protein 746  TP53INP1 - tumor protein p53 inducible nuclear protein 1  MEPCE - methylphosphate capping enzyme  RNF20 - ring finger protein 20, e3 ubiquitin protein ligase  FOSL2 - fos-like antigen 2  FOSB - fbj murine osteosarcoma viral oncogene homolog b  FOS - fbj murine osteosarcoma viral oncogene homolog  KAT6B - k(lysine) acetyltransferase 6b  METTL3 - methyltransferase like 3  LPIN2 - lipin 2  THRA - thyroid hormone receptor, alpha |
| GO:0071840 | cellular component organization or biogenesis | 6.07E-5 | 5.95E-3 | 1.04 (10334,3573,6996,2506) | [+] Show genes  AFG3L2 - afg3 atpase family member 3-like 2 (s. cerevisiae)  AGAP2 - arfgap with gtpase domain, ankyrin repeat and ph domain 2  MSL3 - male-specific lethal 3 homolog (drosophila)  SF3A3 - splicing factor 3a, subunit 3, 60kda  TADA1 - transcriptional adaptor 1  STARD3 - star-related lipid transfer (start) domain containing 3  LRRC16A - leucine rich repeat containing 16a  ABCA1 - atp-binding cassette, sub-family a (abc1), member 1  PPP1R9A - protein phosphatase 1, regulatory subunit 9a  ABCA2 - atp-binding cassette, sub-family a (abc1), member 2  HAUS7 - haus augmin-like complex, subunit 7  PLXNB1 - plexin b1  ABL1 - c-abl oncogene 1, non-receptor tyrosine kinase  SSX2IP - synovial sarcoma, x breakpoint 2 interacting protein  TMED2 - transmembrane emp24 domain trafficking protein 2  ABR - active bcr-related  PML - promyelocytic leukemia  ACOT2 - acyl-coa thioesterase 2  ACACA - acetyl-coa carboxylase alpha  EBNA1BP2 - ebna1 binding protein 2  PMP22 - peripheral myelin protein 22  YWHAQ - tyrosine 3-monooxygenase/tryptophan 5-monooxygenase activation protein, theta polypeptide  LURAP1 - leucine rich adaptor protein 1  KIF27 - kinesin family member 27  ASCC3 - activating signal cointegrator 1 complex subunit 3  TMED10 - transmembrane emp24-like trafficking protein 10 (yeast)  BLCAP - bladder cancer associated protein  SMPD4 - sphingomyelin phosphodiesterase 4, neutral membrane (neutral sphingomyelinase-3)  BRD8 - bromodomain containing 8  TXNL4A - thioredoxin-like 4a  ACHE - acetylcholinesterase  TRAFD1 - traf-type zinc finger domain containing 1  ACOX1 - acyl-coa oxidase 1, palmitoyl  SYBU - syntabulin (syntaxin-interacting)  PLS3 - plastin 3  CHD7 - chromodomain helicase dna binding protein 7  PLTP - phospholipid transfer protein  ACTB - actin, beta  EHMT2 - euchromatic histone-lysine n-methyltransferase 2  SPIN1 - spindlin 1  ACTG1 - actin, gamma 1  MORF4L1 - mortality factor 4 like 1  PLD2 - phospholipase d2  DOCK10 - dedicator of cytokinesis 10  EHD1 - eh-domain containing 1  AMBRA1 - autophagy/beclin-1 regulator 1  CHCHD1 - coiled-coil-helix-coiled-coil-helix domain containing 1  PPID - peptidylprolyl isomerase d  ACTL6A - actin-like 6a  ACTN1 - actinin, alpha 1  AP1AR - adaptor-related protein complex 1 associated regulatory protein  PPIA - peptidylprolyl isomerase a (cyclophilin a)  ACTN4 - actinin, alpha 4  NDUFAF7 - nadh dehydrogenase (ubiquinone) complex i, assembly factor 7  ADAR - adenosine deaminase, rna-specific  RAB10 - rab10, member ras oncogene family  ADAM10 - adam metallopeptidase domain 10  ADARB1 - adenosine deaminase, rna-specific, b1  MRPS30 - mitochondrial ribosomal protein s30  MED1 - mediator complex subunit 1  ADCY1 - adenylate cyclase 1 (brain)  MMP24 - matrix metallopeptidase 24 (membrane-inserted)  RAB39B - rab39b, member ras oncogene family  ADD2 - adducin 2 (beta)  POLRMT - polymerase (rna) mitochondrial (dna directed)  ADD3 - adducin 3 (gamma)  ADD1 - adducin 1 (alpha)  PARP1 - poly (adp-ribose) polymerase 1  H2AFY2 - h2a histone family, member y2  NOP10 - nop10 ribonucleoprotein  CNTROB - centrobin, centrosomal brca2 interacting protein  POLD2 - polymerase (dna directed), delta 2, accessory subunit  SNAP47 - synaptosomal-associated protein, 47kda  POLD1 - polymerase (dna directed), delta 1, catalytic subunit  TUBGCP2 - tubulin, gamma complex associated protein 2  SMPD3 - sphingomyelin phosphodiesterase 3, neutral membrane (neutral sphingomyelinase ii)  POLR2D - polymerase (rna) ii (dna directed) polypeptide d  RUVBL2 - ruvb-like 2 (e. coli)  AP2A2 - adaptor-related protein complex 2, alpha 2 subunit  TMEM30A - transmembrane protein 30a  AP2A1 - adaptor-related protein complex 2, alpha 1 subunit  AP2B1 - adaptor-related protein complex 2, beta 1 subunit  CDK5RAP2 - cdk5 regulatory subunit associated protein 2  KATNA1 - katanin p60 (atpase containing) subunit a 1  RCOR3 - rest corepressor 3  PRKACB - protein kinase, camp-dependent, catalytic, beta  AP1G1 - adaptor-related protein complex 1, gamma 1 subunit  PRKACA - protein kinase, camp-dependent, catalytic, alpha  WDR12 - wd repeat domain 12  NUP133 - nucleoporin 133kda  SRGN - serglycin  AGK - acylglycerol kinase  KCTD12 - potassium channel tetramerization domain containing 12  PRIM2 - primase, dna, polypeptide 2 (58kda)  PRIM1 - primase, dna, polypeptide 1 (49kda)  VPS35 - vacuolar protein sorting 35 homolog (s. cerevisiae)  ENAH - enabled homolog (drosophila)  KIF3A - kinesin family member 3a  COA5 - cytochrome c oxidase assembly factor 5  PARVA - parvin, alpha  ZWINT - zw10 interacting kinetochore protein  COA1 - cytochrome c oxidase assembly factor 1 homolog (s. cerevisiae)  PRDM4 - pr domain containing 4  PPP3R1 - protein phosphatase 3, regulatory subunit b, alpha  CIT - citron (rho-interacting, serine/threonine kinase 21)  FGFR1OP - fgfr1 oncogene partner  PPP5C - protein phosphatase 5, catalytic subunit  PPP6C - protein phosphatase 6, catalytic subunit  PPT1 - palmitoyl-protein thioesterase 1  DNAJC11 - dnaj (hsp40) homolog, subfamily c, member 11  CSGALNACT1 - chondroitin sulfate n-acetylgalactosaminyltransferase 1  AKT1 - v-akt murine thymoma viral oncogene homolog 1  PCID2 - pci domain containing 2  DDX28 - dead (asp-glu-ala-asp) box polypeptide 28  PPP2R2A - protein phosphatase 2, regulatory subunit b, alpha  CPSF6 - cleavage and polyadenylation specific factor 6, 68kda  RIOK2 - rio kinase 2  PPP2R1A - protein phosphatase 2, regulatory subunit a, alpha  NUDT21 - nudix (nucleoside diphosphate linked moiety x)-type motif 21  TBC1D23 - tbc1 domain family, member 23  TRIOBP - trio and f-actin binding protein  PPP2CA - protein phosphatase 2, catalytic subunit, alpha isozyme  PPP2CB - protein phosphatase 2, catalytic subunit, beta isozyme  IFT122 - intraflagellar transport 122 homolog (chlamydomonas)  RMI2 - recq mediated genome instability 2  TTC17 - tetratricopeptide repeat domain 17  STMN2 - stathmin-like 2  LYSMD3 - lysm, putative peptidoglycan-binding, domain containing 3  ESCO1 - establishment of sister chromatid cohesion n-acetyltransferase 1  KANSL3 - kat8 regulatory nsl complex subunit 3  TDRKH - tudor and kh domain containing  TUBGCP5 - tubulin, gamma complex associated protein 5  PEX26 - peroxisomal biogenesis factor 26  RAB35 - rab35, member ras oncogene family  IFT27 - intraflagellar transport 27 homolog (chlamydomonas)  FMNL2 - formin-like 2  PRNP - prion protein  GPRIN1 - g protein regulated inducer of neurite outgrowth 1  NPLOC4 - nuclear protein localization 4 homolog (s. cerevisiae)  PRPF40A - prp40 pre-mrna processing factor 40 homolog a (s. cerevisiae)  PAPD7 - pap associated domain containing 7  TMEM127 - transmembrane protein 127  PIM2 - pim-2 oncogene  RAB31 - rab31, member ras oncogene family  DSTN - destrin (actin depolymerizing factor)  MAPK10 - mitogen-activated protein kinase 10  IMMT - inner membrane protein, mitochondrial  MAPK8 - mitogen-activated protein kinase 8  MAPK3 - mitogen-activated protein kinase 3  WDR11 - wd repeat domain 11  CISD2 - cdgsh iron sulfur domain 2  MAPRE2 - microtubule-associated protein, rp/eb family, member 2  AMPH - amphiphysin  COPS6 - cop9 signalosome subunit 6  CLP1 - cleavage and polyadenylation factor i subunit 1  OSBPL8 - oxysterol binding protein-like 8  OSBPL5 - oxysterol binding protein-like 5  NDC1 - ndc1 transmembrane nucleoporin  PRKD1 - protein kinase d1  HAUS1 - haus augmin-like complex, subunit 1  CCDC88A - coiled-coil domain containing 88a  PKN2 - protein kinase n2  TLK2 - tousled-like kinase 2  PRKCI - protein kinase c, iota  ANXA2 - annexin a2  PRKCE - protein kinase c, epsilon  PRKCB - protein kinase c, beta  RADIL - ras association and dil domains  PRKCD - protein kinase c, delta  SLC25A5 - solute carrier family 25 (mitochondrial carrier; adenine nucleotide translocator), member 5  PRKCA - protein kinase c, alpha  PRKAR1A - protein kinase, camp-dependent, regulatory, type i, alpha  LUC7L - luc7-like (s. cerevisiae)  YEATS2 - yeats domain containing 2  APEX1 - apex nuclease (multifunctional dna repair enzyme) 1  CCDC78 - coiled-coil domain containing 78  MSTO1 - misato 1, mitochondrial distribution and morphology regulator  XIAP - x-linked inhibitor of apoptosis  SDAD1 - sda1 domain containing 1  ATXN2L - ataxin 2-like  NOXO1 - nadph oxidase organizer 1  RFWD3 - ring finger and wd repeat domain 3  TREX1 - three prime repair exonuclease 1  SHQ1 - shq1, h/aca ribonucleoprotein assembly factor  PDCL - phosducin-like  TMEM33 - transmembrane protein 33  PRMT6 - protein arginine methyltransferase 6  MSL2 - male-specific lethal 2 homolog (drosophila)  MRPS10 - mitochondrial ribosomal protein s10  TBCCD1 - tbcc domain containing 1  PACSIN2 - protein kinase c and casein kinase substrate in neurons 2  FOPNL - fgfr1op n-terminal like  APBA1 - amyloid beta (a4) precursor protein-binding, family a, member 1  APBB2 - amyloid beta (a4) precursor protein-binding, family b, member 2  APC - adenomatous polyposis coli  APBB1 - amyloid beta (a4) precursor protein-binding, family b, member 1 (fe65)  CHP1 - calcineurin-like ef-hand protein 1  RIF1 - rap1 interacting factor homolog (yeast)  VPS13D - vacuolar protein sorting 13 homolog d (s. cerevisiae)  SLC25A36 - solute carrier family 25 (pyrimidine nucleotide carrier ), member 36  HPS5 - hermansky-pudlak syndrome 5  POLG2 - polymerase (dna directed), gamma 2, accessory subunit  PDCD10 - programmed cell death 10  PBRM1 - polybromo 1  P4HA1 - prolyl 4-hydroxylase, alpha polypeptide i  APPL2 - adaptor protein, phosphotyrosine interaction, ph domain and leucine zipper containing 2  ARF1 - adp-ribosylation factor 1  MAP1S - microtubule-associated protein 1s  PAFAH1B1 - platelet-activating factor acetylhydrolase 1b, regulatory subunit 1 (45kda)  GOLPH3L - golgi phosphoprotein 3-like  APOD - apolipoprotein d  TMEM170A - transmembrane protein 170a  SETD5 - set domain containing 5  AKAP11 - a kinase (prka) anchor protein 11  APP - amyloid beta (a4) precursor protein  BBS7 - bardet-biedl syndrome 7  DDX20 - dead (asp-glu-ala-asp) box polypeptide 20  PAK2 - p21 protein (cdc42/rac)-activated kinase 2  RCBTB1 - regulator of chromosome condensation (rcc1) and btb (poz) domain containing protein 1  PAK3 - p21 protein (cdc42/rac)-activated kinase 3  PALM - paralemmin  MRPL3 - mitochondrial ribosomal protein l3  CEP250 - centrosomal protein 250kda  PDGFA - platelet-derived growth factor alpha polypeptide  CELF3 - cugbp, elav-like family member 3  ARL3 - adp-ribosylation factor-like 3  NISCH - nischarin  TRAPPC6B - trafficking protein particle complex 6b  ARL2 - adp-ribosylation factor-like 2  ARL1 - adp-ribosylation factor-like 1  NSMCE2 - non-smc element 2, mms21 homolog (s. cerevisiae)  SUPT16H - suppressor of ty 16 homolog (s. cerevisiae)  SEC23IP - sec23 interacting protein  ARSB - arylsulfatase b  PDGFRB - platelet-derived growth factor receptor, beta polypeptide  IFT57 - intraflagellar transport 57 homolog (chlamydomonas)  ARRB2 - arrestin, beta 2  ARRB1 - arrestin, beta 1  PDGFRA - platelet-derived growth factor receptor, alpha polypeptide  PDGFB - platelet-derived growth factor beta polypeptide  RHOB - ras homolog family member b  FAM83H - family with sequence similarity 83, member h  RHOC - ras homolog family member c  PDPK1 - 3-phosphoinositide dependent protein kinase-1  FAM107A - family with sequence similarity 107, member a  STRAP - serine/threonine kinase receptor associated protein  RHOA - ras homolog family member a  PSIP1 - pc4 and sfrs1 interacting protein 1  ARF6 - adp-ribosylation factor 6  PECAM1 - platelet/endothelial cell adhesion molecule 1  ATG2B - autophagy related 2b  RND3 - rho family gtpase 3  BAZ2A - bromodomain adjacent to zinc finger domain, 2a  WDR60 - wd repeat domain 60  PCNA - proliferating cell nuclear antigen  CHMP1A - charged multivesicular body protein 1a  ARHGAP17 - rho gtpase activating protein 17  ISCA2 - iron-sulfur cluster assembly 2  PLEKHJ1 - pleckstrin homology domain containing, family j member 1  TTC8 - tetratricopeptide repeat domain 8  PCM1 - pericentriolar material 1  CDK16 - cyclin-dependent kinase 16  LEO1 - leo1, paf1/rna polymerase ii complex component, homolog (s. cerevisiae)  RABL2B - rab, member of ras oncogene family-like 2b  FRMD6 - ferm domain containing 6  PWP1 - pwp1 homolog (s. cerevisiae)  WRAP53 - wd repeat containing, antisense to tp53  CAPN10 - calpain 10  KPTN - kaptin (actin binding protein)  PLA2G16 - phospholipase a2, group xvi  KAT7 - k(lysine) acetyltransferase 7  HAUS2 - haus augmin-like complex, subunit 2  EPN2 - epsin 2  RUFY3 - run and fyve domain containing 3  BTBD3 - btb (poz) domain containing 3  ATP1A3 - atpase, na+/k+ transporting, alpha 3 polypeptide  ZBTB1 - zinc finger and btb domain containing 1  ZNF365 - zinc finger protein 365  PFN2 - profilin 2  FBXW7 - f-box and wd repeat domain containing 7, e3 ubiquitin protein ligase  ATP1B2 - atpase, na+/k+ transporting, beta 2 polypeptide  BRIX1 - brx1, biogenesis of ribosomes, homolog (s. cerevisiae)  BAHD1 - bromo adjacent homology domain containing 1  ATP1B1 - atpase, na+/k+ transporting, beta 1 polypeptide  MORC2 - morc family cw-type zinc finger 2  PHF2 - phd finger protein 2  PHF1 - phd finger protein 1  LRRK2 - leucine-rich repeat kinase 2  BLOC1S4 - biogenesis of lysosomal organelles complex-1, subunit 4, cappuccino  RERE - arginine-glutamic acid dipeptide (re) repeats  MNS1 - meiosis-specific nuclear structural 1  CLSTN1 - calsyntenin 1  DZIP1 - daz interacting zinc finger protein 1  SEC31A - sec31 homolog a (s. cerevisiae)  PEX6 - peroxisomal biogenesis factor 6  ATG14 - autophagy related 14  PEX7 - peroxisomal biogenesis factor 7  SH3PXD2B - sh3 and px domains 2b  PEX12 - peroxisomal biogenesis factor 12  CNKSR2 - connector enhancer of kinase suppressor of ras 2  PEX13 - peroxisomal biogenesis factor 13  ATP7A - atpase, cu++ transporting, alpha polypeptide  PEX14 - peroxisomal biogenesis factor 14  PPP6R1 - protein phosphatase 6, regulatory subunit 1  FRYL - fry-like  LMTK2 - lemur tyrosine kinase 2  AEBP2 - ae binding protein 2  ICK - intestinal cell (mak-like) kinase  PEX1 - peroxisomal biogenesis factor 1  PFKL - phosphofructokinase, liver  PI4K2A - phosphatidylinositol 4-kinase type 2 alpha  AAK1 - ap2 associated kinase 1  PFKP - phosphofructokinase, platelet  COPRS - coordinator of prmt5, differentiation stimulator  BAX - bcl2-associated x protein  PKD1 - polycystic kidney disease 1 (autosomal dominant)  PKD2 - polycystic kidney disease 2 (autosomal dominant)  PUF60 - poly-u binding splicing factor 60kda  MTF2 - metal response element binding transcription factor 2  PITPNA - phosphatidylinositol transfer protein, alpha  BAK1 - bcl2-antagonist/killer 1  BAD - bcl2-associated agonist of cell death  PIN1 - peptidylprolyl cis/trans isomerase, nima-interacting 1  PI4KA - phosphatidylinositol 4-kinase, catalytic, alpha  PIK3R1 - phosphoinositide-3-kinase, regulatory subunit 1 (alpha)  PDZD8 - pdz domain containing 8  MRAS - muscle ras oncogene homolog  COX18 - cox18 cytochrome c oxidase assembly factor  AXL - axl receptor tyrosine kinase  ZFYVE27 - zinc finger, fyve domain containing 27  PKP2 - plakophilin 2  PEF1 - penta-ef-hand domain containing 1  ATRX - alpha thalassemia/mental retardation syndrome x-linked  COG2 - component of oligomeric golgi complex 2  OGFOD1 - 2-oxoglutarate and iron-dependent oxygenase domain containing 1  GABARAPL2 - gaba(a) receptor-associated protein-like 2  TWF2 - twinfilin actin-binding protein 2  PIH1D2 - pih1 domain containing 2  PHF10 - phd finger protein 10  SERPINE2 - serpin peptidase inhibitor, clade e (nexin, plasminogen activator inhibitor type 1), member 2  GABARAP - gaba(a) receptor-associated protein  BCR - breakpoint cluster region  MRGBP - mrg/morf4l binding protein  BCL9 - b-cell cll/lymphoma 9  RHOT1 - ras homolog family member t1  PPP6R3 - protein phosphatase 6, regulatory subunit 3  BRF2 - brf2, rna polymerase iii transcription initiation factor 50 kda subunit  PIK3C2B - phosphatidylinositol-4-phosphate 3-kinase, catalytic subunit type 2 beta  BCL2 - b-cell cll/lymphoma 2  BCL2L2 - bcl2-like 2  PIK3C3 - phosphatidylinositol 3-kinase, catalytic subunit type 3  BCL2L1 - bcl2-like 1  CCDC66 - coiled-coil domain containing 66  PARK7 - parkinson protein 7  B4GALT7 - xylosylprotein beta 1,4-galactosyltransferase, polypeptide 7  BBS4 - bardet-biedl syndrome 4  BBS2 - bardet-biedl syndrome 2  DST - dystonin  TBC1D20 - tbc1 domain family, member 20  TRIM27 - tripartite motif containing 27  BNIP3L - bcl2/adenovirus e1b 19kda interacting protein 3-like  RFC5 - replication factor c (activator 1) 5, 36.5kda  BNIP1 - bcl2/adenovirus e1b 19kda interacting protein 1  RFC2 - replication factor c (activator 1) 2, 40kda  RFC3 - replication factor c (activator 1) 3, 38kda  BET1 - bet1 golgi vesicular membrane trafficking protein  MAEA - macrophage erythroblast attacher  SF3A1 - splicing factor 3a, subunit 1, 120kda  WHSC1L1 - wolf-hirschhorn syndrome candidate 1-like 1  TTC19 - tetratricopeptide repeat domain 19  MKS1 - meckel syndrome, type 1  RELA - v-rel avian reticuloendotheliosis viral oncogene homolog a  BCOR - bcl6 corepressor  KATNB1 - katanin p80 (wd repeat containing) subunit b 1  RECQL - recq protein-like (dna helicase q1-like)  PAK4 - p21 protein (cdc42/rac)-activated kinase 4  RDX - radixin  RET - ret proto-oncogene  DPF2 - d4, zinc and double phd fingers family 2  RTN3 - reticulon 3  UPF1 - upf1 regulator of nonsense transcripts homolog (yeast)  RLF - rearranged l-myc fusion  RING1 - ring finger protein 1  MTX3 - metaxin 3  FNBP1L - formin binding protein 1-like  HIST3H2BB - histone cluster 3, h2bb  FBLL1 - fibrillarin-like 1  BMP1 - bone morphogenetic protein 1  TFG - trk-fused gene  BMI1 - bmi1 polycomb ring finger oncogene  PCGF3 - polycomb group ring finger 3  BMP7 - bone morphogenetic protein 7  FITM2 - fat storage-inducing transmembrane protein 2  ABCA7 - atp-binding cassette, sub-family a (abc1), member 7  MRPL49 - mitochondrial ribosomal protein l49  TRPM7 - transient receptor potential cation channel, subfamily m, member 7  FLOT1 - flotillin 1  NDE1 - nude neurodevelopment protein 1  VPS51 - vacuolar protein sorting 51 homolog (s. cerevisiae)  BCAS3 - breast carcinoma amplified sequence 3  ABCE1 - atp-binding cassette, sub-family e (oabp), member 1  VPS13C - vacuolar protein sorting 13 homolog c (s. cerevisiae)  FMNL1 - formin-like 1  LRRC49 - leucine rich repeat containing 49  HAUS6 - haus augmin-like complex, subunit 6  STX6 - syntaxin 6  UHMK1 - u2af homology motif (uhm) kinase 1  DYM - dymeclin  AHI1 - abelson helper integration site 1  LIX1L - lix1 homolog (mouse)-like  BRD2 - bromodomain containing 2  RNF2 - ring finger protein 2  GPHN - gephyrin  ROCK1 - rho-associated, coiled-coil containing protein kinase 1  BYSL - bystin-like  TSPO - translocator protein (18kda)  SPRY1 - sprouty homolog 1, antagonist of fgf signaling (drosophila)  DNAJB12 - dnaj (hsp40) homolog, subfamily b, member 12  SPRY2 - sprouty homolog 2 (drosophila)  TET2 - tet methylcytosine dioxygenase 2  STAM2 - signal transducing adaptor molecule (sh3 domain and itam motif) 2  TRPM4 - transient receptor potential cation channel, subfamily m, member 4  MBTD1 - mbt domain containing 1  RAMP2 - receptor (g protein-coupled) activity modifying protein 2  FBLIM1 - filamin binding lim protein 1  AKAP8 - a kinase (prka) anchor protein 8  ZMPSTE24 - zinc metallopeptidase ste24  C5AR1 - complement component 5a receptor 1  GRAMD1C - gram domain containing 1c  STAG1 - stromal antigen 1  EFS - embryonal fyn-associated substrate  UBE4B - ubiquitination factor e4b  CCSAP - centriole, cilia and spindle-associated protein  CAPZA1 - capping protein (actin filament) muscle z-line, alpha 1  VAV3 - vav 3 guanine nucleotide exchange factor  CAPNS1 - calpain, small subunit 1  CAPN2 - calpain 2, (m/ii) large subunit  CAPN1 - calpain 1, (mu/i) large subunit  MCRS1 - microspherule protein 1  ATG16L1 - autophagy related 16-like 1 (s. cerevisiae)  MRPL20 - mitochondrial ribosomal protein l20  NRDE2 - nrde-2, necessary for rna interference, domain containing  VPS37C - vacuolar protein sorting 37 homolog c (s. cerevisiae)  RPL11 - ribosomal protein l11  PTCD3 - pentatricopeptide repeat domain 3  CASP8 - caspase 8, apoptosis-related cysteine peptidase  RPL7 - ribosomal protein l7  PIBF1 - progesterone immunomodulatory binding factor 1  PPIH - peptidylprolyl isomerase h (cyclophilin h)  CASP3 - caspase 3, apoptosis-related cysteine peptidase  HEATR3 - heat repeat containing 3  RPL3 - ribosomal protein l3  BAIAP2 - bai1-associated protein 2  RPA3 - replication protein a3, 14kda  MAD2L2 - mad2 mitotic arrest deficient-like 2 (yeast)  RPA1 - replication protein a1, 70kda  PID1 - phosphotyrosine interaction domain containing 1  CAPZB - capping protein (actin filament) muscle z-line, beta  RPA2 - replication protein a2, 32kda  PHIP - pleckstrin homology domain interacting protein  SEC23A - sec23 homolog a (s. cerevisiae)  CAP1 - cap, adenylate cyclase-associated protein 1 (yeast)  PPP2R3C - protein phosphatase 2, regulatory subunit b'', gamma  SEC23B - sec23 homolog b (s. cerevisiae)  PRPF39 - prp39 pre-mrna processing factor 39 homolog (s. cerevisiae)  STX17 - syntaxin 17  CACNB3 - calcium channel, voltage-dependent, beta 3 subunit  SLC9A6 - solute carrier family 9, subfamily a (nhe6, cation proton antiporter 6), member 6  CACNB2 - calcium channel, voltage-dependent, beta 2 subunit  LAMTOR1 - late endosomal/lysosomal adaptor, mapk and mtor activator 1  DDR1 - discoidin domain receptor tyrosine kinase 1  TADA3 - transcriptional adaptor 3  CALR - calreticulin  PAK1IP1 - pak1 interacting protein 1  UNC13B - unc-13 homolog b (c. elegans)  CALM2 - calmodulin 2 (phosphorylase kinase, delta)  WTIP - wilms tumor 1 interacting protein  STK25 - serine/threonine kinase 25  GLDN - gliomedin  VTI1B - vesicle transport through interaction with t-snares 1b  CRTAP - cartilage associated protein  RPS23 - ribosomal protein s23  SLC35F6 - solute carrier family 35, member f6  MICU1 - mitochondrial calcium uptake 1  PINX1 - pin2/terf1 interacting, telomerase inhibitor 1  CLN6 - ceroid-lipofuscinosis, neuronal 6, late infantile, variant  TUBA1B - tubulin, alpha 1b  NPM3 - nucleophosmin/nucleoplasmin 3  TMEM70 - transmembrane protein 70  INO80C - ino80 complex subunit c  THG1L - trna-histidine guanylyltransferase 1-like (s. cerevisiae)  CCNH - cyclin h  HMG20A - high mobility group 20a  BANP - btg3 associated nuclear protein  GEMIN8 - gem (nuclear organelle) associated protein 8  SSH3 - slingshot protein phosphatase 3  CORO2B - coronin, actin binding protein, 2b  ATP8A1 - atpase, aminophospholipid transporter (aplt), class i, type 8a, member 1  NDRG1 - n-myc downstream regulated 1  RPS15 - ribosomal protein s15  CD9 - cd9 molecule  DLC1 - deleted in liver cancer 1  TUBB4B - tubulin, beta 4b class ivb  TUBB3 - tubulin, beta 3 class iii  C17orf49 - chromosome 17 open reading frame 49  FAM206A - family with sequence similarity 206, member a  CBL - cbl proto-oncogene, e3 ubiquitin protein ligase  SERPINH1 - serpin peptidase inhibitor, clade h (heat shock protein 47), member 1, (collagen binding protein 1)  MRPL10 - mitochondrial ribosomal protein l10  LPCAT2 - lysophosphatidylcholine acyltransferase 2  CAV2 - caveolin 2  CAV1 - caveolin 1, caveolae protein, 22kda  KANSL2 - kat8 regulatory nsl complex subunit 2  SEZ6 - seizure related 6 homolog (mouse)  TUBGCP3 - tubulin, gamma complex associated protein 3  SEC24B - sec24 family, member b (s. cerevisiae)  RBM14 - rna binding motif protein 14  PRMT5 - protein arginine methyltransferase 5  CD2BP2 - cd2 (cytoplasmic tail) binding protein 2  CHTF8 - ctf8, chromosome transmission fidelity factor 8 homolog (s. cerevisiae)  RRM1 - ribonucleotide reductase m1  TGOLN2 - trans-golgi network protein 2  HTRA1 - htra serine peptidase 1  PSAP - prosaposin  CDH3 - cadherin 3, type 1, p-cadherin (placental)  CDH2 - cadherin 2, type 1, n-cadherin (neuronal)  KCTD5 - potassium channel tetramerization domain containing 5  MRPS21 - mitochondrial ribosomal protein s21  POMT1 - protein-o-mannosyltransferase 1  PSEN1 - presenilin 1  CCSER2 - coiled-coil serine-rich protein 2  PSEN2 - presenilin 2 (alzheimer disease 4)  CD151 - cd151 molecule (raph blood group)  USP16 - ubiquitin specific peptidase 16  NLE1 - notchless homolog 1 (drosophila)  CDC42EP3 - cdc42 effector protein (rho gtpase binding) 3  SRSF12 - serine/arginine-rich splicing factor 12  PRPF8 - pre-mrna processing factor 8  SMCR7L - smith-magenis syndrome chromosome region, candidate 7-like  STARD4 - star-related lipid transfer (start) domain containing 4  SCARB2 - scavenger receptor class b, member 2  EXOC5 - exocyst complex component 5  PSMB10 - proteasome (prosome, macropain) subunit, beta type, 10  CD47 - cd47 molecule  CD44 - cd44 molecule (indian blood group)  SPINT2 - serine peptidase inhibitor, kunitz type, 2  YKT6 - ykt6 v-snare homolog (s. cerevisiae)  MTX2 - metaxin 2  SLMO1 - slowmo homolog 1 (drosophila)  TAF6L - taf6-like rna polymerase ii, p300/cbp-associated factor (pcaf)-associated factor, 65kda  GDAP1 - ganglioside induced differentiation associated protein 1  PSMD9 - proteasome (prosome, macropain) 26s subunit, non-atpase, 9  GAR1 - gar1 ribonucleoprotein  PSMD10 - proteasome (prosome, macropain) 26s subunit, non-atpase, 10  CD34 - cd34 molecule  TSPAN33 - tetraspanin 33  PSMD5 - proteasome (prosome, macropain) 26s subunit, non-atpase, 5  GAS2L1 - growth arrest-specific 2 like 1  TERF2IP - telomeric repeat binding factor 2, interacting protein  PSMD13 - proteasome (prosome, macropain) 26s subunit, non-atpase, 13  ATG7 - autophagy related 7  CENPC - centromere protein c  KLHL17 - kelch-like family member 17  DCTN2 - dynactin 2 (p50)  GLRX3 - glutaredoxin 3  PTEN - phosphatase and tensin homolog  LAMTOR5 - late endosomal/lysosomal adaptor, mapk and mtor activator 5  CETN2 - centrin, ef-hand protein, 2  ANP32B - acidic (leucine-rich) nuclear phosphoprotein 32 family, member b  JMY - junction mediating and regulatory protein, p53 cofactor  CETN3 - centrin, ef-hand protein, 3  PRDX4 - peroxiredoxin 4  CFL1 - cofilin 1 (non-muscle)  MYBBP1A - myb binding protein (p160) 1a  FBLN5 - fibulin 5  PTGFRN - prostaglandin f2 receptor inhibitor  CHRAC1 - chromatin accessibility complex 1  CIB1 - calcium and integrin binding 1 (calmyrin)  CLGN - calmegin  KAT5 - k(lysine) acetyltransferase 5  PTK2 - protein tyrosine kinase 2  PTK7 - protein tyrosine kinase 7  SLU7 - slu7 splicing factor homolog (s. cerevisiae)  CDK5 - cyclin-dependent kinase 5  PTMA - prothymosin, alpha  CDK4 - cyclin-dependent kinase 4  TWF1 - twinfilin actin-binding protein 1  PTN - pleiotrophin  CDK7 - cyclin-dependent kinase 7  NPC2 - niemann-pick disease, type c2  CCT2 - chaperonin containing tcp1, subunit 2 (beta)  PTPN1 - protein tyrosine phosphatase, non-receptor type 1  ARPC1A - actin related protein 2/3 complex, subunit 1a, 41kda  CDH6 - cadherin 6, type 2, k-cadherin (fetal kidney)  PTPN11 - protein tyrosine phosphatase, non-receptor type 11  CDH11 - cadherin 11, type 2, ob-cadherin (osteoblast)  CDH8 - cadherin 8, type 2  PTPRD - protein tyrosine phosphatase, receptor type, d  SCLT1 - sodium channel and clathrin linker 1  ARFGEF2 - adp-ribosylation factor guanine nucleotide-exchange factor 2 (brefeldin a-inhibited)  CDK2 - cyclin-dependent kinase 2  ARFGEF1 - adp-ribosylation factor guanine nucleotide-exchange factor 1 (brefeldin a-inhibited)  PTPRF - protein tyrosine phosphatase, receptor type, f  PURA - purine-rich element binding protein a  RRN3 - rrn3 rna polymerase i transcription factor homolog (s. cerevisiae)  NCKAP1 - nck-associated protein 1  ZNHIT6 - zinc finger, hit-type containing 6  CHRNB2 - cholinergic receptor, nicotinic, beta 2 (neuronal)  TMEM106B - transmembrane protein 106b  RSBN1 - round spermatid basic protein 1  ZMYND11 - zinc finger, mynd-type containing 11  PTPRK - protein tyrosine phosphatase, receptor type, k  SRSF10 - serine/arginine-rich splicing factor 10  PTPRM - protein tyrosine phosphatase, receptor type, m  PTPRN - protein tyrosine phosphatase, receptor type, n  LYST - lysosomal trafficking regulator  PLK2 - polo-like kinase 2  PEX2 - peroxisomal biogenesis factor 2  CHCHD4 - coiled-coil-helix-coiled-coil-helix domain containing 4  KDM5B - lysine (k)-specific demethylase 5b  PXN - paxillin  ABCD3 - atp-binding cassette, sub-family d (ald), member 3  PVRL1 - poliovirus receptor-related 1 (herpesvirus entry mediator c)  CHGA - chromogranin a (parathyroid secretory protein 1)  PVRL2 - poliovirus receptor-related 2 (herpesvirus entry mediator b)  RAB5A - rab5a, member ras oncogene family  CHD4 - chromodomain helicase dna binding protein 4  RAB5B - rab5b, member ras oncogene family  CHD2 - chromodomain helicase dna binding protein 2  CHD3 - chromodomain helicase dna binding protein 3  RAB3A - rab3a, member ras oncogene family  RAB3B - rab3b, member ras oncogene family  MTERFD2 - mterf domain containing 2  CHD1 - chromodomain helicase dna binding protein 1  RAB2A - rab2a, member ras oncogene family  RAB1A - rab1a, member ras oncogene family  WASF3 - was protein family, member 3  SDCCAG3 - serologically defined colon cancer antigen 3  RAD21 - rad21 homolog (s. pombe)  SEC24A - sec24 family, member a (s. cerevisiae)  RAC3 - ras-related c3 botulinum toxin substrate 3 (rho family, small gtp binding protein rac3)  PAF1 - paf1, rna polymerase ii associated factor, homolog (s. cerevisiae)  RAC1 - ras-related c3 botulinum toxin substrate 1 (rho family, small gtp binding protein rac1)  RABIF - rab interacting factor  MSL1 - male-specific lethal 1 homolog (drosophila)  INO80 - ino80 complex subunit  RAB27A - rab27a, member ras oncogene family  KCTD18 - potassium channel tetramerization domain containing 18  RALA - v-ral simian leukemia viral oncogene homolog a (ras related)  CCT8 - chaperonin containing tcp1, subunit 8 (theta)  CLTC - clathrin, heavy chain (hc)  MIER2 - mesoderm induction early response 1, family member 2  RARG - retinoic acid receptor, gamma  CLN5 - ceroid-lipofuscinosis, neuronal 5  CLNS1A - chloride channel, nucleotide-sensitive, 1a  BBS5 - bardet-biedl syndrome 5  DCTN6 - dynactin 6  CLTB - clathrin, light chain b  RASA1 - ras p21 protein activator (gtpase activating protein) 1  CLTA - clathrin, light chain a  CELF1 - cugbp, elav-like family member 1  CLU - clusterin  RAP1GDS1 - rap1, gtp-gdp dissociation stimulator 1  GNL3L - guanine nucleotide binding protein-like 3 (nucleolar)-like  MAGEL2 - mage-like 2  CELF2 - cugbp, elav-like family member 2  RAP2A - rap2a, member of ras oncogene family  ING3 - inhibitor of growth family, member 3  TPP1 - tripeptidyl peptidase i  CTCF - ccctc-binding factor (zinc finger protein)  PRMT7 - protein arginine methyltransferase 7  MITD1 - mit, microtubule interacting and transport, domain containing 1  RBL2 - retinoblastoma-like 2 (p130)  RBBP7 - retinoblastoma binding protein 7  CLCN3 - chloride channel, voltage-sensitive 3  CLCN6 - chloride channel, voltage-sensitive 6  RB1 - retinoblastoma 1  ARID4A - at rich interactive domain 4a (rbp1-like)  STAG2 - stromal antigen 2  AP2M1 - adaptor-related protein complex 2, mu 1 subunit  RBBP5 - retinoblastoma binding protein 5  KDM5A - lysine (k)-specific demethylase 5a  RBBP4 - retinoblastoma binding protein 4  NUP35 - nucleoporin 35kda  ERCC8 - excision repair cross-complementing rodent repair deficiency, complementation group 8  MTRF1L - mitochondrial translational release factor 1-like  SLC12A7 - solute carrier family 12 (potassium/chloride transporter), member 7  YME1L1 - yme1-like 1 atpase  PTGES3 - prostaglandin e synthase 3 (cytosolic)  CIRBP - cold inducible rna binding protein  CHMP4B - charged multivesicular body protein 4b  POLD3 - polymerase (dna-directed), delta 3, accessory subunit  DHX29 - deah (asp-glu-ala-his) box polypeptide 29  USP39 - ubiquitin specific peptidase 39  SPTAN1 - spectrin, alpha, non-erythrocytic 1  CEP89 - centrosomal protein 89kda  WDR19 - wd repeat domain 19  SPTBN4 - spectrin, beta, non-erythrocytic 4  CD2AP - cd2-associated protein  FBXO18 - f-box protein, helicase, 18  SPP1 - secreted phosphoprotein 1  EPG5 - ectopic p-granules autophagy protein 5 homolog (c. elegans)  CORO1C - coronin, actin binding protein, 1c  SPRYD3 - spry domain containing 3  SPG7 - spastic paraplegia 7 (pure and complicated autosomal recessive)  COX15 - cytochrome c oxidase assembly homolog 15 (yeast)  SPARC - secreted protein, acidic, cysteine-rich (osteonectin)  COX7A2 - cytochrome c oxidase subunit viia polypeptide 2 (liver)  SPAST - spastin  MIER1 - mesoderm induction early response 1, transcriptional regulator  SPAG1 - sperm associated antigen 1  NCKAP5L - nck-associated protein 5-like  IQSEC3 - iq motif and sec7 domain 3  GBA2 - glucosidase, beta (bile acid) 2  CADM3 - cell adhesion molecule 3  LTV1 - ltv1 homolog (s. cerevisiae)  WDR73 - wd repeat domain 73  MUM1 - melanoma associated antigen (mutated) 1  LSM2 - lsm2 homolog, u6 small nuclear rna associated (s. cerevisiae)  SOS1 - son of sevenless homolog 1 (drosophila)  TMEM41B - transmembrane protein 41b  ORC3 - origin recognition complex, subunit 3  SON - son dna binding protein  HEBP2 - heme binding protein 2  LEMD3 - lem domain containing 3  GATAD1 - gata zinc finger domain containing 1  SOAT1 - sterol o-acyltransferase 1  CREBBP - creb binding protein  ATF2 - activating transcription factor 2  CREB1 - camp responsive element binding protein 1  CGNL1 - cingulin-like 1  SNX2 - sorting nexin 2  SNX1 - sorting nexin 1  SERAC1 - serine active site containing 1  MARK4 - map/microtubule affinity-regulating kinase 4  CADM1 - cell adhesion molecule 1  SNRPD1 - small nuclear ribonucleoprotein d1 polypeptide 16kda  CEP19 - centrosomal protein 19kda  GABARAPL1 - gaba(a) receptor-associated protein like 1  SNRPF - small nuclear ribonucleoprotein polypeptide f  PLK3 - polo-like kinase 3  CNN1 - calponin 1, basic, smooth muscle  RHOU - ras homolog family member u  SNCA - synuclein, alpha (non a4 component of amyloid precursor)  SNCG - synuclein, gamma (breast cancer-specific protein 1)  FSCN1 - fascin homolog 1, actin-bundling protein (strongylocentrotus purpuratus)  TRAPPC1 - trafficking protein particle complex 1  SNRPB - small nuclear ribonucleoprotein polypeptides b and b1  SNAP25 - synaptosomal-associated protein, 25kda  SNCB - synuclein, beta  TP53INP2 - tumor protein p53 inducible nuclear protein 2  PTBP2 - polypyrimidine tract binding protein 2  SMN1 - survival of motor neuron 1, telomeric  CNN3 - calponin 3, acidic  SMARCE1 - swi/snf related, matrix associated, actin dependent regulator of chromatin, subfamily e, member 1  CNN2 - calponin 2  CNR1 - cannabinoid receptor 1 (brain)  CNP - 2',3'-cyclic nucleotide 3' phosphodiesterase  CNTN1 - contactin 1  SMARCB1 - swi/snf related, matrix associated, actin dependent regulator of chromatin, subfamily b, member 1  SMARCC1 - swi/snf related, matrix associated, actin dependent regulator of chromatin, subfamily c, member 1  HLTF - helicase-like transcription factor  ARFIP2 - adp-ribosylation factor interacting protein 2  SMARCA4 - swi/snf related, matrix associated, actin dependent regulator of chromatin, subfamily a, member 4  SSBP3 - single stranded dna binding protein 3  SMARCD2 - swi/snf related, matrix associated, actin dependent regulator of chromatin, subfamily d, member 2  ATCAY - ataxia, cerebellar, cayman type  SMARCD3 - swi/snf related, matrix associated, actin dependent regulator of chromatin, subfamily d, member 3  SMARCC2 - swi/snf related, matrix associated, actin dependent regulator of chromatin, subfamily c, member 2  SMARCD1 - swi/snf related, matrix associated, actin dependent regulator of chromatin, subfamily d, member 1  COL4A4 - collagen, type iv, alpha 4  MID1IP1 - mid1 interacting protein 1  REPS1 - ralbp1 associated eps domain containing 1  COL4A2 - collagen, type iv, alpha 2  SMARCA1 - swi/snf related, matrix associated, actin dependent regulator of chromatin, subfamily a, member 1  COL5A2 - collagen, type v, alpha 2  SMARCA2 - swi/snf related, matrix associated, actin dependent regulator of chromatin, subfamily a, member 2  KMT2C - lysine (k)-specific methyltransferase 2c  EPS15L1 - epidermal growth factor receptor pathway substrate 15-like 1  RAB38 - rab38, member ras oncogene family  COL12A1 - collagen, type xii, alpha 1  ZNF462 - zinc finger protein 462  FBF1 - fas (tnfrsf6) binding factor 1  NAP1L5 - nucleosome assembly protein 1-like 5  STX12 - syntaxin 12  TMEFF2 - transmembrane protein with egf-like and two follistatin-like domains 2  COL16A1 - collagen, type xvi, alpha 1  SYN1 - synapsin i  TFIP11 - tuftelin interacting protein 11  SYP - synaptophysin  PLEKHA1 - pleckstrin homology domain containing, family a (phosphoinositide binding specific) member 1  CST3 - cystatin c  EML2 - echinoderm microtubule associated protein like 2  NAT6 - n-acetyltransferase 6 (gcn5-related)  SYT1 - synaptotagmin i  PRPF6 - pre-mrna processing factor 6  KLHL12 - kelch-like family member 12  KDM2B - lysine (k)-specific demethylase 2b  SYT4 - synaptotagmin iv  SUPT6H - suppressor of ty 6 homolog (s. cerevisiae)  SUPV3L1 - suppressor of var1, 3-like 1 (s. cerevisiae)  CBY1 - chibby homolog 1 (drosophila)  SUN2 - sad1 and unc84 domain containing 2  CACNG7 - calcium channel, voltage-dependent, gamma subunit 7  CTNNA1 - catenin (cadherin-associated protein), alpha 1, 102kda  SLC24A2 - solute carrier family 24 (sodium/potassium/calcium exchanger), member 2  CTBP1 - c-terminal binding protein 1  SUPT4H1 - suppressor of ty 4 homolog 1 (s. cerevisiae)  CTBP2 - c-terminal binding protein 2  EML3 - echinoderm microtubule associated protein like 3  CTNND1 - catenin (cadherin-associated protein), delta 1  CTNND2 - catenin (cadherin-associated protein), delta 2  VAMP2 - vesicle-associated membrane protein 2 (synaptobrevin 2)  CTNNA2 - catenin (cadherin-associated protein), alpha 2  MFSD8 - major facilitator superfamily domain containing 8  SURF6 - surfeit 6  CTNNB1 - catenin (cadherin-associated protein), beta 1, 88kda  PITPNB - phosphatidylinositol transfer protein, beta  STXBP1 - syntaxin binding protein 1  STX5 - syntaxin 5  STX4 - syntaxin 4  STX3 - syntaxin 3  STX1A - syntaxin 1a (brain)  STRN - striatin, calmodulin binding protein  USP30 - ubiquitin specific peptidase 30  FLRT2 - fibronectin leucine rich transmembrane protein 2  UNC119B - unc-119 homolog b (c. elegans)  CTSS - cathepsin s  CTSV - cathepsin v  MPV17L2 - mpv17 mitochondrial membrane protein-like 2  TCTEX1D2 - tctex1 domain containing 2  BRD1 - bromodomain containing 1  PCGF1 - polycomb group ring finger 1  NPNT - nephronectin  STAU1 - staufen double-stranded rna binding protein 1  STC1 - stanniocalcin 1  CXADR - coxsackie virus and adenovirus receptor  AURKC - aurora kinase c  TUBA1C - tubulin, alpha 1c  MTCH1 - mitochondrial carrier 1  SUV420H2 - suppressor of variegation 4-20 homolog 2 (drosophila)  CYLD - cylindromatosis (turban tumor syndrome)  EXOC4 - exocyst complex component 4  STK4 - serine/threonine kinase 4  CNEP1R1 - ctd nuclear envelope phosphatase 1 regulatory subunit 1  C12orf44 - chromosome 12 open reading frame 44  ST13 - suppression of tumorigenicity 13 (colon carcinoma) (hsp70 interacting protein)  CRKL - v-crk avian sarcoma virus ct10 oncogene homolog-like  CRK - v-crk avian sarcoma virus ct10 oncogene homolog  CELF6 - cugbp, elav-like family member 6  CRMP1 - collapsin response mediator protein 1  NIPBL - nipped-b homolog (drosophila)  TRAPPC11 - trafficking protein particle complex 11  COG4 - component of oligomeric golgi complex 4  STAT3 - signal transducer and activator of transcription 3 (acute-phase response factor)  ASF1A - anti-silencing function 1a histone chaperone  BRMS1 - breast cancer metastasis suppressor 1  DFNB31 - deafness, autosomal recessive 31  SS18 - synovial sarcoma translocation, chromosome 18  RIC8A - ric8 guanine nucleotide exchange factor a  NAPB - n-ethylmaleimide-sensitive factor attachment protein, beta  CSF1R - colony stimulating factor 1 receptor  SNX33 - sorting nexin 33  SRP54 - signal recognition particle 54kda  FBXO7 - f-box protein 7  SRP19 - signal recognition particle 19kda  ELMO2 - engulfment and cell motility 2  BLOC1S5 - biogenesis of lysosomal organelles complex-1, subunit 5, muted  SRPK2 - srsf protein kinase 2  LRCH3 - leucine-rich repeats and calponin homology (ch) domain containing 3  SRPK1 - srsf protein kinase 1  TROVE2 - trove domain family, member 2  KLHL22 - kelch-like family member 22  GCA - grancalcin, ef-hand calcium binding protein  GALNT11 - udp-n-acetyl-alpha-d-galactosamine:polypeptide n-acetylgalactosaminyltransferase 11 (galnac-t11)  SSBP1 - single-stranded dna binding protein 1, mitochondrial  ZNF335 - zinc finger protein 335  CSK - c-src tyrosine kinase  SQLE - squalene epoxidase  ATXN10 - ataxin 10  TTLL1 - tubulin tyrosine ligase-like family, member 1  CSNK1A1 - casein kinase 1, alpha 1  SPTBN1 - spectrin, beta, non-erythrocytic 1  CSNK1D - casein kinase 1, delta  SRC - v-src avian sarcoma (schmidt-ruppin a-2) viral oncogene homolog  MRPL17 - mitochondrial ribosomal protein l17  VIPAS39 - vps33b interacting protein, apical-basolateral polarity regulator, spe-39 homolog  CSRP1 - cysteine and glycine-rich protein 1  SHPRH - snf2 histone linker phd ring helicase, e3 ubiquitin protein ligase  CSRP2 - cysteine and glycine-rich protein 2  DHX9 - deah (asp-glu-ala-his) box helicase 9  DHX8 - deah (asp-glu-ala-his) box polypeptide 8  TRAPPC2 - trafficking protein particle complex 2  DDX6 - dead (asp-glu-ala-asp) box helicase 6  SEC13 - sec13 homolog (s. cerevisiae)  DDX3X - dead (asp-glu-ala-asp) box helicase 3, x-linked  DDX1 - dead (asp-glu-ala-asp) box helicase 1  SNX14 - sorting nexin 14  CCDC120 - coiled-coil domain containing 120  SIN3A - sin3 transcription regulator family member a  KIAA1244 - kiaa1244  SDCBP - syndecan binding protein (syntenin)  SAMHD1 - sam domain and hd domain 1  SET - set nuclear oncogene  WWTR1 - ww domain containing transcription regulator 1  SETMAR - set domain and mariner transposase fusion gene  DDB1 - damage-specific dna binding protein 1, 127kda  RHOT2 - ras homolog family member t2  ZNF304 - zinc finger protein 304  DCTN1 - dynactin 1  PTPN23 - protein tyrosine phosphatase, non-receptor type 23  CLIC4 - chloride intracellular channel 4  MCM9 - minichromosome maintenance complex component 9  WDR34 - wd repeat domain 34  GEMIN5 - gem (nuclear organelle) associated protein 5  CSRP2BP - csrp2 binding protein  DBN1 - drebrin 1  ATL3 - atlastin gtpase 3  POT1 - protection of telomeres 1  AHCTF1 - at hook containing transcription factor 1  RAB22A - rab22a, member ras oncogene family  RNF19A - ring finger protein 19a, e3 ubiquitin protein ligase  UNC5A - unc-5 homolog a (c. elegans)  CADM2 - cell adhesion molecule 2  GATAD2B - gata zinc finger domain containing 2b  KCTD13 - potassium channel tetramerization domain containing 13  GRAMD1B - gram domain containing 1b  DNM3 - dynamin 3  ATXN2 - ataxin 2  SIPA1L1 - signal-induced proliferation-associated 1 like 1  AMIGO1 - adhesion molecule with ig-like domain 1  ZNF622 - zinc finger protein 622  SS18L1 - synovial sarcoma translocation gene on chromosome 18-like 1  TBC1D24 - tbc1 domain family, member 24  CHD5 - chromodomain helicase dna binding protein 5  MCFD2 - multiple coagulation factor deficiency 2  ARID1B - at rich interactive domain 1b (swi1-like)  SCO1 - sco1 cytochrome c oxidase assembly protein  ODF2L - outer dense fiber of sperm tails 2-like  HEG1 - heart development protein with egf-like domains 1  KNSTRN - kinetochore-localized astrin/spag5 binding protein  PYGO2 - pygopus homolog 2 (drosophila)  DARS - aspartyl-trna synthetase  DAPK3 - death-associated protein kinase 3  S100B - s100 calcium binding protein b  GORASP2 - golgi reassembly stacking protein 2, 55kda  DAXX - death-domain associated protein  C2CD3 - c2 calcium-dependent domain containing 3  SERINC1 - serine incorporator 1  ZZZ3 - zinc finger, zz-type containing 3  KIDINS220 - kinase d-interacting substrate, 220kda  DAG1 - dystroglycan 1 (dystrophin-associated glycoprotein 1)  MTA3 - metastasis associated 1 family, member 3  DGKQ - diacylglycerol kinase, theta 110kda  S100A10 - s100 calcium binding protein a10  CLIP3 - cap-gly domain containing linker protein 3  STARD13 - star-related lipid transfer (start) domain containing 13  NECAP1 - necap endocytosis associated 1  HACE1 - hect domain and ankyrin repeat containing e3 ubiquitin protein ligase 1  CHMP2B - charged multivesicular body protein 2b  AAR2 - aar2 splicing factor homolog (s. cerevisiae)  SATB1 - satb homeobox 1  COX19 - cytochrome c oxidase assembly homolog 19 (s. cerevisiae)  RPTOR - regulatory associated protein of mtor, complex 1  SAFB - scaffold attachment factor b  SH2B1 - sh2b adaptor protein 1  LYRM7 - lyr motif containing 7  SERBP1 - serpine1 mrna binding protein 1  GAPVD1 - gtpase activating protein and vps9 domains 1  TUBGCP6 - tubulin, gamma complex associated protein 6  MICALL1 - mical-like 1  EPC2 - enhancer of polycomb homolog 2 (drosophila)  DRD2 - dopamine receptor d2  TCTN3 - tectonic family member 3  PHF19 - phd finger protein 19  NLGN2 - neuroligin 2  SLC9A1 - solute carrier family 9, subfamily a (nhe1, cation proton antiporter 1), member 1  NKD2 - naked cuticle homolog 2 (drosophila)  MICAL3 - microtubule associated monooxygenase, calponin and lim domain containing 3  TRAF3IP1 - tnf receptor-associated factor 3 interacting protein 1  TAOK1 - tao kinase 1  SLC8A2 - solute carrier family 8 (sodium/calcium exchanger), member 2  SYDE1 - synapse defective 1, rho gtpase, homolog 1 (c. elegans)  SHANK3 - sh3 and multiple ankyrin repeat domains 3  WIPI2 - wd repeat domain, phosphoinositide interacting 2  SEMA6A - sema domain, transmembrane domain (tm), and cytoplasmic domain, (semaphorin) 6a  PRPF31 - pre-mrna processing factor 31  STRIP1 - striatin interacting protein 1  DR1 - down-regulator of transcription 1, tbp-binding (negative cofactor 2)  DPYSL3 - dihydropyrimidinase-like 3  DPYSL2 - dihydropyrimidinase-like 2  SLC12A4 - solute carrier family 12 (potassium/chloride transporter), member 4  SLC12A2 - solute carrier family 12 (sodium/potassium/chloride transporter), member 2  PREX1 - phosphatidylinositol-3,4,5-trisphosphate-dependent rac exchange factor 1  TNKS1BP1 - tankyrase 1 binding protein 1, 182kda  MAPK15 - mitogen-activated protein kinase 15  NYAP1 - neuronal tyrosine-phosphorylated phosphoinositide-3-kinase adaptor 1  UNK - unkempt homolog (drosophila)  SKP1 - s-phase kinase-associated protein 1  SLC1A5 - solute carrier family 1 (neutral amino acid transporter), member 5  TOR1A - torsin family 1, member a (torsin a)  MKL1 - megakaryoblastic leukemia (translocation) 1  SLC2A1 - solute carrier family 2 (facilitated glucose transporter), member 1  ARHGAP21 - rho gtpase activating protein 21  TANC1 - tetratricopeptide repeat, ankyrin repeat and coiled-coil containing 1  DVL2 - dishevelled segment polarity protein 2  SYT13 - synaptotagmin xiii  DVL3 - dishevelled segment polarity protein 3  TOR1AIP1 - torsin a interacting protein 1  USP36 - ubiquitin specific peptidase 36  DOCK7 - dedicator of cytokinesis 7  RANBP10 - ran binding protein 10  ECM2 - extracellular matrix protein 2, female organ and adipocyte specific  HBEGF - heparin-binding egf-like growth factor  LSM14A - lsm14a, scd6 homolog a (s. cerevisiae)  GTPBP10 - gtp-binding protein 10 (putative)  DKC1 - dyskeratosis congenita 1, dyskerin  DLG3 - discs, large homolog 3 (drosophila)  DLG4 - discs, large homolog 4 (drosophila)  PNPT1 - polyribonucleotide nucleotidyltransferase 1  VPS18 - vacuolar protein sorting 18 homolog (s. cerevisiae)  DLG1 - discs, large homolog 1 (drosophila)  BLOC1S6 - biogenesis of lysosomal organelles complex-1, subunit 6, pallidin  RSPH1 - radial spoke head 1 homolog (chlamydomonas)  FBXW8 - f-box and wd repeat domain containing 8  PHGDH - phosphoglycerate dehydrogenase  DHCR24 - 24-dehydrocholesterol reductase  SCIN - scinderin  EP400 - e1a binding protein p400  DHFR - dihydrofolate reductase  RICTOR - rptor independent companion of mtor, complex 2  DHX37 - deah (asp-glu-ala-his) box polypeptide 37  GRAMD1A - gram domain containing 1a  SIPA1 - signal-induced proliferation-associated 1  SFPQ - splicing factor proline/glutamine-rich  DYNC1H1 - dynein, cytoplasmic 1, heavy chain 1  SFRP2 - secreted frizzled-related protein 2  DYNC1LI2 - dynein, cytoplasmic 1, light intermediate chain 2  SRSF1 - serine/arginine-rich splicing factor 1  DNM2 - dynamin 2  ATG16L2 - autophagy related 16-like 2 (s. cerevisiae)  EPB41L5 - erythrocyte membrane protein band 4.1 like 5  DNMT1 - dna (cytosine-5-)-methyltransferase 1  SRSF5 - serine/arginine-rich splicing factor 5  DNMT3A - dna (cytosine-5-)-methyltransferase 3 alpha  SRSF6 - serine/arginine-rich splicing factor 6  SFSWAP - splicing factor, suppressor of white-apricot homolog (drosophila)  DOCK1 - dedicator of cytokinesis 1  ALS2 - amyotrophic lateral sclerosis 2 (juvenile)  CHD8 - chromodomain helicase dna binding protein 8  ZSWIM6 - zinc finger, swim-type containing 6  HPS4 - hermansky-pudlak syndrome 4  SGK1 - serum/glucocorticoid regulated kinase 1  NOC2L - nucleolar complex associated 2 homolog (s. cerevisiae)  IFT172 - intraflagellar transport 172 homolog (chlamydomonas)  MZT1 - mitotic spindle organizing protein 1  NAV1 - neuron navigator 1  ITSN1 - intersectin 1 (sh3 domain protein)  SH3GL1 - sh3-domain grb2-like 1  SMARCAD1 - swi/snf-related, matrix-associated actin-dependent regulator of chromatin, subfamily a, containing dead/h box 1  EMP1 - epithelial membrane protein 1  MAPRE1 - microtubule-associated protein, rp/eb family, member 1  EMD - emerin  MARK2 - map/microtubule affinity-regulating kinase 2  CHMP4C - charged multivesicular body protein 4c  MAPRE3 - microtubule-associated protein, rp/eb family, member 3  TSC1 - tuberous sclerosis 1  CTTN - cortactin  KIFAP3 - kinesin-associated protein 3  SPIRE1 - spire actin nucleation factor 1  NAF1 - nuclear assembly factor 1 ribonucleoprotein  DHX30 - deah (asp-glu-ala-his) box helicase 30  GORAB - golgin, rab6-interacting  IFT46 - intraflagellar transport 46 homolog (chlamydomonas)  ELN - elastin  EML1 - echinoderm microtubule associated protein like 1  WDR47 - wd repeat domain 47  ELK4 - elk4, ets-domain protein (srf accessory protein 1)  SCAP - srebf chaperone  WRNIP1 - werner helicase interacting protein 1  MGME1 - mitochondrial genome maintenance exonuclease 1  EPAS1 - endothelial pas domain protein 1  EP300 - e1a binding protein p300  HABP4 - hyaluronan binding protein 4  RAB18 - rab18, member ras oncogene family  TIMM50 - translocase of inner mitochondrial membrane 50 homolog (s. cerevisiae)  RAB3GAP1 - rab3 gtpase activating protein subunit 1 (catalytic)  MTSS1L - metastasis suppressor 1-like  GRIPAP1 - grip1 associated protein 1  TUBB2A - tubulin, beta 2a class iia  CELF4 - cugbp, elav-like family member 4  TUBG1 - tubulin, gamma 1  EIF2S1 - eukaryotic translation initiation factor 2, subunit 1 alpha, 35kda  TRIM32 - tripartite motif containing 32  SPHK2 - sphingosine kinase 2  EIF2S3 - eukaryotic translation initiation factor 2, subunit 3 gamma, 52kda  TSPYL1 - tspy-like 1  SAR1A - sar1 homolog a (s. cerevisiae)  KDM2A - lysine (k)-specific demethylase 2a  ELAVL1 - elav (embryonic lethal, abnormal vision, drosophila)-like 1 (hu antigen r)  AZI1 - 5-azacytidine induced 1  BTBD8 - btb (poz) domain containing 8  TSG101 - tumor susceptibility gene 101  AKIP1 - a kinase (prka) interacting protein 1  MAST1 - microtubule associated serine/threonine kinase 1  ACIN1 - apoptotic chromatin condensation inducer 1  CCDC47 - coiled-coil domain containing 47  EFNA1 - ephrin-a1  MVB12A - multivesicular body subunit 12a  TPM4 - tropomyosin 4  EIF2D - eukaryotic translation initiation factor 2d  SLC12A9 - solute carrier family 12, member 9  DAAM1 - dishevelled associated activator of morphogenesis 1  TP53BP1 - tumor protein p53 binding protein 1  PHC1 - polyhomeotic homolog 1 (drosophila)  TP53BP2 - tumor protein p53 binding protein, 2  TOP3A - topoisomerase (dna) iii alpha  TOP2B - topoisomerase (dna) ii beta 180kda  EGFR - epidermal growth factor receptor  MEGF9 - multiple egf-like-domains 9  MEGF8 - multiple egf-like-domains 8  CELSR2 - cadherin, egf lag seven-pass g-type receptor 2  MAST3 - microtubule associated serine/threonine kinase 3  TOP1 - topoisomerase (dna) i  KDM4B - lysine (k)-specific demethylase 4b  KDM1A - lysine (k)-specific demethylase 1a  ATXN7L3 - ataxin 7-like 3  EFNB2 - ephrin-b2  TNS1 - tensin 1  UNC13A - unc-13 homolog a (c. elegans)  EFNA5 - ephrin-a5  SNRNP200 - small nuclear ribonucleoprotein 200kda (u5)  CLUAP1 - clusterin associated protein 1  CCT3 - chaperonin containing tcp1, subunit 3 (gamma)  FNBP1 - formin binding protein 1  TRIP6 - thyroid hormone receptor interactor 6  MFF - mitochondrial fission factor  TNIK - traf2 and nck interacting kinase  XAB2 - xpa binding protein 2  SHKBP1 - sh3kbp1 binding protein 1  E2F4 - e2f transcription factor 4, p107/p130-binding  E2F5 - e2f transcription factor 5, p130-binding  PDS5B - pds5, regulator of cohesion maintenance, homolog b (s. cerevisiae)  ENY2 - enhancer of yellow 2 homolog (drosophila)  DNER - delta/notch-like egf repeat containing  MRPS22 - mitochondrial ribosomal protein s22  C11orf30 - chromosome 11 open reading frame 30  MYCBP2 - myc binding protein 2, e3 ubiquitin protein ligase  TRAF1 - tnf receptor-associated factor 1  LPAR1 - lysophosphatidic acid receptor 1  HSP90B1 - heat shock protein 90kda beta (grp94), member 1  S1PR1 - sphingosine-1-phosphate receptor 1  TRAF2 - tnf receptor-associated factor 2  SETX - senataxin  SETD1B - set domain containing 1b  TPR - translocated promoter region, nuclear basket protein  PPRC1 - peroxisome proliferator-activated receptor gamma, coactivator-related 1  KDM4C - lysine (k)-specific demethylase 4c  BOD1 - biorientation of chromosomes in cell division 1  SIPA1L3 - signal-induced proliferation-associated 1 like 3  TTLL5 - tubulin tyrosine ligase-like family, member 5  ARHGAP26 - rho gtpase activating protein 26  NUP107 - nucleoporin 107kda  UTRN - utrophin  GOPC - golgi-associated pdz and coiled-coil motif containing  IQSEC2 - iq motif and sec7 domain 2  PLSCR4 - phospholipid scramblase 4  PTK2B - protein tyrosine kinase 2 beta  MRPS27 - mitochondrial ribosomal protein s27  ACSL3 - acyl-coa synthetase long-chain family member 3  RAP1GAP2 - rap1 gtpase activating protein 2  PARP11 - poly (adp-ribose) polymerase family, member 11  NFASC - neurofascin  BPTF - bromodomain phd finger transcription factor  CUL9 - cullin 9  KANSL1 - kat8 regulatory nsl complex subunit 1  FARSA - phenylalanyl-trna synthetase, alpha subunit  USP4 - ubiquitin specific peptidase 4 (proto-oncogene)  FBLN1 - fibulin 1  CLASP2 - cytoplasmic linker associated protein 2  POGZ - pogo transposable element with znf domain  PLSCR3 - phospholipid scramblase 3  LAMA1 - laminin, alpha 1  PLSCR2 - phospholipid scramblase 2  ETV6 - ets variant 6  PLXND1 - plexin d1  RAD54L2 - rad54-like 2 (s. cerevisiae)  VHL - von hippel-lindau tumor suppressor, e3 ubiquitin protein ligase  UTP3 - utp3, small subunit (ssu) processome component, homolog (s. cerevisiae)  VIM - vimentin  KDM6B - lysine (k)-specific demethylase 6b  EZR - ezrin  PHF8 - phd finger protein 8  SMC5 - structural maintenance of chromosomes 5  ESAM - endothelial cell adhesion molecule  EPB41L3 - erythrocyte membrane protein band 4.1-like 3  KCTD1 - potassium channel tetramerization domain containing 1  ANKLE2 - ankyrin repeat and lem domain containing 2  KIFC2 - kinesin family member c2  KDM6A - lysine (k)-specific demethylase 6a  FMNL3 - formin-like 3  UVRAG - uv radiation resistance associated  VASP - vasodilator-stimulated phosphoprotein  EYA3 - eyes absent homolog 3 (drosophila)  VAV2 - vav 2 guanine nucleotide exchange factor  EZH1 - enhancer of zeste homolog 1 (drosophila)  VBP1 - von hippel-lindau binding protein 1  VCAM1 - vascular cell adhesion molecule 1  CIAPIN1 - cytokine induced apoptosis inhibitor 1  NCDN - neurochondrin  F2R - coagulation factor ii (thrombin) receptor  VCL - vinculin  SLC25A46 - solute carrier family 25, member 46  PDXP - pyridoxal (pyridoxine, vitamin b6) phosphatase  VCP - valosin containing protein  MAPK8IP3 - mitogen-activated protein kinase 8 interacting protein 3  ATP8B2 - atpase, aminophospholipid transporter, class i, type 8b, member 2  TTLL12 - tubulin tyrosine ligase-like family, member 12  ATP10A - atpase, class v, type 10a  MCOLN1 - mucolipin 1  ERCC5 - excision repair cross-complementing rodent repair deficiency, complementation group 5  NEK9 - nima-related kinase 9  ERCC4 - excision repair cross-complementing rodent repair deficiency, complementation group 4  ERCC3 - excision repair cross-complementing rodent repair deficiency, complementation group 3  NUP205 - nucleoporin 205kda  YTHDC1 - yth domain containing 1  MPRIP - myosin phosphatase rho interacting protein  THOC2 - tho complex 2  ELMSAN1 - elm2 and myb/sant-like domain containing 1  CEP68 - centrosomal protein 68kda  UBE2A - ubiquitin-conjugating enzyme e2a  CHMP7 - charged multivesicular body protein 7  ATP10D - atpase, class v, type 10d  UBB - ubiquitin b  CORO1B - coronin, actin binding protein, 1b  KANK1 - kn motif and ankyrin repeat domains 1  ARRDC4 - arrestin domain containing 4  NDUFAF2 - nadh dehydrogenase (ubiquinone) complex i, assembly factor 2  RCOR1 - rest corepressor 1  ZNFX1 - zinc finger, nfx1-type containing 1  HIRA - histone cell cycle regulator  RFT1 - rft1 homolog (s. cerevisiae)  TYRO3 - tyro3 protein tyrosine kinase  FAF2 - fas associated factor family member 2  MDN1 - mdn1, midasin homolog (yeast)  TPGS1 - tubulin polyglutamylase complex subunit 1  ETS1 - v-ets avian erythroblastosis virus e26 oncogene homolog 1  ZMIZ1 - zinc finger, miz-type containing 1  ETF1 - eukaryotic translation termination factor 1  CYFIP1 - cytoplasmic fmr1 interacting protein 1  COG7 - component of oligomeric golgi complex 7  ARL6IP1 - adp-ribosylation factor-like 6 interacting protein 1  DMTN - dematin actin binding protein  EPB41L1 - erythrocyte membrane protein band 4.1-like 1  SUMO1 - small ubiquitin-like modifier 1  EPB41 - erythrocyte membrane protein band 4.1 (elliptocytosis 1, rh-linked)  UBTF - upstream binding transcription factor, rna polymerase i  ATP11B - atpase, class vi, type 11b  EPB41L2 - erythrocyte membrane protein band 4.1-like 2  UGCG - udp-glucose ceramide glucosyltransferase  ZNF830 - zinc finger protein 830  RRS1 - rrs1 ribosome biogenesis regulator homolog (s. cerevisiae)  PRRC2C - proline-rich coiled-coil 2c  SMURF1 - smad specific e3 ubiquitin protein ligase 1  C12orf65 - chromosome 12 open reading frame 65  SYT11 - synaptotagmin xi  JMJD6 - jumonji domain containing 6  MRPL47 - mitochondrial ribosomal protein l47  MYADM - myeloid-associated differentiation marker  EPRS - glutamyl-prolyl-trna synthetase  UBE2D2 - ubiquitin-conjugating enzyme e2d 2  TBC1D1 - tbc1 (tre-2/usp6, bub2, cdc16) domain family, member 1  EPHB6 - eph receptor b6  STX2 - syntaxin 2  ADCK1 - aarf domain containing kinase 1  ERCC2 - excision repair cross-complementing rodent repair deficiency, complementation group 2  NUP210 - nucleoporin 210kda  EPS8 - epidermal growth factor receptor pathway substrate 8  EPS15 - epidermal growth factor receptor pathway substrate 15  UBE2N - ubiquitin-conjugating enzyme e2n  FMOD - fibromodulin  VPS72 - vacuolar protein sorting 72 homolog (s. cerevisiae)  CAMSAP2 - calmodulin regulated spectrin-associated protein family, member 2  PLK1S1 - polo-like kinase 1 substrate 1  TCP1 - t-complex 1  TSPYL4 - tspy-like 4  BRK1 - brick1, scar/wave actin-nucleating complex subunit  SCFD1 - sec1 family domain containing 1  DDHD2 - ddhd domain containing 2  PDS5A - pds5, regulator of cohesion maintenance, homolog a (s. cerevisiae)  BOP1 - block of proliferation 1  FLII - flightless i homolog (drosophila)  FLNA - filamin a, alpha  ATP11A - atpase, class vi, type 11a  FLNB - filamin b, beta  ARC - activity-regulated cytoskeleton-associated protein  TMEM126B - transmembrane protein 126b  PACS2 - phosphofurin acidic cluster sorting protein 2  HDAC8 - histone deacetylase 8  LARP4 - la ribonucleoprotein domain family, member 4  ASH1L - ash1 (absent, small, or homeotic)-like (drosophila)  ANKRD28 - ankyrin repeat domain 28  SPATA7 - spermatogenesis associated 7  SIN3B - sin3 transcription regulator family member b  BDP1 - b double prime 1, subunit of rna polymerase iii transcription initiation factor iiib  NCAPD3 - non-smc condensin ii complex, subunit d3  FKBP4 - fk506 binding protein 4, 59kda  EHBP1 - eh domain binding protein 1  BICD2 - bicaudal d homolog 2 (drosophila)  HR - hair growth associated  CAND1 - cullin-associated and neddylation-dissociated 1  FKBP1A - fk506 binding protein 1a, 12kda  TDG - thymine-dna glycosylase  FBXW11 - f-box and wd repeat domain containing 11  VPS11 - vacuolar protein sorting 11 homolog (s. cerevisiae)  KDM3A - lysine (k)-specific demethylase 3a  DYNLT1 - dynein, light chain, tctex-type 1  STX1B - syntaxin 1b  IFT43 - intraflagellar transport 43 homolog (chlamydomonas)  TAF9 - taf9 rna polymerase ii, tata box binding protein (tbp)-associated factor, 32kda  FGFR2 - fibroblast growth factor receptor 2  TAF7 - taf7 rna polymerase ii, tata box binding protein (tbp)-associated factor, 55kda  VPS8 - vacuolar protein sorting 8 homolog (s. cerevisiae)  HAUS5 - haus augmin-like complex, subunit 5  FGF13 - fibroblast growth factor 13  TAF6 - taf6 rna polymerase ii, tata box binding protein (tbp)-associated factor, 80kda  TADA2A - transcriptional adaptor 2a  SMCHD1 - structural maintenance of chromosomes flexible hinge domain containing 1  SYNE1 - spectrin repeat containing, nuclear envelope 1  TACC1 - transforming, acidic coiled-coil containing protein 1  VPS39 - vacuolar protein sorting 39 homolog (s. cerevisiae)  NSFL1C - nsfl1 (p97) cofactor (p47)  PHF15 - phd finger protein 15  FGF2 - fibroblast growth factor 2 (basic)  CLASP1 - cytoplasmic linker associated protein 1  SZT2 - seizure threshold 2 homolog (mouse)  TAP2 - transporter 2, atp-binding cassette, sub-family b (mdr/tap)  USP22 - ubiquitin specific peptidase 22  TAPBP - tap binding protein (tapasin)  FGD1 - fyve, rhogef and ph domain containing 1  FEN1 - flap structure-specific endonuclease 1  FER - fer (fps/fes related) tyrosine kinase  PDGFC - platelet derived growth factor c  MAP3K7 - mitogen-activated protein kinase kinase kinase 7  DNAJC13 - dnaj (hsp40) homolog, subfamily c, member 13  EFHC1 - ef-hand domain (c-terminal) containing 1  PIP5K1C - phosphatidylinositol-4-phosphate 5-kinase, type i, gamma  INO80E - ino80 complex subunit e  ZC4H2 - zinc finger, c4h2 domain containing  KMT2E - lysine (k)-specific methyltransferase 2e  SIK3 - sik family kinase 3  NUDCD3 - nudc domain containing 3  TBCE - tubulin folding cofactor e  SPECC1L - sperm antigen with calponin homology and coiled-coil domains 1-like  TBCD - tubulin folding cofactor d  TBCC - tubulin folding cofactor c  TBCA - tubulin folding cofactor a  TAZ - tafazzin  SULF2 - sulfatase 2  CRTC1 - creb regulated transcription coactivator 1  UFL1 - ufm1-specific ligase 1  PPP1R13B - protein phosphatase 1, regulatory subunit 13b  PUM2 - pumilio homolog 2 (drosophila)  DMAP1 - dna methyltransferase 1 associated protein 1  TCF4 - transcription factor 4  OBSL1 - obscurin-like 1  FBN1 - fibrillin 1  RCC2 - regulator of chromosome condensation 2  FBLN2 - fibulin 2  NUDT7 - nudix (nucleoside diphosphate linked moiety x)-type motif 7  FAT1 - fat atypical cadherin 1  ERBB2IP - erbb2 interacting protein  TIE1 - tyrosine kinase with immunoglobulin-like and egf-like domains 1  TIMP1 - timp metallopeptidase inhibitor 1  TIMP2 - timp metallopeptidase inhibitor 2  ITGB3BP - integrin beta 3 binding protein (beta3-endonexin)  POC1B - poc1 centriolar protein b  TMED3 - transmembrane emp24 protein transport domain containing 3  TJP1 - tight junction protein 1  TK1 - thymidine kinase 1, soluble  RYBP - ring1 and yy1 binding protein  MTOR - mechanistic target of rapamycin (serine/threonine kinase)  TLE1 - transducin-like enhancer of split 1 (e(sp1) homolog, drosophila)  TLE3 - transducin-like enhancer of split 3 (e(sp1) homolog, drosophila)  BLOC1S2 - biogenesis of lysosomal organelles complex-1, subunit 2  TLE4 - transducin-like enhancer of split 4 (e(sp1) homolog, drosophila)  TLN1 - talin 1  ATP13A2 - atpase type 13a2  SIRT1 - sirtuin 1  TRAPPC10 - trafficking protein particle complex 10  TMF1 - tata element modulatory factor 1  ABCA5 - atp-binding cassette, sub-family a (abc1), member 5  CADPS2 - ca++-dependent secretion activator 2  TMSB4X - thymosin beta 4, x-linked  ACTR8 - arp8 actin-related protein 8 homolog (yeast)  CBX6 - chromobox homolog 6  BRD4 - bromodomain containing 4  TNFAIP1 - tumor necrosis factor, alpha-induced protein 1 (endothelial)  BCL9L - b-cell cll/lymphoma 9-like  TARDBP - tar dna binding protein  TNFRSF1A - tumor necrosis factor receptor superfamily, member 1a  TBCK - tbc1 domain containing kinase  TJAP1 - tight junction associated protein 1 (peripheral)  ARHGAP18 - rho gtpase activating protein 18  SF3B1 - splicing factor 3b, subunit 1, 155kda  KCTD21 - potassium channel tetramerization domain containing 21  PRKCDBP - protein kinase c, delta binding protein  KCTD2 - potassium channel tetramerization domain containing 2  TEK - tek tyrosine kinase, endothelial  SUZ12 - suz12 polycomb repressive complex 2 subunit  RCOR2 - rest corepressor 2  TFAM - transcription factor a, mitochondrial  TERF1 - telomeric repeat binding factor (nima-interacting) 1  TERF2 - telomeric repeat binding factor 2  TMEM80 - transmembrane protein 80  SNX18 - sorting nexin 18  TESK1 - testis-specific kinase 1  TFDP1 - transcription factor dp-1  CBX7 - chromobox homolog 7  BCCIP - brca2 and cdkn1a interacting protein  ISCU - iron-sulfur cluster assembly enzyme  PES1 - pescadillo ribosomal biogenesis factor 1  FXN - frataxin  TGS1 - trimethylguanosine synthase 1  TGFA - transforming growth factor, alpha  CEP41 - centrosomal protein 41kda  POLE4 - polymerase (dna-directed), epsilon 4, accessory subunit  HEY2 - hairy/enhancer-of-split related with yrpw motif 2  MACF1 - microtubule-actin crosslinking factor 1  TGFBR1 - transforming growth factor, beta receptor 1  TGFB2 - transforming growth factor, beta 2  RBFOX2 - rna binding protein, fox-1 homolog (c. elegans) 2  TGFB3 - transforming growth factor, beta 3  TGFB1 - transforming growth factor, beta 1  MAPK8IP2 - mitogen-activated protein kinase 8 interacting protein 2  LRRC8A - leucine rich repeat containing 8 family, member a  TP53INP1 - tumor protein p53 inducible nuclear protein 1  H2AFV - h2a histone family, member v  RNF20 - ring finger protein 20, e3 ubiquitin protein ligase  TGFBR3 - transforming growth factor, beta receptor iii  WDR45B - wd repeat domain 45b  ARHGAP12 - rho gtpase activating protein 12  THBS1 - thrombospondin 1  CABIN1 - calcineurin binding protein 1  SYTL4 - synaptotagmin-like 4  KAT6B - k(lysine) acetyltransferase 6b  THY1 - thy-1 cell surface antigen  METTL3 - methyltransferase like 3  DNAJB14 - dnaj (hsp40) homolog, subfamily b, member 14  COIL - coilin  ATAT1 - alpha tubulin acetyltransferase 1  SF3A2 - splicing factor 3a, subunit 2, 66kda  GEM - gtp binding protein overexpressed in skeletal muscle  NUDT19 - nudix (nucleoside diphosphate linked moiety x)-type motif 19  OBFC1 - oligonucleotide/oligosaccharide-binding fold containing 1  MKKS - mckusick-kaufman syndrome  KAT2A - k(lysine) acetyltransferase 2a  NCOA3 - nuclear receptor coactivator 3  PANK2 - pantothenate kinase 2  GJA5 - gap junction protein, alpha 5, 40kda  GJA1 - gap junction protein, alpha 1, 43kda  RND2 - rho family gtpase 2  AXIN1 - axin 1  TRRAP - transformation/transcription domain-associated protein  MCMBP - minichromosome maintenance complex binding protein  PICALM - phosphatidylinositol binding clathrin assembly protein  HDAC11 - histone deacetylase 11  GLS - glutaminase  SERF1A - small edrk-rich factor 1a (telomeric)  MAP9 - microtubule-associated protein 9  ARID1A - at rich interactive domain 1a (swi-like)  SETD6 - set domain containing 6  GCLM - glutamate-cysteine ligase, modifier subunit  ACTR5 - arp5 actin-related protein 5 homolog (yeast)  CDC45 - cell division cycle 45  NAA60 - n(alpha)-acetyltransferase 60, natf catalytic subunit  FZD4 - frizzled family receptor 4  GCLC - glutamate-cysteine ligase, catalytic subunit  FZD1 - frizzled family receptor 1  BAP1 - brca1 associated protein-1 (ubiquitin carboxy-terminal hydrolase)  AXIN2 - axin 2  NUP85 - nucleoporin 85kda  KDM5C - lysine (k)-specific demethylase 5c  SMC1A - structural maintenance of chromosomes 1a  USP9X - ubiquitin specific peptidase 9, x-linked  GNAI3 - guanine nucleotide binding protein (g protein), alpha inhibiting activity polypeptide 3  PHF17 - phd finger protein 17  GMFB - glia maturation factor, beta  EHMT1 - euchromatic histone-lysine n-methyltransferase 1  CAMKMT - calmodulin-lysine n-methyltransferase  FUS - fused in sarcoma  NAA40 - n(alpha)-acetyltransferase 40, natd catalytic subunit, homolog (s. cerevisiae)  TOR1AIP2 - torsin a interacting protein 2  GEMIN6 - gem (nuclear organelle) associated protein 6  RAB7A - rab7a, member ras oncogene family  GAB1 - grb2-associated binding protein 1  USP7 - ubiquitin specific peptidase 7 (herpes virus-associated)  GAA - glucosidase, alpha; acid  MANF - mesencephalic astrocyte-derived neurotrophic factor  XRCC6 - x-ray repair complementing defective repair in chinese hamster cells 6  RNF168 - ring finger protein 168, e3 ubiquitin protein ligase  BBS12 - bardet-biedl syndrome 12  GABPB1 - ga binding protein transcription factor, beta subunit 1  GABPA - ga binding protein transcription factor, alpha subunit 60kda  CPSF7 - cleavage and polyadenylation specific factor 7, 59kda  TCTN2 - tectonic family member 2  FYN - fyn oncogene related to src, fgr, yes  UBA5 - ubiquitin-like modifier activating enzyme 5  DDX39B - dead (asp-glu-ala-asp) box polypeptide 39b  GAK - cyclin g associated kinase  TTLL7 - tubulin tyrosine ligase-like family, member 7  KCTD17 - potassium channel tetramerization domain containing 17  KMT2D - lysine (k)-specific methyltransferase 2d  AAAS - achalasia, adrenocortical insufficiency, alacrimia  GABRG2 - gamma-aminobutyric acid (gaba) a receptor, gamma 2  GABRB3 - gamma-aminobutyric acid (gaba) a receptor, beta 3  IFT88 - intraflagellar transport 88 homolog (chlamydomonas)  YEATS4 - yeats domain containing 4  ZFAND1 - zinc finger, an1-type domain 1  ZFPM1 - zinc finger protein, fog family member 1  EID2 - ep300 interacting inhibitor of differentiation 2  TXNDC15 - thioredoxin domain containing 15  GBA - glucosidase, beta, acid  ST7 - suppression of tumorigenicity 7  GEMIN7 - gem (nuclear organelle) associated protein 7  KAT6A - k(lysine) acetyltransferase 6a  MICALL2 - mical-like 2  STAM - signal transducing adaptor molecule (sh3 domain and itam motif) 1  BRD3 - bromodomain containing 3  GAS6 - growth arrest-specific 6  CCDC6 - coiled-coil domain containing 6  DEDD2 - death effector domain containing 2  DHX36 - deah (asp-glu-ala-his) box polypeptide 36  SMC6 - structural maintenance of chromosomes 6  RNF112 - ring finger protein 112  GSPT1 - g1 to s phase transition 1  GSN - gelsolin  GTF2F2 - general transcription factor iif, polypeptide 2, 30kda  ZNF148 - zinc finger protein 148  GTF2E1 - general transcription factor iie, polypeptide 1, alpha 56kda  GTF2H2 - general transcription factor iih, polypeptide 2, 44kda  GTF2H3 - general transcription factor iih, polypeptide 3, 34kda  GTF2H1 - general transcription factor iih, polypeptide 1, 62kda  MCPH1 - microcephalin 1  GTF2A2 - general transcription factor iia, 2, 12kda  GTF2B - general transcription factor iib  PCGF2 - polycomb group ring finger 2  MAP7D3 - map7 domain containing 3  TBL1XR1 - transducin (beta)-like 1 x-linked receptor 1  GTF2H4 - general transcription factor iih, polypeptide 4, 52kda  GTF3A - general transcription factor iiia  BRF1 - brf1, rna polymerase iii transcription initiation factor 90 kda subunit  IPO4 - importin 4  MLF1IP - mlf1 interacting protein  H3F3A - h3 histone, family 3a  TCTN1 - tectonic family member 1  HIST1H2BB - histone cluster 1, h2bb  BRPF1 - bromodomain and phd finger containing, 1  H2AFZ - h2a histone family, member z  FZD5 - frizzled family receptor 5  H2AFX - h2a histone family, member x  CXCR4 - chemokine (c-x-c motif) receptor 4  MUL1 - mitochondrial e3 ubiquitin protein ligase 1  TUBA1A - tubulin, alpha 1a  CORO7 - coronin 7  TMEM231 - transmembrane protein 231  CDC73 - cell division cycle 73  DAP3 - death associated protein 3  HCFC1 - host cell factor c1 (vp16-accessory protein)  MAP3K12 - mitogen-activated protein kinase kinase kinase 12  ZYX - zyxin  NDNF - neuron-derived neurotrophic factor  HDAC2 - histone deacetylase 2  HTT - huntingtin  HDAC1 - histone deacetylase 1  ZNF205 - zinc finger protein 205  HCLS1 - hematopoietic cell-specific lyn substrate 1  ZNF207 - zinc finger protein 207  RIC3 - ric3 acetylcholine receptor chaperone  XPA - xeroderma pigmentosum, complementation group a  XPC - xeroderma pigmentosum, complementation group c  GOLGA2 - golgin a2  HAUS3 - haus augmin-like complex, subunit 3  XPO1 - exportin 1 (crm1 homolog, yeast)  GOLGB1 - golgin b1  XRCC1 - x-ray repair complementing defective repair in chinese hamster cells 1  HIGD2A - hig1 hypoxia inducible domain family, member 2a  PAGR1 - paxip1 associated glutamate-rich protein 1  RSG1 - rem2 and rab-like small gtpase 1  WRB - tryptophan rich basic protein  GPM6B - glycoprotein m6b  EIF4H - eukaryotic translation initiation factor 4h  CLIP2 - cap-gly domain containing linker protein 2  MPZL3 - myelin protein zero-like 3  WEE1 - wee1 homolog (s. pombe)  RILPL2 - rab interacting lysosomal protein-like 2  WHSC1 - wolf-hirschhorn syndrome candidate 1  VRK1 - vaccinia related kinase 1  VWF - von willebrand factor  ARID2 - at rich interactive domain 2 (arid, rfx-like)  PAOX - polyamine oxidase (exo-n4-amino)  TMEM43 - transmembrane protein 43  BRCC3 - brca1/brca2-containing complex, subunit 3  FSD1 - fibronectin type iii and spry domain containing 1  GRB2 - growth factor receptor-bound protein 2  GNPTAB - n-acetylglucosamine-1-phosphate transferase, alpha and beta subunits  ASXL1 - additional sex combs like 1 (drosophila)  CENPO - centromere protein o  SF1 - splicing factor 1  ZNF711 - zinc finger protein 711  ARHGAP35 - rho gtpase activating protein 35  XRCC5 - x-ray repair complementing defective repair in chinese hamster cells 5 (double-strand-break rejoining)  YWHAG - tyrosine 3-monooxygenase/tryptophan 5-monooxygenase activation protein, gamma polypeptide  GSK3B - glycogen synthase kinase 3 beta  YWHAZ - tyrosine 3-monooxygenase/tryptophan 5-monooxygenase activation protein, zeta polypeptide  GSK3A - glycogen synthase kinase 3 alpha  YWHAE - tyrosine 3-monooxygenase/tryptophan 5-monooxygenase activation protein, epsilon polypeptide  YWHAB - tyrosine 3-monooxygenase/tryptophan 5-monooxygenase activation protein, beta polypeptide  NDUFAF5 - nadh dehydrogenase (ubiquinone) complex i, assembly factor 5  MAPKAP1 - mitogen-activated protein kinase associated protein 1  RNF26 - ring finger protein 26  SAMD14 - sterile alpha motif domain containing 14  PEX11B - peroxisomal biogenesis factor 11 beta  PEX11A - peroxisomal biogenesis factor 11 alpha  FLCN - folliculin  CENPV - centromere protein v  NRP1 - neuropilin 1  PRMT1 - protein arginine methyltransferase 1  PRMT2 - protein arginine methyltransferase 2  LIN7A - lin-7 homolog a (c. elegans)  KLHDC8B - kelch domain containing 8b  FBXO45 - f-box protein 45  ARL13B - adp-ribosylation factor-like 13b  AGFG1 - arfgap with fg repeats 1  CCDC28B - coiled-coil domain containing 28b  APOO - apolipoprotein o  KCTD6 - potassium channel tetramerization domain containing 6  ALKBH1 - alkb, alkylation repair homolog 1 (e. coli)  HPRT1 - hypoxanthine phosphoribosyltransferase 1  RNU1-1 - rna, u1 small nuclear 1  TMEM17 - transmembrane protein 17  FAM174B - family with sequence similarity 174, member b  CFLAR - casp8 and fadd-like apoptosis regulator  SYNGAP1 - synaptic ras gtpase activating protein 1  KCTD15 - potassium channel tetramerization domain containing 15  SYNJ1 - synaptojanin 1  LDB1 - lim domain binding 1  WDR77 - wd repeat domain 77  ALDH1A2 - aldehyde dehydrogenase 1 family, member a2  IBA57 - iba57, iron-sulfur cluster assembly homolog (s. cerevisiae)  DSCC1 - dna replication and sister chromatid cohesion 1  KAT2B - k(lysine) acetyltransferase 2b  AKAP4 - a kinase (prka) anchor protein 4  CRTC2 - creb regulated transcription coactivator 2  ZNF259 - zinc finger protein 259  CENPM - centromere protein m  CDC123 - cell division cycle 123  ARHGEF7 - rho guanine nucleotide exchange factor (gef) 7  MIS12 - mis12 kinetochore complex component  MTMR2 - myotubularin related protein 2  AP1M1 - adaptor-related protein complex 1, mu 1 subunit  HUS1 - hus1 checkpoint homolog (s. pombe)  MCM3AP - minichromosome maintenance complex component 3 associated protein  EIF2S2 - eukaryotic translation initiation factor 2, subunit 2 beta, 38kda  TMEM108 - transmembrane protein 108  HERC1 - hect and rld domain containing e3 ubiquitin protein ligase family member 1  TRAK2 - trafficking protein, kinesin binding 2  SNX19 - sorting nexin 19  HSPD1 - heat shock 60kda protein 1 (chaperonin)  HSP90AA1 - heat shock protein 90kda alpha (cytosolic), class a member 1  AP3D1 - adaptor-related protein complex 3, delta 1 subunit  HSPA4 - heat shock 70kda protein 4  SKAP2 - src kinase associated phosphoprotein 2  BAIAP3 - bai1-associated protein 3  HSPA9 - heat shock 70kda protein 9 (mortalin)  WASF1 - was protein family, member 1  HSPA8 - heat shock 70kda protein 8  HMGN1 - high mobility group nucleosome binding domain 1  HMGB3 - high mobility group box 3  NUMB - numb homolog (drosophila)  HMGB2 - high mobility group box 2  HMGB1 - high mobility group box 1  MTIF3 - mitochondrial translational initiation factor 3  HLCS - holocarboxylase synthetase (biotin-(proprionyl-coa-carboxylase (atp-hydrolysing)) ligase)  PHACTR4 - phosphatase and actin regulator 4  EIF3C - eukaryotic translation initiation factor 3, subunit c  BRD9 - bromodomain containing 9  EIF3D - eukaryotic translation initiation factor 3, subunit d  PLEKHA3 - pleckstrin homology domain containing, family a (phosphoinositide binding specific) member 3  EIF3A - eukaryotic translation initiation factor 3, subunit a  EIF3B - eukaryotic translation initiation factor 3, subunit b  DYNLL1 - dynein, light chain, lc8-type 1  TNKS - tankyrase, trf1-interacting ankyrin-related adp-ribose polymerase  AGO1 - argonaute risc catalytic component 1  EIF3F - eukaryotic translation initiation factor 3, subunit f  TIMM9 - translocase of inner mitochondrial membrane 9 homolog (yeast)  EIF3G - eukaryotic translation initiation factor 3, subunit g  BECN1 - beclin 1, autophagy related  ZBTB48 - zinc finger and btb domain containing 48  SRSF9 - serine/arginine-rich splicing factor 9  MRPL1 - mitochondrial ribosomal protein l1  VAMP4 - vesicle-associated membrane protein 4  VMA21 - vma21 vacuolar h+-atpase homolog (s. cerevisiae)  TMEM237 - transmembrane protein 237  HIPK1 - homeodomain interacting protein kinase 1  HYAL2 - hyaluronoglucosaminidase 2  ACD - adrenocortical dysplasia homolog (mouse)  HLA-DMA - major histocompatibility complex, class ii, dm alpha  TMEM135 - transmembrane protein 135  NAIF1 - nuclear apoptosis inducing factor 1  DGAT1 - diacylglycerol o-acyltransferase 1  BSCL2 - berardinelli-seip congenital lipodystrophy 2 (seipin)  HFE - hemochromatosis  MRPL44 - mitochondrial ribosomal protein l44  VPS33A - vacuolar protein sorting 33 homolog a (s. cerevisiae)  HIP1 - huntingtin interacting protein 1  UBE2K - ubiquitin-conjugating enzyme e2k  MARCKSL1 - marcks-like 1  MBTPS1 - membrane-bound transcription factor peptidase, site 1  ARFGAP3 - adp-ribosylation factor gtpase activating protein 3  TRADD - tnfrsf1a-associated via death domain  ERAL1 - era-like 12s mitochondrial rrna chaperone 1  SNX3 - sorting nexin 3  TSNARE1 - t-snare domain containing 1  TUBB - tubulin, beta class i  EED - embryonic ectoderm development  GBF1 - golgi brefeldin a resistant guanine nucleotide exchange factor 1  RIPK1 - receptor (tnfrsf)-interacting serine-threonine kinase 1  HNRNPU - heterogeneous nuclear ribonucleoprotein u (scaffold attachment factor a)  MRPS24 - mitochondrial ribosomal protein s24  MRPS6 - mitochondrial ribosomal protein s6  MRPS11 - mitochondrial ribosomal protein s11  VPS33B - vacuolar protein sorting 33 homolog b (yeast)  TINF2 - terf1 (trf1)-interacting nuclear factor 2  ADAM15 - adam metallopeptidase domain 15  MRPL32 - mitochondrial ribosomal protein l32  CDS2 - cdp-diacylglycerol synthase (phosphatidate cytidylyltransferase) 2  ARV1 - arv1 homolog (s. cerevisiae)  P2RY12 - purinergic receptor p2y, g-protein coupled, 12  RAB11A - rab11a, member ras oncogene family  HNRNPA2B1 - heterogeneous nuclear ribonucleoprotein a2/b1  HNRNPC - heterogeneous nuclear ribonucleoprotein c (c1/c2)  NAPA - n-ethylmaleimide-sensitive factor attachment protein, alpha  NAPG - n-ethylmaleimide-sensitive factor attachment protein, gamma  HMGA1 - high mobility group at-hook 1  VWA1 - von willebrand factor a domain containing 1  DDX31 - dead (asp-glu-ala-asp) box polypeptide 31  DEPTOR - dep domain containing mtor-interacting protein  HAT1 - histone acetyltransferase 1  EIF3K - eukaryotic translation initiation factor 3, subunit k  CRTC3 - creb regulated transcription coactivator 3  RAB30 - rab30, member ras oncogene family  RBMX - rna binding motif protein, x-linked  PKP4 - plakophilin 4  PEX3 - peroxisomal biogenesis factor 3  HTRA2 - htra serine peptidase 2  MEAF6 - myst/esa1-associated factor 6  PPFIA2 - protein tyrosine phosphatase, receptor type, f polypeptide (ptprf), interacting protein (liprin), alpha 2  PPFIA1 - protein tyrosine phosphatase, receptor type, f polypeptide (ptprf), interacting protein (liprin), alpha 1  TOR1B - torsin family 1, member b (torsin b)  ING2 - inhibitor of growth family, member 2  PPM1D - protein phosphatase, mg2+/mn2+ dependent, 1d  UBE2S - ubiquitin-conjugating enzyme e2s  PRPF19 - pre-mrna processing factor 19  ILK - integrin-linked kinase  CDC14B - cell division cycle 14b  PIAS1 - protein inhibitor of activated stat, 1  SMYD3 - set and mynd domain containing 3  BLZF1 - basic leucine zipper nuclear factor 1  AP3B1 - adaptor-related protein complex 3, beta 1 subunit  METTL17 - methyltransferase like 17  PATL1 - protein associated with topoisomerase ii homolog 1 (yeast)  EML4 - echinoderm microtubule associated protein like 4  PPFIA3 - protein tyrosine phosphatase, receptor type, f polypeptide (ptprf), interacting protein (liprin), alpha 3  AGPS - alkylglycerone phosphate synthase  COPS7B - cop9 signalosome subunit 7b  CBX4 - chromobox homolog 4  COPS3 - cop9 signalosome subunit 3  GORASP1 - golgi reassembly stacking protein 1, 65kda  GAS7 - growth arrest-specific 7  HIAT1 - hippocampus abundant transcript 1  ORAOV1 - oral cancer overexpressed 1  SERP1 - stress-associated endoplasmic reticulum protein 1  ITGAE - integrin, alpha e (antigen cd103, human mucosal lymphocyte antigen 1; alpha polypeptide)  TUBGCP4 - tubulin, gamma complex associated protein 4  VPS16 - vacuolar protein sorting 16 homolog (s. cerevisiae)  ITGA1 - integrin, alpha 1  ITGA3 - integrin, alpha 3 (antigen cd49c, alpha 3 subunit of vla-3 receptor)  ITGB5 - integrin, beta 5  NOM1 - nucleolar protein with mif4g domain 1  NFU1 - nfu1 iron-sulfur cluster scaffold homolog (s. cerevisiae)  PDLIM4 - pdz and lim domain 4  ITGAV - integrin, alpha v  POLR1E - polymerase (rna) i polypeptide e, 53kda  CDK10 - cyclin-dependent kinase 10  SUDS3 - suppressor of defective silencing 3 homolog (s. cerevisiae)  TBCEL - tubulin folding cofactor e-like  ARFIP1 - adp-ribosylation factor interacting protein 1  PRPF18 - pre-mrna processing factor 18  ITGB1 - integrin, beta 1 (fibronectin receptor, beta polypeptide, antigen cd29 includes mdf2, msk12)  DEGS1 - delta(4)-desaturase, sphingolipid 1  DENR - density-regulated protein  ITGB4 - integrin, beta 4  ACTR6 - arp6 actin-related protein 6 homolog (yeast)  EIF6 - eukaryotic translation initiation factor 6  BBS9 - bardet-biedl syndrome 9  TPCN2 - two pore segment channel 2  TEKT2 - tektin 2 (testicular)  ARAP3 - arfgap with rhogap domain, ankyrin repeat and ph domain 3  INPPL1 - inositol polyphosphate phosphatase-like 1  ATG3 - autophagy related 3  RND1 - rho family gtpase 1  INSR - insulin receptor  PCDH17 - protocadherin 17  AKTIP - akt interacting protein  KLHL20 - kelch-like family member 20  MPP5 - membrane protein, palmitoylated 5 (maguk p55 subfamily member 5)  LSM3 - lsm3 homolog, u6 small nuclear rna associated (s. cerevisiae)  PSMG1 - proteasome (prosome, macropain) assembly chaperone 1  KLF7 - kruppel-like factor 7 (ubiquitous)  RUVBL1 - ruvb-like 1 (e. coli)  USO1 - uso1 vesicle transport factor  ITGA6 - integrin, alpha 6  NOP14 - nop14 nucleolar protein  ARHGEF28 - rho guanine nucleotide exchange factor (gef) 28  LRWD1 - leucine-rich repeats and wd repeat domain containing 1  TRAPPC3 - trafficking protein particle complex 3  NSD1 - nuclear receptor binding set domain protein 1  MMS19 - mms19 nucleotide excision repair homolog (s. cerevisiae)  PDLIM2 - pdz and lim domain 2 (mystique)  ID2 - inhibitor of dna binding 2, dominant negative helix-loop-helix protein  PIP5K1A - phosphatidylinositol-4-phosphate 5-kinase, type i, alpha  BRPF3 - bromodomain and phd finger containing, 3  C17orf75 - chromosome 17 open reading frame 75  STK36 - serine/threonine kinase 36  NDOR1 - nadph dependent diflavin oxidoreductase 1  THSD7A - thrombospondin, type i, domain containing 7a  TUBG2 - tubulin, gamma 2  NME5 - nme/nm23 family member 5  TMED4 - transmembrane emp24 protein transport domain containing 4  VPS4A - vacuolar protein sorting 4 homolog a (s. cerevisiae)  IGHMBP2 - immunoglobulin mu binding protein 2  RSPH9 - radial spoke head 9 homolog (chlamydomonas)  EPB41L4A - erythrocyte membrane protein band 4.1 like 4a  LRRC4 - leucine rich repeat containing 4  NCK2 - nck adaptor protein 2  GNPAT - glyceronephosphate o-acyltransferase  DYRK3 - dual-specificity tyrosine-(y)-phosphorylation regulated kinase 3  CYFIP2 - cytoplasmic fmr1 interacting protein 2  USP21 - ubiquitin specific peptidase 21  ULK1 - unc-51 like autophagy activating kinase 1  EEA1 - early endosome antigen 1  STX7 - syntaxin 7  GOLPH3 - golgi phosphoprotein 3 (coat-protein)  PHACTR1 - phosphatase and actin regulator 1  NPTN - neuroplastin  MLH3 - mutl homolog 3 (e. coli)  SORBS2 - sorbin and sh3 domain containing 2  JMJD1C - jumonji domain containing 1c  REEP3 - receptor accessory protein 3  SMARCA5 - swi/snf related, matrix associated, actin dependent regulator of chromatin, subfamily a, member 5  ATP2C1 - atpase, ca++ transporting, type 2c, member 1  CDC42BPA - cdc42 binding protein kinase alpha (dmpk-like)  IGF1R - insulin-like growth factor 1 receptor  FAM171A1 - family with sequence similarity 171, member a1  OGT - o-linked n-acetylglucosamine (glcnac) transferase  HIRIP3 - hira interacting protein 3  IGF2R - insulin-like growth factor 2 receptor  RAE1 - rae1 rna export 1 homolog (s. pombe)  GEMIN2 - gem (nuclear organelle) associated protein 2  NSG1 - neuron specific gene family member 1  TSPYL2 - tspy-like 2  CUL4B - cullin 4b  VPS41 - vacuolar protein sorting 41 homolog (s. cerevisiae)  CUL1 - cullin 1  B9D1 - b9 protein domain 1  CUL3 - cullin 3  ATRN - attractin  SUPT3H - suppressor of ty 3 homolog (s. cerevisiae)  CACNG4 - calcium channel, voltage-dependent, gamma subunit 4  PCGF5 - polycomb group ring finger 5  SDHAF1 - succinate dehydrogenase complex assembly factor 1  WAC - ww domain containing adaptor with coiled-coil  PHF21A - phd finger protein 21a  HPS3 - hermansky-pudlak syndrome 3  LIG4 - ligase iv, dna, atp-dependent  PITPNM1 - phosphatidylinositol transfer protein, membrane-associated 1  MKI67IP - mki67 (fha domain) interacting nucleolar phosphoprotein  ABLIM1 - actin binding lim protein 1  LIMK1 - lim domain kinase 1  VPS37A - vacuolar protein sorting 37 homolog a (s. cerevisiae)  NCOR1 - nuclear receptor corepressor 1  LDLR - low density lipoprotein receptor  MTPN - myotrophin  TIMMDC1 - translocase of inner mitochondrial membrane domain containing 1  HOOK3 - hook microtubule-tethering protein 3  LZTS2 - leucine zipper, putative tumor suppressor 2  DOT1L - dot1-like histone h3k79 methyltransferase  REEP2 - receptor accessory protein 2  ABCG1 - atp-binding cassette, sub-family g (white), member 1  LETM1 - leucine zipper-ef-hand containing transmembrane protein 1  SYCE1L - synaptonemal complex central element protein 1-like  L3MBTL3 - l(3)mbt-like 3 (drosophila)  LOXL1 - lysyl oxidase-like 1  TICAM2 - toll-like receptor adaptor molecule 2  BRSK1 - br serine/threonine kinase 1  SLX4 - slx4 structure-specific endonuclease subunit  CHD1L - chromodomain helicase dna binding protein 1-like  SEC22B - sec22 vesicle trafficking protein homolog b (s. cerevisiae) (gene/pseudogene)  PTPDC1 - protein tyrosine phosphatase domain containing 1  H2AFY - h2a histone family, member y  GOSR2 - golgi snap receptor complex member 2  BET1L - bet1 golgi vesicular membrane trafficking protein-like  UBXN2B - ubx domain protein 2b  BCAR1 - breast cancer anti-estrogen resistance 1  LIPA - lipase a, lysosomal acid, cholesterol esterase  SPIRE2 - spire actin nucleation factor 2  LIMK2 - lim domain kinase 2  HOPX - hop homeobox  GOLM1 - golgi membrane protein 1  CDC42BPB - cdc42 binding protein kinase beta (dmpk-like)  JAGN1 - jagunal homolog 1 (drosophila)  NACC2 - nacc family member 2, ben and btb (poz) domain containing  LMNA - lamin a/c  CLOCK - clock circadian regulator  HGSNAT - heparan-alpha-glucosaminide n-acetyltransferase  LMAN1 - lectin, mannose-binding, 1  LLGL1 - lethal giant larvae homolog 1 (drosophila)  ADAMTS4 - adam metallopeptidase with thrombospondin type 1 motif, 4  ADAMTS1 - adam metallopeptidase with thrombospondin type 1 motif, 1  MRPL43 - mitochondrial ribosomal protein l43  TBPL1 - tbp-like 1  YTHDF2 - yth domain family, member 2  VPS4B - vacuolar protein sorting 4 homolog b (s. cerevisiae)  MAP1LC3A - microtubule-associated protein 1 light chain 3 alpha  GOSR1 - golgi snap receptor complex member 1  NIP7 - nip7, nucleolar pre-rrna processing protein  EIF3L - eukaryotic translation initiation factor 3, subunit l  BAG5 - bcl2-associated athanogene 5  KRT10 - keratin 10  BAG4 - bcl2-associated athanogene 4  TRAPPC4 - trafficking protein particle complex 4  ACTL6B - actin-like 6b  LAMP2 - lysosomal-associated membrane protein 2  POMP - proteasome maturation protein  STMN1 - stathmin 1  TEX264 - testis expressed 264  ATG5 - autophagy related 5  ROCK2 - rho-associated, coiled-coil containing protein kinase 2  LCP1 - lymphocyte cytosolic protein 1 (l-plastin)  ATP6V1D - atpase, h+ transporting, lysosomal 34kda, v1 subunit d  ANGPT4 - angiopoietin 4  STX8 - syntaxin 8  FUNDC1 - fun14 domain containing 1  L1CAM - l1 cell adhesion molecule  TAOK3 - tao kinase 3  ZBTB7A - zinc finger and btb domain containing 7a  CRNKL1 - crooked neck pre-mrna splicing factor-like 1 (drosophila)  DPY30 - dpy-30 homolog (c. elegans)  LAMA5 - laminin, alpha 5  LAMA4 - laminin, alpha 4  LAMC1 - laminin, gamma 1 (formerly lamb2)  NAGPA - n-acetylglucosamine-1-phosphodiester alpha-n-acetylglucosaminidase  CHD6 - chromodomain helicase dna binding protein 6  POLR1B - polymerase (rna) i polypeptide b, 128kda  KIFC3 - kinesin family member c3  VPS28 - vacuolar protein sorting 28 homolog (s. cerevisiae)  KIF5C - kinesin family member 5c  ANTXR1 - anthrax toxin receptor 1  MRTO4 - mrna turnover 4 homolog (s. cerevisiae)  RPF2 - ribosome production factor 2 homolog (s. cerevisiae)  KIF2A - kinesin heavy chain member 2a  KIF5B - kinesin family member 5b  ARHGEF6 - rac/cdc42 guanine nucleotide exchange factor (gef) 6  KDR - kinase insert domain receptor (a type iii receptor tyrosine kinase)  PICK1 - protein interacting with prkca 1  HSD17B12 - hydroxysteroid (17-beta) dehydrogenase 12  ING4 - inhibitor of growth family, member 4  EIF2AK3 - eukaryotic translation initiation factor 2-alpha kinase 3  DYNC1LI1 - dynein, cytoplasmic 1, light intermediate chain 1  KCTD3 - potassium channel tetramerization domain containing 3  CCDC41 - coiled-coil domain containing 41  KCNMA1 - potassium large conductance calcium-activated channel, subfamily m, alpha member 1  MAP4K4 - mitogen-activated protein kinase kinase kinase kinase 4  TRAF7 - tnf receptor-associated factor 7, e3 ubiquitin protein ligase  RECQL5 - recq protein-like 5  LRRC8C - leucine rich repeat containing 8 family, member c  KRAS - kirsten rat sarcoma viral oncogene homolog  TGFBRAP1 - transforming growth factor, beta receptor associated protein 1  CIAO1 - cytosolic iron-sulfur protein assembly 1  RPL26L1 - ribosomal protein l26-like 1  KPNB1 - karyopherin (importin) beta 1  SBDS - shwachman-bodian-diamond syndrome  RMDN1 - regulator of microtubule dynamics 1  SUV420H1 - suppressor of variegation 4-20 homolog 1 (drosophila)  KLC1 - kinesin light chain 1  TRAPPC12 - trafficking protein particle complex 12  CRIPT - cysteine-rich pdz-binding protein  DDX23 - dead (asp-glu-ala-asp) box polypeptide 23  PHF20L1 - phd finger protein 20-like 1  TJP2 - tight junction protein 2  TFB1M - transcription factor b1, mitochondrial  NDUFAF1 - nadh dehydrogenase (ubiquinone) complex i, assembly factor 1  PEX16 - peroxisomal biogenesis factor 16  KIT - v-kit hardy-zuckerman 4 feline sarcoma viral oncogene homolog  SH3GLB1 - sh3-domain grb2-like endophilin b1  LONP1 - lon peptidase 1, mitochondrial  TMEM216 - transmembrane protein 216  MRPL51 - mitochondrial ribosomal protein l51  MRPL30 - mitochondrial ribosomal protein l30  KCNA5 - potassium voltage-gated channel, shaker-related subfamily, member 5  TCHP - trichoplein, keratin filament binding  PSMG3 - proteasome (prosome, macropain) assembly chaperone 3  PDZD11 - pdz domain containing 11  SLIT2 - slit homolog 2 (drosophila)  JUP - junction plakoglobin  TMEM175 - transmembrane protein 175  COG1 - component of oligomeric golgi complex 1  JAK2 - janus kinase 2  ING5 - inhibitor of growth family, member 5  COX16 - cox16 cytochrome c oxidase assembly homolog (s. cerevisiae)  SLC25A33 - solute carrier family 25 (pyrimidine nucleotide carrier), member 33  KIF3B - kinesin family member 3b  ITPA - inosine triphosphatase (nucleoside triphosphate pyrophosphatase)  MAST4 - microtubule associated serine/threonine kinase family member 4  GLTP - glycolipid transfer protein  PHF20 - phd finger protein 20  CHCHD6 - coiled-coil-helix-coiled-coil-helix domain containing 6  ZNHIT3 - zinc finger, hit-type containing 3  HMGN3 - high mobility group nucleosomal binding domain 3  COPS2 - cop9 signalosome subunit 2  ATP9B - atpase, class ii, type 9b  ZDHHC2 - zinc finger, dhhc-type containing 2  SNAP29 - synaptosomal-associated protein, 29kda  RSL24D1 - ribosomal l24 domain containing 1  VAMP3 - vesicle-associated membrane protein 3  KCND3 - potassium voltage-gated channel, shal-related subfamily, member 3  MIA3 - melanoma inhibitory activity family, member 3  TAOK2 - tao kinase 2  BRMS1L - breast cancer metastasis-suppressor 1-like  B4GALT6 - udp-gal:betaglcnac beta 1,4- galactosyltransferase, polypeptide 6  LEF1 - lymphoid enhancer-binding factor 1  VPS25 - vacuolar protein sorting 25 homolog (s. cerevisiae)  PLEKHO1 - pleckstrin homology domain containing, family o member 1  GTF3C4 - general transcription factor iiic, polypeptide 4, 90kda  MRPL45 - mitochondrial ribosomal protein l45  TUBE1 - tubulin, epsilon 1  KCNC4 - potassium voltage-gated channel, shaw-related subfamily, member 4  CCDC115 - coiled-coil domain containing 115  TMEM107 - transmembrane protein 107  B4GALT5 - udp-gal:betaglcnac beta 1,4- galactosyltransferase, polypeptide 5  S1PR2 - sphingosine-1-phosphate receptor 2  LUC7L3 - luc7-like 3 (s. cerevisiae)  KCTD10 - potassium channel tetramerization domain containing 10  ARID4B - at rich interactive domain 4b (rbp1-like)  STARD3NL - stard3 n-terminal like  MIF - macrophage migration inhibitory factor (glycosylation-inhibiting factor)  RAB8B - rab8b, member ras oncogene family  ATP8A2 - atpase, aminophospholipid transporter, class i, type 8a, member 2  MLH1 - mutl homolog 1, colon cancer, nonpolyposis type 2 (e. coli)  MKLN1 - muskelin 1, intracellular mediator containing kelch motifs  ITGB1BP1 - integrin beta 1 binding protein 1  KDM3B - lysine (k)-specific demethylase 3b  RSF1 - remodeling and spacing factor 1  CYTH2 - cytohesin 2  KMT2A - lysine (k)-specific methyltransferase 2a  MMP2 - matrix metallopeptidase 2 (gelatinase a, 72kda gelatinase, 72kda type iv collagenase)  NUMBL - numb homolog (drosophila)-like  RPS6KA5 - ribosomal protein s6 kinase, 90kda, polypeptide 5  TRAPPC2L - trafficking protein particle complex 2-like  CCDC8 - coiled-coil domain containing 8  KATNAL1 - katanin p60 subunit a-like 1  MMP14 - matrix metallopeptidase 14 (membrane-inserted)  CACNA2D2 - calcium channel, voltage-dependent, alpha 2/delta subunit 2  MNAT1 - mnat cdk-activating kinase assembly factor 1  UIMC1 - ubiquitin interaction motif containing 1  MTA2 - metastasis associated 1 family, member 2  SYT9 - synaptotagmin ix  NOLC1 - nucleolar and coiled-body phosphoprotein 1  RAB23 - rab23, member ras oncogene family  EIF2A - eukaryotic translation initiation factor 2a, 65kda  MAGI1 - membrane associated guanylate kinase, ww and pdz domain containing 1  DLG5 - discs, large homolog 5 (drosophila)  ZMYM3 - zinc finger, mym-type 3  ZMYM4 - zinc finger, mym-type 4  MCM7 - minichromosome maintenance complex component 7  ANKRD27 - ankyrin repeat domain 27 (vps9 domain)  PLEKHA7 - pleckstrin homology domain containing, family a member 7  VAPB - vamp (vesicle-associated membrane protein)-associated protein b and c  SPTY2D1 - spt2, suppressor of ty, domain containing 1 (s. cerevisiae)  BUB3 - bub3 mitotic checkpoint protein  PELO - pelota homolog (drosophila)  MECP2 - methyl cpg binding protein 2 (rett syndrome)  REPS2 - ralbp1 associated eps domain containing 2  MEF2A - myocyte enhancer factor 2a  ZW10 - zw10 kinetochore protein  BRWD1 - bromodomain and wd repeat domain containing 1  A4GALT - alpha 1,4-galactosyltransferase  FAM160A2 - family with sequence similarity 160, member a2  DCLK1 - doublecortin-like kinase 1  MEN1 - multiple endocrine neoplasia i  RAB8A - rab8a, member ras oncogene family  CEP78 - centrosomal protein 78kda  COX7A2L - cytochrome c oxidase subunit viia polypeptide 2 like  SCAF11 - sr-related ctd-associated factor 11  FAM161A - family with sequence similarity 161, member a  ZFYVE1 - zinc finger, fyve domain containing 1  KAT8 - k(lysine) acetyltransferase 8  MFAP2 - microfibrillar-associated protein 2  ARHGEF2 - rho/rac guanine nucleotide exchange factor (gef) 2  MFGE8 - milk fat globule-egf factor 8 protein  TUBB2B - tubulin, beta 2b class iib  MFAP4 - microfibrillar-associated protein 4  ATG12 - autophagy related 12  ARL6 - adp-ribosylation factor-like 6  STX18 - syntaxin 18  SYNGR1 - synaptogyrin 1  HGS - hepatocyte growth factor-regulated tyrosine kinase substrate  MBD3 - methyl-cpg binding domain protein 3  VTA1 - vesicle (multivesicular body) trafficking 1  RABEP1 - rabaptin, rab gtpase binding effector protein 1  COG3 - component of oligomeric golgi complex 3  MAL - mal, t-cell differentiation protein  DNAL1 - dynein, axonemal, light chain 1  PRPF3 - pre-mrna processing factor 3  FAM50A - family with sequence similarity 50, member a  TMEM138 - transmembrane protein 138  LARS - leucyl-trna synthetase  SMC3 - structural maintenance of chromosomes 3  NCKIPSD - nck interacting protein with sh3 domain  MARK3 - map/microtubule affinity-regulating kinase 3  MARK1 - map/microtubule affinity-regulating kinase 1  MAPT - microtubule-associated protein tau  INA - internexin neuronal intermediate filament protein, alpha  MAP6 - microtubule-associated protein 6  ATP6V0D1 - atpase, h+ transporting, lysosomal 38kda, v0 subunit d1  RAB14 - rab14, member ras oncogene family  MAP2 - microtubule-associated protein 2  LATS1 - large tumor suppressor kinase 1  MAP1B - microtubule-associated protein 1b  MTA1 - metastasis associated 1  SIRT6 - sirtuin 6  MAP1A - microtubule-associated protein 1a  RAB33B - rab33b, member ras oncogene family  VPS54 - vacuolar protein sorting 54 homolog (s. cerevisiae)  FAM161B - family with sequence similarity 161, member b  MAN2A1 - mannosidase, alpha, class 2a, member 1  USP8 - ubiquitin specific peptidase 8  LIMA1 - lim domain and actin binding 1  EVL - enah/vasp-like  MATN2 - matrilin 2  SFMBT1 - scm-like with four mbt domains 1  SART1 - squamous cell carcinoma antigen recognized by t cells  MAX - myc associated factor x  ZMIZ2 - zinc finger, miz-type containing 2  MAT2A - methionine adenosyltransferase ii, alpha  GULP1 - gulp, engulfment adaptor ptb domain containing 1  UFC1 - ubiquitin-fold modifier conjugating enzyme 1  MCM3 - minichromosome maintenance complex component 3  TRMT112 - trna methyltransferase 11-2 homolog (s. cerevisiae)  MCM6 - minichromosome maintenance complex component 6  CHMP5 - charged multivesicular body protein 5  MCM5 - minichromosome maintenance complex component 5  TTBK2 - tau tubulin kinase 2  MCF2 - mcf.2 cell line derived transforming sequence  CDAN1 - codanin 1  MCM2 - minichromosome maintenance complex component 2  ASH2L - ash2 (absent, small, or homeotic)-like (drosophila)  NOP16 - nop16 nucleolar protein  ANKFY1 - ankyrin repeat and fyve domain containing 1  SYT7 - synaptotagmin vii  MAP7 - microtubule-associated protein 7  NFS1 - nfs1 cysteine desulfurase  MRPL48 - mitochondrial ribosomal protein l48  MRPS23 - mitochondrial ribosomal protein s23  L3MBTL2 - l(3)mbt-like 2 (drosophila)  MRPS33 - mitochondrial ribosomal protein s33  FAM96B - family with sequence similarity 96, member b  RPL14 - ribosomal protein l14  RIOK1 - rio kinase 1  LRP6 - low density lipoprotein receptor-related protein 6  OTUD6B - otu domain containing 6b  SEMA5A - sema domain, seven thrombospondin repeats (type 1 and type 1-like), transmembrane domain (tm) and short cytoplasmic domain, (semaphorin) 5a  TRAPPC9 - trafficking protein particle complex 9  LUM - lumican  LYN - v-yes-1 yamaguchi sarcoma viral related oncogene homolog  TPPP3 - tubulin polymerization-promoting protein family member 3  JAM3 - junctional adhesion molecule 3  HIP1R - huntingtin interacting protein 1 related  LTBP3 - latent transforming growth factor beta binding protein 3  RNF8 - ring finger protein 8, e3 ubiquitin protein ligase  TMED6 - transmembrane emp24 protein transport domain containing 6  CHMP3 - charged multivesicular body protein 3  BAZ1B - bromodomain adjacent to zinc finger domain, 1b  TAF1B - tata box binding protein (tbp)-associated factor, rna polymerase i, b, 63kda  MARCKS - myristoylated alanine-rich protein kinase c substrate  RAB34 - rab34, member ras oncogene family  SMAD2 - smad family member 2  RTN4RL1 - reticulon 4 receptor-like 1  MBIP - map3k12 binding inhibitory protein 1  HDAC7 - histone deacetylase 7  FAM49B - family with sequence similarity 49, member b  M6PR - mannose-6-phosphate receptor (cation dependent)  TAF1C - tata box binding protein (tbp)-associated factor, rna polymerase i, c, 110kda  RPS6KA4 - ribosomal protein s6 kinase, 90kda, polypeptide 4  KCTD11 - potassium channel tetramerization domain containing 11  PLOD3 - procollagen-lysine, 2-oxoglutarate 5-dioxygenase 3  SMAD4 - smad family member 4  H1FX - h1 histone family, member x  LSR - lipolysis stimulated lipoprotein receptor  SMAD7 - smad family member 7  TMEM199 - transmembrane protein 199  WASL - wiskott-aldrich syndrome-like  ANP32E - acidic (leucine-rich) nuclear phosphoprotein 32 family, member e  NCBP1 - nuclear cap binding protein subunit 1, 80kda  NDN - necdin, melanoma antigen (mage) family member  G3BP1 - gtpase activating protein (sh3 domain) binding protein 1  AKAP9 - a kinase (prka) anchor protein (yotiao) 9  NCK1 - nck adaptor protein 1  CEBPZ - ccaat/enhancer binding protein (c/ebp), zeta  ABI2 - abl-interactor 2  RACGAP1 - rac gtpase activating protein 1  VMP1 - vacuole membrane protein 1  NAGLU - n-acetylglucosaminidase, alpha  TRIM28 - tripartite motif containing 28  DROSHA - drosha, ribonuclease type iii  ISCA1 - iron-sulfur cluster assembly 1  NAP1L1 - nucleosome assembly protein 1-like 1  NASP - nuclear autoantigenic sperm protein (histone-binding)  WASF2 - was protein family, member 2  NAP1L4 - nucleosome assembly protein 1-like 4  NBN - nibrin  NUBP1 - nucleotide binding protein 1  TDRD3 - tudor domain containing 3  NCAPH2 - non-smc condensin ii complex, subunit h2  FAM49A - family with sequence similarity 49, member a  CNIH - cornichon homolog (drosophila)  RBM5 - rna binding motif protein 5  ZDHHC8 - zinc finger, dhhc-type containing 8  PPP1R12A - protein phosphatase 1, regulatory subunit 12a  LMAN2L - lectin, mannose-binding 2-like  NDEL1 - nude neurodevelopment protein 1-like 1  MYLK - myosin light chain kinase  TMOD2 - tropomodulin 2 (neuronal)  GFOD2 - glucose-fructose oxidoreductase domain containing 2  TNFAIP8L3 - tumor necrosis factor, alpha-induced protein 8-like 3  MYO1D - myosin id  MYO5A - myosin va (heavy chain 12, myoxin)  MYO1E - myosin ie  USPL1 - ubiquitin specific peptidase like 1  MYO6 - myosin vi  INADL - inad-like (drosophila)  ABT1 - activator of basal transcription 1  TOPORS - topoisomerase i binding, arginine/serine-rich, e3 ubiquitin protein ligase  RAB1B - rab1b, member ras oncogene family  ARPC5L - actin related protein 2/3 complex, subunit 5-like  SEH1L - seh1-like (s. cerevisiae)  MYH10 - myosin, heavy chain 10, non-muscle  ATP9A - atpase, class ii, type 9a  MYH11 - myosin, heavy chain 11, smooth muscle  SNX11 - sorting nexin 11  MYH9 - myosin, heavy chain 9, non-muscle  SNUPN - snurportin 1  HUWE1 - hect, uba and wwe domain containing 1, e3 ubiquitin protein ligase  HCFC2 - host cell factor c2  ARPC5 - actin related protein 2/3 complex, subunit 5, 16kda  SNX10 - sorting nexin 10  CNOT7 - ccr4-not transcription complex, subunit 7  COL4A3BP - collagen, type iv, alpha 3 (goodpasture antigen) binding protein  PQBP1 - polyglutamine binding protein 1  MX1 - myxovirus (influenza virus) resistance 1, interferon-inducible protein p78 (mouse)  UBN1 - ubinuclein 1  TSPAN2 - tetraspanin 2  GPSM2 - g-protein signaling modulator 2  INO80B - ino80 complex subunit b  ACTR2 - arp2 actin-related protein 2 homolog (yeast)  ACTR3 - arp3 actin-related protein 3 homolog (yeast)  ARPC3 - actin related protein 2/3 complex, subunit 3, 21kda  GNL2 - guanine nucleotide binding protein-like 2 (nucleolar)  ARPC4 - actin related protein 2/3 complex, subunit 4, 20kda  ARPC2 - actin related protein 2/3 complex, subunit 2, 34kda  NUBP2 - nucleotide binding protein 2  MTX1 - metaxin 1  PHF13 - phd finger protein 13  RAD50 - rad50 homolog (s. cerevisiae)  TRIM37 - tripartite motif containing 37  CDC42EP5 - cdc42 effector protein (rho gtpase binding) 5  TIMM22 - translocase of inner mitochondrial membrane 22 homolog (yeast)  SEC61A1 - sec61 alpha 1 subunit (s. cerevisiae)  SHARPIN - shank-associated rh domain interactor  BLOC1S3 - biogenesis of lysosomal organelles complex-1, subunit 3  AKT3 - v-akt murine thymoma viral oncogene homolog 3  SCO2 - sco2 cytochrome c oxidase assembly protein  ABI1 - abl-interactor 1  SLC12A6 - solute carrier family 12 (potassium/chloride transporter), member 6  MTIF2 - mitochondrial translational initiation factor 2  PDCD6 - programmed cell death 6  MTM1 - myotubularin 1  HDAC5 - histone deacetylase 5  PDCD6IP - programmed cell death 6 interacting protein  OCLN - occludin  HDAC6 - histone deacetylase 6  MT3 - metallothionein 3  CHAF1A - chromatin assembly factor 1, subunit a (p150)  FARSB - phenylalanyl-trna synthetase, beta subunit  EXOC8 - exocyst complex component 8  DNM1L - dynamin 1-like  RANBP9 - ran binding protein 9  DNAJB6 - dnaj (hsp40) homolog, subfamily b, member 6  SMC4 - structural maintenance of chromosomes 4  MSN - moesin  KANSL1L - kat8 regulatory nsl complex subunit 1-like  MSH3 - muts homolog 3 (e. coli)  MSH2 - muts homolog 2, colon cancer, nonpolyposis type 1 (e. coli)  SEC16A - sec16 homolog a (s. cerevisiae)  NCAPD2 - non-smc condensin i complex, subunit d2  DBNL - drebrin-like  SUPT7L - suppressor of ty 7 (s. cerevisiae)-like  WDR1 - wd repeat domain 1  IFT81 - intraflagellar transport 81 homolog (chlamydomonas)  TMEM14A - transmembrane protein 14a  TIMM21 - translocase of inner mitochondrial membrane 21 homolog (yeast)  USP3 - ubiquitin specific peptidase 3  BABAM1 - brisc and brca1 a complex member 1  MRPL15 - mitochondrial ribosomal protein l15  CHMP4A - charged multivesicular body protein 4a  USP15 - ubiquitin specific peptidase 15  TTL - tubulin tyrosine ligase  GOLGA5 - golgin a5  NDUFAF4 - nadh dehydrogenase (ubiquinone) complex i, assembly factor 4  C1GALT1C1 - c1galt1-specific chaperone 1  SETD2 - set domain containing 2  MRPL18 - mitochondrial ribosomal protein l18  RBX1 - ring-box 1, e3 ubiquitin protein ligase  MRE11A - mre11 meiotic recombination 11 homolog a (s. cerevisiae)  HSCB - hscb iron-sulfur cluster co-chaperone homolog (e. coli)  NUP153 - nucleoporin 153kda  MPP2 - membrane protein, palmitoylated 2 (maguk p55 subfamily member 2)  GLTPD1 - glycolipid transfer protein domain containing 1  DTX3L - deltex 3-like (drosophila)  B9D2 - b9 protein domain 2  SETDB1 - set domain, bifurcated 1  FCHSD2 - fch and double sh3 domains 2  SEC24D - sec24 family, member d (s. cerevisiae)  PARD6A - par-6 partitioning defective 6 homolog alpha (c. elegans)  TLK1 - tousled-like kinase 1  STMN3 - stathmin-like 3  OSBP - oxysterol binding protein  ELMO1 - engulfment and cell motility 1  C2CD5 - c2 calcium-dependent domain containing 5  CLDN11 - claudin 11  CCDC80 - coiled-coil domain containing 80  AGRN - agrin  C2CD2L - c2cd2-like  SHANK1 - sh3 and multiple ankyrin repeat domains 1  KIAA0319 - kiaa0319  CEP350 - centrosomal protein 350kda  SV2A - synaptic vesicle glycoprotein 2a  UBAP2L - ubiquitin associated protein 2-like  G3BP2 - gtpase activating protein (sh3 domain) binding protein 2  ARHGAP39 - rho gtpase activating protein 39  OCRL - oculocerebrorenal syndrome of lowe  ARHGAP44 - rho gtpase activating protein 44  ODF2 - outer dense fiber of sperm tails 2  TMED5 - transmembrane emp24 protein transport domain containing 5  OSBPL2 - oxysterol binding protein-like 2  REEP4 - receptor accessory protein 4  FAM84A - family with sequence similarity 84, member a  WDR61 - wd repeat domain 61  OPA1 - optic atrophy 1 (autosomal dominant)  TMED7 - transmembrane emp24 protein transport domain containing 7  FIG4 - fig4 homolog, sac1 lipid phosphatase domain containing (s. cerevisiae)  SNAP91 - synaptosomal-associated protein, 91kda  SLMO2 - slowmo homolog 2 (drosophila)  APITD1 - apoptosis-inducing, taf9-like domain 1  CKAP5 - cytoskeleton associated protein 5  FIS1 - fission 1 (mitochondrial outer membrane) homolog (s. cerevisiae)  KIAA0586 - kiaa0586  NSF - n-ethylmaleimide-sensitive factor  DDR2 - discoidin domain receptor tyrosine kinase 2  TOMM20 - translocase of outer mitochondrial membrane 20 homolog (yeast)  IST1 - increased sodium tolerance 1 homolog (yeast)  NUMA1 - nuclear mitotic apparatus protein 1  DAB2IP - dab2 interacting protein  ATG13 - autophagy related 13  NUP98 - nucleoporin 98kda  GMNN - geminin, dna replication inhibitor  TM9SF4 - transmembrane 9 superfamily protein member 4  NR4A2 - nuclear receptor subfamily 4, group a, member 2  ATL1 - atlastin gtpase 1  NVL - nuclear vcp-like  CEP120 - centrosomal protein 120kda  DNAJC6 - dnaj (hsp40) homolog, subfamily c, member 6  ARHGEF17 - rho guanine nucleotide exchange factor (gef) 17  NPM1 - nucleophosmin (nucleolar phosphoprotein b23, numatrin)  ARHGEF11 - rho guanine nucleotide exchange factor (gef) 11  NPHP1 - nephronophthisis 1 (juvenile)  ZEB2 - zinc finger e-box binding homeobox 2  SPICE1 - spindle and centriole associated protein 1  SLC11A2 - solute carrier family 11 (proton-coupled divalent metal ion transporter), member 2  CNTN4 - contactin 4  RNF40 - ring finger protein 40, e3 ubiquitin protein ligase  SPOCK2 - sparc/osteonectin, cwcv and kazal-like domains proteoglycan (testican) 2  FGD5 - fyve, rhogef and ph domain containing 5  NPTX1 - neuronal pentraxin i  RB1CC1 - rb1-inducible coiled-coil 1  IFT52 - intraflagellar transport 52 homolog (chlamydomonas)  CUL7 - cullin 7  GRIP2 - glutamate receptor interacting protein 2  NRF1 - nuclear respiratory factor 1  SH3D19 - sh3 domain containing 19  NRCAM - neuronal cell adhesion molecule  SIDT2 - sid1 transmembrane family, member 2  CHD9 - chromodomain helicase dna binding protein 9  UBQLN1 - ubiquilin 1  NMT1 - n-myristoyltransferase 1  SPG11 - spastic paraplegia 11 (autosomal recessive)  NOP2 - nop2 nucleolar protein  SLC39A3 - solute carrier family 39 (zinc transporter), member 3  ARMC9 - armadillo repeat containing 9  VPRBP - vpr (hiv-1) binding protein  SLC2A8 - solute carrier family 2 (facilitated glucose transporter), member 8  LRP12 - low density lipoprotein receptor-related protein 12  UBQLN2 - ubiquilin 2  IFT74 - intraflagellar transport 74 homolog (chlamydomonas)  NOTCH4 - notch 4  EFEMP2 - egf containing fibulin-like extracellular matrix protein 2  ERO1L - ero1-like (s. cerevisiae)  USP6NL - usp6 n-terminal like  MYO19 - myosin xix  KCTD7 - potassium channel tetramerization domain containing 7  CEP290 - centrosomal protein 290kda  NOS3 - nitric oxide synthase 3 (endothelial cell)  PACSIN1 - protein kinase c and casein kinase substrate in neurons 1  NPY - neuropeptide y  CEP57 - centrosomal protein 57kda  NOTCH1 - notch 1  BAZ2B - bromodomain adjacent to zinc finger domain, 2b  CTC1 - cts telomere maintenance complex component 1  AMOTL1 - angiomotin like 1  PQLC1 - pq loop repeat containing 1  KCNIP2 - kv channel interacting protein 2  HDAC4 - histone deacetylase 4  TOX - thymocyte selection-associated high mobility group box  EDC3 - enhancer of mrna decapping 3  ZFYVE16 - zinc finger, fyve domain containing 16  CENPT - centromere protein t  CXXC1 - cxxc finger protein 1  KMT2B - lysine (k)-specific methyltransferase 2b  IFT140 - intraflagellar transport 140 homolog (chlamydomonas)  PHACTR2 - phosphatase and actin regulator 2  SLK - ste20-like kinase  CLSTN3 - calsyntenin 3  VCPIP1 - valosin containing protein (p97)/p47 complex interacting protein 1  NHP2L1 - nhp2 non-histone chromosome protein 2-like 1 (s. cerevisiae)  HDAC9 - histone deacetylase 9  SART3 - squamous cell carcinoma antigen recognized by t cells 3  EHD2 - eh-domain containing 2  SETD1A - set domain containing 1a  EHD3 - eh-domain containing 3  CCP110 - centriolar coiled coil protein 110kda  EHD4 - eh-domain containing 4  NID1 - nidogen 1  IQCB1 - iq motif containing b1  NEK3 - nima-related kinase 3  MINK1 - misshapen-like kinase 1  TTLL4 - tubulin tyrosine ligase-like family, member 4  GCC2 - grip and coiled-coil domain containing 2  NEFL - neurofilament, light polypeptide  MTFR1 - mitochondrial fission regulator 1  SMARCAL1 - swi/snf related, matrix associated, actin dependent regulator of chromatin, subfamily a-like 1  NEK1 - nima-related kinase 1  C8orf37 - chromosome 8 open reading frame 37  NEDD9 - neural precursor cell expressed, developmentally down-regulated 9  CTR9 - ctr9, paf1/rna polymerase ii complex component  WRAP73 - wd repeat containing, antisense to tp73  NEFM - neurofilament, medium polypeptide  PPM1F - protein phosphatase, mg2+/mn2+ dependent, 1f  ARHGEF10 - rho guanine nucleotide exchange factor (gef) 10  EPC1 - enhancer of polycomb homolog 1 (drosophila)  MORF4L2 - mortality factor 4 like 2  FEZ1 - fasciculation and elongation protein zeta 1 (zygin i)  CAMSAP1 - calmodulin regulated spectrin-associated protein 1  NUP155 - nucleoporin 155kda  NFIA - nuclear factor i/a  SEC24C - sec24 family, member c (s. cerevisiae)  NF1 - neurofibromin 1  FAM91A1 - family with sequence similarity 91, member a1  CDK5RAP3 - cdk5 regulatory subunit associated protein 3  CROCC - ciliary rootlet coiled-coil, rootletin  VPS37D - vacuolar protein sorting 37 homolog d (s. cerevisiae)  RAPGEF2 - rap guanine nucleotide exchange factor (gef) 2  ARHGEF4 - rho guanine nucleotide exchange factor (gef) 4  NDUFB2 - nadh dehydrogenase (ubiquinone) 1 beta subcomplex, 2, 8kda  NUP93 - nucleoporin 93kda  CLINT1 - clathrin interactor 1  CEP63 - centrosomal protein 63kda  CALY - calcyon neuron-specific vesicular protein  GEMIN4 - gem (nuclear organelle) associated protein 4  KDM4A - lysine (k)-specific demethylase 4a  PHF14 - phd finger protein 14  COPS7A - cop9 signalosome subunit 7a  HP1BP3 - heterochromatin protein 1, binding protein 3  ASAP1 - arfgap with sh3 domain, ankyrin repeat and ph domain 1  KIAA0430 - kiaa0430  NDUFC2 - nadh dehydrogenase (ubiquinone) 1, subcomplex unknown, 2, 14.5kda |
| GO:0006310 | DNA recombination | 6.16E-5 | 5.99E-3 | 1.24 (10334,117,6996,98) | [+] Show genes  APEX1 - apex nuclease (multifunctional dna repair enzyme) 1  TSN - translin  SMC6 - structural maintenance of chromosomes 6  LIG4 - ligase iv, dna, atp-dependent  RFWD3 - ring finger and wd repeat domain 3  FAN1 - fancd2/fanci-associated nuclease 1  TREX1 - three prime repair exonuclease 1  FBXO18 - f-box protein, helicase, 18  SAMHD1 - sam domain and hd domain 1  LIG3 - ligase iii, dna, atp-dependent  MLH1 - mutl homolog 1, colon cancer, nonpolyposis type 2 (e. coli)  RECQL - recq protein-like (dna helicase q1-like)  KIN - kin, antigenic determinant of reca protein homolog (mouse)  MCM9 - minichromosome maintenance complex component 9  POLL - polymerase (dna directed), lambda  NBN - nibrin  MSH6 - muts homolog 6 (e. coli)  AP5Z1 - adaptor-related protein complex 5, zeta 1 subunit  SLX4 - slx4 structure-specific endonuclease subunit  CNTD1 - cyclin n-terminal domain containing 1  TFPT - tcf3 (e2a) fusion partner (in childhood leukemia)  EXOSC3 - exosome component 3  MORF4L1 - mortality factor 4 like 1  PSMD14 - proteasome (prosome, macropain) 26s subunit, non-atpase, 14  ACTL6A - actin-like 6a  MCM7 - minichromosome maintenance complex component 7  H2AFX - h2a histone family, member x  NSMCE2 - non-smc element 2, mms21 homolog (s. cerevisiae)  RNF138 - ring finger protein 138, e3 ubiquitin protein ligase  NABP2 - nucleic acid binding protein 2  HUS1 - hus1 checkpoint homolog (s. pombe)  ACTR5 - arp5 actin-related protein 5 homolog (yeast)  FEN1 - flap structure-specific endonuclease 1  CDC45 - cell division cycle 45  INO80B - ino80 complex subunit b  CDC7 - cell division cycle 7  INO80E - ino80 complex subunit e  UCHL5 - ubiquitin carboxyl-terminal hydrolase l5  HSPD1 - heat shock 60kda protein 1 (chaperonin)  NSMCE1 - non-smc element 1 homolog (s. cerevisiae)  RAD50 - rad50 homolog (s. cerevisiae)  PARP1 - poly (adp-ribose) polymerase 1  APEX2 - apex nuclease (apurinic/apyrimidinic endonuclease) 2  RUVBL1 - ruvb-like 1 (e. coli)  TCF3 - transcription factor 3  SETX - senataxin  PGBD5 - piggybac transposable element derived 5  RUVBL2 - ruvb-like 2 (e. coli)  VPRBP - vpr (hiv-1) binding protein  NONO - non-pou domain containing, octamer-binding  MCRS1 - microspherule protein 1  HMGB3 - high mobility group box 3  HMGB2 - high mobility group box 2  MUS81 - mus81 structure-specific endonuclease subunit  HMGB1 - high mobility group box 1  XRCC1 - x-ray repair complementing defective repair in chinese hamster cells 1  PAGR1 - paxip1 associated glutamate-rich protein 1  SUPV3L1 - suppressor of var1, 3-like 1 (s. cerevisiae)  NDNL2 - necdin-like 2  AP5S1 - adaptor-related protein complex 5, sigma 1 subunit  UNG - uracil-dna glycosylase  RPA3 - replication protein a3, 14kda  RPA1 - replication protein a1, 70kda  RPA2 - replication protein a2, 32kda  NFRKB - nuclear factor related to kappab binding protein  RECQL5 - recq protein-like 5  ACTR8 - arp8 actin-related protein 8 homolog (yeast)  XRCC6 - x-ray repair complementing defective repair in chinese hamster cells 6  RNF168 - ring finger protein 168, e3 ubiquitin protein ligase  SMC5 - structural maintenance of chromosomes 5  WDR48 - wd repeat domain 48  MCM3 - minichromosome maintenance complex component 3  MCM6 - minichromosome maintenance complex component 6  RAD21 - rad21 homolog (s. pombe)  MCM5 - minichromosome maintenance complex component 5  MCM2 - minichromosome maintenance complex component 2  INO80 - ino80 complex subunit  RPAIN - rpa interacting protein  MSH3 - muts homolog 3 (e. coli)  IGHMBP2 - immunoglobulin mu binding protein 2  MSH2 - muts homolog 2, colon cancer, nonpolyposis type 1 (e. coli)  ERCC4 - excision repair cross-complementing rodent repair deficiency, complementation group 4  NIPBL - nipped-b homolog (drosophila)  INO80C - ino80 complex subunit c  EXD2 - exonuclease 3'-5' domain containing 2  SWSAP1 - swim-type zinc finger 7 associated protein 1  ZFYVE26 - zinc finger, fyve domain containing 26  RNF8 - ring finger protein 8, e3 ubiquitin protein ligase  MLH3 - mutl homolog 3 (e. coli)  SFPQ - splicing factor proline/glutamine-rich  RBBP8 - retinoblastoma binding protein 8  ZSWIM7 - zinc finger, swim-type containing 7  XRCC5 - x-ray repair complementing defective repair in chinese hamster cells 5 (double-strand-break rejoining)  XRCC4 - x-ray repair complementing defective repair in chinese hamster cells 4  MRE11A - mre11 meiotic recombination 11 homolog a (s. cerevisiae)  LEF1 - lymphoid enhancer-binding factor 1  NABP1 - nucleic acid binding protein 1  UBE2N - ubiquitin-conjugating enzyme e2n |
| GO:0017156 | calcium ion regulated exocytosis | 6.39E-5 | 6.17E-3 | 1.40 (10334,38,6996,36) | [+] Show genes  SYN2 - synapsin ii  DNAJC5 - dnaj (hsp40) homolog, subfamily c, member 5  SNCA - synuclein, alpha (non a4 component of amyloid precursor)  SYT1 - synaptotagmin i  SYT5 - synaptotagmin v  SYT17 - synaptotagmin xvii  SYT4 - synaptotagmin iv  SNAP25 - synaptosomal-associated protein, 25kda  SDF4 - stromal cell derived factor 4  SCIN - scinderin  RIMS2 - regulating synaptic membrane exocytosis 2  RIMS3 - regulating synaptic membrane exocytosis 3  UNC13A - unc-13 homolog a (c. elegans)  VAMP2 - vesicle-associated membrane protein 2 (synaptobrevin 2)  CHP1 - calcineurin-like ef-hand protein 1  PIP5K1C - phosphatidylinositol-4-phosphate 5-kinase, type i, gamma  CDK5 - cyclin-dependent kinase 5  STXBP1 - syntaxin binding protein 1  ARHGAP17 - rho gtpase activating protein 17  CADPS2 - ca++-dependent secretion activator 2  STX1A - syntaxin 1a (brain)  RAB3A - rab3a, member ras oncogene family  CADPS - ca++-dependent secretion activator  CPLX2 - complexin 2  SYT11 - synaptotagmin xi  SYT13 - synaptotagmin xiii  SYT3 - synaptotagmin iii  DOC2B - double c2-like domains, beta  TRIM9 - tripartite motif containing 9  VAMP3 - vesicle-associated membrane protein 3  SYT9 - synaptotagmin ix  UNC13B - unc-13 homolog b (c. elegans)  CPLX3 - complexin 3  RAPGEF4 - rap guanine nucleotide exchange factor (gef) 4  STX1B - syntaxin 1b  SYT7 - synaptotagmin vii |
| GO:0051253 | negative regulation of RNA metabolic process | 7.21E-5 | 6.92E-3 | 1.09 (10334,863,6996,634) | [+] Show genes  ZMYND8 - zinc finger, mynd-type containing 8  RBAK - rb-associated krab zinc finger  ZNF239 - zinc finger protein 239  MSL3 - male-specific lethal 3 homolog (drosophila)  PHF21A - phd finger protein 21a  HNRNPA0 - heterogeneous nuclear ribonucleoprotein a0  CBX1 - chromobox homolog 1  PATZ1 - poz (btb) and at hook containing zinc finger 1  NCOR1 - nuclear receptor corepressor 1  NCOR2 - nuclear receptor corepressor 2  PML - promyelocytic leukemia  PHC3 - polyhomeotic homolog 3 (drosophila)  MIER1 - mesoderm induction early response 1, transcriptional regulator  DOT1L - dot1-like histone h3k79 methyltransferase  YWHAQ - tyrosine 3-monooxygenase/tryptophan 5-monooxygenase activation protein, theta polypeptide  NRIP1 - nuclear receptor interacting protein 1  ZNF705A - zinc finger protein 705a  SP3 - sp3 transcription factor  L3MBTL3 - l(3)mbt-like 3 (drosophila)  SP2 - sp2 transcription factor  CIR1 - corepressor interacting with rbpj, 1  WWC2 - ww and c2 domain containing 2  LYAR - ly1 antibody reactive  H2AFY - h2a histone family, member y  TCERG1 - transcription elongation regulator 1  ZNF354B - zinc finger protein 354b  TAF15 - taf15 rna polymerase ii, tata box binding protein (tbp)-associated factor, 68kda  RNPS1 - rna binding protein s1, serine-rich domain  GTF2IRD1 - gtf2i repeat domain containing 1  EHMT2 - euchromatic histone-lysine n-methyltransferase 2  MORF4L1 - mortality factor 4 like 1  CREBBP - creb binding protein  SOX13 - sry (sex determining region y)-box 13  ZC3H8 - zinc finger ccch-type containing 8  ATF2 - activating transcription factor 2  CREB1 - camp responsive element binding protein 1  LMO1 - lim domain only 1 (rhombotin 1)  NACC2 - nacc family member 2, ben and btb (poz) domain containing  CLOCK - clock circadian regulator  KLF3 - kruppel-like factor 3 (basic)  PPID - peptidylprolyl isomerase d  PLK3 - polo-like kinase 3  SNCA - synuclein, alpha (non a4 component of amyloid precursor)  ZC3H14 - zinc finger ccch-type containing 14  CREBZF - creb/atf bzip transcription factor  ARID1A - at rich interactive domain 1a (swi-like)  EID1 - ep300 interacting inhibitor of differentiation 1  PTBP2 - polypyrimidine tract binding protein 2  MED1 - mediator complex subunit 1  SMARCE1 - swi/snf related, matrix associated, actin dependent regulator of chromatin, subfamily e, member 1  GCLC - glutamate-cysteine ligase, catalytic subunit  SMO - smoothened, frizzled family receptor  FZD1 - frizzled family receptor 1  BAP1 - brca1 associated protein-1 (ubiquitin carboxy-terminal hydrolase)  ZGPAT - zinc finger, ccch-type with g patch domain  AXIN2 - axin 2  PPARD - peroxisome proliferator-activated receptor delta  KDM5C - lysine (k)-specific demethylase 5c  USP9X - ubiquitin specific peptidase 9, x-linked  RBM10 - rna binding motif protein 10  SMARCA4 - swi/snf related, matrix associated, actin dependent regulator of chromatin, subfamily a, member 4  SNX6 - sorting nexin 6  SMARCC2 - swi/snf related, matrix associated, actin dependent regulator of chromatin, subfamily c, member 2  SMARCA2 - swi/snf related, matrix associated, actin dependent regulator of chromatin, subfamily a, member 2  PARP1 - poly (adp-ribose) polymerase 1  H2AFY2 - h2a histone family, member y2  PPP1R13L - protein phosphatase 1, regulatory subunit 13 like  ZBTB7A - zinc finger and btb domain containing 7a  DPY30 - dpy-30 homolog (c. elegans)  RUVBL2 - ruvb-like 2 (e. coli)  IRF2BP2 - interferon regulatory factor 2 binding protein 2  GLIS2 - glis family zinc finger 2  MAGED1 - melanoma antigen family d, 1  RCOR3 - rest corepressor 3  NELFE - negative elongation factor complex member e  EHMT1 - euchromatic histone-lysine n-methyltransferase 1  AES - amino-terminal enhancer of split  ZNF318 - zinc finger protein 318  CDYL - chromodomain protein, y-like  KDM2B - lysine (k)-specific demethylase 2b  FUS - fused in sarcoma  FAM46B - family with sequence similarity 46, member b  CBY1 - chibby homolog 1 (drosophila)  CTBP1 - c-terminal binding protein 1  CTBP2 - c-terminal binding protein 2  SUPT4H1 - suppressor of ty 4 homolog 1 (s. cerevisiae)  ING4 - inhibitor of growth family, member 4  SUPT5H - suppressor of ty 5 homolog (s. cerevisiae)  ATF7IP - activating transcription factor 7 interacting protein  CTNNB1 - catenin (cadherin-associated protein), beta 1, 88kda  RLIM - ring finger protein, lim domain interacting  RECQL5 - recq protein-like 5  ZNF653 - zinc finger protein 653  XRCC6 - x-ray repair complementing defective repair in chinese hamster cells 6  RNF168 - ring finger protein 168, e3 ubiquitin protein ligase  CUX1 - cut-like homeobox 1  ZNF706 - zinc finger protein 706  GABPA - ga binding protein transcription factor, alpha subunit 60kda  WWP2 - ww domain containing e3 ubiquitin protein ligase 2  AHR - aryl hydrocarbon receptor  NIF3L1 - nif3 ngg1 interacting factor 3-like 1 (s. cerevisiae)  FOXP4 - forkhead box p4  PCGF1 - polycomb group ring finger 1  TGIF2 - tgfb-induced factor homeobox 2  BCL6B - b-cell cll/lymphoma 6, member b  NIPBL - nipped-b homolog (drosophila)  CRY1 - cryptochrome 1 (photolyase-like)  STAT1 - signal transducer and activator of transcription 1, 91kda  CRY2 - cryptochrome 2 (photolyase-like)  STAT3 - signal transducer and activator of transcription 3 (acute-phase response factor)  STAT6 - signal transducer and activator of transcription 6, interleukin-4 induced  KMT2D - lysine (k)-specific methyltransferase 2d  PAPD7 - pap associated domain containing 7  BRMS1 - breast cancer metastasis suppressor 1  MAP2K5 - mitogen-activated protein kinase kinase 5  ZNF219 - zinc finger protein 219  ZNF692 - zinc finger protein 692  CRYM - crystallin, mu  ZFPM1 - zinc finger protein, fog family member 1  MAPK10 - mitogen-activated protein kinase 10  EID2 - ep300 interacting inhibitor of differentiation 2  GATA2 - gata binding protein 2  PHF6 - phd finger protein 6  KAT6A - k(lysine) acetyltransferase 6a  COPS2 - cop9 signalosome subunit 2  ZC3H10 - zinc finger ccch-type containing 10  CCDC85B - coiled-coil domain containing 85b  ZNF639 - zinc finger protein 639  NR4A3 - nuclear receptor subfamily 4, group a, member 3  SRC - v-src avian sarcoma (schmidt-ruppin a-2) viral oncogene homolog  SARNP - sap domain containing ribonucleoprotein  BRMS1L - breast cancer metastasis-suppressor 1-like  LEF1 - lymphoid enhancer-binding factor 1  DNAJB5 - dnaj (hsp40) homolog, subfamily b, member 5  SREBF2 - sterol regulatory element binding transcription factor 2  GAS6 - growth arrest-specific 6  DEDD2 - death effector domain containing 2  YEATS2 - yeats domain containing 2  PRDX5 - peroxiredoxin 5  APEX1 - apex nuclease (multifunctional dna repair enzyme) 1  DHX9 - deah (asp-glu-ala-his) box helicase 9  DHX36 - deah (asp-glu-ala-his) box polypeptide 36  KANK2 - kn motif and ankyrin repeat domains 2  DDX5 - dead (asp-glu-ala-asp) box helicase 5  PARN - poly(a)-specific ribonuclease  ZNF177 - zinc finger protein 177  KLF12 - kruppel-like factor 12  ZNF175 - zinc finger protein 175  ZNF174 - zinc finger protein 174  SIN3A - sin3 transcription regulator family member a  SHQ1 - shq1, h/aca ribonucleoprotein assembly factor  MAP3K10 - mitogen-activated protein kinase kinase kinase 10  SET - set nuclear oncogene  WWTR1 - ww domain containing transcription regulator 1  PRMT6 - protein arginine methyltransferase 6  ZNF148 - zinc finger protein 148  BEND5 - ben domain containing 5  RSF1 - remodeling and spacing factor 1  PBXIP1 - pre-b-cell leukemia homeobox interacting protein 1  MCPH1 - microcephalin 1  ZNF140 - zinc finger protein 140  PCGF2 - polycomb group ring finger 2  APBB1 - amyloid beta (a4) precursor protein-binding, family b, member 1 (fe65)  NELFB - negative elongation factor complex member b  RIF1 - rap1 interacting factor homolog (yeast)  ZNF133 - zinc finger protein 133  RPS6KA5 - ribosomal protein s6 kinase, 90kda, polypeptide 5  TBL1XR1 - transducin (beta)-like 1 x-linked receptor 1  SUFU - suppressor of fused homolog (drosophila)  PA2G4 - proliferation-associated 2g4, 38kda  ZBTB38 - zinc finger and btb domain containing 38  ZHX1 - zinc fingers and homeoboxes 1  ZNF79 - zinc finger protein 79  UIMC1 - ubiquitin interaction motif containing 1  GATAD2B - gata zinc finger domain containing 2b  MTA2 - metastasis associated 1 family, member 2  DDX20 - dead (asp-glu-ala-asp) box polypeptide 20  WWOX - ww domain containing oxidoreductase  MNT - mnt, max dimerization protein  H3F3A - h3 histone, family 3a  ARNTL - aryl hydrocarbon receptor nuclear translocator-like  H2AFZ - h2a histone family, member z  H2AFX - h2a histone family, member x  SAP130 - sin3a-associated protein, 130kda  PAPD4 - pap associated domain containing 4  ZNF451 - zinc finger protein 451  MIER3 - mesoderm induction early response 1, family member 3  SCAF4 - sr-related ctd-associated factor 4  CDC73 - cell division cycle 73  PDGFB - platelet-derived growth factor beta polypeptide  MECP2 - methyl cpg binding protein 2 (rett syndrome)  MEF2A - myocyte enhancer factor 2a  NKAP - nfkb activating protein  PRICKLE1 - prickle homolog 1 (drosophila)  STRAP - serine/threonine kinase receptor associated protein  THAP5 - thap domain containing 5  BCDIN3D - bcdin3 domain containing  MEIS2 - meis homeobox 2  BAZ2A - bromodomain adjacent to zinc finger domain, 2a  MEN1 - multiple endocrine neoplasia i  HCFC1 - host cell factor c1 (vp16-accessory protein)  PCNA - proliferating cell nuclear antigen  CHMP1A - charged multivesicular body protein 1a  PRDM2 - pr domain containing 2, with znf domain  DAXX - death-domain associated protein  ANGEL2 - angel homolog 2 (drosophila)  JDP2 - jun dimerization protein 2  HINFP - histone h4 transcription factor  AKIRIN2 - akirin 2  KAT8 - k(lysine) acetyltransferase 8  MTA3 - metastasis associated 1 family, member 3  ZNF608 - zinc finger protein 608  DAP - death-associated protein  SARS - seryl-trna synthetase  PDE2A - phosphodiesterase 2a, cgmp-stimulated  HDAC2 - histone deacetylase 2  SATB1 - satb homeobox 1  HMBOX1 - homeobox containing 1  HDGF - hepatoma-derived growth factor  MBD3 - methyl-cpg binding domain protein 3  THAP1 - thap domain containing, apoptosis associated protein 1  HDAC1 - histone deacetylase 1  HCLS1 - hematopoietic cell-specific lyn substrate 1  ZNF205 - zinc finger protein 205  PKIG - protein kinase (camp-dependent, catalytic) inhibitor gamma  SALL2 - sal-like 2 (drosophila)  KAT7 - k(lysine) acetyltransferase 7  ASCL1 - achaete-scute complex homolog 1 (drosophila)  CBFA2T2 - core-binding factor, runt domain, alpha subunit 2; translocated to, 2  SBNO2 - strawberry notch homolog 2 (drosophila)  CXXC5 - cxxc finger protein 5  ZBTB1 - zinc finger and btb domain containing 1  IRF2BP1 - interferon regulatory factor 2 binding protein 1  XPO1 - exportin 1 (crm1 homolog, yeast)  BAHD1 - bromo adjacent homology domain containing 1  ATN1 - atrophin 1  ZHX2 - zinc fingers and homeoboxes 2  RERE - arginine-glutamic acid dipeptide (re) repeats  SIRT6 - sirtuin 6  MTA1 - metastasis associated 1  DR1 - down-regulator of transcription 1, tbp-binding (negative cofactor 2)  PPHLN1 - periphilin 1  BHLHE41 - basic helix-loop-helix family, member e41  ZFHX3 - zinc finger homeobox 3  SKI - v-ski avian sarcoma viral oncogene homolog  SKIL - ski-like oncogene  PEX14 - peroxisomal biogenesis factor 14  MBD1 - methyl-cpg binding domain protein 1  WFS1 - wolfram syndrome 1 (wolframin)  DYRK1A - dual-specificity tyrosine-(y)-phosphorylation regulated kinase 1a  WHSC1 - wolf-hirschhorn syndrome candidate 1  NELFA - negative elongation factor complex member a  SFMBT1 - scm-like with four mbt domains 1  MAX - myc associated factor x  AEBP2 - ae binding protein 2  LDB2 - lim domain binding 2  ZFP90 - zfp90 zinc finger protein  FNIP2 - folliculin interacting protein 2  IMPACT - impact rwd domain protein  NELFCD - negative elongation factor complex member c/d  LGR4 - leucine-rich repeat containing g protein-coupled receptor 4  ZFHX2 - zinc finger homeobox 2  SCAF8 - sr-related ctd-associated factor 8  DKC1 - dyskeratosis congenita 1, dyskerin  MTF2 - metal response element binding transcription factor 2  L3MBTL2 - l(3)mbt-like 2 (drosophila)  DLG1 - discs, large homolog 1 (drosophila)  BACH1 - btb and cnc homology 1, basic leucine zipper transcription factor 1  MZF1 - myeloid zinc finger 1  SIAH2 - siah e3 ubiquitin protein ligase 2  BTAF1 - btaf1 rna polymerase ii, b-tfiid transcription factor-associated, 170kda  UBA3 - ubiquitin-like modifier activating enzyme 3  PARP9 - poly (adp-ribose) polymerase family, member 9  PHF12 - phd finger protein 12  RNF8 - ring finger protein 8, e3 ubiquitin protein ligase  ATRX - alpha thalassemia/mental retardation syndrome x-linked  RBM42 - rna binding motif protein 42  ZNF24 - zinc finger protein 24  ZBTB4 - zinc finger and btb domain containing 4  SFPQ - splicing factor proline/glutamine-rich  SFRP1 - secreted frizzled-related protein 1  PHF10 - phd finger protein 10  SFRP2 - secreted frizzled-related protein 2  MXD1 - max dimerization protein 1  SF1 - splicing factor 1  PIAS4 - protein inhibitor of activated stat, 4  TRIM33 - tripartite motif containing 33  PSPC1 - paraspeckle component 1  U2AF2 - u2 small nuclear rna auxiliary factor 2  SMAD2 - smad family member 2  SRSF2 - serine/arginine-rich splicing factor 2  ZBTB14 - zinc finger and btb domain containing 14  DNMT1 - dna (cytosine-5-)-methyltransferase 1  KLF16 - kruppel-like factor 16  HDAC7 - histone deacetylase 7  SRSF4 - serine/arginine-rich splicing factor 4  TRDMT1 - trna aspartic acid methyltransferase 1  DNMT3A - dna (cytosine-5-)-methyltransferase 3 alpha  ZNF8 - zinc finger protein 8  TDP2 - tyrosyl-dna phosphodiesterase 2  SRSF6 - serine/arginine-rich splicing factor 6  SRSF7 - serine/arginine-rich splicing factor 7  SFSWAP - splicing factor, suppressor of white-apricot homolog (drosophila)  BCL7A - b-cell cll/lymphoma 7a  CHD8 - chromodomain helicase dna binding protein 8  ZNF3 - zinc finger protein 3  TAF9B - taf9b rna polymerase ii, tata box binding protein (tbp)-associated factor, 31kda  XRCC5 - x-ray repair complementing defective repair in chinese hamster cells 5 (double-strand-break rejoining)  NOC2L - nucleolar complex associated 2 homolog (s. cerevisiae)  SMAD4 - smad family member 4  NBAS - neuroblastoma amplified sequence  CCND1 - cyclin d1  SMAD5 - smad family member 5  SMAD7 - smad family member 7  YWHAB - tyrosine 3-monooxygenase/tryptophan 5-monooxygenase activation protein, beta polypeptide  TRIM27 - tripartite motif containing 27  YAF2 - yy1 associated factor 2  SIX5 - six homeobox 5  TAGLN3 - transgelin 3  ZNF582 - zinc finger protein 582  SAP18 - sin3a-associated protein, 18kda  BRD7 - bromodomain containing 7  NCK1 - nck adaptor protein 1  TRIM24 - tripartite motif containing 24  CREG1 - cellular repressor of e1a-stimulated genes 1  NAF1 - nuclear assembly factor 1 ribonucleoprotein  RFX5 - regulatory factor x, 5 (influences hla class ii expression)  ELK3 - elk3, ets-domain protein (srf accessory protein 2)  HSBP1 - heat shock factor binding protein 1  FLCN - folliculin  ELK4 - elk4, ets-domain protein (srf accessory protein 1)  SNW1 - snw domain containing 1  RELA - v-rel avian reticuloendotheliosis viral oncogene homolog a  TRIM28 - tripartite motif containing 28  BCOR - bcl6 corepressor  PRMT2 - protein arginine methyltransferase 2  EP300 - e1a binding protein p300  HESX1 - hesx homeobox 1  SAP30 - sin3a-associated protein, 30kda  DPF2 - d4, zinc and double phd fingers family 2  NSUN2 - nop2/sun rna methyltransferase family, member 2  ENO1 - enolase 1, (alpha)  CC2D1A - coiled-coil and c2 domain containing 1a  EIF4A2 - eukaryotic translation initiation factor 4a2  CELF4 - cugbp, elav-like family member 4  RING1 - ring finger protein 1  TRIM11 - tripartite motif containing 11  NAB1 - ngfi-a binding protein 1 (egr1 binding protein 1)  SECISBP2 - secis binding protein 2  NAB2 - ngfi-a binding protein 2 (egr1 binding protein 2)  BMI1 - bmi1 polycomb ring finger oncogene  KDM2A - lysine (k)-specific demethylase 2a  ELAVL1 - elav (embryonic lethal, abnormal vision, drosophila)-like 1 (hu antigen r)  ELAVL4 - elav (embryonic lethal, abnormal vision, drosophila)-like 4  LDB1 - lim domain binding 1  TCF25 - transcription factor 25 (basic helix-loop-helix)  TSG101 - tumor susceptibility gene 101  GON4L - gon-4-like (c. elegans)  KAT2B - k(lysine) acetyltransferase 2b  LBH - limb bud and heart development  BMP7 - bone morphogenetic protein 7  CNOT1 - ccr4-not transcription complex, subunit 1  EFNA1 - ephrin-a1  SPEN - spen homolog, transcriptional regulator (drosophila)  CTNNBIP1 - catenin, beta interacting protein 1  MYD88 - myeloid differentiation primary response 88  ABCE1 - atp-binding cassette, sub-family e (oabp), member 1  BBX - bobby sox homolog (drosophila)  PHC1 - polyhomeotic homolog 1 (drosophila)  CC2D1B - coiled-coil and c2 domain containing 1b  HCFC2 - host cell factor c2  PHC2 - polyhomeotic homolog 2 (drosophila)  EGR1 - early growth response 1  CNOT7 - ccr4-not transcription complex, subunit 7  MXI1 - max interactor 1, dimerization protein  KDM1A - lysine (k)-specific demethylase 1a  RNF2 - ring finger protein 2  GATAD2A - gata zinc finger domain containing 2a  E2F6 - e2f transcription factor 6  E4F1 - e4f transcription factor 1  ZHX3 - zinc fingers and homeoboxes 3  E2F4 - e2f transcription factor 4, p107/p130-binding  E2F5 - e2f transcription factor 5, p130-binding  DNAJB1 - dnaj (hsp40) homolog, subfamily b, member 1  TRIM37 - tripartite motif containing 37  MBTD1 - mbt domain containing 1  ZNF558 - zinc finger protein 558  EDNRB - endothelin receptor type b  LRPPRC - leucine-rich pentatricopeptide repeat containing  ZNF263 - zinc finger protein 263  BTRC - beta-transducin repeat containing e3 ubiquitin protein ligase  ZMPSTE24 - zinc metallopeptidase ste24  NR2C1 - nuclear receptor subfamily 2, group c, member 1  PURG - purine-rich element binding protein g  NR2C2 - nuclear receptor subfamily 2, group c, member 2  MED25 - mediator complex subunit 25  TPR - translocated promoter region, nuclear basket protein  HSPA8 - heat shock 70kda protein 8  ZNF423 - zinc finger protein 423  PTBP3 - polypyrimidine tract binding protein 3  NRDE2 - nrde-2, necessary for rna interference, domain containing  HMGB2 - high mobility group box 2  HMGB1 - high mobility group box 1  C1D - c1d nuclear receptor corepressor  ZBTB33 - zinc finger and btb domain containing 33  BPTF - bromodomain phd finger transcription factor  HDAC5 - histone deacetylase 5  MAD2L2 - mad2 mitotic arrest deficient-like 2 (yeast)  NR1H2 - nuclear receptor subfamily 1, group h, member 2  HDAC6 - histone deacetylase 6  ETV3 - ets variant 3  SRSF9 - serine/arginine-rich splicing factor 9  ETV6 - ets variant 6  MT3 - metallothionein 3  VHL - von hippel-lindau tumor suppressor, e3 ubiquitin protein ligase  EZR - ezrin  NRARP - notch-regulated ankyrin repeat protein  TBX20 - t-box 20  KCTD1 - potassium channel tetramerization domain containing 1  PCBP4 - poly(rc) binding protein 4  CREB3L1 - camp responsive element binding protein 3-like 1  CALR - calreticulin  WTIP - wilms tumor 1 interacting protein  EZH1 - enhancer of zeste homolog 1 (drosophila)  NCOA2 - nuclear receptor coactivator 2  CIC - capicua transcriptional repressor  SYNCRIP - synaptotagmin binding, cytoplasmic rna interacting protein  DNAJB6 - dnaj (hsp40) homolog, subfamily b, member 6  HIVEP1 - human immunodeficiency virus type i enhancer binding protein 1  ERF - ets2 repressor factor  CITED2 - cbp/p300-interacting transactivator, with glu/asp-rich carboxy-terminal domain, 2  CITED1 - cbp/p300-interacting transactivator, with glu/asp-rich carboxy-terminal domain, 1  ZBTB5 - zinc finger and btb domain containing 5  ELMSAN1 - elm2 and myb/sant-like domain containing 1  THAP11 - thap domain containing 11  EED - embryonic ectoderm development  URI1 - uri1, prefoldin-like chaperone  CCND3 - cyclin d3  CCNT1 - cyclin t1  HMG20A - high mobility group 20a  ZNF248 - zinc finger protein 248  UBB - ubiquitin b  HNRNPU - heterogeneous nuclear ribonucleoprotein u (scaffold attachment factor a)  HNRNPK - heterogeneous nuclear ribonucleoprotein k  RCOR1 - rest corepressor 1  HNRNPL - heterogeneous nuclear ribonucleoprotein l  ZNFX1 - zinc finger, nfx1-type containing 1  HIRA - histone cell cycle regulator  CBFA2T3 - core-binding factor, runt domain, alpha subunit 2; translocated to, 3  CBFB - core-binding factor, beta subunit  USP3 - ubiquitin specific peptidase 3  SUMO1 - small ubiquitin-like modifier 1  HNRNPA2B1 - heterogeneous nuclear ribonucleoprotein a2/b1  HNRNPC - heterogeneous nuclear ribonucleoprotein c (c1/c2)  PIAS3 - protein inhibitor of activated stat, 3  HNRNPD - heterogeneous nuclear ribonucleoprotein d (au-rich element rna binding protein 1, 37kda)  CAV1 - caveolin 1, caveolae protein, 22kda  ZC3H4 - zinc finger ccch-type containing 4  UBE2I - ubiquitin-conjugating enzyme e2i  HMGA1 - high mobility group at-hook 1  FAM129B - family with sequence similarity 129, member b  PRMT5 - protein arginine methyltransferase 5  THRAP3 - thyroid hormone receptor associated protein 3  VPS72 - vacuolar protein sorting 72 homolog (s. cerevisiae)  FOXK2 - forkhead box k2  FMR1 - fragile x mental retardation 1  HAT1 - histone acetyltransferase 1  MGA - mga, max dimerization protein  ZC3H6 - zinc finger ccch-type containing 6  MXD4 - max dimerization protein 4  ZEB1 - zinc finger e-box binding homeobox 1  WWC3 - wwc family member 3  ORC2 - origin recognition complex, subunit 2  HEXIM1 - hexamethylene bis-acetamide inducible 1  ZNF436 - zinc finger protein 436  RBMX - rna binding motif protein, x-linked  FLNA - filamin a, alpha  PSEN1 - presenilin 1  PPM1D - protein phosphatase, mg2+/mn2+ dependent, 1d  PLCB1 - phospholipase c, beta 1 (phosphoinositide-specific)  SRSF12 - serine/arginine-rich splicing factor 12  FOXS1 - forkhead box s1  FOXO3 - forkhead box o3  ILF3 - interleukin enhancer binding factor 3, 90kda  HDAC8 - histone deacetylase 8  SIN3B - sin3 transcription regulator family member b  PIAS1 - protein inhibitor of activated stat, 1  SMURF2 - smad specific e3 ubiquitin protein ligase 2  NACC1 - nucleus accumbens associated 1, ben and btb (poz) domain containing  RIPPLY2 - ripply transcriptional repressor 2  CGGBP1 - cgg triplet repeat binding protein 1  HR - hair growth associated  TDG - thymine-dna glycosylase  SOX18 - sry (sex determining region y)-box 18  CBX4 - chromobox homolog 4  EAPP - e2f-associated phosphoprotein  FBXW11 - f-box and wd repeat domain containing 11  PSMD10 - proteasome (prosome, macropain) 26s subunit, non-atpase, 10  FHL2 - four and a half lim domains 2  FGFR2 - fibroblast growth factor receptor 2  TAF7 - taf7 rna polymerase ii, tata box binding protein (tbp)-associated factor, 55kda  FGFR1 - fibroblast growth factor receptor 1  FGF9 - fibroblast growth factor 9  PTBP1 - polypyrimidine tract binding protein 1  ZNF25 - zinc finger protein 25  DAB2IP - dab2 interacting protein  BCLAF1 - bcl2-associated transcription factor 1  TIRAP - toll-interleukin 1 receptor (tir) domain containing adaptor protein  GMNN - geminin, dna replication inhibitor  MYBBP1A - myb binding protein (p160) 1a  NR4A2 - nuclear receptor subfamily 4, group a, member 2  FBLN5 - fibulin 5  ZBTB7B - zinc finger and btb domain containing 7b  SUDS3 - suppressor of defective silencing 3 homolog (s. cerevisiae)  KAT5 - k(lysine) acetyltransferase 5  ZFP69 - zfp69 zinc finger protein  CDK5 - cyclin-dependent kinase 5  GZF1 - gdnf-inducible zinc finger protein 1  CDK9 - cyclin-dependent kinase 9  INSM1 - insulinoma-associated 1  CDKN1C - cyclin-dependent kinase inhibitor 1c (p57, kip2)  ZEB2 - zinc finger e-box binding homeobox 2  CDKN1B - cyclin-dependent kinase inhibitor 1b (p27, kip1)  PDCD4 - programmed cell death 4 (neoplastic transformation inhibitor)  PCBP3 - poly(rc) binding protein 3  PTPN2 - protein tyrosine phosphatase, non-receptor type 2  IRF2 - interferon regulatory factor 2  KLF7 - kruppel-like factor 7 (ubiquitous)  IRF3 - interferon regulatory factor 3  IKZF5 - ikaros family zinc finger 5 (pegasus)  TCF3 - transcription factor 3  RNF40 - ring finger protein 40, e3 ubiquitin protein ligase  DMAP1 - dna methyltransferase 1 associated protein 1  LARP1 - la ribonucleoprotein domain family, member 1  NKRF - nfkb repressing factor  LANCL2 - lanc lantibiotic synthetase component c-like 2 (bacterial)  CDK2 - cyclin-dependent kinase 2  PURA - purine-rich element binding protein a  PURB - purine-rich element binding protein b  VPRBP - vpr (hiv-1) binding protein  NONO - non-pou domain containing, octamer-binding  PAPD5 - pap associated domain containing 5  NSD1 - nuclear receptor binding set domain protein 1  NUDT16 - nudix (nucleoside diphosphate linked moiety x)-type motif 16  RYBP - ring1 and yy1 binding protein  MTOR - mechanistic target of rapamycin (serine/threonine kinase)  PAXBP1 - pax3 and pax7 binding protein 1  MDFIC - myod family inhibitor domain containing  ZMYND11 - zinc finger, mynd-type containing 11  TLE1 - transducin-like enhancer of split 1 (e(sp1) homolog, drosophila)  SRSF10 - serine/arginine-rich splicing factor 10  PTPRK - protein tyrosine phosphatase, receptor type, k  TLE3 - transducin-like enhancer of split 3 (e(sp1) homolog, drosophila)  TLE4 - transducin-like enhancer of split 4 (e(sp1) homolog, drosophila)  NOTCH4 - notch 4  DKK3 - dickkopf wnt signaling pathway inhibitor 3  PEX2 - peroxisomal biogenesis factor 2  NPAT - nuclear protein, ataxia-telangiectasia locus  KDM5B - lysine (k)-specific demethylase 5b  MPHOSPH8 - m-phase phosphoprotein 8  CNOT2 - ccr4-not transcription complex, subunit 2  SIRT1 - sirtuin 1  NOTCH1 - notch 1  NOTCH3 - notch 3  MKX - mohawk homeobox  NOTCH2 - notch 2  DHX34 - deah (asp-glu-ala-his) box polypeptide 34  ID2 - inhibitor of dna binding 2, dominant negative helix-loop-helix protein  ID3 - inhibitor of dna binding 3, dominant negative helix-loop-helix protein  HDAC4 - histone deacetylase 4  CBX6 - chromobox homolog 6  HEY1 - hairy/enhancer-of-split related with yrpw motif 1  IRF2BPL - interferon regulatory factor 2 binding protein-like  JAZF1 - jazf zinc finger 1  CBX5 - chromobox homolog 5  NFKB1 - nuclear factor of kappa light polypeptide gene enhancer in b-cells 1  QARS - glutaminyl-trna synthetase  MYPOP - myb-related transcription factor, partner of profilin  TARDBP - tar dna binding protein  FOXK1 - forkhead box k1  PARP14 - poly (adp-ribose) polymerase family, member 14  PAF1 - paf1, rna polymerase ii associated factor, homolog (s. cerevisiae)  HDAC9 - histone deacetylase 9  ZNF268 - zinc finger protein 268  ZNF614 - zinc finger protein 614  TMBIM6 - transmembrane bax inhibitor motif containing 6  IGHMBP2 - immunoglobulin mu binding protein 2  ZNF282 - zinc finger protein 282  SUZ12 - suz12 polycomb repressive complex 2 subunit  RBPJ - recombination signal binding protein for immunoglobulin kappa j region  RCOR2 - rest corepressor 2  MIER2 - mesoderm induction early response 1, family member 2  PABPC1 - poly(a) binding protein, cytoplasmic 1  NCK2 - nck adaptor protein 2  CTR9 - ctr9, paf1/rna polymerase ii complex component  PPM1F - protein phosphatase, mg2+/mn2+ dependent, 1f  RARG - retinoic acid receptor, gamma  NR2F2 - nuclear receptor subfamily 2, group f, member 2  EPC1 - enhancer of polycomb homolog 1 (drosophila)  RARB - retinoic acid receptor, beta  MORF4L2 - mortality factor 4 like 2  CBX7 - chromobox homolog 7  DLL4 - delta-like 4 (drosophila)  NFATC4 - nuclear factor of activated t-cells, cytoplasmic, calcineurin-dependent 4  NR2F1 - nuclear receptor subfamily 2, group f, member 1  KHDRBS1 - kh domain containing, rna binding, signal transduction associated 1  NFIA - nuclear factor i/a  MAGEL2 - mage-like 2  HEY2 - hairy/enhancer-of-split related with yrpw motif 2  FNIP1 - folliculin interacting protein 1  CTCF - ccctc-binding factor (zinc finger protein)  IGBP1 - immunoglobulin (cd79a) binding protein 1  RBBP7 - retinoblastoma binding protein 7  RBBP8 - retinoblastoma binding protein 8  SMARCA5 - swi/snf related, matrix associated, actin dependent regulator of chromatin, subfamily a, member 5  RBFOX2 - rna binding protein, fox-1 homolog (c. elegans) 2  TGFB1 - transforming growth factor, beta 1  RB1 - retinoblastoma 1  ARID4A - at rich interactive domain 4a (rbp1-like)  ZNF746 - zinc finger protein 746  VGLL4 - vestigial like 4 (drosophila)  H2AFV - h2a histone family, member v  MEPCE - methylphosphate capping enzyme  RNF20 - ring finger protein 20, e3 ubiquitin protein ligase  KDM5A - lysine (k)-specific demethylase 5a  KDM4A - lysine (k)-specific demethylase 4a  PHF14 - phd finger protein 14  FOSB - fbj murine osteosarcoma viral oncogene homolog b  CUL3 - cullin 3  KAT6B - k(lysine) acetyltransferase 6b  CIRBP - cold inducible rna binding protein  FOXP1 - forkhead box p1  BEND6 - ben domain containing 6  THRA - thyroid hormone receptor, alpha |
| GO:0016043 | cellular component organization | 7.28E-5 | 6.94E-3 | 1.04 (10334,3532,6996,2477) | [+] Show genes  AFG3L2 - afg3 atpase family member 3-like 2 (s. cerevisiae)  AGAP2 - arfgap with gtpase domain, ankyrin repeat and ph domain 2  MSL3 - male-specific lethal 3 homolog (drosophila)  SF3A3 - splicing factor 3a, subunit 3, 60kda  TADA1 - transcriptional adaptor 1  STARD3 - star-related lipid transfer (start) domain containing 3  LRRC16A - leucine rich repeat containing 16a  ABCA1 - atp-binding cassette, sub-family a (abc1), member 1  PPP1R9A - protein phosphatase 1, regulatory subunit 9a  ABCA2 - atp-binding cassette, sub-family a (abc1), member 2  HAUS7 - haus augmin-like complex, subunit 7  PLXNB1 - plexin b1  ABL1 - c-abl oncogene 1, non-receptor tyrosine kinase  SSX2IP - synovial sarcoma, x breakpoint 2 interacting protein  TMED2 - transmembrane emp24 domain trafficking protein 2  ABR - active bcr-related  PML - promyelocytic leukemia  ACACA - acetyl-coa carboxylase alpha  ACOT2 - acyl-coa thioesterase 2  PMP22 - peripheral myelin protein 22  YWHAQ - tyrosine 3-monooxygenase/tryptophan 5-monooxygenase activation protein, theta polypeptide  LURAP1 - leucine rich adaptor protein 1  KIF27 - kinesin family member 27  ASCC3 - activating signal cointegrator 1 complex subunit 3  TMED10 - transmembrane emp24-like trafficking protein 10 (yeast)  BLCAP - bladder cancer associated protein  SMPD4 - sphingomyelin phosphodiesterase 4, neutral membrane (neutral sphingomyelinase-3)  BRD8 - bromodomain containing 8  TXNL4A - thioredoxin-like 4a  ACHE - acetylcholinesterase  TRAFD1 - traf-type zinc finger domain containing 1  ACOX1 - acyl-coa oxidase 1, palmitoyl  SYBU - syntabulin (syntaxin-interacting)  PLS3 - plastin 3  CHD7 - chromodomain helicase dna binding protein 7  PLTP - phospholipid transfer protein  ACTB - actin, beta  EHMT2 - euchromatic histone-lysine n-methyltransferase 2  SPIN1 - spindlin 1  ACTG1 - actin, gamma 1  MORF4L1 - mortality factor 4 like 1  PLD2 - phospholipase d2  DOCK10 - dedicator of cytokinesis 10  EHD1 - eh-domain containing 1  AMBRA1 - autophagy/beclin-1 regulator 1  CHCHD1 - coiled-coil-helix-coiled-coil-helix domain containing 1  PPID - peptidylprolyl isomerase d  ACTL6A - actin-like 6a  ACTN1 - actinin, alpha 1  AP1AR - adaptor-related protein complex 1 associated regulatory protein  PPIA - peptidylprolyl isomerase a (cyclophilin a)  ACTN4 - actinin, alpha 4  NDUFAF7 - nadh dehydrogenase (ubiquinone) complex i, assembly factor 7  RAB10 - rab10, member ras oncogene family  ADAR - adenosine deaminase, rna-specific  ADAM10 - adam metallopeptidase domain 10  ADARB1 - adenosine deaminase, rna-specific, b1  MRPS30 - mitochondrial ribosomal protein s30  MED1 - mediator complex subunit 1  ADCY1 - adenylate cyclase 1 (brain)  MMP24 - matrix metallopeptidase 24 (membrane-inserted)  RAB39B - rab39b, member ras oncogene family  ADD2 - adducin 2 (beta)  POLRMT - polymerase (rna) mitochondrial (dna directed)  ADD3 - adducin 3 (gamma)  ADD1 - adducin 1 (alpha)  PARP1 - poly (adp-ribose) polymerase 1  H2AFY2 - h2a histone family, member y2  NOP10 - nop10 ribonucleoprotein  CNTROB - centrobin, centrosomal brca2 interacting protein  POLD2 - polymerase (dna directed), delta 2, accessory subunit  SNAP47 - synaptosomal-associated protein, 47kda  POLD1 - polymerase (dna directed), delta 1, catalytic subunit  TUBGCP2 - tubulin, gamma complex associated protein 2  SMPD3 - sphingomyelin phosphodiesterase 3, neutral membrane (neutral sphingomyelinase ii)  POLR2D - polymerase (rna) ii (dna directed) polypeptide d  RUVBL2 - ruvb-like 2 (e. coli)  AP2A2 - adaptor-related protein complex 2, alpha 2 subunit  TMEM30A - transmembrane protein 30a  AP2A1 - adaptor-related protein complex 2, alpha 1 subunit  AP2B1 - adaptor-related protein complex 2, beta 1 subunit  CDK5RAP2 - cdk5 regulatory subunit associated protein 2  KATNA1 - katanin p60 (atpase containing) subunit a 1  RCOR3 - rest corepressor 3  PRKACB - protein kinase, camp-dependent, catalytic, beta  AP1G1 - adaptor-related protein complex 1, gamma 1 subunit  PRKACA - protein kinase, camp-dependent, catalytic, alpha  NUP133 - nucleoporin 133kda  SRGN - serglycin  AGK - acylglycerol kinase  KCTD12 - potassium channel tetramerization domain containing 12  PRIM2 - primase, dna, polypeptide 2 (58kda)  PRIM1 - primase, dna, polypeptide 1 (49kda)  VPS35 - vacuolar protein sorting 35 homolog (s. cerevisiae)  ENAH - enabled homolog (drosophila)  KIF3A - kinesin family member 3a  COA5 - cytochrome c oxidase assembly factor 5  PARVA - parvin, alpha  ZWINT - zw10 interacting kinetochore protein  COA1 - cytochrome c oxidase assembly factor 1 homolog (s. cerevisiae)  PRDM4 - pr domain containing 4  PPP3R1 - protein phosphatase 3, regulatory subunit b, alpha  CIT - citron (rho-interacting, serine/threonine kinase 21)  FGFR1OP - fgfr1 oncogene partner  PPP5C - protein phosphatase 5, catalytic subunit  PPP6C - protein phosphatase 6, catalytic subunit  PPT1 - palmitoyl-protein thioesterase 1  DNAJC11 - dnaj (hsp40) homolog, subfamily c, member 11  CSGALNACT1 - chondroitin sulfate n-acetylgalactosaminyltransferase 1  AKT1 - v-akt murine thymoma viral oncogene homolog 1  PCID2 - pci domain containing 2  DDX28 - dead (asp-glu-ala-asp) box polypeptide 28  PPP2R2A - protein phosphatase 2, regulatory subunit b, alpha  CPSF6 - cleavage and polyadenylation specific factor 6, 68kda  PPP2R1A - protein phosphatase 2, regulatory subunit a, alpha  NUDT21 - nudix (nucleoside diphosphate linked moiety x)-type motif 21  TBC1D23 - tbc1 domain family, member 23  TRIOBP - trio and f-actin binding protein  PPP2CA - protein phosphatase 2, catalytic subunit, alpha isozyme  PPP2CB - protein phosphatase 2, catalytic subunit, beta isozyme  IFT122 - intraflagellar transport 122 homolog (chlamydomonas)  RMI2 - recq mediated genome instability 2  TTC17 - tetratricopeptide repeat domain 17  STMN2 - stathmin-like 2  LYSMD3 - lysm, putative peptidoglycan-binding, domain containing 3  ESCO1 - establishment of sister chromatid cohesion n-acetyltransferase 1  KANSL3 - kat8 regulatory nsl complex subunit 3  TDRKH - tudor and kh domain containing  TUBGCP5 - tubulin, gamma complex associated protein 5  PEX26 - peroxisomal biogenesis factor 26  RAB35 - rab35, member ras oncogene family  IFT27 - intraflagellar transport 27 homolog (chlamydomonas)  FMNL2 - formin-like 2  PRNP - prion protein  GPRIN1 - g protein regulated inducer of neurite outgrowth 1  NPLOC4 - nuclear protein localization 4 homolog (s. cerevisiae)  PRPF40A - prp40 pre-mrna processing factor 40 homolog a (s. cerevisiae)  PAPD7 - pap associated domain containing 7  TMEM127 - transmembrane protein 127  PIM2 - pim-2 oncogene  RAB31 - rab31, member ras oncogene family  DSTN - destrin (actin depolymerizing factor)  MAPK10 - mitogen-activated protein kinase 10  IMMT - inner membrane protein, mitochondrial  MAPK8 - mitogen-activated protein kinase 8  MAPK3 - mitogen-activated protein kinase 3  WDR11 - wd repeat domain 11  CISD2 - cdgsh iron sulfur domain 2  MAPRE2 - microtubule-associated protein, rp/eb family, member 2  AMPH - amphiphysin  COPS6 - cop9 signalosome subunit 6  CLP1 - cleavage and polyadenylation factor i subunit 1  OSBPL8 - oxysterol binding protein-like 8  OSBPL5 - oxysterol binding protein-like 5  NDC1 - ndc1 transmembrane nucleoporin  PRKD1 - protein kinase d1  HAUS1 - haus augmin-like complex, subunit 1  CCDC88A - coiled-coil domain containing 88a  PKN2 - protein kinase n2  TLK2 - tousled-like kinase 2  PRKCI - protein kinase c, iota  ANXA2 - annexin a2  PRKCE - protein kinase c, epsilon  PRKCB - protein kinase c, beta  RADIL - ras association and dil domains  PRKCD - protein kinase c, delta  SLC25A5 - solute carrier family 25 (mitochondrial carrier; adenine nucleotide translocator), member 5  PRKCA - protein kinase c, alpha  PRKAR1A - protein kinase, camp-dependent, regulatory, type i, alpha  LUC7L - luc7-like (s. cerevisiae)  YEATS2 - yeats domain containing 2  APEX1 - apex nuclease (multifunctional dna repair enzyme) 1  CCDC78 - coiled-coil domain containing 78  MSTO1 - misato 1, mitochondrial distribution and morphology regulator  XIAP - x-linked inhibitor of apoptosis  SDAD1 - sda1 domain containing 1  ATXN2L - ataxin 2-like  NOXO1 - nadph oxidase organizer 1  RFWD3 - ring finger and wd repeat domain 3  TREX1 - three prime repair exonuclease 1  SHQ1 - shq1, h/aca ribonucleoprotein assembly factor  PDCL - phosducin-like  TMEM33 - transmembrane protein 33  PRMT6 - protein arginine methyltransferase 6  MSL2 - male-specific lethal 2 homolog (drosophila)  MRPS10 - mitochondrial ribosomal protein s10  TBCCD1 - tbcc domain containing 1  PACSIN2 - protein kinase c and casein kinase substrate in neurons 2  FOPNL - fgfr1op n-terminal like  APBA1 - amyloid beta (a4) precursor protein-binding, family a, member 1  APBB2 - amyloid beta (a4) precursor protein-binding, family b, member 2  APC - adenomatous polyposis coli  APBB1 - amyloid beta (a4) precursor protein-binding, family b, member 1 (fe65)  CHP1 - calcineurin-like ef-hand protein 1  RIF1 - rap1 interacting factor homolog (yeast)  VPS13D - vacuolar protein sorting 13 homolog d (s. cerevisiae)  SLC25A36 - solute carrier family 25 (pyrimidine nucleotide carrier ), member 36  HPS5 - hermansky-pudlak syndrome 5  POLG2 - polymerase (dna directed), gamma 2, accessory subunit  PDCD10 - programmed cell death 10  PBRM1 - polybromo 1  P4HA1 - prolyl 4-hydroxylase, alpha polypeptide i  APPL2 - adaptor protein, phosphotyrosine interaction, ph domain and leucine zipper containing 2  ARF1 - adp-ribosylation factor 1  MAP1S - microtubule-associated protein 1s  PAFAH1B1 - platelet-activating factor acetylhydrolase 1b, regulatory subunit 1 (45kda)  GOLPH3L - golgi phosphoprotein 3-like  APOD - apolipoprotein d  TMEM170A - transmembrane protein 170a  SETD5 - set domain containing 5  AKAP11 - a kinase (prka) anchor protein 11  APP - amyloid beta (a4) precursor protein  BBS7 - bardet-biedl syndrome 7  DDX20 - dead (asp-glu-ala-asp) box polypeptide 20  PAK2 - p21 protein (cdc42/rac)-activated kinase 2  RCBTB1 - regulator of chromosome condensation (rcc1) and btb (poz) domain containing protein 1  PAK3 - p21 protein (cdc42/rac)-activated kinase 3  PALM - paralemmin  MRPL3 - mitochondrial ribosomal protein l3  CEP250 - centrosomal protein 250kda  PDGFA - platelet-derived growth factor alpha polypeptide  CELF3 - cugbp, elav-like family member 3  ARL3 - adp-ribosylation factor-like 3  NISCH - nischarin  TRAPPC6B - trafficking protein particle complex 6b  ARL2 - adp-ribosylation factor-like 2  ARL1 - adp-ribosylation factor-like 1  NSMCE2 - non-smc element 2, mms21 homolog (s. cerevisiae)  SUPT16H - suppressor of ty 16 homolog (s. cerevisiae)  ARSB - arylsulfatase b  SEC23IP - sec23 interacting protein  PDGFRB - platelet-derived growth factor receptor, beta polypeptide  IFT57 - intraflagellar transport 57 homolog (chlamydomonas)  ARRB2 - arrestin, beta 2  ARRB1 - arrestin, beta 1  PDGFRA - platelet-derived growth factor receptor, alpha polypeptide  PDGFB - platelet-derived growth factor beta polypeptide  RHOB - ras homolog family member b  FAM83H - family with sequence similarity 83, member h  RHOC - ras homolog family member c  PDPK1 - 3-phosphoinositide dependent protein kinase-1  FAM107A - family with sequence similarity 107, member a  RHOA - ras homolog family member a  STRAP - serine/threonine kinase receptor associated protein  ARF6 - adp-ribosylation factor 6  PSIP1 - pc4 and sfrs1 interacting protein 1  PECAM1 - platelet/endothelial cell adhesion molecule 1  ATG2B - autophagy related 2b  RND3 - rho family gtpase 3  BAZ2A - bromodomain adjacent to zinc finger domain, 2a  WDR60 - wd repeat domain 60  PCNA - proliferating cell nuclear antigen  CHMP1A - charged multivesicular body protein 1a  ARHGAP17 - rho gtpase activating protein 17  ISCA2 - iron-sulfur cluster assembly 2  PLEKHJ1 - pleckstrin homology domain containing, family j member 1  TTC8 - tetratricopeptide repeat domain 8  PCM1 - pericentriolar material 1  CDK16 - cyclin-dependent kinase 16  RABL2B - rab, member of ras oncogene family-like 2b  LEO1 - leo1, paf1/rna polymerase ii complex component, homolog (s. cerevisiae)  FRMD6 - ferm domain containing 6  PWP1 - pwp1 homolog (s. cerevisiae)  WRAP53 - wd repeat containing, antisense to tp53  CAPN10 - calpain 10  KPTN - kaptin (actin binding protein)  PLA2G16 - phospholipase a2, group xvi  KAT7 - k(lysine) acetyltransferase 7  HAUS2 - haus augmin-like complex, subunit 2  EPN2 - epsin 2  RUFY3 - run and fyve domain containing 3  BTBD3 - btb (poz) domain containing 3  ZBTB1 - zinc finger and btb domain containing 1  ATP1A3 - atpase, na+/k+ transporting, alpha 3 polypeptide  ZNF365 - zinc finger protein 365  PFN2 - profilin 2  FBXW7 - f-box and wd repeat domain containing 7, e3 ubiquitin protein ligase  ATP1B2 - atpase, na+/k+ transporting, beta 2 polypeptide  BRIX1 - brx1, biogenesis of ribosomes, homolog (s. cerevisiae)  BAHD1 - bromo adjacent homology domain containing 1  ATP1B1 - atpase, na+/k+ transporting, beta 1 polypeptide  MORC2 - morc family cw-type zinc finger 2  PHF2 - phd finger protein 2  PHF1 - phd finger protein 1  LRRK2 - leucine-rich repeat kinase 2  BLOC1S4 - biogenesis of lysosomal organelles complex-1, subunit 4, cappuccino  RERE - arginine-glutamic acid dipeptide (re) repeats  MNS1 - meiosis-specific nuclear structural 1  CLSTN1 - calsyntenin 1  DZIP1 - daz interacting zinc finger protein 1  SEC31A - sec31 homolog a (s. cerevisiae)  PEX6 - peroxisomal biogenesis factor 6  ATG14 - autophagy related 14  PEX7 - peroxisomal biogenesis factor 7  SH3PXD2B - sh3 and px domains 2b  PEX12 - peroxisomal biogenesis factor 12  CNKSR2 - connector enhancer of kinase suppressor of ras 2  PEX13 - peroxisomal biogenesis factor 13  ATP7A - atpase, cu++ transporting, alpha polypeptide  PEX14 - peroxisomal biogenesis factor 14  PPP6R1 - protein phosphatase 6, regulatory subunit 1  FRYL - fry-like  LMTK2 - lemur tyrosine kinase 2  AEBP2 - ae binding protein 2  ICK - intestinal cell (mak-like) kinase  PEX1 - peroxisomal biogenesis factor 1  PFKL - phosphofructokinase, liver  PI4K2A - phosphatidylinositol 4-kinase type 2 alpha  AAK1 - ap2 associated kinase 1  PFKP - phosphofructokinase, platelet  COPRS - coordinator of prmt5, differentiation stimulator  BAX - bcl2-associated x protein  PKD1 - polycystic kidney disease 1 (autosomal dominant)  PKD2 - polycystic kidney disease 2 (autosomal dominant)  PUF60 - poly-u binding splicing factor 60kda  MTF2 - metal response element binding transcription factor 2  PITPNA - phosphatidylinositol transfer protein, alpha  BAK1 - bcl2-antagonist/killer 1  BAD - bcl2-associated agonist of cell death  PIN1 - peptidylprolyl cis/trans isomerase, nima-interacting 1  PI4KA - phosphatidylinositol 4-kinase, catalytic, alpha  PIK3R1 - phosphoinositide-3-kinase, regulatory subunit 1 (alpha)  PDZD8 - pdz domain containing 8  MRAS - muscle ras oncogene homolog  COX18 - cox18 cytochrome c oxidase assembly factor  AXL - axl receptor tyrosine kinase  ZFYVE27 - zinc finger, fyve domain containing 27  PKP2 - plakophilin 2  COG2 - component of oligomeric golgi complex 2  ATRX - alpha thalassemia/mental retardation syndrome x-linked  PEF1 - penta-ef-hand domain containing 1  OGFOD1 - 2-oxoglutarate and iron-dependent oxygenase domain containing 1  GABARAPL2 - gaba(a) receptor-associated protein-like 2  TWF2 - twinfilin actin-binding protein 2  PIH1D2 - pih1 domain containing 2  PHF10 - phd finger protein 10  SERPINE2 - serpin peptidase inhibitor, clade e (nexin, plasminogen activator inhibitor type 1), member 2  GABARAP - gaba(a) receptor-associated protein  BCR - breakpoint cluster region  MRGBP - mrg/morf4l binding protein  BCL9 - b-cell cll/lymphoma 9  RHOT1 - ras homolog family member t1  PPP6R3 - protein phosphatase 6, regulatory subunit 3  BRF2 - brf2, rna polymerase iii transcription initiation factor 50 kda subunit  PIK3C2B - phosphatidylinositol-4-phosphate 3-kinase, catalytic subunit type 2 beta  BCL2 - b-cell cll/lymphoma 2  BCL2L2 - bcl2-like 2  PIK3C3 - phosphatidylinositol 3-kinase, catalytic subunit type 3  BCL2L1 - bcl2-like 1  CCDC66 - coiled-coil domain containing 66  PARK7 - parkinson protein 7  B4GALT7 - xylosylprotein beta 1,4-galactosyltransferase, polypeptide 7  BBS4 - bardet-biedl syndrome 4  BBS2 - bardet-biedl syndrome 2  TBC1D20 - tbc1 domain family, member 20  DST - dystonin  TRIM27 - tripartite motif containing 27  BNIP3L - bcl2/adenovirus e1b 19kda interacting protein 3-like  RFC5 - replication factor c (activator 1) 5, 36.5kda  BNIP1 - bcl2/adenovirus e1b 19kda interacting protein 1  RFC2 - replication factor c (activator 1) 2, 40kda  RFC3 - replication factor c (activator 1) 3, 38kda  BET1 - bet1 golgi vesicular membrane trafficking protein  MAEA - macrophage erythroblast attacher  SF3A1 - splicing factor 3a, subunit 1, 120kda  WHSC1L1 - wolf-hirschhorn syndrome candidate 1-like 1  TTC19 - tetratricopeptide repeat domain 19  MKS1 - meckel syndrome, type 1  RELA - v-rel avian reticuloendotheliosis viral oncogene homolog a  BCOR - bcl6 corepressor  KATNB1 - katanin p80 (wd repeat containing) subunit b 1  RECQL - recq protein-like (dna helicase q1-like)  PAK4 - p21 protein (cdc42/rac)-activated kinase 4  RDX - radixin  RET - ret proto-oncogene  DPF2 - d4, zinc and double phd fingers family 2  RTN3 - reticulon 3  UPF1 - upf1 regulator of nonsense transcripts homolog (yeast)  RLF - rearranged l-myc fusion  RING1 - ring finger protein 1  MTX3 - metaxin 3  FNBP1L - formin binding protein 1-like  HIST3H2BB - histone cluster 3, h2bb  FBLL1 - fibrillarin-like 1  TFG - trk-fused gene  BMP1 - bone morphogenetic protein 1  BMI1 - bmi1 polycomb ring finger oncogene  PCGF3 - polycomb group ring finger 3  BMP7 - bone morphogenetic protein 7  ABCA7 - atp-binding cassette, sub-family a (abc1), member 7  FITM2 - fat storage-inducing transmembrane protein 2  MRPL49 - mitochondrial ribosomal protein l49  TRPM7 - transient receptor potential cation channel, subfamily m, member 7  FLOT1 - flotillin 1  NDE1 - nude neurodevelopment protein 1  VPS51 - vacuolar protein sorting 51 homolog (s. cerevisiae)  BCAS3 - breast carcinoma amplified sequence 3  ABCE1 - atp-binding cassette, sub-family e (oabp), member 1  VPS13C - vacuolar protein sorting 13 homolog c (s. cerevisiae)  FMNL1 - formin-like 1  LRRC49 - leucine rich repeat containing 49  HAUS6 - haus augmin-like complex, subunit 6  STX6 - syntaxin 6  UHMK1 - u2af homology motif (uhm) kinase 1  DYM - dymeclin  AHI1 - abelson helper integration site 1  LIX1L - lix1 homolog (mouse)-like  BRD2 - bromodomain containing 2  RNF2 - ring finger protein 2  GPHN - gephyrin  ROCK1 - rho-associated, coiled-coil containing protein kinase 1  TSPO - translocator protein (18kda)  SPRY1 - sprouty homolog 1, antagonist of fgf signaling (drosophila)  DNAJB12 - dnaj (hsp40) homolog, subfamily b, member 12  SPRY2 - sprouty homolog 2 (drosophila)  TET2 - tet methylcytosine dioxygenase 2  STAM2 - signal transducing adaptor molecule (sh3 domain and itam motif) 2  TRPM4 - transient receptor potential cation channel, subfamily m, member 4  MBTD1 - mbt domain containing 1  RAMP2 - receptor (g protein-coupled) activity modifying protein 2  FBLIM1 - filamin binding lim protein 1  AKAP8 - a kinase (prka) anchor protein 8  ZMPSTE24 - zinc metallopeptidase ste24  C5AR1 - complement component 5a receptor 1  GRAMD1C - gram domain containing 1c  STAG1 - stromal antigen 1  EFS - embryonal fyn-associated substrate  UBE4B - ubiquitination factor e4b  CAPZA1 - capping protein (actin filament) muscle z-line, alpha 1  CCSAP - centriole, cilia and spindle-associated protein  VAV3 - vav 3 guanine nucleotide exchange factor  CAPNS1 - calpain, small subunit 1  CAPN2 - calpain 2, (m/ii) large subunit  CAPN1 - calpain 1, (mu/i) large subunit  MCRS1 - microspherule protein 1  ATG16L1 - autophagy related 16-like 1 (s. cerevisiae)  MRPL20 - mitochondrial ribosomal protein l20  NRDE2 - nrde-2, necessary for rna interference, domain containing  VPS37C - vacuolar protein sorting 37 homolog c (s. cerevisiae)  RPL11 - ribosomal protein l11  PTCD3 - pentatricopeptide repeat domain 3  CASP8 - caspase 8, apoptosis-related cysteine peptidase  PIBF1 - progesterone immunomodulatory binding factor 1  PPIH - peptidylprolyl isomerase h (cyclophilin h)  CASP3 - caspase 3, apoptosis-related cysteine peptidase  RPL3 - ribosomal protein l3  BAIAP2 - bai1-associated protein 2  RPA3 - replication protein a3, 14kda  MAD2L2 - mad2 mitotic arrest deficient-like 2 (yeast)  RPA1 - replication protein a1, 70kda  PID1 - phosphotyrosine interaction domain containing 1  CAPZB - capping protein (actin filament) muscle z-line, beta  RPA2 - replication protein a2, 32kda  PHIP - pleckstrin homology domain interacting protein  SEC23A - sec23 homolog a (s. cerevisiae)  CAP1 - cap, adenylate cyclase-associated protein 1 (yeast)  PPP2R3C - protein phosphatase 2, regulatory subunit b'', gamma  SEC23B - sec23 homolog b (s. cerevisiae)  PRPF39 - prp39 pre-mrna processing factor 39 homolog (s. cerevisiae)  STX17 - syntaxin 17  CACNB3 - calcium channel, voltage-dependent, beta 3 subunit  SLC9A6 - solute carrier family 9, subfamily a (nhe6, cation proton antiporter 6), member 6  CACNB2 - calcium channel, voltage-dependent, beta 2 subunit  LAMTOR1 - late endosomal/lysosomal adaptor, mapk and mtor activator 1  DDR1 - discoidin domain receptor tyrosine kinase 1  TADA3 - transcriptional adaptor 3  CALR - calreticulin  UNC13B - unc-13 homolog b (c. elegans)  CALM2 - calmodulin 2 (phosphorylase kinase, delta)  WTIP - wilms tumor 1 interacting protein  STK25 - serine/threonine kinase 25  GLDN - gliomedin  VTI1B - vesicle transport through interaction with t-snares 1b  CRTAP - cartilage associated protein  RPS23 - ribosomal protein s23  SLC35F6 - solute carrier family 35, member f6  MICU1 - mitochondrial calcium uptake 1  PINX1 - pin2/terf1 interacting, telomerase inhibitor 1  CLN6 - ceroid-lipofuscinosis, neuronal 6, late infantile, variant  TUBA1B - tubulin, alpha 1b  NPM3 - nucleophosmin/nucleoplasmin 3  TMEM70 - transmembrane protein 70  INO80C - ino80 complex subunit c  THG1L - trna-histidine guanylyltransferase 1-like (s. cerevisiae)  CCNH - cyclin h  HMG20A - high mobility group 20a  BANP - btg3 associated nuclear protein  GEMIN8 - gem (nuclear organelle) associated protein 8  SSH3 - slingshot protein phosphatase 3  CORO2B - coronin, actin binding protein, 2b  ATP8A1 - atpase, aminophospholipid transporter (aplt), class i, type 8a, member 1  NDRG1 - n-myc downstream regulated 1  RPS15 - ribosomal protein s15  CD9 - cd9 molecule  DLC1 - deleted in liver cancer 1  TUBB4B - tubulin, beta 4b class ivb  TUBB3 - tubulin, beta 3 class iii  C17orf49 - chromosome 17 open reading frame 49  FAM206A - family with sequence similarity 206, member a  CBL - cbl proto-oncogene, e3 ubiquitin protein ligase  SERPINH1 - serpin peptidase inhibitor, clade h (heat shock protein 47), member 1, (collagen binding protein 1)  MRPL10 - mitochondrial ribosomal protein l10  LPCAT2 - lysophosphatidylcholine acyltransferase 2  CAV2 - caveolin 2  CAV1 - caveolin 1, caveolae protein, 22kda  KANSL2 - kat8 regulatory nsl complex subunit 2  SEZ6 - seizure related 6 homolog (mouse)  TUBGCP3 - tubulin, gamma complex associated protein 3  SEC24B - sec24 family, member b (s. cerevisiae)  RBM14 - rna binding motif protein 14  PRMT5 - protein arginine methyltransferase 5  CD2BP2 - cd2 (cytoplasmic tail) binding protein 2  RRM1 - ribonucleotide reductase m1  CHTF8 - ctf8, chromosome transmission fidelity factor 8 homolog (s. cerevisiae)  TGOLN2 - trans-golgi network protein 2  HTRA1 - htra serine peptidase 1  CDH3 - cadherin 3, type 1, p-cadherin (placental)  PSAP - prosaposin  CDH2 - cadherin 2, type 1, n-cadherin (neuronal)  KCTD5 - potassium channel tetramerization domain containing 5  POMT1 - protein-o-mannosyltransferase 1  MRPS21 - mitochondrial ribosomal protein s21  CCSER2 - coiled-coil serine-rich protein 2  PSEN1 - presenilin 1  PSEN2 - presenilin 2 (alzheimer disease 4)  CD151 - cd151 molecule (raph blood group)  USP16 - ubiquitin specific peptidase 16  CDC42EP3 - cdc42 effector protein (rho gtpase binding) 3  SRSF12 - serine/arginine-rich splicing factor 12  NLE1 - notchless homolog 1 (drosophila)  PRPF8 - pre-mrna processing factor 8  SMCR7L - smith-magenis syndrome chromosome region, candidate 7-like  STARD4 - star-related lipid transfer (start) domain containing 4  SCARB2 - scavenger receptor class b, member 2  EXOC5 - exocyst complex component 5  PSMB10 - proteasome (prosome, macropain) subunit, beta type, 10  CD47 - cd47 molecule  CD44 - cd44 molecule (indian blood group)  SPINT2 - serine peptidase inhibitor, kunitz type, 2  YKT6 - ykt6 v-snare homolog (s. cerevisiae)  MTX2 - metaxin 2  SLMO1 - slowmo homolog 1 (drosophila)  TAF6L - taf6-like rna polymerase ii, p300/cbp-associated factor (pcaf)-associated factor, 65kda  GDAP1 - ganglioside induced differentiation associated protein 1  PSMD9 - proteasome (prosome, macropain) 26s subunit, non-atpase, 9  CD34 - cd34 molecule  PSMD10 - proteasome (prosome, macropain) 26s subunit, non-atpase, 10  GAR1 - gar1 ribonucleoprotein  TSPAN33 - tetraspanin 33  GAS2L1 - growth arrest-specific 2 like 1  PSMD5 - proteasome (prosome, macropain) 26s subunit, non-atpase, 5  TERF2IP - telomeric repeat binding factor 2, interacting protein  PSMD13 - proteasome (prosome, macropain) 26s subunit, non-atpase, 13  ATG7 - autophagy related 7  CENPC - centromere protein c  KLHL17 - kelch-like family member 17  DCTN2 - dynactin 2 (p50)  GLRX3 - glutaredoxin 3  PTEN - phosphatase and tensin homolog  LAMTOR5 - late endosomal/lysosomal adaptor, mapk and mtor activator 5  CETN2 - centrin, ef-hand protein, 2  ANP32B - acidic (leucine-rich) nuclear phosphoprotein 32 family, member b  JMY - junction mediating and regulatory protein, p53 cofactor  CETN3 - centrin, ef-hand protein, 3  PRDX4 - peroxiredoxin 4  CFL1 - cofilin 1 (non-muscle)  FBLN5 - fibulin 5  PTGFRN - prostaglandin f2 receptor inhibitor  CHRAC1 - chromatin accessibility complex 1  CIB1 - calcium and integrin binding 1 (calmyrin)  CLGN - calmegin  KAT5 - k(lysine) acetyltransferase 5  PTK2 - protein tyrosine kinase 2  PTK7 - protein tyrosine kinase 7  SLU7 - slu7 splicing factor homolog (s. cerevisiae)  CDK5 - cyclin-dependent kinase 5  PTMA - prothymosin, alpha  CDK4 - cyclin-dependent kinase 4  TWF1 - twinfilin actin-binding protein 1  PTN - pleiotrophin  CDK7 - cyclin-dependent kinase 7  NPC2 - niemann-pick disease, type c2  CCT2 - chaperonin containing tcp1, subunit 2 (beta)  PTPN1 - protein tyrosine phosphatase, non-receptor type 1  ARPC1A - actin related protein 2/3 complex, subunit 1a, 41kda  CDH6 - cadherin 6, type 2, k-cadherin (fetal kidney)  PTPN11 - protein tyrosine phosphatase, non-receptor type 11  CDH11 - cadherin 11, type 2, ob-cadherin (osteoblast)  CDH8 - cadherin 8, type 2  PTPRD - protein tyrosine phosphatase, receptor type, d  SCLT1 - sodium channel and clathrin linker 1  ARFGEF2 - adp-ribosylation factor guanine nucleotide-exchange factor 2 (brefeldin a-inhibited)  CDK2 - cyclin-dependent kinase 2  ARFGEF1 - adp-ribosylation factor guanine nucleotide-exchange factor 1 (brefeldin a-inhibited)  PTPRF - protein tyrosine phosphatase, receptor type, f  PURA - purine-rich element binding protein a  RRN3 - rrn3 rna polymerase i transcription factor homolog (s. cerevisiae)  NCKAP1 - nck-associated protein 1  ZNHIT6 - zinc finger, hit-type containing 6  CHRNB2 - cholinergic receptor, nicotinic, beta 2 (neuronal)  TMEM106B - transmembrane protein 106b  RSBN1 - round spermatid basic protein 1  ZMYND11 - zinc finger, mynd-type containing 11  PTPRK - protein tyrosine phosphatase, receptor type, k  SRSF10 - serine/arginine-rich splicing factor 10  PTPRM - protein tyrosine phosphatase, receptor type, m  PTPRN - protein tyrosine phosphatase, receptor type, n  LYST - lysosomal trafficking regulator  PLK2 - polo-like kinase 2  PEX2 - peroxisomal biogenesis factor 2  CHCHD4 - coiled-coil-helix-coiled-coil-helix domain containing 4  KDM5B - lysine (k)-specific demethylase 5b  PXN - paxillin  ABCD3 - atp-binding cassette, sub-family d (ald), member 3  PVRL1 - poliovirus receptor-related 1 (herpesvirus entry mediator c)  CHGA - chromogranin a (parathyroid secretory protein 1)  PVRL2 - poliovirus receptor-related 2 (herpesvirus entry mediator b)  RAB5A - rab5a, member ras oncogene family  CHD4 - chromodomain helicase dna binding protein 4  RAB5B - rab5b, member ras oncogene family  CHD2 - chromodomain helicase dna binding protein 2  CHD3 - chromodomain helicase dna binding protein 3  RAB3A - rab3a, member ras oncogene family  RAB3B - rab3b, member ras oncogene family  MTERFD2 - mterf domain containing 2  CHD1 - chromodomain helicase dna binding protein 1  RAB2A - rab2a, member ras oncogene family  RAB1A - rab1a, member ras oncogene family  WASF3 - was protein family, member 3  SDCCAG3 - serologically defined colon cancer antigen 3  RAD21 - rad21 homolog (s. pombe)  SEC24A - sec24 family, member a (s. cerevisiae)  RAC3 - ras-related c3 botulinum toxin substrate 3 (rho family, small gtp binding protein rac3)  PAF1 - paf1, rna polymerase ii associated factor, homolog (s. cerevisiae)  RAC1 - ras-related c3 botulinum toxin substrate 1 (rho family, small gtp binding protein rac1)  RABIF - rab interacting factor  MSL1 - male-specific lethal 1 homolog (drosophila)  INO80 - ino80 complex subunit  RAB27A - rab27a, member ras oncogene family  KCTD18 - potassium channel tetramerization domain containing 18  RALA - v-ral simian leukemia viral oncogene homolog a (ras related)  CCT8 - chaperonin containing tcp1, subunit 8 (theta)  CLTC - clathrin, heavy chain (hc)  MIER2 - mesoderm induction early response 1, family member 2  RARG - retinoic acid receptor, gamma  CLN5 - ceroid-lipofuscinosis, neuronal 5  CLNS1A - chloride channel, nucleotide-sensitive, 1a  BBS5 - bardet-biedl syndrome 5  DCTN6 - dynactin 6  CLTB - clathrin, light chain b  RASA1 - ras p21 protein activator (gtpase activating protein) 1  CLTA - clathrin, light chain a  CELF1 - cugbp, elav-like family member 1  CLU - clusterin  RAP1GDS1 - rap1, gtp-gdp dissociation stimulator 1  MAGEL2 - mage-like 2  CELF2 - cugbp, elav-like family member 2  RAP2A - rap2a, member of ras oncogene family  ING3 - inhibitor of growth family, member 3  TPP1 - tripeptidyl peptidase i  CTCF - ccctc-binding factor (zinc finger protein)  PRMT7 - protein arginine methyltransferase 7  MITD1 - mit, microtubule interacting and transport, domain containing 1  RBL2 - retinoblastoma-like 2 (p130)  RBBP7 - retinoblastoma binding protein 7  CLCN3 - chloride channel, voltage-sensitive 3  CLCN6 - chloride channel, voltage-sensitive 6  RB1 - retinoblastoma 1  ARID4A - at rich interactive domain 4a (rbp1-like)  STAG2 - stromal antigen 2  AP2M1 - adaptor-related protein complex 2, mu 1 subunit  RBBP5 - retinoblastoma binding protein 5  KDM5A - lysine (k)-specific demethylase 5a  RBBP4 - retinoblastoma binding protein 4  NUP35 - nucleoporin 35kda  ERCC8 - excision repair cross-complementing rodent repair deficiency, complementation group 8  MTRF1L - mitochondrial translational release factor 1-like  SLC12A7 - solute carrier family 12 (potassium/chloride transporter), member 7  YME1L1 - yme1-like 1 atpase  PTGES3 - prostaglandin e synthase 3 (cytosolic)  CIRBP - cold inducible rna binding protein  CHMP4B - charged multivesicular body protein 4b  POLD3 - polymerase (dna-directed), delta 3, accessory subunit  DHX29 - deah (asp-glu-ala-his) box polypeptide 29  USP39 - ubiquitin specific peptidase 39  SPTAN1 - spectrin, alpha, non-erythrocytic 1  CEP89 - centrosomal protein 89kda  WDR19 - wd repeat domain 19  SPTBN4 - spectrin, beta, non-erythrocytic 4  CD2AP - cd2-associated protein  FBXO18 - f-box protein, helicase, 18  SPP1 - secreted phosphoprotein 1  EPG5 - ectopic p-granules autophagy protein 5 homolog (c. elegans)  CORO1C - coronin, actin binding protein, 1c  SPRYD3 - spry domain containing 3  SPG7 - spastic paraplegia 7 (pure and complicated autosomal recessive)  COX15 - cytochrome c oxidase assembly homolog 15 (yeast)  SPARC - secreted protein, acidic, cysteine-rich (osteonectin)  COX7A2 - cytochrome c oxidase subunit viia polypeptide 2 (liver)  SPAST - spastin  MIER1 - mesoderm induction early response 1, transcriptional regulator  SPAG1 - sperm associated antigen 1  NCKAP5L - nck-associated protein 5-like  IQSEC3 - iq motif and sec7 domain 3  GBA2 - glucosidase, beta (bile acid) 2  CADM3 - cell adhesion molecule 3  WDR73 - wd repeat domain 73  MUM1 - melanoma associated antigen (mutated) 1  LSM2 - lsm2 homolog, u6 small nuclear rna associated (s. cerevisiae)  SOS1 - son of sevenless homolog 1 (drosophila)  TMEM41B - transmembrane protein 41b  ORC3 - origin recognition complex, subunit 3  SON - son dna binding protein  HEBP2 - heme binding protein 2  LEMD3 - lem domain containing 3  GATAD1 - gata zinc finger domain containing 1  SOAT1 - sterol o-acyltransferase 1  CREBBP - creb binding protein  ATF2 - activating transcription factor 2  CREB1 - camp responsive element binding protein 1  CGNL1 - cingulin-like 1  SNX2 - sorting nexin 2  SNX1 - sorting nexin 1  SERAC1 - serine active site containing 1  MARK4 - map/microtubule affinity-regulating kinase 4  CADM1 - cell adhesion molecule 1  SNRPD1 - small nuclear ribonucleoprotein d1 polypeptide 16kda  CEP19 - centrosomal protein 19kda  GABARAPL1 - gaba(a) receptor-associated protein like 1  SNRPF - small nuclear ribonucleoprotein polypeptide f  PLK3 - polo-like kinase 3  CNN1 - calponin 1, basic, smooth muscle  RHOU - ras homolog family member u  SNCA - synuclein, alpha (non a4 component of amyloid precursor)  SNCG - synuclein, gamma (breast cancer-specific protein 1)  FSCN1 - fascin homolog 1, actin-bundling protein (strongylocentrotus purpuratus)  TRAPPC1 - trafficking protein particle complex 1  SNRPB - small nuclear ribonucleoprotein polypeptides b and b1  SNAP25 - synaptosomal-associated protein, 25kda  SNCB - synuclein, beta  TP53INP2 - tumor protein p53 inducible nuclear protein 2  PTBP2 - polypyrimidine tract binding protein 2  SMN1 - survival of motor neuron 1, telomeric  CNN3 - calponin 3, acidic  SMARCE1 - swi/snf related, matrix associated, actin dependent regulator of chromatin, subfamily e, member 1  CNN2 - calponin 2  CNR1 - cannabinoid receptor 1 (brain)  CNP - 2',3'-cyclic nucleotide 3' phosphodiesterase  CNTN1 - contactin 1  SMARCB1 - swi/snf related, matrix associated, actin dependent regulator of chromatin, subfamily b, member 1  SMARCC1 - swi/snf related, matrix associated, actin dependent regulator of chromatin, subfamily c, member 1  HLTF - helicase-like transcription factor  ARFIP2 - adp-ribosylation factor interacting protein 2  SMARCA4 - swi/snf related, matrix associated, actin dependent regulator of chromatin, subfamily a, member 4  SSBP3 - single stranded dna binding protein 3  SMARCD2 - swi/snf related, matrix associated, actin dependent regulator of chromatin, subfamily d, member 2  ATCAY - ataxia, cerebellar, cayman type  SMARCD3 - swi/snf related, matrix associated, actin dependent regulator of chromatin, subfamily d, member 3  SMARCC2 - swi/snf related, matrix associated, actin dependent regulator of chromatin, subfamily c, member 2  SMARCD1 - swi/snf related, matrix associated, actin dependent regulator of chromatin, subfamily d, member 1  COL4A4 - collagen, type iv, alpha 4  MID1IP1 - mid1 interacting protein 1  REPS1 - ralbp1 associated eps domain containing 1  COL4A2 - collagen, type iv, alpha 2  SMARCA1 - swi/snf related, matrix associated, actin dependent regulator of chromatin, subfamily a, member 1  COL5A2 - collagen, type v, alpha 2  SMARCA2 - swi/snf related, matrix associated, actin dependent regulator of chromatin, subfamily a, member 2  KMT2C - lysine (k)-specific methyltransferase 2c  EPS15L1 - epidermal growth factor receptor pathway substrate 15-like 1  RAB38 - rab38, member ras oncogene family  COL12A1 - collagen, type xii, alpha 1  ZNF462 - zinc finger protein 462  FBF1 - fas (tnfrsf6) binding factor 1  NAP1L5 - nucleosome assembly protein 1-like 5  STX12 - syntaxin 12  TMEFF2 - transmembrane protein with egf-like and two follistatin-like domains 2  COL16A1 - collagen, type xvi, alpha 1  SYN1 - synapsin i  TFIP11 - tuftelin interacting protein 11  SYP - synaptophysin  PLEKHA1 - pleckstrin homology domain containing, family a (phosphoinositide binding specific) member 1  CST3 - cystatin c  EML2 - echinoderm microtubule associated protein like 2  NAT6 - n-acetyltransferase 6 (gcn5-related)  SYT1 - synaptotagmin i  PRPF6 - pre-mrna processing factor 6  KLHL12 - kelch-like family member 12  KDM2B - lysine (k)-specific demethylase 2b  SYT4 - synaptotagmin iv  SUPT6H - suppressor of ty 6 homolog (s. cerevisiae)  SUPV3L1 - suppressor of var1, 3-like 1 (s. cerevisiae)  CBY1 - chibby homolog 1 (drosophila)  SUN2 - sad1 and unc84 domain containing 2  CACNG7 - calcium channel, voltage-dependent, gamma subunit 7  CTNNA1 - catenin (cadherin-associated protein), alpha 1, 102kda  SLC24A2 - solute carrier family 24 (sodium/potassium/calcium exchanger), member 2  CTBP1 - c-terminal binding protein 1  SUPT4H1 - suppressor of ty 4 homolog 1 (s. cerevisiae)  CTBP2 - c-terminal binding protein 2  EML3 - echinoderm microtubule associated protein like 3  CTNND1 - catenin (cadherin-associated protein), delta 1  CTNND2 - catenin (cadherin-associated protein), delta 2  VAMP2 - vesicle-associated membrane protein 2 (synaptobrevin 2)  CTNNA2 - catenin (cadherin-associated protein), alpha 2  MFSD8 - major facilitator superfamily domain containing 8  CTNNB1 - catenin (cadherin-associated protein), beta 1, 88kda  PITPNB - phosphatidylinositol transfer protein, beta  STXBP1 - syntaxin binding protein 1  STX5 - syntaxin 5  STX4 - syntaxin 4  STX3 - syntaxin 3  STX1A - syntaxin 1a (brain)  STRN - striatin, calmodulin binding protein  USP30 - ubiquitin specific peptidase 30  FLRT2 - fibronectin leucine rich transmembrane protein 2  UNC119B - unc-119 homolog b (c. elegans)  CTSS - cathepsin s  CTSV - cathepsin v  MPV17L2 - mpv17 mitochondrial membrane protein-like 2  TCTEX1D2 - tctex1 domain containing 2  BRD1 - bromodomain containing 1  PCGF1 - polycomb group ring finger 1  NPNT - nephronectin  STAU1 - staufen double-stranded rna binding protein 1  STC1 - stanniocalcin 1  CXADR - coxsackie virus and adenovirus receptor  AURKC - aurora kinase c  TUBA1C - tubulin, alpha 1c  MTCH1 - mitochondrial carrier 1  SUV420H2 - suppressor of variegation 4-20 homolog 2 (drosophila)  CYLD - cylindromatosis (turban tumor syndrome)  EXOC4 - exocyst complex component 4  STK4 - serine/threonine kinase 4  CNEP1R1 - ctd nuclear envelope phosphatase 1 regulatory subunit 1  C12orf44 - chromosome 12 open reading frame 44  ST13 - suppression of tumorigenicity 13 (colon carcinoma) (hsp70 interacting protein)  CRKL - v-crk avian sarcoma virus ct10 oncogene homolog-like  CRK - v-crk avian sarcoma virus ct10 oncogene homolog  CELF6 - cugbp, elav-like family member 6  CRMP1 - collapsin response mediator protein 1  NIPBL - nipped-b homolog (drosophila)  TRAPPC11 - trafficking protein particle complex 11  COG4 - component of oligomeric golgi complex 4  STAT3 - signal transducer and activator of transcription 3 (acute-phase response factor)  ASF1A - anti-silencing function 1a histone chaperone  BRMS1 - breast cancer metastasis suppressor 1  DFNB31 - deafness, autosomal recessive 31  SS18 - synovial sarcoma translocation, chromosome 18  RIC8A - ric8 guanine nucleotide exchange factor a  NAPB - n-ethylmaleimide-sensitive factor attachment protein, beta  CSF1R - colony stimulating factor 1 receptor  SNX33 - sorting nexin 33  SRP54 - signal recognition particle 54kda  FBXO7 - f-box protein 7  SRP19 - signal recognition particle 19kda  ELMO2 - engulfment and cell motility 2  BLOC1S5 - biogenesis of lysosomal organelles complex-1, subunit 5, muted  SRPK2 - srsf protein kinase 2  LRCH3 - leucine-rich repeats and calponin homology (ch) domain containing 3  SRPK1 - srsf protein kinase 1  TROVE2 - trove domain family, member 2  KLHL22 - kelch-like family member 22  GCA - grancalcin, ef-hand calcium binding protein  GALNT11 - udp-n-acetyl-alpha-d-galactosamine:polypeptide n-acetylgalactosaminyltransferase 11 (galnac-t11)  SSBP1 - single-stranded dna binding protein 1, mitochondrial  ZNF335 - zinc finger protein 335  CSK - c-src tyrosine kinase  SQLE - squalene epoxidase  ATXN10 - ataxin 10  TTLL1 - tubulin tyrosine ligase-like family, member 1  CSNK1A1 - casein kinase 1, alpha 1  SPTBN1 - spectrin, beta, non-erythrocytic 1  CSNK1D - casein kinase 1, delta  SRC - v-src avian sarcoma (schmidt-ruppin a-2) viral oncogene homolog  MRPL17 - mitochondrial ribosomal protein l17  VIPAS39 - vps33b interacting protein, apical-basolateral polarity regulator, spe-39 homolog  CSRP1 - cysteine and glycine-rich protein 1  SHPRH - snf2 histone linker phd ring helicase, e3 ubiquitin protein ligase  CSRP2 - cysteine and glycine-rich protein 2  DHX9 - deah (asp-glu-ala-his) box helicase 9  DHX8 - deah (asp-glu-ala-his) box polypeptide 8  TRAPPC2 - trafficking protein particle complex 2  DDX6 - dead (asp-glu-ala-asp) box helicase 6  SEC13 - sec13 homolog (s. cerevisiae)  DDX3X - dead (asp-glu-ala-asp) box helicase 3, x-linked  DDX1 - dead (asp-glu-ala-asp) box helicase 1  SNX14 - sorting nexin 14  CCDC120 - coiled-coil domain containing 120  SIN3A - sin3 transcription regulator family member a  KIAA1244 - kiaa1244  SDCBP - syndecan binding protein (syntenin)  SAMHD1 - sam domain and hd domain 1  SET - set nuclear oncogene  WWTR1 - ww domain containing transcription regulator 1  SETMAR - set domain and mariner transposase fusion gene  DDB1 - damage-specific dna binding protein 1, 127kda  RHOT2 - ras homolog family member t2  ZNF304 - zinc finger protein 304  DCTN1 - dynactin 1  PTPN23 - protein tyrosine phosphatase, non-receptor type 23  CLIC4 - chloride intracellular channel 4  MCM9 - minichromosome maintenance complex component 9  WDR34 - wd repeat domain 34  GEMIN5 - gem (nuclear organelle) associated protein 5  CSRP2BP - csrp2 binding protein  DBN1 - drebrin 1  ATL3 - atlastin gtpase 3  POT1 - protection of telomeres 1  AHCTF1 - at hook containing transcription factor 1  RAB22A - rab22a, member ras oncogene family  RNF19A - ring finger protein 19a, e3 ubiquitin protein ligase  UNC5A - unc-5 homolog a (c. elegans)  CADM2 - cell adhesion molecule 2  GATAD2B - gata zinc finger domain containing 2b  KCTD13 - potassium channel tetramerization domain containing 13  GRAMD1B - gram domain containing 1b  DNM3 - dynamin 3  ATXN2 - ataxin 2  SIPA1L1 - signal-induced proliferation-associated 1 like 1  AMIGO1 - adhesion molecule with ig-like domain 1  SS18L1 - synovial sarcoma translocation gene on chromosome 18-like 1  TBC1D24 - tbc1 domain family, member 24  CHD5 - chromodomain helicase dna binding protein 5  MCFD2 - multiple coagulation factor deficiency 2  ARID1B - at rich interactive domain 1b (swi1-like)  SCO1 - sco1 cytochrome c oxidase assembly protein  ODF2L - outer dense fiber of sperm tails 2-like  HEG1 - heart development protein with egf-like domains 1  KNSTRN - kinetochore-localized astrin/spag5 binding protein  PYGO2 - pygopus homolog 2 (drosophila)  DARS - aspartyl-trna synthetase  DAPK3 - death-associated protein kinase 3  S100B - s100 calcium binding protein b  GORASP2 - golgi reassembly stacking protein 2, 55kda  DAXX - death-domain associated protein  C2CD3 - c2 calcium-dependent domain containing 3  ZZZ3 - zinc finger, zz-type containing 3  KIDINS220 - kinase d-interacting substrate, 220kda  DAG1 - dystroglycan 1 (dystrophin-associated glycoprotein 1)  MTA3 - metastasis associated 1 family, member 3  DGKQ - diacylglycerol kinase, theta 110kda  S100A10 - s100 calcium binding protein a10  CLIP3 - cap-gly domain containing linker protein 3  STARD13 - star-related lipid transfer (start) domain containing 13  NECAP1 - necap endocytosis associated 1  HACE1 - hect domain and ankyrin repeat containing e3 ubiquitin protein ligase 1  CHMP2B - charged multivesicular body protein 2b  AAR2 - aar2 splicing factor homolog (s. cerevisiae)  SATB1 - satb homeobox 1  COX19 - cytochrome c oxidase assembly homolog 19 (s. cerevisiae)  RPTOR - regulatory associated protein of mtor, complex 1  SAFB - scaffold attachment factor b  SH2B1 - sh2b adaptor protein 1  LYRM7 - lyr motif containing 7  SERBP1 - serpine1 mrna binding protein 1  GAPVD1 - gtpase activating protein and vps9 domains 1  TUBGCP6 - tubulin, gamma complex associated protein 6  MICALL1 - mical-like 1  DRD2 - dopamine receptor d2  EPC2 - enhancer of polycomb homolog 2 (drosophila)  TCTN3 - tectonic family member 3  PHF19 - phd finger protein 19  NLGN2 - neuroligin 2  SLC9A1 - solute carrier family 9, subfamily a (nhe1, cation proton antiporter 1), member 1  NKD2 - naked cuticle homolog 2 (drosophila)  MICAL3 - microtubule associated monooxygenase, calponin and lim domain containing 3  TRAF3IP1 - tnf receptor-associated factor 3 interacting protein 1  TAOK1 - tao kinase 1  SLC8A2 - solute carrier family 8 (sodium/calcium exchanger), member 2  SYDE1 - synapse defective 1, rho gtpase, homolog 1 (c. elegans)  SHANK3 - sh3 and multiple ankyrin repeat domains 3  WIPI2 - wd repeat domain, phosphoinositide interacting 2  SEMA6A - sema domain, transmembrane domain (tm), and cytoplasmic domain, (semaphorin) 6a  STRIP1 - striatin interacting protein 1  PRPF31 - pre-mrna processing factor 31  DR1 - down-regulator of transcription 1, tbp-binding (negative cofactor 2)  DPYSL3 - dihydropyrimidinase-like 3  DPYSL2 - dihydropyrimidinase-like 2  SLC12A4 - solute carrier family 12 (potassium/chloride transporter), member 4  SLC12A2 - solute carrier family 12 (sodium/potassium/chloride transporter), member 2  PREX1 - phosphatidylinositol-3,4,5-trisphosphate-dependent rac exchange factor 1  TNKS1BP1 - tankyrase 1 binding protein 1, 182kda  MAPK15 - mitogen-activated protein kinase 15  NYAP1 - neuronal tyrosine-phosphorylated phosphoinositide-3-kinase adaptor 1  UNK - unkempt homolog (drosophila)  SKP1 - s-phase kinase-associated protein 1  SLC1A5 - solute carrier family 1 (neutral amino acid transporter), member 5  TOR1A - torsin family 1, member a (torsin a)  MKL1 - megakaryoblastic leukemia (translocation) 1  SLC2A1 - solute carrier family 2 (facilitated glucose transporter), member 1  ARHGAP21 - rho gtpase activating protein 21  TANC1 - tetratricopeptide repeat, ankyrin repeat and coiled-coil containing 1  DVL2 - dishevelled segment polarity protein 2  SYT13 - synaptotagmin xiii  DVL3 - dishevelled segment polarity protein 3  TOR1AIP1 - torsin a interacting protein 1  USP36 - ubiquitin specific peptidase 36  DOCK7 - dedicator of cytokinesis 7  RANBP10 - ran binding protein 10  ECM2 - extracellular matrix protein 2, female organ and adipocyte specific  HBEGF - heparin-binding egf-like growth factor  LSM14A - lsm14a, scd6 homolog a (s. cerevisiae)  DKC1 - dyskeratosis congenita 1, dyskerin  DLG3 - discs, large homolog 3 (drosophila)  PNPT1 - polyribonucleotide nucleotidyltransferase 1  DLG4 - discs, large homolog 4 (drosophila)  VPS18 - vacuolar protein sorting 18 homolog (s. cerevisiae)  DLG1 - discs, large homolog 1 (drosophila)  BLOC1S6 - biogenesis of lysosomal organelles complex-1, subunit 6, pallidin  RSPH1 - radial spoke head 1 homolog (chlamydomonas)  FBXW8 - f-box and wd repeat domain containing 8  DHCR24 - 24-dehydrocholesterol reductase  PHGDH - phosphoglycerate dehydrogenase  SCIN - scinderin  EP400 - e1a binding protein p400  DHFR - dihydrofolate reductase  RICTOR - rptor independent companion of mtor, complex 2  DHX37 - deah (asp-glu-ala-his) box polypeptide 37  GRAMD1A - gram domain containing 1a  SIPA1 - signal-induced proliferation-associated 1  SFPQ - splicing factor proline/glutamine-rich  DYNC1H1 - dynein, cytoplasmic 1, heavy chain 1  SFRP2 - secreted frizzled-related protein 2  DYNC1LI2 - dynein, cytoplasmic 1, light intermediate chain 2  SRSF1 - serine/arginine-rich splicing factor 1  ATG16L2 - autophagy related 16-like 2 (s. cerevisiae)  DNM2 - dynamin 2  EPB41L5 - erythrocyte membrane protein band 4.1 like 5  DNMT1 - dna (cytosine-5-)-methyltransferase 1  SRSF5 - serine/arginine-rich splicing factor 5  DNMT3A - dna (cytosine-5-)-methyltransferase 3 alpha  SRSF6 - serine/arginine-rich splicing factor 6  SFSWAP - splicing factor, suppressor of white-apricot homolog (drosophila)  DOCK1 - dedicator of cytokinesis 1  ALS2 - amyotrophic lateral sclerosis 2 (juvenile)  CHD8 - chromodomain helicase dna binding protein 8  ZSWIM6 - zinc finger, swim-type containing 6  HPS4 - hermansky-pudlak syndrome 4  SGK1 - serum/glucocorticoid regulated kinase 1  NOC2L - nucleolar complex associated 2 homolog (s. cerevisiae)  MZT1 - mitotic spindle organizing protein 1  IFT172 - intraflagellar transport 172 homolog (chlamydomonas)  NAV1 - neuron navigator 1  ITSN1 - intersectin 1 (sh3 domain protein)  SH3GL1 - sh3-domain grb2-like 1  SMARCAD1 - swi/snf-related, matrix-associated actin-dependent regulator of chromatin, subfamily a, containing dead/h box 1  EMP1 - epithelial membrane protein 1  MAPRE1 - microtubule-associated protein, rp/eb family, member 1  EMD - emerin  CHMP4C - charged multivesicular body protein 4c  MARK2 - map/microtubule affinity-regulating kinase 2  MAPRE3 - microtubule-associated protein, rp/eb family, member 3  TSC1 - tuberous sclerosis 1  CTTN - cortactin  KIFAP3 - kinesin-associated protein 3  SPIRE1 - spire actin nucleation factor 1  NAF1 - nuclear assembly factor 1 ribonucleoprotein  GORAB - golgin, rab6-interacting  DHX30 - deah (asp-glu-ala-his) box helicase 30  IFT46 - intraflagellar transport 46 homolog (chlamydomonas)  ELN - elastin  EML1 - echinoderm microtubule associated protein like 1  WDR47 - wd repeat domain 47  ELK4 - elk4, ets-domain protein (srf accessory protein 1)  SCAP - srebf chaperone  WRNIP1 - werner helicase interacting protein 1  MGME1 - mitochondrial genome maintenance exonuclease 1  EPAS1 - endothelial pas domain protein 1  EP300 - e1a binding protein p300  HABP4 - hyaluronan binding protein 4  RAB18 - rab18, member ras oncogene family  RAB3GAP1 - rab3 gtpase activating protein subunit 1 (catalytic)  TIMM50 - translocase of inner mitochondrial membrane 50 homolog (s. cerevisiae)  MTSS1L - metastasis suppressor 1-like  GRIPAP1 - grip1 associated protein 1  TUBB2A - tubulin, beta 2a class iia  CELF4 - cugbp, elav-like family member 4  TUBG1 - tubulin, gamma 1  EIF2S1 - eukaryotic translation initiation factor 2, subunit 1 alpha, 35kda  TRIM32 - tripartite motif containing 32  SPHK2 - sphingosine kinase 2  EIF2S3 - eukaryotic translation initiation factor 2, subunit 3 gamma, 52kda  TSPYL1 - tspy-like 1  SAR1A - sar1 homolog a (s. cerevisiae)  KDM2A - lysine (k)-specific demethylase 2a  ELAVL1 - elav (embryonic lethal, abnormal vision, drosophila)-like 1 (hu antigen r)  AZI1 - 5-azacytidine induced 1  BTBD8 - btb (poz) domain containing 8  TSG101 - tumor susceptibility gene 101  AKIP1 - a kinase (prka) interacting protein 1  MAST1 - microtubule associated serine/threonine kinase 1  ACIN1 - apoptotic chromatin condensation inducer 1  CCDC47 - coiled-coil domain containing 47  MVB12A - multivesicular body subunit 12a  EFNA1 - ephrin-a1  TPM4 - tropomyosin 4  EIF2D - eukaryotic translation initiation factor 2d  SLC12A9 - solute carrier family 12, member 9  DAAM1 - dishevelled associated activator of morphogenesis 1  TP53BP1 - tumor protein p53 binding protein 1  PHC1 - polyhomeotic homolog 1 (drosophila)  TP53BP2 - tumor protein p53 binding protein, 2  TOP3A - topoisomerase (dna) iii alpha  TOP2B - topoisomerase (dna) ii beta 180kda  EGFR - epidermal growth factor receptor  MEGF9 - multiple egf-like-domains 9  MEGF8 - multiple egf-like-domains 8  MAST3 - microtubule associated serine/threonine kinase 3  CELSR2 - cadherin, egf lag seven-pass g-type receptor 2  TOP1 - topoisomerase (dna) i  KDM4B - lysine (k)-specific demethylase 4b  KDM1A - lysine (k)-specific demethylase 1a  ATXN7L3 - ataxin 7-like 3  EFNB2 - ephrin-b2  TNS1 - tensin 1  UNC13A - unc-13 homolog a (c. elegans)  EFNA5 - ephrin-a5  SNRNP200 - small nuclear ribonucleoprotein 200kda (u5)  CLUAP1 - clusterin associated protein 1  CCT3 - chaperonin containing tcp1, subunit 3 (gamma)  FNBP1 - formin binding protein 1  TRIP6 - thyroid hormone receptor interactor 6  MFF - mitochondrial fission factor  TNIK - traf2 and nck interacting kinase  XAB2 - xpa binding protein 2  E2F4 - e2f transcription factor 4, p107/p130-binding  SHKBP1 - sh3kbp1 binding protein 1  PDS5B - pds5, regulator of cohesion maintenance, homolog b (s. cerevisiae)  E2F5 - e2f transcription factor 5, p130-binding  ENY2 - enhancer of yellow 2 homolog (drosophila)  DNER - delta/notch-like egf repeat containing  MRPS22 - mitochondrial ribosomal protein s22  C11orf30 - chromosome 11 open reading frame 30  MYCBP2 - myc binding protein 2, e3 ubiquitin protein ligase  TRAF1 - tnf receptor-associated factor 1  LPAR1 - lysophosphatidic acid receptor 1  HSP90B1 - heat shock protein 90kda beta (grp94), member 1  S1PR1 - sphingosine-1-phosphate receptor 1  TRAF2 - tnf receptor-associated factor 2  SETX - senataxin  SETD1B - set domain containing 1b  TPR - translocated promoter region, nuclear basket protein  PPRC1 - peroxisome proliferator-activated receptor gamma, coactivator-related 1  KDM4C - lysine (k)-specific demethylase 4c  BOD1 - biorientation of chromosomes in cell division 1  SIPA1L3 - signal-induced proliferation-associated 1 like 3  TTLL5 - tubulin tyrosine ligase-like family, member 5  ARHGAP26 - rho gtpase activating protein 26  NUP107 - nucleoporin 107kda  UTRN - utrophin  GOPC - golgi-associated pdz and coiled-coil motif containing  IQSEC2 - iq motif and sec7 domain 2  PLSCR4 - phospholipid scramblase 4  PTK2B - protein tyrosine kinase 2 beta  ACSL3 - acyl-coa synthetase long-chain family member 3  MRPS27 - mitochondrial ribosomal protein s27  RAP1GAP2 - rap1 gtpase activating protein 2  PARP11 - poly (adp-ribose) polymerase family, member 11  NFASC - neurofascin  BPTF - bromodomain phd finger transcription factor  CUL9 - cullin 9  KANSL1 - kat8 regulatory nsl complex subunit 1  FARSA - phenylalanyl-trna synthetase, alpha subunit  USP4 - ubiquitin specific peptidase 4 (proto-oncogene)  FBLN1 - fibulin 1  CLASP2 - cytoplasmic linker associated protein 2  POGZ - pogo transposable element with znf domain  PLSCR3 - phospholipid scramblase 3  LAMA1 - laminin, alpha 1  PLSCR2 - phospholipid scramblase 2  PLXND1 - plexin d1  ETV6 - ets variant 6  RAD54L2 - rad54-like 2 (s. cerevisiae)  VHL - von hippel-lindau tumor suppressor, e3 ubiquitin protein ligase  UTP3 - utp3, small subunit (ssu) processome component, homolog (s. cerevisiae)  VIM - vimentin  KDM6B - lysine (k)-specific demethylase 6b  EZR - ezrin  PHF8 - phd finger protein 8  SMC5 - structural maintenance of chromosomes 5  EPB41L3 - erythrocyte membrane protein band 4.1-like 3  ESAM - endothelial cell adhesion molecule  KCTD1 - potassium channel tetramerization domain containing 1  ANKLE2 - ankyrin repeat and lem domain containing 2  KIFC2 - kinesin family member c2  KDM6A - lysine (k)-specific demethylase 6a  FMNL3 - formin-like 3  UVRAG - uv radiation resistance associated  VASP - vasodilator-stimulated phosphoprotein  EYA3 - eyes absent homolog 3 (drosophila)  VAV2 - vav 2 guanine nucleotide exchange factor  EZH1 - enhancer of zeste homolog 1 (drosophila)  VBP1 - von hippel-lindau binding protein 1  VCAM1 - vascular cell adhesion molecule 1  CIAPIN1 - cytokine induced apoptosis inhibitor 1  F2R - coagulation factor ii (thrombin) receptor  NCDN - neurochondrin  VCL - vinculin  SLC25A46 - solute carrier family 25, member 46  PDXP - pyridoxal (pyridoxine, vitamin b6) phosphatase  VCP - valosin containing protein  MAPK8IP3 - mitogen-activated protein kinase 8 interacting protein 3  ATP8B2 - atpase, aminophospholipid transporter, class i, type 8b, member 2  TTLL12 - tubulin tyrosine ligase-like family, member 12  ATP10A - atpase, class v, type 10a  MCOLN1 - mucolipin 1  NEK9 - nima-related kinase 9  ERCC5 - excision repair cross-complementing rodent repair deficiency, complementation group 5  ERCC4 - excision repair cross-complementing rodent repair deficiency, complementation group 4  NUP205 - nucleoporin 205kda  ERCC3 - excision repair cross-complementing rodent repair deficiency, complementation group 3  MPRIP - myosin phosphatase rho interacting protein  YTHDC1 - yth domain containing 1  THOC2 - tho complex 2  ELMSAN1 - elm2 and myb/sant-like domain containing 1  CEP68 - centrosomal protein 68kda  UBE2A - ubiquitin-conjugating enzyme e2a  CHMP7 - charged multivesicular body protein 7  ATP10D - atpase, class v, type 10d  UBB - ubiquitin b  CORO1B - coronin, actin binding protein, 1b  KANK1 - kn motif and ankyrin repeat domains 1  NDUFAF2 - nadh dehydrogenase (ubiquinone) complex i, assembly factor 2  RCOR1 - rest corepressor 1  ZNFX1 - zinc finger, nfx1-type containing 1  HIRA - histone cell cycle regulator  RFT1 - rft1 homolog (s. cerevisiae)  TYRO3 - tyro3 protein tyrosine kinase  FAF2 - fas associated factor family member 2  ETS1 - v-ets avian erythroblastosis virus e26 oncogene homolog 1  TPGS1 - tubulin polyglutamylase complex subunit 1  MDN1 - mdn1, midasin homolog (yeast)  ZMIZ1 - zinc finger, miz-type containing 1  ETF1 - eukaryotic translation termination factor 1  COG7 - component of oligomeric golgi complex 7  CYFIP1 - cytoplasmic fmr1 interacting protein 1  ARL6IP1 - adp-ribosylation factor-like 6 interacting protein 1  DMTN - dematin actin binding protein  EPB41L1 - erythrocyte membrane protein band 4.1-like 1  SUMO1 - small ubiquitin-like modifier 1  EPB41 - erythrocyte membrane protein band 4.1 (elliptocytosis 1, rh-linked)  UBTF - upstream binding transcription factor, rna polymerase i  EPB41L2 - erythrocyte membrane protein band 4.1-like 2  ATP11B - atpase, class vi, type 11b  UGCG - udp-glucose ceramide glucosyltransferase  ZNF830 - zinc finger protein 830  RRS1 - rrs1 ribosome biogenesis regulator homolog (s. cerevisiae)  PRRC2C - proline-rich coiled-coil 2c  SMURF1 - smad specific e3 ubiquitin protein ligase 1  SYT11 - synaptotagmin xi  C12orf65 - chromosome 12 open reading frame 65  JMJD6 - jumonji domain containing 6  MRPL47 - mitochondrial ribosomal protein l47  MYADM - myeloid-associated differentiation marker  EPRS - glutamyl-prolyl-trna synthetase  UBE2D2 - ubiquitin-conjugating enzyme e2d 2  EPHB6 - eph receptor b6  TBC1D1 - tbc1 (tre-2/usp6, bub2, cdc16) domain family, member 1  STX2 - syntaxin 2  ADCK1 - aarf domain containing kinase 1  ERCC2 - excision repair cross-complementing rodent repair deficiency, complementation group 2  EPS8 - epidermal growth factor receptor pathway substrate 8  NUP210 - nucleoporin 210kda  EPS15 - epidermal growth factor receptor pathway substrate 15  UBE2N - ubiquitin-conjugating enzyme e2n  FMOD - fibromodulin  VPS72 - vacuolar protein sorting 72 homolog (s. cerevisiae)  CAMSAP2 - calmodulin regulated spectrin-associated protein family, member 2  PLK1S1 - polo-like kinase 1 substrate 1  TCP1 - t-complex 1  TSPYL4 - tspy-like 4  BRK1 - brick1, scar/wave actin-nucleating complex subunit  SCFD1 - sec1 family domain containing 1  DDHD2 - ddhd domain containing 2  PDS5A - pds5, regulator of cohesion maintenance, homolog a (s. cerevisiae)  FLII - flightless i homolog (drosophila)  BOP1 - block of proliferation 1  FLNA - filamin a, alpha  FLNB - filamin b, beta  ATP11A - atpase, class vi, type 11a  ARC - activity-regulated cytoskeleton-associated protein  TMEM126B - transmembrane protein 126b  PACS2 - phosphofurin acidic cluster sorting protein 2  HDAC8 - histone deacetylase 8  LARP4 - la ribonucleoprotein domain family, member 4  ASH1L - ash1 (absent, small, or homeotic)-like (drosophila)  ANKRD28 - ankyrin repeat domain 28  SPATA7 - spermatogenesis associated 7  SIN3B - sin3 transcription regulator family member b  BDP1 - b double prime 1, subunit of rna polymerase iii transcription initiation factor iiib  NCAPD3 - non-smc condensin ii complex, subunit d3  EHBP1 - eh domain binding protein 1  FKBP4 - fk506 binding protein 4, 59kda  BICD2 - bicaudal d homolog 2 (drosophila)  HR - hair growth associated  CAND1 - cullin-associated and neddylation-dissociated 1  FKBP1A - fk506 binding protein 1a, 12kda  TDG - thymine-dna glycosylase  FBXW11 - f-box and wd repeat domain containing 11  VPS11 - vacuolar protein sorting 11 homolog (s. cerevisiae)  KDM3A - lysine (k)-specific demethylase 3a  DYNLT1 - dynein, light chain, tctex-type 1  STX1B - syntaxin 1b  IFT43 - intraflagellar transport 43 homolog (chlamydomonas)  TAF9 - taf9 rna polymerase ii, tata box binding protein (tbp)-associated factor, 32kda  FGFR2 - fibroblast growth factor receptor 2  TAF7 - taf7 rna polymerase ii, tata box binding protein (tbp)-associated factor, 55kda  VPS8 - vacuolar protein sorting 8 homolog (s. cerevisiae)  HAUS5 - haus augmin-like complex, subunit 5  FGF13 - fibroblast growth factor 13  TAF6 - taf6 rna polymerase ii, tata box binding protein (tbp)-associated factor, 80kda  TADA2A - transcriptional adaptor 2a  SMCHD1 - structural maintenance of chromosomes flexible hinge domain containing 1  SYNE1 - spectrin repeat containing, nuclear envelope 1  TACC1 - transforming, acidic coiled-coil containing protein 1  VPS39 - vacuolar protein sorting 39 homolog (s. cerevisiae)  NSFL1C - nsfl1 (p97) cofactor (p47)  PHF15 - phd finger protein 15  CLASP1 - cytoplasmic linker associated protein 1  FGF2 - fibroblast growth factor 2 (basic)  SZT2 - seizure threshold 2 homolog (mouse)  TAP2 - transporter 2, atp-binding cassette, sub-family b (mdr/tap)  USP22 - ubiquitin specific peptidase 22  TAPBP - tap binding protein (tapasin)  FGD1 - fyve, rhogef and ph domain containing 1  FEN1 - flap structure-specific endonuclease 1  FER - fer (fps/fes related) tyrosine kinase  PDGFC - platelet derived growth factor c  MAP3K7 - mitogen-activated protein kinase kinase kinase 7  EFHC1 - ef-hand domain (c-terminal) containing 1  DNAJC13 - dnaj (hsp40) homolog, subfamily c, member 13  PIP5K1C - phosphatidylinositol-4-phosphate 5-kinase, type i, gamma  INO80E - ino80 complex subunit e  ZC4H2 - zinc finger, c4h2 domain containing  KMT2E - lysine (k)-specific methyltransferase 2e  SIK3 - sik family kinase 3  NUDCD3 - nudc domain containing 3  TBCE - tubulin folding cofactor e  SPECC1L - sperm antigen with calponin homology and coiled-coil domains 1-like  TBCD - tubulin folding cofactor d  TBCC - tubulin folding cofactor c  TBCA - tubulin folding cofactor a  TAZ - tafazzin  SULF2 - sulfatase 2  CRTC1 - creb regulated transcription coactivator 1  UFL1 - ufm1-specific ligase 1  PPP1R13B - protein phosphatase 1, regulatory subunit 13b  PUM2 - pumilio homolog 2 (drosophila)  DMAP1 - dna methyltransferase 1 associated protein 1  TCF4 - transcription factor 4  OBSL1 - obscurin-like 1  FBN1 - fibrillin 1  RCC2 - regulator of chromosome condensation 2  FBLN2 - fibulin 2  NUDT7 - nudix (nucleoside diphosphate linked moiety x)-type motif 7  FAT1 - fat atypical cadherin 1  ERBB2IP - erbb2 interacting protein  TIE1 - tyrosine kinase with immunoglobulin-like and egf-like domains 1  TIMP1 - timp metallopeptidase inhibitor 1  TIMP2 - timp metallopeptidase inhibitor 2  ITGB3BP - integrin beta 3 binding protein (beta3-endonexin)  POC1B - poc1 centriolar protein b  TMED3 - transmembrane emp24 protein transport domain containing 3  TJP1 - tight junction protein 1  TK1 - thymidine kinase 1, soluble  RYBP - ring1 and yy1 binding protein  MTOR - mechanistic target of rapamycin (serine/threonine kinase)  TLE1 - transducin-like enhancer of split 1 (e(sp1) homolog, drosophila)  TLE3 - transducin-like enhancer of split 3 (e(sp1) homolog, drosophila)  BLOC1S2 - biogenesis of lysosomal organelles complex-1, subunit 2  TLE4 - transducin-like enhancer of split 4 (e(sp1) homolog, drosophila)  TLN1 - talin 1  ATP13A2 - atpase type 13a2  SIRT1 - sirtuin 1  TRAPPC10 - trafficking protein particle complex 10  TMF1 - tata element modulatory factor 1  ABCA5 - atp-binding cassette, sub-family a (abc1), member 5  CADPS2 - ca++-dependent secretion activator 2  TMSB4X - thymosin beta 4, x-linked  CBX6 - chromobox homolog 6  ACTR8 - arp8 actin-related protein 8 homolog (yeast)  BRD4 - bromodomain containing 4  TNFAIP1 - tumor necrosis factor, alpha-induced protein 1 (endothelial)  BCL9L - b-cell cll/lymphoma 9-like  TARDBP - tar dna binding protein  TNFRSF1A - tumor necrosis factor receptor superfamily, member 1a  TBCK - tbc1 domain containing kinase  TJAP1 - tight junction associated protein 1 (peripheral)  ARHGAP18 - rho gtpase activating protein 18  SF3B1 - splicing factor 3b, subunit 1, 155kda  KCTD21 - potassium channel tetramerization domain containing 21  KCTD2 - potassium channel tetramerization domain containing 2  PRKCDBP - protein kinase c, delta binding protein  TEK - tek tyrosine kinase, endothelial  SUZ12 - suz12 polycomb repressive complex 2 subunit  RCOR2 - rest corepressor 2  TFAM - transcription factor a, mitochondrial  TERF1 - telomeric repeat binding factor (nima-interacting) 1  TERF2 - telomeric repeat binding factor 2  TMEM80 - transmembrane protein 80  SNX18 - sorting nexin 18  TESK1 - testis-specific kinase 1  TFDP1 - transcription factor dp-1  CBX7 - chromobox homolog 7  BCCIP - brca2 and cdkn1a interacting protein  ISCU - iron-sulfur cluster assembly enzyme  PES1 - pescadillo ribosomal biogenesis factor 1  FXN - frataxin  TGS1 - trimethylguanosine synthase 1  TGFA - transforming growth factor, alpha  CEP41 - centrosomal protein 41kda  POLE4 - polymerase (dna-directed), epsilon 4, accessory subunit  HEY2 - hairy/enhancer-of-split related with yrpw motif 2  MACF1 - microtubule-actin crosslinking factor 1  TGFBR1 - transforming growth factor, beta receptor 1  TGFB2 - transforming growth factor, beta 2  RBFOX2 - rna binding protein, fox-1 homolog (c. elegans) 2  TGFB3 - transforming growth factor, beta 3  TGFB1 - transforming growth factor, beta 1  MAPK8IP2 - mitogen-activated protein kinase 8 interacting protein 2  LRRC8A - leucine rich repeat containing 8 family, member a  TP53INP1 - tumor protein p53 inducible nuclear protein 1  H2AFV - h2a histone family, member v  RNF20 - ring finger protein 20, e3 ubiquitin protein ligase  TGFBR3 - transforming growth factor, beta receptor iii  WDR45B - wd repeat domain 45b  ARHGAP12 - rho gtpase activating protein 12  THBS1 - thrombospondin 1  CABIN1 - calcineurin binding protein 1  KAT6B - k(lysine) acetyltransferase 6b  SYTL4 - synaptotagmin-like 4  THY1 - thy-1 cell surface antigen  METTL3 - methyltransferase like 3  DNAJB14 - dnaj (hsp40) homolog, subfamily b, member 14  COIL - coilin  ATAT1 - alpha tubulin acetyltransferase 1  SF3A2 - splicing factor 3a, subunit 2, 66kda  GEM - gtp binding protein overexpressed in skeletal muscle  NUDT19 - nudix (nucleoside diphosphate linked moiety x)-type motif 19  OBFC1 - oligonucleotide/oligosaccharide-binding fold containing 1  MKKS - mckusick-kaufman syndrome  KAT2A - k(lysine) acetyltransferase 2a  NCOA3 - nuclear receptor coactivator 3  PANK2 - pantothenate kinase 2  GJA5 - gap junction protein, alpha 5, 40kda  GJA1 - gap junction protein, alpha 1, 43kda  RND2 - rho family gtpase 2  AXIN1 - axin 1  TRRAP - transformation/transcription domain-associated protein  MCMBP - minichromosome maintenance complex binding protein  PICALM - phosphatidylinositol binding clathrin assembly protein  HDAC11 - histone deacetylase 11  GLS - glutaminase  SERF1A - small edrk-rich factor 1a (telomeric)  MAP9 - microtubule-associated protein 9  ARID1A - at rich interactive domain 1a (swi-like)  SETD6 - set domain containing 6  GCLM - glutamate-cysteine ligase, modifier subunit  ACTR5 - arp5 actin-related protein 5 homolog (yeast)  CDC45 - cell division cycle 45  NAA60 - n(alpha)-acetyltransferase 60, natf catalytic subunit  FZD4 - frizzled family receptor 4  GCLC - glutamate-cysteine ligase, catalytic subunit  FZD1 - frizzled family receptor 1  BAP1 - brca1 associated protein-1 (ubiquitin carboxy-terminal hydrolase)  AXIN2 - axin 2  NUP85 - nucleoporin 85kda  KDM5C - lysine (k)-specific demethylase 5c  SMC1A - structural maintenance of chromosomes 1a  USP9X - ubiquitin specific peptidase 9, x-linked  GNAI3 - guanine nucleotide binding protein (g protein), alpha inhibiting activity polypeptide 3  PHF17 - phd finger protein 17  GMFB - glia maturation factor, beta  EHMT1 - euchromatic histone-lysine n-methyltransferase 1  CAMKMT - calmodulin-lysine n-methyltransferase  FUS - fused in sarcoma  NAA40 - n(alpha)-acetyltransferase 40, natd catalytic subunit, homolog (s. cerevisiae)  TOR1AIP2 - torsin a interacting protein 2  GEMIN6 - gem (nuclear organelle) associated protein 6  RAB7A - rab7a, member ras oncogene family  GAB1 - grb2-associated binding protein 1  USP7 - ubiquitin specific peptidase 7 (herpes virus-associated)  GAA - glucosidase, alpha; acid  MANF - mesencephalic astrocyte-derived neurotrophic factor  RNF168 - ring finger protein 168, e3 ubiquitin protein ligase  XRCC6 - x-ray repair complementing defective repair in chinese hamster cells 6  BBS12 - bardet-biedl syndrome 12  GABPB1 - ga binding protein transcription factor, beta subunit 1  GABPA - ga binding protein transcription factor, alpha subunit 60kda  CPSF7 - cleavage and polyadenylation specific factor 7, 59kda  TCTN2 - tectonic family member 2  FYN - fyn oncogene related to src, fgr, yes  UBA5 - ubiquitin-like modifier activating enzyme 5  DDX39B - dead (asp-glu-ala-asp) box polypeptide 39b  GAK - cyclin g associated kinase  TTLL7 - tubulin tyrosine ligase-like family, member 7  KCTD17 - potassium channel tetramerization domain containing 17  KMT2D - lysine (k)-specific methyltransferase 2d  AAAS - achalasia, adrenocortical insufficiency, alacrimia  GABRG2 - gamma-aminobutyric acid (gaba) a receptor, gamma 2  GABRB3 - gamma-aminobutyric acid (gaba) a receptor, beta 3  IFT88 - intraflagellar transport 88 homolog (chlamydomonas)  YEATS4 - yeats domain containing 4  ZFAND1 - zinc finger, an1-type domain 1  ZFPM1 - zinc finger protein, fog family member 1  EID2 - ep300 interacting inhibitor of differentiation 2  TXNDC15 - thioredoxin domain containing 15  GBA - glucosidase, beta, acid  ST7 - suppression of tumorigenicity 7  GEMIN7 - gem (nuclear organelle) associated protein 7  KAT6A - k(lysine) acetyltransferase 6a  MICALL2 - mical-like 2  STAM - signal transducing adaptor molecule (sh3 domain and itam motif) 1  BRD3 - bromodomain containing 3  GAS6 - growth arrest-specific 6  CCDC6 - coiled-coil domain containing 6  DEDD2 - death effector domain containing 2  DHX36 - deah (asp-glu-ala-his) box polypeptide 36  SMC6 - structural maintenance of chromosomes 6  RNF112 - ring finger protein 112  GSPT1 - g1 to s phase transition 1  GSN - gelsolin  GTF2F2 - general transcription factor iif, polypeptide 2, 30kda  ZNF148 - zinc finger protein 148  GTF2E1 - general transcription factor iie, polypeptide 1, alpha 56kda  GTF2H2 - general transcription factor iih, polypeptide 2, 44kda  GTF2H3 - general transcription factor iih, polypeptide 3, 34kda  GTF2H1 - general transcription factor iih, polypeptide 1, 62kda  MCPH1 - microcephalin 1  GTF2A2 - general transcription factor iia, 2, 12kda  GTF2B - general transcription factor iib  PCGF2 - polycomb group ring finger 2  MAP7D3 - map7 domain containing 3  TBL1XR1 - transducin (beta)-like 1 x-linked receptor 1  GTF2H4 - general transcription factor iih, polypeptide 4, 52kda  BRF1 - brf1, rna polymerase iii transcription initiation factor 90 kda subunit  IPO4 - importin 4  MLF1IP - mlf1 interacting protein  H3F3A - h3 histone, family 3a  TCTN1 - tectonic family member 1  HIST1H2BB - histone cluster 1, h2bb  BRPF1 - bromodomain and phd finger containing, 1  H2AFZ - h2a histone family, member z  FZD5 - frizzled family receptor 5  H2AFX - h2a histone family, member x  CXCR4 - chemokine (c-x-c motif) receptor 4  MUL1 - mitochondrial e3 ubiquitin protein ligase 1  TUBA1A - tubulin, alpha 1a  CORO7 - coronin 7  TMEM231 - transmembrane protein 231  CDC73 - cell division cycle 73  DAP3 - death associated protein 3  HCFC1 - host cell factor c1 (vp16-accessory protein)  MAP3K12 - mitogen-activated protein kinase kinase kinase 12  ZYX - zyxin  NDNF - neuron-derived neurotrophic factor  HDAC2 - histone deacetylase 2  HTT - huntingtin  HDAC1 - histone deacetylase 1  ZNF205 - zinc finger protein 205  HCLS1 - hematopoietic cell-specific lyn substrate 1  ZNF207 - zinc finger protein 207  RIC3 - ric3 acetylcholine receptor chaperone  XPA - xeroderma pigmentosum, complementation group a  XPC - xeroderma pigmentosum, complementation group c  GOLGA2 - golgin a2  HAUS3 - haus augmin-like complex, subunit 3  GOLGB1 - golgin b1  XRCC1 - x-ray repair complementing defective repair in chinese hamster cells 1  HIGD2A - hig1 hypoxia inducible domain family, member 2a  PAGR1 - paxip1 associated glutamate-rich protein 1  RSG1 - rem2 and rab-like small gtpase 1  WRB - tryptophan rich basic protein  GPM6B - glycoprotein m6b  EIF4H - eukaryotic translation initiation factor 4h  CLIP2 - cap-gly domain containing linker protein 2  MPZL3 - myelin protein zero-like 3  WEE1 - wee1 homolog (s. pombe)  RILPL2 - rab interacting lysosomal protein-like 2  WHSC1 - wolf-hirschhorn syndrome candidate 1  VRK1 - vaccinia related kinase 1  VWF - von willebrand factor  ARID2 - at rich interactive domain 2 (arid, rfx-like)  PAOX - polyamine oxidase (exo-n4-amino)  TMEM43 - transmembrane protein 43  BRCC3 - brca1/brca2-containing complex, subunit 3  FSD1 - fibronectin type iii and spry domain containing 1  GRB2 - growth factor receptor-bound protein 2  GNPTAB - n-acetylglucosamine-1-phosphate transferase, alpha and beta subunits  ASXL1 - additional sex combs like 1 (drosophila)  CENPO - centromere protein o  SF1 - splicing factor 1  ZNF711 - zinc finger protein 711  ARHGAP35 - rho gtpase activating protein 35  XRCC5 - x-ray repair complementing defective repair in chinese hamster cells 5 (double-strand-break rejoining)  YWHAG - tyrosine 3-monooxygenase/tryptophan 5-monooxygenase activation protein, gamma polypeptide  GSK3B - glycogen synthase kinase 3 beta  YWHAZ - tyrosine 3-monooxygenase/tryptophan 5-monooxygenase activation protein, zeta polypeptide  GSK3A - glycogen synthase kinase 3 alpha  YWHAE - tyrosine 3-monooxygenase/tryptophan 5-monooxygenase activation protein, epsilon polypeptide  YWHAB - tyrosine 3-monooxygenase/tryptophan 5-monooxygenase activation protein, beta polypeptide  NDUFAF5 - nadh dehydrogenase (ubiquinone) complex i, assembly factor 5  MAPKAP1 - mitogen-activated protein kinase associated protein 1  RNF26 - ring finger protein 26  SAMD14 - sterile alpha motif domain containing 14  PEX11B - peroxisomal biogenesis factor 11 beta  PEX11A - peroxisomal biogenesis factor 11 alpha  FLCN - folliculin  CENPV - centromere protein v  NRP1 - neuropilin 1  PRMT1 - protein arginine methyltransferase 1  PRMT2 - protein arginine methyltransferase 2  LIN7A - lin-7 homolog a (c. elegans)  KLHDC8B - kelch domain containing 8b  FBXO45 - f-box protein 45  AGFG1 - arfgap with fg repeats 1  ARL13B - adp-ribosylation factor-like 13b  CCDC28B - coiled-coil domain containing 28b  APOO - apolipoprotein o  KCTD6 - potassium channel tetramerization domain containing 6  ALKBH1 - alkb, alkylation repair homolog 1 (e. coli)  HPRT1 - hypoxanthine phosphoribosyltransferase 1  RNU1-1 - rna, u1 small nuclear 1  TMEM17 - transmembrane protein 17  FAM174B - family with sequence similarity 174, member b  CFLAR - casp8 and fadd-like apoptosis regulator  SYNGAP1 - synaptic ras gtpase activating protein 1  KCTD15 - potassium channel tetramerization domain containing 15  SYNJ1 - synaptojanin 1  LDB1 - lim domain binding 1  WDR77 - wd repeat domain 77  ALDH1A2 - aldehyde dehydrogenase 1 family, member a2  IBA57 - iba57, iron-sulfur cluster assembly homolog (s. cerevisiae)  DSCC1 - dna replication and sister chromatid cohesion 1  KAT2B - k(lysine) acetyltransferase 2b  AKAP4 - a kinase (prka) anchor protein 4  CRTC2 - creb regulated transcription coactivator 2  ZNF259 - zinc finger protein 259  CENPM - centromere protein m  CDC123 - cell division cycle 123  ARHGEF7 - rho guanine nucleotide exchange factor (gef) 7  MIS12 - mis12 kinetochore complex component  MTMR2 - myotubularin related protein 2  AP1M1 - adaptor-related protein complex 1, mu 1 subunit  HUS1 - hus1 checkpoint homolog (s. pombe)  MCM3AP - minichromosome maintenance complex component 3 associated protein  EIF2S2 - eukaryotic translation initiation factor 2, subunit 2 beta, 38kda  TMEM108 - transmembrane protein 108  HERC1 - hect and rld domain containing e3 ubiquitin protein ligase family member 1  TRAK2 - trafficking protein, kinesin binding 2  SNX19 - sorting nexin 19  HSPD1 - heat shock 60kda protein 1 (chaperonin)  HSP90AA1 - heat shock protein 90kda alpha (cytosolic), class a member 1  AP3D1 - adaptor-related protein complex 3, delta 1 subunit  HSPA4 - heat shock 70kda protein 4  SKAP2 - src kinase associated phosphoprotein 2  BAIAP3 - bai1-associated protein 3  HSPA9 - heat shock 70kda protein 9 (mortalin)  WASF1 - was protein family, member 1  HSPA8 - heat shock 70kda protein 8  HMGN1 - high mobility group nucleosome binding domain 1  HMGB3 - high mobility group box 3  NUMB - numb homolog (drosophila)  HMGB2 - high mobility group box 2  HMGB1 - high mobility group box 1  MTIF3 - mitochondrial translational initiation factor 3  HLCS - holocarboxylase synthetase (biotin-(proprionyl-coa-carboxylase (atp-hydrolysing)) ligase)  PHACTR4 - phosphatase and actin regulator 4  EIF3C - eukaryotic translation initiation factor 3, subunit c  BRD9 - bromodomain containing 9  EIF3D - eukaryotic translation initiation factor 3, subunit d  PLEKHA3 - pleckstrin homology domain containing, family a (phosphoinositide binding specific) member 3  EIF3A - eukaryotic translation initiation factor 3, subunit a  EIF3B - eukaryotic translation initiation factor 3, subunit b  DYNLL1 - dynein, light chain, lc8-type 1  TNKS - tankyrase, trf1-interacting ankyrin-related adp-ribose polymerase  AGO1 - argonaute risc catalytic component 1  EIF3F - eukaryotic translation initiation factor 3, subunit f  TIMM9 - translocase of inner mitochondrial membrane 9 homolog (yeast)  EIF3G - eukaryotic translation initiation factor 3, subunit g  BECN1 - beclin 1, autophagy related  ZBTB48 - zinc finger and btb domain containing 48  SRSF9 - serine/arginine-rich splicing factor 9  MRPL1 - mitochondrial ribosomal protein l1  VAMP4 - vesicle-associated membrane protein 4  VMA21 - vma21 vacuolar h+-atpase homolog (s. cerevisiae)  TMEM237 - transmembrane protein 237  HIPK1 - homeodomain interacting protein kinase 1  HYAL2 - hyaluronoglucosaminidase 2  ACD - adrenocortical dysplasia homolog (mouse)  HLA-DMA - major histocompatibility complex, class ii, dm alpha  TMEM135 - transmembrane protein 135  NAIF1 - nuclear apoptosis inducing factor 1  DGAT1 - diacylglycerol o-acyltransferase 1  HFE - hemochromatosis  BSCL2 - berardinelli-seip congenital lipodystrophy 2 (seipin)  MRPL44 - mitochondrial ribosomal protein l44  VPS33A - vacuolar protein sorting 33 homolog a (s. cerevisiae)  HIP1 - huntingtin interacting protein 1  UBE2K - ubiquitin-conjugating enzyme e2k  MARCKSL1 - marcks-like 1  MBTPS1 - membrane-bound transcription factor peptidase, site 1  ARFGAP3 - adp-ribosylation factor gtpase activating protein 3  TRADD - tnfrsf1a-associated via death domain  ERAL1 - era-like 12s mitochondrial rrna chaperone 1  SNX3 - sorting nexin 3  TSNARE1 - t-snare domain containing 1  TUBB - tubulin, beta class i  EED - embryonic ectoderm development  GBF1 - golgi brefeldin a resistant guanine nucleotide exchange factor 1  RIPK1 - receptor (tnfrsf)-interacting serine-threonine kinase 1  HNRNPU - heterogeneous nuclear ribonucleoprotein u (scaffold attachment factor a)  MRPS24 - mitochondrial ribosomal protein s24  MRPS6 - mitochondrial ribosomal protein s6  MRPS11 - mitochondrial ribosomal protein s11  VPS33B - vacuolar protein sorting 33 homolog b (yeast)  TINF2 - terf1 (trf1)-interacting nuclear factor 2  ADAM15 - adam metallopeptidase domain 15  MRPL32 - mitochondrial ribosomal protein l32  CDS2 - cdp-diacylglycerol synthase (phosphatidate cytidylyltransferase) 2  ARV1 - arv1 homolog (s. cerevisiae)  P2RY12 - purinergic receptor p2y, g-protein coupled, 12  RAB11A - rab11a, member ras oncogene family  HNRNPA2B1 - heterogeneous nuclear ribonucleoprotein a2/b1  HNRNPC - heterogeneous nuclear ribonucleoprotein c (c1/c2)  NAPA - n-ethylmaleimide-sensitive factor attachment protein, alpha  NAPG - n-ethylmaleimide-sensitive factor attachment protein, gamma  HMGA1 - high mobility group at-hook 1  VWA1 - von willebrand factor a domain containing 1  DEPTOR - dep domain containing mtor-interacting protein  HAT1 - histone acetyltransferase 1  EIF3K - eukaryotic translation initiation factor 3, subunit k  CRTC3 - creb regulated transcription coactivator 3  RAB30 - rab30, member ras oncogene family  RBMX - rna binding motif protein, x-linked  PKP4 - plakophilin 4  PEX3 - peroxisomal biogenesis factor 3  HTRA2 - htra serine peptidase 2  MEAF6 - myst/esa1-associated factor 6  PPFIA2 - protein tyrosine phosphatase, receptor type, f polypeptide (ptprf), interacting protein (liprin), alpha 2  PPFIA1 - protein tyrosine phosphatase, receptor type, f polypeptide (ptprf), interacting protein (liprin), alpha 1  TOR1B - torsin family 1, member b (torsin b)  ING2 - inhibitor of growth family, member 2  PPM1D - protein phosphatase, mg2+/mn2+ dependent, 1d  UBE2S - ubiquitin-conjugating enzyme e2s  PRPF19 - pre-mrna processing factor 19  ILK - integrin-linked kinase  CDC14B - cell division cycle 14b  PIAS1 - protein inhibitor of activated stat, 1  SMYD3 - set and mynd domain containing 3  BLZF1 - basic leucine zipper nuclear factor 1  AP3B1 - adaptor-related protein complex 3, beta 1 subunit  PATL1 - protein associated with topoisomerase ii homolog 1 (yeast)  EML4 - echinoderm microtubule associated protein like 4  PPFIA3 - protein tyrosine phosphatase, receptor type, f polypeptide (ptprf), interacting protein (liprin), alpha 3  AGPS - alkylglycerone phosphate synthase  COPS7B - cop9 signalosome subunit 7b  CBX4 - chromobox homolog 4  COPS3 - cop9 signalosome subunit 3  GORASP1 - golgi reassembly stacking protein 1, 65kda  GAS7 - growth arrest-specific 7  HIAT1 - hippocampus abundant transcript 1  ORAOV1 - oral cancer overexpressed 1  ITGAE - integrin, alpha e (antigen cd103, human mucosal lymphocyte antigen 1; alpha polypeptide)  SERP1 - stress-associated endoplasmic reticulum protein 1  TUBGCP4 - tubulin, gamma complex associated protein 4  VPS16 - vacuolar protein sorting 16 homolog (s. cerevisiae)  ITGA1 - integrin, alpha 1  ITGA3 - integrin, alpha 3 (antigen cd49c, alpha 3 subunit of vla-3 receptor)  ITGB5 - integrin, beta 5  NFU1 - nfu1 iron-sulfur cluster scaffold homolog (s. cerevisiae)  PDLIM4 - pdz and lim domain 4  ITGAV - integrin, alpha v  POLR1E - polymerase (rna) i polypeptide e, 53kda  SUDS3 - suppressor of defective silencing 3 homolog (s. cerevisiae)  CDK10 - cyclin-dependent kinase 10  ARFIP1 - adp-ribosylation factor interacting protein 1  TBCEL - tubulin folding cofactor e-like  PRPF18 - pre-mrna processing factor 18  ITGB1 - integrin, beta 1 (fibronectin receptor, beta polypeptide, antigen cd29 includes mdf2, msk12)  DEGS1 - delta(4)-desaturase, sphingolipid 1  DENR - density-regulated protein  ITGB4 - integrin, beta 4  ACTR6 - arp6 actin-related protein 6 homolog (yeast)  TPCN2 - two pore segment channel 2  BBS9 - bardet-biedl syndrome 9  EIF6 - eukaryotic translation initiation factor 6  TEKT2 - tektin 2 (testicular)  ARAP3 - arfgap with rhogap domain, ankyrin repeat and ph domain 3  INPPL1 - inositol polyphosphate phosphatase-like 1  ATG3 - autophagy related 3  RND1 - rho family gtpase 1  INSR - insulin receptor  PCDH17 - protocadherin 17  AKTIP - akt interacting protein  KLHL20 - kelch-like family member 20  MPP5 - membrane protein, palmitoylated 5 (maguk p55 subfamily member 5)  LSM3 - lsm3 homolog, u6 small nuclear rna associated (s. cerevisiae)  PSMG1 - proteasome (prosome, macropain) assembly chaperone 1  KLF7 - kruppel-like factor 7 (ubiquitous)  RUVBL1 - ruvb-like 1 (e. coli)  USO1 - uso1 vesicle transport factor  ITGA6 - integrin, alpha 6  ARHGEF28 - rho guanine nucleotide exchange factor (gef) 28  TRAPPC3 - trafficking protein particle complex 3  LRWD1 - leucine-rich repeats and wd repeat domain containing 1  NSD1 - nuclear receptor binding set domain protein 1  MMS19 - mms19 nucleotide excision repair homolog (s. cerevisiae)  PDLIM2 - pdz and lim domain 2 (mystique)  ID2 - inhibitor of dna binding 2, dominant negative helix-loop-helix protein  PIP5K1A - phosphatidylinositol-4-phosphate 5-kinase, type i, alpha  BRPF3 - bromodomain and phd finger containing, 3  C17orf75 - chromosome 17 open reading frame 75  STK36 - serine/threonine kinase 36  NDOR1 - nadph dependent diflavin oxidoreductase 1  THSD7A - thrombospondin, type i, domain containing 7a  TUBG2 - tubulin, gamma 2  NME5 - nme/nm23 family member 5  TMED4 - transmembrane emp24 protein transport domain containing 4  VPS4A - vacuolar protein sorting 4 homolog a (s. cerevisiae)  IGHMBP2 - immunoglobulin mu binding protein 2  RSPH9 - radial spoke head 9 homolog (chlamydomonas)  EPB41L4A - erythrocyte membrane protein band 4.1 like 4a  LRRC4 - leucine rich repeat containing 4  NCK2 - nck adaptor protein 2  GNPAT - glyceronephosphate o-acyltransferase  DYRK3 - dual-specificity tyrosine-(y)-phosphorylation regulated kinase 3  CYFIP2 - cytoplasmic fmr1 interacting protein 2  USP21 - ubiquitin specific peptidase 21  ULK1 - unc-51 like autophagy activating kinase 1  EEA1 - early endosome antigen 1  STX7 - syntaxin 7  GOLPH3 - golgi phosphoprotein 3 (coat-protein)  NPTN - neuroplastin  PHACTR1 - phosphatase and actin regulator 1  MLH3 - mutl homolog 3 (e. coli)  SORBS2 - sorbin and sh3 domain containing 2  JMJD1C - jumonji domain containing 1c  REEP3 - receptor accessory protein 3  SMARCA5 - swi/snf related, matrix associated, actin dependent regulator of chromatin, subfamily a, member 5  ATP2C1 - atpase, ca++ transporting, type 2c, member 1  CDC42BPA - cdc42 binding protein kinase alpha (dmpk-like)  IGF1R - insulin-like growth factor 1 receptor  FAM171A1 - family with sequence similarity 171, member a1  OGT - o-linked n-acetylglucosamine (glcnac) transferase  HIRIP3 - hira interacting protein 3  IGF2R - insulin-like growth factor 2 receptor  RAE1 - rae1 rna export 1 homolog (s. pombe)  GEMIN2 - gem (nuclear organelle) associated protein 2  NSG1 - neuron specific gene family member 1  TSPYL2 - tspy-like 2  CUL4B - cullin 4b  VPS41 - vacuolar protein sorting 41 homolog (s. cerevisiae)  CUL1 - cullin 1  B9D1 - b9 protein domain 1  CUL3 - cullin 3  ATRN - attractin  SUPT3H - suppressor of ty 3 homolog (s. cerevisiae)  CACNG4 - calcium channel, voltage-dependent, gamma subunit 4  PCGF5 - polycomb group ring finger 5  SDHAF1 - succinate dehydrogenase complex assembly factor 1  WAC - ww domain containing adaptor with coiled-coil  PHF21A - phd finger protein 21a  HPS3 - hermansky-pudlak syndrome 3  LIG4 - ligase iv, dna, atp-dependent  PITPNM1 - phosphatidylinositol transfer protein, membrane-associated 1  MKI67IP - mki67 (fha domain) interacting nucleolar phosphoprotein  ABLIM1 - actin binding lim protein 1  LIMK1 - lim domain kinase 1  VPS37A - vacuolar protein sorting 37 homolog a (s. cerevisiae)  NCOR1 - nuclear receptor corepressor 1  LDLR - low density lipoprotein receptor  MTPN - myotrophin  TIMMDC1 - translocase of inner mitochondrial membrane domain containing 1  HOOK3 - hook microtubule-tethering protein 3  LZTS2 - leucine zipper, putative tumor suppressor 2  DOT1L - dot1-like histone h3k79 methyltransferase  REEP2 - receptor accessory protein 2  ABCG1 - atp-binding cassette, sub-family g (white), member 1  LETM1 - leucine zipper-ef-hand containing transmembrane protein 1  SYCE1L - synaptonemal complex central element protein 1-like  L3MBTL3 - l(3)mbt-like 3 (drosophila)  LOXL1 - lysyl oxidase-like 1  TICAM2 - toll-like receptor adaptor molecule 2  BRSK1 - br serine/threonine kinase 1  SLX4 - slx4 structure-specific endonuclease subunit  CHD1L - chromodomain helicase dna binding protein 1-like  SEC22B - sec22 vesicle trafficking protein homolog b (s. cerevisiae) (gene/pseudogene)  PTPDC1 - protein tyrosine phosphatase domain containing 1  H2AFY - h2a histone family, member y  GOSR2 - golgi snap receptor complex member 2  BET1L - bet1 golgi vesicular membrane trafficking protein-like  UBXN2B - ubx domain protein 2b  BCAR1 - breast cancer anti-estrogen resistance 1  LIPA - lipase a, lysosomal acid, cholesterol esterase  SPIRE2 - spire actin nucleation factor 2  LIMK2 - lim domain kinase 2  HOPX - hop homeobox  GOLM1 - golgi membrane protein 1  JAGN1 - jagunal homolog 1 (drosophila)  CDC42BPB - cdc42 binding protein kinase beta (dmpk-like)  NACC2 - nacc family member 2, ben and btb (poz) domain containing  LMNA - lamin a/c  CLOCK - clock circadian regulator  LMAN1 - lectin, mannose-binding, 1  HGSNAT - heparan-alpha-glucosaminide n-acetyltransferase  LLGL1 - lethal giant larvae homolog 1 (drosophila)  ADAMTS4 - adam metallopeptidase with thrombospondin type 1 motif, 4  ADAMTS1 - adam metallopeptidase with thrombospondin type 1 motif, 1  MRPL43 - mitochondrial ribosomal protein l43  TBPL1 - tbp-like 1  YTHDF2 - yth domain family, member 2  VPS4B - vacuolar protein sorting 4 homolog b (s. cerevisiae)  MAP1LC3A - microtubule-associated protein 1 light chain 3 alpha  GOSR1 - golgi snap receptor complex member 1  NIP7 - nip7, nucleolar pre-rrna processing protein  EIF3L - eukaryotic translation initiation factor 3, subunit l  BAG5 - bcl2-associated athanogene 5  KRT10 - keratin 10  BAG4 - bcl2-associated athanogene 4  TRAPPC4 - trafficking protein particle complex 4  ACTL6B - actin-like 6b  LAMP2 - lysosomal-associated membrane protein 2  STMN1 - stathmin 1  POMP - proteasome maturation protein  TEX264 - testis expressed 264  ATG5 - autophagy related 5  ROCK2 - rho-associated, coiled-coil containing protein kinase 2  ATP6V1D - atpase, h+ transporting, lysosomal 34kda, v1 subunit d  LCP1 - lymphocyte cytosolic protein 1 (l-plastin)  ANGPT4 - angiopoietin 4  STX8 - syntaxin 8  L1CAM - l1 cell adhesion molecule  FUNDC1 - fun14 domain containing 1  TAOK3 - tao kinase 3  ZBTB7A - zinc finger and btb domain containing 7a  CRNKL1 - crooked neck pre-mrna splicing factor-like 1 (drosophila)  DPY30 - dpy-30 homolog (c. elegans)  LAMA5 - laminin, alpha 5  LAMA4 - laminin, alpha 4  LAMC1 - laminin, gamma 1 (formerly lamb2)  NAGPA - n-acetylglucosamine-1-phosphodiester alpha-n-acetylglucosaminidase  CHD6 - chromodomain helicase dna binding protein 6  POLR1B - polymerase (rna) i polypeptide b, 128kda  VPS28 - vacuolar protein sorting 28 homolog (s. cerevisiae)  KIFC3 - kinesin family member c3  KIF5C - kinesin family member 5c  ANTXR1 - anthrax toxin receptor 1  MRTO4 - mrna turnover 4 homolog (s. cerevisiae)  RPF2 - ribosome production factor 2 homolog (s. cerevisiae)  KIF2A - kinesin heavy chain member 2a  KIF5B - kinesin family member 5b  ARHGEF6 - rac/cdc42 guanine nucleotide exchange factor (gef) 6  KDR - kinase insert domain receptor (a type iii receptor tyrosine kinase)  PICK1 - protein interacting with prkca 1  HSD17B12 - hydroxysteroid (17-beta) dehydrogenase 12  ING4 - inhibitor of growth family, member 4  EIF2AK3 - eukaryotic translation initiation factor 2-alpha kinase 3  DYNC1LI1 - dynein, cytoplasmic 1, light intermediate chain 1  KCTD3 - potassium channel tetramerization domain containing 3  KCNMA1 - potassium large conductance calcium-activated channel, subfamily m, alpha member 1  CCDC41 - coiled-coil domain containing 41  MAP4K4 - mitogen-activated protein kinase kinase kinase kinase 4  TRAF7 - tnf receptor-associated factor 7, e3 ubiquitin protein ligase  LRRC8C - leucine rich repeat containing 8 family, member c  RECQL5 - recq protein-like 5  KRAS - kirsten rat sarcoma viral oncogene homolog  TGFBRAP1 - transforming growth factor, beta receptor associated protein 1  CIAO1 - cytosolic iron-sulfur protein assembly 1  SBDS - shwachman-bodian-diamond syndrome  KPNB1 - karyopherin (importin) beta 1  RMDN1 - regulator of microtubule dynamics 1  SUV420H1 - suppressor of variegation 4-20 homolog 1 (drosophila)  TRAPPC12 - trafficking protein particle complex 12  KLC1 - kinesin light chain 1  CRIPT - cysteine-rich pdz-binding protein  DDX23 - dead (asp-glu-ala-asp) box polypeptide 23  PHF20L1 - phd finger protein 20-like 1  TJP2 - tight junction protein 2  TFB1M - transcription factor b1, mitochondrial  NDUFAF1 - nadh dehydrogenase (ubiquinone) complex i, assembly factor 1  PEX16 - peroxisomal biogenesis factor 16  KIT - v-kit hardy-zuckerman 4 feline sarcoma viral oncogene homolog  SH3GLB1 - sh3-domain grb2-like endophilin b1  LONP1 - lon peptidase 1, mitochondrial  TMEM216 - transmembrane protein 216  MRPL51 - mitochondrial ribosomal protein l51  MRPL30 - mitochondrial ribosomal protein l30  KCNA5 - potassium voltage-gated channel, shaker-related subfamily, member 5  TCHP - trichoplein, keratin filament binding  PSMG3 - proteasome (prosome, macropain) assembly chaperone 3  PDZD11 - pdz domain containing 11  SLIT2 - slit homolog 2 (drosophila)  JUP - junction plakoglobin  TMEM175 - transmembrane protein 175  COG1 - component of oligomeric golgi complex 1  JAK2 - janus kinase 2  ING5 - inhibitor of growth family, member 5  COX16 - cox16 cytochrome c oxidase assembly homolog (s. cerevisiae)  SLC25A33 - solute carrier family 25 (pyrimidine nucleotide carrier), member 33  KIF3B - kinesin family member 3b  ITPA - inosine triphosphatase (nucleoside triphosphate pyrophosphatase)  MAST4 - microtubule associated serine/threonine kinase family member 4  GLTP - glycolipid transfer protein  PHF20 - phd finger protein 20  CHCHD6 - coiled-coil-helix-coiled-coil-helix domain containing 6  ZNHIT3 - zinc finger, hit-type containing 3  HMGN3 - high mobility group nucleosomal binding domain 3  ATP9B - atpase, class ii, type 9b  COPS2 - cop9 signalosome subunit 2  ZDHHC2 - zinc finger, dhhc-type containing 2  SNAP29 - synaptosomal-associated protein, 29kda  RSL24D1 - ribosomal l24 domain containing 1  VAMP3 - vesicle-associated membrane protein 3  KCND3 - potassium voltage-gated channel, shal-related subfamily, member 3  MIA3 - melanoma inhibitory activity family, member 3  TAOK2 - tao kinase 2  B4GALT6 - udp-gal:betaglcnac beta 1,4- galactosyltransferase, polypeptide 6  BRMS1L - breast cancer metastasis-suppressor 1-like  LEF1 - lymphoid enhancer-binding factor 1  VPS25 - vacuolar protein sorting 25 homolog (s. cerevisiae)  PLEKHO1 - pleckstrin homology domain containing, family o member 1  GTF3C4 - general transcription factor iiic, polypeptide 4, 90kda  MRPL45 - mitochondrial ribosomal protein l45  TUBE1 - tubulin, epsilon 1  KCNC4 - potassium voltage-gated channel, shaw-related subfamily, member 4  CCDC115 - coiled-coil domain containing 115  TMEM107 - transmembrane protein 107  B4GALT5 - udp-gal:betaglcnac beta 1,4- galactosyltransferase, polypeptide 5  S1PR2 - sphingosine-1-phosphate receptor 2  LUC7L3 - luc7-like 3 (s. cerevisiae)  KCTD10 - potassium channel tetramerization domain containing 10  ARID4B - at rich interactive domain 4b (rbp1-like)  STARD3NL - stard3 n-terminal like  MIF - macrophage migration inhibitory factor (glycosylation-inhibiting factor)  RAB8B - rab8b, member ras oncogene family  ATP8A2 - atpase, aminophospholipid transporter, class i, type 8a, member 2  MLH1 - mutl homolog 1, colon cancer, nonpolyposis type 2 (e. coli)  MKLN1 - muskelin 1, intracellular mediator containing kelch motifs  ITGB1BP1 - integrin beta 1 binding protein 1  KDM3B - lysine (k)-specific demethylase 3b  RSF1 - remodeling and spacing factor 1  CYTH2 - cytohesin 2  KMT2A - lysine (k)-specific methyltransferase 2a  MMP2 - matrix metallopeptidase 2 (gelatinase a, 72kda gelatinase, 72kda type iv collagenase)  NUMBL - numb homolog (drosophila)-like  RPS6KA5 - ribosomal protein s6 kinase, 90kda, polypeptide 5  TRAPPC2L - trafficking protein particle complex 2-like  CCDC8 - coiled-coil domain containing 8  KATNAL1 - katanin p60 subunit a-like 1  MMP14 - matrix metallopeptidase 14 (membrane-inserted)  CACNA2D2 - calcium channel, voltage-dependent, alpha 2/delta subunit 2  MNAT1 - mnat cdk-activating kinase assembly factor 1  UIMC1 - ubiquitin interaction motif containing 1  MTA2 - metastasis associated 1 family, member 2  SYT9 - synaptotagmin ix  NOLC1 - nucleolar and coiled-body phosphoprotein 1  RAB23 - rab23, member ras oncogene family  MAGI1 - membrane associated guanylate kinase, ww and pdz domain containing 1  EIF2A - eukaryotic translation initiation factor 2a, 65kda  DLG5 - discs, large homolog 5 (drosophila)  ZMYM3 - zinc finger, mym-type 3  ZMYM4 - zinc finger, mym-type 4  MCM7 - minichromosome maintenance complex component 7  ANKRD27 - ankyrin repeat domain 27 (vps9 domain)  PLEKHA7 - pleckstrin homology domain containing, family a member 7  VAPB - vamp (vesicle-associated membrane protein)-associated protein b and c  SPTY2D1 - spt2, suppressor of ty, domain containing 1 (s. cerevisiae)  BUB3 - bub3 mitotic checkpoint protein  PELO - pelota homolog (drosophila)  MECP2 - methyl cpg binding protein 2 (rett syndrome)  REPS2 - ralbp1 associated eps domain containing 2  MEF2A - myocyte enhancer factor 2a  ZW10 - zw10 kinetochore protein  BRWD1 - bromodomain and wd repeat domain containing 1  A4GALT - alpha 1,4-galactosyltransferase  FAM160A2 - family with sequence similarity 160, member a2  DCLK1 - doublecortin-like kinase 1  MEN1 - multiple endocrine neoplasia i  RAB8A - rab8a, member ras oncogene family  CEP78 - centrosomal protein 78kda  COX7A2L - cytochrome c oxidase subunit viia polypeptide 2 like  SCAF11 - sr-related ctd-associated factor 11  FAM161A - family with sequence similarity 161, member a  ZFYVE1 - zinc finger, fyve domain containing 1  KAT8 - k(lysine) acetyltransferase 8  MFAP2 - microfibrillar-associated protein 2  ARHGEF2 - rho/rac guanine nucleotide exchange factor (gef) 2  MFGE8 - milk fat globule-egf factor 8 protein  TUBB2B - tubulin, beta 2b class iib  MFAP4 - microfibrillar-associated protein 4  ATG12 - autophagy related 12  ARL6 - adp-ribosylation factor-like 6  STX18 - syntaxin 18  SYNGR1 - synaptogyrin 1  HGS - hepatocyte growth factor-regulated tyrosine kinase substrate  MBD3 - methyl-cpg binding domain protein 3  VTA1 - vesicle (multivesicular body) trafficking 1  RABEP1 - rabaptin, rab gtpase binding effector protein 1  COG3 - component of oligomeric golgi complex 3  MAL - mal, t-cell differentiation protein  DNAL1 - dynein, axonemal, light chain 1  PRPF3 - pre-mrna processing factor 3  FAM50A - family with sequence similarity 50, member a  TMEM138 - transmembrane protein 138  LARS - leucyl-trna synthetase  SMC3 - structural maintenance of chromosomes 3  NCKIPSD - nck interacting protein with sh3 domain  MARK3 - map/microtubule affinity-regulating kinase 3  MARK1 - map/microtubule affinity-regulating kinase 1  MAPT - microtubule-associated protein tau  INA - internexin neuronal intermediate filament protein, alpha  MAP6 - microtubule-associated protein 6  ATP6V0D1 - atpase, h+ transporting, lysosomal 38kda, v0 subunit d1  RAB14 - rab14, member ras oncogene family  MAP2 - microtubule-associated protein 2  LATS1 - large tumor suppressor kinase 1  MAP1B - microtubule-associated protein 1b  MTA1 - metastasis associated 1  SIRT6 - sirtuin 6  MAP1A - microtubule-associated protein 1a  RAB33B - rab33b, member ras oncogene family  VPS54 - vacuolar protein sorting 54 homolog (s. cerevisiae)  FAM161B - family with sequence similarity 161, member b  MAN2A1 - mannosidase, alpha, class 2a, member 1  USP8 - ubiquitin specific peptidase 8  LIMA1 - lim domain and actin binding 1  EVL - enah/vasp-like  MATN2 - matrilin 2  SFMBT1 - scm-like with four mbt domains 1  SART1 - squamous cell carcinoma antigen recognized by t cells  MAX - myc associated factor x  ZMIZ2 - zinc finger, miz-type containing 2  MAT2A - methionine adenosyltransferase ii, alpha  GULP1 - gulp, engulfment adaptor ptb domain containing 1  UFC1 - ubiquitin-fold modifier conjugating enzyme 1  MCM3 - minichromosome maintenance complex component 3  TRMT112 - trna methyltransferase 11-2 homolog (s. cerevisiae)  MCM6 - minichromosome maintenance complex component 6  CHMP5 - charged multivesicular body protein 5  MCM5 - minichromosome maintenance complex component 5  TTBK2 - tau tubulin kinase 2  MCF2 - mcf.2 cell line derived transforming sequence  CDAN1 - codanin 1  MCM2 - minichromosome maintenance complex component 2  ASH2L - ash2 (absent, small, or homeotic)-like (drosophila)  ANKFY1 - ankyrin repeat and fyve domain containing 1  SYT7 - synaptotagmin vii  MAP7 - microtubule-associated protein 7  NFS1 - nfs1 cysteine desulfurase  MRPL48 - mitochondrial ribosomal protein l48  MRPS23 - mitochondrial ribosomal protein s23  L3MBTL2 - l(3)mbt-like 2 (drosophila)  MRPS33 - mitochondrial ribosomal protein s33  FAM96B - family with sequence similarity 96, member b  LRP6 - low density lipoprotein receptor-related protein 6  OTUD6B - otu domain containing 6b  SEMA5A - sema domain, seven thrombospondin repeats (type 1 and type 1-like), transmembrane domain (tm) and short cytoplasmic domain, (semaphorin) 5a  TRAPPC9 - trafficking protein particle complex 9  LUM - lumican  LYN - v-yes-1 yamaguchi sarcoma viral related oncogene homolog  TPPP3 - tubulin polymerization-promoting protein family member 3  JAM3 - junctional adhesion molecule 3  HIP1R - huntingtin interacting protein 1 related  LTBP3 - latent transforming growth factor beta binding protein 3  RNF8 - ring finger protein 8, e3 ubiquitin protein ligase  TMED6 - transmembrane emp24 protein transport domain containing 6  CHMP3 - charged multivesicular body protein 3  BAZ1B - bromodomain adjacent to zinc finger domain, 1b  TAF1B - tata box binding protein (tbp)-associated factor, rna polymerase i, b, 63kda  MARCKS - myristoylated alanine-rich protein kinase c substrate  RAB34 - rab34, member ras oncogene family  SMAD2 - smad family member 2  RTN4RL1 - reticulon 4 receptor-like 1  MBIP - map3k12 binding inhibitory protein 1  HDAC7 - histone deacetylase 7  FAM49B - family with sequence similarity 49, member b  M6PR - mannose-6-phosphate receptor (cation dependent)  TAF1C - tata box binding protein (tbp)-associated factor, rna polymerase i, c, 110kda  RPS6KA4 - ribosomal protein s6 kinase, 90kda, polypeptide 4  KCTD11 - potassium channel tetramerization domain containing 11  PLOD3 - procollagen-lysine, 2-oxoglutarate 5-dioxygenase 3  SMAD4 - smad family member 4  H1FX - h1 histone family, member x  LSR - lipolysis stimulated lipoprotein receptor  SMAD7 - smad family member 7  TMEM199 - transmembrane protein 199  WASL - wiskott-aldrich syndrome-like  ANP32E - acidic (leucine-rich) nuclear phosphoprotein 32 family, member e  NCBP1 - nuclear cap binding protein subunit 1, 80kda  NDN - necdin, melanoma antigen (mage) family member  G3BP1 - gtpase activating protein (sh3 domain) binding protein 1  AKAP9 - a kinase (prka) anchor protein (yotiao) 9  NCK1 - nck adaptor protein 1  ABI2 - abl-interactor 2  RACGAP1 - rac gtpase activating protein 1  VMP1 - vacuole membrane protein 1  NAGLU - n-acetylglucosaminidase, alpha  TRIM28 - tripartite motif containing 28  ISCA1 - iron-sulfur cluster assembly 1  NAP1L1 - nucleosome assembly protein 1-like 1  NASP - nuclear autoantigenic sperm protein (histone-binding)  WASF2 - was protein family, member 2  NAP1L4 - nucleosome assembly protein 1-like 4  NBN - nibrin  NUBP1 - nucleotide binding protein 1  TDRD3 - tudor domain containing 3  NCAPH2 - non-smc condensin ii complex, subunit h2  CNIH - cornichon homolog (drosophila)  FAM49A - family with sequence similarity 49, member a  RBM5 - rna binding motif protein 5  ZDHHC8 - zinc finger, dhhc-type containing 8  PPP1R12A - protein phosphatase 1, regulatory subunit 12a  LMAN2L - lectin, mannose-binding 2-like  NDEL1 - nude neurodevelopment protein 1-like 1  MYLK - myosin light chain kinase  TMOD2 - tropomodulin 2 (neuronal)  TNFAIP8L3 - tumor necrosis factor, alpha-induced protein 8-like 3  GFOD2 - glucose-fructose oxidoreductase domain containing 2  MYO1D - myosin id  MYO5A - myosin va (heavy chain 12, myoxin)  MYO1E - myosin ie  USPL1 - ubiquitin specific peptidase like 1  MYO6 - myosin vi  INADL - inad-like (drosophila)  ABT1 - activator of basal transcription 1  TOPORS - topoisomerase i binding, arginine/serine-rich, e3 ubiquitin protein ligase  RAB1B - rab1b, member ras oncogene family  ARPC5L - actin related protein 2/3 complex, subunit 5-like  SEH1L - seh1-like (s. cerevisiae)  MYH10 - myosin, heavy chain 10, non-muscle  ATP9A - atpase, class ii, type 9a  MYH11 - myosin, heavy chain 11, smooth muscle  SNX11 - sorting nexin 11  MYH9 - myosin, heavy chain 9, non-muscle  SNUPN - snurportin 1  HUWE1 - hect, uba and wwe domain containing 1, e3 ubiquitin protein ligase  HCFC2 - host cell factor c2  ARPC5 - actin related protein 2/3 complex, subunit 5, 16kda  SNX10 - sorting nexin 10  CNOT7 - ccr4-not transcription complex, subunit 7  COL4A3BP - collagen, type iv, alpha 3 (goodpasture antigen) binding protein  PQBP1 - polyglutamine binding protein 1  MX1 - myxovirus (influenza virus) resistance 1, interferon-inducible protein p78 (mouse)  UBN1 - ubinuclein 1  TSPAN2 - tetraspanin 2  GPSM2 - g-protein signaling modulator 2  INO80B - ino80 complex subunit b  ACTR2 - arp2 actin-related protein 2 homolog (yeast)  ACTR3 - arp3 actin-related protein 3 homolog (yeast)  ARPC3 - actin related protein 2/3 complex, subunit 3, 21kda  ARPC4 - actin related protein 2/3 complex, subunit 4, 20kda  ARPC2 - actin related protein 2/3 complex, subunit 2, 34kda  NUBP2 - nucleotide binding protein 2  MTX1 - metaxin 1  PHF13 - phd finger protein 13  RAD50 - rad50 homolog (s. cerevisiae)  TRIM37 - tripartite motif containing 37  CDC42EP5 - cdc42 effector protein (rho gtpase binding) 5  TIMM22 - translocase of inner mitochondrial membrane 22 homolog (yeast)  SEC61A1 - sec61 alpha 1 subunit (s. cerevisiae)  SHARPIN - shank-associated rh domain interactor  BLOC1S3 - biogenesis of lysosomal organelles complex-1, subunit 3  AKT3 - v-akt murine thymoma viral oncogene homolog 3  SCO2 - sco2 cytochrome c oxidase assembly protein  ABI1 - abl-interactor 1  SLC12A6 - solute carrier family 12 (potassium/chloride transporter), member 6  MTIF2 - mitochondrial translational initiation factor 2  PDCD6 - programmed cell death 6  MTM1 - myotubularin 1  HDAC5 - histone deacetylase 5  PDCD6IP - programmed cell death 6 interacting protein  HDAC6 - histone deacetylase 6  OCLN - occludin  MT3 - metallothionein 3  CHAF1A - chromatin assembly factor 1, subunit a (p150)  FARSB - phenylalanyl-trna synthetase, beta subunit  EXOC8 - exocyst complex component 8  DNM1L - dynamin 1-like  RANBP9 - ran binding protein 9  DNAJB6 - dnaj (hsp40) homolog, subfamily b, member 6  SMC4 - structural maintenance of chromosomes 4  MSN - moesin  KANSL1L - kat8 regulatory nsl complex subunit 1-like  MSH3 - muts homolog 3 (e. coli)  MSH2 - muts homolog 2, colon cancer, nonpolyposis type 1 (e. coli)  SEC16A - sec16 homolog a (s. cerevisiae)  NCAPD2 - non-smc condensin i complex, subunit d2  DBNL - drebrin-like  SUPT7L - suppressor of ty 7 (s. cerevisiae)-like  WDR1 - wd repeat domain 1  IFT81 - intraflagellar transport 81 homolog (chlamydomonas)  TMEM14A - transmembrane protein 14a  TIMM21 - translocase of inner mitochondrial membrane 21 homolog (yeast)  USP3 - ubiquitin specific peptidase 3  BABAM1 - brisc and brca1 a complex member 1  MRPL15 - mitochondrial ribosomal protein l15  CHMP4A - charged multivesicular body protein 4a  USP15 - ubiquitin specific peptidase 15  TTL - tubulin tyrosine ligase  GOLGA5 - golgin a5  NDUFAF4 - nadh dehydrogenase (ubiquinone) complex i, assembly factor 4  C1GALT1C1 - c1galt1-specific chaperone 1  SETD2 - set domain containing 2  MRPL18 - mitochondrial ribosomal protein l18  RBX1 - ring-box 1, e3 ubiquitin protein ligase  MRE11A - mre11 meiotic recombination 11 homolog a (s. cerevisiae)  HSCB - hscb iron-sulfur cluster co-chaperone homolog (e. coli)  NUP153 - nucleoporin 153kda  MPP2 - membrane protein, palmitoylated 2 (maguk p55 subfamily member 2)  GLTPD1 - glycolipid transfer protein domain containing 1  DTX3L - deltex 3-like (drosophila)  B9D2 - b9 protein domain 2  SETDB1 - set domain, bifurcated 1  FCHSD2 - fch and double sh3 domains 2  SEC24D - sec24 family, member d (s. cerevisiae)  PARD6A - par-6 partitioning defective 6 homolog alpha (c. elegans)  TLK1 - tousled-like kinase 1  STMN3 - stathmin-like 3  OSBP - oxysterol binding protein  ELMO1 - engulfment and cell motility 1  C2CD5 - c2 calcium-dependent domain containing 5  CLDN11 - claudin 11  CCDC80 - coiled-coil domain containing 80  AGRN - agrin  C2CD2L - c2cd2-like  SHANK1 - sh3 and multiple ankyrin repeat domains 1  KIAA0319 - kiaa0319  CEP350 - centrosomal protein 350kda  SV2A - synaptic vesicle glycoprotein 2a  UBAP2L - ubiquitin associated protein 2-like  ARHGAP39 - rho gtpase activating protein 39  G3BP2 - gtpase activating protein (sh3 domain) binding protein 2  OCRL - oculocerebrorenal syndrome of lowe  ARHGAP44 - rho gtpase activating protein 44  ODF2 - outer dense fiber of sperm tails 2  TMED5 - transmembrane emp24 protein transport domain containing 5  REEP4 - receptor accessory protein 4  OSBPL2 - oxysterol binding protein-like 2  FAM84A - family with sequence similarity 84, member a  WDR61 - wd repeat domain 61  OPA1 - optic atrophy 1 (autosomal dominant)  TMED7 - transmembrane emp24 protein transport domain containing 7  FIG4 - fig4 homolog, sac1 lipid phosphatase domain containing (s. cerevisiae)  SNAP91 - synaptosomal-associated protein, 91kda  SLMO2 - slowmo homolog 2 (drosophila)  APITD1 - apoptosis-inducing, taf9-like domain 1  CKAP5 - cytoskeleton associated protein 5  FIS1 - fission 1 (mitochondrial outer membrane) homolog (s. cerevisiae)  KIAA0586 - kiaa0586  NSF - n-ethylmaleimide-sensitive factor  DDR2 - discoidin domain receptor tyrosine kinase 2  TOMM20 - translocase of outer mitochondrial membrane 20 homolog (yeast)  IST1 - increased sodium tolerance 1 homolog (yeast)  NUMA1 - nuclear mitotic apparatus protein 1  DAB2IP - dab2 interacting protein  ATG13 - autophagy related 13  NUP98 - nucleoporin 98kda  GMNN - geminin, dna replication inhibitor  TM9SF4 - transmembrane 9 superfamily protein member 4  NR4A2 - nuclear receptor subfamily 4, group a, member 2  ATL1 - atlastin gtpase 1  CEP120 - centrosomal protein 120kda  DNAJC6 - dnaj (hsp40) homolog, subfamily c, member 6  ARHGEF17 - rho guanine nucleotide exchange factor (gef) 17  NPM1 - nucleophosmin (nucleolar phosphoprotein b23, numatrin)  ARHGEF11 - rho guanine nucleotide exchange factor (gef) 11  NPHP1 - nephronophthisis 1 (juvenile)  ZEB2 - zinc finger e-box binding homeobox 2  SPICE1 - spindle and centriole associated protein 1  SLC11A2 - solute carrier family 11 (proton-coupled divalent metal ion transporter), member 2  CNTN4 - contactin 4  RNF40 - ring finger protein 40, e3 ubiquitin protein ligase  SPOCK2 - sparc/osteonectin, cwcv and kazal-like domains proteoglycan (testican) 2  FGD5 - fyve, rhogef and ph domain containing 5  NPTX1 - neuronal pentraxin i  RB1CC1 - rb1-inducible coiled-coil 1  IFT52 - intraflagellar transport 52 homolog (chlamydomonas)  CUL7 - cullin 7  GRIP2 - glutamate receptor interacting protein 2  NRF1 - nuclear respiratory factor 1  SH3D19 - sh3 domain containing 19  NRCAM - neuronal cell adhesion molecule  SIDT2 - sid1 transmembrane family, member 2  CHD9 - chromodomain helicase dna binding protein 9  UBQLN1 - ubiquilin 1  NMT1 - n-myristoyltransferase 1  SPG11 - spastic paraplegia 11 (autosomal recessive)  NOP2 - nop2 nucleolar protein  SLC39A3 - solute carrier family 39 (zinc transporter), member 3  VPRBP - vpr (hiv-1) binding protein  ARMC9 - armadillo repeat containing 9  SLC2A8 - solute carrier family 2 (facilitated glucose transporter), member 8  LRP12 - low density lipoprotein receptor-related protein 12  UBQLN2 - ubiquilin 2  IFT74 - intraflagellar transport 74 homolog (chlamydomonas)  NOTCH4 - notch 4  EFEMP2 - egf containing fibulin-like extracellular matrix protein 2  ERO1L - ero1-like (s. cerevisiae)  MYO19 - myosin xix  USP6NL - usp6 n-terminal like  KCTD7 - potassium channel tetramerization domain containing 7  CEP290 - centrosomal protein 290kda  NOS3 - nitric oxide synthase 3 (endothelial cell)  PACSIN1 - protein kinase c and casein kinase substrate in neurons 1  NPY - neuropeptide y  CEP57 - centrosomal protein 57kda  NOTCH1 - notch 1  BAZ2B - bromodomain adjacent to zinc finger domain, 2b  CTC1 - cts telomere maintenance complex component 1  AMOTL1 - angiomotin like 1  PQLC1 - pq loop repeat containing 1  KCNIP2 - kv channel interacting protein 2  HDAC4 - histone deacetylase 4  TOX - thymocyte selection-associated high mobility group box  EDC3 - enhancer of mrna decapping 3  CENPT - centromere protein t  ZFYVE16 - zinc finger, fyve domain containing 16  CXXC1 - cxxc finger protein 1  KMT2B - lysine (k)-specific methyltransferase 2b  IFT140 - intraflagellar transport 140 homolog (chlamydomonas)  PHACTR2 - phosphatase and actin regulator 2  SLK - ste20-like kinase  VCPIP1 - valosin containing protein (p97)/p47 complex interacting protein 1  CLSTN3 - calsyntenin 3  NHP2L1 - nhp2 non-histone chromosome protein 2-like 1 (s. cerevisiae)  HDAC9 - histone deacetylase 9  SART3 - squamous cell carcinoma antigen recognized by t cells 3  EHD2 - eh-domain containing 2  SETD1A - set domain containing 1a  EHD3 - eh-domain containing 3  CCP110 - centriolar coiled coil protein 110kda  EHD4 - eh-domain containing 4  NID1 - nidogen 1  IQCB1 - iq motif containing b1  NEK3 - nima-related kinase 3  MINK1 - misshapen-like kinase 1  TTLL4 - tubulin tyrosine ligase-like family, member 4  GCC2 - grip and coiled-coil domain containing 2  NEFL - neurofilament, light polypeptide  MTFR1 - mitochondrial fission regulator 1  SMARCAL1 - swi/snf related, matrix associated, actin dependent regulator of chromatin, subfamily a-like 1  NEK1 - nima-related kinase 1  C8orf37 - chromosome 8 open reading frame 37  NEDD9 - neural precursor cell expressed, developmentally down-regulated 9  CTR9 - ctr9, paf1/rna polymerase ii complex component  WRAP73 - wd repeat containing, antisense to tp73  NEFM - neurofilament, medium polypeptide  PPM1F - protein phosphatase, mg2+/mn2+ dependent, 1f  EPC1 - enhancer of polycomb homolog 1 (drosophila)  ARHGEF10 - rho guanine nucleotide exchange factor (gef) 10  MORF4L2 - mortality factor 4 like 2  FEZ1 - fasciculation and elongation protein zeta 1 (zygin i)  CAMSAP1 - calmodulin regulated spectrin-associated protein 1  NUP155 - nucleoporin 155kda  NFIA - nuclear factor i/a  SEC24C - sec24 family, member c (s. cerevisiae)  NF1 - neurofibromin 1  FAM91A1 - family with sequence similarity 91, member a1  CROCC - ciliary rootlet coiled-coil, rootletin  CDK5RAP3 - cdk5 regulatory subunit associated protein 3  VPS37D - vacuolar protein sorting 37 homolog d (s. cerevisiae)  RAPGEF2 - rap guanine nucleotide exchange factor (gef) 2  ARHGEF4 - rho guanine nucleotide exchange factor (gef) 4  NDUFB2 - nadh dehydrogenase (ubiquinone) 1 beta subcomplex, 2, 8kda  NUP93 - nucleoporin 93kda  CLINT1 - clathrin interactor 1  CEP63 - centrosomal protein 63kda  CALY - calcyon neuron-specific vesicular protein  GEMIN4 - gem (nuclear organelle) associated protein 4  KDM4A - lysine (k)-specific demethylase 4a  PHF14 - phd finger protein 14  COPS7A - cop9 signalosome subunit 7a  HP1BP3 - heterochromatin protein 1, binding protein 3  ASAP1 - arfgap with sh3 domain, ankyrin repeat and ph domain 1  KIAA0430 - kiaa0430  NDUFC2 - nadh dehydrogenase (ubiquinone) 1, subcomplex unknown, 2, 14.5kda |
| GO:0043414 | macromolecule methylation | 7.52E-5 | 7.12E-3 | 1.19 (10334,180,6996,145) | [+] Show genes  WTAP - wilms tumor 1 associated protein  HENMT1 - hen1 methyltransferase homolog 1 (arabidopsis)  ARID4B - at rich interactive domain 4b (rbp1-like)  SETDB1 - set domain, bifurcated 1  GSPT1 - g1 to s phase transition 1  RBM15 - rna binding motif protein 15  WHSC1L1 - wolf-hirschhorn syndrome candidate 1-like 1  TRMT1L - trna methyltransferase 1 homolog (s. cerevisiae)-like  METTL14 - methyltransferase like 14  FAM98A - family with sequence similarity 98, member a  METTL22 - methyltransferase like 22  SETMAR - set domain and mariner transposase fusion gene  PRMT6 - protein arginine methyltransferase 6  PRMT1 - protein arginine methyltransferase 1  THUMPD2 - thump domain containing 2  PRMT2 - protein arginine methyltransferase 2  TRIM28 - tripartite motif containing 28  PPM1D - protein phosphatase, mg2+/mn2+ dependent, 1d  NSUN2 - nop2/sun rna methyltransferase family, member 2  DOT1L - dot1-like histone h3k79 methyltransferase  THUMPD3 - thump domain containing 3  FTSJ3 - ftsj homolog 3 (e. coli)  KMT2A - lysine (k)-specific methyltransferase 2a  ASH1L - ash1 (absent, small, or homeotic)-like (drosophila)  METTL16 - methyltransferase like 16  RLF - rearranged l-myc fusion  SMYD3 - set and mynd domain containing 3  METTL12 - methyltransferase like 12  RAB3D - rab3d, member ras oncogene family  METTL21A - methyltransferase like 21a  FBLL1 - fibrillarin-like 1  PRMT3 - protein arginine methyltransferase 3  FAM173B - family with sequence similarity 173, member b  EHMT2 - euchromatic histone-lysine n-methyltransferase 2  MTA2 - metastasis associated 1 family, member 2  SETD5 - set domain containing 5  METTL18 - methyltransferase like 18  CREBBP - creb binding protein  WDR61 - wd repeat domain 61  KDM3A - lysine (k)-specific demethylase 3a  FAM98B - family with sequence similarity 98, member b  SETD4 - set domain containing 4  SNRPB - small nuclear ribonucleoprotein polypeptides b and b1  CHD5 - chromodomain helicase dna binding protein 5  NDUFAF7 - nadh dehydrogenase (ubiquinone) complex i, assembly factor 7  TRMT10B - trna methyltransferase 10 homolog b (s. cerevisiae)  SETD6 - set domain containing 6  MECP2 - methyl cpg binding protein 2 (rett syndrome)  TRMT10A - trna methyltransferase 10 homolog a (s. cerevisiae)  BCDIN3D - bcdin3 domain containing  TARBP1 - tar (hiv-1) rna binding protein 1  RBM15B - rna binding motif protein 15b  HEMK1 - hemk methyltransferase family member 1  GATAD2A - gata zinc finger domain containing 2a  BAZ2A - bromodomain adjacent to zinc finger domain, 2a  MEN1 - multiple endocrine neoplasia i  KMT2E - lysine (k)-specific methyltransferase 2e  PCMT1 - protein-l-isoaspartate (d-aspartate) o-methyltransferase  METTL1 - methyltransferase like 1  TET2 - tet methylcytosine dioxygenase 2  GNAS - gnas complex locus  TYW3 - trna-yw synthesizing protein 3 homolog (s. cerevisiae)  PWP1 - pwp1 homolog (s. cerevisiae)  KMT2C - lysine (k)-specific methyltransferase 2c  CMTR1 - cap methyltransferase 1  SATB1 - satb homeobox 1  DMAP1 - dna methyltransferase 1 associated protein 1  DIMT1 - dim1 dimethyladenosine transferase 1 homolog (s. cerevisiae)  DPY30 - dpy-30 homolog (c. elegans)  SETD1B - set domain containing 1b  RRNAD1 - ribosomal rna adenine dimethylase domain containing 1  WDR4 - wd repeat domain 4  NOP2 - nop2 nucleolar protein  FTSJ1 - ftsj rna methyltransferase homolog 1 (e. coli)  METTL6 - methyltransferase like 6  MTRR - 5-methyltetrahydrofolate-homocysteine methyltransferase reductase  EHMT1 - euchromatic histone-lysine n-methyltransferase 1  NSD1 - nuclear receptor binding set domain protein 1  ZC3H13 - zinc finger ccch-type containing 13  CAMKMT - calmodulin-lysine n-methyltransferase  TRMT12 - trna methyltransferase 12 homolog (s. cerevisiae)  PAGR1 - paxip1 associated glutamate-rich protein 1  FBXO11 - f-box protein 11  METTL8 - methyltransferase like 8  PICK1 - protein interacting with prkca 1  GTPBP3 - gtp binding protein 3 (mitochondrial)  PRDM4 - pr domain containing 4  ATF7IP - activating transcription factor 7 interacting protein  TRMT5 - trna methyltransferase 5  RAB6A - rab6a, member ras oncogene family  TRMT11 - trna methyltransferase 11 homolog (s. cerevisiae)  MTERFD2 - mterf domain containing 2  CXXC1 - cxxc finger protein 1  RAB3B - rab3b, member ras oncogene family  ICMT - isoprenylcysteine carboxyl methyltransferase  WHSC1 - wolf-hirschhorn syndrome candidate 1  CMTR2 - cap methyltransferase 2  KMT2B - lysine (k)-specific methyltransferase 2b  METTL2A - methyltransferase like 2a  KDM6A - lysine (k)-specific demethylase 6a  TRMT112 - trna methyltransferase 11-2 homolog (s. cerevisiae)  SUV420H1 - suppressor of variegation 4-20 homolog 1 (drosophila)  EZH1 - enhancer of zeste homolog 1 (drosophila)  ASH2L - ash2 (absent, small, or homeotic)-like (drosophila)  CBLL1 - cbl proto-oncogene-like 1, e3 ubiquitin protein ligase  TFB1M - transcription factor b1, mitochondrial  COPRS - coordinator of prmt5, differentiation stimulator  SUV420H2 - suppressor of variegation 4-20 homolog 2 (drosophila)  SETD1A - set domain containing 1a  TTLL12 - tubulin tyrosine ligase-like family, member 12  SUZ12 - suz12 polycomb repressive complex 2 subunit  TDRKH - tudor and kh domain containing  EED - embryonic ectoderm development  CTR9 - ctr9, paf1/rna polymerase ii complex component  RNMT - rna (guanine-7-) methyltransferase  KMT2D - lysine (k)-specific methyltransferase 2d  PCMTD2 - protein-l-isoaspartate (d-aspartate) o-methyltransferase domain containing 2  TGS1 - trimethylguanosine synthase 1  ATRX - alpha thalassemia/mental retardation syndrome x-linked  ETF1 - eukaryotic translation termination factor 1  CTCF - ccctc-binding factor (zinc finger protein)  PRMT7 - protein arginine methyltransferase 7  KIAA0391 - kiaa0391  OGT - o-linked n-acetylglucosamine (glcnac) transferase  DNMT1 - dna (cytosine-5-)-methyltransferase 1  ZNF711 - zinc finger protein 711  ARID4A - at rich interactive domain 4a (rbp1-like)  METTL5 - methyltransferase like 5  TRDMT1 - trna aspartic acid methyltransferase 1  DNMT3A - dna (cytosine-5-)-methyltransferase 3 alpha  RBBP5 - retinoblastoma binding protein 5  MEPCE - methylphosphate capping enzyme  ZNF335 - zinc finger protein 335  LARP7 - la ribonucleoprotein domain family, member 7  FOS - fbj murine osteosarcoma viral oncogene homolog  SETD2 - set domain containing 2  PCIF1 - pdx1 c-terminal inhibiting factor 1  TRMT10C - trna methyltransferase 10 homolog c (s. cerevisiae)  PRMT5 - protein arginine methyltransferase 5  THADA - thyroid adenoma associated  METTL3 - methyltransferase like 3  NSUN5 - nop2/sun domain family, member 5  MTO1 - mitochondrial trna translation optimization 1  NSUN3 - nop2/sun domain family, member 3  TRMT6 - trna methyltransferase 6 homolog (s. cerevisiae) |
| GO:1903311 | regulation of mRNA metabolic process | 7.84E-5 | 7.37E-3 | 1.15 (10334,285,6996,222) | [+] Show genes  NCBP2 - nuclear cap binding protein subunit 2, 20kda  EXOSC9 - exosome component 9  WTAP - wilms tumor 1 associated protein  NCBP1 - nuclear cap binding protein subunit 1, 80kda  SAP18 - sin3a-associated protein, 18kda  RBM39 - rna binding motif protein 39  NCL - nucleolin  MBNL2 - muscleblind-like splicing regulator 2  HNRNPA0 - heterogeneous nuclear ribonucleoprotein a0  METTL14 - methyltransferase like 14  SNW1 - snw domain containing 1  HNRNPM - heterogeneous nuclear ribonucleoprotein m  ZFP36L2 - zfp36 ring finger protein-like 2  ZFP36L1 - zfp36 ring finger protein-like 1  ALKBH5 - alkb, alkylation repair homolog 5 (e. coli)  UPF1 - upf1 regulator of nonsense transcripts homolog (yeast)  CWC22 - cwc22 spliceosome-associated protein homolog (s. cerevisiae)  METTL16 - methyltransferase like 16  FTO - fat mass and obesity associated  CELF4 - cugbp, elav-like family member 4  ANP32A - acidic (leucine-rich) nuclear phosphoprotein 32 family, member a  RBM7 - rna binding motif protein 7  RBM5 - rna binding motif protein 5  ZC3HAV1 - zinc finger ccch-type, antiviral 1  SECISBP2 - secis binding protein 2  PAPOLA - poly(a) polymerase alpha  PABPN1 - poly(a) binding protein, nuclear 1  TAF15 - taf15 rna polymerase ii, tata box binding protein (tbp)-associated factor, 68kda  RNPS1 - rna binding protein s1, serine-rich domain  SON - son dna binding protein  PSME3 - proteasome (prosome, macropain) activator subunit 3 (pa28 gamma; ki)  ELAVL1 - elav (embryonic lethal, abnormal vision, drosophila)-like 1 (hu antigen r)  GTPBP1 - gtp binding protein 1  ELAVL4 - elav (embryonic lethal, abnormal vision, drosophila)-like 4  PSMD14 - proteasome (prosome, macropain) 26s subunit, non-atpase, 14  CNOT1 - ccr4-not transcription complex, subunit 1  EXOSC7 - exosome component 7  MYD88 - myeloid differentiation primary response 88  FXR2 - fragile x mental retardation, autosomal homolog 2  SNRNP70 - small nuclear ribonucleoprotein 70kda (u1)  YTHDF2 - yth domain family, member 2  SNRPA - small nuclear ribonucleoprotein polypeptide a  ZC3H14 - zinc finger ccch-type containing 14  CNOT7 - ccr4-not transcription complex, subunit 7  CPSF4 - cleavage and polyadenylation specific factor 4, 30kda  TRA2A - transformer 2 alpha homolog (drosophila)  RBM17 - rna binding motif protein 17  HNRNPR - heterogeneous nuclear ribonucleoprotein r  AXIN2 - axin 2  RBM15B - rna binding motif protein 15b  ROCK1 - rho-associated, coiled-coil containing protein kinase 1  RBM10 - rna binding motif protein 10  RBM25 - rna binding motif protein 25  ROCK2 - rho-associated, coiled-coil containing protein kinase 2  SF3B4 - splicing factor 3b, subunit 4, 49kda  ZBTB7A - zinc finger and btb domain containing 7a  POLR2G - polymerase (rna) ii (dna directed) polypeptide g  HSPA8 - heat shock 70kda protein 8  SLTM - safb-like, transcription modulator  NELFE - negative elongation factor complex member e  PTBP3 - polypyrimidine tract binding protein 3  FUS - fused in sarcoma  FAM46B - family with sequence similarity 46, member b  SUPT6H - suppressor of ty 6 homolog (s. cerevisiae)  CACNG7 - calcium channel, voltage-dependent, gamma subunit 7  SUPT5H - suppressor of ty 5 homolog (s. cerevisiae)  DAZAP1 - daz associated protein 1  TNRC6B - trinucleotide repeat containing 6b  SRSF9 - serine/arginine-rich splicing factor 9  AKT1 - v-akt murine thymoma viral oncogene homolog 1  PCID2 - pci domain containing 2  TNPO1 - transportin 1  VIM - vimentin  CPSF6 - cleavage and polyadenylation specific factor 6, 68kda  NUDT21 - nudix (nucleoside diphosphate linked moiety x)-type motif 21  PCBP4 - poly(rc) binding protein 4  ZC3H3 - zinc finger ccch-type containing 3  CPSF7 - cleavage and polyadenylation specific factor 7, 59kda  SYNCRIP - synaptotagmin binding, cytoplasmic rna interacting protein  IWS1 - iws1 homolog (s. cerevisiae)  CELF6 - cugbp, elav-like family member 6  YTHDC1 - yth domain containing 1  CCNT1 - cyclin t1  UBB - ubiquitin b  HNRNPU - heterogeneous nuclear ribonucleoprotein u (scaffold attachment factor a)  HNRNPK - heterogeneous nuclear ribonucleoprotein k  HNRNPL - heterogeneous nuclear ribonucleoprotein l  TNFSF13 - tumor necrosis factor (ligand) superfamily, member 13  PAPD7 - pap associated domain containing 7  RBM8A - rna binding motif protein 8a  SRPK2 - srsf protein kinase 2  HNRNPA2B1 - heterogeneous nuclear ribonucleoprotein a2/b1  SRPK1 - srsf protein kinase 1  HNRNPA1 - heterogeneous nuclear ribonucleoprotein a1  HNRNPC - heterogeneous nuclear ribonucleoprotein c (c1/c2)  HNRNPD - heterogeneous nuclear ribonucleoprotein d (au-rich element rna binding protein 1, 37kda)  JMJD6 - jumonji domain containing 6  ZC3H10 - zinc finger ccch-type containing 10  NUP214 - nucleoporin 214kda  PRKCD - protein kinase c, delta  PRKCA - protein kinase c, alpha  CNOT8 - ccr4-not transcription complex, subunit 8  PDE12 - phosphodiesterase 12  THRAP3 - thyroid hormone receptor associated protein 3  DHX9 - deah (asp-glu-ala-his) box helicase 9  FASTKD1 - fast kinase domains 1  APEX1 - apex nuclease (multifunctional dna repair enzyme) 1  PSMD6 - proteasome (prosome, macropain) 26s subunit, non-atpase, 6  DHX36 - deah (asp-glu-ala-his) box polypeptide 36  FMR1 - fragile x mental retardation 1  RBM23 - rna binding motif protein 23  CDK11B - cyclin-dependent kinase 11b  DDX5 - dead (asp-glu-ala-asp) box helicase 5  PARN - poly(a)-specific ribonuclease  RBM15 - rna binding motif protein 15  TNRC6A - trinucleotide repeat containing 6a  RBMX - rna binding motif protein, x-linked  SET - set nuclear oncogene  PSMA1 - proteasome (prosome, macropain) subunit, alpha type, 1  SRSF12 - serine/arginine-rich splicing factor 12  PSMA2 - proteasome (prosome, macropain) subunit, alpha type, 2  PRPF19 - pre-mrna processing factor 19  PSMB1 - proteasome (prosome, macropain) subunit, beta type, 1  RBM19 - rna binding motif protein 19  PSMA5 - proteasome (prosome, macropain) subunit, alpha type, 5  PSMA3 - proteasome (prosome, macropain) subunit, alpha type, 3  KHDRBS3 - kh domain containing, rna binding, signal transduction associated 3  PSMB10 - proteasome (prosome, macropain) subunit, beta type, 10  DCP1A - decapping mrna 1a  PSMC6 - proteasome (prosome, macropain) 26s subunit, atpase, 6  PSMD1 - proteasome (prosome, macropain) 26s subunit, non-atpase, 1  PSMC4 - proteasome (prosome, macropain) 26s subunit, atpase, 4  PSMC2 - proteasome (prosome, macropain) 26s subunit, atpase, 2  PSMD9 - proteasome (prosome, macropain) 26s subunit, non-atpase, 9  EXOSC3 - exosome component 3  PSMD10 - proteasome (prosome, macropain) 26s subunit, non-atpase, 10  PSMD7 - proteasome (prosome, macropain) 26s subunit, non-atpase, 7  YTHDF3 - yth domain family, member 3  PSMD8 - proteasome (prosome, macropain) 26s subunit, non-atpase, 8  PSMD5 - proteasome (prosome, macropain) 26s subunit, non-atpase, 5  PSMD2 - proteasome (prosome, macropain) 26s subunit, non-atpase, 2  EXOSC1 - exosome component 1  PSMD12 - proteasome (prosome, macropain) 26s subunit, non-atpase, 12  CELF3 - cugbp, elav-like family member 3  PSMD13 - proteasome (prosome, macropain) 26s subunit, non-atpase, 13  NSRP1 - nuclear speckle splicing regulatory protein 1  DCP2 - decapping mrna 2  PTBP1 - polypyrimidine tract binding protein 1  CDC73 - cell division cycle 73  TIRAP - toll-interleukin 1 receptor (tir) domain containing adaptor protein  SAMD4B - sterile alpha motif domain containing 4b  NUP98 - nucleoporin 98kda  KHSRP - kh-type splicing regulatory protein  DDX17 - dead (asp-glu-ala-asp) box helicase 17  ANGEL2 - angel homolog 2 (drosophila)  CDK9 - cyclin-dependent kinase 9  NPM1 - nucleophosmin (nucleolar phosphoprotein b23, numatrin)  LEO1 - leo1, paf1/rna polymerase ii complex component, homolog (s. cerevisiae)  CSDC2 - cold shock domain containing c2, rna binding  RNF40 - ring finger protein 40, e3 ubiquitin protein ligase  PUM2 - pumilio homolog 2 (drosophila)  LARP1 - la ribonucleoprotein domain family, member 1  SAFB - scaffold attachment factor b  SERBP1 - serpine1 mrna binding protein 1  MAGOH - mago-nashi homolog, proliferation-associated (drosophila)  PAPD5 - pap associated domain containing 5  MTOR - mechanistic target of rapamycin (serine/threonine kinase)  CNOT6L - ccr4-not transcription complex, subunit 6-like  XPO1 - exportin 1 (crm1 homolog, yeast)  SRSF10 - serine/arginine-rich splicing factor 10  DIS3 - dis3 mitotic control homolog (s. cerevisiae)  NOVA2 - neuro-oncological ventral antigen 2  NOVA1 - neuro-oncological ventral antigen 1  AHCYL1 - adenosylhomocysteinase-like 1  EXOSC2 - exosome component 2  PUM1 - pumilio homolog 1 (drosophila)  DHX34 - deah (asp-glu-ala-his) box polypeptide 34  DYRK1A - dual-specificity tyrosine-(y)-phosphorylation regulated kinase 1a  GIGYF2 - grb10 interacting gyf protein 2  TARDBP - tar dna binding protein  PAF1 - paf1, rna polymerase ii associated factor, homolog (s. cerevisiae)  SART3 - squamous cell carcinoma antigen recognized by t cells 3  SMU1 - smu-1 suppressor of mec-8 and unc-52 homolog (c. elegans)  PUF60 - poly-u binding splicing factor 60kda  RC3H2 - ring finger and ccch-type domains 2  PNPT1 - polyribonucleotide nucleotidyltransferase 1  PABPC1 - poly(a) binding protein, cytoplasmic 1  CTR9 - ctr9, paf1/rna polymerase ii complex component  RBFOX3 - rna binding protein, fox-1 homolog (c. elegans) 3  CELF1 - cugbp, elav-like family member 1  KHDRBS1 - kh domain containing, rna binding, signal transduction associated 1  RBM42 - rna binding motif protein 42  CELF2 - cugbp, elav-like family member 2  IK - ik cytokine, down-regulator of hla ii  EXOSC8 - exosome component 8  RBFOX2 - rna binding protein, fox-1 homolog (c. elegans) 2  SF1 - splicing factor 1  U2AF2 - u2 small nuclear rna auxiliary factor 2  SRSF1 - serine/arginine-rich splicing factor 1  RBM3 - rna binding motif (rnp1, rrm) protein 3  RBM4 - rna binding motif protein 4  SRSF2 - serine/arginine-rich splicing factor 2  SRSF3 - serine/arginine-rich splicing factor 3  SRSF4 - serine/arginine-rich splicing factor 4  SRSF5 - serine/arginine-rich splicing factor 5  SRSF6 - serine/arginine-rich splicing factor 6  SRSF7 - serine/arginine-rich splicing factor 7  SFSWAP - splicing factor, suppressor of white-apricot homolog (drosophila)  RNF20 - ring finger protein 20, e3 ubiquitin protein ligase  TRA2B - transformer 2 beta homolog (drosophila)  LARP7 - la ribonucleoprotein domain family, member 7  EXOSC4 - exosome component 4  TNRC6C - trinucleotide repeat containing 6c  CIRBP - cold inducible rna binding protein  NBAS - neuroblastoma amplified sequence  TIA1 - tia1 cytotoxic granule-associated rna binding protein  YWHAZ - tyrosine 3-monooxygenase/tryptophan 5-monooxygenase activation protein, zeta polypeptide  METTL3 - methyltransferase like 3  SAFB2 - scaffold attachment factor b2  MYEF2 - myelin expression factor 2  YWHAB - tyrosine 3-monooxygenase/tryptophan 5-monooxygenase activation protein, beta polypeptide  RBM4B - rna binding motif protein 4b |
| GO:0007034 | vacuolar transport | 8.11E-5 | 7.57E-3 | 1.23 (10334,124,6996,103) | [+] Show genes  CHMP4C - charged multivesicular body protein 4c  DTX3L - deltex 3-like (drosophila)  PSAP - prosaposin  USE1 - unconventional snare in the er 1 homolog (s. cerevisiae)  EPG5 - ectopic p-granules autophagy protein 5 homolog (c. elegans)  VPS37A - vacuolar protein sorting 37 homolog a (s. cerevisiae)  HOOK3 - hook microtubule-tethering protein 3  NDFIP1 - nedd4 family interacting protein 1  VPS13D - vacuolar protein sorting 13 homolog d (s. cerevisiae)  SCARB2 - scavenger receptor class b, member 2  AP3B1 - adaptor-related protein complex 3, beta 1 subunit  MGRN1 - mahogunin ring finger 1, e3 ubiquitin protein ligase  GOSR2 - golgi snap receptor complex member 2  RNF215 - ring finger protein 215  TSG101 - tumor susceptibility gene 101  VPS11 - vacuolar protein sorting 11 homolog (s. cerevisiae)  CLEC16A - c-type lectin domain family 16, member a  TMEM50A - transmembrane protein 50a  UBXN6 - ubx domain protein 6  HGSNAT - heparan-alpha-glucosaminide n-acetyltransferase  SNX27 - sorting nexin family member 27  MVB12A - multivesicular body subunit 12a  VPS51 - vacuolar protein sorting 51 homolog (s. cerevisiae)  VPS16 - vacuolar protein sorting 16 homolog (s. cerevisiae)  VPS13C - vacuolar protein sorting 13 homolog c (s. cerevisiae)  ARSB - arylsulfatase b  VPS39 - vacuolar protein sorting 39 homolog (s. cerevisiae)  TMEM50B - transmembrane protein 50b  NUMA1 - nuclear mitotic apparatus protein 1  RHOB - ras homolog family member b  VPS4B - vacuolar protein sorting 4 homolog b (s. cerevisiae)  AP1G2 - adaptor-related protein complex 1, gamma 2 subunit  FAM160A2 - family with sequence similarity 160, member a2  LAMP2 - lysosomal-associated membrane protein 2  CHMP1A - charged multivesicular body protein 1a  TRAK2 - trafficking protein, kinesin binding 2  AKTIP - akt interacting protein  AP4M1 - adaptor-related protein complex 4, mu 1 subunit  CHMP2B - charged multivesicular body protein 2b  STX8 - syntaxin 8  AP3D1 - adaptor-related protein complex 3, delta 1 subunit  HGS - hepatocyte growth factor-regulated tyrosine kinase substrate  VPS52 - vacuolar protein sorting 52 homolog (s. cerevisiae)  HSPA8 - heat shock 70kda protein 8  VTA1 - vesicle (multivesicular body) trafficking 1  DENND3 - denn/madd domain containing 3  NAGPA - n-acetylglucosamine-1-phosphodiester alpha-n-acetylglucosaminidase  AP1G1 - adaptor-related protein complex 1, gamma 1 subunit  VPS28 - vacuolar protein sorting 28 homolog (s. cerevisiae)  TMEM106B - transmembrane protein 106b  VPS37C - vacuolar protein sorting 37 homolog c (s. cerevisiae)  CCDC91 - coiled-coil domain containing 91  VPS35 - vacuolar protein sorting 35 homolog (s. cerevisiae)  ATP13A2 - atpase type 13a2  LYST - lysosomal trafficking regulator  MON1B - mon1 secretory trafficking family member b  LEPROT - leptin receptor overlapping transcript  CACNG7 - calcium channel, voltage-dependent, gamma subunit 7  ATP6V0D1 - atpase, h+ transporting, lysosomal 38kda, v0 subunit d1  MTM1 - myotubularin 1  VPS54 - vacuolar protein sorting 54 homolog (s. cerevisiae)  BECN1 - beclin 1, autophagy related  ATG14 - autophagy related 14  HMGXB4 - hmg box domain containing 4  RAB7A - rab7a, member ras oncogene family  ZFYVE16 - zinc finger, fyve domain containing 16  TGFBRAP1 - transforming growth factor, beta receptor associated protein 1  PIK3R4 - phosphoinositide-3-kinase, regulatory subunit 4  CHMP5 - charged multivesicular body protein 5  VPS33A - vacuolar protein sorting 33 homolog a (s. cerevisiae)  VCP - valosin containing protein  VPS4A - vacuolar protein sorting 4 homolog a (s. cerevisiae)  ANKFY1 - ankyrin repeat and fyve domain containing 1  EHD3 - eh-domain containing 3  NDFIP2 - nedd4 family interacting protein 2  VTI1B - vesicle transport through interaction with t-snares 1b  SNX16 - sorting nexin 16  VPS18 - vacuolar protein sorting 18 homolog (s. cerevisiae)  SCYL2 - scy1-like 2 (s. cerevisiae)  GAK - cyclin g associated kinase  LRP1 - low density lipoprotein receptor-related protein 1  GCC2 - grip and coiled-coil domain containing 2  AP3M1 - adaptor-related protein complex 3, mu 1 subunit  CHMP7 - charged multivesicular body protein 7  LEPROTL1 - leptin receptor overlapping transcript-like 1  CLU - clusterin  CHMP3 - charged multivesicular body protein 3  RNF157 - ring finger protein 157  VPS37D - vacuolar protein sorting 37 homolog d (s. cerevisiae)  VPS53 - vacuolar protein sorting 53 homolog (s. cerevisiae)  CHMP4A - charged multivesicular body protein 4a  IGF2R - insulin-like growth factor 2 receptor  M6PR - mannose-6-phosphate receptor (cation dependent)  SMURF1 - smad specific e3 ubiquitin protein ligase 1  ALS2 - amyotrophic lateral sclerosis 2 (juvenile)  VPS41 - vacuolar protein sorting 41 homolog (s. cerevisiae)  STAM - signal transducing adaptor molecule (sh3 domain and itam motif) 1  CHMP4B - charged multivesicular body protein 4b  VIPAS39 - vps33b interacting protein, apical-basolateral polarity regulator, spe-39 homolog  VPS25 - vacuolar protein sorting 25 homolog (s. cerevisiae)  CACNG4 - calcium channel, voltage-dependent, gamma subunit 4  NCOA4 - nuclear receptor coactivator 4  MON1A - mon1 secretory trafficking family member a |
| GO:1904356 | regulation of telomere maintenance via telomere lengthening | 9.04E-5 | 8.39E-3 | 1.34 (10334,53,6996,48) | [+] Show genes  DHX36 - deah (asp-glu-ala-his) box polypeptide 36  CCT6A - chaperonin containing tcp1, subunit 6a (zeta 1)  DKC1 - dyskeratosis congenita 1, dyskerin  EXOSC10 - exosome component 10  ERCC4 - excision repair cross-complementing rodent repair deficiency, complementation group 4  DCP2 - decapping mrna 2  PINX1 - pin2/terf1 interacting, telomerase inhibitor 1  CCT8 - chaperonin containing tcp1, subunit 8 (theta)  PARN - poly(a)-specific ribonuclease  TCP1 - t-complex 1  PAPD5 - pap associated domain containing 5  NAT10 - n-acetyltransferase 10 (gcn5-related)  NAF1 - nuclear assembly factor 1 ribonucleoprotein  TERF1 - telomeric repeat binding factor (nima-interacting) 1  TERF2 - telomeric repeat binding factor 2  HNRNPU - heterogeneous nuclear ribonucleoprotein u (scaffold attachment factor a)  FBXO4 - f-box protein 4  TNKS - tankyrase, trf1-interacting ankyrin-related adp-ribose polymerase  OBFC1 - oligonucleotide/oligosaccharide-binding fold containing 1  TINF2 - terf1 (trf1)-interacting nuclear factor 2  MAP2K7 - mitogen-activated protein kinase kinase 7  GNL3L - guanine nucleotide binding protein-like 3 (nucleolar)-like  MAP3K4 - mitogen-activated protein kinase kinase kinase 4  CTC1 - cts telomere maintenance complex component 1  CTNNB1 - catenin (cadherin-associated protein), beta 1, 88kda  POT1 - protection of telomeres 1  MAPK15 - mitogen-activated protein kinase 15  CCT3 - chaperonin containing tcp1, subunit 3 (gamma)  CCT7 - chaperonin containing tcp1, subunit 7 (eta)  MAPKAPK5 - mitogen-activated protein kinase-activated protein kinase 5  HNRNPA2B1 - heterogeneous nuclear ribonucleoprotein a2/b1  MAPK3 - mitogen-activated protein kinase 3  HNRNPA1 - heterogeneous nuclear ribonucleoprotein a1  MAPK1 - mitogen-activated protein kinase 1  HNRNPC - heterogeneous nuclear ribonucleoprotein c (c1/c2)  CCT5 - chaperonin containing tcp1, subunit 5 (epsilon)  CCT2 - chaperonin containing tcp1, subunit 2 (beta)  SLX4 - slx4 structure-specific endonuclease subunit  CCT4 - chaperonin containing tcp1, subunit 4 (delta)  ACD - adrenocortical dysplasia homolog (mouse)  PARP1 - poly (adp-ribose) polymerase 1  SMG6 - smg6 nonsense mediated mrna decay factor  HMBOX1 - homeobox containing 1  XRCC5 - x-ray repair complementing defective repair in chinese hamster cells 5 (double-strand-break rejoining)  SRC - v-src avian sarcoma (schmidt-ruppin a-2) viral oncogene homolog  TNKS2 - tankyrase, trf1-interacting ankyrin-related adp-ribose polymerase 2  PNKP - polynucleotide kinase 3'-phosphatase  PKIB - protein kinase (camp-dependent, catalytic) inhibitor beta |
| GO:0016925 | protein sumoylation | 9.04E-5 | 8.33E-3 | 1.34 (10334,53,6996,48) | [+] Show genes  NUP188 - nucleoporin 188kda  SENP2 - sumo1/sentrin/smt3 specific peptidase 2  RANBP2 - ran binding protein 2  NUP205 - nucleoporin 205kda  MUL1 - mitochondrial e3 ubiquitin protein ligase 1  RANGAP1 - ran gtpase activating protein 1  SEH1L - seh1-like (s. cerevisiae)  NUP133 - nucleoporin 133kda  NSMCE2 - non-smc element 2, mms21 homolog (s. cerevisiae)  ZNF451 - zinc finger protein 451  NUP107 - nucleoporin 107kda  NUPL2 - nucleoporin like 2  SUMO2 - small ubiquitin-like modifier 2  NUP88 - nucleoporin 88kda  NUP98 - nucleoporin 98kda  AAAS - achalasia, adrenocortical insufficiency, alacrimia  TRIM28 - tripartite motif containing 28  SENP5 - sumo1/sentrin specific peptidase 5  NUP50 - nucleoporin 50kda  ZBED1 - zinc finger, bed-type containing 1  NUP155 - nucleoporin 155kda  NUP85 - nucleoporin 85kda  SUMO3 - small ubiquitin-like modifier 3  PIAS1 - protein inhibitor of activated stat, 1  HDAC4 - histone deacetylase 4  PIAS4 - protein inhibitor of activated stat, 4  SUMO1 - small ubiquitin-like modifier 1  STX1A - syntaxin 1a (brain)  NUP43 - nucleoporin 43kda  EGR2 - early growth response 2  NUP93 - nucleoporin 93kda  PIAS3 - protein inhibitor of activated stat, 3  TRPM4 - transient receptor potential cation channel, subfamily m, member 4  RAE1 - rae1 rna export 1 homolog (s. pombe)  SENP1 - sumo1/sentrin specific peptidase 1  NDC1 - ndc1 transmembrane nucleoporin  NUP35 - nucleoporin 35kda  SAE1 - sumo1 activating enzyme subunit 1  UBE2I - ubiquitin-conjugating enzyme e2i  NUP214 - nucleoporin 214kda  SENP6 - sumo1/sentrin specific peptidase 6  CBX4 - chromobox homolog 4  NUP153 - nucleoporin 153kda  NUP210 - nucleoporin 210kda  TPR - translocated promoter region, nuclear basket protein  TOPORS - topoisomerase i binding, arginine/serine-rich, e3 ubiquitin protein ligase  UBA2 - ubiquitin-like modifier activating enzyme 2  NUP160 - nucleoporin 160kda |

Species used: Homo sapiens

The system has recognized 17559 genes out of 19484 gene terms entered by the user.  
 17559 genes were recognized by gene symbol and 0 genes by other gene IDs .  
7130 duplicate genes were removed (keeping the highest ranking instance of each gene) leaving a total of 10429 genes.  
Only 10334 of these genes are associated with a GO term.

The GOrilla database is periodically updated using the GO database and other sources.  
The GOrilla database was last updated on Aug 29, 2020

This results page will be available on this site for one month from now (until
Oct 1, 2020
). You can bookmark this page and come back to it later.

  
**'P-value'** is the enrichment p-value computed according to the mHG or HG model. This p-value is not corrected for multiple testing of 13915 GO terms.  
  
**'FDR q-value'** is the correction of the above p-value for multiple testing using the Benjamini and Hochberg (1995) method.   
Namely, for the i^th^ term (ranked according to p-value) the FDR q-value is (p-value \* number of GO terms) / i.   
  
**Enrichment (N, B, n, b)** is defined as follows:  
N - is the total number of genes  
B - is the total number of genes associated with a specific GO term  
n - is the number of genes in the top of the user's input list or in the target set when appropriate  
b - is the number of genes in the intersection  
Enrichment = (b/n) / (B/N)  
  
**Genes:** For each GO term you can see the list of associated genes that appear in the optimal top of the list.  
Each gene name is specified by gene symbol followed by a short description of the gene   

Back to the GOrilla main page
